# Supplementary material for: Extracellular Vesicles Bearing Vimentin Drive Epithelial–Mesenchymal Transition
Source: Mol Cell Proteomics. 2025 Jul 4;24(12):101028. doi: 10.1016/j.mcpro.2025.101028 (PMC12719745; doi:10.1016/j.mcpro.2025.101028)
Supplement: Supplemental Data 4 [file mmc7.pdf]

sheet 4

HDF_EV_1 MaxLFQ Intensity	HDF_EV_2 MaxLFQ Intensity	HDF_EV_3 MaxLFQ Intensity	HDF_EV_4 MaxLFQ Intensity	HDF_EV_KO_1 MaxLFQ Intensity	HDF_EV_KO_2 MaxLFQ Intensity	HDF_EV_KO_3 MaxLFQ Intensity	HDF_EV_KO_4 MaxLFQ Intensity	HDF_KO_1 MaxLFQ Intensity	HDF_KO_2 MaxLFQ Intensity	HDF_KO_3 MaxLFQ Intensity	HDF_KO_4 MaxLFQ Intensity
26.9412	25.966	26.0724	25.7915	26.9966	27.6296	26.2629	26.4974	25.107	25.288	24.6004	23.9907
25.9084	25.7907	27.2019	24.9153	25.2474	27.6649	26.7033	25.5943	27.88	27.7384	27.5876	27.7207
25.8105	27.5541	27.4209	27.6397	26.0446	28.0496	26.8105	26.1203	27.0526	27.0613	27.2797	27.216
26.2795	26.2099	26.3246	25.3932	26.7721	27.8254	26.0435	26.4965	24.3157	25.7148	25.5573	26.1082
27.3401	25.8927	26.1676	25.5656	26.7992	25.7994	27.2018	25.0445	26.4476	26.3629	26.625	26.4573
25.2574	25.9864	24.97	26.4563	26.6445	27.6989	27.4223	25.6907	23.8098	26.3176	25.844	25.9156
26.2854	26.9111	26.3737	25.4323	27.5631	26.3631	26.2047	25.6764	26.9788	27.1134	27.0061	27.5635
26.2813	26.4532	25.9019	26.6208	26.9818	25.2217	26.3961	24.8605	26.7347	25.2036	23.7956	25.2204
26.7638	26.5659	26.6585	24.9907	25.6872	25.3825	26.408	26.1085	26.9817	27.1971	26.9073	26.7285
26.0838	25.6746	25.9096	25.7925	25.9078	26.0158	27.0703	26.5307	27.4139	27.2177	27.6075	27.3229
25.3544	25.3149	26.2332	27.7033	27.3798	23.9721	27.7587	24.925	26.924	26.6679	26.0647	26.2
26.0418	24.7189	27.8839	26.831	26.639	25.8927	26.6198	26.0036	29.8877	29.6911	29.3701	29.8653
25.9563	29.9198	25.8838	25.1495	25.9793	26.1343	27.1997	26.0315	29.7103	29.6704	30.1032	29.735
26.9322	25.9458	24.8959	26.4258	26.4675	26.9337	26.8153	25.9334	25.1616	25.7058	26.1523	25.8501
25.682	26.9582	27.802	25.7307	27.1185	26.2276	26.0487	25.5627	24.5515	24.098	24.7455	24.4869
27.0323	25.2126	28.1337	24.9527	27.2984	27.8575	27.2112	27.6179	26.0343	24.2383	26.4063	26.0219
26.5007	25.5086	25.3104	24.5857	27.1648	27.2391	27.2101	25.1758	26.355	26.5031	27.0798	27.1641
27.4588	25.6562	27.0783	26.574	26.0857	26.5897	26.3675	24.986	28.0037	27.8624	28.1311	28.2309
29.0979	28.5581	29.0641	29.1528	28.1539	28.5505	28.2835	29.1629	28.6151	28.3446	28.8566	28.8524
28.3996	28.1209	28.6346	28.1178	28.4876	28.3449	26.4476	27.6494	28.1794	28.0137	27.996	27.9741
30.1942	30.3308	30.65	30.5936	30.4832	29.9958	30.405	29.8667	28.3459	28.4903	28.3775	28.4249
26.083	24.4809	25.5891	27.4515	26.4478	26.3321	26.8035	25.771	26.5846	26.6334	26.4467	26.2562
25.0811	25.9067	26.3187	26.2641	25.3098	26.6049	27.0654	25.8004	27.0027	24.4726	24.0559	26.9734
28.8923	29.1767	29.431	29.2462	29.0841	29.1571	29.4539	28.7242	26.8377	26.4286	26.648	26.3862
25.8766	26.5218	26.7025	25.6817	25.3542	27.0572	27.8095	26.9242	29.6207	29.3816	28.9891	28.9998
26.0881	25.1147	26.2299	26.2353	26.0426	26.7866	27.8631	25.5195	27.223	28.0335	27.3266	27.6411
27.6425	26.5965	28.5555	26.8396	25.7362	26.9425	26.6575	27.0356	26.6676	27.1265	27.3962	26.6391
28.521	28.6068	28.3309	28.3571	26.863	29.3547	25.9989	28.4234	28.4162	28.4507	28.7079	28.5893
27.9065	28.0208	27.6508	27.5754	28.6183	28.3094	28.0755	27.8572	28.1481	28.1388	28.4276	28.4306
27.3819	25.7044	27.8061	25.0442	26.2555	27.546	26.0523	27.0364	28.2474	27.9747	28.0108	27.9726
26.9847	27.2088	27.5901	27.5686	28.6392	25.1755	26.9327	25.2291	28.1485	27.6728	27.385	27.6442
26.0216	27.5332	25.0867	25.96	26.2822	27.4962	26.98	26.9993	27.8858	27.8382	28.1266	28.4979
25.5409	26.1159	25.7838	24.6406	26.1358	26.1707	27.0005	26.2611	25.0687	23.9714	24.2523	26.7066
26.9778	27.0761	27.2841	26.0503	26.1231	25.3586	27.1959	27.6048	26.9225	26.7987	26.8308	26.8641
25.9086	25.3783	27.3006	26.3607	25.4732	25.7959	27.0536	27.3425	26.3693	26.6729	26.6456	26.7036
31.1732	31.2113	31.2673	31.1759	30.2769	30.8068	30.6333	30.3964	30.8798	31.062	31.2218	31.1082
28.409	28.3521	27.3234	27.918	26.9292	28.5108	27.3437	28.9609	28.5265	28.5636	29.1207	28.8835
26.3886	26.1187	27.0146	27.2973	26.6257	25.9918	27.1365	26.7031	25.7126	25.4594	25.4464	26.1333
29.397	29.6128	29.2153	29.1302	26.8258	28.6723	28.2476	28.2044	29.2479	29.2015	29.5954	29.5874
26.7712	27.1302	25.5209	24.791	26.8735	26.9872	26.0826	28.3071	27.4457	27.497	27.4207	27.6385
26.2152	26.313	26.7161	25.026	28.6352	26.9482	27.2159	26.2554	27.3208	27.4592	27.6674	27.4131
27.5343	27.5385	26.4901	26.7285	27.0147	28.1568	26.9361	28.5497	26.7963	26.9621	27.2812	27.0388
26.4388	26.2648	26.5841	25.654	27.1646	27.1769	26.3747	27.0309	25.3515	27.4326	24.4516	24.3004
26.8234	24.8665	26.1343	25.8132	27.4161	26.5403	26.3484	27.0015	24.5131	23.8283	24.7379	26.0814
26.5591	25.9563	26.7719	26.1657	26.5524	26.7046	27.6745	26.361	26.7416	26.5079	26.6546	26.853
29.8423	29.9867	27.5921	27.6843	28.6797	29.5779	28.052	28.7248	23.8618	25.0276	24.1524	24.3381
30.5162	26.9075	24.9014	25.6465	24.9508	26.4372	27.5856	25.7882	28.0312	27.8724	28.0558	28.0234
26.2414	26.4103	26.5661	25.9026	26.5859	27.1631	27.5157	24.7756	25.0372	25.1264	25.5253	25.696
26.581	24.9788	25.9299	25.5251	28.3483	27.1168	26.3337	26.8544	27.0785	27.3423	27.4495	27.4674
27.5472	25.4685	25.713	28.0081	27.5483	25.2954	27.8745	26.0719	29.6986	28.8221	28.3847	28.74
25.9392	28.6131	28.0702	27.9884	26.6455	28.7564	25.6825	26.792	26.9932	27.227	27.0578	27.2371
26.3579	26.584	27.1864	26.1279	26.4467	27.7805	26.1645	26.1941	27.581	27.5241	27.2914	27.3273
26.5122	24.6595	26.0407	26.0142	26.5962	27.001	26.8693	27.1038	26.4799	26.7243	26.4868	26.455
31.3594	31.5673	30.2576	30.1329	31.9676	32.4075	31.9497	30.927	27.3615	27.2596	27.512	27.3489
25.8914	24.8063	25.8571	26.9017	26.4843	26.0428	26.5171	25.5143	25.8399	23.8547	24.2694	25.8871
26.5637	26.9589	26.2676	25.3104	25.6846	28.6754	28.1888	28.2799	26.5508	26.7338	26.742	26.8797
27.9028	27.674	28.1454	27.9995	27.6806	28.7659	28.1249	28.7076	28.6433	28.8407	29.1438	29.0354
28.1981	28.3816	28.3077	28.0455	28.1768	27.2595	26.5729	27.8693	27.2052	26.9584	27.3167	27.6758
29.6982	29.8642	30.9394	30.6149	31.6871	33.2923	31.6039	33.4287	27.0087	27.2118	26.4714	25.6744
26.3597	26.4471	25.7248	25.7363	26.347	26.0042	27.3512	24.201	27.2762	27.8982	24.4489	24.4626
27.5988	27.569	25.9108	27.4131	27.259	27.5667	27.0903	27.2902	26.6321	26.8595	23.9009	27.4088
27.8643	26.3013	25.5053	25.4836	28.0491	27.763	27.3626	26.8069	27.9094	27.6725	27.276	27.2596
27.1625	25.2405	26.4843	25.8652	26.5421	26.3951	27.337	26.2889	27.6968	27.3	27.5262	27.5689
27.3461	25.5478	27.6889	27.3275	27.0927	26.4874	25.8425	27.6067	27.7894	27.4162	25.015	27.3638
26.4613	26.1638	27.8493	27.045	25.972	26.889	26.7015	25.9833	24.6041	25.895	25.7499	25.4121
25.398	26.2883	26.7094	25.8253	26.2195	27.26	26.9051	26.3486	25.686	25.572	25.7544	25.8748
26.6245	26.5532	26.244	25.3285	28.8204	26.8592	27.5459	26.7301	24.0091	24.8636	25.2483	26.1959
25.1496	25.811	26.2324	26.2153	28.5299	26.7591	27.1099	25.3768	26.5579	26.0321	25.6884	25.7939
28.0033	27.7342	27.9488	27.8163	27.5374	26.499	27.5329	26.8769	28.4618	28.4632	28.3826	28.3232
27.1872	25.0783	25.12	25.1006	26.8967	25.5641	26.5473	26.3003	27.0559	27.3885	27.4425	27.4117
25.2791	26.5926	26.0695	25.6463	26.7178	27.0655	25.6895	26.3158	26.5936	26.751	26.6947	27.03
26.094	27.0592	27.0966	26.1834	26.6013	25.7941	26.3064	25.3085	24.4135	25.3385	24.7361	25.0441
29.2024	28.6592	27.8619	28.875	28.7331	28.5803	28.5636	28.6604	26.3667	25.3233	25.9793	26.0909
25.6878	25.8233	25.2937	25.1412	26.4063	26.4318	27.1981	27.008	28.4854	27.7025	27.3155	27.0519
26.8389	25.0455	25.6164	25.286	26.886	25.7291	25.6493	26.6222	24.9041	26.1992	24.3012	26.4536
27.8141	27.6506	27.9913	28.1439	27.8011	26.3323	25.8369	24.8222	27.9893	27.4347	28.1828	27.9809
26.9092	25.4161	25.7784	27.1981	27.303	27.1348	27.4441	25.6665	27.1212	26.9388	27.1044	26.968
28.4765	28.5026	28.814	28.5434	27.6077	25.0597	27.3096	27.2901	24.9343	25.9352	24.9548	24.2105
27.6983	25.9336	25.3543	26.2896	25.8844	26.1247	26.3847	26.9327	28.5004	28.5919	28.5105	28.6275
26.8915	24.7659	26.0698	26.2629	26.6977	27.739	25.6943	25.2334	25.3649	25.7588	24.355	25.4057
25.3656	25.0901	26.5766	26.1965	27.0866	26.9056	26.2177	27.1393	26.4732	23.1463	24.045	24.3551
28.0755	29.8275	25.4946	25.7439	26.2562	27.5804	26.7684	27.3202	29.2885	29.2569	29.1162	28.4951
26.0371	25.9953	25.6744	25.3582	26.6898	26.2515	27.06	26.7768	26.6984	26.5053	26.8268	27.0425
25.9982	24.691	25.9155	26.7334	26.4324	27.0803	26.7447	26.9882	24.1893	26.5083	24.4756	24.6892
29.5228	29.3872	28.6066	28.5882	29.0805	28.8871	29.0466	28.8525	24.0209	27.9366	26.9767	27.4938
29.8609	29.8999	28.565	28.4969	26.7528	30.2499	26.9351	24.6511	25.9596	23.7179	25.3814	24.4509
28.9037	28.949	28.7166	28.3805	28.0796	28.2218	28.3472	28.5139	29.0966	29.0733	29.4081	29.2449
29.2655	29.7086	29.2931	28.6006	27.0943	28.6901	25.6614	25.4963	27.4931	27.7019	27.9417	28.1851
27.1568	27.3441	28.6249	28.6023	27.3668	26.8917	27.5276	26.6063	25.1925	25.4586	25.5527	25.8941
26.9722	24.4614	27.2903	26.4692	26.8509	25.7987	27.4254	26.9565	27.7549	27.1229	27.3584	26.7327
28.1048	28.3627	28.9221	28.6062	28.4487	28.4903	28.4042	26.5323	27.1788	26.8881	24.4889	26.9305
25.3906	26.8794	25.8222	24.7754	26.4167	26.2088	27.1706	25.4929	27.4543	25.3102	27.7681	27.8468
24.8852	25.734	26.0368	27.8063	26.2019	27.5604	27.1085	26.7661	25.639	25.5819	24.1741	24.408
25.6826	25.9084	26.5675	26.711	25.936	26.8463	26.548	26.4196	23.9702	25.2193	24.9346	25.2904
25.2853	24.8509	25.7616	25.2972	27.1881	26.044	26.5856	26.8515	27.8016	27.8864	27.6416	27.6021
29.6559	29.2738	29.2304	29.6049	26.491	29.2669	26.4047	29.048	30.9137	30.895	30.4657	30.0032
28.5758	28.4762	28.1567	27.9505	29.218	29.1332	28.9653	28.5745	27.4196	27.3544	27.4588	27.2333
26.9425	25.7061	25.0311	26.3365	25.7152	25.2559	27.4064	27.4968	24.9791	25.752	25.491	25.0435
26.1462	26.5953	25.5425	25.7008	28.0634	25.9555	27.9162	25.9416	27.4166	27.2514	26.8761	26.6579
25.0842	26.9664	27.6768	25.3969	27.2616	27.4095	26.1573	26.0358	28.7557	28.6783	28.4744	28.4016
26.4749	25.4928	26.6384	24.8781	26.6463	27.6038	26.648	25.9237	24.1194	24.9808	26.0194	25.5055
28.8712	28.568	29.1441	29.0282	27.7852	28.2152	27.1969	24.0898	30.2992	30.0839	30.3141	30.1853
28.1966	28.2075	27.3255	27.8168	28.3443	27.9379	27.524	28.0049	28.1657	28.3604	28.6645	28.7716
24.8045	23.9985	27.2935	25.4121	27.2905	25.978	27.6236	26.5316	26.018	25.9452	26.2591	25.3598
26.5506	26.2497	25.5726	26.4332	27.4262	25.233	27.9188	27.5183	27.4053	27.3889	26.8746	27.1974
28.7117	28.84	28.9914	29.1933	29.6451	29.2056	29.66	28.5909	26.0331	26.6408	26.1399	26.2855
25.3162	24.636	25.9948	25.6794	26.868	27.9915	27.9655	27.0048	26.4062	26.615	26.6359	26.9473
27.4924	27.5473	28.039	28.192	26.9267	27.8973	26.7147	26.3731	27.871	27.8674	27.7516	27.8281
27.3465	27.3281	26.4444	25.8819	25.6684	26.0071	26.3228	26.8292	25.8025	25.4278	26.869	26.7474
27.955	28.2687	25.62	25.5897	28.167	27.314	26.6378	26.807	27.5171	27.7876	28.2419	27.9895
25.0948	26.6924	25.5856	25.2775	24.7794	27.1088	25.3968	25.9022	26.4003	25.5107	25.0695	23.6271
24.9669	24.9206	25.12	24.3893	27.3614	27.2762	26.4915	26.8957	24.7606	24.2562	23.9577	24.3308
28.2084	28.3015	27.7003	27.712	27.96	27.8722	28.1278	27.669	23.5898	27.1877	24.7473	27.4351
28.9091	29.0848	28.8072	28.9748	28.3461	29.0445	28.4085	28.6483	28.5713	28.5948	28.8339	29.0171
28.9262	28.8554	28.6397	28.8658	26.1575	28.4589	27.8403	28.2485	28.5855	28.3911	28.7097	28.747
29.3711	29.3042	29.3682	29.3101	28.5429	28.7596	28.3944	28.6787	28.7698	29.1908	29.0763	28.9515
30.1808	30.0395	30.3952	30.7635	28.4037	28.6427	28.7494	28.2941	23.1128	23.3171	24.4869	24.1492
27.0774	27.2836	26.3915	25.6852	26.1956	26.4874	25.9536	25.045	27.1086	27.369	27.3841	27.558
26.0962	26.7059	26.8612	27.0164	26.9072	26.1644	26.8538	25.2759	27.7109	27.5456	28.0029	27.9739
26.5031	26.7553	26.42	25.3432	27.1132	26.9468	26.9594	24.7995	28.1438	28.0279	27.648	27.8232
25.875	26.7084	25.8174	25.236	26.949	26.0635	26.2468	26.37	24.7079	25.6958	24.9356	24.1321
26.1235	26.4178	26.3588	26.4056	26.5266	26.125	24.9082	27.2377	28.2423	28.4534	27.589	27.9926
26.4406	26.8254	25.6331	26.1939	27.5469	25.0306	27.0426	25.1028	26.4427	26.8163	26.7799	26.8158
26	24.5996	25.3861	24.8942	27.3405	26.1297	25.4144	26.943	25.2007	25.7679	25.4576	25.4839
25.251	24.9411	26.2632	26.2878	26.0543	26.3377	25.724	27.0287	25.408	25.9068	26.5065	24.7298
25.8874	25.3828	27.3718	26.7395	27.1009	25.3761	25.9088	25.8831	28.302	27.899	28.617	28.1714

27.24	27.115	26.6893	25.0001	26.9235	23.8261	26.8684	26.2553	28.1427	28.1679	28.1405	28.0491
26.179	27.1782	25.8487	26.9542	27.3	25.521	28.1859	25.2207	25.3566	25.0886	25.4414	25.364
29.0538	28.837	28.907	28.8845	28.8877	29.835	26.2908	29.793	29.2884	29.5051	29.6485	29.6024
27.5904	27.9319	25.7287	28.127	27.3942	28.0826	25.8153	26.0048	28.4689	28.4198	28.5251	28.3207
27.6599	25.2623	26.1072	25.2156	26.7292	25.1716	25.9388	25.5824	25.279	24.0487	25.6689	25.2828
26.2052	25.402	25.9882	25.5662	27.1299	26.3602	27.6621	26.2581	24.4964	25.4168	25.6753	26.4365
27.4531	25.9987	25.9799	26.4799	26.5883	25.5966	25.7933	26.3722	26.8876	26.8434	26.9311	26.8192
30.9477	30.8986	30.0841	30.1567	30.5155	31.2596	30.3714	31.2903	28.4114	28.625	28.641	28.553
27.1913	25.4481	26.9983	25.8384	26.6596	27.7936	27.4746	26.0612	23.8715	25.359	24.003	23.4719
24.6836	25.6843	25.5588	26.2403	26.174	26.9517	27.3944	24.5434	28.259	28.5045	28.3486	28.4106
28.4559	28.3908	28.0073	27.8212	28.3799	28.5285	28.3442	28.6726	24.9895	24.199	24.0665	25.7852
25.3004	23.5765	25.9932	26.6662	28.4999	27.163	27.4626	26.2477	25.9583	26.0972	26.7938	26.5455
29.2762	29.4165	29.9025	29.2754	26.4942	28.2497	28.671	24.9577	29.2907	29.6033	29.3082	29.4085
25.5074	26.7415	25.786	26.4398	26.5675	25.6519	27.5984	24.0928	23.1926	24.5427	26.0497	24.9099
26.1307	26.3913	25.2447	25.9872	26.3947	26.1293	27.5578	25.5958	27.8818	27.5726	27.6182	27.4269
26.0831	25.659	26.2644	25.1384	26.7305	24.4367	26.5439	26.7591	24.9848	24.1889	24.8489	24.1878
27.4958	27.9557	27.3602	27.1468	26.2059	27.9728	26.6932	27.9585	27.9274	28.268	28.1923	28.1688
25.9347	24.5997	27.2492	25.5024	27.6803	26.4528	27.1558	26.2119	26.0306	23.9264	24.8992	23.5023
26.3538	25.9199	25.7341	26.9709	25.8391	26.2806	25.7792	26.6295	25.7922	25.3832	25.0931	24.844
25.2104	26.5326	25.6717	26.061	26.5102	25.5091	27.391	24.7484	25.7025	24.7129	25.8026	25.8124
26.5456	26.4593	26.5497	27.1287	25.9578	26.7421	27.0962	27.1976	26.6834	26.8093	27.2108	26.7524
29.9701	29.8679	29.5734	29.7251	30.0195	29.5887	30.0195	29.6283	30.2356	30.2139	30.0585	30.1257
27.8264	26.6083	25.4317	26.9528	28.0125	25.887	26.8419	26.4007	27.2386	27.2741	27.057	27.0618
28.3907	28.2481	27.7079	27.8295	27.9831	28.5509	26.944	28.5169	28.1108	28.1729	28.2444	28.2668
27	25.8757	26.2774	26.1198	26.3372	25.9495	26.0021	26.2779	26.2039	26.4511	26.6974	26.2935
26.9618	25.8233	25.7953	26.1922	26.7301	27.6304	26.0225	26.6049	27.3472	27.6104	27.3851	27.1962
26.8292	25.9459	25.3414	25.9937	26.4509	27.4051	27.7302	26.8571	26.8016	27.1482	26.4388	25.7671
26.7862	26.6734	25.2195	26.9509	27.0175	26.2766	26.9481	25.8416	27.2697	27.5588	24.9947	24.0979
27.8904	27.9249	27.9432	26.5289	25.5816	28.7551	26.8752	25.7273	27.3505	27.2844	27.0743	26.7834
26.0752	25.144	26.4029	26.3428	25.8144	26.0794	26.0208	27.8263	23.9955	24.917	25.2707	25.0814
26.4176	27.937	26.6728	25.6495	25.9243	25.7418	26.8622	27.3857	28.0211	28.1301	28.2144	27.7563
25.1826	26.9145	27.1061	27.7339	27.8802	26.3906	25.735	26.7001	25.1993	25.0563	24.4074	24.8948
25.3776	27.054	25.5994	26.7944	26.5682	27.7837	25.4442	24.6268	29.4515	29.4001	29.4039	29.354
26.9728	27.2142	26.345	26.1998	28.1568	25.3936	27.1667	26.9464	27.4911	27.3365	27.1627	27.6179
28.9631	29.0671	29.2834	28.9943	25.9371	28.8231	25.0827	28.1141	29.6133	29.5366	29.7922	29.7935
28.4609	27.7643	27.9543	28.0094	29.0254	28.3893	28.8389	26.6181	29.8903	29.9169	29.6381	29.6534
26.0466	26.2233	26.2785	25.8063	27.1386	25.2293	27.5004	26.5295	27.2665	27.3309	23.6266	27.3835
26.9105	26.284	26.5289	26.0655	27.1895	26.661	26.0585	26.2209	24.0615	23.5328	24.6093	24.7452
26.0049	28.0166	25.6252	26.6541	26.7793	26.5432	26.5134	26.9847	24.4644	25.3046	26.3248	27.1815
25.3001	26.8719	26.3099	24.8157	25.8012	26.2856	26.3085	25.7356	26.8531	26.8291	26.9068	26.46
27.3573	27.5385	27.8648	27.7324	26.0128	27.1801	26.2638	26.1626	27.8987	27.7691	27.7764	27.804
29.0113	28.7639	29.5141	29.1145	29.4936	29.3136	29.4726	29.0989	27.2576	27.1281	27.0257	27.4121
26.7182	26.7948	25.3792	26.1433	25.396	26.3469	25.386	26.7626	25.7567	25.8582	26.1448	26.1458
25.8345	25.9544	25.5866	24.0332	25.4396	25.8148	27.3153	26.2251	27.5405	27.5687	27.02	26.8571
25.8743	25.9701	26.2864	25.116	27.4791	25.9754	26.6894	25.4302	26.7007	27.1082	26.7128	26.6872
26.4561	25.7969	26.9906	23.7448	26.864	26.5427	26.8011	27.6556	27.3442	27.6525	28.4101	27.5844
26.225	27.1257	25.6127	25.9518	27.1506	26.0058	26.4037	27.0087	26.3387	26.6779	26.4036	26.4209
27.3366	27.0887	25.7533	27.1931	27.2983	27.1517	26.4141	27.0582	27.2609	27.0112	27.459	27.3443
28.6112	28.5181	28.0538	28.0121	28.7158	29.0311	28.1629	28.4516	24.392	27.2882	26.6505	26.5604
29.3337	29.4571	27.6337	27.3222	26.0983	29.6243	27.1174	27.1053	25.4449	24.2115	24.4707	25.5215
26.1981	26.676	25.8781	25.876	27.125	25.9371	26.384	25.9603	25.4606	23.6985	24.753	24.8598
26.537	24.9548	26.3326	25.0544	28.3874	26.8311	26.4895	26.1275	26.1613	26.2014	26.1647	26.3267
26.537	26.836	25.4183	24.9313	26.272	26.7114	26.66	24.9933	24.9408	25.2784	26.3695	26.2726
25.6183	26.5532	26.5592	26.7573	26.0224	25.823	26.479	26.1186	28.9364	23.3646	28.1158	27.9704
26.0705	26.6748	27.3507	26.1217	26.1644	25.8084	27.7822	25.7265	26.7478	25.2368	26.7786	26.7378
26.3284	25.8886	25.4796	24.9415	26.6035	27.1888	26.1071	26.7019	24.123	23.5319	25.3011	26.0632
27.5144	27.5795	27.3431	27.3791	27.0701	27.3346	28.0658	27.5482	27.5587	27.7763	28.4546	28.3223
28.1845	28.3749	27.9893	27.9222	27.8167	28.1824	27.9269	26.2304	28.5918	28.5629	28.5534	28.2729
30.4784	30.5223	30.2812	30.8954	29.6115	29.8163	29.5176	29.6033	30.813	30.6893	30.8926	30.8403
26.3955	25.2305	26.3864	25.9761	28.3701	27.9038	26.1738	25.2802	26.047	26.3743	25.8037	25.6982
28.411	28.0202	27.17	28.0719	26.542	25.9764	27.5002	27.8364	27.7774	27.8106	27.7002	27.6327
25.1499	26.7924	26.3541	26.7341	25.65	26.943	26.6956	27.3485	27.4383	26.5327	27.634	27.3926
25.5361	24.3669	24.8956	26.9619	26.833	26.1847	26.1488	26.3151	28.3419	27.7418	27.8722	27.5134
28.4026	28.2189	28.0796	28.3712	27.909	28.6578	28.0822	28.7359	28.6519	28.6175	29.1774	28.9047
29.5139	29.3757	29.3951	29.6367	29.0115	28.5595	28.6806	28.4659	26.0849	26.7213	26.8927	26.8146
27.1997	26.8266	26.2815	26.4167	27.306	25.7877	26.0652	25.7729	27.6049	27.6669	27.4644	27.4571
26.3149	24.6196	27.035	25.5859	26.2918	25.5783	28.7542	26.6089	25.0683	26.1793	26.6706	26.7678
26.016	26.6524	27.1944	24.8162	26.7007	26.408	26.3358	27.1959	26.0565	25.8857	26.4291	26.2454
27.1484	26.5981	26.4591	26.6633	27.4534	26.9361	26.0474	26.7588	25.0568	26.1621	25.877	26.1555
25.7177	24.6048	26.6256	25.0956	26.1002	26.0411	25.5665	25.9464	25.5652	26.6969	26.7393	26.8737
26.1921	25.5506	25.4681	27.6159	26.3313	26.6461	26.011	27.3272	25.9673	24.8965	25.629	25.5661
26.9982	27.4976	25.8126	28.2194	27.0606	25.643	27.0051	27.0926	31.4635	31.2519	31.3927	31.4782
28.4245	28.4358	25.907	25.0315	29.1336	31.0933	25.5886	29.9779	23.1449	24.0158	24.0632	24.3565
26.8836	28.682	25.5243	26.7987	26.741	28.7859	26.2471	27.1294	28.8323	28.87	29.4124	29.2579
26.7249	24.9853	26.3	25.6266	27.7936	26.7384	26.728	25.1051	26.1289	24.5938	25.8572	22.8812
27.7343	25.5504	27.2433	25.6773	26.2826	27.0963	26.0743	28.212	24.0208	24.7966	25.478	24.9439
26.9059	26.5222	26.6852	26.8379	26.434	25.6709	27.3919	26.1095	27.4795	27.0729	27.3286	27.2817
25.9599	26.6712	26.6189	25.9409	27.1682	25.1752	26.2927	26.7691	26.6654	26.3376	26.5947	26.7429
24.0684	24.7384	25.529	25.1874	28.1074	25.931	26.5352	27.8425	27.2919	27.5071	27.654	25.7478
26.1369	27.4568	24.7768	24.9434	27.2202	28.1599	27.7531	25.2705	27.2337	27.2886	27.3624	27.3464
25.6451	25.6369	26.8377	27.516	27.0254	26.287	26.348	26.8397	26.9759	26.7495	26.8193	27.0295
26.6838	26.7271	27.017	25.8234	26.2149	26.3807	26.7824	26.8384	26.9206	27.1019	26.69	27.1676
26.2907	26.4052	26.1679	25.2762	27.4621	26.0595	27.0794	25.774	24.4004	24.1055	26.5882	25.9974
26.6186	26.6768	26.8041	27.109	26.5406	26.2856	27.3778	25.9042	26.2907	26.5518	26.4487	26.3035
27.0514	26.1939	26.9111	26.9356	26.4105	26.5875	26.9204	25.6876	24.9482	24.2441	25.6915	24.2085
26.0573	28.1194	26.0311	26.9533	27.6922	27.6342	27.334	24.9045	24.13	25.5402	26.0921	25.9603
27.989	25.3801	27.1199	26.1873	26.3265	26.2104	25.4519	25.7124	23.6269	26.2853	26.4877	26.5517
28.3324	28.5174	28.6452	28.534	28.4874	28.832	28.5694	26.2917	27.2272	27.042	27.4698	27.4261
27.726	27.984	27.9737	27.9482	27.6918	27.8148	26.2958	28.1013	27.7664	27.7822	28.3306	28.2635
26.0959	27.6655	27.0886	26.3671	26.4149	25.9375	26.5935	27.0843	26.7195	26.6555	26.611	26.7109
29.2105	29.1251	28.658	29.0022	28.5607	29.4689	28.7138	29.3976	30.2066	30.2084	30.2865	30.2214
27.379	27.9983	25.9198	26.4406	25.0038	25.4568	26.9818	26.0378	24.9886	25.3097	22.8805	24.8728
25.4331	26.7223	24.9799	25.8359	27.4529	25.2768	26.5291	26.3982	24.4453	24.5913	24.7092	24.1573
27.2669	26.1451	27.1196	26.0669	26.4046	25.0979	27.3995	26.144	25.6404	25.9998	23.9522	24.8853
31.8978	31.7235	31.2601	31.6222	34.6377	33.1749	34.4664	34.1056	28.6509	27.9737	28.2698	28.7143
25.5565	26.947	24.0559	27.5732	27.3472	26.039	27.3769	26.2549	28.345	28.1216	28.0437	28.1882
26.6089	25.4262	24.9697	25.4132	26.9119	25.3572	26.2606	25.6888	25.7068	25.5097	26.5851	24.1664
27.9387	27.8919	25.7179	27.4828	26.0658	28.5455	26.9981	26.5811	27.4962	27.4923	27.9235	28.0002
28.3782	28.4919	28.1961	28.5781	26.4341	28.385	26.5396	27.5641	29.4027	29.2326	29.6039	29.6307
25.2614	25.3422	25.4709	26.0257	26.1051	26.9039	26.4499	25.4849	28.2947	27.6695	27.8957	27.6655
28.847	28.7639	28.9389	28.7847	28.7267	29.0946	28.4497	29.4263	28.852	28.7703	28.9812	28.9589
26.4785	25.4972	26.2495	25.7576	26.6882	27.676	27.5576	25.4309	24.3373	27.3743	24.6392	24.4822
28.4513	28.5135	29.2169	29.321	29.1276	28.9816	29.1059	28.7093	27.3238	27.2874	27.2215	27.442
27.8352	26.5496	28.1642	27.7482	28.2129	28.3237	27.0305	25.3258	27.9219	27.7506	27.3308	27.5823
26.8756	24.4303	26.6905	25.9548	26.5936	27.2542	28.0031	26.9265	26.442	26.3859	26.8756	26.7562
28.1443	28.175	27.7784	27.4477	25.8361	27.934	26.9627	27.6875	27.8391	27.8952	28.1493	28.3783
26.7787	26.9512	27.1981	26.4312	27.3145	27.1542	26.9797	25.7001	27.3736	27.2003	27.4124	27.2311
26.2792	25.5621	26.6209	26.6326	26.6497	27.0275	26.3477	27.4181	26.16	25.1208	25.6658	25.7484
27.0458	25.1914	26.1127	23.678	26.2124	26.2245	26.8862	25.1332	28.5111	28.116	27.9181	27.417
25.9391	26.8282	26.3547	24.6834	26.4972	26.679	27.1546	26.638	25.9775	27.6555	26.7718	26.7929
26.6588	26.2125	26.8857	25.6273	27.8121	24.9904	26.9851	27.6673	26.1132	26.5099	26.3734	26.1813
27.9746	27.9354	27.9462	27.9411	26.5811	28.0789	28.316	28.0269	27.8822	27.6704	27.7449	27.7507
25.793	26.2421	26.3738	25.7564	27.5947	26.2397	26.6565	27.1095	28.2429	28.2518	28.4621	28.4195
25.979	26.2954	27.1708	25.6173	27.1481	26.5499	26.8791	27.0364	26.1109	23.9278	24.3577	26.096
27.7853	27.543	27.5503	27.3393	26.8874	28.4838	28.2145	27.3984	27.2006	26.9416	26.8526	27.5857
25.709	26.0149	25.1738	27.1033	27.2112	26.1326	26.7291	26.3343	26.1036	26.1132	26.448	26.3009
27.4038	28.7126	29.0625	28.9604	25.9869	26.5055	26.6143	26.2815	28.9314	28.6591	28.6176	28.6204
26.3871	25.2209	26.0322	25.9258	26.9733	25.8056	28.163	26.8168	24.8003	25.6773	25.314	24.1498
25.4117	26.1914	26.0803	26.0676	26.5423	26.2313	26.7898	26.6843	25.3786	24.8836	24.6013	22.6282
26.3671	27.8562	27.0486	25.4157	26.4192	26.6063	26.4905	25.7834	24.8429	25.0451	26.0626	26.1222
30.8171	30.8249	30.8881	30.9687	30.3158	30.4629	30.2033	30.7864	30.566	30.6752	30.8951	30.8181
24.4842	26.8731	25.9457	27.394	25.9633	27.2946	26.411	25.851	26.1155	26.2059	26.5655	26.886
26.3831	26.9947	26.3273	26.2954	26.838	24.7857	27.1042	26.5912	25.2369	23.5121	24.5117	24.8552
29.4804	29.5434	28.4532	28.7503	28.8232	28.6918	28.783	28.5337	26.8771	26.6308	26.7699	27.1154
26.6157	24.3906	25.5515	25.6008	27.1413	26.0329	25.7353	26.6179	24.0964	26.4977	24.3775	24.4175
26.1015	25.8178	26.8287	25.987	28.6551	25.2486	26.562	25.9381	26.0781	26.1716	25.5575	25.6318
25.9227	27.0499	24.7492	26.3056	25.8309	26.6818	27.9137	24.2674	25.2769	27.5524	24.5021	23.8982
24.5012	26.0598	27.1372	25.8409	26.2447	25.4	25.991	27.3249	26.8085	26.7092	26.5215	26.8523
25.0335	25.0153	26.573	27.2203	26.8496	26.8395	28.1265	26.2474	25.6666	25.6295	25.8104	25.5055

24.8304	24.0109	25.8823	27.1871	27.4878	25.1888	25.8202	26.3069	27.7961	27.384	23.1889	27.1821
27.157	25.9106	26.0646	26.2578	26.9961	26.9403	23.9443	25.7386	24.4312	25.8121	24.644	25.8303
27.1735	26.0399	25.9564	28.6311	26.4202	27.7053	26.6044	27.1766	28.7035	29.0008	29.2477	29.2929
25.2597	25.8357	26.9474	25.8641	26.007	26.6111	26.5593	26.5012	26.1355	27.1486	27.6184	27.4621
25.9761	26.4438	26.0538	26.4681	26.0375	25.8221	27.3619	26.6999	26.0286	26.0893	23.9383	26.0824
25.7495	26.7071	24.9343	26.0227	28.5656	27.3285	26.1962	24.9624	25.7123	26.2859	26.1932	26.0377
25.9902	25.497	25.8781	27.2485	26.9019	25.1779	26.2492	27.4928	25.0788	24.4346	25.2299	26.0022
30.0559	29.7546	28.0376	28.6222	28.7767	29.6702	29.0407	29.3866	27.0597	23.6973	28.1193	28.7491
29.2319	29.1554	29.0317	29.1091	28.2884	28.5434	28.5228	28.2479	29.8164	29.6799	30.0278	29.9893
28.6987	28.7157	28.425	28.2227	26.8571	25.7191	28.0471	27.9171	27.8558	27.4746	27.9275	27.1921
27.9375	27.3944	26.7058	27.661	26.8975	28.9124	27.3779	26.983	26.8658	26.7061	26.9697	26.7846
26.1273	24.3342	24.9479	25.3643	26.8717	26.4778	26.6518	26.6927	24.8038	24.9303	26.066	25.6472
28.4996	28.457	28.4629	28.7061	28.8261	28.0956	28.125	28.3343	28.9292	29.036	29.2783	29.327
25.5588	25.6953	26.1285	25.5805	28.4326	25.5784	26.6758	26.071	25.2657	25.8324	25.5388	25.8601
27.2472	27.4165	26.6037	27.1634	27.7205	27.1526	26.6466	26.9123	29.1621	29.2543	29.3336	29.6049
27.9915	28.2474	28.0421	27.9387	26.0296	27.1431	27.3735	26.8034	29.172	28.9135	29.2815	29.0445
26.7915	25.3774	25.968	24.6639	25.6384	25.3703	27.1089	28.8216	26.2744	25.9963	26.7232	26.7993
25.51	26.3144	26.1549	25.5239	26.2077	26.4545	25.6854	25.5084	26.9982	26.7379	26.5642	26.6122
25.3627	25.2855	27.1199	27.1979	25.3448	25.8862	28.1686	25.45	23.8906	25.1586	25.8531	25.6431
25.7034	28.0775	27.4857	26.1503	27.5762	25.9779	25.9092	26.6107	28.4721	28.0723	28.2494	27.6877
26.1351	26.7385	26.4219	25.5265	27.0198	25.8974	26.8056	25.1887	27.0043	24.5494	27.5623	27.4495
25.9895	26.3883	26.3998	25.48	26.8031	25.6668	27.0748	26.0369	28.2433	28.2361	28.4071	28.3385
27.0197	27.1569	27.5159	27.1731	26.773	26.3163	27.6083	25.6811	28.3345	28.3762	28.5235	28.5305
26.2558	24.6407	25.8758	26.0985	27.4077	26.5211	26.312	25.8128	25.3448	22.7829	27.0499	26.6016
26.2455	24.3765	26.9958	25.1882	26.6722	29.1899	25.7188	25.7328	27.4428	27.7274	28.2759	27.9613
31.1974	31.3607	30.1007	29.9934	29.9396	31.4355	30.6201	30.6528	27.5333	27.9847	27.0779	27.696
26.0835	24.2823	27.2325	25.9721	25.9268	25.7115	27.3906	26.4927	27.3185	27.5756	27.5344	27.6051
28.028	24.1075	27.3469	28.7742	26.6471	25.9877	26.5211	26.8482	28.3891	28.3241	28.5801	28.9317
27.9646	27.7639	27.0841	26.726	27.1477	27.8819	27.5338	28.2287	27.9524	28.0647	28.2487	28.376
26.9857	27.3211	27.7784	27.5308	25.5026	26.5904	25.9618	25.9035	27.6633	27.8895	27.7629	27.8773
25.5332	25.6942	25.5447	25.6974	27.3337	27.0329	26.0691	26.4707	26.1332	26.7983	24.3461	25.6695
25.8998	26.989	26.2184	26.2261	26.491	25.0475	27.47	25.3431	27.4272	24.1928	27.658	27.7785
25.0561	27.3117	26.9098	27.307	27.1805	28.0258	26.6505	28.6356	28.2217	28.2874	28.3382	28.2291
27.4823	23.7612	26.6956	27.2306	27.8983	26.0774	25.5461	26.5748	27.3989	27.7882	23.8393	27.4669
27.1249	25.0618	25.4176	26.5669	26.2001	26.2276	26.3425	27.5815	25.2546	25.0715	25.3344	25.9094
26.1051	26.0111	26.35	25.2564	27.1937	26.4866	27.2498	27.0328	25.8135	25.8606	25.8218	25.694
25.8575	26.2115	26.336	27.219	27.1355	26.9725	26.1734	26.582	25.1734	25.9804	26.5762	26.3762
25.1337	26.1334	25.2115	25.0283	27.7296	25.7411	25.485	25.6952	26.782	26.9158	27.1783	27.11
28.4742	28.6131	28.6135	28.5179	26.5411	28.5711	29.0334	27.8417	25.7749	25.9949	26.1729	25.9268
25.9998	25.2931	27.0691	26.5787	28.3223	23.8591	27.0569	25.9044	27.1148	27.515	27.3723	26.7436
26.8572	25.8529	26.7271	25.9857	26.1638	27.115	27.5501	26.2513	23.032	26.6447	26.9531	26.9963
25.4963	26.1516	26.2417	25.969	26.1484	23.9608	26.3252	25.3915	23.1036	23.5108	24.4905	25.1976
26.0722	26.2583	26.6208	27.2381	26.8766	25.6561	25.2176	27.0703	26.5621	24.0319	25.1783	25.6547
26.4837	26.067	25.307	26.8408	25.9226	26.0942	26.8666	26.5965	25.9829	25.2058	24.9052	23.9301
25.5583	28.1922	25.5746	25.9314	25.6491	28.7227	27.0762	25.6952	29.3619	29.321	29.2211	29.1084
26.5622	26.5086	27.5388	26.1773	27.2666	25.6283	26.8531	27.2	23.458	23.9289	25.6951	26.0794
27.0581	26.7898	27.978	27.5494	27.9606	26.915	27.2438	26.9092	27.2703	25.0628	27.0259	26.7516
24.9031	26.6991	26.4466	27.1437	28.1345	27.0985	26.0711	24.8502	26.424	28.0865	28.1554	27.6983
26.9878	26.4486	25.749	25.5407	27.4115	25.5693	26.8173	25.5563	27.7306	27.5506	27.959	27.8771
27.9133	27.9884	27.7243	27.9426	27.6871	28.6693	27.5629	28.8034	27.3225	27.7917	27.5032	27.4105
27.0876	24.2458	26.6387	25.9523	26.4107	26.4912	27.0632	24.9852	28.3972	28.651	28.2331	28.3825
25.1865	27.7752	26.3696	26.0953	26.5429	27.0157	25.3189	26.9192	26.3451	26.62	26.6647	26.6937
26.7586	25.7392	26.5752	26.9006	28.0777	27.7685	27.5152	28.1273	26.2885	25.572	27.1813	27.1321
24.7529	24.5948	24.7173	25.6738	26.5517	25.529	25.7568	26.0939	26.819	26.7801	26.6537	26.7435
24.1778	25.7748	25.6509	26.1301	26.6205	27.34	27.4376	26.906	26.8906	26.8971	26.9439	27.1762
25.8846	27.6924	26.6183	24.5823	27.7582	25.4828	26.8321	27.3124	26.7281	26.5241	27.1844	27.4993
26.6345	25.6408	26.5139	25.8166	26.6186	25.4505	27.5434	26.1739	26.9209	25.1028	23.7353	25.3354
26.227	23.5337	24.9411	25.2455	27.2417	26.1473	27.9894	25.9266	28.5405	28.4607	28.5361	28.496
28.6221	28.717	28.1434	26.5084	24.2545	27.6406	27.5084	25.4366	23.6419	25.1901	25.4649	25.4056
28.9534	28.9967	28.9951	28.4794	28.6731	29.1922	28.9323	28.6531	26.7666	26.2632	26.1358	26.1156
26.5247	26.7838	26.2394	28.3382	26.5898	25.4605	27.7456	25.5906	25.8233	26.6148	27.2186	26.027
26.8592	24.995	25.9166	25.6654	28.8219	26.2134	26.7448	25.8611	28.4145	24.861	28.178	29.0908
25.4678	24.6701	26.0033	25.4236	27.0359	26.5257	25.7703	27.2922	26.1225	25.4642	26.0964	26.029
27.3945	24.1127	26.4891	27.0576	26.8762	26.8211	26.42	25.8947	30.4658	29.1981	28.5825	28.6173
26.1799	26.2397	25.3176	27.2432	25.1144	25.6197	27.3673	25.4165	26.7661	26.7961	26.6459	26.862
25.0084	25.7772	25.9367	25.8776	26.7148	28.3132	27.4513	28.3017	25.9657	26.5599	26.7329	26.946
27.9928	26.3027	26.3194	26.2497	26.5713	26.0003	27.1859	26.1198	25.9046	26.1241	27.3391	27.0073
26.3344	25.0334	26.5628	25.6713	27.1084	25.9195	26.7841	25.9857	26.9193	27.464	27.5756	27.7017
30.2508	30.2948	30.8109	30.5612	25.641	26.1366	29.245	25.59	25.6821	27.2978	26.8648	27.0318
26.0349	24.3225	24.5583	25.2031	25.9684	26.0395	25.9202	27.1434	25.1393	24.3928	24.0024	24.1137
26.3638	27.0654	26.3912	26.778	26.979	27.9085	25.8457	26.6152	27.3119	27.204	27.0672	26.9944
26.2688	27.7559	25.4301	26.4099	26.7511	27.1484	27.034	27.7986	26.0769	26.1194	26.4721	26.2403
25.2627	25.3093	27.1865	26.4996	25.0093	26.113	26.3213	24.9562	24.0102	25.1963	23.389	24.3349
26.7073	26.831	26.5712	26.5637	27.806	28.1009	27.0238	27.9486	24.1174	23.5376	24.817	23.5077
25.1412	26.9774	26.3505	25.2994	27.1071	26.1046	28.5337	26.2464	25.9081	25.2583	25.1271	25.0368
25.7991	25.4222	25.6886	25.9728	27.137	26.6021	26.283	25.0225	27.443	27.261	27.6306	27.6337
27.1889	27.622	28.5238	28.4257	26.3995	27.3232	26.6122	27.7671	23.3276	25.4625	23.3731	23.1406
25.8741	25.9574	25.8132	26.8522	26.0082	26.7894	27.4882	24.9713	27.2058	27.2355	27.3237	26.8016
25.7621	25.4714	25.0919	25.6101	26.2377	26.055	27.9964	27.4543	27.1565	27.295	27.0918	27.1262
27.2162	25.411	26.0392	26.4046	26.9112	26.2993	27.5565	24.8969	26.1392	24.7717	22.5703	25.4003
27.5236	25.5949	26.9445	26.1388	25.5017	26.7741	25.8461	26.043	26.0912	26.0068	26.1384	26.7579
28.3417	25.3504	26.7721	26.4068	25.9107	25.5294	26.4375	26.9355	26.7438	25.6921	23.6043	23.8675
25.8412	25.0824	24.4784	26.765	27.4978	26.8371	26.6547	27.4128	27.621	27.7391	27.8574	27.4129
26.426	27.4176	27.2704	25.7947	26.9225	26.8398	27.3373	27.6772	27.368	27.3768	27.3248	27.2266
28.9724	28.9074	29.571	29.0725	31.8403	30.9517	31.7854	31.4034	25.4071	23.5314	24.5463	23.946
26.1734	24.1096	25.9088	26.0986	27.2847	27.5458	26.975	26.2282	26.0461	24.6832	25.0664	25.1324
26.6794	25.2659	25.3007	26.2016	27.8175	27.0163	27.8656	26.1442	27.1108	27.4877	27.8734	28.0012
26.8657	24.8773	26.3895	26.2182	26.5729	25.9063	27.5893	27.0066	27.1518	27.5385	27.4191	27.7052
28.1457	28.4871	28.4452	28.4703	27.3789	27.8143	27.7492	26.2338	28.2156	28.304	28.5592	28.4859
24.3623	24.9368	26.9939	26.5726	26.6387	26.9621	26.3925	24.9287	24.0173	25.5461	25.764	25.7901
27.4029	25.6219	25.0959	25.3835	25.9172	26.7977	27.1708	26.5953	25.142	23.5994	24.7601	24.1769
26.1255	25.8367	25.6655	26.3142	26.633	25.5407	26.4296	26.0576	24.9928	23.419	23.6255	25.7871
29.2624	29.5986	28.576	28.3064	26.6184	29.6567	27.1482	28.711	23.8577	23.2232	23.769	23.8985
26.1825	26.6379	24.7566	26.1073	26.7667	25.6358	27.2622	27.2355	26.1849	25.9186	26.2575	26.059
26.8242	27.0098	25.7848	26.2999	26.5277	26.6257	27.7661	27.2307	25.3037	25.2744	25.0781	24.487
25.5972	25.2623	27.2796	25.2631	27.7008	27.2582	27.0249	25.701	24.7389	24.9217	25.6545	25.4863
25.0691	26.2046	26.5587	25.3693	26.1659	26.8415	24.8543	26.4775	23.9991	23.7295	23.3029	24.959
25.8888	25.5639	26.9331	25.6984	26.7943	25.9412	25.6422	25.988	25.0275	23.5387	25.5658	24.7221
25.964	26.3081	25.2119	25.9117	25.9595	25.5432	27.3438	27.2235	26.5794	26.92	27.1315	27.1348
26.3903	26.3666	26.2403	25.9763	27.2617	25.5042	25.9963	26.3284	25.7308	23.1753	26.0833	26.828
25.0551	25.494	26.8078	24.3209	25.5637	26.4701	27.582	25.8602	24.7151	24.6749	25.0175	24.1911
25.8485	25.824	26.8726	24.7379	26.4194	25.5893	26.9771	26.5871	27.4598	27.5586	27.3275	27.1527
29.5711	29.585	28.8283	28.6073	28.0306	28.3763	26.3179	27.179	24.794	27.2884	24.9188	27.6996
26.8106	26.3381	25.4858	25.6995	26.7596	27.3982	27.0753	26.728	26.7549	26.5305	26.9955	26.9487
26.4052	24.9113	25.4335	26.2757	26.5221	25.9972	27.3678	26.1992	27.5007	27.3145	27.2166	26.9478
25.4855	24.9543	25.2083	25.0505	25.334	25.9782	26.3486	26.9248	25.4985	24.2458	26.9197	23.6766
25.5712	26.2203	27.0706	26.1985	26.5452	26.6412	27.8577	26.3395	25.4633	27.5141	24.3682	27.3625
25.9872	27.4449	26.8542	26.8553	28.325	27.1704	26.8139	26.8793	24.6136	23.6828	25.2247	23.8934
25.9174	24.7614	26.8544	26.9688	27.8585	28.4438	27.7228	26.0979	26.4037	26.4567	26.4538	26.1432
26.2192	25.5971	25.9596	26.975	25.9111	25.9582	26.4852	24.823	23.9971	25.456	24.5176	23.8102
25.7147	28.074	25.8302	28.1203	27.3686	26.7811	27.2365	26.9631	28.2093	28.3563	28.8466	28.7423
29.6212	29.6151	29.8595	29.484	30.0462	29.2426	27.5875	28.8083	24.3649	23.5437	23.9757	24.8836
25.2563	25.5075	26.41	26.4409	26.2303	27.4749	27.0536	25.9005	24.4097	25.0834	26.9341	27.3639
27.3017	26.5525	26.7274	26.8966	26.0556	25.2472	27.2726	27.8034	27.9638	28.1225	28.0551	28.1384
27.7363	27.9968	27.8534	28.0744	26.4816	27.4239	26.9239	27.1039	28.3045	28.1504	28.5093	28.6132
28.4815	28.4403	28.2202	28.3738	27.7728	28.1356	26.1114	27.9006	27.9829	28.2418	27.9292	28.0256
26.656	26.2278	26.6389	27.2479	27.4019	27.0804	27.6459	25.9131	27.3214	27.6097	27.8633	27.8828
25.5474	24.4891	26.6896	25.692	25.5508	25.9682	25.6751	27.3173	26.3331	26.5571	26.3442	25.8674
24.4968	24.7613	26.9991	25.3239	26.1743	25.5929	26.7211	25.2698	28.2281	27.9901	27.0049	28.1294
26.5821	26.0248	25.6811	26.0764	26.5149	27.6826	26.9695	26.9046	25.3124	24.1497	24.9002	25.3306
28.5417	28.1299	25.1277	27.9807	25.4539	28.3778	27.8401	28.6787	28.168	28.0731	28.4715	28.5634
25.8451	26.4531	26.917	24.906	27.263	26.3725	26.8274	25.1475	25.199	24.5315	24.9872	26.0153
28.1358	28.0776	24.3581	28.0228	27.6098	27.8568	25.8958	27.3181	28.414	28.4154	28.6898	28.6047
25.0014	26.2797	25.7503	27.233	25.669	25.639	27.4393	27.3183	24.7576	25.1665	25.3664	23.7279
27.7435	27.0055	25.8091	27.4199	27.3718	27.1557	27.0418	26.6423	28.1467	28.0321	28.3142	28.2765
25.456	25.4688	26.196	25.8648	27.301	26.0061	28.2304	26.162	26.3184	26.4515	26.356	26.3525
25.7455	24.5152	26.5623	26.1534	27.0915	26.4417	25.7362	26.5818	25.0644	25.5761	25.7034	25.9657
26.3017	24.4228	24.8951	25.6349	26.6022	25.8285	27.2545	26.6681	24.8284	25.4822	24.5044	25.1481
26.3502	25.5919	27.891	26.9207	26.3328	26.3287	25.5929	26.4192	25.3543	24.9485	25.5401	25.815

27.6719	24.4478	26.2065	26.2741	27.3138	27.1839	26.2773	27.9679	26.9901	27.0165	26.6131	26.9878
26.3723	24.4159	27.4036	25.5871	26.5783	27.4663	25.2885	27.856	24.5173	23.4789	22.7016	25.4955
25.1882	27.0371	26.7547	26.6525	25.9655	25.9431	26.253	27.2099	29.4339	29.5778	29.609	29.6309
27.3345	25.2817	25.8156	24.9346	24.8977	26.5728	26.6998	28.0589	27.0587	26.739	26.9025	27.1596
25.4066	26.6195	27.3566	27.3833	28.0035	26.3511	25.9303	25.8409	27.066	27.2227	27.2996	27.0606
26.6794	24.49	24.9002	26.8246	27.19	25.9737	26.947	26.7284	25.1311	24.3681	27.2571	25.9483
25.98	26.3761	26.0429	28.0137	27.6989	26.8666	25.1748	25.9101	25.5201	24.2711	25.6415	26.71
26.0175	27.7645	27.2397	26.2715	24.9359	25.9721	27.5847	27.0879	27.181	24.1854	25.9401	24.527
24.941	23.5696	25.6641	25.3901	27.9759	25.2888	27.177	27.1296	26.6883	26.2253	26.8986	26.7522
26.2332	25.2131	26.4478	26.5801	26.7726	26.972	27.0063	26.4438	27.6711	27.8615	27.13	23.463
25.2057	26.2083	25.0479	27.0685	26.0965	25.8558	25.8112	27.3843	25.4045	25.5984	25.6718	25.4033
25.9048	27.0979	26.3531	25.0294	26.9471	25.6336	26.4677	27.459	26.4718	26.1471	23.5534	24.3219
26.6032	25.1258	25.6171	24.182	26.8501	26.401	26.7416	26.0262	26.8214	27.0407	27.4155	27.1183
29.2278	29.3117	29.1555	29.1935	28.6474	29.0579	28.5274	29.1314	28.0324	28.0485	28.3762	28.3764
25.433	27.5777	25.332	25.6733	28.3404	26.3441	27.7594	26.7766	27.3094	27.0479	26.8293	27.0165
25.8143	26.6842	25.0308	26.2329	26.1918	26.5436	25.6749	25.5068	25.0664	27.1116	23.9647	23.5314
25.5301	25.7986	26.5981	26.9629	26.45	26.5523	27.437	27.2047	24.7329	24.3332	24.6676	24.4393
26.1001	27.7527	27.6319	27.8337	27.7567	27.2459	28.076	26.4333	28.1816	27.8322	25.8174	27.7065
26.8321	24.93	26.4106	25.138	26.7801	26.3609	26.0993	26.5789	27.0666	26.3175	26.8411	27.4885
27.5924	28.3046	30.0439	28.3328	26.6507	27.8401	28.2252	27.4963	25.5583	25.2068	25.6994	25.6958
27.4135	27.9174	26.5111	25.3959	26.9535	26.8979	27.5128	25.6596	24.7762	26.169	26.2504	26.0155
27.6782	25.5437	27.1243	25.4628	25.8576	24.7274	27.3376	26.6466	27.3486	27.4826	27.4386	27.6002
28.9581	27.5555	27.4385	27.0135	27.6307	29.3526	26.6056	29.9345	24.6425	24.8796	25.9239	26.346
26.106	28.0586	28.188	26.8184	26.9663	27.0247	26.8533	26.3038	27.8054	27.9297	27.666	27.7506
28.3743	28.3015	28.8787	28.6029	28.5727	27.5749	28.2024	28.0862	26.4432	27.0119	26.5287	26.6559
26.8211	26.3487	25.932	25.8184	27.0128	26.8781	26.7873	26.0776	28.7518	28.6065	28.6997	28.5908
28.6948	28.7555	26.7805	27.096	26.7604	27.7977	27.5198	27.1192	25.127	24.8858	24.6272	23.5803
26.4137	24.7615	26.2119	25.7305	27.0515	26.4645	27.0856	25.8515	25.441	24.9932	24.3178	23.0203
26.6365	26.6826	24.9909	25.4569	28.3976	26.8296	25.8038	25.5249	25.4209	24.6183	24.1072	23.9181
26.8961	24.6832	25.8766	25.9904	28.3704	26.7321	26.0721	26.262	24.0176	25.2202	23.5338	23.1626
25.3282	26.3892	25.9933	25.8159	26.4742	26.287	26.9778	25.5122	27.0628	26.7446	27.0506	26.7645
24.6479	25.9415	26.791	25.9634	25.854	26.2459	26.3332	25.9025	25.1289	27.0079	26.0955	26.5853
25.6071	26.2349	26.6973	25.7339	27.06	26.6515	25.0856	27.9298	27.9609	27.6705	27.4567	27.5757
25.8834	24.8374	26.4688	24.8462	28.1354	25.4499	24.8923	26.6157	24.9971	25.3842	24.1365	25.5171
25.7157	26.4953	25.2949	26.8583	26.8593	25.7316	27.6193	27.0004	25.3839	25.061	24.0793	24.1401
25.9763	26.4804	27.3143	26.1286	27.5756	26.1358	27.7113	25.5273	27.835	27.6689	24.0979	27.466
32.5164	32.6354	31.923	32.123	31.6402	31.8504	31.541	31.103	32.4614	32.4942	32.7146	32.7015
26.1872	25.4266	26.0771	26.6824	26.9213	27.6636	25.4042	26.4743	28.9617	28.91	29.2143	29.2425
26.9856	27.0169	25.7036	27.4034	28.2785	26.6939	26.8008	25.2741	26.447	26.7572	26.6852	26.7813
28.4183	28.1882	27.6993	28.1146	25.6965	27.5529	27.4354	28.9456	28.8538	29.2234	29.5526	29.8438
27.1191	26.9262	27.3516	27.1451	27.0881	26.0577	25.1478	25.7714	27.168	27.7629	27.5892	27.4189
25.2684	24.9094	25.8342	26.8811	26.281	26.3579	26.0478	26.6981	27.9336	28.1132	28.5074	28.6052
30.4607	30.2671	30.9658	30.9726	30.2672	29.9539	30.2104	28.3747	31.2357	30.4954	31.3301	31.1424
28.3116	28.7344	27.0283	25.3197	26.6895	29.9196	25.7653	30.02	25.3448	25.2133	24.5779	25.5296
24.5754	25.8066	25.4902	24.9864	26.8473	26.122	25.9346	27.1027	26.4539	26.636	26.6071	26.6109
28.5882	28.2236	27.8377	28.2756	27.624	27.2292	27.7697	27.339	28.3124	28.2205	28.5554	28.3747
26.9426	25.813	27.3757	24.3766	25.8855	26.9663	28.0119	26.4449	27.8923	28.5286	28.9343	28.7948
29.5699	29.5375	29.8631	29.6961	30.5155	29.6884	30.1919	29.4633	27.3376	27.5401	27.3954	27.3944
31.8808	31.731	31.0731	31.4655	31.2409	31.3564	31.4335	31.5606	31.8483	31.8768	32.0072	32.1165
29.0227	28.5799	28.2369	28.7218	27.753	27.3444	25.8561	28.334	28.2399	27.91	28.1273	28.1452
32.6356	32.9616	30.1893	30.5223	32.1206	32.1534	31.8909	33.4556	31.5785	31.0223	31.1228	31.1258
28.7587	28.8205	27.935	27.5258	28.0162	27.5772	27.7955	27.269	23.883	24.2133	25.2956	25.6195
31.2691	31.5743	29.0672	28.9748	30.421	32.0321	30.5657	32.3085	23.9858	24.1051	25.8982	25.3663
31.8193	32.0006	29.106	29.2493	30.9692	31.7579	30.9388	33.1734	30.7834	30.6567	30.9678	30.9132
28.2269	28.3056	27.9388	27.6313	31.4758	30.8902	31.3488	31.4609	27.5221	27.2121	26.7525	26.6907
24.7376	26.3391	25.979	26.0776	25.5422	25.6176	27.5473	26.7358	24.4285	24.7587	24.8783	24.1302
25.1443	25.348	25.7396	26.8047	28.0888	26.7668	26.2964	26.8617	24.8648	26.1682	25.9634	26.5049
25.4761	26.9965	26.8282	26.1481	27.8046	27.0785	27.608	27.0154	24.6989	25.0927	27.7321	25.9743
28.9582	28.5205	27.4936	27.0173	27.6159	29.0346	27.033	29.2814	27.138	26.9559	26.6307	27.0666
28.8803	29.1536	27.2787	26.8299	29.2313	28.8128	26.9863	29.6903	31.3184	31.1907	31.0927	31.3673
36.9736	37.3332	34.3876	34.2857	35.7328	36.0178	35.6369	37.1499	34.4065	34.3566	34.4118	34.3254
33.0432	33.176	28.7841	28.9023	29.3408	33.3616	29.4557	33.9989	30.8692	30.5611	30.6738	30.8082
30.1805	30.2747	28.3228	28.2863	29.5131	31.3196	29.1939	31.8343	28.1014	28.0725	28.3017	28.8069
27.5138	26.617	26.5076	28.8122	31.0511	26.0813	30.249	27.974	27.2506	23.9964	26.8252	26.5835
29.2748	29.1828	28.8132	28.8271	28.7874	29.008	28.7961	28.8174	26.5676	24.9422	24.734	25.6738
29.8485	29.8153	30.0843	29.7637	29.9946	29.8224	30.1449	29.7797	27.4371	27.1432	27.3706	27.7242
25.1781	25.8101	26.1777	25.9954	25.664	25.814	27.0659	26.5334	24.658	25.0368	24.475	24.3975
32.8339	33.1957	33.2471	32.9058	32.8799	32.3067	32.9591	31.8809	30.0911	30.434	30.7771	30.9602
31.8952	32.0778	32.604	32.5638	27.7395	31.9425	27.6375	26.2365	30.1757	30.0691	29.8066	30.0183
31.4805	31.523	28.6471	28.6934	30.0487	31.0446	29.4684	31.7495	28.8749	29.2068	28.7002	28.952
32.5886	32.5602	29.9374	30.0962	31.6532	33.6834	31.5863	33.1301	29.8006	29.6636	29.9924	30.1935
25.4734	28.1201	28.0366	25.4079	26.5915	26.653	27.0152	27.8197	27.9525	27.5752	27.7575	24.873
31.0388	30.2278	30.2792	30.574	31.8287	33.2569	31.1002	32.1872	30.0326	29.2342	29.4714	31.026
31.3675	30.5251	30.9435	31.2807	31.558	33.0503	30.9499	32.0787	29.2191	28.9417	28.9002	29.8719
31.3087	30.9568	31.1251	30.8965	30.5383	30.4196	30.0027	29.0392	32.0668	32.0351	32.1482	32.1234
32.5417	32.6955	29.2895	28.8191	31.0816	32.4768	30.9024	32.7448	28.6492	29.2095	29.1411	29.0542
32.2035	32.5207	28.3793	28.838	29.4197	32.6808	29.7191	33.1247	28.7063	28.7707	28.6601	28.6751
31.1976	31.0523	28.4857	28.2007	29.4634	31.1189	29.327	31.8285	24.6327	23.737	24.6343	22.9423
30.9181	31.267	29.9478	30.1507	26.301	30.6236	27.5595	32.0537	32.6825	32.756	32.4974	32.5298
32.76	32.7154	29.8281	29.8141	31.162	33.1936	31.2499	33.4078	28.6067	28.2662	28.2328	28.3821
28.6425	28.2475	28.7168	28.5218	29.8716	29.1444	29.4234	28.7527	31.7654	31.6603	31.1251	30.8533
36.3244	36.3428	35.4974	35.5324	37.2688	36.3314	37.0717	37.1692	38.5449	38.2374	38.6438	38.5435
31.8283	32.1278	31.1816	30.9608	32.5418	31.3197	32.4029	32.3994	33.5742	33.4826	31.0998	33.4021
32.0953	31.6424	30.5922	31.153	31.7653	31.9275	31.1212	32.8633	34.4706	34.4805	34.6857	34.5823
29.7699	29.9051	29.0272	28.3137	29.7049	29.1852	29.3312	28.46	27.8706	27.9769	28.2409	28.2095
30.4479	30.2451	29.1047	29.2349	29.0185	29.7424	28.4175	30.5344	32.675	32.2458	31.797	31.9823
33.6847	33.7934	32.655	32.7835	33.4821	33.732	33.2383	34.3091	35.3959	34.5915	35.0012	35.3434
33.7348	33.1379	32.7618	32.6216	33.7723	33.0001	33.131	32.8987	34.4562	33.7563	33.5889	33.1928
25.2977	28.3788	25.061	25.6824	28.3534	28.3995	27.0158	29.1687	28.5952	28.5856	28.397	28.5403
30.3084	30.4529	29.5785	29.6382	31.2746	26.239	31.4635	28.8568	24.2373	25.9204	26.3538	24.7346
32.5427	32.3924	28.7563	29.898	30.0613	35.1765	30.3322	34.3696	30.6099	31.0252	31.35	30.9559
28.5506	28.5223	25.0105	25.5386	27.0017	27.4277	26.6671	26.5174	28.2203	28.4044	28.1393	28.0144
25.7978	27.1	25.3095	25.9343	27.9538	25.6051	26.0965	26.2597	25.3708	24.6284	26.1471	26.4153
24.911	24.8515	26.5228	25.6474	27.2403	27.1351	26.9426	27.3022	27.3359	27.5214	28.1179	27.878
25.6447	26.6351	26.6774	26.5607	26.5244	25.5007	26.3902	26.6893	25.6626	25.6618	25.8132	25.6003
32.85	32.9521	32.8929	33.0879	32.7623	32.4686	32.6788	32.237	33.1376	33.1194	33.2829	33.2168
26.6334	25.7035	26.8422	26.1223	26.3005	25.3504	26.0672	28.2937	27.8382	25.0934	27.5677	27.7865
33.6316	33.5719	32.0886	32.5312	31.5394	31.7815	31.6601	31.4014	29.8863	29.8697	30.3979	30.4203
27.7619	27.4933	26.7374	27.2828	25.8276	25.7899	27.3138	28.3761	27.2529	27.6265	27.3076	27.2141
27.2114	26.5792	26.2092	25.2861	26.7127	26.0805	25.6904	27.918	27.5249	27.6507	28.4436	24.2789
26.2832	25.8977	25.9113	26.8273	27.5038	27.6247	25.7039	26.5743	28.2315	28.2848	28.896	28.7977
28.3037	28.1611	28.2361	28.3044	27.7531	27.1998	27.2039	26.6273	28.1417	28.2763	27.7192	27.9343
29.7171	29.2601	28.68	29.5456	27.7143	27.5464	27.6204	27.73	26.2483	26.4047	24.7122	26.3217
26.0314	27.0416	24.8413	24.7619	27.1028	26.094	25.5135	26.6855	28.7223	29.367	30.6944	31.0392
32.715	31.2633	31.273	31.8014	32.8036	34.3563	32.1708	32.7717	30.0913	29.1807	29.7737	31.0382
35.0241	34.922	34.274	34.3442	34.3238	35.1976	34.081	35.1622	34.7829	34.5793	34.9829	34.795
24.5688	27.705	25.4617	26.2507	27.2189	26.7799	27.3994	28.4762	26.0742	26.5685	25.8301	26.0827
32.515	32.5354	32.3032	32.5944	32.7263	32.309	32.8019	31.9998	30.5672	30.6045	30.7422	30.5832
28.3532	28.4237	28.1047	28.5786	26.489	28.7436	26.6638	28.8609	28.4538	28.2882	28.4299	28.4305
25.9949	26.1582	26.987	26.4814	28.2901	26.6544	28.4402	26.7604	28.7008	28.6296	28.9717	28.944
29.8462	29.2167	26.2318	27.0533	26.7099	25.9912	26.8543	26.9805	30.676	30.2068	28.4987	28.2244
31.7935	32.1936	32.7602	32.6614	32.2722	32.6688	32.0973	31.3231	33.2123	33.023	33.1487	33.1869
26.2647	25.1573	26.1301	26.4134	26.8716	26.6123	25.0041	26.4113	27.0343	26.981	26.8752	26.8427
27.5019	27.2747	27.6483	27.5176	26.2108	27.7509	27.8427	27.1309	29.2461	29.3256	29.5193	29.5334
26.1454	25.607	26.0173	25.4249	26.7608	26.1516	26.3542	26.2007	28.7682	28.7237	28.9567	28.814
31.4307	31.4927	31.3553	31.6125	31.7271	31.3926	31.8797	30.9931	28.4167	28.5422	28.8836	28.7642
28.4588	27.6633	26.2804	25.1094	26.8712	26.8407	26.127	25.63	29.145	29.7299	29.4488	29.4137
31.7325	32.0497	31.8728	31.6288	32.3928	31.5625	32.3888	31.1392	29.0786	29.2951	29.0191	29.1706
28.0364	28.2958	26.6475	27.159	27.4683	28.4249	27.2314	28.6708	25.3793	26.3191	26.3915	26.5029
26.7886	26.6696	26.144	26.7137	25.1543	26.6919	26.488	27.2491	24.7335	23.691	25.5552	25.2809
26.3704	26.4369	27.0571	26.2644	26.0983	24.8834	26.0764	26.1641	25.2073	24.7638	26.6374	25.4309
30.5088	30.1087	29.1765	29.106	30.8548	31.1236	30.7981	31.3619	24.1288	25.4718	26.1784	26.5342
28.0248	27.0318	26.1788	28.0017	28.1936	26.1107	27.8697	25.3939	28.5115	28.1115	24.1442	28.2623
28.8717	28.6846	28.2022	28.0306	26.9849	27.1039	27.4832	25.5701	28.1472	28.1982	28.4357	28.1622
29.8997	30.0575	25.4163	24.748	26.4256	29.4435	26.8713	28.3735	25.4384	23.6558	25.3053	24.9828
29.5571	25.4639	27.7349	29.3403	28.1014	28.1197	28.3202	27.5517	31.6318	31.8165	31.8494	31.7358
28.8032	28.8939	29.6456	29.6217	26.9547	27.1377	25.9511	26.6638	27.3771	27.2042	27.6035	27.9291
28.1283	27.7041	26.6147	26.9149	25.7818	28.4617	27.6949	27.9905	28.5837	28.5177	28.7865	28.6557
26.3294	28.3518	25.713	27.0648	26.0736	25.7385	25.6873	28.349	28.6112	28.4112	28.0371	27.7878

31.4577	31.4841	31.4594	31.344	30.0333	29.8074	29.8361	29.055	26.4672	26.6593	26.9879	27.1553
29.014	28.6445	28.0661	28.986	28.6395	27.7522	30.5119	28.3866	29.7185	29.4189	29.962	29.8364
29.4521	29.4451	28.3973	28.5982	26.208	28.7912	28.513	28.5483	30.3604	30.3003	30.7204	30.7089
27.8024	27.4369	26.9025	27.5124	25.971	28.2193	25.8656	28.6572	29.8158	29.7262	29.6702	29.6654
28.5748	28.523	27.0603	27.4851	25.4383	25.0779	27.476	28.8487	28.2735	28.4474	28.5307	28.8052
29.334	28.9714	26.3421	27.7393	26.8648	29.0546	27.9526	29.6309	24.7863	28.715	28.6954	28.3621
31.2106	31.0317	29.1167	28.9144	30.5909	30.9316	30.2776	31.6343	29.9427	30.0578	29.9822	30.1174
33.158	33.3388	33.0836	33.2235	33.8598	33.2495	33.8556	32.8829	30.2853	30.2098	30.6877	30.7814
25.1271	25.4564	26.6441	25.2515	26.0416	27.1688	27.2187	26.1006	26.5063	26.5291	25.7755	22.2415
31.8447	29.7021	29.6099	30.0292	31.2876	32.6949	30.7422	31.2348	29.8049	29.3045	28.3222	29.8288
27.6633	27.7136	27.0223	26.9874	28.2603	27.1797	28.4662	25.9853	25.2475	24.4077	26.3905	24.4543
27.8738	27.7746	26.9833	27.0563	25.2009	28.0632	28.666	28.1249	27.3918	26.9847	27.3223	27.1961
26.1353	26.9613	27.1203	27.5865	26.6038	26.0828	27.104	27.1155	24.0691	23.7538	24.1826	24.9378
32.6465	32.7187	30.5873	30.4645	31.1276	32.8267	31.0846	33.8718	30.8913	30.72	30.6919	30.8499
24.9345	27.3099	26.2831	24.5655	27.388	26.8331	27.4668	26.7105	31.3313	30.8742	30.5348	30.3628
29.2873	29.4266	29.1968	29.2895	29.4492	29.7375	29.2503	29.3664	29.6054	29.612	29.9348	30.0196
27.3277	28.6805	27.6924	25.9525	26.7727	29.7174	27.0986	29.9012	31.6163	31.6804	31.6028	31.5705
31.9305	31.7739	31.7918	31.8307	31.3506	31.5567	31.4762	31.1646	30.9802	30.3646	30.4789	30.4069
28.4992	28.3917	28.1006	28.0755	26.6721	28.2459	26.6723	28.5065	28.2769	28.4214	28.6077	28.3766
33.4995	33.5538	33.3076	33.3347	33.0751	33.1227	33.0904	32.8979	33.8304	33.5701	33.4102	33.3495
28.916	28.8305	26.943	27.2944	27.7526	29.6942	25.4895	30.1289	26.8806	27.1249	27.1157	27.0346
29.6629	29.8902	29.5339	29.6822	28.7602	28.6746	28.7547	28.0453	29.1699	29.1982	29.5319	29.4781
30.3204	30.0157	29.6734	29.5101	29.1415	30.2812	29.2816	29.5645	33.1329	33.0884	33.1805	33.1542
29.5807	29.463	29.508	29.7703	28.3502	28.3982	29.0649	27.7356	30.7965	30.2174	30.2746	30.061
29.762	29.7633	29.7023	29.7537	30.1281	29.7954	30.1539	29.4175	27.3305	27.0157	27.0303	27.1198
26.0089	26.075	25.0499	26.4044	27.209	25.3537	26.8918	26.5024	26.3749	24.7969	23.8113	26.3426
30.6203	30.6913	27.9025	27.5181	28.8807	30.7287	28.89	30.2802	25.2606	25.3658	22.2508	24.2552
25.1321	26.1033	25.136	25.229	26.555	27.2913	26.3053	25.3193	27.384	27.5717	27.7794	27.5456
31.2457	31.3294	30.9202	31.1873	30.2211	30.6044	30.3888	31.0026	31.4526	31.6053	31.5379	31.7659
26.8444	26.1328	26.614	26.0194	26.2603	26.8696	27.3476	25.1546	24.5408	26.8921	27.5739	27.2267
28.6116	28.5442	28.3272	28.2381	28.5373	28.4376	28.1713	27.8907	31.1164	31.1446	31.3082	31.3678
25.9552	25.1886	26.817	26.3884	26.629	26.564	26.5088	27.9798	27.5059	27.3681	27.2396	27.1453
26.1434	27.7448	27.4927	27.7506	26.2397	26.9701	26.9498	25.8865	29.9874	29.6853	29.8561	29.9244
35.0563	34.9607	33.8207	34.3313	33.7815	33.7153	33.8427	32.9508	30.0627	30.1855	30.4337	30.4803
28.3251	28.7419	28.159	28.776	25.0468	27.9578	27.744	28.0775	27.5795	27.7117	28.0318	27.7844
34.0452	34.1935	33.7175	33.9012	33.3725	34.1562	33.4035	34.237	34.6976	34.718	34.8478	34.7961
27.415	27.2071	27.3125	27.0812	26.9747	27.2194	26.1144	25.818	29.0841	29.0227	28.9641	28.7934
26.8876	25.8176	24.9661	26.6196	27.6211	25.9784	28.286	27.0033	29.4344	29.4252	29.6047	29.5909
24.1854	26.8824	26.6326	25.7337	28.5904	26.4157	26.6625	25.7545	27.3354	26.9019	27.1676	27.4296
32.8663	32.8211	32.471	32.8919	32.329	32.5481	32.0465	31.9584	32.9961	33.0179	32.9906	32.9498
27.624	25.7124	25.4812	27.2102	28.3599	27.9852	25.988	27.5027	25.7891	23.3792	24.8961	28.0941
28.7745	28.738	28.5503	28.6148	26.8767	28.8562	26.7171	28.3883	29.0856	29.0251	29.1873	29.2005
27.9777	28.4652	25.335	26.782	27.2651	26.5549	26.686	26.7712	30.567	30.6234	30.7998	30.6913
32.1399	32.1645	31.5689	31.6897	31.3459	31.9873	31.4792	31.7907	32.7414	32.8207	33.0334	32.9606
29.0794	28.967	28.2977	28.4956	27.9482	30.2352	26.2713	29.2954	31.2424	31.1795	31.4848	31.3176
25.7332	24.1657	28.2487	26.2504	27.0311	26.0315	26.0044	25.3877	28.504	28.2021	28.5621	28.3289
29.513	29.6094	26.7702	27.0505	28.5271	28.8524	28.446	29.0322	26.9132	27.0014	27.3141	27.0276
29.0617	29.2518	29.1716	29.0947	29.4914	29.7841	29.7187	29.1404	24.8966	24.8483	24.2396	24.7138
29.4424	29.4035	30.3388	30.1396	29.162	29.453	29.0796	28.2985	31.8496	31.647	31.5718	32.0048
30.4105	28.8959	28.0908	28.4615	29.9792	29.3027	27.4607	29.1165	29.7829	29.7939	29.8485	29.8822
32.5088	32.7659	31.5166	31.5592	33.9794	32.691	33.7648	33.7495	30.2038	30.306	30.1829	30.1634
30.5712	30.4809	28.488	28.6254	29.8375	31.3588	29.7452	30.8913	27.9555	27.8446	27.9871	28.2802
33.0921	33.1457	31.5416	31.7862	30.7928	30.7118	30.8854	30.4441	27.0011	26.84	27.2855	27.0225
29.9268	29.7857	29.2306	29.7545	29.2008	29.8225	29.1815	29.8393	28.9701	29.0901	28.7367	28.5715
29.397	29.643	28.9212	29.038	29.2622	29.955	29.5276	29.1783	26.6815	25.8412	24.392	25.2352
30.5329	30.7331	29.9335	29.9813	30.8809	31.2065	30.9548	30.7824	28.3313	28.3455	28.864	28.5851
28.2951	27.0541	25.57	27.1378	25.7039	27.6243	26.6019	28.0225	25.6296	26.1342	26.2966	26.6795
32.4283	32.5379	31.8508	32.003	31.5484	31.8545	31.5469	31.6814	33.0352	33.0296	33.1486	33.0807
24.8809	26.1306	25.5767	25.5474	27.7131	26.7509	27.1212	29.9668	26.7027	26.9105	26.9613	27.3343
27.7379	27.7552	27.3794	26.7042	27.4544	27.5077	26.7259	25.8794	27.4772	27.7245	27.3602	27.4064
28.604	28.6774	27.4456	27.5086	26.5939	27.6168	28.4226	28.034	25.0264	23.8148	25.7842	23.6679
25.8745	25.7141	26.6266	25.902	24.8911	25.0166	27.0715	26.6554	25.5694	25.5967	25.5452	25.295
29.7345	29.804	28.8532	28.5942	29.1834	29.1066	29.1452	28.3258	25.6355	24.7756	25.5208	25.5863
25.4652	23.8423	25.8762	25.2903	26.9755	26.1912	27.6606	26.1468	27.8247	27.6895	27.3316	27.6064
26.0203	26.9582	25.9072	25.32	27.5188	25.9668	27.3461	26.6918	29.0979	29.0803	29.1912	29.0803
25.822	24.2566	26.3928	26.3248	28.4281	26.0622	26.4547	26.9264	27.6515	27.8895	27.9189	27.847
28.0076	28.3845	27.7344	27.0326	27.5162	28.8049	27.054	28.1403	28.4163	28.5184	28.2708	28.2401
31.6235	31.9224	31.8802	32.065	32.0083	31.558	32.0595	31.0208	28.7701	28.9078	29.1656	29.2394
34.5785	34.2065	34.0099	34.5587	27.1071	29.066	27.8986	26.3105	29.5522	29.2548	29.6115	29.4528
31.6316	31.7265	29.8632	29.7106	31.4038	30.8642	31.3731	31.4971	24.0018	25.1312	26.4801	24.0258
29.505	29.5534	29.0036	28.8974	27.8959	29.7013	27.4805	24.1617	30.4797	30.6964	30.3465	30.2265
25.2115	25.1368	27.1227	24.2743	26.5749	25.6695	26.6216	25.9007	24.4932	25.2452	25.9756	28.4177
24.3525	25.4314	26.9885	24.9861	26.2781	26.8345	25.7989	27.2336	25.6751	24.3454	24.178	24.4367
29.6488	29.712	29.6252	29.7164	29.7132	29.6173	29.8684	29.1412	26.2322	26.2211	26.8172	26.5541
33.7547	33.5878	32.6556	32.9848	32.6535	32.4613	32.9507	31.9359	31.1961	31.2871	31.5737	31.5925
27.4201	26.866	26.8552	26.6586	28.5712	29.6198	26.5517	29.0744	25.9596	25.1972	25.2296	24.8902
30.8494	30.6981	29.9749	29.9353	29.2289	30.7211	28.9417	30.0533	31.7794	31.8311	31.962	31.7474
31.8368	32.0224	30.3492	30.0636	29.7343	31.149	29.5192	29.6951	29.0457	28.6093	28.8945	28.7288
27.1742	26.6123	26.3454	25.3824	26.7657	26.1542	25.5668	26.2242	26.079	26.0538	26.641	26.4237
28.9846	28.6886	26.1158	28.2893	27.6442	29.095	27.3957	29.5644	29.0301	28.9595	28.9511	29.1643
26.3194	26.4067	26.9405	25.1854	26.4723	26.7532	26.6215	25.6071	24.8869	23.8934	23.941	25.4415
28.799	28.7797	28.835	29.051	28.6786	28.9083	29.7218	25.8714	29.7064	29.627	29.168	29.4829
26.2156	26.133	24.6905	24.2132	26.1774	25.9725	27.1308	26.3153	27.0534	23.1909	26.8064	27.024
26.0513	25.2322	25.8514	26.1656	26.6165	26.3436	26.6688	26.9742	25.9992	26.6965	25.9923	25.4514
30.5345	30.9796	31.1189	31.3162	29.934	29.3342	29.3293	28.782	30.4331	30.4165	31.0474	30.978
26.6946	25.9908	26.5204	25.5279	27.5149	24.7386	26.3668	27.0105	24.5361	28.4774	27.8497	22.8421
33.6821	33.5471	33.6324	34.0182	33.2586	32.5261	33.1263	32.2734	34.1328	34.0892	34.4266	34.255
27.4706	27.2728	26.8069	25.2914	26.8991	25.3393	25.3974	26.9256	26.4639	26.3831	26.5492	26.4367
28.4162	28.2877	27.8	28.0796	27.5845	28.8712	27.9011	29.0584	30.5884	30.6046	30.9201	31.0113
27.5387	26.7473	27.6357	28.2681	26.3884	27.4607	26.9724	28.0284	27.5544	27.2838	27.8232	27.8305
29.2534	28.9564	29.958	30.0643	28.8474	29.03	29.6	24.7906	31.5568	31.0221	31.3158	31.3124
30.4744	30.155	29.4204	29.5486	29.3562	29.4219	29.4627	29.1489	29.9744	29.7041	29.9842	29.8849
29.207	28.9039	28.0873	28.5868	27.1165	27.2896	26.9444	26.5381	27.8915	27.7384	27.5879	27.5697
30.2156	30.3638	29.5593	29.619	29.2091	29.6548	29.5286	29.4422	25.7275	25.747	25.3245	24.9861
29.6496	29.9282	29.846	30.1408	29.799	29.5195	29.8025	29.1986	27.7231	27.6168	27.5939	27.6073
29.9978	29.9094	30.162	30.3104	30.4149	29.8564	30.3781	29.4177	26.9776	26.7924	27.3653	26.9751
26.9952	24.9328	26.0365	25.8411	27.5599	26.1607	26.5742	25.9123	28.169	28.1523	28.1803	28.0732
30.115	30.0559	30.1175	30.1871	29.9536	30.2672	30.6855	29.4749	31.6521	31.4832	31.8166	31.6482
25.5972	24.3274	25.9195	25.6884	26.5347	26.6828	27.1705	24.519	26.8748	26.7956	27.0388	26.9927
26.7418	27.0792	26.4719	26.3038	26.3774	25.6811	26.9503	25.6972	28.6086	29.132	29.3688	29.0152
27.615	27.7331	26.3215	28.9722	27.463	26.2027	27.5581	26.3506	28.3209	28.399	28.4904	28.5164
26.0665	26.1796	26.4532	24.7184	26.001	25.4905	26.4375	25.7778	24.5168	23.7331	24.388	24.6444
27.052	25.2326	25.9419	26.3259	27.2548	26.5976	27.0302	25.5838	25.3087	26.0619	25.2146	24.6505
27.5785	26.813	26.6852	28.4815	25.7936	26.335	27.0905	26.4993	23.72	24.0351	24.6217	25.4695
29.757	29.8372	29.7265	29.8464	28.6992	28.579	28.7154	27.9108	29.6472	29.6852	30.0387	30.088
25.6226	28.0464	26.0563	27.0852	27.3247	26.4893	27.7219	27.4342	27.9839	27.9883	28.3125	28.284
27.1128	24.6938	26.4638	25.7843	27.0555	26.7641	26.0605	26.8267	28.7495	28.4475	28.8947	28.8944
30.1462	30.3473	28.7905	28.8757	30.0178	31.0957	29.9824	30.9948	29.0745	29.0405	29.6771	29.6767
25.5747	25.5329	26.9382	27.0878	27.8767	27.4773	27.4657	26.1784	27.5176	24.8118	24.6016	28.0066
31.8445	32.4532	31.1055	31.0901	33.5082	34.3195	33.5332	34.5769	31.9997	32.1488	32.3291	32.0954
31.3625	31.4088	30.565	30.7444	31.0929	31.7692	31.1788	31.8066	30.8288	30.8159	31.181	31.0892
33.1082	32.5791	33.3105	34.2379	31.999	31.8809	32.2963	31.7836	30.1985	29.7112	28.8138	28.4838
30.1326	30.213	28.2427	28.1259	29.0163	30.4967	29.443	30.8209	27.3489	27.1892	28.0269	27.2422
27.5362	26.4852	24.9477	25.3807	26.3566	25.9926	26.2659	26.1262	25.8363	26.5665	26.6696	26.7761
25.7637	25.5275	26.1216	27.8807	25.1869	26.1178	25.5415	27.8983	26.9578	25.1739	26.5029	26.445
29.6053	29.826	29.3553	29.4417	29.0373	29.0472	29.0459	28.6025	26.2601	26.6638	26.6807	26.6283
30.7173	31.0557	30.9136	32.0684	32.0088	30.9619	32.1418	30.5835	29.2446	29.1976	29.8138	29.6404
26.5366	26.8192	26.8039	26.0389	26.2395	28.7135	26.9754	27.7827	32.3261	32.2315	31.657	31.889
25.168	25.5048	25.3545	25.9215	25.7353	25.7994	26.7538	27.1563	27.1157	26.9747	26.6398	26.9492
30.9053	30.8966	30.5379	30.7883	30.0766	30.0442	29.883	29.4571	31.9151	31.8318	32.5787	32.2977
27.4598	27.098	25.127	27.4656	27.048	25.581	26.8119	26.5192	29.9366	29.8943	29.031	29.6115
24.982	25.7204	26.417	27.4902	25.6773	24.0315	26.7858	27.0354	27.7582	27.8402	28.1892	28.1191
25.4381	24.9562	25.9853	27.1512	27.2192	26.1427	27.3934	27.149	29.16	28.7923	28.3103	28.184
31.4291	31.7313	28.6248	28.7305	29.9627	31.2326	29.8087	32.1404	29.1622	28.9124	29.2085	28.9282
29.7318	29.6037	29.5554	29.5781	29.0075	30.0325	28.8502	29.99	29.3256	29.0954	29.2102	29.0689
29.259	28.8914	26.9522	27.8926	29.263	30.9493	29.1668	31.0482	24.7353	27.3379	23.9726	23.9387
29.2383	29.4515	29.1892	29.1177	28.8972	29.1008	28.9706	28.754	29.7719	29.6848	29.423	29.5588
26.5804	26.4696	27.112	26.7172	27.4104	27.5301	27.5738	26.7349	31.5242	31.4393	31.7159	31.722
27.7023	27.6537	25.7607	25.1344	27.7622	29.7223	27.6171	28.5798	25.3529	23.7587	25.035	25.7259

28.9317	28.7504	28.9508	28.8113	28.5216	28.9811	28.8017	28.9113	32.4182	32.3517	32.3872	32.3699
30.8312	30.9453	28.4999	28.3117	29.4816	30.3237	29.3254	30.0638	28.0369	28.1031	28.2809	28.2137
27.1237	27.0082	26.747	26.0503	26.5772	27.3076	26.4543	27.4773	22.9893	25.9293	25.3133	23.6021
32.8888	33.0397	32.3554	32.4956	32.2073	32.8727	32.1685	32.3642	32.96	32.8469	32.7254	32.7107
30.9469	31.0691	30.306	30.8344	31.9473	31.9856	32.1012	31.4325	29.1796	29.0713	29.0583	29.123
29.7351	29.7821	28.5438	29.005	29.2897	30.0747	29.0309	29.9336	26.2812	26.6827	27.2351	24.9306
26.0367	26.0662	25.7134	26.1146	26.3694	26.0139	27.2555	25.7792	27.7597	27.4255	27.4977	27.705
26.5221	26.1288	26.303	26.2367	25.8388	27.2205	26.1296	26.6177	29.2994	29.1956	29.0608	29.2686
28.6341	28.4737	27.3749	27.6426	28.1264	29.3335	27.9494	29.3294	28.1508	28.0819	28.5066	28.4284
30.9399	31.1393	30.3348	30.8265	30.6301	30.7376	30.8269	30.5023	28.4032	28.452	28.8019	28.3701
29.0946	29.5872	29.3085	29.6465	29.8021	28.9858	30.3743	29.0176	23.4334	26.6261	25.1039	24.8687
31.2154	31.3767	30.7486	30.4545	30.0964	30.3033	29.9984	29.539	29.6633	29.7063	29.9916	29.8595
25.6682	27.4068	26.7847	24.8936	26.0193	27.3244	27.0152	25.9107	27.0801	26.9677	26.9058	26.8014
27.2619	25.5454	24.1872	25.9802	25.9995	27.7807	27.5125	26.5825	27.4457	27.4853	27.5355	27.5263
27.4291	27.3932	26.3445	25.7704	27.529	26.4138	26.6277	25.9836	25.9614	26.0856	26.4519	26.6265
29.4551	29.4644	29.6877	29.7559	29.6271	29.5141	29.7821	29.5556	30.4673	30.494	30.7027	30.6218
27.6544	25.6037	26.9823	24.9639	27.5809	26.56	28.0182	26.0971	24.8588	26.2962	25.7893	23.9292
26.6681	25.511	28.3937	25.7379	27.1519	24.9538	26.106	25.1908	25.1837	25.0725	25.3025	23.2064
29.3804	29.7151	29.4447	29.4113	29.2067	29.8202	29.3517	29.775	29.7414	29.6799	29.6488	29.684
28.7679	28.8345	27.751	27.8838	26.6543	28.5369	28.6482	29.01	28.2906	28.3849	28.4004	28.463
28.7603	28.7947	28.0726	28.086	28.3424	28.0978	26.7046	28.6436	28.2811	28.2882	28.594	28.4847
25.4503	26.2919	26.1861	25.5856	26.2198	27.5947	28.8693	27.1909	27.5312	27.6305	27.0208	27.3446
25.8613	26.345	27.2879	26.7082	27.3631	25.6202	26.4419	26.1618	27.0527	26.7745	24.5961	26.7953
28.9848	28.967	28.8279	28.884	28.5185	29.6492	28.2421	29.935	29.2658	29.1991	29.7242	29.5715
30.5436	30.5892	30.1521	30.4932	30.3195	30.0647	29.9003	29.3434	31.5303	31.4164	31.5409	31.5063
27.4356	27.6162	27.8935	27.3314	27.1775	26.9555	25.098	26.8165	27.2645	27.1654	27.7461	27.5578
26.7821	26.6181	26.469	26.4614	26.8021	26.9476	26.9319	25.6627	26.5406	26.6531	27.1712	23.8363
32.3241	32.4688	31.8248	31.8865	34.2918	32.4929	34.5441	33.3038	29.3429	29.5862	29.6705	29.6399
30.87	30.8854	30.7134	30.9745	32.9782	30.9438	33.0003	31.6684	27.5115	27.4903	27.9981	28.0331
31.6239	31.7945	31.5425	31.5603	33.9329	31.6346	33.9237	32.3837	28.2606	28.4557	28.5759	28.6114
24.9843	25.6497	26.1566	25.5805	25.6784	26.4598	25.7132	27.6461	28.8604	28.5887	29.101	28.989
30.8871	31.0171	28.7175	28.766	30.203	32.1041	29.8173	31.9165	23.0153	25.0836	23.885	25.6214
28.2655	28.1313	28.3331	28.4511	27.9623	28.7544	27.7501	28.7058	29.282	29.169	29.4404	29.3116
26.7734	27.2722	26.6431	26.0834	27.5358	27.0675	26.0561	27.784	28.5557	28.5733	28.8316	28.8881
25.0352	27.4467	27.6171	27.5165	26.5269	26.4558	27.2208	26.4088	26.7035	26.3547	26.7596	26.7455
25.6067	26.1956	25.6042	26.8628	26.2532	26.2022	27.5022	27.2987	24.459	25.0743	25.3416	23.8823
24.9545	24.4539	27.5608	26.08	27.0533	27.4328	26.4957	24.4176	24.8939	23.8953	24.8316	25.1734
30.4383	30.4795	30.2004	31.0458	29.5768	29.7275	29.6313	29.85	31.6257	31.6303	32.0846	31.9382
27.6128	27.5249	27.7134	27.7052	25.8299	26.6177	26.2071	26.2207	27.1738	26.4595	27.0816	27.0933
28.7747	28.7503	28.3491	24.5596	27.6507	29.3399	27.1229	29.4257	30.8469	30.7382	30.9618	31.0039
27.811	27.8151	26.8744	27.4763	28.0735	27.59	27.6843	28.3582	29.4254	29.6134	29.8651	29.9759
24.5716	26.3753	25.4984	26.5209	25.7073	25.7439	27.5161	25.6636	29.2514	29.2236	29.6479	29.5717
31.9374	31.991	32.9524	33.8527	29.7596	26.4811	29.4699	26.8805	25.0287	25.3354	22.7678	24.3864
28.8433	28.8996	28.3563	28.6728	27.3253	24.7535	28.9207	27.0704	27.9472	28.0712	28.251	27.9589
30.0421	29.9607	29.9611	29.7928	29.2923	28.8177	29.2668	28.6106	29.7124	29.636	30	30.0009
27.8463	27.7983	27.3042	27.1061	28.6003	29.7093	28.5205	28.9233	26.0724	26.0851	23.7287	24.5019
27.1149	26.8248	26.2043	27.0141	26.9377	27.5655	26.7783	26.5464	27.6858	26.972	27.0921	26.9962
29.736	29.6918	29.2994	28.9954	29.4975	29.9942	29.4479	29.3162	25.5203	24.3113	24.5084	25.7276
31.2896	31.4007	31.0945	31.2629	30.5548	30.6116	30.5017	30.585	31.7652	31.861	31.9459	31.9034
32.252	30.5495	29.9619	31.8335	31.4185	33.2616	30.8568	31.6956	30.1394	29.2595	29.1799	30.9533
28.9334	28.7999	29.5462	28.7359	30.8355	31.3542	30.5095	30.6862	27.7206	24.8079	23.2283	28.1404
25.6036	26.7379	27.5444	26.9739	27.1871	26.7448	26.3864	24.5767	30.6381	30.5891	30.7176	30.7289
26.9807	27.2373	25.8744	26.3897	26.9	28.6075	27.2029	28.2377	27.4914	27.973	28.1541	28.1036
30.2514	30.1286	30.0601	29.969	29.475	29.5137	29.3873	29.524	31.213	31.0722	31.37	31.194
30.156	30.3756	29.2439	29.9162	31.265	30.694	30.9582	30.3721	25.0697	24.9843	28.9566	28.6922
27.2072	28.0579	27.4161	25.5783	27.3632	27.3309	26.4184	27.0076	28.0112	27.7704	27.9363	27.8463
29.0948	28.8303	29.0871	28.8239	28.3916	28.4689	28.3777	28.0972	28.9908	29.2462	29.4124	29.3005
29.0593	30.3153	28.3809	28.8097	27.507	27.567	27.2225	23.8522	27.4624	27.4092	27.0213	26.9836
26.9205	27.1642	25.1792	25.5357	25.4519	25.3185	26.9054	25.4706	29.346	28.1317	28.7073	28.2235
25.7578	27.3536	27.3674	24.5447	27.599	27.0254	27.1745	26.3081	24.1083	26.4581	25.5033	26.4232
28.725	28.4872	28.459	28.0959	25.4066	28.557	25.8474	26.1901	28.6433	28.3877	28.4987	28.5789
26.4125	25.5945	26.9146	25.872	27.3454	26.427	26.906	27.3587	27.3917	27.3242	26.8283	27.1302
32.9311	33.1074	32.5562	32.9403	32.5007	31.9638	32.7092	30.8285	28.9154	28.8799	28.4863	29.0896
26.6528	26.6117	25.7196	25.4923	26.7002	26.1901	27.4908	26.904	26.8016	27.1881	27.0425	27.0927
32.7018	32.4487	31.6739	32.6175	31.8417	31.7443	31.5622	26.8407	32.1705	32.1146	31.0318	30.8641
30.0681	30.1752	29.8876	29.8559	30.8009	30.2711	30.6371	29.6173	29.2158	29.0051	29.1633	29.0347
27.2914	28.088	27.541	28.2108	25.4759	25.4195	26.9982	25.451	30.5018	30.501	30.2811	30.2405
28.5408	27.8506	27.8724	28.1254	26.4257	27.6467	26.2863	27.3499	27.6856	27.6566	28.2211	27.9567
30.0726	30.255	27.955	28.0752	29.2936	29.0603	28.9767	28.6527	23.982	25.4009	24.8644	24.4913
26.3507	27.2708	27.3899	26.5515	26.1304	26.3825	26.7427	25.257	27.7337	27.6835	27.963	27.9352
34.0993	34.0382	33.8275	33.9445	33.9897	34.2646	33.7151	34.5937	34.0087	34.0898	34.1707	33.9914
29.0938	29.3138	29.1983	29.2332	29.2421	29.5968	29.1703	29.4037	32.5323	32.6564	32.8433	32.8373
25.8901	26.2538	26.096	26.223	26.2234	25.4353	27.2051	26.3619	26.5174	26.3273	26.3268	25.5883
29.0462	28.9243	28.1627	28.0623	28.0857	29.8286	28.1462	29.2048	29.416	29.1998	29.1485	29.4984
26.0788	27.572	25.5457	26.3575	27.1528	27.7211	25.4834	26.6773	26.3297	26.2424	26.9541	26.6488
26.9209	24.6883	26.0372	27.0394	27.2425	27.9902	25.9191	26.2194	29.003	28.557	28.3566	28.1715
28.5555	28.5147	28.5707	28.5942	25.7912	28.5647	25.421	28.3149	29.7808	29.853	30.2226	29.9371
28.2973	28.4891	28.1992	28.0022	26.0997	28.4239	28.1107	28.0705	28.4881	28.3869	28.689	28.6948
27.5514	27.9691	27.9615	27.3515	28.6458	28.3992	28.2906	28.709	26.4609	25.8978	25.2435	25.9705
27.6407	26.2998	25.8064	24.095	26.5979	27.9123	26.6914	25.8008	23.475	28.5669	27.8568	26.554
25.569	26.4435	26.041	26.5822	26.4062	27.8927	25.2366	26.2574	24.7085	24.5226	25.4181	25.8658
29.7107	29.7077	29.2492	29.5382	29.8388	29.3582	29.7436	29.4806	31.0894	30.9714	31.3663	31.3155
31.9651	32.0785	31.3853	31.467	32.146	31.493	32.0813	31.2899	28.0644	28.245	28.2869	28.3699
28.6512	29.087	28.368	28.2459	28.1185	28.0494	28.078	27.3487	24.4502	25.912	24.7183	25.33
29.4234	28.7221	29.0071	28.9024	27.2168	29.6785	25.5072	30.296	29.1161	29.0457	28.9373	28.9467
27.7434	27.0638	27.7667	26.6069	25.6729	25.6173	26.2118	29.7569	25.7897	26.5564	27.2366	26.8623
30.5988	30.622	30.8186	30.9609	30.1009	30.4987	30.2213	29.8372	29.8533	29.9197	29.8956	29.8507
26.2098	26.2474	25.9688	26.3288	26.3487	25.7572	26.23	25.4715	26.904	26.6884	26.5971	26.5194
24.203	25.2348	26.6873	25.8726	26.6391	26.2339	26.0384	24.4523	23.7805	23.7125	24.8116	23.5082
26.3836	25.3835	25.8093	27.8781	26.3035	25.7461	27.5361	26.0276	27.8059	28.304	23.728	27.4163
29.9669	30.161	29.7171	29.6891	29.5031	29.8815	29.5108	29.7829	31.2913	31.3033	31.642	31.4544
25.7837	28.4433	26.6457	26.2153	26.6949	28.6304	26.5776	26.0706	28.6085	28.5595	28.7917	28.5776
30.9281	30.8294	30.0571	29.5812	29.56	30.8475	29.6912	30.5362	30.7257	30.9197	31.0945	31.1201
26.8285	25.7834	26.109	26.2284	27.7144	25.5697	25.4953	26.0104	27.3856	27.8385	27.5817	27.7035
26.3757	26.0684	25.6278	26.6599	27.1727	25.9906	27.2968	26.8038	25.8186	24.8422	26.212	26.2039
28.6577	28.6565	28.1925	28.2154	29.1607	28.9224	25.82	28.2034	26.7111	26.523	26.2357	25.0705
33.2458	33.4571	33.0575	33.2402	34.0055	33.4191	33.9586	33.2017	31.4659	31.4032	31.3692	31.4177
27.0542	25.3235	25.6785	26.8081	27.3413	25.6685	26.1644	25.8154	24.4933	24.7731	24.4637	24.5228
29.4549	29.1159	28.7771	29.1498	28.7001	29.0237	28.9189	29.4551	29.4481	29.4646	29.5586	29.4352
26.4427	26.7533	27.2153	25.616	25.8233	26.2299	27.711	27.4251	24.4571	24.404	27.0874	24.3182
28.9308	28.7253	24.995	25.5738	27.0743	30.5415	26.5821	29.4071	32.89	32.8142	32.5524	32.4759
28.1493	24.0598	27.0621	27.314	26.0952	25.882	26.875	25.1646	30.7918	25.3884	30.4198	30.5961
27.5771	27.1182	26.1646	26.5385	25.9846	25.0636	26.6012	27.6994	27.7044	27.8046	28.0273	28.012
27.7698	28.0032	27.9485	27.8752	26.9301	27.1977	27.5685	25.2555	29.4128	29.6941	29.8647	29.7571
26.2431	26.0249	26.695	26.8619	27.0205	27.7374	24.8419	26.6317	27.561	27.4659	27.5626	27.2797
30.0957	30.1095	30.6987	30.6565	29.9257	30.3386	29.9628	29.106	31.4251	31.0083	31.4298	31.3737
27.7079	26.3936	28.3022	26.0599	26.6573	26.5645	27.4728	26.6394	28.0733	26.8729	26.2531	26.3875
27.8578	24.3203	27.4162	25.0557	26.9494	26.4779	26.9851	25.3206	28.7855	28.3915	28.6746	28.4181
28.0058	28.1364	28.5421	28.1885	27.9658	28.252	28.0438	27.3497	29.0137	28.6969	28.7989	28.9852
27.351	27.4456	27.5561	27.7701	29.7309	27.9553	29.1338	27.7988	26.8169	26.8087	26.7051	26.8782
25.0232	25.5773	25.3409	25.4191	25.769	26.4431	24.8787	25.9627	25.2647	24.9843	25.0457	25.3178
29.0807	29.1883	29.2151	29.2154	30.5468	29.2717	30.7658	29.9324	28.2293	28.0111	28.4253	28.278
28.7678	28.828	28.2609	26.4229	26.4837	29.6121	27.3198	28.1872	26.6603	24.6969	23.9618	23.9845
26.1921	24.5214	26.2696	26.3134	26.0082	25.784	24.8118	26.6768	27.5006	27.557	27.4525	28.1085
25.2836	26.4977	26.7738	25.0766	28.3703	25.4892	25.2194	26.0808	26.2077	25.8828	24.2883	25.9696
28.3254	28.1744	28.1613	27.9271	26.4221	28.4877	27.7209	28.7562	27.1548	27.3071	27.3659	27.7398
29.4133	29.2994	29.0675	29.2033	29.0715	29.1061	29.0919	29.1636	29.1006	28.9498	29.2353	29.2692
27.8345	27.7816	27.6351	27.6387	27.9031	28.0517	27.9375	28.236	28.2328	28.3986	28.8975	28.7104
29.9393	29.9736	30.1629	30.2378	30.3535	29.1375	29.9727	28.9445	26.4915	26.6195	26.6237	26.553
28.4996	28.3654	27.8164	28.3859	25.9855	29.0831	27.8616	28.8903	28.8597	29.0623	29.0889	29.0413
27.9829	27.2192	27.232	28.1332	26.5035	27.2004	26.2681	28.8784	28.1978	28.202	28.3891	28.2707
25.5897	25.7191	25.7472	27.0454	27.0612	25.775	27.3351	25.2293	23.6075	26.9914	26.3027	23.6388
29.1927	28.6823	29.0779	28.5882	26.2522	29.8763	26.3619	27.4205	28.4831	28.736	29.0043	28.9824
29.052	28.9314	28.7869	29.0399	28.9978	29.4306	28.7676	29.1023	29.5135	29.3251	29.4429	29.3714
29.7116	29.7545	29.3756	29.3331	29.2894	29.7391	29.3533	29.4308	29.9176	29.9931	30.2944	30.34
26.9841	27.1233	27.8473	25.269	26.8007	27.1049	26.3775	25.9284	27.5703	27.759	27.4454	27.5459
29.4547	29.8398	28.6905	28.8765	28.7799	29.3328	28.6337	29.588	30.2098	30.1368	29.4922	29.6512
26.992	27.3023	26.872	28.49	25.705	27.3858	27.0444	26.1437	25.0638	26.4045	26.1329	26.227
30.0715	30.1695	29.7626	29.4931	29.1869	29.6096	29.0165	29.2045	30.2487	30.0844	30.1588	30.5839

30.3094	30.5184	29.1289	29.1142	29.2812	30.1809	29.0846	29.8083	30.7069	30.8967	30.7882	30.9686
29.5476	29.5208	29.4991	29.5622	29.5083	29.5554	29.4345	29.4992	29.8563	29.9643	30.0749	30.1312
26.2421	26.4846	26.0952	25.0997	26.8941	26.9033	26.2614	26.0724	27.4398	27.5567	27.1634	27.2481
26.4896	26.4067	26.4416	23.6927	26.7647	26.504	26.6113	26.1651	26.3688	26.5129	26.7616	27.0647
29.1321	29.4732	28.7696	28.4433	28.1004	28.7892	27.961	28.2316	29.9479	30.0312	29.7262	29.5105
32.2577	32.1759	31.5767	32.274	31.5903	31.6141	31.7064	31.1302	32.3859	32.25	32.2902	32.1898
26.0983	25.7642	26.6725	26.4057	27.0977	26.8155	26.1696	26.4979	28.8106	28.5111	28.8935	28.4951
26.6478	24.9172	27.3611	26.0044	26.5654	28.0172	25.9301	26.257	25.9436	23.8085	26.4082	26.6406
25.342	25.337	25.4907	24.5451	25.994	23.484	27.2106	28.2794	25.314	25.3751	25.5686	24.5735
26.509	26.4015	26.5208	26.2075	27.7305	26.7843	27.7814	27.0712	28.137	27.812	28.572	28.6631
26.913	26.9835	25.8042	26.0564	27.0301	27.358	27.4281	26.5333	25.3541	24.76	24.443	24.4771
25.3333	25.2528	26.3984	25.6141	27.1439	24.6033	28.629	28.6981	26.9071	26.7963	27.2134	26.9823
25.7028	26.1378	25.7171	26.8874	25.8226	25.9593	26.6911	26.1136	27.5654	26.9945	27.39	26.8316
30.3533	30.2478	29.4671	29.2764	28.9398	30.6912	29.6316	30.8775	31.6227	31.4742	31.6611	31.5694
28.3086	27.2641	25.5308	26.1303	27.9692	26.5742	27.8219	25.8504	28.6765	28.6605	28.786	28.846
25.8007	24.7885	26.7792	25.9972	26.0795	25.465	27.8948	27.2081	24.3108	23.8981	24.2227	23.5595
26.1935	26.6817	27.2894	26.8595	26.3686	24.6205	27.0834	25.2854	26.9214	26.4216	25.8901	26.155
25.1587	24.0583	27.792	27.0055	26.9756	26.1245	26.7135	26.0711	24.8237	24.4301	24.7679	24.793
27.2165	26.9081	26.8652	27.3152	26.838	25.9665	28.2633	24.6125	26.3873	26.3921	26.3302	26.5237
28.5007	28.5247	28.3739	28.1806	27.6088	27.7178	27.6957	28.5457	28.1627	28.325	27.7675	27.5267
25.3814	26.3826	26.223	26.3396	27.2287	26.3163	26.867	25.8315	28.2123	28.3243	28.5723	28.5671
33.9507	34.1915	32.6017	32.7239	34.8238	33.5234	34.4964	34.9952	33.384	33.2427	33.3241	33.3287
30.2379	30.5312	28.6734	28.2646	29.5569	29.7106	29.4601	30.7291	29.9308	29.9433	29.2497	29.7115
25.9349	26.4246	26.277	26.4229	27.0114	27.1926	26.3385	25.5891	25.0789	23.7018	24.6002	25.4723
27.2336	27.0186	27.789	27.793	31.4144	29.3202	31.1589	29.9341	25.6462	25.7438	25.0204	25.3283
29.3812	29.8809	29.5937	29.7771	30.5468	29.3074	30.2864	29.0659	27.9613	27.9401	27.9965	28.1736
27.3002	27.3581	26.949	27.2248	27.6323	25.1895	26.9438	25.2571	28.0372	28.3996	28.4013	28.4843
30.0655	30.0718	28.8178	29.0196	29.0027	29.7174	29.1864	29.484	27.8645	27.779	27.9713	28.0515
27.7827	27.751	27.7781	24.4193	27.4347	27.8648	25.7665	26.3694	29.9979	29.8928	30.0687	29.7978
25.0262	25.0046	26.6746	26.0271	26.6402	27.3015	25.6327	26.5459	24.9719	26.8944	28.2517	28.0983
25.8105	26.5262	25.6337	25.5784	27.0683	26.2277	27.0647	26.9683	26.8487	24.6556	26.5893	26.5971
28.0241	27.9921	27.31	27.2837	26.4699	27.6495	26.5813	27.6125	23.9426	23.5561	22.688	24.1502
27.5233	27.4038	28.8812	29.0569	25.2662	25.9058	27.3182	26.5327	24.0974	24.674	24.521	24.9911
29.8068	29.39	28.0315	29.0591	25.726	28.7829	27.5951	28.2844	29.0888	28.8894	29.7556	29.5767
29.1885	29.1625	27.9906	28.5139	28.5195	29.1289	28.4991	28.776	28.4412	27.825	27.4416	27.4851
25.7925	25.0976	26.4453	25.5939	26.8838	26.796	26.9781	27.3667	29.5986	29.5516	29.7829	29.6487
28.9349	28.7393	28.5257	28.8376	26.4828	28.4122	26.572	26.684	29.9033	29.8673	29.9375	30.0114
31.8517	31.4667	28.8829	26.3723	27.3355	27.1559	25.8084	32.0789	23.2192	26.1721	24.4742	24.6796
28.7416	28.7067	26.0577	26.7387	26.9946	28.7976	25.5382	29.6546	23.459	26.1322	24.066	24.619
26.5701	26.7049	26.7524	26.7482	25.7346	26.8105	27.5055	28.0745	28.0159	28.0963	28.0133	27.9543
25.9893	25.005	27.0341	25.0472	26.9571	26.5702	25.8061	27.0326	23.9325	24.1636	25.7582	25.5495
28.4452	28.0112	26.911	27.0758	28.7178	28.5009	26.6652	29.1692	27.7328	27.4376	27.4352	27.4193
26.1005	27.6347	26.6511	26.2826	26.5744	25.5668	26.6597	25.6883	26.0502	25.8292	25.9583	25.92
28.9684	29.0262	29.4986	29.3076	26.9591	29.5518	28.4136	26.2671	30.7565	30.3038	30.4172	30.2873
25.7869	24.9184	25.8536	25.7625	25.5728	25.4481	27.947	25.545	25.1987	25.3536	25.5019	25.6531
28.2002	28.1203	28.1566	27.869	28.0112	27.8123	26.6349	25.9034	28.2749	28.6892	28.8748	28.8216
28.9894	29.0111	28.6762	28.6482	28.1442	28.4123	28.081	28.2027	28.2323	28.0274	28.2659	28.0581
27.6178	27.3262	26.333	26.8851	26.353	26.9034	26.5956	28.5045	27.0285	26.9603	27.039	27.0591
30.5852	30.5295	30.2074	30.0848	29.3185	29.9602	29.4992	29.2434	30.8776	30.6931	30.3226	30.2198
31.6429	31.6022	30.9688	31.2288	30.6201	31.706	30.6226	31.8395	31.825	31.9493	32.1225	32.1049
28.6696	28.7212	27.3606	27.4979	28.3108	29.5806	28.2698	29.7598	28.3759	28.4796	28.751	28.7597
31.1944	31.3919	30.6973	31.0583	30.6341	30.2385	30.8162	30.6756	27.0387	27.7683	27.4805	27.15
26.1488	25.3693	27.5439	27.7	26.9493	27.2027	26.6866	25.945	29.9765	30.0945	30.185	30.078
24.9817	26.4318	26.6663	25.3819	26.4718	25.842	26.2336	26.0105	27.7777	27.4732	27.5849	27.5908
32.9729	33.5015	32.4697	32.358	33.2504	32.9871	33.3183	33.5433	25.5596	25.653	28.9153	28.8103
27.6425	27.5311	27.0354	28.0148	25.2773	26.9939	26.7101	25.3553	29.0118	29.1764	29.3036	29.5003
25.5364	24.9225	25.8597	24.5034	26.3892	26.5867	26.2222	27.4109	28.4557	27.999	28.1299	28.2538
25.2869	27.008	24.9291	26.7176	28.6433	26.3948	27.1425	26.9531	25.1617	25.451	25.572	25.4189
27.4204	27.1903	26.147	26.5291	27.9756	25.3575	26.571	27.6058	26.698	26.2843	27.164	26.5193
28.9015	28.775	28.4496	28.6872	28.2441	28.5011	27.3907	28.0936	29.2236	29.1477	29.441	29.4322
27.6463	27.7634	27.0597	27.0993	26.757	28.6346	27.5051	28.523	27.8196	27.6127	27.4059	27.1154
27.6076	27.607	27.0449	26.9036	26.2509	26.8498	25.5268	25.9026	26.7116	26.8851	26.9035	27.1377
29.0978	29.1129	29.199	29.2494	27.2926	28.8011	28.9307	28.4763	29.9276	29.5459	29.6463	29.7266
28.651	28.5918	28.6	28.5891	26.9954	28.739	26.7115	28.6368	28.7543	28.6557	28.8438	28.9267
27.4106	25.6781	25.0762	25.7327	26.148	27.0889	26.8068	26.4192	28.2149	27.9397	29.0542	28.8691
29.3518	29.4568	29.0106	28.9826	28.9912	29.2428	28.7503	29.6291	29.4901	29.4798	29.6097	29.6237
32.7389	32.7711	32.2899	32.2292	31.959	31.778	31.3961	31.45	33.0627	33.0545	33.0346	33.0811
26.8135	24.9519	27.9169	24.7601	27.5141	26.2883	27.5605	25.4952	25.0444	25.6703	25.3232	25.3311
25.8577	25.7398	27.3382	25.9541	27.5597	25.177	27.3944	26.5546	23.8454	26.2048	25.0189	25.4593
29.0096	29.1546	29.4536	29.6839	28.2499	29.0396	28.3186	28.3376	31.3736	31.1731	31.4601	31.1563
25.7886	24.2875	26.0405	25.598	25.7194	25.951	28.1331	25.7807	24.8292	23.187	24.0572	25.1839
26.196	26.4462	28.9279	26.5095	27.6715	26.0258	25.6275	25.6815	28.5906	28.9575	28.9017	28.912
26.269	26.1853	26.2675	25.6099	28.1254	25.7171	27.0774	27.8764	27.5473	26.7608	27.6742	26.773
30.9145	31.0564	28.6654	28.8952	30.058	30.3857	29.6513	31.351	31.2071	30.9112	30.4665	30.3968
28.018	28.9876	24.8884	25.5631	27.1912	31.2721	27.1514	26.1823	28.0538	27.9797	27.6962	27.7339
26.8378	26.0449	27.0155	25.923	26.0125	25.9175	26.4651	27.7947	26.2791	26.5058	24.9179	26.3459
29.1531	29.2969	29.0163	28.9108	28.9582	29.2761	28.9147	28.7696	25.434	26.0909	26.2274	25.6451
28.9913	28.8868	28.3607	28.5738	27.1549	30.0272	27.7787	29.8176	30.38	30.2831	30.4277	30.5244
26.1769	27.0159	25.6818	24.1815	26.6772	28.0374	27.3221	28.1573	27.5016	27.3619	27.8053	27.8358
28.9521	28.5114	28.1833	28.7281	28.0004	28.5065	28.3724	28.2287	31.3662	31.2274	31.497	31.5101
26.2343	26.1332	26.0608	26.2409	28.336	27.5396	25.4652	25.4871	27.0226	26.7452	27.0663	27.0237
28.7905	28.7837	28.9199	29.6139	28.8891	28.2522	28.5196	28.6604	29.3165	29.4367	29.6583	29.5572
30.2308	30.3633	29.1564	29.1449	29.0406	30.6989	29.8837	30.2945	30.5248	30.662	30.9133	30.9781
26.6744	27.1703	25.8335	27.4532	28.867	25.8372	26.3142	25.5482	25.0213	25.0955	24.9217	24.8487
26.2398	24.6821	26.5308	25.4804	26.7585	26.5105	26.7912	27.0107	24.789	24.7046	24.7537	24.4597
30.335	30.3759	28.9775	28.7963	29.4963	30.9343	29.2065	31.2024	28.9674	29.202	29.1602	29.317
32.0323	32.2715	32.8354	32.4457	30.9053	31.4061	30.8695	30.6124	31.7971	31.4057	31.8079	31.6669
27.8375	27.7887	28.4476	27.9607	25.8824	28.2168	27.7669	27.3339	29.8179	29.6441	29.4877	29.4539
30.8895	31.3029	31.0662	31.0491	31.35	30.4734	31.0674	30.1369	28.6999	28.703	28.5391	28.3397
26.8581	26.3629	26.9256	26.0838	26.7098	25.8259	25.9873	27.0596	24.6217	23.4839	24.6919	24.8163
26.114	26.1981	26.3396	25.3253	27.5283	26.6567	27.3934	27.8325	25.6866	23.1631	26.1855	26.2012
25.7424	25.9379	27.139	27.0012	26.6528	26.7023	26.5727	27.7771	26.8438	26.8385	26.9116	26.7658
27.6136	27.7864	27.7735	27.2426	26.368	27.6383	26.9808	27.7632	26.6166	27.1554	26.6779	26.9572
29.8617	29.7304	29.723	29.2447	28.2781	29.0781	26.6345	25.8028	30.5056	30.4857	30.4597	30.2849
27.7993	26.8614	26.1114	26.4143	25.6979	26.5314	26.9228	24.6328	28.3521	28.3805	28.5742	28.5235
28.1549	27.9315	27.6562	27.2762	29.5396	31.4642	29.583	30.6877	23.0769	24.4597	24.511	24.6201
26.7666	24.8766	25.8534	24.5185	25.8984	26.8572	26.1471	27.3211	23.7708	27.3937	24.6062	27.0089
25.7413	27.2134	25.0969	27.086	25.9363	25.542	26.9657	24.6608	27.2217	27.2757	27.1798	27.3301
31.8945	31.7442	28.7687	29.0968	30.5443	32.3787	30.4515	31.661	26.7752	26.8561	26.9937	26.7553
27.7464	24.9353	26.4871	28.2049	26.3094	27.9152	26.7769	25.0338	29.2022	28.5572	30.2081	29.3425
25.6902	25.5371	25.6568	27.3156	27.0946	26.042	24.7717	27.3318	23.8455	25.7551	25.612	26.5499
25.9919	25.977	25.7405	26.2134	26.4944	25.6177	27.2879	25.5609	27.1215	26.5631	24.4294	24.1792
27.2287	27.3948	27.7159	24.6457	25.1984	28.7164	26.7542	26.1287	29.0624	29.1007	29.1305	29.0579
26.3378	25.2784	26.1169	26.0723	27.0779	26.3968	25.837	25.3132	25.1264	25.7387	24.3653	26.385
28.9313	29.2302	28.7413	28.9663	28.1921	28.4177	27.5997	28.1671	30.0594	29.8509	30.4634	30.6213
26.4087	24.9675	26.0195	26.0944	26.027	25.7545	26.2738	24.7726	25.0251	25.2763	24.7351	24.9718
26.4543	25.7533	26.2286	25.7534	27.5922	25.6978	26.4489	25.6603	26.3944	26.7339	26.6689	26.6521
27.7747	27.4721	28.7141	28.0005	26.6547	28.5496	25.6374	26.0723	27.7198	27.8237	27.3317	27.0231
29.3206	29.1713	32.4452	32.3137	27.0448	29.4317	33.139	33.46	31.3418	31.24	31.5295	31.5534
26.8418	25.9713	25.7582	26.3877	26.1296	26.0562	27.4331	26.7299	26.6345	23.9546	25.8409	26.0245
29.7462	29.738	29.2847	28.997	29.5516	29.4001	29.3863	29.4076	29.772	29.7696	29.4682	29.5659
30.3786	30.2883	29.4958	29.6855	29.4546	29.6869	29.4336	29.9872	29.7953	29.7114	30.2945	29.8166
29.4874	29.5784	28.6077	28.8529	28.7896	29.0461	28.9935	28.7665	29.4968	29.5347	29.6781	29.6465
29.8337	29.7197	28.6931	28.8382	28.873	29.3641	29.0331	29.6853	29.8733	29.6995	29.9894	29.838
30.7187	30.892	30.7435	30.5939	31.4473	31.2943	31.4697	30.7371	27.936	27.988	27.7921	27.8602
32.7809	32.8149	30.5259	30.7858	29.5029	30.4658	29.4408	29.2213	25.2383	25.7802	24.7166	24.7485
32.7893	32.7655	32.6854	32.8802	32.0154	32.5509	31.9602	31.9921	31.6331	31.7545	32.1577	32.1728
25.9714	27.803	26.6268	27.5962	26.7778	28.1882	25.9146	28.5056	27.8606	27.8797	28.2323	28.255
26.8978	26.1461	25.9989	26.2982	26.9153	26.0812	27.9361	25.6674	27.5049	27.5824	27.5447	27.4101
28.4531	28.8085	25.9878	25.7127	24.6089	26.3349	25.103	26.8325	29.0575	29.0818	28.889	28.7417
30.0764	30.3817	29.2796	29.1507	28.8538	30.0495	28.8154	29.5015	31.2429	30.8953	30.9279	30.8353
25.6406	25.3763	27.1724	25.712	28.1144	27.3961	26.9102	25.2565	26.2222	26.2162	26.3946	26.301
28.3742	28.1413	28.661	28.5761	28.3799	28.0441	28.2697	27.3219	28.3253	28.4983	28.4076	28.4416
27.9626	27.3039	26.8843	26.1551	26.798	28.6009	27.2526	28.8976	29.5448	29.3455	29.7843	29.721
28.504	28.4151	28.4494	28.4536	26.6795	27.5529	26.7238	27.8739	29.5665	29.7166	29.5882	29.5573
28.2581	28.6616	28.4813	28.4501	27.6791	28.5092	25.976	28.4793	28.3049	28.5411	28.7253	28.6402
27.32	27.5307	27.862	27.1239	26.7605	28.6644	26.7773	28.8838	28.1527	28.1433	28.3229	28.2017
30.2733	30.2238	29.4237	29.3861	28.8836	29.7508	29.2037	29.6408	30.0105	30.1567	30.4075	30.4075
25.7837	26.2391	26.5062	24.811	27.7626	26.7384	27.7214	26.2717	27.5326	27.3379	26.9547	26.934

31.9689	32.2549	32.4343	32.0219	32.5295	31.7241	32.265	31.5616	27.7647	27.5794	27.772	27.8069
31.9127	31.8776	31.7747	31.8861	31.7103	31.2924	31.6735	31.3097	31.8819	31.9116	31.6981	31.9414
26.1875	26.7607	26.9499	26.4085	27.519	26.7857	25.0302	24.7723	27.1509	27.0208	24.6469	24.9629
28.4918	28.5126	27.9786	28.0956	28.5105	27.7459	28.3261	27.3711	24.1029	26.2619	26.119	25.2684
29.4764	29.407	28.8915	29.0739	28.7923	29.8009	28.9206	29.2719	30.6206	30.7325	30.6512	30.6941
25.3675	27.1507	27.2225	26.3209	27.3352	25.2	26.0704	27.5162	27.906	28.1748	28.2623	28.0697
27.4816	27.9325	27.4056	27.4117	26.4751	27.3259	26.0235	27.1492	29.2077	29.2134	29.2988	29.2281
29.5793	29.4914	27.6963	28.3861	26.2265	28.9382	26.6974	29.259	24.9901	24.6232	24.5783	24.3012
27.7562	25.6642	26.417	25.5599	26.7058	26.6064	27.5365	27.161	24.3515	25.639	24.5066	24.6351
26.5597	27.7433	27.9235	26.9604	26.9243	26.2637	26.644	28.8837	27.8946	28.11	27.9381	28.0245
29.216	29.3885	29.1352	29.276	29.087	28.7331	29.6488	29.6263	32.3633	32.3087	32.5126	32.7689
30.0371	28.4485	28.7608	28.3583	26.262	28.121	26.838	27.3792	29.5874	29.5804	29.5719	29.4116
28.4036	28.5125	28.3663	28.8674	28.5958	27.6042	28.2803	27.4324	31.0875	31.0088	31.3114	31.1808
26.4264	26.9592	24.5149	24.852	27.7618	27.4727	26.6352	25.3019	26.8973	23.4068	27.4722	27.2339
25.1645	24.3701	27.3277	25.1079	26.6357	26.9957	26.2591	27.1316	24.6515	25.7086	28.0556	28.1602
30.2842	30.4073	30.2882	29.9586	30.3237	30.0171	30.1623	29.9704	30.4617	30.2905	29.9591	29.8115
28.799	28.6156	27.6633	27.6245	26.5447	27.7362	26.6174	28.3643	28.3736	28.8217	28.9022	28.7342
29.0745	29.3303	28.8609	28.4251	28.5624	29.1033	28.7837	28.507	29.3938	29.6045	29.822	30.0702
28.177	28.0087	26.1376	27.5526	27.9342	28.0816	26.2835	26.3966	28.2185	28.226	28.1567	28.3193
27.8551	25.3674	25.5685	27.0966	27.239	25.8002	27.2567	26.3486	27.1604	27.1392	27.2208	27.2208
24.4156	28.3009	25.8332	27.0787	27.6091	24.9762	27.0374	25.3307	24.9212	26.0413	24.3435	26.5946
24.9017	25.0011	27.4199	25.1805	26.2004	26.6154	27.3909	25.9672	27.1185	27.308	26.8872	27.2422
27.3569	26.0234	25.3411	25.8078	25.428	26.3034	26.9868	27.1481	26.41	25.7811	26.2108	26.1598
25.3323	25.5789	26.4036	26.1047	25.6187	25.6912	26.9058	26.9305	32.1778	32.534	29.1872	29.2274
29.0557	29.1372	29.28	29.2482	29.0276	29.0469	29.1624	28.9366	29.1109	29.179	29.3214	29.4057
26.0608	25.2739	26.2634	25.0032	27.3696	25.4068	26.8771	26.7077	25.1686	25.3328	25.1027	24.5922
28.5722	27.7699	28.6487	28.5046	26.7055	28.1091	29.2355	27.6134	29.3518	29.1826	28.9741	28.8635
28.1028	28.0076	28.4946	28.7601	27.3263	27.4593	26.2065	27.3913	27.0103	26.9878	26.6058	26.6617
28.7001	28.8925	27.645	27.5454	28.1957	28.0694	27.9553	27.5526	26.711	26.8535	27.0796	27.5442
26.7856	26.4715	26.7822	25.2372	26.5204	26.7321	26.6822	26.0321	27.6819	27.3153	26.6992	26.6995
26.7675	25.3362	25.5131	25.8663	28.1471	28.5699	28.1017	28.7329	24.3911	25.355	25.8327	23.468
29.927	29.8433	30.1146	29.9441	30.1484	30.4616	30.1952	30.1559	27.8705	27.7864	28.3126	28.0011
27.8035	27.9284	28.2642	28.0267	28.9098	27.8707	28.7036	25.71	26.0166	26.153	25.7426	26.0431
25.1675	25.6617	26.9304	26.1998	27.8359	25.1177	27.044	25.6474	27.645	27.5549	26.3199	23.5856
25.696	28.7145	28.4683	28.1387	26.7412	28.0931	26.8123	25.8233	28.3122	28.4644	28.6615	28.5214
29.8032	30.0671	29.5216	29.7693	29.1008	29.4362	29.4603	29.0481	30.927	30.8253	30.9084	31.0505
25.7185	25.5319	26.5885	25.5987	25.074	25.5086	26.3202	25.8792	24.1026	26.304	26.3092	22.5205
25.0314	25.0525	25.3281	25.1647	26.0623	25.7206	25.629	26.59	27.1344	26.9337	26.2093	26.636
30.0969	30.4947	30.4233	29.8858	28.7199	29.2708	28.6912	28.6274	31.0617	30.7916	31.256	31.0159
31.2817	31.2123	31.221	31.2246	30.0326	30.4513	29.9788	29.8311	28.8753	28.7063	28.6004	28.7043
28.8854	29.0556	29.0303	28.4404	28.8822	29.161	28.8233	28.7421	25.8288	26.0982	26.0121	26.5591
26.1967	26.8011	27.1102	26.8786	27.9135	26.1459	26.3081	26.8065	28.8588	28.889	29.3602	29.2661
31.0029	30.9168	30.2672	30.4304	29.3924	29.5874	29.4505	29.9432	30.493	30.4811	30.8843	30.8849
28.3142	28.1531	28.7598	28.6734	25.2948	26.2006	26.6849	25.9288	28.1846	28.2059	28.1075	28.0657
26.6108	28.1382	26.9558	28.7694	27.7841	27.39	28.3669	27.667	28.1098	28.5712	28.0037	27.9156
25.3471	27.7103	26.134	27.1059	26.748	25.6109	25.3072	26.8469	29.5895	28.7951	28.6521	28.6299
25.6869	26.5622	26.64	27.0776	25.656	26.3686	26.1155	26.4278	28.8064	28.4838	29.4786	29.0975
25.1286	26.0733	26.1641	26.2061	26.8714	25.9246	26.4048	24.326	24.8953	24.1757	29.7259	23.9472
29.4347	29.4399	28.1467	28.8569	29.2447	29.3408	29.0521	29.3595	30.1114	30.088	30.0726	29.9993
24.6161	26.0798	26.1724	26.7181	27.3231	26.1436	26.4455	26.3604	28.5547	28.6444	28.8876	28.6717
28.6148	28.1746	28.3696	28.0654	27.6765	28.0014	27.0631	26.3934	28.2501	28.2863	28.6942	28.6555
28.9693	28.8139	29.1202	28.9966	28.4395	28.7182	28.4459	28.7343	29.1269	28.9538	29.315	29.0071
29.1542	29.3432	29.4539	29.5486	29.0913	29.3917	28.864	29.3274	31.849	31.8839	31.9609	31.8892
29.5748	29.7954	29.4172	29.4285	28.8608	29.4428	28.88	29.3477	29.8111	29.7113	29.8187	29.7111
26.1673	26.2196	26.2034	25.9985	26.9606	26.4433	27.4921	26.1898	26.8672	27.1051	25.3609	23.2591
25.816	25.9349	25.9161	26.9246	27.3914	27.5418	26.6255	25.9909	30.1573	30.1305	30.1436	29.909
25.8411	24.4877	26.767	26.2265	25.1276	26.5768	26.7427	27.2454	29.0799	27.4415	25.4542	28.2054
26.4379	26.398	26.1102	26.5051	27.6333	28.6417	26.7017	28.4697	27.0687	27.1902	27.3502	27.6396
27.4965	26.0204	26.6658	26.5514	26.1187	25.3188	27.8974	26.422	27.0628	27.0331	27.1519	26.8866
25.8739	27.4483	25.1289	27.4603	26.4123	26.9401	27.1392	27.0793	27.3665	27.1531	27.3561	27.4396
27.9318	26.8572	26.884	27.5846	26.9422	28.0203	27.285	25.9605	24.4455	24.7964	25.304	24.8429
25.1503	26.3022	25.8318	24.5082	26.5964	26.7663	27.9046	25.4334	27.4337	27.2473	26.9931	26.8903
27.1242	25.4338	26.8	25.8633	26.3146	25.5794	27.9052	27.4572	27.3091	27.2313	27.2118	27.8896
28.3277	28.3037	28.6172	25.6872	26.8865	28.674	28.6678	25.8466	28.122	28.434	28.1137	28.0783
26.7725	25.8921	26.7226	24.1056	27.6175	26.3882	26.0268	25.8033	27.061	27.01	26.829	26.7853
30.2405	30.1988	28.8042	28.832	29.1414	29.3054	29.2251	29.4688	26.9569	27.4601	27.6565	27.6598
25.7906	26.1034	27.3011	25.1951	27.244	24.5252	27.7725	25.4738	28.6623	28.1409	28.5727	28.8588
29.6882	29.8116	28.9569	28.654	29.3225	29.3914	29.0334	29.3648	26.5701	27.2781	27.4239	27.5817
28.1415	25.4732	25.6939	24.9884	25.348	25.7279	25.6765	25.9701	26.545	26.7647	26.6297	26.5658
27.6001	25.7906	26.6627	25.6817	26.6967	26.962	27.0869	27.4755	26.4475	26.6879	26.8069	26.476
28.7195	26.3543	27.748	27.4143	27.4725	26.6513	27.8635	26.4901	28.0625	27.952	27.8373	27.8728
30.0593	30.48	27.6355	26.3438	26.1805	31.1739	28.9856	31.5536	24.1784	24.6844	24.1659	23.6399
28.4085	28.1602	27.9529	28.2617	27.9585	28.157	26.0924	27.7361	28.311	28.5944	28.3668	28.3181
28.7684	28.5652	28.7951	28.5473	28.7457	28.7632	29.199	27.7975	29.0573	28.9337	29.4482	29.1984
26.7373	24.2539	26.922	25.7452	25.471	25.9376	26.7749	25.6088	23.8171	29.4186	23.6082	25.3797
24.6262	26.8281	25.3217	27.2283	26.8148	25.9173	27.0479	26.5451	27.0259	27.1708	27.4916	27.3709
29.5547	29.4524	29.3267	29.558	29.766	30.1558	29.8592	29.0828	29.2579	29.3567	29.3159	29.264
26.6437	25.6632	26.967	26.3371	26.7788	27.3615	26.2406	27.5089	25.885	25.9504	26.6353	26.4786
27.2347	26.1714	26.4464	25.8019	26.4998	24.7459	26.8953	29.0618	24.7737	26.4252	26.9114	24.5903
25.6897	25.4433	26.0991	25.8169	26.5716	25.9687	27.1092	26.9236	29.5279	29.6585	29.5381	29.5176
26.1928	25.1204	26.3667	24.8745	27.4176	25.3109	26.1753	28.1145	26.4858	23.6044	26.7188	26.4703
29.4085	29.3098	28.9421	29.2125	28.2716	28.2429	28.7834	28.4003	29.1078	29.0762	29.1206	28.9069
28.3906	28.2314	28.0358	28.058	26.2526	26.0628	26.7057	27.2125	28.9439	29.0568	29.3933	29.3255
28.4297	28.4694	28.9318	28.9521	28.6993	29.3798	28.1181	28.3325	29.8697	29.6782	29.8244	29.9854
30.3425	30.5695	30.5057	30.6532	30.5674	30.2715	30.0778	30.0721	30.601	30.3981	30.514	30.3901
29.5083	29.3903	29.4982	29.2996	28.8794	28.6418	28.8249	28.4745	29.604	29.435	29.4274	29.5491
32.9713	33.0552	32.4461	32.2191	30.9351	30.6702	30.9	30.0726	30.8934	30.8189	31.1974	31.3639
26.2033	26.8922	25.6697	25.0305	26.165	26.6453	27.7475	26.4686	26.2712	24.3951	26.1531	25.574
29.381	29.2779	29.3252	29.4033	29.1928	29.0589	29.3184	28.5399	29.4266	29.6541	29.549	29.4292
26.8212	27.158	27.4154	26.8526	25.5329	25.9501	25.4016	23.8584	27.1022	27.3854	27.0625	27.24
24.9436	26.3525	26.1359	26.9429	26.7337	25.8916	26.6462	26.9258	27.5235	27.659	27.3147	27.5906
27.8644	27.8486	28.5198	28.5305	28.1651	26.9735	26.3483	26.8598	27.5998	27.5798	27.6457	27.5873
29.302	29.1763	27.8526	27.5705	28.4257	28.99	28.2208	28.8851	29.1747	29.073	29.5727	29.3447
27.2303	24.2702	27.0944	25.5485	26.846	25.7122	26.9759	25.4625	25.4693	25.7063	26.4539	26.525
27.9198	28.1764	28.1131	28.3016	27.9557	28.0568	28.1451	28.4199	27.9566	28.0276	28.3144	28.3462
25.4227	26.2252	25.9203	25.5312	26.9607	27.5135	25.9435	26.4702	27.6029	27.7639	27.4046	27.5217
28.033	28.5602	28.8839	28.6206	27.9011	28.0276	28.1822	26.8017	28.7058	28.5876	28.7257	28.385
28.4206	28.372	28.0824	27.9852	28.66	28.7603	28.742	28.5199	24.3685	24.8578	26.2002	25.7573
26.5749	25.1081	26.5632	25.8936	26.5785	25.3359	26.6238	26.741	26.1211	23.7164	24.4551	26.3577
30.0836	30.0204	29.766	30.2607	32.2573	31.2316	32.1596	32.1881	31.2611	30.8377	28.6705	28.3941
25.0015	25.7448	26.836	24.9786	26.669	26.1553	27.297	26.6272	23.419	24.0464	23.683	24.2283
27.288	26.9917	26.7438	27.3544	26.9513	25.6462	26.6522	27.1994	28.6598	28.7394	28.7168	28.7781
27.6712	28.1155	27.6053	27.7533	28.2357	28.9093	28.3961	28.5651	28.8229	28.9076	29.1901	29.0207
24.9332	27.009	26.4368	28.4033	25.2876	27.998	28.045	27.7064	28.1732	28.1498	28.3523	28.3903
25.8434	27.8592	25.6564	27.7974	26.2211	25.0037	26.4034	26.6898	27.5709	27.6945	27.4379	28.0874
27.0996	27.4244	26.6509	25.6994	28.0056	28.6172	28.0675	29.0423	29.6526	29.5752	29.5052	29.5172
28.7929	28.9519	28.9341	28.7615	28.581	28.6233	28.5867	28.4787	29.1332	29.122	29.3138	29.2511
25.8077	24.6829	26.2703	25.7893	26.4175	26.287	25.8451	25.8837	25.0518	27.4387	26.2913	26.3158
26.6039	25.6423	25.6876	25.0989	28.301	25.7858	26.6244	25.923	27.1005	26.8371	26.9351	26.7897
29.9617	30.0288	29.989	30.0251	28.8091	26.5004	28.762	28.0619	29.6787	29.338	29.2279	29.8275
29.6611	29.7238	29.988	30.3091	29.9664	29.6005	30.061	29.5727	28.7543	28.6857	28.8609	28.813
28.5762	28.2862	29.5504	29.4337	29.2662	29.2372	29.3082	28.7779	27.5096	27.5898	27.4849	27.5235
26.0129	26.8335	26.798	26.2972	26.2665	27.47	26.1636	27.7571	29.0858	29.1457	29.2243	29.2367
26.8668	27.2932	26.135	27.15	27.3504	25.6104	26.5607	26.4156	27.4929	27.4621	28.278	27.9284
29.0677	29.2861	29.9146	29.7095	25.2001	29.002	26.6641	29.1837	27.7167	27.513	27.5985	27.6822
24.8771	26.0285	26.111	25.9122	27.3026	27.024	26.6045	26.1785	27.3753	27.212	28.2881	27.4227
26.9946	25.3212	26.9881	25.8057	28.5888	27.9695	25.4853	25.7306	26.9901	27.1224	27.1631	27.2179
24.8985	25.6386	26.2952	24.651	27.0875	25.1668	26.98	26.3826	25.5116	26.8311	27.1061	26.9512
26.7066	26.4297	26.7749	25.3324	26.4449	26.8044	25.7539	26.8791	25.0649	24.2578	25.1214	25.1146
28.5788	29.0774	27.2158	28.1938	26.4348	29.3783	27.6032	28.8767	29.8716	29.7959	29.9715	29.8806
27.2248	27.17	26.4676	26.6461	26.5756	26.7558	26.3786	27.6917	27.8201	27.651	27.4851	27.3584
25.4589	26.1935	26.6129	26.2483	27.2962	26.69	27.8047	25.6295	27.9763	28.0012	27.8207	28.1454
28.5065	28.5153	28.8407	28.4852	31.7779	29.9079	31.6522	30.7564	24.7406	23.8359	24.9216	25.6677
31.3953	31.7109	29.0453	28.9955	29.7106	31.3778	29.4832	31.8613	29.4821	29.5239	29.3415	29.3725
26.6908	25.2879	25.8067	26.5818	26.8281	27.1594	27.0808	25.7664	24.1995	25.5759	26.2767	26.502
31.5018	30.4774	30.9836	30.5657	32.5448	33.8475	31.7558	31.8914	28.7638	27.5832	28.9615	29.5537
29.3345	29.3126	28.3398	28.3122	30.4484	31.1075	30.3621	28.987	26.6764	26.7907	26.5266	26.4664
29.882	29.9689	28.8571	28.5396	28.4923	30.161	28.757	28.7897	27.9255	26.7277	26.5736	27.9396
27.2991	25.6196	26.1863	25.5266	26.6972	26.3897	26.6657	26.3923	25.433	25.9602	26.1533	25.9894

31.113	31.2494	31.268	31.3437	31.0805	31.9405	31.0633	31.6626	32.3178	32.306	32.6835	32.646
27.9275	28.4679	28.0261	28.3448	30.0343	28.772	28.5037	29.5638	28.1531	28.1853	28.6065	28.6664
28.532	28.6182	28.4849	28.337	28.0892	28.2763	27.712	28.1938	28.6321	28.865	28.8915	28.9295
26.131	26.6183	26.4866	25.7397	26.8739	26.1857	25.8346	25.9551	26.7296	26.945	27.0948	27.1749
25.9596	25.2656	27.3329	26.4239	26.5139	26.9984	26.4328	25.4883	26.2558	26.3936	26.6612	25.7517
32.4717	32.4024	31.4866	31.5678	32.1978	32.0294	32.1007	31.9046	29.7117	30.0588	29.806	30.0774
28.1171	28.3554	27.8619	27.8517	29.4355	30.8337	29.5727	30.6009	25.1787	24.1054	22.8754	25.6483
27.0401	29.024	29.9259	29.3289	25.1188	30.6022	27.9471	28.3363	29.9919	29.8334	29.857	29.7985
26.5764	26.0195	24.9994	25.4973	26.0644	26.3941	26.711	26.2687	26.8958	26.8655	26.4776	26.5019
27.8298	27.4063	27.4326	26.0756	26.9868	28.5944	28.3888	28.8451	28.7351	28.9316	29.2501	29.318
28.9762	28.8421	28.9575	28.3114	26.4564	25.9122	26.4586	26.3865	25.4361	27.8759	26.9617	27.2526
24.9788	26.4001	24.779	25.7543	27.747	26.7064	26.5725	26.021	25.2699	24.1733	26.5359	26.4507
30.9679	29.578	30.1783	30.2546	31.7215	32.5599	31.1934	31.7672	29.233	28.2237	28.1898	30.034
24.4801	26.6249	26.9114	26.8968	28.3046	25.5467	25.1418	26.8946	26.7475	26.7593	26.5705	26.827
28.607	28.6947	28.4115	28.5	28.3172	28.9004	28.456	28.6918	28.9217	29.0429	28.8144	29.0674
26.6219	28.1859	28.4426	28.323	27.234	28.5402	26.0017	25.3882	27.9252	27.8367	28.3021	27.7719
22.9093	25.2038	25.9941	27.0964	26.3864	27.0071	26.9342	26.3107	27.1769	27.0461	27.232	27.2644
25.6636	25.1794	24.7722	26.5876	26.4698	27.2616	25.9542	26.5237	27.7356	27.911	28.0587	28.0994
27.7764	27.3915	25.5624	25.7223	27.266	25.3413	25.9537	26.9125	26.8759	26.4875	26.5526	26.7449
24.7862	25.79	27.4575	27.0123	27.235	26.3601	27.979	26.4093	26.8211	26.5084	26.9049	26.5628
30.3233	30.398	28.9971	29.1395	28.8055	29.7035	28.6343	29.5565	30.8498	30.8125	30.658	30.5715
26.2585	26.2688	26.0074	25.9964	27.2566	26.9916	27.3624	26.9624	27.9474	28.0547	28.2781	28.2434
28.1474	28.3437	27.1525	24.436	25.9099	28.7363	26.2711	29.6983	27.551	27.8998	27.9345	28.1377
25.6979	27.0036	26.2856	26.3658	26.6624	28.2061	26.0355	28.0076	27.8372	27.9458	27.5955	27.8413
24.5548	26.1669	26.662	27.0739	26.8232	26.668	25.8685	24.7498	27.1174	27.4465	27.0483	26.8674
31.169	31.2994	28.1934	28.3838	30.0216	31.7244	29.844	31.5252	28.9863	29.148	29.319	29.3617
26.1527	25.6022	27.2658	26.2658	27.5344	25.1653	26.8151	25.291	28.4288	28.3071	28.3271	28.3174
26.9216	25.0796	27.5349	26.121	26.2657	27.115	26.0577	26.3309	24.6138	26.4307	26.4663	26.5539
27.2498	27.3249	26.0327	27.6475	25.9908	27.5129	28.6707	27.7273	28.6293	28.4298	28.2742	28.4866
25.0444	26.8632	26.8398	26.6371	27.9943	26.1244	27.6684	25.6655	26.748	24.7244	27.0706	27.2852
27.6592	27.4937	27.8956	28.0795	27.9779	27.7464	28.3431	27.7209	26.7738	26.7154	26.8607	26.9749
25.6912	25.4093	25.6141	25.871	27.2626	25.9507	25.8077	23.932	24.1612	25.1799	24.8364	26.5839
32.467	32.1199	31.9681	32.0866	31.5706	32.2548	31.0382	32.4009	32.7738	32.9229	33.0707	32.7249
29.353	29.3942	29.0643	29.1029	28.7761	29.0727	28.9385	28.5905	30.3983	30.4908	30.4666	30.5226
25.1889	28.1999	27.3378	26.363	26.9437	26.7619	26.3994	26.699	27.9339	28.0795	28.2952	28.4496
25.1743	27.5156	27.1708	26.945	26.1492	27.124	25.7345	25.9961	28.7912	28.7297	28.8073	28.85
28.4658	28.5745	28.278	27.8698	27.8491	28.0349	27.8496	28.1311	27.9168	27.9252	28.052	27.9602
27.5895	28.1369	27.5145	27.5805	28.4395	28.3189	27.8329	24.9809	30.7442	30.5508	30.4424	30.3906
28.0918	27.8035	25.8722	27.324	27.6418	28.4596	27.4063	28.2474	28.7297	28.8254	29.0207	29.0066
31.1596	31.3577	30.0002	29.962	29.3166	30.5403	30.1036	29.1669	31.7766	31.5602	31.4631	31.4238
30.2164	30.5481	29.4722	29.193	29.0826	30.2431	29.2111	29.4863	30.9009	30.932	30.7996	30.9281
27.714	27.4784	26.581	26.6558	28.5081	28.8627	28.49	29.2082	23.692	23.1402	25.198	24.7511
25.8761	25.4539	26.2081	27.9046	26.2993	27.2065	25.5475	25.9083	29.406	29.0434	29.6816	29.8572
25.2886	28.2378	26.8522	27.1955	26.78	26.0301	26.6943	25.5643	29.2604	29.2308	28.6892	29.0329
27.8559	27.7768	27.4212	27.7119	26.6452	27.7565	28.353	29.2473	28.2117	28.3015	27.9114	28.1161
27.8896	28.1002	28.4927	28.3502	26.5214	26.2255	28.455	26.0085	26.3902	26.7427	26.8933	26.3901
25.5641	24.3435	24.9728	26.3861	27.4	26.4408	27.2453	26.8992	25.6282	25.0522	27.7797	27.9663
25.7444	26.516	27.7662	24.5422	26.7689	29.3029	27.4497	26.3028	28.506	28.557	28.7211	28.7224
29.6249	29.7048	29.4699	29.5657	29.3394	29.4967	29.2613	29.1868	30.238	30.4057	30.4056	30.4166
29.5036	29.3584	28.5853	28.4432	26.5373	24.6492	26.7965	27.8442	27.781	27.4848	27.6204	27.9845
26.5727	25.5463	26.1956	27.0216	26.2597	26.1683	27.4569	27.5301	25.0137	24.8005	24.728	26.0475
29.415	29.4054	28.2572	27.8395	28.206	29.8649	28.1926	28.6338	29.5306	29.4886	29.6307	30.1222
25.9572	25.9046	25.6347	24.6007	27.4915	25.6326	27.3025	26.2446	23.2587	24.4024	27.2565	26.6437
25.3552	29.1066	26.1291	26.5587	25.9669	26.5776	26.6911	25.9213	27.1025	27.1555	27.5152	27.7469
30.0316	30.1412	29.9367	30.3716	29.6474	29.2146	29.922	28.8242	30.1113	30.311	29.9604	29.947
28.1862	28.1774	27.7137	27.8724	28.3104	28.1896	27.938	27.4228	30.8234	30.5952	30.8652	30.7906
25.3208	26.5941	24.1275	26.9856	27.204	26.0877	26.2512	25.352	26.5167	26.7889	26.9832	27.2361
26.6292	26.4588	25.4905	26.5519	26.6795	25.8997	24.6039	25.5145	23.314	26.5269	26.7444	26.5669
26.6376	24.3934	24.9298	25.7065	25.3257	25.2168	26.4079	26.7834	27.9816	27.9228	28.1691	28.1163
28.1262	27.9471	27.9875	27.9092	25.9932	28.2736	28.2621	28.3402	28.4083	28.8113	28.6742	28.6716
27.2792	26.2348	26.1536	27.0414	27.6923	25.3265	27.0902	27.6199	24.4419	22.5963	24.7268	24.3019
29.3958	29.3484	28.2588	28.2019	29.027	30.067	29.201	29.8442	26.477	26.5041	26.127	25.9203
27.4371	25.4486	26.345	25.6163	25.7586	27.5136	25.8889	26.5272	27.281	27.4674	27.0587	24.8781
27.3277	27.3386	26.0036	27.3725	26.2409	27.7202	28.0574	26.4955	27.6063	27.6453	27.975	27.7756
27.264	26.3681	25.8933	27.7842	25.9759	25.9629	26.8437	26.6214	25.0459	25.4694	25.5545	24.1204
26.1717	26.2357	26.6546	25.5907	26.3355	28.149	25.888	26.3176	24.8955	25.7339	25.6931	24.3347
28.7229	28.9114	28.3414	28.4071	27.9864	28.3123	27.916	27.8188	28.4896	28.7846	28.6569	28.6639
28.5368	28.5591	28.4869	28.4695	26.242	28.589	26.8775	27.5744	28.7845	28.7358	29.1991	29.0257
24.7028	26.4546	25.616	26.4832	26.3062	25.7141	29.0218	26.4354	28.5769	28.3838	28.633	28.6415
26.14	27.1071	27.2807	26.0334	26.377	25.2087	25.9317	26.9362	24.3855	24.5585	25.2498	25.7219
26.4824	26.336	27.9617	26.015	26.3803	25.7657	26.5177	27.2509	26.7522	26.5658	27.289	26.586
26.504	27.1472	24.1981	26.0052	26.8288	25.2738	27.2202	27.1697	28.8245	28.5561	28.2741	28.6272
26.7765	27.1319	26.3097	26.3098	26.5032	25.666	28.3495	26.128	29.0723	28.973	29.4258	29.3367
28.3885	28.1877	28.94	29.1834	27.5164	27.2995	26.2623	27.0207	27.2865	27.406	27.6112	27.5474
25.82	26.0885	25.8309	25.2421	26.4553	26.3287	27.5768	26.382	25.5939	25.4208	26.0221	25.9564
26.4804	25.6213	25.4948	26.2879	25.7399	25.5784	27.1915	26.5794	26.6207	27.2073	27.3611	27.1327
25.668	26.4863	25.6982	27.6121	25.5252	27.1264	25.9433	27.2731	25.4658	26.2658	26.626	26.4706
24.9436	25.6129	25.1375	26.1171	26.1229	25.7989	27.2378	26.9467	24.2106	24.8737	25.2239	24.9032
26.0692	26.3136	27.6655	25.6631	27.249	25.7225	26.7515	26.4845	27.4901	26.9787	26.3084	27.0917
26.7096	30.3456	25.4199	24.1773	27.9916	26.6835	25.4514	26.7503	31.5318	31.128	30.6083	30.8281
25.831	25.6532	27.2796	26.2912	26.299	27.9437	28.1146	26.5894	28.3128	28.3507	28.5716	28.6739
27.8581	24.5997	25.4808	25.2914	27.2017	27.3062	26.0091	24.9125	27.3437	27.4831	26.9245	27.1383
29.4325	29.5516	28.4002	28.695	26.7829	29.7126	27.7848	28.9157	29.9586	29.7421	29.8176	29.8288
26.1624	26.0152	25.924	28.2661	27.5095	27.1175	27.135	26.2154	27.9308	27.8635	28.2913	27.9725
25.4264	26.9204	25.5966	26.9249	28.1021	25.1602	27.6342	24.3569	24.8877	25.5402	25.6863	25.3657
26.7825	25.5543	26.5952	26.9929	26.9542	27.4533	26.5211	25.4342	26.166	24.8257	24.2318	25.5627
29.3978	28.7941	28.2828	28.4788	27.1148	27.4681	27.8367	27.1166	25.3429	23.1747	26.0527	25.9117
29.1055	28.8825	24.5328	28.8199	26.2384	29.2796	25.6616	29.0225	28.544	28.7949	29.0923	29.2981
29.527	29.6674	29.3803	29.5392	28.8389	28.5257	28.7634	27.7992	25.8161	25.6032	25.9868	25.8708
26.6349	27.6232	25.1476	25.3716	26.4591	25.7093	25.8235	27.762	30.0466	30.0379	30.0283	30.236
26.8999	25.805	27.1422	26.7347	26.2832	25.6665	27.004	26.2579	26.9941	27.1045	27.138	27.3639
25.8659	26.0003	26.2472	26.711	26.5491	26.7456	27.4834	26.4454	27.254	27.0627	27.4733	27.5332
27.884	24.9327	26.5678	26.119	27.1766	25.3728	27.2099	26.3522	24.9629	24.0572	25.3344	24.8841
29.6518	29.7727	29.7815	29.6259	29.8251	29.6548	29.8199	29.1058	31.0944	30.9182	31.1525	31.1663
29.7682	29.7546	29.3058	29.4081	29.318	28.6092	29.2172	28.4408	29.6317	29.5077	30.0605	29.9861
26.8851	27.0173	26.1681	26.4576	26.4082	29.0989	28.2917	26.5744	31.1463	30.9426	31.2335	31.1768
27.8431	28.021	28.1496	27.7529	28.1164	28.3925	28.0585	28.4133	28.483	28.3705	28.5083	28.6555
24.9616	24.2125	24.5703	25.1291	28.1683	26.3981	26.3014	27.6783	27.1746	24.2802	27.8193	28.2448
27.0734	27.1445	27.237	27.056	26.524	25.844	27.3637	26.5074	24.3874	24.757	24.6604	25.6477
27.0592	26.5738	27.6597	27.6183	26.3896	27.9575	27.9884	27.6033	30.1098	30.1597	30.2261	30.2251
26.343	26.4769	26.4768	26.4667	26.7391	26.9345	26.8734	24.828	28.2938	28.5415	28.3816	28.4292
28.0085	28.4036	28.2921	28.0931	25.7462	28.3433	26.1472	26.9037	28.493	28.7153	28.6018	28.6353
26.9367	25.9265	26.3838	25.6755	26.2514	25.0865	27.1857	26.4377	27.8006	27.9645	24.951	24.9232
27.3536	25.8346	26.4488	25.6868	26.6533	26.6768	27.0007	27.1193	26.5278	26.3979	25.8443	26.7165
26.842	26.1698	26.8739	25.1616	27.4858	25.8353	25.9588	26.2138	26.5309	25.3729	26.8987	24.5074
28.0518	27.9333	27.7419	27.9049	26.7065	27.7432	30.6209	26.0226	28.4403	28.5286	28.7466	28.7781
26.0524	26.9275	25.9929	25.8996	28.7746	27.6132	26.677	27.2622	27.8211	28.0364	28.2259	28.1866
26.3868	26.7687	26.5902	26.6234	26.6314	28.1074	27.0781	26.3852	26.8599	26.427	26.3658	26.5049
29.1057	28.0224	27.4625	27.5873	26.4668	32.0331	27.1054	32.0812	27.6222	27.8275	27.2156	27.5739
27.5966	25.9103	26.0535	26.2462	27.291	27.2295	26.5715	26.419	27.4832	27.6136	27.585	27.5902
27.7369	28.0384	27.8945	27.797	27.6661	28.2457	27.6677	28.3068	27.8208	27.817	28.0474	27.9426
27.1555	26.855	26.7805	25.8746	27.9285	25.8385	25.9234	27.1011	27.4731	27.1402	26.8508	27.2133
27.7868	26.2506	26.907	27.1898	27.368	25.9114	27.2614	25.5054	24.1402	24.064	25.3953	27.0016
30.2794	30.3342	28.7062	29.0733	27.4074	29.9067	28.8088	29.0305	30.8768	30.6348	30.0836	30.0117
30.0893	30.0489	29.1	29.5223	29.1685	30.3995	29.6626	30.2815	30.6481	30.551	30.5944	30.491
29.09	29.3305	27.9771	27.982	28.7886	28.759	28.3953	28.0989	30.2895	30.1634	29.919	29.858
30.2569	30.3126	29.8373	29.5663	29.736	30.2774	29.4702	29.7442	30.8389	30.9919	30.7202	30.6112
30.4032	30.7207	29.5019	29.1839	28.8979	30.4741	29.1366	30.1574	31.0453	30.8393	30.9655	30.8825
29.3021	29.6313	28.8382	28.5798	28.5966	29.6335	28.1699	29.0277	30.1649	30.1752	29.8408	29.9593
29.1721	29.0905	28.5947	28.4799	28.0881	29.3873	27.0949	27.0923	30.5704	30.4548	30.234	30.158
28.5964	25.9334	27.3841	26.3864	26.1535	26.392	26.0829	25.7692	28.5601	28.7343	28.5128	28.5563
27.16	27.8232	26.0184	23.857	26.6477	26.0724	26.3234	25.2734	27.4339	26.9879	28.2354	27.709
26.3277	26.9345	26.7522	25.9269	28.2466	26.2204	27.8142	25.9336	27.5803	27.6691	27.1515	27.3732
27.9779	27.1821	28.4193	28.067	26.6951	27.6543	26.8877	26.9918	26.8282	26.5638	26.5585	26.7716
30.3711	30.3374	30.6813	30.7385	29.8188	30.4324	29.7538	30.473	29.3341	29.325	29.4417	29.4671
24.1111	26.8111	26.6142	24.102	26.4275	25.7137	28.1362	27.2503	26.7325	26.938	22.0954	26.9152
25.0048	26.942	25.9915	25.7415	26.4539	25.9445	27.2124	28.0075	26.9866	27.0519	27.3558	27.4237
26.8493	26.8901	25.7625	27.6915	27.3376	25.5574	26.1356	26.7836	25.6367	25.8306	24.1641	25.0324
29.1612	28.8689	28.9956	29.4752	27.05	28.7422	24.6885	28.5081	29.5408	29.5146	30.0176	29.8244

30.2164	30.3414	30.2651	30.4379	29.4669	29.8955	29.6219	29.4255	29.8786	29.8622	29.9426	30.1957
26.0042	27.8205	27.8362	26.1001	25.9674	28.0184	26.4497	26.1084	28.0033	27.7705	27.8548	27.9996
27.7662	28.0358	27.967	27.7795	26.8984	27.702	26.197	28.3453	28.5514	28.4776	28.5894	28.5194
27.8203	28.4466	28.3315	26.2664	26.8381	29.2711	27.0326	27.635	29.1684	29.2225	28.5288	28.3363
26.9861	25.6064	26.8409	25.4615	27.1198	26.6942	27.0225	27.2417	27.7836	27.5604	27.5227	27.7992
27.6409	27.2912	26.1679	26.2263	26.4886	25.4667	25.9152	26.9554	28.4453	28.1948	27.9244	27.7913
28.8682	29.2648	29.3305	28.1426	29.2034	29.5371	28.7488	29.9762	27.4991	27.2712	27.4656	27.198
29.6928	29.3511	25.6879	26.8799	29.2499	30.6793	29.029	29.6022	24.1698	26.0675	23.9433	24.1389
25.0132	25.7634	27.0427	25.9453	27.5455	27.4398	25.401	25.7301	26.2992	26.8165	26.9156	27.0399
27.8264	25.5865	26.7238	27.5761	26.4344	24.7291	25.6113	25.7312	23.8245	25.2213	26.4726	24.0119
29.3701	29.3656	27.6921	28.6212	30.3158	29.6896	30.0013	30.0645	27.9783	27.4901	26.8313	27.0632
28.3492	28.4676	28.0506	28.2613	25.5147	27.6826	26.6084	27.2171	28.7206	29.0023	29.0024	29.1203
27.5059	27.6042	26.543	26.2099	26.2654	26.1317	25.5768	27.8335	27.3901	27.134	27.2634	27.365
27.3928	26.3237	25.772	27.0198	27.2305	25.454	27.3162	26.3073	27.2852	27.4012	27.0948	27.2614
32.2191	32.499	31.9112	31.6105	32.0919	32.3198	31.9956	31.7386	29.2197	28.9312	28.8365	28.8212
28.0291	28.259	27.5991	27.5102	27.1658	28.0522	26.3338	26.4806	28.2496	28.3665	28.5971	28.3265
31.8212	32.12	29.7423	29.3115	26.8928	31.4804	30.481	32.2068	26.2736	26.6261	26.6767	26.82
25.4127	25.8568	26.8202	26.0153	27.4546	27.0346	26.0069	27.5756	27.327	27.0216	26.5011	27.3467
29.9296	29.7947	29.437	29.3667	29.2817	29.5651	29.243	29.7899	30.2533	30.2678	30.4182	30.5086
27.1781	25.5338	26.2733	27.0349	26.4228	26.0298	27.0189	25.2768	25.2727	26.4712	25.4862	24.2322
25.716	25.4505	25.9044	27.7459	26.9669	24.6527	26.4661	26.8874	24.0347	26.8156	26.7963	26.8321
24.9493	25.746	26.6346	25.5763	25.4877	26.0855	26.1211	27.7289	26.6864	27.0325	26.6438	27.0769
26.0911	24.9898	25.787	24.7351	28.1174	27.9098	26.1997	27.6484	28.3474	28.4782	28.7272	28.6199
27.6614	27.9096	28.0001	27.814	25.7559	25.5834	26.861	25.8915	28.5514	27.9668	27.8754	28.0985
29.1817	29.2851	28.9955	29.3245	28.5545	28.4037	28.6283	28.0993	23.9597	23.8918	26.6224	26.5031
26.0668	25.0979	26.5773	27.196	27.4705	25.3282	26.297	27.4909	23.9563	26.2205	24.328	25.1092
29.6088	29.7072	29.7771	29.2493	29.7778	30.3344	29.6602	29.488	28.0129	24.4108	27.4711	27.4107
27.8188	27.5834	27.897	27.1081	28.3938	27.5061	26.8031	26.5874	27.775	27.8478	27.7744	27.8826
27.5135	25.9717	26.3537	25.8294	27.0238	25.5911	26.054	24.9271	25.1689	26.5799	24.241	24.9641
28.8549	28.8657	28.2578	28.3166	28.3852	29.2942	27.9225	29.5132	28.0006	28.2867	28.1464	28.04
29.7894	29.8984	29.2153	28.7707	28.4587	29.864	28.4511	29.2749	30.8243	30.8755	30.9351	30.7175
27.6077	27.5569	27.1538	27.2632	27.9154	27.6718	27.8174	27.4354	29.0054	29.1424	29.1863	29.0299
28.1559	28.0318	28.4564	27.8074	26.2341	24.9765	27.8515	26.1361	29.0132	29.0772	28.9922	29.1381
29.2969	29.3701	29.7325	29.6959	29.492	29.467	29.3546	28.9689	29.0208	29.0541	29.1596	29.1549
26.617	24.3718	26.2857	26.0366	26.7327	25.2427	27.2772	27.9568	25.3572	23.1071	25.784	25.0739
30.4714	30.3111	29.815	30.2888	29.8797	30.3231	30.0417	30.5764	30.5144	30.6503	30.5626	30.5481
25.4086	25.5637	26.4214	25.8066	26.4012	27.5335	26.2029	26.4206	26.4276	26.2258	26.5652	26.4258
26.9469	27.2844	25.1687	27.3435	25.165	27.4377	26.6097	26.7896	29.8736	29.8744	29.7309	29.9663
27.5878	28.6287	26.7993	28.7736	27.1838	22.8903	25.2627	27.7171	28.429	28.5749	28.6427	28.8054
24.6353	26.2245	24.7462	27.0361	27.0624	28.7885	26.0117	25.382	23.7087	24.1929	23.6443	23.0723
28.3856	27.9664	29.1054	28.5146	27.1103	27.8265	27.9617	25.9338	28.7462	28.5943	28.7852	28.6435
25.251	26.2098	26.2583	25.9416	26.1081	26.1149	26.5819	25.9966	27.6704	27.3359	27.3322	27.1231
28.4317	28.6549	28.6275	28.5477	27.0129	28.8949	26.4849	26.4774	29.0972	29.0694	29.2586	29.2365
28.2988	28.0016	26.5356	27.7238	26.4084	25.2147	26.8138	26.5917	27.9478	27.8279	28.2926	28.0077
27.7876	28.4379	28.0622	27.9674	27.3584	28.3933	28.1528	28.635	28.2663	28.2262	28.5764	28.4524
26.6551	27.3455	27.0016	25.4826	27.4989	26.4682	27.0075	26.631	25.8735	27.2471	26.7573	27.2407
25.3783	26.6272	25.0895	24.6827	25.4836	26.5808	26.0234	26.6958	24.3974	24.0368	25.4613	25.2859
24.9154	25.2906	26.2595	25.4002	27.1743	26.0835	27.3131	26.5848	25.2933	25.7288	24.7344	24.7156
24.3018	24.8246	26.2107	25.4154	25.5312	25.9172	25.5854	27.5976	23.6681	25.3886	24.8346	25.5705
26.6892	25.8775	25.0668	26.7295	26.2559	25.1739	27.2158	26.2394	25.4247	25.6358	24.0531	25.4814
27.8031	27.8872	26.1643	24.7825	26.9854	27.279	25.2875	27.4313	27.7733	27.7295	27.7345	27.7286
28.1872	28.2216	28.9145	27.3892	26.8432	25.8229	26.4761	26.1131	29.0081	29.1379	29.1575	28.9489
26.7343	27.3765	25.944	26.4731	25.5781	27.3183	27.3654	27.595	27.9566	28.133	28.181	28.1824
29.8564	30.3397	27.6339	25.8585	28.2001	29.8828	28.336	30.6208	28.7965	28.8389	28.9532	29.1935
26.1643	25.8854	24.534	24.7575	27.9324	27.0755	26.6724	27.7268	28.7508	28.7805	29.0435	29.0628
26.4565	26.5742	27.036	25.3636	26.6132	28.0745	26.5418	25.2029	26.0431	25.9186	26.0381	26.3543
25.903	26.2795	27.8184	26.4902	26.8413	25.737	26.8155	24.0731	29.5956	29.3571	29.5744	29.6278
25.9686	25.6964	25.4974	26.9054	27.6349	27.3004	27.5149	27.2681	29.1933	29.2835	29.5842	29.5811
24.9701	25.917	27.0232	25.5677	26.7988	27.1796	27.2841	27.4334	27.094	27.2785	27.3696	27.3301
25.4777	24.0632	25.677	25.9188	27.1558	28.1751	25.7197	26.718	24.4761	24.4336	25.539	25.5812
26.4269	26.7844	27.7904	25.4115	26.8882	26.6455	26.5768	26.2054	25.4282	25.4561	25.7755	25.8032
29.4299	30.0915	30.784	29.8408	30.7121	28.6387	30.6116	28.7174	30.6347	30.6157	30.3656	30.2819
26.8712	27.4932	26.8737	25.9738	26.949	25.8063	25.6089	26.7843	27.4364	26.3937	26.7733	26.6915
27.0322	25.1756	28.1077	30.0594	26.8156	26.0201	25.1839	26.5784	27.579	27.6591	27.7339	27.6361
26.3271	27.044	26.332	25.6499	26.893	27.292	27.2192	27.899	25.9813	24.8705	27.5652	27.131
25.2446	25.1854	26.8831	25.7738	27.781	26.5599	25.8957	24.8988	25.2188	25.9061	26.2918	26.3639
27.3547	27.501	27.4741	25.4437	27.737	27.7809	26.6016	26.7	26.7555	26.8665	27.0473	27.0849
27.6755	26.8343	26.4907	26.7559	26.2003	26.8405	26.1931	26.7564	27.0532	27.0608	27.4478	27.6355
26.2778	25.8688	27.2528	25.7031	26.9778	25.8702	25.646	26.0729	26.2657	26.2816	26.5906	26.332
28.1918	28.096	27.6155	27.7492	26.4289	27.8745	27.752	27.9769	28.481	28.5037	28.6973	28.4524
25.9967	24.7714	26.9974	27.2344	25.8696	26.6498	26.4891	26.1575	27.0133	26.7419	26.4131	26.6139
25.966	25.966	25.9838	26.0837	27.3993	26.7017	27.6763	25.9782	28.4118	28.1739	28.3472	28.3052
25.6605	24.3806	26.8686	25.6155	27.1312	27.2175	26.5514	25.9028	27.7414	27.3469	27.3274	27.8335
28.8064	28.8829	28.9472	29.026	29.8403	29.3932	29.7182	28.7699	27.5383	27.4878	27.4756	27.4892
26.5181	25.044	25.7948	27.1008	26.8134	25.4186	26.9291	26.535	27.0217	24.8808	25.8668	24.3945
30.7797	30.7992	30.4007	30.6031	29.6587	30.1986	29.6675	30.1746	29.5936	29.3539	29.6272	29.508
25.9234	24.4387	26.9564	25.6996	25.53	25.4795	26.5502	25.5051	28.9448	28.9312	28.7562	28.9868
25.7536	25.3142	26.2825	25.9644	26.122	25.5349	26.1333	27.2202	27.5307	27.6914	27.4645	27.4996
25.5993	26.2246	27.5462	25.571	24.9407	27.0847	26.3683	26.1202	27.5334	27.3094	27.5324	27.5039
26.9932	27.6295	25.6007	26.3853	26.5874	25.8185	26.6808	26.2034	24.5426	25.1465	26.0121	26.0864
29.1546	28.9065	28.9143	29.2279	30.2847	28.5616	27.389	29.621	30.2774	30.3497	30.4751	30.4539
24.9142	26.033	25.9922	24.9731	27.9541	26.9301	25.3581	26.0968	27.255	27.3844	27.3866	27.4688
29.6123	29.5952	29.0378	29.3931	29.5897	29.7732	29.4508	29.7048	29.7708	29.6788	29.557	29.6712
29.9615	29.2803	28.9535	29.0295	29.1137	30.9821	29.0092	30.1646	28.1506	28.1893	28.2062	28.1575
31.177	31.1563	31.0162	30.7821	30.409	31.6522	30.6286	31.5044	30.1348	30.2584	30.6824	30.6372
26.1845	26.5868	26.7606	26.3407	26.5317	25.3496	27.678	26.2869	27.9113	27.8603	27.6234	28.0234
24.0903	25.2668	26.2146	27.6125	26.1536	26.4558	27.2564	25.4615	25.26	24.8364	25.8051	25.6063
27.5343	25.0274	26.8451	26.694	27.5834	26.1336	26.9116	27.1312	25.1306	24.0984	23.5062	23.3686
26.2683	25.6267	25.2851	26.4257	26.4331	25.6224	26.6254	27.3858	27.5617	28.4737	28.4588	28.4928
26.3219	25.6084	26.0407	26.1823	26.3789	25.6605	26.5886	26.0881	24.8003	24.6317	25.0046	25.0682
24.8842	26.463	26.9416	26.3601	25.6594	26.2393	26.4242	27.2216	26.4903	25.1116	25.7452	25.9017
27.121	24.9888	25.999	27.193	26.488	26.9605	26.2959	26.4377	28.4088	28.4086	27.8391	27.8485
29.9137	29.9025	28.5076	28.8408	28.5976	29.6704	28.2169	29.2238	30.3347	30.4148	30.5352	30.4932
29.8915	29.9266	29.6051	29.6191	29.0693	29.5546	29.1448	29.3858	30.365	30.4768	30.3742	30.4307
29.5863	29.8296	29.5288	29.362	29.1743	29.4691	29.2565	29.6063	30.3371	30.2559	30.2626	30.2309
30.2488	30.3328	29.6781	29.5736	29.4724	29.6815	29.4704	29.6334	25.7837	25.2876	26.1256	25.5129
26.6474	24.1144	25.1831	26.8395	27.4663	25.6493	26.0433	27.2698	24.8834	26.7987	25.3408	24.4131
27.4651	26.8995	27.2144	27.2034	27.0285	27.3912	27.8428	27.1827	28.5077	28.3179	28.5703	28.6302
26.6853	25.5242	26.2209	26.4261	26.9159	26.8691	26.7305	26.7103	26.7043	27.0177	27.4163	27.3531
30.9337	31.0013	30.1619	30.3996	30.1143	30.5469	30.1311	29.9863	29.7453	29.8893	29.9315	29.8728
31.1355	31.0895	30.4507	30.4511	30.07	30.1764	29.7993	30.204	29.3697	29.5181	29.534	29.6008
25.0861	25.9732	26.4041	25.1332	27.3233	25.6335	27.2592	27.2279	26.5733	24.5759	23.7884	23.5423
28.7985	28.9747	29.1558	28.7869	28.9495	28.8234	29.4813	26.1809	27.405	27.8256	27.6801	27.2494
27.7125	27.4783	26.3556	26.4682	28.027	28.347	27.5142	28.6052	23.6752	25.5796	26.3859	23.7604
26.2125	25.4801	25.8949	25.5268	27.5285	26.2118	26.5186	25.8296	30.0727	29.0673	27.7087	27.5451
27.1095	25.147	26.88	25.3915	26.9056	26.3517	26.6379	27.3384	24.3599	25.0669	26.8292	24.6911
27.4004	26.4442	26.7779	25.3532	27.3212	27.0265	27.8398	26.5423	26.4286	23.6171	26.8424	26.3156
24.7064	26.1105	26.0723	25.3087	26.2588	25.3354	26.6567	25.9216	25.5797	24.8233	26.8761	27.2033
28.1723	28.1309	27.6684	27.2161	28.3149	28.939	27.9269	29.1586	26.9085	26.9276	26.4955	25.5977
26.7698	26.6435	27.4489	24.8174	25.963	24.9473	27.1864	25.9747	28.8479	29.1098	29.4617	29.0698
30.8121	25.2467	26.0998	29.4077	26.1803	31.412	25.3253	25.9898	29.389	29.5585	29.816	29.9181
26.1717	25.6803	25.8537	25.461	26.554	26.1433	26.3688	27.6561	23.7131	24.0613	24.3582	23.6823
25.5827	26.461	25.4403	26.1528	27.4626	26.838	25.6988	26.6147	27.0952	27.3474	26.9705	27.2002
26.6269	26.5595	26.9172	26.7926	26.748	26.495	27.1554	26.3068	24.5985	23.9684	25.1156	25.0795
26.2419	25.2395	26.5609	25.8883	28.7824	26.7742	26.2022	25.9825	28.1196	27.9572	28.01	28.1421
27.9368	27.8756	25.2775	28.3375	27.3799	28.628	28.7744	27.4664	27.9909	27.9246	28.3922	28.3593
24.9829	24.4804	25.4052	26.681	25.6591	26.8978	26.7083	25.3475	26.5243	26.4301	26.8591	26.9504
27.4159	27.4909	26.3367	27.2371	26.2199	25.9563	25.4402	27.0902	29.1553	28.578	28.968	29.0125
33.3299	33.3399	30.7954	31.0486	32.3259	32.9856	32.0535	33.8069	31.291	30.8564	31.0321	31.1068
25.3855	25.1762	26.9504	26.3665	28.4943	25.9163	27.4795	27.1542	25.4618	25.7555	25.6372	25.6764
25.5046	24.909	26.6464	26.3058	27.0521	25.298	26.3932	25.5926	25.1593	24.9825	25.0874	24.3886
26.3384	26.388	25.3705	26.1859	27.4816	26.7105	27.1753	27.9641	24.204	23.266	26.9471	26.992
28.327	27.9486	28.5911	28.794	27.3817	28.9692	27.2073	28.6923	30.1515	30.0897	30.1543	30.2419
29.9662	29.8153	29.43	29.3788	29.1166	29.2461	29.0937	29.8297	29.4744	29.3757	29.582	29.4542
28.5771	28.5848	29.4818	29.195	28.2471	28.7666	28.8005	27.6647	31.0489	30.9642	30.762	30.8162
29.0447	29.07	28.6588	28.6253	26.366	28.6893	28.7098	28.1879	28.9787	29.0069	29.2659	29.2261
25.627	25.9887	26.0053	26.6162	26.8571	26.6166	26.9181	26.6479	27.3238	27.0351	26.9284	27.0968
25.7301	24.9997	26.6642	26.9063	24.5812	24.759	26.2379	25.8948	26.2315	25.9591	26.0721	26.6578
27.0995	27.0288	25.9395	25.9819	26.2657	27.5071	27.9285	25.9158	26.1502	25.9756	26.5832	26.6625

29.8884	29.6619	29.4248	29.8115	29.1999	29.6625	29.1964	29.0978	30.0678	29.8609	30.2088	30.0404
26.0368	26.9119	27.5842	27.0537	25.2384	26.1703	28.3704	25.807	24.3898	27.0644	26.6574	26.678
25.8133	25.1714	26.4585	24.7863	27.1668	26.1765	27.419	25.4596	27.5945	27.6178	27.7137	27.9212
27.1801	27.4293	27.8755	27.4607	27.2996	26.5499	26.0626	25.8458	28.688	28.9091	29.2022	29.2434
26.1588	24.9001	25.5869	26.1307	27.7422	25.4158	25.3873	26.1511	24.8571	24.2424	25.6693	25.8685
25.615	25.3927	26.2659	27.0658	27.3276	25.9287	27.7863	25.2071	26.6862	26.917	26.9201	26.7446
26.0124	26.2048	25.1652	27.1372	26.8314	27.5407	26.9351	26.5113	27.2306	27.5262	27.2432	27.2712
25.048	26.2638	25.4907	26.3323	26.1947	25.9034	26.4096	25.2249	25.6088	25.7963	25.5814	25.5165
27.4437	27.2169	26.9727	27.2941	27.8292	27.3892	26.0842	25.5585	28.0613	28.0719	28.4725	28.2328
26.0133	26.6756	26.7177	25.5408	27.0263	26.1802	26.5218	27.7448	26.7725	26.0345	25.5358	26.056
26.0994	27.0561	27.5226	25.6914	26.33	26.1199	26.4496	26.3194	27.5612	27.2386	27.6301	27.5099
28.1214	28.2302	26.1918	26.9347	31.4224	29.2969	31.0851	29.5488	27.2988	27.0817	26.9077	26.8643
25.8614	28.8622	25.7849	28.2173	27.6525	26.1524	27.3799	28.047	27.835	28.0348	28.1242	28.0248
30.8575	31.17	31.3797	31.1979	30.4428	30.7876	30.4562	29.9506	30.9871	31.2626	30.8261	30.8627
26.0313	25.6237	25.9134	26.1368	27.0583	26.8017	26.8659	26.7791	28.9109	28.7968	28.6445	28.4103
25.4858	25.9017	24.9532	25.7854	26.7711	26.4926	27.0533	25.7025	27.6709	27.1707	27.4713	27.3622
26.6571	29.2038	26.3887	27.5526	27.4028	25.313	26.3972	27.0286	26.9887	26.954	27.309	27.2933
26.1238	26.8716	26.7791	25.6634	26.1226	27.4471	24.9599	25.5961	27.2431	27.1429	27.3957	27.1731
27.7441	26.1263	26.6197	25.6543	27.9847	27.5457	25.9288	26.8639	28.135	28.1042	28.2413	28.1878
25.6536	25.5656	26.5587	27.2858	27.191	25.7141	26.7403	26.5158	26.8327	26.7084	26.4779	26.837
27.4839	24.8334	25.4693	26.0122	26.8262	26.8305	26.7768	27.5837	25.1441	25.6603	24.6334	23.7997
24.6658	26.2137	27.4915	24.5095	26.2129	26.9169	27.267	26.0679	25.9325	24.7214	24.8362	25.3161
26.1707	26.1341	26.1976	25.2163	26.5667	26.2283	26.2471	26.7077	24.0439	26.3442	23.2435	24.4723
27.3424	25.504	26.389	25.5972	25.5351	27.611	26.4717	27.1748	27.1641	27.0112	27.4304	27.3424
29.1771	29.3139	29.0234	29.0582	28.9242	29.4493	28.9767	29.6312	29.1943	29.2757	29.5195	29.4736
25.0335	24.7838	25.6027	24.6481	26.0395	26.1148	26.7747	26.0636	27.2394	27.4547	27.2703	27.2775
26.6145	26.266	26.4969	25.9426	26.6908	26.3105	27.1434	26.7479	27.2	27.1159	26.7936	27.1552
26.3161	25.919	27.2544	25.0451	26.5541	25.6931	28.3227	27.0821	25.6195	25.6651	26.2105	26.6803
28.3829	28.1485	26.1363	28.3228	26.5533	26.2351	26.9254	26.2334	28.4165	28.5524	28.8874	28.773
28.8231	29.0063	29.0214	28.7908	28.349	28.8696	28.1218	28.9184	29.0561	29.0974	29.1468	29.094
27.7475	25.4671	27.0322	26.4462	26.0133	27.3977	25.8916	25.7898	25.0263	26.0985	26.7024	26.6802
28.3625	28.3922	28.4124	28.3301	26.5721	28.0474	25.9971	27.4607	24.5595	24.7785	24.8224	23.1587
26.2273	25.7955	26.0478	25.4959	26.9236	26.5442	26.4342	26.4853	26.3573	26.5308	26.0008	26.1995
29.1217	28.9511	28.066	28.6443	28.0584	27.3062	28.0064	27.4257	25.8896	26.1527	24.6943	24.8319
24.8528	26.2877	25.7597	25.5428	25.7507	30.2218	26.8275	28.311	27.5889	27.3202	28.2807	28.3005
28.8856	28.8059	26.8571	25.6301	27.7617	28.9515	26.0449	28.6513	24.5664	25.5409	24.9047	25.7283
28.33	28.6681	27.9475	27.8129	28.3697	29.3198	28.0992	28.619	25.2534	24.5237	23.5202	25.7682
25.9686	26.0749	25.1618	29.3866	27.8992	26.8664	27.1793	25.5124	28.6967	28.5357	28.7689	28.628
26.2954	25.4941	25.4848	25.9147	26.3407	25.6605	27.7164	28.1646	29.7549	29.0903	28.1175	28.2803
26.8844	25.577	26.3698	26.6572	26.1419	27.1541	26.4239	26.2976	25.2333	24.4283	24.9422	22.9671
28.1313	27.8982	27.2593	27.4442	28.1077	27.8007	26.2411	28.1428	27.9997	27.9036	28.3001	28.2957
28.0245	27.784	27.7577	27.5454	27.5931	27.2425	27.4014	27.2588	26.5261	25.3794	25.5005	25.4591
28.5219	28.6125	28.7983	28.4347	27.4342	28.6336	28.941	28.5863	29.2031	29.2898	29.3777	29.4592
28.25	28.2613	28.0212	28.0495	26.9235	28.212	26.0154	27.4353	28.4228	28.4727	28.6831	28.682
28.7195	28.7247	28.4578	28.4268	27.4002	28.159	25.3498	25.392	26.9246	27.1838	26.8945	27.038
31.0728	31.101	30.2997	30.6763	30.8179	30.1254	30.8345	29.999	28.2679	28.3999	28.3368	28.0334
27.7973	27.3048	27.5835	27.1003	25.5892	26.1286	27.08	26.3924	28.3841	28.521	27.8515	28.0607
28.1597	28.1203	28.02	28.5159	26.0098	26.6385	25.8047	26.4302	29.0739	28.8696	28.7045	28.6963
25.9303	23.7463	26.7956	26.2983	28.1921	26.7437	27.8949	25.6736	28.0121	27.9893	27.8682	28.1205
28.4312	28.3643	27.8675	27.8682	26.8384	28.1618	27.0639	28.004	26.8388	26.5876	27.1163	26.9598
25.9243	28.1116	28.0632	26.3443	26.8774	28.387	27.4882	25.084	29.1325	29.1816	28.9485	29.029
28.3342	27.6569	27.7751	26.363	26.9184	27.7296	26.0477	26.3118	28.8777	29.088	29.34	29.1231
25.2174	26.313	26.4052	25.5153	27.1353	25.8222	25.8831	25.9498	25.0684	26.5797	26.2254	23.396
27.1809	27.1762	26.2477	26.1333	26.9686	25.0708	27.3223	26.3658	26.8552	26.8546	27.2514	27.4052
29.0599	29.074	28.8389	28.7656	28.2475	28.5484	28.1211	28.454	29.3747	29.6156	29.6594	29.7753
25.4689	25.6435	25.3496	25.6709	26.7338	26.8644	26.2779	27.8654	25.5736	25.0975	25.5579	25.4215
30.2314	30.3462	29.9079	30.1462	30.0835	29.9483	29.8319	30.0967	30.7251	30.7391	30.8466	30.856
26.6222	26.6746	26.5887	25.8526	26.1423	25.7636	26.0745	27.433	26.5456	26.4557	25.6063	25.0714
24.8516	26.5705	26.7241	26.2822	27.8142	25.5762	26.4913	26.0172	28.0213	28.1247	28.179	28.4299
26.0052	26.8618	26.9065	26.6672	27.8948	26.3305	25.7647	25.3536	29.3483	29.3854	29.5254	29.4033
26.7382	25.6158	27.1493	25.881	26.173	25.8281	27.6996	26.1694	26.4375	26.885	26.3715	26.3948
29.9778	29.9198	29.0442	29.7685	28.8866	29.8826	28.868	29.627	30.15	30.2488	30.7678	30.5348
26.4774	25.4017	24.8503	25.3835	26.0432	26.3732	27.3395	26.0562	26.1327	25.1836	25.1635	24.4605
28.4613	28.5209	27.9317	28.0668	28.5211	27.6258	28.5878	29.0218	28.1703	27.8823	28.289	28.1792
26.5837	26.0646	26.7335	26.3954	25.1725	26.0408	28.0629	26.5892	27.2939	27.4321	27.5642	27.6922
28.5269	28.1301	27.7955	27.3257	26.6457	26.9928	26.5474	27.0431	25.4754	25.3536	26.2186	26.0781
25.1766	29.0809	25.7865	29.7953	28.9972	24.759	27.5515	28.643	25.9679	27.9277	28.2999	28.3136
29.2971	29.4669	29.3159	29.1117	30.3404	30.1344	30.2906	29.3347	26.7985	27.1396	26.608	26.8645
25.3871	25.4882	28.499	28.6301	27.2832	26.4748	27.0768	27.4669	29.0519	28.5234	29.3329	29.2467
25.6071	26.9432	26.6599	26.2882	27.5531	27.5533	27.7184	27.0981	28.0936	27.5254	25.5326	24.0147
28.3096	28.3975	28.1371	28.0993	26.8546	27.8605	26.7735	28.0089	27.8659	28.0204	28.019	28.1996
25.7005	25.0361	25.2862	25.1082	27.5461	25.381	25.748	24.7095	27.5581	27.2658	27.6763	27.8817
26.37	27.3066	26.7351	25.9478	25.9846	26.9051	25.8162	26.6047	26.9644	27.1928	27.0981	26.9864
28.6438	28.6164	28.1348	28.6205	27.7782	29.0315	27.9689	29.2947	29.4389	29.4377	29.9371	29.9103
25.6709	25.3728	26.2667	25.7458	27.7217	29.0216	27.0915	26.41	27.6885	27.8488	27.3619	27.7952
25.684	24.4047	26.1946	24.6783	24.7554	25.4788	26.7551	26.6073	23.9486	24.6534	24.3369	23.7655
25.3342	24.8897	25.803	27.6893	26.1225	26.4799	26.2496	26.0153	26.2073	26.1336	25.6662	25.695
28.2216	28.127	26.0229	27.5992	26.8413	24.9126	27.5477	26.9125	28.6435	28.6951	28.7038	28.7135
28.892	28.9562	29.677	29.5705	29.2353	28.8272	29.2318	28.4	26.3511	26.4937	26.1629	26.195
27.2266	25.6622	26.7361	25.7285	27.9258	26.5873	26.9273	25.5242	26.9609	25.0386	24.9897	25.1976
25.8156	24.3658	26.5315	25.9679	24.5099	26.9308	27.1126	26.7462	27.9716	28.3849	27.988	27.9397
25.8408	25.2288	26.3404	26.8029	28.0717	26.0995	26.72	27.6664	30.1922	29.6017	28.9876	28.6989
28.477	27.1697	26.5071	28.1308	26.8516	26.2162	26.6281	27.1066	29.4175	29.4285	29.7935	29.9321
27.763	26.7714	26.6562	27.3378	25.5054	24.7101	26.8728	25.1602	27.5086	27.903	27.3341	27.4516
27.8553	27.6099	25.8436	27.1861	26.1494	26.5014	26.7041	25.4812	25.0693	24.9347	24.2242	24.7841
26.8253	26.0385	25.9319	25.0033	26.7616	26.3665	26.6909	27.1516	24.1395	25.2398	25.7194	25.836
31.9339	31.9288	31.1072	31.2428	33.4299	34.1598	33.3712	34.2137	32.5014	32.5414	31.8083	31.7982
27.1882	24.6353	25.4316	25.303	26.1812	25.9976	25.8185	27.9694	27.4943	26.9925	27.5878	27.4935
27.6754	26.8609	26.1228	26.2177	28.5065	26.8071	27.227	27.0061	25.3021	25.67	25.6142	25.7976
27.7307	23.8907	24.8564	25.9275	26.3025	26.6746	26.9875	24.9921	24.577	24.0696	23.4812	22.5538
25.1056	25.9897	27.551	24.9499	26.9397	27.2593	27.0581	25.8847	25.2236	25.5988	25.0542	24.7352
25.4831	24.9959	26.0306	26.8107	26.1402	26.1166	26.5505	25.4413	26.7143	27.3992	27.2763	27.2817
26.6918	24.9774	26.0418	25.3354	26.5064	25.0157	26.8559	27.2773	25.0479	26.2393	25.862	26.1644
28.2027	28.0958	27.801	27.447	27.5429	26.1557	26.8477	26.6929	27.9277	25.0163	29.0064	28.9269
28.7258	28.76	28.3835	28.5138	27.7415	28.4679	27.5365	28.1782	27.8334	27.973	28.4036	27.8652
32.928	32.6718	31.0378	31.2576	32.6357	33.1382	32.5038	32.554	25.9887	25.191	24.9314	22.7541
31.845	31.8562	32.0071	31.9658	31.7272	32.0684	31.8007	31.1715	32.7457	32.6247	32.7832	32.6036
28.4835	28.6673	28.1453	28.0097	28.1777	28.8879	27.5959	29.2608	28.909	28.8859	29.2203	29.2524
27.6672	25.769	27.9499	27.5417	25.6665	26.5354	26.5512	25.5075	29.9846	30.0012	30.0532	30.2546
25.9698	26.955	25.3748	25.959	25.3256	26.6876	26.0803	25.7445	27.7307	27.6787	27.8479	27.6013
25.4699	25.6904	25.0463	26.5509	27.7227	24.2651	27.4309	27.627	23.8785	27.6186	27.5467	27.6869
26.3589	25.1317	26.6089	25.4245	26.8236	25.9842	26.5776	26.9792	25.0813	25.2446	25.1109	23.8372
31.2801	31.0481	31.5053	31.6887	30.7131	31.3233	30.6032	30.9404	32.1555	31.8727	32.2352	32.2737
36.6093	36.9217	37.0289	36.78	36.4219	36.3973	36.2458	35.8455	36.6231	36.5445	36.6945	36.6605
31.2394	31.3581	30.93	31.1127	30.4245	31.3529	30.6236	30.9748	32.1731	32.2656	32.4797	32.4803
30.7195	30.6389	29.7884	29.7736	30.4502	30.8196	29.9888	29.8893	31.2671	31.2904	30.9802	31.1269
28.2927	28.49	28.6197	27.3601	27.542	28.31	27.0982	29.5459	28.7304	28.9328	29.2723	29.0888
29.1726	29.1521	28.6444	28.6596	28.4487	28.5592	28.4639	28.6843	29.1673	29.3166	29.2866	29.078
31.3172	31.1451	30.4326	30.8089	29.4734	29.8999	29.3439	29	27.1558	27.4271	27.4815	27.2398
31.6787	31.9992	31.6107	31.4853	31.5519	31.2897	31.5066	31.0207	30.3814	30.5189	30.5322	30.5526
29.1292	29.1334	29.8591	29.6828	28.5345	28.9039	28.7833	28.4462	29.6162	29.7203	29.4276	29.3614
27.7304	27.9655	27.7553	27.9858	26.7491	26.1428	26.947	24.8865	27.1407	27.1343	28.1344	27.7945
30.252	30.3305	29.9683	29.7553	29.8185	30.3047	29.9285	30.1813	27.7194	28.0847	28.2767	28.2381
26.3368	26.6063	25.4009	27.7742	26.4056	25.6651	28.2551	25.9103	27.6307	27.9405	27.8085	28.1627
25.4699	26.7799	26.488	25.9763	27.7896	27.2992	27.9064	27.324	27.435	27.7109	27.7164	27.5275
28.8645	28.8757	27.6731	28.0621	27.8087	28.386	27.9467	28.6155	27.774	27.8161	28.5447	28.4812
26.0323	25.7484	25.5217	27.0216	26.6645	25.315	26.2422	26.8298	24.8528	25.9599	27.3452	27.6354
25.0977	26.4702	26.7038	25.3452	27.3347	26.0288	26.6399	25.059	27.9563	27.6467	27.5364	25.8619
29.9535	30.127	29.8032	29.8676	30.0479	30.1293	30.0154	30.0709	28.8115	28.9052	29.1872	29.4203
29.0545	29.1161	26.5296	28.4107	28.0681	28.6946	26.3271	28.8933	29.4436	29.6591	29.6855	29.665
28.9978	29.0906	28.7443	28.7586	27.6712	28.1295	27.472	27.906	28.4952	28.1883	28.6485	28.2856
26.8601	27.6517	25.7405	27.9054	27.9741	26.7946	26.1631	26.064	27.3157	26.8141	26.8901	26.6524
29.1948	28.7319	28.2004	28.3024	26.2267	27.6385	26.661	27.1139	28.4186	28.2166	28.211	28.1857
30.1209	29.9901	29.2135	29.2281	29.5865	30.1135	29.7115	29.7447	29.2317	28.7857	29.1819	29.2746
29.4766	29.477	29.6836	29.551	28.8875	28.9897	29.0119	28.9472	28.9753	29.0475	29.1347	29.1861
28.832	28.892	29.2862	29.1413	26.481	28.4411	28.1521	28.3425	28.5691	28.7178	29.01	29.0082
29.4561	29.3217	28.7742	28.9947	28.2615	29.4214	28.7338	29.4026	29.2882	29.1254	29.3167	29.2334
24.7855	26.6502	27.1515	25.3219	27.8075	26.6161	25.6558	27.7673	24.8016	23.8021	23.6212	24.5251
27.4852	27.5077	24.7118	25.9542	26.0227	25.0784	27.4153	27.1349	27.6237	27.6229	28.082	27.9793

31.3422	30.9589	29.6622	30.6213	29.3753	30.3884	29.3495	30.9514	30.4149	30.4594	31.1063	31.0281
27.8946	27.8125	27.6268	27.9695	28.2538	28.5309	28.779	28.2635	28.4238	27.9811	27.9428	27.8231
32.6678	32.9134	31.701	32.1558	32.3299	32.0016	32.2857	32.85	29.0957	29.3237	28.7071	28.7894
30.157	30.1413	29.4125	29.4513	28.9682	30.1438	28.747	29.6375	30.8111	30.7499	30.9813	30.9071
30.2001	30.2933	29.2601	29.6767	27.8211	30.8392	27.6476	30.1446	30.804	30.7736	30.5693	30.5899
26.7647	27.2823	26.471	26.1557	27.0579	26.3941	26.6537	26.3626	27.7753	27.7811	27.9228	27.9395
30.1608	30.264	29.1737	29.0282	28.7576	29.6865	28.6645	29.393	30.9725	31.0598	30.8076	30.9546
28.3294	27.9574	27.3308	28.0858	25.7514	27.5971	26.8278	26.3684	28.997	29.2169	29.3176	29.2139
29.5525	29.702	28.2655	28.5646	27.8242	29.6322	27.8615	28.5306	30.4098	30.4863	30.5482	30.5653
28.8477	28.8291	25.9218	25.5345	27.3996	25.3861	26.8728	26.4208	25.8182	23.4162	25.3333	24.1463
29.5523	29.6469	29.486	28.5057	27.0439	29.5177	27.7183	27.7278	30.6423	30.7861	30.6924	30.4213
32.1912	32.3649	31.5532	32.1061	31.6953	32.0782	32.0313	31.6725	31.2268	31.3022	31.2033	31.2253
27.351	27.4899	27.9153	27.4713	27.4364	27.8117	26.8745	26.5727	25.5209	22.49	25.0547	22.7712
27.686	27.4856	26.7581	26.677	26.2201	27.2459	27.0531	26.2438	30.3975	30.0298	30.4438	30.5304
25.9092	25.8942	25.5342	27.3084	27.0393	26.8575	26.359	26.8887	28.657	28.7818	28.7285	29.0023
26.2829	25.0219	27.4644	25.4736	26.4037	26.8993	26.7907	25.3443	28.2883	28.3386	27.8912	27.6286
28.1316	28.016	26.4218	25.5309	26.0032	28.3741	26.1195	25.4898	26.9512	27.3449	26.9605	27.5184
32.4835	32.4875	33.9584	33.6028	33.6872	32.9069	33.5388	31.437	30.841	30.4479	30.3021	30.5889
26.5689	26.8392	26.2327	27.08	26.8896	27.2199	26.3944	26.4123	25.4576	24.7164	23.287	25.253
26.8777	25.8775	26.7318	25.4271	27.5303	27.2914	27.1068	25.8152	27.0182	25.7994	26.4659	25.5289
26.3359	26.0615	26.5627	25.5473	27.9914	24.3176	25.7183	26.4451	28.0433	27.639	28.1334	28.2532
26.7634	24.7194	26.5099	24.7415	27.4547	26.4388	26.4339	27.4586	29.4241	29.3105	28.212	28.0746
27.7844	27.8905	25.9144	26.6047	27.1229	27.2858	26.8491	26.7822	29.1779	28.9209	28.9764	28.6626
27.1557	26.6237	25.7024	27.1345	25.8671	27.1862	26.2439	26.2781	25.0196	26.0676	25.482	26.1098
25.5309	25.211	27.0975	25.581	28.2536	24.8326	26.8909	27.1149	26.3147	26.1063	24.1969	25.0983
29.1914	25.568	29.0711	28.7326	28.5166	28.9026	28.2916	28.3923	29.2043	29.1765	29.0805	28.6676
29.441	29.612	29.5439	29.478	28.9649	30.1747	28.9055	29.1483	30.9428	30.8548	31.1473	31.1738
32.1623	32.3171	32.5575	32.2565	32.319	32.3474	32.3235	31.5405	32.4584	32.3138	32.3443	32.4034
29.8106	29.7588	29.4681	29.408	29.3479	29.6717	29.4114	29.177	26.6	25.4827	26.7621	26.7651
26.0025	25.3781	27.2165	24.9735	26.989	27.2994	27.2039	25.5724	23.7268	24.7384	26.836	24.3221
28.9636	29.2487	28.3329	28.3982	25.5654	26.8888	26.7439	27.7842	25.7002	22.4053	25.2154	23.9928
29.3301	29.2874	29.3885	28.8607	25.5662	29.6188	26.6608	29.4619	30.2352	30.1947	30.2312	30.2023
29.2289	29.2362	28.8024	29.0876	28.4142	29.0091	28.5218	28.8086	29.4326	29.0794	29.5266	29.286
28.3307	28.2004	28.3673	28.3991	27.8175	28.6204	28.2921	28.221	28.6782	28.8034	28.8638	28.8573
29.0039	29.1126	28.951	28.7351	28.6388	29.5146	28.6406	29.1415	29.146	29.1913	29.2524	29.2487
28.2666	28.4043	28.4956	28.4636	26.9141	28.5373	26.6654	28.0383	28.2749	28.5686	28.4338	28.4971
31.1367	31.1385	30.0759	30.1634	29.7094	30.5427	29.5319	30.0191	31.4076	31.4068	31.3852	31.2858
29.2712	29.5009	28.3526	28.2881	28.1885	29.1661	26.7713	28.862	30.5246	30.2569	30.8062	30.8787
30.8432	31.0454	29.8678	29.704	29.2521	30.8916	29.2979	30.2483	30.8188	31.0513	31.1619	31.1964
31.1153	31.0379	30.6181	30.9464	30.7454	30.8357	30.7584	30.5806	30.9604	30.9078	30.8846	31.1273
29.9163	29.8837	29.5584	29.5392	29.0377	29.5409	29.1401	29.1304	30.6366	30.6297	30.4172	30.5594
25.5875	29.9172	28.5935	28.5328	28.3309	29.2013	26.1503	25.6659	29.7369	29.9148	29.6328	29.6083
30.4952	30.6341	28.9472	29.3221	29.0873	30.5469	28.9451	29.8433	30.1414	30.4376	30.879	30.779
29.8596	29.9422	29.2313	28.8291	26.4631	29.3231	27.719	26.1557	29.8598	29.7002	29.2232	29.248
29.4127	29.5452	28.0802	28.3309	27.2746	29.3449	27.8982	28.6577	29.2771	29.3627	29.8295	29.5569
30.0472	30.0993	29.0187	29.0096	28.6568	30.0052	28.8255	29.1158	30.2915	30.0855	30.1301	30.1453
26.2609	25.2535	26.0706	26.4056	27.2241	27.5781	25.5364	27.0883	27.2715	27.76	27.2889	26.6572
25.6992	26.7937	26.3739	28.3463	26.2314	26.5814	26.9876	27.2553	24.6029	24.7107	24.0267	24.7077
25.3177	27.2701	26.0741	26.0844	27.951	27.7381	26.9364	25.5507	27.3674	27.0419	28.0781	26.8542
27.2848	26.0797	25.5761	24.7241	27.6438	26.5003	26.8605	26.1455	27.3039	27.1201	27.6414	27.5382
29.2729	29.1733	25.4657	25.2319	26.1353	29.4234	25.8945	29.2745	28.1862	28.4641	28.825	28.9659
29.2258	29.0953	28.6705	28.6304	26.9933	29.4261	28.9579	29.6366	29.4725	29.1357	29.5724	29.5381
29.3911	30.0106	29.3844	28.1168	28.3246	30.0857	28.2026	29.3018	29.7905	29.4443	30.0694	29.958
33.1416	32.7231	32.8193	33.0574	34.5112	33.4708	33.4899	32.6474	34.5239	34.1311	33.7163	33.8835
28.665	28.4084	29.3142	28.8527	28.6048	29.9296	28.5374	29.0156	26.0346	22.7998	28.5363	28.3964
28.2673	28.2361	27.6986	28.1636	26.3282	28.6504	28.2913	28.4747	28.086	28.2007	28.6851	28.5333
30.5092	30.478	29.3233	29.2489	29.1814	30.571	29.042	29.9999	30.924	30.9176	30.9352	30.9926
29.4992	29.5785	28.8235	28.7844	28.2253	29.2523	28.4188	29.1012	24.1335	25.224	23.099	24.0652
27.9341	28.1257	27.4698	27.8963	26.0415	28.0077	27.7961	28.0768	28.3163	28.4467	28.8887	28.4704
28.6225	28.9195	29.5011	28.9382	29.1891	28.7134	29.6275	27.6496	29.8503	29.7221	29.6829	29.8038
30.2541	30.5332	29.4993	29.4219	29.0717	30.3458	28.9642	29.8281	30.6402	30.7497	30.9921	30.8866
27.9945	28.3792	28.101	28.3301	27.2059	29.2666	27.2142	29.2523	29.4601	29.3608	29.4727	29.4713
30.636	30.6695	29.7605	29.8851	29.0749	30.2144	29.1659	29.2844	31.279	31.349	31.1755	31.215
31.5913	31.5848	30.5762	30.5934	32.0789	33.4989	31.9601	32.8245	30.3067	30.2015	30.5951	30.5981
30.5305	30.487	29.9672	30.2323	29.4672	29.8996	29.6767	29.9193	29.2332	29.4006	29.3845	29.0844
32.4954	32.6508	31.4108	31.7253	31.0353	32.6953	31.0046	32.8937	31.941	31.9664	32.0508	32.0574
28.1914	28.2613	27.7096	27.998	26.7009	28.6386	27.2047	26.7136	29.5666	29.3238	29.4385	29.4226
32.1353	32.5687	31.5149	31.4793	31.7281	32.1105	31.7173	32.2798	29.7853	29.2775	29.6067	29.5468
24.2778	25.8873	27.1875	25.7068	26.5481	24.2946	26.8382	26.909	29.4744	29.2979	30.0648	29.703
28.4772	28.4648	27.9254	27.7864	27.0239	28.4935	26.769	27.9263	28.8002	28.716	29.03	28.6787
30.6656	30.8324	30.2474	29.96	28.8299	30.3669	29.0692	30.198	31.3099	31.5321	31.3503	31.505
29.4064	29.4292	28.5543	28.2625	27.3558	27.379	25.4656	27.3155	30.1652	30.0425	30.0582	25.6461
29.21	29.443	29.319	29.2709	26.4604	28.1963	29.7075	28.4902	30.4925	30.2971	30.4149	30.4967
29.0772	28.931	28.6012	28.4776	27.8783	28.1555	27.923	27.8031	30.1169	30.3415	29.6309	29.6636
32.61	32.8402	32.7867	32.6355	32.6909	32.5501	32.653	31.9727	29.5609	29.8743	30.226	29.8187
25.8994	24.8605	28.0338	26.9709	26.2562	26.2725	26.0867	27.4284	26.1399	27.3109	27.7309	24.4732
32.531	32.5755	32.6777	32.9785	33.1887	32.416	33.1935	32.3229	29.6164	29.8375	30.0096	29.9672
30.1426	30.1652	29.1177	28.8675	29.1373	30.2191	28.8893	29.8564	30.7901	30.6454	30.6507	30.3348
27.2874	26.2368	26.4503	26.4222	25.9195	25.7479	26.781	27.1315	24.2614	24.856	24.7508	22.6796
28.9321	29.0776	28.7354	28.5406	28.019	30.0426	27.9984	29.0895	30.0947	30.1158	30.156	30.187
29.5441	29.6475	29.1252	28.9436	28.8954	29.8045	28.8453	29.3446	30.6586	30.6512	30.8599	30.8097
29.6783	29.7787	29.6007	28.6283	28.9547	29.5874	25.8912	29.1595	30.0836	30.0974	29.8813	30.0104
30.3655	30.1244	29.4798	29.6416	29.1665	30.175	28.8348	30.13	30.8669	30.9504	30.6136	30.8545
29.9061	30.0153	29.2731	29.0314	28.7017	29.4624	28.2996	29.0315	30.4285	30.4708	30.2271	30.2766
33.5445	33.4281	33.0895	33.5175	33.0488	33.1764	32.99	32.9233	33.9319	33.7776	33.7487	33.5586
29.6854	29.289	27.4769	29.1948	29.0195	27.7569	28.8172	27.9124	30.0945	29.9053	29.9564	30.1476
29.4141	29.4937	29.4777	30.4342	32.1156	31.7167	30.8366	31.0538	29.0599	28.9911	28.8968	28.7768
25.2774	27.3661	24.8078	27.4476	29.3619	27.9257	26.2876	25.8642	29.0029	29.0929	27.6742	27.4528
25.8058	24.8245	25.3115	26.4993	26.4758	26.9532	26.9674	25.2896	27.0706	27.008	26.9411	26.9367
29.4216	29.4743	25.847	26.2802	26.6058	29.2746	27.2867	26.9949	29.1894	29.3962	29.3459	29.4745
31.6125	31.8269	31.4119	31.5871	31.7008	31.7432	31.7322	31.2971	29.8287	29.9205	29.7991	30.1373
29.3926	29.4886	29.5885	29.2011	28.962	28.8734	28.9684	29.5051	28.5732	28.737	28.4847	28.8254
30.1917	30.4126	30.8356	30.6065	31.337	30.4468	31.2038	29.8568	27.8066	27.6701	27.2077	27.239
29.1665	29.1115	28.9172	28.6975	29.0294	25.4892	29.2107	25.5783	25.4934	25.4876	23.3514	26.7446
33.549	33.5014	33.4711	33.4914	33.5024	33.3663	33.5798	33.0768	33.5591	33.4295	33.5783	33.5292
25.857	25.8362	26.3178	24.9924	26.1511	26.9163	27.1506	25.038	27.2403	27.4484	27.7521	27.2426
28.4744	28.2171	28.7034	28.4546	26.5159	25.5103	26.8367	26.4425	28.493	28.4783	27.5598	27.7067
26.1219	28.7539	28.0625	28.5799	26.5225	28.4022	27.4104	27.0579	30.4223	30.2924	30.6449	30.2389
28.3502	28.6807	26.3423	26.177	27.1086	28.519	27.524	26.767	29.9017	29.82	29.8382	30.1266
28.3128	28.217	25.664	28.2085	27.0938	28.1805	26.3166	24.9605	29.2054	29.2683	29.353	28.9869
29.9091	29.8478	29.8107	30.0147	29.5746	29.1823	29.6597	29.1638	26.4855	26.056	26.4007	27.1958
29.5397	29.718	29.7651	29.9266	27.0066	28.4583	28.7783	27.3071	31.007	30.9431	30.711	30.7017
31.3128	31.4714	30.8004	30.9761	30.4662	30.6663	30.5736	29.9832	32.1733	32.0867	32.3355	32.4281
30.4823	30.5589	29.1508	29.2887	29.057	30.1672	29.0797	29.9655	30.5992	30.7972	31.0971	30.9788
26.0369	25.8021	26.7396	25.6619	25.0093	26.3017	26.3102	26.4783	26.7785	26.466	26.2502	24.1181
33.0804	32.4611	33.2471	33.4522	33.5904	32.8081	33.4286	32.0579	34.9548	34.1891	34.4221	34.2748
29.5171	29.4913	29.2228	29.0618	27.4161	29.5179	28.9997	29.0785	29.1403	29.3301	29.1741	29.15
30.6965	30.6332	29.6263	30.0032	29.2352	31.1582	29.3899	30.749	30.7357	30.4198	30.5507	30.6233
26.2437	24.2739	25.1646	27.8929	26.6435	27.3276	27.2409	26.0419	28.0253	27.8558	28.3679	28.2254
25.7382	25.1959	25.9435	26.9625	26.1436	24.8944	25.9568	26.0826	27.9099	23.3342	28.0357	28.1418
32.49	32.3853	32.5826	32.9377	31.5106	31.7226	31.4423	30.7185	33.5611	33.2787	33.417	33.4774
33.4643	34.1212	33.2862	33.4987	32.8119	31.8989	32.3863	32.9808	33.5268	33.7783	34.2769	34.2021
28.1857	27.9366	27.8185	27.3628	26.1199	28.055	26.1466	25.7658	27.9153	27.5505	28.1873	27.8073
33.9179	33.9851	33.5504	33.6022	33.4214	34.0757	33.3861	34.0149	34.5162	34.4625	34.694	34.7692
34.2378	34.0303	32.8249	32.9674	33.2719	35.1271	33.0657	35.4442	32.4672	32.4199	32.9297	32.7105
31.4251	31.5517	31.411	31.6877	30.3506	31.1646	30.7606	31.2138	31.9536	31.9891	32.5692	32.1792
28.2935	28.7411	25.2094	28.6833	26.6329	28.2801	27.4709	26.9017	29.1869	29.0148	29.2514	28.9809
28.4942	25.7737	25.4964	28.3582	25.8953	23.6327	25.5735	25.0935	28.1417	27.9657	28.6727	28.322
24.7216	26.2019	26.0954	26.0648	26.667	26.2885	26.0244	28.0564	27.3039	27.7059	27.479	27.4952
36.9841	37.07	33.5492	33.7015	35.1384	37.5294	34.8385	37.6305	34.1606	34.1208	34.3577	34.2615
24.8627	25.115	26.8613	24.9739	25.9758	25.5159	27.0069	25.8805	26.5021	26.2398	26.3015	26.2863
25.4478	26.8861	26.0144	26.9212	26.8408	26.6109	25.8807	26.3671	24.308	24.0656	26.2275	25.4146
26.4639	26.2659	26.9256	25.8552	27.1623	27.1617	27.3605	26.9564	25.1841	24.248	24.9205	24.041
27.1618	27.1205	26.4119	27.4614	27.3391	27.513	26.6325	25.8961	28.0885	28.151	28.2206	28.3038
24.7286	25.0317	26.1563	26.3148	27.5302	26.3102	26.8834	25.4777	27.3638	27.1315	27.1574	25.1688
24.7695	25.9787	27.5691	26.1666	25.8286	27.3409	27.1701	25.4874	27.0357	27.0387	27.367	27.4731
26.9884	25.4393	26.8988	25.7208	27.6887	25.4967	26.7912	24.2346	25.5971	23.5787	26.1403	24.6537
30.0714	30.2364	29.6354	29.82	29.3195	29.8885	29.5267	29.9797	30.0398	30.3004	30.4855	30.3727
26.4117	26.4317	26.5494	25.9815	26.1273	27.9382	28.0141	26.5355	28.0622	28.0303	27.6209	27.6698

29.23	29.2074	28.4872	28.8159	28.3459	27.9253	28.278	28.125	29.4549	29.4958	29.8509	29.77
27.7412	27.8412	27.3549	27.5771	27.9267	28.6974	26.8859	28.1184	29.6529	29.7201	29.9671	30.0138
27.8542	28.0068	26.6888	26.8547	28.9517	29.9593	28.9653	29.7944	25.6254	24.0669	23.2964	25.2383
30.0135	25.9176	26.6416	27.4768	26.8974	29.548	25.8772	25.2972	30.9662	30.6828	30.666	30.5662
27.3152	25.0634	26.1707	25.2061	27.2614	29.1384	25.4412	26.8005	29.5736	25.4671	26.5392	26.0801
26.9552	26.2353	26.1087	25.5154	25.3162	25.3293	27.6224	24.3943	27.6596	27.3874	27.8986	27.7728
31.7235	31.8899	31.3984	31.3902	30.9874	31.0819	30.9754	31.0028	29.54	29.2196	28.8622	28.9343
28.7503	27.0325	27.5908	28.9765	28.4381	29.6735	27.7026	27.3237	25.8958	24.8714	25.1777	25.1061
26.4397	24.9142	25.0707	26.6348	27.4483	25.6735	25.6931	26.8728	32.3511	31.5886	27.0029	26.9458
25.7381	25.8632	27.0426	24.7822	25.2607	24.4486	25.7513	26.2567	25.4654	24.3001	23.1952	24.7507
27.5341	25.1079	27.4599	27.2284	27.0159	25.6898	27.2944	27.2024	25.6937	26.1569	22.4063	25.0796
26.6827	25.7912	26.1779	26.4174	26.0689	25.4806	26.3933	25.6735	25.4069	25.7607	26.5529	26.4449
26.6327	26.092	25.7301	25.9661	26.7958	26.9313	26.6212	26.7115	24.9677	25.3431	25.1044	25.2035
26.6125	25.2968	26.1329	25.5287	25.6184	25.3907	25.7248	26.6249	26.3014	26.4153	26.3096	26.4466
26.1398	26.517	26.1353	25.718	27.1312	24.75	26.3696	25.3578	24.7144	23.9279	26.1453	24.2489
26.7168	26.7291	27.0833	26.6008	28.0873	27.1424	27.7526	25.3574	28.3817	28.5602	28.5283	28.3772
25.5117	26.6612	25.8594	25.616	27.5526	26.0734	26.9742	26.4877	26.2819	26.338	26.0439	26.0515
29.2308	29.2838	28.4818	28.6159	28.3412	28.8844	28.4726	28.7071	29.6864	29.6722	29.6219	29.6156
29.6768	29.6212	28.8376	28.9362	28.9752	29.5063	29.4787	29.0934	30.5502	30.438	30.0928	29.9386
25.5449	25.7532	25.9161	26.7233	26.77	26.4589	26.3897	25.9558	26.8173	27.2027	27.7931	27.5598
25.4547	24.26	25.3705	26.2167	27.5057	26.5088	27.0334	25.0708	27.894	27.6603	28.5247	28.5255
29.1859	29.1031	28.4856	28.5912	27.2609	28.3173	26.1536	25.6781	31.2521	31.0629	30.9147	30.814
29.5988	30.1108	29.5901	29.7515	28.9563	28.9975	28.639	29.1785	27.7378	27.7351	27.2442	27.415
29.4164	29.3726	28.6745	28.857	28.1448	29.3051	28.735	28.8397	29.6061	29.7907	29.3902	29.3742
27.1485	25.0613	28.073	25.0137	27.4615	26.5424	27.6786	27.897	28.2356	28.4573	27.7908	27.528
29.4167	29.5904	28.5296	28.4558	29.2753	30.23	26.9992	29.6936	30.162	30.2996	30.6081	30.5499
26.55	25.6779	26.2837	25.9954	27.742	27.1408	25.622	26.61	26.4536	26.3276	26.2942	26.3284
26.3772	27.3271	25.921	24.457	27.091	27.611	28.4563	25.6427	25.939	25.3245	25.6799	25.1168
29.9545	29.9201	28.4524	28.3658	29.419	30.4511	29.4716	30.3546	25.8951	26.375	25.8598	25.9826
25.4818	24.6796	25.8337	25.6933	27.4731	26.5826	26.7877	26.0242	26.9631	26.916	26.7091	26.574
28.1503	27.676	29.2299	28.7984	27.9511	29.5706	25.8429	26.0633	31.3083	31.0673	31.2015	30.9631
27.2721	25.1951	26.3125	26.418	26.9675	26.8313	26.0927	26.45	27.425	27.1834	27.5622	27.5536
24.5972	26.5377	25.9003	26.3193	27.0704	25.5909	27.2969	26.5651	30.0513	30.2013	30.3972	30.6217
27.8	27.7798	27.6502	27.8618	27.1521	27.8982	27.0042	27.7987	28.5699	28.602	28.8285	28.8178
26.2668	25.3803	25.3717	26.2895	27.4395	27.9461	27.1346	25.383	27.6445	27.7284	27.5046	27.4524
25.8067	25.4997	26.6993	25.7799	26.5639	26.3516	27.9648	27.3965	26.974	26.9382	26.7261	26.7924
32.1693	32.2968	30.5942	30.6679	30.5152	31.3512	30.5903	30.9199	30.5212	30.5509	30.7596	30.6973
26.1025	25.3974	26.6355	27.3553	25.2131	27.1704	27.8695	26.6662	25.7205	25.7627	25.7748	26.1478
26.0692	27.0009	26.1762	26.4771	25.3083	27.675	27.1174	25.4683	28.0903	27.6741	28.1734	28.1654
26.2013	25.6642	26.1632	26.6508	27.0037	26.7942	26.0845	28.2223	27.4447	27.4898	27.5615	27.6665
27.3498	26.4686	26.6356	25.3777	27.1023	26.5702	26.4301	25.8995	25.9962	25.7916	25.8548	26.2877
29.033	28.8676	28.2646	28.1351	28.8231	29.6112	28.1536	29.1039	30.8262	30.7603	30.9331	30.9082
27.7988	27.9016	27.6279	26.126	27.2856	28.1734	28.8405	25.9727	28.4304	28.295	28.5897	28.3393
27.8479	27.9512	28.5102	28.5834	27.3109	27.5908	25.2938	26.2594	27.5644	27.5886	27.7971	27.7728
24.6788	25.2401	26.4643	26.0885	26.6622	26.2764	27.6054	26.1368	27.1523	27.3524	27.2302	27.3035
29.5294	28.7211	28.5147	28.7484	27.0649	30.9943	29.6091	26.5333	30.5825	30.324	30.5204	30.4069
29.5839	29.0841	28.8348	28.9407	26.7978	30.3059	27.9395	29.8849	29.4146	29.4725	29.7938	29.6826
25.88	27.2261	25.2676	24.3453	27.4082	28.5255	24.9728	26.0813	27.0642	27.2872	23.2914	27.7015
28.8398	28.4999	27.7749	28.3461	28.3273	28.2668	26.1589	27.8498	28.0948	28.1762	28.1372	28.3159
30.1052	30.1366	30.0273	29.9076	29.4943	29.4579	29.5426	29.6888	29.9322	30.055	30.2617	30.4583
27.4924	25.2075	26.3731	25.5797	26.2603	26.9562	25.7566	28.2683	24.4357	24.6748	24.3119	25.3748
30.767	30.7625	30.1862	29.9865	31.0011	31.4937	31.094	30.844	28.5666	28.185	28.208	28.3751
26.1292	27.4853	27.0234	24.6523	27.5224	26.8066	26.362	25.4928	27.2338	27.1075	27.1325	26.988
25.8581	25.3534	27.589	25.8932	24.9032	25.9178	25.8576	26.3398	24.1767	24.0843	24.5805	24.5363
29.4826	29.3763	29.5198	29.667	29.3832	30.086	29.4092	30.0977	29.1793	29.0506	29.307	29.2514
27.9157	28.0993	27.7102	28.1174	25.9283	28.6244	27.7201	29.3179	28.3825	27.8669	27.8757	27.888
26.8189	28.3539	28.9492	26.4689	26.9741	25.7631	26.9903	27.5209	28.6043	27.2127	27.1091	26.8436
29.4824	29.1241	29.4836	29.2129	28.5077	29.5329	25.9582	28.9918	30.0197	29.899	30.0399	29.9184
26.261	24.5781	26.1383	26.8913	26.1875	26.9077	25.5742	25.7976	23.9075	25.3949	23.6097	24.3688
26.1278	26.069	26.4579	26.9073	26.5977	26.5827	26.2811	28.2544	28.9831	29.1019	29.0893	28.9776
27.564	26.7283	25.8305	25.1561	27.6634	25.6653	26.3327	25.9056	24.761	30.0624	24.9218	23.7111
25.1344	27.2033	26.7165	26.9895	26.7319	28.2585	27.8175	26.1351	26.3103	25.316	25.4096	24.5765
25.6909	26.9688	26.7523	26.3514	27.3924	25.2327	27.8071	26.9454	25.0769	25.7923	26.2921	25.314
27.4412	25.2241	25.3082	24.7516	27.5352	26.9043	26.8988	26.8504	23.3617	23.9886	25.2479	24.7785
25.253	25.8639	26.6745	25.4019	27.3462	27.3888	26.9945	26.7892	26.337	26.1018	25.8868	26.0838
25.8423	26.0914	25.5594	24.7812	26.288	25.3481	27.2852	26.5011	31.7754	31.3554	31.1736	31.3175
28.8516	28.8102	28.007	28.2126	28.0568	28.696	28.6907	28.4201	29.1944	29.2813	29.3908	29.4389
27.597	27.6975	27.0431	27.0155	27.6623	27.8762	26.0265	26.2549	27.5195	27.6509	27.9301	27.8747
28.6338	28.988	28.6436	28.7354	28.3611	28.6859	28.6012	28.1312	29.9426	29.8854	30.2104	30.0772
28.373	28.517	27.2074	27.4671	27.653	29.5859	27.9051	29.5412	27.6799	27.5904	27.9943	27.8918
25.1637	26.089	27.6301	27.001	26.876	24.94	25.7947	26.2013	27.4938	27.3657	27.4437	27.6586
31.3466	31.4861	30.3561	30.2824	29.6489	31.0368	29.4869	30.6393	31.8134	31.8086	31.5236	31.6456
26.869	27.0339	25.6344	25.746	27.3608	28.1083	26.575	25.1555	27.5041	27.4695	27.5522	27.7191
24.9702	25.4554	26.5751	26.6093	27.1667	26.8267	26.8272	25.6279	27.5068	27.592	27.9157	27.8861
26.1998	28.439	26.3795	26.4025	26.965	27.2145	27.2677	25.5404	27.9077	27.9423	27.8218	27.607
30.4418	30.3497	29.3355	28.9339	26.541	28.9226	28.5263	28.4897	28.4765	28.7491	28.3431	28.722
27.566	26.9032	24.8228	26.0211	27.9631	27.7174	26.198	25.3113	26.819	26.7606	26.6088	26.649
28.2742	27.9712	28.0457	28.3186	26.3826	27.6638	28.6448	26.0805	29.3178	29.2996	29.5507	29.6907
29.4685	29.5186	29.9484	30.0975	29.6668	28.3666	29.8517	28.5167	24.8817	24.0718	25.3413	25.4455
26.2758	25.4876	27.3698	26.2671	27.4354	24.8522	26.6677	25.7226	25.9557	24.8973	27.2439	26.207
25.5325	26.3187	24.9669	25.0055	27.0964	25.2637	26.6445	25.6339	26.2998	27.2458	26.7196	26.7296
25.7869	27.2085	25.8412	25.8198	27.6581	26.6708	27.4115	25.9603	22.8201	22.8394	24.268	25.0129
24.1515	26.7171	25.409	27.404	26.4501	26.8859	26.0687	26.6577	27.0789	24.139	24.3324	24.8828
25.9492	24.1207	26.9591	26.0803	25.8942	25.5859	28.1264	26.1184	27.9639	27.7783	27.4395	27.4341
27.8266	27.8168	28.0436	28.0601	25.9365	27.9436	27.7919	27.0997	28.1441	28.2381	28.4705	28.5951
29.0925	29.1015	28.7485	28.9445	27.2135	28.7102	28.3161	29.2742	29.8633	29.842	29.8746	29.9509
31.6984	30.5838	30.449	30.6149	31.4275	33.4495	30.2297	31.9489	29.248	28.1303	28.15	29.6358
28.7464	29.1373	28.6894	28.2764	29.0707	28.8738	28.7907	27.9083	26.577	26.604	26.1142	26.2806
25.0159	24.578	26.4153	26.5447	28.8238	26.0747	25.6749	27.4923	22.9872	24.2187	24.4648	24.2639
26.7108	25.8374	26.9881	25.7107	26.7758	24.8433	26.8144	27.607	28.034	28.2232	27.6765	27.5833
29.163	29.2242	29.3827	29.1376	28.7653	29.1618	28.7623	28.3229	29.4501	29.4137	29.6522	29.8524
26.4931	25.5303	25.6455	26.5727	26.5555	28.6327	26.0323	26.5239	29.3973	28.9794	29.0774	28.9041
31.0554	31.0883	30.9422	31.1058	30.8831	30.7248	30.872	30.6274	31.0297	31.1265	31.1036	31.1104
28.2365	25.49	27.3329	26.3399	25.0966	24.5833	27.2934	27.8773	27.9493	27.1529	26.9188	27.2474
25.2606	27.0576	26.6566	26.3966	26.0672	26.7506	25.6677	26.8504	24.6583	23.1351	26.2664	26.2841
26.6307	25.9106	26.9534	26.1736	25.8932	26.2776	27.3278	26.1061	24.728	26.7369	24.0625	26.0956
26.471	25.3983	25.9285	26.3789	27.5222	26.8755	27.1537	25.0502	23.2169	25.4811	26.0488	26.1554
27.0559	26.8382	27.9282	26.1846	27.1519	27.0558	26.7498	25.4387	28.7841	28.5055	28.3456	28.1974
27.0191	24.7494	25.9508	26.3148	25.4996	26.4803	27.4283	27.3857	27.2918	27.4298	27.8681	24.2965
26.2879	25.7412	24.7374	25.4383	27.8517	24.7789	27.1838	27.4184	24.0668	24.3739	23.7985	24.9077
27.9636	27.6736	28.6722	28.7533	26.5554	28.8366	26.2522	27.1363	30.1318	29.9778	30.0348	30.0762
25.6331	26.1574	26.8314	25.6707	26.1377	26.6595	26.3256	27.3135	25.101	25.1363	23.8623	24.3299
31.993	31.9748	29.4894	29.2865	29.8397	31.6552	30.1402	32.5451	31.3111	31.0595	30.9288	31.3428
27.6975	27.3666	26.0009	27.2353	25.4017	28.1641	25.9762	28.1831	27.3676	27.3625	27.7699	27.8381
27.0874	25.4582	25.1478	25.1301	27.241	25.806	26.9102	27.4159	24.2556	25.8492	26.116	23.8347
28.6354	28.6729	28.2626	28.4466	27.409	28.2603	27.6144	28.6973	28.5916	28.8003	28.638	28.7228
27.3689	25.721	25.4034	26.3695	27.9748	27.0652	25.6595	26.1538	26.3155	26.5998	26.4292	25.9681
28.9259	29.0478	28.6522	28.7487	26.5313	27.9169	27.7119	28.2757	28.7258	28.7999	29.325	29.1871
26.5777	24.8395	26.2076	26.1163	26.769	25.4343	26.468	26.7434	25.8775	23.1255	25.4219	25.5748
25.781	27.4209	26.2894	26.1442	26.6943	26.5935	26.4396	27.2257	26.2705	26.2853	26.4519	26.5578
29.7484	29.782	27.7791	27.7435	28.9453	29.6916	28.6814	30.0884	27.5541	27.9594	27.3594	27.2292
31.9735	31.9837	31.7138	32.0731	31.3076	31.453	31.2451	31.0031	32.4477	32.4554	32.8787	32.5921
30.009	30.0569	28.7408	28.6516	28.5924	30.1997	29.0368	29.7759	30.1842	30.2525	30.0652	29.8982
27.2175	27.4813	27.7491	27.8433	26.4651	27.6768	27.7839	26.6662	30.5606	30.4744	30.8644	30.8452
26.4252	26.9883	25.5954	27.8365	26.3369	26.2934	27.2535	26.4445	27.8021	27.5795	27.2346	27.5212
28.2159	28.4335	27.9947	27.8555	26.7166	28.6622	27.9487	23.6141	29.7724	29.6728	29.5776	29.7788
27.8214	28.0633	25.3511	28.2914	26.0885	29.1539	25.8247	26.6766	27.831	27.923	27.8197	27.851
30.9101	31.1216	29.7802	29.8551	29.8093	30.3499	29.6907	30.7432	28.346	28.5158	28.6834	28.5834
28.6345	28.5377	27.2667	27.3695	27.3883	28.9381	26.6689	28.6923	29.4679	29.4961	29.5269	29.6033
27.8525	28.1179	25.8675	27.7311	26.2725	26.3178	26.8733	25.2021	28.028	27.8522	27.9926	27.6849
25.8775	26.7546	25.5627	25.1662	27.5939	27.4587	25.8165	24.8921	27.5195	27.7111	27.0603	27.1806
27.2903	27.426	25.673	24.9243	26.947	24.5694	26.3689	26.7852	26.6409	26.9063	26.9112	27.1117
29.1859	29.1153	28.7781	28.8635	29.3259	30.3465	29.0475	30.1349	29.8979	29.8678	29.8359	30.0005
27.0077	25.6009	26.6971	25.5185	27.1503	25.3488	26.5043	25.9851	26.8103	26.6883	26.9212	27.1865
26.7194	25.1419	26.5671	26.889	27.2234	27.444	27.1123	27.5819	26.2996	26.6552	26.257	26.2195
24.6125	25.8506	23.6137	25.6444	25.5722	25.4237	26.1025	26.2475	26.5771	26.2974	26.2431	26.0893
31.527	31.788	30.8504	31.076	30.9386	30.6341	30.9508	30.4057	25.6056	23.111	25.9097	25.6542
33.5439	33.438	32.6849	32.6006	33.3448	33.9052	32.9284	32.5371	24.3456	25.8529	24.7539	24.8964
26.4887	29.668	29.688	29.4116	29.606	25.0951	29.5456	25.2248	27.5153	25.8403	27.2761	26.9169

27.6823	27.9341	25.6014	24.9899	27.8477	27.9626	27.2395	26.1753	27.7621	27.7822	27.9613	28.1773
26.6559	26.5231	25.5517	26.2937	27.1915	26.4259	25.8901	24.967	27.3886	27.6024	27.7152	27.7515
26.0117	23.8949	26.0351	26.9005	27.187	28.1151	26.2606	27.4359	24.4959	26.5903	25.9782	25.8699
26.4365	26.3259	26.2908	26.1455	27.4786	25.914	25.9368	26.8957	27.0915	27.2253	27.4683	27.4833
26.0508	25.5328	27.2848	27.2017	26.4604	26.3045	27.8171	27.499	27.2482	27.3193	27.4267	27.2114
25.5803	27.4173	26.2105	25.8206	26.7444	27.2571	27.3862	26.9786	28.4614	28.0132	28.8212	28.5323
24.5885	27.3837	26.5703	26.8038	26.6668	27.0982	25.3415	25.9586	27.2341	27.0664	27.5239	27.3764
31.22	31.0009	31.3015	31.2684	30.9343	30.3036	30.6305	29.8798	29.3217	29.2303	29.483	29.4301
25.4119	25.892	27.0518	25.9378	26.2503	26.4852	26.8389	27.596	27.9372	27.5244	27.9044	27.6763
26.3285	25.0005	24.6157	26.3119	27.7837	27.7042	26.2493	24.7964	26.5381	26.5516	26.5419	26.6409
27.7755	27.942	27.8999	27.166	27.1217	27.8469	27.6313	28.2129	28.138	28.0254	27.822	27.707
26.4431	27.8756	26.6492	25.461	27.0117	25.6706	28.1488	25.7634	27.5399	27.5727	27.6277	27.7868
26.927	26.6702	27.1832	26.5303	24.8396	26.974	26.2885	25.076	26.1956	24.2033	25.3903	25.5545
28.0155	28.2589	29.0453	28.8751	28.0704	28.038	28.009	26.8225	25.3082	23.2545	24.5883	25.2227
27.0038	25.1185	28.5183	28.4674	27.3313	27.4053	26.8356	26.783	25.1029	24.1055	25.6125	24.3095
25.6345	26.3764	26.2677	24.6079	27.0487	26.6253	26.9589	26.1192	27.0244	27.2328	27.1895	27.1771
27.8179	27.6434	28.5023	28.2445	26.7805	27.9604	27.3685	27.4926	28.7346	28.5599	28.5138	28.5472
26.3207	24.9239	28.0827	25.6669	26.9424	25.7813	26.2929	24.5864	25.7873	25.9002	25.8461	25.7174
25.9895	26.4264	25.7858	25.1578	27.0429	25.2768	26.2691	26.9494	24.3338	24.2268	26.3011	24.0218
26.4349	26.936	27.1082	25.811	26.8698	27.3651	28.0834	26.6115	24.8525	25.3114	25.116	25.744
28.1892	28.1056	28.424	28.2035	26.12	28.3395	27.7126	28.0139	28.4446	28.2102	28.2529	28.3419
25.3702	28.4003	26.8356	25.5472	27.3623	27.4221	26.8627	26.3977	28.622	28.6143	28.5254	28.3515
25.96	25.8161	25.9679	25.3941	25.4328	25.7809	29.127	26.6514	25.4776	25.3659	25.8272	26.2638
26.5889	25.7183	26.0992	26.8007	27.7861	26.273	27.5322	25.6221	27.3317	27.9938	27.7561	27.5579
26.9096	27.0327	24.8575	26.1462	27.5246	26.7022	26.2808	25.4525	26.7324	26.9115	25.9303	26.1587
29.1345	28.7711	27.9853	28.3354	25.3684	28.5525	28.2684	28.4376	26.912	26.7358	27.5479	27.7233
26.6554	28.6356	26.5398	25.8896	27.4448	28.1617	26.4596	27.1008	27.0283	27.107	23.8617	26.6633
26.254	24.8274	26.3353	26.4742	26.4257	24.6002	27.0076	27.6943	27.6652	27.7791	27.0407	27.4641
27.6117	27.5734	25.8485	27.318	26.959	27.8184	29.3284	28.4218	26.5997	26.4423	26.3273	26.8019
25.5494	26.6656	25.4879	27.6884	25.7031	26.1057	26.8047	26.2537	23.7706	22.9288	24.963	24.4754
27.9728	27.4568	26.9033	26.6056	26.1011	25.0936	26.5009	26.7959	28.1646	28.0613	28.4525	28.4341
26.0109	26.4832	25.1163	25.8352	26.3215	25.6022	26.1314	26.3511	24.4276	24.8317	24.7838	24.7772
27.8343	27.8777	26.8901	27.658	26.5946	28.0213	27.4646	27.6153	25.1948	22.5544	26.1258	26.0498
27.2419	27.3852	25.4309	25.9192	25.8575	26.8322	26.9421	27.8962	23.3408	27.0954	27.2363	27.1312
27.5308	27.5755	28.1795	27.9912	26.9171	26.9459	25.673	27.3012	28.9554	28.8502	28.9569	28.801
29.0893	29.1174	27.8705	28.2198	28.945	29.5294	28.7873	30.2866	30.1508	29.8637	30.1327	30.0641
27.8036	27.9331	27.1407	27.487	28.3576	28.3909	28.4125	28.7305	29.2491	29.2477	29.2647	29.4293
26.0411	28.9573	26.9556	27.2782	27.1791	26.6813	26.5205	26.9303	29.6688	29.6852	30.0884	29.9656
24.1875	26.586	25.1964	25.3065	27.278	26.1865	26.3676	25.8486	25.2484	24.4607	24.2956	26.2134
26.5408	25.7604	25.5199	26.3151	27.8606	26.5613	28.2185	26.0532	27.7701	27.7872	28.1744	27.8958
26.1833	25.8965	26.0361	27.2412	25.8706	25.994	28.3118	25.1255	28.2723	24.6026	28.5942	28.596
26.1995	25.6673	25.7128	25.6738	27.2888	26.8731	27.0792	25.708	31.9238	27.3552	27.405	31.3064
27.0523	25.2859	25.2605	26.3398	26.3491	25.7074	27.6259	25.0227	25.3515	25.5043	26.0805	25.0518
26.4067	26.0869	26.7813	25.1104	26.4904	26.4887	26.5424	28.4537	23.6631	24.1778	24.6507	25.6788
25.5521	26.5275	26.4322	26.106	26.4581	27.1095	26.8907	25.7353	24.6965	26.2783	25.4178	25.6499
27.4755	25.2299	26.6256	24.9803	26.8845	25.3119	26.0973	27.8555	26.1105	25.9114	26.3992	26.8366
26.1336	26.77	26.8523	26.6968	25.6357	26.9077	26.298	26.6284	26.8	26.8171	26.8082	26.8072
25.6351	26.0699	26.6459	26.3108	26.2439	25.9652	27.712	27.3274	26.385	24.3733	25.6049	25.3212
27.0426	26.9289	28.228	27.5731	24.6562	28.0923	26.5744	27.9589	27.0707	27.2237	27.3027	27.2276
26.8524	24.2873	26.0241	26.4827	27.868	26.8396	26.1102	25.2067	27.0821	27.138	27.2079	27.5787
26.9331	26.6337	25.1221	25.9285	27.3074	25.573	26.9224	27.1584	24.8938	25.305	24.8085	24.7607
27.0019	27.6276	26.3297	26.1045	27.652	26.5522	26.2684	26.2688	27.2309	27.0845	27.2609	27.2789
25.3305	25.0717	27.4883	26.2412	27.3029	26.4837	25.1982	26.9943	28.9841	29.287	29.191	29.0058
27.6921	27.3058	27.1168	27.6555	26.4873	26.3235	25.8524	24.9593	28.1601	28.032	28.3632	27.9204
26.5576	25.687	26.6514	25.8275	28.1843	27.1318	26.9042	26.0066	26.9091	26.9468	27.158	26.6361
26.3464	25.5455	25.6252	26.4651	26.3278	24.7802	27.8915	25.3326	24.8235	24.8979	25.1944	24.6305
25.3637	26.1436	26.2418	26.1077	29.1985	26.447	27.2298	25.5431	28.0311	27.9273	28.0045	28.0837
24.7632	26.2003	28.2754	25.9873	26.8608	26.2356	27.2078	26.3588	29.2188	29.1501	29.0319	28.6931
27.5112	27.7162	27.6158	27.1028	26.2829	27.7928	28.0071	27.1531	28.1051	28.2821	28.1144	28.1239
26.6161	25.8653	25.1754	25.2536	25.3638	25.1424	27.2423	25.6763	28.5779	28.6281	28.7027	28.8119
28.2376	28.1782	26.1769	28.5149	26.7533	26.7773	26.5048	28.4243	28.2238	28.299	28.3463	28.4121
28.0032	27.9471	28.5389	25.7753	24.9681	26.9768	26.9528	26.3868	29.312	28.8814	29.3319	29.3915
26.3534	26.6663	26.3092	26.1395	26.0284	26.3895	27.4871	25.6971	25.2233	25.2763	27.3929	27.0929
28.8217	28.9355	28.3236	28.5207	27.4384	29.12	27.6813	29.0276	28.5108	28.5803	28.9545	28.9061
26.9181	27.1191	25.7406	27.5853	27.6303	29.0076	26.4066	28.5632	26.1048	24.1376	24.9224	25.2743
25.8412	25.1983	25.8469	26.1524	27.1347	26.338	26.9975	27.2483	27.7253	28.2808	27.9849	28.1635
26.5673	27.2673	26.1203	26.7627	27.6386	25.3233	26.5654	26.2538	25.5418	24.6809	23.3001	24.268
25.9289	25.7165	26.631	26.4327	26.3367	27.0056	25.9534	27.0313	27.6479	27.2888	28.2632	28.2011
26.1108	25.3362	27.4006	26.8613	25.8913	24.6393	26.3593	25.8048	27.3895	27.7641	27.927	28.0057
28.4502	28.4409	25.8654	26.018	26.9481	29.6002	26.7229	28.7372	29.9131	30.0139	29.6473	29.6516
26.7	25.1362	26.6329	25.0376	25.2447	26.6551	27.2292	27.69	26.941	27.4379	27.2537	27.6419
28.3188	28.4027	27.8857	27.8449	26.7629	28.8964	27.3205	25.1412	30.0455	29.7992	29.9392	29.8337
28.0522	27.5863	28.1873	27.9846	27.6756	28.0789	27.8005	27.5731	29.2191	29.3269	29.7388	29.5389
29.4885	29.9287	29.5936	30.0374	29.7802	30.0192	29.8819	29.5909	27.5724	27.8774	28.3131	28.5401
25.3214	26.2193	26.4736	27.1653	27.1601	27.8032	26.2782	28.0778	27.5186	27.5606	28.0051	27.7428
26.4555	26.4107	25.7879	26.5253	26.1236	26.2564	26.2955	26.4487	24.3108	25.7061	24.7278	24.4802
25.2307	26.1934	28.0531	26.9027	27.6223	26.1747	26.6082	26.825	24.6142	25.5947	23.0927	24.6332
28.5154	28.5486	28.467	28.4503	28.2345	28.2351	27.9915	27.8107	29.3752	29.4431	29.5318	29.5674
25.8962	26.3134	26.5091	25.9266	27.7091	27.0112	26.3332	26.9794	24.6523	26.9604	26.8529	27.1369
26.9992	26.0478	25.613	25.3742	26.1304	27.0887	27.0435	27.7317	25.9118	25.1901	25.289	25.3415
26.3139	25.7553	25.9819	27.753	27.2755	26.7649	25.9604	25.3204	26.0299	24.258	26.1671	24.8881
28.8016	28.357	28.4954	28.3035	27.8698	26.6376	27.8322	27.8586	28.8441	28.7539	28.8605	28.9027
30.1498	30.1735	28.9621	29.2888	29.4827	30.8626	29.2644	31.0943	27.8889	28.1208	27.9775	28.3072
26.6621	26.7209	27.02	25.9514	26.5872	26.8328	25.4028	26.3266	27.4785	26.9072	27.2959	27.3581
27.5363	27.5787	27.3248	27.9776	27.2254	27.7476	27.5357	27.2584	25.3985	24.6665	26.0819	26.2514
25.9896	25.1814	26.2535	26.115	28.0082	28.5322	27.0024	26.2677	25.7238	26.0781	26.0769	26.4205
27.5495	26.9034	25.8082	25.3697	27.2231	26.6826	26.9565	26.5793	27.4306	27.6056	27.4509	27.5024
23.8628	27.5605	26.1352	27.6219	26.3976	27.0827	27.5318	25.7346	28.3878	28.4649	28.5282	28.6401
26.4163	26.0962	25.7928	26.8419	27.0722	26.9955	26.2013	24.9795	23.8725	25.5716	25.4412	24.2055
24.1477	25.6809	27.9577	26.1468	26.1112	26.9594	26.7271	26.1389	25.3481	25.513	26.1701	26.2424
27.0742	25.4435	25.2671	26.7258	26.458	26.0487	27.0307	27.1614	28.2963	28.2734	28.3394	28.1448
29.1266	29.0916	28.8908	28.4585	28.6917	28.3272	28.5375	28.0434	26.6362	26.4887	26.5756	26.7386
25.7597	26.1575	25.7782	25.5611	27.5521	26.9536	27.1481	26.2253	25.4465	27.0893	25.5666	25.7177
25.8275	26.0633	28.1805	26.4032	26.6676	25.9058	26.2169	26.2464	24.6875	25.1616	25.8169	25.1833
25.7586	28.1221	26.9423	27.1011	26.4715	26.024	27.3625	26.224	25.0897	24.5211	25.3459	23.0358
24.9489	27.1614	26.2087	26.3041	25.5508	26.9557	26.1207	26.4451	26.42	26.3399	26.555	26.801
28.2791	28.2959	28.3368	28.3336	28.0087	28.666	28.4597	28.1364	30.2576	30.21	30.2013	29.9333
25.988	26.1064	26.0733	26.136	25.2837	25.5621	26.1671	25.9405	25.1288	24.46	26.1085	26.2519
27.3963	27.7974	26.898	27.9961	28.2457	25.8921	25.2021	25.6774	28.4267	28.4532	28.3354	28.2193
24.9756	25.8673	26.8847	25.8547	26.6837	28.0332	26.1294	26.1647	24.9826	26.3462	26.2233	25.8897
28.0241	27.736	28.1572	28.5075	27.9588	27.7693	26.3518	27.4601	28.1065	28.1881	28.0709	27.842
26.9772	27.1073	26.527	27.2365	27.7986	26.4557	27.1511	25.3608	25.8134	25.2232	22.8946	23.5334
26.6899	26.0443	25.4908	26.5351	27.0902	26.3804	27.1115	27.089	26.9174	27.4456	26.894	26.898
25.8388	27.5409	27.4205	26.9735	25.6564	27.7106	27.0774	27.4994	28.7326	28.9716	28.6961	28.7751
27.2824	26.9824	26.2835	27.4189	27.6987	26.7463	26.5976	26.7521	23.9011	26.4433	26.5422	26.606
29.2139	28.6413	27.0234	26.4503	27.414	29.0709	25.7419	29.8018	30.408	30.3265	30.6642	30.6241
25.9874	26.6179	26.4525	26.2374	25.4524	26.3432	25.4107	27.6497	26.9624	26.7458	27.5654	27.2926
25.1131	25.2972	27.2077	26.5235	28.0836	26.5839	26.3135	26.6833	27.4944	27.6204	27.7039	27.4971
26.4899	24.5429	26.576	24.954	27.1069	25.3087	26.853	25	26.0849	25.9467	24.5122	24.8805
25.566	27.5798	25.5498	27.6841	26.8883	26.7619	27.262	26.2079	26.7162	27.0135	27.2908	27.1618
25.1912	25.2151	27.0312	26.5102	25.9486	25.5579	26.4362	26.4559	24.8594	25.8504	23.2967	25.7407
26.3646	28.9109	25.9894	26.0079	27.0772	25.4983	26.0622	28.0217	27.145	26.204	26.2715	26.4654
26.4736	26.6154	26.5445	24.4945	27.4954	25.7513	27.199	25.3343	25.434	27.2948	27.5681	27.5752
25.6526	25.2374	26.8195	25.4867	27.9836	27.3546	27.0531	26.2114	26.3533	26.2119	22.1235	23.7224
26.1269	24.8242	25.6948	26.9255	25.7954	25.9152	27.0645	26.1657	27.8623	27.5498	27.1794	27.4648
25.8202	25.5456	26.742	26.7331	28.3904	25.821	27.1606	25.2383	25.791	26.0846	25.9873	26.273
25.19	27.4814	25.7577	27.4932	25.1867	27.0569	26.1431	26.4707	27.9334	27.9722	28.131	28.1775
26.2204	26.3989	25.7999	26.1455	26.708	26.687	26.3426	25.7051	25.982	25.5194	26.1704	25.9444
25.4271	26.2964	26.343	25.5863	27.8719	26.1934	27.3969	26.1957	24.0662	25.6246	27.4845	25.7552
28.8676	28.9567	28.5261	28.497	29.4271	28.8023	29.4793	28.4422	24.0807	22.1154	24.3388	25.1113
27.8093	27.0188	27.4637	27.617	27.7643	29.7272	26.6026	30.3743	26.6625	26.7576	26.5763	26.5566
23.877	26.2195	27.6932	26.4897	27.4135	26.676	25.8129	26.4667	26.9403	26.3559	26.6244	26.7417
26.4078	25.7176	25.5655	25.923	27.773	26.8502	26.0645	26.9785	26.3576	26.2783	26.3478	26.0086
27.4795	25.6534	27.6599	26.2228	27.4635	26.6967	26.4144	26.8485	27.0492	27.3727	27.714	27.8663
30.0526	30.1981	30.0484	29.976	29.6633	29.4556	29.6822	28.8172	27.7975	27.6956	27.6597	27.5785
27.6867	27.7683	28.4396	28.0158	26.3723	26.5376	27.3763	26.8107	27.5812	27.5333	27.5863	27.8185
25.8977	25.9226	27.3621	26.0043	26.9895	27.5297	27.374	26.0211	25.3443	25.7976	24.8102	24.8582
28.972	28.9659	28.8521	28.394	28.3477	28.9167	28.5937	28.3109	30.0117	29.9853	30.4049	30.3083
26.3881	26.8389	26.0649	25.4092	27.3222	26.0912	26.0841	26.2541	27.9488	27.4541	27.5388	27.5538

27.1507	25.6793	26.7111	25.5623	26.5202	25.6544	27.2796	27.0369	25.2645	26.2147	27.0928	26.8401
29.0814	28.6903	29.1399	29.5366	27.406	28.4	28.6419	28.2024	29.3834	29.7358	29.4542	28.9886
26.2239	26.0743	26.9758	25.4869	27.4618	25.8452	27.2214	25.6902	26.3169	26.0057	25.5945	26.1323
24.5689	25.9839	26.566	26.8648	26.8622	25.8233	26.9326	26.1353	24.2922	23.2969	26.4972	26.3778
26.4965	26.2221	26.5345	27.3258	26.8071	25.69	25.9466	26.7916	29.1626	25.2259	28.7235	28.6424
27.5341	27.413	27.2897	27.6172	27.1374	27.6351	26.9587	27.7556	27.6838	27.6185	27.7755	27.58
26.0356	27.5574	28.0436	27.2691	26.9167	28.578	27.0202	26.3108	28.5455	28.2412	28.23	28.2274
31.0744	30.9522	30.5776	30.4562	30.4695	30.579	30.3452	31.1042	31.2863	31.3581	31.1535	31.2202
27.9216	27.2206	25.607	25.7149	26.1358	25.7279	26.8657	25.7301	24.7747	25.7125	26.2952	26.9806
29.5645	29.4649	28.5141	29.3358	28.8576	29.9393	29.0412	29.7448	30.2048	30.2862	30.5877	30.404
29.1006	29.4062	28.5471	28.3894	28.2469	28.3031	28.141	28.1052	27.7163	27.6718	28.4203	28.505
28.6735	28.754	26.0328	26.9577	28.3704	29.175	28.0266	28.8899	26.0046	23.1348	24.5215	24.0094
26.4011	24.4557	27.2734	25.2492	25.997	24.724	26.5052	27.38	25.3286	25.088	25.51	25.1447
24.6332	26.0804	26.8543	26.3056	25.7839	27.3216	27.9008	24.6714	26.3897	25.1784	24.018	24.6949
26.6339	24.858	25.5848	25.446	25.7582	29.6079	29.7384	27.3557	30.811	30.7551	30.8233	31.0246
25.6995	26.1457	25.7817	26.7629	26.0903	25.4928	27.2367	25.9098	24.8555	24.1617	25.7848	25.8371
29.3551	29.3423	29.1951	29.151	29.1797	30.0768	29.2082	30.1494	30.0766	30.1132	30.1209	30.103
25.6989	25.027	26.1297	26.128	25.8238	26.5891	26.727	26.7799	26.0843	25.8589	26.4054	26.4037
27.5155	25.2242	24.7568	25.6915	27.3274	26.8294	26.4768	29.1005	28.0552	28.0515	28.0332	27.9699
24.0619	25.6017	26.3874	25.3147	27.3246	27.1243	28.9899	26.4507	26.0947	26.2547	25.9965	26.0942
27.37	27.4978	26.3365	25.7175	27.0657	25.6958	26.8049	26.0933	25.2312	24.7694	24.8525	25.0179
25.9396	26.0756	26.8552	26.1959	27.1389	26.9745	27.1809	26.0165	24.6269	24.7583	27.5148	27.1555
25.7197	25.226	27.0723	26.7651	26.6101	26.72	27.2539	26.6611	27.3804	26.8555	27.0661	27.0309
28.3442	28.1789	28.9156	28.5052	29.1496	28.2244	28.9724	28.4456	28.2845	28.3903	28.8165	28.9096
28.7872	28.8323	28.93	29.1549	28.8952	28.6487	28.8794	28.2528	29.1638	29.0003	29.1238	28.9207
26.2115	26.1885	26.3398	26.1157	26.2856	26.5293	28.2009	27.1294	24.882	23.9076	23.6154	24.2905
27.281	27.1642	27.2837	27.5274	26.7488	27.2661	27.2783	25.6765	27.5106	27.5378	27.4684	27.6644
29.9753	30.0928	29.5508	29.6023	29.4632	30.4806	29.4261	30.6358	29.6242	29.7048	29.9095	29.7938
26.2615	25.6954	27.3157	27.3889	28.4453	26.0318	27.1321	27.0068	25.0923	26.7153	26.2134	24.1601
25.3601	25.116	26.3429	25.9428	27.0963	27.0175	27.0024	25.8162	26.7986	23.8734	26.1855	26.3158
25.4814	25.9166	26.688	28.2206	25.9208	26.5372	27.2075	27.067	25.3472	22.9646	24.9585	23.6039
27.1529	27.1951	28.2945	28.4737	27.1817	27.0595	26.7795	26.2566	29.3182	29.0736	29.1639	29.139
28.9679	29.2178	28.6123	28.0677	28.6278	27.8502	28.8076	29.4002	26.3514	26.1627	26.5933	26.1958
26.7377	26.4408	25.2498	25.0769	26.9393	26.5841	27.3425	24.6733	28.1963	28.1755	27.751	27.4996
25.4775	25.1539	26.2147	25.7248	25.843	25.9618	26.9792	26.6844	27.8821	27.6905	28.0032	27.9217
29.2337	29.196	29.2759	29.0342	28.4194	29.0268	28.2895	28.4779	29.875	29.7948	30.1584	30.2227
27.2569	26.3244	26.2945	25.8934	26.033	26.4018	25.9476	27.2578	24.6175	23.4365	26.5504	26.7355
24.9093	25.728	26.1846	24.07	26.3839	25.6019	27.0456	25.9708	28.2794	28.2601	28.0984	28.3322
25.3695	25.2193	26.2107	24.8575	26.3701	26.237	26.7528	24.6896	25.1073	24.2323	25.7776	24.039
28.6018	28.7502	28.6269	28.4288	29.411	29.285	29.4075	28.6226	26.3156	26.6972	26.7573	26.7602
29.5416	29.5322	29.8769	29.6565	29.8794	29.7744	29.8926	29.453	25.3917	23.9247	25.2758	25.0197
26.4227	26.2633	25.8832	26.0016	27.7482	26.218	26.7305	26.0339	26.124	26.7142	28.1726	27.6526
26.0908	26.1135	25.5091	26.6372	27.9629	25.3895	26.3039	25.6173	27.3104	27.4672	27.7587	27.5552
29.4797	27.4937	29.3769	28.1847	26.088	29.6069	27.6935	25.6071	28.5558	28.4005	28.7253	28.8831
25.5625	26.5184	27.1728	27.6569	25.5293	25.8711	26.3942	27.1509	27.6247	27.536	27.6405	27.9965
28.6346	28.748	26.0215	27.1177	29.0856	29.8767	29.3675	30.7796	26.6203	26.5532	26.2529	26.2113
24.8992	26.3492	26.4526	25.2228	27.1291	25.215	26.407	26.95	24.9488	24.9796	25.4433	25.3724
26.9644	25.7257	26.2555	26.6584	27.6401	26.3487	26.8445	25.9973	25.1907	24.2762	25.4106	24.9555
25.8179	27.5061	27.9477	26.6756	26.7201	27.2663	27.8661	26.615	26.4445	26.6536	26.72	26.7198
27.0869	27.0884	26.4318	26.6822	26.845	27.084	27.4354	27.0596	28.2705	28.204	28.53	28.5341
27.3157	26.1458	25.6602	26.3395	27.4012	26.2334	27.7244	27.8837	28.4376	22.5732	24.1287	29.0073
32.1899	32.442	29.5003	29.374	30.8428	31.704	30.4176	32.723	31.1412	30.9905	30.9208	31.5052
25.1405	26.237	26.4043	26.5351	26.2709	26.7617	25.4181	26.9878	24.6941	26.1113	26.3323	22.7085
26.0761	26.8725	28.2729	26.5469	26.9824	27.4694	27.8814	25.2043	26.0572	25.3576	24.9482	25.1157
25.8263	25.0705	26.4293	26.4898	27.4448	26.1779	26.5257	26.6659	26.1534	26.3276	26.4875	26.5708
26.652	26.3235	24.4243	27.3785	26.7829	25.9	27.7678	27.1864	24.9133	26.2293	25.1299	25.2741
28.1654	25.9745	25.0451	26.6433	25.4886	27.4833	26.2625	26.0296	27.0289	26.7049	27.0622	27.0277
28.1094	26.7374	26.458	24.9547	28.0521	26.9228	27.4771	25.6102	27.2395	27.2547	27.319	27.4803
27.9871	26.631	25.7253	26.2352	25.7628	26.5155	25.3171	25.9448	24.9455	24.0853	23.8641	27.4211
27.2358	26.6475	26.4509	25.6158	27.8851	26.3625	25.2123	25.2595	23.2004	25.0125	25.4181	24.7162
25.6277	25.4511	26.8858	26.3558	27.3937	25.7731	28.0149	26.9919	25.7765	25.9514	25.5551	25.5726
25.6681	26.685	27.2141	25.9594	26.8181	26.5725	26.4284	25.3351	27.2701	27.2304	27.2369	27.5002
25.7728	25.4576	25.2476	26.6662	26.4746	26.6077	26.5705	25.519	26.7966	27.2106	27.4108	27.5884
28.1647	28.3991	27.875	28.207	28.3385	28.4056	28.5758	28.1547	29.6913	29.722	29.9376	29.9518
27.9989	28.075	28.4013	28.3482	28.7856	28.6104	28.775	28.1238	26.3241	26.0713	26.0063	26.2976
24.4476	25.1178	26.4051	25.6486	26.8993	26.2424	25.5497	25.3395	26.1519	24.3504	27.0826	26.5964
25.5697	25.0671	25.9005	26.1892	26.2406	25.223	26.4358	26.5469	27.6253	27.6752	27.9267	27.758
25.9165	26.1766	26.3925	26.1428	27.4925	26.2759	27.142	26.4672	24.9744	25.3452	24.9815	25.4489
29.7366	29.7834	28.9966	28.9847	30.3121	29.7487	30.423	30.1762	28.7682	28.8049	28.7441	28.9493
26.6326	26.8092	26.5901	25.8901	27.8675	29.4464	28.1718	28.9466	24.3468	26.1235	23.7868	25.13
26.0439	27.4657	26.7367	27.4698	26.0275	26.8133	27.3929	27.3781	26.3339	26.5497	26.5201	26.5626
26.5401	25.3682	25.431	25.7686	27.424	25.8709	26.8539	27.4182	24.6237	24.9178	23.8579	25.8157
25.963	26.9511	26.4251	25.4583	27.2454	26.9149	26.7673	25.9629	27.4374	27.9485	27.8551	27.7397
28.9583	28.9981	28.8774	29.3478	28.6772	28.8191	28.671	28.1324	30.2269	30.1787	30.2662	30.2153
27.7766	27.9559	27.042	27.8215	27.5269	27.2664	26.5612	28.4813	29.026	29.0226	28.8183	26.6071
28.1447	28.565	27.4573	27.0333	30.0462	30.2341	30.1899	28.7126	30.6701	30.3622	30.1066	30.7989
30.7625	30.814	30.4844	30.4981	30.384	30.3973	30.3977	30.2402	31.1692	31.0802	31.2567	31.4244
27.3499	26.0353	26.2646	25.7575	26.9815	27.6756	27.5945	25.9597	27.2412	27.3743	27.2685	27.0475
26.8005	26.6313	27.2324	25.7236	26.6739	26.2341	27.2505	25.3496	27.5214	27.4182	27.559	27.5934
25.3269	26.471	27.0381	26.8595	28.3119	25.456	27.8675	25.8799	25.805	26.0234	25.5531	25.8461
24.8554	28.0263	26.7991	25.7845	25.6427	26.7689	27.3278	25.9537	27.6215	27.3664	27.5769	27.5454
27.332	25.9657	26.0254	26.2686	27.3661	27.7826	26.1059	26.1184	25.5832	25.9215	26.1703	26.2673
25.9676	23.7734	26.3176	26.9665	27.1245	27.4825	27.6599	26.609	27.6854	27.6035	27.782	27.7569
24.9459	26.1204	25.709	26.6921	28.4083	26.064	26.1913	26.1883	24.849	24.8247	23.6253	24.1916
26.2017	26.3869	26.8437	27.0669	27.1132	26.6531	27.6273	27.2049	27.9139	28.6405	28.1722	28.2258
26.6738	26.5306	26.1293	26.2425	27.3629	26.8578	25.9563	27.4328	28.5167	28.6429	28.2863	28.4692
26.0055	26.594	26.7977	25.7475	25.565	26.7528	27.2793	27.3755	26.2485	26.4439	26.3495	26.5173
27.407	27.4658	25.6078	27.88	26.6265	27.4632	27.2503	26.6146	27.3719	27.4411	27.489	27.4525
26.0146	25.7872	26.0643	25.1368	26.632	25.435	26.5616	25.8984	25.0107	23.376	22.5366	23.2344
25.5564	26.3175	24.6385	26.2856	27.9273	26.3075	26.5803	26.5109	25.7907	25.8985	25.3848	25.6307
28.8603	28.6244	28.6077	28.9517	27.9094	28.8551	26.2203	28.3107	28.9834	28.9722	28.966	28.9639
27.5461	24.9116	27.2309	25.5584	26.9769	26.0617	26.8328	26.2523	27.4361	27.3372	27.1803	27.24
26.8403	26.7278	26.752	26.9816	27.8348	26.3063	27.7777	26.2125	27.0659	27.3529	27.1484	27.2147
26.7	25.7541	24.4882	26.2755	27.3529	27.2599	26.4639	26.4124	24.1315	26.3629	26.33	23.5798
29.0717	29.0055	30.0029	29.819	28.3631	28.6302	26.3772	26.0194	30.0626	29.9319	29.7319	29.6637
25.5391	27.1031	25.4594	26.3989	27.415	26.8693	26.5153	28.4939	28.0986	28.1352	28.2822	28.2832
29.8222	30.0465	29.2101	29.3337	28.4568	28.745	28.5793	28.4054	28.6183	29.0345	28.9493	29.0746
26.0486	26.4106	26.1519	26.1862	26.8952	27.3731	26.1933	26.8206	26.3559	24.6705	26.1415	26.0254
27.0267	26.2911	27.2912	27.1315	25.8683	26.5199	27.4802	26.9337	25.9132	26.0083	24.8291	25.858
27.8125	27.6981	27.3567	27.2705	27.3006	27.7998	27.9035	26.569	28.6971	28.3478	28.7019	28.7345
25.6284	26.6931	26.677	27.0578	25.6482	26.0009	26.9907	24.4013	26.2276	24.8237	25.9867	26.2646
27.7192	27.5959	28.6746	28.5929	26.0898	28.8667	27.9639	26.9353	30.2973	30.3739	29.9279	29.8923
29.4044	29.1773	27.7223	28.0094	28.2463	29.1756	28.6425	29.0165	27.8172	27.8623	28.4893	28.3328
26.5795	25.2721	26.9024	25.8562	25.8319	26.6706	26.4616	26.5107	24.2972	25.9919	26.1273	26.3655
26.9733	27.053	25.7916	26.3073	26.9771	25.5904	27.8802	26.3912	26.7561	26.7154	26.911	26.835
25.5842	27.6456	28.3875	28.666	29.5635	26.8818	25.6977	26.2514	30.4245	30.5234	30.608	30.5234
27.9143	27.9227	28.0127	27.7529	27.756	27.9376	26.9176	27.4719	27.2153	26.5623	26.6931	27.2183
30.5733	30.5608	28.2246	28.3115	29.6612	30.6768	29.6252	30.7706	27.6688	27.0575	27.0678	26.8373
24.2615	24.83	25.448	26.6097	27.0915	26.0474	27.2598	27.0246	27.8192	27.8146	27.8465	27.6661
26.6042	26.406	24.8574	26.3287	26.7309	25.9266	26.0933	26.7034	28.2821	28.1732	28.6982	28.167
26.3495	24.9219	26.5756	25.7177	24.9949	28.7155	27.0842	26.346	23.9468	24.675	25.0002	26.2023
29.2451	29.1689	29.6379	29.6046	28.7502	29.2478	29.1367	28.5244	30.2254	30.2613	30.4326	30.4223
25.0461	25.4632	25.7376	26.0877	26.7397	26.7051	26.0561	26.8467	26.6938	26.8667	26.5526	26.4907
28.8376	29.1131	28.8843	28.9516	26.6477	28.4946	28.3149	27.4522	29.5622	29.2394	29.7592	29.3397
30.0545	30.078	30.0483	30.1514	30.3701	29.5517	29.622	29.8515	31.1035	30.6933	31.1639	31.0543
28.2729	27.9173	28.0603	27.5195	26.1143	28.2009	27.4881	27.5033	29.5347	29.5182	30.0568	29.9873
25.881	27.7921	26.2034	27.5055	26.9564	26.3697	26.3522	24.6855	27.5259	27.5103	27.9511	27.9936
26.3133	26.9943	25.8732	25.7586	26.9459	26.1156	26.6402	27.1641	26.074	26.2955	26.2228	25.8692
26.11	26.7691	25.9466	26.0774	26.8413	26.2615	26.5575	27.1247	25.3856	24.3495	25.5661	26.0376
27.0136	25.3008	25.5357	27.1268	26.8234	25.5395	28.3091	27.6109	25.8729	25.7844	25.9738	26.0576
30.4284	30.6617	30.4651	30.7202	30.951	30.9552	31.377	30.2276	28.0291	28.3804	27.9816	28.3605
26.0545	27.629	26.3403	25.5599	27.7686	27.1706	25.93	26.5305	29.378	29.3495	29.4916	29.1707
25.8498	24.0889	26.3422	26.1441	27.2274	25.9906	26.8378	27.4155	25.8669	26.4308	25.3511	23.1374
25.9035	26.819	26.342	26.8366	27.7458	26.8474	26.2862	26.1353	29.1428	29.006	29.1293	29.0644
26.5301	25.7913	26.397	26.3187	26.7967	26.4511	27.3071	26.1911	24.6138	25.777	24.9985	23.6322
27.2374	27.3445	28.0675	28.0388	28.9883	28.2526	28.6865	26.951	26.2372	26.2453	26.0243	27.0259
25.6316	25.9231	26.2363	25.4731	27.2649	26.02	25.2646	26.4552	28.4036	29.1348	28.6542	28.639
30.2545	30.2336	30.4465	30.4777	29.7679	29.8304	29.9564	29.5201	31.1228	31.0343	31.295	31.3114
29.3104	28.9473	29.0299	29.1168	29.0005	29.0393	29.1987	28.3924	30.2104	30.2525	30.2021	30.3886

28.0726	28.2636	28.4419	28.5683	28.0891	28.1456	28.035	27.7515	29.1292	29.2281	29.6208	29.6507
27.7624	27.5538	27.5614	27.7573	27.2487	25.7444	26.1981	27.4652	28.2446	28.3301	28.2172	28.0901
27.9176	28.2119	26.1839	28.3324	27.63	28.4541	26.3718	26.4227	27.869	27.9179	28.1997	28.0042
25.597	26.7214	27.0134	26.8936	26.2296	25.7325	25.1643	26.2416	25.3428	25.5013	25.7445	25.698
26.8133	27.7824	27.0828	25.8395	26.504	28.1862	26.6383	26.7418	27.9991	28.2818	28.3867	28.4434
26.8515	25.0206	26.6616	26.0015	27.6962	27.6998	26.7431	27.2309	24.2437	24.4732	24.8393	24.77
26.8133	24.9477	27.2181	25.3979	26.5834	25.9333	26.6612	27.3363	25.6578	25.9598	26.3332	26.1006
29.0903	28.9547	26.0489	27.9095	27.4264	28.9639	25.1567	29.5564	26.6158	26.9061	26.7499	26.6311
29.0585	28.7845	29.1455	28.5378	26.8122	25.7279	27.4951	26.3143	30.0636	29.7671	29.8389	30.1161
25.2761	25.2926	27.3438	27.2903	26.6296	25.9936	25.9108	26.1882	25.2833	25.715	25.9766	26.3917
24.2934	25.6577	25.684	28.0254	25.2428	25.3473	27.5627	26.7841	28.4017	28.2493	28.4761	28.3778
25.4003	26.1592	25.5981	26.0938	29.3145	27.2331	28.3127	27.1988	28.0425	28.0477	27.8345	27.7832
26.2297	26.2862	27.3236	26.0053	27.4666	26.4737	28.377	25.5375	23.9892	26.1291	26.4542	25.7108
26.9623	27.0232	25.5502	26.8988	27.7249	26.154	27.2444	25.7264	24.3309	25.8547	27.0415	25.6266
29.2382	29.1893	29.1546	29.2679	26.557	28.6808	27.023	27.6774	29.2761	29.3107	29.5058	29.6195
26.4978	25.2457	27.3271	25.1862	26.0688	26.3287	27.8115	27.4833	26.3954	26.9141	26.7403	26.9354
27.1587	27.1061	26.9239	27.526	27.7703	25.1565	27.0066	26.9086	28.3989	28.1878	28.5501	28.566
25.8355	25.354	26.6266	25.632	26.6683	24.4596	27.6752	26.5357	25.0684	23.0707	23.9165	23.2893
29.1211	29.1567	27.2541	27.3635	27.4888	29.6388	27.5777	29.7181	24.693	24.9999	25.4536	26.8741
31.4747	31.258	29.5366	29.6451	32.2645	34.1537	32.3313	32.9569	27.8584	27.9881	28.3302	28.271
27.5737	27.456	27.3657	27.1453	27.3123	26.9392	27.9295	25.6835	27.8267	27.579	27.9349	27.9554
27.1666	26.8644	27.2296	27.0213	27.2628	25.8199	27.2519	26.5857	28.5705	28.4004	28.377	28.5013
26.0141	24.8309	26.9699	25.9378	27.7182	26.6967	27.3899	25.8174	25.1137	25.3245	25.6332	25.1952
25.186	24.337	26.1482	27.0132	26.92	24.7013	26.6254	26.061	25.4707	25.7247	26.9438	25.6658
26.8832	24.7951	26.8267	26.0478	26.2266	24.4418	26.952	27.1045	26.9896	27.4135	27.8232	27.7945
25.822	25.7307	24.4085	26.108	28.0518	25.4819	26.7147	25.4238	25.746	25.0261	25.918	24.3492
27.4472	26.5437	27.8906	28.4071	28.1467	26.1691	26.6063	25.5505	28.3344	28.0537	28.5177	28.1576
25.8279	25.4825	26.5014	26.4564	27.3189	27.0821	26.7576	27.3193	25.3516	23.5006	25.7029	24.1047
28.6207	28.5656	28.5656	28.561	27.9077	28.5214	27.1242	29.1157	29.2773	29.4241	29.7921	29.6429
27.5305	27.4051	27.2418	26.2043	27.502	27.1333	26.8778	26.6775	27.9307	28.5437	28.376	28.4973
27.646	24.4452	27.4892	25.943	27.4426	26.7772	28.0557	26.3551	26.5563	26.2599	26.2583	26.3749
26.6499	27.7888	25.6483	26.1796	26.5866	28.1742	26.0708	26.4953	28.1536	27.8687	27.6463	27.7847
31.8822	32.1616	31.6572	31.5352	31.5509	31.2429	31.295	30.3086	28.2581	28.148	27.8355	27.7651
25.6037	27.2583	26.4693	24.2237	25.643	27.4053	27.4573	26.5901	27.4014	27.3513	27.2014	27.1603
27.1401	26.9716	26.5306	25.6199	26.4744	25.9055	26.2144	25.5901	25.572	24.8854	24.6923	24.6938
27.0779	27.1279	26.6774	27.2896	26.6245	25.7325	28.1404	28.0935	25.943	26.8828	26.5685	26.4513
25.7807	27.1501	26.5016	26.1719	27.8677	26.4836	26.2475	24.9344	25.3413	25.5867	25.4324	25.4477
29.5109	29.5805	29.6358	29.4265	28.9523	29.0306	28.6918	28.8287	29.9459	29.9131	30.2577	30.1793
25.7689	26.256	27.1581	25.3495	26.6885	26.709	24.9908	27.098	25.8038	23.4208	24.7585	29.7491
29.9082	29.8185	29.3911	29.5732	26.528	29.8015	27.2371	26.2569	29.0645	24.7985	29.713	28.4804
28.127	27.8608	27.8025	28.0877	28.2172	26.8589	26.2915	26.4205	28.5539	28.1862	28.2738	28.246
30.8784	30.9467	30.72	30.569	30.3723	30.6765	30.283	30.4273	29.4157	29.808	29.6504	29.568
27.2176	25.722	27.601	25.7374	27.8901	27.4876	27.5254	26.799	25.4816	23.8987	24.8169	25.2256
29.2755	29.3873	29.7989	29.5095	29.3969	29.815	29.3475	29.2405	30.8116	30.6068	30.7892	30.8188
28.9647	28.9717	29.172	29.2285	28.092	29.1727	28.0083	28.5962	28.3079	28.3493	28.5125	28.4963
26.3476	25.2016	27.4037	24.7546	26.7396	26.1264	26.6168	27.396	28.5411	27.1885	27.9932	27.4647
25.3705	25.9117	26.9123	26.5243	26.137	26.8538	26.1378	26.7087	26.6582	26.3434	26.2219	26.2466
28.1236	28.3515	27.4847	27.6078	26.5991	27.5958	27.1606	26.3136	27.3808	27.2743	27.7373	27.7094
27.266	27.2315	28.7179	28.7291	27.1715	27.5347	28.8741	27.9861	27.4028	27.1028	26.8086	26.6643
30.1019	28.5506	27.3851	27.4155	28.462	29.1935	28.172	28.9249	25.5666	25.5467	25.2169	24.9841
27.9144	27.9013	26.546	26.2385	27.679	29.0603	27.5637	29.1745	24.6213	24.6847	23.9455	26.6716
26.3784	27.7411	27.3007	26.0033	25.9245	24.9337	27.227	26.5246	27.033	27.3761	27.9462	28.1303
26.1327	24.9651	26.0224	25.6229	26.9017	26.3087	27.1015	26.0254	24.0273	24.7959	25.3919	24.1656
27.8947	24.7307	25.9363	25.4133	26.3072	26.0773	27.3846	29.5047	26.2899	24.9175	28.6351	28.0365
27.8525	27.4129	28.4925	28.2392	28.0567	28.2639	25.6332	26.4256	27.9406	27.9457	27.9932	28.3326
27.1015	27.3546	27.3256	27.0754	26.3491	26.8414	25.8662	26.3768	27.4993	27.8142	28.0127	28.0207
27.6212	27.8102	27.3398	27.4917	26.6329	27.9586	27.4738	27.9712	27.7657	27.574	28.4163	28.0882
28.2873	28.1122	28.399	28.5705	25.1187	25.8286	27.8812	27.4612	28.4116	28.5392	28.8114	28.8233
29.3916	29.3369	29.3235	29.7484	29.0198	29.3603	28.8519	29.2321	29.4842	29.4066	29.3223	29.3611
28.0645	27.9703	27.2747	27.7472	26.1826	27.26	26.511	24.9103	29.2312	29.1048	29.28	29.0213
28.7849	28.4928	27.9201	27.9381	29.0971	29.3173	29.0014	30.1027	26.4031	26.437	26.4551	26.5241
28.189	28.4258	24.7604	25.6232	27.4371	29.1922	25.7656	28.8795	29.0333	29.0039	29.4864	29.2802
26.3258	26.4433	27.6218	24.8558	25.8574	27.1157	26.469	25.8962	27.0119	26.7575	27.0097	27.1077
26.528	27.5552	25.5316	28.056	25.9909	27.2409	26.869	25.8449	27.9054	28.0496	27.9518	27.9752
29.9667	29.8791	30.063	30.0702	28.7689	29.1934	29.0489	29.0725	29.5541	29.4955	29.5274	29.4785
24.305	26.1399	26.9604	26.3148	27.4019	26.2637	26.5567	28.207	27.557	27.5238	27.4383	27.6735
25.7252	26.3277	26.8293	25.3715	26.3069	27.3804	27.6231	25.9908	28.1368	28.0763	27.9573	28.0911
26.1032	25.6524	26.8379	25.8002	27.5693	26.8183	26.6633	26.0272	25.9562	26.5285	27.1214	26.6498
26.872	24.4296	27.2655	27.0432	25.5728	24.0623	26.5697	25.7225	25.9872	26.0497	26.367	26.3801
26.7833	27.5144	25.7012	26.7109	27.1655	25.4887	26.6779	25.9393	26.1678	25.0725	24.7585	25.7921
27.2372	26.211	25.4484	25.7681	26.2092	27.0194	26.5561	25.9267	22.1346	24.64	24.741	25.5317
27.0707	26.9927	24.7894	25.7592	26.5296	26.5504	27.2443	27.042	24.4862	26.4456	23.2168	25.7515
25.8016	25.4138	25.2959	26.0247	26.4735	26.775	26.8559	27.3195	26.8888	26.4077	26.9126	26.8493
25.5982	25.8628	25.8266	26.0108	26.2514	26.3492	26.1384	27.1247	24.4142	25.4208	25.7174	25.693
26.8297	25.0245	25.2709	25.9656	25.8992	25.7041	25.593	26.8313	28.5739	28.9592	28.4819	28.3624
24.4749	24.482	26.385	25.5759	27.5687	25.8297	27.4953	27.1849	28.2767	27.7749	27.5227	27.5822
26.5927	26.1724	26.6509	26.4004	27.7501	25.624	27.1085	26.4071	27.7379	27.7834	28.2289	28.2342
27.6023	26.4162	26.2487	26.0952	27.1801	25.4158	26.6277	25.8763	25.1548	26.16	24.6458	25.7272
29.1587	29.0912	28.6964	28.6651	27.8951	28.6644	28.2644	28.8953	28.2455	28.3963	28.4069	28.5664
28.7326	28.6701	28.9175	28.9059	28.5106	28.2725	29.0231	28.2216	30.1181	30.1805	30.3301	30.2788
25.6347	24.1326	25.6041	25.9452	27.1515	25.5829	26.0257	26.3714	27.4069	27.5124	27.9388	27.9886
25.8684	27.6837	26.3733	26.8203	27.095	26.6628	26.9822	26.1473	28.4168	28.1898	28.2462	28.1954
26.1875	26.1234	27.4412	26.9025	26.1467	25.6626	27.953	27.8433	27.0106	27.2126	27.2146	27.1962
27.2273	26.2712	26.5024	25.9594	27.4604	25.1439	27.5939	26.9098	26.4408	26.6664	26.7495	26.8583
27.8838	25.038	26.9027	26.2569	26.9985	28.2289	27.1168	26.9875	25.6	25.7718	25.955	25.9706
27.2964	27.1725	26.9502	27.0472	27.1688	24.6935	26.7916	26.7289	26.442	26.2738	26.3362	26.3796
26.2086	26.3597	26.7823	26.9421	27.4661	25.1246	26.1707	27.4824	24.8387	24.8602	25.3062	24.0741
26.7459	25.7262	25.8966	25.9521	27.341	26.5684	26.9443	27.8427	24.2578	27.3473	27.0446	26.9763
25.749	24.5065	27.5257	26.6556	26.7446	26.0826	26.4966	26.5399	26.1067	26.2936	26.1796	26.4374
27.5245	26.3595	25.4378	25.6216	26.2964	27.2923	25.7577	26.5238	27.3895	27.5495	27.6323	27.7029
25.1426	25.5887	26.7199	25.4835	27.2169	25.8078	27.4182	27.5532	26.3096	25.0533	25.7407	24.0973
26.5278	25.8485	26.3189	24.9299	26.825	25.7855	27.7192	25.483	24.9811	24.0987	23.5099	22.5727
25.7821	24.7271	26.4132	26.7042	25.1764	26.6923	26.7228	27.3694	28.1223	28.0263	27.9082	27.8977
26.9263	26.9818	26.7306	26.4578	27.3495	26.6096	26.8801	27.2925	27.563	27.6193	27.8293	27.9232
26.5569	26.6893	26.7667	25.5016	26.5277	26.4684	27.9186	26.0729	26.4768	26.6776	26.6413	26.5585
25.3097	25.2023	25.9654	25.4851	27.9478	26.2213	26.7814	25.6332	26.6712	26.8704	27.7258	27.5644
27.9799	24.643	25.903	25.8003	26.4181	26.4318	26.1516	26.7014	25.1318	24.8495	23.4606	24.4968
26.4408	25.7266	27.8106	25.5511	25.8338	25.7087	25.027	27.2506	23.5888	23.5876	24.5855	24.4599
29.6152	29.5466	26.4545	25.4527	25.6004	29.4581	27.4275	28.9438	28.3522	27.8647	27.01	27.1231
24.8467	26.694	25.7703	24.2994	27.7429	26.3715	27.3254	26.7203	25.2985	24.971	23.4331	24.3382
25.2719	25.5999	25.96	28.2083	27.2634	26.2551	26.0066	25.8988	26.2971	26.6928	25.9278	25.765
27.5806	25.7628	25.8942	25.1682	27.3237	26.3176	27.4986	26.7574	25.0958	24.897	24.2719	25.3668
26.4231	25.6655	27.5583	26.5105	27.0188	25.1664	27.2347	26.666	23.9731	25.3422	25.1844	25.4804
27.7373	27.8779	28.3154	27.5192	28.2299	28.2708	28.2168	27.3364	25.1994	25.4788	24.8932	24.8795
25.2756	25.8823	26.0617	27.4692	27.4413	25.4708	24.8266	26.9006	25.7504	25.8357	25.5996	25.4101
25.0518	25.8097	26.1848	26.0806	27.4238	26.3651	25.1876	26.1541	28.0287	27.7835	28.4981	28.9123
28.1773	28.2239	28.8003	28.661	29.1407	28.2816	29.0911	25.9619	25.3404	26.1329	24.591	23.6834
27.2508	26.0251	26.9312	25.8137	27.2978	27.3124	26.8129	25.7553	26.2433	25.7924	26.3267	25.8803
25.7586	26.5176	27.0022	26.8409	26.4167	25.464	26.4031	26.3851	27.4162	27.1186	27.3792	27.3604
26.2852	25.1437	26.6035	26.7472	26.2478	26.9334	26.6402	27.0102	25.8286	28.7597	24.3279	26.122
25.2455	25.3897	26.8777	24.8904	28.0073	26.5169	25.6338	26.9307	22.7437	24.8549	25.1819	24.8367
26.6853	25.5976	26.7393	26.5591	26.5628	24.4986	28.0554	25.9288	26.2034	26.6903	26.3764	26.6351
26.9097	27.0538	27.3015	27.1858	26.7162	28.8202	25.9629	27.1396	24.4287	25.3315	24.578	23.2341
25.8421	26.637	26.1209	27.0706	28.6722	27.5459	26.4229	26.1955	25.9654	25.1089	25.0443	26.0228
26.7378	26.042	27.4155	25.623	26.0006	24.896	27.0863	27.7157	27.0046	26.9159	26.9124	26.9432
26.3583	26.1005	26.6824	26.2936	26.3118	27.4677	27.3226	26.146	26.1567	26.3651	27.2747	27.194
27.7303	25.2323	25.2697	24.8594	28.36	26.0755	25.7008	25.9487	23.015	26.5698	26.9015	26.8359
26.9169	25.8912	25.0558	26.1621	24.9886	26.485	26.7052	26.3795	26.0275	26.7293	22.7641	22.403
29.6026	29.7446	29.1825	29.1066	29.1078	28.2486	29.0007	28.1616	25.5852	25.1502	25.3843	25.5963
25.9546	25.5946	26.1019	26.8327	26.3793	26.3978	27.0665	26.3345	27.0561	24.4325	24.3134	25.507
26.7834	26.6486	26.542	25.5012	25.9497	26.3969	25.9436	26.9559	25.3058	22.4643	24.4286	23.4382
26.5861	24.2283	25.9194	26.884	27.2195	26.1029	25.7021	27.4947	23.0992	24.4716	24.4612	24.0359
25.3058	25.5537	24.3715	26.1993	27.8419	25.4031	27.7584	25.7588	26.8318	27.0378	27.043	27.1598
28.0444	25.1378	25.5695	26.6689	26.2079	25.1245	27.1788	26.5066	24.1963	24.667	24.3517	24.2892
24.7986	25.005	25.2455	26.0425	25.7362	25.3592	26.3137	25.3342	27.1933	27.3475	27.603	27.8795
26.3958	25.2674	24.8816	26.3609	27.9416	26.0095	25.9515	24.93	26.4846	23.8259	25.0576	23.634
26.5723	25.8368	25.9563	27.2856	27.0567	26.5842	24.6637	27.2478	27.3355	26.96	26.5876	25.7673
25.9729	27.057	25.9263	25.7948	25.4853	26.2812	25.8066	25.3072	26.0655	25.9572	25.1202	25.413
25.5532	26.451	26.4142	26.7827	26.8299	26.4214	26.01	26.5276	26.0196	25.7121	26.429	26.0396

26.9261	26.0348	26.1921	26.4349	26.6518	26.4244	27.0135	27.2516	26.2155	26.2285	26.3257	26.7053
26.1255	26.3022	27.1781	25.8566	26.5694	26.8401	27.2624	25.0664	24.2377	24.5164	25.8251	25.2553
26.4042	25.696	25.7174	25.6334	26.6264	26.5626	27.7668	26.5529	24.1027	24.84	24.8434	24.6591
25.8929	26.8151	26.4094	26.4876	25.7727	26.1442	26.5747	26.9961	26.5374	26.8539	27.0012	27.2062
26.1806	27.0624	25.248	25.4612	25.7179	27.9194	27.4848	27.0844	27.9726	27.7762	27.9871	27.943
26.3642	26.6397	26.5232	26.5828	26.1547	26.7569	27.0958	26.8554	26.6656	26.2396	23.5113	26.4802
25.6173	27.0406	27.911	25.8892	27.7631	26.4313	26.676	26.4747	26.1936	24.7787	24.2603	23.9317
25.7466	26.4799	27.7829	24.2654	27.2335	26.1408	26.9698	28.2764	22.8432	23.4265	25.9305	23.3651
25.6956	25.8607	26.5405	27.0601	26.6918	25.7578	26.5986	27.1056	26.9093	26.2379	25.8552	26.1907
24.0531	25.1732	26.4917	24.6982	26.8536	28.3743	26.4772	26.4271	25.7888	23.9075	24.5155	25.3718
26.8204	25.9	25.3372	25.9497	26.4431	24.6376	26.4402	25.4681	27.7048	27.6406	27.5484	27.5023
26.5449	27.6001	26.4778	25.2237	26.6675	25.0136	26.835	26.0355	25.7706	25.1259	25.733	24.5245
25.0142	26.6629	27.3499	26.0178	26.8603	27.4242	26.5929	28.2677	28.0086	28.1209	28.1048	28.2419
25.325	28.6344	28.2638	28.3192	26.3966	25.5103	26.242	26.3958	26.7325	26.6551	26.4083	26.2073
28.7909	25.6932	27.4291	26.0442	28.1951	27.1783	27.0571	25.9784	22.685	24.8971	25.7748	23.2105
25.2945	24.0896	25.5458	24.979	26.4263	26.2331	26.8919	26.0307	24.7006	24.6748	26.2923	26.6641
25.6297	25.3282	25.4017	24.1886	26.6775	26.813	26.4072	24.422	26.5284	26.8946	26.6353	26.5091
26.5321	26.0125	25.5385	28.0043	25.8265	26.7464	27.1033	25.2074	28.7149	24.265	28.4851	28.1679
27.2544	27.3237	26.9752	25.9299	25.6325	27.4592	27.1203	28.3479	27.031	27.0789	27.1808	27.0209
27.5803	27.0162	26.9083	25.7779	25.6722	26.8333	26.9336	25.8169	23.5091	24.749	25.6567	25.5637
25.8996	26.3255	27.2058	24.1677	26.305	24.6965	26.3765	26.9634	26.4087	26.3778	26.1189	26.2399
26.0191	26.6428	26.4059	27.6438	25.7436	25.99	26.5971	27.1823	27.0844	26.8041	26.9533	26.5029
27.6311	27.2634	26.8565	24.6339	29.691	28.9283	28.8222	29.0672	23.9074	25.872	24.7076	25.6971
24.8815	26.7367	25.3835	24.3251	26.2069	27.0893	25.4951	28.4444	23.9261	24.3308	26.1352	25.4705
26.6474	25.2185	26.7447	26.0049	27.2915	27.1735	25.7589	25.6469	26.8574	26.8514	25.6219	26.1265
26.3342	25.3166	25.1371	26.6065	26.8449	26.1641	26.9013	26.1431	25.0739	25.1691	25.8992	24.0484
27.4061	23.5417	26.8315	27.1804	26.8125	25.8558	26.7268	24.6193	25.8938	24.5722	24.0815	24.7297
31.0896	31.3303	30.8715	30.5107	32.2953	33.7134	32.1638	32.224	32.0908	31.9711	31.6522	31.6735
26.511	26.7506	26.7962	25.6496	26.2199	25.4516	28.695	28.6062	24.7992	25.6253	22.8846	24.7419
27.7834	25.5553	26.0625	26.2173	27.2152	26.1504	26.5536	26.7259	25.6642	27.5602	24.116	25.016
25.6988	26.7607	25.639	25.4113	25.2409	28.1986	26.7428	26.0386	24.3281	24.6338	24.5219	24.8362
27.0049	25.3475	26.1398	25.7719	26.3312	25.4738	27.2122	25.8704	24.3658	24.8589	24.8995	26.4764
26.7952	25.5072	26.0733	26.4203	26.4637	26.0157	27.9394	25.9895	26.9942	26.7315	27.147	27.5184
26.0097	25.0488	26.7186	25.7929	27.1486	26.4519	26.5832	26.9349	23.5663	23.8282	22.7572	24.0609
27.5122	27.5917	26.1573	25.2146	26.7672	27.9723	26.8293	26.7544	27.3061	26.9959	27.8047	27.4721
25.6401	25.453	26.0772	26.7541	26.7955	27.6121	27.5241	25.7307	25.4457	23.3865	25.0969	25.1811
26.2319	26.8643	25.6539	25.8876	26.2148	25.8373	26.427	26.0583	24.3258	24.334	25.3563	25.1449
26.8173	24.5681	26.6219	27.1304	25.5705	25.277	27.9927	25.5741	24.7838	22.9989	25.6839	24.9754
28.5392	28.6069	28.2856	28.3492	28.4283	27.8924	28.2806	28.0898	23.8884	25.0263	25.8807	23.3179
26.823	24.7931	25.9413	26.1203	26.564	26.5981	26.5876	27.0092	24.6602	25.7143	23.5363	24.4785
26.8602	25.2324	26.6471	26.9362	27.051	25.4608	27.7277	27.6642	25.7146	25.3771	24.6731	25.2462
26.1673	26.8494	27.6734	27.4146	26.7997	27.1716	26.0996	27.6867	26.1631	26.5269	26.1506	26.415
25.1128	26.5479	26.2269	26.3892	26.4354	26.5423	26.8098	25.0389	26.9662	26.3924	26.9465	26.8102
27.2258	25.4008	27.9869	26.5505	26.9761	26.4702	28.343	26.5036	25.168	25.0362	25.4887	25.5329
26.7178	25.8583	24.9362	26.4504	26.8954	27.2591	27.2286	25.7163	26.4689	23.6364	24.9214	25.0695
25.0348	25.7895	26.4103	27.2687	26.3622	27.5771	27.9417	27.6327	25.027	24.1662	25.3684	24.2713
25.1104	26.3141	25.2423	25.3453	25.5021	27.0788	28.4104	26.8207	25.6337	24.2956	26.3389	26.6768
25.6554	24.9235	25.8382	26.4817	26.6868	25.7067	26.8048	26.6118	23.7959	23.1246	25.2783	25.414
25.6125	24.6462	26.4566	25.8834	27.9276	26.15	27.1793	26.7976	26.7405	26.8795	26.881	26.8955
26.5416	25.9934	25.7562	25.6023	27.3054	24.2484	26.7028	27.1665	24.2904	26.1335	25.6132	25.4511
26.9061	25.9717	26.8858	26.4546	25.5045	28.1369	26.5029	26.9073	26.4279	26.1409	25.9251	25.576
25.9167	25.3487	26.7141	25.3309	26.9825	26.3183	27.3655	24.1141	25.2961	25.8032	24.2715	25.9562
26.4405	25.8959	25.047	24.6415	27.4791	27.4942	27.0211	26.3877	30.7243	30.532	24.268	30.4178
24.8884	26.732	25.8391	26.0936	25.9587	28.1305	26.3255	26.6106	25.1855	24.8615	25.3108	24.9407
24.9248	26.0861	25.0143	26.4153	27.4591	24.9146	26.5821	27.0783	23.2411	25.2055	25.4154	26.0464
28.1766	28.1743	25.6195	25.7234	26.8495	26.7623	25.3094	27.2496	26.7189	26.9779	27.0028	27.1659
27.076	25.2855	25.348	27.1981	26.7132	24.5948	26.5251	27.1351	24.4959	24.5131	24.0018	25.3154
24.9318	26.6766	25.7095	27.2335	26.029	26.2264	27.1713	26.7466	25.532	26.878	27.3355	27.7898
25.8964	26.1919	26.7316	28.5863	26.4056	26.8219	27.3009	27.0778	28.4727	28.8687	28.4572	28.5388
26.4866	25.1747	27.779	26.1984	28.0298	25.7521	25.9296	28.3957	27.444	27.4696	27.5957	27.6687
26.8163	24.9818	26.3686	25.2527	27.4241	26.82	27.464	27.1985	23.6863	23.594	24.6497	25.249
26.1479	25.4656	26.9457	26.6798	27.4338	26.2254	27.3195	25.05	24.2511	24.9903	24.4379	23.6046
26.4375	27.123	26.2688	25.2243	27.3471	26.1283	26.2567	25.9469	25.94	23.9155	26.6399	26.7638
26.9805	26.3392	26.5397	26.4391	26.6788	27.1741	25.1546	26.1811	24.7663	23.9393	25.5357	25.5827
26.8673	28.2414	28.7068	28.9224	26.0173	26.4386	25.6039	27.0653	27.8875	27.8563	27.7178	27.7146
27.4155	24.8165	27.2424	26.2874	27.9624	25.906	26.7246	25.0198	28.0937	23.5482	28.2256	27.7905
25.0112	26.6208	27.2842	26.193	26.4514	26.1682	27.6029	26.7037	27.6003	27.7587	27.7045	27.6285
26.8838	26.191	25.5721	26.7766	25.7573	27.2098	27.1467	27.7869	23.3216	23.2839	24.2024	24.657
25.4583	26.066	26.7896	26.3437	26.527	25.6713	26.4798	26.6508	25.1463	25.0243	25.4911	25.3884
25.9398	27.3895	26.7103	26.853	27.8133	26.3655	27.5049	26.1647	27.0137	26.9725	26.6811	26.6826
26.9755	25.5431	26.4424	29.694	26.1147	27.6709	27.0028	25.9994	27.5831	24.9714	27.8574	27.9747
26.1126	25.5424	26.5944	26.8246	27.6446	27.4015	25.8419	25.458	25.4669	25.2716	23.3369	25.4179
25.8056	25.1075	27.6299	27.5825	27.1515	26.4448	27.3275	27.0472	27.3793	27.4494	27.1441	27.0876
29.2948	29.2499	29.4563	29.6033	28.7762	28.9625	28.5588	28.3798	30.1426	29.8103	29.6348	29.4825
28.1567	26.9925	26.4571	26.37	26.6936	25.932	26.9598	26.3873	26.289	23.1658	26.7474	26.408
25.7114	26.4173	26.7341	25.3802	26.9623	26.2086	26.7574	27.361	26.4227	25.9562	24.6262	25.859
26.0219	27.4177	26.275	27.1977	26.3658	26.235	26.5088	26.1909	25.3301	24.2293	25.7706	25.5157
25.4071	26.602	25.5935	26.8838	26.1869	26.4784	27.5674	25.4812	27.555	27.6567	27.3362	27.3319
26.8865	26.9535	26.9408	26.1249	27.7733	27.3398	26.777	27.6978	27.9184	27.8444	27.8711	27.8882
25.8976	26.3257	25.1068	26.7792	26.1821	26.4643	27.3711	27.3757	26.4139	25.6527	26.0566	25.9928
25.1315	26.1754	26.1722	25.8951	27.0412	26.3282	27.1268	26.3066	25.3962	26.2315	26.1348	26.4965
27.1134	25.951	26.148	25.4498	26.6795	25.7845	27.2912	26.4366	26.4424	23.8665	25.5034	23.1348
24.5809	25.5484	26.3787	25.9234	27.2179	26.8708	26.3041	24.7214	27.7482	27.6772	28.2025	27.7091
28.1307	28.3043	28.6735	28.1628	29.0389	28.3853	28.9733	28.2808	27.2591	27.0614	24.2329	26.8092
25.2601	26.2406	26.0872	27.3772	25.0637	27.0591	26.7317	26.3087	24.5936	25.9184	25.8799	26.0452
25.9796	25.9712	25.5156	25.4056	26.7677	26.5534	26.9692	26.3744	22.767	25.5028	24.1628	25.7426
26.4991	25.5813	26.3949	27.1374	25.7281	25.8595	26.4744	26.0745	25.7975	25.1171	26.0311	24.5372
25.6253	25.3849	26.0246	26.5416	25.9607	26.1695	26.2402	25.7664	26.7962	26.7986	27.1159	27.1633
26.2995	28.7125	27.3611	24.5471	25.7346	25.6353	28.2104	26.263	22.4166	26.7539	26.7606	26.9231
26.4755	25.446	26.7602	27.3186	26.8184	25.578	27.2546	26.6858	26.4342	26.39	26.2778	26.3848
25.7447	25.7423	26.9609	25.9308	26.2892	28.1581	26.4849	26.1924	25.8258	25.9861	25.5291	25.4362
26.332	24.4641	26.9624	26.055	27.779	26.5901	26.4981	26.5249	26.6961	27.3064	27.1434	26.8016
27.7484	25.8355	26.4147	26.7296	27.6126	24.0899	26.6146	26.8767	24.8465	25.161	25.3877	25.3684
27.3426	24.0627	26.0386	26.5765	25.8008	27.4852	26.7194	25.9061	25.8441	26.4633	26.3179	26.4156
27.2837	26.2222	25.4625	25.6126	27.1849	26.2754	25.9921	25.7148	26.3131	26.2548	26.8561	26.5111
30.9114	30.9971	28.6162	28.3139	28.9763	30.5669	29.0904	29.8368	27.5525	27.8225	27.6592	27.6772
28.7022	28.7733	27.8918	27.8535	28.5337	28.4915	28.283	28.1611	25.6654	23.8638	26.2354	25.7368
25.0316	27.6778	25.7806	25.83	26.3122	26.6222	25.8596	25.3769	27.411	24.6468	25.5676	24.8757
26.203	25.3643	27.1069	25.9727	26.9201	25.4776	25.9796	26.2292	24.6447	24.6617	23.7293	24.6286
24.5528	25.2514	25.5132	25.3331	27.5627	27.3554	25.9739	27.477	25.2311	24.3465	26.9889	27.0737
25.9556	24.3301	26.1172	26.4923	26.0924	26.9574	26.4332	26.6815	25.6037	25.3425	24.3353	25.3858
26.0536	26.5283	26.2108	25.9756	25.7708	27.0069	26.9336	27.4056	25.9552	25.8327	26.0828	26.1075
27.1167	25.2963	25.388	24.8711	26.7451	26.9382	27.6776	26.584	25.2385	25.0241	25.1572	25.7471
26.9912	26.9771	28.3962	26.8728	27.9703	27.9019	27.7834	27.8694	23.764	25.775	25.5869	24.841
27.4215	25.4576	27.0283	26.1406	26.5563	25.5598	26.409	27.5792	26.8571	26.1889	26.5373	26.2051
25.8972	26.4189	26.8308	26.758	25.9957	27.0603	27.0026	25.8855	26.9402	26.6826	26.5804	26.1968
26.775	25.3855	24.3089	26.9507	27.9682	27.4752	26.1444	25.3307	25.5654	24.8082	25.7869	25.8526
26.8166	25.5621	26.4007	25.841	25.4206	27.9836	27.238	26.3907	26.7384	26.732	26.5891	26.6309
28.291	28.4333	28.5461	27.7704	28.4081	28.4015	28.4858	28.2671	26.774	25.2669	26.1237	23.5157
25.9901	25.5539	25.5745	25.5075	26.6223	26.31	26.5132	25.8186	24.958	24.5758	24.2617	23.3886
28.1326	26.9034	26.8627	25.3327	26.2998	26.0651	25.7872	26.8148	27.3709	27.7674	27.6923	27.2123
25.2938	26.7239	26.4392	25.8271	26.7093	26.9475	27.2012	26.5461	24.3022	26.3641	26.9929	24.3689
26.74	26.3047	24.9145	26.2494	25.1029	26.8513	26.8538	26.3238	23.393	24.6974	25.056	24.6293
25.4597	25.0773	26.7055	27.1542	27.0897	26.9154	27.8659	26.6787	26.3484	24.6382	23.9007	23.9977
26.8818	26.4223	25.8577	25.8445	27.7312	25.383	25.8044	27.7931	25.6843	23.1045	24.9875	23.6688
27.4295	25.9922	27.059	25.5859	26.0665	25.3008	26.0878	26.6646	26.1452	26.2375	26.0908	25.9613
26.5762	25.2557	27.0888	25.4686	28.3183	25.3384	27.6083	27.0487	25.2157	23.9213	25.8852	25.3121
26.2943	25.9245	25.3884	25.104	26.3177	26.2379	26.6514	26.2955	23.4235	24.2408	25.1558	23.2639
26.9618	25.8645	26.727	25.3194	25.822	24.9868	25.3496	26.9138	26.8508	26.9733	27.1936	26.9599
29.5604	29.7127	28.6092	29.0898	31.4264	31.525	31.1776	31.2444	29.4173	29.1159	29.4465	29.6269
27.0339	25.6697	27.1568	26.338	25.4677	25.3382	26.4407	26.0804	27.1119	26.9439	27.2342	27.1607
28.5008	28.7401	26.7628	26.8176	28.2084	29.0742	25.6027	29.9463	25.4728	25.1164	25.3522	23.2538
25.0219	27.6138	26.7484	26.9123	25.9359	27.0252	28.6271	26.4605	24.1571	25.2058	23.627	24.3979
26.2367	25.2742	26.4983	24.6551	26.9373	24.2586	27.0325	26.3635	23.873	25.3766	25.8491	25.9801
29.187	29.1693	29.2455	29.2122	28.7826	29.2055	28.7474	29.3378	30.0531	30.0198	30.0289	30.0475
25.96	22.9314	27.0702	25.7164	26.1937	28.1627	26.8468	26.0515	26.6112	24.3392	25.4489	24.37
25.691	26.9645	27.0857	25.7973	25.9024	25.6981	26.6832	27.719	27.129	27.3733	27.267	27.3771
25.4845	27.0067	26.3534	25.7655	27.7798	26.1198	26.6753	25.7532	28.6316	25.1479	28.4638	29.2331
29.0108	28.8647	28.6125	28.5801	25.8878	28.5996	28.2722	28.7525	28.997	29.1983	29.3497	29.2697

26.0629	27.0702	25.8946	25.9225	26.5665	26.4017	27.3298	27.2857	27.0164	27.117	27.183	27.134
25.7784	27.4824	25.4676	26.4136	26.3465	26.9404	26.5848	27.6836	26.9929	27.3324	27.5669	27.7098
26.7801	26.8384	26.8841	25.671	26.9499	27.0185	26.5232	27.366	26.1933	26.3374	26.4146	26.318
27.092	27.7239	26.9765	25.437	27.9271	25.1092	26.1364	27.007	25.5543	24.4403	24.5769	25.9522
29.3468	29.4284	29.5906	29.3752	29.0577	29.603	29.113	29.2994	27.737	27.5853	27.7306	27.845
26.1157	26.6806	26.0779	25.4325	26.6065	27.1607	25.4555	27.1929	26.5298	26.7815	27.0634	26.8815
26.38	26.1599	25.4145	26.786	25.8469	26.5676	27.9574	27.2929	25.6617	24.7832	24.8765	26.2856
27.1812	25.3707	27.8627	25.8321	27.0027	26.425	28.4068	26.6253	24.3285	23.0986	24.0956	24.8062
27.3768	25.8397	25.6013	25.2204	26.0066	24.9656	26.6844	25.6145	25.47	26.1976	26.1683	25.7584
28.6874	28.6995	28.4544	28.2347	29.0638	29.6389	29.0295	28.6351	27.6629	28.0471	27.5393	27.7981
26.8471	25.1926	26.5263	26.0966	27.2367	25.839	27.4762	27.2081	27.1665	27.2341	27.2899	27.0812
27.6701	27.8062	28.4877	28.1933	28.8247	27.8132	26.1798	28.117	27.2137	27.2659	26.924	26.9421
26.0743	25.0959	24.986	25.6848	27.2874	26.4539	26.6932	25.8977	25.4025	25.9565	25.0517	25.3172
26.7274	27.282	26.0029	25.6865	25.892	26.4684	25.2476	27.7318	25.191	25.4544	25.4548	25.4064
26.061	26.3051	26.915	25.0164	26.6146	26.4819	27.0003	26.9797	25.1395	24.1838	22.8023	24.8219
26.0675	26.3808	28.6546	25.5534	27.1918	26.0685	26.7183	25.7486	25.2017	24.8618	25.7006	24.7435
26.6564	26.7251	26.2309	26.3588	27.7618	28.8513	26.8122	27.5459	24.3773	24.2059	24.2272	25.2197
27.2336	26.8483	26.2393	26.3435	27.7683	26.8297	26.5404	26.869	26.8235	24.8591	26.8853	24.4951
26.1312	24.9215	26.7957	26.8359	26.2637	27.7001	27.111	26.6361	24.1745	23.3301	24.3527	24.8922
26.9208	27.6355	26.5509	25.9949	25.4775	26.3977	26.2384	25.7779	24.0779	24.7932	25.8551	25.9449
26.6956	24.4452	26.3449	25.3837	28.4553	27.1714	27.7884	25.5906	26.0038	26.1407	25.6593	25.6485
25.1043	25.6021	27.1225	25.1422	26.833	26.0006	27.5115	26.4914	25.2171	25.4711	25.309	25.2446
26.2514	27.4041	26.7743	26.5156	26.8478	26.596	26.8076	26.7517	24.6451	24.3016	26.452	25.7144
25.7145	26.8916	26.186	25.7443	27.9108	25.9262	27.2516	25.8396	24.516	25.6953	25.4689	25.2353
25.7013	25.3057	25.9586	27.4827	26.7595	26.1337	26.3268	26.0275	24.6681	24.6223	25.4576	26.301
25.0783	26.8121	25.2219	26.8156	26.8887	26.5033	27.2648	26.1532	27.0727	27.1201	27.0929	27.217
27.4387	27.0435	26.5564	26.5663	27.5103	28.2807	27.2439	28.5919	27.2875	26.3004	26.2703	26.309
28.2407	28.4935	28.4342	28.1975	26.9785	26.0919	26.5958	28.3758	24.4261	25.2811	24.396	25.0017
27.8705	27.6189	25.905	25.9434	27.8152	28.2756	26.1915	27.2946	31.5538	30.7771	30.5119	30.8791
27.5398	26.2496	26.9657	24.4418	27.2889	27.9633	26.9101	27.7038	25.4934	24.0532	24.6709	24.9111
27.6975	27.1423	26.6904	24.8178	26.7285	25.644	26.2823	28.3008	27.2046	27.5831	27.4201	27.5375
25.8101	28.0317	27.4958	26.8952	27.4125	25.5969	27.1027	25.2766	26.5839	26.7288	26.8164	26.8906
26.3357	26.1328	25.6622	26.4742	27.8443	25.4883	25.8566	26.5609	28.287	28.0139	28.2621	28.5111
25.9011	25.8622	25.7987	26.5551	26.5293	25.4865	25.8801	25.1715	25.0465	25.0469	24.3833	23.6306
27.2359	24.7411	26.0813	26.9073	27.7306	25.5144	26.9163	25.6054	24.798	25.8293	26.0157	25.885
25.4954	25.3159	25.9066	26.0401	28.3941	26.9102	26.4405	25.3015	23.9842	25.7567	24.146	24.5849
24.7651	25.6043	26.2895	24.976	26.903	28.2637	26.3538	26.5029	25.5151	25.0584	26.1232	26.049
27.6441	27.8297	27.6861	27.6067	25.5347	26.1498	26.3684	25.5308	25.9679	27.1465	24.3309	25.1189
26.4503	25.4696	26.498	25.2147	27.9379	26.9518	26.8327	27.3379	24.8407	23.6787	24.686	24.311
26.5434	25.8783	25.8419	25.7268	26.4728	26.7996	25.8408	24.4762	25.1489	26.2668	26.4323	23.3541
26.9025	27.3163	26.276	24.9256	27.0149	25.4982	26.9714	26.5121	27.9982	27.6945	28.2894	28.1314
26.1582	25.4824	24.8002	26.7921	26.6567	26.6888	26.2735	26.1346	25.6747	25.592	25.5061	25.1971
26.0073	25.5753	25.8657	26.7678	26.4811	27.1236	26.8452	25.7239	27.7183	27.7863	27.8678	27.7918
27.4664	24.2561	28.0824	27.5269	25.816	27.0162	27.8374	26.2958	28.9607	28.9622	29.138	29.0986
26.127	25.3409	27.2402	24.919	25.7659	26.6104	27.4472	26.9195	24.2246	24.8959	24.4128	23.0095
29.6868	29.791	28.1292	25.8631	27.1202	30.3431	28.8328	30.8272	25.78	24.3403	24.611	24.4787
26.8563	26.7716	26.0692	25.4016	27.1633	25.3711	26.8838	26.5719	24.822	26.0811	25.6779	25.9081
25.7938	25.4884	27.4823	24.7871	26.0105	25.8937	26.0034	26.2699	25.1675	24.2773	25.7429	25.8492
25.2171	25.6252	27.2743	27.8887	26.9149	26.3546	26.7219	26.6845	29.7602	29.4942	29.4585	29.3008
26.2918	25.5981	27.6587	26.6376	27.1462	27.0002	27.8862	26.7525	24.9335	24.9298	24.8787	24.8777
26.0471	25.6013	25.7984	25.7283	27.1857	27.0946	27.4523	25.6339	26.2287	26.2521	26.1956	26.1188
28.8775	28.8537	28.3948	28.4012	27.5751	27.7856	26.1821	27.9218	28.4932	28.8167	28.6065	28.7828
25.4404	26.0601	26.1075	27.002	26.3105	25.8882	26.2776	24.9945	26.2834	26.4615	26.8637	26.5427
26.3695	26.457	26.8228	25.5241	26.5015	25.9192	26.6667	26.6741	24.9739	27.1572	26.0135	25.3609
26.285	25.7132	26.0177	27.6094	25.9599	26.5525	27.3901	26.4628	25.9377	25.8606	26.7358	26.3613
27.5511	24.4911	26.0249	25.7759	28.192	25.827	24.8252	27.2172	24.6892	24.1622	24.5395	24.5704
25.3245	26.9707	26.1547	25.9203	27.3703	26.4887	26.0096	26.0924	24.92	26.2519	24.7467	25.7467
27.2681	25.8183	27.2474	25.292	27.0619	25.7529	26.2984	26.8203	25.7973	22.9021	22.9398	26.078
24.8857	26.1622	25.4911	26.6174	27.819	25.2018	25.3147	26.9971	26.5505	26.4469	27.2929	27.1095
26.4992	25.7408	26.6433	25.167	26.4863	26.5696	26.7026	25.3857	25.6285	26.1352	26.3271	26.1606
26.4154	25.2846	26.9872	24.6791	26.7685	25.6796	27.3268	25.3418	23.5815	26.5794	24.304	24.412
27.3529	25.0425	26.0993	26.6834	27.7353	25.2604	26.3862	26.4806	25.595	24.1588	25.9451	26.0174
26.2395	28.3016	26.9113	25.7484	26.3249	25.2436	26.8249	27.4938	27.5451	27.5155	27.4727	27.1767
25.6892	26.6977	26.7376	25.6264	26.1276	26.9205	26.9196	25.9811	25.4198	25.0707	24.922	23.6098
26.6672	25.0032	25.2819	26.402	27.5627	25.6964	26.6509	27.6742	25.1618	25.2257	25.0466	25.2043
27.4976	27.7527	27.3296	26.5408	27.2358	28.0254	27.9532	26.401	27.3229	27.1651	27.3078	27.2652
27.6115	24.5213	25.9786	25.4771	25.9496	26.604	27.0796	25.9408	22.6267	24.6737	25.3161	25.158
26.2611	24.3603	26.7745	26.8754	27.0008	26.514	27.5339	26.267	25.237	24.2019	24.3388	24.8917
28.1431	27.7082	28.8302	26.262	30.1441	30.571	27.7391	26.5527	26.4257	24.7365	25.8928	25.8912
27.2561	26.7091	26.4388	25.6819	27.6064	25.6114	27.5381	26.8131	27.8913	28.215	27.1318	26.9694
25.8088	25.3748	25.6843	25.1498	28.2706	25.264	26.8993	27.4258	26.0374	26.0572	26.4488	26.4219
26.2515	25.3412	26.0732	26.5429	27.8029	26.6059	28.5953	26.6805	23.577	25.5479	24.476	25.4948
26.2074	24.9615	26.718	26.8634	28.2718	25.7668	26.641	25.8499	24.2957	24.76	24.5869	24.3674
27.5912	24.688	25.85	26.4801	27.828	27.2929	26.7413	26.5574	24.4966	24.9862	24.9575	25.5526
26.2848	26.6619	26.7467	27.0857	27.4346	26.5356	27.432	26.3132	24.8587	25.7937	26.1645	26.6769
26.7886	25.0039	25.7875	25.8799	26.4181	27.4724	26.0975	27.9432	25.5188	26.2752	25.8391	24.5368
26.568	25.5313	27.0135	26.1994	27.2911	25.2067	26.4922	25.5801	26.5465	26.6167	26.7483	26.5482
29.0062	28.6799	29.2165	28.9066	29.0964	29.7775	29.4227	29.3186	24.4024	24.2554	23.8963	25.507
27.2375	26.1858	26.9861	25.5154	25.6309	27.0514	25.9644	24.0479	24.3179	24.6081	24.3538	24.1697
25.1126	25.8541	25.4653	26.581	27.3189	26.4722	26.9082	27.0975	27.4528	26.6656	26.6568	26.5119
26.8162	27.1684	27.3237	27.3556	26.3714	27.1779	25.5335	26.7181	27.9965	27.9792	28.4309	28.1569
25.3735	24.8239	26.2882	26.9138	27.3713	26.2033	27.2841	27.6004	26.7406	26.5249	26.4898	26.2795
25.9444	26.2047	26.2296	24.9576	26.3422	24.1723	27.5072	25.0585	26.2271	26.1178	26.3404	26.4412
26.3927	25.4476	25.1911	25.9671	26.0761	26.1479	25.8047	26.7265	25.481	25.2229	24.4001	25.0628
25.6117	25.5795	25.6958	25.333	26.4955	26.7037	25.8348	26.663	25.3428	25.5323	26.0668	25.547
27.1371	26.0911	26.4015	26.6788	26.4559	26.4376	24.3306	27.0017	24.1678	25.2999	25.649	25.1916
27.1413	25.6792	25.4284	24.5222	26.5442	26.9885	27.7902	26.6769	25.5343	24.7381	24.9624	24.4093
27.2585	25.0654	25.9699	26.8506	27.4456	25.5966	27.073	26.474	25.0223	25.9235	24.8261	25.0819
25.6291	25.4257	26.5078	25.994	27.0284	24.8585	26.5097	28.1298	26.6226	26.6034	26.7321	26.6721
26.5317	25.5478	26.5941	25.4862	28.9027	26.7133	26.9641	26.059	26.2555	26.2218	25.9904	26.2868
26.2212	26.4616	25.8962	26.5158	26.141	27.0632	28.1479	27.3248	24.9591	25.0903	24.0171	26.1021
25.7263	25.1591	23.7751	25.7664	25.1881	25.4377	25.492	25.7761	25.8303	25.8169	23.9812	26.3319
25.4144	28.0678	26.3488	26.0456	26.9332	27.0392	26.8611	25.5868	25.4184	25.3532	25.0473	24.9095
26.3506	26.6365	26.9651	26.9618	26.4844	27.1955	26.9578	25.3771	26.4385	26.8452	26.6036	26.4416
25.3481	24.929	27.4184	25.566	26.6848	26.5866	27.1137	27.1077	25.3588	25.7123	25.3581	26.1287
25.1488	25.1063	26.5726	26.3599	25.2436	27.1619	26.9171	26.7572	26.8784	27.185	26.9964	26.9291
27.1028	25.2118	24.8613	25.0804	28.4058	26.053	27.1491	26.1141	25.7274	24.5422	25.9578	26.4544
25.7473	25.8256	27.0504	26.1397	26.5132	27.5535	26.1123	27.0857	28.3431	23.3564	24.4496	27.4219
24.6376	26.3128	27.4408	27.5173	26.1797	24.7101	27.1693	26.3998	26.0592	25.9061	25.8955	25.8524
26.5758	25.9386	26.2876	26.6555	26.7712	27.608	26.9463	25.6797	24.7303	23.877	23.9041	25.4067
26.2986	27.2856	24.827	27.0435	26.7125	25.3874	27.8173	24.1375	24.5251	24.2323	23.5423	24.0834
24.8826	25.8748	26.6979	27.0631	26.8534	26.2979	26.9823	27.388	25.0766	24.8435	24.5841	24.2718
26.6494	25.7537	25.2409	26.7693	26.2088	26.3184	26.3787	28.6439	25.4576	23.823	25.2582	25.0571
27.5772	27.6462	27.6557	27.6031	27.985	27.7105	27.9097	28.2424	26.3232	26.2386	24.9909	26.6384
27.0328	26.8426	25.2424	26.6612	26.2712	26.0345	27.2216	25.9323	24.6825	26.7253	26.5148	26.8493
26.6293	28.6577	25.8737	28.8842	28.0963	27.0372	26.6578	26.5452	25.9778	25.9302	25.5943	25.4824
25.6745	26.2424	27.1744	25.9772	27.0782	25.4593	29.1725	26.4818	26.5134	26.4293	26.7247	26.9623
25.1195	27.7658	26.4442	25.5351	25.7701	26.861	27.0372	26.5132	25.6357	27.7841	26.7322	27.2233
27.886	27.674	25.8704	25.4685	27.5797	27.7539	28.4978	27.6478	28.4501	28.5634	28.4468	28.3747
25.9849	26.735	26.0418	25.1005	27.1821	25.3438	26.5932	26.7082	23.736	24.6847	25.3619	23.9943
26.6358	26.923	26.3064	26.8505	26.9394	25.7161	26.5377	26.1404	27.223	27.2498	27.6486	27.615
26.4379	25.8658	25.3951	26.7171	27.0703	26.183	26.2943	25.1754	25.5079	25.5307	25.7507	25.5939
27.7839	26.8327	27.0267	26.0471	25.5075	25.0941	27.1462	27.5239	26.9446	26.917	26.6335	26.7138
24.2343	26.157	25.8428	24.3936	26.0186	26.4948	27.5826	25.4945	24.033	25.5768	25.2914	24.0541
24.8903	25.5002	27.1693	26.41	26.6976	26.5061	26.856	26.9173	26.3246	26.3823	27.2564	26.6863
25.198	25.5888	26.9166	26.0327	26.7328	26.564	26.0326	26.8704	26.0935	26.2074	25.8367	26.0399
27.5849	25.4081	26.495	26.2864	26.2895	28.1547	26.0901	26.4004	25.5697	25.4393	25.7027	25.9216
26.8536	26.0893	26.3496	26.3858	27.2546	26.739	27.101	25.6685	24.4623	25.1442	25.5561	25.6613
27.3758	25.6282	25.3894	26.1318	27.4691	26.6703	28.3013	25.2311	24.7921	24.2096	24.771	23.5445
26.4707	25.4721	26.6561	27.1438	26.8107	27.136	25.7088	24.9032	24.073	24.8064	25.9253	23.7263
26.9708	25.4193	26.7315	26.9966	26.2321	26.1746	27.1219	26.7468	25.1811	25.3991	25.4518	25.6801
27.8242	27.8629	26.944	27.438	29.0269	29.1073	28.8437	29.0804	26.4217	24.31	24.4946	25.1555
27.4056	26.1317	26.928	26.0016	26.4085	26.1609	27.5948	26.9187	25.761	24.8647	25.3849	25.2949
26.2643	26.0106	25.4539	26.4035	27.9583	25.5805	26.9057	26.443	25.1115	25.0844	24.6326	24.2051
27.8626	26.0131	25.8933	26.7799	27.0311	26.8199	26.9413	27.211	23.9103	25.8186	26.253	25.8312
24.8652	26.9794	25.8333	24.8529	27.4847	25.4321	27.1863	24.8803	25.0586	24.1894	24.8858	25.961
27.4064	27.6202	26.5389	26.6577	26.3351	26.1013	25.8464	27.3132	25.5488	24.3713	23.6175	23.7341
26.5218	28.2801	25.7357	25.3847	27.8465	26.2164	26.1842	25.1078	26.9216	27.6806	27.1367	26.9198
26.9753	25.748	26.4762	25.745	26.403	26.3805	27.3162	24.8271	25.6014	25.5218	25.837	25.2225

24.397	25.3422	27.4217	25.0786	26.5677	27.1526	26.3975	26.6738	27.5004	27.6234	27.4168	27.6155
28.2433	28.2923	26.3161	26.2507	26.1158	29.0218	27.0457	28.4738	24.0389	24.5954	24.8683	22.79
27.4164	25.6272	25.7727	26.6648	25.7339	26.3326	27.6691	25.0847	26.6076	26.4381	26.7309	26.8836
25.3682	25.7132	26.2595	25.7454	26.5689	27.0269	27.2727	27.0499	25.7367	24.4846	23.6735	26.9841
26.0422	24.6009	26.0905	26.2485	26.6906	26.4696	27.9487	26.2292	26.0407	24.6396	24.8058	25.2228
26.5145	25.7268	26.8119	25.3477	25.4921	26.6654	28.4815	26.3075	27.18	27.125	26.8836	26.9443
26.86	26.122	26.7298	25.6413	26.7779	26.1649	26.2867	26.2513	27.1103	26.9104	26.5028	26.9725
26.4668	25.1522	25.7438	26.6197	26.3702	25.9373	27.8398	26.7016	24.1853	24.5367	24.2517	24.074
27.4982	27.2789	26.6453	25.952	25.4883	26.2525	26.508	25.3879	27.1705	27.2336	27.6718	27.7503
25.0284	25.9009	25.5376	26.526	28.4946	24.4224	25.6449	26.8308	28.3919	28.4957	28.0759	27.8853
25.0031	24.4414	26.2166	24.5266	26.7485	24.4451	26.987	25.6405	25.455	26.7117	26.7075	27.2449
29.9416	30.1653	29.1211	29.3119	29.5516	29.9855	29.3641	29.4458	24.3634	25.0717	23.4825	25.7438
24.7619	24.6778	25.401	23.9253	25.9796	25.973	27.342	26.66	26.633	24.8749	24.8464	24.4929
26.2334	26.8237	26.3696	25.7794	27.4517	28.0751	27.8365	28.352	27.3779	27.5733	27.7975	27.7229
26.2409	26.5626	26.0781	24.9628	25.7544	25.7452	25.8414	27.9225	24.9401	26.2344	24.1869	25.4447
27.7472	25.6128	26.0266	27.0116	25.749	26.749	26.064	27.2684	24.4997	25.2679	24.6324	24.5473
25.2962	24.087	26.85	25.6916	26.0508	25.4567	26.4222	26.3997	26.8151	26.8561	25.7726	26.0774
26.3994	27.1905	25.6332	25.7399	27.574	26.6307	26.3848	26.962	22.6199	23.363	23.4209	24.0553
26.651	25.8263	26.4425	26.4543	26.9148	28.1886	26.8459	25.0707	29.8749	29.7979	29.8569	29.9876
25.644	24.9031	26.8084	24.8433	25.7571	26.3799	27.0203	26.0211	25.4602	26.888	26.7531	26.5618
26.4533	25.7794	26.4258	26.1666	27.57	26.3639	27.3972	26.3841	26.8851	26.8627	26.4924	24.4985
23.8054	24.7017	27.5622	25.8993	25.6472	25.9049	26.9298	27.3319	25.3195	26.8213	27.1149	27.4012
28.0375	27.7028	28.0289	27.4858	26.302	28.7902	27.7054	28.8434	29.9582	29.7861	30.0547	29.9045
27.1832	25.9765	27.1902	25.9433	27.4207	26.8369	25.0397	26.7822	26.6169	26.5424	26.7719	26.7444
25.8867	26.0781	25.833	25.3386	26.4202	25.6978	26.8544	25.584	26.0875	25.9933	26.0041	25.8583
26.4463	26.9	26.9334	26.7082	28.3078	28.4862	27.8135	28.5846	24.8342	23.8856	24.2441	25.9757
25.6122	26.3019	25.9144	26.7307	27.4888	25.3134	27.6253	26.6688	25.2839	25.1832	25.728	25.6572
25.6703	26.5363	25.5068	26.2195	27.0624	25.8959	26.3926	28.8919	24.601	24.2534	25.8231	23.092
25.6654	27.0328	25.8621	25.2471	28.1166	28.0912	24.6326	28.4989	27.2545	27.5456	27.7525	27.826
25.8066	26.6758	26.381	27.4256	27.9051	24.9652	26.142	25.0495	25.229	26.7198	25.8481	25.4073
25.17	27.1871	26.8586	27.306	27.1386	26.9947	26.8773	25.5195	23.3628	24.1137	25.8134	24.7047
26.4337	25.5749	26.5772	25.4103	26.5205	26.4193	27.5548	26.1182	25.8344	25.8895	26.5056	26.113
27.4246	25.5845	26.0897	24.9239	27.6295	26.8738	26.033	28.1495	25.1944	24.4522	25.6569	24.9604
26.1622	25.3789	27.1573	26.626	28.2137	24.3068	24.7996	26.4602	24.4652	24.8759	24.335	24.8643
24.9324	26.2064	27.7042	27.0017	27.0117	25.1225	27.8098	28.3879	23.7782	25.2921	25.814	26.0067
25.734	27.3833	26.5495	26.701	27.0879	24.4069	27.7078	26.0189	23.9103	26.7256	26.4848	25.514
26.4419	27.0298	27.4827	26.5595	26.1009	27.3608	26.1248	26.8903	25.2464	25.1105	23.6042	26.9093
26.1519	25.3432	25.9639	25.646	26.9606	28.3484	26.0297	26.0322	26.3212	26.3548	26.7894	26.7884
26.0017	25.5337	25.0208	26.0537	25.7849	26.4852	26.2414	27.0861	24.189	25.7777	25.0494	25.1322
29.6973	29.9831	29.9519	29.4866	27.5644	29.4712	27.3516	29.6143	25.5184	24.7306	24.8639	26.4981
26.3847	25.6845	26.0384	25.3379	27.7149	25.3479	26.2816	27.1816	26.5229	26.4061	26.243	26.4786
25.6829	27.6409	25.4858	25.1933	27.8638	25.534	26.281	24.7022	25.8622	25.5542	25.7783	25.9504
26.0184	25.8327	27.3965	25.8354	26.9839	27.572	26.3758	24.355	25.3493	26.5708	24.8453	24.3581
25.5141	26.9788	26.3539	27.1215	28.2721	25.7056	27.6382	28.1732	25.0263	25.2893	24.1955	24.3791
25.4339	26.228	26.0712	24.8393	28.0005	24.5931	26.0817	28.087	24.3031	26.3582	26.6592	26.6167
27.2413	28.2172	26.3082	26.017	29.201	26.1546	26.303	27.6305	25.5532	24.4127	23.7265	25.6523
26.8177	25.059	26.9775	25.2099	26.2411	25.9926	26.4097	26.2357	25.8047	25.5941	24.166	24.9059
25.7002	25.3406	28.0713	26.5248	27.1277	26.199	26.4174	25.318	24.1608	24.6554	25.2661	25.8536
26.8702	26.9591	26.1948	27.157	26.4046	26.3556	27.0101	26.5636	24.1671	24.9386	24.9094	24.6519
29.4518	29.4427	28.9646	29.3312	28.7168	29.6886	25.9014	28.9588	25.0927	24.578	25.0655	24.8097
26.4766	26.8861	25.852	26.2134	26.4016	25.4097	26.4074	26.4768	26.8122	25.9005	26.8655	26.4515
25.529	28.3605	27.3722	27.1568	27.1783	28.4748	26.7026	27.3227	27.2266	27.6017	27.1907	27.1982
25.723	25.9497	26.1279	26.5658	27.4824	25.4356	26.1696	26.6795	26.3655	26.1479	25.7143	25.6896
26.7595	25.3014	26.4016	24.4358	25.4995	26.6675	26.1075	25.5985	23.9722	22.3799	27.027	27.0164
26.5539	26.0089	27.1833	26.8024	27.0261	24.9168	26.2885	25.9161	25.6826	25.9871	25.7141	25.9291
25.3295	26.0333	25.0994	27.781	27.0519	26.9129	27.5221	25.2237	27.0017	27.497	27.3289	27.1551
26.9632	25.4281	26.3391	26.4712	27.4273	26.0773	26.6404	25.5842	25.7571	25.9869	26.6118	26.0451
25.1489	23.4608	26.6767	26.3451	27.4385	27.5365	27.0689	25.6651	25.9428	26.3766	26.2719	26.2751
25.9955	28.4483	28.9944	25.7814	26.6865	25.8908	28.1117	26.033	26.9068	27.1847	27.0749	27.1907
25.871	26.5716	25.6443	26.2518	27.766	25.0113	26.6056	26.4648	27.153	27.4787	27.2792	26.988
26.0798	24.56	26.3812	25.667	26.257	25.2147	26.4616	26.2846	26.3456	24.1246	25.7193	24.1871
25.2572	26.751	26.9953	26.816	27.1049	26.5732	27.8591	25.5155	26.687	26.9446	26.9069	26.9125
25.0132	25.6378	25.778	25.275	25.134	25.7135	25.6079	27.5134	23.3915	24.8168	24.5731	25.0971
26.7154	24.9863	26.5886	26.2885	29.0869	26.4514	26.438	26.2086	24.809	24.9956	24.3185	26.3619
25.1075	26.1625	26.6039	26.1416	26.1416	26.1584	25.9728	27.1961	26.4812	28.4395	28.0724	28.5108
25.9009	26.6103	27.4098	27.173	26.1781	25.4784	24.5771	28.502	27.5787	26.612	26.9233	26.7279
29.0165	28.8296	28.0121	26.0655	27.8695	29.0202	27.2639	29.7178	28.8574	28.8096	25.4987	26.408
26.1231	25.8009	27.5452	26.4286	27.0066	26.3159	26.2527	26.2272	27.61	27.6805	27.1522	27.6681
27.3549	27.2513	25.6923	24.9741	28.204	28.1241	26.5411	26.2722	25.7986	25.9044	25.9969	25.9369
26.1459	26.7531	27.0931	24.5327	27.6049	26.1027	27.3663	27.0601	24.2505	24.6555	28.0639	24.5248
25.6515	26.6139	25.4212	27.4747	26.6448	25.608	26.3378	25.2935	26.0324	26.1816	25.7463	25.9208
26.544	26.7613	26.7501	25.3622	27.8635	24.5025	27.3936	26.5311	26.4942	26.4219	26.6159	26.5224
26.0439	27.9122	27.684	26.6993	26.4769	27.4751	26.8761	25.5182	25.0494	23.4895	25.7939	25.4855
27.3153	27.5739	25.8605	26.2759	27.9029	28.3314	27.5989	27.7728	24.1854	24.5874	23.826	23.8307
25.1672	24.0822	26.0674	23.9108	25.9079	26.5507	25.497	26.0575	26.1631	26.2088	26.346	26.1461
25.5566	25.8644	27.8901	26.6956	26.2958	27.0178	26.4913	25.6932	25.7618	25.1648	24.1954	24.5646
27.3316	25.3437	26.0894	25.719	26.6007	26.6251	26.5726	26.6175	24.3205	23.8485	23.3798	26.4498
26.648	25.8425	26.183	26.6604	25.9975	26.5229	28.005	26.3487	25.5738	26.8819	26.3547	26.094
27.9203	27.9083	27.4475	27.5933	28.602	28.3419	28.4833	28.5984	25.2953	25.5762	24.5326	24.8101
26.368	25.6452	26.4938	27.0713	27.2168	27.0057	26.4265	27.2957	25.6124	25.5366	26.6118	26.7372
30.7177	30.8032	29.6116	29.825	30.5334	31.0313	30.7467	30.5575	28.476	28.4869	28.4848	28.7131
26.772	27.1592	27.8157	28.113	26.8058	25.2154	26.991	26.4989	27.492	27.4915	26.9859	26.9841
28.4724	28.3637	29.2792	29.0928	28.7588	28.403	28.6228	26.1653	27.3621	22.3725	26.1764	25.4794
26.2403	24.6285	25.9502	26.3102	25.8762	26.4442	27.4151	25.0443	25.3631	25.1373	26.5653	26.1488
25.4027	27.1677	25.3821	25.2299	27.1659	28.0866	28.3881	25.9233	26.6243	26.6063	26.5527	26.6138
28.7227	28.2288	27.544	27.4489	27.4628	28.7011	26.6082	28.0044	24.1987	25.1845	24.152	24.5302
26.788	25.8354	26.4024	26.4071	25.1653	25.5401	27.2304	24.8931	24.7729	26.5609	26.0518	25.4019
24.4538	27.4746	25.5513	25.2312	27.8021	24.7787	26.7516	27.0977	24.9255	24.6689	26.3135	24.0546
27.0088	28.9486	26.177	28.8044	26.6451	27.6929	26.45	26.7165	29.1955	29.3915	29.2093	29.1204
25.0053	26.1985	24.7542	24.6901	26.7656	25.7568	27.2835	26.4014	26.5528	26.5712	27.4869	27.3472
25.804	26.2492	26.6035	26.4255	26.5001	27.2162	26.3673	27.1559	28.3741	27.9558	27.812	27.6329
25.7337	25.7057	27.2014	27.0817	26.571	26.2296	27.2646	27.1276	27.0711	27.1603	27.3032	27.3351
29.5819	29.55	29.4293	29.4823	29.7754	29.2989	29.8097	29.4184	24.8485	25.1701	23.8354	24.681
25.9109	25.3546	27.279	26.5224	27.5742	25.6607	27.5288	26.1011	25.4079	25.555	25.0493	24.831
26.7594	26.5991	27.8649	25.7588	26.8099	27.4271	27.5733	26.1091	28.1659	28.2639	27.983	28.1566
25.8084	26.4903	26.818	26.397	25.7968	26.5401	26.9041	28.5224	26.2889	26.2811	26.4274	25.9192
25.336	26.6105	26.083	25.5394	26.9301	26.196	25.8003	26.2562	27.1203	26.742	26.7972	26.9592
26.2686	28.1961	27.9738	25.6604	28.3055	27.8345	27.5081	28.1214	28.5881	28.5846	28.2557	28.7368
28.4416	27.9874	26.6428	26.0213	29.9091	28.4111	29.9495	25.7207	30.6911	30.3983	31.6041	31.4333
25.4306	25.2008	27.5803	24.7255	26.7959	26.8399	26.2495	25.877	26.822	26.8348	26.3954	26.8215
27.2144	25.6022	26.2865	25.5763	27.5607	25.4947	27.4231	26.2002	24.9635	26.8771	28.0679	25.4507
26.6567	24.8779	28.1646	25.7321	24.938	26.8179	26.7379	26.5559	24.9823	23.8914	25.1603	24.521
25.1486	27.1243	27.7271	27.0298	25.6452	26.9418	26.9004	25.8974	23.9139	27.7579	27.9677	27.8535
26.3934	26.915	26.0772	27.2909	25.6211	26.6438	28.5324	25.82	26.3299	26.0082	26.452	26.0755
25.5899	26.9328	27.9515	27.599	25.8125	26.3293	25.6525	26.8821	25.0817	24.7182	25.0672	25.1063
25.7377	26.5922	26.5343	26.0479	26.8486	26.3622	26.747	27.6188	23.9974	25.3063	24.8309	25.0486
27.5475	28.2074	28.7182	27.8984	27.2313	28.7731	26.1746	24.7852	28.5225	28.6327	28.5682	28.5543
25.8617	26.4011	24.8992	26.8215	27.1339	26.4089	27.5962	24.1565	26.8943	26.7591	26.6654	26.5797
27.5538	27.9753	25.5421	26.7698	26.7539	26.2365	26.3375	26.8781	28.4176	28.8288	28.0992	27.9972
26.4631	25.5804	26.7537	26.1162	26.9704	27.1141	27.015	25.0107	27.446	26.9491	26.8367	27.1154
26.847	25.8248	25.4409	26.3964	25.8946	26.3749	26.4013	26.3695	26.6077	27.3252	27.3277	27.3359
27.8551	26.0197	25.5883	25.4483	27.6739	26.6121	27.7124	26.1494	26.2142	26.4134	26.834	26.9602
26.0132	26.0149	25.3142	25.6926	26.9458	25.3449	27.8718	27.3113	25.6531	26.2858	26.3148	26.6474
27.7549	26.0495	26.4983	26.9692	26.0507	25.7382	25.4472	27.0708	25.4818	25.401	25.9285	25.8614
25.5555	25.8542	25.3717	26.7506	27.3183	24.8156	27.0933	27.3055	26.4112	26.4692	27.1074	27.0979
25.2826	26.32	26.5048	25.6914	28.1811	25.557	26.8328	26.745	23.9949	25.8557	26.3323	26.385
27.1289	26.288	26.362	25.9667	28.2236	25.9242	26.949	27.4316	27.8465	27.7118	27.5878	27.4918
24.4626	26.6378	27.4405	24.8476	26.2903	26.4923	25.7759	26.8844	24.5361	24.3905	24.581	24.9543
27.8839	27.5397	27.4141	28.0085	28.0523	28.393	28.3654	27.5703	27.7439	27.485	27.3947	27.4388
28.6538	28.6378	28.7802	28.8626	28.4786	28.8649	28.4728	28.4176	29.6403	29.5945	29.9479	29.9856
26.3287	25.0087	26.6343	25.281	25.7501	27.041	26.5983	27.1298	27.544	27.7478	27.6743	27.5454
26.2607	26.9079	25.7048	26.8032	27.2791	27.0937	27.5589	27.5505	26.5421	26.3283	23.6219	26.317
26.0537	25.6509	26.4701	26.2283	26.3377	25.7109	26.6237	27.1135	24.7532	26.3063	26.0641	25.8468
26.2512	26.1628	25.6481	26.8668	25.9165	27.8518	26.7938	25.7843	26.4554	26.1649	25.975	25.9006
24.23	26.3654	24.9399	25.9325	27.9176	25.5186	26.0571	27.6394	24.7932	23.6135	23.7598	24.7508
28.9979	27.0091	26.9543	26.8622	27.0985	26.7243	25.8838	26.5098	27.7639	27.6239	27.8516	27.8252
26.6228	26.3466	26.1905	27.365	26.9774	26.8431	27.0279	25.9133	28.1371	28.3626	27.8938	27.9397
26.409	24.3767	26.285	24.8458	26.8463	26.1874	27.5659	27.9339	26.6103	26.8711	27.0382	27.2076
30.0716	30.217	29.1692	29.4527	32.0403	33.068	32.0256	32.9889	27.1986	27.2834	27.3307	27.3158

29.3779	29.6736	29.5526	28.9073	29.1082	23.8922	28.9444	28.416	23.9005	24.8205	24.2335	26.2188
27.6695	27.7084	26.8587	27.0832	25.3096	25.7512	25.2793	26.3075	28.9082	28.8243	29.262	29.2601
28.7541	28.7163	28.9197	28.745	28.2121	28.488	28.0013	27.7262	23.5285	23.944	25.2978	25.2442
25.5054	28.0159	25.7819	25.5404	27.1104	26.3704	26.3736	26.3096	27.4649	25.088	24.1372	24.6393
28.3599	28.1889	26.2527	25.3066	27.8797	29.0107	27.9313	28.9909	25.6465	24.7734	24.7429	22.9453
26.6388	28.5674	28.7016	28.4718	27.2607	28.2294	28.5776	26.6223	27.9182	27.6631	27.7396	27.8101
26.6316	25.5989	27.2001	26.5241	25.3496	26.8493	26.4665	25.4141	26.8735	26.7974	26.8612	26.9448
26.013	25.5483	26.3388	25.8695	25.6426	25.7614	28.3297	26.8634	25.3862	24.8064	24.7222	26.0744
25.916	25.0223	25.8025	24.5445	25.5192	27.1614	26.4948	28.8275	24.5322	24.4818	24.8418	24.191
25.9952	24.8519	26.6262	26.1485	28.706	25.7154	26.0297	26.6532	25.6344	25.5628	25.8931	25.7071
25.0784	26.0722	25.1914	26.7766	26.5778	30.1558	26.5896	25.9991	28.2066	27.9048	28.4584	28.1267
28.406	28.6367	26.1259	26.274	26.022	26.2723	26.2368	28.0562	25.279	23.9858	25.1534	24.9488
26.3187	26.0185	25.5052	25.5577	26.3096	26.3284	27.0777	24.7325	26.9286	26.7787	27.9571	27.5841
29.1255	28.9015	28.5231	28.5611	30.0493	29.985	28.5211	29.8124	29.5122	29.4568	29.3712	29.4514
26.2641	27.1518	25.0775	24.9622	26.339	26.5664	25.5911	24.976	26.275	26.4286	26.4866	26.4211
25.4037	26.6877	26.0602	27.0583	26.178	26.7965	26.6336	26.0235	27.5557	27.4652	27.5396	26.9426
25.1649	24.8102	26.7594	25.7878	27.0358	28.4979	26.5845	24.6347	25.6376	26.4681	25.4379	24.4822
24.245	26.2488	25.2498	25.5682	26.2939	25.9551	26.7963	27.1127	26.9901	28.0868	26.2227	26.2594
24.3906	26.7719	26.0539	26.9678	26.1582	26.345	28.4691	26.334	26.2383	26.8326	26.2149	26.4155
27.4079	27.3317	26.9061	29.3443	27.5541	27.9141	27.3339	28.1542	27.8361	27.8231	28.0456	28.0482
25.0727	27.9223	28.5696	28.2425	26.8562	28.7416	24.8045	28.9158	28.3834	28.4284	28.6341	28.4298
26.5436	26.1477	25.8909	25.9998	27.202	25.8839	27.0464	27.2829	27.0022	25.5428	26.7685	27.0138
25.4729	26.5561	27.0513	26.0774	26.261	27.0292	26.9195	27.3552	27.3958	27.2598	26.931	26.8265
26.3091	25.3613	26.8928	25.944	26.2879	23.5813	25.7198	26.194	26.4375	26.6702	26.9897	26.5823
25.6845	24.7807	26.5292	26.9642	27.7818	26.3696	24.926	24.9043	26.2051	24.3816	25.5294	25.3212
29.4758	29.6325	29.639	29.3342	27.1667	29.4395	29.2574	28.8358	26.4938	26.364	26.596	26.7957
28.8985	28.8918	28.6411	28.8417	28.2893	27.6697	27.9095	26.3716	29.8205	29.8078	29.9715	29.8992
28.0908	28.2345	26.2847	27.5299	26.5142	27.9543	26.64	27.9498	28.6098	28.5507	28.9405	28.9109
28.3639	28.4113	28.8701	28.389	27.5967	28.1512	25.8967	28.7393	28.6759	28.6503	28.3645	28.1616
27.0899	24.1188	25.4955	25.9118	27.0897	25.5442	25.9174	27.28	24.2716	22.8019	24.2821	24.0682
27.8736	27.0374	26.392	25.582	25.7852	28.5247	27.5618	25.1506	26.8551	27.037	27.2027	27.2592
26.6042	26.5925	26.4885	27.016	26.5705	26.231	27.4239	25.9049	26.5988	26.656	27.2923	26.972
25.9159	25.5694	26.428	25.9987	26.9243	24.6447	26.8658	26.2991	23.9272	23.9645	25.6777	25.6502
26.6886	25.5569	26.1738	24.5187	25.5337	25.333	26.4943	26.1022	25.1244	25.1378	25.2032	24.867
25.4982	26.895	26.4486	27.4073	28.7545	27.5413	25.2795	26.6914	27.5131	27.3064	27.3156	27.4061
26.2606	26.7073	26.5588	26.4932	27.7201	27.5039	27.7492	25.946	23.8884	24.107	25.4228	24.9719
26.6462	26.1471	23.9852	27.2439	25.6882	27.0483	24.9148	25.5767	24.7446	25.7032	26.0559	24.5765
27.3112	25.639	28.6896	27.9023	26.2916	24.8905	26.7582	26.2699	27.8979	27.7072	27.6386	27.4066
27.4824	27.2052	26.7385	25.6734	25.5252	25.7901	26.7491	26.5779	29.5935	29.3392	29.5895	29.6597
26.7842	24.726	26.5175	25.4889	26.7146	26.2153	27.0293	26.2931	25.6175	25.7781	26.8389	25.7933
29.557	29.8364	28.6611	28.4104	29.3761	30.4337	29.4605	29.2088	24.1092	24.3433	22.9895	24.9685
26.6689	25.3559	28.0099	28.1237	26.4917	28.2993	26.0477	26.9377	28.803	28.7285	28.5635	28.7305
24.3659	26.3215	26.8288	26.6983	27.3248	26.4013	26.1219	26.8856	23.1111	25.1028	24.8674	26.1554
25.6115	25.7973	26.0672	25.7831	27.44	26.8757	26.8353	25.5123	26.4818	26.5952	25.9954	27.8722
26.0131	26.7749	24.1141	24.8358	26.9707	26.6791	27.0768	26.8266	23.8958	25.4824	23.5996	24.6757
27.0155	26.1945	26.5094	26.5285	26.4277	25.9957	26.9795	26.0316	27.1509	27.4384	27.4192	27.5158
24.9512	27.1456	26.6677	24.7431	27.1866	25.8335	27.3563	25.4875	24.9241	24.3781	26.0157	26.4403
26.5269	25.9449	27.3696	25.825	25.8147	28.0819	27.8644	25.5244	26.4402	27.4095	26.0201	26.184
27.4251	25.9162	26.1055	26.5528	26.7588	26.9787	26.6378	26.0195	24.1274	24.9936	24.3805	25.2724
26.1062	27.6942	26.0886	25.1893	26.819	25.2702	25.9203	27.0651	27.7988	27.8515	27.6105	27.782
25.9641	25.3293	26.0968	26.0603	27.4384	27.1684	27.8644	27.7223	28.0901	28.025	27.5643	27.9411
24.9598	25.774	26.7441	25.3719	26.6951	27.6078	27.213	27.0415	25.7532	26.0496	24.5797	25.6381
26.3034	26.3957	25.9566	25.6959	27.7262	26.6728	27.6508	26.1487	27.2517	27.1705	26.9211	26.7537
28.4779	28.4779	28.5334	28.9633	28.3461	28.446	28.447	28.1281	27.6438	27.5894	27.9602	27.9086
28.1504	28.0297	28.3178	27.9991	26.4644	27.5801	26.0164	26.6005	29.1269	28.9694	29.0515	29.1228
27.6307	27.3608	27.819	27.2851	27.3714	27.1053	27.57	25.4313	30.0852	30.08	30.176	30.0245
26.2352	26.0451	26.5876	26.7524	25.2035	25.6743	27.2448	26.2312	26.9688	26.6711	24.9482	26.0624
25.8763	25.5658	26.179	25.8476	26.3152	27.4003	26.3404	25.5576	25.3311	25.8161	26.042	26.47
25.5518	25.468	27.3646	25.8875	26.2614	26.0822	27.591	25.8583	29.7557	29.56	29.7588	29.7558
26.7616	24.7329	26.8494	27.8917	27.1667	26.6084	27.3339	27.0039	26.3392	26.6556	26.545	26.8121
27.0714	26.3201	25.6658	25.3436	27.902	27.0148	25.847	24.8785	26.4933	25.7748	24.6333	24.3971
27.1835	25.7416	26.814	25.3236	27.4227	26.0152	26.4819	26.521	26.2651	26.311	26.5642	26.7558
24.1952	25.287	26.683	25.5586	26.8197	25.3453	27.0151	27.3168	25.6884	24.5133	25.8361	24.0739
26.3969	26.7089	25.8123	24.8736	27.4959	24.9506	27.1208	25.3469	24.6137	25.5651	23.7626	24.5201
26.0587	25.3189	26.7782	26.9612	25.6824	25.6369	26.7167	26.7284	24.709	25.68	25.9388	24.5544
24.9114	25.4597	24.2878	25.6951	26.1198	25.4685	26.5869	24.5935	25.9335	25.5523	25.9203	25.1884
26.6764	27.0559	25.9753	25.9919	26.5426	25.7092	26.2803	27.6961	26.916	25.173	24.1694	23.2073
26.1389	25.3152	27.1287	25.8597	26.8021	26.5398	26.159	25.6226	26.8186	26.6103	27.0444	26.8982
25.5856	27.1379	26.33	30.5368	26.6979	25.2065	26.6176	26.3585	27.9242	27.4012	27.2153	27.61
27.0569	25.8916	26.5074	26.0174	27.7423	26.0688	25.4635	25.0396	24.5028	24.7427	25.2682	25.891
27.7093	25.4244	25.9432	26.1749	26.8941	25.5787	25.9605	26.7241	29.2936	29.0745	29.3637	29.2959
25.295	27.0003	26.7174	25.9459	26.52	25.7901	26.5584	26.4907	26.4586	24.7057	26.3263	26.877
27.0204	25.5705	26.807	26.3831	27.0384	27.1446	26.8042	26.3312	24.4041	24.0738	23.7706	24.2318
27.5531	25.8893	25.6934	25.444	26.4661	26.7355	26.4596	24.7486	22.7659	26.5979	24.7779	24.5256
26.7694	24.9715	27.2531	26.8741	25.9023	25.9379	26.3081	26.0243	26.9893	26.713	26.932	22.0619
26.7562	26.7795	26.4163	26.4254	26.5136	26.1108	28.0805	25.8859	27.8584	28.01	27.8853	28.0211
24.2604	26.2684	27.3736	26.6878	26.9623	26.6775	27.9818	24.7834	27.1029	26.9587	26.7591	27.0179
27.4704	25.7181	26.97	26.4942	27.0162	26.1735	27.2754	27.8064	25.8824	25.7424	25.4811	25.7778
29.2172	29.2713	29.5242	29.3418	28.724	28.9929	28.604	28.9925	28.354	28.3168	28.6374	28.5012
25.5616	26.6152	25.6787	26.55	26.5594	25.5845	25.5803	26.919	26.2032	26.3474	26.4135	26.4891
26.9314	25.157	26.0182	26.5315	27.6554	25.8917	27.226	26.2143	27.8814	27.671	27.7355	27.7357
26.4804	25.3207	27.1881	26.428	27.5177	26.7554	26.094	28.3304	24.7043	25.2237	25.2176	25.4734
25.3289	25.534	26.5281	25.5962	27.0072	26.2729	27.185	26.0175	26.6979	26.0566	26.4331	26.3002
27.263	25.7782	26.2638	26.5327	26.5015	26.7434	26.7145	26.6682	25.2988	25.6981	25.4495	25.8728
26.7395	25.1515	26.3393	27.2194	25.5855	25.2379	25.7757	26.4814	27.7461	27.4588	27.3122	27.365
24.5982	24.5731	26.086	27.1075	26.7823	26.904	27.0853	26.7916	26.3144	26.5586	26.8526	26.6591
26.0074	24.7999	25.5706	26.0272	26.0431	25.8457	26.4836	26.3569	24.8224	25.7653	25.2845	24.9112
26.9571	24.8084	24.9331	26.3969	26.752	26.713	27.899	26.4235	25.5344	23.1532	24.3951	24.3067
26.192	24.6387	27.0085	25.3761	27.2232	28.2358	26.8926	27.3622	25.107	25.4354	23.5978	24.7235
26.54	25.9216	26.6756	26.5429	26.2886	26.1784	26.9918	27.0702	23.8729	23.287	24.5637	24.9364
25.9721	25.3701	26.5388	24.6512	26.7721	26.9584	27.4069	26.3659	26.5329	26.5585	26.603	26.6537
26.2921	26.5241	26.4078	26.205	26.8206	26.8121	25.6518	26.1633	26.8642	27.0984	27.5479	27.2308
26.2851	24.8119	26.5889	26.2143	26.3727	25.8898	26.1237	26.6834	26.2496	24.0993	25.1046	26.3927
26.7958	26.4683	25.9441	27.6508	26.4542	27.1622	26.9979	25.9615	26.943	26.8199	26.1186	25.7678
26.315	27.8161	26.0519	26.4979	28.03	26.0101	27.0812	25.8841	28.6196	28.8911	28.1163	27.9809
26.6626	25.2216	26.338	27.0705	25.309	24.6287	26.3284	27.1512	23.5179	24.8839	25.3513	25.1886
27.1455	26.392	25.6475	26.0775	27.4731	27.314	26.5934	23.5939	26.6018	25.6419	23.4231	25.2605
26.1255	26.7545	25.4522	25.2148	25.9037	26.8753	26.8799	26.2365	26.0378	26.6114	25.0623	25.9177
25.4943	27.4803	26.2539	25.3381	27.4246	26.3658	26.5833	26.5465	24.9695	23.4173	24.7135	24.0731
27.9746	27.3525	26.0101	27.7165	25.0701	27.0369	27.5729	24.5932	26.3606	23.9883	24.8813	24.1152
27.8356	24.5327	26.3838	25.3184	25.6743	27.1913	27.7471	25.5174	26.0294	26.1303	25.6594	26.1754
27.8567	27.567	25.8448	27.465	28.1658	27.3337	28.1334	27.5601	27.0684	27.0075	27.0947	26.5211
27.2402	25.9777	26.5581	25.7458	26.3769	25.7048	26.3745	26.7419	24.4938	26.8209	27.1816	27.1701
27.4365	26.102	26.0427	27.1988	26.8351	25.0177	26.9068	26.4091	27.2937	27.5658	27.805	27.4514
25.3504	26.1765	27.7359	24.9995	27.3863	26.3717	25.6591	25.487	28.6366	27.7659	24.8803	28.1697
26.8741	26.2412	26.3644	26.3568	26.258	27.3814	26.9193	26.3829	25.7445	25.041	24.6681	24.2565
25.7906	25.8036	26.6863	25.3997	27.3389	25.7213	26.896	26.2379	24.284	25.334	25.6185	24.3398
25.4289	25.7625	25.1582	26.8775	27.2379	25.2087	25.9063	25.9007	26.8772	27.4752	26.7114	26.3189
25.3285	25.5241	26.6409	25.7754	28.3602	26.1585	27.6588	26.588	24.6895	23.1991	24.7141	25.0735
25.567	26.594	26.0182	25.8491	26.6195	25.6163	26.27	26.088	26.0591	25.8551	24.8843	24.9966
26.0096	26.0435	26.2859	24.8694	28.2359	26.1102	26.3716	26.2204	25.9697	26.24	26.5273	23.8362
26.2201	26.3766	27.5618	27.2485	26.3166	26.8568	28.1172	26.8984	25.3255	25.6779	25.5865	25.763
25.9012	26.8515	27.0711	25.3572	27.0624	25.1621	27.4473	26.6967	26.5759	24.6062	25.0699	25.6553
26.7599	25.8346	25.9365	25.4631	25.6025	25.7801	26.5902	26.588	23.5673	24.0622	25.2746	25.2086
26.303	25.6122	24.708	24.7551	28.8893	27.6857	27.1716	26.0575	24.8137	23.7697	24.4089	24.4622
27.4399	27.0589	27.3988	27.4635	27.2048	27.1365	26.8432	27.2725	28.4665	28.2881	28.5789	28.9452
25.8294	25.8907	25.4845	26.5505	27.8018	25.0356	27.1695	27.1057	27.8761	27.7405	28.0941	28.0876
26.139	26.6906	25.2265	24.2688	27.2298	25.6288	26.1161	26.8027	27.0493	27.1974	27.6593	27.8906
25.5477	26.0887	27.0642	25.7297	26.7045	26.8989	26.4852	24.7568	26.3333	26.0648	26.4731	25.0591
25.5233	27.9989	27.7694	26.6498	26.2145	25.8039	26.7233	26.1811	28.6435	28.6863	28.9237	28.97
27.5635	25.1531	25.9899	25.7611	26.2067	27.5891	26.6702	26.5162	24.9162	23.9439	25.6476	25.5959
26.5884	26.8066	26.6517	26.219	28.2973	25.0689	26.975	26.1576	27.2507	27.6405	27.6263	27.6204
27.0625	26.866	27.6444	24.7889	27.0552	26.0513	27.4853	27.5967	26.6926	26.8094	26.6164	26.6886
26.0731	27.0973	28.7144	27.3446	27.4112	27.9182	27.3464	27.1149	27.8282	27.7193	28.1457	28.0769
25.5271	27.849	25.0083	25.2615	27.3146	25.3951	25.4784	25.8527	27.8058	23.9791	23.0819	25.7011
27.0415	25.6015	25.3196	26.2418	27.9753	25.4184	26.574	26.7314	25.1033	25.4786	25.5302	25.8356
26.1949	24.5776	25.7782	26.3342	25.88	25.9353	26.8698	26.0013	26.8806	26.5914	27.6332	27.7118
26.343	24.5852	26.6483	27.1804	26.6675	26.9202	27.0616	25.7589	25.9718	23.8207	23.6705	23.0983
27.225	25.2772	26.2911	25.3913	27.0793	27.4497	28.0442	27.4487	24.843	26.2812	25.1489	24.9051

26.9013	25.0935	25.9627	28.9013	26.267	26.298	27.2548	26.3775	26.4727	26.3463	26.9049	26.5672
27.0164	25.3339	27.0085	25.8687	26.6137	25.5171	27.399	26.0716	24.6037	23.3321	24.8294	25.1759
24.3479	25.5647	27.4093	25.5313	25.567	24.8285	26.4653	27.5631	25.2907	25.2347	25.2114	25.4623
26.6538	25.2986	25.4829	25.1095	25.823	24.8181	26.1705	25.3887	26.2941	26.2545	26.0364	25.4349
26.2375	26.8342	26.3859	27.2556	25.6774	27.9396	26.7485	27.984	28.4555	28.4368	28.2108	28.5062
26.172	25.6053	25.3493	26.2675	26.0398	26.981	26.3019	26.8461	26.6256	25.3381	25.2077	25.1449
27.3179	26.9978	27.0281	25.8721	26.2391	26.3261	27.7015	27.7341	23.4581	24.4576	26.5977	26.5483
26.5985	26.2402	25.6701	25.7015	26.9571	26.3342	26.4121	26.9729	27.1477	27.3379	27.1292	26.9359
27.9728	27.8089	27.5965	27.8331	28.0433	28.0021	27.3569	26.3114	27.2103	27.2991	27.1092	27.4313
25.9602	27.4447	26.3381	27.0455	26.9123	27.1189	26.5486	25.3929	23.5308	26.2583	25.9606	25.3585
27.8744	28.0344	27.7855	27.6227	26.5032	27.6548	27.3944	26.2261	28.277	28.348	28.5649	28.5213
25.8063	26.3564	26.0573	25.1742	26.6572	26.0087	27.5809	26.1252	22.8367	23.9149	25.7778	24.3244
25.793	26.5123	24.9208	24.6214	26.468	25.3769	27.5309	25.9396	26.2979	26.5539	26.2405	26.2505
26.6913	25.6803	26.5814	26.0332	27.8673	26.8835	27.0847	26.8782	25.4467	26.0451	26.1957	26.0059
26.9217	25.9573	25.7387	25.5908	26.2045	25.5261	25.7911	25.6668	25.5839	24.6877	25.2349	24.9609
27.6545	24.6219	25.8158	25.7809	26.9953	27.7043	26.6802	26.4498	28.245	28.1833	28.3981	28.3369
26.918	26.3561	26.2564	26.5152	25.8983	25.8149	26.9916	27.6236	26.6283	26.2296	26.0942	25.9297
26.4643	24.641	26.8678	26.5488	26.2205	26.557	26.3835	27.3037	27.1812	26.286	26.3414	25.8855
26.1668	25.9573	27.4667	25.7848	28.1193	26.0426	26.1004	27.1244	23.5851	24.7536	24.4519	23.9476
26.0225	24.9843	26.4875	24.6171	26.0716	26.5921	25.0373	25.7696	25.0567	25.7313	25.4738	25.207
25.339	25.7143	26.4061	26.4304	27.2129	25.6293	26.6744	25.4985	25.8293	25.9801	26.2099	26.3873
25.8487	24.6207	26.1454	25.6518	26.6341	27.3736	26.6051	26.3758	24.5315	23.9276	27.7526	28.2283
26.3661	25.7147	25.6797	27.5734	26.6918	25.696	26.1475	26.3463	25.7263	23.7682	25.3705	23.8656
25.4024	25.9795	26.7242	25.7285	27.8462	25.2092	27.459	27.0815	24.6616	26.3753	25.6431	24.9647
26.9706	27.6183	26.0363	26.8705	26.5894	25.7533	26.4225	26.2941	27.6187	27.6257	27.8782	27.9074
26.2006	24.7866	27.6113	26.7147	26.0209	26.1099	25.8165	28.4196	28.649	28.6666	28.5345	28.6223
26.4988	25.4742	26.4081	25.4641	26.4372	26.8868	27.5866	25.5611	27.5486	27.9069	28.0629	27.8405
25.7653	24.295	24.5719	26.4738	26.4037	26.0192	26.914	26.9209	24.2362	24.152	24.184	24.529
26.547	26.3603	26.9494	26.7952	27.7533	26.4801	26.7936	26.1229	27.4961	27.4793	24.8197	27.0078
29.6325	29.4085	28.7519	28.9307	29.5791	29.3898	29.5093	25.9925	24.6601	24.3707	25.0039	27.1061
28.5233	28.5066	28.1528	28.2771	27.9963	28.2667	28.1859	28.1838	28.0058	28.1915	28.4349	28.4667
27.4945	27.6288	27.709	27.3661	26.5838	28.3044	25.3842	28.4047	27.6841	27.7986	27.49	27.3227
27.6702	25.34	27.6382	26.6564	26.3682	26.5049	26.3102	27.7815	26.0177	24.6366	25.7139	25.7055
26.589	25.9067	26.5898	27.1505	26.9993	26.9694	27.4146	25.7555	25.1214	24.8833	24.9627	23.648
27.84	26.4357	26.8277	25.108	26.5674	25.6439	26.2487	26.1635	23.7522	24.8929	25.4741	27.6524
26.5795	27.4386	25.6438	25.2415	26.6862	26.8212	27.1271	26.6285	25.7204	26.3088	25.5654	25.6687
26.2054	24.3949	26.3664	26.1355	26.4718	26.48	27.5754	25.804	24.1011	24.9392	25.974	26.637
25.9458	25.5252	26.0013	26.6432	27.2276	25.8427	28.0257	25.9564	23.2165	26.7316	26.8202	26.7058
27.8464	27.3564	27.5735	28.371	28.6869	27.3688	27.0562	27.4647	25.975	26.0702	25.7394	25.6999
27.1184	25.7293	24.9424	25.7028	27.0704	25.4175	27.9373	25.7808	34.6064	34.7814	35.3812	35.4528
25.3395	24.5347	25.507	27.5787	25.078	25.5679	25.6909	26.1126	25.2668	25.1606	23.6963	23.8009
24.8956	27.3655	25.8442	27.0696	28.0683	25.7607	26.9303	25.9621	27.7051	24.679	25.5656	24.4794
25.4043	24.763	25.9763	24.9816	27.061	25.4083	27.3371	26.2084	25.8933	25.8821	25.7598	25.7603
26.0384	26.0379	24.7802	25.3521	24.4745	26.704	27.355	26.3539	27.1325	26.4481	26.0718	26.8494
25.8979	26.1904	26.4432	25.326	26.7382	27.2455	27.0677	25.2004	25.5188	25.044	24.5331	25.2202
25.2891	25.3931	26.165	25.5639	25.1245	25.7024	26.1026	25.459	24.0534	24.7415	26.2213	26.419
25.614	25.424	25.4145	26.7591	26.139	25.7431	26.931	26.8168	24.5819	25.6803	26.0518	26.2032
24.5507	25.2418	26.5935	25.5489	25.5038	25.4387	27.3068	25.9691	25.7356	25.029	25.7644	25.8558
25.4331	25.5826	25.5697	26.4615	25.9012	25.2708	26.8363	26.5548	26.3887	25.7314	26.3527	26.2526
26.5556	25.4623	25.6677	25.3362	27.1858	26.8137	26.4198	26.7764	22.821	24.6066	24.0901	25.3856
26.9626	25.7444	25.6833	24.729	27.4248	25.5152	26.191	25.9854	27.0917	26.9515	27.125	26.9735
29.6689	29.6219	30.0008	30.2406	29.121	29.6546	29.7168	29.0776	28.5534	28.585	28.9589	28.8555
25.041	25.8238	24.3535	27.5977	27.0722	26.8021	27.8601	26.3706	26.1392	24.9213	25.6678	24.3674
26.3394	25.5924	27.7346	24.5241	28.7531	27.0421	28.165	27.5217	27.4422	27.4199	27.4498	27.2904
28.6166	28.392	28.2561	28.9948	27.4051	27.4211	27.1708	25.1774	27.8794	28.0587	28.0944	28.2648
29.3582	29.2095	28.4808	28.5145	27.459	28.9569	27.4033	28.5698	29.3993	29.2915	29.7183	29.6118
29.4459	29.1414	28.9158	28.9954	26.3956	28.9617	27.2795	29.1261	29.7535	29.6742	29.8152	29.8679
25.2733	27.0146	25.7761	26.627	25.6129	24.9139	26.5204	27.4903	26.9591	27.1024	26.6178	26.5013
24.8405	25.0357	26.7356	26.2245	26.3919	26.9893	26.1118	26.7835	25.1641	25.6881	25.03	25.7149
26.2655	25.4981	26.3779	26.8739	27.3539	26.1103	26.4766	25.6673	26.8632	27.0114	26.938	26.9607
27.8721	27.9922	28.1596	27.8216	28.8379	27.4859	28.9138	28.0496	28.2873	28.4328	28.6054	28.3135
27.3621	27.8751	26.4822	24.6039	26.5677	27.5837	27.4854	28.3075	25.0152	25.4275	22.7579	25.0881
27.7762	25.3159	25.1169	24.8424	24.595	26.0713	24.8799	26.9707	28.1562	27.8452	28.572	28.5918
25.7135	25.4412	26.4399	26.5945	27.4057	26.072	27.4236	26.2115	25.9579	26.1228	26.0259	26.2749
29.7719	29.6654	29.7881	29.8674	29.3297	29.0955	29.2751	28.7457	30.4569	30.5865	30.4086	30.4371
30.3649	30.582	30.5608	30.465	30.5488	29.5203	30.4192	29.6895	29.6424	29.544	29.6479	29.802
25.2342	24.8614	25.7218	26.0718	26.995	28.522	27.4539	27.1799	26.1442	24.8667	23.31	26.1864
25.054	25.4667	25.7543	26.2182	27.6532	26.8734	27.5255	28.1475	27.789	28.0926	27.9441	28.0245
25.9758	25.5162	26.2048	25.8972	26.8264	26.7958	26.6555	27.9388	25.883	26.1621	26.2277	26.04
25.2591	25.1307	26.8396	25.0549	26.4329	26.3281	26.0385	25.7296	23.6012	22.7563	24.3452	22.869
25.7289	25.5402	27.5189	26.5514	27.5574	26.9662	27.236	26.4941	24.7568	25.2531	24.3907	27.3643
26.8439	26.9486	26.0575	27.1353	27.2772	26.338	26.7865	26.5614	25.8687	25.9327	25.9841	25.8808
32.1523	32.2482	29.9835	30.2667	31.1821	31.8314	31.0797	32.0891	29.2481	28.634	29.5465	29.0422
24.985	26.2419	27.1695	25.9089	26.5113	26.9661	25.8675	26.4063	25.1212	25.3555	25.9	23.5737
27.572	25.3929	26.4155	25.7108	27.0853	26.1802	25.4864	27.4614	25.3194	23.0452	24.0248	24.2997
26.7289	25.2737	24.956	25.9806	25.6431	26.1288	27.425	26.9197	26.9824	26.5683	26.3076	26.6655
28.0036	28.0167	28.0845	28.106	27.9387	28.7625	28.231	28.7965	28.3218	28.4643	28.3448	28.4224
27.3174	27.2084	27.7876	27.2339	27.3154	26.0796	26.888	28.6602	27.5413	27.042	27.3034	27.5714
26.6409	25.3032	26.4311	25.8815	26.6192	26.8214	26.4122	25.1545	27.1744	27.2945	27.6877	27.4668
27.7564	24.2201	26.8525	25.9546	26.761	26.5957	27.946	24.8007	29.8505	29.7306	29.7709	29.6672
26.6122	24.6861	25.2991	26.8033	27.5265	25.8733	27.9091	25.6511	24.3406	24.2021	26.7944	26.9614
26.0647	26.505	25.8748	26.695	27.527	25.4775	25.4243	25.7732	26.1081	23.863	25.0955	22.973
25.2748	25.2108	26.5015	26.0311	26.7692	26.9506	26.1162	26.4119	26.0432	26.6233	26.5429	26.6828
25.0431	24.3403	26.4212	26.7316	27.8529	26.8604	27.106	26.4605	25.1589	24.3121	26.39	25.7227
25.2052	26.683	24.9714	25.8395	27.7282	25.2139	25.3011	28.2564	27.4746	27.4305	27.2206	26.9736
25.8278	25.9814	27.0767	25.8352	26.7141	25.8569	27.1178	26.1463	24.2558	25.5975	24.7603	25.2802
25.3265	27.2344	24.6772	26.1566	26.549	26.1697	26.5822	26.2008	27.8766	27.9487	28.1261	28.1723
31.7712	31.8367	29.6986	29.694	30.9527	30.4612	30.8611	31.0078	27.7954	27.6141	27.7604	28.1165
27.2119	27.9805	27.6654	27.6123	27.6326	28.1783	25.4615	26.0159	29.2951	29.2919	29.2955	29.068
29.2926	29.7279	29.2229	28.9228	29.8822	29.7107	29.8853	29.077	24.9126	24.8179	23.5442	24.5797
28.0665	28.2359	27.0364	28.2414	27.136	25.6223	26.7215	25.6278	29.1229	29.234	29.1911	29.4062
27.7156	27.6708	26.4214	27.8783	26.9985	26.8697	27.8225	27.394	26.0447	26.4118	26.2381	25.0525
25.3498	25.5048	26.904	25.7561	25.7085	26.1693	27.0791	26.4205	28.962	28.6344	28.7925	28.7036
25.8252	27.5134	25.1358	26.7143	27.4069	26.753	26.2821	26.6142	24.2552	26.9481	27.5281	27.5081
28.6736	28.6113	27.4757	27.5832	27.9858	29.1676	28.1761	28.3834	23.6668	24.349	22.1011	24.5849
25.8138	26.5114	25.8842	25.3392	26.5058	26.3393	27.2945	27.3129	24.7201	24.3705	24.2493	24.9725
27.9432	28.1673	27.115	26.7699	25.3394	28.2999	27.4289	27.9362	25.1629	23.5776	25.0591	24.5309
30.1428	30.1784	29.7198	29.6636	29.5959	29.3868	29.4127	29.4935	28.5491	28.6495	28.8747	28.6635
29.5217	29.6694	29.4712	29.8005	29.1424	29.1392	29.2374	29.3285	29.9017	29.7969	30.1387	30.2425
26.0874	24.3782	26.0608	25.055	25.323	27.6584	27.2462	28.2114	26.7326	26.9003	26.6504	26.4998
28.5699	28.4744	27.0441	27.4836	26.5317	27.6278	26.598	26.3101	28.0554	27.9053	28.256	28.4903
26.0539	24.9007	25.9148	25.8552	27.8395	25.3156	27.7524	25.5508	26.4558	25.1908	27.1395	24.5535
26.1756	28.0194	28.6896	28.5315	28.7334	28.667	28.6945	25.7054	28.589	28.6062	28.0889	28.4661
27.3721	27.5131	27.265	26.807	29.5267	31.4946	29.455	31.0882	24.7202	24.8174	25.3816	25.1586
26.0181	24.8324	26.1021	25.9389	27.6558	28.5021	27.7973	25.5331	26.7677	26.6862	26.4217	26.79
29.7494	29.7128	27.5311	27.5897	29.2744	29.8327	29.3196	30.1594	25.1795	26.3819	26.4295	26.0461
26.7652	27.5147	25.3049	25.677	26.8108	26.1683	25.9941	25.4688	27.4084	27.4762	27.3658	27.3776
26.145	26.2752	26.4424	26.1983	27.9082	27.3312	27.0929	26.5679	26.4581	26.911	26.4614	26.4436
27.6698	25.7714	25.7225	25.5657	25.712	26.2332	26.242	25.4419	25.3864	25.2542	26.2921	24.8182
26.192	26.361	25.686	24.4805	28.6621	25.1591	27.0425	27.3745	24.9506	26.5513	25.3556	26.9094
25.4813	24.8522	25.1958	25.5465	25.3707	25.7348	26.7302	26.0321	26.0998	26.223	25.2734	26.167
25.0207	26.1672	26.3953	25.6681	27.9933	27.118	26.5982	26.4705	28.4562	28.216	28.3587	27.6963
25.782	25.9159	25.8587	26.316	27.6985	25.5326	27.2619	27.1707	26.5898	24.9795	27.0132	26.8093
25.0439	25.6786	26.2197	26.2371	24.4059	26.3955	27.6516	27.3168	24.8091	24.5707	24.5568	25.4288
25.7232	25.289	25.7055	26.4625	26.9632	25.5563	26.0185	26.2711	26.6079	26.7912	26.4847	26.5579
26.7458	24.5297	27.4811	26.9811	27.6597	25.8694	27.8087	25.8857	24.4703	25.9379	25.5666	26.1622
26.7601	25.6581	26.7406	24.6191	26.75	25.9926	26.9109	25.9517	24.3859	24.5608	27.4386	27.5521
34.8956	34.8172	34.3965	34.6407	34.1815	35.0054	34.2092	35.3947	34.9326	34.9455	35.3663	35.1262
26.2706	26.4584	25.2009	26.9721	28.002	26.8475	26.7398	24.4244	23.9766	27.0674	26.5399	26.9491
25.3943	26.3758	25.4226	25.979	26.9328	26.3398	27.854	26.4868	27.2473	27.1092	27.4045	27.465
27.8509	25.8931	25.8426	24.3482	26.8548	25.6955	27.0221	28.0353	25.7928	24.6927	25.5726	25.9314
28.1241	28.0709	27.9843	28.3837	26.1639	28.6902	26.8408	28.8791	26.9713	27.2552	26.4928	26.9211
28.4956	28.5252	28.5986	28.4835	27.9201	27.4545	27.5144	26.8141	28.3294	28.2551	28.5468	28.3527
24.5397	24.9397	26.0293	25.6293	26.1965	26.1075	26.7815	26.5168	24.6839	23.6684	24.6529	25.7702
25.5471	25.2285	25.1555	26.003	26.0001	25.4882	26.3414	24.7916	23.4815	25.2937	26.3846	26.4148
26.4883	26.3341	25.7009	25.4142	27.6673	25.0796	26.6495	25.7127	24.7158	27.2095	27.2985	26.6246
26.0175	25.7622	26.5962	27.9729	26.3982	26.67	28.4291	25.9203	25.3328	25.2644	26.6071	25.8084
26.6422	26.2537	25.934	24.6741	26.1047	25.7102	27.3164	27.1993	28.4239	27.6973	27.358	27.473
27.054	26.102	26.0036	24.5637	27.143	25.2495	26.5302	25.8853	25.6248	24.0321	26.1479	24.43

25.1887	24.5344	27.3638	25.9503	27.3455	26.548	27.2819	27.1654	27.0745	26.7917	27.1109	27.6
26.4411	24.7418	26.1452	27.1821	28.2184	26.4298	26.6905	25.9209	25.9868	26.0138	25.738	25.6086
26.4779	26.8161	26.1292	26.3205	27.3829	27.7898	27.1964	27.1672	26.8262	27.0025	26.7347	26.8139
26.487	25.0401	27.0201	25.2722	26.6874	26.5047	26.0468	25.7105	23.5177	23.2723	25.0779	25.1138
25.1528	25.2526	25.5336	25.8707	26.4786	26.4047	25.9472	25.9021	24.8139	27.3985	27.4221	27.6107
25.7157	28.1908	27.6119	27.0508	26.3777	28.2481	28.042	25.7975	28.5857	28.6089	28.6712	28.5008
25.898	26.1903	27.3644	26.7199	27.4162	26.0373	26.7719	25.482	25.8001	25.7165	25.6988	25.6761
27.3918	26.6479	26.5899	25.3181	25.931	27.149	27.0532	26.117	25.7433	25.7492	26.3083	26.047
27.4723	25.5727	25.6017	24.7012	26.2348	28.2583	28.8534	26.1942	25.7696	24.6359	25.7693	25.5707
29.2204	29.0085	28.8535	28.5816	28.173	28.0601	27.8698	27.5147	28.2302	28.1276	28.3559	28.418
24.8954	26.159	26.3879	26.053	27.2605	26.6231	26.6266	27.1564	25.1364	26.9711	26.6756	26.4891
26.9511	27.937	26.7258	26.0016	27.2869	27.4184	25.9305	25.4588	23.5616	25.7003	25.8893	25.3913
26.1486	25.7033	26.0032	26.4564	26.4133	26.5721	27.26	26.1715	25.8582	25.8841	25.0887	24.3979
27.7344	27.7533	25.4698	27.1828	28.7275	27.4244	27.5359	26.5415	27.3695	27.3987	27.5985	28.1218
25.8613	25.4116	25.9432	25.1797	26.8812	27.525	26.8034	27.0318	25.5078	25.758	23.8289	25.2646
27.3326	25.877	25.4307	26.329	27.5805	25.6828	28.0384	26.7883	24.9517	24.7873	26.2131	24.6488
26.6031	27.9149	25.1322	25.5257	28.6869	27.7272	27.1495	26.7731	27.8275	27.9595	26.9472	24.5247
26.3317	26.406	26.8389	25.4372	27.2041	25.5026	26.8204	27.1441	27.6305	27.6684	26.908	26.5918
28.4698	28.2661	26.7144	27.9041	27.6461	26.2379	26.9463	27.9664	26.6447	25.6636	26.8378	26.9964
26.1777	27.1858	25.5731	27.1882	25.9474	25.6371	26.9689	26.665	28.0011	28.1328	28.5971	28.6082
24.9175	25.7001	27.0434	27.3254	26.2197	25.6483	27.7536	27.0397	24.7873	24.5922	25.3088	25.4213
27.1359	26.6549	26.0744	26.8121	27.1831	27.1309	26.6297	26.9619	27.2831	27.2473	26.9455	27.2247
26.6039	25.2252	26.9147	24.7395	28.5241	26.2709	27.321	26.6052	26.5372	27.0253	26.7745	25.2978
26.7315	25.3313	25.7423	25.6255	27.9711	25.7912	27.4896	25.587	27.215	26.9041	26.4876	26.7738
26.4092	24.744	25.8815	26.297	27.3465	25.9674	26.5059	27.4671	27.3839	27.642	27.5229	27.6504
26.1603	27.9097	25.3561	26.1174	27.8343	26.5415	26.4772	26.3259	26.5248	25.8069	24.6153	26.288
29.9502	29.8259	29.5846	30.2652	29.3299	29.4377	29.3927	28.9117	30.3215	30.3626	30.4838	30.3354
29.4382	28.9469	28.6412	28.5926	26.1335	29.1823	26.9944	28.9063	27.9139	27.688	27.5289	27.2533
25.976	27.161	26.9726	28.2245	27.5152	28.3908	27.0257	28.6535	26.8404	26.5912	27.1894	27.0682
25.468	26.1121	26.5428	25.871	26.2959	26.192	26.149	26.2172	23.8976	25.0518	25.6088	25.9967
25.979	26.8604	27.2632	27.5197	26.4166	26.119	26.4349	26.4626	27.1336	27.1456	26.9639	27.1361
26.8977	26.733	26.64	26.3082	26.1078	25.2561	26.4219	26.5561	23.2979	23.7207	23.9266	22.7904
24.8782	26.382	26.9971	26.6947	27.0867	26.046	26.4864	27.5444	24.8501	26.057	26.0332	26.6994
26.7856	26.8183	25.7693	25.3962	27.5955	25.3815	27.0581	26.8958	25.278	25.3445	23.1534	25.6706
26.9238	26.4566	25.3667	25.5107	26.7781	25.6019	26.4581	27.6337	26.5562	26.9407	26.6982	27.0363
26.0195	24.3191	26.4056	26.1817	26.2124	25.3158	26.7818	26.6624	23.9178	24.2679	23.8031	24.9415
26.1757	26.6698	24.3531	26.8584	26.5725	26.7637	27.2314	26.9475	25.7318	24.8729	23.3423	24.6054
25.9253	26.4554	25.6884	27.0633	28.2118	26.6248	28.192	25.9352	25.0252	25.013	24.4165	25.0778
26.8698	26.8007	26.6556	25.1941	26.3927	26.7194	26.7979	26.697	27.0871	24.6563	24.0099	25.1119
26.2061	25.4016	26.2249	26.4218	26.3083	26.6348	26.5512	25.6529	26.8242	26.2125	26.7715	25.9792
25.852	27.9195	26.0601	27.7723	25.4814	26.1845	27.512	26.6088	28.6016	28.9201	28.555	28.3768
25.813	26.6697	27.1507	26.0401	27.8917	25.9192	27.5643	24.6243	27.1602	26.4529	27.4519	27.198
25.3378	25.3015	25.418	25.9991	27.4275	27.5265	26.0665	27.1272	25.9368	26.7453	25.9579	25.6734
25.5612	28.4661	26.0344	25.5291	26.7547	26.4488	25.7523	26.3669	29.8974	29.7029	29.5767	29.7701
27.2671	26.1976	26.6382	25.2742	27.2423	26.667	25.7181	27.3491	25.6301	26.4456	26.8655	26.5945
26.8942	25.3353	25.8135	26.5903	25.694	24.3681	26.5127	26.4666	23.8485	26.8589	25.8176	24.4372
26.0524	26.3403	26.9334	25.6741	27.423	24.9748	28.1828	26.3939	26.3898	26.4103	26.6853	27.0372
28.3114	25.6275	26.7499	26.0033	27.0177	25.5339	26.7851	27.7005	25.0854	25.2897	25.1103	24.6633
26.6755	24.8618	25.902	27.6857	27.9287	27.5616	27.6038	25.9408	24.5557	25.292	24.8821	25.0396
28.3095	27.8694	28.5029	28.5914	27.3716	28.0098	26.3108	27.9582	28.4952	28.294	28.3123	28.2485
26.0886	25.2385	27.2543	25.0156	26.3361	27.314	27.4464	24.6024	28.0747	28.0233	28.1019	28.1749
25.1861	25.596	26.6588	25.3827	25.32	27.9648	27.0346	26.1272	27.6025	25.1651	28.2619	28.0648
25.7774	25.1331	27.4477	25.1953	25.8378	25.8634	27.5042	25.3776	26.7968	26.8018	26.6301	26.674
25.1219	25.9592	25.0152	25.2902	25.5841	26.3037	26.4827	26.2752	25.0415	24.9673	24.2558	24.3058
27.2479	26.6243	26.9798	26.1168	25.4277	26.936	26.1921	25.8668	26.1135	26.4303	26.4242	26.5753
27.107	26.4774	27.0326	25.8529	26.7384	28.1714	24.9631	25.9916	24.3368	26.001	25.3227	24.3277
26.4387	25.7649	26.0098	26.031	25.9389	26.8487	25.4819	26.4775	26.8812	27.0791	27.3664	27.2238
25.7962	26.4613	25.4151	26.2512	25.7805	25.2541	25.9004	25.904	24.9226	25.3001	25.2044	25.157
25.9487	25.8457	25.7007	25.2985	27.1146	26.4355	27.2337	24.8893	24.908	24.7983	24.5747	25.087
26.3559	27.9489	27.1193	26.0917	26.2795	25.9968	25.225	26.4548	27.0575	27.205	26.5723	26.3504
27.1467	27.6355	27.2832	27.3355	26.8251	28.8777	26.7253	28.56	27.5299	27.5242	27.4386	27.5983
27.5231	27.315	27.3231	27.0213	26.7229	28.5064	26.365	25.719	27.9599	28.0146	27.92	27.9731
25.9649	25.2415	25.8151	23.4811	25.9462	24.8951	25.4161	28.2137	27.0749	24.4788	27.1653	25.6431
27.5673	27.8499	26.2477	27.854	26.5313	26.805	27.352	26.4835	27.072	27.156	27.4726	27.2291
24.2275	25.6068	25.9488	26.4364	25.7536	27.9272	26.4399	26.5729	25.3236	25.0123	25.1626	24.7098
26.1473	25.5731	26.9518	26.8965	26.4468	27.0752	27.1937	25.3117	25.8944	25.5319	27.4186	26.353
26.1123	25.7046	26.9789	26.2054	25.9851	26.6276	27.9886	25.6221	24.0379	24.7257	24.3452	23.698
26.6581	25.8207	26.6891	25.9549	27.1795	25.388	27.1403	26.1297	24.123	24.6853	27.3119	25.9342
26.0489	27.866	25.9104	25.9017	27.049	26.8267	27.303	26.1841	26.6915	26.3679	25.0292	27.3657
26.7471	27.0215	25.0645	27.5027	26.7658	27.1386	27.0186	27.2726	27.1296	27.2686	27.4979	27.8462
24.8177	26.7537	25.7252	26.2819	27.3509	27.4269	26.0499	25.4485	27.3258	27.426	26.9842	26.8174
26.4347	26.2402	27.3386	25.8505	28.0865	27.1769	27.4592	25.7454	26.5589	26.9471	26.6137	26.2623
26.0334	25.0164	27.1824	25.5112	28.1521	28.0932	26.3783	27.0854	26.2321	25.6507	24.8554	23.5555
26.8136	25.8771	27.3292	25.7451	28.3523	24.7684	26.9847	27.6698	25.6361	24.8827	25.7044	25.6049
27.2055	26.8304	25.4814	24.5019	26.6712	27.6698	27.1795	26.7163	25.4377	25.4973	25.2581	25.068
25.2791	26.913	24.6895	26.6865	26.3007	26.5474	27.5157	25.7353	25.2613	25.0532	24.511	25.0075
27.5592	25.2687	24.5503	25.0397	27.0621	26.5207	27.2652	27.2327	26.2912	26.3124	26.3572	26.595
26.6972	23.2014	26.5985	27.3019	27.38	26.3295	27.0297	26.4321	26.5691	26.1906	24.3501	25.925
27.4016	25.9297	25.4208	25.931	26.7701	27.3292	27.4978	24.7181	25.9422	25.974	25.458	25.4061
25.9405	27.7683	26.9217	26.8173	26.9383	26.3986	26.3836	26.0205	28.3448	28.4818	27.9011	27.9398
27.0254	26.7293	26.6464	26.462	27.5171	26.7513	26.5515	26.2873	25.2801	24.8217	25.3848	25.6263
26.5284	26.0115	26.336	25.9366	26.1226	25.0459	27.5199	25.446	25.3638	24.8803	25.0281	24.4338
25.2499	25.2734	25.3481	25.3692	27.6797	24.8385	27.1352	28.1172	26.599	26.4081	26.7283	26.5741
26.0643	25.207	25.1798	26.8801	26.0461	26.8763	26.2881	26.4817	26.9229	26.8855	27.0839	27.2639
26.2523	27.46	29.5678	29.9751	26.4256	25.7764	26.1136	25.7276	28.6705	28.5947	28.1925	28.0405
27.9763	27.7589	26.9881	26.3049	26.7382	26.2114	27.7169	27.4598	25.8725	25.9281	26.4179	26.371
25.3339	24.468	26.783	25.3134	26.9806	27.1251	27.0402	25.6723	24.5245	25.0102	24.7792	24.4296
26.1029	27.3881	26.3522	25.543	27.471	25.7632	26.6188	25.5099	26.1032	23.6882	25.4697	24.5104
26.9534	26.5246	27.055	26.303	26.6093	24.8123	26.5905	26.7286	25.9965	26.1283	26.2148	26.4966
26.8028	26.4805	25.1346	25.3491	26.8435	25.9156	25.7193	25.1781	24.462	25.6306	24.2326	24.4271
29.1298	29.2456	28.9471	29.1378	28.8435	28.5446	28.8007	28.3833	27.061	27.0168	27.2346	27.3103
26.9869	26.1648	25.9348	26.8101	26.2321	27.1748	26.2533	27.4155	23.8448	24.309	24.621	23.9332
26.5513	25.4645	27.4473	26.0185	26.0982	26.9075	27.6192	24.0181	26.1222	24.2736	25.7293	25.8919
26.3292	27.0701	26.7501	25.5568	25.5258	26.2931	27.3044	26.4294	28.0606	27.9735	28.2445	28.2279
25.4477	24.9937	26.8262	26.0243	25.9893	27.2228	26.4668	26.4237	24.791	25.8506	28.0347	27.8504
25.6092	24.7122	27.6264	26.612	26.8686	26.8821	28.1574	26.4379	26.3612	26.2631	26.1463	26.1805
26.4436	26.8989	27.0153	27.2844	27.2428	25.5847	26.1186	26.4282	26.7105	26.6171	26.4673	26.9267
27.5335	24.9655	26.0138	25.0243	26.9047	26.2593	26.6666	25.9635	24.4431	26.1034	25.9457	26.3836
27.3575	24.701	26.3351	26.0867	25.4089	24.3313	26.9174	26.464	23.5068	23.7325	24.339	24.4187
29.9062	30.0651	29.0532	28.87	29.4256	29.8734	29.1715	28.6637	23.7343	24.3801	24.0373	24.3594
27.3464	25.4898	26.1139	25.4003	27.2461	28.4534	24.5681	24.8182	27.0505	26.7241	26.7911	26.9669
26.2961	25.8254	26.4264	25.2823	26.0759	26.5017	27.1241	27.5053	23.1908	23.9021	24.2488	26.8649
25.9111	24.5946	26.38	26.3701	27.5743	25.1305	26.5849	26.065	24.6865	24.3363	25.2061	24.6276
24.4176	25.6966	27.5956	24.6917	27.1955	27.9447	27.1248	27.2553	25.0772	27.7451	25.5692	24.3231
26.9476	25.443	26.6678	25.0994	27.0711	26.2532	26.8862	25.5303	25.6777	25.3405	24.0625	23.9976
27.2304	24.4149	25.4036	27.3281	25.4487	26.854	26.4892	26.6085	24.6997	26.7004	26.7032	27.0479
27.5412	26.1134	27.3361	26.3695	26.8893	25.3694	26.7971	26.3078	25.1492	27.585	24.9229	27.597
26.7596	26.5711	25.3324	25.4909	25.7092	26.9712	28.2671	24.9825	24.3597	23.6575	23.792	26.3457
25.5581	23.6273	27.109	25.4094	26.6219	25.9233	26.7439	26.9949	25.3523	25.155	25.1241	25.1785
25.5854	24.9465	26.2642	26.3021	27.4179	26.3756	26.6471	26.0246	26.0272	26.3208	26.4535	26.5061
26.5275	26.506	27.1266	26.1182	25.8471	26.0883	27.9057	26.766	26.4194	25.3721	26.5437	26.9233
25.768	25.1594	25.2557	27.0138	26.7876	25.3639	25.6627	26.7935	23.8177	23.4707	26.6416	25.4916
28.0386	28.2406	26.7014	28.0274	27.0274	25.7397	26.6556	27.0257	29.0478	28.9989	28.388	28.3803
25.8074	24.5931	25.5353	26.5155	27.2244	27.0253	27.1951	25.3799	23.9296	25.7625	25.6329	24.5298
24.7563	26.0289	26.8349	25.8554	26.5271	24.9098	26.6786	25.7371	23.6013	21.4063	26.9322	25.1886
26.2116	26.1448	26.556	26.1669	27.4105	26.3972	27.1832	26.9719	25.2803	25.4756	25.7166	26.1292
25.8201	26.0374	27.1132	25.9948	27.3539	25.93	26.9371	25.4591	24.6364	26.1102	26.053	26.4387
27.0221	24.1777	26.0063	26.5293	26.937	26.647	27.0721	26.5416	23.4109	25.3449	25.8759	23.7075
27.8787	26.2892	25.1121	25.5314	25.7153	26.3345	26.2385	26.4965	26.979	26.5898	26.9234	26.7806
25.8648	27.9905	26.4804	26.7711	26.0501	27.7874	27.1856	26.0705	27.5192	28.3317	28.2966	27.9526
26.8971	25.6294	27.0374	26.5128	27.0109	26.5772	26.9015	25.6968	23.6654	25.4039	24.7648	22.6496
26.9662	26.8835	27.728	27.2211	26.3348	29.3289	28.2755	27.5715	26.1966	25.8195	25.2749	25.85
27.2111	26.8827	26.4016	25.7562	27.1844	25.5903	26.4277	25.373	24.9484	23.4804	25.6319	24.8379
27.0363	25.9387	26.5338	25.303	27.0508	26.5387	25.9839	27.4827	24.7624	28.5239	27.4871	28.9319
27.3781	26.6089	26.5431	25.6255	26.9261	25.4946	27.2182	25.8255	26.8389	27.8151	27.9189	27.879
31.088	31.1479	30.1926	30.6212	30.4551	30.607	30.2559	30.7394	29.6123	29.8577	29.8768	29.9399
26.4002	25.4811	26.7783	25.4645	26.997	25.3677	26.2317	25.6313	25.2075	25.8312	26.1434	23.367
25.5411	26.6491	25.3405	26.6315	26.7641	25.6991	27.283	26.8746	25.0299	24.9291	25.8519	24.7747
26.3256	25.5623	26.119	26.8411	27.3092	27.2109	28.1184	25.2852	25.883	26.0773	26.1997	25.6838

28.521	28.9769	27.2091	27.292	26.7683	26.3622	26.9774	26.3045	28.9726	28.6937	28.5745	28.7052
27.9309	27.9686	26.5642	25.668	26.7678	28.8216	28.146	28.5153	23.7391	24.9259	24.0304	25.8567
26.4976	25.8654	25.908	27.0482	26.5746	26.5359	27.209	27.3513	26.9506	23.3195	26.5523	26.8714
26.2652	26.4705	26.4017	25.9767	26.3447	27.2176	26.3096	26.7166	25.7168	24.8874	25.2526	25.1764
25.7136	25.3576	26.8626	26.1621	26.8753	26.7807	26.8852	26.0711	25.3927	25.8345	23.8984	25.1293
29.7805	29.7848	30.5565	30.6298	29.4828	29.4043	29.3898	29.2902	27.664	27.3799	27.8762	27.8172
33.2335	33.4789	29.1955	25.824	29.7628	33.5544	26.7618	25.5596	26.2679	29.5628	30.7153	30.314
25.1539	26.7183	25.1881	25.9036	25.5739	26.4158	27.4417	25.9857	27.002	26.4194	26.5904	26.3852
25.3182	25.2158	26.2688	26.6828	25.4381	26.1534	27.2889	25.3792	25.5747	25.5074	24.8819	26.0159
30.7853	30.7592	31.7749	31.3718	30.0475	30.6442	30.0032	29.2985	31.7322	31.6469	31.6099	31.2882
28.9593	28.4889	28.5932	28.4289	28.2285	29.5506	25.5237	29.6952	26.9848	27.3281	27.46	27.8091
25.4094	27.349	27.5525	27.8375	26.9898	27.3864	27.265	27.3831	26.7815	26.725	26.7106	26.9025
27.4619	25.611	26.1538	26.5231	25.8618	26.524	26.0866	26.0381	25.5719	23.3177	23.2547	25.5862
25.9084	25.9527	26.1271	26.3896	28.1025	26.9937	27.8187	25.6483	23.9136	25.2598	25.1237	25.4539
25.9556	25.4838	26.4082	25.0898	27.8077	27.3699	26.4911	25.7376	26.1664	26.7726	26.3615	26.5757
25.6776	24.9321	27.1184	26.3872	26.9522	26.4107	26.3458	24.8885	24.7915	25.3844	25.6544	25.7851
24.4356	25.2348	26.5187	24.7774	26.4877	25.4908	27.1427	25.1148	26.3749	26.2696	25.3786	24.9688
24.7434	24.8846	26.4663	26.5645	26.1377	27.0886	27.337	25.0781	25.4779	25.9961	24.9151	24.6467
26.8566	26.0053	26.0375	25.4749	27.5669	26.4742	26.6397	25.6809	23.9074	24.3013	27.0365	25.0059
26.7051	26.3883	25.9397	26.5417	26.1537	26.595	26.4248	26.6957	26.299	26.4795	25.9523	25.8388
25.6427	27.3205	25.9474	27.1819	26.2659	27.9293	27.8284	27.5688	27.4422	27.3584	26.9521	26.9079
27.4453	25.9582	25.666	25.7222	25.2704	24.9836	26.6464	25.4234	26.5856	26.5603	26.5426	26.0246
25.5278	28.8521	28.2591	28.6559	28.8741	26.298	28.1691	26.9935	28.8276	28.2659	28.2355	28.5996
26.5811	27.282	26.5062	25.387	26.9111	26.4571	25.4277	26.8961	29.7325	25.3607	29.3379	29.481
25.9061	26.5629	27.0236	25.481	26.6988	27.7852	24.6705	25.277	26.6856	26.8824	27.0224	27.121
26.2548	25.0338	25.606	26.9484	26.2103	26.2215	24.6851	27.1017	25.303	25.3118	25.3359	25.5433
26.1893	26.3728	25.0481	25.9835	28.0295	29.0555	27.3289	26.948	24.7903	25.9857	26.0184	26.0085
26.3017	27.2324	27.4284	26.949	28.9	25.5721	27.3499	27.5845	27.1264	27.3106	27.2797	27.4005
26.7483	26.8727	25.3701	25.5333	26.1801	25.6059	26.1252	25.8616	25.4335	24.5459	24.1768	24.4123
26.8087	22.9669	26.0077	25.2962	27.4845	25.8917	26.4864	26.3151	23.7056	24.2864	25.8016	25.5255
29.5602	29.4556	29.3101	29.347	29.5762	30.6613	29.6473	29.3237	28.4939	28.2593	28.3568	28.5151
26.9634	25.9758	26.0288	26.0314	27.8389	27.6153	25.7882	27.3191	27.0561	27.2035	27.658	27.4555
26.9005	25.1487	26.4984	25.6052	26.7143	26.6689	26.7995	26.6479	25.3789	24.4584	25.547	25.5951
28.0632	28.2242	28.0752	27.8987	27.6364	27.5895	27.1263	27.7817	27.7681	28.4123	28.2313	28.0193
24.8128	25.9413	26.1292	25.3868	25.9767	26.9767	27.3517	27.2696	26.6224	26.221	26.5165	26.1138
31.3305	31.5265	29.1037	29.5008	30.3703	32.1818	29.8696	32.7109	25.0644	24.9376	26.8507	28.1761
26.0615	25.1903	25.8654	25.4833	27.8019	25.3946	27.2029	26.0148	26.4832	26.2303	25.0677	24.0919
29.0328	29.199	29.1714	29.0457	30.57	26.2021	30.4733	29.4134	22.4536	24.8561	24.1505	24.5877
25.7598	27.8605	27.658	26.6337	26.3482	26.4622	27.1975	26.0537	24.3591	25.0336	24.309	24.1888
26.1081	25.8783	27.7183	27.4264	26.6687	26.1938	27.6463	27.0641	25.7283	24.846	25.3815	25.2115
29.9208	29.9106	29.7462	30.3325	28.6586	29.7169	28.7494	29.9223	27.4765	27.0995	27.8411	27.736
27.2805	25.7471	26.8954	25.6087	26.7048	26.1559	26.7351	26.5921	22.8807	25.6632	23.4149	23.92
28.5683	28.6198	27.8258	27.7846	27.3469	27.2192	27.5132	27.2724	23.5853	22.9687	23.7918	25.7845
25.9994	26.6242	27.4048	25.5426	25.7192	26.3792	26.0763	25.589	24.9102	24.8704	24.87	24.9215
25.3701	25.9482	26.8667	25.1327	25.5956	26.3417	26.4213	25.4019	24.8352	25.2933	25.6139	24.9882
25.1364	25.2032	27.5495	25.0335	26.9493	24.662	27.2387	26.9135	25.0535	24.506	27.1974	28.2106
25.6891	24.6173	26.8063	24.7939	26.8324	25.5049	27.0583	26.7896	25.0518	27.4041	27.0039	26.6764
25.7005	26.1925	26.6076	25.9078	26.6968	25.2623	26.6777	24.6997	25.3901	24.3308	24.2588	24.8428
26.3515	25.3973	26.6797	25.3536	27.1996	25.6459	27.4683	25.4967	24.8113	27.641	26.5013	25.6143
28.0824	24.8659	25.8134	24.5072	27.8871	26.4823	26.6155	26.2382	26.0482	26.0751	26.0568	26.2871
25.4017	26.1108	26.3336	26.1824	27.6781	26.436	27.4414	26.1679	25.8139	25.4048	26.0969	25.741
27.8324	26.4049	25.2733	26.317	26.6866	26.0965	26.1314	26.7542	25.1649	26.3939	25.7854	23.564
25.9813	25.4324	26.7043	26.2418	26.2797	26.5254	25.4542	27.2854	25.666	25.891	25.1808	25.0388
26.1437	25.7096	25.9616	25.2848	27.7405	25.7606	25.9145	26.9973	21.5241	24.1497	25.1735	24.1122
26.4841	25.1811	26.7495	25.8471	28.3308	24.7105	26.2692	26.0206	24.6925	25.5623	24.8236	24.0381
24.7482	25.7357	28.4096	26.5201	27.6551	26.9891	26.7724	26.2901	24.7736	26.0245	25.74	25.6293
26.9221	25.6098	26.6553	26.3684	26.6458	25.0545	26.5508	26.6629	23.8015	23.9065	26.5578	23.6376
27.8913	27.7396	25.7415	27.7109	27.8366	27.5275	26.6541	26.4831	28.2588	28.1939	28.0606	28.2495
27.2104	25.6054	25.7565	25.867	26.9344	25.4183	26.5097	25.721	27.0499	27.1534	27.321	27.2544
28.022	28.3668	27.6667	27.9181	26.2325	28.2826	26.541	25.9604	24.86	27.2315	27.7656	27.1509
25.9239	24.9342	25.4583	25.2784	26.5716	25.0676	26.4899	27.2236	24.9991	23.4607	23.8606	22.4998
26.6449	24.205	25.1544	24.9256	26.481	26.9055	27.1173	26.4827	23.6993	24.0466	23.2686	24.5574
25.4752	24.6399	27.861	26.0399	25.8885	26.0714	27.2674	26.0851	24.7992	23.1805	25.7039	23.2454
25.5841	25.4531	25.9707	26.4779	25.2439	24.8427	26.5171	24.6921	26.9146	26.9981	27.3326	26.8793
25.1813	26.2068	27.2578	27.5789	25.9204	26.2348	28.0228	26.6554	29.0203	28.6112	28.2879	28.3966
25.9912	24.382	27.2591	25.5712	26.834	25.8426	27.4933	26.4226	24.4954	24.2389	24.4274	26.7011
26.0389	25.8532	25.5804	26.1675	25.6239	25.7644	26.8611	25.6677	24.4621	24.2038	24.6619	24.4781
26.5251	25.4522	26.4709	26.3712	25.7564	25.2384	27.1651	25.7957	28.2429	28.0748	28.2517	28.2623
27.3532	25.7037	26.7393	25.9437	25.9411	25.7544	27.144	27.1998	24.4324	25.1657	25.4998	25.4688
25.0574	25.0822	26.5503	24.258	26.8492	25.7284	26.3279	27.0655	23.9952	25.019	23.7911	24.6559
26.2903	25.0188	25.536	26.9932	26.4699	26.6456	25.9937	27.3294	23.5184	25.0886	25.0915	26.1615
25.5569	27.9176	26.3084	26.8852	25.9929	25.6906	26.1448	25.7561	27.5345	27.3146	27.0192	27.0371
26.251	24.7281	28.234	26.4248	26.5227	26.9584	25.5453	25.7451	26.1921	26.3141	25.2305	25.986
28.9551	29.2186	29.5097	29.5177	28.2015	28.6286	28.2974	28.2262	25.7801	24.8164	24.883	26.2114
26.0056	24.6024	26.0677	26.1649	26.7177	26.3359	25.7256	26.2617	26.5652	26.2118	27.2607	27.4614
26.3976	27.0794	25.9902	26.425	26.2726	25.9449	25.7744	27.6561	27.4266	27.3826	27.8935	27.9929
26.1909	26.5908	26.8121	26.9645	26.9711	26.6329	27.5347	25.6303	27.4044	27.6969	27.3062	27.2923
25.9468	27.3192	27.7809	27.4135	27.8483	28.2157	26.4942	29.1663	26.0979	23.6345	24.8541	25.0073
25.329	27.6935	27.4423	28.085	26.6752	26.6449	26.2897	27.4561	28.6169	28.8247	28.5593	28.7261
25.774	26.5936	26.7029	25.9783	26.1227	26.8267	25.8321	26.9499	24.6658	25.3738	28.7979	28.4588
26.3625	26.6462	26.3246	26.8266	26.5306	27.1198	25.9689	24.5285	25.3664	25.6095	25.3574	25.7061
27.3668	25.329	25.3871	26.707	25.8735	26.7519	26.4408	25.865	27.062	25.2836	27.5259	27.7837
25.5777	26.3853	26.3556	25.4459	26.7338	26.2782	26.338	25.3063	25.2184	23.2831	26.3523	26.7014
27.422	27.6613	26.4341	26.0947	27.284	27.2633	26.6069	25.8073	28.7054	28.5779	28.7229	28.9053
26.065	24.1587	26.8593	26.8869	26.2655	26.6264	26.1655	27.7204	23.8151	23.777	25.762	24.7506
24.8147	25.1032	25.9555	25.9514	27.4726	27.3713	27.7418	25.6201	25.283	24.6703	25.7359	24.3334
26.0112	25.8989	26.4955	25.8236	26.67	25.9578	26.22	26.2969	26.9117	26.8367	26.8806	26.9195
25.2659	26.2531	25.8888	24.7486	25.9635	25.7854	27.0047	27.0253	26.3771	27.627	26.4259	27.1572
25.6353	25.6532	26.2859	26.5666	25.1858	26.4097	26.7436	24.9934	26.5876	23.9859	24.5942	25.12
27.0416	25.6489	26.1684	25.551	26.9629	26.0399	26.8917	26.6343	25.1336	24.9597	24.2467	24.3317
26.8966	25.6709	25.8095	25.5766	25.8393	25.5877	26.4129	26.6168	24.8802	26.3593	25.5487	25.0544
26.3773	25.6763	27.4285	25.7119	27.4509	27.0535	26.3874	26.7589	28.6047	28.4028	27.6785	27.5753
25.3287	25.3665	25.8577	25.1079	26.6952	26.0861	26.3732	26.2457	25.2076	24.9142	25.2999	25.0461
25.9143	26.3232	26.3792	26.727	27.4182	26.1063	25.8905	25.7455	24.2095	24.7664	24.9203	23.8985
26.1417	25.7654	27.7217	26.2599	27.2245	25.6234	26.7603	25.7777	25.0513	26.3403	26.1414	25.9269
25.8375	26.3501	26.676	26.3714	27.9048	26.6813	27.4693	26.1684	25.4612	26.842	25.0185	24.866
27.0283	26.5006	26.2472	26.6046	27.8494	27.1681	26.0621	26.7672	25.3994	23.1294	24.2289	25.2992
27.3789	25.9742	26.9192	26.7139	26.3653	25.1618	26.6429	27.4814	26.7383	27.6423	27.9723	28.1049
26.8828	25.9491	25.9932	25.0577	26.6615	25.646	26.9056	26.7508	26.414	26.6793	26.7471	26.8954
28.7263	28.795	28.6151	28.4637	28.5893	29.1154	28.5452	28.3413	27.6712	27.4916	26.994	27.3838
27.5885	25.0382	25.7664	26.2916	27.1742	28.3203	28.1518	25.4049	28.6377	28.368	28.3029	28.4666
27.6032	29.1528	27.0591	28.0638	26.6051	29.1431	27.6066	26.3509	28.6897	28.7108	28.9702	29.0482
26.2334	27.2605	26.4112	25.1197	25.9551	26.1309	27.4174	25.8184	25.1886	25.0644	24.9332	23.7142
24.0124	26.1558	27.6715	24.7852	26.4073	26.2423	27.0543	26.2456	27.5671	27.7129	27.8169	27.6223
28.3314	28.2686	25.2981	28.0961	27.0898	25.7492	27.51	27.167	26.9801	27.2446	26.8834	27.3141
25.9492	27.2067	26.4986	26.1236	25.148	26.3812	26.6677	26.3476	25.7589	24.0547	25.0018	24.4142
28.7601	28.7064	28.5952	28.653	28.5913	29.1052	26.8264	28.5963	27.1478	28.4086	24.7499	27.8003
26.3554	26.3921	25.6301	26.0175	24.6787	26.5477	26.5116	25.6201	26.454	23.6269	23.9971	24.0281
24.9864	25.6925	26.6216	26.7895	27.0511	26.9179	26.1704	27.3972	26.1932	25.5522	26.0294	23.9278
28.776	28.1447	27.5598	28.7491	26.4419	27.3758	27.875	27.0885	29.1031	28.6219	29.1046	28.8409
26.6479	24.767	25.9048	26.3873	27.2861	25.4221	28.0724	25.8522	28.9097	28.5068	27.8154	28.0378
26.4099	26.0483	25.7151	26.1537	26.5529	26.5366	28.1222	25.6361	26.0318	25.9843	26.1917	25.8436
27.664	26.7287	27.1136	25.7794	26.8545	25.5554	26.2061	26.6553	25.0277	25.5202	24.5423	25.6293
26.34	25.2456	26.196	26.1347	25.1098	26.8774	26.2186	25.7603	24.1027	25.319	23.9507	23.0441
25.6786	24.639	25.9858	25.5595	26.856	25.4067	26.348	25.7908	24.6287	27.1902	24.429	25.1394
25.5443	25.6306	26.626	26.502	26.5983	26.9169	27.0694	25.4644	25.7522	24.9919	26.7155	26.7748
26.6323	25.2884	26.4177	25.6681	25.8014	27.1238	26.8603	26.4501	27.8813	26.3561	27.9286	27.9531
26.4324	25.5169	26.1271	25.7728	26.7009	25.9284	26.0781	25.7902	27.5557	26.9171	26.7616	26.7207
26.1139	26.043	27.2999	25.6747	26.1455	26.259	25.3273	27.7889	25.5609	25.7748	25.0239	25.0838
26.3929	26.0252	26.7886	27.1269	26.9061	27.094	27.7375	26.7103	24.7287	28.0263	27.3975	29.5633
31.6136	32	30.188	30.4504	29.414	29.8647	29.3998	29.9064	29.8693	30.2498	30.4428	30.5753
25.9245	24.9225	26.7424	26.1237	27.3387	25.8955	26.7985	26.0782	27.2978	25.6577	26.8901	27.2321
26.766	24.6503	27.5262	25.2887	27.7759	26.6738	27.5478	27.2291	24.395	23.9524	25.0757	23.821
27.1126	26.3522	27.2969	26.1759	26.5443	26.5288	26.7237	26.5132	24.3163	24.9056	24.0422	26.3822
27.631	25.6638	25.0268	25.6684	26.1924	26.6207	26.4483	26.2023	25.2696	22.9576	24.5025	25.9297
25.8185	26.9419	26.733	26.4289	27.1407	25.4631	26.4628	26.6089	25.6039	26.6263	26.7831	24.5588
27.1258	24.8984	27.3581	24.4082	26.2196	25.615	26.6601	26.0236	25.0105	24.458	23.9231	23.9409
28.9034	28.3792	29.0595	28.6224	28.0463	29.5594	26.288	29.4087	28.7564	28.8623	28.504	28.7277
27.9686	27.4821	27.4828	27.092	26.2391	27.8566	26.8093	28.4606	26.7715	26.7278	26.961	27.1317

26.4499	27.2626	26.9915	25.8902	25.7728	26.8669	26.2822	26.8714	24.2323	25.3594	24.6889	24.3525
26.285	24.9535	27.265	26.3785	27.7639	25.3128	28.3805	26.5087	26.2334	27.3506	26.5908	27.3655
28.5077	27.983	28.7705	27.924	26.6728	28.6992	26.2241	27.7827	29.3199	29.2438	29.1835	29.2383
25.8851	25.0337	25.7011	25.701	27.2609	24.9103	26.5273	26.1714	25.2027	25.17	25.497	25.5642
25.1921	26.2388	27.4948	26.4381	27.2016	27.0279	26.8067	26.8155	23.5458	26.036	25.9912	26.2851
25.9796	26.1164	25.8111	25.6535	26.2758	23.9306	26.0905	25.4888	28.0514	28.1142	28.0775	28.0314
28.124	28.652	26.1341	26.2678	27.5088	26.933	25.8842	27.5305	28.3646	28.7427	28.8796	28.8206
28.5499	29.0235	28.6345	28.2716	25.583	29.1394	26.9141	28.3602	28.9172	29.0342	29.0624	29.0624
26.5883	25.2546	26.8603	26.4848	26.4295	26.1744	28.0977	27.0233	26.297	26.8138	26.5348	26.2919
26.3734	25.2283	28.5624	26.4886	27.6849	27.4931	27.7656	28.5127	28.281	28.0134	28.2123	28.5099
25.0908	26.7249	25.5891	27.4457	26.9572	26.7448	26.0116	26.3252	27.7687	27.8258	28.2131	27.8215
27.5141	26.017	27.3684	25.3873	26.8856	26.8139	26.7453	26.0308	24.9441	26.4051	24.889	24.0265
26.4506	26.4996	24.9502	25.8254	26.957	28.1736	28.2232	26.4096	27.1588	24.4049	24.6896	25.5275
26.3492	27.2192	27.4529	25.6361	26.0915	25.831	27.2946	26.496	23.1777	25.032	24.4892	24.2739
26.2731	24.6131	26.351	26.4746	27.2632	27.6807	28.1597	27.4345	25.7388	26.1623	23.3984	26.0918
25.8469	25.9502	25.9385	25.771	27.6024	25.6335	26.7014	25.9145	25.2501	26.1682	25.272	25.2833
25.0299	25.4443	26.6893	26.5002	25.942	25.5433	28.3137	27.6885	26.5704	26.2969	25.8629	25.9481
29.0269	29.1613	28.3131	28.7241	29.7081	30.5833	29.788	30.8145	23.4241	25.4821	24.4499	22.8789
27.2713	25.7055	26.4554	25.7831	25.8813	27.2901	27.1511	26.2421	24.0165	27.3405	23.2011	23.9171
28.4041	28.5683	28.4378	28.2002	25.8639	27.0578	26.9591	27.3615	27.3188	27.5674	27.362	27.5325
25.828	26.756	25.5379	27.8445	27.2537	26.2291	26.391	26.1581	24.9666	24.5331	24.5717	24.1319
26.5358	26.1785	27.2599	26.3837	27.4144	26.2294	26.6828	26.9661	26.9162	25.0202	26.0069	25.9314
24.9653	26.3535	28.0395	25.9607	27.5571	25.5491	25.8348	25.7754	22.0376	26.0499	25.6296	24.077
27.3476	27.2296	28.0742	28.3489	27.3719	27.5929	27.0421	26.265	28.6476	28.3245	28.7881	28.9605
29.0469	29.0638	26.1719	25.0463	26.4786	26.5345	27.5741	26.5571	26.0877	26.3617	26.794	26.8387
26.4718	26.6627	26.3223	26.3942	28.5279	25.8779	27.345	27.0614	23.7403	24.2704	25.5006	25.6106
25.6061	26.126	25.3234	25.3567	26.2983	27.1202	26.2849	26.1399	26.7198	24.7891	26.0251	25.8316
32.042	31.911	30.3228	30.4024	30.9222	31.2156	31.0791	30.3099	29.4273	25.9189	24.9296	30.2755
26.282	26.0079	26.17	26.6449	26.303	25.8363	28.3069	25.9994	25.5441	25.5973	23.1567	25.8622
25.6112	26.2196	26.5349	26.5296	27.3606	27.1301	26.4063	26.4579	25.7071	26.2548	25.9079	25.9624
26.6863	26.6148	25.7687	27.7435	26.7499	26.3367	26.2934	25.5182	27.1721	25.2769	25.3475	25.4714
27.9136	27.824	27.659	27.4813	26.207	27.4939	27.0998	28.0792	28.0887	28.0247	28.19	28.086
27.4666	25.7058	27.4902	26.8204	26.4328	25.9821	26.3672	26.8049	27.9324	27.9493	28.086	27.7995
27.1739	25.6171	27.0619	24.8362	26.8904	26.1532	26.5244	26.5586	23.1662	25.653	25.3404	25.2735
26.4446	27.3446	25.7078	27.6197	26.7867	26.3589	26.5084	25.2393	26.2931	26.1768	26.3202	26.0002
26.4944	25.7852	25.4165	25.3101	27.2906	26.0937	26.4149	26.6214	26.5667	26.5277	26.8808	27.042
27.4674	27.5983	26.1046	26.2884	27.3039	27.1739	27.2671	27.3282	28.1831	28.2176	28.3741	28.2794
26.6123	25.3811	25.6216	26.9286	27.4234	26.6747	26.8739	26.2767	26.8197	26.8965	26.5526	26.8565
28.7278	28.7082	28.6118	28.4699	25.9217	29.4737	27.4747	27.4353	28.4477	28.7905	28.7221	28.7043
25.6716	23.1374	25.786	24.769	27.5659	26.4814	26.438	26.3729	25.4632	24.1608	26.977	27.2105
26.4468	26.5416	26.1926	26.1692	26.8831	26.6655	27.4969	26.266	25.5289	27.1501	27.4887	27.1427
26.5415	25.5123	26.0835	27.3793	27.7948	26.9063	27.355	26.1125	25.8442	25.0062	25.8309	25.6745
26.4619	26.1738	27.4005	26.2504	27.703	25.8458	26.6623	26.8987	26.4244	24.8859	25.799	26.3147
25.4164	27.5065	26.6768	25.1561	26.8286	25.7116	27.1986	27.4411	24.0334	26.0757	25.9503	26.0118
25.8102	25.141	26.5977	25.852	26.0059	25.731	26.926	27.1449	25.3518	23.8175	22.5622	26.268
25.0397	26.0464	26.3007	26.1135	25.6301	27.2695	27.3619	27.0105	26.4451	26.3243	26.235	26.2509
24.8534	25.3299	25.5388	26.0622	26.9836	26.3644	27.7356	26.7007	24.4106	24.4838	25.2757	25.1604
28.5992	28.4517	28.9433	24.6756	26.3649	28.5063	26.849	28.1219	28.1099	27.7833	27.854	27.8258
26.179	25.2966	26.3649	25.5141	27.3905	26.8814	26.881	27.2158	25.6128	25.3574	25.7279	24.9672
25.8495	28.39	23.6975	25.9436	26.3598	28.4227	26.3751	27.0934	26.8918	27.2932	27.0322	27.2154
28.9774	28.8479	29.2693	29.6516	26.0228	28.9617	30.2743	28.3934	28.5883	28.5847	28.9811	28.9766
27.6625	28.0041	27.9741	27.849	27.2423	28.681	27.9781	27.5392	27.549	27.4886	27.6499	27.9301
25.3899	24.6942	26.6981	26.8511	27.7861	26.1745	27.9586	25.7072	27.3212	27.4014	26.3836	26.6519
26.0793	26.1616	26.2377	26.5583	27.923	26.8589	25.829	26.7331	24.569	25.5429	24.7272	23.8713
25.8582	28.0384	26.5146	25.7265	27.0705	24.8345	26.9004	25.4989	29.1403	29.0989	28.8251	28.8434
27.994	28.4589	28.09	27.8484	27.8548	27.7078	27.1551	27.0639	27.5135	27.0145	27.5601	26.8603
29.5494	29.3977	30.0303	30.0901	27.5379	30.0922	30.8899	29.7571	25.6775	26.0414	26.9954	25.8211
25.782	26.7661	26.3352	26.383	25.9979	26.0553	28.6011	25.755	23.6781	25.0762	24.9241	24.3213
25.9894	26.2967	25.9368	25.9371	26.9536	26.2382	27.1062	26.9179	26.6786	26.4211	26.9339	26.8195
27.2512	26.4912	26.7116	26.57	24.824	25.5003	25.6245	25.4555	24.626	26.928	26.5668	26.6295
25.1042	26.1243	25.8353	26.8544	28.4427	26.4787	26.2561	27.4968	26.1005	26.2573	26.3948	26.6141
25.19	24.7917	26.6157	25.3402	26.2032	27.2502	27.3344	26.0123	23.4861	24.7685	23.6918	23.769
27.3901	26.3028	26.4595	27.9723	26.1223	26.2664	27.2596	26.475	26.7744	26.7267	26.397	26.3953
27.5376	26.401	25.9508	26.5117	27.2776	26.4286	25.844	27.2281	26.2657	26.9066	23.3024	25.26
24.7871	24.926	27.1516	26.8064	27.6036	25.1603	25.2891	27.0519	26.3016	26.1588	23.8075	26.7493
27.3462	24.8238	28.1773	26.2028	25.6412	25.8924	25.3929	26.3894	26.0703	26.3746	26.1752	26.2177
26.9674	26.9395	26.4487	25.3954	27.904	27.0524	25.9382	26.4002	25.8894	24.0916	24.4182	23.4904
26.4797	24.9959	27.6427	26.5399	26.0064	24.5805	25.4864	25.8434	24.6003	25.5623	24.2752	25.1077
26.9852	27.2349	27.4688	26.9073	26.2579	26.1163	27.4089	26.993	28.1937	27.9841	28.1173	27.8585
25.3244	26.72	25.5085	26.2324	25.1557	26.9231	25.7425	27.0143	28.0696	28.2884	28.2707	28.1724
25.7066	27.1633	25.4831	25.1698	26.4935	26.2742	25.9449	25.3336	26.2758	24.8682	24.5613	23.1269
25.1047	25.9583	26.574	26.7763	27.5614	27.3444	26.1649	25.8761	26.2622	26.1016	26.2654	26.0563
26.457	25.7758	26.0589	24.7994	25.6494	26.7468	27.3154	26.3862	28.8295	28.5709	28.5214	28.5869
26.8543	25.841	25.8973	26.8391	27.2592	26.2626	26.7938	24.2149	24.9422	24.8013	26.4873	26.5522
27.8359	26.4131	26.2278	26.0796	26.8717	26.0174	27.0327	26.8932	27.3554	23.7341	27.8878	27.3648
25.8671	25.4373	27.1533	26.3886	25.7844	26.7295	27.5567	25.7325	26.758	26.554	26.8064	26.8999
26.3046	25.7845	26.2538	26.6499	25.837	27.1568	27.8832	26.6152	26.5473	26.9433	26.1548	26.1585
27.2209	26.1603	26.7346	25.677	27.363	27.0123	27.3479	27.0787	29.0836	29.0042	28.9303	29.0153
25.2055	27.1232	25.5835	26.159	25.5794	26.3667	25.6754	25.3197	25.9812	23.8858	26.8473	25.4204
25.1148	25.5022	25.8744	25.7579	26.8002	25.4401	26.0672	26.2974	27.0584	24.7576	24.38	24.3777
26.4269	26.3623	25.2206	25.7884	27.2107	27.2791	27.038	26.0025	24.2519	23.9484	25.311	25.1459
26.5163	25.1911	26.2245	26.1339	27.2362	24.9672	27.2823	26.2468	27.9068	27.2824	27.5308	27.4902
27.5173	26.4985	25.8944	26.6494	27.6187	26.3413	27.8649	28.2254	24.9153	25.2019	27.4205	25.6657
27.1539	25.7278	28.0702	26.7591	26.815	25.9771	26.8038	26.8153	26.0217	26.536	26.1233	25.1847
26.8056	25.1236	25.2788	26.8423	25.5196	26.0795	25.8083	27.1416	24.6424	23.7962	25.1076	25.6795
25.9685	25.4331	25.7219	26.1871	25.5557	27.0276	27.0727	27.7599	26.3356	25.9806	25.9103	26.045
27.3876	26.5177	27.3557	25.5297	26.5258	27.255	26.8858	25.7015	25.351	25.7039	24.2531	25.9716
26.0257	28.0345	25.635	28.072	26.1477	26.9916	26.3406	25.3875	27.7291	27.3529	28.1282	27.7144
24.7836	25.8203	27.793	26.1456	25.4247	27.8545	26.8407	25.5982	24.7996	23.8411	24.6577	27.1195
26.6806	26.2638	26.9084	25.2413	28.409	26.0719	26.9538	26.2394	23.5771	24.0073	25.049	26.0447
26.2288	26.7386	24.5284	26.4624	27.0604	26.1485	24.9346	26.0595	27.5963	27.9124	27.6514	27.804
31.5813	32.1132	28.2754	28.8134	30.5871	30.6202	31.111	26.6594	25.4148	24.8798	24.3994	26.2814
28.952	25.9424	24.8693	25.7616	25.9962	25.3763	26.8612	25.9171	25.1023	25.2577	25.6801	25.6497
26.9164	26.6257	25.4199	24.8557	28.597	25.3566	27.3178	26.1519	25.6162	25.645	24.2875	25.7009
28.0051	28.0532	28.5601	29.0683	26.7433	26.1637	25.5205	26.8325	28.7248	28.7086	28.6674	28.7895
27.6977	26.2115	27.1441	25.9987	26.7925	25.6158	26.712	27.0527	27.1426	27.3334	27.9094	27.9026
27.1932	26.4508	24.8137	25.8502	27.3069	26.1604	27.4717	28.0668	27.976	28.2224	28.255	28.531
25.7156	25.3388	25.9226	25.9544	26.6199	25.9639	25.7	26.0471	27.5289	27.647	26.8937	27.6894
25.9526	25.054	27.6107	26.2998	27.0338	27.3767	26.5478	26.1745	28.0742	28.2203	28.1942	29.2199
26.3255	26.5966	27.1542	26.8569	26.7003	27.4831	27.3015	26.6668	27.2564	27.4022	27.2974	27.3016
26.3377	25.5621	26.087	25.6765	27.1543	26.4206	27.7239	26.2021	23.5001	25.9759	26.5579	26.429
26.4149	26.2708	25.6254	25.9107	26.4711	25.1756	27.2026	25.9065	25.6179	25.5638	26.3015	26.4729
25.5405	26.2933	25.9812	26.138	27.4693	25.8226	27.5487	25.5209	28.4609	25.7432	27.9853	27.3834
25.7396	25.576	26.7387	26.7519	26.2524	27.6579	26.1876	25.974	26.3287	26.4858	26.932	26.7113
27.4328	24.9591	26.3381	26.4766	27.2976	27.2598	26.3575	27.9445	24.3009	27.1379	26.7535	26.2483
30.652	30.8151	30.5861	30.5524	30.0759	29.7949	30.222	29.4317	28.6115	28.7605	28.9104	28.9492
27.1401	27.3194	26.6842	27.6085	27.6373	26.5298	26.2154	26.3969	24.2192	23.9764	25.2134	24.4717
30.0761	30.1559	30.2804	30.503	29.665	30.0535	29.8413	29.3934	27.8137	28.0361	27.9223	28.1223
27.5769	26.1331	26.8613	25.0862	26.887	25.8253	27.5787	27.5715	24.4007	25.9748	24.1893	26.8267
25.9347	26.0016	26.2135	24.7915	26.4906	26.3282	27.2573	26.7461	26.4136	26.2452	25.2762	25.1697
27.2344	25.0238	27.9749	26.1	27.1632	25.8283	26.014	25.0036	32.0465	24.1911	25.1521	25.8197
25.5806	26.1818	28.56	25.1026	26.9191	26.6741	27.0254	26.6593	25.9644	26.1695	26.1702	26.0346
26.4043	25.3568	26.468	25.1661	25.9362	26.4142	25.9427	26.3462	26.3337	26.2449	24.4522	24.7175
29.051	28.9717	28.5255	28.2126	26.5474	27.7108	26.5639	26.009	25.9086	25.5559	25.1216	24.0603
25.8002	28.5926	26.174	28.3111	26.4398	26.8652	25.469	26.195	27.5605	23.4807	24.4989	24.9978
27.1701	25.4937	27.3989	25.0697	27.196	27.0706	27.3662	26.4338	24.4318	25.6489	24.1076	24.9978
26.2213	26.4116	27.599	26.0679	26.846	25.6239	27.3045	26.7874	25.7532	23.8303	23.1306	23.5497
26.7649	26.1525	25.3098	27.1099	26.5911	25.903	26.3822	26.944	24.7225	26.8482	25.31	25.9235
25.6169	25.3342	27.4919	25.8887	26.823	26.7134	26.7257	26.7977	24.5895	25.016	23.3317	24.429
25.3892	26.6342	25.8329	26.4372	26.6221	26.8124	27.7994	24.5292	27.3969	27.046	26.6491	23.739
25.7047	25.8014	25.6886	25.6062	27.5089	24.9901	25.9599	24.6232	24.9818	26.3115	26.4891	26.3873
25.2921	23.9833	25.1791	26.6856	26.9747	27.4539	26.9316	25.8872	26.2696	26.5561	26.6498	26.4771
25.4919	26.1356	25.8753	26.9945	28.0666	26.1763	26.338	26.3437	26.3741	26.678	26.407	26.1827
28.5544	28.4359	28.402	28.4451	25.8856	27.5626	26.9231	27.4161	29.2134	29.1687	29.1982	29.2065
24.564	26.2256	27.225	26.2314	26.9941	25.4249	27.0511	27.6124	25.5833	23.6458	24.2788	24.9233
26.2412	26.9715	27.7528	25.5923	25.767	27.7122	27.4994	27.0374	25.5427	25.8611	26.1489	25.9787
27.2419	26.4565	26.6163	26.4709	26.0725	27.327	26.8139	24.9218	27.1729	27.2034	27.2285	27.3604
26.0705	26.9328	27.5184	25.2102	26.4136	26.3494	28.4898	26.5315	24.9394	25.5446	25.8906	24.2839
27.3178	27.7198	24.6449	28.2926	25.5143	27.9532	27.1307	27.6224	27.6836	27.8373	27.7693	27.8415

25.7133	25.7463	25.633	25.3702	26.8973	26.1828	25.2779	25.7462	25.5915	24.3464	25.3185	25.8435
26.6946	25.9205	28.0165	24.1723	26.9956	27.7081	25.6469	26.2547	26.4507	26.5368	26.3688	26.6213
30.6521	30.5908	26.0795	26.4719	25.2928	30.2878	28.0152	27.2785	33.3498	32.0339	30.6775	32.4819
28.0129	27.9311	28.4058	28.1466	30.778	31.0287	30.8808	29.8892	25.5636	24.1048	25.3184	25.6725
26.602	25.0332	25.6516	24.8669	26.6003	26.7949	27.4621	25.8729	26.1515	25.1032	25.3783	24.6811
25.8284	25.3177	26.5666	26.4853	26.0821	25.2489	26.4719	25.9031	23.7577	24.7991	24.1402	24.6463
25.5799	26.8186	26.8009	27.7719	27.9633	25.0001	27.6421	27.2817	29.1725	29.1888	29.3851	29.3582
25.8671	25.326	26.5077	25.2161	26.4626	27.3724	27.2909	25.9969	26.6583	26.3306	26.6275	26.3542
26.5582	26.9445	25.9665	27.2832	27.2652	28.109	26.3325	26.6148	27.6366	27.6318	27.6446	27.6413
24.8807	26.1195	26.0991	24.732	25.0687	27.883	27.085	25.4102	24.9571	26.5602	25.614	25.4172
26.8268	24.1825	27.1262	26.6744	26.6748	27.3464	26.6378	26.1993	25.4383	25.1386	25.2146	24.6618
28.8673	28.5299	25.4263	27.2291	27.43	28.6151	26.936	26.3478	27.0132	26.5016	27.1484	26.9738
26.5865	27.58	26.7624	26.2221	25.4636	25.5938	27.2751	26.4698	27.3292	27.2734	27.3407	27.4972
27.1505	27.2868	27.4394	27.088	28.9925	23.999	26.6373	24.2011	26.7584	26.6591	26.9334	26.8172
27.4035	26.9309	26.6326	25.5972	26.9928	27.4821	28.0452	25.8848	25.6605	25.916	25.622	25.5648
26.7487	26.7369	26.5965	25.9292	27.6123	26.8075	27.9494	26.3508	27.0456	26.6289	27.4942	27.6392
26.5608	26.2456	24.3195	25.94	26.7809	26.611	25.4341	26.2133	24.6089	25.0099	24.9447	23.9972
24.833	26.3731	26.6982	25.0717	28.0009	26.3657	27.4611	25.9246	25.8793	25.8648	25.739	26.3326
27.0628	27.1854	26.2244	26.6813	25.8734	25.3531	26.2849	26.3012	27.7494	27.9006	28.0102	28.0116
25.539	27.2333	27.1613	25.5742	27.1095	25.6061	26.6653	25.9354	26.3084	26.2177	26.9294	26.9315
25.5998	25.4436	26.9431	25.515	27.0765	26.5247	25.8743	26.0931	25.86	25.511	25.4758	24.5234
27.4548	27.3779	27.3689	26.9051	25.2145	27.2513	26.7571	26.5769	26.3763	26.6554	26.5365	26.5272
31.6821	31.5477	31.1199	31.4088	31.3504	31.2394	31.5125	30.9428	28.175	28.1568	28.116	28.0074
25.8904	24.8459	26.0684	26.2271	26.0577	26.1184	26.3437	26.6293	25.3519	25.6479	25.1067	23.8496
25.6443	24.1971	28.0786	25.5126	27.6119	26.6313	26.4483	26.4582	25.1972	25.3082	25.6863	25.8035
27.9742	27.7523	24.7823	26.9792	25.9426	27.9909	27.0708	27.2465	27.3973	27.007	28.0525	27.8917
27.3819	26.1735	26.8779	26.9014	27.5733	27.0039	25.4552	26.1725	27.8219	27.6936	27.4991	27.57
28.3358	28.5279	28.1646	28.1234	28.3467	28.4741	28.0687	28.8367	28.8421	28.7416	28.7569	28.8835
29.6054	29.8339	25.5576	27.5546	25.8849	24.9915	25.9017	30.1922	29.3002	29.2204	28.5957	29.1049
25.8121	26.0509	26.0647	26.502	26.7802	27.2212	27.1031	26.6275	27.9787	27.9899	27.9492	27.8175
27.7734	27.8064	27.2692	26.0322	26.8672	24.9862	28.4219	26.3824	28.1686	28.0926	27.9963	27.8889
26.0079	27.7713	25.9434	25.7496	27.7676	26.8869	26.5549	25.1256	24.7242	24.6418	25.6244	24.3268
27.4142	25.0604	27.4133	26.6844	27.0814	25.6703	25.6666	26.6997	25.9466	26.2941	25.9048	26.0811
27.9031	26.8856	26.8122	26.9617	25.7448	26.683	26.691	26.294	26.1946	26.1123	26.5633	26.4384
25.0323	25.8264	27.0384	25.0111	26.9078	25.4224	26.9598	24.6838	25.2074	27.1587	27.3924	27.4678
25.4072	25.092	25.3323	25.3105	26.5476	27.9815	27.8265	27.0018	27.1673	27.5668	26.7051	26.9089
26.4849	26.151	25.4255	26.5046	26.246	25.9967	27.9006	25.8702	26.6522	27.3832	27.311	27.4675
25.1567	24.1173	27.036	27.184	28.8729	25.8128	26.7397	25.9503	27.0514	26.7895	26.6376	26.7087
25.9584	24.2228	25.606	26.7286	28.0367	26.0715	26.0801	26.0818	24.7333	26.5275	24.4761	23.946
26.8741	27.2689	26.1213	27.5867	26.9836	25.4531	25.9601	26.7926	28.5622	28.3623	27.6473	28.0318
26.7199	27.3346	27.4967	27.8866	26.1913	28.1093	26.9951	28.6655	27.4305	27.4841	27.5272	27.6013
26.1733	25.3109	25.6378	28.4689	25.7327	27.2111	26.3707	26.2855	25.0064	24.6466	24.7514	24.3641
25.1694	25.6535	26.6336	26.2553	26.7125	27.369	26.041	25.6658	26.418	26.3932	26.7935	26.7278
26.565	25.1949	26.3719	25.9031	26.8317	25.8098	28.2152	26.3061	25.8107	25.73	25.128	24.9862
28.1509	28.1341	26.7574	24.124	26.702	26.3951	27.3544	26.4887	24.4428	25.1602	24.7324	26.6133
24.5351	24.71	27.4932	26.1081	27.3612	26.6482	27.5704	24.1767	25.6512	24.8492	26.3388	26.3997
27.5172	27.3521	24.7155	27.4524	27.6117	27.074	25.4952	26.8896	27.2552	27.3528	27.4936	27.2896
24.1964	27.0693	26.431	25.8971	25.6795	25.9578	26.5981	24.0345	27.0586	27.0054	27.5961	27.2715
29.3237	29.1436	29.0144	29.5772	27.4072	28.1448	29.1199	29.2563	29.4112	29.2903	29.4015	29.2369
26.3875	26.6153	26.0588	26.2697	26.1989	27.7358	26.6903	27.751	27.707	27.6937	27.2931	27.4194
26.7597	25.1379	26.7709	25.1517	26.9672	26.728	26.5421	26.246	26.6402	26.6662	26.629	26.7275
25.2469	26.382	25.8263	26.6387	26.3286	26.219	26.4359	25.9357	26.0321	26.7568	25.7728	25.9457
28.9157	29.2127	29.2501	29.1429	28.7116	29.2929	28.4618	29.3844	28.7467	28.71	28.9037	28.9331
26.946	25.4806	25.0458	26.4714	26.5398	25.7349	27.5667	26.4902	28.8459	28.4086	27.9183	28.4703
26.9454	29.5458	25.6117	29.1565	26.824	26.45	26.8989	26.3509	23.5398	26.2921	26.8698	26.4121
24.7121	25.209	25.0358	25.1238	28.7152	28.3969	28.7794	28.5324	26.2192	23.1632	24.2049	23.7399
25.4917	26.7331	24.9611	25.9582	25.2261	26.5295	25.9019	24.7051	25.6521	25.5968	23.1024	22.9129
25.2957	25.7672	27.2065	25.9528	24.4377	24.3684	26.9924	27.7083	25.8388	24.748	25.0126	25.6957
26.0041	25.552	26.2313	26.5335	25.9945	25.0209	28.5993	27.2608	26.1936	27.2137	26.5952	26.3278
27.8797	26.0303	26.2928	27.9485	27.9077	26.4246	27.9403	26.0469	29.0172	28.768	29.0611	29.1346
27.1237	28.5073	25.4646	25.2999	27.4989	26.7157	26.2402	26.4237	28.8449	29.0756	29.2056	29.1042
26.4454	27.2227	27.945	25.537	26.3618	26.3107	26.9964	26.1713	26.7322	26.9688	27.093	26.8671
27.1088	26.6275	25.9081	24.5037	26.1838	25.7158	25.9642	27.9433	25.2278	26.3982	26.5262	26.1371
26.3679	26.3237	25.7476	24.931	26.9054	27.7542	27.2074	25.9208	24.3424	23.9147	25.5677	25.3545
26.016	25.6304	26.6359	26.3821	27.2066	25.762	28.4428	25.6901	25.2826	25.7502	26.0676	25.1555
25.7145	25.46	27.4565	27.0656	25.4367	27.1072	26.9582	25.7811	26.7645	26.7355	26.8057	27.1676
26.5878	26.7808	27.1879	26.1164	26.7374	26.0033	27.7662	25.8949	24.5032	22.8679	25.8798	24.2948
26.5512	25.3822	24.4769	26.588	25.9657	26.619	27.5556	28.132	24.6423	24.9974	24.3196	24.1009
26.4246	25.9089	25.6472	27.8581	26.7668	27.1386	25.1157	27.5253	23.8628	25.0814	27.2251	23.0672
26.2735	27.2665	26.314	26.8761	27.5812	24.7933	26.0887	26.7171	26.3112	26.3344	27.0646	26.7857
25.9181	26.3121	25.4261	26.9578	27.3255	26.7308	26.4114	25.2846	24.8782	24.0259	24.4528	25.43
25.2669	25.6862	25.9525	25.2165	26.5552	26.5804	26.9844	24.5789	26.2601	26.0912	26.7658	26.874
27.5922	26.9845	26.7248	26.6073	26.1395	27.5108	27.8449	25.071	28.2834	27.8382	28.0159	27.679
25.4817	25.2289	26.4528	25.0072	25.7319	24.953	26.8087	26.4793	22.8528	26.7216	26.9214	25.7256
24.8147	25.3029	25.7719	25.8016	26.4409	26.5584	26.0015	26.3079	25.8749	25.2718	25.0795	24.265
25.67	25.7932	24.6411	25.6417	26.9808	26.0393	25.2266	27.8455	24.4291	25.2317	25.2925	25.1384
27.3046	26.6854	27.5478	27.6504	26.8695	25.402	26.5184	26.3228	26.9464	27.0026	27.209	27.368
27.1889	27.1964	26.4662	25.5766	27.1487	26.9783	26.7816	24.3766	24.1461	25.3764	23.5628	24.8915
26.8775	25.11	25.7023	26.4275	26.7596	25.2724	25.3421	25.7828	26.5854	26.4627	26.6609	26.4207
27.9906	26.4289	27.7354	26.7534	25.9342	26.0195	26.7511	24.2493	25.9787	26.0589	26.9006	26.7458
26.519	28.4685	28.9696	28.8702	26.3944	28.3214	26.1292	30.212	29.0386	29.1212	29.0499	28.7338
27.3891	27.3347	26.9818	26.9724	26.794	27.1645	26.5115	27.7895	27.1771	24.6961	27.3307	27.3706
27.0404	24.8215	25.8626	26.4282	26.6915	28.1322	27.3787	29.999	23.9149	26.2258	26.5981	26.8677
26.4786	25.3038	26.3855	26.7325	26.619	26.4034	26.8051	25.7161	24.9489	26.6383	26.561	26.4622
26.6777	28.4949	26.2274	26.331	27.8787	28.9841	27.524	25.7884	28.2411	27.9211	28.3792	28.665
29.5723	29.5857	29.0908	29.0059	29.7074	29.5	29.5623	28.6909	27.5636	27.8727	27.7304	27.6828
25.6173	26.967	25.9108	25.5187	26.1767	27.2337	25.6983	26.832	27.8037	27.6039	27.6605	27.6374
26.231	25.4811	26.5704	27.3121	26.9118	27.4165	26.5732	26.5457	25.5254	25.3185	25.5378	25.5784
28.1804	27.9194	26.0944	28.7155	26.394	26.0735	26.5529	27.8687	28.6124	28.0261	27.9784	27.8137
25.5422	25.0262	26.1637	26.5636	27.7043	26.4387	27.365	27.4736	27.8479	27.8809	28.0218	27.8944
26.959	25.2453	27.2094	25.1747	25.7536	26.9896	27.0085	27.1186	27.212	22.4737	24.4101	26.6367
26.2691	25.2206	25.0908	25.5031	27.1404	26.5055	26.6473	25.3291	26.9419	26.8219	27.1673	26.7721
25.1473	25.9389	27.0354	26.8755	26.8728	25.7134	26.7055	26.9866	25.6983	26.0292	26.4029	26.2182
27.9451	28.422	26.1265	29.047	25.7363	26.7171	26.5058	26.0875	27.8232	27.7988	27.6304	27.3485
26.295	26.5988	26.8331	26.5549	26.4555	27.6446	26.7729	26.688	28.7615	28.9371	29.0769	28.7439
25.6614	25.2544	27.8716	26.2673	25.892	25.5209	27.2254	28.2018	22.785	24.1752	25.6216	25.4163
25.3056	25.2555	24.8034	24.9201	26.9705	26.9717	26.766	25.8472	26.1855	26.7844	23.4636	24.3638
26.2822	25.5678	26.3796	25.037	26.0251	26.5733	26.3717	25.913	27.3932	27.5318	27.375	27.3827
26.6535	24.7571	26.3936	25.934	26.5489	27.0947	26.9273	25.5923	26.1541	26.2672	26.7894	26.7165
27.2536	23.8511	27.853	25.9617	26.3432	25.894	26.02	26.7526	29.242	29.0647	28.9787	28.7015
28.9085	29.3293	26.8404	28.5936	29.1484	29.5561	28.9947	28.7155	28.0132	24.8714	28.3706	28.2636
25.5545	26.5847	26.5249	26.8364	27.8669	25.1646	26.9194	26.9652	25.5917	24.7636	25.65	22.932
30.6359	30.7148	30.6961	30.9297	29.8529	29.6096	29.5728	29.7122	31.0527	30.82	31.0786	31.2079
27.586	27.6055	27.434	27.3708	26.5573	27.4443	27.4056	27.2687	24.8555	24.4953	24.4579	24.6132
28.044	28.3101	28.2427	28.3686	28.8328	28.9971	28.8785	28.6845	28.2985	27.7005	27.8945	27.9133
26.477	27.4066	27.0062	26.5785	27.4255	27.2942	27.5786	26.4043	27.4514	27.2943	27.4555	27.5655
25.6797	24.0901	25.3759	25.2635	26.4853	27.2741	27.2309	25.8612	25.3459	25.2981	25.3331	25.3953
28.6579	28.9843	27.786	28.0536	28.3764	27.6047	28.2434	27.5515	25.7372	24.7015	24.3066	23.8554
26.506	27.5201	26.142	26.6456	27.4035	26.2237	26.5473	26.6325	23.8571	25.2626	25.6569	26.1177
26.505	26.2734	26.9833	27.0342	26.18	26.8418	26.4832	26.9869	26.1064	26.0006	25.7607	25.8839
30.3966	30.5399	30.5523	30.3995	29.5097	30.1734	29.1479	30.2234	30.2034	30.2425	30.0831	30.0488
26.3492	25.1225	25.8893	25.2981	26.0309	25.6415	27.9795	26.5203	24.0833	23.9965	24.9327	26.9566
27.5467	25.1319	27.0468	24.6658	27.3884	27.1471	27.7858	26.2253	26.0149	26.299	25.936	26.33
25.943	25.5953	25.7245	26.0779	27.6822	26.8462	26.0188	26.9616	26.3165	26.2962	26.2705	26.0635
26.1193	27.1304	26.4324	26.5902	28.1114	24.5933	27.333	27.1105	26.1486	24.1874	26.7032	26.7108
25.5496	25.1028	27.0421	27.0546	25.8225	26.3433	27.0273	26.1598	28.1674	28.0938	27.7609	27.7072
26.2135	25.6105	26.1728	26.8525	26.9724	26.6783	25.3093	27.5718	26.5751	25.8223	25.8386	25.8877
25.4891	25.4862	27.9289	27.4098	26.0378	27.9301	26.8669	25.4347	27.9063	27.8326	28.0974	28.0125
27.939	28.0862	28.1458	27.876	27.1141	27.7339	25.7139	27.2356	28.5495	28.598	28.5872	28.4809
26.9714	24.8273	25.8031	27.5036	27.5865	27.0198	26.9704	26.2109	25.8748	26.5651	26.5619	26.4493
25.9127	24.4961	25.7807	23.8265	26.7776	24.9037	28.1446	26.7792	28.7046	28.4921	28.1321	28.2285
27.8317	27.9329	27.9826	28.0744	26.7923	26.5387	27.8748	26.181	27.5683	27.1212	27.0322	26.7652
28.531	28.3181	28.6845	28.7271	28.5635	28.7297	28.5945	27.8547	27.7335	28.0131	28.1823	28.2352
28.0331	25.4708	28.7814	27.8568	27.2747	28.9418	26.1798	29.1082	27.5086	27.8628	27.8712	27.8861
25.2824	25.7068	27.9853	25.9464	26.0946	26.2937	26.6997	26.7186	27.5141	27.8603	27.7076	28.3098
26.1745	26.4855	26.5847	27.0698	26.236	28.7494	27.0006	26.646	25.6246	25.609	26.1324	25.8064
28.3164	28.5784	27.4787	27.9961	26.9295	28.2583	27.6117	28.4573	28.4853	28.3193	28.8907	28.7669
26.4013	26.335	26.5088	26.2409	27.2888	25.3249	28.6668	25.4778	24.7041	23.5173	24.5669	24.2237
25.2349	25.2717	26.7629	26.9969	26.9454	26.7578	26.6974	27.5958	24.6764	24.4582	25.7247	25.5851

27.3126	25.6599	26.6887	25.1211	27.1127	26.0694	26.5651	27.64	24.2741	23.8632	25.0834	25.2452
27.2216	26.5548	26.6717	25.5858	26.3097	26.253	26.4832	26.9462	27.6578	27.7054	28.2415	28.1049
25.9793	26.6479	25.6207	25.7489	27.2937	26.0124	25.8225	25.217	24.9745	25.4483	25.4496	25.7526
24.9871	26.9572	27.9374	27.4669	28.4087	27.9272	25.8068	25.9572	28.417	28.3361	28.6572	28.795
27.349	26.7781	25.8306	26.9358	27.1195	25.9188	28.8318	26.718	24.4075	24.3771	23.6144	23.9645
24.3151	26.4015	26.0792	24.8463	27.2865	26.5689	26.4782	24.1139	26.4402	26.2072	26.2621	26.4915
27.0657	26.501	26.3813	26.3002	26.424	26.6915	27.6686	27.4131	25.1065	24.2375	24.0377	24.7247
26.2539	25.7696	27.3093	23.7459	26.7112	26.4014	24.527	25.2726	26.7476	26.5143	26.5898	26.7452
25.1184	26.6519	27.4466	26.4377	26.8138	25.4449	26.4559	27.2565	24.6216	22.9329	23.6742	24.4611
25.858	26.7412	26.1115	26.837	26.6967	25.102	25.9264	27.3154	26.575	26.4863	26.6892	26.8444
25.7596	26.4491	25.7397	25.1453	26.7349	26.9715	26.4406	27.3596	24.1819	24.831	24.9352	24.6236
25.4856	25.352	25.9154	24.7142	25.8701	25.3599	26.6026	26.4653	25.9365	23.791	26.0532	25.8195
26.5604	25.7659	26.439	26.3069	27.445	25.4181	26.8174	25.8417	24.7993	25.6006	26.1948	24.3332
25.8334	26.1035	26.0792	26.0749	27.4564	27.1008	25.9569	26.5379	23.9592	25.2279	24.8119	23.9123
26.1422	26.5113	24.7481	25.7785	27.7064	27.3117	27.3585	28.4011	24.9295	23.8733	23.8975	25.1705
25.0281	27.9304	25.9457	25.5397	26.9486	26.9154	27.0906	25.9429	27.1081	27.5711	27.6587	27.5859
26.4955	26.2185	26.4827	25.0661	26.963	26.182	27.2852	27.371	28.4424	28.438	28.3104	28.4394
29.2292	29.2273	29.0272	28.9909	27.1263	29.2283	26.9357	29.0476	30.0997	30.1121	30.2309	30.1378
25.9344	26.2528	28.0529	28.4943	27.5666	27.3608	27.3959	27.0306	27.276	27.418	27.4314	27.3678
28.0978	27.8974	28.1192	27.9688	28.346	28.6131	28.2319	28.3088	27.4453	27.407	27.1067	27.0691
24.5966	25.7899	24.6753	26.5865	26.8755	26.6266	26.485	24.5833	24.7588	22.7692	23.7503	25.2738
24.9119	27.5099	26.938	25.7957	26.5718	26.0263	26.5847	26.528	25.1813	26.6933	25.9256	26.4762
26.4626	26.3443	25.5366	27.717	26.5751	24.5424	26.5302	25.5051	28.8161	28.8375	29.1969	29.1331
27.897	27.8731	27.5554	27.4458	26.1566	27.972	28.5685	27.4799	27.8988	27.8551	27.8944	28.0958
27.2337	27.7611	25.3035	25.9244	27.1855	25.2289	25.8409	25.6079	27.4318	27.0407	27.4489	27.4524
27.5437	28.0613	27.3695	27.5346	27.585	28.2341	26.8226	27.9909	27.9705	27.8904	28.301	28.3276
25.943	26.2323	26.9451	25.2404	27.1622	26.7435	27.3147	26.9127	25.9641	26.5737	26.6453	26.6198
28.2454	28.3564	28.122	27.9035	25.6562	28.2637	27.5442	27.9657	28.4529	28.4925	28.6427	28.5318
28.7822	28.3127	28.1337	28.1931	26.9461	28.3196	27.2114	27.7999	29.6128	29.6231	29.7082	29.9913
25.8283	24.8597	25.8801	24.6845	26.8388	27.2802	26.7417	25.1697	28.0965	27.933	28.7033	28.5591
29.4324	29.3135	29.3805	29.1902	25.8493	29.2714	28.5869	27.9975	28.763	28.8842	29.48	29.4663
26.8306	25.9251	26.7622	27.3248	26.4488	25.453	26.7172	25.7107	26.2582	26.9294	23.7508	27.311
27.7877	27.6121	26.5015	27.5299	27.5595	28.6435	27.3193	28.1727	27.2171	27.2239	27.3414	27.3853
26.6891	24.6896	26.6166	27.9937	26.3748	25.9249	26.6629	26.4341	26.5341	27.7189	27.731	27.5561
28.4003	28.5922	28.6602	28.9393	26.5536	28.3279	26.7371	28.3313	27.0707	27.059	27.1971	27.0856
24.991	25.5854	26.0101	25.8783	26.3806	25.8696	27.1418	25.8391	28.8746	28.5857	28.604	28.5961
26.5583	27.3899	27.248	27.5797	27.0317	26.0353	27.0512	25.4557	28.3929	28.0213	27.7111	27.8639
27.9589	23.3748	25.7815	26.2913	26.9462	26.9582	27.4488	26.6413	25.1248	23.8206	25.3917	25.2119
26.2036	25.249	26.7793	26.5647	25.7758	25.6499	27.1014	25.715	26.869	27.0962	26.7216	27.0045
25.2992	26.8827	25.4669	25.396	26.8209	28.1097	27.1122	25.4045	27.8966	27.9066	27.857	28.0793
27.7097	26.6585	26.4336	26.1091	26.9496	26.4787	25.8168	26.8728	26.8507	27.0361	26.8203	26.5878
25.375	26.9678	26.4737	26.1133	25.978	26.6041	24.8626	26.9223	27.7386	27.4041	27.3029	26.788
25.9206	26.1475	26.9805	25.1098	26.9361	25.7439	25.6439	26.1808	27.6569	24.3526	24.391	24.1194
26.895	26.2375	25.6982	27.6643	27.3762	25.5278	26.3881	26.3324	27.1204	26.9445	27.1908	27.1381
25.5808	26.0141	25.3304	25.2867	26.5252	25.7526	25.6522	25.7799	25.421	26.1056	26.5325	25.9084
26.0193	25.9641	26.6153	26.7863	26.6293	26.9405	27.0537	25.3383	24.8551	25.097	25.9548	26.5055
26.556	27.5584	26.3797	25.0769	25.7028	27.0483	26.5303	25.8655	23.8918	25.6181	25.5443	25.0297
28.7832	28.7717	29.7941	29.3798	27.2614	28.5869	26.003	27.2935	28.697	28.4465	28.5744	28.2812
28.9049	27.336	26.1093	25.8055	28.5578	27.3025	27.6052	28.8182	28.4853	28.3886	28.3947	28.4254
26.4975	24.8105	27.5954	25.9793	24.3769	27.0868	27.2257	26.0852	29.9525	29.7946	29.7737	29.5762
25.3936	25.455	26.2965	25.855	27.8796	25.9154	26.624	26.9234	26.5624	27.0765	27.1242	27.3935
27.3694	24.0672	25.2838	28.0923	27.1534	25.0317	27.2638	25.3456	28.088	27.9991	28.2849	27.9639
26.1839	26.6849	26.1159	26.7738	27.4117	25.3655	27.9839	27.1185	27.2983	27.2196	27.2673	27.3069
26.7593	28.1242	25.4469	27.0314	26.3369	26.0856	27.112	27.1019	27.2614	27.3711	27.6083	27.4809
24.5303	24.7191	27.1508	25.9376	26.6733	28.0508	26.5959	26.5003	26.6171	26.5449	26.3439	26.6873
28.2311	25.2845	26.2096	26.5051	27.773	26.3923	26.6335	26.4755	26.4875	24.9093	23.8357	25.1435
25.9293	26.1856	26.0061	23.9591	26.4455	27.6228	26.1691	26.8522	24.0165	24.1138	23.8091	24.3932
24.6856	27.8863	25.5505	24.9878	28.4748	28.858	26.9053	28.8512	28.0184	27.9362	28.1327	28.1163
25.7389	27.8817	25.3305	25.7469	26.1919	27.8017	25.4955	26.5833	27.052	26.8	27.3314	26.877
27.7182	27.7783	26.1969	25.8774	25.8407	28.2764	28.4032	26.8644	28.019	28.0232	28.1448	28.195
26.3952	26.7736	26.4998	26.2459	28.1503	27.2075	26.5352	25.8945	27.7507	27.8923	27.5874	27.5869
25.3605	27.8631	26.7897	26.0534	27.4891	24.7572	26.4834	26.1675	27.815	27.9565	27.7756	27.7357
27.2598	24.596	25.0232	26.8251	26.7475	24.9519	27.576	25.8644	23.0381	26.2637	24.3426	26.5987
26.515	25.7023	25.9291	25.1062	25.177	27.928	27.0195	28.3525	27.749	27.7707	27.8754	27.6613
26.4206	27.7551	26.6734	25.4079	25.4163	27.4301	27.143	27.1648	28.2967	25.1782	27.9972	27.8004
27.3001	25.2307	25.8498	25.6733	26.9597	27.2855	26.6579	25.7718	26.4944	26.4947	25.3628	26.1587
28.8063	28.4247	29.0085	25.6504	28.6055	25.5924	27.4565	25.9105	26.7771	26.5853	26.5538	26.4799
26.484	25.9148	25.7135	26.5514	26.9232	25.0182	25.1168	29.0911	25.9059	26.0231	25.6327	25.4846
26.8896	25.2546	26.2737	25.8884	27.4854	26.2831	27.1502	26.6309	24.5611	25.4626	23.4764	23.395
28.4605	28.456	27.5524	27.7029	28.0394	28.1268	27.1119	25.0007	28.67	28.8198	29.0861	29.2683
26.8366	26.8037	26.2618	26.9998	28.1727	25.3644	26.0321	26.2119	27.3425	27.1704	26.8786	26.6179
25.0888	25.4182	26.7132	25.8927	27.1689	26.282	27.1536	26.4125	25.3958	26.7847	24.2364	25.216
26.0189	25.7781	26.1918	25.8186	27.2615	24.7013	27.2858	27.8158	25.0117	24.796	23.7359	23.4265
26.6353	25.2181	25.7096	25.798	26.9099	26.713	27.686	24.7274	27.2339	27.081	27.0005	26.6444
25.6306	24.5993	25.8943	25.8747	27.4096	26.3708	26.689	24.8023	25.379	24.4811	24.6852	24.9803
27.9526	27.9235	27.5885	27.7361	27.2403	27.1644	26.9811	27.1337	28.4586	28.4117	28.7686	28.7228
28.7809	28.684	28.6304	29.1738	26.5362	28.8173	28.9113	28.932	28.9651	29.3565	29.1469	29.0332
26.8501	27.2273	26.5872	26.1367	27.3204	24.4445	26.4005	26.7711	25.5412	25.4557	25.5761	25.567
24.9222	26.1438	26.1127	23.3997	27.137	26.641	25.7023	27.079	24.5773	23.7171	25.0764	24.1525
26.891	26.3858	26.8958	26.6046	27.1751	25.6851	26.6996	27.1598	25.8235	25.801	25.4104	25.3285
29.5342	29.6582	28.5202	28.8377	28.2765	28.9949	28.4906	28.3987	30.0363	29.9927	29.7382	30.0142
24.6689	24.7547	26.4334	26.1946	26.0637	26.188	26.8183	26.0416	28.075	28.096	28.016	29.4001
26.6015	26.026	25.9749	26.1164	27.0048	26.564	27.0421	26.3495	25.4777	26.2536	26.2617	26.7084
26.3103	25.556	27.8191	25.1958	26.5165	27.5287	27.5441	26.2693	26.8008	26.7713	27.0059	27.4278
31.3581	31.4895	30.2776	30.2542	30.369	31.6851	30.2779	32.0345	30.4311	30.5286	30.5606	30.5217
25.5783	26.0173	26.1116	26.5249	26.4937	26.0365	27.545	26.1791	25.2577	24.1853	25.0362	23.9544
27.4162	25.2388	26.5536	26.1704	27.4586	26.2903	26.7943	26.1302	26.1653	26.1781	26.1635	23.7462
28.959	29.7145	29.6471	29.2172	29.0604	29.2981	28.8401	28.6022	27.9493	27.9884	25.5436	23.7457
27.1466	25.3589	27.4219	26.4483	26.7797	26.5502	25.6551	27.8167	25.011	25.3742	23.4721	25.0074
26.0056	25.7923	26.1802	25.1734	28.0665	26.0677	27.5764	26.7824	26.2491	24.0261	25.3197	24.2029
25.7726	24.4259	26.1964	24.9622	26.8535	25.9331	26.5565	26.8327	24.91	25.2901	27.4398	27.4733
29.9045	29.9574	27.6202	28.0518	29.6392	30.8454	29.4175	30.6633	28.1784	27.91	27.9794	27.7831
26.2357	25.2527	27.1693	26.3896	28.302	26.805	27.3324	26.2386	24.7891	25.9021	24.6408	25.4789
25.8172	26.0657	25.0981	25.7443	26.7961	27.1281	27.0948	25.097	28.364	28.2918	28.3065	28.3855
25.8538	24.7953	27.4454	27.7427	26.798	25.7453	27.7822	25.3877	27.5564	27.3104	27.2523	27.4678
26.9698	26.2028	26.2756	25.8298	26.9238	25.9983	26.0212	26.0081	26.308	26.079	25.8084	25.7592
26.2147	25.1103	25.6743	26.2643	25.4911	27.6863	27.7529	27.2873	25.8695	24.9779	24.9722	24.7076
24.6738	26.1879	25.5573	26.3724	27.2055	26.3554	27.5929	26.8841	26.971	27.0576	27.2832	27.135
25.4193	27.7488	27.5105	24.9928	26.6872	27.784	27.2757	27.4144	27.4811	27.1159	27.3564	26.643
26.5284	25.8322	26.4353	25.4467	26.0191	26.7752	25.7525	26.4711	27.2516	26.9759	26.5638	26.6461
26.4776	26.7399	27.1386	25.4654	28.0865	26.0228	27.4627	26.5347	26.9487	27.44	27.6034	26.9256
25.3559	26.0233	26.2505	26.6585	25.6574	25.7755	26.3254	27.1219	27.313	27.2628	27.0529	27.1682
27.1465	27.146	26.1832	27.7024	27.0135	28.0232	24.8842	25.6985	26.3053	26.6218	27.1296	26.7707
25.7482	25.6554	26.4923	26.7779	26.5382	26.0498	26.0736	25.5475	24.7499	24.6883	25.0254	25.3806
25.2771	24.7871	26.1629	24.5097	27.3109	27.503	25.7413	27.2768	26.3757	24.055	26.1239	25.8673
27.4321	27.6156	25.9935	26.0864	27.3849	27.4072	27.9255	26.0234	28.7148	28.6927	28.8256	28.7275
24.6185	26.5587	25.8453	25.8955	26.1191	25.8576	26.1446	26.4776	26.442	26.4176	26.4303	26.0706
26.808	26.9057	26.2916	26.3075	27.7128	26.9923	26.8647	28.7765	25.1182	24.7317	25.4644	26.7945
26.4667	25.9531	26.2205	26.1585	28.2355	24.7875	26.0748	26.4329	26.1665	26.2591	26.1793	26.0547
27.473	27.4423	25.0715	26.9973	27.3901	27.3859	25.5148	25.7809	26.1255	26.1986	25.8864	26.0012
26.4751	26.2921	25.7051	26.0633	26.0815	25.7327	26.8228	25.8148	26.2041	26.558	25.698	25.3472
27.5725	25.7582	24.7139	25.0036	28.2447	26.3725	27.1513	26.2578	27.3188	26.8836	27.0439	27.1223
26.2045	25.2855	25.3339	27.1904	27.1652	26.0835	26.3335	27.7262	27.3704	27.0054	27.7157	27.6197
26.6175	28.0444	26.121	25.5245	26.6797	25.4155	26.041	27.1104	26.5575	22.8756	26.8554	26.9846
26.2407	24.1292	26.7288	24.482	26.1804	26.0031	27.1419	26.3944	23.9855	24.1478	26.8682	26.3908
29.0538	29.0448	26.8472	26.5882	25.4249	25.8895	27.4275	24.6206	29.7251	30.3993	29.0954	29.7696
26.4024	26.6618	25.066	26.4207	27.1951	25.7756	27.1053	26.8572	25.7629	25.9891	25.6459	25.6078
26.0544	25.0139	25.0849	26.0404	27.4098	25.2869	25.3839	25.9388	27.9181	28.9891	28.0527	27.7609
25.5829	27.8727	25.0264	25.9811	28.3413	24.8178	27.2774	25.973	25.4588	28.8771	31.1904	30.8223
27.5061	27.3765	27.1012	25.6732	26.2207	27.3782	28.7554	26.2657	26.5588	26.5755	26.8723	26.7267
26.8784	27.175	24.5382	26.265	27.3336	25.9151	27.609	25.194	22.7009	25.3172	25.009	26.463
25.7756	26.0344	25.2104	26.522	26.6091	26.6874	27.5687	26.1204	24.8016	26.4139	27.0686	27.1517
25.9673	25.384	26.3535	28.0606	26.7527	26.8461	26.251	25.893	25.3187	25.6854	26.0432	24.5203
25.9146	24.2918	27.1066	24.7377	26.7225	26.7327	27.4098	26.3662	26.1543	26.1298	26.4995	26.7991
28.6818	28.7736	27.927	27.9341	28.2687	28.1082	28.2099	24.665	28.6764	28.482	28.8874	29.0471
27.2473	25.8212	25.6159	26.0968	26.3651	26.0852	26.4566	25.3146	23.9797	24.0189	23.3021	24.2106
29.3613	29.5717	29.2438	29.4357	30.0346	29.5139	29.8681	29.2518	27.7726	27.6858	27.7615	27.3785
26.1704	25.9876	27.479	25.6156	26.3718	25.7368	26.6485	25.2224	26.7012	26.3808	24.5543	24.2031
25.2754	25.3681	26.763	25.6306	25.9043	25.3173	26.5293	26.6747	26.7811	26.8848	26.6684	26.6822

27.3485	26.7026	25.8787	26.5749	25.8971	27.1384	28.4401	26.4706	26.2898	27.0641	26.4607	26.785
28.2246	28.5108	28.4859	28.6073	26.9736	27.9959	27.714	28.1021	28.5306	28.5565	28.505	28.79
26.2497	24.8335	26.3372	27.0671	27.373	26.6612	26.1379	26.8921	25.9145	26.1249	26.4059	26.497
25.065	25.6613	25.9248	26.9629	25.5604	27.4353	26.795	27.1047	25.4425	25.8571	25.2088	25.3694
29.9699	30.0676	30.2503	30.7062	29.7177	29.5511	29.6321	29.1835	29.9101	30.1711	29.6022	29.818
25.2888	26.1101	25.847	24.0259	25.6884	26.4238	25.4899	25.677	25.5828	26.9335	25.8828	26.0757
26.3671	25.6798	26.4391	24.7057	26.4167	25.9648	26.2104	27.2509	25.5612	25.6799	25.9915	25.6308
28.0971	25.6235	26.4617	25.8909	26.4514	25.0897	26.7499	27.8449	24.9091	27.5349	24.8814	27.4944
30.6337	30.8368	29.1797	29.1311	30.5727	31.0809	30.3345	30.2969	26.3601	26.2047	25.8412	25.9169
25.5703	26.8001	25.3679	26.2116	27.7886	25.7997	27.0751	26.811	27.1261	27.3799	27.6704	27.5125
26.2578	26.5784	25.8928	24.8631	26.1533	25.9734	26.5811	25.3743	24.9836	25.3293	25.3052	25.1031
25.579	26.5646	26.8138	26.0419	26.2579	24.9267	24.9455	26.1728	24.0642	23.9092	25.1898	25.4672
25.8354	25.4519	25.6732	27.4688	26.5949	26.9922	26.6798	26.8308	25.5413	23.5114	24.5121	24.4404
26.2117	25.7921	25.6608	26.1499	27.6224	25.4306	26.2252	25.2375	26.4823	26.6892	27.402	26.9999
28.3694	28.2428	26.4432	25.0246	25.0798	28.9817	28.2	28.5358	27.371	27.3663	27.8166	27.7777
26.5764	23.6594	26.8133	25.1074	27.784	26.168	27.3262	26.3072	29.5181	29.1736	23.1588	27.9524
25.5258	25.6427	25.9621	25.213	25.3237	25.857	26.9605	25.9412	25.4199	24.4832	24.2688	23.1992
25.6108	25.3371	26.5062	26.6456	27.4661	27.6421	26.0291	25.16	26.6658	24.6953	27.1489	27.1796
24.6701	26.6827	25.414	25.6792	27.1424	26.6149	24.7751	27.2337	26.0972	26.5308	26.3135	26.3381
26.0304	27.9064	26.1155	26.2022	26.6653	27.1912	25.5795	25.9488	27.5268	27.5451	27.6985	27.5207
25.2711	26.7094	26.5824	24.7663	27.7765	25.6201	26.1788	27.493	23.78	23.6921	27.8873	27.1361
26.3965	25.0174	26.9407	25.5864	26.5666	24.7718	26.5292	25.2508	27.0815	26.7116	27.0774	27.1121
27.4633	26.0918	27.0243	26.0689	25.0589	25.2473	27.0501	27.2021	25.9074	26.2704	25.5889	25.4185
27.6783	26.4976	25.7663	26.5	26.1458	26.6905	26.341	25.6298	24.4978	23.0578	25.3314	24.2955
27.6348	26.1232	26.0204	27.3645	26.2176	26.3147	26.899	26.4743	26.3343	25.5226	24.6043	24.3369
27.6066	26.9747	27.7955	25.9519	27.008	26.4792	27.4009	25.0915	27.9062	27.884	28.0821	28.1009

HDF_WT_1 MaxLFQ Intensity	HDF_WT_2 MaxLFQ Intensity	HDF_WT_3 MaxLFQ Intensity	HDF_WT_4 MaxLFQ Intensity	C: Protein Existence	C: GOBP name
25.931	24.0963	26.2404	25.944	3:Protein inferred from homology	
26.7282	27.1847	27.6292	27.694	5:Protein uncertain	
27.6258	27.7963	27.5704	27.6337	1:Experimental evidence at protein level	catabolic process;cellular catabolic process;cellular macromolecule catabolic process;cellular macromolecule metabolic process;cellular metabolic process;cellular process;cellular protein metabolic process;macromolecule
25.8279	25.7258	25.5864	25.7672	1:Experimental evidence at protein level	cellular component organization;cellular component organization or biogenesis;cellular membrane organization;cellular process;endocytosis;establishment of localization;lipid transport;membrane invagination;membrane orga
26.4053	26.6415	26.1321	26.1983	1:Experimental evidence at protein level	
26.3063	26.416	23.8004	26.1755	1:Experimental evidence at protein level	adipose tissue development;anatomical structure development;biological regulation;bone development;cell differentiation;cellular component assembly;cellular component disassembly;cellular component disassembly at cell
27.3257	27.1354	27.0685	26.9609	1:Experimental evidence at protein level	biological regulation;biosynthetic process;catabolic process;cellular biosynthetic process;cellular catabolic process;cellular macromolecule biosynthetic process;cellular macromolecule catabolic process;cellular macromolec
26.5594	26.7512	26.516	26.5617	1:Experimental evidence at protein level	autophagic vacuole assembly;autophagy;catabolic process;cell communication;cellular catabolic process;cellular component assembly;cellular component assembly at cellular level;cellular component organization;cellular c
26.4956	27.2006	26.831	26.8824	1:Experimental evidence at protein level	carbohydrate metabolic process;cellular macromolecule metabolic process;cellular metabolic process;cellular process;cellular protein metabolic process;dephosphorylation;macromolecule metabolic process;macromolecule
27.2888	26.6555	26.8032	26.633	1:Experimental evidence at protein level	biological regulation;cellular component organization;cellular component organization at cellular level;cellular component organization or biogenesis;cellular component organization or biogenesis at cellular level;cellular proce
26.0504	26.3903	25.8521	26.1041	1:Experimental evidence at protein level	establishment of localization;establishment of protein localization;establishment of RNA localization;mRNA transport;nucleic acid transport;nucleobase-containing compound transport;protein transport;RNA transport;transpo
29.4833	25.5068	29.6365	29.8322	5:Protein uncertain	cellular macromolecule metabolic process;cellular metabolic process;cellular nitrogen compound metabolic process;cellular process;macromolecule metabolic process;metabolic process;mRNA metabolic process;mRNA pro
30.0576	30.1588	30.0062	30.4091	1:Experimental evidence at protein level	biological regulation;biosynthetic process;cellular biosynthetic process;cellular macromolecule biosynthetic process;cellular macromolecule metabolic process;cellular metabolic process;cellular nitrogen compound metabolic
26.7565	26.6623	27.1426	27.0541	4:Protein predicted	
24.9407	22.0403	24.8916	24.4676	3:Protein inferred from homology	
26.853	27.1869	27.5009	27.5802	1:Experimental evidence at protein level	catabolic process;cell differentiation;cellular catabolic process;cellular developmental process;cellular macromolecule catabolic process;cellular macromolecule metabolic process;cellular metabolic process;cellular nitrogen c
26.9156	26.7884	26.7673	26.6213	1:Experimental evidence at protein level	biosynthetic process;cellular biosynthetic process;cellular lipid metabolic process;cellular metabolic process;cellular process;ether lipid biosynthetic process;ether lipid metabolic process;glycerol ether biosynthetic process;g
28.3066	27.8678	28.1019	27.8962	1:Experimental evidence at protein level	biological regulation;cellular macromolecule metabolic process;cellular metabolic process;cellular nitrogen compound metabolic process;cellular process;cellular response to stimulus;cellular response to stress;establishment
28.8744	29.2505	29.595	29.4852	1:Experimental evidence at protein level	biological regulation;regulation of biological process;regulation of biosynthetic process;regulation of cellular biosynthetic process;regulation of cellular macromolecule biosynthetic process;regulation of cellular metabolic proc
27.9594	28.1826	28.1721	28.2461	1:Experimental evidence at protein level	biosynthetic process;carboxylic acid biosynthetic process;carboxylic acid catabolic process;carboxylic acid metabolic process;catabolic process;cellular biosynthetic process;cellular catabolic process;cellular ketone metabo
28.8444	28.6694	28.4651	28.5184	1:Experimental evidence at protein level	biological regulation;cellular component organization;cellular component organization or biogenesis;defense response;establishment of localization;establishment of localization in cell;establishment of protein localization;esta
26.8496	27.2129	27.001	27.2645	1:Experimental evidence at protein level	biological regulation;cellular macromolecule metabolic process;cellular metabolic process;cellular process;cellular protein metabolic process;cellular response to stimulus;establishment of localization;establishment of localiza
26.912	27.4472	26.8999	27.2124	1:Experimental evidence at protein level	biological regulation;cellular process;cellular response to stimulus;immune response;immune system process;metabolic process;positive regulation of biological process;positive regulation of catabolic process;positive regula
26.8877	26.844	27.2092	26.8856	1:Experimental evidence at protein level	anatomical structure development;biological adhesion;biological regulation;blood coagulation;brain development;carbohydrate transport;cell activation;cell activation involved in immune response;cell adhesion;cell-cell adhes
29.3306	29.3839	29.6918	29.5657	1:Experimental evidence at protein level	
27.4929	27.4706	27.5142	27.1988	1:Experimental evidence at protein level	anterograde axon cargo transport;anterograde synaptic vesicle transport;antigen processing and presentation;antigen processing and presentation of exogenous antigen;antigen processing and presentation of lipid antigen v
26.8933	26.17	26.6868	26.518	1:Experimental evidence at protein level	cellular component assembly;cellular component assembly at cellular level;cellular component biogenesis;cellular component organization;cellular component organization at cellular level;cellular component organization or b
28.7951	28.6291	28.6615	28.5234	1:Experimental evidence at protein level	activation of immune response;activation of innate immune response;anaphase-promoting complex-dependent proteasomal ubiquitin-dependent protein catabolic process;antigen processing and presentation;antigen proces
28.5477	28.3924	28.2593	28.1238	1:Experimental evidence at protein level	activation of immune response;activation of innate immune response;anaphase-promoting complex-dependent proteasomal ubiquitin-dependent protein catabolic process;antigen processing and presentation;antigen proces
27.912	28.1521	28.4944	28.4216	1:Experimental evidence at protein level	activation of immune response;activation of innate immune response;anaphase-promoting complex-dependent proteasomal ubiquitin-dependent protein catabolic process;antigen processing and presentation;antigen proces
26.906	28.1565	28.1394	28.1222	1:Experimental evidence at protein level	biological regulation;cation homeostasis;cation transport;cellular cation homeostasis;cellular chemical homeostasis;cellular copper ion homeostasis;cellular homeostasis;cellular ion homeostasis;cellular process;chemical hor
28.3152	28.0551	28.3476	28.1368	1:Experimental evidence at protein level	axon guidance;chemotaxis;locomotion;response to chemical stimulus;response to external stimulus;response to stimulus;taxis
27.0761	26.9692	26.838	26.6744	1:Experimental evidence at protein level	biological regulation;biosynthetic process;cell cycle;cellular biosynthetic process;cellular component organization;cellular component organization at cellular level;cellular component organization or biogenesis;cellular compo
27.1266	27.0694	27.5363	27.577	1:Experimental evidence at protein level	apoptosis;biological regulation;catabolic process;cell death;cellular catabolic process;cellular component disassembly;cellular component disassembly at cellular level;cellular component disassembly involved in apoptosis;c
26.8456	26.8958	26.6645	26.2358	1:Experimental evidence at protein level	activation of caspase activity;apoptosis;biological regulation;biosynthetic process;cell death;cell differentiation;cellular biosynthetic process;cellular component assembly;cellular component assembly at cellular level;cellular c
30.5667	31.1896	30.9893	30.9766	1:Experimental evidence at protein level	anion transport;biological adhesion;biological regulation;cell adhesion;cell-cell adhesion;cellular chemical homeostasis;cellular homeostasis;cellular ion homeostasis;cellular process;cellular response to stimulus;chemical hor
28.9832	28.9132	28.9099	28.8685	1:Experimental evidence at protein level	biological regulation;biosynthetic process;cellular biosynthetic process;cellular component assembly;cellular component assembly at cellular level;cellular component organization;cellular component organization at cellular le
25.858	25.4304	25.4284	25.3558	1:Experimental evidence at protein level	biological regulation;carboxylic acid metabolic process;cellular ketone metabolic process;cellular metabolic process;cellular process;metabolic process;monocarboxylic acid metabolic process;organic acid metabolic process
29.6731	29.726	29.5296	29.5436	1:Experimental evidence at protein level	biological regulation;cellular process;cellular response to acid;cellular response to amine stimulus;cellular response to amino acid stimulus;cellular response to chemical stimulus;cellular response to endogenous stimulus;cell
27.1208	25.845	26.9926	26.98	1:Experimental evidence at protein level	biological regulation;biosynthetic process;cellular biosynthetic process;cellular component organization;cellular component organization at cellular level;cellular component organization or biogenesis;cellular component orga
27.2963	27.2147	27.2765	27.2703	1:Experimental evidence at protein level	anatomical structure morphogenesis;biological regulation;biosynthetic process;cellular biosynthetic process;cellular macromolecule biosynthetic process;cellular macromolecule metabolic process;cellular metabolic process;
27.1299	27.0351	26.9478	27.0336	1:Experimental evidence at protein level	anatomical structure morphogenesis;apoptosis;apoptotic mitochondrial changes;biological regulation;cell death;cell part morphogenesis;cellular component assembly;cellular component assembly at cellular level;cellular cor
27.3054	27.2174	27.0315	24.8993	1:Experimental evidence at protein level	cellular macromolecule metabolic process;cellular metabolic process;cellular nitrogen compound metabolic process;cellular process;macromolecule metabolic process;metabolic process;nitrogen compound metabolic proce
24.9911	24.7068	24.9468	24.4357	1:Experimental evidence at protein level	adaptive immune response;biological regulation;biosynthetic process;cell activation;cell chemotaxis;cell migration;cell motility;cell surface receptor linked signaling pathway;cellular biosynthetic process;cellular component as
27.0031	26.9769	27.1452	26.8442	1:Experimental evidence at protein level	establishment of localization;transport
25.8679	25.554	25.5168	25.315	1:Experimental evidence at protein level	amine metabolic process;aminoglycan biosynthetic process;aminoglycan catabolic process;aminoglycan metabolic process;axon guidance;biological regulation;biosynthetic process;carbohydrate biosynthetic process;carbo
29.1044	29.1307	29.0926	29.094	1:Experimental evidence at protein level	cellular component organization;cellular component organization at cellular level;cellular component organization or biogenesis;cellular component organization or biogenesis at cellular level;cellular macromolecule metabolic
25.5149	25.1627	25.3891	25.0368	1:Experimental evidence at protein level	cellular component organization;cellular component organization at cellular level;cellular component organization or biogenesis;cellular component organization or biogenesis at cellular level;cellular macromolecule metabolic
27.8352	27.8185	27.7381	27.3672	1:Experimental evidence at protein level	cellular metabolic process;cellular process;electron transport chain;generation of precursor metabolites and energy;metabolic process;mitochondrial electron transport, NADH to ubiquinone;oxidation-reduction process;respi
28.9676	28.4889	28.7136	28.6358	1:Experimental evidence at protein level	activation of immune response;activation of innate immune response;anaphase-promoting complex-dependent proteasomal ubiquitin-dependent protein catabolic process;antigen processing and presentation;antigen proces
26.672	26.5216	26.8338	26.8439	1:Experimental evidence at protein level	biological regulation;cell differentiation;cell proliferation;cellular component assembly;cellular component assembly at cellular level;cellular component organization;cellular component organization at cellular level;cellular com
27.5336	27.2684	27.4892	27.7187	1:Experimental evidence at protein level	biological regulation;cell surface receptor linked signaling pathway;cellular component assembly;cellular component organization;cellular component organization or biogenesis;cellular process;cellular response to chemical s
26.8875	26.3911	26.5994	26.4056	1:Experimental evidence at protein level	biological regulation;cell proliferation;cellular component biogenesis;cellular component biogenesis at cellular level;cellular component organization;cellular component organization at cellular level;cellular component organiza
27.5648	27.1957	27.8797	27.9411	1:Experimental evidence at protein level	actin cytoskeleton organization;actin filament-based process;axon guidance;biological regulation;cell communication;cell projection assembly;cell projection organization;cell surface receptor linked signaling pathway;cell-cel
24.4986	24.2134	24.3963	23.7647	1:Experimental evidence at protein level	biological regulation;cellular macromolecule metabolic process;cellular metabolic process;cellular nitrogen compound metabolic process;cellular process;macromolecule metabolic process;maturation of SSU-rRNA;metabolic
27.2845	26.6932	26.8116	26.7378	1:Experimental evidence at protein level	cellular macromolecule metabolic process;cellular metabolic process;cellular nitrogen compound metabolic process;cellular process;macromolecule metabolic process;macromolecule modification;metabolic process;ncRNA
28.7617	28.8515	28.7244	28.694	1:Experimental evidence at protein level	biological regulation;biosynthetic process;cellular biosynthetic process;cellular component assembly;cellular component assembly at cellular level;cellular component organization;cellular component organization at cellular le
26.1332	25.2068	24.1183	26.0143	1:Experimental evidence at protein level	anatomical structure development;anatomical structure formation involved in morphogenesis;biological adhesion;biological regulation;cell adhesion;cell development;cell migration;cell motility;cellular component movement;
27.6555	27.8873	27.8154	27.6728	1:Experimental evidence at protein level	anatomical structure development;anatomical structure formation involved in morphogenesis;anatomical structure morphogenesis;angiogenesis;apoptosis;apoptosis involved in heart morphogenesis;apoptosis involved in mo
27.1492	27.3332	26.8026	27.2735	1:Experimental evidence at protein level	biological regulation;cell surface receptor linked signaling pathway;cellular process;cellular response to chemical stimulus;cellular response to cytokine stimulus;cellular response to organic substance;cellular response to stim
27.0854	27.5369	27.7919	27.7282	1:Experimental evidence at protein level	cell cycle process;cellular macromolecule metabolic process;cellular metabolic process;cellular process;cellular protein metabolic process;dephosphorylation;G1/S transition of mitotic cell cycle;macromolecule metabolic pro
26.9593	27.3414	27.2689	27.4233	1:Experimental evidence at protein level	alcohol metabolic process;carbohydrate metabolic process;cellular carbohydrate metabolic process;cellular macromolecule metabolic process;cellular metabolic process;cellular process;cellular protein metabolic process;he
27.8815	28.5442	28.311	28.4306	1:Experimental evidence at protein level	activation of anaphase-promoting complex activity;anaphase-promoting complex-dependent proteasomal ubiquitin-dependent protein catabolic process;biological regulation;catabolic process;cell cycle;cell cycle checkpoint
27.0414	27.2528	27.5419	27.52	1:Experimental evidence at protein level	biosynthetic process;cell proliferation;cellular biosynthetic process;cellular metabolic compound salvage;cellular metabolic process;cellular process;coenzyme biosynthetic process;coenzyme metabolic process;cofactor bios
25.9223	25.8332	25.8816	26.0563	1:Experimental evidence at protein level	anatomical structure development;anatomical structure formation involved in morphogenesis;androgen receptor signaling pathway;ATP-dependent chromatin remodeling;biological regulation;biosynthetic process;blood vess
25.9302	25.9057	25.9205	24.6442	1:Experimental evidence at protein level	
26.9532	27.3753	27.1419	27.3517	1:Experimental evidence at protein level	biological regulation;regulation of biological process;regulation of multicellular organismal process;regulation of muscle contraction;regulation of muscle system process;regulation of system process
26.3277	26.2044	26.4167	26.1131	1:Experimental evidence at protein level	anatomical structure development;biological regulation;cell cycle phase;cell cycle process;cell differentiation;cell projection organization;cellular component organization;cellular component organization at cellular level;cellula
28.8466	28.588	28.8258	28.6232	1:Experimental evidence at protein level	cellular component organization;cellular component organization at cellular level;cellular component organization or biogenesis;cellular component organization or biogenesis at cellular level;cellular process;COPI coating of
27.591	27.2915	27.3672	27.397	1:Experimental evidence at protein level	anterograde axon cargo transport;anterograde synaptic vesicle transport;antigen processing and presentation;antigen processing and presentation of exogenous antigen;antigen processing and presentation of lipid antigen v
26.5538	27.12	27.2437	27.2817	1:Experimental evidence at protein level	biological regulation;cation transport;cellular metabolic process;cellular process;cellular response to chemical stimulus;cellular response to oxidative stress;cellular response to oxygen radical;cellular response to reactive oxy
24.5429	24.7283	24.2555	24.5832	1:Experimental evidence at protein level	cellular component assembly;cellular component organization;cellular component organization at cellular level;cellular component organization or biogenesis;cellular component organization or biogenesis at cellular level;cellu
25.7237	26.6564	25.8605	26.3239	1:Experimental evidence at protein level	axon guidance;biological regulation;cell activation;cell communication;cell surface receptor linked signaling pathway;cell-cell signaling;cellular component disassembly;cellular component disassembly at cellular level;cellular
26.5243	24.1594	26.0783	26.5072	1:Experimental evidence at protein level	acute inflammatory response;arachidonic acid metabolic process;biological regulation;biosynthetic process;carboxylic acid biosynthetic process;carboxylic acid metabolic process;cellular biosynthetic process;cellular ketone
26.4975	26.783	27.0219	26.4864	1:Experimental evidence at protein level	acylglycerol metabolic process;biosynthetic process;CDP-diacylglycerol metabolic process;cellular biosynthetic process;cellular lipid metabolic process;cellular metabolic process;cellular process;diacylglycerol metabolic pro
28.2566	28.5085	29.0208	28.7219	1:Experimental evidence at protein level	biological regulation;cellular process;cellular response to chemical stimulus;cellular response to endogenous stimulus;cellular response to growth factor stimulus;cellular response to organic substance;cellular response to sti
27.2382	27.0791	27.0982	27.0698	1:Experimental evidence at protein level	biological regulation;biosynthetic process;cell activation;cell proliferation;cellular biosynthetic process;cellular component assembly;cellular component assembly at cellular level;cellular component organization;cellular comp
27.0862	26.9229	27.5741	27.2387	1:Experimental evidence at protein level	acid secretion;actin cytoskeleton organization;actin filament-based process;activation of adenylate cyclase activity;activation of adenylate cyclase activity by dopamine receptor signaling pathway;activation of adenylate cycla
24.3449	24.2078	24.4308	23.1983	1:Experimental evidence at protein level	aging;anatomical structure homeostasis;biological regulation;biosynthetic process;cell aging;cellular biosynthetic process;cellular component organization;cellular component organization at cellular level;cellular component
24.82	24.6781	24.7729	24.4815	1:Experimental evidence at protein level	activation of caspase activity;activation of NF-kappaB-inducing kinase activity;activation of protein kinase activity;apoptosis;apoptosis in response to endoplasmic reticulum stress;biological regulation;cell death;cell surface
25.4486	25.9214	24.4852	25.7278	1:Experimental evidence at protein level	anatomical structure development;biological regulation;biosynthetic process;cellular biosynthetic process;cellular macromolecule biosynthetic process;cellular macromolecule metabolic process;cellular metabolic process;ce
28.581	28.78	28.801	29.0217	1:Experimental evidence at protein level	anatomical structure development;anatomical structure homeostasis;biological regulation;bone resorption;catabolic process;cell differentiation;cellular catabolic process;cellular component organization;cellular component or
27.0878	27.0368	26.8159	26.9856	1:Experimental evidence at protein level	biological regulation;biosynthetic process;cellular biosynthetic process;cellular macromolecule biosynthetic process;cellular macromolecule metabolic process;cellular metabolic process;cellular nitrogen compound metabolic
26.7848	26.9352	27.2822	26.3251	1:Experimental evidence at protein level	attachment of spindle microtubules to chromosome;attachment of spindle microtubules to kinetochore;attachment of spindle microtubules to kinetochore involved in mitotic sister chromatid segregation;biological regulation;
26.3621	26.1913	26.3086	26.2764	1:Experimental evidence at protein level	anatomical structure arrangement;anatomical structure development;anatomical structure formation involved in morphogenesis;anatomical structure morphogenesis;angiogenesis;angiogenesis involved in coronary vascular
24.3707	24.6136	23.925	23.7271	1:Experimental evidence at protein level	cellular component assembly;cellular component organization;cellular component organization or biogenesis;macromolecular complex assembly;macromolecular complex subunit organization;protein complex assembly;prot
28.9409	29.0678	29.3273	29.2831	1:Experimental evidence at protein level	activation of immune response;activation of innate immune response;anaphase-promoting complex-dependent proteasomal ubiquitin-dependent protein catabolic process;antigen processing and presentation;antigen proces
27.6873	27.7229	27.7862	27.7763	1:Experimental evidence at protein level	cellular process;establishment of localization;establishment of localization in cell;establishment of protein localization;Golgi vesicle transport;intracellular transport;post-Golgi vesicle-mediated transport;protein transport;resp
26.7157	26.658	26.8307	26.7102	1:Experimental evidence at protein level	biological regulation;cell surface receptor linked signaling pathway;cellular process;cellular response to chemical stimulus;cellular response to cytokine stimulus;cellular response to interferon-alpha;cellular response to organi
26.8658	26.4947	26.7592	27.2305	1:Experimental evidence at protein level	biological regulation;cellular metabolic process;cellular process;cellular response to stimulus;lipid metabolic process;metabolic process;oxidation-reduction process;primary metabolic process;regulation of biological process
27.7217	27.9612	27.8388	28.1766	1:Experimental evidence at protein level	biological regulation;cell communication;cell migration;cell motility;cell surface receptor linked signaling pathway;cell-cell signaling;cellular component movement;cellular process;cellular response to stimulus;endothelial cell
28.1325	28.0633	28.2654	28.0421	1:Experimental evidence at protein level	biological regulation;cellular component assembly;cellular component assembly at cellular level;cellular component organization;cellular component organization at cellular level;cellular component organization or biogenesis;
27.1218	27.2504	26.9505	27.0746	1:Experimental evidence at protein level	biological regulation;biosynthetic process;catabolic process;cell surface receptor linked signaling pathway;cellular biosynthetic process;cellular catabolic process;cellular macromolecule biosynthetic process;cellular macrom
24.7957	24.1879	25.2212	24.8703	1:Experimental evidence at protein level	biological adhesion;biological regulation;calcium ion import;calcium ion transmembrane transport;calcium ion transport;cation transport;cell adhesion;cell communication;cell-cell signaling;cellular component organization;cel
27.7491	27.6951	26.9276	27.4905	1:Experimental evidence at protein level	anatomical structure development;cell development;cellular developmental process;cellular metabolic process;cellular process;central nervous system neuron development;cerebellar Purkinje cell layer development;develop
29.6658	30.1992	29.7916	30.1651	1:Experimental evidence at protein level	axon guidance;biological regulation;cell surface receptor linked signaling pathway;cellular process;cellular response to stimulus;chemotaxis;enzyme linked receptor protein signaling pathway;ephrin receptor signaling pathwa
27.7212	27.8218	27.6466	27.6719	1:Experimental evidence at protein level	biological regulation;cell surface receptor linked signaling pathway;cellular component organization;cellular component organization or biogenesis;cellular localization;cellular macromolecule localization;cellular membrane org
25.8134	26.9108	25.6362	25.5869	1:Experimental evidence at protein level	anaphase-promoting complex-dependent proteasomal ubiquitin-dependent protein catabolic process;anterior/posterior axis specification;axis specification;biological regulation;catabolic process;cell cycle;cell cycle phase;c
25.3171	24.4385	25.7336	25.0775	1:Experimental evidence at protein level	binding of sperm to zona pellucida;cell recognition;cell-cell recognition;cellular component assembly;cellular component organization;cellular component organization or biogenesis;cellular macromolecule metabolic process;
28.2951	28.5153	28.3499	28.5059	1:Experimental evidence at protein level	biological regulation;cell cycle;cell cycle phase;cell cycle process;cellular component organization;cellular component organization at cellular level;cellular component organization or biogenesis;cellular component organizatio
25.4779	25.279	25.1568	24.7859	1:Experimental evidence at protein level	anatomical structure development;anatomical structure morphogenesis;biological regulation;cell cycle;cell development;cell differentiation;cellular component disassembly;cellular component disassembly at cellular level;cell
30.4488	30.0723	30.8062	30.5605	1:Experimental evidence at protein level	biological regulation;biosynthetic process;cellular biosynthetic process;cellular macromolecule biosynthetic process;cellular macromolecule metabolic process;cellular metabolic process;cellular nitrogen compound metabolic
28.392	28.3155	28.1927	28.1679	1:Experimental evidence at protein level	biological regulation;cell cycle;cell surface receptor linked signaling pathway;cellular localization;cellular macromolecule localization;cellular process;cellular protein localization;cellular response to stimulus;enzyme linked rece
25.8515	25.8008	24.3759	25.7291	1:Experimental evidence at protein level	axon ensheathment;biological regulation;cell cycle process;cellular component assembly;cellular component assembly at cellular level;cellular component organization;cellular component organization at cellular level;cellular
26.8943	26.948	26.8873	27.201	1:Experimental evidence at protein level	anatomical structure development;cellular component organization;cellular component organization at cellular level;cellular component organization or biogenesis;cellular component organization or biogenesis at cellular leve
26.6379	26.5966	26.5201	26.5109	1:Experimental evidence at protein level	anatomical structure development;anatomical structure formation involved in morphogenesis;biological regulation;brain development;cell proliferation;cell surface receptor linked signaling pathway;cellular process;cellular res
27.1888	26.9749	27.2826	26.8556	1:Experimental evidence at protein level	cellular macromolecule metabolic process;cellular metabolic process;cellular nitrogen compound metabolic process;cellular process;macromolecule metabolic process;metabolic process;nitrogen compound metabolic proce
27.7823	27.9952	27.946	27.9204	1:Experimental evidence at protein level	amine metabolic process;biosynthetic process;carboxylic acid metabolic process;cellular amine metabolic process;cellular amino acid metabolic process;cellular aromatic compound metabolic process;cellular biosynthetic p
26.1802	26.4495	26.6989	26.4822	1:Experimental evidence at protein level	catabolic process;cellular catabolic process;cellular macromolecule catabolic process;cellular macromolecule metabolic process;cellular metabolic process;cellular nitrogen compound metabolic process;cellular process;dea
26.9672	26.4719	26.8799	26.5241	1:Experimental evidence at protein level	adult behavior;adult locomotory behavior;adult walking behavior;alcohol metabolic process;autophagy;behavior;bile acid metabolic process;biological regulation;carbohydrate metabolic process;carboxylic acid metabolic pro
26.6322	26.2556	26.7772	26.5206	1:Experimental evidence at protein level	biosynthetic process;carboxylic acid biosynthetic process;carboxylic acid metabolic process;cellular biosynthetic process;cellular ketone metabolic process;cellular lipid metabolic process;cellular metabolic process;cellular
25.8943	26.0598	25.9066	25.9981	1:Experimental evidence at protein level	cellular component organization;cellular component organization or biogenesis;cellular membrane organization;cellular process;endocytosis;establishment of localization;establishment of localization in cell;establishment of p
27.2716	24.7064	24.7897	27.2364	1:Experimental evidence at protein level	cellular process;establishment of localization;establishment of localization in cell;establishment of protein localization;Golgi vesicle transport;intracellular transport;post-Golgi vesicle-mediated transport;protein transport;trans
29.0307	28.7581	28.8965	28.5955	1:Experimental evidence at protein level	actin cytoskeleton organization;actin filament organization;actin filament-based process;actin nucleation;Arp2/3 complex-mediated actin nucleation;biological regulation;cellular component movement;cellular component orga
28.6894	28.7862	28.7663	28.6705	1:Experimental evidence at protein level	actin cytoskeleton organization;actin filament organization;actin filament-based process;actin nucleation;Arp2/3 complex-mediated actin nucleation;axon guidance;biological regulation;cell surface receptor linked signaling pa
29.3494	28.9748	28.8813	28.7597	1:Experimental evidence at protein level	actin cytoskeleton organization;actin filament organization;actin filament-based process;actin nucleation;Arp2/3 complex-mediated actin nucleation;axon guidance;biological regulation;cell surface receptor linked signaling pa
22.8758	24.4252	24.9281	24.303	1:Experimental evidence at protein level	acute inflammatory response;acute-phase response;apoptosis;biological regulation;biosynthetic process;cell activation;cell death;cellular biosynthetic process;cellular component organization;cellular component organization
27.6754	27.1989	27.3803	27.1429	1:Experimental evidence at protein level	biological regulation;cellular process;cellular response to chemical stimulus;cellular response to endogenous stimulus;cellular response to hormone stimulus;cellular response to organic substance;cellular response to steroid
27.0127	27.1898	27.3746	27.3889	1:Experimental evidence at protein level	cellular component assembly;cellular component assembly at cellular level;cellular component organization;cellular component organization at cellular level;cellular component organization or biogenesis;cellular component
26.6391	26.3848	26.6394	26.7661	1:Experimental evidence at protein level	
26.175	26.0816	25.9291	26.3536	1:Experimental evidence at protein level	biosynthetic process;cellular biosynthetic process;cellular component disassembly;cellular component disassembly at cellular level;cellular component organization;cellular component organization at cellular level;cellular cor
28.2149	28.1755	28.1002	28.3063	1:Experimental evidence at protein level	biological regulation;cellular component organization;cellular component organization or biogenesis;cellular localization;cellular macromolecule localization;cellular membrane organization;cellular process;cellular protein local
27.2492	26.913	26.7558	26.8842	1:Experimental evidence at protein level	alcohol biosynthetic process;alcohol metabolic process;amine metabolic process;biosynthetic process;cellular biosynthetic process;cellular lipid metabolic process;cellular metabolic process;cellular nitrogen compound bios
26.3059	25.9756	25.854	25.8573	1:Experimental evidence at protein level	alcohol biosynthetic process;alcohol metabolic process;amine metabolic process;biosynthetic process;cellular biosynthetic process;cellular lipid metabolic process;cellular metabolic process;cellular nitrogen compound bios
26.8678	26.614	26.4454	26.3844	1:Experimental evidence at protein level	apoptosis;biological regulation;carbohydrate metabolic process;cell communication;cell death;cellular carbohydrate metabolic process;cellular component organization;cellular component organization at cellular level;cellular
27.909	27.614	28.1585	28.0488	1:Experimental evidence at protein level	biological regulation;cellular component organization;cellular component organization at cellular level;cellular component organization or biogenesis;cellular component organization or biogenesis at cellular level;cellular macr

28.3598	28.7352	28.4488	28.4067	1:Experimental evidence at protein level	biological regulation;cell cycle arrest;cell cycle process;cellular macromolecule metabolic process;cellular metabolic process;cellular process;cellular protein metabolic process;dephosphorylation;macromolecule metabolic pr
25.4728	25.5279	25.2051	25.2762	1:Experimental evidence at protein level	actin cytoskeleton organization;actin filament organization;actin filament-based process;alcohol metabolic process;biological adhesion;biological regulation;biosynthetic process;carbohydrate metabolic process;cell adhesion
29.5568	29.5897	29.4745	29.3958	1:Experimental evidence at protein level	biological regulation;biosynthetic process;cellular biosynthetic process;cellular component assembly;cellular component assembly at cellular level;cellular component organization;cellular component organization at cellular le
28.9368	28.601	28.871	28.8229	1:Experimental evidence at protein level	biological regulation;biosynthetic process;cellular biosynthetic process;cellular component assembly;cellular component assembly at cellular level;cellular component organization;cellular component organization at cellular le
26.8268	27.4676	27.0688	27.3478	1:Experimental evidence at protein level	amine biosynthetic process;amine catabolic process;amine metabolic process;biological regulation;biosynthetic process;branched chain family amino acid biosynthetic process;branched chain family amino acid catabolic pro
25.5177	25.9326	24.3771	26.5898	1:Experimental evidence at protein level	biological regulation;biosynthetic process;cell cycle;cell cycle checkpoint;cell cycle phase;cell cycle process;cell division;cellular biosynthetic process;cellular component assembly;cellular component assembly at cellular lev
26.5635	26.0883	26.8275	26.4971	1:Experimental evidence at protein level	biological regulation;cellular component assembly;cellular component assembly at cellular level;cellular component organization;cellular component organization at cellular level;cellular component organization or biogenesis;
28.4099	28.4108	28.1719	28.1973	1:Experimental evidence at protein level	anion transport;biological regulation;blood coagulation;carboxylic acid metabolic process;carboxylic acid transport;cell migration;cell motility;cellular component movement;cellular ketone metabolic process;cellular metaboli
26.8612	26.0588	26.6959	26.9226	1:Experimental evidence at protein level	biological regulation;biosynthetic process;cell surface receptor linked signaling pathway;cellular biosynthetic process;cellular macromolecule biosynthetic process;cellular macromolecule metabolic process;cellular metabolic
28.9847	28.8679	28.9959	29.0241	1:Experimental evidence at protein level	4-hydroxyproline metabolic process;amine metabolic process;carboxylic acid metabolic process;cellular amine metabolic process;cellular amino acid metabolic process;cellular ketone metabolic process;cellular macromolec
25.5843	24.7887	23.5581	24.8651	1:Experimental evidence at protein level	biological regulation;cellular process;cellular response to stimulus;macromolecule metabolic process;metabolic process;primary metabolic process;protein metabolic process;proteolysis;regulation of biological process;regula
25.9197	26.0717	26.259	25.9708	1:Experimental evidence at protein level	cellular component organization;cellular component organization at cellular level;cellular component organization or biogenesis;cellular component organization or biogenesis at cellular level;cellular membrane fusion;cellular
29.7062	29.4068	29.7342	29.615	1:Experimental evidence at protein level	actin cytoskeleton organization;actin filament organization;actin filament-based process;actin nucleation;Arp2/3 complex-mediated actin nucleation;axon guidance;biological regulation;cell migration;cell motility;cell surface re
24.8329	25.3379	25.3007	25.038	1:Experimental evidence at protein level	biological regulation;biosynthetic process;catabolic process;cellular biosynthetic process;cellular catabolic process;cellular component assembly;cellular component assembly at cellular level;cellular component organization;
26.8785	27.0705	27.0349	27.107	1:Experimental evidence at protein level	amide transport;antigen processing and presentation;antigen processing and presentation of endogenous antigen;antigen processing and presentation of endogenous peptide antigen;antigen processing and presentation of
24.0856	25.6782	25.4922	25.6679	1:Experimental evidence at protein level	
28.3747	28.306	28.2228	28.0333	1:Experimental evidence at protein level	cellular macromolecule metabolic process;cellular metabolic process;cellular nitrogen compound metabolic process;cellular process;macromolecule metabolic process;metabolic process;mRNA metabolic process;mRNA pro
24.8397	25.8122	25.3589	24.839	1:Experimental evidence at protein level	biosynthetic process;cellular biosynthetic process;cellular macromolecule biosynthetic process;cellular macromolecule metabolic process;cellular metabolic process;cellular nitrogen compound metabolic process;cellular pro
24.8394	25.1212	24.632	24.8812	1:Experimental evidence at protein level	
28.1523	28.1681	27.8968	28.0691	1:Experimental evidence at protein level	metabolic process;oxidation-reduction process
27.0839	26.748	26.9066	26.6491	1:Experimental evidence at protein level	cellular macromolecule metabolic process;cellular metabolic process;cellular nitrogen compound metabolic process;cellular process;gene expression;macromolecule metabolic process;metabolic process;mRNA metabolic p
29.089	29.5237	29.0361	29.2467	1:Experimental evidence at protein level	amine biosynthetic process;amine metabolic process;anatomical structure development;aspartate family amino acid metabolic process;biological regulation;biosynthetic process;brain development;carboxylic acid biosynthet
26.8741	26.9342	26.632	26.7479	1:Experimental evidence at protein level	antigen processing and presentation;antigen processing and presentation of exogenous antigen;antigen processing and presentation of exogenous peptide antigen;antigen processing and presentation of exogenous peptide
28.3834	28.1714	28.145	28.0565	1:Experimental evidence at protein level	activation of immune response;activation of innate immune response;anaphase-promoting complex-dependent proteasomal ubiquitin-dependent protein catabolic process;antigen processing and presentation;antigen proces
26.5962	26.8428	26.8032	26.5899	1:Experimental evidence at protein level	3'-phosphoadenosine 5'-phosphosulfate biosynthetic process;3'-phosphoadenosine 5'-phosphosulfate metabolic process;amine metabolic process;aminoglycan metabolic process;anatomical structure development;biosynt
27.6501	28.0005	27.6912	27.5985	1:Experimental evidence at protein level	biological regulation;cell cycle;cell cycle checkpoint;cell cycle phase;cell cycle process;cell division;cellular component assembly;cellular component organization;cellular component organization at cellular level;cellular comp
27.3167	27.1205	27.5279	27.3503	1:Experimental evidence at protein level	biological regulation;cell cycle arrest;cell cycle process;cellular component assembly;cellular component assembly at cellular level;cellular component organization;cellular component organization at cellular level;cellular com
27.4102	27.684	27.2758	27.0659	1:Experimental evidence at protein level	apoptosis;biological regulation;biosynthetic process;cell death;cell differentiation;cellular biosynthetic process;cellular component organization;cellular component organization at cellular level;cellular component organization
26.3723	25.303	23.5617	27.1115	1:Experimental evidence at protein level	anatomical structure morphogenesis;androgen receptor signaling pathway;biological adhesion;biological regulation;biosynthetic process;catabolic process;cell adhesion;cell differentiation;cell fate commitment;cell surface re
25.2356	25.2344	25.5192	25.273	1:Experimental evidence at protein level	activation of immune response;activation of innate immune response;activation of MAPK activity;activation of MAPKK activity;activation of NF-kappaB-inducing kinase activity;activation of protein kinase activity;anatomical s
27.7749	27.8676	28.0549	27.7247	1:Experimental evidence at protein level	amine metabolic process;amino acid activation;biological regulation;carboxylic acid metabolic process;cellular amine metabolic process;cellular amino acid metabolic process;cellular ketone metabolic process;cellular macro
23.6927	24.7079	24.4652	23.6278	1:Experimental evidence at protein level	activation of immune response;activation of innate immune response;activation of MAPK activity;adaptive immune response;antigen receptor-mediated signaling pathway;apoptosis;biological regulation;cell activation;cell dea
29.6866	29.5686	29.5959	29.5336	1:Experimental evidence at protein level	cellular macromolecule metabolic process;cellular metabolic process;cellular nitrogen compound metabolic process;cellular process;gene expression;macromolecule metabolic process;metabolic process;mRNA metabolic p
27.3559	27.2077	27.2703	27.4562	1:Experimental evidence at protein level	assembly of spliceosomal tri-snRNP;cellular component assembly;cellular component assembly at cellular level;cellular component organization;cellular component organization at cellular level;cellular component organizatio
29.3132	29.4672	29.5034	29.6582	1:Experimental evidence at protein level	biological regulation;cell redox homeostasis;cellular homeostasis;cellular macromolecule metabolic process;cellular metabolic process;cellular process;cellular protein metabolic process;cellular response to chemical stimulus
29.2152	29.2444	29.4023	29.4507	1:Experimental evidence at protein level	biological regulation;regulation of biological process;regulation of cell proliferation;regulation of cellular process
27.1123	27.1016	27.1108	27.4494	1:Experimental evidence at protein level	
26.0534	26.6669	26.4615	24.5609	1:Experimental evidence at protein level	biological adhesion;biological regulation;cell adhesion;cell surface receptor linked signaling pathway;cell-cell adhesion;cellular process;cellular response to chemical stimulus;cellular response to fibroblast growth factor stimu
25.8981	25.9813	25.9812	26.0363	1:Experimental evidence at protein level	biological regulation;cell surface receptor linked signaling pathway;cellular process;cellular response to chemical stimulus;cellular response to cytokine stimulus;cellular response to organic substance;cellular response to stim
26.1883	24.3969	26.7884	27.1317	1:Experimental evidence at protein level	biological regulation;cellular component assembly;cellular component organization;cellular component organization or biogenesis;cellular macromolecule metabolic process;cellular metabolic process;cellular nitrogen compo
27.2931	27.6639	27.7714	27.5754	1:Experimental evidence at protein level	carbohydrate metabolic process;cellular aldehyde metabolic process;cellular metabolic process;cellular process;metabolic process;primary metabolic process;small molecule metabolic process;xenobiotic metabolic process
27.5727	27.6614	27.4603	27.5304	1:Experimental evidence at protein level	actin cytoskeleton organization;actin filament-based process;cellular component organization;cellular component organization at cellular level;cellular component organization or biogenesis;cellular component organization or
25.7814	24.2857	25.5193	25.3542	1:Experimental evidence at protein level	
25.9938	26.5568	26.8427	27.1154	1:Experimental evidence at protein level	cap-independent translational initiation;cellular component assembly;cellular component assembly at cellular level;cellular component disassembly;cellular component disassembly at cellular level;cellular component organiza
26.0694	24.4293	25.7371	25.4279	1:Experimental evidence at protein level	establishment of localization;establishment of localization in cell;establishment of protein localization;establishment of RNA localization;intracellular protein transport;intracellular transport;nuclear export;nuclear transport;nucl
26.0736	26.557	26.6212	26.8905	1:Experimental evidence at protein level	biological regulation;catabolic process;cell differentiation;cell proliferation;cellular catabolic process;cellular developmental process;cellular metabolic process;cellular nitrogen compound catabolic process;cellular nitrogen co
26.3236	26.6553	26.28	26.4477	1:Experimental evidence at protein level	cellular macromolecule metabolic process;cellular metabolic process;cellular process;cellular protein metabolic process;establishment of localization;establishment of localization in cell;establishment of protein localization;es
27.5156	27.9316	27.8168	27.7984	1:Experimental evidence at protein level	cellular process;establishment of localization;transport;vesicle-mediated transport
26.5588	26.8434	26.5833	26.5512	1:Experimental evidence at protein level	biological regulation;cell cycle process;cellular component assembly;cellular component assembly at cellular level;cellular component organization;cellular component organization at cellular level;cellular component organiza
25.5767	24.6143	25.3206	25.4034	1:Experimental evidence at protein level	biological regulation;cellular process;cellular response to stimulus;positive regulation of biological process;positive regulation of cell communication;positive regulation of cellular process;positive regulation of I-kappaB kinase
26.9269	25.9536	26.437	26.4292	1:Experimental evidence at protein level	biological regulation;cellular localization;cellular macromolecule localization;cellular macromolecule metabolic process;cellular metabolic process;cellular nitrogen compound metabolic process;cellular process;cellular protein
26.646	26.6615	26.3681	26.3879	1:Experimental evidence at protein level	biological regulation;cell cycle process;cellular process;cellular response to stimulus;cytokinesis;intracellular signal transduction;microtubule-based process;mitotic spindle elongation;regulation of biological process;regulatio
26.6771	26.7029	27.2647	26.9039	1:Experimental evidence at protein level	biological regulation;negative regulation of biological process;negative regulation of cell communication;negative regulation of cellular process;negative regulation of G-protein coupled receptor protein signaling pathway;nega
27.6891	27.8421	28.0072	28.0813	1:Experimental evidence at protein level	attachment of spindle microtubules to chromosome;attachment of spindle microtubules to kinetochore;biological regulation;cell cycle checkpoint;cell cycle process;cell division;cellular component organization;cellular compo
26.7522	26.5686	26.7191	26.5819	1:Experimental evidence at protein level	cellular metabolic process;cellular process;electron transport chain;generation of precursor metabolites and energy;metabolic process;mitochondrial electron transport, NADH to ubiquinone;oxidation-reduction process;respi
26.3114	26.8418	27.0131	27.0157	1:Experimental evidence at protein level	cellular metabolic process;cellular process;electron transport chain;generation of precursor metabolites and energy;metabolic process;mitochondrial electron transport, NADH to ubiquinone;oxidation-reduction process;respi
28.0643	27.7908	28.1412	27.9201	1:Experimental evidence at protein level	anion transport;biological regulation;cellular localization;cellular macromolecule localization;cellular macromolecule metabolic process;cellular metabolic process;cellular process;cellular protein localization;cellular protein me
28.2536	28.5387	28.4975	28.5242	1:Experimental evidence at protein level	anaphase-promoting complex-dependent proteasomal ubiquitin-dependent protein catabolic process;attachment of spindle microtubules to chromosome;attachment of spindle microtubules to kinetochore;biological regulati
30.8823	30.7169	30.7075	30.7956	1:Experimental evidence at protein level	actin cytoskeleton organization;actin filament bundle assembly;actin filament organization;actin filament-based process;biological regulation;blood coagulation;cell activation;cell junction assembly;cell junction organization;c
25.8918	26.4743	26.1244	25.9691	1:Experimental evidence at protein level	biological regulation;biosynthetic process;cellular biosynthetic process;cellular macromolecule biosynthetic process;cellular macromolecule metabolic process;cellular metabolic process;cellular nitrogen compound metabolic
27.7712	27.886	27.5624	27.4361	1:Experimental evidence at protein level	antigen processing and presentation;antigen processing and presentation of exogenous antigen;antigen processing and presentation of exogenous peptide antigen;antigen processing and presentation of exogenous peptide
27.0507	27.6166	27.7478	27.7648	1:Experimental evidence at protein level	viral reproduction
26.7891	28.1021	28.2913	28.4602	1:Experimental evidence at protein level	biological regulation;cell cycle;cell cycle phase;cell cycle process;cell division;cellular component organization;cellular component organization at cellular level;cellular component organization or biogenesis;cellular componen
28.5398	28.6588	28.5156	28.437	1:Experimental evidence at protein level	amine metabolic process;amino acid activation;asparaginyl-tRNA aminoacylation;carboxylic acid metabolic process;cellular amine metabolic process;cellular amino acid metabolic process;cellular ketone metabolic process;c
28.0273	27.8735	27.7902	27.8036	1:Experimental evidence at protein level	actin cytoskeleton organization;actin filament bundle assembly;actin filament organization;actin filament-based movement;actin filament-based process;cellular component assembly;cellular component assembly at cellular le
27.5353	27.8406	27.7036	27.8104	1:Experimental evidence at protein level	biosynthetic process;cellular biosynthetic process;cellular component assembly;cellular component organization;cellular component organization or biogenesis;cellular macromolecule biosynthetic process;cellular macromole
26.5327	26.532	26.6633	26.5363	1:Experimental evidence at protein level	biological regulation;cell surface receptor linked signaling pathway;cellular process;cellular response to stimulus;G-protein coupled receptor protein signaling pathway;regulation of biological process;regulation of cellular proc
26.3046	26.0899	26.3035	26.0947	1:Experimental evidence at protein level	cellular macromolecule metabolic process;cellular metabolic process;cellular nitrogen compound metabolic process;cellular process;macromolecule metabolic process;metabolic process;ncRNA metabolic process;ncRNA pr
26.1902	26.4372	26.5453	26.4447	1:Experimental evidence at protein level	biological regulation;cell cycle phase;cell cycle process;cellular component organization;cellular component organization at cellular level;cellular component organization or biogenesis;cellular component organization or biog
26.6928	26.4451	26.5792	26.4875	1:Experimental evidence at protein level	2-oxoglutarate metabolic process;acetyl-CoA catabolic process;acetyl-CoA metabolic process;carboxylic acid metabolic process;catabolic process;cellular catabolic process;cellular ketone metabolic process;cellular metab
26.3961	26.3343	26.5278	26.1081	1:Experimental evidence at protein level	biological regulation;cell migration;cell motility;cell proliferation;cellular component movement;cellular component organization;cellular component organization or biogenesis;cellular process;locomotion;macromolecule metab
30.5414	30.8527	31.1164	31.0908	1:Experimental evidence at protein level	biological regulation;blood coagulation;cell activation;cellular process;coagulation;establishment of localization;establishment of localization in cell;exocytosis;hemostasis;multicellular organismal process;platelet activation;pl
23.8706	26.8062	24.323	24.076	1:Experimental evidence at protein level	biological adhesion;biological regulation;cell adhesion;cellular process;developmental process;multicellular organismal development;multicellular organismal process;positive regulation of biological process;positive regulation
28.9052	28.9506	29.0615	28.7607	1:Experimental evidence at protein level	amine metabolic process;biological regulation;carboxylic acid metabolic process;cellular amine metabolic process;cellular amino acid metabolic process;cellular ketone metabolic process;cellular macromolecule metabolic pr
25.151	24.1168	25.1115	24.8544	1:Experimental evidence at protein level	cellular component assembly;cellular component assembly at cellular level;cellular component biogenesis;cellular component organization;cellular component organization at cellular level;cellular component organization or b
26.1656	25.8307	25.8449	25.8974	1:Experimental evidence at protein level	apoptosis;biological regulation;cell cycle;cell cycle phase;cell cycle process;cell death;cell division;cellular component organization;cellular component organization at cellular level;cellular component organization or biogenes
25.4865	26.6714	26.8876	27.0052	1:Experimental evidence at protein level	anatomical structure development;cellular macromolecule metabolic process;cellular metabolic process;cellular process;cellular protein metabolic process;chaperone-mediated protein transport;developmental process;estab
26.5314	26.3228	26.5674	26.1843	1:Experimental evidence at protein level	cellular macromolecule metabolic process;cellular metabolic process;cellular nitrogen compound metabolic process;cellular process;macromolecule metabolic process;metabolic process;mRNA metabolic process;mRNA pro
26.6343	27.6635	27.5215	27.8386	1:Experimental evidence at protein level	cell cycle phase;cell cycle process;cell division;cellular component organization;cellular component organization at cellular level;cellular component organization or biogenesis;cellular component organization or biogenesis at
27.0671	26.7292	26.9297	26.598	1:Experimental evidence at protein level	anatomical structure development;biological regulation;biosynthetic process;bone development;cellular biosynthetic process;cellular metabolic process;cellular nitrogen compound biosynthetic process;cellular nitrogen comp
27.4355	27.1079	27.2088	27.0809	1:Experimental evidence at protein level	ATP-dependent chromatin remodeling;biological regulation;biosynthetic process;cellular biosynthetic process;cellular component assembly;cellular component assembly at cellular level;cellular component organization;cellula
27.148	26.9838	27.0253	27.0601	1:Experimental evidence at protein level	activation of JUN kinase activity;activation of MAPK activity;biological regulation;cell differentiation;cellular component assembly;cellular component organization;cellular component organization or biogenesis;cellular develop
26.4798	25.9061	25.8813	25.3756	1:Experimental evidence at protein level	cellular macromolecule metabolic process;cellular metabolic process;cellular nitrogen compound metabolic process;cellular process;macromolecule metabolic process;metabolic process;mRNA metabolic process;mRNA pro
26.4551	26.3068	26.3976	26.3815	1:Experimental evidence at protein level	aging;anatomical structure formation involved in morphogenesis;apoptosis;axon cargo transport;axon transport of mitochondrion;biological regulation;cell aging;cell death;cellular component movement;cellular component o
25.3408	24.8306	25.4007	22.5833	1:Experimental evidence at protein level	anterograde axon cargo transport;apoptosis;axon cargo transport;cell communication;cell death;cell-cell signaling;cellular component movement;cellular process;cytoskeleton-dependent intracellular transport;death;establish
25.9961	25.749	25.9771	26.0423	1:Experimental evidence at protein level	anatomical structure development;biological regulation;biosynthetic process;blood coagulation;cell development;cell differentiation;cell proliferation;cellular biosynthetic process;cellular component organization;cellular comp
23.0291	24.7548	24.6628	24.8484	1:Experimental evidence at protein level	apoptosis;biological regulation;cell death;cell differentiation;cellular developmental process;cellular process;death;developmental process;inner ear receptor cell differentiation;mechanoreceptor differentiation;multicellular org
27.7136	27.487	27.6168	27.4744	1:Experimental evidence at protein level	acyl-CoA biosynthetic process;acyl-CoA metabolic process;acylglycerol biosynthetic process;acylglycerol metabolic process;anatomical structure development;biological regulation;biosynthetic process;carboxylic acid meta
28.0768	27.975	28.5042	28.1844	1:Experimental evidence at protein level	biological regulation;biosynthetic process;cation transport;cellular biosynthetic process;cellular component organization;cellular component organization at cellular level;cellular component organization or biogenesis;cellular c
26.6447	26.2607	26.5818	26.2287	1:Experimental evidence at protein level	2'-deoxyribonucleotide metabolic process;aging;alcohol metabolic process;amino sugar metabolic process;biological regulation;carbohydrate metabolic process;catabolic process;cell death;cellular carbohydrate metabolic p
30.3238	30.2762	30.3092	30.3763	1:Experimental evidence at protein level	biological regulation;cell differentiation;cellular developmental process;cellular macromolecule metabolic process;cellular metabolic process;cellular nitrogen compound metabolic process;cellular process;cellular response to
25.9546	25.6876	25.4479	24.313	1:Experimental evidence at protein level	biosynthetic process;cellular biosynthetic process;cellular macromolecule biosynthetic process;cellular macromolecule metabolic process;cellular metabolic process;cellular nitrogen compound metabolic process;cellular pro
25.249	25.6971	25.4581	25.7341	1:Experimental evidence at protein level	establishment of localization;establishment of protein localization;protein transport;transport
25.3071	25.8043	24.6644	25.2379	1:Experimental evidence at protein level	alcohol biosynthetic process;alcohol metabolic process;biological regulation;biosynthetic process;carbohydrate biosynthetic process;carbohydrate metabolic process;cell surface receptor linked signaling pathway;cellular bio
29.3216	29.0341	29.5107	29.4497	1:Experimental evidence at protein level	anatomical structure development;anatomical structure formation involved in morphogenesis;anatomical structure morphogenesis;apoptosis;appendage development;appendage morphogenesis;biological regulation;blood v
28.5116	28.4264	28.577	28.4552	1:Experimental evidence at protein level	anatomical structure development;anatomical structure morphogenesis;basement membrane assembly;basement membrane organization;biological regulation;cell morphogenesis;cell morphogenesis involved in differentiatio
26.5152	26.8811	26.8508	26.6304	1:Experimental evidence at protein level	biological regulation;cell surface receptor linked signaling pathway;cellular process;cellular response to chemical stimulus;cellular response to cytokine stimulus;cellular response to organic substance;cellular response to stim
27.5893	27.3398	27.2012	27.139	1:Experimental evidence at protein level	actin cytoskeleton organization;actin filament organization;actin filament polymerization;actin filament-based process;actin polymerization or depolymerization;biological regulation;cell projection organization;cellular compone
30.155	30.2741	30.5134	30.3799	1:Experimental evidence at protein level	cellular process;establishment of localization;transport;vesicle-mediated transport
28.0045	28.1784	28.0893	27.8432	1:Experimental evidence at protein level	biological regulation;establishment of localization;establishment of localization in cell;establishment of protein localization;intracellular protein transport;intracellular transport;maternal process involved in female pregnancy;mu
29.0504	29.084	29.0891	28.9291	1:Experimental evidence at protein level	alcohol metabolic process;amine metabolic process;aminoglycan biosynthetic process;aminoglycan metabolic process;anatomical structure morphogenesis;biosynthetic process;carbohydrate biosynthetic process;carbohyd
28.1676	28.2807	28.4406	28.535	1:Experimental evidence at protein level	biological adhesion;biological regulation;biosynthetic process;cell adhesion;cellular biosynthetic process;cellular component assembly;cellular component organization;cellular component organization or biogenesis;cellular m
28.0021	28.2269	28.0268	28.0502	1:Experimental evidence at protein level	adherens junction organization;anatomical structure development;biological adhesion;biological regulation;biosynthetic process;brain development;cell adhesion;cell junction assembly;cell junction organization;cell surface re
27.7897	28.0397	27.9235	27.8309	1:Experimental evidence at protein level	anatomical structure morphogenesis;cell part morphogenesis;cell projection morphogenesis;cell projection organization;cellular component assembly;cellular component morphogenesis;cellular component organization;cellu
26.8322	26.605	26.6156	26.4925	1:Experimental evidence at protein level	alcohol metabolic process;biosynthetic process;carbohydrate biosynthetic process;carbohydrate metabolic process;cellular biosynthetic process;cellular carbohydrate metabolic process;cellular lipid metabolic process;cellul
28.0671	27.8976	27.7533	27.6543	1:Experimental evidence at protein level	cell cycle;cellular component organization;cellular component organization at cellular level;cellular component organization or biogenesis;cellular component organization or biogenesis at cellular level;cellular membrane fusio
26.5584	26.7724	26.8706	26.9732	1:Experimental evidence at protein level	cellular component organization;cellular component organization or biogenesis;cellular membrane organization;cellular process;endocytosis;endosome transport;establishment of localization;establishment of localization in c
25.965	26.4359	26.2868	25.9343	1:Experimental evidence at protein level	biological regulation;cation homeostasis;cellular cation homeostasis;cellular chemical homeostasis;cellular copper ion homeostasis;cellular homeostasis;cellular ion homeostasis;cellular process;chemical homeostasis;coppe
28.0631	27.9939	28.3384	28.3093	1:Experimental evidence at protein level	alternative nuclear mRNA splicing, via spliceosome;biological regulation;biosynthetic process;cell projection organization;cellular biosynthetic process;cellular component organization;cellular component organization at cellu
27.1273	27.3319	27.4902	26.9698	1:Experimental evidence at protein level	acidic amino acid transport;amine transport;amino acid transport;anion transport;carboxylic acid transport;establishment of localization;establishment of protein localization;ion transport;L-glutamate transport;nitrogen comp
26.548	26.0445	26.0486	26.0413	1:Experimental evidence at protein level	anatomical structure homeostasis;biological regulation;biosynthetic process;box H/ACA snoRNA 3'-end processing;box H/ACA snoRNA metabolic process;box H/ACA snoRNA processing;cell proliferation;cellular biosynthet
28.9335	28.7164	28.6984	28.5025	1:Experimental evidence at protein level	biological regulation;biosynthetic process;cellular biosynthetic process;cellular macromolecule biosynthetic process;cellular macromolecule metabolic process;cellular metabolic process;cellular process;cellular protein metab
28.8018	28.2997	28.7178	28.6401	1:Experimental evidence at protein level	biological regulation;biosynthetic process;cell differentiation;cellular biosynthetic process;cellular developmental process;cellular macromolecule biosynthetic process;cellular macromolecule metabolic process;cellular metab
26.5468	25.7661	26.1382	24.7318	1:Experimental evidence at protein level	actin cytoskeleton organization;actin filament organization;actin filament polymerization;actin filament-based process;actin polymerization or depolymerization;biological regulation;cellular component assembly;cellular compo
27.3408	26.9831	27.4393	27.3776	1:Experimental evidence at protein level	biological regulation;cellular macromolecule metabolic process;cellular metabolic process;cellular process;cellular protein metabolic process;macromolecule metabolic process;metabolic process;positive regulation of biologi
26.8964	26.8776	26.8563	26.779	1:Experimental evidence at protein level	biological regulation;biosynthetic process;cell cycle process;cell proliferation;cellular biosynthetic process;cellular component organization;cellular component organization at cellular level;cellular component organization or b
27.8687	27.9536	28.2481	28.3568	1:Experimental evidence at protein level	actin cytoskeleton organization;actin filament-based process;anatomical structure development;B cell activation;biological regulation;cell activation;cell cycle;cellular component organization;cellular component organization a
24.9402	22.6645	24.8935	24.972	1:Experimental evidence at protein level	anatomical structure homeostasis;B cell activation;B cell activation involved in immune response;biological regulation;blastocyst growth;cell activation;cell activation involved in immune response;cell cycle;cell cycle arrest;ce
24.9719	24.3806	24.0695	24.2358	1:Experimental evidence at protein level	biological regulation;cation homeostasis;cellular cation homeostasis;cellular chemical homeostasis;cellular homeostasis;cellular ion homeostasis;cellular iron ion homeostasis;cellular metal ion homeostasis;cellular process;ch
26.0247	26.3947	26.0735	25.8005	1:Experimental evidence at protein level	actin filament severing;actin filament-based process;anatomical structure development;anatomical structure morphogenesis;axon guidance;biological adhesion;biological regulation;cell adhesion;cell morphogenesis;cell morp
30.7192	30.8107	30.7038	30.7061	1:Experimental evidence at protein level	biological regulation;blood coagulation;cell activation;cellular process;coagulation;establishment of localization;establishment of localization in cell;exocytosis;hemostasis;multicellular organismal process;neurological system
26.9431	26.7851	26.973	26.4838	1:Experimental evidence at protein level	actin cytoskeleton organization;actin filament-based process;anatomical structure formation involved in morphogenesis;anatomical structure morphogenesis;axon guidance;biological regulation;cell part morphogenesis;cell p
25.804	26.7677	25.8339	25.7716	1:Experimental evidence at protein level	axon guidance;biological regulation;cell cycle;cell cycle phase;cell cycle process;cell division;cellular component organization;cellular component organization at cellular level;cellular component organization or biogenesis;cel
27.1528	27.3256	27.4554	27.4674	1:Experimental evidence at protein level	biosynthetic process;cellular biosynthetic process;cellular lipid metabolic process;cellular macromolecule metabolic process;cellular metabolic process;cellular process;cellular protein metabolic process;cellular response to c
24.3568	23.6565	23.8229	24.3859	1:Experimental evidence at protein level	apoptosis;cell death;cellular component organization;cellular component organization or biogenesis;cellular membrane organization;cellular process;clathrin-mediated endocytosis;death;endocytosis;establishment of localiza
26.2536	26.258	26.3105	26.423	1:Experimental evidence at protein level	catabolic process;cellular catabolic process;cellular component organization;cellular component organization at cellular level;cellular component organization or biogenesis;cellular component organization or biogenesis at ce
26.9331	23.9867	26.9297	27.0429	1:Experimental evidence at protein level	establishment of localization;establishment of localization in cell;establishment of RNA localization;intracellular transport;mRNA export from nucleus;mRNA transport;nuclear export;nuclear transport;nucleic acid transport;nuc
26.6658	26.6507	26.6224	26.6824	1:Experimental evidence at protein level	cellular component organization;cellular component organization at cellular level;cellular component organization or biogenesis;cellular component organization or biogenesis at cellular level;cellular localization;cellular proces
26.0585	25.8522	25.788	25.7681	1:Experimental evidence at protein level	biological regulation;cell differentiation;cellular component organization;cellular component organization at cellular level;cellular component organization or biogenesis;cellular component organization or biogenesis at cellular

26.7878	23.7821	26.3195	26.4446	1:Experimental evidence at protein level	biological regulation;biosynthetic process;catabolic process;cell differentiation;cellular biosynthetic process;cellular catabolic process;cellular developmental process;cellular macromolecule biosynthetic process;cellular macr
25.3485	25.2091	25.0695	24.7174	1:Experimental evidence at protein level	anatomical structure maturation;biological regulation;blood vessel maturation;defense response;defense response to bacterium;developmental maturation;developmental process;immune response;immune system process;i
28.8412	29.1164	28.6905	29.0258	1:Experimental evidence at protein level	amine biosynthetic process;amine metabolic process;apoptotic mitochondrial changes;biosynthetic process;carboxylic acid biosynthetic process;carboxylic acid metabolic process;cellular amine metabolic process;cellular a
27.3046	27.1611	27.1464	26.9461	1:Experimental evidence at protein level	cellular metabolic process;cellular process;electron transport chain;generation of precursor metabolites and energy;metabolic process;mitochondrial electron transport, NADH to ubiquinone;oxidation-reduction process;respi
26.3657	26.7064	27.1006	26.8428	1:Experimental evidence at protein level	anatomical structure development;biological regulation;biosynthetic process;Cajal body organization;cell cycle process;cell proliferation;cellular biosynthetic process;cellular component organization;cellular component organ
25.7237	26.0463	25.9079	25.7053	1:Experimental evidence at protein level	ATP biosynthetic process;ATP metabolic process;biological regulation;biosynthetic process;cellular biosynthetic process;cellular metabolic process;cellular nitrogen compound biosynthetic process;cellular nitrogen compoun
25.159	26.0265	25.4433	24.9952	1:Experimental evidence at protein level	amine metabolic process;aminoglycan catabolic process;aminoglycan metabolic process;carbohydrate catabolic process;carbohydrate metabolic process;catabolic process;glycosaminoglycan catabolic process;glycosamin
26.8526	27.0914	27.0896	27.0337	1:Experimental evidence at protein level	activation of caspase activity;anatomical structure formation involved in morphogenesis;angiogenesis;biological regulation;cellular process;cellular response to heat;cellular response to stimulus;cellular response to stress;dev
29.1291	29.8923	30.2219	30.1296	1:Experimental evidence at protein level	cellular component assembly;cellular component assembly at cellular level;cellular component organization;cellular component organization at cellular level;cellular component organization or biogenesis;cellular component c
27.3874	27.563	27.7241	27.8455	1:Experimental evidence at protein level	biological regulation;cation homeostasis;cation transport;cell surface receptor linked signaling pathway;cellular cation homeostasis;cellular chemical homeostasis;cellular component organization;cellular component organizat
26.6028	27.0246	27.006	27.0564	1:Experimental evidence at protein level	biological regulation;catabolic process;cation transport;cell cycle process;cellular catabolic process;cellular component disassembly;cellular component disassembly at cellular level;cellular component organization;cellular co
27.5782	24.2171	25.8091	26.1382	1:Experimental evidence at protein level	biological regulation;cellular component assembly;cellular component assembly at cellular level;cellular component organization;cellular component organization at cellular level;cellular component organization or biogenesis;
29.1776	28.7704	28.9809	28.8602	1:Experimental evidence at protein level	actin cytoskeleton organization;actin filament-based process;anatomical structure development;biological regulation;cell differentiation;cell surface receptor linked signaling pathway;cellular component organization;cellular c
25.5342	25.4792	24.0197	25.705	1:Experimental evidence at protein level	biological regulation;cellular component assembly;cellular component organization;cellular component organization at cellular level;cellular component organization or biogenesis;cellular component organization or biogenesis
29.2041	29.4385	29.286	29.1811	1:Experimental evidence at protein level	acetyl-CoA catabolic process;acetyl-CoA metabolic process;carbohydrate metabolic process;catabolic process;cellular carbohydrate metabolic process;cellular catabolic process;cellular metabolic process;cellular process;c
28.9668	29.0402	28.9565	28.7364	1:Experimental evidence at protein level	biological regulation;cellular process;ER to Golgi vesicle-mediated transport;establishment of localization;establishment of localization in cell;establishment of protein localization;Golgi vesicle transport;intracellular transport;p
27.0258	26.4633	26.7187	26.5864	1:Experimental evidence at protein level	biological regulation;cell cycle;cell division;cell migration;cell motility;cellular component movement;cellular component organization;cellular component organization at cellular level;cellular component organization or biogene
25.9108	25.933	26.0574	26.2086	1:Experimental evidence at protein level	anatomical structure development;biological regulation;cell cycle;cell differentiation;cell division;cell proliferation;cellular component organization;cellular component organization at cellular level;cellular component organizatio
26.3584	24.8132	25.9789	25.9897	1:Experimental evidence at protein level	cellular macromolecule metabolic process;cellular metabolic process;cellular process;cellular protein metabolic process;establishment of localization;establishment of localization in cell;establishment of protein localization;es
27.6956	27.9584	27.9598	27.8714	1:Experimental evidence at protein level	cellular process;endosome transport;establishment of localization;establishment of localization in cell;establishment of protein localization;intracellular protein transport;intracellular transport;protein transport;retrograde transp
27.2385	27.2197	27.0718	27.0948	1:Experimental evidence at protein level	cellular macromolecule metabolic process;cellular metabolic process;cellular process;cellular protein metabolic process;establishment of localization;establishment of localization in cell;establishment of protein localization;es
28.8931	28.722	28.8365	28.8325	1:Experimental evidence at protein level	biological regulation;biosynthetic process;cellular biosynthetic process;cellular component assembly;cellular component assembly at cellular level;cellular component organization;cellular component organization at cellular le
28.7425	28.3814	28.6361	28.2564	1:Experimental evidence at protein level	catabolic process;cellular catabolic process;cellular macromolecule catabolic process;cellular macromolecule metabolic process;cellular metabolic process;cellular process;ER-associated protein catabolic process;macromo
26.4848	26.3308	24.7255	26.4744	1:Experimental evidence at protein level	anatomical structure development;biological regulation;cellular metabolic process;cellular process;developmental process;electron transport chain;generation of precursor metabolites and energy;metabolic process;mitochon
27.5377	27.2113	27.5022	27.0094	1:Experimental evidence at protein level	assembly of spliceosomal tri-snRNP;biological regulation;cellular component assembly;cellular component assembly at cellular level;cellular component organization;cellular component organization at cellular level;cellular co
28.1196	23.8191	27.6359	27.681	1:Experimental evidence at protein level	axon ensheathment;biological adhesion;calcium-independent cell-cell adhesion;cell adhesion;cell-cell adhesion;cellular process;ensheathment of neurons;gamete generation;male gamete generation;multicellular organismal
27.1467	27.1704	26.4905	27.0093	1:Experimental evidence at protein level	carboxylic acid catabolic process;carboxylic acid metabolic process;catabolic process;cellular catabolic process;cellular ketone metabolic process;cellular lipid catabolic process;cellular lipid metabolic process;cellular metab
28.442	28.5865	28.7174	28.7319	1:Experimental evidence at protein level	cell cycle;cell cycle process;cellular component disassembly;cellular component disassembly at cellular level;cellular component organization;cellular component organization at cellular level;cellular component organization
28.6003	28.3053	28.2875	28.2534	1:Experimental evidence at protein level	anterior/posterior pattern specification;cellular macromolecule metabolic process;cellular metabolic process;cellular nitrogen compound metabolic process;cellular process;developmental process;gene expression;macromol
28.1125	28.0052	28.1061	28.1636	1:Experimental evidence at protein level	anatomical structure development;biological regulation;catabolic process;cellular catabolic process;cellular macromolecule catabolic process;cellular macromolecule metabolic process;cellular metabolic process;cellular nitro
26.7074	26.7295	26.4025	26.6082	1:Experimental evidence at protein level	anatomical structure morphogenesis;biological regulation;cellular macromolecule metabolic process;cellular metabolic process;cellular nitrogen compound metabolic process;cellular process;cellular protein metabolic proces
26.5601	27.3102	27.224	27.5835	1:Experimental evidence at protein level	biological regulation;carboxylic acid metabolic process;catabolic process;cellular catabolic process;cellular ketone metabolic process;cellular lipid metabolic process;cellular macromolecule catabolic process;cellular macrom
28.4445	28.0321	28.1253	28.0424	1:Experimental evidence at protein level	cell differentiation;cellular component assembly;cellular component assembly at cellular level;cellular component organization;cellular component organization at cellular level;cellular component organization or biogenesis;ce
27.6986	27.5169	27.7351	27.6782	1:Experimental evidence at protein level	biological regulation;cell cycle checkpoint;cellular process;cellular response to stimulus;cellular response to stress;DNA damage checkpoint;DNA integrity checkpoint;negative regulation of catalytic activity;negative regulation
25.3814	24.0773	25.4426	25.3092	1:Experimental evidence at protein level	cellular component biogenesis;cellular component biogenesis at cellular level;cellular component organization or biogenesis;cellular component organization or biogenesis at cellular level;cellular process;ribonucleoprotein co
25.534	26.148	26.039	26.071	1:Experimental evidence at protein level	biological regulation;cell surface receptor linked signaling pathway;cellular macromolecule metabolic process;cellular metabolic process;cellular process;cellular protein metabolic process;cellular response to chemical stimul
26.3295	24.4487	25.5552	25.7652	1:Experimental evidence at protein level	biological regulation;cellular macromolecule metabolic process;cellular metabolic process;cellular nitrogen compound metabolic process;cellular process;cleavage involved in rRNA processing;endonucleolytic cleavage in 5'-
27.1753	26.8398	26.8885	26.6819	1:Experimental evidence at protein level	biological regulation;biosynthetic process;carbohydrate metabolic process;carbohydrate transport;cell cycle;cell cycle process;cell surface receptor linked signaling pathway;cellular biosynthetic process;cellular component c
25.8305	26.0479	25.8249	25.7689	1:Experimental evidence at protein level	anatomical structure morphogenesis;biological regulation;biosynthetic process;cell morphogenesis;cellular biosynthetic process;cellular component morphogenesis;cellular component organization;cellular component organi
27.7473	27.7364	27.6631	27.5113	1:Experimental evidence at protein level	biological regulation;cellular component maintenance;cellular component maintenance at cellular level;cellular component organization;cellular component organization at cellular level;cellular component organization or biog
26.9555	26.5902	26.7204	26.7295	1:Experimental evidence at protein level	biological regulation;cellular component organization;cellular component organization at cellular level;cellular component organization or biogenesis;cellular component organization or biogenesis at cellular level;cellular macr
25.4533	25.0165	25.2718	25.3788	1:Experimental evidence at protein level	anatomical structure morphogenesis;angiotensin maturation;anterior/posterior pattern specification;biological regulation;cellular macromolecule metabolic process;cellular metabolic process;cellular process;cellular protein m
24.8571	25.2626	24.5464	24.5792	1:Experimental evidence at protein level	biological regulation;cell cycle arrest;cell cycle process;cell division;cellular process;negative regulation of biological process;negative regulation of cell cycle;negative regulation of cellular process;positive regulation of biolog
25.9945	26.6821	26.4944	26.0493	1:Experimental evidence at protein level	cellular macromolecule metabolic process;cellular metabolic process;cellular nitrogen compound metabolic process;cellular process;macromolecule metabolic process;metabolic process;ncRNA metabolic process;ncRNA pr
29.5401	29.0662	29.2592	29.4056	1:Experimental evidence at protein level	biological regulation;biosynthetic process;cellular biosynthetic process;cellular component assembly;cellular component assembly at cellular level;cellular component organization;cellular component organization at cellular le
26.0622	25.9298	26.1229	25.9799	1:Experimental evidence at protein level	biological regulation;biosynthetic process;cellular biosynthetic process;cellular component assembly;cellular component assembly at cellular level;cellular component organization;cellular component organization at cellular le
27.0116	27.299	27.0586	27.4028	1:Experimental evidence at protein level	catabolic process;cellular catabolic process;cellular ketone metabolic process;cellular metabolic process;cellular process;coenzyme catabolic process;coenzyme metabolic process;cofactor catabolic process;cofactor metab
26.6733	27.0698	27.3528	27.0186	1:Experimental evidence at protein level	activation of immune response;activation of innate immune response;anaphase-promoting complex-dependent proteasomal ubiquitin-dependent protein catabolic process;antigen processing and presentation;antigen proces
27.8036	27.895	27.6821	27.7682	1:Experimental evidence at protein level	CAAX-box protein processing;catabolic process;cellular catabolic process;cellular component organization;cellular component organization or biogenesis;cellular macromolecule catabolic process;cellular macromolecule me
27.2887	27.5255	27.4494	27.2409	1:Experimental evidence at protein level	2-oxoglutarate metabolic process;acetyl-CoA catabolic process;acetyl-CoA metabolic process;amine metabolic process;anatomical structure development;carbohydrate metabolic process;carboxylic acid metabolic process
28.3334	28.4807	28.3458	28.3587	1:Experimental evidence at protein level	biological regulation;cation homeostasis;cation transport;cellular cation homeostasis;cellular chemical homeostasis;cellular component assembly;cellular component assembly at cellular level;cellular component organization;
26.2868	25.8415	26.2286	26.3903	1:Experimental evidence at protein level	biological regulation;regulation of biological process;regulation of cell proliferation;regulation of cellular process
27.4001	27.4671	27.3123	26.9952	1:Experimental evidence at protein level	acidic amino acid transport;amine transport;amino acid transport;anion transport;biological regulation;carboxylic acid transport;cellular chemical homeostasis;cellular homeostasis;cellular ion homeostasis;cellular process;che
27.0656	26.855	27.1121	27.1741	1:Experimental evidence at protein level	cellular macromolecule metabolic process;cellular metabolic process;cellular nitrogen compound metabolic process;cellular process;gene expression;macromolecule metabolic process;metabolic process;mRNA metabolic p
26.6185	26.4839	26.4519	26.3501	1:Experimental evidence at protein level	antigen processing and presentation;antigen processing and presentation of exogenous antigen;antigen processing and presentation of exogenous peptide antigen;antigen processing and presentation of exogenous peptide
27.2648	27.241	27.6956	27.2895	1:Experimental evidence at protein level	cellular macromolecule metabolic process;cellular metabolic process;cellular nitrogen compound metabolic process;cellular process;gene expression;macromolecule metabolic process;metabolic process;mRNA metabolic p
27.9167	27.828	27.9758	27.8576	1:Experimental evidence at protein level	apoptosis;cell death;cellular component assembly;cellular component assembly at cellular level;cellular component organization;cellular component organization at cellular level;cellular component organization or biogenesis;
28.4599	28.5022	28.6538	28.6105	1:Experimental evidence at protein level	ATP biosynthetic process;ATP metabolic process;ATP synthesis coupled proton transport;biosynthetic process;cation transport;cellular biosynthetic process;cellular metabolic process;cellular nitrogen compound biosyntheti
24.3983	23.6239	23.5714	24.4687	1:Experimental evidence at protein level	
26.4153	25.5764	25.6621	25.942	1:Experimental evidence at protein level	anatomical structure morphogenesis;axonogenesis;biological regulation;cell part morphogenesis;cell projection morphogenesis;cell projection organization;cellular component assembly;cellular component assembly at cellula
25.7226	25.4423	25.5105	25.3225	1:Experimental evidence at protein level	axon guidance;biological regulation;cell surface receptor linked signaling pathway;cellular process;cellular response to stimulus;chemotaxis;enzyme linked receptor protein signaling pathway;intracellular signal transduction;l
28.7747	28.9417	28.8527	28.6008	1:Experimental evidence at protein level	ATP biosynthetic process;ATP metabolic process;ATP synthesis coupled proton transport;biosynthetic process;cation transport;cellular biosynthetic process;cellular metabolic process;cellular nitrogen compound biosyntheti
25.9987	25.5127	25.3842	25.0233	1:Experimental evidence at protein level	
28.2326	27.8297	28.4518	28.3061	1:Experimental evidence at protein level	biological regulation;cell redox homeostasis;cellular homeostasis;cellular process;homeostatic process;negative regulation of biological process;negative regulation of cardiac muscle hypertrophy;negative regulation of multic
27.525	26.7691	27.0076	27.1415	1:Experimental evidence at protein level	biological regulation;cell differentiation;cellular developmental process;cellular process;developmental process;osteoblast differentiation;regulation of apoptosis;regulation of biological process;regulation of cell aging;regulati
24.0193	24.788	23.9488	25.463	1:Experimental evidence at protein level	anatomical structure development;biological regulation;calcium ion homeostasis;cation homeostasis;cellular calcium ion homeostasis;cellular cation homeostasis;cellular chemical homeostasis;cellular divalent inorganic catio
26.1843	25.8465	24.2539	26.715	1:Experimental evidence at protein level	biological regulation;biosynthetic process;cellular biosynthetic process;cellular metabolic process;cellular process;chromosome segregation;cofactor biosynthetic process;cofactor metabolic process;iron-sulfur cluster assem
27.2123	26.9546	27.1786	26.7729	1:Experimental evidence at protein level	biosynthetic process;cellular biosynthetic process;cellular macromolecule biosynthetic process;cellular macromolecule metabolic process;cellular metabolic process;cellular process;cellular protein metabolic process;cotrans
28.6112	28.9053	28.8081	29.1941	1:Experimental evidence at protein level	amine catabolic process;amine metabolic process;arginine catabolic process;arginine metabolic process;biological regulation;carboxylic acid catabolic process;carboxylic acid metabolic process;catabolic process;cellular am
25.4407	24.8626	25.5274	25.0159	1:Experimental evidence at protein level	biological regulation;cellular process;cellular response to chemical stimulus;cellular response to endogenous stimulus;cellular response to growth factor stimulus;cellular response to hormone stimulus;cellular response to org
27.9455	27.8662	27.8485	27.8249	1:Experimental evidence at protein level	ATP-dependent chromatin remodeling;biological regulation;cellular component organization;cellular component organization at cellular level;cellular component organization or biogenesis;cellular component organization or b
26.7773	26.6753	26.957	26.7925	1:Experimental evidence at protein level	activation of protein kinase activity;biological adhesion;biological regulation;cell adhesion;cell cycle;cell-cell adhesion;cellular macromolecule metabolic process;cellular metabolic process;cellular process;cellular protein meta
29.3103	29.2844	29.6852	28.7756	1:Experimental evidence at protein level	alcohol biosynthetic process;alcohol metabolic process;amine metabolic process;amino sugar biosynthetic process;amino sugar metabolic process;biosynthetic process;carbohydrate biosynthetic process;carbohydrate meta
25.1058	25.6657	25.4024	25.7051	1:Experimental evidence at protein level	anatomical structure development;anatomical structure morphogenesis;apoptosis;apoptosis involved in luteolysis;axon extension;axon extension involved in axon guidance;axon guidance;biological adhesion;biological regul
25.4073	25.8736	25.6176	25.7804	1:Experimental evidence at protein level	cellular macromolecule metabolic process;cellular metabolic process;cellular process;cellular protein metabolic process;macromolecule metabolic process;macromolecule modification;metabolic process;primary metabolic p
27.7379	27.5194	27.6201	27.5278	1:Experimental evidence at protein level	cellular macromolecule metabolic process;cellular metabolic process;cellular process;cellular protein metabolic process;establishment of localization;establishment of localization in cell;establishment of protein localization;es
25.7412	25.569	25.7453	25.117	1:Experimental evidence at protein level	biological regulation;cellular localization;cellular process;localization;negative regulation of catalytic activity;negative regulation of hydrolase activity;negative regulation of molecular function;negative regulation of phosphatase
27.5595	27.4849	27.5724	27.313	1:Experimental evidence at protein level	antigen processing and presentation;antigen processing and presentation of exogenous antigen;antigen processing and presentation of exogenous peptide antigen;antigen processing and presentation of exogenous peptide
26.8869	26.7398	26.6333	26.4872	1:Experimental evidence at protein level	biological regulation;cell differentiation;cellular developmental process;cellular macromolecule metabolic process;cellular metabolic process;cellular process;cellular protein metabolic process;cellular response to stimulus;cell
25.4682	25.5626	25.3121	25.4519	1:Experimental evidence at protein level	biological regulation;cellular macromolecule metabolic process;cellular metabolic process;cellular process;cellular protein metabolic process;macromolecule metabolic process;macromolecule modification;metabolic process
26.5641	23.2518	26.807	26.609	1:Experimental evidence at protein level	
26.0421	24.3569	26.4543	26.8517	1:Experimental evidence at protein level	
27.5484	26.7004	27.5377	27.0894	1:Experimental evidence at protein level	catabolic process;cellular catabolic process;cellular macromolecule catabolic process;cellular macromolecule metabolic process;cellular metabolic process;cellular process;ER-associated protein catabolic process;macromo
27.2734	27.1642	27.0673	27.0927	1:Experimental evidence at protein level	assembly of spliceosomal tri-snRNP;biological regulation;cellular component assembly;cellular component assembly at cellular level;cellular component organization;cellular component organization at cellular level;cellular co
25.8641	25.3729	25.2782	23.8915	1:Experimental evidence at protein level	anatomical structure development;anatomical structure formation involved in morphogenesis;anatomical structure morphogenesis;appendage morphogenesis;biological regulation;cell morphogenesis;cell morphogenesis invo
25.9235	25.4035	23.4033	25.6125	1:Experimental evidence at protein level	biological regulation;biosynthetic process;cellular biosynthetic process;cellular macromolecule biosynthetic process;cellular macromolecule metabolic process;cellular metabolic process;cellular nitrogen compound metaboli
28.1124	28.0048	28.1562	27.9207	1:Experimental evidence at protein level	amine biosynthetic process;amine catabolic process;amine metabolic process;behavior;behavioral interaction between organisms;biological regulation;biosynthetic process;carboxylic acid biosynthetic process;carboxylic aci
27.8721	27.5829	27.5692	27.4933	1:Experimental evidence at protein level	antigen processing and presentation;antigen processing and presentation of exogenous antigen;antigen processing and presentation of exogenous peptide antigen;antigen processing and presentation of exogenous peptide
28.6972	28.5611	28.5168	28.537	1:Experimental evidence at protein level	antigen processing and presentation;antigen processing and presentation of exogenous antigen;antigen processing and presentation of exogenous peptide antigen;antigen processing and presentation of exogenous peptide
25.8723	25.4208	26.0826	26.0594	1:Experimental evidence at protein level	anatomical structure development;biological regulation;biosynthetic process;cellular biosynthetic process;cellular macromolecule biosynthetic process;cellular macromolecule metabolic process;cellular metabolic process;ce
25.4513	25.1997	25.3144	25.1486	1:Experimental evidence at protein level	anatomical structure development;biological regulation;cell cycle;cell cycle phase;cell cycle process;cell division;cellular component disassembly;cellular component disassembly at cellular level;cellular component organizati
26.0783	25.9741	26.1651	25.8191	1:Experimental evidence at protein level	biological regulation;catabolic process;cell proliferation;cellular catabolic process;cellular macromolecule catabolic process;cellular macromolecule metabolic process;cellular metabolic process;cellular nitrogen compound m
24.9947	24.2465	23.9903	24.8811	1:Experimental evidence at protein level	
26.2314	25.9927	25.9302	26.0789	1:Experimental evidence at protein level	
27.1764	27.8419	25.0027	27.0278	1:Experimental evidence at protein level	cellular metabolic process;cellular process;electron transport chain;generation of precursor metabolites and energy;metabolic process;mitochondrial electron transport, NADH to ubiquinone;oxidation-reduction process;respi
25.3551	24.9793	25.3575	25.0273	1:Experimental evidence at protein level	anatomical structure formation involved in morphogenesis;anatomical structure morphogenesis;apoptosis;autophagy;biological regulation;blastocyst formation;blood coagulation;camera-type eye morphogenesis;catabolic pr
24.8077	24.7728	25.3042	25.0538	1:Experimental evidence at protein level	cellular component assembly;cellular component assembly at cellular level;cellular component organization;cellular component organization at cellular level;cellular component organization or biogenesis;cellular component c
25.7376	25.9323	26.0605	26.1037	1:Experimental evidence at protein level	biological regulation;catabolic process;cellular catabolic process;cellular macromolecule catabolic process;cellular macromolecule metabolic process;cellular metabolic process;cellular process;cellular protein metabolic proc
26.4411	26.1961	26.2872	26.1812	1:Experimental evidence at protein level	biological regulation;cellular process;establishment of localization;regulation of biological process;regulation of biosynthetic process;regulation of cellular biosynthetic process;regulation of cellular macromolecule biosynthe
26.5414	26.3865	26.3674	26.53	1:Experimental evidence at protein level	biological regulation;biosynthetic process;cellular biosynthetic process;cellular component assembly;cellular component organization;cellular component organization at cellular level;cellular component organization or bioger
27.4766	26.8482	27.157	27.0579	1:Experimental evidence at protein level	cellular metabolic process;cellular process;electron transport chain;generation of precursor metabolites and energy;metabolic process;mitochondrial electron transport, NADH to ubiquinone;oxidation-reduction process;respi
27.5106	27.4165	27.2056	27.1983	1:Experimental evidence at protein level	cellular metabolic process;cellular process;electron transport chain;generation of precursor metabolites and energy;metabolic process;mitochondrial electron transport, NADH to ubiquinone;oxidation-reduction process;respi
28.3151	28.0009	27.6748	27.9055	1:Experimental evidence at protein level	apoptosis;cell death;cellular component organization;cellular component organization at cellular level;cellular component organization or biogenesis;cellular component organization or biogenesis at cellular level;cellular proce
26.7984	26.3485	26.7816	26.4938	1:Experimental evidence at protein level	cellular component organization;cellular component organization at cellular level;cellular component organization or biogenesis;cellular component organization or biogenesis at cellular level;cellular membrane organization;ce
27.4667	27.5229	27.3634	27.4038	1:Experimental evidence at protein level	biological regulation;cellular macromolecule metabolic process;cellular metabolic process;cellular nitrogen compound metabolic process;cellular process;macromolecule metabolic process;metabolic process;mRNA metaboli
27.1565	27.6909	27.3188	27.1585	1:Experimental evidence at protein level	cellular component organization;cellular component organization at cellular level;cellular component organization or biogenesis;cellular component organization or biogenesis at cellular level;cellular process;endocytic recyclin
26.98	26.2937	27.1738	26.7114	1:Experimental evidence at protein level	cellular component assembly;cellular component assembly at cellular level;cellular component organization;cellular component organization at cellular level;cellular component organization or biogenesis;cellular component c
25.72	25.7411	26.2925	26.7128	1:Experimental evidence at protein level	cell cycle;cell cycle cytokinesis;cell cycle process;cellular component movement;cellular component organization;cellular component organization at cellular level;cellular component organization or biogenesis;cellular compo
26.6328	26.7222	26.8435	26.6783	1:Experimental evidence at protein level	anterograde axon cargo transport;antigen processing and presentation;antigen processing and presentation of exogenous antigen;antigen processing and presentation of exogenous peptide antigen;antigen processing and p
26.4965	23.95	25.9693	26.3389	1:Experimental evidence at protein level	cellular component organization;cellular component organization or biogenesis;cellular process;endosome transport;ER to Golgi vesicle-mediated transport;establishment of localization;establishment of localization in cell;est
28.362	27.9025	28.0747	27.9717	1:Experimental evidence at protein level	activation of signaling protein activity involved in unfolded protein response;biological regulation;biosynthetic process;calcium ion homeostasis;cation homeostasis;cellular biosynthetic process;cellular calcium ion homeostas
23.9345	24.7281	24.3851	24.2104	1:Experimental evidence at protein level	biological regulation;cell communication;cell surface receptor linked signaling pathway;cell-cell signaling;cellular process;cellular response to stimulus;enzyme linked receptor protein signaling pathway;regulation of biological
27.2247	27.3722	26.6255	26.7098	1:Experimental evidence at protein level	cellular metabolic process;cellular process;electron transport chain;generation of precursor metabolites and energy;metabolic process;mitochondrial electron transport, NADH to ubiquinone;oxidation-reduction process;respi
28.073	28.0648	28.2117	28.0227	1:Experimental evidence at protein level	cellular macromolecule metabolic process;cellular metabolic process;cellular process;cellular protein metabolic process;chaperone-mediated protein folding;macromolecule metabolic process;macromolecule modification;me
27.7887	27.8923	28.0378	27.9638	1:Experimental evidence at protein level	alcohol catabolic process;alcohol metabolic process;carbohydrate catabolic process;carbohydrate metabolic process;catabolic process;cellular carbohydrate catabolic process;cellular carbohydrate metabolic process;cellula
28.9219	29.2648	28.9866	29.018	1:Experimental evidence at protein level	3'-phosphoadenosine 5'-phosphosulfate biosynthetic process;3'-phosphoadenosine 5'-phosphosulfate metabolic process;amine metabolic process;aminoglycan metabolic process;anatomical structure development;biologi
28.0555	27.8138	27.8826	27.7246	1:Experimental evidence at protein level	cell cycle;cell cycle process;cell division;cellular component organization;cellular component organization at cellular level;cellular component organization or biogenesis;cellular component organization or biogenesis at cellula
25.7465	25.5751	25.8504	25.9784	1:Experimental evidence at protein level	adult behavior;adult locomotory behavior;adult walking behavior;amine metabolic process;anatomical structure development;anatomical structure morphogenesis;autophagy;axonogenesis;behavior;biological regulation;carbo
27.0149	26.9135	23.5491	25.1715	1:Experimental evidence at protein level	biological regulation;cellular component organization;cellular component organization at cellular level;cellular component organization or biogenesis;cellular component organization or biogenesis at cellular level;cellular macr
27.1337	27.4732	27.3653	27.842	1:Experimental evidence at protein level	carboxylic acid metabolic process;cellular ketone metabolic process;cellular lipid metabolic process;cellular metabolic process;cellular process;fatty acid metabolic process;lipid metabolic process;metabolic process;monoca
28.4789	28.2956	28.4022	28.3121	1:Experimental evidence at protein level	biological regulation;cellular process;cellular response to stimulus;defense response;establishment of localization;establishment of localization in cell;establishment of protein localization;immune response;immune system pro
26.4901	26.5933	26.2374	26.4986	1:Experimental evidence at protein level	cell growth;cell proliferation;cellular macromolecule metabolic process;cellular metabolic process;cellular process;cellular protein metabolic process;developmental cell growth;developmental growth;developmental process;g
28.2228	28.1214	28.1467	28.0962	1:Experimental evidence at protein level	alcohol biosynthetic process;alcohol metabolic process;amino sugar biosynthetic process;amino sugar metabolic process;anatomical structure development;biosynthetic process;carbohydrate biosynthetic process;carbohyd
26.639	26.2933	26.8041	26.3126	1:Experimental evidence at protein level	assembly of spliceosomal tri-snRNP;biological regulation;cellular component assembly;cellular component assembly at cellular level;cellular component organization;cellular component organization at cellular level;cellular co
28.0248	28.0873	28.0212	28.0046	1:Experimental evidence at protein level	biological regulation;positive regulation of ATPase activity;positive regulation of catalytic activity;positive regulation of hydrolase activity;positive regulation of molecular function;regulation of ATPase activity;regulation of biolo
26.387	25.1884	26.5148	26.916	1:Experimental evidence at protein level	cell proliferation;cell proliferation in external granule layer;cell proliferation in hindbrain;cellular component assembly;cellular component assembly at cellular level;cellular component organization;cellular component organizat
26.0764	25.9583	25.8545	25.8165	1:Experimental evidence at protein level	ameboidal cell migration;anatomical structure development;anatomical structure morphogenesis;androgen metabolic process;biological regulation;biosynthetic process;carboxylic acid metabolic process;catabolic process;ce
25.9993	25.4542	25.4441	25.609	1:Experimental evidence at protein level	cellular macromolecule metabolic process;cellular metabolic process;cellular nitrogen compound metabolic process;cellular process;macromolecule metabolic process;metabolic process;ncRNA metabolic process;ncRNA pr
23.1276	25.2842	24.9307	25.3186	1:Experimental evidence at protein level	alcohol catabolic process;alcohol metabolic process;carbohydrate catabolic process;carbohydrate metabolic process;catabolic process;cellular carbohydrate catabolic process;cellular carbohydrate metabolic process;cellula

27.0116	27.0348	27.0585	26.9885	1:Experimental evidence at protein level	antigen processing and presentation;antigen processing and presentation of exogenous antigen;antigen processing and presentation of exogenous peptide antigen;antigen processing and presentation of exogenous peptide
25.5096	25.4716	25.6192	23.56	1:Experimental evidence at protein level	anatomical structure development;anatomical structure formation involved in morphogenesis;anatomical structure morphogenesis;antigen processing and presentation;antigen processing and presentation of exogenous anti
24.0586	26.6175	24.2592	24.3081	1:Experimental evidence at protein level	acute inflammatory response;amine metabolic process;biological adhesion;biological regulation;carboxylic acid metabolic process;cell adhesion;cell-cell adhesion;cellular amine metabolic process;cellular amino acid metabo
26.9359	27.7737	27.8796	28.1654	1:Experimental evidence at protein level	amine catabolic process;amine metabolic process;carboxylic acid catabolic process;carboxylic acid metabolic process;catabolic process;cellular amine metabolic process;cellular amino acid catabolic process;cellular amino
27.558	27.4148	27.5697	27.3084	1:Experimental evidence at protein level	acyl-CoA biosynthetic process;acyl-CoA metabolic process;acylglycerol biosynthetic process;acylglycerol metabolic process;anatomical structure development;biological regulation;biosynthetic process;brain development;ca
26.2794	26.3648	26.3452	26.5444	1:Experimental evidence at protein level	biological regulation;cell cycle cytokinesis;cell cycle process;cellular macromolecule metabolic process;cellular metabolic process;cellular process;cellular protein metabolic process;cellular response to stimulus;cytokinesis;c
27.7101	27.6557	27.7671	27.7555	1:Experimental evidence at protein level	
27.8651	27.8693	28.1331	27.9813	1:Experimental evidence at protein level	biological regulation;cellular macromolecule metabolic process;cellular metabolic process;cellular process;cellular protein metabolic process;cellular response to stimulus;establishment of localization;establishment of localiza
26.7915	26.702	27.0158	26.9461	1:Experimental evidence at protein level	autophagic vacuole fusion;cellular component assembly;cellular component assembly at cellular level;cellular component organization;cellular component organization at cellular level;cellular component organization or bioge
27.2979	27.4233	27.022	27.1202	1:Experimental evidence at protein level	activation of protein kinase activity;biological regulation;cellular hypotonic response;cellular macromolecule metabolic process;cellular metabolic process;cellular process;cellular protein metabolic process;cellular response to
25.2986	25.2746	25.5569	24.8087	1:Experimental evidence at protein level	cellular macromolecule metabolic process;cellular metabolic process;cellular process;cellular protein metabolic process;macromolecule metabolic process;metabolic process;primary metabolic process;protein folding;protein
26.4137	26.4164	26.3	26.2892	1:Experimental evidence at protein level	anatomical structure morphogenesis;biological regulation;cell maturation;cellular developmental process;cellular macromolecule metabolic process;cellular metabolic process;cellular nitrogen compound metabolic process;c
26.479	26.7168	27.1112	27.4636	1:Experimental evidence at protein level	cellular macromolecule metabolic process;cellular metabolic process;cellular nitrogen compound metabolic process;cellular process;macromolecule metabolic process;metabolic process;mRNA metabolic process;mRNA pro
29.0229	28.9119	28.7754	28.8391	1:Experimental evidence at protein level	antigen processing and presentation;antigen processing and presentation of exogenous antigen;antigen processing and presentation of exogenous peptide antigen;antigen processing and presentation of exogenous peptide
27.6087	27.2475	27.2546	27.6686	1:Experimental evidence at protein level	biological regulation;cellular localization;cellular macromolecule localization;cellular process;chemical homeostasis;homeostatic process;interaction with host;interspecies interaction between organisms;intracellular mRNA lo
27.4578	27.1937	26.97	26.9219	1:Experimental evidence at protein level	
26.0992	25.5311	23.6083	26.0785	1:Experimental evidence at protein level	
27.8951	27.8861	28.2846	28.3484	1:Experimental evidence at protein level	cellular macromolecule metabolic process;cellular metabolic process;cellular process;cellular protein metabolic process;cellular response to heat;cellular response to stimulus;cellular response to stress;macromolecule metab
27.2227	27.5274	27.5501	27.5332	1:Experimental evidence at protein level	anatomical structure development;biological regulation;brain development;cellular macromolecule metabolic process;cellular metabolic process;cellular process;cellular protein metabolic process;cellular response to abiotic s
26.4007	26.2289	26.3396	26.2353	1:Experimental evidence at protein level	activation of protein kinase activity;anatomical structure morphogenesis;biological regulation;cell part morphogenesis;cell projection morphogenesis;cell projection organization;cellular component morphogenesis;cellular con
26.7354	26.5834	26.976	26.6485	1:Experimental evidence at protein level	
26.8548	26.9267	26.9802	26.7403	1:Experimental evidence at protein level	activation of caspase activity;apoptosis;apoptosis in response to endoplasmic reticulum stress;biological regulation;catabolic process;cell death;cell differentiation;cell redox homeostasis;cellular catabolic process;cellular co
25.8338	25.8877	25.9811	25.3492	1:Experimental evidence at protein level	biological regulation;multicellular organismal process;negative regulation of biological process;negative regulation of cellular component organization;negative regulation of cellular process;negative regulation of cytoskeleton
26.9808	27.9304	27.716	27.8719	1:Experimental evidence at protein level	3'-phosphoadenosine 5'-phosphosulfate metabolic process;anatomical structure development;cellular lipid metabolic process;cellular metabolic process;cellular nitrogen compound metabolic process;cellular process;depho
26.3797	26.8175	26.7471	27.0624	1:Experimental evidence at protein level	amine catabolic process;amine metabolic process;arginine catabolic process;arginine metabolic process;biological regulation;biosynthetic process;carboxylic acid catabolic process;carboxylic acid metabolic process;catabo
28.2771	29.0804	28.9957	29.1698	1:Experimental evidence at protein level	biological regulation;cell redox homeostasis;cellular homeostasis;cellular process;homeostatic process;regulation of biological process;regulation of biological quality;regulation of cellular process
24.0327	24.194	24.3247	25.4213	1:Experimental evidence at protein level	cellular component organization;cellular component organization at cellular level;cellular component organization or biogenesis;cellular component organization or biogenesis at cellular level;cellular process;extracellular matr
26.1218	24.3563	25.844	26.2107	1:Experimental evidence at protein level	anatomical structure development;ATP-dependent chromatin remodeling;biological regulation;biosynthetic process;cellular biosynthetic process;cellular component organization;cellular component organization at cellular lev
24.4694	24.921	24.5803	24.4864	1:Experimental evidence at protein level	bis(5'-nucleosidyl) oligophosphate catabolic process;bis(5'-nucleosidyl) oligophosphate metabolic process;catabolic process;cell communication;cell-cell signaling;cellular catabolic process;cellular metabolic process;cellular
25.6258	25.8473	25.8537	26.1588	1:Experimental evidence at protein level	activation of immune response;activation of innate immune response;adaptive immune response;adaptive immune response based on somatic recombination of immune receptors built from immunoglobulin superfamily dom
26.7243	26.3663	26.5933	26.7344	1:Experimental evidence at protein level	cellular metabolic process;cellular process;electron transport chain;generation of precursor metabolites and energy;metabolic process;mitochondrial electron transport, NADH to ubiquinone;oxidation-reduction process;respi
27.3619	27.9978	27.6319	27.8085	1:Experimental evidence at protein level	biological regulation;cell differentiation;cellular developmental process;cellular process;developmental process;multicellular organismal development;multicellular organismal process;regulation of biological process;regulation
28.334	27.7276	28.1044	27.9434	1:Experimental evidence at protein level	cellular macromolecule metabolic process;cellular metabolic process;cellular process;cellular protein metabolic process;establishment of localization;establishment of localization in cell;establishment of protein localization;es
26.7974	26.5195	26.5922	26.4022	1:Experimental evidence at protein level	actin cytoskeleton organization;actin filament-based process;activation of protein kinase activity;apoptosis;biological regulation;cell cycle;cell death;cell growth;cell migration;cell motility;cell proliferation;cellular component m
27.4964	27.3849	27.5075	27.3463	1:Experimental evidence at protein level	anatomical structure development;ATP-dependent chromatin remodeling;biological regulation;biosynthetic process;cellular biosynthetic process;cellular component organization;cellular component organization at cellular lev
28.0503	28.1333	28.0185	28.2003	1:Experimental evidence at protein level	carboxylic acid metabolic process;cellular ketone metabolic process;cellular metabolic process;cellular process;L-ascorbic acid metabolic process;metabolic process;organic acid metabolic process;oxoacid metabolic proce
32.9514	33.1976	32.9447	32.8818	1:Experimental evidence at protein level	alcohol catabolic process;alcohol metabolic process;anatomical structure development;carbohydrate catabolic process;carbohydrate metabolic process;carboxylic acid metabolic process;catabolic process;cell communicati
28.984	29.1124	28.9449	29.0212	1:Experimental evidence at protein level	amine biosynthetic process;amine catabolic process;amine metabolic process;anatomical structure development;biological regulation;biosynthetic process;carboxylic acid biosynthetic process;carboxylic acid catabolic proc
27.025	27.2797	27.0811	27.4241	1:Experimental evidence at protein level	amine biosynthetic process;amine metabolic process;aromatic compound biosynthetic process;biological regulation;biosynthetic process;carboxylic acid biosynthetic process;carboxylic acid metabolic process;cell cycle;cell
28.988	29.0576	29.0246	28.8803	1:Experimental evidence at protein level	alcohol metabolic process;biosynthetic process;blood circulation;carboxylic acid metabolic process;cellular ketone metabolic process;cellular metabolic process;cellular process;cholesterol biosynthetic process;cholesterol m
26.2675	26.5624	26.9488	26.8443	1:Experimental evidence at protein level	amine metabolic process;biological regulation;biosynthetic process;carboxylic acid metabolic process;cell redox homeostasis;cellular amine metabolic process;cellular amino acid metabolic process;cellular biosynthetic proc
28.4735	28.2357	28.3917	28.2172	1:Experimental evidence at protein level	biosynthetic process;cellular biosynthetic process;cellular macromolecule metabolic process;cellular metabolic process;cellular nitrogen compound metabolic process;cellular process;electron transport chain;gene expressio
30.3248	30.9723	31.3742	31.2918	1:Experimental evidence at protein level	activation of MAPK activity;aging;amine metabolic process;anatomical structure development;anatomical structure homeostasis;anterograde axon cargo transport;auditory receptor cell stereocilium organization;axon cargo tr
23.1593	23.3057	24.5338	25.8323	1:Experimental evidence at protein level	biological regulation;blood coagulation;cell activation;cellular macromolecule metabolic process;cellular metabolic process;cellular process;cellular protein metabolic process;coagulation;establishment of localization;establis
25.4787	26.0123	26.2335	26.1535	1:Experimental evidence at protein level	biological regulation;biosynthetic process;catabolic process;cellular aromatic compound metabolic process;cellular biosynthetic process;cellular catabolic process;cellular metabolic compound salvage;cellular metabolic proc
28.2115	28.3967	28.6264	28.5996	1:Experimental evidence at protein level	adenine biosynthetic process;adenine metabolic process;adenine salvage;alcohol metabolic process;amine metabolic process;anatomical structure development;anatomical structure morphogenesis;behavior;benzene-conta
26.884	27.9214	27.2594	27.2944	1:Experimental evidence at protein level	2-oxoglutarate metabolic process;4-hydroxyproline catabolic process;4-hydroxyproline metabolic process;alcohol biosynthetic process;alcohol metabolic process;amine biosynthetic process;amine catabolic process;amine m
27.56	27.5738	27.4637	27.4157	1:Experimental evidence at protein level	activation of phospholipase A2 activity;activation of phospholipase A2 activity by calcium-mediated signaling;activation of phospholipase C activity;alkanesulfonate metabolic process;anatomical structure development;anato
30.9155	31.5268	31.7311	31.7489	1:Experimental evidence at protein level	alcohol biosynthetic process;alcohol metabolic process;biosynthetic process;carbohydrate biosynthetic process;carbohydrate metabolic process;cell differentiation;cellular biosynthetic process;cellular carbohydrate biosynth
27.7002	28.1904	28.0894	28.0231	1:Experimental evidence at protein level	ADP biosynthetic process;ADP metabolic process;AMP metabolic process;ATP metabolic process;biological regulation;biosynthetic process;cell cycle arrest;cell cycle process;cellular biosynthetic process;cellular metabolic
23.946	27.607	25.4122	26.3327	1:Experimental evidence at protein level	acute inflammatory response;acute-phase response;biological regulation;blood coagulation;blood coagulation, intrinsic pathway;calcium ion homeostasis;cation homeostasis;cell activation;cell migration;cell motility;cell surfa
23.4835	25.2345	24.3262	24.2604	1:Experimental evidence at protein level	activation of immune response;biological regulation;complement activation;complement activation, classical pathway;defense response;humoral immune response;immune effector process;immune response;immune system
22.7659	23.9454	23.6453	23.2481	1:Experimental evidence at protein level	biological regulation;blood coagulation;blood coagulation, extrinsic pathway;blood coagulation, intrinsic pathway;cellular macromolecule metabolic process;cellular metabolic process;cellular process;cellular protein metaboli
25.3235	24.3002	23.7257	24.7159	1:Experimental evidence at protein level	biological regulation;blood coagulation;cell activation;cellular component disassembly;cellular component disassembly at cellular level;cellular component organization;cellular component organization at cellular level;cellular
25.479	25.5106	25.5024	25.5279	1:Experimental evidence at protein level	biological regulation;blood coagulation;cell migration;cell motility;cellular component movement;cellular process;cellular response to stimulus;chemotaxis;coagulation;fibrinolysis;hemostasis;locomotion;macromolecule metab
25.3017	25.4409	25.3338	25.2645	1:Experimental evidence at protein level	biological regulation;blood coagulation;cell communication;cell migration;cell motility;cell surface receptor linked signaling pathway;cell-cell signaling;cellular component movement;cellular macromolecule metabolic process;
26.1431	25.8824	26.0946	25.9461	1:Experimental evidence at protein level	2'-deoxyribonucleotide metabolic process;adenosine catabolic process;adenosine metabolic process;aging;amine transport;anatomical structure development;B cell activation;B cell activation involved in immune response;E
27.8587	24.2602	27.7486	27.0598	1:Experimental evidence at protein level	ATP biosynthetic process;ATP metabolic process;ATP synthesis coupled proton transport;biosynthetic process;cation transport;cellular biosynthetic process;cellular metabolic process;cellular nitrogen compound biosynthetic
27.3763	27.4041	27.216	27.2916	1:Experimental evidence at protein level	acute inflammatory response;acute-phase response;aging;amide biosynthetic process;amine biosynthetic process;amine metabolic process;anatomical structure development;arginine biosynthetic process;arginine metabolic
24.6566	23.9076	24.4984	23.9132	1:Experimental evidence at protein level	biological regulation;blood coagulation;coagulation;hemostasis;multicellular organismal process;negative regulation of biological process;negative regulation of catalytic activity;negative regulation of defense response;negati
30.78	31.9857	31.6841	31.5231	1:Experimental evidence at protein level	biological regulation;blood coagulation;blood coagulation, intrinsic pathway;cell activation;cell differentiation;cellular component disassembly;cellular component disassembly at cellular level;cellular component organization;c
26.9417	27.4678	27.6252	27.2485	1:Experimental evidence at protein level	activation of immune response;biological regulation;carboxylic acid metabolic process;cell surface receptor linked signaling pathway;cellular ketone metabolic process;cellular lipid metabolic process;cellular metabolic proces
23.4313	24.1555	25.5188	25.0259	1:Experimental evidence at protein level	activation of immune response;activation of MAPK activity;biological regulation;calcium ion homeostasis;carbohydrate homeostasis;cation homeostasis;cell chemotaxis;cell death;cell migration;cell motility;cell surface recept
24.0231	25.5774	25.1322	23.7779	1:Experimental evidence at protein level	aging;biological regulation;blood coagulation;cell activation;cellular component disassembly;cellular component disassembly at cellular level;cellular component organization;cellular component organization at cellular level;c
23.2094	23.3831	24.1147	26.9292	1:Experimental evidence at protein level	actin cytoskeleton organization;actin filament-based process;activation of immune response;activation of innate immune response;activation of MAPKK activity;activation of protein kinase activity;anatomical structure morph
27.1213	27.2443	27.3648	27.388	1:Experimental evidence at protein level	activation of immune response;activation of innate immune response;activation of MAPKK activity;activation of protein kinase activity;axon guidance;behavior;behavioral interaction between organisms;biological regulation;bl
24.808	25.1239	24.674	25.4344	1:Experimental evidence at protein level	alcohol metabolic process;biological regulation;catabolic process;cellular catabolic process;cellular component organization;cellular component organization or biogenesis;cellular lipid metabolic process;cellular macromolec
30.1768	30.2009	30.1507	29.9779	1:Experimental evidence at protein level	antigen processing and presentation;antigen processing and presentation of exogenous antigen;antigen processing and presentation of exogenous peptide antigen;antigen processing and presentation of exogenous peptide
29.3678	23.8259	23.925	29.5424	5:Protein uncertain	antigen processing and presentation;antigen processing and presentation of peptide antigen;antigen processing and presentation of peptide antigen via MHC class I;biological regulation;immune response;immune system pr
25.2768	23.1489	25.4198	24.9754	1:Experimental evidence at protein level	biological regulation;blood coagulation;coagulation;hemostasis;multicellular organismal process;regulation of biological quality;regulation of body fluid levels
29.685	29.2059	29.4605	29.4804	1:Experimental evidence at protein level	anatomical structure development;anatomical structure formation involved in morphogenesis;anatomical structure morphogenesis;biological regulation;biomineral tissue development;biosynthetic process;blood coagulation;b
27.2358	26.7769	27.1007	26.9774	1:Experimental evidence at protein level	aging;anatomical structure development;anatomical structure morphogenesis;aorta smooth muscle tissue morphogenesis;axon guidance;biological adhesion;biological regulation;cell activation;cell adhesion;cell surface rece
24.044	29.2905	29.5772	28.9075	1:Experimental evidence at protein level	aging;anatomical structure development;cell differentiation;cell junction assembly;cell junction organization;cell-substrate junction assembly;cellular component assembly;cellular component assembly at cellular level;cellular
28.2678	28.6331	29.1878	28.5825	1:Experimental evidence at protein level	anatomical structure morphogenesis;biological regulation;cell differentiation;cellular developmental process;cellular process;developmental process;morphogenesis of an epithelium;positive regulation of biological process;po
31.7384	31.6119	31.7544	31.7293	1:Experimental evidence at protein level	anatomical structure development;apoptosis;biological regulation;cardiac cell development;cardiac muscle cell development;cell cycle;cell cycle process;cell death;cell development;cellular component disassembly;cellular c
25.0179	25.0261	25.5535	25.4273	1:Experimental evidence at protein level	acylglycerol catabolic process;acylglycerol metabolic process;aging;alcohol metabolic process;alpha-amino-3-hydroxy-5-methyl-4-isoxazole propionate receptor clustering;anatomical structure morphogenesis;artery morpho
23.7665	24.0402	23.7616	25.156	1:Experimental evidence at protein level	antibacterial humoral response;antimicrobial humoral response;biological adhesion;biological regulation;blood coagulation;cell activation;cell adhesion;cell-cell adhesion;cell-matrix adhesion;cell-substrate adhesion;cellular c
24.6596	24.9825	24.947	24.4228	1:Experimental evidence at protein level	activation of immune response;biological regulation;cell killing;complement activation;complement activation, alternative pathway;complement activation, classical pathway;cytolysis by symbiont of host cells;cytolysis in othe
24.4887	25.8513	27.9538	28.1832	1:Experimental evidence at protein level	acylglycerol metabolic process;acylglycerol transport;biological regulation;blood coagulation, intrinsic pathway;cellular lipid metabolic process;cellular metabolic process;cellular process;establishment of localization;glycerol
27.0322	26.6186	27.0047	26.7607	1:Experimental evidence at protein level	acute inflammatory response;acute-phase response;anatomical structure formation involved in morphogenesis;anatomical structure morphogenesis;angiogenesis;biological adhesion;biological regulation;blood coagulation;c
25.9973	27.671	27.299	27.6708	1:Experimental evidence at protein level	alcohol biosynthetic process;alcohol metabolic process;anatomical structure development;anatomical structure formation involved in morphogenesis;anatomical structure homeostasis;anatomical structure morphogenesis;ap
33.92	34.561	34.0701	34.9252	1:Experimental evidence at protein level	acute inflammatory response;acute-phase response;anatomical structure development;biological regulation;cellular component organization;cellular component organization or biogenesis;cellular membrane organization;cell
28.9996	29.3852	29.6117	29.6756	1:Experimental evidence at protein level	biological regulation;C21-steroid hormone metabolic process;cellular hormone metabolic process;cellular metabolic process;cellular process;cellular response to stimulus;establishment of localization;hormone metabolic proc
30.1811	30.2521	30.4379	30.3584	1:Experimental evidence at protein level	establishment of localization;fat-soluble vitamin metabolic process;lipid metabolic process;metabolic process;primary metabolic process;small molecule metabolic process;steroid metabolic process;transport;vitamin D meta
28.4286	28.5167	28.2974	28.2292	1:Experimental evidence at protein level	biological regulation;cation homeostasis;cation transport;cell differentiation;cellular cation homeostasis;cellular chemical homeostasis;cellular developmental process;cellular homeostasis;cellular ion homeostasis;cellular iron
27.6379	28.237	28.1556	28.5028	1:Experimental evidence at protein level	anatomical structure homeostasis;biological regulation;blood coagulation;cation homeostasis;cation transport;cell activation;cellular cation homeostasis;cellular chemical homeostasis;cellular homeostasis;cellular ion homeos
30.131	31.6412	31.931	31.5764	1:Experimental evidence at protein level	anatomical structure homeostasis;anatomical structure morphogenesis;antibacterial humoral response;antifungal humoral response;antimicrobial humoral response;biological regulation;biosynthetic process;bone morphogen
30.0775	31.5905	31.5248	32.829	1:Experimental evidence at protein level	biological regulation;cation homeostasis;cell surface receptor linked signaling pathway;cellular cation homeostasis;cellular chemical homeostasis;cellular copper ion homeostasis;cellular homeostasis;cellular ion homeostasis
22.7879	24.4836	24.849	24.6728	1:Experimental evidence at protein level	biological regulation;blood coagulation;blood coagulation, intrinsic pathway;coagulation;hemostasis;macromolecule metabolic process;metabolic process;multicellular organismal process;negative regulation of biological pro
27.0296	26.931	27.2506	26.7948	1:Experimental evidence at protein level	biological regulation;blood coagulation;cell migration;cell motility;cellular component disassembly;cellular component disassembly at cellular level;cellular component movement;cellular component organization;cellular comp
26.9124	22.7427	23.744	27.121	1:Experimental evidence at protein level	biological adhesion;biological regulation;cell adhesion;cell adhesion mediated by integrin;cell differentiation;cell-matrix adhesion;cell-substrate adhesion;cellular component organization;cellular component organization at cel
27.4941	27.8294	27.8841	27.9746	1:Experimental evidence at protein level	acylglycerol metabolic process;aerobic respiration;aging;alcohol metabolic process;anatomical structure development;biological regulation;catabolic process;cell differentiation;cellular aromatic compound metabolic process;
26.3943	26.5057	26.3791	25.9222	1:Experimental evidence at protein level	activation of adenylate cyclase activity;activation of immune response;activation of innate immune response;activation of MAPKK activity;activation of protein kinase activity;anatomical structure development;apoptosis;axon
27.1906	27.3025	26.9888	26.9508	1:Experimental evidence at protein level	alcohol biosynthetic process;alcohol metabolic process;amine metabolic process;anatomical structure morphogenesis;biological regulation;biosynthetic process;carbohydrate metabolic process;catabolic process;cellular bio
24.5567	24.2989	24.4812	24.1972	1:Experimental evidence at protein level	alcohol metabolic process;amine metabolic process;aminoglycan catabolic process;aminoglycan metabolic process;carbohydrate catabolic process;carbohydrate metabolic process;catabolic process;cellular carbohydrate c
32.8734	33.1008	33.0756	33.0969	1:Experimental evidence at protein level	actin cytoskeleton organization;actin filament organization;actin filament-based process;alcohol biosynthetic process;alcohol catabolic process;alcohol metabolic process;anatomical structure homeostasis;ATP biosynthetic p
27.4085	28.2694	28.165	28.4714	1:Experimental evidence at protein level	adult behavior;adult locomotory behavior;behavior;biological regulation;locomotory behavior;multicellular organismal process;negative regulation of biological process;negative regulation of catalytic activity;negative regulatio
31.6516	31.649	31.5931	31.5703	1:Experimental evidence at protein level	acid secretion;alpha-beta T cell activation;alpha-beta T cell differentiation;anatomical structure development;arachidonic acid secretion;biological regulation;carboxylic acid transport;cell activation;cell differentiation;cell surfa
29.4021	25.0205	27.0371	23.5824	1:Experimental evidence at protein level	acylglycerol catabolic process;acylglycerol metabolic process;alcohol metabolic process;anatomical structure development;anatomical structure morphogenesis;artery morphogenesis;biological regulation;biosynthetic proces
27.4526	28.0498	28.2755	28.1767	1:Experimental evidence at protein level	age-dependent response to oxidative stress;age-dependent response to reactive oxygen species;amine metabolic process;anatomical structure development;apoptotic mitochondrial changes;behavior;biological regulation;b
28.2801	28.2991	28.4243	28.3075	1:Experimental evidence at protein level	amine biosynthetic process;amine metabolic process;biosynthetic process;carboxylic acid biosynthetic process;carboxylic acid metabolic process;cellular amine metabolic process;cellular amino acid biosynthetic process;ce
28.722	29.255	28.9518	29.078	1:Experimental evidence at protein level	anatomical structure development;biosynthetic process;cell proliferation;cellular aromatic compound metabolic process;cellular biosynthetic process;cellular component assembly;cellular component organization;cellular com
28.1255	27.9712	27.7081	27.8693	1:Experimental evidence at protein level	activation of immune response;anatomical structure development;anatomical structure formation involved in morphogenesis;angiogenesis;antigen receptor-mediated signaling pathway;biological adhesion;biological regulatio
23.4037	26.0812	23.7345	26.7947	1:Experimental evidence at protein level	
27.012	29.5292	29.9256	29.0588	1:Experimental evidence at protein level	activation of immune response;anatomical structure homeostasis;biological regulation;complement activation;complement activation, lectin pathway;defense response;fibrinolysis;homeostatic process;humoral immune respo
34.0916	34.5939	34.6794	34.6187	1:Experimental evidence at protein level	alcohol biosynthetic process;alcohol metabolic process;apoptosis;biological regulation;biosynthetic process;carbohydrate biosynthetic process;carbohydrate metabolic process;cell death;cellular biosynthetic process;cellular
26.1761	26.3015	25.939	26.3226	1:Experimental evidence at protein level	amide biosynthetic process;amine biosynthetic process;amine catabolic process;amine metabolic process;arginine biosynthetic process;arginine biosynthetic process via ornithine;arginine catabolic process;arginine metabol
29.2609	29.9478	29.8829	29.6815	1:Experimental evidence at protein level	antigen processing and presentation;antigen processing and presentation of exogenous antigen;antigen processing and presentation of exogenous peptide antigen;antigen processing and presentation of exogenous peptide
28.9821	28.9202	29.2693	28.8994	1:Experimental evidence at protein level	biological regulation;cellular component disassembly;cellular component disassembly at cellular level;cellular component organization;cellular component organization at cellular level;cellular component organization or bioge
28.361	28.2416	28.4118	28.3478	1:Experimental evidence at protein level	aging;anatomical structure development;anatomical structure formation involved in morphogenesis;anatomical structure morphogenesis;anterior/posterior pattern specification;apoptosis;apoptotic mitochondrial changes;B c
26.1582	24.7991	27.3556	29.4889	1:Experimental evidence at protein level	biological regulation;cellular process;cellular response to cadmium ion;cellular response to chemical stimulus;cellular response to inorganic substance;cellular response to metal ion;cellular response to stimulus;cellular respo
32.7038	32.4722	33.056	33.1086	1:Experimental evidence at protein level	anatomical structure homeostasis;biological adhesion;biological regulation;cell adhesion;cell surface receptor linked signaling pathway;cell-cell adhesion;cellular component movement;cellular process;cellular response to ch
27.2466	27.4807	27.5614	27.3607	1:Experimental evidence at protein level	2'-deoxyribonucleotide biosynthetic process;2'-deoxyribonucleotide metabolic process;aging;anatomical structure development;anatomical structure morphogenesis;biological regulation;biosynthetic process;cartilage develo
29.777	29.6775	29.6187	29.5007	1:Experimental evidence at protein level	biosynthetic process;carbohydrate metabolic process;cellular biosynthetic process;cellular carbohydrate metabolic process;cellular macromolecule biosynthetic process;cellular macromolecule metabolic process;cellular met
29.1933	29.0959	29.0043	28.8517	1:Experimental evidence at protein level	aging;biosynthetic process;carbohydrate metabolic process;cellular biosynthetic process;cellular carbohydrate metabolic process;cellular macromolecule biosynthetic process;cellular macromolecule metabolic process;cellul
29.2564	29.3139	29.0496	29.106	1:Experimental evidence at protein level	activation of MAPKK activity;activation of protein kinase activity;adenosine receptor signaling pathway;biological regulation;blood coagulation;cAMP-mediated signaling;cell activation;cell communication;cell cycle;cell divisio
31.9683	29.3258	30.4809	30.6572	1:Experimental evidence at protein level	biological regulation;negative regulation of biological process;negative regulation of cell proliferation;negative regulation of cellular process;regulation of biological process;regulation of cell proliferation;regulation of cellular pr
29.4683	29.6275	29.3972	29.3654	1:Experimental evidence at protein level	biological regulation;cardiac muscle contraction;cation homeostasis;cation transport;cellular cation homeostasis;cellular chemical homeostasis;cellular homeostasis;cellular ion homeostasis;cellular metal ion homeostasis;cell
25.5675	24.4959	26.0462	25.69	1:Experimental evidence at protein level	biological adhesion;biological regulation;blood coagulation;calcium ion homeostasis;calcium-mediated signaling;cardiac muscle contraction;cation homeostasis;cation transport;cell adhesion;cell migration;cell motility;cellula
25.4629	25.7774	25.5464	25.7328	1:Experimental evidence at protein level	adult behavior;adult locomotory behavior;alcohol metabolic process;anatomical structure development;anatomical structure morphogenesis;apoptosis;associative learning;axon cargo transport;axon choice point recognition;
25.5842	25.7562	26.266	24.2151	1:Experimental evidence at protein level	alcohol catabolic process;alcohol metabolic process;biological regulation;biosynthetic process;carbohydrate metabolic process;catabolic process;cell communication;cell-cell signaling;cellular biosynthetic process;cellular m
25.8458	26.3823	26.0327	25.5815	1:Experimental evidence at protein level	activation of protein kinase activity;anatomical structure development;anatomical structure formation involved in morphogenesis;anatomical structure morphogenesis;angiogenesis;angiogenesis involved in wound healing;apo
28.2639	24.4067	23.9303	23.8462	1:Experimental evidence at protein level	anatomical structure morphogenesis;biological regulation;cellular component organization;cellular component organization at cellular level;cellular component organization or biogenesis;cellular component organization or bio
28.31	28.7506	28.5788	28.4249	1:Experimental evidence at protein level	biological regulation;blood coagulation;coagulation;fibrinolysis;hemostasis;multicellular organismal process;negative regulation of apoptosis;negative regulation of biological process;negative regulation of blood coagulation;n
25.6912	25.2853	25.0371	25.7293	1:Experimental evidence at protein level	aging;anatomical structure formation involved in morphogenesis;angiogenesis;biological regulation;biosynthetic process;blood coagulation;cell activation;cell aging;cell surface receptor linked signaling pathway;cellular biosy
31.4237	31.657	31.448	31.4266	1:Experimental evidence at protein level	adenine transport;biological regulation;cellular metabolic process;cellular process;chromosome segregation;energy derivation by oxidation of organic compounds;energy reserve metabolic process;establishment of localizatio
28.2394	28.1746	28.0105	28.1065	1:Experimental evidence at protein level	biological regulation;biosynthetic process;catabolic process;cell surface receptor linked signaling pathway;cellular biosynthetic process;cellular catabolic process;cellular macromolecule biosynthetic process;cellular macrom
28.7911	28.5347	28.5405	28.4429	1:Experimental evidence at protein level	biological regulation;biosynthetic process;cellular biosynthetic process;cellular macromolecule biosynthetic process;cellular macromolecule metabolic process;cellular metabolic process;cellular process;cellular protein metab
27.3564	24.4859	24.8983	27.6079	1:Experimental evidence at protein level	

27.8679	28.1169	28.211	28.1073	1:Experimental evidence at protein level	acute inflammatory response;acute inflammatory response to antigenic stimulus;adhesion to host;adhesion to other organism involved in symbiotic interaction;aging;anatomical structure development;antigen processing and
29.476	29.4656	30.0146	29.7614	1:Experimental evidence at protein level	biosynthetic process;catabolic process;cellular biosynthetic process;cellular catabolic process;cellular component disassembly;cellular component disassembly at cellular level;cellular component organization;cellular compo
30.6909	30.5221	30.587	30.4335	1:Experimental evidence at protein level	biosynthetic process;catabolic process;cellular biosynthetic process;cellular catabolic process;cellular component biogenesis;cellular component biogenesis at cellular level;cellular component disassembly;cellular componen
23.8432	25.9087	24.1936	26.6713	1:Experimental evidence at protein level	anatomical structure development;biological regulation;biomineral tissue development;bone mineralization;cellular process;cellular response to chemical stimulus;cellular response to endogenous stimulus;cellular response to
29.1047	29.381	29.2694	29.2061	1:Experimental evidence at protein level	cellular macromolecule metabolic process;cellular metabolic process;cellular nitrogen compound metabolic process;cellular process;histone mRNA metabolic process;macromolecule metabolic process;macromolecule modi
24.7974	25.8603	24.7377	24.8325	1:Experimental evidence at protein level	aging;amine transport;amino acid transport;biological regulation;carboxylic acid transport;developmental process;establishment of localization;hormone transport;modified amino acid transport;negative regulation of catalytic
24.4883	23.321	26.5067	26.5362	1:Experimental evidence at protein level	biological regulation;blood coagulation;chemotaxis;coagulation;hemostasis;locomotion;multicellular organismal process;negative regulation of catalytic activity;negative regulation of endopeptidase activity;negative regulation
30.7516	30.7125	30.7749	30.5295	1:Experimental evidence at protein level	actin cytoskeleton organization;actin filament-based process;actomyosin structure organization;anatomical structure homeostasis;anatomical structure morphogenesis;associative learning;axon extension;axon guidance;B ce
28.1005	27.1237	27.6182	27.6491	1:Experimental evidence at protein level	anatomical structure morphogenesis;biological regulation;cell cycle;cell surface receptor linked signaling pathway;cellular component organization;cellular component organization at cellular level;cellular component organiza
26.7118	29.2755	29.7166	29.6992	1:Experimental evidence at protein level	actin cytoskeleton organization;actin filament-based process;actomyosin structure organization;biological regulation;cell differentiation;cell differentiation involved in embryonic placenta development;cell surface receptor link
26.8044	26.912	27.1992	27.1889	1:Experimental evidence at protein level	anatomical structure development;anatomical structure morphogenesis;axon guidance;cellular component disassembly;cellular component disassembly at cellular level;cellular component organization;cellular component org
27.4627	27.6573	28.0038	28.1221	1:Experimental evidence at protein level	biosynthetic process;cellular aromatic compound metabolic process;cellular biosynthetic process;cellular metabolic process;cellular nitrogen compound biosynthetic process;cellular nitrogen compound metabolic process;ce
23.9496	25.8977	25.2624	25.2251	1:Experimental evidence at protein level	biological regulation;carbohydrate catabolic process;carbohydrate metabolic process;catabolic process;cellular carbohydrate catabolic process;cellular carbohydrate metabolic process;cellular catabolic process;cellular lipid
29.2606	29.2641	29.6024	29.3949	1:Experimental evidence at protein level	actin cytoskeleton organization;actin filament capping;actin filament organization;actin filament polymerization;actin filament severing;actin filament-based process;actin nucleation;actin polymerization or depolymerization;ag
30.2049	30.2942	30.4812	31.0398	1:Experimental evidence at protein level	biosynthetic process;cellular biosynthetic process;cellular macromolecule biosynthetic process;cellular macromolecule metabolic process;cellular metabolic process;cellular nitrogen compound metabolic process;cellular pro
29.8754	29.9025	29.8361	29.825	1:Experimental evidence at protein level	activation of immune response;activation of innate immune response;activation of MAPK activity;activation of MAPKK activity;activation of protein kinase activity;aging;anaphase-promoting complex-dependent proteasomal
31.5575	31.7566	31.7066	31.6546	1:Experimental evidence at protein level	anatomical structure formation involved in morphogenesis;angiogenesis;ATP biosynthetic process;ATP hydrolysis coupled proton transport;ATP metabolic process;ATP synthesis coupled proton transport;biological regulation
31.654	32.0393	32.3475	32.1606	1:Experimental evidence at protein level	anatomical structure morphogenesis;axonogenesis;biological regulation;cell part morphogenesis;cell projection morphogenesis;cell projection organization;cellular component morphogenesis;cellular component organization
28.2642	28.0217	28.1691	27.9666	1:Experimental evidence at protein level	anatomical structure development;biological regulation;biosynthetic process;catabolic process;cell cycle process;cell surface receptor linked signaling pathway;cellular biosynthetic process;cellular catabolic process;cellular
32.7315	33.6432	33.591	33.8356	1:Experimental evidence at protein level	alcohol biosynthetic process;alcohol metabolic process;biological regulation;biosynthetic process;carbohydrate biosynthetic process;carbohydrate metabolic process;cellular biosynthetic process;cellular carbohydrate biosy
27.3483	27.2148	27.2048	27.0611	1:Experimental evidence at protein level	5-phosphoribose 1-diphosphate biosynthetic process;5-phosphoribose 1-diphosphate metabolic process;alcohol biosynthetic process;alcohol metabolic process;biological regulation;biosynthetic process;carbohydrate bios
30.1422	30.4016	30.4227	30.4175	1:Experimental evidence at protein level	alcohol biosynthetic process;alcohol metabolic process;aldehyde biosynthetic process;aldehyde catabolic process;anatomical structure formation involved in morphogenesis;angiogenesis;behavior;biological regulation;biosy
32.6332	33.0135	33.0984	33.2222	1:Experimental evidence at protein level	aging;ATP-dependent chromatin remodeling;biological regulation;cell aging;cell cycle process;cellular component assembly;cellular component assembly at cellular level;cellular component organization;cellular component o
30.0292	30.3868	30.336	30.7655	1:Experimental evidence at protein level	actin filament-based movement;actin filament-based process;actin-mediated cell contraction;actin-myosin filament sliding;cellular component movement;cellular process;multicellular organismal process;muscle contraction;
26.9833	26.8485	26.8414	26.8954	1:Experimental evidence at protein level	anatomical structure formation involved in morphogenesis;anatomical structure morphogenesis;angiogenesis;antigen processing and presentation;antigen processing and presentation of exogenous antigen;antigen processi
26.5055	26.6961	26.584	26.6207	1:Experimental evidence at protein level	amine metabolic process;aminoglycan catabolic process;aminoglycan metabolic process;carbohydrate catabolic process;carbohydrate metabolic process;catabolic process;cellular catabolic process;cellular lipid metabolic p
27.3565	27.2451	27.1841	27.403	1:Experimental evidence at protein level	anatomical structure development;anatomical structure morphogenesis;behavior;biological regulation;blood coagulation;cellular component organization;cellular component organization at cellular level;cellular component or
26.5058	26.6553	26.3184	25.9866	1:Experimental evidence at protein level	aromatic compound catabolic process;catabolic process;cellular aromatic compound metabolic process;cellular catabolic process;cellular metabolic process;cellular process;metabolic process;response to chemical stimulus
31.8134	31.7597	31.6326	31.6769	1:Experimental evidence at protein level	carbohydrate metabolic process;carboxylic acid metabolic process;cellular ketone metabolic process;cellular metabolic process;cellular nitrogen compound metabolic process;cellular process;coenzyme metabolic process;c
25.5071	26.0281	26.1118	25.6737	1:Experimental evidence at protein level	acylglycerol metabolic process;aging;amine metabolic process;anatomical structure development;anatomical structure formation involved in morphogenesis;anatomical structure morphogenesis;angiogenesis;angiogenesis in
30.9555	31.0687	31.1665	31.0839	1:Experimental evidence at protein level	4-hydroxyproline metabolic process;amine metabolic process;biological regulation;carboxylic acid metabolic process;cell redox homeostasis;cellular amine metabolic process;cellular amino acid metabolic process;cellular co
29.0719	28.0707	28.117	28.2542	1:Experimental evidence at protein level	apoptosis;catabolic process;cell death;cellular catabolic process;cellular component assembly;cellular component assembly at cellular level;cellular component disassembly;cellular component disassembly at cellular level;ce
29.0245	29.2877	29.3907	29.531	1:Experimental evidence at protein level	antigen processing and presentation;antigen processing and presentation of exogenous antigen;antigen processing and presentation of exogenous peptide antigen;antigen processing and presentation of exogenous peptide
32.5601	32.101	32.2073	32.0503	1:Experimental evidence at protein level	anatomical structure formation involved in morphogenesis;angiogenesis;biological regulation;body fluid secretion;cellular component assembly;cellular component assembly at cellular level;cellular component organization;ce
28.5959	28.4819	28.5118	28.4338	1:Experimental evidence at protein level	biological regulation;catabolic process;cellular catabolic process;cellular component disassembly;cellular component disassembly at cellular level;cellular component organization;cellular component organization at cellular le
34.6864	34.9419	34.8376	34.7998	1:Experimental evidence at protein level	biological regulation;cell cycle;cell cycle process;cell division;cell killing;cellular component assembly;cellular component assembly at cellular level;cellular component movement;cellular component organization;cellular comp
28.3677	28.9336	29.1523	29.1293	1:Experimental evidence at protein level	biological regulation;blood coagulation;cell activation;cell differentiation;cellular developmental process;cellular lipid metabolic process;cellular metabolic process;cellular process;cellular process involved in reproduction;cellu
28.5686	28.4252	28.5961	28.5423	1:Experimental evidence at protein level	amine metabolic process;aminoglycan catabolic process;aminoglycan metabolic process;anatomical structure development;astrocyte cell migration;axon ensheathment;behavior;biological regulation;biosynthetic process;cal
26.6154	28.0561	28.0285	27.7967	1:Experimental evidence at protein level	activation of immune response;activation of innate immune response;adaptive immune response;antigen processing and presentation;antigen processing and presentation of exogenous antigen;antigen processing and prese
31.8685	32.8494	32.7412	32.9945	1:Experimental evidence at protein level	actin cytoskeleton organization;actin filament-based process;anatomical structure formation involved in morphogenesis;biological regulation;blood coagulation;cell activation;cellular component organization;cellular compone
27.514	27.8224	27.9311	25.9769	1:Experimental evidence at protein level	adenine biosynthetic process;adenine metabolic process;adenine salvage;AMP biosynthetic process;AMP metabolic process;AMP salvage;behavior;biological regulation;biosynthetic process;body fluid secretion;cellular aron
29.2768	29.1245	29.098	29.0364	1:Experimental evidence at protein level	amine metabolic process;amino acid activation;biological regulation;carboxylic acid metabolic process;cellular amine metabolic process;cellular amino acid metabolic process;cellular component assembly;cellular componen
30.5836	30.8561	31.1655	31.0152	1:Experimental evidence at protein level	activation of immune response;activation of innate immune response;anatomical structure development;biological regulation;cell differentiation;cellular component disassembly;cellular component disassembly at cellular leve
32.4357	32.5273	32.4314	32.3847	1:Experimental evidence at protein level	apoptosis;axon guidance;biological regulation;cardiac muscle cell apoptosis;cell cycle;cell cycle process;cell death;cell migration;cell motility;cell surface receptor linked signaling pathway;cellular component assembly;cellula
31.5289	30.8108	31.5296	31.2079	1:Experimental evidence at protein level	3'-UTR-mediated mRNA stabilization;ATP-dependent chromatin remodeling;biological regulation;cell differentiation;cellular component organization;cellular component organization at cellular level;cellular component organiz
27.4048	27.6579	28.2921	28.1544	1:Experimental evidence at protein level	aerobic respiration;cellular metabolic process;cellular process;cellular respiration;electron transport chain;energy derivation by oxidation of organic compounds;generation of precursor metabolites and energy;metabolic proce
27.2058	26.9281	27.1237	27.0668	1:Experimental evidence at protein level	anatomical structure morphogenesis;axon guidance;biological adhesion;biological regulation;cell adhesion;cell differentiation;cell morphogenesis;cell morphogenesis involved in differentiation;cell projection organization;cell-
26.8972	26.606	27.3951	24	1:Experimental evidence at protein level	axon guidance;biological regulation;blood coagulation;carbohydrate transport;cell differentiation;cell migration;cell motility;cell surface receptor linked signaling pathway;cellular component movement;cellular developmental
31.0324	31.3883	31.8418	31.5008	1:Experimental evidence at protein level	actin filament-based movement;actin filament-based process;actin-mediated cell contraction;actin-myosin filament sliding;biological regulation;cellular component movement;cellular process;multicellular organismal process
29.0626	29.3656	29.3374	29.4604	1:Experimental evidence at protein level	acetyl-CoA catabolic process;acetyl-CoA metabolic process;anatomical structure homeostasis;biological regulation;carboxylic acid metabolic process;catabolic process;cellular catabolic process;cellular ketone metabolic pr
30.9503	31.2364	31.1444	30.8554	1:Experimental evidence at protein level	activation of MAPK activity;anatomical structure development;anatomical structure formation involved in morphogenesis;anatomical structure morphogenesis;angiogenesis;behavior;behavioral response to pain;biological adh
28.2163	27.9845	28.4332	28.3212	1:Experimental evidence at protein level	anatomical structure development;anatomical structure morphogenesis;biological regulation;blood coagulation;blood vessel development;cell activation;cell migration;cell motility;cell surface receptor linked signaling pathwa
29.7209	29.0441	29.2415	29.0837	1:Experimental evidence at protein level	biological regulation;calcium ion transport;cation transport;cellular component assembly;cellular component organization;cellular component organization or biogenesis;cellular process;divalent inorganic cation transport;diva
29.197	29.5096	29.0148	29.3158	1:Experimental evidence at protein level	apical junction assembly;axon guidance;biological regulation;cell junction assembly;cell junction organization;cell migration;cell motility;cell-cell junction assembly;cell-cell junction organization;cellular component assembly;c
25.6798	25.4868	25.9225	25.5921	1:Experimental evidence at protein level	activation of immune response;adaptive immune response;adaptive immune response based on somatic recombination of immune receptors built from immunoglobulin superfamily domains;biological regulation;calcium ion h
27.6678	27.8189	27.6622	27.7316	1:Experimental evidence at protein level	amine transport;amino acid import;amino acid transport;aromatic amino acid transport;biological regulation;blood coagulation;branched-chain aliphatic amino acid transport;calcium ion transport;carbohydrate metabolic proc
26.6038	26.7615	26.9344	26.4841	1:Experimental evidence at protein level	alcohol catabolic process;alcohol metabolic process;anatomical structure homeostasis;biological regulation;carbohydrate catabolic process;carbohydrate homeostasis;carbohydrate metabolic process;carbohydrate phospho
32.9676	33.1179	32.8996	32.8925	1:Experimental evidence at protein level	anatomical structure development;axon guidance;biological regulation;cellular macromolecule metabolic process;cellular metabolic process;cellular process;cellular protein metabolic process;cellular response to chemical sti
28.2422	28.2535	28.2371	28.036	1:Experimental evidence at protein level	biological regulation;biosynthetic process;cellular biosynthetic process;cellular macromolecule biosynthetic process;cellular macromolecule metabolic process;cellular metabolic process;cellular process;cellular protein metab
26.736	26.5458	26.5162	26.6445	1:Experimental evidence at protein level	amine biosynthetic process;amine metabolic process;anatomical structure development;asparagine biosynthetic process;asparagine metabolic process;aspartate family amino acid biosynthetic process;aspartate family amin
25.6158	24.1649	24.194	24.9361	1:Experimental evidence at protein level	anatomical structure formation involved in morphogenesis;anatomical structure maturation;anatomical structure morphogenesis;angiogenesis;axon guidance;biological regulation;blood vessel maturation;bone trabecula form
25.0676	25.5705	25.9369	25.8817	1:Experimental evidence at protein level	biosynthetic process;cellular aromatic compound metabolic process;cellular biosynthetic process;cellular macromolecule metabolic process;cellular metabolic process;cellular nitrogen compound biosynthetic process;cellula
26.1105	26.5714	26.3492	26.13	1:Experimental evidence at protein level	aging;amine metabolic process;anatomical structure development;angiotensin maturation;beta-amyloid metabolic process;biological regulation;carboxylic acid metabolic process;cell aging;cellular amide metabolic process;c
27.696	27.5614	27.6009	27.6492	1:Experimental evidence at protein level	acetyl-CoA biosynthetic process;acetyl-CoA biosynthetic process from pyruvate;acetyl-CoA catabolic process;acetyl-CoA metabolic process;alcohol catabolic process;alcohol metabolic process;biological regulation;biosynt
28.442	28.4608	28.3361	28.3477	1:Experimental evidence at protein level	cellular metabolic process;cellular process;electron transport chain;generation of precursor metabolites and energy;metabolic process;oxidation-reduction process;respiratory electron transport chain;response to chemical sti
28.5183	28.5392	28.3864	28.2256	1:Experimental evidence at protein level	biological regulation;cellular macromolecule metabolic process;cellular metabolic process;cellular nitrogen compound metabolic process;cellular process;gene expression;macromolecule metabolic process;metabolic proces
28.5121	28.318	28.5685	28.7147	1:Experimental evidence at protein level	biological regulation;cellular macromolecule metabolic process;cellular metabolic process;cellular nitrogen compound metabolic process;cellular process;gene expression;macromolecule metabolic process;metabolic proces
29.6016	29.4466	29.3827	29.3091	1:Experimental evidence at protein level	anatomical structure formation involved in morphogenesis;angiogenesis;axon guidance;behavior;biological adhesion;biological regulation;blood coagulation;cell adhesion;cell adhesion mediated by integrin;cell differentiation;
36.1741	35.2508	35.7348	35.7223	1:Experimental evidence at protein level	actin filament-based movement;actin filament-based process;actin-mediated cell contraction;actin-myosin filament sliding;anatomical structure development;apoptosis;astrocyte development;astrocyte differentiation;Bergma
24.3906	26.522	23.0462	24.6241	1:Experimental evidence at protein level	acute inflammatory response;acute-phase response;anatomical structure morphogenesis;biological regulation;blood coagulation;blood vessel morphogenesis;cell activation;cellular component organization;cellular componer
30.5543	30.7757	30.6198	30.576	1:Experimental evidence at protein level	biological regulation;biosynthetic process;catabolic process;cellular biosynthetic process;cellular catabolic process;cellular component assembly;cellular component assembly at cellular level;cellular component biogenesis;c
26.8067	24.5967	24.819	24.4265	1:Experimental evidence at protein level	actin cytoskeleton organization;actin filament-based process;actomyosin structure organization;cell differentiation;cell differentiation involved in embryonic placenta development;cellular component organization;cellular comp
27.5123	26.7753	26.791	26.9625	1:Experimental evidence at protein level	viral reproduction
27.2467	27.2351	26.9458	26.7377	1:Experimental evidence at protein level	biological regulation;blood coagulation;cAMP-mediated signaling;cell activation;cell communication;cell cycle;cell division;cell surface receptor linked signaling pathway;cell-cell signaling;cellular component organization;cellu
31.9411	32.0541	32.039	31.9045	1:Experimental evidence at protein level	biological regulation;blood coagulation;cellular process;cellular response to stimulus;coagulation;hemostasis;multicellular organismal process;negative regulation of apoptosis;negative regulation of biological process;negative
25.1868	24.0296	24.0252	23.7764	1:Experimental evidence at protein level	aging;anatomical structure development;anatomical structure morphogenesis;biological regulation;cell differentiation;cell migration;cell motility;cell proliferation;cellular component movement;cellular component organization;
31.8324	31.7963	32.1029	32.0058	1:Experimental evidence at protein level	biological adhesion;biosynthetic process;catabolic process;cell adhesion;cellular biosynthetic process;cellular catabolic process;cellular component assembly;cellular component assembly at cellular level;cellular component
28.1647	28.1629	27.5797	27.7752	1:Experimental evidence at protein level	biological adhesion;biological regulation;blood coagulation;cell activation;cell adhesion;cell migration;cell motility;cell-matrix adhesion;cell-substrate adhesion;cellular component movement;cellular localization;cellular macro
26.5933	26.2712	26.296	26.1692	1:Experimental evidence at protein level	biosynthetic process;cellular biosynthetic process;cellular component disassembly;cellular component disassembly at cellular level;cellular component organization;cellular component organization at cellular level;cellular cor
29.273	29.0857	29.4563	29.122	1:Experimental evidence at protein level	cellular macromolecule metabolic process;cellular metabolic process;cellular nitrogen compound metabolic process;cellular process;gene expression;macromolecule metabolic process;metabolic process;mRNA metabolic p
25.6954	24.6013	25.8472	25.9845	1:Experimental evidence at protein level	activation of MAPK activity;amine metabolic process;aminoglycan catabolic process;aminoglycan metabolic process;anatomical structure development;anatomical structure morphogenesis;biological regulation;biosynthetic
28.3544	29.0434	29.0627	29.1414	1:Experimental evidence at protein level	alcohol biosynthetic process;alcohol metabolic process;biosynthetic process;carbohydrate biosynthetic process;carbohydrate metabolic process;cellular biosynthetic process;cellular carbohydrate biosynthetic process;cellul
26.6365	26.7829	26.6661	26.7877	1:Experimental evidence at protein level	bile acid metabolic process;carboxylic acid catabolic process;carboxylic acid metabolic process;catabolic process;cellular catabolic process;cellular ketone metabolic process;cellular lipid catabolic process;cellular lipid meta
26.5267	26.6695	26.5272	26.5683	1:Experimental evidence at protein level	biosynthetic process;cellular biosynthetic process;cellular macromolecule biosynthetic process;cellular macromolecule metabolic process;cellular metabolic process;cellular process;cellular protein metabolic process;cotrans
30.0789	31.4845	31.0352	30.994	1:Experimental evidence at protein level	amine metabolic process;anatomical structure development;biological regulation;carboxylic acid metabolic process;cell proliferation;cellular amine metabolic process;cellular amino acid metabolic process;cellular ketone met
27.7989	28.2934	27.8837	28.5527	1:Experimental evidence at protein level	cellular component assembly;cellular component assembly at cellular level;cellular component organization;cellular component organization at cellular level;cellular component organization or biogenesis;cellular component c
33.816	33.9303	34.2803	34.2173	1:Experimental evidence at protein level	apoptosis;B cell activation;B cell activation involved in immune response;B cell differentiation;biological regulation;carbohydrate homeostasis;cell activation;cell activation involved in immune response;cell death;cell differenti
26.1369	26.5516	26.7391	26.7352	1:Experimental evidence at protein level	amine catabolic process;amine metabolic process;anatomical structure development;aromatic amino acid family catabolic process;aromatic amino acid family metabolic process;aromatic compound biosynthetic process;aro
30.9437	30.8627	31.2546	31.0282	1:Experimental evidence at protein level	apoptosis;base-excision repair, DNA ligation;biological regulation;catabolic process;cell activation;cell chemotaxis;cell death;cell migration;cell motility;cell projection organization;cellular catabolic process;cellular component
27.0583	27.2681	27.6701	27.3004	1:Experimental evidence at protein level	anatomical structure development;biological regulation;blood coagulation;bone development;cell activation;cellular component organization;cellular component organization at cellular level;cellular component organization or
30.9013	30.7207	31.3093	31.1198	1:Experimental evidence at protein level	actin cytoskeleton organization;actin filament-based movement;actin filament-based process;actin-mediated cell contraction;actin-myosin filament sliding;actomyosin structure organization;anatomical structure morphogene
29.3775	29.1966	29.5128	29.5553	1:Experimental evidence at protein level	antigen processing and presentation;antigen processing and presentation of exogenous antigen;antigen processing and presentation of exogenous peptide antigen;antigen processing and presentation of exogenous peptide
27.9309	28.181	28.3021	28.564	1:Experimental evidence at protein level	cellular component organization;cellular component organization or biogenesis;cellular membrane organization;cellular process;clathrin-mediated endocytosis;endocytosis;establishment of localization;establishment of localiz
27.5851	27.5561	27.6273	27.5701	1:Experimental evidence at protein level	biological regulation;cell differentiation;cellular developmental process;cellular process;cellular response to stimulus;developmental process;epithelial cell differentiation;negative regulation of apoptosis;negative regulation of
28.1826	28.0145	28.1193	27.9157	1:Experimental evidence at protein level	adult behavior;adult locomotory behavior;aging;anatomical structure development;anatomical structure morphogenesis;axonogenesis;behavior;biological regulation;catabolic process;cell communication;cell differentiation;ce
27.7901	27.9047	27.7306	27.6286	1:Experimental evidence at protein level	actin cytoskeleton organization;actin filament-based process;actomyosin structure organization;adrenal gland development;amine metabolic process;aminoglycan biosynthetic process;aminoglycan metabolic process;anator
27.6142	27.6314	27.8348	27.9049	1:Experimental evidence at protein level	acetyl-CoA catabolic process;acetyl-CoA metabolic process;amine catabolic process;amine metabolic process;anatomical structure morphogenesis;aspartate family amino acid catabolic process;aspartate family amino acid
31.5154	31.5967	31.7707	31.5878	1:Experimental evidence at protein level	alternative nuclear mRNA splicing, via spliceosome;cellular macromolecule metabolic process;cellular metabolic process;cellular nitrogen compound metabolic process;cellular process;establishment of localization;establishm
27.5009	27.0113	27.1356	27.0155	1:Experimental evidence at protein level	cellular macromolecule metabolic process;cellular metabolic process;cellular nitrogen compound metabolic process;cellular process;gene expression;macromolecule metabolic process;metabolic process;mRNA metabolic p
28.9897	28.7651	28.6433	28.5501	1:Experimental evidence at protein level	biosynthetic process;cellular biosynthetic process;cellular macromolecule metabolic process;cellular metabolic process;cellular nitrogen compound metabolic process;cellular process;electron transport chain;gene expressio
29.133	29.0184	28.9499	28.8407	1:Experimental evidence at protein level	anatomical structure homeostasis;base-excision repair;biological regulation;biosynthetic process;cell differentiation;cell surface receptor linked signaling pathway;cellular biosynthetic process;cellular component organization
25.9234	25.8645	26.0202	25.9583	1:Experimental evidence at protein level	anatomical structure homeostasis;biological regulation;biosynthetic process;cell cycle;cell cycle process;cell proliferation;cellular biosynthetic process;cellular component organization;cellular component organization at cellul
24.8846	25.0868	24.7784	24.6798	1:Experimental evidence at protein level	apoptotic mitochondrial changes;biological regulation;cell surface receptor linked signaling pathway;cellular component organization;cellular component organization at cellular level;cellular component organization or biogen
25.8191	26.3748	26.0667	26.1107	1:Experimental evidence at protein level	biological regulation;cell surface receptor linked signaling pathway;cellular process;cellular response to chemical stimulus;cellular response to cytokine stimulus;cellular response to dsRNA;cellular response to exogenous dsR
30.1258	30.228	30.4785	30.2158	1:Experimental evidence at protein level	adult behavior;adult locomotory behavior;adult walking behavior;anatomical structure development;axon cargo transport;axon target recognition;axon transport of mitochondrion;behavior;biological regulation;catabolic proce
27.9073	28.1104	27.8236	27.9686	1:Experimental evidence at protein level	alkene biosynthetic process;arachidonic acid metabolic process;biosynthetic process;carboxylic acid biosynthetic process;carboxylic acid metabolic process;catabolic process;cellular alkene metabolic process;cellular biosy
27.9637	28.6188	28.1728	28.4191	1:Experimental evidence at protein level	aging;alcohol biosynthetic process;alcohol metabolic process;anatomical structure morphogenesis;apoptosis;biosynthetic process;carbohydrate biosynthetic process;carbohydrate metabolic process;cell death;cell differentia
25.4081	24.9099	25.5814	23.0409	1:Experimental evidence at protein level	activation of immune response;biological regulation;complement activation;complement activation, classical pathway;defense response;humoral immune response;immune effector process;immune response;immune system
29.6357	24.4353	28.067	28.1831	1:Experimental evidence at protein level	biological regulation;cellular process;cellular response to chemical stimulus;cellular response to endogenous stimulus;cellular response to estradiol stimulus;cellular response to estrogen stimulus;cellular response to hormone
33.9323	31.6909	32.5956	33.1868	1:Experimental evidence at protein level	
30.3689	30.4603	30.912	30.7237	1:Experimental evidence at protein level	
28.7958	30.6257	30.3843	31.0147	1:Experimental evidence at protein level	
22.8398	25.4522	24.2083	25.4612	1:Experimental evidence at protein level	
26.2285	26.0542	26.1729	26.0061	1:Experimental evidence at protein level	anatomical structure development;anatomical structure morphogenesis;biological regulation;biosynthetic process;cell part morphogenesis;cell projection morphogenesis;cell projection organization;cell surface receptor linke
23.7561	26.0324	25.9034	26.4695	1:Experimental evidence at protein level	alcohol metabolic process;anatomical structure development;anatomical structure homeostasis;anatomical structure morphogenesis;behavior;biological regulation;carbohydrate catabolic process;carbohydrate metabolic pro
27.2889	27.1628	27.3588	27.2998	1:Experimental evidence at protein level	anatomical structure morphogenesis;axon guidance;biological regulation;cell communication;cell differentiation;cell-cell signaling;cellular developmental process;cellular process;cellular response to stimulus;chemotaxis;deve
28.8868	28.8394	28.9319	28.6403	1:Experimental evidence at protein level	antigen processing and presentation;antigen processing and presentation of exogenous antigen;antigen processing and presentation of exogenous peptide antigen;antigen processing and presentation of exogenous peptide
32.3722	31.999	31.826	32.0489	1:Experimental evidence at protein level	cellular component assembly;cellular component assembly at cellular level;cellular component organization;cellular component organization at cellular level;cellular component organization or biogenesis;cellular component c
27.3214	26.8222	26.7417	26.7762	1:Experimental evidence at protein level	acetyl-CoA catabolic process;acetyl-CoA metabolic process;alcohol metabolic process;biological regulation;carbohydrate metabolic process;carboxylic acid metabolic process;catabolic process;cellular carbohydrate metabo
30.3624	31.2627	31.7513	31.6501	1:Experimental evidence at protein level	activation of protein kinase activity;activation of protein kinase B activity;biological regulation;biosynthetic process;cell communication;cell proliferation;cell redox homeostasis;cell-cell signaling;cellular biosynthetic process;c
28.5558	29.2499	29.342	29.4929	1:Experimental evidence at protein level	biosynthetic process;cellular biosynthetic process;cellular macromolecule metabolic process;cellular metabolic process;cellular nitrogen compound metabolic process;cellular process;electron transport chain;gene expressio
27.8504	27.5818	27.7808	27.7453	1:Experimental evidence at protein level	biological regulation;biosynthetic process;carbohydrate biosynthetic process;carbohydrate metabolic process;cellular carbohydrate metabolic process;cellular lipid metabolic process;cellular macromolecule metabolic proces
27.5084	27.1991	27.3513	27.2985	1:Experimental evidence at protein level	amine metabolic process;carboxylic acid metabolic process;cell differentiation;cellular amine metabolic process;cellular amino acid metabolic process;cellular component assembly;cellular component organization;cellular co
24.2847	25.4632	23.8656	24.2215	1:Experimental evidence at protein level	activation of immune response;biological regulation;cation homeostasis;cell death;cellular cation homeostasis;cellular chemical homeostasis;cellular homeostasis;cellular ion homeostasis;cellular metal ion homeostasis;cellula
29.0184	29.0949	29.2466	29.2696	1:Experimental evidence at protein level	actin cytoskeleton organization;actin filament-based process;activation of phospholipase C activity;activation of protein kinase A activity;activation of protein kinase activity;actomyosin structure organization;anatomical struc
25.263	23.6295	24.7229	23.9302	1:Experimental evidence at protein level	biological regulation;blood coagulation;blood coagulation, extrinsic pathway;coagulation;hemostasis;macromolecule metabolic process;metabolic process;multicellular organismal process;negative regulation of catalytic activ
28.8166	29.2926	29.0596	29.1233	1:Experimental evidence at protein level	aldehyde catabolic process;catabolic process;cellular aldehyde metabolic process;cellular catabolic process;cellular metabolic process;cellular process;formaldehyde catabolic process;formaldehyde metabolic process;meta
31.4618	31.5834	31.7079	31.5429	1:Experimental evidence at protein level	activation of caspase activity;activation of immune response;activation of innate immune response;adaptive immune response;adaptive immune response based on somatic recombination of immune receptors built from imm
25.055	24.4649	25.5686	24.6278	1:Experimental evidence at protein level	activation of immune response;anatomical structure morphogenesis;apoptotic mitochondrial changes;biological regulation;blood coagulation;cell activation;cell morphogenesis;cellular component assembly;cellular compone

31.3556	31.4732	31.6865	31.785	1:Experimental evidence at protein level	activation of signaling protein activity involved in unfolded protein response;anatomical structure arrangement;anatomical structure development;biological regulation;blood coagulation;catabolic process;cell activation;cell co
28.5834	28.3559	28.5325	28.4501	1:Experimental evidence at protein level	anatomical structure development;anatomical structure morphogenesis;axon guidance;biological adhesion;biological regulation;cell adhesion;cell junction assembly;cell junction organization;cell migration;cell morphogenesis
26.4542	26.1556	24.8737	26.4517	1:Experimental evidence at protein level	anatomical structure development;cellular component organization;cellular component organization at cellular level;cellular component organization or biogenesis;cellular component organization or biogenesis at cellular leve
32.6932	32.9916	33.1397	33.2093	1:Experimental evidence at protein level	axon guidance;biological regulation;biosynthetic process;cell communication;cell-cell signaling;cellular biosynthetic process;cellular component disassembly;cellular component disassembly at cellular level;cellular componen
28.5076	28.4736	28.3966	28.1909	1:Experimental evidence at protein level	biological regulation;biosynthetic process;carbohydrate biosynthetic process;carbohydrate metabolic process;carbohydrate transmembrane transport;carbohydrate transport;carboxylic acid metabolic process;cation transpo
27.5312	27.4959	27.5831	27.237	1:Experimental evidence at protein level	carbohydrate metabolic process;carbohydrate transmembrane transport;carbohydrate transport;carboxylic acid metabolic process;cation transport;cellular ketone metabolic process;cellular metabolic process;cellular proces
27.6661	27.5988	27.5416	27.5677	1:Experimental evidence at protein level	biological regulation;biosynthetic process;body fluid secretion;cellular aromatic compound metabolic process;cellular biosynthetic process;cellular metabolic process;cellular nitrogen compound biosynthetic process;cellular
29.6283	29.476	29.5515	29.5699	1:Experimental evidence at protein level	acetyl-CoA biosynthetic process;acetyl-CoA biosynthetic process from pyruvate;acetyl-CoA catabolic process;acetyl-CoA metabolic process;alcohol metabolic process;biological regulation;biosynthetic process;carbohydrat
27.7629	27.6572	27.6386	27.7042	1:Experimental evidence at protein level	alcohol metabolic process;carbohydrate catabolic process;carbohydrate metabolic process;catabolic process;cellular carbohydrate catabolic process;cellular carbohydrate metabolic process;cellular glucan metabolic proces
28.4522	28.1835	28.4127	28.3772	1:Experimental evidence at protein level	actin cytoskeleton organization;actin cytoskeleton reorganization;actin filament-based process;biological regulation;cell surface receptor linked signaling pathway;cellular component organization;cellular component organiza
27.6247	26.7797	26.8574	27.1738	1:Experimental evidence at protein level	apoptosis;biological regulation;cell communication;cell death;cell surface receptor linked signaling pathway;cellular process;cellular response to chemical stimulus;cellular response to dsRNA;cellular response to exogenous
28.7696	29.3286	29.2556	29.3441	1:Experimental evidence at protein level	autophagic cell death;autophagy;biological regulation;catabolic process;cell death;cellular catabolic process;cellular metabolic process;cellular process;death;establishment of localization;establishment of localization in cell;
26.6257	27.0557	27.0864	26.9506	1:Experimental evidence at protein level	amine biosynthetic process;amine metabolic process;betaine biosynthetic process;betaine metabolic process;biosynthetic process;carboxylic acid biosynthetic process;carboxylic acid catabolic process;carboxylic acid meta
28.0897	27.4327	27.6236	27.4104	1:Experimental evidence at protein level	biological regulation;biosynthetic process;cell death;cell division;cellular biosynthetic process;cellular component organization;cellular component organization at cellular level;cellular component organization or biogenesis;ce
27.8342	27.1382	27.2241	27.1316	1:Experimental evidence at protein level	apoptotic chromosome condensation;biological regulation;cell cycle;cell cycle process;cell differentiation;cell division;cellular component organization;cellular component organization at cellular level;cellular component orga
29.6102	29.5968	29.562	29.4043	1:Experimental evidence at protein level	alcohol biosynthetic process;alcohol catabolic process;alcohol metabolic process;amine metabolic process;anatomical structure development;biological regulation;biosynthetic process;carbohydrate biosynthetic process;car
25.4437	22.936	25.3425	26.04	1:Experimental evidence at protein level	cellular localization;cellular macromolecule localization;cellular macromolecule metabolic process;cellular metabolic process;cellular process;cellular protein localization;cellular protein metabolic process;establishment of loca
25.9606	25.6676	25.4173	25.6809	1:Experimental evidence at protein level	alcohol biosynthetic process;alcohol metabolic process;biological regulation;biosynthetic process;biotin metabolic process;carbohydrate biosynthetic process;carbohydrate metabolic process;carboxylic acid metabolic proce
29.6611	29.8177	29.6734	29.6814	1:Experimental evidence at protein level	10-formyltetrahydrofolate biosynthetic process;10-formyltetrahydrofolate metabolic process;amine biosynthetic process;amine metabolic process;anatomical structure development;anatomical structure formation involved in
28.313	28.256	28.1697	28.2211	1:Experimental evidence at protein level	anatomical structure development;anatomical structure morphogenesis;biological regulation;cell surface receptor linked signaling pathway;cellular component organization;cellular component organization or biogenesis;cellul
28.5681	28.7604	28.5568	28.6018	1:Experimental evidence at protein level	aging;alcohol catabolic process;alcohol metabolic process;aldehyde catabolic process;biological regulation;catabolic process;cellular aldehyde metabolic process;cellular catabolic process;cellular lipid metabolic process;cel
27.601	27.8611	27.8514	27.8669	1:Experimental evidence at protein level	anatomical structure development;anatomical structure morphogenesis;biological regulation;cell cycle;cell cycle process;cell division;cellular component organization;cellular component organization at cellular level;cellular co
26.3514	26.9779	26.6635	26.6378	1:Experimental evidence at protein level	5-phosphoribose 1-diphosphate biosynthetic process;5-phosphoribose 1-diphosphate metabolic process;alcohol biosynthetic process;alcohol metabolic process;AMP biosynthetic process;AMP metabolic process;anatomic
29.5568	29.2892	29.2931	29.4059	1:Experimental evidence at protein level	biological regulation;biosynthetic process;catabolic process;cellular biosynthetic process;cellular catabolic process;cellular macromolecule biosynthetic process;cellular macromolecule catabolic process;cellular macromolec
30.9021	31.2151	31.1505	31.22	1:Experimental evidence at protein level	anatomical structure development;anatomical structure homeostasis;base-excision repair;biological regulation;biosynthetic process;cell cycle;cell cycle process;cell differentiation;cell proliferation;cellular biosynthetic process
27.6377	27.385	27.5104	27.4771	1:Experimental evidence at protein level	amine metabolic process;amino acid activation;biosynthetic process;carboxylic acid metabolic process;cellular amine metabolic process;cellular amino acid metabolic process;cellular biosynthetic process;cellular componen
25.9721	23.4221	23.99	24.7313	1:Experimental evidence at protein level	anatomical structure development;anatomical structure morphogenesis;biological adhesion;cardiac muscle tissue morphogenesis;cartilage condensation;cell adhesion;cell development;cell differentiation;cell-cell adhesion;c
28.945	28.4226	28.6459	28.5801	1:Experimental evidence at protein level	axon guidance;biological adhesion;cell adhesion;cell differentiation;cellular component assembly;cellular component disassembly;cellular component disassembly at cellular level;cellular component organization;cellular com
28.1352	27.7861	28.147	27.8193	1:Experimental evidence at protein level	axon guidance;biological adhesion;cell adhesion;cellular component assembly;cellular component disassembly;cellular component disassembly at cellular level;cellular component organization;cellular component organizatio
28.6092	28.2416	28.2915	28.1544	1:Experimental evidence at protein level	anatomical structure development;axon guidance;biological adhesion;cell adhesion;cellular component disassembly;cellular component disassembly at cellular level;cellular component organization;cellular component organ
28.0952	28.6565	28.4726	28.4766	1:Experimental evidence at protein level	active induction of host immune response by virus;adenine nucleotide transport;ADP transport;apoptosis;ATP transport;biological regulation;cell death;cellular macromolecule metabolic process;cellular metabolic process;cel
24.8686	24.6691	25.023	25.5429	1:Experimental evidence at protein level	biological regulation;blood circulation;blood coagulation;cell activation;cellular process;circulatory system process;coagulation;establishment of localization;establishment of localization in cell;exocytosis;hemostasis;multicell
29.5885	29.4341	29.7584	29.647	1:Experimental evidence at protein level	anatomical structure development;biosynthetic process;cell activation;cell proliferation;cellular aromatic compound metabolic process;cellular biosynthetic process;cellular component assembly;cellular component organizati
29.073	28.5797	28.9566	28.7932	1:Experimental evidence at protein level	biological regulation;biosynthetic process;carbohydrate metabolic process;carbohydrate transport;cell cycle;cell cycle checkpoint;cell cycle phase;cell cycle process;cell division;cell surface receptor linked signaling pathway
26.5639	27.1784	27.5451	27.5239	1:Experimental evidence at protein level	amine metabolic process;anatomical structure development;anion homeostasis;biological regulation;carboxylic acid metabolic process;cellular amine metabolic process;cellular amino acid metabolic process;cellular anion h
25.7246	26.3689	25.968	25.9895	1:Experimental evidence at protein level	biological regulation;cell activation;cell activation involved in immune response;cellular component organization;cellular component organization or biogenesis;cellular membrane organization;cellular process;defense respons
25.5679	25.3189	25.7202	25.8783	1:Experimental evidence at protein level	amine catabolic process;amine metabolic process;branched chain family amino acid catabolic process;branched chain family amino acid metabolic process;carboxylic acid catabolic process;carboxylic acid metabolic proce
31.5601	31.4329	31.4962	31.403	1:Experimental evidence at protein level	actin crosslink formation;actin cytoskeleton organization;actin filament bundle assembly;actin filament organization;actin filament-based process;biological regulation;blood coagulation;cell activation;cell junction assembly;c
26.5712	26.9087	27.0439	27.1687	1:Experimental evidence at protein level	amine metabolic process;carboxylic acid metabolic process;cellular amine metabolic process;cellular amino acid metabolic process;cellular ketone metabolic process;cellular metabolic process;cellular nitrogen compound m
30.9814	30.9041	30.8354	30.7451	1:Experimental evidence at protein level	anatomical structure development;anatomical structure homeostasis;biological regulation;biosynthetic process;brain development;cellular biosynthetic process;cellular component organization;cellular component organizatio
29.9728	29.8251	29.7345	29.7021	1:Experimental evidence at protein level	anatomical structure development;anatomical structure homeostasis;biological regulation;biosynthetic process;brain development;cell differentiation;cell proliferation;cellular biosynthetic process;cellular component organizat
29.2044	29.1926	29.3118	28.9611	1:Experimental evidence at protein level	biosynthetic process;cellular biosynthetic process;cellular macromolecule metabolic process;cellular metabolic process;cellular nitrogen compound metabolic process;cellular process;electron transport chain;gene expressio
24.972	29.0542	23.9298	26.1136	1:Experimental evidence at protein level	biological regulation;cell surface receptor linked signaling pathway;cellular process;cellular response to chemical stimulus;cellular response to cytokine stimulus;cellular response to organic substance;cellular response to stim
26.7987	27.0933	27.0407	27.2636	1:Experimental evidence at protein level	biological regulation;blood coagulation;cell activation;cellular process;coagulation;establishment of localization;establishment of localization in cell;exocytosis;hemostasis;multicellular organismal process;platelet activation;pl
30.4245	30.5059	30.5797	30.4442	1:Experimental evidence at protein level	biological regulation;catabolic process;cellular catabolic process;cellular macromolecule catabolic process;cellular macromolecule metabolic process;cellular metabolic process;cellular nitrogen compound metabolic process
24.3875	25.1276	24.0571	25.0275	1:Experimental evidence at protein level	anatomical structure development;biological adhesion;biological regulation;cartilage condensation;cell adhesion;cell differentiation;cell-cell adhesion;cellular component disassembly;cellular component disassembly at cellula
23.6663	23.8575	25.0601	25.4459	1:Experimental evidence at protein level	axon guidance;biological adhesion;biological regulation;cell adhesion;cell surface receptor linked signaling pathway;cellular component organization;cellular component organization at cellular level;cellular component organiz
25.9651	25.8288	25.2442	25.515	1:Experimental evidence at protein level	anatomical structure development;anatomical structure morphogenesis;B cell activation;B cell differentiation;biological adhesion;biological regulation;blood coagulation;blood vessel remodeling;cell activation;cell adhesion;c
32.3263	32.4683	32.2696	32.2839	1:Experimental evidence at protein level	biological regulation;biosynthetic process;cell differentiation;cellular biosynthetic process;cellular developmental process;cellular macromolecule biosynthetic process;cellular macromolecule metabolic process;cellular metab
27.0705	29.3635	29.9535	29.1681	1:Experimental evidence at protein level	cell differentiation;cellular developmental process;cellular process;cellular response to calcium ion;cellular response to chemical stimulus;cellular response to inorganic substance;cellular response to metal ion;cellular respons
24.364	26.6285	27.1165	24.7277	1:Experimental evidence at protein level	anatomical structure development;cell junction assembly;cell junction organization;cell-substrate junction assembly;cellular component assembly;cellular component assembly at cellular level;cellular component organization
29.7353	29.8652	29.7632	29.8443	1:Experimental evidence at protein level	biological regulation;cell redox homeostasis;cellular homeostasis;cellular macromolecule metabolic process;cellular metabolic process;cellular process;cellular protein metabolic process;cellular response to stimulus;cellular
28.0355	27.9272	28.1342	28.0273	1:Experimental evidence at protein level	4-hydroxyproline metabolic process;amine metabolic process;carboxylic acid metabolic process;cellular amine metabolic process;cellular amino acid metabolic process;cellular component organization;cellular component or
30.9123	30.9628	31.4738	31.2199	1:Experimental evidence at protein level	biological regulation;calcium ion homeostasis;calcium ion transport;cation homeostasis;cation transport;cellular calcium ion homeostasis;cellular cation homeostasis;cellular chemical homeostasis;cellular divalent inorganic c
23.1876	28.9975	29.2696	24.1052	1:Experimental evidence at protein level	activation of blood coagulation via clotting cascade;activation of caspase activity;activation of plasma proteins involved in acute inflammatory response;biological regulation;blood coagulation;blood coagulation, extrinsic pat
24.1952	25.764	23.0818	23.2922	1:Experimental evidence at protein level	actin cytoskeleton organization;actin filament bundle assembly;actin filament organization;actin filament-based process;anatomical structure morphogenesis;biological regulation;cell activation;cell activation involved in immu
29.0413	29.1275	28.9996	28.9067	1:Experimental evidence at protein level	anatomical structure development;bone development;developmental process
26.9261	27.5491	27.4729	27.5009	1:Experimental evidence at protein level	beta-amyloid metabolic process;cellular macromolecule metabolic process;cellular metabolic process;cellular process;glycoprotein metabolic process;macromolecule metabolic process;metabolic process;primary metabolic
27.735	28.0228	27.747	27.8308	1:Experimental evidence at protein level	cellular metabolic process;cellular process;electron transport chain;generation of precursor metabolites and energy;metabolic process;oxidation-reduction process;respiratory electron transport chain;small molecule metaboli
26.4026	26.1341	26.0901	25.8645	1:Experimental evidence at protein level	alcohol metabolic process;anatomical structure development;biosynthetic process;carbohydrate biosynthetic process;carbohydrate metabolic process;cellular biosynthetic process;cellular carbohydrate biosynthetic process;
28.4288	28.4895	28.5276	28.5328	1:Experimental evidence at protein level	activation of phospholipase C activity;activation of protein kinase A activity;activation of protein kinase activity;biological regulation;blood coagulation;cell surface receptor linked signaling pathway;cellular metabolic process;
27.1431	26.8237	27.0045	27.2028	1:Experimental evidence at protein level	biological regulation;biosynthetic process;cellular biosynthetic process;cellular macromolecule biosynthetic process;cellular macromolecule metabolic process;cellular metabolic process;cellular nitrogen compound metabolic
30.2362	30.2313	29.9801	30.2265	1:Experimental evidence at protein level	biological regulation;blood coagulation;cell activation;cell surface receptor linked signaling pathway;cellular process;cellular response to stimulus;coagulation;defense response;hemostasis;immune response;immune system
27.0447	27.1716	27.2779	27.1267	1:Experimental evidence at protein level	aromatic compound biosynthetic process;biosynthetic process;cellular aromatic compound metabolic process;cellular biosynthetic process;cellular metabolic process;cellular nitrogen compound biosynthetic process;cellular
28.5826	31.8067	30.8509	31.7138	1:Experimental evidence at protein level	aging;biological regulation;biosynthetic process;carboxylic acid biosynthetic process;carboxylic acid metabolic process;cell aging;cell proliferation;cell surface receptor linked signaling pathway;cellular biosynthetic process;
29.0386	29.5079	29.2743	29.5426	1:Experimental evidence at protein level	biological adhesion;cell adhesion;cellular process
30.4241	30.4258	30.6324	30.5814	1:Experimental evidence at protein level	biological regulation;carbohydrate metabolic process;cellular carbohydrate metabolic process;cellular macromolecule metabolic process;cellular metabolic process;cellular process;cellular protein metabolic process;cellular
28.1538	27.8304	27.8108	27.5914	1:Experimental evidence at protein level	alcohol biosynthetic process;alcohol metabolic process;biosynthetic process;cellular biosynthetic process;cellular lipid metabolic process;cellular metabolic process;cellular process;cholesterol biosynthetic process;choleste
26.8739	26.4423	26.4785	26.2896	1:Experimental evidence at protein level	basement membrane organization;biological adhesion;biological regulation;cell adhesion;cell-matrix adhesion;cell-substrate adhesion;cellular component disassembly;cellular component disassembly at cellular level;cellular
26.5304	27.1138	26.9456	27.1376	1:Experimental evidence at protein level	alcohol metabolic process;aldehyde catabolic process;biosynthetic process;carbohydrate catabolic process;carbohydrate metabolic process;carboxylic acid biosynthetic process;carboxylic acid catabolic process;carboxylic
33.8512	34.1674	33.9947	33.9051	1:Experimental evidence at protein level	alcohol metabolic process;carbohydrate metabolic process;cell death;cellular carbohydrate metabolic process;cellular metabolic process;cellular process;death;glucose metabolic process;hexose metabolic process;metaboli
32.1824	32.1989	32.2171	32.0459	1:Experimental evidence at protein level	actin cytoskeleton organization;actin filament organization;actin filament-based process;actin rod assembly;activation of immune response;activation of innate immune response;biological regulation;catabolic process;cellular
27.2198	27.0646	27.1869	26.7235	1:Experimental evidence at protein level	anaphase-promoting complex-dependent proteasomal ubiquitin-dependent protein catabolic process;anatomical structure development;anatomical structure morphogenesis;biological regulation;cardiac cell development;ca
29.0173	29.0653	29.0376	29.0436	1:Experimental evidence at protein level	biological regulation;biosynthetic process;cellular biosynthetic process;cellular component assembly;cellular component assembly at cellular level;cellular component organization;cellular component organization at cellular le
26.5134	26.5344	26.2947	26.26	1:Experimental evidence at protein level	beta-amyloid metabolic process;biological regulation;bradykinin catabolic process;catabolic process;cell surface receptor linked signaling pathway;cellular catabolic process;cellular component assembly;cellular component
26.3172	27.9719	27.6186	28.7509	1:Experimental evidence at protein level	anatomical structure development;biosynthetic process;cellular biosynthetic process;cellular macromolecule metabolic process;cellular metabolic process;cellular nitrogen compound metabolic process;cellular process;deve
30.1249	29.6623	29.9265	29.926	1:Experimental evidence at protein level	cellular macromolecule metabolic process;cellular metabolic process;cellular nitrogen compound metabolic process;cellular process;gene expression;macromolecule metabolic process;metabolic process;mRNA metabolic p
28.4599	28.2863	28.3187	28.2386	1:Experimental evidence at protein level	amine metabolic process;amino acid activation;aspartyl-tRNA aminoacylation;biosynthetic process;carboxylic acid metabolic process;cellular amine metabolic process;cellular amino acid metabolic process;cellular biosynthe
25.7797	24.6757	25.9603	26.37	1:Experimental evidence at protein level	adherens junction assembly;adherens junction organization;anatomical structure development;biological adhesion;biological regulation;cell adhesion;cell junction assembly;cell junction organization;cell migration;cell motility;
27.187	27.2431	27.2448	27.4951	1:Experimental evidence at protein level	aerobic respiration;cellular metabolic process;cellular process;cellular respiration;electron transport chain;energy derivation by oxidation of organic compounds;generation of precursor metabolites and energy;metabolic proce
26.5592	26.6591	26.5525	27.0206	1:Experimental evidence at protein level	amine biosynthetic process;amine catabolic process;amine metabolic process;biological regulation;biosynthetic process;carboxylic acid biosynthetic process;carboxylic acid catabolic process;carboxylic acid metabolic proc
28.6044	29.8213	29.4829	29.7644	1:Experimental evidence at protein level	alcohol biosynthetic process;alcohol metabolic process;biological regulation;biosynthetic process;C21-steroid hormone biosynthetic process;C21-steroid hormone metabolic process;carbohydrate biosynthetic process;carb
28.4215	27.7407	27.8331	27.8335	1:Experimental evidence at protein level	anatomical structure formation involved in morphogenesis;angiogenesis;angiotensin maturation;biological regulation;cell differentiation;cellular developmental process;cellular macromolecule metabolic process;cellular metab
26.9754	26.9234	27.0734	26.9254	1:Experimental evidence at protein level	adherens junction organization;biological adhesion;biological regulation;cell adhesion;cell junction assembly;cell junction organization;cell-cell junction organization;cellular component assembly;cellular component assembly
28.9759	29.0505	29.0494	29.081	1:Experimental evidence at protein level	catabolic process;cell cycle process;cellular catabolic process;cellular component disassembly;cellular component disassembly at cellular level;cellular component organization;cellular component organization at cellular leve
26.4965	25.8177	26.0035	25.7373	1:Experimental evidence at protein level	acute inflammatory response;alcohol metabolic process;amine metabolic process;aminoglycan biosynthetic process;aminoglycan metabolic process;anatomical structure development;anatomical structure formation involved
29.9808	30.1178	30.1287	30.0374	1:Experimental evidence at protein level	actin cytoskeleton organization;actin cytoskeleton reorganization;actin filament bundle assembly;actin filament organization;actin filament-based process;anatomical structure development;axon guidance;biological adhesion
26.5317	26.8442	26.918	26.8229	1:Experimental evidence at protein level	catabolic process;cellular catabolic process;cellular macromolecule catabolic process;cellular macromolecule metabolic process;cellular metabolic process;cellular process;cellular protein metabolic process;macromolecule
25.3358	24.1349	24.5145	25.0205	1:Experimental evidence at protein level	biological regulation;biosynthetic process;cell death;cellular biosynthetic process;cellular macromolecule biosynthetic process;cellular macromolecule metabolic process;cellular metabolic process;cellular nitrogen compounc
27.7644	27.7673	27.5282	27.9411	1:Experimental evidence at protein level	anatomical structure development;biological regulation;biosynthetic process;carbohydrate homeostasis;cellular biosynthetic process;cellular chemical homeostasis;cellular component organization;cellular component organiz
30.5386	30.668	30.5878	30.5156	1:Experimental evidence at protein level	aging;biological regulation;biosynthetic process;cell communication;cell-cell signaling;cellular biosynthetic process;cellular metabolic process;cellular nitrogen compound biosynthetic process;cellular nitrogen compound met
28.5109	28.7871	29.1351	29.0043	1:Experimental evidence at protein level	amine metabolic process;aminoglycan catabolic process;aminoglycan metabolic process;carbohydrate catabolic process;carbohydrate metabolic process;catabolic process;cellular catabolic process;cellular metabolic proce
31.118	30.7291	30.8193	30.806	1:Experimental evidence at protein level	biological regulation;biosynthetic process;catabolic process;cellular biosynthetic process;cellular catabolic process;cellular component disassembly;cellular component disassembly at cellular level;cellular component organiz
27.354	27.0797	27.4731	27.1254	1:Experimental evidence at protein level	anatomical structure homeostasis;base-excision repair;biological regulation;biosynthetic process;catabolic process;cell cycle;cell cycle checkpoint;cell cycle process;cellular biosynthetic process;cellular catabolic process;ce
26.5309	25.903	24.3967	26.1373	1:Experimental evidence at protein level	biosynthetic process;cellular biosynthetic process;cellular macromolecule metabolic process;cellular metabolic process;cellular nitrogen compound metabolic process;cellular process;electron transport chain;gene expressio
25.762	24.1074	26.0635	26.3934	1:Experimental evidence at protein level	aging;anatomical structure development;biological regulation;cellular component disassembly;cellular component disassembly at cellular level;cellular component organization;cellular component organization at cellular level;
31.1614	31.1477	31.1539	30.9338	1:Experimental evidence at protein level	amine metabolic process;aminoglycan catabolic process;aminoglycan metabolic process;anatomical structure development;anatomical structure morphogenesis;biological adhesion;biological regulation;blood coagulation;br
25.7399	24.9996	25.5409	24.8258	1:Experimental evidence at protein level	biological regulation;cell cycle;cell cycle checkpoint;cellular component assembly;cellular component assembly at cellular level;cellular component organization;cellular component organization at cellular level;cellular compo
28.4007	29.4282	29.5168	29.6281	1:Experimental evidence at protein level	arachidonic acid metabolic process;carboxylic acid metabolic process;cell differentiation;cellular developmental process;cellular ketone metabolic process;cellular lipid metabolic process;cellular metabolic process;cellular pr
27.8086	26.1807	26.6626	26.4534	1:Experimental evidence at protein level	actin cytoskeleton organization;actin filament organization;actin filament-based process;activation of immune response;antigen receptor-mediated signaling pathway;axon guidance;biological regulation;cell activation;cell pro
32.8678	32.5503	32.5569	32.6709	1:Experimental evidence at protein level	biological regulation;cellular component assembly;cellular component assembly at cellular level;cellular component organization;cellular component organization at cellular level;cellular component organization or biogenesis;
31.2075	31.0425	24.3406	31.3336	1:Experimental evidence at protein level	cellular component assembly;cellular component assembly at cellular level;cellular component organization;cellular component organization at cellular level;cellular component organization or biogenesis;cellular component c
27.46	27.7538	27.3459	27.3923	1:Experimental evidence at protein level	amine metabolic process;betaine metabolic process;biological regulation;carboxylic acid metabolic process;carnitine metabolic process;catabolic process;cellular amine metabolic process;cellular amino acid metabolic proce
29.1702	29.2022	28.9676	28.9127	1:Experimental evidence at protein level	anatomical structure development;biological adhesion;biological regulation;blood coagulation;calcium ion homeostasis;calcium ion transmembrane transport;calcium ion transport;calcium-mediated signaling;cation homeost
25.839	26.5995	26.6193	26.7304	1:Experimental evidence at protein level	amine catabolic process;amine metabolic process;arginine catabolic process;arginine metabolic process;aromatic amino acid family catabolic process;aromatic amino acid family metabolic process;aromatic compound catal
31.4316	31.5025	32.1784	31.9405	1:Experimental evidence at protein level	anatomical structure development;anatomical structure morphogenesis;axonogenesis;biological regulation;brain development;cell cycle process;cell part morphogenesis;cell projection morphogenesis;cell projection organiza
23.2352	26.7581	26.4104	26.8362	1:Experimental evidence at protein level	3'-UTR-mediated mRNA stabilization;anatomical structure development;biological regulation;cellular hyperosmotic response;cellular macromolecule metabolic process;cellular metabolic process;cellular nitrogen compound
27.9647	25.1608	23.1187	27.8385	1:Experimental evidence at protein level	aging;base-excision repair;biological regulation;biosynthetic process;cell aging;cellular biosynthetic process;cellular component assembly;cellular component assembly at cellular level;cellular component disassembly;cellula
28.1209	28.319	28.4024	28.4719	1:Experimental evidence at protein level	2-oxoglutarate metabolic process;alcohol biosynthetic process;alcohol metabolic process;alditol biosynthetic process;alditol metabolic process;amine biosynthetic process;amine catabolic process;amine metabolic process;
26.6243	26.738	26.863	26.7593	1:Experimental evidence at protein level	activation of adenylate cyclase activity;activation of phospholipase C activity;anatomical structure formation involved in morphogenesis;angiogenesis;biological adhesion;biological regulation;blood coagulation;calcium ion h
25.8063	24.0751	23.7217	24.5126	1:Experimental evidence at protein level	anatomical structure development;biological regulation;biosynthetic process;blood vessel development;cell differentiation;cell proliferation;cell surface receptor linked signaling pathway;cellular biosynthetic process;cellular d
26.4268	26.4114	26.3675	26.1553	1:Experimental evidence at protein level	anatomical structure development;anatomical structure morphogenesis;axon guidance;biological adhesion;biological regulation;blood coagulation;cell adhesion;cell adhesion mediated by integrin;cell differentiation;cell migra
26.3081	25.5374	25.1598	25.7624	1:Experimental evidence at protein level	adenine nucleotide transport;adult heart development;anatomical structure development;anatomical structure morphogenesis;apoptosis;ATP transport;atrial ventricular junction remodeling;biological regulation;blood vessel m
27.6011	26.9792	27.1297	27.0169	1:Experimental evidence at protein level	biological regulation;biosynthetic process;cellular biosynthetic process;cellular component organization;cellular component organization at cellular level;cellular component organization or biogenesis;cellular component orga
26.2318	23.4208	25.8603	25.7457	1:Experimental evidence at protein level	cellular metabolic process;cellular process;electron transport chain;generation of precursor metabolites and energy;metabolic process;mitochondrial electron transport, NADH to ubiquinone;oxidation-reduction process;respi
27.732	27.6234	27.5356	27.4051	1:Experimental evidence at protein level	activation of immune response;activation of innate immune response;activation of phospholipase C activity;activation of protein kinase A activity;activation of protein kinase activity;acylglycerol catabolic process;acylglycerol
29.2722	29.2667	29.2018	29.1818	1:Experimental evidence at protein level	anatomical structure development;anatomical structure formation involved in morphogenesis;biological regulation;blastocyst development;cellular process;cellular response to acid;cellular response to amine stimulus;cellular
28.3954	28.1777	28.0724	28.0202	1:Experimental evidence at protein level	amine metabolic process;B cell activation;B cell proliferation;biosynthetic process;carboxylic acid metabolic process;cell activation;cell proliferation;cellular amine metabolic process;cellular amino acid metabolic process;cell
27.3193	27.8033	27.3863	27.5863	1:Experimental evidence at protein level	aging;anatomical structure development;anatomical structure morphogenesis;artery morphogenesis;biological adhesion;biological regulation;blood vessel morphogenesis;BMP signaling pathway;bone development;branchin
29.4031	29.1188	29.1167	29.1528	1:Experimental evidence at protein level	biological regulation;biosynthetic process;cell growth;cellular biosynthetic process;cellular macromolecule biosynthetic process;cellular macromolecule metabolic process;cellular metabolic process;cellular nitrogen compoun
27.9522	27.9323	27.7244	27.7874	1:Experimental evidence at protein level	alcohol catabolic process;alcohol metabolic process;biological regulation;carbohydrate catabolic process;carbohydrate metabolic process;carbohydrate phosphorylation;catabolic process;cellular carbohydrate catabolic pro
25.3468	23.9229	26.2715	26.4505	1:Experimental evidence at protein level	behavior;biological regulation;carbohydrate catabolic process;carbohydrate metabolic process;catabolic process;cellular catabolic process;cellular lipid catabolic process;cellular lipid metabolic process;cellular metabolic pro
28.4132	28.5257	28.7037	28.7703	1:Experimental evidence at protein level	anatomical structure development;biological regulation;cell chemotaxis;cell differentiation;cell migration;cell motility;cellular component movement;cellular component organization;cellular component organization at cellular l
29.2225	29.5068	29.6587	29.4932	1:Experimental evidence at protein level	activation of immune response;activation of innate immune response;anaphase-promoting complex-dependent proteasomal ubiquitin-dependent protein catabolic process;anatomical structure development;antigen processi
30.2039	29.9816	30.0501	29.8952	1:Experimental evidence at protein level	binding of sperm to zona pellucida;biological regulation;cell recognition;cell-cell recognition;cellular component assembly;cellular component assembly at cellular level;cellular component organization;cellular component orga
27.0485	27.0123	26.7044	26.8785	1:Experimental evidence at protein level	actin cytoskeleton organization;actin cytoskeleton reorganization;actin filament-based process;activation of signaling protein activity involved in unfolded protein response;biological regulation;blood coagulation;cell activatio
30.0908	30.0702	29.4434	29.9214	1:Experimental evidence at protein level	biosynthetic process;catabolic process;cellular biosynthetic process;cellular catabolic process;cellular component biogenesis;cellular component biogenesis at cellular level;cellular component disassembly;cellular componen
26.0722	26.8193	26.1998	26.1427	1:Experimental evidence at protein level	actin cytoskeleton organization;actin filament bundle assembly;actin filament organization;actin filament-based process;antigen processing and presentation;antigen processing and presentation of exogenous antigen;antiger
30.2084	29.9723	29.9774	30.143	1:Experimental evidence at protein level	activation of phospholipase D activity;anatomical structure development;biological regulation;brain development;cell migration;cell motility;cell surface receptor linked signaling pathway;cellular component movement;cellular

31.3179	30.7886	30.9995	30.876	1:Experimental evidence at protein level	biosynthetic process;catabolic process;cellular biosynthetic process;cellular catabolic process;cellular component biogenesis;cellular component biogenesis at cellular level;cellular component disassembly;cellular componer
29.9959	29.9056	29.8767	29.8503	1:Experimental evidence at protein level	adherens junction assembly;adherens junction organization;anatomical structure morphogenesis;apical junction assembly;axon extension;biological adhesion;biological regulation;blood coagulation;cell activation;cell adhesio
27.7823	27.5904	27.4905	27.6038	1:Experimental evidence at protein level	biological regulation;cellular component organization;cellular component organization at cellular level;cellular component organization or biogenesis;cellular component organization or biogenesis at cellular level;cellular macro
26.7428	26.676	27.2621	26.981	1:Experimental evidence at protein level	biological regulation;biosynthetic process;cellular biosynthetic process;cellular macromolecule biosynthetic process;cellular macromolecule metabolic process;cellular metabolic process;cellular nitrogen compound metabolic
30.2615	30.0714	30.1309	30.1964	1:Experimental evidence at protein level	biosynthetic process;catabolic process;cellular biosynthetic process;cellular catabolic process;cellular component disassembly;cellular component disassembly at cellular level;cellular component organization;cellular compo
31.9193	32.4311	32.3151	32.3994	1:Experimental evidence at protein level	alcohol biosynthetic process;alcohol catabolic process;alcohol metabolic process;biological regulation;biosynthetic process;carbohydrate biosynthetic process;carbohydrate catabolic process;carbohydrate metabolic proces
28.2378	28.2288	28.3145	28.4155	1:Experimental evidence at protein level	biological regulation;cell cycle phase;cell cycle process;cell division;cellular component assembly;cellular component assembly at cellular level;cellular component organization;cellular component organization at cellular level
26.6311	26.1962	26.7276	26.6357	1:Experimental evidence at protein level	activation of immune response;activation of innate immune response;biological regulation;cell surface receptor linked signaling pathway;cellular component assembly;cellular component assembly at cellular level;cellular com
25.9443	26.5445	26.2007	26.2949	1:Experimental evidence at protein level	anatomical structure homeostasis;anatomical structure morphogenesis;base-excision repair;biological regulation;biosynthetic process;cell cycle;cell division;cellular biosynthetic process;cellular component organization;cellu
26.7567	27.8597	28.0817	28.3164	1:Experimental evidence at protein level	anatomical structure development;ATP biosynthetic process;ATP metabolic process;ATP synthesis coupled proton transport;biosynthetic process;cation transport;cellular biosynthetic process;cellular metabolic process;cellu
25.9006	26.1148	25.6721	25.5721	1:Experimental evidence at protein level	anatomical structure development;base-excision repair;cellular macromolecule metabolic process;cellular metabolic process;cellular nitrogen compound metabolic process;cellular process;cellular response to stimulus;cellula
23.7785	26.5361	23.0238	22.5517	1:Experimental evidence at protein level	adherens junction organization;anatomical structure morphogenesis;biological adhesion;biological regulation;blood vessel morphogenesis;calcium-dependent cell-cell adhesion;cell adhesion;cell adhesion mediated by integri
27.0325	26.8008	26.6472	26.6586	1:Experimental evidence at protein level	activation of immune response;activation of MAPKK activity;activation of phospholipase C activity;activation of protein kinase activity;antigen receptor-mediated signaling pathway;axon guidance;biological regulation;blood c
31.7103	31.7138	31.671	31.5719	1:Experimental evidence at protein level	anatomical structure formation involved in morphogenesis;angiogenesis;developmental process
28.7891	28.2102	28.368	28.1987	1:Experimental evidence at protein level	alcohol catabolic process;alcohol metabolic process;biological regulation;carbohydrate catabolic process;carbohydrate homeostasis;carbohydrate metabolic process;carbohydrate phosphorylation;carbohydrate transport;ca
26.6244	26.37	26.8609	26.7398	1:Experimental evidence at protein level	biological regulation;biosynthetic process;cellular biosynthetic process;cellular macromolecule biosynthetic process;cellular macromolecule metabolic process;cellular metabolic process;cellular nitrogen compound metabolic
27.2341	23.6253	26.6311	23.3426	1:Experimental evidence at protein level	biological regulation;biosynthetic process;cellular biosynthetic process;cellular macromolecule biosynthetic process;cellular macromolecule metabolic process;cellular metabolic process;cellular nitrogen compound metabolic
25.4597	24.3415	24.0868	23.4737	1:Experimental evidence at protein level	apoptosis;biological regulation;biosynthetic process;catabolic process;cell death;cell differentiation;cellular biosynthetic process;cellular catabolic process;cellular developmental process;cellular macromolecule biosynthetic
26.7824	26.5837	26.8599	26.6253	1:Experimental evidence at protein level	activation of MAPKK activity;activation of protein kinase activity;avoidance of defenses of other organism involved in symbiotic interaction;avoidance of host defenses;biological regulation;biosynthetic process;cellular biosyn
28.2908	28.5572	28.5764	28.4543	1:Experimental evidence at protein level	amine biosynthetic process;amine metabolic process;biosynthetic process;cellular amine metabolic process;cellular biogenic amine biosynthetic process;cellular biogenic amine metabolic process;cellular biosynthetic process
28.0794	28.2423	28.2079	27.9132	1:Experimental evidence at protein level	apoptosis;axon guidance;biological regulation;biosynthetic process;cell cycle;cell cycle checkpoint;cell death;cell surface receptor linked signaling pathway;cellular biosynthetic process;cellular macromolecule biosynthetic pr
28.9469	29.0868	29.2168	29.1486	1:Experimental evidence at protein level	amine metabolic process;aminoglycan metabolic process;biological regulation;carbohydrate metabolic process;glycosaminoglycan metabolic process;hyaluronan metabolic process;macromolecule metabolic process;metabo
24.0505	26.0471	25.6295	25.8082	1:Experimental evidence at protein level	amine metabolic process;aminoglycan metabolic process;carbohydrate metabolic process;glycosaminoglycan metabolic process;hyaluronan metabolic process;macromolecule metabolic process;metabolic process;nitrogen
26.46	26.2014	26.113	25.9816	1:Experimental evidence at protein level	activation of immune response;activation of innate immune response;antigen receptor-mediated signaling pathway;apoptosis;biological regulation;biosynthetic process;cell death;cell surface receptor linked signaling pathway
23.7398	23.93	23.18	25.1815	1:Experimental evidence at protein level	anatomical structure development;anatomical structure morphogenesis;biological regulation;BMP signaling pathway;cell differentiation;cell proliferation;cell surface receptor linked signaling pathway;cellular developmental pr
28.212	28.0666	27.6936	27.7715	1:Experimental evidence at protein level	biological regulation;blood coagulation;cellular process;coagulation;establishment of localization;hemostasis;ion transmembrane transport;ion transport;multicellular organismal process;regulation of biological quality;regulati
28.0997	28.1525	28.0773	27.9494	1:Experimental evidence at protein level	anatomical structure development;biosynthetic process;cell proliferation;cellular biosynthetic process;cellular macromolecule biosynthetic process;cellular macromolecule metabolic process;cellular metabolic process;cellular
28.476	28.2317	28.4642	28.3487	1:Experimental evidence at protein level	autophagy;behavior;behavioral interaction between organisms;biological regulation;calcium ion homeostasis;catabolic process;cation homeostasis;cell differentiation;cell proliferation;cell volume homeostasis;cellular calcium
30.0161	30.1001	30.5244	30.2304	1:Experimental evidence at protein level	biological regulation;biosynthetic process;cellular biosynthetic process;cellular macromolecule biosynthetic process;cellular macromolecule metabolic process;cellular metabolic process;cellular nitrogen compound metabolic
28.3745	28.115	28.2681	28.1036	1:Experimental evidence at protein level	antigen processing and presentation;biological regulation;cellular macromolecule metabolic process;cellular metabolic process;cellular process;cellular protein metabolic process;cellular response to stimulus;establishment o
26.9722	27.7678	27.4762	27.0476	1:Experimental evidence at protein level	biological regulation;blood coagulation;cellular component organization;cellular component organization or biogenesis;cellular macromolecule metabolic process;cellular membrane organization;cellular metabolic process;cel
26.6636	26.6017	26.496	26.6053	1:Experimental evidence at protein level	antigen processing and presentation;biological regulation;cellular component movement;cellular localization;cellular macromolecule localization;cellular macromolecule metabolic process;cellular metabolic process;cellular pr
26.4454	26.1968	25.9578	26.0264	1:Experimental evidence at protein level	apoptosis;biological regulation;cell death;cell surface receptor linked signaling pathway;cellular process;cellular response to chemical stimulus;cellular response to cytokine stimulus;cellular response to organic substance;cel
29.197	29.3583	29.6765	29.4713	1:Experimental evidence at protein level	activation of immune response;activation of innate immune response;anaphase-promoting complex-dependent proteasomal ubiquitin-dependent protein catabolic process;antigen processing and presentation;antigen proces
27.6094	28.0771	27.7633	27.8457	1:Experimental evidence at protein level	biological regulation;cellular component organization;cellular component organization or biogenesis;cellular membrane organization;cellular process;cellular response to stimulus;endocytosis;endosome to lysosome transport
28.3601	29.1798	29.4292	29.228	1:Experimental evidence at protein level	biosynthetic process;cellular biosynthetic process;cellular macromolecule metabolic process;cellular metabolic process;cellular nitrogen compound metabolic process;cellular process;electron transport chain;gene expressio
30.1571	29.4317	29.9305	29.8606	1:Experimental evidence at protein level	apoptosis;cell death;cellular component disassembly;cellular component disassembly at cellular level;cellular component disassembly involved in apoptosis;cellular component organization;cellular component organization a
25.4951	24.9176	23.3056	24.4441	1:Experimental evidence at protein level	biological regulation;female pregnancy;multi-organism process;negative regulation of catalytic activity;negative regulation of endopeptidase activity;negative regulation of hydrolase activity;negative regulation of molecular fur
23.7941	24.3443	24.3936	23.702	1:Experimental evidence at protein level	amine metabolic process;aminoglycan biosynthetic process;aminoglycan catabolic process;aminoglycan metabolic process;biological regulation;biosynthetic process;carbohydrate biosynthetic process;carbohydrate catabol
28.0627	28.113	28.248	28.351	1:Experimental evidence at protein level	biological regulation;negative regulation of apoptosis;negative regulation of biological process;negative regulation of catalytic activity;negative regulation of cell death;negative regulation of cellular process;negative regulation
25.8098	24.7544	25.6242	25.7458	1:Experimental evidence at protein level	biosynthetic process;cell activation;cell proliferation;cellular aromatic compound metabolic process;cellular biosynthetic process;cellular metabolic process;cellular nitrogen compound biosynthetic process;cellular nitrogen co
27.413	27.1271	27.3969	27.4139	1:Experimental evidence at protein level	anatomical structure development;anatomical structure morphogenesis;axon guidance;biological adhesion;biological regulation;biosynthetic process;blood vessel development;cell adhesion;cell migration;cell motility;cellular
25.9029	25.5016	25.1891	24.9837	1:Experimental evidence at protein level	anatomical structure morphogenesis;axon guidance;biological regulation;blood vessel morphogenesis;cell cycle cytokinesis;cell cycle process;cell differentiation;cell surface receptor linked signaling pathway;cellular compon
29.9501	30.1323	30.7729	30.427	1:Experimental evidence at protein level	biosynthetic process;cellular biosynthetic process;cellular macromolecule biosynthetic process;cellular macromolecule metabolic process;cellular metabolic process;cellular nitrogen compound metabolic process;cellular pro
26.0113	25.8354	25.969	25.8721	1:Experimental evidence at protein level	apoptosis;biological regulation;cell cycle phase;cell cycle process;cell death;cell proliferation;cellular component organization;cellular component organization at cellular level;cellular component organization or biogenesis;cel
28.0553	28.277	27.9937	28.0379	1:Experimental evidence at protein level	amine metabolic process;anatomical structure development;benzene-containing compound metabolic process;carboxylic acid metabolic process;catabolic process;cell development;cellular amine metabolic process;cellular
28.2004	28.101	28.0057	28.1894	1:Experimental evidence at protein level	ATP hydrolysis coupled proton transport;ATP metabolic process;biological regulation;cation homeostasis;cation transport;cell surface receptor linked signaling pathway;cellular cation homeostasis;cellular chemical homeosta
27.134	26.9641	26.7885	26.8252	1:Experimental evidence at protein level	ATP hydrolysis coupled proton transport;biological regulation;cation homeostasis;cation transport;cell surface receptor linked signaling pathway;cellular cation homeostasis;cellular chemical homeostasis;cellular component c
29.6478	30.3986	30.1913	30.381	1:Experimental evidence at protein level	biological adhesion;cell adhesion;cell-cell adhesion;cellular process;homotypic cell-cell adhesion;platelet aggregation
31.6705	31.5633	31.4446	31.4579	1:Experimental evidence at protein level	actin crosslink formation;actin cytoskeleton organization;actin cytoskeleton reorganization;actin filament organization;actin filament-based process;anatomical structure formation involved in morphogenesis;anatomical structu
27.7409	27.7416	27.7303	27.7185	1:Experimental evidence at protein level	acetyl-CoA catabolic process;acetyl-CoA metabolic process;biological regulation;carboxylic acid metabolic process;catabolic process;cation homeostasis;cellular catabolic process;cellular cation homeostasis;cellular chemic
27.1337	26.253	26.5444	26.6338	1:Experimental evidence at protein level	adenosine biosynthetic process;adenosine metabolic process;AMP catabolic process;AMP metabolic process;biological adhesion;biological regulation;biosynthetic process;catabolic process;cell adhesion;cell-cell adhesion;
29.7357	29.261	29.4658	29.4914	1:Experimental evidence at protein level	anion transport;apoptosis;behavior;behavioral defense response;behavioral fear response;cell communication;cell death;cell differentiation;cell-cell signaling;cellular developmental process;cellular process;cognition;death;de
27.5151	27.6439	27.6474	27.5625	1:Experimental evidence at protein level	acetyl-CoA catabolic process;acetyl-CoA metabolic process;aerobic respiration;carboxylic acid metabolic process;catabolic process;cellular catabolic process;cellular ketone metabolic process;cellular metabolic process;cel
29.4364	25.8621	23.0866	29.1776	1:Experimental evidence at protein level	anatomical structure development;anatomical structure formation involved in morphogenesis;biological adhesion;biological regulation;blood coagulation;brain development;cell activation;cell adhesion;cell development;cell ju
28.7442	28.9999	28.8547	28.726	1:Experimental evidence at protein level	alcohol catabolic process;alcohol metabolic process;amine catabolic process;amine metabolic process;behavior;benzene-containing compound metabolic process;biological regulation;biosynthetic process;catabolic process
26.5338	26.3871	26.7631	26.7897	1:Experimental evidence at protein level	anatomical structure formation involved in morphogenesis;anatomical structure morphogenesis;apoptotic cell clearance;biological regulation;blood vessel remodeling;branching involved in salivary gland morphogenesis;calci
25.7582	25.3775	25.7128	25.2391	1:Experimental evidence at protein level	amine metabolic process;carboxylic acid catabolic process;carboxylic acid metabolic process;catabolic process;cellular amine metabolic process;cellular amino acid metabolic process;cellular catabolic process;cellular keto
26.9142	26.8314	26.9861	26.8303	1:Experimental evidence at protein level	biological regulation;establishment of localization;lipid transport;modulation of growth of symbiont involved in interaction with host;organic substance transport;positive regulation of biological process;positive regulation of gr
28.9688	29.6985	29.6685	29.6511	1:Experimental evidence at protein level	cellular macromolecule metabolic process;cellular metabolic process;cellular process;cellular protein metabolic process;macromolecule metabolic process;macromolecule methylation;macromolecule modification;metabolic
28.2215	27.7498	27.6373	27.8223	1:Experimental evidence at protein level	box C/D snoRNA 3'-end processing;box C/D snoRNA metabolic process;box C/D snoRNA processing;cell differentiation;cellular developmental process;cellular macromolecule metabolic process;cellular metabolic process;c
28.2124	27.5585	27.6423	27.6614	1:Experimental evidence at protein level	biosynthetic process;catabolic process;cellular biosynthetic process;cellular catabolic process;cellular component disassembly;cellular component disassembly at cellular level;cellular component organization;cellular compo
29.7401	29.7085	29.7153	29.6729	1:Experimental evidence at protein level	amine metabolic process;anatomical structure development;aromatic compound biosynthetic process;biosynthetic process;brainstem development;carboxylic acid metabolic process;cellular amine metabolic process;cellula
29.2574	29.3435	29.2162	29.2405	1:Experimental evidence at protein level	biosynthetic process;cellular aromatic compound metabolic process;cellular biosynthetic process;cellular metabolic process;cellular nitrogen compound biosynthetic process;cellular nitrogen compound metabolic process;ce
28.3852	28.7156	28.6974	28.8921	1:Experimental evidence at protein level	bile acid biosynthetic process;bile acid metabolic process;biological regulation;biosynthetic process;C21-steroid hormone biosynthetic process;C21-steroid hormone metabolic process;carboxylic acid biosynthetic process;c
29.3735	29.5524	29.3657	29.3446	1:Experimental evidence at protein level	catabolic process;cellular catabolic process;cellular macromolecule catabolic process;cellular macromolecule metabolic process;cellular metabolic process;cellular process;cellular protein metabolic process;cellular response
32.7399	33.2547	33.1215	33.4633	1:Experimental evidence at protein level	biological adhesion;biological regulation;biosynthetic process;cell adhesion;cell surface receptor linked signaling pathway;cellular biosynthetic process;cellular macromolecule biosynthetic process;cellular macromolecule me
25.5645	25.2384	25.1354	25.1037	1:Experimental evidence at protein level	3'-phosphoadenosine 5'-phosphosulfate metabolic process;anatomical structure development;anion homeostasis;anion transport;biological regulation;biomineral tissue development;bone remodeling;catabolic process;cellul
25.277	25.5098	25.4186	25.3746	1:Experimental evidence at protein level	alcohol metabolic process;biological regulation;biosynthetic process;C21-steroid hormone biosynthetic process;C21-steroid hormone metabolic process;cellular biosynthetic process;cellular hormone metabolic process;cellu
30.9205	31.1643	31.091	31.1871	1:Experimental evidence at protein level	biological regulation;cellular macromolecule metabolic process;cellular metabolic process;cellular nitrogen compound metabolic process;cellular process;establishment of localization;establishment of RNA localization;gene e
26.0845	26.1061	26.1396	25.8826	1:Experimental evidence at protein level	biological regulation;cell surface receptor linked signaling pathway;cellular macromolecule metabolic process;cellular metabolic process;cellular process;cellular protein metabolic process;cellular response to chemical stimul
28.682	28.3618	28.5409	28.3163	1:Experimental evidence at protein level	aerobic respiration;cellular metabolic process;cellular process;cellular respiration;electron transport chain;energy derivation by oxidation of organic compounds;generation of precursor metabolites and energy;metabolic proce
26.7636	26.8701	26.8104	26.888	1:Experimental evidence at protein level	alcohol metabolic process;biological regulation;biosynthetic process;cation homeostasis;cell differentiation;cellular aromatic compound metabolic process;cellular biosynthetic process;cellular component assembly;cellular c
27.6066	28.2645	27.8439	28.2856	1:Experimental evidence at protein level	cellular component organization;cellular component organization at cellular level;cellular component organization or biogenesis;cellular component organization or biogenesis at cellular level;cellular process;embryo implantat
27.6753	27.9802	28.0243	27.8755	1:Experimental evidence at protein level	biological regulation;biosynthetic process;cell differentiation;cellular biosynthetic process;cellular developmental process;cellular macromolecule biosynthetic process;cellular macromolecule metabolic process;cellular metab
27.001	27.215	26.8887	27.0548	1:Experimental evidence at protein level	arachidonic acid metabolic process;biological regulation;biosynthetic process;carboxylic acid biosynthetic process;carboxylic acid metabolic process;cellular biosynthetic process;cellular ketone metabolic process;cellular lip
25.9245	25.9878	25.8498	25.824	1:Experimental evidence at protein level	anatomical structure development;biological adhesion;biological regulation;blood coagulation;brown fat cell differentiation;cell adhesion;cell adhesion mediated by integrin;cell communication;cell differentiation;cell junction a
30.5124	30.4811	30.5544	30.4819	1:Experimental evidence at protein level	alternative nuclear mRNA splicing, via spliceosome;biological regulation;biosynthetic process;cellular biosynthetic process;cellular component organization;cellular component organization at cellular level;cellular component
27.7493	27.6189	27.6911	27.5023	1:Experimental evidence at protein level	cell cycle;cell cycle process;cellular component organization;cellular component organization at cellular level;cellular component organization or biogenesis;cellular component organization or biogenesis at cellular level;cellula
31.1621	31.3314	31.4727	31.5058	1:Experimental evidence at protein level	anatomical structure development;biological regulation;bone development;cellular component organization;cellular component organization at cellular level;cellular component organization or biogenesis;cellular component o
26.8617	26.8281	26.8287	26.655	1:Experimental evidence at protein level	carboxylic acid metabolic process;cellular ketone metabolic process;cellular metabolic process;cellular process;dicarboxylic acid metabolic process;malate metabolic process;metabolic process;organic acid metabolic proce
28.9469	29.0834	29.1181	28.9302	1:Experimental evidence at protein level	amine metabolic process;amino acid activation;anatomical structure formation involved in morphogenesis;angiogenesis;biological regulation;biosynthetic process;carboxylic acid metabolic process;cellular amine metabolic p
30.8803	30.4905	30.5116	30.5543	1:Experimental evidence at protein level	apoptosis;biological regulation;biosynthetic process;catabolic process;cell cycle phase;cell cycle process;cell death;cell division;cellular biosynthetic process;cellular catabolic process;cellular component disassembly;cellula
24.7239	26.1375	25.6223	25.6866	1:Experimental evidence at protein level	biological regulation;cell differentiation;cell migration;cell motility;cell surface receptor linked signaling pathway;cellular component movement;cellular developmental process;cellular macromolecule metabolic process;cellula
25.394	25.294	25.855	25.2259	1:Experimental evidence at protein level	anatomical structure homeostasis;biological regulation;biosynthetic process;cell surface receptor linked signaling pathway;cellular biosynthetic process;cellular component organization;cellular component organization at cell
28.7147	28.8813	28.8385	28.7802	1:Experimental evidence at protein level	amine metabolic process;carboxylic acid metabolic process;cellular amine metabolic process;cellular amino acid metabolic process;cellular ketone metabolic process;cellular metabolic process;cellular modified amino acid n
31.7223	32.0843	32.3269	32.3219	1:Experimental evidence at protein level	actin cytoskeleton organization;actin filament depolymerization;actin filament organization;actin filament-based process;actin polymerization or depolymerization;ameboidal cell migration;anatomical structure morphogenesis;
29.4598	29.2212	29.6293	29.5803	1:Experimental evidence at protein level	biological regulation;biosynthetic process;catabolic process;cell surface receptor linked signaling pathway;cellular biosynthetic process;cellular catabolic process;cellular macromolecule biosynthetic process;cellular macrom
28.9543	28.883	28.6743	28.9383	1:Experimental evidence at protein level	biological regulation;blood coagulation;calcium ion homeostasis;calcium ion transmembrane transport;calcium ion transport;cation homeostasis;cation transport;cellular calcium ion homeostasis;cellular cation homeostasis;c
24.9524	25.2942	24.8222	24.9845	1:Experimental evidence at protein level	activation of protein kinase activity;activation of protein kinase C activity by G-protein coupled receptor protein signaling pathway;biological regulation;blood coagulation;cell activation;cell surface receptor linked signaling pa
25.5889	25.4038	25.3288	25.5771	1:Experimental evidence at protein level	amine transport;amino acid transmembrane transport;amino acid transport;betaine transport;carboxylic acid catabolic process;carboxylic acid metabolic process;carboxylic acid transport;carnitine shuttle;carnitine transport;
27.0511	27.0734	27.1787	27.1752	1:Experimental evidence at protein level	2'-deoxyribonucleotide biosynthetic process;2'-deoxyribonucleotide metabolic process;biosynthetic process;cell cycle;cell differentiation;cell proliferation;cellular biosynthetic process;cellular developmental process;cellular r
28.1588	28.1358	27.9619	27.9339	1:Experimental evidence at protein level	anatomical structure development;biosynthetic process;cell cycle;cell proliferation;cell proliferation in forebrain;cellular aromatic compound metabolic process;cellular biosynthetic process;cellular component assembly;cellula
30.644	30.824	31.0329	31.0335	1:Experimental evidence at protein level	biosynthetic process;cellular biosynthetic process;cellular macromolecule biosynthetic process;cellular macromolecule metabolic process;cellular metabolic process;cellular process;cellular protein metabolic process;gene ex
28.7197	28.6947	28.6444	28.4814	1:Experimental evidence at protein level	anatomical structure development;ATP biosynthetic process;ATP metabolic process;ATP synthesis coupled proton transport;biosynthetic process;cation transport;cellular biosynthetic process;cellular metabolic process;cellu
24.6272	23.7463	25.8507	24.7274	1:Experimental evidence at protein level	alcohol metabolic process;anatomical structure morphogenesis;biological regulation;carbohydrate homeostasis;carbohydrate metabolic process;cell differentiation;cell proliferation;cellular carbohydrate metabolic process;ce
26.1601	26.937	26.7237	27.0917	1:Experimental evidence at protein level	
27.9986	28.348	28.2822	28.3825	1:Experimental evidence at protein level	adipose tissue development;amine catabolic process;amine metabolic process;anatomical structure development;biosynthetic process;brain development;branched chain family amino acid catabolic process;branched chain
26.0991	25.9339	26.3157	25.8562	1:Experimental evidence at protein level	anatomical structure morphogenesis;axis elongation;axon regeneration;axonogenesis;biological adhesion;biological regulation;branch elongation of an epithelium;bud outgrowth involved in lung branching;cell adhesion;cell c
28.3144	28.5342	29.1345	28.5616	1:Experimental evidence at protein level	axon guidance;biological adhesion;biological regulation;cell adhesion;cell surface receptor linked signaling pathway;cell-cell adhesion;cellular process;cellular response to stimulus;chemotaxis;enzyme linked receptor protein
26.22	25.3182	25.7346	25.7945	1:Experimental evidence at protein level	biological regulation;biosynthetic process;cellular biosynthetic process;cellular macromolecule biosynthetic process;cellular macromolecule metabolic process;cellular metabolic process;cellular nitrogen compound metabolic
26.8824	26.9925	26.9059	26.6553	1:Experimental evidence at protein level	anaphase-promoting complex-dependent proteasomal ubiquitin-dependent protein catabolic process;biological regulation;biosynthetic process;blood coagulation;catabolic process;cation transport;cell cycle;cell cycle check
28.9256	28.7609	28.6792	28.779	1:Experimental evidence at protein level	biosynthetic process;cell cycle;cell cycle process;cellular biosynthetic process;cellular macromolecule biosynthetic process;cellular macromolecule metabolic process;cellular metabolic process;cellular nitrogen compound m
26.4697	26.3934	26.508	26.4944	1:Experimental evidence at protein level	biosynthetic process;catabolic process;cellular biosynthetic process;cellular catabolic process;cellular metabolic process;cellular nitrogen compound catabolic process;cellular nitrogen compound metabolic process;cellular p
30.558	29.971	30.5915	30.3581	1:Experimental evidence at protein level	biosynthetic process;catabolic process;cellular biosynthetic process;cellular catabolic process;cellular component disassembly;cellular component disassembly at cellular level;cellular component organization;cellular compo
25.0817	24.8066	24.67	25.2932	1:Experimental evidence at protein level	biological regulation;biosynthetic process;cellular biosynthetic process;cellular component assembly;cellular component assembly at cellular level;cellular component organization;cellular component organization at cellular le
26.5945	27.1922	27.1215	27.2159	1:Experimental evidence at protein level	anatomical structure morphogenesis;anterior/posterior pattern specification;biological regulation;camera-type eye morphogenesis;cell differentiation;cellular component organization;cellular component organization at cellula
27.4662	27.5113	27.5838	27.7203	1:Experimental evidence at protein level	biological regulation;cellular macromolecule metabolic process;cellular metabolic process;cellular process;cellular protein metabolic process;cellular response to heat;cellular response to stimulus;cellular response to stress;c
31.4642	31.4886	31.366	31.306	1:Experimental evidence at protein level	ATP biosynthetic process;ATP hydrolysis coupled proton transport;ATP metabolic process;ATP synthesis coupled proton transport;biological regulation;biosynthetic process;cation transport;cellular biosynthetic process;cellu
25.5448	24.3973	25.6828	24.1292	1:Experimental evidence at protein level	activation of immune response;activation of innate immune response;adaptive immune response;antigen processing and presentation;antigen processing and presentation of exogenous antigen;antigen processing and prese
29.0737	29.2571	29.491	29.6743	1:Experimental evidence at protein level	activation of immune response;activation of innate immune response;anaphase-promoting complex-dependent proteasomal ubiquitin-dependent protein catabolic process;antigen processing and presentation;antigen proces
29.3024	29.7946	29.5801	29.6188	1:Experimental evidence at protein level	activation of immune response;activation of innate immune response;anaphase-promoting complex-dependent proteasomal ubiquitin-dependent protein catabolic process;antigen processing and presentation;antigen proces
29.3757	29.2252	29.7045	29.5826	1:Experimental evidence at protein level	activation of immune response;activation of innate immune response;anaphase-promoting complex-dependent proteasomal ubiquitin-dependent protein catabolic process;antigen processing and presentation;antigen proces
29.5514	29.2676	29.6826	29.5885	1:Experimental evidence at protein level	activation of immune response;activation of innate immune response;anaphase-promoting complex-dependent proteasomal ubiquitin-dependent protein catabolic process;antigen processing and presentation;antigen proces
27.5649	27.5354	27.0814	27.3347	1:Experimental evidence at protein level	anatomical structure development;behavior;biological adhesion;biological regulation;blood coagulation;cell adhesion;cell differentiation;cell migration;cell motility;cell surface receptor linked signaling pathway;cell-matrix adhe
27.3852	27.3434	27.2881	27.1866	1:Experimental evidence at protein level	biological regulation;defense response;immune effector process;immune response;immune system process;inflammatory response;innate immune response;multi-organism process;negative regulation of biological process;n
31.9492	31.9314	32.0226	31.8563	1:Experimental evidence at protein level	anatomical structure development;biological adhesion;biological regulation;cell adhesion;cell development;cell migration;cell motility;cell-cell adhesion;cellular component movement;cellular developmental process;cellular pr
28.4476	28.2893	28.0305	28.1005	1:Experimental evidence at protein level	biological regulation;catabolic process;cellular catabolic process;cellular component assembly;cellular component assembly at cellular level;cellular component organization;cellular component organization at cellular level;ce
28.1041	28.1144	27.9792	28.1885	1:Experimental evidence at protein level	biological regulation;biosynthetic process;cellular biosynthetic process;cellular component organization;cellular component organization at cellular level;cellular component organization or biogenesis;cellular component orga
29.2104	29.4553	29.7042	29.4546	1:Experimental evidence at protein level	biological regulation;biosynthetic process;cellular biosynthetic process;cellular macromolecule biosynthetic process;cellular macromolecule metabolic process;cellular metabolic process;cellular nitrogen compound metabolic
31.1329	31.0188	31.0043	31.1905	1:Experimental evidence at protein level	biosynthetic process;catabolic process;cellular biosynthetic process;cellular catabolic process;cellular component disassembly;cellular component disassembly at cellular level;cellular component organization;cellular compo
26.1199	26.2802	26.5613	26.4266	1:Experimental evidence at protein level	amine catabolic process;amine metabolic process;branched chain family amino acid catabolic process;branched chain family amino acid metabolic process;carboxylic acid catabolic process;carboxylic acid metabolic proces
28.782	28.4384	28.8024	28.9182	1:Experimental evidence at protein level	anatomical structure morphogenesis;biological regulation;cell morphogenesis;cell morphogenesis involved in differentiation;cellular component morphogenesis;cellular component organization;cellular component organizatio
29.4956	29.6504	29.9394	29.7483	1:Experimental evidence at protein level	anatomical structure development;apoptosis;base-excision repair, DNA ligation;biological regulation;catabolic process;cell chemotaxis;cell death;cell migration;cell motility;cellular catabolic process;cellular component assen
30.0043	29.8718	29.9089	29.9061	1:Experimental evidence at protein level	alternative nuclear mRNA splicing, via spliceosome;biological regulation;cellular macromolecule metabolic process;cellular metabolic process;cellular nitrogen compound metabolic process;cellular process;gene expression;r
28.2938	28.3367	28.3608	28.2795	1:Experimental evidence at protein level	amine metabolic process;amino acid activation;biosynthetic process;carboxylic acid metabolic process;cellular amine metabolic process;cellular amino acid metabolic process;cellular biosynthetic process;cellular ketone me
27.7523	27.7375	27.5847	27.6056	1:Experimental evidence at protein level	amine metabolic process;amino acid activation;carboxylic acid metabolic process;cellular amine metabolic process;cellular amino acid metabolic process;cellular ketone metabolic process;cellular macromolecule metabolic p
30.7003	30.7134	30.6613	30.5155	1:Experimental evidence at protein level	biosynthetic process;cellular biosynthetic process;cellular macromolecule biosynthetic process;cellular macromolecule metabolic process;cellular metabolic process;cellular process;cellular protein metabolic process;gene ex
26.4847	27.2208	27.7923	27.7032	1:Experimental evidence at protein level	cellular macromolecule metabolic process;cellular metabolic process;cellular process;cellular protein metabolic process;chaperone-mediated protein folding;macromolecule metabolic process;macromolecule modification;me

28.2815	27.947	28.0948	28.1259	1:Experimental evidence at protein level	biological regulation;cellular component assembly;cellular component organization;cellular component organization or biogenesis;macromolecular complex assembly;macromolecular complex subunit organization;positive re
31.9894	32.1856	31.8449	31.926	1:Experimental evidence at protein level	anatomical structure development;apoptosis;biological regulation;biosynthetic process;cell death;cellular biosynthetic process;cellular component organization;cellular component organization or biogenesis;cellular macromo
26.6147	27.0467	26.5789	26.8997	1:Experimental evidence at protein level	activation of immune response;activation of innate immune response;activation of MAPK activity;activation of MAPKK activity;activation of protein kinase activity;anatomical structure development;anatomical structure morph
25.4543	24.4558	25.9674	25.6569	1:Experimental evidence at protein level	biological regulation;cell activation;cell migration;cell motility;cellular component movement;cellular process;endothelial cell migration;entry into cell of other organism involved in symbiotic interaction;entry into host;entry into
31.0515	30.729	30.7545	30.7621	1:Experimental evidence at protein level	biosynthetic process;catabolic process;cellular biosynthetic process;cellular catabolic process;cellular component disassembly;cellular component disassembly at cellular level;cellular component organization;cellular compo
28.3394	28.0999	27.817	28.0076	1:Experimental evidence at protein level	anatomical structure development;anatomical structure homeostasis;base-excision repair;biological regulation;biosynthetic process;catabolic process;cell cycle;cell cycle checkpoint;cell cycle phase;cell cycle process;cellula
29.0901	29.1785	29.0032	29.1067	1:Experimental evidence at protein level	aging;anatomical structure homeostasis;base-excision repair;biological regulation;biosynthetic process;cell cycle;cell redox homeostasis;cellular biosynthetic process;cellular component organization;cellular component orga
23.3412	25.7872	25.8974	23.3412	1:Experimental evidence at protein level	
26.3823	26.665	27.1823	26.7815	1:Experimental evidence at protein level	biosynthetic process;cellular aromatic compound metabolic process;cellular biosynthetic process;cellular metabolic compound salvage;cellular metabolic process;cellular nitrogen compound biosynthetic process;cellular nitro
28.268	27.93	27.8973	27.8915	1:Experimental evidence at protein level	amide biosynthetic process;amine biosynthetic process;amine catabolic process;amine metabolic process;anatomical structure development;anatomical structure morphogenesis;arginine biosynthetic process;arginine metab
32.5741	32.6808	32.9827	32.8448	1:Experimental evidence at protein level	actin cytoskeleton organization;actin filament-based process;aging;antigen processing and presentation;antigen processing and presentation of exogenous antigen;antigen processing and presentation of exogenous peptide
29.8798	29.8429	29.8116	30.0049	1:Experimental evidence at protein level	cell cycle process;cell division;cellular component movement;cellular component organization;cellular component organization at cellular level;cellular component organization or biogenesis;cellular component organization or
30.6784	30.8563	30.7166	30.5893	1:Experimental evidence at protein level	aging;antigen processing and presentation;antigen processing and presentation of exogenous antigen;antigen processing and presentation of exogenous peptide antigen;antigen processing and presentation of exogenous pe
24.4783	24.4428	26.941	27.0101	1:Experimental evidence at protein level	activation of immune response;activation of innate immune response;anaphase-promoting complex-dependent proteasomal ubiquitin-dependent protein catabolic process;antigen processing and presentation;antigen proces
26.9411	23.8562	27.3528	27.1418	1:Experimental evidence at protein level	activation of immune response;activation of innate immune response;anaphase-promoting complex-dependent proteasomal ubiquitin-dependent protein catabolic process;antigen processing and presentation;antigen proces
29.9722	30.3087	30.3536	30.6054	1:Experimental evidence at protein level	activation of immune response;activation of innate immune response;anaphase-promoting complex-dependent proteasomal ubiquitin-dependent protein catabolic process;antigen processing and presentation;antigen proces
28.0412	28.2384	28.423	28.5901	1:Experimental evidence at protein level	activation of immune response;activation of innate immune response;anaphase-promoting complex-dependent proteasomal ubiquitin-dependent protein catabolic process;antigen processing and presentation;antigen proces
29.1694	29.2218	29.6727	29.3672	1:Experimental evidence at protein level	activation of immune response;activation of innate immune response;anaphase-promoting complex-dependent proteasomal ubiquitin-dependent protein catabolic process;antigen processing and presentation;antigen proces
27.8332	27.8361	28.4056	28.1475	1:Experimental evidence at protein level	activation of immune response;activation of innate immune response;anaphase-promoting complex-dependent proteasomal ubiquitin-dependent protein catabolic process;antigen processing and presentation;antigen proces
26.7751	26.7805	26.8317	26.4769	1:Experimental evidence at protein level	carboxylic acid catabolic process;carboxylic acid metabolic process;catabolic process;cellular catabolic process;cellular component organization;cellular component organization at cellular level;cellular component organizati
26.8874	27.6377	27.0859	27.1207	1:Experimental evidence at protein level	anatomical structure development;blood vessel development;cellular component assembly;cellular component assembly at cellular level;cellular component organization;cellular component organization at cellular level;cellula
27.0211	27.2466	27.1056	27.0588	1:Experimental evidence at protein level	apoptotic mitochondrial changes;ATP metabolic process;biological regulation;cellular chemical homeostasis;cellular component organization;cellular component organization at cellular level;cellular component organization o
27.1237	27.111	27.1135	27.0408	1:Experimental evidence at protein level	anatomical structure homeostasis;base-excision repair;base-excision repair, gap-filling;biological regulation;biosynthetic process;cell cycle;cellular biosynthetic process;cellular component organization;cellular component org
30.5248	30.4049	30.3378	30.41	1:Experimental evidence at protein level	anatomical structure development;ATP-dependent chromatin remodeling;biological regulation;biosynthetic process;brain development;cell differentiation;cellular biosynthetic process;cellular component organization;cellular c
29.1142	29.2187	29.321	29.3211	1:Experimental evidence at protein level	activation of immune response;activation of innate immune response;activation of MAPK activity;activation of MAPKK activity;activation of protein kinase activity;anatomical structure development;anatomical structure morph
24.9105	23.1675	24.1761	24.2266	1:Experimental evidence at protein level	anatomical structure development;biological regulation;biosynthetic process;cardiac cell differentiation;cardiac muscle cell differentiation;cardiac muscle cell proliferation;cell communication;cell differentiation;cell proliferation
28.3086	28.3678	28.5239	28.6322	1:Experimental evidence at protein level	biological regulation;blastocyst hatching;cellular process;cellular response to stimulus;developmental process;embryo implantation;hatching;organism emergence from protective structure;positive regulation of biological proc
25.6542	26.6019	26.3486	26.4063	1:Experimental evidence at protein level	macromolecule metabolic process;metabolic process;primary metabolic process;protein metabolic process;proteolysis
27.4204	26.8859	27.0645	26.9595	1:Experimental evidence at protein level	antigen processing and presentation;antigen processing and presentation of peptide antigen;antigen processing and presentation of peptide antigen via MHC class I;cellular macromolecule metabolic process;cellular metabo
26.2572	26.837	27.0511	27.117	1:Experimental evidence at protein level	alcohol biosynthetic process;alcohol metabolic process;biological regulation;biosynthetic process;carbohydrate biosynthetic process;carbohydrate metabolic process;catabolic process;cellular biosynthetic process;cellular ca
25.0728	24.5065	24.6774	25.2518	1:Experimental evidence at protein level	aging;anatomical structure development;anatomical structure formation involved in morphogenesis;anatomical structure homeostasis;angiogenesis;biological adhesion;biological regulation;biosynthetic process;calcium ion tr
27.2311	27.228	27.3069	27.1434	1:Experimental evidence at protein level	anatomical structure development;anatomical structure formation involved in morphogenesis;anatomical structure morphogenesis;angiogenesis;axial mesoderm formation;axon guidance;biological adhesion;biological regulat
25.3902	25.1887	25.3757	25.3732	1:Experimental evidence at protein level	anatomical structure development;anatomical structure formation involved in morphogenesis;anatomical structure morphogenesis;angiogenesis;axon guidance;axonal fasciculation;axonogenesis;behavior;biological regulation
27.8468	28.1209	28.044	28.2333	1:Experimental evidence at protein level	2'-deoxyribonucleotide metabolic process;base-excision repair;base-excision repair, AP site formation;catabolic process;cellular catabolic process;cellular macromolecule catabolic process;cellular macromolecule metabolic
27.5835	27.9755	27.9126	28.0142	1:Experimental evidence at protein level	anatomical structure development;anatomical structure morphogenesis;appendage morphogenesis;biological regulation;biosynthetic process;carboxylic acid metabolic process;cellular biosynthetic process;cellular hormone
29.7668	29.4998	30.3863	29.6387	1:Experimental evidence at protein level	alcohol biosynthetic process;alcohol catabolic process;alcohol metabolic process;aldehyde biosynthetic process;biological regulation;biosynthetic process;carbohydrate biosynthetic process;carbohydrate catabolic process;
26.6831	26.9207	26.9087	27.0885	1:Experimental evidence at protein level	activation of caspase activity;apoptosis;biological regulation;cell death;cellular chemical homeostasis;cellular homeostasis;cellular ion homeostasis;cellular process;cellular response to abiotic stimulus;cellular response to ch
27.1826	27.2914	27.2157	27.3779	1:Experimental evidence at protein level	activation of caspase activity;aging;anatomical structure morphogenesis;apoptosis;apoptosis in response to endoplasmic reticulum stress;biological regulation;biosynthetic process;branching involved in mammary gland duc
30.7738	31.2116	31.3136	31.2036	1:Experimental evidence at protein level	biological regulation;biosynthetic process;cellular biosynthetic process;cellular macromolecule biosynthetic process;cellular macromolecule metabolic process;cellular metabolic process;cellular nitrogen compound metabolic
28.7187	29.5362	29.1443	29.5497	1:Experimental evidence at protein level	biological regulation;cellular metabolic process;cellular process;energy derivation by oxidation of organic compounds;energy reserve metabolic process;generation of precursor metabolites and energy;metabolic process;oxid
26.6403	26.3999	26.1527	26.2307	1:Experimental evidence at protein level	activation of phospholipase C activity;activation of phospholipase C activity by dopamine receptor signaling pathway;activation of phospholipase C activity by G-protein coupled receptor protein signaling pathway coupled to
28.8225	28.77	28.9917	28.8818	1:Experimental evidence at protein level	activation of MAPK activity;biological regulation;cellular macromolecule metabolic process;cellular metabolic process;cellular process;cellular protein metabolic process;establishment of localization;establishment of localizati
31.1038	31.5307	31.3535	31.2993	1:Experimental evidence at protein level	catabolic process;cellular catabolic process;cellular lipid catabolic process;cellular lipid metabolic process;cellular metabolic process;cellular process;cellular response to chemical stimulus;cellular response to hydrogen pero
28.2353	29.0124	29.0056	28.9769	1:Experimental evidence at protein level	catabolic process;cellular catabolic process;cellular metabolic process;cellular nitrogen compound catabolic process;cellular nitrogen compound metabolic process;cellular process;cofactor catabolic process;cofactor metab
28.5372	28.7267	29.1159	28.9402	1:Experimental evidence at protein level	biological regulation;biosynthetic process;catabolic process;cellular biosynthetic process;cellular catabolic process;cellular macromolecule metabolic process;cellular metabolic process;cellular nitrogen compound metabolic
27.8609	29.1721	29.1958	29.4052	1:Experimental evidence at protein level	biosynthetic process;cellular biosynthetic process;cellular metabolic process;cellular process;melanin biosynthetic process;melanin metabolic process;metabolic process;pigment biosynthetic process;pigment metabolic proc
28.2652	28.4706	28.6735	28.4895	1:Experimental evidence at protein level	anatomical structure development;biological regulation;catabolic process;cell differentiation;cellular catabolic process;cellular chemical homeostasis;cellular component organization;cellular component organization at cellular
28.6541	28.9305	29.4405	29.1837	1:Experimental evidence at protein level	ATP biosynthetic process;ATP metabolic process;ATP synthesis coupled proton transport;biosynthetic process;cation transport;cellular biosynthetic process;cellular metabolic process;cellular nitrogen compound biosynthetic
30.1805	30.3392	30.068	30.0528	1:Experimental evidence at protein level	biosynthetic process;catabolic process;cellular biosynthetic process;cellular catabolic process;cellular component disassembly;cellular component disassembly at cellular level;cellular component organization;cellular compo
28.3304	28.3053	28.7591	28.3287	1:Experimental evidence at protein level	carboxylic acid catabolic process;carboxylic acid metabolic process;catabolic process;cellular catabolic process;cellular ketone metabolic process;cellular lipid catabolic process;cellular lipid metabolic process;cellular metab
28.4023	28.4687	28.6619	28.4821	1:Experimental evidence at protein level	2'-deoxyribonucleotide biosynthetic process;2'-deoxyribonucleotide metabolic process;benzene-containing compound metabolic process;biosynthetic process;carboxylic acid metabolic process;CDP biosynthetic process;C
27.8969	28.9911	29.3147	29.3595	1:Experimental evidence at protein level	
31.6549	31.7931	31.8771	31.8788	1:Experimental evidence at protein level	antigen processing and presentation;antigen processing and presentation of exogenous antigen;antigen processing and presentation of exogenous peptide antigen;antigen processing and presentation of exogenous peptide
29.764	30.0916	29.8624	29.8324	1:Experimental evidence at protein level	apoptosis;biological regulation;catabolic process;cell cycle;cell cycle process;cell death;cell surface receptor linked signaling pathway;cellular catabolic process;cellular component assembly;cellular component organization;
27.8147	27.7158	27.463	27.7173	1:Experimental evidence at protein level	apoptosis;apoptosis involved in morphogenesis;cell death;cellular developmental process;cellular process;death;developmental process;developmental programmed cell death;programmed cell death
27.7542	28.9589	28.8078	29.1012	1:Experimental evidence at protein level	apoptotic mitochondrial changes;biological regulation;cell death;cellular component organization;cellular component organization at cellular level;cellular component organization or biogenesis;cellular component organization
28.1021	28.4976	28.2004	28.4368	1:Experimental evidence at protein level	cellular macromolecule metabolic process;cellular metabolic process;cellular process;cellular protein metabolic process;macromolecule metabolic process;macromolecule modification;metabolic process;peptidyl-amino acid
27.3088	27.0024	27.3319	26.9233	1:Experimental evidence at protein level	apoptosis;biological regulation;cell death;cell surface receptor linked signaling pathway;cellular macromolecule metabolic process;cellular metabolic process;cellular process;cellular protein metabolic process;cellular respons
27.2258	27.3884	27.1391	27.0751	1:Experimental evidence at protein level	biological regulation;catabolic process;cation homeostasis;cellular catabolic process;cellular cation homeostasis;cellular chemical homeostasis;cellular homeostasis;cellular ion homeostasis;cellular iron ion homeostasis;cellu
27.3834	27.7094	27.4866	27.6308	1:Experimental evidence at protein level	amine metabolic process;AMP biosynthetic process;AMP metabolic process;aspartate family amino acid metabolic process;aspartate metabolic process;biosynthetic process;carboxylic acid metabolic process;cellular amine
26.621	26.5161	26.5749	26.4238	1:Experimental evidence at protein level	anatomical structure development;anatomical structure morphogenesis;apoptotic cell clearance;biological adhesion;biological regulation;blood vessel remodeling;cell activation;cell adhesion;cell communication;cell differenti
26.8968	26.8774	27.218	27.4385	1:Experimental evidence at protein level	biological regulation;cellular component organization;cellular component organization or biogenesis;cellular macromolecule metabolic process;cellular membrane organization;cellular metabolic process;cellular process;cellul
27.4282	26.3559	26.4364	26.93	1:Experimental evidence at protein level	adrenal gland development;aging;anatomical structure development;anatomical structure morphogenesis;anion transport;apoptosis;axon regeneration;axonogenesis;behavior;behavioral response to pain;biological regulation
27.64	28.031	27.8493	28.0287	1:Experimental evidence at protein level	aerobic respiration;AMP biosynthetic process;AMP metabolic process;biosynthetic process;cellular aromatic compound metabolic process;cellular biosynthetic process;cellular component assembly;cellular component orga
26.7146	26.6187	26.6325	26.784	1:Experimental evidence at protein level	biological regulation;cell cycle;cell cycle phase;cell cycle process;cellular component organization;cellular component organization at cellular level;cellular component organization or biogenesis;cellular component organizatio
27.0708	27.6767	27.5101	27.9066	1:Experimental evidence at protein level	anatomical structure development;biological regulation;calcium ion transport;cation transport;cellular chemical homeostasis;cellular homeostasis;cellular ion homeostasis;cellular process;cellular response to stimulus;chemica
27.9484	28.0554	28.0873	27.74	1:Experimental evidence at protein level	biological regulation;negative regulation of catalytic activity;negative regulation of endopeptidase activity;negative regulation of hydrolase activity;negative regulation of molecular function;negative regulation of peptidase acti
27.4914	27.0484	27.0125	27.2599	1:Experimental evidence at protein level	amine transport;amino acid transport;carboxylic acid transport;cellular process;establishment of localization;ion transport;nitrogen compound transport;organic acid transport;organic substance transport;transmembrane tran
25.8355	26.3975	26.1293	26.1285	1:Experimental evidence at protein level	alcohol catabolic process;alcohol metabolic process;carbohydrate metabolic process;catabolic process;ethanol catabolic process;ethanol metabolic process;metabolic process;primary alcohol catabolic process;primary alco
27.0874	26.8068	26.6935	26.6212	1:Experimental evidence at protein level	biological regulation;biosynthetic process;cellular biosynthetic process;cellular macromolecule biosynthetic process;cellular macromolecule metabolic process;cellular metabolic process;cellular nitrogen compound metabolic
27.804	28.1243	28.0832	27.9978	1:Experimental evidence at protein level	acetyl-CoA catabolic process;acetyl-CoA metabolic process;anatomical structure development;carboxylic acid metabolic process;catabolic process;cellular catabolic process;cellular ketone metabolic process;cellular metab
26.2968	24.4437	26.4655	24.7514	1:Experimental evidence at protein level	actin cytoskeleton organization;actin filament organization;actin filament-based process;anatomical structure homeostasis;biological adhesion;biological regulation;calcium ion transport;cation transport;cell activation;cell act
27.9046	28.3007	28.0708	27.9355	1:Experimental evidence at protein level	biological regulation;cellular process;cellular response to stimulus;establishment of localization;establishment of protein localization;intracellular signal transduction;negative regulation of axonogenesis;negative regulation of b
29.0675	29.3223	29.3599	29.2513	1:Experimental evidence at protein level	amine biosynthetic process;amine metabolic process;biosynthetic process;carboxylic acid biosynthetic process;carboxylic acid metabolic process;cellular amine metabolic process;cellular amino acid biosynthetic process;ce
27.6221	27.2963	26.7954	27.2203	1:Experimental evidence at protein level	acylglycerol catabolic process;acylglycerol metabolic process;amide biosynthetic process;amine biosynthetic process;amine catabolic process;amine metabolic process;anatomical structure development;anion homeostasis;
28.0199	28.0456	27.858	27.8964	1:Experimental evidence at protein level	biological regulation;biosynthetic process;cell cycle;cell cycle process;cellular biosynthetic process;cellular component assembly;cellular component organization;cellular component organization or biogenesis;cellular macror
29.4753	29.3099	29.5087	29.5175	1:Experimental evidence at protein level	androgen receptor signaling pathway;biological regulation;cell motility;cellular component movement;cellular localization;cellular macromolecule localization;cellular macromolecule metabolic process;cellular metabolic proce
26.3663	25.9797	26.352	26.1194	1:Experimental evidence at protein level	activation-induced cell death of T cells;aging;alcohol metabolic process;amine metabolic process;aminoglycan metabolic process;anatomical structure development;apoptosis;apoptotic mitochondrial changes;biological regu
25.2045	23.9345	25.0108	24.447	1:Experimental evidence at protein level	alcohol metabolic process;apoptosis;biological regulation;biosynthetic process;carbohydrate biosynthetic process;carbohydrate metabolic process;carbohydrate transport;cell death;cell differentiation;cell surface receptor lin
29.4165	29.4151	29.5359	29.3785	1:Experimental evidence at protein level	aerobic respiration;cellular metabolic process;cellular process;cellular respiration;electron transport chain;energy derivation by oxidation of organic compounds;generation of precursor metabolites and energy;metabolic proce
25.6716	26.0946	25.8692	26.0181	1:Experimental evidence at protein level	alcohol catabolic process;alcohol metabolic process;amine catabolic process;amine metabolic process;branched chain family amino acid catabolic process;branched chain family amino acid metabolic process;carbohydrate
29.0765	29.5436	29.5461	29.616	1:Experimental evidence at protein level	anatomical structure development;anatomical structure morphogenesis;aromatic compound biosynthetic process;biosynthetic process;brainstem development;cellular aromatic compound metabolic process;cellular biosynth
29.0286	28.899	29.2687	29.1595	1:Experimental evidence at protein level	cell differentiation;cellular developmental process;cellular macromolecule metabolic process;cellular metabolic process;cellular nitrogen compound metabolic process;cellular process;developmental process;epithelial cell diff
29.9305	29.6411	29.9222	29.7171	1:Experimental evidence at protein level	biological regulation;cellular macromolecule metabolic process;cellular metabolic process;cellular nitrogen compound metabolic process;cellular process;gene expression;macromolecule metabolic process;metabolic proces
30.3438	30.54	30.4289	30.448	1:Experimental evidence at protein level	activation of MAPKK activity;activation of protein kinase activity;apoptosis;axon guidance;biological regulation;biosynthetic process;cell death;cell surface receptor linked signaling pathway;cellular biosynthetic process;cellul
29.7355	29.9251	30.0078	30.056	1:Experimental evidence at protein level	response to stimulus;response to stress
30.6279	31.5688	31.5899	31.688	1:Experimental evidence at protein level	biological regulation;cellular process;cellular response to stimulus;negative regulation of biological process;negative regulation of biosynthetic process;negative regulation of cell proliferation;negative regulation of cellular bios
25.2812	25.207	25.162	22.1538	1:Experimental evidence at protein level	anatomical structure development;axon guidance;biological adhesion;biological regulation;blood coagulation;cell adhesion;cell adhesion mediated by integrin;cell migration;cell motility;cell surface receptor linked signaling pa
29.0048	29.2056	29.2808	29.301	1:Experimental evidence at protein level	biological regulation;biosynthetic process;catabolic process;cellular biosynthetic process;cellular catabolic process;cellular macromolecule metabolic process;cellular metabolic process;cellular nitrogen compound metabolic
26.8126	27.176	26.9348	27.2886	1:Experimental evidence at protein level	biosynthetic process;cellular aromatic compound metabolic process;cellular biosynthetic process;cellular metabolic process;cellular nitrogen compound biosynthetic process;cellular nitrogen compound metabolic process;ce
26.4288	26.5934	26.5392	26.7396	1:Experimental evidence at protein level	amine biosynthetic process;amine metabolic process;biological regulation;biosynthetic process;carboxylic acid biosynthetic process;carboxylic acid metabolic process;cellular amine metabolic process;cellular amino acid bio
27.4719	27.7987	27.5789	27.4774	1:Experimental evidence at protein level	biological regulation;cell surface receptor linked signaling pathway;cellular process;cellular response to chemical stimulus;cellular response to cytokine stimulus;cellular response to interferon-gamma;cellular response to orga
29.8815	29.2009	29.2514	29.3087	1:Experimental evidence at protein level	biosynthetic process;catabolic process;cellular biosynthetic process;cellular catabolic process;cellular component disassembly;cellular component disassembly at cellular level;cellular component organization;cellular compo
25.706	25.5919	25.6561	25.7088	1:Experimental evidence at protein level	acyl-CoA biosynthetic process;acyl-CoA metabolic process;acylglycerol biosynthetic process;acylglycerol metabolic process;adiponectin-mediated signaling pathway;biological regulation;biosynthetic process;carboxylic acid
28.4043	28.299	28.423	28.2818	1:Experimental evidence at protein level	axon guidance;biological regulation;cellular chemical homeostasis;cellular component disassembly;cellular component disassembly at cellular level;cellular component movement;cellular component organization;cellular com
26.7931	27.0333	27.3699	27.5173	1:Experimental evidence at protein level	biosynthetic process;cellular biosynthetic process;cellular macromolecule biosynthetic process;cellular macromolecule metabolic process;cellular metabolic process;cellular nitrogen compound metabolic process;cellular pro
29.0072	29.9059	29.7582	29.7966	1:Experimental evidence at protein level	2'-deoxyribonucleotide biosynthetic process;2'-deoxyribonucleotide metabolic process;biosynthetic process;catabolic process;cellular aromatic compound metabolic process;cellular biosynthetic process;cellular catabolic p
25.6417	25.8155	25.2357	25.6284	1:Experimental evidence at protein level	arachidonic acid metabolic process;carboxylic acid metabolic process;cellular alkene metabolic process;cellular ketone metabolic process;cellular lipid metabolic process;cellular metabolic process;cellular process;cobalami
26.6485	27.023	27.3794	27.4169	1:Experimental evidence at protein level	biological regulation;cell cycle phase;cell cycle process;cell division;cell proliferation;cellular process;meiosis I;regulation of biological process;regulation of catalytic activity;regulation of cell cycle;regulation of cellular metabo
31.1118	28.7029	30.2292	30.996	1:Experimental evidence at protein level	cellular component assembly;cellular component assembly at cellular level;cellular component organization;cellular component organization at cellular level;cellular component organization or biogenesis;cellular component c
25.1626	24.3512	24.8481	24.21	1:Experimental evidence at protein level	biological regulation;cell cycle checkpoint;cell cycle process;cellular component organization;cellular component organization at cellular level;cellular component organization or biogenesis;cellular component organization or
28.816	28.5956	28.5992	28.6064	1:Experimental evidence at protein level	biosynthetic process;cell cycle;cell cycle process;cellular biosynthetic process;cellular macromolecule biosynthetic process;cellular macromolecule metabolic process;cellular metabolic process;cellular nitrogen compound m
29.0665	28.8003	28.7932	28.6333	1:Experimental evidence at protein level	biosynthetic process;cell cycle;cell cycle process;cellular biosynthetic process;cellular macromolecule biosynthetic process;cellular macromolecule metabolic process;cellular metabolic process;cellular nitrogen compound m
28.4171	28.1545	28.2819	28.1353	1:Experimental evidence at protein level	biological regulation;biosynthetic process;cell cycle;cell cycle process;cell proliferation;cellular biosynthetic process;cellular macromolecule biosynthetic process;cellular macromolecule metabolic process;cellular metabolic p
27.2939	27.3022	27.4558	27.2757	1:Experimental evidence at protein level	amine biosynthetic process;amine catabolic process;amine metabolic process;betaine biosynthetic process;betaine metabolic process;biosynthetic process;carboxylic acid biosynthetic process;carboxylic acid catabolic proc
29.1082	29.2562	29.1829	29.1397	1:Experimental evidence at protein level	amine biosynthetic process;amine metabolic process;biological regulation;biosynthetic process;carboxylic acid biosynthetic process;carboxylic acid metabolic process;cellular amine metabolic process;cellular amino acid bio
29.2773	29.3655	29.2873	29.2615	1:Experimental evidence at protein level	cellular component assembly;cellular component assembly at cellular level;cellular component organization;cellular component organization at cellular level;cellular component organization or biogenesis;cellular component c
26.3861	26.6393	26.4028	26.3541	1:Experimental evidence at protein level	alcohol biosynthetic process;alcohol metabolic process;biosynthetic process;carbohydrate biosynthetic process;carbohydrate metabolic process;cellular biosynthetic process;cellular carbohydrate biosynthetic process;cellul
26.8159	26.3193	26.3094	26.556	1:Experimental evidence at protein level	amine metabolic process;aminoglycan biosynthetic process;aminoglycan catabolic process;aminoglycan metabolic process;anatomical structure formation involved in morphogenesis;axon guidance;biological regulation;bios
29.0852	29.948	29.6966	30.0168	1:Experimental evidence at protein level	actin cytoskeleton organization;actin filament-based process;biological regulation;cellular component organization;cellular component organization at cellular level;cellular component organization or biogenesis;cellular compo
29.0402	28.91	29.0399	28.8572	1:Experimental evidence at protein level	actin cytoskeleton organization;actin filament organization;actin filament-based process;adherens junction organization;aging;anatomical structure development;anatomical structure morphogenesis;apical junction assembly;a
27.6769	27.7585	27.5434	27.761	1:Experimental evidence at protein level	adherens junction assembly;adherens junction organization;anatomical structure development;anatomical structure formation involved in morphogenesis;anatomical structure homeostasis;anatomical structure morphogenesi
28.832	28.8612	28.8451	28.8188	1:Experimental evidence at protein level	biological regulation;biosynthetic process;cell differentiation;cellular biosynthetic process;cellular component organization;cellular component organization at cellular level;cellular component organization or biogenesis;cellula
28.0334	28.1024	28.07	27.8974	1:Experimental evidence at protein level	biological regulation;cellular process;cellular response to abiotic stimulus;cellular response to osmotic stress;cellular response to stimulus;cellular response to stress;multicellular organismal process;negative regulation of cata
28.1947	28.0886	28.358	28.3927	1:Experimental evidence at protein level	actin filament capping;anatomical structure development;apical protein localization;asymmetric protein localization;biological regulation;cell development;cell projection assembly;cell projection organization;cellular compone
27.5139	27.4137	28.0936	27.7023	1:Experimental evidence at protein level	anatomical structure homeostasis;base-excision repair;biological regulation;biosynthetic process;catabolic process;cell cycle;cell cycle process;cellular biosynthetic process;cellular catabolic process;cellular component asse
27.3202	27.1437	27.2729	27.0921	1:Experimental evidence at protein level	anatomical structure homeostasis;base-excision repair;biological regulation;biosynthetic process;cell cycle;cellular biosynthetic process;cellular component organization;cellular component organization at cellular level;cellula
26.5139	26.5129	26.848	26.1949	1:Experimental evidence at protein level	anatomical structure homeostasis;base-excision repair;biological regulation;biosynthetic process;cell cycle;cellular biosynthetic process;cellular component organization;cellular component organization at cellular level;cellula
26.2009	25.9838	25.964	25.6515	1:Experimental evidence at protein level	anatomical structure homeostasis;base-excision repair;biological regulation;biosynthetic process;cell cycle;cellular biosynthetic process;cellular component organization;cellular component organization at cellular level;cellula
30.3621	29.6737	30.1142	30.0513	1:Experimental evidence at protein level	alpha-beta T cell activation;alpha-beta T cell differentiation;biosynthetic process;catabolic process;cell activation;cell differentiation;cellular biosynthetic process;cellular catabolic process;cellular component disassembly;cell
27.6235	27.8642	27.5464	27.7655	1:Experimental evidence at protein level	biological regulation;biosynthetic process;cellular biosynthetic process;cellular macromolecule biosynthetic process;cellular macromolecule metabolic process;cellular metabolic process;cellular nitrogen compound metabolic
27.4496	24.3659	23.8978	24.0482	1:Experimental evidence at protein level	alkaloid metabolic process;anatomical structure development;anatomical structure formation involved in morphogenesis;angiogenesis;arachidonic acid metabolic process;behavior;biological regulation;biomineral tissue deve
25.5608	25.5163	25.3093	25.3862	1:Experimental evidence at protein level	biological adhesion;biological regulation;cell adhesion;cellular process;negative regulation of angiogenesis;negative regulation of biological process;negative regulation of developmental process;positive regulation of biologic
25.3651	24.5911	24.3596	23.8291	1:Experimental evidence at protein level	behavior;behavioral response to pain;biological adhesion;biological regulation;cell adhesion;cell migration;cell motility;cell-cell adhesion;cellular component movement;cellular process;cellular response to stimulus;cellular res
27.0634	27.2405	27.0414	27.0988	1:Experimental evidence at protein level	amine biosynthetic process;amine catabolic process;amine metabolic process;biosynthetic process;carboxylic acid biosynthetic process;carboxylic acid catabolic process;carboxylic acid metabolic process;catabolic process
25.8386	28.1155	27.7557	27.1657	1:Experimental evidence at protein level	anatomical structure development;cellular component organization;cellular component organization at cellular level;cellular component organization or biogenesis;cellular component organization or biogenesis at cellular leve
27.1208	26.8541	27.082	26.959	1:Experimental evidence at protein level	anatomical structure development;anatomical structure morphogenesis;biological regulation;cellular component disassembly;cellular component disassembly at cellular level;cellular component organization;cellular compone
27.8934	27.2856	27.9053	27.6294	1:Experimental evidence at protein level	anatomical structure formation involved in morphogenesis;anatomical structure morphogenesis;appendage morphogenesis;biological regulation;bone trabecula formation;cellular component disassembly;cellular component
25.3027	25.6506	25.8344	25.1261	1:Experimental evidence at protein level	alcohol metabolic process;biosynthetic process;carbohydrate biosynthetic process;carbohydrate catabolic process;carbohydrate metabolic process;catabolic process;cellular biosynthetic process;cellular carbohydrate biosy

32.2794	32.0055	32.0687	31.9136	1:Experimental evidence at protein level	actin cytoskeleton organization;actin cytoskeleton reorganization;actin filament-based movement;actin filament-based process;actomyosin structure organization;anatomical structure formation involved in morphogenesis;an
28.2998	27.8773	28.0945	27.8148	1:Experimental evidence at protein level	actin cytoskeleton organization;actin filament-based movement;actin filament-based process;actomyosin structure organization;adult heart development;anatomical structure development;anatomical structure formation invo
29.2048	29.1115	29.0229	29.0123	1:Experimental evidence at protein level	cellular component organization;cellular component organization at cellular level;cellular component organization or biogenesis;cellular component organization or biogenesis at cellular level;cellular process;COPI coating of G
27.0583	26.7217	26.744	26.7586	1:Experimental evidence at protein level	alcohol metabolic process;biological regulation;cell differentiation;cellular component assembly;cellular component organization;cellular component organization or biogenesis;cellular developmental process;cellular lipid met
27.205	26.9364	27.0105	27.0885	1:Experimental evidence at protein level	actin cytoskeleton organization;actin filament bundle assembly;actin filament capping;actin filament organization;actin filament-based process;anatomical structure homeostasis;anatomical structure morphogenesis;apoptosi
29.6561	29.7754	29.78	29.819	1:Experimental evidence at protein level	anatomical structure development;anatomical structure morphogenesis;biological regulation;blood coagulation;carboxylic acid metabolic process;cell migration;cell motility;cell surface receptor linked signaling pathway;cellul
24.4673	24.0022	24.3041	23.379	1:Experimental evidence at protein level	biological regulation;cellular process;cellular response to chemical stimulus;cellular response to organic substance;cellular response to stimulus;multicellular organismal process;negative regulation of biological process;negat
30.0266	30.2512	30.4013	30.2805	1:Experimental evidence at protein level	cellular macromolecule metabolic process;cellular metabolic process;cellular nitrogen compound metabolic process;cellular process;gene expression;macromolecule metabolic process;metabolic process;mRNA metabolic p
27.1329	26.8932	26.7776	26.843	1:Experimental evidence at protein level	biological regulation;biosynthetic process;carbohydrate metabolic process;carbohydrate transport;cell cycle;cell cycle process;cell surface receptor linked signaling pathway;cellular biosynthetic process;cellular component c
29.2467	28.9859	29.186	28.8213	1:Experimental evidence at protein level	biological regulation;biosynthetic process;cellular biosynthetic process;cellular component organization;cellular component organization at cellular level;cellular component organization or biogenesis;cellular component orga
27.289	28.86	28.29	29.3059	1:Experimental evidence at protein level	biological regulation;cell redox homeostasis;cellular chemical homeostasis;cellular homeostasis;cellular ion homeostasis;cellular macromolecule metabolic process;cellular metabolic process;cellular nitrogen compound meta
25.8704	25.9029	25.8242	26.1081	1:Experimental evidence at protein level	biological regulation;biosynthetic process;cell cycle arrest;cell cycle process;cell surface receptor linked signaling pathway;cellular biosynthetic process;cellular macromolecule biosynthetic process;cellular macromolecule me
26.2487	28.3351	28.6999	28.1389	1:Experimental evidence at protein level	anatomical structure development;cell activation;cell migration;cell motility;cell proliferation;cellular component movement;cellular process;developmental process;epidermis development;keratinization;keratinocyte activation
25.3306	25.8507	25.8514	26.0048	1:Experimental evidence at protein level	acyl-CoA metabolic process;amine catabolic process;amine metabolic process;anatomical structure development;biosynthetic process;branched chain family amino acid catabolic process;branched chain family amino acid r
29.1083	28.944	28.9793	28.939	1:Experimental evidence at protein level	activation of immune response;activation of innate immune response;anaphase-promoting complex-dependent proteasomal ubiquitin-dependent protein catabolic process;antigen processing and presentation;antigen proces
27.6469	27.9757	28.4829	28.2872	1:Experimental evidence at protein level	anatomical structure development;anatomical structure morphogenesis;biological regulation;cell development;cell part morphogenesis;cell projection morphogenesis;cell projection organization;cell surface receptor linked sig
27.1625	27.3382	27.1194	27.1627	1:Experimental evidence at protein level	activation of immune response;activation of innate immune response;activation of MAPK activity;activation of MAPKK activity;activation of protein kinase activity;axon guidance;biological regulation;cell surface receptor linke
28.2857	28.3893	28.3131	28.069	1:Experimental evidence at protein level	ATP biosynthetic process;ATP metabolic process;ATP synthesis coupled proton transport;biosynthetic process;cation transport;cellular biosynthetic process;cellular metabolic process;cellular nitrogen compound biosyntheti
26.3269	26.058	26.2127	26.1167	1:Experimental evidence at protein level	ATP hydrolysis coupled proton transport;biological regulation;cation homeostasis;cation transport;cell surface receptor linked signaling pathway;cellular cation homeostasis;cellular chemical homeostasis;cellular component c
26.2478	26.3801	26.4396	26.4921	1:Experimental evidence at protein level	biosynthetic process;cellular aromatic compound metabolic process;cellular biosynthetic process;cellular metabolic process;cellular nitrogen compound biosynthetic process;cellular nitrogen compound metabolic process;ce
31.0529	30.8107	30.7692	30.9125	1:Experimental evidence at protein level	biosynthetic process;catabolic process;cellular biosynthetic process;cellular catabolic process;cellular component disassembly;cellular component disassembly at cellular level;cellular component organization;cellular compo
27.6867	27.8452	27.7692	27.619	1:Experimental evidence at protein level	aging;biological regulation;catabolic process;cellular catabolic process;cellular component assembly;cellular component assembly at cellular level;cellular component organization;cellular component organization at cellular le
26.7054	26.9245	26.9444	27.0514	1:Experimental evidence at protein level	alcohol biosynthetic process;alcohol catabolic process;alcohol metabolic process;biosynthetic process;carbohydrate biosynthetic process;carbohydrate catabolic process;carbohydrate metabolic process;catabolic process;c
27.796	25.3205	28.0735	27.8638	1:Experimental evidence at protein level	acylglycerol catabolic process;acylglycerol metabolic process;biological regulation;carbohydrate metabolic process;catabolic process;cell cycle;cell differentiation;cell division;cell surface receptor linked signaling pathway;ce
27.07	27.3596	27.0778	27.2817	1:Experimental evidence at protein level	adaptive immune response;adaptive immune response based on somatic recombination of immune receptors built from immunoglobulin superfamily domains;biological regulation;cellular component biogenesis;cellular comp
25.3331	24.2458	24.4086	23.4244	1:Experimental evidence at protein level	aging;anatomical structure development;behavior;biological regulation;carbohydrate homeostasis;cell communication;cell proliferation;cellular chemical homeostasis;cellular glucose homeostasis;cellular homeostasis;cellular
27.0983	27.2674	27.1783	27.3526	1:Experimental evidence at protein level	acetyl-CoA catabolic process;acetyl-CoA metabolic process;amine catabolic process;amine metabolic process;aspartate family amino acid catabolic process;aspartate family amino acid metabolic process;carboxylic acid ca
25.6857	25.735	25.5488	25.7627	1:Experimental evidence at protein level	aging;amine metabolic process;arachidonic acid metabolic process;biological regulation;carboxylic acid metabolic process;catabolic process;cellular amine metabolic process;cellular amino acid metabolic process;cellular ca
28.671	29.1541	28.821	28.8339	1:Experimental evidence at protein level	biosynthetic process;cellular biosynthetic process;cellular macromolecule biosynthetic process;cellular macromolecule metabolic process;cellular metabolic process;cellular process;cellular protein metabolic process;cotrans
27.3549	27.244	27.4108	27.5334	1:Experimental evidence at protein level	aging;biological regulation;biosynthetic process;carbohydrate metabolic process;carbohydrate transport;cell aging;cell cycle;cell cycle process;cell death;cell surface receptor linked signaling pathway;cellular biosynthetic pro
24.1977	24.6848	25.2436	24.4874	1:Experimental evidence at protein level	biological regulation;cellular process;cellular response to stimulus;regulation of biological process;regulation of cellular process;response to stimulus;signal transduction
26.0915	26.3022	25.8438	25.8445	1:Experimental evidence at protein level	alcohol metabolic process;biosynthetic process;cellular biosynthetic process;cellular lipid metabolic process;cellular metabolic process;cellular process;cholesterol biosynthetic process;cholesterol metabolic process;farnesyl
32.4066	32.7444	32.6993	32.7007	1:Experimental evidence at protein level	cell differentiation;cellular developmental process;cellular process;developmental process;epithelial cell differentiation
29.763	29.8683	29.8265	29.8972	1:Experimental evidence at protein level	alcohol biosynthetic process;alcohol catabolic process;alcohol metabolic process;biosynthetic process;carbohydrate biosynthetic process;carbohydrate catabolic process;carbohydrate metabolic process;catabolic process;c
27.9847	27.739	27.7764	27.8566	1:Experimental evidence at protein level	cellular metabolic process;cellular process;electron transport chain;generation of precursor metabolites and energy;metabolic process;oxidation-reduction process;respiratory electron transport chain;small molecule metaboli
29.2651	28.4709	28.7776	28.7618	1:Experimental evidence at protein level	biological regulation;biosynthetic process;cell differentiation;cellular biosynthetic process;cellular component assembly;cellular component assembly at cellular level;cellular component organization;cellular component organi
27.7283	27.4387	27.4399	27.4476	1:Experimental evidence at protein level	ATP hydrolysis coupled proton transport;ATP metabolic process;biological regulation;cation homeostasis;cation transport;cell surface receptor linked signaling pathway;cellular cation homeostasis;cellular chemical homeosta
30.1889	30.474	30.5955	30.539	1:Experimental evidence at protein level	biological regulation;cellular macromolecule metabolic process;cellular metabolic process;cellular process;cellular protein metabolic process;establishment of localization;establishment of localization in cell;establishment of p
29.1386	28.8593	28.8864	28.8355	1:Experimental evidence at protein level	anatomical structure morphogenesis;biological regulation;catabolic process;cell surface receptor linked signaling pathway;cellular catabolic process;cellular macromolecule catabolic process;cellular macromolecule metaboli
31.8204	31.6495	31.6932	31.8208	1:Experimental evidence at protein level	biological regulation;biosynthetic process;catabolic process;cell chemotaxis;cell differentiation;cell migration;cell motility;cellular biosynthetic process;cellular catabolic process;cellular component assembly;cellular compone
31.1067	30.8321	30.8313	30.9406	1:Experimental evidence at protein level	biosynthetic process;catabolic process;cellular biosynthetic process;cellular catabolic process;cellular component disassembly;cellular component disassembly at cellular level;cellular component organization;cellular compo
24.3558	24.2881	24.1451	22.9083	1:Experimental evidence at protein level	anatomical structure formation involved in morphogenesis;anatomical structure morphogenesis;angiogenesis;biological adhesion;biological regulation;cell adhesion;cell morphogenesis;cell morphogenesis involved in differen
29.7399	29.5717	29.6175	29.4902	1:Experimental evidence at protein level	biosynthetic process;carbohydrate metabolic process;cell activation;cellular biosynthetic process;cellular carbohydrate metabolic process;cellular macromolecule biosynthetic process;cellular macromolecule metabolic proce
28.7873	28.7242	28.9857	29.0773	1:Experimental evidence at protein level	biological regulation;biosynthetic process;cellular biosynthetic process;cellular macromolecule biosynthetic process;cellular macromolecule metabolic process;cellular metabolic process;cellular nitrogen compound metabolic
29.1017	29.0158	28.9686	28.9102	1:Experimental evidence at protein level	anatomical structure homeostasis;base-excision repair;behavior;biological regulation;biosynthetic process;catabolic process;cell cycle;cellular biosynthetic process;cellular catabolic process;cellular component organization;c
27.2096	27.7597	27.9654	27.6917	1:Experimental evidence at protein level	actin filament capping;barbed-end actin filament capping;biological regulation;cell projection assembly;cell projection organization;cellular component assembly;cellular component assembly at cellular level;cellular compone
27.5992	27.4935	27.4718	27.2798	1:Experimental evidence at protein level	activation of adenylate cyclase activity;axon guidance;biological regulation;cellular component organization;cellular component organization at cellular level;cellular component organization or biogenesis;cellular component o
28.4702	28.2398	28.7281	28.7369	1:Experimental evidence at protein level	B cell activation;cell activation;cell proliferation;cellular process;establishment of localization;establishment of localization in cell;exocytosis;immune system process;leukocyte activation;lymphocyte activation;secretion;secret
30.4246	30.3105	30.2022	30.3273	1:Experimental evidence at protein level	binding of sperm to zona pellucida;cell recognition;cell-cell recognition;cellular macromolecule metabolic process;cellular metabolic process;cellular process;cellular process involved in reproduction;cellular protein metabolic
30.0437	29.9038	29.8622	29.8011	1:Experimental evidence at protein level	anatomical structure morphogenesis;cellular metabolic process;cellular process;developmental process;metabolic process;methylation;one-carbon metabolic process;organ morphogenesis;organ regeneration;regeneration;re
25.1233	26.2457	25.4174	25.9553	1:Experimental evidence at protein level	activation of immune response;activation of innate immune response;anaphase-promoting complex-dependent proteasomal ubiquitin-dependent protein catabolic process;anatomical structure morphogenesis;antigen proces
30.0161	29.6439	29.7326	29.5533	1:Experimental evidence at protein level	biological regulation;biosynthetic process;catabolic process;cellular biosynthetic process;cellular catabolic process;cellular component disassembly;cellular component disassembly at cellular level;cellular component organiz
26.5217	26.2294	26.1361	24.5713	1:Experimental evidence at protein level	activation of phospholipase D activity;biological regulation;cellular component organization;cellular component organization at cellular level;cellular component organization or biogenesis;cellular component organization or bi
28.1222	27.8065	27.7539	27.7135	1:Experimental evidence at protein level	acute inflammatory response;acute-phase response;anatomical structure development;astrocyte differentiation;behavior;biological regulation;carbohydrate homeostasis;cell differentiation;cell proliferation;cell surface recepto
30.2685	30.5013	30.165	30.4142	1:Experimental evidence at protein level	acetyl-CoA catabolic process;acetyl-CoA metabolic process;alcohol biosynthetic process;alcohol metabolic process;biological regulation;biosynthetic process;carbohydrate biosynthetic process;carbohydrate metabolic proc
30.0657	30.3887	30.778	30.6793	1:Experimental evidence at protein level	acetyl-CoA catabolic process;acetyl-CoA metabolic process;alcohol biosynthetic process;alcohol metabolic process;biosynthetic process;carbohydrate biosynthetic process;carbohydrate metabolic process;carboxylic acid r
27.1829	26.8057	26.8311	26.7846	1:Experimental evidence at protein level	anatomical structure homeostasis;base-excision repair;biological regulation;biosynthetic process;cell cycle;cellular biosynthetic process;cellular component organization;cellular component organization at cellular level;cellula
26.8081	26.5481	26.6362	26.5655	1:Experimental evidence at protein level	anatomical structure homeostasis;base-excision repair;biological regulation;biosynthetic process;cell cycle;cellular biosynthetic process;cellular component organization;cellular component organization at cellular level;cellula
28.432	28.3999	28.1976	28.1218	1:Experimental evidence at protein level	biosynthetic process;carboxylic acid catabolic process;carboxylic acid metabolic process;cardiolipin acyl-chain remodeling;cardiolipin metabolic process;catabolic process;cellular biosynthetic process;cellular catabolic proc
28.7321	28.7431	28.7217	28.6733	1:Experimental evidence at protein level	biosynthetic process;cellular biosynthetic process;cellular macromolecule biosynthetic process;cellular macromolecule metabolic process;cellular metabolic process;cellular process;cellular protein metabolic process;establis
25.0543	24.5964	24.783	24.6152	1:Experimental evidence at protein level	cap-independent translational initiation;cellular component assembly;cellular component assembly at cellular level;cellular component disassembly;cellular component disassembly at cellular level;cellular component organiza
26.4849	26.2684	26.271	26.2088	1:Experimental evidence at protein level	activation of JUN kinase activity;activation of MAPK activity;activation of protein kinase activity;activation of protein kinase B activity;ameboidal cell migration;anatomical structure development;anatomical structure formation
27.6898	27.3095	27.1798	27.2758	1:Experimental evidence at protein level	biological regulation;cellular macromolecule metabolic process;cellular metabolic process;cellular nitrogen compound metabolic process;cellular process;macromolecule metabolic process;metabolic process;mRNA metabol
27.6723	27.7465	27.7957	27.6922	1:Experimental evidence at protein level	cellular component organization;cellular component organization at cellular level;cellular component organization or biogenesis;cellular component organization or biogenesis at cellular level;cellular macromolecule metabolic
26.1027	26.3243	26.6104	26.6722	1:Experimental evidence at protein level	biological regulation;carbohydrate metabolic process;cellular carbohydrate metabolic process;cellular glucan metabolic process;cellular macromolecule metabolic process;cellular metabolic process;cellular polysaccharide m
25.5529	25.6068	25.6431	25.4343	1:Experimental evidence at protein level	activation of immune response;adherens junction organization;anatomical structure development;anatomical structure morphogenesis;antigen receptor-mediated signaling pathway;biological regulation;blood coagulation;bra
28.6777	28.6496	28.6133	28.606	1:Experimental evidence at protein level	amine metabolic process;amino acid activation;biosynthetic process;bis(5'-nucleosidyl) oligophosphate biosynthetic process;bis(5'-nucleosidyl) oligophosphate metabolic process;carboxylic acid metabolic process;cellular a
28.7555	28.4797	28.4915	28.2541	1:Experimental evidence at protein level	amine metabolic process;amino acid activation;carboxylic acid metabolic process;cell differentiation;cellular amine metabolic process;cellular amino acid metabolic process;cellular developmental process;cellular ketone met
28.5294	27.823	28.5337	28.5786	1:Experimental evidence at protein level	biological regulation;dosage compensation;dosage compensation, by inactivation of X chromosome;posttranscriptional regulation of gene expression;regulation of biological process;regulation of biosynthetic process;regulat
26.0375	26.3449	25.3904	25.5255	1:Experimental evidence at protein level	actin cytoskeleton organization;actin filament organization;actin filament-based process;anatomical structure development;biological regulation;cell development;cell junction assembly;cell junction organization;cell migration;
26.9477	27.1082	26.9516	26.9542	1:Experimental evidence at protein level	carboxylic acid catabolic process;carboxylic acid metabolic process;catabolic process;cellular catabolic process;cellular ketone metabolic process;cellular lipid catabolic process;cellular lipid metabolic process;cellular metab
28.8923	28.7258	29.0663	29.0432	1:Experimental evidence at protein level	biological regulation;regulation of biological process;regulation of biosynthetic process;regulation of cellular biosynthetic process;regulation of cellular macromolecule biosynthetic process;regulation of cellular metabolic proc
29.4262	29.1006	29.2655	29.2225	1:Experimental evidence at protein level	
28.461	28.3942	28.3309	28.2323	1:Experimental evidence at protein level	anatomical structure development;apoptosis;biological regulation;biosynthetic process;blood circulation;cell death;cell differentiation;cell differentiation involved in kidney development;cell differentiation involved in metaneph
26.279	26.1414	26.2772	25.8673	1:Experimental evidence at protein level	anatomical structure morphogenesis;biological regulation;biosynthetic process;cell fate commitment;cell proliferation;cell surface receptor linked signaling pathway;cellular biosynthetic process;cellular developmental process
27.2996	27.0159	26.9584	26.968	1:Experimental evidence at protein level	cellular macromolecule metabolic process;cellular metabolic process;cellular nitrogen compound metabolic process;cellular process;macromolecule metabolic process;maturation of 5.8S rRNA;metabolic process;mRNA met
25.4561	25.587	25.0653	25.5592	1:Experimental evidence at protein level	alcohol catabolic process;alcohol metabolic process;anatomical structure development;androgen biosynthetic process;androgen metabolic process;apocarotenoid metabolic process;arachidonic acid metabolic process;biolo
25.0094	25.1394	24.8823	24.6026	1:Experimental evidence at protein level	anatomical structure development;biological regulation;biosynthetic process;catabolic process;cell communication;cell cycle arrest;cell cycle process;cell development;cell growth;cell projection organization;cell surface rece
28.053	27.8999	27.8798	27.6635	1:Experimental evidence at protein level	activation of caspase activity;activation of caspase activity by cytochrome c;anatomical structure development;anatomical structure formation involved in morphogenesis;apoptosis;apoptotic mitochondrial changes;B cell ho
31.3077	31.0464	30.9412	31.3628	1:Experimental evidence at protein level	biological regulation;biosynthetic process;catabolic process;cell cycle;cell proliferation;cellular biosynthetic process;cellular catabolic process;cellular component disassembly;cellular component disassembly at cellular level;
28.6966	28.502	28.4117	28.4413	1:Experimental evidence at protein level	biological regulation;biosynthetic process;cellular biosynthetic process;cellular component movement;cellular macromolecule biosynthetic process;cellular macromolecule metabolic process;cellular metabolic process;cellula
26.6585	26.8258	26.7937	26.8616	1:Experimental evidence at protein level	alcohol metabolic process;biosynthetic process;carboxylic acid metabolic process;cellular ketone metabolic process;cellular lipid metabolic process;cellular metabolic process;cellular process;cellular response to chemical st
30.249	30.1465	30.4194	30.2109	1:Experimental evidence at protein level	biosynthetic process;catabolic process;cellular biosynthetic process;cellular catabolic process;cellular component disassembly;cellular component disassembly at cellular level;cellular component organization;cellular compo
28.3272	28.9629	29.0264	29.1132	1:Experimental evidence at protein level	aging;biological regulation;cell aging;cell cycle;cell cycle arrest;cell cycle process;cellular component assembly;cellular component assembly at cellular level;cellular component organization;cellular component organization a
26.7092	26.7243	26.7939	26.5275	1:Experimental evidence at protein level	acute inflammatory response;anatomical structure formation involved in morphogenesis;angiogenesis;angiogenesis involved in wound healing;biological regulation;blood coagulation;blood coagulation, intrinsic pathway;carb
25.8099	24.8903	25.783	25.6659	1:Experimental evidence at protein level	acidic amino acid transport;aging;amide biosynthetic process;amine metabolic process;amine transport;amino acid import;amino acid transport;anatomical structure development;anatomical structure formation involved in m
26.9842	27.0508	26.9663	26.7688	1:Experimental evidence at protein level	anatomical structure development;anatomical structure morphogenesis;biological regulation;bradykinin catabolic process;calcitonin catabolic process;catabolic process;cellular catabolic process;cellular hormone metabolic p
28.4783	28.5476	28.557	28.5395	1:Experimental evidence at protein level	acrosome assembly;actin cytoskeleton organization;actin filament-based process;adult behavior;adult locomotory behavior;alcohol metabolic process;ameboidal cell migration;amine metabolic process;anatomical structure c
27.3579	27.1801	26.972	26.9503	1:Experimental evidence at protein level	anatomical structure formation involved in morphogenesis;anatomical structure morphogenesis;angiogenesis;angiogenesis involved in wound healing;biological adhesion;biological regulation;cell adhesion;cellular process;de
30.0824	30.058	29.9852	29.9601	1:Experimental evidence at protein level	biological regulation;posttranscriptional regulation of gene expression;regulation of biological process;regulation of gene expression;regulation of macromolecule metabolic process;regulation of metabolic process
27.4136	27.2024	27.1735	27.1291	1:Experimental evidence at protein level	adaptive immune response;adaptive immune response based on somatic recombination of immune receptors built from immunoglobulin superfamily domains;anatomical structure development;B cell activation;B cell activati
27.5165	27.3175	27.4457	27.3613	1:Experimental evidence at protein level	alcohol biosynthetic process;alcohol catabolic process;alcohol metabolic process;alditol catabolic process;alditol metabolic process;anatomical structure development;biosynthetic process;camera-type eye development;ca
28.4826	28.4283	28.0849	28.8551	1:Experimental evidence at protein level	biological regulation;biosynthetic process;cellular biosynthetic process;cellular macromolecule biosynthetic process;cellular macromolecule metabolic process;cellular metabolic process;cellular process;cellular protein metab
30.5767	30.8071	31.1485	31.0314	1:Experimental evidence at protein level	biological regulation;cellular process;cellular response to stimulus;establishment of localization;establishment of localization in cell;intracellular transport;regulation of biological process;regulation of catalytic activity;regulation
29.6767	29.5949	29.6792	29.6065	1:Experimental evidence at protein level	alkaloid metabolic process;biological regulation;biosynthetic process;cell communication;cell-cell signaling;cellular amide metabolic process;cellular biosynthetic process;cellular metabolic process;cellular nitrogen compoun
24.5691	25.8471	24.4154	24.4021	1:Experimental evidence at protein level	establishment of localization;transport;vitamin transport
28.6175	28.6251	28.9368	28.714	1:Experimental evidence at protein level	activation of immune response;activation of innate immune response;anaphase-promoting complex-dependent proteasomal ubiquitin-dependent protein catabolic process;anatomical structure development;antigen processi
27.7553	27.7256	27.7532	27.6755	1:Experimental evidence at protein level	biological regulation;biosynthetic process;cellular biosynthetic process;cellular component organization;cellular component organization at cellular level;cellular component organization or biogenesis;cellular component orga
23.7447	25.315	25.4724	25.8869	1:Experimental evidence at protein level	cellular macromolecule metabolic process;cellular metabolic process;cellular process;cellular protein metabolic process;macromolecule metabolic process;macromolecule modification;metabolic process;peptidyl-amino acid
30.2042	29.4205	29.7954	29.7142	1:Experimental evidence at protein level	anion transport;biological regulation;establishment of localization;ion transport;negative regulation of biological process;negative regulation of cellular component organization;negative regulation of cellular process;negative r
27.8103	27.7417	27.7139	27.6676	1:Experimental evidence at protein level	biological regulation;blood coagulation;coagulation;hemostasis;multicellular organismal process;negative regulation of biological process;negative regulation of biosynthetic process;negative regulation of cellular biosynthetic
28.3566	28.5685	28.4071	28.4383	1:Experimental evidence at protein level	biological regulation;catabolic process;cellular catabolic process;cellular macromolecule catabolic process;cellular macromolecule metabolic process;cellular metabolic process;cellular process;cellular protein metabolic proc
27.3749	25.375	23.8768	28.609	1:Experimental evidence at protein level	activation of immune response;activation of innate immune response;anatomical structure development;apoptotic mitochondrial changes;biological regulation;cell projection organization;cellular component organization;cellu
27.8892	27.5015	27.7517	27.5657	1:Experimental evidence at protein level	cell cycle phase;cell cycle process;cell proliferation;cellular process;meiosis
25.9591	23.683	26.1152	25.685	1:Experimental evidence at protein level	alcohol metabolic process;carbohydrate catabolic process;carbohydrate metabolic process;catabolic process;cellular carbohydrate catabolic process;cellular carbohydrate metabolic process;cellular glucan metabolic proces
28.8048	28.6035	28.6167	28.4918	1:Experimental evidence at protein level	biological regulation;cell cycle;cellular process;cellular response to stimulus;intracellular signal transduction;mitotic cell cycle;negative regulation of biological process;negative regulation of intracellular protein transport;negat
27.9746	27.5749	27.7536	27.4691	1:Experimental evidence at protein level	biosynthetic process;cellular biosynthetic process;cellular macromolecule biosynthetic process;cellular macromolecule metabolic process;cellular metabolic process;cellular nitrogen compound metabolic process;cellular pro
26.7953	26.2269	26.4614	26.4413	1:Experimental evidence at protein level	biological regulation;cellular macromolecule metabolic process;cellular metabolic process;cellular nitrogen compound metabolic process;cellular process;macromolecule metabolic process;metabolic process;ncRNA metabo
27.253	27.2475	27.3144	27.3653	1:Experimental evidence at protein level	activation of MAPKK activity;activation of protein kinase activity;biological regulation;blood coagulation;cell activation;cell surface receptor linked signaling pathway;cellular macromolecule metabolic process;cellular metaboli
27.661	27.4782	27.537	27.5996	1:Experimental evidence at protein level	anatomical structure development;apoptosis;apoptosis in response to endoplasmic reticulum stress;biological regulation;brain development;catabolic process;cell cycle process;cell death;cell differentiation;cellular catabolic
27.8882	27.8602	27.6875	27.7081	1:Experimental evidence at protein level	ATP metabolic process;biological regulation;cation transport;cell communication;cell-cell signaling;cellular component organization;cellular component organization or biogenesis;cellular membrane fusion;cellular membrane
27.0162	27.0129	26.9624	26.8074	1:Experimental evidence at protein level	activation of immune response;activation of innate immune response;activation of MAPK activity;biological regulation;cardiac muscle contraction;cellular macromolecule metabolic process;cellular metabolic process;cellular p
23.1225	25.7881	26.0424	25.9706	1:Experimental evidence at protein level	biological regulation;cell cycle checkpoint;cellular component organization;cellular component organization at cellular level;cellular component organization or biogenesis;cellular component organization or biogenesis at cellu
30.8103	30.9152	30.6508	31.0962	1:Experimental evidence at protein level	biosynthetic process;catabolic process;cellular biosynthetic process;cellular catabolic process;cellular component disassembly;cellular component disassembly at cellular level;cellular component organization;cellular compo
30.7905	30.3895	30.5998	30.6467	1:Experimental evidence at protein level	biosynthetic process;catabolic process;cellular biosynthetic process;cellular catabolic process;cellular component biogenesis;cellular component biogenesis at cellular level;cellular component disassembly;cellular componen
30.4125	30.0718	30.1968	30.398	1:Experimental evidence at protein level	biosynthetic process;catabolic process;cellular biosynthetic process;cellular catabolic process;cellular component disassembly;cellular component disassembly at cellular level;cellular component organization;cellular compo
31.0729	31.0027	30.7311	30.971	1:Experimental evidence at protein level	biosynthetic process;catabolic process;cellular biosynthetic process;cellular catabolic process;cellular component disassembly;cellular component disassembly at cellular level;cellular component organization;cellular compo
31.0814	30.6698	30.721	30.7443	1:Experimental evidence at protein level	biological regulation;biosynthetic process;catabolic process;cellular biosynthetic process;cellular catabolic process;cellular component disassembly;cellular component disassembly at cellular level;cellular component organiz
29.8723	29.9571	29.5443	29.8572	1:Experimental evidence at protein level	biological regulation;biosynthetic process;catabolic process;cellular biosynthetic process;cellular catabolic process;cellular component disassembly;cellular component disassembly at cellular level;cellular component organiz
30.9	30.5396	30.7141	30.5657	1:Experimental evidence at protein level	biosynthetic process;catabolic process;cellular biosynthetic process;cellular catabolic process;cellular component disassembly;cellular component disassembly at cellular level;cellular component organization;cellular compo
28.7085	28.2914	28.556	28.5932	1:Experimental evidence at protein level	anatomical structure development;axon extension;biological regulation;cell growth;cell projection organization;cellular component movement;cellular component organization;cellular component organization at cellular level;c
26.9505	27.3634	27.4441	27.3811	1:Experimental evidence at protein level	alcohol catabolic process;alcohol metabolic process;amino sugar catabolic process;amino sugar metabolic process;carbohydrate catabolic process;carbohydrate metabolic process;catabolic process;cellular carbohydrate c
27.757	28.5004	28.369	28.6115	1:Experimental evidence at protein level	anatomical structure development;anatomical structure morphogenesis;biological regulation;biosynthetic process;cell differentiation;cell morphogenesis;cell proliferation;cellular biosynthetic process;cellular component morp
27.4922	27.37	27.2512	27.0643	1:Experimental evidence at protein level	aging;anatomical structure development;anatomical structure morphogenesis;biological regulation;cell differentiation;cell part morphogenesis;cell projection morphogenesis;cell projection organization;cellular component mo
29.6786	29.4171	29.376	29.3377	1:Experimental evidence at protein level	anatomical structure development;biological regulation;cell development;cell surface receptor linked signaling pathway;cellular developmental process;cellular metabolic process;cellular process;cellular response to calcium i
26.9684	26.8218	27.0881	27.0802	1:Experimental evidence at protein level	alcohol metabolic process;biosynthetic process;carbohydrate biosynthetic process;carbohydrate catabolic process;carbohydrate metabolic process;catabolic process;cellular biosynthetic process;cellular carbohydrate biosy
28.078	27.766	27.825	27.6293	1:Experimental evidence at protein level	carbohydrate metabolic process;cellular carbohydrate metabolic process;cellular macromolecule metabolic process;cellular metabolic process;cellular process;cellular protein metabolic process;co-translational protein modif
25.1776	26.5594	26.9433	26.8197	1:Experimental evidence at protein level	biological regulation;cellular component organization;cellular component organization or biogenesis;cellular process;cellular response to stimulus;establishment of localization;establishment of protein localization;intracellular
29.8697	29.4596	29.6751	29.5233	1:Experimental evidence at protein level	actin filament capping;barbed-end actin filament capping;biological regulation;blood coagulation;cellular component assembly;cellular component movement;cellular component organization;cellular component organization

30.2663	30.1611	30.1038	30.1744	1:Experimental evidence at protein level	actin cytoskeleton organization;actin filament capping;actin filament-based process;barbed-end actin filament capping;biological regulation;blood coagulation;cellular component movement;cellular component organization;c
28.0524	28.1189	28.3486	28.4152	1:Experimental evidence at protein level	biosynthetic process;cellular biosynthetic process;cellular macromolecule biosynthetic process;cellular macromolecule metabolic process;cellular metabolic process;cellular process;cellular protein metabolic process;gene ex
28.5051	28.4736	28.3883	28.3943	1:Experimental evidence at protein level	amine metabolic process;amino acid activation;anatomical structure development;brain development;carboxylic acid metabolic process;cellular amine metabolic process;cellular amino acid metabolic process;cellular ketone
28.8719	29.4117	29.1294	29.5083	1:Experimental evidence at protein level	biosynthetic process;catabolic process;cellular biosynthetic process;cellular catabolic process;cellular component disassembly;cellular component disassembly at cellular level;cellular component organization;cellular compo
28.0442	27.7626	28.1211	28.1219	1:Experimental evidence at protein level	cellular metabolic process;cellular process;electron transport chain;generation of precursor metabolites and energy;metabolic process;oxidation-reduction process;respiratory electron transport chain;response to antibiotic;re
28.3364	28.4371	28.2453	28.4531	1:Experimental evidence at protein level	ATP biosynthetic process;ATP metabolic process;ATP synthesis coupled proton transport;biosynthetic process;cation transport;cellular biosynthetic process;cellular metabolic process;cellular nitrogen compound biosynthetic
27.049	27.2203	27.632	27.4139	1:Experimental evidence at protein level	aging;biological regulation;cell aging;cell junction assembly;cell junction organization;cellular component assembly;cellular component assembly at cellular level;cellular component organization;cellular component organizatio
23.463	23.7548	25.0391	22.7765	1:Experimental evidence at protein level	adult behavior;adult locomotory behavior;ameboidal cell migration;anatomical structure development;anatomical structure morphogenesis;axon guidance;behavior;biological adhesion;biological regulation;blood circulation;br
26.6509	26.8847	26.8718	26.8451	1:Experimental evidence at protein level	macromolecule metabolic process;metabolic process;primary metabolic process;protein metabolic process;proteolysis
25.0494	23.9787	25.3814	25.1505	1:Experimental evidence at protein level	biosynthetic process;carbohydrate metabolic process;carboxylic acid metabolic process;cellular biosynthetic process;cellular component assembly;cellular component organization;cellular component organization or biogen
26.127	25.9979	26.143	26.5219	1:Experimental evidence at protein level	biological regulation;blood coagulation;coagulation;hemostasis;multicellular organismal process;regulation of biological quality;regulation of body fluid levels
29.4048	29.4082	29.3418	29.2306	1:Experimental evidence at protein level	adult behavior;adult locomotory behavior;anatomical structure maturation;behavior;cellular component organization;cellular component organization at cellular level;cellular component organization or biogenesis;cellular comp
27.2111	27.2258	27.0526	27.0143	1:Experimental evidence at protein level	alcohol metabolic process;biosynthetic process;cholesterol biosynthetic process;cholesterol metabolic process;lipid biosynthetic process;lipid metabolic process;metabolic process;primary metabolic process;small molecule
26.8634	25.1784	26.7013	26.7634	1:Experimental evidence at protein level	amine biosynthetic process;amine metabolic process;apoptotic mitochondrial changes;biological regulation;biosynthetic process;carboxylic acid biosynthetic process;carboxylic acid metabolic process;cellular amine metabo
29.1751	24.6151	29.0485	29.5054	1:Experimental evidence at protein level	biological adhesion;cell activation;cell adhesion;cell junction assembly;cell junction organization;cell migration;cell motility;cell proliferation;cell-substrate junction assembly;cellular component assembly;cellular component as
28.3311	28.2664	28.307	28.0641	1:Experimental evidence at protein level	activation of immune response;activation of innate immune response;anaphase-promoting complex-dependent proteasomal ubiquitin-dependent protein catabolic process;antigen processing and presentation;antigen proces
26.3085	26.1563	26.4474	26.3403	1:Experimental evidence at protein level	
26.0291	26.1248	26.6024	26.5668	1:Experimental evidence at protein level	aging;amine biosynthetic process;amine metabolic process;anatomical structure development;biosynthetic process;carboxylic acid biosynthetic process;carboxylic acid metabolic process;cellular amine metabolic process;ce
30.0759	30.224	30.2616	30.1988	1:Experimental evidence at protein level	binding of sperm to zona pellucida;cell recognition;cell-cell recognition;cellular macromolecule metabolic process;cellular metabolic process;cellular process;cellular process involved in reproduction;cellular protein metabolic
28.0729	27.0736	27.6425	27.598	1:Experimental evidence at protein level	anatomical structure development;anatomical structure morphogenesis;biological regulation;brain development;camera-type eye development;cell cycle process;cell part morphogenesis;cell projection morphogenesis;cell pr
26.8112	25.994	25.8367	23.106	1:Experimental evidence at protein level	
27.3849	27.2533	27.1122	26.8516	1:Experimental evidence at protein level	biological regulation;cell cycle phase;cell cycle process;cell division;cell surface receptor linked signaling pathway;cellular component organization;cellular component organization at cellular level;cellular component organiza
28.0271	28.2263	27.9907	28.0907	1:Experimental evidence at protein level	2-oxoglutarate metabolic process;acetyl-CoA catabolic process;acetyl-CoA metabolic process;carbohydrate metabolic process;carboxylic acid metabolic process;catabolic process;cellular aldehyde metabolic process;cellul
27.2212	27.933	27.4818	27.6478	1:Experimental evidence at protein level	biosynthetic process;cellular biosynthetic process;cellular lipid metabolic process;cellular metabolic process;cellular process;chordate embryonic development;developmental process;embryo development;embryo developm
27.3987	26.9936	27.1599	27.2578	1:Experimental evidence at protein level	biological adhesion;biological regulation;cell adhesion;cell communication;cell surface receptor linked signaling pathway;cell-cell signaling;cellular component movement;cellular process;cellular response to stimulus;defense
26.3797	25.8961	26.1918	26.1276	1:Experimental evidence at protein level	anatomical structure homeostasis;base-excision repair;biological regulation;biosynthetic process;cell cycle;cellular biosynthetic process;cellular component organization;cellular component organization at cellular level;cellula
26.9103	27.2615	27.4233	27.7324	1:Experimental evidence at protein level	biological regulation;positive regulation of biological process;positive regulation of cell proliferation;positive regulation of cellular process;regulation of biological process;regulation of cell proliferation;regulation of cellular proc
27.5468	27.6515	27.8795	27.8215	1:Experimental evidence at protein level	activation of MAPK activity;anatomical structure morphogenesis;biological adhesion;biological regulation;branching morphogenesis of a tube;cell adhesion;cell junction assembly;cell junction organization;cell morphogenesis
26.181	26.1027	25.6162	25.9684	1:Experimental evidence at protein level	3'-UTR-mediated mRNA stabilization;activation of immune response;activation of innate immune response;activation of MAPK activity;anatomical structure development;arachidonic acid metabolic process;biological regulati
28.3396	28.4097	28.444	28.2658	1:Experimental evidence at protein level	amine biosynthetic process;amine metabolic process;anatomical structure development;betaine biosynthetic process;betaine metabolic process;biological regulation;biosynthetic process;carboxylic acid biosynthetic process
31.2287	30.7976	30.9521	31.0048	1:Experimental evidence at protein level	biosynthetic process;catabolic process;cellular biosynthetic process;cellular catabolic process;cellular component disassembly;cellular component disassembly at cellular level;cellular component organization;cellular compo
28.8587	28.7232	28.709	28.5563	1:Experimental evidence at protein level	biological regulation;blood coagulation;carbohydrate metabolic process;cellular carbohydrate metabolic process;cellular component organization;cellular component organization at cellular level;cellular component organizati
29.1847	29.3796	29.3653	29.4316	1:Experimental evidence at protein level	anatomical structure development;ATP-dependent chromatin remodeling;biosynthetic process;blastocyst development;cell cycle;cell proliferation;cellular biosynthetic process;cellular component assembly;cellular componen
29.3563	29.392	29.3128	29.2131	1:Experimental evidence at protein level	acetyl-CoA metabolic process;acyl-CoA biosynthetic process;acyl-CoA metabolic process;acylglycerol biosynthetic process;acylglycerol metabolic process;amine metabolic process;biological regulation;biosynthetic process
25.4653	25.9083	26.0293	25.9813	1:Experimental evidence at protein level	amine biosynthetic process;amine catabolic process;amine metabolic process;biological regulation;biosynthetic process;carbohydrate homeostasis;carboxylic acid biosynthetic process;carboxylic acid metabolic process;cat
30.5361	30.5926	30.3827	30.3592	1:Experimental evidence at protein level	binding of sperm to zona pellucida;cell recognition;cell-cell recognition;cellular component assembly;cellular component assembly at cellular level;cellular component organization;cellular component organization at cellular le
26.2803	26.331	26.0906	26.2643	1:Experimental evidence at protein level	biosynthetic process;cellular biosynthetic process;cellular component disassembly;cellular component disassembly at cellular level;cellular component organization;cellular component organization at cellular level;cellular cor
29.6451	29.6474	29.7002	29.6541	1:Experimental evidence at protein level	biosynthetic process;cellular biosynthetic process;cellular component organization;cellular component organization at cellular level;cellular component organization or biogenesis;cellular component organization or biogenesis
28.991	28.9695	28.8519	28.8319	1:Experimental evidence at protein level	alcohol metabolic process;amine biosynthetic process;amine catabolic process;amine metabolic process;aspartate family amino acid catabolic process;aspartate family amino acid metabolic process;betaine biosynthetic pro
26.3005	25.4562	25.8639	25.4513	1:Experimental evidence at protein level	anatomical structure development;biological regulation;biosynthetic process;cell cycle;cell cycle checkpoint;cell cycle phase;cell cycle process;cell differentiation;cell division;cell proliferation;cellular biosynthetic process;cell
28.5473	28.6983	29.1082	28.7837	1:Experimental evidence at protein level	biological regulation;biosynthetic process;cellular biosynthetic process;cellular macromolecule biosynthetic process;cellular macromolecule metabolic process;cellular metabolic process;cellular process;cellular protein metab
27.6493	28.0811	27.9869	27.9911	1:Experimental evidence at protein level	alcohol metabolic process;amine metabolic process;biosynthetic process;CDP-choline pathway;cellular amine metabolic process;cellular biogenic amine metabolic process;cellular biosynthetic process;cellular lipid metabolic
28.795	28.8597	28.8896	28.6421	1:Experimental evidence at protein level	alanyl-tRNA aminoacylation;amine metabolic process;amino acid activation;anatomical structure development;biological regulation;carboxylic acid metabolic process;cellular amine metabolic process;cellular amino acid meta
28.0245	28.2272	28.258	28.0147	1:Experimental evidence at protein level	amine metabolic process;amino acid activation;carboxylic acid metabolic process;cellular amine metabolic process;cellular amino acid metabolic process;cellular ketone metabolic process;cellular macromolecule metabolic p
27.9987	28.0437	27.88	27.9044	1:Experimental evidence at protein level	amine metabolic process;amino acid activation;biosynthetic process;carboxylic acid metabolic process;cellular amine metabolic process;cellular amino acid metabolic process;cellular biosynthetic process;cellular ketone me
27.3439	27.2511	27.2873	27.4869	1:Experimental evidence at protein level	biological regulation;cellular component organization;cellular component organization at cellular level;cellular component organization or biogenesis;cellular component organization or biogenesis at cellular level;cellular macr
25.5402	25.6035	25.1438	25.1073	1:Experimental evidence at protein level	anatomical structure homeostasis;biological regulation;biosynthetic process;cell cycle;cell cycle process;cellular biosynthetic process;cellular component organization;cellular component organization at cellular level;cellular c
26.0524	25.9732	25.6883	25.699	1:Experimental evidence at protein level	anatomical structure homeostasis;biological regulation;biosynthetic process;cell cycle;cell cycle process;cellular biosynthetic process;cellular component organization;cellular component organization at cellular level;cellular c
26.2122	25.5598	25.3693	25.5987	1:Experimental evidence at protein level	apoptosis;apoptosis in response to endoplasmic reticulum stress;biological regulation;cell death;cellular process;death;macromolecule metabolic process;metabolic process;primary metabolic process;programmed cell death
25.6708	25.6727	25.8967	25.86	1:Experimental evidence at protein level	biological regulation;cellular component assembly;cellular component assembly at cellular level;cellular component organization;cellular component organization at cellular level;cellular component organization or biogenesis;
27.6303	27.4583	27.9503	27.8728	1:Experimental evidence at protein level	activation of immune response;activation of innate immune response;anaphase-promoting complex-dependent proteasomal ubiquitin-dependent protein catabolic process;antigen processing and presentation;antigen proces
28.9863	28.7322	29.0829	29.0234	1:Experimental evidence at protein level	activation of immune response;activation of innate immune response;anaphase-promoting complex-dependent proteasomal ubiquitin-dependent protein catabolic process;antigen processing and presentation;antigen proces
28.4556	27.9494	28.1006	27.9124	1:Experimental evidence at protein level	biosynthetic process;cell cycle;cell cycle process;cellular biosynthetic process;cellular component assembly;cellular component assembly at cellular level;cellular component organization;cellular component organization at c
24.719	24.8918	25.0796	25.1018	1:Experimental evidence at protein level	anatomical structure development;anatomical structure morphogenesis;apoptosis;appendage development;biological adhesion;biological regulation;cartilage development;cartilage development involved in endochondral bo
27.423	27.7382	27.6267	27.737	1:Experimental evidence at protein level	biological regulation;carboxylic acid catabolic process;carboxylic acid metabolic process;catabolic process;cell differentiation;cellular catabolic process;cellular developmental process;cellular ketone metabolic process;cellu
26.2252	26.3548	26.4314	26.1203	1:Experimental evidence at protein level	biological regulation;regulation of biological process;regulation of cellular component organization;regulation of cellular metabolic process;regulation of cellular process;regulation of chromosome organization;regulation of DN
29.3321	29.1902	28.4182	29.2422	1:Experimental evidence at protein level	acyl-CoA metabolic process;carboxylic acid metabolic process;cellular ketone metabolic process;cellular lipid metabolic process;cellular metabolic process;cellular process;coenzyme metabolic process;cofactor metabolic p
28.907	28.9039	29.0006	28.6859	1:Experimental evidence at protein level	anatomical structure development;cargo loading into vesicle;cellular component assembly;cellular component assembly at cellular level;cellular component organization;cellular component organization at cellular level;cellula
27.2553	27.2461	27.3016	27.1368	1:Experimental evidence at protein level	biological regulation;cellular macromolecule metabolic process;cellular metabolic process;cellular nitrogen compound metabolic process;cellular process;macromolecule metabolic process;metabolic process;mRNA metaboli
25.9443	25.6233	25.5777	25.9802	1:Experimental evidence at protein level	adherens junction organization;anatomical structure development;axon guidance;biological regulation;cell differentiation;cell division;cell junction organization;cell surface receptor linked signaling pathway;cell-cell junction or
26.143	26.3466	26.2208	26.0069	1:Experimental evidence at protein level	anatomical structure development;axon ensheathment;biological regulation;biosynthetic process;cell development;cellular biosynthetic process;cellular developmental process;cellular macromolecule biosynthetic process;ce
28.1953	30.1827	29.394	30.25	1:Experimental evidence at protein level	biological regulation;biosynthetic process;catabolic process;cellular biosynthetic process;cellular catabolic process;cellular macromolecule biosynthetic process;cellular macromolecule metabolic process;cellular metabolic p
26.6419	26.4158	26.535	26.5008	1:Experimental evidence at protein level	biological regulation;biosynthetic process;carbohydrate metabolic process;carbohydrate transport;cell cycle;cell cycle process;cell surface receptor linked signaling pathway;cellular biosynthetic process;cellular component a
27.8052	27.5165	27.6415	27.386	1:Experimental evidence at protein level	biological regulation;biosynthetic process;carbohydrate metabolic process;carbohydrate transport;cell cycle;cell cycle process;cell surface receptor linked signaling pathway;cellular biosynthetic process;cellular component c
27.6222	27.1425	27.5395	27.0844	1:Experimental evidence at protein level	cellular metabolic process;cellular process;electron transport chain;generation of precursor metabolites and energy;metabolic process;mitochondrial electron transport, NADH to ubiquinone;oxidation-reduction process;respi
26.8727	26.9595	26.9416	26.8657	1:Experimental evidence at protein level	anatomical structure development;anatomical structure morphogenesis;biological regulation;carbohydrate metabolic process;cardiac chamber morphogenesis;cardiac left ventricle morphogenesis;cardiac ventricle morphoge
27.1794	27.2546	27.0673	26.9481	1:Experimental evidence at protein level	anatomical structure development;anatomical structure formation involved in morphogenesis;anatomical structure morphogenesis;axon guidance;biological regulation;canonical Wnt receptor signaling pathway;canonical Wnt
27.1157	27.12	27.071	27.0677	1:Experimental evidence at protein level	adenosine metabolic process;catabolic process;cellular aromatic compound metabolic process;cellular catabolic process;cellular metabolic process;cellular nitrogen compound catabolic process;cellular nitrogen compound
26.6715	26.9848	26.9481	26.9464	1:Experimental evidence at protein level	cellular macromolecule metabolic process;cellular metabolic process;cellular process;cellular protein metabolic process;macromolecule metabolic process;macromolecule modification;metabolic process;primary metabolic p
28.8458	28.9737	28.819	28.938	1:Experimental evidence at protein level	amine metabolic process;biosynthetic process;carboxylic acid metabolic process;cellular amine metabolic process;cellular amino acid metabolic process;cellular aromatic compound metabolic process;cellular biosynthetic p
27.3301	27.3814	27.4512	27.5058	1:Experimental evidence at protein level	anatomical structure homeostasis;base-excision repair;biological regulation;biosynthetic process;cell cycle checkpoint;cell cycle process;cell proliferation;cellular biosynthetic process;cellular component organization;cellular
27.8936	27.726	27.8396	27.8424	1:Experimental evidence at protein level	acetyl-CoA catabolic process;acetyl-CoA metabolic process;carbohydrate metabolic process;catabolic process;cellular catabolic process;cellular metabolic process;cellular process;coenzyme catabolic process;coenzyme m
27.3268	28.5436	28.4815	29.0676	1:Experimental evidence at protein level	anatomical structure development;anatomical structure morphogenesis;biological regulation;cell proliferation;cellular process;cellular response to abiotic stimulus;cellular response to antibiotic;cellular response to chemical st
27.1181	26.4238	26.504	26.3897	1:Experimental evidence at protein level	anatomical structure development;anatomical structure formation involved in morphogenesis;anatomical structure morphogenesis;angiogenesis;astrocyte cell migration;biological regulation;branching morphogenesis of a tub
24.5451	24.761	24.9646	25.3501	1:Experimental evidence at protein level	biosynthetic process;cellular aromatic compound metabolic process;cellular biosynthetic process;cellular metabolic process;cellular nitrogen compound biosynthetic process;cellular nitrogen compound metabolic process;ce
30.1631	30.1698	30.1189	30.0806	1:Experimental evidence at protein level	biological regulation;cellular process;cellular response to stimulus;establishment of localization;establishment of protein localization;intracellular signal transduction;protein transport;regulation of biological process;regulation
29.0951	28.9572	28.8738	28.8403	1:Experimental evidence at protein level	anatomical structure development;biological regulation;cell cycle;cell cycle process;cell differentiation;cellular component disassembly;cellular component disassembly at cellular level;cellular component organization;cellular
27.4653	27.7782	27.6052	27.5115	1:Experimental evidence at protein level	acylglycerol metabolic process;alcohol metabolic process;amine metabolic process;amine transport;amino acid transmembrane transport;amino acid transport;behavior;betaine metabolic process;betaine transport;biological
26.7562	26.5634	26.9659	26.2529	1:Experimental evidence at protein level	biological regulation;negative regulation of catalytic activity;negative regulation of endopeptidase activity;negative regulation of hydrolase activity;negative regulation of molecular function;negative regulation of peptidase acti
26.9961	26.7274	26.8074	26.6911	1:Experimental evidence at protein level	biological regulation;cellular process;cellular response to chemical stimulus;cellular response to endogenous stimulus;cellular response to estrogen stimulus;cellular response to hormone stimulus;cellular response to organic
31.6129	31.5301	31.5097	31.3723	1:Experimental evidence at protein level	anatomical structure development;biological regulation;biosynthetic process;cell development;cellular component organization;cellular component organization at cellular level;cellular component organization or biogenesis;c
25.71	26.2543	26.5296	26.1257	1:Experimental evidence at protein level	
29.4922	29.6577	29.5813	29.7429	1:Experimental evidence at protein level	biological regulation;cellular component assembly;cellular component organization;cellular component organization or biogenesis;cellular macromolecule metabolic process;cellular metabolic process;cellular process;cellular
28.7544	29.1745	28.7563	28.7158	1:Experimental evidence at protein level	actin cytoskeleton organization;actin filament organization;actin filament-based process;actin polymerization or depolymerization;activation of immune response;anatomical structure formation involved in morphogenesis;anti
30.4259	30.5527	30.5929	30.449	1:Experimental evidence at protein level	anatomical structure morphogenesis;antigen processing and presentation;antigen processing and presentation of exogenous antigen;antigen processing and presentation of exogenous peptide antigen;antigen processing an
27.501	27.5428	27.7743	27.7327	1:Experimental evidence at protein level	biological regulation;cell surface receptor linked signaling pathway;cellular macromolecule metabolic process;cellular metabolic process;cellular process;cellular protein metabolic process;cellular response to stimulus;detecti
24.2671	24.4966	24.1474	24.5392	1:Experimental evidence at protein level	apoptosis;cell death;cellular metabolic process;cellular nitrogen compound metabolic process;cellular process;death;metabolic process;nitrogen compound metabolic process;nucleobase-containing compound metabolic pr
25.785	23.3388	26.056	26.2885	1:Experimental evidence at protein level	androgen receptor signaling pathway;biological regulation;biosynthetic process;catabolic process;cell cycle;cell cycle arrest;cell cycle process;cell division;cell proliferation;cellular biosynthetic process;cellular catabolic proce
28.3487	28.0019	28.3829	27.7759	1:Experimental evidence at protein level	biological regulation;cell cycle;cell cycle checkpoint;cell cycle phase;cell cycle process;cell division;cellular component assembly;cellular component organization;cellular component organization at cellular level;cellular comp
24.3665	23.8223	24.3695	24.0653	1:Experimental evidence at protein level	cellular process;endosome transport;establishment of localization;establishment of localization in cell;intracellular transport;transport;vesicle-mediated transport
26.3717	26.3614	26.6306	26.4621	1:Experimental evidence at protein level	biological adhesion;biological regulation;cell adhesion;cell-matrix adhesion;cell-substrate adhesion;cellular process;cellular response to stimulus;regulation of biological process;regulation of cellular process;response to stimu
27.9742	28.3345	28.4027	28.3121	1:Experimental evidence at protein level	adult behavior;adult locomotory behavior;anatomical structure development;associative learning;behavior;biological regulation;brain development;catabolic process;cation homeostasis;cell development;cellular catabolic pro
30.7398	30.4889	30.6187	30.453	1:Experimental evidence at protein level	biosynthetic process;catabolic process;cellular biosynthetic process;cellular catabolic process;cellular component biogenesis;cellular component biogenesis at cellular level;cellular component disassembly;cellular componen
30.3133	30.4507	30.3002	30.3313	1:Experimental evidence at protein level	binding of sperm to zona pellucida;cell recognition;cell-cell recognition;cellular component assembly;cellular component assembly at cellular level;cellular component organization;cellular component organization at cellular le
30.1889	30.272	30.136	30.1259	1:Experimental evidence at protein level	binding of sperm to zona pellucida;cell recognition;cell-cell recognition;cellular macromolecule metabolic process;cellular metabolic process;cellular process;cellular process involved in reproduction;cellular protein metabolic
27.7772	27.8387	27.9882	27.835	1:Experimental evidence at protein level	cell cycle;cell division;cellular component organization;cellular component organization or biogenesis;cellular membrane organization;cellular process;endocytosis;establishment of localization;membrane invagination;membra
26.1359	24.8241	25.9878	25.7842	1:Experimental evidence at protein level	biosynthetic process;cellular biosynthetic process;cellular macromolecule biosynthetic process;cellular macromolecule metabolic process;cellular metabolic process;cellular nitrogen compound metabolic process;cellular pro
29.0406	28.6906	28.8477	28.7343	1:Experimental evidence at protein level	anatomical structure development;apoptosis;biological regulation;cell death;cell differentiation;cellular developmental process;cellular process;death;developmental process;muscle organ development;muscle structure devel
27.1281	26.765	26.8755	26.8354	1:Experimental evidence at protein level	biological regulation;negative regulation of biological process;negative regulation of biosynthetic process;negative regulation of cellular biosynthetic process;negative regulation of cellular macromolecule biosynthetic process
29.7894	29.903	29.7794	29.8461	1:Experimental evidence at protein level	biological regulation;cellular process;cellular response to stimulus;establishment of localization;establishment of localization in cell;establishment of protein localization;intracellular protein transport;intracellular signal transdu
29.3928	29.4897	29.3278	29.3325	1:Experimental evidence at protein level	anatomical structure homeostasis;antigen processing and presentation;antigen processing and presentation of exogenous antigen;antigen processing and presentation of exogenous peptide antigen;antigen processing and p
25.119	25.4812	25.371	25.3886	1:Experimental evidence at protein level	biological regulation;cellular process;cellular response to stimulus;endosome transport;establishment of localization;establishment of localization in cell;establishment of protein localization;intracellular protein transport;intrac
27.6453	27.845	27.6548	27.4809	1:Experimental evidence at protein level	actin cytoskeleton organization;actin filament-based process;biological regulation;cell chemotaxis;cell junction assembly;cell junction organization;cell migration;cell motility;cell projection organization;cell-cell junction assem
25.6273	25.6775	25.1917	25.2424	1:Experimental evidence at protein level	antigen processing and presentation;biological regulation;blood coagulation;cell activation;cell activation involved in immune response;cell differentiation;cellular developmental process;cellular localization;cellular macromole
26.8455	24.8478	25.07	28.045	1:Experimental evidence at protein level	activation of caspase activity;apoptosis;autophagy;biological regulation;catabolic process;cell communication;cell death;cellular catabolic process;cellular metabolic process;cellular process;cellular response to amino acid s
22.9363	26.8948	26.8115	26.84	1:Experimental evidence at protein level	activation of immune response;activation of innate immune response;biological regulation;cell surface receptor linked signaling pathway;cellular macromolecule metabolic process;cellular metabolic process;cellular process;c
27.1357	26.6819	26.8424	26.6521	1:Experimental evidence at protein level	anatomical structure development;anatomical structure formation involved in morphogenesis;anatomical structure morphogenesis;aortic smooth muscle cell differentiation;appendage morphogenesis;ATP-dependent chroma
25.7535	26.268	26.0346	25.8859	1:Experimental evidence at protein level	acetyl-CoA catabolic process;acetyl-CoA metabolic process;carbohydrate metabolic process;carboxylic acid metabolic process;catabolic process;cellular catabolic process;cellular ketone metabolic process;cellular metabol
26.6313	26.8157	26.7559	26.8148	1:Experimental evidence at protein level	alcohol catabolic process;alcohol metabolic process;alditol metabolic process;carbohydrate catabolic process;carbohydrate metabolic process;catabolic process;cellular carbohydrate catabolic process;cellular carbohydrate
28.6629	28.9311	28.8956	28.8562	1:Experimental evidence at protein level	biosynthetic process;cellular biosynthetic process;cellular macromolecule biosynthetic process;cellular macromolecule metabolic process;cellular metabolic process;cellular process;cellular protein metabolic process;cotrans
29.289	29.0782	29.1471	29.0522	1:Experimental evidence at protein level	antigen processing and presentation;antigen processing and presentation of peptide antigen;antigen processing and presentation of peptide antigen via MHC class I;apoptosis;biological regulation;calcium ion homeostasis;c
25.8058	25.8771	25.9328	25.8897	1:Experimental evidence at protein level	cellular metabolic process;cellular nitrogen compound metabolic process;cellular process;metabolic process;methylation;nitrogen compound metabolic process;nucleobase-containing compound metabolic process;one-carb
26.9949	26.8806	26.661	26.7049	1:Experimental evidence at protein level	biological regulation;biosynthetic process;cell cycle;cellular biosynthetic process;cellular component organization;cellular component organization at cellular level;cellular component organization or biogenesis;cellular compo
24.7044	25.163	26.0594	24.5469	1:Experimental evidence at protein level	alcohol metabolic process;anatomical structure development;cellular aldehyde metabolic process;cellular lipid metabolic process;cellular metabolic process;cellular process;central nervous system development;development
27.9708	27.6705	27.7386	27.6628	1:Experimental evidence at protein level	anatomical structure development;androgen metabolic process;bile acid biosynthetic process;bile acid metabolic process;biological regulation;biosynthetic process;carboxylic acid biosynthetic process;carboxylic acid catab
28.4097	28.2064	28.2233	28.0175	1:Experimental evidence at protein level	activation of immune response;activation of innate immune response;anaphase-promoting complex-dependent proteasomal ubiquitin-dependent protein catabolic process;antigen processing and presentation;antigen proces
26.6206	26.5399	26.2397	26.3955	1:Experimental evidence at protein level	activation of immune response;activation of innate immune response;anatomical structure development;axon guidance;biological regulation;cell communication;cell cycle;cell surface receptor linked signaling pathway;cell-ce
28.8358	29.2097	29.3884	29.3061	1:Experimental evidence at protein level	biological regulation;cell proliferation;cellular macromolecule metabolic process;cellular metabolic process;cellular process;cellular protein metabolic process;cellular response to chemical stimulus;cellular response to organic
24.8205	24.4377	23.7272	25.2579	1:Experimental evidence at protein level	amine metabolic process;aminoglycan biosynthetic process;aminoglycan catabolic process;aminoglycan metabolic process;anatomical structure development;biological regulation;biosynthetic process;carbohydrate biosynth
25.666	25.7541	25.5873	25.7265	1:Experimental evidence at protein level	biological regulation;biosynthetic process;catabolic process;cell cycle;cell cycle process;cellular biosynthetic process;cellular catabolic process;cellular macromolecule biosynthetic process;cellular macromolecule catabolic p
25.9381	26.0759	26.0273	26.1862	1:Experimental evidence at protein level	adult heart development;anatomical structure development;biological regulation;biosynthetic process;catabolic process;cell cycle;cell cycle process;cell proliferation;cellular biosynthetic process;cellular catabolic process;cel
26.0077	24.7318	26.7893	26.4198	1:Experimental evidence at protein level	cellular metabolic process;cellular process;electron transport chain;generation of precursor metabolites and energy;metabolic process;mitochondrial electron transport, NADH to ubiquinone;oxidation-reduction process;respi
29.9568	29.5918	29.656	29.8621	1:Experimental evidence at protein level	cellular macromolecule metabolic process;cellular metabolic process;cellular nitrogen compound metabolic process;cellular process;gene expression;macromolecule metabolic process;metabolic process;mRNA metabolic p
29.8986	29.7463	29.8073	29.8228	1:Experimental evidence at protein level	alcohol biosynthetic process;alcohol catabolic process;alcohol metabolic process;aldonic acid metabolic process;biosynthetic process;carbohydrate biosynthetic process;carbohydrate catabolic process;carbohydrate metab
30.8998	31.0729	30.9667	30.9647	1:Experimental evidence at protein level	alternative nuclear mRNA splicing, via spliceosome;cellular macromolecule metabolic process;cellular metabolic process;cellular nitrogen compound metabolic process;cellular process;gene expression;macromolecule metab
29.96	29.9204	29.8836	29.8531	1:Experimental evidence at protein level	biological regulation;cell surface receptor linked signaling pathway;cellular macromolecule metabolic process;cellular metabolic process;cellular nitrogen compound metabolic process;cellular process;cellular response to che
27.4198	28.0383	27.5769	27.6968	1:Experimental evidence at protein level	apoptosis;biological regulation;catabolic process;cell death;cell surface receptor linked signaling pathway;cellular catabolic process;cellular component disassembly;cellular component disassembly at cellular level;cellular co
25.9924	25.8332	26.1639	26.0379	1:Experimental evidence at protein level	biological regulation;biosynthetic process;catabolic process;cellular biosynthetic process;cellular catabolic process;cellular component assembly;cellular component assembly at cellular level;cellular component organization;
27.1922	27.1094	27.0472	27.0615	1:Experimental evidence at protein level	actin cytoskeleton organization;actin filament-based process;biological regulation;calcium ion homeostasis;cation homeostasis;cellular calcium ion homeostasis;cellular cation homeostasis;cellular chemical homeostasis;cellu

30.0673	30.2006	30.4269	30.2343	1:Experimental evidence at protein level	biological regulation;cell surface receptor linked signaling pathway;cellular component movement;cellular process;cellular response to stimulus;enzyme linked receptor protein signaling pathway;intracellular signal transductio
26.0565	26.3547	26.7165	26.509	1:Experimental evidence at protein level	actin cytoskeleton organization;actin filament-based process;biological regulation;cellular component movement;cellular component organization;cellular component organization at cellular level;cellular component organizati
28.045	24.0518	28.3609	28.219	1:Experimental evidence at protein level	acrosome assembly;anatomical structure formation involved in morphogenesis;cellular component assembly;cellular component assembly at cellular level;cellular component assembly involved in morphogenesis;cellular com
28.8201	28.7347	28.9568	28.7751	1:Experimental evidence at protein level	biological regulation;cellular macromolecule metabolic process;cellular metabolic process;cellular nitrogen compound metabolic process;cellular process;gene expression;macromolecule metabolic process;metabolic process
26.6975	26.8056	26.7979	26.6419	1:Experimental evidence at protein level	biological regulation;biosynthetic process;cell surface receptor linked signaling pathway;cellular biosynthetic process;cellular macromolecule biosynthetic process;cellular macromolecule metabolic process;cellular metabolic
27.0405	27.1257	26.9573	26.8	1:Experimental evidence at protein level	B cell activation;B cell activation involved in immune response;biological regulation;cell activation;cell activation involved in immune response;cell cycle process;cellular macromolecule metabolic process;cellular metabolic pr
27.6856	27.7408	27.622	27.5924	1:Experimental evidence at protein level	antigen processing and presentation;antigen processing and presentation of exogenous antigen;antigen processing and presentation of exogenous peptide antigen;antigen processing and presentation of exogenous peptide
25.5449	25.2982	25.4784	25.1479	1:Experimental evidence at protein level	anatomical structure formation involved in morphogenesis;angiogenesis;axon guidance;biological regulation;blood coagulation;cell activation;cell migration;cell motility;cell projection assembly;cell projection organization;cell
28.0429	28.0054	28.2697	27.923	1:Experimental evidence at protein level	amine biosynthetic process;amine metabolic process;aspartate family amino acid metabolic process;biosynthetic process;carboxylic acid metabolic process;cellular amine metabolic process;cellular amino acid metabolic pro
27.0254	26.4714	26.5392	26.7875	1:Experimental evidence at protein level	alcohol catabolic process;alcohol metabolic process;apoptotic mitochondrial changes;biological regulation;body fluid secretion;carbohydrate catabolic process;carbohydrate homeostasis;carbohydrate metabolic process;car
27.2447	27.3426	27.7376	27.6201	1:Experimental evidence at protein level	biological regulation;biosynthetic process;cellular biosynthetic process;cellular component disassembly;cellular component disassembly at cellular level;cellular component organization;cellular component organization at cell
25.0698	25.9718	25.088	25.5764	1:Experimental evidence at protein level	anatomical structure development;anatomical structure morphogenesis;axis specification;biological regulation;bone development;calcium ion homeostasis;cation homeostasis;cell morphogenesis;cell morphogenesis involved
27.5355	27.8682	27.68	27.9364	1:Experimental evidence at protein level	biological regulation;cellular metabolic process;cellular process;cellular response to stimulus;intracellular signal transduction;metabolic process;peptide metabolic process;regulation of biological process;regulation of cellular
31.0896	31.204	31.2523	31.0897	1:Experimental evidence at protein level	actin filament capping;barbed-end actin filament capping;biological regulation;blood coagulation;cellular component assembly;cellular component movement;cellular component organization;cellular component organization
27.8791	23.9166	27.6391	27.7405	1:Experimental evidence at protein level	aging;anatomical structure morphogenesis;base-excision repair;biological regulation;cell aging;cell communication;cell cycle checkpoint;cell cycle phase;cell cycle process;cell differentiation;cell division;cell morphogenesis;c
25.3808	25.1097	26.5232	26.3091	1:Experimental evidence at protein level	anatomical structure development;biological regulation;developmental process;hemopoiesis;hemopoietic or lymphoid organ development;organ development;positive regulation of biological process;positive regulation of cell
27.4623	27.2359	27.3383	27.3567	1:Experimental evidence at protein level	biological regulation;biosynthetic process;carbohydrate metabolic process;carbohydrate transport;cell cycle;cell cycle process;cell surface receptor linked signaling pathway;cellular biosynthetic process;cellular component a
27.1672	27.1022	27.0376	27.0286	1:Experimental evidence at protein level	catabolic process;cellular catabolic process;cellular metabolic process;cellular nitrogen compound catabolic process;cellular nitrogen compound metabolic process;cellular process;cofactor catabolic process;cofactor metab
27.9757	27.5648	27.6762	27.6891	1:Experimental evidence at protein level	acyl-CoA biosynthetic process;acyl-CoA metabolic process;acylglycerol biosynthetic process;acylglycerol metabolic process;alcohol biosynthetic process;alcohol metabolic process;anion transport;biosynthetic process;carb
27.0354	27.3118	27.6085	27.5237	1:Experimental evidence at protein level	biological regulation;biosynthetic process;cell cycle phase;cell cycle process;cellular biosynthetic process;cellular component assembly;cellular component organization;cellular component organization at cellular level;cellula
26.1817	25.7281	25.8705	25.769	1:Experimental evidence at protein level	activation of anaphase-promoting complex activity;activation of mitotic anaphase-promoting complex activity;anaphase-promoting complex-dependent proteasomal ubiquitin-dependent protein catabolic process;biological r
25.1793	25.7815	25.5979	25.4169	1:Experimental evidence at protein level	actin cytoskeleton organization;actin filament-based process;biological regulation;cell projection assembly;cell projection organization;cellular component assembly;cellular component assembly at cellular level;cellular comp
26.6334	26.174	26.0722	26.5001	1:Experimental evidence at protein level	biological regulation;establishment of localization;establishment of localization in cell;establishment of protein localization;intracellular protein transport;intracellular transport;protein transport;regulation of actin cytoskeleton o
26.6775	26.9525	26.8767	26.9995	1:Experimental evidence at protein level	biological regulation;biosynthetic process;cation homeostasis;cell growth;cellular biosynthetic process;cellular cation homeostasis;cellular chemical homeostasis;cellular homeostasis;cellular ion homeostasis;cellular iron ion h
29.3446	29.4074	29.3159	29.2679	1:Experimental evidence at protein level	acyl-CoA biosynthetic process;acyl-CoA metabolic process;acylglycerol biosynthetic process;acylglycerol metabolic process;biological regulation;biosynthetic process;carbohydrate metabolic process;carboxylic acid metabo
27.4741	27.6814	27.588	27.6764	1:Experimental evidence at protein level	biological adhesion;biological regulation;cell adhesion;cell surface receptor linked signaling pathway;cell-cell adhesion;cellular macromolecule metabolic process;cellular metabolic process;cellular process;cellular protein me
26.8681	27.0471	27.0773	26.9794	1:Experimental evidence at protein level	acetyl-CoA catabolic process;acetyl-CoA metabolic process;carboxylic acid metabolic process;catabolic process;cellular catabolic process;cellular ketone metabolic process;cellular metabolic process;cellular process;coenz
26.6119	26.7661	26.3681	26.762	1:Experimental evidence at protein level	cellular macromolecule metabolic process;cellular metabolic process;cellular process;cellular protein metabolic process;macromolecule metabolic process;macromolecule modification;metabolic process;multicellular organis
29.1437	28.9786	28.9061	28.7867	1:Experimental evidence at protein level	cellular component organization;cellular component organization at cellular level;cellular component organization or biogenesis;cellular component organization or biogenesis at cellular level;cellular process;COPI coating of G
29.4298	29.3448	29.2251	29.207	1:Experimental evidence at protein level	biological regulation;body fluid secretion;cellular component organization;cellular component organization at cellular level;cellular component organization or biogenesis;cellular component organization or biogenesis at cellula
27.6931	27.6415	27.9135	27.862	1:Experimental evidence at protein level	activation of caspase activity;adaptive immune response;adaptive immune response based on somatic recombination of immune receptors built from immunoglobulin superfamily domains;aging;biological regulation;cell killin
28.2891	27.7849	27.8778	27.6516	1:Experimental evidence at protein level	antigen processing and presentation;antigen processing and presentation of exogenous antigen;antigen processing and presentation of exogenous peptide antigen;antigen processing and presentation of exogenous peptide
26.0765	26.3803	26.0354	26.2096	1:Experimental evidence at protein level	anatomical structure morphogenesis;developmental process;metabolic process;organ morphogenesis;oxidation-reduction process
25.4082	27.6132	27.1841	27.2459	1:Experimental evidence at protein level	developmental process;establishment of localization;establishment of localization in cell;establishment of protein localization;intracellular protein transport;intracellular transport;multicellular organismal development;multicellu
28.5823	28.3496	28.357	28.4031	1:Experimental evidence at protein level	anatomical structure development;developmental process;multicellular organismal process;muscle contraction;muscle organ development;muscle structure development;muscle system process;organ development;smooth m
26.4207	26.4193	26.5557	26.67	1:Experimental evidence at protein level	anion transport;behavioral response to nutrient;biological regulation;blood coagulation;carbohydrate homeostasis;carboxylic acid metabolic process;carboxylic acid transport;cell migration;cell motility;cellular component mo
26.8254	26.6116	26.5759	26.6275	1:Experimental evidence at protein level	abscission;biological regulation;cell division;cellular process;cytokinesis;developmental process;establishment of localization;establishment of protein localization;localization;macromolecule localization;positive regulation of a
28.8646	28.7604	28.6723	28.6997	1:Experimental evidence at protein level	antigen processing and presentation;antigen processing and presentation of exogenous antigen;antigen processing and presentation of exogenous peptide antigen;antigen processing and presentation of exogenous peptide
28.2906	28.9337	28.475	28.6009	1:Experimental evidence at protein level	biological regulation;cellular process;cellular response to stimulus;regulation of biological process;regulation of biosynthetic process;regulation of cellular biosynthetic process;regulation of cellular macromolecule biosynthetic
26.5821	26.255	26.8667	26.4585	1:Experimental evidence at protein level	anion transport;biological regulation;cell volume homeostasis;cellular component assembly;cellular component assembly at cellular level;cellular component organization;cellular component organization at cellular level;cellula
28.2141	28.0818	27.9953	27.9475	1:Experimental evidence at protein level	amine metabolic process;amino acid activation;arginyl-tRNA aminoacylation;carboxylic acid metabolic process;cellular amine metabolic process;cellular amino acid metabolic process;cellular ketone metabolic process;cellula
23.9287	22.8629	24.9948	23.9202	1:Experimental evidence at protein level	biological regulation;calcium ion transmembrane transport;calcium ion transport;cation transport;cellular process;divalent inorganic cation transport;divalent metal ion transport;establishment of localization;ion transmembran
28.8697	28.9826	28.7923	28.869	1:Experimental evidence at protein level	amine metabolic process;amino acid activation;apoptosis;biological regulation;carboxylic acid metabolic process;cell death;cellular amine metabolic process;cellular amino acid metabolic process;cellular ketone metabolic pr
28.3703	28.4724	28.4756	28.4407	1:Experimental evidence at protein level	biological regulation;catabolic process;cell communication;cell-cell signaling;cellular catabolic process;cellular macromolecule catabolic process;cellular macromolecule metabolic process;cellular metabolic process;cellular p
28.8543	28.8309	29.1992	29.0716	1:Experimental evidence at protein level	anatomical structure development;biological regulation;cell cycle phase;cell cycle process;cell development;cellular component disassembly;cellular component disassembly at cellular level;cellular component organization;c
28.8813	28.4867	28.6053	28.8127	1:Experimental evidence at protein level	biological regulation;blood coagulation;cation homeostasis;cation transport;cell migration;cell motility;cellular cation homeostasis;cellular chemical homeostasis;cellular component movement;cellular component organization
27.8796	28.5592	28.0182	28.6164	1:Experimental evidence at protein level	biological regulation;catabolic process;cellular catabolic process;cellular component organization;cellular component organization at cellular level;cellular component organization or biogenesis;cellular component organizatio
27.7923	28.714	28.597	28.6316	1:Experimental evidence at protein level	2'-deoxyribonucleotide metabolic process;ADP biosynthetic process;ADP metabolic process;AMP metabolic process;anatomical structure development;biosynthetic process;brain development;cellular biosynthetic process;c
28.091	27.8312	27.7905	27.7146	1:Experimental evidence at protein level	amine biosynthetic process;amine metabolic process;biosynthetic process;carboxylic acid biosynthetic process;carboxylic acid metabolic process;cellular amine metabolic process;cellular amino acid biosynthetic process;ce
27.1168	27.2324	27.2119	27.0391	1:Experimental evidence at protein level	anatomical structure development;apical protein localization;asymmetric protein localization;biological regulation;brain development;cell communication;cell differentiation;cell-cell signaling;cellular component disassembly;ce
28.9337	29.1842	29.0559	28.9428	1:Experimental evidence at protein level	biological regulation;biosynthetic process;cellular biosynthetic process;cellular macromolecule biosynthetic process;cellular macromolecule metabolic process;cellular metabolic process;cellular process;cellular protein metab
29.0408	28.5303	29.0605	29.0405	1:Experimental evidence at protein level	activation of immune response;activation of innate immune response;anaphase-promoting complex-dependent proteasomal ubiquitin-dependent protein catabolic process;antigen processing and presentation;antigen proces
26.5051	26.1323	26.1585	26.3302	1:Experimental evidence at protein level	biological regulation;cellular process;cellular response to stimulus;regulation of biological process;regulation of cellular process;response to stimulus;signal transduction
23.6899	25.2664	24.4516	23.3967	1:Experimental evidence at protein level	biological regulation;biosynthetic process;cell motility;cellular biosynthetic process;cellular component movement;cellular component organization;cellular component organization or biogenesis;cellular metabolic process;cell
29.495	29.4549	29.4199	29.3911	1:Experimental evidence at protein level	apoptosis;cell death;cell proliferation;cellular process;death;establishment of localization;establishment of localization in cell;establishment of protein localization;intracellular protein transport;intracellular transport;nuclear exp
24.7364	23.0468	24.0686	24.2928	1:Experimental evidence at protein level	apoptosis;apoptosis in response to endoplasmic reticulum stress;biological regulation;calcium ion homeostasis;cation homeostasis;cell death;cellular calcium ion homeostasis;cellular cation homeostasis;cellular chemical ho
30.3214	30.3904	30.5583	30.5009	1:Experimental evidence at protein level	activation of caspase activity;aggresome assembly;biological regulation;biosynthetic process;carbohydrate metabolic process;catabolic process;cellular biosynthetic process;cellular carbohydrate metabolic process;cellular c
26.7073	25.0739	26.4411	26.3829	1:Experimental evidence at protein level	cellular component organization;cellular component organization at cellular level;cellular component organization or biogenesis;cellular component organization or biogenesis at cellular level;cellular process;extracellular matr
28.5931	28.3601	28.2914	28.3279	1:Experimental evidence at protein level	biosynthetic process;carboxylic acid catabolic process;carboxylic acid metabolic process;cardiolipin acyl-chain remodeling;cardiolipin metabolic process;catabolic process;cellular biosynthetic process;cellular catabolic proc
28.4252	28.6093	28.8543	28.8609	1:Experimental evidence at protein level	biological regulation;circulatory system process;multicellular organismal process;positive regulation of blood pressure;positive regulation of systemic arterial blood pressure;regulation of anatomical structure size;regulation of
26.2127	26.4823	26.1992	25.8823	1:Experimental evidence at protein level	adherens junction organization;biological adhesion;biological regulation;cell adhesion;cell communication;cell junction assembly;cell junction organization;cell-cell junction organization;cell-cell signaling;cellular component as
30.2197	30.3577	30.7778	30.5252	1:Experimental evidence at protein level	biological regulation;biosynthetic process;cellular biosynthetic process;cellular component assembly;cellular component assembly at cellular level;cellular component organization;cellular component organization at cellular le
25.259	25.682	25.7724	25.8334	1:Experimental evidence at protein level	activation of caspase activity;activation of caspase activity by cytochrome c;apoptosis;biological regulation;cell death;cellular component disassembly;cellular component disassembly at cellular level;cellular component disa
27.6401	28.0751	28.0434	28.1629	1:Experimental evidence at protein level	2'-deoxyribonucleotide biosynthetic process;2'-deoxyribonucleotide metabolic process;adenosine metabolic process;AMP biosynthetic process;AMP metabolic process;AMP salvage;biological regulation;biosynthetic proces
27.7527	27.6822	27.542	27.4485	1:Experimental evidence at protein level	adenosine to inosine editing;anatomical structure development;base conversion or substitution editing;biological regulation;cell differentiation;cell surface receptor linked signaling pathway;cellular component assembly;cellul
26.2114	26.055	26.2696	26.2282	1:Experimental evidence at protein level	anatomical structure development;astrocyte development;axon extension;axon extension involved in development;axon extension involved in regeneration;axon guidance;biological adhesion;cell adhesion;cell development;c
28.075	28.1072	28.0095	28.0762	1:Experimental evidence at protein level	adherens junction organization;anatomical structure development;anatomical structure morphogenesis;axonogenesis;biological adhesion;cell adhesion;cell junction assembly;cell junction organization;cell part morphogenesis
25.6344	23.296	23.5382	23.6163	1:Experimental evidence at protein level	adherens junction organization;anatomical structure formation involved in morphogenesis;angiogenesis;biological adhesion;biological regulation;calcium-dependent cell-cell adhesion;cell adhesion;cell junction assembly;cell
28.7303	28.8176	29.0481	28.7595	1:Experimental evidence at protein level	antigen processing and presentation;antigen processing and presentation of exogenous antigen;antigen processing and presentation of exogenous peptide antigen;antigen processing and presentation of exogenous peptide
26.6331	26.7382	27.2358	27.2151	1:Experimental evidence at protein level	cellular component biogenesis;cellular component biogenesis at cellular level;cellular component organization or biogenesis;cellular component organization or biogenesis at cellular level;cellular macromolecule metabolic pro
28.2008	28.1003	28.0173	28.0133	1:Experimental evidence at protein level	antigen processing and presentation;antigen processing and presentation of peptide antigen;antigen processing and presentation of peptide antigen via MHC class I;cellular macromolecule metabolic process;cellular metabo
27.4521	27.1151	27.6256	27.376	1:Experimental evidence at protein level	cellular macromolecule metabolic process;cellular metabolic process;cellular nitrogen compound metabolic process;cellular process;gene expression;macromolecule metabolic process;metabolic process;mRNA metabolic pr
26.7498	27.0254	27.1654	27.0657	1:Experimental evidence at protein level	adipose tissue development;anatomical structure development;biological regulation;brain development;catabolic process;cellular catabolic process;cellular ketone body metabolic process;cellular ketone metabolic process;c
29.8729	29.6415	29.6796	29.5328	1:Experimental evidence at protein level	biological regulation;biosynthetic process;cellular biosynthetic process;cellular component assembly;cellular component assembly at cellular level;cellular component organization;cellular component organization at cellular le
27.2582	27.1966	27.4017	27.4539	1:Experimental evidence at protein level	activation of caspase activity;anatomical structure development;apoptosis;apoptotic mitochondrial changes;biological regulation;brain development;cell death;cellular component assembly;cellular component organization;ce
28.5527	28.6298	28.7304	28.5455	1:Experimental evidence at protein level	ATP biosynthetic process;ATP metabolic process;ATP synthesis coupled proton transport;biosynthetic process;cation transport;cellular biosynthetic process;cellular metabolic process;cellular nitrogen compound biosynthetic
26.2772	26.2904	26.0329	25.9222	1:Experimental evidence at protein level	cellular macromolecule metabolic process;cellular metabolic process;cellular nitrogen compound metabolic process;cellular process;macromolecule metabolic process;metabolic process;ncRNA metabolic process;ncRNA pr
28.7522	28.6494	28.5798	28.5796	1:Experimental evidence at protein level	amine metabolic process;amino acid activation;carboxylic acid metabolic process;cellular amine metabolic process;cellular amino acid metabolic process;cellular ketone metabolic process;cellular macromolecule metabolic p
26.4718	26.1127	26.1968	26.1994	1:Experimental evidence at protein level	activation of MAPK activity;axon guidance;biological adhesion;biological regulation;blood coagulation;cell adhesion;cell chemotaxis;cell migration;cell motility;cell surface receptor linked signaling pathway;cell-matrix adhesio
23.705	26.5732	26.1611	26.7329	1:Experimental evidence at protein level	biological regulation;cell cycle;cell cycle phase;cell cycle process;cell division;cell surface receptor linked signaling pathway;cellular component organization;cellular component organization at cellular level;cellular componen
28.0314	27.682	27.7112	27.7742	1:Experimental evidence at protein level	ATP biosynthetic process;ATP metabolic process;ATP synthesis coupled proton transport;biosynthetic process;cation transport;cellular biosynthetic process;cellular metabolic process;cellular nitrogen compound biosynthetic
28.3689	29.3449	28.5589	28.8564	1:Experimental evidence at protein level	ATP biosynthetic process;ATP metabolic process;ATP synthesis coupled proton transport;biosynthetic process;cation transport;cellular biosynthetic process;cellular metabolic process;cellular nitrogen compound biosynthetic
29.5268	29.258	29.5908	29.5491	1:Experimental evidence at protein level	cellular component assembly;cellular component assembly at cellular level;cellular component biogenesis;cellular component biogenesis at cellular level;cellular component organization;cellular component organization at ce
27.8846	27.9889	27.7046	27.9126	1:Experimental evidence at protein level	biological regulation;biosynthetic process;cell differentiation;cellular biosynthetic process;cellular developmental process;cellular macromolecule biosynthetic process;cellular macromolecule metabolic process;cellular metab
25.8358	26.348	25.0945	25.9964	1:Experimental evidence at protein level	cellular metabolic process;cellular process;electron transport chain;generation of precursor metabolites and energy;metabolic process;mitochondrial electron transport, NADH to ubiquinone;oxidation-reduction process;respi
24.5387	24.7771	24.6565	24.8412	1:Experimental evidence at protein level	actin cytoskeleton organization;actin filament organization;actin filament-based process;activation of immune response;antigen receptor-mediated signaling pathway;B cell receptor signaling pathway;biological adhesion;biolo
33.6674	31.5357	32.4627	32.9192	1:Experimental evidence at protein level	anatomical structure development;antibacterial humoral response;antimicrobial humoral response;defense response;defense response to bacterium;defense response to Gram-positive bacterium;developmental process;hum
27.2805	27.2218	27.0825	27.1536	1:Experimental evidence at protein level	cellular component assembly;cellular component assembly at cellular level;cellular component organization;cellular component organization at cellular level;cellular component organization or biogenesis;cellular component o
25.5766	25.6992	25.4345	25.3718	1:Experimental evidence at protein level	
28.5306	27.669	28.0486	28.1166	1:Experimental evidence at protein level	cellular component assembly;cellular component assembly at cellular level;cellular component organization;cellular component organization at cellular level;cellular component organization or biogenesis;cellular component o
24.9698	24.8767	25.0695	24.8296	1:Experimental evidence at protein level	actin cytoskeleton organization;actin filament organization;actin filament polymerization;actin filament-based process;actin polymerization or depolymerization;cellular component assembly;cellular component assembly at ce
27.4586	27.07	27.0865	27.0359	1:Experimental evidence at protein level	biological regulation;biosynthetic process;carbohydrate metabolic process;carbohydrate transport;cell cycle;cell cycle process;cell surface receptor linked signaling pathway;cellular biosynthetic process;cellular component a
26.1288	26.4501	26.2082	26.187	1:Experimental evidence at protein level	biological regulation;biosynthetic process;cellular biosynthetic process;cellular macromolecule biosynthetic process;cellular macromolecule metabolic process;cellular metabolic process;cellular process;macromolecule biosy
28.2171	28.5368	29.0665	28.9803	1:Experimental evidence at protein level	alcohol metabolic process;amine metabolic process;anatomical structure development;anatomical structure morphogenesis;benzene-containing compound metabolic process;biological regulation;catecholamine metabolic p
28.3454	28.3878	28.1567	27.9852	1:Experimental evidence at protein level	actin cytoskeleton organization;actin filament organization;actin filament polymerization;actin filament-based process;actin nucleation;actin polymerization or depolymerization;Arp2/3 complex-mediated actin nucleation;axon
23.9782	23.9744	25.7313	23.8759	1:Experimental evidence at protein level	activation of MAPK activity;biological regulation;biosynthetic process;cell proliferation;cellular biosynthetic process;cellular lipid metabolic process;cellular macromolecule metabolic process;cellular metabolic process;cellular
31.7887	32.4937	33.0673	32.7094	1:Experimental evidence at protein level	alcohol biosynthetic process;alcohol catabolic process;alcohol metabolic process;biosynthetic process;carbohydrate biosynthetic process;carbohydrate catabolic process;carbohydrate metabolic process;catabolic process;c
29.0384	28.9541	28.9808	28.9271	1:Experimental evidence at protein level	biological regulation;biosynthetic process;catabolic process;cellular biosynthetic process;cellular catabolic process;cellular component assembly;cellular component assembly at cellular level;cellular component organization;
29.354	30.4692	29.1896	29.4543	1:Experimental evidence at protein level	antigen processing and presentation;antigen processing and presentation of exogenous antigen;antigen processing and presentation of exogenous peptide antigen;antigen processing and presentation of exogenous peptide
27.429	27.6831	27.9812	27.9046	1:Experimental evidence at protein level	biological regulation;cellular metabolic process;cellular process;dephosphorylation;metabolic process;phosphate-containing compound metabolic process;phosphorus metabolic process;regulation of biological process;regu
27.7498	25.5448	27.4526	27.3927	1:Experimental evidence at protein level	aging;biological regulation;cell aging;cellular process;cellular response to chemical stimulus;cellular response to oxidative stress;cellular response to reactive oxygen species;cellular response to stimulus;cellular response to s
25.7856	26.5397	26.6461	26.5944	1:Experimental evidence at protein level	carbohydrate metabolic process;catabolic process;cellular carbohydrate metabolic process;cellular catabolic process;cellular macromolecule catabolic process;cellular macromolecule metabolic process;cellular metabolic pr
31.6966	31.5917	31.9215	31.9323	1:Experimental evidence at protein level	actin filament-based movement;actin filament-based process;actin-mediated cell contraction;actin-myosin filament sliding;anatomical structure development;axon guidance;biological regulation;cell surface receptor linked si
36.3358	36.6328	36.4478	36.5915	1:Experimental evidence at protein level	adherens junction organization;anatomical structure development;anatomical structure homeostasis;ATP-dependent chromatin remodeling;axon guidance;biological adhesion;biological regulation;blood coagulation;cell adhe
32.5021	32.4598	32.4189	32.3035	1:Experimental evidence at protein level	anatomical structure morphogenesis;biological regulation;biosynthetic process;catabolic process;cell surface receptor linked signaling pathway;cellular biosynthetic process;cellular catabolic process;cellular macromolecule b
31.506	31.0863	31.3577	31.4395	1:Experimental evidence at protein level	biosynthetic process;catabolic process;cellular biosynthetic process;cellular catabolic process;cellular component disassembly;cellular component disassembly at cellular level;cellular component organization;cellular compo
28.3712	28.3926	28.4545	28.1691	1:Experimental evidence at protein level	5-phosphoribose 1-diphosphate biosynthetic process;5-phosphoribose 1-diphosphate metabolic process;alcohol biosynthetic process;alcohol metabolic process;AMP biosynthetic process;AMP metabolic process;anatomic
28.8178	28.9925	29.2145	29.1807	1:Experimental evidence at protein level	activation of immune response;activation of innate immune response;anaphase-promoting complex-dependent proteasomal ubiquitin-dependent protein catabolic process;antigen processing and presentation;antigen proces
28.5385	27.8482	27.1927	27.4321	1:Experimental evidence at protein level	biological regulation;cellular component assembly;cellular component assembly at cellular level;cellular component organization;cellular component organization at cellular level;cellular component organization or biogenesis;
30.0015	30.2408	29.938	30.2487	1:Experimental evidence at protein level	actin cytoskeleton organization;actin filament branching;actin filament bundle assembly;actin filament organization;actin filament-based process;adherens junction organization;anatomical structure development;anatomical s
28.7719	29.0962	28.9846	29.0574	1:Experimental evidence at protein level	actin cytoskeleton organization;actin filament depolymerization;actin filament organization;actin filament severing;actin filament-based process;actin polymerization or depolymerization;biological regulation;cellular componen
27.7811	27.6899	28.0117	28.0125	1:Experimental evidence at protein level	anatomical structure development;behavior;biological regulation;cellular macromolecule metabolic process;cellular metabolic process;cellular process;cellular protein metabolic process;cellular response to stimulus;cognition
27.9597	28.3022	28.1227	27.9519	1:Experimental evidence at protein level	anatomical structure formation involved in morphogenesis;anatomical structure morphogenesis;axonogenesis;biological regulation;cell cycle;cell cycle process;cell part morphogenesis;cell projection assembly;cell projection
28.1952	28.117	28.1667	28.0962	1:Experimental evidence at protein level	biological regulation;biosynthetic process;cellular biosynthetic process;cellular macromolecule biosynthetic process;cellular macromolecule metabolic process;cellular metabolic process;cellular process;cellular protein metab
27.5065	27.3811	27.388	27.3073	1:Experimental evidence at protein level	biosynthetic process;cellular biosynthetic process;cellular component assembly;cellular component assembly at cellular level;cellular component organization;cellular component organization at cellular level;cellular compone
28.015	27.9552	27.9082	27.8627	1:Experimental evidence at protein level	biological regulation;cell cycle;cellular component organization;cellular component organization at cellular level;cellular component organization or biogenesis;cellular component organization or biogenesis at cellular level;cell
27.1611	27.0846	27.6523	27.6385	1:Experimental evidence at protein level	antigen processing and presentation;biological regulation;cellular component organization;cellular component organization at cellular level;cellular component organization or biogenesis;cellular component organization or bio
27.2516	27.709	27.698	27.9587	1:Experimental evidence at protein level	biological regulation;cell cycle;cell cycle process;cell division;cell proliferation;cellular process;G1/S transition of mitotic cell cycle;mitotic cell cycle;regulation of biological process;regulation of catalytic activity;regulation of c
28.8008	28.7147	28.667	28.5936	1:Experimental evidence at protein level	anatomical structure morphogenesis;antigen processing and presentation;axonogenesis;biological regulation;cell differentiation;cell part morphogenesis;cell projection morphogenesis;cell projection organization;cellular comp
29.8992	29.8766	29.6091	29.8564	1:Experimental evidence at protein level	activation of immune response;activation of innate immune response;apoptosis;biological regulation;biosynthetic process;BMP signaling pathway;catabolic process;cell death;cell surface receptor linked signaling pathway;ce
28.3732	28.7564	28.2752	28.6716	1:Experimental evidence at protein level	activation of immune response;activation of innate immune response;biological regulation;cell surface receptor linked signaling pathway;cellular macromolecule metabolic process;cellular metabolic process;cellular process;c
27.7721	27.5804	27.567	27.7435	1:Experimental evidence at protein level	apoptosis;apoptosis in response to endoplasmic reticulum stress;biological regulation;catabolic process;cell death;cellular catabolic process;cellular macromolecule catabolic process;cellular macromolecule metabolic proce
28.7291	29.0092	28.6622	28.7129	1:Experimental evidence at protein level	activation of immune response;activation of innate immune response;antigen receptor-mediated signaling pathway;biological regulation;catabolic process;cell surface receptor linked signaling pathway;cellular catabolic proce
28.7927	28.9659	28.9966	28.8524	1:Experimental evidence at protein level	biological regulation;cell surface receptor linked signaling pathway;cellular component assembly;cellular component assembly at cellular level;cellular component organization;cellular component organization at cellular level;c
29.4601	29.3588	29.3707	29.1592	1:Experimental evidence at protein level	actin cytoskeleton organization;actin filament organization;actin filament-based process;actin nucleation;anatomical structure morphogenesis;Arp2/3 complex-mediated actin nucleation;asymmetric cell division;axon guidanc
29.0782	28.9698	29.1247	28.8572	1:Experimental evidence at protein level	actin cytoskeleton organization;actin filament organization;actin filament-based process;actin nucleation;anatomical structure formation involved in morphogenesis;Arp2/3 complex-mediated actin nucleation;asymmetric cell d
29.6149	29.2111	29.3924	29.2152	1:Experimental evidence at protein level	antigen processing and presentation;antigen processing and presentation of exogenous antigen;antigen processing and presentation of exogenous peptide antigen;antigen processing and presentation of exogenous peptide
26.6935	26.9444	26.9111	26.6367	1:Experimental evidence at protein level	
27.9764	27.6083	27.9139	27.7271	1:Experimental evidence at protein level	biological regulation;biosynthetic process;cell differentiation;cell proliferation;cellular biosynthetic process;cellular developmental process;cellular macromolecule biosynthetic process;cellular macromolecule metabolic proces

30.6866	30.5476	30.6459	30.3571	1:Experimental evidence at protein level	biological regulation;biosynthetic process;cellular biosynthetic process;cellular lipid metabolic process;cellular metabolic process;cellular process;cellular response to stimulus;establishment of localization;establishment of pr
28.2938	28.4267	28.3683	28.4093	1:Experimental evidence at protein level	biological regulation;catabolic process;cellular catabolic process;cellular macromolecule catabolic process;cellular macromolecule metabolic process;cellular metabolic process;cellular nitrogen compound metabolic process
24.1669	25.7696	25.8229	24.3016	1:Experimental evidence at protein level	anatomical structure development;biological regulation;blood coagulation;cell activation;cell development;cell proliferation;cellular developmental process;cellular process;cellular response to cAMP;cellular response to chem
31.2641	30.7997	31.0851	30.9814	1:Experimental evidence at protein level	biological regulation;biosynthetic process;catabolic process;cell differentiation;cellular biosynthetic process;cellular catabolic process;cellular component disassembly;cellular component disassembly at cellular level;cellular c
31.049	30.7304	30.8318	31.0292	1:Experimental evidence at protein level	biosynthetic process;catabolic process;cellular biosynthetic process;cellular catabolic process;cellular component biogenesis;cellular component biogenesis at cellular level;cellular component disassembly;cellular componer
28.0045	27.9357	28.022	27.9132	1:Experimental evidence at protein level	activation of immune response;activation of innate immune response;anaphase-promoting complex-dependent proteasomal ubiquitin-dependent protein catabolic process;antigen processing and presentation;antigen proces
31.0873	31.0482	30.8306	30.9519	1:Experimental evidence at protein level	biosynthetic process;catabolic process;cellular biosynthetic process;cellular catabolic process;cellular component disassembly;cellular component disassembly at cellular level;cellular component organization;cellular compo
29.1054	28.965	28.8882	28.8836	1:Experimental evidence at protein level	biological regulation;biosynthetic process;catabolic process;cellular biosynthetic process;cellular catabolic process;cellular macromolecule biosynthetic process;cellular macromolecule catabolic process;cellular macromolec
30.8243	30.4206	30.6569	30.8081	1:Experimental evidence at protein level	biosynthetic process;catabolic process;cellular biosynthetic process;cellular catabolic process;cellular component disassembly;cellular component disassembly at cellular level;cellular component organization;cellular compo
27.1369	27.079	27.3891	26.8735	1:Experimental evidence at protein level	anatomical structure development;anatomical structure formation involved in morphogenesis;ATP hydrolysis coupled proton transport;biological regulation;brain development;cation homeostasis;cation transport;cell projectio
31.0166	30.705	30.7999	30.922	1:Experimental evidence at protein level	biosynthetic process;catabolic process;cellular biosynthetic process;cellular catabolic process;cellular component disassembly;cellular component disassembly at cellular level;cellular component organization;cellular compo
31.1374	31.2656	31.0102	31.1108	1:Experimental evidence at protein level	actin cytoskeleton organization;actin filament-based process;anatomical structure development;anatomical structure morphogenesis;apical junction assembly;apolipoprotein A-I-mediated signaling pathway;axon guidance;bi
25.6009	25.5925	25.2038	25.4923	1:Experimental evidence at protein level	actin cytoskeleton organization;actin filament-based process;biological adhesion;biological regulation;cell adhesion;cellular component organization;cellular component organization at cellular level;cellular component organiz
29.7157	30.0998	30.3761	30.3417	1:Experimental evidence at protein level	activation of caspase activity;biological regulation;cell differentiation;cellular developmental process;cellular macromolecule metabolic process;cellular metabolic process;cellular process;cellular protein metabolic process;de
29.1623	28.8589	28.917	28.9255	1:Experimental evidence at protein level	antigen processing and presentation;antigen processing and presentation of exogenous antigen;antigen processing and presentation of exogenous peptide antigen;antigen processing and presentation of exogenous peptide
27.6365	27.6994	27.9705	28.0968	1:Experimental evidence at protein level	cellular macromolecule metabolic process;cellular metabolic process;cellular process;cellular protein metabolic process;'de novo' posttranslational protein folding;'de novo' protein folding;macromolecule metabolic process;r
26.9094	26.7399	26.6831	26.6759	1:Experimental evidence at protein level	axon target recognition;biological adhesion;biological regulation;cell adhesion;cell communication;cell-cell adhesion;cell-cell signaling;cellular component organization;cellular component organization at cellular level;cellular c
29.5623	29.7252	30.5993	30.1869	1:Experimental evidence at protein level	anatomical structure homeostasis;antigen processing and presentation;antigen processing and presentation of exogenous antigen;antigen processing and presentation of exogenous peptide antigen;antigen processing and p
27.6205	27.9786	27.7353	27.5133	1:Experimental evidence at protein level	anatomical structure development;apoptosis;biological regulation;blastocyst development;carbohydrate metabolic process;cell death;cellular carbohydrate metabolic process;cellular macromolecule metabolic process;cellula
24.3672	26.683	26.4668	26.4558	1:Experimental evidence at protein level	alcohol metabolic process;biological regulation;chemical homeostasis;cholesterol efflux;cholesterol homeostasis;cholesterol metabolic process;cholesterol transport;establishment of localization;establishment of localization
28.5057	28.2107	28.1762	27.9508	1:Experimental evidence at protein level	cellular component organization;cellular component organization at cellular level;cellular component organization or biogenesis;cellular component organization or biogenesis at cellular level;cellular process;COPI coating of G
28.8696	24.3993	29.3086	29.3288	1:Experimental evidence at protein level	biosynthetic process;catabolic process;cellular biosynthetic process;cellular catabolic process;cellular component disassembly;cellular component disassembly at cellular level;cellular component organization;cellular compo
28.2203	29.7959	27.7323	28.809	1:Experimental evidence at protein level	biological regulation;cellular macromolecule metabolic process;cellular metabolic process;cellular process;cellular protein metabolic process;macromolecule metabolic process;macromolecule modification;metabolic process
26.0369	26.4218	26.356	26.332	1:Experimental evidence at protein level	cellular macromolecule metabolic process;cellular metabolic process;cellular process;cellular protein metabolic process;developmental process;macromolecule metabolic process;macromolecule modification;metabolic proc
25.1108	26.6548	26.9397	26.2687	1:Experimental evidence at protein level	anatomical structure development;biological regulation;biosynthetic process;cellular biosynthetic process;cellular component organization;cellular component organization at cellular level;cellular component organization or b
27.7952	29.3703	29.1148	28.9015	1:Experimental evidence at protein level	establishment of localization;establishment of localization in cell;establishment of protein localization;intracellular protein transport;intracellular transport;nuclear export;nuclear transport;nucleocytoplasmic transport;protein e
30.9824	30.9382	31.0947	31.0138	1:Experimental evidence at protein level	biological regulation;cellular macromolecule metabolic process;cellular metabolic process;cellular nitrogen compound metabolic process;cellular process;cellular response to stimulus;gene expression;macromolecule metabo
32.205	32.4619	32.3328	32.1981	1:Experimental evidence at protein level	apoptosis;biological regulation;biosynthetic process;cell cycle;cell cycle process;cell death;cellular biosynthetic process;cellular component organization;cellular component organization at cellular level;cellular component or
27.3109	27.5304	27.3933	27.0609	1:Experimental evidence at protein level	biological regulation;cell differentiation;cellular developmental process;cellular process;cellular response to stimulus;developmental process;intracellular signal transduction;metabolic process;osteoblast differentiation;positive
25.706	25.6874	26.4336	26.3504	1:Experimental evidence at protein level	cellular component organization;cellular component organization at cellular level;cellular component organization or biogenesis;cellular component organization or biogenesis at cellular level;cellular macromolecule metabolic
26.279	24.8147	25.6933	25.251	1:Experimental evidence at protein level	biological regulation;establishment of localization;establishment of protein localization;establishment of protein localization in membrane;establishment of protein localization in plasma membrane;macromolecule metabolic pr
30.5661	30.1478	30.4341	30.3568	1:Experimental evidence at protein level	biosynthetic process;catabolic process;cellular biosynthetic process;cellular catabolic process;cellular component biogenesis;cellular component biogenesis at cellular level;cellular component disassembly;cellular componer
29.6721	29.6401	29.7681	29.916	1:Experimental evidence at protein level	acylglycerol catabolic process;acylglycerol metabolic process;anatomical structure development;anatomical structure morphogenesis;biological regulation;branching morphogenesis of a tube;carbohydrate metabolic process
28.7252	28.7959	28.6833	28.7413	1:Experimental evidence at protein level	acylglycerol catabolic process;acylglycerol metabolic process;biological regulation;carbohydrate metabolic process;catabolic process;cell cycle;cell cycle process;cell division;cell surface receptor linked signaling pathway;ce
29.2206	29.2076	29.2288	29.148	1:Experimental evidence at protein level	activation of immune response;activation of innate immune response;anaphase-promoting complex-dependent proteasomal ubiquitin-dependent protein catabolic process;antigen processing and presentation;antigen proces
28.2973	28.3418	28.4822	28.2684	1:Experimental evidence at protein level	activation of immune response;activation of innate immune response;anaphase-promoting complex-dependent proteasomal ubiquitin-dependent protein catabolic process;antigen processing and presentation;antigen proces
31.5208	31.5042	31.5075	31.5534	1:Experimental evidence at protein level	biosynthetic process;catabolic process;cellular biosynthetic process;cellular catabolic process;cellular component disassembly;cellular component disassembly at cellular level;cellular component organization;cellular compo
30.7465	30.2253	30.4899	30.2056	1:Experimental evidence at protein level	biological regulation;biosynthetic process;catabolic process;cellular biosynthetic process;cellular catabolic process;cellular component disassembly;cellular component disassembly at cellular level;cellular component organiz
31.3412	30.8071	30.8808	30.8687	1:Experimental evidence at protein level	biosynthetic process;catabolic process;cellular biosynthetic process;cellular catabolic process;cellular component biogenesis;cellular component biogenesis at cellular level;cellular component disassembly;cellular componer
30.7521	30.8737	30.9125	30.8765	1:Experimental evidence at protein level	anatomical structure development;apoptosis;biological regulation;biosynthetic process;cell cycle;cell cycle process;cell death;cell migration;cell motility;cell surface receptor linked signaling pathway;cellular biosynthetic proc
30.9793	30.7633	30.9837	30.9864	1:Experimental evidence at protein level	biological regulation;biosynthetic process;catabolic process;cell differentiation;cellular biosynthetic process;cellular catabolic process;cellular component assembly;cellular component assembly at cellular level;cellular compo
30.0872	29.9672	29.7243	30.0287	1:Experimental evidence at protein level	biosynthetic process;catabolic process;cellular biosynthetic process;cellular catabolic process;cellular component disassembly;cellular component disassembly at cellular level;cellular component organization;cellular compo
31.1845	30.6788	30.672	30.6626	1:Experimental evidence at protein level	biosynthetic process;catabolic process;cellular biosynthetic process;cellular catabolic process;cellular component biogenesis;cellular component biogenesis at cellular level;cellular component disassembly;cellular componer
30.1236	29.6771	29.6917	30.0634	1:Experimental evidence at protein level	biosynthetic process;catabolic process;cellular biosynthetic process;cellular catabolic process;cellular component disassembly;cellular component disassembly at cellular level;cellular component organization;cellular compo
30.4985	29.9782	30.1294	29.9799	1:Experimental evidence at protein level	biological regulation;biosynthetic process;catabolic process;cellular biosynthetic process;cellular catabolic process;cellular component disassembly;cellular component disassembly at cellular level;cellular component organiz
30.5077	30.2994	30.1011	30.1861	1:Experimental evidence at protein level	biosynthetic process;catabolic process;cellular biosynthetic process;cellular catabolic process;cellular component disassembly;cellular component disassembly at cellular level;cellular component organization;cellular compo
27.7352	27.7665	27.7899	27.1774	1:Experimental evidence at protein level	biosynthetic process;cellular biosynthetic process;cellular component assembly;cellular component assembly at cellular level;cellular component organization;cellular component organization at cellular level;cellular compone
29.2735	28.842	28.9844	28.9328	1:Experimental evidence at protein level	biosynthetic process;cellular biosynthetic process;cellular component assembly;cellular component assembly at cellular level;cellular component organization;cellular component organization at cellular level;cellular compone
27.3005	27.5153	27.5534	27.7325	1:Experimental evidence at protein level	catabolic process;cellular catabolic process;cellular macromolecule catabolic process;cellular macromolecule metabolic process;cellular metabolic process;cellular nitrogen compound metabolic process;cellular process;exo
27.4936	27.2768	27.6192	27.5014	1:Experimental evidence at protein level	catabolic process;cellular catabolic process;cellular macromolecule catabolic process;cellular macromolecule metabolic process;cellular metabolic process;cellular nitrogen compound metabolic process;cellular process;exo
29.2314	28.8664	28.67	29.0041	1:Experimental evidence at protein level	biological regulation;cellular component assembly;cellular component assembly at cellular level;cellular component organization;cellular component organization at cellular level;cellular component organization or biogenesis;
29.1642	29.319	29.4622	29.2661	1:Experimental evidence at protein level	cellular component assembly;cellular component assembly at cellular level;cellular component organization;cellular component organization at cellular level;cellular component organization or biogenesis;cellular component c
29.8654	29.8332	30.0869	29.8224	1:Experimental evidence at protein level	biosynthetic process;cellular biosynthetic process;cellular component assembly;cellular component assembly at cellular level;cellular component organization;cellular component organization at cellular level;cellular compone
32.0968	34.3164	33.6284	34.8092	1:Experimental evidence at protein level	actin cytoskeleton organization;actin filament organization;actin filament-based process;biological regulation;blood coagulation;cell activation;cellular component organization;cellular component organization at cellular level;c
27.8102	27.5045	27.5742	27.5826	1:Experimental evidence at protein level	actin cytoskeleton organization;actin filament-based process;anatomical structure development;apoptosis;biological adhesion;biological regulation;cell adhesion;cell cycle;cell death;cell division;cell projection organization;ce
28.3358	28.1233	28.2681	28.2361	1:Experimental evidence at protein level	activation of immune response;activation of innate immune response;anaphase-promoting complex-dependent proteasomal ubiquitin-dependent protein catabolic process;antigen processing and presentation;antigen proces
31.3514	30.9178	30.9292	30.9897	1:Experimental evidence at protein level	biosynthetic process;catabolic process;cellular biosynthetic process;cellular catabolic process;cellular component biogenesis;cellular component biogenesis at cellular level;cellular component disassembly;cellular componer
24.775	23.8999	24.1887	27.245	1:Experimental evidence at protein level	astral microtubule organization;biological regulation;cell cycle process;cell projection organization;cellular component assembly;cellular component assembly at cellular level;cellular component organization;cellular compone
28.5503	28.6075	28.7684	28.3883	1:Experimental evidence at protein level	biological regulation;biosynthetic process;catabolic process;cellular biosynthetic process;cellular catabolic process;cellular component disassembly;cellular component disassembly at cellular level;cellular component organiz
29.0936	29.5742	29.9261	29.6427	1:Experimental evidence at protein level	alcohol metabolic process;biological regulation;biosynthetic process;cellular biosynthetic process;cellular macromolecule biosynthetic process;cellular macromolecule metabolic process;cellular metabolic process;cellular nitr
30.9153	30.5452	30.4297	30.4712	1:Experimental evidence at protein level	biological regulation;biosynthetic process;catabolic process;cellular biosynthetic process;cellular catabolic process;cellular component disassembly;cellular component disassembly at cellular level;cellular component organiz
29.8965	29.2802	29.8526	29.5932	1:Experimental evidence at protein level	biosynthetic process;catabolic process;cell proliferation;cellular biosynthetic process;cellular catabolic process;cellular component disassembly;cellular component disassembly at cellular level;cellular component organizatio
31.7482	31.3176	31.5692	31.485	1:Experimental evidence at protein level	activation-induced cell death of T cells;anatomical structure development;anatomical structure morphogenesis;apoptosis;biological regulation;biosynthetic process;carbohydrate homeostasis;catabolic process;cell activation
31.7124	29.7597	30.0677	30.514	1:Experimental evidence at protein level	anatomical structure homeostasis;ATP-dependent chromatin remodeling;biological regulation;cell cycle;cellular component assembly;cellular component assembly at cellular level;cellular component organization;cellular com
29.2108	29.6979	29.4395	29.308	1:Experimental evidence at protein level	anatomical structure morphogenesis;autophagic vacuole assembly;autophagy;biological adhesion;biological regulation;cargo loading into COPII-coated vesicle;cargo loading into vesicle;catabolic process;cell adhesion;cell c
32.0495	32.3518	32.3459	32.2276	1:Experimental evidence at protein level	actin cytoskeleton organization;actin filament-based process;anatomical structure development;androgen receptor signaling pathway;biological regulation;cell cycle phase;cell cycle process;cell development;cell division;cell
29.9183	29.7755	29.8089	29.7466	1:Experimental evidence at protein level	biosynthetic process;catabolic process;cellular biosynthetic process;cellular catabolic process;cellular component disassembly;cellular component disassembly at cellular level;cellular component organization;cellular compo
28.895	28.8222	28.6965	28.8387	1:Experimental evidence at protein level	activation of MAPKK activity;activation of protein kinase activity;anatomical structure development;biological regulation;blood coagulation;cell activation;cell development;cell surface receptor linked signaling pathway;cellula
29.9473	29.4327	30.2424	29.6751	1:Experimental evidence at protein level	biosynthetic process;catabolic process;cell differentiation;cellular biosynthetic process;cellular catabolic process;cellular component assembly;cellular component assembly at cellular level;cellular component biogenesis;cell
29.5208	29.1172	29.2768	29.3859	1:Experimental evidence at protein level	biological regulation;biosynthetic process;catabolic process;cellular biosynthetic process;cellular catabolic process;cellular component biogenesis;cellular component biogenesis at cellular level;cellular component disassemb
31.5269	31.1393	31.1873	31.4925	1:Experimental evidence at protein level	biosynthetic process;catabolic process;cellular biosynthetic process;cellular catabolic process;cellular component assembly;cellular component assembly at cellular level;cellular component disassembly;cellular component c
29.0982	25.3231	24.5597	30.2555	1:Experimental evidence at protein level	biological regulation;biosynthetic process;catabolic process;cellular biosynthetic process;cellular catabolic process;cellular component disassembly;cellular component disassembly at cellular level;cellular component organiz
30.2284	30.0741	30.5132	30.5109	1:Experimental evidence at protein level	biosynthetic process;catabolic process;cellular biosynthetic process;cellular catabolic process;cellular component biogenesis;cellular component biogenesis at cellular level;cellular component disassembly;cellular componer
30.4195	30.1668	30.2559	30.4698	1:Experimental evidence at protein level	antibacterial humoral response;antimicrobial humoral response;biosynthetic process;catabolic process;cellular biosynthetic process;cellular catabolic process;cellular component disassembly;cellular component disassembly
30.4175	30.5764	30.5403	30.2907	1:Experimental evidence at protein level	activation of adenylate cyclase activity;activation of adenylate cyclase activity by dopamine receptor signaling pathway;activation of adenylate cyclase activity by G-protein signaling pathway;activation of phospholipase C ac
26.9635	27.5983	27.4725	27.4324	1:Experimental evidence at protein level	biological regulation;biosynthetic process;catabolic process;cell surface receptor linked signaling pathway;cellular biosynthetic process;cellular catabolic process;cellular macromolecule biosynthetic process;cellular macrom
30.0202	30.1781	30.0321	29.9196	1:Experimental evidence at protein level	biological regulation;cell communication;cell surface receptor linked signaling pathway;cell-cell signaling;cellular metabolic process;cellular process;cellular response to chemical stimulus;cellular response to endogenous stin
30.782	30.3832	30.5618	30.5488	1:Experimental evidence at protein level	biosynthetic process;catabolic process;cellular biosynthetic process;cellular catabolic process;cellular component disassembly;cellular component disassembly at cellular level;cellular component organization;cellular compo
28.242	28.9328	28.7967	28.4804	1:Experimental evidence at protein level	antibacterial humoral response;antimicrobial humoral response;biosynthetic process;catabolic process;cellular biosynthetic process;cellular catabolic process;cellular component disassembly;cellular component disassembly
30.355	29.9827	29.9431	30.1896	1:Experimental evidence at protein level	biosynthetic process;catabolic process;cellular biosynthetic process;cellular catabolic process;cellular component disassembly;cellular component disassembly at cellular level;cellular component organization;cellular compo
31.121	30.7167	30.9347	30.7465	1:Experimental evidence at protein level	anatomical structure morphogenesis;biosynthetic process;catabolic process;cellular biosynthetic process;cellular catabolic process;cellular component disassembly;cellular component disassembly at cellular level;cellular co
30.6046	30.078	30.3565	30.4378	1:Experimental evidence at protein level	biosynthetic process;catabolic process;cellular biosynthetic process;cellular catabolic process;cellular component disassembly;cellular component disassembly at cellular level;cellular component organization;cellular compo
31.131	30.7675	30.7981	30.8873	1:Experimental evidence at protein level	biosynthetic process;catabolic process;cellular biosynthetic process;cellular catabolic process;cellular component biogenesis;cellular component biogenesis at cellular level;cellular component disassembly;cellular componer
30.5197	30.2275	30.4169	30.5036	1:Experimental evidence at protein level	biosynthetic process;catabolic process;cellular biosynthetic process;cellular catabolic process;cellular component disassembly;cellular component disassembly at cellular level;cellular component organization;cellular compo
31.8383	33.6628	33.3652	33.9176	1:Experimental evidence at protein level	biological regulation;biosynthetic process;blood coagulation;cell activation;cell migration;cell motility;cellular biosynthetic process;cellular component assembly;cellular component assembly at cellular level;cellular componer
26.4314	28.6265	28.079	29.2958	1:Experimental evidence at protein level	anatomical structure formation involved in morphogenesis;anatomical structure morphogenesis;biological regulation;calcium ion transmembrane transport;calcium ion transport;cardiac muscle tissue morphogenesis;cation tr
29.2922	28.9254	29.0855	29.1679	1:Experimental evidence at protein level	activation of immune response;activation of innate immune response;activation of MAPK activity;alcohol metabolic process;anaphase-promoting complex-dependent proteasomal ubiquitin-dependent protein catabolic proce
28.598	29.2626	28.9475	29.0591	1:Experimental evidence at protein level	activation of immune response;activation of innate immune response;activation of MAPK activity;alcohol metabolic process;anaphase-promoting complex-dependent proteasomal ubiquitin-dependent protein catabolic proce
27.391	27.4005	27.5607	27.7626	1:Experimental evidence at protein level	aging;anatomical structure formation involved in morphogenesis;anatomical structure morphogenesis;axon guidance;biological regulation;blood coagulation;branching involved in embryonic placenta morphogenesis;cell activ
29.5005	29.0137	29.1017	29.2025	1:Experimental evidence at protein level	biological regulation;cellular macromolecule metabolic process;cellular metabolic process;cellular nitrogen compound metabolic process;cellular process;macromolecule metabolic process;metabolic process;mRNA metabol
29.7124	30.1605	29.6769	29.79	1:Experimental evidence at protein level	actin cytoskeleton organization;actin filament organization;actin filament polymerization;actin filament-based process;actin polymerization or depolymerization;anatomical structure arrangement;anatomical structure homeosta
28.9771	28.9708	28.8478	28.838	1:Experimental evidence at protein level	antigen processing and presentation;antigen processing and presentation of exogenous antigen;antigen processing and presentation of exogenous peptide antigen;antigen processing and presentation of exogenous peptide
27.6758	28.127	27.6913	27.9102	1:Experimental evidence at protein level	activation of adenylate cyclase activity;activation of adenylate cyclase activity by adrenergic receptor signaling pathway;activation of adenylate cyclase activity by dopamine receptor signaling pathway;activation of adenylate
26.9255	26.8425	26.7203	26.686	1:Experimental evidence at protein level	biological regulation;blood coagulation;cAMP-mediated signaling;cell activation;cell communication;cell cycle;cell division;cell surface receptor linked signaling pathway;cell-cell signaling;cellular process;cellular response to
33.1871	33.4981	33.3664	33.2881	1:Experimental evidence at protein level	amine transport;apoptosis;biological regulation;biosynthetic process;blood coagulation;cell activation;cell death;cellular biosynthetic process;cellular component assembly;cellular component assembly at cellular level;cellula
26.7234	26.6803	27.026	26.3251	1:Experimental evidence at protein level	biological regulation;catabolic process;cell cycle;cell cycle process;cellular catabolic process;cellular component organization;cellular component organization or biogenesis;cellular macromolecule catabolic process;cellular r
28.5716	28.6376	28.8679	29.2372	1:Experimental evidence at protein level	anatomical structure development;biological regulation;cell surface receptor linked signaling pathway;cellular component organization;cellular component organization at cellular level;cellular component organization or bioge
30.637	30.1799	30.3764	30.5581	1:Experimental evidence at protein level	actin cytoskeleton organization;actin filament-based process;anatomical structure development;anatomical structure morphogenesis;antigen processing and presentation;antigen processing and presentation of exogenous a
30.7864	29.9914	30.621	30.5077	1:Experimental evidence at protein level	90S preribosome assembly;anatomical structure development;anatomical structure morphogenesis;axial mesoderm development;biological regulation;biosynthetic process;catabolic process;cellular biosynthetic process;cell
29.0161	28.5224	29.2229	29.6209	1:Experimental evidence at protein level	activation of immune response;activation of innate immune response;anaphase-promoting complex-dependent proteasomal ubiquitin-dependent protein catabolic process;biological regulation;catabolic process;cell cycle;ce
27.2989	27.1623	27.1184	26.9556	1:Experimental evidence at protein level	biological regulation;cell communication;cell surface receptor linked signaling pathway;cell-cell signaling;cellular metabolic process;cellular process;cellular response to chemical stimulus;cellular response to endogenous stin
30.8273	30.6268	30.9996	31.0962	1:Experimental evidence at protein level	biosynthetic process;catabolic process;cellular biosynthetic process;cellular catabolic process;cellular component biogenesis;cellular component biogenesis at cellular level;cellular component disassembly;cellular componer
32.3665	32.6668	32.6539	32.768	1:Experimental evidence at protein level	amine biosynthetic process;amine metabolic process;apoptosis;biological regulation;biosynthetic process;carboxylic acid biosynthetic process;carboxylic acid metabolic process;cell death;cellular amine metabolic process;c
31.2019	30.5947	30.8366	30.7561	1:Experimental evidence at protein level	activation of caspase activity;anatomical structure morphogenesis;biological regulation;carbohydrate homeostasis;cell cycle;cell differentiation;cellular chemical homeostasis;cellular developmental process;cellular glucose ho
25.931	26.2144	26.1675	26.0381	1:Experimental evidence at protein level	biological regulation;biosynthetic process;cellular biosynthetic process;cellular component organization;cellular component organization at cellular level;cellular component organization or biogenesis;cellular component orga
32.3449	34.838	34.2779	35.0306	1:Experimental evidence at protein level	actin cytoskeleton organization;actin filament organization;actin filament-based process;biological regulation;cellular component organization;cellular component organization at cellular level;cellular component organization c
29.2252	29.5071	29.5044	29.4192	1:Experimental evidence at protein level	anatomical structure development;apoptosis;biological regulation;catabolic process;cell cycle;cell cycle process;cell death;cell surface receptor linked signaling pathway;cellular catabolic process;cellular component assemb
30.4036	30.4303	30.6989	30.8448	1:Experimental evidence at protein level	biological regulation;biosynthetic process;cellular biosynthetic process;cellular macromolecule biosynthetic process;cellular macromolecule metabolic process;cellular metabolic process;cellular nitrogen compound metabolic
28.5463	28.5682	28.6205	28.4432	1:Experimental evidence at protein level	biological regulation;biosynthetic process;cellular biosynthetic process;cellular macromolecule biosynthetic process;cellular macromolecule metabolic process;cellular metabolic process;cellular process;cellular protein metab
28.1458	27.7544	27.9412	27.4878	1:Experimental evidence at protein level	adiponectin-mediated signaling pathway;anatomical structure morphogenesis;axon guidance;biological regulation;cell cycle;cell surface receptor linked signaling pathway;cellular component assembly;cellular component ass
33.1534	33.3493	33.8496	33.7822	1:Experimental evidence at protein level	actin filament-based movement;actin filament-based process;actin-mediated cell contraction;actin-myosin filament sliding;cell differentiation;cellular component movement;cellular developmental process;cellular process;dev
34.003	33.6998	33.7575	33.7642	1:Experimental evidence at protein level	actin cytoskeleton organization;actin filament organization;actin filament-based movement;actin filament-based process;actin-mediated cell contraction;actin-myosin filament sliding;actomyosin structure organization;anatom
27.9268	28.3784	28.1798	28.1013	1:Experimental evidence at protein level	biological regulation;biosynthetic process;catabolic process;cell proliferation;cellular biosynthetic process;cellular catabolic process;cellular macromolecule biosynthetic process;cellular macromolecule catabolic process;cellu
34.4746	34.7702	34.5852	34.6	1:Experimental evidence at protein level	biological regulation;biosynthetic process;cellular biosynthetic process;cellular macromolecule biosynthetic process;cellular macromolecule metabolic process;cellular metabolic process;cellular nitrogen compound metabolic
32.9122	33.0312	32.9281	32.6684	1:Experimental evidence at protein level	biological regulation;blood coagulation;cell activation;cell cycle;cell cycle process;cellular component assembly;cellular component assembly at cellular level;cellular component organization;cellular component organization a
32.051	32.4642	32.4072	32.1502	1:Experimental evidence at protein level	biological regulation;cell cycle;cell cycle process;cell killing;cellular component assembly;cellular component assembly at cellular level;cellular component movement;cellular component organization;cellular component orgar
29.4256	29.1693	29.3826	29.2692	1:Experimental evidence at protein level	axon guidance;biological regulation;biosynthetic process;cell cycle;cell cycle checkpoint;cell surface receptor linked signaling pathway;cellular biosynthetic process;cellular macromolecule biosynthetic process;cellular macro
28.4451	28.7863	29.0319	28.8028	1:Experimental evidence at protein level	anatomical structure development;biological regulation;brain development;catabolic process;developmental process;gamete generation;lipid catabolic process;lipid metabolic process;male gamete generation;metabolic proc
27.7571	27.708	27.6663	27.721	1:Experimental evidence at protein level	
29.7931	30.3555	30.1529	30.2459	1:Experimental evidence at protein level	anion transport;bicarbonate transport;biological regulation;catabolic process;cellular catabolic process;cellular component assembly;cellular component organization;cellular component organization or biogenesis;cellular me
26.3359	26.2213	26.6426	26.4985	1:Experimental evidence at protein level	B cell activation;biological regulation;cell activation;cellular process;cellular response to stimulus;immune system process;leukocyte activation;lymphocyte activation;negative regulation of biological process;negative regulatio
25.8199	26.0038	26.1117	26.0719	1:Experimental evidence at protein level	amine biosynthetic process;amine metabolic process;biosynthetic process;carboxylic acid biosynthetic process;carboxylic acid metabolic process;cellular amine metabolic process;cellular amino acid biosynthetic process;ce
24.6497	24.9769	24.669	24.4675	1:Experimental evidence at protein level	cellular macromolecule metabolic process;cellular metabolic process;cellular nitrogen compound metabolic process;cellular process;macromolecule metabolic process;metabolic process;nitrogen compound metabolic proce
28.3341	28.0663	28.0983	27.8496	1:Experimental evidence at protein level	biological regulation;cell cycle arrest;cell cycle process;cell death;cell surface receptor linked signaling pathway;cellular process;cellular response to chemical stimulus;cellular response to cytokine stimulus;cellular response t
27.0176	26.7578	23.2445	27.0384	1:Experimental evidence at protein level	cellular macromolecule metabolic process;cellular metabolic process;cellular nitrogen compound metabolic process;cellular process;macromolecule metabolic process;metabolic process;ncRNA metabolic process;ncRNA pr
27.25	27.1952	27.2211	27.0944	1:Experimental evidence at protein level	biological regulation;biosynthetic process;calcium ion homeostasis;cation homeostasis;cellular biosynthetic process;cellular calcium ion homeostasis;cellular cation homeostasis;cellular chemical homeostasis;cellular divalen
25.5521	25.5857	25.2333	25.2102	1:Experimental evidence at protein level	anatomical structure formation involved in morphogenesis;anatomical structure morphogenesis;axon cargo transport;axon guidance;biological adhesion;biological regulation;cell adhesion;cell junction assembly;cell junction c
30.3549	30.2999	30.447	30.3465	1:Experimental evidence at protein level	binding of sperm to zona pellucida;cell recognition;cell-cell recognition;cellular component assembly;cellular component assembly at cellular level;cellular component organization;cellular component organization at cellular le
27.5295	27.7474	27.9162	27.8682	1:Experimental evidence at protein level	biological regulation;biosynthetic process;carbohydrate metabolic process;carbohydrate transport;cell cycle;cell cycle process;cell surface receptor linked signaling pathway;cellular biosynthetic process;cellular component c

28.8929	29.2195	29.1936	29.3564	1:Experimental evidence at protein level	biological regulation;calcium-mediated signaling;carboxylic acid metabolic process;catabolic process;cellular catabolic process;cellular ketone metabolic process;cellular metabolic process;cellular process;cellular response
29.7094	29.5058	29.4991	29.3865	1:Experimental evidence at protein level	anatomical structure development;anatomical structure formation involved in morphogenesis;anatomical structure homeostasis;anterior/posterior pattern specification;B cell lineage commitment;biological regulation;brain dev
25.7201	24.288	24.4574	24.6207	1:Experimental evidence at protein level	autophagy;biological adhesion;biological regulation;catabolic process;cell adhesion;cellular catabolic process;cellular component assembly;cellular component assembly at cellular level;cellular component organization;cellul
31.0938	30.6395	30.8558	30.868	1:Experimental evidence at protein level	biological regulation;cellular component organization;cellular component organization at cellular level;cellular component organization or biogenesis;cellular component organization or biogenesis at cellular level;cellular proce
23.765	26.9452	24.6104	27.1031	1:Experimental evidence at protein level	biological regulation;cation homeostasis;cellular cation homeostasis;cellular chemical homeostasis;cellular divalent inorganic cation homeostasis;cellular homeostasis;cellular ion homeostasis;cellular process;cellular respons
26.9584	27.0777	27.5353	27.3848	1:Experimental evidence at protein level	
29.2456	29.2981	29.2022	29.4368	1:Experimental evidence at protein level	anatomical structure development;anatomical structure morphogenesis;anterior/posterior pattern specification;biological regulation;cell differentiation;cell differentiation involved in kidney development;cell morphogenesis;cel
25.2792	24.5266	25.011	24.7207	1:Experimental evidence at protein level	cell killing;defense response;defense response to bacterium;defense response to fungus;killing of cells of other organism;multi-organism process;response to bacterium;response to biotic stimulus;response to fungus;respons
27.726	28.7324	25.7048	29.1267	1:Experimental evidence at protein level	acrosome assembly;anatomical structure formation involved in morphogenesis;biological regulation;biosynthetic process;cell differentiation;cell motility;cellular biosynthetic process;cellular component assembly;cellular comp
25.9664	26.2249	26.3964	26.5661	1:Experimental evidence at protein level	biosynthetic process;cellular biosynthetic process;cellular component disassembly;cellular component disassembly at cellular level;cellular component organization;cellular component organization at cellular level;cellular cor
25.7425	26.4407	25.4697	25.5745	1:Experimental evidence at protein level	biosynthetic process;cellular biosynthetic process;cellular component disassembly;cellular component disassembly at cellular level;cellular component organization;cellular component organization at cellular level;cellular cor
26.6875	26.261	26.4994	26.2853	1:Experimental evidence at protein level	biosynthetic process;cellular biosynthetic process;cellular component disassembly;cellular component disassembly at cellular level;cellular component organization;cellular component organization at cellular level;cellular cor
25.2064	23.4425	25.3008	24.8912	1:Experimental evidence at protein level	biosynthetic process;cellular biosynthetic process;cellular component disassembly;cellular component disassembly at cellular level;cellular component organization;cellular component organization at cellular level;cellular cor
25.8378	25.7428	25.7375	25.6902	1:Experimental evidence at protein level	biosynthetic process;cellular biosynthetic process;cellular component disassembly;cellular component disassembly at cellular level;cellular component organization;cellular component organization at cellular level;cellular cor
25.8968	25.4618	25.6907	25.5092	1:Experimental evidence at protein level	biosynthetic process;cellular biosynthetic process;cellular component disassembly;cellular component disassembly at cellular level;cellular component organization;cellular component organization at cellular level;cellular cor
28.6864	28.3926	28.5912	28.6961	1:Experimental evidence at protein level	biological regulation;biosynthetic process;cellular biosynthetic process;cellular macromolecule biosynthetic process;cellular macromolecule metabolic process;cellular metabolic process;cellular nitrogen compound metabolic
25.7531	25.3031	25.596	25.9369	1:Experimental evidence at protein level	
29.9366	29.7914	29.7768	29.7994	1:Experimental evidence at protein level	anatomical structure development;axon guidance;biological regulation;biosynthetic process;catabolic process;cell cycle checkpoint;cell cycle process;cellular biosynthetic process;cellular catabolic process;cellular compone
30.5198	30.5867	30.3149	30.6204	1:Experimental evidence at protein level	biosynthetic process;catabolic process;cellular biosynthetic process;cellular catabolic process;cellular component disassembly;cellular component disassembly at cellular level;cellular component organization;cellular compo
27.2238	27.1715	27.657	27.2685	1:Experimental evidence at protein level	biological regulation;negative regulation of biological process;negative regulation of biosynthetic process;negative regulation of cellular biosynthetic process;negative regulation of cellular macromolecule biosynthetic process
27.3264	24.1042	28.0903	27.357	1:Experimental evidence at protein level	biological regulation;cellular process;cellular response to stimulus;establishment of localization;establishment of protein localization;intracellular signal transduction;metabolic process;protein transport;regulation of biological
31.0308	30.9648	30.9378	31.1955	1:Experimental evidence at protein level	biological regulation;biosynthetic process;cell cycle;cell differentiation;cellular biosynthetic process;cellular developmental process;cellular metabolic process;cellular nitrogen compound biosynthetic process;cellular nitrogen
27.646	28.6198	28.1117	28.301	1:Experimental evidence at protein level	actin cytoskeleton organization;actin filament-based process;axon guidance;biological regulation;blood coagulation;cell activation;cell chemotaxis;cell migration;cell motility;cellular component movement;cellular component
30.0361	29.4526	29.5967	29.833	1:Experimental evidence at protein level	biosynthetic process;catabolic process;cellular biosynthetic process;cellular catabolic process;cellular component disassembly;cellular component disassembly at cellular level;cellular component organization;cellular compo
28.3338	24.3707	28.246	28.7662	1:Experimental evidence at protein level	
30.0744	30.12	30.1241	29.9513	1:Experimental evidence at protein level	biosynthetic process;cellular biosynthetic process;cellular macromolecule biosynthetic process;cellular macromolecule metabolic process;cellular metabolic process;cellular nitrogen compound metabolic process;cellular pro
26.4692	26.2786	26.566	26.3094	1:Experimental evidence at protein level	anatomical structure development;anatomical structure morphogenesis;biological regulation;cell differentiation;cellular developmental process;cellular process;developmental process;muscle organ development;muscle struc
27.5827	27.7919	27.9632	27.6652	1:Experimental evidence at protein level	activation of JUN kinase activity;activation of MAPK activity;anatomical structure development;anatomical structure morphogenesis;apoptosis;biological regulation;cell death;cell differentiation;cell morphogenesis;cell morpho
26.0466	26.0176	25.6276	25.9558	1:Experimental evidence at protein level	amine metabolic process;aminoglycan biosynthetic process;aminoglycan catabolic process;aminoglycan metabolic process;anatomical structure development;anatomical structure formation involved in morphogenesis;anato
26.8497	26.9893	27.2426	27.1617	1:Experimental evidence at protein level	cellular macromolecule metabolic process;cellular metabolic process;cellular nitrogen compound metabolic process;cellular process;macromolecule metabolic process;metabolic process;mRNA metabolic process;mRNA pro
30.2807	30.7985	30.94	31.0657	1:Experimental evidence at protein level	biological regulation;biosynthetic process;cellular biosynthetic process;cellular macromolecule biosynthetic process;cellular macromolecule metabolic process;cellular metabolic process;cellular nitrogen compound metabolic
27.2856	27.1489	27.293	27.3803	1:Experimental evidence at protein level	biological regulation;biosynthetic process;cellular biosynthetic process;cellular component assembly;cellular component assembly at cellular level;cellular component organization;cellular component organization at cellular le
30.1301	29.9668	29.9264	29.9093	1:Experimental evidence at protein level	anion transport;cellular metabolic process;cellular process;establishment of localization;generation of precursor metabolites and energy;inorganic anion transport;ion transmembrane transport;ion transport;metabolic process
29.2695	29.0213	29.1277	29.0426	1:Experimental evidence at protein level	alcohol metabolic process;cholesterol metabolic process;establishment of localization;lipid metabolic process;lipid transport;metabolic process;organic substance transport;primary metabolic process;small molecule metabo
27.4172	27.5196	27.2735	27.5438	1:Experimental evidence at protein level	anatomical structure development;biological regulation;biosynthetic process;cell differentiation;cellular biosynthetic process;cellular developmental process;cellular macromolecule biosynthetic process;cellular macromolecul
26.0069	26.1486	26.4354	26.5246	1:Experimental evidence at protein level	biological regulation;cellular component organization;cellular component organization or biogenesis;cellular process;cellular response to stimulus;intracellular signal transduction;positive regulation of actin filament polymeriz
30.6949	30.4237	30.4989	30.3767	1:Experimental evidence at protein level	antigen processing and presentation;antigen processing and presentation of exogenous antigen;antigen processing and presentation of exogenous peptide antigen;antigen processing and presentation of exogenous peptide
25.9718	25.9064	25.8836	25.8595	1:Experimental evidence at protein level	activation of immune response;activation of innate immune response;adaptive immune response;adaptive immune response based on somatic recombination of immune receptors built from immunoglobulin superfamily dom
28.1421	27.846	28.1063	28.1426	1:Experimental evidence at protein level	cellular macromolecule metabolic process;cellular metabolic process;cellular process;cellular protein metabolic process;chaperone-mediated protein folding;macromolecule metabolic process;macromolecule modification;me
27.2965	27.4589	27.0219	27.2528	1:Experimental evidence at protein level	
25.7865	26.1082	26.0187	25.982	1:Experimental evidence at protein level	alcohol biosynthetic process;alcohol catabolic process;alcohol metabolic process;alditol catabolic process;alditol metabolic process;biosynthetic process;carbohydrate biosynthetic process;carbohydrate catabolic process;c
30.912	31.0494	30.9774	30.8889	1:Experimental evidence at protein level	biological regulation;cell differentiation;cellular developmental process;cellular macromolecule metabolic process;cellular metabolic process;cellular nitrogen compound metabolic process;cellular process;circadian regulation
28.4681	28.7663	28.6441	28.7067	1:Experimental evidence at protein level	biosynthetic process;cellular biosynthetic process;cellular macromolecule biosynthetic process;cellular macromolecule metabolic process;cellular metabolic process;cellular nitrogen compound metabolic process;cellular pro
28.4025	27.845	28.0534	27.8731	1:Experimental evidence at protein level	actin filament capping;axon guidance;biological regulation;cell cycle cytokinesis;cell cycle process;cellular component assembly;cellular component assembly at cellular level;cellular component organization;cellular compone
27.4677	27.433	27.1719	27.5656	1:Experimental evidence at protein level	anatomical structure development;apoptosis;biological regulation;cell death;cell development;cell division;cellular developmental process;cellular process;cellular process involved in reproduction;death;defense response;dev
30.0249	30.177	30.378	30.3639	1:Experimental evidence at protein level	biological regulation;biosynthetic process;cell cycle;cellular biosynthetic process;cellular component assembly;cellular component assembly at cellular level;cellular component disassembly;cellular component disassembly a
29.6694	29.7803	29.6722	29.3702	1:Experimental evidence at protein level	biosynthetic process;cellular biosynthetic process;cellular macromolecule biosynthetic process;cellular macromolecule metabolic process;cellular metabolic process;cellular nitrogen compound metabolic process;cellular pro
26.8601	27.1255	26.8008	27.0689	1:Experimental evidence at protein level	biological regulation;biosynthetic process;cellular aromatic compound metabolic process;cellular biosynthetic process;cellular metabolic compound salvage;cellular metabolic process;cellular nitrogen compound biosynthetic
28.1545	28.7189	28.8657	28.9081	1:Experimental evidence at protein level	acylglycerol catabolic process;acylglycerol metabolic process;alcohol metabolic process;amine metabolic process;anatomical structure development;biosynthetic process;carbohydrate metabolic process;carbohydrate trans
30.2925	30.2812	30.1657	30.2091	1:Experimental evidence at protein level	actin cytoskeleton organization;actin filament-based process;activation of adenylate cyclase activity;ameboidal cell migration;axon guidance;biological regulation;blood coagulation;cell activation;cell migration;cell motility;cel
26.6595	27.8606	25.814	24.7244	1:Experimental evidence at protein level	alcohol metabolic process;anatomical structure development;biosynthetic process;brain development;cellular biosynthetic process;cellular lipid metabolic process;cellular metabolic process;cellular process;cellular response
27.1527	27.428	27.4643	27.138	1:Experimental evidence at protein level	amine metabolic process;amine transport;amino acid transmembrane transport;amino acid transport;anatomical structure development;aromatic amino acid transport;biological regulation;blood coagulation;branched-chain a
27.5814	27.2499	27.4051	27.4213	1:Experimental evidence at protein level	biological regulation;catabolic process;cellular catabolic process;cellular localization;cellular macromolecule catabolic process;cellular macromolecule localization;cellular macromolecule metabolic process;cellular metabolic
24.7075	25.1968	22.6612	24.605	1:Experimental evidence at protein level	cellular macromolecule metabolic process;cellular metabolic process;cellular process;cellular protein metabolic process;macromolecule metabolic process;macromolecule modification;metabolic process;primary metabolic p
29.3788	29.4541	29.39	29.2148	1:Experimental evidence at protein level	alcohol metabolic process;carbohydrate metabolic process;carbohydrate phosphorylation;cellular carbohydrate metabolic process;cellular metabolic process;cellular process;fructose 6-phosphate metabolic process;fructose
27.9876	28.1501	28.2114	28.3729	1:Experimental evidence at protein level	biological regulation;biosynthetic process;cellular biosynthetic process;cellular macromolecule biosynthetic process;cellular macromolecule metabolic process;cellular metabolic process;cellular nitrogen compound metabolic
27.4586	27.4322	27.4411	27.4917	1:Experimental evidence at protein level	biological regulation;catabolic process;cell communication;cell-cell signaling;cellular process;cellular response to stimulus;inositol phosphate metabolic process;intracellular signal transduction;lipid catabolic process;lipid me
29.8783	30.1914	30.2823	30.1036	1:Experimental evidence at protein level	anatomical structure development;cell differentiation;cellular developmental process;cellular process;developmental process;epithelial cell differentiation;muscle organ development;muscle structure development;organ devel
23.2577	24.515	25.0549	24.2434	1:Experimental evidence at protein level	biological regulation;biosynthetic process;body fluid secretion;cellular aromatic compound metabolic process;cellular biosynthetic process;cellular metabolic process;cellular nitrogen compound biosynthetic process;cellular
28.812	28.7846	28.7608	28.5714	1:Experimental evidence at protein level	2-oxoglutarate metabolic process;acetyl-CoA catabolic process;acetyl-CoA metabolic process;alcohol catabolic process;alcohol metabolic process;amine catabolic process;amine metabolic process;anatomical structure dev
29.3032	29.6562	24.6624	29.8325	1:Experimental evidence at protein level	antigen processing and presentation;antigen processing and presentation of exogenous antigen;antigen processing and presentation of exogenous peptide antigen;antigen processing and presentation of exogenous peptide
27.0252	26.8213	26.7011	26.7803	1:Experimental evidence at protein level	antigen processing and presentation;antigen processing and presentation of exogenous antigen;antigen processing and presentation of exogenous peptide antigen;antigen processing and presentation of exogenous peptide
22.5521	25.4143	25.2471	24.9114	1:Experimental evidence at protein level	amine catabolic process;amine metabolic process;branched chain family amino acid catabolic process;branched chain family amino acid metabolic process;brown fat cell differentiation;carboxylic acid catabolic process;carb
24.277	24.1699	24.4248	24.8448	1:Experimental evidence at protein level	anatomical structure development;biological adhesion;cell adhesion;cell differentiation;cellular component disassembly;cellular component disassembly at cellular level;cellular component organization;cellular component org
26.0461	25.4616	26.043	26.2886	1:Experimental evidence at protein level	anatomical structure development;anatomical structure morphogenesis;B cell activation;B cell differentiation;biological regulation;biosynthetic process;cell activation;cell differentiation;cellular biosynthetic process;cellular dev
31.8757	31.8162	31.8424	31.9332	1:Experimental evidence at protein level	cellular component assembly;cellular component assembly at cellular level;cellular component organization;cellular component organization at cellular level;cellular component organization or biogenesis;cellular component o
29.8322	29.474	29.5431	29.4971	1:Experimental evidence at protein level	biosynthetic process;catabolic process;cellular biosynthetic process;cellular catabolic process;cellular component disassembly;cellular component disassembly at cellular level;cellular component organization;cellular compo
28.0655	27.6202	27.8239	27.7293	1:Experimental evidence at protein level	activation of immune response;activation of innate immune response;activation of MAPK activity;activation of MAPKK activity;activation of protein kinase activity;aging;anatomical structure development;axon guidance;biolog
29.5488	29.7252	29.7407	29.7752	1:Experimental evidence at protein level	anatomical structure development;androgen receptor signaling pathway;biological regulation;cation transport;cellular component assembly;cellular component assembly at cellular level;cellular component organization;cellula
28.6066	28.3185	28.443	28.3456	1:Experimental evidence at protein level	amine biosynthetic process;amine metabolic process;anatomical structure development;aspartate family amino acid biosynthetic process;aspartate family amino acid metabolic process;biosynthetic process;carboxylic acid b
27.3149	27.3419	27.528	27.3923	1:Experimental evidence at protein level	
31.9385	31.8376	31.7386	31.8184	1:Experimental evidence at protein level	biological regulation;biosynthetic process;catabolic process;cellular biosynthetic process;cellular catabolic process;cellular component disassembly;cellular component disassembly at cellular level;cellular component organiz
27.243	27.1389	27.5093	27.2993	1:Experimental evidence at protein level	biological regulation;cell surface receptor linked signaling pathway;cellular process;cellular response to stimulus;establishment of localization;establishment of localization in cell;establishment of protein localization;G-protein
27.3829	27.4726	27.338	27.0532	1:Experimental evidence at protein level	alcohol biosynthetic process;alcohol metabolic process;alpha-ketoglutarate transport;anion transport;biosynthetic process;carbohydrate biosynthetic process;carbohydrate metabolic process;carboxylic acid transport;cellula
28.5525	28.1098	28.0004	27.992	1:Experimental evidence at protein level	biological adhesion;biological regulation;cell adhesion;cell cycle arrest;cell cycle process;cell junction assembly;cell junction organization;cell motility;cell surface receptor linked signaling pathway;cell-substrate junction asse
29.0723	28.827	28.7284	28.58	1:Experimental evidence at protein level	acylglycerol metabolic process;anatomical structure development;anatomical structure formation involved in morphogenesis;angiogenesis;biological regulation;blood coagulation;body fluid secretion;calcium ion homeostasis
26.4818	26.082	26.2974	26.5443	1:Experimental evidence at protein level	amine metabolic process;carboxylic acid metabolic process;cellular amine metabolic process;cellular amino acid metabolic process;cellular ketone metabolic process;cellular metabolic process;cellular nitrogen compound m
29.8269	29.0911	29.5158	29.4567	1:Experimental evidence at protein level	
26.5633	26.5911	26.5472	26.537	1:Experimental evidence at protein level	attachment of GPI anchor to protein;biological regulation;blood coagulation;cellular component movement;cellular macromolecule metabolic process;cellular metabolic process;cellular process;cellular protein metabolic proc
26.9034	27.3011	26.9567	26.7733	1:Experimental evidence at protein level	antigen processing and presentation;antigen processing and presentation of endogenous antigen;antigen processing and presentation of endogenous peptide antigen;antigen processing and presentation of endogenous pep
26.6046	26.3472	26.0481	26.1317	1:Experimental evidence at protein level	antigen processing and presentation;antigen processing and presentation of endogenous antigen;antigen processing and presentation of endogenous peptide antigen;antigen processing and presentation of endogenous pep
26.9705	26.8811	26.4987	26.6652	1:Experimental evidence at protein level	biological regulation;biosynthetic process;cellular biosynthetic process;cellular macromolecule biosynthetic process;cellular macromolecule metabolic process;cellular metabolic process;cellular nitrogen compound metabolic
27.6345	27.5873	27.7885	27.8602	1:Experimental evidence at protein level	acetaldehyde metabolic process;activation of immune response;activation of innate immune response;aging;anatomical structure development;anatomical structure morphogenesis;antigen receptor-mediated signaling pathw
28.0693	28.2662	28.1741	28.3923	1:Experimental evidence at protein level	biological regulation;catabolic process;cellular catabolic process;cellular macromolecule catabolic process;cellular macromolecule metabolic process;cellular metabolic process;cellular process;macromolecule catabolic proc
28.0634	28.1026	28.1486	27.9063	1:Experimental evidence at protein level	alcohol metabolic process;biosynthetic process;carbohydrate biosynthetic process;carbohydrate metabolic process;cellular biosynthetic process;cellular carbohydrate biosynthetic process;cellular carbohydrate metabolic pr
30.1359	30.0718	29.9402	29.8987	1:Experimental evidence at protein level	biological regulation;biosynthetic process;catabolic process;cell surface receptor linked signaling pathway;cellular biosynthetic process;cellular catabolic process;cellular macromolecule biosynthetic process;cellular macrom
27.092	28.1959	28.0006	28.5866	1:Experimental evidence at protein level	anatomical structure development;anatomical structure morphogenesis;biological regulation;cellular component organization;cellular component organization at cellular level;cellular component organization or biogenesis;cell
26.7869	26.8311	27.0452	26.8646	1:Experimental evidence at protein level	anatomical structure development;anatomical structure morphogenesis;apoptosis;appendage morphogenesis;atrial septum morphogenesis;biological regulation;biosynthetic process;blood vessel development;bone remodel
25.5285	25.4162	26.0235	24.6713	1:Experimental evidence at protein level	anatomical structure morphogenesis;biological regulation;biosynthetic process;cell surface receptor linked signaling pathway;cellular biosynthetic process;cellular macromolecule biosynthetic process;cellular macromolecule
26.6075	24.297	25.1363	24.2101	1:Experimental evidence at protein level	macromolecule metabolic process;metabolic process;primary metabolic process;protein metabolic process;proteolysis
28.6372	29.3661	30.0185	29.8548	1:Experimental evidence at protein level	amine metabolic process;biological regulation;carbohydrate metabolic process;carboxylic acid metabolic process;cell differentiation;cellular aldehyde metabolic process;cellular amine metabolic process;cellular amino acid m
27.01	28.5385	27.9698	28.2536	1:Experimental evidence at protein level	anatomical structure morphogenesis;biological regulation;biosynthetic process;cell part morphogenesis;cellular biosynthetic process;cellular component morphogenesis;cellular component organization;cellular component or
31.181	31.2224	31.0676	31.0737	1:Experimental evidence at protein level	anatomical structure development;apoptosis;biological regulation;biosynthetic process;C21-steroid hormone catabolic process;C21-steroid hormone metabolic process;catabolic process;cell death;cellular biosynthetic proce
26.8945	26.9355	27.215	26.9403	1:Experimental evidence at protein level	biosynthetic process;cellular biosynthetic process;cellular macromolecule biosynthetic process;cellular macromolecule metabolic process;cellular metabolic process;cellular nitrogen compound metabolic process;cellular pro
25.6818	24.9313	26.0575	25.4397	1:Experimental evidence at protein level	anatomical structure development;androgen receptor signaling pathway;biological regulation;brain development;catabolic process;cellular catabolic process;cellular component organization;cellular component organization o
25.9039	23.7719	25.5455	25.2332	1:Experimental evidence at protein level	axon guidance;biological regulation;cell surface receptor linked signaling pathway;cellular component organization;cellular component organization at cellular level;cellular component organization or biogenesis;cellular comp
25.8187	25.7759	25.7354	25.5469	1:Experimental evidence at protein level	cellular macromolecule metabolic process;cellular metabolic process;cellular process;cellular protein metabolic process;dephosphorylation;macromolecule metabolic process;macromolecule modification;metabolic process;p
27.9787	28.0506	27.6285	27.6907	1:Experimental evidence at protein level	anatomical structure development;anatomical structure formation involved in morphogenesis;anatomical structure morphogenesis;angiogenesis;apoptosis;axon guidance;axonogenesis;biological regulation;blood coagulation
28.0419	27.959	27.8519	27.5718	1:Experimental evidence at protein level	biosynthetic process;cellular biosynthetic process;cellular macromolecule biosynthetic process;cellular macromolecule metabolic process;cellular metabolic process;cellular nitrogen compound metabolic process;cellular pro
25.3869	25.4434	25.4233	25.4705	1:Experimental evidence at protein level	activation of immune response;activation of innate immune response;activation of phospholipase C activity;adaptive immune response;adaptive immune response based on somatic recombination of immune receptors built f
29.8596	29.9433	30.2139	30.1793	1:Experimental evidence at protein level	cellular component movement;cellular process;multicellular organismal process;muscle contraction;muscle system process;system process
25.252	25.2863	24.9557	24.8903	1:Experimental evidence at protein level	amine metabolic process;anatomical structure development;anatomical structure morphogenesis;brain development;carboxylic acid metabolic process;cellular amine metabolic process;cellular amino acid metabolic process
27.6369	27.4337	28.0758	27.7867	1:Experimental evidence at protein level	amine metabolic process;aminoglycan metabolic process;biological regulation;carbohydrate metabolic process;glycosaminoglycan metabolic process;hyaluronan metabolic process;macromolecule metabolic process;metab
27.3476	27.3912	27.2714	27.2448	1:Experimental evidence at protein level	abortive mitotic cell cycle;activation of MAPK activity;acylglycerol metabolic process;anatomical structure development;anatomical structure formation involved in morphogenesis;anatomical structure morphogenesis;axon gu
26.3725	26.2395	26.2074	24.1233	1:Experimental evidence at protein level	amine catabolic process;amine metabolic process;anatomical structure development;anatomical structure morphogenesis;biological regulation;biosynthetic process;body fluid secretion;carboxylic acid catabolic process;carb
28.8089	28.6037	28.7028	28.6738	1:Experimental evidence at protein level	alcohol biosynthetic process;alcohol metabolic process;amine metabolic process;amino sugar biosynthetic process;amino sugar metabolic process;biological regulation;biosynthetic process;carbohydrate biosynthetic proce
26.4176	26.4387	26.4708	26.6156	1:Experimental evidence at protein level	biological regulation;catabolic process;cellular catabolic process;cellular macromolecule catabolic process;cellular macromolecule metabolic process;cellular metabolic process;cellular nitrogen compound metabolic process
28.6771	28.8401	28.9437	28.8433	1:Experimental evidence at protein level	activation of immune response;activation of innate immune response;anaphase-promoting complex-dependent proteasomal ubiquitin-dependent protein catabolic process;antigen processing and presentation;antigen proces
25.1573	25.3234	25.3903	25.1393	1:Experimental evidence at protein level	alcohol metabolic process;anatomical structure development;behavior;behavioral interaction between organisms;biological regulation;cation homeostasis;cell surface receptor linked signaling pathway;cellular cation homeos
26.2413	26.2392	26.2857	26.2746	1:Experimental evidence at protein level	anatomical structure development;biological regulation;central nervous system development;developmental process;establishment of localization;establishment of RNA localization;mRNA transport;negative regulation of biol
25.7741	23.6908	26.2849	23.8769	1:Experimental evidence at protein level	amine metabolic process;aminoglycan biosynthetic process;aminoglycan catabolic process;aminoglycan metabolic process;biosynthetic process;carbohydrate biosynthetic process;carbohydrate catabolic process;carbohyd
32.2727	32.6662	32.6115	32.5695	1:Experimental evidence at protein level	anatomical structure development;anatomical structure homeostasis;biological regulation;biosynthetic process;catabolic process;cell killing;cell proliferation;cellular biosynthetic process;cellular catabolic process;cellular mac
30.676	30.1193	30.2644	30.4415	1:Experimental evidence at protein level	biosynthetic process;catabolic process;cellular biosynthetic process;cellular catabolic process;cellular component disassembly;cellular component disassembly at cellular level;cellular component organization;cellular compo
30.6322	30.5203	30.7574	30.5562	1:Experimental evidence at protein level	
27.6988	27.6158	27.7961	27.7611	1:Experimental evidence at protein level	anatomical structure formation involved in morphogenesis;apoptosis;biological regulation;blastocyst formation;carbohydrate homeostasis;cell death;cell junction assembly;cell junction organization;cell-cell junction assembly
29.7578	30.0378	29.6604	29.9136	1:Experimental evidence at protein level	biological regulation;biosynthetic process;cell cycle arrest;cell cycle process;cell proliferation;cell surface receptor linked signaling pathway;cellular biosynthetic process;cellular macromolecule biosynthetic process;cellular m
28.1391	28.0259	28.1199	28.1104	1:Experimental evidence at protein level	antigen processing and presentation;antigen processing and presentation of exogenous antigen;antigen processing and presentation of exogenous peptide antigen;antigen processing and presentation of exogenous peptide
29.0273	28.7195	28.7993	28.7011	1:Experimental evidence at protein level	activation of protein kinase activity;activation of protein kinase C activity by G-protein coupled receptor protein signaling pathway;aging;alcohol metabolic process;anatomical structure morphogenesis;aorta morphogenesis;a
29.4693	29.1223	29.5114	29.3202	1:Experimental evidence at protein level	biological regulation;biosynthetic process;cardiac muscle contraction;cellular biosynthetic process;cellular component assembly;cellular component assembly at cellular level;cellular component organization;cellular compone
28.1793	28.1709	28.1642	28.1907	1:Experimental evidence at protein level	biological regulation;cellular process;cellular response to stimulus;intracellular signal transduction;positive regulation of biological process;positive regulation of catalytic activity;positive regulation of cell communication;positi
27.4374	27.8252	27.2503	27.2113	1:Experimental evidence at protein level	biological regulation;biosynthetic process;cell differentiation;cellular biosynthetic process;cellular developmental process;cellular macromolecule biosynthetic process;cellular macromolecule metabolic process;cellular metab
26.8841	26.7963	27.2023	27.0533	1:Experimental evidence at protein level	activation of immune response;activation of innate immune response;anatomical structure development;biological regulation;calcineurin-NFAT signaling pathway;calcium ion transport;calcium-mediated signaling;carbohydrat
30.1113	29.8996	29.9256	29.7784	1:Experimental evidence at protein level	biological regulation;cell differentiation;cellular developmental process;cellular macromolecule metabolic process;cellular metabolic process;cellular nitrogen compound metabolic process;cellular process;cellular response to
27.0217	26.7506	26.9219	26.7365	1:Experimental evidence at protein level	catabolic process;cellular catabolic process;cellular component assembly;cellular component organization;cellular component organization or biogenesis;cellular metabolic process;cellular process;macromolecular complex a
26.2913	26.039	26.2511	26.2028	1:Experimental evidence at protein level	cellular process;establishment of localization;establishment of localization in cell;Golgi vesicle transport;intracellular transport;intra-Golgi vesicle-mediated transport;transport;vesicle-mediated transport
26.2229	26.5015	26.6281	26.45	1:Experimental evidence at protein level	biological regulation;cell cycle;cellular process;mitotic cell cycle;negative regulation of binding;negative regulation of molecular function;negative regulation of protein binding;regulation of binding;regulation of molecular funct
24.9776	24.1507	25.148	24.7827	1:Experimental evidence at protein level	biological adhesion;biological regulation;cell adhesion;cellular component organization;cellular component organization or biogenesis;cellular defense response;cellular membrane organization;cellular process;cellular respons
25.6231	25.8025	26.0068	25.6544	1:Experimental evidence at protein level	anatomical structure formation involved in morphogenesis;angiogenesis;biological adhesion;biological regulation;cell adhesion;cell recognition;cellular component organization;cellular component organization or biogenesis;c
26.54	23.3299	26.8643	26.7407	1:Experimental evidence at protein level	biological adhesion;biological regulation;blood coagulation;cell adhesion;cell migration;cell motility;cell surface receptor linked signaling pathway;cellular component movement;cellular component organization;cellular compo

27.6367	27.5386	27.4951	27.8245	1:Experimental evidence at protein level	apoptosis;biological regulation;cell death;cellular component assembly;cellular component organization;cellular component organization at cellular level;cellular component organization or biogenesis;cellular component orga
28.3464	28.2058	28.1084	28.0962	1:Experimental evidence at protein level	biological regulation;biosynthetic process;cellular biosynthetic process;cellular component organization;cellular component organization at cellular level;cellular component organization or biogenesis;cellular component orga
25.1898	24.7166	25.8172	25.2655	1:Experimental evidence at protein level	cell differentiation;cellular developmental process;cellular process;developmental process
27.0821	26.9869	26.8095	27.0379	1:Experimental evidence at protein level	biological regulation;biosynthetic process;cellular biosynthetic process;cellular lipid metabolic process;cellular metabolic process;cellular process;cellular response to stimulus;glycerolipid biosynthetic process;glycerolipid me
27.4277	27.4631	27.2702	27.2659	1:Experimental evidence at protein level	cell cycle phase;cell cycle process;cell division;cellular component organization;cellular component organization at cellular level;cellular component organization or biogenesis;cellular component organization or biogenesis at
28.7679	28.9962	29.2326	29.1439	1:Experimental evidence at protein level	ATP-dependent chromatin remodeling;biological regulation;biosynthetic process;cell cycle;cell cycle process;cellular biosynthetic process;cellular component assembly;cellular component assembly at cellular level;cellular co
27.4738	27.3395	27.2602	26.9537	1:Experimental evidence at protein level	biological regulation;biosynthetic process;catabolic process;cellular biosynthetic process;cellular catabolic process;cellular component assembly;cellular component assembly at cellular level;cellular component organization;
31.1972	30.6811	30.9727	30.9663	1:Experimental evidence at protein level	biological regulation;cellular component assembly;cellular component organization;cellular component organization or biogenesis;macromolecular complex assembly;macromolecular complex subunit organization;protein co
28.2691	28.0628	28.2168	27.9796	1:Experimental evidence at protein level	cellular macromolecule metabolic process;cellular metabolic process;cellular process;cellular protein metabolic process;'de novo' cotranslational protein folding;'de novo' protein folding;macromolecule metabolic process;me
26.3752	25.4066	26.0777	26.0714	1:Experimental evidence at protein level	anatomical structure development;biological regulation;cardiac muscle fiber development;cell development;cellular developmental process;cellular macromolecule metabolic process;cellular metabolic process;cellular proces
27.971	27.9543	27.8824	28.0247	1:Experimental evidence at protein level	biosynthetic process;carbohydrate metabolic process;cellular carbohydrate metabolic process;cellular macromolecule metabolic process;cellular metabolic process;cellular process;cellular protein metabolic process;glycosyl
27.9255	27.565	27.6608	27.6082	1:Experimental evidence at protein level	antigen processing and presentation;antigen processing and presentation of exogenous antigen;antigen processing and presentation of exogenous peptide antigen;antigen processing and presentation of exogenous peptide
25.6778	24.9553	25.322	25.4733	1:Experimental evidence at protein level	biosynthetic process;cellular biosynthetic process;cellular macromolecule biosynthetic process;cellular macromolecule metabolic process;cellular metabolic process;cellular nitrogen compound metabolic process;cellular pro
23.9129	24.4905	24.6764	23.9909	1:Experimental evidence at protein level	biological regulation;developmental process;humoral immune response;immune response;immune system process;metabolic process;multicellular organismal development;multicellular organismal process;positive regulation
24.5552	26.3314	23.3321	23.8728	1:Experimental evidence at protein level	B cell activation;biological regulation;cell activation;cell communication;cell proliferation;cell surface receptor linked signaling pathway;cell-cell signaling;cellular process;cellular response to chemical stimulus;cellular respons
26.7742	27.1524	27.0163	26.9049	1:Experimental evidence at protein level	cellular macromolecule metabolic process;cellular metabolic process;cellular process;cellular protein metabolic process;establishment of localization;establishment of localization in cell;establishment of protein localization;es
28.2399	28.6847	28.7499	28.6935	1:Experimental evidence at protein level	cellular process;establishment of localization;establishment of localization in cell;exocytosis;secretion;secretion by cell;transport;vesicle-mediated transport
26.0804	25.8632	25.8307	25.5749	1:Experimental evidence at protein level	cellular process;endosome transport;establishment of localization;establishment of localization in cell;establishment of protein localization;intracellular transport;protein transport;transport;vesicle-mediated transport
26.1811	25.8164	27.0369	25.4249	1:Experimental evidence at protein level	biological regulation;biosynthetic process;carbohydrate metabolic process;carbohydrate transport;cell cycle;cell cycle process;cell surface receptor linked signaling pathway;cellular biosynthetic process;cellular component c
25.8002	23.7473	25.0405	25.2744	1:Experimental evidence at protein level	biological regulation;cell surface receptor linked signaling pathway;cellular macromolecule metabolic process;cellular metabolic process;cellular nitrogen compound metabolic process;cellular process;cellular response to stin
28.6351	28.6457	28.5611	28.5258	1:Experimental evidence at protein level	biological regulation;cellular component organization;cellular component organization or biogenesis;maintenance of location;maintenance of protein location;negative regulation of actin filament polymerization;negative regula
28.7249	28.4834	28.3605	28.5989	1:Experimental evidence at protein level	activation of store-operated calcium channel activity;anatomical structure development;anatomical structure morphogenesis;appendage morphogenesis;biological regulation;calcium ion transmembrane transport;calcium ion
26.2732	25.5417	26.1507	25.9641	1:Experimental evidence at protein level	biological regulation;cell surface receptor linked signaling pathway;cellular macromolecule metabolic process;cellular metabolic process;cellular process;cellular protein metabolic process;cellular response to stimulus;enzym
22.5353	24.3824	25.1089	24.9585	1:Experimental evidence at protein level	biological regulation;cell surface receptor linked signaling pathway;cellular component organization;cellular component organization at cellular level;cellular component organization or biogenesis;cellular component organizat
27.0955	23.0204	26.5281	27.0426	1:Experimental evidence at protein level	activation of anaphase-promoting complex activity;anaphase-promoting complex-dependent proteasomal ubiquitin-dependent protein catabolic process;biological regulation;catabolic process;cell cycle;cell cycle checkpoint
27.8894	27.614	28.3231	28.0885	1:Experimental evidence at protein level	biological regulation;BMP signaling pathway;cell surface receptor linked signaling pathway;cellular process;cellular response to stimulus;enzyme linked receptor protein signaling pathway;regulation of biological process;regu
26.9434	27.0122	27.229	26.9973	1:Experimental evidence at protein level	biological regulation;blood coagulation;cell activation;cellular component assembly;cellular component assembly at cellular level;cellular component organization;cellular component organization at cellular level;cellular comp
27.554	27.0609	27.524	27.4688	1:Experimental evidence at protein level	anatomical structure morphogenesis;anterior/posterior pattern specification;cellular macromolecule metabolic process;cellular metabolic process;cellular nitrogen compound metabolic process;cellular process;developmenta
24.4198	24.9165	24.2739	24.4082	1:Experimental evidence at protein level	anatomical structure development;axon guidance;biological adhesion;biological regulation;cell adhesion;cell surface receptor linked signaling pathway;cellular process;cellular response to stimulus;cerebellum development;c
23.9177	23.9298	23.9426	23.2536	1:Experimental evidence at protein level	biological regulation;biosynthetic process;cellular biosynthetic process;cellular component organization;cellular component organization at cellular level;cellular component organization or biogenesis;cellular component orga
28.2233	27.982	28.3178	28.0877	1:Experimental evidence at protein level	cellular component assembly;cellular component assembly at cellular level;cellular component organization;cellular component organization at cellular level;cellular component organization or biogenesis;cellular component c
25.4941	25.3072	25.7486	25.4556	1:Experimental evidence at protein level	amine biosynthetic process;amine metabolic process;beta-alanine biosynthetic process;beta-alanine metabolic process;biosynthetic process;carboxylic acid biosynthetic process;carboxylic acid metabolic process;catabolic
24.6245	25.0785	23.3844	23.0562	1:Experimental evidence at protein level	anatomical structure formation involved in morphogenesis;angiogenesis;biological adhesion;biological regulation;cell adhesion;cell cycle arrest;cell cycle process;cell migration;cell motility;cellular component movement;cellu
27.7283	27.0415	27.3355	26.9699	1:Experimental evidence at protein level	biological regulation;biosynthetic process;cellular biosynthetic process;cellular macromolecule biosynthetic process;cellular macromolecule metabolic process;cellular metabolic process;cellular nitrogen compound metabolic
28.5991	28.7193	28.5317	28.5027	1:Experimental evidence at protein level	alcohol metabolic process;amine metabolic process;amino acid activation;anatomical structure formation involved in morphogenesis;angiogenesis;apoptosis;biological adhesion;biological regulation;carbohydrate metabolic
30.1775	29.7439	29.9564	29.9114	1:Experimental evidence at protein level	biological regulation;biosynthetic process;cellular biosynthetic process;cellular macromolecule biosynthetic process;cellular macromolecule metabolic process;cellular metabolic process;cellular nitrogen compound metabolic
29.532	29.1764	29.2885	29.3225	1:Experimental evidence at protein level	biological regulation;biosynthetic process;cellular biosynthetic process;cellular macromolecule biosynthetic process;cellular macromolecule metabolic process;cellular metabolic process;cellular nitrogen compound metabolic
29.2368	29.2036	29.0464	28.9518	1:Experimental evidence at protein level	biological regulation;cellular process;establishment of localization;establishment of localization in cell;establishment of protein localization;Golgi vesicle transport;intracellular transport;positive regulation of biological process;
26.0254	26.0177	26.1326	26.0128	1:Experimental evidence at protein level	actin crosslink formation;actin cytoskeleton organization;actin cytoskeleton reorganization;actin filament bundle assembly;actin filament capping;actin filament organization;actin filament-based process;actin polymerization-d
27.5047	27.2884	27.2941	27.1943	1:Experimental evidence at protein level	biological regulation;cellular macromolecule metabolic process;cellular metabolic process;cellular process;cellular protein metabolic process;chaperone-mediated protein folding;macromolecule metabolic process;metabolic
27.0595	27.4107	27.0481	26.9247	1:Experimental evidence at protein level	activation of caspase activity;activation of NF-kappaB-inducing kinase activity;activation of protein kinase activity;apoptosis;apoptosis in response to endoplasmic reticulum stress;biological regulation;catabolic process;cell
27.3139	27.4686	27.4456	27.3151	1:Experimental evidence at protein level	actin filament-based movement;actin filament-based process;anatomical structure development;biological regulation;cell development;cell differentiation;cell surface receptor linked signaling pathway;cellular component mov
26.1746	26.2996	26.5082	26.5189	1:Experimental evidence at protein level	biological regulation;biosynthetic process;catabolic process;cell proliferation;cellular biosynthetic process;cellular catabolic process;cellular macromolecule biosynthetic process;cellular macromolecule catabolic process;cellu
25.3462	26.0198	25.7044	26.0115	1:Experimental evidence at protein level	cellular macromolecule metabolic process;cellular metabolic process;cellular process;cellular protein metabolic process;dephosphorylation;macromolecule metabolic process;macromolecule modification;metabolic process;
25.4533	25.1749	25.4551	24.6566	1:Experimental evidence at protein level	actin cytoskeleton organization;actin filament-based process;anatomical structure development;anatomical structure morphogenesis;biological regulation;brain development;cell surface receptor linked signaling pathway;cell
26.2545	25.9903	26.0192	25.8614	1:Experimental evidence at protein level	biosynthetic process;cellular biosynthetic process;cellular macromolecule biosynthetic process;cellular macromolecule metabolic process;cellular metabolic process;cellular nitrogen compound metabolic process;cellular pro
26.6435	26.8958	26.4695	26.5171	1:Experimental evidence at protein level	carboxylic acid catabolic process;carboxylic acid metabolic process;catabolic process;cellular catabolic process;cellular ketone metabolic process;cellular lipid catabolic process;cellular lipid metabolic process;cellular metal
26.0651	26.4896	25.9861	23.3307	1:Experimental evidence at protein level	activation of MAPKK activity;activation of protein kinase activity;anatomical structure development;anatomical structure formation involved in morphogenesis;anatomical structure morphogenesis;apoptosis;biological regulati
27.6951	27.6139	27.4158	27.4343	1:Experimental evidence at protein level	actin cytoskeleton organization;actin filament severing;actin filament-based process;biological regulation;biosynthetic process;cellular biosynthetic process;cellular component organization;cellular component organization at
26.9609	26.7881	26.7635	26.8231	1:Experimental evidence at protein level	amine metabolic process;biosynthetic process;carboxylic acid metabolic process;cellular amine metabolic process;cellular amino acid metabolic process;cellular biosynthetic process;cellular ketone metabolic process;cellula
25.8556	25.9758	25.6524	25.7227	1:Experimental evidence at protein level	acetyl-CoA metabolic process;acyl-CoA biosynthetic process;acyl-CoA metabolic process;acylglycerol biosynthetic process;acylglycerol metabolic process;amine transport;amino acid transmembrane transport;amino acid t
27.4271	27.3871	27.398	27.2717	1:Experimental evidence at protein level	biological regulation;cell cycle;cellular macromolecule metabolic process;cellular metabolic process;cellular process;cellular protein metabolic process;cellular response to stimulus;cellular response to stress;cullin deneddyla
29.0933	29.1359	29.343	29.0798	1:Experimental evidence at protein level	cell communication;cell-cell signaling;cellular process;immune response;immune system process;response to stimulus;signaling
27.3786	27.9518	28.36	28.1476	1:Experimental evidence at protein level	amine biosynthetic process;amine metabolic process;amino acid salvage;aspartate family amino acid biosynthetic process;aspartate family amino acid metabolic process;biosynthetic process;carboxylic acid biosynthetic pro
26.5688	26.4165	26.5847	26.4622	1:Experimental evidence at protein level	activation of MAPK activity;alcohol metabolic process;autophagy;biological regulation;biosynthetic process;carbohydrate homeostasis;carbohydrate metabolic process;carboxylic acid biosynthetic process;carboxylic acid me
25.6264	25.664	25.7317	25.2309	1:Experimental evidence at protein level	biological adhesion;biological regulation;cell adhesion;cell communication;cell-cell signaling;cell-matrix adhesion;cell-substrate adhesion;cellular process;cellular response to stimulus;establishment of localization;establishme
28.0178	28.0599	28.1252	28.0941	1:Experimental evidence at protein level	3'-UTR-mediated mRNA stabilization;biological regulation;biosynthetic process;cellular biosynthetic process;cellular component organization;cellular component organization or biogenesis;cellular macromolecule biosyntheti
28.8703	28.8897	28.5915	28.7143	1:Experimental evidence at protein level	3'-UTR-mediated mRNA stabilization;biological regulation;cellular macromolecule metabolic process;cellular metabolic process;cellular nitrogen compound metabolic process;cellular process;defense response;gene express
28.1965	27.8402	27.8039	27.7221	1:Experimental evidence at protein level	amine metabolic process;amino acid activation;apoptosis;biological regulation;carboxylic acid metabolic process;cell death;cell differentiation;cellular amine metabolic process;cellular amino acid metabolic process;cellular d
28.6639	28.4252	28.289	28.3682	1:Experimental evidence at protein level	4-hydroxyproline metabolic process;amine metabolic process;anatomical structure development;biological regulation;carboxylic acid metabolic process;cell redox homeostasis;cellular amine metabolic process;cellular amino
28.9954	28.678	28.7727	28.6982	1:Experimental evidence at protein level	actin cytoskeleton organization;actin filament-based process;activation of immune response;activation of innate immune response;activation of protein kinase activity;antigen receptor-mediated signaling pathway;apoptosis;
29.4465	29.5094	29.8449	29.6478	1:Experimental evidence at protein level	biological regulation;biosynthetic process;cellular biosynthetic process;cellular component organization;cellular component organization at cellular level;cellular component organization or biogenesis;cellular component orga
26.2984	26.1434	26.1339	26.3883	1:Experimental evidence at protein level	biological regulation;cellular component disassembly;cellular component disassembly at cellular level;cellular component organization;cellular component organization at cellular level;cellular component organization or bioge
29.237	28.9876	29.0196	28.9711	1:Experimental evidence at protein level	activation of immune response;activation of innate immune response;anaphase-promoting complex-dependent proteasomal ubiquitin-dependent protein catabolic process;antigen processing and presentation;antigen proces
24.2026	23.8005	24.6693	22.6687	1:Experimental evidence at protein level	axon guidance;cell communication;cell-cell signaling;cellular process;chemotaxis;locomotion;response to chemical stimulus;response to external stimulus;response to stimulus;signaling;taxis
27.9466	28.0886	27.6826	27.8482	1:Experimental evidence at protein level	biological regulation;cellular macromolecule metabolic process;cellular metabolic process;cellular process;cellular protein metabolic process;cellular response to chemical stimulus;cellular response to organic substance;cellu
26.3517	27.1863	27.2641	27.0686	1:Experimental evidence at protein level	establishment of localization;establishment of protein localization;protein transport;transport
27.5458	27.2035	27.7897	27.6211	1:Experimental evidence at protein level	biological regulation;biosynthetic process;cellular biosynthetic process;cellular component assembly;cellular component assembly at cellular level;cellular component organization;cellular component organization at cellular le
27.81	27.894	27.6901	27.6523	1:Experimental evidence at protein level	biological regulation;biosynthetic process;cellular biosynthetic process;cellular component assembly;cellular component assembly at cellular level;cellular component organization;cellular component organization at cellular le
29.8465	29.7494	29.5717	29.7329	1:Experimental evidence at protein level	alternative nuclear mRNA splicing, via spliceosome;biological regulation;biosynthetic process;cellular biosynthetic process;cellular component assembly;cellular component assembly at cellular level;cellular component orga
27.6164	27.6904	27.2898	27.7015	1:Experimental evidence at protein level	anaphase-promoting complex-dependent proteasomal ubiquitin-dependent protein catabolic process;biological regulation;catabolic process;cell cycle;cell cycle checkpoint;cell cycle process;cell division;cellular catabolic pr
30.1727	30.1443	30.1883	30.1642	1:Experimental evidence at protein level	anatomical structure morphogenesis;biological regulation;biosynthetic process;cell morphogenesis;cell morphogenesis involved in differentiation;cellular biosynthetic process;cellular component assembly;cellular component
29.4824	29.5234	29.7453	29.5645	1:Experimental evidence at protein level	biological regulation;cellular macromolecule metabolic process;cellular metabolic process;cellular nitrogen compound metabolic process;cellular process;cellular response to stimulus;DNA conformation change;DNA duplex u
28.4799	27.9657	28.1423	28.1155	1:Experimental evidence at protein level	actin cytoskeleton organization;actin cytoskeleton reorganization;actin filament-based process;anatomical structure development;anatomical structure formation involved in morphogenesis;anatomical structure morphogenes
27.5641	27.3815	27.5585	27.3845	1:Experimental evidence at protein level	biological regulation;biosynthetic process;blood coagulation;catabolic process;cellular biosynthetic process;cellular catabolic process;cellular macromolecule biosynthetic process;cellular macromolecule catabolic process;ce
26.3595	23.874	26.3095	26.097	1:Experimental evidence at protein level	defense response;defense response to virus;immune effector process;immune response;immune system process;innate immune response;multi-organism process;response to biotic stimulus;response to other organism;resp
25.4313	25.0496	25.0928	25.2339	1:Experimental evidence at protein level	behavior;biological regulation;biosynthetic process;catabolic process;cellular biosynthetic process;cellular catabolic process;cellular component organization;cellular component organization at cellular level;cellular componen
29.3157	29.4574	29.4929	29.4411	1:Experimental evidence at protein level	biological regulation;biosynthetic process;cellular biosynthetic process;cellular component assembly;cellular component assembly at cellular level;cellular component organization;cellular component organization at cellular le
26.9485	27.0278	26.853	27.0663	1:Experimental evidence at protein level	biological regulation;cell differentiation;cellular component organization;cellular component organization at cellular level;cellular component organization or biogenesis;cellular component organization or biogenesis at cellular
25.0223	24.1897	24.2364	25.5775	1:Experimental evidence at protein level	biological regulation;cellular macromolecule metabolic process;cellular metabolic process;cellular process;cellular protein metabolic process;cellular response to stimulus;intracellular signal transduction;macromolecule metal
25.6387	26.4428	26.5811	25.8603	1:Experimental evidence at protein level	activation of immune response;activation of innate immune response;antigen receptor-mediated signaling pathway;biological regulation;cell differentiation;cell surface receptor linked signaling pathway;cellular developmental
29.0297	29.0229	29.0965	29.0094	1:Experimental evidence at protein level	antigen processing and presentation;antigen processing and presentation of exogenous antigen;antigen processing and presentation of exogenous peptide antigen;antigen processing and presentation of exogenous peptide
28.3583	28.2421	28.1886	27.9767	1:Experimental evidence at protein level	aging;ameboidal cell migration;anatomical structure development;anatomical structure formation involved in morphogenesis;anatomical structure morphogenesis;axon ensheathment;biological adhesion;biological regulation;
26.9846	26.9145	26.6538	26.7002	1:Experimental evidence at protein level	acetyl-CoA catabolic process;acetyl-CoA metabolic process;biological regulation;catabolic process;cation transport;cell redox homeostasis;cellular catabolic process;cellular homeostasis;cellular metabolic process;cellular p
26.4944	26.0661	26.2474	26.1283	1:Experimental evidence at protein level	
26.3317	26.1446	26.3392	26.2674	1:Experimental evidence at protein level	cellular macromolecule metabolic process;cellular metabolic process;cellular nitrogen compound metabolic process;cellular process;cellular protein metabolic process;macromolecule metabolic process;macromolecule modi
27.639	27.3755	27.1959	27.2781	1:Experimental evidence at protein level	anatomical structure development;biosynthetic process;cellular biosynthetic process;cellular macromolecule biosynthetic process;cellular macromolecule metabolic process;cellular metabolic process;cellular nitrogen compo
28.9946	28.7178	29.0641	28.8951	1:Experimental evidence at protein level	cellular macromolecule metabolic process;cellular metabolic process;cellular nitrogen compound metabolic process;cellular process;gene expression;macromolecule metabolic process;metabolic process;mRNA metabolic p
23.5844	26.134	26.6267	26.4982	1:Experimental evidence at protein level	biological regulation;catabolic process;cellular catabolic process;cellular macromolecule catabolic process;cellular macromolecule metabolic process;cellular metabolic process;cellular process;cellular protein metabolic proc
25.6109	25.2737	25.829	25.5168	1:Experimental evidence at protein level	biological regulation;cellular process;establishment of localization;establishment of localization in cell;establishment of protein localization;establishment of protein localization in Golgi;establishment of protein localization to c
28.2246	28.0942	28.6201	28.4192	1:Experimental evidence at protein level	biological regulation;cell proliferation;cellular process;cellular response to stimulus;regulation of biological process;regulation of cellular process;response to stimulus;signal transduction
27.2868	27.1116	27.1353	27.0575	1:Experimental evidence at protein level	activation of MAPKK activity;activation of protein kinase activity;biological adhesion;biological regulation;cell activation;cell adhesion;cell adhesion mediated by integrin;cell differentiation;cell surface receptor linked signaling
26.6201	26.702	26.447	26.731	1:Experimental evidence at protein level	cellular macromolecule metabolic process;cellular metabolic process;cellular process;cellular protein metabolic process;chaperone-mediated protein folding;macromolecule metabolic process;macromolecule modification;me
25.7167	26.4686	26.2964	25.8161	1:Experimental evidence at protein level	actin filament-based movement;actin filament-based process;anatomical structure morphogenesis;biological regulation;cell chemotaxis;cell migration;cell motility;cell part morphogenesis;cell projection morphogenesis;cell pr
26.448	25.3881	26.302	26.0691	1:Experimental evidence at protein level	actin cytoskeleton organization;actin filament-based process;actin-mediated cell contraction;apical constriction;apoptosis;axon guidance;biological adhesion;biological regulation;bleb assembly;cell adhesion;cell death;cell m
27.5024	27.2706	27.0735	27.2597	1:Experimental evidence at protein level	anatomical structure development;anatomical structure morphogenesis;axonogenesis;biological regulation;cargo loading into vesicle;cation homeostasis;cell part morphogenesis;cell projection morphogenesis;cell projection
29.1646	28.9893	29.3712	29.1846	1:Experimental evidence at protein level	autophagy;biological regulation;catabolic process;cell communication;cell differentiation;cell surface receptor linked signaling pathway;cellular catabolic process;cellular component assembly;cellular component organization;
26.6605	26.4294	26.2761	26.3961	1:Experimental evidence at protein level	cellular macromolecule metabolic process;cellular metabolic process;cellular process;cellular protein metabolic process;establishment of localization;establishment of localization in cell;establishment of protein localization;es
28.5775	28.8678	28.7839	28.7469	1:Experimental evidence at protein level	axon guidance;cell cycle phase;cell cycle process;cellular component assembly;cellular component assembly at cellular level;cellular component organization;cellular component organization at cellular level;cellular compone
26.258	25.7985	26.0159	25.8428	1:Experimental evidence at protein level	cellular macromolecule metabolic process;cellular metabolic process;cellular nitrogen compound metabolic process;cellular process;cellular protein metabolic process;macromolecule metabolic process;macromolecule modi
27.8466	28.5346	28.6674	28.5799	1:Experimental evidence at protein level	biological regulation;cell cycle;cell surface receptor linked signaling pathway;cellular macromolecule metabolic process;cellular metabolic process;cellular process;cellular protein metabolic process;cellular response to chemi
23.9791	27.2829	27.3918	27.7395	1:Experimental evidence at protein level	anatomical structure development;biological regulation;biosynthetic process;cap-independent translational initiation;cell cycle process;cell surface receptor linked signaling pathway;cellular biosynthetic process;cellular macr
26.6283	26.2185	26.2876	26.3241	1:Experimental evidence at protein level	activation of caspase activity;activation of immune response;activation of innate immune response;activation of JUN kinase activity;activation of MAPK activity;apoptosis;biological regulation;catabolic process;cell death;cell
29.1175	28.8574	28.8018	28.7402	1:Experimental evidence at protein level	anatomical structure development;anatomical structure formation involved in morphogenesis;anatomical structure morphogenesis;ATP-dependent chromatin remodeling;biological regulation;biosynthetic process;blood coagu
27.4311	27.228	27.3347	27.2965	1:Experimental evidence at protein level	biological regulation;calcium ion homeostasis;calcium ion transport;calcium-mediated signaling;cation homeostasis;cation transport;cell communication;cell cycle process;cell growth;cell growth involved in cardiac muscle ce
30.4242	30.2852	30.7319	30.5167	1:Experimental evidence at protein level	antigen processing and presentation;antigen processing and presentation of exogenous antigen;antigen processing and presentation of exogenous peptide antigen;antigen processing and presentation of exogenous peptide
27.2607	27.0008	27.2855	27.0488	1:Experimental evidence at protein level	apoptosis;biological regulation;cell cycle checkpoint;cell death;cellular macromolecule metabolic process;cellular metabolic process;cellular process;cellular protein metabolic process;cellular response to stimulus;death;DNA
27.9649	27.57	28.0001	27.8884	1:Experimental evidence at protein level	biological regulation;biosynthetic process;cell communication;cell surface receptor linked signaling pathway;cellular biosynthetic process;cellular macromolecule biosynthetic process;cellular macromolecule metabolic proces
25.8288	25.3039	25.5402	25.6012	1:Experimental evidence at protein level	cellular macromolecule metabolic process;cellular metabolic process;cellular nitrogen compound metabolic process;cellular process;macromolecule metabolic process;metabolic process;mRNA metabolic process;mRNA pro
27.5764	27.3505	27.4227	27.1789	1:Experimental evidence at protein level	anatomical structure morphogenesis;biological regulation;cell part morphogenesis;cell projection morphogenesis;cell projection organization;cellular component morphogenesis;cellular component organization;cellular comp
25.9387	26.1632	25.3376	26.5412	1:Experimental evidence at protein level	cellular macromolecule metabolic process;cellular metabolic process;cellular nitrogen compound metabolic process;cellular process;macromolecule metabolic process;maturation of SSU-rRNA;maturation of SSU-rRNA from
26.7317	27.0371	27.1123	27.3028	1:Experimental evidence at protein level	biosynthetic process;cellular biosynthetic process;cellular macromolecule biosynthetic process;cellular macromolecule metabolic process;cellular metabolic process;cellular nitrogen compound metabolic process;cellular pro
27.5653	27.2531	27.1986	27.3436	1:Experimental evidence at protein level	activation of immune response;activation of innate immune response;anaphase-promoting complex-dependent proteasomal ubiquitin-dependent protein catabolic process;anatomical structure morphogenesis;biological regu
26.0934	26.5883	26.3575	26.4194	1:Experimental evidence at protein level	biological regulation;cell cycle arrest;cell cycle process;cellular macromolecule metabolic process;cellular metabolic process;cellular process;cellular protein metabolic process;cellular response to chemical stimulus;cellular r
27.7125	27.6303	27.5416	27.5264	1:Experimental evidence at protein level	actin cytoskeleton organization;actin filament bundle assembly;actin filament organization;actin filament-based process;anatomical structure morphogenesis;biological regulation;catabolic process;cell cycle arrest;cell cycle p
25.9288	25.8983	25.8359	25.6133	1:Experimental evidence at protein level	anatomical structure development;biological regulation;biosynthetic process;catabolic process;cell cycle arrest;cell cycle process;cellular biosynthetic process;cellular catabolic process;cellular macromolecule biosynthetic p
28.3172	28.0243	28.1367	27.9752	1:Experimental evidence at protein level	biological regulation;biosynthetic process;cell cycle;cellular biosynthetic process;cellular component organization;cellular component organization at cellular level;cellular component organization or biogenesis;cellular compo
26.5512	26.7285	26.5219	26.4751	1:Experimental evidence at protein level	alcohol biosynthetic process;alcohol metabolic process;biological adhesion;biosynthetic process;carbohydrate biosynthetic process;carbohydrate metabolic process;cell adhesion;cell death;cell-cell adhesion;cellular biosynt
26.9	26.9065	26.9498	26.8812	1:Experimental evidence at protein level	antigen processing and presentation;biological regulation;cellular component organization;cellular component organization at cellular level;cellular component organization or biogenesis;cellular component organization or bio
27.1286	26.6006	26.7075	26.5579	1:Experimental evidence at protein level	biological adhesion;cell adhesion;cellular process
26.5985	26.6694	26.8806	26.8409	1:Experimental evidence at protein level	anatomical structure development;anatomical structure morphogenesis;biological regulation;cell differentiation;cellular developmental process;cellular process;developmental process;muscle organ development;muscle struc
26.1738	26.3485	26.7137	26.7902	1:Experimental evidence at protein level	actin cytoskeleton organization;actin filament-based process;anatomical structure development;cellular component organization;cellular component organization at cellular level;cellular component organization or biogenesis;
26.5789	26.7623	26.7995	26.6496	1:Experimental evidence at protein level	anatomical structure formation involved in morphogenesis;angiogenesis;biological regulation;cell differentiation;cell migration;cell motility;cellular component movement;cellular developmental process;cellular process;develo
27.7403	27.6529	27.576	27.6107	1:Experimental evidence at protein level	carbohydrate metabolic process;cellular carbohydrate metabolic process;cellular macromolecule metabolic process;cellular metabolic process;cellular process;cellular protein metabolic process;glycosylation;macromolecule
28.2693	28.3623	28.0174	28.1379	1:Experimental evidence at protein level	axon guidance;biological adhesion;biological regulation;cell adhesion;cellular process;cellular response to stimulus;chemotaxis;locomotion;motor axon guidance;regulation of biological process;regulation of cellular process;
28.1171	27.6988	28.0263	27.7651	1:Experimental evidence at protein level	actin filament capping;apoptosis;axon guidance;biological regulation;cell death;cellular component disassembly;cellular component disassembly at cellular level;cellular component disassembly involved in apoptosis;cellular
25.9956	25.5407	25.5311	25.6705	1:Experimental evidence at protein level	cellular component biogenesis;cellular component biogenesis at cellular level;cellular component organization or biogenesis;cellular component organization or biogenesis at cellular level;cellular process;metabolic process;ri
30.0086	29.8865	29.9108	29.7812	1:Experimental evidence at protein level	biological regulation;cellular component assembly;cellular component assembly at cellular level;cellular component organization;cellular component organization at cellular level;cellular component organization or biogenesis;
27.0228	27.2109	27.3806	27.4054	1:Experimental evidence at protein level	antigen processing and presentation;antigen processing and presentation of peptide antigen;antigen processing and presentation of peptide antigen via MHC class I;cellular macromolecule metabolic process;cellular metabo

26.5456	24.8687	27.5871	26.8769	1:Experimental evidence at protein level	biological regulation;catabolic process;cellular catabolic process;cellular localization;cellular macromolecule catabolic process;cellular macromolecule localization;cellular macromolecule metabolic process;cellular metabolic
29.9639	30.0562	30.0961	29.9611	1:Experimental evidence at protein level	cellular component assembly;cellular component assembly at cellular level;cellular component organization;cellular component organization at cellular level;cellular component organization or biogenesis;cellular component c
26.7266	26.2146	26.3771	26.4282	1:Experimental evidence at protein level	biological adhesion;cell adhesion;cell differentiation;cell proliferation;cellular developmental process;cellular macromolecule metabolic process;cellular metabolic process;cellular nitrogen compound metabolic process;cellula
24.0576	27.2989	26.8323	27.5046	1:Experimental evidence at protein level	alcohol biosynthetic process;alcohol metabolic process;biosynthetic process;cellular biosynthetic process;cellular lipid metabolic process;cellular metabolic process;cellular process;cholesterol biosynthetic process;choleste
27.6609	27.9401	27.9644	28.0035	1:Experimental evidence at protein level	anatomical structure development;biological regulation;biosynthetic process;cell activation;cell differentiation;cell maturation;cellular biosynthetic process;cellular developmental process;cellular macromolecule biosynthetic p
28.125	27.8792	27.9622	27.8914	1:Experimental evidence at protein level	biological regulation;cell cycle;cell cycle phase;cell cycle process;cell division;cellular component organization;cellular component organization at cellular level;cellular component organization or biogenesis;cellular componen
28.2933	28.5894	28.8802	28.6896	1:Experimental evidence at protein level	biological regulation;cellular component assembly;cellular component assembly at cellular level;cellular component organization;cellular component organization at cellular level;cellular component organization or biogenesis;
30.5974	30.9076	30.6804	30.7536	1:Experimental evidence at protein level	alkene biosynthetic process;biosynthetic process;carboxylic acid biosynthetic process;carboxylic acid metabolic process;cellular alkene metabolic process;cellular biosynthetic process;cellular ketone metabolic process;cellu
24.4761	27.0564	26.5956	26.5599	1:Experimental evidence at protein level	anatomical structure development;brain development;cation transport;cellular metabolic process;cellular process;copper ion transport;developmental process;establishment of localization;generation of precursor metabolites
30.6506	30.7326	30.9239	30.7771	1:Experimental evidence at protein level	biological regulation;biosynthetic process;catabolic process;cellular biosynthetic process;cellular catabolic process;cellular macromolecule biosynthetic process;cellular macromolecule catabolic process;cellular macromolec
28.0968	27.633	28.0084	27.7294	1:Experimental evidence at protein level	biological adhesion;cell adhesion;cellular process;establishment of localization;establishment of localization in cell;establishment of protein localization;establishment of protein localization to organelle;establishment of protei
24.5086	23.4762	24.0136	24.4624	1:Experimental evidence at protein level	basement membrane organization;biological adhesion;cell adhesion;cell-matrix adhesion;cell-substrate adhesion;cellular component organization;cellular component organization at cellular level;cellular component organizat
25.8824	25.5747	25.3514	25.5482	1:Experimental evidence at protein level	biological regulation;cell proliferation;cellular component biogenesis;cellular component biogenesis at cellular level;cellular component organization or biogenesis;cellular component organization or biogenesis at cellular level
25.2169	25.2957	25.0189	25.0781	1:Experimental evidence at protein level	catabolic process;cellular catabolic process;cellular macromolecule catabolic process;cellular macromolecule metabolic process;cellular metabolic process;cellular process;cellular protein metabolic process;macromolecule c
29.9868	29.7426	29.9732	30.0629	1:Experimental evidence at protein level	biological regulation;biosynthetic process;cellular biosynthetic process;cellular macromolecule biosynthetic process;cellular macromolecule metabolic process;cellular metabolic process;cellular nitrogen compound metabolic
26.2636	26.3787	26.5998	26.4333	1:Experimental evidence at protein level	biological regulation;biosynthetic process;cellular biosynthetic process;cellular macromolecule biosynthetic process;cellular macromolecule metabolic process;cellular metabolic process;cellular nitrogen compound metabolic
30.3891	30.095	30.1672	30.0654	1:Experimental evidence at protein level	biological regulation;biosynthetic process;cellular biosynthetic process;cellular component assembly;cellular component assembly at cellular level;cellular component organization;cellular component organization at cellular le
26.1905	25.2065	26.03	25.9801	1:Experimental evidence at protein level	axon guidance;biological regulation;cell differentiation;cell junction assembly;cell junction organization;cell projection assembly;cell projection organization;cell surface receptor linked signaling pathway;cell-substrate junction
27.9688	28.0728	28.2813	28.2639	1:Experimental evidence at protein level	binding of sperm to zona pellucida;cell recognition;cell-cell recognition;cellular process;cellular process involved in reproduction;reproductive process;sperm-egg recognition
26.3476	26.0055	25.9707	26.1391	1:Experimental evidence at protein level	anatomical structure development;anatomical structure formation involved in morphogenesis;anatomical structure morphogenesis;apoptosis;apoptosis involved in morphogenesis;astrocyte cell migration;asymmetric protein l
26.9083	27.1022	26.8511	26.9264	1:Experimental evidence at protein level	behavior;behavioral response to pain;biological regulation;multicellular organismal process;multicellular organismal response to stress;regulation of biological process;regulation of cellular process;regulation of G-protein coup
27.0595	26.8974	27.0224	26.9107	1:Experimental evidence at protein level	carbohydrate metabolic process;cellular carbohydrate metabolic process;cellular macromolecule metabolic process;cellular metabolic process;cellular process;cellular protein metabolic process;glycosylation;macromolecule
26.5753	26.4629	26.4335	26.5625	1:Experimental evidence at protein level	cellular macromolecule metabolic process;cellular metabolic process;cellular process;cellular protein metabolic process;macromolecule metabolic process;macromolecule modification;metabolic process;primary metabolic p
28.7375	28.714	29.1608	29.0314	1:Experimental evidence at protein level	anatomical structure development;anatomical structure formation involved in morphogenesis;androgen receptor signaling pathway;atrial cardiac muscle cell development;biological regulation;biosynthetic process;cardiac cel
28.8535	29.0614	29.12	28.9744	1:Experimental evidence at protein level	actin crosslink formation;actin cytoskeleton organization;actin filament bundle assembly;actin filament organization;actin filament-based process;axon guidance;biological regulation;cellular component assembly;cellular com
25.7113	23.9123	25.4116	26.0046	1:Experimental evidence at protein level	biosynthetic process;cellular biosynthetic process;cellular component disassembly;cellular component disassembly at cellular level;cellular component organization;cellular component organization at cellular level;cellular co
27.8236	27.5389	27.6501	27.5445	1:Experimental evidence at protein level	anatomical structure development;antigen processing and presentation;antigen processing and presentation of exogenous antigen;antigen processing and presentation of exogenous peptide antigen;antigen processing and
30.0409	29.7984	29.8203	29.6898	1:Experimental evidence at protein level	antigen processing and presentation;antigen processing and presentation of exogenous antigen;antigen processing and presentation of exogenous peptide antigen;antigen processing and presentation of exogenous peptide
27.0543	23.9361	26.7292	26.6714	1:Experimental evidence at protein level	anatomical structure development;biological regulation;biosynthetic process;cell development;cellular biosynthetic process;cellular developmental process;cellular macromolecule biosynthetic process;cellular macromolecule
27.1407	27.0298	27.1971	26.9817	1:Experimental evidence at protein level	biological regulation;biosynthetic process;catabolic process;cell surface receptor linked signaling pathway;cellular biosynthetic process;cellular catabolic process;cellular macromolecule biosynthetic process;cellular macrom
25.7386	25.4987	25.7265	25.7037	1:Experimental evidence at protein level	biological regulation;biosynthetic process;cellular biosynthetic process;cellular macromolecule biosynthetic process;cellular macromolecule metabolic process;cellular metabolic process;cellular nitrogen compound metabolic
29.7499	29.5787	29.9117	29.8378	1:Experimental evidence at protein level	actin cytoskeleton organization;actin cytoskeleton reorganization;actin filament organization;actin filament polymerization;actin filament-based process;actin polymerization or depolymerization;anatomical structure morphoge
26.6304	23.8502	24.795	25.8147	1:Experimental evidence at protein level	anatomical structure development;biological adhesion;biological regulation;cell adhesion;cellular component assembly;cellular component assembly at cellular level;cellular component organization;cellular component organi
27.4754	27.8325	27.9739	28.0263	1:Experimental evidence at protein level	
27.5222	27.4262	27.6432	27.4095	1:Experimental evidence at protein level	biological regulation;biosynthetic process;cell surface receptor linked signaling pathway;cellular biosynthetic process;cellular macromolecule biosynthetic process;cellular macromolecule metabolic process;cellular metabolic
29.937	29.6724	29.6128	29.7682	1:Experimental evidence at protein level	anatomical structure development;cell development;cell junction assembly;cell junction organization;cellular component assembly;cellular component assembly at cellular level;cellular component organization;cellular compo
27.3189	27.4485	27.1747	27.134	1:Experimental evidence at protein level	anatomical structure development;apoptosis;biological regulation;camera-type eye development;cell death;cell fate specification;cell surface receptor linked signaling pathway;cellular developmental process;cellular macrom
27.9347	27.8409	28.0339	28.0175	1:Experimental evidence at protein level	gamete generation;male gamete generation;multicellular organismal process;multicellular organismal reproductive process;reproductive process;spermatogenesis
24.7685	24.0842	25.3832	25.1386	1:Experimental evidence at protein level	anatomical structure development;cellular macromolecule metabolic process;cellular metabolic process;cellular nitrogen compound metabolic process;cellular process;developmental process;macromolecule metabolic proce
26.8474	27.0323	26.8301	26.8571	1:Experimental evidence at protein level	anatomical structure development;anatomical structure morphogenesis;biological regulation;canonical Wnt receptor signaling pathway;cell communication;cell development;cell differentiation;cell surface receptor linked sign
27.2396	28.0459	27.6932	27.3857	1:Experimental evidence at protein level	activation of adenylate cyclase activity;activation of adenylate cyclase activity by G-protein signaling pathway;activation of phospholipase D activity;anatomical structure morphogenesis;biological regulation;blood coagulatio
26.7711	25.3632	27.1126	26.675	1:Experimental evidence at protein level	amine biosynthetic process;amine metabolic process;anatomical structure morphogenesis;biological regulation;biosynthetic process;carboxylic acid biosynthetic process;carboxylic acid metabolic process;cellular amine met
26.6081	26.6187	26.4601	26.3835	1:Experimental evidence at protein level	alcohol catabolic process;alcohol metabolic process;carbohydrate catabolic process;carbohydrate metabolic process;catabolic process;cellular carbohydrate catabolic process;cellular carbohydrate metabolic process;cellula
28.8122	28.6782	28.9729	28.8612	1:Experimental evidence at protein level	biological regulation;negative regulation of biological process;negative regulation of biosynthetic process;negative regulation of cellular biosynthetic process;negative regulation of cellular macromolecule biosynthetic process
27.443	27.6038	27.5902	27.3314	1:Experimental evidence at protein level	biological regulation;biosynthetic process;cellular biosynthetic process;cellular macromolecule biosynthetic process;cellular macromolecule metabolic process;cellular metabolic process;cellular nitrogen compound metabolic
25.1847	22.8214	25.1555	23.9796	1:Experimental evidence at protein level	biological adhesion;cell adhesion;cellular process
26.0393	25.7432	25.8604	25.735	1:Experimental evidence at protein level	biological regulation;biosynthetic process;cellular biosynthetic process;cellular component organization;cellular component organization at cellular level;cellular component organization or biogenesis;cellular component orga
26.108	26.1444	25.8008	25.7769	1:Experimental evidence at protein level	biological regulation;cell redox homeostasis;cellular homeostasis;cellular macromolecule metabolic process;cellular metabolic process;cellular process;cellular protein metabolic process;cellular response to chemical stimulus
26.517	26.7409	26.9385	26.6239	1:Experimental evidence at protein level	biological regulation;biosynthetic process;cellular biosynthetic process;cellular metabolic process;cellular nitrogen compound biosynthetic process;cellular nitrogen compound metabolic process;cellular process;metabolic pr
28.4195	28.2266	27.9993	28.0665	1:Experimental evidence at protein level	biosynthetic process;cell cycle;cell cycle process;cellular biosynthetic process;cellular macromolecule biosynthetic process;cellular macromolecule metabolic process;cellular metabolic process;cellular nitrogen compound m
27.9564	27.2567	27.4711	27.3916	1:Experimental evidence at protein level	activation of immune response;activation of innate immune response;activation of phospholipase C activity;behavior;biological regulation;blood coagulation;calcium ion homeostasis;calcium ion transport;calcium ion transpo
25.105	25.1078	25.0415	24.3428	1:Experimental evidence at protein level	acute inflammatory response;acute-phase response;amine metabolic process;aminoglycan metabolic process;biological regulation;carbohydrate metabolic process;defense response;glycosaminoglycan metabolic process;h
27.1975	26.9253	26.969	26.7938	1:Experimental evidence at protein level	activation of immune response;activation of innate immune response;apoptosis;biological regulation;biosynthetic process;cell death;cell surface receptor linked signaling pathway;cellular biosynthetic process;cellular macrom
25.9784	26.3923	26.1434	26.3841	1:Experimental evidence at protein level	biological regulation;cellular macromolecule metabolic process;cellular metabolic process;cellular nitrogen compound metabolic process;cellular process;cellular protein metabolic process;cellular response to stimulus;cellula
26.7815	26.5111	26.7719	26.5455	1:Experimental evidence at protein level	biological regulation;cellular component organization;cellular component organization or biogenesis;cellular process;establishment of localization;establishment of localization in cell;Golgi vesicle transport;intracellular transpo
26.591	26.087	26.3413	26.402	1:Experimental evidence at protein level	biological regulation;cell cycle checkpoint;cellular macromolecule metabolic process;cellular metabolic process;cellular nitrogen compound metabolic process;cellular process;cellular protein metabolic process;cellular respo
27.2813	27.0839	27.3959	27.1569	1:Experimental evidence at protein level	cellular component assembly;cellular component assembly at cellular level;cellular component organization;cellular component organization at cellular level;cellular component organization or biogenesis;cellular component c
27.701	27.2791	27.4971	27.413	1:Experimental evidence at protein level	biological regulation;cell cycle;cell cycle checkpoint;cell cycle phase;cell cycle process;cell division;cellular component organization;cellular component organization at cellular level;cellular component organization or biogene
26.202	26.5229	26.4449	26.1118	1:Experimental evidence at protein level	biological regulation;cellular macromolecule metabolic process;cellular metabolic process;cellular nitrogen compound metabolic process;cellular process;macromolecule metabolic process;metabolic process;ncRNA metabo
24.7051	24.7735	25.0885	22.1625	1:Experimental evidence at protein level	biological regulation;developmental process;multicellular organismal development;multicellular organismal process;negative regulation of biological process;negative regulation of gene expression;negative regulation of macro
25.9056	25.7755	25.3616	25.037	1:Experimental evidence at protein level	cellular component assembly;cellular component assembly at cellular level;cellular component organization;cellular component organization at cellular level;cellular component organization or biogenesis;cellular component c
27.1348	27.0792	27.1251	27.0735	1:Experimental evidence at protein level	autophagy;biological regulation;biosynthetic process;catabolic process;cellular biosynthetic process;cellular catabolic process;cellular macromolecule biosynthetic process;cellular macromolecule catabolic process;cellular m
27.3377	27.0757	27.46	27.5146	1:Experimental evidence at protein level	anatomical structure development;biological regulation;cell surface receptor linked signaling pathway;cellular localization;cellular macromolecule localization;cellular macromolecule metabolic process;cellular metabolic proce
30.1799	30.0945	30.0505	30.0204	1:Experimental evidence at protein level	carbohydrate metabolic process;cellular carbohydrate metabolic process;cellular macromolecule metabolic process;cellular metabolic process;cellular process;cellular protein metabolic process;glycosylation;macromolecule
26.3473	26.075	26.1539	26.2043	1:Experimental evidence at protein level	activation of immune response;activation of innate immune response;antigen processing and presentation;antigen receptor-mediated signaling pathway;B cell receptor signaling pathway;biological regulation;cell surface rece
27.0383	26.6984	26.5913	26.5371	1:Experimental evidence at protein level	activation of immune response;activation of innate immune response;alcohol metabolic process;anatomical structure development;biological regulation;carbohydrate metabolic process;cell surface receptor linked signaling p
28.0297	27.7911	27.9744	28.0342	1:Experimental evidence at protein level	alcohol metabolic process;biosynthetic process;cholesterol biosynthetic process;cholesterol metabolic process;lipid biosynthetic process;lipid metabolic process;metabolic process;primary metabolic process;small molecule
24.6812	24.739	26.1259	24.055	1:Experimental evidence at protein level	biosynthetic process;carbohydrate biosynthetic process;carbohydrate metabolic process;cellular carbohydrate metabolic process;cellular component organization;cellular component organization at cellular level;cellular com
29.3033	28.6209	28.7667	28.7735	1:Experimental evidence at protein level	biological regulation;cell proliferation;establishment of localization;establishment of protein localization;establishment of RNA localization;macromolecule metabolic process;metabolic process;mRNA transport;negative regula
25.0916	24.7635	25.0733	24.3622	1:Experimental evidence at protein level	biological regulation;cell surface receptor linked signaling pathway;cellular component organization;cellular component organization at cellular level;cellular component organization or biogenesis;cellular component organizat
26.4732	26.1695	26.18	26.057	1:Experimental evidence at protein level	cellular component organization;cellular component organization at cellular level;cellular component organization or biogenesis;cellular component organization or biogenesis at cellular level;cellular process;Golgi organizatior
25.139	25.5855	25.5302	25.2944	1:Experimental evidence at protein level	activation of caspase activity;activation of immune response;activation of innate immune response;anatomical structure development;anatomical structure formation involved in morphogenesis;angiogenesis;apoptosis;B cell a
28.4485	28.2406	28.1495	28.1213	1:Experimental evidence at protein level	ATP-dependent chromatin remodeling;biological regulation;biosynthetic process;cellular biosynthetic process;cellular component assembly;cellular component assembly at cellular level;cellular component organization;cellula
29.6287	30.3349	30.5349	30.4051	1:Experimental evidence at protein level	biological regulation;establishment of localization;ion transport;positive regulation of biological process;positive regulation of cell communication;positive regulation of cellular process;positive regulation of response to stimulu
27.9609	28.4008	28.4476	28.5965	1:Experimental evidence at protein level	arachidonic acid metabolic process;carboxylic acid metabolic process;cellular alkene metabolic process;cellular ketone metabolic process;cellular lipid metabolic process;cellular metabolic process;cellular process;cyclooxy
30.1088	30.0462	30.2565	30.0093	1:Experimental evidence at protein level	biological regulation;biosynthetic process;cellular biosynthetic process;cellular macromolecule biosynthetic process;cellular macromolecule metabolic process;cellular metabolic process;cellular nitrogen compound metabolic
31.3415	31.2378	31.1989	31.1263	1:Experimental evidence at protein level	apoptosis;biological regulation;catabolic process;cell death;cell surface receptor linked signaling pathway;cellular catabolic process;cellular component disassembly;cellular component disassembly at cellular level;cellular co
27.5539	27.2126	27.1036	27.007	1:Experimental evidence at protein level	cell cycle;cell cycle phase;cell cycle process;cellular component organization;cellular component organization at cellular level;cellular component organization or biogenesis;cellular component organization or biogenesis at ce
29.0725	28.6002	28.7473	28.6548	1:Experimental evidence at protein level	cell cycle;cell cycle phase;cell cycle process;cell differentiation;cell division;cellular component organization;cellular component organization at cellular level;cellular component organization or biogenesis;cellular component c
26.5379	26.1336	26.2407	25.9669	1:Experimental evidence at protein level	activation of immune response;activation of innate immune response;anaphase-promoting complex-dependent proteasomal ubiquitin-dependent protein catabolic process;antigen processing and presentation;antigen proces
26.6535	26.7316	26.84	26.7151	1:Experimental evidence at protein level	biological regulation;cellular component organization;cellular component organization or biogenesis;cellular membrane organization;cellular process;cellular response to stimulus;endocytosis;establishment of localization;mer
25.8227	25.8486	25.6321	25.2565	1:Experimental evidence at protein level	
28.1382	28.0995	28.2734	28.019	1:Experimental evidence at protein level	cell cycle;cell cycle process;cell division;cellular component organization;cellular component organization at cellular level;cellular component organization or biogenesis;cellular component organization or biogenesis at cellula
27.7287	27.3886	27.6081	27.694	1:Experimental evidence at protein level	biological regulation;biosynthetic process;cellular biosynthetic process;cellular component organization;cellular component organization at cellular level;cellular component organization or biogenesis;cellular component orga
28.344	28.5423	28.5327	28.2538	1:Experimental evidence at protein level	biological regulation;biosynthetic process;cellular biosynthetic process;cellular macromolecule biosynthetic process;cellular macromolecule metabolic process;cellular metabolic process;cellular process;cellular protein metab
28.0707	28.2863	27.9971	28.2453	1:Experimental evidence at protein level	
26.7045	26.664	26.604	26.6232	1:Experimental evidence at protein level	biological regulation;cell cycle;cellular macromolecule metabolic process;cellular metabolic process;cellular nitrogen compound metabolic process;cellular process;macromolecule metabolic process;macromolecule methylat
27.6871	27.4566	27.283	27.3778	1:Experimental evidence at protein level	activation of immune response;activation of innate immune response;anaphase-promoting complex-dependent proteasomal ubiquitin-dependent protein catabolic process;antigen processing and presentation;antigen proces
26.0936	25.8749	25.875	25.901	1:Experimental evidence at protein level	biological regulation;biosynthetic process;cellular biosynthetic process;cellular component organization;cellular component organization at cellular level;cellular component organization or biogenesis;cellular component orga
25.5206	26.0764	25.2948	25.2034	1:Experimental evidence at protein level	cellular process;cellular response to cold;cellular response to freezing;cellular response to stimulus;cellular response to stress;response to abiotic stimulus;response to cold;response to freezing;response to stimulus;response
28.9101	29.0118	29.1214	29.1688	1:Experimental evidence at protein level	anatomical structure formation involved in morphogenesis;biological regulation;cell cycle phase;cell cycle process;cell division;cell projection assembly;cell projection organization;cell surface receptor linked signaling pathwa
27.4012	26.9874	27.0709	26.9389	1:Experimental evidence at protein level	anatomical structure morphogenesis;assembly of spliceosomal tri-snRNP;biological regulation;cell morphogenesis;cell proliferation;cellular component assembly;cellular component assembly at cellular level;cellular compone
27.5933	27.6927	27.4716	27.4549	1:Experimental evidence at protein level	cell cycle;cell cycle process;cell division;cellular component organization;cellular component organization at cellular level;cellular component organization or biogenesis;cellular component organization or biogenesis at cellula
26.5642	26.1763	26.9177	26.9554	1:Experimental evidence at protein level	catabolic process;cellular catabolic process;cellular macromolecule catabolic process;cellular macromolecule metabolic process;cellular metabolic process;cellular nitrogen compound metabolic process;cellular process;exo
29.6676	29.9415	30.1536	30.4561	1:Experimental evidence at protein level	establishment of localization;establishment of protein localization;protein transport;transport
28.3844	28.0922	28.171	28.0308	1:Experimental evidence at protein level	cellular macromolecule metabolic process;cellular metabolic process;cellular nitrogen compound metabolic process;cellular process;gene expression;macromolecule metabolic process;metabolic process;mRNA metabolic p
28.5375	28.6096	28.3194	28.2517	1:Experimental evidence at protein level	cotranslational protein targeting to membrane;establishment of localization;establishment of localization in cell;establishment of protein localization;intracellular protein transport;intracellular transport;protein targeting;protein
26.3963	26.4518	26.3253	26.4524	1:Experimental evidence at protein level	anatomical structure development;anatomical structure morphogenesis;biological regulation;brain development;camera-type eye development;cellular chemical homeostasis;cellular component organization;cellular compone
26.4914	26.2188	25.8968	25.8084	1:Experimental evidence at protein level	biological regulation;cation homeostasis;cation transport;cellular cation homeostasis;cellular chemical homeostasis;cellular divalent inorganic cation homeostasis;cellular homeostasis;cellular ion homeostasis;cellular process
28.6146	28.3177	28.4363	28.2636	1:Experimental evidence at protein level	amine metabolic process;amino acid activation;biosynthetic process;bis(5'-nucleosidyl) oligophosphate biosynthetic process;bis(5'-nucleosidyl) oligophosphate metabolic process;carboxylic acid metabolic process;cellular a
26.1838	25.8837	25.9535	26.0152	1:Experimental evidence at protein level	cell cycle process;cell differentiation;cellular component biogenesis;cellular component biogenesis at cellular level;cellular component organization or biogenesis;cellular component organization or biogenesis at cellular level;
29.6842	29.6962	30.0285	30.2261	1:Experimental evidence at protein level	biological regulation;biosynthetic process;cellular biosynthetic process;cellular macromolecule biosynthetic process;cellular macromolecule metabolic process;cellular metabolic process;cellular process;cellular protein metab
26.5759	24.6075	26.0036	25.8249	1:Experimental evidence at protein level	anatomical structure development;biological adhesion;biological regulation;cell adhesion;cellular component organization;cellular component organization at cellular level;cellular component organization or biogenesis;cellula
25.6122	25.4209	25.8965	25.7648	1:Experimental evidence at protein level	biological regulation;carboxylic acid catabolic process;carboxylic acid metabolic process;catabolic process;cellular catabolic process;cellular component organization;cellular component organization at cellular level;cellular c
27.013	26.8826	27.3118	27.0565	1:Experimental evidence at protein level	cellular component organization;cellular component organization at cellular level;cellular component organization or biogenesis;cellular component organization or biogenesis at cellular level;cellular membrane fusion;cellular
30.521	30.764	30.7223	30.5662	1:Experimental evidence at protein level	apoptotic cell clearance;biological regulation;cell redox homeostasis;cellular component organization;cellular component organization or biogenesis;cellular homeostasis;cellular macromolecule metabolic process;cellular me
27.2342	27.6906	27.5527	27.7648	1:Experimental evidence at protein level	anatomical structure development;brain development;catabolic process;developmental process;gamete generation;lipid catabolic process;lipid metabolic process;male gamete generation;metabolic process;multicellular orga
27.3331	27.5611	27.192	27.2297	1:Experimental evidence at protein level	biological regulation;developmental process;macromolecule metabolic process;metabolic process;multicellular organismal development;multicellular organismal process;positive regulation of catalytic activity;positive regulati
27.6863	28.0229	28.4092	28.3481	1:Experimental evidence at protein level	apoptosis;biological regulation;carbohydrate transport;cell cycle checkpoint;cell death;cellular process;cellular response to stimulus;cellular response to stress;death;DNA damage checkpoint;DNA integrity checkpoint;establi
28.5022	27.5718	26.678	28.596	1:Experimental evidence at protein level	alcohol metabolic process;anatomical structure development;biological regulation;biosynthetic process;cellular process;cellular response to stimulus;cholesterol biosynthetic process;cholesterol metabolic process;developme
25.7146	26.2052	26.07	26.1137	1:Experimental evidence at protein level	acetyl-CoA metabolic process;alcohol metabolic process;biosynthetic process;cellular biosynthetic process;cellular lipid metabolic process;cellular metabolic process;cellular process;cholesterol biosynthetic process;cholest
30.3754	29.1932	29.6261	29.451	1:Experimental evidence at protein level	apoptosis;cell death;cell junction assembly;cell junction organization;cell-substrate junction assembly;cellular component assembly;cellular component assembly at cellular level;cellular component disassembly;cellular comp
26.1854	25.7614	25.9992	25.9557	1:Experimental evidence at protein level	anatomical structure formation involved in morphogenesis;biological regulation;cell cycle;cell cycle process;cell projection assembly;cell projection organization;cellular component assembly;cellular component assembly at c
29.3317	30.0572	30.0209	30.0819	1:Experimental evidence at protein level	amine metabolic process;amino acid activation;carboxylic acid metabolic process;cellular amine metabolic process;cellular amino acid metabolic process;cellular ketone metabolic process;cellular macromolecule metabolic p
30.6088	30.9445	31.0744	31.1531	1:Experimental evidence at protein level	anatomical structure homeostasis;arachidonic acid metabolic process;biological regulation;biosynthetic process;carboxylic acid biosynthetic process;carboxylic acid metabolic process;cellular biosynthetic process;cellular co
29.7875	29.532	29.7146	29.4218	1:Experimental evidence at protein level	biological regulation;biosynthetic process;cellular biosynthetic process;cellular macromolecule biosynthetic process;cellular macromolecule metabolic process;cellular metabolic process;cellular nitrogen compound metabolic
27.4649	28.0819	27.6901	27.5156	1:Experimental evidence at protein level	biological regulation;cell cycle process;cellular component organization;cellular component organization at cellular level;cellular component organization or biogenesis;cellular component organization or biogenesis at cellular
25.3085	25.9702	23.4422	25.4501	1:Experimental evidence at protein level	biological adhesion;biological regulation;cell adhesion;cell junction assembly;cell junction organization;cell migration;cell motility;cell surface receptor linked signaling pathway;cell-substrate junction assembly;cellular compor
25.7455	24.6646	24.7863	24.7925	1:Experimental evidence at protein level	cellular component assembly;cellular component assembly at cellular level;cellular component organization;cellular component organization at cellular level;cellular component organization or biogenesis;cellular component c
25.406	25.6951	25.6863	25.8296	1:Experimental evidence at protein level	apoptosis;cell death;cellular component organization;cellular component organization or biogenesis;cellular membrane organization;cellular process;death;endocytosis;establishment of localization;establishment of protein lo
28.507	28.6058	28.5652	28.5887	1:Experimental evidence at protein level	antigen processing and presentation;biological regulation;cell projection organization;cellular component organization;cellular component organization at cellular level;cellular component organization or biogenesis;cellular co
29.5866	29.0897	28.9998	29.0523	1:Experimental evidence at protein level	biological regulation;biosynthetic process;catabolic process;cellular biosynthetic process;cellular catabolic process;cellular macromolecule biosynthetic process;cellular macromolecule catabolic process;cellular macromolec
25.9404	27.1131	25.527	25.563	1:Experimental evidence at protein level	biological regulation;biosynthetic process;cellular biosynthetic process;cellular component organization;cellular component organization at cellular level;cellular component organization or biogenesis;cellular component orga
28.7327	29.0806	29.4748	29.3704	1:Experimental evidence at protein level	anatomical structure development;camera-type eye development;chordate embryonic development;developmental process;embryo development;embryo development ending in birth or egg hatching;eye development;in uter
25.6204	24.392	25.295	25.3109	1:Experimental evidence at protein level	biological regulation;cellular process;cellular response to stimulus;chemotaxis;establishment of localization;intracellular signal transduction;locomotion;metabolic process;positive regulation of catalytic activity;positive regulat
26.1211	25.8448	25.8575	26.1118	1:Experimental evidence at protein level	actin cytoskeleton organization;actin filament-based process;anatomical structure morphogenesis;axonogenesis;biological regulation;cell part morphogenesis;cell projection morphogenesis;cell projection organization;cellula
28.2374	27.9828	27.9608	27.8221	1:Experimental evidence at protein level	anatomical structure development;anatomical structure morphogenesis;biological regulation;cargo loading into vesicle;cellular component assembly;cellular component assembly at cellular level;cellular component organizati
31.3538	31.3635	31.3845	31.3616	1:Experimental evidence at protein level	cellular macromolecule metabolic process;cellular metabolic process;cellular nitrogen compound metabolic process;cellular process;gene expression;macromolecule metabolic process;metabolic process;mRNA metabolic p
30.1167	30.5724	30.2377	30.4506	1:Experimental evidence at protein level	biological regulation;catabolic process;cellular catabolic process;cellular macromolecule catabolic process;cellular macromolecule metabolic process;cellular metabolic process;cellular nitrogen compound metabolic process

28.9816	29.2126	29.2418	29.0243	1:Experimental evidence at protein level	biological regulation;biosynthetic process;cellular biosynthetic process;cellular macromolecule biosynthetic process;cellular macromolecule metabolic process;cellular metabolic process;cellular nitrogen compound metabolic
27.948	28.0116	27.9566	28.1052	1:Experimental evidence at protein level	biological regulation;biosynthetic process;cellular biosynthetic process;cellular component assembly;cellular component organization;cellular component organization or biogenesis;cellular macromolecule biosynthetic proces
28.0975	28.1679	28.0899	28.0405	1:Experimental evidence at protein level	biological regulation;biosynthetic process;cell cycle arrest;cell cycle process;cell surface receptor linked signaling pathway;cellular biosynthetic process;cellular macromolecule metabolic process;cellular metabolic process;c
27.0446	27.3708	27.2375	26.956	1:Experimental evidence at protein level	alcohol metabolic process;amyloid precursor protein catabolic process;amyloid precursor protein metabolic process;anatomical structure development;apoptosis;biological regulation;biosynthetic process;catabolic process;
28.6903	28.3094	28.4348	28.2658	1:Experimental evidence at protein level	biological regulation;cellular component assembly;cellular component organization;cellular component organization or biogenesis;cellular macromolecule metabolic process;cellular metabolic process;cellular nitrogen compo
25.7448	25.3083	25.015	25.1438	1:Experimental evidence at protein level	biological regulation;posttranscriptional regulation of gene expression;regulation of biological process;regulation of biosynthetic process;regulation of cellular biosynthetic process;regulation of cellular macromolecule biosynt
26.4628	26.2938	26.5792	26.3578	1:Experimental evidence at protein level	biological regulation;cell cycle process;cell proliferation;cellular component movement;cellular metabolic process;cellular process;chromosome movement towards spindle pole;dephosphorylation;establishment of chromoso
27.2432	27.1542	26.9911	27.3413	1:Experimental evidence at protein level	biological regulation;cell junction assembly;cell junction organization;cellular component assembly;cellular component assembly at cellular level;cellular component organization;cellular component organization at cellular leve
30.3048	30.5801	30.6292	30.6219	1:Experimental evidence at protein level	actin cytoskeleton organization;actin filament-based process;actomyosin structure organization;cell differentiation;cellular component organization;cellular component organization at cellular level;cellular component organiza
26.3617	25.9759	26.1504	25.5256	1:Experimental evidence at protein level	activation of immune response;activation of innate immune response;axon guidance;biological regulation;cell communication;cell cycle;cell proliferation;cell surface receptor linked signaling pathway;cell-cell signaling;cellular
28.4291	28.275	28.5164	28.4454	1:Experimental evidence at protein level	biological regulation;biosynthetic process;cellular biosynthetic process;cellular component organization;cellular component organization at cellular level;cellular component organization or biogenesis;cellular component orga
28.2737	27.6717	28.0941	28.0426	1:Experimental evidence at protein level	cellular component assembly;cellular component assembly at cellular level;cellular component organization;cellular component organization at cellular level;cellular component organization or biogenesis;cellular component c
27.0968	26.0905	26.6992	26.9804	1:Experimental evidence at protein level	cellular macromolecule metabolic process;cellular metabolic process;cellular nitrogen compound metabolic process;cellular process;macromolecule metabolic process;metabolic process;nitrogen compound metabolic proce
26.7242	26.7689	26.9248	27.0524	1:Experimental evidence at protein level	biological regulation;cellular process;chromosome segregation;positive regulation of biological process;positive regulation of cellular metabolic process;positive regulation of cellular process;positive regulation of cellular prote
29.4725	29.322	29.2167	29.2677	1:Experimental evidence at protein level	antigen processing and presentation;antigen processing and presentation of exogenous antigen;antigen processing and presentation of exogenous peptide antigen;antigen processing and presentation of exogenous peptide
26.6283	26.3554	26.5546	26.5423	1:Experimental evidence at protein level	cellular process;ER to Golgi vesicle-mediated transport;establishment of localization;establishment of localization in cell;establishment of protein localization;Golgi vesicle transport;intracellular protein transport;intracellular tr
28.3892	28.3821	28.5302	28.4383	1:Experimental evidence at protein level	biological regulation;cellular component assembly;cellular component assembly at cellular level;cellular component organization;cellular component organization at cellular level;cellular component organization or biogenesis;
25.1099	24.1394	25.5355	24.8552	1:Experimental evidence at protein level	
27.5498	27.1554	27.1007	27.2474	1:Experimental evidence at protein level	biological regulation;cell cycle phase;cell cycle process;cell division;cell proliferation;cellular component organization;cellular component organization at cellular level;cellular component organization or biogenesis;cellular com
26.3913	26.1822	26.6023	26.3549	1:Experimental evidence at protein level	anatomical structure formation involved in morphogenesis;angiogenesis;biological adhesion;biological regulation;cell adhesion;cell differentiation;cell proliferation;cellular component organization;cellular component organizat
27.5096	27.8222	27.8112	27.7538	1:Experimental evidence at protein level	cellular macromolecule metabolic process;cellular metabolic process;cellular nitrogen compound metabolic process;cellular process;DNA metabolic process;DNA recombination;gene expression;macromolecule metabolic pr
28.5803	28.7591	28.883	28.7965	1:Experimental evidence at protein level	biological regulation;biosynthetic process;cell differentiation;cellular biosynthetic process;cellular component assembly;cellular component assembly at cellular level;cellular component organization;cellular component organi
26.415	26.5978	26.4434	26.4359	1:Experimental evidence at protein level	actin cytoskeleton organization;actin filament-based process;biological regulation;cell communication;cellular component organization;cellular component organization at cellular level;cellular component organization or bioge
25.7036	24.6392	25.6239	25.2078	1:Experimental evidence at protein level	anatomical structure development;biosynthetic process;cardiac septum development;cellular biosynthetic process;cellular component organization;cellular component organization at cellular level;cellular component organiza
28.1656	27.4078	27.7585	27.4215	1:Experimental evidence at protein level	anatomical structure development;biosynthetic process;cell cycle phase;cell cycle process;cell development;cell maturation;cellular biosynthetic process;cellular component assembly;cellular component assembly at cellular
25.2097	24.4423	23.6596	24.5668	1:Experimental evidence at protein level	biological regulation;cell surface receptor linked signaling pathway;cellular process;cellular response to chemical stimulus;cellular response to cytokine stimulus;cellular response to interferon-gamma;cellular response to orga
28.1075	28.4951	28.4596	28.6586	1:Experimental evidence at protein level	biological regulation;biosynthetic process;cell junction assembly;cell junction organization;cell-substrate junction assembly;cellular biosynthetic process;cellular component assembly;cellular component assembly at cellular l
26.6814	26.6035	26.3425	26.4061	1:Experimental evidence at protein level	anatomical structure morphogenesis;biological regulation;biosynthetic process;cellular biosynthetic process;cellular macromolecule biosynthetic process;cellular macromolecule metabolic process;cellular metabolic process;
29.4912	29.5036	29.6134	29.5624	1:Experimental evidence at protein level	biological regulation;cell cycle;cell cycle phase;cell cycle process;cell division;cell proliferation;cellular component organization;cellular component organization at cellular level;cellular component organization or biogenesis;c
28.942	28.7092	28.8538	28.7575	1:Experimental evidence at protein level	3'-UTR-mediated mRNA stabilization;biological regulation;cellular macromolecule metabolic process;cellular metabolic process;cellular nitrogen compound metabolic process;cellular process;developmental process;gene ex
26.2869	26.3308	26.356	26.2101	1:Experimental evidence at protein level	alcohol metabolic process;anatomical structure development;biological regulation;biosynthetic process;blood vessel development;cell surface receptor linked signaling pathway;cellular process;cellular response to stimulus;
27.3452	27.1318	27.0999	27.0943	1:Experimental evidence at protein level	actin cytoskeleton organization;actin filament organization;actin filament-based process;anatomical structure morphogenesis;aorta smooth muscle tissue morphogenesis;biological regulation;bleb assembly;cell projection ass
29.1096	29.4093	29.115	29.0079	1:Experimental evidence at protein level	amine transport;amino acid transport;carboxylic acid transport;cellular process;establishment of localization;extracellular amino acid transport;extracellular transport;glutamine transport;ion transport;neutral amino acid trans
26.3131	26.3213	26.6003	26.4007	1:Experimental evidence at protein level	establishment of localization;establishment of localization in cell;establishment of protein localization;establishment of protein localization in mitochondrion;establishment of protein localization to organelle;intracellular protein
26.3718	24.4624	25.9204	26.3203	1:Experimental evidence at protein level	activin receptor signaling pathway;anatomical structure development;anatomical structure formation involved in morphogenesis;anatomical structure morphogenesis;anterior/posterior pattern specification;axis specification;
26.8134	26.6753	26.3977	26.5985	1:Experimental evidence at protein level	adult behavior;adult locomotory behavior;anatomical structure morphogenesis;axonogenesis;behavior;cell part morphogenesis;cell projection morphogenesis;cell projection organization;cellular component morphogenesis;
26.3727	26.2499	25.9282	26.0878	1:Experimental evidence at protein level	anatomical structure morphogenesis;cell morphogenesis;cellular component morphogenesis;cellular component organization;cellular component organization or biogenesis;cellular developmental process;cellular macromole
28.8436	30.0912	29.4867	29.8222	1:Experimental evidence at protein level	biological regulation;catabolic process;cell proliferation;cellular catabolic process;cellular macromolecule catabolic process;cellular macromolecule metabolic process;cellular metabolic process;cellular nitrogen compound m
27.8149	28.0824	28.118	28.3659	1:Experimental evidence at protein level	biological regulation;cell activation;cell activation involved in immune response;cell killing;cellular process;establishment of localization;establishment of localization in cell;establishment of protein localization;exocytosis;immu
29.0474	29.4	29.0635	29.0968	1:Experimental evidence at protein level	anatomical structure morphogenesis;biological adhesion;biological regulation;calcium ion-dependent exocytosis;cell adhesion;cell morphogenesis;cell morphogenesis involved in differentiation;cell-substrate adhesion;cellula
28.2276	28.4308	28.4352	28.5475	1:Experimental evidence at protein level	anatomical structure morphogenesis;biological regulation;catabolic process;cell surface receptor linked signaling pathway;cellular catabolic process;cellular macromolecule catabolic process;cellular macromolecule metaboli
29.6695	29.5698	29.57	29.6261	1:Experimental evidence at protein level	biological regulation;cation transport;cellular macromolecule metabolic process;cellular metabolic process;cellular process;cellular response to abiotic stimulus;cellular response to acidity;cellular response to pH;cellular resp
25.3902	26.2173	25.0604	24.8449	1:Experimental evidence at protein level	anatomical structure development;biological regulation;biosynthetic process;cell cycle arrest;cell cycle process;cellular biosynthetic process;cellular component organization;cellular component organization at cellular level;ce
30.2975	30.5898	30.6845	30.6921	1:Experimental evidence at protein level	actin cytoskeleton organization;actin filament bundle assembly;actin filament organization;actin filament-based process;biological adhesion;biological regulation;cell adhesion;cell communication;cell surface receptor linked s
28.781	28.6464	28.8904	28.5044	1:Experimental evidence at protein level	anatomical structure morphogenesis;biological regulation;cell cycle phase;cell cycle process;cell part morphogenesis;cell projection morphogenesis;cell projection organization;cellular component assembly;cellular compone
27.0207	27.3196	27.692	27.3734	1:Experimental evidence at protein level	biological regulation;biosynthetic process;cellular biosynthetic process;cellular component assembly;cellular component assembly at cellular level;cellular component organization;cellular component organization at cellular le
26.758	26.601	27.0984	26.9082	1:Experimental evidence at protein level	
27.8915	28.0719	28.1429	27.9511	1:Experimental evidence at protein level	alcohol biosynthetic process;alcohol metabolic process;amino sugar biosynthetic process;amino sugar metabolic process;biosynthetic process;carbohydrate biosynthetic process;carbohydrate metabolic process;cellular bio
26.5233	26.6632	26.9614	26.9368	1:Experimental evidence at protein level	anatomical structure development;biological adhesion;biological regulation;cell adhesion;cellular process;cellular response to chemical stimulus;cellular response to endogenous stimulus;cellular response to hormone stimulu
26.8144	26.5085	26.2686	26.444	1:Experimental evidence at protein level	biological adhesion;biological regulation;cell adhesion;cellular component organization;cellular component organization at cellular level;cellular component organization or biogenesis;cellular component organization or bioger
25.4996	24.8918	23.6081	25.939	1:Experimental evidence at protein level	amine metabolic process;aminoglycan biosynthetic process;aminoglycan metabolic process;anatomical structure development;anatomical structure morphogenesis;axon guidance;biological regulation;biosynthetic process;
27.604	27.4542	27.473	27.4164	1:Experimental evidence at protein level	activation of immune response;activation of innate immune response;anaphase-promoting complex-dependent proteasomal ubiquitin-dependent protein catabolic process;antigen processing and presentation;antigen proces
25.5198	25.6222	25.6055	25.4716	1:Experimental evidence at protein level	activation of JUN kinase activity;activation of MAPK activity;ameboidal cell migration;biological regulation;biosynthetic process;cell migration;cell motility;cellular biosynthetic process;cellular component movement;cellular c
27.875	27.8992	27.5477	27.8757	1:Experimental evidence at protein level	ameboidal cell migration;apical junction assembly;apoptosis;biological adhesion;biological regulation;biosynthetic process;cell adhesion;cell cycle;cell death;cell division;cell junction assembly;cell junction organization;cell m
27.9251	28.0274	28.541	28.3956	1:Experimental evidence at protein level	cell differentiation;cellular developmental process;cellular process;developmental process;multicellular organismal development;multicellular organismal process
28.2397	28.0618	28.0467	27.9064	1:Experimental evidence at protein level	biological regulation;biosynthetic process;catabolic process;cell surface receptor linked signaling pathway;cellular biosynthetic process;cellular catabolic process;cellular component organization;cellular component organizat
27.8073	27.6126	27.6992	27.5562	1:Experimental evidence at protein level	3'-UTR-mediated mRNA stabilization;activation of immune response;activation of innate immune response;activation of MAPK activity;aging;alcohol metabolic process;anatomical structure development;anatomical structure
29.0544	29.0539	29.1122	28.9972	1:Experimental evidence at protein level	biological regulation;cellular macromolecule metabolic process;cellular metabolic process;cellular process;cellular protein metabolic process;establishment of localization;establishment of localization in cell;establishment of p
29.3914	29.6703	29.7946	29.6672	1:Experimental evidence at protein level	anatomical structure development;axon guidance;biological regulation;cellular component organization;cellular component organization at cellular level;cellular component organization or biogenesis;cellular component orga
28.9143	29.1616	29.486	29.4208	1:Experimental evidence at protein level	ATP-dependent chromatin remodeling;biological regulation;biosynthetic process;cell proliferation;cellular biosynthetic process;cellular component assembly;cellular component assembly at cellular level;cellular component or
26.7001	26.928	27.2082	27.2839	1:Experimental evidence at protein level	anatomical structure development;anatomical structure formation involved in morphogenesis;angiogenesis;biological regulation;biomineral tissue development;cellular process;cellular response to stimulus;defense response;
29.2231	28.6525	28.8769	28.8971	1:Experimental evidence at protein level	biological regulation;biosynthetic process;cellular biosynthetic process;cellular macromolecule biosynthetic process;cellular macromolecule metabolic process;cellular metabolic process;cellular nitrogen compound metabolic
26.9772	27.1328	27.4741	27.284	1:Experimental evidence at protein level	cellular component assembly;cellular component organization;cellular component organization or biogenesis;cellular macromolecule metabolic process;cellular metabolic process;cellular nitrogen compound metabolic proce
28.5408	28.1322	28.4295	28.3696	1:Experimental evidence at protein level	actin cytoskeleton organization;actin filament organization;actin filament-based process;biological regulation;cell communication;cell communication by chemical coupling;cell communication by electrical coupling;cell prolife
29.5538	30.1014	29.622	29.7121	1:Experimental evidence at protein level	actin cytoskeleton organization;actin filament bundle assembly;actin filament organization;actin filament-based process;cell migration;cell motility;cell proliferation;cellular component assembly;cellular component assembly a
28.571	28.55	28.3726	28.341	1:Experimental evidence at protein level	activation of immune response;activation of innate immune response;anatomical structure development;autophagy;biological regulation;catabolic process;cell communication;cell differentiation;cell proliferation;cellular catabo
27.7935	27.7789	27.6901	27.6209	1:Experimental evidence at protein level	carboxylic acid catabolic process;carboxylic acid metabolic process;catabolic process;cellular catabolic process;cellular component assembly;cellular component organization;cellular component organization or biogenesis;
26.3281	26.6543	26.4241	26.2845	1:Experimental evidence at protein level	alcohol metabolic process;anatomical structure development;anatomical structure morphogenesis;biological regulation;carbohydrate metabolic process;cellular carbohydrate metabolic process;cellular component organizati
26.2629	26.0564	26.4396	26.2976	1:Experimental evidence at protein level	cellular metabolic process;cellular process;electron transport chain;generation of precursor metabolites and energy;metabolic process;mitochondrial electron transport, NADH to ubiquinone;oxidation-reduction process;respi
26.3332	26.2307	25.9665	25.8459	1:Experimental evidence at protein level	amine catabolic process;amine metabolic process;carboxylic acid catabolic process;carboxylic acid metabolic process;catabolic process;cell differentiation;cellular amine metabolic process;cellular amino acid catabolic proc
25.8772	26.6854	26.4412	26.6294	1:Experimental evidence at protein level	activation of anaphase-promoting complex activity;anaphase-promoting complex-dependent proteasomal ubiquitin-dependent protein catabolic process;biological regulation;catabolic process;cell cycle process;cell division;
23.8341	25.5692	25.825	25.4953	1:Experimental evidence at protein level	2'-deoxyribonucleotide biosynthetic process;2'-deoxyribonucleotide metabolic process;alcohol metabolic process;ATP metabolic process;biosynthetic process;carbohydrate metabolic process;cellular biosynthetic process;
26.2187	23.954	26.7957	26.7867	1:Experimental evidence at protein level	amine biosynthetic process;amine metabolic process;biosynthetic process;carboxylic acid biosynthetic process;carboxylic acid metabolic process;cellular amine metabolic process;cellular amino acid biosynthetic process;ce
25.9742	25.4203	25.6456	24.9489	1:Experimental evidence at protein level	cation transport;cellular metabolic process;cellular process;electron transport chain;establishment of localization;generation of precursor metabolites and energy;ion transport;metabolic process;metal ion transport;mitochon
28.566	28.3298	28.1882	28.4631	1:Experimental evidence at protein level	alcohol biosynthetic process;alcohol metabolic process;biological regulation;biosynthetic process;carbohydrate biosynthetic process;carbohydrate homeostasis;carbohydrate metabolic process;carboxylic acid metabolic pro
27.1447	27.5546	27.2495	27.5599	1:Experimental evidence at protein level	biosynthetic process;catabolic process;cell communication;cellular aromatic compound metabolic process;cellular biosynthetic process;cellular catabolic process;cellular metabolic compound salvage;cellular metabolic proc
27.5695	27.6266	27.7101	27.6384	1:Experimental evidence at protein level	biological regulation;carboxylic acid catabolic process;carboxylic acid metabolic process;catabolic process;cellular catabolic process;cellular ketone metabolic process;cellular lipid catabolic process;cellular lipid metabolic p
26.35	26.5301	26.2215	26.3302	1:Experimental evidence at protein level	alcohol metabolic process;biosynthetic process;cellular metabolic process;cellular process;cholesterol biosynthetic process;cholesterol biosynthetic process via 24,25-dihydrolanosterol;cholesterol metabolic process;demeth
28.9749	28.9658	28.8891	28.8369	1:Experimental evidence at protein level	alcohol metabolic process;biosynthetic process;carbohydrate biosynthetic process;carbohydrate metabolic process;carboxylic acid biosynthetic process;carboxylic acid metabolic process;cellular biosynthetic process;cellula
28.7113	28.9001	29.0374	28.9714	1:Experimental evidence at protein level	anatomical structure formation involved in morphogenesis;biological regulation;biosynthetic process;cell proliferation;cell redox homeostasis;cellular biosynthetic process;cellular homeostasis;cellular lipid metabolic process;
27.9156	27.2281	27.5828	27.3077	1:Experimental evidence at protein level	biological regulation;calcium ion homeostasis;cation homeostasis;cellular calcium ion homeostasis;cellular cation homeostasis;cellular chemical homeostasis;cellular divalent inorganic cation homeostasis;cellular homeostasi
28.9337	28.8805	28.9796	28.9894	1:Experimental evidence at protein level	
27.2673	27.1607	27.2367	27.0538	1:Experimental evidence at protein level	actin cytoskeleton organization;actin filament-based process;biological regulation;cellular component organization;cellular component organization at cellular level;cellular component organization or biogenesis;cellular comp
26.7244	26.629	26.4516	26.5024	1:Experimental evidence at protein level	biological regulation;cell cycle;cell cycle process;cell division;cellular component organization;cellular component organization at cellular level;cellular component organization or biogenesis;cellular component organization or
26.0169	25.7041	25.7255	25.7244	1:Experimental evidence at protein level	biological regulation;cellular component organization;cellular component organization or biogenesis;cellular macromolecule metabolic process;cellular membrane organization;cellular metabolic process;cellular process;cellula
26.572	23.9734	26.5688	26.3761	1:Experimental evidence at protein level	cellular component organization;cellular component organization at cellular level;cellular component organization or biogenesis;cellular component organization or biogenesis at cellular level;cellular process;endosome organi
26.2534	26.1808	26.1246	26.1574	1:Experimental evidence at protein level	biological regulation;cell cycle;cell cycle phase;cell cycle process;cell division;cellular component organization;cellular component organization at cellular level;cellular component organization or biogenesis;cellular componer
27.7127	27.5067	27.575	27.5964	1:Experimental evidence at protein level	cellular component assembly;cellular component assembly at cellular level;cellular component organization;cellular component organization at cellular level;cellular component organization or biogenesis;cellular component c
26.4659	26.1996	26.494	26.4054	1:Experimental evidence at protein level	
27.8135	27.1321	27.3848	27.2513	1:Experimental evidence at protein level	
24.3014	25.3308	24.0698	24.9166	1:Experimental evidence at protein level	autophagic vacuole assembly;autophagy;catabolic process;cell communication;cellular catabolic process;cellular component assembly;cellular component assembly at cellular level;cellular component organization;cellular c
23.9452	23.7397	23.9899	23.9843	1:Experimental evidence at protein level	antigen processing and presentation;antigen processing and presentation of peptide antigen;antigen processing and presentation of peptide antigen via MHC class I;biological regulation;biosynthetic process;BMP signaling p
28.198	27.9967	28.4134	28.3907	1:Experimental evidence at protein level	biological regulation;biosynthetic process;cellular biosynthetic process;cellular macromolecule biosynthetic process;cellular macromolecule metabolic process;cellular metabolic process;cellular nitrogen compound metabolic
28.1677	27.8629	28.0028	27.8973	1:Experimental evidence at protein level	anatomical structure development;biological regulation;bone development;cell growth;cellular component organization;cellular component organization at cellular level;cellular component organization or biogenesis;cellular c
26.9155	26.8876	27.0379	26.9281	1:Experimental evidence at protein level	cellular component organization;cellular component organization at cellular level;cellular component organization or biogenesis;cellular component organization or biogenesis at cellular level;cellular process;cytoskeleton orga
26.7734	27.1243	27.0667	27.0157	1:Experimental evidence at protein level	
24.953	25.0621	24.6485	25.0102	1:Experimental evidence at protein level	alcohol metabolic process;alditol metabolic process;biological regulation;carbohydrate metabolic process;carbohydrate phosphorylation;cellular carbohydrate metabolic process;cellular metabolic process;cellular process;de
25.6193	25.1145	25.4989	25.9516	1:Experimental evidence at protein level	
26.2646	26.2191	23.7253	23.2867	1:Experimental evidence at protein level	10-formyltetrahydrofolate catabolic process;10-formyltetrahydrofolate metabolic process;aromatic compound catabolic process;biosynthetic process;catabolic process;cellular aromatic compound metabolic process;cellular
25.6821	25.357	25.5797	25.3031	1:Experimental evidence at protein level	activation of protein kinase activity;activation of protein kinase B activity;biological regulation;biosynthetic process;cell migration;cell motility;cell projection assembly;cell projection organization;cellular biosynthetic process;c
26.6909	26.5796	26.261	26.6218	1:Experimental evidence at protein level	apoptotic mitochondrial changes;cellular component organization;cellular component organization at cellular level;cellular component organization or biogenesis;cellular component organization or biogenesis at cellular level;
25.6267	25.7717	25.2692	25.3377	1:Experimental evidence at protein level	alcohol catabolic process;alcohol metabolic process;carbohydrate catabolic process;carbohydrate metabolic process;catabolic process;cellular carbohydrate catabolic process;cellular carbohydrate metabolic process;cellula
25.662	24.5317	25.6068	25.5137	1:Experimental evidence at protein level	biological regulation;cellular process;endosome transport;establishment of localization;establishment of localization in cell;intracellular transport;positive regulation of catalytic activity;positive regulation of GTPase activity;pos
25.3443	25.5789	25.5231	25.323	1:Experimental evidence at protein level	activation of blood coagulation via clotting cascade;anatomical structure development;anion transport;biological regulation;biomineral tissue development;blood coagulation;bone mineralization;bone mineralization involved i
25.9755	25.5862	26.1996	25.9761	1:Experimental evidence at protein level	
26.5675	27.1066	27.1186	26.6559	1:Experimental evidence at protein level	
26.6983	24.607	26.3213	26.8715	1:Experimental evidence at protein level	cell differentiation;cellular developmental process;cellular process;developmental process;osteoblast differentiation
25.3424	26.063	26.1797	26.2418	1:Experimental evidence at protein level	
27.2034	26.8649	26.9727	27.0781	1:Experimental evidence at protein level	
29.1147	24.875	28.6357	28.6418	1:Experimental evidence at protein level	biological regulation;cellular process;cellular response to stimulus;intracellular signal transduction;positive regulation of catalytic activity;positive regulation of GTPase activity;positive regulation of hydrolase activity;positive re
25.9217	26.0378	25.6105	25.8267	1:Experimental evidence at protein level	aging;apoptosis;biological regulation;cell aging;cell death;cellular process;death;developmental process;negative regulation of biological process;negative regulation of biosynthetic process;negative regulation of catalytic act
27.1028	27.2378	26.8449	26.8606	1:Experimental evidence at protein level	
26.1602	25.8071	26.258	26.127	1:Experimental evidence at protein level	cell cycle cytokinesis;cell cycle phase;cell cycle process;cellular component organization;cellular component organization at cellular level;cellular component organization or biogenesis;cellular component organization or biog
25.7762	25.9024	26.0144	26.0501	1:Experimental evidence at protein level	
26.6179	27.0412	26.4461	26.2439	1:Experimental evidence at protein level	acyl-CoA biosynthetic process;acyl-CoA metabolic process;acylglycerol biosynthetic process;acylglycerol metabolic process;biological regulation;biosynthetic process;carboxylic acid biosynthetic process;carboxylic acid me
26.9754	26.9332	26.9467	26.6578	1:Experimental evidence at protein level	catabolic process;cell cycle;cell division;cellular catabolic process;cellular component assembly;cellular component assembly at cellular level;cellular component organization;cellular component organization at cellular level;
26.1788	24.9709	26.4473	26.6958	1:Experimental evidence at protein level	
26.8016	27.0447	26.8856	26.9719	1:Experimental evidence at protein level	amine biosynthetic process;amine metabolic process;biosynthetic process;carboxylic acid biosynthetic process;carboxylic acid metabolic process;cellular amine metabolic process;cellular amino acid biosynthetic process;ce
25.6667	24.8495	23.7943	25.3368	1:Experimental evidence at protein level	biological regulation;cation homeostasis;cellular cation homeostasis;cellular chemical homeostasis;cellular homeostasis;cellular ion homeostasis;cellular iron ion homeostasis;cellular metal ion homeostasis;cellular process;ch
25.7395	25.5188	25.1429	25.4706	1:Experimental evidence at protein level	cellular process;establishment of localization;establishment of localization in cell;establishment of protein localization;Golgi to plasma membrane transport;Golgi vesicle transport;intracellular transport;post-Golgi vesicle-med
27.5521	28.9086	27.6516	28.4317	5:Protein uncertain	cellular macromolecule metabolic process;cellular metabolic process;cellular process;cellular protein metabolic process;macromolecule metabolic process;metabolic process;primary metabolic process;protein folding;protein
26.4071	27.1364	26.8316	26.9335	1:Experimental evidence at protein level	
26.9303	26.9473	27.1247	26.9517	1:Experimental evidence at protein level	biological regulation;biosynthetic process;cellular biosynthetic process;cellular macromolecule biosynthetic process;cellular macromolecule metabolic process;cellular metabolic process;cellular nitrogen compound metabolic
25.0164	26.2109	24.7717	24.7255	1:Experimental evidence at protein level	
28.0065	27.7928	27.8796	27.754	1:Experimental evidence at protein level	cell differentiation;cellular developmental process;cellular macromolecule metabolic process;cellular metabolic process;cellular process;cellular protein metabolic process;developmental process;gamete generation;macromo
26.8497	24.7138	27.6626	27.5142	1:Experimental evidence at protein level	biological regulation;biosynthetic process;cellular biosynthetic process;cellular macromolecule biosynthetic process;cellular macromolecule metabolic process;cellular metabolic process;cellular nitrogen compound metabolic
27.6508	27.7067	27.6185	27.703	1:Experimental evidence at protein level	biological regulation;regulation of biological process;regulation of catalytic activity;regulation of cellular metabolic process;regulation of cellular process;regulation of dephosphorylation;regulation of hydrolase activity;regulatio
25.0153	26.2009	25.3222	25.3643	1:Experimental evidence at protein level	biological regulation;cellular process;cellular response to stimulus;establishment of localization;establishment of localization in cell;establishment of protein localization;intracellular protein transport;intracellular signal transdu
25.6407	25.1672	25.2658	23.9302	1:Experimental evidence at protein level	amine metabolic process;amino acid activation;carboxylic acid metabolic process;cellular amine metabolic process;cellular amino acid metabolic process;cellular ketone metabolic process;cellular macromolecule metabolic

26.8465	27.1139	26.5118	26.7309	1:Experimental evidence at protein level	anatomical structure development;biological regulation;cell development;cellular component organization;cellular component organization at cellular level;cellular component organization or biogenesis;cellular component org
26.6649	26.8341	26.9257	26.7765	1:Experimental evidence at protein level	biological regulation;macromolecule metabolic process;metabolic process;positive regulation of catalytic activity;positive regulation of molecular function;primary metabolic process;protein metabolic process;proteolysis;regu
25.3464	25.2378	25.4444	25.0332	1:Experimental evidence at protein level	
26.8098	26.5295	26.5033	26.5391	1:Experimental evidence at protein level	
28.0996	28.1327	27.9835	28.0009	1:Experimental evidence at protein level	biological regulation;cellular component organization;cellular component organization or biogenesis;cellular localization;cellular macromolecule localization;cellular membrane organization;cellular process;cellular protein local
26.9314	27.0424	26.7755	26.5724	1:Experimental evidence at protein level	alanyl-tRNA aminoacylation;amine metabolic process;amino acid activation;carboxylic acid metabolic process;cellular amine metabolic process;cellular amino acid metabolic process;cellular component assembly;cellular co
26.4084	26.9302	26.3686	26.4521	1:Experimental evidence at protein level	anatomical structure development;biological regulation;cellular macromolecule metabolic process;cellular metabolic process;cellular nitrogen compound metabolic process;cellular process;developmental process;hemopoiet
25.9779	25.8821	26.3125	26.0566	1:Experimental evidence at protein level	activation of immune response;activation of innate immune response;biological regulation;carbohydrate mediated signaling;cell recognition;cellular component assembly;cellular component organization;cellular component o
22.9632	24.3868	23.5759	24.8951	1:Experimental evidence at protein level	biological adhesion;cell adhesion;cellular process
25.6852	25.4026	26.0599	26.1211	1:Experimental evidence at protein level	autophagic vacuole assembly;autophagy;catabolic process;cell communication;cellular catabolic process;cellular component assembly;cellular component assembly at cellular level;cellular component organization;cellular co
27.4069	27.2193	27.1875	27.5215	1:Experimental evidence at protein level	B cell activation;B cell activation involved in immune response;catabolic process;cell activation;cell activation involved in immune response;cellular catabolic process;cellular macromolecule catabolic process;cellular macrom
25.6836	25.6757	25.0756	25.1813	1:Experimental evidence at protein level	biological regulation;biosynthetic process;carbohydrate metabolic process;carbohydrate transport;cell cycle;cell cycle process;cell surface receptor linked signaling pathway;cellular biosynthetic process;cellular component c
28.5689	28.3936	28.1924	28.2736	1:Experimental evidence at protein level	biological regulation;cellular component assembly;cellular component assembly at cellular level;cellular component organization;cellular component organization at cellular level;cellular component organization or biogenesis;
26.7802	26.9386	26.6695	26.7795	1:Experimental evidence at protein level	
26.1663	24.7917	26.0525	25.9367	1:Experimental evidence at protein level	cellular macromolecule metabolic process;cellular metabolic process;cellular nitrogen compound metabolic process;cellular process;cleavage in ITS2 between 5.8S rRNA and LSU-rRNA of tricistronic rRNA transcript (SSU-rF
26.0936	26.2675	25.874	26.2001	1:Experimental evidence at protein level	actin filament-based movement;actin filament-based process;anatomical structure formation involved in morphogenesis;autophagy;biological regulation;catabolic process;cell projection assembly;cell projection organization;
26.5851	26.7538	26.9877	26.6779	1:Experimental evidence at protein level	biological regulation;cellular component organization;cellular component organization or biogenesis;cellular macromolecule metabolic process;cellular membrane organization;cellular metabolic process;cellular process;cellula
26.4525	24.4075	27.5576	27.2469	1:Experimental evidence at protein level	
26.9861	26.8935	26.8203	26.8352	1:Experimental evidence at protein level	cellular macromolecule metabolic process;cellular metabolic process;cellular process;cellular protein metabolic process;macromolecule metabolic process;macromolecule modification;metabolic process;primary metabolic p
25.7822	25.8144	25.9889	25.8393	1:Experimental evidence at protein level	anatomical structure development;developmental process;embryonic skeletal system development;skeletal system development;system development
25.6532	24.6715	25.4678	25.8398	1:Experimental evidence at protein level	
26.8398	27.1808	26.7774	27.0264	1:Experimental evidence at protein level	
23.8374	24.3829	25.9087	24.3501	1:Experimental evidence at protein level	
26.516	25.6479	25.8623	26.4014	1:Experimental evidence at protein level	biological regulation;cellular macromolecule metabolic process;cellular metabolic process;cellular nitrogen compound metabolic process;cellular process;macromolecule metabolic process;metabolic process;mRNA metabol
27.6166	27.3821	27.2347	27.372	1:Experimental evidence at protein level	
25.869	26.1569	25.9639	24.9008	1:Experimental evidence at protein level	anatomical structure formation involved in morphogenesis;anterior/posterior axis specification;axis specification;biological regulation;cell surface receptor linked signaling pathway;cellular process;cellular response to stimulu
29.1948	24.6263	29.7715	29.4277	1:Experimental evidence at protein level	cellular component assembly;cellular component organization;cellular component organization or biogenesis;macromolecular complex assembly;macromolecular complex subunit organization;protein complex assembly;prot
32.9897	30.8618	31.5045	31.9293	1:Experimental evidence at protein level	
25.3357	24.7301	25.5527	25.4643	1:Experimental evidence at protein level	cellular metabolic process;cellular process;dephosphorylation;metabolic process;phosphate-containing compound metabolic process;phosphorus metabolic process
25.5159	25.3472	25.4135	25.1748	1:Experimental evidence at protein level	biological regulation;cell cycle;cellular process;cellular response to stimulus;cellular response to stress;developmental process;negative regulation of biological process;negative regulation of cell differentiation;negative regula
24.5733	25.5326	24.6559	25.1435	1:Experimental evidence at protein level	actin cytoskeleton organization;actin filament-based process;biological regulation;cellular component organization;cellular component organization at cellular level;cellular component organization or biogenesis;cellular compo
26.6865	26.1596	26.5426	26.1858	1:Experimental evidence at protein level	biological regulation;catabolic process;cellular catabolic process;cellular component organization;cellular component organization at cellular level;cellular component organization or biogenesis;cellular component organizatio
27.1326	26.8701	27.1463	27.0643	1:Experimental evidence at protein level	
24.6711	24.5283	24.3535	24.3305	1:Experimental evidence at protein level	
27.4689	27.4013	27.2879	27.1818	1:Experimental evidence at protein level	catabolic process;cellular catabolic process;cellular macromolecule catabolic process;cellular macromolecule metabolic process;cellular metabolic process;cellular process;ER-associated protein catabolic process;macromo
26.0865	26.0003	25.9602	25.8668	1:Experimental evidence at protein level	biological regulation;cell proliferation;muscle cell proliferation;negative regulation of apoptosis;negative regulation of biological process;negative regulation of cell death;negative regulation of cellular process;negative regulatio
25.6488	25.6034	25.5322	25.4486	1:Experimental evidence at protein level	
24.5574	23.9648	24.4544	24.5066	1:Experimental evidence at protein level	cellular macromolecule metabolic process;cellular metabolic process;cellular process;cellular protein metabolic process;macromolecule metabolic process;macromolecule modification;metabolic process;primary metabolic p
26.1568	25.8395	25.7802	25.8266	1:Experimental evidence at protein level	biological regulation;cell activation;cell proliferation;cellular process;immune response;immune system process;leukocyte activation;lymphocyte activation;negative regulation of biological process;negative regulation of biosy
25.329	24.995	24.686	25.6639	1:Experimental evidence at protein level	cellular macromolecule metabolic process;cellular metabolic process;cellular process;cellular protein metabolic process;macromolecule metabolic process;macromolecule modification;metabolic process;primary metabolic p
26.0646	24.7007	24.7714	25.3617	1:Experimental evidence at protein level	anatomical structure development;biological regulation;cellular homeostasis;cellular process;cellular response to stimulus;collagen metabolic process;developmental process;growth;homeostatic process;intracellular signal tr
26.6147	26.4517	26.4823	26.2827	1:Experimental evidence at protein level	apoptosis;biological regulation;biosynthetic process;cell death;cellular biosynthetic process;cellular macromolecule biosynthetic process;cellular macromolecule metabolic process;cellular metabolic process;cellular nitrogen
26.7887	26.4629	27.0792	26.7637	1:Experimental evidence at protein level	cellular process;endosome transport;establishment of localization;establishment of localization in cell;establishment of protein localization;intracellular transport;protein transport;retrograde transport, endosome to Golgi;trans
25.5239	25.2982	25.2969	25.4745	1:Experimental evidence at protein level	
25.3525	24.9328	25.1724	24.3974	1:Experimental evidence at protein level	
26.2308	23.7031	26.0655	26.0662	1:Experimental evidence at protein level	anatomical structure development;anatomical structure morphogenesis;apoptosis;autophagy;biological regulation;brain development;catabolic process;cell death;cell part morphogenesis;cell projection morphogenesis;cell p
26.4542	26.5226	26.2577	26.304	1:Experimental evidence at protein level	cellular macromolecule metabolic process;cellular metabolic process;cellular nitrogen compound metabolic process;cellular process;macromolecule metabolic process;metabolic process;mRNA metabolic process;mRNA pro
25.3876	25.1273	25.2188	25.5445	1:Experimental evidence at protein level	cellular macromolecule metabolic process;cellular metabolic process;cellular nitrogen compound metabolic process;cellular process;developmental process;macromolecule metabolic process;maturation of SSU-rRNA;matur
27.381	27.198	27.2769	27.3963	1:Experimental evidence at protein level	anatomical structure development;biological regulation;cell migration;cell motility;cellular component movement;cellular process;developmental process;locomotion;lung alveolus development;positive regulation of biological
25.6526	25.5327	25.3183	25.7148	1:Experimental evidence at protein level	biological regulation;cell cycle checkpoint;cellular macromolecule metabolic process;cellular metabolic process;cellular nitrogen compound metabolic process;cellular process;cellular response to stimulus;cellular response to
25.9281	26.5659	26.0285	26.211	1:Experimental evidence at protein level	biological regulation;cellular process;cellular response to stimulus;intracellular signal transduction;regulation of biological process;regulation of cellular process;regulation of response to stimulus;regulation of signal transducti
26.2636	26.4951	26.0024	26.3469	1:Experimental evidence at protein level	
28.6608	24.9101	28.2786	23.3333	1:Experimental evidence at protein level	cellular macromolecule metabolic process;cellular metabolic process;cellular nitrogen compound metabolic process;cellular process;macromolecule metabolic process;macromolecule methylation;macromolecule modificatio
26.5985	26.5664	26.7981	26.4398	1:Experimental evidence at protein level	alcohol metabolic process;biosynthetic process;lipid biosynthetic process;lipid metabolic process;metabolic process;oxidation-reduction process;primary metabolic process;small molecule metabolic process;steroid biosynth
26.0546	26.2048	26.4323	26.2533	1:Experimental evidence at protein level	biological regulation;biosynthetic process;cellular biosynthetic process;cellular macromolecule biosynthetic process;cellular macromolecule metabolic process;cellular metabolic process;cellular nitrogen compound metabolic
27.3093	26.9662	26.9497	27.2886	1:Experimental evidence at protein level	cellular component assembly;cellular component organization;cellular component organization at cellular level;cellular component organization or biogenesis;cellular component organization or biogenesis at cellular level;cellu
25.7714	25.3838	24.9026	24.5561	1:Experimental evidence at protein level	
26.6468	26.4587	26.3482	26.8985	1:Experimental evidence at protein level	anatomical structure development;biological regulation;cardiovascular system development;cell differentiation;cell surface receptor linked signaling pathway;cellular developmental process;cellular process;cellular response to
28.534	28.3549	28.4266	28.6684	1:Experimental evidence at protein level	biological regulation;cellular process;cellular response to chemical stimulus;cellular response to hypoxia;cellular response to oxygen levels;cellular response to redox state;cellular response to stimulus;cellular response to stre
27.3812	27.1609	27.4434	27.5701	1:Experimental evidence at protein level	anatomical structure development;apoptosis;biological regulation;biosynthetic process;cell death;cellular biosynthetic process;cellular metabolic process;cellular process;cofactor biosynthetic process;cofactor metabolic proc
25.4749	25.2574	26.0295	26.3173	1:Experimental evidence at protein level	apoptosis;biological regulation;cell death;cellular process;cellular response to chemical stimulus;cellular response to cytokine stimulus;cellular response to organic substance;cellular response to stimulus;cellular response to
25.3227	25.8799	25.5061	26.0194	1:Experimental evidence at protein level	cellular macromolecule metabolic process;cellular metabolic process;cellular nitrogen compound metabolic process;cellular process;establishment of localization;establishment of localization in cell;establishment of RNA loca
26.6663	26.5613	25.0589	26.7823	1:Experimental evidence at protein level	biological regulation;biosynthetic process;cellular biosynthetic process;cellular component organization;cellular component organization at cellular level;cellular component organization or biogenesis;cellular component orga
23.5905	24.6709	24.3078	24.6258	1:Experimental evidence at protein level	
27.8312	27.8886	27.9446	27.7368	1:Experimental evidence at protein level	actin filament capping;barbed-end actin filament capping;biological regulation;cell communication;cell projection organization;cellular component organization;cellular component organization at cellular level;cellular compone
27.5853	27.1891	26.9272	27.3565	1:Experimental evidence at protein level	cellular macromolecule metabolic process;cellular metabolic process;cellular nitrogen compound metabolic process;cellular process;macromolecule metabolic process;metabolic process;mRNA metabolic process;mRNA pro
27.6295	27.6369	27.2693	27.4806	1:Experimental evidence at protein level	adenine nucleotide transport;ATP transport;biological regulation;cellular process;cellular response to calcium ion;cellular response to chemical stimulus;cellular response to inorganic substance;cellular response to metal ion;c
25.6485	26.1225	25.838	25.9908	1:Experimental evidence at protein level	cell cycle phase;cell cycle process;cell division;cellular component organization;cellular component organization at cellular level;cellular component organization or biogenesis;cellular component organization or biogenesis at
25.5389	25.1591	25.4678	25.211	1:Experimental evidence at protein level	biological regulation;cellular hormone metabolic process;cellular lipid metabolic process;cellular metabolic process;cellular process;diterpenoid metabolic process;fat-soluble vitamin metabolic process;hormone metabolic pr
27.3977	24.7621	27.2168	27.2809	1:Experimental evidence at protein level	apoptosis;cell death;cellular process;death;programmed cell death
27.778	27.91	27.8886	27.5873	1:Experimental evidence at protein level	amine catabolic process;amine metabolic process;branched chain family amino acid catabolic process;branched chain family amino acid metabolic process;carboxylic acid catabolic process;carboxylic acid metabolic proces
26.0693	25.5493	25.7026	25.5772	1:Experimental evidence at protein level	cell differentiation;cellular developmental process;cellular process;developmental process;hemopoietic progenitor cell differentiation
26.8035	26.7226	26.8247	27.0346	1:Experimental evidence at protein level	
30.3167	30.3487	30.5895	30.77	1:Experimental evidence at protein level	biological regulation;biosynthetic process;cellular biosynthetic process;cellular macromolecule biosynthetic process;cellular macromolecule metabolic process;cellular metabolic process;cellular nitrogen compound metabolic
26.1913	26.1867	26.5335	26.4811	1:Experimental evidence at protein level	cellular macromolecule metabolic process;cellular metabolic process;cellular nitrogen compound metabolic process;cellular process;macromolecule metabolic process;metabolic process;mRNA metabolic process;mRNA pro
27.1059	26.3463	26.8448	26.4761	1:Experimental evidence at protein level	biological regulation;biosynthetic process;cell cycle;cell fate commitment;cell fate commitment involved in formation of primary germ layers;cell surface receptor linked signaling pathway;cellular biosynthetic process;cellular c
26.2401	25.8846	25.3967	25.8712	1:Experimental evidence at protein level	biological regulation;biosynthetic process;cellular biosynthetic process;cellular macromolecule biosynthetic process;cellular macromolecule metabolic process;cellular metabolic process;cellular nitrogen compound metabolic
27.3283	26.965	27.3766	27.1045	1:Experimental evidence at protein level	catabolic process;cellular catabolic process;cellular macromolecule catabolic process;cellular macromolecule metabolic process;cellular metabolic process;cellular nitrogen compound metabolic process;cellular process;exo
27.9314	27.4657	27.5581	27.4294	1:Experimental evidence at protein level	assembly of spliceosomal tri-snRNP;cellular component assembly;cellular component assembly at cellular level;cellular component organization;cellular component organization at cellular level;cellular component organizatio
26.3308	26.272	26.0037	26.3539	1:Experimental evidence at protein level	
26.6835	26.4672	25.4141	23.0121	1:Experimental evidence at protein level	biosynthetic process;cellular biosynthetic process;cellular macromolecule biosynthetic process;cellular macromolecule metabolic process;cellular metabolic process;cellular process;cellular protein metabolic process;macron
26.357	26.4453	26.5806	26.7195	1:Experimental evidence at protein level	biological regulation;cell differentiation;cellular developmental process;cellular macromolecule metabolic process;cellular metabolic process;cellular nitrogen compound metabolic process;cellular process;demethylation;deve
27.2982	27.1749	27.1469	27.1614	1:Experimental evidence at protein level	carboxylic acid metabolic process;cellular ketone metabolic process;cellular metabolic process;cellular process;metabolic process;organic acid metabolic process;oxoacid metabolic process;small molecule metabolic proces
26.0448	26.4101	25.988	26.4865	1:Experimental evidence at protein level	
27.0083	26.5968	26.703	26.3462	1:Experimental evidence at protein level	biological regulation;biosynthetic process;cell fate commitment;cell fate commitment involved in formation of primary germ layers;cell surface receptor linked signaling pathway;cellular biosynthetic process;cellular componen
25.9501	22.729	25.8167	25.7011	1:Experimental evidence at protein level	alcohol metabolic process;carbohydrate metabolic process;cellular carbohydrate metabolic process;cellular metabolic process;cellular process;inositol metabolic process;inositol phosphate metabolic process;metabolic proc
25.1049	24.8455	25.3017	25.303	1:Experimental evidence at protein level	
27.0887	26.9882	27.0143	26.9545	1:Experimental evidence at protein level	amine metabolic process;amino acid activation;asparaginyl-tRNA aminoacylation;carboxylic acid metabolic process;cellular amine metabolic process;cellular amino acid metabolic process;cellular ketone metabolic process;c
27.6676	28.0508	27.625	27.7	1:Experimental evidence at protein level	catabolic process;lipid catabolic process;lipid metabolic process;metabolic process;primary metabolic process
26.6076	26.1159	26.228	26.3658	1:Experimental evidence at protein level	biological regulation;cellular macromolecule metabolic process;cellular metabolic process;cellular nitrogen compound metabolic process;cellular process;macromolecule metabolic process;metabolic process;mRNA metabol
26.5449	25.5209	25.2176	25.9211	1:Experimental evidence at protein level	
27.3126	27.0425	27.3014	26.9949	1:Experimental evidence at protein level	biological regulation;cell proliferation;cellular process;cellular response to stimulus;intracellular signal transduction;positive regulation of autophagy;positive regulation of biological process;positive regulation of biosynthetic p
25.1825	24.4673	24.579	24.4314	1:Experimental evidence at protein level	actin cytoskeleton organization;actin cytoskeleton reorganization;actin filament-based process;biological regulation;cell surface receptor linked signaling pathway;cellular component organization;cellular component organizat
26.161	26.1688	26.2241	26.3077	1:Experimental evidence at protein level	aromatic compound biosynthetic process;biosynthetic process;carboxylic acid metabolic process;cellular aromatic compound metabolic process;cellular biosynthetic process;cellular ketone metabolic process;cellular metab
26.6136	24.5365	26.9698	27.0426	1:Experimental evidence at protein level	cellular macromolecule metabolic process;cellular metabolic process;cellular nitrogen compound metabolic process;cellular process;macromolecule metabolic process;metabolic process;mRNA metabolic process;mRNA pro
29.0143	29.092	28.9574	28.9122	1:Experimental evidence at protein level	activation of MAPK activity;amine metabolic process;aminoglycan biosynthetic process;aminoglycan catabolic process;aminoglycan metabolic process;anatomical structure formation involved in morphogenesis;angiogenesis
23.9089	22.9639	23.7645	25.8219	1:Experimental evidence at protein level	
27.625	27.7873	24.3614	27.4158	1:Experimental evidence at protein level	
24.9793	24.3269	25.2897	25.6046	1:Experimental evidence at protein level	cellular macromolecule metabolic process;cellular metabolic process;cellular nitrogen compound metabolic process;cellular process;cellular protein metabolic process;macromolecule metabolic process;macromolecule modi
26.4947	26.4585	26.63	26.7621	1:Experimental evidence at protein level	
25.3963	25.3049	25.7544	25.3322	1:Experimental evidence at protein level	anatomical structure formation involved in morphogenesis;anatomical structure morphogenesis;angiogenesis;biological regulation;blood vessel morphogenesis;developmental process;lymphangiogenesis;positive regulation c
26.3541	26.2178	25.8839	26.0119	1:Experimental evidence at protein level	cellular component organization;cellular component organization at cellular level;cellular component organization or biogenesis;cellular component organization or biogenesis at cellular level;cellular macromolecule metabolic
25.2331	24.7021	24.6829	24.9204	1:Experimental evidence at protein level	
24.0531	25.1364	23.3342	25.0508	1:Experimental evidence at protein level	apoptosis;biological regulation;carbohydrate metabolic process;cell death;cellular carbohydrate metabolic process;cellular component organization;cellular component organization at cellular level;cellular component organiza
26.8412	26.2379	26.5054	26.4126	1:Experimental evidence at protein level	
26.0814	27.2434	27.181	27.4434	1:Experimental evidence at protein level	alkaloid metabolic process;biosynthetic process;cellular amide metabolic process;cellular biosynthetic process;cellular metabolic compound salvage;cellular metabolic process;cellular nitrogen compound biosynthetic proces
26.305	25.8821	25.9841	26.2864	1:Experimental evidence at protein level	biological regulation;cellular process;cellular response to stimulus;intracellular signal transduction;regulation of biological process;regulation of cellular process;response to stimulus;signal transduction
27.0316	27.1285	26.8773	26.9297	1:Experimental evidence at protein level	adult behavior;adult locomotory behavior;anatomical structure homeostasis;behavior;biological regulation;cell cycle checkpoint;cell differentiation;cell differentiation in spinal cord;cell surface receptor linked signaling pathway
25.5624	24.6254	26.0635	25.1052	1:Experimental evidence at protein level	anatomical structure development;anatomical structure formation involved in morphogenesis;biological regulation;cellular process;developmental process;epidermis development;hair cycle process;hair follicle development;n
26.6761	25.2363	27.0683	26.7011	2:Experimental evidence at transcript leve	biological regulation;calcium ion homeostasis;cation homeostasis;cellular calcium ion homeostasis;cellular cation homeostasis;cellular chemical homeostasis;cellular divalent inorganic cation homeostasis;cellular homeostasi
26.9886	27.6811	27.6238	27.2362	1:Experimental evidence at protein level	
27.2987	27.1574	27.0317	27.0629	1:Experimental evidence at protein level	2-oxoglutarate metabolic process;amine catabolic process;amine metabolic process;aromatic amino acid family catabolic process;aromatic amino acid family metabolic process;aromatic compound catabolic process;biosyn
24.1532	25.0183	23.911	24.4925	1:Experimental evidence at protein level	biological regulation;biosynthetic process;cellular biosynthetic process;cellular macromolecule biosynthetic process;cellular macromolecule metabolic process;cellular metabolic process;cellular nitrogen compound metabolic
25.8682	26.0162	25.8669	26.0775	1:Experimental evidence at protein level	biological regulation;cell redox homeostasis;cellular homeostasis;cellular macromolecule metabolic process;cellular metabolic process;cellular process;cellular protein metabolic process;homeostatic process;macromolecule
25.852	25.633	25.8483	25.8318	1:Experimental evidence at protein level	
26.4584	26.3518	26.3583	26.3186	2:Experimental evidence at transcript level	
25.58	25.3772	25.8342	25.9346	1:Experimental evidence at protein level	anatomical structure development;axis elongation;biological regulation;branch elongation of an epithelium;bud outgrowth involved in lung branching;cell development;cellular developmental process;cellular process;developm
25.7172	25.7589	26.042	26.5127	1:Experimental evidence at protein level	axon extension;biological adhesion;biological regulation;cell adhesion;cell growth;cell-matrix adhesion;cell-substrate adhesion;cellular process;cellular response to stimulus;developmental cell growth;developmental growth;d
27.4222	27.4897	27.5976	27.4179	1:Experimental evidence at protein level	response to abiotic stimulus;response to ionizing radiation;response to radiation;response to stimulus;response to X-ray
29.8771	28.5739	28.6983	28.8341	1:Experimental evidence at protein level	biological regulation;blood coagulation;cellular component assembly;cellular component assembly at cellular level;cellular component organization;cellular component organization at cellular level;cellular component organiza
26.5918	26.9278	26.6275	26.8028	1:Experimental evidence at protein level	biological regulation;cellular component organization;cellular component organization at cellular level;cellular component organization or biogenesis;cellular component organization or biogenesis at cellular level;cellular proce
25.5784	24.3877	23.6609	24.4269	1:Experimental evidence at protein level	biological adhesion;biological regulation;carbohydrate metabolic process;catabolic process;cell activation;cell adhesion;cell surface receptor linked signaling pathway;cell-matrix adhesion;cell-substrate adhesion;cellular carb
24.5197	25.4798	25.5746	25.0496	1:Experimental evidence at protein level	lipid metabolic process;metabolic process;primary metabolic process
26.568	26.2903	26.3447	26.1617	1:Experimental evidence at protein level	biological regulation;biosynthetic process;cellular biosynthetic process;cellular component organization;cellular component organization at cellular level;cellular component organization or biogenesis;cellular component orga
30.0504	29.7452	29.8062	29.7482	1:Experimental evidence at protein level	biological regulation;biosynthetic process;cell differentiation;cellular biosynthetic process;cellular developmental process;cellular macromolecule biosynthetic process;cellular macromolecule metabolic process;cellular metab
25.4287	24.776	25.4574	25.5918	1:Experimental evidence at protein level	activation of protein kinase activity;autophagy;biological regulation;catabolic process;cell migration;cell motility;cell surface receptor linked signaling pathway;cellular catabolic process;cellular component movement;cellular l
27.5851	27.4953	27.5027	27.4422	1:Experimental evidence at protein level	biological regulation;cellular macromolecule metabolic process;cellular metabolic process;cellular nitrogen compound metabolic process;cellular process;macromolecule metabolic process;metabolic process;mRNA metabol
28.1583	28.2126	28.1496	28.5345	1:Experimental evidence at protein level	cellular component organization;cellular component organization at cellular level;cellular component organization or biogenesis;cellular component organization or biogenesis at cellular level;cellular macromolecule metabolic
29.9746	29.6812	29.737	29.5959	1:Experimental evidence at protein level	biological regulation;biosynthetic process;cellular biosynthetic process;cellular macromolecule biosynthetic process;cellular macromolecule metabolic process;cellular metabolic process;cellular nitrogen compound metabolic

27.0886	26.6954	26.6426	26.6087	1:Experimental evidence at protein level	cellular macromolecule metabolic process;cellular metabolic process;cellular nitrogen compound metabolic process;cellular process;macromolecule metabolic process;metabolic process;nitrogen compound metabolic proce
27.6605	27.4146	27.3386	27.2846	1:Experimental evidence at protein level	biological regulation;cellular component assembly;cellular component assembly at cellular level;cellular component organization;cellular component organization at cellular level;cellular component organization or biogenesis;
25.9728	25.4789	26.1352	25.9884	1:Experimental evidence at protein level	biological regulation;cellular macromolecule metabolic process;cellular metabolic process;cellular nitrogen compound metabolic process;cellular process;macromolecule metabolic process;macromolecule methylation;macro
25.6402	24.0382	25.7779	26.0702	1:Experimental evidence at protein level	apoptosis;biological regulation;cell communication;cell death;cellular localization;cellular macromolecule localization;cellular process;cellular protein localization;cellular response to acid;cellular response to amine stimulus;ce
28.3475	28.267	28.1622	28.0631	1:Experimental evidence at protein level	axon extension;biological regulation;cell growth;cell projection assembly;cell projection organization;cell surface receptor linked signaling pathway;cellular component assembly;cellular component assembly at cellular level;c
27.138	27.0867	27.148	27.1096	1:Experimental evidence at protein level	cellular macromolecule metabolic process;cellular metabolic process;cellular process;cellular protein metabolic process;cullin deneddylation;macromolecule metabolic process;macromolecule modification;metabolic process
25.656	25.8044	25.7736	24.5356	1:Experimental evidence at protein level	alcohol metabolic process;amine metabolic process;biosynthetic process;cellular amine metabolic process;cellular biogenic amine metabolic process;cellular biosynthetic process;cellular component organization;cellular com
24.5802	25.0141	24.613	24.1821	1:Experimental evidence at protein level	anatomical structure development;biological adhesion;biological regulation;cell adhesion;cell cycle;cell development;cell division;cell maturation;cellular component organization;cellular component organization at cellular leve
26.3348	26.2751	25.4263	25.8945	1:Experimental evidence at protein level	biological regulation;biosynthetic process;cellular biosynthetic process;cellular component organization;cellular component organization at cellular level;cellular component organization or biogenesis;cellular component orga
27.0803	27.4185	27.1429	27.2909	1:Experimental evidence at protein level	biological regulation;cell cycle process;cell division;cellular component organization;cellular component organization at cellular level;cellular component organization or biogenesis;cellular component organization or biogenes
27.4191	27.055	27.5659	27.781	1:Experimental evidence at protein level	biological regulation;biosynthetic process;cellular biosynthetic process;cellular macromolecule biosynthetic process;cellular macromolecule metabolic process;cellular metabolic process;cellular nitrogen compound metabolic
27.5547	27.4777	27.435	27.3737	1:Experimental evidence at protein level	biological regulation;defense response;defense response to virus;immune effector process;immune response;immune system process;innate immune response;multi-organism process;negative regulation of biological proces
25.5545	25.6433	25.5098	25.5609	1:Experimental evidence at protein level	cellular component assembly;cellular component assembly at cellular level;cellular component organization;cellular component organization at cellular level;cellular component organization or biogenesis;cellular component c
26.4739	23.8388	26.4211	26.181	1:Experimental evidence at protein level	biological regulation;biosynthetic process;carbohydrate metabolic process;carbohydrate transport;cell cycle;cell cycle process;cell surface receptor linked signaling pathway;cellular biosynthetic process;cellular component c
24.6884	24.9627	24.8134	24.6911	1:Experimental evidence at protein level	cell cycle process;cell division;cellular component assembly;cellular component assembly at cellular level;cellular component organization;cellular component organization at cellular level;cellular component organization or b
26.3828	23.9167	26.0731	25.7249	1:Experimental evidence at protein level	actin cytoskeleton organization;actin filament-based process;biological regulation;cellular component organization;cellular component organization at cellular level;cellular component organization or biogenesis;cellular comp
23.9128	23.9725	26.4449	22.7662	1:Experimental evidence at protein level	anatomical structure morphogenesis;developmental process;hair cycle process;hair follicle morphogenesis;molting cycle;molting cycle process;multicellular organismal process
26.6375	26.664	26.9567	26.7557	1:Experimental evidence at protein level	
26.6377	25.4613	26.9487	26.4592	1:Experimental evidence at protein level	
24.334	25.5594	25.1984	25.4265	1:Experimental evidence at protein level	activation of immune response;activation of innate immune response;biological regulation;cellular process;cellular response to chemical stimulus;cellular response to dsRNA;cellular response to exogenous dsRNA;cellular res
26.001	25.8062	25.9957	25.7318	1:Experimental evidence at protein level	axon guidance;biological regulation;cell cycle;cell cycle process;cell division;cellular component organization;cellular component organization at cellular level;cellular component organization or biogenesis;cellular component
26.633	26.6347	26.5687	26.5632	1:Experimental evidence at protein level	
25.0624	25.1189	25.1863	25.256	1:Experimental evidence at protein level	biological adhesion;biological regulation;cell adhesion;cell junction assembly;cell junction organization;cell-cell adhesion;cell-cell junction organization;cellular component assembly;cellular component assembly at cellular lev
25.2729	25.1114	24.9073	25.3041	1:Experimental evidence at protein level	
26.5242	25.9452	26.2004	25.599	1:Experimental evidence at protein level	apoptosis;cell death;cellular process;death;programmed cell death
27.5151	27.673	27.4919	27.5793	1:Experimental evidence at protein level	biological regulation;biosynthetic process;cellular biosynthetic process;cellular macromolecule biosynthetic process;cellular macromolecule metabolic process;cellular metabolic process;cellular nitrogen compound metabolic
25.4979	25.951	25.8426	24.462	1:Experimental evidence at protein level	alcohol metabolic process;carbohydrate metabolic process;cellular carbohydrate metabolic process;cellular component assembly;cellular component organization;cellular component organization or biogenesis;cellular metab
25.2906	24.0289	25.3217	25.4681	1:Experimental evidence at protein level	biological regulation;cellular macromolecule metabolic process;cellular metabolic process;cellular process;cellular protein metabolic process;establishment of localization;establishment of localization in cell;establishment of p
29.0402	29.5188	29.0937	29.5861	2:Experimental evidence at transcript lev	response to chemical stimulus;response to organic substance;response to pheromone;response to stimulus
25.6061	24.7047	25.8905	25.7221	1:Experimental evidence at protein level	biological regulation;directional locomotion;locomotion;negative regulation of actin nucleation;negative regulation of biological process;negative regulation of cell migration;negative regulation of cell motility;negative regulatio
27.2077	27.2547	27.1182	27.0281	1:Experimental evidence at protein level	base-excision repair;cell differentiation;cellular component organization;cellular component organization at cellular level;cellular component organization or biogenesis;cellular component organization or biogenesis at cellular
26.9297	25.9818	26.5247	26.2907	1:Experimental evidence at protein level	
27.7043	27.1771	27.42	27.5635	1:Experimental evidence at protein level	biological regulation;cellular process;cellular response to stimulus;establishment of localization;establishment of protein localization;positive regulation of biological process;positive regulation of cell communication;positive r
24.5951	26.5239	26.4495	26.5716	1:Experimental evidence at protein level	
25.9724	25.9604	25.9389	25.3808	1:Experimental evidence at protein level	biological regulation;defense response;defense response to virus;immune effector process;immune system process;multi-organism process;negative regulation of biological process;negative regulation of cell cycle process;n
24.9204	25.5615	25.1174	25.2122	1:Experimental evidence at protein level	
25.4239	25.7389	26.772	25.9044	1:Experimental evidence at protein level	anatomical structure development;biological regulation;cell redox homeostasis;cellular homeostasis;cellular process;developmental process;hemopoiesis;hemopoietic or lymphoid organ development;homeostatic process;or
22.2831	26.1737	25.8651	26.2577	1:Experimental evidence at protein level	
25.5152	25.2578	25.6255	26.0005	1:Experimental evidence at protein level	catabolic process;cellular catabolic process;cellular component assembly;cellular component assembly at cellular level;cellular component organization;cellular component organization at cellular level;cellular component org
26.1552	26.1066	25.9959	25.8644	1:Experimental evidence at protein level	
27.7142	27.5936	28.2425	27.9865	1:Experimental evidence at protein level	biological regulation;biosynthetic process;cellular biosynthetic process;cellular macromolecule biosynthetic process;cellular macromolecule metabolic process;cellular metabolic process;cellular nitrogen compound metabolic
25.6968	25.2106	25.213	25.7485	1:Experimental evidence at protein level	anatomical structure development;biological regulation;biosynthetic process;cell cycle phase;cell cycle process;cellular biosynthetic process;cellular component organization;cellular component organization at cellular level;c
28.0072	27.726	27.7619	27.8107	1:Experimental evidence at protein level	biological regulation;cell junction assembly;cell junction organization;cell surface receptor linked signaling pathway;cell-cell junction assembly;cell-cell junction organization;cellular component assembly;cellular component as
28.7815	28.4455	28.7062	28.5465	1:Experimental evidence at protein level	biological regulation;cellular component movement;cellular process;cellular response to stimulus;intracellular signal transduction;microtubule-based movement;microtubule-based process;regulation of biological process;reg
26.1759	25.6997	25.6596	25.8524	1:Experimental evidence at protein level	biological regulation;catabolic process;cellular catabolic process;cellular macromolecule catabolic process;cellular macromolecule metabolic process;cellular metabolic process;cellular process;cellular protein metabolic proc
23.8072	25.3493	24.464	23.9953	1:Experimental evidence at protein level	anatomical structure morphogenesis;biological adhesion;biological regulation;cell adhesion;cell morphogenesis;cell morphogenesis involved in differentiation;cell surface receptor linked signaling pathway;cell-cell adhesion;c
26.4793	26.3145	26.3238	26.4686	1:Experimental evidence at protein level	adipose tissue development;anatomical structure development;biological regulation;carbohydrate homeostasis;carboxylic acid metabolic process;cell differentiation;cellular chemical homeostasis;cellular developmental proc
26.7674	26.4951	26.5978	26.6335	1:Experimental evidence at protein level	anatomical structure development;anatomical structure morphogenesis;artery development;blood vessel development;cardiac septum development;developmental process;embryonic morphogenesis;morphogenesis of an e
29.3226	29.1925	29.576	29.5351	1:Experimental evidence at protein level	biological regulation;biosynthetic process;cell differentiation;cellular biosynthetic process;cellular developmental process;cellular macromolecule biosynthetic process;cellular macromolecule metabolic process;cellular metab
25.1409	23.9052	24.7913	24.6512	1:Experimental evidence at protein level	activation of MAPK activity;biological regulation;cell cycle process;cellular component organization;cellular component organization or biogenesis;cellular macromolecule metabolic process;cellular metabolic process;cellular
26.3613	25.9255	26.3219	25.9095	1:Experimental evidence at protein level	
28.6276	28.7344	28.5238	28.4365	1:Experimental evidence at protein level	biological regulation;cell differentiation;cellular component assembly;cellular component assembly at cellular level;cellular component organization;cellular component organization at cellular level;cellular component organiza
26.5284	26.4933	26.4254	26.3039	1:Experimental evidence at protein level	biological regulation;cellular component organization;cellular component organization at cellular level;cellular component organization or biogenesis;cellular component organization or biogenesis at cellular level;cellular locali
26.6805	26.7674	27.1251	26.5498	1:Experimental evidence at protein level	biological regulation;cellular component assembly;cellular component assembly at cellular level;cellular component organization;cellular component organization at cellular level;cellular component organization or biogenesis;
26.3172	26.2265	26.6356	26.1524	1:Experimental evidence at protein level	biological adhesion;cell adhesion;cellular process
26.224	24.0583	26.9006	27.1021	1:Experimental evidence at protein level	biosynthetic process;cellular biosynthetic process;cellular macromolecule metabolic process;cellular metabolic process;cellular nitrogen compound metabolic process;cellular process;gene expression;macromolecule metabo
26.0554	24.6798	26.0877	26.0396	1:Experimental evidence at protein level	
25.6826	25.0856	25.8391	25.6836	1:Experimental evidence at protein level	activation of immune response;activation of innate immune response;apoptosis;biological regulation;cell death;cellular process;cellular response to chemical stimulus;cellular response to cytokine stimulus;cellular response to
27.2199	27.2959	27.5957	27.0954	1:Experimental evidence at protein level	anatomical structure morphogenesis;biological regulation;biosynthetic process;bone morphogenesis;cellular biosynthetic process;cellular component organization;cellular component organization at cellular level;cellular com
25.0374	26.1784	26.1981	26.0706	1:Experimental evidence at protein level	metabolic process;nitrogen compound metabolic process
25.8974	26.4917	25.7396	26.3342	1:Experimental evidence at protein level	cell cycle;cell cycle phase;cell cycle process;cell division;cell proliferation;cellular component organization;cellular component organization at cellular level;cellular component organization or biogenesis;cellular component or
25.6764	25.842	25.6961	25.6645	1:Experimental evidence at protein level	biological regulation;cell cycle;cell cycle phase;cell cycle process;cell division;cellular component organization;cellular component organization at cellular level;cellular component organization or biogenesis;cellular componen
27.4064	27.2997	27.4146	27.2375	1:Experimental evidence at protein level	cellular macromolecule metabolic process;cellular metabolic process;cellular nitrogen compound metabolic process;cellular process;localization;macromolecule localization;macromolecule metabolic process;metabolic proce
25.5106	25.5082	25.6366	25.6651	1:Experimental evidence at protein level	
25.3406	25.4922	25.3552	25.2119	1:Experimental evidence at protein level	cell projection organization;cellular component movement;cellular component organization;cellular component organization at cellular level;cellular component organization or biogenesis;cellular component organization or bi
26.7119	26.7862	26.8767	26.9293	1:Experimental evidence at protein level	biological regulation;cellular component organization;cellular component organization at cellular level;cellular component organization or biogenesis;cellular component organization or biogenesis at cellular level;cellular mem
25.5172	25.5173	25.5218	25.0415	1:Experimental evidence at protein level	anatomical structure development;biological regulation;cell differentiation;cellular developmental process;cellular process;developmental process;nervous system development;regulation of biological process;regulation of ce
26.1827	27.1937	26.0627	25.9459	1:Experimental evidence at protein level	biological regulation;cellular component assembly;cellular component assembly at cellular level;cellular component organization;cellular component organization at cellular level;cellular component organization or biogenesis;
23.4769	24.14	25.5384	24.4553	1:Experimental evidence at protein level	cell differentiation;cell envelope organization;cellular component organization;cellular component organization or biogenesis;cellular developmental process;cellular process;developmental process;external encapsulating stru
26.5191	27.2949	27.0355	26.228	1:Experimental evidence at protein level	biological regulation;biosynthetic process;cellular biosynthetic process;cellular macromolecule biosynthetic process;cellular macromolecule metabolic process;cellular metabolic process;cellular nitrogen compound metabolic
26.8713	26.534	26.7441	26.7251	1:Experimental evidence at protein level	biological regulation;cellular macromolecule metabolic process;cellular metabolic process;cellular process;cellular protein metabolic process;developmental process;endosome transport;establishment of localization;establish
25.3027	25.1354	25.3897	25.0694	1:Experimental evidence at protein level	cellular process;establishment of localization;transport;vesicle-mediated transport
26.6027	26.8859	27.2031	26.9346	1:Experimental evidence at protein level	biosynthetic process;catabolic process;cellular biosynthetic process;cellular lipid metabolic process;cellular metabolic process;cellular process;glycerolipid biosynthetic process;glycerolipid metabolic process;glycerophosph
25.2409	23.8455	25.3587	25.6523	1:Experimental evidence at protein level	biological regulation;catabolic process;cellular catabolic process;cellular macromolecule catabolic process;cellular macromolecule metabolic process;cellular metabolic process;cellular nitrogen compound metabolic process
26.205	25.3583	25.5341	25.6518	1:Experimental evidence at protein level	
26.2804	26.4804	26.4805	26.6325	1:Experimental evidence at protein level	biological regulation;cellular component organization;cellular component organization at cellular level;cellular component organization or biogenesis;cellular component organization or biogenesis at cellular level;cellular proce
26.619	26.7716	26.7266	26.9425	1:Experimental evidence at protein level	
25.2496	24.9524	25.6567	24.0379	1:Experimental evidence at protein level	alcohol metabolic process;amine metabolic process;amine transport;biological regulation;biosynthetic process;cation transport;cellular amine metabolic process;cellular biogenic amine metabolic process;cellular biosynthetic
25.3435	25.0255	25.101	25.0158	1:Experimental evidence at protein level	
26.8595	26.9709	24.5724	27.2006	1:Experimental evidence at protein level	cellular component assembly;cellular component assembly at cellular level;cellular component organization;cellular component organization at cellular level;cellular component organization or biogenesis;cellular component c
28.0329	28.0671	28.2263	28.1407	1:Experimental evidence at protein level	
26.5217	24.2329	26.6113	26.2232	1:Experimental evidence at protein level	
26.8338	26.8559	27.0156	26.9019	1:Experimental evidence at protein level	biological regulation;biosynthetic process;cellular biosynthetic process;cellular macromolecule biosynthetic process;cellular macromolecule metabolic process;cellular metabolic process;cellular nitrogen compound metabolic
25.008	24.5666	24.6138	24.6211	1:Experimental evidence at protein level	anatomical structure morphogenesis;biological regulation;cellular process;cellular response to stimulus;developmental process;intracellular signal transduction;morphogenesis of an epithelial sheet;morphogenesis of an epith
26.1968	26.1243	26.3608	26.3085	1:Experimental evidence at protein level	anatomical structure development;biological regulation;calcium ion homeostasis;calcium ion transport;calcium ion transport into cytosol;cation homeostasis;cation transport;cellular calcium ion homeostasis;cellular cation ho
26.4441	24.3703	24.4435	22.9818	1:Experimental evidence at protein level	
25.1275	25.2025	25.3937	24.2262	1:Experimental evidence at protein level	cellular macromolecule metabolic process;cellular metabolic process;cellular nitrogen compound metabolic process;cellular process;gene expression;macromolecule metabolic process;metabolic process;mRNA metabolic p
24.8806	24.2321	24.4327	24.7678	1:Experimental evidence at protein level	cellular macromolecule metabolic process;cellular metabolic process;cellular nitrogen compound metabolic process;cellular process;macromolecule metabolic process;metabolic process;mRNA metabolic process;mRNA pro
26.9832	26.5099	26.8288	26.6744	1:Experimental evidence at protein level	apoptosis;biological regulation;biosynthetic process;cell cycle;cell death;cellular biosynthetic process;cellular macromolecule biosynthetic process;cellular macromolecule metabolic process;cellular metabolic process;cellula
26.0284	25.9402	25.7039	25.8081	1:Experimental evidence at protein level	apoptosis;apoptosis in response to endoplasmic reticulum stress;biological regulation;catabolic process;cell death;cell redox homeostasis;cellular catabolic process;cellular homeostasis;cellular macromolecule catabolic proc
24.6574	25.4968	24.0768	24.5444	1:Experimental evidence at protein level	biological regulation;biosynthetic process;cellular biosynthetic process;cellular macromolecule biosynthetic process;cellular macromolecule metabolic process;cellular metabolic process;cellular nitrogen compound metabolic
26.3826	26.2535	26.2912	26.4497	1:Experimental evidence at protein level	biological regulation;cellular component movement;cellular homeostasis;cellular process;cellular response to stimulus;cytoskeleton-dependent intracellular transport;establishment of localization;establishment of localization
23.7699	25.231	24.8257	24.6391	1:Experimental evidence at protein level	
26.9169	23.6873	23.0068	26.5089	1:Experimental evidence at protein level	
25.1448	24.5092	24.9556	25.2279	1:Experimental evidence at protein level	alcohol metabolic process;amine metabolic process;anatomical structure formation involved in morphogenesis;anatomical structure morphogenesis;angiogenesis;catabolic process;cellular amine metabolic process;cellular b
27.0072	27.3181	26.8371	27.0715	1:Experimental evidence at protein level	
26.3907	26.0896	26.1373	26.1337	1:Experimental evidence at protein level	cellular macromolecule metabolic process;cellular metabolic process;cellular nitrogen compound metabolic process;cellular process;enzyme-directed rRNA 2'-O-methylation;macromolecule metabolic process;macromolecul
28.1732	24.2	28.0092	27.997	1:Experimental evidence at protein level	biosynthetic process;cellular biosynthetic process;cellular macromolecule biosynthetic process;cellular macromolecule metabolic process;cellular metabolic process;cellular nitrogen compound metabolic process;cellular pro
25.8755	25.9946	25.8001	25.7114	1:Experimental evidence at protein level	cellular component assembly;cellular component assembly at cellular level;cellular component disassembly;cellular component disassembly at cellular level;cellular component organization;cellular component organization at
25.7687	24.4662	25.2542	25.5315	1:Experimental evidence at protein level	
25.547	25.5799	25.4338	25.5219	1:Experimental evidence at protein level	biological regulation;calcium ion homeostasis;calcium ion transport;cation homeostasis;cation transport;cellular calcium ion homeostasis;cellular cation homeostasis;cellular chemical homeostasis;cellular divalent inorganic c
25.021	25.1091	24.8633	25.4313	1:Experimental evidence at protein level	
24.9875	24.3533	24.9863	25.0777	1:Experimental evidence at protein level	cellular macromolecule metabolic process;cellular metabolic process;cellular nitrogen compound metabolic process;cellular process;macromolecule metabolic process;macromolecule modification;metabolic process;nitroge
27.249	27.123	27.0406	27.0611	1:Experimental evidence at protein level	
26.077	23.9128	25.4323	25.2007	1:Experimental evidence at protein level	biological regulation;biosynthetic process;cellular biosynthetic process;cellular macromolecule biosynthetic process;cellular macromolecule metabolic process;cellular metabolic process;cellular nitrogen compound metabolic
26.2308	26.4806	26.3904	26.649	1:Experimental evidence at protein level	actin cytoskeleton organization;actin filament organization;actin filament-based process;actin polymerization or depolymerization;anatomical structure development;anatomical structure formation involved in morphogenesis;
26.9574	27.0592	27.0568	26.9375	1:Experimental evidence at protein level	abscission;adipose tissue development;anatomical structure development;biological regulation;cell division;cellular chemical homeostasis;cellular component organization;cellular component organization at cellular level;cellu
26.4248	23.8191	26.6297	23.8506	1:Experimental evidence at protein level	biological regulation;biosynthetic process;cell communication;cell cycle arrest;cell cycle process;cell growth;cell surface receptor linked signaling pathway;cellular biosynthetic process;cellular component organization;cellular
28.3633	28.6019	28.4345	28.4932	1:Experimental evidence at protein level	apoptotic mitochondrial changes;biological regulation;cell cycle;cellular component organization;cellular component organization at cellular level;cellular component organization or biogenesis;cellular component organization
25.3826	25.6527	25.5135	24.2183	1:Experimental evidence at protein level	biological regulation;cellular metabolic process;cellular process;electron transport chain;generation of precursor metabolites and energy;metabolic process;negative regulation of biological process;negative regulation of cellu
27.6176	27.351	27.4299	27.3943	1:Experimental evidence at protein level	biological regulation;biosynthetic process;carbohydrate metabolic process;carbohydrate transport;cell cycle;cell cycle process;cell surface receptor linked signaling pathway;cellular biosynthetic process;cellular component a
25.3581	25.0793	25.2612	25.131	1:Experimental evidence at protein level	cellular macromolecule metabolic process;cellular metabolic process;cellular nitrogen compound metabolic process;cellular process;macromolecule metabolic process;macromolecule methylation;macromolecule modificatio
26.7153	27.139	26.8939	26.9232	1:Experimental evidence at protein level	
24.9612	24.9494	24.5992	24.8796	1:Experimental evidence at protein level	
26.451	26.1492	26.2473	26.2437	1:Experimental evidence at protein level	acylglycerol catabolic process;acylglycerol metabolic process;adult behavior;adult locomotory behavior;adult walking behavior;behavior;catabolic process;cellular catabolic process;cellular lipid catabolic process;cellular lipid
26.173	24.7139	25.2777	23.35	1:Experimental evidence at protein level	biological regulation;cell surface receptor linked signaling pathway;cellular component organization;cellular component organization at cellular level;cellular component organization or biogenesis;cellular component organizat
26.5457	26.0039	26.2875	25.8991	1:Experimental evidence at protein level	biological regulation;cellular process;cellular response to stimulus;intracellular signal transduction;regulation of biological process;regulation of cellular process;regulation of response to stimulus;regulation of signal transducti
25.7029	25.5415	25.5009	25.3461	1:Experimental evidence at protein level	biological regulation;biosynthetic process;cell proliferation;cellular biosynthetic process;cellular macromolecule biosynthetic process;cellular macromolecule metabolic process;cellular metabolic process;cellular nitrogen com
25.2207	25.1136	24.9917	25.3726	1:Experimental evidence at protein level	
25.7573	24.9711	25.6631	25.3973	1:Experimental evidence at protein level	cell projection organization;cellular component organization;cellular component organization at cellular level;cellular component organization or biogenesis;cellular component organization or biogenesis at cellular level;cellula
25.9756	25.5026	25.6086	25.4643	1:Experimental evidence at protein level	biological regulation;cell cycle;cell cycle phase;cell cycle process;cell division;cellular component organization;cellular component organization at cellular level;cellular component organization or biogenesis;cellular componen
24.7231	22.3108	25.3796	23.9339	1:Experimental evidence at protein level	anatomical structure development;anatomical structure formation involved in morphogenesis;anatomical structure morphogenesis;axis specification;biological regulation;bone trabecula formation;branching involved in prosta
25.7697	25.8067	25.5738	24.1185	1:Experimental evidence at protein level	actin cytoskeleton organization;actin cytoskeleton reorganization;actin filament-based process;activation of protein kinase activity;anatomical structure morphogenesis;biological regulation;cell communication;cell part morph
27.5293	27.4648	27.6442	26.972	1:Experimental evidence at protein level	cation transport;cellular process;divalent inorganic cation transport;divalent metal ion transport;establishment of localization;ion transport;magnesium ion transport;metal ion transport;transmembrane transport;transport
25.6842	25.8053	25.5051	25.2747	1:Experimental evidence at protein level	biological regulation;cell proliferation;cellular component assembly;cellular component assembly at cellular level;cellular component organization;cellular component organization at cellular level;cellular component organizatio
26.2555	24.8178	25.6934	25.1441	1:Experimental evidence at protein level	biological regulation;regulation of biological process;regulation of cellular component organization;regulation of cellular process;regulation of cytoskeleton organization;regulation of organelle organization;regulation of respons
24.6678	24.4892	25.3773	24.0212	1:Experimental evidence at protein level	
26.7898	27.408	26.0407	26.8414	1:Experimental evidence at protein level	aging;autophagy;biological regulation;catabolic process;cellular catabolic process;cellular metabolic process;cellular process;developmental process;metabolic process;mitochondrion degradation;multicellular organismal ag
26.483	26.9316	26.6122	26.7736	1:Experimental evidence at protein level	

27.7629	28.0184	27.8967	27.9323	1:Experimental evidence at protein level	biosynthetic process;cellular biosynthetic process;cellular component assembly;cellular component organization;cellular component organization or biogenesis;cellular macromolecule biosynthetic process;cellular macromole
24.1709	25.32	25.6836	25.7088	1:Experimental evidence at protein level	carbohydrate metabolic process;cellular carbohydrate metabolic process;cellular macromolecule metabolic process;cellular metabolic process;cellular process;cellular protein metabolic process;glycosylation;macromolecule
26.6595	26.6612	26.5702	26.5434	1:Experimental evidence at protein level	cellular process;establishment of localization;establishment of protein localization;protein transport;transport;vesicle-mediated transport
27.0843	26.9924	27.011	27.2743	1:Experimental evidence at protein level	
25.3978	25.4011	25.2099	24.9555	1:Experimental evidence at protein level	
26.5322	26.3812	26.3942	26.5573	1:Experimental evidence at protein level	biological regulation;cellular component organization;cellular component organization at cellular level;cellular component organization or biogenesis;cellular component organization or biogenesis at cellular level;cellular macr
27.1334	26.9028	26.7471	26.7869	1:Experimental evidence at protein level	
26.498	26.4972	26.9976	26.4766	1:Experimental evidence at protein level	biological regulation;biosynthetic process;cell fate commitment;cell fate commitment involved in formation of primary germ layers;cell surface receptor linked signaling pathway;cellular biosynthetic process;cellular componen
27.2487	27.2906	27.4252	27.3299	1:Experimental evidence at protein level	actin cytoskeleton organization;actin filament organization;actin filament-based process;actin polymerization or depolymerization;activation of immune response;anatomical structure formation involved in morphogenesis;anti
27.8152	28.2676	27.2658	27.7627	1:Experimental evidence at protein level	cell differentiation;cellular developmental process;cellular process;developmental process;gamete generation;male gamete generation;multicellular organismal development;multicellular organismal process;multicellular organ
26.2549	26.6261	26.5644	26.3716	1:Experimental evidence at protein level	
27.0991	26.513	26.7645	26.6036	1:Experimental evidence at protein level	cellular process;establishment of localization;transmembrane transport;transport
26.7381	26.7085	26.3813	26.7592	1:Experimental evidence at protein level	biological regulation;cellular component organization;cellular component organization at cellular level;cellular component organization or biogenesis;cellular component organization or biogenesis at cellular level;cellular proce
28.4163	28.1307	28.132	27.9503	1:Experimental evidence at protein level	cellular component organization;cellular component organization at cellular level;cellular component organization or biogenesis;cellular component organization or biogenesis at cellular level;cellular process;extracellular matr
25.9804	26.9215	26.7349	26.8226	1:Experimental evidence at protein level	cellular macromolecule metabolic process;cellular metabolic process;cellular process;cellular protein metabolic process;macromolecule metabolic process;macromolecule modification;metabolic process;post-translational pr
25.4944	25.5587	25.2509	25.2523	1:Experimental evidence at protein level	anatomical structure development;biological regulation;carbohydrate metabolic process;cardiovascular system development;cell surface receptor linked signaling pathway;cellular carbohydrate metabolic process;cellular ma
25.5529	25.3685	25.5806	23.424	1:Experimental evidence at protein level	cellular localization;cellular macromolecule localization;cellular process;cellular protein localization;localization;macromolecule localization;protein localization;protein localization in endoplasmic reticulum;protein localization t
24.38	24.9844	24.647	24.8309	1:Experimental evidence at protein level	androgen catabolic process;androgen metabolic process;biological regulation;biosynthetic process;catabolic process;cellular hormone metabolic process;cellular metabolic process;cellular process;hormone catabolic proces
29.689	29.8659	30.0291	29.9824	1:Experimental evidence at protein level	apoptotic cell clearance;biological regulation;cell redox homeostasis;cellular component organization;cellular component organization or biogenesis;cellular homeostasis;cellular macromolecule metabolic process;cellular me
27.3034	27.3124	27.6088	27.4166	1:Experimental evidence at protein level	biological regulation;cell cycle;cell cycle phase;cell cycle process;cell division;cellular component organization;cellular component organization at cellular level;cellular component organization or biogenesis;cellular componen
25.7955	25.6166	25.6358	25.6606	1:Experimental evidence at protein level	behavior;biological regulation;cellular component organization;cellular component organization at cellular level;cellular component organization or biogenesis;cellular component organization or biogenesis at cellular level;cellu
26.6957	26.7761	26.7999	26.4703	1:Experimental evidence at protein level	
30.0431	29.7934	30.0817	30.2264	1:Experimental evidence at protein level	biological regulation;cellular macromolecule metabolic process;cellular metabolic process;cellular nitrogen compound metabolic process;cellular process;macromolecule metabolic process;metabolic process;mRNA metaboli
26.2153	26.3078	26.5084	26.0467	1:Experimental evidence at protein level	biological regulation;cell differentiation;cellular developmental process;cellular process;developmental process;muscle cell differentiation;negative regulation of biological process;negative regulation of cell communication;neg
26.3297	24.0037	26.0302	26.1108	1:Experimental evidence at protein level	
24.9883	23.4492	24.8638	25.2057	1:Experimental evidence at protein level	amine metabolic process;aminoglycan biosynthetic process;aminoglycan metabolic process;biosynthetic process;carbohydrate biosynthetic process;carbohydrate metabolic process;cellular biosynthetic process;cellular mac
25.8313	25.6573	25.7503	25.5514	1:Experimental evidence at protein level	biological regulation;carboxylic acid metabolic process;cellular ketone metabolic process;cellular metabolic process;cellular process;metabolic process;monocarboxylic acid metabolic process;organic acid metabolic process
27.7837	27.5818	27.6856	27.4369	1:Experimental evidence at protein level	cellular component assembly;cellular component organization;cellular component organization or biogenesis;cellular metabolic process;cellular nitrogen compound metabolic process;cellular process;coenzyme metabolic pro
27.9722	27.8951	27.9759	27.7476	1:Experimental evidence at protein level	biological regulation;biosynthetic process;cellular biosynthetic process;cellular macromolecule biosynthetic process;cellular macromolecule metabolic process;cellular metabolic process;cellular process;cellular protein metab
25.457	25.4083	25.4567	25.0602	1:Experimental evidence at protein level	biological regulation;calcium ion homeostasis;calcium ion transport;calcium-mediated signaling;carbohydrate homeostasis;cation homeostasis;cation transport;cellular calcium ion homeostasis;cellular cation homeostasis;ce
26.4985	26.1703	26.2576	26.1697	1:Experimental evidence at protein level	biological regulation;cell surface receptor linked signaling pathway;cellular process;cellular response to chemical stimulus;cellular response to fibroblast growth factor stimulus;cellular response to growth factor stimulus;cellu
25.4296	25.6326	25.4528	25.3532	1:Experimental evidence at protein level	biological regulation;cation transport;cellular macromolecule metabolic process;cellular metabolic process;cellular process;cellular protein metabolic process;divalent inorganic cation transport;divalent metal ion transport;est
25.5101	25.6055	26.1058	25.6298	1:Experimental evidence at protein level	developmental process;keratinization
24.3621	24.4803	24.9546	24.6808	1:Experimental evidence at protein level	catabolic process;cellular catabolic process;cellular component assembly;cellular component assembly at cellular level;cellular component organization;cellular component organization at cellular level;cellular component org
26.2966	23.5125	26.6408	26.1949	1:Experimental evidence at protein level	acylglycerol biosynthetic process;acylglycerol metabolic process;anatomical structure development;anatomical structure homeostasis;biological regulation;biosynthetic process;carboxylic acid biosynthetic process;carboxylic
26.6838	26.2583	26.2794	26.7318	1:Experimental evidence at protein level	
26.1776	27.3348	28.0368	27.0334	1:Experimental evidence at protein level	biological regulation;biosynthetic process;carbohydrate metabolic process;carbohydrate transport;cell cycle;cell cycle phase;cell cycle process;cell division;cell surface receptor linked signaling pathway;cellular biosynthetic p
26.3648	25.7741	25.8794	25.9421	1:Experimental evidence at protein level	biological regulation;biosynthetic process;carbohydrate metabolic process;carbohydrate transport;cell cycle;cell cycle phase;cell cycle process;cell division;cell surface receptor linked signaling pathway;cellular biosynthetic p
25.5662	25.4464	25.8397	25.7269	1:Experimental evidence at protein level	biological regulation;biosynthetic process;carbohydrate metabolic process;carbohydrate transport;cell cycle;cell cycle process;cell surface receptor linked signaling pathway;cellular biosynthetic process;cellular component c
24.6668	24.5239	23.4433	24.0254	1:Experimental evidence at protein level	biological regulation;cellular process;cellular response to stimulus;regulation of biological process;regulation of cellular process;response to stimulus;signal transduction
27.2377	27.2497	27.2933	27.2701	1:Experimental evidence at protein level	biological regulation;cellular component organization;cellular component organization at cellular level;cellular component organization or biogenesis;cellular component organization or biogenesis at cellular level;cellular proce
25.7189	25.8654	25.5086	25.5627	1:Experimental evidence at protein level	
25.1342	26.2135	23.8125	25.23	1:Experimental evidence at protein level	alcohol metabolic process;amino sugar metabolic process;biosynthetic process;carbohydrate biosynthetic process;carbohydrate metabolic process;carboxylic acid metabolic process;cellular carbohydrate metabolic process
25.6731	26.0994	25.8409	25.7661	1:Experimental evidence at protein level	biological regulation;cellular localization;cellular macromolecule localization;cellular process;cellular protein complex localization;cellular protein localization;establishment of localization;establishment of localization in cell;exo
26.6165	26.8845	26.9852	27.094	1:Experimental evidence at protein level	catabolic process;lipid catabolic process;lipid metabolic process;metabolic process;primary metabolic process
26.747	24.9735	26.8161	25.8379	1:Experimental evidence at protein level	cellular macromolecule metabolic process;cellular metabolic process;cellular nitrogen compound metabolic process;cellular process;macromolecule metabolic process;metabolic process;nitrogen compound metabolic proce
26.366	24.2037	26.2248	25.0613	1:Experimental evidence at protein level	cell cycle;cell cycle phase;cell cycle process;cell division;cellular component organization;cellular component organization at cellular level;cellular component organization or biogenesis;cellular component organization or bio
25.4349	24.361	24.5106	24.8101	1:Experimental evidence at protein level	cellular macromolecule metabolic process;cellular metabolic process;cellular nitrogen compound metabolic process;cellular process;establishment of localization;establishment of localization in cell;establishment of RNA loca
25.3705	24.8195	24.8429	24.8287	1:Experimental evidence at protein level	anatomical structure homeostasis;biological regulation;cellular macromolecule metabolic process;cellular metabolic process;cellular nitrogen compound metabolic process;cellular process;homeostatic process;macromolecu
26.9639	24.0626	23.6852	27.0058	1:Experimental evidence at protein level	
27.298	27.2255	27.1873	27.0035	1:Experimental evidence at protein level	ATP-dependent chromatin remodeling;biological regulation;biosynthetic process;cellular biosynthetic process;cellular component disassembly;cellular component disassembly at cellular level;cellular component organization
26.9831	27.3913	27.0637	27.1919	1:Experimental evidence at protein level	biosynthetic process;catabolic process;cellular biosynthetic process;cellular catabolic process;cellular component organization;cellular component organization at cellular level;cellular component organization or biogenesis;c
25.3465	26.0192	25.6336	25.9548	1:Experimental evidence at protein level	
27.0327	25.1219	26.6579	26.8467	1:Experimental evidence at protein level	3'-phosphoadenosine 5'-phosphosulfate biosynthetic process;3'-phosphoadenosine 5'-phosphosulfate metabolic process;3'-phosphoadenosine 5'-phosphosulfate transport;amine metabolic process;aminoglycan metabolic
25.648	25.2317	22.4351	26.2788	1:Experimental evidence at protein level	cellular component organization;cellular component organization at cellular level;cellular component organization or biogenesis;cellular component organization or biogenesis at cellular level;cellular process;establishment of l
27.267	27.1959	27.1895	27.3747	1:Experimental evidence at protein level	activation of immune response;activation of innate immune response;biological regulation;cell cycle;cell surface receptor linked signaling pathway;cellular macromolecule metabolic process;cellular metabolic process;cellular
25.3643	24.7069	26.0726	25.5526	1:Experimental evidence at protein level	biological regulation;positive regulation of catalytic activity;positive regulation of GTPase activity;positive regulation of hydrolase activity;positive regulation of molecular function;regulation of biological process;regulation of ca
26.6788	26.1864	26.4011	25.8826	1:Experimental evidence at protein level	adaptation of rhodopsin mediated signaling;adaptation of signaling pathway;apocarotenoid metabolic process;biological regulation;cellular aldehyde metabolic process;cellular hormone metabolic process;cellular lipid metab
28.1625	27.7486	27.9152	27.6344	1:Experimental evidence at protein level	biological regulation;gamete generation;male gamete generation;multicellular organismal process;multicellular organismal reproductive process;positive regulation of biological process;positive regulation of cell proliferation;p
27.3379	27.2017	27.1387	27.01	1:Experimental evidence at protein level	carbohydrate metabolic process;catabolic process;cellular carbohydrate metabolic process;cellular catabolic process;cellular macromolecule catabolic process;cellular macromolecule metabolic process;cellular metabolic pr
26.2259	26.1483	26.1714	26.2135	1:Experimental evidence at protein level	anatomical structure morphogenesis;biological regulation;catabolic process;cell cycle arrest;cell cycle process;cell part morphogenesis;cellular catabolic process;cellular component assembly;cellular component morphoge
26.5226	26.789	26.1844	26.2244	1:Experimental evidence at protein level	cell cycle;cell cycle phase;cell cycle process;cell division;cellular component disassembly;cellular component disassembly at cellular level;cellular component organization;cellular component organization at cellular level;cellu
25.8746	25.9638	25.3125	25.6558	1:Experimental evidence at protein level	cellular component organization;cellular component organization at cellular level;cellular component organization or biogenesis;cellular component organization or biogenesis at cellular level;cellular macromolecule metabolic
26.8169	25.7855	23.7924	26.2046	1:Experimental evidence at protein level	biological regulation;biosynthetic process;cellular biosynthetic process;cellular macromolecule biosynthetic process;cellular macromolecule metabolic process;cellular metabolic process;cellular nitrogen compound metabolic
28.7264	28.204	28.1606	28.2427	1:Experimental evidence at protein level	cellular component assembly;cellular component assembly at cellular level;cellular component organization;cellular component organization at cellular level;cellular component organization or biogenesis;cellular component c
28.1251	27.9879	28.0514	27.8532	1:Experimental evidence at protein level	biological regulation;cellular component assembly;cellular component assembly at cellular level;cellular component organization;cellular component organization at cellular level;cellular component organization or biogenesis;
29.174	29.0907	28.6013	29.1132	1:Experimental evidence at protein level	actin cytoskeleton organization;actin filament depolymerization;actin filament organization;actin filament-based process;actin polymerization or depolymerization;biological regulation;cellular component disassembly;cellular c
27.6539	28.0372	27.7034	27.8466	1:Experimental evidence at protein level	response to chemical stimulus;response to inorganic substance;response to oxidative stress;response to reactive oxygen species;response to stimulus;response to stress
26.4198	26.2359	26.303	26.2334	1:Experimental evidence at protein level	cellular component assembly;cellular component assembly at cellular level;cellular component organization;cellular component organization at cellular level;cellular component organization or biogenesis;cellular component c
27.3663	27.4095	27.5724	27.6617	1:Experimental evidence at protein level	cellular component assembly;cellular component assembly at cellular level;cellular component organization;cellular component organization at cellular level;cellular component organization or biogenesis;cellular component c
26.0864	26.0201	25.9698	25.7955	1:Experimental evidence at protein level	biological regulation;cellular macromolecule metabolic process;cellular metabolic process;cellular process;cellular protein metabolic process;cellular response to stimulus;dephosphorylation;macromolecule metabolic process
26.0258	26.5545	26.8234	26.7847	1:Experimental evidence at protein level	biological regulation;biosynthetic process;cellular biosynthetic process;cellular component organization;cellular component organization at cellular level;cellular component organization or biogenesis;cellular component orga
25.7155	24.542	25.3957	24.6782	1:Experimental evidence at protein level	cell differentiation;cellular developmental process;cellular process;developmental process;fat cell differentiation
24.1212	24.1341	25.3174	23.7388	1:Experimental evidence at protein level	adhesion to host;adhesion to other organism involved in symbiotic interaction;alcohol metabolic process;androgen biosynthetic process;androgen metabolic process;biological adhesion;biological regulation;biosynthetic proc
27.0478	27.0784	27.1995	27.2794	1:Experimental evidence at protein level	biological regulation;cell surface receptor linked signaling pathway;cellular process;cellular response to chemical stimulus;cellular response to cytokine stimulus;cellular response to organic substance;cellular response to stim
25.3006	25.7984	26.0346	25.9216	1:Experimental evidence at protein level	cellular macromolecule metabolic process;cellular metabolic process;cellular process;cellular protein metabolic process;macromolecule metabolic process;macromolecule modification;metabolic process;peptidyl-amino acid
25.6138	25.5464	25.314	25.6226	1:Experimental evidence at protein level	anatomical structure development;anatomical structure morphogenesis;apoptosis;biological regulation;biosynthetic process;camera-type eye development;cardiac chamber morphogenesis;cardiac muscle contraction;cardia
26.0246	24.4185	26.279	26.5799	1:Experimental evidence at protein level	biological regulation;biosynthetic process;cell differentiation;cell junction assembly;cell junction organization;cell surface receptor linked signaling pathway;cell-cell junction assembly;cell-cell junction organization;cellular bios
25.4598	25.1224	24.862	25.2476	1:Experimental evidence at protein level	amine metabolic process;aminoglycan catabolic process;aminoglycan metabolic process;biological regulation;carbohydrate catabolic process;carbohydrate metabolic process;catabolic process;glycosaminoglycan catabolic
26.6759	26.5776	26.42	26.657	1:Experimental evidence at protein level	anatomical structure development;biological regulation;biosynthetic process;carbohydrate metabolic process;carbohydrate transport;cell cycle;cell cycle process;cell surface receptor linked signaling pathway;cellular biosynt
28.5762	28.5229	28.4404	28.5008	1:Experimental evidence at protein level	apoptosis;biological regulation;cell cycle;cell death;cellular process;cytokinetic cell separation;cytokinetic process;death;establishment of localization;establishment of protein localization;programmed cell death;protein transp
27.3155	27.4711	27.4249	27.6525	1:Experimental evidence at protein level	biological adhesion;biological regulation;cell adhesion;cell junction assembly;cell junction organization;cell-cell adhesion;cellular component assembly;cellular component assembly at cellular level;cellular component organiza
26.5846	27.4794	27.1635	28.0109	1:Experimental evidence at protein level	actin cytoskeleton organization;actin filament-based process;biological regulation;cell motility;cell surface receptor linked signaling pathway;cellular component assembly;cellular component movement;cellular component org
26.0597	26.3704	26.2523	26.1003	1:Experimental evidence at protein level	
25.9109	25.5169	25.7863	25.811	1:Experimental evidence at protein level	apoptosis;biological regulation;biosynthetic process;cell death;cell differentiation;cellular biosynthetic process;cellular developmental process;cellular macromolecule biosynthetic process;cellular macromolecule metabolic pr
23.7795	26.2439	24.4147	24.6561	1:Experimental evidence at protein level	biological regulation;cell cycle cytokinesis;cell cycle process;cellular process;cytokinesis;cytokinesis after mitosis;cytokinetic cell separation;cytokinetic process;establishment of localization;negative regulation of binding;neg
26.1018	26.3061	26.3412	26.3318	1:Experimental evidence at protein level	biological regulation;biosynthetic process;cell fate commitment;cell fate commitment involved in formation of primary germ layers;cell surface receptor linked signaling pathway;cellular biosynthetic process;cellular componen
25.8262	26.0899	26.4737	25.5466	1:Experimental evidence at protein level	
29.1302	28.9131	28.9504	28.7266	1:Experimental evidence at protein level	anatomical structure morphogenesis;biological regulation;cell morphogenesis;cellular component morphogenesis;cellular component organization;cellular component organization or biogenesis;cellular developmental proces
26.8285	26.882	27.0685	26.5681	1:Experimental evidence at protein level	
27.7378	28.2809	28.0989	28.3243	1:Experimental evidence at protein level	catabolic process;cell cycle;cellular catabolic process;cellular macromolecule catabolic process;cellular macromolecule metabolic process;cellular metabolic process;cellular process;cellular protein metabolic process;macro
27.7952	27.6218	27.774	27.8149	1:Experimental evidence at protein level	biological regulation;cellular macromolecule metabolic process;cellular metabolic process;cellular process;cellular protein metabolic process;macromolecule metabolic process;macromolecule modification;metabolic process
23.6832	25.4164	26.0536	25.8055	1:Experimental evidence at protein level	alcohol metabolic process;amine metabolic process;amine transport;biosynthetic process;cation transport;cellular amine metabolic process;cellular biogenic amine metabolic process;cellular biosynthetic process;cellular lipid
25.9763	24.4127	25.7487	26.0053	1:Experimental evidence at protein level	apoptosis;biological regulation;cell cycle cytokinesis;cell cycle process;cell death;cellular process;cytokinesis;cytokinesis after mitosis;death;negative regulation of biological process;negative regulation of cellular component
27.8547	28.0668	28.1031	28.0658	1:Experimental evidence at protein level	biological regulation;cellular component assembly;cellular component assembly at cellular level;cellular component organization;cellular component organization at cellular level;cellular component organization or biogenesis;
26.8981	26.6221	26.6187	26.4013	1:Experimental evidence at protein level	assembly of spliceosomal tri-snRNP;cellular component assembly;cellular component assembly at cellular level;cellular component organization;cellular component organization at cellular level;cellular component organizatio
26.9673	26.3782	26.6766	26.4	1:Experimental evidence at protein level	biological regulation;biosynthetic process;cellular biosynthetic process;cellular macromolecule biosynthetic process;cellular macromolecule metabolic process;cellular metabolic process;cellular nitrogen compound metabolic
28.0468	28.009	28.1447	28.1443	1:Experimental evidence at protein level	cellular component organization;cellular component organization at cellular level;cellular component organization or biogenesis;cellular component organization or biogenesis at cellular level;cellular process;cytoskeleton orga
30.4849	30.0793	30.0097	30.1609	1:Experimental evidence at protein level	biological regulation;cell differentiation;cellular component organization;cellular component organization or biogenesis;cellular developmental process;cellular membrane organization;cellular process;developmental process;e
27.5716	27.2686	27.7758	27.6174	1:Experimental evidence at protein level	biological regulation;biosynthetic process;cellular biosynthetic process;cellular macromolecule biosynthetic process;cellular macromolecule metabolic process;cellular metabolic process;cellular nitrogen compound metabolic
26.103	26.569	26.2207	27.2115	1:Experimental evidence at protein level	actin filament-based movement;actin filament-based process;ameboidal cell migration;biological regulation;cell migration;cell motility;cellular component movement;cellular component organization;cellular component organi
25.9145	25.5933	25.4748	25.6416	1:Experimental evidence at protein level	ATP-dependent chromatin remodeling;biosynthetic process;cellular biosynthetic process;cellular component organization;cellular component organization at cellular level;cellular component organization or biogenesis;cellular
28.0343	28.2319	28.2896	28.1701	1:Experimental evidence at protein level	behavior;behavioral interaction between organisms;multi-organism process;response to stimulus;social behavior
26.4549	26.5756	26.36	26.3187	1:Experimental evidence at protein level	apoptosis;biological regulation;cell death;cellular macromolecule metabolic process;cellular metabolic process;cellular nitrogen compound metabolic process;cellular process;death;gene expression;immune system process;
25.947	24.9154	26.164	25.6881	1:Experimental evidence at protein level	biological regulation;cell cycle;cellular component assembly;cellular component assembly at cellular level;cellular component organization;cellular component organization at cellular level;cellular component organization or bi
25.743	26.4784	26.162	26.1557	1:Experimental evidence at protein level	
28.3436	28.5011	28.3548	28.5319	1:Experimental evidence at protein level	biological regulation;biosynthetic process;cellular biosynthetic process;cellular component assembly;cellular component assembly at cellular level;cellular component organization;cellular component organization at cellular le
26.9626	24.9533	24.1153	27.3086	1:Experimental evidence at protein level	cation transport;cellular process;divalent inorganic cation transport;divalent metal ion transport;establishment of localization;ion transport;metal ion transport;transition metal ion transport;transmembrane transport;transport;z
27.8415	28.799	27.8398	27.9685	1:Experimental evidence at protein level	developmental process;multicellular organismal development;multicellular organismal process
27.9528	27.306	27.5352	27.622	1:Experimental evidence at protein level	cellular component assembly;cellular component assembly at cellular level;cellular component organization;cellular component organization at cellular level;cellular component organization or biogenesis;cellular component c
27.3411	27.7179	27.2209	27.3725	1:Experimental evidence at protein level	activation of immune response;activation of innate immune response;anaphase-promoting complex-dependent proteasomal ubiquitin-dependent protein catabolic process;antigen processing and presentation;antigen proces
26.8201	26.708	26.791	26.5943	1:Experimental evidence at protein level	biological regulation;cell activation;cell activation involved in immune response;cell chemotaxis;cell migration;cell motility;cellular component disassembly;cellular component disassembly at cellular level;cellular component m
26.5921	26.2185	26.4302	26.021	1:Experimental evidence at protein level	biological regulation;biosynthetic process;cell fate commitment;cell fate commitment involved in formation of primary germ layers;cell surface receptor linked signaling pathway;cellular biosynthetic process;cellular componen
25.2794	24.5402	25.8277	25.5883	1:Experimental evidence at protein level	amyloid precursor protein catabolic process;amyloid precursor protein metabolic process;axon guidance;beta-amyloid metabolic process;biological regulation;catabolic process;cell activation;cell proliferation;cell surface rec
26.5963	26.4377	26.5437	26.3937	1:Experimental evidence at protein level	
26.3296	26.1034	26.1352	25.9285	1:Experimental evidence at protein level	biosynthetic process;cellular biosynthetic process;cellular component disassembly;cellular component disassembly at cellular level;cellular component organization;cellular component organization at cellular level;cellular com
27.5644	27.3613	27.4242	27.4396	1:Experimental evidence at protein level	catabolic process;cellular catabolic process;cellular macromolecule catabolic process;cellular macromolecule metabolic process;cellular metabolic process;cellular process;ER-associated protein catabolic process;macromo
24.3085	23.6558	24.8159	24.5702	1:Experimental evidence at protein level	biosynthetic process;cellular biosynthetic process;cellular macromolecule biosynthetic process;cellular macromolecule metabolic process;cellular metabolic process;cellular nitrogen compound metabolic process;cellular pro
28.1517	28.177	28.3909	28.3527	1:Experimental evidence at protein level	biological regulation;cell activation;cellular component organization;cellular component organization or biogenesis;cellular membrane organization;cellular process;cellular response to chemical stimulus;cellular response to hy
29.4601	29.5428	29.5964	29.4507	1:Experimental evidence at protein level	biological regulation;cellular component organization;cellular component organization or biogenesis;cellular macromolecule metabolic process;cellular membrane organization;cellular metabolic process;cellular process;cellul
27.458	27.1024	27.462	27.2788	1:Experimental evidence at protein level	
26.6526	26.3883	26.1509	26.2343	1:Experimental evidence at protein level	biological regulation;biosynthetic process;catabolic process;cell surface receptor linked signaling pathway;cellular biosynthetic process;cellular catabolic process;cellular macromolecule biosynthetic process;cellular macrom
25.6013	24.8685	24.5497	25.3956	1:Experimental evidence at protein level	autophagy;biological regulation;catabolic process;cellular catabolic process;cellular metabolic process;cellular process;endosome transport;establishment of localization;establishment of localization in cell;establishment of p
26.3407	25.5447	25.7622	25.449	1:Experimental evidence at protein level	actin cytoskeleton organization;actin filament-based process;actomyosin structure organization;asymmetric Golgi ribbon formation;biological regulation;cell migration;cell motility;cellular component movement;cellular compo
25.7742	24.1246	25.7276	25.9651	1:Experimental evidence at protein level	biological regulation;positive regulation of biological process;positive regulation of biosynthetic process;positive regulation of cellular biosynthetic process;positive regulation of cellular metabolic process;positive regulation of
27.8851	27.607	27.7141	27.6443	1:Experimental evidence at protein level	biological regulation;biosynthetic process;cellular biosynthetic process;cellular macromolecule biosynthetic process;cellular macromolecule metabolic process;cellular metabolic process;cellular process;cellular protein metab
27.9594	27.9659	27.8925	28.065	1:Experimental evidence at protein level	biosynthetic process;cellular biosynthetic process;cellular macromolecule biosynthetic process;cellular macromolecule metabolic process;cellular metabolic process;cellular nitrogen compound metabolic process;cellular pro
27.2655	26.6101	27.0249	26.6707	1:Experimental evidence at protein level	biological regulation;biosynthetic process;carbohydrate metabolic process;carbohydrate transport;cell cycle;cell cycle process;cell surface receptor linked signaling pathway;cellular biosynthetic process;cellular component a
26.7575	26.7919	26.7319	26.8933	1:Experimental evidence at protein level	biological regulation;catabolic process;cellular catabolic process;cellular component organization;cellular component organization at cellular level;cellular component organization or biogenesis;cellular component organizatio

25.7568	24.9193	26.1475	25.1745	1:Experimental evidence at protein level	activation of MAPK activity;activation of phospholipase C activity;axon ensheathment;biological regulation;bleb assembly;calcium ion homeostasis;cation homeostasis;cell projection assembly;cell projection organization;cell
29.0338	28.8574	29.1862	28.9975	1:Experimental evidence at protein level	activation of caspase activity;anatomical structure development;biological regulation;cellular component assembly;cellular component assembly at cellular level;cellular component organization;cellular component organizatio
26.8748	27.137	26.7602	26.9136	1:Experimental evidence at protein level	acrosome assembly;adherens junction organization;adhesion to host;adhesion to other organism involved in symbiotic interaction;anatomical structure development;anatomical structure formation involved in morphogenesis;
27.4807	27.5411	27.5727	27.4937	1:Experimental evidence at protein level	biological regulation;cellular process;cellular response to stimulus;positive regulation of biological process;positive regulation of cell communication;positive regulation of cellular process;positive regulation of I-kappaB kinase
24.7031	24.7667	23.8089	25.9136	1:Experimental evidence at protein level	biological regulation;macromolecule metabolic process;metabolic process;negative regulation of biological process;negative regulation of BMP signaling pathway;negative regulation of cell communication;negative regulation
28.1891	28.5331	28.3137	28.2046	1:Experimental evidence at protein level	actin cytoskeleton organization;actin filament organization;actin filament-based process;actin nucleation;Arp2/3 complex-mediated actin nucleation;axon guidance;biological regulation;cell surface receptor linked signaling pa
27.3987	27.1674	27.1926	27.0411	1:Experimental evidence at protein level	anatomical structure development;anatomical structure formation involved in morphogenesis;anatomical structure morphogenesis;ATP-dependent chromatin remodeling;biological regulation;biosynthetic process;blood coagu
25.7305	25.893	26.0436	26.1573	1:Experimental evidence at protein level	apoptosis;biological regulation;biosynthetic process;cell death;cellular biosynthetic process;cellular macromolecule biosynthetic process;cellular macromolecule metabolic process;cellular metabolic process;cellular nitrogen
25.8372	25.643	24.5739	25.8248	1:Experimental evidence at protein level	activation of immune response;activation of innate immune response;anatomical structure morphogenesis;biological regulation;biosynthetic process;cell surface receptor linked signaling pathway;cellular biosynthetic process
26.0809	24.2062	24.3053	26.0748	1:Experimental evidence at protein level	biological adhesion;biological regulation;cell adhesion;cellular macromolecule metabolic process;cellular metabolic process;cellular nitrogen compound metabolic process;cellular process;macromolecule metabolic process;
27.887	28.2617	28.4923	28.1381	1:Experimental evidence at protein level	biological regulation;positive regulation of biological process;positive regulation of biosynthetic process;positive regulation of cellular biosynthetic process;positive regulation of cellular metabolic process;positive regulation of
24.3176	24.2307	24.2836	24.7698	1:Experimental evidence at protein level	amine metabolic process;aminoglycan biosynthetic process;aminoglycan metabolic process;anatomical structure development;anatomical structure morphogenesis;biological regulation;biosynthetic process;bone morphoge
27.3024	27.1136	27.7101	27.2242	1:Experimental evidence at protein level	amine metabolic process;carboxylic acid metabolic process;cellular amine metabolic process;cellular amino acid metabolic process;cellular ketone metabolic process;cellular metabolic process;cellular nitrogen compound m
29.5187	29.4222	29.5159	29.4679	1:Experimental evidence at protein level	biological regulation;biosynthetic process;cellular biosynthetic process;cellular macromolecule biosynthetic process;cellular macromolecule metabolic process;cellular metabolic process;cellular nitrogen compound metabolic
26.6911	26.5407	26.5649	26.5181	1:Experimental evidence at protein level	anatomical structure homeostasis;biological regulation;biosynthetic process;cell cycle process;cellular biosynthetic process;cellular component organization;cellular component organization at cellular level;cellular componen
27.2663	27.1771	27.5566	27.4376	1:Experimental evidence at protein level	anatomical structure development;biological regulation;cell development;cellular component assembly;cellular component assembly at cellular level;cellular component organization;cellular component organization at cellular
27.048	28.4034	26.999	28.102	1:Experimental evidence at protein level	biological regulation;cellular process;cellular response to stimulus;multicellular organismal process;ossification;regulation of biological process;regulation of cellular process;response to stimulus;signal transduction
27.3479	27.5534	27.3237	27.2047	1:Experimental evidence at protein level	biological regulation;cell proliferation;cell surface receptor linked signaling pathway;cellular process;cellular response to stimulus;enzyme linked receptor protein signaling pathway;intracellular signal transduction;negative reg
26.4581	26.931	26.6029	26.6504	1:Experimental evidence at protein level	anatomical structure development;biosynthetic process;catabolic process;cellular biosynthetic process;cellular catabolic process;cellular macromolecule biosynthetic process;cellular macromolecule catabolic process;cellula
28.0819	27.9221	27.9925	27.8312	1:Experimental evidence at protein level	anatomical structure morphogenesis;biological regulation;blood coagulation;bone morphogenesis;cell migration;cell motility;cellular component movement;cellular process;coagulation;developmental process;hemostasis;imn
29.001	28.8083	28.8605	28.8027	1:Experimental evidence at protein level	biological regulation;biosynthetic process;catabolic process;cellular biosynthetic process;cellular catabolic process;cellular macromolecule biosynthetic process;cellular macromolecule catabolic process;cellular macromolecu
27.0582	27.0274	26.868	26.7928	1:Experimental evidence at protein level	biological regulation;biosynthetic process;cellular biosynthetic process;cellular macromolecule biosynthetic process;cellular macromolecule metabolic process;cellular metabolic process;cellular nitrogen compound metabolic
26.9848	27.2087	27.057	26.7693	1:Experimental evidence at protein level	
27.0494	26.7992	26.9269	26.9794	1:Experimental evidence at protein level	anatomical structure development;anatomical structure morphogenesis;ATP-dependent chromatin remodeling;biological regulation;biosynthetic process;cell surface receptor linked signaling pathway;cellular biosynthetic proc
25.8477	25.8828	25.4079	25.7283	1:Experimental evidence at protein level	ATP-dependent chromatin remodeling;biological regulation;biosynthetic process;cellular biosynthetic process;cellular component disassembly;cellular component disassembly at cellular level;cellular component organization
27.3583	27.3365	27.1595	27.1198	1:Experimental evidence at protein level	adherens junction organization;antigen processing and presentation;biological regulation;cell junction organization;cell-cell junction organization;cellular component organization;cellular component organization at cellular leve
29.5626	29.9279	30.0975	30.0469	1:Experimental evidence at protein level	biological regulation;biosynthetic process;catabolic process;cellular biosynthetic process;cellular catabolic process;cellular macromolecule biosynthetic process;cellular macromolecule catabolic process;cellular macromolecu
28.7347	28.7834	28.7176	28.6436	1:Experimental evidence at protein level	cellular component organization;cellular component organization at cellular level;cellular component organization or biogenesis;cellular component organization or biogenesis at cellular level;cellular process;establishment of l
28.6099	28.6958	28.4205	28.6046	1:Experimental evidence at protein level	actin cytoskeleton organization;actin filament organization;actin filament-based process;anatomical structure morphogenesis;biological regulation;cell cycle phase;cell cycle process;cell division;cell morphogenesis;cell surfac
25.393	24.6624	25.1846	25.8618	1:Experimental evidence at protein level	anatomical structure formation involved in morphogenesis;biological regulation;cell differentiation;cellular developmental process;cellular process;developmental process;muscle cell differentiation;negative regulation of biolog
27.3342	27.1138	27.2098	27.0108	1:Experimental evidence at protein level	anatomical structure arrangement;anatomical structure development;axon extension;biological regulation;biosynthetic process;BMP signaling pathway;catabolic process;cell cycle phase;cell cycle process;cell division;cell gr
26.8621	26.7125	26.8812	26.7501	1:Experimental evidence at protein level	biological regulation;catabolic process;cellular catabolic process;cellular component organization;cellular component organization at cellular level;cellular component organization or biogenesis;cellular component organizatio
25.5608	25.2636	25.4967	24.9924	1:Experimental evidence at protein level	biological regulation;calcium ion homeostasis;calcium ion transmembrane transport;calcium ion transport;cation homeostasis;cation transport;cell cycle arrest;cell cycle process;cell proliferation;cellular calcium ion homeosta
25.6145	25.0135	24.0413	25.2006	1:Experimental evidence at protein level	ATP biosynthetic process;ATP hydrolysis coupled proton transport;ATP metabolic process;ATP synthesis coupled proton transport;biological regulation;biosynthetic process;cation homeostasis;cation transport;cell surface re
26.9123	27.2541	27.4397	27.565	1:Experimental evidence at protein level	biological adhesion;cell adhesion;cellular process
24.6896	24.6486	25.4388	25.075	1:Experimental evidence at protein level	biological regulation;biosynthetic process;cellular biosynthetic process;cellular macromolecule biosynthetic process;cellular macromolecule metabolic process;cellular metabolic process;cellular nitrogen compound metabolic
26.9714	26.7992	27.0572	27.739	1:Experimental evidence at protein level	anatomical structure development;ATP-dependent chromatin remodeling;biological regulation;cellular component disassembly;cellular component disassembly at cellular level;cellular component organization;cellular compor
27.9261	27.8516	27.8128	28.2784	1:Experimental evidence at protein level	actin cytoskeleton organization;actin filament-based process;biological regulation;cellular component organization;cellular component organization at cellular level;cellular component organization or biogenesis;cellular compo
28.754	29.3084	29.6451	29.6381	1:Experimental evidence at protein level	anatomical structure formation involved in morphogenesis;angiogenesis;apoptosis;biological regulation;cell death;cellular macromolecule metabolic process;cellular metabolic process;cellular process;cellular protein metabol
25.7734	25.4084	25.9848	25.7042	1:Experimental evidence at protein level	apoptosis;attachment of GPI anchor to protein;cell death;cell differentiation;cellular developmental process;cellular macromolecule metabolic process;cellular metabolic process;cellular process;cellular protein metabolic proc
25.8558	26.0021	26.1116	25.6195	1:Experimental evidence at protein level	anatomical structure development;biological regulation;cell proliferation;cellular component movement;cellular process;developmental process;fertilization;muscle tissue development;nervous system development;regulation
28.4947	24.6045	29.3043	28.1597	1:Experimental evidence at protein level	biosynthetic process;cellular biosynthetic process;cellular macromolecule biosynthetic process;cellular macromolecule metabolic process;cellular metabolic process;cellular process;cellular protein metabolic process;macron
25.4471	24.9058	25.284	25.309	1:Experimental evidence at protein level	biological regulation;induction of apoptosis;induction of apoptosis by intracellular signals;induction of apoptosis by oxidative stress;induction of programmed cell death;positive regulation of apoptosis;positive regulation of bi
25.2064	24.7668	26.708	26.2984	1:Experimental evidence at protein level	biosynthetic process;cellular biosynthetic process;cellular component disassembly;cellular component disassembly at cellular level;cellular component organization;cellular component organization at cellular level;cellular cor
25.8608	27.3926	26.6879	26.3266	1:Experimental evidence at protein level	biological regulation;cell cycle checkpoint;cellular component assembly;cellular component assembly at cellular level;cellular component organization;cellular component organization at cellular level;cellular component orgar
27.6407	27.6345	27.449	27.4011	1:Experimental evidence at protein level	biological regulation;posttranscriptional regulation of gene expression;protein destabilization;regulation of biological process;regulation of biological quality;regulation of cellular component biogenesis;regulation of cellular cor
27.016	26.6673	26.7414	26.5781	1:Experimental evidence at protein level	cellular process;ER to Golgi vesicle-mediated transport;establishment of localization;establishment of localization in cell;Golgi vesicle transport;intracellular transport;transport;vesicle-mediated transport
26.3506	24.5575	26.8581	26.0911	1:Experimental evidence at protein level	biological regulation;cell cycle arrest;cell cycle process;cellular metabolic process;cellular process;cellular respiration;energy derivation by oxidation of organic compounds;generation of precursor metabolites and energy;met
25.5525	26.086	26.0391	26.0735	1:Experimental evidence at protein level	activation of caspase activity;apoptosis;biological regulation;cell death;cellular process;cellular response to chemical stimulus;cellular response to hypoxia;cellular response to oxygen levels;cellular response to stimulus;cellu
28.2215	27.9944	28.0228	28.1483	1:Experimental evidence at protein level	biological regulation;calcium ion homeostasis;cation homeostasis;cell differentiation;cellular developmental process;cellular process;cellular response to biotic stimulus;cellular response to stimulus;cellular response to stress
27.8384	27.6053	27.8161	27.6956	1:Experimental evidence at protein level	
25.6643	25.6408	25.6319	25.6615	1:Experimental evidence at protein level	cellular component organization;cellular component organization at cellular level;cellular component organization or biogenesis;cellular component organization or biogenesis at cellular level;cellular macromolecule metabolic
26.9868	27.6368	27.5656	27.7809	1:Experimental evidence at protein level	biological regulation;posttranscriptional regulation of gene expression;protein destabilization;regulation of biological process;regulation of biological quality;regulation of gene expression;regulation of macromolecule metaboli
28.3887	28.1631	28.1505	28.2635	1:Experimental evidence at protein level	anatomical structure morphogenesis;biological adhesion;biological regulation;cell adhesion;cell junction assembly;cell junction organization;cell morphogenesis;cell morphogenesis involved in differentiation;cell surface recep
28.6912	29.1603	29.5192	29.4676	1:Experimental evidence at protein level	biological regulation;biosynthetic process;cellular biosynthetic process;cellular macromolecule biosynthetic process;cellular macromolecule metabolic process;cellular metabolic process;cellular nitrogen compound metabolic
29.8946	30.1089	29.8803	29.8665	1:Experimental evidence at protein level	
26.013	27.0363	26.7053	26.9651	1:Experimental evidence at protein level	alcohol catabolic process;alcohol metabolic process;carbohydrate catabolic process;carbohydrate metabolic process;catabolic process;cellular carbohydrate catabolic process;cellular carbohydrate metabolic process;cellula
26.2649	26.4237	26.2407	26.2575	1:Experimental evidence at protein level	anatomical structure formation involved in morphogenesis;biological regulation;cell differentiation;cellular developmental process;cellular localization;cellular process;developmental process;establishment of localization;estab
29.5584	29.5795	29.6932	29.7584	1:Experimental evidence at protein level	cellular macromolecule metabolic process;cellular metabolic process;cellular process;cellular protein metabolic process;chaperone-mediated protein folding;macromolecule metabolic process;macromolecule modification;me
26.5198	24.474	26.4934	26.6102	1:Experimental evidence at protein level	catabolic process;cellular catabolic process;cellular macromolecule catabolic process;cellular macromolecule metabolic process;cellular metabolic process;cellular nitrogen compound metabolic process;cellular process;exo
25.4182	23.6883	25.4153	25.8952	1:Experimental evidence at protein level	cellular macromolecule metabolic process;cellular metabolic process;cellular process;cellular protein metabolic process;establishment of localization;establishment of localization in cell;establishment of protein localization;es
25.5096	25.4215	25.7141	25.6288	1:Experimental evidence at protein level	apoptosis;biological regulation;cell communication;cell death;cell migration;cell motility;cell surface receptor linked signaling pathway;cell-cell signaling;cellular component movement;cellular component organization;cellular
24.4579	25.1358	25.1352	24.6042	1:Experimental evidence at protein level	biological regulation;cellular macromolecule metabolic process;cellular metabolic process;cellular process;cellular protein metabolic process;macromolecule metabolic process;macromolecule modification;metabolic process
26.3938	26.0701	26.5442	26.415	1:Experimental evidence at protein level	biological regulation;developmental process;dorsal/ventral pattern formation;negative regulation of biological process;negative regulation of catalytic activity;negative regulation of cell communication;negative regulation of ce
23.7596	25.1959	25.313	25.1014	1:Experimental evidence at protein level	activation of immune response;activation of innate immune response;anatomical structure formation involved in morphogenesis;angiogenesis;biological regulation;canonical Wnt receptor signaling pathway;cell surface recept
25.3664	24.0055	26.3637	26.3309	1:Experimental evidence at protein level	
25.3858	26.051	25.9736	25.9123	1:Experimental evidence at protein level	biological regulation;cellular process;cellular response to stimulus;intracellular signal transduction;regulation of biological process;regulation of cell migration;regulation of cell motility;regulation of cellular component moveme
26.3559	26.7841	26.9354	27.0682	1:Experimental evidence at protein level	
27.5718	27.978	27.9023	28.1106	1:Experimental evidence at protein level	
25.5398	26.1613	25.7529	25.7817	1:Experimental evidence at protein level	alcohol metabolic process;carbohydrate metabolic process;cellular carbohydrate metabolic process;cellular metabolic process;cellular process;galactose metabolic process;glucose metabolic process;hexose metabolic proc
28.5339	28.274	28.5022	28.3291	1:Experimental evidence at protein level	amine biosynthetic process;amine metabolic process;biosynthetic process;carboxylic acid biosynthetic process;carboxylic acid metabolic process;cellular amine metabolic process;cellular amino acid biosynthetic process;ce
27.4531	26.927	26.9959	27.1348	1:Experimental evidence at protein level	biological regulation;catabolic process;cellular catabolic process;cellular macromolecule catabolic process;cellular macromolecule metabolic process;cellular metabolic process;cellular nitrogen compound metabolic process
28.4471	29.192	29.5497	29.5595	1:Experimental evidence at protein level	biological regulation;defense response;immune response;immune system process;innate immune response;regulation of biological process;regulation of cellular metabolic process;regulation of cellular process;regulation of m
23.9828	26.4144	26.5448	26.6315	1:Experimental evidence at protein level	carboxylic acid metabolic process;cellular ketone metabolic process;cellular lipid metabolic process;cellular metabolic process;cellular process;fatty acid metabolic process;lipid metabolic process;metabolic process;monoca
26.2945	26.2447	26.6092	26.3902	1:Experimental evidence at protein level	
27.7636	27.7579	27.708	27.6477	1:Experimental evidence at protein level	biological regulation;cellular component organization;cellular component organization at cellular level;cellular component organization or biogenesis;cellular component organization or biogenesis at cellular level;cellular proce
27.176	27.6385	27.6999	27.4245	1:Experimental evidence at protein level	cell cycle;cell division;cellular process
26.7561	26.6655	27.1343	27.0833	1:Experimental evidence at protein level	autophagy;biological regulation;catabolic process;cell communication;cell cycle;cell cycle process;cell death;cellular catabolic process;cellular component organization;cellular component organization at cellular level;cellular
28.785	28.761	28.7173	28.6354	1:Experimental evidence at protein level	antigen processing and presentation;antigen processing and presentation of exogenous antigen;antigen processing and presentation of exogenous peptide antigen;antigen processing and presentation of exogenous peptide
26.2893	26.1444	25.7703	26.0095	1:Experimental evidence at protein level	cell cycle;cell cycle process;cellular component organization;cellular component organization at cellular level;cellular component organization or biogenesis;cellular component organization or biogenesis at cellular level;cellula
28.7237	28.9282	29.0722	29.1341	1:Experimental evidence at protein level	
26.2743	26.3007	26.1249	26.0943	1:Experimental evidence at protein level	cellular localization;cellular process;establishment of localization;establishment of localization in cell;establishment of organelle localization;establishment of protein localization;establishment of ribosome localization;intracellu
26.0722	25.734	26.2751	25.4449	1:Experimental evidence at protein level	
23.7378	24.387	25.2805	24.607	1:Experimental evidence at protein level	
27.2243	27.3487	27.168	27.426	1:Experimental evidence at protein level	cellular macromolecule metabolic process;cellular metabolic process;cellular nitrogen compound metabolic process;cellular process;gene expression;macromolecule metabolic process;metabolic process;mRNA metabolic p
26.1641	26.0524	26.0299	25.9218	1:Experimental evidence at protein level	biosynthetic process;cellular biosynthetic process;cellular component disassembly;cellular component disassembly at cellular level;cellular component organization;cellular component organization at cellular level;cellular cor
25.8216	24.6554	25.8194	25.2941	1:Experimental evidence at protein level	biosynthetic process;cellular biosynthetic process;cellular component disassembly;cellular component disassembly at cellular level;cellular component organization;cellular component organization at cellular level;cellular cor
26.1912	25.7126	26.1031	25.6281	1:Experimental evidence at protein level	biological regulation;cell cycle;cell cycle checkpoint;cell cycle process;cell division;cellular component organization;cellular component organization at cellular level;cellular component organization or biogenesis;cellular comp
25.5845	25.9285	25.7988	24.9092	1:Experimental evidence at protein level	biological regulation;biosynthetic process;cellular biosynthetic process;cellular component organization;cellular component organization at cellular level;cellular component organization or biogenesis;cellular component orgar
24.9814	23.1979	25.0582	25.2126	1:Experimental evidence at protein level	acylglycerol metabolic process;aging;anatomical structure development;anatomical structure formation involved in morphogenesis;angiogenesis;behavioral response to starvation;biological regulation;biosynthetic process;ca
26.6149	26.8679	26.4463	26.6238	1:Experimental evidence at protein level	attachment of spindle microtubules to chromosome;attachment of spindle microtubules to kinetochore;attachment of spindle microtubules to kinetochore involved in mitotic sister chromatid segregation;biological regulation;
27.4609	27.3337	27.236	27.231	1:Experimental evidence at protein level	anatomical structure development;cell differentiation;cellular developmental process;cellular process;developmental process;establishment of localization;establishment of localization in cell;intracellular transport;mitochondri
26.4975	26.1374	25.8241	25.8371	1:Experimental evidence at protein level	biosynthetic process;cellular biosynthetic process;cellular component disassembly;cellular component disassembly at cellular level;cellular component organization;cellular component organization at cellular level;cellular cor
26.1818	26.3852	24.9255	26.4512	1:Experimental evidence at protein level	biosynthetic process;cellular biosynthetic process;cellular component disassembly;cellular component disassembly at cellular level;cellular component organization;cellular component organization at cellular level;cellular cor
28.0823	28.8387	28.2692	28.467	1:Experimental evidence at protein level	anatomical structure development;biological regulation;cell differentiation;cell proliferation;cellular developmental process;cellular process;developmental process;gamete generation;male gamete generation;maternal placent
23.8925	23.4531	25.474	22.61	1:Experimental evidence at protein level	
26.2481	25.5902	25.9576	25.5828	1:Experimental evidence at protein level	activation of caspase activity;activation-induced cell death of T cells;aging;apoptosis;biological regulation;biosynthetic process;cell activation;cell aging;cell death;cell differentiation;cellular biosynthetic process;cellular comp
26.4071	25.9841	26.1593	25.795	1:Experimental evidence at protein level	biological regulation;biosynthetic process;cellular biosynthetic process;cellular component disassembly;cellular component disassembly at cellular level;cellular component organization;cellular component organization at cell
26.0537	26.3477	26.1672	25.9946	1:Experimental evidence at protein level	catabolic process;cellular catabolic process;cellular macromolecule catabolic process;cellular macromolecule metabolic process;cellular metabolic process;cellular nitrogen compound metabolic process;cellular process;exo
27.1292	27.3649	27.3584	27.1328	1:Experimental evidence at protein level	response to calcium ion;response to chemical stimulus;response to inorganic substance;response to metal ion;response to stimulus
25.6671	25.3036	25.5101	25.6177	1:Experimental evidence at protein level	apoptosis;biological regulation;biosynthetic process;cell death;cellular biosynthetic process;cellular macromolecule biosynthetic process;cellular macromolecule metabolic process;cellular metabolic process;cellular nitrogen
26.9181	27.539	27.2414	27.4442	1:Experimental evidence at protein level	biological regulation;cellular macromolecule metabolic process;cellular metabolic process;cellular nitrogen compound metabolic process;cellular process;cellular protein metabolic process;cellular response to stimulus;cellula
27.0268	26.9063	27.0767	26.9557	1:Experimental evidence at protein level	cellular macromolecule metabolic process;cellular metabolic process;cellular nitrogen compound metabolic process;cellular process;macromolecule metabolic process;macromolecule methylation;macromolecule modificatio
27.5243	27.6452	27.689	27.8216	1:Experimental evidence at protein level	alcohol catabolic process;alcohol metabolic process;biosynthetic process;carbohydrate biosynthetic process;carbohydrate catabolic process;carbohydrate metabolic process;catabolic process;cellular biosynthetic process;
27.8522	28.0665	27.7963	27.9427	1:Experimental evidence at protein level	biosynthetic process;cellular biosynthetic process;cellular lipid metabolic process;cellular metabolic process;cellular process;ceramide biosynthetic process;ceramide metabolic process;lipid biosynthetic process;lipid metabo
25.7154	25.3514	25.719	25.7606	1:Experimental evidence at protein level	
26.4325	26.6562	26.581	26.6275	1:Experimental evidence at protein level	
25.2409	25.303	22.7202	25.1369	1:Experimental evidence at protein level	amine catabolic process;amine metabolic process;aspartate family amino acid catabolic process;aspartate family amino acid metabolic process;carboxylic acid catabolic process;carboxylic acid metabolic process;catabolic
25.4954	24.5946	25.3489	25.5525	1:Experimental evidence at protein level	biosynthetic process;cellular biosynthetic process;cellular component disassembly;cellular component disassembly at cellular level;cellular component organization;cellular component organization at cellular level;cellular cor
25.7832	23.8168	24.1659	25.8575	1:Experimental evidence at protein level	autophagy;biological adhesion;biological regulation;catabolic process;cell adhesion;cell junction assembly;cell junction organization;cell-cell adhesion;cellular catabolic process;cellular component assembly;cellular compone
27.0217	27.024	26.8742	26.7438	1:Experimental evidence at protein level	abscission;aging;anaphase-promoting complex-dependent proteasomal ubiquitin-dependent protein catabolic process;attachment of spindle microtubules to chromosome;attachment of spindle microtubules to kinetochore;
26.4135	24.5086	25.7337	25.7817	1:Experimental evidence at protein level	
26.565	25.6897	26.0656	25.9656	1:Experimental evidence at protein level	cellular macromolecule metabolic process;cellular metabolic process;cellular nitrogen compound metabolic process;cellular process;macromolecule metabolic process;metabolic process;nitrogen compound metabolic proce
26.0414	25.9988	26.0257	26.1679	1:Experimental evidence at protein level	
23.7463	25.4986	25.1151	25.2824	1:Experimental evidence at protein level	biological regulation;cellular macromolecule metabolic process;cellular metabolic process;cellular nitrogen compound metabolic process;cellular process;cellular response to stimulus;cellular response to stress;cytoplasmic s
28.2356	28.1046	28.1511	28.2663	1:Experimental evidence at protein level	biological regulation;regulation of anatomical structure morphogenesis;regulation of biological process;regulation of cell development;regulation of cell differentiation;regulation of cell morphogenesis;regulation of cell morpho
27.407	27.4335	27.2923	27.3652	1:Experimental evidence at protein level	4-hydroxyproline metabolic process;amine metabolic process;apoptosis;apoptosis in response to endoplasmic reticulum stress;biological regulation;brown fat cell differentiation;calcium ion homeostasis;calcium ion transport
27.3581	27.1794	27.0992	27.0468	1:Experimental evidence at protein level	biological regulation;cell death;cellular metabolic process;cellular process;cellular response to stimulus;death;dephosphorylation;intracellular signal transduction;metabolic process;necroptosis;necrotic cell death;phosphate-c
25.6837	24.7163	24.7233	25.6158	1:Experimental evidence at protein level	biological regulation;cellular process;cellular response to stimulus;cellular response to stress;positive regulation of molecular function;positive regulation of NF-kappaB transcription factor activity;positive regulation of sequen
28.5776	28.2507	28.5251	28.5482	1:Experimental evidence at protein level	biological regulation;biosynthetic process;cellular biosynthetic process;cellular macromolecule biosynthetic process;cellular macromolecule metabolic process;cellular metabolic process;cellular nitrogen compound metabolic
26.1498	26.2188	26.3053	26.1765	1:Experimental evidence at protein level	alternative nuclear mRNA splicing, via spliceosome;cellular macromolecule metabolic process;cellular metabolic process;cellular nitrogen compound metabolic process;cellular process;macromolecule metabolic process;met
27.3472	27.3801	27.5216	27.4728	1:Experimental evidence at protein level	acetyl-CoA catabolic process;acetyl-CoA metabolic process;carboxylic acid metabolic process;catabolic process;cellular catabolic process;cellular ketone metabolic process;cellular metabolic process;cellular process;coenz
26.5699	26.7892	26.5841	26.4778	1:Experimental evidence at protein level	alcohol biosynthetic process;alcohol metabolic process;biosynthetic process;carbohydrate biosynthetic process;carbohydrate metabolic process;cellular biosynthetic process;cellular carbohydrate biosynthetic process;cellul
27.2029	27.0434	27.2861	27.204	1:Experimental evidence at protein level	biological regulation;metabolic process;positive regulation of biological process;positive regulation of biosynthetic process;positive regulation of cellular biosynthetic process;positive regulation of cellular metabolic process;p
26.5079	26.8997	27.0706	27.1036	1:Experimental evidence at protein level	actin cytoskeleton organization;actin filament bundle assembly;actin filament organization;actin filament-based process;apoptosis;biological regulation;biosynthetic process;cell death;cellular biosynthetic process;cellular con
25.6565	25.3201	25.3709	25.2842	1:Experimental evidence at protein level	biological adhesion;biological regulation;cell adhesion;cell-cell adhesion;cellular component organization;cellular component organization or biogenesis;cellular process;establishment of localization;excretion;multicellular orga
26.9973	26.7439	26.9206	26.6448	1:Experimental evidence at protein level	anatomical structure development;biological regulation;brain development;cell cycle checkpoint;cell cycle process;cell proliferation;cellular macromolecule metabolic process;cellular metabolic process;cellular process;cellula
26.0601	27.1343	28.0993	26.197	1:Experimental evidence at protein level	cell cycle phase;cell cycle process;cellular component assembly;cellular component assembly at cellular level;cellular component organization;cellular component organization at cellular level;cellular component organization
24.9492	24.7126	24.7087	24.7527	1:Experimental evidence at protein level	apoptosis;biological regulation;cell chemotaxis;cell death;cell migration;cell motility;cell surface receptor linked signaling pathway;cellular component movement;cellular process;cellular response to chemical stimulus;cellular

26.7779	27.313	26.8527	26.9935	1:Experimental evidence at protein level	biological regulation;cell redox homeostasis;cellular homeostasis;cellular macromolecule metabolic process;cellular metabolic process;cellular process;cellular protein metabolic process;cellular response to stimulus;cellular r
25.595	24.664	25.8226	25.3331	1:Experimental evidence at protein level	attachment of spindle microtubules to chromosome;attachment of spindle microtubules to kinetochore;attachment of spindle microtubules to kinetochore involved in mitotic sister chromatid segregation;biological regulation;c
25.7379	25.8148	26.0938	25.9406	1:Experimental evidence at protein level	activation of NF-kappaB-inducing kinase activity;activation of protein kinase activity;biological regulation;cellular macromolecule metabolic process;cellular metabolic process;cellular process;cellular protein metabolic proces
25.9229	26.8092	26.8816	27.0419	1:Experimental evidence at protein level	
28.2571	28.5058	28.6927	28.7763	1:Experimental evidence at protein level	
25.4889	22.7946	25.6777	25.6287	1:Experimental evidence at protein level	abscission;biological regulation;cell cycle;cell division;cellular process;developmental process;negative regulation of biological process;negative regulation of cell division;negative regulation of cellular process;negative regula
26.5349	26.3597	26.5994	26.4513	1:Experimental evidence at protein level	base-excision repair;biological regulation;catabolic process;cellular catabolic process;cellular macromolecule catabolic process;cellular macromolecule metabolic process;cellular metabolic process;cellular nitrogen compoun
27.4599	27.8462	27.5842	27.8562	1:Experimental evidence at protein level	biological regulation;cell cycle phase;cell cycle process;cellular component organization;cellular component organization at cellular level;cellular component organization or biogenesis;cellular component organization or bioge
27.9851	28.0865	27.9481	27.8848	1:Experimental evidence at protein level	biological regulation;biosynthetic process;cellular biosynthetic process;cellular macromolecule biosynthetic process;cellular macromolecule metabolic process;cellular metabolic process;cellular nitrogen compound metabolic
25.8966	25.2537	25.4752	25.3032	1:Experimental evidence at protein level	cellular component organization;cellular component organization at cellular level;cellular component organization or biogenesis;cellular component organization or biogenesis at cellular level;cellular macromolecule metabolic
27.6756	28.0013	28.356	28.1216	1:Experimental evidence at protein level	amine biosynthetic process;amine metabolic process;biosynthetic process;carboxylic acid biosynthetic process;carboxylic acid metabolic process;cellular amine metabolic process;cellular amino acid biosynthetic process;ce
24.6594	24.6087	27.6174	24.2304	1:Experimental evidence at protein level	biological regulation;cellular component organization;cellular component organization at cellular level;cellular component organization or biogenesis;cellular component organization or biogenesis at cellular level;cellular macro
26.4845	25.9047	26.2882	26.0997	1:Experimental evidence at protein level	developmental process;multicellular organismal development;multicellular organismal process
26.3006	25.9	26.1907	25.74	1:Experimental evidence at protein level	biological regulation;cellular process;cellular response to stimulus;endosome to lysosome transport;endosome transport;establishment of cell polarity;establishment of localization;establishment of localization in cell;establish
25.7454	25.6392	25.8906	25.836	1:Experimental evidence at protein level	
27.5205	27.6392	28.0303	27.8323	1:Experimental evidence at protein level	
25.9783	24.6647	26.1156	26.1085	1:Experimental evidence at protein level	cellular component assembly;cellular component assembly at cellular level;cellular component organization;cellular component organization at cellular level;cellular component organization or biogenesis;cellular component c
26.3358	26.2949	26.5707	26.6619	1:Experimental evidence at protein level	
28.2581	28.3831	27.9668	28.0404	1:Experimental evidence at protein level	anatomical structure development;anatomical structure formation involved in morphogenesis;biosynthetic process;cellular biosynthetic process;cellular lipid metabolic process;cellular metabolic process;cellular process;deve
25.9381	25.9774	25.7761	25.4735	1:Experimental evidence at protein level	anatomical structure morphogenesis;axonogenesis;biological regulation;cell differentiation;cell part morphogenesis;cell projection morphogenesis;cell projection organization;cellular component morphogenesis;cellular comp
25.9651	25.2009	26.2529	25.7541	1:Experimental evidence at protein level	
25.5111	26.0779	26.1477	26.1532	1:Experimental evidence at protein level	
25.0825	24.8717	24.5998	24.9739	1:Experimental evidence at protein level	cellular macromolecule metabolic process;cellular metabolic process;cellular nitrogen compound metabolic process;cellular process;macromolecule metabolic process;metabolic process;ncRNA metabolic process;ncRNA pr
25.549	26.0167	25.6291	25.9413	1:Experimental evidence at protein level	actin cytoskeleton organization;actin cytoskeleton reorganization;actin filament organization;actin filament reorganization involved in cell cycle;actin filament-based process;biological regulation;cell cycle process;cellular com
27.2232	27.5567	27.252	27.3358	1:Experimental evidence at protein level	establishment of localization;establishment of localization in cell;establishment of protein localization;intracellular protein transport;intracellular transport;nuclear import;nuclear transport;nucleocytoplasmic transport;protein in
28.6731	28.5762	28.5508	28.6805	1:Experimental evidence at protein level	biological regulation;biosynthetic process;cellular biosynthetic process;cellular component organization;cellular component organization at cellular level;cellular component organization or biogenesis;cellular component orga
27.8896	28.0184	28.3717	28.1542	1:Experimental evidence at protein level	3'-UTR-mediated mRNA destabilization;anatomical structure development;axon ensheathment;biological regulation;biosynthetic process;carboxylic acid biosynthetic process;carboxylic acid metabolic process;cell developm
25.8746	25.946	25.7232	25.3557	1:Experimental evidence at protein level	cellular macromolecule metabolic process;cellular metabolic process;cellular nitrogen compound metabolic process;cellular process;macromolecule metabolic process;macromolecule modification;metabolic process;ncRNA
24.3982	24.1289	24.549	27.5397	1:Experimental evidence at protein level	catabolic process;cellular catabolic process;cellular lipid metabolic process;cellular macromolecule catabolic process;cellular macromolecule metabolic process;cellular metabolic process;cellular nitrogen compound metabo
26.9357	24.4265	23.7642	23.6455	1:Experimental evidence at protein level	amine transport;amino acid transmembrane transport;amino acid transport;biological regulation;carboxylic acid transport;cation transport;cell communication;cell-cell signaling;cellular process;establishment of localization;es
28.2549	28.2328	28.1327	27.9367	1:Experimental evidence at protein level	cellular process;endosome transport;establishment of localization;establishment of localization in cell;establishment of protein localization;intracellular protein transport;intracellular transport;protein transport;retrograde trans
26.802	27.2391	26.9974	27.0001	1:Experimental evidence at protein level	biological regulation;biosynthetic process;cell differentiation;cell proliferation;cellular biosynthetic process;cellular developmental process;cellular macromolecule biosynthetic process;cellular macromolecule metabolic proces
26.2411	25.8156	25.8835	26.304	1:Experimental evidence at protein level	establishment of localization;establishment of localization in cell;establishment of protein localization;intracellular protein transport;intracellular transport;nuclear export;nuclear transport;nucleocytoplasmic transport;protein e
25.9352	26.0308	25.7622	25.6379	1:Experimental evidence at protein level	biological regulation;cell cycle process;cell division;cellular component organization;cellular component organization at cellular level;cellular component organization or biogenesis;cellular component organization or biogenes
27.8231	27.8374	27.7061	27.9602	1:Experimental evidence at protein level	biological regulation;cell cycle cytokinesis;cell cycle phase;cell cycle process;cellular component organization;cellular component organization at cellular level;cellular component organization or biogenesis;cellular componen
25.9861	25.7072	25.7507	26.2901	1:Experimental evidence at protein level	biological regulation;biosynthetic process;cellular biosynthetic process;cellular macromolecule metabolic process;cellular metabolic process;cellular nitrogen compound metabolic process;cellular process;developmental pro
26.6177	26.5915	26.6835	26.5731	1:Experimental evidence at protein level	biosynthetic process;cellular biosynthetic process;cellular component organization;cellular component organization at cellular level;cellular component organization or biogenesis;cellular component organization or biogenesis
27.164	27.1265	27.3465	26.9262	1:Experimental evidence at protein level	immune system process
26.6212	26.5392	26.4323	26.6102	1:Experimental evidence at protein level	asymmetric protein localization;basal protein localization;biological adhesion;biological regulation;cell adhesion;cell cycle;cell growth;cell surface receptor linked signaling pathway;cellular component organization;cellular con
28.955	26.4233	24.5292	25.266	1:Experimental evidence at protein level	multicellular organismal process;neurological system process;response to stimulus;sensory perception;sensory perception of light stimulus;system process;visual perception
24.2887	25.8075	24.0575	26.0072	1:Experimental evidence at protein level	
25.7907	26.0113	25.8923	25.7251	1:Experimental evidence at protein level	axon guidance;biological regulation;cellular component assembly;cellular component organization;cellular component organization at cellular level;cellular component organization or biogenesis;cellular component organizatio
25.9132	25.9498	25.971	25.8184	1:Experimental evidence at protein level	anatomical structure development;biological regulation;cell cycle arrest;cell cycle process;cell differentiation;cell migration;cell motility;cell projection assembly;cell projection organization;cellular component assembly;cellular
26.1952	25.9091	25.8465	25.9934	1:Experimental evidence at protein level	biological regulation;cellular component organization;cellular component organization at cellular level;cellular component organization or biogenesis;cellular component organization or biogenesis at cellular level;cellular macro
25.0953	25.6503	22.3792	25.4489	1:Experimental evidence at protein level	
26.8129	26.5132	26.6686	26.2541	1:Experimental evidence at protein level	biological regulation;biosynthetic process;cellular biosynthetic process;cellular macromolecule biosynthetic process;cellular macromolecule metabolic process;cellular metabolic process;cellular nitrogen compound metabolic
26.4943	26.3996	26.5244	25.7916	1:Experimental evidence at protein level	activation of immune response;activation of innate immune response;aging;biological regulation;biosynthetic process;blood coagulation;carbohydrate homeostasis;cell differentiation;cellular biosynthetic process;cellular chem
26.0273	25.7435	25.7313	25.8265	1:Experimental evidence at protein level	anatomical structure development;anatomical structure morphogenesis;biological regulation;blood vessel development;branching morphogenesis of a tube;cardiac septum morphogenesis;developmental process;hemopoieti
26.0802	25.8369	25.7081	25.6937	1:Experimental evidence at protein level	biological regulation;cellular component organization;cellular component organization or biogenesis;cellular membrane organization;cellular process;endocytosis;establishment of localization;membrane invagination;membran
26.2839	26.16	26.2911	25.9326	1:Experimental evidence at protein level	biological regulation;biosynthetic process;cellular biosynthetic process;cellular macromolecule biosynthetic process;cellular macromolecule metabolic process;cellular metabolic process;cellular nitrogen compound metabolic
27.6184	27.8107	27.7375	27.647	1:Experimental evidence at protein level	biological regulation;biosynthetic process;cell cycle;cell proliferation;cellular biosynthetic process;cellular component organization;cellular component organization at cellular level;cellular component organization or biogenesi
29.165	28.9739	28.9173	28.8228	1:Experimental evidence at protein level	biological regulation;negative regulation of apoptosis;negative regulation of biological process;negative regulation of cell death;negative regulation of cellular process;negative regulation of programmed cell death;regulation o
25.5711	25.2781	25.5649	25.4903	1:Experimental evidence at protein level	anatomical structure development;biological regulation;developmental process;immune response;immune system process;muscle organ development;muscle structure development;organ development;positive regulation of
27.1167	27.0954	27.5753	27.5521	1:Experimental evidence at protein level	biological regulation;biosynthetic process;cellular biosynthetic process;cellular macromolecule biosynthetic process;cellular macromolecule metabolic process;cellular metabolic process;cellular nitrogen compound metabolic
28.672	28.8565	29.0394	28.8866	1:Experimental evidence at protein level	anatomical structure development;cell differentiation;cellular developmental process;cellular macromolecule metabolic process;cellular metabolic process;cellular process;cellular protein metabolic process;'de novo' posttran
29.3534	29.4356	29.6719	29.5781	1:Experimental evidence at protein level	activation of immune response;activation of innate immune response;anaphase-promoting complex-dependent proteasomal ubiquitin-dependent protein catabolic process;antigen processing and presentation;antigen proces
30.1515	30.1844	30.3417	30.3555	1:Experimental evidence at protein level	actin cytoskeleton organization;actin filament-based process;actomyosin structure organization;anatomical structure development;biological regulation;cellular component organization;cellular component organization at cellu
27.4384	27.4069	27.3953	27.3722	1:Experimental evidence at protein level	biological regulation;cellular macromolecule metabolic process;cellular metabolic process;cellular process;cellular protein metabolic process;cellular response to chemical stimulus;cellular response to organic substance;cellu
25.2803	24.0505	25.7183	25.9326	1:Experimental evidence at protein level	alcohol metabolic process;amine biosynthetic process;amine metabolic process;biosynthetic process;cellular amine metabolic process;cellular biogenic amine biosynthetic process;cellular biogenic amine metabolic process;
27.1469	26.9053	27.0101	27.0438	1:Experimental evidence at protein level	biological regulation;biosynthetic process;cellular biosynthetic process;cellular macromolecule biosynthetic process;cellular macromolecule metabolic process;cellular metabolic process;cellular nitrogen compound metabolic
28.6891	28.442	28.4502	28.5249	1:Experimental evidence at protein level	activation of immune response;activation of innate immune response;anaphase-promoting complex-dependent proteasomal ubiquitin-dependent protein catabolic process;antigen processing and presentation;antigen proces
25.0557	26.4553	26.5225	26.6205	1:Experimental evidence at protein level	carbohydrate metabolic process;cellular carbohydrate metabolic process;cellular macromolecule metabolic process;cellular metabolic process;cellular process;cellular protein metabolic process;glycosylation;macromolecule
27.5142	27.5269	27.959	27.9454	1:Experimental evidence at protein level	anatomical structure development;biological regulation;cellular macromolecule metabolic process;cellular metabolic process;cellular process;cellular protein metabolic process;'de novo' posttranslational protein folding;'de n
26.4388	26.3701	26.6195	26.8935	1:Experimental evidence at protein level	anatomical structure morphogenesis;anterior/posterior axis specification;axis specification;biological regulation;biosynthetic process;cell cycle;cellular biosynthetic process;cellular component organization;cellular componen
30.2018	30.4301	30.4053	30.5923	1:Experimental evidence at protein level	activation of protein kinase activity;activation of protein kinase B activity;adult behavior;adult locomotory behavior;alcohol biosynthetic process;alcohol metabolic process;aldehyde catabolic process;amine transport;autopha
29.5485	29.7253	29.6055	29.4272	1:Experimental evidence at protein level	biological regulation;negative regulation of biological process;negative regulation of cellular component organization;negative regulation of cellular process;negative regulation of developmental process;negative regulation of
27.3578	27.2864	27.3488	27.266	1:Experimental evidence at protein level	activation of immune response;activation of innate immune response;antigen processing and presentation;antigen processing and presentation of exogenous antigen;antigen processing and presentation of exogenous peptic
25.1604	25.6689	25.5819	25.7596	1:Experimental evidence at protein level	carboxylic acid transport;cellular lipid metabolic process;cellular metabolic process;cellular process;establishment of localization;fatty acid transport;lipid localization;lipid metabolic process;lipid storage;lipid transport;localiza
26.5584	26.2228	26.4269	26.4798	1:Experimental evidence at protein level	biological regulation;biosynthetic process;cellular biosynthetic process;cellular component organization;cellular component organization at cellular level;cellular component organization or biogenesis;cellular component orga
25.4676	25.3451	25.9155	25.9178	1:Experimental evidence at protein level	cellular macromolecule metabolic process;cellular metabolic process;cellular nitrogen compound metabolic process;cellular process;macromolecule metabolic process;maturation of 5.8S rRNA;metabolic process;ncRNA me
27.7286	27.611	27.5969	27.4026	1:Experimental evidence at protein level	biological regulation;biosynthetic process;carbohydrate metabolic process;carbohydrate transport;cell cycle;cell cycle process;cell surface receptor linked signaling pathway;cellular biosynthetic process;cellular component c
26.4884	26.5973	26.4357	26.1652	1:Experimental evidence at protein level	catabolic process;cellular catabolic process;cellular macromolecule catabolic process;cellular macromolecule metabolic process;cellular metabolic process;cellular nitrogen compound metabolic process;cellular process;mac
27.8574	28.4396	28.5399	28.6144	1:Experimental evidence at protein level	biological regulation;cell activation;cell activation involved in immune response;cellular process;cytokine secretion;establishment of localization;establishment of localization in cell;establishment of protein localization;exocyto
25.4762	25.2643	25.5239	25.4386	1:Experimental evidence at protein level	cellular component assembly;cellular component assembly at cellular level;cellular component organization;cellular component organization at cellular level;cellular component organization or biogenesis;cellular component c
25.7036	26.4027	25.4301	26.2909	1:Experimental evidence at protein level	biological regulation;biosynthetic process;cellular biosynthetic process;cellular macromolecule metabolic process;cellular metabolic process;cellular nitrogen compound metabolic process;cellular process;cellular response to
26.632	26.7768	26.9991	26.7934	1:Experimental evidence at protein level	cell differentiation;cellular developmental process;cellular process;developmental process;establishment of localization;establishment of protein localization;gamete generation;gene expression;macromolecule metabolic proc
28.6172	28.3991	28.3202	28.2391	1:Experimental evidence at protein level	biological regulation;biosynthetic process;cellular biosynthetic process;cellular component assembly;cellular component assembly at cellular level;cellular component organization;cellular component organization at cellular le
27.1122	27.6356	27.7115	27.8989	1:Experimental evidence at protein level	cellular macromolecule metabolic process;cellular metabolic process;cellular process;cellular protein metabolic process;macromolecule metabolic process;metabolic process;primary metabolic process;protein folding;protein
27.4353	27.3904	27.5211	27.2654	1:Experimental evidence at protein level	cellular macromolecule metabolic process;cellular metabolic process;cellular process;cellular protein metabolic process;cellular response to heat;cellular response to stimulus;cellular response to stress;chaperone cofactor-d
29.8305	29.5678	29.6486	29.5841	1:Experimental evidence at protein level	anatomical structure development;anatomical structure morphogenesis;biological regulation;biosynthetic process;branching involved in mammary gland duct morphogenesis;branching morphogenesis of a tube;cellular biosy
27.1322	27.1625	26.9332	26.8442	1:Experimental evidence at protein level	activation of NF-kappaB-inducing kinase activity;activation of protein kinase activity;biological regulation;cellular macromolecule metabolic process;cellular metabolic process;cellular process;cellular protein metabolic proces
26.1303	24.386	26.4326	25.8014	1:Experimental evidence at protein level	cellular macromolecule metabolic process;cellular metabolic process;cellular nitrogen compound metabolic process;cellular process;macromolecule metabolic process;metabolic process;mRNA metabolic process;mRNA pro
27.2671	27.2888	27.1768	27.0042	1:Experimental evidence at protein level	antigen processing and presentation;antigen processing and presentation of exogenous antigen;antigen processing and presentation of exogenous peptide antigen;antigen processing and presentation of exogenous peptide
26.614	26.8471	26.7524	27.362	1:Experimental evidence at protein level	acylglycerol catabolic process;acylglycerol metabolic process;arachidonic acid metabolic process;biological regulation;biosynthetic process;blood coagulation;carboxylic acid biosynthetic process;carboxylic acid metabolic p
27.3695	27.6742	27.495	27.3888	1:Experimental evidence at protein level	anatomical structure morphogenesis;biological regulation;cell differentiation;cell differentiation in hindbrain;cell part morphogenesis;cell projection morphogenesis;cell projection organization;cellular component assembly;cell
25.0589	24.5178	24.6917	24.8328	1:Experimental evidence at protein level	amine biosynthetic process;amine metabolic process;anatomical structure development;aspartate family amino acid biosynthetic process;aspartate family amino acid metabolic process;biosynthetic process;carboxylic acid b
27.9947	28.0981	28.1222	28.1145	1:Experimental evidence at protein level	amine catabolic process;amine metabolic process;branched chain family amino acid catabolic process;branched chain family amino acid metabolic process;carboxylic acid catabolic process;carboxylic acid metabolic proces
30.0168	29.6596	29.76	29.445	1:Experimental evidence at protein level	anatomical structure development;biological adhesion;cell adhesion;cell differentiation;cellular component disassembly;cellular component disassembly at cellular level;cellular component organization;cellular component org
29.1961	28.5258	29.1499	29.0457	1:Experimental evidence at protein level	anatomical structure morphogenesis;biological regulation;cell morphogenesis;cell morphogenesis involved in differentiation;cellular component morphogenesis;cellular component organization;cellular component organizatio
24.5175	26.4303	25.8988	24.5086	1:Experimental evidence at protein level	aging;apoptosis;biological regulation;biosynthetic process;cell death;cellular biosynthetic process;cellular macromolecule biosynthetic process;cellular macromolecule metabolic process;cellular metabolic process;cellular nit
29.1729	29.528	29.6132	29.6447	1:Experimental evidence at protein level	cellular component assembly;cellular component assembly at cellular level;cellular component organization;cellular component organization at cellular level;cellular component organization or biogenesis;cellular component c
26.7451	26.8049	26.9222	27.0281	1:Experimental evidence at protein level	biological regulation;cellular component assembly;cellular component organization;cellular component organization or biogenesis;cellular process;establishment of localization;establishment of localization in cell;establishmen
28.1837	28.2182	28.2386	28.2107	1:Experimental evidence at protein level	acetyl-CoA catabolic process;acetyl-CoA metabolic process;carboxylic acid metabolic process;catabolic process;cellular catabolic process;cellular ketone metabolic process;cellular metabolic process;cellular process;citrate
27.61	27.6694	27.4262	27.3767	1:Experimental evidence at protein level	establishment of localization;transport
26.2458	26.0958	26.0528	25.8313	1:Experimental evidence at protein level	behavior;biological regulation;body fluid secretion;carbohydrate homeostasis;cellular chemical homeostasis;cellular glucose homeostasis;cellular homeostasis;cellular ion homeostasis;cellular metabolic process;cellular nitrog
25.0674	23.3064	25.0132	25.3434	2:Experimental evidence at transcript lev	anatomical structure development;anatomical structure morphogenesis;appendage morphogenesis;artery morphogenesis;biological regulation;blood vessel morphogenesis;cartilage development;developmental process;emb
24.631	26.7061	25.103	24.5092	1:Experimental evidence at protein level	biological regulation;catabolic process;cell cycle arrest;cell cycle process;cell differentiation;cell division;cellular catabolic process;cellular component assembly;cellular component assembly at cellular level;cellular componen
28.9229	29.0531	28.8087	28.9538	1:Experimental evidence at protein level	biological regulation;biosynthetic process;cellular biosynthetic process;cellular lipid metabolic process;cellular metabolic process;cellular process;cellular response to calcium ion;cellular response to chemical stimulus;cellula
29.9914	30.0183	30.0064	29.9267	1:Experimental evidence at protein level	binding of sperm to zona pellucida;cell recognition;cell-cell recognition;cellular macromolecule metabolic process;cellular metabolic process;cellular process;cellular process involved in reproduction;cellular protein metabolic
27.1072	26.3586	26.7545	26.6992	1:Experimental evidence at protein level	cellular component biogenesis;cellular component biogenesis at cellular level;cellular component organization or biogenesis;cellular component organization or biogenesis at cellular level;cellular process;ribonucleoprotein co
28.9899	28.6797	28.6574	28.651	1:Experimental evidence at protein level	biological regulation;cell projection organization;cell surface receptor linked signaling pathway;cellular component organization;cellular component organization at cellular level;cellular component organization or biogenesis;c
25.908	23.5698	25.7868	25.6902	1:Experimental evidence at protein level	acylglycerol biosynthetic process;acylglycerol metabolic process;biological regulation;biosynthetic process;carboxylic acid biosynthetic process;carboxylic acid metabolic process;CDP-diacylglycerol biosynthetic process;CD
28.6243	28.6072	28.8986	28.9728	1:Experimental evidence at protein level	anatomical structure development;biological regulation;cellular component organization;cellular component organization or biogenesis;cellular membrane organization;cellular process;cellular response to stimulus;central nerv
26.055	24.7613	24.2341	24.0348	2:Experimental evidence at transcript lev	ameboidal cell migration;anatomical structure development;anatomical structure formation involved in morphogenesis;anatomical structure morphogenesis;anterior/posterior pattern specification;axon guidance;blood vessel
26.7391	26.3261	26.5028	26.0994	1:Experimental evidence at protein level	biological regulation;cell cycle;cell cycle phase;cell cycle process;cell division;cell surface receptor linked signaling pathway;cellular component disassembly;cellular component disassembly at cellular level;cellular componen
24.249	25.4338	24.5441	25.1658	1:Experimental evidence at protein level	anatomical structure development;biological regulation;cell communication;cell development;cell surface receptor linked signaling pathway;cell-cell signaling;cellular developmental process;cellular process;cellular response t
27.0749	27.0299	27.0429	26.8205	1:Experimental evidence at protein level	multicellular organismal process;neurological system process;sensory perception;sensory perception of pain;system process
26.907	26.8266	27.0294	26.8571	1:Experimental evidence at protein level	cell cycle;cell cycle process;cell division;cellular component organization;cellular component organization at cellular level;cellular component organization or biogenesis;cellular component organization or biogenesis at cellula
25.357	25.009	24.8976	24.7707	1:Experimental evidence at protein level	biological regulation;calcium ion homeostasis;calcium ion import;calcium ion transmembrane transport;calcium ion transport;cation homeostasis;cation transport;cellular calcium ion homeostasis;cellular cation homeostasis;c
26.5236	26.368	26.1937	26.1372	1:Experimental evidence at protein level	cellular macromolecule metabolic process;cellular metabolic process;cellular nitrogen compound metabolic process;cellular process;macromolecule metabolic process;metabolic process;nitrogen compound metabolic proce
25.9853	25.9473	25.8894	26.018	1:Experimental evidence at protein level	cellular component organization;cellular component organization at cellular level;cellular component organization or biogenesis;cellular component organization or biogenesis at cellular level;cellular macromolecule metabolic
28.221	28.3873	28.7021	28.7824	1:Experimental evidence at protein level	
26.9667	27.1383	27.4661	27.3002	1:Experimental evidence at protein level	
25.3839	25.8356	25.6298	24.6527	1:Experimental evidence at protein level	cellular macromolecule metabolic process;cellular metabolic process;cellular nitrogen compound metabolic process;cellular process;cellular protein metabolic process;cellular response to stimulus;cellular response to stress;
26.4258	26.7528	26.3333	26.5771	1:Experimental evidence at protein level	anatomical structure development;biological regulation;biosynthetic process;cellular biosynthetic process;cellular macromolecule biosynthetic process;cellular macromolecule metabolic process;cellular metabolic process;cel
25.2704	24.7681	24.8061	24.6311	1:Experimental evidence at protein level	
26.4966	26.4933	26.705	26.5209	1:Experimental evidence at protein level	biological regulation;cell differentiation;cellular component assembly;cellular component assembly at cellular level;cellular component organization;cellular component organization at cellular level;cellular component organiza
34.9699	35.1733	35.0422	34.9501	1:Experimental evidence at protein level	cell division;cellular component assembly;cellular component assembly at cellular level;cellular component organization;cellular component organization at cellular level;cellular component organization or biogenesis;cellular c
27.5269	27.6729	27.2294	27.5521	1:Experimental evidence at protein level	acute inflammatory response;acute-phase response;alcohol metabolic process;cellular macromolecule metabolic process;cellular metabolic process;cellular process;cholesterol metabolic process;defense response;developm
27.5112	27.3768	27.5575	27.0784	1:Experimental evidence at protein level	biological regulation;biosynthetic process;cell communication;cell differentiation;cellular biosynthetic process;cellular developmental process;cellular macromolecule biosynthetic process;cellular macromolecule metabolic pro
26.1114	25.7618	25.5646	25.7451	1:Experimental evidence at protein level	cellular component movement;cellular localization;cellular process;cytoskeleton-dependent intracellular transport;establishment of localization;establishment of localization in cell;intracellular transport;localization;microtubule
27.8096	27.8026	27.6991	27.7656	1:Experimental evidence at protein level	actin cytoskeleton organization;actin filament branching;actin filament bundle assembly;actin filament organization;actin filament-based process;biological regulation;cell chemotaxis;cell migration;cell motility;cell projection o
28.7111	28.787	29.1331	28.8249	1:Experimental evidence at protein level	biological regulation;cell surface receptor linked signaling pathway;cellular process;cellular response to chemical stimulus;cellular response to cytokine stimulus;cellular response to organic substance;cellular response to stim
26.7508	26.3877	24.0816	26.5804	1:Experimental evidence at protein level	biological regulation;biosynthetic process;cellular biosynthetic process;cellular component organization;cellular component organization or biogenesis;cellular macromolecule biosynthetic process;cellular macromolecule met
26.0925	26.1786	26.2722	25.986	1:Experimental evidence at protein level	biosynthetic process;cellular biosynthetic process;cellular component disassembly;cellular component disassembly at cellular level;cellular component organization;cellular component organization at cellular level;cellular con
26.8119	26.9036	27.2552	27.1566	1:Experimental evidence at protein level	anatomical structure development;calcium ion-dependent exocytosis;cell differentiation;cellular developmental process;cellular process;cerebellum development;developmental process;establishment of localization;establish
25.744	25.3155	25.622	25.6627	1:Experimental evidence at protein level	viral reproduction
27.5888	27.2558	27.4399	27.7329	1:Experimental evidence at protein level	biological regulation;catabolic process;cellular catabolic process;cellular macromolecule catabolic process;cellular macromolecule metabolic process;cellular metabolic process;cellular nitrogen compound metabolic process
24.7878	25.9806	25.9588	25.8816	1:Experimental evidence at protein level	apoptotic mitochondrial changes;biological regulation;cellular component organization;cellular component organization at cellular level;cellular component organization or biogenesis;cellular component organization or bioger

27.0377	27.1931	27.0881	27.1562	1:Experimental evidence at protein level	alcohol catabolic process;alcohol metabolic process;carbohydrate catabolic process;carbohydrate metabolic process;catabolic process;cellular carbohydrate catabolic process;cellular carbohydrate metabolic process;cellula
24.9953	24.6278	26.4086	25.3055	1:Experimental evidence at protein level	biological regulation;cellular component assembly;cellular component assembly at cellular level;cellular component biogenesis;cellular component organization;cellular component organization at cellular level;cellular compor
27.0439	26.855	26.7475	26.3891	1:Experimental evidence at protein level	catabolic process;cell cycle;cell division;cell proliferation;cellular catabolic process;cellular component organization;cellular component organization at cellular level;cellular component organization or biogenesis;cellular comp
24.9051	26.0425	25.7734	26.2948	1:Experimental evidence at protein level	biosynthetic process;cellular biosynthetic process;cellular macromolecule biosynthetic process;cellular macromolecule metabolic process;cellular metabolic process;cellular nitrogen compound metabolic process;cellular pro
27.7821	27.5074	27.6038	27.3905	1:Experimental evidence at protein level	biological regulation;cellular macromolecule metabolic process;cellular metabolic process;cellular process;cellular protein metabolic process;cytokine production;defense response;defense response to virus;immune effector
27.9204	28.4085	28.4231	28.4109	1:Experimental evidence at protein level	biological regulation;cell redox homeostasis;cellular homeostasis;cellular macromolecule metabolic process;cellular metabolic process;cellular process;cellular protein metabolic process;cellular response to stimulus;cellular r
25.6789	25.5882	26.0382	26.0394	1:Experimental evidence at protein level	
25.74	25.714	25.743	26.0746	1:Experimental evidence at protein level	biological regulation;posttranscriptional regulation of gene expression;regulation of biological process;regulation of biosynthetic process;regulation of cellular biosynthetic process;regulation of cellular macromolecule biosynth
25.8215	25.8927	25.1638	25.3833	1:Experimental evidence at protein level	cell cycle;cell cycle process;cellular component assembly;cellular component organization;cellular component organization at cellular level;cellular component organization or biogenesis;cellular component organization or bio
28.8598	28.6311	28.6442	28.6222	1:Experimental evidence at protein level	establishment of localization;lipid transport;organic substance transport;transport
26.3561	26.6371	26.712	26.8336	1:Experimental evidence at protein level	activation of immune response;activation of innate immune response;biological regulation;cellular process;cellular response to stimulus;defense response;immune response;immune response-activating signal transduction;im
25.2519	25.3863	25.5923	25.3801	1:Experimental evidence at protein level	biosynthetic process;carbohydrate biosynthetic process;carbohydrate metabolic process;cellular biosynthetic process;cellular carbohydrate biosynthetic process;cellular carbohydrate metabolic process;cellular lipid metabol
26.1245	26.413	23.5583	26.2305	1:Experimental evidence at protein level	cell cycle phase;cell cycle process;cell division;cellular component assembly;cellular component assembly at cellular level;cellular component organization;cellular component organization at cellular level;cellular component
27.9846	27.7214	27.7438	27.5566	1:Experimental evidence at protein level	cellular macromolecule metabolic process;cellular metabolic process;cellular process;cellular protein metabolic process;cullin deneddylation;macromolecule metabolic process;macromolecule modification;metabolic process
25.8153	25.5454	25.704	25.5094	1:Experimental evidence at protein level	biosynthetic process;cellular biosynthetic process;cellular macromolecule biosynthetic process;cellular macromolecule metabolic process;cellular metabolic process;cellular nitrogen compound metabolic process;cellular pro
25.5854	24.7283	25.5741	24.5373	1:Experimental evidence at protein level	biological regulation;cellular component organization;cellular component organization at cellular level;cellular component organization or biogenesis;cellular component organization or biogenesis at cellular level;cellular proce
27.9198	28.0913	28.0298	27.9964	1:Experimental evidence at protein level	biosynthetic process;cell cycle phase;cell cycle process;cell division;cellular biosynthetic process;cellular component organization;cellular component organization at cellular level;cellular component organization or biogenesi
27.294	27.668	27.6469	27.7292	1:Experimental evidence at protein level	ATP-dependent chromatin remodeling;cellular component organization;cellular component organization at cellular level;cellular component organization or biogenesis;cellular component organization or biogenesis at cellular l
26.8496	26.3938	26.38	26.2724	1:Experimental evidence at protein level	biosynthetic process;cellular biosynthetic process;cellular lipid metabolic process;cellular metabolic process;cellular process;glycerolipid biosynthetic process;glycerolipid metabolic process;glycerophospholipid biosynthetic
28.1329	28.2081	27.7786	28.1398	1:Experimental evidence at protein level	cellular component organization;cellular component organization or biogenesis;cellular membrane organization;cellular process;membrane organization;nuclear membrane organization
24.5264	24.6015	24.3115	24.5401	1:Experimental evidence at protein level	cell cycle phase;cell cycle process;cell division;cellular component organization;cellular component organization at cellular level;cellular component organization or biogenesis;cellular component organization or biogenesis at
26.5442	26.3054	26.2452	26.4897	1:Experimental evidence at protein level	adherens junction assembly;adherens junction organization;biological regulation;cell junction assembly;cell junction organization;cell-cell junction assembly;cell-cell junction organization;cellular component assembly;cellular
26.2584	26.6556	26.249	26.8364	1:Experimental evidence at protein level	
25.7684	25.0269	26.5446	26.2847	1:Experimental evidence at protein level	alcohol metabolic process;cellular component assembly;cellular component organization;cellular component organization or biogenesis;cellular ketone metabolic process;cellular metabolic process;cellular process;lipid meta
27.7472	27.4216	27.5234	27.2399	1:Experimental evidence at protein level	
25.6609	24.0009	25.8495	26.4151	1:Experimental evidence at protein level	
30.7915	30.9732	30.8396	30.7486	1:Experimental evidence at protein level	cellular component assembly;cellular component assembly at cellular level;cellular component organization;cellular component organization at cellular level;cellular component organization or biogenesis;cellular component c
27.4584	27.9334	27.9757	28.1432	1:Experimental evidence at protein level	biological regulation;biosynthetic process;cellular biosynthetic process;cellular macromolecule biosynthetic process;cellular macromolecule metabolic process;cellular metabolic process;cellular nitrogen compound metabolic
26.9351	27.0554	27.0577	27.0461	1:Experimental evidence at protein level	anatomical structure formation involved in morphogenesis;angiogenesis;biological regulation;cell migration;cell motility;cellular component assembly;cellular component assembly at cellular level;cellular component movemen
25.2109	24.4283	24.822	24.5052	1:Experimental evidence at protein level	anatomical structure formation involved in morphogenesis;angiogenesis;apoptosis;biological regulation;cell death;cell differentiation;cellular developmental process;cellular process;death;developmental process;establishmen
27.1037	26.8921	26.9396	26.8599	1:Experimental evidence at protein level	cellular component assembly;cellular component assembly at cellular level;cellular component organization;cellular component organization at cellular level;cellular component organization or biogenesis;cellular component c
26.1438	26.2457	26.1263	26.3047	1:Experimental evidence at protein level	establishment of localization;lipid transport;organic substance transport;transport
26.6257	26.2938	25.8901	26.0555	1:Experimental evidence at protein level	biological regulation;biosynthetic process;carboxylic acid catabolic process;carboxylic acid metabolic process;catabolic process;cation homeostasis;cell differentiation;cellular biosynthetic process;cellular catabolic process;c
26.0712	26.2938	25.7767	26.0883	1:Experimental evidence at protein level	amine biosynthetic process;amine metabolic process;amino acid salvage;aspartate family amino acid biosynthetic process;aspartate family amino acid metabolic process;biosynthetic process;carboxylic acid biosynthetic pro
26.8271	26.6883	26.8372	26.4678	1:Experimental evidence at protein level	developmental process;multicellular organismal development;multicellular organismal process
26.414	26.6752	26.6227	26.6525	1:Experimental evidence at protein level	
25.9435	26.8457	26.6436	26.8673	1:Experimental evidence at protein level	amine biosynthetic process;amine metabolic process;amino acid salvage;aspartate family amino acid biosynthetic process;aspartate family amino acid metabolic process;biosynthetic process;carboxylic acid biosynthetic pro
26.7775	25.9836	27.184	27.2727	1:Experimental evidence at protein level	biological regulation;catabolic process;cellular catabolic process;cellular macromolecule catabolic process;cellular macromolecule metabolic process;cellular metabolic process;cellular process;cellular protein metabolic proc
25.582	25.0078	25.0875	23.4935	1:Experimental evidence at protein level	biosynthetic process;carboxylic acid biosynthetic process;carboxylic acid metabolic process;cellular biosynthetic process;cellular ketone metabolic process;cellular lipid metabolic process;cellular metabolic process;cellular p
26.5999	26.9623	26.9766	26.8178	1:Experimental evidence at protein level	cellular component organization;cellular component organization at cellular level;cellular component organization or biogenesis;cellular component organization or biogenesis at cellular level;cellular macromolecule metabolic
29.4463	29.6249	29.6676	29.4377	1:Experimental evidence at protein level	biological regulation;cellular process;cellular response to abiotic stimulus;cellular response to gamma radiation;cellular response to ionizing radiation;cellular response to radiation;cellular response to stimulus;cellular respons
26.7926	27.1181	27.4448	27.3042	1:Experimental evidence at protein level	
25.7972	25.2101	25.2495	25.3815	1:Experimental evidence at protein level	cellular macromolecule metabolic process;cellular metabolic process;cellular nitrogen compound metabolic process;cellular process;macromolecule metabolic process;maturation of SSU-rRNA;maturation of SSU-rRNA from
29.5806	29.6238	29.6078	29.6096	1:Experimental evidence at protein level	biological regulation;cellular component organization;cellular component organization at cellular level;cellular component organization or biogenesis;cellular component organization or biogenesis at cellular level;cellular proce
27.4236	26.5823	26.8927	26.8904	1:Experimental evidence at protein level	biological regulation;cell proliferation;cellular component biogenesis;cellular component biogenesis at cellular level;cellular component organization or biogenesis;cellular component organization or biogenesis at cellular level
25.9548	26.0302	25.8057	25.5637	1:Experimental evidence at protein level	
26.5969	26.3123	26.4688	26.3883	1:Experimental evidence at protein level	biological regulation;biosynthetic process;carbohydrate metabolic process;carbohydrate transport;cell chemotaxis;cell cycle;cell cycle process;cell migration;cell motility;cell projection assembly;cell projection organization;ce
23.3788	25.4994	22.7242	24.0869	1:Experimental evidence at protein level	biological regulation;cell surface receptor linked signaling pathway;cellular component movement;cellular component organization;cellular component organization at cellular level;cellular component organization or biogenesi
25.2011	24.4618	25.2591	25.1042	1:Experimental evidence at protein level	amine metabolic process;amino acid activation;carboxylic acid metabolic process;cellular amine metabolic process;cellular amino acid metabolic process;cellular ketone metabolic process;cellular macromolecule metabolic p
27.8259	28.4013	28.4672	28.3424	1:Experimental evidence at protein level	lipid metabolic process;metabolic process;primary metabolic process
28.3165	28.2409	28.4271	28.5134	1:Experimental evidence at protein level	biological regulation;cap-independent translational initiation;cell differentiation;cellular developmental process;cellular macromolecule metabolic process;cellular metabolic process;cellular nitrogen compound metabolic proce
28.4071	27.7947	28.3241	28.6175	1:Experimental evidence at protein level	cellular macromolecule metabolic process;cellular metabolic process;cellular nitrogen compound metabolic process;cellular process;gene expression;macromolecule metabolic process;metabolic process;mRNA metabolic pr
26.5638	26.2318	26.2555	26.1868	1:Experimental evidence at protein level	biological regulation;cation homeostasis;chemical homeostasis;homeostatic process;ion homeostasis;iron ion homeostasis;regulation of biological quality
25.3295	25.1292	25.3234	23.486	1:Experimental evidence at protein level	amine metabolic process;aminoglycan catabolic process;aminoglycan metabolic process;carbohydrate catabolic process;carbohydrate metabolic process;catabolic process;chitin catabolic process;chitin metabolic process;
27.0227	26.9011	26.9348	26.8999	1:Experimental evidence at protein level	
26.311	23.2118	26.2332	26.5075	1:Experimental evidence at protein level	biological regulation;developmental process;multicellular organismal development;multicellular organismal process;posttranscriptional regulation of gene expression;regulation of biological process;regulation of biosynthetic p
24.3319	26.4343	25.7673	26.2841	1:Experimental evidence at protein level	apoptosis;biosynthetic process;cell death;cellular process;death;lipid biosynthetic process;lipid metabolic process;metabolic process;primary metabolic process;programmed cell death;steroid biosynthetic process;steroid m
25.0856	24.6656	25.3474	24.2018	1:Experimental evidence at protein level	establishment of localization;lipid transport;organic substance transport;transport
25.5787	23.5275	25.1884	25.4947	1:Experimental evidence at protein level	biological regulation;cellular process;cellular response to abiotic stimulus;cellular response to acidity;cellular response to pH;cellular response to stimulus;establishment of localization;establishment of localization in cell;exoc
23.7643	26.595	23.9862	23.73	1:Experimental evidence at protein level	biological regulation;cell differentiation;cellular chemical homeostasis;cellular component assembly;cellular component organization;cellular component organization or biogenesis;cellular developmental process;cellular home
27.8008	27.7515	27.6918	27.7567	1:Experimental evidence at protein level	anatomical structure formation involved in morphogenesis;angiogenesis;biological regulation;biosynthetic process;cell differentiation;cellular biosynthetic process;cellular developmental process;cellular macromolecule biosyn
28.0676	27.9482	28.0269	28.1194	1:Experimental evidence at protein level	biological regulation;biosynthetic process;cell proliferation;cellular biosynthetic process;cellular macromolecule biosynthetic process;cellular macromolecule metabolic process;cellular metabolic process;cellular nitrogen com
25.475	24.8888	25.6136	25.8448	1:Experimental evidence at protein level	7-methylguanosine biosynthetic process;7-methylguanosine metabolic process;biosynthetic process;cellular biosynthetic process;cellular macromolecule metabolic process;cellular metabolic process;cellular nitrogen compo
27.5194	27.4908	27.398	27.3583	1:Experimental evidence at protein level	antigen processing and presentation;antigen processing and presentation of exogenous antigen;antigen processing and presentation of exogenous peptide antigen;antigen processing and presentation of exogenous peptide
25.8573	26.1595	26.3522	26.0744	1:Experimental evidence at protein level	biological regulation;cell cycle cytokinesis;cell cycle process;cellular component organization;cellular component organization at cellular level;cellular component organization or biogenesis;cellular component organization or
26.401	26.7885	23.9521	26.8817	1:Experimental evidence at protein level	
26.1001	26.143	25.8939	26.1734	1:Experimental evidence at protein level	
26.7231	23.542	26.4908	26.6726	1:Experimental evidence at protein level	catabolic process;cellular catabolic process;cellular component organization;cellular component organization at cellular level;cellular component organization or biogenesis;cellular component organization or biogenesis at ce
26.7723	27.1769	27.0844	27.3388	1:Experimental evidence at protein level	
27.9596	27.8252	27.7365	27.7347	1:Experimental evidence at protein level	biological regulation;cellular component assembly;cellular component assembly at cellular level;cellular component organization;cellular component organization at cellular level;cellular component organization or biogenesis;
26.9847	26.4013	26.6887	26.7697	1:Experimental evidence at protein level	biological regulation;establishment of localization;establishment of localization in cell;establishment of RNA localization;intracellular transport;mRNA export from nucleus;mRNA transport;nuclear export;nuclear transport;nucle
26.2958	25.7204	25.1237	25.8259	1:Experimental evidence at protein level	alcohol catabolic process;alcohol metabolic process;biological regulation;biosynthetic process;carbohydrate biosynthetic process;carbohydrate catabolic process;carbohydrate metabolic process;catabolic process;cell migra
26.3406	26.4449	25.9986	26.6313	1:Experimental evidence at protein level	biosynthetic process;cellular biosynthetic process;cellular component disassembly;cellular component disassembly at cellular level;cellular component organization;cellular component organization at cellular level;cellular cor
26.0909	25.5245	25.9676	25.1828	1:Experimental evidence at protein level	biosynthetic process;cellular biosynthetic process;cellular component disassembly;cellular component disassembly at cellular level;cellular component organization;cellular component organization at cellular level;cellular cor
24.9998	24.1275	24.6527	25.0916	1:Experimental evidence at protein level	biosynthetic process;cellular biosynthetic process;cellular component disassembly;cellular component disassembly at cellular level;cellular component organization;cellular component organization at cellular level;cellular cor
26.1446	25.5575	24.221	25.3224	1:Experimental evidence at protein level	biosynthetic process;cellular biosynthetic process;cellular component disassembly;cellular component disassembly at cellular level;cellular component organization;cellular component organization at cellular level;cellular cor
26.013	25.8349	25.6356	25.9299	1:Experimental evidence at protein level	biosynthetic process;cellular biosynthetic process;cellular component disassembly;cellular component disassembly at cellular level;cellular component organization;cellular component organization at cellular level;cellular cor
26.1872	26.3356	26.2757	26.0962	1:Experimental evidence at protein level	
24.8503	25.0484	24.9833	25.0843	1:Experimental evidence at protein level	anatomical structure formation involved in morphogenesis;cell cycle;cell cycle process;cell projection assembly;cell projection organization;cellular component assembly;cellular component assembly at cellular level;cellular c
27.8591	22.4345	25.0196	23.4503	1:Experimental evidence at protein level	biological regulation;positive regulation of catalytic activity;positive regulation of GTPase activity;positive regulation of hydrolase activity;positive regulation of molecular function;regulation of biological process;regulation of ca
26.5332	26.2153	26.0522	26.109	1:Experimental evidence at protein level	biological regulation;cell cycle;cell cycle phase;cell cycle process;cell division;cellular component organization;cellular component organization at cellular level;cellular component organization or biogenesis;cellular componen
25.4187	25.5463	25.6513	25.7906	1:Experimental evidence at protein level	biosynthetic process;cellular biosynthetic process;cellular component disassembly;cellular component disassembly at cellular level;cellular component organization;cellular component organization at cellular level;cellular cor
27.2564	27.0166	27.0453	26.995	1:Experimental evidence at protein level	biological regulation;cell differentiation;cellular component biogenesis;cellular component biogenesis at cellular level;cellular component organization or biogenesis;cellular component organization or biogenesis at cellular lev
26.9729	26.7109	26.9096	26.8668	1:Experimental evidence at protein level	biological regulation;cell differentiation;cellular developmental process;cellular localization;cellular macromolecule localization;cellular process;cellular protein localization;developmental process;establishment of localization;fa
27.8273	27.6658	27.5653	27.8564	1:Experimental evidence at protein level	biological regulation;cellular process;cellular response to stimulus;cellular response to stress;DNA damage response, signal transduction resulting in induction of apoptosis;induction of apoptosis;induction of apoptosis by int
25.625	25.8138	25.3365	25.6731	1:Experimental evidence at protein level	antigen processing and presentation;biological regulation;cellular component organization;cellular component organization at cellular level;cellular component organization or biogenesis;cellular component organization or bio
25.5623	25.0919	25.2365	25.1373	1:Experimental evidence at protein level	biological regulation;cellular component assembly;cellular component assembly at cellular level;cellular component organization;cellular component organization at cellular level;cellular component organization or biogenesis;
25.9188	25.4596	25.8786	25.5927	1:Experimental evidence at protein level	biological regulation;biosynthetic process;canonical Wnt receptor signaling pathway;catabolic process;cell surface receptor linked signaling pathway;cellular biosynthetic process;cellular catabolic process;cellular component
26.3437	26.7067	26.0934	26.439	1:Experimental evidence at protein level	cellular macromolecule metabolic process;cellular metabolic process;cellular nitrogen compound metabolic process;cellular process;cellular protein metabolic process;macromolecule metabolic process;macromolecule modi
25.7623	25.0023	25.876	25.9121	1:Experimental evidence at protein level	activation of immune response;anatomical structure formation involved in morphogenesis;anatomical structure morphogenesis;angiogenesis;antigen receptor-mediated signaling pathway;biological adhesion;biological regula
27.4492	27.5371	27.1838	27.3561	1:Experimental evidence at protein level	biological regulation;cellular process;cellular response to stimulus;cellular response to stress;negative regulation of biological process;negative regulation of cellular metabolic process;negative regulation of cellular process;ne
23.1967	25.4041	25.0984	25.2711	1:Experimental evidence at protein level	
26.3254	26.3176	26.3859	25.9375	1:Experimental evidence at protein level	behavior;biosynthetic process;cellular aromatic compound metabolic process;cellular biosynthetic process;cellular metabolic compound salvage;cellular metabolic process;cellular nitrogen compound biosynthetic process;ce
28.4704	28.4455	28.3374	28.404	1:Experimental evidence at protein level	apoptosis;biological regulation;cell death;cellular process;death;negative regulation of apoptosis;negative regulation of biological process;negative regulation of cell death;negative regulation of cellular process;negative regula
26.8724	24.7936	27.4009	27.9516	1:Experimental evidence at protein level	biological regulation;biosynthetic process;cellular biosynthetic process;cellular component organization;cellular component organization at cellular level;cellular component organization or biogenesis;cellular component orga
25.7332	24.5965	25.6347	26.1111	1:Experimental evidence at protein level	adipose tissue development;anatomical structure development;biological regulation;cellular macromolecule metabolic process;cellular metabolic process;cellular nitrogen compound metabolic process;cellular process;deme
27.3512	27.2201	27.6586	27.4988	1:Experimental evidence at protein level	anatomical structure homeostasis;biological regulation;biosynthetic process;catabolic process;cellular biosynthetic process;cellular catabolic process;cellular component organization;cellular component organization at cellul
26.0091	26.0373	25.8553	25.7725	1:Experimental evidence at protein level	biological regulation;cellular macromolecule metabolic process;cellular metabolic process;cellular process;cellular protein metabolic process;endosome transport;establishment of localization;establishment of localization in c
24.3466	23.83	24.0078	23.8485	1:Experimental evidence at protein level	
26.9301	24.4507	27.1001	24.3388	1:Experimental evidence at protein level	anion transport;cellular process;chloride transport;establishment of localization;inorganic anion transport;ion transmembrane transport;ion transport;transmembrane transport;transport
26.9709	24.4751	27.1209	27.0021	1:Experimental evidence at protein level	biological regulation;cell proliferation;cell surface receptor linked signaling pathway;cellular component biogenesis;cellular component biogenesis at cellular level;cellular component organization or biogenesis;cellular compor
26.3711	26.4652	26.3739	26.374	1:Experimental evidence at protein level	cell differentiation;cellular developmental process;cellular process;developmental process
25.616	25.5807	24.8731	25.7611	1:Experimental evidence at protein level	biological regulation;regulation of biological process;regulation of biosynthetic process;regulation of cellular biosynthetic process;regulation of cellular macromolecule biosynthetic process;regulation of cellular metabolic proc
25.7301	25.2068	25.1429	24.7577	1:Experimental evidence at protein level	cellular macromolecule metabolic process;cellular metabolic process;cellular nitrogen compound metabolic process;cellular process;macromolecule metabolic process;metabolic process;nitrogen compound metabolic proce
26.2772	25.7894	25.9337	25.8821	1:Experimental evidence at protein level	biological regulation;biosynthetic process;cellular biosynthetic process;cellular component assembly;cellular component assembly at cellular level;cellular component organization;cellular component organization at cellular le
26.3904	26.5015	26.6818	26.7972	1:Experimental evidence at protein level	biological regulation;biosynthetic process;catabolic process;cell surface receptor linked signaling pathway;cellular biosynthetic process;cellular catabolic process;cellular component organization;cellular component organizat
27.5473	28.0328	28.0733	28.1842	1:Experimental evidence at protein level	anatomical structure development;anatomical structure morphogenesis;biological regulation;biosynthetic process;cell development;cell motility;cell part morphogenesis;cellular biosynthetic process;cellular component morph
26.1622	26.6275	26.5781	26.4651	1:Experimental evidence at protein level	biological regulation;cell differentiation;cellular developmental process;cellular process;developmental process;neuron differentiation;positive regulation of biological process;positive regulation of biosynthetic process;positive
25.9179	25.4831	25.7739	24.5176	1:Experimental evidence at protein level	4-hydroxyproline metabolic process;amine metabolic process;anatomical structure development;anatomical structure formation involved in morphogenesis;anatomical structure morphogenesis;biological regulation;carboxylic
26.3293	26.0776	26.56	26.0677	1:Experimental evidence at protein level	
23.7011	23.516	26.5033	26.5266	1:Experimental evidence at protein level	anatomical structure development;anatomical structure formation involved in morphogenesis;anatomical structure morphogenesis;biological regulation;BMP signaling pathway;camera-type eye development;cell differentiatio
26.2528	26.5388	26.1817	26.3681	1:Experimental evidence at protein level	anatomical structure morphogenesis;apoptotic mitochondrial changes;biological regulation;cell part morphogenesis;cellular component assembly;cellular component morphogenesis;cellular component organization;cellular c
28.928	29.131	28.729	28.9829	1:Experimental evidence at protein level	cell cycle process;cellular component organization;cellular component organization at cellular level;cellular component organization or biogenesis;cellular component organization or biogenesis at cellular level;cellular macrom
26.4276	26.2855	26.8248	26.892	1:Experimental evidence at protein level	anatomical structure development;cranial nerve development;developmental process;nerve development;optic nerve development
26.1417	24.7977	26.1588	26.5251	1:Experimental evidence at protein level	biological regulation;catabolic process;cellular catabolic process;cellular macromolecule catabolic process;cellular macromolecule metabolic process;cellular metabolic process;cellular nitrogen compound metabolic process
26.3058	25.9665	26.2698	25.9409	1:Experimental evidence at protein level	cellular process;cellular response to heat;cellular response to stimulus;cellular response to stress;response to abiotic stimulus;response to heat;response to stimulus;response to stress;response to temperature stimulus
26.9708	26.8087	26.7175	26.4583	1:Experimental evidence at protein level	
26.1512	26.1159	26.2905	26.0811	1:Experimental evidence at protein level	antigen processing and presentation;antigen processing and presentation of exogenous antigen;antigen processing and presentation of exogenous peptide antigen;antigen processing and presentation of exogenous peptide
27.1696	27.0481	27.0188	27.2514	1:Experimental evidence at protein level	biological regulation;cellular macromolecule metabolic process;cellular metabolic process;cellular process;cellular protein metabolic process;dephosphorylation;macromolecule metabolic process;macromolecule modification
28.022	28.0218	27.8091	27.8186	1:Experimental evidence at protein level	biological regulation;biosynthetic process;catabolic process;cell growth;cellular biosynthetic process;cellular catabolic process;cellular macromolecule catabolic process;cellular macromolecule metabolic process;cellular met
25.9958	25.9713	26.1555	26.4324	1:Experimental evidence at protein level	biological regulation;cellular macromolecule metabolic process;cellular metabolic process;cellular nitrogen compound metabolic process;cellular process;developmental process;establishment of localization;establishment of
26.8196	26.7496	26.5805	26.5555	1:Experimental evidence at protein level	actin cytoskeleton organization;actin filament-based process;actomyosin structure organization;anion transport;antigen processing and presentation;antigen processing and presentation of exogenous antigen;antigen proces
26.2505	25.2054	26.2846	26.392	1:Experimental evidence at protein level	cellular macromolecule metabolic process;cellular metabolic process;cellular nitrogen compound metabolic process;cellular process;macromolecule metabolic process;metabolic process;mRNA metabolic process;mRNA pro
26.9887	26.7907	26.9608	26.4423	1:Experimental evidence at protein level	biological regulation;cellular macromolecule metabolic process;cellular metabolic process;cellular nitrogen compound metabolic process;cellular process;induction of apoptosis;induction of apoptosis by extracellular signals;i
27.9607	27.6333	27.5607	27.4489	1:Experimental evidence at protein level	carbohydrate metabolic process;cation transport;cellular carbohydrate metabolic process;cellular macromolecule metabolic process;cellular metabolic process;cellular process;cellular protein metabolic process;cognition;div
30.1897	30.2396	30.0606	30.0422	1:Experimental evidence at protein level	autophagy;biological regulation;catabolic process;cellular catabolic process;cellular component assembly;cellular component assembly at cellular level;cellular component organization;cellular component organization at cellu
25.757	26.0848	25.738	23.1422	1:Experimental evidence at protein level	biosynthetic process;cellular biosynthetic process;cellular component disassembly;cellular component disassembly at cellular level;cellular component organization;cellular component organization at cellular level;cellular cor
24.8192	24.9028	25.606	25.5521	1:Experimental evidence at protein level	metabolic process
25.1039	24.9763	25.7506	24.8538	1:Experimental evidence at protein level	biological regulation;development of secondary female sexual characteristics;development of secondary sexual characteristics;developmental process;developmental process involved in reproduction;negative regulation of b

28.5172	28.5296	28.3468	28.6941	1:Experimental evidence at protein level	
23.9658	24.4865	22.9976	24.3845	2:Experimental evidence at transcript lev	anatomical structure development;biological regulation;cell communication;cell development;cell differentiation;cell fate commitment;cell surface receptor linked signaling pathway;cellular developmental process;cellular proc
26.2628	24.8002	25.6013	26.4388	1:Experimental evidence at protein level	biological regulation;biosynthetic process;cation homeostasis;cellular biosynthetic process;cellular cation homeostasis;cellular chemical homeostasis;cellular homeostasis;cellular ion homeostasis;cellular iron ion homeostasis
24.7356	24.4233	24.4306	24.2486	1:Experimental evidence at protein level	anatomical structure development;apoptosis;autophagic vacuole assembly;autophagy;biological regulation;blood vessel remodeling;cardiac cell development;cardiac muscle cell development;catabolic process;cell communi
24.5002	23.6448	24.082	24.0669	1:Experimental evidence at protein level	
28.0826	28.026	28.1581	27.9771	1:Experimental evidence at protein level	biological regulation;cellular component assembly;cellular component organization;cellular component organization or biogenesis;cellular membrane organization;cellular process;cellular response to chemical stimulus;cellula
25.0096	23.7718	25.0901	27.1449	1:Experimental evidence at protein level	biological adhesion;biological regulation;calcium ion homeostasis;calcium ion transport;calcium-dependent cell-cell adhesion;cation homeostasis;cation transport;cell adhesion;cell-cell adhesion;cellular calcium ion homeosta
26.0891	26.2708	26.2649	26.1431	1:Experimental evidence at protein level	cellular component organization;cellular component organization at cellular level;cellular component organization or biogenesis;cellular component organization or biogenesis at cellular level;cellular localization;cellular proces
26.12	25.7987	25.7731	25.9465	1:Experimental evidence at protein level	cellular process;establishment of localization;establishment of localization in cell;establishment of protein localization;intracellular protein transport;intracellular transport;protein transport;transport;vesicle-mediated transport
31.0464	31.8904	31.7785	31.9226	1:Experimental evidence at protein level	biological regulation;cell redox homeostasis;cellular component organization;cellular component organization or biogenesis;cellular homeostasis;cellular process;homeostatic process;positive regulation of catalytic activity;po
27.703	27.1823	27.55	27.3671	1:Experimental evidence at protein level	actin cytoskeleton organization;actin filament capping;actin filament-based process;actin modification;barbed-end actin filament capping;biological regulation;cell cycle phase;cell cycle process;cell division;cellular componer
26.7741	26.7694	26.9777	26.822	1:Experimental evidence at protein level	activation of protein kinase activity;apoptosis;biological regulation;cell death;cellular macromolecule metabolic process;cellular metabolic process;cellular process;cellular protein metabolic process;cellular response to stimul
27.8902	27.7067	28.4961	28.6744	1:Experimental evidence at protein level	cellular macromolecule metabolic process;cellular metabolic process;cellular nitrogen compound metabolic process;cellular process;cellular protein metabolic process;macromolecule metabolic process;macromolecule modi
25.07	25.479	24.8819	25.4422	1:Experimental evidence at protein level	activation of MAPKK activity;activation of protein kinase activity;anatomical structure development;biological regulation;cell cycle checkpoint;cell cycle process;cellular macromolecule metabolic process;cellular metabolic pro
26.8868	26.7122	26.5598	26.5572	1:Experimental evidence at protein level	biological regulation;establishment of localization;establishment of localization in cell;establishment of protein localization;establishment of protein localization in endoplasmic reticulum membrane;establishment of protein loc
25.7972	24.4643	25.6407	25.481	1:Experimental evidence at protein level	biological regulation;biosynthetic process;cellular biosynthetic process;cellular macromolecule biosynthetic process;cellular macromolecule metabolic process;cellular metabolic process;cellular nitrogen compound metabolic
25.324	24.3113	26.1881	23.5225	1:Experimental evidence at protein level	biosynthetic process;cellular biosynthetic process;cellular macromolecule metabolic process;cellular metabolic process;cellular process;cellular protein metabolic process;macromolecule metabolic process;macromolecule m
26.1366	26.1818	25.8843	25.9732	1:Experimental evidence at protein level	biological regulation;biosynthetic process;cellular biosynthetic process;cellular macromolecule biosynthetic process;cellular macromolecule metabolic process;cellular metabolic process;cellular nitrogen compound metabolic
25.8291	25.2668	26.5777	25.6736	1:Experimental evidence at protein level	amine metabolic process;amino acid activation;carboxylic acid metabolic process;cellular amine metabolic process;cellular amino acid metabolic process;cellular ketone metabolic process;cellular macromolecule metabolic p
26.0532	25.6173	26.1348	25.8428	1:Experimental evidence at protein level	biosynthetic process;cellular biosynthetic process;cellular component disassembly;cellular component disassembly at cellular level;cellular component organization;cellular component organization at cellular level;cellular cor
27.3118	27.1688	27.0559	27.2159	1:Experimental evidence at protein level	biological adhesion;biological regulation;biosynthetic process;cell adhesion;cellular biosynthetic process;cellular macromolecule biosynthetic process;cellular macromolecule metabolic process;cellular metabolic process;cell
26.0752	23.9941	26.3251	26.1908	1:Experimental evidence at protein level	
28.3408	28.8617	29.0065	29.1547	1:Experimental evidence at protein level	
28.4738	29.242	28.6844	28.8031	1:Experimental evidence at protein level	biological regulation;cell redox homeostasis;cellular homeostasis;cellular macromolecule metabolic process;cellular metabolic process;cellular process;cellular protein metabolic process;cellular response to stimulus;cellular r
26.905	26.7617	27.0037	26.8382	1:Experimental evidence at protein level	biosynthetic process;establishment of localization;lipid biosynthetic process;lipid metabolic process;metabolic process;primary metabolic process;steroid biosynthetic process;steroid metabolic process;transport
25.5467	25.8935	26.2249	25.9922	1:Experimental evidence at protein level	biological regulation;cellular component organization;cellular component organization or biogenesis;cellular process;cellular response to stimulus;intracellular signal transduction;positive regulation of actin filament polymeriza
26.4296	26.025	26.1237	25.8804	1:Experimental evidence at protein level	anatomical structure morphogenesis;biological regulation;catabolic process;cell part morphogenesis;cell projection morphogenesis;cell projection organization;cellular catabolic process;cellular component morphogenesis;ce
27.3715	27.2366	27.1718	27.1253	1:Experimental evidence at protein level	anatomical structure development;cell differentiation;cellular developmental process;cellular macromolecule metabolic process;cellular metabolic process;cellular process;cellular protein metabolic process;chaperone-mediat
24.266	24.9743	24.657	24.1218	1:Experimental evidence at protein level	biological regulation;cell cycle;cell cycle phase;cell cycle process;cell division;cellular component organization;cellular component organization at cellular level;cellular component organization or biogenesis;cellular componer
26.5509	26.5113	26.7168	26.4562	1:Experimental evidence at protein level	
28.3024	28.7016	28.8273	28.8206	1:Experimental evidence at protein level	biological regulation;cell cycle process;cellular component assembly;cellular component organization;cellular component organization at cellular level;cellular component organization or biogenesis;cellular component organiz
27.7544	27.6701	27.8596	27.5963	1:Experimental evidence at protein level	alcohol metabolic process;anatomical structure development;anatomical structure formation involved in morphogenesis;angiogenesis;anterior/posterior pattern specification;biological regulation;carbohydrate metabolic proc
25.5653	25.6817	25.1586	25.517	1:Experimental evidence at protein level	anatomical structure development;biological regulation;cell development;cellular developmental process;cellular macromolecule metabolic process;cellular metabolic process;cellular process;cellular protein metabolic proces
28.0216	28.373	28.185	28.1708	1:Experimental evidence at protein level	alkene biosynthetic process;biosynthetic process;carboxylic acid biosynthetic process;carboxylic acid metabolic process;cellular alkene metabolic process;cellular biosynthetic process;cellular ketone metabolic process;cellu
26.5401	26.2477	26.0959	26.2444	1:Experimental evidence at protein level	asymmetric Golgi ribbon formation;biological adhesion;biological regulation;biosynthetic process;cell adhesion;cell adhesion molecule production;cell migration;cell motility;cell projection assembly;cell projection organization
24.4171	25.7122	24.7831	24.0477	1:Experimental evidence at protein level	cellular component assembly;cellular component assembly at cellular level;cellular component organization;cellular component organization at cellular level;cellular component organization or biogenesis;cellular component c
25.5129	25.2175	25.4615	25.7716	1:Experimental evidence at protein level	actin cytoskeleton organization;actin filament-based process;cell communication;cell-cell signaling;cellular component organization;cellular component organization at cellular level;cellular component organization or biogene
25.1277	24.8543	26.5964	24.9952	1:Experimental evidence at protein level	biological regulation;positive regulation of biological process;positive regulation of cell communication;positive regulation of cell development;positive regulation of cell differentiation;positive regulation of cell migration;positiv
24.1058	25.7233	24.6507	24.1424	1:Experimental evidence at protein level	
26.2119	25.9772	25.9982	25.9265	1:Experimental evidence at protein level	establishment of localization;lipid transport;organic substance transport;transport
28.1305	28.0223	28.0038	27.8892	1:Experimental evidence at protein level	biological regulation;blood coagulation;cell projection organization;cellular component assembly;cellular component organization;cellular component organization at cellular level;cellular component organization or biogenesis
25.4169	25.3817	25.0979	25.4094	1:Experimental evidence at protein level	biosynthetic process;carbohydrate biosynthetic process;carbohydrate metabolic process;cellular carbohydrate metabolic process;cellular macromolecule metabolic process;cellular metabolic process;cellular process;cellular
25.3912	25.4589	25.0511	25.1507	1:Experimental evidence at protein level	
26.1404	25.7595	26.1671	26.1211	1:Experimental evidence at protein level	viral reproduction
25.4531	24.7214	25.0626	24.8648	1:Experimental evidence at protein level	cellular macromolecule metabolic process;cellular metabolic process;cellular nitrogen compound metabolic process;cellular process;establishment of localization;establishment of localization in cell;establishment of RNA loca
25.5507	25.0596	25.4374	25.3527	1:Experimental evidence at protein level	cellular macromolecule metabolic process;cellular metabolic process;cellular nitrogen compound metabolic process;cellular process;macromolecule metabolic process;metabolic process;nitrogen compound metabolic proce
26.5755	26.3291	26.4827	26.2593	1:Experimental evidence at protein level	
27.1834	27.1578	27.0473	27.4617	1:Experimental evidence at protein level	establishment of localization;establishment of localization in cell;establishment of protein localization;intracellular transport;protein transport;transport
27.1984	27.7597	28.0037	28.0896	1:Experimental evidence at protein level	catabolic process;cellular catabolic process;cellular component assembly;cellular component organization;cellular component organization or biogenesis;cellular metabolic process;cellular nitrogen compound catabolic proce
27.3974	26.8523	26.4662	26.7069	1:Experimental evidence at protein level	biological regulation;negative regulation of catalytic activity;negative regulation of hydrolase activity;negative regulation of molecular function;negative regulation of phosphatase activity;regulation of biological process;regulat
26.4054	24.8418	26.1811	26.0666	1:Experimental evidence at protein level	cellular component assembly;cellular component assembly at cellular level;cellular component organization;cellular component organization at cellular level;cellular component organization or biogenesis;cellular component c
25.9112	25.7427	25.4782	23.727	1:Experimental evidence at protein level	cellular component organization;cellular component organization at cellular level;cellular component organization or biogenesis;cellular component organization or biogenesis at cellular level;cellular process;chromatin organiz
26.1707	25.6053	25.9256	25.8208	1:Experimental evidence at protein level	
26.983	24.1662	26.4387	26.5304	1:Experimental evidence at protein level	
25.0015	24.1095	24.7959	25.0122	1:Experimental evidence at protein level	biosynthetic process;cellular biosynthetic process;cellular macromolecule biosynthetic process;cellular macromolecule metabolic process;cellular metabolic process;cellular nitrogen compound metabolic process;cellular pro
25.2814	25.2331	26.2846	25.6059	1:Experimental evidence at protein level	biological regulation;biosynthetic process;carboxylic acid biosynthetic process;carboxylic acid metabolic process;cell redox homeostasis;cellular biosynthetic process;cellular homeostasis;cellular ketone metabolic process;c
26.6061	26.7691	27.2017	27.3205	1:Experimental evidence at protein level	cellular component assembly;cellular component assembly at cellular level;cellular component organization;cellular component organization at cellular level;cellular component organization or biogenesis;cellular component c
28.2122	28.151	28.0603	28.1062	1:Experimental evidence at protein level	apoptosis;cell death;cellular macromolecule metabolic process;cellular metabolic process;cellular process;cellular protein metabolic process;death;macromolecule metabolic process;macromolecule modification;metabolic p
27.2032	27.0356	27.158	27.2607	1:Experimental evidence at protein level	cellular component assembly;cellular component assembly at cellular level;cellular component biogenesis;cellular component organization;cellular component organization at cellular level;cellular component organization or b
27.4038	27.592	27.5499	27.5508	1:Experimental evidence at protein level	biological regulation;cellular process;cellular response to stimulus;hippo signaling cascade;intracellular signal transduction;regulation of biological process;regulation of cellular process;response to stimulus;signal transductio
25.4827	24.9971	25.7149	25.641	1:Experimental evidence at protein level	
24.8128	24.2674	24.6521	24.3854	1:Experimental evidence at protein level	activation of protein kinase activity;anatomical structure morphogenesis;biological regulation;cell junction assembly;cell junction organization;cell morphogenesis;cell surface receptor linked signaling pathway;cell-cell junction
23.2545	24.1342	24.0911	24.6009	1:Experimental evidence at protein level	
26.8636	26.7816	27.2186	26.9656	1:Experimental evidence at protein level	cell cycle;cellular component organization;cellular component organization at cellular level;cellular component organization or biogenesis;cellular component organization or biogenesis at cellular level;cellular process;mitotic
27.714	28.6109	28.5789	29.1177	1:Experimental evidence at protein level	
26.6333	26.9902	26.2303	26.0581	1:Experimental evidence at protein level	acidic amino acid transport;amine transport;amino acid transport;anion transport;carboxylic acid transport;establishment of localization;ion transport;L-glutamate transport;nitrogen compound transport;organic acid transpor
26.104	26.1386	24.1847	26.0434	1:Experimental evidence at protein level	
28.0865	28.0756	27.9517	27.9961	1:Experimental evidence at protein level	biological regulation;cation homeostasis;cation transport;cell differentiation;cellular developmental process;cellular process;chemical homeostasis;developmental process;erythrocyte differentiation;establishment of localizatio
26.1367	24.8062	25.4199	25.2938	1:Experimental evidence at protein level	cellular component assembly;cellular component assembly at cellular level;cellular component organization;cellular component organization at cellular level;cellular component organization or biogenesis;cellular component c
26.4088	25.9022	26.1308	26.2279	1:Experimental evidence at protein level	cellular macromolecule metabolic process;cellular metabolic process;cellular process;cellular protein metabolic process;cullin deneddylation;macromolecule metabolic process;macromolecule modification;metabolic process
25.2688	25.0197	25.105	24.9264	1:Experimental evidence at protein level	anatomical structure development;biological regulation;biosynthetic process;cell migration;cell motility;cellular biosynthetic process;cellular component movement;cellular component organization;cellular component organiza
27.3903	27.3077	27.3341	27.3932	1:Experimental evidence at protein level	cellular metabolic process;cellular process;metabolic process;phosphate-containing compound metabolic process;phosphorus metabolic process;phosphorylation
25.8273	25.9894	26.1202	26.1739	1:Experimental evidence at protein level	amine metabolic process;biosynthetic process;carboxylic acid metabolic process;cellular amine metabolic process;cellular amino acid metabolic process;cellular biosynthetic process;cellular ketone metabolic process;cellula
27.0443	24.4866	26.8059	27.2693	1:Experimental evidence at protein level	anatomical structure development;cellular metabolic process;cellular process;cellular response to chemical stimulus;cellular response to endogenous stimulus;cellular response to glucagon stimulus;cellular response to horm
27.0914	26.9642	26.7828	26.7313	1:Experimental evidence at protein level	establishment of localization;establishment of localization in cell;establishment of protein localization;establishment of RNA localization;gene expression;intracellular protein transport;intracellular transport;macromolecule met
27.4682	27.5112	27.6567	27.6925	1:Experimental evidence at protein level	cellular macromolecule metabolic process;cellular metabolic process;cellular process;cellular protein metabolic process;establishment of localization;establishment of localization in cell;establishment of protein localization;es
26.9917	27.6709	27.5968	27.7312	1:Experimental evidence at protein level	behavior;exploration behavior;locomotory behavior;locomotory exploration behavior;pigmentation;response to stimulus
24.9535	26.7077	24.5411	24.5124	1:Experimental evidence at protein level	axon guidance;biological regulation;cell communication;cell maturation;cellular component organization;cellular component organization at cellular level;cellular component organization or biogenesis;cellular component orga
28.836	28.9592	28.8705	28.8813	1:Experimental evidence at protein level	aging;biological regulation;cardiac cell differentiation;cardiac muscle cell differentiation;cell differentiation;cellular developmental process;cellular process;cellular response to calcium ion;cellular response to chemical stimulus
28.479	25.0349	29.3123	28.8216	1:Experimental evidence at protein level	apoptosis;biological regulation;biosynthetic process;cell communication;cell death;cell growth;cellular biosynthetic process;cellular localization;cellular macromolecule biosynthetic process;cellular macromolecule localization
25.4712	24.6232	24.9543	25.6486	1:Experimental evidence at protein level	cell differentiation;cellular developmental process;cellular process;developmental process;osteoblast differentiation
27.2735	27.557	27.4713	27.2985	1:Experimental evidence at protein level	biological regulation;calcium ion homeostasis;calcium ion transport;carbohydrate metabolic process;cation homeostasis;cation transport;cellular calcium ion homeostasis;cellular carbohydrate metabolic process;cellular catio
27.3377	27.1511	26.9908	26.9434	1:Experimental evidence at protein level	biological regulation;cell cycle phase;cell cycle process;cellular component organization;cellular component organization at cellular level;cellular component organization or biogenesis;cellular component organization or bioge
27.6633	28.5899	28.3451	28.4725	1:Experimental evidence at protein level	
24.4651	25.0684	24.6995	24.5007	1:Experimental evidence at protein level	biological regulation;biosynthetic process;cellular biosynthetic process;cellular component organization;cellular component organization at cellular level;cellular component organization or biogenesis;cellular component orga
25.1453	25.0391	25.2598	25.4677	1:Experimental evidence at protein level	amine catabolic process;amine metabolic process;biotin metabolic process;branched chain family amino acid catabolic process;branched chain family amino acid metabolic process;carboxylic acid catabolic process;carbox
26.1536	26.2808	26.3816	26.3609	1:Experimental evidence at protein level	biological regulation;biosynthetic process;carbohydrate homeostasis;cellular biosynthetic process;cellular macromolecule biosynthetic process;cellular macromolecule metabolic process;cellular metabolic process;cellular nit
26.9662	26.5223	26.6648	26.6708	1:Experimental evidence at protein level	biological regulation;biosynthetic process;cell surface receptor linked signaling pathway;cellular biosynthetic process;cellular macromolecule biosynthetic process;cellular macromolecule metabolic process;cellular metabolic
25.3087	24.3899	24.7046	24.382	1:Experimental evidence at protein level	biological regulation;cellular macromolecule metabolic process;cellular metabolic process;cellular nitrogen compound metabolic process;cellular process;macromolecule metabolic process;metabolic process;mRNA metaboli
25.0661	25.8046	25.2982	25.2282	1:Experimental evidence at protein level	anatomical structure development;ATP-dependent chromatin remodeling;biological regulation;biosynthetic process;brain development;canonical Wnt receptor signaling pathway;cell surface receptor linked signaling pathway
25.9006	26.2247	24.4289	25.4862	1:Experimental evidence at protein level	
27.4521	27.667	27.6047	27.6623	1:Experimental evidence at protein level	biological regulation;catabolic process;cellular catabolic process;cellular macromolecule catabolic process;cellular macromolecule metabolic process;cellular metabolic process;cellular process;cellular response to chemical s
26.3551	25.6135	26.1311	25.6879	1:Experimental evidence at protein level	anatomical structure development;biosynthetic process;blastocyst development;cellular biosynthetic process;cellular macromolecule biosynthetic process;cellular macromolecule metabolic process;cellular metabolic process
26.215	26.7764	26.5203	26.651	1:Experimental evidence at protein level	anatomical structure development;anatomical structure regression;biological regulation;cell migration;cell motility;cellular component movement;cellular process;developmental process;hemopoietic or lymphoid organ develo
25.9064	26.2413	25.5597	25.6756	1:Experimental evidence at protein level	biological regulation;biosynthetic process;carbohydrate metabolic process;cellular biosynthetic process;cellular carbohydrate metabolic process;cellular component organization;cellular component organization at cellular lev
25.9347	26.2574	23.968	26.3261	1:Experimental evidence at protein level	apoptosis;biological regulation;biosynthetic process;cell death;cell differentiation;cell proliferation;cellular biosynthetic process;cellular developmental process;cellular macromolecule biosynthetic process;cellular macromolec
26.3521	26.1624	24.9485	26.1679	1:Experimental evidence at protein level	cation transport;establishment of localization;ion transport;transport
26.6085	26.7466	26.9928	26.5087	1:Experimental evidence at protein level	apical protein localization;asymmetric protein localization;biological regulation;cellular component assembly;cellular component organization;cellular component organization at cellular level;cellular component organization or
26.6731	26.3534	26.3402	26.2392	1:Experimental evidence at protein level	biosynthetic process;cellular biosynthetic process;cellular component disassembly;cellular component disassembly at cellular level;cellular component organization;cellular component organization at cellular level;cellular cor
27.7875	28.063	27.8317	28.0644	1:Experimental evidence at protein level	biological regulation;biosynthetic process;carbon catabolite regulation of transcription;carbon catabolite repression of transcription;cell communication;cell cycle process;cell division;cellular biosynthetic process;cellular com
28.2056	28.2257	28.1711	28.0355	1:Experimental evidence at protein level	
28.4912	28.2982	28.3991	28.3728	1:Experimental evidence at protein level	biosynthetic process;metabolic process
25.4709	25.1462	25.382	25.0922	1:Experimental evidence at protein level	biological regulation;cell cycle arrest;cell cycle process;cellular process;negative regulation of autophagy;negative regulation of biological process;negative regulation of catabolic process;negative regulation of cell cycle;nega
27.4419	27.395	27.3373	27.4378	1:Experimental evidence at protein level	biological regulation;cellular process;establishment of localization;establishment of localization in cell;establishment of protein localization;intracellular protein transport;intracellular transport;positive regulation of catalytic acti
27.6353	27.6406	27.4914	27.8186	1:Experimental evidence at protein level	anatomical structure development;biological regulation;brain development;cellular component organization;cellular component organization at cellular level;cellular component organization or biogenesis;cellular component o
26.0013	26.3491	26.6017	26.9797	1:Experimental evidence at protein level	biological regulation;cellular macromolecule metabolic process;cellular metabolic process;cellular nitrogen compound metabolic process;cellular process;cellular protein metabolic process;dephosphorylation;dephosphorylati
27.8528	28.0151	28.1106	28.0374	1:Experimental evidence at protein level	cellular component organization;cellular component organization or biogenesis;cellular process;endosome transport;establishment of localization;establishment of localization in cell;establishment of protein localization;intrac
24.9449	24.9907	25.0016	24.8758	1:Experimental evidence at protein level	amine metabolic process;amino acid activation;carboxylic acid metabolic process;cellular amine metabolic process;cellular amino acid metabolic process;cellular ketone metabolic process;cellular macromolecule metabolic p
25.9451	26.323	25.0464	25.2925	1:Experimental evidence at protein level	apoptosis;biosynthetic process;cell death;cellular biosynthetic process;cellular component disassembly;cellular component disassembly at cellular level;cellular component organization;cellular component organization at cel
28.1808	28.8098	29.0065	28.8667	1:Experimental evidence at protein level	behavior;cellular component movement;cellular component organization;cellular component organization at cellular level;cellular component organization or biogenesis;cellular component organization or biogenesis at cellula
28.0507	27.7805	27.7353	27.6607	1:Experimental evidence at protein level	
25.8179	25.6416	25.8325	25.9956	1:Experimental evidence at protein level	biological regulation;catabolic process;cellular catabolic process;cellular macromolecule catabolic process;cellular macromolecule metabolic process;cellular metabolic process;cellular nitrogen compound metabolic process
25.7437	24.8135	25.9265	25.4818	1:Experimental evidence at protein level	cellular macromolecule metabolic process;cellular metabolic process;cellular nitrogen compound metabolic process;cellular process;macromolecule metabolic process;macromolecule modification;metabolic process;ncRNA
27.237	28.2113	27.3254	22.4639	1:Experimental evidence at protein level	adenosine metabolic process;cellular macromolecule metabolic process;cellular metabolic process;cellular nitrogen compound metabolic process;cellular process;heterocycle metabolic process;macromolecule metabolic pro
25.8776	26.1002	26.2308	26.1135	1:Experimental evidence at protein level	cellular component assembly;cellular component organization;cellular component organization or biogenesis;macromolecular complex assembly;macromolecular complex subunit organization;metabolic process;protein com
26.9177	27.0287	26.7215	26.851	1:Experimental evidence at protein level	ameboidal cell migration;anatomical structure development;associative learning;basement membrane organization;behavior;biological adhesion;biological regulation;cAMP-mediated signaling;cell adhesion;cell migration;cell
26.8475	27.3845	27.5912	27.6084	1:Experimental evidence at protein level	biological regulation;cellular component assembly;cellular component assembly at cellular level;cellular component organization;cellular component organization at cellular level;cellular component organization or biogenesis;
26.633	27.2284	26.9105	26.609	1:Experimental evidence at protein level	cellular component organization;cellular component organization at cellular level;cellular component organization or biogenesis;cellular component organization or biogenesis at cellular level;cellular process;establishment of l
26.2887	24.117	24.4038	26.0116	2:Experimental evidence at transcript level	
27.2981	27.2864	27.8799	27.7843	1:Experimental evidence at protein level	anatomical structure development;biological regulation;biosynthetic process;cell development;cellular biosynthetic process;cellular developmental process;cellular macromolecule metabolic process;cellular metabolic proces
29.9171	29.9743	29.664	29.9283	1:Experimental evidence at protein level	anatomical structure formation involved in morphogenesis;anatomical structure morphogenesis;angiogenesis;apoptosis;axonal fasciculation;biological regulation;cardiac epithelial to mesenchymal transition;cell death;cell mig
26.3156	26.165	26.315	26.3139	1:Experimental evidence at protein level	biological regulation;biosynthetic process;cellular biosynthetic process;cellular macromolecule biosynthetic process;cellular macromolecule metabolic process;cellular metabolic process;cellular nitrogen compound metabolic
25.1235	25.2937	25.3144	25.129	1:Experimental evidence at protein level	glomerular filtration;macromolecule metabolic process;metabolic process;multicellular organismal process;primary metabolic process;protein maturation;protein metabolic process;protein processing;renal system process;sy
26.64	26.9678	26.8839	27.0332	1:Experimental evidence at protein level	amine metabolic process;asparagine metabolic process;aspartate family amino acid metabolic process;carboxylic acid metabolic process;cellular amine metabolic process;cellular amino acid metabolic process;cellular ketor
26.5898	26.0676	26.2138	26.4154	1:Experimental evidence at protein level	adherens junction organization;anatomical structure morphogenesis;biological adhesion;cell adhesion;cell junction assembly;cell junction organization;cell-cell adhesion;cell-cell junction organization;cellular component asser
26.689	23.9346	26.4466	26.5143	1:Experimental evidence at protein level	catabolic process;cellular catabolic process;cellular macromolecule catabolic process;cellular macromolecule metabolic process;cellular metabolic process;cellular nitrogen compound metabolic process;cellular process;defe
24.6307	24.6772	24.7856	24.4949	1:Experimental evidence at protein level	biological regulation;cell activation;cellular component movement;cellular process;cellular response to stimulus;establishment of localization;establishment of localization in cell;establishment of protein localization;immune sy
29.316	29.3846	29.3509	29.3117	1:Experimental evidence at protein level	biological regulation;cell cycle cytokinesis;cell cycle phase;cell cycle process;cell differentiation;cellular component assembly;cellular component assembly at cellular level;cellular component organization;cellular component
26.9603	27.1332	26.9249	26.9175	1:Experimental evidence at protein level	bradykinin catabolic process;catabolic process;cellular catabolic process;cellular metabolic process;cellular process;macromolecule metabolic process;metabolic process;peptide catabolic process;peptide metabolic proces

24.7426	24.8566	24.7269	24.6766	1:Experimental evidence at protein level	actin cytoskeleton organization;actin filament organization;actin filament-based process;anatomical structure development;anatomical structure morphogenesis;barrier septum formation;biological regulation;cell development
25.2132	24.9681	24.8641	24.7978	1:Experimental evidence at protein level	anatomical structure development;apoptosis;biological regulation;cell cycle phase;cell cycle process;cell death;cell division;cellular component organization;cellular component organization at cellular level;cellular component
28.5924	28.6285	28.7901	28.8277	1:Experimental evidence at protein level	actin cytoskeleton organization;actin filament-based process;biological regulation;cell differentiation;cellular component organization;cellular component organization at cellular level;cellular component organization or biogen
24.9657	25.036	24.9451	24.1713	1:Experimental evidence at protein level	acetate biosynthetic process;acetate metabolic process;acetyl-CoA biosynthetic process;acetyl-CoA biosynthetic process from acetate;acetyl-CoA metabolic process;alcohol metabolic process;biosynthetic process;carboxy
25.9685	25.9938	26.0949	26.1787	1:Experimental evidence at protein level	activation of caspase activity;activation of caspase activity by cytochrome c;apoptosis;biological regulation;cell death;cellular process;death;induction of apoptosis;induction of apoptosis by extracellular signals;induction of a
28.9434	28.4603	28.5112	28.5142	1:Experimental evidence at protein level	biosynthetic process;cell differentiation;cellular biosynthetic process;cellular developmental process;cellular macromolecule biosynthetic process;cellular macromolecule metabolic process;cellular metabolic process;cellular
28.9618	29.2073	29.058	29.2594	1:Experimental evidence at protein level	cellular process;establishment of localization;establishment of localization in cell;establishment of protein localization;intracellular protein transport;intracellular transport;protein transport;transport;vesicle-mediated transport
28.6462	28.7178	28.9953	28.8638	1:Experimental evidence at protein level	biosynthetic process;carbohydrate biosynthetic process;carbohydrate metabolic process;cellular biosynthetic process;cellular carbohydrate biosynthetic process;cellular carbohydrate metabolic process;cellular lipid metabol
26.299	26.457	26.3845	26.6339	1:Experimental evidence at protein level	
28.1476	28.3895	28.1497	28.1971	1:Experimental evidence at protein level	anatomical structure development;biological regulation;biosynthetic process;cell development;cellular biosynthetic process;cellular developmental process;cellular macromolecule biosynthetic process;cellular macromolecule
27.5751	27.8155	27.7569	27.9742	1:Experimental evidence at protein level	alternative nuclear mRNA splicing, via spliceosome;anatomical structure development;anatomical structure morphogenesis;appendage morphogenesis;biological regulation;cell differentiation;cellular component assembly;ce
28.026	24.2907	27.8471	28.1663	1:Experimental evidence at protein level	amine metabolic process;biosynthetic process;carboxylic acid metabolic process;cellular amine metabolic process;cellular amino acid metabolic process;cellular biosynthetic process;cellular ketone metabolic process;cellula
26.9424	26.507	27.1613	26.9909	1:Experimental evidence at protein level	biosynthetic process;cellular biosynthetic process;cellular component organization;cellular component organization at cellular level;cellular component organization or biogenesis;cellular component organization or biogenesis
26.663	27.4061	26.8153	27.2762	1:Experimental evidence at protein level	biosynthetic process;cellular biosynthetic process;cellular component organization;cellular component organization at cellular level;cellular component organization or biogenesis;cellular component organization or biogenesis
26.6404	26.1399	26.3271	26.1614	1:Experimental evidence at protein level	behavior;biological regulation;biosynthetic process;carbohydrate metabolic process;carbohydrate transport;cell cycle;cell cycle process;cell surface receptor linked signaling pathway;cellular biosynthetic process;cellular com
25.3819	25.4129	25.2414	25.4053	1:Experimental evidence at protein level	biological regulation;biosynthetic process;cellular biosynthetic process;cellular component organization;cellular component organization at cellular level;cellular component organization or biogenesis;cellular component orga
26.4407	25.153	26.4501	26.7083	1:Experimental evidence at protein level	
24.8419	24.6114	26.7002	25.4672	2:Experimental evidence at transcript lev	developmental process;multicellular organismal development;multicellular organismal process
26.8407	27.1728	27.4527	27.3592	1:Experimental evidence at protein level	biological regulation;regulation of biological process;regulation of catabolic process;regulation of cellular catabolic process;regulation of cellular metabolic process;regulation of cellular process;regulation of cellular protein me
26.9618	27.5577	27.7615	27.4653	1:Experimental evidence at protein level	circadian rhythm;rhythmic process
26.1842	25.143	26.6607	25.9869	1:Experimental evidence at protein level	biological regulation;catabolic process;cellular catabolic process;cellular macromolecule metabolic process;cellular metabolic process;cellular nitrogen compound catabolic process;cellular nitrogen compound metabolic pro
25.9742	25.5769	25.6482	25.4617	1:Experimental evidence at protein level	
26.2096	24.0117	26.2008	25.887	1:Experimental evidence at protein level	biosynthetic process;cellular biosynthetic process;cellular component disassembly;cellular component disassembly at cellular level;cellular component organization;cellular component organization at cellular level;cellular co
26.9101	27.8872	27.9895	28.1653	1:Experimental evidence at protein level	biological regulation;cellular macromolecule metabolic process;cellular metabolic process;cellular process;cellular protein metabolic process;dephosphorylation;macromolecule metabolic process;macromolecule modification
24.7369	24.818	27.017	25.1516	1:Experimental evidence at protein level	amine transport;amino acid transport;biological regulation;biosynthetic process;carboxylic acid transport;cellular biosynthetic process;cellular lipid metabolic process;cellular metabolic process;cellular process;establishment
24.3348	24.6418	24.8631	24.5079	1:Experimental evidence at protein level	anatomical structure development;anatomical structure formation involved in morphogenesis;axon guidance;biological regulation;biosynthetic process;camera-type eye development;cellular biosynthetic process;cellular mac
26.0304	24.838	24.6194	24.1673	1:Experimental evidence at protein level	
27.8255	27.7665	27.9942	27.9626	1:Experimental evidence at protein level	apoptosis;biological regulation;carbohydrate homeostasis;cell death;cellular component organization;cellular component organization or biogenesis;cellular membrane organization;cellular process;cellular response to biotic
25.6984	25.7372	25.736	25.4102	1:Experimental evidence at protein level	biological regulation;biosynthetic process;cell activation;cell cycle phase;cell cycle process;cell division;cell proliferation;cellular biosynthetic process;cellular component assembly;cellular component assembly at cellular leve
26.6298	26.8118	26.7056	26.3674	1:Experimental evidence at protein level	antigen processing and presentation;antigen processing and presentation of exogenous antigen;antigen processing and presentation of exogenous peptide antigen;antigen processing and presentation of exogenous peptide
25.3257	24.1648	24.2553	25.0826	1:Experimental evidence at protein level	biological regulation;cell surface receptor linked signaling pathway;cellular process;cellular response to stimulus;establishment of localization;establishment of localization in cell;establishment of protein localization;glutamate
28.3029	28.4337	28.3219	28.1714	1:Experimental evidence at protein level	amine metabolic process;amino acid activation;biosynthetic process;carboxylic acid metabolic process;cellular amine metabolic process;cellular amino acid metabolic process;cellular biosynthetic process;cellular ketone me
27.7623	27.7041	27.6122	27.7039	1:Experimental evidence at protein level	amine metabolic process;amino acid activation;biosynthetic process;carboxylic acid metabolic process;cellular amine metabolic process;cellular amino acid metabolic process;cellular biosynthetic process;cellular componen
26.3524	26.078	26.2663	26.2299	1:Experimental evidence at protein level	actin cytoskeleton organization;actin filament-based process;biological regulation;cellular component organization;cellular component organization at cellular level;cellular component organization or biogenesis;cellular comp
26.3446	27.0515	26.7537	26.8358	1:Experimental evidence at protein level	apoptotic mitochondrial changes;autophagic vacuole assembly;autophagy;catabolic process;cellular catabolic process;cellular component assembly;cellular component assembly at cellular level;cellular component organiza
27.0978	26.5766	26.8883	26.5874	1:Experimental evidence at protein level	biological regulation;cell cycle;cell cycle process;cell division;cell proliferation;cellular component organization;cellular component organization at cellular level;cellular component organization or biogenesis;cellular componen
28.7477	28.3223	28.5347	28.4614	1:Experimental evidence at protein level	cell cycle;cell cycle process;cell division;cellular component organization;cellular component organization at cellular level;cellular component organization or biogenesis;cellular component organization or biogenesis at cellula
26.9316	26.8067	26.7005	26.5158	1:Experimental evidence at protein level	biosynthetic process;cellular biosynthetic process;cellular lipid metabolic process;cellular metabolic process;cellular process;dephosphorylation;glycerolipid biosynthetic process;glycerolipid metabolic process;glycerophosph
28.6697	28.8931	28.9752	28.8301	1:Experimental evidence at protein level	
26.5999	26.3758	26.1429	26.5034	1:Experimental evidence at protein level	
27.0257	26.8711	27.1298	27.1368	1:Experimental evidence at protein level	
24.4243	25.0477	23.2733	25.1366	1:Experimental evidence at protein level	
25.456	25.4359	25.2426	25.5045	1:Experimental evidence at protein level	biosynthetic process;carboxylic acid biosynthetic process;carboxylic acid metabolic process;cellular biosynthetic process;cellular ketone metabolic process;cellular lipid metabolic process;cellular metabolic process;cellular
25.7452	25.7995	26.2198	26.0118	1:Experimental evidence at protein level	carbohydrate catabolic process;carbohydrate metabolic process;catabolic process;cellular carbohydrate catabolic process;cellular carbohydrate metabolic process;cellular metabolic process;cellular process;glucuronoside c
25.078	26.178	25.5857	25.844	1:Experimental evidence at protein level	anatomical structure morphogenesis;asymmetric protein localization;biological regulation;cell communication;cell-cell signaling;cellular process;developmental process;establishment of localization;establishment of localizatio
27.3112	27.5471	27.3368	27.3828	1:Experimental evidence at protein level	
25.0831	24.9025	24.9742	24.599	1:Experimental evidence at protein level	defense response;defense response to virus;immune effector process;immune system process;multi-organism process;response to biotic stimulus;response to other organism;response to stimulus;response to stress;respons
27.3473	27.5739	27.2818	27.4315	1:Experimental evidence at protein level	
26.3195	26.3596	26.2018	26.0964	1:Experimental evidence at protein level	biological regulation;positive regulation of biological process;positive regulation of cytokine production;positive regulation of interleukin-6 production;positive regulation of multicellular organismal process;regulation of biologi
27.3962	27.0823	26.8198	27.1583	1:Experimental evidence at protein level	biological regulation;cellular component organization;cellular component organization or biogenesis;cellular membrane organization;cellular process;drug transmembrane transport;drug transport;establishment of localization
29.0289	29.3649	29.5295	29.3371	1:Experimental evidence at protein level	cell cycle;cell division;cellular component assembly;cellular component organization;cellular component organization or biogenesis;cellular process;macromolecular complex assembly;macromolecular complex subunit organ
28.3134	27.9024	27.8032	27.8027	1:Experimental evidence at protein level	actin cytoskeleton organization;actin cytoskeleton reorganization;actin filament-based process;actin-mediated cell contraction;anatomical structure formation involved in morphogenesis;anatomical structure morphogenesis;
26.1572	26.2721	26.3015	26.1201	1:Experimental evidence at protein level	
25.0227	25.503	25.0448	24.5492	1:Experimental evidence at protein level	biological regulation;cell cycle;cellular macromolecule metabolic process;cellular metabolic process;cellular nitrogen compound metabolic process;cellular process;cellular response to stimulus;cellular response to stress;DN
29.2255	29.1235	29.0489	28.9938	1:Experimental evidence at protein level	biological regulation;cell growth;cellular process;growth;negative regulation of apoptosis;negative regulation of biological process;negative regulation of cell death;negative regulation of cellular process;negative regulation of
23.9107	26.358	26.266	26.1987	1:Experimental evidence at protein level	biological regulation;cell cycle;cell division;cellular process;cellular response to stimulus;chromosome segregation;intracellular signal transduction;metabolic process;regulation of biological process;regulation of cellular proce
26.5382	27.2409	23.6372	26.6448	1:Experimental evidence at protein level	
24.8799	25.0726	24.8365	24.8777	1:Experimental evidence at protein level	biological regulation;biosynthetic process;cell differentiation;cellular biosynthetic process;cellular component assembly;cellular component assembly at cellular level;cellular component organization;cellular component organi
27.8755	27.2915	27.3992	27.1731	1:Experimental evidence at protein level	cellular macromolecule metabolic process;cellular metabolic process;cellular nitrogen compound metabolic process;cellular process;macromolecule metabolic process;metabolic process;nitrogen compound metabolic proce
27.1568	26.6121	26.8184	26.2446	1:Experimental evidence at protein level	cation transport;establishment of localization;ion transport;metal ion transport;transport
26.9935	27.0918	26.9659	26.9518	1:Experimental evidence at protein level	cellular component organization;cellular component organization or biogenesis;cellular membrane organization;cellular process;endocytosis;establishment of localization;establishment of protein localization;membrane invagi
25.9826	25.7056	25.446	25.4436	1:Experimental evidence at protein level	cellular macromolecule metabolic process;cellular metabolic process;cellular nitrogen compound metabolic process;cellular process;macromolecule metabolic process;metabolic process;mRNA metabolic process;mRNA pro
26.7085	26.3143	26.5425	26.7371	1:Experimental evidence at protein level	biological regulation;cellular macromolecule metabolic process;cellular metabolic process;cellular nitrogen compound metabolic process;cellular process;cellular response to chemical stimulus;cellular response to drug;cellul
26.6081	26.3486	26.5654	26.683	1:Experimental evidence at protein level	
26.8734	26.6319	26.7135	26.6095	1:Experimental evidence at protein level	biological regulation;biosynthetic process;cellular biosynthetic process;cellular macromolecule biosynthetic process;cellular macromolecule metabolic process;cellular metabolic process;cellular nitrogen compound metabolic
26.0735	26.4322	25.9848	26.3131	1:Experimental evidence at protein level	apoptosis;biological regulation;biosynthetic process;cell death;cellular biosynthetic process;cellular macromolecule biosynthetic process;cellular macromolecule metabolic process;cellular metabolic process;cellular nitrogen
25.5418	25.4473	25.9092	25.565	1:Experimental evidence at protein level	box C/D snoRNP assembly;cell differentiation;cellular component assembly;cellular component assembly at cellular level;cellular component organization;cellular component organization at cellular level;cellular component o
25.856	25.6155	25.4441	25.7699	1:Experimental evidence at protein level	anatomical structure development;biological regulation;cell proliferation;developmental process;negative regulation of biological process;negative regulation of cell communication;negative regulation of cellular process;negat
27.274	27.6663	27.7765	27.8598	1:Experimental evidence at protein level	
28.0591	27.7176	28.3602	28.2879	1:Experimental evidence at protein level	biological regulation;cell cycle checkpoint;cellular component organization;cellular component organization at cellular level;cellular component organization or biogenesis;cellular component organization or biogenesis at cellu
26.5772	26.3506	26.431	26.529	1:Experimental evidence at protein level	biological regulation;biosynthetic process;cellular biosynthetic process;cellular macromolecule biosynthetic process;cellular macromolecule metabolic process;cellular metabolic process;cellular nitrogen compound metabolic
26.8309	26.8538	26.8381	26.5055	1:Experimental evidence at protein level	cellular macromolecule metabolic process;cellular metabolic process;cellular nitrogen compound metabolic process;cellular process;macromolecule metabolic process;macromolecule modification;metabolic process;ncRNA
28.2362	28.3853	28.0085	28.323	1:Experimental evidence at protein level	
26.4004	25.5966	26.1862	26.2457	1:Experimental evidence at protein level	cellular process;cellular response to chemical stimulus;cellular response to oxidative stress;cellular response to oxygen radical;cellular response to reactive oxygen species;cellular response to stimulus;cellular response to st
27.3536	27.6632	28.0347	27.9113	1:Experimental evidence at protein level	
26.7078	26.9304	27.0214	26.8406	1:Experimental evidence at protein level	
25.9807	24.56	25.3166	25.9287	1:Experimental evidence at protein level	alcohol biosynthetic process;alcohol metabolic process;amine metabolic process;aminoglycan metabolic process;anatomical structure development;anatomical structure morphogenesis;biosynthetic process;carbohydrate bi
28.888	28.5804	28.6156	28.5802	1:Experimental evidence at protein level	biological regulation;cellular component organization;cellular component organization at cellular level;cellular component organization or biogenesis;cellular component organization or biogenesis at cellular level;cellular memb
25.6278	25.2651	25.5391	25.8374	1:Experimental evidence at protein level	biological regulation;biosynthetic process;cellular biosynthetic process;cellular macromolecule biosynthetic process;cellular macromolecule metabolic process;cellular metabolic process;cellular nitrogen compound metabolic
24.8706	25.6331	24.4621	24.8125	1:Experimental evidence at protein level	biological regulation;negative regulation of biological process;negative regulation of catalytic activity;negative regulation of cell death;negative regulation of cellular process;negative regulation of kinase activity;negative regula
25.7477	24.7881	25.1322	25.0072	1:Experimental evidence at protein level	biosynthetic process;catabolic process;cellular biosynthetic process;cellular catabolic process;cellular lipid catabolic process;cellular lipid metabolic process;cellular metabolic process;cellular process;cellular response to ch
26.7751	26.5447	26.7455	26.8031	1:Experimental evidence at protein level	
25.3266	24.6948	23.7214	24.3109	1:Experimental evidence at protein level	
25.9107	25.8058	25.5213	25.4294	1:Experimental evidence at protein level	
25.6184	25.4403	24.919	25.277	1:Experimental evidence at protein level	biological regulation;cellular process;cellular response to stimulus;cellular response to stress;negative regulation of biological process;negative regulation of cell growth;negative regulation of cellular process;negative regulati
26.4066	26.7315	26.5906	26.2085	1:Experimental evidence at protein level	
26.5039	26.3439	26.1557	26.3662	1:Experimental evidence at protein level	cation transport;cellular macromolecule metabolic process;cellular metabolic process;cellular nitrogen compound metabolic process;cellular process;establishment of localization;ion transport;macromolecule metabolic proce
27.3833	27.525	27.2916	27.3171	1:Experimental evidence at protein level	macromolecule metabolic process;metabolic process;primary metabolic process;protein metabolic process;proteolysis
26.4361	24.0272	26.9531	26.538	1:Experimental evidence at protein level	anatomical structure homeostasis;biological regulation;biosynthetic process;cellular biosynthetic process;cellular component organization;cellular component organization at cellular level;cellular component organization or bi
26.1014	25.9166	26.1858	26.1224	1:Experimental evidence at protein level	actin cytoskeleton organization;actin filament organization;actin filament-based process;actin polymerization or depolymerization;biological regulation;cell migration;cell motility;cellular component movement;cellular compon
27.9068	27.7034	27.7366	27.633	1:Experimental evidence at protein level	apoptosis;biological regulation;biosynthetic process;cell death;cellular biosynthetic process;cellular macromolecule biosynthetic process;cellular macromolecule metabolic process;cellular metabolic process;cellular nitrogen
25.288	24.6876	24.9445	24.7853	1:Experimental evidence at protein level	cellular macromolecule metabolic process;cellular metabolic process;cellular nitrogen compound metabolic process;cellular process;macromolecule metabolic process;maturation of SSU-rRNA;maturation of SSU-rRNA from
26.546	26.7293	26.6179	26.3071	1:Experimental evidence at protein level	activation of JUN kinase activity;activation of MAPK activity;activation of MAPKK activity;activation of protein kinase activity;biological regulation;cell cycle arrest;cell cycle checkpoint;cell cycle process;cell death;cell differen
24.7331	26.4203	26.8914	26.536	1:Experimental evidence at protein level	cellular macromolecule metabolic process;cellular metabolic process;cellular process;cellular protein metabolic process;chaperone-mediated protein folding;macromolecule metabolic process;macromolecule modification;me
29.1577	28.9331	29.317	29.1259	1:Experimental evidence at protein level	actin cytoskeleton organization;actin filament capping;actin filament organization;actin filament-based process;actomyosin structure organization;anatomical structure development;anatomical structure formation involved in
27.6111	27.4216	27.4771	27.2924	1:Experimental evidence at protein level	carbohydrate metabolic process;cellular carbohydrate metabolic process;cellular macromolecule metabolic process;cellular metabolic process;cellular process;cellular protein metabolic process;'de novo' posttranslational pr
28.0331	27.6957	27.6111	27.6863	1:Experimental evidence at protein level	acyl-CoA biosynthetic process;acyl-CoA metabolic process;acylglycerol biosynthetic process;acylglycerol metabolic process;biosynthetic process;carboxylic acid biosynthetic process;carboxylic acid metabolic process;cellu
27.3418	26.8811	26.7641	26.6155	1:Experimental evidence at protein level	anatomical structure formation involved in morphogenesis;angiogenesis;antigen processing and presentation;antigen processing and presentation of endogenous antigen;antigen processing and presentation of endogenous
27.322	27.9348	27.1841	27.0528	1:Experimental evidence at protein level	biological regulation;regulation of biological process;regulation of cellular metabolic process;regulation of cellular process;regulation of cellular respiration;regulation of generation of precursor metabolites and energy;regulatio
27.343	26.9546	27.1151	27.2038	1:Experimental evidence at protein level	
26.8636	26.5417	26.8886	26.7134	1:Experimental evidence at protein level	anatomical structure formation involved in morphogenesis;angiogenesis;biological regulation;biosynthetic process;cellular biosynthetic process;cellular macromolecule biosynthetic process;cellular macromolecule metabolic
26.1965	22.9548	25.8946	25.9748	1:Experimental evidence at protein level	biological regulation;cellular macromolecule metabolic process;cellular metabolic process;cellular nitrogen compound metabolic process;cellular process;CRD-mediated mRNA stabilization;establishment of localization;estab
27.743	27.5992	27.5892	27.6821	1:Experimental evidence at protein level	activation of caspase activity;biological regulation;cellular component organization;cellular component organization or biogenesis;cellular membrane organization;cellular process;establishment of localization;membrane orga
27.3298	27.1117	26.985	26.8665	1:Experimental evidence at protein level	biological regulation;cellular macromolecule metabolic process;cellular metabolic process;cellular process;cellular protein metabolic process;macromolecule metabolic process;metabolic process;negative regulation of cataly
26.5292	26.8516	27.0253	26.8005	1:Experimental evidence at protein level	amine biosynthetic process;amine metabolic process;biological regulation;biosynthetic process;carboxylic acid biosynthetic process;carboxylic acid metabolic process;cellular amine metabolic process;cellular amino acid bio
28.79	28.6128	28.5983	28.4982	1:Experimental evidence at protein level	anatomical structure formation involved in morphogenesis;biological regulation;blood circulation;cellular component organization;cellular component organization or biogenesis;cellular membrane organization;cellular process
23.343	25.6444	24.3088	24.7774	1:Experimental evidence at protein level	biological regulation;blood coagulation;cellular component assembly;cellular component organization;cellular component organization or biogenesis;cellular process;coagulation;endocytic recycling;endosome transport;estab
29.3359	28.8715	29.0193	28.8926	1:Experimental evidence at protein level	actin cytoskeleton organization;actin filament-based process;biological regulation;blood coagulation;cellular component organization;cellular component organization at cellular level;cellular component organization or biogen
24.941	25.4176	25.6122	23.6208	3:Protein inferred from homology	biological regulation;cell surface receptor linked signaling pathway;cellular process;cellular response to stimulus;detection of chemical stimulus;detection of chemical stimulus involved in sensory perception;detection of chem
25.495	24.4161	24.3324	25.0322	1:Experimental evidence at protein level	biological regulation;cellular component organization;cellular component organization at cellular level;cellular component organization or biogenesis;cellular component organization or biogenesis at cellular level;cellular proce
29.2151	27.7846	27.9635	28.4006	1:Experimental evidence at protein level	actin cytoskeleton organization;actin filament capping;actin filament organization;actin filament-based process;actomyosin structure organization;anatomical structure development;anatomical structure formation involved in
26.4832	26.3992	26.3252	26.2768	1:Experimental evidence at protein level	biological regulation;cell surface receptor linked signaling pathway;cellular process;cellular response to stimulus;G-protein coupled receptor protein signaling pathway;opioid receptor signaling pathway;regulation of biologica
26.3125	26.3232	26.7401	26.8916	1:Experimental evidence at protein level	biological regulation;biosynthetic process;cellular biosynthetic process;cellular macromolecule biosynthetic process;cellular macromolecule metabolic process;cellular metabolic process;cellular nitrogen compound metabolic
26.4453	24.8957	26.1933	26.339	1:Experimental evidence at protein level	anatomical structure development;biological regulation;cell surface receptor linked signaling pathway;cellular process;cellular response to stimulus;developmental process;enzyme linked receptor protein signaling pathway;in
26.7988	27.3818	27.1277	27.6946	1:Experimental evidence at protein level	biological regulation;cell cycle process;cellular component organization;cellular component organization at cellular level;cellular component organization or biogenesis;cellular component organization or biogenesis at cellular
24.8833	25.7975	25.5642	25.637	1:Experimental evidence at protein level	biological regulation;biosynthetic process;cation transport;cellular biosynthetic process;cellular macromolecule biosynthetic process;cellular macromolecule metabolic process;cellular metabolic process;cellular nitrogen com
26.3775	25.0614	26.5753	26.4826	1:Experimental evidence at protein level	cellular macromolecule metabolic process;cellular metabolic process;cellular nitrogen compound metabolic process;cellular process;gene expression;macromolecule metabolic process;metabolic process;mRNA metabolic p
25.9083	26.6286	26.3326	26.5103	1:Experimental evidence at protein level	biosynthetic process;cellular biosynthetic process;cellular component disassembly;cellular component disassembly at cellular level;cellular component organization;cellular component organization at cellular level;cellular co
25.5812	21.9602	25.5978	25.7454	1:Experimental evidence at protein level	
27.1808	27.0655	26.875	27.0545	1:Experimental evidence at protein level	activation of JUN kinase activity;activation of MAPK activity;biological regulation;biosynthetic process;carboxylic acid biosynthetic process;carboxylic acid metabolic process;cellular biosynthetic process;cellular ketone meta
26.7649	26.5642	26.5959	26.535	1:Experimental evidence at protein level	biological regulation;cell communication;cellular component organization;cellular component organization at cellular level;cellular component organization or biogenesis;cellular component organization or biogenesis at cellula
26.3563	26.0977	26.5958	26.308	1:Experimental evidence at protein level	
26.856	26.7805	26.9232	26.7377	1:Experimental evidence at protein level	
28.9758	28.9266	28.9691	28.9246	1:Experimental evidence at protein level	biological regulation;biosynthetic process;cell death;cell projection organization;cellular biosynthetic process;cellular component organization;cellular component organization at cellular level;cellular component organization
25.5174	26.1243	26.3081	26.3909	1:Experimental evidence at protein level	cellular macromolecule metabolic process;cellular metabolic process;cellular process;cellular protein metabolic process;macromolecule metabolic process;macromolecule modification;metabolic process;primary metabolic p
26.256	23.4067	24.5535	25.7474	1:Experimental evidence at protein level	biological regulation;cellular component organization;cellular component organization or biogenesis;cellular membrane organization;cellular process;cellular response to acid;cellular response to amine stimulus;cellular respo
26.5663	26.8444	26.5286	26.5979	1:Experimental evidence at protein level	cell cycle;cell division;cellular process
24.4397	24.4244	23.1225	24.05	1:Experimental evidence at protein level	biological regulation;cell surface receptor linked signaling pathway;cellular component assembly;cellular component organization;cellular component organization or biogenesis;cellular process;cellular response to stimulus;c
28.0172	28.0348	27.9496	27.8105	1:Experimental evidence at protein level	biological regulation;cell cycle;cell cycle phase;cell cycle process;cell division;cell junction assembly;cell junction organization;cell surface receptor linked signaling pathway;cell-substrate junction assembly;cellular componen

24.159	23.3777	23.9881	23.8667	1:Experimental evidence at protein level	
25.7051	26.3967	26.1578	26.0626	1:Experimental evidence at protein level	biological regulation;cellular component organization;cellular component organization at cellular level;cellular component organization or biogenesis;cellular component organization or biogenesis at cellular level;cellular proce
28.0461	27.3706	27.6954	27.9404	1:Experimental evidence at protein level	biological regulation;cell cycle;cell differentiation;cellular developmental process;cellular macromolecule metabolic process;cellular metabolic process;cellular nitrogen compound metabolic process;cellular process;cellular re
24.792	26.3825	25.0951	24.4636	1:Experimental evidence at protein level	cellular component organization;cellular component organization at cellular level;cellular component organization or biogenesis;cellular component organization or biogenesis at cellular level;cellular process;lipid particle orga
26.1716	25.4674	26.0907	25.8716	1:Experimental evidence at protein level	biological regulation;cellular macromolecule metabolic process;cellular metabolic process;cellular process;cellular protein metabolic process;dephosphorylation;macromolecule metabolic process;macromolecule modification
25.249	25.3617	23.3702	25.0845	1:Experimental evidence at protein level	
29.4276	28.9133	29.2385	29.003	1:Experimental evidence at protein level	biosynthetic process;cell differentiation;cellular biosynthetic process;cellular developmental process;cellular macromolecule biosynthetic process;cellular macromolecule metabolic process;cellular metabolic process;cellular
26.5443	26.5775	26.5789	26.5895	1:Experimental evidence at protein level	biosynthetic process;cellular biosynthetic process;cellular macromolecule biosynthetic process;cellular macromolecule metabolic process;cellular metabolic process;cellular nitrogen compound metabolic process;cellular pro
27.7242	27.3892	27.436	27.3583	1:Experimental evidence at protein level	amine metabolic process;amino acid activation;carboxylic acid metabolic process;cellular amine metabolic process;cellular amino acid metabolic process;cellular ketone metabolic process;cellular macromolecule metabolic
25.9766	25.9159	22.3232	27.0946	1:Experimental evidence at protein level	biosynthetic process;cell differentiation;cellular biosynthetic process;cellular developmental process;cellular macromolecule biosynthetic process;cellular macromolecule metabolic process;cellular metabolic process;cellular
25.8456	26.0445	25.4143	25.6968	1:Experimental evidence at protein level	cellular macromolecule metabolic process;cellular metabolic process;cellular nitrogen compound metabolic process;cellular process;macromolecule metabolic process;metabolic process;mRNA metabolic process;mRNA pro
26.4124	25.8199	25.9946	26.1379	1:Experimental evidence at protein level	biological regulation;cellular component organization;cellular component organization at cellular level;cellular component organization or biogenesis;cellular component organization or biogenesis at cellular level;cellular locali
27.0548	26.7664	27.0231	26.9507	1:Experimental evidence at protein level	acetyl-CoA catabolic process;acetyl-CoA metabolic process;biosynthetic process;carboxylic acid metabolic process;catabolic process;cellular biosynthetic process;cellular catabolic process;cellular ketone metabolic proces
26.6765	26.613	26.5908	26.5419	1:Experimental evidence at protein level	biosynthetic process;cellular aromatic compound metabolic process;cellular biosynthetic process;cellular metabolic compound salvage;cellular metabolic process;cellular nitrogen compound biosynthetic process;cellular nitr
25.8879	23.772	24.3697	25.8188	1:Experimental evidence at protein level	cellular component organization;cellular component organization or biogenesis;cellular membrane organization;cellular process;endoplasmic reticulum membrane organization;establishment of localization;establishment of lo
27.3259	27.1783	27.0987	27.0869	1:Experimental evidence at protein level	anatomical structure development;cell projection organization;cellular component organization;cellular component organization at cellular level;cellular component organization or biogenesis;cellular component organization o
25.1602	24.9486	24.8996	25.0169	1:Experimental evidence at protein level	anatomical structure homeostasis;biological regulation;bone resorption;cell projection organization;cellular component organization;cellular component organization at cellular level;cellular component organization or biogene
26.5724	26.6539	26.3454	26.7104	1:Experimental evidence at protein level	biological regulation;cell surface receptor linked signaling pathway;cellular component organization;cellular component organization or biogenesis;cellular membrane organization;cellular process;cellular response to stimulus
28.4881	28.4031	28.3609	28.4502	1:Experimental evidence at protein level	biological regulation;cellular macromolecule metabolic process;cellular metabolic process;cellular process;cellular protein metabolic process;macromolecule metabolic process;macromolecule modification;metabolic process
26.5589	26.2604	26.5649	26.25	1:Experimental evidence at protein level	cellular process;establishment of localization;establishment of localization in cell;establishment of protein localization;Golgi vesicle transport;intracellular protein transport;intracellular transport;intra-Golgi vesicle-mediated tra
25.6548	25.7822	26.0774	26.2718	1:Experimental evidence at protein level	catabolic process;cellular catabolic process;cellular macromolecule catabolic process;cellular macromolecule metabolic process;cellular metabolic process;cellular process;cellular protein metabolic process;macromolecule
26.7905	26.7932	26.8683	26.6703	1:Experimental evidence at protein level	cell differentiation;cellular component organization;cellular component organization or biogenesis;cellular developmental process;cellular membrane organization;cellular process;collagen catabolic process;collagen metabolic
29.2743	29.0178	29.06	28.9131	1:Experimental evidence at protein level	anatomical structure development;biological regulation;cell communication;cell surface receptor linked signaling pathway;cell-cell signaling;cellular metabolic process;cellular process;cellular response to chemical stimulus;ce
26.4558	24.3668	26.1417	25.7397	1:Experimental evidence at protein level	alcohol metabolic process;anatomical structure development;biological regulation;biosynthetic process;blood vessel development;cell differentiation;cellular developmental process;cellular process;cholesterol biosynthetic pr
25.4307	24.9603	25.2409	25.0882	1:Experimental evidence at protein level	aggresome assembly;autophagy;biological regulation;biosynthetic process;catabolic process;cell communication;cellular biosynthetic process;cellular catabolic process;cellular component assembly;cellular component asse
26.1267	27.3778	27.3417	27.5637	1:Experimental evidence at protein level	cellular process;endosome transport;establishment of localization;establishment of localization in cell;establishment of protein localization;intracellular protein transport;intracellular transport;protein transport;retrograde trans
27.6139	27.9143	27.7587	27.7921	1:Experimental evidence at protein level	biological regulation;biosynthetic process;cellular biosynthetic process;cellular component assembly;cellular component assembly at cellular level;cellular component organization;cellular component organization at cellular le
28.2825	28.6545	28.5025	28.5065	1:Experimental evidence at protein level	carboxylic acid metabolic process;cellular aldehyde metabolic process;cellular component assembly;cellular component organization;cellular component organization or biogenesis;cellular ketone metabolic process;cellular r
28.883	29.027	29.1287	29.3805	1:Experimental evidence at protein level	anatomical structure morphogenesis;angiotensin maturation;biological regulation;branching morphogenesis of a tube;cellular macromolecule metabolic process;cellular metabolic process;cellular process;cellular protein meta
27.7761	28.0499	27.8922	28.0331	1:Experimental evidence at protein level	biological regulation;cellular macromolecule metabolic process;cellular metabolic process;cellular nitrogen compound metabolic process;cellular process;cellular protein metabolic process;cellular response to chemical stimu
28.2071	28.2747	28.3669	28.3615	1:Experimental evidence at protein level	biological regulation;cellular macromolecule metabolic process;cellular metabolic process;cellular process;cellular protein metabolic process;macromolecule metabolic process;macromolecule modification;metabolic process
25.1141	25.0359	26.3452	25.1982	1:Experimental evidence at protein level	biological regulation;biosynthetic process;cell proliferation;cellular biosynthetic process;cellular component organization;cellular component organization at cellular level;cellular component organization or biogenesis;cellular
26.1921	26.4293	26.3019	26.2355	1:Experimental evidence at protein level	establishment of localization;establishment of localization in cell;establishment of RNA localization;gene expression;intracellular transport;macromolecule metabolic process;metabolic process;mRNA export from nucleus;mR
26.4802	26.6676	27.1559	26.7936	1:Experimental evidence at protein level	biological regulation;catabolic process;cell surface receptor linked signaling pathway;cellular catabolic process;cellular macromolecule catabolic process;cellular macromolecule metabolic process;cellular metabolic process;
26.4485	24.5427	25.7147	24.853	1:Experimental evidence at protein level	macromolecule metabolic process;metabolic process;primary metabolic process;protein metabolic process;proteolysis;response to calcium ion;response to chemical stimulus;response to inorganic substance;response to me
26.6386	26.0558	26.7585	26.5406	1:Experimental evidence at protein level	alcohol biosynthetic process;alcohol metabolic process;amine catabolic process;amine metabolic process;biosynthetic process;carbohydrate biosynthetic process;carbohydrate metabolic process;carboxylic acid catabolic p
26.2789	26.2059	26.1793	26.3785	1:Experimental evidence at protein level	cellular metabolic process;cellular process;electron transport chain;generation of precursor metabolites and energy;metabolic process;mitochondrial electron transport, ubiquinol to cytochrome c;oxidation-reduction process;
27.1578	26.9986	27.3112	27.2289	1:Experimental evidence at protein level	apoptosis;biological regulation;cell death;cellular component disassembly;cellular component disassembly at cellular level;cellular component disassembly involved in apoptosis;cellular component organization;cellular comp
26.8155	26.9853	26.9669	26.6235	1:Experimental evidence at protein level	cellular process;establishment of localization;transmembrane transport;transport
28.5101	28.6596	28.538	28.653	1:Experimental evidence at protein level	establishment of localization;transport
26.9542	26.9391	27.048	27.0525	1:Experimental evidence at protein level	biological regulation;negative regulation of biological process;negative regulation of cell proliferation;negative regulation of cellular process;regulation of biological process;regulation of cell proliferation;regulation of cellular pr
25.3142	25.8359	25.7603	25.869	1:Experimental evidence at protein level	anatomical structure development;biological regulation;biosynthetic process;cell development;cell migration;cell motility;cellular biosynthetic process;cellular component assembly;cellular component assembly at cellular leve
26.8884	26.72	26.7084	26.4653	1:Experimental evidence at protein level	aging;anatomical structure development;biological regulation;cellular macromolecule metabolic process;cellular metabolic process;cellular process;cellular protein metabolic process;cellular response to chemical stimulus;cel
24.9129	25.4645	23.5827	25.5406	1:Experimental evidence at protein level	viral reproduction
26.7592	26.6851	26.6876	26.8001	1:Experimental evidence at protein level	biological regulation;cell differentiation;cellular developmental process;cellular process;cellular response to stimulus;developmental process;gamete generation;male gamete generation;multicellular organismal process;multic
26.1712	25.1096	26.4902	26.0926	1:Experimental evidence at protein level	biological regulation;cellular localization;cellular macromolecule localization;cellular process;cellular protein localization;localization;macromolecule localization;positive regulation of biological process;positive regulation of bio
28.0648	27.6835	27.7091	27.7091	1:Experimental evidence at protein level	B cell activation;B cell activation involved in immune response;cell activation;cell activation involved in immune response;cellular macromolecule metabolic process;cellular metabolic process;cellular nitrogen compound meta
28.1607	28.014	28.1366	27.922	1:Experimental evidence at protein level	actin filament-based movement;actin filament-based process;biological regulation;cell cycle process;cellular component movement;cellular component organization;cellular component organization at cellular level;cellular co
30.0606	30.0149	30.1563	30.2266	1:Experimental evidence at protein level	actin cytoskeleton organization;actin filament bundle assembly;actin filament organization;actin filament-based process;biological regulation;cell projection organization;cellular component assembly;cellular component asser
27.4114	27.3384	27.2411	27.1594	1:Experimental evidence at protein level	biosynthetic process;cellular biosynthetic process;cellular macromolecule biosynthetic process;cellular macromolecule metabolic process;cellular metabolic process;cellular process;cellular protein metabolic process;cotrans
27.0618	27.1425	26.9475	27.2582	1:Experimental evidence at protein level	biological regulation;cellular macromolecule metabolic process;cellular metabolic process;cellular process;cellular protein metabolic process;chaperone-mediated protein folding;macromolecule metabolic process;metabolic
26.1901	26.2144	26.0643	26.3172	1:Experimental evidence at protein level	activation of immune response;activation of innate immune response;biological regulation;cellular process;cellular response to stimulus;cytokine production;defense response;defense response to bacterium;defense respons
29.1364	29.1672	29.1707	29.0702	1:Experimental evidence at protein level	cell cycle;cell division;cellular component assembly;cellular component organization;cellular component organization or biogenesis;cellular process;macromolecular complex assembly;macromolecular complex subunit organ
27.931	27.8934	28.3849	28.1028	1:Experimental evidence at protein level	biological regulation;regulation of autophagic vacuole assembly;regulation of autophagy;regulation of biological process;regulation of catabolic process;regulation of cell communication;regulation of cellular catabolic process
27.1237	26.9837	27.0002	26.8584	1:Experimental evidence at protein level	amine catabolic process;amine metabolic process;carboxylic acid catabolic process;carboxylic acid metabolic process;catabolic process;cellular amine metabolic process;cellular amino acid catabolic process;cellular amino
24.3486	24.3619	24.3383	24.9784	1:Experimental evidence at protein level	anatomical structure development;anatomical structure formation involved in morphogenesis;biological regulation;cell surface receptor linked signaling pathway;cellular process;cellular response to stimulus;developmental pr
26.0876	25.3368	25.4953	25.7611	1:Experimental evidence at protein level	
26.6816	24.9246	26.0818	26.1269	1:Experimental evidence at protein level	apoptosis;cell death;cell differentiation;cellular developmental process;cellular localization;cellular macromolecule localization;cellular process;cellular protein localization;death;developmental process;ER to Golgi vesicle-med
26.848	26.6552	27.0119	26.37	1:Experimental evidence at protein level	alcohol metabolic process;biosynthetic process;lipid biosynthetic process;lipid metabolic process;metabolic process;primary metabolic process;small molecule metabolic process;steroid biosynthetic process;steroid metabo
28.9046	28.7035	29.2348	29.1216	1:Experimental evidence at protein level	biological regulation;cellular macromolecule metabolic process;cellular metabolic process;cellular process;cellular protein metabolic process;'de novo' posttranslational protein folding;'de novo' protein folding;macromolecule
29.3174	29.0418	28.9964	29.0971	1:Experimental evidence at protein level	apoptosis;biological regulation;biosynthetic process;cell death;cellular biosynthetic process;cellular macromolecule biosynthetic process;cellular macromolecule metabolic process;cellular metabolic process;cellular nitrogen
27.3226	26.998	27.2436	26.7486	1:Experimental evidence at protein level	biosynthetic process;cellular biosynthetic process;cellular macromolecule metabolic process;cellular metabolic process;cellular nitrogen compound metabolic process;cellular process;ER to Golgi vesicle-mediated transport;
26.1249	27.1186	26.2913	26.2341	1:Experimental evidence at protein level	amine biosynthetic process;amine metabolic process;amino acid salvage;aspartate family amino acid biosynthetic process;aspartate family amino acid metabolic process;biosynthetic process;carboxylic acid biosynthetic pro
24.9402	24.2815	24.423	24.5413	1:Experimental evidence at protein level	cellular metabolic process;cellular process;electron transport chain;generation of precursor metabolites and energy;metabolic process;multicellular organismal process;oxidation-reduction process;respiratory electron transpo
26.4452	26.303	25.6574	25.7078	1:Experimental evidence at protein level	anatomical structure development;axon ensheathment;biological regulation;biosynthetic process;cell development;cellular biosynthetic process;cellular developmental process;cellular macromolecule biosynthetic process;ce
26.7075	26.6885	26.3884	26.4022	1:Experimental evidence at protein level	ATP hydrolysis coupled proton transport;biological regulation;cation homeostasis;cation transport;cell surface receptor linked signaling pathway;cellular cation homeostasis;cellular chemical homeostasis;cellular component
26.6732	26.7234	26.2637	26.6629	1:Experimental evidence at protein level	
25.826	25.9259	26.2627	25.7908	1:Experimental evidence at protein level	establishment of localization;establishment of localization in cell;establishment of protein localization;intracellular protein transport;intracellular transport;nuclear import;nuclear transport;nucleocytoplasmic transport;protein in
26.9181	24.4093	27.4239	26.9553	1:Experimental evidence at protein level	cellular macromolecule metabolic process;cellular metabolic process;cellular process;cellular protein metabolic process;macromolecule metabolic process;macromolecule methylation;macromolecule modification;metabolic
26.6248	26.4111	26.4744	26.4737	1:Experimental evidence at protein level	establishment of localization;establishment of localization in cell;establishment of protein localization;establishment of RNA localization;intracellular protein transport;intracellular transport;mRNA transport;nuclear export;nucle
25.2608	24.0621	25.1164	25.039	1:Experimental evidence at protein level	autophagy;catabolic process;cellular catabolic process;cellular metabolic process;cellular process;endosome transport;establishment of localization;establishment of localization in cell;establishment of protein localization;int
26.9194	26.728	26.6405	26.5064	1:Experimental evidence at protein level	anatomical structure morphogenesis;biological regulation;biosynthetic process;cellular biosynthetic process;cellular component organization;cellular component organization at cellular level;cellular component organization o
27.8863	24.0232	28.5961	28.2746	1:Experimental evidence at protein level	anatomical structure formation involved in morphogenesis;angiogenesis;biological regulation;biosynthetic process;cell differentiation;cellular biosynthetic process;cellular chemical homeostasis;cellular component assembly;
26.1973	26.5094	26.4041	26.5768	1:Experimental evidence at protein level	ADP biosynthetic process;ADP metabolic process;AMP metabolic process;biological regulation;biosynthetic process;blood coagulation;cellular biosynthetic process;cellular metabolic process;cellular nitrogen compound bio
25.0125	24.5696	24.2981	24.4004	1:Experimental evidence at protein level	biological regulation;cellular component organization;cellular component organization or biogenesis;cellular membrane organization;cellular process;endocytosis;establishment of localization;establishment of localization in ce
24.675	24.2997	25.2953	24.8836	1:Experimental evidence at protein level	amine metabolic process;aspartate family amino acid metabolic process;carboxylic acid metabolic process;cellular amine metabolic process;cellular amino acid metabolic process;cellular ketone metabolic process;cellular m
27.1645	27.3884	27.3277	27.2933	1:Experimental evidence at protein level	alcohol catabolic process;alcohol metabolic process;amino sugar catabolic process;amino sugar metabolic process;carbohydrate catabolic process;carbohydrate metabolic process;carbohydrate phosphorylation;carboxylic
24.47	24.7027	24.6973	24.4859	1:Experimental evidence at protein level	carboxylic acid catabolic process;carboxylic acid metabolic process;catabolic process;cellular catabolic process;cellular component assembly;cellular component organization;cellular component organization or biogenesis;
26.6418	26.6134	26.4885	26.5588	1:Experimental evidence at protein level	cellular macromolecule metabolic process;cellular metabolic process;cellular nitrogen compound metabolic process;cellular process;macromolecule metabolic process;macromolecule methylation;macromolecule modificatio
26.1532	25.628	26.0891	25.8073	1:Experimental evidence at protein level	acidic amino acid transport;alcohol biosynthetic process;alcohol metabolic process;amine transport;amino acid transport;anion transport;aspartate transport;ATP biosynthetic process;ATP metabolic process;biosynthetic pro
29.2079	29.2143	29.4526	29.3612	1:Experimental evidence at protein level	activation of JUN kinase activity;activation of MAPK activity;anatomical structure morphogenesis;apoptosis;biological regulation;cell death;cell part morphogenesis;cell projection morphogenesis;cell projection organization;
27.4039	27.0103	27.1126	27.388	1:Experimental evidence at protein level	antigen processing and presentation;antigen processing and presentation of exogenous antigen;antigen processing and presentation of exogenous peptide antigen;antigen processing and presentation of exogenous peptide
26.4447	22.9184	25.9111	26.6194	1:Experimental evidence at protein level	anaphase-promoting complex-dependent proteasomal ubiquitin-dependent protein catabolic process;biological regulation;catabolic process;cell cycle;cell cycle checkpoint;cell cycle phase;cell cycle process;cell division;cel
26.878	26.4081	26.367	26.4144	1:Experimental evidence at protein level	anaphase-promoting complex-dependent proteasomal ubiquitin-dependent protein catabolic process;biological regulation;catabolic process;cell cycle;cell cycle checkpoint;cell cycle phase;cell cycle process;cell division;cel
27.8417	27.6697	28.0177	27.7308	1:Experimental evidence at protein level	activation of immune response;alpha-beta T cell activation;antigen receptor-mediated signaling pathway;ATP biosynthetic process;ATP metabolic process;ATP synthesis coupled proton transport;biological regulation;biosynth
28.0326	27.7592	27.9683	27.6898	1:Experimental evidence at protein level	biological regulation;catabolic process;cellular catabolic process;cellular component assembly;cellular component assembly at cellular level;cellular component organization;cellular component organization at cellular level;ce
27.4877	26.9113	27.6948	27.6183	1:Experimental evidence at protein level	catabolic process;cellular catabolic process;cellular macromolecule catabolic process;cellular macromolecule metabolic process;cellular metabolic process;cellular nitrogen compound metabolic process;cellular process;exo
25.5045	25.5969	25.5463	25.5476	1:Experimental evidence at protein level	cellular macromolecule metabolic process;cellular metabolic process;cellular nitrogen compound metabolic process;cellular process;macromolecule metabolic process;metabolic process;mRNA metabolic process;mRNA pro
26.351	28.609	28.3349	28.9684	1:Experimental evidence at protein level	developmental process
28.5304	28.3418	28.108	28.12	1:Experimental evidence at protein level	cellular component biogenesis;cellular component biogenesis at cellular level;cellular component organization or biogenesis;cellular component organization or biogenesis at cellular level;cellular process;ribonucleoprotein co
26.2978	26.158	26.3563	25.8466	1:Experimental evidence at protein level	biosynthetic process;cellular biosynthetic process;cellular macromolecule biosynthetic process;cellular macromolecule metabolic process;cellular metabolic process;cellular nitrogen compound metabolic process;cellular pro
26.8097	26.7802	26.8962	26.8021	1:Experimental evidence at protein level	apoptosis;biological regulation;cell cycle;cell death;cell proliferation;cell surface receptor linked signaling pathway;cellular process;cellular response to chemical stimulus;cellular response to endogenous stimulus;cellular resp
26.3479	25.9629	26.337	26.076	1:Experimental evidence at protein level	cell death;cellular macromolecule metabolic process;cellular metabolic process;cellular nitrogen compound metabolic process;cellular process;cellular protein metabolic process;cellular response to stimulus;cellular response
27.9032	28.6566	28.9493	29.0483	1:Experimental evidence at protein level	alcohol catabolic process;alcohol metabolic process;carbohydrate catabolic process;carbohydrate metabolic process;catabolic process;cellular carbohydrate catabolic process;cellular carbohydrate metabolic process;cellula
28.6534	28.1774	28.1806	28.2786	1:Experimental evidence at protein level	cellular macromolecule metabolic process;cellular metabolic process;cellular nitrogen compound metabolic process;cellular process;macromolecule metabolic process;metabolic process;mRNA metabolic process;mRNA pro
25.84	23.7584	25.4592	25.2864	1:Experimental evidence at protein level	5S class rRNA transcription from RNA polymerase III type 1 promoter;biological regulation;biosynthetic process;cellular biosynthetic process;cellular component organization;cellular component organization at cellular level;c
26.0996	25.7418	26.2731	26.6449	1:Experimental evidence at protein level	biological regulation;cellular component organization;cellular component organization at cellular level;cellular component organization or biogenesis;cellular component organization or biogenesis at cellular level;cellular meml
27.4766	27.0599	27.3745	27.2836	1:Experimental evidence at protein level	apoptosis;apoptotic chromosome condensation;biological regulation;cell death;cell differentiation;cellular component disassembly;cellular component disassembly at cellular level;cellular component disassembly involved in
26.703	26.1683	26.7171	26.4987	1:Experimental evidence at protein level	biological regulation;biosynthetic process;carbohydrate metabolic process;carbohydrate transport;cell cycle;cell cycle process;cell surface receptor linked signaling pathway;cellular biosynthetic process;cellular component c
29.1102	29.7273	29.4686	29.6628	1:Experimental evidence at protein level	cell proliferation
27.5957	27.975	27.7395	27.8079	1:Experimental evidence at protein level	biological regulation;cellular process;cellular response to stimulus;establishment of localization;establishment of localization in cell;establishment of protein localization;intracellular protein transport;intracellular signal transdu
25.4386	24.7961	25.0511	25.4146	1:Experimental evidence at protein level	biological regulation;cellular component organization;cellular component organization at cellular level;cellular component organization or biogenesis;cellular component organization or biogenesis at cellular level;cellular meml
30.8615	30.6552	30.782	30.8102	1:Experimental evidence at protein level	activation of immune response;activation of innate immune response;anaphase-promoting complex-dependent proteasomal ubiquitin-dependent protein catabolic process;antigen processing and presentation;antigen proces
26.3621	24.1324	27.2644	24.0714	1:Experimental evidence at protein level	cellular metabolic process;cellular process;metabolic process;peptide metabolic process
27.6061	26.9891	26.8395	26.4148	1:Experimental evidence at protein level	anatomical structure formation involved in morphogenesis;anatomical structure morphogenesis;autophagic vacuole assembly;biological regulation;cell projection assembly;cell projection organization;cellular component asse
26.8984	27.3231	27.3822	27.4196	1:Experimental evidence at protein level	biological regulation;biosynthetic process;cap-independent translational initiation;cell cycle;cellular biosynthetic process;cellular component assembly;cellular component assembly at cellular level;cellular component disasse
25.4633	25.6269	25.6446	25.3763	1:Experimental evidence at protein level	anatomical structure morphogenesis;cell part morphogenesis;cell projection morphogenesis;cell projection organization;cellular component morphogenesis;cellular component organization;cellular component organization at
25.0743	24.3586	25.0144	25.0384	1:Experimental evidence at protein level	anatomical structure development;anatomical structure morphogenesis;biological regulation;blood vessel morphogenesis;cardiac atrium morphogenesis;cardiac chamber morphogenesis;cardiac muscle tissue growth;cardiac
25.7242	23.3027	25.2986	25.1523	1:Experimental evidence at protein level	cellular macromolecule metabolic process;cellular metabolic process;cellular nitrogen compound metabolic process;cellular process;macromolecule metabolic process;metabolic process;mRNA metabolic process;mRNA pro
26.2673	25.9345	25.8442	25.9399	1:Experimental evidence at protein level	anatomical structure formation involved in morphogenesis;cellular macromolecule metabolic process;cellular metabolic process;cellular process;cellular protein metabolic process;developmental process;macromolecule meta
30.4952	30.1984	30.0439	30.1449	1:Experimental evidence at protein level	actin cytoskeleton organization;actin filament-based process;ameboidal cell migration;biological regulation;cell migration;cell motility;cellular component movement;cellular component organization;cellular component organiz
25.9932	25.5264	26.1376	25.7698	1:Experimental evidence at protein level	activation of protein kinase activity;apoptosis;biological regulation;cell cycle phase;cell cycle process;cell death;cell division;cell proliferation;cellular component organization;cellular component organization at cellular level;ce
26.5245	26.6051	26.5621	26.7735	1:Experimental evidence at protein level	multicellular organismal process;neurological system process;sensory perception;sensory perception of light stimulus;system process;visual perception
25.8633	26.1785	26.1166	26.0841	1:Experimental evidence at protein level	
26.629	24.8564	26.6962	26.6287	1:Experimental evidence at protein level	biological regulation;biosynthetic process;blood coagulation;cell differentiation;cellular biosynthetic process;cellular developmental process;cellular macromolecule biosynthetic process;cellular macromolecule metabolic proc
27.5379	27.6221	27.3742	27.516	1:Experimental evidence at protein level	
25.9867	25.5648	25.8236	25.6631	1:Experimental evidence at protein level	actin filament-based movement;actin filament-based process;anatomical structure development;anatomical structure morphogenesis;auditory receptor cell differentiation;behavior;biological regulation;cell communication;cell
28.1231	28.1415	28.0729	27.9275	1:Experimental evidence at protein level	cellular macromolecule metabolic process;cellular metabolic process;cellular nitrogen compound metabolic process;cellular process;establishment of localization;establishment of localization in cell;establishment of protein lo
28.8051	28.5344	28.5577	28.6101	1:Experimental evidence at protein level	assembly of spliceosomal tri-snRNP;biological regulation;biosynthetic process;cell proliferation;cellular component assembly;cellular component assembly at cellular level;cellular component organization;cellular component
26.4133	26.7302	26.9117	26.8815	1:Experimental evidence at protein level	biological regulation;cellular component organization;cellular component organization at cellular level;cellular component organization or biogenesis;cellular component organization or biogenesis at cellular level;cellular meml
28.7076	28.6056	29.0421	28.8661	1:Experimental evidence at protein level	biological regulation;cellular process;cellular response to stimulus;cytoplasmic sequestering of NF-kappaB;cytoplasmic sequestering of protein;cytoplasmic sequestering of transcription factor;establishment of localization;es
26.7696	27.2355	26.8307	26.8193	1:Experimental evidence at protein level	biological regulation;catabolic process;cell surface receptor linked signaling pathway;cellular catabolic process;cellular macromolecule catabolic process;cellular macromolecule metabolic process;cellular metabolic process;
27.8186	27.6412	27.7406	27.7143	1:Experimental evidence at protein level	actin cytoskeleton organization;actin filament-based process;anatomical structure morphogenesis;biological regulation;caveola assembly;caveolin-mediated endocytosis;cell part morphogenesis;cell projection morphogenesi
27.9846	27.8471	27.6645	27.5751	1:Experimental evidence at protein level	
28.0894	28.045	28.1162	27.9241	1:Experimental evidence at protein level	biological regulation;cellular component organization;cellular component organization at cellular level;cellular component organization or biogenesis;cellular component organization or biogenesis at cellular level;cellular meml
25.6155	25.9675	22.5468	25.5316	1:Experimental evidence at protein level	biosynthetic process;cellular biosynthetic process;cellular macromolecule biosynthetic process;cellular macromolecule metabolic process;cellular metabolic process;cellular process;cellular protein metabolic process;cotrans
28.8621	28.6194	28.542	28.5071	1:Experimental evidence at protein level	activation of immune response;activation of innate immune response;anaphase-promoting complex-dependent proteasomal ubiquitin-dependent protein catabolic process;antigen processing and presentation;antigen proces
27.4843	27.0138	27.3046	27.0637	1:Experimental evidence at protein level	apoptosis;biological regulation;catabolic process;cell death;cellular catabolic process;cellular macromolecule catabolic process;cellular macromolecule metabolic process;cellular metabolic process;cellular process;cytoplasn
24.8359	25.0978	25.6734	24.4125	1:Experimental evidence at protein level	antigen processing and presentation;biological regulation;blood coagulation;cell migration;cell motility;cellular component movement;cellular process;coagulation;hemostasis;immune response;immune system process;leuko

26.5072	25.6437	25.5323	25.8581	1:Experimental evidence at protein level	
27.96	27.8719	27.8832	27.8449	1:Experimental evidence at protein level	biological regulation;catabolic process;cellular catabolic process;cellular macromolecule catabolic process;cellular macromolecule metabolic process;cellular metabolic process;cellular process;cellular protein metabolic proc
26.0482	25.1639	25.2846	24.9932	1:Experimental evidence at protein level	cellular macromolecule metabolic process;cellular metabolic process;cellular nitrogen compound metabolic process;cellular process;macromolecule metabolic process;maturation of SSU-rRNA;maturation of SSU-rRNA from
28.3412	28.2289	28.6536	28.2691	1:Experimental evidence at protein level	autophagic vacuole assembly;biological regulation;catabolic process;cellular catabolic process;cellular component assembly;cellular component assembly at cellular level;cellular component organization;cellular component
26.8137	24.2925	26.7843	27.2407	1:Experimental evidence at protein level	
25.9165	26.3152	25.7847	26.0487	1:Experimental evidence at protein level	anion transport;biological regulation;cation transport;cell volume homeostasis;cellular homeostasis;cellular process;chloride transport;establishment of localization;homeostatic process;inorganic anion transport;ion transport;
25.0772	23.3369	24.892	24.7696	1:Experimental evidence at protein level	cellular component organization;cellular component organization or biogenesis;cellular process;establishment of localization;establishment of localization in cell;establishment of protein localization;Golgi vesicle transport;intra
27.0041	27.0348	27.06	27.0808	1:Experimental evidence at protein level	anatomical structure formation involved in morphogenesis;biological regulation;cell cycle arrest;cell cycle process;cell surface receptor linked signaling pathway;cellular process;cellular response to stimulus;developmental pr
26.6683	27.1987	24.8866	26.6827	1:Experimental evidence at protein level	biological regulation;cell cycle;cell cycle process;cell differentiation;cellular component movement;cellular component organization;cellular component organization at cellular level;cellular component organization or biogenes
26.8397	26.8718	26.7038	26.5949	1:Experimental evidence at protein level	biological regulation;regulation of biological process;regulation of catalytic activity;regulation of cellular metabolic process;regulation of cellular process;regulation of dephosphorylation;regulation of hydrolase activity;regulatio
24.9491	25.9745	25.6055	22.9963	1:Experimental evidence at protein level	biological regulation;biosynthetic process;cell surface receptor linked signaling pathway;cellular biosynthetic process;cellular macromolecule biosynthetic process;cellular macromolecule metabolic process;cellular metabolic
25.541	25.891	25.6437	25.5689	1:Experimental evidence at protein level	biological regulation;cell surface receptor linked signaling pathway;cellular process;cellular response to chemical stimulus;cellular response to fibroblast growth factor stimulus;cellular response to growth factor stimulus;cellul
25.6926	25.1438	25.5458	25.7309	1:Experimental evidence at protein level	cellular component organization;cellular component organization at cellular level;cellular component organization or biogenesis;cellular component organization or biogenesis at cellular level;cellular macromolecule metabolic
25.1947	25.5412	25.3337	25.2001	1:Experimental evidence at protein level	biological regulation;catabolic process;cellular catabolic process;cellular macromolecule catabolic process;cellular macromolecule metabolic process;cellular metabolic process;cellular process;cellular protein metabolic proc
25.0219	26.3332	25.3192	25.0422	1:Experimental evidence at protein level	anatomical structure morphogenesis;biological regulation;biosynthetic process;cell differentiation;cellular biosynthetic process;cellular developmental process;cellular macromolecule biosynthetic process;cellular macromolec
23.998	24.8906	25.0038	25.5493	1:Experimental evidence at protein level	amine transport;amino acid transport;anatomical structure development;biological adhesion;biological regulation;blood coagulation;brain development;carboxylic acid transport;cell adhesion;cell differentiation;cell migration;
28.4449	28.2634	28.326	28.247	1:Experimental evidence at protein level	cellular macromolecule metabolic process;cellular metabolic process;cellular nitrogen compound metabolic process;cellular process;macromolecule metabolic process;metabolic process;mRNA metabolic process;mRNA pro
30.2099	30.082	30.437	30.1947	1:Experimental evidence at protein level	biological regulation;biosynthetic process;cell cycle arrest;cell cycle process;cell proliferation;cellular biosynthetic process;cellular macromolecule biosynthetic process;cellular macromolecule metabolic process;cellular meta
27.6783	27.3589	27.4364	27.3678	1:Experimental evidence at protein level	biological regulation;cell cycle;cell cycle phase;cell cycle process;cell division;cellular component organization;cellular component organization at cellular level;cellular component organization or biogenesis;cellular componer
26.9027	26.9705	27.4332	27.1185	1:Experimental evidence at protein level	anatomical structure homeostasis;biological regulation;cell cycle process;cellular component organization;cellular component organization at cellular level;cellular component organization or biogenesis;cellular component org
25.7108	25.6992	25.5513	25.0523	1:Experimental evidence at protein level	biosynthetic process;cation transport;cellular biosynthetic process;cellular lipid metabolic process;cellular macromolecule metabolic process;cellular metabolic process;cellular potassium ion transport;cellular process;cellula
25.8336	24.5958	25.7555	25.6574	1:Experimental evidence at protein level	alcohol biosynthetic process;alcohol metabolic process;amino sugar biosynthetic process;amino sugar metabolic process;biological adhesion;biosynthetic process;carbohydrate biosynthetic process;carbohydrate metabolic
28.6379	28.6439	28.721	28.2357	1:Experimental evidence at protein level	biological regulation;biosynthetic process;cellular biosynthetic process;cellular macromolecule biosynthetic process;cellular macromolecule metabolic process;cellular metabolic process;cellular nitrogen compound metabolic
28.131	28.1188	28.199	27.9969	1:Experimental evidence at protein level	biological regulation;biosynthetic process;cellular biosynthetic process;cellular component organization;cellular component organization at cellular level;cellular component organization or biogenesis;cellular component orga
27.4484	27.7884	27.7439	27.4776	1:Experimental evidence at protein level	cellular macromolecule metabolic process;cellular metabolic process;cellular nitrogen compound metabolic process;cellular process;macromolecule metabolic process;metabolic process;ncRNA metabolic process;ncRNA pr
28.428	28.2258	28.2864	28.0746	1:Experimental evidence at protein level	biological regulation;cellular component assembly;cellular component assembly at cellular level;cellular component organization;cellular component organization at cellular level;cellular component organization or biogenesis;
26.4159	26.5842	26.1653	26.1754	1:Experimental evidence at protein level	biological regulation;cellular lipid metabolic process;cellular metabolic process;cellular process;cellular response to stimulus;defense response;inflammatory response;lipid metabolic process;metabolic process;organophosph
28.9039	28.8653	28.9068	28.7925	1:Experimental evidence at protein level	ATP-dependent chromatin remodeling;biological regulation;biosynthetic process;cell cycle phase;cell cycle process;cell division;cellular biosynthetic process;cellular component assembly;cellular component assembly at cell
29.3583	29.2536	29.4255	29.4021	1:Experimental evidence at protein level	biological regulation;cell cycle;cell cycle phase;cell cycle process;cell division;cell proliferation;cellular component organization;cellular component organization at cellular level;cellular component organization or biogenesis;c
28.3898	27.5522	28.1053	27.8614	1:Experimental evidence at protein level	adenine transport;cellular process;establishment of localization;nitrogen compound transport;nucleobase transport;nucleobase-containing compound transport;purine base transport;purine-containing compound transmemb
28.3113	28.3811	28.7245	28.5958	1:Experimental evidence at protein level	actin cytoskeleton organization;actin filament depolymerization;actin filament organization;actin filament-based process;actin polymerization or depolymerization;actomyosin structure organization;anatomical structure homeo
26.7961	26.3687	26.6373	26.286	1:Experimental evidence at protein level	cellular process;establishment of localization;transport;vesicle-mediated transport
27.8173	27.5027	27.5808	27.4302	1:Experimental evidence at protein level	amine metabolic process;amino acid activation;carboxylic acid metabolic process;cellular amine metabolic process;cellular amino acid metabolic process;cellular ketone metabolic process;cellular macromolecule metabolic p
27.8058	27.7116	27.4029	27.6309	1:Experimental evidence at protein level	biosynthetic process;cellular biosynthetic process;cellular macromolecule biosynthetic process;cellular macromolecule metabolic process;cellular metabolic process;cellular nitrogen compound metabolic process;cellular pro
27.7327	27.4566	27.6495	27.3483	1:Experimental evidence at protein level	actin cytoskeleton organization;actin filament-based process;ameboidal cell migration;anatomical structure development;anatomical structure formation involved in morphogenesis;anatomical structure morphogenesis;anteric
27.3711	27.8728	28.1775	28.3539	1:Experimental evidence at protein level	biological regulation;negative regulation of biological process;negative regulation of gene expression;negative regulation of macromolecule metabolic process;negative regulation of metabolic process;positive regulation of bio
28.3388	28.5764	28.5373	28.7915	1:Experimental evidence at protein level	
25.0691	25.6087	25.8151	25.552	1:Experimental evidence at protein level	biological regulation;cell surface receptor linked signaling pathway;cellular process;cellular response to stimulus;hippo signaling cascade;intracellular signal transduction;regulation of biological process;regulation of cellular p
27.0637	26.7923	26.9532	26.7663	1:Experimental evidence at protein level	biological regulation;catabolic process;cellular catabolic process;cellular macromolecule catabolic process;cellular macromolecule metabolic process;cellular metabolic process;cellular nitrogen compound metabolic process
28.1379	28.0938	27.9108	28.1547	1:Experimental evidence at protein level	amine metabolic process;carboxylic acid metabolic process;cell differentiation;cellular amine metabolic process;cellular amino acid metabolic process;cellular developmental process;cellular ketone metabolic process;cellula
25.9925	26.2745	26.396	26.2863	1:Experimental evidence at protein level	biosynthetic process;cellular biosynthetic process;cellular component disassembly;cellular component disassembly at cellular level;cellular component organization;cellular component organization at cellular level;cellular com
27.4118	27.6638	27.3468	27.625	1:Experimental evidence at protein level	biological regulation;cellular component assembly;cellular component assembly at cellular level;cellular component biogenesis;cellular component organization;cellular component organization at cellular level;cellular compor
25.9014	26.3032	25.2044	26.4182	1:Experimental evidence at protein level	cellular macromolecule metabolic process;cellular metabolic process;cellular nitrogen compound metabolic process;cellular process;macromolecule metabolic process;metabolic process;ncRNA metabolic process;ncRNA pr
27.3113	27.4007	27.9655	27.6783	1:Experimental evidence at protein level	
26.6045	26.595	26.2926	26.5656	1:Experimental evidence at protein level	anatomical structure morphogenesis;cell part morphogenesis;cellular component morphogenesis;cellular component organization;cellular component organization at cellular level;cellular component organization or biogenesi
26.6875	26.3927	26.7599	26.4511	1:Experimental evidence at protein level	anterograde axon cargo transport;anterograde synaptic vesicle transport;axon cargo transport;cellular component movement;cellular process;cytoskeleton-dependent intracellular transport;establishment of localization;estab
24.8959	24.3251	24.8262	25.4198	1:Experimental evidence at protein level	biological regulation;blood vessel endothelial cell migration;blood vessel endothelial cell migration involved in intussusceptive angiogenesis;cell differentiation;cell migration;cell motility;cellular component movement;cellular c
28.0127	29.0774	29.0709	29.1921	1:Experimental evidence at protein level	biological regulation;cellular macromolecule metabolic process;cellular metabolic process;cellular nitrogen compound metabolic process;cellular process;cellular response to stimulus;intracellular signal transduction;macromo
28.4149	28.5237	28.3605	28.1864	1:Experimental evidence at protein level	androgen receptor signaling pathway;biological regulation;biosynthetic process;catabolic process;cellular biosynthetic process;cellular catabolic process;cellular macromolecule catabolic process;cellular macromolecule meta
29.8886	29.7468	29.8492	29.5444	1:Experimental evidence at protein level	cellular macromolecule metabolic process;cellular metabolic process;cellular nitrogen compound metabolic process;cellular process;macromolecule metabolic process;metabolic process;mRNA metabolic process;mRNA pro
27.6302	26.682	27.1422	27.0203	1:Experimental evidence at protein level	cell growth;cellular macromolecule metabolic process;cellular metabolic process;cellular nitrogen compound metabolic process;cellular process;establishment of localization;establishment of localization in cell;establishment
28.3904	28.3917	28.4637	28.5037	1:Experimental evidence at protein level	biological regulation;cell cycle phase;cell cycle process;cellular component organization;cellular component organization at cellular level;cellular component organization or biogenesis;cellular component organization or bioge
25.3235	25.0596	23.4381	24.3758	1:Experimental evidence at protein level	adenosine metabolic process;cellular component organization;cellular component organization at cellular level;cellular component organization or biogenesis;cellular component organization or biogenesis at cellular level;cellu
27.1105	27.3149	27.1511	26.8841	1:Experimental evidence at protein level	acyl-CoA metabolic process;cellular metabolic process;cellular process;coenzyme metabolic process;cofactor metabolic process;metabolic process;thioester metabolic process
27.3432	26.9404	27.1585	27.2791	1:Experimental evidence at protein level	biological regulation;metabolic process;negative regulation of catalytic activity;negative regulation of molecular function;negative regulation of monooxygenase activity;negative regulation of nitric-oxide synthase activity;nega
26.7099	27.2501	27.1234	26.9513	1:Experimental evidence at protein level	biological regulation;cell redox homeostasis;cellular homeostasis;cellular process;homeostatic process;regulation of biological process;regulation of biological quality;regulation of cellular process
26.9261	26.9792	27.1208	26.8946	1:Experimental evidence at protein level	catabolic process;cellular catabolic process;cellular macromolecule catabolic process;cellular macromolecule metabolic process;cellular metabolic process;cellular nitrogen compound metabolic process;cellular process;exo
27.76	27.7961	28.0135	27.7991	1:Experimental evidence at protein level	apoptosis;autophagic cell death;biological regulation;catabolic process;cell communication;cell death;cellular catabolic process;cellular component assembly;cellular component organization;cellular component organization
27.0528	26.9225	27.0231	26.7653	1:Experimental evidence at protein level	activation of protein kinase activity;biological regulation;cell cycle arrest;cell cycle process;cell surface receptor linked signaling pathway;cellular component assembly;cellular component organization;cellular component orga
28.2333	27.9474	28.1644	28.0966	1:Experimental evidence at protein level	cellular component assembly;cellular component assembly at cellular level;cellular component organization;cellular component organization at cellular level;cellular component organization or biogenesis;cellular component c
27.2688	27.3998	27.1323	27.1145	1:Experimental evidence at protein level	biological regulation;biosynthetic process;cellular biosynthetic process;cellular macromolecule biosynthetic process;cellular macromolecule metabolic process;cellular metabolic process;cellular process;cellular protein metab
27.9053	28.0402	28.0709	27.7969	1:Experimental evidence at protein level	anatomical structure development;biological regulation;biomineral tissue development;bone marrow development;bone mineralization;cell chemotaxis;cell cycle process;cell migration;cell motility;cell proliferation;cellular com
26.1221	26.0674	25.9708	26.1199	1:Experimental evidence at protein level	catabolic process;cellular catabolic process;cellular macromolecule catabolic process;cellular macromolecule metabolic process;cellular metabolic process;cellular nitrogen compound metabolic process;cellular process;exo
27.4025	26.7476	24.6702	26.8001	1:Experimental evidence at protein level	establishment of localization;establishment of protein localization;protein transport;transport
28.4534	28.4495	28.1701	28.2747	1:Experimental evidence at protein level	cellular macromolecule metabolic process;cellular metabolic process;cellular nitrogen compound metabolic process;cellular process;gene expression;macromolecule metabolic process;metabolic process;mRNA metabolic p
26.4472	23.3098	26.5806	26.6841	1:Experimental evidence at protein level	biosynthetic process;cellular biosynthetic process;cellular component disassembly;cellular component disassembly at cellular level;cellular component organization;cellular component organization at cellular level;cellular com
27.0791	28.1954	28.4015	28.4187	1:Experimental evidence at protein level	cellular metabolic process;cellular nitrogen compound metabolic process;cellular process;metabolic process;nitrogen compound metabolic process;nucleic acid metabolic process;nucleic acid phosphodiester bond hydrolys
26.3052	26.0106	25.8994	25.9471	1:Experimental evidence at protein level	
26.601	25.5131	26.5476	26.4514	1:Experimental evidence at protein level	cellular component biogenesis;cellular component biogenesis at cellular level;cellular component organization or biogenesis;cellular component organization or biogenesis at cellular level;cellular process;ribonucleoprotein co
28.5672	28.4276	28.2513	28.2556	1:Experimental evidence at protein level	cellular macromolecule metabolic process;cellular metabolic process;cellular nitrogen compound metabolic process;cellular process;cellular protein metabolic process;macromolecule metabolic process;macromolecule modi
26.7833	26.9456	26.5749	26.8615	1:Experimental evidence at protein level	cellular macromolecule metabolic process;cellular metabolic process;cellular process;cellular protein metabolic process;cellular response to stimulus;cellular response to stress;macromolecule metabolic process;macromolec
25.4337	25.2061	25.522	25.2208	1:Experimental evidence at protein level	cellular metabolic process;cellular nitrogen compound metabolic process;cellular process;metabolic process;nitrogen compound metabolic process;nucleobase-containing compound metabolic process;nucleobase-containi
26.0913	25.8581	23.441	25.9265	1:Experimental evidence at protein level	biosynthetic process;cellular biosynthetic process;cellular component disassembly;cellular component disassembly at cellular level;cellular component organization;cellular component organization at cellular level;cellular com
26.5573	26.7542	26.818	26.4535	1:Experimental evidence at protein level	apoptosis;biological regulation;cell death;cellular process;death;metabolic process;negative regulation of anoikis;negative regulation of apoptosis;negative regulation of biological process;negative regulation of cell death;neg
26.8579	26.1311	26.2862	23.7145	1:Experimental evidence at protein level	apoptosis;biological regulation;cell cycle;cell death;cellular component assembly;cellular component assembly at cellular level;cellular component organization;cellular component organization at cellular level;cellular compon
28.4631	28.3895	28.7567	28.6718	1:Experimental evidence at protein level	anatomical structure homeostasis;biological regulation;cell surface receptor linked signaling pathway;cellular component assembly;cellular component assembly at cellular level;cellular component organization;cellular compo
28.7142	28.8238	28.7063	28.9103	1:Experimental evidence at protein level	anatomical structure development;cellular macromolecule metabolic process;cellular metabolic process;cellular nitrogen compound metabolic process;cellular process;chordate embryonic development;developmental proce
25.6553	25.5718	25.1972	25.3969	1:Experimental evidence at protein level	biological regulation;cell cycle;cellular process;positive regulation of catalytic activity;positive regulation of GTPase activity;positive regulation of hydrolase activity;positive regulation of molecular function;regulation of biologic
25.0011	24.5438	24.9919	24.6621	1:Experimental evidence at protein level	biological regulation;biosynthetic process;cellular biosynthetic process;cellular macromolecule biosynthetic process;cellular macromolecule metabolic process;cellular metabolic process;cellular nitrogen compound metabolic
25.711	25.3872	25.5033	25.7333	1:Experimental evidence at protein level	apoptosis;biological regulation;biosynthetic process;cell death;cellular biosynthetic process;cellular component assembly;cellular component assembly at cellular level;cellular component organization;cellular component orga
30.3195	30.1117	30.0686	30.1923	1:Experimental evidence at protein level	biosynthetic process;catabolic process;cellular biosynthetic process;cellular catabolic process;cellular component disassembly;cellular component disassembly at cellular level;cellular component organization;cellular compo
28.3969	27.9884	29.1306	27.9471	1:Experimental evidence at protein level	biological regulation;biosynthetic process;cellular biosynthetic process;cellular macromolecule biosynthetic process;cellular macromolecule metabolic process;cellular metabolic process;cellular nitrogen compound metabolic
26.6947	26.7307	26.2538	26.8796	1:Experimental evidence at protein level	2'-deoxyribonucleotide metabolic process;biological regulation;catabolic process;cell surface receptor linked signaling pathway;cellular catabolic process;cellular component assembly;cellular component organization;cellula
27.0154	26.9153	27.0736	26.8001	1:Experimental evidence at protein level	biological regulation;biosynthetic process;cellular biosynthetic process;cellular macromolecule biosynthetic process;cellular macromolecule metabolic process;cellular metabolic process;cellular process;cellular protein metab
30.4753	30.419	30.3715	30.3304	1:Experimental evidence at protein level	actin cytoskeleton organization;actin filament-based process;axon guidance;biological adhesion;biological regulation;blood coagulation;cell activation;cell adhesion;cell junction assembly;cell junction organization;cell-cell ad
24.2462	25.871	24.0564	23.6763	1:Experimental evidence at protein level	biological regulation;biosynthetic process;cell cycle checkpoint;cellular biosynthetic process;cellular component organization;cellular component organization at cellular level;cellular component organization or biogenesis;cell
26.3774	26.4486	26.7447	26.3328	1:Experimental evidence at protein level	biological regulation;BMP signaling pathway;catabolic process;cell surface receptor linked signaling pathway;cellular catabolic process;cellular component organization;cellular component organization at cellular level;cellular
27.0018	26.9907	23.9691	26.7975	1:Experimental evidence at protein level	anatomical structure morphogenesis;biological regulation;cell part morphogenesis;cell projection morphogenesis;cell projection organization;cellular component assembly;cellular component assembly at cellular level;cellular
26.092	25.8161	26.0055	25.6739	1:Experimental evidence at protein level	biological adhesion;biological regulation;cell adhesion;cell junction assembly;cell junction organization;cell-cell junction assembly;cell-cell junction organization;cellular component assembly;cellular component assembly at ce
25.7859	26.3122	25.9103	25.9768	1:Experimental evidence at protein level	alcohol metabolic process;anatomical structure development;biological regulation;brain development;carbohydrate homeostasis;carbohydrate metabolic process;cell proliferation;cell surface receptor linked signaling pathwa
27.6081	27.6099	26.0367	27.1416	1:Experimental evidence at protein level	actin filament-based movement;actin filament-based process;anatomical structure morphogenesis;axon ensheathment;biological regulation;biosynthetic process;carboxylic acid biosynthetic process;carboxylic acid metaboli
27.8437	27.9245	27.924	27.8526	1:Experimental evidence at protein level	aging;anatomical structure formation involved in morphogenesis;anatomical structure morphogenesis;angiogenesis;biological adhesion;biological regulation;biosynthetic process;cell adhesion;cell migration;cell morphogenes
26.7007	27.1098	26.8008	27.0208	1:Experimental evidence at protein level	activation of JUN kinase activity;activation of MAPK activity;activation of MAPKKK activity;activation of protein kinase activity;biological regulation;cellular macromolecule metabolic process;cellular metabolic process;cellular
28.64	28.7736	28.7717	28.7214	1:Experimental evidence at protein level	biological regulation;cellular component organization;cellular component organization or biogenesis;cellular macromolecule metabolic process;cellular membrane organization;cellular metabolic process;cellular process;cellul
27.0889	26.8297	27.0742	26.867	1:Experimental evidence at protein level	biological regulation;cell communication;cellular process;cellular response to chemical stimulus;cellular response to external stimulus;cellular response to extracellular stimulus;cellular response to glucose starvation;cellular r
25.8444	24.0285	24.9681	25.7297	1:Experimental evidence at protein level	biological regulation;regulation of biological process;regulation of cellular process;regulation of response to stimulus;regulation of signal transduction;regulation of signaling;regulation of TOR signaling cascade
26.0709	26.0452	26.0813	24.4969	1:Experimental evidence at protein level	cellular macromolecule metabolic process;cellular metabolic process;cellular nitrogen compound metabolic process;cellular process;macromolecule metabolic process;metabolic process;ncRNA metabolic process;ncRNA pr
26.5159	27.2328	26.9934	26.8263	1:Experimental evidence at protein level	anatomical structure development;anatomical structure morphogenesis;axon ensheathment;axonogenesis;biological regulation;cell development;cell part morphogenesis;cell projection morphogenesis;cell projection organiza
27.2781	27.5417	27.5853	27.6077	1:Experimental evidence at protein level	biological regulation;catabolic process;cellular catabolic process;cellular macromolecule catabolic process;cellular macromolecule metabolic process;cellular metabolic process;cellular nitrogen compound metabolic process
26.7087	27.3298	27.4077	27.5262	1:Experimental evidence at protein level	cell differentiation;cellular developmental process;cellular macromolecule metabolic process;cellular metabolic process;cellular process;cellular protein metabolic process;developmental process;gamete generation;macromo
27.2125	27.1125	27.1057	26.9805	1:Experimental evidence at protein level	cellular component organization;cellular component organization at cellular level;cellular component organization or biogenesis;cellular component organization or biogenesis at cellular level;cellular macromolecule metabolic
27.3107	27.2646	27.4148	27.3172	1:Experimental evidence at protein level	cell differentiation;cellular developmental process;cellular process;developmental process;hemopoietic progenitor cell differentiation
27.5758	27.6955	27.5278	27.4337	1:Experimental evidence at protein level	biological regulation;cellular macromolecule metabolic process;cellular metabolic process;cellular process;cellular protein metabolic process;demethylation;macromolecule metabolic process;macromolecule modification;met
25.9366	24.8586	25.0828	25.9547	1:Experimental evidence at protein level	cell cycle;cellular process;meiotic cell cycle
26.2377	25.5821	24.1804	26.1711	1:Experimental evidence at protein level	biological regulation;cellular macromolecule metabolic process;cellular metabolic process;cellular nitrogen compound metabolic process;cellular process;humoral immune response;immune response;immune system process
29.2066	29.0633	28.9615	29.0061	1:Experimental evidence at protein level	biological regulation;biosynthetic process;cellular biosynthetic process;cellular component disassembly;cellular component disassembly at cellular level;cellular component organization;cellular component organization at cell
25.4134	26.2446	24.2874	26.5882	1:Experimental evidence at protein level	cellular macromolecule metabolic process;cellular metabolic process;cellular process;cellular protein metabolic process;chaperone-mediated protein transport;establishment of localization;establishment of localization in cell;
26.5408	26.5961	26.7453	26.7176	1:Experimental evidence at protein level	anatomical structure development;anatomical structure morphogenesis;biological regulation;biosynthetic process;catabolic process;cell surface receptor linked signaling pathway;cellular biosynthetic process;cellular catabol
25.7695	26.0674	26.2726	26.289	1:Experimental evidence at protein level	biological regulation;cell cycle phase;cell cycle process;cell division;cell projection assembly;cell projection organization;cellular component assembly;cellular component assembly at cellular level;cellular component organiza
26.8783	25.9498	26.4025	26.4253	1:Experimental evidence at protein level	anatomical structure formation involved in morphogenesis;biological regulation;cation homeostasis;cation transport;cell projection assembly;cell projection organization;cell surface receptor linked signaling pathway;cellular c
25.4894	26.1235	25.5864	25.5361	1:Experimental evidence at protein level	establishment of localization;establishment of localization in cell;establishment of protein localization;intracellular protein transport;intracellular transport;nuclear import;nuclear transport;nucleocytoplasmic transport;protein in
25.6465	26.3832	26.5898	26.6901	1:Experimental evidence at protein level	cellular macromolecule metabolic process;cellular metabolic process;cellular process;cellular protein metabolic process;chaperone-mediated protein transport;establishment of localization;establishment of localization in cell;
27.8064	27.4816	27.8002	27.5511	1:Experimental evidence at protein level	biological regulation;biosynthetic process;cellular biosynthetic process;cellular macromolecule biosynthetic process;cellular macromolecule metabolic process;cellular metabolic process;cellular process;cellular protein metab
26.8836	26.6935	26.426	26.6316	1:Experimental evidence at protein level	alcohol biosynthetic process;alcohol metabolic process;biosynthetic process;carbohydrate biosynthetic process;carbohydrate metabolic process;cellular biosynthetic process;cellular carbohydrate biosynthetic process;cellul
27.1009	26.9813	26.9045	26.9532	1:Experimental evidence at protein level	actin cytoskeleton organization;actin cytoskeleton reorganization;actin filament-based process;actomyosin structure organization;biological regulation;cell migration;cell motility;cellular component movement;cellular compon
29.4611	29.0935	29.8471	29.5494	1:Experimental evidence at protein level	biological regulation;biosynthetic process;catabolic process;cellular biosynthetic process;cellular catabolic process;cellular macromolecule biosynthetic process;cellular macromolecule catabolic process;cellular macromolecu
25.8249	24.8496	23.8577	25.793	1:Experimental evidence at protein level	apoptosis;biological regulation;cell death;cell surface receptor linked signaling pathway;cellular process;cellular response to stimulus;circadian regulation of gene expression;circadian rhythm;death;enzyme linked receptor pro
27.4378	27.7947	27.4316	27.6819	1:Experimental evidence at protein level	biological regulation;cell cycle cytokinesis;cell cycle phase;cell cycle process;cellular component assembly;cellular component assembly at cellular level;cellular component organization;cellular component organization at cel
27.4569	27.2386	27.1004	27.2985	1:Experimental evidence at protein level	cellular component organization;cellular component organization at cellular level;cellular component organization or biogenesis;cellular component organization or biogenesis at cellular level;cellular membrane organization;ce
27.0026	26.9051	26.9245	26.7531	1:Experimental evidence at protein level	cellular component organization;cellular component organization at cellular level;cellular component organization or biogenesis;cellular component organization or biogenesis at cellular level;cellular membrane organization;ce
26.6691	27.4561	24.3411	26.6067	1:Experimental evidence at protein level	biosynthetic process;cellular biosynthetic process;cellular metabolic process;cellular process;cofactor biosynthetic process;cofactor metabolic process;iron-sulfur cluster assembly;metabolic process;metallo-sulfur cluster as
27.4877	27.5421	28.1334	27.8298	1:Experimental evidence at protein level	
25.2015	25.0336	25.2764	24.9964	1:Experimental evidence at protein level	biological regulation;cellular macromolecule metabolic process;cellular metabolic process;cellular nitrogen compound metabolic process;cellular process;macromolecule metabolic process;macromolecule modification;metab
27.2532	26.868	27.4903	27.286	1:Experimental evidence at protein level	biological regulation;cell surface receptor linked signaling pathway;cellular process;cellular response to stimulus;regulation of biological process;regulation of cellular process;response to stimulus;signal transduction;Wnt rece
27.7493	28.1269	28.2888	28.2805	1:Experimental evidence at protein level	amine biosynthetic process;amine metabolic process;biosynthetic process;carboxylic acid biosynthetic process;carboxylic acid metabolic process;cellular amine metabolic process;cellular amino acid biosynthetic process;ce
24.6685	24.2073	24.9057	24.6888	1:Experimental evidence at protein level	biological regulation;biosynthetic process;cell surface receptor linked signaling pathway;cellular biosynthetic process;cellular component organization;cellular component organization at cellular level;cellular component orga
27.7825	27.5242	27.3525	27.7126	1:Experimental evidence at protein level	biological adhesion;biological regulation;calcium ion homeostasis;cation homeostasis;cell adhesion;cell surface receptor linked signaling pathway;cell-cell adhesion;cellular calcium ion homeostasis;cellular cation homeostasi
26.0818	23.7724	25.7502	26.21	1:Experimental evidence at protein level	biological regulation;biosynthetic process;cell cycle;cell surface receptor linked signaling pathway;cellular biosynthetic process;cellular component organization;cellular component organization at cellular level;cellular compor
26.456	26.4758	26.4475	26.1698	1:Experimental evidence at protein level	biosynthetic process;carbohydrate biosynthetic process;carbohydrate metabolic process;cellular carbohydrate metabolic process;cellular macromolecule metabolic process;cellular metabolic process;cellular process;cellular

26.534	26.3565	26.6034	26.3365	1:Experimental evidence at protein level	biosynthetic process;cellular biosynthetic process;cellular component disassembly;cellular component disassembly at cellular level;cellular component organization;cellular component organization at cellular level;cellular cor
29.1446	29.0691	28.9712	28.8517	1:Experimental evidence at protein level	cellular component movement;cellular component organization;cellular component organization at cellular level;cellular component organization or biogenesis;cellular component organization or biogenesis at cellular level;cel
26.2068	26.2191	25.9927	25.7288	1:Experimental evidence at protein level	
25.1848	26.0642	26.3043	26.4922	1:Experimental evidence at protein level	cellular macromolecule metabolic process;cellular metabolic process;cellular process;cellular protein metabolic process;chaperone-mediated protein folding;macromolecule metabolic process;macromolecule modification;me
29.8115	30.1402	29.8979	30.0584	1:Experimental evidence at protein level	anatomical structure formation involved in morphogenesis;anatomical structure morphogenesis;angiogenesis;anion transport;apoptosis;biological regulation;branching morphogenesis of a tube;cation homeostasis;cell death;
25.1683	26.4755	25.7565	25.6292	1:Experimental evidence at protein level	amine metabolic process;aromatic compound biosynthetic process;biosynthetic process;carboxylic acid metabolic process;cellular amine metabolic process;cellular amino acid metabolic process;cellular aromatic compounc
26.8316	26.6674	26.9901	26.7361	1:Experimental evidence at protein level	anatomical structure development;astral microtubule organization;biological regulation;cell differentiation;cell proliferation;cellular component organization;cellular component organization at cellular level;cellular component o
27.771	27.8558	27.5065	27.3537	1:Experimental evidence at protein level	biological regulation;biosynthetic process;cellular biosynthetic process;cellular macromolecule biosynthetic process;cellular macromolecule metabolic process;cellular metabolic process;cellular process;cellular protein metab
25.7327	25.4177	25.5548	25.5429	1:Experimental evidence at protein level	biological adhesion;biological regulation;cell adhesion;cellular component organization;cellular component organization at cellular level;cellular component organization or biogenesis;cellular component organization or bioger
27.5275	27.1489	27.2219	27.2506	1:Experimental evidence at protein level	cellular localization;cellular macromolecule localization;cellular process;cellular protein localization;localization;macromolecule localization;protein localization;protein localization in mitochondrion;protein localization to organe
25.2035	24.7154	24.6099	24.5967	1:Experimental evidence at protein level	biological regulation;cellular component organization;cellular component organization at cellular level;cellular component organization or biogenesis;cellular component organization or biogenesis at cellular level;cellular meml
25.1959	24.7813	25.3248	25.2094	1:Experimental evidence at protein level	biological regulation;cell cycle;cell cycle checkpoint;cell cycle phase;cell cycle process;cell division;cellular component organization;cellular component organization at cellular level;cellular component organization or biogene
26.321	25.9334	26.1358	26.1824	1:Experimental evidence at protein level	activation of protein kinase activity;apoptosis;biological regulation;cell death;cellular component disassembly;cellular component disassembly at cellular level;cellular component disassembly involved in apoptosis;cellular cor
27.0955	27.1451	26.9259	27.0421	1:Experimental evidence at protein level	anatomical structure development;cell differentiation;cellular developmental process;cellular process;developmental process;nervous system development;system development
27.867	27.7916	27.8977	27.7489	1:Experimental evidence at protein level	biological regulation;cell cycle phase;cell cycle process;cell division;cellular component movement;cellular component organization;cellular component organization at cellular level;cellular component organization or biogene
27.6187	28.2723	28.1276	28.712	1:Experimental evidence at protein level	biological regulation;biosynthetic process;cellular biosynthetic process;cellular macromolecule biosynthetic process;cellular macromolecule metabolic process;cellular metabolic process;cellular nitrogen compound metabolic
26.2432	26.039	25.7924	26.3451	1:Experimental evidence at protein level	anatomical structure development;biological regulation;cell surface receptor linked signaling pathway;cellular component organization;cellular component organization or biogenesis;cellular membrane organization;cellular pro
26.1867	26.5439	26.5517	25.856	1:Experimental evidence at protein level	
26.3457	26.3082	26.5664	26.4435	1:Experimental evidence at protein level	activation of immune response;activation of innate immune response;activation of MAPK activity;activation of NF-kappaB-inducing kinase activity;activation of protein kinase activity;antigen receptor-mediated signaling pathv
28.1613	27.8672	27.8792	27.7754	1:Experimental evidence at protein level	anatomical structure morphogenesis;biological regulation;developmental process;establishment of localization;establishment of RNA localization;gene expression;macromolecule metabolic process;metabolic process;mRNA
27.337	26.7469	27.0267	26.9796	1:Experimental evidence at protein level	cellular metabolic process;cellular process;electron transport chain;generation of precursor metabolites and energy;metabolic process;mitochondrial electron transport, NADH to ubiquinone;multicellular organismal process;n
27.4587	27.2586	27.2365	27.1034	1:Experimental evidence at protein level	amine catabolic process;amine metabolic process;carboxylic acid catabolic process;carboxylic acid metabolic process;catabolic process;cellular amine metabolic process;cellular amino acid catabolic process;cellular amino
26.05	26.3753	26.6126	26.5822	1:Experimental evidence at protein level	actin cytoskeleton organization;actin filament-based movement;actin filament-based process;ameboidal cell migration;anatomical structure development;anatomical structure formation involved in morphogenesis;anatomical
26.5039	24.1763	26.4747	26.5045	1:Experimental evidence at protein level	carboxylic acid metabolic process;cellular ketone metabolic process;cellular lipid metabolic process;cellular metabolic process;cellular process;fatty acid metabolic process;lipid metabolic process;metabolic process;monoca
25.6865	25.9972	25.7813	25.8203	1:Experimental evidence at protein level	biosynthetic process;cellular biosynthetic process;cellular macromolecule biosynthetic process;cellular macromolecule metabolic process;cellular metabolic process;cellular nitrogen compound metabolic process;cellular pro
27.917	27.8553	27.8593	27.7163	1:Experimental evidence at protein level	acrosome assembly;anatomical structure formation involved in morphogenesis;cellular component assembly;cellular component assembly at cellular level;cellular component assembly involved in morphogenesis;cellular com

C: GOMF name
binding;catalytic activity;hydrolase activity;nucleic acid binding;RNA binding;snoRNA binding
adenyl nucleotide binding;adenyl ribonucleotide binding;ATP binding;binding;catalytic activity;FAT10 activating enzyme activity;nucleotide binding;purine nucleotide binding;purine ribonucleoside triphosphate binding;purine r
alcohol binding;amine binding;binding;calcium ion binding;calcium-dependent phospholipid binding;cation binding;identical protein binding;ion binding;lipid binding;metal ion binding;phosphatidylcholine binding;phosphatidy
binding;catalytic activity;cation binding;coenzyme binding;cofactor binding;ion binding;magnesium ion binding;metal ion binding;thiamine pyrophosphate binding;transferase activity;vitamin binding
binding;lipid binding;phosphatidylinositol binding;phosphatidylinositol-3,5-bisphosphate binding;phosphatidylinositol-3-phosphate binding;phosphatidylinositol-4-phosphate binding;phosphatidylinositol-5-phosphate binding
armadillo repeat domain binding;binding;estrogen receptor binding;hormone receptor binding;nuclear hormone receptor binding;protein binding;protein complex scaffold;protein domain specific binding;receptor binding;retin
binding;cytoskeletal protein binding;microtubule binding;protein binding;tubulin binding
binding;catalytic activity;cation binding;hydrolase activity;hydrolase activity, acting on ester bonds;ion binding;magnesium ion binding;metal ion binding;phosphatase activity;phosphoglycolate phosphatase activity;phosphop
adenyl nucleotide binding;adenyl ribonucleotide binding;ATP binding;binding;nucleotide binding;purine nucleotide binding;purine ribonucleoside triphosphate binding;purine ribonucleotide binding;ribonucleotide binding
binding;nucleic acid binding;RNA binding
binding;DNA binding;nucleic acid binding
binding;catalytic activity;deoxyribonuclease activity;deoxyribonuclease II activity;DNA binding;endodeoxyribonuclease activity;endodeoxyribonuclease activity, producing 3'-phosphomonoesters;endonuclease activity;endonu
alkylglycerone-phosphate synthase activity;binding;catalytic activity;coenzyme binding;cofactor binding;FAD binding;flavin adenine dinucleotide binding;oxidoreductase activity;oxidoreductase activity, acting on CH-OH grou
adenyl nucleotide binding;adenyl ribonucleotide binding;ATP binding;ATPase activity;ATPase activity, coupled;ATP-dependent helicase activity;ATP-dependent RNA helicase activity;binding;catalytic activity;helicase activity;h
binding;cation binding;ion binding;metal ion binding;protein binding transcription factor activity;transcription coactivator activity;transcription cofactor activity;transcription factor binding transcription factor activity;transition r
acyl-CoA thioesterase activity;binding;carboxylic acid binding;carboxylic ester hydrolase activity;catalytic activity;CoA hydrolase activity;coenzyme binding;cofactor binding;fatty acid binding;fatty-acyl-CoA binding;hydrolase
actin-dependent ATPase activity;adenyl nucleotide binding;adenyl ribonucleotide binding;ATP binding;ATPase activity;ATPase activity, coupled;binding;catalytic activity;enzyme binding;GTPase binding;hydrolase activity;hydr
binding;GAF domain binding;molecular transducer activity;protein binding;protein binding transcription factor activity;protein domain specific binding;signal transducer activity;transcription coactivator activity;transcription co
binding;catalytic activity;GTP binding;GTPase activity;guanyl nucleotide binding;guanyl ribonucleotide binding;hydrolase activity;hydrolase activity, acting on acid anhydrides;hydrolase activity, acting on acid anhydrides, in pl
binding;protein binding;SNARE binding;syntaxin binding
binding;enzyme binding;GTP-dependent protein binding;phosphatase binding;protein binding;protein phosphatase binding
4 iron, 4 sulfur cluster binding;binding;catalytic activity;cation binding;ion binding;iron-sulfur cluster binding;metal cluster binding;metal ion binding;NADH dehydrogenase (quinone) activity;NADH dehydrogenase (ubiquinone)
bHLH transcription factor binding;binding;protein binding;protein binding transcription factor activity;transcription coactivator activity;transcription cofactor activity;transcription factor binding;transcription factor binding trans
binding;cation binding;cation transmembrane transporter activity;copper chaperone activity;copper ion binding;copper ion transmembrane transporter activity;copper-dependent protein binding;inorganic cation transmembra
binding;cation binding;heme binding;ion binding;iron ion binding;lipid binding;metal ion binding;steroid binding;tetrapyrrole binding;transition metal ion binding
binding;chromatin binding;enzyme binding;protein binding;protein dimerization activity;protein heterodimerization activity
binding;clathrin binding;lipid binding;phosphatidylinositol binding;phospholipid binding;protein binding;structural constituent of cytoskeleton;structural molecule activity
anion channel activity;anion transmembrane transporter activity;channel activity;chloride channel activity;gated channel activity;ion channel activity;ion transmembrane transporter activity;passive transmembrane transporter
binding;catalytic activity;cysteine-type peptidase activity;hydrolase activity;nucleic acid binding;peptidase activity;peptidase activity, acting on L-amino acid peptides;RNA binding;small conjugating protein-specific protease a
catalytic activity;transferase activity;transferase activity, transferring acyl groups
binding;enzyme binding;enzyme inhibitor activity;enzyme regulator activity;GTPase binding;GTPase inhibitor activity;GTPase regulator activity;nucleoside-triphosphatase regulator activity;protein binding;protein transporter ac
protein binding transcription factor activity;transcription cofactor activity;transcription corepressor activity;transcription factor binding transcription factor activity
binding;mRNA 3'-UTR binding;mRNA 5'-UTR binding;mRNA binding;nucleic acid binding;nucleotide binding;RNA binding;translation regulator activity
binding;catalytic activity;enzyme binding;GTP binding;GTPase activity;guanyl nucleotide binding;guanyl ribonucleotide binding;hydrolase activity;hydrolase activity, acting on acid anhydrides;hydrolase activity, acting on acid
adenyl nucleotide binding;adenyl ribonucleotide binding;ATP binding;binding;catalytic activity;cyclase activity;ligase activity;ligase activity, forming phosphoric ester bonds;nucleic acid binding;nucleotide binding;purine nucle
1-phosphatidylinositol-3-kinase activity;adenyl nucleotide binding;adenyl ribonucleotide binding;ATP binding;binding;catalytic activity;inositol or phosphatidylinositol kinase activity;kinase activity;lipid binding;lipid kinase activ
acetylcholine receptor regulator activity;binding;calcium ion binding;carbohydrate binding;cation binding;chondroitin sulfate binding;dystroglycan binding;extracellular matrix binding;glycoprotein binding;glycosaminoglycan b
binding;carboxylic acid binding;catalytic activity;cation binding;ion binding;iron ion binding;L-ascorbic acid binding;metal ion binding;oxidoreductase activity;oxidoreductase activity, acting on paired donors, with incorporatio
binding;enzyme binding;GTPase binding;protein binding;Ral GTPase binding;Ras GTPase binding;small GTPase binding
binding;catalytic activity;NADH dehydrogenase (quinone) activity;NADH dehydrogenase (ubiquinone) activity;NADH dehydrogenase activity;oxidoreductase activity;oxidoreductase activity, acting on NADH or NADPH;oxidored
binding;catalytic activity;cation binding;endopeptidase activator activity;endopeptidase regulator activity;enzyme activator activity;enzyme regulator activity;hydrolase activity;ion binding;metal ion binding;metallopeptidase ac
binding;cytoskeletal protein binding;enzyme binding;identical protein binding;protein binding;RNA polymerase binding;tau protein binding
binding;nuclear localization sequence binding;peptide binding;protein transporter activity;signal sequence binding;substrate-specific transporter activity;transporter activity
binding;ribonucleoprotein binding
binding;binding, bridging;cytokine receptor binding;cytoskeletal adaptor activity;cytoskeletal protein binding;frizzled binding;G-protein-coupled receptor binding;growth factor receptor binding;identical protein binding;interleu
binding;nucleic acid binding;RNA binding;snoRNA binding
adenyl nucleotide binding;adenyl ribonucleotide binding;ATP binding;ATPase activity;ATPase activity, coupled;ATP-dependent DNA helicase activity;ATP-dependent helicase activity;ATP-dependent RNA helicase activity;bind
binding;carbohydrate binding;extracellular matrix binding;glycosaminoglycan binding;heparin binding;integrin binding;pattern binding;polysaccharide binding;protein binding;protein complex binding;receptor binding
protein transporter activity;substrate-specific transporter activity;transporter activity
binding;catalytic activity;cation binding;hydrolase activity;hydrolase activity, acting on ester bonds;ion binding;metal ion binding;phosphatase activity;phosphoprotein phosphatase activity;phosphoric ester hydrolase activity;p
alpha-mannosidase activity;binding;carbohydrate binding;catalytic activity;cation binding;hydrolase activity;hydrolase activity, acting on glycosyl bonds;hydrolase activity, hydrolyzing O-glycosyl compounds;ion binding;mann
acid-amino acid ligase activity;adenyl nucleotide binding;adenyl ribonucleotide binding;ATP binding;binding;catalytic activity;enzyme binding;ligase activity;ligase activity, forming carbon-nitrogen bonds;nucleotide binding;pr
adenyl nucleotide binding;adenyl ribonucleotide binding;alkali metal ion binding;ATP binding;binding;catalytic activity;cation binding;cofactor binding;identical protein binding;ion binding;kinase activity;lithium ion binding;mag
binding;DNA binding;ligand-dependent nuclear receptor binding;nucleic acid binding;protein binding;protein binding transcription factor activity;receptor binding;transcription coactivator activity;transcription cofactor activity;
binding;identical protein binding;protein binding;protein dimerization activity;protein homodimerization activity;structural constituent of eye lens;structural molecule activity
adenyl nucleotide binding;adenyl ribonucleotide binding;ATP binding;binding;catalytic activity;cation binding;ion binding;kinase activity;metal ion binding;nucleotide binding;PDZ domain binding;phosphotransferase activity, a
structural molecule activity
transporter activity
antioxidant activity;binding;catalytic activity;cation binding;cation transmembrane transporter activity;copper ion binding;copper ion transmembrane transporter activity;disulfide oxidoreductase activity;inorganic cation transr
adenyl nucleotide binding;adenyl ribonucleotide binding;ATP binding;ATPase activity;binding;catalytic activity;hydrolase activity;hydrolase activity, acting on acid anhydrides;hydrolase activity, acting on acid anhydrides, in ph
binding;catalytic activity;cation binding;endopeptidase activity;enzyme binding;hydrolase activity;identical protein binding;integrin binding;ion binding;kinase binding;metal ion binding;metalloendopeptidase activity;metallope
amine binding;amino acid binding;binding;carboxylic acid binding;catalytic activity;glutathione binding;intramolecular oxidoreductase activity;isomerase activity;modified amino acid binding;peptide binding;prostaglandin-E sy
alcohol binding;binding;carbohydrate binding;catalytic activity;cation binding;CDP-alcohol phosphatidyltransferase activity;CDP-diacylglycerol-inositol 3-phosphatidyltransferase activity;diacylglycerol binding;ion binding;lipic
acetyltransferase activator activity;beta-tubulin binding;binding;carbohydrate binding;cytoskeletal protein binding;DNA binding;enzyme activator activity;enzyme regulator activity;glycosaminoglycan binding;heparin binding;n
arginine N-methyltransferase activity;binding;catalytic activity;chromatin binding;core promoter binding;core promoter sequence-specific DNA binding;DNA binding;histone methyltransferase activity;histone-arginine N-methy
adrenergic receptor binding;beta-2 adrenergic receptor binding;beta-catenin binding;binding;channel regulator activity;chloride channel regulator activity;enzyme binding;G-protein-coupled receptor binding;growth factor rece
binding;catalytic activity;cation binding;DNA binding;DNA polymerase activity;identical protein binding;ion binding;metal ion binding;nucleic acid binding;nucleotidyltransferase activity;protein binding;protein dimerization acti
binding;protein binding;receptor activity;TRAIL binding
binding;core promoter proximal region DNA binding;core promoter proximal region sequence-specific DNA binding;DNA binding;nucleic acid binding;nucleic acid binding transcription factor activity;protein binding;protein bir
binding;catalytic activity;cation binding;endopeptidase activity;exopeptidase activity;hydrolase activity;ion binding;metal ion binding;peptidase activity;peptidase activity, acting on L-amino acid peptides;peptide binding;serin
binding;protein binding;protein binding transcription factor activity;repressing transcription factor binding;RNA polymerase II repressing transcription factor binding;RNA polymerase II transcription cofactor activity;RNA polym
binding;identical protein binding;protein binding;structural constituent of cytoskeleton;structural molecule activity
binding;carbohydrate binding;catalytic activity;cation binding;coreceptor activity;cytokine binding;glycosaminoglycan binding;growth factor binding;heparin binding;ion binding;kinase activity;metal ion binding;molecular trans
antigen binding;binding;integrin binding;protein binding;protein complex binding;receptor binding
binding;catalytic activity;endopeptidase activity;hydrolase activity;identical protein binding;peptidase activity;peptidase activity, acting on L-amino acid peptides;protein binding;threonine-type endopeptidase activity;threonin
binding;enzyme binding;protein binding;ubiquitin protein ligase binding
binding;identical protein binding;protein binding
antioxidant activity;catalytic activity;glutathione peroxidase activity;glutathione transferase activity;oxidoreductase activity;oxidoreductase activity, acting on peroxide as acceptor;peroxidase activity;transferase activity;transfe
actin binding;binding;cytoskeletal protein binding;identical protein binding;myosin binding;protein binding;protein dimerization activity;protein homodimerization activity;receptor binding
acetyltransferase activity;catalytic activity;H4 histone acetyltransferase activity;histone acetyltransferase activity;lysine N-acetyltransferase activity;N-acetyltransferase activity;N-acyltransferase activity;transferase activity;trar
acid-amino acid ligase activity;catalytic activity;ISG15 ligase activity;ligase activity;ligase activity, forming carbon-nitrogen bonds;small conjugating protein ligase activity;ubiquitin-protein ligase activity
adenyl nucleotide binding;adenyl ribonucleotide binding;ATP binding;binding;catalytic activity;guanylate kinase activity;kinase activity;nucleobase-containing compound kinase activity;nucleotide binding;nucleotide kinase ac
catalytic activity;cation transmembrane transporter activity;hydrogen ion transmembrane transporter activity;inorganic cation transmembrane transporter activity;ion transmembrane transporter activity;monovalent inorganic c
binding;calcium ion binding;cation binding;ion binding;metal ion binding
binding;cation binding;ion binding;metal ion binding;protein binding;protein domain specific binding
adenyl nucleotide binding;adenyl ribonucleotide binding;ATP binding;binding;catalytic activity;enzyme binding;histone kinase activity;histone serine kinase activity;kinase activity;kinase binding;nucleotide binding;phosphotrar
binding;calcium ion binding;cation binding;ion binding;metal ion binding;protein binding;unfolded protein binding
14-3-3 protein binding;binding;enzyme binding;enzyme inhibitor activity;enzyme regulator activity;kinase binding;molecular transducer activity;phosphatase regulator activity;protein binding;protein kinase binding;signal trans
adenyl nucleotide binding;adenyl ribonucleotide binding;ATP binding;binding;catalytic activity;kinase activity;nucleotide binding;phosphotransferase activity, alcohol group as acceptor;protein kinase activity;protein serine/thre
binding;DNA binding;double-stranded DNA binding;mRNA binding;nucleic acid binding;nucleotide binding;poly(A) RNA binding;poly(G) RNA binding;poly-purine tract binding;RNA binding;single-stranded DNA binding;single-
binding;nucleic acid binding;nucleocytoplasmic transporter activity;protein transporter activity;RNA binding;substrate-specific transporter activity;transporter activity
binding;cytoskeletal protein binding;enzyme regulator activity;GTPase regulator activity;guanyl-nucleotide exchange factor activity;kinesin binding;nucleoside-triphosphatase regulator activity;protein binding;Ras guanyl-nucle
molecular transducer activity;receptor activity;semaphorin receptor activity;signal transducer activity;signaling receptor activity;transmembrane signaling receptor activity
binding;nucleotide binding
adenyl nucleotide binding;adenyl ribonucleotide binding;ATP binding;binding;carbon-nitrogen ligase activity, with glutamine as amido-N-donor;catalytic activity;cation binding;ion binding;ligase activity;ligase activity, forming c
binding;nucleic acid binding;RNA binding;RNA cap binding
binding;cholesterol binding;hedgehog receptor activity;lipid binding;lipid transporter activity;molecular transducer activity;receptor activity;signal transducer activity;signaling receptor activity;steroid binding;sterol binding;ster
catalytic activity;electron carrier activity;oxidoreductase activity;oxidoreductase activity, acting on paired donors, with incorporation or reduction of molecular oxygen;sphingolipid delta-4 desaturase activity
structural constituent of cytoskeleton;structural molecule activity
structural constituent of cytoskeleton;structural molecule activity
structural constituent of cytoskeleton;structural molecule activity
binding;calcium ion binding;cation binding;CD4 receptor binding;DNA binding;enzyme binding;epidermal growth factor receptor binding;growth factor receptor binding;ion binding;lipid transporter activity;metal ion binding;nu
binding;cation binding;heme binding;ion binding;iron ion binding;ligand-dependent nuclear receptor activity;lipid binding;metal ion binding;molecular transducer activity;receptor activity;signal transducer activity;signaling rec
binding;chaperone binding;protein binding;unfolded protein binding
binding;cation binding;ion binding;metal ion binding;transition metal ion binding;zinc ion binding
structural constituent of ribosome;structural molecule activity
binding;C-acyltransferase activity;catalytic activity;cofactor binding;C-palmitoyltransferase activity;palmitoyltransferase activity;pyridoxal phosphate binding;serine C-palmitoyltransferase activity;transferase activity;transferas
binding;C-acyltransferase activity;catalytic activity;cofactor binding;C-palmitoyltransferase activity;palmitoyltransferase activity;pyridoxal phosphate binding;serine C-palmitoyltransferase activity;transferase activity;transferas
acetylglucosaminyltransferase activity;binding;catalytic activity;enzyme activator activity;enzyme regulator activity;lipid binding;phosphatidylinositol binding;phosphatidylinositol-3,4,5-trisphosphate binding;phospholipid bindi
binding;chromatin binding;DNA bending activity;DNA binding;double-stranded DNA binding;nucleic acid binding;structure-specific DNA binding

binding;catalytic activity;cation binding;hydrolase activity;hydrolase activity, acting on ester bonds;ion binding;metal ion binding;phosphatase activity;phosphoprotein phosphatase activity;phosphoric ester hydrolase activity;p
binding;catalytic activity;hydrolase activity;protein binding;protein domain specific binding;SH2 domain binding
binding;nucleic acid binding;RNA binding;translation factor activity, nucleic acid binding;translation initiation factor activity
binding;nucleic acid binding;RNA binding;translation factor activity, nucleic acid binding;translation initiation factor activity
branched-chain-amino-acid transaminase activity;catalytic activity;L-isoleucine transaminase activity;L-leucine transaminase activity;L-valine transaminase activity;transaminase activity;transferase activity;transferase activity
acid-amino acid ligase activity;binding;caspase inhibitor activity;caspase regulator activity;catalytic activity;cation binding;chaperone binding;cobalt ion binding;cofactor binding;cysteine-type endopeptidase inhibitor activity;
binding;channel inhibitor activity;channel regulator activity;chloride channel inhibitor activity;chloride channel regulator activity;ion channel inhibitor activity;protein binding;SNAP receptor activity;SNARE binding;syntaxin bind
active transmembrane transporter activity;carboxylic acid transmembrane transporter activity;lactate transmembrane transporter activity;monocarboxylic acid transmembrane transporter activity;organic acid transmembrane
catalytic activity;DNA-directed RNA polymerase activity;nucleotidyltransferase activity;RNA polymerase activity;transferase activity;transferase activity, transferring phosphorus-containing groups
binding;carboxylic acid binding;catalytic activity;cation binding;dioxygenase activity;electron carrier activity;ion binding;iron ion binding;L-ascorbic acid binding;metal ion binding;oxidoreductase activity;oxidoreductase activit
calcium-dependent cysteine-type endopeptidase activity;catalytic activity;cysteine-type endopeptidase activity;cysteine-type peptidase activity;endopeptidase activity;hydrolase activity;peptidase activity;peptidase activity, a
binding;catalytic activity;palmitoyltransferase activity;protein binding;protein-cysteine S-acyltransferase activity;protein-cysteine S-palmitoleyltransferase activity;S-acyltransferase activity;SNAP receptor activity;SNARE bindi
structural constituent of cytoskeleton;structural molecule activity
binding;catalytic activity;DNA-directed RNA polymerase activity;nucleotide binding;nucleotidyltransferase activity;protein binding;RNA polymerase activity;transferase activity;transferase activity, transferring phosphorus-cont
active transmembrane transporter activity;antigen binding;ATPase activity;ATPase activity, coupled;ATPase activity, coupled to movement of substances;ATPase activity, coupled to transmembrane movement of substances;
binding;cation binding;ion binding;metal ion binding;transition metal ion binding;zinc ion binding
adenyl nucleotide binding;adenyl ribonucleotide binding;ATP binding;ATPase activity;ATPase activity, coupled;ATP-dependent helicase activity;ATP-dependent RNA helicase activity;binding;catalytic activity;double-stranded
binding;catalytic activity;methyltransferase activity;mRNA (guanine-N7-)-methyltransferase activity;mRNA methyltransferase activity;N-methyltransferase activity;nucleic acid binding;RNA binding;RNA methyltransferase activ
binding;calcium ion binding;cation binding;ion binding;metal ion binding;transition metal ion binding;zinc ion binding
binding;cation binding;heme binding;ion binding;iron ion binding;metal ion binding;tetrapyrrole binding;transition metal ion binding
binding;nucleic acid binding;RNA binding;snRNA binding;U4 snRNA binding;U6 snRNA binding
binding;catalytic activity;coenzyme binding;cofactor binding;electron carrier activity;NAD binding;nucleotide binding;oxidoreductase activity;oxidoreductase activity, acting on CH-OH group of donors;oxidoreductase activity,
adenyl nucleotide binding;adenyl ribonucleotide binding;ATP binding;binding;catalytic activity;hydrolase activity;hydrolase activity, acting on acid anhydrides;hydrolase activity, acting on acid anhydrides, in phosphorus-conta
enzyme regulator activity
adenyl nucleotide binding;adenyl ribonucleotide binding;adenylylsulfate kinase activity;adenylyltransferase activity;ATP binding;binding;catalytic activity;kinase activity;nucleotide binding;nucleotidyltransferase activity;phosph
binding;centromeric DNA binding;DNA binding;nucleic acid binding;sequence-specific DNA binding
adenyl nucleotide binding;adenyl ribonucleotide binding;ATP binding;binding;calmodulin-dependent protein kinase activity;cAMP response element binding protein binding;catalytic activity;enzyme binding;GTPase binding;id
androgen receptor binding;binding;cation binding;hormone receptor binding;ion binding;I-SMAD binding;metal ion binding;nuclear hormone receptor binding;protein binding;protein binding transcription factor activity;recepto
adenyl nucleotide binding;adenyl ribonucleotide binding;ATP binding;binding;catalytic activity;cation binding;ion binding;kinase activity;magnesium ion binding;MAP kinase kinase activity;MAP kinase kinase kinase activity;me
adenyl nucleotide binding;adenyl ribonucleotide binding;ATP binding;binding;CARD domain binding;catalytic activity;identical protein binding;kinase activity;LIM domain binding;molecular transducer activity;non-membrane s
binding;nucleic acid binding;nucleotide binding;RNA binding
binding;identical protein binding;protein binding
catalytic activity;disulfide oxidoreductase activity;oxidoreductase activity;oxidoreductase activity, acting on a sulfur group of donors;oxidoreductase activity, acting on a sulfur group of donors, disulfide as acceptor;protein dis
binding;identical protein binding;protein binding;protein dimerization activity;protein heterodimerization activity;protein homodimerization activity
binding;fibroblast growth factor binding;growth factor binding;protein binding
binding;nucleic acid binding;RNA binding;RNA cap binding;translation factor activity, nucleic acid binding;translation initiation factor activity
binding;catalytic activity;cis-trans isomerase activity;cyclosporin A binding;drug binding;isomerase activity;peptide binding;peptidyl-prolyl cis-trans isomerase activity;ribonucleoprotein binding
alcohol dehydrogenase (NADP+) activity;alditol:NADP+ 1-oxidoreductase activity;aldo-keto reductase (NADP) activity;catalytic activity;electron carrier activity;epoxide hydrolase activity;ether hydrolase activity;hydrolase activ
structural molecule activity
binding;nucleic acid binding;RNA binding;translation factor activity, nucleic acid binding;translation initiation factor activity
binding;nucleic acid binding;RNA binding;tRNA binding
catalytic activity;deoxyribonucleoside 5'-monophosphate N-glycosidase activity;hydrolase activity;hydrolase activity, acting on glycosyl bonds;hydrolase activity, hydrolyzing N-glycosyl compounds;nucleoside deoxyribosyltra
active transmembrane transporter activity;adenyl nucleotide binding;adenyl ribonucleotide binding;ATP binding;binding;chaperone binding;macromolecule transmembrane transporter activity;nucleotide binding;P-P-bond-hy
alcohol binding;amine binding;binding;cation binding;ion binding;lipid binding;phosphatidylcholine binding;phospholipid binding;protein binding;protein domain specific binding;quaternary ammonium group binding
molecular transducer activity;signal transducer activity
molecular transducer activity;protein binding transcription factor activity;signal transducer activity;transcription cofactor activity;transcription corepressor activity;transcription factor binding transcription factor activity
binding;cytoskeletal protein binding;enzyme binding;identical protein binding;kinase binding;kinesin binding;protein binding;protein kinase binding
enzyme activator activity;enzyme regulator activity;GTPase activator activity;GTPase regulator activity;nucleoside-triphosphatase regulator activity
binding;carbohydrate binding;cation binding;cytoskeletal protein binding;DNA binding;glycosaminoglycan binding;heparin binding;ion binding;metal ion binding;microtubule binding;nucleic acid binding;nucleic acid binding tr
catalytic activity;NADH dehydrogenase (quinone) activity;NADH dehydrogenase (ubiquinone) activity;NADH dehydrogenase activity;oxidoreductase activity;oxidoreductase activity, acting on NADH or NADPH;oxidoreductase
catalytic activity;NADH dehydrogenase (quinone) activity;NADH dehydrogenase (ubiquinone) activity;NADH dehydrogenase activity;oxidoreductase activity;oxidoreductase activity, acting on NADH or NADPH;oxidoreductase
adenyl nucleotide binding;adenyl ribonucleotide binding;anion transmembrane transporter activity;arsenite transmembrane transporter activity;ATP binding;ATPase activity;binding;catalytic activity;cation binding;hydrolase ac
actin binding;actin filament binding;binding;calcium ion binding;cation binding;cytoskeletal protein binding;hormone receptor binding;identical protein binding;integrin binding;ion binding;ligand-dependent nuclear receptor tra
binding;nucleotide binding
binding;cytoskeletal protein binding;enzyme binding;GTPase binding;GTP-dependent protein binding;kinesin binding;protein binding;protein transporter activity;Rab GTPase binding;Ras GTPase binding;small GTPase bindin
binding;channel inhibitor activity;channel regulator activity;enzyme binding;enzyme inhibitor activity;enzyme regulator activity;ion channel inhibitor activity;phosphatase binding;phosphatase inhibitor activity;phosphatase regu
adenyl nucleotide binding;adenyl ribonucleotide binding;aminoacyl-tRNA ligase activity;asparagine-tRNA ligase activity;ATP binding;binding;catalytic activity;ligase activity;ligase activity, forming aminoacyl-tRNA and related c
actin binding;actin filament binding;adenyl nucleotide binding;adenyl ribonucleotide binding;ATP binding;binding;catalytic activity;cytoskeletal protein binding;hydrolase activity;hydrolase activity, acting on acid anhydrides;hy
AU-rich element binding;binding;catalytic activity;enzyme binding;histone deacetylase binding;hydrolase activity;identical protein binding;mRNA binding;nucleic acid binding;protein binding;protein dimerization activity;protei
amine binding;amino acid binding;binding;carboxylic acid binding;catalytic activity;cation binding;glutathione binding;G-protein coupled receptor activity;ion binding;lipoprotein particle receptor binding;low-density lipoprotei
binding;nucleic acid binding;RNA binding;snoRNA binding
binding;cation binding;DNA binding;double-stranded DNA binding;ion binding;metal ion binding;nucleic acid binding;structure-specific DNA binding;transition metal ion binding;zinc ion binding
binding;catalytic activity;cation binding;coenzyme binding;cofactor binding;electron carrier activity;ion binding;isocitrate dehydrogenase (NAD+) activity;isocitrate dehydrogenase activity;magnesium ion binding;metal ion binc
binding;catalytic activity;cation binding;endopeptidase activity;epidermal growth factor binding;growth factor binding;hormone binding;hydrolase activity;ion binding;metal ion binding;metalloendopeptidase activity;metallope
binding;calcium ion binding;cation binding;ion binding;metal ion binding
binding;calcium ion binding;cation binding;integrin binding;ion binding;metal ion binding;protein binding;protein complex binding;receptor binding
catalytic activity;hydrolase activity
catalytic activity;NADH dehydrogenase (quinone) activity;NADH dehydrogenase (ubiquinone) activity;NADH dehydrogenase activity;oxidoreductase activity;oxidoreductase activity, acting on NADH or NADPH;oxidoreductase
binding;cation binding;ion binding;metal ion binding
adenyl nucleotide binding;adenyl ribonucleotide binding;ATP binding;ATPase activity;ATPase activity, coupled;ATP-dependent helicase activity;ATP-dependent RNA helicase activity;binding;catalytic activity;helicase activity;h
binding;catalytic activity;cation binding;diphosphotransferase activity;enzyme inhibitor activity;enzyme regulator activity;ion binding;magnesium ion binding;metal ion binding;ribose phosphate diphosphokinase activity;transfe
adenyl nucleotide binding;adenyl ribonucleotide binding;ATP binding;ATPase activity;binding;catalytic activity;chromatin binding;DNA binding;helicase activity;hydrolase activity;hydrolase activity, acting on acid anhydrides;hy
binding;binding, bridging;cytoskeletal protein binding;enzyme binding;JUN kinase binding;kinase binding;kinesin binding;MAP-kinase scaffold activity;protein binding;protein complex scaffold;protein kinase binding;receptor
binding;catalytic activity;cation binding;GTP binding;GTPase activity;guanyl nucleotide binding;guanyl ribonucleotide binding;hydrolase activity;hydrolase activity, acting on acid anhydrides;hydrolase activity, acting on acid a
adenyl nucleotide binding;adenyl ribonucleotide binding;ATP binding;ATPase activity;binding;catalytic activity;cytoskeletal protein binding;hydrolase activity;hydrolase activity, acting on acid anhydrides;hydrolase activity, acti
androgen receptor binding;bHLH transcription factor binding;binding;catalytic activity;chromatin binding;coenzyme binding;cofactor binding;demethylase activity;DNA binding;enzyme binding;flavin adenine dinucleotide bind
acid-thiol ligase activity;adenyl nucleotide binding;adenyl ribonucleotide binding;arachidonate-CoA ligase activity;ATP binding;binding;catalytic activity;fatty acid ligase activity;ligase activity;ligase activity, forming carbon-sul
binding;enzyme binding;lipid binding;phosphatase binding;phosphatidylinositol binding;phosphatidylinositol-3,5-bisphosphate binding;phosphatidylinositol-3-phosphate binding;phosphatidylinositol-4-phosphate binding;pho
acetyltransferase activity;beta-N-acetylglucosaminidase activity;beta-N-acetylhexosaminidase activity;catalytic activity;hexosaminidase activity;histone acetyltransferase activity;hyaluronoglucosaminidase activity;hydrolase
binding;mRNA binding;nucleic acid binding;nucleotide binding;poly(A) RNA binding;poly-purine tract binding;RNA binding;single-stranded RNA binding
binding;carbon-oxygen lyase activity;catalytic activity;coenzyme binding;cofactor binding;GDP-mannose 4,6-dehydratase activity;hydro-lyase activity;lyase activity;NADP binding;NADP+ binding;nucleotide binding
binding;cytokine receptor binding;enzyme activator activity;enzyme regulator activity;growth factor receptor binding;kinase activator activity;kinase regulator activity;morphogen activity;protein binding;protein kinase activator
binding;carboxylic acid binding;catalytic activity;cation binding;galactosyltransferase activity;glucosyltransferase activity;ion binding;iron ion binding;L-ascorbic acid binding;metal ion binding;oxidoreductase activity;oxidored
binding;enzyme binding;nucleic acid binding;protein binding;RNA binding;RNA cap binding;translation factor activity, nucleic acid binding;translation initiation factor activity;ubiquitin protein ligase binding
binding;ion channel binding;protein binding;receptor binding
protein transporter activity;substrate-specific transporter activity;transporter activity
binding;catalytic activity;coenzyme binding;cofactor binding;electron carrier activity;NAD binding;nucleotide binding;oxidoreductase activity;oxidoreductase activity, acting on CH-OH group of donors;oxidoreductase activity,
binding;cation binding;ion binding;metal ion binding;protein binding transcription factor activity;transcription cofactor activity;transcription factor binding transcription factor activity;transition metal ion binding;zinc ion binding
binding;cadherin binding;cell adhesion molecule binding;protein binding;receptor binding
binding;epidermal growth factor receptor binding;growth factor receptor binding;insulin receptor binding;lipid binding;phosphatidylinositol binding;phospholipid binding;protein binding;protein complex binding;receptor bindi
alcohol binding;binding;carbohydrate binding;catalytic activity;dolichyl-phosphate beta-D-mannosyltransferase activity;dolichyl-phosphate-mannose-protein mannosyltransferase activity;mannose binding;mannosyltransferas
protein transporter activity;substrate-specific transporter activity;transporter activity
binding;clathrin binding;protein binding
binding;DNA binding;nucleic acid binding;protein binding transcription factor activity;ribonucleoprotein binding;transcription coactivator activity;transcription cofactor activity;transcription factor binding transcription factor ac
binding;catalytic activity;DNA polymerase activity;intramolecular transferase activity;isomerase activity;nucleic acid binding;nucleotidyltransferase activity;pseudouridine synthase activity;RNA binding;RNA-directed DNA poly
binding;catalytic activity;GTP binding;GTPase activity;guanyl nucleotide binding;guanyl ribonucleotide binding;hydrolase activity;hydrolase activity, acting on acid anhydrides;hydrolase activity, acting on acid anhydrides, in ph
binding;DNA binding;nucleic acid binding;protein binding transcription factor activity;sequence-specific DNA binding;transcription coactivator activity;transcription cofactor activity;transcription factor binding transcription fac
binding;protein binding;receptor binding
adenyl nucleotide binding;adenyl ribonucleotide binding;ATP binding;binding;cation binding;chaperone binding;ion binding;metal ion binding;nucleotide binding;protein binding;purine nucleotide binding;purine ribonucleoside
binding;chromatin binding;DNA binding;histone acetyl-lysine binding;histone binding;nucleic acid binding;p53 binding;protein binding
binding;nucleic acid binding transcription factor activity;protein binding;protein binding involved in protein folding;sequence-specific DNA binding transcription factor activity
binding;damaged DNA binding;DNA binding;nucleic acid binding;protein binding;protein N-terminus binding;transcription factor binding
active transmembrane transporter activity;adenyl nucleotide binding;adenyl ribonucleotide binding;ATP binding;ATPase activity;ATPase activity, coupled;ATPase activity, coupled to movement of substances;ATPase activity, c
binding;enzyme activator activity;enzyme binding;enzyme regulator activity;GTPase activator activity;GTPase binding;GTPase regulator activity;identical protein binding;nucleoside-triphosphatase regulator activity;protein bin
adenyl nucleotide binding;adenyl ribonucleotide binding;ATP binding;binding;catalytic activity;cation binding;ion binding;kinase activity;metal ion binding;nucleotide binding;phosphotransferase activity, alcohol group as acce
alpha(1,2)-fucosyltransferase activity;binding;catalytic activity;cytoskeletal protein binding;fucosyltransferase activity;galactoside 2-alpha-L-fucosyltransferase activity;microtubule binding;microtubule plus-end binding;protei
binding;calcium-dependent phospholipid binding;catalytic activity;kinase activity;lipid binding;phospholipid binding;phosphotransferase activity, alcohol group as acceptor;protein kinase activity;protein serine/threonine kinas
binding;lipid binding;phosphatidylinositol binding;phospholipid binding
acid-amino acid ligase activity;binding;catalytic activity;cation binding;enzyme binding;identical protein binding;ion binding;ligase activity;ligase activity, forming carbon-nitrogen bonds;metal ion binding;mRNA 3'-UTR binding
binding;cation binding;ion binding;metal ion binding
binding;mRNA binding;nucleic acid binding;RNA binding

binding;chromatin binding
binding;catalytic activity;gamma-glutamylcyclotransferase activity;identical protein binding;protein binding;protein dimerization activity;protein homodimerization activity;transferase activity;transferase activity, transferring acy
4 iron, 4 sulfur cluster binding;binding;catalytic activity;cation binding;coenzyme binding;cofactor binding;electron carrier activity;enzyme binding;ion binding;iron-sulfur cluster binding;metal cluster binding;metal ion binding;
binding;cation binding;ion binding;metal ion binding;protein binding;transition metal ion binding;translation initiation factor binding;zinc ion binding
binding;carbohydrate binding;glycosaminoglycan binding;hyaluronic acid binding;pattern binding;polysaccharide binding
binding;binding, bridging;calcium ion binding;calcium-dependent cysteine-type endopeptidase activity;calcium-dependent protein binding;catalytic activity;cation binding;cysteine-type endopeptidase activity;cysteine-type p
binding;chaperone binding;protein binding
active transmembrane transporter activity;ATPase activity;ATPase activity, coupled;ATPase activity, coupled to movement of substances;ATPase activity, coupled to transmembrane movement of ions;ATPase activity, couplec
adenyl nucleotide binding;adenyl ribonucleotide binding;ATP binding;ATPase activity;ATPase activity, coupled;binding;catalytic activity;hydrolase activity;hydrolase activity, acting on acid anhydrides;hydrolase activity, acting
binding;chromatin binding;chromatin DNA binding;core promoter binding;core promoter sequence-specific DNA binding;DNA binding;double-stranded DNA binding;double-stranded methylated DNA binding;enzyme binding;
actin binding;binding;cytoskeletal protein binding;identical protein binding;protein binding
beta-tubulin binding;binding;cytoskeletal protein binding;microtubule binding;protein binding;protein binding transcription factor activity;protein N-terminus binding;receptor binding;transcription cofactor activity;transcription
catalytic activity;citrate (Si)-synthase activity;transferase activity;transferase activity, transferring acyl groups;transferase activity, transferring acyl groups, acyl groups converted into alkyl on transfer
protein transporter activity;substrate-specific transporter activity;transporter activity
binding;catalytic activity;cation binding;endopeptidase activity;hydrolase activity;ion binding;metal ion binding;metalloendopeptidase activity;metallopeptidase activity;peptidase activity;peptidase activity, acting on L-amino a
activating transcription factor binding;binding;chromatin binding;DNA binding;nucleic acid binding;protein binding;protein binding transcription factor activity;RNA polymerase II transcription coactivator activity;RNA polymera
catalytic activity;electron carrier activity;NADH dehydrogenase (quinone) activity;NADH dehydrogenase (ubiquinone) activity;NADH dehydrogenase activity;oxidoreductase activity;oxidoreductase activity, acting on NADH or N
binding;nucleic acid binding;nucleotide binding;protein binding;protein domain specific binding;RNA binding;RS domain binding;unfolded protein binding
binding;identical protein binding;protein binding;structural molecule activity
binding;carboxylic acid binding;catalytic activity;coenzyme binding;cofactor binding;dodecenoyl-CoA delta-isomerase activity;fatty acid binding;fatty-acyl-CoA binding;intramolecular oxidoreductase activity;intramolecular ox
binding;DNA binding;nucleic acid binding
binding;chromatin binding
binding;DNA binding;nucleic acid binding
binding;double-stranded RNA binding;enzyme activator activity;enzyme binding;enzyme regulator activity;identical protein binding;nucleic acid binding;protein binding;protein dimerization activity;protein homodimerization ac
carboxylic ester hydrolase activity;catalytic activity;hydrolase activity;hydrolase activity, acting on ester bonds;lipase activity;lysophospholipase activity;palmitoyl-(protein) hydrolase activity;phospholipase activity;thiolester hy
adenyl nucleotide binding;adenyl ribonucleotide binding;ATP binding;ATPase activity;ATPase activity, coupled;ATP-dependent helicase activity;ATP-dependent RNA helicase activity;binding;catalytic activity;helicase activity;h
binding;DNA binding;nucleic acid binding;RNA binding
binding;catalytic activity;cation binding;hydrolase activity;hydrolase activity, acting on ester bonds;ion binding;magnesium ion binding;manganese ion binding;metal ion binding;phosphatase activity;phosphoprotein phosphat
structural constituent of nuclear pore;structural molecule activity;transporter activity
adenyl nucleotide binding;adenyl ribonucleotide binding;ATP binding;binding;catalytic activity;enzyme activator activity;enzyme regulator activity;GTP binding;GTPase activator activity;GTPase regulator activity;guanyl nucleo
binding;chromatin binding;DNA binding;nucleic acid binding;RNA binding
binding;protein binding;protein complex binding
aspartic-type endopeptidase activity;aspartic-type peptidase activity;catalytic activity;endopeptidase activity;hydrolase activity;peptidase activity;peptidase activity, acting on L-amino acid peptides;receptor activity
catalytic activity;endonuclease activity;endonuclease activity, active with either ribo- or deoxyribonucleic acids and producing 5'-phosphomonoesters;endoribonuclease activity;endoribonuclease activity, producing 5'-phosph
binding;nucleic acid binding;nucleotide binding;RNA binding;translation factor activity, nucleic acid binding;translation initiation factor activity
binding;nucleic acid binding;RNA binding;translation factor activity, nucleic acid binding;translation initiation factor activity
3-keto sterol reductase activity;binding;carbonyl reductase (NADPH) activity;catalytic activity;coenzyme binding;cofactor binding;NADP binding;NADPH binding;nucleotide binding;oxidoreductase activity;oxidoreductase acti
binding;protein binding;transcription factor binding
binding;catalytic activity;cation binding;endopeptidase activity;exopeptidase activity;hydrolase activity;ion binding;metal ion binding;metalloendopeptidase activity;metalloexopeptidase activity;metallopeptidase activity;peptic
binding;catalytic activity;cation binding;coenzyme binding;cofactor binding;identical protein binding;ion binding;isocitrate dehydrogenase (NADP+) activity;isocitrate dehydrogenase activity;magnesium ion binding;metal ion b
binding;cation binding;copper ion binding;ion binding;metal ion binding;transition metal ion binding
catalytic activity;hydrolase activity
structural molecule activity
cation transmembrane transporter activity;hydrogen ion transmembrane transporter activity;inorganic cation transmembrane transporter activity;ion transmembrane transporter activity;monovalent inorganic cation transmemb
binding;enzyme binding;protease binding;protein binding
adenyl nucleotide binding;adenyl ribonucleotide binding;ATP binding;binding;catalytic activity;enzyme regulator activity;GTPase regulator activity;guanyl-nucleotide exchange factor activity;kinase activity;nucleoside-triphosp
cation transmembrane transporter activity;hydrogen ion transmembrane transporter activity;inorganic cation transmembrane transporter activity;ion transmembrane transporter activity;monovalent inorganic cation transmemb
binding;carboxypeptidase activity;catalytic activity;cation binding;exopeptidase activity;hydrolase activity;ion binding;metal ion binding;metallocarboxypeptidase activity;metalloexopeptidase activity;metallopeptidase activity;
binding;catalytic activity;cation binding;disulfide oxidoreductase activity;electron carrier activity;ion binding;iron-sulfur cluster binding;metal cluster binding;metal ion binding;oxidoreductase activity;oxidoreductase activity, ac
binding;cation binding;enzyme binding;heme binding;identical protein binding;ion binding;iron ion binding;metal ion binding;protein binding;protein dimerization activity;protein homodimerization activity;tetrapyrrole binding;tr
7S RNA binding;binding;nucleic acid binding;ribonucleoprotein binding;RNA binding;signal recognition particle binding
amine binding;amino acid binding;binding;carboxylic acid binding;catalytic activity;cation binding;dimethylargininase activity;hydrolase activity;hydrolase activity, acting on carbon-nitrogen (but not peptide) bonds;hydrolase a
binding;chromatin binding;enzyme inhibitor activity;enzyme regulator activity;phosphatase inhibitor activity;phosphatase regulator activity;protein binding transcription factor activity;protein phosphatase inhibitor activity;prote
binding;catalytic activity;cation binding;chromatin binding;deacetylase activity;DNA binding;histone deacetylase activity;hydrolase activity;hydrolase activity, acting on carbon-nitrogen (but not peptide) bonds;hydrolase activit
adenyl nucleotide binding;adenyl ribonucleotide binding;ATP binding;binding;catalytic activity;identical protein binding;kinase activity;molecular transducer activity;nucleotide binding;phosphotransferase activity, alcohol grou
binding;carbohydrate binding;catalytic activity;glutamine-fructose-6-phosphate transaminase (isomerizing) activity;transaminase activity;transferase activity;transferase activity, transferring nitrogenous groups
binding;calcium ion binding;carbohydrate binding;cation binding;chemorepellent activity;enzyme inhibitor activity;enzyme regulator activity;extracellular matrix binding;glycoprotein binding;glycosaminoglycan binding;GTPase
acid-amino acid ligase activity;binding;catalytic activity;cation binding;ion binding;ligase activity;ligase activity, forming carbon-nitrogen bonds;metal ion binding;small conjugating protein ligase activity;transition metal ion bin
macromolecule transmembrane transporter activity;protein transmembrane transporter activity;protein transporter activity;substrate-specific transmembrane transporter activity;substrate-specific transporter activity;transmem
actin binding;actin filament binding;actin-dependent ATPase activity;adenyl nucleotide binding;adenyl ribonucleotide binding;ATP binding;ATPase activity;ATPase activity, coupled;binding;calmodulin binding;catalytic activity;
binding;cation binding;ion binding;metal ion binding;transition metal ion binding;zinc ion binding
acid-amino acid ligase activity;catalytic activity;ligase activity;ligase activity, forming carbon-nitrogen bonds;small conjugating protein ligase activity;UFM1 conjugating enzyme activity
binding;enzyme binding;kinase binding;mitogen-activated protein kinase kinase kinase binding;protein binding;protein complex scaffold;protein C-terminus binding;protein kinase binding;structural molecule activity
binding;enzyme binding;protein binding;small conjugating protein binding;transcription factor binding;ubiquitin binding;ubiquitin protein ligase binding
binding;cofactor binding;pyridoxal phosphate binding;vitamin B6 binding;vitamin binding
androgen receptor binding;binding;hormone receptor binding;nuclear hormone receptor binding;nucleic acid binding;protein binding;protein binding transcription factor activity;receptor binding;ribonucleoprotein binding;RNA
binding;growth factor activity;lipoprotein particle receptor binding;low-density lipoprotein particle receptor binding;molecular transducer activity;protein binding;receptor antagonist activity;receptor binding;receptor inhibitor a
catalytic activity;glutaminase activity;hydrolase activity;hydrolase activity, acting on carbon-nitrogen (but not peptide) bonds;hydrolase activity, acting on carbon-nitrogen (but not peptide) bonds, in linear amides
binding;enzyme binding;kinase binding;lipid binding;protein binding;protein kinase binding;protein transporter activity;substrate-specific transporter activity;transporter activity
binding;calcium-dependent protein binding;protein binding
binding;cyclin-dependent protein kinase inhibitor activity;cyclin-dependent protein kinase regulator activity;enzyme inhibitor activity;enzyme regulator activity;kinase inhibitor activity;kinase regulator activity;nucleic acid bindir
acid-amino acid ligase activity;binding;catalytic activity;cation binding;ion binding;ligase activity;ligase activity, forming carbon-nitrogen bonds;metal ion binding;nucleic acid binding;RNA binding;small conjugating protein liga
catalytic activity;endopeptidase activity;hydrolase activity;peptidase activity;peptidase activity, acting on L-amino acid peptides;serine hydrolase activity;serine-type endopeptidase activity;serine-type peptidase activity
binding;nucleotide binding
catalytic activity;NADH dehydrogenase (quinone) activity;NADH dehydrogenase (ubiquinone) activity;NADH dehydrogenase activity;oxidoreductase activity;oxidoreductase activity, acting on NADH or NADPH;oxidoreductase
binding;catalytic activity;enzyme binding;GTP binding;GTPase activity;guanyl nucleotide binding;guanyl ribonucleotide binding;hydrolase activity;hydrolase activity, acting on acid anhydrides;hydrolase activity, acting on acid
binding;nucleic acid binding;protein transporter activity;RNA binding;RNA cap binding;substrate-specific transporter activity;transporter activity
acid-amino acid ligase activity;binding;catalytic activity;enzyme binding;ligase activity;ligase activity, forming carbon-nitrogen bonds;protein binding;small conjugating protein ligase activity;ubiquitin-protein ligase activity;ubic
binding;cation binding;DNA binding;ion binding;metal ion binding;nucleic acid binding;transition metal ion binding;zinc ion binding
enzyme regulator activity;kinase regulator activity;molecular transducer activity;phosphorylase kinase regulator activity;protein kinase regulator activity;signal transducer activity
catalytic activity;NADH dehydrogenase (quinone) activity;NADH dehydrogenase (ubiquinone) activity;NADH dehydrogenase activity;oxidoreductase activity;oxidoreductase activity, acting on NADH or NADPH;oxidoreductase
catalytic activity;NADH dehydrogenase (quinone) activity;NADH dehydrogenase (ubiquinone) activity;NADH dehydrogenase activity;oxidoreductase activity;oxidoreductase activity, acting on NADH or NADPH;oxidoreductase
binding;calcium ion binding;cation binding;ion binding;metal ion binding
binding;cation binding;ion binding;metal ion binding;nucleic acid binding;nucleic acid binding transcription factor activity;RNA binding;sequence-specific DNA binding transcription factor activity;transition metal ion binding;zi
binding;epidermal growth factor receptor binding;growth factor receptor binding;insulin receptor binding;lipid binding;phosphatidylinositol binding;phospholipid binding;protein binding;protein complex binding;receptor bindir
binding;DNA binding;mRNA binding;nucleic acid binding;RNA binding
adenyl nucleotide binding;adenyl ribonucleotide binding;ATP binding;ATPase activity;binding;catalytic activity;enzyme binding;hydrolase activity;hydrolase activity, acting on acid anhydrides;hydrolase activity, acting on acid a
adenyl nucleotide binding;adenyl ribonucleotide binding;ATP binding;ATPase activity;binding;catalytic activity;DNA binding;hydrolase activity;hydrolase activity, acting on acid anhydrides;hydrolase activity, acting on acid anhy
binding;protein binding;SNAP receptor activity
beta-tubulin binding;binding;cytoskeletal protein binding;enzyme binding;FFAT motif binding;identical protein binding;microtubule binding;protein binding;protein dimerization activity;protein domain specific binding;protein he
structural molecule activity
adenyl nucleotide binding;adenyl ribonucleotide binding;ATP binding;binding;catalytic activity;kinase activity;NADH dehydrogenase (quinone) activity;NADH dehydrogenase (ubiquinone) activity;NADH dehydrogenase activity;
binding;calcium ion binding;catalytic activity;cation binding;cis-trans isomerase activity;drug binding;FK506 binding;ion binding;isomerase activity;macrolide binding;metal ion binding;peptidyl-prolyl cis-trans isomerase activi
6-phosphogluconolactonase activity;binding;carbohydrate binding;carboxylic ester hydrolase activity;catalytic activity;hydrolase activity;hydrolase activity, acting on ester bonds;monosaccharide binding;sugar binding
adenyl nucleotide binding;adenyl ribonucleotide binding;adenylylsulfate kinase activity;adenylyltransferase activity;ATP binding;binding;catalytic activity;kinase activity;nucleotide binding;nucleotidyltransferase activity;phosph
adenyl nucleotide binding;adenyl ribonucleotide binding;ATP binding;binding;nucleotide binding;protein binding;protein dimerization activity;protein heterodimerization activity;purine nucleotide binding;purine ribonucleoside t
APG12 activating enzyme activity;APG8 activating enzyme activity;binding;catalytic activity;identical protein binding;protein binding;protein dimerization activity;protein homodimerization activity;small protein activating enzyn
binding;cation binding;cytokine binding;DNA binding;growth factor binding;interleukin-1 binding;ion binding;metal ion binding;NACHT domain binding;nucleic acid binding;protein binding;protein domain specific binding;trans
catalytic activity;hydrolase activity
binding;enzyme binding;enzyme regulator activity;GTPase binding;GTPase regulator activity;nucleoside-triphosphatase regulator activity;protein binding;Ran GTPase binding;Ras GTPase binding;small GTPase binding;transp
acid-amino acid ligase activity;binding;catalytic activity;cation binding;ion binding;ligase activity;ligase activity, forming carbon-nitrogen bonds;metal ion binding;nucleic acid binding;small conjugating protein ligase activity;tra
binding;catalytic activity;cation binding;intramolecular transferase activity;intramolecular transferase activity, phosphotransferases;ion binding;isomerase activity;magnesium ion binding;metal ion binding;phosphoacetylgluco
binding;ribonucleoprotein binding
ATPase activator activity;ATPase regulator activity;binding;chaperone binding;enzyme activator activity;enzyme regulator activity;nucleoside-triphosphatase regulator activity;protein binding
aldehyde-lyase activity;binding;carbon-carbon lyase activity;carboxy-lyase activity;catalytic activity;cofactor binding;lyase activity;pyridoxal phosphate binding;sphinganine-1-phosphate aldolase activity;vitamin B6 binding;vi
6-phosphogluconolactonase activity;binding;carboxylic ester hydrolase activity;catalytic activity;coenzyme binding;cofactor binding;glucose 1-dehydrogenase [NAD(P)] activity;glucose-6-phosphate dehydrogenase activity;hy

binding;cation binding;ion binding;metal ion binding;transition metal ion binding;zinc ion binding
binding;cation binding;ion binding;metal ion binding;transition metal ion binding;transporter activity;zinc ion binding
binding;catalytic activity;GPI anchor binding;hydrolase activity;hydrolase activity, acting on carbon-nitrogen (but not peptide) bonds;hydrolase activity, acting on carbon-nitrogen (but not peptide) bonds, in linear amides;lipid b
binding;catalytic activity;cation binding;dioxygenase activity;ion binding;iron ion binding;metal ion binding;oxidoreductase activity;oxidoreductase activity, acting on single donors with incorporation of molecular oxygen;oxido
adenyl nucleotide binding;adenyl ribonucleotide binding;ATP binding;binding;catalytic activity;enzyme binding;fatty acid ligase activity;kinase binding;ligase activity;ligase activity, forming carbon-sulfur bonds;long-chain fatty
binding;catalytic activity;cation binding;cysteine-type peptidase activity;hydrolase activity;ion binding;metal ion binding;metallopeptidase activity;peptidase activity;peptidase activity, acting on L-amino acid peptides;protein b
catalytic activity;methyltransferase activity;O-methyltransferase activity;transferase activity;transferase activity, transferring one-carbon groups
binding;catalytic activity;cytoskeletal protein binding;GTP binding;GTPase activity;guanyl nucleotide binding;guanyl ribonucleotide binding;hydrolase activity;hydrolase activity, acting on acid anhydrides;hydrolase activity, act
binding;protein binding;SNAP receptor activity;SNARE binding;syntaxin binding
adenyl nucleotide binding;adenyl ribonucleotide binding;ATP binding;binding;catalytic activity;cation binding;ion binding;kinase activity;magnesium ion binding;metal ion binding;molecular transducer activity;nucleotide bindin
adenyl nucleotide binding;adenyl ribonucleotide binding;ATP binding;binding;nucleotide binding;purine nucleotide binding;purine ribonucleoside triphosphate binding;purine ribonucleotide binding;ribonucleotide binding
binding;nucleic acid binding;nucleotide binding;RNA binding
binding;nucleic acid binding;RNA binding;snRNA binding;U6 snRNA 3'-end binding;U6 snRNA binding
binding;enzyme binding;kinase binding;protein binding;protein kinase binding;protein transporter activity;substrate-specific transporter activity;transporter activity
binding;double-stranded RNA binding;nucleic acid binding;RNA binding
binding;enzyme binding;kinase binding;lipid binding;phosphatidylserine binding;phospholipid binding;protein binding;protein kinase binding;protein kinase C binding
binding;identical protein binding;protein binding
adenyl nucleotide binding;adenyl ribonucleotide binding;ATP binding;binding;catalytic activity;creatine kinase activity;kinase activity;nucleotide binding;phosphotransferase activity, alcohol group as acceptor;phosphotransfer
binding;catalytic activity;cation binding;coenzyme binding;cofactor binding;ion binding;metal ion binding;NADP binding;NADPH:quinone reductase activity;nucleotide binding;oxidoreductase activity;oxidoreductase activity, a
binding;catalytic activity;coenzyme binding;cofactor binding;DNA binding;electron carrier activity;FAD binding;flavin adenine dinucleotide binding;NAD(P)H oxidase activity;nucleic acid binding;oxidoreductase activity;oxidore
binding;cytoskeletal protein binding;microtubule binding;protein binding;tubulin binding
3'(2'),5'-bisphosphate nucleotidase activity;binding;catalytic activity;cation binding;hydrolase activity;hydrolase activity, acting on ester bonds;inositol bisphosphate phosphatase activity;inositol or phosphatidylinositol phosph
amine binding;amino acid binding;binding;carboxylic acid binding;catalytic activity;dimethylargininase activity;hydrolase activity;hydrolase activity, acting on carbon-nitrogen (but not peptide) bonds;hydrolase activity, acting c
catalytic activity;oxidoreductase activity;oxidoreductase activity, acting on a sulfur group of donors;oxidoreductase activity, acting on a sulfur group of donors, disulfide as acceptor;protein-disulfide reductase (glutathione) act
binding;calcium ion binding;cation binding;extracellular matrix structural constituent;ion binding;metal ion binding;structural molecule activity
binding;chromatin binding;DNA binding;methyl-CpG binding;nucleic acid binding;nucleotide binding;sequence-specific DNA binding
binding;catalytic activity;cation binding;diphosphoinositol-polyphosphate diphosphatase activity;hydrolase activity;hydrolase activity, acting on acid anhydrides;hydrolase activity, acting on acid anhydrides, in phosphorus-co
binding;enzyme binding;kinase binding;NF-kappaB binding;protease binding;protein binding;protein binding transcription factor activity;protein C-terminus binding;protein kinase B binding;protein kinase binding;protein self-a
catalytic activity;NADH dehydrogenase (quinone) activity;NADH dehydrogenase (ubiquinone) activity;NADH dehydrogenase activity;oxidoreductase activity;oxidoreductase activity, acting on NADH or NADPH;oxidoreductase
channel activity;macromolecule transmembrane transporter activity;passive transmembrane transporter activity;porin activity;protein transmembrane transporter activity;protein transporter activity;substrate-specific transmen
adenyl nucleotide binding;adenyl ribonucleotide binding;ATP binding;binding;catalytic activity;enzyme binding;GTPase binding;kinase activity;nucleotide binding;phosphotransferase activity, alcohol group as acceptor;protein
binding;chromatin binding;protein binding transcription factor activity;transcription coactivator activity;transcription cofactor activity;transcription factor binding transcription factor activity
aldo-keto reductase (NADP) activity;binding;catalytic activity;cation binding;cation transmembrane transporter activity;cytochrome-c oxidase activity;enzyme binding;heme binding;heme-copper terminal oxidase activity;hydr
catalytic activity;lactate dehydrogenase activity;L-lactate dehydrogenase activity;oxidoreductase activity;oxidoreductase activity, acting on CH-OH group of donors;oxidoreductase activity, acting on the CH-OH group of dono
adenyl nucleotide binding;adenyl ribonucleotide binding;ADP binding;amine binding;amino acid binding;ATP binding;binding;carboxylic acid binding;catalytic activity;coenzyme binding;cofactor binding;glutamate dehydrogen
binding;catalytic activity;coenzyme binding;cofactor binding;dihydrofolate reductase activity;drug binding;mRNA binding;NADP binding;nucleic acid binding;nucleotide binding;oxidoreductase activity;oxidoreductase activity,
adenyl nucleotide binding;adenyl ribonucleotide binding;ADP binding;AMP binding;binding;catalytic activity;coenzyme binding;cofactor binding;cytochrome-b5 reductase activity;FAD binding;flavin adenine dinucleotide bindi
antioxidant activity;binding;catalytic activity;coenzyme binding;cofactor binding;disulfide oxidoreductase activity;electron carrier activity;flavin adenine dinucleotide binding;glutathione disulfide oxidoreductase activity;glutathi
binding;catalytic activity;cation binding;cation transmembrane transporter activity;copper ion binding;cytochrome-c oxidase activity;heme-copper terminal oxidase activity;hydrogen ion transmembrane transporter activity;inc
antioxidant activity;binding;catalytic activity;cation binding;chaperone binding;copper ion binding;enzyme binding;GTPase binding;identical protein binding;ion binding;metal ion binding;oxidoreductase activity;oxidoreductas
binding;catalytic activity;cation binding;ion binding;metal ion binding;protein-glutamine gamma-glutamyltransferase activity;transferase activity;transferase activity, transferring acyl groups;transferase activity, transferring ami
anion binding;binding;catalytic activity;drug binding;ion binding;nucleobase binding;nucleoside binding;phosphate ion binding;purine base binding;purine-nucleoside phosphorylase activity;transferase activity;transferase act
binding;catalytic activity;cation binding;guanine phosphoribosyltransferase activity;hypoxanthine phosphoribosyltransferase activity;identical protein binding;ion binding;magnesium ion binding;metal ion binding;nucleotide bi
binding;catalytic activity;cofactor binding;kynurenine-oxoglutarate transaminase activity;L-aspartate:2-oxoglutarate aminotransferase activity;L-phenylalanine aminotransferase activity;L-phenylalanine:2-oxoglutarate aminotra
actin binding;actin filament binding;adenyl nucleotide binding;adenyl ribonucleotide binding;ATP binding;binding;catalytic activity;chromatin binding;cytoskeletal protein binding;DNA binding;double-stranded DNA binding;enz
adenyl nucleotide binding;adenyl ribonucleotide binding;ATP binding;binding;catalytic activity;kinase activity;nucleotide binding;phosphoglycerate kinase activity;phosphotransferase activity, carboxyl group as acceptor;purin
adenyl nucleotide binding;adenyl ribonucleotide binding;adenylate kinase activity;ATP binding;binding;catalytic activity;kinase activity;nucleobase-containing compound kinase activity;nucleoside diphosphate kinase activity;r
binding;calcium ion binding;catalytic activity;cation binding;endopeptidase activity;growth factor activity;hydrolase activity;ion binding;metal ion binding;molecular transducer activity;peptidase activity;peptidase activity, actin
binding;calcium ion binding;catalytic activity;cation binding;endopeptidase activity;hydrolase activity;ion binding;metal ion binding;peptidase activity;peptidase activity, acting on L-amino acid peptides;serine hydrolase activit
binding;calcium ion binding;catalytic activity;cation binding;endopeptidase activity;hydrolase activity;ion binding;lipid binding;metal ion binding;peptidase activity;peptidase activity, acting on L-amino acid peptides;phospholi
apolipoprotein binding;binding;catalytic activity;endopeptidase activity;hydrolase activity;peptidase activity;peptidase activity, acting on L-amino acid peptides;protein binding;protein domain specific binding;receptor binding
catalytic activity;endopeptidase activity;hydrolase activity;peptidase activity;peptidase activity, acting on L-amino acid peptides;serine hydrolase activity;serine-type endopeptidase activity;serine-type peptidase activity
catalytic activity;endopeptidase activity;hydrolase activity;peptidase activity;peptidase activity, acting on L-amino acid peptides;serine hydrolase activity;serine-type endopeptidase activity;serine-type peptidase activity
adenosine deaminase activity;binding;catalytic activity;cation binding;deaminase activity;hydrolase activity;hydrolase activity, acting on carbon-nitrogen (but not peptide) bonds;hydrolase activity, acting on carbon-nitrogen (b
cation transmembrane transporter activity;hydrogen ion transmembrane transporter activity;inorganic cation transmembrane transporter activity;ion transmembrane transporter activity;monovalent inorganic cation transmemb
adenyl nucleotide binding;adenyl ribonucleotide binding;amine binding;amino acid binding;argininosuccinate synthase activity;ATP binding;binding;carboxylic acid binding;catalytic activity;identical protein binding;ligase activ
binding;carbohydrate binding;endopeptidase inhibitor activity;endopeptidase regulator activity;enzyme binding;enzyme inhibitor activity;enzyme regulator activity;glycosaminoglycan binding;heparin binding;pattern binding;pe
binding;calcium-dependent protein binding;chemokine binding;C-X-C chemokine binding;cytokine binding;endopeptidase inhibitor activity;endopeptidase regulator activity;enzyme binding;enzyme inhibitor activity;enzyme re
binding;C5a anaphylatoxin chemotactic receptor binding;C5L2 anaphylatoxin chemotactic receptor binding;endopeptidase inhibitor activity;endopeptidase regulator activity;enzyme inhibitor activity;enzyme regulator activity;
binding;chemokine activity;chemokine receptor binding;cytokine activity;cytokine receptor binding;endopeptidase inhibitor activity;endopeptidase regulator activity;enzyme inhibitor activity;enzyme regulator activity;G-protein
binding;cation binding;cytokine activity;endopeptidase inhibitor activity;endopeptidase regulator activity;enzyme binding;enzyme inhibitor activity;enzyme regulator activity;ion binding;metal ion binding;metalloendopeptidase
binding;GTP binding;guanyl nucleotide binding;guanyl ribonucleotide binding;nucleotide binding;protein binding;protein complex binding;purine nucleotide binding;purine ribonucleoside triphosphate binding;purine ribonucle
binding;GDP binding;GMP binding;GTP binding;guanyl nucleotide binding;guanyl ribonucleotide binding;nucleotide binding;protein binding;protein complex binding;purine nucleotide binding;purine ribonucleoside triphospha
binding;calcium ion binding;cargo receptor activity;cation binding;clathrin binding;clathrin heavy chain binding;glycoprotein binding;ion binding;lipoprotein particle binding;lipoprotein particle receptor activity;low-density lipop
antigen binding;binding;peptide antigen binding;peptide binding;protein binding;receptor binding
antigen binding;beta-2-microglobulin binding;binding;peptide antigen binding;peptide binding;protein binding
binding;cation binding;heme binding;ion binding;iron ion binding;metal ion binding;oxygen binding;oxygen transporter activity;substrate-specific transporter activity;tetrapyrrole binding;transition metal ion binding;transporter
binding;cation binding;extracellular matrix structural constituent;growth factor binding;identical protein binding;ion binding;metal ion binding;platelet-derived growth factor binding;protein binding;structural molecule activity
binding;cation binding;extracellular matrix structural constituent;growth factor binding;integrin binding;ion binding;metal ion binding;platelet-derived growth factor binding;protein binding;protein complex binding;receptor bin
structural constituent of cytoskeleton;structural molecule activity
structural constituent of cytoskeleton;structural molecule activity
structural molecule activity
antioxidant activity;beta-amyloid binding;binding;carbohydrate binding;cation binding;cholesterol binding;cholesterol transporter activity;cytoskeletal protein binding;enzyme activator activity;enzyme regulator activity;glycosa
binding;chaperone binding;protein binding;structural molecule activity
binding;carbohydrate binding;enzyme activator activity;enzyme regulator activity;glycoprotein binding;glycosaminoglycan binding;heparin binding;identical protein binding;lipase activator activity;lipid binding;lipoprotein lipas
binding;carbohydrate binding;collagen binding;enzyme activator activity;enzyme binding;enzyme regulator activity;glycosaminoglycan binding;heparin binding;integrin binding;pattern binding;peptidase activator activity;pepti
binding;isoprenoid binding;lipid binding;retinal binding;retinoid binding;retinol binding;retinol transporter activity;transmembrane transporter activity;transporter activity;vitamin binding;vitamin transporter activity
cysteine-type endopeptidase inhibitor activity;endopeptidase inhibitor activity;endopeptidase regulator activity;enzyme inhibitor activity;enzyme regulator activity;kinase inhibitor activity;kinase regulator activity;peptidase inhil
binding;cation binding;ion binding;metal ion binding
actin binding;binding;cytoskeletal protein binding;lipid binding;protein binding;steroid binding;transmembrane transporter activity;transporter activity;vitamin binding;vitamin D binding;vitamin transporter activity
binding;cargo receptor activity;double-stranded RNA binding;identical protein binding;nucleic acid binding;protein binding;receptor activity;RNA binding;transferrin receptor activity
binding;cation binding;cation transmembrane transporter activity;ferric iron binding;ferric iron transmembrane transporter activity;inorganic cation transmembrane transporter activity;ion binding;ion transmembrane transporte
binding;carbohydrate binding;catalytic activity;cation binding;DNA binding;endopeptidase activity;enzyme activator activity;enzyme regulator activity;glycosaminoglycan binding;heparin binding;hydrolase activity;ion binding;i
binding;cation binding;drug binding;ion binding;metal ion binding;transition metal ion binding;zinc ion binding
aminopeptidase activity;binding;carbohydrate binding;catalytic activity;endopeptidase activity;exopeptidase activity;glycosaminoglycan binding;heparin binding;hydrolase activity;pattern binding;peptidase activity;peptidase a
binding;calcium ion binding;catalytic activity;cation binding;endopeptidase activity;hydrolase activity;ion binding;metal ion binding;metalloendopeptidase activity;metallopeptidase activity;peptidase activity;peptidase activity,
binding;carbohydrate binding;cargo receptor activity;extracellular matrix binding;glycosaminoglycan binding;heparin binding;integrin binding;pattern binding;polysaccharide binding;protein binding;protein complex binding;re
aminoacylase activity;antioxidant activity;binding;catalase activity;catalytic activity;cation binding;coenzyme binding;cofactor binding;enzyme binding;heme binding;hydrolase activity;hydrolase activity, acting on carbon-nitro
adenyl nucleotide binding;adenyl ribonucleotide binding;ATP binding;binding;catalytic activity;cation binding;identical protein binding;ion binding;kinase activity;MAP kinase kinase kinase activity;metal ion binding;molecular t
binding;catalytic activity;ceramidase activity;glucosylceramidase activity;hydrolase activity;hydrolase activity, acting on carbon-nitrogen (but not peptide) bonds;hydrolase activity, acting on carbon-nitrogen (but not peptide) b
alpha-L-fucosidase activity;binding;carbohydrate binding;catalytic activity;fucose binding;fucosidase activity;hydrolase activity;hydrolase activity, acting on glycosyl bonds;hydrolase activity, hydrolyzing O-glycosyl compound
actin binding;aldehyde-lyase activity;binding;carbohydrate binding;carbon-carbon lyase activity;catalytic activity;cytoskeletal protein binding;fructose binding;fructose-bisphosphate aldolase activity;identical protein binding;l
binding;cysteine-type endopeptidase inhibitor activity;endopeptidase inhibitor activity;endopeptidase regulator activity;enzyme binding;enzyme inhibitor activity;enzyme regulator activity;peptidase inhibitor activity;peptidase
ATPase activity;ATPase activity, coupled;binding;binding, bridging;calcium ion binding;calcium-dependent phospholipid binding;calcium-dependent protein binding;catalytic activity;cation binding;DNA binding;DNA-depende
binding;carbohydrate binding;cholesterol transporter activity;enzyme binding;glycosaminoglycan binding;heparin binding;lipase binding;lipid binding;lipid transporter activity;lipoprotein particle receptor binding;low-density lip
antioxidant activity;binding;catalytic activity;cation binding;DNA binding;identical protein binding;ion binding;manganese ion binding;metal ion binding;nucleic acid binding;oxidoreductase activity;oxidoreductase activity, actin
binding;catalytic activity;cofactor binding;ornithine-oxo-acid transaminase activity;pyridoxal phosphate binding;transaminase activity;transferase activity;transferase activity, transferring nitrogenous groups;vitamin B6 binding
adenyl nucleotide binding;adenyl ribonucleotide binding;ATP binding;binding;catalytic activity;cation binding;deoxynucleoside kinase activity;identical protein binding;ion binding;kinase activity;metal ion binding;nucleobase-c
binding;enzyme activator activity;enzyme regulator activity;GPI anchor binding;GTPase activator activity;GTPase regulator activity;integrin binding;lipid binding;nucleoside-triphosphatase regulator activity;phosphatidylinosito
binding;carbohydrate binding;receptor activity;structural molecule activity
binding;catalytic activity;coenzyme binding;cofactor binding;cytoskeletal protein binding;glyceraldehyde-3-phosphate dehydrogenase (NAD+) (phosphorylating) activity;identical protein binding;microtubule binding;NAD bindi
amidine-lyase activity;argininosuccinate lyase activity;carbon-nitrogen lyase activity;catalytic activity;lyase activity
antigen binding;beta-2-microglobulin binding;binding;peptide antigen binding;peptide binding;protein binding;TAP binding
binding;calcium ion binding;calcium-dependent cysteine-type endopeptidase activity;catalytic activity;cation binding;cysteine-type endopeptidase activity;cysteine-type peptidase activity;endopeptidase activity;hydrolase ac
adenyl nucleotide binding;adenyl ribonucleotide binding;ATP binding;binding;cation binding;chaperone binding;chromatin binding;copper ion binding;core promoter binding;core promoter sequence-specific DNA binding;dam
binding;cation binding;ion binding;metal ion binding;transition metal ion binding;zinc ion binding
binding;enzyme binding;enzyme inhibitor activity;enzyme regulator activity;identical protein binding;kinase binding;kinase inhibitor activity;kinase regulator activity;protein binding;protein kinase binding;protein kinase C bindir
5,10-methylenetetrahydrofolate-dependent methyltransferase activity;amine binding;amino acid binding;binding;carboxylic acid binding;catalytic activity;cofactor binding;drug binding;folic acid binding;methyltransferase acti
catalytic activity;dolichyl-diphosphooligosaccharide-protein glycotransferase activity;oligosaccharyl transferase activity;transferase activity;transferase activity, transferring glycosyl groups;transferase activity, transferring hex
binding;catalytic activity;ribonucleoprotein binding;ribosome binding;transferase activity;transferase activity, transferring glycosyl groups
binding;catalytic activity;cation binding;G-protein beta/gamma-subunit complex binding;G-protein-coupled receptor binding;GTP binding;GTPase activity;guanyl nucleotide binding;guanyl ribonucleotide binding;hydrolase ac
binding;DNA binding;nucleic acid binding
active transmembrane transporter activity;adenyl nucleotide binding;adenyl ribonucleotide binding;ADP binding;alkali metal ion binding;ATP binding;ATPase activity;ATPase activity, coupled;ATPase activity, coupled to moven
active transmembrane transporter activity;antigen binding;ATPase activator activity;ATPase activity;ATPase activity, coupled;ATPase activity, coupled to movement of substances;ATPase activity, coupled to transmembrane m
acetylcholine receptor binding;binding;carbohydrate binding;cation binding;DNA binding;endopeptidase inhibitor activity;endopeptidase regulator activity;enzyme activator activity;enzyme binding;enzyme inhibitor activity;enz
aldehyde dehydrogenase (NAD) activity;aldehyde dehydrogenase [NAD(P)+] activity;catalytic activity;electron carrier activity;oxidoreductase activity;oxidoreductase activity, acting on the aldehyde or oxo group of donors;oxid
binding;catalytic activity;cell adhesion molecule binding;cytokine receptor binding;enzyme binding;extracellular matrix binding;fibronectin binding;growth factor receptor binding;identical protein binding;intramolecular oxidore
binding;chromatin binding;DNA binding;nucleic acid binding
endopeptidase inhibitor activity;endopeptidase regulator activity;enzyme inhibitor activity;enzyme regulator activity;peptidase inhibitor activity;peptidase regulator activity;serine-type endopeptidase inhibitor activity
binding;endopeptidase inhibitor activity;endopeptidase regulator activity;enzyme binding;enzyme inhibitor activity;enzyme regulator activity;peptidase inhibitor activity;peptidase regulator activity;protease binding;protein binc
adenine transmembrane transporter activity;binding;enzyme binding;nucleobase transmembrane transporter activity;nucleobase-containing compound transmembrane transporter activity;protein binding;purine base transme
protein tag
binding;nucleic acid binding;ribonucleoprotein binding;ribosome binding;RNA binding;translation factor activity, nucleic acid binding;translation initiation factor activity
binding;DNA binding;nucleic acid binding

binding;integrin binding;molecular transducer activity;protein binding;protein complex binding;receptor activity;receptor binding;signal transducer activity;signaling receptor activity;transmembrane signaling receptor activity;v
binding;nucleic acid binding;RNA binding;structural constituent of ribosome;structural molecule activity
binding;nucleic acid binding;RNA binding;structural constituent of ribosome;structural molecule activity
binding;calcium ion binding;carbohydrate binding;cation binding;glycosaminoglycan binding;heparin binding;ion binding;metal ion binding;pattern binding;polysaccharide binding
binding;mRNA binding;nucleic acid binding;nucleotide binding;RNA binding;tRNA binding
binding;endopeptidase inhibitor activity;endopeptidase regulator activity;enzyme inhibitor activity;enzyme regulator activity;hormone binding;peptidase inhibitor activity;peptidase regulator activity;serine-type endopeptidase i
binding;carbohydrate binding;endopeptidase inhibitor activity;endopeptidase regulator activity;enzyme inhibitor activity;enzyme regulator activity;glycosaminoglycan binding;heparin binding;pattern binding;peptidase inhibitor
actin binding;binding;cation binding;cell adhesion molecule binding;cytoskeletal protein binding;enzyme binding;ion binding;metal ion binding;peptide binding;protease binding;protein binding;protein dimerization activity;pro
binding;protein binding;scaffold protein binding;structural molecule activity
binding;protein binding;scaffold protein binding;structural molecule activity
binding;cation binding;extracellular matrix structural constituent;ion binding;metal ion binding;structural molecule activity
carbon-carbon lyase activity;carboxy-lyase activity;catalytic activity;lyase activity;uroporphyrinogen decarboxylase activity
alpha-galactosidase activity;binding;carbohydrate binding;catalytic activity;galactosidase activity;galactoside binding;hydrolase activity;hydrolase activity, acting on glycosyl bonds;hydrolase activity, hydrolyzing O-glycosyl co
actin binding;binding;calcium ion binding;cation binding;cytoskeletal protein binding;ion binding;metal ion binding;myosin binding;myosin II binding;protein binding;protein domain specific binding
adenyl nucleotide binding;adenyl ribonucleotide binding;ATP binding;binding;catalytic activity;cyclin-dependent protein kinase activity;histone kinase activity;kinase activity;nucleotide binding;phosphotransferase activity, alco
active transmembrane transporter activity;adenyl nucleotide binding;adenyl ribonucleotide binding;ATP binding;ATPase activity;ATPase activity, coupled;ATPase activity, coupled to movement of substances;ATPase activity, c
binding;calcium ion binding;calcium-dependent protein binding;cation binding;cytoskeletal protein binding;identical protein binding;ion binding;ion transmembrane transporter activity;metal ion binding;protein binding;protein
binding;nucleic acid binding;RNA binding;RNA cap binding;translation factor activity, nucleic acid binding;translation initiation factor activity
binding;carbon-oxygen lyase activity;catalytic activity;cation binding;DNA binding;enzyme binding;GTPase binding;hydro-lyase activity;ion binding;lyase activity;magnesium ion binding;metal ion binding;nucleic acid binding;r
adenyl nucleotide binding;adenyl ribonucleotide binding;AMP binding;ATP binding;bile acid binding;binding;carbohydrate binding;carboxylic acid binding;catalytic activity;cofactor binding;drug binding;glucose binding;glycog
binding;carbohydrate binding;catalytic activity;glucose-6-phosphate isomerase activity;intramolecular oxidoreductase activity;intramolecular oxidoreductase activity, interconverting aldoses and ketoses;intramolecular transfe
binding;enzyme binding;enzyme inhibitor activity;enzyme regulator activity;histone binding;identical protein binding;kinase binding;kinase inhibitor activity;kinase regulator activity;NF-kappaB binding;nucleic acid binding;prot
binding;calcium channel activity;cation binding;cation channel activity;cation transmembrane transporter activity;channel activity;cytokine binding;enzyme binding;extracellular matrix binding;fibronectin binding;gated channe
beta-N-acetylhexosaminidase activity;binding;catalytic activity;hexosaminidase activity;hydrolase activity;hydrolase activity, acting on glycosyl bonds;hydrolase activity, hydrolyzing O-glycosyl compounds;protein binding;pro
binding;carbohydrate binding;endopeptidase inhibitor activity;endopeptidase regulator activity;enzyme inhibitor activity;enzyme regulator activity;glycosaminoglycan binding;heparin binding;pattern binding;peptidase inhibitor
catalytic activity;cis-stilbene-oxide hydrolase activity;epoxide hydrolase activity;ether hydrolase activity;hydrolase activity;hydrolase activity, acting on ether bonds
binding;catalytic activity;coenzyme binding;cofactor binding;lactate dehydrogenase activity;L-lactate dehydrogenase activity;NAD binding;nucleotide binding;oxidoreductase activity;oxidoreductase activity, acting on CH-OH
amine binding;amino acid binding;antioxidant activity;binding;carboxylic acid binding;catalytic activity;glutathione binding;glutathione peroxidase activity;modified amino acid binding;oxidoreductase activity;oxidoreductase a
catalytic activity;endopeptidase activity;hydrolase activity;intramolecular oxidoreductase activity;intramolecular oxidoreductase activity, interconverting keto- and enol-groups;intramolecular oxidoreductase activity, transposin
binding;chromatin binding;chromatin DNA binding;DNA binding;nucleic acid binding;structure-specific DNA binding
aspartic-type endopeptidase activity;aspartic-type peptidase activity;catalytic activity;endopeptidase activity;hydrolase activity;peptidase activity;peptidase activity, acting on L-amino acid peptides
binding;calcium ion binding;calcium-dependent phospholipid binding;calcium-dependent protein binding;cation binding;enzyme inhibitor activity;enzyme regulator activity;ion binding;lipase inhibitor activity;lipid binding;meta
binding;calcium ion binding;calcium-dependent cysteine-type endopeptidase activity;catalytic activity;cation binding;cysteine-type endopeptidase activity;cysteine-type peptidase activity;endopeptidase activity;hydrolase ac
binding;catalytic activity;enzyme binding;GTP binding;GTPase activity;guanyl nucleotide binding;guanyl ribonucleotide binding;hydrolase activity;hydrolase activity, acting on acid anhydrides;hydrolase activity, acting on acid
binding;enzyme activator activity;enzyme regulator activity;lipid binding
beta-N-acetylhexosaminidase activity;binding;catalytic activity;hexosaminidase activity;hydrolase activity;hydrolase activity, acting on glycosyl bonds;hydrolase activity, hydrolyzing O-glycosyl compounds;identical protein bin
binding;catalytic activity;collagen binding;cysteine-type endopeptidase activity;cysteine-type peptidase activity;endopeptidase activity;fibronectin binding;glycoprotein binding;histone binding;hydrolase activity;peptidase acti
actin binding;actin monomer binding;adenyl nucleotide binding;adenyl-nucleotide exchange factor activity;ATPase regulator activity;binding;cytoskeletal protein binding;enzyme regulator activity;lipid binding;nucleoside-triph
adenine binding;adenine phosphoribosyltransferase activity;adenyl nucleotide binding;adenyl ribonucleotide binding;AMP binding;binding;catalytic activity;nucleobase binding;nucleotide binding;purine base binding;purine nu
adenyl nucleotide binding;adenyl ribonucleotide binding;aminoacyl-tRNA ligase activity;ATP binding;binding;catalytic activity;enzyme binding;glutamate-tRNA ligase activity;GTPase binding;ligase activity;ligase activity, formi
binding;catalytic activity;collagen binding;cysteine-type endopeptidase activity;cysteine-type peptidase activity;endopeptidase activity;glycoprotein binding;hydrolase activity;peptidase activity;peptidase activity, acting on L-
adenyl deoxyribonucleotide binding;adenyl nucleotide binding;adenyl ribonucleotide binding;antigen binding;ATP binding;ATPase activity;binding;catalytic activity;CTP binding;dATP binding;deoxyribonucleotide binding;enzy
binding;chromatin binding;chromatin DNA binding;core promoter proximal region DNA binding;core promoter proximal region sequence-specific DNA binding;DNA binding;enhancer binding;enhancer sequence-specific DNA
catalytic activity;cation transmembrane transporter activity;hydrogen ion transmembrane transporter activity;inorganic cation transmembrane transporter activity;ion transmembrane transporter activity;monovalent inorganic c
binding;extracellular matrix structural constituent;glycolipid binding;glycosphingolipid binding;lipid binding;sphingolipid binding;structural molecule activity
adenyl nucleotide binding;adenyl ribonucleotide binding;ATP binding;binding;catalytic activity;enzyme binding;ion channel binding;kinase activity;non-membrane spanning protein tyrosine kinase activity;nucleotide binding;ph
actin binding;binding;cytoskeletal protein binding;protein binding;structural constituent of muscle;structural molecule activity
carbon-oxygen lyase activity;catalytic activity;fumarate hydratase activity;hydro-lyase activity;lyase activity
binding;calcium ion binding;carbohydrate binding;cation binding;collagen binding;collagen V binding;cytokine binding;extracellular matrix binding;fibrinogen binding;fibroblast growth factor binding;fibronectin binding;glycopr
binding;binding, bridging;cation binding;extracellular matrix structural constituent;growth factor binding;identical protein binding;ion binding;metal ion binding;platelet-derived growth factor binding;protein binding;protein bin
binding;calcium ion binding;calcium-dependent phospholipid binding;calcium-dependent protein binding;cation binding;channel activity;cholesterol binding;gated channel activity;GTP binding;guanyl nucleotide binding;guan
binding;GTP binding;guanyl nucleotide binding;guanyl ribonucleotide binding;molecular transducer activity;nucleotide binding;purine nucleotide binding;purine ribonucleoside triphosphate binding;purine ribonucleotide bindi
binding;lipid binding;receptor activity;viral receptor activity
active transmembrane transporter activity;amine transmembrane transporter activity;amino acid transmembrane transporter activity;antiporter activity;binding;calcium ion transmembrane transporter activity;calcium:cation an
6-phosphofructokinase activity;adenyl nucleotide binding;adenyl ribonucleotide binding;ATP binding;binding;carbohydrate binding;carbohydrate kinase activity;catalytic activity;cation binding;enzyme binding;fructose binding
adenyl nucleotide binding;adenyl ribonucleotide binding;antigen binding;ATP binding;binding;double-stranded RNA binding;enzyme binding;enzyme regulator activity;kinase binding;MHC class II protein complex binding;MH
binding;catalytic activity;GTP binding;GTPase activity;guanyl nucleotide binding;guanyl ribonucleotide binding;hydrolase activity;hydrolase activity, acting on acid anhydrides;hydrolase activity, acting on acid anhydrides, in ph
adenyl nucleotide binding;adenyl ribonucleotide binding;asparagine synthase (glutamine-hydrolyzing) activity;ATP binding;binding;carbon-nitrogen ligase activity, with glutamine as amido-N-donor;catalytic activity;cofactor bi
binding;catalytic activity;cation binding;endopeptidase activity;hydrolase activity;ion binding;metal ion binding;metalloendopeptidase activity;metallopeptidase activity;peptidase activity;peptidase activity, acting on L-amino a
catalytic activity;hydroxymethylbilane synthase activity;transferase activity;transferase activity, transferring alkyl or aryl (other than methyl) groups
binding;catalytic activity;cation binding;endopeptidase activity;exopeptidase activity;hydrolase activity;ion binding;metal ion binding;metalloendopeptidase activity;metallopeptidase activity;peptidase activity;peptidase activit
catalytic activity;oxidoreductase activity;oxidoreductase activity, acting on the aldehyde or oxo group of donors;oxidoreductase activity, acting on the aldehyde or oxo group of donors, disulfide as acceptor;pyruvate dehydro
binding;cation binding;electron carrier activity;electron transporter, transferring electrons from CoQH2-cytochrome c reductase complex and cytochrome c oxidase complex activity;heme binding;ion binding;iron ion binding;r
binding;nucleic acid binding;nucleotide binding;RNA binding;snRNA binding
binding;nucleic acid binding;nucleotide binding;RNA binding
binding;cation binding;cytokine receptor binding;growth factor receptor binding;ion binding;metal ion binding;platelet-derived growth factor receptor binding;protein binding;receptor binding;vascular endothelial growth facto
binding;double-stranded RNA binding;glycoprotein binding;identical protein binding;nucleic acid binding;protein binding;protein C-terminus binding;RNA binding;scaffold protein binding;structural constituent of cytoskeleton;
binding;endopeptidase inhibitor activity;endopeptidase regulator activity;enzyme binding;enzyme inhibitor activity;enzyme regulator activity;identical protein binding;peptidase inhibitor activity;peptidase regulator activity;prot
structural constituent of ribosome;structural molecule activity
structural constituent of cytoskeleton;structural constituent of muscle;structural molecule activity
structural molecule activity
binding;catalytic activity;cation binding;G-protein beta/gamma-subunit complex binding;G-protein-coupled receptor binding;GTP binding;GTPase activity;guanyl nucleotide binding;guanyl ribonucleotide binding;hydrolase ac
binding;calcium ion binding;calcium-dependent phospholipid binding;cation binding;enzyme inhibitor activity;enzyme regulator activity;ion binding;lipase inhibitor activity;lipid binding;metal ion binding;phospholipase inhibito
structural constituent of cytoskeleton;structural molecule activity
binding;laminin receptor activity;receptor activity;ribonucleoprotein binding;ribosome binding;structural constituent of ribosome;structural molecule activity
binding;nucleic acid binding;RNA binding;structural constituent of ribosome;structural molecule activity
binding;nucleic acid binding;nucleotide binding;RNA binding;snRNA binding
binding;carbohydrate binding;chemoattractant activity;cytokine activity;fibroblast growth factor receptor binding;glycosaminoglycan binding;growth factor activity;growth factor receptor binding;heparin binding;ligand-depend
binding;carbon-oxygen lyase activity;catalytic activity;cation binding;hydro-lyase activity;ion binding;lyase activity;magnesium ion binding;metal ion binding;phosphopyruvate hydratase activity
acetyl-CoA C-acyltransferase activity;acyl-CoA oxidase activity;C-acyltransferase activity;catalytic activity;oxidoreductase activity;oxidoreductase activity, acting on the CH-CH group of donors;oxidoreductase activity, acting
7S RNA binding;binding;nucleic acid binding;RNA binding
binding;catalytic activity;dinitrosyl-iron complex binding;enzyme binding;enzyme regulator activity;glutathione transferase activity;JUN kinase binding;kinase binding;kinase regulator activity;nitric oxide binding;protein binding
binding;cation binding;identical protein binding;ion binding;metal ion binding;mRNA binding;nucleic acid binding;protein binding;protein dimerization activity;protein homodimerization activity;RNA binding;single-stranded RN
binding;carbohydrate binding;disaccharide binding;galactoside binding;lactose binding;molecular transducer activity;monosaccharide binding;signal transducer activity;sugar binding
6,7-dihydropteridine reductase activity;binding;catalytic activity;coenzyme binding;cofactor binding;electron carrier activity;NAD binding;NADH binding;NADP binding;NADPH binding;nucleotide binding;oxidoreductase activi
binding;chemoattractant activity;chromatin binding;cytokine activity;damaged DNA binding;DNA bending activity;DNA binding;double-stranded DNA binding;nucleic acid binding;nucleic acid binding transcription factor activi
binding;calcium ion binding;cation binding;collagen binding;extracellular matrix binding;ion binding;metal ion binding;protein binding
actin binding;binding;cytoskeletal protein binding;protein binding;structural constituent of cytoskeleton;structural constituent of muscle;structural molecule activity
binding;clathrin binding;clathrin heavy chain binding;peptide binding;protein binding;structural molecule activity
binding;clathrin binding;clathrin heavy chain binding;peptide binding;protein binding;structural molecule activity
binding;calcium ion binding;calcium-dependent phospholipid binding;calcium-dependent protein binding;cation binding;enzyme inhibitor activity;enzyme regulator activity;identical protein binding;ion binding;lipase inhibitor a
2',3'-cyclic-nucleotide 3'-phosphodiesterase activity;binding;catalytic activity;cyclic nucleotide binding;cyclic-nucleotide phosphodiesterase activity;hydrolase activity;hydrolase activity, acting on ester bonds;nucleic acid bin
adenyl nucleotide binding;adenyl ribonucleotide binding;ATP binding;binding;catalytic activity;enzyme binding;G-protein coupled receptor activity;growth factor binding;growth factor receptor binding;kinase activity;kinase bi
binding;catalytic activity;coenzyme binding;cofactor binding;dihydrolipoyl dehydrogenase activity;flavin adenine dinucleotide binding;oxidoreductase activity;oxidoreductase activity, acting on a sulfur group of donors;oxidore
binding;DNA binding;nucleic acid binding;nucleotide binding;RNA binding;single-stranded DNA binding;single-stranded RNA binding;structure-specific DNA binding
catalytic activity;cation transmembrane transporter activity;cytochrome-c oxidase activity;heme-copper terminal oxidase activity;hydrogen ion transmembrane transporter activity;inorganic cation transmembrane transporter a
binding;catalytic activity;cation binding;coenzyme binding;cofactor binding;DNA binding;enzyme binding;identical protein binding;ion binding;kinase binding;metal ion binding;NAD binding;NAD+ ADP-ribosyltransferase activi
3'-5' exonuclease activity;4 iron, 4 sulfur cluster binding;binding;catalytic activity;cation binding;chromatin binding;DNA binding;DNA polymerase activity;DNA-directed DNA polymerase activity;enzyme binding;exonuclease a
binding;nucleic acid binding;RNA binding
adrenergic receptor binding;alpha-2A adrenergic receptor binding;binding;catalytic activity;cysteine-type endopeptidase activity;cysteine-type peptidase activity;endopeptidase activity;enzyme binding;exopeptidase activity;C
aminopeptidase activity;binding;catalytic activity;cation binding;epoxide hydrolase activity;ether hydrolase activity;exopeptidase activity;hydrolase activity;hydrolase activity, acting on ether bonds;ion binding;leukotriene-A4 h
aldehyde-lyase activity;binding;carbon-carbon lyase activity;catalytic activity;cytoskeletal protein binding;fructose-bisphosphate aldolase activity;lyase activity;protein binding
binding;complement binding;complement component C1q binding;endopeptidase inhibitor activity;endopeptidase regulator activity;enzyme inhibitor activity;enzyme regulator activity;opsonin binding;peptidase inhibitor activi
binding;chromatin binding;chromatin DNA binding;core promoter binding;core promoter proximal region DNA binding;core promoter proximal region sequence-specific DNA binding;core promoter sequence-specific DNA bin
binding;DNA binding;enzyme binding;nucleic acid binding;protein binding
binding;cation binding;ion binding;metal ion binding;nucleic acid binding;RNA binding;snRNA binding;U2 snRNA binding
alpha-glucosidase activity;binding;carbohydrate binding;catalytic activity;glucosidase activity;hydrolase activity;hydrolase activity, acting on glycosyl bonds;hydrolase activity, hydrolyzing O-glycosyl compounds;maltose alph
binding;catalytic activity;GDP binding;GTP binding;GTPase activity;guanyl nucleotide binding;guanyl ribonucleotide binding;hydrolase activity;hydrolase activity, acting on acid anhydrides;hydrolase activity, acting on acid anh
antigen binding;binding;peptide antigen binding;peptide binding
binding;chromatin binding;chromatin DNA binding;DNA binding;nucleic acid binding;structure-specific DNA binding
acetyltransferase activity;catalytic activity;dihydrolipoamide S-acyltransferase activity;dihydrolipoyllysine-residue acetyltransferase activity;S-acetyltransferase activity;S-acyltransferase activity;transferase activity;transferase
catalytic activity;disulfide oxidoreductase activity;oxidoreductase activity;oxidoreductase activity, acting on a sulfur group of donors;oxidoreductase activity, acting on a sulfur group of donors, disulfide as acceptor;peptide di
binding;catalytic activity;cation binding;cation transmembrane transporter activity;cytochrome-c oxidase activity;heme-copper terminal oxidase activity;hydrogen ion transmembrane transporter activity;inorganic cation transm
carboxypeptidase activity;catalytic activity;enzyme activator activity;enzyme regulator activity;exopeptidase activity;hydrolase activity;peptidase activity;peptidase activity, acting on L-amino acid peptides;serine hydrolase ac
amine binding;amino acid binding;antioxidant activity;binding;carboxylic acid binding;catalytic activity;glutathione binding;glutathione peroxidase activity;glutathione transferase activity;modified amino acid binding;oxidoredu
adenyl nucleotide binding;adenyl ribonucleotide binding;AMP binding;binding;cAMP binding;cAMP-dependent protein kinase inhibitor activity;cAMP-dependent protein kinase regulator activity;cyclic nucleotide binding;enzy
endopeptidase inhibitor activity;endopeptidase regulator activity;enzyme inhibitor activity;enzyme regulator activity;peptidase inhibitor activity;peptidase regulator activity;serine-type endopeptidase inhibitor activity
carboxylic ester hydrolase activity;catalytic activity;deacetylase activity;hydrolase activity;hydrolase activity, acting on ester bonds;methylumbelliferyl-acetate deacetylase activity;S-formylglutathione hydrolase activity;thioles
adenyl nucleotide binding;adenyl ribonucleotide binding;ATP binding;ATPase activity;binding;catalytic activity;chaperone binding;DNA binding;DNA replication origin binding;double-stranded RNA binding;enzyme binding;hy
binding;chaperone binding;enzyme binding;misfolded protein binding;protein binding;ubiquitin protein ligase binding

adenyl nucleotide binding;adenyl ribonucleotide binding;ATP binding;ATPase activity;binding;calcium ion binding;catalytic activity;cation binding;chaperone binding;enzyme binding;glycoprotein binding;hydrolase activity;hyd
binding;extracellular matrix structural constituent;glycolipid binding;glycosphingolipid binding;lipid binding;sphingolipid binding;structural molecule activity
acid phosphatase activity;catalytic activity;hydrolase activity;hydrolase activity, acting on ester bonds;phosphatase activity;phosphoric ester hydrolase activity
adenyl nucleotide binding;adenyl ribonucleotide binding;antigen binding;ATP binding;ATPase activity;ATPase activity, coupled;binding;C3HC4-type RING finger domain binding;catalytic activity;enzyme binding;G-protein-cou
active transmembrane transporter activity;binding;carbohydrate transmembrane transporter activity;cation transmembrane transporter activity;cation:sugar symporter activity;dehydroascorbic acid transporter activity;D-gluco
active transmembrane transporter activity;carbohydrate transmembrane transporter activity;cation transmembrane transporter activity;cation:sugar symporter activity;glucose transmembrane transporter activity;hexose trans
carbon-carbon lyase activity;carboxy-lyase activity;catalytic activity;lyase activity;orotate phosphoribosyltransferase activity;orotidine-5'-phosphate decarboxylase activity;transferase activity;transferase activity, transferring g
catalytic activity;oxidoreductase activity;oxidoreductase activity, acting on the aldehyde or oxo group of donors;oxidoreductase activity, acting on the aldehyde or oxo group of donors, disulfide as acceptor;pyruvate dehydro
binding;catalytic activity;cofactor binding;glycogen phosphorylase activity;phosphorylase activity;pyridoxal phosphate binding;transferase activity;transferase activity, transferring glycosyl groups;transferase activity, transferri
ATPase binding;binding;catalytic activity;cytoskeletal protein binding;Edg-2 lysophosphatidic acid receptor binding;endothelial differentiation G-protein coupled receptor binding;enzyme binding;GDP binding;G-protein-coupl
ATPase binding;binding;catalytic activity;enzyme binding;GDP binding;GTP binding;GTPase activity;guanyl nucleotide binding;guanyl ribonucleotide binding;hydrolase activity;hydrolase activity, acting on acid anhydrides;hyd
binding;enzyme binding;protein binding
acyl-CoA dehydrogenase activity;binding;catalytic activity;coenzyme binding;cofactor binding;flavin adenine dinucleotide binding;identical protein binding;medium-chain-acyl-CoA dehydrogenase activity;oxidoreductase acti
ATPase activity;ATPase activity, coupled;binding;catalytic activity;chromatin binding;core promoter binding;core promoter sequence-specific DNA binding;DNA binding;DNA topoisomerase (ATP-hydrolyzing) activity;DNA topo
adenyl nucleotide binding;adenyl ribonucleotide binding;ATP binding;ATPase activity;ATPase activity, coupled;binding;catalytic activity;cation binding;chromatin binding;DNA bending activity;DNA binding;DNA topoisomerase
binding;carbohydrate binding;catalytic activity;coenzyme binding;cofactor binding;glucose binding;glucose-6-phosphate dehydrogenase activity;identical protein binding;monosaccharide binding;NADP binding;nucleotide bi
acid-amino acid ligase activity;catalytic activity;ligase activity;ligase activity, forming carbon-nitrogen bonds;small conjugating protein ligase activity
adenyl nucleotide binding;adenyl ribonucleotide binding;ATP binding;binding;biotin binding;biotin carboxylase activity;carboxylic acid binding;catalytic activity;cation binding;DNA binding;ion binding;ligase activity;ligase activ
adenyl nucleotide binding;adenyl ribonucleotide binding;ATP binding;binding;catalytic activity;cyclohydrolase activity;formate-tetrahydrofolate ligase activity;hydrolase activity;hydrolase activity, acting on carbon-nitrogen (but
binding;carbohydrate binding;carboxylic acid binding;catalytic activity;glycoprotein binding;G-protein coupled receptor activity;identical protein binding;insulin-like growth factor-activated receptor activity;isoprenoid binding;
alcohol dehydrogenase (NAD) activity;binding;carboxylic acid binding;catalytic activity;cation binding;electron carrier activity;fatty acid binding;formaldehyde dehydrogenase activity;ion binding;lipid binding;metal ion binding;
adenyl nucleotide binding;adenyl ribonucleotide binding;ATP binding;binding;catalytic activity;cyclin binding;cyclin-dependent protein kinase activity;cyclin-dependent protein kinase regulator activity;enzyme regulator activity
adenyl nucleotide binding;adenyl ribonucleotide binding;ADP binding;AMP binding;ATP binding;binding;carbohydrate binding;catalytic activity;cation binding;diphosphotransferase activity;GDP binding;guanyl nucleotide binc
binding;mRNA binding;nucleic acid binding;nucleotide binding;poly(A) RNA binding;poly(U) RNA binding;poly-purine tract binding;poly-pyrimidine tract binding;protein binding;protein C-terminus binding;RNA binding;single-s
binding;catalytic activity;chromatin binding;damaged DNA binding;dinucleotide insertion or deletion binding;DNA binding;DNA insertion or deletion binding;DNA N-glycosylase activity;DNA polymerase binding;DNA polymera
adenyl nucleotide binding;adenyl ribonucleotide binding;aminoacyl-tRNA ligase activity;ATP binding;binding;catalytic activity;histidine-tRNA ligase activity;ligase activity;ligase activity, forming aminoacyl-tRNA and related cor
binding;binding, bridging;cation binding;extracellular matrix binding;extracellular matrix structural constituent;ion binding;metal ion binding;protein binding;protein binding, bridging;structural molecule activity
binding;growth factor binding;platelet-derived growth factor binding;protein binding
endopeptidase inhibitor activity;endopeptidase regulator activity;enzyme inhibitor activity;enzyme regulator activity;peptidase inhibitor activity;peptidase regulator activity;serine-type endopeptidase inhibitor activity
active transmembrane transporter activity;antiporter activity;ATP:ADP antiporter activity;secondary active transmembrane transporter activity;solute:solute antiporter activity;transmembrane transporter activity;transporter act
binding;catalytic activity;cation binding;copper ion binding;endopeptidase activity;hydrolase activity;ion binding;metal ion binding;peptidase activity;peptidase activity, acting on L-amino acid peptides;serine hydrolase activity
binding;catalytic activity;cation binding;DNA binding;IMP dehydrogenase activity;ion binding;metal ion binding;nucleic acid binding;nucleotide binding;oxidoreductase activity;oxidoreductase activity, acting on CH-OH group
binding;chromatin binding;cytoskeletal protein binding;dynein complex binding;enzyme binding;heat shock protein binding;identical protein binding;kinase binding;mitogen-activated protein kinase binding;mRNA binding;nuc
adenyl nucleotide binding;adenyl ribonucleotide binding;ATP binding;binding;catalytic activity;creatine kinase activity;enzyme binding;kinase activity;nucleotide binding;phosphotransferase activity, nitrogenous group as acce
binding;calcium ion binding;calcium-dependent phospholipid binding;calcium-dependent protein binding;cation binding;enzyme inhibitor activity;enzyme regulator activity;ion binding;lipase inhibitor activity;lipid binding;meta
3-methyl-2-oxobutanoate dehydrogenase (2-methylpropanoyl-transferring) activity;alpha-ketoacid dehydrogenase activity;binding;carbon-carbon lyase activity;carboxy-lyase activity;catalytic activity;cation binding;ion binding
binding;calcium ion binding;cation binding;cytoskeletal protein binding;double-stranded RNA binding;integrin binding;ion binding;ion channel binding;ligand-dependent nuclear receptor transcription coactivator activity;metal
aminopeptidase activity;binding;carboxypeptidase activity;catalytic activity;cation binding;dipeptidase activity;exopeptidase activity;hydrolase activity;ion binding;manganese ion binding;metal ion binding;metallocarboxypept
5'-deoxyribose-5-phosphate lyase activity;adenyl nucleotide binding;adenyl ribonucleotide binding;ATP binding;ATPase activity;ATPase activity, coupled;ATP-dependent DNA helicase activity;ATP-dependent helicase activity;
adenyl nucleotide binding;adenyl ribonucleotide binding;ATP binding;ATPase activity;ATPase activity, coupled;ATP-dependent DNA helicase activity;ATP-dependent helicase activity;binding;catalytic activity;damaged DNA bir
catalytic activity;cation transmembrane transporter activity;cytochrome-c oxidase activity;heme-copper terminal oxidase activity;hydrogen ion transmembrane transporter activity;inorganic cation transmembrane transporter a
molecular transducer activity;receptor signaling protein activity;signal transducer activity
binding;enzyme binding;protein binding
enzyme inhibitor activity;enzyme regulator activity;ribonuclease inhibitor activity
binding;calcium ion binding;catalytic activity;cation binding;endopeptidase activity;hydrolase activity;ion binding;metal ion binding;metalloendopeptidase activity;metallopeptidase activity;peptidase activity;peptidase activity,
binding;identical protein binding;protein binding
binding;cation binding;cell adhesion molecule binding;ion binding;metal ion binding;protein binding
binding;catalytic activity;enzyme binding;GTP binding;GTPase activity;guanyl nucleotide binding;guanyl ribonucleotide binding;hydrolase activity;hydrolase activity, acting on acid anhydrides;hydrolase activity, acting on acid
structural constituent of epidermis;structural molecule activity
binding;protein binding;scaffold protein binding;structural constituent of cytoskeleton;structural molecule activity
catalytic activity;intramolecular oxidoreductase activity;intramolecular oxidoreductase activity, interconverting keto- and enol-groups;intramolecular oxidoreductase activity, transposing S-S bonds;isomerase activity;protein d
binding;carboxylic acid binding;catalytic activity;cation binding;dioxygenase activity;ion binding;iron ion binding;L-ascorbic acid binding;metal ion binding;oxidoreductase activity;oxidoreductase activity, acting on paired don
binding;calcium ion binding;cation binding;ion binding;metal ion binding
binding;cytokine binding;cytokine receptor activity;enzyme binding;lipid binding;molecular transducer activity;phospholipid binding;protease binding;protein binding;receptor activity;signal transducer activity;signaling recepto
actin binding;actin filament binding;binding;calcium ion binding;cation binding;cytoskeletal protein binding;enzyme binding;GTPase binding;ion binding;metal ion binding;protein binding
binding;calcium ion binding;cation binding;ion binding;metal ion binding
catalytic activity;endopeptidase activity;exopeptidase activity;hydrolase activity;omega peptidase activity;peptidase activity;peptidase activity, acting on L-amino acid peptides;serine hydrolase activity;serine-type endopeptic
binding;catalytic activity;coenzyme binding;cofactor binding;electron carrier activity;flavin adenine dinucleotide binding;oxidoreductase activity
binding;carbohydrate binding;catalytic activity;enzyme binding;glucose binding;glucosyltransferase activity;glycogen (starch) synthase activity;kinase binding;monosaccharide binding;protein binding;protein kinase binding;su
adenyl nucleotide binding;adenyl ribonucleotide binding;AMP binding;binding;cAMP binding;cAMP-dependent protein kinase inhibitor activity;cAMP-dependent protein kinase regulator activity;cyclic nucleotide binding;enzyr
adenyl nucleotide binding;adenyl ribonucleotide binding;ATP binding;binding;catalytic activity;DNA binding;helicase activity;hydrolase activity;hydrolase activity, acting on acid anhydrides;hydrolase activity, acting on acid anh
binding;complement binding;protein binding
anion binding;binding;catalytic activity;cation binding;cyclohydrolase activity;hydrolase activity;hydrolase activity, acting on carbon-nitrogen (but not peptide) bonds;hydrolase activity, acting on carbon-nitrogen (but not peptic
binding;catalytic activity;chemoattractant activity;cytokine activity;cytokine receptor binding;dopachrome isomerase activity;intramolecular oxidoreductase activity;intramolecular oxidoreductase activity, interconverting keto-
binding;calcium ion binding;cation binding;enzyme binding;ion binding;ion channel binding;kinase binding;metal ion binding;phosphoprotein binding;protein binding;protein kinase binding;protein kinase C binding
binding;catalytic activity;cation binding;dimethylallyltranstransferase activity;geranyltranstransferase activity;ion binding;metal ion binding;prenyltransferase activity;transferase activity;transferase activity, transferring alkyl or a
binding;calcium ion binding;cation binding;collagen binding;extracellular matrix binding;glycoprotein binding;ion binding;laminin binding;metal ion binding;protein binding;proteoglycan binding
alcohol dehydrogenase (NADP+) activity;alditol:NADP+ 1-oxidoreductase activity;aldo-keto reductase (NADP) activity;catalytic activity;electron carrier activity;L-glucuronate reductase activity;oxidoreductase activity;oxidored
adenyl nucleotide binding;adenyl ribonucleotide binding;alkali metal ion binding;antigen binding;ATP binding;binding;catalytic activity;cation binding;ion binding;kinase activity;magnesium ion binding;metal ion binding;MHC c
adenyl nucleotide binding;adenyl ribonucleotide binding;ATP binding;binding;calcium ion binding;cation binding;enzyme binding;ion binding;lipoprotein particle receptor binding;low-density lipoprotein particle receptor bindin
binding;catalytic activity;enzyme binding;histone kinase activity;kinase activity;kinase binding;patched binding;phosphotransferase activity, alcohol group as acceptor;protein binding;protein kinase activity;protein kinase bind
binding;histone pre-mRNA DCP binding;nucleic acid binding;RNA binding
adenyl nucleotide binding;adenyl ribonucleotide binding;ATP binding;ATPase activity;beta-amyloid binding;beta-endorphin binding;binding;catalytic activity;cation binding;endopeptidase activity;glycoprotein binding;hormone
catalytic activity;cation transmembrane transporter activity;cytochrome-c oxidase activity;heme-copper terminal oxidase activity;hydrogen ion transmembrane transporter activity;inorganic cation transmembrane transporter a
binding;DNA binding;nucleic acid binding;nucleotide binding;regulatory region DNA binding;regulatory region nucleic acid binding;RNA binding;transcription regulatory region DNA binding
adenyl nucleotide binding;adenyl ribonucleotide binding;aminoacylase activity;aminoacyl-tRNA ligase activity;aspartate-tRNA ligase activity;ATP binding;binding;catalytic activity;hydrolase activity;hydrolase activity, acting on
alpha-catenin binding;binding;cadherin binding;cell adhesion molecule binding;enzyme binding;identical protein binding;molecular transducer activity;phosphatase binding;protein binding;protein binding transcription factor a
acid-ammonia (or amide) ligase activity;adenyl nucleotide binding;adenyl ribonucleotide binding;amine binding;amino acid binding;ammonia ligase activity;ATP binding;binding;carbon-carbon lyase activity;carboxylic acid bin
alcohol dehydrogenase (NADP+) activity;alditol:NADP+ 1-oxidoreductase activity;aldo-keto reductase (NADP) activity;catalytic activity;electron carrier activity;glyceraldehyde oxidoreductase activity;oxidoreductase activity;ox
aminopeptidase activity;binding;catalytic activity;cation binding;exopeptidase activity;hydrolase activity;ion binding;metal ion binding;metallopeptidase activity;peptidase activity;peptidase activity, acting on L-amino acid pep
binding;cell adhesion molecule binding;protein binding;receptor activity
binding;catalytic activity;GTP binding;GTPase activity;guanyl nucleotide binding;guanyl ribonucleotide binding;hydrolase activity;hydrolase activity, acting on acid anhydrides;hydrolase activity, acting on acid anhydrides, in pl
alpha-tubulin binding;beta-N-acetylglucosaminylglycopeptide beta-1,4-galactosyltransferase activity;beta-tubulin binding;binding;catalytic activity;cation binding;cytoskeletal protein binding;galactosyltransferase activity;ider
actin binding;actin filament binding;binding;cell adhesion molecule binding;cytoskeletal protein binding;protein binding
binding;catalytic activity;cysteine-type peptidase activity;hydrolase activity;peptidase activity;peptidase activity, acting on L-amino acid peptides;protein binding;small conjugating protein binding;small conjugating protein-sp
binding;DNA binding;double-stranded DNA binding;nucleic acid binding;nucleic acid binding transcription factor activity;regulatory region DNA binding;regulatory region nucleic acid binding;RNA polymerase II regulatory regi
adenyl nucleotide binding;adenyl ribonucleotide binding;ATP binding;binding;catalytic activity;cation binding;deoxyribonuclease activity;DNA binding;GTP binding;guanyl nucleotide binding;guanyl ribonucleotide binding;hydr
antioxidant activity;binding;catalytic activity;cytochrome-b5 reductase activity;identical protein binding;NAD(P)H dehydrogenase (quinone) activity;oxidoreductase activity;oxidoreductase activity, acting on NADH or NADPH;o
binding;catalytic activity;cation binding;hydrolase activity;hydrolase activity, acting on ester bonds;ion binding;metal ion binding;N-acetylglucosamine-6-sulfatase activity;sulfuric ester hydrolase activity
binding;enzyme binding;fibroblast growth factor binding;growth factor binding;mRNA binding;nucleic acid binding;protein binding;RNA binding;structural constituent of ribosome;structural molecule activity
binding;damaged DNA binding;DNA binding;enzyme binding;nucleic acid binding;phosphatase binding;protein binding;protein phosphatase binding;single-stranded DNA binding;structure-specific DNA binding;ubiquitin prote
catalytic activity;cation transmembrane transporter activity;cytochrome-c oxidase activity;heme-copper terminal oxidase activity;hydrogen ion transmembrane transporter activity;inorganic cation transmembrane transporter a
binding;cation binding;endopeptidase inhibitor activity;endopeptidase regulator activity;enzyme activator activity;enzyme binding;enzyme inhibitor activity;enzyme regulator activity;ion binding;metal ion binding;metalloendop
binding;carbohydrate binding;catalytic activity;collagen binding;glycosaminoglycan binding;hexosaminidase activity;hyaluronic acid binding;hyalurononglucosaminidase activity;hydrolase activity;hydrolase activity, acting on g
binding;damaged DNA binding;DNA binding;enzyme binding;histone binding;nucleic acid binding;protein binding
15-hydroxyprostaglandin dehydrogenase (NADP+) activity;carbonyl reductase (NADPH) activity;catalytic activity;oxidoreductase activity;oxidoreductase activity, acting on CH-OH group of donors;oxidoreductase activity, acti
binding;binding, bridging;cytoskeletal adaptor activity;cytoskeletal protein binding;enzyme inhibitor activity;enzyme regulator activity;eukaryotic initiation factor eIF2 binding;kinase inhibitor activity;kinase regulator activity;pro
binding;chromatin binding;chromatin DNA binding;DNA binding;enzyme binding;histone deacetylase binding;nucleic acid binding;protein binding;structure-specific DNA binding
binding;chromatin binding;chromatin DNA binding;DNA binding;nucleic acid binding;structure-specific DNA binding
binding;catalytic activity;cation binding;coenzyme binding;cofactor binding;cytochrome-b5 reductase activity;dioxygenase activity;electron carrier activity;flavin adenine dinucleotide binding;FMN binding;hydrolase activity;ior
active transmembrane transporter activity;adenyl nucleotide binding;adenyl ribonucleotide binding;ATP binding;ATPase activity;ATPase activity, coupled;ATPase activity, coupled to movement of substances;ATPase activity, c
binding;catalytic activity;cation binding;fumarylacetoacetase activity;hydrolase activity;hydrolase activity, acting on acid carbon-carbon bonds;hydrolase activity, acting on acid carbon-carbon bonds, in ketonic substances;ior
binding;cytoskeletal protein binding;molecular transducer activity;protein binding;signal transducer activity;tubulin binding
binding;DNA binding;double-stranded DNA binding;enzyme binding;GTPase binding;mRNA 3'-UTR binding;mRNA binding;nucleic acid binding;nucleic acid binding transcription factor activity;protein binding;protein binding
5'-deoxyribose-5-phosphate lyase activity;AT DNA binding;binding;carbon-oxygen lyase activity;catalytic activity;DNA binding;DNA-(apurinic or apyrimidinic site) lyase activity;enzyme binding;hormone receptor binding;ligand
binding;carbon-carbon lyase activity;carboxylic acid binding;carboxy-lyase activity;catalytic activity;cofactor binding;L-aspartate:2-oxoglutarate aminotransferase activity;L-cysteine:2-oxoglutarate aminotransferase activity;L
adenyl nucleotide binding;adenyl ribonucleotide binding;ATP binding;binding;calcium-dependent protein kinase activity;calcium-dependent protein kinase C activity;calcium-dependent protein serine/threonine kinase activity;
binding;core promoter proximal region DNA binding;core promoter proximal region sequence-specific DNA binding;DNA binding;double-stranded DNA binding;nucleic acid binding;nucleic acid binding transcription factor act
binding;cation binding;collagen binding;ion binding;metal ion binding;protein binding;receptor activity;viral receptor activity
channel activity;gap junction channel activity;ion transmembrane transporter activity;molecular transducer activity;passive transmembrane transporter activity;signal transducer activity;substrate-specific transmembrane trans
binding;chromatin binding;DNA binding;nucleic acid binding
catalytic activity;NADH dehydrogenase (quinone) activity;NADH dehydrogenase (ubiquinone) activity;NADH dehydrogenase activity;oxidoreductase activity;oxidoreductase activity, acting on NADH or NADPH;oxidoreductase
adenyl nucleotide binding;adenyl ribonucleotide binding;ATP binding;binding;cAMP-dependent protein kinase activity;catalytic activity;cyclic nucleotide-dependent protein kinase activity;enzyme binding;kinase activity;kinase
binding;calcium ion binding;calcium-dependent cysteine-type endopeptidase activity;catalytic activity;cation binding;cysteine-type endopeptidase activity;cysteine-type peptidase activity;cytoskeletal protein binding;endope
adenyl nucleotide binding;adenyl ribonucleotide binding;ATP binding;binding;catalytic activity;CTP synthase activity;ligase activity;ligase activity, forming carbon-nitrogen bonds;nucleotide binding;purine nucleotide binding;p
activin binding;binding;carbohydrate binding;catalytic activity;cytokine binding;cytokine receptor binding;galactose binding;glycosaminoglycan binding;growth factor binding;identical protein binding;kinase activity;molecular
adenyl nucleotide binding;adenyl ribonucleotide binding;androgen receptor binding;ATP binding;ATPase activity;ATPase activity, coupled;ATP-dependent helicase activity;ATP-dependent RNA helicase activity;binding;catalyti
6-phosphofructokinase activity;adenyl nucleotide binding;adenyl ribonucleotide binding;ATP binding;binding;carbohydrate binding;carbohydrate kinase activity;catalytic activity;cation binding;enzyme binding;fructose binding
beta-N-acetylgalactosaminidase activity;beta-N-acetylhexosaminidase activity;catalytic activity;enzyme activator activity;enzyme regulator activity;hexosaminidase activity;hydrolase activity;hydrolase activity, acting on glyco
binding;carbohydrate binding;chemoattractant activity;extracellular matrix binding;IgE binding;immunoglobulin binding;laminin binding;protein binding;protein complex binding
adenyl nucleotide binding;adenyl ribonucleotide binding;ATP binding;ATPase activity;binding;catalytic activity;hydrolase activity;hydrolase activity, acting on acid anhydrides;hydrolase activity, acting on acid anhydrides, in phi
adenyl nucleotide binding;adenyl ribonucleotide binding;ATP binding;binding;enzyme binding;nucleotide binding;protein binding;purine nucleotide binding;purine ribonucleoside triphosphate binding;purine ribonucleotide binc
binding;catalytic activity;cation binding;enzyme binding;ephrin receptor binding;hydrolase activity;hydrolase activity, acting on ester bonds;ion binding;kinase binding;metal ion binding;phosphatase activity;phosphoprotein pl
binding;nucleic acid binding;RNA binding;structural constituent of ribosome;structural molecule activity;tRNA binding
receptor activity
binding;catalytic activity;epidermal growth factor receptor binding;growth factor receptor binding;GTP binding;GTPase activity;guanyl nucleotide binding;guanyl ribonucleotide binding;hydrolase activity;hydrolase activity, acti

binding;DNA binding;identical protein binding;mRNA binding;nucleic acid binding;protein binding;protein dimerization activity;protein homodimerization activity;RNA binding;structural constituent of ribosome;structural molec
actin binding;alpha-catenin binding;beta-catenin binding;binding;cadherin binding;cell adhesion molecule binding;cytoskeletal protein binding;dystroglycan binding;enzyme binding;glycoprotein binding;protein binding;structu
binding;DNA binding;nucleic acid binding;RNA binding
binding;nucleic acid binding;nucleotide binding;RNA binding
structural constituent of ribosome;structural molecule activity
binding;bisphosphoglycerate 2-phosphatase activity;bisphosphoglycerate mutase activity;bisphosphoglycerate phosphatase activity;catalytic activity;enzyme binding;hydrolase activity;hydrolase activity, acting on ester bonc
binding;chromatin binding;chromatin DNA binding;DNA binding;enzyme regulator activity;GTPase regulator activity;guanyl-nucleotide exchange factor activity;histone binding;nucleic acid binding;nucleoside-triphosphatase r
binding;DNA binding;nucleic acid binding;nucleic acid binding transcription factor activity;regulatory region DNA binding;regulatory region nucleic acid binding;RNA polymerase II regulatory region DNA binding;RNA polymera
adenyl nucleotide binding;adenyl ribonucleotide binding;ATP binding;binding;catalytic activity;cation binding;DNA binding;DNA ligase (ATP) activity;DNA ligase activity;ion binding;ligase activity;ligase activity, forming phospho
cation transmembrane transporter activity;hydrogen ion transmembrane transporter activity;inorganic cation transmembrane transporter activity;ion transmembrane transporter activity;monovalent inorganic cation transmemb
binding;damaged DNA binding;DNA binding;enzyme binding;nucleic acid binding;protein binding
alpha-catenin binding;beta-catenin binding;binding;calcium ion binding;cation binding;gamma-catenin binding;ion binding;metal ion binding;protein binding
binding;calcium ion binding;catalytic activity;cation binding;cytokine receptor binding;enzyme binding;hydrolase activity;hydrolase activity, acting on ester bonds;ion binding;kinase binding;lipase activity;metal ion binding;mo
binding;DNA binding;identical protein binding;nucleic acid binding;nucleotide binding;protein binding;protein C-terminus binding;RNA binding;sequence-specific DNA binding;telomeric DNA binding
adenyl nucleotide binding;adenyl ribonucleotide binding;ATP binding;binding;carbohydrate kinase activity;catalytic activity;fructokinase activity;glucokinase activity;hexokinase activity;kinase activity;mannokinase activity;nuc
binding;catalytic activity;DNA binding;DNA-directed RNA polymerase activity;nucleic acid binding;nucleotidyltransferase activity;RNA polymerase activity;transferase activity;transferase activity, transferring phosphorus-conta
binding;catalytic activity;DNA binding;DNA-directed RNA polymerase activity;nucleic acid binding;nucleotidyltransferase activity;RNA polymerase activity;transferase activity;transferase activity, transferring phosphorus-conta
3'-5' DNA helicase activity;adenyl deoxyribonucleotide binding;adenyl nucleotide binding;adenyl ribonucleotide binding;ATP binding;ATPase activity;ATPase activity, coupled;ATP-dependent DNA helicase activity;ATP-depend
adenyl nucleotide binding;adenyl ribonucleotide binding;ATP binding;binding;catalytic activity;double-stranded RNA binding;enzyme regulator activity;eukaryotic translation initiation factor 2alpha kinase activity;kinase activity
binding;catalytic activity;identical protein binding;protein binding;protein dimerization activity;protein homodimerization activity;spermidine synthase activity;transferase activity;transferase activity, transferring alkyl or aryl (oth
adenyl nucleotide binding;adenyl ribonucleotide binding;ATP binding;binding;catalytic activity;kinase activity;nucleotide binding;phosphotransferase activity, alcohol group as acceptor;protein binding;protein kinase activity;pr
endopeptidase inhibitor activity;endopeptidase regulator activity;enzyme inhibitor activity;enzyme regulator activity;peptidase inhibitor activity;peptidase regulator activity;serine-type endopeptidase inhibitor activity
binding;calcium ion binding;cation binding;endopeptidase inhibitor activity;endopeptidase regulator activity;enzyme inhibitor activity;enzyme regulator activity;ion binding;metal ion binding;peptidase inhibitor activity;peptidas
binding;chromatin binding;DNA binding;double-stranded DNA binding;enhancer binding;enhancer sequence-specific DNA binding;identical protein binding;nucleic acid binding;nucleic acid binding transcription factor activity
activin binding;binding;molecular transducer activity;protein binding;protein complex binding;signal transducer activity
active transmembrane transporter activity;adenyl nucleotide binding;adenyl ribonucleotide binding;ATP binding;ATPase activity;ATPase activity, coupled;ATPase activity, coupled to movement of substances;ATPase activity, c
binding;cation binding;ion binding;metal ion binding;nucleic acid binding;RNA binding;translation factor activity, nucleic acid binding;translation initiation factor activity
binding;calcium ion binding;calcium-dependent phospholipid binding;calcium-dependent protein binding;cation binding;integrin binding;ion binding;lipid binding;metal ion binding;phospholipid binding;protein binding;protein
binding;catalytic activity;cytoskeletal protein binding;GDP binding;GTP binding;GTPase activity;guanyl nucleotide binding;guanyl ribonucleotide binding;hydrolase activity;hydrolase activity, acting on acid anhydrides;hydrolas
binding;catalytic activity;GDP binding;GTP binding;GTPase activity;guanyl nucleotide binding;guanyl ribonucleotide binding;hydrolase activity;hydrolase activity, acting on acid anhydrides;hydrolase activity, acting on acid anh
binding;catalytic activity;cytoskeletal protein binding;GTP binding;GTPase activity;guanyl nucleotide binding;guanyl ribonucleotide binding;hydrolase activity;hydrolase activity, acting on acid anhydrides;hydrolase activity, act
binding;catalytic activity;GTP binding;GTPase activity;guanyl nucleotide binding;guanyl ribonucleotide binding;hydrolase activity;hydrolase activity, acting on acid anhydrides;hydrolase activity, acting on acid anhydrides, in pl
catalytic activity;endopeptidase activity;hydrolase activity;peptidase activity;peptidase activity, acting on L-amino acid peptides;threonine-type endopeptidase activity;threonine-type peptidase activity
binding;carbohydrate binding;carbohydrate transmembrane transporter activity;hexose transmembrane transporter activity;mannose binding;mannose transmembrane transporter activity;molecular transducer activity;monos
binding;catalytic activity;cation binding;cation transmembrane transporter activity;cytochrome-c oxidase activity;electron carrier activity;heme-copper terminal oxidase activity;hydrogen ion transmembrane transporter activity
structural molecule activity
endopeptidase inhibitor activity;endopeptidase regulator activity;enzyme inhibitor activity;enzyme regulator activity;peptidase inhibitor activity;peptidase regulator activity;serine-type endopeptidase inhibitor activity
cysteine-type endopeptidase inhibitor activity;endopeptidase inhibitor activity;endopeptidase regulator activity;enzyme inhibitor activity;enzyme regulator activity;peptidase inhibitor activity;peptidase regulator activity
binding;catalytic activity;cation binding;DNA binding;IMP dehydrogenase activity;ion binding;metal ion binding;nucleic acid binding;nucleotide binding;oxidoreductase activity;oxidoreductase activity, acting on CH-OH group
binding;carbohydrate binding;cation binding;extracellular matrix structural constituent;glycoprotein binding;glycosaminoglycan binding;growth factor binding;heparin binding;integrin binding;ion binding;metal ion binding;patt
binding;channel inhibitor activity;channel regulator activity;enzyme activator activity;enzyme binding;enzyme regulator activity;glycoprotein binding;GTPase activator activity;GTPase binding;GTPase regulator activity;ion char
adenyl nucleotide binding;adenyl ribonucleotide binding;ATP binding;binding;catalytic activity;cyclin-dependent protein kinase activity;kinase activity;nucleotide binding;phosphotransferase activity, alcohol group as acceptor
amine binding;amino acid binding;binding;carboxylic acid binding;catalytic activity;enzyme binding;glutathione binding;glutathione transferase activity;identical protein binding;modified amino acid binding;peptide binding;pro
active transmembrane transporter activity;adenyl nucleotide binding;adenyl ribonucleotide binding;ATP binding;ATPase activity;ATPase activity, coupled;ATPase activity, coupled to movement of substances;ATPase activity, c
active transmembrane transporter activity;ATPase activity;ATPase activity, coupled;ATPase activity, coupled to movement of substances;ATPase activity, coupled to transmembrane movement of ions;ATPase activity, couplec
binding;cation binding;ion binding;metal ion binding;transition metal ion binding;zinc ion binding
actin binding;actin filament binding;binding;cytoskeletal protein binding;enzyme binding;Fc-gamma receptor I complex binding;glycoprotein binding;GTPase binding;identical protein binding;immunoglobulin receptor binding;
4 iron, 4 sulfur cluster binding;aconitate hydratase activity;binding;carbon-oxygen lyase activity;catalytic activity;cation binding;hydro-lyase activity;ion binding;iron-responsive element binding;iron-sulfur cluster binding;lyase
5'-nucleotidase activity;binding;catalytic activity;cation binding;hydrolase activity;hydrolase activity, acting on ester bonds;ion binding;metal ion binding;nucleotidase activity;nucleotide binding;phosphatase activity;phosphori
anion channel activity;anion transmembrane transporter activity;binding;channel activity;enzyme binding;gated channel activity;ion channel activity;ion transmembrane transporter activity;kinase binding;passive transmembra
2 iron, 2 sulfur cluster binding;3 iron, 4 sulfur cluster binding;4 iron, 4 sulfur cluster binding;binding;catalytic activity;cation binding;cofactor binding;electron carrier activity;ion binding;iron-sulfur cluster binding;metal cluster b
binding;catalytic activity;catechol O-methyltransferase activity;cation binding;ion binding;magnesium ion binding;metal ion binding;methyltransferase activity;O-methyltransferase activity;S-adenosylmethionine-dependent me
binding;catalytic activity;cation binding;GTP binding;guanyl nucleotide binding;guanyl ribonucleotide binding;ion binding;metal ion binding;nucleotide binding;protein-glutamine gamma-glutamyltransferase activity;purine nuc
amine binding;amino acid binding;binding;carboxylic acid binding;catalytic activity;cation binding;cobalamin binding;intramolecular transferase activity;ion binding;isomerase activity;metal ion binding;methylmalonyl-CoA mut
binding;lipid binding;oxysterol binding;protein binding;protein domain specific binding;steroid binding;sterol binding
carboxyl-O-methyltransferase activity;catalytic activity;methyltransferase activity;O-methyltransferase activity;protein carboxyl O-methyltransferase activity;protein methyltransferase activity;protein-L-isoaspartate (D-aspartat
binding;catalytic activity;methyltransferase activity;nucleic acid binding;RNA binding;RNA methyltransferase activity;rRNA methyltransferase activity;S-adenosylmethionine-dependent methyltransferase activity;transferase ac
binding;nucleic acid binding;RNA binding;rRNA binding;structural constituent of ribosome;structural molecule activity
adenyl nucleotide binding;adenyl ribonucleotide binding;ATP binding;binding;catalytic activity;cation binding;cyclo-ligase activity;hydroxymethyl-, formyl- and related transferase activity;ion binding;ligase activity;ligase activit
acid-amino acid ligase activity;adenyl nucleotide binding;adenyl ribonucleotide binding;ATP binding;binding;carbon-carbon lyase activity;carboxy-lyase activity;catalytic activity;identical protein binding;ligase activity;ligase ac
binding;C-acyltransferase activity;carboxylic acid binding;catalytic activity;cholesterol binding;coenzyme binding;cofactor binding;fatty acid binding;fatty-acyl-CoA binding;lipid binding;lipid transporter activity;monocarboxyli
acid-amino acid ligase activity;adenyl nucleotide binding;adenyl ribonucleotide binding;ATP binding;binding;catalytic activity;ligase activity;ligase activity, forming carbon-nitrogen bonds;nucleotide binding;purine nucleotide b
adenyl nucleotide binding;adenyl ribonucleotide binding;ATP binding;binding;catalytic activity;cation binding;DNA binding;ion binding;kinase activity;metal ion binding;nucleic acid binding;nucleic acid binding transcription fac
3'-phosphoadenosine 5'-phosphosulfate binding;adenyl nucleotide binding;adenyl ribonucleotide binding;ATP binding;binding;calcium ion binding;carbohydrate binding;cargo receptor activity;catalytic activity;cation binding;
catalytic activity;ferredoxin-NADP+ reductase activity;NADPH-adrenodoxin reductase activity;oxidoreductase activity;oxidoreductase activity, acting on iron-sulfur proteins as donors;oxidoreductase activity, acting on iron-sul
binding;DNA binding;nucleic acid binding;nucleotide binding;RNA binding;sequence-specific DNA binding;single-stranded DNA binding;single-stranded telomeric DNA binding;structure-specific DNA binding;telomeric DNA b
acid-amino acid ligase activity;binding;calcium ion binding;catalytic activity;cation binding;ephrin receptor binding;ion binding;ligase activity;ligase activity, forming carbon-nitrogen bonds;metal ion binding;molecular transduc
binding;catalytic activity;cation binding;endopeptidase activity;hydrolase activity;ion binding;metal ion binding;metalloendopeptidase activity;metallopeptidase activity;peptidase activity;peptidase activity, acting on L-amino a
2 iron, 2 sulfur cluster binding;binding;catalytic activity;cation binding;ferrochelatase activity;ferrous iron binding;heme binding;ion binding;iron ion binding;iron-responsive element binding;iron-sulfur cluster binding;lyase activ
binding;calcium ion binding;cation binding;enzyme activator activity;enzyme regulator activity;extracellular matrix structural constituent;ion binding;metal ion binding;peptidase activator activity;peptidase regulator activity;stru
binding;cation binding;DNA binding;ion binding;metal ion binding;nucleic acid binding;transition metal ion binding;zinc ion binding
antioxidant activity;binding;catalytic activity;cation binding;dioxygenase activity;heme binding;ion binding;iron ion binding;metal ion binding;oxidoreductase activity;oxidoreductase activity, acting on paired donors, with incorp
binding;cation binding;ion binding;metal ion binding
binding;core promoter binding;DNA binding;enzyme binding;histone deacetylase binding;nucleic acid binding;nucleotide binding;protein binding;regulatory region DNA binding;regulatory region nucleic acid binding;sequence
binding;catalytic activity;GTP binding;GTPase activity;guanyl nucleotide binding;guanyl ribonucleotide binding;hydrolase activity;hydrolase activity, acting on acid anhydrides;hydrolase activity, acting on acid anhydrides, in pl
binding;catalytic activity;cis-trans isomerase activity;enzyme binding;isomerase activity;peptide binding;peptidyl-prolyl cis-trans isomerase activity;protein binding;protein complex binding;RNA polymerase binding;unfolded p
binding;carbon-carbon lyase activity;carboxy-lyase activity;catalytic activity;cation binding;coenzyme binding;cofactor binding;electron carrier activity;ion binding;lyase activity;malate dehydrogenase (decarboxylating) activity
adenyl nucleotide binding;adenyl ribonucleotide binding;aminoacyl-tRNA ligase activity;ATP binding;binding;catalytic activity;ligase activity;ligase activity, forming aminoacyl-tRNA and related compounds;ligase activity, formi
binding;carbon-oxygen lyase activity;catalytic activity;damaged DNA binding;DNA binding;DNA N-glycosylase activity;DNA-(apurinic or apyrimidinic site) lyase activity;enzyme binding;hydrolase activity;hydrolase activity, acti
adenyl nucleotide binding;adenyl ribonucleotide binding;ATP binding;binding;catalytic activity;cytokine receptor binding;enzyme binding;growth hormone receptor binding;hormone receptor binding;kinase activity;non-memb
binding;chromatin binding;chromo shadow domain binding;DNA binding;enzyme binding;identical protein binding;kinase binding;nucleic acid binding;protein binding;protein binding transcription factor activity;protein dimeriz
adenosylhomocysteinase activity;catalytic activity;hydrolase activity;hydrolase activity, acting on ether bonds;trialkylsulfonium hydrolase activity
binding;nucleic acid binding;nucleotide binding;RNA binding;translation factor activity, nucleic acid binding;translation initiation factor activity
active transmembrane transporter activity;adenyl nucleotide binding;adenyl ribonucleotide binding;ATP binding;ATPase activity;ATPase activity, coupled;ATPase activity, coupled to movement of substances;ATPase activity, c
adenyl nucleotide binding;adenyl ribonucleotide binding;ATP binding;binding;calcium ion binding;catalytic activity;cation binding;diacylglycerol kinase activity;ion binding;kinase activity;lipid binding;metal ion binding;NAD+ ki
carnitine O-acyltransferase activity;carnitine O-palmitoyltransferase activity;catalytic activity;O-acyltransferase activity;O-palmitoyltransferase activity;palmitoyltransferase activity;transferase activity;transferase activity, transf
adenyl nucleotide binding;adenyl ribonucleotide binding;ATP binding;binding;catalytic activity;kinase activity;nucleobase-containing compound kinase activity;nucleoside phosphate kinase activity;nucleotide binding;nucleoti
adenyl nucleotide binding;adenyl ribonucleotide binding;ATP binding;binding;catalytic activity;nucleotide binding;oxidoreductase activity;oxidoreductase activity, acting on CH or CH2 groups;oxidoreductase activity, acting o
binding;nucleic acid binding;RNA binding;translation elongation factor activity;translation factor activity, nucleic acid binding
cation transmembrane transporter activity;hydrogen ion transmembrane transporter activity;inorganic cation transmembrane transporter activity;ion transmembrane transporter activity;monovalent inorganic cation transmemb
binding;fibronectin binding;growth factor binding;insulin-like growth factor binding;insulin-like growth factor I binding;insulin-like growth factor II binding;protein binding
acid phosphatase activity;catalytic activity;hydrolase activity;hydrolase activity, acting on ester bonds;non-membrane spanning protein tyrosine phosphatase activity;phosphatase activity;phosphoprotein phosphatase activity
acetyl-CoA C-acetyltransferase activity;acetyl-CoA C-acyltransferase activity;acetyltransferase activity;binding;C-acetyltransferase activity;C-acyltransferase activity;catalytic activity;cation binding;coenzyme binding;cofactor
binding;protein binding;syndecan binding
binding;calcium ion binding;cation binding;ion binding;metal ion binding;structural constituent of muscle;structural molecule activity
binding;catalytic activity;cation binding;DNA binding;DNA-directed RNA polymerase activity;enzyme binding;ion binding;metal ion binding;nucleic acid binding;nucleotidyltransferase activity;protein binding;RNA polymerase a
adenyl nucleotide binding;adenyl ribonucleotide binding;ATP binding;binding;catalytic activity;cation binding;cyclin binding;cyclin-dependent protein kinase activity;ion binding;kinase activity;metal ion binding;nucleotide bind
adenyl nucleotide binding;adenyl ribonucleotide binding;ATP binding;binding;catalytic activity;DNA binding;DNA helicase activity;helicase activity;hydrolase activity;hydrolase activity, acting on acid anhydrides;hydrolase activ
3-mercaptopyruvate sulfurtransferase activity;catalytic activity;sulfurtransferase activity;thiosulfate sulfurtransferase activity;transferase activity;transferase activity, transferring sulfur-containing groups
structural constituent of ribosome;structural molecule activity
binding;chromatin binding;histone acetyl-lysine binding;histone binding;protein binding
binding;cation binding;DNA binding;DNA secondary structure binding;enhancer binding;enhancer sequence-specific DNA binding;four-way junction DNA binding;ion binding;metal ion binding;nucleic acid binding;nucleic acic
ATPase activator activity;ATPase binding;ATPase regulator activity;binding;chaperone binding;enzyme activator activity;enzyme binding;enzyme regulator activity;heat shock protein binding;Hsp70 protein binding;nucleoside-
active transmembrane transporter activity;adenyl nucleotide binding;adenyl ribonucleotide binding;ATP binding;ATPase activity;ATPase activity, coupled;ATPase activity, coupled to movement of substances;ATPase activity, c
binding;catalytic activity;collagen binding;cysteine-type endopeptidase activity;cysteine-type peptidase activity;endopeptidase activity;extracellular matrix binding;fibronectin binding;glycoprotein binding;hydrolase activity;lar
binding;catalytic activity;endopeptidase activity;hydrolase activity;nucleic acid binding;peptidase activity;peptidase activity, acting on L-amino acid peptides;RNA binding;threonine-type endopeptidase activity;threonine-type
catalytic activity;endopeptidase activity;hydrolase activity;peptidase activity;peptidase activity, acting on L-amino acid peptides;threonine-type endopeptidase activity;threonine-type peptidase activity
catalytic activity;endopeptidase activity;hydrolase activity;peptidase activity;peptidase activity, acting on L-amino acid peptides;threonine-type endopeptidase activity;threonine-type peptidase activity
catalytic activity;endopeptidase activity;hydrolase activity;peptidase activity;peptidase activity, acting on L-amino acid peptides;threonine-type endopeptidase activity;threonine-type peptidase activity
binding;cation binding;enzyme binding;glycoprotein binding;ion binding;metal ion binding;protease binding;protein binding;protein dimerization activity;protein heterodimerization activity
1,3-beta-D-glucan binding;binding;carbohydrate binding;complement binding;complement component C1q binding;opsonin binding;pattern binding;polysaccharide binding;protein binding;virion binding
binding;cell adhesion molecule binding;double-stranded RNA binding;enzyme binding;kinase binding;nucleic acid binding;protein binding;protein kinase binding;receptor binding;RNA binding;structural constituent of cytoske
adenyl nucleotide binding;adenyl ribonucleotide binding;ATP binding;ATPase activity;ATPase activity, coupled;ATP-dependent helicase activity;ATP-dependent RNA helicase activity;binding;catalytic activity;helicase activity;h
binding;catalytic activity;cation binding;chromatin binding;DNA (cytosine-5-)-methyltransferase activity;DNA binding;DNA-methyltransferase activity;ion binding;metal ion binding;methyl-CpG binding;methyltransferase activit
binding;enzyme binding;nucleotide binding;protein binding
binding;nucleic acid binding;RNA binding;structural constituent of ribosome;structural molecule activity
acyl-CoA dehydrogenase activity;binding;catalytic activity;coenzyme binding;cofactor binding;flavin adenine dinucleotide binding;isovaleryl-CoA dehydrogenase activity;oxidoreductase activity;oxidoreductase activity, acting
binding;calcium ion binding;cation binding;ion binding;metal ion binding;protein binding;RAGE receptor binding;receptor binding
binding;chemoattractant activity;chromatin binding;damaged DNA binding;DNA bending activity;DNA binding;double-stranded DNA binding;nucleic acid binding;nucleic acid binding transcription factor activity;protein bindin
binding;nucleic acid binding;nucleotide binding;poly-pyrimidine tract binding;pre-mRNA binding;RNA binding;single-stranded RNA binding
adenyl nucleotide binding;adenyl ribonucleotide binding;aminoacyl-tRNA ligase activity;ATP binding;binding;catalytic activity;identical protein binding;ligase activity;ligase activity, forming aminoacyl-tRNA and related compou
adenyl nucleotide binding;adenyl ribonucleotide binding;aminoacyl-tRNA editing activity;aminoacyl-tRNA ligase activity;ATP binding;binding;carboxylic ester hydrolase activity;catalytic activity;hydrolase activity;hydrolase acti
binding;nucleic acid binding;RNA binding;translation elongation factor activity;translation factor activity, nucleic acid binding
binding;catalytic activity;cis-trans isomerase activity;drug binding;FK506 binding;isomerase activity;macrolide binding;peptidyl-prolyl cis-trans isomerase activity

binding;enzyme binding;protein binding;RNA polymerase binding
binding;protein binding;protein N-terminus binding
adenyl nucleotide binding;adenyl ribonucleotide binding;ATP binding;binding;catalytic activity;enzyme binding;kinase activity;MAP kinase activity;molecular transducer activity;nucleotide binding;phosphatase binding;phosph
binding;catalytic activity;dipeptidyl-peptidase activity;endopeptidase activity;enzyme binding;exopeptidase activity;hydrolase activity;identical protein binding;peptidase activity;peptidase activity, acting on L-amino acid pept
structural constituent of ribosome;structural molecule activity
binding;cation binding;chromatin binding;damaged DNA binding;DNA binding;ion binding;metal ion binding;nucleic acid binding;single-stranded DNA binding;structure-specific DNA binding
3'-5' exonuclease activity;3'-5'-exodeoxyribonuclease activity;binding;carbon-oxygen lyase activity;catalytic activity;cation binding;chromatin binding;chromatin DNA binding;damaged DNA binding;deoxyribonuclease activity
adenyl nucleotide binding;adenyl ribonucleotide binding;ATP binding;binding;catalytic activity;deoxycytidine kinase activity;deoxynucleoside kinase activity;drug binding;identical protein binding;kinase activity;nucleobase-co
adenyl nucleotide binding;adenyl ribonucleotide binding;amine binding;amino acid binding;aspartate binding;aspartate carbamoyltransferase activity;ATP binding;binding;carbamoyl-phosphate synthase (ammonia) activity;ca
androgen receptor binding;binding;calcium ion binding;carbohydrate binding;cation binding;chaperone binding;complement binding;complement component C1q binding;DNA binding;enzyme binding;glycoprotein binding;ho
structural molecule activity
binding;calcium ion binding;carbohydrate binding;cation binding;ion binding;metal ion binding
catalytic activity;endopeptidase activity;hydrolase activity;peptidase activity;peptidase activity, acting on L-amino acid peptides;threonine-type endopeptidase activity;threonine-type peptidase activity
catalytic activity;endopeptidase activity;hydrolase activity;peptidase activity;peptidase activity, acting on L-amino acid peptides;threonine-type endopeptidase activity;threonine-type peptidase activity
catalytic activity;endopeptidase activity;hydrolase activity;peptidase activity;peptidase activity, acting on L-amino acid peptides;threonine-type endopeptidase activity;threonine-type peptidase activity
binding;catalytic activity;endopeptidase activity;hydrolase activity;lipopolysaccharide binding;peptidase activity;peptidase activity, acting on L-amino acid peptides;threonine-type endopeptidase activity;threonine-type peptic
catalytic activity;endopeptidase activity;hydrolase activity;peptidase activity;peptidase activity, acting on L-amino acid peptides;threonine-type endopeptidase activity;threonine-type peptidase activity
catalytic activity;endopeptidase activity;hydrolase activity;peptidase activity;peptidase activity, acting on L-amino acid peptides;threonine-type endopeptidase activity;threonine-type peptidase activity
active transmembrane transporter activity;adenyl nucleotide binding;adenyl ribonucleotide binding;ATP binding;ATPase activity;ATPase activity, coupled;ATPase activity, coupled to movement of substances;ATPase activity, c
binding;catalytic activity;cation binding;copper ion binding;ion binding;metal ion binding;oxidoreductase activity;oxidoreductase activity, acting on the CH-NH2 group of donors;oxidoreductase activity, acting on the CH-NH2
2 iron, 2 sulfur cluster binding;4 iron, 4 sulfur cluster binding;binding;catalytic activity;cation binding;electron carrier activity;ion binding;iron-sulfur cluster binding;metal cluster binding;metal ion binding;NADH dehydrogenase
3'-5' exonuclease activity;3'-5'-exodeoxyribonuclease activity;4 iron, 4 sulfur cluster binding;binding;catalytic activity;cation binding;chromatin binding;damaged DNA binding;deoxyribonuclease activity;DNA binding;DNA pol
adenyl nucleotide binding;adenyl ribonucleotide binding;ATP binding;ATPase activity;binding;catalytic activity;chromatin binding;DNA binding;helicase activity;hydrolase activity;hydrolase activity, acting on acid anhydrides;hy
adenyl nucleotide binding;adenyl ribonucleotide binding;ATP binding;binding;catalytic activity;DNA binding;enzyme binding;kinase activity;MAP kinase activity;molecular transducer activity;nucleic acid binding;nucleotide bin
binding;cation binding;DNA binding;enhancer binding;enhancer sequence-specific DNA binding;ion binding;ligand-dependent nuclear receptor activity;metal ion binding;molecular transducer activity;nucleic acid binding;nucl
binding;growth factor activity;protein binding;receptor binding
aminopeptidase activity;binding;catalytic activity;cation binding;exopeptidase activity;hydrolase activity;ion binding;manganese ion binding;metal ion binding;metalloexopeptidase activity;metallopeptidase activity;peptidase a
aminopeptidase activity;binding;catalytic activity;endopeptidase activity;exopeptidase activity;hydrolase activity;peptidase activity;peptidase activity, acting on L-amino acid peptides;peptide binding;serine hydrolase activity;
alkali metal ion binding;binding;catalytic activity;cation binding;hydrolase activity;hydrolase activity, acting on ester bonds;identical protein binding;inositol monophosphate phosphatase activity;inositol or phosphatidylinositol
binding;carbohydrate binding;glycosaminoglycan binding;growth factor binding;heparin binding;insulin-like growth factor binding;integrin binding;pattern binding;polysaccharide binding;protein binding;protein complex bindi
adenyl nucleotide binding;adenyl ribonucleotide binding;ATP binding;binding;catalytic activity;ephrin receptor activity;kinase activity;molecular transducer activity;nucleotide binding;phosphotransferase activity, alcohol group
adenyl nucleotide binding;adenyl ribonucleotide binding;ATP binding;axon guidance receptor activity;binding;catalytic activity;ephrin receptor activity;kinase activity;molecular transducer activity;nucleotide binding;phosphotr
alkylbase DNA N-glycosylase activity;binding;catalytic activity;damaged DNA binding;DNA binding;DNA N-glycosylase activity;DNA-3-methyladenine glycosylase I activity;DNA-7-methylguanine glycosylase activity;hydrolase
binding;carboxylic acid binding;isoprenoid binding;lipid binding;monocarboxylic acid binding;retinal binding;retinoic acid binding;retinoid binding;retinol binding;transporter activity;vitamin binding
binding;catalytic activity;cation binding;cofactor binding;identical protein binding;ion binding;metal ion binding;protein binding;protein dimerization activity;protein homodimerization activity;transferase activity;transferase acti
apoptotic protease activator activity;caspase activator activity;caspase regulator activity;catalytic activity;cysteine-type endopeptidase activity;cysteine-type peptidase activity;endopeptidase activity;enzyme activator activity
binding;cation binding;cobalt ion binding;DNA binding;enzyme binding;identical protein binding;ion binding;metal ion binding;nucleic acid binding;protein binding;protein binding transcription factor activity;protein dimerizatio
activating transcription factor binding;binding;DNA binding;heat shock protein binding;molecular transducer activity;nucleic acid binding;protein binding;RNA binding;signal transducer activity;transcription factor binding;trans
actin binding;actin filament binding;binding;calmodulin binding;cytoskeletal protein binding;protein binding
binding;catalytic activity;cation binding;G-protein beta/gamma-subunit complex binding;G-protein-coupled receptor binding;GTP binding;GTPase activity;guanyl nucleotide binding;guanyl ribonucleotide binding;hydrolase ac
binding;chaperone binding;identical protein binding;protein binding;protein dimerization activity;protein homodimerization activity
antioxidant activity;binding;carboxylic ester hydrolase activity;catalytic activity;enzyme binding;glutathione peroxidase activity;hydrolase activity;hydrolase activity, acting on ester bonds;lipase activity;oxidoreductase activity;
biliverdin reductase activity;catalytic activity;flavin reductase activity;oxidoreductase activity;oxidoreductase activity, acting on the CH-CH group of donors;oxidoreductase activity, acting on the CH-CH group of donors, NAD
antioxidant activity;binding;caspase inhibitor activity;caspase regulator activity;catalytic activity;cysteine-type endopeptidase inhibitor activity;DNA binding;endopeptidase inhibitor activity;endopeptidase regulator activity;enz
carbon-carbon lyase activity;carboxy-lyase activity;catalytic activity;D-dopachrome decarboxylase activity;dopachrome isomerase activity;intramolecular oxidoreductase activity;intramolecular oxidoreductase activity, transpo
alkyl hydroperoxide reductase activity;antioxidant activity;binding;caspase inhibitor activity;caspase regulator activity;catalytic activity;cysteine-type endopeptidase inhibitor activity;endopeptidase inhibitor activity;endopeptic
active transmembrane transporter activity;ATPase activity;ATPase activity, coupled;ATPase activity, coupled to movement of substances;ATPase activity, coupled to transmembrane movement of ions;ATPase activity, couplec
structural constituent of ribosome;structural molecule activity
carbon-oxygen lyase activity;catalytic activity;enoyl-CoA hydratase activity;hydro-lyase activity;lyase activity
adenyl nucleotide binding;adenyl ribonucleotide binding;ATP binding;binding;catalytic activity;cytidylate kinase activity;kinase activity;nucleobase-containing compound kinase activity;nucleoside diphosphate kinase activity;n
adenyl nucleotide binding;adenyl ribonucleotide binding;ATP binding;binding;endopeptidase inhibitor activity;endopeptidase regulator activity;enzyme binding;enzyme inhibitor activity;enzyme regulator activity;kinase binding
catalytic activity;cysteine-type endopeptidase activity;cysteine-type peptidase activity;disulfide oxidoreductase activity;endopeptidase activity;hydrolase activity;hydrolase activity, acting on ester bonds;intramolecular oxidore
antigen binding;binding;catalytic activity;enzyme regulator activity;hydrolase activity;hydrolase activity, acting on ester bonds;phosphatase activity;phosphatase regulator activity;phosphoprotein phosphatase activity;phospho
binding;catalytic activity;cis-trans isomerase activity;cyclosporin A binding;drug binding;isomerase activity;peptide binding;peptidyl-prolyl cis-trans isomerase activity
binding;catalytic activity;cis-trans isomerase activity;cyclosporin A binding;drug binding;isomerase activity;peptide binding;peptidyl-prolyl cis-trans isomerase activity
catalytic activity;glycylpeptide N-tetradecanoyltransferase activity;myristoyltransferase activity;N-acyltransferase activity;transferase activity;transferase activity, transferring acyl groups;transferase activity, transferring acyl gr
binding;catalytic activity;cation binding;heme oxygenase (decyclizing) activity;ion binding;metal ion binding;oxidoreductase activity;oxidoreductase activity, acting on paired donors, with incorporation or reduction of molecula
adenylosuccinate synthase activity;anion binding;binding;catalytic activity;cation binding;GTP binding;guanyl nucleotide binding;guanyl ribonucleotide binding;ion binding;ligase activity;ligase activity, forming carbon-nitrogen
adenyl nucleotide binding;adenyl ribonucleotide binding;ATP binding;binding;catalytic activity;kinase activity;lipid binding;molecular transducer activity;nucleotide binding;phosphatidylserine binding;phospholipid binding;pho
asialoglycoprotein receptor activity;binding;carbohydrate binding;cargo receptor activity;glycosaminoglycan binding;heparin binding;lipoprotein particle receptor binding;low-density lipoprotein particle receptor binding;patter
androgen binding;benzodiazepine receptor activity;binding;cholesterol binding;hormone binding;lipid binding;molecular transducer activity;neurotransmitter binding;neurotransmitter receptor activity;receptor activity;signal tra
(S)-2-(5-amino-1-(5-phospho-D-ribosyl)imidazole-4-carboxamido)succinate AMP-lyase (fumarate-forming) activity;amidine-lyase activity;carbon-nitrogen lyase activity;catalytic activity;lyase activity;N6-(1,2-dicarboxyethyl)AM
binding;cation binding;cytoskeletal protein binding;identical protein binding;ion binding;metal ion binding;microtubule binding;microtubule plus-end binding;nucleic acid binding;protein binding;protein dimerization activity;pro
binding;calcium channel regulator activity;calcium ion binding;calcium-dependent cysteine-type endopeptidase activity;catalytic activity;cation binding;channel regulator activity;cysteine-type endopeptidase activity;cysteine-
endopeptidase inhibitor activity;endopeptidase regulator activity;enzyme inhibitor activity;enzyme regulator activity;peptidase inhibitor activity;peptidase regulator activity;serine-type endopeptidase inhibitor activity
active transmembrane transporter activity;amine transmembrane transporter activity;amino acid transmembrane transporter activity;antiporter activity;arginine transmembrane transporter activity;basic amino acid transmemb
aldehyde dehydrogenase (NAD) activity;catalytic activity;oxidoreductase activity;oxidoreductase activity, acting on the aldehyde or oxo group of donors;oxidoreductase activity, acting on the aldehyde or oxo group of donors,
binding;catalytic activity;cation binding;chromatin binding;DNA binding;DNA-directed RNA polymerase activity;ion binding;metal ion binding;nucleic acid binding;nucleoside binding;nucleotidyltransferase activity;ribonucleosi
binding;catalytic activity;coenzyme binding;cofactor binding;flavin adenine dinucleotide binding;oxidoreductase activity;oxidoreductase activity, acting on the CH-CH group of donors;oxidoreductase activity, acting on the CH
actin binding;actin filament binding;actin monomer binding;binding;cytoskeletal protein binding;enzyme binding;identical protein binding;kinase binding;myosin binding;myosin heavy chain binding;phosphatidylinositol 3-kina
enzyme activator activity;enzyme regulator activity;GDP-dissociation inhibitor activity;GTPase activator activity;GTPase regulator activity;nucleoside-triphosphatase regulator activity;Rab GDP-dissociation inhibitor activity;sm
adenyl nucleotide binding;adenyl ribonucleotide binding;ATP binding;binding;catalytic activity;cation binding;ion binding;metal ion binding;methionine adenosyltransferase activity;nucleotide binding;purine nucleotide binding;
adenyl nucleotide binding;adenyl ribonucleotide binding;amine binding;amino acid binding;ATP binding;binding;calcium ion binding;carbamoyl-phosphate synthase (ammonia) activity;carbamoyl-phosphate synthase (glutami
binding;catalytic activity;cation binding;ion binding;metal ion binding;oxidoreductase activity;oxidoreductase activity, acting on CH or CH2 groups;oxidoreductase activity, acting on CH or CH2 groups, disulfide as acceptor;ri
adenyl nucleotide binding;adenyl ribonucleotide binding;ATP binding;binding;C3HC4-type RING finger domain binding;cation binding;chaperone binding;enzyme binding;G-protein-coupled receptor binding;heat shock protei
14-3-3 protein binding;adenyl nucleotide binding;adenyl ribonucleotide binding;ATP binding;binding;catalytic activity;enzyme binding;enzyme regulator activity;identical protein binding;kinase activity;lipid binding;nitric-oxide
adenyl nucleotide binding;adenyl ribonucleotide binding;ATP binding;binding;catalytic activity;kinase activity;nucleotide binding;phosphotransferase activity, alcohol group as acceptor;protein kinase activity;protein serine/thre
binding;catalytic activity;cation binding;cation transmembrane transporter activity;enzyme binding;hydrogen ion transmembrane transporter activity;inorganic cation transmembrane transporter activity;ion binding;ion transme
3-hydroxyisobutyrate dehydrogenase activity;binding;catalytic activity;coenzyme binding;cofactor binding;NAD binding;nucleotide binding;oxidoreductase activity;oxidoreductase activity, acting on CH-OH group of donors;ox
binding;catalytic activity;cyclohydrolase activity;hydrolase activity;hydrolase activity, acting on carbon-nitrogen (but not peptide) bonds;hydrolase activity, acting on carbon-nitrogen (but not peptide) bonds, in cyclic amidines;
binding;nucleic acid binding;nucleotide binding;RNA binding
binding;nucleic acid binding;nucleotide binding;poly(U) RNA binding;poly-pyrimidine tract binding;RNA binding;single-stranded RNA binding
amine binding;amino acid binding;binding;carboxylic acid binding;enzyme binding;histone deacetylase binding;phosphoprotein binding;phosphoserine binding;protein binding;protein binding transcription factor activity;prote
binding;calcium ion binding;calcium-dependent protein binding;cation binding;identical protein binding;ion binding;metal ion binding;protein binding;protein dimerization activity;protein homodimerization activity
binding;carbohydrate binding;monosaccharide binding;sialic acid binding;sugar binding
antioxidant activity;catalytic activity;oxidoreductase activity;oxidoreductase activity, acting on paired donors, with incorporation or reduction of molecular oxygen;oxidoreductase activity, acting on peroxide as acceptor;perox
binding;catalytic activity;cation binding;dCMP deaminase activity;deaminase activity;hydrolase activity;hydrolase activity, acting on carbon-nitrogen (but not peptide) bonds;hydrolase activity, acting on carbon-nitrogen (but n
binding;catalytic activity;identical protein binding;oxidoreductase activity;oxidoreductase activity, acting on the CH-NH group of donors;oxidoreductase activity, acting on the CH-NH group of donors, NAD or NADP as accept
actin binding;binding;catalytic activity;cytokine binding;cytoskeletal protein binding;enzyme binding;GTP binding;GTPase activity;guanyl nucleotide binding;guanyl ribonucleotide binding;heat shock protein binding;Hsp90 pro
binding;nucleic acid binding;RNA binding;rRNA binding;structural constituent of ribosome;structural molecule activity
adenyl nucleotide binding;adenyl ribonucleotide binding;ATP binding;binding;catalytic activity;fatty acid ligase activity;ligase activity;ligase activity, forming carbon-sulfur bonds;long-chain fatty acid-CoA ligase activity;nucleot
adenyl nucleotide binding;adenyl ribonucleotide binding;ATP binding;ATPase activity;binding;catalytic activity;cytoskeletal protein binding;hydrolase activity;hydrolase activity, acting on acid anhydrides;hydrolase activity, acti
binding;nucleic acid binding;nucleotide binding;RNA binding
binding;catalytic activity;cation binding;dUTP diphosphatase activity;hydrolase activity;hydrolase activity, acting on acid anhydrides;hydrolase activity, acting on acid anhydrides, in phosphorus-containing anhydrides;ion bind
active transmembrane transporter activity;adenyl nucleotide binding;adenyl ribonucleotide binding;ATP binding;ATPase activity;ATPase activity, coupled;ATPase activity, coupled to movement of substances;ATPase activity, c
cyclin-dependent protein kinase regulator activity;enzyme regulator activity;kinase regulator activity;protein kinase regulator activity
binding;DNA binding;nucleic acid binding
adenyl nucleotide binding;adenyl ribonucleotide binding;ATP binding;binding;catalytic activity;kinase activity;nucleotide binding;phosphotransferase activity, alcohol group as acceptor;protein kinase activity;protein serine/thre
adenyl nucleotide binding;adenyl ribonucleotide binding;ATP binding;binding;catalytic activity;DNA binding;DNA helicase activity;helicase activity;hydrolase activity;hydrolase activity, acting on acid anhydrides;hydrolase activ
adenyl nucleotide binding;adenyl ribonucleotide binding;ATP binding;binding;catalytic activity;chromatin binding;DNA binding;DNA helicase activity;DNA replication origin binding;helicase activity;hydrolase activity;hydrolase
adenyl nucleotide binding;adenyl ribonucleotide binding;ATP binding;binding;catalytic activity;DNA binding;DNA helicase activity;helicase activity;hydrolase activity;hydrolase activity, acting on acid anhydrides;hydrolase activ
aldehyde-lyase activity;amine binding;amino acid binding;binding;carbon-carbon lyase activity;carboxylic acid binding;catalytic activity;cofactor binding;glycine hydroxymethyltransferase activity;hydroxymethyl-, formyl- and
aldehyde-lyase activity;amine binding;amino acid binding;binding;carbon-carbon lyase activity;carboxylic acid binding;catalytic activity;chromatin binding;cofactor binding;glycine hydroxymethyltransferase activity;hydroxyme
adenyl nucleotide binding;adenyl ribonucleotide binding;ATP binding;binding;nucleotide binding;purine nucleotide binding;purine ribonucleoside triphosphate binding;purine ribonucleotide binding;ribonucleotide binding
binding;catalytic activity;cation binding;intramolecular oxidoreductase activity;intramolecular oxidoreductase activity, interconverting aldoses and ketoses;ion binding;isomerase activity;mannose-6-phosphate isomerase activ
binding;cation binding;copper ion binding;extracellular matrix binding;fibroblast growth factor binding;growth factor binding;ion binding;laminin binding;metal ion binding;protein binding;transition metal ion binding
actin binding;actin monomer binding;adenyl nucleotide binding;adenyl-nucleotide exchange factor activity;ATPase regulator activity;binding;cytoskeletal protein binding;enzyme regulator activity;lipid binding;nucleoside-triph
beta-catenin binding;binding;cadherin binding;cell adhesion molecule binding;cytoskeletal protein binding;gamma-catenin binding;protein binding;structural molecule activity;vinculin binding
activating transcription factor binding;alpha-catenin binding;androgen receptor binding;binding;cadherin binding;cell adhesion molecule binding;DNA binding;double-stranded DNA binding;enzyme binding;estrogen receptor
binding;complement binding;complement component C3a binding;complement component C3b binding;DNA binding;enzyme binding;G-protein-coupled receptor binding;histone deacetylase binding;nucleic acid binding;nu
binding;endopeptidase inhibitor activity;endopeptidase regulator activity;enzyme binding;enzyme inhibitor activity;enzyme regulator activity;peptidase inhibitor activity;peptidase regulator activity;protease binding;protein binc
binding;damaged DNA binding;DNA binding;nucleic acid binding;single-stranded DNA binding;structure-specific DNA binding
adenyl nucleotide binding;adenyl ribonucleotide binding;ATP binding;binding;DNA binding;nucleic acid binding;nucleotide binding;purine nucleotide binding;purine ribonucleoside triphosphate binding;purine ribonucleotide bi
adenyl nucleotide binding;adenyl ribonucleotide binding;ATP binding;binding;DNA binding;nucleic acid binding;nucleotide binding;purine nucleotide binding;purine ribonucleoside triphosphate binding;purine ribonucleotide bi
adenyl nucleotide binding;adenyl ribonucleotide binding;ATP binding;ATPase activity;ATPase activity, coupled;binding;catalytic activity;DNA binding;DNA clamp loader activity;DNA-dependent ATPase activity;double-strandec
binding;carbohydrate binding;glycosaminoglycan binding;heparin binding;nucleic acid binding;pattern binding;polysaccharide binding;RNA binding;structural constituent of ribosome;structural molecule activity
binding;catalytic activity;DNA binding;enzyme activator activity;enzyme regulator activity;nucleic acid binding;phosphatase activator activity;phosphatase regulator activity;protein binding;protein binding transcription factor a
antioxidant activity;arachidonate 15-lipoxygenase activity;binding;catalytic activity;cation binding;dioxygenase activity;enzyme binding;heme binding;ion binding;iron ion binding;lipid binding;metal ion binding;oxidoreductase
binding;calcium ion binding;carbohydrate binding;cation binding;glycosaminoglycan binding;heparin binding;ion binding;metal ion binding;pattern binding;polysaccharide binding
binding;calcium ion binding;carbohydrate binding;cation binding;glycosaminoglycan binding;heparin binding;integrin binding;ion binding;metal ion binding;pattern binding;polysaccharide binding;protein binding;protein comp
amine binding;amino acid binding;binding;carbon monoxide binding;carbon-oxygen lyase activity;carboxylic acid binding;catalytic activity;cation binding;cofactor binding;cystathionine beta-synthase activity;enzyme binding;
structural constituent of cytoskeleton;structural molecule activity
binding;calcium ion binding;cation binding;extracellular matrix structural constituent;ion binding;metal ion binding;structural molecule activity
binding;calcium ion binding;cation binding;extracellular matrix structural constituent;ion binding;metal ion binding;structural molecule activity
4-alpha-glucanotransferase activity;alpha-glucosidase activity;amylo-alpha-1,6-glucosidase activity;binding;carbohydrate binding;catalytic activity;glucosidase activity;glycogen debranching enzyme activity;hydrolase activity

actin binding;actin filament binding;actin-dependent ATPase activity;adenyl nucleotide binding;adenyl ribonucleotide binding;ADP binding;ATP binding;ATPase activity;ATPase activity, coupled;binding;catalytic activity;cytosk
actin binding;actin filament binding;actin-dependent ATPase activity;adenyl nucleotide binding;adenyl ribonucleotide binding;ADP binding;ATP binding;ATPase activity;ATPase activity, coupled;binding;catalytic activity;cytosk
structural molecule activity
binding;carboxylic acid binding;catalytic activity;cholesterol binding;cholesterol O-acyltransferase activity;coenzyme binding;cofactor binding;fatty acid binding;fatty-acyl-CoA binding;lipid binding;monocarboxylic acid bindin
actin binding;actin filament binding;binding;cytoskeletal protein binding;identical protein binding;protein binding;protein dimerization activity;protein heterodimerization activity;protein homodimerization activity;spectrin bindin
binding;carbohydrate binding;mannose binding;monosaccharide binding;sugar binding
binding;cation binding;endopeptidase inhibitor activity;endopeptidase regulator activity;enzyme binding;enzyme inhibitor activity;enzyme regulator activity;ion binding;metal ion binding;metalloendopeptidase inhibitor activity;
binding;cation binding;DNA binding;identical protein binding;ion binding;metal ion binding;nucleic acid binding;nucleotide binding;protein binding;protein binding transcription factor activity;RNA binding;transcription coactiva
nucleocytoplasmic transporter activity;transporter activity
binding;DNA binding;histone binding;nucleic acid binding;protein binding
binding;catalytic activity;disulfide oxidoreductase activity;electron carrier activity;glutathione disulfide oxidoreductase activity;oxidoreductase activity;oxidoreductase activity, acting on a sulfur group of donors;peptide disulfid
binding;calcium-dependent protein serine/threonine phosphatase activity;calmodulin-dependent protein phosphatase activity;catalytic activity;cation binding;hydrolase activity;hydrolase activity, acting on ester bonds;ion bin
structural constituent of cytoskeleton;structural molecule activity
binding;carbon-carbon lyase activity;carboxylic acid binding;catalytic activity;cation binding;coenzyme binding;cofactor binding;fatty acid binding;fatty-acyl-CoA binding;hydroxymethylglutaryl-CoA lyase activity;identical pro
adenyl nucleotide binding;adenyl ribonucleotide binding;ATP binding;ATPase activity;binding;catalytic activity;hydrolase activity;hydrolase activity, acting on acid anhydrides;hydrolase activity, acting on acid anhydrides, in ph
binding;catalytic activity;cation binding;cytoskeletal protein binding;GDP binding;GTP binding;GTPase activity;guanyl nucleotide binding;guanyl ribonucleotide binding;hydrolase activity;hydrolase activity, acting on acid anhy
adenyl nucleotide binding;adenyl ribonucleotide binding;ATP binding;binding;catalytic activity;enzyme activator activity;enzyme regulator activity;kinase activator activity;kinase activity;kinase regulator activity;MAP kinase kin
active transmembrane transporter activity;ATPase activity;ATPase activity, coupled;ATPase activity, coupled to movement of substances;ATPase activity, coupled to transmembrane movement of ions;ATPase activity, couplec
active transmembrane transporter activity;ATPase activity;ATPase activity, coupled;ATPase activity, coupled to movement of substances;ATPase activity, coupled to transmembrane movement of ions;ATPase activity, couplec
binding;catalytic activity;coproporphyrinogen oxidase activity;identical protein binding;oxidoreductase activity;oxidoreductase activity, acting on the CH-CH group of donors;oxidoreductase activity, acting on the CH-CH grou
binding;nucleic acid binding;RNA binding;structural constituent of ribosome;structural molecule activity
adenyl nucleotide binding;adenyl ribonucleotide binding;ADP binding;ATP binding;ATPase activity;ATPase activity, coupled;ATP-dependent peptidase activity;binding;catalytic activity;DNA binding;DNA polymerase binding;en
binding;catalytic activity;cation binding;intramolecular transferase activity;intramolecular transferase activity, phosphotransferases;ion binding;isomerase activity;magnesium ion binding;metal ion binding;phosphoglucomutas
binding;catalytic activity;cation binding;enzyme binding;hydrolase activity;hydrolase activity, acting on ester bonds;ion binding;kinase binding;metal ion binding;phosphatase activity;phosphoprotein phosphatase activity;phos
binding;catalytic activity;GTP binding;GTPase activity;guanyl nucleotide binding;guanyl ribonucleotide binding;hydrolase activity;hydrolase activity, acting on acid anhydrides;hydrolase activity, acting on acid anhydrides, in ph
endopeptidase inhibitor activity;endopeptidase regulator activity;enzyme inhibitor activity;enzyme regulator activity;peptidase inhibitor activity;peptidase regulator activity;serine-type endopeptidase inhibitor activity
catalytic activity;dihydrolipoyllysine-residue succinyltransferase activity;S-acyltransferase activity;S-succinyltransferase activity;succinyltransferase activity;transferase activity;transferase activity, transferring acyl groups;trans
amine binding;amino acid binding;antioxidant activity;binding;carboxylic acid binding;catalytic activity;glutathione binding;glutathione peroxidase activity;modified amino acid binding;oxidoreductase activity;oxidoreductase a
7S RNA binding;binding;endoplasmic reticulum signal peptide binding;nucleic acid binding;peptide binding;RNA binding;signal sequence binding
binding;chromatin binding;hormone receptor binding;nuclear hormone receptor binding;protein binding;protein complex scaffold;protein domain specific binding;receptor binding;receptor signaling complex scaffold activity;S
binding;calcium ion binding;cation binding;ion binding;metal ion binding
catalytic activity;farnesyl-diphosphate farnesyltransferase activity;farnesyltranstransferase activity;oxidoreductase activity;prenyltransferase activity;squalene synthase activity;transferase activity;transferase activity, transferri
catalytic activity;sedoheptulose-7-phosphate:D-glyceraldehyde-3-phosphate glyceronetransferase activity;transferase activity;transferase activity, transferring aldehyde or ketonic groups
electron carrier activity
binding;chromatin binding;core promoter binding;DNA binding;mRNA binding;nucleic acid binding;nucleotide binding;regulatory region DNA binding;regulatory region nucleic acid binding;RNA binding;single-stranded RNA bi
active transmembrane transporter activity;adenyl nucleotide binding;adenyl ribonucleotide binding;ATP binding;ATPase activity;ATPase activity, coupled;ATPase activity, coupled to movement of substances;ATPase activity, c
adenyl nucleotide binding;adenyl ribonucleotide binding;ATP binding;binding;enzyme binding;nucleotide binding;protein binding;purine nucleotide binding;purine ribonucleoside triphosphate binding;purine ribonucleotide bin
adenyl nucleotide binding;adenyl ribonucleotide binding;ATP binding;ATPase activity;ATPase activity, coupled;ATP-dependent helicase activity;ATP-dependent RNA helicase activity;binding;catalytic activity;helicase activity;h
binding;enzyme binding;fibroblast growth factor binding;growth factor binding;identical protein binding;kinase binding;protein binding;protein dimerization activity;protein homodimerization activity;protein kinase binding;struc
binding;nucleic acid binding;RNA binding;structural constituent of ribosome;structural molecule activity
binding;cation binding;identical protein binding;ion binding;metal ion binding;protein binding;structural molecule activity
catalytic activity;dolichyl-diphosphooligosaccharide-protein glycotransferase activity;oligosaccharyl transferase activity;transferase activity;transferase activity, transferring glycosyl groups;transferase activity, transferring hex
5'-3' exonuclease activity;5'-flap endonuclease activity;binding;catalytic activity;cation binding;damaged DNA binding;deoxyribonuclease activity;DNA binding;double-stranded DNA binding;double-stranded DNA specific ex
binding;protein binding;protein complex binding;protein domain specific binding
binding;cytokine activity;growth factor receptor binding;high molecular weight B cell growth factor receptor binding;protein binding;receptor binding
adenyl nucleotide binding;adenyl ribonucleotide binding;ATP binding;binding;nucleotide binding;protein binding;purine nucleotide binding;purine ribonucleoside triphosphate binding;purine ribonucleotide binding;ribonucleoti
catalytic activity;methyltransferase activity;nicotinamide N-methyltransferase activity;N-methyltransferase activity;S-adenosylmethionine-dependent methyltransferase activity;transferase activity;transferase activity, transferri
catalytic activity;endopeptidase activity;hydrolase activity;peptidase activity;peptidase activity, acting on L-amino acid peptides;threonine-type endopeptidase activity;threonine-type peptidase activity
structural constituent of ribosome;structural molecule activity
binding;catalytic activity;cation binding;enzyme activator activity;enzyme regulator activity;GTP binding;GTPase activity;guanyl nucleotide binding;guanyl ribonucleotide binding;hydrolase activity;hydrolase activity, acting on a
binding;core promoter proximal region DNA binding;core promoter proximal region sequence-specific DNA binding;DNA binding;enzyme binding;kinase binding;ligand-dependent nuclear receptor activity;molecular transduce
binding;catalytic activity;coenzyme binding;cofactor binding;diiodophenylpyruvate reductase activity;L-malate dehydrogenase activity;malate dehydrogenase activity;malic enzyme activity;NAD binding;nucleotide binding;oxi
catalytic activity;L-malate dehydrogenase activity;malate dehydrogenase (NADP+) activity;malate dehydrogenase activity;oxidoreductase activity;oxidoreductase activity, acting on CH-OH group of donors;oxidoreductase act
adenyl nucleotide binding;adenyl ribonucleotide binding;ATP binding;binding;DNA binding;enzyme binding;nucleic acid binding;nucleotide binding;protein binding;purine nucleotide binding;purine ribonucleoside triphosphate
ATPase activity;ATPase activity, coupled;binding;catalytic activity;DNA binding;DNA clamp loader activity;DNA-dependent ATPase activity;hydrolase activity;hydrolase activity, acting on acid anhydrides;hydrolase activity, acti
3-hydroxyacyl-CoA dehydrogenase activity;acetyl-CoA C-acetyltransferase activity;acetyl-CoA C-acyltransferase activity;acetyltransferase activity;binding;C-acetyltransferase activity;C-acyltransferase activity;carbon-oxyge
binding;catalytic activity;GTP binding;GTPase activity;guanyl nucleotide binding;guanyl ribonucleotide binding;hydrolase activity;hydrolase activity, acting on acid anhydrides;hydrolase activity, acting on acid anhydrides, in p
binding;nucleic acid binding;receptor activity;RNA binding;translation factor activity, nucleic acid binding;translation initiation factor activity
binding;DNA binding;frizzled binding;G-protein-coupled receptor binding;nucleic acid binding;nucleic acid binding transcription factor activity;protein binding;receptor activator activity;receptor agonist activity;receptor bindin
nucleic acid binding transcription factor activity;sequence-specific DNA binding transcription factor activity
acetyltransferase activity;catalytic activity;N-acetyltransferase activity;N-acyltransferase activity;transferase activity;transferase activity, transferring acyl groups;transferase activity, transferring acyl groups other than amino-a
enzyme inhibitor activity;enzyme regulator activity;phosphatase inhibitor activity;phosphatase regulator activity;protein phosphatase inhibitor activity;protein phosphatase regulator activity;protein serine/threonine phosphatas
adenyl nucleotide binding;adenyl ribonucleotide binding;ATP binding;binding;catalytic activity;identical protein binding;kinase activity;non-membrane spanning protein tyrosine kinase activity;nucleotide binding;phosphotrans
adenyl nucleotide binding;adenyl ribonucleotide binding;aminoacyl-tRNA ligase activity;ATP binding;binding;catalytic activity;glycine-tRNA ligase activity;ligase activity;ligase activity, forming aminoacyl-tRNA and related com
adenyl nucleotide binding;adenyl ribonucleotide binding;aminoacyl-tRNA editing activity;aminoacyl-tRNA ligase activity;ATP binding;binding;carboxylic ester hydrolase activity;catalytic activity;enzyme binding;GTPase binding
binding;nucleic acid binding;RNA binding;translation factor activity, nucleic acid binding;translation initiation factor activity
adenyl nucleotide binding;adenyl ribonucleotide binding;ATP binding;binding;catalytic activity;cation binding;ion binding;kinase activity;lipid binding;metal ion binding;nucleotide binding;phospholipid binding;phosphotransfer
catalytic activity;dodecenoyl-CoA delta-isomerase activity;intramolecular oxidoreductase activity;intramolecular oxidoreductase activity, transposing C=C bonds;isomerase activity
binding;DNA binding;lamin binding;nucleic acid binding;protein binding
binding;DNA binding;lamin binding;nucleic acid binding;protein binding
binding;core promoter binding;core promoter proximal region DNA binding;core promoter proximal region sequence-specific DNA binding;core promoter sequence-specific DNA binding;cytokine receptor binding;DNA bindin
binding;core promoter binding;core promoter sequence-specific DNA binding;DNA binding;enzyme binding;identical protein binding;molecular transducer activity;nucleic acid binding;nucleic acid binding transcription factor a
adenyl nucleotide binding;adenyl ribonucleotide binding;ATP binding;binding;catalytic activity;helicase activity;hydrolase activity;hydrolase activity, acting on acid anhydrides;hydrolase activity, acting on acid anhydrides, in ph
15-hydroxyprostaglandin-D dehydrogenase (NADP+) activity;alcohol dehydrogenase (NADP+) activity;alditol:NADP+ 1-oxidoreductase activity;aldo-keto reductase (NADP) activity;androsterone dehydrogenase activity;catalyt
5S rDNA binding;adenyl nucleotide binding;adenyl ribonucleotide binding;ATP binding;binding;catalytic activity;DNA binding;drug binding;kinase activity;nucleic acid binding;nucleotide binding;phosphoprotein binding;phosp
aspartic-type endopeptidase activity;aspartic-type peptidase activity;catalytic activity;cyclin-dependent protein kinase inhibitor activity;cyclin-dependent protein kinase regulator activity;cysteine-type endopeptidase activity;c
binding;cation binding;DNA binding;ion binding;metal ion binding;nucleic acid binding;structural constituent of ribosome;structural molecule activity;transition metal ion binding;zinc ion binding
beta-tubulin binding;binding;cytoskeletal protein binding;DNA binding;enzyme binding;microtubule binding;nucleic acid binding;protein binding;RNA binding;single-stranded DNA binding;structure-specific DNA binding;tubuli
acetyl-CoA C-acyltransferase activity;C-acyltransferase activity;catalytic activity;transferase activity;transferase activity, transferring acyl groups;transferase activity, transferring acyl groups other than amino-acyl groups
binding;mRNA binding;nucleic acid binding;RNA binding;structural constituent of ribosome;structural molecule activity
binding;cyclin-dependent protein kinase inhibitor activity;cyclin-dependent protein kinase regulator activity;enzyme binding;enzyme inhibitor activity;enzyme regulator activity;kinase binding;kinase inhibitor activity;kinase regu
carboxypeptidase activity;catalytic activity;exopeptidase activity;hydrolase activity;peptidase activity;peptidase activity, acting on L-amino acid peptides;serine hydrolase activity;serine-type carboxypeptidase activity;serine-t
beta-tubulin binding;binding;cytoskeletal protein binding;diazepam binding;drug binding;dynactin binding;dynein binding;dynein intermediate chain binding;identical protein binding;ion channel binding;p53 binding;profilin bin
binding;catalytic activity;cation binding;endopeptidase activity;hormone binding;hydrolase activity;identical protein binding;ion binding;metal ion binding;metalloendopeptidase activity;metallopeptidase activity;peptidase acti
binding;carbohydrate binding;cytoskeletal protein binding;dynactin binding;dynein binding;enzyme binding;glycosaminoglycan binding;heparin binding;identical protein binding;microtubule binding;pattern binding;phospholip
binding;cation binding;ion binding;metal ion binding;nucleotide binding;structural molecule activity;transition metal ion binding;zinc ion binding
adenyl nucleotide binding;adenyl ribonucleotide binding;ATP binding;ATPase activity;ATPase activity, coupled;binding;catalytic activity;centromeric DNA binding;damaged DNA binding;DNA binding;DNA insertion or deletion
binding;calcium ion binding;catalytic activity;cation binding;glycerol-3-phosphate dehydrogenase activity;ion binding;metal ion binding;oxidoreductase activity;oxidoreductase activity, acting on CH-OH group of donors;oxido
binding;enzyme activator activity;enzyme binding;enzyme regulator activity;GDP-dissociation inhibitor activity;GTPase activator activity;GTPase binding;GTPase regulator activity;nucleoside-triphosphatase regulator activity;
binding;catalytic activity;cytokine activity;drug binding;nicotinamide phosphoribosyltransferase activity;nicotinate-nucleotide diphosphorylase (carboxylating) activity;protein binding;receptor binding;transferase activity;transf
binding;vitamin binding;vitamin E binding
adenyl nucleotide binding;adenyl ribonucleotide binding;ATP binding;ATPase activity;binding;catalytic activity;hydrolase activity;hydrolase activity, acting on acid anhydrides;hydrolase activity, acting on acid anhydrides, in ph
binding;nucleic acid binding;RNA binding;translation elongation factor activity;translation factor activity, nucleic acid binding
binding;catalytic activity;cis-trans isomerase activity;cyclosporin A binding;drug binding;isomerase activity;peptide binding;peptidyl-prolyl cis-trans isomerase activity
anion channel activity;anion transmembrane transporter activity;binding;channel activity;gated channel activity;ion channel activity;ion transmembrane transporter activity;nucleotide binding;passive transmembrane transport
binding;binding, bridging;chromatin binding;histone binding;methylated histone residue binding;protein binding;protein binding, bridging;repressing transcription factor binding;transcription factor binding
binding;catalytic activity;cation binding;cysteine-type endopeptidase activity;cysteine-type peptidase activity;endopeptidase activity;exopeptidase activity;hydrolase activity;ion binding;metal ion binding;omega peptidase ac
adenyl nucleotide binding;adenyl ribonucleotide binding;apoptotic protease activator activity;ATP binding;binding;caspase activator activity;caspase regulator activity;catalytic activity;enzyme activator activity;enzyme regulat
adenyl nucleotide binding;adenyl ribonucleotide binding;ATP binding;binding;nucleotide binding;protein binding;protein C-terminus binding;purine nucleotide binding;purine ribonucleoside triphosphate binding;purine ribonuc
calmodulin-dependent protein kinase activity;catalytic activity;hydrolase activity;hydrolase activity, acting on glycosyl bonds;hydrolase activity, hydrolyzing O-glycosyl compounds;kinase activity;phosphorylase kinase activity;
enzyme activator activity;enzyme regulator activity;GTPase activator activity;GTPase regulator activity;nucleoside-triphosphatase regulator activity
3'-5' DNA helicase activity;adenyl nucleotide binding;adenyl ribonucleotide binding;ATP binding;ATPase activity;ATPase activity, coupled;ATP-dependent 3'-5' DNA helicase activity;ATP-dependent DNA helicase activity;ATP-
catalytic activity;methyltransferase activity;S-adenosylmethionine-dependent methyltransferase activity;transferase activity;transferase activity, transferring one-carbon groups
binding;ephrin receptor binding;protein binding;protein domain specific binding;receptor binding;SH2 domain binding
binding;enzyme binding;polyubiquitin binding;proteasome binding;protein binding;protein complex binding;ribonucleoprotein binding;ribosome binding;small conjugating protein binding;ubiquitin binding;ubiquitin protein liga
adenyl nucleotide binding;adenyl ribonucleotide binding;ATP binding;ATPase activity;ATPase activity, coupled;binding;catalytic activity;cation binding;enzyme binding;GTPase binding;hydrolase activity;hydrolase activity, acti
adenyl nucleotide binding;adenyl ribonucleotide binding;ATP binding;binding;catalytic activity;enzyme binding;kinase activity;kinase binding;MAP kinase kinase activity;nucleotide binding;phosphotransferase activity, alcohol
binding;catalytic activity;cation binding;cysteine-type peptidase activity;enzyme regulator activity;hydrolase activity;ion binding;metal ion binding;metallopeptidase activity;peptidase activity;peptidase activity, acting on L-ami
binding;nucleic acid binding;RNA binding;structural constituent of ribosome;structural molecule activity
5S rRNA binding;binding;nucleic acid binding;RNA binding;rRNA binding;structural constituent of ribosome;structural molecule activity
binding;nucleic acid binding;RNA binding;structural constituent of ribosome;structural molecule activity
binding;nucleic acid binding;RNA binding;structural constituent of ribosome;structural molecule activity
binding;nucleic acid binding;RNA binding;rRNA binding;structural constituent of ribosome;structural molecule activity;translation regulator activity
binding;mRNA binding;nucleic acid binding;RNA binding;structural constituent of ribosome;structural molecule activity
structural molecule activity
catalytic activity;deaminase activity;glucosamine-6-phosphate deaminase activity;hydrolase activity;intramolecular oxidoreductase activity;intramolecular oxidoreductase activity, interconverting aldoses and ketoses;isomeras
binding;chromatin binding;core promoter binding;core promoter sequence-specific DNA binding;DNA binding;nucleic acid binding;protein binding transcription factor activity;regulatory region DNA binding;regulatory region n
actin binding;binding;cation binding;cytoskeletal protein binding;enzyme binding;integrin binding;ion binding;kinase binding;metal ion binding;protein binding;protein complex binding;protein kinase binding;receptor binding;t
binding;calcium ion binding;calmodulin binding;cation binding;enzyme activator activity;enzyme binding;enzyme inhibitor activity;enzyme regulator activity;GTPase activator activity;GTPase inhibitor activity;GTPase regulator
binding;carbohydrate binding;catalytic activity;cation binding;glucose binding;glucosyltransferase activity;glycogenin glucosyltransferase activity;ion binding;metal ion binding;monosaccharide binding;sugar binding;transfera
catalytic activity;dolichyl-diphosphooligosaccharide-protein glycotransferase activity;oligosaccharyl transferase activity;transferase activity;transferase activity, transferring glycosyl groups;transferase activity, transferring hex
binding;cation binding;enzyme regulator activity;GTPase regulator activity;guanyl-nucleotide exchange factor activity;ion binding;metal ion binding;nucleoside-triphosphatase regulator activity;transition metal ion binding;zinc

actin binding;actin filament binding;binding;cytoskeletal protein binding;protein binding
binding;nucleic acid binding;RNA binding;translation factor activity, nucleic acid binding;translation initiation factor activity
adenyl nucleotide binding;adenyl ribonucleotide binding;aminoacyl-tRNA ligase activity;ATP binding;binding;catalytic activity;glutamine-tRNA ligase activity;ligase activity;ligase activity, forming aminoacyl-tRNA and related co
binding;carbohydrate binding;glycosaminoglycan binding;heparin binding;nucleic acid binding;pattern binding;polysaccharide binding;RNA binding;structural constituent of ribosome;structural molecule activity
2 iron, 2 sulfur cluster binding;binding;catalytic activity;cation binding;cation transmembrane transporter activity;hydrogen ion transmembrane transporter activity;inorganic cation transmembrane transporter activity;ion bindi
binding;cation transmembrane transporter activity;drug binding;hydrogen ion transmembrane transporter activity;hydrogen ion transporting ATP synthase activity, rotational mechanism;inorganic cation transmembrane transp
binding;cation binding;enzyme binding;ion binding;kinase binding;metal ion binding;protein binding;protein kinase binding;transition metal ion binding;zinc ion binding
binding;chemokine activity;chemokine receptor binding;CXCR chemokine receptor binding;cytokine activity;cytokine receptor binding;G-protein-coupled receptor binding;protein binding;receptor binding
catalytic activity;endopeptidase activity;exopeptidase activity;hydrolase activity;peptidase activity;peptidase activity, acting on L-amino acid peptides;serine hydrolase activity;serine-type endopeptidase activity;serine-type ex
adenyl nucleotide binding;adenyl ribonucleotide binding;ADP binding;binding;carbon-carbon lyase activity;carboxy-lyase activity;catalytic activity;cation binding;coenzyme binding;cofactor binding;electron carrier activity;ion
endopeptidase inhibitor activity;endopeptidase regulator activity;enzyme inhibitor activity;enzyme regulator activity;extracellular matrix structural constituent;peptidase inhibitor activity;peptidase regulator activity;serine-type
catalytic activity;intramolecular transferase activity;isomerase activity;lanosterol synthase activity;oxidosqualene cyclase activity
acid-amino acid ligase activity;binding;catalytic activity;enzyme binding;enzyme regulator activity;glutamate-cysteine ligase activity;glutamate-cysteine ligase catalytic subunit binding;ligase activity;ligase activity, forming carb
binding;integrin binding;protein binding;protein complex binding;receptor binding
acid-amino acid ligase activity;adenyl nucleotide binding;adenyl ribonucleotide binding;amine binding;amino acid binding;ATP binding;binding;carboxylic acid binding;catalytic activity;cation binding;glutathione binding;glutat
adenyl nucleotide binding;adenyl ribonucleotide binding;ATP binding;beta-tubulin binding;binding;cytoskeletal protein binding;G-protein beta-subunit binding;nucleotide binding;protein binding;purine nucleotide binding;purin
binding;intermediate filament binding;protein binding;protein complex binding;structural molecule activity
adenyl nucleotide binding;adenyl ribonucleotide binding;ATP binding;binding;nucleotide binding;purine nucleotide binding;purine ribonucleoside triphosphate binding;purine ribonucleotide binding;ribonucleotide binding
adenyl nucleotide binding;adenyl ribonucleotide binding;ATP binding;binding;catalytic activity;kinase activity;nucleotide binding;phosphotransferase activity, alcohol group as acceptor;protein kinase activity;protein serine/thre
binding;catalytic activity;cation binding;coenzyme binding;cofactor binding;ion binding;isocitrate dehydrogenase (NADP+) activity;isocitrate dehydrogenase activity;magnesium ion binding;metal ion binding;NAD binding;nucle
binding;lipid binding
binding;calcium ion binding;cation binding;G-protein coupled receptor activity;ion binding;metal ion binding;molecular transducer activity;receptor activity;signal transducer activity;signaling receptor activity;transmembrane s
binding;catalytic activity;DNA binding;DNA polymerase activity;DNA-directed DNA polymerase activity;nucleic acid binding;nucleotidyltransferase activity;transferase activity;transferase activity, transferring phosphorus-conta
beta-catenin binding;binding;cation binding;cytoskeletal protein binding;ion binding;metal ion binding;protein binding;transition metal ion binding;vinculin binding;zinc ion binding
adenyl nucleotide binding;adenyl ribonucleotide binding;ATP binding;binding;calcium-dependent protein kinase activity;calcium-dependent protein serine/threonine kinase activity;calmodulin binding;calmodulin-dependent pr
1-pyrroline dehydrogenase activity;3-chloroallyl aldehyde dehydrogenase activity;4-trimethylammoniobutyraldehyde dehydrogenase activity;aldehyde dehydrogenase (NAD) activity;amine binding;aminobutyraldehyde dehydro
binding;nucleic acid binding;RNA binding;structural constituent of ribosome;structural molecule activity
binding;carbohydrate binding;cation binding;ion binding;mannose binding;metal ion binding;monosaccharide binding;protein binding;sugar binding;unfolded protein binding
binding;heat shock protein binding;Hsp90 protein binding;protein binding;protein complex binding
[acyl-carrier-protein] S-acetyltransferase activity;[acyl-carrier-protein] S-malonyltransferase activity;3-hydroxyacyl-[acyl-carrier-protein] dehydratase activity;3-hydroxyoctanoyl-[acyl-carrier-protein] dehydratase activity;3-hydro
catalytic activity;deoxyhypusine synthase activity;transferase activity;transferase activity, transferring alkyl or aryl (other than methyl) groups
adenyl nucleotide binding;adenyl ribonucleotide binding;ATP binding;binding;nucleotide binding;protein binding;purine nucleotide binding;purine ribonucleoside triphosphate binding;purine ribonucleotide binding;ribonucleoti
structural constituent of ribosome;structural molecule activity
binding;catalytic activity;GTP binding;GTPase activity;guanyl nucleotide binding;guanyl ribonucleotide binding;hydrolase activity;hydrolase activity, acting on acid anhydrides;hydrolase activity, acting on acid anhydrides, in ph
aldehyde dehydrogenase (NAD) activity;betaine-aldehyde dehydrogenase activity;catalytic activity;L-aminoadipate-semialdehyde dehydrogenase activity;oxidoreductase activity;oxidoreductase activity, acting on the aldehyde
binding;chromatin binding;dynein binding;identical protein binding;protein binding;protein C-terminus binding;protein dimerization activity;protein homodimerization activity;transcription factor binding
7S RNA binding;binding;nucleic acid binding;ribonucleoprotein binding;RNA binding;signal recognition particle binding
binding;catalytic activity;choline-phosphate cytidylyltransferase activity;cytidylyltransferase activity;lipid binding;nucleotidyltransferase activity;transferase activity;transferase activity, transferring phosphorus-containing group
adenyl nucleotide binding;adenyl ribonucleotide binding;alanine-tRNA ligase activity;amine binding;amino acid binding;aminoacyl-tRNA editing activity;aminoacyl-tRNA ligase activity;ATP binding;binding;carboxylic acid bindi
adenyl nucleotide binding;adenyl ribonucleotide binding;aminoacyl-tRNA ligase activity;ATP binding;binding;catalytic activity;cation binding;cysteine-tRNA ligase activity;identical protein binding;ion binding;ligase activity;liga
adenyl nucleotide binding;adenyl ribonucleotide binding;aminoacyl-tRNA ligase activity;ATP binding;binding;catalytic activity;ligase activity;ligase activity, forming aminoacyl-tRNA and related compounds;ligase activity, formi
binding;calcium-dependent protein serine/threonine phosphatase activity;calmodulin-dependent protein phosphatase activity;catalytic activity;cation binding;hydrolase activity;hydrolase activity, acting on ester bonds;ion bin
binding;catalytic activity;cation binding;DNA primase activity;DNA-directed RNA polymerase activity;ion binding;metal ion binding;nucleotidyltransferase activity;RNA polymerase activity;transferase activity;transferase activity
4 iron, 4 sulfur cluster binding;binding;catalytic activity;cation binding;DNA binding;DNA primase activity;DNA-directed RNA polymerase activity;ion binding;iron-sulfur cluster binding;metal cluster binding;metal ion binding;nu
catalytic activity;cysteine-type endopeptidase activity;cysteine-type peptidase activity;endopeptidase activity;hydrolase activity;peptidase activity;peptidase activity, acting on L-amino acid peptides
binding;cation binding;chromatin binding;chromatin DNA binding;chromatin insulator sequence binding;core promoter proximal region DNA binding;core promoter proximal region sequence-specific DNA binding;DNA binding
catalytic activity;endopeptidase activity;hydrolase activity;peptidase activity;peptidase activity, acting on L-amino acid peptides;threonine-type endopeptidase activity;threonine-type peptidase activity
catalytic activity;endopeptidase activity;hydrolase activity;peptidase activity;peptidase activity, acting on L-amino acid peptides;threonine-type endopeptidase activity;threonine-type peptidase activity
adenyl nucleotide binding;adenyl ribonucleotide binding;ATP binding;binding;catalytic activity;cation binding;DNA binding;DNA helicase activity;DNA replication origin binding;helicase activity;hydrolase activity;hydrolase activ
binding;calcium ion binding;carbohydrate binding;cation binding;collagen binding;enzyme binding;extracellular matrix structural constituent;glycoprotein binding;glycosaminoglycan binding;heparan sulfate proteoglycan bind
acyl-CoA dehydrogenase activity;binding;catalytic activity;coenzyme binding;cofactor binding;flavin adenine dinucleotide binding;long-chain-acyl-CoA dehydrogenase activity;oxidoreductase activity;oxidoreductase activity, a
nucleic acid binding transcription factor activity;RNA polymerase II core promoter proximal region sequence-specific DNA binding transcription factor activity;RNA polymerase II core promoter proximal region sequence-spec
acyl-CoA hydrolase activity;acyl-CoA thioesterase activity;binding;carboxylic ester hydrolase activity;catalytic activity;CoA hydrolase activity;hydrolase activity;hydrolase activity, acting on ester bonds;palmitoyl-CoA hydrolase
binding;protein binding;SNARE binding;syntaxin binding
binding;mRNA binding;nucleic acid binding;nucleotide binding;RNA binding
adenyl nucleotide binding;adenyl ribonucleotide binding;ATP binding;binding;GTP binding;guanyl nucleotide binding;guanyl ribonucleotide binding;nucleic acid binding;nucleotide binding;purine nucleotide binding;purine ribo
binding;catalytic activity;enzyme binding;hydrolase activity;kinase binding;nucleotide binding;protein binding;protein kinase binding;protein kinase C binding
binding;cation binding;DNA binding;identical protein binding;ion binding;metal ion binding;nucleic acid binding;nucleocytoplasmic transporter activity;protein anchor;protein binding;structural constituent of nuclear pore;struc
binding;catalytic activity;cation binding;cis-trans isomerase activity;enzyme binding;GTPase binding;ion binding;isomerase activity;ligase activity;metal ion binding;nucleic acid binding;peptidyl-prolyl cis-trans isomerase activ
4 iron, 4 sulfur cluster binding;binding;catalytic activity;cation binding;coenzyme binding;cofactor binding;FMN binding;ion binding;iron-sulfur cluster binding;metal cluster binding;metal ion binding;NAD binding;NADH dehyd
adenyl nucleotide binding;adenyl ribonucleotide binding;ATP binding;binding;catalytic activity;enzyme binding;kinase activity;kinase binding;nucleotide binding;phosphotransferase activity, alcohol group as acceptor;protein b
adenyl nucleotide binding;adenyl ribonucleotide binding;ATP binding;beta-catenin binding;binding;catalytic activity;enzyme binding;kinase activity;kinase binding;NF-kappaB binding;nucleotide binding;p53 binding;phosphot
5'-nucleotidase activity;binding;catalytic activity;cation binding;hydrolase activity;hydrolase activity, acting on ester bonds;ion binding;kinase activity;metal ion binding;nucleobase-containing compound kinase activity;nucleos
adenyl nucleotide binding;adenyl ribonucleotide binding;ATP binding;binding;catalytic activity;GTP binding;guanyl nucleotide binding;guanyl ribonucleotide binding;identical protein binding;kinase activity;nucleotide binding;p
adenyl nucleotide binding;adenyl ribonucleotide binding;ATP binding;binding;carbon-nitrogen ligase activity, with glutamine as amido-N-donor;catalytic activity;GMP synthase (glutamine-hydrolyzing) activity;GMP synthase ac
3'-5' exonuclease activity;binding;catalytic activity;cation binding;deoxyribonuclease activity;DNA binding;double-stranded DNA binding;endodeoxyribonuclease activity;endonuclease activity;exonuclease activity;hydrolase a
binding;catalytic activity;cation binding;coenzyme binding;cofactor binding;ion binding;isocitrate dehydrogenase (NAD+) activity;isocitrate dehydrogenase activity;magnesium ion binding;metal ion binding;NAD binding;nucleo
AT DNA binding;binding;cation binding;DNA bending activity;DNA binding;ion binding;metal ion binding;nucleic acid binding;peptide binding;sequence-specific DNA binding;transition metal ion binding;zinc ion binding
binding;calcium ion binding;catalytic activity;cation binding;endopeptidase activity;enzyme activator activity;enzyme regulator activity;hydrolase activity;ion binding;metal ion binding;metalloendopeptidase activity;metallopep
binding;catalytic activity;coenzyme binding;cofactor binding;flavin adenine dinucleotide binding;oxidoreductase activity;oxidoreductase activity, acting on the CH-CH group of donors;oxidoreductase activity, acting on the CH
enzyme activator activity;enzyme regulator activity;GDP-dissociation inhibitor activity;GTPase activator activity;GTPase regulator activity;nucleoside-triphosphatase regulator activity;Rab GDP-dissociation inhibitor activity;sm
actin binding;beta-tubulin binding;binding;cytoskeletal protein binding;protein binding;tubulin binding
carnitine O-acyltransferase activity;carnitine O-palmitoyltransferase activity;catalytic activity;O-acyltransferase activity;O-palmitoyltransferase activity;palmitoyltransferase activity;transferase activity;transferase activity, transf
endopeptidase inhibitor activity;endopeptidase regulator activity;enzyme inhibitor activity;enzyme regulator activity;peptidase inhibitor activity;peptidase regulator activity;serine-type endopeptidase inhibitor activity
binding;caspase inhibitor activity;caspase regulator activity;cysteine-type endopeptidase inhibitor activity;endopeptidase inhibitor activity;endopeptidase regulator activity;enzyme binding;enzyme inhibitor activity;enzyme reg
binding;collagen binding;endopeptidase inhibitor activity;endopeptidase regulator activity;enzyme inhibitor activity;enzyme regulator activity;peptidase inhibitor activity;peptidase regulator activity;protein binding;serine-type e
binding;cation binding;ion binding;metal ion binding;transition metal ion binding;zinc ion binding
adenyl deoxyribonucleotide binding;adenyl nucleotide binding;binding;binding, bridging;dATP binding;deoxyribonucleotide binding;nucleotide binding;protein binding;protein binding, bridging;purine deoxyribonucleotide bind
binding;profilin binding;protein binding
binding;catalytic activity;cytoskeletal protein binding;enzyme binding;GTP binding;GTPase activity;guanyl nucleotide binding;guanyl ribonucleotide binding;hydrolase activity;hydrolase activity, acting on acid anhydrides;hydro
aminopeptidase activity;binding;catalytic activity;cation binding;exopeptidase activity;hydrolase activity;ion binding;metal ion binding;metalloaminopeptidase activity;metalloexopeptidase activity;metallopeptidase activity;pep
binding;bis(5'-nucleosyl)-tetraphosphatase (asymmetrical) activity;bis(5'-nucleosyl)-tetraphosphatase (symmetrical) activity;bis(5'-nucleosyl)-tetraphosphatase activity;catalytic activity;GTP binding;guanyl nucleotide binding;g
adenyl nucleotide binding;adenyl ribonucleotide binding;androgen receptor binding;ATP binding;ATPase activity;ATPase activity, coupled;binding;catalytic activity;cyclin-dependent protein kinase activity;DNA-dependent ATP
binding;lipid binding;phospholipid binding
binding;extracellular matrix binding;laminin binding;laminin receptor activity;molecular transducer activity;protein binding;protein C-terminus binding;receptor activity;signal transducer activity;signaling receptor activity;transm
acyl-CoA thioesterase activity;catalytic activity;CoA hydrolase activity;hydrolase activity;hydrolase activity, acting on ester bonds;palmitoyl-(protein) hydrolase activity;palmitoyl-CoA hydrolase activity;thiolester hydrolase activ
binding;nucleic acid binding;RNA binding;structural constituent of ribosome;structural molecule activity
adenyl nucleotide binding;adenyl ribonucleotide binding;ATP binding;ATPase activity;ATPase activity, coupled;binding;catalytic activity;hydrolase activity;hydrolase activity, acting on acid anhydrides;hydrolase activity, acting
adenyl nucleotide binding;adenyl ribonucleotide binding;ATP binding;binding;nucleotide binding;protein binding;purine nucleotide binding;purine ribonucleoside triphosphate binding;purine ribonucleotide binding;ribonucleoti
antigen binding;binding;calcium ion binding;calcium-dependent phospholipid binding;calcium-dependent protein binding;cation binding;ion binding;lipid binding;metal ion binding;MHC class II protein complex binding;MHC
adenyl nucleotide binding;adenyl ribonucleotide binding;adenylyltransferase activity;ATP binding;binding;catalytic activity;cation binding;ion binding;magnesium ion binding;manganese ion binding;metal ion binding;nucleic ac
binding;G-quadruplex RNA binding;mRNA 3'-UTR binding;mRNA binding;nucleic acid binding;RNA binding
binding;identical protein binding;mRNA binding;nucleic acid binding;protein binding;RNA binding
binding;catalytic activity;GDP binding;GTP binding;GTPase activity;guanyl nucleotide binding;guanyl ribonucleotide binding;hydrolase activity;hydrolase activity, acting on acid anhydrides;hydrolase activity, acting on acid anh
binding;catalytic activity;GDP binding;GTP binding;GTPase activity;guanyl nucleotide binding;guanyl ribonucleotide binding;hydrolase activity;hydrolase activity, acting on acid anhydrides;hydrolase activity, acting on acid anh
binding;catalytic activity;GDP binding;GTP binding;GTPase activity;guanyl nucleotide binding;guanyl ribonucleotide binding;hydrolase activity;hydrolase activity, acting on acid anhydrides;hydrolase activity, acting on acid anh
binding;catalytic activity;GTP binding;GTPase activity;guanyl nucleotide binding;guanyl ribonucleotide binding;hydrolase activity;hydrolase activity, acting on acid anhydrides;hydrolase activity, acting on acid anhydrides, in ph
binding;catalytic activity;GDP binding;GTP binding;GTPase activity;guanyl nucleotide binding;guanyl ribonucleotide binding;hydrolase activity;hydrolase activity, acting on acid anhydrides;hydrolase activity, acting on acid anh
binding;death domain binding;protein binding;protein domain specific binding
binding;catalytic activity;enzyme binding;hydrolase activity;hydrolase activity, acting on ester bonds;kinase binding;MAP kinase phosphatase activity;phosphatase activity;phosphoprotein phosphatase activity;phosphoric est
adenyl nucleotide binding;adenyl ribonucleotide binding;androgen receptor binding;ATP binding;ATPase activity;ATPase activity, coupled;binding;catalytic activity;chromatin binding;DNA binding;DNA polymerase binding;DNA
adenyl nucleotide binding;adenyl ribonucleotide binding;ATP binding;binding;catalytic activity;cation binding;coenzyme binding;cofactor binding;ion binding;isocitrate dehydrogenase (NAD+) activity;isocitrate dehydrogenase
adenyl nucleotide binding;adenyl ribonucleotide binding;ATP binding;binding;carbohydrate binding;carbohydrate kinase activity;catalytic activity;galactokinase activity;galactose binding;kinase activity;monosaccharide bindin
catalytic activity;methyltransferase activity;S-adenosylmethionine-dependent methyltransferase activity;S-methyltransferase activity;thiopurine S-methyltransferase activity;transferase activity;transferase activity, transferring c
binding;chromatin binding;identical protein binding;nucleic acid binding transcription factor activity;protein binding;protein binding transcription factor activity;sequence-specific DNA binding transcription factor activity;transc
3-chloroallyl aldehyde dehydrogenase activity;aldehyde dehydrogenase (NAD) activity;aldehyde dehydrogenase [NAD(P)+] activity;catalytic activity;long-chain-alcohol oxidase activity;long-chain-aldehyde dehydrogenase acti
3alpha,7alpha,12alpha-trihydroxy-5beta-cholest-24-enoyl-CoA hydratase activity;3-hydroxyacyl-CoA dehydrogenase activity;binding;carbon-oxygen lyase activity;catalytic activity;hydro-lyase activity;identical protein binding
binding;identical protein binding;protein binding;protein dimerization activity;protein homodimerization activity
adenyl nucleotide binding;adenyl ribonucleotide binding;ATP binding;binding;caspase inhibitor activity;caspase regulator activity;catalytic activity;cation binding;cysteine-type endopeptidase inhibitor activity;endopeptidase in
binding;carbohydrate binding;DNA binding;glycosaminoglycan binding;heparin binding;nucleic acid binding;nucleotide binding;pattern binding;polysaccharide binding;protein binding transcription factor activity;RNA polymer
binding;collagen binding;extracellular matrix structural constituent;protein binding;structural molecule activity
catalytic activity;cyclin-dependent protein kinase regulator activity;enzyme regulator activity;kinase activity;kinase regulator activity;protein kinase regulator activity;transferase activity;transferase activity, transferring phospho
binding;cation binding;ion binding;metal ion binding;protein binding;protein N-terminus binding;transition metal ion binding;zinc ion binding
binding;catalytic activity;NADH dehydrogenase (quinone) activity;NADH dehydrogenase (ubiquinone) activity;NADH dehydrogenase activity;oxidoreductase activity;oxidoreductase activity, acting on NADH or NADPH;oxidore
binding;nucleic acid binding;nucleotide binding;RNA binding
binding;catalytic activity;coenzyme binding;cofactor binding;NADP binding;nucleotide binding;oxidoreductase activity;oxidoreductase activity, acting on CH-OH group of donors;oxidoreductase activity, acting on the CH-OH
binding;nucleic acid binding;nucleotide binding;protein binding;protein domain specific binding;RNA binding
binding;enzyme binding;histone deacetylase binding;nuclear localization sequence binding;peptide binding;protein binding;protein transporter activity;signal sequence binding;substrate-specific transporter activity;transporte
binding;nuclear localization sequence binding;peptide binding;protein transporter activity;signal sequence binding;substrate-specific transporter activity;transporter activity
binding;nucleic acid binding;nucleotide binding;RNA 7-methylguanosine cap binding;RNA binding;RNA cap binding
enzyme activator activity;enzyme regulator activity;GTPase activator activity;GTPase regulator activity;nucleoside-triphosphatase regulator activity

enzyme activator activity;enzyme regulator activity;GDP-dissociation inhibitor activity;GTPase activator activity;GTPase regulator activity;nucleoside-triphosphatase regulator activity;Rho GDP-dissociation inhibitor activity;sm
catalytic activity;enzyme activator activity;enzyme regulator activity;GDP-dissociation inhibitor activity;GTPase activator activity;GTPase activity;GTPase regulator activity;hydrolase activity;hydrolase activity, acting on acid an
binding;cation binding;DNA binding;ion binding;metal ion binding;nucleic acid binding;RNA binding;transition metal ion binding;zinc ion binding
binding;nucleic acid binding;nucleotide binding;RNA binding;single-stranded RNA binding
binding;DNA binding;identical protein binding;molecular transducer activity;nucleic acid binding;nucleic acid binding transcription factor activity;protein binding;sequence-specific DNA binding transcription factor activity;sign
adenyl nucleotide binding;adenyl ribonucleotide binding;ATP binding;ATPase activity;ATPase activity, coupled;binding;catalytic activity;chromatin binding;damaged DNA binding;DNA binding;DNA-dependent ATPase activity;
adenyl nucleotide binding;adenyl ribonucleotide binding;ATP binding;ATPase activity;binding;catalytic activity;enzyme binding;hydrolase activity;hydrolase activity, acting on acid anhydrides;hydrolase activity, acting on acid a
binding;cation binding;enzyme regulator activity;GTPase regulator activity;guanyl-nucleotide exchange factor activity;ion binding;metal ion binding;nucleoside-triphosphatase regulator activity;Ras guanyl-nucleotide exchange
catalytic activity;spermine synthase activity;transferase activity;transferase activity, transferring alkyl or aryl (other than methyl) groups
adenyl nucleotide binding;adenyl ribonucleotide binding;ATP binding;binding;carbohydrate binding;carbohydrate kinase activity;catalytic activity;fructokinase activity;glucokinase activity;glucose binding;hexokinase activity;ki
binding;nucleic acid binding;RNA binding;structural constituent of ribosome;structural molecule activity
binding;catalytic activity;cation binding;endopeptidase activity;hydrolase activity;ion binding;metal ion binding;metalloendopeptidase activity;metallopeptidase activity;peptidase activity;peptidase activity, acting on L-amino a
actin binding;binding;cytoskeletal protein binding;protein binding
5'-deoxyribose-5-phosphate lyase activity;AT DNA binding;binding;C2H2 zinc finger domain binding;cAMP response element binding;carbon-oxygen lyase activity;catalytic activity;chromatin binding;chromatin DNA binding;
binding;cation binding;ion binding;metal ion binding;transition metal ion binding;zinc ion binding
binding;peptide binding;structural constituent of nuclear pore;structural molecule activity;transporter activity
biliverdin reductase activity;binding;catalytic activity;cation binding;ion binding;metal ion binding;oxidoreductase activity;oxidoreductase activity, acting on the CH-CH group of donors;oxidoreductase activity, acting on the C
carboxylic acid transmembrane transporter activity;citrate transmembrane transporter activity;organic acid transmembrane transporter activity;substrate-specific transmembrane transporter activity;substrate-specific transpo
binding;catalytic activity;cation binding;hydrolase activity;hydrolase activity, acting on ester bonds;identical protein binding;ion binding;lipid binding;metal ion binding;molecular transducer activity;nucleic acid binding;phosph
adenyl nucleotide binding;adenyl ribonucleotide binding;anaphase-promoting complex binding;ATP binding;binding;catalytic activity;cytoskeletal protein binding;enzyme binding;kinase activity;kinase binding;microtubule bin
binding;enzyme binding;GTP binding;GTPase binding;GTP-dependent protein binding;guanyl nucleotide binding;guanyl ribonucleotide binding;lipid binding;nucleotide binding;phosphatidylinositol binding;phosphatidylinosito
binding;lipid binding;phosphatidylinositol binding;phosphatidylinositol-4-phosphate binding;phospholipid binding
4 iron, 4 sulfur cluster binding;adenyl nucleotide binding;adenyl ribonucleotide binding;ATP binding;binding;cation binding;ion binding;iron-sulfur cluster binding;metal cluster binding;metal ion binding;nucleotide binding;puri
acid-thiol ligase activity;adenyl nucleotide binding;adenyl ribonucleotide binding;ATP binding;ATP citrate synthase activity;binding;catalytic activity;cation binding;CoA-ligase activity;cofactor binding;ion binding;ligase activity
aminopeptidase activity;binding;catalytic activity;cation binding;exopeptidase activity;hydrolase activity;ion binding;metal ion binding;metalloaminopeptidase activity;metalloexopeptidase activity;metallopeptidase activity;pep
acid-thiol ligase activity;ATP citrate synthase activity;binding;catalytic activity;CoA-ligase activity;cofactor binding;GDP binding;GTP binding;guanyl nucleotide binding;guanyl ribonucleotide binding;ligase activity;ligase activit
binding;catalytic activity;cation binding;enzyme binding;GTPase binding;ion binding;metal ion binding;prenyltransferase activity;protein binding;protein geranylgeranyltransferase activity;protein prenyltransferase activity;Rab
structural molecule activity
structural molecule activity
anion binding;apoptotic protease activator activity;binding;caspase activator activity;caspase regulator activity;catalytic activity;chaperone binding;chloride ion binding;cysteine-type endopeptidase activity;cysteine-type pept
protein transporter activity;substrate-specific transporter activity;transporter activity
binding;carbon-sulfur lyase activity;catalytic activity;cation binding;holocytochrome-c synthase activity;ion binding;lyase activity;metal ion binding
receptor activity
actin binding;binding;cytoskeletal protein binding;protein binding;structural constituent of muscle;structural molecule activity
active transmembrane transporter activity;carboxylic acid transmembrane transporter activity;lactate transmembrane transporter activity;mevalonate transmembrane transporter activity;monocarboxylic acid transmembrane
binding;protein binding;protein complex binding;protein domain specific binding
binding;cation binding;ion binding;metal ion binding;transition metal ion binding;zinc ion binding
binding;DNA binding;nucleic acid binding;protein binding transcription factor activity;single-stranded DNA binding;structure-specific DNA binding;transcription coactivator activity;transcription cofactor activity;transcription fa
binding;protein binding;protein dimerization activity;protein heterodimerization activity
adenyl nucleotide binding;adenyl ribonucleotide binding;amine binding;amino acid binding;aminoacyl-tRNA ligase activity;arginine binding;arginine-tRNA ligase activity;ATP binding;binding;carboxylic acid binding;catalytic ac
binding;calcium channel activity;calcium channel regulator activity;cation binding;cation channel activity;cation transmembrane transporter activity;channel activity;channel regulator activity;gated channel activity;ion binding;
adenyl nucleotide binding;adenyl ribonucleotide binding;aminoacyl-tRNA ligase activity;ATP binding;binding;catalytic activity;chemokine receptor binding;CXCR chemokine receptor binding;cytokine receptor binding;G-prote
binding;catalytic activity;cysteine-type endopeptidase activity;cysteine-type peptidase activity;endopeptidase activity;endopeptidase inhibitor activity;endopeptidase regulator activity;enzyme inhibitor activity;enzyme regulat
adenyl nucleotide binding;adenyl ribonucleotide binding;ATP binding;binding;enzyme binding;glycolipid binding;lipid binding;nucleotide binding;protein binding;purine nucleotide binding;purine ribonucleoside triphosphate bi
active transmembrane transporter activity;ATPase activator activity;ATPase activity;ATPase activity, coupled;ATPase activity, coupled to movement of substances;ATPase activity, coupled to transmembrane movement of ions
binding;damaged DNA binding;DNA binding;nucleic acid binding;polyubiquitin binding;protein binding;single-stranded DNA binding;small conjugating protein binding;structure-specific DNA binding;ubiquitin binding
adenyl nucleotide binding;adenyl ribonucleotide binding;adenylate kinase activity;ATP binding;binding;catalytic activity;kinase activity;nucleobase-containing compound kinase activity;nucleotide binding;nucleotide kinase ac
adenyl nucleotide binding;adenyl ribonucleotide binding;amino acid kinase activity;ATP binding;binding;catalytic activity;coenzyme binding;cofactor binding;glutamate 5-kinase activity;glutamate-5-semialdehyde dehydrogen
binding;protein binding;SNARE binding;soluble NSF attachment protein activity;syntaxin binding
binding;catalytic activity;GTP binding;GTPase activity;guanyl nucleotide binding;guanyl ribonucleotide binding;hydrolase activity;hydrolase activity, acting on acid anhydrides;hydrolase activity, acting on acid anhydrides, in p
binding;identical protein binding;protein binding
binding;GTP binding;guanyl nucleotide binding;guanyl ribonucleotide binding;nucleotide binding;purine nucleotide binding;purine ribonucleoside triphosphate binding;purine ribonucleotide binding;ribonucleotide binding
binding;lipid binding
binding;importin-alpha export receptor activity;nuclear export signal receptor activity;protein binding;protein transporter activity;substrate-specific transporter activity;transporter activity
binding;endoribonuclease inhibitor activity;enzyme binding;enzyme inhibitor activity;enzyme regulator activity;protein binding;ribonuclease inhibitor activity;ubiquitin protein ligase binding
adenyl nucleotide binding;adenyl ribonucleotide binding;ADP binding;ATP binding;ATPase activity;binding;catalytic activity;enzyme activator activity;enzyme binding;enzyme regulator activity;hydrolase activity;hydrolase activ
3-hydroxyacyl-CoA dehydrogenase activity;acetyl-CoA C-acyltransferase activity;binding;C-acyltransferase activity;carbon-oxygen lyase activity;carboxylic acid binding;catalytic activity;coenzyme binding;cofactor binding;e
binding;enzyme binding;GTPase binding;protein binding;protein C-terminus binding;Ras GTPase binding;small GTPase binding
catalytic activity;cysteine-type endopeptidase activity;cysteine-type peptidase activity;endopeptidase activity;hydrolase activity;peptidase activity;peptidase activity, acting on L-amino acid peptides
adenosine kinase activity;adenyl nucleotide binding;adenyl ribonucleotide binding;ATP binding;binding;catalytic activity;cation binding;ion binding;kinase activity;metal ion binding;nucleobase-containing compound kinase ac
adenosine deaminase activity;binding;catalytic activity;cation binding;deaminase activity;DNA binding;double-stranded RNA adenosine deaminase activity;hydrolase activity;hydrolase activity, acting on carbon-nitrogen (but
structural molecule activity
binding;calcium ion binding;cation binding;ion binding;metal ion binding
adiponectin binding;binding;cadherin binding;calcium ion binding;cation binding;cell adhesion molecule binding;hormone binding;ion binding;lipoprotein particle binding;low-density lipoprotein particle binding;metal ion bindi
binding;box C/D snoRNA binding;nucleic acid binding;RNA binding;snoRNA binding;snRNA binding;U3 snoRNA binding;U4 snRNA binding
aminopeptidase activity;binding;catalytic activity;cation binding;exopeptidase activity;hydrolase activity;ion binding;metal ion binding;metallopeptidase activity;peptidase activity;peptidase activity, acting on L-amino acid pep
binding;nucleic acid binding;nucleotide binding;RNA binding
3-oxoacid CoA-transferase activity;binding;catalytic activity;CoA-transferase activity;identical protein binding;protein binding;protein dimerization activity;protein homodimerization activity;transferase activity;transferase activ
binding;nucleic acid binding;nucleotide binding;protein binding;protein complex scaffold;RNA binding;structural molecule activity;translation factor activity, nucleic acid binding;translation initiation factor activity
binding;cytokine receptor binding;death receptor binding;protein binding;receptor binding;tumor necrosis factor receptor superfamily binding
transmembrane transporter activity;transporter activity
adenyl nucleotide binding;adenyl ribonucleotide binding;aminoacyl-tRNA ligase activity;ATP binding;binding;catalytic activity;ligase activity;ligase activity, forming aminoacyl-tRNA and related compounds;ligase activity, formi
binding;cation binding;collagen binding;enzyme binding;ion binding;metal ion binding;phosphatase binding;protein binding;protein phosphatase binding
binding;channel regulator activity;enzyme binding;enzyme inhibitor activity;enzyme regulator activity;phosphatase binding;phosphatase inhibitor activity;phosphatase regulator activity;potassium channel regulator activity;pro
active transmembrane transporter activity;ATPase activity;ATPase activity, coupled;ATPase activity, coupled to movement of substances;ATPase activity, coupled to transmembrane movement of ions;ATPase activity, couple
cation transmembrane transporter activity;hydrogen ion transmembrane transporter activity;inorganic cation transmembrane transporter activity;ion transmembrane transporter activity;monovalent inorganic cation transmem
binding;nucleic acid binding;ribonucleoprotein binding;ribosomal large subunit binding;ribosome binding;RNA binding;translation factor activity, nucleic acid binding;translation initiation factor activity
binding;catalytic activity;chromatin binding;coenzyme binding;cofactor binding;NAD binding;nucleotide binding;oxidoreductase activity;oxidoreductase activity, acting on CH-OH group of donors;oxidoreductase activity, acti
catalytic activity;NADH dehydrogenase (quinone) activity;NADH dehydrogenase (ubiquinone) activity;NADH dehydrogenase activity;oxidoreductase activity;oxidoreductase activity, acting on NADH or NADPH;oxidoreductase
binding;enzyme binding;kinase binding;molecular transducer activity;protein binding;protein kinase binding;signal transducer activity
binding;DNA binding;nucleic acid binding
structural constituent of nuclear pore;structural molecule activity
actin binding;binding;cytoskeletal protein binding;protein binding
nucleocytoplasmic transporter activity;structural constituent of nuclear pore;structural molecule activity;transporter activity
binding;catalytic activity;GTP binding;GTPase activity;guanyl nucleotide binding;guanyl ribonucleotide binding;hydrolase activity;hydrolase activity, acting on acid anhydrides;hydrolase activity, acting on acid anhydrides, in p
binding;enzyme binding;protein binding;structural constituent of cytoskeleton;structural molecule activity
antigen binding;binding;MHC class II protein complex binding;MHC protein complex binding
binding;catalytic activity;enzyme binding;intramolecular oxidoreductase activity;intramolecular oxidoreductase activity, interconverting aldoses and ketoses;isomerase activity;protein binding;triose-phosphate isomerase acti
binding;nucleic acid binding;protein binding;protein N-terminus binding;RNA binding;translation factor activity, nucleic acid binding;translation initiation factor activity
binding;epidermal growth factor binding;growth factor binding;hormone binding;protein binding
binding;catalytic activity;cation binding;hydrolase activity;hydrolase activity, acting on ester bonds;ion binding;kinase activity;metal ion binding;molecular transducer activity;NF-kappaB-inducing kinase activity;phosphatase a
acid-amino acid ligase activity;adenyl nucleotide binding;adenyl ribonucleotide binding;ATP binding;binding;catalytic activity;enzyme binding;ligase activity;ligase activity, forming carbon-nitrogen bonds;nucleotide binding;pr
actin-dependent ATPase activity;ATPase activity;ATPase activity, coupled;binding;calcium ion binding;catalytic activity;cation binding;hydrolase activity;hydrolase activity, acting on acid anhydrides;hydrolase activity, acting o
adenyl nucleotide binding;adenyl ribonucleotide binding;ATP binding;binding;cytoskeletal protein binding;enzyme binding;identical protein binding;kinesin binding;nitric-oxide synthase binding;nucleotide binding;protein bindi
adenyl nucleotide binding;adenyl ribonucleotide binding;ATP binding;ATPase activity;ATPase activity, coupled;ATP-dependent helicase activity;ATP-dependent RNA helicase activity;binding;catalytic activity;double-stranded
structural constituent of ribosome;structural molecule activity
adenyl nucleotide binding;adenyl ribonucleotide binding;ADP binding;AMP binding;ATP binding;binding;carbohydrate binding;catalytic activity;cation binding;diphosphotransferase activity;GDP binding;guanyl nucleotide bin
binding;catalytic activity;endopeptidase activity;hydrolase activity;NF-kappaB binding;nucleic acid binding;nucleotide binding;peptidase activity;peptidase activity, acting on L-amino acid peptides;protein binding;purine nucle
binding;calcium ion binding;cation binding;identical protein binding;ion binding;ion channel binding;metal ion binding;protein binding;protein dimerization activity;protein homodimerization activity
apolipoprotein A-I receptor binding;apolipoprotein receptor binding;binding;catalytic activity;enzyme binding;GTP binding;GTPase activity;guanyl nucleotide binding;guanyl ribonucleotide binding;hydrolase activity;hydrolase
enzyme activator activity;enzyme inhibitor activity;enzyme regulator activity;kinase inhibitor activity;kinase regulator activity;molecular transducer activity;protein kinase inhibitor activity;protein kinase regulator activity;signal t
binding;catalytic activity;cytoskeletal protein binding;enzyme binding;GDP binding;GTP binding;GTPase activity;GTPase binding;guanyl nucleotide binding;guanyl ribonucleotide binding;hydrolase activity;hydrolase activity, a
catalytic activity;hydrolase activity;peptidase activity
7S RNA binding;binding;catalytic activity;drug binding;endoplasmic reticulum signal peptide binding;GDP binding;GTP binding;GTPase activity;guanyl nucleotide binding;guanyl ribonucleotide binding;hydrolase activity;hydro
binding;catalytic activity;GDP binding;GTP binding;GTPase activity;guanyl nucleotide binding;guanyl ribonucleotide binding;hydrolase activity;hydrolase activity, acting on acid anhydrides;hydrolase activity, acting on acid an
binding;catalytic activity;GDP binding;GTP binding;GTPase activity;GTP-dependent protein binding;guanyl nucleotide binding;guanyl ribonucleotide binding;hydrolase activity;hydrolase activity, acting on acid anhydrides;hyd
cyclin-dependent protein kinase regulator activity;enzyme regulator activity;kinase regulator activity;protein kinase regulator activity
binding;catalytic activity;cytoskeletal protein binding;GDP binding;GTP binding;GTPase activity;guanyl nucleotide binding;guanyl ribonucleotide binding;hydrolase activity;hydrolase activity, acting on acid anhydrides;hydrola
acid-amino acid ligase activity;adenyl nucleotide binding;adenyl ribonucleotide binding;ATP binding;binding;catalytic activity;ligase activity;ligase activity, forming carbon-nitrogen bonds;nucleotide binding;purine nucleotide b
acid-amino acid ligase activity;adenyl nucleotide binding;adenyl ribonucleotide binding;ATP binding;binding;catalytic activity;ligase activity;ligase activity, forming carbon-nitrogen bonds;NEDD8 ligase activity;nucleotide bindi
acid-amino acid ligase activity;adenyl nucleotide binding;adenyl ribonucleotide binding;ATP binding;binding;catalytic activity;enzyme binding;ligase activity;ligase activity, forming carbon-nitrogen bonds;nucleotide binding;pr
acid-amino acid ligase activity;adenyl nucleotide binding;adenyl ribonucleotide binding;ATP binding;binding;catalytic activity;enzyme binding;ligase activity;ligase activity, forming carbon-nitrogen bonds;nucleotide binding;pr
binding;catalytic activity;cytoskeletal protein binding;GDP binding;GTP binding;GTPase activity;guanyl nucleotide binding;guanyl ribonucleotide binding;hydrolase activity;hydrolase activity, acting on acid anhydrides;hydrola
adenyl nucleotide binding;adenyl ribonucleotide binding;ATP binding;binding;nucleotide binding;purine nucleotide binding;purine ribonucleoside triphosphate binding;purine ribonucleotide binding;ribonucleotide binding;stru
adenyl nucleotide binding;adenyl ribonucleotide binding;ATP binding;binding;nucleotide binding;purine nucleotide binding;purine ribonucleoside triphosphate binding;purine ribonucleotide binding;ribonucleotide binding;stru
adenyl nucleotide binding;adenyl ribonucleotide binding;ATP binding;binding;nucleotide binding;purine nucleotide binding;purine ribonucleoside triphosphate binding;purine ribonucleotide binding;ribonucleotide binding
molecular transducer activity;protein binding transcription factor activity;signal transducer activity;transcription cofactor activity;transcription corepressor activity;transcription factor binding transcription factor activity

binding;catalytic activity;GTP binding;GTPase activity;guanyl nucleotide binding;guanyl ribonucleotide binding;hydrolase activity;hydrolase activity, acting on acid anhydrides;hydrolase activity, acting on acid anhydrides, in pl
adenyl nucleotide binding;adenyl ribonucleotide binding;ATP binding;ATPase activity;binding;catalytic activity;enzyme inhibitor activity;enzyme regulator activity;hydrolase activity;hydrolase activity, acting on acid anhydrides;
binding;catalytic activity;GDP binding;GTP binding;GTPase activity;guanyl nucleotide binding;guanyl ribonucleotide binding;hydrolase activity;hydrolase activity, acting on acid anhydrides;hydrolase activity, acting on acid anh
binding;nucleic acid binding;RNA binding;structural constituent of ribosome;structural molecule activity
binding;nucleic acid binding;RNA binding;structural constituent of ribosome;structural molecule activity
binding;endopeptidase activator activity;endopeptidase regulator activity;enzyme activator activity;enzyme regulator activity;identical protein binding;p53 binding;peptidase activator activity;peptidase regulator activity;protei
binding;nucleic acid binding;RNA binding;structural constituent of ribosome;structural molecule activity
structural constituent of ribosome;structural molecule activity
active transmembrane transporter activity;ATPase activity;ATPase activity, coupled;ATPase activity, coupled to movement of substances;ATPase activity, coupled to transmembrane movement of ions;ATPase activity, couplec
binding;cation binding;ion binding;metal ion binding;structural constituent of ribosome;structural molecule activity
binding;catalytic activity;cytoskeletal protein binding;GTP binding;GTPase activity;guanyl nucleotide binding;guanyl ribonucleotide binding;hydrolase activity;hydrolase activity, acting on acid anhydrides;hydrolase activity, act
binding;catalytic activity;GTP binding;GTPase activity;guanyl nucleotide binding;guanyl ribonucleotide binding;hydrolase activity;hydrolase activity, acting on acid anhydrides;hydrolase activity, acting on acid anhydrides, in pl
adenyl nucleotide binding;adenyl ribonucleotide binding;ATP binding;binding;chaperone binding;nucleotide binding;protein binding;purine nucleotide binding;purine ribonucleoside triphosphate binding;purine ribonucleotide b
binding;ribonucleoprotein binding;ribosome binding
binding;identical protein binding;protein binding;SNARE binding;syntaxin binding;syntaxin-1 binding
binding;identical protein binding;protein binding
catalytic activity;dolichyl-diphosphooligosaccharide-protein glycotransferase activity;oligosaccharyl transferase activity;transferase activity;transferase activity, transferring glycosyl groups;transferase activity, transferring hex
binding;cholesterol binding;enzyme binding;lipid binding;protein binding;steroid binding;sterol binding
binding;cation binding;ion binding;metal ion binding;nucleic acid binding;RNA binding;rRNA binding;structural constituent of ribosome;structural molecule activity
acid-amino acid ligase activity;binding;catalytic activity;enzyme binding;ligase activity;ligase activity, forming carbon-nitrogen bonds;protein binding;small conjugating protein ligase activity;SUMO ligase activity;ubiquitin prot
binding;enzyme binding;GTPase binding;protein binding;Ran GTPase binding;Ras GTPase binding;small GTPase binding;transporter activity
binding;core promoter proximal region DNA binding;core promoter proximal region sequence-specific DNA binding;DNA binding;nucleic acid binding;nucleic acid binding transcription factor activity;regulatory region DNA bin
binding;enzyme binding;enzyme inhibitor activity;enzyme regulator activity;insulin-like growth factor receptor binding;kinase binding;kinase inhibitor activity;kinase regulator activity;protein binding;protein kinase binding;prote
binding;catalytic activity;GTP binding;GTPase activity;guanyl nucleotide binding;guanyl ribonucleotide binding;hydrolase activity;hydrolase activity, acting on acid anhydrides;hydrolase activity, acting on acid anhydrides, in pl
binding;cation binding;chaperone binding;identical protein binding;ion binding;metal ion binding;protein binding;protein dimerization activity;protein homodimerization activity;protein transporter activity;substrate-specific tran
binding;enzyme binding;protein binding
binding;nucleic acid binding;RNA binding;structural constituent of ribosome;structural molecule activity
binding;catalytic activity;cation binding;hydrolase activity;hydrolase activity, acting on ester bonds;ion binding;metal ion binding;phosphatase activity;phosphoprotein phosphatase activity;phosphoric ester hydrolase activity;p
binding;catalytic activity;cation binding;enzyme binding;hydrolase activity;hydrolase activity, acting on ester bonds;ion binding;kinase binding;metal ion binding;myosin phosphatase activity;myosin-light-chain-phosphatase a
adenyl nucleotide binding;adenyl ribonucleotide binding;ATP binding;ATPase activity;binding;catalytic activity;hydrolase activity;hydrolase activity, acting on acid anhydrides;hydrolase activity, acting on acid anhydrides, in ph
adenyl nucleotide binding;adenyl ribonucleotide binding;ATP binding;ATPase activity;binding;catalytic activity;G-protein-coupled receptor binding;hormone receptor binding;hydrolase activity;hydrolase activity, acting on acid
structural constituent of ribosome;structural molecule activity
binding;nucleic acid binding;RNA binding;structural constituent of ribosome;structural molecule activity
binding;nucleic acid binding;RNA binding;structural constituent of ribosome;structural molecule activity
amine binding;amino acid binding;antigen binding;binding;carboxylic acid binding;channel regulator activity;enzyme binding;histone deacetylase binding;ion channel binding;MHC class II protein complex binding;MHC protei
binding;mRNA 5'-UTR binding;mRNA binding;nucleic acid binding;RNA binding;structural constituent of ribosome;structural molecule activity;translation regulator activity
structural constituent of ribosome;structural molecule activity
binding;nucleic acid binding;RNA binding;rRNA binding;structural constituent of ribosome;structural molecule activity
binding;cation binding;ion binding;metal ion binding;structural constituent of ribosome;structural molecule activity;transition metal ion binding;zinc ion binding
binding;mRNA binding;nucleic acid binding;RNA binding;rRNA binding;SSU rRNA binding;structural constituent of ribosome;structural molecule activity
binding;nucleic acid binding;RNA binding;rRNA binding;structural constituent of ribosome;structural molecule activity
binding;nucleic acid binding;RNA binding
binding;nucleic acid binding;RNA binding
binding;nucleic acid binding;RNA binding
binding;enzyme binding;histone pre-mRNA DCP binding;nucleic acid binding;protein binding;RNA binding
binding;catalytic activity;enzyme binding;GTP binding;GTPase activity;guanyl nucleotide binding;guanyl ribonucleotide binding;hydrolase activity;hydrolase activity, acting on acid anhydrides;hydrolase activity, acting on acid
adenyl nucleotide binding;adenyl ribonucleotide binding;ATP binding;ATPase activity;binding;binding, bridging;catalytic activity;hydrolase activity;hydrolase activity, acting on acid anhydrides;hydrolase activity, acting on acid
binding;nucleic acid binding;RNA binding;structural constituent of ribosome;structural molecule activity
binding;catalytic activity;cytoskeletal protein binding;GTP binding;GTPase activity;guanyl nucleotide binding;guanyl ribonucleotide binding;hydrolase activity;hydrolase activity, acting on acid anhydrides;hydrolase activity, act
binding;nucleic acid binding;ribonucleoprotein binding;ribosome binding;RNA binding;translation factor activity, nucleic acid binding;translation release factor activity;translation release factor activity, codon specific;translatic
binding;cation binding;DNA binding;ion binding;metal ion binding;nucleic acid binding;nucleic acid binding transcription factor activity;RNA binding;sequence-specific DNA binding transcription factor activity;single-stranded
binding;nucleic acid binding;RNA binding;rRNA binding;structural constituent of ribosome;structural molecule activity
binding;nucleic acid binding;nucleotide binding;RNA binding;rRNA binding;structural constituent of ribosome;structural molecule activity
binding;enzyme binding;kinase binding;protein binding;protein kinase binding;structural constituent of ribosome;structural molecule activity
binding;catalytic activity;demethylase activity;DNA binding;histone binding;histone demethylase activity;histone demethylase activity (H4-K20 specific);nucleic acid binding;protein binding
binding;catalytic activity;GTP binding;GTPase activity;guanyl nucleotide binding;guanyl ribonucleotide binding;hydrolase activity;hydrolase activity, acting on acid anhydrides;hydrolase activity, acting on acid anhydrides, in pl
androgen receptor binding;binding;catalytic activity;chromatin binding;GDP binding;GTP binding;GTPase activity;guanyl nucleotide binding;guanyl ribonucleotide binding;hormone receptor binding;hydrolase activity;hydrolas
structural constituent of ribosome;structural molecule activity
binding;catalytic activity;enzyme regulator activity;GTP binding;GTPase activity;GTPase regulator activity;guanyl nucleotide binding;guanyl ribonucleotide binding;guanyl-nucleotide exchange factor activity;hydrolase activity;
binding;DNA binding;nucleic acid binding;structural constituent of ribosome;structural molecule activity
binding;nucleotide binding;protein binding;structural constituent of ribosome;structural molecule activity;translation initiation factor binding
binding;nucleic acid binding;RNA binding;structural constituent of ribosome;structural molecule activity
binding;mRNA binding;nucleic acid binding;RNA binding;structural constituent of ribosome;structural molecule activity
structural constituent of ribosome;structural molecule activity
binding;nucleic acid binding;RNA binding;structural constituent of ribosome;structural molecule activity
binding;catalytic activity;enzyme binding;GTPase activity;GTPase binding;hydrolase activity;hydrolase activity, acting on acid anhydrides;hydrolase activity, acting on acid anhydrides, in phosphorus-containing anhydrides;mc
acid-amino acid ligase activity;binding;catalytic activity;cation binding;enzyme binding;ion binding;ligase activity;ligase activity, forming carbon-nitrogen bonds;metal ion binding;NEDD8 ligase activity;protein binding;small co
binding;calcium channel regulator activity;catalytic activity;channel regulator activity;enzyme binding;GTPase activity;GTPase binding;hydrolase activity;hydrolase activity, acting on acid anhydrides;hydrolase activity, acting o
binding;nucleic acid binding;RNA binding;structural constituent of ribosome;structural molecule activity
binding;nucleic acid binding;RNA binding;structural constituent of ribosome;structural molecule activity
binding;nucleic acid binding;RNA binding;structural constituent of ribosome;structural molecule activity
structural constituent of ribosome;structural molecule activity
structural constituent of ribosome;structural molecule activity
binding;nucleic acid binding;RNA binding;rRNA binding;structural constituent of ribosome;structural molecule activity
binding;nucleic acid binding;RNA binding;rRNA binding;structural constituent of ribosome;structural molecule activity
binding;catalytic activity;cis-trans isomerase activity;isomerase activity;peptide binding;peptidyl-prolyl cis-trans isomerase activity;protein binding;unfolded protein binding;virion binding
activin binding;binding;catalytic activity;cis-trans isomerase activity;cytokine receptor binding;drug binding;FK506 binding;ion channel binding;isomerase activity;macrolide binding;molecular transducer activity;peptidyl-prolyl
binding;cation binding;ion binding;metal ion binding;structural constituent of ribosome;structural molecule activity
structural constituent of ribosome;structural molecule activity
binding;binding, bridging;cytokine receptor binding;enzyme binding;ephrin receptor binding;epidermal growth factor receptor binding;growth factor receptor binding;identical protein binding;insulin receptor substrate binding;
binding;mRNA binding;nucleic acid binding;nucleotide binding;RNA binding
binding;catalytic activity;enzyme binding;GDP-dissociation inhibitor binding;GTP binding;GTPase activity;guanyl nucleotide binding;guanyl ribonucleotide binding;hydrolase activity;hydrolase activity, acting on acid anhydride
binding;clathrin binding;peptide binding;protein binding;protein transporter activity;signal sequence binding;substrate-specific transporter activity;transporter activity
adenylate cyclase activity;binding;catalytic activity;cation binding;cyclase activity;GTP binding;GTPase activity;guanyl nucleotide binding;guanyl ribonucleotide binding;hydrolase activity;hydrolase activity, acting on acid anhy
binding;catalytic activity;cation binding;G-protein beta/gamma-subunit complex binding;G-protein-coupled receptor binding;GTP binding;GTPase activity;guanyl nucleotide binding;guanyl ribonucleotide binding;hydrolase ac
binding;enzyme binding;identical protein binding;kinase binding;protein binding;protein kinase binding;transcription factor binding;ubiquitin protein ligase binding
catalytic activity;enzyme regulator activity;hydrolase activity;hydrolase activity, acting on ester bonds;phosphatase activity;phosphatase regulator activity;phosphoprotein phosphatase activity;phosphoric ester hydrolase activ
acid-amino acid ligase activity;binding;catalytic activity;enzyme binding;ligase activity;ligase activity, forming carbon-nitrogen bonds;protein binding;small conjugating protein ligase activity;SUMO ligase activity;transcription f
catalytic activity;enzyme regulator activity;hydrolase activity;hydrolase activity, acting on acid anhydrides;hydrolase activity, acting on acid anhydrides, in phosphorus-containing anhydrides;motor activity;nitric-oxide synthase
binding;nucleic acid binding;RNA binding;structural constituent of ribosome;structural molecule activity
acid-amino acid ligase activity;catalytic activity;ligase activity;ligase activity, forming carbon-nitrogen bonds;small conjugating protein ligase activity;ubiquitin-protein ligase activity
binding;catalytic activity;GTPase activity;hydrolase activity;hydrolase activity, acting on acid anhydrides;hydrolase activity, acting on acid anhydrides, in phosphorus-containing anhydrides;molecular transducer activity;nuclec
binding;protein binding;protein N-terminus binding;structural constituent of ribosome;structural molecule activity
binding;nucleic acid binding;protein binding;protein N-terminus binding;ribonucleoprotein binding;ribosome binding;RNA binding;snRNA binding;translation elongation factor activity;translation factor activity, nucleic acid binc
apoptotic protease activator activity;binding;binding, bridging;caspase activator activity;caspase regulator activity;channel inhibitor activity;channel regulator activity;enzyme activator activity;enzyme binding;enzyme inhibitor
binding;cation binding;ion binding;metal ion binding;nucleic acid binding transcription factor activity;protein binding;protein dimerization activity;protein heterodimerization activity;sequence-specific DNA binding transcription
binding;catalytic activity;cation binding;hydrolase activity;hydrolase activity, acting on ester bonds;ion binding;metal ion binding;phosphatase activity;phosphoprotein phosphatase activity;phosphoric ester hydrolase activity;p
binding;core promoter proximal region DNA binding;core promoter proximal region sequence-specific DNA binding;DNA binding;double-stranded DNA binding;enzyme binding;GTPase binding;nucleic acid binding;nucleic ac
catalytic activity;hydrolase activity;peptidase activity;peptidase activity, acting on L-amino acid peptides;serine hydrolase activity;serine-type peptidase activity
binding;cation binding;chromatin binding;enzyme regulator activity;identical protein binding;ion binding;kinase regulator activity;metal ion binding;protein binding;protein domain specific binding;protein kinase regulator activit
binding;calcium ion binding;cation binding;ion binding;metal ion binding;structural constituent of muscle;structural molecule activity
adenyl nucleotide binding;adenyl ribonucleotide binding;ATP binding;ATPase activity;binding;catalytic activity;cytoskeletal protein binding;hydrolase activity;hydrolase activity, acting on acid anhydrides;hydrolase activity, acti
acid-amino acid ligase activity;adenyl nucleotide binding;adenyl ribonucleotide binding;ATP binding;binding;catalytic activity;enzyme binding;enzyme regulator activity;ligase activity;ligase activity, forming carbon-nitrogen bo
binding;catalytic activity;enzyme binding;GTP binding;GTPase activity;guanyl nucleotide binding;guanyl ribonucleotide binding;hydrolase activity;hydrolase activity, acting on acid anhydrides;hydrolase activity, acting on acid
binding;catalytic activity;GTP binding;GTPase activity;guanyl nucleotide binding;guanyl ribonucleotide binding;hydrolase activity;hydrolase activity, acting on acid anhydrides;hydrolase activity, acting on acid anhydrides, in pl
binding;catalytic activity;double-stranded RNA binding;GTP binding;GTPase activity;guanyl nucleotide binding;guanyl ribonucleotide binding;hydrolase activity;hydrolase activity, acting on acid anhydrides;hydrolase activity, a
adenyl nucleotide binding;adenyl ribonucleotide binding;ATP binding;binding;catalytic activity;heat shock protein binding;Hsp90 protein binding;kinase activity;nucleotide binding;phosphotransferase activity, alcohol group as
1-alkyl-2-acetylglycerophosphocholine esterase activity;acetyltransferase activity;carboxylic ester hydrolase activity;catalytic activity;hydrolase activity;hydrolase activity, acting on ester bonds;platelet-activating factor acetylt
binding;carbohydrate binding
binding;cation binding;heme binding;ion binding;iron ion binding;metal ion binding;oxygen binding;oxygen transporter activity;substrate-specific transporter activity;tetrapyrrole binding;transition metal ion binding;transporter
binding;enzyme binding;enzyme regulator activity;phosphatase binding;phosphatase regulator activity;protein binding;protein phosphatase 2A binding;protein phosphatase binding;protein phosphatase regulator activity;prote
binding;calcium ion binding;catalytic activity;cation binding;hydrolase activity;hydrolase activity, acting on ester bonds;identical protein binding;ion binding;magnesium ion binding;metal ion binding;phosphatase activity;phos
binding;DNA binding;nucleic acid binding;nucleotide binding;RNA binding
binding;nucleic acid binding;RNA binding;translation factor activity, nucleic acid binding;translation initiation factor activity
catalytic activity;endonuclease activity;endonuclease activity, active with either ribo- or deoxyribonucleic acids and producing 5'-phosphomonoesters;endoribonuclease activity;endoribonuclease activity, producing 5'-phosph
binding;DNA binding;nucleic acid binding;nucleic acid binding transcription factor activity;sequence-specific DNA binding transcription factor activity
binding;binding, bridging;protein binding;protein binding, bridging;protein domain specific binding;receptor activity;SH3 domain binding;SH3/SH2 adaptor activity;signaling adaptor activity
adenyl nucleotide binding;adenyl ribonucleotide binding;ATP binding;binding;enzyme binding;nucleotide binding;protein binding;purine nucleotide binding;purine ribonucleoside triphosphate binding;purine ribonucleotide bin
binding;cytoskeletal protein binding;microtubule binding;nucleic acid binding;protein binding;RNA binding;tubulin binding

antioxidant activity;catalytic activity;disulfide oxidoreductase activity;glutathione dehydrogenase (ascorbate) activity;glutathione disulfide oxidoreductase activity;glutathione transferase activity;methylarsonate reductase activ
adenyl nucleotide binding;adenyl ribonucleotide binding;ATP binding;binding;catalytic activity;DNA binding;DNA-dependent protein kinase activity;kinase activity;nucleic acid binding;nucleotide binding;phosphotransferase a
structural molecule activity
binding;cation binding;ion binding;metal ion binding;transition metal ion binding;zinc ion binding
binding;calcium ion binding;cation binding;DNA binding;ion binding;metal ion binding;nucleic acid binding
binding;DNA binding;nucleic acid binding;protein binding;protein binding transcription factor activity;protein domain specific binding;regulatory region DNA binding;regulatory region nucleic acid binding;transcription cofactor
catalytic activity;hydrolase activity;peptidase activity
binding;DNA binding;nucleic acid binding;protein binding transcription factor activity;transcription cofactor activity;transcription factor binding transcription factor activity
structural constituent of ribosome;structural molecule activity
structural constituent of ribosome;structural molecule activity
structural constituent of ribosome;structural molecule activity
structural constituent of ribosome;structural molecule activity
binding;DNA binding;nucleic acid binding
catalytic activity;hydrolase activity
binding;nucleic acid binding;RNA binding;structural constituent of ribosome;structural molecule activity
structural constituent of ribosome;structural molecule activity
binding;chromatin binding;enzyme binding;protein binding
binding;catalytic activity;GTP binding;GTPase activity;guanyl nucleotide binding;guanyl ribonucleotide binding;hydrolase activity;hydrolase activity, acting on acid anhydrides;hydrolase activity, acting on acid anhydrides, in ph
binding;catalytic activity;GTP binding;GTPase activity;guanyl nucleotide binding;guanyl ribonucleotide binding;hydrolase activity;hydrolase activity, acting on acid anhydrides;hydrolase activity, acting on acid anhydrides, in ph
binding;nucleic acid binding;RNA binding;structural constituent of ribosome;structural molecule activity
binding;nucleic acid binding;nucleotide binding;RNA binding
binding;DNA binding;nucleic acid binding;nucleic acid binding transcription factor activity;regulatory region DNA binding;regulatory region nucleic acid binding;RNA polymerase II regulatory region DNA binding;RNA polymera
binding;binding, bridging;cargo receptor activity;clathrin adaptor activity;clathrin binding;lipid binding;phosphatidylinositol binding;phosphatidylinositol-4,5-bisphosphate binding;phospholipid binding;protein binding;protein b
binding;cation binding;ion binding;metal ion binding;protein binding;protein C-terminus binding
binding;cation binding;identical protein binding;ion binding;metal ion binding;nucleotide binding;protein binding;protein complex binding;transition metal ion binding;zinc ion binding
binding;nucleic acid binding;nucleotide binding;ribonucleoprotein binding;ribosomal large subunit binding;RNA binding
binding;chromatin binding;core promoter proximal region DNA binding;core promoter proximal region sequence-specific DNA binding;DNA bending activity;DNA binding;mitochondrial light strand promoter sense binding;nuc
active transmembrane transporter activity;anion transmembrane transporter activity;binding;inorganic anion transmembrane transporter activity;ion transmembrane transporter activity;phosphate ion carrier activity;phosphate
binding;lipid binding
binding;DNA binding;double-stranded DNA binding;double-stranded telomeric DNA binding;nucleic acid binding;nucleic acid binding transcription factor activity;protein binding;purine-rich negative regulatory element binding
binding;enzyme binding;GTPase binding;GTP-Rho binding;protein binding;Ras GTPase binding;Rho GTPase binding;small GTPase binding
binding;clathrin binding;clathrin light chain binding;double-stranded RNA binding;enzyme binding;kinase binding;nucleic acid binding;protein binding;protein kinase binding;RNA binding;structural molecule activity
binding;chromatin binding;core promoter proximal region DNA binding;core promoter proximal region sequence-specific DNA binding;DNA binding;nucleic acid binding;nucleic acid binding transcription factor activity;protein
binding;catalytic activity;cis-trans isomerase activity;drug binding;FK506 binding;isomerase activity;macrolide binding;peptidyl-prolyl cis-trans isomerase activity;receptor activity
binding;carbohydrate binding;catalytic activity;cation binding;coenzyme binding;cofactor binding;ion binding;L-iditol 2-dehydrogenase activity;metal ion binding;NAD binding;nucleotide binding;oxidoreductase activity;oxidor
adenyl nucleotide binding;adenyl ribonucleotide binding;ATP binding;binding;core promoter binding;DNA binding;nucleic acid binding;nucleotide binding;purine nucleotide binding;purine ribonucleoside triphosphate binding;
binding;cation binding;ion binding;metal ion binding;nucleotide binding
actin binding;ankyrin binding;binding;cytoskeletal protein binding;enzyme binding;GTPase binding;lipid binding;phospholipid binding;protein binding;structural constituent of cytoskeleton;structural molecule activity
AU-rich element binding;binding;nucleic acid binding;nucleotide binding;RNA binding
binding;DNA binding;enzyme inhibitor activity;enzyme regulator activity;histone binding;nucleic acid binding;phosphatase inhibitor activity;phosphatase regulator activity;protein binding;protein phosphatase inhibitor activity;
binding;nucleotide binding;protein binding transcription factor activity;transcription cofactor activity;transcription corepressor activity;transcription factor binding transcription factor activity
AMP deaminase activity;binding;catalytic activity;cation binding;deaminase activity;hydrolase activity;hydrolase activity, acting on carbon-nitrogen (but not peptide) bonds;hydrolase activity, acting on carbon-nitrogen (but not
binding;carboxylic acid binding;fatty acid binding;lipid binding;monocarboxylic acid binding;transporter activity
binding;catalytic activity;drug binding;hydroxymethylglutaryl-CoA synthase activity;isomerase activity;organic acid binding;transferase activity;transferase activity, transferring acyl groups;transferase activity, transferring acyl
active transmembrane transporter activity;amine transmembrane transporter activity;amino acid transmembrane transporter activity;antigen binding;antiporter activity;aromatic amino acid transmembrane transporter activity;
3'-5' exonuclease activity;binding;catalytic activity;exonuclease activity;exoribonuclease activity;hydrolase activity;hydrolase activity, acting on ester bonds;nuclease activity;nucleotide binding;ribonuclease activity
catalytic activity;cysteine-type peptidase activity;hydrolase activity;peptidase activity;peptidase activity, acting on L-amino acid peptides;small conjugating protein-specific protease activity;ubiquitin-specific protease activity
6-phosphofructokinase activity;adenyl nucleotide binding;adenyl ribonucleotide binding;ATP binding;binding;carbohydrate kinase activity;catalytic activity;cation binding;ion binding;kinase activity;metal ion binding;nucleotide
binding;cation binding;identical protein binding;ion binding;metal ion binding;nucleic acid binding;nucleotide binding;protein binding;RNA binding;transition metal ion binding;zinc ion binding
binding;calcium ion binding;calmodulin binding;catalytic activity;cation binding;hydrolase activity;hydrolase activity, acting on ester bonds;ion binding;lipase activity;metal ion binding;molecular transducer activity;phosphatidy
binding;catalytic activity;coenzyme binding;cofactor binding;dihydroorotate dehydrogenase activity;drug binding;FMN binding;nucleotide binding;oxidoreductase activity;oxidoreductase activity, acting on the CH-CH group o
binding;catalytic activity;cation binding;coenzyme binding;cofactor binding;ion binding;metal ion binding;oxidoreductase activity;oxidoreductase activity, acting on the aldehyde or oxo group of donors;oxidoreductase activity
adenyl nucleotide binding;adenyl ribonucleotide binding;ATP binding;ATPase activity;binding;catalytic activity;hydrolase activity;hydrolase activity, acting on acid anhydrides;hydrolase activity, acting on acid anhydrides, in ph
adenyl nucleotide binding;adenyl ribonucleotide binding;ATP binding;ATPase activity;binding;catalytic activity;cytoskeletal protein binding;hydrolase activity;hydrolase activity, acting on acid anhydrides;hydrolase activity, acti
aldehyde dehydrogenase (NAD) activity;binding;carboxylic acid binding;catalytic activity;coenzyme binding;cofactor binding;fatty acid binding;fatty-acyl-CoA binding;lipid binding;malonate-semialdehyde dehydrogenase (ace
endopeptidase inhibitor activity;endopeptidase regulator activity;enzyme inhibitor activity;enzyme regulator activity;peptidase inhibitor activity;peptidase regulator activity;serine-type endopeptidase inhibitor activity
binding;cation binding;chromatin binding;core promoter binding;core promoter proximal region DNA binding;core promoter proximal region sequence-specific DNA binding;core promoter sequence-specific DNA binding;DNA
binding;chromatin binding;chromatin DNA binding;DNA binding;nucleic acid binding;structure-specific DNA binding
binding;nucleic acid binding;RNA binding;structural constituent of ribosome;structural molecule activity
adenyl nucleotide binding;adenyl ribonucleotide binding;ATP binding;binding;catalytic activity;enzyme activator activity;enzyme regulator activity;hydrolase activity;hydrolase activity, acting on ester bonds;kinase activator act
adenyl nucleotide binding;adenyl ribonucleotide binding;ATP binding;binding;binding, bridging;catalytic activity;cis-trans isomerase activity;drug binding;FK506 binding;GTP binding;guanyl nucleotide binding;guanyl ribonucle
binding;carboxylic acid binding;catalytic activity;cation binding;identical protein binding;ion binding;iron ion binding;L-ascorbic acid binding;metal ion binding;oxidoreductase activity;oxidoreductase activity, acting on paired
binding;calcium ion binding;cation binding;DNA binding;ion binding;metal ion binding;nucleic acid binding
binding;DNA binding;nucleic acid binding;RNA binding;structural constituent of ribosome;structural molecule activity
adenylate cyclase binding;binding;enzyme binding;protein binding;protein kinase A binding
active transmembrane transporter activity;anion transmembrane transporter activity;anion:anion antiporter activity;antiporter activity;C4-dicarboxylate transmembrane transporter activity;carboxylic acid transmembrane trans
binding;calcium ion binding;cation binding;cytoskeletal protein binding;identical protein binding;integrin binding;ion binding;metal ion binding;microtubule binding;microtubule plus-end binding;protein binding;protein complex
binding;channel inhibitor activity;channel regulator activity;cholesterol binding;enzyme activator activity;enzyme binding;enzyme regulator activity;identical protein binding;inward rectifier potassium channel inhibitor activity;io
aminoacylase activity;binding;catalytic activity;cation binding;hydrolase activity;hydrolase activity, acting on carbon-nitrogen (but not peptide) bonds;hydrolase activity, acting on carbon-nitrogen (but not peptide) bonds, in lin
structural molecule activity
binding;enzyme binding;molecular transducer activity;protein binding;protein domain specific binding;receptor activity;receptor binding;signal transducer activity;signaling receptor activity;U-plasminogen activator receptor ac
active transmembrane transporter activity;adenyl nucleotide binding;adenyl ribonucleotide binding;ADP binding;antigen binding;ATP binding;ATPase activity;ATPase activity, coupled;ATPase activity, coupled to movement of
active transmembrane transporter activity;adenyl nucleotide binding;adenyl ribonucleotide binding;ATP binding;ATPase activity;ATPase activity, coupled;ATPase activity, coupled to movement of substances;ATPase activity, c
binding;core promoter proximal region DNA binding;core promoter proximal region sequence-specific DNA binding;DNA binding;nucleic acid binding;nucleic acid binding transcription factor activity;regulatory region DNA bin
activating transcription factor binding;anion binding;binding;chromatin binding;core promoter proximal region DNA binding;core promoter proximal region sequence-specific DNA binding;DNA binding;enhancer binding;enhan
ATPase binding;binding;enzyme binding;K6-linked polyubiquitin binding;polyubiquitin binding;protein binding;small conjugating protein binding;ubiquitin binding;ubiquitin protein ligase binding
1,4-alpha-glucan branching enzyme activity;binding;catalytic activity;cation binding;hydrolase activity;hydrolase activity, acting on glycosyl bonds;hydrolase activity, hydrolyzing O-glycosyl compounds;ion binding;transferase
binding;nucleic acid binding;RNA binding;translation factor activity, nucleic acid binding;translation initiation factor activity
binding;MHC class II protein binding;MHC class II receptor activity;MHC protein binding;molecular transducer activity;protein binding;receptor activity;receptor binding;signal transducer activity;signaling receptor activity;stru
binding;calcium ion binding;cation binding;ion binding;metal ion binding;receptor activity
catalytic activity;endopeptidase activity;hydrolase activity;peptidase activity;peptidase activity, acting on L-amino acid peptides;serine hydrolase activity;serine-type endopeptidase activity;serine-type peptidase activity
binding;carbon-sulfur lyase activity;catalytic activity;cation binding;ion binding;lactoylglutathione lyase activity;lyase activity;metal ion binding;transition metal ion binding;zinc ion binding
binding;chromatin binding;DNA binding;nucleic acid binding;single-stranded DNA binding;structure-specific DNA binding
binding;channel regulator activity;enzyme binding;glucocorticoid receptor binding;hormone receptor binding;insulin-like growth factor receptor binding;ion channel binding;nuclear hormone receptor binding;protein binding;pr
binding;nucleic acid binding;RNA binding
acid-amino acid ligase activity;binding;catalytic activity;cation binding;ion binding;ligase activity;ligase activity, forming carbon-nitrogen bonds;metal ion binding;protein binding transcription factor activity;small conjugating p
binding;catalytic activity;enzyme binding;GTP binding;GTPase activity;guanyl nucleotide binding;guanyl ribonucleotide binding;hydrolase activity;hydrolase activity, acting on acid anhydrides;hydrolase activity, acting on acid
binding;catalytic activity;hydrolase activity;hydrolase activity, acting on ester bonds;non-membrane spanning protein tyrosine phosphatase activity;phosphatase activity;phosphoprotein phosphatase activity;phosphoric ester
actin binding;adenyl nucleotide binding;adenyl ribonucleotide binding;ATP binding;binding;catalytic activity;cytoskeletal protein binding;enzyme binding;JUN kinase binding;kinase activity;kinase binding;molecular transducer
binding;nucleotide binding
adenyl nucleotide binding;adenyl ribonucleotide binding;ATP binding;binding;calcium-independent protein kinase C activity;catalytic activity;cation binding;enzyme activator activity;enzyme binding;enzyme regulator activity;i
actin binding;binding;calmodulin binding;cytoskeletal protein binding;protein binding;tropomyosin binding
acid-amino acid ligase activity;adenyl nucleotide binding;adenyl ribonucleotide binding;ATP binding;binding;catalytic activity;ligase activity;ligase activity, forming carbon-nitrogen bonds;nucleotide binding;purine nucleotide b
endopeptidase inhibitor activity;endopeptidase regulator activity;enzyme inhibitor activity;enzyme regulator activity;peptidase inhibitor activity;peptidase regulator activity;serine-type endopeptidase inhibitor activity
binding;binding, bridging;catalytic activity;hydrolase activity;hydrolase activity, acting on ester bonds;insulin receptor binding;non-membrane spanning protein tyrosine phosphatase activity;phosphatase activity;phosphoprote
4 iron, 4 sulfur cluster binding;amidophosphoribosyltransferase activity;binding;catalytic activity;cation binding;ion binding;iron-sulfur cluster binding;metal cluster binding;metal ion binding;transferase activity;transferase acti
amine binding;amino acid binding;binding;carbohydrate binding;carboxylic acid binding;catalytic activity;glutamine-fructose-6-phosphate transaminase (isomerizing) activity;transaminase activity;transferase activity;transfera
3'-5' exonuclease activity;3'-5'-exoribonuclease activity;AU-rich element binding;binding;catalytic activity;exonuclease activity;exonuclease activity, active with either ribo- or deoxyribonucleic acids and producing 5'-phosph
endopeptidase activator activity;endopeptidase regulator activity;enzyme activator activity;enzyme regulator activity;peptidase activator activity;peptidase regulator activity
binding;carbohydrate binding;cation binding;DNA binding;endopeptidase inhibitor activity;endopeptidase regulator activity;enzyme inhibitor activity;enzyme regulator activity;glycosaminoglycan binding;heparin binding;identi
binding;mRNA binding;nucleic acid binding;RNA binding
antioxidant activity;binding;catalytic activity;cation binding;heme binding;ion binding;iron ion binding;metal ion binding;oxidoreductase activity;oxidoreductase activity, acting on paired donors, with incorporation or reduction
binding;nucleic acid binding;RNA binding;structural constituent of ribosome;structural molecule activity
binding;binding, bridging;DNA binding;identical protein binding;mRNA binding;nucleic acid binding;poly(A) RNA binding;poly(U) RNA binding;poly-purine tract binding;poly-pyrimidine tract binding;protein binding;protein bind
catalytic activity;hydrolase activity;hydrolase activity, acting on acid anhydrides;hydrolase activity, acting on acid anhydrides, in phosphorus-containing anhydrides;microtubule motor activity;motor activity;nucleoside-triphosp
apolipoprotein binding;binding;calcium ion binding;cation binding;ion binding;lipoprotein particle receptor binding;lipoprotein transporter activity;metal ion binding;protein binding;protein complex binding;protein transporter a
binding;mRNA binding;nucleic acid binding;nucleotide binding;RNA binding
binding;binding, bridging;enzyme activator activity;enzyme regulator activity;GTPase activator activity;GTPase regulator activity;nucleoside-triphosphatase regulator activity;protein binding;protein binding, bridging;SH3/SH2
binding;nucleotide binding
binding;calcium ion binding;calcium-dependent protein serine/threonine phosphatase activity;calmodulin binding;calmodulin-dependent protein phosphatase activity;catalytic activity;cation binding;drug binding;enzyme bind
adenyl nucleotide binding;adenyl ribonucleotide binding;ATP binding;ATPase activity;ATPase activity, coupled;ATP-dependent DNA helicase activity;ATP-dependent helicase activity;ATP-dependent RNA helicase activity;bind
binding;catalytic activity;cation binding;coenzyme binding;cofactor binding;ion binding;metal ion binding;mRNA 3'-UTR binding;mRNA binding;NADP binding;NADPH binding;NADPH:quinone reductase activity;nucleic acid b
transporter activity
binding;enzyme binding;kinase binding;protein binding;protein kinase binding;SNARE binding;syntaxin binding
cargo receptor activity;receptor activity;scavenger receptor activity
binding;lipid binding;phosphatidylethanolamine binding;phosphatidylserine binding;phospholipid binding
molecular transducer activity;receptor activity;signal transducer activity;signaling receptor activity;thrombospondin receptor activity

binding;catalytic activity;cis-trans isomerase activity;cyclosporin A binding;drug binding;estrogen receptor binding;heat shock protein binding;hormone receptor binding;Hsp70 protein binding;Hsp90 protein binding;isomeras
binding;chromatin binding;DNA binding;nucleic acid binding
adenyl nucleotide binding;adenyl ribonucleotide binding;ATP binding;binding;nucleotide binding;purine nucleotide binding;purine ribonucleoside triphosphate binding;purine ribonucleotide binding;ribonucleotide binding
receptor activity
binding;catalytic activity;methyltransferase activity;nucleic acid binding;RNA binding;RNA methyltransferase activity;S-adenosylmethionine-dependent methyltransferase activity;transferase activity;transferase activity, transfe
binding;enzyme binding;histone binding;histone deacetylase binding;protein binding
binding;nucleic acid binding;RNA binding;RNA cap binding
adenyl nucleotide binding;adenyl ribonucleotide binding;ATP binding;binding;nucleotide binding;purine nucleotide binding;purine ribonucleoside triphosphate binding;purine ribonucleotide binding;ribonucleotide binding
actin binding;actin filament binding;binding;calmodulin-dependent protein kinase activity;catalytic activity;cytoskeletal protein binding;kinase activity;phosphotransferase activity, alcohol group as acceptor;protein binding;pro
acetylgalactosaminyltransferase activity;binding;carbohydrate binding;catalytic activity;cation binding;ion binding;manganese ion binding;metal ion binding;polypeptide N-acetylgalactosaminyltransferase activity;transferase a
binding;enzyme binding;kinase binding;protein binding;protein kinase binding;protein transporter activity;substrate-specific transporter activity;transporter activity
binding;mRNA 3'-UTR binding;mRNA binding;nucleic acid binding;RNA binding
catalytic activity;hydrolase activity;hydrolase activity, acting on glycosyl bonds;hydrolase activity, hydrolyzing N-glycosyl compounds;lyase activity;NAD(P)+ nucleosidase activity;NAD+ nucleosidase activity;phosphorus-oxyg
binding;endopeptidase inhibitor activity;endopeptidase regulator activity;enzyme inhibitor activity;enzyme regulator activity;identical protein binding;metalloendopeptidase inhibitor activity;metalloenzyme inhibitor activity;meta
binding;catalytic activity;cation binding;endopeptidase activity;hydrolase activity;ion binding;metal ion binding;metalloendopeptidase activity;metallopeptidase activity;peptidase activity;peptidase activity, acting on L-amino a
catalytic activity;dipeptidase activity;exopeptidase activity;hydrolase activity;peptidase activity;peptidase activity, acting on L-amino acid peptides
nucleocytoplasmic transporter activity;transporter activity
binding;molecular transducer activity;nucleic acid binding;receptor signaling protein activity;RNA binding;signal transducer activity;snoRNA binding
actin binding;actin monomer binding;adenyl nucleotide binding;adenyl ribonucleotide binding;ATP binding;binding;cytoskeletal protein binding;lipid binding;nucleotide binding;phosphatidylinositol binding;phosphatidylinositol
binding;calcium ion binding;catalytic activity;cation binding;electron carrier activity;ion binding;ion channel binding;metal ion binding;oxidoreductase activity;oxidoreductase activity, acting on paired donors, with incorporation
binding;cAMP-dependent protein kinase activity;catalytic activity;cation binding;cyclic nucleotide-dependent protein kinase activity;enzyme regulator activity;GTPase regulator activity;guanyl-nucleotide exchange factor activ
binding;calcium ion binding;catalytic activity;cation binding;epidermal growth factor receptor binding;epidermal growth factor-activated receptor activity;growth factor receptor binding;ion binding;kinase activity;metal ion bind
binding;enzyme binding;protein binding;protein C-terminus binding
binding;calcium ion binding;carbohydrate binding;cation binding;glycosaminoglycan binding;heparin binding;ion binding;metal ion binding;pattern binding;polysaccharide binding
binding;enzyme activator activity;enzyme regulator activity;lipase activator activity;phospholipase activator activity;protein binding;SNAP receptor activity;SNARE binding;sphingomyelin phosphodiesterase activator activity
binding;mRNA binding;nucleic acid binding;nucleotide binding;RNA binding
binding;carbohydrate binding
adenyl nucleotide binding;adenyl ribonucleotide binding;ATP binding;ATPase activity;ATPase activity, coupled;ATP-dependent DNA helicase activity;ATP-dependent helicase activity;binding;catalytic activity;cation binding;DN
binding;cation binding;ion binding;metal ion binding;transition metal ion binding;zinc ion binding
4 iron, 4 sulfur cluster binding;binding;catalytic activity;cation binding;coenzyme binding;cofactor binding;dihydroorotate dehydrogenase activity;dihydropyrimidine dehydrogenase (NADP+) activity;flavin adenine dinucleotide
binding;catalytic activity;dipeptidyl-peptidase activity;endopeptidase activity;enzyme binding;exopeptidase activity;hydrolase activity;identical protein binding;integrin binding;metalloendopeptidase activity;metallopeptidase a
activating transcription factor binding;binding;damaged DNA binding;DNA binding;histone binding;methylated histone residue binding;nucleic acid binding;p53 binding;protein binding;protein binding transcription factor activ
binding;cytokine activity;enzyme binding;GTPase binding;identical protein binding;nucleic acid binding;protein binding;protein dimerization activity;protein homodimerization activity;receptor binding;RNA binding;tRNA bindin
adenyl nucleotide binding;adenyl ribonucleotide binding;ATP binding;binding;catalytic activity;DNA binding;double-stranded RNA binding;nucleic acid binding;nucleotide binding;purine nucleotide binding;purine ribonucleosi
binding;DNA binding;double-stranded RNA binding;nucleic acid binding;RNA binding
binding;carbohydrate binding;cation binding;glycoprotein binding;heat shock protein binding;ion binding;mannose binding;metal ion binding;monosaccharide binding;protein binding;sugar binding
actin binding;binding;binding, bridging;cytoskeletal protein binding;enzyme binding;GTPase binding;protein binding;protein binding, bridging;Rac GTPase binding;Ras GTPase binding;Rho GTPase binding;SH3/SH2 adaptor
adenyl nucleotide binding;adenyl ribonucleotide binding;ATP binding;binding;cytokine receptor binding;enzyme binding;kinase binding;nucleotide binding;protein binding;protein kinase binding;purine nucleotide binding;purin
acid-amino acid ligase activity;binding;catalytic activity;cation binding;CD40 receptor binding;cytokine receptor binding;enzyme binding;identical protein binding;ion binding;kinase binding;ligase activity;ligase activity, forming
actin binding;actin filament binding;adenyl nucleotide binding;adenyl ribonucleotide binding;ATP binding;ATPase activity;ATPase activity, coupled;binding;calmodulin binding;catalytic activity;cytoskeletal protein binding;hydro
binding;catalytic activity;DNA binding;endonuclease activity;enzyme inhibitor activity;enzyme regulator activity;hydrolase activity;hydrolase activity, acting on ester bonds;nuclease activity;nucleic acid binding;phosphatase in
catalytic activity;hydrolase activity;hydrolase activity, acting on ester bonds;phosphatase activity;phosphoprotein phosphatase activity;phosphoric ester hydrolase activity;prenylated protein tyrosine phosphatase activity;prote
enzyme activator activity;enzyme regulator activity;GTPase activator activity;GTPase regulator activity;guanyl-nucleotide exchange factor activity;nucleoside-triphosphatase regulator activity;Ras guanyl-nucleotide exchange
binding;mRNA binding;nucleic acid binding;RNA binding
binding;catalytic activity;isomerase activity;protein binding;receptor binding
adenyl nucleotide binding;adenyl ribonucleotide binding;ATP binding;binding;catalytic activity;cation binding;enzyme activator activity;enzyme regulator activity;identical protein binding;ion binding;kinase activator activity;kin
actin binding;binding;cytoskeletal protein binding;protein binding
adenyl nucleotide binding;adenyl ribonucleotide binding;adenylyltransferase activity;ATP binding;binding;catalytic activity;dephospho-CoA kinase activity;kinase activity;nucleotide binding;nucleotidyltransferase activity;pante
acetyl-CoA carboxylase activity;adenyl nucleotide binding;adenyl ribonucleotide binding;ATP binding;binding;biotin carboxylase activity;catalytic activity;cation binding;CoA carboxylase activity;ion binding;ligase activity;ligas
enzyme inhibitor activity;enzyme regulator activity;GTPase inhibitor activity;GTPase regulator activity;nucleoside-triphosphatase regulator activity
binding;identical protein binding;protein binding
catalytic activity;phosphorylase activity;purine-nucleoside phosphorylase activity;S-methyl-5-thioadenosine phosphorylase activity;transferase activity;transferase activity, transferring glycosyl groups;transferase activity, trans
[acetyl-CoA carboxylase] kinase activity;[hydroxymethylglutaryl-CoA reductase (NADPH)] kinase activity;adenyl nucleotide binding;adenyl ribonucleotide binding;AMP-activated protein kinase activity;ATP binding;binding;cAM
molecular transducer activity;signal transducer activity
binding;DNA binding;double-stranded DNA binding;identical protein binding;mRNA 3'-UTR binding;mRNA binding;nucleic acid binding;nucleic acid binding transcription factor activity;nucleotide binding;protein binding;RNA
AU-rich element binding;binding;enzyme binding;kinase binding;nucleic acid binding;nucleotide binding;protein binding;protein kinase binding;RNA binding
antioxidant activity;catalytic activity;oxidoreductase activity;oxidoreductase activity, acting on paired donors, with incorporation or reduction of molecular oxygen;oxidoreductase activity, acting on peroxide as acceptor;perox
adenyl nucleotide binding;adenyl ribonucleotide binding;ATP binding;binding;catalytic activity;enzyme activator activity;enzyme binding;enzyme regulator activity;GTPase binding;identical protein binding;kinase activator activ
binding;enzyme binding;identical protein binding;protein binding;protein domain specific binding
binding;protein binding;protein N-terminus binding;SNAP receptor activity;SNARE binding
enzyme regulator activity
receptor activity
enzyme inhibitor activity;enzyme regulator activity;kinase inhibitor activity;kinase regulator activity;protein kinase inhibitor activity;protein kinase regulator activity
binding;selenium binding
binding;nucleotide binding
binding;nucleotide binding
binding;nucleic acid binding;nucleotide binding;pre-mRNA binding;RNA binding
binding;identical protein binding;protein binding;protein dimerization activity;protein homodimerization activity
acid-amino acid ligase activity;binding;catalytic activity;cation binding;chromo shadow domain binding;DNA binding;enzyme binding;ion binding;kinase activity;Krueppel-associated box domain binding;ligase activity;ligase a
adenyl nucleotide binding;adenyl ribonucleotide binding;ATP binding;ATPase activity;ATPase activity, coupled;ATP-dependent DNA helicase activity;ATP-dependent helicase activity;ATP-dependent RNA helicase activity;bind
adenyl nucleotide binding;adenyl ribonucleotide binding;ATP binding;binding;catalytic activity;kinase activity;nucleotide binding;phosphotransferase activity, alcohol group as acceptor;protein kinase activity;protein tyrosine ki
binding;mRNA binding;nucleic acid binding;nucleotide binding;poly(A) RNA binding;poly(U) RNA binding;poly-purine tract binding;poly-pyrimidine tract binding;RNA binding;single-stranded RNA binding
binding;nucleic acid binding;RNA binding;single-stranded RNA binding;tRNA binding
binding;cation binding;chromatin binding;core promoter binding;core promoter sequence-specific DNA binding;DNA binding;ion binding;metal ion binding;nucleic acid binding;nucleic acid binding transcription factor activity;
binding;nucleic acid binding;RNA binding;translation factor activity, nucleic acid binding;translation initiation factor activity
binding;catalytic activity;coenzyme binding;cofactor binding;NAD binding;nucleic acid binding transcription factor activity;nucleotide binding;oxidoreductase activity;oxidoreductase activity, acting on CH-OH group of donors
molecular transducer activity;receptor signaling protein activity;signal transducer activity
acid-amino acid ligase activity;binding;catalytic activity;enzyme binding;ligase activity;ligase activity, forming carbon-nitrogen bonds;protein binding;small conjugating protein ligase activity;ubiquitin protein ligase binding
catalytic activity;hydrolase activity;hydrolase activity, acting on acid anhydrides;hydrolase activity, acting on acid anhydrides, in phosphorus-containing anhydrides;microtubule motor activity;motor activity;nucleoside-triphosp
adenyl nucleotide binding;adenyl ribonucleotide binding;ATP binding;binding;catalytic activity;enzyme binding;kinase activity;kinase binding;molecular transducer activity;nucleotide binding;phosphotransferase activity, alcoh
binding;catalytic activity;cation transmembrane transporter activity;coenzyme binding;cofactor binding;hydrogen ion transmembrane transporter activity;inorganic cation transmembrane transporter activity;ion transmembran
binding;catalytic activity;cis-trans isomerase activity;cyclosporin A binding;drug binding;isomerase activity;peptide binding;peptidyl-prolyl cis-trans isomerase activity
transporter activity
binding;carbohydrate binding;glycoprotein binding;protein binding
binding;enzyme binding;GTPase binding;protein binding
binding;catalytic activity;cation binding;collagen binding;endopeptidase activity;enzyme binding;extracellular matrix binding;hydrolase activity;integrin binding;ion binding;kinase binding;laminin binding;metal ion binding;meta
binding;catalytic activity;cis-trans isomerase activity;drug binding;FK506 binding;heat shock protein binding;isomerase activity;macrolide binding;peptidyl-prolyl cis-trans isomerase activity;protein binding
actin binding;actin-dependent ATPase activity;adenyl nucleotide binding;adenyl ribonucleotide binding;ADP binding;ATP binding;ATPase activity;ATPase activity, coupled;binding;calmodulin binding;catalytic activity;cation bin
adenyl nucleotide binding;adenyl ribonucleotide binding;ATP binding;binding;catalytic activity;cation binding;ion binding;kinase activity;metal ion binding;nucleotide binding;phosphotransferase activity, alcohol group as accep
1-phosphatidylinositol binding;binding;binding, bridging;clathrin adaptor activity;clathrin binding;clathrin heavy chain binding;lipid binding;phosphatidylinositol binding;phospholipid binding;protein binding;protein binding, bri
binding;catalytic activity;cation binding;enzyme binding;identical protein binding;ion binding;kinase activity;kinase binding;metal ion binding;phosphotransferase activity, alcohol group as acceptor;protein binding;protein dom
binding;catalytic activity;GTP binding;GTPase activity;guanyl nucleotide binding;guanyl ribonucleotide binding;hydrolase activity;hydrolase activity, acting on acid anhydrides;hydrolase activity, acting on acid anhydrides, in ph
adenyl nucleotide binding;adenyl ribonucleotide binding;ATP binding;binding;catalytic activity;kinase activity;nucleotide binding;phosphotransferase activity, alcohol group as acceptor;protein kinase activity;protein serine/thre
amine binding;amino acid binding;binding;carboxylic acid binding;catalytic activity;cis-trans isomerase activity;enzyme binding;GTPase activating protein binding;isomerase activity;kinase binding;mitogen-activated protein k
binding;eukaryotic initiation factor 4E binding;protein binding;translation initiation factor binding;translation regulator activity;translation repressor activity
adenyl nucleotide binding;adenyl ribonucleotide binding;ATP binding;binding;catalytic activity;cytokine receptor binding;death domain binding;death receptor binding;enzyme binding;identical protein binding;kinase activity;nu
activating transcription factor binding;binding;catalytic activity;core promoter binding;deacetylase activity;DNA binding;enzyme binding;histone deacetylase activity;histone deacetylase activity (H3-K14 specific);histone deac
adenyl nucleotide binding;adenyl ribonucleotide binding;ATP binding;binding;calmodulin binding;calmodulin-dependent protein kinase activity;catalytic activity;channel inhibitor activity;channel regulator activity;cytoskeletal p
binding;catalytic activity;cytoskeletal protein binding;hydrolase activity;hydrolase activity, acting on acid anhydrides;hydrolase activity, acting on acid anhydrides, in phosphorus-containing anhydrides;motor activity;nucleosid
binding;catalytic activity;enzyme binding;NEDD8 activating enzyme activity;protein binding;protein dimerization activity;protein heterodimerization activity;small protein activating enzyme activity;ubiquitin protein ligase bindin
binding;hormone receptor binding;Notch binding;nuclear hormone receptor binding;protein binding;protein binding transcription factor activity;receptor binding;retinoic acid receptor binding;SMAD binding;transcription coact
binding;nucleotide binding
binding;epidermal growth factor receptor binding;growth factor receptor binding;identical protein binding;insulin receptor binding;lipid binding;phosphatidylinositol binding;phospholipid binding;protein binding;protein comple
binding;enzyme binding;protein binding;ubiquitin protein ligase binding
binding;POZ domain binding;protein binding;protein domain specific binding
binding;catalytic activity;coenzyme binding;cofactor binding;electron carrier activity;GDP-4-dehydro-D-rhamnose reductase activity;GDP-L-fucose synthase activity;isomerase activity;oxidoreductase activity;oxidoreductase a
AP-1 adaptor complex binding;AP-3 adaptor complex binding;binding;catalytic activity;GTP binding;GTPase activity;GTP-dependent protein binding;guanyl nucleotide binding;guanyl ribonucleotide binding;hydrolase activity
binding;cation binding;ion binding;ion channel binding;metal ion binding;protein binding;transition metal ion binding;zinc ion binding
binding;cation binding;ion binding;metal ion binding;transition metal ion binding;zinc ion binding
binding;carbohydrate binding;glycosaminoglycan binding;heparin binding;pattern binding;polysaccharide binding
catalytic activity;glucosidase activity;hydrolase activity;hydrolase activity, acting on glycosyl bonds;hydrolase activity, hydrolyzing O-glycosyl compounds;mannosyl-oligosaccharide glucosidase activity
binding;protein binding;receptor binding
actin binding;binding;calcium ion binding;cation binding;cytoskeletal protein binding;ion binding;metal ion binding;protein binding;structural constituent of cytoskeleton;structural molecule activity
binding;catalytic activity;GTP binding;GTPase activity;guanyl nucleotide binding;guanyl ribonucleotide binding;hydrolase activity;hydrolase activity, acting on acid anhydrides;hydrolase activity, acting on acid anhydrides, in pl
adenyl nucleotide binding;adenyl ribonucleotide binding;ATP binding;ATPase activity;ATPase activity, coupled;ATP-dependent helicase activity;ATP-dependent protein binding;ATP-dependent RNA helicase activity;binding;ca
aminopeptidase activity;carboxypeptidase activity;catalytic activity;cysteine-type endopeptidase activity;cysteine-type peptidase activity;endopeptidase activity;exopeptidase activity;hydrolase activity;peptidase activity;pept

3'-5' exonuclease activity;3'-5'-exoribonuclease activity;7S RNA binding;binding;catalytic activity;exonuclease activity;exonuclease activity, active with either ribo- or deoxyribonucleic acids and producing 5'-phosphomonoes
binding;catalytic activity;GTP binding;GTPase activity;guanyl nucleotide binding;guanyl ribonucleotide binding;hydrolase activity;hydrolase activity, acting on acid anhydrides;hydrolase activity, acting on acid anhydrides, in ph
binding;catalytic activity;cation binding;hydrolase activity;intramolecular oxidoreductase activity;intramolecular oxidoreductase activity, transposing C=C bonds;ion binding;isomerase activity;isopentenyl-diphosphate delta-is
binding;DNA binding;nucleic acid binding;nucleic acid binding transcription factor activity;protein binding transcription factor activity;sequence-specific DNA binding transcription factor activity;transcription coactivator activit
binding;mRNA 3'-UTR binding;mRNA binding;nucleic acid binding;nucleotide binding;RNA binding;rRNA binding;SSU rRNA binding;translation regulator activity;translation repressor activity
actin binding;binding;cytoskeletal protein binding;enzyme binding;protein binding
binding;cation binding;copper chaperone activity;copper ion binding;ion binding;metal ion binding;metallochaperone activity;transition metal ion binding
binding;DNA binding;nucleic acid binding;nucleotide binding;RNA binding;sequence-specific DNA binding;telomeric DNA binding
binding;enzyme binding;protein binding;receptor activity
binding;calcium ion binding;cation binding;collagen binding;ion binding;metal ion binding;protein binding
binding;ribonucleoprotein binding
acid-amino acid ligase activity;catalytic activity;ligase activity;ligase activity, forming carbon-nitrogen bonds;small conjugating protein ligase activity;ubiquitin-protein ligase activity;ubiquitin-ubiquitin ligase activity
binding;protein binding;transcription factor binding
binding;DNA binding;identical protein binding;nucleic acid binding;nucleotide binding;protein binding
binding;nucleic acid binding;RNA binding;structural molecule activity;translation factor activity, nucleic acid binding;translation initiation factor activity
binding;enzyme binding;enzyme regulator activity;GTPase regulator activity;guanyl-nucleotide exchange factor activity;kinase binding;nucleoside-triphosphatase regulator activity;protein binding;protein kinase binding;Ras gu
binding;cation binding;enzyme activator activity;enzyme regulator activity;GTPase activator activity;GTPase regulator activity;ion binding;metal ion binding;nucleoside-triphosphatase regulator activity;transition metal ion bind
binding;carbohydrate binding;enzyme binding;protein binding
androgen receptor binding;binding;cation binding;hormone receptor binding;identical protein binding;ion binding;metal ion binding;nuclear hormone receptor binding;protein binding;protein binding transcription factor activity
binding;carbohydrate binding;catalytic activity;chondroitin sulfate binding;glycosaminoglycan binding;hydrolase activity;hydrolase activity, acting on carbon-nitrogen (but not peptide) bonds;pattern binding;polysaccharide bi
aminoacyl-tRNA hydrolase activity;binding;carboxylic ester hydrolase activity;catalytic activity;hydrolase activity;hydrolase activity, acting on ester bonds;nucleic acid binding;RNA binding;translation factor activity, nucleic aci
catalytic activity;hydrolase activity;hydrolase activity, acting on acid anhydrides;hydrolase activity, acting on acid anhydrides, in phosphorus-containing anhydrides;motor activity;nucleoside-triphosphatase activity;pyrophosp
adenyl nucleotide binding;adenyl ribonucleotide binding;ATP binding;ATPase activity;binding;catalytic activity;hydrolase activity;hydrolase activity, acting on acid anhydrides;hydrolase activity, acting on acid anhydrides, in ph
binding;enzyme regulator activity;GDP binding;GTP binding;guanyl nucleotide binding;guanyl ribonucleotide binding;nucleic acid binding;nucleotide binding;purine nucleotide binding;purine ribonucleoside triphosphate bindi
adenyl nucleotide binding;adenyl ribonucleotide binding;ATP binding;ATPase activity;ATPase activity, coupled;ATP-dependent helicase activity;ATP-dependent RNA helicase activity;binding;catalytic activity;helicase activity;h
binding;DNA binding;nucleic acid binding
binding;profilin binding;protein binding
binding;calcium ion binding;cation binding;ion binding;metal ion binding
acid-amino acid ligase activity;binding;catalytic activity;cation binding;ion binding;ligase activity;ligase activity, forming carbon-nitrogen bonds;metal ion binding;nucleic acid binding transcription factor activity;sequence-spec
ankyrin binding;binding;cytoskeletal protein binding;protein binding
binding;catalytic activity;cation binding;cis-trans isomerase activity;drug binding;FK506 binding;identical protein binding;ion binding;isomerase activity;macrolide binding;metal ion binding;peptidyl-prolyl cis-trans isomerase a
binding;G-protein coupled receptor activity;molecular transducer activity;PDZ domain binding;protein binding;protein domain specific binding;receptor activity;signal transducer activity;signaling receptor activity;transmembra
angiotensin receptor binding;binding;catalytic activity;cation binding;D5 dopamine receptor binding;dopamine receptor binding;G-protein beta/gamma-subunit complex binding;G-protein-coupled receptor binding;GTP bindi
catalytic activity;guanidinoacetate N-methyltransferase activity;methyltransferase activity;S-adenosylmethionine-dependent methyltransferase activity;transferase activity;transferase activity, transferring one-carbon groups
binding;catalytic activity;coenzyme binding;cofactor binding;identical protein binding;isomerase activity;protein binding;protein dimerization activity;protein homodimerization activity;racemase and epimerase activity;racemas
binding;nucleic acid binding;RNA binding
binding;nucleotide binding
binding;carbohydrate binding;catalytic activity;endopeptidase activity;glycosaminoglycan binding;hydrolase activity;pattern binding;peptidase activity;peptidase activity, acting on L-amino acid peptides;polysaccharide bindi
adenyl nucleotide binding;adenyl ribonucleotide binding;ATP binding;ATPase activity;binding;catalytic activity;cation binding;DNA binding;helicase activity;hydrolase activity;hydrolase activity, acting on acid anhydrides;hydrol
catalytic activity;intramolecular oxidoreductase activity;intramolecular oxidoreductase activity, interconverting keto- and enol-groups;intramolecular oxidoreductase activity, transposing S-S bonds;isomerase activity;oxidored
binding;catalytic activity;cation binding;diphosphotransferase activity;enzyme inhibitor activity;enzyme regulator activity;ion binding;magnesium ion binding;metal ion binding;ribose phosphate diphosphokinase activity;transfe
adenyl nucleotide binding;adenyl ribonucleotide binding;ATP binding;binding;catalytic activity;DNA binding;DNA helicase activity;helicase activity;hydrolase activity;hydrolase activity, acting on acid anhydrides;hydrolase activ
alcohol binding;binding;calcium channel activity;calcium-release channel activity;cation channel activity;cation transmembrane transporter activity;channel activity;gated channel activity;inositol 1,3,4,5 tetrakisphosphate bind
endopeptidase inhibitor activity;endopeptidase regulator activity;enzyme inhibitor activity;enzyme regulator activity;peptidase inhibitor activity;peptidase regulator activity;serine-type endopeptidase inhibitor activity
binding;DNA binding;identical protein binding;nucleic acid binding;nucleic acid binding transcription factor activity;protein binding;protein binding transcription factor activity;protein dimerization activity;protein homodimeriza
acid-amino acid ligase activity;binding;catalytic activity;hormone receptor binding;ligase activity;ligase activity, forming carbon-nitrogen bonds;nuclear hormone receptor binding;protein binding;receptor binding;small conjuga
binding;FHA domain binding;protein binding;protein C-terminus binding;protein domain specific binding
binding;clathrin binding;lipid binding;protein binding
adenyl nucleotide binding;adenyl ribonucleotide binding;ATP binding;binding;catalytic activity;chromatin binding;hydrolase activity;hydrolase activity, acting on acid anhydrides;hydrolase activity, acting on acid anhydrides, in
catalytic activity
adenyl nucleotide binding;adenyl ribonucleotide binding;ATP binding;binding;nucleotide binding;purine nucleotide binding;purine ribonucleoside triphosphate binding;purine ribonucleotide binding;ribonucleotide binding
binding;catalytic activity;cysteine-type endopeptidase activity;cysteine-type peptidase activity;endopeptidase activity;hydrolase activity;ion channel binding;p53 binding;peptidase activity;peptidase activity, acting on L-amino
alpha-glucosidase activity;binding;carbohydrate binding;catalytic activity;glucan 1,3-alpha-glucosidase activity;glucosidase activity;hydrolase activity;hydrolase activity, acting on glycosyl bonds;hydrolase activity, hydrolyzing
binding;double-stranded RNA binding;nucleic acid binding;RNA binding
catalytic activity;enzyme regulator activity;hydrolase activity;hydrolase activity, acting on ester bonds;phosphatase activity;phosphatase regulator activity;phosphoprotein phosphatase activity;phosphoric ester hydrolase activ
binding;catalytic activity;chromo shadow domain binding;DNA binding;lamin binding;nucleic acid binding;oxidoreductase activity;oxidoreductase activity, acting on the CH-CH group of donors;oxidoreductase activity, acting
protein transporter activity;substrate-specific transporter activity;transporter activity
binding;enzyme binding;kinase binding;phosphatase binding;protein binding;protein kinase binding;protein phosphatase binding
binding;calcium ion binding;catalytic activity;cation binding;cytokine binding;growth factor binding;ion binding;kinase activity;metal ion binding;microfibril binding;molecular transducer activity;phosphotransferase activity, alco
binding;catalytic activity;cysteine-type endopeptidase activity;cysteine-type endopeptidase activity involved in apoptotic process;cysteine-type peptidase activity;death effector domain binding;endopeptidase activity;enzyme
adenyl nucleotide binding;adenyl ribonucleotide binding;ATP binding;ATPase activity;ATPase activity, coupled;ATP-dependent DNA helicase activity;ATP-dependent helicase activity;binding;catalytic activity;cation binding;DN
binding;binding, bridging;cation binding;ion binding;ion transmembrane transporter activity;metal ion binding;protein binding;protein binding, bridging;SH3/SH2 adaptor activity;signaling adaptor activity;substrate-specific tra
15-oxoprostaglandin 13-oxidase activity;2-alkenal reductase [NAD(P)] activity;binding;catalytic activity;cation binding;ion binding;metal ion binding;oxidoreductase activity;oxidoreductase activity, acting on the CH-CH group
binding;cation binding;DNA binding;double-stranded DNA binding;ion binding;metal ion binding;nucleic acid binding;nucleotide binding;RNA binding;structure-specific DNA binding;transition metal ion binding;zinc ion bindin
binding;cation binding;enzyme binding;ion binding;metal ion binding;nuclear localization sequence binding;peptide binding;protein binding;protein domain specific binding;protein transporter activity;signal sequence binding;
adenyl nucleotide binding;adenyl ribonucleotide binding;ATP binding;binding;GTP binding;guanyl nucleotide binding;guanyl ribonucleotide binding;nucleotide binding;purine nucleotide binding;purine ribonucleoside triphosph
structural molecule activity
binding;enzyme activator activity;enzyme regulator activity;histone acetyl-lysine binding;histone binding;peptidase activator activity;peptidase regulator activity;protein binding
binding;enzyme activator activity;enzyme regulator activity;GTPase activating protein binding;GTPase activator activity;GTPase regulator activity;guanyl-nucleotide exchange factor activity;nucleoside-triphosphatase regulato
binding;chromatin binding
catalytic activity;hydrolase activity;peptidase activity
ATPase activity;catalytic activity;hydrolase activity;hydrolase activity, acting on acid anhydrides;hydrolase activity, acting on acid anhydrides, in phosphorus-containing anhydrides;nucleoside-triphosphatase activity;pyrophos
binding;polyubiquitin binding;protein binding;small conjugating protein binding;ubiquitin binding
binding;enzyme regulator activity;GTP binding;guanyl nucleotide binding;guanyl ribonucleotide binding;nucleotide binding;purine nucleotide binding;purine ribonucleoside triphosphate binding;purine ribonucleotide binding;ri
binding;histone binding;nucleic acid binding;nucleotide binding;protein binding;RNA binding;snRNA binding;U4 snRNA binding;U6 snRNA binding;U6atac snRNA binding
binding;histone binding;protein binding
3'-5' exonuclease activity;3'-5'-exoribonuclease activity;binding;catalytic activity;exonuclease activity;exonuclease activity, active with either ribo- or deoxyribonucleic acids and producing 5'-phosphomonoesters;exoribonucl
binding;cation binding;enzyme activator activity;enzyme regulator activity;GTPase activator activity;GTPase regulator activity;ion binding;metal ion binding;nucleoside-triphosphatase regulator activity;transition metal ion bind
binding;catalytic activity;GTP binding;GTPase activity;guanyl nucleotide binding;guanyl ribonucleotide binding;hydrolase activity;hydrolase activity, acting on acid anhydrides;hydrolase activity, acting on acid anhydrides, in pl
binding;enzyme activator activity;enzyme binding;enzyme regulator activity;GTPase activator activity;GTPase binding;GTPase regulator activity;nucleoside-triphosphatase regulator activity;protein binding;Rab GTPase bindin
cation transmembrane transporter activity;divalent inorganic cation transmembrane transporter activity;ferrous iron transmembrane transporter activity;inorganic cation transmembrane transporter activity;ion transmembrane
adenyl nucleotide binding;adenyl ribonucleotide binding;amine binding;amino acid binding;aminoacyl-tRNA ligase activity;ATP binding;binding;carboxylic acid binding;catalytic activity;cation binding;ion binding;ligase activity
binding;nucleic acid binding;nucleotide binding;RNA binding;translation factor activity, nucleic acid binding;translation initiation factor activity
binding;carbohydrate binding;cell adhesion molecule binding;glycosaminoglycan binding;heparin binding;pattern binding;polysaccharide binding;protein binding
acyl-CoA dehydrogenase activity;acyl-CoA oxidase activity;binding;carboxylic acid binding;catalytic activity;coenzyme binding;cofactor binding;FAD binding;fatty acid binding;flavin adenine dinucleotide binding;lipid binding;
1-phosphatidylinositol binding;binding;calmodulin binding;cation binding;GTP-dependent protein binding;identical protein binding;ion binding;lipid binding;metal ion binding;phosphatidylinositol binding;phospholipid binding;
catalytic activity;intramolecular oxidoreductase activity;intramolecular oxidoreductase activity, interconverting keto- and enol-groups;intramolecular oxidoreductase activity, transposing S-S bonds;isomerase activity;protein d
1-alkyl-2-acetylglycerophosphocholine esterase activity;acetyltransferase activity;binding;carboxylic ester hydrolase activity;catalytic activity;hydrolase activity;hydrolase activity, acting on ester bonds;identical protein binding
binding;carbohydrate binding;collagen binding;enzyme activator activity;enzyme regulator activity;glycosaminoglycan binding;heparin binding;pattern binding;peptidase activator activity;peptidase regulator activity;polysacch
C-8 sterol isomerase activity;catalytic activity;cholestenol delta-isomerase activity;drug transmembrane transporter activity;intramolecular oxidoreductase activity;intramolecular oxidoreductase activity, transposing C=C bond
adenyl nucleotide binding;adenyl ribonucleotide binding;ATP binding;binding;catalytic activity;kinase activity;nucleotide binding;phosphomevalonate kinase activity;phosphotransferase activity, phosphate group as acceptor;
ankyrin binding;binding;cytoskeletal protein binding;protein binding;structural constituent of muscle;structural molecule activity
binding;identical protein binding;protein binding
binding;catalytic activity;cation binding;hydrolase activity;hydrolase activity, acting on acid anhydrides;hydrolase activity, acting on acid anhydrides, in phosphorus-containing anhydrides;inorganic diphosphatase activity;ion b
binding;catalytic activity;DNA polymerase activity;intramolecular oxidoreductase activity;isomerase activity;nucleotidyltransferase activity;prostaglandin-E synthase activity;protein binding;RNA-directed DNA polymerase activ
binding;core promoter binding;DNA binding;identical protein binding;nucleic acid binding;nucleotide binding;protein binding;regulatory region DNA binding;regulatory region nucleic acid binding;transcription regulatory region
adenyl nucleotide binding;adenyl ribonucleotide binding;ATP binding;binding;catalytic activity;cis-trans isomerase activity;enzyme activator activity;enzyme binding;enzyme regulator activity;identical protein binding;isomeras
beta-catenin binding;binding;catalytic activity;enzyme binding;gamma-catenin binding;hydrolase activity;hydrolase activity, acting on ester bonds;kinase binding;molecular transducer activity;phosphatase activity;phosphopro
binding;nucleic acid binding;RNA binding;snoRNA binding
binding;enzyme activator activity;enzyme regulator activity;GTPase activator activity;GTPase regulator activity;identical protein binding;nucleoside-triphosphatase regulator activity;protein binding;protein dimerization activity;
binding;catalytic activity;GDP binding;GTP binding;GTPase activity;guanyl nucleotide binding;guanyl ribonucleotide binding;hydrolase activity;hydrolase activity, acting on acid anhydrides;hydrolase activity, acting on acid anh
binding;mRNA 3'-UTR binding;mRNA binding;nucleic acid binding;nucleotide binding;RNA binding
binding;DNA binding;histone binding;methylated histone residue binding;nucleic acid binding;protein binding;regulatory region DNA binding;regulatory region nucleic acid binding;transcription regulatory region DNA binding
binding;calcium ion binding;cation binding;ion binding;metal ion binding
ATPase activity;ATPase activity, coupled;ATPase activity, coupled to movement of substances;binding;catalytic activity;enzyme activator activity;enzyme binding;enzyme regulator activity;GTPase activator activity;GTPase bir
binding;enzyme activator activity;enzyme binding;enzyme regulator activity;GTPase activator activity;GTPase regulator activity;kinase binding;nucleoside-triphosphatase regulator activity;protein binding;protein kinase binding
binding;DNA binding;nucleic acid binding;RNA binding;single-stranded DNA binding;structure-specific DNA binding
binding;DNA binding;enzyme binding;nucleic acid binding;protein binding;RNA binding;ubiquitin protein ligase binding

binding;enzyme binding;protein binding;ubiquitin protein ligase binding
binding;catalytic activity;cation binding;GDP binding;GTP binding;GTPase activity;guanyl nucleotide binding;guanyl ribonucleotide binding;hydrolase activity;hydrolase activity, acting on acid anhydrides;hydrolase activity, act
antigen binding;binding;catalytic activity;coenzyme binding;cofactor binding;delta24(24-1) sterol reductase activity;delta24-sterol reductase activity;enzyme binding;flavin adenine dinucleotide binding;oxidoreductase activity;
binding;nucleic acid binding;RNA binding
binding;DNA binding;mRNA binding;nucleic acid binding;RNA binding
catalytic activity;hydrolase activity;hydrolase activity, acting on ester bonds;phosphatase activity;phosphoprotein phosphatase activity;phosphoric ester hydrolase activity
adenyl nucleotide binding;adenyl ribonucleotide binding;ATP binding;binding;caspase inhibitor activity;caspase regulator activity;catalytic activity;cation binding;cysteine-type endopeptidase inhibitor activity;endopeptidase ir
binding;chromatin binding;core promoter binding;DNA binding;double-stranded DNA binding;nucleic acid binding;nucleotide binding;regulatory region DNA binding;regulatory region nucleic acid binding;structure-specific DN
binding;cation binding;ion binding;metal ion binding;transition metal ion binding;zinc ion binding
binding;nucleic acid binding;nucleotide binding;RNA binding
enzyme regulator activity;phosphatase regulator activity;protein phosphatase regulator activity;protein phosphatase type 1 regulator activity
binding;cation binding;ion binding;metal ion binding;transition metal ion binding;zinc ion binding
binding;cation binding;ion binding;metal ion binding;transition metal ion binding;zinc ion binding
binding;nucleic acid binding;RNA binding
protein binding transcription factor activity;RNA polymerase II transcription cofactor activity;RNA polymerase II transcription factor binding transcription factor activity;transcription cofactor activity;transcription factor binding
binding;collagen binding;extracellular matrix binding;integrin binding;protein binding;protein complex binding;receptor binding
binding;catalytic activity;DNA binding;endonuclease activity;endoribonuclease activity;hydrolase activity;hydrolase activity, acting on ester bonds;mRNA binding;nuclease activity;nucleic acid binding;ribonuclease activity;RN
binding;cation binding;ion binding;metal ion binding;nucleic acid binding;protein binding transcription factor activity;RNA binding;transcription cofactor activity;transcription corepressor activity;transcription factor binding tra
binding;identical protein binding;lipid binding;protein binding
protein binding transcription factor activity;transcription coactivator activity;transcription cofactor activity;transcription factor binding transcription factor activity
adenyl nucleotide binding;adenyl ribonucleotide binding;ATP binding;binding;identical protein binding;nucleotide binding;protein binding;protein binding transcription factor activity;purine nucleotide binding;purine ribonucleo
adenyl nucleotide binding;adenyl ribonucleotide binding;ATP binding;binding;catalytic activity;DNA binding;double-stranded RNA binding;hormone receptor binding;nuclear hormone receptor binding;nucleic acid binding;nuc
binding;cation binding;cytokine receptor binding;enzyme binding;growth factor receptor binding;hormone receptor binding;interleukin-1 receptor binding;ion binding;kinase binding;metal ion binding;nuclear hormone recepto
binding;catalytic activity;hydrolase activity;hydrolase activity, acting on ester bonds;phosphatase activity;phosphoprotein phosphatase activity;phosphoric ester hydrolase activity;protein binding;protein binding transcription f
binding;cytoskeletal protein binding;microtubule binding;microtubule plus-end binding;protein binding;protein C-terminus binding;tubulin binding
AU-rich element binding;binding;double-stranded RNA binding;enzyme binding;kinase binding;mRNA 3'-UTR AU-rich region binding;mRNA 3'-UTR binding;mRNA binding;nucleic acid binding;nucleotide binding;protein bind
3-beta-hydroxy-delta5-steroid dehydrogenase activity;catalytic activity;oxidoreductase activity;oxidoreductase activity, acting on CH-OH group of donors;oxidoreductase activity, acting on the CH-OH group of donors, NAD c
adenyl nucleotide binding;adenyl ribonucleotide binding;ATP binding;binding;calmodulin-dependent protein kinase activity;catalytic activity;cation binding;ion binding;kinase activity;metal ion binding;myosin light chain kinase
active transmembrane transporter activity;amine transmembrane transporter activity;amino acid transmembrane transporter activity;anion transmembrane transporter activity;anion:cation symporter activity;carboxylic acid tra
binding;heat shock protein binding;protein binding
activating transcription factor binding;binding;cation binding;chromatin binding;core promoter proximal region DNA binding;core promoter proximal region sequence-specific DNA binding;co-SMAD binding;cytokine receptor
binding;chaperone binding;protein binding
binding;catalytic activity;chaperone binding;GTPase activity;hydrolase activity;hydrolase activity, acting on acid anhydrides;hydrolase activity, acting on acid anhydrides, in phosphorus-containing anhydrides;nucleoside-triph
acid-amino acid ligase activity;binding;catalytic activity;enzyme binding;ligase activity;ligase activity, forming carbon-nitrogen bonds;protein binding;small conjugating protein ligase activity;ubiquitin protein ligase binding
binding;protein binding;SNARE binding;syntaxin binding;syntaxin-3 binding
binding;protein binding;SNAP receptor activity;SNARE binding
binding;enzyme binding;protein binding;ubiquitin protein ligase binding
binding;catalytic activity;cytoskeletal protein binding;GDP binding;GTP binding;GTPase activity;guanyl nucleotide binding;guanyl ribonucleotide binding;hydrolase activity;hydrolase activity, acting on acid anhydrides;hydrolas
binding;catalytic activity;chromatin binding;chromatin DNA binding;core promoter binding;DNA binding;histone methyltransferase activity;histone methyltransferase activity (H3-K27 specific);histone-lysine N-methyltransferas
binding;cation binding;ion binding;metal ion binding;transition metal ion binding;zinc ion binding
binding;GTP binding;guanyl nucleotide binding;guanyl ribonucleotide binding;identical protein binding;nucleotide binding;protein binding;purine nucleotide binding;purine ribonucleoside triphosphate binding;purine ribonucle
binding;endopeptidase activator activity;endopeptidase regulator activity;enzyme activator activity;enzyme binding;enzyme regulator activity;peptidase activator activity;peptidase regulator activity;protease binding;proteaso
structural constituent of cytoskeleton;structural molecule activity
binding;carbohydrate binding;catalytic activity;identical protein binding;nucleotidyltransferase activity;protein binding;transferase activity;transferase activity, transferring phosphorus-containing groups;UDP-N-acetylglucosam
extracellular matrix structural constituent;structural molecule activity
acetylglucosaminyltransferase activity;binding;catalytic activity;cation binding;glucuronosyl-N-acetylglucosaminyl-proteoglycan 4-alpha-N-acetylglucosaminyltransferase activity;glucuronosyltransferase activity;heparan sulfa
adenyl nucleotide binding;adenyl ribonucleotide binding;androgen receptor binding;ATP binding;binding;catalytic activity;chromatin binding;enzyme binding;GTPase binding;GTP-Rho binding;histone binding;histone deacetyl
adenyl nucleotide binding;adenyl ribonucleotide binding;ATP binding;binding;catalytic activity;enzyme binding;histone deacetylase binding;kinase activity;nucleotide binding;phosphotransferase activity, alcohol group as acce
binding;cation binding;ion binding;metal ion binding;transition metal ion binding;zinc ion binding
binding;damaged DNA binding;DNA binding;nucleic acid binding
adenyl nucleotide binding;adenyl ribonucleotide binding;ATP binding;binding;catalytic activity;kinase activity;MAP kinase activity;MAP kinase kinase activity;molecular transducer activity;NFAT protein binding;nucleotide bindi
binding;chaperone binding;enzyme binding;heat shock protein binding;kinase binding;protein binding;unfolded protein binding
catalytic activity;dihydropyrimidinase activity;hydrolase activity;hydrolase activity, acting on carbon-nitrogen (but not peptide) bonds;hydrolase activity, acting on carbon-nitrogen (but not peptide) bonds, in cyclic amides
binding;nucleic acid binding;RNA binding
binding;enzyme binding;extracellular matrix binding;laminin binding;molecular transducer activity;protease binding;protein binding;protein C-terminus binding;signal transducer activity
binding;cation binding;ion binding;metal ion binding;nucleotide binding;transition metal ion binding;zinc ion binding
binding;mRNA binding;nucleic acid binding;nucleotide binding;RNA binding
actin binding;binding;cytoskeletal protein binding;profilin binding;protein binding
actin binding;actin filament binding;binding;cytoskeletal protein binding;drug binding;protein binding
binding;core promoter binding;core promoter proximal region DNA binding;core promoter proximal region sequence-specific DNA binding;DNA binding;double-stranded DNA binding;identical protein binding;nucleic acid binc
2,4-dienoyl-CoA reductase (NADPH) activity;binding;catalytic activity;coenzyme binding;cofactor binding;NADP binding;NADPH binding;nucleotide binding;oxidoreductase activity;oxidoreductase activity, acting on NADH or
binding;carbohydrate binding;catalytic activity;cation binding;hydrolase activity;hydrolase activity, acting on glycosyl bonds;hydrolase activity, hydrolyzing N-glycosyl compounds;hydrolase activity, hydrolyzing O-glycosyl cor
catalytic activity;NADH dehydrogenase (quinone) activity;NADH dehydrogenase (ubiquinone) activity;NADH dehydrogenase activity;oxidoreductase activity;oxidoreductase activity, acting on NADH or NADPH;oxidoreductase
5S rRNA binding;binding;catalytic activity;nucleic acid binding;RNA binding;rRNA binding;sulfurtransferase activity;thiosulfate sulfurtransferase activity;transferase activity;transferase activity, transferring sulfur-containing gro
acid-amino acid ligase activity;adenyl nucleotide binding;adenyl ribonucleotide binding;ATP binding;binding;catalytic activity;enzyme binding;ligase activity;ligase activity, forming carbon-nitrogen bonds;nucleotide binding;pr
adenyl nucleotide binding;adenyl ribonucleotide binding;ATP binding;binding;catalytic activity;guanylate kinase activity;kinase activity;nucleobase-containing compound kinase activity;nucleotide binding;nucleotide kinase ac
binding;catalytic activity;cation binding;hydrolase activity;hydrolase activity, acting on ester bonds;hydroxyacylglutathione hydrolase activity;ion binding;metal ion binding;thiolester hydrolase activity;transition metal ion bindin
binding;catalytic activity;coenzyme binding;cofactor binding;NADH dehydrogenase (quinone) activity;NADH dehydrogenase (ubiquinone) activity;NADH dehydrogenase activity;oxidoreductase activity;oxidoreductase activity,
binding;carbon-carbon lyase activity;carboxy-lyase activity;catalytic activity;cation binding;GTP binding;guanyl nucleotide binding;guanyl ribonucleotide binding;ion binding;lyase activity;metal ion binding;nucleotide binding;p
catalytic activity;transferase activity;transferase activity, transferring glycosyl groups;transferase activity, transferring pentosyl groups;uridine phosphorylase activity
3-hydroxyacyl-CoA dehydrogenase activity;binding;catalytic activity;coenzyme binding;cofactor binding;NAD binding;NAD+ binding;nucleotide binding;oxidoreductase activity;oxidoreductase activity, acting on CH-OH group
binding;catalytic activity;cation binding;demethylase activity;heme binding;ion binding;iron ion binding;metal ion binding;monooxygenase activity;oxidoreductase activity;oxidoreductase activity, acting on paired donors, with
binding;carbohydrate binding;catalytic activity;cation binding;glucose binding;identical protein binding;ion binding;metal ion binding;monosaccharide binding;nucleotide binding;nucleotidyltransferase activity;protein binding;p
antioxidant activity;binding;catalytic activity;coenzyme binding;cofactor binding;disulfide oxidoreductase activity;electron carrier activity;flavin adenine dinucleotide binding;oxidoreductase activity;oxidoreductase activity, acti
adenyl nucleotide binding;adenyl ribonucleotide binding;AP-2 adaptor complex binding;ATP binding;binding;catalytic activity;kinase activity;Notch binding;nucleotide binding;phosphotransferase activity, alcohol group as acc
adenyl nucleotide binding;adenyl ribonucleotide binding;ATP binding;binding;catalytic activity;DNA binding;helicase activity;hydrolase activity;hydrolase activity, acting on acid anhydrides;hydrolase activity, acting on acid anh
antigen binding;binding;peptide antigen binding;peptide binding;protein binding;receptor binding
binding;DNA binding;double-stranded RNA binding;identical protein binding;nucleic acid binding;protein binding;protein dimerization activity;protein homodimerization activity;RNA binding
binding;carboxylic acid binding;catalytic activity;cation binding;ion binding;iron ion binding;L-ascorbic acid binding;metal ion binding;oxidoreductase activity;oxidoreductase activity, acting on paired donors, with incorporatio
structural molecule activity
catalytic activity;nucleotidyltransferase activity;transferase activity;transferase activity, transferring phosphorus-containing groups;uridylyltransferase activity
adenyl nucleotide binding;adenyl ribonucleotide binding;ATP binding;binding;catalytic activity;cation binding;cyclase activity;FAD-AMP lyase (cyclizing) activity;glycerone kinase activity;ion binding;kinase activity;lyase activity;
aldehyde dehydrogenase (NAD) activity;catalytic activity;formyltetrahydrofolate dehydrogenase activity;hydroxymethyl-, formyl- and related transferase activity;methyltransferase activity;oxidoreductase activity;oxidoreductas
actin binding;binding;cytoskeletal protein binding;enzyme binding;identical protein binding;kinase binding;lipid binding;microtubule binding;phosphatidylinositol binding;phospholipid binding;protein binding;protein dimerizatio
binding;catalytic activity;cytokine receptor binding;growth factor receptor binding;hydrolase activity;hydrolase activity, acting on ester bonds;interleukin-2 receptor binding;nucleic acid binding;phosphatase activity;phospho
binding;catalytic activity;coenzyme binding;cofactor binding;DNA binding;histone binding;methylated histone residue binding;NAD binding;nucleic acid binding;nucleotide binding;oxidoreductase activity;oxidoreductase activ
enzyme activator activity;enzyme regulator activity;GTPase activator activity;GTPase regulator activity;nucleoside-triphosphatase regulator activity
anion channel activity;anion transmembrane transporter activity;calcium activated cation channel activity;cation channel activity;cation transmembrane transporter activity;channel activity;chloride channel activity;gated chann
binding;cation binding;enzyme activator activity;enzyme regulator activity;GTPase activator activity;GTPase regulator activity;ion binding;metal ion binding;nucleoside-triphosphatase regulator activity;PDZ domain binding;pro
binding;nucleic acid binding;RNA binding
binding;nucleotide binding
binding;carbohydrate binding;catalytic activity;estradiol 17-beta-dehydrogenase activity;glycosaminoglycan binding;heparin binding;oxidoreductase activity;oxidoreductase activity, acting on CH-OH group of donors;oxidored
binding;cation binding;ion binding;metal ion binding;transition metal ion binding;zinc ion binding
binding;catalytic activity;cation binding;hydrolase activity;ion binding;metal ion binding
catalytic activity;oxidoreductase activity;oxidoreductase activity, acting on the CH-NH group of donors;oxidoreductase activity, acting on the CH-NH group of donors, NAD or NADP as acceptor;pyrroline-5-carboxylate reduc
binding;catalytic activity;cation binding;ferric-chelate reductase activity;ion binding;metal ion binding;oxidoreductase activity;oxidoreductase activity, oxidizing metal ions;oxidoreductase activity, oxidizing metal ions, NAD or
adenyl nucleotide binding;adenyl ribonucleotide binding;ATP binding;binding;nucleotide binding;purine nucleotide binding;purine ribonucleoside triphosphate binding;purine ribonucleotide binding;ribonucleotide binding
basal RNA polymerase II transcription machinery binding;basal transcription machinery binding;binding;cation binding;DNA binding;enzyme binding;ion binding;metal ion binding;nucleic acid binding;protein binding;RNA poly
acid-amino acid ligase activity;catalytic activity;ligase activity;ligase activity, forming carbon-nitrogen bonds;small conjugating protein ligase activity;ubiquitin-protein ligase activity
binding;enzyme binding;phosphatase binding;protein binding;protein phosphatase binding
binding;catalytic activity;GTP binding;GTPase activity;guanyl nucleotide binding;guanyl ribonucleotide binding;hydrolase activity;hydrolase activity, acting on acid anhydrides;hydrolase activity, acting on acid anhydrides, in ph
adenyl nucleotide binding;adenyl ribonucleotide binding;aminoacyl-tRNA ligase activity;ATP binding;binding;catalytic activity;glutamate-tRNA ligase activity;glutamate-tRNA(Gln) ligase activity;ligase activity;ligase activity, for

binding;catalytic activity;cation binding;endopeptidase activity;enzyme activator activity;enzyme regulator activity;hydrolase activity;ion binding;metal ion binding;metalloendopeptidase activity;metallopeptidase activity;peptic
ATPase activator activity;ATPase binding;ATPase regulator activity;binding;cytoskeletal protein binding;enzyme activator activity;enzyme binding;enzyme regulator activity;nucleoside-triphosphatase regulator activity;protein b
adenyl nucleotide binding;adenyl ribonucleotide binding;alanine-tRNA ligase activity;aminoacyl-tRNA ligase activity;ATP binding;binding;catalytic activity;cation binding;ion binding;ligase activity;ligase activity, forming aminoa
binding;carbohydrate binding;cargo receptor activity;cation binding;galactose binding;ion binding;lipoprotein particle binding;low-density lipoprotein particle binding;metal ion binding;molecular transducer activity;monosacch
binding;lipid binding;phosphatidylinositol binding;phosphatidylinositol-3,5-bisphosphate binding;phosphatidylinositol-3-phosphate binding;phospholipid binding
binding;chromatin binding;DNA binding;nucleic acid binding;nucleosome binding
adenyl nucleotide binding;adenyl ribonucleotide binding;ATP binding;binding;catalytic activity;kinase activity;nucleic acid binding;nucleobase-containing compound kinase activity;nucleotide binding;phosphotransferase activ
binding;lipid binding
binding;cation binding;ion binding;metal ion binding
acid-amino acid ligase activity;binding;catalytic activity;cation binding;ion binding;ligase activity;ligase activity, forming carbon-nitrogen bonds;metal ion binding;small conjugating protein ligase activity;transition metal ion bin
binding;cation binding;ion binding;metal ion binding;nucleotide binding
adenyl nucleotide binding;adenyl ribonucleotide binding;ATP binding;binding;nucleotide binding;purine nucleotide binding;purine ribonucleoside triphosphate binding;purine ribonucleotide binding;ribonucleotide binding
molecular transducer activity;signal transducer activity
binding;DNA binding;nucleic acid binding
5'-nucleotidase activity;binding;catalytic activity;cation binding;hydrolase activity;hydrolase activity, acting on ester bonds;ion binding;metal ion binding;nucleotidase activity;phosphatase activity;phosphoric ester hydrolase a
acid-amino acid ligase activity;binding;catalytic activity;cation binding;chromatin binding;enzyme binding;histone binding;ion binding;ligase activity;ligase activity, forming carbon-nitrogen bonds;metal ion binding;mRNA 3'-U
binding;catalytic activity;cation binding;ion binding;ligase activity;metal ion binding;transition metal ion binding;zinc ion binding
binding;protein binding;receptor binding
acid-amino acid ligase activity;ATPase activity;binding;catalytic activity;cation binding;hydrolase activity;hydrolase activity, acting on acid anhydrides;hydrolase activity, acting on acid anhydrides, in phosphorus-containing an
binding;catalytic activity;cation binding;enzyme binding;hydrolase activity;hydrolase activity, acting on ester bonds;ion binding;kinase binding;metal ion binding;phosphatase activity;phosphoprotein phosphatase activity;phos
binding;lipid binding
binding;calcium ion binding;cation binding;enzyme activator activity;enzyme regulator activity;GTPase activator activity;GTPase regulator activity;ion binding;metal ion binding;nucleoside-triphosphatase regulator activity
actin binding;actin filament binding;beta-tubulin binding;binding;cytoskeletal protein binding;DNA binding;microtubule binding;nucleic acid binding;protein binding;tubulin binding
binding;nucleic acid binding;RNA binding;rRNA binding;snoRNA binding;U3 snoRNA binding
enzyme activator activity;enzyme regulator activity;GTPase activator activity;GTPase regulator activity;nucleoside-triphosphatase regulator activity
catalytic activity
catalytic activity;cytochrome-b5 reductase activity;oxidoreductase activity;oxidoreductase activity, acting on NADH or NADPH;oxidoreductase activity, acting on NADH or NADPH, heme protein as acceptor
binding;cation binding;DNA binding;ion binding;metal ion binding;nucleic acid binding
binding;catalytic activity;GTP binding;GTPase activity;guanyl nucleotide binding;guanyl ribonucleotide binding;hydrolase activity;hydrolase activity, acting on acid anhydrides;hydrolase activity, acting on acid anhydrides, in pl
antioxidant activity;catalytic activity;oxidoreductase activity;oxidoreductase activity, acting on a sulfur group of donors;oxidoreductase activity, acting on a sulfur group of donors, NAD or NADP as acceptor;oxidoreductase ac
binding;cytokine binding;growth factor binding;protein binding;transforming growth factor beta binding
2 iron, 2 sulfur cluster binding;binding;cation binding;electron carrier activity;ion binding;iron-sulfur cluster binding;metal cluster binding;metal ion binding
binding;cation binding;DNA binding;ion binding;metal ion binding;nucleic acid binding;polyubiquitin binding;protein binding;small conjugating protein binding;transition metal ion binding;ubiquitin binding;zinc ion binding
binding;nucleic acid binding;RNA binding
catalytic activity;oxidoreductase activity
actin binding;actin monomer binding;adenyl nucleotide binding;adenyl ribonucleotide binding;ATP binding;binding;cytoskeletal protein binding;enzyme binding;kinase binding;lipid binding;nucleotide binding;phosphatidylinos
3'-5' exonuclease activity;3'-5'-exoribonuclease activity;binding;catalytic activity;cation binding;exonuclease activity;exonuclease activity, active with either ribo- or deoxyribonucleic acids and producing 5'-phosphomonoeste
adenine nucleotide transmembrane transporter activity;ATP transmembrane transporter activity;binding;calcium ion binding;cation binding;ion binding;metal ion binding;nucleobase-containing compound transmembrane tran
all-trans-retinol 13,14-reductase activity;catalytic activity;oxidoreductase activity;oxidoreductase activity, acting on the CH-CH group of donors
3-hydroxyisobutyryl-CoA hydrolase activity;catalytic activity;CoA hydrolase activity;hydrolase activity;hydrolase activity, acting on ester bonds;thiolester hydrolase activity
binding;nucleic acid binding;RNA binding;rRNA binding;rRNA primary transcript binding
binding;cation binding;ion binding;metal ion binding;transition metal ion binding;zinc ion binding
basal RNA polymerase II transcription machinery binding;basal transcription machinery binding;binding;enzyme binding;protein binding;RNA polymerase binding;RNA polymerase core enzyme binding;RNA polymerase II core
binding;core promoter proximal region DNA binding;core promoter proximal region sequence-specific DNA binding;DNA binding;molecular transducer activity;nucleic acid binding;nucleic acid binding transcription factor activ
binding;catalytic activity;K63-linked polyubiquitin binding;nucleic acid binding;polyubiquitin binding;protein binding;RNA binding;second spliceosomal transesterification activity;small conjugating protein binding;snRNA bindi
structural constituent of ribosome;structural molecule activity
binding;catalytic activity;cation binding;demethylase activity;ion binding;metal ion binding;oxidative RNA demethylase activity;oxidoreductase activity;oxidoreductase activity, acting on paired donors, with incorporation or red
binding;carbon-carbon lyase activity;carboxy-lyase activity;catalytic activity;cofactor binding;lyase activity;pyridoxal phosphate binding;vitamin B6 binding;vitamin binding
basal RNA polymerase II transcription machinery binding;basal transcription machinery binding;binding;enzyme binding;protein binding;RNA polymerase binding;RNA polymerase core enzyme binding;RNA polymerase II core
acid phosphatase activity;adenyl nucleotide binding;adenyl ribonucleotide binding;ATP binding;binding;catalytic activity;diphosphoinositol-pentakisphosphate kinase activity;hydrolase activity;hydrolase activity, acting on este
adenyl nucleotide binding;adenyl ribonucleotide binding;aminoacyl-tRNA ligase activity;aspartate-tRNA ligase activity;aspartate-tRNA(Asn) ligase activity;ATP binding;binding;catalytic activity;identical protein binding;ligase ac
carboxylic ester hydrolase activity;catalytic activity;hydrolase activity;hydrolase activity, acting on ester bonds
binding;cation binding;ion binding;metal ion binding;mRNA binding;nucleic acid binding;poly(A) RNA binding;poly-purine tract binding;RNA binding;single-stranded RNA binding
binding;eukaryotic initiation factor 4E binding;mRNA 3'-UTR binding;mRNA 5'-UTR binding;mRNA binding;nucleic acid binding;protein binding;RNA binding;RNA cap binding;translation activator activity;translation initiation f
binding;enzyme binding;kinase binding;protein binding;protein kinase binding;ribonucleoprotein binding;ribosome binding
adenyl nucleotide binding;adenyl ribonucleotide binding;ATP binding;binding;catalytic activity;formate-tetrahydrofolate ligase activity;identical protein binding;ligase activity;ligase activity, forming carbon-nitrogen bonds;nucle
binding;enzyme binding;kinase binding;molecular transducer activity;protein binding;protein kinase binding;signal transducer activity
binding;catalytic activity;cation binding;copper ion binding;ion binding;metal ion binding;monooxygenase activity;oxidoreductase activity;oxidoreductase activity, acting on paired donors, with incorporation or reduction of mo
catalytic activity;cis-trans isomerase activity;isomerase activity;peptidyl-prolyl cis-trans isomerase activity
binding;calcium ion binding;cation binding;ion binding;metal ion binding
binding;calcium ion binding;cation binding;collagen binding;enzyme binding;ion binding;metal ion binding;protease binding;protein binding
binding;chromatin binding
binding;catalytic activity;endopeptidase activity;enzyme binding;hydrolase activity;metalloendopeptidase activity;metallopeptidase activity;peptidase activity;peptidase activity, acting on L-amino acid peptides;protease bindi
catalytic activity;ligase activity;nicotinate phosphoribosyltransferase activity;nicotinate-nucleotide diphosphorylase (carboxylating) activity;transferase activity;transferase activity, transferring glycosyl groups;transferase activit
enzyme regulator activity;GTPase regulator activity;guanyl-nucleotide exchange factor activity;nucleoside-triphosphatase regulator activity;Ras guanyl-nucleotide exchange factor activity;Rho guanyl-nucleotide exchange fac
binding;cytokine binding;endopeptidase inhibitor activity;endopeptidase regulator activity;enzyme inhibitor activity;enzyme regulator activity;growth factor binding;peptidase inhibitor activity;peptidase regulator activity;protei
catalytic activity;oxidoreductase activity
binding;carbon-sulfur lyase activity;catalytic activity;cofactor binding;cysteine-S-conjugate beta-lyase activity;kynurenine-glyoxylate transaminase activity;kynurenine-oxoglutarate transaminase activity;lyase activity;pyridoxal
binding;cation binding;DNA binding;ion binding;metal ion binding;nucleic acid binding;nucleic acid binding transcription factor activity;regulatory region DNA binding;regulatory region nucleic acid binding;RNA polymerase II c
catalytic activity;flavin-linked sulfhydryl oxidase activity;intramolecular oxidoreductase activity;intramolecular oxidoreductase activity, interconverting keto- and enol-groups;intramolecular oxidoreductase activity, transposing
binding;DNA binding;histone binding;nucleic acid binding;protein binding
binding;calcium ion binding;catalytic activity;cation binding;endopeptidase activity;hydrolase activity;integrin binding;ion binding;metal ion binding;metalloendopeptidase activity;metallopeptidase activity;peptidase activity;pe
binding;DNA binding;histone binding;nucleic acid binding;nucleic acid binding transcription factor activity;protein binding;sequence-specific DNA binding transcription factor activity
catalytic activity;hydrolase activity;hydrolase activity, acting on ester bonds;nuclease activity;protein binding transcription factor activity;transcription cofactor activity;transcription factor binding transcription factor activity
adenyl nucleotide binding;adenyl ribonucleotide binding;ATP binding;binding;catalytic activity;cation binding;enzyme activator activity;enzyme regulator activity;ion binding;kinase activator activity;kinase activity;kinase regula
adenyl nucleotide binding;adenyl ribonucleotide binding;ATP binding;ATPase activity;ATPase activity, coupled;ATP-dependent helicase activity;ATP-dependent RNA helicase activity;binding;catalytic activity;helicase activity;h
catalytic activity;methyltransferase activity;transferase activity;transferase activity, transferring one-carbon groups

adenyl nucleotide binding;adenyl ribonucleotide binding;ATP binding;ATPase activity;ATPase activity, coupled;ATP-dependent helicase activity;ATP-dependent RNA helicase activity;binding;catalytic activity;chromatin binding
binding;nucleic acid binding;RNA binding;translation factor activity, nucleic acid binding;translation initiation factor activity
catalytic activity;methyltransferase activity;RNA methyltransferase activity;S-adenosylmethionine-dependent methyltransferase activity;transferase activity;transferase activity, transferring one-carbon groups
binding;catalytic activity;GTP binding;GTPase activity;guanyl nucleotide binding;guanyl ribonucleotide binding;hydrolase activity;hydrolase activity, acting on acid anhydrides;hydrolase activity, acting on acid anhydrides, in pl
actin binding;actin filament binding;binding;cytoskeletal protein binding;enzyme binding;GTPase binding;protein binding;protein complex binding;Rac GTPase binding;Ras GTPase binding;Rho GTPase binding;small GTPase
1-acylglycerol-3-phosphate O-acyltransferase activity;1-acylglycerophosphocholine O-acyltransferase activity;1-alkylglycerophosphocholine O-acetyltransferase activity;acetyltransferase activity;acylglycerol O-acyltransferas
actin binding;binding;cytoskeletal protein binding;protein binding
binding;catalytic activity;cation binding;dioxygenase activity;ion binding;metal ion binding;oxidoreductase activity
binding;protein binding;protein domain specific binding
binding;DNA binding;nucleic acid binding;nucleic acid binding transcription factor activity;sequence-specific DNA binding transcription factor activity
binding;catalytic activity;cation binding;ion binding;metal ion binding;NAD+ ADP-ribosyltransferase activity;transferase activity;transferase activity, transferring glycosyl groups;transferase activity, transferring pentosyl groups
binding;catalytic activity;GTP binding;GTPase activity;guanyl nucleotide binding;guanyl ribonucleotide binding;hydrolase activity;hydrolase activity, acting on acid anhydrides;hydrolase activity, acting on acid anhydrides, in pl
nucleocytoplasmic transporter activity;transporter activity
binding;cation binding;DNA binding;ion binding;metal ion binding;nucleic acid binding
ARF guanyl-nucleotide exchange factor activity;enzyme regulator activity;GTPase regulator activity;guanyl-nucleotide exchange factor activity;nucleoside-triphosphatase regulator activity;small GTPase regulator activity
structural molecule activity
binding;nucleic acid binding;RNA binding
binding;CARD domain binding;enzyme binding;kinase binding;molecular transducer activity;protein binding;protein domain specific binding;protein kinase binding;signal transducer activity
binding;cytoskeletal protein binding;kinetochore binding;microtubule binding;microtubule plus-end binding;protein binding;tubulin binding
binding;cation binding;ion binding;metal ion binding;transition metal ion binding;zinc ion binding
binding;chromatin binding;DNA binding;nucleic acid binding
catalytic activity;L-xylulose reductase (NADP+) activity;oxidoreductase activity;oxidoreductase activity, acting on CH-OH group of donors;oxidoreductase activity, acting on NADH or NADPH;oxidoreductase activity, acting on
G-protein coupled receptor activity;molecular transducer activity;pheromone receptor activity;receptor activity;signal transducer activity;signaling receptor activity;transmembrane signaling receptor activity
acid-amino acid ligase activity;binding;catalytic activity;DNA binding;ligase activity;ligase activity, forming carbon-nitrogen bonds;nucleic acid binding;small conjugating protein ligase activity;ubiquitin-protein ligase activity
molecular transducer activity;signal transducer activity
binding;cation binding;ion binding;metal ion binding
adenyl nucleotide binding;adenyl ribonucleotide binding;ATP binding;binding;nucleic acid binding;nucleotide binding;purine nucleotide binding;purine ribonucleoside triphosphate binding;purine ribonucleotide binding;ribonuc
2 iron, 2 sulfur cluster binding;binding;catalytic activity;cation binding;disulfide oxidoreductase activity;electron carrier activity;ion binding;iron-sulfur cluster binding;metal cluster binding;metal ion binding;oxidoreductase acti
catalytic activity;hydrolase activity
binding;nucleic acid binding;poly(G) RNA binding;poly(U) RNA binding;poly-purine tract binding;poly-pyrimidine tract binding;RNA binding;single-stranded RNA binding
aminopeptidase activity;binding;catalytic activity;exopeptidase activity;hydrolase activity;identical protein binding;peptidase activity;peptidase activity, acting on L-amino acid peptides;protein binding;serine hydrolase activity
binding;nucleic acid binding;nucleotide binding;RNA binding
binding;chromatin binding;DNA binding;nucleic acid binding
binding;double-stranded RNA binding;NF-kappaB binding;nucleic acid binding;protein binding;protein binding transcription factor activity;RNA binding;RNA polymerase II transcription factor binding;transcription coactivator
catalytic activity;cysteine-type endopeptidase activity;cysteine-type peptidase activity;endopeptidase activity;hydrolase activity;peptidase activity;peptidase activity, acting on L-amino acid peptides;small conjugating protein-
binding;integrin binding;protein binding;protein complex binding;receptor binding
acetoacetate-CoA ligase activity;acid-thiol ligase activity;adenyl nucleotide binding;adenyl ribonucleotide binding;ATP binding;binding;butyrate-CoA ligase activity;catalytic activity;ligase activity;ligase activity, forming carbon
binding;nucleotide binding
binding;calmodulin binding;cytoskeletal protein binding;myosin binding;myosin light chain binding;myosin VI binding;myosin VI light chain binding;protein binding
binding;cation binding;ion binding;metal ion binding
binding;cytoskeletal protein binding;identical protein binding;microtubule binding;protein binding;tubulin binding
binding;cyclic nucleotide binding;cyclic-di-GMP binding;enzyme binding;guanyl nucleotide binding;guanyl ribonucleotide binding;identical protein binding;kinase binding;nucleotide binding;protein binding;protein dimerizatio
arginine N-methyltransferase activity;beta-catenin binding;binding;catalytic activity;DNA binding;histone acetyl-lysine binding;histone binding;histone methyltransferase activity;histone methyltransferase activity (H3-R17 spec
catalytic activity;hydrolase activity;hydrolase activity, acting on carbon-nitrogen (but not peptide) bonds;hydrolase activity, acting on carbon-nitrogen (but not peptide) bonds, in nitriles;nitrilase activity
binding;histone binding;methylated histone residue binding;protein binding
binding;enzyme binding;enzyme regulator activity;phosphatase binding;phosphatase regulator activity;protein binding;protein phosphatase 2A binding;protein phosphatase binding;protein phosphatase regulator activity;prote
adenyl nucleotide binding;adenyl ribonucleotide binding;ATP binding;ATPase activity;ATPase activity, coupled;ATP-dependent helicase activity;ATP-dependent RNA helicase activity;binding;catalytic activity;helicase activity;h
binding;dynein binding;dynein intermediate chain binding;protein binding
binding;protein binding;SNAP receptor activity;SNARE binding
enzyme regulator activity;GDP-dissociation inhibitor activity;GTPase regulator activity;nucleoside-triphosphatase regulator activity;small GTPase regulator activity
binding;catalytic activity;GTP binding;GTPase activity;guanyl nucleotide binding;guanyl ribonucleotide binding;hydrolase activity;hydrolase activity, acting on acid anhydrides;hydrolase activity, acting on acid anhydrides, in pl
binding;calcium ion binding;cation binding;ion binding;metal ion binding
binding;catalytic activity;cation binding;ion binding;ligase activity;metal ion binding
binding;enzyme binding;GTPase binding;leucine zipper domain binding;LRR domain binding;PDZ domain binding;protein binding;protein domain specific binding;Rab GTPase binding;Ras GTPase binding;small GTPase bind
catalytic activity;hydrolase activity;hydrolase activity, acting on ester bonds;lipase activity;NAPE-specific phospholipase D activity;phospholipase activity;phospholipase D activity;phosphoric diester hydrolase activity;phosph
3'-5' exonuclease activity;3'-5'-exoribonuclease activity;binding;catalytic activity;cation binding;exonuclease activity;exonuclease activity, active with either ribo- or deoxyribonucleic acids and producing 5'-phosphomonoeste
binding;carboxylic acid binding;catalytic activity;cation binding;ion binding;iron ion binding;L-ascorbic acid binding;metal ion binding;oxidoreductase activity;oxidoreductase activity, acting on paired donors, with incorporatio
binding;enzyme binding;protein binding;ubiquitin protein ligase binding
active transmembrane transporter activity;amine transmembrane transporter activity;choline transmembrane transporter activity;molecular transducer activity;signal transducer activity;substrate-specific transmembrane trans
binding;cytoskeletal protein binding;microtubule binding;protein binding;structural molecule activity;tubulin binding
binding;cation binding;ion binding;metal ion binding
binding;cation binding;cytoskeletal protein binding;DNA binding;enzyme binding;histone binding;histone deacetylase binding;ion binding;metal ion binding;nucleic acid binding;phosphoprotein binding;protein binding;ribonuc
enzyme activator activity;enzyme regulator activity;GTPase activator activity;GTPase regulator activity;nucleoside-triphosphatase regulator activity
binding;ion channel binding;protein binding
ligand-dependent nuclear receptor transcription coactivator activity;protein binding transcription factor activity;transcription coactivator activity;transcription cofactor activity;transcription factor binding transcription factor act
ATPase activator activity;ATPase binding;ATPase regulator activity;binding;catalytic activity;chaperone binding;disulfide oxidoreductase activity;enzyme activator activity;enzyme binding;enzyme regulator activity;heat shock p
binding;calcium ion binding;catalytic activity;cation binding;GTP binding;GTPase activity;guanyl nucleotide binding;guanyl ribonucleotide binding;hydrolase activity;hydrolase activity, acting on acid anhydrides;hydrolase activ
binding;nucleotide binding
carboxylic ester hydrolase activity;catalytic activity;hydrolase activity;hydrolase activity, acting on ester bonds;lipase activity;lysophospholipase activity;phospholipase activity
binding;nucleotide binding
catalytic activity;methyltransferase activity;RNA methyltransferase activity;rRNA methyltransferase activity;S-adenosylmethionine-dependent methyltransferase activity;transferase activity;transferase activity, transferring one-
binding;DNA binding;nucleic acid binding
binding;enzyme binding;GTPase binding;protein binding;Ral GTPase binding;Ras GTPase binding;small GTPase binding
binding;calcium ion binding;cation binding;ion binding;metal ion binding;protein binding;protein dimerization activity;protein heterodimerization activity
catalytic activity;intramolecular transferase activity;isomerase activity;pseudouridine synthase activity
aldehyde dehydrogenase (NAD) activity;catalytic activity;oxidoreductase activity;oxidoreductase activity, acting on the aldehyde or oxo group of donors;oxidoreductase activity, acting on the aldehyde or oxo group of donors,
binding;chromatin binding;protein binding;transcription factor binding
binding;cytoskeletal protein binding;enzyme activator activity;enzyme regulator activity;kinase activator activity;kinase regulator activity;protein binding;protein complex binding;protein kinase activator activity;protein kinase r
binding;enzyme binding;protein binding;ubiquitin protein ligase binding
14-3-3 protein binding;5S rDNA binding;binding;DNA binding;enzyme binding;kinase binding;nucleic acid binding;protein binding;protein complex binding;protein kinase binding;rDNA binding;regulatory region DNA binding;r
basal RNA polymerase II transcription machinery binding;basal transcription machinery binding;binding;enzyme binding;enzyme inhibitor activity;enzyme regulator activity;protein binding;RNA polymerase binding;RNA polym
catalytic activity;NADH dehydrogenase (quinone) activity;NADH dehydrogenase (ubiquinone) activity;NADH dehydrogenase activity;oxidoreductase activity;oxidoreductase activity, acting on NADH or NADPH;oxidoreductase
structural constituent of nuclear pore;structural molecule activity
binding;catalytic activity;methyltransferase activity;mRNA (nucleoside-2'-O-)-methyltransferase activity;mRNA methyltransferase activity;nucleic acid binding;O-methyltransferase activity;RNA methyltransferase activity;S-ade
acylglycerol lipase activity;carboxylic ester hydrolase activity;catalytic activity;hydrolase activity;hydrolase activity, acting on ester bonds;lipase activity
binding;calcium ion binding;carbohydrate binding;catalytic activity;cation binding;cytokine binding;glycosaminoglycan binding;growth factor binding;integrin binding;ion binding;kinase activity;metal ion binding;molecular tran
enzyme activator activity;enzyme regulator activity;GTPase activator activity;GTPase regulator activity;nucleoside-triphosphatase regulator activity
3'-5' DNA helicase activity;adenyl nucleotide binding;adenyl ribonucleotide binding;ATP binding;ATPase activity;ATPase activity, coupled;ATP-dependent 3'-5' DNA helicase activity;ATP-dependent DNA helicase activity;ATP-
binding;cation binding;enzyme binding;GTPase binding;identical protein binding;ion binding;lipid binding;metal ion binding;phosphatidic acid binding;phospholipid binding;protein binding;Rab GTPase binding;Ras GTPase bi
binding;chromatin binding
binding;carbohydrate binding;catalytic activity;cysteine-type endopeptidase activity;cysteine-type peptidase activity;drug binding;endopeptidase activity;frizzled binding;glycosaminoglycan binding;G-protein-coupled recepto
adenyl nucleotide binding;adenyl ribonucleotide binding;ATP binding;binding;catalytic activity;kinase activity;nucleotide binding;phosphotransferase activity, alcohol group as acceptor;protein kinase activity;protein serine/thre
cation transmembrane transporter activity;divalent inorganic cation transmembrane transporter activity;inorganic cation transmembrane transporter activity;ion transmembrane transporter activity;magnesium ion transmembra
binding;carboxylic acid binding;catalytic activity;cation binding;ion binding;iron ion binding;L-ascorbic acid binding;metal ion binding;oxidoreductase activity;oxidoreductase activity, acting on paired donors, with incorporatio
2 iron, 2 sulfur cluster binding;binding;cation binding;identical protein binding;ion binding;iron-sulfur cluster binding;metal cluster binding;metal ion binding;protein binding;protein dimerization activity;protein homodimerizatio

binding;nucleotide binding
binding;catalytic activity;cation binding;hydrolase activity;ion binding;metal ion binding;metallopeptidase activity;peptidase activity;peptidase activity, acting on L-amino acid peptides;transition metal ion binding;zinc ion bind
binding;cation binding;enzyme activator activity;enzyme regulator activity;GTPase activator activity;GTPase regulator activity;ion binding;metal ion binding;nucleoside-triphosphatase regulator activity;transition metal ion bind
catalytic activity;cysteine-type peptidase activity;hydrolase activity;peptidase activity;peptidase activity, acting on L-amino acid peptides
catalytic activity;methyltransferase activity;transferase activity;transferase activity, transferring one-carbon groups
binding;cation binding;enzyme activator activity;enzyme regulator activity;GTPase activator activity;GTPase regulator activity;ion binding;metal ion binding;nucleoside-triphosphatase regulator activity;transition metal ion bind
basal RNA polymerase II transcription machinery binding;basal transcription machinery binding;binding;chromatin binding;enzyme binding;protein binding;RNA polymerase binding;RNA polymerase core enzyme binding;RNA
binding;protein binding;protein domain specific binding;WW domain binding
adenyl nucleotide binding;adenyl ribonucleotide binding;ATP binding;binding;nucleotide binding;purine nucleotide binding;purine ribonucleoside triphosphate binding;purine ribonucleotide binding;ribonucleotide binding
catalytic activity;galactosyltransferase activity;procollagen galactosyltransferase activity;transferase activity;transferase activity, transferring glycosyl groups;transferase activity, transferring hexosyl groups;UDP-galactosyltran
binding;cation binding;ion binding;metal ion binding
catalytic activity;glucosyltransferase activity;protein xylosyltransferase activity;transferase activity;transferase activity, transferring glycosyl groups;transferase activity, transferring hexosyl groups;transferase activity, transferrin
catalytic activity;estradiol 17-beta-dehydrogenase activity;oxidoreductase activity;oxidoreductase activity, acting on CH-OH group of donors;oxidoreductase activity, acting on the CH-OH group of donors, NAD or NADP as a
catalytic activity;intramolecular oxidoreductase activity;intramolecular oxidoreductase activity, interconverting keto- and enol-groups;intramolecular oxidoreductase activity, transposing S-S bonds;isomerase activity;protein d
adenyl nucleotide binding;adenyl ribonucleotide binding;ATP binding;ATPase activity;ATPase activity, coupled;binding;catalytic activity;hydrolase activity;hydrolase activity, acting on acid anhydrides;hydrolase activity, acting
catalytic activity;oxidoreductase activity
binding;mRNA 3'-UTR binding;mRNA binding;nucleic acid binding;RNA binding
anion binding;binding;catalytic activity;ion binding;N-acetylgalactosamine 4-O-sulfotransferase activity;phosphate ion binding;sulfotransferase activity;transferase activity;transferase activity, transferring sulfur-containing grou
catalytic activity;oxidoreductase activity
binding;cation binding;ion binding;metal ion binding;nucleotide binding
adenyl nucleotide binding;adenyl ribonucleotide binding;ATP binding;ATPase activity;binding;catalytic activity;hydrolase activity;hydrolase activity, acting on acid anhydrides;hydrolase activity, acting on acid anhydrides, in ph
active transmembrane transporter activity;calcium channel activity;cation channel activity;cation transmembrane transporter activity;channel activity;ion channel activity;ion transmembrane transporter activity;passive transme
cation transmembrane transporter activity;ion transmembrane transporter activity;substrate-specific transmembrane transporter activity;substrate-specific transporter activity;transmembrane transporter activity;transporter ac
1-acylglycerophosphocholine O-acyltransferase activity;1-alkenylglycerophosphocholine O-acyltransferase activity;1-alkylglycerophosphocholine O-acetyltransferase activity;1-alkylglycerophosphocholine O-acyltransferase a
binding;DNA binding;nucleic acid binding
G-protein coupled receptor activity;molecular transducer activity;receptor activity;signal transducer activity;signaling receptor activity;transmembrane signaling receptor activity
ATPase activator activity;ATPase binding;ATPase regulator activity;binding;enzyme activator activity;enzyme binding;enzyme regulator activity;nucleoside-triphosphatase regulator activity;protein binding
catalytic activity;hydrolase activity
catalytic activity;cytidylyltransferase activity;N-acylneuraminate cytidylyltransferase activity;nucleotidyltransferase activity;transferase activity;transferase activity, transferring phosphorus-containing groups
binding;enzyme binding;GTPase binding;GTP-Rho binding;lipid binding;phosphatidylinositol binding;phosphatidylinositol-4,5-bisphosphate binding;phospholipid binding;protein binding;Ras GTPase binding;Rho GTPase bin
catalytic activity;hydrolase activity
adenyl nucleotide binding;adenyl ribonucleotide binding;ATP binding;ATPase activity;ATPase activity, coupled;ATP-dependent helicase activity;ATP-dependent RNA helicase activity;binding;catalytic activity;helicase activity;h
binding;nucleic acid binding;RNA binding
binding;chromatin binding;DNA binding;nucleic acid binding;protein binding transcription factor activity;transcription coactivator activity;transcription cofactor activity;transcription factor binding transcription factor activity
binding;cation binding;enzyme binding;ion binding;metal ion binding;protein binding;small conjugating protein binding;transition metal ion binding;ubiquitin binding;ubiquitin protein ligase binding;zinc ion binding
acid-amino acid ligase activity;binding;catalytic activity;cation binding;ion binding;ligase activity;ligase activity, forming carbon-nitrogen bonds;metal ion binding;small conjugating protein ligase activity;transition metal ion bin
3'-phosphoadenosine 5'-phosphosulfate transmembrane transporter activity;molecular transducer activity;nucleobase-containing compound transmembrane transporter activity;nucleoside transmembrane transporter activity
binding;enzyme binding;GTPase binding;identical protein binding;protein binding;protein dimerization activity;protein homodimerization activity;Rab GTPase binding;Ras GTPase binding;small GTPase binding
acid-amino acid ligase activity;adenyl nucleotide binding;adenyl ribonucleotide binding;ATP binding;binding;catalytic activity;ligase activity;ligase activity, forming carbon-nitrogen bonds;NEDD8 activating enzyme activity;nuc
enzyme activator activity;enzyme regulator activity;GTPase activator activity;GTPase regulator activity;nucleoside-triphosphatase regulator activity
alcohol dehydrogenase (NADP+) activity;aldo-keto reductase (NADP) activity;catalytic activity;NADP-retinol dehydrogenase activity;oxidoreductase activity;oxidoreductase activity, acting on CH-OH group of donors;oxidoredu
catalytic activity;dolichyl-diphosphooligosaccharide-protein glycotransferase activity;oligosaccharyl transferase activity;transferase activity;transferase activity, transferring glycosyl groups;transferase activity, transferring hex
3'-5' exonuclease activity;3'-5'-exoribonuclease activity;binding;catalytic activity;exonuclease activity;exonuclease activity, active with either ribo- or deoxyribonucleic acids and producing 5'-phosphomonoesters;exoribonucl
adenyl nucleotide binding;adenyl ribonucleotide binding;ATP binding;binding;catalytic activity;cation binding;enzyme binding;ion binding;kinase activity;kinase binding;metal ion binding;nucleotide binding;phosphotransferase
acid-amino acid ligase activity;binding;catalytic activity;cation binding;histone binding;ion binding;ligase activity;ligase activity, forming carbon-nitrogen bonds;metal ion binding;protein binding;small conjugating protein ligase
adenyl nucleotide binding;adenyl ribonucleotide binding;ATP binding;ATPase activity;ATPase activity, coupled;ATP-dependent helicase activity;ATP-dependent RNA helicase activity;binding;catalytic activity;estrogen receptor
adenyl nucleotide binding;adenyl ribonucleotide binding;ATP binding;binding;catalytic activity;cation binding;ion binding;kinase activity;metal ion binding;nucleotide binding;phosphotransferase activity, alcohol group as acce
actin binding;binding;catalytic activity;cation binding;coenzyme binding;cofactor binding;cytoskeletal protein binding;FAD binding;flavin adenine dinucleotide binding;ion binding;metal ion binding;monooxygenase activity;oxi
antioxidant activity;catalytic activity;glutathione peroxidase activity;oxidoreductase activity;oxidoreductase activity, acting on peroxide as acceptor;peroxidase activity
binding;nucleic acid binding;RNA binding;snRNA binding
enzyme regulator activity;phosphatase regulator activity;protein phosphatase regulator activity;protein phosphatase type 4 regulator activity
binding;catalytic activity;histone methyltransferase activity;histone-lysine N-methyltransferase activity;lysine N-methyltransferase activity;methyltransferase activity;N-methyltransferase activity;p53 binding;protein binding;pro
binding;chromatin binding
1-phosphatidylinositol binding;apolipoprotein A-I binding;apolipoprotein binding;binding;cargo receptor activity;high-density lipoprotein particle binding;high-density lipoprotein particle receptor activity;lipid binding;lipopolysa
binding;cation binding;ion binding;metal ion binding
binding;catalytic activity;cis-trans isomerase activity;isomerase activity;nucleotide binding;peptidyl-prolyl cis-trans isomerase activity
binding;identical protein binding;protein binding;protein binding transcription factor activity;transcription cofactor activity;transcription corepressor activity;transcription factor binding;transcription factor binding transcription
14-3-3 protein binding;activating transcription factor binding;binding;catalytic activity;cation binding;chromatin binding;deacetylase activity;enzyme binding;histone deacetylase activity;histone deacetylase activity (H3-K14 sp
binding;carbohydrate binding;catalytic activity;clathrin binding;clathrin heavy chain binding;ER retention sequence binding;glycosaminoglycan binding;hexosaminidase activity;hyaluronic acid binding;hyaluronoglucosaminid
nucleocytoplasmic transporter activity;transporter activity
binding;calcium-dependent protein binding;G-protein-coupled receptor binding;identical protein binding;protein binding;protein dimerization activity;protein homodimerization activity;proteinase activated receptor binding;rec
binding;cation binding;cytoskeletal protein binding;filamin binding;ion binding;metal ion binding;protein binding;transition metal ion binding;zinc ion binding
binding;protein binding;protein complex binding
binding;identical protein binding;lipid binding;phosphatidylinositol binding;phospholipid binding;protein binding;protein dimerization activity;protein domain specific binding;protein homodimerization activity
binding;protein binding;SNARE binding;syntaxin binding
catalytic activity;hydrolase activity;hydrolase activity, acting on ester bonds;phosphatase activity;phosphoprotein phosphatase activity;phosphoric ester hydrolase activity
acid-amino acid ligase activity;binding;catalytic activity;cation binding;ion binding;ligase activity;ligase activity, forming carbon-nitrogen bonds;metal ion binding;small conjugating protein ligase activity;transition metal ion bin
active transmembrane transporter activity;amine transmembrane transporter activity;choline transmembrane transporter activity;substrate-specific transmembrane transporter activity;substrate-specific transporter activity;tra
binding;ribonucleoprotein binding;snRNP binding
actinin binding;alpha-actinin binding;binding;cytoskeletal protein binding;muscle alpha-actinin binding;protein binding
binding;core promoter binding;DNA binding;nucleic acid binding;nucleotide binding;regulatory region DNA binding;regulatory region nucleic acid binding;transcription regulatory region DNA binding
actin binding;binding;cytoskeletal protein binding;protein binding
binding;cation binding;DNA binding;ion binding;metal ion binding;nucleic acid binding;nucleic acid binding transcription factor activity;sequence-specific DNA binding;sequence-specific DNA binding transcription factor activ
binding;enzyme binding;protein binding
binding;DNA binding;nucleic acid binding;nucleic acid binding transcription factor activity;sequence-specific DNA binding transcription factor activity
binding;GTP binding;guanyl nucleotide binding;guanyl ribonucleotide binding;nucleotide binding;purine nucleotide binding;purine ribonucleoside triphosphate binding;purine ribonucleotide binding;ribonucleotide binding
adenyl nucleotide binding;adenyl ribonucleotide binding;ATP binding;ATPase activity;ATPase activity, coupled;ATP-dependent helicase activity;ATP-dependent RNA helicase activity;binding;catalytic activity;chromatin binding
cation transmembrane transporter activity;ion transmembrane transporter activity;metal ion transmembrane transporter activity;substrate-specific transmembrane transporter activity;substrate-specific transporter activity;tran
binding;cytokine activity;protein binding;receptor binding
binding;DNA binding;nucleic acid binding
binding;endopeptidase inhibitor activity;endopeptidase regulator activity;enzyme inhibitor activity;enzyme regulator activity;peptidase inhibitor activity;peptidase regulator activity;proteasome binding;protein binding;protein co
ARF guanyl-nucleotide exchange factor activity;binding;enzyme regulator activity;GTPase regulator activity;guanyl-nucleotide exchange factor activity;lipid binding;nucleoside-triphosphatase regulator activity;phosphatidylino
binding;DNA binding;nucleic acid binding;single-stranded DNA binding;structure-specific DNA binding
catalytic activity;endopeptidase activity;hydrolase activity;peptidase activity;peptidase activity, acting on L-amino acid peptides
binding;cation binding;ion binding;metal ion binding;transition metal ion binding;zinc ion binding
binding;cadherin binding;cell adhesion molecule binding;cytoskeletal protein binding;enzyme binding;gamma-tubulin binding;GTPase binding;microtubule binding;protein binding;Rab GTPase binding;Ras GTPase binding;sm
adenyl nucleotide binding;adenyl ribonucleotide binding;ATP binding;binding;nucleotide binding;purine nucleotide binding;purine ribonucleoside triphosphate binding;purine ribonucleotide binding;ribonucleotide binding
binding;GTP binding;guanyl nucleotide binding;guanyl ribonucleotide binding;nucleotide binding;purine nucleotide binding;purine ribonucleoside triphosphate binding;purine ribonucleotide binding;ribonucleotide binding
binding;identical protein binding;protein binding;protein dimerization activity;protein domain specific binding;protein homodimerization activity
AP-2 adaptor complex binding;binding;enzyme activator activity;enzyme regulator activity;GTPase activator activity;GTPase regulator activity;nucleoside-triphosphatase regulator activity;protein binding;protein complex bind
actin binding;actin filament binding;adenyl nucleotide binding;adenyl ribonucleotide binding;ADP binding;ATP binding;binding;catalytic activity;cytoskeletal protein binding;DNA binding;hydrolase activity;hydrolase activity, ac
binding;nucleic acid binding;RNA binding;translation factor activity, nucleic acid binding
adenyl nucleotide binding;adenyl ribonucleotide binding;ATP binding;ATPase activity;ATPase activity, coupled;ATP-dependent helicase activity;ATP-dependent RNA helicase activity;binding;catalytic activity;helicase activity;h
structural constituent of nuclear pore;structural molecule activity
antioxidant activity;binding;catalytic activity;cation binding;cytokine receptor binding;extracellular matrix structural constituent;heme binding;interleukin-1 receptor antagonist activity;ion binding;iron ion binding;metal ion bind

binding;bioactive lipid receptor activity;G-protein coupled receptor activity;lipid binding;lysophosphatidic acid receptor activity;molecular transducer activity;phospholipid binding;receptor activity;signal transducer activity;sig
binding;enzyme binding;histone binding;protein binding;RNA polymerase binding
binding;cell adhesion molecule binding;coreceptor activity;identical protein binding;molecular transducer activity;protein binding;protein dimerization activity;protein homodimerization activity;receptor activity;signal transduce
molecular transducer activity;signal transducer activity
catalytic activity;endopeptidase activity;hydrolase activity;peptidase activity;peptidase activity, acting on L-amino acid peptides;serine hydrolase activity;serine-type endopeptidase activity;serine-type peptidase activity
actin binding;actin filament binding;binding;cytoskeletal protein binding;protein binding
binding;catalytic activity;chromatin binding;chromatin DNA binding;core promoter binding;deacetylase activity;DNA binding;enzyme binding;histone deacetylase activity;histone deacetylase activity (H3-K14 specific);histone
binding;cation binding;core promoter proximal region DNA binding;core promoter proximal region sequence-specific DNA binding;DNA binding;ion binding;metal ion binding;nucleic acid binding;regulatory region DNA binding
acetyltransferase activity;activating transcription factor binding;bHLH transcription factor binding;binding;catalytic activity;cation binding;chromatin binding;core promoter proximal region DNA binding;core promoter proximal
binding;cation binding;DNA binding;ion binding;metal ion binding;nucleic acid binding;nucleotide binding;transition metal ion binding;zinc ion binding
catalytic activity;hyaluronan synthase activity;transferase activity;transferase activity, transferring glycosyl groups;transferase activity, transferring hexosyl groups;UDP-glycosyltransferase activity
catalytic activity;exopeptidase activity;gamma-glutamyl-peptidase activity;hydrolase activity;omega peptidase activity;peptidase activity;peptidase activity, acting on L-amino acid peptides
adenyl nucleotide binding;adenyl ribonucleotide binding;ATP binding;ATPase activity;ATPase activity, coupled;ATP-dependent helicase activity;ATP-dependent RNA helicase activity;binding;catalytic activity;estrogen receptor
adenyl nucleotide binding;adenyl ribonucleotide binding;ATP binding;ATPase activity;binding;binding, bridging;catalytic activity;cation binding;DNA binding;hydrolase activity;hydrolase activity, acting on acid anhydrides;hydro
binding;BRE binding;mRNA binding;nucleic acid binding;nucleotide binding;RNA binding;translation regulator activity;translation regulator activity, nucleic acid binding;translation repressor activity;translation repressor activity
enzyme activator activity;enzyme regulator activity;GTPase activator activity;GTPase regulator activity;guanyl-nucleotide exchange factor activity;nucleoside-triphosphatase regulator activity;Ras guanyl-nucleotide exchange
catalytic activity;cysteine-type peptidase activity;hydrolase activity;peptidase activity;peptidase activity, acting on L-amino acid peptides;small conjugating protein-specific protease activity;ubiquitin-specific protease activity
binding;protein binding;receptor binding
adenyl nucleotide binding;adenyl ribonucleotide binding;ATP binding;ATPase activity;ATPase activity, coupled;ATP-dependent helicase activity;ATP-dependent RNA helicase activity;binding;catalytic activity;cation binding;chr
binding;catalytic activity;cation binding;cysteine-type peptidase activity;hydrolase activity;ion binding;metal ion binding;metallopeptidase activity;nucleic acid binding;peptidase activity;peptidase activity, acting on L-amino ac
binding;nucleic acid binding
binding;chromatin binding;DNA binding;nucleic acid binding;protein binding;protein binding transcription factor activity;protein N-terminus binding;transcription coactivator activity;transcription cofactor activity;transcription f
protein binding transcription factor activity;transcription coactivator activity;transcription cofactor activity;transcription factor binding transcription factor activity
binding;catalytic activity;GDP binding;GTP binding;GTPase activity;guanyl nucleotide binding;guanyl ribonucleotide binding;hydrolase activity;hydrolase activity, acting on acid anhydrides;hydrolase activity, acting on acid anh
binding;DNA binding;mRNA binding;nucleic acid binding;RNA binding
binding;nuclear localization sequence binding;peptide binding;signal sequence binding
binding;cation binding;cytoskeletal protein binding;enzyme binding;enzyme regulator activity;GTPase binding;GTPase regulator activity;guanyl-nucleotide exchange factor activity;ion binding;metal ion binding;microtubule bin
binding;enzyme binding;enzyme inhibitor activity;enzyme regulator activity;growth factor receptor binding;hepatocyte growth factor receptor binding;ligase inhibitor activity;ligase regulator activity;protein binding;receptor bin
binding;catalytic activity;co-SMAD binding;cysteine-type endopeptidase activity;cysteine-type peptidase activity;endopeptidase activity;hydrolase activity;peptidase activity;peptidase activity, acting on L-amino acid peptides
binding;catalytic activity;cysteine-type endopeptidase activity;cysteine-type peptidase activity;endopeptidase activity;enzyme binding;hydrolase activity;p53 binding;peptidase activity;peptidase activity, acting on L-amino aci
binding;calcium channel activity;cation channel activity;cation transmembrane transporter activity;channel activity;enzyme binding;ion channel activity;ion transmembrane transporter activity;passive transmembrane transport
active transmembrane transporter activity;ATPase activity;ATPase activity, coupled;ATPase activity, coupled to movement of substances;ATPase activity, coupled to transmembrane movement of ions;ATPase activity, coupled
binding;cation binding;ion binding;metal ion binding;transition metal ion binding;zinc ion binding
binding;mRNA binding;nucleic acid binding;nucleotide binding;poly(A) RNA binding;poly-purine tract binding;protein binding transcription factor activity;RNA binding;single-stranded RNA binding;transcription coactivator acti
acetyltransferase activity;binding;catalytic activity;chromatin binding;DNA binding;ligand-dependent nuclear receptor binding;N-acetyltransferase activity;N-acyltransferase activity;nucleic acid binding;protein binding;protein
binding;enzyme binding;kinase binding;protein binding;protein kinase binding;protein kinase C binding
catalytic activity;GPI-anchor transamidase activity;hydrolase activity
structural constituent of ribosome;structural molecule activity
binding;cation binding;ion binding;metal ion binding;transition metal ion binding;zinc ion binding
binding;catalytic activity;GTP binding;GTPase activity;guanyl nucleotide binding;guanyl ribonucleotide binding;hydrolase activity;hydrolase activity, acting on acid anhydrides;hydrolase activity, acting on acid anhydrides, in ph
catalytic activity;kinase activity;phosphotransferase activity, alcohol group as acceptor;protein kinase activity;transferase activity;transferase activity, transferring phosphorus-containing groups
binding;calcium ion binding;cation binding;ion binding;metal ion binding
binding;enzyme binding;GTPase binding;protein binding;protein N-terminus binding;Ral GTPase binding;Ras GTPase binding;small GTPase binding
catalytic activity
binding;lipid binding;phosphatidylinositol binding;phosphatidylinositol-3,4,5-trisphosphate binding;phospholipid binding
binding;DNA binding;nucleic acid binding;nucleic acid binding transcription factor activity;sequence-specific DNA binding transcription factor activity;single-stranded DNA binding;structure-specific DNA binding
binding;catalytic activity;cation binding;identical protein binding;ion binding;isomerase activity;metal ion binding;protein binding;protein dimerization activity;protein homodimerization activity;racemase and epimerase activity;
binding;calcium ion binding;catalytic activity;cation binding;cis-trans isomerase activity;drug binding;FK506 binding;ion binding;isomerase activity;macrolide binding;metal ion binding;peptidyl-prolyl cis-trans isomerase activi
AU-rich element binding;binding;nucleic acid binding;RNA binding
acid-amino acid ligase activity;binding;catalytic activity;cation binding;ion binding;ligase activity;ligase activity, forming carbon-nitrogen bonds;metal ion binding;NF-kappaB binding;protein binding;small conjugating protein li
catalytic activity;cysteine-type peptidase activity;hydrolase activity;peptidase activity;peptidase activity, acting on L-amino acid peptides;small conjugating protein-specific protease activity;ubiquitin-specific protease activity
enzyme regulator activity;GTPase regulator activity;guanyl-nucleotide exchange factor activity;nucleoside-triphosphatase regulator activity;Ras guanyl-nucleotide exchange factor activity;Rho guanyl-nucleotide exchange fac
binding;calcium ion binding;cation binding;ion binding;metal ion binding
aldose 1-epimerase activity;binding;carbohydrate binding;catalytic activity;isomerase activity;racemase and epimerase activity;racemase and epimerase activity, acting on carbohydrates and derivatives
catalytic activity;oxidoreductase activity;oxidoreductase activity, acting on the CH-NH group of donors;oxidoreductase activity, acting on the CH-NH group of donors, NAD or NADP as acceptor;pyrroline-5-carboxylate reduc
binding;catalytic activity;hydrolase activity;hydrolase activity, acting on acid anhydrides;hydrolase activity, acting on acid anhydrides, in phosphorus-containing anhydrides;m7G(5')pppN diphosphatase activity;nucleic acid bin
enzyme inhibitor activity;enzyme regulator activity;phosphatase inhibitor activity;phosphatase regulator activity;protein phosphatase inhibitor activity;protein phosphatase regulator activity
adenyl nucleotide binding;adenyl ribonucleotide binding;ATP binding;binding;catalytic activity;ligase activity;nucleotide binding;purine nucleotide binding;purine ribonucleoside triphosphate binding;purine ribonucleotide bindi
binding;enzyme binding;enzyme inhibitor activity;enzyme regulator activity;lipase binding;lipase inhibitor activity;protein binding;small conjugating protein binding;ubiquitin binding;ubiquitin protein ligase binding
binding;enzyme binding;GTPase binding;identical protein binding;K63-linked polyubiquitin binding;polyubiquitin binding;protein binding;protein C-terminus binding;Rab GTPase binding;Ras GTPase binding;small conjugating
binding;lipid binding;lipoprotein particle receptor binding;low-density lipoprotein particle receptor binding;peptide binding;protein binding;receptor binding;signal sequence binding;transporter activity
binding;cytoskeletal protein binding;gamma-tubulin binding;protein binding;structural constituent of cytoskeleton;structural molecule activity;tubulin binding
binding;calcium ion binding;cation binding;ion binding;metal ion binding
binding;binding, bridging;protein binding;protein binding, bridging;ribonucleoprotein binding;ribosomal large subunit binding
catalytic activity;hydrolase activity
binding;enzyme binding;kinetochore binding;protein binding
enzyme regulator activity;kinase regulator activity;phosphorylase kinase regulator activity;protein kinase regulator activity
bHLH transcription factor binding;binding;catalytic activity;cation binding;coenzyme binding;cofactor binding;core promoter binding;core promoter sequence-specific DNA binding;deacetylase activity;DNA binding;enzyme bi
binding;cytoskeletal protein binding;kinesin binding;protein binding
structural constituent of ribosome;structural molecule activity
binding;nucleic acid binding;nucleotide binding;RNA binding;RNA stem-loop binding
adenyl nucleotide binding;adenyl ribonucleotide binding;ATP binding;binding;cation binding;chaperone binding;cytokine receptor binding;enzyme binding;interferon-gamma receptor binding;ion binding;kinase binding;metal i
binding;nucleic acid binding;ribonucleoprotein binding;ribosomal small subunit binding;RNA binding;rRNA binding
binding;identical protein binding;nucleic acid binding;protein binding;RNA binding
binding;calcium ion binding;cation binding;identical protein binding;ion binding;metal ion binding;protein binding;protein dimerization activity;protein homodimerization activity
binding;DNA binding;nucleic acid binding;RNA binding
binding;catalytic activity;cysteine-type peptidase activity;enzyme binding;hydrolase activity;NEDD8-specific protease activity;peptidase activity;peptidase activity, acting on L-amino acid peptides;protein binding;small conjug
catalytic activity;methyltransferase activity;RNA methyltransferase activity;S-adenosylmethionine-dependent methyltransferase activity;transferase activity;transferase activity, transferring one-carbon groups;tRNA (adenine) m
binding;catalytic activity;cation binding;intramolecular transferase activity;intramolecular transferase activity, phosphotransferases;ion binding;isomerase activity;magnesium ion binding;metal ion binding;phosphoglucomutas
binding;catalytic activity;DNA binding;N-acyltransferase activity;nucleic acid binding;sphingosine N-acyltransferase activity;transferase activity;transferase activity, transferring acyl groups;transferase activity, transferring acyl
binding;catalytic activity;cation binding;coenzyme binding;cofactor binding;flavin adenine dinucleotide binding;ion binding;metal ion binding;oxidoreductase activity;oxidoreductase activity, acting on the CH-CH group of don
ammonia-lyase activity;binding;carbon-nitrogen lyase activity;catalytic activity;cofactor binding;L-serine ammonia-lyase activity;L-threonine ammonia-lyase activity;lyase activity;pyridoxal phosphate binding;vitamin B6 bindin
adenyl nucleotide binding;adenyl ribonucleotide binding;ATP binding;binding;catalytic activity;cation binding;histone kinase activity;histone serine kinase activity;ion binding;kinase activity;metal ion binding;nucleotide binding
binding;cation binding;ion binding;metal ion binding;nucleic acid binding
adenyl nucleotide binding;adenyl ribonucleotide binding;ATP binding;ATPase activity;ATPase activity, coupled;ATP-dependent helicase activity;ATP-dependent RNA helicase activity;binding;catalytic activity;helicase activity;h
binding;histone binding;protein binding;protein binding transcription factor activity;transcription cofactor activity;transcription corepressor activity;transcription factor binding transcription factor activity
actin binding;actinin binding;binding;cation binding;cytoskeletal protein binding;enzyme binding;ion binding;kinase binding;metal ion binding;protein binding;protein kinase binding;protein kinase C binding;transition metal ion
catalytic activity;disulfide oxidoreductase activity;intramolecular oxidoreductase activity;intramolecular oxidoreductase activity, interconverting keto- and enol-groups;intramolecular oxidoreductase activity, transposing S-S b
binding;catalytic activity;enzyme activator activity;enzyme regulator activity;GTPase activator activity;GTPase regulator activity;hydrolase activity;hydrolase activity, acting on ester bonds;molecular transducer activity;nucleos
binding;core promoter proximal region DNA binding;core promoter proximal region sequence-specific DNA binding;DNA binding;nucleic acid binding;nucleic acid binding transcription factor activity;regulatory region DNA bin
binding;nucleic acid binding;nucleotide binding;RNA binding
acid-thiol ligase activity;adenyl nucleotide binding;adenyl ribonucleotide binding;ATP binding;binding;catalytic activity;CoA-ligase activity;GDP binding;GTP binding;guanyl nucleotide binding;guanyl ribonucleotide binding;liga
catalytic activity;nucleotidyltransferase activity;transferase activity;transferase activity, transferring phosphorus-containing groups
catalytic activity;hydrolase activity
actin binding;binding;cytoskeletal protein binding;enzyme binding;leucine zipper domain binding;LRR domain binding;protein binding;protein binding transcription factor activity;protein domain specific binding;transcription c
binding;cytoskeletal protein binding;myosin binding;protein binding
binding;cyclin binding;enzyme binding;kinase binding;NF-kappaB binding;protein binding;protein kinase binding;transcription factor binding
catalytic activity;cysteine-type peptidase activity;hydrolase activity;peptidase activity;peptidase activity, acting on L-amino acid peptides;small conjugating protein-specific protease activity;ubiquitin-specific protease activity
binding;protein binding;receptor tyrosine kinase binding

catalytic activity;intramolecular oxidoreductase activity;intramolecular oxidoreductase activity, interconverting keto- and enol-groups;intramolecular oxidoreductase activity, transposing S-S bonds;isomerase activity;protein d
binding;cation binding;ion binding;metal ion binding;nucleic acid binding
acid-amino acid ligase activity;binding;catalytic activity;cation binding;ion binding;ligase activity;ligase activity, forming carbon-nitrogen bonds;metal ion binding;nucleic acid binding;small conjugating protein ligase activity;ub
binding;cation binding;ion binding;metal ion binding;transition metal ion binding;zinc ion binding
binding;cation binding;ion binding;lipid binding;metal ion binding;phosphatidylinositol binding;phosphatidylinositol-3-phosphate binding;phospholipid binding;transition metal ion binding;zinc ion binding
binding;catalytic activity;cysteine-type endopeptidase activity;cysteine-type peptidase activity;endopeptidase activity;hydrolase activity;peptidase activity;peptidase activity, acting on L-amino acid peptides;protein binding;pr
adenyl nucleotide binding;adenyl ribonucleotide binding;ATP binding;binding;catalytic activity;kinase activity;nucleotide binding;phosphotransferase activity, alcohol group as acceptor;protein kinase activity;protein serine/thre
adenyl nucleotide binding;adenyl ribonucleotide binding;ATP binding;binding;catalytic activity;DNA binding;kinase activity;nucleic acid binding;nucleotide binding;phosphotransferase activity, alcohol group as acceptor;protei
binding;enzyme binding;GTPase binding;kinase binding;protein binding;protein kinase binding;Ral GTPase binding;Ras GTPase binding;small GTPase binding
binding;carboxypeptidase activity;catalytic activity;cation binding;dipeptidase activity;exopeptidase activity;hydrolase activity;ion binding;metal ion binding;metallopeptidase activity;peptidase activity;peptidase activity, acting
binding;C2H2 zinc finger domain binding;catalytic activity;cation binding;histone methyltransferase activity;histone methyltransferase activity (H3-K27 specific);histone methyltransferase activity (H3-K9 specific);histone-lysine
binding;cation binding;DNA binding;ion binding;metal ion binding;nucleic acid binding;transition metal ion binding;zinc ion binding
binding;lipid binding;phosphatidylinositol binding;phosphatidylinositol-3-phosphate binding;phospholipid binding
catalytic activity;oxidoreductase activity
binding;nucleic acid binding;RNA binding
catalytic activity;lysophospholipid acyltransferase activity;transferase activity;transferase activity, transferring acyl groups;transferase activity, transferring acyl groups other than amino-acyl groups
binding;enzyme binding;enzyme regulator activity;GTPase binding;GTPase regulator activity;guanyl-nucleotide exchange factor activity;nucleoside-triphosphatase regulator activity;protein binding;Rac GTPase binding;Ras G
binding;cation binding;DNA binding;ion binding;metal ion binding;nucleic acid binding;transition metal ion binding;zinc ion binding
catalytic activity;methyltransferase activity;transferase activity;transferase activity, transferring one-carbon groups
binding;cation binding;enzyme activator activity;enzyme regulator activity;GTPase activator activity;GTPase regulator activity;ion binding;lipid binding;metal ion binding;nucleoside-triphosphatase regulator activity;phosphatid
binding;histone binding;protein binding;protein transporter activity;substrate-specific transporter activity;transporter activity
binding;binding, bridging;ligand-dependent nuclear receptor transcription coactivator activity;nucleic acid binding;nucleotide binding;protein binding;protein binding transcription factor activity;protein binding, bridging;RNA b
binding;mRNA binding;nucleic acid binding;RNA binding
binding;catalytic activity;enzyme binding;intramolecular transferase activity;isomerase activity;protein binding;pseudouridine synthase activity
adenyl nucleotide binding;adenyl ribonucleotide binding;ATP binding;binding;catalytic activity;cation binding;ion binding;kinase activity;metal ion binding;nucleotide binding;phosphotransferase activity, alcohol group as acce
active transmembrane transporter activity;amine transmembrane transporter activity;amino acid transmembrane transporter activity;carboxylic acid transmembrane transporter activity;organic acid transmembrane transporte
protein transporter activity;substrate-specific transporter activity;transporter activity
binding;DNA binding;double-stranded DNA binding;mRNA binding;nucleic acid binding;nucleic acid binding transcription factor activity;protein binding;RNA binding;sequence-specific DNA binding transcription factor activity
protein transporter activity;substrate-specific transporter activity;transporter activity
binding;lipid binding;phosphatidylinositol binding;phosphatidylinositol-4,5-bisphosphate binding;phospholipid binding
protein binding transcription factor activity;RNA polymerase II transcription cofactor activity;RNA polymerase II transcription factor binding transcription factor activity;transcription cofactor activity;transcription factor binding
binding;catalytic activity;GTP binding;GTPase activity;guanyl nucleotide binding;guanyl ribonucleotide binding;hydrolase activity;hydrolase activity, acting on acid anhydrides;hydrolase activity, acting on acid anhydrides, in p
binding;ErbB-2 class receptor binding;integrin binding;protein binding;protein complex binding;receptor binding;structural constituent of cytoskeleton;structural molecule activity
binding;calcium ion binding;cation binding;ion binding;metal ion binding
binding;enzyme binding;GTPase binding;protein binding;Ran GTPase binding;Ras GTPase binding;small GTPase binding
binding;enzyme binding;enzyme inhibitor activity;enzyme regulator activity;phosphatase binding;phosphatase inhibitor activity;phosphatase regulator activity;protein binding;protein phosphatase 1 binding;protein phosphatas
adenyl nucleotide binding;adenyl ribonucleotide binding;ATP binding;binding;catalytic activity;cation binding;ion binding;kinase activity;magnesium ion binding;metal ion binding;nucleotide binding;phosphotransferase activity
binding;DNA binding;nucleic acid binding
activating transcription factor binding;binding;catalytic activity;chromatin binding;deacetylase activity;DNA binding;hydrolase activity;nucleic acid binding;nucleic acid binding transcription factor activity;protein binding;protei
binding;nucleotide binding
binding;cation binding;ion binding;lipid binding;metal ion binding;protein transporter activity;substrate-specific transporter activity;transition metal ion binding;transporter activity;zinc ion binding
binding;binding, bridging;estrogen receptor binding;hormone receptor binding;nuclear hormone receptor binding;protein binding;protein binding transcription factor activity;protein binding, bridging;protein complex scaffold;r
acid-amino acid ligase activity;binding;catalytic activity;cation binding;chromatin binding;core promoter proximal region DNA binding;core promoter proximal region sequence-specific DNA binding;DNA binding;histone bindi
bHLH transcription factor binding;binding;core promoter proximal region DNA binding;core promoter proximal region sequence-specific DNA binding;DNA binding;E-box binding;enhancer binding;identical protein binding;nuc
protein binding transcription factor activity;transcription coactivator activity;transcription cofactor activity;transcription factor binding transcription factor activity
catalytic activity;endopeptidase activity;hydrolase activity;peptidase activity;peptidase activity, acting on L-amino acid peptides;threonine-type endopeptidase activity;threonine-type peptidase activity
actin binding;binding;cytoskeletal protein binding;protein binding
protein transporter activity;receptor activity;substrate-specific transporter activity;transporter activity
catalytic activity;cytidylyltransferase activity;ethanolamine-phosphate cytidylyltransferase activity;nucleotidyltransferase activity;transferase activity;transferase activity, transferring phosphorus-containing groups
binding;chromatin binding;DNA binding;nucleic acid binding;protein binding;protein domain specific binding;WD40-repeat domain binding
enzyme regulator activity
catalytic activity;dolichyl-phosphate-mannose-protein mannosyltransferase activity;mannosyltransferase activity;transferase activity;transferase activity, transferring glycosyl groups;transferase activity, transferring hexosyl gro
protein binding transcription factor activity;transcription cofactor activity;transcription corepressor activity;transcription factor binding transcription factor activity
acid-amino acid ligase activity;binding;catalytic activity;cation binding;chromatin binding;ion binding;ligase activity;ligase activity, forming carbon-nitrogen bonds;metal ion binding;protein binding;protein domain specific bind
androgen receptor binding;binding;catalytic activity;cation binding;copper chaperone activity;copper ion binding;cytokine binding;enzyme binding;glyoxalase III activity;hormone receptor binding;hydrolase activity;identical pr
binding;catalytic activity;cation binding;ion binding;metal ion binding;oxidoreductase activity;transition metal ion binding;zinc ion binding
catalytic activity;cysteine-type endopeptidase activity;cysteine-type peptidase activity;endopeptidase activity;hydrolase activity;peptidase activity;peptidase activity, acting on L-amino acid peptides
binding;chromatin binding;DNA binding;heat shock protein binding;histone binding;Hsp70 protein binding;nucleic acid binding;protein binding;small conjugating protein binding;ubiquitin binding
binding;nucleic acid binding;RNA binding
transporter activity
catalytic activity;endonuclease activity;endonuclease activity, active with either ribo- or deoxyribonucleic acids and producing 5'-phosphomonoesters;endoribonuclease activity;endoribonuclease activity, producing 5'-phosph
binding;calcium ion binding;cation binding;copper ion binding;fibroblast growth factor binding;growth factor binding;identical protein binding;ion binding;lipid binding;metal ion binding;protein binding;protein dimerization acti
binding;cation binding;ion binding;metal ion binding;transition metal ion binding;zinc ion binding
binding;DNA binding;nucleic acid binding;nucleic acid binding transcription factor activity;protein binding;RNA polymerase II transcription factor binding;sequence-specific DNA binding transcription factor activity;transcriptio
binding;catalytic activity;cation binding;DNA binding;endonuclease activity;endoribonuclease activity;hydrolase activity;hydrolase activity, acting on ester bonds;ion binding;metal ion binding;nuclease activity;nucleic acid bin
binding;nucleic acid binding;RNA binding;translation factor activity, nucleic acid binding;translation initiation factor activity
binding;protein binding;unfolded protein binding
binding;heat shock protein binding;protein binding
binding;estrogen receptor binding;hormone receptor binding;nuclear hormone receptor binding;protein binding;protein N-terminus binding;receptor binding;steroid hormone receptor binding
channel inhibitor activity;channel regulator activity;ion channel inhibitor activity;potassium channel inhibitor activity;potassium channel regulator activity
adenyl nucleotide binding;adenyl ribonucleotide binding;ATP binding;ATPase activity;binding;catalytic activity;centromeric DNA binding;cytoskeletal protein binding;DNA binding;hydrolase activity;hydrolase activity, acting on
acylglycerol lipase activity;binding;carboxylic ester hydrolase activity;catalytic activity;hydrolase activity;hydrolase activity, acting on ester bonds;identical protein binding;lipase activity;lipid binding;lysophospholipase activity;
binding;epidermal growth factor receptor binding;growth factor receptor binding;nucleic acid binding;protein binding;protein C-terminus binding;receptor binding;RNA binding
5-methyltetrahydrofolate-dependent methyltransferase activity;binding;catalytic activity;cation binding;cobalamin binding;homocysteine S-methyltransferase activity;ion binding;metal ion binding;methionine synthase activity;
3-hydroxy-2-methylbutyryl-CoA dehydrogenase activity;3-hydroxyacyl-CoA dehydrogenase activity;catalytic activity;cholate 7-alpha-dehydrogenase activity;oxidoreductase activity;oxidoreductase activity, acting on CH-OH
extracellular matrix structural constituent;extracellular matrix structural constituent conferring tensile strength;structural molecule activity
binding;mRNA binding;nucleic acid binding;nucleotide binding;RNA binding
binding;molecular transducer activity;protein binding;protein domain specific binding;signal transducer activity;WW domain binding
binding;protein binding;unfolded protein binding
binding;protein binding;SNARE binding;soluble NSF attachment protein activity;syntaxin binding
3 iron, 4 sulfur cluster binding;4 iron, 4 sulfur cluster binding;aconitate hydratase activity;binding;carbon-oxygen lyase activity;catalytic activity;cation binding;hydro-lyase activity;ion binding;iron ion binding;iron-sulfur cluster
nucleobase-containing compound transmembrane transporter activity;nucleoside transmembrane transporter activity;substrate-specific transmembrane transporter activity;substrate-specific transporter activity;transmembra
binding;DNA binding;nucleic acid binding;nucleic acid binding transcription factor activity;sequence-specific DNA binding;sequence-specific DNA binding transcription factor activity
binding;calcium-dependent protein binding;DNA binding;enzyme binding;identical protein binding;nucleic acid binding;protein binding;protein binding transcription factor activity;protein dimerization activity;protein homodime
binding;calcium ion binding;calcium-dependent phospholipid binding;catalytic activity;cation binding;endopeptidase activity;hydrolase activity;identical protein binding;ion binding;lipid binding;metal ion binding;NF-kappaB b
adenyl nucleotide binding;adenyl ribonucleotide binding;ATP binding;binding;nucleotide binding;protein binding;purine nucleotide binding;purine ribonucleoside triphosphate binding;purine ribonucleotide binding;ribonucleoti
arginine N-methyltransferase activity;binding;catalytic activity;histone methyltransferase activity;histone methyltransferase activity (H4-R3 specific);histone-arginine N-methyltransferase activity;identical protein binding;methyl
1-acylglycerol-3-phosphate O-acyltransferase activity;acylglycerol O-acyltransferase activity;catalytic activity;O-acyltransferase activity;transferase activity;transferase activity, transferring acyl groups;transferase activity, tran
binding;identical protein binding;lipid binding;protein binding
receptor activity
adenyl nucleotide binding;adenyl ribonucleotide binding;ATP binding;binding;catalytic activity;chromatin binding;enzyme binding;histone binding;histone kinase activity;histone kinase activity (H3-S10 specific);histone kinase
binding;cytokine activity;cytokine receptor binding;protein binding;receptor binding;transforming growth factor beta receptor binding
binding;neurotransmitter binding
binding;calcium ion binding;cation binding;identical protein binding;ion binding;metal ion binding;protein binding;protein dimerization activity;protein heterodimerization activity
adenyl nucleotide binding;adenyl ribonucleotide binding;ATP binding;ATPase activity;ATPase activity, coupled;ATP-dependent helicase activity;ATP-dependent RNA helicase activity;binding;catalytic activity;helicase activity;h
3'-tRNA processing endoribonuclease activity;binding;catalytic activity;cation binding;endonuclease activity;endonuclease activity, active with either ribo- or deoxyribonucleic acids and producing 5'-phosphomonoesters;end
catalytic activity;deacetylase activity;hydrolase activity;hydrolase activity, acting on glycosyl bonds
binding;DNA binding;nucleic acid binding;nucleic acid binding transcription factor activity;sequence-specific DNA binding transcription factor activity
ligand-dependent nuclear receptor transcription coactivator activity;protein binding transcription factor activity;transcription coactivator activity;transcription cofactor activity;transcription factor binding transcription factor act
binding;catalytic activity;GTP binding;GTPase activity;guanyl nucleotide binding;guanyl ribonucleotide binding;hydrolase activity;hydrolase activity, acting on acid anhydrides;hydrolase activity, acting on acid anhydrides, in p
binding;high-density lipoprotein particle binding;lipid binding;lipoprotein particle binding;protein binding;protein-lipid complex binding;receptor binding
binding;catalytic activity;core promoter binding;DNA binding;DNA polymerase activity;DNA-directed DNA polymerase activity;nucleic acid binding;nucleotidyltransferase activity;protein binding;protein binding transcription fa
binding;cation binding;ion binding;metal ion binding
actin binding;actin filament binding;Arp2/3 complex binding;binding;cytoskeletal protein binding;identical protein binding;protein binding;protein complex binding
antioxidant activity;catalytic activity;electron carrier activity;oxidoreductase activity;oxidoreductase activity, acting on a sulfur group of donors;oxidoreductase activity, acting on a sulfur group of donors, NAD or NADP as acce
binding;identical protein binding;protein binding;protein dimerization activity;protein homodimerization activity;protein N-terminus binding;structural molecule activity
binding;calcium ion binding;cation binding;identical protein binding;ion binding;metal ion binding;protein binding
binding;ribonucleoprotein binding;ribosome binding
binding;catalytic activity;coenzyme binding;cofactor binding;DNA binding;electron-transferring-flavoprotein dehydrogenase activity;flavin adenine dinucleotide binding;NADH dehydrogenase (quinone) activity;NADH dehydrog

ADP-specific glucokinase activity;binding;catalytic activity;cation binding;ion binding;kinase activity;metal ion binding;phosphotransferase activity, alcohol group as acceptor;transferase activity;transferase activity, transferring
binding;catalytic activity;cation binding;endonuclease activity;hydrolase activity;hydrolase activity, acting on ester bonds;ion binding;metal ion binding;nuclease activity
acid-amino acid ligase activity;binding;catalytic activity;cation binding;ion binding;ligase activity;ligase activity, forming carbon-nitrogen bonds;metal ion binding;small conjugating protein ligase activity;transition metal ion bin
catalytic activity;intramolecular oxidoreductase activity;intramolecular oxidoreductase activity, interconverting keto- and enol-groups;intramolecular oxidoreductase activity, transposing S-S bonds;isomerase activity;protein d
adenyl nucleotide binding;adenyl ribonucleotide binding;ATP binding;binding;catalytic activity;hydrolase activity;hydrolase activity, acting on acid anhydrides;hydrolase activity, acting on acid anhydrides, in phosphorus-conta
binding;cation binding;ion binding;lipid binding;metal ion binding;phospholipid binding
beta-1,4-mannosyltransferase activity;catalytic activity;chitobiosyldiphosphodolichol beta-mannosyltransferase activity;mannosyltransferase activity;transferase activity;transferase activity, transferring glycosyl groups;transfe
binding;cation binding;ion binding;metal ion binding;transition metal ion binding;zinc ion binding
binding;cation binding;chromatin binding;DNA binding;ion binding;metal ion binding;nucleic acid binding;nucleic acid binding transcription factor activity;sequence-specific DNA binding;sequence-specific DNA binding transc
binding;chromatin binding
binding;enzyme inhibitor activity;enzyme regulator activity;histone binding;phosphatase inhibitor activity;phosphatase regulator activity;protein binding
1-phosphatidylinositol 4-kinase activity;adenyl nucleotide binding;adenyl ribonucleotide binding;AP-3 adaptor complex binding;ATP binding;binding;catalytic activity;cation binding;inositol or phosphatidylinositol kinase activi
beta-tubulin binding;binding;chaperone binding;cytoskeletal protein binding;enzyme activator activity;enzyme regulator activity;GTPase activator activity;GTPase regulator activity;nucleoside-triphosphatase regulator activity;
3-keto sterol reductase activity;alcohol dehydrogenase [NAD(P)+] activity;binding;carbonyl reductase (NADPH) activity;catalytic activity;oxidoreductase activity;oxidoreductase activity, acting on CH-OH group of donors;oxido
binding;catalytic activity;GTP binding;GTPase activity;guanyl nucleotide binding;guanyl ribonucleotide binding;hydrolase activity;hydrolase activity, acting on acid anhydrides;hydrolase activity, acting on acid anhydrides, in pl
binding;enzyme binding;nucleic acid binding;protein binding;RNA binding
binding;enzyme binding;identical protein binding;kinase binding;protein binding;protein dimerization activity;protein homodimerization activity;protein kinase binding;protein N-terminus binding
catalytic activity;oxidoreductase activity;protein binding transcription factor activity;transcription coactivator activity;transcription cofactor activity;transcription factor binding transcription factor activity
adenyl nucleotide binding;adenyl ribonucleotide binding;ATP binding;ATPase activity;ATPase activity, coupled;ATP-dependent helicase activity;ATP-dependent RNA helicase activity;binding;catalytic activity;helicase activity;h
3-hydroxybutyrate dehydrogenase activity;binding;catalytic activity;coenzyme binding;cofactor binding;NAD binding;nucleotide binding;oxidoreductase activity;oxidoreductase activity, acting on CH-OH group of donors;oxid
binding;catalytic activity;identical protein binding;intramolecular oxidoreductase activity;intramolecular oxidoreductase activity, interconverting aldoses and ketoses;isomerase activity;protein binding;S-methyl-5-thioribose-1-
binding;catalytic activity;methyltransferase activity;nucleic acid binding;RNA binding;transferase activity;transferase activity, transferring one-carbon groups
acireductone dioxygenase [iron(II)-requiring] activity;binding;catalytic activity;cation binding;dioxygenase activity;ion binding;iron ion binding;metal ion binding;oxidoreductase activity;oxidoreductase activity, acting on single
binding;catalytic activity;cation binding;ion binding;ligase activity;metal ion binding;transition metal ion binding;zinc ion binding
binding;catalytic activity;cation binding;ion binding;metal ion binding;oxidoreductase activity;oxidoreductase activity, acting on the CH-CH group of donors;oxidoreductase activity, acting on the CH-CH group of donors, NAD
catalytic activity;methyltransferase activity;N-terminal protein N-methyltransferase activity;protein methyltransferase activity;transferase activity;transferase activity, transferring one-carbon groups
binding;protein binding;SNARE binding;syntaxin binding
binding;catalytic activity;GTP binding;GTPase activity;guanyl nucleotide binding;guanyl ribonucleotide binding;hydrolase activity;hydrolase activity, acting on acid anhydrides;hydrolase activity, acting on acid anhydrides, in pl
adenyl nucleotide binding;adenyl ribonucleotide binding;ATP binding;binding;catalytic activity;kinase activity;nucleotide binding;phosphotransferase activity, alcohol group as acceptor;protein kinase activity;protein serine/thre
binding;catalytic activity;GTP binding;GTPase activity;guanyl nucleotide binding;guanyl ribonucleotide binding;hydrolase activity;hydrolase activity, acting on acid anhydrides;hydrolase activity, acting on acid anhydrides, in pl
adenyl nucleotide binding;adenyl ribonucleotide binding;aminoacyl-tRNA ligase activity;ATP binding;binding;catalytic activity;ligase activity;ligase activity, forming aminoacyl-tRNA and related compounds;ligase activity, formi
acetyl-CoA C-acetyltransferase activity;acetyl-CoA C-acyltransferase activity;acetyltransferase activity;C-acetyltransferase activity;C-acyltransferase activity;catalytic activity;transferase activity;transferase activity, transferring
binding;cation binding;ion binding;metal ion binding;miRNA binding;mRNA 3'-UTR binding;mRNA binding;nucleic acid binding;nucleotide binding;RNA binding;transition metal ion binding;zinc ion binding
ion transmembrane transporter activity;substrate-specific transmembrane transporter activity;substrate-specific transporter activity;transmembrane transporter activity;transporter activity
binding;carbohydrate binding;catalytic activity;chitin binding;chitinase activity;hydrolase activity;hydrolase activity, acting on glycosyl bonds;hydrolase activity, hydrolyzing O-glycosyl compounds;oligosaccharide binding;patt
binding;catalytic activity;hydrolase activity;nucleotide binding
binding;cholesterol binding;lipid binding;steroid binding;sterol binding
binding;cytoskeletal protein binding;enzyme binding;gamma-tubulin binding;GTPase binding;protein binding;Rab GTPase binding;Ras GTPase binding;small GTPase binding;tubulin binding
acetyltransferase activity;binding;catalytic activity;N-acetyltransferase activity;N-acyltransferase activity;ribonucleoprotein binding;ribosome binding;transferase activity;transferase activity, transferring acyl groups;transferase
binding;DNA binding;nucleic acid binding
binding;catalytic activity;cation binding;ion binding;metal ion binding;queuine tRNA-ribosyltransferase activity;transferase activity;transferase activity, transferring glycosyl groups;transferase activity, transferring pentosyl grou
binding;DNA binding;nucleic acid binding
binding;catalytic activity;cation binding;dITP diphosphatase activity;hydrolase activity;hydrolase activity, acting on acid anhydrides;hydrolase activity, acting on acid anhydrides, in phosphorus-containing anhydrides;ion bindi
binding;nucleic acid binding;ribonucleoprotein binding;ribosome binding;RNA binding;translation factor activity, nucleic acid binding;translation initiation factor activity;tRNA binding
binding;nucleotide binding
alpha(1,6)-fucosyltransferase activity;catalytic activity;fucosyltransferase activity;glycoprotein 6-alpha-L-fucosyltransferase activity;transferase activity;transferase activity, transferring glycosyl groups;transferase activity, trans
binding;nucleic acid binding;RNA binding;rRNA binding;structural constituent of ribosome;structural molecule activity
structural constituent of ribosome;structural molecule activity
structural constituent of ribosome;structural molecule activity
structural constituent of ribosome;structural molecule activity
binding;catalytic activity;cation binding;endopeptidase activity;hydrolase activity;ion binding;metal ion binding;metalloendopeptidase activity;metallopeptidase activity;peptidase activity;peptidase activity, acting on L-amino a
binding;cadherin binding;cell adhesion molecule binding;enzyme activator activity;enzyme regulator activity;GTPase activator activity;GTPase regulator activity;nucleoside-triphosphatase regulator activity;protein binding
structural constituent of ribosome;structural molecule activity
binding;catalytic activity;GTP binding;GTPase activity;guanyl nucleotide binding;guanyl ribonucleotide binding;hydrolase activity;hydrolase activity, acting on acid anhydrides;hydrolase activity, acting on acid anhydrides, in pl
binding;cholesterol binding;lipid binding;steroid binding;sterol binding
binding;catalytic activity;enzyme binding;GTP binding;GTPase activity;GTPase binding;guanyl nucleotide binding;guanyl ribonucleotide binding;hydrolase activity;hydrolase activity, acting on acid anhydrides;hydrolase activity
binding;nucleic acid binding;RNA binding
beta-catenin binding;binding;DNA binding;histone binding;nucleic acid binding;protein binding;protein binding transcription factor activity;protein N-terminus binding;regulatory region DNA binding;regulatory region nucleic ac
protein tag
adenyl nucleotide binding;adenyl ribonucleotide binding;ATP binding;binding;catalytic activity;cation binding;ion binding;kinase activity;metal ion binding;nucleotide binding;phosphotransferase activity, alcohol group as acce
adenyl nucleotide binding;adenyl ribonucleotide binding;ATP binding;binding;catalytic activity;kinase activity;nucleobase-containing compound kinase activity;nucleoside kinase activity;nucleotide binding;purine nucleotide bi
binding;fibroblast growth factor binding;growth factor binding;protein binding
binding;identical protein binding;protein binding;protein dimerization activity;protein homodimerization activity
binding;catalytic activity;cation binding;demethylase activity;DNA demethylase activity;DNA-N1-methyladenine dioxygenase activity;ferrous iron binding;ion binding;iron ion binding;metal ion binding;oxidative DNA demethyla
ankyrin binding;binding;cytoskeletal protein binding;enzyme binding;protein binding
acid-amino acid ligase activity;adenyl nucleotide binding;adenyl ribonucleotide binding;ATP binding;binding;catalytic activity;enzyme binding;ligase activity;ligase activity, forming carbon-nitrogen bonds;nucleotide binding;pr
anion channel activity;anion transmembrane transporter activity;channel activity;chloride channel activity;gated channel activity;intracellular calcium activated chloride channel activity;ion channel activity;ion gated channel ac
binding;ribonucleoprotein binding
catalytic activity;exonuclease activity;hydrolase activity;hydrolase activity, acting on ester bonds;nuclease activity;nucleic acid binding transcription factor activity;sequence-specific DNA binding transcription factor activity
adenyl nucleotide binding;adenyl ribonucleotide binding;ATP binding;ATPase activity;ATPase activity, coupled;ATP-dependent helicase activity;ATP-dependent RNA helicase activity;binding;catalytic activity;helicase activity;h
binding;catalytic activity;DNA binding;DNA-directed RNA polymerase activity;nucleic acid binding;nucleotidyltransferase activity;RNA polymerase activity;transferase activity;transferase activity, transferring phosphorus-conta
binding;nucleotide binding
binding;identical protein binding;protein binding;transcription factor binding
binding;carboxylic acid binding;catalytic activity;cation binding;enzyme binding;ion binding;iron ion binding;L-ascorbic acid binding;metal ion binding;oxidoreductase activity;oxidoreductase activity, acting on paired donors, v
binding;identical protein binding;protein binding;protein dimerization activity;protein homodimerization activity
acetyltransferase activity;catalytic activity;H4 histone acetyltransferase activity;histone acetyltransferase activity;lysine N-acetyltransferase activity;N-acetyltransferase activity;N-acyltransferase activity;peptide alpha-N-acetyl
binding;nucleic acid binding;RNA binding;translation activator activity;translation regulator activity;translation regulator activity, nucleic acid binding
adenyl nucleotide binding;adenyl ribonucleotide binding;ATP binding;binding;nucleotide binding;purine nucleotide binding;purine ribonucleoside triphosphate binding;purine ribonucleotide binding;ribonucleotide binding
acetyltransferase activity;adenyl nucleotide binding;adenyl ribonucleotide binding;ATP binding;binding;catalytic activity;N-acetyltransferase activity;N-acyltransferase activity;nucleotide binding;purine nucleotide binding;purin
binding;catalytic activity;cytoskeletal protein binding;hydrolase activity;hydrolase activity, acting on acid anhydrides;hydrolase activity, acting on acid anhydrides, in phosphorus-containing anhydrides;kinesin binding;microtub
binding;catalytic activity;cation binding;hydrolase activity;hydrolase activity, acting on ester bonds;ion binding;metal ion binding;phosphatase activity;phosphoprotein phosphatase activity;phosphoric ester hydrolase activity;p
5'-3' exonuclease activity;5'-3' exoribonuclease activity;binding;catalytic activity;cation binding;DNA binding;exonuclease activity;exonuclease activity, active with either ribo- or deoxyribonucleic acids and producing 5'-phos
binding;mRNA binding;nucleic acid binding;RNA binding
alpha-tubulin binding;beta-tubulin binding;binding;cation binding;cytoskeletal protein binding;enzyme activator activity;enzyme binding;enzyme regulator activity;gamma-tubulin binding;GTPase activator activity;GTPase regu
binding;nucleotide binding
adenyl nucleotide binding;adenyl ribonucleotide binding;ATP binding;ATPase activity;ATPase activity, coupled;ATP-dependent helicase activity;ATP-dependent RNA helicase activity;binding;catalytic activity;helicase activity;h
cation transmembrane transporter activity;divalent inorganic cation transmembrane transporter activity;inorganic cation transmembrane transporter activity;ion transmembrane transporter activity;magnesium ion transmembra
binding;catalytic activity;GTP binding;GTPase activity;guanyl nucleotide binding;guanyl ribonucleotide binding;hydrolase activity;hydrolase activity, acting on acid anhydrides;hydrolase activity, acting on acid anhydrides, in pl
5S rRNA binding;binding;nucleic acid binding;RNA binding;rRNA binding;structural constituent of ribosome;structural molecule activity
binding;catalytic activity;cation binding;hydrolase activity;hydrolase activity, acting on ester bonds;ion binding;metal ion binding;transition metal ion binding;zinc ion binding
binding;cation binding;ion binding;metal ion binding

binding;frizzled binding;G-protein-coupled receptor binding;protein binding;receptor binding
2 iron, 2 sulfur cluster binding;4 iron, 4 sulfur cluster binding;binding;cation binding;ferrous iron binding;ion binding;iron ion binding;iron-sulfur cluster binding;metal cluster binding;metal ion binding;protein binding;protein cor
adenyl nucleotide binding;adenyl ribonucleotide binding;ATP binding;binding;calcium ion binding;cation binding;GTP binding;guanyl nucleotide binding;guanyl ribonucleotide binding;ion binding;metal ion binding;nucleic acid
binding;calcium ion binding;cation binding;ion binding;metal ion binding
binding;cation binding;ion binding;metal ion binding;nucleotide binding;protein binding;SNARE binding;syntaxin binding;transition metal ion binding;zinc ion binding
binding;catalytic activity;disulfide oxidoreductase activity;electron carrier activity;enzyme activator activity;enzyme regulator activity;GTPase activator activity;GTPase regulator activity;nucleoside-triphosphatase regulator act
actin binding;actin filament binding;binding;cytoskeletal protein binding;enzyme binding;GTPase binding;GTP-Rho binding;myosin binding;myosin II binding;protein binding;Ras GTPase binding;Rho GTPase binding;small GT
adenyl nucleotide binding;adenyl ribonucleotide binding;ATP binding;binding;catalytic activity;identical protein binding;kinase activity;nucleotide binding;phosphotransferase activity, alcohol group as acceptor;protein binding;
catalytic activity;cis-trans isomerase activity;isomerase activity;peptidyl-prolyl cis-trans isomerase activity
adenyl nucleotide binding;adenyl ribonucleotide binding;ATP binding;binding;catalytic activity;enzyme inhibitor activity;enzyme regulator activity;kinase activity;kinase inhibitor activity;kinase regulator activity;MAP kinase kina
binding;enzyme activator activity;enzyme binding;enzyme regulator activity;GTPase activator activity;GTPase binding;GTPase regulator activity;nucleoside-triphosphatase regulator activity;protein binding;protein dimerization
binding;cation binding;chromatin binding;DNA binding;ion binding;metal ion binding;nucleic acid binding
catalytic activity;diphthine synthase activity;methyltransferase activity;S-adenosylmethionine-dependent methyltransferase activity;transferase activity;transferase activity, transferring one-carbon groups
adenyl nucleotide binding;adenyl ribonucleotide binding;ATP binding;ATPase activity;ATPase activity, coupled;ATP-dependent helicase activity;ATP-dependent RNA helicase activity;binding;catalytic activity;core promoter bin
binding;catalytic activity;cation binding;hydrolase activity;hydrolase activity, acting on acid anhydrides;hydrolase activity, acting on acid anhydrides, in phosphorus-containing anhydrides;hydrolase activity, acting on ester bor
catalytic activity;hydrolase activity
binding;DNA binding;nucleic acid binding;structural molecule activity
catalytic activity;disulfide oxidoreductase activity;intramolecular oxidoreductase activity;intramolecular oxidoreductase activity, interconverting keto- and enol-groups;intramolecular oxidoreductase activity, transposing S-S bo
binding;carboxylic acid binding;coenzyme binding;cofactor binding;fatty acid binding;fatty-acyl-CoA binding;lipid binding;monocarboxylic acid binding
binding;enzyme binding;GTPase binding;GTP-Rho binding;protein binding;Ras GTPase binding;Rho GTPase binding;small GTPase binding
binding;catalytic activity;enzyme binding;hydrolase activity;hydrolase activity, acting on ester bonds;kinase binding;phosphatase activity;phosphoprotein phosphatase activity;phosphoric ester hydrolase activity;protein bindin
binding;identical protein binding;protein binding;protein dimerization activity;protein homodimerization activity
catalytic activity;fucosyltransferase activity;peptide-O-fucosyltransferase activity;transferase activity;transferase activity, transferring glycosyl groups;transferase activity, transferring hexosyl groups
adenyl nucleotide binding;adenyl ribonucleotide binding;ATP binding;binding;catalytic activity;channel inhibitor activity;channel regulator activity;chloride channel inhibitor activity;chloride channel regulator activity;enzyme bin
aminopeptidase activity;binding;catalytic activity;cation binding;epoxide hydrolase activity;ether hydrolase activity;exopeptidase activity;hydrolase activity;hydrolase activity, acting on ether bonds;ion binding;metal ion binding
binding;enzyme binding;lipid binding;phosphatidylinositol binding;phosphatidylinositol-4-phosphate binding;phospholipid binding;protein binding
binding;catalytic activity;GTP binding;GTPase activity;guanyl nucleotide binding;guanyl ribonucleotide binding;hydrolase activity;hydrolase activity, acting on acid anhydrides;hydrolase activity, acting on acid anhydrides, in ph
structural molecule activity
binding;identical protein binding;protein binding;protein dimerization activity;protein homodimerization activity
catalytic activity;cysteine-type peptidase activity;hydrolase activity;peptidase activity;peptidase activity, acting on L-amino acid peptides
binding;cholesterol binding;lipid binding;steroid binding;sterol binding
adenyl nucleotide binding;adenyl ribonucleotide binding;ATP binding;binding;calcium ion binding;cation binding;enzyme binding;GTP binding;GTPase binding;guanyl nucleotide binding;guanyl ribonucleotide binding;ion bindi
alpha-1,3-mannosyltransferase activity;alpha-1,6-mannosyltransferase activity;binding;calcium-dependent protein binding;catalytic activity;glycolipid 3-alpha-mannosyltransferase activity;glycolipid 6-alpha-mannosyltransfera
adenyl nucleotide binding;adenyl ribonucleotide binding;ATP binding;ATPase activity;ATPase activity, coupled;ATP-dependent helicase activity;ATP-dependent RNA helicase activity;binding;catalytic activity;enzyme binding;h
binding;enzyme binding;GTPase binding;protein binding;Ran GTPase binding;Ras GTPase binding;R-SMAD binding;SMAD binding;small GTPase binding
binding;catalytic activity;cation binding;dCTP diphosphatase activity;deoxyribonucleotide binding;hydrolase activity;hydrolase activity, acting on acid anhydrides;hydrolase activity, acting on acid anhydrides, in phosphorus-co
binding;enzyme binding;phosphatase binding;protein binding
binding;nucleic acid binding;RNA binding;rRNA binding
binding;catalytic activity;cation binding;histone methyltransferase activity;histone-lysine N-methyltransferase activity;ion binding;lysine N-methyltransferase activity;metal ion binding;methyltransferase activity;N-methyltransfe
binding;nucleic acid binding;RNA binding
amine binding;amino acid binding;binding;carboxylic acid binding;catalytic activity;cation binding;disulfide oxidoreductase activity;DNA binding;electron carrier activity;glutathione binding;heme binding;intramolecular oxidore
binding;nucleic acid binding;RNA binding;toxin binding
acid-amino acid ligase activity;adenyl nucleotide binding;adenyl ribonucleotide binding;ATP binding;binding;catalytic activity;enzyme binding;ligase activity;ligase activity, forming carbon-nitrogen bonds;nucleotide binding;pro
acyl-CoA dehydrogenase activity;binding;catalytic activity;coenzyme binding;cofactor binding;flavin adenine dinucleotide binding;oxidoreductase activity;oxidoreductase activity, acting on the CH-CH group of donors
binding;cation binding;ion binding;metal ion binding
binding;cation binding;DNA binding;ion binding;metal ion binding;nucleic acid binding;transition metal ion binding;zinc ion binding
binding;enzyme activator activity;enzyme regulator activity;GTPase activator activity;GTPase regulator activity;guanyl-nucleotide exchange factor activity;identical protein binding;molecular transducer activity;nucleoside-triph
binding;cation binding;ion binding;metal ion binding
acidic amino acid transmembrane transporter activity;active transmembrane transporter activity;amine transmembrane transporter activity;amino acid transmembrane transporter activity;carboxylic acid transmembrane trans
ion transmembrane transporter activity;substrate-specific transmembrane transporter activity;substrate-specific transporter activity;transmembrane transporter activity;transporter activity
acetyltransferase activity;binding;catalytic activity;cation binding;enzyme regulator activity;H3 histone acetyltransferase activity;H4 histone acetyltransferase activity;histone acetyltransferase activity;ion binding;iron-sulfur clus
catalytic activity;kinase activity;transferase activity;transferase activity, transferring phosphorus-containing groups
acid-amino acid ligase activity;catalytic activity;ligase activity;ligase activity, forming carbon-nitrogen bonds;phosphopantothenate--cysteine ligase activity
binding;molecular transducer activity;protein binding;protein complex binding;signal transducer activity
binding;enzyme binding;GTPase binding;mRNA binding;nucleic acid binding;pre-miRNA binding;protein binding;protein transporter activity;Ran GTPase binding;Ras GTPase binding;RNA binding;small GTPase binding;subst
adenyl nucleotide binding;adenyl-nucleotide exchange factor activity;ATPase regulator activity;binding;enzyme regulator activity;nucleoside-triphosphatase regulator activity;nucleotide binding;protein binding;purine nucleoti
binding;identical protein binding;protein binding;protein dimerization activity;protein homodimerization activity
binding;catalytic activity;cation binding;GDP binding;GTP binding;GTPase activity;guanyl nucleotide binding;guanyl ribonucleotide binding;hydrolase activity;hydrolase activity, acting on acid anhydrides;hydrolase activity, act
catalytic activity;oxidoreductase activity
acid-amino acid ligase activity;binding;catalytic activity;histone binding;ligase activity;ligase activity, forming carbon-nitrogen bonds;methylated histone residue binding;nucleic acid binding;protein binding;RNA binding;single
adenyl nucleotide binding;adenyl ribonucleotide binding;ATP binding;binding;catalytic activity;CoA carboxylase activity;ligase activity;ligase activity, forming carbon-carbon bonds;methylcrotonoyl-CoA carboxylase activity;nu
binding;chromatin binding
adenyl nucleotide binding;adenyl ribonucleotide binding;ATP binding;binding;catalytic activity;helicase activity;hydrolase activity;hydrolase activity, acting on acid anhydrides;hydrolase activity, acting on acid anhydrides, in ph
binding;nucleic acid binding;RNA binding
adenyl nucleotide binding;adenyl ribonucleotide binding;ATP binding;ATPase activity;ATPase activity, coupled;beta-catenin binding;binding;catalytic activity;chromatin binding;DNA binding;DNA helicase activity;DNA-depend
binding;catalytic activity;GTP binding;GTPase activity;guanyl nucleotide binding;guanyl ribonucleotide binding;hydrolase activity;hydrolase activity, acting on acid anhydrides;hydrolase activity, acting on acid anhydrides, in ph
binding;calcium ion binding;carbohydrate binding;cation binding;extracellular matrix binding;ion binding;metal ion binding
binding;DNA binding;nucleic acid binding
ligand-dependent nuclear receptor transcription coactivator activity;protein binding transcription factor activity;transcription coactivator activity;transcription cofactor activity;transcription factor binding transcription factor ac
adenyl nucleotide binding;adenyl ribonucleotide binding;ATP binding;ATPase activity;binding;catalytic activity;cation binding;hydrolase activity;hydrolase activity, acting on acid anhydrides;hydrolase activity, acting on acid an
binding;ion channel binding;protein binding
structural constituent of ribosome;structural molecule activity
binding;catalytic activity;cation binding;hydrolase activity;identical protein binding;ion binding;metal ion binding;metallopeptidase activity;peptidase activity;peptidase activity, acting on L-amino acid peptides;protein binding;
amine-lyase activity;arylesterase activity;carbon-nitrogen lyase activity;carboxylic ester hydrolase activity;catalytic activity;hydrolase activity;hydrolase activity, acting on ester bonds;lyase activity;strictosidine synthase activity
binding;cation binding;enzyme activator activity;enzyme regulator activity;GTPase activator activity;GTPase regulator activity;ion binding;metal ion binding;nucleoside-triphosphatase regulator activity;protein transporter activ
binding;catalytic activity;GDP binding;GTP binding;GTPase activity;guanyl nucleotide binding;guanyl ribonucleotide binding;hydrolase activity;hydrolase activity, acting on acid anhydrides;hydrolase activity, acting on acid anh
catalytic activity;CTD phosphatase activity;hydrolase activity;hydrolase activity, acting on ester bonds;phosphatase activity;phosphoprotein phosphatase activity;phosphoric ester hydrolase activity;protein serine/threonine ph
binding;protein binding;protein C-terminus binding
adenyl nucleotide binding;adenyl ribonucleotide binding;aminoacyl-tRNA ligase activity;ATP binding;binding;catalytic activity;ligase activity;ligase activity, forming aminoacyl-tRNA and related compounds;ligase activity, formi
structural constituent of ribosome;structural molecule activity
catalytic activity;hydrolase activity;hydrolase activity, acting on acid anhydrides;hydrolase activity, acting on acid anhydrides, in phosphorus-containing anhydrides;microtubule motor activity;motor activity;nucleoside-triphos
binding;carbohydrate binding
3'-5' exonuclease activity;3'-5'-exoribonuclease activity;AU-rich element binding;binding;catalytic activity;exonuclease activity;exonuclease activity, active with either ribo- or deoxyribonucleic acids and producing 5'-phosph
binding;nucleic acid binding;RNA binding;snoRNA binding
binding;catalytic activity;cation binding;ion binding;metal ion binding;transferase activity;transferase activity, transferring acyl groups
acyl-CoA hydrolase activity;catalytic activity;CoA hydrolase activity;hydrolase activity;hydrolase activity, acting on ester bonds;thiolester hydrolase activity
binding;enzyme activator activity;enzyme regulator activity;G-protein alpha-subunit binding;GTPase activator activity;GTPase regulator activity;guanyl-nucleotide exchange factor activity;nucleoside-triphosphatase regulator a
binding;identical protein binding;mRNA binding;nucleic acid binding;protein binding;RNA binding
binding;identical protein binding;protein binding;protein complex binding
carbohydrate phosphatase activity;catalytic activity;fructose-2,6-bisphosphate 2-phosphatase activity;hydrolase activity;hydrolase activity, acting on ester bonds;phosphatase activity;phosphoric ester hydrolase activity;suga
basal RNA polymerase II transcription machinery binding;basal transcription machinery binding;binding;enzyme binding;protein binding;RNA polymerase binding;RNA polymerase core enzyme binding;RNA polymerase II core
aminopeptidase activity;binding;catalytic activity;cation binding;exopeptidase activity;hydrolase activity;ion binding;manganese ion binding;metal ion binding;metallopeptidase activity;peptidase activity;peptidase activity, acti
catalytic activity;hydrolase activity;hydrolase activity, acting on carbon-nitrogen (but not peptide) bonds;hydrolase activity, acting on carbon-nitrogen (but not peptide) bonds, in linear amides;omega-amidase activity
binding;cell adhesion molecule binding;identical protein binding;protein binding;protein dimerization activity;protein homodimerization activity
3'-5' exonuclease activity;3'-5'-exoribonuclease activity;binding;catalytic activity;exonuclease activity;exonuclease activity, active with either ribo- or deoxyribonucleic acids and producing 5'-phosphomonoesters;exoribonucl
14-3-3 protein binding;adenyl nucleotide binding;adenyl ribonucleotide binding;ATP binding;binding;catalytic activity;enzyme binding;hydrolase activity;hydrolase activity, acting on acid anhydrides;hydrolase activity, acting o
actin binding;binding;cytoskeletal protein binding;protein binding
aminopeptidase activity;binding;catalytic activity;cation binding;exopeptidase activity;hydrolase activity;identical protein binding;ion binding;manganese ion binding;metal ion binding;metalloaminopeptidase activity;metalloex

binding;binding, bridging;cytoskeletal adaptor activity;cytoskeletal protein binding;protein binding;protein binding, bridging
acid-amino acid ligase activity;catalytic activity;cysteine-type endopeptidase inhibitor activity;endopeptidase inhibitor activity;endopeptidase regulator activity;enzyme inhibitor activity;enzyme regulator activity;ligase activity;
binding;cation binding;ion binding;metal ion binding;transition metal ion binding;zinc ion binding
acetate-CoA ligase activity;acid-thiol ligase activity;adenyl nucleotide binding;adenyl ribonucleotide binding;AMP binding;ATP binding;binding;catalytic activity;CoA-ligase activity;ligase activity;ligase activity, forming carbon-
adenyl nucleotide binding;adenyl ribonucleotide binding;ATP binding;ATPase activity;ATPase activity, coupled;ATP-dependent helicase activity;ATP-dependent RNA helicase activity;binding;catalytic activity;double-stranded
binding;GTP binding;guanyl nucleotide binding;guanyl ribonucleotide binding;nucleotide binding;purine nucleotide binding;purine ribonucleoside triphosphate binding;purine ribonucleotide binding;ribonucleotide binding
catalytic activity;cytidylyltransferase activity;N-acetylneuraminate synthase activity;N-acylneuraminate cytidylyltransferase activity;N-acylneuraminate-9-phosphate synthase activity;nucleotidyltransferase activity;transferase a
binding;catalytic activity;enzyme regulator activity;GTPase regulator activity;guanyl-nucleotide exchange factor activity;nucleic acid binding;nucleoside-triphosphatase regulator activity;nucleotidyltransferase activity;RNA bin
binding;cation binding;double-stranded RNA binding;ion binding;metal ion binding;nucleic acid binding;RNA binding
adenyl nucleotide binding;adenyl ribonucleotide binding;ATP binding;binding;catalytic activity;CTP synthase activity;ligase activity;ligase activity, forming carbon-nitrogen bonds;nucleotide binding;purine nucleotide binding;p
binding;catalytic activity;DNA binding;DNA polymerase activity;DNA-directed DNA polymerase activity;nucleic acid binding;nucleotidyltransferase activity;sequence-specific DNA binding;transferase activity;transferase activit
binding;catalytic activity;DNA binding;DNA polymerase activity;DNA-directed DNA polymerase activity;nucleic acid binding;nucleotidyltransferase activity;sequence-specific DNA binding;transferase activity;transferase activit
binding;cation binding;ion binding;metal ion binding;transition metal ion binding;zinc ion binding
armadillo repeat domain binding;binding;calmodulin binding;enzyme binding;phosphatase binding;protein binding;protein complex binding;protein domain specific binding;protein phosphatase 2A binding;protein phosphatas
binding;identical protein binding;polyubiquitin binding;protein binding;small conjugating protein binding;ubiquitin binding
binding;cation binding;heme binding;ion binding;iron ion binding;metal ion binding;tetrapyrrole binding;transition metal ion binding
binding;catalytic activity;cation binding;hydrolase activity;hydrolase activity, acting on carbon-nitrogen (but not peptide) bonds;hydrolase activity, acting on carbon-nitrogen (but not peptide) bonds, in cyclic amidines;ion bindi
binding;protein binding;protein domain specific binding;structural constituent of ribosome;structural molecule activity
binding;calcium channel inhibitor activity;calcium channel regulator activity;catalytic activity;channel inhibitor activity;channel regulator activity;hydrolase activity;hydrolase activity, acting on ester bonds;ion channel binding;io
active transmembrane transporter activity;amine transmembrane transporter activity;amino acid transmembrane transporter activity;carboxylic acid transmembrane transporter activity;L-amino acid transmembrane transporte
binding;DNA binding;enzyme activator activity;enzyme regulator activity;GTP binding;GTPase activator activity;GTPase regulator activity;guanyl nucleotide binding;guanyl ribonucleotide binding;nucleic acid binding;nucleosic
binding;nucleotide binding;purine nucleotide binding
binding;calcium ion binding;calcium-dependent protein binding;cation binding;ion binding;lipid transporter activity;metal ion binding;phospholipid scramblase activity;phospholipid transporter activity;protein binding;substrate
adenyl nucleotide binding;adenyl ribonucleotide binding;ATP binding;binding;catalytic activity;DNA binding;helicase activity;hydrolase activity;hydrolase activity, acting on acid anhydrides;hydrolase activity, acting on acid anh
adenyl nucleotide binding;adenyl ribonucleotide binding;ATP binding;ATPase activity;binding;catalytic activity;DNA binding;hydrolase activity;hydrolase activity, acting on acid anhydrides;hydrolase activity, acting on acid anhy
binding;glutamate receptor binding;G-protein-coupled receptor binding;metabotropic glutamate receptor binding;protein binding;receptor binding
adenyl nucleotide binding;adenyl ribonucleotide binding;aminoacyl-tRNA ligase activity;ATP binding;binding;catalytic activity;cation binding;ion binding;ligase activity;ligase activity, forming aminoacyl-tRNA and related compo
adenyl nucleotide binding;adenyl ribonucleotide binding;aminoacyl-tRNA editing activity;aminoacyl-tRNA ligase activity;ATP binding;binding;carboxylic ester hydrolase activity;catalytic activity;hydrolase activity;hydrolase acti
acid-amino acid ligase activity;Atg12 ligase activity;Atg8 ligase activity;binding;catalytic activity;enzyme binding;ligase activity;ligase activity, forming carbon-nitrogen bonds;protein binding;small conjugating protein ligase ac
adenyl nucleotide binding;adenyl ribonucleotide binding;ATP binding;binding;DNA binding;nucleic acid binding;nucleotide binding;purine nucleotide binding;purine ribonucleoside triphosphate binding;purine ribonucleotide bi
adenyl nucleotide binding;adenyl ribonucleotide binding;ATP binding;binding;nucleotide binding;protein binding;protein dimerization activity;protein heterodimerization activity;purine nucleotide binding;purine ribonucleoside t
catalytic activity;hydrolase activity;hydrolase activity, acting on ester bonds;inositol or phosphatidylinositol phosphatase activity;lipid phosphatase activity;phosphatase activity;phosphatidylinositol bisphosphate phosphatase
adenyl nucleotide binding;adenyl ribonucleotide binding;ATP binding;ATPase activity;binding;catalytic activity;cation binding;GTP binding;guanyl nucleotide binding;guanyl ribonucleotide binding;hydrolase activity;hydrolase a
carbon-carbon lyase activity;carboxy-lyase activity;catalytic activity;lyase activity;methylmalonyl-CoA decarboxylase activity
binding;nucleotide binding
binding;cation binding;ion binding;metal ion binding;transition metal ion binding;zinc ion binding
2,4-dienoyl-CoA reductase (NADPH) activity;binding;catalytic activity;oxidoreductase activity;oxidoreductase activity, acting on the CH-CH group of donors;oxidoreductase activity, acting on the CH-CH group of donors, NAD
catalytic activity;hydrolase activity;hydrolase activity, acting on glycosyl bonds;hydrolase activity, hydrolyzing O-glycosyl compounds
binding;cytoskeletal protein binding;L27 domain binding;protein binding;protein domain specific binding
adenyl nucleotide binding;adenyl ribonucleotide binding;ATP binding;ATPase activity;binding;catalytic activity;hydrolase activity;hydrolase activity, acting on acid anhydrides;hydrolase activity, acting on acid anhydrides, in ph
binding;GTP binding;guanyl nucleotide binding;guanyl ribonucleotide binding;nucleotide binding;purine nucleotide binding;purine ribonucleoside triphosphate binding;purine ribonucleotide binding;ribonucleotide binding
enzyme activator activity;enzyme regulator activity;GTPase activator activity;GTPase regulator activity;nucleoside-triphosphatase regulator activity
binding;DNA binding;DNA polymerase binding;enzyme binding;nucleic acid binding;protein binding
adenyl nucleotide binding;adenyl ribonucleotide binding;ATP binding;binding;nucleotide binding;purine nucleotide binding;purine ribonucleoside triphosphate binding;purine ribonucleotide binding;ribonucleotide binding
alpha-tubulin binding;beta-tubulin binding;binding;catalytic activity;cytoskeletal protein binding;GDP binding;GTP binding;GTPase activity;guanyl nucleotide binding;guanyl ribonucleotide binding;hydrolase activity;hydrolase
[myelin basic protein]-arginine N-methyltransferase activity;arginine N-methyltransferase activity;binding;catalytic activity;histone binding;histone methyltransferase activity;histone methyltransferase activity (H4-R3 specific);h
adenyl nucleotide binding;adenyl ribonucleotide binding;ATP binding;ATPase activity;ATPase activity, coupled;ATP-dependent helicase activity;ATP-dependent RNA helicase activity;binding;catalytic activity;helicase activity;h
binding;cation binding;ion binding;metal ion binding
binding;nucleotide binding
binding;calcium-dependent protein binding;cation binding;ion binding;metal ion binding;nucleic acid binding;nucleocytoplasmic transporter activity;nucleotide binding;pre-mRNA binding;protein binding;RNA binding;snRNA b
binding;enzyme binding;protein binding
binding;nucleotide binding
binding;nucleic acid binding;RNA binding;snoRNA binding
binding;catalytic activity;cation binding;hydrolase activity;hydrolase activity, acting on glycosyl bonds;hydrolase activity, hydrolyzing O-glycosyl compounds;ion binding;metal ion binding;poly(ADP-ribose) glycohydrolase activ
binding;cation binding;ion binding;metal ion binding
3'(2'),5'-bisphosphate nucleotidase activity;3'-nucleotidase activity;binding;catalytic activity;cation binding;hydrolase activity;hydrolase activity, acting on ester bonds;inositol monophosphate phosphatase activity;inositol or p
binding;enzyme binding;phosphatase binding;protein binding;protein complex scaffold;structural molecule activity
binding;catalytic activity;coenzyme binding;cofactor binding;double-stranded RNA binding;enzyme inhibitor activity;enzyme regulator activity;flavin adenine dinucleotide binding;kinase inhibitor activity;kinase regulator activity
binding;catalytic activity;cation binding;hydrolase activity;hydrolase activity, acting on ester bonds;ion binding;lipase activity;metal ion binding;phospholipase activity;phosphoric diester hydrolase activity;phosphoric ester hyd
adenyl nucleotide binding;adenyl ribonucleotide binding;ATP binding;binding;nucleotide binding;purine nucleotide binding;purine ribonucleoside triphosphate binding;purine ribonucleotide binding;ribonucleotide binding
binding;catalytic activity;cation binding;ion binding;metal ion binding;methyltransferase activity;N-methyltransferase activity;nucleic acid binding;RNA binding;RNA methyltransferase activity;S-adenosylmethionine-dependent
binding;nucleic acid binding;p53 binding;protein binding;RNA binding
cation channel activity;cation transmembrane transporter activity;channel activity;ion channel activity;ion transmembrane transporter activity;passive transmembrane transporter activity;substrate-specific channel activity;sub
binding;catalytic activity;cation binding;dipeptidyl-peptidase activity;exopeptidase activity;hydrolase activity;ion binding;metal ion binding;metallopeptidase activity;peptidase activity;peptidase activity, acting on L-amino acid
binding;DNA binding;nucleic acid binding
binding;binding, bridging;cytoskeletal adaptor activity;cytoskeletal protein binding;DNA binding;enzyme binding;kinase binding;nucleic acid binding;proline-rich region binding;protein binding;protein binding, bridging;protein
binding;DNA binding;nucleic acid binding
binding;nucleic acid binding;RNA binding;snoRNA binding
adenyl nucleotide binding;adenyl ribonucleotide binding;ATP binding;binding;catalytic activity;cation binding;ion binding;kinase activity;magnesium ion binding;MAP kinase kinase kinase activity;metal ion binding;molecular tr
binding;catalytic activity;cis-trans isomerase activity;drug binding;FK506 binding;isomerase activity;macrolide binding;peptidyl-prolyl cis-trans isomerase activity
binding;cytoskeletal protein binding;protein binding;tropomyosin binding
binding;catalytic activity;glucosyltransferase activity;protein binding;transferase activity;transferase activity, transferring glycosyl groups;transferase activity, transferring hexosyl groups;UDP-glucose:glycoprotein glucosyltrans
catalytic activity;oxidoreductase activity;oxidoreductase activity, acting on the CH-CH group of donors
aminopeptidase activity;binding;catalytic activity;cation binding;cytokine receptor binding;exopeptidase activity;growth factor receptor binding;hydrolase activity;interleukin-1 receptor binding;interleukin-1, Type II receptor bi
2 iron, 2 sulfur cluster binding;binding;cation binding;ion binding;iron-sulfur cluster binding;metal cluster binding;metal ion binding
binding;DNA binding;nucleic acid binding;nucleic acid binding transcription factor activity;protein binding transcription factor activity;sequence-specific DNA binding;sequence-specific DNA binding transcription factor activit
binding;mRNA 3'-UTR binding;mRNA 5'-UTR binding;mRNA binding;nucleic acid binding;nucleotide binding;RNA binding;translation regulator activity
enzyme inhibitor activity;enzyme regulator activity
binding;enzyme binding;enzyme regulator activity;methionine adenosyltransferase regulator activity;protein binding
binding;lipid binding;phospholipid binding
adenyl nucleotide binding;adenyl ribonucleotide binding;ATP binding;binding;calcium ion binding;cation binding;GTP binding;guanyl nucleotide binding;guanyl ribonucleotide binding;ion binding;metal ion binding;nucleic acid
adenyl nucleotide binding;adenyl ribonucleotide binding;ATP binding;binding;calcium ion binding;catalytic activity;cation binding;GTP binding;guanyl nucleotide binding;guanyl ribonucleotide binding;hydrolase activity;ion bin
binding;G-protein coupled receptor activity;molecular transducer activity;odorant binding;olfactory receptor activity;receptor activity;signal transducer activity;signaling receptor activity;transmembrane signaling receptor acti
binding;cytoskeletal protein binding;protein binding
binding;cytoskeletal protein binding;protein binding;tropomyosin binding
binding;G-protein coupled receptor activity;molecular transducer activity;opioid receptor activity;peptide binding;peptide receptor activity;peptide receptor activity, G-protein coupled;receptor activity;signal transducer activity
binding;cation binding;ion binding;metal ion binding;protein binding transcription factor activity;transcription cofactor activity;transcription corepressor activity;transcription factor binding transcription factor activity;transition
binding;catalytic activity;endopeptidase inhibitor activity;endopeptidase regulator activity;enzyme inhibitor activity;enzyme regulator activity;insulin-like growth factor-activated receptor activity;kinase activity;molecular transd
binding;nucleic acid binding;RNA binding
structural constituent of ribosome;structural molecule activity
3-hydroxyacyl-CoA dehydratase activity;binding;carbon-oxygen lyase activity;catalytic activity;enzyme activator activity;enzyme binding;enzyme regulator activity;GTPase activator activity;GTPase regulator activity;hydro-lyas
adenyl nucleotide binding;adenyl ribonucleotide binding;ATP binding;binding;catalytic activity;NADH dehydrogenase (quinone) activity;NADH dehydrogenase (ubiquinone) activity;NADH dehydrogenase activity;nucleotide bin
binding;catalytic activity;cation binding;ion binding;ligase activity;metal ion binding;transition metal ion binding;zinc ion binding
binding;cytoskeletal protein binding;FFAT motif binding;microtubule binding;molecular transducer activity;protein binding;protein dimerization activity;protein domain specific binding;protein heterodimerization activity;signal t
acid-amino acid ligase activity;binding;catalytic activity;cation binding;ion binding;ligase activity;ligase activity, forming carbon-nitrogen bonds;metal ion binding;small conjugating protein ligase activity;transition metal ion bin
binding;enzyme binding;enzyme regulator activity;GDP-dissociation inhibitor activity;GTPase binding;GTPase regulator activity;identical protein binding;nucleoside-triphosphatase regulator activity;protein binding;Ras GTPas
binding;GTP binding;guanyl nucleotide binding;guanyl ribonucleotide binding;nucleotide binding;purine nucleotide binding;purine ribonucleoside triphosphate binding;purine ribonucleotide binding;ribonucleotide binding
binding;PDZ domain binding;protein binding;protein domain specific binding;protein self-association
binding;cytoskeletal protein binding;enzyme binding;GTPase binding;kinase binding;microtubule binding;protein binding;protein domain specific binding;protein kinase binding;Rac GTPase binding;Ras GTPase binding;Rho

catalytic activity
enzyme regulator activity;kinase regulator activity
binding;enzyme binding;phosphatase binding;protein binding;protein phosphatase 2A binding;protein phosphatase binding
receptor activity
binding;nucleic acid binding;RNA binding
adenyl nucleotide binding;adenyl ribonucleotide binding;aminoacyl-tRNA editing activity;aminoacyl-tRNA ligase activity;ATP binding;binding;carboxylic ester hydrolase activity;catalytic activity;hydrolase activity;hydrolase acti
binding;DNA binding;nucleic acid binding;nucleotide binding
binding;cation binding;ion binding;metal ion binding;nucleotide binding
binding;cation binding;enzyme binding;GTPase binding;ion binding;metal ion binding;protein binding;Rab GTPase binding;Ras GTPase binding;small GTPase binding
acid-thiol ligase activity;adenyl nucleotide binding;adenyl ribonucleotide binding;ATP binding;binding;catalytic activity;cation binding;CoA-ligase activity;ion binding;ligase activity;ligase activity, forming carbon-sulfur bonds;m
binding;catalytic activity;cation binding;GMP reductase activity;ion binding;metal ion binding;oxidoreductase activity;oxidoreductase activity, acting on NADH or NADPH;oxidoreductase activity, acting on NADH or NADPH, ni
binding;protein binding;SNAP receptor activity
binding;calcium ion binding;cation binding;ion binding;metal ion binding
ATP-dependent protein binding;binding;catalytic activity;enzyme activator activity;enzyme regulator activity;protein binding;protein C-terminus binding;protein dimerization activity;protein heterodimerization activity;small prot
structural molecule activity
acid-amino acid ligase activity;binding;catalytic activity;cation binding;copper ion binding;ion binding;ligase activity;ligase activity, forming carbon-nitrogen bonds;metal ion binding;NEDD8 ligase activity;small conjugating pro
binding;carbohydrate binding;collagen binding;protein binding
anion binding;binding;ion binding;molecular transducer activity;PDZ domain binding;phosphate ion binding;protein binding;protein domain specific binding;signal transducer activity
7-dehydrocholesterol reductase activity;catalytic activity;oxidoreductase activity;oxidoreductase activity, acting on the CH-CH group of donors;oxidoreductase activity, acting on the CH-CH group of donors, NAD or NADP as
alpha-tubulin binding;beta-catenin binding;binding;catalytic activity;cation binding;core promoter binding;cytoskeletal protein binding;deacetylase activity;DNA binding;dynein complex binding;enzyme binding;heat shock pro
binding;cation binding;ion binding;metal ion binding;transition metal ion binding;zinc ion binding
binding;nucleic acid binding;ribonucleoprotein binding;ribosome binding;RNA binding;translation factor activity, nucleic acid binding;translation initiation factor activity
binding;carboxylic acid binding;catalytic activity;coenzyme binding;cofactor binding;glycerate dehydrogenase activity;glyoxylate reductase (NADP) activity;hydroxypyruvate reductase activity;identical protein binding;NAD bin
catalytic activity;cysteine-type endopeptidase activity;cysteine-type peptidase activity;endopeptidase activity;hydrolase activity;peptidase activity;peptidase activity, acting on L-amino acid peptides
adenyl nucleotide binding;adenyl ribonucleotide binding;ATP binding;binding;catalytic activity;cation binding;enzyme activator activity;enzyme regulator activity;ion binding;metal ion binding;nucleotide binding;protein binding
binding;chromatin binding;protein binding;protein N-terminus binding
binding;mRNA binding;nucleic acid binding;nucleocytoplasmic transporter activity;nucleotide binding;RNA binding;transporter activity
binding;calcium ion binding;calcium-dependent cysteine-type endopeptidase activity;catalytic activity;cation binding;cysteine-type endopeptidase activity;cysteine-type peptidase activity;endopeptidase activity;hydrolase ac
carboxylic acid transmembrane transporter activity;dicarboxylic acid transmembrane transporter activity;organic acid transmembrane transporter activity;substrate-specific transmembrane transporter activity;substrate-speci
catalytic activity;cation transmembrane transporter activity;hydrogen ion transmembrane transporter activity;inorganic cation transmembrane transporter activity;ion transmembrane transporter activity;monovalent inorganic c
binding;binding, bridging;catalytic activity;guanylate kinase activity;kinase activity;nucleobase-containing compound kinase activity;nucleotide kinase activity;phosphotransferase activity, phosphate group as acceptor;protein
structural constituent of cytoskeleton;structural molecule activity
adenyl nucleotide binding;adenyl ribonucleotide binding;ATP binding;ATPase activity;binding;catalytic activity;hydrolase activity;hydrolase activity, acting on acid anhydrides;hydrolase activity, acting on acid anhydrides, in ph
binding;cation binding;ion binding;metal ion binding;transition metal ion binding;zinc ion binding
binding;cation binding;ion binding;metal ion binding;protein binding transcription factor activity;transcription cofactor activity;transcription corepressor activity;transcription factor binding transcription factor activity;transition
protein transporter activity;receptor activity;substrate-specific transporter activity;transporter activity
binding;cation binding;ion binding;metal ion binding
adenyl nucleotide binding;adenyl ribonucleotide binding;ATP binding;binding;calcium ion binding;cation binding;DNA binding;ion binding;metal ion binding;nucleic acid binding;nucleotide binding;purine nucleotide binding;pu
binding;cytoskeletal protein binding;identical protein binding;lamin binding;microtubule binding;protein binding;tubulin binding
actin binding;actin filament binding;actin monomer binding;binding;cation binding;cytoskeletal protein binding;ion binding;metal ion binding;protein binding;transition metal ion binding;zinc ion binding
7S RNA binding;binding;endoplasmic reticulum signal peptide binding;nucleic acid binding;peptide binding;ribonucleoprotein binding;RNA binding;signal recognition particle binding;signal sequence binding
binding;cation binding;heat shock protein binding;Hsp90 protein binding;ion binding;metal ion binding;protein binding;transition metal ion binding;zinc ion binding
adenyl nucleotide binding;adenyl ribonucleotide binding;ATP binding;binding;catalytic activity;kinase activity;nucleic acid binding;nucleotide binding;phosphoprotein binding;phosphotransferase activity, alcohol group as acce
binding;catalytic activity;GTP binding;GTPase activity;guanyl nucleotide binding;guanyl ribonucleotide binding;hydrolase activity;hydrolase activity, acting on acid anhydrides;hydrolase activity, acting on acid anhydrides, in pl
active transmembrane transporter activity;anion transmembrane transporter activity;ATPase activity;ATPase activity, coupled;ATPase activity, coupled to movement of substances;ATPase activity, coupled to transmembrane r
binding;carbohydrate binding;catalytic activity;cation binding;endopeptidase activity;glycosaminoglycan binding;heparin binding;hydrolase activity;ion binding;metal ion binding;metalloendopeptidase activity;metallopeptidas
catalytic activity;dipeptidyl-peptidase activity;exopeptidase activity;hydrolase activity;peptidase activity;peptidase activity, acting on L-amino acid peptides;serine hydrolase activity;serine-type peptidase activity
catalytic activity;cytochrome-b5 reductase activity;oxidoreductase activity;oxidoreductase activity, acting on NADH or NADPH;oxidoreductase activity, acting on NADH or NADPH, heme protein as acceptor
binding;protein binding;protein binding involved in protein folding;unfolded protein binding
binding;DNA binding;identical protein binding;nucleic acid binding;nucleotide binding;protein binding
adenyl nucleotide binding;adenyl ribonucleotide binding;ATP binding;binding;catalytic activity;identical protein binding;kinase activity;nucleotide binding;phosphotransferase activity, alcohol group as acceptor;protein binding
acireductone synthase activity;binding;catalytic activity;cation binding;hydrolase activity;hydrolase activity, acting on ester bonds;ion binding;magnesium ion binding;metal ion binding;phosphatase activity;phosphoric ester h
catalytic activity;electron carrier activity;NADH dehydrogenase (quinone) activity;NADH dehydrogenase (ubiquinone) activity;NADH dehydrogenase activity;oxidoreductase activity;oxidoreductase activity, acting on NADH or N
binding;nucleic acid binding;protein binding;RNA binding;translation factor activity, nucleic acid binding;translation initiation factor activity;translation initiation factor binding
active transmembrane transporter activity;ATPase activity;ATPase activity, coupled;ATPase activity, coupled to movement of substances;ATPase activity, coupled to transmembrane movement of ions;ATPase activity, couplec
binding;identical protein binding;protein binding
protein transporter activity;substrate-specific transporter activity;transporter activity
catalytic activity;methyltransferase activity;protein methyltransferase activity;transferase activity;transferase activity, transferring one-carbon groups
binding;nuclear export signal receptor activity;protein binding
acetylcholine receptor regulator activity;adenyl nucleotide binding;adenyl ribonucleotide binding;ATP binding;binding;catalytic activity;cation binding;chromatin binding;histone acetyl-lysine binding;histone binding;histone kin
angiostatin binding;ATPase binding;ATPase inhibitor activity;ATPase regulator activity;binding;calmodulin binding;enzyme binding;enzyme inhibitor activity;enzyme regulator activity;identical protein binding;nucleoside-triphos
adenyl nucleotide binding;adenyl ribonucleotide binding;ATP binding;binding;catalytic activity;GTP binding;guanyl nucleotide binding;guanyl ribonucleotide binding;kinase activity;nucleobase-containing compound kinase act
acid-amino acid ligase activity;binding;catalytic activity;cation binding;DNA binding;enzyme binding;enzyme regulator activity;GTPase binding;GTPase regulator activity;guanyl-nucleotide exchange factor activity;ion binding;
catalytic activity;oxidoreductase activity;oxidoreductase activity, acting on a sulfur group of donors;oxidoreductase activity, acting on a sulfur group of donors, disulfide as acceptor;peptide-methionine-(S)-S-oxide reductase
adenyl nucleotide binding;adenyl ribonucleotide binding;ATP binding;binding;carbohydrate kinase activity;catalytic activity;kinase activity;N-acetylglucosamine kinase activity;N-acylmannosamine kinase activity;nucleotide bir
binding;carbon-carbon lyase activity;catalytic activity;cation binding;coenzyme binding;cofactor binding;identical protein binding;ion binding;lyase activity;magnesium ion binding;metal ion binding;protein binding;receptor bir
acidic amino acid transmembrane transporter activity;active transmembrane transporter activity;amine transmembrane transporter activity;amino acid transmembrane transporter activity;binding;calcium ion binding;carboxyli
actin binding;actin filament binding;binding;cytoskeletal protein binding;enzyme activator activity;enzyme regulator activity;protein binding
binding;protein binding;protein N-terminus binding
acid-amino acid ligase activity;catalytic activity;ligase activity;ligase activity, forming carbon-nitrogen bonds;small conjugating protein ligase activity;ubiquitin-protein ligase activity
binding;enzyme binding;phosphatase binding;protein binding;protein phosphatase binding
binding;enzyme binding;GTPase binding;protein binding;receptor binding
binding;protein binding;protein complex binding;small conjugating protein binding;ubiquitin binding
binding;nucleic acid binding;RNA binding;snRNA binding;U6 snRNA binding
binding;catalytic activity;cation binding;endonuclease activity;endonuclease activity, active with either ribo- or deoxyribonucleic acids and producing 5'-phosphomonoesters;endoribonuclease activity;endoribonuclease activit
5'-3' exonuclease activity;binding;catalytic activity;cation binding;endonuclease activity;endoribonuclease activity;exonuclease activity;hydrolase activity;hydrolase activity, acting on ester bonds;ion binding;metal ion binding;
binding;enzyme binding;identical protein binding;kinase binding;protein binding;protein kinase B binding;protein kinase binding
binding;catalytic activity;DNA binding;enzyme binding;NAD+ ADP-ribosyltransferase activity;nucleic acid binding;protein binding;transferase activity;transferase activity, transferring glycosyl groups;transferase activity, transfe
ADP-ribose diphosphatase activity;ADP-sugar diphosphatase activity;binding;catalytic activity;cation binding;hydrolase activity;hydrolase activity, acting on acid anhydrides;hydrolase activity, acting on acid anhydrides, in pho
binding;nucleic acid binding;nucleotide binding;RNA binding
acetyltransferase activity;binding;catalytic activity;DNA binding;enzyme activator activity;enzyme regulator activity;histone acetyltransferase activity;lysine N-acetyltransferase activity;N-acetyltransferase activity;N-acyltransfe
binding;calcium channel inhibitor activity;calcium channel regulator activity;channel inhibitor activity;channel regulator activity;cytoskeletal protein binding;ion channel inhibitor activity;lipid binding;phospholipid binding;protei
ATPase activity;binding;catalytic activity;enzyme binding;hydrolase activity;hydrolase activity, acting on acid anhydrides;hydrolase activity, acting on acid anhydrides, in phosphorus-containing anhydrides;nucleic acid binding
binding;catalytic activity;GDP binding;GTP binding;GTPase activity;guanyl nucleotide binding;guanyl ribonucleotide binding;hydrolase activity;hydrolase activity, acting on acid anhydrides;hydrolase activity, acting on acid anh
binding;catalytic activity;GDP binding;GTP binding;GTPase activity;guanyl nucleotide binding;guanyl ribonucleotide binding;hydrolase activity;hydrolase activity, acting on acid anhydrides;hydrolase activity, acting on acid anh
aminopeptidase activity;binding;catalytic activity;cation binding;exopeptidase activity;hydrolase activity;ion binding;metal ion binding;metallopeptidase activity;peptidase activity;peptidase activity, acting on L-amino acid pep
binding;catalytic activity;GTP binding;GTPase activity;guanyl nucleotide binding;guanyl ribonucleotide binding;hydrolase activity;hydrolase activity, acting on acid anhydrides;hydrolase activity, acting on acid anhydrides, in pl
binding;nucleic acid binding;RNA binding;translation factor activity, nucleic acid binding;translation initiation factor activity
binding;cation binding;enzyme activator activity;enzyme regulator activity;GTPase activator activity;GTPase regulator activity;ion binding;lipid binding;metal ion binding;nucleoside-triphosphatase regulator activity;phosphatid
binding;calcium ion binding;cation binding;ion binding;metal ion binding
acid-amino acid ligase activity;binding;catalytic activity;cation binding;ion binding;ligase activity;ligase activity, forming carbon-nitrogen bonds;metal ion binding;small conjugating protein ligase activity;ubiquitin-protein ligase
actin binding;actin filament binding;binding;cytoskeletal protein binding;protein binding
adenyl nucleotide binding;adenyl ribonucleotide binding;ATP binding;binding;enzyme binding;GTP binding;guanyl nucleotide binding;guanyl ribonucleotide binding;kinase binding;nucleotide binding;protein binding;protein kir
binding;cation binding;ion binding;metal ion binding
binding;cation binding;DEAD/H-box RNA helicase binding;DNA binding;enzyme binding;ion binding;metal ion binding;nucleic acid binding;protein binding
binding;DNA binding;nucleic acid binding;nucleic acid binding transcription factor activity;sequence-specific DNA binding;sequence-specific DNA binding transcription factor activity
actin binding;actin filament binding;adenyl nucleotide binding;adenyl ribonucleotide binding;ADP binding;ATP binding;binding;calmodulin binding;catalytic activity;cytoskeletal protein binding;hydrolase activity;hydrolase activ
adenyl nucleotide binding;adenyl ribonucleotide binding;ATP binding;ATPase activity;ATPase activity, coupled;ATP-dependent helicase activity;ATP-dependent RNA helicase activity;binding;catalytic activity;helicase activity;h
acid-amino acid ligase activity;binding;catalytic activity;identical protein binding;ligase activity;ligase activity, forming carbon-nitrogen bonds;protein binding;small conjugating protein ligase activity;ubiquitin-protein ligase acti
binding;enzyme binding;lipid binding;phosphatidylinositol binding;phospholipid binding;protein binding
binding;nucleotide binding;protein binding;protein complex scaffold;receptor signaling complex scaffold activity;structural molecule activity
acid-amino acid ligase activity;binding;binding, bridging;catalytic activity;enzyme binding;G-protein-coupled receptor binding;heat shock protein binding;Hsp70 protein binding;Hsp90 protein binding;identical protein binding;
binding;cytoskeletal protein binding;identical protein binding;lipid binding;phosphatidic acid binding;phospholipid binding;protein binding;transporter activity
binding;cytoskeletal protein binding;dynactin binding;identical protein binding;lipid binding;phosphatidylinositol binding;phospholipid binding;protein binding;protein dimerization activity;protein homodimerization activity
binding;enzyme binding;enzyme regulator activity;heat shock protein binding;kinase binding;kinase regulator activity;NF-kappaB binding;protein binding;protein kinase binding;protein kinase regulator activity;small conjugatin
receptor activity

catalytic activity;methyltransferase activity;N-methyltransferase activity;RNA methyltransferase activity;rRNA (adenine) methyltransferase activity;rRNA (adenine-N6,N6-)-dimethyltransferase activity;rRNA methyltransferase ac
binding;nucleic acid binding;RNA binding;snoRNA binding
binding;enzyme regulator activity;lipid binding;phosphatase regulator activity;protein binding;protein phosphatase regulator activity;protein phosphatase type 1 regulator activity;small conjugating protein binding;ubiquitin binc
active transmembrane transporter activity;anion transmembrane transporter activity;anion:cation symporter activity;binding;cation transmembrane transporter activity;cation:chloride symporter activity;chloride transmembran
actin binding;actin filament binding;ATPase activity;binding;calcium ion binding;catalytic activity;cation binding;cytoskeletal protein binding;hydrolase activity;hydrolase activity, acting on acid anhydrides;hydrolase activity, ac
binding;identical protein binding;protein binding;protein complex binding;protein dimerization activity;protein homodimerization activity
binding;enzyme binding;phosphatase binding;protein binding;protein phosphatase binding
acid-amino acid ligase activity;binding;catalytic activity;cation binding;co-SMAD binding;DNA binding;ion binding;ligase activity;ligase activity, forming carbon-nitrogen bonds;metal ion binding;nucleic acid binding;protein bir
binding;nucleotide binding
catalytic activity;cysteine-type endopeptidase activity;cysteine-type peptidase activity;endopeptidase activity;hydrolase activity;peptidase activity;peptidase activity, acting on L-amino acid peptides;small conjugating protein-
binding;DNA binding;nucleic acid binding;nucleic acid binding transcription factor activity;sequence-specific DNA binding;sequence-specific DNA binding RNA polymerase II transcription factor activity;sequence-specific DN
acidic amino acid transmembrane transporter activity;active transmembrane transporter activity;amine transmembrane transporter activity;amino acid transmembrane transporter activity;antiporter activity;carboxylic acid trar
binding;C2H2 zinc finger domain binding;protein binding;protein domain specific binding;protein N-terminus binding
binding;DNA binding;enzyme binding;nucleic acid binding;nucleic acid binding transcription factor activity;protein binding;sequence-specific DNA binding transcription factor activity;ubiquitin protein ligase binding
adenyl nucleotide binding;adenyl ribonucleotide binding;ATP binding;binding;catalytic activity;chromatin binding;dynein binding;hydrolase activity;hydrolase activity, acting on acid anhydrides;hydrolase activity, acting on acid
binding;protein binding;protein domain specific binding
calcium activated cation channel activity;calcium-activated potassium channel activity;catalytic activity;cation channel activity;cation transmembrane transporter activity;channel activity;gated channel activity;hydrolase activi
adenyl nucleotide binding;adenyl ribonucleotide binding;ATP binding;binding;carbohydrate kinase activity;catalytic activity;cation binding;hydrolase activity;hydrolase activity, acting on glycosyl bonds;hydrolase activity, hydro
basal RNA polymerase II transcription machinery binding;basal transcription machinery binding;binding;enzyme binding;identical protein binding;nucleic acid binding;protein binding;RNA binding;RNA polymerase binding;RN
5'-3' DNA helicase activity;adenyl nucleotide binding;adenyl ribonucleotide binding;ATP binding;ATPase activity;ATPase activity, coupled;ATP-dependent 5'-3' DNA helicase activity;ATP-dependent DNA helicase activity;ATP-
bent DNA binding;binding;catalytic activity;cis-trans isomerase activity;DNA binding;double-stranded DNA binding;isomerase activity;nucleic acid binding;peptidyl-prolyl cis-trans isomerase activity;structure-specific DNA bir
binding;nucleic acid binding;RNA binding;translation factor activity, nucleic acid binding;translation initiation factor activity
enzyme activator activity;enzyme regulator activity;lipase activator activity;phospholipase A2 activator activity;phospholipase activator activity
adenyl nucleotide binding;adenyl ribonucleotide binding;ATP binding;binding;catalytic activity;DNA helicase activity;helicase activity;hydrolase activity;hydrolase activity, acting on acid anhydrides;hydrolase activity, acting on
anion channel activity;anion transmembrane transporter activity;binding;channel activity;gated channel activity;ion channel activity;ion transmembrane transporter activity;nucleotide binding;passive transmembrane transporte
adenyl nucleotide binding;adenyl ribonucleotide binding;aminoacyl-tRNA ligase activity;ATP binding;binding;catalytic activity;ligase activity;ligase activity, forming aminoacyl-tRNA and related compounds;ligase activity, formi
binding;GTP binding;guanyl nucleotide binding;guanyl ribonucleotide binding;identical protein binding;nucleotide binding;protein binding;purine nucleotide binding;purine ribonucleoside triphosphate binding;purine ribonucle
binding;protein binding;protein complex binding
3'-5' exonuclease activity;3'-5'-exoribonuclease activity;binding;catalytic activity;endonuclease activity;enzyme regulator activity;exonuclease activity;exonuclease activity, active with either ribo- or deoxyribonucleic acids anc
antioxidant activity;binding;catalytic activity;disulfide oxidoreductase activity;glutathione peroxidase activity;glutathione transferase activity;oxidoreductase activity;oxidoreductase activity, acting on a sulfur group of donors;o
adenyl nucleotide binding;adenyl ribonucleotide binding;ATP binding;ATPase activity;ATPase activity, coupled;ATP-dependent helicase activity;ATP-dependent RNA helicase activity;binding;catalytic activity;helicase activity;h
binding;DNA binding;nucleic acid binding
binding;enzyme binding;GTPase binding;protein binding;Rab GTPase binding;Ras GTPase binding;small GTPase binding
binding;DNA binding;nucleic acid binding;nucleotide binding
binding;DNA binding;enzyme binding;mRNA 3'-UTR binding;mRNA binding;nucleic acid binding;phosphatase binding;protein binding;RNA binding
adenyl nucleotide binding;adenyl ribonucleotide binding;ATP binding;binding;core promoter binding;core promoter sequence-specific DNA binding;DNA binding;hormone receptor binding;ligand-dependent nuclear receptor t
binding;DNA binding;enzyme regulator activity;nucleic acid binding;phosphatase regulator activity;protein binding;protein domain specific binding;protein phosphatase regulator activity;protein phosphatase type 1 regulator a
binding;nucleic acid binding;RNA binding;snoRNA binding
binding;coenzyme binding;cofactor binding;flavin adenine dinucleotide binding
acetyl-CoA hydrolase activity;carboxylic ester hydrolase activity;catalytic activity;CoA hydrolase activity;hydrolase activity;hydrolase activity, acting on ester bonds;thiolester hydrolase activity
binding;enzyme binding;GTPase binding;nucleic acid binding;protein binding;Ral GTPase binding;Ras GTPase binding;RNA binding;small GTPase binding;snRNA binding;U6 snRNA binding
binding;identical protein binding;lipid binding;protein binding;protein dimerization activity;protein homodimerization activity
binding;catalytic activity;enzyme activator activity;enzyme binding;enzyme regulator activity;kinase activator activity;kinase activity;kinase binding;kinase regulator activity;phosphotransferase activity, alcohol group as accept
binding;enzyme binding;mRNA binding;nucleic acid binding;protein binding;RNA binding
structural constituent of ribosome;structural molecule activity
binding;cytoskeletal protein binding;microtubule binding;nucleic acid binding;protein binding;ribonucleoprotein binding;ribosome binding;RNA binding;rRNA binding;tubulin binding
binding;nucleic acid binding;RNA binding
binding;nucleotide binding
structural constituent of ribosome;structural molecule activity
3'-5' exonuclease activity;3'-5'-exoribonuclease activity;binding;catalytic activity;exonuclease activity;exonuclease activity, active with either ribo- or deoxyribonucleic acids and producing 5'-phosphomonoesters;exoribonucl
catalytic activity;cis-trans isomerase activity;isomerase activity;peptidyl-prolyl cis-trans isomerase activity
acid-amino acid ligase activity;catalytic activity;ligase activity;ligase activity, forming carbon-nitrogen bonds;small conjugating protein ligase activity;UFM1 conjugating enzyme activity
adenyl nucleotide binding;adenyl ribonucleotide binding;adenylate kinase activity;ATP binding;ATPase activity;binding;catalytic activity;hydrolase activity;hydrolase activity, acting on acid anhydrides;hydrolase activity, acting
structural constituent of ribosome;structural molecule activity
aminoacyl-tRNA hydrolase activity;carboxylic ester hydrolase activity;catalytic activity;hydrolase activity;hydrolase activity, acting on ester bonds
alcohol binding;amine binding;binding;cation binding;identical protein binding;ion binding;lipid binding;phosphatidylcholine binding;phospholipid binding;protein binding;protein dimerization activity;protein homodimerization
adenyl nucleotide binding;adenyl ribonucleotide binding;ATP binding;binding;catalytic activity;cation binding;cytoskeletal protein binding;ion binding;ligase activity;ligase activity, forming phosphoric ester bonds;metal ion binc
binding;cytoskeletal protein binding;DNA binding;enzyme activator activity;enzyme binding;enzyme regulator activity;GTPase activator activity;GTPase binding;GTPase regulator activity;nucleic acid binding;nucleoside-tripho
nucleic acid binding transcription factor activity;sequence-specific DNA binding transcription factor activity
binding;chromatin binding;histone binding;nucleosome binding;protein binding;protein binding transcription factor activity;repressing transcription factor binding;transcription cofactor activity;transcription corepressor activity
structural constituent of ribosome;structural molecule activity
binding;catalytic activity;cation binding;deoxyribonucleotide binding;dGTP binding;dGTPase activity;guanyl deoxyribonucleotide binding;guanyl nucleotide binding;hydrolase activity;hydrolase activity, acting on ester bonds;ic
binding;catalytic activity;GTP binding;GTPase activity;guanyl nucleotide binding;guanyl ribonucleotide binding;hydrolase activity;hydrolase activity, acting on acid anhydrides;hydrolase activity, acting on acid anhydrides, in pl
binding;cytoskeletal protein binding;integrin binding;LIM domain binding;protein binding;protein complex binding;protein domain specific binding;receptor binding;structural constituent of cytoskeleton;structural molecule act
catalytic activity;kinase activity;phosphotransferase activity, alcohol group as acceptor;protein binding transcription factor activity;transcription coactivator activity;transcription cofactor activity;transcription factor binding trar
binding;catalytic activity;cysteine-type endopeptidase activity;cysteine-type peptidase activity;cytokine receptor binding;endopeptidase activity;hydrolase activity;identical protein binding;peptidase activity;peptidase activity,
enzyme regulator activity;GTPase regulator activity;guanyl-nucleotide exchange factor activity;nucleoside-triphosphatase regulator activity;Rac guanyl-nucleotide exchange factor activity;Ras guanyl-nucleotide exchange fact
actin binding;binding;cytoskeletal protein binding;protein binding;structural constituent of cytoskeleton;structural molecule activity
binding;enzyme binding;insulin receptor binding;kinase binding;molecular transducer activity;phosphatidylinositol 3-kinase binding;protein binding;protein complex binding;receptor binding;signal transducer activity
adenyl nucleotide binding;adenyl ribonucleotide binding;ATP binding;binding;calcium ion binding;catalytic activity;cation binding;enzyme binding;GTPase binding;hydrolase activity;hydrolase activity, acting on acid anhydride
binding;carbohydrate binding;cargo receptor activity;catalytic activity;cation binding;chromatin binding;copper ion binding;electron carrier activity;histone binding;ion binding;metal ion binding;methylated histone residue binc
adenyl nucleotide binding;adenyl ribonucleotide binding;ATP binding;binding;catalytic activity;kinase activity;MAP kinase kinase kinase kinase activity;molecular transducer activity;nucleotide binding;phosphotransferase acti
adenyl nucleotide binding;adenyl ribonucleotide binding;ATP binding;binding;chaperone binding;nucleotide binding;protein binding;purine nucleotide binding;purine ribonucleoside triphosphate binding;purine ribonucleotide b
binding;enzyme binding;kinase binding;phosphoprotein binding;protein binding;protein kinase binding;translation initiation factor binding
binding;protein binding;protein complex binding
adenyl nucleotide binding;adenyl ribonucleotide binding;ATP binding;binding;catalytic activity;cation binding;endopeptidase activity;hydrolase activity;ion binding;metal ion binding;metalloendopeptidase activity;metallopeptic
binding;catalytic activity;cation binding;ion binding;ligase activity;metal ion binding;transition metal ion binding;zinc ion binding
binding;protein binding;protein C-terminus binding
binding;carboxylic ester hydrolase activity;catalytic activity;enzyme binding;enzyme inhibitor activity;enzyme regulator activity;hydrolase activity;hydrolase activity, acting on ester bonds;phosphatase binding;phosphatase inh
binding;nucleotide binding
binding;cation binding;ion binding;metal ion binding;transition metal ion binding;zinc ion binding
binding;catalytic activity;cysteine-type peptidase activity;endopeptidase inhibitor activity;endopeptidase regulator activity;enzyme inhibitor activity;enzyme regulator activity;exopeptidase activity;hydrolase activity;omega pep
binding;protein binding;protein domain specific binding;SH3 domain binding;structural constituent of cytoskeleton;structural molecule activity
active transmembrane transporter activity;ATPase activity;ATPase activity, coupled;ATPase activity, coupled to movement of substances;ATPase activity, coupled to transmembrane movement of substances;catalytic activity;
receptor activity
binding;cation binding;ion binding;metal ion binding;transition metal ion binding;zinc ion binding
binding;GTP binding;guanyl nucleotide binding;guanyl ribonucleotide binding;nucleotide binding;purine nucleotide binding;purine ribonucleoside triphosphate binding;purine ribonucleotide binding;ribonucleotide binding
binding;catalytic activity;GTP binding;guanyl nucleotide binding;guanyl ribonucleotide binding;guanylyltransferase activity;mannose-1-phosphate guanylyltransferase activity;mannose-phosphate guanylyltransferase activity;r
adenyl nucleotide binding;adenyl ribonucleotide binding;ATP binding;binding;catalytic activity;cation binding;ion binding;kinase activity;magnesium ion binding;metal ion binding;nucleotide binding;phosphotransferase activity
binding;mRNA binding;nucleic acid binding;nucleotide binding;RNA binding
protein binding transcription factor activity;transcription coactivator activity;transcription cofactor activity;transcription factor binding transcription factor activity
1-phosphatidylinositol binding;Arp2/3 complex binding;binding;enzyme binding;identical protein binding;lipid binding;phosphatidylinositol binding;phospholipid binding;protein binding;protein complex binding;protein dimeriz
binding;identical protein binding;lipid binding;phosphatidylinositol binding;phospholipid binding;protein binding
binding;cytoskeletal protein binding;dynactin binding;lipid binding;phosphatidylinositol binding;phospholipid binding;protein binding
4 iron, 4 sulfur cluster binding;adenyl nucleotide binding;adenyl ribonucleotide binding;ATP binding;binding;cation binding;ion binding;iron-sulfur cluster binding;metal cluster binding;metal ion binding;nucleotide binding;purir
carbon-oxygen lyase activity;catalytic activity;hydro-lyase activity;intramolecular transferase activity;isomerase activity;ligand-dependent nuclear receptor transcription coactivator activity;lyase activity;protein binding transcri
binding;LRR domain binding;protein binding;protein domain specific binding
binding;catalytic activity;cofactor binding;O-phospho-L-serine:2-oxoglutarate aminotransferase activity;pyridoxal phosphate binding;transaminase activity;transferase activity;transferase activity, transferring nitrogenous grou
binding;chromatin binding;DNA binding;enzyme binding;histone deacetylase binding;Notch binding;nucleic acid binding;protein binding;protein binding transcription factor activity;protein N-terminus binding;receptor binding
binding;catalytic activity;cell adhesion molecule binding;fibroblast growth factor receptor binding;growth factor receptor binding;kinase activity;molecular transducer activity;phosphotransferase activity, alcohol group as acce
binding;histone binding;methylated histone residue binding;protein binding
catalytic activity;dolichyl-phosphate beta-glucosyltransferase activity;glucosyltransferase activity;oligosaccharyl transferase activity;transferase activity;transferase activity, transferring glycosyl groups;transferase activity, tran

structural constituent of ribosome;structural molecule activity
structural molecule activity
binding;calcium ion binding;catalytic activity;cation binding;cis-trans isomerase activity;drug binding;FK506 binding;ion binding;isomerase activity;macrolide binding;metal ion binding;peptidyl-prolyl cis-trans isomerase activi
anion channel activity;anion transmembrane transporter activity;channel activity;chloride channel activity;gated channel activity;ion channel activity;ion transmembrane transporter activity;passive transmembrane transporter
binding;catalytic activity;cation binding;cofactor binding;cysteine desulfurase activity;identical protein binding;ion binding;iron-sulfur cluster binding;metal cluster binding;metal ion binding;protein binding;protein dimerization
catalytic activity;hydrolase activity;peptidase activity
extracellular matrix constituent conferring elasticity;extracellular matrix structural constituent;structural molecule activity
ARF guanyl-nucleotide exchange factor activity;binding;cytoskeletal protein binding;enzyme regulator activity;GTPase regulator activity;guanyl-nucleotide exchange factor activity;myosin binding;nucleoside-triphosphatase re
adenyl nucleotide binding;adenyl ribonucleotide binding;ATP binding;binding;catalytic activity;cation binding;ion binding;kinase activity;metal ion binding;nucleotide binding;phosphotransferase activity, alcohol group as acce
adenyl nucleotide binding;adenyl ribonucleotide binding;ATP binding;binding;catalytic activity;hydrolase activity;hydrolase activity, acting on acid anhydrides;hydrolase activity, acting on acid anhydrides, in phosphorus-conta
binding;DNA binding;nucleic acid binding;protein binding;sequence-specific DNA binding;transcription factor binding
binding;lipid binding
binding;cation binding;enzyme binding;identical protein binding;ion binding;K63-linked polyubiquitin binding;metal ion binding;molecular transducer activity;polyubiquitin binding;protein binding;protein dimerization activity;pr
binding;mRNA 3'-UTR binding;mRNA 5'-UTR binding;mRNA binding;nucleic acid binding;nucleotide binding;RNA binding;translation regulator activity
catalytic activity;NADH dehydrogenase (quinone) activity;NADH dehydrogenase (ubiquinone) activity;NADH dehydrogenase activity;oxidoreductase activity;oxidoreductase activity, acting on NADH or NADPH;oxidoreductase
binding;catalytic activity;cofactor binding;oxidoreductase activity;oxidoreductase activity, acting on a sulfur group of donors;oxidoreductase activity, acting on a sulfur group of donors, quinone or similar compound as accept
actin binding;binding;cytoskeletal protein binding;protein binding;protein complex binding
binding;cation binding;ion binding;metal ion binding;transition metal ion binding;zinc ion binding
binding;cation binding;ion binding;metal ion binding

C: GOCC name	C: KEGG name	C: Organism	C: ANOVA Significant
		Homo sapiens	+
		Homo sapiens	+
cell part;cytoplasm;cytoplasmic part;cytosol;intracellular part	Ubiquitin mediated proteolysis	Homo sapiens	
cell part;cytoplasmic part;endoplasmic reticulum part;extrinsic to internal side of plasma membrane;extrinsic to membrane;extrinsic to plasma membrane;integral to membrane;integral to plasma membrane;intracellular organelle part;intracellular part;intrinsic to endoplasmic reticulum membrane;intrinsic to membrane;intrinsic to organelle membrane;intrinsic to plasma m		Homo sapiens	+
cell part;integral to membrane;intrinsic to membrane;membrane;membrane part		Homo sapiens	
cell junction;cell part;cell projection;cytoplasm;cytoskeletal part;intracellular non-membrane-bounded organelle;intracellular organelle;intracellular organelle part;intracellular part;macromolecular complex;non-membrane-bounded organelle;organelle;organelle part;podosome;protein complex		Homo sapiens	
CCR4-NOT complex;cell part;cytoplasmic mRNA processing body;cytoplasmic part;cytosol;extracellular region part;extracellular space;intracellular membrane-bounded organelle;intracellular non-membrane-bounded organe	RNA degradation	Homo sapiens	+
autophagic vacuole membrane;cell part;cytoplasmic part;cytoplasmic vesicle;cytoskeletal part;cytosol;endomembrane system;extrinsic to membrane;intracellular organelle;intracellular organelle part;intracellular part;macromolecular complex;membrane;membrane part;microtubule;organelle;organelle membrane;organelle part;pre-autophagosomal structure;protein comp		Homo sapiens	+
	Glyoxylate and dicarboxylate metabolism	Homo sapiens	+
Barr body;cell part;chromosome;intracellular non-membrane-bounded organelle;intracellular organelle;intracellular organelle part;intracellular part;non-membrane-bounded organelle;nuclear chromosome;nuclear part;organelle;organelle part;sex chromosome;X chromosome		Homo sapiens	+
cell part;cytoplasmic part;endoplasmic reticulum membrane;endoplasmic reticulum part;intracellular organelle part;intracellular part;macromolecular complex;membrane;membrane part;nuclear membrane;nuclear part;nuclear pore;organelle membrane;organelle part;pore complex;protein complex		Homo sapiens	
cell part;intracellular organelle part;intracellular part;macromolecular complex;nuclear part;organelle part;ribonucleoprotein complex;small nucleolar ribonucleoprotein complex;spliceosomal complex		Homo sapiens	+
cell part;cytoplasm;intracellular membrane-bounded organelle;intracellular organelle;intracellular part;membrane-bounded organelle;nucleus;organelle		Homo sapiens	+
		Homo sapiens	+
		Homo sapiens	+
cell part;cytoplasmic part;extracellular membrane-bounded organelle;extracellular organelle;extracellular region part;extracellular vesicular exosome;intracellular;intracellular membrane-bounded organelle;intracellular organel	Lysosome	Homo sapiens	+
cell part;cytoplasmic part;intracellular membrane-bounded organelle;intracellular non-membrane-bounded organelle;intracellular organelle;intracellular organelle lumen;intracellular organelle part;intracellular part;membrane;m	Ether lipid metabolism;Peroxisome	Homo sapiens	+
cell part;cytoplasm;intracellular membrane-bounded organelle;intracellular non-membrane-bounded organelle;intracellular organelle;intracellular organelle part;intracellular part;macromolecular complex;membrane;membrane-bounded organelle;non-membrane-bounded organelle;nuclear part;nucleolus;nucleus;organelle;organelle part;ribonucleoprotein complex;spliceos		Homo sapiens	+
adherens junction;anchoring junction;cell junction;cell part;cell-substrate adherens junction;cell-substrate junction;cytoplasm;cytoskeleton;focal adhesion;intracellular non-membrane-bounded organelle;intracellular organelle;intracellular organelle part;intracellular part;macromolecular complex;non-membrane-bounded organelle;nuclear part;nucleoplasm part;organelle;o		Homo sapiens	+
cell body;cell part;cell projection;cytoplasm;cytoplasmic part;cytosol;extracellular membrane-bounded organelle;extracellular organelle;extracellular region part;extracellular vesicular exosome;intracellular membrane-bounde	Biosynthesis of unsaturated fatty acids	Homo sapiens	
basal plasma membrane;brush border;cell part;cell projection;cell projection membrane;cell projection part;cytoplasm;cytoplasmic membrane-bounded vesicle;cytoplasmic part;cytoplasmic vesicle;cytoskeletal part;cytosol;extracellular membrane-bounded organelle;extracellular organelle;extracellular region part;extracellular vesicular exosome;filamentous actin;intracell		Homo sapiens	+
cell part;cytoplasm;cytoplasmic part;cytosol;intracellular organelle part;intracellular part;membrane;nuclear part;nucleoplasm;organelle part		Homo sapiens	+
cell part;cytoplasmic exosome (RNase complex);cytoplasmic part;cytosol;exosome (RNase complex);intracellular part;macromolecular complex;membrane;protein complex		Homo sapiens	
apical plasma membrane;basolateral plasma membrane;cell part;cytoplasmic membrane-bounded vesicle;cytoplasmic part;cytoplasmic vesicle;cytosol;extracellular membrane-bounded organelle;extracellular organelle;extracellular region part;extracellular vesicular exosome;intracellular membrane-bounded organelle;intracellular organelle;intracellular part;membrane;me		Homo sapiens	+
		Homo sapiens	+
AP-3 adaptor complex;AP-type membrane coat adaptor complex;cell part;clathrin coated vesicle membrane;coated vesicle membrane;cytoplasmic part;cytoplasmic vesicle membrane;cytoplasmic vesicle part;Golgi apparatu	Lysosome	Homo sapiens	+
cell part;cytoplasmic part;intracellular membrane-bounded organelle;intracellular organelle;intracellular organelle part;intracellular part;macromolecular complex;membrane;membrane part;membrane-bounded organelle;mitoc	Alzheimer's disease;Huntington's disease;Oxidative phosphorylation;Parkinson's disease	Homo sapiens	
cell part;cytoplasmic part;cytosol;extracellular membrane-bounded organelle;extracellular organelle;extracellular region part;extracellular vesicular exosome;intracellular membrane-bounded organelle;intracellular organelle;int	Proteasome	Homo sapiens	
cell part;cytoplasmic part;cytosol;extracellular membrane-bounded organelle;extracellular organelle;extracellular region part;extracellular vesicular exosome;intracellular organelle part;intracellular part;macromolecular comple	Proteasome	Homo sapiens	+
cell part;cytoplasm;cytoplasmic part;cytosol;intracellular membrane-bounded organelle;intracellular organelle;intracellular organelle part;intracellular part;macromolecular complex;membrane-bounded organelle;nuclear part;nucleoplasm;nucleus;organelle;organelle part;proteasome regulatory particle;protein complex		Homo sapiens	+
cell part;cytoplasmic part;cytosol;intracellular part	Mineral absorption	Homo sapiens	
cell part;cytoplasmic part;endoplasmic reticulum;endoplasmic reticulum membrane;endoplasmic reticulum part;extracellular membrane-bounded organelle;extracellular organelle;extracellular region part;extracellular vesicular exosome;integral to membrane;intracellular membrane-bounded organelle;intracellular non-membrane-bounded organelle;intracellular organelle;ir		Homo sapiens	+
cell part;DSIF complex;intracellular membrane-bounded organelle;intracellular organelle;intracellular organelle part;intracellular part;macromolecular complex;membrane-bounded organelle;nuclear part;nucleoplasm;nucleoplasm part;nucleus;organelle;organelle part;protein complex;transcription elongation factor complex		Homo sapiens	+
cell part;chromatin;chromosomal part;cytoplasm;cytoplasmic part;cytosol;intracellular membrane-bounded organelle;intracellular organelle;intracellular organelle part;intracellular part;membrane-bounded organelle;nuclear ch	Apoptosis	Homo sapiens	
cell part;clathrin coated vesicle membrane;clathrin-coated vesicle;coated vesicle;coated vesicle membrane;cytoplasm;cytoplasmic membrane-bounded vesicle;cytoplasmic part;cytoplasmic vesicle;cytoplasmic vesicle meml	Huntington's disease	Homo sapiens	
blood microparticle;brush border;cell part;cell projection;chloride channel complex;cytoplasm;cytoplasmic part;envelope;extracellular membrane-bounded organelle;extracellular organelle;extracellular region part;extracellular space;extracellular vesicular exosome;intracellular membrane-bounded organelle;intracellular organelle;intracellular organelle part;intracellular par		Homo sapiens	+
cell part;cytoplasmic part;cytosol;eukaryotic 43S preinitiation complex;eukaryotic 48S preinitiation complex;eukaryotic translation initiation factor 3 complex;intracellular part;macromolecular complex;membrane;protein comp	RNA transport	Homo sapiens	+
cell part;cytoplasmic part;intracellular organelle lumen;intracellular organelle part;intracellular part;membrane-enclosed lumen;mitochondrial matrix;mitochondrial part;organelle lumen;organelle part	Citrate cycle (TCA cycle);Glycolysis / Gluconeogenesis;Pyruvate metabolism	Homo sapiens	+
cell part;cytoplasm;cytoplasmic part;Golgi apparatus;intracellular membrane-bounded organelle;intracellular non-membrane-bounded organelle;intracellular organelle;intracellular organelle part;intracellular part;macromolecular complex;membrane;membrane part;membrane-bounded organelle;non-membrane-bounded organelle;nuclear membrane;nuclear part;nuclear p		Homo sapiens	+
cell part;chromatin remodeling complex;cytoplasm;histone deacetylase complex;intracellular organelle part;intracellular part;macromolecular complex;nuclear body;nuclear part;nuclear speck;nucleoplasm;nucleoplasm part;c	mRNA surveillance pathway;RNA transport	Homo sapiens	+
cell part;cytoplasm;cytoplasmic part;cytosol;intracellular membrane-bounded organelle;intracellular organelle;intracellular part;membrane-bounded organelle;nucleus;organelle		Homo sapiens	+
cell junction;cell part;clathrin coated vesicle membrane;coated pit;coated vesicle membrane;cytoplasm;cytoplasmic part;cytoplasmic vesicle membrane;cytoplasmic vesicle part;cytoskeletal part;cytosol;Golgi apparatus;intra	Bacterial invasion of epithelial cells;Endocrine and other factor-regulated calcium reabsorption;Endocytosis;Fc gamma R-mediated phagocytosis	Homo sapiens	
cell part;intracellular membrane-bounded organelle;intracellular organelle;intracellular organelle part;intracellular part;membrane-bounded organelle;nuclear part;nucleoplasm;nucleus;organelle;organelle part		Homo sapiens	
cell part;clathrin-coated vesicle;coated vesicle;cytoplasm;cytoplasmic membrane-bounded vesicle;cytoplasmic part;cytoplasmic vesicle;cytosol;cytosolic part;extracellular membrane-bounded organelle;extracellular organell	Inositol phosphate metabolism;Phosphatidylinositol signaling system	Homo sapiens	+
cell part;cis-Golgi network;cytoplasmic membrane-bounded vesicle;cytoplasmic part;cytoplasmic vesicle;endocytic vesicle;endosomal part;endosome membrane;Golgi apparatus;Golgi apparatus part;Golgi cisterna membrane;Golgi lumen;Golgi membrane;integral to membrane;intracellular membrane-bounded organelle;intracellular organelle;intracellular organelle lume		Homo sapiens	+
basal lamina;cell junction;cell part;cell surface;cytoplasm;cytoplasmic part;extracellular matrix;extracellular matrix part;extracellular membrane-bounded organelle;extracellular organelle;extracellular region;extracellular region	ECM-receptor interaction	Homo sapiens	+
cell part;cytoplasmic part;endoplasmic reticulum;endoplasmic reticulum membrane;endoplasmic reticulum part;extracellular membrane-bounded organelle;extracellular organelle;extracellular region part;extracellular vesicula	Lysine degradation	Homo sapiens	+
cell part;cytoplasm;cytoplasmic part;cytosol;intracellular part		Homo sapiens	+
cell part;cytoplasmic part;extracellular membrane-bounded organelle;extracellular organelle;extracellular region part;extracellular vesicular exosome;intracellular membrane-bounded organelle;intracellular organelle;intracellula	Alzheimer's disease;Huntington's disease;Oxidative phosphorylation;Parkinson's disease	Homo sapiens	+
cell part;cytoplasmic part;cytosol;extracellular membrane-bounded organelle;extracellular organelle;extracellular region part;extracellular vesicular exosome;intracellular membrane-bounded organelle;intracellular organelle;int	Proteasome	Homo sapiens	+
actin cytoskeleton;axon;axon part;axon terminus;cell part;cell projection;cell projection part;cerebellar mossy fiber;clathrin-coated vesicle;coated vesicle;contractile fiber part;cytoplasm;cytoplasmic membrane-bounded vesicle;cytoplasmic part;cytoplasmic vesicle;cytoskeleton;I band;initial segment;intracellular membrane-bounded organelle;intracellular non-membrane-		Homo sapiens	
cell part;cytoplasmic part;cytosol;intracellular organelle part;intracellular part;macromolecular complex;membrane part;nuclear part;nuclear pore;nucleoplasm;organelle part;pore complex;protein complex		Homo sapiens	+
cell part;chromosome;condensed chromosome;cytoplasm;intracellular membrane-bounded organelle;intracellular non-membrane-bounded organelle;intracellular organelle;intracellular organelle part;intracellular part;macromolecular complex;membrane;membrane-bounded organelle;non-membrane-bounded organelle;nuclear part;nucleolar part;nucleolus;nucleoplasm;r		Homo sapiens	+
adherens junction;anchoring junction;blood microparticle;cell junction;cell part;cell-substrate adherens junction;cell-substrate junction;cytoplasm;cytoplasmic membrane-bounded vesicle;cytoplasmic part;cytoplasmic vesicle;cytoskeleton;cytosol;endoplasmic reticulum membrane;endoplasmic reticulum part;extracellular membrane-bounded organelle;extracellular orgar		Homo sapiens	+
cell part;chromosome;intracellular non-membrane-bounded organelle;intracellular organelle;intracellular organelle part;intracellular part;macromolecular complex;Mpp10 complex;non-membrane-bounded organelle;nuclear pa	Ribosome biogenesis in eukaryotes	Homo sapiens	+
box C/D snoRNP complex;cell part;cytoplasm;intracellular non-membrane-bounded organelle;intracellular organelle;intracellular organelle part;intracellular part;macromolecular complex;membrane;non-membrane-bounded c	Ribosome biogenesis in eukaryotes	Homo sapiens	+
cell part;cytoplasm;cytoplasmic part;extracellular membrane-bounded organelle;extracellular organelle;extracellular region part;extracellular vesicular exosome;intracellular membrane-bounded organelle;intracellular non-mer	RIG-I-like receptor signaling pathway	Homo sapiens	+
apical plasma membrane;cell body;cell part;cell projection;cell projection membrane;cell projection part;cytoplasm;extracellular membrane-bounded organelle;extracellular organelle;extracellular region part;extracellular space;extracellular vesicular exosome;filopodium;integral to membrane;integral to plasma membrane;intracellular part;intrinsic to membrane;intrinsic to		Homo sapiens	+
extracellular matrix;extracellular region part;proteinaceous extracellular matrix		Homo sapiens	+
cell part;cytoplasmic part;cytosol;extracellular membrane-bounded organelle;extracellular organelle;extracellular region part;extracellular vesicular exosome;intracellular organelle part;intracellular part;membrane-bounded organelle;membrane-bounded vesicle;nuclear part;nucleoplasm;organelle;organelle part;vesicle		Homo sapiens	
cell part;cytoplasmic part;cytosol;intracellular part		Homo sapiens	
cell part;cytoplasmic part;extracellular membrane-bounded organelle;extracellular organelle;extracellular region part;extracellular vesicular exosome;intracellular membrane-bounded organelle;intracellular organelle;intracellula	Lysosome;Other glycan degradation	Homo sapiens	+
anaphase-promoting complex;cell part;cullin-RING ubiquitin ligase complex;cytoplasm;cytoplasmic part;cytosol;intracellular organelle part;intracellular part;macromolecular complex;nuclear part;nuclear ubiquitin ligase comp	Ubiquitin mediated proteolysis	Homo sapiens	+
cell part;cytoplasm;cytoplasmic part;cytosol;extracellular membrane-bounded organelle;extracellular organelle;extracellular region part;extracellular vesicular exosome;intracellular membrane-bounded organelle;intracellular c	Vitamin B6 metabolism	Homo sapiens	
cell part;chromatin;chromatin remodeling complex;chromosomal part;intracellular membrane-bounded organelle;intracellular organelle;intracellular organelle part;intracellular part;macromolecular complex;membrane-bounded organelle;nBAF complex;npBAF complex;nuclear chromatin;nuclear chromosome part;nuclear part;nucleoplasm;nucleus;organelle;organelle part		Homo sapiens	+
cell part;integral to membrane;intrinsic to membrane;membrane part		Homo sapiens	+
cell part;cytoplasm;intracellular membrane-bounded organelle;intracellular organelle;intracellular part;membrane-bounded organelle;nucleus;organelle		Homo sapiens	+
actin cytoskeleton;cell body;cell part;cell projection;cytoplasmic part;cytoskeleton;cytosol;Golgi apparatus part;Golgi cisterna;intracellular membrane-bounded organelle;intracellular non-membrane-bounded organelle;intracellular organelle;intracellular organelle part;intracellular part;membrane;membrane-bounded organelle;neuronal cell body;non-membrane-bounded c		Homo sapiens	
cell part;COPI vesicle coat;cytoplasmic part;cytoplasmic vesicle part;cytosol;Golgi apparatus;Golgi apparatus part;intracellular membrane-bounded organelle;intracellular organelle;intracellular organelle part;intracellular part;macromolecular complex;membrane coat;membrane part;membrane-bounded organelle;nuclear part;nucleoplasm;organelle;organelle part;protein		Homo sapiens	+
axon part;cell part;cell projection part;cytoplasmic part;endosomal part;endosome membrane;Golgi apparatus;Golgi apparatus part;Golgi membrane;intracellular membrane-bounded organelle;intracellular organelle;intracellu	Lysosome	Homo sapiens	+
cell part;cytoplasm;cytoplasmic part;cytosol;intracellular membrane-bounded organelle;intracellular organelle;intracellular part;membrane-bounded organelle;nucleus;organelle	Amyotrophic lateral sclerosis (ALS)	Homo sapiens	+
cell part;cytoplasmic part;endoplasmic reticulum;endoplasmic reticulum lumen;endoplasmic reticulum part;envelope;extracellular membrane-bounded organelle;extracellular organelle;extracellular region part;extracellular vesicular exosome;intracellular membrane-bounded organelle;intracellular organelle;intracellular organelle lumen;intracellular organelle part;intracellula		Homo sapiens	+
adherens junction;anchoring junction;cell junction;cell part;cell surface;cell-substrate adherens junction;cell-substrate junction;cytoplasm;cytoplasmic membrane-bounded vesicle;cytoplasmic part;cytoplasmic vesicle;cytosk	Alzheimer's disease;Epithelial cell signaling in Helicobacter pylori infection	Homo sapiens	+
cell part;cytoplasmic part;endoplasmic reticulum membrane;endoplasmic reticulum part;integral to membrane;intracellular organelle part;intracellular part;intrinsic to membrane;membrane;membrane part;membrane-enclose	Arachidonic acid metabolism	Homo sapiens	+
cell part;cytoplasmic part;endoplasmic reticulum membrane;endoplasmic reticulum part;Golgi apparatus;Golgi apparatus part;Golgi membrane;integral to membrane;intracellular membrane-bounded organelle;intracellular or	Glycerophospholipid metabolism;Inositol phosphate metabolism;Phosphatidylinositol signaling system	Homo sapiens	+
cell part;cytoplasm;extracellular membrane-bounded organelle;extracellular organelle;extracellular region part;extracellular vesicular exosome;intracellular membrane-bounded organelle;intracellular organelle;intracellular part;membrane-bounded organelle;membrane-bounded vesicle;nucleus;organelle;vesicle		Homo sapiens	+
cell part;cytoplasm;cytoplasmic part;cytosol;intracellular membrane-bounded organelle;intracellular organelle;intracellular organelle part;intracellular part;macromolecular complex;membrane-bounded organelle;methylosome	Cell cycle - yeast;RNA transport	Homo sapiens	
actin cytoskeleton;apical plasma membrane;brush border membrane;cell part;cell periphery;cell projection;cell projection membrane;cell projection part;centrosome;cytoplasm;cytoplasmic part;cytoskeletal part;cytoskeleton;endomembrane system;extracellular membrane-bounded organelle;extracellular organelle;extracellular region part;extracellular vesicular exosome;		Homo sapiens	+
cell part;chromosomal part;chromosome, telomeric region;cytoplasmic part;intracellular non-membrane-bounded organelle;intracellular organelle;intracellular organelle part;intracellular part;macromolecular complex;mitochondrial nucleoid;mitochondrial part;non-membrane-bounded organelle;nuclear body;nuclear chromosome part;nuclear part;nuclear telomere cap cor		Homo sapiens	+
cell part;cell surface;integral to membrane;intrinsic to membrane;membrane;membrane part;plasma membrane	Apoptosis;Cytokine-cytokine receptor interaction;Measles;Natural killer cell mediated cytotoxicity	Homo sapiens	+
cell part;intracellular membrane-bounded organelle;intracellular organelle;intracellular part;membrane-bounded organelle;nucleus;organelle		Homo sapiens	+
cell part;cytoplasmic membrane-bounded vesicle;cytoplasmic part;cytoplasmic vesicle;extracellular membrane-bounded organelle;extracellular organelle;extracellular region part;extracellular vesicular exosome;intracellular m	Lysosome	Homo sapiens	+
actin cytoskeleton;cell part;cytoskeleton;intracellular membrane-bounded organelle;intracellular non-membrane-bounded organelle;intracellular organelle;intracellular organelle part;intracellular part;membrane-bounded orgar	Spliceosome	Homo sapiens	+
cell part;chromosomal part;chromosome, centromeric region;condensed chromosome kinetochore;condensed chromosome outer kinetochore;condensed nuclear chromosome outer kinetochore;cytoplasmic part;cytosol;intracellular membrane-bounded organelle;intracellular non-membrane-bounded organelle;intracellular organelle;intracellular organelle part;intracellula		Homo sapiens	+
adherens junction;anchoring junction;axon;axon part;cell body;cell junction;cell part;cell projection;cell projection part;cell surface;cell-substrate adherens junction;cell-substrate junction;cytoplasmic part;cytoplasmic vesicle;	Axon guidance	Homo sapiens	+
adherens junction;anchoring junction;cell junction;cell part;cell-substrate adherens junction;cell-substrate junction;focal adhesion;integral to membrane;intrinsic to membrane;membrane;membrane part;organelle;plasma membrane;vesicle		Homo sapiens	+
cell part;cytoplasm;cytoplasmic part;cytosol;extracellular membrane-bounded organelle;extracellular organelle;extracellular region part;extracellular vesicular exosome;intracellular membrane-bounded organelle;intracellular c	Proteasome	Homo sapiens	+
cell part;cytoplasmic part;extracellular membrane-bounded organelle;extracellular organelle;extracellular region part;extracellular vesicular exosome;Golgi apparatus part;Golgi membrane;integral to membrane;intracellular organelle part;intracellular part;intrinsic to membrane;membrane;membrane part;membrane-bounded organelle;membrane-bounded vesicle;organelle		Homo sapiens	+
cell part;cytoplasm;cytoplasmic part;cytosol;intracellular membrane-bounded organelle;intracellular organelle;intracellular part;membrane-bounded organelle;mitochondrion;organelle		Homo sapiens	+
cell part;cytoplasmic part;endoplasmic reticulum membrane;endoplasmic reticulum part;envelope;extracellular membrane-bounded organelle;extracellular organelle;extracellular region part;extracellular vesicular exosome;int	Drug metabolism - cytochrome P450;Glutathione metabolism;Metabolism of xenobiotics by cytochrome P450	Homo sapiens	
brush border;cell cortex;cell part;cell projection;cell projection part;clathrin-coated vesicle;coated vesicle;cytoplasm;cytoplasmic membrane-bounded vesicle;cytoplasmic part;cytoplasmic vesicle;cytosol;dendritic shaft;dendritic spine;endocytic vesicle;extracellular membrane-bounded organelle;extracellular organelle;extracellular region part;extracellular vesicular exoso		Homo sapiens	+
cell part;chromatin;chromosomal part;cytoplasm;intracellular membrane-bounded organelle;intracellular organelle;intracellular organelle part;intracellular part;macromolecular complex;membrane-bounded organelle;nuclear chromatin;nuclear chromosome part;nuclear matrix;nuclear part;nucleoplasm;nucleus;organelle;organelle part;protein complex		Homo sapiens	+
cell part;cytoplasmic part;cytosol;intracellular organelle part;intracellular part;nuclear part;nucleoplasm;organelle part	Parkinson's disease;Ubiquitin mediated proteolysis	Homo sapiens	+
actin cytoskeleton;adherens junction;anchoring junction;basement membrane;basolateral plasma membrane;cell junction;cell part;cell projection membrane;cell projection part;cell-cell junction;cell-substrate adherens junctio	Tight junction	Homo sapiens	+
cell part;cytoplasmic part;intracellular membrane-bounded organelle;intracellular organelle;intracellular organelle part;intracellular part;membrane;membrane part;membrane-bounded organelle;mitochondrial inner membrane	Alzheimer's disease;Cardiac muscle contraction;Huntington's disease;Oxidative phosphorylation;Parkinson's disease	Homo sapiens	+
actin filament bundle;actomyosin;apical part of cell;cell part;contractile fiber part;cytoplasmic part;cytoskeletal part;cytosol;extracellular membrane-bounded organelle;extracellular organelle;extracellular region part;extracellu	Focal adhesion;Leukocyte transendothelial migration;Regulation of actin cytoskeleton;Tight junction	Homo sapiens	+
cell part;cytoplasm;cytoplasmic membrane-bounded vesicle;cytoplasmic part;cytoplasmic vesicle;cytosol;early endosome;early endosome membrane;endosomal part;endosome;endosome membrane;extracellular membran	Endocytosis;Phagosome	Homo sapiens	+
axon hillock;axon part;cell part;cell projection part;centrosome;chromosomal part;chromosome passenger complex;chromosome, centromeric region;condensed chromosome, centromeric region;condensed nuclear chromos	Oocyte meiosis	Homo sapiens	
cell part;cytoplasmic part;endoplasmic reticulum;endoplasmic reticulum membrane;endoplasmic reticulum part;envelope;integral to membrane;intracellular membrane-bounded organelle;intracellular organelle;intracellular organelle part;intracellular part;intrinsic to membrane;membrane;membrane part;membrane-bounded organelle;nuclear envelope;nuclear part;organel		Homo sapiens	+
actin cytoskeleton;adherens junction;anchoring junction;cell junction;cell part;cell-substrate adherens junction;cell-substrate junction;centrosome;chromosomal part;contractile fiber;cytoplasm;cytoplasmic part;cytoskeletal pa	Focal adhesion;Long-term potentiation;Regulation of actin cytoskeleton;Vascular smooth muscle contraction	Homo sapiens	+
adherens junction;anchoring junction;cell junction;cell part;cell-substrate adherens junction;cell-substrate junction;cytoplasmic part;focal adhesion;Golgi apparatus;intracellular membrane-bounded organelle;intracellular organelle;intracellular part;membrane;membrane-bounded organelle;organelle;perinuclear region of cytoplasm		Homo sapiens	+
cell part;cytoplasm;extracellular membrane-bounded organelle;extracellular organelle;extracellular region part;extracellular vesicular exosome;intracellular membrane-bounded organelle;intracellular organelle;intracellular organelle part;intracellular part;macromolecular complex;membrane-bounded organelle;membrane-bounded vesicle;nuclear part;nucleoplasm;nucleus;		Homo sapiens	+
annulate lamellae;Cajal body;cell part;chromosomal part;cytoplasm;cytoplasmic part;cytosol;envelope;intracellular membrane-bounded organelle;intracellular non-membrane-bounded organelle;intracellular organelle;intracell	Ribosome biogenesis in eukaryotes;RNA transport	Homo sapiens	+
cell part;centrosome;cytoplasmic part;cytoskeletal part;cytosol;intracellular non-membrane-bounded organelle;intracellular organelle;intracellular organelle part;intracellular part;microtubule organizing center;non-membrane-bounded organelle;organelle;organelle part		Homo sapiens	+
cell part;cytoplasm;cytoplasmic part;cytosol;endoplasmic reticulum membrane;endoplasmic reticulum part;Golgi apparatus;Golgi apparatus part;Golgi membrane;intracellular membrane-bounded organelle;intracellular organelle;intracellular organelle part;intracellular part;membrane;membrane part;membrane-bounded organelle;organelle;organelle membrane;organelle p		Homo sapiens	
cell part;cell surface;extracellular membrane-bounded organelle;extracellular organelle;extracellular region part;extracellular vesicular exosome;integral to membrane;integral to plasma membrane;intracellular;intrinsic to mem	Axon guidance	Homo sapiens	+
cell part;intracellular membrane-bounded organelle;intracellular organelle;intracellular organelle part;intracellular part;membrane-bounded organelle;nuclear part;nucleoplasm;nucleus;organelle;organelle part	Spliceosome	Homo sapiens	+
cell part;cytoplasm;cytoplasmic part;cytosol;extracellular membrane-bounded organelle;extracellular organelle;extracellular region part;extracellular vesicular exosome;intracellular part;membrane-bounded organelle;membra	Purine metabolism	Homo sapiens	+
cell part;cytoplasm;cytoplasmic mRNA processing body;cytoplasmic part;cytosol;intracellular membrane-bounded organelle;intracellular non-membrane-bounded organelle;intracellular organelle;intracellular part;macromolec	RNA degradation	Homo sapiens	
cell part;cytoplasmic part;endoplasmic reticulum;endosomal part;endosome membrane;envelope;extracellular membrane-bounded organelle;extracellular organelle;extracellular region;extracellular region part;extracellular ve	Lysosome	Homo sapiens	
cell part;cytoplasmic part;endoplasmic reticulum;endoplasmic reticulum membrane;endoplasmic reticulum part;integral to membrane;integral to plasma membrane;intracellular membrane-bounded organelle;intracellular orga	Sphingolipid metabolism	Homo sapiens	
cell part;clathrin coated vesicle membrane;clathrin-coated vesicle;coated vesicle;coated vesicle membrane;cytoplasmic membrane-bounded vesicle;cytoplasmic part;cytoplasmic vesicle;cytoplasmic vesicle membrane;cytoplasmic vesicle part;endosomal part;endosome membrane;Golgi apparatus part;integral to membrane;intracellular membrane-bounded organelle;in		Homo sapiens	+
cell part;cytoplasmic membrane-bounded vesicle;cytoplasmic part;cytoplasmic vesicle;endosomal part;endosome membrane;extracellular membrane-bounded organelle;extracellular organelle;extracellular region part;extracellular vesicular exosome;Golgi apparatus;Golgi apparatus part;Golgi membrane;integral to membrane;intracellular membrane-bounded organelle;i		Homo sapiens	+
actin cytoskeleton;adherens junction;anchoring junction;Arp2/3 protein complex;cell junction;cell part;cell-substrate adherens junction;cell-substrate junction;cytoplasm;cytoskeletal part;cytoskeleton;extracellular membrane-	Bacterial invasion of epithelial cells;Fc gamma R-mediated phagocytosis;Pathogenic Escherichia coli infection;Regulation of actin cytoskeleton;Sh	Homo sapiens	
actin cytoskeleton;adherens junction;anchoring junction;Arp2/3 protein complex;cell junction;cell leading edge;cell part;cell projection;cell-substrate adherens junction;cell-substrate junction;cytoplasm;cytoplasmic part;cytos	Bacterial invasion of epithelial cells;Fc gamma R-mediated phagocytosis;Pathogenic Escherichia coli infection;Regulation of actin cytoskeleton;Sh	Homo sapiens	+
actin cytoskeleton;adherens junction;anchoring junction;Arp2/3 protein complex;cell junction;cell part;cell projection;cell-substrate adherens junction;cell-substrate junction;cytoplasmic part;cytoskeletal part;cytoskeleton;cyt	Bacterial invasion of epithelial cells;Fc gamma R-mediated phagocytosis;Pathogenic Escherichia coli infection;Regulation of actin cytoskeleton;Sh	Homo sapiens	+
cell part;cytoplasmic part;cytosol;extracellular matrix;extracellular membrane-bounded organelle;extracellular organelle;extracellular region part;extracellular vesicular exosome;Golgi apparatus;integral to membrane;integral to plasma membrane;intracellular membrane-bounded organelle;intracellular non-membrane-bounded organelle;intracellular organelle;intracellular c		Homo sapiens	+
cell part;endomembrane system;integral to membrane;intrinsic to membrane;membrane;membrane part		Homo sapiens	+
cell part;cytoplasm;cytoplasmic part;cytosolic part;intracellular part;macromolecular complex;prefoldin complex;protein complex		Homo sapiens	+
actin cytoskeleton;adherens junction;anchoring junction;cell junction;cell part;cell-substrate adherens junction;cell-substrate junction;cytoplasm;cytoskeleton;focal adhesion;intracellular non-membrane-bounded organelle;intracellular organelle;intracellular part;membrane;non-membrane-bounded organelle;organelle;plasma membrane		Homo sapiens	+
cell part;cytoplasmic part;intracellular non-membrane-bounded organelle;intracellular organelle;intracellular organelle part;intracellular part;macromolecular complex;membrane;mitochondrial inner membrane;mitochondrial membrane;mitochondrial part;mitochondrial ribosome;non-membrane-bounded organelle;organellar ribosome;organelle;organelle inner membrane;o		Homo sapiens	+
cell part;cell surface;cytoplasmic part;endoplasmic reticulum-Golgi intermediate compartment;Golgi apparatus;Golgi apparatus part;integral to Golgi membrane;integral to membrane;integral to organelle membrane;intracellular membrane-bounded organelle;intracellular organelle;intracellular organelle part;intracellular part;intrinsic to Golgi membrane;intrinsic to membra		Homo sapiens	+
cell part;cytoplasmic part;endoplasmic reticulum membrane;endoplasmic reticulum palmitoyltransferase complex;endoplasmic reticulum part;integral to membrane;intracellular organelle part;intracellular part;intrinsic to mem	Sphingolipid metabolism	Homo sapiens	
cell part;cytoplasmic part;endoplasmic reticulum membrane;endoplasmic reticulum palmitoyltransferase complex;endoplasmic reticulum part;integral to membrane;intracellular membrane-bounded organelle;intracellular orga	Sphingolipid metabolism	Homo sapiens	+
cell part;cytoplasm;cytoplasmic part;cytoskeletal part;cytosol;histone acetyltransferase complex;histone methyltransferase complex;intracellular membrane-bounded organelle;intracellular organelle;intracellular organelle part	Other types of O-glycan biosynthesis	Homo sapiens	+
cell part;chromosome;intracellular membrane-bounded organelle;intracellular non-membrane-bounded organelle;intracellular organelle;intracellular part;membrane-bounded organelle;non-membrane-bounded organelle;nucleus;organelle		Homo sapiens	+

cell part;cytoplasm;intracellular membrane-bounded organelle;intracellular organelle;intracellular organelle part;intracellular part;membrane-bounded organelle;nuclear part;nucleoplasm;nucleus;organelle;organelle part		Homo sapiens	+
cell part;cell projection;cytoplasm;cytoplasmic part;cytoskeleton;cytosol;filopodium;Golgi apparatus;intracellular membrane-bounded organelle;intracellular non-membrane-bounded organelle;intracellular organelle;intracellula	Inositol phosphate metabolism;Phosphatidylinositol signaling system	Homo sapiens	+
cell part;cytoplasmic part;cytosol;eukaryotic 43S preinitiation complex;eukaryotic 48S preinitiation complex;eukaryotic translation initiation factor 3 complex;eukaryotic translation initiation factor 3 complex, eIF3m;intracellula	RNA transport	Homo sapiens	
cell part;cytoplasmic part;cytosol;eukaryotic 43S preinitiation complex;eukaryotic 48S preinitiation complex;eukaryotic translation initiation factor 3 complex;eukaryotic translation initiation factor 3 complex, eIF3m;extracellula	Measles;RNA transport	Homo sapiens	+
cell part;cytoplasmic part;intracellular membrane-bounded organelle;intracellular organelle;intracellular organelle lumen;intracellular organelle part;intracellular part;membrane-bounded organelle;membrane-enclosed lumen;m	Pantothenate and CoA biosynthesis;Valine, leucine and isoleucine biosynthesis;Valine, leucine and isoleucine degradation	Homo sapiens	+
cell part;centriole;chromosomal part;chromosome;chromosome passenger complex;chromosome, centromeric region;condensed chromosome kinetochore;cytoplasm;cytoplasmic microtubule;cytoplasmic part;cytoskeletal p	Colorectal cancer;Pathways in cancer	Homo sapiens	+
azurophil granule;cell part;cytoplasmic membrane-bounded vesicle;cytoplasmic part;cytoplasmic vesicle;early endosome;early endosome membrane;endocytic vesicle;endomembrane system;endosomal part;endosome;end	Phagosome;SNARE interactions in vesicular transport	Homo sapiens	+
actin cytoskeleton;cell part;cytoskeleton;integral to membrane;integral to plasma membrane;intracellular non-membrane-bounded organelle;intracellular organelle;intracellular organelle part;intracellular part;intrinsic to membrane;intrinsic to plasma membrane;membrane;membrane part;non-membrane-bounded organelle;nuclear membrane;nuclear part;organelle;organe		Homo sapiens	+
cell part;chromosome;cytoplasm;cytoplasmic part;DNA-directed RNA polymerase complex;DNA-directed RNA polymerase I complex;intracellular membrane-bounded organelle;intracellular non-membrane-bounded organelle;intracellular organelle;intracellular organelle part;intracellular part;macromolecular complex;membrane-bounded organelle;mitochondrion;non-me		Homo sapiens	+
cell part;cytoplasm;cytoplasmic part;endoplasmic reticulum;endoplasmic reticulum lumen;endoplasmic reticulum part;intracellular membrane-bounded organelle;intracellular organelle;intracellular organelle lumen;intracellular	Arginine and proline metabolism	Homo sapiens	+
adherens junction;anchoring junction;cell junction;cell part;cell surface;cell-substrate adherens junction;cell-substrate junction;cytoplasm;extracellular membrane-bounded organelle;extracellular organelle;extracellular region part;extracellular vesicular exosome;focal adhesion;intracellular part;membrane-bounded organelle;membrane-bounded vesicle;organelle;vesicle		Homo sapiens	+
cell part;cytoplasm;cytoplasmic part;cytoplasmic vesicle membrane;cytoplasmic vesicle part;cytosol;endoplasmic reticulum;endosome;extracellular membrane-bounded organelle;extracellular organelle;extracellular region pa	SNARE interactions in vesicular transport	Homo sapiens	+
actin cytoskeleton;adherens junction;anchoring junction;Arp2/3 protein complex;cell junction;cell part;cell projection;cell-substrate adherens junction;cell-substrate junction;cytoplasm;cytoplasmic part;cytoskeletal part;cytosl	Bacterial invasion of epithelial cells;Fc gamma R-mediated phagocytosis;Pathogenic Escherichia coli infection;Regulation of actin cytoskeleton;Sh	Homo sapiens	+
cell part;cytoplasmic mRNA processing body;cytoplasmic part;DNA-directed RNA polymerase complex;DNA-directed RNA polymerase II, core complex;intracellular membrane-bounded organelle;intracellular non-membrane	Huntington's disease;Purine metabolism;Pyrimidine metabolism;RNA polymerase	Homo sapiens	
cell part;cytoplasmic part;endoplasmic reticulum;endoplasmic reticulum membrane;endoplasmic reticulum part;Golgi apparatus part;Golgi membrane;integral to endoplasmic reticulum membrane;integral to lumenal side of e	Antigen processing and presentation	Homo sapiens	+
		Homo sapiens	+
cell part;cytoplasm;intracellular membrane-bounded organelle;intracellular non-membrane-bounded organelle;intracellular organelle;intracellular organelle part;intracellular part;macromolecular complex;membrane-bounded c	Spliceosome	Homo sapiens	+
cell part;intracellular membrane-bounded organelle;intracellular non-membrane-bounded organelle;intracellular organelle;intracellular organelle part;intracellular part;macromolecular complex;membrane-bounded organelle;m	mRNA surveillance pathway	Homo sapiens	+
		Homo sapiens	+
cell part;cytoplasmic part;integral to membrane;intracellular organelle part;intracellular part;intrinsic to membrane;membrane;membrane part;mitochondrial membrane;mitochondrial outer membrane;mitochondrial part;organelle membrane;organelle outer membrane;organelle part;outer membrane		Homo sapiens	+
Cajal body;cell part;intracellular membrane-bounded organelle;intracellular organelle;intracellular organelle part;intracellular part;macromolecular complex;membrane-bounded organelle;nuclear body;nuclear part;nuclear spe	Spliceosome	Homo sapiens	
cell part;cytoplasmic part;cytosol;extracellular membrane-bounded organelle;extracellular organelle;extracellular region part;extracellular vesicular exosome;intracellular part;membrane-bounded organelle;membrane-bounde	Glycine, serine and threonine metabolism;Methane metabolism	Homo sapiens	+
cell part;centrosome;cytoplasmic dynein complex;cytoplasmic part;cytoskeletal part;cytosol;dynein complex;intracellular non-membrane-bounded organelle;intracellular organelle;intracellular organelle part;intracellular part;m	Phagosome;Vasopressin-regulated water reabsorption	Homo sapiens	
cell part;cytoplasm;cytoplasmic part;cytosol;extracellular membrane-bounded organelle;extracellular organelle;extracellular region part;extracellular vesicular exosome;intracellular membrane-bounded organelle;intracellular c	Proteasome	Homo sapiens	
cell part;cytoplasmic part;cytosol;intracellular part	Purine metabolism;Selenocompound metabolism;Sulfur metabolism	Homo sapiens	+
cell part;chromosomal part;condensed chromosome kinetochore;cytoplasm;cytoplasmic part;cytoskeletal part;cytosol;endoplasmic reticulum;endoplasmic reticulum membrane;endoplasmic reticulum part;intracellular membrane-bounded organelle;intracellular non-membrane-bounded organelle;intracellular organelle;intracellular organelle part;intracellular part;kinetocho		Homo sapiens	+
Cajal body;catalytic step 2 spliceosome;cell part;cytoplasm;cytoplasmic part;cytosol;Golgi apparatus;intracellular membrane-bounded organelle;intracellular organelle;intracellular organelle part;intracellular part;macromolecu	Spliceosome	Homo sapiens	+
cell part;cytoplasm;intracellular membrane-bounded organelle;intracellular organelle;intracellular organelle part;intracellular part;membrane part;membrane raft;membrane-bounded organelle;nuclear body;nuclear part;nucleo	Bladder cancer;Pathways in cancer	Homo sapiens	
adherens junction;anchoring junction;cell junction;cell part;cell-substrate adherens junction;cell-substrate junction;cytoplasm;cytoskeleton;focal adhesion;intracellular;intracellular non-membrane-bounded organelle;intracellular organelle;intracellular organelle part;intracellular part;non-membrane-bounded organelle;nuclear matrix;nuclear part;organelle;organelle part		Homo sapiens	+
Ada2/Gcn5/Ada3 transcription activator complex;cell part;cytoplasm;cytoplasmic part;cytosol;endosomal part;endosome membrane;histone acetyltransferase complex;intracellular membrane-bounded organelle;intracellular	Adherens junction;Leishmaniasis;MAPK signaling pathway;Measles;NOD-like receptor signaling pathway;Osteoclast differentiation;RIG-I-like rece	Homo sapiens	+
cell part;cytoplasm;cytoplasmic part;cytosol;extracellular membrane-bounded organelle;extracellular organelle;extracellular region part;extracellular vesicular exosome;intracellular membrane-bounded organelle;intracellular organelle;intracellular part;membrane-bounded organelle;membrane-bounded vesicle;nucleus;organelle;vesicle		Homo sapiens	+
cell part;cytoplasm;cytoplasmic part;cytoskeleton;cytosol;intracellular non-membrane-bounded organelle;intracellular organelle;intracellular part;macromolecular complex;non-membrane-bounded organelle;organelle;protein	ko05152;Neurotrophin signaling pathway;NOD-like receptor signaling pathway;Shigellosis	Homo sapiens	+
catalytic step 2 spliceosome;cell part;cytoplasm;intracellular organelle part;intracellular part;macromolecular complex;nuclear part;nucleoplasm;organelle part;ribonucleoprotein complex;spliceosomal complex		Homo sapiens	+
Cajal body;cell part;cytoplasm;intracellular membrane-bounded organelle;intracellular organelle;intracellular organelle part;intracellular part;macromolecular complex;membrane-bounded organelle;nuclear body;nuclear part;r	Spliceosome	Homo sapiens	
cell part;cytoplasm;extracellular membrane-bounded organelle;extracellular organelle;extracellular region part;extracellular vesicular exosome;intracellular membrane-bounded organelle;intracellular organelle;intracellular part;macromolecular complex;membrane-bounded organelle;membrane-bounded vesicle;nucleus;organelle;proteasome complex;protein complex;vesi		Homo sapiens	+
cell part;cytoplasm;cytoplasmic part;intracellular part;perinuclear region of cytoplasm		Homo sapiens	+
cell part;cytoplasm;cytoplasmic part;ER membrane protein complex;intracellular membrane-bounded organelle;intracellular organelle;intracellular part;macromolecular complex;membrane;membrane-bounded organelle;mitochondrion;nucleus;organelle;protein complex		Homo sapiens	
cell part;cytoplasmic part;endomembrane system;extracellular membrane-bounded organelle;extracellular organelle;extracellular region part;extracellular vesicular exosome;intracellular membrane-bounded organelle;intracellular organelle;intracellular organelle part;intracellular part;membrane;membrane-bounded organelle;membrane-bounded vesicle;mitochondrion;nu		Homo sapiens	+
cell part;cytoplasmic part;cytosol;eukaryotic translation initiation factor 4F complex;intracellular part;macromolecular complex;protein complex	RNA transport;Viral myocarditis	Homo sapiens	
cell part;cytoplasm;intracellular organelle part;intracellular part;macromolecular complex;nuclear body;nuclear part;nuclear speck;nucleoplasm part;organelle part;ribonucleoprotein complex;small nuclear ribonucleoprotein co	Spliceosome	Homo sapiens	
cell part;cytoplasmic part;cytosol;extracellular membrane-bounded organelle;extracellular organelle;extracellular region part;extracellular vesicular exosome;Golgi apparatus;intracellular membrane-bounded organelle;intracellular organelle;intracellular part;membrane-bounded organelle;membrane-bounded vesicle;mitochondrion;organelle;vesicle		Homo sapiens	+
adherens junction;anchoring junction;cell cortex part;cell junction;cell part;cell-substrate adherens junction;cell-substrate junction;cytoplasmic part;cytoskeletal part;extracellular membrane-bounded organelle;extracellular or	Tight junction	Homo sapiens	+
cell part;cytoplasmic membrane-bounded vesicle;cytoplasmic part;cytoplasmic vesicle;Golgi apparatus;Golgi apparatus part;integral to membrane;intracellular membrane-bounded organelle;intracellular organelle;intracellular organelle part;intracellular part;intrinsic to membrane;membrane;membrane part;membrane-bounded organelle;membrane-bounded vesicle;nucle		Homo sapiens	+
		Homo sapiens	+
cell part;cytoplasm;intracellular organelle part;intracellular part;nuclear part;nucleoplasm;organelle part	RNA transport	Homo sapiens	+
cell part;cytoplasm;extracellular membrane-bounded organelle;extracellular organelle;extracellular region part;extracellular vesicular exosome;intracellular membrane-bounded organelle;intracellular organelle;intracellular organelle part;intracellular part;membrane-bounded organelle;membrane-bounded vesicle;nuclear part;nucleoplasm;nucleus;organelle;organelle part;ve		Homo sapiens	+
cell part;cytoplasmic part;intracellular membrane-bounded organelle;intracellular organelle;intracellular organelle lumen;intracellular organelle part;intracellular part;membrane;membrane-bounded organelle;membrane-enclosed lumen;mitochondrial inner membrane;mitochondrial matrix;mitochondrial membrane;mitochondrial part;mitochondrion;organelle;organelle inner		Homo sapiens	
cell part;cytoplasmic part;endoplasmic reticulum;Golgi apparatus;intracellular membrane-bounded organelle;intracellular organelle;intracellular part;membrane-bounded organelle;organelle		Homo sapiens	+
cell part;cytoplasmic part;cytosol;endosomal part;endosome membrane;extracellular membrane-bounded organelle;extracellular organelle;extracellular region part;extracellular vesicular exosome;intracellular organelle part;in	Endocytosis	Homo sapiens	+
cell part;extracellular membrane-bounded organelle;extracellular organelle;extracellular region part;extracellular vesicular exosome;integral to membrane;intrinsic to membrane;membrane part;membrane-bounded organelle;membrane-bounded vesicle;organelle;vesicle		Homo sapiens	+
catalytic step 2 spliceosome;cell part;intracellular membrane-bounded organelle;intracellular organelle;intracellular organelle part;intracellular part;macromolecular complex;membrane-bounded organelle;nuclear body;nuclea	Spliceosome	Homo sapiens	+
cell part;cytoplasm;cytoplasmic part;cytoskeletal part;cytoskeleton;cytosol;intracellular membrane-bounded organelle;intracellular non-membrane-bounded organelle;intracellular organelle;intracellular organelle part;intracellular part;macromolecular complex;membrane;membrane-bounded organelle;microtubule;microtubule cytoskeleton;non-membrane-bounded organe		Homo sapiens	+
axon part;axon terminus;cell body;cell part;cell projection;cell projection part;cytoplasm;dendritic spine;intracellular membrane-bounded organelle;intracellular organelle;intracellular part;membrane;membrane-bounded organelle;neuron projection;neuron projection terminus;neuron spine;neuronal cell body;nucleus;organelle;plasma membrane		Homo sapiens	+
cell part;chromosomal part;condensed chromosome kinetochore;cytoskeletal part;intracellular membrane-bounded organelle;intracellular non-membrane-bounded organelle;intracellular organelle;intracellular organelle part;intracellular part;kinetochore;macromolecular complex;membrane-bounded organelle;microtubule;non-membrane-bounded organelle;nuclear part;n		Homo sapiens	
cell part;cytoplasmic part;integral to membrane;intracellular membrane-bounded organelle;intracellular organelle;intracellular organelle part;intracellular part;intrinsic to membrane;macromolecular complex;membrane;membra	Alzheimer's disease;Huntington's disease;Oxidative phosphorylation;Parkinson's disease	Homo sapiens	
cell part;cytoplasmic part;intracellular organelle part;intracellular part;macromolecular complex;membrane;membrane part;mitochondrial inner membrane;mitochondrial membrane;mitochondrial membrane part;mitochondrial	Alzheimer's disease;Huntington's disease;Oxidative phosphorylation;Parkinson's disease	Homo sapiens	+
cell part;cytoplasm;cytoplasmic part;endoplasmic reticulum membrane;endoplasmic reticulum part;extracellular membrane-bounded organelle;extracellular organelle;extracellular region part;extracellular vesicular exosome;intracellular membrane-bounded organelle;intracellular non-membrane-bounded organelle;intracellular organelle;intracellular organelle part;intracellu		Homo sapiens	+
cell part;chromosomal part;condensed chromosome kinetochore;cytoplasmic part;cytosol;intracellular non-membrane-bounded organelle;intracellular organelle;intracellular organelle part;intracellular part;kinetochore;macron	Cell cycle;Cell cycle - yeast	Homo sapiens	+
actin filament bundle;actomyosin;adherens junction;anchoring junction;cell cortex part;cell junction;cell part;cell projection;cell-cell junction;cell-substrate adherens junction;cell-substrate junction;contractile fiber part;cortical	Adherens junction;Amoebiasis;Arrhythmogenic right ventricular cardiomyopathy (ARVC);Focal adhesion;Leukocyte transendothelial migration;Reg	Homo sapiens	+
cell part;intracellular membrane-bounded organelle;intracellular organelle;intracellular organelle part;intracellular part;membrane-bounded organelle;nuclear part;nucleoplasm;nucleus;organelle;organelle part		Homo sapiens	
AP-type membrane coat adaptor complex;cell part;clathrin adaptor complex;clathrin coated vesicle membrane;clathrin-coated endocytic vesicle membrane;clathrin-coated vesicle;coated vesicle;coated vesicle membrane;cy	Lysosome	Homo sapiens	+
cell part;cytoplasm;intracellular part		Homo sapiens	+
cell part;cytoplasm;intracellular organelle part;intracellular part;nuclear part;nucleoplasm;organelle part		Homo sapiens	+
cell part;cytoplasm;cytoplasmic part;cytosol;extracellular membrane-bounded organelle;extracellular organelle;extracellular region part;extracellular vesicular exosome;intracellular membrane-bounded organelle;intracellular c	Aminoacyl-tRNA biosynthesis	Homo sapiens	+
actin filament;brush border;cell part;cell periphery;cell projection;cytoplasm;cytoplasmic part;cytoskeletal part;early endosome;endosomal part;endosome;endosome membrane;extracellular membrane-bounded organelle;extracellular organelle;extracellular region part;extracellular vesicular exosome;filopodium;intracellular membrane-bounded organelle;intracellular orga		Homo sapiens	+
cell part;centrosome;cytoplasmic part;cytoskeletal part;intracellular membrane-bounded organelle;intracellular non-membrane-bounded organelle;intracellular organelle;intracellular organelle part;intracellular part;macromolec	mRNA surveillance pathway	Homo sapiens	+
cell part;cytoplasm;extracellular membrane-bounded organelle;extracellular organelle;extracellular region part;extracellular vesicular exosome;integral to membrane;integral to plasma membrane;intracellular membrane-bounded organelle;intracellular organelle;intracellular part;intrinsic to membrane;intrinsic to plasma membrane;membrane part;membrane-bounded orga		Homo sapiens	
box C/D snoRNP complex;cell part;intracellular membrane-bounded organelle;intracellular non-membrane-bounded organelle;intracellular organelle;intracellular organelle part;intracellular part;macromolecular complex;membrane-bounded organelle;non-membrane-bounded organelle;nuclear part;nucleolar part;nucleolus;nucleus;organelle;organelle part;preribosome;rib		Homo sapiens	
cell part;chromosome;condensed chromosome;cytoplasmic part;female pronucleus;Golgi apparatus;intracellular membrane-bounded organelle;intracellular non-membrane-bounded organelle;intracellular organelle;intracellular organelle part;intracellular part;membrane;membrane-bounded organelle;mitochondrion;non-membrane-bounded organelle;nuclear matrix;nucle		Homo sapiens	+
cell part;cytoplasmic part;intracellular membrane-bounded organelle;intracellular organelle;intracellular organelle lumen;intracellular organelle part;intracellular part;membrane-bounded organelle;membrane-enclosed lumen;m	Citrate cycle (TCA cycle)	Homo sapiens	+
cell part;cell surface;cytoplasmic part;cytosol;intracellular part		Homo sapiens	+
cell part;cytoplasmic membrane-bounded vesicle;cytoplasmic part;cytoplasmic vesicle;endoplasmic reticulum;endoplasmic reticulum lumen;endoplasmic reticulum part;extracellular region;Golgi apparatus;intracellular membrane-bounded organelle;intracellular organelle;intracellular organelle lumen;intracellular organelle part;intracellular part;melanosome;membrane;me		Homo sapiens	+
extracellular membrane-bounded organelle;extracellular organelle;extracellular region part;extracellular vesicular exosome;membrane-bounded organelle;membrane-bounded vesicle;organelle;vesicle		Homo sapiens	+
cell part;cytoplasmic part;cytosol;endoplasmic reticulum;extracellular membrane-bounded organelle;extracellular organelle;extracellular region part;extracellular vesicular exosome;intracellular membrane-bounded organelle;i	Cysteine and methionine metabolism	Homo sapiens	+
cell part;cytoplasmic part;intracellular membrane-bounded organelle;intracellular organelle;intracellular organelle part;intracellular part;macromolecular complex;membrane;membrane part;membrane-bounded organelle;mem	Alzheimer's disease;Huntington's disease;Oxidative phosphorylation;Parkinson's disease	Homo sapiens	+
cell part;chromatin;chromosomal part;chromosome;chromosome, centromeric region;cohesin complex;cytoplasmic part;cytosol;intracellular non-membrane-bounded organelle;intracellular organelle;intracellular organelle par	Cell cycle;Cell cycle - yeast	Homo sapiens	+
cell part;cytoplasmic part;intracellular membrane-bounded organelle;intracellular organelle;intracellular organelle part;intracellular part;membrane;membrane-bounded organelle;membrane-enclosed lumen;mitochondrial inner membrane;mitochondrial intermembrane space;mitochondrial membrane;mitochondrial part;mitochondrion;organelle;organelle envelope lumen;or		Homo sapiens	+
cell part;cytoplasm;intracellular membrane-bounded organelle;intracellular organelle;intracellular organelle part;intracellular part;macromolecular complex;membrane-bounded organelle;nuclear part;nucleoplasm;nucleus;orga	Spliceosome	Homo sapiens	
		Homo sapiens	+
macromolecular complex;protein complex;ribose phosphate diphosphokinase complex		Homo sapiens	+
cell part;chromatin remodeling complex;chromatin silencing complex;chromosome;condensed chromosome;intracellular membrane-bounded organelle;intracellular non-membrane-bounded organelle;intracellular organelle;intracellular organelle part;intracellular part;ISWI complex;macromolecular complex;membrane-bounded organelle;non-membrane-bounded organell		Homo sapiens	
acrosomal vesicle;cell part;cytoplasm;cytoplasmic membrane-bounded vesicle;cytoplasmic part;cytoplasmic vesicle;cytosol;extracellular membrane-bounded organelle;extracellular organelle;extracellular region part;extracellular vesicular exosome;integral to membrane;intracellular membrane-bounded organelle;intracellular organelle;intracellular part;intrinsic to membra		Homo sapiens	+
catalytic step 2 spliceosome;cell part;intracellular membrane-bounded organelle;intracellular organelle;intracellular organelle part;intracellular part;macromolecular complex;membrane;membrane-bounded organelle;nuclear p	Spliceosome	Homo sapiens	
cell part;cell projection;cytoplasm;cytoplasmic part;dendrite;integral to membrane;intracellular membrane-bounded organelle;intracellular organelle;intracellular organelle part;intracellular part;intrinsic to membrane;membrane;membrane part;membrane-bounded organelle;membrane-enclosed lumen;mitochondrial crista;mitochondrial inner membrane;mitochondrial inter		Homo sapiens	
cell part;cell projection;cytoplasmic part;cytoplasmic vesicle;cytoplasmic vesicle membrane;cytoplasmic vesicle part;cytoskeletal part;intracellular membrane-bounded organelle;intracellular organelle;intracellular organelle part;intracellular part;kinesin complex;macromolecular complex;membrane;membrane-bounded organelle;microtubule;microtubule associated comp		Homo sapiens	+
cell part;chromatin;chromosomal part;intracellular membrane-bounded organelle;intracellular organelle;intracellular organelle part;intracellular part;macromolecular complex;membrane-bounded organelle;nuclear chromatin;nuclear chromosome part;nuclear part;nucleoplasm;nucleoplasm part;nucleus;organelle;organelle part;protein complex;transcription factor complex		Homo sapiens	+
cell part;cytoplasm;intracellular part		Homo sapiens	+
cell body;cell part;cytoplasm;cytoplasmic part;endoplasmic reticulum membrane;endoplasmic reticulum part;ER-mitochondrion membrane contact site;extracellular membrane-bounded organelle;extracellular organelle;extra	Adipocytokine signaling pathway;Fatty acid metabolism;Peroxisome;PPAR signaling pathway	Homo sapiens	+
cell part;clathrin-coated vesicle;coated vesicle;cytoplasm;cytoplasmic membrane-bounded vesicle;cytoplasmic part;cytoplasmic vesicle;cytosol;early endosome;early endosome membrane;early phagosome;endocytic vesicle;endosomal part;endosome;endosome membrane;extracellular membrane-bounded organelle;extracellular organelle;extracellular region part;extra		Homo sapiens	
cell part;cytoplasmic part;cytosol;intracellular membrane-bounded organelle;intracellular organelle;intracellular part;membrane;membrane-bounded organelle;nucleus;organelle		Homo sapiens	
catalytic step 2 spliceosome;cell part;CRD-mediated mRNA stability complex;cytoplasmic part;endoplasmic reticulum;histone pre-mRNA 3'end processing complex;intracellular membrane-bounded organelle;intracellular organelle;intracellular organelle part;intracellular part;macromolecular complex;membrane;membrane-bounded organelle;nuclear part;nucleoplasm;nu		Homo sapiens	+
catalytic step 2 spliceosome;cell part;intracellular organelle part;intracellular part;macromolecular complex;nuclear part;nucleoplasm;organelle part;ribonucleoprotein complex;spliceosomal complex	Spliceosome	Homo sapiens	+
cell part;cytoplasm;intracellular membrane-bounded organelle;intracellular organelle;intracellular part;membrane-bounded organelle;nucleus;organelle		Homo sapiens	+
cell part;cytoplasm;extracellular membrane-bounded organelle;extracellular organelle;extracellular region part;extracellular vesicular exosome;intracellular part;membrane-bounded organelle;membrane-bounded vesicle;orga	Amino sugar and nucleotide sugar metabolism;Fructose and mannose metabolism	Homo sapiens	+
cell part;cell surface;extracellular region part;extracellular space		Homo sapiens	+
cell part;cytoplasmic part;endoplasmic reticulum;endoplasmic reticulum membrane;endoplasmic reticulum part;extracellular membrane-bounded organelle;extracellular organelle;extracellular region part;extracellular vesicula	Lysine degradation;Other types of O-glycan biosynthesis	Homo sapiens	+
cell part;cytoplasmic part;cytosol;intracellular part;macromolecular complex;mRNA cap binding complex;protein complex;RNA cap binding complex	Insulin signaling pathway;mTOR signaling pathway;RNA transport	Homo sapiens	+
cell part;cell projection;cell projection membrane;cell projection part;cytoplasmic part;cytoskeletal part;cytosol;intracellular non-membrane-bounded organelle;intracellular organelle;intracellular organelle part;intracellular part;	Focal adhesion;Regulation of actin cytoskeleton;Shigellosis	Homo sapiens	
cell part;cytoplasm;cytoplasmic part;endosomal part;endosome;endosome membrane;Golgi apparatus;intracellular membrane-bounded organelle;intracellular organelle;intracellular organelle part;intracellular part;lipid particle;membrane;membrane-bounded organelle;organelle;organelle membrane;organelle part		Homo sapiens	+
cell part;cytoplasm;intracellular membrane-bounded organelle;intracellular organelle;intracellular part;membrane;membrane-bounded organelle;nucleus;organelle		Homo sapiens	+
cell part;cytoplasmic part;cytosol;extracellular membrane-bounded organelle;extracellular organelle;extracellular region part;extracellular vesicular exosome;intracellular organelle part;intracellular part;membrane-bounded org	Amino sugar and nucleotide sugar metabolism;Ascorbate and aldarate metabolism;Pentose and glucuronate interconversions;Starch and sucrose	Homo sapiens	
adherens junction;anchoring junction;cell junction;cell part;cell projection;cell-substrate adherens junction;cell-substrate junction;cytoplasm;cytoplasmic part;cytoskeletal part;focal adhesion;intracellular membrane-bounded organelle;intracellular non-membrane-bounded organelle;intracellular organelle;intracellular organelle part;intracellular part;macromolecular comple		Homo sapiens	+
adherens junction;anchoring junction;cell junction;cell part;cell projection;cell projection part;cell-cell adherens junction;cell-cell junction;cytoplasm;cytoplasmic part;cytosol;dendritic spine;extracellular membrane-bounded o	Adherens junction;Leukocyte transendothelial migration	Homo sapiens	+
cell part;cell projection;cytoplasm;cytoplasmic part;early endosome membrane;endosomal part;endosome membrane;extracellular membrane-bounded organelle;extracellular organelle;extracellular region part;extracellular vesicular exosome;extrinsic to membrane;intracellular membrane-bounded organelle;intracellular organelle;intracellular organelle part;intracellular pa		Homo sapiens	
cell part;cytoplasmic part;dolichol-phosphate-mannose synthase complex;endoplasmic reticulum;endoplasmic reticulum membrane;endoplasmic reticulum part;intracellular membrane-bounded organelle;intracellular organel	N-Glycan biosynthesis	Homo sapiens	+
cell part;coated vesicle membrane;cytoplasmic part;cytoplasmic vesicle membrane;cytoplasmic vesicle part;cytosol;endoplasmic reticulum;ER to Golgi transport vesicle membrane;Golgi apparatus;Golgi apparatus part;Golgi membrane;Golgi stack;intracellular membrane-bounded organelle;intracellular non-membrane-bounded organelle;intracellular organelle;intracellul		Homo sapiens	+
cell part;cytoplasm;cytoplasmic part;cytosol;early endosome;endosome;extracellular membrane-bounded organelle;extracellular organelle;extracellular region part;extracellular vesicular exosome;intracellular membrane-bounded organelle;intracellular organelle;intracellular part;membrane;membrane-bounded organelle;membrane-bounded vesicle;organelle;vesicle		Homo sapiens	
cell part;cytoplasmic part;endosome;intracellular membrane-bounded organelle;intracellular organelle;intracellular part;membrane-bounded organelle;organelle		Homo sapiens	+
cell part;cell projection part;cytoplasm;cytoplasmic part;intracellular membrane-bounded organelle;intracellular non-membrane-bounded organelle;intracellular organelle;intracellular organelle part;intracellular part;macromole	Spliceosome	Homo sapiens	+
cell part;cytoplasmic part;endosomal part;endosome membrane;integral to membrane;intracellular organelle part;intracellular part;intrinsic to membrane;membrane;membrane part;organelle membrane;organelle part		Homo sapiens	+
box H/ACA RNP complex;box H/ACA snoRNP complex;Cajal body;cell part;cytoplasm;intracellular membrane-bounded organelle;intracellular non-membrane-bounded organelle;intracellular organelle;intracellular organelle p	Ribosome biogenesis in eukaryotes	Homo sapiens	
cell part;cytoplasm;cytoplasmic part;cytosol;intracellular part	RNA transport	Homo sapiens	+
cell part;cytoplasm;extracellular membrane-bounded organelle;extracellular organelle;extracellular region part;extracellular vesicular exosome;intracellular;intracellular membrane-bounded organelle;intracellular non-membrane-bounded organelle;intracellular organelle;intracellular organelle part;intracellular part;membrane-bounded organelle;membrane-bounded vesicle;		Homo sapiens	+
cell part;cytoplasmic part;cytosol;early endosome;endosome;Golgi apparatus;intracellular membrane-bounded organelle;intracellular non-membrane-bounded organelle;intracellular organelle;intracellular organelle part;intrac	Regulation of actin cytoskeleton	Homo sapiens	+
cell part;cytoplasmic part;cytosol;extracellular membrane-bounded organelle;extracellular organelle;extracellular region part;extracellular vesicular exosome;intracellular part;membrane;membrane-bounded organelle;membra	Protein processing in endoplasmic reticulum	Homo sapiens	
carboxy-terminal domain protein kinase complex;cell part;chromatin;chromosomal part;chromosome;condensed chromosome;condensed nuclear chromosome;cytoplasm;intracellular membrane-bounded organelle;intracellular non-membrane-bounded organelle;intracellular organelle;intracellular organelle part;intracellular part;macromolecular complex;membrane-bour		Homo sapiens	+
cell part;cytoplasmic part;cytosolic part;intracellular part;macromolecular complex;prefoldin complex;protein complex		Homo sapiens	+
cell part;chromosomal part;chromosome, telomeric region;inclusion body;intracellular membrane-bounded organelle;intracellular non-membrane-bounded organelle;intracellular organelle;intracellular organelle part;intracellula	Homologous recombination	Homo sapiens	+
cell part;cytoplasmic part;integral to membrane;intracellular membrane-bounded organelle;intracellular organelle;intracellular organelle part;intracellular part;intrinsic to membrane;membrane;membrane part;membrane-bounc	ABC transporters	Homo sapiens	+
cell junction;cell part;cell projection;cell projection part;cytoplasm;cytoplasmic membrane-bounded vesicle;cytoplasmic part;cytoplasmic vesicle;cytoskeletal part;cytosol;dendritic spine head;endocytic vesicle;intracellular me	Axon guidance	Homo sapiens	+
actin filament;cell junction;cell part;cell projection;cytoplasm;cytoplasmic part;cytoskeletal part;cytosol;extracellular membrane-bounded organelle;extracellular organelle;extracellular region;extracellular region part;extracellular vesicular exosome;intracellular non-membrane-bounded organelle;intracellular organelle;intracellular organelle part;intracellular part;macromolec		Homo sapiens	+
cell part;centrosome;cytoplasmic part;cytoskeletal part;cytosol;intracellular membrane-bounded organelle;intracellular non-membrane-bounded organelle;intracellular organelle;intracellular organelle part;intracellular part;mer	Axon guidance;Chemokine signaling pathway;Focal adhesion;Leukocyte transendothelial migration;Pathogenic Escherichia coli infection;Regulati	Homo sapiens	
cell cortex;cell part;cell projection membrane;cell projection part;chromosomal part;condensed chromosome kinetochore;cytoplasm;cytoplasmic microtubule;cytoplasmic part;cytoskeletal part;cytosol;Golgi apparatus;Golgi apparatus part;intracellular membrane-bounded organelle;intracellular non-membrane-bounded organelle;intracellular organelle;intracellular organe		Homo sapiens	+
cell part;cytoplasm;cytoplasmic part;cytosol;extracellular membrane-bounded organelle;extracellular organelle;extracellular region part;extracellular vesicular exosome;intracellular membrane-bounded organelle;intracellular organelle;intracellular part;membrane;membrane-bounded organelle;membrane-bounded vesicle;nucleus;organelle;plasma membrane;vesicle		Homo sapiens	+
cell part;clathrin coated vesicle membrane;clathrin-coated vesicle;coated pit;coated vesicle;coated vesicle membrane;cytoplasm;cytoplasmic membrane-bounded vesicle;cytoplasmic part;cytoplasmic vesicle;cytoplasmic vesicle membrane;cytoplasmic vesicle part;cytoskeleton;intracellular membrane-bounded organelle;intracellular non-membrane-bounded organelle;i		Homo sapiens	+
cell part;cell projection;chromosomal part;HULC complex;intracellular membrane-bounded organelle;intracellular organelle;intracellular organelle part;intracellular part;macromolecular complex;membrane;membrane-bounded organelle;neuron projection;nuclear part;nucleoplasm;nucleus;organelle;organelle part;protein complex;ubiquitin conjugating enzyme complex;ub		Homo sapiens	
		Homo sapiens	
cell part;cytoplasm;intracellular part		Homo sapiens	
cell part;cytoplasmic part;early endosome membrane;endosomal part;endosome membrane;extracellular membrane-bounded organelle;extracellular organelle;extracellular region part;extracellular vesicular exosome;intracellular organelle part;intracellular part;lysosomal membrane;membrane;membrane-bounded organelle;membrane-bounded vesicle;organelle;organelle		Homo sapiens	+

CCR4-NOT complex;cell part;cytoplasmic mRNA processing body;cytoplasmic part;cytosol;intracellular membrane-bounded organelle;intracellular non-membrane-bounded organelle;intracellular organelle;intracellular organe	RNA degradation	Homo sapiens	
cell part;chromatin;chromosomal part;cytoplasm;intracellular membrane-bounded organelle;intracellular organelle;intracellular organelle part;intracellular part;membrane;membrane-bounded organelle;nucleus;organelle;organelle part		Homo sapiens	+
cell part;cytoplasmic part;cytosol;extracellular membrane-bounded organelle;extracellular organelle;extracellular region part;extracellular vesicular exosome;intracellular part;membrane-bounded organelle;membrane-bounde	Glutathione metabolism	Homo sapiens	+
cell part;cytoplasmic part;intracellular membrane-bounded organelle;intracellular organelle;intracellular organelle part;intracellular part;macromolecular complex;membrane;membrane part;membrane-bounded organelle;mito	Alzheimer's disease;Huntington's disease;Oxidative phosphorylation;Parkinson's disease	Homo sapiens	+
axon;Cajal body;cell body;cell part;cell projection;cell projection part;cytoplasm;cytoplasmic part;growth cone;intracellular membrane-bounded organelle;intracellular non-membrane-bounded organelle;intracellular organelle;intracellular organelle part;intracellular part;macromolecular complex;membrane-bounded organelle;neuron projection;neuronal cell body;non-mem		Homo sapiens	+
cell part;cytoplasmic part;integral to membrane;integral to plasma membrane;intracellular membrane-bounded organelle;intracellular organelle;intracellular part;intrinsic to membrane;intrinsic to plasma membrane;membrane;membrane part;membrane-bounded organelle;mitochondrion;organelle;plasma membrane part		Homo sapiens	
cell part;cell surface;cytoplasm;intracellular part;membrane;plasma membrane	ECM-receptor interaction	Homo sapiens	+
cell part;cytoplasm;cytoplasmic part;cytoplasmic vesicle;endoplasmic reticulum;endoplasmic reticulum exit site;endoplasmic reticulum membrane;endoplasmic reticulum part;endosome;extracellular membrane-bounded organelle;extracellular organelle;extracellular region part;extracellular vesicular exosome;intracellular membrane-bounded organelle;intracellular organe		Homo sapiens	+
cell part;cytoplasm;cytoskeletal part;cytoskeleton;extracellular membrane-bounded organelle;extracellular organelle;extracellular region part;extracellular vesicular exosome;intracellular non-membrane-bounded organelle;intracellular organelle;intracellular organelle part;intracellular part;macromolecular complex;membrane-bounded organelle;membrane-bounded vesicle		Homo sapiens	+
cell part;cytoplasmic part;cytosol;extracellular membrane-bounded organelle;extracellular organelle;extracellular region part;extracellular vesicular exosome;intracellular organelle part;intracellular part;lysosomal membrane;m	Collecting duct acid secretion;Epithelial cell signaling in Helicobacter pylori infection;Oxidative phosphorylation;Phagosome;Rheumatoid arthritis;\	Homo sapiens	+
cell part;centrosome;cytoplasm;cytoplasmic part;cytoskeletal part;cytosol;endosomal part;endosome;endosome membrane;extracellular membrane-bounded organelle;extracellular organelle;extracellular region part;extracell	Endocytosis	Homo sapiens	
Barr body;cell part;centromeric heterochromatin;chromatin;chromosomal part;chromosome;condensed chromosome;extracellular membrane-bounded organelle;extracellular organelle;extracellular region part;extracellular ve	Systemic lupus erythematosus	Homo sapiens	+
actin cytoskeleton;actin filament bundle;actomyosin;adherens junction;anchoring junction;cell cortex;cell junction;cell part;cell-substrate adherens junction;cell-substrate junction;contractile fiber part;cytoplasm;cytoplasmic p	Focal adhesion;MAPK signaling pathway	Homo sapiens	+
cell part;cytoplasmic part;integral to membrane;intracellular;intracellular membrane-bounded organelle;intracellular non-membrane-bounded organelle;intracellular organelle;intracellular organelle part;intracellular part;intrinsic	Peroxisome	Homo sapiens	+
cell part;cytoplasmic part;extracellular membrane-bounded organelle;extracellular organelle;extracellular region part;extracellular vesicular exosome;intracellular membrane-bounded organelle;intracellular organelle;intracellula	Citrate cycle (TCA cycle);Glyoxylate and dicarboxylate metabolism	Homo sapiens	+
cell part;cytoplasmic membrane-bounded vesicle;cytoplasmic part;cytoplasmic vesicle;endoplasmic reticulum membrane;endoplasmic reticulum part;endoplasmic reticulum-Golgi intermediate compartment;endoplasmic reti	Phagosome;SNARE interactions in vesicular transport	Homo sapiens	+
cell part;cytoplasm;intracellular organelle part;intracellular part;membrane;nuclear body;nuclear matrix;nuclear part;nuclear speck;nucleoplasm;nucleoplasm part;organelle part	Spliceosome	Homo sapiens	
cell part;cytoplasm;cytoplasmic part;cytoskeletal part;cytoskeleton;intermediate filament cytoskeleton;intracellular membrane-bounded organelle;intracellular non-membrane-bounded organelle;intracellular organelle;intracellular organelle part;intracellular part;membrane-bounded organelle;microtubule cytoskeleton;microtubule organizing center;non-membrane-bounde		Homo sapiens	+
cell part;cytoplasmic part;intracellular organelle part;intracellular part;membrane;mitochondrial membrane;mitochondrial outer membrane;mitochondrial part;organelle membrane;organelle outer membrane;organelle part;outer membrane		Homo sapiens	
cell part;cytoplasmic part;cytosol;early endosome;endosomal part;endosome;endosome membrane;extracellular membrane-bounded organelle;extracellular organelle;extracellular region part;extracellular vesicular exosome;intracellular membrane-bounded organelle;intracellular organelle;intracellular organelle part;intracellular part;macromolecular complex;membrane;m		Homo sapiens	+
cell part;cytoplasmic part;intracellular organelle lumen;intracellular organelle part;intracellular part;membrane;membrane-enclosed lumen;mitochondrial inner membrane;mitochondrial matrix;mitochondrial membrane;mitochondrial part;organelle inner membrane;organelle lumen;organelle membrane;organelle part		Homo sapiens	
cell part;chromatin;chromosomal part;cytoplasmic part;cytosol;heterochromatin;intracellular membrane-bounded organelle;intracellular organelle;intracellular organelle part;intracellular part;membrane-bounded organelle;nuclear chromatin;nuclear chromosome part;nuclear heterochromatin;nuclear part;nuclear periphery;nucleoplasm;nucleus;organelle;organelle part;tran		Homo sapiens	+
cell part;cytoplasmic part;endoplasmic reticulum;endoplasmic reticulum membrane;endoplasmic reticulum part;integral to membrane;intracellular membrane-bounded organelle;intracellular organelle;intracellular organelle part;intracellular part;intrinsic to membrane;macromolecular complex;membrane;membrane part;membrane-bounded organelle;organelle;organelle m		Homo sapiens	+
cell part;cytoplasmic part;intracellular membrane-bounded organelle;intracellular organelle;intracellular organelle part;intracellular part;macromolecular complex;membrane;membrane part;membrane-bounded organelle;mito	Alzheimer's disease;Huntington's disease;Oxidative phosphorylation;Parkinson's disease	Homo sapiens	
cell part;cytoplasm;intracellular membrane-bounded organelle;intracellular organelle;intracellular organelle part;intracellular part;membrane-bounded organelle;nuclear body;nuclear part;nuclear speck;nucleoplasm;nucleopla	Spliceosome	Homo sapiens	+
basal part of cell;cell junction;cell part;cell-cell junction;extracellular membrane-bounded organelle;extracellular organelle;extracellular region part;extracellular vesicular exosome;integral to membrane;intrinsic to membrane;n	Cell adhesion molecules (CAMs);Hepatitis C;Leukocyte transendothelial migration;Tight junction	Homo sapiens	+
cell part;cytoplasmic part;intracellular membrane-bounded organelle;intracellular organelle;intracellular organelle lumen;intracellular organelle part;intracellular part;membrane;membrane-bounded organelle;membrane-enclos	Fatty acid metabolism;Peroxisome	Homo sapiens	+
cell part;chromosome;cytoplasm;cytoplasmic part;cytosol;extracellular membrane-bounded organelle;extracellular organelle;extracellular region part;extracellular vesicular exosome;intracellular membrane-bounded organelle;intracellular non-membrane-bounded organelle;intracellular organelle;intracellular organelle part;intracellular part;membrane-bounded organelle;m		Homo sapiens	+
catalytic step 2 spliceosome;cell part;intracellular membrane-bounded organelle;intracellular organelle;intracellular organelle part;intracellular part;macromolecular complex;membrane-bounded organelle;nuclear body;nuclea	Spliceosome	Homo sapiens	+
cell part;CRD-mediated mRNA stability complex;cytoplasm;cytoplasmic part;Golgi apparatus;intracellular membrane-bounded organelle;intracellular organelle;intracellular organelle part;intracellular part;macromolecular complex;membrane;membrane-bounded organelle;mitochondrial inner membrane;mitochondrial membrane;mitochondrial part;organelle;organelle inne		Homo sapiens	+
cell part;cytoplasm;cytoplasmic part;cytosol;intracellular organelle part;intracellular part;membrane;nuclear part;nucleoplasm;organelle part;perinuclear region of cytoplasm		Homo sapiens	+
cell part;cytoplasmic part;cytosol;extracellular membrane-bounded organelle;extracellular organelle;extracellular region part;extracellular vesicular exosome;intracellular membrane-bounded organelle;intracellular organelle;int	Glycerophospholipid metabolism	Homo sapiens	
catalytic step 2 spliceosome;cell part;intracellular membrane-bounded organelle;intracellular organelle;intracellular organelle part;intracellular part;macromolecular complex;membrane;membrane-bounded organelle;nuclear p	Spliceosome	Homo sapiens	+
cell part;cytoplasm;intracellular membrane-bounded organelle;intracellular organelle;intracellular part;membrane-bounded organelle;organelle		Homo sapiens	
cell part;granular component;intracellular non-membrane-bounded organelle;intracellular organelle;intracellular organelle part;intracellular part;non-membrane-bounded organelle;nuclear part;nucleolar part;nucleolus;nucleoplasm;organelle;organelle part		Homo sapiens	+
cell part;cytoplasmic part;cytosol;intracellular part;membrane	MAPK signaling pathway	Homo sapiens	+
90S preribosome;cell part;cytoplasm;intracellular non-membrane-bounded organelle;intracellular organelle;intracellular organelle part;intracellular part;macromolecular complex;non-membrane-bounded organelle;nuclear part;nucleolus;nucleoplasm;organelle;organelle part;preribosome;preribosome, small subunit precursor;ribonucleoprotein complex;small-subunit proce		Homo sapiens	+
cell part;envelope;intracellular organelle part;intracellular part;macromolecular complex;membrane;membrane part;nuclear envelope;nuclear membrane;nuclear part;nuclear pore;organelle envelope;organelle membrane;orgar	RNA transport	Homo sapiens	+
cell part;cell projection;centriole;cilium;cytoplasm;cytoplasmic part;cytoplasmic vesicle;cytoskeletal part;extracellular membrane-bounded organelle;extracellular organelle;extracellular region part;extracellular vesicular exosome;Golgi apparatus;intracellular membrane-bounded organelle;intracellular non-membrane-bounded organelle;intracellular organelle;intracellular o		Homo sapiens	+
cell part;chromosomal part;chromosome, centromeric region;cytoplasm;intracellular organelle part;intracellular part;nuclear part;nucleoplasm;organelle part		Homo sapiens	
cell part;cytoplasmic part;endoplasmic reticulum;endoplasmic reticulum lumen;endoplasmic reticulum part;extracellular matrix;extracellular region part;extracellular space;intracellular membrane-bounded organelle;intracellular organelle;intracellular organelle lumen;intracellular organelle part;intracellular part;macromolecular complex;membrane-bounded organelle;memb		Homo sapiens	
cell body;cell part;cell projection;external side of plasma membrane;extracellular membrane-bounded organelle;extracellular organelle;extracellular region part;extracellular vesicular exosome;integral to membrane;intrinsic to membrane;membrane;membrane part;membrane-bounded organelle;membrane-bounded vesicle;neuron projection;organelle;plasma membrane;p		Homo sapiens	+
cell part;cytoplasm;intracellular part		Homo sapiens	+
cell part;cytoplasmic part;intracellular membrane-bounded organelle;intracellular organelle;intracellular organelle part;intracellular part;macromolecular complex;membrane-bounded organelle;mitochondrial part;mitochondria	Ribosome biogenesis in eukaryotes;RNA transport	Homo sapiens	+
cell part;cytoplasm;cytoplasmic part;cytosol;eukaryotic 43S preinitiation complex;eukaryotic 48S preinitiation complex;eukaryotic translation initiation factor 3 complex;intracellular part;macromolecular complex;perinuclear re	RNA transport	Homo sapiens	+
cell part;cytoplasmic part;cytosol;eukaryotic 43S preinitiation complex;eukaryotic 48S preinitiation complex;eukaryotic translation initiation factor 3 complex;intracellular part;macromolecular complex;protein complex;ribonuc	RNA transport	Homo sapiens	+
cell part;cytoplasm;cytoplasmic part;cytosol;extracellular region part;extracellular space;intracellular organelle part;intracellular part;nuclear part;nucleoplasm;organelle part	Arachidonic acid metabolism	Homo sapiens	
cell part;cytoplasm;cytoplasmic part;cytoskeleton;cytosol;intermediate filament cytoskeleton;intracellular membrane-bounded organelle;intracellular non-membrane-bounded organelle;intracellular organelle;intracellular organelle part;intracellular part;macromolecular complex;membrane-bounded organelle;non-membrane-bounded organelle;nuclear part;nucleoplasm;nu		Homo sapiens	
cell part;cytoplasmic part;endoplasmic reticulum membrane;endoplasmic reticulum part;extracellular membrane-bounded organelle;extracellular organelle;extracellular region part;extracellular vesicular exosome;integral to membrane;intracellular organelle part;intracellular part;intrinsic to membrane;membrane;membrane part;membrane-bounded organelle;membrane-b		Homo sapiens	+
cell part;cytoplasm;cytoplasmic part;cytosol;extracellular membrane-bounded organelle;extracellular organelle;extracellular region part;extracellular vesicular exosome;intracellular membrane-bounded organelle;intracellular c	Carbon fixation pathways in prokaryotes;Citrate cycle (TCA cycle);Glutathione metabolism;Peroxisome	Homo sapiens	+
cell part;contractile fiber;cytoplasmic part;intracellular membrane-bounded organelle;intracellular non-membrane-bounded organelle;intracellular organelle;intracellular organelle part;intracellular part;membrane;membrane-bounded organelle;mitochondrial inner membrane;mitochondrial membrane;mitochondrial part;mitochondrion;myofibril;non-membrane-bounded orga		Homo sapiens	+
cell part;cytoplasm;extracellular membrane-bounded organelle;extracellular organelle;extracellular region part;extracellular vesicular exosome;intracellular membrane-bounded organelle;intracellular non-membrane-bounded organelle;intracellular organelle;intracellular organelle part;intracellular part;membrane-bounded organelle;membrane-bounded vesicle;non-membra		Homo sapiens	
cell part;cytoplasmic part;endoplasmic reticulum;endoplasmic reticulum membrane;endoplasmic reticulum part;extracellular membrane-bounded organelle;extracellular organelle;extracellular region part;extracellular vesicular exosome;integral to membrane;intracellular membrane-bounded organelle;intracellular organelle;intracellular organelle part;intracellular part;intrins		Homo sapiens	+
cell junction;cell part;intracellular membrane-bounded organelle;intracellular non-membrane-bounded organelle;intracellular organelle;intracellular organelle part;intracellular part;macromolecular complex;membrane-boundec	Spliceosome	Homo sapiens	+
cell division site part;cell part;centrosome;chromosomal part;cleavage furrow;condensed chromosome kinetochore;cytoplasmic part;cytoskeletal part;cytosol;dynactin complex;intracellular non-membrane-bounded organelle;intracellular organelle;intracellular organelle part;intracellular part;kinetochore;macromolecular complex;microtubule associated complex;microtubu		Homo sapiens	+
cell part;intracellular organelle part;intracellular part;nuclear part;nucleoplasm;organelle part		Homo sapiens	
Cajal body;cell part;cytoplasm;cytoskeleton;intermediate filament cytoskeleton;intracellular membrane-bounded organelle;intracellular non-membrane-bounded organelle;intracellular organelle;intracellular organelle part;intra	Spliceosome	Homo sapiens	+
cell part;cytoplasm;cytoplasmic part;extracellular membrane-bounded organelle;extracellular organelle;extracellular region part;extracellular vesicular exosome;intracellular membrane-bounded organelle;intracellular organelle	Alzheimer's disease;Huntington's disease;Oxidative phosphorylation;Parkinson's disease	Homo sapiens	+
adherens junction;anchoring junction;cell junction;cell part;cell-substrate adherens junction;cell-substrate junction;focal adhesion;integral to membrane;intrinsic to membrane;membrane;membrane part;plasma membrane		Homo sapiens	+
adherens junction;anchoring junction;caveola;cell junction;cell part;cell-substrate adherens junction;cell-substrate junction;centriolar satellite;cytoplasmic membrane-bounded vesicle;cytoplasmic part;cytoplasmic vesicle;cyt	Insulin signaling pathway	Homo sapiens	+
cell part;cytoplasmic part;cytosol;intracellular part		Homo sapiens	+
cell part;cytoplasmic part;extracellular membrane-bounded organelle;extracellular organelle;extracellular region part;extracellular vesicular exosome;intracellular membrane-bounded organelle;intracellular organelle;intracellul	Oxidative phosphorylation	Homo sapiens	+
cell part;extracellular membrane-bounded organelle;extracellular organelle;extracellular region part;extracellular vesicular exosome;integral to membrane;intrinsic to membrane;membrane;membrane part;membrane-bounded organelle;membrane-bounded vesicle;organelle;vesicle		Homo sapiens	+
cell cortex;cell part;contractile fiber part;cytoplasmic part;extracellular membrane-bounded organelle;extracellular organelle;extracellular region part;extracellular vesicular exosome;intracellular part;membrane-bounded organelle;membrane-bounded vesicle;organelle;organelle part;vesicle;Z disc		Homo sapiens	+
cell part;intracellular non-membrane-bounded organelle;intracellular organelle;intracellular organelle part;intracellular part;membrane;non-membrane-bounded organelle;nuclear part;nucleolus;organelle;organelle part		Homo sapiens	+
cell part;cytoplasmic part;endoplasmic reticulum;extracellular region part;extracellular space;Golgi apparatus;intracellular membrane-bounded organelle;intracellular organelle;intracellular part;membrane-bounded organelle;organelle;perinuclear region of cytoplasm		Homo sapiens	+
cell part;cytoskeletal part;intracellular organelle part;intracellular part;macromolecular complex;MMXD complex;organelle part;protein complex		Homo sapiens	
cell part;cytoplasm;cytoplasmic part;cytosol;endoplasmic reticulum;intracellular membrane-bounded organelle;intracellular non-membrane-bounded organelle;intracellular organelle;intracellular organelle part;intracellular part	Protein export	Homo sapiens	+
cell part;cytoplasmic part;extracellular membrane-bounded organelle;extracellular organelle;extracellular region part;extracellular vesicular exosome;intracellular membrane-bounded organelle;intracellular organelle;intracellular part;membrane-bounded organelle;membrane-bounded vesicle;mitochondrion;organelle;vesicle		Homo sapiens	+
cell part;cell projection;cytoplasm;cytoplasmic part;dendrite;DNA-directed RNA polymerase complex;DNA-directed RNA polymerase II, core complex;intracellular membrane-bounded organelle;intracellular organelle;intracellular organelle part;intracellular part;macromolecular complex;membrane-bounded organelle;mitochondrion;neuron projection;nuclear DNA-directed		Homo sapiens	+
cell part;chromatin;chromatin remodeling complex;chromosomal part;histone deacetylase complex;intracellular organelle part;intracellular part;macromolecular complex;membrane;nuclear chromatin;nuclear chromosome part;nuclear part;nucleoplasm;nucleoplasm part;NuRD complex;organelle part;protein complex;transcription factor complex;transcriptional repressor		Homo sapiens	+
cell part;cytoplasm;extracellular membrane-bounded organelle;extracellular organelle;extracellular region part;extracellular vesicular exosome;intracellular part;membrane;membrane-bounded organelle;membrane-bounded vesicle;organelle;plasma membrane;vesicle		Homo sapiens	+
cell part;cytoplasmic part;cytosol;intracellular part	Alanine, aspartate and glutamate metabolism;Amino sugar and nucleotide sugar metabolism	Homo sapiens	+
cell part;cell surface;cytoplasm;extracellular membrane-bounded organelle;extracellular organelle;extracellular region;extracellular region part;extracellular space;extracellular vesicular exosome;intracellular part;membrane;m	Axon guidance	Homo sapiens	+
		Homo sapiens	+
cell part;cytoplasmic part;extracellular membrane-bounded organelle;extracellular organelle;extracellular region part;extracellular vesicular exosome;integral to membrane;intracellular membrane-bounded organelle;intracellular organelle;intracellular organelle part;intracellular part;intrinsic to membrane;macromolecular complex;membrane;membrane part;membrane-bou		Homo sapiens	+
axolemma;axon;axon part;basolateral plasma membrane;brush border;cell body;cell part;cell projection;cell projection membrane;cell projection part;cytoplasmic part;cytoplasmic vesicle;cytoskeletal part;endoplasmic reticulum;endosome;extracellular membrane-bounded organelle;extracellular organelle;extracellular region part;extracellular vesicular exosome;intracellu		Homo sapiens	+
cell part;coated vesicle membrane;COPII vesicle coat;cytoplasmic part;cytoplasmic vesicle membrane;cytoplasmic vesicle part;cytosol;endoplasmic reticulum membrane;endoplasmic reticulum part;ER to Golgi transport ves	Protein processing in endoplasmic reticulum	Homo sapiens	+
cell part;cytoplasm;cytoplasmic part;cytosol;endoplasmic reticulum;endoplasmic reticulum membrane;endoplasmic reticulum part;intracellular membrane-bounded organelle;intracellular organelle;intracellular organelle part;intracellular part;macromolecular complex;membrane;membrane part;membrane-bounded organelle;nuclear part;nucleoplasm;nucleus;organelle;org		Homo sapiens	+
cell part;macromolecular complex;membrane;protein complex		Homo sapiens	+
Cdc48p-Npl4p-Ufd1p AAA ATPase complex;cell part;cytoplasmic part;endoplasmic reticulum part;intracellular organelle part;intracellular part;macromolecular complex;membrane part;nuclear part;nucleoplasm;organelle part;protein complex		Homo sapiens	
cell part;cytoplasm;cytoplasmic part;extracellular membrane-bounded organelle;extracellular organelle;extracellular region part;extracellular vesicular exosome;intracellular;intracellular membrane-bounded organelle;intracellular organelle;intracellular part;membrane-bounded organelle;membrane-bounded vesicle;mitochondrion;organelle;vesicle		Homo sapiens	
cell part;cytoplasm;cytoplasmic part;endoplasmic reticulum;endoplasmic reticulum membrane;endoplasmic reticulum part;extracellular membrane-bounded organelle;extracellular organelle;extracellular region part;extracellular vesicular exosome;integral to membrane;intracellular membrane-bounded organelle;intracellular organelle;intracellular organelle part;intracellular		Homo sapiens	+
catalytic step 2 spliceosome;cell part;intracellular membrane-bounded organelle;intracellular organelle;intracellular organelle part;intracellular part;macromolecular complex;membrane;membrane-bounded organelle;nuclear b	Spliceosome	Homo sapiens	
cell part;cytoplasmic part;early endosome membrane;endosomal part;endosome membrane;extracellular region;extracellular region part;extracellular space;intracellular organelle part;intracellular part;membrane;organelle me	Wnt signaling pathway	Homo sapiens	+
		Homo sapiens	+
cell part;cytoplasmic part;cytosol;intracellular membrane-bounded organelle;intracellular organelle;intracellular organelle lumen;intracellular organelle part;intracellular part;membrane-bounded organelle;membrane-enclosed l	Alanine, aspartate and glutamate metabolism;Arginine and proline metabolism;D-Glutamine and D-glutamate metabolism;Nitrogen metabolism;Pr	Homo sapiens	+
AP-2 adaptor complex;AP-type membrane coat adaptor complex;cell part;clathrin adaptor complex;clathrin coated vesicle membrane;clathrin-coated endocytic vesicle membrane;coated vesicle membrane;cytoplasmic mem	Endocrine and other factor-regulated calcium reabsorption;Endocytosis;Huntington's disease	Homo sapiens	+
cell part;coated vesicle;coated vesicle membrane;COPII vesicle coat;cytoplasm;cytoplasmic membrane-bounded vesicle;cytoplasmic part;cytoplasmic vesicle;cytoplasmic vesicle membrane;cytoplasmic vesicle part;cytosol;	Protein processing in endoplasmic reticulum	Homo sapiens	+
cell part;cytoplasm;intracellular membrane-bounded organelle;intracellular organelle;intracellular organelle part;intracellular part;membrane-bounded organelle;nuclear part;nucleoplasm;nucleus;organelle;organelle part		Homo sapiens	
cell part;centrosome;cytoplasmic part;cytoskeletal part;cytoskeleton;cytosol;intracellular non-membrane-bounded organelle;intracellular organelle;intracellular organelle part;intracellular part;membrane;microtubule cytoskele	Cell cycle;p53 signaling pathway;Progesterone-mediated oocyte maturation	Homo sapiens	+
cell part;cytoplasm;intracellular membrane-bounded organelle;intracellular organelle;intracellular organelle part;intracellular part;membrane;membrane-bounded organelle;nuclear part;nucleoplasm;nucleus;organelle;organelle	Ubiquitin mediated proteolysis	Homo sapiens	+
cell part;extracellular membrane-bounded organelle;extracellular organelle;extracellular region part;extracellular vesicular exosome;intracellular membrane-bounded organelle;intracellular organelle;intracellular part;membrane-bounded organelle;membrane-bounded vesicle;nucleus;organelle;vesicle		Homo sapiens	+
cell part;intracellular organelle part;intracellular part;nuclear part;nucleoplasm;organelle part		Homo sapiens	
cell part;cytoplasmic part;integral to membrane;intracellular membrane-bounded organelle;intracellular organelle;intracellular organelle part;intracellular part;intrinsic to membrane;macromolecular complex;membrane;membra	Alzheimer's disease;Huntington's disease;Oxidative phosphorylation;Parkinson's disease	Homo sapiens	+
cell part;cytoplasmic part;cytoskeleton;cytosol;integral to membrane;intracellular membrane-bounded organelle;intracellular non-membrane-bounded organelle;intracellular organelle;intracellular organelle part;intracellular part;intrinsic to membrane;intrinsic to mitochondrial outer membrane;intrinsic to organelle membrane;membrane;membrane part;membrane-bounded		Homo sapiens	+
cell part;cytoplasmic part;cytosol;extracellular membrane-bounded organelle;extracellular organelle;extracellular region part;extracellular vesicular exosome;intracellular organelle part;intracellular part;macromolecular comple	RNA transport	Homo sapiens	+
cell part;cytoplasm;intracellular membrane-bounded organelle;intracellular organelle;intracellular part;macromolecular complex;membrane-bounded organelle;nucleus;organelle;protein complex;ubiquitin ligase complex	Protein processing in endoplasmic reticulum;Ubiquitin mediated proteolysis	Homo sapiens	+
cell part;cytoplasmic part;Golgi apparatus;integral to membrane;intracellular membrane-bounded organelle;intracellular organelle;intracellular part;intrinsic to membrane;membrane part;membrane-bounded organelle;nucleus;organelle		Homo sapiens	+
cell part;cytoplasm;Elongator holoenzyme complex;histone acetyltransferase complex;intracellular non-membrane-bounded organelle;intracellular organelle;intracellular organelle part;intracellular part;macromolecular complex;non-membrane-bounded organelle;nuclear part;nucleolus;nucleoplasm part;organelle;organelle part;protein complex;transcription elongation fac		Homo sapiens	
cell part;cytoplasmic part;extracellular membrane-bounded organelle;extracellular organelle;extracellular region part;extracellular vesicular exosome;integral to membrane;intracellular membrane-bounded organelle;intracellul	Alzheimer's disease;Huntington's disease;Oxidative phosphorylation;Parkinson's disease	Homo sapiens	+
cell part;cytoplasmic part;endoplasmic reticulum;integral to membrane;intracellular membrane-bounded organelle;intracellular organelle;intracellular organelle part;intracellular part;intrinsic to membrane;macromolecular com	Alzheimer's disease;Huntington's disease;Oxidative phosphorylation;Parkinson's disease	Homo sapiens	+
cell part;cytoplasmic part;endoplasmic reticulum;endoplasmic reticulum membrane;endoplasmic reticulum part;extracellular membrane-bounded organelle;extracellular organelle;extracellular region part;extracellular space;extracellular vesicular exosome;Golgi apparatus part;Golgi membrane;integral to membrane;intracellular membrane-bounded organelle;intracellular c		Homo sapiens	+
cell part;cytoplasmic part;integral to membrane;intracellular membrane-bounded organelle;intracellular organelle;intracellular organelle part;intracellular part;intrinsic to membrane;membrane;membrane part;membrane-bounded organelle;mitochondrial inner membrane;mitochondrial membrane;mitochondrial part;mitochondrion;organelle;organelle inner membrane;organ		Homo sapiens	+
cell part;intracellular membrane-bounded organelle;intracellular organelle;intracellular organelle part;intracellular part;membrane-bounded organelle;nuclear part;nucleoplasm;nucleus;organelle;organelle part		Homo sapiens	+
cell part;cytoplasm;cytoplasmic dynein complex;cytoplasmic part;cytoskeletal part;dynein complex;early endosome membrane;endosomal part;endosome membrane;extrinsic to membrane;intracellular organelle part;intracellular part;macromolecular complex;membrane;membrane part;microtubule associated complex;organelle membrane;organelle part;plasma membr		Homo sapiens	+
cell part;intracellular membrane-bounded organelle;intracellular organelle;intracellular organelle part;intracellular part;macromolecular complex;membrane-bounded organelle;nuclear body;nuclear part;nuclear speck;nucleoplasm;nucleoplasm part;nucleus;organelle;organelle part;ribonucleoprotein complex;small nuclear ribonucleoprotein complex;U1 snRNP		Homo sapiens	
cell part;cytoplasmic part;cytoskeletal part;Golgi apparatus;intracellular membrane-bounded organelle;intracellular non-membrane-bounded organelle;intracellular organelle;intracellular organelle part;intracellular part;kinesin complex;macromolecular complex;membrane-bounded organelle;microtubule;microtubule associated complex;midbody;non-membrane-bounded		Homo sapiens	+
cell part;chromosome;cytoplasm;cytoplasmic part;cytoskeletal part;cytosol;intracellular non-membrane-bounded organelle;intracellular organelle;intracellular organelle part;intracellular part;kinesin complex;macromolecular complex;membrane;microtubule;microtubule associated complex;midbody;non-membrane-bounded organelle;nuclear matrix;nuclear part;nucleopla		Homo sapiens	+
cell part;cis-Golgi network;cytoplasmic part;Golgi apparatus;Golgi apparatus part;Golgi membrane;integral to membrane;intracellular membrane-bounded organelle;intracellular organelle;intracellular organelle part;intracellula	SNARE interactions in vesicular transport	Homo sapiens	+
cell part;cytoplasmic part;endoplasmic reticulum;endoplasmic reticulum membrane;endoplasmic reticulum part;Golgi apparatus;Golgi apparatus part;Golgi membrane;integral to membrane;intracellular membrane-bounded organelle;intracellular organelle;intracellular organelle part;intracellular part;intrinsic to membrane;membrane;membrane part;membrane-bounded or		Homo sapiens	+
adherens junction;anchoring junction;cell junction;cell part;cell surface;cell-substrate adherens junction;cell-substrate junction;focal adhesion;integral to membrane;integral to plasma membrane;intrinsic to membrane;intrinsic	Cell adhesion molecules (CAMs)	Homo sapiens	+
cell part;cytoplasmic part;intracellular organelle lumen;intracellular organelle part;intracellular part;macromolecular complex;membrane;membrane part;membrane-enclosed lumen;mitochondrial inner membrane;mitochondria	Alzheimer's disease;Huntington's disease;Oxidative phosphorylation;Parkinson's disease	Homo sapiens	
cell part;cytoplasmic part;endoplasmic reticulum;endoplasmic reticulum membrane;endoplasmic reticulum part;intracellular membrane-bounded organelle;intracellular organelle;intracellular organelle part;intracellular part;membrane;membrane part;membrane-bounded organelle;organelle;organelle membrane;organelle part		Homo sapiens	+
cell part;cytoplasmic part;cytosol;extracellular membrane-bounded organelle;extracellular organelle;extracellular region part;extracellular vesicular exosome;intracellular part;membrane-bounded organelle;membrane-bounde	Pentose phosphate pathway	Homo sapiens	+
cell part;cytoplasmic part;cytosol;intracellular part	Purine metabolism;Selenocompound metabolism;Sulfur metabolism	Homo sapiens	+
cell part;chromosomal part;chromosome;condensed chromosome;condensin complex;cytoplasm;cytoplasmic part;cytosol;extracellular membrane-bounded organelle;extracellular organelle;extracellular region part;extracellu	Cell cycle - yeast	Homo sapiens	+
axoneme;cell part;cell projection part;cytoplasm;cytoplasmic part;cytosol;intracellular part;organelle part;pre-autophagosomal structure	Regulation of autophagy	Homo sapiens	
cell part;cytoplasm;intracellular organelle part;intracellular part;nuclear body;nuclear part;nucleoplasm part;organelle part;PML body		Homo sapiens	+
cell part;cytoplasm;extracellular membrane-bounded organelle;extracellular organelle;extracellular region part;extracellular vesicular exosome;intracellular part;membrane-bounded organelle;membrane-bounded vesicle;orgai	Glycerophospholipid metabolism	Homo sapiens	+
cell part;cytoplasm;intracellular organelle part;intracellular part;macromolecular complex;membrane;membrane part;nuclear part;nuclear pore;nucleoplasm;organelle part;pore complex;protein complex		Homo sapiens	
cell part;cytoplasm;intracellular membrane-bounded organelle;intracellular organelle;intracellular part;membrane-bounded organelle;nucleus;organelle		Homo sapiens	+
cell part;cytoplasmic part;cytosol;intracellular part	Amino sugar and nucleotide sugar metabolism	Homo sapiens	
cell part;cytoplasm;intracellular membrane-bounded organelle;intracellular organelle;intracellular organelle part;intracellular part;macromolecular complex;membrane-bounded organelle;nuclear part;nucleoplasm;nucleus;organelle;organelle part;ribonucleoprotein complex;small nuclear ribonucleoprotein complex;U5 snRNP		Homo sapiens	+
cell part;cytoplasm;cytoplasmic part;cytosol;endoplasmic reticulum;extracellular membrane-bounded organelle;extracellular organelle;extracellular region part;extracellular vesicular exosome;intracellular membrane-bounded organelle;intracellular organelle;intracellular part;membrane-bounded organelle;membrane-bounded vesicle;organelle;vesicle		Homo sapiens	+
cell part;cytoplasm;cytoplasmic part;endoplasmic reticulum;intracellular membrane-bounded organelle;intracellular organelle;intracellular part;membrane-bounded organelle;nucleus;organelle		Homo sapiens	+
cell part;cytoplasmic part;endoplasmic reticulum;endoplasmic reticulum membrane;endoplasmic reticulum part;integral to endoplasmic reticulum membrane;integral to membrane;integral to organelle membrane;intracellular	Sphingolipid metabolism	Homo sapiens	
cell part;intracellular membrane-bounded organelle;intracellular non-membrane-bounded organelle;intracellular organelle;intracellular organelle part;intracellular part;macromolecular complex;membrane-bounded organelle;non-membrane-bounded organelle;nuclear part;nucleolus;nucleus;organelle;organelle part;ribonucleoprotein complex		Homo sapiens	+
cell part;cytoplasmic part;endoplasmic reticulum lumen;endoplasmic reticulum part;intracellular organelle lumen;intracellular organelle part;intracellular part;membrane-enclosed lumen;organelle lumen;organelle part	Pentose phosphate pathway	Homo sapiens	+

cell part;coated vesicle membrane;COPII vesicle coat;cytoplasmic part;cytoplasmic vesicle membrane;cytoplasmic vesicle part;cytosol;endoplasmic reticulum membrane;endoplasmic reticulum part;ER to Golgi transport ves	Protein processing in endoplasmic reticulum	Homo sapiens	
cell part;coated vesicle membrane;COPII vesicle coat;cytoplasmic part;cytoplasmic vesicle membrane;cytoplasmic vesicle part;cytosol;endoplasmic reticulum membrane;endoplasmic reticulum part;ER to Golgi transport ves	Protein processing in endoplasmic reticulum	Homo sapiens	+
anchored to membrane;cell part;extracellular membrane-bounded organelle;extracellular organelle;extracellular region part;extracellular vesicular exosome;integral to membrane;intrinsic to membrane;membrane;membrane p	Pantothenate and CoA biosynthesis	Homo sapiens	+
cell part;cytoplasm;cytoplasmic part;intracellular membrane-bounded organelle;intracellular organelle;intracellular organelle lumen;intracellular organelle part;intracellular part;membrane-bounded organelle;membrane-enclosed lumen;mitochondrial matrix;mitochondrial part;mitochondrion;nuclear part;nucleoplasm;organelle;organelle lumen;organelle part		Homo sapiens	+
cell part;cytoplasmic part;endoplasmic reticulum;endoplasmic reticulum membrane;endoplasmic reticulum part;Golgi apparatus;integral to membrane;intracellular membrane-bounded organelle;intracellular organelle;intracell	Adipocytokine signaling pathway;Fatty acid metabolism;Peroxisome;PPAR signaling pathway	Homo sapiens	
cell division site part;cell part;cleavage furrow;cytoplasm;cytoplasmic part;early endosome;endosome;extracellular membrane-bounded organelle;extracellular organelle;extracellular region part;extracellular vesicular exosome	Endocytosis	Homo sapiens	
cell part;cytoplasm;intracellular part		Homo sapiens	+
cell part;clathrin-coated vesicle;coated vesicle;cytoplasmic membrane-bounded vesicle;cytoplasmic part;cytoplasmic vesicle;cytoplasmic vesicle membrane;cytoplasmic vesicle part;endosome;extracellular membrane-bound	Pancreatic secretion	Homo sapiens	+
cell junction;cell part;cell projection;centrosome;cytoplasm;cytoplasmic part;cytoskeletal part;intracellular non-membrane-bounded organelle;intracellular organelle;intracellular organelle part;intracellular part;macromolecular	SNARE interactions in vesicular transport	Homo sapiens	+
cell part;cytoplasm;extracellular membrane-bounded organelle;extracellular organelle;extracellular region part;extracellular vesicular exosome;intracellular part;membrane-bounded organelle;membrane-bounded vesicle;organelle;vesicle		Homo sapiens	
cell part;cytoplasm;cytoplasmic part;cytosol;intracellular membrane-bounded organelle;intracellular organelle;intracellular part;membrane-bounded organelle;nucleus;organelle	Protein processing in endoplasmic reticulum	Homo sapiens	+
cell part;intracellular membrane-bounded organelle;intracellular organelle;intracellular part;membrane-bounded organelle;nucleus;organelle		Homo sapiens	+
cell part;intracellular organelle part;intracellular part;macromolecular complex;nuclear part;organelle part;ribonucleoprotein complex;small nuclear ribonucleoprotein complex;spliceosomal complex;U4/U6 x U5 tri-snRNP com	RNA degradation;Spliceosome	Homo sapiens	+
AP-2 adaptor complex;apical plasma membrane;AP-type membrane coat adaptor complex;basolateral plasma membrane;cell part;clathrin adaptor complex;clathrin coat;clathrin coat of trans-Golgi network vesicle;clathrin co	Endocrine and other factor-regulated calcium reabsorption;Endocytosis;Huntington's disease	Homo sapiens	+
cell body;cell part;cell projection;cytoplasm;cytoplasmic part;cytoskeletal part;dendrite;endoplasmic reticulum;extracellular membrane-bounded organelle;extracellular organelle;extracellular region part;extracellular vesicular exosome;intracellular membrane-bounded organelle;intracellular non-membrane-bounded organelle;intracellular organelle;intracellular organelle pa		Homo sapiens	+
		Homo sapiens	+
caveola;cell part;cytoplasm;cytoplasmic part;cytosol;intracellular part;membrane part;membrane raft;plasma membrane part		Homo sapiens	+
cell part;cytoplasmic part;cytosol;intracellular part	Protein processing in endoplasmic reticulum	Homo sapiens	
cell part;cell projection;contractile fiber part;cytoplasm;cytoplasmic part;cytosol;intracellular part;membrane;neuron projection;organelle part;plasma membrane;Z disc		Homo sapiens	+
cell part;cytoplasm;intracellular part	MAPK signaling pathway	Homo sapiens	+
cell part;cytoplasmic part;cytosol;intracellular part		Homo sapiens	
cell part;cytoplasmic part;cytosol;intracellular membrane-bounded organelle;intracellular organelle;intracellular organelle part;intracellular part;membrane;membrane-bounded organelle;membrane-enclosed lumen;mitochond	Apoptosis	Homo sapiens	+
cell part;cytoplasm;cytoskeletal part;intracellular non-membrane-bounded organelle;intracellular organelle;intracellular organelle part;intracellular part;macromolecular complex;microtubule;microtubule associated complex;non-membrane-bounded organelle;organelle;organelle part;protein complex;spindle		Homo sapiens	+
cell part;cytoplasmic part;cytosol;extracellular membrane-bounded organelle;extracellular organelle;extracellular region part;extracellular vesicular exosome;intracellular part;membrane-bounded organelle;membrane-bounde	Sulfur metabolism	Homo sapiens	+
cell part;centrosome;cytoplasm;cytoplasmic part;cytoskeletal part;extracellular membrane-bounded organelle;extracellular organelle;extracellular region part;extracellular vesicular exosome;intracellular membrane-bounded organelle;intracellular non-membrane-bounded organelle;intracellular organelle;intracellular organelle part;intracellular part;membrane-bounded orga		Homo sapiens	+
cell part;cytoplasmic part;endoplasmic reticulum lumen;endoplasmic reticulum part;intracellular organelle lumen;intracellular organelle part;intracellular part;membrane-enclosed lumen;organelle lumen;organelle part	Glutathione metabolism	Homo sapiens	+
basement membrane;extracellular matrix part;extracellular membrane-bounded organelle;extracellular organelle;extracellular region;extracellular region part;extracellular vesicular exosome;membrane-bounded organelle;membrane-bounded vesicle;organelle;vesicle		Homo sapiens	+
cell part;chromatin;chromatin remodeling complex;chromosomal part;cytoplasm;heterochromatin;histone deacetylase complex;intracellular organelle part;intracellular part;macromolecular complex;nuclear chromatin;nuclear chromosome part;nuclear part;nucleoplasm;nucleoplasm part;NuRD complex;organelle part;protein complex;transcriptional repressor complex		Homo sapiens	+
cell part;cytoplasmic part;cytosol;extracellular membrane-bounded organelle;extracellular organelle;extracellular region part;extracellular vesicular exosome;intracellular part;membrane-bounded organelle;membrane-bounded vesicle;organelle;vesicle		Homo sapiens	+
CBM complex;cell part;cytoplasm;cytoplasmic microtubule;cytoplasmic part;cytoskeletal part;cytosol;immunological synapse;intracellular membrane-bounded organelle;intracellular organelle;intracellular organelle part;intrac	B cell receptor signaling pathway;ko05152;T cell receptor signaling pathway	Homo sapiens	+
cell part;cytoplasmic part;extracellular membrane-bounded organelle;extracellular organelle;extracellular region part;extracellular vesicular exosome;intracellular organelle part;intracellular part;macromolecular complex;memb	Alzheimer's disease;Huntington's disease;Oxidative phosphorylation;Parkinson's disease	Homo sapiens	+
cell part;external side of plasma membrane;integral to membrane;integral to plasma membrane;intrinsic to membrane;intrinsic to plasma membrane;membrane;membrane part;plasma membrane part		Homo sapiens	+
cell part;cytoplasm;cytoplasmic part;extracellular membrane-bounded organelle;extracellular organelle;extracellular region part;extracellular vesicular exosome;integral to membrane;integral to mitochondrial membrane;integr	Amyotrophic lateral sclerosis (ALS)	Homo sapiens	+
adherens junction;anchoring junction;cell junction;cell part;cell-substrate adherens junction;cell-substrate junction;cytoplasmic part;focal adhesion;Golgi apparatus;intracellular membrane-bounded organelle;intracellular orga	Axon guidance;ErbB signaling pathway;Focal adhesion;Regulation of actin cytoskeleton;Renal cell carcinoma;T cell receptor signaling pathway	Homo sapiens	+
cell part;chromatin;chromatin remodeling complex;chromosomal part;DNA helicase complex;H4/H2A histone acetyltransferase complex;histone acetyltransferase complex;Ino80 complex;intracellular membrane-bounded organelle;intracellular organelle;intracellular organelle part;intracellular part;macromolecular complex;membrane;membrane-bounded organelle;npBAF		Homo sapiens	+
cell part;cytoplasmic part;endoplasmic reticulum membrane;endoplasmic reticulum part;extracellular membrane-bounded organelle;extracellular organelle;extracellular region part;extracellular vesicular exosome;integral to membrane;intracellular organelle part;intracellular part;intrinsic to membrane;membrane;membrane part;membrane-bounded organelle;membrane-b		Homo sapiens	
cell part;cell projection part;cytoplasmic part;cytosol;extracellular membrane-bounded organelle;extracellular organelle;extracellular region part;extracellular vesicular exosome;flagellar fibrous sheath;flagellum part;intracellula	Cysteine and methionine metabolism;Glycolysis / Gluconeogenesis;Propanoate metabolism;Pyruvate metabolism	Homo sapiens	+
cell part;cytoplasm;cytoplasmic part;intracellular membrane-bounded organelle;intracellular organelle;intracellular organelle lumen;intracellular organelle part;intracellular part;membrane-bounded organelle;membrane-enclose	Alanine, aspartate and glutamate metabolism;Arginine and proline metabolism;D-Glutamine and D-glutamate metabolism;Nitrogen metabolism;Pr	Homo sapiens	+
cell part;cytoplasmic part;cytosol;intracellular organelle part;intracellular part;nuclear part;nucleoplasm;organelle part	Folate biosynthesis;One carbon pool by folate	Homo sapiens	
cell part;cytoplasm;cytoplasmic part;cytosolic part;endoplasmic reticulum;endoplasmic reticulum membrane;endoplasmic reticulum part;extracellular membrane-bounded organelle;extracellular organelle;extracellular region p	Amino sugar and nucleotide sugar metabolism	Homo sapiens	+
cell part;cytoplasmic part;cytosol;external side of plasma membrane;extracellular membrane-bounded organelle;extracellular organelle;extracellular region part;extracellular vesicular exosome;intracellular organelle lumen;intr	Glutathione metabolism	Homo sapiens	+
cell part;cytoplasmic part;extracellular membrane-bounded organelle;extracellular organelle;extracellular region part;extracellular vesicular exosome;integral to membrane;intracellular membrane-bounded organelle;intracellul	Alzheimer's disease;Cardiac muscle contraction;Huntington's disease;Oxidative phosphorylation;Parkinson's disease	Homo sapiens	+
cell body;cell part;cell projection cytoplasm;cell projection part;cytoplasm;cytoplasmic membrane-bounded vesicle;cytoplasmic part;cytoplasmic vesicle;cytosol;dendrite cytoplasm;dense core granule;extracellular matrix;ext	Amyotrophic lateral sclerosis (ALS);Huntington's disease;Peroxisome;Prion diseases	Homo sapiens	+
blood microparticle;cell part;cytoplasmic membrane-bounded vesicle lumen;cytoplasmic part;cytoplasmic vesicle part;extracellular region;extracellular region part;intracellular organelle part;intracellular part;membrane-enclos	Complement and coagulation cascades	Homo sapiens	+
cell part;cytoplasm;cytoplasmic part;cytoskeleton;cytosol;extracellular membrane-bounded organelle;extracellular organelle;extracellular region part;extracellular vesicular exosome;intracellular;intracellular non-membrane-bo	Nicotinate and nicotinamide metabolism;Purine metabolism;Pyrimidine metabolism	Homo sapiens	+
cell part;cytoplasm;cytoplasmic part;cytosol;extracellular membrane-bounded organelle;extracellular organelle;extracellular region part;extracellular vesicular exosome;intracellular part;membrane-bounded organelle;membra	Drug metabolism - other enzymes;Purine metabolism	Homo sapiens	+
cell part;cytoplasmic part;extracellular membrane-bounded organelle;extracellular organelle;extracellular region part;extracellular vesicular exosome;intracellular membrane-bounded organelle;intracellular organelle;intracellul	Alanine, aspartate and glutamate metabolism;Arginine and proline metabolism;Cysteine and methionine metabolism;Fat digestion and absorption;	Homo sapiens	+
adherens junction;anchoring junction;apical plasma membrane;basolateral plasma membrane;cell junction;cell part;cell surface;cell-substrate adherens junction;cell-substrate junction;cytoplasm;cytoplasmic membrane-boun	Adherens junction;Bladder cancer;Calcium signaling pathway;Cytokine-cytokine receptor interaction;Dorso-ventral axis formation;Endocytosis;En	Homo sapiens	+
cell part;cytoplasmic part;cytosol;extracellular membrane-bounded organelle;extracellular organelle;extracellular region part;extracellular vesicular exosome;intracellular part;membrane;membrane part;membrane raft;membra	Carbon fixation in photosynthetic organisms;Glycolysis / Gluconeogenesis	Homo sapiens	+
axoneme part;cell part;cell projection part;cytoplasm;cytoplasmic part;cytosol;extracellular membrane-bounded organelle;extracellular organelle;extracellular region part;extracellular vesicular exosome;intracellular part;memb	Purine metabolism	Homo sapiens	+
blood microparticle;cell part;cytoplasmic part;endoplasmic reticulum lumen;endoplasmic reticulum part;extracellular membrane-bounded organelle;extracellular organelle;extracellular region;extracellular region part;extracellu	Complement and coagulation cascades;Neuroactive ligand-receptor interaction;Regulation of actin cytoskeleton	Homo sapiens	+
blood microparticle;extracellular membrane-bounded organelle;extracellular organelle;extracellular region;extracellular region part;extracellular vesicular exosome;membrane-bounded organelle;membrane-bounded vesicle;or	Complement and coagulation cascades;Phagosome;Staphylococcus aureus infection;Systemic lupus erythematosus	Homo sapiens	+
cell part;cytoplasmic part;endoplasmic reticulum lumen;endoplasmic reticulum part;extracellular region;Golgi apparatus part;Golgi lumen;intracellular organelle lumen;intracellular organelle part;intracellular part;intrinsic to ext	Complement and coagulation cascades	Homo sapiens	+
blood microparticle;cell part;cell surface;cytoplasmic membrane-bounded vesicle lumen;cytoplasmic part;cytoplasmic vesicle part;extracellular membrane-bounded organelle;extracellular organelle;extracellular region;extrac	Complement and coagulation cascades;Neuroactive ligand-receptor interaction;Staphylococcus aureus infection	Homo sapiens	+
adherens junction;anchoring junction;cell junction;cell part;cell surface;cell-substrate adherens junction;cell-substrate junction;extracellular membrane-bounded organelle;extracellular organelle;extracellular region;extracellula	Complement and coagulation cascades	Homo sapiens	+
apical part of cell;cell part;cell surface;cytoplasm;cytoplasmic membrane-bounded vesicle;cytoplasmic part;cytoplasmic vesicle;extracellular membrane-bounded organelle;extracellular organelle;extracellular region;extracellu	Complement and coagulation cascades	Homo sapiens	+
cell body;cell junction;cell part;cell projection cytoplasm;cell projection part;cell surface;cytoplasm;cytoplasmic membrane-bounded vesicle lumen;cytoplasmic part;cytoplasmic vesicle part;cytosol;dendrite cytoplasm;extern	Primary immunodeficiency;Purine metabolism	Homo sapiens	+
cell part;cytoplasmic part;extracellular membrane-bounded organelle;extracellular organelle;extracellular region part;extracellular vesicular exosome;integral to membrane;intracellular organelle part;intracellular part;intrinsic to	Alzheimer's disease;Huntington's disease;Oxidative phosphorylation;Parkinson's disease	Homo sapiens	
cell body fiber;cell part;cell projection;cytoplasm;cytoplasmic part;cytosol;endoplasmic reticulum;extracellular membrane-bounded organelle;extracellular organelle;extracellular region part;extracellular vesicular exosome;intr	Alanine, aspartate and glutamate metabolism;Arginine and proline metabolism	Homo sapiens	+
blood microparticle;cell part;extracellular membrane-bounded organelle;extracellular organelle;extracellular region;extracellular region part;extracellular space;extracellular vesicular exosome;membrane;membrane-bounded o	Complement and coagulation cascades	Homo sapiens	+
blood microparticle;cell part;cytoplasmic membrane-bounded vesicle lumen;cytoplasmic part;cytoplasmic vesicle part;cytosol;extracellular membrane-bounded organelle;extracellular organelle;extracellular region;extracellula	Complement and coagulation cascades	Homo sapiens	+
blood microparticle;cell part;extracellular membrane-bounded organelle;extracellular organelle;extracellular region;extracellular region part;extracellular space;extracellular vesicular exosome;membrane;membrane-bounded o	Chagas disease (American trypanosomiasis);Complement and coagulation cascades;ko05152;Leishmaniasis;Phagosome;Staphylococcus aureus	Homo sapiens	+
cell part;extracellular membrane-bounded organelle;extracellular organelle;extracellular region;extracellular region part;extracellular space;extracellular vesicular exosome;macromolecular complex;membrane attack complex;	Complement and coagulation cascades;Prion diseases;Staphylococcus aureus infection;Systemic lupus erythematosus	Homo sapiens	+
basement membrane;cell part;cytoplasmic membrane-bounded vesicle lumen;cytoplasmic part;cytoplasmic vesicle part;extracellular matrix;extracellular matrix part;extracellular membrane-bounded organelle;extracellular organelle;extracellular region;extracellular region part;extracellular space;extracellular vesicular exosome;intracellular organelle part;intracellular part;r		Homo sapiens	+
cell part;cytoplasmic part;extracellular membrane-bounded organelle;extracellular organelle;extracellular region part;extracellular vesicular exosome;Golgi apparatus;Golgi apparatus part;Golgi membrane;intracellular membra	Acute myeloid leukemia;Axon guidance;B cell receptor signaling pathway;Bladder cancer;Chemokine signaling pathway;Chronic myeloid leukemia	Homo sapiens	+
adherens junction;anchoring junction;cell junction;cell part;cell-substrate adherens junction;cell-substrate junction;cytoplasm;cytoplasmic part;cytosol;extrinsic to internal side of plasma membrane;extrinsic to membrane;ext	Acute myeloid leukemia;Aldosterone-regulated sodium reabsorption;Axon guidance;B cell receptor signaling pathway;Bladder cancer;Chemokine	Homo sapiens	+
apical part of cell;basolateral plasma membrane;cell part;cell surface;clathrin coated vesicle membrane;clathrin-coated endocytic vesicle membrane;coated pit;coated vesicle membrane;cytoplasmic part;cytoplasmic vesicle	Bile secretion;Endocytosis;Hepatitis C;Toxoplasmosis	Homo sapiens	+
cell part;cell surface;coated vesicle membrane;cytoplasmic part;cytoplasmic vesicle membrane;cytoplasmic vesicle part;early endosome membrane;endocytic vesicle membrane;endoplasmic reticulum;endoplasmic reticulum	Allograft rejection;Antigen processing and presentation;Autoimmune thyroid disease;Cell adhesion molecules (CAMs);Endocytosis;Graft-versus-ho	Homo sapiens	+
cell part;cell surface;integral to membrane;integral to plasma membrane;intrinsic to membrane;intrinsic to plasma membrane;macromolecular complex;membrane part;MHC class I protein complex;MHC protein complex;plasma membrane part;protein complex		Homo sapiens	+
blood microparticle;cell part;cytoplasmic part;cytosol;cytosolic part;extracellular region part;hemoglobin complex;intracellular part;macromolecular complex;protein complex	African trypanosomiasis;Malaria	Homo sapiens	+
cell part;collagen;collagen type I;cytoplasmic membrane-bounded vesicle;cytoplasmic part;cytoplasmic vesicle;endoplasmic reticulum lumen;endoplasmic reticulum part;extracellular matrix;extracellular matrix part;extracellu	Amoebiasis;ECM-receptor interaction;Focal adhesion;Protein digestion and absorption	Homo sapiens	+
cell part;collagen;collagen type III;cytoplasmic part;endoplasmic reticulum lumen;endoplasmic reticulum part;extracellular matrix;extracellular matrix part;extracellular region;extracellular region part;extracellular space;fibrillar	Amoebiasis;ECM-receptor interaction;Focal adhesion;Protein digestion and absorption	Homo sapiens	
cell part;cell periphery;cytoplasm;cytoplasmic part;cytoskeletal part;cytosol;extracellular membrane-bounded organelle;extracellular organelle;extracellular region part;extracellular vesicular exosome;intermediate filament;intracellular membrane-bounded organelle;intracellular organelle;intracellular organelle part;intracellular part;keratin filament;macromolecular complex;		Homo sapiens	+
cell part;cytoskeletal part;extracellular membrane-bounded organelle;extracellular organelle;extracellular region part;extracellular vesicular exosome;intermediate filament;intracellular membrane-bounded organelle;intracellular organelle;intracellular organelle part;intracellular part;keratin filament;macromolecular complex;membrane;membrane-bounded organelle;membra		Homo sapiens	+
cell part;cytoplasm;cytoplasmic part;cytoskeletal part;cytosol;envelope;intermediate filament;intracellular membrane-bounded organelle;intracellular organelle;intracellular organelle part;intracellular part;lamin filament;macron	Arrhythmogenic right ventricular cardiomyopathy (ARVC);Dilated cardiomyopathy;Hypertrophic cardiomyopathy (HCM)	Homo sapiens	+
blood microparticle;cell body;cell part;cell projection;chylomicron;cytoplasm;cytoplasmic membrane-bounded vesicle lumen;cytoplasmic part;cytoplasmic vesicle part;cytoskeletal part;dendrite;discoidal high-density lipoprot	Alzheimer's disease	Homo sapiens	+
blood microparticle;cell cortex;cell part;cell surface;cytoplasmic membrane-bounded vesicle;cytoplasmic membrane-bounded vesicle lumen;cytoplasmic part;cytoplasmic vesicle;cytoplasmic vesicle part;external side of plas	Complement and coagulation cascades	Homo sapiens	+
blood microparticle;cell part;cytoplasm;extracellular membrane-bounded organelle;extracellular organelle;extracellular region;extracellular region part;extracellular vesicular exosome;integral to membrane;integral to plasma m	Amoebiasis;Complement and coagulation cascades;Prion diseases;Systemic lupus erythematosus	Homo sapiens	+
cell part;cell surface;chylomicron;extracellular membrane-bounded organelle;extracellular organelle;extracellular region part;extracellular space;extracellular vesicular exosome;high-density lipoprotein particle;macromolecular complex;membrane-bounded organelle;membrane-bounded vesicle;organelle;plasma lipoprotein particle;protein-lipid complex;triglyceride-rich lip		Homo sapiens	+
apical plasma membrane;basal lamina;blood microparticle;cell part;cytoplasmic membrane-bounded vesicle lumen;cytoplasmic part;cytoplasmic vesicle part;endoplasmic reticulum-Golgi intermediate compartment;extracellu	Amoebiasis;Bacterial invasion of epithelial cells;ECM-receptor interaction;Focal adhesion;Pathways in cancer;Regulation of actin cytoskeleton;Sm	Homo sapiens	+
cell part;cytoplasmic part;cytosol;extracellular membrane-bounded organelle;extracellular organelle;extracellular region;extracellular region part;extracellular space;extracellular vesicular exosome;intracellular part;macromolecular complex;membrane-bounded organelle;membrane-bounded vesicle;organelle;protein complex;vesicle		Homo sapiens	+
blood microparticle;extracellular membrane-bounded organelle;extracellular organelle;extracellular region;extracellular region part;extracellular space;extracellular vesicular exosome;membrane-bounded organelle;membrane-bounded vesicle;organelle;vesicle		Homo sapiens	+
cell part;cytoplasm;extracellular region part;extracellular space;intracellular part		Homo sapiens	+
blood microparticle;cell part;cytoplasmic part;cytosol;extracellular membrane-bounded organelle;extracellular organelle;extracellular region;extracellular region part;extracellular space;extracellular vesicular exosome;intracellular organelle lumen;intracellular organelle part;intracellular part;lysosomal lumen;membrane-bounded organelle;membrane-bounded vesicle;memb		Homo sapiens	+
blood microparticle;cell part;cell surface;coated pit;cytoplasmic membrane-bounded vesicle;cytoplasmic part;cytoplasmic vesicle;endosome;external side of plasma membrane;extracellular membrane-bounded organelle;ext	Endocytosis;Hematopoietic cell lineage;Phagosome	Homo sapiens	+
apical plasma membrane;basal part of cell;basal plasma membrane;blood microparticle;cell part;cell surface;coated pit;cytoplasmic membrane-bounded vesicle;cytoplasmic membrane-bounded vesicle lumen;cytoplasmic p	Mineral absorption	Homo sapiens	+
cell part;cytoplasm;cytoplasmic membrane-bounded vesicle;cytoplasmic membrane-bounded vesicle lumen;cytoplasmic part;cytoplasmic vesicle;cytoplasmic vesicle part;endocytic vesicle lumen;extracellular membrane-bounded organelle;extracellular organelle;extracellular region;extracellular region part;extracellular space;extracellular vesicular exosome;intracellular		Homo sapiens	+
cell part;cytoplasm;cytoplasmic part;cytosol;intracellular membrane-bounded organelle;intracellular organelle;intracellular part;membrane-bounded organelle;nucleus;organelle;perinuclear region of cytoplasm	Mineral absorption	Homo sapiens	+
cell part;extracellular membrane-bounded organelle;extracellular organelle;extracellular region;extracellular region part;extracellular space;extracellular vesicular exosome;membrane;membrane-bounded organelle;membrane	Complement and coagulation cascades	Homo sapiens	+
extracellular matrix;extracellular region;extracellular region part;proteinaceous extracellular matrix	Bladder cancer;Pathways in cancer;PPAR signaling pathway;Rheumatoid arthritis	Homo sapiens	+
alphav-beta3 integrin-vitronectin complex;blood microparticle;cell part;extracellular matrix;extracellular membrane-bounded organelle;extracellular organelle;extracellular region;extracellular region part;extracellular space;ext	ECM-receptor interaction;Focal adhesion	Homo sapiens	+
adherens junction;anchoring junction;cell junction;cell part;cell-substrate adherens junction;cell-substrate junction;cytoplasmic part;cytosol;endoplasmic reticulum;extracellular membrane-bounded organelle;extracellular orga	Amyotrophic lateral sclerosis (ALS);Methane metabolism;Peroxisome;Tryptophan metabolism	Homo sapiens	
cell part;cell projection;cytoplasm;cytoplasmic part;cytosol;Golgi apparatus;intracellular membrane-bounded organelle;intracellular organelle;intracellular organelle part;intracellular part;membrane;membrane-bounded organe	Acute myeloid leukemia;B cell receptor signaling pathway;Bladder cancer;Chemokine signaling pathway;Chronic myeloid leukemia;Colorectal can	Homo sapiens	
cell part;cytoplasmic part;extracellular membrane-bounded organelle;extracellular organelle;extracellular region part;extracellular vesicular exosome;intracellular organelle lumen;intracellular organelle part;intracellular part;lyso	Lysosome;Other glycan degradation;Sphingolipid metabolism	Homo sapiens	+
cell part;cytoplasm;cytoplasmic part;extracellular membrane-bounded organelle;extracellular organelle;extracellular region part;extracellular vesicular exosome;intracellular membrane-bounded organelle;intracellular organelle	Other glycan degradation	Homo sapiens	+
actin cytoskeleton;cell part;contractile fiber part;cytoplasmic membrane-bounded vesicle lumen;cytoplasmic part;cytoplasmic vesicle part;cytoskeleton;cytosol;extracellular membrane-bounded organelle;extracellular organe	Carbon fixation in photosynthetic organisms;Fructose and mannose metabolism;Glycolysis / Gluconeogenesis;Pentose phosphate pathway	Homo sapiens	+
cell part;cytoplasm;extracellular membrane-bounded organelle;extracellular organelle;extracellular region part;extracellular space;extracellular vesicular exosome;intracellular non-membrane-bounded organelle;intracellular organelle;intracellular organelle part;intracellular part;membrane-bounded organelle;membrane-bounded vesicle;non-membrane-bounded organelle;r		Homo sapiens	+
adherens junction;anchoring junction;basolateral plasma membrane;cell junction;cell part;cell projection;cell surface;cell-substrate adherens junction;cell-substrate junction;cilium;cornified envelope;cytoplasm;cytoplasmic part;cytoskeleton;endosome;extracellular membrane-bounded organelle;extracellular organelle;extracellular region;extracellular region part;extracellu		Homo sapiens	+
actin cytoskeleton;cell body;cell part;chylomicron;chylomicron remnant;clathrin coated vesicle membrane;clathrin-coated endocytic vesicle membrane;coated vesicle membrane;cytoplasm;cytoplasmic membrane-bounded v	Fat digestion and absorption;Vitamin digestion and absorption	Homo sapiens	
cell part;cytoplasmic part;extracellular membrane-bounded organelle;extracellular organelle;extracellular region part;extracellular vesicular exosome;intracellular membrane-bounded organelle;intracellular non-membrane-bou	Huntington's disease;Peroxisome	Homo sapiens	
cell part;cytoplasmic part;intracellular membrane-bounded organelle;intracellular organelle;intracellular organelle lumen;intracellular organelle part;intracellular part;membrane-bounded organelle;membrane-enclosed lumen;m	Arginine and proline metabolism	Homo sapiens	+
cell part;cytoplasmic part;cytosol;intracellular part	Drug metabolism - other enzymes;Pyrimidine metabolism	Homo sapiens	+
adherens junction;anchored to external side of plasma membrane;anchored to membrane;anchored to plasma membrane;anchoring junction;apical plasma membrane;cell junction;cell part;cell projection;cell projection part;c	Leukocyte transendothelial migration	Homo sapiens	+
blood microparticle;extracellular membrane-bounded organelle;extracellular organelle;extracellular region;extracellular region part;extracellular space;extracellular vesicular exosome;membrane-bounded organelle;membrane-bounded vesicle;organelle;vesicle		Homo sapiens	+
blood microparticle;cell part;cytoskeletal part;cytoskeleton;extracellular matrix;extracellular membrane-bounded organelle;extracellular organelle;extracellular region part;extracellular space;extracellular vesicular exosome;intermediate filament;intracellular membrane-bounded organelle;intracellular non-membrane-bounded organelle;intracellular organelle;intracellular org		Homo sapiens	+
cell part;cytoplasm;cytoplasmic part;cytoskeleton;cytosol;extracellular membrane-bounded organelle;extracellular organelle;extracellular region part;extracellular vesicular exosome;intracellular membrane-bounded organelle	Alzheimer's disease;Glycolysis / Gluconeogenesis	Homo sapiens	
cell part;cytoplasm;cytoplasmic part;cytosol;extracellular membrane-bounded organelle;extracellular organelle;extracellular region part;extracellular vesicular exosome;intracellular part;membrane-bounded organelle;membra	Alanine, aspartate and glutamate metabolism;Arginine and proline metabolism	Homo sapiens	+
cell part;cell surface;coated vesicle membrane;cytoplasmic part;cytoplasmic vesicle membrane;cytoplasmic vesicle part;early endosome membrane;endocytic vesicle membrane;endoplasmic reticulum;endoplasmic reticulun	Allograft rejection;Antigen processing and presentation;Autoimmune thyroid disease;Cell adhesion molecules (CAMs);Endocytosis;Graft-versus-ho	Homo sapiens	+
cell part;cytoplasm;cytoplasmic part;cytosol;extracellular membrane-bounded organelle;extracellular organelle;extracellular region part;extracellular vesicular exosome;intracellular part;membrane;membrane-bounded organelle;membrane-bounded vesicle;organelle;plasma membrane;vesicle		Homo sapiens	+
cell part;chromatin;chromosomal part;cytoplasm;cytoplasmic part;cytosol;endoplasmic reticulum;intracellular membrane-bounded organelle;intracellular non-membrane-bounded organelle;intracellular organelle;intracellular c	Amyotrophic lateral sclerosis (ALS);Apoptosis;Basal cell carcinoma;Bladder cancer;Cell cycle;Chronic myeloid leukemia;Colorectal cancer;Endom	Homo sapiens	+
cell part;cytoplasm;cytoplasmic part;intracellular membrane-bounded organelle;intracellular organelle;intracellular part;membrane-bounded organelle;nucleus;organelle;perinuclear region of cytoplasm	Mineral absorption	Homo sapiens	+
adherens junction;anchoring junction;cell junction;cell part;cell-substrate adherens junction;cell-substrate junction;contractile fiber part;cytoplasm;cytoplasmic part;cytoskeletal part;cytoskeleton;cytosol;extracellular membra	Amoebiasis;MAPK signaling pathway;VEGF signaling pathway	Homo sapiens	+
cell part;cytoplasm;cytoplasmic part;cytosol;intracellular membrane-bounded organelle;intracellular non-membrane-bounded organelle;intracellular organelle;intracellular organelle lumen;intracellular organelle part;intracellula	One carbon pool by folate;Pyrimidine metabolism	Homo sapiens	+
cell part;cytoplasmic membrane-bounded vesicle;cytoplasmic part;cytoplasmic vesicle;endoplasmic reticulum;endoplasmic reticulum membrane;endoplasmic reticulum part;integral to membrane;intracellular membrane-bou	N-Glycan biosynthesis;Protein processing in endoplasmic reticulum;Various types of N-glycan biosynthesis	Homo sapiens	+
autophagic vacuole membrane;cell part;cytoplasmic part;endoplasmic reticulum;endoplasmic reticulum membrane;endoplasmic reticulum part;integral to membrane;intracellular membrane-bounded organelle;intracellular org	N-Glycan biosynthesis;Protein processing in endoplasmic reticulum;Various types of N-glycan biosynthesis	Homo sapiens	+
cell part;centrosome;cytoplasm;cytoplasmic part;cytoskeletal part;cytosol;extracellular membrane-bounded organelle;extracellular organelle;extracellular region part;extracellular vesicular exosome;extrinsic to internal side of	Axon guidance;Chagas disease (American trypanosomiasis);Chemokine signaling pathway;Gap junction;Gastric acid secretion;Leukocyte transen	Homo sapiens	+
cell part;chromosomal part;extracellular membrane-bounded organelle;extracellular organelle;extracellular region part;extracellular vesicular exosome;intracellular membrane-bounded organelle;intracellular organelle;intracellu	Systemic lupus erythematosus	Homo sapiens	+
apical plasma membrane;basolateral plasma membrane;caveola;cell part;cytoplasmic membrane-bounded vesicle;cytoplasmic part;cytoplasmic vesicle;endoplasmic reticulum;endosome;extracellular membrane-bounded or	Aldosterone-regulated sodium reabsorption;Bile secretion;Carbohydrate digestion and absorption;Cardiac muscle contraction;Endocrine and othe	Homo sapiens	+
apical plasma membrane;basolateral plasma membrane;caveola;cell junction;cell part;cell-cell contact zone;cell-cell junction;extracellular membrane-bounded organelle;extracellular organelle;extracellular region part;extracel	Aldosterone-regulated sodium reabsorption;Bile secretion;Carbohydrate digestion and absorption;Cardiac muscle contraction;Endocrine and othe	Homo sapiens	+
apical part of cell;axon;cell junction;cell part;cell projection;cell projection part;cell surface;cell-cell junction;ciliary rootlet;cilium part;coated pit;coated vesicle;cytoplasm;cytoplasmic membrane-bounded vesicle;cytoplasmic r	Alzheimer's disease	Homo sapiens	+
cell part;cytoplasmic part;extracellular membrane-bounded organelle;extracellular organelle;extracellular region part;extracellular vesicular exosome;intracellular organelle lumen;intracellular organelle part;intracellular part;me	Arginine and proline metabolism;Ascorbate and aldarate metabolism;beta-Alanine metabolism;Chloroalkane and chloroalkene degradation;Fatty a	Homo sapiens	+
adherens junction;alphav-beta3 integrin complex;alphav-beta3 integrin-vitronectin complex;anchoring junction;cell junction;cell part;cell projection membrane;cell projection part;cell surface;cell-substrate adherens junction;c	Arrhythmogenic right ventricular cardiomyopathy (ARVC);Dilated cardiomyopathy;ECM-receptor interaction;Focal adhesion;Hematopoietic cell line	Homo sapiens	+
cell part;chromatin;chromosomal part;cytoplasm;intracellular membrane-bounded organelle;intracellular organelle;intracellular organelle part;intracellular part;membrane-bounded organelle;nucleus;organelle;organelle part		Homo sapiens	
cell part;cytoplasm;extracellular region;extracellular region part;extracellular space;intracellular part;membrane;plasma membrane	Amoebiasis	Homo sapiens	+
cell part;cytoplasmic membrane-bounded vesicle lumen;cytoplasmic part;cytoplasmic vesicle part;extracellular matrix;extracellular membrane-bounded organelle;extracellular organelle;extracellular region;extracellular region	Chagas disease (American trypanosomiasis);Complement and coagulation cascades;p53 signaling pathway	Homo sapiens	+
cell part;cytoplasmic part;cytoskeletal part;extracellular membrane-bounded organelle;extracellular organelle;extracellular region part;extracellular vesicular exosome;integral to membrane;integral to plasma membrane;intrac	Calcium signaling pathway;Huntington's disease;Parkinson's disease	Homo sapiens	+
cell part;cytoplasmic part;cytosol;extracellular region;intracellular organelle part;intracellular part;nuclear part;nucleoplasm;organelle part	RIG-I-like receptor signaling pathway	Homo sapiens	+
cell part;cytoplasmic part;cytosol;eukaryotic 48S preinitiation complex;eukaryotic translation initiation factor 2 complex;extracellular membrane-bounded organelle;extracellular organelle;extracellular region part;extracellular v	Hepatitis C;Measles;Protein processing in endoplasmic reticulum;RNA transport	Homo sapiens	+
cell part;chromatin;chromosomal part;cytoplasm;intracellular membrane-bounded organelle;intracellular organelle;intracellular organelle part;intracellular part;membrane-bounded organelle;nucleus;organelle;organelle part		Homo sapiens	+

adherens junction;anchoring junction;cell junction;cell part;cell surface;cell-substrate adherens junction;cell-substrate junction;external side of plasma membrane;extracellular membrane-bounded organelle;extracellular orgar	African trypanosomiasis;Cell adhesion molecules (CAMs);Leukocyte transendothelial migration;Malaria;Natural killer cell mediated cytotoxicity;Rhe	Homo sapiens	+
adherens junction;anchoring junction;cell junction;cell part;cell-substrate adherens junction;cell-substrate junction;cytoplasmic part;cytosol;cytosolic large ribosomal subunit;extracellular membrane-bounded organelle;extrac	Ribosome	Homo sapiens	+
adherens junction;anchoring junction;cell junction;cell part;cell-substrate adherens junction;cell-substrate junction;cytoplasm;cytoplasmic part;cytosol;cytosolic large ribosomal subunit;extracellular membrane-bounded orgar	Ribosome	Homo sapiens	+
cell part;cytoplasm;extracellular membrane-bounded organelle;extracellular organelle;extracellular region;extracellular region part;extracellular space;extracellular vesicular exosome;granular component;intracellular organelle part;intracellular part;membrane-bounded organelle;membrane-bounded vesicle;nuclear part;nucleolar part;organelle;organelle part;vesicle		Homo sapiens	+
cell part;intracellular membrane-bounded organelle;intracellular organelle;intracellular part;macromolecular complex;membrane-bounded organelle;nucleus;organelle;ribonucleoprotein complex	Systemic lupus erythematosus	Homo sapiens	+
extracellular membrane-bounded organelle;extracellular organelle;extracellular region;extracellular region part;extracellular space;extracellular vesicular exosome;membrane-bounded organelle;membrane-bounded vesicle;organelle;vesicle		Homo sapiens	+
extracellular membrane-bounded organelle;extracellular organelle;extracellular region;extracellular region part;extracellular space;extracellular vesicular exosome;membrane-bounded organelle;membrane-bounded vesicle;or	Complement and coagulation cascades	Homo sapiens	+
acrosomal vesicle;adherens junction;alpha10-beta1 integrin complex;alpha11-beta1 integrin complex;alpha1-beta1 integrin complex;alpha2-beta1 integrin complex;alpha3-beta1 integrin complex;alpha7-beta1 integrin comp	Arrhythmogenic right ventricular cardiomyopathy (ARVC);Axon guidance;Bacterial invasion of epithelial cells;Cell adhesion molecules (CAMs);Dilat	Homo sapiens	+
cell part;cell periphery;centriolar satellite;cytoplasm;cytoplasmic part;cytoskeletal part;extracellular membrane-bounded organelle;extracellular organelle;extracellular region part;extracellular vesicular exosome;intermediate filament;intracellular non-membrane-bounded organelle;intracellular organelle;intracellular organelle part;intracellular part;keratin filament;macromole		Homo sapiens	+
cell junction;cell part;cell-cell junction;contractile fiber part;costamere;cytoplasm;cytoplasmic part;cytoskeletal part;cytoskeleton;dystrophin-associated glycoprotein complex;extracellular membrane-bounded organelle;extracellular organelle;extracellular region part;extracellular vesicular exosome;intermediate filament;intermediate filament cytoskeleton;intracellular mem		Homo sapiens	+
cell part;collagen;collagen type V;cytoplasmic part;endoplasmic reticulum lumen;endoplasmic reticulum part;extracellular matrix;extracellular matrix part;extracellular region;extracellular region part;fibrillar collagen;intracellula	Amoebiasis;ECM-receptor interaction;Focal adhesion;Protein digestion and absorption	Homo sapiens	+
cell part;cytoplasm;cytoplasmic part;cytosol;intracellular organelle part;intracellular part;nuclear part;nucleoplasm;organelle part	Porphyrin and chlorophyll metabolism	Homo sapiens	
cell part;cytoplasm;cytoplasmic part;extracellular membrane-bounded organelle;extracellular organelle;extracellular region;extracellular region part;extracellular vesicular exosome;Golgi apparatus;intracellular membrane-bou	Galactose metabolism;Glycerolipid metabolism;Glycosphingolipid biosynthesis - globo series;Lysosome;Sphingolipid metabolism	Homo sapiens	+
actin cap;actin cytoskeleton;adherens junction;anchoring junction;blood microparticle;cell cortex part;cell junction;cell part;cell projection;cell-substrate adherens junction;cell-substrate junction;cortical actin cytoskeleton;cor	Fc gamma R-mediated phagocytosis;Regulation of actin cytoskeleton	Homo sapiens	+
cell part;cytoplasm;extracellular membrane-bounded organelle;extracellular organelle;extracellular region part;extracellular vesicular exosome;intracellular membrane-bounded organelle;intracellular organelle;intracellular organelle part;intracellular part;membrane-bounded organelle;membrane-bounded vesicle;nuclear part;nucleoplasm;nucleus;organelle;organelle part;ve		Homo sapiens	+
cell part;centrosome;cytoplasm;cytoplasmic part;cytoskeletal part;cytosol;extracellular membrane-bounded organelle;extracellular organelle;extracellular region part;extracellular vesicular exosome;intracellular membrane-bo	Cell cycle;Gap junction;Oocyte meiosis;p53 signaling pathway;Progesterone-mediated oocyte maturation	Homo sapiens	+
cell part;cell surface;cytoplasmic part;extracellular membrane-bounded organelle;extracellular organelle;extracellular region part;extracellular vesicular exosome;intracellular membrane-bounded organelle;intracellular non-me	Alzheimer's disease;Huntington's disease;Oxidative phosphorylation;Parkinson's disease	Homo sapiens	+
cell part;cell projection;cytoplasm;cytoplasmic part;cytosol;envelope;extracellular membrane-bounded organelle;extracellular organelle;extracellular region part;extracellular vesicular exosome;extrinsic to internal side of plasma membrane;extrinsic to membrane;extrinsic to plasma membrane;intracellular membrane-bounded organelle;intracellular organelle;intracellular o		Homo sapiens	+
cell part;chromatoid body;cytoplasm;cytoplasmic mRNA processing body;cytoplasmic part;cytosol;eukaryotic translation initiation factor 4F complex;extracellular membrane-bounded organelle;extracellular organelle;extrace	Insulin signaling pathway;mTOR signaling pathway;RNA transport	Homo sapiens	+
cell part;contractile fiber part;cytoplasm;cytoplasmic part;cytosol;cytosolic part;extracellular membrane-bounded organelle;extracellular organelle;extracellular region part;extracellular space;extracellular vesicular exosome;in	Glycolysis / Gluconeogenesis;Methane metabolism;RNA degradation	Homo sapiens	+
cell part;cytoplasm;cytoplasmic part;cytosol;extracellular membrane-bounded organelle;extracellular organelle;extracellular region part;extracellular vesicular exosome;intracellular part;membrane;membrane-bounded organe	Insulin signaling pathway;Starch and sucrose metabolism	Homo sapiens	
cell part;cell projection;cytoplasm;cytoplasmic part;cytosol;extracellular membrane-bounded organelle;extracellular organelle;extracellular region part;extracellular space;extracellular vesicular exosome;intracellular organelle	Amino sugar and nucleotide sugar metabolism;Glycolysis / Gluconeogenesis;Pentose phosphate pathway;Starch and sucrose metabolism	Homo sapiens	+
adherens junction;anchoring junction;cell junction;cell part;cell-substrate adherens junction;cell-substrate junction;centrosome;cytoplasm;cytoplasmic part;cytoskeletal part;cytosol;focal adhesion;intracellular membrane-bounded organelle;intracellular non-membrane-bounded organelle;intracellular organelle;intracellular organelle part;intracellular part;macromolecular co		Homo sapiens	+
actin filament bundle;actomyosin;cell part;contractile fiber part;cytoplasmic part;cytoskeletal part;cytoskeleton;cytosol;extracellular membrane-bounded organelle;extracellular organelle;extracellular region part;extracellular v	Cardiac muscle contraction;Dilated cardiomyopathy;Hypertrophic cardiomyopathy (HCM);Pathways in cancer;Thyroid cancer	Homo sapiens	+
adherens junction;alphav-beta3 integrin complex;alphav-beta3 integrin-IGF-1-IGF1R complex;alphav-beta5 integrin complex;alphav-beta8 integrin complex;anchoring junction;cell junction;cell part;cell projection membrane;	Arrhythmogenic right ventricular cardiomyopathy (ARVC);Cell adhesion molecules (CAMs);Dilated cardiomyopathy;ECM-receptor interaction;Foca	Homo sapiens	+
cell part;cytoplasmic part;extracellular membrane-bounded organelle;extracellular organelle;extracellular region part;extracellular vesicular exosome;intracellular organelle lumen;intracellular organelle part;intracellular part;lyso	Amino sugar and nucleotide sugar metabolism;Glycosaminoglycan degradation;Glycosphingolipid biosynthesis - ganglio series;Glycosphingolipid	Homo sapiens	+
cell part;cytoplasmic membrane-bounded vesicle;cytoplasmic part;cytoplasmic vesicle;cytosol;extracellular matrix;extracellular region;extracellular region part;extracellular space;extrinsic to external side of plasma membrane;extrinsic to membrane;extrinsic to plasma membrane;intracellular membrane-bounded organelle;intracellular organelle;intracellular part;membrar		Homo sapiens	+
cell part;cytoplasmic part;endoplasmic reticulum membrane;endoplasmic reticulum part;integral to membrane;intracellular organelle part;intracellular part;intrinsic to membrane;membrane;membrane part;organelle membrane	Bile secretion;Metabolism of xenobiotics by cytochrome P450	Homo sapiens	+
cell part;cytoplasm;cytoplasmic part;cytosol;extracellular membrane-bounded organelle;extracellular organelle;extracellular region part;extracellular vesicular exosome;intracellular membrane-bounded organelle;intracellular c	Cysteine and methionine metabolism;Glycolysis / Gluconeogenesis;Propanoate metabolism;Pyruvate metabolism	Homo sapiens	+
cell part;cytoplasm;cytoplasmic part;cytosol;extracellular membrane-bounded organelle;extracellular organelle;extracellular region part;extracellular vesicular exosome;intracellular membrane-bounded organelle;intracellular c	Arachidonic acid metabolism;Glutathione metabolism	Homo sapiens	
adherens junction;anchoring junction;cell junction;cell part;cell-substrate adherens junction;cell-substrate junction;cytoplasmic membrane-bounded vesicle;cytoplasmic part;cytoplasmic vesicle;endoplasmic reticulum;endopl	Protein processing in endoplasmic reticulum	Homo sapiens	+
actin cytoskeleton;cell part;chromatin;chromosomal part;cytoplasmic part;cytoskeleton;euchromatin;Golgi apparatus;intracellular membrane-bounded organelle;intracellular non-membrane-bounded organelle;intracellular organelle;intracellular organelle part;intracellular part;macromolecular complex;membrane-bounded organelle;non-membrane-bounded organelle;nucl		Homo sapiens	+
cell part;cytoplasmic membrane-bounded vesicle;cytoplasmic part;cytoplasmic vesicle;extracellular membrane-bounded organelle;extracellular organelle;extracellular region;extracellular region part;extracellular space;extrac	ko05152;Lysosome	Homo sapiens	+
basement membrane;basolateral plasma membrane;cell cortex;cell part;cell projection;cell surface;cytoplasmic membrane-bounded vesicle;cytoplasmic part;cytoplasmic vesicle;early endosome;endosomal part;endosome;endosome membrane;extracellular matrix part;extracellular membrane-bounded organelle;extracellular organelle;extracellular region part;extracellula		Homo sapiens	+
adherens junction;anchoring junction;cell junction;cell part;cell-substrate adherens junction;cell-substrate junction;cytoplasm;cytoplasmic part;cytosol;extracellular membrane-bounded organelle;extracellular organelle;extrac	Alzheimer's disease;Apoptosis;Protein processing in endoplasmic reticulum	Homo sapiens	+
cell body;cell part;cytoplasmic part;cytoskeletal part;cytoskeleton;cytosol;extracellular membrane-bounded organelle;extracellular organelle;extracellular region part;extracellular vesicular exosome;intracellular membrane-bou	Gap junction;Pathogenic Escherichia coli infection;Phagosome	Homo sapiens	+
cell part;cytoplasmic part;extracellular membrane-bounded organelle;extracellular organelle;extracellular region;extracellular region part;extracellular space;extracellular vesicular exosome;Golgi apparatus;integral to membra	Lysosome	Homo sapiens	+
acrosomal vesicle;cell part;cytoplasmic membrane-bounded vesicle;cytoplasmic part;cytoplasmic vesicle;extracellular membrane-bounded organelle;extracellular organelle;extracellular region part;extracellular vesicular exos	Amino sugar and nucleotide sugar metabolism;Glycosaminoglycan degradation;Glycosphingolipid biosynthesis - ganglio series;Glycosphingolipid	Homo sapiens	+
cell part;cytoplasmic part;extracellular membrane-bounded organelle;extracellular organelle;extracellular region;extracellular region part;extracellular space;extracellular vesicular exosome;intracellular membrane-bounded or	Antigen processing and presentation;Lysosome;Phagosome;Rheumatoid arthritis	Homo sapiens	+
adherens junction;anchoring junction;blood microparticle;cell cortex;cell junction;cell part;cell projection;cell-substrate adherens junction;cell-substrate junction;cytoplasm;cytoplasmic part;cytoskeleton;cytosol;extracellular n	Regulation of actin cytoskeleton;Shigellosis	Homo sapiens	+
cell part;cytoplasm;cytoplasmic part;cytosol;extracellular membrane-bounded organelle;extracellular organelle;extracellular region part;extracellular vesicular exosome;intracellular organelle part;intracellular part;membrane-b	Purine metabolism	Homo sapiens	
aminoacyl-tRNA synthetase multienzyme complex;cell part;cytoplasm;cytoplasmic part;cytosol;intracellular part;macromolecular complex;membrane;protein complex;ribonucleoprotein complex	Aminoacyl-tRNA biosynthesis;Porphyrin and chlorophyll metabolism	Homo sapiens	+
cell part;cytoplasmic membrane-bounded vesicle;cytoplasmic part;cytoplasmic vesicle;extracellular membrane-bounded organelle;extracellular organelle;extracellular region;extracellular region part;extracellular space;extrac	Antigen processing and presentation;Lysosome	Homo sapiens	+
apical plasma membrane;basolateral plasma membrane;brush border membrane;cell body;cell part;cell projection;cell projection membrane;cell projection part;cell surface;cytoplasm;cytoplasmic membrane-bounded vesicle	Antigen processing and presentation;NOD-like receptor signaling pathway;Pathways in cancer;Plant-pathogen interaction;Progesterone-mediated	Homo sapiens	+
catalytic step 2 spliceosome;cell part;chromatin;chromosomal part;cytoplasmic part;cytosol;extracellular membrane-bounded organelle;extracellular organelle;extracellular region part;extracellular vesicular exosome;intracellu	Spliceosome	Homo sapiens	+
cell part;cytoplasmic part;intracellular membrane-bounded organelle;intracellular organelle;intracellular organelle part;intracellular part;membrane;membrane part;membrane-bounded organelle;mitochondrial inner membrane	Alzheimer's disease;Cardiac muscle contraction;Huntington's disease;Oxidative phosphorylation;Parkinson's disease	Homo sapiens	+
basement membrane;cell part;cytoplasmic part;extracellular matrix;extracellular matrix part;extracellular membrane-bounded organelle;extracellular organelle;extracellular region;extracellular region part;extracellular space;ex	Amoebiasis;ECM-receptor interaction;Focal adhesion;Pathways in cancer;Small cell lung cancer;Toxoplasmosis	Homo sapiens	+
actin filament;adherens junction;anchoring junction;cell junction;cell part;cell-substrate adherens junction;cell-substrate junction;cytoplasm;cytoplasmic part;cytoskeletal part;cytosol;extracellular membrane-bounded organel	Adherens junction;Tight junction	Homo sapiens	+
cell part;contractile fiber part;cytoplasmic part;cytoskeletal part;cytosol;intracellular organelle part;intracellular part;muscle thin filament tropomyosin;organelle part	Cardiac muscle contraction;Dilated cardiomyopathy;Hypertrophic cardiomyopathy (HCM)	Homo sapiens	+
cell part;cytoplasm;cytoplasmic part;extracellular membrane-bounded organelle;extracellular organelle;extracellular region part;extracellular vesicular exosome;intracellular membrane-bounded organelle;intracellular organelle	Carbon fixation pathways in prokaryotes;Citrate cycle (TCA cycle);Pathways in cancer;Renal cell carcinoma	Homo sapiens	
cell part;cell surface;cytoplasmic membrane-bounded vesicle;cytoplasmic membrane-bounded vesicle lumen;cytoplasmic part;cytoplasmic vesicle;cytoplasmic vesicle part;endoplasmic reticulum;endoplasmic reticulum lume	ECM-receptor interaction;Focal adhesion;Malaria;Phagosome;TGF-beta signaling pathway	Homo sapiens	+
cell part;collagen;collagen type I;cytoplasmic part;endoplasmic reticulum lumen;endoplasmic reticulum part;extracellular matrix;extracellular matrix part;extracellular membrane-bounded organelle;extracellular organelle;extra	Amoebiasis;ECM-receptor interaction;Focal adhesion;Protein digestion and absorption	Homo sapiens	+
adherens junction;anchoring junction;cell junction;cell part;cell-substrate adherens junction;cell-substrate junction;cytoplasmic membrane-bounded vesicle;cytoplasmic part;cytoplasmic vesicle;endosomal part;endosome membrane;extracellular membrane-bounded organelle;extracellular organelle;extracellular region part;extracellular vesicular exosome;focal adhesion;		Homo sapiens	+
cell division site part;cell part;cleavage furrow;cytoplasmic part;cytosol;extracellular membrane-bounded organelle;extracellular organelle;extracellular region part;extracellular vesicular exosome;intracellular membrane-bounded organelle;intracellular organelle;intracellular part;membrane;membrane-bounded organelle;membrane-bounded vesicle;nucleus;organelle;plasm		Homo sapiens	+
anchored to membrane;cell part;cell surface;extracellular membrane-bounded organelle;extracellular organelle;extracellular region;extracellular region part;extracellular vesicular exosome;integral to membrane;integral to plas	Complement and coagulation cascades;Hematopoietic cell lineage;Viral myocarditis	Homo sapiens	+
apical plasma membrane;cell part;cell surface;cytoplasm;cytoplasmic membrane-bounded vesicle;cytoplasmic part;cytoplasmic vesicle;extracellular membrane-bounded organelle;extracellular organelle;extracellular region p	Protein digestion and absorption	Homo sapiens	+
6-phosphofructokinase complex;apical plasma membrane;cell part;cytoplasmic part;cytosol;cytosolic part;extracellular membrane-bounded organelle;extracellular organelle;extracellular region part;extracellular vesicular exo	Fructose and mannose metabolism;Galactose metabolism;Glycolysis / Gluconeogenesis;Methane metabolism;Pentose phosphate pathway	Homo sapiens	
cell part;cytoplasm;cytoplasmic membrane-bounded vesicle;cytoplasmic part;cytoplasmic vesicle;cytosol;extracellular membrane-bounded organelle;extracellular organelle;extracellular region part;extracellular vesicular exos	Antigen processing and presentation;NOD-like receptor signaling pathway;Pathways in cancer;Plant-pathogen interaction;Progesterone-mediated	Homo sapiens	+
cell part;cytoplasmic part;endoplasmic reticulum;endoplasmic reticulum membrane;endoplasmic reticulum part;extracellular membrane-bounded organelle;extracellular organelle;extracellular region part;extracellular vesicula	Protein export	Homo sapiens	+
cell part;cytoplasmic part;cytosol;intracellular part	Alanine, aspartate and glutamate metabolism;Nitrogen metabolism	Homo sapiens	+
cell part;contractile fiber part;cytoplasmic part;extracellular matrix;extracellular region;extracellular region part;extracellular space;intracellular membrane-bounded organelle;intracellular organelle;intracellular part;membrane;r	Bladder cancer;GnRH signaling pathway;Leukocyte transendothelial migration;Pathways in cancer	Homo sapiens	+
cell part;cytoplasmic part;cytosol;intracellular part	Porphyrin and chlorophyll metabolism	Homo sapiens	
adherens junction;anchoring junction;axon;brush border;cell junction;cell part;cell projection;cell projection part;cell-substrate adherens junction;cell-substrate junction;clathrin-coated vesicle;coated vesicle;cytoplasm;cytopla	Alzheimer's disease;Hematopoietic cell lineage;Protein digestion and absorption;Renin-angiotensin system	Homo sapiens	+
cell part;cytoplasmic part;intracellular membrane-bounded organelle;intracellular organelle;intracellular organelle lumen;intracellular organelle part;intracellular part;macromolecular complex;membrane-bounded organelle;mer	Butanoate metabolism;Citrate cycle (TCA cycle);Glycolysis / Gluconeogenesis;Pyruvate metabolism;Valine, leucine and isoleucine biosynthesis	Homo sapiens	+
cell part;cytoplasmic part;integral to membrane;intracellular membrane-bounded organelle;intracellular organelle;intracellular organelle part;intracellular part;intrinsic to membrane;membrane;membrane part;membrane-bounc	Alzheimer's disease;Cardiac muscle contraction;Huntington's disease;Oxidative phosphorylation;Parkinson's disease	Homo sapiens	+
catalytic step 2 spliceosome;cell part;intracellular non-membrane-bounded organelle;intracellular organelle;intracellular organelle part;intracellular part;macromolecular complex;non-membrane-bounded organelle;nuclear par	Spliceosome	Homo sapiens	+
cell part;intracellular membrane-bounded organelle;intracellular organelle;intracellular organelle part;intracellular part;macromolecular complex;membrane-bounded organelle;nuclear body;nuclear part;nuclear speck;nucleopl	Spliceosome	Homo sapiens	+
adherens junction;alphav-beta3 integrin-vitronectin complex;anchoring junction;cell junction;cell part;cell projection;cell surface;cell-substrate adherens junction;cell-substrate junction;cytoplasm;external side of plasma mem	Arrhythmogenic right ventricular cardiomyopathy (ARVC);Bacterial invasion of epithelial cells;Dilated cardiomyopathy;ECM-receptor interaction;Fo	Homo sapiens	+
adherens junction;anchoring junction;cell junction;cell leading edge;cell part;cell projection;cell-substrate adherens junction;cell-substrate junction;cytoplasm;cytoplasmic part;cytoskeletal part;cytoskeleton;cytosol;extracellular membrane-bounded organelle;extracellular organelle;extracellular region part;extracellular vesicular exosome;focal adhesion;intermediate filame		Homo sapiens	+
blood microparticle;cell part;cell surface;cytoplasmic membrane-bounded vesicle lumen;cytoplasmic part;cytoplasmic vesicle part;extracellular membrane-bounded organelle;extracellular organelle;extracellular region;extrac	Complement and coagulation cascades	Homo sapiens	+
adherens junction;anchoring junction;cell junction;cell part;cell-substrate adherens junction;cell-substrate junction;cytoplasmic part;cytosol;cytosolic small ribosomal subunit;extracellular membrane-bounded organelle;extrac	Ribosome	Homo sapiens	+
cell part;cell periphery;contractile fiber part;costamere;cytoplasmic part;cytoskeletal part;dystrophin-associated glycoprotein complex;extracellular membrane-bounded organelle;extracellular organelle;extracellular region part;extracellular vesicular exosome;intermediate filament;intracellular organelle part;intracellular part;macromolecular complex;membrane;membrane		Homo sapiens	
cell part;cytoplasm;cytoskeletal part;extracellular membrane-bounded organelle;extracellular organelle;extracellular region part;extracellular vesicular exosome;intermediate filament;intracellular membrane-bounded organelle;intracellular organelle;intracellular organelle part;intracellular part;keratin filament;macromolecular complex;membrane-bounded organelle;membra		Homo sapiens	+
cell part;centrosome;cytoplasm;cytoplasmic membrane-bounded vesicle;cytoplasmic part;cytoplasmic vesicle;cytoskeletal part;extracellular membrane-bounded organelle;extracellular organelle;extracellular region part;extra	Axon guidance;Chagas disease (American trypanosomiasis);Chemokine signaling pathway;Gap junction;Gastric acid secretion;Leukocyte transen	Homo sapiens	+
adherens junction;anchoring junction;blood microparticle;cell junction;cell part;cell-substrate adherens junction;cell-substrate junction;cytoplasm;endothelial microparticle;external side of plasma membrane;extracellular membrane-bounded organelle;extracellular organelle;extracellular region part;extracellular vesicular exosome;focal adhesion;intracellular;intracellular pa		Homo sapiens	+
cell part;cytoskeletal part;cytoskeleton;extracellular membrane-bounded organelle;extracellular organelle;extracellular region part;extracellular vesicular exosome;intermediate filament;intracellular membrane-bounded organelle;intracellular non-membrane-bounded organelle;intracellular organelle;intracellular organelle part;intracellular part;macromolecular complex;mem		Homo sapiens	+
90S preribosome;cell part;cytoplasm;cytoplasmic part;cytosol;cytosolic small ribosomal subunit;extracellular membrane-bounded organelle;extracellular organelle;extracellular region part;extracellular vesicular exosome;intra	Ribosome	Homo sapiens	+
cell part;cell surface;cytoplasmic membrane-bounded vesicle;cytoplasmic part;cytoplasmic vesicle;cytoplasmic vesicle membrane;cytoplasmic vesicle part;endosomal part;endosome lumen;endosome membrane;extracellula	Lysosome	Homo sapiens	+
cell part;cytoplasmic part;intracellular organelle part;intracellular part;large ribosomal subunit;macromolecular complex;membrane;mitochondrial inner membrane;mitochondrial large ribosomal subunit;mitochondrial membrane;mitochondrial part;organellar large ribosomal subunit;organelle inner membrane;organelle membrane;organelle part;ribonucleoprotein complex		Homo sapiens	
cell part;cytoplasm;intracellular non-membrane-bounded organelle;intracellular organelle;intracellular organelle part;intracellular part;macromolecular complex;non-membrane-bounded organelle;nuclear part;nucleolus;nucleo	Spliceosome	Homo sapiens	
cell part;extracellular region;extracellular region part;extracellular space;intracellular membrane-bounded organelle;intracellular organelle;intracellular part;membrane-bounded organelle;nucleus;organelle	MAPK signaling pathway;Melanoma;Pathways in cancer;Regulation of actin cytoskeleton	Homo sapiens	+
cell part;cytoplasmic part;cytosol;cytosolic part;extracellular membrane-bounded organelle;extracellular organelle;extracellular region part;extracellular space;extracellular vesicular exosome;intracellular part;macromolecular	Glycolysis / Gluconeogenesis;Methane metabolism;RNA degradation	Homo sapiens	
cell part;cytoplasmic part;intracellular membrane-bounded organelle;intracellular organelle;intracellular organelle lumen;intracellular organelle part;intracellular part;membrane;membrane-bounded organelle;membrane-enclos	Benzoate degradation;Biosynthesis of unsaturated fatty acids;Fatty acid metabolism;Peroxisome;PPAR signaling pathway;Valine, leucine and isole	Homo sapiens	
cell part;cytoplasm;cytoplasmic part;cytosol;intracellular membrane-bounded organelle;intracellular non-membrane-bounded organelle;intracellular organelle;intracellular organelle part;intracellular part;macromolecular comp	Protein export	Homo sapiens	+
cell part;cytoplasm;cytoplasmic part;cytosol;extracellular membrane-bounded organelle;extracellular organelle;extracellular region part;extracellular space;extracellular vesicular exosome;intracellular;intracellular membrane-b	Drug metabolism - cytochrome P450;Glutathione metabolism;Metabolism of xenobiotics by cytochrome P450	Homo sapiens	+
Cajal body;cell part;commitment complex;intracellular organelle part;intracellular part;macromolecular complex;nuclear body;nuclear part;nucleoplasm;nucleoplasm part;organelle part;prespliceosome;ribonucleoprotein comp	Spliceosome	Homo sapiens	
cell part;cell surface;cytoplasm;extracellular matrix;extracellular membrane-bounded organelle;extracellular organelle;extracellular region part;extracellular space;extracellular vesicular exosome;intracellular;intracellular membrane-bounded organelle;intracellular organelle;intracellular part;membrane-bounded organelle;membrane-bounded vesicle;nucleus;organelle;prote		Homo sapiens	+
cell part;cell projection;cytoplasm;cytoplasmic part;cytosol;extracellular membrane-bounded organelle;extracellular organelle;extracellular region part;extracellular vesicular exosome;intracellular membrane-bounded organell	Folate biosynthesis	Homo sapiens	
cell part;cell surface;chromosome;condensed chromosome;extracellular region;extracellular region part;extracellular space;intracellular membrane-bounded organelle;intracellular non-membrane-bounded organelle;intracellu	Base excision repair	Homo sapiens	+
basement membrane;cell part;cell surface;cytoplasm;cytoplasmic membrane-bounded vesicle;cytoplasmic membrane-bounded vesicle lumen;cytoplasmic part;cytoplasmic vesicle;cytoplasmic vesicle membrane;cytoplasmic vesicle part;endocytic vesicle lumen;extracellular matrix part;extracellular region;extracellular region part;extracellular space;intracellular membra		Homo sapiens	
actin filament bundle;actomyosin;bleb;cell part;cell projection;cell projection membrane;cell projection part;contractile fiber part;cytoplasmic part;cytoskeletal part;cytoskeleton;cytosol;filamentous actin;intracellular non-mem	Cardiac muscle contraction;Dilated cardiomyopathy;Hypertrophic cardiomyopathy (HCM)	Homo sapiens	+
cell part;clathrin coat;clathrin coat of coated pit;clathrin coat of trans-Golgi network vesicle;clathrin coated vesicle membrane;clathrin complex;clathrin vesicle coat;clathrin-coated endocytic vesicle membrane;coated vesicle	Bacterial invasion of epithelial cells;Endocrine and other factor-regulated calcium reabsorption;Endocytosis;Huntington's disease;Lysosome	Homo sapiens	+
cell part;cell projection membrane;cell projection part;cilium membrane;cilium part;clathrin coat;clathrin coat of coated pit;clathrin coat of trans-Golgi network vesicle;clathrin vesicle coat;cytoplasm;cytoplasmic part;cytoplas	Bacterial invasion of epithelial cells;Endocrine and other factor-regulated calcium reabsorption;Endocytosis;Huntington's disease;Lysosome	Homo sapiens	+
cell part;cell surface;cytoplasm;cytoplasmic part;extracellular membrane-bounded organelle;extracellular organelle;extracellular region part;extracellular vesicular exosome;intracellular membrane-bounded organelle;intracellular organelle;intracellular organelle part;intracellular part;membrane;membrane-bounded organelle;membrane-bounded vesicle;nuclear membrane;		Homo sapiens	+
cell part;cell projection;cytoplasm;cytoplasmic membrane-bounded vesicle;cytoplasmic part;cytoplasmic vesicle;cytoskeletal part;extracellular membrane-bounded organelle;extracellular organelle;extracellular region part;extracellular space;extracellular vesicular exosome;intracellular membrane-bounded organelle;intracellular organelle;intracellular organelle part;intrace		Homo sapiens	+
adherens junction;anchoring junction;apical plasma membrane;cell junction;cell part;cell surface;cell-substrate adherens junction;cell-substrate junction;cytoplasm;cytoplasmic membrane-bounded vesicle;cytoplasmic part;c	Calcium signaling pathway;Cytokine-cytokine receptor interaction;Focal adhesion;Gap junction;Glioma;MAPK signaling pathway;Melanoma;Pathw	Homo sapiens	+
acrosomal matrix;cell part;cell projection;cilium;cytoplasmic part;cytoplasmic vesicle part;intracellular membrane-bounded organelle;intracellular organelle;intracellular organelle lumen;intracellular organelle part;intracellular p	Citrate cycle (TCA cycle);Glycine, serine and threonine metabolism;Glycolysis / Gluconeogenesis;Pyruvate metabolism;Valine, leucine and isoleuci	Homo sapiens	+
catalytic step 2 spliceosome;cell part;cytoplasm;cytoskeleton;extracellular membrane-bounded organelle;extracellular organelle;extracellular region part;extracellular vesicular exosome;intermediate filament cytoskeleton;intra	Spliceosome	Homo sapiens	+
catalytic step 2 spliceosome;cell part;intracellular organelle part;intracellular part;macromolecular complex;nuclear part;nucleoplasm;organelle part;ribonucleoprotein complex;small nuclear ribonucleoprotein complex;spliceo	Spliceosome	Homo sapiens	+
cell part;cytoplasmic part;integral to membrane;intracellular membrane-bounded organelle;intracellular organelle;intracellular organelle part;intracellular part;intrinsic to membrane;membrane;membrane part;membrane-bounc	Alzheimer's disease;Cardiac muscle contraction;Huntington's disease;Oxidative phosphorylation;Parkinson's disease	Homo sapiens	+
cell part;envelope;intracellular membrane-bounded organelle;intracellular non-membrane-bounded organelle;intracellular organelle;intracellular organelle part;intracellular part;macromolecular complex;membrane;membrane-	Base excision repair	Homo sapiens	+
alpha DNA polymerase:primase complex;cell part;chromosomal part;cytoplasm;envelope;intracellular membrane-bounded organelle;intracellular non-membrane-bounded organelle;intracellular organelle;intracellular organelle	DNA replication;Purine metabolism;Pyrimidine metabolism	Homo sapiens	+
cell part;cytoplasm;cytoplasmic part;cytosol;endoplasmic reticulum;intracellular membrane-bounded organelle;intracellular organelle;intracellular part;membrane-bounded organelle;organelle		Homo sapiens	+
cell part;cytoplasm;cytoplasmic part;cytosol;intracellular part	Hepatitis C	Homo sapiens	+
axon;cell body;cell part;cell projection;cytoplasm;cytoplasmic part;cytosol;endoplasmic reticulum membrane;endoplasmic reticulum part;extracellular membrane-bounded organelle;extracellular organelle;extracellular region	Parkinson's disease	Homo sapiens	+
cell part;cytoplasm;cytoplasmic part;cytosol;extracellular membrane-bounded organelle;extracellular organelle;extracellular region part;extracellular vesicular exosome;intracellular organelle part;intracellular part;membrane-b	Arachidonic acid metabolism	Homo sapiens	+
axon;cell part;cell projection;cytoplasmic part;cytoskeleton;cytosol;extracellular membrane-bounded organelle;extracellular organelle;extracellular region part;extracellular vesicular exosome;intracellular membrane-bounded	Carbon fixation in photosynthetic organisms;Fructose and mannose metabolism;Glycolysis / Gluconeogenesis;Pentose phosphate pathway	Homo sapiens	+
blood microparticle;cell part;extracellular membrane-bounded organelle;extracellular organelle;extracellular region;extracellular region part;extracellular vesicular exosome;membrane;membrane-bounded organelle;membrane	Complement and coagulation cascades;Staphylococcus aureus infection;Systemic lupus erythematosus	Homo sapiens	+
cell part;chromatin;chromosomal part;euchromatin;extracellular membrane-bounded organelle;extracellular organelle;extracellular region part;extracellular vesicular exosome;heterochromatin;intracellular membrane-bounded	Systemic lupus erythematosus	Homo sapiens	
cell part;chromosomal part;extracellular membrane-bounded organelle;extracellular organelle;extracellular region part;extracellular vesicular exosome;intracellular membrane-bounded organelle;intracellular organelle;intracellu	Systemic lupus erythematosus	Homo sapiens	+
		Homo sapiens	+
		Homo sapiens	+
		Homo sapiens	+
cell part;cytoplasm;intracellular organelle part;intracellular part;macromolecular complex;nuclear part;nucleoplasm;organelle part;ribonucleoprotein complex	Systemic lupus erythematosus	Homo sapiens	
cell part;cytoplasmic part;extracellular membrane-bounded organelle;extracellular organelle;extracellular region part;extracellular vesicular exosome;intracellular membrane-bounded organelle;intracellular organelle;intracellula	Galactose metabolism;Lysosome;Starch and sucrose metabolism	Homo sapiens	
adherens junction;anchoring junction;cell junction;cell part;cell-substrate adherens junction;cell-substrate junction;extracellular membrane-bounded organelle;extracellular organelle;extracellular region part;extracellular vesicu	MAPK signaling pathway;Regulation of actin cytoskeleton;Tight junction	Homo sapiens	+
cell part;cell surface;coated vesicle membrane;cytoplasmic part;cytoplasmic vesicle membrane;cytoplasmic vesicle part;early endosome membrane;endocytic vesicle membrane;endoplasmic reticulum;endoplasmic reticulun	Allograft rejection;Antigen processing and presentation;Autoimmune thyroid disease;Cell adhesion molecules (CAMs);Endocytosis;Graft-versus-hc	Homo sapiens	+
cell part;chromatin;chromosomal part;extracellular membrane-bounded organelle;extracellular organelle;extracellular region part;extracellular vesicular exosome;heterochromatin;intracellular membrane-bounded organelle;intracellular organelle;intracellular organelle part;intracellular part;macromolecular complex;membrane-bounded organelle;membrane-bounded vesicle		Homo sapiens	+
cell part;cytoplasmic part;intracellular membrane-bounded organelle;intracellular organelle;intracellular organelle lumen;intracellular organelle part;intracellular part;macromolecular complex;membrane-bounded organelle;mer	Citrate cycle (TCA cycle);Glycolysis / Gluconeogenesis;Pyruvate metabolism	Homo sapiens	+
cell part;cytoplasm;cytoplasmic part;cytosol;extracellular membrane-bounded organelle;extracellular organelle;extracellular region part;extracellular vesicular exosome;intracellular membrane-bounded organelle;intracellular organelle;intracellular organelle part;intracellular part;membrane-bounded organelle;membrane-bounded vesicle;mitochondrion;nuclear part;nucleo		Homo sapiens	+
cell part;cytoplasmic part;extracellular membrane-bounded organelle;extracellular organelle;extracellular region part;extracellular vesicular exosome;intracellular membrane-bounded organelle;intracellular organelle;intracellula	Alzheimer's disease;Cardiac muscle contraction;Huntington's disease;Oxidative phosphorylation;Parkinson's disease	Homo sapiens	+
cell part;cytoplasmic part;endoplasmic reticulum;extracellular membrane-bounded organelle;extracellular organelle;extracellular region part;extracellular vesicular exosome;intracellular membrane-bounded organelle;intracellu	Lysosome;Renin-angiotensin system	Homo sapiens	+
apical part of cell;cell part;cytoplasmic part;endoplasmic reticulum;endoplasmic reticulum membrane;endoplasmic reticulum part;integral to membrane;intracellular membrane-bounded organelle;intracellular organelle;intrace	Drug metabolism - cytochrome P450;Glutathione metabolism;Metabolism of xenobiotics by cytochrome P450	Homo sapiens	+
cell part;extracellular membrane-bounded organelle;extracellular organelle;extracellular region;extracellular region part;extracellular vesicular exosome;macromolecular complex;membrane attack complex;membrane part;me	Complement and coagulation cascades;Prion diseases;Systemic lupus erythematosus	Homo sapiens	+
AMP-activated protein kinase complex;cAMP-dependent protein kinase complex;cell part;cytoplasmic part;cytosol;intracellular part;macromolecular complex;membrane;neuromuscular junction;plasma membrane;protein co	Apoptosis;Insulin signaling pathway	Homo sapiens	
anchored to membrane;cell part;cell surface;cytoplasmic part;endoplasmic reticulum;extracellular region;extracellular region part;extracellular space;intracellular membrane-bounded organelle;intracellular organelle;intracellu	Complement and coagulation cascades	Homo sapiens	+
cell part;cytoplasm;cytoplasmic membrane-bounded vesicle;cytoplasmic part;cytoplasmic vesicle;extracellular membrane-bounded organelle;extracellular organelle;extracellular region part;extracellular vesicular exosome;Go	Methane metabolism	Homo sapiens	+
cell part;cell surface;coated pit;coated vesicle;cytoplasm;cytoplasmic membrane-bounded vesicle;cytoplasmic part;cytoplasmic vesicle;cytosol;early endosome;endosome;extracellular membrane-bounded organelle;extrace	ko05152;RNA degradation;Type I diabetes mellitus	Homo sapiens	+
blood microparticle;cell part;chromaffin granule;cytoplasm;cytoplasmic membrane-bounded vesicle;cytoplasmic membrane-bounded vesicle lumen;cytoplasmic part;cytoplasmic vesicle;cytoplasmic vesicle part;cytosol;endoplasmic reticulum;extracellular matrix;extracellular membrane-bounded organelle;extracellular organelle;extracellular region;extracellular region par		Homo sapiens	+

adherens junction;anchoring junction;cell junction;cell part;cell surface;cell-substrate adherens junction;cell-substrate junction;cytoplasmic membrane-bounded vesicle;cytoplasmic part;cytoplasmic vesicle;endoplasmic retic	Prion diseases;Protein export;Protein processing in endoplasmic reticulum	Homo sapiens	+
basement membrane;extracellular matrix;extracellular matrix part;extracellular membrane-bounded organelle;extracellular organelle;extracellular region;extracellular region part;extracellular space;extracellular vesicular exoso	Amoebiasis;ECM-receptor interaction;Focal adhesion;Pathways in cancer;Prion diseases;Small cell lung cancer;Toxoplasmosis	Homo sapiens	+
cell part;cytoplasmic part;extracellular membrane-bounded organelle;extracellular organelle;extracellular region part;extracellular vesicular exosome;integral to membrane;intracellular membrane-bounded organelle;intracellula	Riboflavin metabolism	Homo sapiens	+
adherens junction;anchoring junction;blood microparticle;cell junction;cell part;cell-substrate adherens junction;cell-substrate junction;clathrin coated vesicle membrane;clathrin sculpted gamma-aminobutyric acid transport v	Antigen processing and presentation;Endocytosis;MAPK signaling pathway;Measles;Protein processing in endoplasmic reticulum;Spliceosome;To	Homo sapiens	+
basolateral plasma membrane;blood microparticle;caveola;cell cortex part;cell junction;cell part;cell-cell junction;cortical actin cytoskeleton;cortical cytoskeleton;cytoplasmic membrane-bounded vesicle;cytoplasmic part;cyto	Adipocytokine signaling pathway;Bile secretion;Pathways in cancer;Renal cell carcinoma	Homo sapiens	+
cell part;extracellular membrane-bounded organelle;extracellular organelle;extracellular region part;extracellular vesicular exosome;integral to membrane;integral to plasma membrane;intrinsic to membrane;intrinsic to plasma membrane;membrane;membrane part;membrane-bounded organelle;membrane-bounded vesicle;organelle;plasma membrane;plasma membrane		Homo sapiens	+
cell part;cytoplasm;cytoplasmic part;cytosol;intracellular membrane-bounded organelle;intracellular organelle;intracellular part;membrane-bounded organelle;nucleus;organelle	Drug metabolism - other enzymes;Pyrimidine metabolism	Homo sapiens	+
cell part;cytoplasmic part;extracellular membrane-bounded organelle;extracellular organelle;extracellular region part;extracellular vesicular exosome;intracellular membrane-bounded organelle;intracellular organelle;intracellula	Butanoate metabolism;Citrate cycle (TCA cycle);Glycolysis / Gluconeogenesis;Pyruvate metabolism;Valine, leucine and isoleucine biosynthesis	Homo sapiens	+
cell part;cytoplasm;extracellular membrane-bounded organelle;extracellular organelle;extracellular region part;extracellular vesicular exosome;intracellular part;membrane;membrane-bounded organelle;membrane-bounded v	Insulin signaling pathway;Starch and sucrose metabolism	Homo sapiens	+
adherens junction;anchoring junction;cell division site part;cell junction;cell part;cell surface;cell-substrate adherens junction;cell-substrate junction;cleavage furrow;cytoplasmic part;cytoplasmic vesicle membrane;cytoplasm	Pancreatic cancer;Pathways in cancer	Homo sapiens	+
cell part;extracellular membrane-bounded organelle;extracellular organelle;extracellular region part;extracellular vesicular exosome;membrane;membrane-bounded organelle;membrane-bounded vesicle;midbody;organelle;pl	Pancreatic cancer;Pathways in cancer	Homo sapiens	+
cell body;cell part;cell projection;cytolytic granule;cytoplasm;cytoplasmic membrane-bounded vesicle;cytoplasmic part;cytoplasmic vesicle;dendrite;endosomal part;endosome;endosome membrane;external side of plasma r	ko05152;Lysosome;Phagosome	Homo sapiens	+
axon;cell part;cell projection;cytoplasmic part;extracellular membrane-bounded organelle;extracellular organelle;extracellular region part;extracellular vesicular exosome;intracellular membrane-bounded organelle;intracellular	beta-Alanine metabolism;Fatty acid metabolism;PPAR signaling pathway;Propanoate metabolism;Valine, leucine and isoleucine degradation	Homo sapiens	
cell part;chromosomal part;cytoplasmic mRNA processing body;cytoplasmic part;intracellular membrane-bounded organelle;intracellular non-membrane-bounded organelle;intracellular organelle;intracellular organelle part;intracellular part;macromolecular complex;membrane-bounded organelle;non-membrane-bounded organelle;nuclear chromosome part;nuclear part;r		Homo sapiens	+
cell part;chromosome;condensed chromosome;cytoplasm;DNA topoisomerase complex (ATP-hydrolyzing);intracellular membrane-bounded organelle;intracellular non-membrane-bounded organelle;intracellular organelle;intracellular organelle part;intracellular part;macromolecular complex;membrane-bounded organelle;non-membrane-bounded organelle;nuclear part;nu		Homo sapiens	+
cell part;centrosome;cytoplasm;cytoplasmic part;cytoskeletal part;cytosol;extracellular membrane-bounded organelle;extracellular organelle;extracellular region part;extracellular vesicular exosome;internal side of plasma me	Glutathione metabolism;Pentose phosphate pathway	Homo sapiens	+
BAT3 complex;cell part;cytoplasmic part;cytosol;cytosolic part;ER membrane insertion complex;intracellular part;macromolecular complex;protein complex		Homo sapiens	+
cell part;cytoplasm;cytoplasmic part;cytosol;intracellular membrane-bounded organelle;intracellular organelle;intracellular organelle lumen;intracellular organelle part;intracellular part;membrane;membrane-bounded organelle	Citrate cycle (TCA cycle);Pyruvate metabolism	Homo sapiens	+
cell part;cytoplasmic part;cytosol;extracellular membrane-bounded organelle;extracellular organelle;extracellular region part;extracellular vesicular exosome;intracellular membrane-bounded organelle;intracellular organelle;int	One carbon pool by folate	Homo sapiens	
adherens junction;anchoring junction;cell junction;cell part;cell surface;cell-substrate adherens junction;cell-substrate junction;clathrin coat;clathrin-coated vesicle;coated vesicle;cytoplasmic membrane-bounded vesicle;cyto	Lysosome	Homo sapiens	
cell part;cytoplasmic part;extracellular membrane-bounded organelle;extracellular organelle;extracellular region part;extracellular vesicular exosome;intracellular membrane-bounded organelle;intracellular organelle;intracellula	Chloroalkane and chloroalkene degradation;Drug metabolism - cytochrome P450;Fatty acid metabolism;Glycolysis / Gluconeogenesis;Metabolism	Homo sapiens	
cell junction;cell part;cell-cell junction;chromatin;chromosomal part;cyclin-dependent protein kinase holoenzyme complex;cytoplasmic part;cytosol;intracellular membrane-bounded organelle;intracellular non-membrane-bour	Bladder cancer;Cell cycle;Chronic myeloid leukemia;Glioma;Measles;Melanoma;Non-small cell lung cancer;p53 signaling pathway;Pancreatic can	Homo sapiens	+
extracellular membrane-bounded organelle;extracellular organelle;extracellular region part;extracellular vesicular exosome;macromolecular complex;membrane-bounded organelle;membrane-bounded vesicle;organelle;protei	Pentose phosphate pathway;Purine metabolism	Homo sapiens	
adherens junction;anchoring junction;catalytic step 2 spliceosome;cell junction;cell part;cell-substrate adherens junction;cell-substrate junction;cytoplasm;cytoplasmic part;cytosol;extracellular membrane-bounded organelle;	mRNA surveillance pathway;RNA degradation;RNA transport	Homo sapiens	
cell part;centrosome;chromosomal part;cytoplasm;cytoplasmic part;cytoskeletal part;DNA replication factor C complex;extracellular membrane-bounded organelle;extracellular organelle;extracellular region part;extracellular v	Base excision repair;Cell cycle;DNA replication;Mismatch repair;Nucleotide excision repair	Homo sapiens	+
cell part;cytoplasm;cytoplasmic part;cytosol;intracellular membrane-bounded organelle;intracellular organelle;intracellular part;membrane-bounded organelle;mitochondrion;organelle	Aminoacyl-tRNA biosynthesis	Homo sapiens	+
cell part;collagen;collagen type XI;cytoplasmic part;endoplasmic reticulum lumen;endoplasmic reticulum part;extracellular matrix part;extracellular region;extracellular region part;fibrillar collagen;intracellular organelle lumen;i	Amoebiasis;ECM-receptor interaction;Focal adhesion;Protein digestion and absorption	Homo sapiens	+
anchoring collagen;cell part;collagen;collagen type VI;cytoplasmic part;endoplasmic reticulum lumen;endoplasmic reticulum part;extracellular matrix;extracellular matrix part;extracellular membrane-bounded organelle;extrac	ECM-receptor interaction;Focal adhesion;Protein digestion and absorption	Homo sapiens	+
cell part;collagen;cytoplasmic part;endoplasmic reticulum lumen;endoplasmic reticulum part;extracellular matrix;extracellular matrix part;extracellular membrane-bounded organelle;extracellular organelle;extracellular region;e	ECM-receptor interaction;Focal adhesion;Protein digestion and absorption	Homo sapiens	+
anchoring collagen;cell part;collagen;collagen type VI;cytoplasmic part;endoplasmic reticulum lumen;endoplasmic reticulum part;extracellular matrix;extracellular matrix part;extracellular membrane-bounded organelle;extrac	ECM-receptor interaction;Focal adhesion;Protein digestion and absorption	Homo sapiens	+
cell part;cytoplasmic part;integral to membrane;intracellular membrane-bounded organelle;intracellular organelle;intracellular organelle part;intracellular part;intrinsic to membrane;macromolecular complex;membrane;membra	Calcium signaling pathway;Huntington's disease;Parkinson's disease	Homo sapiens	+
cell part;cytoplasmic membrane-bounded vesicle lumen;cytoplasmic part;cytoplasmic vesicle part;endoplasmic reticulum;extracellular region;extracellular region part;extracellular space;Golgi apparatus;intracellular membrar	Complement and coagulation cascades	Homo sapiens	+
cell part;cytoplasm;cytoplasmic part;cytosol;extracellular membrane-bounded organelle;extracellular organelle;extracellular region part;extracellular vesicular exosome;intracellular membrane-bounded organelle;intracellular c	Drug metabolism - other enzymes;Purine metabolism	Homo sapiens	+
cell part;chromosomal part;cytoplasm;cytoplasmic dynein complex;cytoplasmic part;cytoskeletal part;dynein complex;envelope;extrinsic to membrane;inclusion body;intracellular membrane-bounded organelle;intracellular no	MAPK signaling pathway - yeast;Pathways in cancer;RNA transport;Thyroid cancer	Homo sapiens	+
cell part;cytoplasmic part;cytosol;extracellular membrane-bounded organelle;extracellular organelle;extracellular region part;extracellular vesicular exosome;intracellular membrane-bounded organelle;intracellular organelle;int	Arginine and proline metabolism	Homo sapiens	
cell part;cytoplasm;cytoplasmic membrane-bounded vesicle;cytoplasmic part;cytoplasmic vesicle;cytoplasmic vesicle membrane;cytoplasmic vesicle part;endocytic vesicle membrane;extracellular membrane-bounded organelle;extracellular organelle;extracellular region part;extracellular vesicular exosome;intracellular membrane-bounded organelle;intracellular organell		Homo sapiens	+
cell part;cytoplasmic part;dihydrolipoyl dehydrogenase complex;intracellular membrane-bounded organelle;intracellular organelle;intracellular organelle lumen;intracellular organelle part;intracellular part;macromolecular comp	Valine, leucine and isoleucine degradation	Homo sapiens	
actin filament bundle;actomyosin;adherens junction;anchoring junction;cell cortex part;cell junction;cell part;cell projection;cell projection part;cell-cell adherens junction;cell-cell junction;cell-substrate adherens junction;cell-s	Adherens junction;Amoebiasis;Arrhythmogenic right ventricular cardiomyopathy (ARVC);Focal adhesion;Leukocyte transendothelial migration;Reg	Homo sapiens	+
extracellular membrane-bounded organelle;extracellular organelle;extracellular region part;extracellular vesicular exosome;membrane-bounded organelle;membrane-bounded vesicle;organelle;vesicle		Homo sapiens	+
cell part;chromosomal part;cytoplasmic part;cytosol;intracellular membrane-bounded organelle;intracellular non-membrane-bounded organelle;intracellular organelle;intracellular organelle part;intracellular part;Ku70:Ku80 cor	Non-homologous end-joining	Homo sapiens	+
cell part;chromosomal part;chromosome, telomeric region;cytoplasmic part;cytosol;intracellular membrane-bounded organelle;intracellular non-membrane-bounded organelle;intracellular organelle;intracellular organelle part;	Non-homologous end-joining	Homo sapiens	+
cell part;cytoplasmic part;extracellular membrane-bounded organelle;extracellular organelle;extracellular region part;extracellular vesicular exosome;intracellular membrane-bounded organelle;intracellular organelle;intracellula	Alzheimer's disease;Cardiac muscle contraction;Huntington's disease;Oxidative phosphorylation;Parkinson's disease	Homo sapiens	+
cell part;integral to membrane;intrinsic to membrane;membrane;membrane part;plasma membrane		Homo sapiens	+
cell part;cytoplasmic part;cytoplasmic vesicle membrane;cytoplasmic vesicle part;endocytic vesicle membrane;endosomal part;endosome;endosome membrane;extracellular membrane-bounded organelle;extracellular orgar	ko05152;Lysosome;Phagosome	Homo sapiens	+
angiogenin-PRI complex;cell part;cytoplasm;extracellular membrane-bounded organelle;extracellular organelle;extracellular region part;extracellular vesicular exosome;intracellular part;macromolecular complex;membrane-bounded organelle;membrane-bounded vesicle;organelle;protein complex;vesicle		Homo sapiens	+
cell part;cytoplasmic part;extracellular matrix;extracellular region;extracellular region part;extracellular space;Golgi apparatus;intracellular membrane-bounded organelle;intracellular organelle;intracellular part;membrane-bounded organelle;membrane-bounded vesicle;organelle;proteinaceous extracellular matrix;vesicle		Homo sapiens	+
anchored to membrane;cell part;cell surface;cytoplasmic part;external side of plasma membrane;extracellular membrane-bounded organelle;extracellular organelle;extracellular region part;extracellular vesicular exosome;Gol	Cell adhesion molecules (CAMs);Prion diseases	Homo sapiens	+
adherens junction;alpha4-beta7 integrin complex;anchoring junction;cell junction;cell part;cell surface;cell-cell junction;cell-substrate adherens junction;cell-substrate junction;external side of plasma membrane;extracellular r	Arrhythmogenic right ventricular cardiomyopathy (ARVC);Cell adhesion molecules (CAMs);Dilated cardiomyopathy;ECM-receptor interaction;Foca	Homo sapiens	+
aggresome;cell part;cytoplasm;cytoplasmic part;cytosol;extracellular membrane-bounded organelle;extracellular organelle;extracellular region part;extracellular vesicular exosome;inclusion body;intracellular membrane-bounded organelle;intracellular organelle;intracellular part;macromolecular complex;membrane;membrane-bounded organelle;membrane-bounded vesi		Homo sapiens	+
cell part;cytoplasm;cytoskeletal part;extracellular membrane-bounded organelle;extracellular organelle;extracellular region part;extracellular space;extracellular vesicular exosome;intermediate filament;intracellular membrane-bounded organelle;intracellular organelle;intracellular organelle part;intracellular part;keratin filament;macromolecular complex;membrane;membra		Homo sapiens	+
cell part;cytoplasm;cytoplasmic part;cytoskeletal part;cytosol;extracellular membrane-bounded organelle;extracellular organelle;extracellular region part;extracellular vesicular exosome;intermediate filament;intracellular membrane-bounded organelle;intracellular organelle;intracellular organelle part;intracellular part;keratin filament;macromolecular complex;membrane;me		Homo sapiens	+
cell part;cell surface;cytoplasmic membrane-bounded vesicle;cytoplasmic part;cytoplasmic vesicle;endoplasmic reticulum;endoplasmic reticulum lumen;endoplasmic reticulum part;intracellular membrane-bounded organelle	Protein processing in endoplasmic reticulum;Vibrio cholerae infection	Homo sapiens	+
cell part;cytoplasmic part;endoplasmic reticulum;endoplasmic reticulum lumen;endoplasmic reticulum part;intracellular membrane-bounded organelle;intracellular organelle;intracellular organelle lumen;intracellular organelle p	Arginine and proline metabolism	Homo sapiens	+
cell part;cytoplasm;cytoplasmic part;endosome;extracellular membrane-bounded organelle;extracellular organelle;extracellular region part;extracellular space;extracellular vesicular exosome;intracellular membrane-bounded organelle;intracellular organelle;intracellular part;late endosome;membrane-bounded organelle;membrane-bounded vesicle;multivesicular body;nuc		Homo sapiens	+
cell part;cell surface;extracellular matrix;extracellular membrane-bounded organelle;extracellular organelle;extracellular region part;extracellular space;extracellular vesicular exosome;integral to membrane;intrinsic to external	Complement and coagulation cascades	Homo sapiens	+
actin cytoskeleton;actin filament;actin filament bundle;adherens junction;anchoring junction;cell junction;cell part;cell projection;cell projection membrane;cell projection part;cell-substrate adherens junction;cell-substrate junction;cytoplasm;cytoplasmic part;cytoskeletal part;cytoskeleton;cytosol;extracellular membrane-bounded organelle;extracellular organelle;extracell		Homo sapiens	+
cell part;cytoplasm;intracellular part		Homo sapiens	+
cell part;cytoplasm;extracellular membrane-bounded organelle;extracellular organelle;extracellular region part;extracellular vesicular exosome;intracellular organelle part;intracellular part;membrane;membrane-bounded organelle;membrane-bounded vesicle;nuclear membrane;nuclear part;organelle;organelle membrane;organelle part;vesicle		Homo sapiens	+
cell part;cytoplasmic part;extracellular membrane-bounded organelle;extracellular organelle;extracellular region part;extracellular vesicular exosome;intracellular membrane-bounded organelle;intracellular organelle;intracellular organelle lumen;intracellular organelle part;intracellular part;membrane-bounded organelle;membrane-bounded vesicle;membrane-enclosed lum		Homo sapiens	+
cell part;cytoplasm;cytoplasmic part;cytosol;inclusion body;intracellular part;membrane	Insulin signaling pathway;Starch and sucrose metabolism	Homo sapiens	
adherens junction;AMP-activated protein kinase complex;anchoring junction;cAMP-dependent protein kinase complex;cell junction;cell part;cell-substrate adherens junction;cell-substrate junction;centrosome;cytoplasm;cyto	Apoptosis;Insulin signaling pathway	Homo sapiens	+
cell part;cytoskeleton;intracellular membrane-bounded organelle;intracellular non-membrane-bounded organelle;intracellular organelle;intracellular organelle part;intracellular part;macromolecular complex;membrane-bounde	Basal transcription factors	Homo sapiens	+
adherens junction;anchored to external side of plasma membrane;anchored to membrane;anchored to plasma membrane;anchoring junction;cell junction;cell part;cell surface;cell-substrate adherens junction;cell-substrate ju	Complement and coagulation cascades;Hematopoietic cell lineage	Homo sapiens	+
cell part;cytoplasmic part;extracellular region part;extracellular space;intracellular membrane-bounded organelle;intracellular organelle;intracellular part;membrane-bounded organelle;mitochondrion;organelle	One carbon pool by folate	Homo sapiens	+
cell part;cell surface;cytoplasm;extracellular membrane-bounded organelle;extracellular organelle;extracellular region;extracellular region part;extracellular space;extracellular vesicular exosome;intracellular organelle part;intra	Phenylalanine metabolism;Tyrosine metabolism	Homo sapiens	
adherens junction;anchoring junction;cell junction;cell part;cell-substrate adherens junction;cell-substrate junction;cytoplasm;focal adhesion;integral to membrane;integral to plasma membrane;intracellular part;intrinsic to me	Cell adhesion molecules (CAMs);Leukocyte transendothelial migration	Homo sapiens	+
cell part;cytoplasmic part;endoplasmic reticulum;endoplasmic reticulum lumen;endoplasmic reticulum part;intracellular;intracellular membrane-bounded organelle;intracellular organelle;intracellular organelle lumen;intracellula	Protein processing in endoplasmic reticulum	Homo sapiens	+
cell part;cytoplasm;cytoplasmic part;cytosol;intracellular membrane-bounded organelle;intracellular organelle;intracellular organelle part;intracellular part;membrane-bounded organelle;mitochondrion;nuclear part;nucleoplasr	Terpenoid backbone biosynthesis	Homo sapiens	+
basal lamina;basement membrane;cell part;cell periphery;extracellular matrix;extracellular matrix part;extracellular membrane-bounded organelle;extracellular organelle;extracellular region;extracellular region part;extracellular vesicular exosome;membrane-bounded organelle;membrane-bounded vesicle;organelle;vesicle		Homo sapiens	+
apical plasma membrane;cell part;cytoplasmic part;cytosol;extracellular membrane-bounded organelle;extracellular organelle;extracellular region part;extracellular space;extracellular vesicular exosome;intracellular part;mem	Caprolactam degradation;Glycerolipid metabolism;Glycolysis / Gluconeogenesis	Homo sapiens	+
cell part;cell projection;cilium;cytoplasm;cytoplasmic part;cytosol;extracellular matrix;extracellular membrane-bounded organelle;extracellular organelle;extracellular region part;extracellular vesicular exosome;intracellular me	Carbon fixation in photosynthetic organisms;Glycolysis / Gluconeogenesis;Purine metabolism;Pyruvate metabolism;Type II diabetes mellitus	Homo sapiens	
adherens junction;anchoring junction;cell junction;cell part;cell-substrate adherens junction;cell-substrate junction;cytoplasmic membrane-bounded vesicle;cytoplasmic membrane-bounded vesicle lumen;cytoplasmic part;cy	NOD-like receptor signaling pathway;Pathways in cancer;Plant-pathogen interaction;Prostate cancer;Protein processing in endoplasmic reticulum	Homo sapiens	+
cell part;centrosome;chromosomal part;condensed chromosome outer kinetochore;condensed nuclear chromosome outer kinetochore;cytoplasm;cytoplasmic part;cytoskeletal part;cytosol;intracellular membrane-bounded c	Cell cycle;p53 signaling pathway;Progesterone-mediated oocyte maturation	Homo sapiens	+
catalytic step 2 spliceosome;cell part;cytoplasmic part;cytosol;extracellular membrane-bounded organelle;extracellular organelle;extracellular region part;extracellular vesicular exosome;histone pre-mRNA 3'end processing c	Spliceosome;Systemic lupus erythematosus	Homo sapiens	
cell part;cell surface;cytoplasm;cytoplasmic part;cytosol;cytosolic part;cytosolic proteasome complex;extracellular region part;extracellular space;intracellular membrane-bounded organelle;intracellular organelle;intracellular c	Alzheimer's disease	Homo sapiens	
cell part;cytoplasmic part;intracellular membrane-bounded organelle;intracellular organelle;intracellular organelle part;intracellular part;membrane;membrane-bounded organelle;membrane-enclosed lumen;mitochondrial inner	Alzheimer's disease;Cardiac muscle contraction;Huntington's disease;Oxidative phosphorylation;Parkinson's disease	Homo sapiens	+
cell part;cytoplasm;extracellular membrane-bounded organelle;extracellular organelle;extracellular region part;extracellular vesicular exosome;intracellular membrane-bounded organelle;intracellular organelle;intracellular organelle part;intracellular part;macromolecular complex;membrane;membrane-bounded organelle;membrane-bounded vesicle;nuclear part;nucleoplas		Homo sapiens	+
cell part;cytoplasm;cytoplasmic part;cytosol;extracellular membrane-bounded organelle;extracellular organelle;extracellular region part;extracellular vesicular exosome;intracellular part;membrane;membrane-bounded organe	Aminoacyl-tRNA biosynthesis	Homo sapiens	
actin cytoskeleton;adherens junction;anchoring junction;apicolateral plasma membrane;catenin complex;catenin-TCF7L2 complex;cell junction;cell part;cell-cell adherens junction;cell-cell contact zone;cell-cell junction;cell-s	Acute myeloid leukemia;Arrhythmogenic right ventricular cardiomyopathy (ARVC);Pathways in cancer	Homo sapiens	+
cell part;cytoplasmic part;intracellular organelle part;intracellular part;macromolecular complex;membrane;membrane part;mitochondrial inner membrane;mitochondrial membrane;mitochondrial membrane part;mitochondrial	Alzheimer's disease;Cardiac muscle contraction;Huntington's disease;Oxidative phosphorylation;Parkinson's disease	Homo sapiens	
axon part;axon terminus;cell part;cell projection part;cytoplasm;cytoplasmic part;cytosol;endoplasmic reticulum;extracellular membrane-bounded organelle;extracellular organelle;extracellular region part;extracellular vesicula	Alanine, aspartate and glutamate metabolism;Arginine and proline metabolism;Nitrogen metabolism;Two-component system	Homo sapiens	+
cell part;cytoplasm;cytoplasmic part;cytosol;extracellular membrane-bounded organelle;extracellular organelle;extracellular region part;extracellular space;extracellular vesicular exosome;intracellular organelle part;intracellula	Fructose and mannose metabolism;Galactose metabolism;Glycerolipid metabolism;Pentose and glucuronate interconversions;Pyruvate metabolis	Homo sapiens	+
cell part;cytoplasmic part;cytosol;endoplasmic reticulum-Golgi intermediate compartment;external side of plasma membrane;extracellular membrane-bounded organelle;extracellular organelle;extracellular region part;extrace	Glutathione metabolism;Hematopoietic cell lineage;Renin-angiotensin system	Homo sapiens	+
adherens junction;anchoring junction;cell junction;cell part;cell surface;cell-substrate adherens junction;cell-substrate junction;cytoplasm;extracellular membrane-bounded organelle;extracellular organelle;extracellular region	Cell adhesion molecules (CAMs)	Homo sapiens	+
cell part;intracellular	mRNA surveillance pathway	Homo sapiens	
anchoring junction;basolateral plasma membrane;brush border membrane;cell junction;cell part;cell projection;cell projection membrane;cell projection part;cell-cell junction;cytoplasmic part;desmosome;external encapsulati	Galactose metabolism;Glycosaminoglycan biosynthesis - keratan sulfate;Glycosphingolipid biosynthesis - lacto and neolacto series;N-Glycan bio	Homo sapiens	
actin cytoskeleton;actin filament;adherens junction;anchoring junction;apical part of cell;apical plasma membrane;basolateral plasma membrane;brush border;cell body;cell cortex part;cell junction;cell part;cell periphery;cell	Gastric acid secretion;Leukocyte transendothelial migration;Pathogenic Escherichia coli infection;Regulation of actin cytoskeleton	Homo sapiens	+
cell part;cytoplasm;extracellular membrane-bounded organelle;extracellular organelle;extracellular region part;extracellular vesicular exosome;intracellular membrane-bounded organelle;intracellular organelle;intracellular organelle part;intracellular part;membrane-bounded organelle;membrane-bounded vesicle;nuclear part;nucleoplasm;nucleus;organelle;organelle part;ve		Homo sapiens	+
cell part;intracellular membrane-bounded organelle;intracellular organelle;intracellular organelle part;intracellular part;membrane-bounded organelle;nuclear part;nucleoplasm;nucleus;organelle;organelle part	Osteoclast differentiation	Homo sapiens	+
cell part;cell projection membrane;cell projection part;centrosome;cytoplasm;cytoplasmic part;cytoskeletal part;cytosol;extracellular membrane-bounded organelle;extracellular organelle;extracellular region part;extracellular v	Purine metabolism;Pyrimidine metabolism	Homo sapiens	
cell body;cell part;cytoplasm;cytoplasmic part;cytosol;extracellular membrane-bounded organelle;extracellular organelle;extracellular region part;extracellular vesicular exosome;intracellular part;membrane-bounded organelle;membrane-bounded vesicle;neuronal cell body;organelle;vesicle		Homo sapiens	+
cell part;cytoplasmic part;extracellular membrane-bounded organelle;extracellular organelle;extracellular region part;extracellular vesicular exosome;intracellular organelle lumen;intracellular organelle part;intracellular part;lyso	Glycosaminoglycan degradation;Lysosome	Homo sapiens	+
adherens junction;anchoring junction;cell junction;cell part;cell-substrate adherens junction;cell-substrate junction;cytoplasm;cytoplasmic part;cytosol;cytosolic small ribosomal subunit;extracellular membrane-bounded orga	Ribosome	Homo sapiens	+
cell part;chromatin;chromosomal part;DNA replication factor A complex;intracellular membrane-bounded organelle;intracellular organelle;intracellular organelle part;intracellular part;macromolecular complex;membrane-bounc	DNA replication;Homologous recombination;Mismatch repair;Nucleotide excision repair	Homo sapiens	+
cell part;cytoplasmic part;integral to membrane;intracellular membrane-bounded organelle;intracellular organelle;intracellular organelle part;intracellular part;intrinsic to membrane;membrane;membrane part;membrane-bounc	Alzheimer's disease;Cardiac muscle contraction;Huntington's disease;Oxidative phosphorylation;Parkinson's disease	Homo sapiens	+
basement membrane;cell body;cell part;cell projection part;cell surface;extracellular matrix;extracellular matrix part;extracellular membrane-bounded organelle;extracellular organelle;extracellular region;extracellular region part;extracellular space;extracellular vesicular exosome;growth cone;membrane-bounded organelle;membrane-bounded vesicle;neuronal cell body;o		Homo sapiens	+
adherens junction;anchoring junction;basolateral plasma membrane;cell junction;cell part;cell surface;cell-substrate adherens junction;cell-substrate junction;cytoplasm;cytoplasmic part;external side of plasma membrane;ex	ECM-receptor interaction;Hematopoietic cell lineage;Shigellosis	Homo sapiens	+
cell part;chromatin;chromosomal part;chromosome;condensed chromosome;condensed nuclear chromosome;extracellular membrane-bounded organelle;extracellular organelle;extracellular region part;extracellular vesicular	Systemic lupus erythematosus	Homo sapiens	+
cell part;cytoplasmic part;cytosol;extracellular membrane-bounded organelle;extracellular organelle;extracellular region part;extracellular vesicular exosome;intracellular part;membrane-bounded organelle;membrane-bounde	Arachidonic acid metabolism	Homo sapiens	
cell junction;cell part;cell-cell junction;cytoplasm;cytoplasmic part;cytosol;endoplasmic reticulum;intracellular membrane-bounded organelle;intracellular non-membrane-bounded organelle;intracellular organelle;intracellular p	ErbB signaling pathway;Pathogenic Escherichia coli infection;T cell receptor signaling pathway	Homo sapiens	+
cell part;chromatin;chromosomal part;extracellular membrane-bounded organelle;extracellular organelle;extracellular region part;extracellular vesicular exosome;heterochromatin;intracellular organelle part;intracellular part;macromolecular complex;membrane-bounded organelle;membrane-bounded vesicle;nuclear chromatin;nuclear chromosome part;nuclear heterochro		Homo sapiens	+
cell part;chromatin;chromosomal part;euchromatin;intracellular membrane-bounded organelle;intracellular organelle;intracellular organelle part;intracellular part;macromolecular complex;membrane-bounded organelle;nuclear chromatin;nuclear chromosome part;nuclear euchromatin;nuclear part;nucleosome;nucleus;organelle;organelle part;protein-DNA complex		Homo sapiens	+
cell part;cytoplasmic part;endoplasmic reticulum membrane;endoplasmic reticulum part;intracellular membrane-bounded organelle;intracellular organelle;intracellular organelle part;intracellular part;membrane;membrane part;membrane-bounded organelle;mitochondrion;organelle;organelle membrane;organelle part		Homo sapiens	+
cell part;cytoplasmic part;endoplasmic reticulum;endoplasmic reticulum membrane;endoplasmic reticulum part;integral to membrane;integral to plasma membrane;intracellular membrane-bounded organelle;intracellular orga	Alzheimer's disease;Calcium signaling pathway;Pancreatic secretion	Homo sapiens	+
cell part;cytoplasmic part;cytosol;extracellular membrane-bounded organelle;extracellular organelle;extracellular region part;extracellular vesicular exosome;intracellular part;membrane-bounded organelle;membrane-bounde	Styrene degradation;Tyrosine metabolism	Homo sapiens	+
cell part;cell projection;cytoplasm;cytoplasmic part;cytoskeletal part;cytosol;extracellular membrane-bounded organelle;extracellular organelle;extracellular region part;extracellular vesicular exosome;intracellular;intracellular	MAPK signaling pathway	Homo sapiens	+
cell junction;cell part;cell-cell junction;cytoplasm;cytoplasmic part;intracellular membrane-bounded organelle;intracellular organelle;intracellular part;macromolecular complex;membrane-bounded organelle;nucleus;occluding	Tight junction	Homo sapiens	
adherens junction;anchoring junction;cell junction;cell part;cell-substrate adherens junction;cell-substrate junction;chromatin;chromosomal part;cytoplasmic part;cytosol;focal adhesion;heterochromatin;intracellular membrane-bounded organelle;intracellular organelle;intracellular organelle part;intracellular part;macromolecular complex;membrane-bounded organelle;nuc		Homo sapiens	+
axon part;axon terminus;cell part;cell projection part;cytoplasm;cytoplasmic part;cytosol;extracellular membrane-bounded organelle;extracellular organelle;extracellular region part;extracellular vesicular exosome;intracellular	Alanine, aspartate and glutamate metabolism;Arginine and proline metabolism;Cysteine and methionine metabolism;Phenylalanine metabolism;Ph	Homo sapiens	+
apical part of cell;cell body;cell part;cell projection;cilium;cytoplasm;cytoplasmic part;cytosol;dendrite;endoplasmic reticulum;extracellular membrane-bounded organelle;extracellular organelle;extracellular region part;extrace	African trypanosomiasis;Aldosterone-regulated sodium reabsorption;Amoebiasis;Calcium signaling pathway;Endocrine and other factor-regulated	Homo sapiens	+
cell part;chromatin;chromosomal part;intracellular organelle part;intracellular part;macromolecular complex;nuclear chromatin;nuclear chromosome part;nuclear part;nucleoplasm;nucleoplasm part;organelle part;protein comp	Osteoclast differentiation	Homo sapiens	+
adherens junction;alpha2-beta1 integrin complex;anchoring junction;axon part;axon terminus;basal part of cell;cell junction;cell outer membrane;cell part;cell projection part;cell surface;cell-substrate adherens junction;cell-su	Arrhythmogenic right ventricular cardiomyopathy (ARVC);Dilated cardiomyopathy;ECM-receptor interaction;Focal adhesion;Hematopoietic cell line	Homo sapiens	+
adherens junction;anchoring junction;apical plasma membrane;cell junction;cell part;cell-cell adherens junction;cell-cell contact zone;cell-cell junction;cell-substrate adherens junction;cell-substrate junction;connexon comple	Arrhythmogenic right ventricular cardiomyopathy (ARVC);Gap junction	Homo sapiens	+
cell part;intracellular non-membrane-bounded organelle;intracellular organelle;intracellular organelle part;intracellular part;non-membrane-bounded organelle;nuclear part;nucleolus;nucleoplasm;organelle;organelle part		Homo sapiens	+
cell part;cytoplasmic part;intracellular membrane-bounded organelle;intracellular organelle;intracellular organelle part;intracellular part;macromolecular complex;membrane;membrane part;membrane-bounded organelle;mem	Alzheimer's disease;Huntington's disease;Oxidative phosphorylation;Parkinson's disease	Homo sapiens	
AMP-activated protein kinase complex;calcium channel complex;cAMP-dependent protein kinase complex;cation channel complex;cell part;cell projection;centrosome;cilium;cytoplasmic part;cytoskeletal part;cytosol;extrac	Amoebiasis;Apoptosis;Bile secretion;Calcium signaling pathway;Chemokine signaling pathway;Dilated cardiomyopathy;Endocrine and other facto	Homo sapiens	
adherens junction;anchoring junction;cell cortex part;cell junction;cell part;cell projection;cell-substrate adherens junction;cell-substrate junction;chromatin;chromosomal part;cortical actin cytoskeleton;cortical cytoskeleton;c	Alzheimer's disease;Apoptosis;Focal adhesion;Protein processing in endoplasmic reticulum	Homo sapiens	
cell part;cytoplasmic part;cytosol;intracellular part;membrane	Pyrimidine metabolism	Homo sapiens	+
adherens junction;anchoring junction;blood microparticle;cell junction;cell part;cell surface;cell-substrate adherens junction;cell-substrate junction;cytoplasm;endothelial microparticle;external side of plasma membrane;extracellular region part;extracellular space;focal adhesion;intracellular organelle part;intracellular part;macromolecular complex;membrane part;nuclear		Homo sapiens	+
catalytic step 2 spliceosome;cell part;cytoplasm;extracellular membrane-bounded organelle;extracellular organelle;extracellular region part;extracellular vesicular exosome;intracellular membrane-bounded organelle;intracellu	Spliceosome	Homo sapiens	+
6-phosphofructokinase complex;cell part;cytoplasmic part;cytosol;cytosolic part;extracellular membrane-bounded organelle;extracellular organelle;extracellular region part;extracellular vesicular exosome;intracellular part;ma	Fructose and mannose metabolism;Galactose metabolism;Glycolysis / Gluconeogenesis;Methane metabolism;Pentose phosphate pathway	Homo sapiens	
apical cortex;cell cortex part;cell part;cytoplasmic part;extracellular membrane-bounded organelle;extracellular organelle;extracellular region part;extracellular vesicular exosome;hydrogen:potassium-exchanging ATPase com	Lysosome	Homo sapiens	
cell part;cytoplasm;cytoplasmic part;external side of plasma membrane;extracellular matrix;extracellular membrane-bounded organelle;extracellular organelle;extracellular region part;extracellular space;extracellular vesicular exosome;immunological synapse;intracellular membrane-bounded organelle;intracellular organelle;intracellular organelle part;intracellular part;mac		Homo sapiens	+
cell part;cytoplasmic mRNA processing body;cytoplasmic part;cytosol;cytosolic part;cytosolic proteasome complex;intracellular membrane-bounded organelle;intracellular non-membrane-bounded organelle;intracellular orga	Proteasome	Homo sapiens	+
acrosomal vesicle;cell body;cell part;centrosome;chaperonin-containing T-complex;chromatin;chromosomal part;cytoplasmic membrane-bounded vesicle;cytoplasmic part;cytoplasmic vesicle;cytoskeletal part;cytosol;cytosolic part;extracellular membrane-bounded organelle;extracellular organelle;extracellular region part;extracellular vesicular exosome;Golgi apparatus;		Homo sapiens	+
cell part;cytoplasmic part;cytoplasmic vesicle;cytosol;early endosome;endoplasmic reticulum;endosome;intracellular membrane-bounded organelle;intracellular organelle;intracellular part;membrane-bounded organelle;organ	Adherens junction;Insulin signaling pathway	Homo sapiens	+
cell part;cytoplasmic part;cytosol;cytosolic large ribosomal subunit;extracellular membrane-bounded organelle;extracellular organelle;extracellular region part;extracellular vesicular exosome;intracellular membrane-bounded	Ribosome	Homo sapiens	+
adherens junction;alphav-beta5 integrin complex;anchoring junction;cell junction;cell leading edge;cell part;cell surface;cell-substrate adherens junction;cell-substrate junction;cytoplasmic membrane-bounded vesicle;cytopla	Arrhythmogenic right ventricular cardiomyopathy (ARVC);Dilated cardiomyopathy;ECM-receptor interaction;Focal adhesion;Hypertrophic cardiomy	Homo sapiens	+
cell part;cell projection membrane;cell projection part;cytoplasmic part;cytosol;extracellular membrane-bounded organelle;extracellular organelle;extracellular region part;extracellular vesicular exosome;Golgi apparatus;intracellular membrane-bounded organelle;intracellular organelle;intracellular part;leading edge membrane;membrane;membrane part;membrane-bound		Homo sapiens	+

adherens junction;anchoring junction;cell junction;cell part;cell-substrate adherens junction;cell-substrate junction;cytoplasm;cytoplasmic part;cytosol;cytosolic large ribosomal subunit;extracellular membrane-bounded orgar	Ribosome	Homo sapiens	+
actin cytoskeleton;adherens junction;anchoring junction;cell junction;cell part;cell-cell adherens junction;cell-cell junction;cell-substrate adherens junction;cell-substrate junction;contractile fiber part;costamere;cytoplasmic pa	Adherens junction;Amoebiasis;Bacterial invasion of epithelial cells;Focal adhesion;Leukocyte transendothelial migration;Regulation of actin cytosk	Homo sapiens	+
cell part;intracellular organelle part;intracellular part;nuclear body;nuclear part;nuclear speck;nucleoplasm;nucleoplasm part;organelle part		Homo sapiens	+
cell part;cytoplasmic part;intracellular membrane-bounded organelle;intracellular organelle;intracellular organelle part;intracellular part;macromolecular complex;membrane-bounded organelle;mitochondrion;NELF complex;nuclear part;nucleoplasm;nucleoplasm part;nucleus;organelle;organelle part;protein complex;transcription elongation factor complex		Homo sapiens	
cell part;cytoplasmic part;cytosol;cytosolic large ribosomal subunit;intracellular membrane-bounded organelle;intracellular organelle;intracellular organelle part;intracellular part;large ribosomal subunit;macromolecular comple	Ribosome	Homo sapiens	+
cell part;cytoplasm;cytoplasmic part;cytosol;extracellular membrane-bounded organelle;extracellular organelle;extracellular region part;extracellular vesicular exosome;intracellular part;membrane;membrane-bounded organe	Glycolysis / Gluconeogenesis;Methane metabolism	Homo sapiens	+
cell part;chromatin;chromosomal part;chromosome;condensed chromosome;condensed nuclear chromosome;cytoplasm;intracellular membrane-bounded organelle;intracellular non-membrane-bounded organelle;intracellular organelle;intracellular organelle part;intracellular part;membrane;membrane-bounded organelle;non-membrane-bounded organelle;nuclear chrom		Homo sapiens	+
cell part;intracellular membrane-bounded organelle;intracellular organelle;intracellular organelle part;intracellular part;membrane-bounded organelle;nuclear part;nucleoplasm;nucleus;organelle;organelle part		Homo sapiens	
cell part;cytoplasmic part;Golgi apparatus;intracellular membrane-bounded organelle;intracellular organelle;intracellular organelle part;intracellular part;membrane-bounded organelle;mitochondrion;nuclear part;nucleoplasm;r	Base excision repair;DNA replication;Mismatch repair;Nucleotide excision repair	Homo sapiens	
cell part;cytoplasmic part;intracellular membrane-bounded organelle;intracellular organelle;intracellular organelle part;intracellular part;macromolecular complex;membrane;membrane part;membrane-bounded organelle;mitoc	Alzheimer's disease;Huntington's disease;Oxidative phosphorylation;Parkinson's disease	Homo sapiens	+
cell part;intracellular membrane-bounded organelle;intracellular organelle;intracellular organelle part;intracellular part;membrane-bounded organelle;nuclear part;nucleoplasm;nucleus;organelle;organelle part	Base excision repair	Homo sapiens	+
adherens junction;anchoring junction;apical plasma membrane;catenin complex;cell junction;cell part;cell projection;cell-cell adherens junction;cell-cell contact zone;cell-cell junction;cell-substrate adherens junction;cell-subs	Arrhythmogenic right ventricular cardiomyopathy (ARVC);Cell adhesion molecules (CAMs)	Homo sapiens	+
cell junction;cell part;cell projection;cell-cell junction;cytoplasm;cytoplasmic part;cytosol;intracellular organelle part;intracellular part;lamellipodium;macromolecular complex;membrane;nuclear part;organelle part;plasma mem	Calcium signaling pathway;Epithelial cell signaling in Helicobacter pylori infection;ErbB signaling pathway;Fc epsilon RI signaling pathway;Fc gam	Homo sapiens	+
cell cortex;cell part;cytoplasmic part;extracellular membrane-bounded organelle;extracellular organelle;extracellular region part;extracellular vesicular exosome;intracellular membrane-bounded organelle;intracellular non-mem	Pathogenic Escherichia coli infection	Homo sapiens	+
cell part;cytoplasmic part;cytosol;intracellular membrane-bounded organelle;intracellular organelle;intracellular organelle part;intracellular part;membrane;membrane part;membrane raft;membrane-bounded organelle;mitocho	Amino sugar and nucleotide sugar metabolism;Butirosin and neomycin biosynthesis;Carbohydrate digestion and absorption;Fructose and mannos	Homo sapiens	+
cell part;cytoplasm;cytoskeleton;DNA-directed RNA polymerase complex;DNA-directed RNA polymerase II, core complex;intracellular membrane-bounded organelle;intracellular non-membrane-bounded organelle;intracellula	Huntington's disease;Purine metabolism;Pyrimidine metabolism;RNA polymerase	Homo sapiens	+
cell part;cytoplasmic part;cytosol;DNA-directed RNA polymerase complex;DNA-directed RNA polymerase I complex;DNA-directed RNA polymerase II, core complex;DNA-directed RNA polymerase III complex;intracellular me	Cytosolic DNA-sensing pathway;Huntington's disease;Purine metabolism;Pyrimidine metabolism;RNA polymerase	Homo sapiens	
carboxy-terminal domain protein kinase complex;cell part;core TFIIH complex;holo TFIIH complex;intracellular membrane-bounded organelle;intracellular organelle;intracellular organelle part;intracellular part;macromolecular	Basal transcription factors;Nucleotide excision repair	Homo sapiens	+
cell part;cytoplasm;cytoplasmic part;cytosol;intracellular membrane-bounded organelle;intracellular non-membrane-bounded organelle;intracellular organelle;intracellular part;macromolecular complex;membrane;membrane-	Hepatitis C;Measles;Protein processing in endoplasmic reticulum	Homo sapiens	
cell part;cytoplasmic part;cytosol;intracellular part	Arginine and proline metabolism;beta-Alanine metabolism;Cysteine and methionine metabolism;Glutathione metabolism	Homo sapiens	+
cell part;cytoplasmic part;cytosol;intracellular membrane-bounded organelle;intracellular organelle;intracellular part;membrane-bounded organelle;nucleus;organelle	Adherens junction;Circadian rhythm - plant;Measles;Ribosome biogenesis in eukaryotes;Tight junction;Wnt signaling pathway	Homo sapiens	+
blood microparticle;extracellular membrane-bounded organelle;extracellular organelle;extracellular region;extracellular region part;extracellular vesicular exosome;membrane-bounded organelle;membrane-bounded vesicle;organelle;vesicle		Homo sapiens	+
blood microparticle;extracellular membrane-bounded organelle;extracellular organelle;extracellular region;extracellular region part;extracellular vesicular exosome;membrane-bounded organelle;membrane-bounded vesicle;organelle;vesicle		Homo sapiens	+
cell part;cytoplasm;cytoplasmic part;cytosol;I-kappaB/NF-kappaB complex;intracellular membrane-bounded organelle;intracellular organelle;intracellular organelle part;intracellular part;macromolecular complex;membrane-b	Acute myeloid leukemia;Adipocytokine signaling pathway;Amoebiasis;Apoptosis;B cell receptor signaling pathway;Chagas disease (American tryp	Homo sapiens	+
cell part;cytoplasm;extracellular region;intracellular membrane-bounded organelle;intracellular organelle;intracellular part;membrane-bounded organelle;nucleus;organelle	TGF-beta signaling pathway	Homo sapiens	+
cell part;extracellular membrane-bounded organelle;extracellular organelle;extracellular region part;extracellular vesicular exosome;integral to membrane;integral to plasma membrane;intracellular membrane-bounded organe	Calcium signaling pathway;Pancreatic secretion;Salivary secretion	Homo sapiens	+
cell part;cytoplasm;cytoplasmic part;cytosol;eukaryotic translation initiation factor 2 complex;intracellular part;macromolecular complex;protein complex	RNA transport	Homo sapiens	+
cell part;cytoplasmic part;cytosol;endoplasmic reticulum membrane;endoplasmic reticulum part;envelope;extracellular membrane-bounded organelle;extracellular organelle;extracellular region part;extracellular vesicular exosome;intracellular membrane-bounded organelle;intracellular organelle;intracellular organelle part;intracellular part;membrane;membrane part;memb		Homo sapiens	+
cell part;cytoplasm;intracellular membrane-bounded organelle;intracellular organelle;intracellular part;membrane-bounded organelle;nucleus;organelle		Homo sapiens	+
cell part;clathrin-coated vesicle;coated vesicle;cytoplasm;cytoplasmic membrane-bounded vesicle;cytoplasmic part;cytoplasmic vesicle;cytoplasmic vesicle membrane;cytoplasmic vesicle part;cytosol;endosome;extracellula	Tight junction	Homo sapiens	+
actin cytoskeleton;axon;axon part;axon terminus;cell body;cell part;cell projection;cell projection part;clathrin-coated vesicle;coated vesicle;cytoplasm;cytoplasmic membrane-bounded vesicle;cytoplasmic part;cytoplasmic v	Amoebiasis;Amyotrophic lateral sclerosis (ALS);Endocytosis;ko05152;Phagosome;Vasopressin-regulated water reabsorption	Homo sapiens	+
cell part;cytoplasmic part;cytoplasmic vesicle;cytosol;extracellular membrane-bounded organelle;extracellular organelle;extracellular region part;extracellular vesicular exosome;Golgi apparatus;Golgi apparatus part;Golgi membrane;intracellular membrane-bounded organelle;intracellular organelle;intracellular organelle part;intracellular part;membrane;membrane-bounde		Homo sapiens	+
cell part;cytoplasm;cytoplasmic part;cytosol;endoplasmic reticulum membrane;endoplasmic reticulum part;intracellular organelle part;intracellular part;membrane;membrane part;nuclear membrane;nuclear part;organelle mer	Measles	Homo sapiens	+
cell part;cytoplasm;cytoplasmic part;cytosol;extracellular membrane-bounded organelle;extracellular organelle;extracellular region part;extracellular vesicular exosome;intracellular membrane-bounded organelle;intracellular c	Proteasome	Homo sapiens	+
cell part;cytoplasmic part;endosome;integral to membrane;integral to plasma membrane;intracellular membrane-bounded organelle;intracellular organelle;intracellular organelle part;intracellular part;intrinsic to membrane;intri	Lysosome;Phagosome	Homo sapiens	+
cell part;cytoplasmic part;extracellular membrane-bounded organelle;extracellular organelle;extracellular region part;extracellular vesicular exosome;intracellular organelle part;intracellular part;membrane;membrane-boundec	Alzheimer's disease;Cardiac muscle contraction;Huntington's disease;Oxidative phosphorylation;Parkinson's disease	Homo sapiens	+
cell part;cytoskeletal part;envelope;intermediate filament;intracellular organelle part;intracellular part;lamin filament;macromolecular complex;membrane;nuclear envelope;nuclear inner membrane;nuclear membrane;nuclear part;nucleoplasm;organelle envelope;organelle inner membrane;organelle membrane;organelle part;protein complex		Homo sapiens	+
blood microparticle;extracellular membrane-bounded organelle;extracellular organelle;extracellular region;extracellular region part;extracellular vesicular exosome;membrane-bounded organelle;membrane-bounded vesicle;organelle;vesicle		Homo sapiens	+
cell part;cytoplasmic part;extracellular matrix;extracellular membrane-bounded organelle;extracellular organelle;extracellular region;extracellular region part;extracellular space;extracellular vesicular exosome;Golgi apparatus part;Golgi lumen;intracellular organelle lumen;intracellular organelle part;intracellular part;lysosomal lumen;membrane-bounded organelle;membrar		Homo sapiens	+
cell part;cytoplasm;cytoplasmic part;cytosol;intracellular part;membrane		Homo sapiens	+
cell junction;cell part;cytoplasm;cytoplasmic part;cytosol;intracellular membrane-bounded organelle;intracellular organelle;intracellular organelle part;intracellular part;membrane-bounded organelle;nuclear part;nucleoplasm;r	Drug metabolism - other enzymes;Purine metabolism	Homo sapiens	+
basement membrane;cell part;collagen;collagen type V;cytoplasmic part;endoplasmic reticulum lumen;endoplasmic reticulum part;extracellular matrix;extracellular matrix part;extracellular membrane-bounded organelle;extra	Amoebiasis;ECM-receptor interaction;Focal adhesion;Protein digestion and absorption	Homo sapiens	
cell part;cell projection;cytoplasm;cytoplasmic part;cytosol;intracellular part;intrinsic to internal side of plasma membrane;intrinsic to membrane;intrinsic to plasma membrane;membrane part;plasma membrane part;ruffle	Axon guidance;MAPK signaling pathway	Homo sapiens	+
cell part;intracellular membrane-bounded organelle;intracellular organelle;intracellular part;membrane-bounded organelle;nucleus;organelle		Homo sapiens	+
cell part;cytoplasm;intracellular membrane-bounded organelle;intracellular organelle;intracellular part;membrane-bounded organelle;nucleus;organelle		Homo sapiens	
cell part;cell projection part;cytoplasm;cytoplasmic part;cytosol;extracellular membrane-bounded organelle;extracellular organelle;extracellular region part;extracellular vesicular exosome;flagellar fibrous sheath;flagellum part	Drug metabolism - cytochrome P450;Glutathione metabolism;Metabolism of xenobiotics by cytochrome P450	Homo sapiens	+
cell part;cell projection;cytoplasmic membrane-bounded vesicle;cytoplasmic part;cytoplasmic vesicle;cytosol;endomembrane system;extracellular membrane-bounded organelle;extracellular organelle;extracellular region par	Collecting duct acid secretion;Epithelial cell signaling in Helicobacter pylori infection;Oxidative phosphorylation;Phagosome;Rheumatoid arthritis;\	Homo sapiens	+
apical part of cell;cell part;cytoplasmic part;cytoplasmic vesicle;cytosol;extracellular membrane-bounded organelle;extracellular organelle;extracellular region part;extracellular vesicular exosome;intracellular organelle;intracel	Collecting duct acid secretion;Epithelial cell signaling in Helicobacter pylori infection;Oxidative phosphorylation;Phagosome;Rheumatoid arthritis;\	Homo sapiens	
adherens junction;anchoring junction;cell junction;cell part;cell-substrate adherens junction;cell-substrate junction;extracellular membrane-bounded organelle;extracellular organelle;extracellular region part;extracellular vesicular exosome;focal adhesion;intracellular membrane-bounded organelle;intracellular organelle;intracellular part;membrane-bounded organelle;mem		Homo sapiens	+
actin cytoskeleton;actin filament;adherens junction;anchoring junction;cell body;cell cortex part;cell junction;cell part;cell projection part;cell-substrate adherens junction;cell-substrate junction;chromosomal part;cortical cyto:	Focal adhesion;MAPK signaling pathway	Homo sapiens	+
cell part;cytoplasm;cytoplasmic part;cytosol;endoplasmic reticulum;extracellular membrane-bounded organelle;extracellular organelle;extracellular region part;extracellular vesicular exosome;Golgi apparatus;intracellular mer	Carbon fixation pathways in prokaryotes;Citrate cycle (TCA cycle);Glyoxylate and dicarboxylate metabolism	Homo sapiens	+
anchored to membrane;cell part;cell surface;cytoplasm;extracellular membrane-bounded organelle;extracellular organelle;extracellular region part;extracellular vesicular exosome;intracellular part;intrinsic to membrane;memb	Nicotinate and nicotinamide metabolism;Purine metabolism;Pyrimidine metabolism	Homo sapiens	+
cell part;cytoplasmic part;extracellular membrane-bounded organelle;extracellular organelle;extracellular region part;extracellular vesicular exosome;intracellular membrane-bounded organelle;intracellular non-membrane-bou	Calcium signaling pathway;Huntington's disease;Parkinson's disease	Homo sapiens	+
cell part;cytoplasmic part;extracellular membrane-bounded organelle;extracellular organelle;extracellular region part;extracellular vesicular exosome;fumarate reductase complex;intracellular membrane-bounded organelle;int	Alzheimer's disease;Citrate cycle (TCA cycle);Huntington's disease;Oxidative phosphorylation;Parkinson's disease	Homo sapiens	+
adherens junction;anchoring junction;apical plasma membrane;cell junction;cell part;cell-substrate adherens junction;cell-substrate junction;clathrin coated vesicle membrane;clathrin-coated endocytic vesicle membrane;coa	Hematopoietic cell lineage	Homo sapiens	+
axon;cell body;cell part;cell projection;cell projection part;cytoplasmic part;cytosol;dendritic spine;extracellular membrane-bounded organelle;extracellular organelle;extracellular region part;extracellular vesicular exosome;int	Betalain biosynthesis;Steroid hormone biosynthesis;Tyrosine metabolism	Homo sapiens	+
adherens junction;anchoring junction;cell junction;cell part;cell-substrate adherens junction;cell-substrate junction;cytoplasmic part;cytosol;extracellular membrane-bounded organelle;extracellular organelle;extracellular regic	Huntington's disease	Homo sapiens	+
cell part;cytoplasmic part;intracellular membrane-bounded organelle;intracellular organelle;intracellular organelle lumen;intracellular organelle part;intracellular part;membrane-bounded organelle;membrane-enclosed lumen;m	Carbon fixation pathways in prokaryotes;Glyoxylate and dicarboxylate metabolism;Propanoate metabolism;Valine, leucine and isoleucine degrada	Homo sapiens	+
cell junction;cell part;cytoplasm;cytoplasmic part;Golgi apparatus;Golgi apparatus part;Golgi membrane;intracellular membrane-bounded organelle;intracellular non-membrane-bounded organelle;intracellular organelle;intracellular organelle part;intracellular part;membrane;membrane-bounded organelle;non-membrane-bounded organelle;nuclear part;nucleolus;nucleopl		Homo sapiens	
cell part;cytoplasm;cytoplasmic part;endoplasmic reticulum;extracellular membrane-bounded organelle;extracellular organelle;extracellular region part;extracellular vesicular exosome;intracellular membrane-bounded organelle;intracellular organelle;intracellular part;membrane-bounded organelle;membrane-bounded vesicle;organelle;vesicle		Homo sapiens	+
box C/D snoRNP complex;Cajal body;cell part;extracellular membrane-bounded organelle;extracellular organelle;extracellular region part;extracellular vesicular exosome;granular component;intracellular membrane-bounded	Ribosome biogenesis in eukaryotes	Homo sapiens	
cell part;cytoplasmic part;cytosol;cytosolic small ribosomal subunit;intracellular membrane-bounded organelle;intracellular organelle;intracellular organelle part;intracellular part;macromolecular complex;membrane;membrane	Ribosome	Homo sapiens	+
cell part;cytoplasmic part;cytosol;extracellular membrane-bounded organelle;extracellular organelle;extracellular region part;extracellular vesicular exosome;intracellular part;membrane-bounded organelle;membrane-bounde	One carbon pool by folate;Purine metabolism	Homo sapiens	+
cell part;cytoplasmic part;cytosol;extracellular membrane-bounded organelle;extracellular organelle;extracellular region part;extracellular vesicular exosome;intracellular part;membrane;membrane-bounded organelle;membra	Purine metabolism	Homo sapiens	+
cell part;cytoplasm;cytoplasmic part;extracellular membrane-bounded organelle;extracellular organelle;extracellular region part;extracellular vesicular exosome;intracellular membrane-bounded organelle;intracellular organelle	Peroxisome;PPAR signaling pathway;Primary bile acid biosynthesis	Homo sapiens	+
cell part;cytoplasm;cytoplasmic part;cytosol;extracellular membrane-bounded organelle;extracellular organelle;extracellular region part;extracellular vesicular exosome;intracellular membrane-bounded organelle;intracellular c	Parkinson's disease;Ubiquitin mediated proteolysis	Homo sapiens	+
cell part;cell periphery;cell projection;cytoplasm;cytoplasmic part;cytosol;extracellular membrane-bounded organelle;extracellular organelle;extracellular region part;extracellular vesicular exosome;intracellular membrane-bou	Purine metabolism;Pyrimidine metabolism	Homo sapiens	+
basolateral plasma membrane;cell part;cell surface;cytoplasmic part;extracellular region part;extracellular space;integral to membrane;integral to plasma membrane;intracellular organelle part;intracellular part;intrinsic to mem	Nicotinate and nicotinamide metabolism;Pantothenate and CoA biosynthesis;Purine metabolism;Riboflavin metabolism;Starch and sucrose metab	Homo sapiens	+
cell part;cytoplasmic part;intracellular membrane-bounded organelle;intracellular organelle;intracellular organelle lumen;intracellular organelle part;intracellular part;membrane-bounded organelle;membrane-enclosed lumen;mitochondrial matrix;mitochondrial part;mitochondrion;organelle;organelle lumen;organelle part		Homo sapiens	+
catalytic step 2 spliceosome;cell part;cytoplasm;intracellular membrane-bounded organelle;intracellular organelle;intracellular organelle part;intracellular part;macromolecular complex;membrane;membrane-bounded organelle;nuclear part;nucleoplasm;nucleus;organelle;organelle part;ribonucleoprotein complex;spliceosomal complex		Homo sapiens	+
cell part;cytoplasm;cytoplasmic part;cytosol;flotillin complex;intracellular membrane-bounded organelle;intracellular organelle;intracellular part;macromolecular complex;membrane;membrane part;membrane-bounded organ	Bacterial invasion of epithelial cells;Chronic myeloid leukemia;Endocytosis;ErbB signaling pathway;Insulin signaling pathway;Jak-STAT signaling p	Homo sapiens	+
cell part;cytoplasmic part;extracellular membrane-bounded organelle;extracellular organelle;extracellular region part;extracellular vesicular exosome;intracellular membrane-bounded organelle;intracellular organelle;intracellul	Alzheimer's disease;Cardiac muscle contraction;Huntington's disease;Oxidative phosphorylation;Parkinson's disease	Homo sapiens	+
cell part;cytoplasmic part;intracellular membrane-bounded organelle;intracellular organelle;intracellular organelle lumen;intracellular organelle part;intracellular part;membrane;membrane-bounded organelle;membrane-enclos	Porphyrin and chlorophyll metabolism	Homo sapiens	+
basement membrane;extracellular matrix;extracellular matrix part;extracellular membrane-bounded organelle;extracellular organelle;extracellular region;extracellular region part;extracellular space;extracellular vesicular exosome;membrane-bounded organelle;membrane-bounded vesicle;organelle;proteinaceous extracellular matrix;vesicle		Homo sapiens	+
cell part;intracellular membrane-bounded organelle;intracellular non-membrane-bounded organelle;intracellular organelle;intracellular organelle part;intracellular part;membrane-bounded organelle;non-membrane-bounded organelle;nuclear part;nucleolus;nucleoplasm;nucleus;organelle;organelle part		Homo sapiens	
cell part;cell projection;cilium;cytoplasm;cytoplasmic part;endoplasmic reticulum membrane;endoplasmic reticulum part;extracellular membrane-bounded organelle;extracellular organelle;extracellular region part;extracellular	Arachidonic acid metabolism	Homo sapiens	
adherens junction;alpha6-beta4 integrin complex;anchoring junction;basal plasma membrane;basement membrane;cell junction;cell part;cell projection;cell surface;cell-cell adherens junction;cell-cell junction;cell-substrate ac	Arrhythmogenic right ventricular cardiomyopathy (ARVC);Cell adhesion molecules (CAMs);Dilated cardiomyopathy;ECM-receptor interaction;Foca	Homo sapiens	+
cell part;chromatin;chromosomal part;cytoplasm;intracellular membrane-bounded organelle;intracellular organelle;intracellular organelle part;intracellular part;membrane-bounded organelle;nuclear body;nuclear matrix;nuclear part;nucleoplasm;nucleoplasm part;nucleus;organelle;organelle part;paraspeckles		Homo sapiens	+
cell part;centrosome;chromosome;condensed chromosome;condensed nuclear chromosome;cytoplasm;cytoplasmic part;cytoskeletal part;cytosol;endosome;gamma-tubulin complex;intracellular membrane-bounded organelle;intracellular non-membrane-bounded organelle;intracellular organelle;intracellular organelle part;intracellular part;macromolecular complex;mem		Homo sapiens	+
adherens junction;anchoring junction;cell junction;cell part;cell-substrate adherens junction;cell-substrate junction;cytoplasmic membrane-bounded vesicle;cytoplasmic part;cytoplasmic vesicle;endoplasmic reticulum;endoplasmic reticulum lumen;endoplasmic reticulum part;extracellular membrane-bounded organelle;extracellular organelle;extracellular region part;extra		Homo sapiens	+
cell part;cytoplasmic part;intracellular membrane-bounded organelle;intracellular organelle;intracellular organelle lumen;intracellular organelle part;intracellular part;membrane-bounded organelle;membrane-enclosed lumen;m	Pyruvate metabolism;Two-component system	Homo sapiens	
cell part;cytoplasm;cytoplasmic part;cytosol;extracellular membrane-bounded organelle;extracellular organelle;extracellular region part;extracellular vesicular exosome;intracellular membrane-bounded organelle;intracellular c	Aminoacyl-tRNA biosynthesis;Tryptophan metabolism	Homo sapiens	+
adherens junction;anchoring junction;cell junction;cell part;cell projection membrane;cell projection part;cell-substrate adherens junction;cell-substrate junction;cytoplasm;cytoplasmic part;cytoskeletal part;cytosol;cytosolic s	Ribosome	Homo sapiens	+
adherens junction;anchoring junction;cell junction;cell part;cell-substrate adherens junction;cell-substrate junction;cytoplasm;cytoplasmic part;cytoskeleton;cytosol;endomembrane system;extrinsic to internal side of plasma	Hepatitis C;Jak-STAT signaling pathway;ko05152;Leishmaniasis;Measles;Osteoclast differentiation;Pancreatic cancer;Pathways in cancer;Toxopla	Homo sapiens	+
cell part;cytoplasm;intracellular membrane-bounded organelle;intracellular non-membrane-bounded organelle;intracellular organelle;intracellular organelle part;intracellular part;membrane-bounded organelle;non-membrane-bounded organelle;nuclear body;nuclear part;nuclear periphery;nucleolus;nucleoplasm;nucleoplasm part;nucleus;organelle;organelle part;PML body		Homo sapiens	+
cell part;cytoplasm;cytoplasmic membrane-bounded vesicle;cytoplasmic part;cytoplasmic vesicle;cytosol;extracellular membrane-bounded organelle;extracellular organelle;extracellular region part;extracellular vesicular exos	Cysteine and methionine metabolism	Homo sapiens	+
adherens junction;anchoring junction;cell cortex part;cell junction;cell part;cell projection membrane;cell projection part;cell-cell junction;cell-substrate adherens junction;cell-substrate junction;cortical actin cytoskeleton;corti	Axon guidance;Fc gamma R-mediated phagocytosis;Regulation of actin cytoskeleton	Homo sapiens	+
cell part;cytoplasmic part;cytosol;eukaryotic translation initiation factor 4F complex;intracellular part;macromolecular complex;protein complex	mTOR signaling pathway;RNA transport	Homo sapiens	+
caveola;cell part;contractile fiber part;cytoplasmic part;integral to membrane;integral to plasma membrane;intracellular part;intrinsic to membrane;intrinsic to plasma membrane;macromolecular complex;membrane;membran	Calcium signaling pathway;Pancreatic secretion;Salivary secretion	Homo sapiens	+
cell part;cytoplasmic part;cytosol;intracellular part;membrane;plasma membrane	Glycerolipid metabolism;Glycerophospholipid metabolism;Phosphatidylinositol signaling system	Homo sapiens	+
cell part;cytoplasmic part;intracellular membrane-bounded organelle;intracellular non-membrane-bounded organelle;intracellular organelle;intracellular organelle part;intracellular part;membrane;membrane-bounded organelle	Fatty acid metabolism;PPAR signaling pathway	Homo sapiens	+
cell part;cytoplasmic part;cytosol;intracellular membrane-bounded organelle;intracellular organelle;intracellular organelle lumen;intracellular organelle part;intracellular part;membrane-bounded organelle;membrane-enclosed l	Pyrimidine metabolism	Homo sapiens	
cell body;cell part;cell projection;cytoplasm;cytoplasmic part;cytosol;envelope;extracellular membrane-bounded organelle;extracellular organelle;extracellular region part;extracellular vesicular exosome;intracellular organelle	Glutathione metabolism;Purine metabolism;Pyrimidine metabolism	Homo sapiens	+
cell part;cytoplasm;cytoplasmic part;cytosol;eukaryotic translation elongation factor 1 complex;intracellular part;macromolecular complex;protein complex		Homo sapiens	+
cell part;cytoplasmic part;extracellular membrane-bounded organelle;extracellular organelle;extracellular region part;extracellular vesicular exosome;intracellular membrane-bounded organelle;intracellular organelle;intracellul	Alzheimer's disease;Huntington's disease;Oxidative phosphorylation;Parkinson's disease	Homo sapiens	+
extracellular region;extracellular region part;extracellular space;insulin-like growth factor binding protein complex;macromolecular complex;protein complex		Homo sapiens	+
cell part;cytoplasm;extracellular membrane-bounded organelle;extracellular organelle;extracellular region part;extracellular vesicular exosome;internal side of plasma membrane;intracellular part;membrane part;membrane-bo	Riboflavin metabolism	Homo sapiens	
cell part;cytoplasmic part;extracellular membrane-bounded organelle;extracellular organelle;extracellular region part;extracellular vesicular exosome;intracellular membrane-bounded organelle;intracellular organelle;intracellul	Benzoate degradation;Butanoate metabolism;Carbon fixation pathways in prokaryotes;Fatty acid metabolism;Glyoxylate and dicarboxylate metab	Homo sapiens	+
adherens junction;anchoring junction;basement membrane;cell junction;cell part;cell-substrate adherens junction;cell-substrate junction;extracellular matrix;extracellular matrix part;extracellular region;extracellular region part	ECM-receptor interaction;Focal adhesion	Homo sapiens	+
actin filament bundle;actomyosin;cell part;contractile fiber part;cytoplasmic part;cytoskeletal part;cytosol;intracellular organelle part;intracellular part;macromolecular complex;muscle myosin complex;myosin complex;myosir	Focal adhesion;Leukocyte transendothelial migration;Regulation of actin cytoskeleton;Tight junction;Vascular smooth muscle contraction	Homo sapiens	+
cell part;DNA-directed RNA polymerase complex;DNA-directed RNA polymerase II, core complex;intracellular membrane-bounded organelle;intracellular organelle;intracellular organelle part;intracellular part;macromolecular	Huntington's disease;Purine metabolism;Pyrimidine metabolism;RNA polymerase	Homo sapiens	
Cajal body;cell part;centrosome;chromosomal part;chromosome;chromosome, telomeric region;condensed chromosome;cyclin-dependent protein kinase holoenzyme complex;cytoplasm;cytoplasmic part;cytoskeletal part;c	Cell cycle;Measles;Oocyte meiosis;p53 signaling pathway;Pathways in cancer;Progesterone-mediated oocyte maturation;Prostate cancer;Small c	Homo sapiens	
alpha DNA polymerase:primase complex;cell part;centrosome;chromosomal part;cytoplasmic part;cytoskeletal part;intracellular membrane-bounded organelle;intracellular non-membrane-bounded organelle;intracellular orga	Cell cycle;Cell cycle - yeast;DNA replication;Meiosis - yeast	Homo sapiens	+
cell junction;cell part;cell projection;cytoplasmic part;extracellular membrane-bounded organelle;extracellular organelle;extracellular region part;extracellular vesicular exosome;intracellular organelle part;intracellular part;mer	Cysteine and methionine metabolism;Sulfur relay system	Homo sapiens	+
cell part;cytoplasmic part;cytosol;cytosolic small ribosomal subunit;intracellular organelle part;intracellular part;macromolecular complex;membrane;organelle part;ribonucleoprotein complex;small ribosomal subunit	Ribosome	Homo sapiens	+
cell part;cytoplasm;intracellular membrane-bounded organelle;intracellular organelle;intracellular part;membrane-bounded organelle;nucleus;organelle		Homo sapiens	+
cell part;chromatin remodeling complex;chromosomal part;DNA helicase complex;Ino80 complex;intracellular membrane-bounded organelle;intracellular organelle;intracellular organelle part;intracellular part;macromolecular complex;membrane-bounded organelle;nuclear chromosome part;nuclear matrix;nuclear part;nucleoplasm part;nucleus;organelle;organelle part;Pc		Homo sapiens	+
cell part;cytoplasmic part;cytosol;extracellular membrane-bounded organelle;extracellular organelle;extracellular region part;extracellular vesicular exosome;intracellular membrane-bounded organelle;intracellular non-membr	Protein processing in endoplasmic reticulum	Homo sapiens	+
cell part;cytoplasmic part;extracellular membrane-bounded organelle;extracellular organelle;extracellular region part;extracellular vesicular exosome;intracellular membrane-bounded organelle;intracellular organelle;intracellul	Alzheimer's disease;Huntington's disease;Oxidative phosphorylation;Parkinson's disease	Homo sapiens	
cell part;cytoplasmic part;extracellular region;extracellular region part;extracellular space;intracellular membrane-bounded organelle;intracellular organelle;intracellular organelle lumen;intracellular organelle part;intracellular pa	Antigen processing and presentation;ko05152;Lysosome;Phagosome	Homo sapiens	+
cell part;centrosome;cytoplasm;cytoplasmic part;cytoskeletal part;cytosol;extracellular membrane-bounded organelle;extracellular organelle;extracellular region part;extracellular vesicular exosome;intracellular membrane-bo	Proteasome	Homo sapiens	
cell part;cytoplasm;cytoplasmic mRNA processing body;cytoplasmic part;cytosol;extracellular membrane-bounded organelle;extracellular organelle;extracellular region part;extracellular vesicular exosome;intracellular membr	Proteasome	Homo sapiens	
cell part;cytoplasm;cytoplasmic part;cytosol;extracellular membrane-bounded organelle;extracellular organelle;extracellular region part;extracellular vesicular exosome;intracellular membrane-bounded organelle;intracellular c	Proteasome	Homo sapiens	+
cell part;cytoplasm;cytoplasmic mRNA processing body;cytoplasmic part;cytosol;extracellular membrane-bounded organelle;extracellular organelle;extracellular region part;extracellular vesicular exosome;intracellular membr	Proteasome	Homo sapiens	+
adherens junction;alpha3-beta1 integrin complex;anchoring junction;basolateral plasma membrane;cell junction;cell part;cell periphery;cell projection membrane;cell projection part;cell surface;cell-substrate adherens junctio	Arrhythmogenic right ventricular cardiomyopathy (ARVC);Dilated cardiomyopathy;ECM-receptor interaction;Focal adhesion;Hematopoietic cell line	Homo sapiens	+
extracellular region;extracellular region part;extracellular space		Homo sapiens	+
adherens junction;anchoring junction;apical part of cell;apical plasma membrane;basolateral plasma membrane;blood microparticle;cell junction;cell part;cell periphery;cell projection;cell projection membrane;cell projection p	Leukocyte transendothelial migration;Measles;Regulation of actin cytoskeleton	Homo sapiens	+
cell part;cytoplasm;cytoplasmic mRNA processing body;cytoplasmic part;cytosol;intracellular membrane-bounded organelle;intracellular non-membrane-bounded organelle;intracellular organelle;intracellular organelle part;int	RNA degradation	Homo sapiens	+
cell part;centromeric heterochromatin;chromatin;chromosomal part;heterochromatin;intracellular membrane-bounded organelle;intracellular organelle;intracellular organelle part;intracellular part;membrane-bounded organelle	Cysteine and methionine metabolism	Homo sapiens	+
cell part;intracellular organelle part;intracellular part;macromolecular complex;nuclear body;nuclear part;nuclear speck;nucleoplasm;nucleoplasm part;organelle part;ribonucleoprotein complex;spliceosomal complex	Spliceosome	Homo sapiens	+
cell part;cytoplasm;cytoplasmic part;cytosol;cytosolic large ribosomal subunit;cytosolic part;cytosolic ribosome;intracellular membrane-bounded organelle;intracellular non-membrane-bounded organelle;intracellular organelle	Ribosome	Homo sapiens	+
cell part;cytoplasmic part;intracellular membrane-bounded organelle;intracellular organelle;intracellular organelle lumen;intracellular organelle part;intracellular part;membrane-bounded organelle;membrane-enclosed lumen;m	Valine, leucine and isoleucine degradation	Homo sapiens	
cell part;cell projection;cytoplasmic part;extracellular membrane-bounded organelle;extracellular organelle;extracellular region part;extracellular space;extracellular vesicular exosome;intracellular membrane-bounded organelle;intracellular organelle;intracellular part;membrane-bounded organelle;membrane-bounded vesicle;neuron projection;nucleus;organelle;perinuclea		Homo sapiens	+
cell part;chromosome;condensed chromosome;cytoplasm;cytoplasmic part;extracellular region part;extracellular space;intracellular membrane-bounded organelle;intracellular non-membrane-bounded organelle;intracellular organelle;intracellular organelle part;intracellular part;macromolecular complex;membrane-bounded organelle;non-membrane-bounded organelle;nu		Homo sapiens	+
cell part;extracellular membrane-bounded organelle;extracellular organelle;extracellular region part;extracellular vesicular exosome;intracellular non-membrane-bounded organelle;intracellular organelle;intracellular organelle part;intracellular part;membrane;membrane-bounded organelle;membrane-bounded vesicle;non-membrane-bounded organelle;nuclear part;nucleo		Homo sapiens	+
actin cytoskeleton;cell part;cytoplasm;cytoplasmic part;cytoskeleton;cytosol;extracellular membrane-bounded organelle;extracellular organelle;extracellular region part;extracellular vesicular exosome;intracellular non-membr	Aminoacyl-tRNA biosynthesis	Homo sapiens	
cell part;cytoplasmic part;cytosol;intracellular membrane-bounded organelle;intracellular organelle;intracellular part;membrane-bounded organelle;mitochondrion;organelle	Aminoacyl-tRNA biosynthesis;Valine, leucine and isoleucine biosynthesis	Homo sapiens	
cell part;cytoplasm;cytoplasmic part;cytosol;extracellular membrane-bounded organelle;extracellular organelle;extracellular region part;extracellular vesicular exosome;intracellular membrane-bounded organelle;intracellular organelle;intracellular part;membrane;membrane-bounded organelle;membrane-bounded vesicle;nucleus;organelle;vesicle		Homo sapiens	+
cell part;cytoplasmic part;endoplasmic reticulum;endoplasmic reticulum membrane;endoplasmic reticulum part;extracellular membrane-bounded organelle;extracellular organelle;extracellular region part;extracellular vesicular exosome;intracellular membrane-bounded organelle;intracellular organelle;intracellular organelle part;intracellular part;membrane;membrane part;		Homo sapiens	+

blood microparticle;cell part;cytoplasm;cytoplasmic membrane-bounded vesicle;cytoplasmic part;cytoplasmic vesicle;cytoskeleton;endoplasmic reticulum;extracellular membrane-bounded organelle;extracellular organelle;extracellular region part;extracellular space;extracellular vesicular exosome;integral to membrane;integral to plasma membrane;intracellular membrar		Homo sapiens	+
adherens junction;anchoring junction;cell junction;cell part;cell-substrate adherens junction;cell-substrate junction;cytoplasm;cytoplasmic part;cytoplasmic vesicle membrane;cytoplasmic vesicle part;cytosol;extracellular mer	Cell cycle;Neurotrophin signaling pathway;Oocyte meiosis	Homo sapiens	+
adherens junction;anchoring junction;caveola;cell junction;cell part;cell projection;cell-substrate adherens junction;cell-substrate junction;cytoplasmic part;cytoskeleton;cytosol;early endosome;endosome;envelope;extracellu	Acute myeloid leukemia;Adherens junction;Aldosterone-regulated sodium reabsorption;Alzheimer's disease;Axon guidance;B cell receptor signalir	Homo sapiens	
adherens junction;anchoring junction;apical plasma membrane;cell junction;cell part;cell projection;cell projection membrane;cell projection part;cell surface;cell-cell junction;cell-substrate adherens junction;cell-substrate jun	Protein digestion and absorption	Homo sapiens	+
cell part;cytoplasmic part;cytosol;cytosolic large ribosomal subunit;endoplasmic reticulum;intracellular membrane-bounded organelle;intracellular organelle;intracellular organelle part;intracellular part;large ribosomal subunit;r	Ribosome	Homo sapiens	+
cell part;chromosomal part;DNA replication factor A complex;germ cell nucleus;intracellular membrane-bounded organelle;intracellular organelle;intracellular organelle part;intracellular part;lateral element;macromolecular cor	DNA replication;Homologous recombination;Mismatch repair;Nucleotide excision repair	Homo sapiens	+
cell part;centrosome;cytoplasm;cytoplasmic part;cytoskeletal part;endoplasmic reticulum;intracellular membrane-bounded organelle;intracellular non-membrane-bounded organelle;intracellular organelle;intracellular organelle	Base excision repair	Homo sapiens	+
cell part;extracellular membrane-bounded organelle;extracellular organelle;extracellular region part;extracellular vesicular exosome;integral to membrane;integral to plasma membrane;intrinsic to membrane;intrinsic to plasma	p53 signaling pathway	Homo sapiens	+
cell part;cytoplasmic part;cytosol;intracellular membrane-bounded organelle;intracellular organelle;intracellular part;membrane-bounded organelle;nucleus;organelle	Purine metabolism;Pyrimidine metabolism	Homo sapiens	+
axon part;cell body;cell part;cell projection;cell projection part;cytoplasmic part;cytosol;extracellular membrane-bounded organelle;extracellular organelle;extracellular region part;extracellular vesicular exosome;intracellular m	Alanine, aspartate and glutamate metabolism;Pyrimidine metabolism	Homo sapiens	
acrosomal vesicle;adherens junction;anchoring junction;cell junction;cell part;cell surface;cell-substrate adherens junction;cell-substrate junction;cytoplasm;cytoplasmic membrane-bounded vesicle;cytoplasmic membrane-b	Antigen processing and presentation;Chagas disease (American trypanosomiasis);Phagosome;Protein processing in endoplasmic reticulum	Homo sapiens	+
cell part;cytoplasm;cytoskeletal part;cytoskeleton;extracellular membrane-bounded organelle;extracellular organelle;extracellular region part;extracellular vesicular exosome;intracellular non-membrane-bounded organelle;intracellular organelle;intracellular organelle part;intracellular part;macromolecular complex;membrane;membrane-bounded organelle;membrane-bour		Homo sapiens	+
axon;cell body;cell part;cell projection;cell projection cytoplasm;cell projection part;cytoplasmic membrane-bounded vesicle;cytoplasmic part;cytoplasmic vesicle;dendrite cytoplasm;dendritic spine;endoplasmic reticulum;en	Antigen processing and presentation;Phagosome;Protein processing in endoplasmic reticulum	Homo sapiens	+
cell part;cytoplasm;cytoplasmic part;cytosol;extracellular membrane-bounded organelle;extracellular organelle;extracellular region part;extracellular vesicular exosome;intracellular membrane-bounded organelle;intracellular c	Proteasome	Homo sapiens	
cell part;cytoplasm;cytoplasmic part;cytosol;extracellular membrane-bounded organelle;extracellular organelle;extracellular region part;extracellular vesicular exosome;intracellular membrane-bounded organelle;intracellular c	Proteasome	Homo sapiens	
cell part;cytoplasm;cytoplasmic part;cytosol;extracellular membrane-bounded organelle;extracellular organelle;extracellular region part;extracellular vesicular exosome;intracellular membrane-bounded organelle;intracellular c	Proteasome	Homo sapiens	
cell part;cytoplasm;cytoplasmic part;cytosol;extracellular membrane-bounded organelle;extracellular organelle;extracellular region part;extracellular vesicular exosome;intracellular membrane-bounded organelle;intracellular c	Proteasome	Homo sapiens	+
cell part;cytoplasm;cytoplasmic part;cytosol;extracellular membrane-bounded organelle;extracellular organelle;extracellular region part;extracellular vesicular exosome;Golgi apparatus;intracellular membrane-bounded organe	Proteasome	Homo sapiens	+
cell part;centrosome;cytoplasm;cytoplasmic part;cytoskeletal part;cytosol;extracellular membrane-bounded organelle;extracellular organelle;extracellular region part;extracellular vesicular exosome;intracellular membrane-bo	Proteasome	Homo sapiens	+
cell part;cytoplasmic part;cytosol;integral to membrane;intracellular membrane-bounded organelle;intracellular organelle;intracellular organelle lumen;intracellular organelle part;intracellular part;intrinsic to membrane;membra	ABC transporters;Peroxisome	Homo sapiens	
cell part;collagen;extracellular matrix;extracellular matrix part;extracellular region;extracellular region part;extracellular space;intracellular membrane-bounded organelle;intracellular organelle;intracellular part;membrane-bounded organelle;nucleus;organelle;proteinaceous extracellular matrix		Homo sapiens	
cell part;cytoplasmic part;intracellular membrane-bounded organelle;intracellular organelle;intracellular organelle part;intracellular part;macromolecular complex;membrane;membrane part;membrane-bounded organelle;mem	Alzheimer's disease;Huntington's disease;Oxidative phosphorylation;Parkinson's disease	Homo sapiens	+
aggresome;cell part;chromosomal part;cytoplasm;delta DNA polymerase complex;DNA polymerase complex;inclusion body;intracellular membrane-bounded organelle;intracellular organelle;intracellular organelle part;intracel	Base excision repair;DNA replication;Homologous recombination;Mismatch repair;Nucleotide excision repair;Purine metabolism;Pyrimidine metab	Homo sapiens	+
cell part;chromatin remodeling complex;intracellular membrane-bounded organelle;intracellular organelle;intracellular organelle part;intracellular part;ISWI complex;macromolecular complex;membrane-bounded organelle;nuclear part;nucleus;NURF complex;organelle;organelle part;protein complex		Homo sapiens	+
adherens junction;anchoring junction;axon;caveola;cell junction;cell part;cell projection;cell projection cytoplasm;cell projection part;cell-substrate adherens junction;cell-substrate junction;cytoplasm;cytoplasmic part;cytoske	Acute myeloid leukemia;Adherens junction;Aldosterone-regulated sodium reabsorption;Alzheimer's disease;Axon guidance;B cell receptor signalir	Homo sapiens	+
cell part;intracellular membrane-bounded organelle;intracellular organelle;intracellular organelle part;intracellular part;membrane-bounded organelle;nuclear part;nucleoplasm;nucleus;organelle;organelle part	Adipocytokine signaling pathway;Non-small cell lung cancer;Pathways in cancer;PPAR signaling pathway;Small cell lung cancer;Thyroid cancer	Homo sapiens	+
cell part;cytoplasmic part;extracellular membrane-bounded organelle;extracellular organelle;extracellular region part;extracellular space;extracellular vesicular exosome;intracellular membrane-bounded organelle;intracellular organelle;intracellular part;membrane-bounded organelle;membrane-bounded vesicle;mitochondrion;organelle;vesicle		Homo sapiens	+
adherens junction;anchoring junction;cell junction;cell part;cell-substrate adherens junction;cell-substrate junction;cytoplasm;cytoplasmic part;extracellular membrane-bounded organelle;extracellular organelle;extracellular re	Arginine and proline metabolism;Glutathione metabolism	Homo sapiens	+
cell part;cytoplasm;cytoplasmic part;cytosol;intracellular membrane-bounded organelle;intracellular organelle;intracellular organelle part;intracellular part;membrane-bounded organelle;nuclear part;nucleoplasm;nucleus;organelle;organelle part		Homo sapiens	+
cell part;cytoplasm;cytoplasmic part;cytosol;extracellular membrane-bounded organelle;extracellular organelle;extracellular region part;extracellular vesicular exosome;intracellular part;membrane-bounded organelle;membra	Inositol phosphate metabolism;Phosphatidylinositol signaling system;Streptomycin biosynthesis	Homo sapiens	
cell cortex;cell part;cis-Golgi network;cytoplasmic part;cytosol;extracellular matrix;extracellular region;extracellular region part;extracellular space;Golgi apparatus;Golgi apparatus part;intracellular membrane-bounded organelle;intracellular organelle;intracellular organelle part;intracellular part;membrane;membrane-bounded organelle;organelle;organelle part;perinuclear		Homo sapiens	+
adherens junction;anchoring junction;cell junction;cell part;cell projection membrane;cell projection part;cell surface;cell-substrate adherens junction;cell-substrate junction;focal adhesion;integral to membrane;integral to plas	Axon guidance	Homo sapiens	+
axon;cell body;cell part;cell projection;cytoplasmic part;cytosol;dendrite;extracellular region;integral to membrane;integral to plasma membrane;intracellular part;intrinsic to membrane;intrinsic to plasma membrane;membran	Axon guidance	Homo sapiens	+
cell part;cytoplasmic part;intracellular non-membrane-bounded organelle;intracellular organelle;intracellular organelle part;intracellular part;mitochondrial nucleoid;mitochondrial part;non-membrane-bounded organelle;nuclea	Base excision repair	Homo sapiens	+
cell part;cytoplasm;cytoplasmic part;cytosol;endoplasmic reticulum;extracellular membrane-bounded organelle;extracellular organelle;extracellular region part;extracellular vesicular exosome;intracellular membrane-bounded organelle;intracellular organelle;intracellular organelle part;intracellular part;membrane-bounded organelle;membrane-bounded vesicle;nuclear part		Homo sapiens	+
cell part;cytoplasmic part;cytosol;extracellular membrane-bounded organelle;extracellular organelle;extracellular region part;extracellular vesicular exosome;intracellular membrane-bounded organelle;intracellular organelle;int	Biosynthesis of ansamycins;Carbon fixation in photosynthetic organisms;Pentose phosphate pathway	Homo sapiens	+
cell part;cytoplasm;cytoplasmic part;cytosol;extracellular region;intracellular part;IPAF inflammasome complex;macromolecular complex;NALP1 inflammasome complex;NALP3 inflammasome complex;protein complex	Amyotrophic lateral sclerosis (ALS);Cytosolic DNA-sensing pathway;NOD-like receptor signaling pathway	Homo sapiens	+
cell part;cytoplasm;cytoplasmic part;cytosol;early endosome membrane;endoplasmic reticulum part;endosomal part;endosome membrane;extrinsic to endoplasmic reticulum membrane;extrinsic to membrane;extrinsic to org	Acute myeloid leukemia;Endocytosis;Pathways in cancer;Ubiquitin mediated proteolysis	Homo sapiens	+
cell part;cytoplasm;cytoplasmic part;cytosol;eukaryotic translation elongation factor 1 complex;intracellular membrane-bounded organelle;intracellular non-membrane-bounded organelle;intracellular organelle;intracellular organelle part;intracellular part;macromolecular complex;membrane-bounded organelle;non-membrane-bounded organelle;nuclear part;nucleolus;nuc		Homo sapiens	+
actin cytoskeleton;adherens junction;anchoring junction;cell cortex;cell junction;cell part;cell-substrate adherens junction;cell-substrate junction;centrosome;cytoplasmic part;cytoskeletal part;cytoskeleton;extracellular memb	Fc gamma R-mediated phagocytosis	Homo sapiens	+
cell part;cytoplasm;cytoplasmic part;extracellular membrane-bounded organelle;extracellular organelle;extracellular region part;extracellular vesicular exosome;extrinsic to internal side of plasma membrane;extrinsic to memb	Amoebiasis;Calcium signaling pathway;Chagas disease (American trypanosomiasis);Gap junction;GnRH signaling pathway;Long-term depression	Homo sapiens	+
cell part;cell surface;cytoplasmic membrane-bounded vesicle;cytoplasmic part;cytoplasmic vesicle;endoplasmic reticulum;endoplasmic reticulum lumen;endoplasmic reticulum part;extracellular membrane-bounded organelle	Protein processing in endoplasmic reticulum	Homo sapiens	+
cell part;cytoplasm;cytoplasmic membrane-bounded vesicle;cytoplasmic part;cytoplasmic vesicle;cytosol;extracellular membrane-bounded organelle;extracellular organelle;extracellular region part;extracellular space;extrace	Methane metabolism;Phenylalanine metabolism;Phenylpropanoid biosynthesis	Homo sapiens	+
cell part;cytoplasm;cytoplasmic part;cytosol;extracellular membrane-bounded organelle;extracellular organelle;extracellular region part;extracellular vesicular exosome;intracellular organelle part;intracellular part;membrane;m	Porphyrin and chlorophyll metabolism;Riboflavin metabolism	Homo sapiens	+
cell part;cytoplasm;cytoplasmic part;cytoplasmic vesicle;cytosol;extracellular membrane-bounded organelle;extracellular organelle;extracellular region part;extracellular space;extracellular vesicular exosome;intracellular mem	Peroxisome	Homo sapiens	+
cell part;cytoplasm;extracellular membrane-bounded organelle;extracellular organelle;extracellular region part;extracellular vesicular exosome;intracellular part;membrane-bounded organelle;membrane-bounded vesicle;organelle;vesicle		Homo sapiens	+
cell part;cytoplasm;cytoplasmic part;cytosol;early endosome;endosome;extracellular membrane-bounded organelle;extracellular organelle;extracellular region part;extracellular vesicular exosome;intracellular membrane-bounded organelle;intracellular organelle;intracellular organelle lumen;intracellular organelle part;intracellular part;membrane-bounded organelle;membr		Homo sapiens	+
cell part;cytoplasmic part;intracellular membrane-bounded organelle;intracellular organelle;intracellular organelle lumen;intracellular organelle part;intracellular part;macromolecular complex;membrane;membrane part;membr	Alzheimer's disease;Huntington's disease;Oxidative phosphorylation;Parkinson's disease	Homo sapiens	+
adherens junction;anchoring junction;cell junction;cell part;cell-substrate adherens junction;cell-substrate junction;cytoplasmic part;cytosol;cytosolic large ribosomal subunit;extracellular membrane-bounded organelle;extrac	Ribosome	Homo sapiens	+
cell part;cytoplasmic part;extracellular membrane-bounded organelle;extracellular organelle;extracellular region part;extracellular vesicular exosome;intracellular membrane-bounded organelle;intracellular organelle;intracellul	Aminobenzoate degradation;Benzoate degradation;beta-Alanine metabolism;Butanoate metabolism;Caprolactam degradation;Fatty acid elongatio	Homo sapiens	+
cell part;cytoplasm;cytoplasmic part;cytosol;extracellular membrane-bounded organelle;extracellular organelle;extracellular region part;extracellular vesicular exosome;intracellular membrane-bounded organelle;intracellular r	Pyrimidine metabolism	Homo sapiens	+
cell part;cytoplasm;extracellular membrane-bounded organelle;extracellular organelle;extracellular region part;extracellular vesicular exosome;intracellular membrane-bounded organelle;intracellular organelle;intracellular part;membrane-bounded organelle;membrane-bounded vesicle;nucleus;organelle;vesicle		Homo sapiens	
adherens junction;anchoring junction;cell junction;cell part;cell surface;cell-substrate adherens junction;cell-substrate junction;cytoplasmic membrane-bounded vesicle;cytoplasmic part;cytoplasmic vesicle;endoplasmic retic	Antigen processing and presentation;Protein processing in endoplasmic reticulum	Homo sapiens	+
cell part;chromosomal part;chromosome, centromeric region;cytoplasmic part;cytoskeleton;cytosol;extracellular membrane-bounded organelle;extracellular organelle;extracellular region part;extracellular vesicular exosome;i	Cell cycle - yeast;Chagas disease (American trypanosomiasis);Hepatitis C;Long-term depression;Meiosis - yeast;mRNA surveillance pathway;Ooc	Homo sapiens	+
cell part;extracellular membrane-bounded organelle;extracellular organelle;extracellular region part;extracellular vesicular exosome;membrane part;membrane raft;membrane-bounded organelle;membrane-bounded vesicle;o	Cell cycle - yeast;Chagas disease (American trypanosomiasis);Hepatitis C;Long-term depression;Meiosis - yeast;mRNA surveillance pathway;Ooc	Homo sapiens	+
cell part;cytoplasmic part;intracellular organelle lumen;intracellular organelle part;intracellular part;membrane;membrane-enclosed lumen;mitochondrial inner membrane;mitochondrial matrix;mitochondrial membrane;mitocho	Toxoplasmosis	Homo sapiens	+
cell part;membrane		Homo sapiens	+
actin cytoskeleton;cell junction;cell part;cytoplasm;cytoplasmic part;cytoskeleton;cytosol;extrinsic to membrane;intracellular non-membrane-bounded organelle;intracellular organelle;intracellular part;membrane;membrane part;non-membrane-bounded organelle;organelle;plasma membrane		Homo sapiens	+
cell part;cytoplasmic part;endoplasmic reticulum membrane;endoplasmic reticulum part;intracellular organelle part;intracellular part;membrane;membrane part;organelle membrane;organelle part;plasma membrane	Mineral absorption;Porphyrin and chlorophyll metabolism	Homo sapiens	
cell part;cytoplasm;cytoplasmic part;cytosol;extracellular membrane-bounded organelle;extracellular organelle;extracellular region part;extracellular vesicular exosome;intracellular part;membrane;membrane-bounded organe	Alanine, aspartate and glutamate metabolism;Purine metabolism	Homo sapiens	+
cell part;cell surface;extracellular membrane-bounded organelle;extracellular organelle;extracellular region part;extracellular space;extracellular vesicular exosome;host cell part;host cell surface;integral to membrane;integral to plasma membrane;intrinsic to membrane;intrinsic to plasma membrane;membrane;membrane part;membrane-bounded organelle;membrane-bo		Homo sapiens	+
cell part;cell surface;cytoplasmic part;endoplasmic reticulum;endoplasmic reticulum lumen;endoplasmic reticulum part;extracellular region;integral to membrane;intracellular membrane-bounded organelle;intracellular organelle;intracellular organelle lumen;intracellular organelle part;intracellular part;intrinsic to membrane;membrane;membrane part;membrane-bounded o		Homo sapiens	+
cell part;cytoplasm;cytoplasmic part;extracellular membrane-bounded organelle;extracellular organelle;extracellular region part;extracellular vesicular exosome;integral to membrane;intracellular membrane-bounded organelle	Neuroactive ligand-receptor interaction	Homo sapiens	
cell part;cytoplasmic part;cytosol;intracellular membrane-bounded organelle;intracellular organelle;intracellular part;membrane-bounded organelle;mitochondrion;organelle	Alanine, aspartate and glutamate metabolism;Purine metabolism	Homo sapiens	
cell part;cell projection;centrosome;chromosomal part;cytoplasm;cytoplasmic microtubule;cytoplasmic part;cytoplasmic vesicle membrane;cytoplasmic vesicle part;cytoskeletal part;cytoskeleton;cytosol;endosome;intermediate filament;intracellular membrane-bounded organelle;intracellular non-membrane-bounded organelle;intracellular organelle;intracellular organelle		Homo sapiens	
axon part;axon terminus;cell part;cell projection part;contractile fiber part;cytoplasm;cytoplasmic part;cytosol;dendritic spine neck;endoplasmic reticulum;endoplasmic reticulum membrane;endoplasmic reticulum part;extracellular membrane-bounded organelle;extracellular organelle;extracellular region part;extracellular vesicular exosome;intracellular membrane-bounde		Homo sapiens	+
cell part;cytoplasm;extracellular membrane-bounded organelle;extracellular organelle;extracellular region part;extracellular space;extracellular vesicular exosome;intracellular part;membrane;membrane-bounded organelle;me	Amoebiasis	Homo sapiens	+
cell part;integral to membrane;integral to plasma membrane;intrinsic to membrane;intrinsic to plasma membrane;membrane;membrane part;plasma membrane;plasma membrane part		Homo sapiens	+
cell part;cytoplasmic part;intracellular membrane-bounded organelle;intracellular organelle;intracellular organelle lumen;intracellular organelle part;intracellular part;membrane-bounded organelle;membrane-enclosed lumen;m	Arginine and proline metabolism;Ascorbate and aldarate metabolism;beta-Alanine metabolism;Chloroalkane and chloroalkene degradation;Fatty a	Homo sapiens	
cell part;DNA-directed RNA polymerase complex;DNA-directed RNA polymerase II, core complex;intracellular membrane-bounded organelle;intracellular organelle;intracellular organelle part;intracellular part;macromolecular	Huntington's disease;Purine metabolism;Pyrimidine metabolism;RNA polymerase	Homo sapiens	
cell part;cytoplasmic part;fumarate reductase complex;intracellular membrane-bounded organelle;intracellular organelle;intracellular organelle part;intracellular part;macromolecular complex;membrane;membrane part;memb	Alzheimer's disease;Citrate cycle (TCA cycle);Huntington's disease;Oxidative phosphorylation;Parkinson's disease	Homo sapiens	
actin filament;axon;cell cortex part;cell junction;cell part;cell projection;cell-cell junction;cortical actin cytoskeleton;cortical cytoskeleton;cytoplasm;cytoplasmic membrane-bounded vesicle;cytoplasmic part;cytoplasmic vesic	ko05152;Phagosome	Homo sapiens	+
cell part;cell projection;cytoplasm;cytoplasmic part;cytosol;Golgi apparatus;intracellular membrane-bounded organelle;intracellular organelle;intracellular part;macromolecular complex;membrane-bounded organelle;midbody;neuron projection;organelle;protein complex		Homo sapiens	+
cell part;cytoplasmic part;cytosol;intracellular part;macromolecular complex;methionine adenosyltransferase complex;protein complex	Cysteine and methionine metabolism	Homo sapiens	+
cell part;cytoplasmic part;intracellular non-membrane-bounded organelle;intracellular organelle;intracellular organelle lumen;intracellular organelle part;intracellular part;macromolecular complex;membrane;membrane-enclose	Alanine, aspartate and glutamate metabolism;Arginine and proline metabolism;Nitrogen metabolism	Homo sapiens	
cell part;cytoplasm;cytoplasmic part;cytosol;intracellular membrane-bounded organelle;intracellular organelle;intracellular organelle part;intracellular part;membrane-bounded organelle;nuclear part;nucleoplasm;nucleus;orga	Glutathione metabolism;p53 signaling pathway;Purine metabolism;Pyrimidine metabolism	Homo sapiens	+
cell part;cytoplasmic part;cytosol;extracellular membrane-bounded organelle;extracellular organelle;extracellular region part;extracellular vesicular exosome;intracellular membrane-bounded organelle;intracellular organelle;int	Protein processing in endoplasmic reticulum	Homo sapiens	+
cell junction;cell part;cell-cell junction;cytoplasm;cytoplasmic part;cytoskeletal part;cytoskeleton;cytosol;intracellular membrane-bounded organelle;intracellular non-membrane-bounded organelle;intracellular organelle;intrac	Acute myeloid leukemia;Adipocytokine signaling pathway;Apoptosis;B cell receptor signaling pathway;Carbohydrate digestion and absorption;Ch	Homo sapiens	+
cell cortex;cell part;cell projection membrane;cell projection part;cytoplasmic part;cytosol;intracellular membrane-bounded organelle;intracellular organelle;intracellular organelle part;intracellular part;leading edge membrane;	Acute myeloid leukemia;Adipocytokine signaling pathway;Apoptosis;B cell receptor signaling pathway;Carbohydrate digestion and absorption;Ch	Homo sapiens	+
cell part;cytoplasmic part;intracellular membrane-bounded organelle;intracellular organelle;intracellular organelle part;intracellular part;macromolecular complex;membrane;membrane part;membrane-bounded organelle;mitoc	Alzheimer's disease;Cardiac muscle contraction;Huntington's disease;Oxidative phosphorylation;Parkinson's disease	Homo sapiens	+
cell part;cytoplasmic part;intracellular organelle lumen;intracellular organelle part;intracellular part;membrane-enclosed lumen;mitochondrial matrix;mitochondrial part;organelle lumen;organelle part	Valine, leucine and isoleucine degradation	Homo sapiens	
cell part;cytoplasmic part;cytosol;extracellular membrane-bounded organelle;extracellular organelle;extracellular region part;extracellular vesicular exosome;intracellular membrane-bounded organelle;intracellular organelle;int	One carbon pool by folate;Purine metabolism	Homo sapiens	+
cell part;intracellular membrane-bounded organelle;intracellular organelle;intracellular organelle part;intracellular part;macromolecular complex;membrane-bounded organelle;nuclear part;nucleoplasm;nucleus;organelle;organelle part;ribonucleoprotein complex;spliceosomal complex		Homo sapiens	+
catalytic step 2 spliceosome;cell part;intracellular membrane-bounded organelle;intracellular organelle;intracellular organelle part;intracellular part;macromolecular complex;membrane;membrane-bounded organelle;nuclear part;nucleoplasm;nucleus;organelle;organelle part;ribonucleoprotein complex;spliceosomal complex		Homo sapiens	+
adherens junction;anchoring junction;cell junction;cell part;cell-substrate adherens junction;cell-substrate junction;cytoplasm;cytoplasmic membrane-bounded vesicle;cytoplasmic part;cytoplasmic vesicle;cytoplasmic vesicle	Cell cycle;Neurotrophin signaling pathway;Oocyte meiosis	Homo sapiens	+
cell part;cytoplasmic part;Golgi apparatus;intracellular membrane-bounded organelle;intracellular organelle;intracellular part;membrane-bounded organelle;nucleus;organelle	Prion diseases	Homo sapiens	+
cell part;cell projection;cytoplasm;extracellular membrane-bounded organelle;extracellular organelle;extracellular region part;extracellular space;extracellular vesicular exosome;intracellular membrane-bounded organelle;intracellular organelle;intracellular organelle part;intracellular part;membrane-bounded organelle;membrane-bounded vesicle;nuclear part;nucleoplasm;		Homo sapiens	+
adherens junction;anchoring junction;axon part;cell junction;cell part;cell projection part;cell surface;cell-substrate adherens junction;cell-substrate junction;external side of plasma membrane;focal adhesion;integral to memb	Axon guidance;Cell adhesion molecules (CAMs)	Homo sapiens	+
cell part;cytoplasm;cytoplasmic part;cytosol;extracellular membrane-bounded organelle;extracellular organelle;extracellular region part;extracellular vesicular exosome;intracellular part;membrane-bounded organelle;membrane-bounded vesicle;organelle;vesicle		Homo sapiens	+
cell part;cytoplasmic part;cytosol;extracellular membrane-bounded organelle;extracellular organelle;extracellular region part;extracellular vesicular exosome;intracellular part;membrane-bounded organelle;membrane-bounde	Pyrimidine metabolism	Homo sapiens	+
cell part;cytoplasmic part;intracellular membrane-bounded organelle;intracellular organelle;intracellular organelle lumen;intracellular organelle part;intracellular part;membrane-bounded organelle;membrane-enclosed lumen;m	Arginine and proline metabolism	Homo sapiens	+
cell part;cytoplasmic part;cytosol;extracellular region;Golgi apparatus part;Golgi membrane;intracellular organelle part;intracellular part;membrane;organelle membrane;organelle part		Homo sapiens	+
adherens junction;anchoring junction;cell junction;cell part;cell-substrate adherens junction;cell-substrate junction;cytoplasm;cytoplasmic part;cytosol;cytosolic large ribosomal subunit;focal adhesion;intracellular membrane-	Ribosome	Homo sapiens	+
cell part;cytoplasmic part;endoplasmic reticulum membrane;endoplasmic reticulum part;integral to membrane;intracellular membrane-bounded organelle;intracellular organelle;intracellular organelle part;intracellular part;intrir	Adipocytokine signaling pathway;Fatty acid metabolism;Peroxisome;PPAR signaling pathway	Homo sapiens	
cell part;cell projection;cell projection part;ciliary rootlet;cilium part;cytoplasm;cytoplasmic membrane-bounded vesicle;cytoplasmic part;cytoplasmic vesicle;cytoskeletal part;endocytic vesicle;intracellular membrane-bounded organelle;intracellular organelle;intracellular organelle part;intracellular part;kinesin complex;macromolecular complex;membrane;membrane-bou		Homo sapiens	
cell part;cleavage body;intracellular membrane-bounded organelle;intracellular organelle;intracellular organelle part;intracellular part;macromolecular complex;membrane-bounded organelle;mRNA cleavage and polyadenylati	mRNA surveillance pathway	Homo sapiens	+
cell part;cytoplasmic part;extracellular membrane-bounded organelle;extracellular organelle;extracellular region part;extracellular vesicular exosome;intracellular membrane-bounded organelle;intracellular organelle;intracellula	Pyrimidine metabolism	Homo sapiens	+
basolateral plasma membrane;cell part;extracellular membrane-bounded organelle;extracellular organelle;extracellular region part;extracellular vesicular exosome;integral to membrane;integral to plasma membrane;intrinsic to	ABC transporters;Vitamin digestion and absorption	Homo sapiens	+
		Homo sapiens	+
cell part;chromosomal part;intracellular membrane-bounded organelle;intracellular organelle;intracellular organelle part;intracellular part;macromolecular complex;membrane-bounded organelle;nuclear chromosome part;nucl	Systemic lupus erythematosus	Homo sapiens	+
cell part;cytoskeletal part;intracellular non-membrane-bounded organelle;intracellular organelle;intracellular organelle part;intracellular part;membrane;non-membrane-bounded organelle;organelle;organelle part;spindle	Cell cycle;Cell cycle - yeast	Homo sapiens	+
cell part;chromosomal part;intracellular membrane-bounded organelle;intracellular organelle;intracellular organelle part;intracellular part;macromolecular complex;MCM complex;membrane;membrane-bounded organelle;nuc	Cell cycle;Cell cycle - yeast;DNA replication;Meiosis - yeast	Homo sapiens	+
cell part;chromosomal part;intracellular membrane-bounded organelle;intracellular organelle;intracellular organelle part;intracellular part;macromolecular complex;MCM complex;membrane;membrane-bounded organelle;nuc	Cell cycle;Cell cycle - yeast;DNA replication;Meiosis - yeast	Homo sapiens	+
cell part;chromatin;chromosomal part;cytoplasm;cytoplasmic part;cytosol;intracellular organelle part;intracellular part;macromolecular complex;MCM complex;membrane;nuclear chromosome part;nuclear part;nucleoplasm;r	Cell cycle;Cell cycle - yeast;DNA replication;Meiosis - yeast	Homo sapiens	+
cell part;cytoplasm;cytoplasmic part;cytosol;extracellular membrane-bounded organelle;extracellular organelle;extracellular region part;extracellular vesicular exosome;intracellular membrane-bounded organelle;intracellular c	Cyanoamino acid metabolism;Glycine, serine and threonine metabolism;Methane metabolism;One carbon pool by folate	Homo sapiens	+
cell part;cytoplasmic part;cytoskeleton;extracellular membrane-bounded organelle;extracellular organelle;extracellular region part;extracellular vesicular exosome;intracellular membrane-bounded organelle;intracellular non-m	Cyanoamino acid metabolism;Glycine, serine and threonine metabolism;Methane metabolism;One carbon pool by folate	Homo sapiens	+
cell part;cytoplasmic part;cytosol;extracellular membrane-bounded organelle;extracellular organelle;extracellular region part;extracellular vesicular exosome;intracellular membrane-bounded organelle;intracellular organelle;int	Antigen processing and presentation	Homo sapiens	+
cell part;cytoplasmic part;cytosol;extracellular membrane-bounded organelle;extracellular organelle;extracellular region part;extracellular vesicular exosome;intracellular part;membrane-bounded organelle;membrane-bounde	Amino sugar and nucleotide sugar metabolism;Fructose and mannose metabolism	Homo sapiens	
anchored to membrane;cell part;cytoplasmic part;endosome;extracellular matrix;extracellular membrane-bounded organelle;extracellular organelle;extracellular region part;extracellular space;extracellular vesicular exosome;Golgi apparatus part;Golgi lumen;integral to membrane;integral to plasma membrane;intracellular membrane-bounded organelle;intracellular organe		Homo sapiens	+
axon part;cell part;cell projection part;cytoplasm;cytoskeleton;extracellular membrane-bounded organelle;extracellular organelle;extracellular region part;extracellular vesicular exosome;intracellular non-membrane-bounded c	Regulation of actin cytoskeleton;Shigellosis	Homo sapiens	+
acrosomal vesicle;actin cytoskeleton;adherens junction;anchoring junction;catenin complex;cell junction;cell part;cell projection;cell-cell adherens junction;cell-cell contact zone;cell-cell junction;cell-substrate adherens juncti	Adherens junction;Arrhythmogenic right ventricular cardiomyopathy (ARVC);Bacterial invasion of epithelial cells;Endometrial cancer;Leukocyte trar	Homo sapiens	+
adherens junction;anchoring junction;apical part of cell;basolateral plasma membrane;beta-catenin destruction complex;beta-catenin-TCF7L2 complex;catenin complex;catenin-TCF7L2 complex;cell cortex;cell junction;cell p	Adherens junction;Arrhythmogenic right ventricular cardiomyopathy (ARVC);Bacterial invasion of epithelial cells;Basal cell carcinoma;Colorectal ca	Homo sapiens	+
cell part;cell surface;cytoplasm;cytoplasmic part;early endosome;endosome;extracellular membrane-bounded organelle;extracellular organelle;extracellular region part;extracellular vesicular exosome;integral to membrane;integral to plasma membrane;intracellular membrane-bounded organelle;intracellular organelle;intracellular organelle part;intracellular part;intrinsic to		Homo sapiens	+
cell part;cytoplasm;cytoplasmic part;cytosol;extracellular membrane-bounded organelle;extracellular organelle;extracellular region part;extracellular space;extracellular vesicular exosome;intracellular part;macromolecular cor	Amoebiasis	Homo sapiens	+
adherens junction;anchoring junction;apical part of cell;cell division site part;cell junction;cell part;cell periphery;cell pole;cell projection;cell tip;cell-substrate adherens junction;cell-substrate junction;cleavage furrow;cytoplas	Regulation of actin cytoskeleton	Homo sapiens	+
cell part;chromosomal part;DNA replication factor A complex;intracellular organelle part;intracellular part;macromolecular complex;nuclear chromosome part;nuclear part;nucleoplasm;organelle part;protein complex	DNA replication;Homologous recombination;Mismatch repair;Nucleotide excision repair	Homo sapiens	+
cell part;chromosomal part;cytoplasm;DNA replication factor C complex;intracellular membrane-bounded organelle;intracellular organelle;intracellular organelle part;intracellular part;macromolecular complex;membrane-bour	DNA replication;Mismatch repair;Nucleotide excision repair	Homo sapiens	
cell part;chromosomal part;cytoplasmic part;DNA replication factor C complex;Golgi apparatus;intracellular membrane-bounded organelle;intracellular organelle;intracellular organelle part;intracellular part;macromolecular co	DNA replication;Mismatch repair;Nucleotide excision repair	Homo sapiens	+
cell junction;cell part;chromosomal part;cytoplasmic part;DNA replication factor C complex;extracellular membrane-bounded organelle;extracellular organelle;extracellular region part;extracellular vesicular exosome;Golgi app	DNA replication;Mismatch repair;Nucleotide excision repair	Homo sapiens	+
adherens junction;anchoring junction;cell junction;cell part;cell-substrate adherens junction;cell-substrate junction;cytoplasm;cytoplasmic part;cytosol;cytosolic large ribosomal subunit;extracellular membrane-bounded orgar	Ribosome	Homo sapiens	+
cell junction;cell part;intracellular membrane-bounded organelle;intracellular organelle;intracellular organelle part;intracellular part;macromolecular complex;membrane-bounded organelle;nuclear part;nucleoplasm;nucleoplas	Basal transcription factors	Homo sapiens	+
caveola;cell part;cell projection;cytoplasm;cytoplasmic part;endoplasmic reticulum membrane;endoplasmic reticulum part;intracellular membrane-bounded organelle;intracellular organelle;intracellular organelle part;intracellu	Arachidonic acid metabolism;Leishmaniasis;Pathways in cancer;Small cell lung cancer;VEGF signaling pathway	Homo sapiens	+
basement membrane;cell part;cytoplasmic membrane-bounded vesicle;cytoplasmic part;cytoplasmic vesicle;extracellular matrix part;extracellular region;extracellular region part;intracellular membrane-bounded organelle;int	ECM-receptor interaction;Focal adhesion;Malaria;Phagosome;TGF-beta signaling pathway	Homo sapiens	+
basement membrane;cell part;cytoplasmic part;endoplasmic reticulum;extracellular matrix;extracellular matrix part;extracellular membrane-bounded organelle;extracellular organelle;extracellular region;extracellular region pa	ECM-receptor interaction;Focal adhesion;Malaria;Phagosome;TGF-beta signaling pathway	Homo sapiens	+
cell part;cytoplasmic part;cytosol;intracellular membrane-bounded organelle;intracellular non-membrane-bounded organelle;intracellular organelle;intracellular organelle part;intracellular part;membrane-bounded organelle;no	Cysteine and methionine metabolism;Glycine, serine and threonine metabolism	Homo sapiens	+
cell part;cytoskeletal part;extracellular membrane-bounded organelle;extracellular organelle;extracellular region part;extracellular space;extracellular vesicular exosome;intermediate filament;intracellular membrane-bounded organelle;intracellular organelle;intracellular organelle part;intracellular part;macromolecular complex;membrane;membrane-bounded organelle;mer		Homo sapiens	+
basement membrane;extracellular matrix;extracellular matrix part;extracellular membrane-bounded organelle;extracellular organelle;extracellular region;extracellular region part;extracellular space;extracellular vesicular exosome;fibril;membrane-bounded organelle;membrane-bounded vesicle;microfibril;organelle;proteinaceous extracellular matrix;vesicle		Homo sapiens	+
extracellular matrix;extracellular matrix part;extracellular region;extracellular region part;fibril;microfibril;proteinaceous extracellular matrix		Homo sapiens	+
cell part;cytoplasm;cytoplasmic part;cytosol;endoplasmic reticulum;inclusion body;intracellular membrane-bounded organelle;intracellular organelle;intracellular organelle part;intracellular part;isoamylase complex;macromole	Starch and sucrose metabolism	Homo sapiens	+

actin cytoskeleton;actin filament bundle;actomyosin;actomyosin contractile ring;adherens junction;anchoring junction;cell cortex part;cell division site part;cell junction;cell leading edge;cell part;cell projection;cell-cell adhere	Tight junction;Viral myocarditis	Homo sapiens	+
actin cytoskeleton;actin filament bundle;actomyosin;axon;cell body;cell cortex;cell division site part;cell part;cell projection;cell projection part;cleavage furrow;cytoplasm;cytoplasmic part;cytoskeletal part;cytoskeleton;cytos	Tight junction;Viral myocarditis	Homo sapiens	+
cell part;COPI vesicle coat;cytoplasmic part;cytoplasmic vesicle part;cytosol;Golgi apparatus part;intracellular organelle part;intracellular part;macromolecular complex;membrane coat;membrane part;organelle part;protein complex;vesicle coat		Homo sapiens	+
cell part;cytoplasmic part;endoplasmic reticulum;endoplasmic reticulum membrane;endoplasmic reticulum part;integral to membrane;intracellular membrane-bounded organelle;intracellular organelle;intracellular organelle pa	Steroid biosynthesis	Homo sapiens	+
adherens junction;anchoring junction;cell junction;cell part;cell projection;cell projection part;cell-substrate adherens junction;cell-substrate junction;cytoplasmic part;cytoskeletal part;cytoskeleton;cytosol;dendritic spine;F-actin capping protein complex;focal adhesion;intermediate filament cytoskeleton;intracellular membrane-bounded organelle;intracellular non-membr		Homo sapiens	
acrosomal membrane;adherens junction;anchoring junction;cell junction;cell part;cell-substrate adherens junction;cell-substrate junction;cytoplasmic membrane-bounded vesicle;cytoplasmic part;cytoplasmic vesicle;cytoplasmic vesicle membrane;cytoplasmic vesicle part;extracellular membrane-bounded organelle;extracellular organelle;extracellular region part;extrace		Homo sapiens	+
basement membrane;cell part;extracellular matrix;extracellular matrix part;extracellular membrane-bounded organelle;extracellular organelle;extracellular region part;extracellular space;extracellular vesicular exosome;intracellular membrane-bounded organelle;intracellular organelle;intracellular part;membrane-bounded organelle;membrane-bounded vesicle;nucleus;orga		Homo sapiens	+
cell part;cytoplasm;intracellular membrane-bounded organelle;intracellular organelle;intracellular organelle part;intracellular part;membrane-bounded organelle;nuclear part;nucleoplasm;nucleus;organelle;organelle part		Homo sapiens	+
adherens junction;anchoring junction;cell junction;cell part;cell-substrate adherens junction;cell-substrate junction;cytoplasmic part;cytosol;focal adhesion;intracellular membrane-bounded organelle;intracellular organelle;intra	RNA transport	Homo sapiens	+
cell part;contractile fiber;cytoplasmic part;intracellular membrane-bounded organelle;intracellular non-membrane-bounded organelle;intracellular organelle;intracellular part;membrane-bounded organelle;non-membrane-bounded organelle;nucleus;organelle		Homo sapiens	+
cell part;cytoplasmic part;cytosol;extracellular membrane-bounded organelle;extracellular organelle;extracellular region part;extracellular vesicular exosome;intracellular membrane-bounded organelle;intracellular organelle;intracellular part;membrane-bounded organelle;membrane-bounded vesicle;mitochondrion;nucleus;organelle;vesicle		Homo sapiens	+
calcium channel complex;cation channel complex;cell part;cell projection;cytoplasmic part;cytosol;intracellular membrane-bounded organelle;intracellular organelle;intracellular organelle part;intracellular part;ion channel com	MAPK signaling pathway	Homo sapiens	+
cell part;cytoplasm;cytoplasmic part;cytoskeletal part;cytoskeleton;extracellular membrane-bounded organelle;extracellular organelle;extracellular region part;extracellular space;extracellular vesicular exosome;Golgi apparatus;intermediate filament;intermediate filament cytoskeleton;intracellular membrane-bounded organelle;intracellular non-membrane-bounded organe		Homo sapiens	+
cell part;cytoplasmic part;intracellular membrane-bounded organelle;intracellular organelle;intracellular organelle lumen;intracellular organelle part;intracellular part;membrane;membrane-bounded organelle;membrane-enclos	Butanoate metabolism;Geraniol degradation;Peroxisome;Synthesis and degradation of ketone bodies;Valine, leucine and isoleucine degradation	Homo sapiens	
cell part;cytoplasm;cytoplasmic mRNA processing body;cytoplasmic part;cytosol;cytosolic part;cytosolic proteasome complex;intracellular membrane-bounded organelle;intracellular non-membrane-bounded organelle;intrac	Proteasome	Homo sapiens	+
cell part;cell projection;centrosome;cilium;cytoplasmic microtubule;cytoplasmic part;cytoskeletal part;extracellular membrane-bounded organelle;extracellular organelle;extracellular region part;extracellular vesicular exosome;Golgi apparatus;Golgi apparatus part;Golgi membrane;intracellular membrane-bounded organelle;intracellular non-membrane-bounded organelle;		Homo sapiens	+
adherens junction;anchoring junction;cell cortex;cell junction;cell part;cell-cell junction;cell-substrate adherens junction;cell-substrate junction;cytoplasm;cytoplasmic part;cytoskeletal part;cytosol;early endosome;endoplasm	Acute myeloid leukemia;B cell receptor signaling pathway;Bladder cancer;Chronic myeloid leukemia;Endometrial cancer;ErbB signaling pathway;F	Homo sapiens	+
cell part;cytoplasmic part;extracellular membrane-bounded organelle;extracellular organelle;extracellular region part;extracellular vesicular exosome;intracellular membrane-bounded organelle;intracellular organelle;intracellula	Alzheimer's disease;Huntington's disease;Oxidative phosphorylation;Parkinson's disease	Homo sapiens	+
apical plasma membrane;cell part;cell projection;cytoplasmic part;cytosol;endosome;extracellular membrane-bounded organelle;extracellular organelle;extracellular region part;extracellular vesicular exosome;intracellular me	Collecting duct acid secretion;Epithelial cell signaling in Helicobacter pylori infection;Oxidative phosphorylation;Phagosome;Rheumatoid arthritis;V	Homo sapiens	
cell part;cytoplasm;cytoplasmic part;intracellular membrane-bounded organelle;intracellular organelle;intracellular organelle part;intracellular part;membrane;membrane-bounded organelle;membrane-enclosed lumen;mitocho	Porphyrin and chlorophyll metabolism	Homo sapiens	
adherens junction;anchoring junction;cell junction;cell part;cell-substrate adherens junction;cell-substrate junction;cytoplasm;cytoplasmic part;cytosol;cytosolic large ribosomal subunit;extracellular membrane-bounded orga	Ribosome	Homo sapiens	+
cell part;cytoplasm;cytoplasmic part;intracellular membrane-bounded organelle;intracellular non-membrane-bounded organelle;intracellular organelle;intracellular organelle lumen;intracellular organelle part;intracellular part;membrane;membrane-bounded organelle;membrane-enclosed lumen;mitochondrial matrix;mitochondrial nucleoid;mitochondrial part;mitochondrion		Homo sapiens	+
actin cytoskeleton;cell part;cytoplasm;cytoplasmic part;cytoskeleton;cytosol;extracellular membrane-bounded organelle;extracellular organelle;extracellular region part;extracellular vesicular exosome;intracellular non-membr	Amino sugar and nucleotide sugar metabolism;Galactose metabolism;Glycolysis / Gluconeogenesis;Pentose phosphate pathway;Purine metabolis	Homo sapiens	
adherens junction;anchoring junction;cell division site part;cell junction;cell part;cell projection;cell projection part;cell-substrate adherens junction;cell-substrate junction;chromosomal part;cleavage furrow;condensed chromo	Focal adhesion;Insulin signaling pathway;Long-term potentiation;Meiosis - yeast;Oocyte meiosis;Regulation of actin cytoskeleton;Vascular smoot	Homo sapiens	
cell part;extracellular region part;extracellular space;intracellular membrane-bounded organelle;intracellular organelle;intracellular part;membrane-bounded organelle;nucleus;organelle		Homo sapiens	+
axon hillock;axon part;basement membrane;cell part;cell projection part;cytoplasmic membrane-bounded vesicle;cytoplasmic part;cytoplasmic vesicle;extracellular matrix part;extracellular membrane-bounded organelle;extracellular organelle;extracellular region;extracellular region part;extracellular space;extracellular vesicular exosome;intracellular membrane-bounded		Homo sapiens	+
cell part;cytoplasmic part;extracellular membrane-bounded organelle;extracellular organelle;extracellular region part;extracellular vesicular exosome;intracellular membrane-bounded organelle;intracellular organelle;intracellula	Citrate cycle (TCA cycle);Lysine degradation	Homo sapiens	+
cell part;cytoplasmic part;cytosol;extracellular membrane-bounded organelle;extracellular organelle;extracellular region part;extracellular vesicular exosome;intracellular membrane-bounded organelle;intracellular organelle;int	Glutathione metabolism	Homo sapiens	
cell part;cytoplasm;cytoplasmic part;cytosol;extracellular membrane-bounded organelle;extracellular organelle;extracellular region part;extracellular vesicular exosome;intercellular bridge;intracellular membrane-bounded orga	Protein export	Homo sapiens	+
cell part;cytoplasm;cytoskeletal part;envelope;intracellular membrane-bounded organelle;intracellular organelle;intracellular organelle part;intracellular part;macromolecular complex;membrane;membrane part;membrane-bou	RNA transport	Homo sapiens	
extracellular membrane-bounded organelle;extracellular organelle;extracellular region part;extracellular vesicular exosome;membrane-bounded organelle;membrane-bounded vesicle;organelle;vesicle		Homo sapiens	+
cell part;cytoplasmic part;endoplasmic reticulum;endoplasmic reticulum membrane;endoplasmic reticulum part;integral to membrane;intracellular membrane-bounded organelle;intracellular organelle;intracellular organelle pa	Steroid biosynthesis	Homo sapiens	
extracellular membrane-bounded organelle;extracellular organelle;extracellular region part;extracellular vesicular exosome;membrane-bounded organelle;membrane-bounded vesicle;organelle;vesicle		Homo sapiens	+
cell part;cytoplasm;cytoplasmic part;cytosol;extracellular membrane-bounded organelle;extracellular organelle;extracellular region part;extracellular vesicular exosome;intracellular membrane-bounded organelle;intracellular o	Pentose phosphate pathway	Homo sapiens	+
cell part;cytoplasmic part;extracellular membrane-bounded organelle;extracellular organelle;extracellular region part;extracellular vesicular exosome;intracellular membrane-bounded organelle;intracellular organelle;intracellular organelle lumen;intracellular organelle part;intracellular part;membrane-bounded organelle;membrane-bounded vesicle;membrane-enclosed lum		Homo sapiens	+
catalytic step 2 spliceosome;cell part;chromatin;chromosomal part;euchromatin;extracellular membrane-bounded organelle;extracellular organelle;extracellular region part;extracellular space;extracellular vesicular exosome;ir	Spliceosome	Homo sapiens	+
apical plasma membrane;cell part;cell projection;cytoplasmic part;cytosol;extracellular membrane-bounded organelle;extracellular organelle;extracellular region part;extracellular vesicular exosome;integral to membrane;integ	Collecting duct acid secretion;Epithelial cell signaling in Helicobacter pylori infection;Oxidative phosphorylation;Phagosome;Rheumatoid arthritis;V	Homo sapiens	+
adherens junction;anchoring junction;cell junction;cell part;cell-substrate adherens junction;cell-substrate junction;cytoplasm;cytoplasmic part;extracellular membrane-bounded organelle;extracellular organelle;extracellular re	ko05152;RNA degradation	Homo sapiens	+
catalytic step 2 spliceosome;cell part;cytoplasm;cytoplasmic part;cytosol;exon-exon junction complex;intracellular non-membrane-bounded organelle;intracellular organelle;intracellular organelle part;intracellular part;macron	mRNA surveillance pathway;RNA transport;Spliceosome	Homo sapiens	+
adherens junction;anchoring junction;cell junction;cell part;cell-substrate adherens junction;cell-substrate junction;cytoplasm;cytoplasmic part;cytosol;cytosolic small ribosomal subunit;extracellular membrane-bounded orga	Ribosome	Homo sapiens	+
adherens junction;anchoring junction;cell junction;cell part;cell-substrate adherens junction;cell-substrate junction;cytoplasm;cytoplasmic part;cytosol;cytosolic large ribosomal subunit;extracellular membrane-bounded orga	Ribosome	Homo sapiens	+
basement membrane;cell part;collagen;cytoplasmic part;endoplasmic reticulum lumen;endoplasmic reticulum part;extracellular matrix;extracellular matrix part;extracellular membrane-bounded organelle;extracellular organell	Protein digestion and absorption	Homo sapiens	+
cell part;cytoplasmic part;endoplasmic reticulum;endoplasmic reticulum membrane;endoplasmic reticulum part;integral to membrane;intracellular membrane-bounded organelle;intracellular organelle;intracellular organelle pa	N-Glycan biosynthesis;Protein processing in endoplasmic reticulum;Various types of N-glycan biosynthesis	Homo sapiens	+
cell part;cytoplasm;cytoplasmic part;endoplasmic reticulum;intracellular membrane-bounded organelle;intracellular organelle;intracellular organelle part;intracellular part;membrane-bounded organelle;nuclear part;nucleoplasm;nucleus;organelle;organelle part;perinuclear region of cytoplasm		Homo sapiens	+
cell part;cytoplasmic part;intracellular membrane-bounded organelle;intracellular non-membrane-bounded organelle;intracellular organelle;intracellular organelle part;intracellular part;membrane;membrane-bounded organelle	Base excision repair;DNA replication;Non-homologous end-joining	Homo sapiens	+
cell part;centriole;cytoplasm;cytoplasmic membrane-bounded vesicle;cytoplasmic part;cytoplasmic vesicle;cytoskeletal part;extracellular membrane-bounded organelle;extracellular organelle;extracellular region part;extracellular vesicular exosome;F-actin capping protein complex;intracellular membrane-bounded organelle;intracellular non-membrane-bounded organelle		Homo sapiens	+
cell part;membrane;plasma membrane		Homo sapiens	+
cell part;cytoplasm;extracellular region;intracellular part;membrane		Homo sapiens	+
acrosomal vesicle;cell body;cell part;chaperonin-containing T-complex;cytoplasm;cytoplasmic membrane-bounded vesicle;cytoplasmic part;cytoplasmic vesicle;cytoskeletal part;cytosol;cytosolic part;extracellular membrane-bounded organelle;extracellular organelle;extracellular region part;extracellular vesicular exosome;intracellular membrane-bounded organelle;intra		Homo sapiens	+
cell part;cytoplasmic part;cytosol;intracellular part	Nicotinate and nicotinamide metabolism	Homo sapiens	+
cell part;cytoplasm;cytoplasmic part;cytosol;intracellular membrane-bounded organelle;intracellular organelle;intracellular organelle part;intracellular part;macromolecular complex;membrane-bounded organelle;nuclear part;r	Proteasome	Homo sapiens	+
adherens junction;anchoring junction;cell junction;cell part;cell-substrate adherens junction;cell-substrate junction;cytoplasm;cytoplasmic part;cytosol;cytosolic large ribosomal subunit;focal adhesion;intracellular membrane-	Ribosome	Homo sapiens	+
cell part;cytoplasm;cytoplasmic part;extracellular membrane-bounded organelle;extracellular organelle;extracellular region part;extracellular vesicular exosome;Golgi apparatus;Golgi apparatus part;Golgi membrane;intracellular membrane-bounded organelle;intracellular organelle;intracellular organelle part;intracellular part;membrane;membrane-bounded organelle;mem		Homo sapiens	
cell part;chromatin;chromosomal part;cytoplasm;cytoplasmic part;cytosol;intracellular membrane-bounded organelle;intracellular organelle;intracellular organelle part;intracellular part;membrane;membrane-bounded organell	Acute myeloid leukemia;Adipocytokine signaling pathway;Chemokine signaling pathway;Hepatitis C;Jak-STAT signaling pathway;ko05152;Measle	Homo sapiens	+
cell part;centrosome;cytoplasm;cytoplasmic part;cytoskeletal part;cytosol;extracellular membrane-bounded organelle;extracellular organelle;extracellular region part;extracellular space;extracellular vesicular exosome;intrace	Carbon fixation in photosynthetic organisms;Citrate cycle (TCA cycle);Glyoxylate and dicarboxylate metabolism;Proximal tubule bicarbonate recla	Homo sapiens	+
cell part;cytoplasmic part;extracellular membrane-bounded organelle;extracellular organelle;extracellular region part;extracellular vesicular exosome;intracellular membrane-bounded organelle;intracellular organelle;intracellula	Carbon fixation in photosynthetic organisms;Citrate cycle (TCA cycle);Glyoxylate and dicarboxylate metabolism;Pyruvate metabolism	Homo sapiens	+
cell part;chromosomal part;DNA replication factor C complex;intracellular membrane-bounded organelle;intracellular organelle;intracellular organelle part;intracellular part;macromolecular complex;membrane-bounded organe	DNA replication;Mismatch repair;Nucleotide excision repair	Homo sapiens	+
cell part;chromosomal part;DNA replication factor C complex;intracellular membrane-bounded organelle;intracellular organelle;intracellular organelle part;intracellular part;macromolecular complex;membrane-bounded organe	DNA replication;Mismatch repair;Nucleotide excision repair	Homo sapiens	
cell part;cytoplasmic part;fatty acid beta-oxidation multienzyme complex;intracellular membrane-bounded organelle;intracellular non-membrane-bounded organelle;intracellular organelle;intracellular organelle part;intracellula	Aminobenzoate degradation;Benzoate degradation;beta-Alanine metabolism;Biosynthesis of unsaturated fatty acids;Butanoate metabolism;Capro	Homo sapiens	+
cell part;cytoplasm;cytoplasmic part;cytosol;extracellular membrane-bounded organelle;extracellular organelle;extracellular region part;extracellular vesicular exosome;intracellular part;membrane-bounded organelle;membra	RNA transport	Homo sapiens	+
cell part;cytoplasm;intracellular membrane-bounded organelle;intracellular organelle;intracellular organelle part;intracellular part;membrane-bounded organelle;nuclear part;nucleoplasm;nucleus;organelle;organelle part		Homo sapiens	+
cell part;cell surface;clathrin coated vesicle membrane;clathrin-coated endocytic vesicle membrane;coated vesicle membrane;cytoplasmic part;cytoplasmic vesicle membrane;cytoplasmic vesicle part;endocytic vesicle mem	Basal cell carcinoma;Hedgehog signaling pathway;Melanogenesis;Pathways in cancer;Wnt signaling pathway	Homo sapiens	+
cell part;intracellular membrane-bounded organelle;intracellular organelle;intracellular organelle part;intracellular part;macromolecular complex;membrane-bounded organelle;nuclear part;nucleus;organelle;organelle part;ribor	Spliceosome	Homo sapiens	
cell part;cytoplasm;cytoplasmic part;cytosolic part;cytosolic ribosome;intracellular;intracellular membrane-bounded organelle;intracellular non-membrane-bounded organelle;intracellular organelle;intracellular organelle part;intracellular part;macromolecular complex;membrane;membrane-bounded organelle;NatA complex;non-membrane-bounded organelle;N-terminal p		Homo sapiens	
		Homo sapiens	+
cell junction;cell part;cell-cell junction;cytoplasm;cytoplasmic part;cytosol;extracellular membrane-bounded organelle;extracellular organelle;extracellular region part;extracellular vesicular exosome;extrinsic to internal side of	Chemokine signaling pathway;Epithelial cell signaling in Helicobacter pylori infection;Neurotrophin signaling pathway;Regulation of actin cytoske	Homo sapiens	+
axon;cell part;cell projection;cytoplasm;cytoplasmic membrane-bounded vesicle;cytoplasmic part;cytoplasmic vesicle;cytosol;extracellular membrane-bounded organelle;extracellular organelle;extracellular region part;extrac	Aminoacyl-tRNA biosynthesis	Homo sapiens	+
cell part;cytoplasm;cytoplasmic part;cytosol;extracellular membrane-bounded organelle;extracellular organelle;extracellular region part;extracellular vesicular exosome;intracellular organelle part;intracellular part;membrane;m	Aminoacyl-tRNA biosynthesis;Valine, leucine and isoleucine biosynthesis	Homo sapiens	+
cell part;cytoplasm;intracellular membrane-bounded organelle;intracellular organelle;intracellular part;membrane-bounded organelle;nucleus;organelle	RNA transport	Homo sapiens	+
apical plasma membrane;cell junction;cell leading edge;cell part;cell-cell junction;cytoplasm;cytoplasmic part;cytoskeleton;cytosol;endosome;extracellular membrane-bounded organelle;extracellular organelle;extracellular re	Endocytosis;Insulin signaling pathway;Tight junction	Homo sapiens	+
cell part;cytoplasmic part;extracellular membrane-bounded organelle;extracellular organelle;extracellular region part;extracellular vesicular exosome;intracellular membrane-bounded organelle;intracellular organelle;intracellula	Fatty acid metabolism	Homo sapiens	
cell part;chromatin;chromosomal part;envelope;intracellular membrane-bounded organelle;intracellular organelle;intracellular organelle part;intracellular part;membrane;membrane-bounded organelle;nuclear envelope;nuclear inner membrane;nuclear membrane;nuclear part;nucleus;organelle;organelle envelope;organelle inner membrane;organelle membrane;organelle pa		Homo sapiens	+
cell part;cytoplasm;envelope;integral to membrane;intracellular membrane-bounded organelle;intracellular organelle;intracellular organelle part;intracellular part;intrinsic to membrane;membrane;membrane part;membrane-bounded organelle;nuclear envelope;nuclear inner membrane;nuclear membrane;nuclear part;nucleus;organelle;organelle envelope;organelle inner m		Homo sapiens	+
axon;cell part;cell projection;chromatin;chromosomal part;cytoplasm;cytoplasmic part;cytosol;dendrite;intracellular membrane-bounded organelle;intracellular non-membrane-bounded organelle;intracellular organelle;intracel	Chemokine signaling pathway;Hepatitis C;Jak-STAT signaling pathway;ko05152;Leishmaniasis;Measles;Osteoclast differentiation;Pancreatic canc	Homo sapiens	+
cell part;chromatin;chromosomal part;cytoplasm;cytoplasmic part;cytosol;intracellular organelle part;intracellular part;membrane part;membrane raft;nuclear chromatin;nuclear chromosome part;nuclear part;nucleoplasm;org	Jak-STAT signaling pathway;ko05152	Homo sapiens	+
catalytic step 2 spliceosome;cell part;intracellular membrane-bounded organelle;intracellular non-membrane-bounded organelle;intracellular organelle;intracellular organelle part;intracellular part;macromolecular complex;mer	RNA degradation	Homo sapiens	+
cell part;cytoplasm;cytoplasmic part;cytosol;extracellular membrane-bounded organelle;extracellular organelle;extracellular region part;extracellular vesicular exosome;intracellular;intracellular membrane-bounded organelle;i	Arachidonic acid metabolism;Metabolism of xenobiotics by cytochrome P450;Steroid hormone biosynthesis	Homo sapiens	
cell body;cell part;cell projection;cytoplasm;cytoplasmic part;cytosol;cytosolic part;dendrite;endomembrane system;endoplasmic reticulum membrane;endoplasmic reticulum part;Golgi apparatus part;Golgi membrane;intrac	Acute myeloid leukemia;Adipocytokine signaling pathway;ErbB signaling pathway;Glioma;Insulin signaling pathway;mTOR signaling pathway;Path	Homo sapiens	+
cell part;cytoplasmic part;cytosol;intracellular membrane-bounded organelle;intracellular organelle;intracellular organelle part;intracellular part;membrane;membrane-bounded organelle;nuclear part;nucleoplasm;nucleus;orga	Alzheimer's disease;Amoebiasis;Amyotrophic lateral sclerosis (ALS);Apoptosis;Colorectal cancer;Epithelial cell signaling in Helicobacter pylori infe	Homo sapiens	+
cell part;cytoplasmic part;cytosol;cytosolic small ribosomal subunit;intracellular membrane-bounded organelle;intracellular non-membrane-bounded organelle;intracellular organelle;intracellular organelle part;intracellular part	Ribosome	Homo sapiens	+
cell part;chromosome;condensed chromosome;condensed nuclear chromosome;cytoplasmic part;cytoskeletal part;cytoskeleton;intracellular membrane-bounded organelle;intracellular non-membrane-bounded organelle;intracellular organelle;intracellular organelle part;intracellular part;macromolecular complex;membrane;membrane part;membrane-bounded organelle;		Homo sapiens	+
cell part;cytoplasmic part;extracellular membrane-bounded organelle;extracellular organelle;extracellular region part;extracellular vesicular exosome;intracellular membrane-bounded organelle;intracellular organelle;intracellula	Benzoate degradation;Fatty acid elongation in mitochondria;Fatty acid metabolism;Valine, leucine and isoleucine degradation	Homo sapiens	
cell part;cytoplasm;cytoplasmic part;cytosol;cytosolic large ribosomal subunit;intracellular non-membrane-bounded organelle;intracellular organelle;intracellular organelle part;intracellular part;large ribosomal subunit;macrom	Ribosome	Homo sapiens	+
cell part;chromatin;chromosomal part;cytoplasm;cytoplasmic part;cytosol;heterochromatin;intracellular membrane-bounded organelle;intracellular organelle;intracellular organelle part;intracellular part;membrane-bounded or	Bladder cancer;Cell cycle;Chronic myeloid leukemia;Glioma;Melanoma;Non-small cell lung cancer;p53 signaling pathway;Pancreatic cancer;Pathw	Homo sapiens	+
basal part of cell;cell part;cytoplasmic part;extracellular membrane-bounded organelle;extracellular organelle;extracellular region part;extracellular vesicular exosome;intracellular membrane-bounded organelle;intracellular or	Protein digestion and absorption	Homo sapiens	
autophagic vacuole;axon;cell part;cell projection;cytoplasm;cytoplasmic part;cytoplasmic vesicle membrane;cytoplasmic vesicle part;cytosol;dendrite;endoplasmic reticulum;endosome;Golgi apparatus;inclusion body;intrace	Huntington's disease	Homo sapiens	+
cell part;clathrin-coated vesicle;coated vesicle;cytoplasmic membrane-bounded vesicle;cytoplasmic part;cytoplasmic vesicle;early endosome;endosomal part;endosome;external side of plasma membrane;extracellular membrane-bounded organelle;extracellular organelle;extracellular region part;extracellular vesicular exosome;integral to membrane;intracellular membra		Homo sapiens	+
astral microtubule;axon;cell body;cell cortex;cell leading edge;cell part;cell projection;cell projection part;centrosome;chromosomal part;cilium;cytoplasmic microtubule;cytoplasmic part;cytoskeletal part;cytosol;envelope;ext	Ether lipid metabolism	Homo sapiens	
adherens junction;anchoring junction;cell junction;cell part;cell-substrate adherens junction;cell-substrate junction;external side of plasma membrane;extracellular region part;extracellular space;focal adhesion;integral to membrane;intracellular membrane-bounded organelle;intracellular organelle;intracellular part;intrinsic to membrane;membrane;membrane part;membra		Homo sapiens	+
cell part;intracellular organelle part;intracellular part;membrane;nuclear inner membrane;nuclear matrix;nuclear membrane;nuclear part;nucleoplasm;organelle inner membrane;organelle membrane;organelle part		Homo sapiens	+
cell part;chromosome;intracellular non-membrane-bounded organelle;intracellular organelle;intracellular organelle part;intracellular part;macromolecular complex;membrane;mismatch repair complex;MutSalpha complex;Mut	Colorectal cancer;Mismatch repair;Pathways in cancer	Homo sapiens	+
cell part;cytoplasmic part;glycerol-3-phosphate dehydrogenase complex;intracellular organelle part;intracellular part;macromolecular complex;membrane;mitochondrial inner membrane;mitochondrial membrane;mitochondri	Glycerophospholipid metabolism	Homo sapiens	+
cell part;cytoplasmic part;endoplasmic reticulum;endoplasmic reticulum membrane;endoplasmic reticulum part;integral to membrane;intracellular membrane-bounded organelle;intracellular organelle;intracellular organelle pa	Protein processing in endoplasmic reticulum	Homo sapiens	+
cell part;cytoplasm;envelope;intracellular membrane-bounded organelle;intracellular organelle;intracellular organelle part;intracellular part;membrane-bounded organelle;nuclear envelope;nuclear part;nucleus;organelle;organelle envelope;organelle part		Homo sapiens	+
cell part;cytoplasmic part;cytosol;extracellular membrane-bounded organelle;extracellular organelle;extracellular region part;extracellular space;extracellular vesicular exosome;intracellular membrane-bounded organelle;intra	Nicotinate and nicotinamide metabolism	Homo sapiens	+
blood microparticle;extracellular membrane-bounded organelle;extracellular organelle;extracellular region;extracellular region part;extracellular space;extracellular vesicular exosome;membrane-bounded organelle;membrane-bounded vesicle;organelle;vesicle		Homo sapiens	+
cell part;cytoplasm;cytoplasmic part;cytosol;cytosolic part;cytosolic proteasome complex;inclusion body;intracellular membrane-bounded organelle;intracellular organelle;intracellular organelle part;intracellular part;macromo	Proteasome	Homo sapiens	+
cell part;cytoplasmic part;intracellular membrane-bounded organelle;intracellular organelle;intracellular organelle part;intracellular part;membrane-bounded organelle;mitochondrion;nuclear part;nucleoplasm;nucleus;organelle;organelle part		Homo sapiens	+
cell part;cytoplasm;extracellular membrane-bounded organelle;extracellular organelle;extracellular region part;extracellular vesicular exosome;intracellular part;membrane-bounded organelle;membrane-bounded vesicle;organelle;vesicle		Homo sapiens	+
cell part;cytoplasmic part;extracellular membrane-bounded organelle;extracellular organelle;extracellular region part;extracellular vesicular exosome;intracellular membrane-bounded organelle;intracellular non-membrane-bou	Calcium signaling pathway;Huntington's disease;Parkinson's disease	Homo sapiens	+
cell part;centromeric heterochromatin;chromatin;chromatin remodeling complex;chromocenter;chromosomal part;envelope;heterochromatin;histone deacetylase complex;histone methyltransferase complex;intracellular membrane-bounded organelle;intracellular non-membrane-bounded organelle;intracellular organelle;intracellular organelle part;intracellular part;kinetoch		Homo sapiens	+
cell part;cytoplasmic part;intracellular membrane-bounded organelle;intracellular organelle;intracellular part;lysosome;lytic vacuole;membrane-bounded organelle;organelle;vacuole		Homo sapiens	+
cell part;cytoplasmic part;cytosol;intracellular membrane-bounded organelle;intracellular organelle;intracellular organelle part;intracellular part;membrane-bounded organelle;mitochondrion;nuclear part;nucleoplasm;organelle	Adipocytokine signaling pathway;Chagas disease (American trypanosomiasis);Colorectal cancer;Epithelial cell signaling in Helicobacter pylori infe	Homo sapiens	
cell part;chromosomal part;chromosome;chromosome, centromeric region;condensed chromosome;cytoplasm;intracellular membrane-bounded organelle;intracellular non-membrane-bounded organelle;intracellular organelle;intracellular organelle part;intracellular part;membrane;membrane-bounded organelle;non-membrane-bounded organelle;nuclear part;nucleolus;n		Homo sapiens	+
cell part;cytoplasmic part;cytosol;intracellular part;macromolecular complex;membrane;phosphorylase kinase complex;plasma membrane;protein complex	Calcium signaling pathway;Insulin signaling pathway	Homo sapiens	
cell part;chromosomal part;condensed chromosome kinetochore;cytoplasm;cytoplasmic part;cytoskeletal part;cytosol;intracellular non-membrane-bounded organelle;intracellular organelle;intracellular organelle part;intracellu	RNA transport	Homo sapiens	
cell part;intracellular membrane-bounded organelle;intracellular organelle;intracellular organelle part;intracellular part;membrane;membrane-bounded organelle;nuclear part;nucleoplasm;nucleus;organelle;organelle part		Homo sapiens	+
cell part;intracellular non-membrane-bounded organelle;intracellular organelle;intracellular organelle part;intracellular part;non-membrane-bounded organelle;nuclear part;nucleolus;organelle;organelle part		Homo sapiens	
actin cytoskeleton;cell part;cytoplasm;cytoplasmic part;cytoskeleton;cytosol;extracellular membrane-bounded organelle;extracellular organelle;extracellular region part;extracellular vesicular exosome;intracellular membrane-	Bacterial invasion of epithelial cells;Chemokine signaling pathway;Chronic myeloid leukemia;ErbB signaling pathway;Fc gamma R-mediated phag	Homo sapiens	
BAT3 complex;cell part;cytoplasm;cytoplasmic part;cytosol;cytosolic part;ER membrane insertion complex;intracellular membrane-bounded organelle;intracellular organelle;intracellular organelle part;intracellular part;macromolecular complex;membrane-bounded organelle;nuclear part;nucleoplasm;nucleus;organelle;organelle part;protein complex		Homo sapiens	+
cell part;cell projection part;cytoplasm;cytoplasmic part;cytoskeletal part;cytosol;dendritic shaft;extracellular membrane-bounded organelle;extracellular organelle;extracellular region part;extracellular vesicular exosome;Golg	Vasopressin-regulated water reabsorption	Homo sapiens	
cell part;cytoplasm;cytoplasmic part;cytosol;intracellular organelle part;intracellular part;membrane;nuclear part;nucleoplasm;organelle part	Amyotrophic lateral sclerosis (ALS);Fc epsilon RI signaling pathway;GnRH signaling pathway;MAPK signaling pathway;Toll-like receptor signaling	Homo sapiens	
BRCA1-A complex;BRISC complex;cell part;intracellular membrane-bounded organelle;intracellular organelle;intracellular organelle part;intracellular part;macromolecular complex;membrane-bounded organelle;nuclear part;nuclear ubiquitin ligase complex;nucleoplasm;nucleus;organelle;organelle part;protein complex;ubiquitin ligase complex		Homo sapiens	+
cell part;cytoplasmic part;cytosol;cytosolic large ribosomal subunit;intracellular organelle part;intracellular part;large ribosomal subunit;macromolecular complex;membrane;organelle part;ribonucleoprotein complex	Ribosome	Homo sapiens	+
adherens junction;anchoring junction;cell junction;cell part;cell-substrate adherens junction;cell-substrate junction;cytoplasm;cytoplasmic part;cytosol;cytosolic large ribosomal subunit;extracellular membrane-bounded orga	Ribosome	Homo sapiens	+
cell part;cytoplasm;cytoplasmic part;cytosol;cytosolic large ribosomal subunit;intracellular non-membrane-bounded organelle;intracellular organelle;intracellular organelle part;intracellular part;large ribosomal subunit;macrom	Ribosome	Homo sapiens	+
cell body;cell part;cell projection;cytoplasm;cytoplasmic part;cytosol;cytosolic large ribosomal subunit;dendrite;extracellular membrane-bounded organelle;extracellular organelle;extracellular region part;extracellular vesicula	Ribosome	Homo sapiens	+
adherens junction;anchoring junction;cell junction;cell part;cell-substrate adherens junction;cell-substrate junction;cytoplasm;cytoplasmic part;cytosol;cytosolic small ribosomal subunit;extracellular membrane-bounded orga	Ribosome	Homo sapiens	+
adherens junction;anchoring junction;cell junction;cell part;cell-substrate adherens junction;cell-substrate junction;cytoplasmic part;cytosol;cytosolic small ribosomal subunit;extracellular membrane-bounded organelle;extrac	Ribosome	Homo sapiens	+
adherens junction;anchoring junction;cell junction;cell part;cell-substrate adherens junction;cell-substrate junction;cytoplasm;cytoplasmic part;cytosol;cytosolic small ribosomal subunit;extracellular membrane-bounded orga	Ribosome	Homo sapiens	+
cell junction;cell part;cell projection;cell projection part;cytoplasm;cytoplasmic part;cytoskeletal part;cytosol;dendritic spine;intracellular organelle part;intracellular part;macromolecular complex;membrane;microtubule;microtubule associated complex;neuron projection;neuron spine;organelle part;plasma membrane;postsynaptic density;protein complex;synapse part		Homo sapiens	+
cell part;cytoplasm;extracellular membrane-bounded organelle;extracellular organelle;extracellular region part;extracellular vesicular exosome;intracellular part;membrane-bounded organelle;membrane-bounded vesicle;orga	Amino sugar and nucleotide sugar metabolism	Homo sapiens	+
cell junction;cell part;cytoplasm;cytoplasmic part;cytosol;intracellular membrane-bounded organelle;intracellular organelle;intracellular organelle part;intracellular part;macromolecular complex;membrane;membrane-bounded organelle;nuclear part;nucleoplasm;nucleoplasm part;nucleus;organelle;organelle part;protein complex;transcription factor complex		Homo sapiens	+
cell junction;cell part;cell projection;cell projection membrane;cell projection part;contractile fiber;cytoplasm;cytoplasmic part;cytoskeleton;dystrophin-associated glycoprotein complex;extracellular membrane-bounded organelle;extracellular organelle;extracellular region part;extracellular vesicular exosome;filopodium;filopodium membrane;intracellular non-membrane-b		Homo sapiens	+
actin filament;adherens junction;anchoring junction;axon;cell junction;cell leading edge;cell part;cell projection;cell projection part;cell-substrate adherens junction;cell-substrate junction;cytoplasm;cytoplasmic part;cytoskele	Regulation of actin cytoskeleton	Homo sapiens	+
cell part;cytoplasmic part;cytosol;extracellular membrane-bounded organelle;extracellular organelle;extracellular region part;extracellular vesicular exosome;intracellular organelle lumen;intracellular organelle part;intracellular part;lysosomal lumen;membrane;membrane-bounded organelle;membrane-bounded vesicle;membrane-enclosed lumen;organelle;organelle lumen		Homo sapiens	
cell part;cytoplasmic part;endoplasmic reticulum membrane;endoplasmic reticulum part;integral to membrane;intracellular organelle part;intracellular part;intrinsic to membrane;macromolecular complex;membrane;membran	N-Glycan biosynthesis;Protein processing in endoplasmic reticulum;Various types of N-glycan biosynthesis	Homo sapiens	+
		Homo sapiens	+
actin cytoskeleton;cell cortex part;cell part;cortical cytoskeleton;cytoplasmic part;cytoskeletal part;cytoskeleton;cytosol;extracellular membrane-bounded organelle;extracellular organelle;extracellular region;extracellular region part;extracellular vesicular exosome;F-actin capping protein complex;intracellular non-membrane-bounded organelle;intracellular organelle;intrac		Homo sapiens	+

actin cytoskeleton;actin filament;cell part;contractile fiber part;cytoplasmic part;cytoskeletal part;cytoskeleton;cytosol;extracellular membrane-bounded organelle;extracellular organelle;extracellular region part;extracellular vesicular exosome;F-actin capping protein complex;intracellular non-membrane-bounded organelle;intracellular organelle;intracellular organelle part;		Homo sapiens	+
cell part;cytoplasmic part;cytosol;intracellular part	RNA transport	Homo sapiens	+
cell part;cytoplasm;cytoplasmic part;cytosol;intracellular organelle lumen;intracellular organelle part;intracellular part;membrane-enclosed lumen;mitochondrial matrix;mitochondrial part;organelle lumen;organelle part	Aminoacyl-tRNA biosynthesis	Homo sapiens	+
cell part;cytoplasmic part;cytosol;cytosolic large ribosomal subunit;intracellular organelle part;intracellular part;large ribosomal subunit;macromolecular complex;membrane;organelle part;ribonucleoprotein complex	Ribosome	Homo sapiens	+
cell part;cytoplasmic part;intracellular membrane-bounded organelle;intracellular organelle;intracellular organelle part;intracellular part;macromolecular complex;membrane;membrane part;membrane-bounded organelle;mito	Alzheimer's disease;Cardiac muscle contraction;Huntington's disease;Oxidative phosphorylation;Parkinson's disease	Homo sapiens	+
cell part;cytoplasmic part;extracellular membrane-bounded organelle;extracellular organelle;extracellular region part;extracellular vesicular exosome;intracellular membrane-bounded organelle;intracellular organelle;intracellula	Alzheimer's disease;Huntington's disease;Oxidative phosphorylation;Parkinson's disease	Homo sapiens	+
adherens junction;anchoring junction;cell junction;cell part;cell-cell junction;cell-substrate adherens junction;cell-substrate junction;cytoplasmic part;cytosol;focal adhesion;intracellular part;membrane;perinuclear region of cytoplasm;plasma membrane		Homo sapiens	+
cell part;external side of plasma membrane;extracellular membrane-bounded organelle;extracellular organelle;extracellular region;extracellular region part;extracellular space;extracellular vesicular exosome;membrane part;m	Chemokine signaling pathway;Cytokine-cytokine receptor interaction;Intestinal immune network for IgA production;Leukocyte transendothelial mig	Homo sapiens	+
cell part;cytoplasm;intracellular membrane-bounded organelle;intracellular organelle;intracellular part;membrane;membrane-bounded organelle;nucleus;organelle		Homo sapiens	
cell part;cytoplasmic part;cytosol;intracellular membrane-bounded organelle;intracellular organelle;intracellular part;membrane-bounded organelle;mitochondrion;organelle	Carbon fixation in photosynthetic organisms;Pyruvate metabolism	Homo sapiens	+
cell part;extracellular matrix;extracellular region part;intracellular membrane-bounded organelle;intracellular organelle;intracellular part;membrane-bounded organelle;nucleus;organelle;proteinaceous extracellular matrix		Homo sapiens	+
AP-type membrane coat adaptor complex;cell part;clathrin adaptor complex;COPI vesicle coat;cytoplasm;cytoplasmic part;cytoplasmic vesicle part;cytosol;endoplasmic reticulum;Golgi apparatus;Golgi apparatus part;intracellular membrane-bounded organelle;intracellular organelle;intracellular organelle part;intracellular part;macromolecular complex;membrane;membr		Homo sapiens	+
cell part;cytoplasmic part;endoplasmic reticulum membrane;endoplasmic reticulum part;intracellular organelle part;intracellular part;lipid particle;membrane;membrane part;organelle membrane;organelle part	Steroid biosynthesis	Homo sapiens	
cell part;cytoplasmic part;cytosol;glutamate-cysteine ligase complex;intracellular part;macromolecular complex;protein complex	Glutathione metabolism	Homo sapiens	
adherens junction;anchoring junction;basement membrane;cell junction;cell part;cell-substrate adherens junction;cell-substrate junction;cytoplasmic part;cytosol;extracellular matrix part;extracellular region part;focal adhesion;integral to membrane;integral to plasma membrane;intracellular part;intrinsic to membrane;intrinsic to plasma membrane;membrane;membrane p		Homo sapiens	+
cell part;cytoplasmic part;cytosol;extracellular membrane-bounded organelle;extracellular organelle;extracellular region part;extracellular vesicular exosome;intracellular membrane-bounded organelle;intracellular organelle;in	Proteasome	Homo sapiens	+
cell part;cytoplasm;extracellular membrane-bounded organelle;extracellular organelle;extracellular region part;extracellular vesicular exosome;intracellular membrane-bounded organelle;intracellular organelle;intracellular part;membrane;membrane-bounded organelle;membrane-bounded vesicle;nucleus;organelle;vesicle		Homo sapiens	+
cell part;cytoplasmic part;cytosol;extracellular membrane-bounded organelle;extracellular organelle;extracellular region part;extracellular vesicular exosome;intracellular part;membrane-bounded organelle;membrane-bounde	Glutathione metabolism	Homo sapiens	+
cell body;cell part;centrosome;chaperonin-containing T-complex;cytoplasm;cytoplasmic part;cytoskeletal part;cytosol;cytosolic part;extracellular membrane-bounded organelle;extracellular organelle;extracellular region part;extracellular vesicular exosome;intracellular non-membrane-bounded organelle;intracellular organelle;intracellular organelle part;intracellular part;m		Homo sapiens	+
cell part;cytoplasm;cytoskeletal part;cytoskeleton;intermediate filament;intermediate filament cytoskeleton;intracellular non-membrane-bounded organelle;intracellular organelle;intracellular organelle part;intracellular part;macromolecular complex;non-membrane-bounded organelle;organelle;organelle part;protein complex		Homo sapiens	+
cell part;cytoplasmic part;endoplasmic reticulum;extracellular membrane-bounded organelle;extracellular organelle;extracellular region part;extracellular vesicular exosome;intracellular membrane-bounded organelle;intracellular organelle;intracellular part;membrane-bounded organelle;membrane-bounded vesicle;organelle;vesicle		Homo sapiens	
cell part;cell projection;centrosome;chromosomal part;cilium;condensed chromosome kinetochore;cytoplasm;cytoplasmic part;cytoskeletal part;cytosol;intracellular non-membrane-bounded organelle;intracellular organelle;in	Hedgehog signaling pathway;Wnt signaling pathway	Homo sapiens	+
cell part;cytoplasmic part;extracellular membrane-bounded organelle;extracellular organelle;extracellular region part;extracellular vesicular exosome;intracellular membrane-bounded organelle;intracellular organelle;intracellula	Carbon fixation pathways in prokaryotes;Citrate cycle (TCA cycle);Glutathione metabolism;Peroxisome	Homo sapiens	+
cell part;cytoplasmic part;endoplasmic reticulum membrane;endoplasmic reticulum part;extracellular membrane-bounded organelle;extracellular organelle;extracellular region part;extracellular vesicular exosome;Golgi apparatus part;Golgi membrane;intracellular organelle part;intracellular part;membrane;membrane part;membrane-bounded organelle;membrane-bounde		Homo sapiens	+
adherens junction;anchoring junction;cell junction;cell part;cell-substrate adherens junction;cell-substrate junction;extracellular membrane-bounded organelle;extracellular organelle;extracellular region part;extracellular space;extracellular vesicular exosome;focal adhesion;integral to membrane;integral to plasma membrane;intrinsic to membrane;intrinsic to plasma mem		Homo sapiens	+
cell part;intracellular membrane-bounded organelle;intracellular organelle;intracellular organelle part;intracellular part;membrane-bounded organelle;nuclear part;nucleoplasm;nucleus;organelle;organelle part	Base excision repair;DNA replication;Homologous recombination;Mismatch repair;Nucleotide excision repair;Purine metabolism;Pyrimidine metab	Homo sapiens	+
cell part;cytoplasm;extracellular membrane-bounded organelle;extracellular organelle;extracellular region part;extracellular vesicular exosome;intracellular part;membrane;membrane-bounded organelle;membrane-bounded v	Fc gamma R-mediated phagocytosis;Leishmaniasis	Homo sapiens	+
actin filament bundle;actomyosin;adherens junction;anchoring junction;cell cortex;cell junction;cell part;cell projection;cell-substrate adherens junction;cell-substrate junction;cytoplasm;cytoplasmic part;cytoskeletal part;cyto	Bacterial invasion of epithelial cells;Chemokine signaling pathway;Focal adhesion;Leukocyte transendothelial migration;Regulation of actin cytosk	Homo sapiens	
cell part;centrosome;cytoplasm;cytoplasmic part;cytoskeletal part;cytosol;extracellular membrane-bounded organelle;extracellular organelle;extracellular region part;extracellular vesicular exosome;intracellular membrane-bo	MAPK signaling pathway;Neurotrophin signaling pathway;VEGF signaling pathway	Homo sapiens	
cell part;cytoplasm;cytoplasmic part;cytosol;extracellular membrane-bounded organelle;extracellular organelle;extracellular region part;extracellular vesicular exosome;intracellular membrane-bounded organelle;intracellular o	Arginine and proline metabolism;Ascorbate and aldarate metabolism;beta-Alanine metabolism;Fatty acid metabolism;Glycerolipid metabolism;Gly	Homo sapiens	
cell part;cytoplasm;cytoplasmic part;cytosol;cytosolic large ribosomal subunit;extracellular membrane-bounded organelle;extracellular organelle;extracellular region part;extracellular vesicular exosome;intracellular non-memb	Ribosome	Homo sapiens	+
cell part;coated vesicle membrane;contractile fiber part;cytoplasmic part;cytoplasmic vesicle membrane;cytoplasmic vesicle part;endoplasmic reticulum membrane;endoplasmic reticulum part;endoplasmic reticulum-Golgi in	Protein processing in endoplasmic reticulum	Homo sapiens	+
cell part;chromatin;chromosomal part;cytoplasm;intracellular membrane-bounded organelle;intracellular organelle;intracellular organelle part;intracellular part;macromolecular complex;membrane-bounded organelle;nuclear chromatin;nuclear chromosome part;nuclear part;nucleoplasm;nucleus;organelle;organelle part;protein complex		Homo sapiens	+
cell part;cytoplasm;cytoplasmic membrane-bounded vesicle;cytoplasmic part;cytoplasmic vesicle;cytosol;extracellular membrane-bounded organelle;extracellular organelle;extracellular region part;extracellular vesicular exos	Fatty acid biosynthesis;Insulin signaling pathway	Homo sapiens	+
cell part;cytoplasmic part;cytosol;intracellular part		Homo sapiens	+
cell body;cell part;chaperonin-containing T-complex;cytoplasm;cytoplasmic part;cytoskeletal part;cytoskeleton;cytosol;cytosolic part;extracellular membrane-bounded organelle;extracellular organelle;extracellular region part;extracellular vesicular exosome;intracellular non-membrane-bounded organelle;intracellular organelle;intracellular organelle part;intracellular part;m		Homo sapiens	+
cell part;cytoplasmic part;intracellular membrane-bounded organelle;intracellular non-membrane-bounded organelle;intracellular organelle;intracellular organelle part;intracellular part;macromolecular complex;membrane;membrane-bounded organelle;mitochondrial inner membrane;mitochondrial membrane;mitochondrial part;non-membrane-bounded organelle;nuclear		Homo sapiens	+
cell part;cytoplasmic part;extracellular membrane-bounded organelle;extracellular organelle;extracellular region part;extracellular vesicular exosome;intracellular membrane-bounded organelle;intracellular non-membrane-bou	Plant-pathogen interaction	Homo sapiens	+
cell part;cytoplasm;cytoplasmic part;cytosol;extracellular membrane-bounded organelle;extracellular organelle;extracellular region part;extracellular vesicular exosome;intracellular membrane-bounded organelle;intracellular o	Arginine and proline metabolism;Ascorbate and aldarate metabolism;beta-Alanine metabolism;Fatty acid metabolism;Glycerolipid metabolism;Gly	Homo sapiens	+
cell part;chromosomal part;chromosome, centromeric region;condensed chromosome outer kinetochore;cytoplasm;cytoplasmic part;cytoskeletal part;cytosol;envelope;intracellular membrane-bounded organelle;intracellular non-membrane-bounded organelle;intracellular organelle;intracellular organelle part;intracellular part;kinetochore;macromolecular complex;membra		Homo sapiens	+
cell part;cytoplasmic part;cytosol;endoplasmic reticulum part;extracellular membrane-bounded organelle;extracellular organelle;extracellular region part;extracellular vesicular exosome;intracellular organelle part;intracellular p	Protein export	Homo sapiens	+
cell part;cytoplasmic part;cytosol;endoplasmic reticulum membrane;endoplasmic reticulum part;glycogen granule;intracellular organelle part;intracellular part;membrane;membrane part;organelle membrane;organelle part	Glycerophospholipid metabolism;Phosphonate and phosphinate metabolism	Homo sapiens	+
cell part;cytoplasm;cytoplasmic part;cytosol;extracellular membrane-bounded organelle;extracellular organelle;extracellular region part;extracellular vesicular exosome;intracellular part;membrane;membrane-bounded organe	Aminoacyl-tRNA biosynthesis	Homo sapiens	+
cell part;cytoplasm;cytoplasmic part;cytosol;intracellular part	Aminoacyl-tRNA biosynthesis	Homo sapiens	+
cell part;cytoplasm;cytoplasmic part;cytosol;extracellular membrane-bounded organelle;extracellular organelle;extracellular region part;extracellular vesicular exosome;intracellular membrane-bounded organelle;intracellular o	Aminoacyl-tRNA biosynthesis	Homo sapiens	
cell part;cytoplasmic part;cytosol;intracellular part;macromolecular complex;perinuclear region of cytoplasm;protein complex		Homo sapiens	
cell part;intracellular organelle part;intracellular part;membrane;nuclear part;nucleoplasm;organelle part	DNA replication;Purine metabolism;Pyrimidine metabolism	Homo sapiens	+
cell part;intracellular organelle part;intracellular part;nuclear part;nucleoplasm;organelle part	DNA replication;Purine metabolism;Pyrimidine metabolism	Homo sapiens	+
cell part;cytoplasm;cytoplasmic part;endoplasmic reticulum;endoplasmic reticulum membrane;endoplasmic reticulum part;intracellular membrane-bounded organelle;intracellular organelle;intracellular organelle part;intracellular part;IPAF inflammasome complex;macromolecular complex;membrane;membrane part;membrane-bounded organelle;mitochondrion;NALP3 in		Homo sapiens	+
cell part;chromosomal part;chromosome;chromosome, centromeric region;condensed chromosome;intracellular membrane-bounded organelle;intracellular non-membrane-bounded organelle;intracellular organelle;intracellular organelle part;intracellular part;membrane-bounded organelle;non-membrane-bounded organelle;nuclear part;nucleolus;nucleoplasm;nucleus;or		Homo sapiens	+
actin cytoskeleton;cell part;cytoplasm;cytoplasmic part;cytoskeleton;cytosol;extracellular membrane-bounded organelle;extracellular organelle;extracellular region part;extracellular vesicular exosome;Golgi apparatus;intracel	Proteasome	Homo sapiens	
cell part;cytoplasm;cytoplasmic part;cytosol;extracellular membrane-bounded organelle;extracellular organelle;extracellular region part;extracellular vesicular exosome;intracellular membrane-bounded organelle;intracellular o	Proteasome	Homo sapiens	+
cell part;chromatin;chromosomal part;cytoplasm;cytoskeleton;intracellular membrane-bounded organelle;intracellular non-membrane-bounded organelle;intracellular organelle;intracellular organelle part;intracellular part;macr	Cell cycle;Cell cycle - yeast;DNA replication;Meiosis - yeast	Homo sapiens	+
extracellular matrix;extracellular membrane-bounded organelle;extracellular organelle;extracellular region;extracellular region part;extracellular space;extracellular vesicular exosome;membrane-bounded organelle;membrane-	ECM-receptor interaction;Focal adhesion;Malaria;Phagosome;TGF-beta signaling pathway	Homo sapiens	+
cell part;cytoplasm;cytoplasmic part;intracellular membrane-bounded organelle;intracellular non-membrane-bounded organelle;intracellular organelle;intracellular organelle lumen;intracellular organelle part;intracellular part;m	Fatty acid metabolism	Homo sapiens	+
cell part;cytoplasm;intracellular membrane-bounded organelle;intracellular organelle;intracellular organelle part;intracellular part;membrane-bounded organelle;nuclear body;nuclear part;nuclear speck;nucleoplasm;nucleoplasm part;nucleus;organelle;organelle part		Homo sapiens	
cell part;cytoplasmic part;extracellular membrane-bounded organelle;extracellular organelle;extracellular region part;extracellular vesicular exosome;intracellular membrane-bounded organelle;intracellular organelle;intracellula	Biosynthesis of unsaturated fatty acids	Homo sapiens	+
cell part;cis-Golgi network;clathrin-coated vesicle;coated vesicle;COPI-coated vesicle;cytoplasmic membrane-bounded vesicle;cytoplasmic part;cytoplasmic vesicle;cytoplasmic vesicle membrane;cytoplasmic vesicle part;endoplasmic reticulum;endoplasmic reticulum membrane;endoplasmic reticulum part;endoplasmic reticulum-Golgi intermediate compartment;endop		Homo sapiens	+
cell part;cytoplasm;intracellular organelle part;intracellular part;macromolecular complex;nuclear body;nuclear part;nuclear speck;nucleoplasm;nucleoplasm part;organelle part;ribonucleoprotein complex;spliceosomal compl	Spliceosome	Homo sapiens	+
adherens junction;anchoring junction;apical part of cell;basolateral plasma membrane;cell junction;cell part;cell-substrate adherens junction;cell-substrate junction;clathrin-coated vesicle;coated vesicle;cytoplasmic membrar	Notch signaling pathway	Homo sapiens	+
cell part;cytoplasm;cytoplasmic part;cytosol;eukaryotic translation initiation factor 2B complex;intracellular part;macromolecular complex;protein complex	RNA transport	Homo sapiens	+
cell part;chromatin remodeling complex;cytoplasm;cytoskeleton;extracellular membrane-bounded organelle;extracellular organelle;extracellular region part;extracellular vesicular exosome;histone deacetylase complex;intracellular membrane-bounded organelle;intracellular non-membrane-bounded organelle;intracellular organelle;intracellular organelle part;intracellular pa		Homo sapiens	
cell part;cytoplasm;inclusion body;intracellular non-membrane-bounded organelle;intracellular organelle;intracellular organelle part;intracellular part;macromolecular complex;membrane;membrane part;non-membrane-bounc	RNA transport	Homo sapiens	
cell part;cytoplasmic part;cytosol;inclusion body;intracellular membrane-bounded organelle;intracellular organelle;intracellular organelle part;intracellular part;macromolecular complex;membrane;membrane part;membrane-b	RNA transport	Homo sapiens	
cell part;cytoplasmic part;intracellular organelle part;intracellular part;macromolecular complex;membrane;membrane part;mitochondrial inner membrane;mitochondrial membrane;mitochondrial membrane part;mitochondrial	Alzheimer's disease;Huntington's disease;Oxidative phosphorylation;Parkinson's disease	Homo sapiens	+
beta-catenin destruction complex;cell part;cytoplasmic part;cytosol;intracellular part;macromolecular complex;protein complex	Chemokine signaling pathway	Homo sapiens	
beta-catenin destruction complex;cell body;cell part;cell projection;cell projection part;centrosome;cytoplasm;cytoplasmic part;cytoskeletal part;cytosol;dendritic shaft;dendritic spine;growth cone;intracellular membrane-bou	Alzheimer's disease;Axon guidance;B cell receptor signaling pathway;Basal cell carcinoma;Cell cycle;Chemokine signaling pathway;Circadian rhy	Homo sapiens	
cell part;cytoplasmic part;cytosol;intracellular part	Nicotinate and nicotinamide metabolism;Purine metabolism;Pyrimidine metabolism	Homo sapiens	+
cell part;cytoplasm;intracellular organelle part;intracellular part;membrane;nuclear membrane;nuclear part;organelle membrane;organelle part;plasma membrane	Selenocompound metabolism	Homo sapiens	+
cell part;cytoplasm;cytoplasmic part;cytosol;intracellular part	Drug metabolism - other enzymes;Purine metabolism	Homo sapiens	+
cell part;chromatin;chromosomal part;cytoplasmic part;cytosol;intracellular membrane-bounded organelle;intracellular organelle;intracellular organelle part;intracellular part;macromolecular complex;membrane-bounded orga	Homologous recombination;Non-homologous end-joining	Homo sapiens	+
cell part;cytoplasmic part;intracellular membrane-bounded organelle;intracellular organelle;intracellular organelle lumen;intracellular organelle part;intracellular part;membrane-bounded organelle;membrane-enclosed lumen;m	Citrate cycle (TCA cycle)	Homo sapiens	+
cell part;cytoplasm;intracellular part		Homo sapiens	+
adherens junction;anchoring junction;cell junction;cell part;cell-substrate adherens junction;cell-substrate junction;cytoplasmic membrane-bounded vesicle;cytoplasmic part;cytoplasmic vesicle;extracellular matrix;extracellula	GnRH signaling pathway	Homo sapiens	+
cell part;cytoplasmic part;integral to membrane;integral to mitochondrial inner membrane;integral to mitochondrial membrane;integral to organelle membrane;intracellular organelle part;intracellular part;intrinsic to membrane;	Porphyrin and chlorophyll metabolism	Homo sapiens	+
adherens junction;anchoring junction;cell junction;cell part;cell-substrate adherens junction;cell-substrate junction;cytoplasm;cytoplasmic part;cytosol;extracellular membrane-bounded organelle;extracellular organelle;extracellular region part;extracellular vesicular exosome;focal adhesion;Golgi apparatus;intracellular membrane-bounded organelle;intracellular organelle;		Homo sapiens	+
cell part;cytoplasmic part;cytoskeletal part;endoplasmic reticulum;envelope;integral to membrane;intracellular membrane-bounded organelle;intracellular organelle;intracellular organelle part;intracellular part;intrinsic to memb	Arrhythmogenic right ventricular cardiomyopathy (ARVC);Dilated cardiomyopathy;Hypertrophic cardiomyopathy (HCM)	Homo sapiens	+
cell part;cytoplasmic part;integral to membrane;integral to mitochondrial membrane;integral to mitochondrial outer membrane;integral to organelle membrane;intracellular membrane-bounded organelle;intracellular organelle;	Adipocytokine signaling pathway;Fatty acid metabolism;PPAR signaling pathway	Homo sapiens	+
cell part;cytoplasmic part;cytosol;extracellular membrane-bounded organelle;extracellular organelle;extracellular region part;extracellular space;extracellular vesicular exosome;intracellular part;membrane-bounded organelle;membrane-bounded vesicle;organelle;vesicle		Homo sapiens	+
cell part;cytoplasm;cytoplasmic part;cytosol;extracellular membrane-bounded organelle;extracellular organelle;extracellular region part;extracellular space;extracellular vesicular exosome;intracellular;intracellular membrane-b	Amoebiasis	Homo sapiens	+
cell part;cytoplasmic part;endoplasmic reticulum;endoplasmic reticulum lumen;endoplasmic reticulum part;endoplasmic reticulum-Golgi intermediate compartment;extracellular membrane-bounded organelle;extracellular organelle;extracellular region part;extracellular vesicular exosome;intracellular membrane-bounded organelle;intracellular organelle;intracellular organe		Homo sapiens	+
		Homo sapiens	+
cell part;cytoplasm;cytoplasmic part;cytosol;extracellular membrane-bounded organelle;extracellular organelle;extracellular region part;extracellular vesicular exosome;intracellular part;macromolecular complex;membrane-bounded organelle;membrane-bounded vesicle;organelle;protein complex;vesicle		Homo sapiens	+
actin cytoskeleton;adherens junction;anchoring junction;cell junction;cell part;cell projection membrane;cell projection part;cell-cell junction;cell-substrate adherens junction;cell-substrate junction;cytoplasm;cytoplasmic part	Fc gamma R-mediated phagocytosis;Focal adhesion;Leukocyte transendothelial migration	Homo sapiens	+
adherens junction;anchoring junction;cell junction;cell part;cell-substrate adherens junction;cell-substrate junction;coated pit;cytoplasm;cytoplasmic part;cytoplasmic vesicle membrane;cytoplasmic vesicle part;cytoskeletal p	Bacterial invasion of epithelial cells;Endocrine and other factor-regulated calcium reabsorption;Endocytosis;Fc gamma R-mediated phagocytosis	Homo sapiens	+
cell part;cytoplasm;cytoplasmic part;cytosol;intracellular part;membrane;plasma membrane		Homo sapiens	+
cell part;cytoplasmic part;intracellular membrane-bounded organelle;intracellular organelle;intracellular part;membrane-bounded organelle;mitochondrion;organelle	Purine metabolism;Pyrimidine metabolism	Homo sapiens	+
cell part;cytoplasm;cytoplasmic part;holo TFIIH complex;intracellular membrane-bounded organelle;intracellular organelle;intracellular organelle part;intracellular part;macromolecular complex;membrane-bounded organelle;n	Basal transcription factors;Cell cycle;Nucleotide excision repair	Homo sapiens	+
actin cytoskeleton;cell part;chromosomal part;condensed chromosome kinetochore;cytoplasm;cytoplasmic part;cytoskeletal part;cytoskeleton;cytosol;intracellular membrane-bounded organelle;intracellular non-membrane-bounded organelle;intracellular organelle;intracellular organelle part;intracellular part;kinetochore;kinetochore microtubule;macromolecular complex;		Homo sapiens	+
cell part;cytoplasmic membrane-bounded vesicle;cytoplasmic part;cytoplasmic vesicle;endomembrane system;endoplasmic reticulum;extrinsic to membrane;Golgi apparatus;integral to membrane;intracellular membrane-bounded organelle;intracellular organelle;intracellular part;intrinsic to membrane;lysosome;lytic vacuole;membrane;membrane part;membrane-bounde		Homo sapiens	+
cell part;external side of plasma membrane;extracellular membrane-bounded organelle;extracellular organelle;extracellular region part;extracellular vesicular exosome;integral to membrane;integral to plasma membrane;intrinsic to membrane;intrinsic to plasma membrane;membrane;membrane part;membrane-bounded organelle;membrane-bounded vesicle;organelle;pl		Homo sapiens	
axon;cell body;cell part;cell projection;clathrin-coated vesicle;coated vesicle;cytoplasmic membrane-bounded vesicle;cytoplasmic part;cytoplasmic vesicle;cytosol;dendrite;extracellular membrane-bounded organelle;extrace	Fatty acid elongation in mitochondria;Lysosome	Homo sapiens	+
cell part;cytoplasm;cytoplasmic part;cytosol;cytosolic large ribosomal subunit;extracellular membrane-bounded organelle;extracellular organelle;extracellular region part;extracellular vesicular exosome;intracellular organelle p	Ribosome	Homo sapiens	+
aggresome;cell body;cell part;centrosome;chaperonin-containing T-complex;cytoplasm;cytoplasmic part;cytoskeletal part;cytoskeleton;cytosol;cytosolic part;extracellular membrane-bounded organelle;extracellular organelle;extracellular region part;extracellular vesicular exosome;inclusion body;intermediate filament cytoskeleton;intracellular non-membrane-bounded o		Homo sapiens	+
cell body;cell part;centrosome;chaperonin-containing T-complex;cytoplasm;cytoplasmic membrane-bounded vesicle;cytoplasmic part;cytoplasmic vesicle;cytoskeletal part;cytosol;cytosolic part;extracellular membrane-bounded organelle;extracellular organelle;extracellular region part;extracellular vesicular exosome;intracellular membrane-bounded organelle;intracellula		Homo sapiens	+
azurophil granule;cell part;cytoplasm;cytoplasmic membrane-bounded vesicle;cytoplasmic part;cytoplasmic vesicle;cytoskeletal part;endocytic vesicle;envelope;extracellular membrane-bounded organelle;extracellular organelle;extracellular region part;extracellular vesicular exosome;intracellular membrane-bounded organelle;intracellular non-membrane-bounded orga		Homo sapiens	+
cell part;cytoplasm;intracellular membrane-bounded organelle;intracellular organelle;intracellular organelle part;intracellular part;membrane-bounded organelle;nuclear part;nucleoplasm;nucleus;organelle;organelle part	mRNA surveillance pathway	Homo sapiens	
cell part;contractile fiber part;costamere;cytoplasm;cytoplasmic part;intracellular non-membrane-bounded organelle;intracellular organelle;intracellular organelle part;intracellular part;macromolecular complex;membrane;non-membrane-bounded organelle;nuclear part;nucleolus;organelle;organelle part;polysome;ribonucleoprotein complex		Homo sapiens	+
cell part;cytoplasm;cytosolic large ribosomal subunit;extracellular membrane-bounded organelle;extracellular organelle;extracellular region part;extracellular vesicular exosome;intracellular membrane-bounded organelle;intracellular organelle;intracellular organelle part;intracellular part;large ribosomal subunit;macromolecular complex;membrane;membrane-bounded org		Homo sapiens	+
cell part;cytoplasmic membrane-bounded vesicle;cytoplasmic part;cytoplasmic vesicle;early endosome;early endosome membrane;endocytic vesicle;endosomal part;endosome;endosome membrane;extracellular membrane	Amoebiasis;Endocytosis;ko05152;Phagosome;Vasopressin-regulated water reabsorption	Homo sapiens	+
cell part;cytoplasmic membrane-bounded vesicle;cytoplasmic part;cytoplasmic vesicle;cytoplasmic vesicle membrane;cytoplasmic vesicle part;endocytic vesicle;endocytic vesicle membrane;endosome;extracellular membra	Amoebiasis;Endocytosis;ko05152;Phagosome	Homo sapiens	+
cell part;cytoplasmic membrane-bounded vesicle;cytoplasmic part;cytoplasmic vesicle;cytoplasmic vesicle membrane;cytoplasmic vesicle part;endocytic vesicle;endocytic vesicle membrane;endoplasmic reticulum membrar	Measles	Homo sapiens	+
cell junction;cell part;cell projection;cell-cell junction;cytoplasm;cytoplasmic membrane-bounded vesicle;cytoplasmic part;cytoplasmic vesicle;cytoplasmic vesicle membrane;cytoplasmic vesicle part;endocytic vesicle;endos	Tight junction	Homo sapiens	+
apical plasma membrane;cell part;cell projection;cilium;cytoplasmic membrane-bounded vesicle;cytoplasmic part;cytoplasmic vesicle;cytoplasmic vesicle membrane;cytoplasmic vesicle part;dendrite;endosomal part;endosome;endosome membrane;exocytic vesicle;extracellular membrane-bounded organelle;extracellular organelle;extracellular region part;extracellular v		Homo sapiens	+
		Homo sapiens	+
cell part;cytoplasm;cytoplasmic part;cytosol;extracellular membrane-bounded organelle;extracellular organelle;extracellular region part;extracellular vesicular exosome;immunological synapse;intracellular membrane-bounded	MAPK signaling pathway	Homo sapiens	
cell part;chromatin;chromatin remodeling complex;chromosomal part;euchromatin;extracellular region part;extracellular space;heterochromatin;intracellular membrane-bounded organelle;intracellular non-membrane-bounded organelle;intracellular organelle;intracellular organelle part;intracellular part;macromolecular complex;membrane;membrane-bounded organelle;nE		Homo sapiens	
cell part;cytoplasmic part;intracellular membrane-bounded organelle;intracellular non-membrane-bounded organelle;intracellular organelle;intracellular organelle lumen;intracellular organelle part;intracellular part;membrane-b	Citrate cycle (TCA cycle)	Homo sapiens	
cell part;cytoplasm;cytoplasmic part;cytosol;extracellular membrane-bounded organelle;extracellular organelle;extracellular region part;extracellular vesicular exosome;intracellular part;membrane;membrane-bounded organe	Amino sugar and nucleotide sugar metabolism;Galactose metabolism	Homo sapiens	+
cell part;cytoplasmic part;endoplasmic reticulum part;extracellular membrane-bounded organelle;extracellular organelle;extracellular region part;extracellular vesicular exosome;integral to membrane;intracellular organelle par	Protein processing in endoplasmic reticulum	Homo sapiens	+
cell part;clathrin-coated vesicle;coated vesicle;cytoplasmic membrane-bounded vesicle;cytoplasmic part;cytoplasmic vesicle;cytosol;endoplasmic reticulum;endoplasmic reticulum membrane;endoplasmic reticulum part;end	Protein processing in endoplasmic reticulum	Homo sapiens	
cell part;cytoplasmic part;cytosol;extracellular membrane-bounded organelle;extracellular organelle;extracellular region part;extracellular vesicular exosome;intracellular part;membrane-bounded organelle;membrane-bounde	Drug metabolism - other enzymes	Homo sapiens	+
cell body;cell part;chromatin remodeling complex;cytoplasm;cytoplasmic part;histone acetyltransferase complex;histone methyltransferase complex;intracellular membrane-bounded organelle;intracellular organelle;intracellular organelle part;intracellular part;macromolecular complex;membrane;membrane-bounded organelle;methyltransferase complex;mitochondrion;M		Homo sapiens	+
cell part;cytoplasmic part;endoplasmic reticulum membrane;endoplasmic reticulum part;extracellular membrane-bounded organelle;extracellular organelle;extracellular region part;extracellular vesicular exosome;integral to m	Arginine and proline metabolism;Ascorbate and aldarate metabolism;beta-Alanine metabolism;Chloroalkane and chloroalkene degradation;Fatty a	Homo sapiens	+
cell part;cytoplasmic part;intracellular membrane-bounded organelle;intracellular organelle;intracellular organelle lumen;intracellular organelle part;intracellular part;membrane;membrane-bounded organelle;membrane-enclos	Peroxisome;Primary bile acid biosynthesis	Homo sapiens	+
cell part;cytoplasmic part;cytosol;extracellular membrane-bounded organelle;extracellular organelle;extracellular region part;extracellular vesicular exosome;intracellular membrane-bounded organelle;intracellular organelle;in	Proteasome	Homo sapiens	
cell part;cytoplasm;cytoplasmic part;cytosol;intracellular membrane-bounded organelle;intracellular organelle;intracellular organelle part;intracellular part;membrane-bounded organelle;nuclear part;nucleoplasm;nucleus;orga	Long-term potentiation;MAPK signaling pathway;mTOR signaling pathway;Neurotrophin signaling pathway;Oocyte meiosis;Progesterone-mediate	Homo sapiens	+
cell part;cytoplasm;extracellular region part;extracellular space;intracellular organelle part;intracellular part;macromolecular complex;nuclear part;nucleoplasm;nucleoplasm part;organelle part;protein complex;transcriptional repressor complex		Homo sapiens	+
cell part;collagen;cytoplasmic part;extracellular matrix;extracellular matrix part;extracellular membrane-bounded organelle;extracellular organelle;extracellular region;extracellular region part;extracellular space;extracellular vesicular exosome;fibrillar collagen;Golgi apparatus part;Golgi lumen;intracellular organelle lumen;intracellular organelle part;intracellular part;lysosom		Homo sapiens	+
cell part;cyclin-dependent protein kinase activating kinase holoenzyme complex;holo TFIIH complex;intracellular membrane-bounded organelle;intracellular organelle;intracellular organelle part;intracellular part;macromolecul	Basal transcription factors;Cell cycle;Nucleotide excision repair	Homo sapiens	+
cell part;cytoplasm;holo TFIIH complex;intracellular organelle part;intracellular part;macromolecular complex;nuclear part;nucleoplasm;nucleoplasm part;organelle part;protein complex;transcription factor complex	Basal transcription factors;Nucleotide excision repair	Homo sapiens	+
cell part;cytoplasmic part;intracellular membrane-bounded organelle;intracellular organelle;intracellular organelle part;intracellular part;macromolecular complex;membrane;membrane part;membrane-bounded organelle;mem	Alzheimer's disease;Huntington's disease;Oxidative phosphorylation;Parkinson's disease	Homo sapiens	+
catalytic step 2 spliceosome;cell part;intracellular membrane-bounded organelle;intracellular organelle;intracellular organelle part;intracellular part;macromolecular complex;membrane-bounded organelle;nuclear part;nucleop	Spliceosome	Homo sapiens	+
cell part;cytoplasmic part;cytosol;extracellular membrane-bounded organelle;extracellular organelle;extracellular region part;extracellular vesicular exosome;intracellular membrane-bounded organelle;intracellular organelle;in	Glutathione metabolism;Pentose phosphate pathway	Homo sapiens	+
catalytic step 2 spliceosome;cell part;extracellular matrix;extracellular membrane-bounded organelle;extracellular organelle;extracellular region part;extracellular vesicular exosome;integral to membrane;integral to plasma me	Spliceosome	Homo sapiens	+
cell part;cytoplasm;cytoplasmic part;cytosol;intracellular organelle part;intracellular part;membrane;nuclear part;nucleoplasm;organelle part		Homo sapiens	+
cell part;cell projection;cytoplasm;cytoplasmic part;cytosol;dendrite;intracellular membrane-bounded organelle;intracellular organelle;intracellular organelle part;intracellular part;macromolecular complex;membrane part;membrane-bounded organelle;neuron projection;nuclear part;nuclear pore;nucleoplasm;nucleus;organelle;organelle part;pore complex;protein complex		Homo sapiens	+
cell part;cytoplasm;cytoplasmic part;cytosol;intracellular membrane-bounded organelle;intracellular organelle;intracellular organelle part;intracellular part;macromolecular complex;membrane-bounded organelle;mRNA cap bi	mRNA surveillance pathway;RNA transport;Spliceosome	Homo sapiens	
cell part;cytoplasmic part;cytosol;endoplasmic reticulum;extracellular membrane-bounded organelle;extracellular organelle;extracellular region part;extracellular vesicular exosome;intracellular membrane-bounded organelle;intracellular organelle;intracellular part;membrane-bounded organelle;membrane-bounded vesicle;mitochondrion;organelle;vesicle		Homo sapiens	

cell part;cytoplasmic part;cytoskeleton;cytosol;extracellular membrane-bounded organelle;extracellular organelle;extracellular region part;extracellular vesicular exosome;immunological synapse;intracellular non-membrane-b	Neurotrophin signaling pathway;Vasopressin-regulated water reabsorption	Homo sapiens	+
cell part;cytoplasm;cytoplasmic membrane-bounded vesicle;cytoplasmic part;cytoplasmic vesicle;cytoskeleton;cytosol;extracellular membrane-bounded organelle;extracellular organelle;extracellular region part;extracellular v	Neurotrophin signaling pathway;Vasopressin-regulated water reabsorption	Homo sapiens	
cell body;cell part;cell projection;cytoplasmic membrane-bounded vesicle;cytoplasmic part;cytoplasmic vesicle;intracellular membrane-bounded organelle;intracellular organelle;intracellular organelle part;intracellular part;macromolecular complex;membrane part;membrane-bounded organelle;membrane-bounded vesicle;neuronal cell body;nuclear part;nuclear pore;orga		Homo sapiens	+
catalytic step 2 spliceosome;cell part;intracellular membrane-bounded organelle;intracellular organelle;intracellular organelle part;intracellular part;macromolecular complex;membrane;membrane-bounded organelle;nuclear part;nucleoplasm;nucleus;organelle;organelle part;ribonucleoprotein complex;spliceosomal complex		Homo sapiens	+
cell part;cytoplasm;cytoplasmic part;cytosol;intracellular organelle part;intracellular part;membrane;nuclear part;nucleoplasm;organelle part;plasma membrane	Chemokine signaling pathway;Hepatitis C;Jak-STAT signaling pathway;ko05152;Measles;Osteoclast differentiation	Homo sapiens	+
cell part;chromatin;chromosomal part;chromosome;cytoplasm;cytoplasmic part;Golgi apparatus;intracellular membrane-bounded organelle;intracellular non-membrane-bounded organelle;intracellular organelle;intracellular or	Colorectal cancer;Mismatch repair;Pathways in cancer	Homo sapiens	
cell part;cytoplasm;cytoplasmic part;cytoskeletal part;cytosol;intracellular non-membrane-bounded organelle;intracellular organelle;intracellular organelle part;intracellular part;kinesin complex;macromolecular complex;membrane;microtubule;microtubule associated complex;non-membrane-bounded organelle;organelle;organelle part;protein complex;spindle;spindle pol		Homo sapiens	+
cell part;cytoplasmic part;cytosol;intracellular part;membrane;plasma membrane	B cell receptor signaling pathway;Chemokine signaling pathway;Fc epsilon RI signaling pathway;Fc gamma R-mediated phagocytosis;Focal adhe	Homo sapiens	
cell part;cytoplasmic part;cytosol;extracellular membrane-bounded organelle;extracellular organelle;extracellular region part;extracellular vesicular exosome;intracellular part;membrane-bounded organelle;membrane-bounde	Arginine and proline metabolism;beta-Alanine metabolism;Cysteine and methionine metabolism;Glutathione metabolism	Homo sapiens	+
cell part;cytoplasmic part;cytosol;intracellular organelle part;intracellular part;membrane;mitochondrial membrane;mitochondrial outer membrane;mitochondrial part;organelle membrane;organelle outer membrane;organelle p	Amino sugar and nucleotide sugar metabolism;Butirosin and neomycin biosynthesis;Carbohydrate digestion and absorption;Fructose and mannos	Homo sapiens	+
cell part;cytoplasmic part;intracellular membrane-bounded organelle;intracellular organelle;intracellular organelle part;intracellular part;large ribosomal subunit;macromolecular complex;membrane;membrane-bounded organelle;mitochondrial inner membrane;mitochondrial large ribosomal subunit;mitochondrial membrane;mitochondrial part;mitochondrion;organellar larg		Homo sapiens	+
apical plasma membrane;cell part;cytoplasm;extracellular region part;extracellular space;intracellular membrane-bounded organelle;intracellular organelle;intracellular part;membrane part;membrane-bounded organelle;nucleus;organelle;plasma membrane part		Homo sapiens	+
cell part;cytoplasm;intracellular part	African trypanosomiasis;Renin-angiotensin system	Homo sapiens	
actin cytoskeleton;cell part;cytoplasmic part;cytoskeletal part;cytoskeleton;cytosol;extracellular membrane-bounded organelle;extracellular organelle;extracellular region;extracellular region part;extracellular vesicular exosome;F-actin capping protein complex;intracellular non-membrane-bounded organelle;intracellular organelle;intracellular organelle part;intracellular par		Homo sapiens	+
cell part;chromatin;chromosomal part;chromosome;heterochromatin;intracellular membrane-bounded organelle;intracellular non-membrane-bounded organelle;intracellular organelle;intracellular organelle part;intracellular part;macromolecular complex;membrane-bounded organelle;non-membrane-bounded organelle;nuclear chromosome;nuclear part;nucleoplasm;nucle		Homo sapiens	+
cell cortex;cell part;cytoplasmic part;intracellular part		Homo sapiens	+
cell part;cytoplasmic part;cytosol;envelope;inclusion body;intracellular organelle part;intracellular part;macromolecular complex;membrane;membrane part;nuclear envelope;nuclear inclusion body;nuclear membrane;nuclear	RNA transport	Homo sapiens	
cell part;cytoplasmic part;cytosol;extracellular membrane-bounded organelle;extracellular organelle;extracellular region part;extracellular vesicular exosome;intracellular part;membrane-bounded organelle;membrane-bounde	Porphyrin and chlorophyll metabolism	Homo sapiens	+
cell part;cytoplasmic part;extracellular membrane-bounded organelle;extracellular organelle;extracellular region part;extracellular vesicular exosome;integral to membrane;intracellular membrane-bounded organelle;intracellular organelle;intracellular organelle part;intracellular part;intrinsic to membrane;membrane;membrane part;membrane-bounded organelle;membrane		Homo sapiens	+
cell body;cell part;cell projection;cytoplasm;cytoplasmic part;cytosol;intracellular membrane-bounded organelle;intracellular organelle;intracellular organelle part;intracellular part;membrane;membrane-bounded organelle;neu	MAPK signaling pathway	Homo sapiens	+
cell part;centrosome;chromosomal part;condensed chromosome outer kinetochore;condensed nuclear chromosome outer kinetochore;cytoplasm;cytoplasmic part;cytoskeletal part;cytoskeleton;cytosol;intracellular membrar	Cell cycle;Oocyte meiosis;Progesterone-mediated oocyte maturation	Homo sapiens	+
cell cortex;cell part;cell projection;cytoplasm;cytoplasmic part;Golgi apparatus part;Golgi membrane;intracellular organelle part;intracellular part;membrane;organelle membrane;organelle part;plasma membrane;ruffle;trans-Golgi network membrane		Homo sapiens	+
cell part;cytoplasmic part;cytosol;Golgi apparatus part;Golgi membrane;intracellular organelle part;intracellular part;membrane;organelle membrane;organelle part;trans-Golgi network membrane		Homo sapiens	+
cell part;cytoplasm;cytoplasmic part;cytosol;extracellular membrane-bounded organelle;extracellular organelle;extracellular region part;extracellular vesicular exosome;intracellular part;membrane;membrane-bounded organelle;membrane-bounded vesicle;organelle;plasma membrane;vesicle		Homo sapiens	
cell part;citrate lyase complex;cytoplasm;cytoplasmic part;cytosol;extracellular membrane-bounded organelle;extracellular organelle;extracellular region part;extracellular vesicular exosome;intracellular membrane-bounded o	Carbon fixation pathways in prokaryotes;Citrate cycle (TCA cycle)	Homo sapiens	
cell part;cytoplasm;cytoplasmic part;cytosol;cytosolic part;cytosolic ribosome;intracellular membrane-bounded organelle;intracellular non-membrane-bounded organelle;intracellular organelle;intracellular part;macromolecular complex;membrane-bounded organelle;non-membrane-bounded organelle;nucleus;organelle;ribonucleoprotein complex;ribosome		Homo sapiens	+
cell part;cytoplasm;cytoplasmic part;extracellular membrane-bounded organelle;extracellular organelle;extracellular region part;extracellular vesicular exosome;intracellular membrane-bounded organelle;intracellular organelle	Citrate cycle (TCA cycle);Propanoate metabolism	Homo sapiens	+
cell part;cytoplasmic part;intracellular part;macromolecular complex;protein complex;Rab-protein geranylgeranyltransferase complex		Homo sapiens	
cell part;COPI vesicle coat;cytoplasm;cytoplasmic membrane-bounded vesicle;cytoplasmic part;cytoplasmic vesicle;cytoplasmic vesicle part;cytosol;endoplasmic reticulum-Golgi intermediate compartment;Golgi apparatus;Golgi apparatus part;Golgi membrane;Golgi-associated vesicle;intracellular membrane-bounded organelle;intracellular organelle;intracellular organe		Homo sapiens	+
cell part;COPI vesicle coat;cytoplasm;cytoplasmic part;cytoplasmic vesicle part;cytosol;extracellular membrane-bounded organelle;extracellular organelle;extracellular region part;extracellular space;extracellular vesicular exc	Neuroactive ligand-receptor interaction	Homo sapiens	+
cell part;cytoplasmic part;endoplasmic reticulum;extracellular membrane-bounded organelle;extracellular organelle;extracellular region part;extracellular space;extracellular vesicular exosome;Golgi apparatus;intracellular me	Lysosome	Homo sapiens	+
AP-2 adaptor complex;AP-type membrane coat adaptor complex;cell part;clathrin adaptor complex;clathrin coated vesicle membrane;clathrin-coated endocytic vesicle membrane;coated vesicle membrane;cytoplasmic part	Endocrine and other factor-regulated calcium reabsorption;Endocytosis;Huntington's disease	Homo sapiens	+
cell part;cytoplasmic part;intracellular membrane-bounded organelle;intracellular organelle;intracellular organelle part;intracellular part;membrane;membrane-bounded organelle;mitochondrial inner membrane;mitochondrial m	Porphyrin and chlorophyll metabolism	Homo sapiens	+
cell part;cytoplasm;extracellular membrane-bounded organelle;extracellular organelle;extracellular region part;extracellular vesicular exosome;integral to membrane;intracellular membrane-bounded organelle;intracellular organelle;intracellular part;intrinsic to membrane;membrane;membrane part;membrane-bounded organelle;membrane-bounded vesicle;nucleus;organe		Homo sapiens	+
actin cytoskeleton;cell part;cytoplasm;cytoskeleton;intracellular non-membrane-bounded organelle;intracellular organelle;intracellular organelle part;intracellular part;non-membrane-bounded organelle;nuclear part;nucleoplasm;organelle;organelle part		Homo sapiens	+
cell part;centrosome;cytoplasmic part;cytoskeletal part;extracellular membrane-bounded organelle;extracellular organelle;extracellular region part;extracellular vesicular exosome;integral to membrane;integral to plasma membrane;intracellular membrane-bounded organelle;intracellular non-membrane-bounded organelle;intracellular organelle;intracellular organelle part;i		Homo sapiens	+
cell part;centrosome;cytoplasmic membrane-bounded vesicle;cytoplasmic part;cytoplasmic vesicle;cytoskeletal part;cytosol;endoplasmic reticulum-Golgi intermediate compartment;extracellular membrane-bounded organelle;extracellular organelle;extracellular region part;extracellular vesicular exosome;intracellular membrane-bounded organelle;intracellular non-memb		Homo sapiens	+
cell part;coated vesicle membrane;COPII vesicle coat;cytoplasmic part;cytoplasmic vesicle membrane;cytoplasmic vesicle part;cytosol;endoplasmic reticulum membrane;endoplasmic reticulum part;ER to Golgi transport ves	Protein processing in endoplasmic reticulum	Homo sapiens	+
cell part;extracellular membrane-bounded organelle;extracellular organelle;extracellular region part;extracellular vesicular exosome;intracellular membrane-bounded organelle;intracellular non-membrane-bounded organelle;intracellular organelle;intracellular organelle part;intracellular part;macromolecular complex;membrane-bounded organelle;membrane-bounded vesic		Homo sapiens	+
cell part;cytoplasm;cytoplasmic part;cytoskeleton;cytosol;intracellular membrane-bounded organelle;intracellular non-membrane-bounded organelle;intracellular organelle;intracellular organelle part;intracellular part;macromo	RNA transport	Homo sapiens	+
aminoacyl-tRNA synthetase multienzyme complex;cell part;cytoplasm;cytoplasmic part;cytosol;extracellular membrane-bounded organelle;extracellular organelle;extracellular region part;extracellular vesicular exosome;intrac	Aminoacyl-tRNA biosynthesis	Homo sapiens	
calcium channel complex;cation channel complex;cell part;cytoplasmic part;endoplasmic reticulum;extracellular membrane-bounded organelle;extracellular organelle;extracellular region part;extracellular vesicular exosome;ir	Arrhythmogenic right ventricular cardiomyopathy (ARVC);Cardiac muscle contraction;Dilated cardiomyopathy;Hypertrophic cardiomyopathy (HCM	Homo sapiens	+
cell part;cytoplasm;cytoplasmic part;cytosol;extracellular region part;extracellular space;intracellular membrane-bounded organelle;intracellular organelle;intracellular part;membrane-bounded organelle;nucleus;organelle	Aminoacyl-tRNA biosynthesis	Homo sapiens	+
cell part;cell surface;cytoplasm;cytoplasmic membrane-bounded vesicle;cytoplasmic part;cytoplasmic vesicle;extracellular membrane-bounded organelle;extracellular organelle;extracellular region part;extracellular vesicular exosome;intracellular membrane-bounded organelle;intracellular organelle;intracellular part;macromolecular complex;membrane;membrane-bound		Homo sapiens	+
blood microparticle;cell part;cell surface;chromosomal part;cytoplasmic part;cytosol;extracellular membrane-bounded organelle;extracellular organelle;extracellular region part;extracellular vesicular exosome;germ cell nucleu	Antigen processing and presentation;Endocytosis;MAPK signaling pathway;Measles;Protein processing in endoplasmic reticulum;Spliceosome;To	Homo sapiens	+
caveola;cell part;cytoplasmic membrane-bounded vesicle;cytoplasmic part;cytoplasmic vesicle;extracellular membrane-bounded organelle;extracellular organelle;extracellular region part;extracellular vesicular exosome;intrac	Aldosterone-regulated sodium reabsorption;Bile secretion;Carbohydrate digestion and absorption;Cardiac muscle contraction;Endocrine and othe	Homo sapiens	+
cell part;cytoplasm;intracellular membrane-bounded organelle;intracellular organelle;intracellular organelle part;intracellular part;macromolecular complex;membrane-bounded organelle;nuclear part;nucleoplasm;nucleotide-e	Nucleotide excision repair;Protein processing in endoplasmic reticulum	Homo sapiens	+
cell part;cytoplasmic part;cytosol;extracellular membrane-bounded organelle;extracellular organelle;extracellular region part;extracellular vesicular exosome;intracellular organelle part;intracellular part;membrane;membrane-b	Purine metabolism	Homo sapiens	+
cell part;cytoplasm;cytoplasmic part;intracellular membrane-bounded organelle;intracellular organelle;intracellular organelle part;intracellular part;membrane;membrane-bounded organelle;mitochondrial inner membrane;mito	Arginine and proline metabolism	Homo sapiens	+
cell part;cytoplasmic part;cytosol;extracellular membrane-bounded organelle;extracellular organelle;extracellular region part;extracellular vesicular exosome;intracellular organelle part;intracellular part;macromolecular complex;membrane;membrane part;membrane-bounded organelle;membrane-bounded vesicle;organelle;organelle membrane;organelle part;protein comp		Homo sapiens	+
cell part;cytoplasm;cytoplasmic part;cytosol;intracellular part;membrane;plasma membrane	RNA transport	Homo sapiens	+
cell part;cytoplasm;cytoplasmic part;cytosol;intracellular organelle part;intracellular part;macromolecular complex;nuclear part;nucleoplasm;organelle part;proteasome accessory complex;proteasome complex;proteasome re	Proteasome	Homo sapiens	+
cell part;cytoplasm;cytoplasmic part;intracellular membrane-bounded organelle;intracellular organelle;intracellular part;membrane;membrane-bounded organelle;mitochondrion;organelle		Homo sapiens	
extracellular region;extracellular region part;extracellular space	PPAR signaling pathway	Homo sapiens	+
cell part;cytoplasm;extracellular membrane-bounded organelle;extracellular organelle;extracellular region part;extracellular vesicular exosome;intracellular membrane-bounded organelle;intracellular organelle;intracellular organelle part;intracellular part;membrane;membrane-bounded organelle;membrane-bounded vesicle;nuclear part;nucleoplasm;nucleus;organelle;orga		Homo sapiens	+
cell part;cytoplasm;cytoplasmic part;endoplasmic reticulum;endoplasmic reticulum membrane;endoplasmic reticulum part;integral to membrane;integral to plasma membrane;intracellular membrane-bounded organelle;intracellular organelle;intracellular organelle part;intracellular part;intrinsic to membrane;intrinsic to plasma membrane;membrane;membrane part;membr		Homo sapiens	+
cell part;chromosomal part;cytoplasm;cytoplasmic part;cytoplasmic ubiquitin ligase complex;cytosol;endoplasmic reticulum;endoplasmic reticulum membrane;endoplasmic reticulum part;ER ubiquitin ligase complex;extrace	Protein processing in endoplasmic reticulum	Homo sapiens	+
extracellular matrix part;extracellular region;extracellular region part;fibril;microfibril		Homo sapiens	
cell part;cytoplasmic part;endoplasmic reticulum;envelope;extracellular membrane-bounded organelle;extracellular organelle;extracellular region part;extracellular vesicular exosome;fatty acid beta-oxidation multienzyme con	Benzoate degradation;Fatty acid elongation in mitochondria;Fatty acid metabolism;Valine, leucine and isoleucine degradation	Homo sapiens	+
cell part;cytoplasmic part;endoplasmic reticulum;extracellular region part;extracellular space;intracellular membrane-bounded organelle;intracellular organelle;intracellular part;membrane-bounded organelle;nucleus;organelle;perinuclear region of cytoplasm		Homo sapiens	+
adherens junction;anchoring junction;apical part of cell;cell junction;cell part;cell-cell adherens junction;cell-cell junction;cytoplasm;cytoplasmic part;cytosol;intracellular organelle part;intracellular part;membrane;nuclear part	Adherens junction;Leukocyte transendothelial migration;Tight junction	Homo sapiens	
cell part;cytoplasmic membrane-bounded vesicle;cytoplasmic part;cytoplasmic vesicle;intracellular membrane-bounded organelle;intracellular organelle;intracellular part;melanosome;membrane;membrane-bounded organelle;membrane-bounded vesicle;nucleus;organelle;pigment granule;vesicle		Homo sapiens	+
cell part;cytoplasm;cytoplasmic part;cytosol;intracellular membrane-bounded organelle;intracellular organelle;intracellular organelle part;intracellular part;membrane-bounded organelle;nuclear part;nucleoplasm;nucleus;orga	Alzheimer's disease;Apoptosis	Homo sapiens	+
cell part;cytoplasm;cytoplasmic part;cytosol;intracellular organelle part;intracellular part;nuclear part;nucleoplasm;organelle part	Purine metabolism	Homo sapiens	
cell part;cytoplasm;intracellular membrane-bounded organelle;intracellular non-membrane-bounded organelle;intracellular organelle;intracellular organelle part;intracellular part;membrane;membrane-bounded organelle;non-r	Cytosolic DNA-sensing pathway;Measles	Homo sapiens	+
basal lamina;basement membrane;extracellular matrix part;extracellular membrane-bounded organelle;extracellular organelle;extracellular region;extracellular region part;extracellular vesicular exosome;laminin complex;lamir	Amoebiasis;ECM-receptor interaction;Focal adhesion;Pathways in cancer;Small cell lung cancer;Toxoplasmosis	Homo sapiens	+
cell part;cytoplasm;extracellular membrane-bounded organelle;extracellular organelle;extracellular region part;extracellular vesicular exosome;integral to membrane;intracellular part;intrinsic to membrane;membrane;membrane part;membrane-bounded organelle;membrane-bounded vesicle;organelle;plasma membrane;vesicle		Homo sapiens	
adherens junction;anchored to membrane;anchoring junction;caveola;cell junction;cell part;cell projection;cell-substrate adherens junction;cell-substrate junction;cytoplasm;external side of plasma membrane;extracellular membrane-bounded organelle;extracellular organelle;extracellular region part;extracellular space;extracellular vesicular exosome;focal adhesion;intrac		Homo sapiens	+
cell part;coated vesicle membrane;cytoplasmic part;cytoplasmic vesicle membrane;cytoplasmic vesicle part;cytosol;endoplasmic reticulum membrane;endoplasmic reticulum part;envelope;ER to Golgi transport vesicle mem	Protein processing in endoplasmic reticulum;RNA transport	Homo sapiens	+
box C/D snoRNP complex;cell part;intracellular membrane-bounded organelle;intracellular non-membrane-bounded organelle;intracellular organelle;intracellular organelle part;intracellular part;macromolecular complex;memb	Ribosome biogenesis in eukaryotes;Spliceosome	Homo sapiens	
cell part;cytoplasmic part;cytosol;extracellular membrane-bounded organelle;extracellular organelle;extracellular region part;extracellular vesicular exosome;intracellular membrane-bounded organelle;intracellular organelle;intracellular part;membrane-bounded organelle;membrane-bounded vesicle;nucleus;organelle;vesicle		Homo sapiens	+
cell part;intracellular membrane-bounded organelle;intracellular organelle;intracellular organelle part;intracellular part;macromolecular complex;membrane;membrane-bounded organelle;nuclear part;nucleoplasm;nucleus;organelle;organelle part;ribonucleoprotein complex		Homo sapiens	+
cell part;cytoplasmic part;intracellular membrane-bounded organelle;intracellular organelle;intracellular organelle lumen;intracellular organelle part;intracellular part;membrane-bounded organelle;membrane-enclosed lumen;m	Butanoate metabolism;Synthesis and degradation of ketone bodies;Valine, leucine and isoleucine degradation	Homo sapiens	+
cell part;cytoplasm;cytoplasmic part;cytosol;eukaryotic 43S preinitiation complex;eukaryotic 48S preinitiation complex;eukaryotic translation initiation factor 3 complex;eukaryotic translation initiation factor 3 complex, eIF3m	RNA transport	Homo sapiens	+
cell part;cytoplasmic part;cytosol;extracellular membrane-bounded organelle;extracellular organelle;extracellular region part;extracellular vesicular exosome;integral to membrane;integral to mitochondrial membrane;integral t	Alzheimer's disease;Amyotrophic lateral sclerosis (ALS);Apoptosis;ko05152;Natural killer cell mediated cytotoxicity;p53 signaling pathway;Pathwa	Homo sapiens	+
cell part;cytoplasmic part;extracellular membrane-bounded organelle;extracellular organelle;extracellular region part;extracellular vesicular exosome;integral to membrane;intracellular membrane-bounded organelle;intracellul	Oxidative phosphorylation	Homo sapiens	+
cell part;intracellular membrane-bounded organelle;intracellular non-membrane-bounded organelle;intracellular organelle;intracellular organelle part;intracellular part;macromolecular complex;membrane-bounded organelle;non-membrane-bounded organelle;nuclear part;nucleolus;nucleus;organelle;organelle part;preribosome;preribosome, small subunit precursor;ribonu		Homo sapiens	
cell part;cytoplasm;cytoplasmic part;cytosol;extracellular membrane-bounded organelle;extracellular organelle;extracellular region part;extracellular vesicular exosome;intracellular membrane-bounded organelle;intracellular o	Aminoacyl-tRNA biosynthesis;Selenocompound metabolism	Homo sapiens	+
acrosomal vesicle;adherens junction;alpha1-beta1 integrin complex;anchoring junction;basal part of cell;cell junction;cell part;cell projection;cell surface;cell-substrate adherens junction;cell-substrate junction;cytoplasmic me	Arrhythmogenic right ventricular cardiomyopathy (ARVC);Dilated cardiomyopathy;ECM-receptor interaction;Focal adhesion;Hematopoietic cell line	Homo sapiens	+
cell part;cytoplasm;intracellular organelle part;intracellular part;nuclear part;nucleoplasm;organelle part		Homo sapiens	
cell part;cytoplasmic part;intracellular organelle lumen;intracellular organelle part;intracellular part;macromolecular complex;membrane part;membrane-enclosed lumen;mitochondrial matrix;mitochondrial membrane part;mitc	Alzheimer's disease;Huntington's disease;Oxidative phosphorylation;Parkinson's disease	Homo sapiens	+
cell part;cytoplasmic part;intracellular membrane-bounded organelle;intracellular organelle;intracellular organelle part;intracellular part;macromolecular complex;membrane;membrane part;membrane-bounded organelle;mitc	Oxidative phosphorylation	Homo sapiens	+
cell part;cytoplasm;cytoskeletal part;extracellular membrane-bounded organelle;extracellular organelle;extracellular region part;extracellular vesicular exosome;intermediate filament;intracellular membrane-bounded organelle	Ribosome biogenesis in eukaryotes	Homo sapiens	+
cell junction;cell part;intracellular membrane-bounded organelle;intracellular organelle;intracellular organelle part;intracellular part;macromolecular complex;membrane-bounded organelle;nuclear part;nucleoplasm part;nucleu	Chronic myeloid leukemia;Notch signaling pathway;Pathways in cancer;Wnt signaling pathway	Homo sapiens	+
cell part;cytoplasmic part;intracellular organelle part;intracellular part;macromolecular complex;membrane;membrane part;mitochondrial inner membrane;mitochondrial membrane;mitochondrial membrane part;mitochondrial	Alzheimer's disease;Huntington's disease;Oxidative phosphorylation;Parkinson's disease	Homo sapiens	+
actin cytoskeleton;adherens junction;anchoring junction;cell junction;cell part;cell projection;cell-substrate adherens junction;cell-substrate junction;cytoplasm;cytoplasmic part;cytoskeleton;cytosol;focal adhesion;intracellula	Bacterial invasion of epithelial cells;Chemokine signaling pathway;Focal adhesion;Leukocyte transendothelial migration;Regulation of actin cytosk	Homo sapiens	+
cell part;chromosomal part;extracellular region part;extracellular space;intracellular membrane-bounded organelle;intracellular organelle;intracellular organelle part;intracellular part;macromolecular complex;membrane-bounded organelle;nucleosome;nucleus;organelle;organelle part;protein-DNA complex		Homo sapiens	+
cell part;cytoplasmic membrane-bounded vesicle;cytoplasmic part;cytoplasmic vesicle;endoplasmic reticulum;extracellular membrane-bounded organelle;extracellular organelle;extracellular region part;extracellular vesicular exosome;intracellular membrane-bounded organelle;intracellular organelle;intracellular organelle part;intracellular part;macromolecular complex;me		Homo sapiens	+
cell part;cytoplasmic part;integral to membrane;intracellular membrane-bounded organelle;intracellular organelle;intracellular organelle part;intracellular part;intrinsic to membrane;membrane;membrane part;membrane-bounded organelle;mitochondrial membrane;mitochondrial outer membrane;mitochondrial part;mitochondrion;organelle;organelle membrane;organelle o		Homo sapiens	+
cell part;cytoplasm;cytoplasmic part;cytosol;extracellular membrane-bounded organelle;extracellular organelle;extracellular region part;extracellular vesicular exosome;intracellular non-membrane-bounded organelle;intracellu	RNA transport	Homo sapiens	+
cell part;cytoplasmic membrane-bounded vesicle;cytoplasmic part;cytoplasmic vesicle;cytosol;Golgi apparatus;Golgi apparatus part;Golgi membrane;integral to membrane;intracellular membrane-bounded organelle;intracellular organelle;intracellular organelle part;intracellular part;intrinsic to membrane;membrane;membrane part;membrane-bounded organelle;membra		Homo sapiens	+
cell part;centrosome;chromosomal part;condensed chromosome kinetochore;cytoplasmic part;cytoskeletal part;cytosol;envelope;intracellular non-membrane-bounded organelle;intracellular organelle;intracellular organelle pa	RNA transport	Homo sapiens	+
cell part;cytoplasmic part;intracellular membrane-bounded organelle;intracellular organelle;intracellular part;macromolecular complex;membrane-bounded organelle;mitochondrion;nucleus;organelle;ribonucleoprotein complex		Homo sapiens	
axon;cell part;cell projection;cytoplasmic part;cytoskeletal part;cytosol;extracellular membrane-bounded organelle;extracellular organelle;extracellular region part;extracellular vesicular exosome;F-actin capping protein complex;intracellular membrane-bounded organelle;intracellular organelle;intracellular organelle part;intracellular part;macromolecular complex;membrar		Homo sapiens	+
Arp2/3 protein complex;cell part;cell projection;cytoplasmic part;cytoskeletal part;cytosol;extracellular membrane-bounded organelle;extracellular organelle;extracellular region part;extracellular vesicular exosome;intracellula	Bacterial invasion of epithelial cells;Fc gamma R-mediated phagocytosis;Pathogenic Escherichia coli infection;Regulation of actin cytoskeleton;Sh	Homo sapiens	+
adherens junction;anchoring junction;cell junction;cell part;cell-substrate adherens junction;cell-substrate junction;extracellular membrane-bounded organelle;extracellular organelle;extracellular region part;extracellular vesicu	B cell receptor signaling pathway;Hepatitis C;Malaria	Homo sapiens	+
cell part;cytoplasmic part;cytosol;extracellular membrane-bounded organelle;extracellular organelle;extracellular region part;extracellular space;extracellular vesicular exosome;intracellular membrane-bounded organelle;intra	Carbon fixation in photosynthetic organisms;Fructose and mannose metabolism;Glycolysis / Gluconeogenesis;Inositol phosphate metabolism	Homo sapiens	+
cell part;cytoplasm;cytoplasmic part;cytosol;eukaryotic 43S preinitiation complex;eukaryotic 48S preinitiation complex;eukaryotic translation initiation factor 3 complex;extracellular membrane-bounded organelle;extracellular	Hepatitis C;RNA transport	Homo sapiens	+
cell part;cytoplasmic part;cytosol;endoplasmic reticulum;endoplasmic reticulum membrane;endoplasmic reticulum part;endoplasmic reticulum Sec complex;integral to membrane;intracellular membrane-bounded organelle;ir	Phagosome;Protein export;Protein processing in endoplasmic reticulum;Vibrio cholerae infection	Homo sapiens	+
cell part;cytoplasm;cytoplasmic part;cytoskeletal part;intracellular membrane-bounded organelle;intracellular organelle;intracellular organelle part;intracellular part;macromolecular complex;membrane;membrane-bounded organelle;microtubule organizing center;nuclear part;nucleoplasm;nucleus;organelle;organelle part;plasma membrane;protein complex;protein phospł		Homo sapiens	+
cell part;cytoplasmic part;integral to membrane;intracellular membrane-bounded organelle;intracellular organelle;intracellular organelle part;intracellular part;intrinsic to membrane;membrane;membrane part;membrane-bounded organelle;mitochondrial inner membrane;mitochondrial membrane;mitochondrial part;mitochondrion;organelle;organelle inner membrane;organ		Homo sapiens	
cell part;cytoplasmic part;cytosol;endoplasmic reticulum;intracellular membrane-bounded organelle;intracellular organelle;intracellular part;membrane-bounded organelle;organelle	Parkinson's disease;Protein processing in endoplasmic reticulum;Ubiquitin mediated proteolysis	Homo sapiens	+
brush border;cell part;cell projection;cytoplasmic part;cytoskeletal part;cytosol;extracellular membrane-bounded organelle;extracellular organelle;extracellular region part;extracellular vesicular exosome;intracellular organelle	Vascular smooth muscle contraction	Homo sapiens	+
adherens junction;anchoring junction;axon;blood microparticle;cell cortex part;cell junction;cell part;cell projection;cell-substrate adherens junction;cell-substrate junction;chromatin;chromosomal part;cortical cytoskeleton;cy	Adherens junction;Arrhythmogenic right ventricular cardiomyopathy (ARVC);Bacterial invasion of epithelial cells;Dilated cardiomyopathy;Focal adh	Homo sapiens	+
cell part;cytoplasm;cytoplasmic part;cytosol;eukaryotic translation initiation factor 4F complex;extracellular membrane-bounded organelle;extracellular organelle;extracellular region part;extracellular vesicular exosome;intrac	RNA transport	Homo sapiens	+
cell part;cytoplasm;cytoplasmic part;cytosol;cytosolic small ribosomal subunit;extracellular membrane-bounded organelle;extracellular organelle;extracellular region part;extracellular vesicular exosome;intracellular organelle	Ribosome	Homo sapiens	+
cell part;cytoplasmic part;cytosol;intracellular part;macromolecular complex;protein complex;ribose phosphate diphosphokinase complex	Pentose phosphate pathway;Purine metabolism	Homo sapiens	
cell part;contractile fiber;contractile fiber part;cytoplasm;cytoplasmic mRNA processing body;cytoplasmic part;cytosol;extracellular membrane-bounded organelle;extracellular organelle;extracellular region part;extracellular v	Proteasome	Homo sapiens	+
cell part;extracellular membrane-bounded organelle;extracellular organelle;extracellular region part;extracellular vesicular exosome;extrinsic to membrane;extrinsic to plasma membrane;membrane part;membrane-bounded organelle;membrane-bounded vesicle;organelle;plasma membrane part;vesicle		Homo sapiens	+
adherens junction;anchoring junction;apical part of cell;cell body;cell junction;cell part;cell projection;cell-cell junction;cell-substrate adherens junction;cell-substrate junction;cytoplasm;cytoplasmic membrane-bounded vesic	Adherens junction;Axon guidance;Bacterial invasion of epithelial cells;Chemokine signaling pathway;Endocytosis;Epithelial cell signaling in Helicol	Homo sapiens	+
actin cytoskeleton;cell cortex part;cell part;cortical actin cytoskeleton;cortical cytoskeleton;cytoplasmic part;cytoskeletal part;cytoskeleton;extracellular membrane-bounded organelle;extracellular organelle;extracellular region part;extracellular vesicular exosome;intracellular non-membrane-bounded organelle;intracellular organelle;intracellular organelle part;intracellular		Homo sapiens	+
cell part;intracellular		Homo sapiens	+
cell body;cell part;cell projection;centrosome;cilium;clathrin-coated vesicle;coated vesicle;cytoplasmic membrane-bounded vesicle;cytoplasmic part;cytoplasmic vesicle;cytoplasmic vesicle membrane;cytoplasmic vesicle pa	Pancreatic secretion	Homo sapiens	+
cell part;cytoplasmic part;endoplasmic reticulum;endoplasmic reticulum membrane;endoplasmic reticulum part;integral to membrane;intracellular membrane-bounded organelle;intracellular organelle;intracellular organelle pa	Protein export	Homo sapiens	+
cell part;cytoplasm;cytoplasmic part;cytosol;intracellular membrane-bounded organelle;intracellular non-membrane-bounded organelle;intracellular organelle;intracellular organelle part;intracellular part;macromolecular comp	Bacterial secretion system;Protein export	Homo sapiens	+
cell part;cytoplasmic membrane-bounded vesicle;cytoplasmic part;cytoplasmic vesicle;endoplasmic reticulum membrane;endoplasmic reticulum part;endoplasmic reticulum-Golgi intermediate compartment membrane;extracellular membrane-bounded organelle;extracellular organelle;extracellular region part;extracellular vesicular exosome;Golgi apparatus part;Golgi me		Homo sapiens	
cell part;cytoplasmic membrane-bounded vesicle;cytoplasmic part;cytoplasmic vesicle;early endosome;early endosome membrane;endocytic vesicle;endosomal part;endosome;endosome membrane;extracellular membrane	Amoebiasis;Endocytosis;ko05152;Phagosome;Vasopressin-regulated water reabsorption	Homo sapiens	+
cell part;intracellular organelle part;intracellular part;nuclear part;nucleoplasm;organelle part	Cell cycle - yeast;Pathways in cancer;Small cell lung cancer	Homo sapiens	+
adherens junction;anchoring junction;cell junction;cell part;cell projection;cell-substrate adherens junction;cell-substrate junction;cilium;cytoplasmic membrane-bounded vesicle;cytoplasmic part;cytoplasmic vesicle;cytoplasmic vesicle membrane;cytoplasmic vesicle part;endocytic vesicle membrane;endoplasmic reticulum membrane;endoplasmic reticulum part;endopla		Homo sapiens	+
cell part;cytoplasmic part;cytosol;endosomal part;endosome membrane;extracellular membrane-bounded organelle;extracellular organelle;extracellular region part;extracellular vesicular exosome;intracellular organelle part;in	Protein processing in endoplasmic reticulum;Ubiquitin mediated proteolysis	Homo sapiens	+
cell part;cytoplasm;cytoplasmic part;cytosol;extracellular membrane-bounded organelle;extracellular organelle;extracellular region part;extracellular vesicular exosome;intracellular part;membrane-bounded organelle;membra	Ubiquitin mediated proteolysis	Homo sapiens	+
actin cytoskeleton;cell part;cytoplasm;cytoskeleton;extracellular membrane-bounded organelle;extracellular organelle;extracellular region part;extracellular vesicular exosome;intermediate filament cytoskeleton;intracellular m	Ubiquitin mediated proteolysis	Homo sapiens	
cell part;cytoplasm;cytoplasmic part;cytosol;extracellular membrane-bounded organelle;extracellular organelle;extracellular region part;extracellular vesicular exosome;intracellular membrane-bounded organelle;intracellular o	Ubiquitin mediated proteolysis	Homo sapiens	+
cell part;clathrin-coated vesicle;coated vesicle;cytoplasmic membrane-bounded vesicle;cytoplasmic part;cytoplasmic vesicle;cytoplasmic vesicle membrane;cytoplasmic vesicle part;cytosol;early endosome;early endosome membrane;endocytic vesicle;endocytic vesicle membrane;endoplasmic reticulum;endosomal part;endosome;endosome membrane;extracellular m		Homo sapiens	+
actin cytoskeleton;actin filament;adherens junction;anchoring junction;Arp2/3 protein complex;cell junction;cell part;cell projection;cell-cell junction;cell-substrate adherens junction;cell-substrate junction;cytoplasmic part;cytoskeletal part;cytoskeleton;cytosol;excitatory synapse;extracellular membrane-bounded organelle;extracellular organelle;extracellular region part;e		Homo sapiens	+
actin cap;actin cytoskeleton;adherens junction;anchoring junction;Arp2/3 protein complex;cell cortex part;cell junction;cell part;cell projection;cell-substrate adherens junction;cell-substrate junction;cytoplasm;cytoplasmic part;cytoskeletal part;cytoskeleton;cytosol;extracellular membrane-bounded organelle;extracellular organelle;extracellular region part;extracellular ve		Homo sapiens	+
cell part;centrosome;cytoplasm;cytoplasmic part;cytoskeletal part;cytosol;dynactin complex;extracellular membrane-bounded organelle;extracellular organelle;extracellular region part;extracellular vesicular exosome;intracellular non-membrane-bounded organelle;intracellular organelle;intracellular organelle part;intracellular part;macromolecular complex;membrane-bour		Homo sapiens	
cell part;integral to membrane;intrinsic to membrane;membrane part		Homo sapiens	+
cell part;cytoplasm;intracellular organelle part;intracellular part;macromolecular complex;nuclear part;organelle part;protein complex;signalosome		Homo sapiens	+

cell part;cytoplasmic part;extracellular membrane-bounded organelle;extracellular organelle;extracellular region part;extracellular vesicular exosome;Golgi apparatus part;Golgi membrane;intracellular organelle part;intracellular part;membrane;membrane-bounded organelle;membrane-bounded vesicle;organelle;organelle membrane;organelle part;perinuclear region of cy		Homo sapiens	
cell part;cytoplasm;cytoplasmic part;intracellular membrane-bounded organelle;intracellular organelle;intracellular organelle lumen;intracellular organelle part;intracellular part;membrane;membrane-bounded organelle;membrane-enclosed lumen;mitochondrial matrix;mitochondrial part;mitochondrion;organelle;organelle lumen;organelle part		Homo sapiens	+
cell junction;cell part;cell-cell junction;cytoplasmic part;cytosol;extracellular membrane-bounded organelle;extracellular organelle;extracellular region part;extracellular vesicular exosome;intracellular;intracellular part;lipid part	Chemokine signaling pathway;Focal adhesion;Leukocyte transendothelial migration;Long-term potentiation;MAPK signaling pathway;Neurotrophi	Homo sapiens	+
adherens junction;anchoring junction;cell junction;cell part;cell-substrate adherens junction;cell-substrate junction;cytoplasm;cytoplasmic part;cytosol;cytosolic small ribosomal subunit;extracellular membrane-bounded orga	Ribosome	Homo sapiens	+
cell part;cytoplasmic part;cytosol;cytosolic large ribosomal subunit;extracellular membrane-bounded organelle;extracellular organelle;extracellular region part;extracellular vesicular exosome;intracellular organelle part;intrace	Ribosome	Homo sapiens	+
cell part;cytoplasm;cytoplasmic part;cytosol;intracellular membrane-bounded organelle;intracellular organelle;intracellular organelle part;intracellular part;macromolecular complex;membrane;membrane-bounded organelle;nu	Antigen processing and presentation;Hepatitis C;Proteasome	Homo sapiens	+
cell part;cytoplasmic part;cytosol;cytosolic large ribosomal subunit;extracellular membrane-bounded organelle;extracellular organelle;extracellular region part;extracellular vesicular exosome;intracellular membrane-bounded	Ribosome	Homo sapiens	+
catalytic step 2 spliceosome;cell part;cytoplasmic part;cytosol;exon-exon junction complex;intracellular membrane-bounded organelle;intracellular organelle;intracellular organelle part;intracellular part;macromolecular compl	mRNA surveillance pathway;RNA transport;Spliceosome	Homo sapiens	+
adherens junction;anchoring junction;cell junction;cell part;cell-substrate adherens junction;cell-substrate junction;cytoplasmic part;cytosol;cytosolic large ribosomal subunit;extracellular membrane-bounded organelle;extrac	Ribosome	Homo sapiens	+
apical plasma membrane;axon part;axon terminus;cell part;cell projection part;clathrin-coated vesicle;coated vesicle;cytoplasmic membrane-bounded vesicle;cytoplasmic part;cytoplasmic vesicle;cytoplasmic vesicle membr	Collecting duct acid secretion;Epithelial cell signaling in Helicobacter pylori infection;ko05152;Lysosome;Oxidative phosphorylation;Phagosome;R	Homo sapiens	+
adherens junction;anchoring junction;cell junction;cell part;cell-substrate adherens junction;cell-substrate junction;cytoplasmic part;cytosol;cytosolic large ribosomal subunit;extracellular membrane-bounded organelle;extrac	Ribosome	Homo sapiens	+
adherens junction;anchoring junction;apical junction complex;cell cortex;cell division site part;cell junction;cell part;cell projection;cell-cell junction;cell-substrate adherens junction;cell-substrate junction;cleavage furrow;cyto	Adherens junction;Axon guidance;Bacterial invasion of epithelial cells;Chemokine signaling pathway;Colorectal cancer;Endocytosis;Focal adhesio	Homo sapiens	+
adherens junction;anchoring junction;cell junction;cell part;cell-substrate adherens junction;cell-substrate junction;cytoplasmic part;extracellular membrane-bounded organelle;extracellular organelle;extracellular region part;extracellular vesicular exosome;focal adhesion;Golgi apparatus part;Golgi membrane;intracellular organelle part;intracellular part;membrane;membra		Homo sapiens	+
cell part;cytoplasmic part;extracellular membrane-bounded organelle;extracellular organelle;extracellular region part;extracellular vesicular exosome;intracellular membrane-bounded organelle;intracellular organelle;intracellular organelle lumen;intracellular organelle part;intracellular part;membrane;membrane-bounded organelle;membrane-bounded vesicle;membrane-er		Homo sapiens	+
cell part;cytoplasmic part;cytosol;endoplasmic reticulum;endoplasmic reticulum part;integral to endoplasmic reticulum membrane;integral to membrane;integral to organelle membrane;intracellular membrane-bounded organ	Phagosome;Protein export;Protein processing in endoplasmic reticulum;Vibrio cholerae infection	Homo sapiens	+
cell part;cytoplasm;cytoplasmic part;cytosolic part;intracellular membrane-bounded organelle;intracellular organelle;intracellular part;macromolecular complex;membrane-bounded organelle;nucleus;organelle;prefoldin complex;protein complex		Homo sapiens	+
cell part;cytoplasm;cytoplasmic membrane-bounded vesicle;cytoplasmic part;cytoplasmic vesicle;cytosol;extracellular membrane-bounded organelle;extracellular organelle;extracellular region part;extracellular vesicular exosome;intracellular membrane-bounded organelle;intracellular organelle;intracellular organelle part;intracellular part;macromolecular complex;membr		Homo sapiens	
adherens junction;anchoring junction;cell junction;cell part;cell-substrate adherens junction;cell-substrate junction;coated vesicle membrane;cytoplasm;cytoplasmic part;cytoplasmic vesicle membrane;cytoplasmic vesicle pa	Antigen processing and presentation	Homo sapiens	+
cell part;cytoplasmic part;endoplasmic reticulum membrane;endoplasmic reticulum part;extracellular membrane-bounded organelle;extracellular organelle;extracellular region part;extracellular vesicular exosome;integral to m	N-Glycan biosynthesis;Protein processing in endoplasmic reticulum;Various types of N-glycan biosynthesis	Homo sapiens	+
cell part;cytoplasmic part;endoplasmic reticulum;extracellular membrane-bounded organelle;extracellular organelle;extracellular region part;extracellular vesicular exosome;intracellular membrane-bounded organelle;intracellu	Lysosome	Homo sapiens	
cell part;COPI vesicle coat;cytoplasmic part;cytoplasmic vesicle part;cytosol;Golgi apparatus part;intracellular organelle part;intracellular part;macromolecular complex;membrane coat;membrane part;organelle part;protein complex;vesicle coat		Homo sapiens	+
cell part;cytoplasmic part;cytosol;cytosolic large ribosomal subunit;intracellular organelle part;intracellular part;large ribosomal subunit;macromolecular complex;organelle part;ribonucleoprotein complex	Ribosome	Homo sapiens	+
cell part;intracellular membrane-bounded organelle;intracellular organelle;intracellular organelle part;intracellular part;membrane-bounded organelle;nuclear body;nuclear part;nucleoplasm;nucleoplasm part;nucleus;organelle;	RNA transport	Homo sapiens	+
cell part;CUL4 RING ubiquitin ligase complex;cullin-RING ubiquitin ligase complex;cytoplasm;intracellular organelle part;intracellular part;macromolecular complex;nuclear matrix;nuclear part;nucleoplasm;organelle part;protein complex;ubiquitin ligase complex		Homo sapiens	+
Ada2/Gcn5/Ada3 transcription activator complex;cell part;chromatin remodeling complex;histone acetyltransferase complex;histone methyltransferase complex;intracellular membrane-bounded organelle;intracellular organelle;intracellular organelle part;intracellular part;macromolecular complex;membrane-bounded organelle;methyltransferase complex;MLL1 complex;n		Homo sapiens	
cell part;cytoplasmic part;cytosol;extracellular membrane-bounded organelle;extracellular organelle;extracellular region part;extracellular vesicular exosome;intracellular organelle part;intracellular part;macromolecular complex;membrane part;membrane-bounded organelle;membrane-bounded vesicle;nuclear part;nuclear pore;organelle;organelle part;pore complex;prote		Homo sapiens	
adherens junction;anchoring junction;catalytic step 2 spliceosome;cell junction;cell part;cell projection;cell-substrate adherens junction;cell-substrate junction;chromatin;chromosomal part;cytoplasm;cytoskeletal part;extrace	Spliceosome	Homo sapiens	+
adherens junction;anchoring junction;cell junction;cell part;cell-substrate adherens junction;cell-substrate junction;cytoplasmic part;cytoplasmic vesicle membrane;cytoplasmic vesicle part;cytosol;extracellular membrane-bo	Cell cycle;Neurotrophin signaling pathway;Oocyte meiosis	Homo sapiens	
adherens junction;anchoring junction;cell junction;cell part;cell-substrate adherens junction;cell-substrate junction;cytoplasmic part;endoplasmic reticulum;extracellular membrane-bounded organelle;extracellular organelle;ex	MAPK signaling pathway;Regulation of actin cytoskeleton;Tight junction	Homo sapiens	+
cell part;cytoplasmic part;intracellular membrane-bounded organelle;intracellular organelle;intracellular organelle part;intracellular part;macromolecular complex;membrane;membrane part;membrane-bounded organelle;membrane-enclosed lumen;mitochondrial inner membrane;mitochondrial inner membrane presequence translocase complex;mitochondrial intermembr		Homo sapiens	+
cell part;integral to membrane;intrinsic to membrane;membrane;membrane part;plasma membrane		Homo sapiens	+
90S preribosome;adherens junction;anchoring junction;cell junction;cell part;cell-substrate adherens junction;cell-substrate junction;cytoplasm;cytoplasmic part;cytoskeletal part;cytosol;cytosolic small ribosomal subunit;extr	Ribosome	Homo sapiens	+
cell part;cell projection;cell projection part;cytoplasm;cytoplasmic part;cytosol;dendritic spine;extracellular membrane-bounded organelle;extracellular organelle;extracellular region part;extracellular vesicular exosome;glycog	Focal adhesion;Insulin signaling pathway;Long-term potentiation;Meiosis - yeast;Oocyte meiosis;Regulation of actin cytoskeleton;Vascular smoot	Homo sapiens	+
adherens junction;anchoring junction;cell junction;cell part;cell-substrate adherens junction;cell-substrate junction;cytoplasmic part;cytosol;extracellular membrane-bounded organelle;extracellular organelle;extracellular regio	Focal adhesion;Insulin signaling pathway;Long-term potentiation;Meiosis - yeast;Oocyte meiosis;Regulation of actin cytoskeleton;Vascular smoot	Homo sapiens	+
cell junction;cell part;cytoplasm;cytoplasmic part;cytosol;cytosolic part;cytosolic proteasome complex;intracellular membrane-bounded organelle;intracellular organelle;intracellular organelle part;intracellular part;macromolec	Proteasome	Homo sapiens	
blood microparticle;cell part;cytoplasm;cytoplasmic part;cytoplasmic vesicle;cytosol;cytosolic part;cytosolic proteasome complex;extracellular membrane-bounded organelle;extracellular organelle;extracellular region part;ex	Proteasome	Homo sapiens	+
adherens junction;anchoring junction;cell junction;cell part;cell-substrate adherens junction;cell-substrate junction;cytoplasm;cytoplasmic part;cytosol;cytosolic small ribosomal subunit;extracellular membrane-bounded orga	Ribosome	Homo sapiens	+
cell part;cytoplasm;cytoplasmic part;cytosol;cytosolic small ribosomal subunit;extracellular membrane-bounded organelle;extracellular organelle;extracellular region part;extracellular vesicular exosome;intracellular organelle	Ribosome	Homo sapiens	+
adherens junction;anchoring junction;cell junction;cell part;cell-substrate adherens junction;cell-substrate junction;cytoplasmic part;cytosol;cytosolic small ribosomal subunit;extracellular membrane-bounded organelle;extrac	Ribosome	Homo sapiens	+
adherens junction;anchoring junction;axon;cell junction;cell part;cell projection;cell-substrate adherens junction;cell-substrate junction;cytoplasmic membrane-bounded vesicle;cytoplasmic part;cytoplasmic vesicle;cytoplasm	Cell cycle;Neurotrophin signaling pathway;Oocyte meiosis	Homo sapiens	+
adherens junction;anchoring junction;cell junction;cell part;cell-substrate adherens junction;cell-substrate junction;cytoplasm;cytoplasmic part;cytosol;cytosolic small ribosomal subunit;extracellular membrane-bounded orga	Ribosome	Homo sapiens	+
cell part;cytoplasm;cytoplasmic part;cytosol;cytosolic small ribosomal subunit;intracellular non-membrane-bounded organelle;intracellular organelle;intracellular organelle part;intracellular part;macromolecular complex;memb	Ribosome	Homo sapiens	+
adherens junction;anchoring junction;cell junction;cell part;cell-substrate adherens junction;cell-substrate junction;cytoplasm;cytoplasmic part;cytosol;cytosolic small ribosomal subunit;extracellular membrane-bounded orga	Ribosome	Homo sapiens	+
adherens junction;anchoring junction;cell junction;cell part;cell-substrate adherens junction;cell-substrate junction;cytoplasm;cytoplasmic part;cytosol;cytosolic small ribosomal subunit;extracellular membrane-bounded orga	Ribosome	Homo sapiens	+
adherens junction;anchoring junction;cell junction;cell part;cell-substrate adherens junction;cell-substrate junction;cytoplasmic part;cytosol;cytosolic small ribosomal subunit;extracellular membrane-bounded organelle;extrac	Ribosome	Homo sapiens	+
adherens junction;anchoring junction;cell junction;cell part;cell-substrate adherens junction;cell-substrate junction;cytoplasm;cytoplasmic part;cytosol;cytosolic small ribosomal subunit;extracellular membrane-bounded orga	Ribosome	Homo sapiens	+
catalytic step 2 spliceosome;cell part;cytoplasmic part;cytosol;extracellular membrane-bounded organelle;extracellular organelle;extracellular region part;extracellular vesicular exosome;intracellular membrane-bounded orga	Spliceosome	Homo sapiens	+
catalytic step 2 spliceosome;cell part;cytoplasmic part;cytosol;intracellular organelle part;intracellular part;macromolecular complex;methylosome;methyltransferase complex;nuclear part;nucleoplasm;organelle part;pICln-Sm	Spliceosome	Homo sapiens	+
catalytic step 2 spliceosome;cell part;cytoplasmic part;cytosol;intracellular membrane-bounded organelle;intracellular organelle;intracellular organelle part;intracellular part;macromolecular complex;membrane-bounded orga	RNA degradation;Spliceosome	Homo sapiens	+
cell part;cytoplasmic mRNA processing body;cytoplasmic part;cytosol;extracellular membrane-bounded organelle;extracellular organelle;extracellular region part;extracellular vesicular exosome;intracellular non-membrane-b	RNA degradation;Spliceosome	Homo sapiens	+
catalytic step 2 spliceosome;cell part;cytoplasmic part;cytosol;intracellular membrane-bounded organelle;intracellular organelle;intracellular organelle part;intracellular part;macromolecular complex;membrane-bounded orga	Spliceosome;Systemic lupus erythematosus	Homo sapiens	
catalytic step 2 spliceosome;cell part;cytoplasmic part;cytosol;extracellular membrane-bounded organelle;extracellular organelle;extracellular region part;extracellular vesicular exosome;intracellular organelle part;intracellular	Spliceosome	Homo sapiens	
catalytic step 2 spliceosome;cell part;cytoplasm;cytoplasmic part;cytosol;extracellular membrane-bounded organelle;extracellular organelle;extracellular region part;extracellular vesicular exosome;intracellular organelle part;i	Spliceosome;Systemic lupus erythematosus	Homo sapiens	+
cell part;cytoplasmic membrane-bounded vesicle lumen;cytoplasmic part;cytoplasmic vesicle part;cytoskeleton;cytosol;extracellular region;intracellular membrane-bounded organelle;intracellular non-membrane-bounded or	Regulation of actin cytoskeleton	Homo sapiens	
adherens junction;anchoring junction;cell cortex;cell division site part;cell junction;cell part;cell projection;cell projection membrane;cell projection part;cell-substrate adherens junction;cell-substrate junction;cleavage furrow;c	Endocytosis;Fc gamma R-mediated phagocytosis	Homo sapiens	+
cell part;cytoplasmic part;cytosol;cytosolic part;cytosolic proteasome complex;extracellular membrane-bounded organelle;extracellular organelle;extracellular region part;extracellular vesicular exosome;intracellular membran	Proteasome	Homo sapiens	
adherens junction;anchoring junction;cell junction;cell part;cell-substrate adherens junction;cell-substrate junction;cytoplasm;cytoplasmic part;cytosol;cytosolic large ribosomal subunit;extracellular membrane-bounded orga	Ribosome	Homo sapiens	+
axon;cell division site part;cell part;cell projection;cleavage furrow;cytoplasmic membrane-bounded vesicle;cytoplasmic part;cytoplasmic vesicle;cytoplasmic vesicle membrane;cytoplasmic vesicle part;cytoskeletal part;cyto	Endocrine and other factor-regulated calcium reabsorption;Endocytosis;Pancreatic secretion;Vasopressin-regulated water reabsorption	Homo sapiens	+
cell part;cytoplasm;cytoplasmic part;cytosol;intracellular part;macromolecular complex;protein complex;translation release factor complex	mRNA surveillance pathway	Homo sapiens	+
cell part;cytoplasmic part;cytosol;endoplasmic reticulum;intracellular membrane-bounded organelle;intracellular organelle;intracellular part;membrane-bounded organelle;nucleus;organelle		Homo sapiens	+
adherens junction;anchoring junction;cell junction;cell part;cell-substrate adherens junction;cell-substrate junction;cytoplasmic part;cytosol;cytosolic small ribosomal subunit;extracellular membrane-bounded organelle;extrac	Ribosome	Homo sapiens	+
cell part;cytoplasm;cytoplasmic part;cytosol;cytosolic large ribosomal subunit;extracellular membrane-bounded organelle;extracellular organelle;extracellular region part;extracellular vesicular exosome;intracellular membrane	Ribosome	Homo sapiens	+
cell body;cell part;cell projection;cytoplasm;cytoplasmic part;cytosol;cytosolic small ribosomal subunit;dendrite;intracellular membrane-bounded organelle;intracellular non-membrane-bounded organelle;intracellular organelle	Insulin signaling pathway;mTOR signaling pathway;Ribosome	Homo sapiens	+
cell part;chromosomal part;chromosome;extracellular membrane-bounded organelle;extracellular organelle;extracellular region;extracellular region part;extracellular vesicular exosome;intracellular membrane-bounded organe	Systemic lupus erythematosus	Homo sapiens	+
cell part;cytoplasmic membrane-bounded vesicle;cytoplasmic part;cytoplasmic vesicle;cytosol;early endosome;endoplasmic reticulum membrane;endoplasmic reticulum part;endosome;extracellular membrane-bounded organelle;extracellular organelle;extracellular region part;extracellular vesicular exosome;Golgi apparatus;Golgi apparatus part;Golgi membrane;intrace		Homo sapiens	+
cell part;centriole;cytoplasm;cytoplasmic membrane-bounded vesicle;cytoplasmic part;cytoplasmic vesicle;cytoskeletal part;cytosol;endosome;extracellular membrane-bounded organelle;extracellular organelle;extracellular r	Ribosome biogenesis in eukaryotes;RNA transport	Homo sapiens	
adherens junction;anchoring junction;cell junction;cell part;cell-substrate adherens junction;cell-substrate junction;cytoplasm;cytoplasmic part;cytosol;cytosolic large ribosomal subunit;extracellular membrane-bounded orga	Ribosome	Homo sapiens	+
cell junction;cell part;cell projection;cytoplasm;cytoplasmic part;cytosol;early endosome;endosome;extracellular membrane-bounded organelle;extracellular organelle;extracellular region part;extracellular vesicular exosome;g	Chemokine signaling pathway;Focal adhesion;Leukocyte transendothelial migration;Long-term potentiation;MAPK signaling pathway;Neurotrophi	Homo sapiens	+
adherens junction;anchoring junction;cell junction;cell part;cell-substrate adherens junction;cell-substrate junction;cytoplasm;cytoplasmic part;cytosol;cytosolic small ribosomal subunit;focal adhesion;intracellular membrane-	Ribosome	Homo sapiens	+
cell part;cytoplasm;cytoplasmic part;cytosol;cytosolic small ribosomal subunit;intracellular membrane-bounded organelle;intracellular non-membrane-bounded organelle;intracellular organelle;intracellular organelle part;intrac	Ribosome	Homo sapiens	+
cell part;cytoplasm;cytoplasmic part;cytosol;cytosolic small ribosomal subunit;extracellular membrane-bounded organelle;extracellular organelle;extracellular region part;extracellular vesicular exosome;intracellular membrane	Ribosome	Homo sapiens	+
cell part;cytoplasm;cytoplasmic part;cytosol;cytosolic small ribosomal subunit;extracellular membrane-bounded organelle;extracellular organelle;extracellular region part;extracellular vesicular exosome;intracellular non-meml	Ribosome	Homo sapiens	
cell part;cytoplasm;cytoplasmic part;cytosol;cytosolic small ribosomal subunit;extracellular membrane-bounded organelle;extracellular organelle;extracellular region part;extracellular vesicular exosome;intracellular organelle	Ribosome	Homo sapiens	+
cell part;cytoplasmic part;cytosol;cytosolic small ribosomal subunit;extracellular region part;extracellular space;intracellular organelle part;intracellular part;macromolecular complex;organelle part;ribonucleoprotein complex;small ribosomal subunit		Homo sapiens	+
cell body;cell part;cell projection;cell projection part;cilium part;cytoplasmic part;dendrite;extracellular membrane-bounded organelle;extracellular organelle;extracellular region part;extracellular vesicular exosome;extrinsic to	Chemokine signaling pathway;Phototransduction;Taste transduction	Homo sapiens	+
cell part;Cul2-RING ubiquitin ligase complex;Cul3-RING ubiquitin ligase complex;CUL4 RING ubiquitin ligase complex;Cul4A-RING ubiquitin ligase complex;Cul4B-RING ubiquitin ligase complex;Cul5-RING ubiquitin ligase c	Cell cycle;Cell cycle - yeast;Circadian rhythm - mammal;Nucleotide excision repair;Oocyte meiosis;Pathways in cancer;Protein processing in endo	Homo sapiens	
adherens junction;anchoring junction;cell body;cell junction;cell part;cell-substrate adherens junction;cell-substrate junction;cytoplasmic part;extracellular membrane-bounded organelle;extracellular organelle;extracellular reg	Chemokine signaling pathway	Homo sapiens	+
adherens junction;anchoring junction;cell junction;cell part;cell-substrate adherens junction;cell-substrate junction;cytoplasm;cytoplasmic part;cytosol;cytosolic large ribosomal subunit;extracellular membrane-bounded orga	Ribosome	Homo sapiens	+
cell part;cytoplasmic part;cytosol;cytosolic large ribosomal subunit;extracellular region part;extracellular space;intracellular organelle part;intracellular part;large ribosomal subunit;macromolecular complex;organelle part;ribor	Ribosome	Homo sapiens	+
adherens junction;anchoring junction;cell junction;cell part;cell-substrate adherens junction;cell-substrate junction;cytoplasmic part;cytosol;cytosolic large ribosomal subunit;extracellular membrane-bounded organelle;extrac	Ribosome	Homo sapiens	+
adherens junction;anchoring junction;cell junction;cell part;cell-substrate adherens junction;cell-substrate junction;cytoplasm;cytoplasmic part;cytosol;cytosolic large ribosomal subunit;extracellular membrane-bounded orga	Ribosome	Homo sapiens	+
cell part;centrosome;cytoplasm;cytoplasmic part;cytoskeletal part;cytosol;cytosolic large ribosomal subunit;intracellular non-membrane-bounded organelle;intracellular organelle;intracellular organelle part;intracellular part;lar	Ribosome	Homo sapiens	+
cell part;cytoplasm;cytoplasmic part;cytosol;cytosolic large ribosomal subunit;extracellular membrane-bounded organelle;extracellular organelle;extracellular region part;extracellular vesicular exosome;intracellular non-memb	Ribosome	Homo sapiens	+
adherens junction;anchoring junction;cell junction;cell part;cell-substrate adherens junction;cell-substrate junction;cytoplasm;cytoplasmic part;cytosol;cytosolic large ribosomal subunit;focal adhesion;intracellular non-membr	Ribosome	Homo sapiens	+
adherens junction;anchoring junction;cell junction;cell part;cell-substrate adherens junction;cell-substrate junction;cytoplasmic part;cytosol;extracellular membrane-bounded organelle;extracellular organelle;extracellular region;extracellular region part;extracellular space;extracellular vesicular exosome;focal adhesion;intracellular membrane-bounded organelle;intracellula		Homo sapiens	
cell part;contractile fiber part;cytoplasm;cytoplasmic part;cytosol;endoplasmic reticulum membrane;endoplasmic reticulum part;extracellular membrane-bounded organelle;extracellular organelle;extracellular region part;extracellular vesicular exosome;intracellular organelle part;intracellular part;membrane;membrane part;membrane-bounded organelle;membrane-bounc		Homo sapiens	+
cell part;cytoplasm;cytoplasmic part;cytoplasmic vesicle membrane;cytoplasmic vesicle part;cytosol;cytosolic small ribosomal subunit;endocytic vesicle membrane;endosomal part;endosome membrane;extracellular membr	Ribosome	Homo sapiens	+
cell part;cytoplasmic part;cytoplasmic vesicle membrane;cytoplasmic vesicle part;cytosol;cytosolic large ribosomal subunit;endocytic vesicle membrane;endosomal part;endosome membrane;extracellular membrane-bounde	Ribosome	Homo sapiens	+
cell junction;cell part;cell-cell junction;cytoplasm;cytoplasmic part;cytosol;endosome;extracellular membrane-bounded organelle;extracellular organelle;extracellular region part;extracellular vesicular exosome;Golgi apparatus	Acute myeloid leukemia;B cell receptor signaling pathway;Chemokine signaling pathway;Chronic myeloid leukemia;Dorso-ventral axis formation;E	Homo sapiens	+
cell part;intracellular membrane-bounded organelle;intracellular organelle;intracellular part;membrane-bounded organelle;nucleus;organelle	Spliceosome	Homo sapiens	+
adherens junction;anchoring junction;cell junction;cell part;cell projection;cell projection membrane;cell projection part;cell-substrate adherens junction;cell-substrate junction;cytoplasm;cytoplasmic membrane-bounded vesi	Adherens junction;Amyotrophic lateral sclerosis (ALS);Axon guidance;B cell receptor signaling pathway;Bacterial invasion of epithelial cells;Chemo	Homo sapiens	+
AP-2 adaptor complex;AP-type membrane coat adaptor complex;cell part;clathrin adaptor complex;clathrin coated vesicle membrane;clathrin-coated endocytic vesicle membrane;coated vesicle membrane;cytoplasmic part;	Endocrine and other factor-regulated calcium reabsorption;Endocytosis;Huntington's disease	Homo sapiens	+
cell part;cytoplasm;cytoplasmic part;cytosol;extracellular membrane-bounded organelle;extracellular organelle;extracellular region part;extracellular vesicular exosome;extrinsic to internal side of plasma membrane;extrinsic t	Amoebiasis;Bile secretion;Calcium signaling pathway;Chagas disease (American trypanosomiasis);Dilated cardiomyopathy;Endocrine and other fa	Homo sapiens	+
cell part;centrosome;cytoplasm;cytoplasmic part;cytoskeletal part;extracellular membrane-bounded organelle;extracellular organelle;extracellular region part;extracellular vesicular exosome;extrinsic to internal side of plasma	Axon guidance;Chagas disease (American trypanosomiasis);Chemokine signaling pathway;Gap junction;Gastric acid secretion;Leukocyte transen	Homo sapiens	+
adherens junction;anchoring junction;blood microparticle;cell junction;cell leading edge;cell part;cell-substrate adherens junction;cell-substrate junction;cytoplasm;cytoplasmic membrane-bounded vesicle;cytoplasmic part;cy	Cell cycle;Neurotrophin signaling pathway;Oocyte meiosis	Homo sapiens	
cell part;cytoplasmic part;cytosol;intracellular organelle part;intracellular part;macromolecular complex;nuclear part;nucleoplasm;organelle part;protein complex;protein phosphatase type 2A complex;protein serine/threonine	Cell cycle - yeast;Chagas disease (American trypanosomiasis);Hepatitis C;mRNA surveillance pathway;Tight junction	Homo sapiens	+
cell part;cell projection;cytoplasm;dendrite;fibrillar center;intracellular membrane-bounded organelle;intracellular non-membrane-bounded organelle;intracellular organelle;intracellular organelle part;intracellular part;macromol	RNA transport	Homo sapiens	+
cell part;cell projection;centrosome;chromosomal part;cilium;cytoplasm;cytoplasmic dynein complex;cytoplasmic part;cytoskeletal part;cytosol;dynein complex;extracellular membrane-bounded organelle;extracellular organe	Vasopressin-regulated water reabsorption	Homo sapiens	+
adherens junction;anchoring junction;cell junction;cell part;cell-substrate adherens junction;cell-substrate junction;cytoplasmic part;cytosol;cytosolic large ribosomal subunit;eukaryotic 80S initiation complex;focal adhesion;ir	Ribosome	Homo sapiens	+
cell part;Cul7-RING ubiquitin ligase complex;cullin-RING ubiquitin ligase complex;cytoplasmic part;cytosol;extracellular membrane-bounded organelle;extracellular organelle;extracellular region part;extracellular vesicular exo	Cell cycle;Cell cycle - yeast;Circadian rhythm - mammal;Oocyte meiosis;Protein processing in endoplasmic reticulum;TGF-beta signaling pathway	Homo sapiens	+
cell part;extracellular membrane-bounded organelle;extracellular organelle;extracellular region part;extracellular vesicular exosome;extrinsic to internal side of plasma membrane;extrinsic to membrane;extrinsic to plasma me	Chemokine signaling pathway	Homo sapiens	+
cell part;cytoplasm;cytoplasmic part;cytosol;cytosolic small ribosomal subunit;intracellular organelle part;intracellular part;macromolecular complex;organelle part;ribonucleoprotein complex;small ribosomal subunit	Ribosome	Homo sapiens	+
annulate lamellae;cell part;cytoplasm;cytoplasmic part;cytosol;endoplasmic reticulum membrane;endoplasmic reticulum part;extracellular membrane-bounded organelle;extracellular organelle;extracellular region part;extracellular vesicular exosome;intracellular membrane-bounded organelle;intracellular organelle;intracellular organelle part;intracellular part;macromolecul		Homo sapiens	+
cell body;cell part;cell projection;cytoplasm;cytoplasmic part;cytoskeleton;cytosol;dendrite;extracellular membrane-bounded organelle;extracellular organelle;extracellular region part;extracellular vesicular exosome;intracellul	Measles	Homo sapiens	+
cell part;DSIF complex;intracellular membrane-bounded organelle;intracellular organelle;intracellular organelle part;intracellular part;macromolecular complex;membrane-bounded organelle;nuclear part;nucleoplasm;nucleoplasm part;nucleus;organelle;organelle part;protein complex;transcription elongation factor complex		Homo sapiens	
cell part;cytoplasm;cytoskeleton;intracellular non-membrane-bounded organelle;intracellular organelle;intracellular part;non-membrane-bounded organelle;organelle		Homo sapiens	+
cell part;chromosomal part;chromosome, centromeric region;cytoplasmic part;cytoskeletal part;cytoskeleton;cytosol;extracellular membrane-bounded organelle;extracellular organelle;extracellular region part;extracellular ves	Cell cycle - yeast;Chagas disease (American trypanosomiasis);Hepatitis C;Long-term depression;Meiosis - yeast;mRNA surveillance pathway;Ooc	Homo sapiens	
cell part;CRD-mediated mRNA stability complex;cytoplasm;cytoplasmic part;extracellular membrane-bounded organelle;extracellular organelle;extracellular region part;extracellular vesicular exosome;histone pre-mRNA 3'end processing complex;intracellular membrane-bounded organelle;intracellular non-membrane-bounded organelle;intracellular organelle;intracellular		Homo sapiens	
cell part;cytoplasmic part;endoplasmic reticulum membrane;endoplasmic reticulum part;extracellular membrane-bounded organelle;extracellular organelle;extracellular region part;extracellular vesicular exosome;integral to m	Protein export	Homo sapiens	+
cell part;cytoplasm;cytoplasmic part;cytosol;extracellular membrane-bounded organelle;extracellular organelle;extracellular region part;extracellular vesicular exosome;intracellular membrane-bounded organelle;intracellular c	Adherens junction;Circadian rhythm - plant;Measles;Ribosome biogenesis in eukaryotes;Tight junction;Wnt signaling pathway	Homo sapiens	+
actin filament bundle;actomyosin;adherens junction;anchoring junction;cell cortex part;cell junction;cell part;cell-substrate adherens junction;cell-substrate junction;contractile fiber part;cortical cytoskeleton;cytoplasmic part;	Cardiac muscle contraction;Dilated cardiomyopathy;Hypertrophic cardiomyopathy (HCM)	Homo sapiens	+
actin filament;actomyosin, actin part;adherens junction;anchoring junction;blood microparticle;cell junction;cell part;cell-substrate adherens junction;cell-substrate junction;contractile fiber part;cytoplasm;cytoplasmic part;cyt	Cardiac muscle contraction;Dilated cardiomyopathy;Hypertrophic cardiomyopathy (HCM)	Homo sapiens	+
cell part;cytoplasm;extracellular membrane-bounded organelle;extracellular organelle;extracellular region part;extracellular vesicular exosome;intracellular membrane-bounded organelle;intracellular organelle;intracellular part	Parkinson's disease;Ubiquitin mediated proteolysis	Homo sapiens	+
cell cortex part;cell part;cell projection membrane;cell projection part;cortical actin cytoskeleton;cortical cytoskeleton;cytoplasm;cytoplasmic part;cytoskeletal part;cytoskeleton;cytosol;eukaryotic translation elongation factor	RNA transport	Homo sapiens	+
cell part;cytoplasmic part;cytoskeletal part;cytosol;extracellular membrane-bounded organelle;extracellular organelle;extracellular region;extracellular region part;extracellular vesicular exosome;intracellular organelle part;intra	Gap junction;Pathogenic Escherichia coli infection;Phagosome	Homo sapiens	+
cell part;cytoplasmic part;cytoskeletal part;cytoskeleton;cytosol;extracellular membrane-bounded organelle;extracellular organelle;extracellular region part;extracellular vesicular exosome;intracellular membrane-bounded org	Gap junction;Pathogenic Escherichia coli infection;Phagosome	Homo sapiens	+
cell part;chromatin remodeling complex;chromosomal part;cytoplasmic part;cytosol;histone deacetylase complex;intracellular membrane-bounded organelle;intracellular organelle;intracellular organelle part;intracellular part;n	Adherens junction;Circadian rhythm - plant;Measles;Ribosome biogenesis in eukaryotes;Tight junction;Wnt signaling pathway	Homo sapiens	+
cell part;cytoplasm;cytoplasmic part;cytosol;extracellular membrane-bounded organelle;extracellular organelle;extracellular region part;extracellular vesicular exosome;intracellular non-membrane-bounded organelle;intracellu	Ether lipid metabolism	Homo sapiens	+
cell part;integral to membrane;intrinsic to membrane;membrane part		Homo sapiens	+
blood microparticle;cell part;cytoplasmic membrane-bounded vesicle lumen;cytoplasmic part;cytoplasmic vesicle part;cytosol;cytosolic part;cytosolic small ribosomal subunit;endocytic vesicle lumen;extracellular membrane-	African trypanosomiasis;Malaria	Homo sapiens	+
cell part;cytoplasm;cytoplasmic part;cytoskeletal part;cytosol;intracellular organelle part;intracellular part;macromolecular complex;microtubule;organelle part;protein complex		Homo sapiens	+
cell part;cell projection;cytoplasm;cytoplasmic part;cytosol;intracellular part;neuron projection	Glycine, serine and threonine metabolism;Methane metabolism	Homo sapiens	+
cell part;intracellular membrane-bounded organelle;intracellular organelle;intracellular part;membrane-bounded organelle;nucleus;organelle		Homo sapiens	+
cell part;cytoplasmic part;cytosol;eukaryotic translation initiation factor 4F complex;intracellular part;macromolecular complex;membrane;protein complex	RNA transport;Viral myocarditis	Homo sapiens	+
cell part;intracellular membrane-bounded organelle;intracellular organelle;intracellular organelle part;intracellular part;macromolecular complex;membrane-bounded organelle;multimeric ribonuclease P complex;nuclear part;n	Ribosome biogenesis in eukaryotes;RNA transport	Homo sapiens	
cell body;cell part;cell projection;cytoplasm;intracellular membrane-bounded organelle;intracellular organelle;intracellular organelle part;intracellular part;membrane;membrane-bounded organelle;neuronal cell body;nuclear pa	Basal transcription factors	Homo sapiens	+
axon part;cation channel complex;cell part;cell projection part;integral to membrane;integral to plasma membrane;intrinsic to membrane;intrinsic to plasma membrane;ion channel complex;macromolecular complex;membrar	Cell adhesion molecules (CAMs)	Homo sapiens	
cell body;cell part;chaperonin-containing T-complex;cytoplasm;cytoplasmic part;cytoskeletal part;cytosol;cytosolic part;extracellular membrane-bounded organelle;extracellular organelle;extracellular region part;extracellular vesicular exosome;intracellular organelle part;intracellular part;macromolecular complex;membrane-bounded organelle;membrane-bounded vesicle		Homo sapiens	+
cell part;cytoplasm;cytoskeleton;envelope;intracellular membrane-bounded organelle;intracellular non-membrane-bounded organelle;intracellular organelle;intracellular organelle part;intracellular part;macromolecular complex	RNA transport	Homo sapiens	+

cell part;cytoplasm;cytoplasmic part;cytosol;extracellular membrane-bounded organelle;extracellular organelle;extracellular region part;extracellular vesicular exosome;intracellular part;membrane-bounded organelle;membra	Drug metabolism - cytochrome P450;Glutathione metabolism;Metabolism of xenobiotics by cytochrome P450	Homo sapiens	+
cell part;cytoplasmic part;cytosol;DNA-dependent protein kinase-DNA ligase 4 complex;intracellular non-membrane-bounded organelle;intracellular organelle;intracellular organelle part;intracellular part;macromolecular comp	Cell cycle;Non-homologous end-joining	Homo sapiens	+
autophagic vacuole;cell part;cell surface;cytoplasmic part;endoplasmic reticulum;intracellular membrane-bounded organelle;intracellular organelle;intracellular part;membrane;membrane-bounded organelle;organelle;vacuole		Homo sapiens	+
cell part;cytoplasmic part;cytoskeletal part;cytosol;intracellular organelle part;intracellular part;macromolecular complex;microtubule;microtubule associated complex;organelle part;protein complex		Homo sapiens	+
cell part;cytoplasm;cytoplasmic part;intracellular membrane-bounded organelle;intracellular organelle;intracellular part;membrane-bounded organelle;nucleus;organelle;perinuclear region of cytoplasm	Mineral absorption	Homo sapiens	
cell part;cytoplasmic part;cytosol;endoplasmic reticulum;endoplasmic reticulum-Golgi intermediate compartment;envelope;extracellular membrane-bounded organelle;extracellular organelle;extracellular region part;extracellular space;extracellular vesicular exosome;Golgi apparatus;intracellular membrane-bounded organelle;intracellular organelle;intracellular organelle p		Homo sapiens	+
cell junction;cell part;cell projection part;cytoplasm;cytoskeleton;extracellular membrane-bounded organelle;extracellular organelle;extracellular region part;extracellular vesicular exosome;growth cone;intracellular membrane-bounded organelle;intracellular non-membrane-bounded organelle;intracellular organelle;intracellular organelle part;intracellular part;membrane;m		Homo sapiens	+
extracellular membrane-bounded organelle;extracellular organelle;extracellular region;extracellular region part;extracellular vesicular exosome;membrane-bounded organelle;membrane-bounded vesicle;organelle;vesicle		Homo sapiens	+
cell part;cytoplasmic part;endoplasmic reticulum;Golgi apparatus;Golgi apparatus part;Golgi membrane;intracellular membrane-bounded organelle;intracellular organelle;intracellular organelle part;intracellular part;membrane;membrane-bounded organelle;nucleus;organelle;organelle membrane;organelle part		Homo sapiens	+
cell part;cytoplasmic part;intracellular membrane-bounded organelle;intracellular non-membrane-bounded organelle;intracellular organelle;intracellular organelle part;intracellular part;macromolecular complex;membrane;membrane-bounded organelle;mitochondrial inner membrane;mitochondrial membrane;mitochondrial part;mitochondrial ribosome;mitochondrial small		Homo sapiens	+
cell part;cytoplasmic part;intracellular membrane-bounded organelle;intracellular non-membrane-bounded organelle;intracellular organelle;intracellular organelle part;intracellular part;macromolecular complex;membrane;membrane-bounded organelle;mitochondrial inner membrane;mitochondrial membrane;mitochondrial part;mitochondrion;non-membrane-bounded org		Homo sapiens	+
cell part;cytoplasmic part;intracellular membrane-bounded organelle;intracellular organelle;intracellular organelle part;intracellular part;macromolecular complex;membrane;membrane-bounded organelle;mitochondrial inner membrane;mitochondrial membrane;mitochondrial part;mitochondrial small ribosomal subunit;mitochondrion;organellar small ribosomal subunit;org		Homo sapiens	
cell part;cytoplasmic part;intracellular membrane-bounded organelle;intracellular organelle;intracellular organelle part;intracellular part;macromolecular complex;membrane;membrane-bounded organelle;mitochondrial inner membrane;mitochondrial membrane;mitochondrial part;mitochondrial small ribosomal subunit;mitochondrion;organellar small ribosomal subunit;org		Homo sapiens	+
cell part;cytoplasmic part;intracellular membrane-bounded organelle;intracellular non-membrane-bounded organelle;intracellular organelle;intracellular organelle part;intracellular part;macromolecular complex;membrane;membrane-bounded organelle;mitochondrial inner membrane;mitochondrial membrane;mitochondrial part;mitochondrion;non-membrane-bounded org		Homo sapiens	
cell part;cytoplasmic part;intracellular membrane-bounded organelle;intracellular non-membrane-bounded organelle;intracellular organelle;intracellular organelle part;intracellular part;macromolecular complex;membrane;membrane-bounded organelle;mitochondrial inner membrane;mitochondrial membrane;mitochondrial part;mitochondrial small ribosomal subunit;mitoc		Homo sapiens	+
cell part;intracellular membrane-bounded organelle;intracellular organelle;intracellular organelle part;intracellular part;macromolecular complex;membrane-bounded organelle;nuclear body;nuclear part;nuclear speck;nucleoplasm;nucleoplasm part;nucleus;organelle;organelle part;protein complex;transcription export complex		Homo sapiens	+
cell part;cytoplasm;cytoplasmic part;intracellular membrane-bounded organelle;intracellular organelle;intracellular part;membrane-bounded organelle;mitochondrion;organelle		Homo sapiens	+
cell part;cytoplasmic part;cytosol;cytosolic large ribosomal subunit;extracellular membrane-bounded organelle;extracellular organelle;extracellular region part;extracellular vesicular exosome;intracellular organelle part;intrace	Ribosome	Homo sapiens	+
cell part;cytoplasmic part;cytosol;cytosolic large ribosomal subunit;intracellular non-membrane-bounded organelle;intracellular organelle;intracellular organelle part;intracellular part;large ribosomal subunit;macromolecular co	Ribosome	Homo sapiens	+
cell part;centromeric heterochromatin;chromatin;chromocenter;chromosomal part;chromosome, centromeric region;cytoskeletal part;female pronucleus;heterochromatin;intracellular membrane-bounded organelle;intracellular non-membrane-bounded organelle;intracellular organelle;intracellular organelle part;intracellular part;male pronucleus;membrane-bounded organe		Homo sapiens	+
cell part;cytoplasmic part;extracellular membrane-bounded organelle;extracellular organelle;extracellular region part;extracellular vesicular exosome;Golgi apparatus;intracellular membrane-bounded organelle;intracellular organelle;intracellular part;membrane;membrane-bounded organelle;membrane-bounded vesicle;organelle;perinuclear region of cytoplasm;plasma me		Homo sapiens	+
cell part;membrane;midbody		Homo sapiens	+
adherens junction;anchoring junction;cell junction;cell part;cell-substrate adherens junction;cell-substrate junction;cytoplasmic part;cytosol;endoplasmic reticulum membrane;endoplasmic reticulum part;extracellular membra	Bacterial invasion of epithelial cells;Shigellosis	Homo sapiens	+
adherens junction;anchoring junction;cell junction;cell part;cell-substrate adherens junction;cell-substrate junction;cytoplasm;cytoplasmic part;cytosol;cytosolic large ribosomal subunit;focal adhesion;intracellular non-membr	Ribosome	Homo sapiens	+
cell part;cytoplasmic part;cytosol;intracellular membrane-bounded organelle;intracellular organelle;intracellular part;membrane-bounded organelle;nucleus;organelle		Homo sapiens	
cell part;cytoplasm;intracellular organelle part;intracellular part;nuclear part;nucleoplasm;organelle part	Spliceosome	Homo sapiens	+
cell part;intracellular membrane-bounded organelle;intracellular non-membrane-bounded organelle;intracellular organelle;intracellular organelle part;intracellular part;membrane-bounded organelle;non-membrane-bounded organelle;nuclear part;nucleolus;nucleoplasm;nucleus;organelle;organelle part		Homo sapiens	
adherens junction;anchoring junction;apical plasma membrane;cell junction;cell part;cell-substrate adherens junction;cell-substrate junction;clathrin coat;clathrin coat of coated pit;clathrin coated vesicle membrane;clathrin-c	Endocytosis	Homo sapiens	+
adherens junction;anchoring junction;basal lamina;cell junction;cell part;cell-substrate adherens junction;cell-substrate junction;cytoplasmic part;extracellular matrix;extracellular matrix part;extracellular membrane-bounded (	ECM-receptor interaction	Homo sapiens	+
cell part;intracellular membrane-bounded organelle;intracellular organelle;intracellular organelle part;intracellular part;membrane-bounded organelle;nuclear part;nucleoplasm;nucleus;organelle;organelle part		Homo sapiens	+
cell part;cell projection;cytoplasm;dendrite;intracellular membrane-bounded organelle;intracellular organelle;intracellular part;membrane-bounded organelle;neuron projection;nucleus;organelle		Homo sapiens	+
cell part;cytoplasmic part;cytosol;intracellular membrane-bounded organelle;intracellular non-membrane-bounded organelle;intracellular organelle;intracellular organelle lumen;intracellular organelle part;intracellular part;mem	Huntington's disease	Homo sapiens	+
cell part;cytoplasmic part;extracellular membrane-bounded organelle;extracellular organelle;extracellular region part;extracellular vesicular exosome;integral to membrane;integral to plasma membrane;intracellular membrane-bounded organelle;intracellular organelle;intracellular organelle part;intracellular part;intrinsic to membrane;intrinsic to plasma membrane;membrar		Homo sapiens	+
cell part;cytoplasm;extracellular region part;high-density lipoprotein particle;intracellular membrane-bounded organelle;intracellular organelle;intracellular part;macromolecular complex;membrane;membrane-bounded organelle;nucleus;organelle;plasma lipoprotein particle;plasma membrane;protein-lipid complex		Homo sapiens	+
cell body;cell part;cell projection;chromosomal part;chromosome, telomeric region;cytoplasm;dendrite;DNA replication factor A complex;intracellular membrane-bounded organelle;intracellular organelle;intracellular organelle part;intracellular part;macromolecular complex;membrane-bounded organelle;neuron projection;neuronal cell body;nuclear chromosome part;nucl		Homo sapiens	+
actin cytoskeleton;adherens junction;anchoring junction;cell junction;cell part;cell-substrate adherens junction;cell-substrate junction;cytoplasm;cytoplasmic part;cytoskeleton;focal adhesion;Golgi apparatus;intracellular membrane-bounded organelle;intracellular non-membrane-bounded organelle;intracellular organelle;intracellular part;membrane;membrane-bounded o		Homo sapiens	+
adherens junction;anchoring junction;cell junction;cell part;cell-substrate adherens junction;cell-substrate junction;clathrin coat;clathrin coat of coated pit;clathrin coat of trans-Golgi network vesicle;clathrin coated vesicle me	Bacterial invasion of epithelial cells;Endocrine and other factor-regulated calcium reabsorption;Endocytosis;Huntington's disease;Lysosome	Homo sapiens	+
Bcl3/NF-kappaB2 complex;cell part;cytoplasm;cytoplasmic part;cytosol;I-kappaB/NF-kappaB complex;intracellular membrane-bounded organelle;intracellular organelle;intracellular organelle part;intracellular part;macromole	MAPK signaling pathway;Osteoclast differentiation;Pathways in cancer	Homo sapiens	
cell part;cytoplasmic part;endoplasmic reticulum membrane;endoplasmic reticulum part;intracellular membrane-bounded organelle;intracellular organelle;intracellular organelle part;intracellular part;membrane;membrane part;membrane-bounded organelle;nucleus;organelle;organelle membrane;organelle part		Homo sapiens	+
cell part;extracellular membrane-bounded organelle;extracellular organelle;extracellular region part;extracellular vesicular exosome;integral to membrane;intrinsic to membrane;membrane part;membrane-bounded organelle;membrane-bounded vesicle;organelle;vesicle		Homo sapiens	+
cell part;cell projection;cilium;cytoplasmic part;cytosol;extracellular membrane-bounded organelle;extracellular organelle;extracellular region part;extracellular space;extracellular vesicular exosome;intracellular organelle part;	Fructose and mannose metabolism	Homo sapiens	
catalytic step 2 spliceosome;cell part;cell surface;CRD-mediated mRNA stability complex;cytoplasmic part;intracellular membrane-bounded organelle;intracellular organelle;intracellular organelle part;intracellular part;macrom	Spliceosome	Homo sapiens	+
Cajal body;catalytic step 2 spliceosome;cell part;intracellular organelle part;intracellular part;macromolecular complex;nuclear body;nuclear part;nuclear speck;nucleoplasm;nucleoplasm part;organelle part;ribonucleoprotein (	Shigellosis;Spliceosome	Homo sapiens	+
axolemma;axon part;cell cortex part;cell part;cell projection membrane;cell projection part;contractile fiber part;cuticular plate;cytoplasm;cytoplasmic part;cytoskeletal part;cytoskeleton;cytosol;extracellular membrane-bounded organelle;extracellular organelle;extracellular region part;extracellular vesicular exosome;intracellular non-membrane-bounded organelle;intrace		Homo sapiens	+
cell part;cytoplasm;cytoplasmic part;extracellular membrane-bounded organelle;extracellular organelle;extracellular region part;extracellular vesicular exosome;intracellular membrane-bounded organelle;intracellular non-membrane-bounded organelle;intracellular organelle;intracellular part;lysosome;lytic vacuole;macromolecular complex;membrane-bounded organelle;n		Homo sapiens	+
cell part;cytoplasm;cytoplasmic part;cytosol;endoplasmic reticulum;intracellular membrane-bounded organelle;intracellular organelle;intracellular organelle part;intracellular part;macromolecular complex;membrane-bounded organelle;nuclear part;nucleoplasm;nucleus;organelle;organelle part;perinuclear region of cytoplasm;protein complex		Homo sapiens	+
cell part;cytoplasm;extracellular membrane-bounded organelle;extracellular organelle;extracellular region part;extracellular vesicular exosome;intracellular membrane-bounded organelle;intracellular organelle;intracellular orga	Spliceosome	Homo sapiens	
cell part;cytoplasmic part;cytosol;intracellular part	Purine metabolism	Homo sapiens	
cell part;cytoplasm;cytoplasmic part;cytosol;extracellular membrane-bounded organelle;extracellular organelle;extracellular region part;extracellular vesicular exosome;intracellular organelle part;intracellular part;membrane-b	PPAR signaling pathway	Homo sapiens	+
adherens junction;anchoring junction;cell cortex part;cell junction;cell part;cell-substrate adherens junction;cell-substrate junction;cortical actin cytoskeleton;cortical cytoskeleton;cytoplasmic part;cytoskeletal part;cytoskeleton;extracellular membrane-bounded organelle;extracellular organelle;extracellular region part;extracellular vesicular exosome;focal adhesion;intrace		Homo sapiens	+
cell part;cytoplasm;cytoplasmic part;cytosol;intracellular membrane-bounded organelle;intracellular organelle;intracellular organelle part;intracellular part;membrane;membrane-bounded organelle;nuclear part;nucleoplasm;nu	Butanoate metabolism;Synthesis and degradation of ketone bodies;Terpenoid backbone biosynthesis;Valine, leucine and isoleucine degradation	Homo sapiens	+
apical plasma membrane;cell part;cytoplasm;cytoplasmic part;cytosol;extracellular membrane-bounded organelle;extracellular organelle;extracellular region part;extracellular vesicular exosome;integral to membrane;integral to plasma membrane;intracellular membrane-bounded organelle;intracellular non-membrane-bounded organelle;intracellular organelle;intracellular		Homo sapiens	+
cell part;chromatin;chromosomal part;cytoplasm;exosome (RNase complex);intracellular membrane-bounded organelle;intracellular non-membrane-bounded organelle;intracellular organelle;intracellular organelle part;intracel	RNA degradation	Homo sapiens	
		Homo sapiens	+
cell part;cytoplasmic part;cytosol;extracellular membrane-bounded organelle;extracellular organelle;extracellular region part;extracellular vesicular exosome;intracellular membrane-bounded organelle;intracellular organelle;int	Fructose and mannose metabolism;Galactose metabolism;Glycolysis / Gluconeogenesis;Methane metabolism;Pentose phosphate pathway	Homo sapiens	+
cell part;cytoplasm;intracellular membrane-bounded organelle;intracellular non-membrane-bounded organelle;intracellular organelle;intracellular organelle part;intracellular part;membrane;membrane-bounded organelle;non-membrane-bounded organelle;nuclear part;nucleolus;nucleoplasm;nucleus;organelle;organelle part;plasma membrane		Homo sapiens	
cell part;cytoplasmic part;cytosol;intracellular organelle part;intracellular part;macromolecular complex;membrane;nuclear part;nucleoplasm;organelle part;protein complex	African trypanosomiasis;Alzheimer's disease;Amoebiasis;Calcium signaling pathway;Chagas disease (American trypanosomiasis);Chemokine sign	Homo sapiens	
cell part;cytoplasm;intracellular part		Homo sapiens	+
cell body;cell part;cytoplasmic part;integral to membrane;intracellular membrane-bounded organelle;intracellular organelle;intracellular organelle part;intracellular part;intrinsic to membrane;intrinsic to mitochondrial inner men	Pyrimidine metabolism	Homo sapiens	+
cell part;cytoplasmic part;intracellular membrane-bounded organelle;intracellular organelle;intracellular organelle lumen;intracellular organelle part;intracellular part;macromolecular complex;membrane;membrane-bounded or	Citrate cycle (TCA cycle);Lysine degradation;Tryptophan metabolism	Homo sapiens	+
cell part;chromosomal part;chromosome;chromosome, centromeric region;condensed chromosome kinetochore;condensed chromosome outer kinetochore;condensed chromosome, centromeric region;condensed nuclear chromosome kinetochore;cytoplasm;cytoplasmic part;cytoskeletal part;cytoskeleton;cytosol;intracellular membrane-bounded organelle;intracellular		Homo sapiens	
adherens junction;anchoring junction;cell junction;cell part;cell-substrate adherens junction;cell-substrate junction;centralspindlin complex;centrosome;cytoplasmic part;cytoskeletal part;cytosol;extracellular region part;focal adhesion;intercellular bridge;intracellular membrane-bounded organelle;intracellular non-membrane-bounded organelle;intracellular organelle;intrac		Homo sapiens	+
cell part;cytoplasmic part;extracellular membrane-bounded organelle;extracellular organelle;extracellular region part;extracellular vesicular exosome;intracellular membrane-bounded organelle;intracellular organelle;intracellula	Inositol phosphate metabolism;Propanoate metabolism;Valine, leucine and isoleucine degradation	Homo sapiens	+
anchoring collagen;basement membrane;cell part;collagen;collagen type VII;cytoplasmic part;endoplasmic reticulum lumen;endoplasmic reticulum part;extracellular matrix part;extracellular region;extracellular region part;extracellular space;intracellular organelle lumen;intracellular organelle part;intracellular part;membrane-enclosed lumen;organelle lumen;organelle part		Homo sapiens	+
cell part;cytoplasm;cytoplasmic part;Golgi apparatus;intracellular membrane-bounded organelle;intracellular organelle;intracellular organelle part;intracellular part;macromolecular complex;membrane;membrane-bounded organelle;nuclear body;nuclear part;nucleoplasm;nucleoplasm part;nucleus;organelle;organelle part;plasma membrane;PML body;protein complex;tra		Homo sapiens	+
cell part;chromatin;chromosomal part;euchromatin;intracellular organelle part;intracellular part;macromolecular complex;nuclear chromatin;nuclear chromosome part;nuclear euchromatin;nuclear part;nucleosome;organelle part;protein-DNA complex		Homo sapiens	+
cell part;cytoplasmic part;cytosol;cytosolic large ribosomal subunit;intracellular organelle part;intracellular part;large ribosomal subunit;macromolecular complex;membrane;organelle part;ribonucleoprotein complex	Ribosome	Homo sapiens	+
adherens junction;anchoring junction;axon;cell cortex;cell junction;cell part;cell projection;cell projection cytoplasm;cell projection part;cell-substrate adherens junction;cell-substrate junction;cytoplasm;cytoplasmic part;cyto:	Acute myeloid leukemia;B cell receptor signaling pathway;Bladder cancer;Chemokine signaling pathway;Chronic myeloid leukemia;Colorectal can	Homo sapiens	+
axon part;axonal growth cone;cell body;cell part;cell projection part;cytoplasm;cytoplasmic part;cytoskeletal part;cytosol;endoplasmic reticulum membrane;endoplasmic reticulum part;extracellular membrane-bounded organelle;extracellular organelle;extracellular region part;extracellular vesicular exosome;growth cone;intracellular membrane-bounded organelle;intracel		Homo sapiens	+
cell part;cytoplasmic part;endoplasmic reticulum membrane;endoplasmic reticulum part;extracellular membrane-bounded organelle;extracellular organelle;extracellular region part;extracellular vesicular exosome;intracellular (	Lysine degradation	Homo sapiens	
cell part;cytoplasmic part;cytoskeleton;endoplasmic reticulum-Golgi intermediate compartment;extracellular membrane-bounded organelle;extracellular organelle;extracellular region part;extracellular space;extracellular vesicular exosome;Golgi apparatus;intracellular membrane-bounded organelle;intracellular non-membrane-bounded organelle;intracellular organelle;intr		Homo sapiens	+
adherens junction;anchoring junction;cell junction;cell part;cell-substrate adherens junction;cell-substrate junction;cytoplasmic part;cytosol;cytosolic large ribosomal subunit;focal adhesion;intracellular membrane-bounded o	Ribosome	Homo sapiens	+
adherens junction;anchoring junction;cell cortex;cell junction;cell part;cell-substrate adherens junction;cell-substrate junction;cytoplasm;cytoplasmic part;cytoskeleton;focal adhesion;intracellular non-membrane-bounded organelle;intracellular organelle;intracellular part;membrane;non-membrane-bounded organelle;organelle;plasma membrane		Homo sapiens	+
cell part;cytoplasmic part;integral to membrane;integral to plasma membrane;intracellular membrane-bounded organelle;intracellular organelle;intracellular organelle part;intracellular part;intrinsic to membrane;intrinsic to plasma membrane;membrane;membrane part;membrane-bounded organelle;mitochondrial inner membrane;mitochondrial membrane;mitochondrial p		Homo sapiens	+
actin cytoskeleton;adherens junction;anchoring junction;axon;basal plasma membrane;basement membrane;cell cortex;cell junction;cell leading edge;cell part;cell projection;cell-substrate adherens junction;cell-substrate junction;contractile fiber part;cytoplasm;cytoplasmic membrane-bounded vesicle;cytoplasmic part;cytoplasmic vesicle;cytoskeletal part;cytoskeleton		Homo sapiens	+
acrosomal membrane;adherens junction;anchoring junction;apical plasma membrane;basolateral plasma membrane;caveola;cell cortex;cell junction;cell part;cell projection;cell-substrate adherens junction;cell-substrate junc	Bacterial invasion of epithelial cells;Endocytosis;Focal adhesion;Viral myocarditis	Homo sapiens	+
cell part;cytoplasmic part;cytosol;extracellular membrane-bounded organelle;extracellular organelle;extracellular region part;extracellular vesicular exosome;intracellular part;membrane-bounded organelle;membrane-bounde	Arginine and proline metabolism	Homo sapiens	
cell part;cytoskeletal part;intermediate filament;intracellular organelle part;intracellular part;lamin filament;macromolecular complex;membrane;nuclear inner membrane;nuclear membrane;nuclear part;organelle inner membrane;organelle membrane;organelle part;protein complex		Homo sapiens	+
adherens junction;anchored to membrane;anchoring junction;cell junction;cell part;cell projection membrane;cell projection part;cell-substrate adherens junction;cell-substrate junction;cytoplasmic part;endoplasmic reticulum	Complement and coagulation cascades	Homo sapiens	+
cell part;cytoplasmic part;endoplasmic reticulum membrane;endoplasmic reticulum part;integral to endoplasmic reticulum membrane;integral to membrane;integral to organelle membrane;intracellular membrane-bounded or	ABC transporters;Antigen processing and presentation;Phagosome;Primary immunodeficiency	Homo sapiens	
cell part;cytoplasmic part;endoplasmic reticulum membrane;endoplasmic reticulum part;integral to endoplasmic reticulum membrane;integral to membrane;integral to organelle membrane;intracellular organelle part;intracellu	ABC transporters;Antigen processing and presentation;Phagosome;Primary immunodeficiency	Homo sapiens	+
cell part;intracellular membrane-bounded organelle;intracellular organelle;intracellular part;membrane-bounded organelle;nucleus;organelle		Homo sapiens	+
cell part;cytoplasm;cytoplasmic part;cytosol;I-kappaB/NF-kappaB complex;intracellular membrane-bounded organelle;intracellular organelle;intracellular organelle part;intracellular part;macromolecular complex;membrane-b	Acute myeloid leukemia;Adipocytokine signaling pathway;Amoebiasis;Apoptosis;B cell receptor signaling pathway;Chagas disease (American tryp	Homo sapiens	+
Cdc48p-Npl4p-Ufd1p AAA ATPase complex;cell body;cell part;cell projection;cytoplasm;cytoplasmic part;dendrite;endoplasmic reticulum part;intracellular membrane-bounded organelle;intracellular organelle;intracellular organelle part;intracellular part;macromolecular complex;membrane part;membrane-bounded organelle;neuron projection;neuronal cell body;nucleus;(		Homo sapiens	+
cell part;cytoplasmic part;cytosol;extracellular membrane-bounded organelle;extracellular organelle;extracellular region part;extracellular vesicular exosome;intracellular part;membrane-bounded organelle;membrane-bounde	Starch and sucrose metabolism	Homo sapiens	+
cell part;cytoplasmic part;cytosol;eukaryotic translation initiation factor 4F complex;intracellular part;macromolecular complex;membrane;protein complex	RNA transport;Viral myocarditis	Homo sapiens	+
cell part;cell periphery;cytoplasm;cytoskeletal part;extracellular membrane-bounded organelle;extracellular organelle;extracellular region part;extracellular vesicular exosome;intermediate filament;intracellular organelle part;intracellular part;macromolecular complex;membrane-bounded organelle;membrane-bounded vesicle;organelle;organelle part;protein complex;vesic		Homo sapiens	+
cell part;cell projection;cell surface;cilium;cytoplasmic part;endoplasmic reticulum membrane;endoplasmic reticulum part;extracellular region;Golgi apparatus part;Golgi membrane;integral to membrane;integral to plasma me	Dorso-ventral axis formation;Notch signaling pathway	Homo sapiens	+
cell part;intracellular membrane-bounded organelle;intracellular organelle;intracellular organelle part;intracellular part;membrane-bounded organelle;nuclear part;nucleoplasm;nucleus;organelle;organelle part		Homo sapiens	+
extracellular region;extracellular region part;extracellular space		Homo sapiens	+
cell part;cytoplasm;extracellular membrane-bounded organelle;extracellular organelle;extracellular region part;extracellular vesicular exosome;intracellular part;membrane-bounded organelle;membrane-bounded vesicle;orga	MAPK signaling pathway - yeast;Pyruvate metabolism	Homo sapiens	+
cell part;cytoplasmic part;extracellular membrane-bounded organelle;extracellular organelle;extracellular region part;extracellular vesicular exosome;intracellular membrane-bounded organelle;intracellular non-membrane-bou	DNA replication;Homologous recombination;Mismatch repair	Homo sapiens	+
cell junction;cell part;cell-cell contact zone;cell-cell junction;cytoplasm;cytoplasmic part;cytoplasmic vesicle membrane;cytoplasmic vesicle part;cytosol;extracellular membrane-bounded organelle;extracellular organelle;extra	Cell cycle;Neurotrophin signaling pathway;Oocyte meiosis	Homo sapiens	+
cell part;cytoplasm;intracellular membrane-bounded organelle;intracellular organelle;intracellular organelle part;intracellular part;membrane-bounded organelle;nuclear part;nucleoplasm;nucleus;organelle;organelle part	mRNA surveillance pathway	Homo sapiens	
cell part;cytoplasm;cytoplasmic part;cytosol;intracellular membrane-bounded organelle;intracellular organelle;intracellular part;macromolecular complex;membrane-bounded organelle;nucleus;organelle;proteasome complex;	Ubiquitin mediated proteolysis	Homo sapiens	
cell part;cytoplasmic part;cytoskeletal part;extracellular membrane-bounded organelle;extracellular organelle;extracellular region part;extracellular vesicular exosome;intracellular organelle part;intracellular part;macromolecula	Bacterial invasion of epithelial cells;Endocrine and other factor-regulated calcium reabsorption;Endocytosis;Fc gamma R-mediated phagocytosis	Homo sapiens	+
cell junction;cell part;cell projection;cytoplasm;cytoplasmic part;cytoskeletal part;cytosol;intracellular non-membrane-bounded organelle;intracellular organelle;intracellular organelle part;intracellular part;macromolecular complex;membrane;non-membrane-bounded organelle;organelle;organelle part;plasma membrane;podosome;protein complex		Homo sapiens	
actin filament bundle;actomyosin;adherens junction;anchoring junction;apical plasma membrane;cell cortex;cell junction;cell part;cell projection;cell-substrate adherens junction;cell-substrate junction;cytoplasm;cytoplasmic	Amoebiasis;Axon guidance;Bacterial invasion of epithelial cells;Chemokine signaling pathway;ErbB signaling pathway;Focal adhesion;Leukocyte t	Homo sapiens	+
cell part;intracellular membrane-bounded organelle;intracellular organelle;intracellular organelle part;intracellular part;membrane-bounded organelle;nuclear part;nucleoplasm;nucleus;organelle;organelle part		Homo sapiens	+
cell junction;cell part;cell-cell junction;cytoplasm;cytoplasmic part;cytosol;endoplasmic reticulum;extracellular membrane-bounded organelle;extracellular organelle;extracellular region part;extracellular vesicular exosome;intr	Tight junction;Vascular smooth muscle contraction	Homo sapiens	+
actin cap;actin cytoskeleton;cell cortex part;cell part;contractile fiber;cytoplasmic part;cytoskeletal part;cytoskeleton;cytosol;intracellular non-membrane-bounded organelle;intracellular organelle;intracellular organelle part;int	Vascular smooth muscle contraction	Homo sapiens	+
cell part;cytoplasm;cytoplasmic part;cytosol;intracellular membrane-bounded organelle;intracellular organelle;intracellular organelle lumen;intracellular organelle part;intracellular part;membrane;membrane-bounded organelle	Folate biosynthesis	Homo sapiens	+
extracellular membrane-bounded organelle;extracellular organelle;extracellular region;extracellular region part;extracellular vesicular exosome;membrane-bounded organelle;membrane-bounded vesicle;organelle;vesicle		Homo sapiens	+
cell part;cytoplasm;cytoplasmic part;cytosol;intracellular membrane-bounded organelle;intracellular organelle;intracellular part;macromolecular complex;membrane-bounded organelle;mitochondrion;nucleus;organelle;protein	Adipocytokine signaling pathway;Chronic myeloid leukemia;Epithelial cell signaling in Helicobacter pylori infection;Jak-STAT signaling pathway;Le	Homo sapiens	
cell part;cytoplasmic part;cytosol;intracellular part	Alanine, aspartate and glutamate metabolism;Purine metabolism	Homo sapiens	+
cell part;cytoplasmic part;cytosol;extracellular membrane-bounded organelle;extracellular organelle;extracellular region part;extracellular vesicular exosome;intracellular part;membrane-bounded organelle;membrane-bounde	Alanine, aspartate and glutamate metabolism;Amino sugar and nucleotide sugar metabolism	Homo sapiens	+
cell part;chromosome;cytoplasm;cytoplasmic part;cytoskeleton;cytosol;exosome (RNase complex);extracellular membrane-bounded organelle;extracellular organelle;extracellular region part;extracellular vesicular exosome;ir	RNA degradation	Homo sapiens	
cell part;cytoplasm;cytoplasmic part;cytosol;extracellular membrane-bounded organelle;extracellular organelle;extracellular region part;extracellular vesicular exosome;intracellular organelle part;intracellular part;macromolecu	Antigen processing and presentation;Proteasome	Homo sapiens	+
cell part;extracellular membrane-bounded organelle;extracellular organelle;extracellular region part;extracellular vesicular exosome;integral to membrane;intracellular membrane-bounded organelle;intracellular organelle;intracellular part;intrinsic to membrane;membrane;membrane part;membrane-bounded organelle;membrane-bounded vesicle;nucleus;organelle;plasma		Homo sapiens	+
cell body;cell part;cell projection;cell projection part;cytoplasm;cytoplasmic part;cytoskeletal part;dendritic shaft;dendritic spine;intracellular non-membrane-bounded organelle;intracellular organelle;intracellular organelle part;intracellular part;macromolecular complex;membrane;microtubule;mRNA cap binding complex;neuron projection;neuron spine;neuronal RNA gran		Homo sapiens	
cell part;cytoplasmic part;extracellular matrix;extracellular region;extracellular region part;extracellular space;Golgi apparatus part;Golgi lumen;intracellular organelle lumen;intracellular organelle part;intracellular part;lysosomal lumen;membrane-enclosed lumen;organelle lumen;organelle part;proteinaceous extracellular matrix;vacuolar lumen;vacuolar part		Homo sapiens	+
cell part;chromatin;chromosomal part;cytoplasm;cytoplasmic membrane-bounded vesicle;cytoplasmic part;cytoplasmic vesicle;cytosol;euchromatin;extracellular membrane-bounded organelle;extracellular organelle;extracel	Peroxisome	Homo sapiens	+
adherens junction;anchoring junction;cell junction;cell part;cell-substrate adherens junction;cell-substrate junction;cytoplasm;cytoplasmic part;cytosol;cytosolic large ribosomal subunit;focal adhesion;intracellular membrane-	Ribosome	Homo sapiens	+
cell part;cytoplasmic part;cytoskeleton;endoplasmic reticulum;endoplasmic reticulum membrane;endoplasmic reticulum part;extracellular membrane-bounded organelle;extracellular organelle;extracellular region part;extracel	Protein processing in endoplasmic reticulum	Homo sapiens	+
adherens junction;anchoring junction;apical junction complex;apical part of cell;apical plasma membrane;apicolateral plasma membrane;basolateral plasma membrane;cell junction;cell part;cell-cell adherens junction;cell-cel	Adherens junction;Epithelial cell signaling in Helicobacter pylori infection;Gap junction;Tight junction;Vibrio cholerae infection	Homo sapiens	+
cell part;cytoplasm;Grb2-Sos complex;intracellular membrane-bounded organelle;intracellular organelle;intracellular organelle part;intracellular part;macromolecular complex;membrane;membrane part;membrane-bounded organelle;nuclear part;nucleoplasm;nucleus;organelle;organelle part;plasma membrane part;protein complex		Homo sapiens	+
cell part;cell projection part;cytoplasmic membrane-bounded vesicle;cytoplasmic part;cytoplasmic vesicle;cytoskeletal part;cytosol;growth cone;intracellular membrane-bounded organelle;intracellular organelle;intracellular organelle part;intracellular part;kinesin complex;macromolecular complex;membrane;membrane-bounded organelle;membrane-bounded vesicle;mic		Homo sapiens	
adherens junction;anchoring junction;cell body;cell junction;cell part;cell projection;cell-substrate adherens junction;cell-substrate junction;clathrin-coated vesicle;coated pit;coated vesicle;cytoplasm;cytoplasmic membrane-	Alzheimer's disease;Malaria	Homo sapiens	+
catalytic step 2 spliceosome;cell part;cytoplasm;extracellular membrane-bounded organelle;extracellular organelle;extracellular region part;extracellular vesicular exosome;intracellular membrane-bounded organelle;intracellu	Spliceosome	Homo sapiens	+
cell part;cell projection;cytoplasm;cytoplasmic part;cytosol;extracellular membrane-bounded organelle;extracellular organelle;extracellular region part;extracellular vesicular exosome;intracellular part;membrane;membrane-bounded organelle;membrane-bounded vesicle;organelle;plasma membrane;ruffle;vesicle		Homo sapiens	+
cell part;intracellular membrane-bounded organelle;intracellular organelle;intracellular organelle part;intracellular part;membrane-bounded organelle;nuclear body;nuclear part;nuclear speck;nucleoplasm;nucleoplasm part;nuc	Spliceosome	Homo sapiens	+
calcineurin complex;cell part;contractile fiber part;cytoplasm;cytoplasmic part;cytosol;intracellular membrane-bounded organelle;intracellular organelle;intracellular organelle part;intracellular part;macromolecular complex;me	Alzheimer's disease;Amyotrophic lateral sclerosis (ALS);Apoptosis;Axon guidance;B cell receptor signaling pathway;Calcium signaling pathway;Ko	Homo sapiens	
cell part;centrosome;CRD-mediated mRNA stability complex;cytoplasm;cytoplasmic part;cytoskeletal part;cytosol;intracellular membrane-bounded organelle;intracellular non-membrane-bounded organelle;intracellular organelle;intracellular organelle part;intracellular part;macromolecular complex;membrane;membrane-bounded organelle;microtubule organizing center;r		Homo sapiens	+
cell part;cytoplasm;cytoplasmic part;cytosol;extracellular membrane-bounded organelle;extracellular organelle;extracellular region part;extracellular vesicular exosome;Golgi apparatus;intracellular membrane-bounded organelle;intracellular organelle;intracellular part;membrane-bounded organelle;membrane-bounded vesicle;organelle;vesicle		Homo sapiens	
cell part;cytoplasmic part;cytosol;endoplasmic reticulum-Golgi intermediate compartment;Golgi apparatus;Golgi apparatus part;Golgi cisterna membrane;Golgi membrane;Golgi transport complex;intracellular membrane-bounded organelle;intracellular non-membrane-bounded organelle;intracellular organelle;intracellular organelle part;intracellular part;macromolecular c		Homo sapiens	+
cell part;cis-Golgi network;coated vesicle;cytoplasmic membrane-bounded vesicle;cytoplasmic part;cytoplasmic vesicle;ER to Golgi transport vesicle;Golgi apparatus;Golgi apparatus part;Golgi cis cisterna;Golgi cisterna;Golgi cisterna membrane;Golgi membrane;intracellular membrane-bounded organelle;intracellular organelle;intracellular organelle part;intracellular par		Homo sapiens	+
blood microparticle;cell part;extracellular matrix;extracellular membrane-bounded organelle;extracellular organelle;extracellular region part;extracellular space;extracellular vesicular exosome;membrane;membrane-bounded organelle;membrane-bounded vesicle;organelle;proteinaceous extracellular matrix;vesicle		Homo sapiens	+
cell part;external side of plasma membrane;extracellular membrane-bounded organelle;extracellular organelle;extracellular region;extracellular region part;extracellular space;extracellular vesicular exosome;extrinsic to membrane;extrinsic to plasma membrane;membrane;membrane part;membrane-bounded organelle;membrane-bounded vesicle;organelle;plasma memb		Homo sapiens	+
cell part;extracellular membrane-bounded organelle;extracellular organelle;extracellular region part;extracellular vesicular exosome;integral to membrane;integral to plasma membrane;intrinsic to membrane;intrinsic to plasma	ECM-receptor interaction	Homo sapiens	+

cell part;cytoplasm;intracellular membrane-bounded organelle;intracellular non-membrane-bounded organelle;intracellular organelle;intracellular organelle part;intracellular part;membrane-bounded organelle;non-membrane-l	Calcium signaling pathway;Huntington's disease;Parkinson's disease	Homo sapiens	
cell part;chromosome;cytoplasm;intracellular membrane-bounded organelle;intracellular non-membrane-bounded organelle;intracellular organelle;intracellular organelle part;intracellular part;membrane-bounded organelle;non-membrane-bounded organelle;nuclear part;nucleolus;nucleoplasm;nucleus;organelle;organelle part		Homo sapiens	+
cell part;intracellular membrane-bounded organelle;intracellular organelle;intracellular part;membrane-bounded organelle;nucleus;organelle		Homo sapiens	+
cell part;cytoplasmic part;early endosome membrane;endoplasmic reticulum;endosomal part;endosome membrane;Golgi apparatus part;Golgi membrane;intracellular membrane-bounded organelle;intracellular organelle;intracellular organelle part;intracellular part;late endosome membrane;macromolecular complex;membrane;membrane part;membrane-bounded organ		Homo sapiens	+
cell part;cytoplasm;cytoskeletal part;intracellular membrane-bounded organelle;intracellular non-membrane-bounded organelle;intracellular organelle;intracellular organelle part;intracellular part;membrane-bounded organelle;non-membrane-bounded organelle;nuclear part;nucleolus;nucleus;organelle;organelle part;spindle		Homo sapiens	
CAF-1 complex;cell part;chromatin;chromatin assembly complex;chromatin remodeling complex;chromosomal part;ESC/E(Z) complex;histone deacetylase complex;histone methyltransferase complex;intracellular membrane-bounded organelle;intracellular organelle;intracellular organelle part;intracellular part;ISWI complex;macromolecular complex;membrane-bounded		Homo sapiens	+
cell part;cytoplasm;cytoplasmic part;cytosol;intracellular membrane-bounded organelle;intracellular organelle;intracellular organelle part;intracellular part;macromolecular complex;membrane-bounded organelle;mitochondrio	mRNA surveillance pathway;RNA transport;Spliceosome	Homo sapiens	+
actin cytoskeleton;adherens junction;anchoring junction;cell junction;cell part;cell-cell contact zone;cell-cell junction;cell-substrate adherens junction;cell-substrate junction;contractile fiber part;costamere;cytoplasm;cytoplasmic part;cytoskeleton;cytosol;extracellular membrane-bounded organelle;extracellular organelle;extracellular region part;extracellular vesicular exo		Homo sapiens	+
cell part;cytoplasmic part;cytosol;intracellular part;membrane		Homo sapiens	+
adherens junction;anchoring junction;cell junction;cell part;cell-substrate adherens junction;cell-substrate junction;contractile fiber part;cytoplasmic part;cytoskeleton;focal adhesion;intracellular non-membrane-bounded organelle;intracellular organelle;intracellular part;non-membrane-bounded organelle;organelle;organelle part;Z disc		Homo sapiens	
cell part;cytoplasmic part;extracellular membrane-bounded organelle;extracellular organelle;extracellular region part;extracellular vesicular exosome;Golgi apparatus;Golgi apparatus part;Golgi cisterna membrane;Golgi meml	Mucin type O-Glycan biosynthesis	Homo sapiens	
AP-type membrane coat adaptor complex;cell part;clathrin adaptor complex;clathrin coated vesicle membrane;clathrin-coated endocytic vesicle membrane;coated vesicle membrane;cytoplasmic part;cytoplasmic vesicle me	Lysosome	Homo sapiens	+
cell part;intracellular organelle part;intracellular part;macromolecular complex;mRNA cleavage and polyadenylation specificity factor complex;mRNA cleavage factor complex;nuclear part;nucleoplasm;nucleoplasm part;organ	mRNA surveillance pathway	Homo sapiens	+
anchored to membrane;cell part;extracellular membrane-bounded organelle;extracellular organelle;extracellular region part;extracellular vesicular exosome;extrinsic to membrane;intrinsic to membrane;membrane;membrane	Calcium signaling pathway;Nicotinate and nicotinamide metabolism;Pancreatic secretion;Salivary secretion	Homo sapiens	+
anchored to membrane;apical plasma membrane;cell part;cell surface;cytoplasm;cytoplasmic part;endosome;extracellular membrane-bounded organelle;extracellular organelle;extracellular region part;extracellular vesicular exosome;Golgi apparatus;integral to membrane;integral to plasma membrane;intracellular membrane-bounded organelle;intracellular organelle;intra		Homo sapiens	+
cell part;cytoplasmic part;extracellular region part;extracellular space;intracellular membrane-bounded organelle;intracellular organelle;intracellular organelle lumen;intracellular organelle part;intracellular part;membrane;membrane-bounded organelle;membrane-enclosed lumen;mitochondrial inner membrane;mitochondrial matrix;mitochondrial membrane;mitochondrial p		Homo sapiens	+
cell part;cytoplasm;intracellular membrane-bounded organelle;intracellular organelle;intracellular organelle part;intracellular part;membrane;membrane-bounded organelle;nuclear membrane;nuclear part;nucleus;organelle;organelle membrane;organelle part		Homo sapiens	+
cell part;cytoplasmic part;cytosol;early endosome;endoplasmic reticulum;endosome;intracellular membrane-bounded organelle;intracellular organelle;intracellular part;macromolecular complex;membrane-bounded organelle;organelle;protein complex;WASH complex		Homo sapiens	
cell part;cytoplasmic part;cytosol;envelope;intracellular organelle part;intracellular part;macromolecular complex;membrane part;nuclear envelope;nuclear part;nuclear pore;Nup107-160 complex;organelle envelope;organelle	RNA transport	Homo sapiens	+
cell part;intracellular membrane-bounded organelle;intracellular non-membrane-bounded organelle;intracellular organelle;intracellular organelle part;intracellular part;macromolecular complex;membrane-bounded organelle;no	Ribosome biogenesis in eukaryotes	Homo sapiens	+
actin cytoskeleton;adherens junction;anchoring junction;cell junction;cell part;cell projection;cell-cell junction;cell-substrate adherens junction;cell-substrate junction;contractile fiber;cytoplasm;cytoplasmic part;cytoskeleton;filopodium;focal adhesion;intracellular non-membrane-bounded organelle;intracellular organelle;intracellular part;myofibril;non-membrane-bounded		Homo sapiens	+
calcium channel complex;cation channel complex;cell cortex part;cell part;cortical endoplasmic reticulum;cytoplasmic part;endoplasmic reticulum;endoplasmic reticulum lumen;endoplasmic reticulum membrane;endoplasmic reticulum part;integral to endoplasmic reticulum membrane;integral to membrane;integral to organelle membrane;intracellular membrane-bounde		Homo sapiens	+
cell part;cytoplasm;cytoplasmic part;cytosol;intracellular membrane-bounded organelle;intracellular organelle;intracellular part;membrane;membrane-bounded organelle;nucleus;organelle;perinuclear region of cytoplasm		Homo sapiens	
extracellular matrix;extracellular membrane-bounded organelle;extracellular organelle;extracellular region;extracellular region part;extracellular space;extracellular vesicular exosome;membrane-bounded organelle;membrane-bounded vesicle;organelle;proteinaceous extracellular matrix;vesicle		Homo sapiens	+
cell part;centrosome;cytoplasm;cytoplasmic part;cytoskeletal part;cytosol;intracellular membrane-bounded organelle;intracellular non-membrane-bounded organelle;intracellular organelle;intracellular organelle part;intracellul	Cell cycle;Cell cycle - yeast;Meiosis - yeast;Oocyte meiosis;Ubiquitin mediated proteolysis	Homo sapiens	
extracellular membrane-bounded organelle;extracellular organelle;extracellular region;extracellular region part;extracellular space;extracellular vesicular exosome;membrane-bounded organelle;membrane-bounded vesicle;organelle;vesicle		Homo sapiens	
basolateral plasma membrane;cell part;cell projection;cell projection part;cell surface;clathrin-coated vesicle;coated vesicle;cytoplasmic membrane-bounded vesicle;cytoplasmic part;cytoplasmic vesicle;cytosol;dendritic spi	SNARE interactions in vesicular transport;Vasopressin-regulated water reabsorption	Homo sapiens	
cell part;cytoplasm;cytoplasmic part;intracellular membrane-bounded organelle;intracellular non-membrane-bounded organelle;intracellular organelle;intracellular organelle part;intracellular part;membrane-bounded organelle;mitochondrial nucleoid;mitochondrial part;mitochondrion;non-membrane-bounded organelle;nucleoid;organelle;organelle part		Homo sapiens	+
anchored to membrane;cell part;extracellular membrane-bounded organelle;extracellular organelle;extracellular region part;extracellular vesicular exosome;intrinsic to membrane;membrane;membrane part;membrane-bound	Cell adhesion molecules (CAMs)	Homo sapiens	+
cell part;centrosome;chromatin remodeling complex;cytoplasm;cytoplasmic part;cytoskeletal part;cytoskeleton;histone deacetylase complex;intermediate filament cytoskeleton;intracellular membrane-bounded organelle;intracellular non-membrane-bounded organelle;intracellular organelle;intracellular organelle part;intracellular part;macromolecular complex;membrane-		Homo sapiens	+
catalytic step 2 spliceosome;cell part;intracellular organelle part;intracellular part;macromolecular complex;nuclear body;nuclear part;nuclear speck;nucleoplasm;nucleoplasm part;organelle part;ribonucleoprotein complex;sp	Spliceosome	Homo sapiens	+
cell part;cytoplasm;cytoplasmic part;cytosol;intracellular part	beta-Alanine metabolism;Drug metabolism - other enzymes;Pantothenate and CoA biosynthesis;Pyrimidine metabolism	Homo sapiens	+
adherens junction;anchoring junction;apical part of cell;basal part of cell;cell junction;cell part;cell projection;cell projection membrane;cell projection part;cell surface;cell-substrate adherens junction;cell-substrate junction;cytoplasm;extracellular region part;extracellular space;focal adhesion;integral to membrane;intracellular part;intrinsic to membrane;invadopodium me		Homo sapiens	+
cell part;chromosomal part;condensed chromosome kinetochore;cytoplasm;intracellular membrane-bounded organelle;intracellular non-membrane-bounded organelle;intracellular organelle;intracellular organelle part;intracellular part;kinetochore;macromolecular complex;membrane-bounded organelle;non-membrane-bounded organelle;nuclear part;nucleoplasm;nucleu		Homo sapiens	
aminoacyl-tRNA synthetase multienzyme complex;cell part;cell surface;cytoplasm;cytoplasmic membrane-bounded vesicle;cytoplasmic part;cytoplasmic vesicle;cytosol;endoplasmic reticulum;extracellular region part;extracellular space;Golgi apparatus;intracellular membrane-bounded organelle;intracellular organelle;intracellular part;macromolecular complex;membran		Homo sapiens	+
cell part;cytoplasm;intracellular membrane-bounded organelle;intracellular non-membrane-bounded organelle;intracellular organelle;intracellular organelle part;intracellular part;macromolecular complex;membrane;membrane-bounded organelle;non-membrane-bounded organelle;nuclear part;nucleolus;nucleoplasm;nucleus;organelle;organelle part;ribonucleoprotein com		Homo sapiens	+
cell part;cytoplasm;cytoplasmic part;intracellular membrane-bounded organelle;intracellular non-membrane-bounded organelle;intracellular organelle;intracellular organelle part;intracellular part;macromolecular complex;membrane;membrane-bounded organelle;mitochondrion;non-membrane-bounded organelle;nuclear part;nucleolus;nucleoplasm;nucleus;organelle;orga		Homo sapiens	+
cell part;cell surface;cytoplasmic part;endoplasmic reticulum membrane;endoplasmic reticulum part;endoplasmic reticulum-Golgi intermediate compartment;endoplasmic reticulum-Golgi intermediate compartment membran	Protein processing in endoplasmic reticulum	Homo sapiens	+
cell cortex;cell junction;cell part;cell projection;cell projection membrane;cell projection part;cytoplasmic part;cytoskeletal part;extracellular membrane-bounded organelle;extracellular organelle;extracellular region part;extracellular vesicular exosome;growth cone;intracellular organelle part;intracellular part;ionotropic glutamate receptor complex;leading edge membrane;		Homo sapiens	+
cell part;cytoplasmic part;extracellular membrane-bounded organelle;extracellular organelle;extracellular region part;extracellular vesicular exosome;intracellular membrane-bounded organelle;intracellular organelle;intracellular organelle lumen;intracellular organelle part;intracellular part;lipid particle;membrane;membrane-bounded organelle;membrane-bounded vesicle;n		Homo sapiens	+
CD40 receptor complex;cell cortex;cell part;cytoplasm;cytoplasmic part;cytosol;internal side of plasma membrane;intracellular part;macromolecular complex;membrane;membrane part;membrane raft;organelle membrane;or	Adipocytokine signaling pathway;Apoptosis;Hepatitis C;MAPK signaling pathway;Osteoclast differentiation;Pathways in cancer;Protein processing	Homo sapiens	
actin cytoskeleton;adherens junction;anchoring junction;cell junction;cell part;cell-cell junction;clathrin-coated vesicle;coated vesicle;cytoplasm;cytoplasmic membrane-bounded vesicle;cytoplasmic part;cytoplasmic vesicle;cytoskeletal part;cytoskeleton;extracellular membrane-bounded organelle;extracellular organelle;extracellular region part;extracellular vesicular exos		Homo sapiens	+
cell part;cytoplasm;intracellular membrane-bounded organelle;intracellular organelle;intracellular organelle part;intracellular part;macromolecular complex;membrane-bounded organelle;nuclear body;nuclear part;nuclear speck;nucleoplasm;nucleoplasm part;nucleus;organelle;organelle part;ribonucleoprotein complex;spliceosomal complex		Homo sapiens	
cell part;cytoplasm;cytoplasmic part;early endosome;endosome;extracellular membrane-bounded organelle;extracellular organelle;extracellular region part;extracellular vesicular exosome;intracellular membrane-bounded organelle;intracellular organelle;intracellular part;membrane;membrane-bounded organelle;membrane-bounded vesicle;organelle;plasma membrane;v		Homo sapiens	+
cell part;cytoplasmic part;cytosol;intracellular part;membrane;plasma membrane		Homo sapiens	+
cell part;intracellular membrane-bounded organelle;intracellular organelle;intracellular organelle part;intracellular part;membrane-bounded organelle;nuclear part;nucleoplasm;nucleus;organelle;organelle part	mRNA surveillance pathway	Homo sapiens	
cell part;cytoplasmic part;extracellular membrane-bounded organelle;extracellular organelle;extracellular region part;extracellular vesicular exosome;intracellular membrane-bounded organelle;intracellular organelle;intracellula	Peroxisome	Homo sapiens	
cell part;cytoplasm;cytoplasmic part;cytosol;intracellular membrane-bounded organelle;intracellular organelle;intracellular organelle part;intracellular part;membrane-bounded organelle;nuclear part;nucleoplasm;nucleus;orgar	MAPK signaling pathway;Non-small cell lung cancer;Pathways in cancer	Homo sapiens	
adherens junction;anchoring junction;cell junction;cell part;cell-substrate adherens junction;cell-substrate junction;cytoplasm;cytoplasmic part;cytoskeletal part;focal adhesion;intracellular organelle part;intracellular part;microtubule organizing center;nuclear part;nucleoplasm;organelle part		Homo sapiens	
cell part;cytoplasm;cytoplasmic part;extracellular membrane-bounded organelle;extracellular organelle;extracellular region part;extracellular vesicular exosome;intracellular organelle lumen;intracellular organelle part;intracellu	Pantothenate and CoA biosynthesis	Homo sapiens	+
actin cytoskeleton;cell part;cytoplasm;cytoplasmic part;cytoskeleton;cytosol;extracellular membrane-bounded organelle;extracellular organelle;extracellular region part;extracellular vesicular exosome;intracellular membrane-	Fatty acid biosynthesis;Insulin signaling pathway;Propanoate metabolism;Pyruvate metabolism	Homo sapiens	+
cell part;cytoplasm;intracellular membrane-bounded organelle;intracellular organelle;intracellular organelle part;intracellular part;macromolecular complex;membrane-bounded organelle;nuclear part;nucleus;organelle;organelle part;protein complex;signalosome		Homo sapiens	+
cell part;extracellular region part;extracellular space;intracellular non-membrane-bounded organelle;intracellular organelle;intracellular organelle part;intracellular part;non-membrane-bounded organelle;nuclear part;nucleolus;nucleoplasm;organelle;organelle part		Homo sapiens	+
cell part;cytoplasm;cytoplasmic part;cytosol;extracellular membrane-bounded organelle;extracellular organelle;extracellular region part;extracellular vesicular exosome;intracellular membrane-bounded organelle;intracellular c	Cysteine and methionine metabolism	Homo sapiens	+
AMP-activated protein kinase complex;apical plasma membrane;cell part;cytoplasm;cytoplasmic part;cytosol;intracellular;intracellular membrane-bounded organelle;intracellular organelle;intracellular organelle part;intracellul	Adipocytokine signaling pathway;Hypertrophic cardiomyopathy (HCM);Insulin signaling pathway;mTOR signaling pathway;Regulation of autophag	Homo sapiens	+
adherens junction;anchoring junction;cell junction;cell part;cell-substrate adherens junction;cell-substrate junction;cytoplasm;cytoplasmic part;cytosol;focal adhesion;intracellular part;presynaptic active zone;synapse part		Homo sapiens	+
cell part;intracellular membrane-bounded organelle;intracellular organelle;intracellular organelle part;intracellular part;membrane-bounded organelle;nuclear part;nucleoplasm;nucleus;organelle;organelle part		Homo sapiens	+
cell part;intracellular membrane-bounded organelle;intracellular organelle;intracellular organelle part;intracellular part;macromolecular complex;membrane-bounded organelle;nuclear part;nucleoplasm;nucleus;organelle;organelle part;ribonucleoprotein complex		Homo sapiens	+
cell part;cytoplasmic part;cytosol;intracellular membrane-bounded organelle;intracellular organelle;intracellular part;membrane;membrane-bounded organelle;nucleus;organelle		Homo sapiens	+
cell part;cytoplasmic part;cytosol;endoplasmic reticulum;extracellular membrane-bounded organelle;extracellular organelle;extracellular region part;extracellular space;extracellular vesicular exosome;intracellular membrane-bounded organelle;intracellular organelle;intracellular part;membrane-bounded organelle;membrane-bounded vesicle;mitochondrion;nucleus;organe		Homo sapiens	+
cell part;cytoplasm;cytoplasmic part;cytosol;intracellular organelle part;intracellular part;membrane;nuclear part;nucleoplasm;organelle part;perinuclear region of cytoplasm;plasma membrane	Axon guidance;ErbB signaling pathway;Focal adhesion;MAPK signaling pathway;Regulation of actin cytoskeleton;Renal cell carcinoma;T cell rece	Homo sapiens	+
cell part;centromeric heterochromatin;chromatin;chromosomal part;chromosome, centromeric region;condensed chromosome, centromeric region;cytoskeletal part;envelope;euchromatin;heterochromatin;intracellular membrane-bounded organelle;intracellular non-membrane-bounded organelle;intracellular organelle;intracellular organelle part;intracellular part;membrane		Homo sapiens	+
cell part;cytoplasmic part;endoplasmic reticulum;endoplasmic reticulum-Golgi intermediate compartment membrane;Golgi apparatus;Golgi apparatus part;Golgi membrane;integral to membrane;intracellular membrane-boun	SNARE interactions in vesicular transport	Homo sapiens	
cell part;cytoplasmic part;cytosol;extracellular membrane-bounded organelle;extracellular organelle;extracellular region part;extracellular vesicular exosome;intracellular organelle part;intracellular part;macromolecular comple	Proteasome	Homo sapiens	
cell part;cytoplasmic part;endoplasmic reticulum;extracellular membrane-bounded organelle;extracellular organelle;extracellular region part;extracellular vesicular exosome;intracellular membrane-bounded organelle;intracellu	Axon guidance	Homo sapiens	+
cell part;cytoplasm;cytoplasmic part;endoplasmic reticulum;endoplasmic reticulum lumen;endoplasmic reticulum part;endoplasmic reticulum Sec complex;extracellular membrane-bounded organelle;extracellular organelle;ex	Protein processing in endoplasmic reticulum	Homo sapiens	+
cell part;cytoplasmic part;cytosol;extracellular membrane-bounded organelle;extracellular organelle;extracellular region part;extracellular space;extracellular vesicular exosome;intracellular non-membrane-bounded organelle;intracellular organelle;intracellular organelle part;intracellular part;membrane;membrane-bounded organelle;membrane-bounded vesicle;non-memb		Homo sapiens	+
cell part;intracellular non-membrane-bounded organelle;intracellular organelle;intracellular organelle part;intracellular part;non-membrane-bounded organelle;nuclear part;nucleolus;nucleoplasm;organelle;organelle part	Spliceosome	Homo sapiens	+
cell part;cytoplasm;intracellular membrane-bounded organelle;intracellular non-membrane-bounded organelle;intracellular organelle;intracellular organelle part;intracellular part;membrane-bounded organelle;non-membrane-l	Spliceosome	Homo sapiens	+
cell part;intracellular organelle part;intracellular part;nuclear body;nuclear part;nuclear speck;nucleoplasm;nucleoplasm part;organelle part	Spliceosome	Homo sapiens	+
cell part;chromosomal part;condensed chromosome kinetochore;cytoplasmic part;cytoskeletal part;cytosol;intracellular membrane-bounded organelle;intracellular non-membrane-bounded organelle;intracellular organelle;inti	Cell cycle;Cell cycle - yeast;Meiosis - yeast;Oocyte meiosis;Progesterone-mediated oocyte maturation	Homo sapiens	+
cell part;chromatin;chromosomal part;euchromatin;heterochromatin;intracellular membrane-bounded organelle;intracellular organelle;intracellular organelle part;intracellular part;membrane-bounded organelle;nuclear chromatin;nuclear chromosome part;nuclear euchromatin;nuclear heterochromatin;nuclear part;nucleoplasm;nucleus;organelle;organelle part		Homo sapiens	+
adherens junction;anchoring junction;cell junction;cell part;cell-substrate adherens junction;cell-substrate junction;cytoplasm;cytoplasmic part;cytosol;focal adhesion;intracellular membrane-bounded organelle;intracellular non-membrane-bounded organelle;intracellular organelle;intracellular part;macromolecular complex;membrane;membrane-bounded organelle;non-m		Homo sapiens	+
adherens junction;anchoring junction;cell junction;cell part;cell-cell junction;cell-substrate adherens junction;cell-substrate junction;focal adhesion;integral to membrane;integral to plasma membrane;intrinsic to membrane;intrinsic to plasma membrane;membrane part;plasma membrane part		Homo sapiens	+
cell part;cytoplasm;cytoplasmic part;intracellular membrane-bounded organelle;intracellular non-membrane-bounded organelle;intracellular organelle;intracellular part;macromolecular complex;membrane-bounded organelle;	mRNA surveillance pathway;RNA degradation;RNA transport	Homo sapiens	+
actin cytoskeleton;apical part of cell;cell part;cell projection membrane;cell projection part;cytoskeleton;intracellular non-membrane-bounded organelle;intracellular organelle;intracellular part;leading edge membrane;membrane part;non-membrane-bounded organelle;organelle;plasma membrane part;ruffle membrane		Homo sapiens	+
cell part;chromatin remodeling complex;cytoplasm;cytoskeletal part;envelope;histone deacetylase complex;intracellular membrane-bounded organelle;intracellular organelle;intracellular organelle part;intracellular part;macromolecular complex;membrane-bounded organelle;microtubule;nuclear envelope;nuclear part;nucleoplasm;nucleoplasm part;nucleus;NuRD comple		Homo sapiens	+
cell part;cytoplasmic part;cytosol;eukaryotic 43S preinitiation complex;eukaryotic 48S preinitiation complex;eukaryotic translation initiation factor 3 complex;eukaryotic translation initiation factor 3 complex, eIF3m;extracellula	RNA transport	Homo sapiens	+
cell part;cytoplasmic part;cytosol;intracellular membrane-bounded organelle;intracellular organelle;intracellular organelle part;intracellular part;macromolecular complex;membrane-bounded organelle;nuclear part;nucleoplasn	Chronic myeloid leukemia;Notch signaling pathway;Pathways in cancer;Wnt signaling pathway	Homo sapiens	
cell part;cytoplasm;intracellular part		Homo sapiens	+
cell part;cytoplasm;cytoplasmic part;cytosol;extracellular membrane-bounded organelle;extracellular organelle;extracellular region part;extracellular vesicular exosome;intracellular membrane-bounded organelle;intracellular organelle;intracellular part;macromolecular complex;membrane-bounded organelle;membrane-bounded vesicle;nucleus;organelle;protein complex;(		Homo sapiens	
cell part;centrosome;cytoplasm;cytoplasmic dynein complex;cytoplasmic part;cytoskeletal part;cytosol;dynein complex;intracellular non-membrane-bounded organelle;intracellular organelle;intracellular organelle part;intracel	Phagosome;Vasopressin-regulated water reabsorption	Homo sapiens	+
actin filament bundle;actomyosin;adherens junction;anchoring junction;axon part;cell body;cell junction;cell part;cell projection;cell projection part;cell-cell junction;cell-substrate adherens junction;cell-substrate junction;cont	Bacterial invasion of epithelial cells;Endometrial cancer;Focal adhesion;PPAR signaling pathway	Homo sapiens	+
cell part;cytoplasmic part;integral to membrane;intracellular membrane-bounded organelle;intracellular organelle;intracellular organelle part;intracellular part;intrinsic to membrane;membrane;membrane part;membrane-bounc	Nicotinate and nicotinamide metabolism	Homo sapiens	+
adherens junction;anchoring junction;cell junction;cell part;cell-substrate adherens junction;cell-substrate junction;cytoplasm;cytoplasmic part;cytoplasmic vesicle membrane;cytoplasmic vesicle part;cytoskeletal part;dystrophin-associated glycoprotein complex;extracellular membrane-bounded organelle;extracellular organelle;extracellular region part;extracellular vesic		Homo sapiens	+
cell part;cytoplasm;intracellular membrane-bounded organelle;intracellular organelle;intracellular organelle part;intracellular part;membrane-bounded organelle;nuclear body;nuclear matrix;nuclear part;nuclear speck;nucleoplasm;nucleoplasm part;nucleus;organelle;organelle part		Homo sapiens	+
cell part;cytoplasm;intracellular membrane-bounded organelle;intracellular non-membrane-bounded organelle;intracellular organelle;intracellular organelle part;intracellular part;membrane-bounded organelle;non-membrane-l	Ribosome biogenesis in eukaryotes	Homo sapiens	+
catalytic step 2 spliceosome;cell part;intracellular organelle part;intracellular part;macromolecular complex;nuclear part;nucleoplasm;organelle part;ribonucleoprotein complex;spliceosomal complex;U12-type spliceosomal cc	Spliceosome	Homo sapiens	+
cell part;cytoplasmic part;cytoplasmic ubiquitin ligase complex;endoplasmic reticulum lumen;endoplasmic reticulum part;endoplasmic reticulum quality control compartment;ER ubiquitin ligase complex;Hrd1p ubiquitin ligase	Protein processing in endoplasmic reticulum	Homo sapiens	+
cell part;cytoplasm;cytoplasmic part;extracellular membrane-bounded organelle;extracellular organelle;extracellular region part;extracellular vesicular exosome;Golgi apparatus;Golgi apparatus part;Golgi membrane;intracellular membrane-bounded organelle;intracellular organelle;intracellular organelle part;intracellular part;membrane;membrane-bounded organelle;mem		Homo sapiens	
		Homo sapiens	+
adherens junction;anchoring junction;basolateral plasma membrane;cell junction;cell part;cell surface;cell-substrate adherens junction;cell-substrate junction;cytoplasm;extracellular membrane-bounded organelle;extracellular organelle;extracellular region part;extracellular space;extracellular vesicular exosome;focal adhesion;integral to membrane;intracellular part;intrins		Homo sapiens	+
cell part;cytoplasmic part;endoplasmic reticulum membrane;endoplasmic reticulum part;extracellular membrane-bounded organelle;extracellular organelle;extracellular region part;extracellular vesicular exosome;intracellular organelle part;intracellular part;membrane;membrane part;membrane-bounded organelle;membrane-bounded vesicle;nuclear part;nucleoplasm;org		Homo sapiens	+
actin cytoskeleton;cell cortex;cell part;cell projection;cell projection part;cytoplasm;cytoplasmic part;cytoskeletal part;cytoskeleton;cytosol;filamentous actin;filopodium tip;intracellular non-membrane-bounded organelle;intracellular organelle;intracellular organelle part;intracellular part;lamellipodium;macromolecular complex;membrane;myosin complex;non-membrane-b		Homo sapiens	+
bleb;cell part;cell projection;centriole;cytoplasmic part;cytoskeletal part;cytoskeleton;cytosol;Golgi apparatus part;Golgi membrane;intracellular non-membrane-bounded organelle;intracellular organelle;intracellular organelle	Axon guidance;Chemokine signaling pathway;Focal adhesion;Leukocyte transendothelial migration;Pathogenic Escherichia coli infection;Regulati	Homo sapiens	+
cell body;cell part;clathrin-coated vesicle;coated pit;coated vesicle;cytoplasmic membrane-bounded vesicle;cytoplasmic part;cytoplasmic vesicle;Golgi apparatus;intracellular membrane-bounded organelle;intracellular organelle;intracellular part;membrane;membrane part;membrane-bounded organelle;membrane-bounded vesicle;neuronal cell body;nucleus;organelle;p		Homo sapiens	+
aggresome;autophagic vacuole;cell part;cytoplasm;cytoplasmic mRNA processing body;cytoplasmic part;cytoplasmic vesicle;cytosol;endoplasmic reticulum;endosome;extracellular membrane-bounded organelle;extracellula	Osteoclast differentiation	Homo sapiens	+
cell part;cytoplasmic part;integral to membrane;intracellular organelle part;intracellular part;intrinsic to membrane;membrane;membrane part;mitochondrial membrane;mitochondrial outer membrane;mitochondrial part;organelle membrane;organelle outer membrane;organelle part;outer membrane		Homo sapiens	+
axon;cell body;cell part;cell periphery;cell projection;cytoplasm;cytoskeletal part;dendrite;extracellular membrane-bounded organelle;extracellular organelle;extracellular region part;extracellular vesicular exosome;intracellular	Gap junction;Pathogenic Escherichia coli infection;Phagosome	Homo sapiens	+
catalytic step 2 spliceosome;cell part;chromosome;intracellular non-membrane-bounded organelle;intracellular organelle;intracellular organelle part;intracellular part;macromolecular complex;non-membrane-bounded organelle;nuclear part;nucleoplasm;organelle;organelle part;ribonucleoprotein complex;spliceosomal complex		Homo sapiens	
cell part;cytoplasm;intracellular membrane-bounded organelle;intracellular organelle;intracellular organelle part;intracellular part;membrane-bounded organelle;midbody;nuclear body;nuclear part;nuclear speck;nucleoplasm;r	RIG-I-like receptor signaling pathway	Homo sapiens	+
cell part;cytoplasm;cytoplasmic part;cytosol;intracellular organelle part;intracellular part;macromolecular complex;nuclear part;nucleoplasm;organelle part;protein complex	Acute myeloid leukemia;ErbB signaling pathway;Insulin signaling pathway;mTOR signaling pathway;RNA transport	Homo sapiens	+
cell part;cytoplasm;cytoplasmic part;cytosol;death-inducing signaling complex;intracellular membrane-bounded organelle;intracellular organelle;intracellular part;macromolecular complex;membrane part;membrane raft;mem	Apoptosis;Cytosolic DNA-sensing pathway;Hepatitis C;RIG-I-like receptor signaling pathway;Toll-like receptor signaling pathway	Homo sapiens	+
cell part;chromatin;chromatin remodeling complex;chromosomal part;cytoplasm;cytoplasmic part;cytosol;histone deacetylase complex;intracellular membrane-bounded organelle;intracellular organelle;intracellular organelle p	Cell cycle;Chronic myeloid leukemia;Huntington's disease;Notch signaling pathway;Pathways in cancer	Homo sapiens	+
axon part;calcium channel complex;cation channel complex;cell body;cell junction;cell part;cell projection part;cell-cell contact zone;cell-cell junction;cytoplasm;cytoplasmic part;cytoplasmic vesicle membrane;cytoplasmic v	Calcium signaling pathway;ErbB signaling pathway;Gastric acid secretion;Glioma;GnRH signaling pathway;ko05152;Long-term potentiation;Melar	Homo sapiens	+
cell part;cell projection part;centrosome;chromosomal part;cytoplasm;cytoplasmic part;cytoskeletal part;cytosol;dynactin complex;dynein complex;extracellular membrane-bounded organelle;extracellular organelle;extracellu	Huntington's disease;Vasopressin-regulated water reabsorption	Homo sapiens	+
cell part;cytoplasm;cytoplasmic part;cytosol;intracellular membrane-bounded organelle;intracellular organelle;intracellular part;membrane;membrane-bounded organelle;nucleus;organelle;plasma membrane	Alzheimer's disease	Homo sapiens	+
catalytic step 2 spliceosome;cell part;chromatin;chromosomal part;intracellular membrane-bounded organelle;intracellular organelle;intracellular organelle part;intracellular part;macromolecular complex;membrane-bounded c	Notch signaling pathway;Spliceosome	Homo sapiens	+
cell part;intracellular membrane-bounded organelle;intracellular non-membrane-bounded organelle;intracellular organelle;intracellular organelle part;intracellular part;membrane-bounded organelle;non-membrane-bounded or	Spliceosome	Homo sapiens	
cell part;cell projection;cytoplasm;cytoplasmic part;cytosol;early endosome membrane;endosomal part;endosome membrane;extrinsic to membrane;Golgi apparatus;intracellular membrane-bounded organelle;intracellular organelle;intracellular organelle part;intracellular part;lamellipodium;macromolecular complex;membrane;membrane part;membrane-bounded organe		Homo sapiens	
cell part;cytoplasm;extracellular region part;intercellular bridge;intracellular membrane-bounded organelle;intracellular non-membrane-bounded organelle;intracellular organelle;intracellular organelle part;intracellular part;macromolecular complex;membrane;membrane-bounded organelle;non-membrane-bounded organelle;nuclear part;nucleolus;nucleus;organelle;organe		Homo sapiens	
cell part;cytoplasmic part;Golgi apparatus;intracellular membrane-bounded organelle;intracellular non-membrane-bounded organelle;intracellular organelle;intracellular organelle part;intracellular part;membrane-bounded organelle;non-membrane-bounded organelle;nuclear part;nucleolus;nucleus;organelle;organelle part		Homo sapiens	
cell part;cullin-RING ubiquitin ligase complex;cytoplasmic part;cytosol;intracellular organelle part;intracellular part;macromolecular complex;nuclear part;nucleoplasm;organelle part;protein complex;SCF ubiquitin ligase comp	Cell cycle;Cell cycle - yeast;Circadian rhythm - mammal;Oocyte meiosis;Protein processing in endoplasmic reticulum;TGF-beta signaling pathway	Homo sapiens	
cell part;Cul2-RING ubiquitin ligase complex;cullin-RING ubiquitin ligase complex;cytoplasmic part;cytosol;intracellular organelle part;intracellular part;macromolecular complex;nuclear part;nucleoplasm;organelle part;protein	Pathways in cancer;Renal cell carcinoma;Ubiquitin mediated proteolysis	Homo sapiens	+
cell part;Cul3-RING ubiquitin ligase complex;cullin-RING ubiquitin ligase complex;cytoplasmic part;cytoskeletal part;cytosol;extracellular membrane-bounded organelle;extracellular organelle;extracellular region part;extracell	Ubiquitin mediated proteolysis	Homo sapiens	+
cell part;CUL4 RING ubiquitin ligase complex;Cul4A-RING ubiquitin ligase complex;cullin-RING ubiquitin ligase complex;intracellular organelle part;intracellular part;macromolecular complex;nuclear part;nucleoplasm;organel	Nucleotide excision repair;Ubiquitin mediated proteolysis	Homo sapiens	
cell part;CUL4 RING ubiquitin ligase complex;Cul4B-RING ubiquitin ligase complex;cullin-RING ubiquitin ligase complex;cytoplasm;extracellular membrane-bounded organelle;extracellular organelle;extracellular region part;e	Nucleotide excision repair;Ubiquitin mediated proteolysis	Homo sapiens	+
cell part;cytoplasm;extracellular membrane-bounded organelle;extracellular organelle;extracellular region part;extracellular vesicular exosome;intracellular part;membrane-bounded organelle;membrane-bounded vesicle;orga	Amino sugar and nucleotide sugar metabolism;Fructose and mannose metabolism	Homo sapiens	+
cell part;cytoplasmic membrane-bounded vesicle;cytoplasmic part;cytoplasmic vesicle;cytoplasmic vesicle membrane;cytoplasmic vesicle part;early endosome;endocytic vesicle;endocytic vesicle membrane;endosome;Golgi apparatus part;intracellular membrane-bounded organelle;intracellular organelle;intracellular organelle part;intracellular part;melanosome;membra		Homo sapiens	+
cell part;cell surface;cytoplasmic part;endoplasmic reticulum;integral to membrane;integral to plasma membrane;intracellular membrane-bounded organelle;intracellular organelle;intracellular part;intrinsic to membrane;intrinsic to plasma membrane;membrane part;membrane-bounded organelle;organelle;plasma membrane part		Homo sapiens	+
adherens junction;anchoring junction;cell junction;cell part;cell-substrate adherens junction;cell-substrate junction;cytoplasm;cytoplasmic part;cytosol;focal adhesion;intracellular membrane-bounded organelle;intracellular organelle;intracellular part;membrane;membrane-bounded organelle;nucleus;organelle;plasma membrane		Homo sapiens	+
actin filament bundle;actomyosin;adherens junction;anchoring junction;cell junction;cell part;cell-substrate adherens junction;cell-substrate junction;contractile fiber part;cytoplasmic part;cytoskeletal part;focal adhesion;intracellular membrane-bounded organelle;intracellular organelle;intracellular organelle part;intracellular part;membrane-bounded organelle;nucleus;orga		Homo sapiens	
cell part;cell surface;cytoplasm;intracellular part;membrane;plasma membrane		Homo sapiens	+
cell part;cytoplasmic part;endoplasmic reticulum;endoplasmic reticulum membrane;endoplasmic reticulum part;extracellular membrane-bounded organelle;extracellular organelle;extracellular region part;extracellular vesicula	N-Glycan biosynthesis;Protein processing in endoplasmic reticulum	Homo sapiens	+
adherens junction;anchoring junction;axon;cell body;cell junction;cell part;cell projection;cell-substrate adherens junction;cell-substrate junction;external side of plasma membrane;extracellular membrane-bounded organelle;	Cell adhesion molecules (CAMs)	Homo sapiens	+
adherens junction;anchoring junction;cell cortex part;cell junction;cell part;cell-cell adherens junction;cell-cell junction;contractile fiber part;cuticular plate;cytoplasmic part;cytoskeletal part;cytoskeleton;cytosol;extracellular membrane-bounded organelle;extracellular organelle;extracellular region part;extracellular vesicular exosome;fascia adherens;intracellular membran		Homo sapiens	+
cell part;intracellular membrane-bounded organelle;intracellular non-membrane-bounded organelle;intracellular organelle;intracellular organelle part;intracellular part;membrane;membrane-bounded organelle;non-membrane-	Ribosome biogenesis in eukaryotes	Homo sapiens	+
cell part;cytoplasm;intracellular membrane-bounded organelle;intracellular non-membrane-bounded organelle;intracellular organelle;intracellular organelle part;intracellular part;macromolecular complex;membrane-bounded (	mRNA surveillance pathway;RNA transport;Spliceosome	Homo sapiens	+
cell part;cytoplasm;cytoplasmic part;cytosol;extracellular membrane-bounded organelle;extracellular organelle;extracellular region part;extracellular vesicular exosome;intracellular membrane-bounded organelle;intracellular organelle;intracellular organelle part;intracellular part;membrane-bounded organelle;membrane-bounded vesicle;nuclear part;nucleoplasm;nucleus;(		Homo sapiens	+

cell part;cytoplasm;cytoplasmic exosome (RNase complex);cytoplasmic part;cytosol;exosome (RNase complex);intracellular membrane-bounded organelle;intracellular non-membrane-bounded organelle;intracellular organell	RNA degradation	Homo sapiens	
cell part;cytoplasm;cytoskeletal part;extracellular membrane-bounded organelle;extracellular organelle;extracellular region part;extracellular vesicular exosome;intracellular membrane-bounded organelle;intracellular organelle	Gap junction;Pathogenic Escherichia coli infection;Phagosome	Homo sapiens	+
apical part of cell;cell part;cytoplasm;intracellular membrane-bounded organelle;intracellular non-membrane-bounded organelle;intracellular organelle;intracellular organelle part;intracellular part;membrane;membrane-bounded organelle;non-membrane-bounded organelle;nuclear part;nucleolus;nucleus;organelle;organelle part		Homo sapiens	
cell part;cytoplasmic part;cytosol;intracellular membrane-bounded organelle;intracellular organelle;intracellular part;membrane-bounded organelle;microbody;mitochondrion;organelle;peroxisome	Terpenoid backbone biosynthesis	Homo sapiens	
cell part;intracellular membrane-bounded organelle;intracellular organelle;intracellular part;membrane;membrane-bounded organelle;nucleus;organelle		Homo sapiens	+
cell part;centrosome;cytoplasmic part;cytoskeletal part;cytosol;intracellular non-membrane-bounded organelle;intracellular organelle;intracellular organelle part;intracellular part;macromolecular complex;membrane;microtubule organizing center;non-membrane-bounded organelle;organelle;organelle part;protein complex;spindle pole		Homo sapiens	+
cell part;cytoplasm;cytoplasmic part;intracellular membrane-bounded organelle;intracellular non-membrane-bounded organelle;intracellular organelle;intracellular organelle part;intracellular part;macromolecular complex;membrane-bounded organelle;non-membrane-bounded organelle;nuclear part;nucleoplasm;nucleus;organelle;organelle part;ribonucleoprotein comple		Homo sapiens	+
cell part;cytoplasm;cytoskeleton;extracellular membrane-bounded organelle;extracellular organelle;extracellular region part;extracellular vesicular exosome;intracellular non-membrane-bounded organelle;intracellular organelle;intracellular organelle part;intracellular part;membrane;membrane-bounded organelle;membrane-bounded vesicle;non-membrane-bounded orga		Homo sapiens	+
cell part;cytoplasm;cytoplasmic part;intracellular membrane-bounded organelle;intracellular organelle;intracellular organelle part;intracellular part;membrane-bounded organelle;membrane-enclosed lumen;mitochondrial inter	Oxidative phosphorylation	Homo sapiens	
cell part;cytoplasmic part;cytosol;extracellular membrane-bounded organelle;extracellular organelle;extracellular region part;extracellular vesicular exosome;intracellular membrane-bounded organelle;intracellular organelle;intracellular organelle part;intracellular part;macromolecular complex;membrane-bounded organelle;membrane-bounded vesicle;nuclear part;nucleop		Homo sapiens	+
adherens junction;anchoring junction;cell junction;cell part;cell-substrate adherens junction;cell-substrate junction;cytoplasmic part;extracellular membrane-bounded organelle;extracellular organelle;extracellular region part;e	Lysosome	Homo sapiens	+
basement membrane;cell part;cell surface;extracellular matrix;extracellular matrix part;extracellular membrane-bounded organelle;extracellular organelle;extracellular region;extracellular region part;extracellular vesicular exosome;membrane-bounded organelle;membrane-bounded vesicle;organelle;vesicle		Homo sapiens	+
cell part;intracellular organelle part;intracellular part;macromolecular complex;nuclear part;nucleolar part;nucleoplasm;organelle part;PeBoW complex;preribosome;preribosome, large subunit precursor;protein complex;ribonucleoprotein complex		Homo sapiens	
cell part;cytoplasm;intracellular part;macromolecular complex;protein complex;ubiquitin ligase complex	Ubiquitin mediated proteolysis	Homo sapiens	
cell part;Cul3-RING ubiquitin ligase complex;cullin-RING ubiquitin ligase complex;cytoplasm;cytoplasmic part;cytoskeletal part;cytosol;endoplasmic reticulum;intracellular membrane-bounded organelle;intracellular organelle	Ubiquitin mediated proteolysis	Homo sapiens	+
cell part;cytoplasm;extracellular membrane-bounded organelle;extracellular organelle;extracellular region part;extracellular vesicular exosome;intracellular membrane-bounded organelle;intracellular organelle;intracellular organelle part;intracellular part;membrane-bounded organelle;membrane-bounded vesicle;nuclear part;nucleoplasm;organelle;organelle part;vesicle		Homo sapiens	+
cell part;cytoplasm;cytoplasmic part;cytosol;eukaryotic 43S preinitiation complex;eukaryotic 48S preinitiation complex;eukaryotic translation initiation factor 3 complex;eukaryotic translation initiation factor 3 complex, eIF3m	RNA transport	Homo sapiens	+
adherens junction;anchoring junction;cell body;cell cortex;cell junction;cell part;cell projection;cell-substrate adherens junction;cell-substrate junction;cytoplasmic part;cytosol;focal adhesion;intracellular part;lamellipodium;m	Regulation of actin cytoskeleton	Homo sapiens	+
cell part;intracellular organelle part;intracellular part;macromolecular complex;nuclear part;organelle part;PcG protein complex;protein complex		Homo sapiens	+
adherens junction;anchoring junction;basolateral plasma membrane;cell junction;cell part;cell projection;cell-cell adherens junction;cell-cell junction;cytoplasmic part;extracellular membrane-bounded organelle;extracellular organelle;extracellular region part;extracellular vesicular exosome;intracellular organelle part;intracellular part;lamellipodium;macromolecular comple		Homo sapiens	+
adherens junction;anchoring junction;cell junction;cell part;cell-substrate adherens junction;cell-substrate junction;focal adhesion;intracellular organelle part;intracellular part;nuclear part;nucleoplasm;organelle part	Endocytosis	Homo sapiens	+
cell part;cytoplasmic part;endoplasmic reticulum;endoplasmic reticulum membrane;endoplasmic reticulum part;integral to membrane;intracellular membrane-bounded organelle;intracellular organelle;intracellular organelle part;intracellular part;intrinsic to membrane;membrane;membrane part;membrane-bounded organelle;organelle;organelle membrane;organelle part		Homo sapiens	
		Homo sapiens	+
actin cytoskeleton;adherens junction;anchoring junction;cell junction;cell part;cell-substrate adherens junction;cell-substrate junction;contractile fiber part;cytoplasmic part;cytoskeleton;focal adhesion;intracellular membrane-	Osteoclast differentiation	Homo sapiens	
cell body;cell part;cell projection;cell projection part;cytoplasmic membrane-bounded vesicle;cytoplasmic part;cytoplasmic vesicle;cytoskeletal part;cytosol;exocytic vesicle;extracellular region part;extracellular space;filamentous actin;growth cone;intracellular membrane-bounded organelle;intracellular organelle;intracellular organelle part;intracellular part;lamellipodium;		Homo sapiens	+
cell part;cytoplasmic part;intracellular membrane-bounded organelle;intracellular organelle;intracellular organelle lumen;intracellular organelle part;intracellular part;large ribosomal subunit;macromolecular complex;membrane;membrane-bounded organelle;membrane-enclosed lumen;mitochondrial inner membrane;mitochondrial large ribosomal subunit;mitochondrial ma		Homo sapiens	+
cell leading edge;cell part;centrosome;chromosomal part;cytoplasm;cytoplasmic part;cytoskeletal part;cytosol;dynactin complex;dynein complex;intracellular non-membrane-bounded organelle;intracellular organelle;intracell	Huntington's disease;Vasopressin-regulated water reabsorption	Homo sapiens	+
cell part;cell projection;centrosome;cytoplasmic dynein complex;cytoplasmic part;cytoskeletal part;cytosol;dynein complex;extracellular membrane-bounded organelle;extracellular organelle;extracellular region part;extracellu	Phagosome;Vasopressin-regulated water reabsorption	Homo sapiens	
cell part;cytoplasm;cytoplasmic part;cytosol;eukaryotic translation initiation factor 2B complex;intracellular part;macromolecular complex;membrane;plasma membrane;protein complex	RNA transport	Homo sapiens	
cell part;cytoplasmic part;cytosol;eukaryotic translation initiation factor 4F complex;intracellular non-membrane-bounded organelle;intracellular organelle;intracellular organelle part;intracellular part;macromolecular complex;r	RNA transport	Homo sapiens	+
cell part;cytoplasm;elongin complex;extracellular region part;extracellular space;integral to membrane;intracellular membrane-bounded organelle;intracellular organelle;intracellular organelle part;intracellular part;intrinsic to membrane;macromolecular complex;membrane part;membrane-bounded organelle;nuclear part;nucleoplasm;nucleoplasm part;nucleus;organelle;or		Homo sapiens	+
adherens junction;anchoring junction;cell cortex;cell cortex part;cell junction;cell part;cell projection;cell projection part;cell-substrate adherens junction;cell-substrate junction;coated pit;cortical cytoskeleton;cytoplasm;cytop	Bacterial invasion of epithelial cells;Pathogenic Escherichia coli infection;Shigellosis;Tight junction	Homo sapiens	+
acrosomal membrane;adherens junction;anchoring junction;caveola;cell junction;cell part;cell-substrate adherens junction;cell-substrate junction;cytoplasmic membrane-bounded vesicle;cytoplasmic part;cytoplasmic vesicle	Insulin signaling pathway	Homo sapiens	+
cell part;cytoplasmic part;endoplasmic reticulum;endoplasmic reticulum lumen;endoplasmic reticulum part;intracellular membrane-bounded organelle;intracellular non-membrane-bounded organelle;intracellular organelle;intracellular organelle lumen;intracellular organelle part;intracellular part;membrane-bounded organelle;membrane-enclosed lumen;non-membrane-bo		Homo sapiens	+
cell part;cytoplasm;cytoplasmic part;cytosol;intracellular organelle part;intracellular part;nuclear part;nucleoplasm;organelle part	RIG-I-like receptor signaling pathway	Homo sapiens	+
adherens junction;anchoring junction;cell junction;cell part;cell-substrate adherens junction;cell-substrate junction;contractile fiber part;costamere;cytoplasm;cytoplasmic part;cytoskeleton;cytosol;focal adhesion;intracellular	Focal adhesion;MAPK signaling pathway	Homo sapiens	+
cell part;cytoplasmic part;endoplasmic reticulum membrane;endoplasmic reticulum part;integral to endoplasmic reticulum membrane;integral to membrane;integral to organelle membrane;intracellular organelle part;intracellular part;intrinsic to endoplasmic reticulum membrane;intrinsic to membrane;intrinsic to organelle membrane;membrane;membrane part;mitochondr		Homo sapiens	+
cell part;intracellular membrane-bounded organelle;intracellular organelle;intracellular organelle part;intracellular part;membrane-bounded organelle;nuclear part;nucleoplasm;nucleus;organelle;organelle part		Homo sapiens	+
Cajal body;catalytic step 2 spliceosome;cell part;contractile fiber part;cytoplasmic part;intracellular non-membrane-bounded organelle;intracellular organelle;intracellular organelle part;intracellular part;macromolecular complex;non-membrane-bounded organelle;nuclear body;nuclear part;nucleolus;nucleoplasm part;organelle;organelle part;ribonucleoprotein complex;sp		Homo sapiens	+
adherens junction;anchoring junction;cell junction;cell part;cell-substrate adherens junction;cell-substrate junction;clathrin coated vesicle membrane;clathrin-coated endocytic vesicle membrane;coated vesicle membrane;cyt	Basal cell carcinoma;Melanogenesis;Pathways in cancer;Wnt signaling pathway	Homo sapiens	+
adherens junction;anchoring junction;brush border membrane;cell junction;cell part;cell projection membrane;cell projection part;cell-substrate adherens junction;cell-substrate junction;cytoplasmic membrane-bounded vesic	Long-term depression;Regulation of actin cytoskeleton;Vascular smooth muscle contraction	Homo sapiens	+
cell part;cytoplasmic part;cytosol;extracellular membrane-bounded organelle;extracellular organelle;extracellular region part;extracellular vesicular exosome;intracellular part;membrane-bounded organelle;membrane-bounde	Arginine and proline metabolism;Glycine, serine and threonine metabolism	Homo sapiens	
cell part;cytoplasmic part;cytosol;extracellular membrane-bounded organelle;extracellular organelle;extracellular region part;extracellular vesicular exosome;intracellular part;membrane-bounded organelle;membrane-bounde	Amino sugar and nucleotide sugar metabolism;Galactose metabolism	Homo sapiens	+
cell part;cell projection;cytoplasm;cytoplasmic mRNA processing body;cytoplasmic part;cytosol;dendrite;integral to membrane;integral to plasma membrane;intracellular non-membrane-bounded organelle;intracellular organelle;intracellular part;intrinsic to membrane;intrinsic to plasma membrane;macromolecular complex;membrane;membrane part;neuron projection;n		Homo sapiens	+
cell part;cytoplasmic part;cytoskeletal part;cytoskeleton;intracellular non-membrane-bounded organelle;intracellular organelle;intracellular organelle part;intracellular part;microtubule cytoskeleton;microtubule organizing center;non-membrane-bounded organelle;nuclear body;nuclear part;nuclear speck;nucleoplasm;nucleoplasm part;organelle;organelle part		Homo sapiens	+
extracellular region;extracellular region part;extracellular space		Homo sapiens	+
cell part;cytoplasm;intracellular membrane-bounded organelle;intracellular non-membrane-bounded organelle;intracellular organelle;intracellular organelle part;intracellular part;membrane;membrane-bounded organelle;non-membrane-bounded organelle;nuclear part;nucleolus;nucleoplasm;nucleus;organelle;organelle part		Homo sapiens	+
cell part;cytoplasmic part;endoplasmic reticulum;endoplasmic reticulum lumen;endoplasmic reticulum membrane;endoplasmic reticulum part;intracellular membrane-bounded organelle;intracellular organelle;intracellular organelle lumen;intracellular organelle part;intracellular part;membrane;membrane part;membrane-bounded organelle;membrane-enclosed lumen;orga		Homo sapiens	+
macromolecular complex;protein complex;ribose phosphate diphosphokinase complex		Homo sapiens	
cell part;chromosomal part;intracellular membrane-bounded organelle;intracellular organelle;intracellular organelle part;intracellular part;macromolecular complex;MCM complex;membrane-bounded organelle;nuclear chromo	Cell cycle;Cell cycle - yeast;DNA replication;Meiosis - yeast	Homo sapiens	+
apical part of cell;brush border;cell body;cell part;cell projection;cytoplasm;cytoplasmic part;endoplasmic reticulum;endoplasmic reticulum membrane;endoplasmic reticulum part;integral to membrane;integral to plasma men	Alzheimer's disease;Calcium signaling pathway;Gap junction;Gastric acid secretion;GnRH signaling pathway;Long-term depression;Long-term po	Homo sapiens	
blood microparticle;cell part;cytoplasm;extracellular membrane-bounded organelle;extracellular organelle;extracellular region;extracellular region part;extracellular vesicular exosome;intracellular part;membrane;membrane-bounded organelle;membrane-bounded vesicle;organelle;plasma membrane;vesicle		Homo sapiens	+
cell part;cytoplasm;cytoplasmic part;cytosol;intracellular membrane-bounded organelle;intracellular organelle;intracellular organelle part;intracellular part;membrane-bounded organelle;nuclear part;nucleoplasm;nucleus;orga	Cytosolic DNA-sensing pathway;Hepatitis C;Measles;RIG-I-like receptor signaling pathway;Toll-like receptor signaling pathway	Homo sapiens	+
cell part;cytoplasm;intracellular membrane-bounded organelle;intracellular organelle;intracellular organelle part;intracellular part;membrane-bounded organelle;nuclear part;nucleoplasm;nucleus;organelle;organelle part	Ubiquitin mediated proteolysis	Homo sapiens	+
cell part;cytoplasmic part;cytosol;intracellular part		Homo sapiens	+
adherens junction;anchoring junction;cell junction;cell part;cell-substrate adherens junction;cell-substrate junction;chromosome;focal adhesion;intracellular membrane-bounded organelle;intracellular non-membrane-bounded organelle;intracellular organelle;intracellular organelle part;intracellular part;membrane-bounded organelle;non-membrane-bounded organelle;nucl		Homo sapiens	+
cell part;clathrin-coated vesicle;coated vesicle;cytoplasmic membrane-bounded vesicle;cytoplasmic part;cytoplasmic vesicle;cytosol;Golgi apparatus;intracellular membrane-bounded organelle;intracellular organelle;intracellular organelle part;intracellular part;membrane;membrane-bounded organelle;membrane-bounded vesicle;nuclear part;nucleoplasm;organelle;orga		Homo sapiens	
cell part;chromosomal part;chromosome;chromosome, centromeric region;cohesin complex;cohesin core heterodimer;condensed chromosome;condensed chromosome kinetochore;condensed nuclear chromosome;cytopla	Cell cycle;Cell cycle - yeast;Meiosis - yeast;Oocyte meiosis	Homo sapiens	
cell part;chromatin;chromosomal part;cytoplasmic part;cytosol;euchromatin;heterochromatin;intracellular membrane-bounded organelle;intracellular non-membrane-bounded organelle;intracellular organelle;intracellular organelle part;intracellular part;macromolecular complex;membrane-bounded organelle;non-membrane-bounded organelle;nuclear part;nucleolus;nucle		Homo sapiens	
cell part;cell surface;intracellular membrane-bounded organelle;intracellular organelle;intracellular part;membrane-bounded organelle;nucleus;organelle		Homo sapiens	+
cell part;intracellular membrane-bounded organelle;intracellular non-membrane-bounded organelle;intracellular organelle;intracellular organelle part;intracellular part;membrane-bounded organelle;non-membrane-bounded or	Ribosome biogenesis in eukaryotes	Homo sapiens	+
cell part;cytoplasm;cytoplasmic part;early endosome;endosome;intracellular membrane-bounded organelle;intracellular organelle;intracellular organelle part;intracellular part;membrane-bounded organelle;nuclear part;nucleoplasm;nucleus;organelle;organelle part		Homo sapiens	+
cell part;cytoplasmic part;endoplasmic reticulum;intracellular membrane-bounded organelle;intracellular organelle;intracellular part;membrane;membrane-bounded organelle;organelle;plasma membrane		Homo sapiens	+
cell part;cytoplasmic membrane-bounded vesicle;cytoplasmic part;cytoplasmic vesicle;endoplasmic reticulum lumen;endoplasmic reticulum part;extracellular membrane-bounded organelle;extracellular organelle;extracellula	N-Glycan biosynthesis;Protein processing in endoplasmic reticulum	Homo sapiens	+
cell part;cytoplasm;cytoplasmic part;endosome;extracellular membrane-bounded organelle;extracellular organelle;extracellular region part;extracellular vesicular exosome;intracellular membrane-bounded organelle;intracellular organelle;intracellular part;macromolecular complex;membrane;membrane part;membrane raft;membrane-bounded organelle;membrane-bound		Homo sapiens	+
cell part;cytoplasmic part;cytosol;intracellular membrane-bounded organelle;intracellular organelle;intracellular organelle part;intracellular part;macromolecular complex;membrane-bounded organelle;nuclear part;nucleoplasm	Meiosis - yeast;mRNA surveillance pathway;Oocyte meiosis;Wnt signaling pathway	Homo sapiens	
cell part;envelope;integral to membrane;integral to nuclear inner membrane;integral to organelle membrane;intracellular organelle part;intracellular part;intrinsic to membrane;intrinsic to nuclear inner membrane;intrinsic to organelle membrane;membrane;membrane part;nuclear envelope;nuclear membrane;nuclear membrane part;nuclear part;organelle envelope;organell		Homo sapiens	+
cell part;cytoplasmic part;cytosol;Golgi apparatus part;Golgi membrane;Golgi stack;Golgi transport complex;intracellular organelle part;intracellular part;macromolecular complex;membrane;organelle membrane;organelle part;protein complex		Homo sapiens	+
cell part;cytoplasm;cytoplasmic part;cytoskeleton;extracellular membrane-bounded organelle;extracellular organelle;extracellular region part;extracellular vesicular exosome;intracellular membrane-bounded organelle;intracellular non-membrane-bounded organelle;intracellular organelle;intracellular organelle part;intracellular part;macromolecular complex;membrane;me		Homo sapiens	+
extracellular matrix;extracellular matrix part;extracellular region;extracellular region part;fibril;macromolecular complex;microfibril;protein complex;proteinaceous extracellular matrix		Homo sapiens	+
cell part;cis-Golgi network;cytoplasmic part;endoplasmic reticulum-Golgi intermediate compartment;Golgi apparatus;Golgi apparatus part;Golgi membrane;Golgi stack;integral to membrane;intracellular membrane-bounded organelle;intracellular organelle;intracellular organelle part;intracellular part;intrinsic to membrane;membrane;membrane part;membrane-bounded o		Homo sapiens	+
CD95 death-inducing signaling complex;cell body;cell part;cell projection;cytoplasm;cytoplasmic part;cytoskeletal part;cytoskeleton;cytosol;death-inducing signaling complex;intracellular membrane-bounded organelle;intrac	Alzheimer's disease;Apoptosis;Chagas disease (American trypanosomiasis);Huntington's disease;ko05152;NOD-like receptor signaling pathway;p	Homo sapiens	+
cell part;centrosome;chromatin;chromatin remodeling complex;chromosomal part;cytoplasm;cytoplasmic part;cytoskeletal part;histone deacetylase complex;intracellular membrane-bounded organelle;intracellular non-membrane-bounded organelle;intracellular organelle;intracellular organelle part;intracellular part;macromolecular complex;membrane;membrane-bounde		Homo sapiens	+
adherens junction;anchoring junction;cell cortex part;cell junction;cell part;cell-substrate adherens junction;cell-substrate junction;cortical actin cytoskeleton;cortical cytoskeleton;cytoplasmic part;cytoskeletal part;cytoskeleton;extracellular membrane-bounded organelle;extracellular organelle;extracellular region part;extracellular vesicular exosome;focal adhesion;intrace		Homo sapiens	+
cell part;cytoplasm;extracellular membrane-bounded organelle;extracellular organelle;extracellular region part;extracellular vesicular exosome;intracellular part;membrane-bounded organelle;membrane-bounded vesicle;organelle;vesicle		Homo sapiens	+
cell part;cytoplasm;intracellular membrane-bounded organelle;intracellular organelle;intracellular organelle part;intracellular part;membrane-bounded organelle;nuclear body;nuclear part;nuclear speck;nucleoplasm;nucleoplasm part;organelle;organelle part		Homo sapiens	+
cell part;cytoplasm;cytoplasmic part;cytosol;endoplasmic reticulum part;endoplasmic reticulum tubular network;envelope;extracellular membrane-bounded organelle;extracellular organelle;extracellular region part;extracellula	RNA transport	Homo sapiens	+
Cajal body;cell part;cytoplasm;intracellular non-membrane-bounded organelle;intracellular organelle;intracellular organelle part;intracellular part;non-membrane-bounded organelle;nuclear body;nuclear part;nucleolus;nucleoplasm;nucleoplasm part;organelle;organelle part		Homo sapiens	+
apical part of cell;cell body;cell part;cell projection;chromosome;cytoplasm;cytoplasmic part;cytoskeletal part;cytosol;dendrite;extracellular membrane-bounded organelle;extracellular organelle;extracellular region part;extracellular vesicular exosome;Golgi apparatus part;Golgi membrane;intracellular membrane-bounded organelle;intracellular non-membrane-bounded o		Homo sapiens	+
cell part;cytoplasmic part;cytosol;intracellular membrane-bounded organelle;intracellular organelle;intracellular organelle part;intracellular part;membrane-bounded organelle;nuclear body;nuclear part;nuclear speck;nucleopla	Proteasome	Homo sapiens	
cell part;cytoplasmic part;cytosol;endosome;intracellular membrane-bounded organelle;intracellular organelle;intracellular part;membrane;membrane-bounded organelle;organelle		Homo sapiens	
cell part;cytoplasm;cytoplasmic part;Golgi apparatus;intracellular membrane-bounded organelle;intracellular organelle;intracellular organelle part;intracellular part;membrane-bounded organelle;nuclear part;nucleoplasm;organelle;organelle part		Homo sapiens	+
cell part;chromosomal part;condensin complex;cytoplasm;cytoplasmic part;cytosol;intracellular membrane-bounded organelle;intracellular organelle;intracellular organelle part;intracellular part;macromolecular complex;mem	Cell cycle - yeast	Homo sapiens	+
cell part;cytoplasm;cytoplasmic part;intracellular membrane-bounded organelle;intracellular organelle;intracellular organelle part;intracellular part;membrane-bounded organelle;nuclear part;nucleoplasm;nucleus;organelle;organelle part;perinuclear region of cytoplasm		Homo sapiens	+
cell part;cytoplasmic part;endoplasmic reticulum membrane;endoplasmic reticulum part;integral to membrane;intracellular organelle part;intracellular part;intrinsic to membrane;macromolecular complex;membrane;membran	Protein export	Homo sapiens	+
cell part;cytoplasm;cytoplasmic part;endoplasmic reticulum;ER membrane protein complex;intracellular membrane-bounded organelle;intracellular non-membrane-bounded organelle;intracellular organelle;intracellular organelle part;intracellular part;macromolecular complex;membrane-bounded organelle;mitochondrion;non-membrane-bounded organelle;nuclear part;n		Homo sapiens	+
cell part;intracellular membrane-bounded organelle;intracellular organelle;intracellular organelle part;intracellular part;membrane;membrane-bounded organelle;nuclear body;nuclear membrane;nuclear part;nuclear speck;nucleoplasm;nucleoplasm part;nucleus;organelle;organelle membrane;organelle part		Homo sapiens	
cell part;cytoplasmic part;cytosol;extracellular membrane-bounded organelle;extracellular organelle;extracellular region part;extracellular vesicular exosome;intracellular organelle part;intracellular part;macromolecular comple	Proteasome	Homo sapiens	
cell part;intracellular non-membrane-bounded organelle;intracellular organelle;intracellular organelle part;intracellular part;non-membrane-bounded organelle;nuclear part;nucleolus;nucleoplasm;organelle;organelle part		Homo sapiens	+
BRISC complex;cell part;cytoplasm;intracellular organelle part;intracellular part;macromolecular complex;nuclear part;organelle part;protein complex		Homo sapiens	+
actin cytoskeleton;cell cortex part;cell division site part;cell part;cell projection membrane;cell projection part;chromosomal part;ciliary transition zone;cilium membrane;cilium part;cleavage furrow;condensed chromosome kinetochore;cytoplasm;cytoplasmic part;cytoskeletal part;cytoskeleton;exocyst;extracellular membrane-bounded organelle;extracellular organelle;ext		Homo sapiens	+
Cajal body;cell part;cytoplasm;intracellular membrane-bounded organelle;intracellular organelle;intracellular organelle part;intracellular part;membrane-bounded organelle;nuclear body;nuclear part;nuclear speck;nucleoplasm;nucleoplasm part;nucleus;organelle;organelle part		Homo sapiens	
cell part;chromosomal part;chromosome;condensed chromosome;condensin complex;condensin core heterodimer;cytoplasm;cytoplasmic part;cytosol;intracellular membrane-bounded organelle;intracellular non-membrane-	Cell cycle - yeast	Homo sapiens	
cell part;cytoplasmic part;cytosol;exosome (RNase complex);intracellular membrane-bounded organelle;intracellular non-membrane-bounded organelle;intracellular organelle;intracellular organelle part;intracellular part;macro	RNA degradation	Homo sapiens	+
cell part;cytoplasmic part;endosomal part;endosome membrane;intracellular organelle part;intracellular part;membrane;organelle membrane;organelle part;recycling endosome membrane	Endocytosis	Homo sapiens	+
Cajal body;catalytic step 2 spliceosome;cell part;cytoplasm;intracellular organelle part;intracellular part;macromolecular complex;membrane;nuclear body;nuclear part;nuclear speck;nucleoplasm;nucleoplasm part;organelle p	Spliceosome	Homo sapiens	+
cell part;cytoplasmic part;cytosol;endoplasmic reticulum part;integral to membrane;intracellular organelle part;intracellular part;intrinsic to membrane;macromolecular complex;membrane;membrane part;organelle part;protein complex;Sec61 translocon complex;translocon complex		Homo sapiens	+
cell part;cytoplasm;cytoplasmic part;endoplasmic reticulum part;endoplasmic reticulum tubular network;extracellular membrane-bounded organelle;extracellular organelle;extracellular region part;extracellular vesicular exosome;intracellular organelle part;intracellular part;macromolecular complex;membrane-bounded organelle;membrane-bounded vesicle;organelle;orga		Homo sapiens	+
cell part;cell projection;cytoplasmic part;endoplasmic reticulum;Golgi apparatus;integral to membrane;integral to plasma membrane;intracellular membrane-bounded organelle;intracellular organelle;intracellular part;intrinsic to membrane;intrinsic to plasma membrane;lamellipodium;membrane;membrane part;membrane-bounded organelle;organelle;plasma membrane;p		Homo sapiens	+
aminoacyl-tRNA synthetase multienzyme complex;cell part;cytoplasm;cytoplasmic part;cytoskeleton;cytosol;extracellular region;intracellular membrane-bounded organelle;intracellular non-membrane-bounded organelle;intra	Aminoacyl-tRNA biosynthesis	Homo sapiens	+
cell part;chromosome;condensed chromosome;condensed nuclear chromosome;cytoplasmic part;endoplasmic reticulum;intracellular membrane-bounded organelle;intracellular non-membrane-bounded organelle;intracellular organelle;intracellular organelle part;intracellular part;membrane-bounded organelle;non-membrane-bounded organelle;nuclear chromosome;nuc		Homo sapiens	
cell part;cytoplasmic part;cytosol;eukaryotic translation initiation factor 4F complex;intracellular part;macromolecular complex;membrane;perinuclear region of cytoplasm;protein complex		Homo sapiens	+
cell part;cytoplasmic part;extracellular matrix;extracellular region part;extracellular space;Golgi apparatus part;intracellular organelle part;intracellular part;organelle part;proteinaceous extracellular matrix;trans-Golgi network		Homo sapiens	+
cell part;cytoplasmic part;intracellular membrane-bounded organelle;intracellular non-membrane-bounded organelle;intracellular organelle;intracellular organelle lumen;intracellular organelle part;intracellular part;membrane;m	alpha-Linolenic acid metabolism;Biosynthesis of unsaturated fatty acids;Fatty acid metabolism;Peroxisome;PPAR signaling pathway	Homo sapiens	
axon part;axonal spine;cell part;cell projection;cell projection part;cytoplasm;cytoplasmic part;cytoplasmic vesicle;cytosol;early endosome;early endosome membrane;endosomal part;endosome;endosome membrane;extrac	Endocytosis;ko05152;Phagosome	Homo sapiens	
cell part;cytoplasmic membrane-bounded vesicle;cytoplasmic part;cytoplasmic vesicle;endoplasmic reticulum;endoplasmic reticulum chaperone complex;endoplasmic reticulum lumen;endoplasmic reticulum membrane;end	Protein processing in endoplasmic reticulum	Homo sapiens	+
cell part;cytoplasm;cytoplasmic part;cytosol;extracellular membrane-bounded organelle;extracellular organelle;extracellular region part;extracellular vesicular exosome;intracellular part;membrane;membrane-bounded organe	Ether lipid metabolism	Homo sapiens	+
extracellular membrane-bounded organelle;extracellular organelle;extracellular region part;extracellular space;extracellular vesicular exosome;membrane-bounded organelle;membrane-bounded vesicle;organelle;vesicle		Homo sapiens	+
cell part;cytoplasm;cytoskeletal part;intracellular organelle part;intracellular part;macromolecular complex;microtubule associated complex;organelle part;protein complex		Homo sapiens	+
cell part;cytoplasmic part;endoplasmic reticulum;endoplasmic reticulum membrane;endoplasmic reticulum part;integral to membrane;integral to plasma membrane;intracellular membrane-bounded organelle;intracellular orga	Steroid biosynthesis	Homo sapiens	+
cell part;cytoplasmic part;cytosol;extracellular membrane-bounded organelle;extracellular organelle;extracellular region part;extracellular vesicular exosome;intracellular membrane-bounded organelle;intracellular organelle;int	Peroxisome;Terpenoid backbone biosynthesis	Homo sapiens	+
adherens junction;anchoring junction;cell junction;cell part;cell-substrate adherens junction;cell-substrate junction;contractile fiber part;costamere;cytoplasm;cytoplasmic part;cytoskeleton;cytosol;extracellular membrane-bounded organelle;extracellular organelle;extracellular region part;extracellular vesicular exosome;focal adhesion;hemidesmosome;intermediate filame		Homo sapiens	+
cell part;centriolar satellite;centrosome;cytoplasm;cytoplasmic part;cytoskeletal part;cytosol;intracellular non-membrane-bounded organelle;intracellular organelle;intracellular organelle part;intracellular part;macromolecular complex;membrane;microtubule organizing center;microtubule organizing center part;non-membrane-bounded organelle;nuclear membrane;nuclea		Homo sapiens	+
cell part;cytoplasm;cytoplasmic part;cytosol;extracellular membrane-bounded organelle;extracellular organelle;extracellular region part;extracellular vesicular exosome;intracellular part;membrane-bounded organelle;membra	Oxidative phosphorylation	Homo sapiens	+
cell part;chromosomal part;chromosome, telomeric region;cytoplasm;cytoplasmic part;cytosol;extracellular membrane-bounded organelle;extracellular organelle;extracellular region part;extracellular vesicular exosome;intracellular membrane-bounded organelle;intracellular organelle;intracellular organelle part;intracellular part;macromolecular complex;membrane-bound		Homo sapiens	+
cell part;intracellular membrane-bounded organelle;intracellular non-membrane-bounded organelle;intracellular organelle;intracellular organelle part;intracellular part;membrane;membrane-bounded organelle;non-membrane-bounded organelle;nuclear body;nuclear matrix;nuclear part;nuclear speck;nucleolus;nucleoplasm;nucleoplasm part;nucleus;organelle;organelle pa		Homo sapiens	+
calcium channel complex;cation channel complex;cell part;cytoplasm;extracellular membrane-bounded organelle;extracellular organelle;extracellular region part;extracellular vesicular exosome;intracellular membrane-bounded organelle;intracellular organelle;intracellular organelle part;intracellular part;ion channel complex;macromolecular complex;membrane part;memb		Homo sapiens	+
adherens junction;anchoring junction;axon;cell body;cell junction;cell part;cell projection;cell surface;cell-cell junction;cilium;dendrite;integral to membrane;integral to plasma membrane;intrinsic to membrane;intrinsic to plasma membrane;leading edge membrane;membrane part;neuron projection;neuronal cell body;nonmotile primary cilium;photoreceptor outer segment		Homo sapiens	+
cell part;cytoplasm;intracellular non-membrane-bounded organelle;intracellular organelle;intracellular organelle part;intracellular part;macromolecular complex;non-membrane-bounded organelle;nuclear part;nucleolar part;nu	Ribosome biogenesis in eukaryotes	Homo sapiens	+
cell part;cytoplasmic membrane-bounded vesicle;cytoplasmic part;cytoplasmic vesicle;early endosome;endocytic vesicle;endosome;intracellular membrane-bounded organelle;intracellular organelle;intracellular part;membra	Endocytosis	Homo sapiens	+
cell part;cell projection membrane;cell projection part;clathrin-coated endocytic vesicle;clathrin-coated vesicle;coated pit;coated vesicle;cytoplasmic membrane-bounded vesicle;cytoplasmic part;cytoplasmic vesicle;endocytic vesicle;endosomal part;endosome membrane;extracellular membrane-bounded organelle;extracellular organelle;extracellular region part;extrace		Homo sapiens	+
cell part;cytoplasm;cytoplasmic part;cytosol;intracellular membrane-bounded organelle;intracellular organelle;intracellular organelle part;intracellular part;membrane-bounded organelle;nuclear body;nuclear part;nuclear spec	mRNA surveillance pathway;RNA transport	Homo sapiens	+
cell part;chromatin remodeling complex;histone methyltransferase complex;intracellular membrane-bounded organelle;intracellular non-membrane-bounded organelle;intracellular organelle;intracellular organelle part;intracellular part;macromolecular complex;membrane-bounded organelle;methyltransferase complex;MLL1 complex;non-membrane-bounded organelle;nu		Homo sapiens	+
cell part;cytoplasmic part;endoplasmic reticulum;endoplasmic reticulum lumen;endoplasmic reticulum part;intracellular membrane-bounded organelle;intracellular organelle;intracellular organelle lumen;intracellular organelle part;intracellular part;membrane-bounded organelle;membrane-enclosed lumen;organelle;organelle lumen;organelle part		Homo sapiens	+
cell part;cytoplasmic part;cytosol;intracellular part;membrane	Pancreatic cancer;Pathways in cancer	Homo sapiens	+
axon;cell cortex part;cell part;cell projection;cortical actin cytoskeleton;cortical cytoskeleton;cytoplasm;cytoplasmic part;cytoskeletal part;cytoskeleton;early endosome membrane;endosomal part;endosome membrane;Golg	Tight junction	Homo sapiens	+
cell part;coated vesicle;coated vesicle membrane;COPI coated vesicle membrane;COPI-coated vesicle;cytoplasmic membrane-bounded vesicle;cytoplasmic part;cytoplasmic vesicle;cytoplasmic vesicle membrane;cytoplasmic vesicle part;endoplasmic reticulum;endoplasmic reticulum membrane;endoplasmic reticulum part;endoplasmic reticulum-Golgi intermediate co		Homo sapiens	+
cell part;cytoplasm;extracellular membrane-bounded organelle;extracellular organelle;extracellular region part;extracellular vesicular exosome;intracellular membrane-bounded organelle;intracellular organelle;intracellular orga	Spliceosome	Homo sapiens	+
adherens junction;anchoring junction;cell junction;cell part;cell-substrate adherens junction;cell-substrate junction;cytoplasm;cytoplasmic part;cytosol;extracellular membrane-bounded organelle;extracellular organelle;extracellular region part;extracellular vesicular exosome;focal adhesion;intracellular membrane-bounded organelle;intracellular organelle;intracellular orga		Homo sapiens	+

cell part;cytoplasmic part;cytosol;elongin complex;intracellular organelle part;intracellular part;macromolecular complex;nuclear part;nucleoplasm;nucleoplasm part;organelle part;protein complex;transcription elongation fact	Pathways in cancer;Renal cell carcinoma;Ubiquitin mediated proteolysis	Homo sapiens	+
cell part;Cul2-RING ubiquitin ligase complex;Cul5-RING ubiquitin ligase complex;cullin-RING ubiquitin ligase complex;cytoplasmic part;cytoplasmic ubiquitin ligase complex;cytosol;elongin complex;extracellular membrane-b	Pathways in cancer;Renal cell carcinoma;Ubiquitin mediated proteolysis	Homo sapiens	+
cell body;cell part;cell projection;cytoplasmic part;cytosol;dendrite;endoplasmic reticulum membrane;endoplasmic reticulum part;extracellular membrane-bounded organelle;extracellular organelle;extracellular region part;ext	Insulin signaling pathway;mTOR signaling pathway	Homo sapiens	
cell part;cytoplasmic part;cytoskeleton;cytosol;endoplasmic reticulum;endoplasmic reticulum membrane;endoplasmic reticulum part;Golgi apparatus part;Golgi membrane;integral to membrane;intracellular membrane-bounc	Steroid biosynthesis	Homo sapiens	+
catalytic step 2 spliceosome;cell part;intracellular organelle part;intracellular part;macromolecular complex;nuclear part;nucleoplasm;organelle part;ribonucleoprotein complex;small nuclear ribonucleoprotein complex;spliceos	Spliceosome	Homo sapiens	+
cell part;cytoplasmic part;endoplasmic reticulum;intracellular membrane-bounded organelle;intracellular non-membrane-bounded organelle;intracellular organelle;intracellular organelle part;intracellular part;membrane-bounded organelle;non-membrane-bounded organelle;nuclear part;nucleolus;organelle;organelle part		Homo sapiens	+
cell part;centrosome;cytoplasm;cytoplasmic part;cytoskeletal part;intracellular membrane-bounded organelle;intracellular non-membrane-bounded organelle;intracellular organelle;intracellular organelle part;intracellular part;membrane-bounded organelle;microtubule organizing center;non-membrane-bounded organelle;nucleus;organelle;organelle part;spindle pole centr		Homo sapiens	
adherens junction;anchoring junction;cell junction;cell part;cell-substrate adherens junction;cell-substrate junction;cytoplasmic part;cytosol;extracellular membrane-bounded organelle;extracellular organelle;extracellular region part;extracellular vesicular exosome;focal adhesion;intracellular part;membrane-bounded organelle;membrane-bounded vesicle;organelle;vesicle		Homo sapiens	
actin cytoskeleton;adherens junction;anchoring junction;cell junction;cell part;cell-substrate adherens junction;cell-substrate junction;cytoplasm;cytoskeleton;focal adhesion;intracellular non-membrane-bounded organelle;intracellular organelle;intracellular part;non-membrane-bounded organelle;organelle		Homo sapiens	+
cell part;cytoplasm;cytoplasmic part;cytoskeletal part;cytosol;intracellular membrane-bounded organelle;intracellular non-membrane-bounded organelle;intracellular organelle;intracellular organelle part;intracellular part;meml	Long-term potentiation;MAPK signaling pathway;mTOR signaling pathway;Neurotrophin signaling pathway;Oocyte meiosis;Progesterone-mediate	Homo sapiens	
cell part;intracellular membrane-bounded organelle;intracellular organelle;intracellular organelle part;intracellular part;membrane-bounded organelle;nuclear part;nucleoplasm;nucleus;organelle;organelle part		Homo sapiens	+
catalytic step 2 spliceosome;cell part;intracellular organelle part;intracellular part;macromolecular complex;nuclear part;nucleoplasm;organelle part;ribonucleoprotein complex;small nuclear ribonucleoprotein complex;spliceos	Spliceosome	Homo sapiens	+
cell part;intracellular membrane-bounded organelle;intracellular organelle;intracellular part;membrane-bounded organelle;nucleus;organelle		Homo sapiens	
cell part;chromosome;cytoplasm;extracellular membrane-bounded organelle;extracellular organelle;extracellular region part;extracellular vesicular exosome;intracellular membrane-bounded organelle;intracellular non-membrane-bounded organelle;intracellular organelle;intracellular part;membrane-bounded organelle;membrane-bounded vesicle;non-membrane-bounded		Homo sapiens	
cell part;coated vesicle membrane;COPII vesicle coat;cytoplasmic part;cytoplasmic vesicle membrane;cytoplasmic vesicle part;cytosol;endoplasmic reticulum;endoplasmic reticulum membrane;endoplasmic reticulum part;El	Protein processing in endoplasmic reticulum	Homo sapiens	+
cell part;COPII vesicle coat;cytoplasmic part;cytoplasmic vesicle part;endomembrane system;endoplasmic reticulum membrane;endoplasmic reticulum part;endoplasmic reticulum-Golgi intermediate compartment membrane	Protein processing in endoplasmic reticulum	Homo sapiens	
catalytic step 2 spliceosome;cell part;intracellular organelle part;intracellular part;macromolecular complex;nuclear part;nucleoplasm;organelle part;prespliceosome;ribonucleoprotein complex;small nuclear ribonucleoprotein	Spliceosome	Homo sapiens	+
cell part;cytoplasm;intracellular organelle part;intracellular part;macromolecular complex;mediator complex;nuclear part;nucleoplasm part;organelle part;protein complex		Homo sapiens	+
cell part;cytoplasm;cytoskeletal part;cytoskeleton;intracellular non-membrane-bounded organelle;intracellular organelle;intracellular organelle part;intracellular part;microtubule cytoskeleton;microtubule plus end;non-membrane-bounded organelle;organelle;organelle part		Homo sapiens	+
basement membrane;cell part;cytoplasmic part;extracellular matrix;extracellular matrix part;extracellular membrane-bounded organelle;extracellular organelle;extracellular region;extracellular region part;extracellular space;extracellular vesicular exosome;Golgi apparatus part;intracellular organelle part;intracellular part;membrane;membrane-bounded organelle;membrane		Homo sapiens	+
cell part;cytoplasm;cytoplasmic part;cytosol;intracellular membrane-bounded organelle;intracellular organelle;intracellular part;membrane-bounded organelle;nucleus;organelle		Homo sapiens	+
cell part;cytoplasmic part;intracellular membrane-bounded organelle;intracellular non-membrane-bounded organelle;intracellular organelle;intracellular organelle part;intracellular part;macromolecular complex;membrane-bounded organelle;non-membrane-bounded organelle;nuclear part;nucleoplasm;nucleus;organelle;organelle part;ribonucleoprotein complex;ribosome;		Homo sapiens	+
cell cortex;cell part;cell projection;cytoplasm;cytoplasmic part;cytoskeleton;cytosol;extracellular membrane-bounded organelle;extracellular organelle;extracellular region part;extracellular vesicular exosome;Golgi apparatus;i	Insulin signaling pathway	Homo sapiens	+
cell part;cis-Golgi network;cytoplasmic part;cytoplasmic vesicle part;cytoskeleton;Golgi apparatus;Golgi apparatus part;Golgi membrane;inner acrosomal membrane;intracellular membrane-bounded organelle;intracellular non-membrane-bounded organelle;intracellular organelle;intracellular organelle part;intracellular part;membrane;membrane part;membrane-bounded		Homo sapiens	
cell part;germ cell nucleus;intracellular membrane-bounded organelle;intracellular organelle;intracellular part;male germ cell nucleus;membrane-bounded organelle;nucleus;organelle		Homo sapiens	+
cell part;cytoplasm;cytoplasmic part;cytosol;intracellular non-membrane-bounded organelle;intracellular organelle;intracellular organelle part;intracellular part;membrane;non-membrane-bounded organelle;nuclear part;nucleolus;organelle;organelle part		Homo sapiens	+
adherens junction;anchoring junction;cell junction;cell part;cell-substrate adherens junction;cell-substrate junction;cytoplasm;cytoskeleton;focal adhesion;intracellular membrane-bounded organelle;intracellular non-membran	NOD-like receptor signaling pathway	Homo sapiens	+
cell part;cytoplasm;cytoskeleton;intracellular membrane-bounded organelle;intracellular non-membrane-bounded organelle;intracellular organelle;intracellular organelle part;intracellular part;membrane-bounded organelle;non-membrane-bounded organelle;nuclear part;nucleoplasm;nucleus;organelle;organelle part		Homo sapiens	+
cell cortex part;cell part;cell projection membrane;cell projection part;centrosome;cortical cytoskeleton;cortical microtubule cytoskeleton;cytoplasm;cytoplasmic microtubule;cytoplasmic part;cytoskeletal part;cytoskeleton;cytosol;Golgi apparatus;intracellular membrane-bounded organelle;intracellular non-membrane-bounded organelle;intracellular organelle;intracellular		Homo sapiens	+
cell part;cytoplasm;cytoplasmic part;cytosol;intracellular membrane-bounded organelle;intracellular organelle;intracellular organelle part;intracellular part;membrane;membrane-bounded organelle;nuclear part;nucleoplasm;nucleus;organelle;organelle part		Homo sapiens	+
cell part;cytoplasmic part;endoplasmic reticulum;endoplasmic reticulum membrane;endoplasmic reticulum part;integral to membrane;intracellular membrane-bounded organelle;intracellular organelle;intracellular organelle pa	Steroid biosynthesis	Homo sapiens	
actin filament bundle;actomyosin;cell division site part;cell junction;cell part;cell projection;cell-cell junction;cleavage furrow;cytoplasm;cytoplasmic part;cytoskeletal part;cytosol;extracellular membrane-bounded organelle;ex	Calcium signaling pathway;Focal adhesion;Gastric acid secretion;Regulation of actin cytoskeleton;Vascular smooth muscle contraction	Homo sapiens	+
cell part;cytoplasmic membrane-bounded vesicle;cytoplasmic part;cytoplasmic vesicle;extracellular membrane-bounded organelle;extracellular organelle;extracellular region part;extracellular vesicular exosome;Golgi apparat	Protein digestion and absorption	Homo sapiens	+
cell part;cytoplasm;cytoplasmic part;integral to membrane;intracellular membrane-bounded organelle;intracellular organelle;intracellular organelle part;intracellular part;intrinsic to membrane;membrane;membrane part;membrane-bounded organelle;mitochondrial membrane;mitochondrial outer membrane;mitochondrial part;nuclear part;nucleoplasm;nucleus;organelle;or		Homo sapiens	+
activin responsive factor complex;cell part;chromatin;chromosomal part;cytoplasm;cytoplasmic part;cytosol;intracellular membrane-bounded organelle;intracellular organelle;intracellular organelle part;intracellular part;macro	Adherens junction;Cell cycle;Chagas disease (American trypanosomiasis);Colorectal cancer;Endocytosis;Pancreatic cancer;Pathways in cancer;T(	Homo sapiens	+
cell part;cytoplasm;cytoskeletal part;intracellular organelle part;intracellular part;macromolecular complex;microtubule;organelle part;protein complex		Homo sapiens	
cell part;cell projection;cilium;cytoplasm;cytoskeletal part;cytoskeleton;intracellular non-membrane-bounded organelle;intracellular organelle;intracellular organelle part;intracellular part;macromolecular complex;microtubule;non-membrane-bounded organelle;nonmotile primary cilium;organelle;organelle part;photoreceptor connecting cilium;primary cilium;protein comple		Homo sapiens	
cell part;cytoplasm;extracellular membrane-bounded organelle;extracellular organelle;extracellular region part;extracellular vesicular exosome;intracellular membrane-bounded organelle;intracellular organelle;intracellular part;macromolecular complex;membrane-bounded organelle;membrane-bounded vesicle;nucleus;organelle;protein complex;UBC13-MMS2 complex;u		Homo sapiens	+
apical plasma membrane;azurophil granule;cell part;cytolytic granule;cytoplasmic membrane-bounded vesicle;cytoplasmic part;cytoplasmic vesicle;cytosol;extracellular membrane-bounded organelle;extracellular organelle;extracellular region part;extracellular vesicular exosome;intracellular membrane-bounded organelle;intracellular organelle;intracellular part;lysosome		Homo sapiens	
apical plasma membrane;cell junction;cell part;cell projection;cell surface;clathrin coated vesicle membrane;clathrin-coated vesicle;coated vesicle;coated vesicle membrane;cytoplasmic membrane-bounded vesicle;cytoplasr	Phagosome;SNARE interactions in vesicular transport	Homo sapiens	+
cell part;cytoplasmic part;cytosol;extracellular membrane-bounded organelle;extracellular organelle;extracellular region part;extracellular vesicular exosome;intracellular membrane-bounded organelle;intracellular organelle;intracellular part;membrane-bounded organelle;membrane-bounded vesicle;nucleus;organelle;vesicle		Homo sapiens	+
cell junction;cell part;clathrin coated vesicle membrane;clathrin-coated vesicle;coated vesicle;coated vesicle membrane;cytoplasmic membrane-bounded vesicle;cytoplasmic part;cytoplasmic vesicle;cytoplasmic vesicle mer	Endocytosis;Vasopressin-regulated water reabsorption	Homo sapiens	+
cell part;chromatin;chromosomal part;cytoplasm;ESC/E(Z) complex;histone methyltransferase complex;intracellular membrane-bounded organelle;intracellular organelle;intracellular organelle part;intracellular part;macromolecular complex;membrane-bounded organelle;methyltransferase complex;nuclear chromatin;nuclear chromosome part;nuclear part;nucleoplasm;nu		Homo sapiens	+
actin filament bundle;actomyosin;adherens junction;anchoring junction;cell junction;cell part;cell-cell adherens junction;cell-cell junction;cell-substrate adherens junction;cell-substrate junction;cytoplasm;cytoskeletal part;foc:	Focal adhesion	Homo sapiens	+
actin cytoskeleton;actin filament bundle;actomyosin;axon part;axon terminus;axoneme;cell cortex part;cell division site part;cell part;cell projection part;chromosomal part;cleavage furrow;condensed chromosome kinetochore;cytoplasm;cytoplasmic part;cytoskeletal part;cytoskeleton;extracellular membrane-bounded organelle;extracellular organelle;extracellular region		Homo sapiens	+
cell part;cytoplasm;integral to membrane;integral to plasma membrane;intracellular organelle part;intracellular part;intrinsic to membrane;intrinsic to plasma membrane;macromolecular complex;membrane;membrane part;nuclear part;nucleoplasm;organelle part;plasma membrane;plasma membrane part;proteasome complex;protein complex		Homo sapiens	+
cell part;cytoplasm;cytoskeleton;intracellular non-membrane-bounded organelle;intracellular organelle;intracellular part;non-membrane-bounded organelle;organelle	Pathways in cancer;Thyroid cancer	Homo sapiens	
cell part;cytoplasm;cytoplasmic part;cytosol;intracellular organelle part;intracellular part;membrane;nuclear part;nucleoplasm;organelle part;plasma membrane	Amino sugar and nucleotide sugar metabolism	Homo sapiens	+
extracellular membrane-bounded organelle;extracellular organelle;extracellular region;extracellular region part;extracellular space;extracellular vesicular exosome;membrane-bounded organelle;membrane-bounded vesicle;organelle;vesicle		Homo sapiens	+
basal lamina;basement membrane;extracellular matrix part;extracellular membrane-bounded organelle;extracellular organelle;extracellular region;extracellular region part;extracellular vesicular exosome;laminin complex;lamir	African trypanosomiasis;Amoebiasis;ECM-receptor interaction;Focal adhesion;Pathways in cancer;Small cell lung cancer;Toxoplasmosis	Homo sapiens	+
cell part;cytoplasmic part;endoplasmic reticulum;endoplasmic reticulum membrane;endoplasmic reticulum part;Golgi apparatus;Golgi apparatus part;Golgi membrane;integral to endoplasmic reticulum membrane;integral to (	Glycosaminoglycan biosynthesis - heparan sulfate	Homo sapiens	+
cell part;cytoplasmic part;cytosol;intracellular organelle part;intracellular part;macromolecular complex;nuclear part;nucleoplasm;organelle part;proteasome accessory complex;proteasome complex;protein complex		Homo sapiens	+
cell division site part;cell part;cleavage furrow;cytoplasm;cytoplasmic membrane-bounded vesicle;cytoplasmic part;cytoplasmic vesicle;cytosol;endosome;intracellular membrane-bounded organelle;intracellular organelle;intracellular organelle part;intracellular part;membrane;membrane-bounded organelle;membrane-bounded vesicle;midbody;nuclear part;nucleoplasm;		Homo sapiens	+
apical junction complex;cell division site part;cell junction;cell part;cell projection;cell-cell junction;centrosome;cleavage furrow;cytoplasm;cytoplasmic part;cytoskeletal part;cytoskeleton;cytosol;intermediate filament cytoskeleton;intracellular membrane-bounded organelle;intracellular non-membrane-bounded organelle;intracellular organelle;intracellular organelle part;in		Homo sapiens	
adherens junction;anchoring junction;cell junction;cell part;cell-substrate adherens junction;cell-substrate junction;focal adhesion;intracellular membrane-bounded organelle;intracellular organelle;intracellular part;membrane-bounded organelle;nucleus;organelle		Homo sapiens	+
cell part;CUL4 RING ubiquitin ligase complex;Cul4A-RING ubiquitin ligase complex;Cul4B-RING ubiquitin ligase complex;cullin-RING ubiquitin ligase complex;cytoplasm;extracellular membrane-bounded organelle;extracellu	Nucleotide excision repair;Ubiquitin mediated proteolysis	Homo sapiens	+
cell part;cytoplasm;cytoplasmic part;cytoskeletal part;cytosol;extracellular membrane-bounded organelle;extracellular organelle;extracellular region part;extracellular vesicular exosome;intracellular membrane-bounded organ	Amyotrophic lateral sclerosis (ALS);Chagas disease (American trypanosomiasis);Epithelial cell signaling in Helicobacter pylori infection;Fc epsilon	Homo sapiens	
cell part;cell projection membrane;cell projection part;cytoplasm;cytoplasmic part;cytosol;extracellular membrane-bounded organelle;extracellular organelle;extracellular region part;extracellular vesicular exosome;intracellular part;leading edge membrane;macromolecular complex;membrane part;membrane-bounded organelle;membrane-bounded vesicle;organelle;plasi		Homo sapiens	+
axon part;cell body;cell part;cell projection;cell projection part;cytoplasmic part;cytoskeleton;cytosol;dendrite;extracellular membrane-bounded organelle;extracellular organelle;extracellular region part;extracellular vesicular e	Axon guidance	Homo sapiens	+
cell part;chromatin remodeling complex;ESC/E(Z) complex;histone deacetylase complex;histone methyltransferase complex;intracellular membrane-bounded organelle;intracellular organelle;intracellular organelle part;intracellular part;macromolecular complex;membrane-bounded organelle;methyltransferase complex;nuclear part;nucleoplasm;nucleoplasm part;nucleus;		Homo sapiens	+
extracellular matrix;extracellular membrane-bounded organelle;extracellular organelle;extracellular region part;extracellular space;extracellular vesicular exosome;membrane-bounded organelle;membrane-bounded vesicle;organelle;proteinaceous extracellular matrix;vesicle		Homo sapiens	+
cell part;cytoplasm;extracellular membrane-bounded organelle;extracellular organelle;extracellular region part;extracellular vesicular exosome;intracellular membrane-bounded organelle;intracellular organelle;intracellular orga	Spliceosome	Homo sapiens	+
cell part;intracellular membrane-bounded organelle;intracellular organelle;intracellular organelle part;intracellular part;macromolecular complex;membrane;membrane-bounded organelle;mRNA cleavage factor complex;nucle	mRNA surveillance pathway	Homo sapiens	+
actin cytoskeleton;actomyosin;cell cortex;cell junction;cell part;cell projection;cell-cell junction;cytoplasm;cytoplasmic part;cytoskeletal part;cytoskeleton;dendrite;gap junction;intracellular non-membrane-bounded organelle;intracellular organelle;intracellular organelle part;intracellular part;membrane;neuron projection;non-membrane-bounded organelle;organelle;organe		Homo sapiens	+
actin cytoskeleton;actin filament bundle;actomyosin;cell junction;cell part;cell projection;cytoplasm;cytoplasmic part;cytoskeletal part;cytoskeleton;cytosol;extracellular membrane-bounded organelle;extracellular organelle;extracellular region part;extracellular vesicular exosome;filopodium;intracellular non-membrane-bounded organelle;intracellular organelle;intracellular		Homo sapiens	+
cell part;cytoplasm;cytoplasmic part;cytosol;intracellular membrane-bounded organelle;intracellular non-membrane-bounded organelle;intracellular organelle;intracellular organelle part;intracellular part;membrane;membrane-bounded organelle;non-membrane-bounded organelle;nuclear body;nuclear part;nuclear speck;nucleolus;nucleoplasm;nucleoplasm part;nucleus;(		Homo sapiens	+
cell part;cytoplasm;cytoplasmic part;extracellular membrane-bounded organelle;extracellular organelle;extracellular region part;extracellular vesicular exosome;intracellular membrane-bounded organelle;intracellular organelle;intracellular organelle lumen;intracellular organelle part;intracellular part;membrane-bounded organelle;membrane-bounded vesicle;membrane-en		Homo sapiens	+
cell part;cis-Golgi network;cytoplasmic part;extracellular membrane-bounded organelle;extracellular organelle;extracellular region part;extracellular vesicular exosome;Golgi apparatus part;Golgi membrane;integral to membra	N-Glycan biosynthesis;Various types of N-glycan biosynthesis	Homo sapiens	
cell part;cytoplasmic part;intracellular organelle part;intracellular part;macromolecular complex;membrane;membrane part;mitochondrial inner membrane;mitochondrial membrane;mitochondrial membrane part;mitochondrial	Alzheimer's disease;Huntington's disease;Oxidative phosphorylation;Parkinson's disease	Homo sapiens	
cell part;cytoplasmic part;extracellular membrane-bounded organelle;extracellular organelle;extracellular region part;extracellular space;extracellular vesicular exosome;intracellular membrane-bounded organelle;intracellular (	Cysteine and methionine metabolism;Sulfur relay system	Homo sapiens	+
anaphase-promoting complex;cell part;cullin-RING ubiquitin ligase complex;cytoplasm;intracellular organelle part;intracellular part;macromolecular complex;nuclear part;nuclear ubiquitin ligase complex;organelle part;protein	Ubiquitin mediated proteolysis	Homo sapiens	+
cell part;cytoplasmic part;cytosol;intracellular part	Purine metabolism	Homo sapiens	+
cell part;cytoplasm;cytoplasmic part;extracellular membrane-bounded organelle;extracellular organelle;extracellular region part;extracellular vesicular exosome;intracellular organelle lumen;intracellular organelle part;intracellu	Pyruvate metabolism	Homo sapiens	+
cell part;cytoplasmic part;intracellular membrane-bounded organelle;intracellular organelle;intracellular organelle lumen;intracellular organelle part;intracellular part;macromolecular complex;membrane;membrane part;membr	Alzheimer's disease;Huntington's disease;Oxidative phosphorylation;Parkinson's disease	Homo sapiens	+
cell part;cytoplasmic part;extracellular membrane-bounded organelle;extracellular organelle;extracellular region part;extracellular vesicular exosome;intracellular membrane-bounded organelle;intracellular organelle;intracellula	Adipocytokine signaling pathway;Citrate cycle (TCA cycle);Glycolysis / Gluconeogenesis;Insulin signaling pathway;PPAR signaling pathway;Proxin	Homo sapiens	+
cell part;cytoplasmic part;cytosol;intracellular part	Drug metabolism - other enzymes;Pyrimidine metabolism	Homo sapiens	+
cell part;cytoplasm;cytoplasmic part;intracellular membrane-bounded organelle;intracellular organelle;intracellular organelle lumen;intracellular organelle part;intracellular part;membrane;membrane-bounded organelle;membr	Butanoate metabolism;Caprolactam degradation;Fatty acid elongation in mitochondria;Fatty acid metabolism;Geraniol degradation;Lysine degrad	Homo sapiens	+
cell part;cytoplasmic part;endoplasmic reticulum;endoplasmic reticulum membrane;endoplasmic reticulum part;integral to membrane;intracellular membrane-bounded organelle;intracellular organelle;intracellular organelle pa	Steroid biosynthesis	Homo sapiens	+
cell part;cytoplasmic part;cytosol;extracellular membrane-bounded organelle;extracellular organelle;extracellular region part;extracellular vesicular exosome;intracellular membrane-bounded organelle;intracellular organelle;int	Amino sugar and nucleotide sugar metabolism;Galactose metabolism;Pentose and glucuronate interconversions;Starch and sucrose metabolism	Homo sapiens	+
cell part;cytoplasmic part;cytosol;extracellular membrane-bounded organelle;extracellular organelle;extracellular region part;extracellular vesicular exosome;intracellular membrane-bounded organelle;intracellular non-membr	Pyrimidine metabolism;Selenocompound metabolism	Homo sapiens	+
cell part;cytoplasmic part;integral to membrane;intracellular membrane-bounded organelle;intracellular organelle;intracellular organelle part;intracellular part;intrinsic to membrane;membrane;membrane part;membrane-bounded organelle;mitochondrial inner membrane;mitochondrial membrane;mitochondrial part;mitochondrion;organelle;organelle inner membrane;organ		Homo sapiens	+
cell part;intracellular membrane-bounded organelle;intracellular organelle;intracellular part;membrane;membrane-bounded organelle;nucleus;organelle		Homo sapiens	+
cell part;cytoplasm;cytoplasmic part;endoplasmic reticulum;intracellular membrane-bounded organelle;intracellular organelle;intracellular part;membrane-bounded organelle;nucleus;organelle;perinuclear region of cytoplasm		Homo sapiens	
cell part;chromatin;chromosomal part;chromosome;chromosome, centromeric region;cytoplasmic part;cytosol;intracellular non-membrane-bounded organelle;intracellular organelle;intracellular organelle part;intracellular part;membrane;non-membrane-bounded organelle;nuclear part;nucleoplasm;organelle;organelle part;plasma membrane		Homo sapiens	
axon part;cell leading edge;cell part;cell projection part;clathrin-coated vesicle;coated pit;coated vesicle;cytoplasmic membrane-bounded vesicle;cytoplasmic part;cytoplasmic vesicle;extrinsic to membrane;extrinsic to plasma membrane;intracellular membrane-bounded organelle;intracellular organelle;intracellular part;membrane part;membrane-bounded organelle;mer		Homo sapiens	+
cell part;cytoplasmic part;early endosome;endosome;intracellular membrane-bounded organelle;intracellular organelle;intracellular organelle part;intracellular part;macromolecular complex;membrane-bounded organelle;nuclear part;nucleoplasm;organelle;organelle part;protein complex;WASH complex		Homo sapiens	
cell part;centrosome;chromosomal part;condensed chromosome kinetochore;cytoplasm;cytoplasmic part;cytoskeletal part;cytosol;intracellular non-membrane-bounded organelle;intracellular organelle;intracellular organelle part;intracellular part;kinetochore;macromolecular complex;membrane;microtubule organizing center;non-membrane-bounded organelle;nuclear pa		Homo sapiens	+
cell part;intracellular non-membrane-bounded organelle;intracellular organelle;intracellular organelle part;intracellular part;non-membrane-bounded organelle;nuclear part;nucleolus;organelle;organelle part		Homo sapiens	+
cell part;intracellular organelle part;intracellular part;nuclear part;nucleoplasm;organelle part		Homo sapiens	
cell part;cytoplasm;intracellular membrane-bounded organelle;intracellular organelle;intracellular part;membrane-bounded organelle;nucleus;organelle		Homo sapiens	+
cell part;cytoplasmic part;extrinsic to membrane;intracellular part;lipid particle;membrane;membrane part;pre-autophagosomal structure;pre-autophagosomal structure membrane		Homo sapiens	+
apical part of cell;basal part of cell;cell part;cytoplasmic part;cytoplasmic vesicle;early endosome;endosome;external side of plasma membrane;integral to membrane;integral to plasma membrane;intracellular membrane-bounded organelle;intracellular organelle;intracellular part;intrinsic to membrane;intrinsic to plasma membrane;macromolecular complex;membrane;m		Homo sapiens	+
cell part;cytoplasm;cytoplasmic part;cytoskeleton;cytosol;intracellular membrane-bounded organelle;intracellular non-membrane-bounded organelle;intracellular organelle;intracellular part;membrane;membrane-bounded organelle;non-membrane-bounded organelle;nucleus;organelle;plasma membrane		Homo sapiens	+
cell part;cytoplasmic part;endoplasmic reticulum;endoplasmic reticulum lumen;endoplasmic reticulum part;extracellular matrix;extracellular membrane-bounded organelle;extracellular organelle;extracellular region part;extracellular vesicular exosome;intracellular membrane-bounded organelle;intracellular organelle;intracellular organelle lumen;intracellular organelle part;i		Homo sapiens	+
cell part;cytoplasm;cytoskeletal part;intracellular non-membrane-bounded organelle;intracellular organelle;intracellular organelle part;intracellular part;macromolecular complex;microtubule;non-membrane-bounded organelle;organelle;organelle part;protein complex;spindle		Homo sapiens	
	Amino sugar and nucleotide sugar metabolism	Homo sapiens	+
cell part;cytoplasmic part;cytosol;extracellular membrane-bounded organelle;extracellular organelle;extracellular region part;extracellular vesicular exosome;intracellular membrane-bounded organelle;intracellular organelle;int	Glycerolipid metabolism;Methane metabolism;RIG-I-like receptor signaling pathway	Homo sapiens	+
		Homo sapiens	+
cell part;cytoplasmic part;extracellular membrane-bounded organelle;extracellular organelle;extracellular region part;extracellular vesicular exosome;intracellular membrane-bounded organelle;intracellular organelle;intracellula	One carbon pool by folate	Homo sapiens	+
cell part;cell projection;cytoplasmic membrane-bounded vesicle;cytoplasmic part;cytoplasmic vesicle;cytosol;endoplasmic reticulum;Golgi apparatus;intracellular membrane-bounded organelle;intracellular organelle;intracellular part;lamellipodium;membrane;membrane-bounded organelle;membrane-bounded vesicle;organelle;plasma membrane;vesicle		Homo sapiens	+
cell part;cytoplasmic part;integral to membrane;intracellular membrane-bounded organelle;intracellular organelle;intracellular organelle part;intracellular part;intrinsic to membrane;macromolecular complex;membrane;membrane part;membrane-bounded organelle;mitochondrial inner membrane;mitochondrial inner membrane presequence translocase complex;mitochon		Homo sapiens	
cell part;cytoplasm;cytoplasmic part;Golgi apparatus;intracellular membrane-bounded organelle;intracellular organelle;intracellular organelle part;intracellular part;membrane-bounded organelle;nuclear part;nucleoplasm;organelle;organelle part		Homo sapiens	+
cell part;cytoplasm;intracellular part;membrane;plasma membrane		Homo sapiens	+
cell part;cell surface;chloride channel complex;extracellular membrane-bounded organelle;extracellular organelle;extracellular region part;extracellular vesicular exosome;intracellular;ion channel complex;macromolecular complex;membrane;membrane part;membrane-bounded organelle;membrane-bounded vesicle;organelle;plasma membrane;protein complex;vesicle		Homo sapiens	+
cell part;cytoplasm;intracellular membrane-bounded organelle;intracellular organelle;intracellular part;membrane;membrane-bounded organelle;nucleus;organelle		Homo sapiens	
blood microparticle;cell part;cytoplasmic part;early endosome;endosome;extracellular region part;intracellular membrane-bounded organelle;intracellular organelle;intracellular part;membrane-bounded organelle;organelle		Homo sapiens	+
cell part;cytoplasmic part;endoplasmic reticulum;integral to membrane;intracellular membrane-bounded organelle;intracellular organelle;intracellular part;intrinsic to membrane;membrane part;membrane-bounded organelle;organelle		Homo sapiens	+
cell part;cytoplasmic part;intracellular membrane-bounded organelle;intracellular organelle;intracellular part;membrane-bounded organelle;mitochondrion;organelle		Homo sapiens	+
cell part;cytoplasm;intracellular membrane-bounded organelle;intracellular organelle;intracellular part;macromolecular complex;membrane-bounded organelle;nucleus;organelle;protein complex;tRNA-splicing ligase complex		Homo sapiens	+
cell part;cytoplasmic part;cytosol;intracellular part		Homo sapiens	
cell part;cytoplasm;cytoplasmic part;cytosol;intracellular membrane-bounded organelle;intracellular organelle;intracellular organelle part;intracellular part;membrane-bounded organelle;nuclear part;nucleoplasm;nucleus;organelle;organelle part		Homo sapiens	+
cell part;integral to membrane;intrinsic to membrane;membrane part		Homo sapiens	
cell division site part;cell part;centriole;centrosome;cleavage furrow;cytoplasmic part;cytoskeletal part;extracellular region part;intercellular bridge;intracellular non-membrane-bounded organelle;intracellular organelle;intracellular organelle part;intracellular part;membrane;microtubule organizing center;microtubule organizing center part;midbody;non-membrane-bounded		Homo sapiens	+
		Homo sapiens	+
cell part;cytoplasmic part;endoplasmic reticulum membrane;endoplasmic reticulum part;extracellular matrix;extracellular region part;integral to membrane;intracellular organelle part;intracellular part;intrinsic to membrane;me	Biosynthesis of unsaturated fatty acids;Steroid hormone biosynthesis	Homo sapiens	
cell part;intracellular organelle part;intracellular part;macromolecular complex;nuclear part;organelle part;ribonucleoprotein complex;spliceosomal complex	Spliceosome	Homo sapiens	
cell part;cytoplasmic part;intracellular membrane-bounded organelle;intracellular organelle;intracellular part;membrane-bounded organelle;mitochondrion;organelle		Homo sapiens	
	Arginine and proline metabolism	Homo sapiens	+
brush border membrane;cell part;cell projection membrane;cell projection part;extracellular membrane-bounded organelle;extracellular organelle;extracellular region part;extracellular vesicular exosome;integral to membrane;	Mineral absorption	Homo sapiens	+
cell part;cytoplasmic part;early endosome;endosome;intracellular membrane-bounded organelle;intracellular organelle;intracellular part;membrane-bounded organelle;organelle		Homo sapiens	+
cell part;cytoplasm;intracellular part		Homo sapiens	+
cell part;integral to membrane;intrinsic to membrane;membrane part		Homo sapiens	+
cell part;intracellular membrane-bounded organelle;intracellular organelle;intracellular organelle part;intracellular part;membrane-bounded organelle;nuclear matrix;nuclear part;nucleoplasm;organelle;organelle part		Homo sapiens	+
cell part;intracellular membrane-bounded organelle;intracellular non-membrane-bounded organelle;intracellular organelle;intracellular organelle part;intracellular part;membrane-bounded organelle;non-membrane-bounded organelle;nuclear part;nucleolus;nucleus;organelle;organelle part		Homo sapiens	+
cell part;cytoplasm;cytoplasmic part;cytosol;intracellular membrane-bounded organelle;intracellular organelle;intracellular part;membrane-bounded organelle;nucleus;organelle	Ubiquitin mediated proteolysis	Homo sapiens	+
cell part;intracellular membrane-bounded organelle;intracellular organelle;intracellular part;membrane-bounded organelle;nucleus;organelle		Homo sapiens	
cell part;cytoplasm;intracellular organelle part;intracellular part;membrane;nuclear part;nucleoplasm;organelle part;plasma membrane		Homo sapiens	+
cell part;endomembrane system		Homo sapiens	
cell part;cytoplasmic part;intracellular membrane-bounded organelle;intracellular organelle;intracellular organelle lumen;intracellular organelle part;intracellular part;membrane-bounded organelle;membrane-enclosed lumen;m	Aminoacyl-tRNA biosynthesis;Porphyrin and chlorophyll metabolism	Homo sapiens	+

cell part;cytoplasmic part;endoplasmic reticulum membrane;endoplasmic reticulum part;integral to membrane;intracellular membrane-bounded organelle;intracellular organelle;intracellular organelle part;intracellular part;intrinsic to membrane;membrane;membrane part;membrane-bounded organelle;organelle;organelle membrane;organelle part		Homo sapiens	+
cell part;cytoplasmic part;intracellular membrane-bounded organelle;intracellular organelle;intracellular organelle lumen;intracellular organelle part;intracellular part;membrane-bounded organelle;membrane-enclosed lumen;mitochondrial matrix;mitochondrial part;mitochondrion;organelle;organelle lumen;organelle part		Homo sapiens	+
		Homo sapiens	+
cell part;integral to membrane;intracellular non-membrane-bounded organelle;intracellular organelle;intracellular organelle part;intracellular part;intrinsic to membrane;membrane;membrane part;non-membrane-bounded organelle;nuclear membrane;nuclear part;nucleolus;organelle;organelle membrane;organelle part		Homo sapiens	
cell part;integral to membrane;intracellular membrane-bounded organelle;intracellular organelle;intracellular organelle part;intracellular part;intrinsic to membrane;membrane;membrane part;membrane-bounded organelle;nuclear inner membrane;nuclear membrane;nuclear part;nucleus;organelle;organelle inner membrane;organelle membrane;organelle part		Homo sapiens	+
cell part;cytoplasmic part;intracellular membrane-bounded organelle;intracellular organelle;intracellular part;membrane-bounded organelle;mitochondrion;organelle	Aminoacyl-tRNA biosynthesis	Homo sapiens	
		Homo sapiens	+
cell part;collagen;cytoplasmic part;cytoplasmic vesicle membrane;cytoplasmic vesicle part;endocytic vesicle membrane;extracellular matrix part;extracellular membrane-bounded organelle;extracellular organelle;extracellular	Phagosome	Homo sapiens	+
cell part;intracellular membrane-bounded organelle;intracellular organelle;intracellular part;membrane-bounded organelle;nucleus;organelle		Homo sapiens	+
cell part;cytoplasmic part;cytosol;extrinsic to membrane;intracellular part;membrane;membrane part;pre-autophagosomal structure membrane		Homo sapiens	+
cell part;cytoplasmic exosome (RNase complex);cytoplasmic part;cytosol;exosome (RNase complex);intracellular non-membrane-bounded organelle;intracellular organelle;intracellular organelle part;intracellular part;macromo	RNA degradation	Homo sapiens	+
cell part;envelope;intracellular organelle part;intracellular part;macromolecular complex;membrane;membrane part;nuclear envelope;nuclear part;nuclear pore;organelle envelope;organelle part;pore complex;protein complex	RNA transport	Homo sapiens	+
cell part;chromosomal part;chromosome;intracellular membrane-bounded organelle;intracellular non-membrane-bounded organelle;intracellular organelle;intracellular organelle part;intracellular part;macromolecular complex;membrane-bounded organelle;non-membrane-bounded organelle;nucleosome;nucleus;organelle;organelle part;protein-DNA complex		Homo sapiens	+
cell part;centriole;centrosome;cytoplasm;cytoplasmic part;cytoskeletal part;intracellular non-membrane-bounded organelle;intracellular organelle;intracellular organelle part;intracellular part;macromolecular complex;membrane;microtubule;microtubule organizing center;microtubule organizing center part;non-membrane-bounded organelle;nuclear membrane;nuclear par		Homo sapiens	+
cell part;intracellular non-membrane-bounded organelle;intracellular organelle;intracellular organelle part;intracellular part;membrane;non-membrane-bounded organelle;nuclear part;nucleolus;organelle;organelle part		Homo sapiens	+
cell cortex;cell part;cytoplasm;cytoplasmic membrane-bounded vesicle;cytoplasmic part;cytoplasmic vesicle;cytoskeleton;extracellular membrane-bounded organelle;extracellular organelle;extracellular region part;extracellular vesicular exosome;intracellular membrane-bounded organelle;intracellular non-membrane-bounded organelle;intracellular organelle;intracellular		Homo sapiens	+
actin filament;axon;cell part;cell projection;cell projection part;cytoplasmic part;cytoskeletal part;early endosome;endosome;growth cone;intracellular membrane-bounded organelle;intracellular organelle;intracellular organelle part;intracellular part;macromolecular complex;membrane;membrane-bounded organelle;neuron projection;organelle;organelle part;protein compl		Homo sapiens	+
		Homo sapiens	
cell part;centrosome;cytoplasm;cytoplasmic part;cytoskeletal part;integral to membrane;intracellular non-membrane-bounded organelle;intracellular organelle;intracellular organelle part;intracellular part;intrinsic to membrane;membrane;membrane part;microtubule organizing center;non-membrane-bounded organelle;nuclear part;nucleoplasm;organelle;organelle part		Homo sapiens	
cell part;cytoplasm;intracellular part		Homo sapiens	+
		Homo sapiens	
		Homo sapiens	
cell part;cytoplasm;extracellular membrane-bounded organelle;extracellular organelle;extracellular region part;extracellular vesicular exosome;intracellular part;membrane-bounded organelle;membrane-bounded vesicle;organelle;vesicle		Homo sapiens	+
		Homo sapiens	+
cell part;cytoplasmic part;intracellular organelle part;intracellular part;membrane;mitochondrial inner membrane;mitochondrial membrane;mitochondrial part;organelle inner membrane;organelle membrane;organelle part		Homo sapiens	+
cell part;cell projection cytoplasm;cell projection membrane;cell projection part;cytoplasmic part;cytoplasmic vesicle membrane;cytoplasmic vesicle part;dendrite cytoplasm;dendrite membrane;early endosome membrane;endosomal part;endosome membrane;Golgi apparatus part;Golgi membrane;integral to membrane;intracellular organelle part;intracellular part;intrins		Homo sapiens	+
cell part;cytoplasmic part;intracellular membrane-bounded organelle;intracellular organelle;intracellular part;membrane-bounded organelle;mitochondrion;organelle		Homo sapiens	+
cell part;chromosomal part;intracellular membrane-bounded organelle;intracellular organelle;intracellular organelle part;intracellular part;macromolecular complex;membrane-bounded organelle;nucleosome;nucleus;organelle;organelle part;protein-DNA complex		Homo sapiens	+
		Homo sapiens	+
cell part;chromosomal part;chromosome, telomeric region;cytoplasm;cytoskeletal part;female pronucleus;intracellular membrane-bounded organelle;intracellular non-membrane-bounded organelle;intracellular organelle;intracellular organelle part;intracellular part;male pronucleus;membrane;membrane-bounded organelle;non-membrane-bounded organelle;nucleus;orga		Homo sapiens	
cell part;cytoplasm;extracellular membrane-bounded organelle;extracellular organelle;extracellular region part;extracellular vesicular exosome;intracellular membrane-bounded organelle;intracellular organelle;intracellular part;membrane-bounded organelle;membrane-bounded vesicle;nucleus;organelle;vesicle		Homo sapiens	+
cell part;chromosomal part;HULC complex;intracellular membrane-bounded organelle;intracellular non-membrane-bounded organelle;intracellular organelle;intracellular organelle part;intracellular part;macromolecular complex;membrane-bounded organelle;non-membrane-bounded organelle;nuclear part;nucleolus;nucleoplasm;nucleus;organelle;organelle part;protein co		Homo sapiens	+
cell part;extracellular membrane-bounded organelle;extracellular organelle;extracellular region part;extracellular vesicular exosome;membrane;membrane-bounded organelle;membrane-bounded vesicle;organelle;vesicle		Homo sapiens	+
adherens junction;anchoring junction;cell junction;cell part;cell-substrate adherens junction;cell-substrate junction;focal adhesion;integral to membrane;intrinsic to membrane;membrane part		Homo sapiens	+
cell part;centrosome;coated vesicle;cytoplasmic membrane-bounded vesicle;cytoplasmic part;cytoplasmic vesicle;cytoskeletal part;early endosome;endocytic vesicle;endoplasmic reticulum;endoplasmic reticulum-Golgi intermediate compartment;endosome;ER to Golgi transport vesicle;intracellular membrane-bounded organelle;intracellular non-membrane-bounded o		Homo sapiens	
cell part;cytoplasm;cytoplasmic part;intracellular part;macromolecular complex;NatC complex;N-terminal protein acetyltransferase complex;protein acetyltransferase complex;protein complex		Homo sapiens	+
		Homo sapiens	+
cell part;cytoplasm;intracellular part		Homo sapiens	+
cell part;external side of plasma membrane;extracellular membrane-bounded organelle;extracellular organelle;extracellular region part;extracellular vesicular exosome;integral to membrane;intrinsic to membrane;membrane p	Cell adhesion molecules (CAMs)	Homo sapiens	+
cell part;cytoplasm;intracellular non-membrane-bounded organelle;intracellular organelle;intracellular organelle part;intracellular part;membrane;non-membrane-bounded organelle;nuclear part;nucleolus;organelle;organelle part		Homo sapiens	+
adherens junction;anchoring junction;cell junction;cell part;cell-substrate adherens junction;cell-substrate junction;focal adhesion;membrane;plasma membrane		Homo sapiens	+
cell part;cytoplasm;cytoplasmic part;intracellular membrane-bounded organelle;intracellular organelle;intracellular part;membrane-bounded organelle;mitochondrion;organelle		Homo sapiens	+
cell part;cytoplasmic part;early endosome;early endosome membrane;endosomal part;endosome;endosome membrane;intracellular membrane-bounded organelle;intracellular organelle;intracellular organelle part;intracellular part;macromolecular complex;membrane;membrane-bounded organelle;organelle;organelle membrane;organelle part;plasma membrane;protein c		Homo sapiens	
		Homo sapiens	+
cell part;integral to membrane;intrinsic to membrane;membrane part		Homo sapiens	+
cell body;cell junction;cell part;cell projection;cytoplasm;cytoplasmic part;cytoskeletal part;cytosol;dendrite;intracellular membrane-bounded organelle;intracellular non-membrane-bounded organelle;intracellular organelle;intracellular organelle part;intracellular part;macromolecular complex;membrane-bounded organelle;microtubule;neuron projection;neuronal cell body;		Homo sapiens	+
cell part;intracellular membrane-bounded organelle;intracellular non-membrane-bounded organelle;intracellular organelle;intracellular organelle part;intracellular part;membrane-bounded organelle;non-membrane-bounded organelle;nuclear body;nuclear part;nuclear speck;nucleolus;nucleoplasm part;nucleus;organelle;organelle part		Homo sapiens	+
cell part;intracellular non-membrane-bounded organelle;intracellular organelle;intracellular organelle part;intracellular part;macromolecular complex;non-membrane-bounded organelle;nuclear part;nucleolus;organelle;organelle part;preribosome;ribonucleoprotein complex;small-subunit processome		Homo sapiens	+
adherens junction;anchoring junction;cell junction;cell part;cell-substrate adherens junction;cell-substrate junction;cytoplasm;focal adhesion;intracellular part		Homo sapiens	+
cell part;integrator complex;intracellular membrane-bounded organelle;intracellular organelle;intracellular organelle part;intracellular part;macromolecular complex;membrane-bounded organelle;nuclear part;nucleoplasm part;nucleus;organelle;organelle part;protein complex;SOSS complex		Homo sapiens	
cell junction;cell part;cell-cell junction;cytoplasmic part;cytosol;intracellular part;membrane;occluding junction;plasma membrane;tight junction		Homo sapiens	
		Homo sapiens	
cell part;intracellular organelle part;intracellular part;nuclear body;nuclear part;nuclear speck;nucleoplasm part;organelle part		Homo sapiens	+
cell part;cytoplasmic part;intracellular membrane-bounded organelle;intracellular organelle;intracellular part;membrane;membrane-bounded organelle;mitochondrion;nucleus;organelle	Amino sugar and nucleotide sugar metabolism	Homo sapiens	+
cell part;intracellular membrane-bounded organelle;intracellular organelle;intracellular part;membrane-bounded organelle;nucleus;organelle		Homo sapiens	+
cell part;cytoplasmic part;endoplasmic reticulum;endoplasmic reticulum membrane;endoplasmic reticulum part;integral to membrane;intracellular membrane-bounded organelle;intracellular organelle;intracellular organelle part;intracellular part;intrinsic to membrane;membrane;membrane part;membrane-bounded organelle;organelle;organelle membrane;organelle part		Homo sapiens	
cell part;cytoplasmic part;intracellular non-membrane-bounded organelle;intracellular organelle;intracellular part;macromolecular complex;non-membrane-bounded organelle;organelle;ribonucleoprotein complex;ribosome		Homo sapiens	+
cell part;cytoplasmic part;cytosol;intracellular membrane-bounded organelle;intracellular organelle;intracellular part;membrane-bounded organelle;nucleus;organelle		Homo sapiens	
cell part;cell surface;cytoplasmic part;extracellular membrane-bounded organelle;extracellular organelle;extracellular region part;extracellular space;extracellular vesicular exosome;integral to membrane;intracellular membrane-bounded organelle;intracellular organelle;intracellular organelle part;intracellular part;intrinsic to membrane;lysosomal membrane;membrane;mem		Homo sapiens	+
cell part;cytoplasm;cytoplasmic part;intracellular non-membrane-bounded organelle;intracellular organelle;intracellular organelle part;intracellular part;membrane-enclosed lumen;mitochondrial intermembrane space;mitochondrial part;non-membrane-bounded organelle;nuclear part;nucleolus;nucleoplasm;organelle;organelle envelope lumen;organelle part		Homo sapiens	
cell part;cytoplasm;intracellular part		Homo sapiens	+
cell part;cytoplasm;intracellular membrane-bounded organelle;intracellular organelle;intracellular organelle part;intracellular part;macromolecular complex;membrane-bounded organelle;nuclear body;nuclear part;nuclear spec	RNA transport	Homo sapiens	+
cell part;cytoplasm;Elongator holoenzyme complex;histone acetyltransferase complex;intracellular organelle part;intracellular part;macromolecular complex;nuclear part;nucleoplasm part;organelle part;protein complex;transcription elongation factor complex		Homo sapiens	
cell part;cytoplasmic part;endoplasmic reticulum membrane;endoplasmic reticulum part;integral to membrane;intracellular organelle part;intracellular part;intrinsic to membrane;membrane;membrane part;organelle membrane;organelle part		Homo sapiens	+
cell part;cell projection;cell projection part;contractile fiber;cytoplasm;cytoplasmic part;cytoskeleton;extracellular membrane-bounded organelle;extracellular organelle;extracellular region part;extracellular vesicular exosome;filopodium;growth cone;intracellular non-membrane-bounded organelle;intracellular organelle;intracellular part;lamellipodium;membrane-bounded c		Homo sapiens	+
cell part;cytoplasmic part;intracellular organelle lumen;intracellular organelle part;intracellular part;membrane-enclosed lumen;mitochondrial matrix;mitochondrial part;organelle lumen;organelle part		Homo sapiens	
cell part;cytoplasm;cytoplasmic part;integral to membrane;intracellular membrane-bounded organelle;intracellular organelle;intracellular organelle part;intracellular part;intrinsic to membrane;membrane;membrane part;membrane-bounded organelle;mitochondrial inner membrane;mitochondrial membrane;mitochondrial part;mitochondrion;organelle;organelle inner memb		Homo sapiens	+
cell part;cytoplasmic part;cytoskeletal part;endoplasmic reticulum membrane;endoplasmic reticulum part;integral to membrane;intracellular organelle part;intracellular part;intrinsic to membrane;macromolecular complex;membrane;membrane part;microtubule;organelle membrane;organelle part;protein complex		Homo sapiens	+
cell part;cytoplasmic part;endoplasmic reticulum membrane;endoplasmic reticulum part;intracellular organelle part;intracellular part;membrane;membrane part;nuclear membrane;nuclear outer membrane;nuclear part;organe	Retinol metabolism	Homo sapiens	+
cell part;cytoplasm;cytoplasmic microtubule;cytoplasmic part;cytoskeletal part;endoplasmic reticulum;endoplasmic reticulum membrane;endoplasmic reticulum part;Golgi apparatus;integral to membrane;intracellular membrane-bounded organelle;intracellular organelle;intracellular organelle part;intracellular part;intrinsic to membrane;macromolecular complex;membran		Homo sapiens	
cell part;cytoplasmic part;extracellular membrane-bounded organelle;extracellular organelle;extracellular region part;extracellular vesicular exosome;intracellular organelle lumen;intracellular organelle part;intracellular part;me	beta-Alanine metabolism;Propanoate metabolism;Valine, leucine and isoleucine degradation	Homo sapiens	
		Homo sapiens	+
cell part;cytoplasm;cytoskeleton;intracellular non-membrane-bounded organelle;intracellular organelle;intracellular part;non-membrane-bounded organelle;organelle		Homo sapiens	
caveola;cell part;cytoplasm;cytoplasmic part;cytosol;endoplasmic reticulum;intracellular membrane-bounded organelle;intracellular organelle;intracellular organelle part;intracellular part;macromolecular complex;membrane;membrane part;membrane raft;membrane-bounded organelle;mitochondrion;nuclear part;nucleoplasm;nucleus;organelle;organelle part;plasma mem		Homo sapiens	+
catalytic step 2 spliceosome;cell part;intracellular membrane-bounded organelle;intracellular organelle;intracellular organelle part;intracellular part;macromolecular complex;membrane-bounded organelle;nuclear part;nucleoplasm;nucleus;organelle;organelle part;ribonucleoprotein complex;spliceosomal complex		Homo sapiens	
Cdc73/Paf1 complex;cell part;intracellular membrane-bounded organelle;intracellular organelle;intracellular organelle part;intracellular part;macromolecular complex;membrane-bounded organelle;nuclear part;nucleoplasm;nucleoplasm part;nucleus;organelle;organelle part;protein complex;transcription elongation factor complex		Homo sapiens	+
cell part;cytoplasm;cytoplasmic part;cytoskeletal part;extracellular membrane-bounded organelle;extracellular organelle;extracellular region part;extracellular vesicular exosome;intracellular membrane-bounded organelle;intracellular organelle;intracellular organelle part;intracellular part;membrane;membrane-bounded organelle;membrane-bounded vesicle;microtubule or		Homo sapiens	+
cell part;cytoplasm;cytoplasmic mRNA processing body;cytoplasmic part;cytosol;intracellular membrane-bounded organelle;intracellular non-membrane-bounded organelle;intracellular organelle;intracellular organelle part;in	RNA degradation	Homo sapiens	+
catalytic step 2 spliceosome;cell part;intracellular membrane-bounded organelle;intracellular organelle;intracellular organelle part;intracellular part;macromolecular complex;membrane;membrane-bounded organelle;nuclear b	Spliceosome	Homo sapiens	+
		Homo sapiens	+
cell part;cytoplasmic part;intracellular non-membrane-bounded organelle;intracellular organelle;intracellular part;macromolecular complex;non-membrane-bounded organelle;organelle;ribonucleoprotein complex;ribosome	Ribosome	Homo sapiens	
cell part;intracellular membrane-bounded organelle;intracellular organelle;intracellular organelle part;intracellular part;membrane-bounded organelle;nuclear body;nuclear part;nuclear speck;nucleoplasm part;nucleus;organelle;organelle part		Homo sapiens	+
cell part;cytoplasmic part;Golgi apparatus;intracellular membrane-bounded organelle;intracellular organelle;intracellular part;membrane-bounded organelle;organelle		Homo sapiens	+
cell part;cytoplasmic part;intracellular organelle part;intracellular part;lysosomal membrane;membrane;organelle membrane;organelle part;vacuolar membrane;vacuolar part		Homo sapiens	+
Cdc73/Paf1 complex;cell part;chromatin;chromosomal part;intracellular organelle part;intracellular part;macromolecular complex;nuclear body;nuclear part;nuclear speck;nucleoplasm part;organelle part;protein complex;transcription elongation factor complex;transcriptionally active chromatin		Homo sapiens	
cell part;cytoplasm;cytoplasmic part;cytosol;intracellular organelle part;intracellular part;membrane;nuclear part;nucleoplasm;organelle part;plasma membrane		Homo sapiens	+
cell part;chromatin;chromosomal part;cytoplasm;cytoplasmic part;intracellular membrane-bounded organelle;intracellular organelle;intracellular organelle part;intracellular part;macromolecular complex;membrane-bounded o	RNA degradation	Homo sapiens	+
cell part;cytoplasmic part;intracellular membrane-bounded organelle;intracellular organelle;intracellular organelle lumen;intracellular organelle part;intracellular part;membrane-bounded organelle;membrane-enclosed lumen;m	Aminoacyl-tRNA biosynthesis	Homo sapiens	+
cell part;cytoplasmic part;endoplasmic reticulum;integral to membrane;intracellular membrane-bounded organelle;intracellular organelle;intracellular part;intrinsic to membrane;membrane;membrane part;membrane-bounded	Bile secretion	Homo sapiens	
cell part;cytoplasm;intracellular membrane-bounded organelle;intracellular non-membrane-bounded organelle;intracellular organelle;intracellular organelle part;intracellular part;membrane-bounded organelle;non-membrane-bounded organelle;nuclear body;nuclear part;nuclear speck;nucleolus;nucleoplasm;nucleoplasm part;nucleus;organelle;organelle part		Homo sapiens	
cell part;intracellular non-membrane-bounded organelle;intracellular organelle;intracellular organelle part;intracellular part;non-membrane-bounded organelle;nuclear part;nucleolus;organelle;organelle part		Homo sapiens	+
cell part;cytoplasm;intracellular organelle part;intracellular part;membrane;nuclear part;nucleoplasm;organelle part		Homo sapiens	+
cell part;cytoplasmic part;cytosol;intracellular part;macromolecular complex;protein complex;TORC2 complex	mTOR signaling pathway	Homo sapiens	+
cell part;cytoplasmic part;intracellular membrane-bounded organelle;intracellular organelle;intracellular part;membrane;membrane-bounded organelle;mitochondrion;organelle	One carbon pool by folate	Homo sapiens	
cell part;intracellular organelle part;intracellular part;macromolecular complex;mRNA cleavage and polyadenylation specificity factor complex;mRNA cleavage factor complex;nuclear part;nucleoplasm;nucleoplasm part;orgar	mRNA surveillance pathway	Homo sapiens	
adherens junction;anchoring junction;apical plasma membrane;cell junction;cell part;cell projection membrane;cell projection part;cell surface;cell-substrate adherens junction;cell-substrate junction;cytoplasmic part;extracellular membrane-bounded organelle;extracellular organelle;extracellular region;extracellular region part;extracellular vesicular exosome;focal adhesic		Homo sapiens	+
cell part;cytoplasmic part;endoplasmic reticulum membrane;endoplasmic reticulum part;integral to membrane;intracellular organelle part;intracellular part;intrinsic to membrane;membrane;membrane part;organelle membrane;organelle part		Homo sapiens	+
cell part;extracellular membrane-bounded organelle;extracellular organelle;extracellular region part;extracellular vesicular exosome;integral to membrane;intrinsic to membrane;membrane part;membrane-bounded organelle;membrane-bounded vesicle;organelle;vesicle		Homo sapiens	
catalytic step 2 spliceosome;cell part;intracellular organelle part;intracellular part;macromolecular complex;nuclear part;organelle part;ribonucleoprotein complex;spliceosomal complex		Homo sapiens	+
cell part;cytoplasmic part;endoplasmic reticulum;extracellular region part;extracellular space;Golgi apparatus;intracellular membrane-bounded organelle;intracellular organelle;intracellular part;membrane-bounded organelle;organelle		Homo sapiens	+
collagen;extracellular matrix;extracellular matrix part;extracellular region part;extracellular space;proteinaceous extracellular matrix		Homo sapiens	+
cell part;chromatin;chromatin remodeling complex;chromosomal part;histone methyltransferase complex;intracellular organelle part;intracellular part;macromolecular complex;methyltransferase complex;nuclear part;nucleoplasm part;organelle part;protein complex;protein serine/threonine phosphatase complex;PTW/PP1 phosphatase complex;Set1C/COMPASS comple		Homo sapiens	+
cell part;cytoplasmic part;integral to membrane;intracellular organelle part;intracellular part;intrinsic to membrane;membrane;membrane part;mitochondrial inner membrane;mitochondrial membrane;mitochondrial part;organelle inner membrane;organelle membrane;organelle part		Homo sapiens	+
cell part;cytoplasmic part;endoplasmic reticulum lumen;endoplasmic reticulum part;extracellular matrix;extracellular region part;interstitial matrix;intracellular organelle lumen;intracellular organelle part;intracellular part;membrane-enclosed lumen;organelle lumen;organelle part;proteinaceous extracellular matrix		Homo sapiens	+
actin cytoskeleton;adherens junction;anchoring junction;cell junction;cell part;cell-substrate adherens junction;cell-substrate junction;cytoplasm;cytoskeleton;focal adhesion;intracellular non-membrane-bounded organelle;intracellular organelle;intracellular part;non-membrane-bounded organelle;organelle		Homo sapiens	
cell part;cytoplasm;cytoplasmic part;cytosol;extracellular membrane-bounded organelle;extracellular organelle;extracellular region part;extracellular vesicular exosome;Golgi apparatus;intracellular membrane-bounded organe	Nicotinate and nicotinamide metabolism	Homo sapiens	
cell junction;cell part;cytoplasmic part;cytoskeleton;Golgi apparatus part;Golgi stack;intracellular non-membrane-bounded organelle;intracellular organelle;intracellular organelle part;intracellular part;non-membrane-bounded organelle;organelle;organelle part;synapse		Homo sapiens	
cell part;cytoplasm;intracellular part;membrane		Homo sapiens	
anchored to membrane;cell part;cell surface;extracellular region part;extracellular space;intrinsic to membrane;membrane;membrane part;plasma membrane		Homo sapiens	+
cell part;cytoplasmic part;endoplasmic reticulum;integral to membrane;intracellular membrane-bounded organelle;intracellular organelle;intracellular part;intrinsic to membrane;membrane part;membrane-bounded organelle;organelle		Homo sapiens	+
cell part;cytoplasmic part;intracellular membrane-bounded organelle;intracellular organelle;intracellular part;membrane;membrane-bounded organelle;microbody;mitochondrion;organelle;peroxisome		Homo sapiens	+
cell part;cytoplasmic part;intracellular membrane-bounded organelle;intracellular organelle;intracellular part;membrane-bounded organelle;mitochondrion;organelle	Selenocompound metabolism;Tryptophan metabolism	Homo sapiens	+
cell part;integral to membrane;intracellular membrane-bounded organelle;intracellular organelle;intracellular part;intrinsic to membrane;membrane;membrane part;membrane-bounded organelle;nucleus;organelle		Homo sapiens	+
cell part;cytoplasmic part;extracellular region part;extracellular space;Golgi apparatus part;integral to Golgi membrane;integral to membrane;integral to organelle membrane;intracellular organelle part;intracellular part;intrinsic to Golgi membrane;intrinsic to membrane;intrinsic to organelle membrane;membrane;membrane part;nuclear membrane;nuclear part;organelle me		Homo sapiens	+
cell part;cytoplasm;extracellular membrane-bounded organelle;extracellular organelle;extracellular region part;extracellular vesicular exosome;intracellular part;membrane-bounded organelle;membrane-bounded vesicle;organelle;vesicle		Homo sapiens	+
		Homo sapiens	
cell part;integral to membrane;intrinsic to membrane;membrane part		Homo sapiens	+
cell part;cell projection;cytoplasm;cytoskeleton;filopodium;intracellular non-membrane-bounded organelle;intracellular organelle;intracellular part;lamellipodium;membrane;non-membrane-bounded organelle;organelle;plasma membrane		Homo sapiens	+
cell part;cytoplasmic part;endoplasmic reticulum;endoplasmic reticulum membrane;endoplasmic reticulum part;integral to membrane;intracellular membrane-bounded organelle;intracellular organelle;intracellular organelle part;intracellular part;intrinsic to membrane;membrane;membrane part;membrane-bounded organelle;organelle;organelle membrane;organelle part		Homo sapiens	+
cell part;chromosomal part;extracellular membrane-bounded organelle;extracellular organelle;extracellular region;extracellular region part;extracellular vesicular exosome;intracellular membrane-bounded organelle;intracellula	Systemic lupus erythematosus	Homo sapiens	+
cell part;membrane		Homo sapiens	+
cell part;cell surface;cytoplasmic part;endoplasmic reticulum lumen;endoplasmic reticulum part;extracellular matrix;extracellular region part;extracellular space;intracellular organelle lumen;intracellular organelle part;intracellular part;membrane-enclosed lumen;organelle lumen;organelle part;proteinaceous extracellular matrix		Homo sapiens	+
cell part;extracellular membrane-bounded organelle;extracellular organelle;extracellular region part;extracellular vesicular exosome;integral to membrane;integral to plasma membrane;intrinsic to membrane;intrinsic to plasma membrane;membrane part;membrane-bounded organelle;membrane-bounded vesicle;organelle;plasma membrane part;vesicle		Homo sapiens	+
cell part;intracellular membrane-bounded organelle;intracellular organelle;intracellular part;membrane-bounded organelle;nucleus;organelle		Homo sapiens	
cell part;cytoplasm;cytoplasmic membrane-bounded vesicle;cytoplasmic part;cytoplasmic vesicle;extracellular membrane-bounded organelle;extracellular organelle;extracellular region part;extracellular vesicular exosome;intracellular membrane-bounded organelle;intracellular organelle;intracellular part;macromolecular complex;melanosome;membrane;membrane-bour		Homo sapiens	+
actin filament;basal cortex;cell cortex part;cell part;cytoplasmic part;cytoskeletal part;intracellular membrane-bounded organelle;intracellular organelle;intracellular organelle part;intracellular part;lateral plasma membrane;macromolecular complex;membrane;membrane part;membrane-bounded organelle;mitochondrion;nucleus;organelle;organelle part;plasma membrane		Homo sapiens	
Cajal body;cell part;cytoplasm;intracellular membrane-bounded organelle;intracellular non-membrane-bounded organelle;intracellular organelle;intracellular organelle part;intracellular part;membrane-bounded organelle;non-r	Spliceosome	Homo sapiens	+
cell part;cytoplasmic part;intracellular membrane-bounded organelle;intracellular non-membrane-bounded organelle;intracellular organelle;intracellular organelle part;intracellular part;membrane-bounded organelle;mitochondrial nucleoid;mitochondrial part;mitochondrion;non-membrane-bounded organelle;nuclear part;nucleoid;nucleoplasm;organelle;organelle part		Homo sapiens	+
cell part;cytoplasm;intracellular part;membrane		Homo sapiens	+

cell part;cytoplasm;cytoplasmic part;intracellular membrane-bounded organelle;intracellular non-membrane-bounded organelle;intracellular organelle;intracellular organelle part;intracellular part;membrane-bounded organelle;mitochondrial nucleoid;mitochondrial part;mitochondrion;non-membrane-bounded organelle;nucleoid;nucleus;organelle;organelle part		Homo sapiens	+
cell part;cytoplasmic part;eukaryotic 43S preinitiation complex;eukaryotic 48S preinitiation complex;eukaryotic translation initiation factor 3 complex;eukaryotic translation initiation factor 3 complex, eIF3m;intracellular part;macromolecular complex;protein complex;ribonucleoprotein complex;translation preinitiation complex		Homo sapiens	+
		Homo sapiens	+
cell part;cytoplasm;cytoplasmic part;EGO complex;Golgi apparatus;intracellular membrane-bounded organelle;intracellular organelle;intracellular organelle part;intracellular part;lysosomal membrane;lysosome;lytic vacuole;macromolecular complex;membrane;membrane part;membrane-bounded organelle;nucleus;organelle;organelle membrane;organelle part;protein co		Homo sapiens	+
adherens junction;anchoring junction;cell junction;cell part;cell projection;cell-substrate adherens junction;cell-substrate junction;cytoplasmic part;cytosol;extracellular membrane-bounded organelle;extracellular organelle;ext	Regulation of actin cytoskeleton	Homo sapiens	+
cell part;cytoplasm;extracellular membrane-bounded organelle;extracellular organelle;extracellular region part;extracellular vesicular exosome;intracellular organelle part;intracellular part;macromolecular complex;membrane-bounded organelle;membrane-bounded vesicle;nuclear part;nucleoplasm;organelle;organelle part;protein complex;signalosome;vesicle		Homo sapiens	+
cell part;cytoplasmic part;endoplasmic reticulum;endoplasmic reticulum membrane;endoplasmic reticulum part;Golgi apparatus part;Golgi membrane;Golgi stack;integral to membrane;intracellular membrane-bounded organ	Ether lipid metabolism;Glycerophospholipid metabolism	Homo sapiens	+
actomyosin contractile ring;cell cortex part;cell division site part;cell part;contractile ring;cytoplasm;cytoplasmic part;cytoskeletal part;cytoskeleton;integral to membrane;integral to plasma membrane;intracellular membrane-bounded organelle;intracellular non-membrane-bounded organelle;intracellular organelle;intracellular organelle part;intracellular part;intrinsic to mer		Homo sapiens	+
cell part;intracellular organelle part;intracellular part;nuclear part;nucleoplasm;organelle part		Homo sapiens	
cell part;cytoplasmic part;cytosol;endosomal part;endosome membrane;ESCRT III complex;extracellular membrane-bounded organelle;extracellular organelle;extracellular region part;extracellular vesicular exosome;intracellu	Endocytosis	Homo sapiens	+
cell part;intracellular organelle part;intracellular part;macromolecular complex;nuclear body;nuclear matrix;nuclear part;nuclear speck;nucleoplasm;nucleoplasm part;organelle part;ribonucleoprotein complex;small nuclear ribo	Spliceosome	Homo sapiens	+
cell part;cytoplasm;cytoplasmic part;Golgi apparatus;intracellular membrane-bounded organelle;intracellular organelle;intracellular part;membrane-bounded organelle;nucleus;organelle		Homo sapiens	
	Ribosome biogenesis in eukaryotes	Homo sapiens	+
cell part;cytoplasm;envelope;intracellular organelle part;intracellular part;macromolecular complex;membrane;membrane part;nuclear envelope;nuclear membrane;nuclear part;nuclear pore;nucleoplasm;organelle envelope;or	RNA transport	Homo sapiens	
cell part;chromatin;chromosomal part;cytoplasm;intracellular membrane-bounded organelle;intracellular organelle;intracellular organelle part;intracellular part;membrane-bounded organelle;nuclear chromatin;nuclear chromosome part;nuclear part;nucleoplasm;nucleus;organelle;organelle part		Homo sapiens	+
cell part;cytoplasmic part;extracellular membrane-bounded organelle;extracellular organelle;extracellular region part;extracellular vesicular exosome;Golgi apparatus part;intracellular organelle part;intracellular part;membrane-bounded organelle;membrane-bounded vesicle;organelle;organelle part;trans-Golgi network;vesicle		Homo sapiens	+
cell part;cytoplasm;cytoskeletal part;extracellular membrane-bounded organelle;extracellular organelle;extracellular region part;extracellular vesicular exosome;intermediate filament;intracellular organelle part;intracellular part;macromolecular complex;membrane-bounded organelle;membrane-bounded vesicle;organelle;organelle part;protein complex;vesicle		Homo sapiens	+
cell part;cytoplasm;cytoplasmic part;intracellular membrane-bounded organelle;intracellular non-membrane-bounded organelle;intracellular organelle;intracellular part;macromolecular complex;membrane;membrane-bounded organelle;non-membrane-bounded organelle;nucleus;organelle;polysomal ribosome;ribonucleoprotein complex;ribosome		Homo sapiens	+
		Homo sapiens	+
cell part;cytoplasmic part;integral to membrane;intracellular membrane-bounded organelle;intracellular organelle;intracellular organelle part;intracellular part;intrinsic to membrane;membrane;membrane part;membrane-bounc	Cytosolic DNA-sensing pathway;Hepatitis C;Measles;RIG-I-like receptor signaling pathway	Homo sapiens	+
cell cortex;cell cortex part;cell part;centrosomal corona;centrosome;chromosomal part;condensed chromosome kinetochore;cortical cytoskeleton;cortical microtubule cytoskeleton;cytoplasmic microtubule;cytoplasmic part;cytoskeletal part;cytoskeleton;cytosol;extracellular membrane-bounded organelle;extracellular organelle;extracellular region part;extracellular vesicu		Homo sapiens	+
cell part;cytoplasmic part;endoplasmic reticulum lumen;endoplasmic reticulum part;intracellular organelle lumen;intracellular organelle part;intracellular part;membrane-enclosed lumen;organelle lumen;organelle part		Homo sapiens	+
adherens junction;anchoring junction;cell junction;cell part;cell-substrate adherens junction;cell-substrate junction;cytoplasmic part;cytosol;focal adhesion;intracellular membrane-bounded organelle;intracellular organelle;intracellular part;membrane;membrane-bounded organelle;nucleus;organelle;plasma membrane		Homo sapiens	+
		Homo sapiens	+
extracellular membrane-bounded organelle;extracellular organelle;extracellular region part;extracellular vesicular exosome;membrane-bounded organelle;membrane-bounded vesicle;organelle;vesicle		Homo sapiens	
cell part;cytoplasmic part;intracellular membrane-bounded organelle;intracellular organelle;intracellular organelle part;intracellular part;membrane-bounded organelle;mitochondrion;nuclear part;nucleoplasm;nucleus;organelle;organelle part		Homo sapiens	+
brush border;cell part;cell projection;cytoplasmic microtubule;cytoplasmic part;cytoskeletal part;extracellular membrane-bounded organelle;extracellular organelle;extracellular region part;extracellular vesicular exosome;intra	Pentose and glucuronate interconversions	Homo sapiens	+
cell part;cytoplasmic part;extracellular membrane-bounded organelle;extracellular organelle;extracellular region part;extracellular vesicular exosome;Golgi apparatus part;Golgi stack;intracellular organelle part;intracellular part;intrinsic to Golgi membrane;intrinsic to membrane;intrinsic to organelle membrane;macromolecular complex;membrane part;membrane-bounded		Homo sapiens	+
cell part;integral to membrane;intrinsic to membrane;membrane;membrane part;plasma membrane		Homo sapiens	+
cell part;cell projection;lamellipodium		Homo sapiens	+
cell part;cytoplasm;extracellular membrane-bounded organelle;extracellular organelle;extracellular region part;extracellular vesicular exosome;intracellular membrane-bounded organelle;intracellular organelle;intracellular orga	Ubiquitin mediated proteolysis	Homo sapiens	
		Homo sapiens	
cell part;cytoplasmic part;endoplasmic reticulum membrane;endoplasmic reticulum part;extracellular membrane-bounded organelle;extracellular organelle;extracellular region part;extracellular vesicular exosome;integral to membrane;intracellular organelle part;intracellular part;intrinsic to membrane;membrane;membrane part;membrane-bounded organelle;membrane-b		Homo sapiens	+
cell part;cytoplasmic part;intracellular membrane-bounded organelle;intracellular organelle;intracellular part;membrane-bounded organelle;mitochondrion;organelle		Homo sapiens	+
aggresome;cell part;cytoplasm;inclusion body;intracellular membrane-bounded organelle;intracellular organelle;intracellular organelle part;intracellular part;membrane-bounded organelle;nuclear part;nucleoplasm;nucleus;organelle;organelle part		Homo sapiens	
cell part;cytoplasm;cytoskeleton;intermediate filament cytoskeleton;intracellular non-membrane-bounded organelle;intracellular organelle;intracellular part;membrane;non-membrane-bounded organelle;organelle;plasma membrane		Homo sapiens	+
cell part;cytoplasmic part;intracellular membrane-bounded organelle;intracellular organelle;intracellular part;membrane-bounded organelle;mitochondrion;nucleus;organelle		Homo sapiens	+
cell part;cytoplasmic part;endosomal part;endosome membrane;integral to membrane;intracellular organelle part;intracellular part;intrinsic to membrane;late endosome membrane;lysosomal membrane;membrane;membrane part;organelle membrane;organelle part;vacuolar membrane;vacuolar part		Homo sapiens	+
cell part;cytoplasmic mRNA processing body;cytoplasmic part;cytosol;intracellular membrane-bounded organelle;intracellular non-membrane-bounded organelle;intracellular organelle;intracellular organelle part;intracellular p	RNA degradation	Homo sapiens	+
cell part;cytoplasm;cytoplasmic part;cytosol;intracellular membrane-bounded organelle;intracellular organelle;intracellular part;membrane;membrane-bounded organelle;nucleus;organelle		Homo sapiens	
cell part;cytoplasm;intracellular membrane-bounded organelle;intracellular organelle;intracellular organelle part;intracellular part;macromolecular complex;membrane-bounded organelle;nuclear part;nucleoplasm;nucleus;orga	mRNA surveillance pathway	Homo sapiens	+
cell part;chromosomal part;chromosome;intracellular non-membrane-bounded organelle;intracellular organelle;intracellular organelle part;intracellular part;kinetochore;macromolecular complex;non-membrane-bounded organelle;nuclear chromosome;nuclear part;organelle;organelle part;protein complex		Homo sapiens	+
apical plasma membrane;cell junction;cell part;cell-cell junction;cytoplasm;cytoplasmic part;endoplasmic reticulum;endoplasmic reticulum membrane;endoplasmic reticulum part;integral to membrane;intercellular canaliculus;intracellular membrane-bounded organelle;intracellular non-membrane-bounded organelle;intracellular organelle;intracellular organelle part;intrace		Homo sapiens	+
cell part;cytoplasmic part;endoplasmic reticulum;endoplasmic reticulum membrane;endoplasmic reticulum part;integral to membrane;integral to plasma membrane;intracellular membrane-bounded organelle;intracellular organelle;intracellular organelle part;intracellular part;intrinsic to membrane;intrinsic to plasma membrane;membrane;membrane part;membrane-bound		Homo sapiens	+
cell part;cytoplasm;cytoplasmic part;intracellular membrane-bounded organelle;intracellular organelle;intracellular organelle part;intracellular part;membrane-bounded organelle;mitochondrion;nuclear part;nucleoplasm;organelle;organelle part		Homo sapiens	+
cell junction;cell part;cell projection;cytoskeletal part;extracellular membrane-bounded organelle;extracellular organelle;extracellular region part;extracellular vesicular exosome;intracellular non-membrane-bounded organelle;intracellular organelle;intracellular organelle part;intracellular part;macromolecular complex;membrane;membrane-bounded organelle;membrane-bo		Homo sapiens	+
cell part;cytoplasmic part;cytosol;intracellular part	Butanoate metabolism	Homo sapiens	
cell part;extracellular membrane-bounded organelle;extracellular organelle;extracellular region part;extracellular vesicular exosome;intracellular membrane-bounded organelle;intracellular organelle;intracellular part;membrane;membrane-bounded organelle;membrane-bounded vesicle;nucleus;organelle;vesicle		Homo sapiens	+
catalytic step 2 spliceosome;cell part;cytoplasmic part;cytosol;extracellular membrane-bounded organelle;extracellular organelle;extracellular region part;extracellular vesicular exosome;intracellular membrane-bounded orga	mRNA surveillance pathway;RNA transport;Spliceosome	Homo sapiens	+
cell junction;cell part;cell-cell junction;cytoplasmic part;cytosol;intracellular part;lateral plasma membrane;membrane part;plasma membrane part	Regulation of actin cytoskeleton	Homo sapiens	+
cell part;intracellular organelle part;intracellular part;nuclear part;nucleoplasm;organelle part		Homo sapiens	+
cell part;cullin-RING ubiquitin ligase complex;cytoplasm;extracellular membrane-bounded organelle;extracellular organelle;extracellular region part;extracellular vesicular exosome;intracellular membrane-bounded organelle;intracellular organelle;intracellular organelle part;intracellular part;macromolecular complex;membrane;membrane-bounded organelle;membrane-bo		Homo sapiens	+
cell part;centriolar satellite;centrosome;cis-Golgi network;cytoplasm;cytoplasmic part;cytoskeletal part;FHF complex;Golgi apparatus;Golgi apparatus part;intracellular membrane-bounded organelle;intracellular non-membrane-bounded organelle;intracellular organelle;intracellular organelle part;intracellular part;macromolecular complex;membrane-bounded organelle;m		Homo sapiens	
cell part;intracellular membrane-bounded organelle;intracellular organelle;intracellular part;membrane-bounded organelle;nucleus;organelle		Homo sapiens	
adherens junction;anchoring junction;cell junction;cell part;cell-substrate adherens junction;cell-substrate junction;focal adhesion;membrane;plasma membrane		Homo sapiens	
cell part;extracellular membrane-bounded organelle;extracellular organelle;extracellular region part;extracellular vesicular exosome;intracellular organelle part;intracellular part;membrane-bounded organelle;membrane-bounded vesicle;nuclear part;nucleoplasm;organelle;organelle part;vesicle		Homo sapiens	+
extracellular membrane-bounded organelle;extracellular organelle;extracellular region part;extracellular vesicular exosome;membrane-bounded organelle;membrane-bounded vesicle;organelle;vesicle		Homo sapiens	
cell part;cytoplasmic part;cytoplasmic vesicle membrane;cytoplasmic vesicle part;endoplasmic reticulum membrane;endoplasmic reticulum part;Golgi apparatus;integral to membrane;intracellular membrane-bounded organe	Cytosolic DNA-sensing pathway;RIG-I-like receptor signaling pathway	Homo sapiens	+
cell part;cytoplasm;cytoplasmic part;cytosol;intracellular membrane-bounded organelle;intracellular organelle;intracellular organelle part;intracellular part;membrane-bounded organelle;nuclear part;nucleoplasm;nucleus;organelle;organelle part		Homo sapiens	+
cell part;cytoplasmic part;extracellular membrane-bounded organelle;extracellular organelle;extracellular region part;extracellular vesicular exosome;intracellular membrane-bounded organelle;intracellular organelle;intracellular part;membrane-bounded organelle;membrane-bounded vesicle;mitochondrion;nucleus;organelle;vesicle		Homo sapiens	
cell part;intracellular membrane-bounded organelle;intracellular organelle;intracellular organelle part;intracellular part;membrane;membrane-bounded organelle;nuclear part;nucleoplasm;nucleus;organelle;organelle part		Homo sapiens	+
cell part;cytoplasmic part;endoplasmic reticulum membrane;endoplasmic reticulum part;integral to endoplasmic reticulum membrane;integral to membrane;integral to organelle membrane;intracellular organelle part;intracellular part;intrinsic to endoplasmic reticulum membrane;intrinsic to membrane;intrinsic to organelle membrane;membrane;membrane part;organelle m		Homo sapiens	
Cajal body;cell part;cytoplasm;intracellular membrane-bounded organelle;intracellular non-membrane-bounded organelle;intracellular organelle;intracellular organelle part;intracellular part;membrane;membrane-bounded orga	Spliceosome	Homo sapiens	
cell part;cytoplasm;intracellular part		Homo sapiens	+
cell part;cytoplasm;intracellular part		Homo sapiens	+
cell part;cytoplasmic membrane-bounded vesicle;cytoplasmic part;cytoplasmic vesicle;endocytic vesicle;endomembrane system;endosomal part;endosome membrane;extracellular membrane-bounded organelle;extracellula	Phagosome	Homo sapiens	
cell part;cytoplasmic part;cytosol;endoplasmic reticulum membrane;endoplasmic reticulum part;Golgi apparatus;Golgi apparatus part;Golgi membrane;intracellular membrane-bounded organelle;intracellular organelle;intracellular organelle part;intracellular part;membrane;membrane part;membrane-bounded organelle;nuclear part;nucleoplasm;organelle;organelle memb		Homo sapiens	+
cell part;cytoplasmic membrane-bounded vesicle;cytoplasmic part;cytoplasmic vesicle;cytoplasmic vesicle membrane;cytoplasmic vesicle part;endocytic vesicle;endocytic vesicle membrane;endosome;extracellular membrane-bounded organelle;extracellular organelle;extracellular region part;extracellular vesicular exosome;Golgi apparatus;intracellular membrane-boun		Homo sapiens	+
cell envelope;cell part;cornified envelope;cytoplasm;cytoplasmic part;cytoskeleton;envelope;extracellular membrane-bounded organelle;extracellular organelle;extracellular region part;extracellular vesicular exosome;intracellular membrane-bounded organelle;intracellular non-membrane-bounded organelle;intracellular organelle;intracellular part;membrane-bounded orga		Homo sapiens	+
aggresome;cell part;inclusion body;intracellular membrane-bounded organelle;intracellular organelle;intracellular organelle part;intracellular part;membrane-bounded organelle;nuclear part;nucleoplasm;organelle;organelle part		Homo sapiens	
cell part;cytoplasm;cytoplasmic part;cytosolic part;Golgi apparatus part;Golgi membrane;IkappaB kinase complex;intracellular organelle part;intracellular part;macromolecular complex;membrane;organelle membrane;organelle part;presynaptic active zone;presynaptic membrane;protein complex;synapse;synapse part;synaptic membrane		Homo sapiens	+
cell part;cytoplasmic part;endoplasmic reticulum;Golgi apparatus;Golgi apparatus part;intracellular membrane-bounded organelle;intracellular organelle;intracellular organelle part;intracellular part;macromolecular complex;membrane-bounded organelle;organelle;organelle part;protein complex;TRAPP complex		Homo sapiens	+
cell part;cytoplasmic part;endoplasmic reticulum membrane;endoplasmic reticulum part;extracellular membrane-bounded organelle;extracellular organelle;extracellular region part;extracellular vesicular exosome;integral to membrane;intracellular organelle part;intracellular part;intrinsic to membrane;membrane;membrane part;membrane-bounded organelle;membrane-b		Homo sapiens	+
cell part;cytoplasm;histone pre-mRNA 3'end processing complex;intracellular membrane-bounded organelle;intracellular non-membrane-bounded organelle;intracellular organelle;intracellular organelle part;intracellular part;macromolecular complex;membrane-bounded organelle;non-membrane-bounded organelle;nuclear part;nucleolus;nucleus;organelle;organelle part;		Homo sapiens	+
cell part;contractile fiber part;costamere;cytoplasm;cytoplasmic part;cytoplasmic vesicle membrane;cytoplasmic vesicle part;intracellular membrane-bounded organelle;intracellular organelle;intracellular organelle part;intracellular part;membrane;membrane part;membrane-bounded organelle;nucleus;organelle;organelle membrane;organelle part;plasma membrane;plasn		Homo sapiens	+
cell part;cytoplasmic part;endoplasmic reticulum lumen;endoplasmic reticulum part;intracellular organelle lumen;intracellular organelle part;intracellular part;membrane-enclosed lumen;organelle lumen;organelle part		Homo sapiens	+
cell part;cytoplasm;intracellular part		Homo sapiens	
cell part;cytoplasmic part;extracellular membrane-bounded organelle;extracellular organelle;extracellular region part;extracellular vesicular exosome;integral to membrane;intracellular organelle part;intracellular part;intrinsic to membrane;lysosomal membrane;membrane;membrane part;membrane-bounded organelle;membrane-bounded vesicle;organelle;organelle meml		Homo sapiens	+
cell part;membrane;plasma membrane		Homo sapiens	+
actin cytoskeleton;cell part;centrosome;cytoplasm;cytoplasmic part;cytoskeletal part;cytoskeleton;intracellular membrane-bounded organelle;intracellular non-membrane-bounded organelle;intracellular organelle;intracellular organelle part;intracellular part;macromolecular complex;membrane;membrane-bounded organelle;microtubule;microtubule organizing center;non-		Homo sapiens	+
cell part;cytoplasm;intracellular part		Homo sapiens	+
cell part;intracellular membrane-bounded organelle;intracellular organelle;intracellular part;membrane-bounded organelle;nucleus;organelle		Homo sapiens	+
cell part;chromosomal part;condensed chromosome kinetochore;intracellular membrane-bounded organelle;intracellular non-membrane-bounded organelle;intracellular organelle;intracellular organelle part;intracellular part;kinetochore;macromolecular complex;membrane-bounded organelle;non-membrane-bounded organelle;nuclear part;nucleolus;nucleoplasm;nucleus		Homo sapiens	
cell part;cytoplasmic part;cytosol;intracellular part		Homo sapiens	+
cell part;cytoplasm;cytoplasmic part;endoplasmic reticulum membrane;endoplasmic reticulum part;intracellular organelle part;intracellular part;membrane;membrane part;organelle membrane;organelle part;perinuclear regior	Spliceosome	Homo sapiens	+
cell part;cytoplasm;intracellular part		Homo sapiens	+
cell part;intracellular organelle part;intracellular part;macromolecular complex;nuclear part;nucleoplasm;organelle part;ribonucleoprotein complex;spliceosomal complex		Homo sapiens	+
cell part;intracellular organelle part;intracellular part;nuclear part;nucleoplasm;organelle part		Homo sapiens	+
cell part;cytoplasmic part;intracellular organelle part;intracellular part;nuclear part;nucleoplasm;organelle part;perinuclear region of cytoplasm		Homo sapiens	
cell part;cytoplasmic part;endoplasmic reticulum;endoplasmic reticulum chaperone complex;endoplasmic reticulum lumen;endoplasmic reticulum part;intracellular membrane-bounded organelle;intracellular organelle;intracel	Protein processing in endoplasmic reticulum	Homo sapiens	+
cell part;intracellular organelle part;intracellular part;macromolecular complex;membrane;NELF complex;nuclear part;nucleoplasm;nucleoplasm part;organelle part;protein complex;transcription elongation factor complex		Homo sapiens	+
cell part;cytoplasmic part;cytosol;extracellular membrane-bounded organelle;extracellular organelle;extracellular region part;extracellular vesicular exosome;integral to membrane;integral to mitochondrial membrane;integral to mitochondrial outer membrane;integral to organelle membrane;intracellular organelle part;intracellular part;intrinsic to membrane;intrinsic to mitoc		Homo sapiens	+
		Homo sapiens	+
		Homo sapiens	+
cell part;cytoplasmic part;endoplasmic reticulum;endoplasmic reticulum membrane;endoplasmic reticulum part;integral to membrane;intracellular membrane-bounded organelle;intracellular organelle;intracellular organelle pa	Glycerophospholipid metabolism	Homo sapiens	+
cell part;cytoplasm;intracellular membrane-bounded organelle;intracellular organelle;intracellular part;membrane-bounded organelle;nucleus;organelle		Homo sapiens	+
cell part;intracellular membrane-bounded organelle;intracellular non-membrane-bounded organelle;intracellular organelle;intracellular organelle part;intracellular part;macromolecular complex;membrane-bounded organelle;non-membrane-bounded organelle;nuclear part;nucleolus;nucleus;organelle;organelle part;preribosome;preribosome, small subunit precursor;ribonu		Homo sapiens	+
catalytic step 2 spliceosome;cell part;cytoplasmic part;cytosol;intracellular membrane-bounded organelle;intracellular organelle;intracellular organelle part;intracellular part;macromolecular complex;membrane-bounded orgar	mRNA surveillance pathway;RNA transport	Homo sapiens	
cell cortex part;cell part;cell projection part;cytoplasm;cytoplasmic part;cytosol;endosome;exocyst;growth cone;intracellular membrane-bounded organelle;intracellular organelle;intracellular part;late endosome;macromolecular complex;membrane;membrane-bounded organelle;organelle;plasma membrane;protein complex;site of polarized growth		Homo sapiens	
cell part;integral to membrane;intrinsic to membrane;membrane;membrane part		Homo sapiens	+
calcium channel complex;cation channel complex;cell part;cytoplasmic part;intracellular membrane-bounded organelle;intracellular organelle;intracellular organelle part;intracellular part;ion channel complex;macromolecular complex;membrane part;membrane-bounded organelle;membrane-enclosed lumen;mitochondrial intermembrane space;mitochondrial part;mitocho		Homo sapiens	+
cell part;cytoplasmic part;endosome;intracellular membrane-bounded organelle;intracellular organelle;intracellular part;late endosome;membrane;membrane-bounded organelle;organelle;plasma membrane		Homo sapiens	+
		Homo sapiens	+
cell part;extracellular membrane-bounded organelle;extracellular organelle;extracellular region part;extracellular vesicular exosome;membrane;membrane-bounded organelle;membrane-bounded vesicle;organelle;vesicle		Homo sapiens	+
cell part;chromatin;chromosomal part;cytoplasm;histone methyltransferase complex;intracellular membrane-bounded organelle;intracellular non-membrane-bounded organelle;intracellular organelle;intracellular organelle part;intracellular part;macromolecular complex;membrane;membrane-bounded organelle;methyltransferase complex;MLL1 complex;non-membrane-b		Homo sapiens	+
cell junction;cell part;cell projection;cell projection part;cytoplasmic part;cytoskeletal part;cytoskeleton;cytosol;endoplasmic reticulum;extracellular membrane-bounded organelle;extracellular organelle;extracellular region part;extracellular vesicular exosome;filopodium;growth cone;intracellular;intracellular membrane-bounded organelle;intracellular non-membrane-bounc		Homo sapiens	+
cell part;cytoplasm;cytoplasmic part;intracellular organelle part;intracellular part;lipid particle;membrane;midbody;mitochondrial membrane;mitochondrial outer membrane;mitochondrial part;organelle membrane;organelle outer membrane;organelle part;outer membrane;plasma membrane;synapse		Homo sapiens	
cell body;cell part;cell projection;cytoplasm;cytoplasmic part;cytosol;dendrite;intracellular membrane-bounded organelle;intracellular organelle;intracellular organelle part;intracellular part;lysosomal membrane;lysosome;lytic	Insulin signaling pathway;mTOR signaling pathway	Homo sapiens	
cell part;chromatin;chromosomal part;cytoplasm;cytoplasmic part;intracellular membrane-bounded organelle;intracellular organelle;intracellular organelle lumen;intracellular organelle part;intracellular part;membrane-bounded organelle;membrane-enclosed lumen;mitochondrial matrix;mitochondrial part;nuclear chromatin;nuclear chromosome part;nuclear part;nucleopla		Homo sapiens	+
cell part;cytoplasmic part;intracellular membrane-bounded organelle;intracellular organelle;intracellular part;membrane-bounded organelle;mitochondrion;organelle		Homo sapiens	+
cell part;envelope;intracellular organelle part;intracellular part;macromolecular complex;membrane;membrane part;nuclear envelope;nuclear membrane;nuclear part;nuclear periphery;nuclear pore;organelle envelope;organell	RNA transport	Homo sapiens	+
cell part;cytoplasm;intracellular membrane-bounded organelle;intracellular organelle;intracellular organelle part;intracellular part;membrane-bounded organelle;nuclear part;nucleoplasm;nucleus;organelle;organelle part		Homo sapiens	+
		Homo sapiens	
cell part;cytoplasmic part;endoplasmic reticulum;envelope;intracellular membrane-bounded organelle;intracellular organelle;intracellular organelle part;intracellular part;membrane;membrane-bounded organelle;nuclear envelope;nuclear part;organelle;organelle envelope;organelle part		Homo sapiens	+
alpha-amino-3-hydroxy-5-methyl-4-isoxazolepropionic acid selective glutamate receptor complex;cell part;integral to membrane;intrinsic to membrane;ionotropic glutamate receptor complex;macromolecular complex;membrane part;plasma membrane part;protein complex;receptor complex		Homo sapiens	
extracellular matrix;extracellular membrane-bounded organelle;extracellular organelle;extracellular region;extracellular region part;extracellular space;extracellular vesicular exosome;membrane-bounded organelle;membrane-bounded vesicle;organelle;proteinaceous extracellular matrix;vesicle		Homo sapiens	+
cell part;cytoplasmic part;cytosol;intracellular part		Homo sapiens	
cell part;cytoplasm;cytoplasmic part;Golgi apparatus;intracellular;intracellular membrane-bounded organelle;intracellular organelle;intracellular organelle part;intracellular part;membrane;membrane-bounded organelle;nuclear part;nucleoplasm;nucleus;organelle;organelle part		Homo sapiens	+
cell part;membrane		Homo sapiens	+
cell part;cytoplasmic part;endosomal part;endosome;endosome membrane;extrinsic to membrane;intracellular membrane-bounded organelle;intracellular organelle;intracellular organelle part;intracellular part;late endosome;late endosome membrane;membrane;membrane part;membrane-bounded organelle;organelle;organelle membrane;organelle part;recycling endosc		Homo sapiens	+
actin cytoskeleton;cell part;chromatin;chromosomal part;chromosome;chromosome, centromeric region;cytoplasmic part;cytoskeleton;cytosol;intermediate filament cytoskeleton;intracellular membrane-bounded organelle;in	Cell cycle;Cell cycle - yeast;Meiosis - yeast	Homo sapiens	+
cell part;cell surface;cytoplasmic part;cytosol;extracellular matrix;extracellular membrane-bounded organelle;extracellular organelle;extracellular region;extracellular region part;extracellular space;extracellular vesicular exoso	Wnt signaling pathway	Homo sapiens	+
axon;cell junction;cell part;cell projection;cytoplasm;cytoplasmic part;cytoskeletal part;cytosol;dendrite;extracellular membrane-bounded organelle;extracellular organelle;extracellular region part;extracellular vesicular exosome;Golgi apparatus;intracellular membrane-bounded organelle;intracellular organelle;intracellular organelle part;intracellular part;membrane;membra		Homo sapiens	+
cell part;cytoplasmic part;early endosome;early endosome membrane;endosomal part;endosome;endosome membrane;ER membrane protein complex;Golgi apparatus;Golgi apparatus part;Golgi membrane;integral to membrane;intracellular membrane-bounded organelle;intracellular organelle;intracellular organelle part;intracellular part;intrinsic to membrane;macromol		Homo sapiens	+
cell part;cytoplasm;cytoplasmic part;intracellular membrane-bounded organelle;intracellular non-membrane-bounded organelle;intracellular organelle;intracellular part;macromolecular complex;membrane-bounded organelle;non-membrane-bounded organelle;nucleus;organelle;ribonucleoprotein complex;RNA granule;stress granule		Homo sapiens	+
actin cytoskeleton;adherens junction;anchoring junction;cell junction;cell part;cell-substrate adherens junction;cell-substrate junction;cytoplasm;cytoskeleton;focal adhesion;intracellular non-membrane-bounded organelle;intracellular organelle;intracellular part;non-membrane-bounded organelle;organelle		Homo sapiens	
cell part;cytoplasmic part;cytoplasmic vesicle;extracellular membrane-bounded organelle;extracellular organelle;extracellular region part;extracellular vesicular exosome;intracellular organelle;intracellular part;membrane;membrane-bounded organelle;membrane-bounded vesicle;organelle;plasma membrane;vesicle		Homo sapiens	+
cell part;cytoplasmic part;endoplasmic reticulum;endoplasmic reticulum membrane;endoplasmic reticulum part;integral to membrane;intracellular membrane-bounded organelle;intracellular organelle;intracellular organelle part;intracellular part;intrinsic to membrane;macromolecular complex;membrane;membrane part;membrane-bounded organelle;mitochondrial membr		Homo sapiens	
		Homo sapiens	+

cell part;intracellular membrane-bounded organelle;intracellular organelle;intracellular organelle part;intracellular part;macromolecular complex;membrane;membrane-bounded organelle;mRNA cleavage factor complex;nuclea	mRNA surveillance pathway	Homo sapiens	+
cell part;cytoplasmic part;endoplasmic reticulum lumen;endoplasmic reticulum part;extracellular matrix;extracellular region part;intracellular organelle lumen;intracellular organelle part;intracellular part;membrane-enclosed lumen;organelle lumen;organelle part;proteinaceous extracellular matrix		Homo sapiens	+
cell part;cytoplasm;cytoplasmic part;Golgi apparatus;Golgi apparatus part;Golgi membrane;intracellular membrane-bounded organelle;intracellular non-membrane-bounded organelle;intracellular organelle;intracellular organe	Endocytosis	Homo sapiens	
		Homo sapiens	+
		Homo sapiens	+
cell part;cytoplasmic part;cytoplasmic vesicle membrane;cytoplasmic vesicle part;cytosol;Golgi apparatus part;Golgi membrane;Golgi-associated vesicle membrane;intracellular organelle part;intracellular part;membrane;orga	Endocytosis	Homo sapiens	
cell part;ER membrane protein complex;integral to membrane;intrinsic to membrane;macromolecular complex;membrane part;protein complex		Homo sapiens	+
Cdc73/Paf1 complex;cell part;cytoplasm;intracellular organelle part;intracellular part;macromolecular complex;membrane;nuclear part;nucleoplasm part;organelle part;protein complex;transcription elongation factor complex		Homo sapiens	+
actin filament bundle;actomyosin;adherens junction;anchoring junction;cell junction;cell part;cell projection;cell-substrate adherens junction;cell-substrate junction;cytoplasm;cytoplasmic part;cytoskeletal part;cytosol;filopodi	Regulation of actin cytoskeleton	Homo sapiens	+
cell part;cytoplasm;cytoplasmic part;intracellular membrane-bounded organelle;intracellular organelle;intracellular part;membrane-bounded organelle;mitochondrion;organelle	Ribosome biogenesis in eukaryotes	Homo sapiens	+
		Homo sapiens	+
cell part;integral to membrane;intrinsic to membrane;membrane part		Homo sapiens	+
cell part;cytoplasmic part;extracellular membrane-bounded organelle;extracellular organelle;extracellular region part;extracellular space;extracellular vesicular exosome;Golgi apparatus;integral to membrane;integral to plasma membrane;intracellular membrane-bounded organelle;intracellular organelle;intracellular part;intrinsic to membrane;intrinsic to plasma membrane		Homo sapiens	+
cell part;cytoplasmic part;endoplasmic reticulum lumen;endoplasmic reticulum part;intracellular organelle lumen;intracellular organelle part;intracellular part;membrane;membrane-enclosed lumen;organelle lumen;organelle pa	Lysine degradation;Other types of O-glycan biosynthesis	Homo sapiens	+
cell part;cytoplasmic part;endoplasmic reticulum lumen;endoplasmic reticulum part;intracellular organelle lumen;intracellular organelle part;intracellular part;membrane-enclosed lumen;organelle lumen;organelle part		Homo sapiens	+
cell part;cytoplasmic part;endoplasmic reticulum lumen;endoplasmic reticulum part;extracellular membrane-bounded organelle;extracellular organelle;extracellular region part;extracellular vesicular exosome;intracellular orga	Other types of O-glycan biosynthesis	Homo sapiens	+
cell part;cytoplasmic part;endoplasmic reticulum;integral to membrane;intracellular membrane-bounded organelle;intracellular organelle;intracellular part;intrinsic to membrane;membrane part;membrane-bounded organelle;organelle		Homo sapiens	+
cell part;cytoplasm;cytoplasmic part;extracellular region;intracellular part;lipid particle		Homo sapiens	+
cell part;cytoplasmic part;endoplasmic reticulum;endoplasmic reticulum lumen;endoplasmic reticulum part;extracellular membrane-bounded organelle;extracellular organelle;extracellular region part;extracellular vesicular exo	Protein processing in endoplasmic reticulum	Homo sapiens	+
cell part;chromosomal part;condensed chromosome kinetochore;cytoplasmic part;cytosol;intracellular membrane-bounded organelle;intracellular non-membrane-bounded organelle;intracellular organelle;intracellular organelle part;intracellular part;kinetochore;macromolecular complex;membrane-bounded organelle;Ndc80 complex;non-membrane-bounded organelle;nu		Homo sapiens	+
cell junction;cell part;cytoplasmic part;intracellular membrane-bounded organelle;intracellular organelle;intracellular organelle part;intracellular part;membrane;membrane-bounded organelle;microbody;microbody membrane;microbody part;mitochondrion;nucleus;organelle;organelle membrane;organelle part;peroxisomal membrane;peroxisomal part;postsynaptic membr		Homo sapiens	+
cell part;cytoplasmic part;intracellular membrane-bounded organelle;intracellular organelle;intracellular part;lipid particle;membrane;membrane-bounded organelle;midbody;mitochondrion;nucleus;organelle		Homo sapiens	
cell part;cytoplasm;cytoplasmic part;extracellular membrane-bounded organelle;extracellular organelle;extracellular region part;extracellular vesicular exosome;intracellular membrane-bounded organelle;intracellular organelle;intracellular part;membrane;membrane-bounded organelle;membrane-bounded vesicle;nucleus;organelle;perinuclear region of cytoplasm;plasma		Homo sapiens	+
cell part;integral to membrane;integral to nuclear inner membrane;integral to organelle membrane;intracellular organelle part;intracellular part;intrinsic to membrane;intrinsic to nuclear inner membrane;intrinsic to organelle membrane;membrane;membrane part;nuclear membrane;nuclear membrane part;nuclear part;organelle membrane;organelle part		Homo sapiens	
cell part;intracellular part;macromolecular complex;protein complex;tRNA-splicing ligase complex		Homo sapiens	
cell part;cytoplasmic part;extracellular membrane-bounded organelle;extracellular organelle;extracellular region part;extracellular vesicular exosome;Golgi apparatus part;Golgi membrane;integral to membrane;intracellular or	Glycosaminoglycan biosynthesis - chondroitin sulfate	Homo sapiens	+
cell part;cytoplasm;cytoplasmic part;intracellular organelle lumen;intracellular organelle part;intracellular part;membrane-enclosed lumen;mitochondrial matrix;mitochondrial part;organelle lumen;organelle part		Homo sapiens	+
cell body;cell part;cell projection;cilium;cytoplasm;cytoplasmic part;extracellular membrane-bounded organelle;extracellular organelle;extracellular region;extracellular region part;extracellular space;extracellular vesicular exosome;intracellular membrane-bounded organelle;intracellular organelle;intracellular organelle part;intracellular part;membrane-bounded organelle;m		Homo sapiens	+
cell part;cytoplasm;cytoplasmic part;cytosol;envelope;intracellular non-membrane-bounded organelle;intracellular organelle;intracellular organelle part;intracellular part;macromolecular complex;membrane;non-membrane-bounded organelle;nuclear envelope;nuclear part;nucleoplasm;organelle;organelle envelope;organelle part;polysomal ribosome;ribonucleoprotein cor		Homo sapiens	+
calcium channel complex;cation channel complex;cell part;cytoplasmic part;integral to membrane;integral to mitochondrial inner membrane;integral to mitochondrial membrane;integral to organelle membrane;intracellular membrane-bounded organelle;intracellular organelle;intracellular organelle part;intracellular part;intrinsic to membrane;intrinsic to mitochondrial inner		Homo sapiens	
		Homo sapiens	+
cell part;cytoplasm;cytoplasmic membrane-bounded vesicle;cytoplasmic part;cytoplasmic vesicle;extracellular membrane-bounded organelle;extracellular organelle;extracellular region part;extracellular vesicular exosome;Golgi apparatus;integral to membrane;intracellular membrane-bounded organelle;intracellular organelle;intracellular part;intrinsic to membrane;memb		Homo sapiens	+
cell part;cytoplasm;cytoplasmic part;Golgi apparatus;intracellular membrane-bounded organelle;intracellular organelle;intracellular organelle part;intracellular part;membrane-bounded organelle;nuclear part;nucleoplasm;organelle;organelle part		Homo sapiens	+
cell part;centrosome;cytoplasm;cytoplasmic part;cytoskeletal part;endosomal part;endosome membrane;ESCRT I complex;intracellular membrane-bounded organelle;intracellular non-membrane-bounded organelle;intracellu	Endocytosis	Homo sapiens	+
cell part;cytoplasmic part;endoplasmic reticulum;endoplasmic reticulum membrane;endoplasmic reticulum part;Golgi apparatus;Golgi apparatus part;Golgi membrane;integral to membrane;intracellular membrane-bounded c	Ether lipid metabolism;Glycerophospholipid metabolism	Homo sapiens	
cell part;intracellular organelle part;intracellular part;nuclear part;nucleoplasm;organelle part		Homo sapiens	
cell part;chromosomal part;condensed chromosome kinetochore;cytoplasmic part;cytosol;envelope;intracellular non-membrane-bounded organelle;intracellular organelle;intracellular organelle part;intracellular part;kinetocho	RNA transport	Homo sapiens	+
cell part;chromosomal part;condensed chromosome kinetochore;cytoplasmic part;cytosol;envelope;intracellular membrane-bounded organelle;intracellular non-membrane-bounded organelle;intracellular organelle;intracellula	RNA transport	Homo sapiens	+
cell part;cytoskeleton;envelope;intermediate filament cytoskeleton;intracellular membrane-bounded organelle;intracellular non-membrane-bounded organelle;intracellular organelle;intracellular organelle part;intracellular part;r	RNA transport	Homo sapiens	+
cell part;cytoplasmic part;cytoplasmic vesicle membrane;cytoplasmic vesicle part;extracellular membrane-bounded organelle;extracellular organelle;extracellular region part;extracellular vesicular exosome;integral to membrane;integral to plasma membrane;intracellular membrane-bounded organelle;intracellular organelle;intracellular organelle part;intracellular part;intrin		Homo sapiens	+
cell part;cytoplasm;cytoplasmic part;endoplasmic reticulum;endoplasmic reticulum membrane;endoplasmic reticulum part;integral to membrane;intracellular membrane-bounded organelle;intracellular organelle;intracellular organelle part;intracellular part;intrinsic to membrane;membrane;membrane part;membrane-bounded organelle;nuclear membrane;nuclear part;nucl		Homo sapiens	+
cell part;cytoplasmic part;intracellular membrane-bounded organelle;intracellular organelle;intracellular part;membrane-bounded organelle;mitochondrion;organelle		Homo sapiens	
cell part;intracellular membrane-bounded organelle;intracellular organelle;intracellular organelle part;intracellular part;membrane;membrane-bounded organelle;nuclear part;nucleoplasm;nucleus;organelle;organelle part	Amino sugar and nucleotide sugar metabolism	Homo sapiens	
cell cortex part;cell part;cytoplasmic part;exocyst;integral to membrane;intracellular part;intrinsic to membrane;macromolecular complex;membrane;membrane part;plasma membrane;protein complex		Homo sapiens	+
cell part;cytoplasmic part;extracellular membrane-bounded organelle;extracellular organelle;extracellular region part;extracellular vesicular exosome;intracellular organelle lumen;intracellular organelle part;intracellular part;lysosomal lumen;membrane-bounded organelle;membrane-bounded vesicle;membrane-enclosed lumen;organelle;organelle lumen;organelle part;vacu		Homo sapiens	
cell part;cytoplasm;intracellular non-membrane-bounded organelle;intracellular organelle;intracellular organelle part;intracellular part;membrane;non-membrane-bounded organelle;nuclear part;nucleolus;organelle;organelle part		Homo sapiens	+
apical part of cell;cell part;centriole;centrosome;cytoplasmic part;cytoskeletal part;cytosol;intracellular non-membrane-bounded organelle;intracellular organelle;intracellular organelle part;intracellular part;membrane;microtubule organizing center;microtubule organizing center part;non-membrane-bounded organelle;nuclear part;nucleolus;nucleoplasm;organelle;organelle		Homo sapiens	
cell part;intracellular organelle part;intracellular part;macromolecular complex;nuclear body;nuclear part;nuclear speck;nucleoplasm;nucleoplasm part;organelle part;protein complex;THO complex;THO complex part of transc	RNA transport;Spliceosome	Homo sapiens	+
cell part;intracellular non-membrane-bounded organelle;intracellular organelle;intracellular organelle part;intracellular part;macromolecular complex;non-membrane-bounded organelle;nuclear part;nucleolar part;nucleolus;org	Ribosome biogenesis in eukaryotes	Homo sapiens	+
		Homo sapiens	+
cell part;chromatin;chromatin remodeling complex;chromosomal part;intracellular organelle part;intracellular part;macromolecular complex;nBAF complex;npBAF complex;nuclear chromatin;nuclear chromosome part;nuclear part;nucleoplasm;organelle part;protein complex;SWI/SNF complex;SWI/SNF-type complex		Homo sapiens	+
cell part;cytoplasmic part;cytosol;endoplasmic reticulum;intracellular membrane-bounded organelle;intracellular organelle;intracellular organelle part;intracellular part;membrane;membrane part;membrane-bounded organelle;	Protein processing in endoplasmic reticulum	Homo sapiens	
		Homo sapiens	
cell part;cytoplasmic part;Golgi apparatus;Golgi apparatus part;Golgi membrane;integral to membrane;intracellular membrane-bounded organelle;intracellular organelle;intracellular organelle part;intracellular part;intrinsic to membrane;membrane;membrane part;membrane-bounded organelle;organelle;organelle membrane;organelle part		Homo sapiens	
cell part;cis-Golgi network;cytoplasmic part;Golgi apparatus;Golgi apparatus part;Golgi cisterna;Golgi membrane;integral to membrane;intracellular membrane-bounded organelle;intracellular organelle;intracellular organelle part;intracellular part;intrinsic to membrane;membrane;membrane part;membrane-bounded organelle;organelle;organelle membrane;organelle part;		Homo sapiens	+
cell part;cytoplasmic part;cytosol;intracellular membrane-bounded organelle;intracellular organelle;intracellular part;membrane-bounded organelle;nucleus;organelle	Ubiquitin mediated proteolysis	Homo sapiens	+
cell part;cytoplasm;cytoplasmic part;extracellular membrane-bounded organelle;extracellular organelle;extracellular region part;extracellular vesicular exosome;intracellular membrane-bounded organelle;intracellular organelle;intracellular part;membrane-bounded organelle;membrane-bounded vesicle;mitochondrion;organelle;vesicle		Homo sapiens	+
cell part;cytoplasmic part;endoplasmic reticulum membrane;endoplasmic reticulum part;integral to membrane;intracellular;intracellular organelle part;intracellular part;intrinsic to membrane;membrane;membrane part;organel	Retinol metabolism	Homo sapiens	+
cell part;cytoplasm;integral to membrane;intracellular part;intrinsic to membrane;membrane;membrane part;plasma membrane		Homo sapiens	
cell part;cytoplasmic part;endoplasmic reticulum;endoplasmic reticulum part;integral to membrane;intracellular membrane-bounded organelle;intracellular organelle;intracellular organelle part;intracellular part;intrinsic to mem	N-Glycan biosynthesis;Protein processing in endoplasmic reticulum;Various types of N-glycan biosynthesis	Homo sapiens	+
cell part;cytoplasm;cytoplasmic exosome (RNase complex);cytoplasmic part;exosome (RNase complex);intracellular membrane-bounded organelle;intracellular organelle;intracellular organelle part;intracellular part;macromol	Purine metabolism;Pyrimidine metabolism;RNA degradation	Homo sapiens	
cell part;cytoplasmic part;cytosol;intracellular membrane-bounded organelle;intracellular organelle;intracellular part;membrane-bounded organelle;nucleus;organelle		Homo sapiens	
cell part;cytoplasm;intracellular membrane-bounded organelle;intracellular organelle;intracellular organelle part;intracellular part;membrane-bounded organelle;nuclear part;nucleoplasm;nucleus;organelle;organelle part	Notch signaling pathway	Homo sapiens	+
cell part;cytoplasm;intracellular membrane-bounded organelle;intracellular non-membrane-bounded organelle;intracellular organelle;intracellular organelle part;intracellular part;membrane;membrane-bounded organelle;non-membrane-bounded organelle;nuclear part;nucleolus;nucleus;organelle;organelle part		Homo sapiens	+
cell part;intracellular membrane-bounded organelle;intracellular non-membrane-bounded organelle;intracellular organelle;intracellular organelle part;intracellular part;membrane-bounded organelle;non-membrane-bounded organelle;nuclear part;nucleolus;nucleus;organelle;organelle part		Homo sapiens	+
cell part;cytoplasm;cytoplasmic part;cytoskeletal part;intracellular organelle part;intracellular part;macromolecular complex;microtubule;microtubule organizing center;nuclear part;nucleoplasm;organelle part;protein complex;spindle pole		Homo sapiens	+
cell part;cytoplasm;cytoskeletal part;intermediate filament;intracellular organelle part;intracellular part;macromolecular complex;organelle part;protein complex		Homo sapiens	
cell part;cytoplasmic part;endoplasmic reticulum lumen;endoplasmic reticulum part;integral to membrane;intracellular organelle lumen;intracellular organelle part;intracellular part;intrinsic to membrane;membrane part;membrane-enclosed lumen;organelle lumen;organelle part		Homo sapiens	+
cell part;cytoplasm;cytoplasmic part;cytosol;intracellular membrane-bounded organelle;intracellular organelle;intracellular organelle part;intracellular part;macromolecular complex;membrane;membrane-bounded organelle;nu	RNA transport	Homo sapiens	+
cell part;chromatin;chromosomal part;cytoplasm;intracellular organelle part;intracellular part;macromolecular complex;membrane;nuclear chromatin;nuclear chromosome part;nuclear part;organelle part;protein complex		Homo sapiens	+
cell part;intracellular part;macromolecular complex;protein complex;protein phosphatase 4 complex;protein serine/threonine phosphatase complex		Homo sapiens	
cell part;chromosome;intracellular non-membrane-bounded organelle;intracellular organelle;intracellular organelle part;intracellular part;non-membrane-bounded organelle;nuclear part;nucleolus;nucleoplasm;organelle;organe	Lysine degradation	Homo sapiens	
cell part;intracellular membrane-bounded organelle;intracellular non-membrane-bounded organelle;intracellular organelle;intracellular organelle part;intracellular part;membrane-bounded organelle;non-membrane-bounded organelle;nuclear body;nuclear part;nuclear speck;nucleolus;nucleoplasm part;nucleus;organelle;organelle part		Homo sapiens	+
caveola;cell part;cell projection membrane;cell projection part;cell surface;cytoplasmic part;cytoplasmic vesicle membrane;cytoplasmic vesicle part;endocytic vesicle membrane;extracellular membrane-bounded organelle;ex	Bile secretion;Fat digestion and absorption;Hepatitis C;Phagosome;Vitamin digestion and absorption	Homo sapiens	+
cell part;cytoplasm;intracellular non-membrane-bounded organelle;intracellular organelle;intracellular organelle part;intracellular part;non-membrane-bounded organelle;nuclear part;nucleolus;organelle;organelle part		Homo sapiens	+
cell part;intracellular membrane-bounded organelle;intracellular organelle;intracellular part;membrane-bounded organelle;nucleus;organelle		Homo sapiens	+
cell junction;cell part;cytoplasm;extracellular region part;intercellular bridge;intracellular membrane-bounded organelle;intracellular organelle;intracellular part;membrane-bounded organelle;nucleus;organelle		Homo sapiens	+
cell part;chromatin remodeling complex;cytoplasm;histone deacetylase complex;intracellular membrane-bounded organelle;intracellular organelle;intracellular organelle part;intracellular part;macromolecular complex;membrane-bounded organelle;nuclear part;nucleoplasm;nucleoplasm part;nucleus;organelle;organelle part;protein complex		Homo sapiens	
cell part;clathrin coated vesicle membrane;coated pit;coated vesicle membrane;cytoplasm;cytoplasmic part;cytoplasmic vesicle membrane;cytoplasmic vesicle part;endoplasmic reticulum;extracellular region;intracellular membrane-bounded organelle;intracellular organelle;intracellular organelle part;intracellular part;membrane;membrane part;membrane-bounded organ		Homo sapiens	+
cell part;chromosomal part;condensed chromosome kinetochore;cytoplasmic part;cytosol;envelope;intracellular non-membrane-bounded organelle;intracellular organelle;intracellular organelle part;intracellular part;kinetocho	RNA transport	Homo sapiens	+
adherens junction;anchoring junction;cell junction;cell part;cell-substrate adherens junction;cell-substrate junction;cytoplasm;cytoplasmic membrane-bounded vesicle;cytoplasmic part;cytoplasmic vesicle;cytoskeletal part;cy	Endocytosis	Homo sapiens	+
actin filament bundle;actomyosin;adherens junction;anchoring junction;cell cortex;cell junction;cell part;cell-substrate adherens junction;cell-substrate junction;cytoplasmic part;cytoskeletal part;cytosol;focal adhesion;intracellular organelle part;intracellular part;organelle part;stress fiber		Homo sapiens	+
cell part;cell projection;cytoplasmic part;cytoskeleton;cytosol;extracellular membrane-bounded organelle;extracellular organelle;extracellular region part;extracellular vesicular exosome;intracellular non-membrane-bounded c	Regulation of actin cytoskeleton	Homo sapiens	+
extracellular region		Homo sapiens	
cell part;cytoplasmic part;cytoplasmic vesicle membrane;cytoplasmic vesicle part;intracellular non-membrane-bounded organelle;intracellular organelle;intracellular organelle part;intracellular part;membrane;non-membrane-bounded organelle;nuclear part;nucleolus;nucleoplasm;organelle;organelle membrane;organelle part;plasma membrane;vesicle membrane		Homo sapiens	+
cell part;cytoplasmic part;endosomal part;endosome membrane;extracellular membrane-bounded organelle;extracellular organelle;extracellular region part;extracellular vesicular exosome;extrinsic to membrane;intracellular membrane-bounded organelle;intracellular organelle;intracellular organelle part;intracellular part;late endosome membrane;membrane;membrane pa		Homo sapiens	+
Cdc73/Paf1 complex;cell part;intracellular non-membrane-bounded organelle;intracellular organelle;intracellular organelle part;intracellular part;macromolecular complex;non-membrane-bounded organelle;nuclear part;nucleolus;nucleoplasm;nucleoplasm part;organelle;organelle part;protein complex;transcription elongation factor complex		Homo sapiens	
cell part;chromosomal part;condensed chromosome kinetochore;cytoplasm;cytoplasmic part;cytoskeletal part;cytoskeleton;extracellular membrane-bounded organelle;extracellular organelle;extracellular region part;extracellular vesicular exosome;intracellular;intracellular non-membrane-bounded organelle;intracellular organelle;intracellular organelle part;intracellular pa		Homo sapiens	
cell part;cis-Golgi network;cytoplasmic membrane-bounded vesicle;cytoplasmic part;cytoplasmic vesicle;endoplasmic reticulum membrane;endoplasmic reticulum part;Golgi apparatus part;Golgi cisterna membrane;Golgi membrane;Golgi transport complex;Golgi-associated vesicle;intracellular membrane-bounded organelle;intracellular organelle;intracellular organelle		Homo sapiens	+
cell part;intracellular membrane-bounded organelle;intracellular non-membrane-bounded organelle;intracellular organelle;intracellular organelle part;intracellular part;membrane-bounded organelle;non-membrane-bounded organelle;nuclear part;nucleolus;nucleus;organelle;organelle part		Homo sapiens	+
cell part;intracellular membrane-bounded organelle;intracellular organelle;intracellular organelle part;intracellular part;membrane-bounded organelle;nuclear part;nucleoplasm;nucleus;organelle;organelle part		Homo sapiens	+
adherens junction;anchoring junction;cell junction;cell part;cell-substrate adherens junction;cell-substrate junction;cytoplasm;focal adhesion;intracellular membrane-bounded organelle;intracellular organelle;intracellular part;r	Adherens junction	Homo sapiens	+
cell part;cytoplasmic part;extracellular membrane-bounded organelle;extracellular organelle;extracellular region part;extracellular vesicular exosome;integral to membrane;intracellular organelle part;intracellular part;intrinsic to membrane;membrane;membrane part;membrane-bounded organelle;membrane-bounded vesicle;mitochondrial membrane;mitochondrial outer m		Homo sapiens	+
cell part;centrosome;cytoplasm;cytoplasmic microtubule;cytoplasmic part;cytoskeletal part;cytoskeleton;intracellular non-membrane-bounded organelle;intracellular organelle;intracellular organelle part;intracellular part;macromolecular complex;microtubule;microtubule cytoskeleton;microtubule organizing center;non-membrane-bounded organelle;organelle;organelle pa		Homo sapiens	+
cell part;cytoplasm;cytoplasmic part;intracellular non-membrane-bounded organelle;intracellular organelle;intracellular organelle part;intracellular part;macromolecular complex;membrane;non-membrane-bounded organelle;nuclear body;nuclear part;nuclear speck;nucleoplasm part;organelle;organelle part;ribonucleoprotein complex;RNA granule;stress granule		Homo sapiens	+
Cajal body;cell part;histone methyltransferase complex;intracellular membrane-bounded organelle;intracellular organelle;intracellular organelle part;intracellular part;macromolecular complex;membrane-bounded organelle;me	Spliceosome	Homo sapiens	
cell part;cytoplasm;intracellular organelle part;intracellular part;macromolecular complex;NELF complex;nuclear part;nucleoplasm;nucleoplasm part;organelle part;protein complex;transcription elongation factor complex		Homo sapiens	+
actin cytoskeleton;actin filament;adherens junction;anchoring junction;cell junction;cell part;cell projection;cell-substrate adherens junction;cell-substrate junction;contractile fiber part;cytoplasm;cytoplasmic part;cytoskeletal part;cytoskeleton;focal adhesion;intracellular membrane-bounded organelle;intracellular non-membrane-bounded organelle;intracellular organelle;i		Homo sapiens	+
AP-type membrane coat adaptor complex;cell junction;cell part;cell projection;clathrin adaptor complex;clathrin-coated vesicle;coated vesicle;cytoplasm;cytoplasmic membrane-bounded vesicle;cytoplasmic part;cytoplasmic vesicle;intracellular membrane-bounded organelle;intracellular non-membrane-bounded organelle;intracellular organelle;intracellular organelle pa		Homo sapiens	+
cell part;cytoplasm;intracellular non-membrane-bounded organelle;intracellular organelle;intracellular organelle part;intracellular part;non-membrane-bounded organelle;nuclear body;nuclear matrix;nuclear part;nuclear speck;nucleolus;nucleoplasm;nucleoplasm part;organelle;organelle part;paraspeckles		Homo sapiens	+
adherens junction;aggresome;anchoring junction;cell junction;cell part;cell projection membrane;cell projection part;cell-substrate adherens junction;cell-substrate junction;contractile fiber part;cytoplasm;cytoplasmic part;cytoskeleton;endoplasmic reticulum;endoplasmic reticulum membrane;endoplasmic reticulum part;envelope;extracellular membrane-bounded organe		Homo sapiens	
cell part;chromatin;chromosomal part;intracellular organelle part;intracellular part;macromolecular complex;nuclear body;nuclear chromatin;nuclear chromosome part;nuclear part;nuclear speck;nucleoplasm;nucleoplasm part;organelle part;protein complex		Homo sapiens	+
cell part;intracellular membrane-bounded organelle;intracellular organelle;intracellular part;membrane-bounded organelle;nucleus;organelle		Homo sapiens	
cell part;cytoplasm;intracellular membrane-bounded organelle;intracellular organelle;intracellular organelle part;intracellular part;macromolecular complex;membrane;membrane-bounded organelle;nuclear part;nucleoplasm;n	Spliceosome	Homo sapiens	
cell part;chromosomal part;condensed chromosome kinetochore;cytoplasmic part;cytosol;extracellular membrane-bounded organelle;extracellular organelle;extracellular region part;extracellular vesicular exosome;intracellular membrane-bounded organelle;intracellular non-membrane-bounded organelle;intracellular organelle;intracellular organelle part;intracellular part;k		Homo sapiens	+
		Homo sapiens	+
cell part;cleavage body;cytoplasm;cytoplasmic part;intracellular membrane-bounded organelle;intracellular non-membrane-bounded organelle;intracellular organelle;intracellular organelle part;intracellular part;macromolecular complex;membrane;membrane-bounded organelle;non-membrane-bounded organelle;nuclear body;nuclear part;nucleolus;nucleoplasm;nucleop		Homo sapiens	+
cell part;cytoplasmic part;endoplasmic reticulum;endoplasmic reticulum membrane;endoplasmic reticulum part;Golgi apparatus;integral to membrane;intracellular membrane-bounded organelle;intracellular organelle;intracellular organelle part;intracellular part;intrinsic to membrane;membrane;membrane part;membrane-bounded organelle;nuclear part;nucleoplasm;orga		Homo sapiens	
cell part;cytoplasmic membrane-bounded vesicle;cytoplasmic part;cytoplasmic vesicle;extracellular membrane-bounded organelle;extracellular organelle;extracellular region;extracellular region part;extracellular vesicular exosome;Golgi apparatus;intracellular membrane-bounded organelle;intracellular organelle;intracellular part;membrane-bounded organelle;membrane-		Homo sapiens	+
cell part;chromosomal part;intracellular membrane-bounded organelle;intracellular non-membrane-bounded organelle;intracellular organelle;intracellular organelle part;intracellular part;macromolecular complex;membrane-bounded organelle;non-membrane-bounded organelle;nuclear part;nucleolus;nucleosome;nucleus;organelle;organelle part;protein-DNA complex		Homo sapiens	+
cell part;cytoplasm;cytoplasmic part;cytosol;endoplasmic reticulum;intracellular membrane-bounded organelle;intracellular organelle;intracellular organelle part;intracellular part;macromolecular complex;membrane;membran	Proteasome	Homo sapiens	+
cell leading edge;cell part;cis-Golgi network;cytoplasmic part;endoplasmic reticulum lumen;endoplasmic reticulum part;endoplasmic reticulum-Golgi intermediate compartment;Golgi apparatus;Golgi apparatus part;Golgi membrane;Golgi stack;intracellular membrane-bounded organelle;intracellular organelle;intracellular organelle lumen;intracellular organelle part;intrace		Homo sapiens	
Cdc73/Paf1 complex;cell part;intracellular non-membrane-bounded organelle;intracellular organelle;intracellular organelle part;intracellular part;macromolecular complex;non-membrane-bounded organelle;nuclear part;nucleolus;nucleoplasm part;organelle;organelle part;protein complex;transcription elongation factor complex		Homo sapiens	+
adherens junction;anchoring junction;cell junction;cell part;cell-substrate adherens junction;cell-substrate junction;cytoplasmic membrane-bounded vesicle;cytoplasmic part;cytoplasmic vesicle;endoplasmic reticulum;extrac	Alzheimer's disease;Notch signaling pathway	Homo sapiens	+
cell part;integral to membrane;intrinsic to membrane;membrane part		Homo sapiens	
cell part;cytoplasmic part;intracellular non-membrane-bounded organelle;intracellular organelle;intracellular organelle part;intracellular part;macromolecular complex;membrane;mitochondrial inner membrane;mitochondrial membrane;mitochondrial part;non-membrane-bounded organelle;organelle;organelle inner membrane;organelle membrane;organelle part;ribonucleo		Homo sapiens	
cell part;cytoplasm;cytoplasmic part;endoplasmic reticulum;endoplasmic reticulum membrane;endoplasmic reticulum part;envelope;intracellular membrane-bounded organelle;intracellular organelle;intracellular organelle part;intracellular part;membrane;membrane part;membrane-bounded organelle;nuclear envelope;nuclear part;organelle;organelle envelope;organelle m		Homo sapiens	+
cell part;intracellular membrane-bounded organelle;intracellular organelle;intracellular part;membrane-bounded organelle;nucleus;organelle		Homo sapiens	+
adherens junction;anchoring junction;cell junction;cell part;cell-cell adherens junction;cell-cell junction;centrosome;cytoplasm;cytoplasmic part;cytoskeletal part;cytoskeleton;cytosol;endosomal part;endosome membrane;extracellular membrane-bounded organelle;extracellular organelle;extracellular region part;extracellular vesicular exosome;intracellular membrane-bou		Homo sapiens	+
cell part;cytoplasm;cytoplasmic membrane-bounded vesicle lumen;cytoplasmic part;cytoplasmic vesicle part;cytoskeletal part;cytosol;endocytic vesicle lumen;extracellular membrane-bounded organelle;extracellular organel	Protein processing in endoplasmic reticulum	Homo sapiens	+
cell part;cytoplasm;cytoskeleton;intracellular non-membrane-bounded organelle;intracellular organelle;intracellular part;non-membrane-bounded organelle;organelle		Homo sapiens	+
CCR4-NOT complex;cell part;cytoplasmic mRNA processing body;cytoplasmic part;cytosol;intracellular membrane-bounded organelle;intracellular non-membrane-bounded organelle;intracellular organelle;intracellular organe	RNA degradation	Homo sapiens	+
autophagic vacuole;cell part;cytoplasmic part;cytoplasmic vesicle;endosomal part;endosome membrane;intracellular membrane-bounded organelle;intracellular organelle;intracellular organelle part;intracellular part;macromolecular complex;membrane;membrane part;membrane-bounded organelle;organelle;organelle membrane;organelle part;protein complex;retromer c		Homo sapiens	+
actomyosin;cell part;cytoplasmic part;cytoskeletal part;endoplasmic reticulum-Golgi intermediate compartment;Golgi apparatus part;Golgi membrane;intracellular membrane-bounded organelle;intracellular organelle;intracellular organelle part;intracellular part;macromolecular complex;membrane;membrane-bounded organelle;myosin complex;organelle;organelle memb		Homo sapiens	
cell part;cytoplasm;cytoplasmic part;cytosol;intracellular non-membrane-bounded organelle;intracellular organelle;intracellular organelle part;intracellular part;macromolecular complex;membrane;non-membrane-bounded organelle;nuclear part;nucleolus;organelle;organelle part;polysomal ribosome;ribonucleoprotein complex;ribosome;RNA granule;stress granule		Homo sapiens	+
cell part;cytoplasm;cytoplasmic part;intracellular non-membrane-bounded organelle;intracellular organelle;intracellular part;macromolecular complex;membrane;non-membrane-bounded organelle;organelle;ribonucleoprotein complex;ribosome		Homo sapiens	+
catalytic step 2 spliceosome;cell part;cytoplasm;intracellular membrane-bounded organelle;intracellular organelle;intracellular organelle part;intracellular part;macromolecular complex;membrane;membrane-bounded organell	Spliceosome	Homo sapiens	+
cell part;cytoplasm;envelope;intracellular organelle part;intracellular part;macromolecular complex;membrane;membrane part;nuclear envelope;nuclear membrane;nuclear part;nuclear periphery;nuclear pore;nucleoplasm;org	RNA transport	Homo sapiens	+
cell part;cytoplasmic part;endoplasmic reticulum;extracellular matrix;extracellular membrane-bounded organelle;extracellular organelle;extracellular region part;extracellular space;extracellular vesicular exosome;intracellular membrane-bounded organelle;intracellular organelle;intracellular part;membrane-bounded organelle;membrane-bounded vesicle;organelle;proteina		Homo sapiens	+

cell part;cell projection;cell projection part;cell surface;cytoplasm;dendritic shaft;dendritic spine;integral to membrane;integral to plasma membrane;intracellular part;intrinsic to membrane;intrinsic to plasma membrane;memb	Gap junction;Neuroactive ligand-receptor interaction	Homo sapiens	+
cell part;cytoplasm;extracellular membrane-bounded organelle;extracellular organelle;extracellular region part;extracellular vesicular exosome;intracellular membrane-bounded organelle;intracellular organelle;intracellular part;membrane-bounded organelle;membrane-bounded vesicle;nucleus;organelle;vesicle		Homo sapiens	+
adherens junction;anchoring junction;cell junction;cell part;cell surface;cell-cell adherens junction;cell-cell junction;cell-substrate adherens junction;cell-substrate junction;extracellular membrane-bounded organelle;extracellu	Adherens junction;Cell adhesion molecules (CAMs)	Homo sapiens	+
cell part;cytoplasm;extracellular membrane-bounded organelle;extracellular organelle;extracellular region part;extracellular vesicular exosome;intracellular part;membrane-bounded organelle;membrane-bounded vesicle;orga	Pathways in cancer;Thyroid cancer	Homo sapiens	+
cell part;cytoplasmic part;cytosol;extracellular matrix;extracellular membrane-bounded organelle;extracellular organelle;extracellular region part;extracellular space;extracellular vesicular exosome;intracellular part;membrane-bounded organelle;membrane-bounded vesicle;organelle;vesicle		Homo sapiens	+
actin cytoskeleton;Arp2/3 protein complex;cell part;cytoplasmic part;cytoskeletal part;cytoskeleton;cytosol;extracellular membrane-bounded organelle;extracellular organelle;extracellular region part;extracellular vesicular exo	Bacterial invasion of epithelial cells;Fc gamma R-mediated phagocytosis;Pathogenic Escherichia coli infection;Regulation of actin cytoskeleton;Sh	Homo sapiens	
cell part;chromatin;chromatin remodeling complex;chromosomal part;cytoplasm;ESC/E(Z) complex;heterochromatin;histone deacetylase complex;histone methyltransferase complex;intracellular membrane-bounded organell	Cell cycle;Chronic myeloid leukemia;Huntington's disease;Notch signaling pathway;Pathways in cancer	Homo sapiens	+
cell part;centrosome;chromatin;chromosomal part;cytoplasm;cytoplasmic part;cytoskeletal part;intracellular membrane-bounded organelle;intracellular non-membrane-bounded organelle;intracellular organelle;intracellular organelle part;intracellular part;membrane-bounded organelle;microtubule organizing center;non-membrane-bounded organelle;nuclear chromatin;nu		Homo sapiens	+
cell part;chromatin;chromosomal part;condensed chromosome outer kinetochore;cytoplasm;histone acetyltransferase complex;intracellular membrane-bounded organelle;intracellular organelle;intracellular organelle part;intra	Adherens junction;Cell cycle;Huntington's disease;Jak-STAT signaling pathway;ko05152;Long-term potentiation;Melanogenesis;Notch signaling p	Homo sapiens	+
cell junction;cell part;cell-cell junction;cytoplasm;cytoskeleton;intracellular non-membrane-bounded organelle;intracellular organelle;intracellular organelle part;intracellular part;membrane;non-membrane-bounded organelle;n	mRNA surveillance pathway;Tight junction	Homo sapiens	+
cell part;cytoplasm;intracellular membrane-bounded organelle;intracellular organelle;intracellular organelle part;intracellular part;membrane-bounded organelle;nuclear part;nucleoplasm;nucleus;organelle;organelle part	Basal transcription factors	Homo sapiens	+
cell part;integral to membrane;integral to plasma membrane;intrinsic to membrane;intrinsic to plasma membrane;membrane part;plasma membrane part		Homo sapiens	+
cell part;cytoplasmic membrane-bounded vesicle;cytoplasmic part;cytoplasmic vesicle;cytosol;extracellular membrane-bounded organelle;extracellular organelle;extracellular region part;extracellular space;extracellular vesic	Folate biosynthesis	Homo sapiens	+
cell part;cytoplasm;intracellular membrane-bounded organelle;intracellular non-membrane-bounded organelle;intracellular organelle;intracellular organelle part;intracellular part;membrane;membrane-bounded organelle;non-membrane-bounded organelle;nuclear part;nucleolus;nucleoplasm;nucleus;organelle;organelle part		Homo sapiens	+
cell part;chromosomal part;chromosome, telomeric region;intracellular membrane-bounded organelle;intracellular organelle;intracellular organelle part;intracellular part;macromolecular complex;membrane;membrane-bounde	Homologous recombination;Non-homologous end-joining	Homo sapiens	
cell part;cytoplasm;intracellular membrane-bounded organelle;intracellular organelle;intracellular organelle part;intracellular part;macromolecular complex;membrane;membrane-bounded organelle;nuclear part;nucleoplasm;nucleus;organelle;organelle part;ribonucleoprotein complex		Homo sapiens	+
cell part;cytoplasm;extracellular membrane-bounded organelle;extracellular organelle;extracellular region part;extracellular vesicular exosome;intracellular;intracellular part;membrane-bounded organelle;membrane-bounded vesicle;organelle;vesicle		Homo sapiens	+
cell part;cytoplasm;cytoplasmic part;cytosol;intracellular part;membrane;plasma membrane	ko05152;Regulation of actin cytoskeleton;Vascular smooth muscle contraction	Homo sapiens	+
cell part;cytoplasmic part;cytosol;intracellular membrane-bounded organelle;intracellular organelle;intracellular organelle part;intracellular part;membrane-bounded organelle;nuclear part;nucleoplasm;nucleus;organelle;organe	Protein processing in endoplasmic reticulum	Homo sapiens	
cell part;cytoplasmic part;extracellular matrix;extracellular membrane-bounded organelle;extracellular organelle;extracellular region part;extracellular vesicular exosome;Golgi apparatus;Golgi apparatus part;Golgi membrane;	Cell adhesion molecules (CAMs)	Homo sapiens	
cell part;chromatin;chromosomal part;cytoplasm;cytoplasmic mRNA processing body;cytoplasmic part;cytosol;exon-exon junction complex;intracellular membrane-bounded organelle;intracellular non-membrane-bounded o	mRNA surveillance pathway;RNA transport	Homo sapiens	
cell junction;cell part;clathrin-coated vesicle;coated vesicle;cytoplasm;cytoplasmic membrane-bounded vesicle;cytoplasmic part;cytoplasmic vesicle;eukaryotic translation initiation factor 3 complex;intracellular membrane-bounded organelle;intracellular organelle;intracellular organelle part;intracellular part;macromolecular complex;membrane-bounded organelle;membr		Homo sapiens	+
cell part;intracellular organelle part;intracellular part;nuclear part;nucleoplasm;organelle part		Homo sapiens	+
cell part;chromatin;chromatin remodeling complex;chromosomal part;chromosome;intracellular non-membrane-bounded organelle;intracellular organelle;intracellular organelle part;intracellular part;macromolecular complex;nBAF complex;non-membrane-bounded organelle;npBAF complex;nuclear chromatin;nuclear chromosome part;nuclear part;nucleoplasm;organelle		Homo sapiens	+
cell part;chromatin;chromatin remodeling complex;chromosomal part;intracellular membrane-bounded organelle;intracellular organelle;intracellular organelle part;intracellular part;macromolecular complex;membrane-bounded organelle;nuclear chromatin;nuclear chromosome part;nuclear part;nucleus;organelle;organelle part;protein complex;SWI/SNF complex;SWI/SNF		Homo sapiens	
cell part;cell pole;cell tip;clathrin-coated vesicle;coated vesicle;cytoplasmic membrane-bounded vesicle;cytoplasmic part;cytoplasmic vesicle;cytoplasmic vesicle membrane;cytoplasmic vesicle part;endocytic vesicle;endocytic vesicle membrane;endosome;extracellular membrane-bounded organelle;extracellular organelle;extracellular region part;extracellular vesicular e		Homo sapiens	+
cell part;cytoplasmic part;cytosol;intracellular non-membrane-bounded organelle;intracellular organelle;intracellular organelle part;intracellular part;macromolecular complex;membrane;non-membrane-bounded organelle;nuclear part;nucleoplasm;organelle;organelle part;ribonucleoprotein complex;RNA granule;stress granule		Homo sapiens	+
cell part;cell projection;cilium;cytoplasm;cytoplasmic part;cytosol;extracellular membrane-bounded organelle;extracellular organelle;extracellular region part;extracellular vesicular exosome;intracellular membrane-bounded organelle;intracellular organelle;intracellular part;membrane-bounded organelle;membrane-bounded vesicle;nucleus;organelle;primary cilium;vesicle		Homo sapiens	+
adherens junction;anchoring junction;cell body;cell junction;cell part;cell projection membrane;cell projection part;cell-cell junction;cell-substrate adherens junction;cell-substrate junction;cytoplasm;cytoplasmic membrane-bo	Pathogenic Escherichia coli infection	Homo sapiens	+
cell part;Cul2-RING ubiquitin ligase complex;Cul3-RING ubiquitin ligase complex;CUL4 RING ubiquitin ligase complex;Cul4A-RING ubiquitin ligase complex;cullin-RING ubiquitin ligase complex;intracellular;intracellular part;macromolecular complex;protein complex;ubiquitin ligase complex		Homo sapiens	+
apical part of cell;cell part;cell projection part;cytoplasm;cytoplasmic part;cytosol;growth cone;intracellular part;membrane;site of polarized growth		Homo sapiens	
cell part;cytoplasmic part;cytosol;intracellular membrane-bounded organelle;intracellular organelle;intracellular organelle part;intracellular part;membrane-bounded organelle;nuclear body;nuclear part;nucleoplasm part;nucleus;organelle;organelle part;PML body		Homo sapiens	
cell part;Cul5-RING ubiquitin ligase complex;cullin-RING ubiquitin ligase complex;cytoplasmic part;cytosol;intracellular membrane-bounded organelle;intracellular organelle;intracellular part;macromolecular complex;membra	Ubiquitin mediated proteolysis	Homo sapiens	+
cell part;cytoplasm;cytoplasmic membrane-bounded vesicle;cytoplasmic part;cytoplasmic vesicle;cytoplasmic vesicle membrane;cytoplasmic vesicle part;endocytic vesicle membrane;endosomal part;endosome membrane;	Collecting duct acid secretion;Epithelial cell signaling in Helicobacter pylori infection;ko05152;Lysosome;Oxidative phosphorylation;Phagosome;R	Homo sapiens	+
adherens junction;anchoring junction;cell junction;cell part;cell-substrate adherens junction;cell-substrate junction;cytoplasm;focal adhesion;intracellular membrane-bounded organelle;intracellular organelle;intracellular part;membrane;membrane-bounded organelle;nucleus;organelle;plasma membrane		Homo sapiens	
cell part;cytoplasm;intracellular organelle part;intracellular part;nuclear part;nucleoplasm;organelle part		Homo sapiens	+
cell part;chromatin;chromatin remodeling complex;chromosomal part;chromosome;intracellular membrane-bounded organelle;intracellular non-membrane-bounded organelle;intracellular organelle;intracellular organelle part;intracellular part;macromolecular complex;membrane-bounded organelle;nBAF complex;non-membrane-bounded organelle;npBAF complex;nucle		Homo sapiens	+
caveola;cell part;cytoplasm;intracellular part;macromolecular complex;membrane part;membrane raft;plasma membrane part;protein complex		Homo sapiens	+
cell part;cytoplasmic part;endoplasmic reticulum lumen;endoplasmic reticulum part;endoplasmic reticulum-Golgi intermediate compartment;extracellular membrane-bounded organelle;extracellular organelle;extracellular region part;extracellular space;extracellular vesicular exosome;intracellular membrane-bounded organelle;intracellular organelle;intracellular organelle l		Homo sapiens	+
cell part;cytoplasmic membrane-bounded vesicle;cytoplasmic part;cytoplasmic vesicle;endoplasmic reticulum membrane;endoplasmic reticulum part;GPI-anchor transamidase complex;integral to endoplasmic reticulum mer	Glycosylphosphatidylinositol(GPI)-anchor biosynthesis	Homo sapiens	
axon;cell part;cell projection;extracellular membrane-bounded organelle;extracellular organelle;extracellular region part;extracellular vesicular exosome;integral to membrane;intrinsic to membrane;membrane;membrane part;membrane-bounded organelle;membrane-bounded vesicle;neuron projection;organelle;plasma membrane;synapse;vesicle		Homo sapiens	+
cell part;cytosolic large ribosomal subunit;intracellular organelle part;intracellular part;large ribosomal subunit;macromolecular complex;organelle part;ribonucleoprotein complex	Ribosome	Homo sapiens	
cell part;cytoplasm;cytoplasmic part;Golgi apparatus;intracellular membrane-bounded organelle;intracellular non-membrane-bounded organelle;intracellular organelle;intracellular organelle part;intracellular part;membrane-bounded organelle;non-membrane-bounded organelle;nuclear part;nucleolus;organelle;organelle part		Homo sapiens	+
cell part;cytoplasmic part;intracellular organelle lumen;intracellular organelle part;intracellular part;membrane-enclosed lumen;mitochondrial matrix;mitochondrial part;organelle lumen;organelle part		Homo sapiens	+
cell part;intracellular membrane-bounded organelle;intracellular organelle;intracellular part;membrane-bounded organelle;nucleus;organelle		Homo sapiens	+
cell part;cytoplasmic part;endoplasmic reticulum membrane;endoplasmic reticulum part;integral to membrane;intracellular organelle part;intracellular part;intrinsic to membrane;membrane;membrane part;organelle membrane;organelle part		Homo sapiens	+
cell part;cytoplasmic part;endoplasmic reticulum membrane;endoplasmic reticulum part;endoplasmic reticulum-Golgi intermediate compartment;endoplasmic reticulum-Golgi intermediate compartment membrane;Golgi apparatus part;Golgi membrane;integral to membrane;intracellular membrane-bounded organelle;intracellular organelle;intracellular organelle part;intra		Homo sapiens	
cell part;cytoplasm;cytoplasmic part;intracellular membrane-bounded organelle;intracellular organelle;intracellular part;membrane-bounded organelle;mitochondrion;organelle		Homo sapiens	
cell part;cytoplasm;cytoplasmic part;extracellular membrane-bounded organelle;extracellular organelle;extracellular region part;extracellular vesicular exosome;integral to membrane;intracellular membrane-bounded organelle;intracellular organelle;intracellular part;intrinsic to membrane;membrane part;membrane-bounded organelle;membrane-bounded vesicle;mitochor		Homo sapiens	+
cell part;cytoplasmic part;endoplasmic reticulum;integral to membrane;intracellular membrane-bounded organelle;intracellular organelle;intracellular part;intrinsic to membrane;membrane;membrane part;membrane-bounded organelle;organelle		Homo sapiens	+
cell part;cytoplasm;cytoplasmic part;extracellular membrane-bounded organelle;extracellular organelle;extracellular region part;extracellular vesicular exosome;Golgi apparatus;intracellular membrane-bounded organelle;intracellular organelle;intracellular organelle part;intracellular part;membrane-bounded organelle;membrane-bounded vesicle;nuclear part;nucleoplasm;		Homo sapiens	+
cell cortex part;cell part;cell projection part;cytoplasm;cytoplasmic part;cytosol;exocyst;growth cone membrane;intracellular part;macromolecular complex;membrane;membrane part;myelin sheath abaxonal region;plasma m	Tight junction	Homo sapiens	+
cell part;cytoplasm;cytoplasmic part;intracellular membrane-bounded organelle;intracellular organelle;intracellular part;membrane-bounded organelle;mitochondrion;nucleus;organelle		Homo sapiens	+
actin filament bundle;actomyosin;adherens junction;anchoring junction;cell cortex;cell junction;cell part;cell projection membrane;cell projection part;cell surface;cell-substrate adherens junction;cell-substrate junction;contractile fiber part;cytoplasm;cytoplasmic part;cytoskeletal part;cytosol;extrinsic to internal side of plasma membrane;extrinsic to membrane;extrinsic to		Homo sapiens	+
cell part;intracellular membrane-bounded organelle;intracellular organelle;intracellular organelle part;intracellular part;membrane-bounded organelle;nuclear part;nucleoplasm;nucleus;organelle;organelle part		Homo sapiens	+
cell part;cytoplasmic part;endoplasmic reticulum;endoplasmic reticulum membrane;endoplasmic reticulum part;envelope;integral to membrane;intracellular membrane-bounded organelle;intracellular non-membrane-bounded organelle;intracellular organelle;intracellular organelle part;intracellular part;intrinsic to membrane;membrane;membrane part;membrane-bounded		Homo sapiens	+
cell part;cytoplasmic part;cytosol;extracellular membrane-bounded organelle;extracellular organelle;extracellular region part;extracellular vesicular exosome;intracellular part;membrane-bounded organelle;membrane-bounde	Carbon fixation in photosynthetic organisms;Pentose and glucuronate interconversions;Pentose phosphate pathway	Homo sapiens	
cell part;cytoplasmic part;early endosome;endosomal part;endosome;endosome membrane;intracellular membrane-bounded organelle;intracellular organelle;intracellular organelle part;intracellular part;late endosome;late endosome membrane;lysosomal membrane;membrane;membrane-bounded organelle;organelle;organelle membrane;organelle part;perinuclear regio		Homo sapiens	
cell part;cytoplasmic part;endoplasmic reticulum lumen;endoplasmic reticulum membrane;endoplasmic reticulum part;intracellular organelle lumen;intracellular organelle part;intracellular part;membrane;membrane part;membrane-enclosed lumen;organelle lumen;organelle membrane;organelle part		Homo sapiens	+
cell part;cytoplasm;cytoplasmic part;cytosol;exosome (RNase complex);intracellular membrane-bounded organelle;intracellular non-membrane-bounded organelle;intracellular organelle;intracellular organelle part;intracellular	RNA degradation	Homo sapiens	
cell part;cytoplasmic part;intracellular membrane-bounded organelle;intracellular organelle;intracellular organelle part;intracellular part;macromolecular complex;membrane part;membrane-bounded organelle;mitochondrial membrane part;mitochondrial outer membrane translocase complex;mitochondrial part;mitochondrion;organelle;organelle part;protein complex		Homo sapiens	
adherens junction;anchoring junction;cell junction;cell part;cell projection;cell-cell junction;cell-substrate adherens junction;cell-substrate junction;cytoplasmic part;cytoplasmic vesicle membrane;cytoplasmic vesicle part;cyto	Endocytosis	Homo sapiens	+
cell part;cytoplasmic part;cytosol;intracellular membrane-bounded organelle;intracellular organelle;intracellular part;membrane-bounded organelle;nucleus;organelle		Homo sapiens	+
cell part;cytoplasm;intracellular part		Homo sapiens	+
cell part;cytoplasm;intracellular part;LUBAC complex;macromolecular complex;protein complex;ubiquitin ligase complex		Homo sapiens	+
cell part;cytoplasmic part;intracellular organelle part;intracellular part;membrane-enclosed lumen;mitochondrial intermembrane space;mitochondrial part;organelle envelope lumen;organelle part		Homo sapiens	
cell part;extracellular membrane-bounded organelle;extracellular organelle;extracellular region part;extracellular vesicular exosome;membrane;membrane-bounded organelle;membrane-bounded vesicle;organelle;vesicle		Homo sapiens	+
cell part;cytoplasm;cytoplasmic part;intracellular membrane-bounded organelle;intracellular organelle;intracellular part;membrane-bounded organelle;mitochondrion;organelle		Homo sapiens	+
cell part;membrane part;membrane raft		Homo sapiens	
cell part;cytoplasm;extracellular membrane-bounded organelle;extracellular organelle;extracellular region part;extracellular vesicular exosome;intracellular part;membrane-bounded organelle;membrane-bounded vesicle;orga	Glycolysis / Gluconeogenesis	Homo sapiens	+
cell part;cytoplasm;intracellular part	Arginine and proline metabolism	Homo sapiens	+
cell part;cytoplasm;cytoplasmic part;cytosol;intracellular membrane-bounded organelle;intracellular organelle;intracellular organelle part;intracellular part;membrane-bounded organelle;mitochondrion;nuclear part;nucleoplasr	RNA degradation	Homo sapiens	+
cell part;cytoplasm;intracellular part		Homo sapiens	+
cell part;cytoplasmic part;intracellular membrane-bounded organelle;intracellular organelle;intracellular part;membrane-bounded organelle;mitochondrion;organelle		Homo sapiens	
		Homo sapiens	
Cdc48p-Npl4p-Ufd1p AAA ATPase complex;cell part;cytoplasmic part;endoplasmic reticulum;endoplasmic reticulum part;intracellular membrane-bounded organelle;intracellular organelle;intracellular organelle part;intracellular part;lipid particle;macromolecular complex;membrane part;membrane-bounded organelle;organelle;organelle part;protein complex		Homo sapiens	+
cell part;cytoplasm;cytoplasmic part;cytoskeletal part;intracellular organelle part;intracellular part;membrane;microtubule organizing center;midbody;organelle part;plasma membrane		Homo sapiens	
autophagic vacuole;cell part;cytoplasm;cytoplasmic membrane-bounded vesicle;cytoplasmic part;cytoplasmic vesicle;cytosol;endosome;Golgi apparatus;Golgi apparatus part;Golgi membrane;intracellular membrane-bounded organelle;intracellular organelle;intracellular organelle part;intracellular part;membrane;membrane-bounded organelle;membrane-bounded vesic		Homo sapiens	+
AP-2 adaptor complex;AP-type membrane coat adaptor complex;cell part;clathrin adaptor complex;clathrin coated vesicle membrane;clathrin-coated endocytic vesicle membrane;coated vesicle membrane;cytoplasmic part	Endocrine and other factor-regulated calcium reabsorption;Endocytosis;Huntington's disease	Homo sapiens	+
cell part;centriole;centrosome;cytoplasm;cytoplasmic part;cytoskeletal part;cytosol;intracellular non-membrane-bounded organelle;intracellular organelle;intracellular organelle part;intracellular part;macromolecular complex;membrane;microtubule;microtubule organizing center;microtubule organizing center part;non-membrane-bounded organelle;organelle;organelle par		Homo sapiens	
cell part;cytoplasmic part;endoplasmic reticulum lumen;endoplasmic reticulum part;intracellular organelle lumen;intracellular organelle part;intracellular part;membrane-enclosed lumen;organelle lumen;organelle part		Homo sapiens	+
cell part;cytoplasm;intracellular membrane-bounded organelle;intracellular non-membrane-bounded organelle;intracellular organelle;intracellular organelle part;intracellular part;membrane;membrane-bounded organelle;non-r	Ribosome biogenesis in eukaryotes;RNA transport	Homo sapiens	+
cell part;centrosome;cytoplasmic part;cytoskeletal part;intracellular membrane-bounded organelle;intracellular non-membrane-bounded organelle;intracellular organelle;intracellular organelle part;intracellular part;macromolecular complex;membrane-bounded organelle;microtubule;microtubule organizing center;mitochondrion;non-membrane-bounded organelle;organell		Homo sapiens	+
cell part;cytoplasmic part;cytosol;extracellular membrane-bounded organelle;extracellular organelle;extracellular region part;extracellular vesicular exosome;intracellular part;membrane-bounded organelle;membrane-bounde	Chlorocyclohexane and chlorobenzene degradation;Fluorobenzoate degradation;Toluene degradation	Homo sapiens	+
catalytic step 2 spliceosome;cell part;cytoplasm;intracellular organelle part;intracellular part;macromolecular complex;nuclear part;nucleoplasm;organelle part;ribonucleoprotein complex;small nuclear ribonucleoprotein comp	Spliceosome	Homo sapiens	+
cell part;cytoplasmic part;intracellular non-membrane-bounded organelle;intracellular organelle;intracellular organelle part;intracellular part;macromolecular complex;membrane;mitochondrial inner membrane;mitochondrial membrane;mitochondrial part;non-membrane-bounded organelle;organelle;organelle inner membrane;organelle membrane;organelle part;ribonucleo		Homo sapiens	+
cell part;cytoplasmic part;intracellular organelle lumen;intracellular organelle part;intracellular part;membrane-enclosed lumen;mitochondrial matrix;mitochondrial part;organelle lumen;organelle part		Homo sapiens	+
cell part;chromosomal part;condensed chromosome outer kinetochore;cytoplasmic part;cytoskeletal part;cytosol;intracellular membrane-bounded organelle;intracellular organelle;intracellular organelle part;intracellular part;macromolecular complex;membrane-bounded organelle;microtubule organizing center;nucleus;organelle;organelle part;protein complex;spindle pole		Homo sapiens	+
cell part;cytoplasm;Elongator holoenzyme complex;histone acetyltransferase complex;intracellular organelle part;intracellular part;macromolecular complex;nuclear part;nucleoplasm;nucleoplasm part;organelle part;protein complex;transcription elongation factor complex		Homo sapiens	+
cell part;chromatin;chromatin remodeling complex;chromatin silencing complex;chromosomal part;cytoplasm;cytoplasmic part;envelope;euchromatin;heterochromatin;intracellular membrane-bounded organelle;intracellular non-membrane-bounded organelle;intracellular organelle;intracellular organelle part;intracellular part;macromolecular complex;membrane;membran		Homo sapiens	+
cell part;chromosomal part;condensed chromosome kinetochore;cytoplasmic part;cytosol;envelope;intracellular non-membrane-bounded organelle;intracellular organelle;intracellular organelle part;intracellular part;kinetocho	RNA transport	Homo sapiens	+
cell part;cytoplasmic part;cytoskeleton;intracellular membrane-bounded organelle;intracellular non-membrane-bounded organelle;intracellular organelle;intracellular part;membrane-bounded organelle;mitochondrion;non-membrane-bounded organelle;organelle		Homo sapiens	+
cell part;cytoplasmic part;intracellular organelle part;intracellular part;large ribosomal subunit;macromolecular complex;membrane;mitochondrial inner membrane;mitochondrial large ribosomal subunit;mitochondrial membrane;mitochondrial part;mitochondrial small ribosomal subunit;organellar large ribosomal subunit;organellar small ribosomal subunit;organelle inner m		Homo sapiens	
cell part;cytoplasmic part;intracellular non-membrane-bounded organelle;intracellular organelle;intracellular organelle part;intracellular part;macromolecular complex;membrane;mitochondrial inner membrane;mitochondrial membrane;mitochondrial part;non-membrane-bounded organelle;organelle;organelle inner membrane;organelle membrane;organelle part;ribonucleo		Homo sapiens	
cell part;cytoplasm;intracellular organelle part;intracellular part;nuclear part;nucleoplasm;organelle part	mRNA surveillance pathway	Homo sapiens	+
cell part;cytoplasmic part;integral to membrane;intracellular membrane-bounded organelle;intracellular organelle;intracellular organelle part;intracellular part;intrinsic to membrane;membrane part;membrane-bounded organelle;mitochondrion;nuclear part;nucleoplasm;organelle;organelle part		Homo sapiens	+
cell junction;cell part;cytoplasm;cytoplasmic part;cytosol;extrinsic to membrane;extrinsic to plasma membrane;intracellular membrane-bounded organelle;intracellular non-membrane-bounded organelle;intracellular organelle;intracellular organelle lumen;intracellular organelle part;intracellular part;membrane;membrane part;membrane-bounded organelle;membrane-encl		Homo sapiens	
cell part;cytoplasmic part;intracellular membrane-bounded organelle;intracellular organelle;intracellular organelle part;intracellular part;membrane;membrane-bounded organelle;mitochondrial inner membrane;mitochondrial membrane;mitochondrial part;mitochondrion;organelle;organelle inner membrane;organelle membrane;organelle part		Homo sapiens	
cell part;cytoplasmic mRNA processing body;cytoplasmic part;cytosol;intracellular non-membrane-bounded organelle;intracellular organelle;intracellular part;macromolecular complex;membrane;non-membrane-bounded org	RNA degradation	Homo sapiens	+
cell part;cytoplasm;cytoplasmic part;cytosol;extracellular membrane-bounded organelle;extracellular organelle;extracellular region part;extracellular vesicular exosome;intracellular non-membrane-bounded organelle;intracellular organelle;intracellular organelle part;intracellular part;membrane;membrane-bounded organelle;membrane-bounded vesicle;non-membrane-bo		Homo sapiens	+
cell part;cytoplasm;extracellular region part;intercellular bridge;intracellular membrane-bounded organelle;intracellular organelle;intracellular organelle part;intracellular part;macromolecular complex;membrane-bounded orga	RNA transport;Spliceosome	Homo sapiens	
cell part;cytoplasm;extracellular membrane-bounded organelle;extracellular organelle;extracellular region part;extracellular vesicular exosome;intracellular part;membrane-bounded organelle;membrane-bounded vesicle;organelle;vesicle		Homo sapiens	
cell part;cytoplasmic part;intracellular membrane-bounded organelle;intracellular organelle;intracellular part;macromolecular complex;membrane-bounded organelle;methyltransferase complex;nucleus;organelle;protein complex;tRNA (m1A) methyltransferase complex;tRNA methyltransferase complex		Homo sapiens	
cell part;cytoplasmic part;cytosol;extracellular membrane-bounded organelle;extracellular organelle;extracellular region part;extracellular vesicular exosome;intracellular part;membrane-bounded organelle;membrane-bounde	Amino sugar and nucleotide sugar metabolism;Galactose metabolism;Glycolysis / Gluconeogenesis;Pentose phosphate pathway;Purine metabolis	Homo sapiens	+
cell part;cytoplasmic part;endoplasmic reticulum;endoplasmic reticulum membrane;endoplasmic reticulum part;integral to membrane;intracellular membrane-bounded organelle;intracellular organelle;intracellular organelle part;intracellular part;intrinsic to membrane;membrane;membrane part;membrane-bounded organelle;nuclear membrane;nuclear part;organelle;organ		Homo sapiens	+
		Homo sapiens	+
cell part;cytoplasm;intracellular organelle part;intracellular part;nuclear part;nucleoplasm;organelle part		Homo sapiens	+
cell part;cytoplasm;cytoplasmic part;extracellular membrane-bounded organelle;extracellular organelle;extracellular region part;extracellular vesicular exosome;intracellular membrane-bounded organelle;intracellular organelle;intracellular part;membrane-bounded organelle;membrane-bounded vesicle;mitochondrion;organelle;vesicle		Homo sapiens	+
cell part;cytoplasmic part;intracellular non-membrane-bounded organelle;intracellular organelle;intracellular organelle part;intracellular part;macromolecular complex;membrane;mitochondrial inner membrane;mitochondrial membrane;mitochondrial part;mitochondrial ribosome;non-membrane-bounded organelle;organellar ribosome;organelle;organelle inner membrane;o		Homo sapiens	+
autophagic vacuole membrane;cell part;cytoplasmic part;endoplasmic reticulum;endoplasmic reticulum-Golgi intermediate compartment membrane;integral to membrane;intracellular membrane-bounded organelle;intracellular organelle;intracellular organelle part;intracellular part;intrinsic to membrane;membrane;membrane part;membrane-bounded organelle;organelle;		Homo sapiens	+
cell part;centrosome;chromocenter;chromosomal part;chromosome passenger complex;chromosome, centromeric region;condensed chromosome, centromeric region;condensed nuclear chromosome, centromeric region;cytoplasmic part;cytoskeletal part;cytosol;intracellular membrane-bounded organelle;intracellular non-membrane-bounded organelle;intracellular org		Homo sapiens	+
cell part;intracellular membrane-bounded organelle;intracellular non-membrane-bounded organelle;intracellular organelle;intracellular organelle part;intracellular part;membrane-bounded organelle;non-membrane-bounded organelle;nuclear body;nuclear part;nuclear speck;nucleolus;nucleoplasm;nucleoplasm part;nucleus;organelle;organelle part		Homo sapiens	+
cell part;cytoplasm;intracellular non-membrane-bounded organelle;intracellular organelle;intracellular organelle part;intracellular part;non-membrane-bounded organelle;nuclear part;nucleolus;organelle;organelle part		Homo sapiens	
		Homo sapiens	+
cell part;chromosomal part;cytoplasm;intracellular organelle part;intracellular part;nuclear chromosome part;nuclear part;nuclear replication fork;nucleoplasm;organelle part;replication fork		Homo sapiens	+
actin cytoskeleton;cell junction;cell part;cell projection;contractile fiber part;cytoplasmic part;cytoskeletal part;cytoskeleton;cytosol;intracellular non-membrane-bounded organelle;intracellular organelle;intracellular organelle part;intracellular part;membrane;neuron projection;non-membrane-bounded organelle;organelle;organelle part;postsynaptic density;postsynaptic m		Homo sapiens	+
cell part;cytoplasmic part;endoplasmic reticulum;endoplasmic reticulum lumen;endoplasmic reticulum membrane;endoplasmic reticulum part;intracellular membrane-bounded organelle;intracellular organelle;intracellular orga	Protein processing in endoplasmic reticulum;Vibrio cholerae infection	Homo sapiens	+
cell part;cytoplasmic part;integral to membrane;intracellular membrane-bounded organelle;intracellular organelle;intracellular organelle part;intracellular part;intrinsic to membrane;membrane;membrane part;membrane-bounded organelle;mitochondrial membrane;mitochondrial outer membrane;mitochondrial part;mitochondrion;organelle;organelle membrane;organelle o		Homo sapiens	+
cell part;cytoplasmic part;endoplasmic reticulum;endoplasmic reticulum membrane;endoplasmic reticulum part;intracellular membrane-bounded organelle;intracellular organelle;intracellular organelle part;intracellular part;membrane;membrane part;membrane-bounded organelle;organelle;organelle membrane;organelle part		Homo sapiens	
cell part;cytoplasm;intracellular membrane-bounded organelle;intracellular organelle;intracellular part;membrane;membrane-bounded organelle;nucleus;organelle		Homo sapiens	+
cell part;intracellular organelle part;intracellular part;macromolecular complex;nuclear part;nucleoplasm;organelle part;ribonucleoprotein complex;spliceosomal complex	Spliceosome	Homo sapiens	+
cell part;cytoplasmic part;intracellular membrane-bounded organelle;intracellular organelle;intracellular organelle lumen;intracellular organelle part;intracellular part;macromolecular complex;membrane;membrane-bounded or	Citrate cycle (TCA cycle);Propanoate metabolism	Homo sapiens	+
cell part;cytoplasm;extracellular membrane-bounded organelle;extracellular organelle;extracellular region part;extracellular vesicular exosome;intracellular part;membrane-bounded organelle;membrane-bounded vesicle;orga	Amino sugar and nucleotide sugar metabolism;Fructose and mannose metabolism	Homo sapiens	
cell part;cytoplasm;cytoplasmic part;cytosol;extracellular membrane-bounded organelle;extracellular organelle;extracellular region part;extracellular vesicular exosome;intracellular membrane-bounded organelle;intracellular non-membrane-bounded organelle;intracellular organelle;intracellular organelle part;intracellular part;membrane-bounded organelle;membrane-bou		Homo sapiens	
actin cytoskeleton;cell part;cytoplasm;cytoskeleton;intracellular membrane-bounded organelle;intracellular non-membrane-bounded organelle;intracellular organelle;intracellular part;membrane;membrane-bounded organelle;non-membrane-bounded organelle;nucleus;organelle;plasma membrane		Homo sapiens	
cell junction;cell part;cell projection membrane;cell projection part;cell-cell junction;cytoplasmic part;dendritic shaft;extracellular membrane-bounded organelle;extracellular organelle;extracellular region part;extracellular vesicular exosome;integral to membrane;intracellular part;intrinsic to membrane;membrane;membrane part;membrane raft;membrane-bounded organe		Homo sapiens	+
cell part;centrosome;cytoplasm;cytoplasmic part;cytoskeletal part;endomembrane system;intracellular membrane-bounded organelle;intracellular non-membrane-bounded organelle;intracellular organelle;intracellular organelle part;intracellular part;macromolecular complex;membrane;membrane-bounded organelle;microtubule organizing center;non-membrane-bounded		Homo sapiens	+
cell part;cytoplasm;cytoplasmic part;endoplasmic reticulum;Golgi apparatus part;Golgi stack;intracellular membrane-bounded organelle;intracellular organelle;intracellular organelle part;intracellular part;membrane-bounded organelle;organelle;organelle part		Homo sapiens	+
cell part;cytoplasm;cytoplasmic part;cytoskeleton;cytosol;intracellular non-membrane-bounded organelle;intracellular organelle;intracellular part;membrane;non-membrane-bounded organelle;organelle		Homo sapiens	+

cell part;cell surface;cytoplasmic part;endoplasmic reticulum;endoplasmic reticulum membrane;endoplasmic reticulum part;integral to membrane;intracellular membrane-bounded organelle;intracellular organelle;intracellular organelle part;intracellular part;intrinsic to membrane;membrane;membrane part;membrane-bounded organelle;organelle;organelle membrane;orga		Homo sapiens	
cell part;chromosomal part;chromosome;condensed chromosome;condensed chromosome kinetochore;cytoplasm;cytoskeletal part;intracellular membrane-bounded organelle;intracellular non-membrane-bounded organelle;intracellular organelle;intracellular organelle part;intracellular part;kinetochore;macromolecular complex;membrane-bounded organelle;non-membr		Homo sapiens	+
cell part;intracellular membrane-bounded organelle;intracellular organelle;intracellular part;membrane-bounded organelle;nucleus;organelle		Homo sapiens	
actin cytoskeleton;actin filament bundle;actomyosin;adherens junction;anchoring junction;cell cortex part;cell junction;cell part;cell-substrate adherens junction;cell-substrate junction;cortical actin cytoskeleton;cortical cytoskeleton;cytoplasmic part;cytoskeletal part;cytoskeleton;extracellular membrane-bounded organelle;extracellular organelle;extracellular region part;e		Homo sapiens	+
		Homo sapiens	+
cell division site part;cell part;centrosome;cleavage furrow;cytoplasm;cytoplasmic part;cytoskeletal part;intracellular non-membrane-bounded organelle;intracellular organelle;intracellular organelle part;intracellular part;microtubule organizing center;midbody;non-membrane-bounded organelle;organelle;organelle part		Homo sapiens	+
cell part;cullin-RING ubiquitin ligase complex;cytoplasm;intracellular membrane-bounded organelle;intracellular organelle;intracellular part;macromolecular complex;membrane-bounded organelle;nucleus;organelle;protein complex;SCF ubiquitin ligase complex;ubiquitin ligase complex		Homo sapiens	+
		Homo sapiens	+
cell part;cis-Golgi network;COPI vesicle coat;cytoplasm;cytoplasmic part;cytoplasmic vesicle part;cytoskeletal part;endoplasmic reticulum-Golgi intermediate compartment;Golgi apparatus;Golgi apparatus part;intracellular membrane-bounded organelle;intracellular organelle;intracellular organelle part;intracellular part;macromolecular complex;membrane;membrane coa		Homo sapiens	+
cell cortex part;cell part;cytoplasmic part;cytosol;exocyst;intracellular membrane-bounded organelle;intracellular organelle;intracellular part;macromolecular complex;membrane;membrane-bounded organelle;organelle;plasma membrane;protein complex		Homo sapiens	+
cell part;cytoplasmic part;cytosol;extracellular membrane-bounded organelle;extracellular organelle;extracellular region part;extracellular vesicular exosome;intracellular part;membrane-bounded organelle;membrane-bounded vesicle;organelle;vesicle		Homo sapiens	+
cell part;chromatin;chromosomal part;cytoskeleton;intermediate filament cytoskeleton;intracellular membrane-bounded organelle;intracellular non-membrane-bounded organelle;intracellular organelle;intracellular organelle pa	Lysine degradation	Homo sapiens	+
cell part;chromosome;cytoplasm;intracellular non-membrane-bounded organelle;intracellular organelle;intracellular organelle part;intracellular part;non-membrane-bounded organelle;nuclear part;nucleoplasm;organelle;organelle part		Homo sapiens	
cell part;cytoplasm;cytoplasmic part;cytosol;early endosome;early endosome membrane;endosomal part;endosome;endosome membrane;immunological synapse;intracellular membrane-bounded organelle;intracellular organelle;intracellular organelle part;intracellular part;membrane;membrane part;membrane-bounded organelle;nuclear part;nucleoplasm;organelle;orga		Homo sapiens	+
cell part;cytoplasmic part;endoplasmic reticulum;intracellular membrane-bounded organelle;intracellular organelle;intracellular organelle part;intracellular part;membrane;membrane-bounded organelle;mitochondrial inner membrane;mitochondrial membrane;mitochondrial part;organelle;organelle inner membrane;organelle membrane;organelle part		Homo sapiens	+
cell part;cytoplasmic part;Golgi apparatus;intracellular membrane-bounded organelle;intracellular organelle;intracellular part;membrane-bounded organelle;organelle		Homo sapiens	+
cell part;intracellular membrane-bounded organelle;intracellular organelle;intracellular organelle part;intracellular part;membrane-bounded organelle;nuclear body;nuclear part;nucleoplasm part;nucleus;organelle;organelle part		Homo sapiens	
		Homo sapiens	
cell part;cytoplasmic part;endoplasmic reticulum membrane;endoplasmic reticulum part;integral to membrane;intracellular organelle part;intracellular part;intrinsic to membrane;membrane;membrane part;organelle membran	Glycerophospholipid metabolism	Homo sapiens	+
adherens junction;anchoring junction;axon;basal part of cell;cell junction;cell part;cell projection;cell projection part;cell-substrate adherens junction;cell-substrate junction;focal adhesion;growth cone;neuron projection;site of polarized growth		Homo sapiens	
cell part;intracellular membrane-bounded organelle;intracellular organelle;intracellular part;membrane-bounded organelle;nucleus;organelle	Spliceosome	Homo sapiens	
cell junction;cell part;cytoplasm;cytoskeletal part;intracellular organelle part;intracellular part;membrane;organelle part;postsynaptic density;postsynaptic membrane;synapse part;synaptic membrane		Homo sapiens	
cell part;intracellular membrane-bounded organelle;intracellular non-membrane-bounded organelle;intracellular organelle;intracellular organelle part;intracellular part;membrane-bounded organelle;non-membrane-bounded organelle;nuclear part;nucleolus;nucleus;organelle;organelle part		Homo sapiens	+
cell part;cytoplasmic part;cytoplasmic vesicle;cytosol;Golgi apparatus;Golgi apparatus part;Golgi cisterna membrane;Golgi membrane;intracellular membrane-bounded organelle;intracellular organelle;intracellular organelle p	Endocytosis	Homo sapiens	+
cell part;cytoplasm;intracellular membrane-bounded organelle;intracellular organelle;intracellular part;membrane;membrane-bounded organelle;nucleus;organelle		Homo sapiens	+
cell part;intracellular non-membrane-bounded organelle;intracellular organelle;intracellular organelle part;intracellular part;macromolecular complex;mediator complex;non-membrane-bounded organelle;nuclear part;nucleolus;nucleoplasm;nucleoplasm part;organelle;organelle part;protein complex;ribonucleoprotein complex;transcription factor complex		Homo sapiens	+
cell part;cytoplasm;intracellular membrane-bounded organelle;intracellular organelle;intracellular part;membrane-bounded organelle;nucleus;organelle		Homo sapiens	+
		Homo sapiens	+
cell part;cytoplasm;cytoplasmic part;cytosol;intracellular membrane-bounded organelle;intracellular organelle;intracellular part;membrane-bounded organelle;nucleus;organelle	mRNA surveillance pathway	Homo sapiens	+
cell part;integral to membrane;integral to plasma membrane;intrinsic to membrane;intrinsic to plasma membrane;membrane;membrane part;plasma membrane;plasma membrane part	Protein digestion and absorption	Homo sapiens	+
cell part;cytoplasmic part;cytosol;early endosome;endosome;extracellular membrane-bounded organelle;extracellular organelle;extracellular region part;extracellular vesicular exosome;intracellular membrane-bounded organelle;intracellular organelle;intracellular organelle part;intracellular part;late endosome;lysosomal membrane;macromolecular complex;membrane;me		Homo sapiens	
cell part;chromosomal part;DNA replication factor A complex;intracellular membrane-bounded organelle;intracellular organelle;intracellular organelle part;intracellular part;macromolecular complex;membrane-bounded organelle;nuclear chromosome part;nuclear part;nucleoplasm;nucleus;organelle;organelle part;protein complex		Homo sapiens	
cell part;cytoplasm;intracellular membrane-bounded organelle;intracellular non-membrane-bounded organelle;intracellular organelle;intracellular organelle part;intracellular part;membrane;membrane-bounded organelle;non-membrane-bounded organelle;nuclear part;nucleolus;nucleoplasm;nucleus;organelle;organelle part;plasma membrane		Homo sapiens	+
cell part;chromosomal part;condensed chromosome kinetochore;cytoplasm;cytoplasmic part;cytoskeletal part;intracellular membrane-bounded organelle;intracellular non-membrane-bounded organelle;intracellular organelle;intracellular organelle part;intracellular part;kinetochore;macromolecular complex;membrane-bounded organelle;microtubule organizing center;mic		Homo sapiens	+
cell body;cell part;cell projection part;cytoplasmic membrane-bounded vesicle;cytoplasmic part;cytoplasmic vesicle;cytoplasmic vesicle membrane;cytoplasmic vesicle part;endosomal part;endosome;endosome membrane;extracellular membrane-bounded organelle;extracellular organelle;extracellular region part;extracellular vesicular exosome;extrinsic to internal side		Homo sapiens	+
cell part;cytoplasm;intracellular membrane-bounded organelle;intracellular organelle;intracellular organelle part;intracellular part;macromolecular complex;mediator complex;membrane;membrane-bounded organelle;nuclear part;nucleoplasm;nucleoplasm part;nucleus;organelle;organelle part;protein complex		Homo sapiens	+
cell part;cytoplasmic part;intracellular organelle lumen;intracellular organelle part;intracellular part;membrane-enclosed lumen;mitochondrial matrix;mitochondrial part;organelle lumen;organelle part		Homo sapiens	+
cell part;cytoplasm;intracellular membrane-bounded organelle;intracellular organelle;intracellular organelle part;intracellular part;membrane-bounded organelle;nuclear part;nucleoplasm;nucleus;organelle;organelle part		Homo sapiens	
basal plasma membrane;basement membrane;cell junction;cell part;cell-substrate junction;cytoplasm;extracellular matrix part;extracellular region part;hemidesmosome;intracellular membrane-bounded organelle;intracellular	NOD-like receptor signaling pathway	Homo sapiens	+
basement membrane;cell cortex;cell junction;cell part;cytoplasmic part;extracellular matrix part;extracellular membrane-bounded organelle;extracellular organelle;extracellular region part;extracellular vesicular exosome;intracellular part;membrane-bounded organelle;membrane-bounded vesicle;organelle;vesicle		Homo sapiens	+
		Homo sapiens	
cell part;cytoplasmic part;cytoskeletal part;cytosol;intracellular membrane-bounded organelle;intracellular organelle;intracellular organelle part;intracellular part;macromolecular complex;membrane-bounded organelle;microtubule associated complex;nucleus;organelle;organelle part;protein complex		Homo sapiens	
adherens junction;anchoring junction;cell junction;cell part;cell projection;cell projection membrane;cell projection part;cytoplasm;cytoplasmic part;cytoskeleton;dendritic spine;filopodium;intracellular non-membrane-bounded organelle;intracellular organelle;intracellular organelle part;intracellular part;lamellipodium;leading edge membrane;macromolecular complex;mem		Homo sapiens	+
cell part;cytoplasm;cytoplasmic part;endoplasmic reticulum;intracellular membrane-bounded organelle;intracellular organelle;intracellular organelle part;intracellular part;membrane;membrane-bounded organelle;nuclear matrix;nuclear part;nucleoplasm;nucleus;organelle;organelle part;plasma membrane		Homo sapiens	
cell part;integral to membrane;intrinsic to membrane;membrane part		Homo sapiens	+
cell part;cytoplasm;intracellular membrane-bounded organelle;intracellular organelle;intracellular organelle part;intracellular part;membrane-bounded organelle;nuclear part;nucleoplasm;nucleus;organelle;organelle part		Homo sapiens	+
cell part;chromatin remodeling complex;chromosomal part;cytoplasm;extracellular region part;histone deacetylase complex;intercellular bridge;intracellular membrane-bounded organelle;intracellular non-membrane-boundec	Huntington's disease	Homo sapiens	
cell part;intracellular organelle part;intracellular part;membrane;nuclear membrane;nuclear part;nucleoplasm;organelle membrane;organelle part		Homo sapiens	
cell part;cytoplasm;cytoplasmic part;early endosome membrane;endosomal part;endosome membrane;intracellular membrane-bounded organelle;intracellular organelle;intracellular organelle part;intracellular part;membrane;	Endocytosis	Homo sapiens	
cell part;cytoplasm;cytoskeletal part;holo TFIIH complex;intracellular membrane-bounded organelle;intracellular non-membrane-bounded organelle;intracellular organelle;intracellular organelle part;intracellular part;macromolecular complex;membrane;membrane-bounded organelle;MMXD complex;non-membrane-bounded organelle;nuclear part;nucleoplasm;nucleoplas		Homo sapiens	+
cell part;chromatin;chromosomal part;euchromatin;heterochromatin;intracellular membrane-bounded organelle;intracellular organelle;intracellular organelle part;intracellular part;membrane-bounded organelle;nuclear chromatin;nuclear chromosome part;nuclear heterochromatin;nuclear matrix;nuclear part;nucleus;organelle;organelle part;replication fork		Homo sapiens	+
actin cytoskeleton;adherens junction;anchoring junction;cell junction;cell part;cytoplasm;cytoplasmic part;cytoskeleton;cytosol;extracellular membrane-bounded organelle;extracellular organelle;extracellular region part;extracellular vesicular exosome;intracellular membrane-bounded organelle;intracellular non-membrane-bounded organelle;intracellular organelle;intracell		Homo sapiens	+
cell part;chromatin;chromosomal part;cytoplasm;intracellular membrane-bounded organelle;intracellular organelle;intracellular organelle part;intracellular part;membrane-bounded organelle;nuclear chromatin;nuclear chromosome part;nuclear part;nucleus;organelle;organelle part		Homo sapiens	+
cell part;centrosome;cytoplasm;cytoplasmic part;cytoskeletal part;intracellular membrane-bounded organelle;intracellular non-membrane-bounded organelle;intracellular organelle;intracellular organelle part;intracellular part;membrane-bounded organelle;microtubule organizing center;mitochondrion;non-membrane-bounded organelle;nucleus;organelle;organelle part		Homo sapiens	+
cell part;cytoplasm;cytoskeletal part;cytoskeleton;intracellular non-membrane-bounded organelle;intracellular organelle;intracellular organelle part;intracellular part;macromolecular complex;microtubule;microtubule cytoskeleton;non-membrane-bounded organelle;nuclear part;nucleoplasm;organelle;organelle part;protein complex		Homo sapiens	+
cell part;cytoplasm;cytoplasmic part;cytosol;extracellular membrane-bounded organelle;extracellular organelle;extracellular region part;extracellular vesicular exosome;intracellular membrane-bounded organelle;intracellular c	Proteasome	Homo sapiens	+
actin filament bundle;actomyosin;adherens junction;anchoring junction;cell junction;cell part;cell-cell junction;cell-substrate adherens junction;cell-substrate junction;cytoskeletal part;cytoskeleton;extracellular membrane-bounded organelle;extracellular organelle;extracellular region part;extracellular vesicular exosome;focal adhesion;intracellular non-membrane-boundec		Homo sapiens	+
aggresome;cell part;cytoplasmic part;cytoskeleton;cytosol;endoplasmic reticulum;endoplasmic reticulum membrane;endoplasmic reticulum part;inclusion body;integral to membrane;intermediate filament cytoskeleton;intrace	Protein export;Protein processing in endoplasmic reticulum	Homo sapiens	+
cell part;cytoplasmic part;endoplasmic reticulum membrane;endoplasmic reticulum part;intracellular organelle part;intracellular part;membrane;membrane part;organelle membrane;organelle part	Glycerophospholipid metabolism;Phosphonate and phosphinate metabolism	Homo sapiens	+
catalytic step 2 spliceosome;cell part;cytoplasm;intracellular membrane-bounded organelle;intracellular non-membrane-bounded organelle;intracellular organelle;intracellular organelle part;intracellular part;macromolecular cc	Spliceosome	Homo sapiens	+
cell part;cytoplasmic part;cytosol;extracellular membrane-bounded organelle;extracellular organelle;extracellular region part;extracellular vesicular exosome;intracellular organelle part;intracellular part;macromolecular comple	Proteasome	Homo sapiens	
cell part;extracellular region part;extracellular space;membrane		Homo sapiens	+
cell part;cytoplasm;cytoplasmic part;cytosolic part;intracellular membrane-bounded organelle;intracellular organelle;intracellular part;macromolecular complex;membrane-bounded organelle;nucleus;organelle;prefoldin complex;protein complex		Homo sapiens	+
cell part;chromatin;chromosomal part;euchromatin;heterochromatin;histone methyltransferase complex;intracellular membrane-bounded organelle;intracellular organelle;intracellular organelle part;intracellular part;macromolecular complex;membrane-bounded organelle;methyltransferase complex;MLL1 complex;nuclear body;nuclear chromatin;nuclear chromosome part		Homo sapiens	+
axon;cell body;cell part;cell projection;chromatin;chromosomal part;cytoplasm;cytoplasmic part;cytosol;extracellular membrane-bounded organelle;extracellular organelle;extracellular region part;extracellular vesicular exosor	Parkinson's disease	Homo sapiens	+
cell part;cytoplasmic part;extracellular membrane-bounded organelle;extracellular organelle;extracellular region part;extracellular vesicular exosome;integral to membrane;intracellular organelle part;intracellular part;intrinsic to membrane;membrane;membrane part;membrane-bounded organelle;membrane-bounded vesicle;mitochondrial membrane;mitochondrial outer n		Homo sapiens	+
apical part of cell;cell part;cytoplasmic part;endosome;extracellular membrane-bounded organelle;extracellular organelle;extracellular region part;extracellular vesicular exosome;intracellular membrane-bounded organelle;intr	Antigen processing and presentation;Lysosome	Homo sapiens	+
cell part;cytoplasmic part;cytosol;endoplasmic reticulum;extracellular region;intracellular membrane-bounded organelle;intracellular organelle;intracellular part;lipid particle;membrane;membrane-bounded organelle;nucleus;organelle;plasma membrane		Homo sapiens	+
cell part;cytoplasm;cytoplasmic part;cytosol;intracellular membrane-bounded organelle;intracellular organelle;intracellular organelle part;intracellular part;membrane;membrane-bounded organelle;nuclear membrane;nuclear part;nucleoplasm;nucleus;organelle;organelle membrane;organelle part		Homo sapiens	+
cell part;cytoplasm;exosome (RNase complex);intracellular membrane-bounded organelle;intracellular non-membrane-bounded organelle;intracellular organelle;intracellular organelle part;intracellular part;macromolecular con	RNA degradation	Homo sapiens	+
cell part;cytoplasmic part;cytosol;intracellular organelle part;intracellular part;macromolecular complex;membrane part;nuclear part;nuclear pore;nucleoplasm;organelle part;pore complex;protein complex	RNA transport	Homo sapiens	+
cell part;extracellular region part;extracellular space;intracellular organelle part;intracellular part;macromolecular complex;multimeric ribonuclease P complex;nuclear part;nucleolar part;nucleolar ribonuclease P complex;orga	Ribosome biogenesis in eukaryotes;RNA transport	Homo sapiens	+
cell part;cytoplasm;cytoplasmic part;cytosol;extracellular membrane-bounded organelle;extracellular organelle;extracellular region part;extracellular space;extracellular vesicular exosome;intracellular membrane-bounded organelle;intracellular organelle;intracellular part;lysosome;lytic vacuole;mast cell granule;membrane-bounded organelle;membrane-bounded vesicle;n		Homo sapiens	+
cell part;intracellular membrane-bounded organelle;intracellular non-membrane-bounded organelle;intracellular organelle;intracellular organelle part;intracellular part;membrane-bounded organelle;non-membrane-bounded organelle;nuclear part;nucleolus;nucleoplasm;nucleus;organelle;organelle part		Homo sapiens	+
cell part;intracellular organelle part;intracellular part;macromolecular complex;nuclear part;nucleoplasm;nucleoplasm part;organelle part;protein complex;transcription factor complex		Homo sapiens	+
cell part;cytoplasm;cytoplasmic part;cytosol;Golgi apparatus;intracellular membrane-bounded organelle;intracellular organelle;intracellular part;membrane-bounded organelle;nucleus;organelle;perinuclear region of cytoplasm		Homo sapiens	+
cell part;cytoplasmic part;cytosol;eukaryotic 43S preinitiation complex;eukaryotic 48S preinitiation complex;eukaryotic translation initiation factor 3 complex;intracellular part;macromolecular complex;protein complex;ribonuc	RNA transport	Homo sapiens	+
cell part;cytoplasm;cytoplasmic part;intracellular membrane-bounded organelle;intracellular organelle;intracellular organelle part;intracellular part;membrane;membrane-bounded organelle;microbody;microbody membrane;microbody part;organelle;organelle membrane;organelle part;peroxisomal membrane;peroxisomal part		Homo sapiens	
cell part;cytoplasm;cytoplasmic part;cytoskeleton;cytosol;extracellular membrane-bounded organelle;extracellular organelle;extracellular region part;extracellular vesicular exosome;intracellular non-membrane-bounded organelle;intracellular organelle;intracellular organelle part;intracellular part;membrane;membrane-bounded organelle;membrane-bounded vesicle;non-r		Homo sapiens	+
cell part;cell surface;cytoplasmic part;extracellular membrane-bounded organelle;extracellular organelle;extracellular region part;extracellular vesicular exosome;intracellular membrane-bounded organelle;intracellular organelle;intracellular organelle part;intracellular part;macromolecular complex;membrane;membrane-bounded organelle;membrane-bounded vesicle;mito		Homo sapiens	+
cell part;cytoplasm;cytoplasmic part;extracellular membrane-bounded organelle;extracellular organelle;extracellular region part;extracellular vesicular exosome;intracellular membrane-bounded organelle;intracellular organelle;intracellular organelle part;intracellular part;macromolecular complex;membrane-bounded organelle;membrane-bounded vesicle;nuclear part;nucl		Homo sapiens	
cell part;intracellular membrane-bounded organelle;intracellular organelle;intracellular organelle part;intracellular part;macromolecular complex;membrane-bounded organelle;nuclear body;nuclear part;nuclear speck;nucleopl	Spliceosome	Homo sapiens	+
cell part;chromosomal part;chromosome, centromeric region;condensed chromosome kinetochore;cytoplasmic part;cytoskeletal part;cytoskeleton;cytosol;intracellular membrane-bounded organelle;intracellular non-membrane-bounded organelle;intracellular organelle;intracellular organelle part;intracellular part;kinesin complex;kinetochore;macromolecular complex;mer		Homo sapiens	+
axon part;cell part;cell projection part;cytoplasmic part;cytosol;endoplasmic reticulum membrane;endoplasmic reticulum part;extrinsic to membrane;intracellular organelle part;intracellular part;membrane;membrane part;nuc	Glycerolipid metabolism	Homo sapiens	+
cell part;cytoplasm;cytoplasmic part;Golgi apparatus;Golgi apparatus part;intracellular membrane-bounded organelle;intracellular non-membrane-bounded organelle;intracellular organelle;intracellular organelle part;intracellular part;macromolecular complex;membrane;membrane-bounded organelle;non-membrane-bounded organelle;nuclear part;nucleoplasm;organelle		Homo sapiens	+
cell part;cytoplasm;cytoplasmic part;cytosol;intracellular part	Cysteine and methionine metabolism;One carbon pool by folate;Selenocompound metabolism	Homo sapiens	+
cell part;cytoplasm;cytoplasmic part;endoplasmic reticulum;intracellular membrane-bounded organelle;intracellular organelle;intracellular organelle lumen;intracellular organelle part;intracellular part;membrane;membrane-bou	Alzheimer's disease;Valine, leucine and isoleucine degradation	Homo sapiens	+
anchoring collagen;cell part;collagen;collagen type XII;cytoplasmic part;endoplasmic reticulum lumen;endoplasmic reticulum part;extracellular matrix;extracellular matrix part;extracellular membrane-bounded organelle;extrac	Protein digestion and absorption	Homo sapiens	+
cell part;cytoplasm;intracellular membrane-bounded organelle;intracellular organelle;intracellular organelle part;intracellular part;macromolecular complex;membrane-bounded organelle;nuclear part;nucleoplasm;nucleus;organelle;organelle part;ribonucleoprotein complex		Homo sapiens	+
cell part;cytoplasm;cytoplasmic part;Golgi apparatus;intracellular membrane-bounded organelle;intracellular organelle;intracellular organelle part;intracellular part;lysosomal membrane;membrane;membrane-bounded organelle;nuclear part;nucleoplasm;organelle;organelle membrane;organelle part;plasma membrane;vacuolar membrane;vacuolar part		Homo sapiens	+
cell part;cytoplasm;intracellular membrane-bounded organelle;intracellular organelle;intracellular part;membrane-bounded organelle;nucleus;organelle		Homo sapiens	+
cell part;cytoplasmic part;extracellular membrane-bounded organelle;extracellular organelle;extracellular region part;extracellular vesicular exosome;intracellular membrane-bounded organelle;intracellular organelle;intracellular organelle part;intracellular part;lysosomal membrane;macromolecular complex;membrane;membrane part;membrane-bounded organelle;membr		Homo sapiens	+
cell part;cytoplasmic part;intracellular membrane-bounded organelle;intracellular organelle;intracellular organelle lumen;intracellular organelle part;intracellular part;membrane-bounded organelle;membrane-enclosed lumen;m	Carbon fixation pathways in prokaryotes;Citrate cycle (TCA cycle);Glyoxylate and dicarboxylate metabolism	Homo sapiens	+
cell part;cytoplasmic part;endosomal part;endosome;endosome membrane;extracellular membrane-bounded organelle;extracellular organelle;extracellular region part;extracellular vesicular exosome;integral to membrane;integral to plasma membrane;intracellular membrane-bounded organelle;intracellular organelle;intracellular organelle part;intracellular part;intrinsic to		Homo sapiens	
apical plasma membrane;basolateral plasma membrane;cell part;integral to membrane;integral to plasma membrane;intrinsic to membrane;intrinsic to plasma membrane;membrane;membrane part;plasma membrane;plasma membrane part		Homo sapiens	+
cell part;intracellular membrane-bounded organelle;intracellular organelle;intracellular part;membrane-bounded organelle;nucleus;organelle		Homo sapiens	+
cell part;cytoplasm;cytoplasmic part;early endosome;endosomal part;endosome;endosome membrane;ESCRT I complex;extracellular membrane-bounded organelle;extracellular organelle;extracellular region part;extracellula	Endocytosis	Homo sapiens	+
cell part;cytoplasm;cytoplasmic part;cytosol;extracellular membrane-bounded organelle;extracellular organelle;extracellular region part;extracellular vesicular exosome;intracellular membrane-bounded organelle;intracellular organelle;intracellular part;membrane;membrane-bounded organelle;membrane-bounded vesicle;nucleus;organelle;plasma membrane;vesicle		Homo sapiens	+
cell body;cell part;chaperonin-containing T-complex;cytoplasm;cytoplasmic part;cytoskeletal part;cytosol;cytosolic part;extracellular membrane-bounded organelle;extracellular organelle;extracellular region part;extracellular vesicular exosome;intracellular membrane-bounded organelle;intracellular organelle;intracellular organelle part;intracellular part;macromolecular co		Homo sapiens	+
cell part;intracellular membrane-bounded organelle;intracellular non-membrane-bounded organelle;intracellular organelle;intracellular organelle part;intracellular part;membrane;membrane-bounded organelle;non-membrane-bounded organelle;nuclear part;nucleolus;nucleus;organelle;organelle part		Homo sapiens	+
cell part;cytoplasm;cytoplasmic part;cytosol;intracellular membrane-bounded organelle;intracellular organelle;intracellular organelle part;intracellular part;membrane-bounded organelle;nuclear part;nucleoplasm;nucleus;organelle;organelle part		Homo sapiens	+
cell part;cytoplasmic part;endoplasmic reticulum;endoplasmic reticulum membrane;endoplasmic reticulum part;integral to membrane;intracellular membrane-bounded organelle;intracellular organelle;intracellular organelle pa	Fat digestion and absorption;Glycerolipid metabolism;Glycerophospholipid metabolism	Homo sapiens	
cell junction;cell part;cell projection;cytoplasm;cytoplasmic part;cytoskeletal part;early endosome membrane;endosomal part;endosome membrane;intracellular non-membrane-bounded organelle;intracellular organelle;intrac	Endocytosis	Homo sapiens	
cell part;extracellular membrane-bounded organelle;extracellular organelle;extracellular region part;extracellular space;extracellular vesicular exosome;membrane;membrane-bounded organelle;membrane-bounded vesicle;or	Axon guidance	Homo sapiens	+
cell part;cytoplasm;cytoplasmic part;cytoskeletal part;cytosol;Golgi apparatus part;Golgi stack;intracellular membrane-bounded organelle;intracellular non-membrane-bounded organelle;intracellular organelle;intracellular organelle part;intracellular part;membrane-bounded organelle;non-membrane-bounded organelle;nuclear part;nucleolus;nucleoplasm;nucleus;organell		Homo sapiens	+
extracellular membrane-bounded organelle;extracellular organelle;extracellular region;extracellular region part;extracellular space;extracellular vesicular exosome;membrane-bounded organelle;membrane-bounded vesicle;organelle;vesicle		Homo sapiens	+
cell part;cytoplasmic part;intracellular membrane-bounded organelle;intracellular organelle;intracellular organelle part;intracellular part;membrane;membrane-bounded organelle;mitochondrial inner membrane;mitochondrial membrane;mitochondrial part;mitochondrion;organelle;organelle inner membrane;organelle membrane;organelle part;synapse part;synaptic membra		Homo sapiens	+
actin cytoskeleton;cell part;centrosome;chromosomal part;condensin complex;cytoplasm;cytoplasmic part;cytoskeletal part;cytoskeleton;cytosol;intracellular membrane-bounded organelle;intracellular non-membrane-bounc	Cell cycle - yeast	Homo sapiens	+
calcium channel complex;cation channel complex;cell part;cytoplasmic part;integral to membrane;integral to mitochondrial membrane;integral to organelle membrane;intracellular;intracellular membrane-bounded organelle;intracellular organelle;intracellular organelle part;intracellular part;intrinsic to membrane;intrinsic to organelle membrane;ion channel complex;macror		Homo sapiens	+
cell part;cytoplasm;intracellular non-membrane-bounded organelle;intracellular organelle;intracellular organelle part;intracellular part;membrane;non-membrane-bounded organelle;nuclear part;nucleolus;organelle;organelle part;plasma membrane		Homo sapiens	
cell part;cytoplasmic part;intracellular membrane-bounded organelle;intracellular organelle;intracellular organelle part;intracellular part;membrane-bounded organelle;mitochondrion;nuclear part;nucleoplasm;nucleus;organelle	RNA transport	Homo sapiens	+
		Homo sapiens	+
cell part;cytoplasm;intracellular membrane-bounded organelle;intracellular non-membrane-bounded organelle;intracellular organelle;intracellular organelle part;intracellular part;membrane-bounded organelle;non-membrane-bounded organelle;nuclear part;nucleolus;nucleus;organelle;organelle part		Homo sapiens	+
cell part;cytoplasmic part;intracellular membrane-bounded organelle;intracellular organelle;intracellular part;membrane-bounded organelle;mitochondrion;nucleus;organelle		Homo sapiens	+
cell part;intracellular membrane-bounded organelle;intracellular organelle;intracellular part;membrane-bounded organelle;nucleus;organelle		Homo sapiens	+
		Homo sapiens	+
cell part;cytoplasm;cytoplasmic part;cytosol;Golgi apparatus;intracellular membrane-bounded organelle;intracellular organelle;intracellular organelle part;intracellular part;macromolecular complex;membrane-bounded organelle;methylosome;methyltransferase complex;nuclear part;nucleoplasm;nucleus;organelle;organelle part;protein complex		Homo sapiens	
cell part;cytoplasmic microtubule;cytoplasmic part;cytoskeletal part;intracellular membrane-bounded organelle;intracellular organelle;intracellular organelle part;intracellular part;macromolecular complex;membrane-bounded	Gap junction;Pathogenic Escherichia coli infection;Phagosome	Homo sapiens	
cell part;cytoplasmic part;endoplasmic reticulum membrane;endoplasmic reticulum part;extracellular region;intracellular organelle part;intracellular part;membrane;membrane part;organelle membrane;organelle part		Homo sapiens	
cell part;cytoplasm;intracellular membrane-bounded organelle;intracellular non-membrane-bounded organelle;intracellular organelle;intracellular organelle part;intracellular part;macromolecular complex;membrane;membrane-bounded organelle;NLS-dependent protein nuclear import complex;non-membrane-bounded organelle;nuclear part;nucleocytoplasmic shuttling c		Homo sapiens	+
autophagic vacuole;cell part;cytoplasmic part;cytoplasmic vesicle;endosome;integral to membrane;intracellular membrane-bounded organelle;intracellular organelle;intracellular part;intrinsic to membrane;late endosome;lysosome;lytic vacuole;membrane;membrane part;membrane-bounded organelle;organelle;vacuole;vesicle		Homo sapiens	
actin cytoskeleton;actin filament;actin filament bundle;actomyosin;adherens junction;anchoring junction;cell junction;cell leading edge;cell part;cell periphery;cell projection;cell-substrate adherens junction;cell-substrate junction;cytoplasm;cytoplasmic part;cytoskeletal part;cytoskeleton;cytosol;extracellular membrane-bounded organelle;extracellular organelle;extracellu		Homo sapiens	+
cell part;cytoplasmic part;cytosol;extracellular membrane-bounded organelle;extracellular organelle;extracellular region part;extracellular vesicular exosome;intracellular part;membrane-bounded organelle;membrane-bounded vesicle;organelle;vesicle		Homo sapiens	+
cell part;cytoplasm;cytoplasmic part;cytosol;endosomal part;endosome membrane;ESCRT II complex;extracellular membrane-bounded organelle;extracellular organelle;extracellular region part;extracellular vesicular exosom	Endocytosis	Homo sapiens	+
cell part;cytoplasmic part;intracellular membrane-bounded organelle;intracellular non-membrane-bounded organelle;intracellular organelle;intracellular organelle part;intracellular part;macromolecular complex;membrane;membrane-bounded organelle;mitochondrial inner membrane;mitochondrial membrane;mitochondrial part;mitochondrion;non-membrane-bounded org		Homo sapiens	
bleb;cell part;cell projection;cytoplasm;cytoplasmic part;endosome;extracellular membrane-bounded organelle;extracellular organelle;extracellular region part;extracellular vesicular exosome;Golgi apparatus;Golgi apparatus part;Golgi lumen;intracellular membrane-bounded organelle;intracellular organelle;intracellular organelle lumen;intracellular organelle part;intracellu		Homo sapiens	
cell part;intracellular part;macromolecular complex;proteasome complex;protein complex		Homo sapiens	+
cell part;cytoplasm;exon-exon junction complex;intracellular non-membrane-bounded organelle;intracellular organelle;intracellular organelle part;intracellular part;macromolecular complex;non-membrane-bounded organelle;	mRNA surveillance pathway;RNA transport	Homo sapiens	+
cell part;cytoplasm;cytoplasmic part;cytosol;extracellular region part;extracellular space;integral to membrane;intracellular membrane-bounded organelle;intracellular organelle;intracellular organelle part;intracellular part;intrinsic to membrane;lipid particle;membrane;membrane part;membrane-bounded organelle;mitochondrial membrane;mitochondrial outer membrane;r		Homo sapiens	

cell part;cytoplasmic part;endoplasmic reticulum;extracellular region;intracellular membrane-bounded organelle;intracellular organelle;intracellular part;membrane;membrane-bounded organelle;organelle	Glycolysis / Gluconeogenesis	Homo sapiens	+
cell part;cytoplasmic part;intracellular membrane-bounded organelle;intracellular non-membrane-bounded organelle;intracellular organelle;intracellular organelle lumen;intracellular organelle part;intracellular part;membrane;membrane-bounded organelle;membrane-enclosed lumen;mitochondrial inner membrane;mitochondrial intermembrane space;mitochondrial matrix;		Homo sapiens	+
cell part;cytoplasm;intracellular membrane-bounded organelle;intracellular organelle;intracellular part;membrane-bounded organelle;nucleus;organelle	mRNA surveillance pathway	Homo sapiens	+
cell part;intracellular organelle part;intracellular part;nuclear part;nucleoplasm;organelle part		Homo sapiens	+
cell part;cytoplasm;cytoplasmic part;cytosol;intracellular part		Homo sapiens	+
cell part;cell surface;cytoplasmic part;endoplasmic reticulum lumen;endoplasmic reticulum membrane;endoplasmic reticulum part;endoplasmic reticulum-Golgi intermediate compartment;extracellular membrane-bounded organelle;extracellular organelle;extracellular region part;extracellular vesicular exosome;intracellular membrane-bounded organelle;intracellular orgar		Homo sapiens	+
cell part;extracellular membrane-bounded organelle;extracellular organelle;extracellular region part;extracellular vesicular exosome;membrane;membrane-bounded organelle;membrane-bounded vesicle;organelle;vesicle	Purine metabolism;Thiamine metabolism	Homo sapiens	+
cell part;cytoplasmic part;intracellular membrane-bounded organelle;intracellular organelle;intracellular organelle part;intracellular part;membrane-bounded organelle;mitochondrion;nuclear part;nucleoplasm;nucleus;organelle;organelle part		Homo sapiens	
cell part;centrosome;cytoplasmic microtubule;cytoplasmic part;cytoskeletal part;cytosol;intracellular non-membrane-bounded organelle;intracellular organelle;intracellular organelle part;intracellular part;macromolecular complex;membrane;microtubule;microtubule organizing center;non-membrane-bounded organelle;nuclear part;nucleoplasm;organelle;organelle part;pro		Homo sapiens	+
cell part;cytoplasmic part;endoplasmic reticulum part;integral to endoplasmic reticulum membrane;integral to membrane;integral to organelle membrane;intracellular organelle part;intracellular part;intrinsic to endoplasmic reticulum membrane;intrinsic to membrane;intrinsic to organelle membrane;membrane;membrane part;organelle membrane contact site;organelle par		Homo sapiens	+
cell part;cytoplasmic part;endoplasmic reticulum lumen;endoplasmic reticulum part;intracellular organelle lumen;intracellular organelle part;intracellular part;membrane-enclosed lumen;organelle lumen;organelle part		Homo sapiens	+
cell part;cytoplasmic part;endoplasmic reticulum;endoplasmic reticulum membrane;endoplasmic reticulum part;integral to membrane;intracellular membrane-bounded organelle;intracellular organelle;intracellular organelle pa	N-Glycan biosynthesis;Various types of N-glycan biosynthesis	Homo sapiens	+
cell part;centrosome;cytoplasm;cytoplasmic part;cytoskeletal part;HAUS complex;intracellular non-membrane-bounded organelle;intracellular organelle;intracellular organelle part;intracellular part;macromolecular complex;microtubule;microtubule associated complex;microtubule organizing center;non-membrane-bounded organelle;organelle;organelle part;protein comp		Homo sapiens	+
cell junction;cell part;clathrin-coated vesicle;coated vesicle;cytoplasmic membrane-bounded vesicle;cytoplasmic part;cytoplasmic vesicle;extracellular membrane-bounded organelle;extracellular organelle;extracellular region part;extracellular vesicular exosome;intracellular membrane-bounded organelle;intracellular organelle;intracellular organelle part;intracellular part;r		Homo sapiens	
cell part;cytoplasm;cytoskeletal part;intracellular membrane-bounded organelle;intracellular non-membrane-bounded organelle;intracellular organelle;intracellular organelle part;intracellular part;membrane-bounded organelle;non-membrane-bounded organelle;nucleus;organelle;organelle part;spindle		Homo sapiens	+
cell part;intracellular membrane-bounded organelle;intracellular organelle;intracellular organelle part;intracellular part;membrane-bounded organelle;nuclear part;nucleoplasm;nucleus;organelle;organelle part		Homo sapiens	+
cell part;cytoplasm;intracellular membrane-bounded organelle;intracellular organelle;intracellular organelle part;intracellular part;membrane;membrane-bounded organelle;nuclear part;nucleoplasm;nucleus;organelle;organelle part;plasma membrane		Homo sapiens	+
cell part;chromatin remodeling complex;chromosomal part;cytoplasmic membrane-bounded vesicle;cytoplasmic part;cytoplasmic vesicle;intracellular membrane-bounded organelle;intracellular organelle;intracellular organelle part;intracellular part;macromolecular complex;membrane-bounded organelle;membrane-bounded vesicle;nuclear chromosome part;nuclear part		Homo sapiens	+
cell body;cell junction;cell part;cell pole;cell projection;cell tip;cytoplasmic membrane-bounded vesicle;cytoplasmic part;cytoplasmic vesicle;cytosol;dendrite;endosome;extracellular region part;Golgi apparatus;growing cell ti	Inositol phosphate metabolism;Phosphatidylinositol signaling system	Homo sapiens	+
cell part;cytoplasmic part;endoplasmic reticulum lumen;endoplasmic reticulum part;Golgi apparatus;integral to membrane;integral to nuclear inner membrane;integral to organelle membrane;intracellular membrane-bounded organelle;intracellular organelle;intracellular organelle lumen;intracellular organelle part;intracellular part;intrinsic to membrane;intrinsic to nuclear in		Homo sapiens	+
cell division site part;cell part;cleavage furrow;cytoplasm;cytoplasmic part;cytoskeletal part;intracellular membrane-bounded organelle;intracellular organelle;intracellular organelle part;intracellular part;macromolecular complex;membrane-bounded organelle;microtubule;microtubule organizing center;nucleus;organelle;organelle part;protein complex		Homo sapiens	+
adherens junction;anchoring junction;cell junction;cell part;cell-cell junction;cytoplasm;cytoskeletal part;intracellular organelle part;intracellular part;lateral plasma membrane;macromolecular complex;membrane part;microtubule;occluding junction;organelle part;plasma membrane part;protein complex;tight junction		Homo sapiens	+
		Homo sapiens	
cell part;cytoplasmic part;endoplasmic reticulum membrane;endoplasmic reticulum part;extracellular membrane-bounded organelle;extracellular organelle;extracellular region part;extracellular vesicular exosome;intracellular	Peroxisome;Retinol metabolism	Homo sapiens	
cell part;cytoplasmic part;endoplasmic reticulum membrane;endoplasmic reticulum part;integral to membrane;intracellular organelle part;intracellular part;intrinsic to membrane;membrane;membrane part;organelle membrane;organelle part		Homo sapiens	+
		Homo sapiens	
cell part;cytoplasm;cytoskeletal part;extracellular membrane-bounded organelle;extracellular organelle;extracellular region part;extracellular vesicular exosome;intracellular membrane-bounded organelle;intracellular organelle	Gap junction;Pathogenic Escherichia coli infection;Phagosome	Homo sapiens	+
cell part;intracellular membrane-bounded organelle;intracellular organelle;intracellular organelle part;intracellular part;macromolecular complex;membrane-bounded organelle;nuclear part;nucleoplasm;nucleus;organelle;organelle part;ribonucleoprotein complex		Homo sapiens	+
cell part;cytoplasm;cytoplasmic part;cytosol;extracellular membrane-bounded organelle;extracellular organelle;extracellular region part;extracellular vesicular exosome;Golgi apparatus;Golgi apparatus part;Golgi membrane;intracellular membrane-bounded organelle;intracellular organelle;intracellular organelle part;intracellular part;membrane;membrane-bounded organe		Homo sapiens	+
cell part;cytoplasm;envelope;intracellular membrane-bounded organelle;intracellular organelle;intracellular organelle part;intracellular part;membrane;membrane-bounded organelle;nuclear envelope;nuclear part;nucleus;organelle;organelle envelope;organelle part		Homo sapiens	+
catalytic step 2 spliceosome;cell part;cytoplasmic part;extracellular membrane-bounded organelle;extracellular organelle;extracellular region part;extracellular vesicular exosome;intracellular membrane-bounded organelle;int	Spliceosome	Homo sapiens	+
cell part;cytoplasmic part;extracellular region part;extracellular space;high-density lipoprotein particle;integral to membrane;intracellular membrane-bounded organelle;intracellular organelle;intracellular part;intrinsic to membrane;low-density lipoprotein particle;macromolecular complex;membrane part;membrane-bounded organelle;mitochondrion;organelle;plasma lipop		Homo sapiens	+
cell part;cytoplasm;extracellular membrane-bounded organelle;extracellular organelle;extracellular region part;extracellular vesicular exosome;intracellular part;membrane-bounded organelle;membrane-bounded vesicle;orga	Butanoate metabolism;Synthesis and degradation of ketone bodies	Homo sapiens	
cell part;cell projection;cytoplasm;cytoplasmic part;cytosol;intracellular non-membrane-bounded organelle;intracellular organelle;intracellular organelle part;intracellular part;non-membrane-bounded organelle;nuclear part;nu	Cysteine and methionine metabolism	Homo sapiens	+
cell part;cytoplasm;intracellular organelle part;intracellular part;nuclear part;nucleoplasm;organelle part		Homo sapiens	
cell part;cytoplasm;intracellular non-membrane-bounded organelle;intracellular organelle;intracellular organelle part;intracellular part;non-membrane-bounded organelle;nuclear part;nucleolus;organelle;organelle part		Homo sapiens	+
cell part;cytoplasm;cytoplasmic part;cytosol;Golgi apparatus;intracellular membrane-bounded organelle;intracellular organelle;intracellular organelle part;intracellular part;membrane;membrane-bounded organelle;nuclear par	Cysteine and methionine metabolism	Homo sapiens	+
cell part;cytoplasm;intracellular membrane-bounded organelle;intracellular organelle;intracellular part;membrane-bounded organelle;nucleus;organelle		Homo sapiens	+
cell part;cytoplasmic part;intracellular membrane-bounded organelle;intracellular organelle;intracellular part;membrane-bounded organelle;mitochondrion;nucleus;organelle	Fatty acid elongation in mitochondria	Homo sapiens	+
cell part;cytoplasm;intracellular membrane-bounded organelle;intracellular organelle;intracellular organelle part;intracellular part;membrane-bounded organelle;nuclear part;nucleoplasm;nucleus;organelle;organelle part		Homo sapiens	+
cell part;cytoplasmic part;endoplasmic reticulum membrane;endoplasmic reticulum part;extracellular membrane-bounded organelle;extracellular organelle;extracellular region part;extracellular vesicular exosome;integral to membrane;intracellular organelle part;intracellular part;intrinsic to membrane;membrane;membrane part;membrane-bounded organelle;membrane-b		Homo sapiens	+
		Homo sapiens	
cell part;cytoplasmic part;integral to membrane;intracellular membrane-bounded organelle;intracellular non-membrane-bounded organelle;intracellular organelle;intracellular organelle part;intracellular part;intrinsic to membrane;macromolecular complex;membrane;membrane part;membrane-bounded organelle;Noc complex;Noc4p-Nop14p complex;non-membrane-bou		Homo sapiens	+
cell part;clathrin-coated vesicle;coated vesicle;cytoplasmic membrane-bounded vesicle;cytoplasmic part;cytoplasmic vesicle;endoplasmic reticulum;endoplasmic reticulum membrane;endoplasmic reticulum part;endoplasmic reticulum-Golgi intermediate compartment;endoplasmic reticulum-Golgi intermediate compartment membrane;extracellular membrane-bounded		Homo sapiens	+
cell part;extracellular region part;extracellular space;intracellular membrane-bounded organelle;intracellular non-membrane-bounded organelle;intracellular organelle;intracellular organelle part;intracellular part;membrane;mer	Ribosome biogenesis in eukaryotes	Homo sapiens	
	Ribosome biogenesis in eukaryotes	Homo sapiens	
cell part;chromosomal part;condensed chromosome kinetochore;cytoplasmic part;cytoskeletal part;cytosol;envelope;intracellular non-membrane-bounded organelle;intracellular organelle;intracellular organelle part;intracellul	RNA transport	Homo sapiens	
axoneme part;cell part;cell projection;cell projection part;centrosome;cilium;cytoplasmic part;cytoskeletal part;endomembrane system;intracellular non-membrane-bounded organelle;intracellular organelle;intracellular organelle part;intracellular part;intraflagellar transport particle B;macromolecular complex;microtubule organizing center;motile cilium;non-membrane-bou		Homo sapiens	+
cell part;cytoplasmic part;intracellular organelle lumen;intracellular organelle part;intracellular part;membrane-enclosed lumen;mitochondrial matrix;mitochondrial part;organelle lumen;organelle part	Aminoacyl-tRNA biosynthesis	Homo sapiens	+
cell part;cytoplasm;extracellular membrane-bounded organelle;extracellular organelle;extracellular region part;extracellular vesicular exosome;intracellular membrane-bounded organelle;intracellular non-membrane-bounded	Benzoate degradation;Butanoate metabolism;Carbon fixation pathways in prokaryotes;Fatty acid metabolism;Glyoxylate and dicarboxylate metab	Homo sapiens	+
cell part;cytoplasm;cytoplasmic part;intracellular membrane-bounded organelle;intracellular non-membrane-bounded organelle;intracellular organelle;intracellular organelle part;intracellular part;macromolecular complex;membrane-bounded organelle;non-membrane-bounded organelle;nuclear body;nuclear part;nuclear speck;nucleolus;nucleoplasm;nucleoplasm part;nu		Homo sapiens	+
cell part;intracellular membrane-bounded organelle;intracellular organelle;intracellular organelle part;intracellular part;macromolecular complex;membrane-bounded organelle;nuclear part;nucleoplasm;nucleus;organelle;organ	Spliceosome	Homo sapiens	+
cell part;cytoplasmic part;integral to membrane;intracellular membrane-bounded organelle;intracellular organelle;intracellular organelle part;intracellular part;intrinsic to membrane;membrane;membrane part;membrane-bounded organelle;mitochondrial membrane;mitochondrial part;mitochondrion;organelle;organelle membrane;organelle part		Homo sapiens	
cell part;cytoplasmic part;endosome;extracellular membrane-bounded organelle;extracellular organelle;extracellular region part;extracellular space;extracellular vesicular exosome;Golgi apparatus part;intracellular membrane-bounded organelle;intracellular organelle;intracellular organelle part;intracellular part;late endosome;lysosome;lytic vacuole;membrane;membrane-		Homo sapiens	+
cell part;cytoplasm;intracellular membrane-bounded organelle;intracellular organelle;intracellular organelle part;intracellular part;membrane;membrane-bounded organelle;nuclear part;nucleoplasm;nucleus;organelle;organelle part;plasma membrane		Homo sapiens	+
cell part;intracellular part;macromolecular complex;ribonucleoprotein complex		Homo sapiens	
cell part;cytoplasmic part;intracellular membrane-bounded organelle;intracellular non-membrane-bounded organelle;intracellular organelle;intracellular organelle part;intracellular part;membrane-bounded organelle;mitochondrion;non-membrane-bounded organelle;nuclear part;nucleolus;organelle;organelle part		Homo sapiens	+
		Homo sapiens	+
cell part;cytoplasmic membrane-bounded vesicle;cytoplasmic part;cytoplasmic vesicle;cytoplasmic vesicle membrane;cytoplasmic vesicle part;cytoskeletal part;early endosome;early endosome membrane;endosomal part;e	Endocytosis	Homo sapiens	+
cell part;collagen;extracellular matrix part;extracellular membrane-bounded organelle;extracellular organelle;extracellular region part;extracellular space;extracellular vesicular exosome;membrane;membrane-bounded organelle;membrane-bounded vesicle;organelle;vesicle		Homo sapiens	+
cell part;cytoplasm;cytoplasmic part;intracellular membrane-bounded organelle;intracellular organelle;intracellular organelle part;intracellular part;macromolecular complex;membrane;membrane-bounded organelle;NatA complex;N-terminal protein acetyltransferase complex;nuclear part;nucleoplasm part;nucleus;organelle;organelle part;protein acetyltransferase comple		Homo sapiens	
cell part;cytoplasm;intracellular membrane-bounded organelle;intracellular organelle;intracellular organelle part;intracellular part;membrane-bounded organelle;nuclear part;nucleoplasm;nucleus;organelle;organelle part		Homo sapiens	+
cell part;cytoplasmic part;intracellular membrane-bounded organelle;intracellular organelle;intracellular part;membrane-bounded organelle;mitochondrion;nucleus;organelle		Homo sapiens	
AP-type membrane coat adaptor complex;cell part;clathrin adaptor complex;clathrin coated vesicle membrane;clathrin-coated endocytic vesicle membrane;coated vesicle membrane;cytoplasmic part;cytoplasmic vesicle me	Lysosome	Homo sapiens	
cell part;chromosome;cytoplasm;cytoskeletal part;intracellular membrane-bounded organelle;intracellular non-membrane-bounded organelle;intracellular organelle;intracellular organelle part;intracellular part;macromolecular complex;membrane-bounded organelle;microtubule;non-membrane-bounded organelle;nuclear part;nucleolus;nucleus;organelle;organelle part;pro		Homo sapiens	+
cell part;cytoplasmic part;intracellular membrane-bounded organelle;intracellular organelle;intracellular part;membrane-bounded organelle;mitochondrion;organelle		Homo sapiens	
cell part;intracellular membrane-bounded organelle;intracellular non-membrane-bounded organelle;intracellular organelle;intracellular organelle part;intracellular part;membrane-bounded organelle;non-membrane-bounded organelle;nuclear part;nucleolus;organelle;organelle part		Homo sapiens	+
cell part;cytoplasm;cytoplasmic part;cytosol;intracellular part	Drug metabolism - other enzymes;Purine metabolism;Pyrimidine metabolism	Homo sapiens	
		Homo sapiens	
blood microparticle;cell part;cytoplasm;cytoplasmic part;eukaryotic translation initiation factor 2 complex;extracellular region part;extracellular space;intracellular part;macromolecular complex;protein complex		Homo sapiens	+
cell part;cytoplasm;intracellular membrane-bounded organelle;intracellular organelle;intracellular organelle part;intracellular part;membrane-bounded organelle;nuclear body;nuclear part;nuclear speck;nucleoplasm;nucleoplasm part;organelle;organelle part		Homo sapiens	+
cell part;cytoplasm;cytoplasmic part;extracellular membrane-bounded organelle;extracellular organelle;extracellular region part;extracellular vesicular exosome;Golgi apparatus;Golgi apparatus part;Golgi cisterna membrane;	Glycosaminoglycan biosynthesis - keratan sulfate;N-Glycan biosynthesis;Various types of N-glycan biosynthesis	Homo sapiens	+
cell part;cytoplasmic part;intracellular non-membrane-bounded organelle;intracellular organelle;intracellular organelle part;intracellular part;macromolecular complex;membrane;mitochondrial inner membrane;mitochondrial membrane;mitochondrial part;mitochondrial ribosome;non-membrane-bounded organelle;organellar ribosome;organelle;organelle inner membrane;o		Homo sapiens	+
cell part;cytoplasmic part;intracellular non-membrane-bounded organelle;intracellular organelle;intracellular organelle part;intracellular part;large ribosomal subunit;macromolecular complex;membrane;mitochondrial inner me	Ribosome	Homo sapiens	+
cell part;cytoplasmic part;intracellular membrane-bounded organelle;intracellular non-membrane-bounded organelle;intracellular organelle;intracellular organelle part;intracellular part;macromolecular complex;membrane;membrane-bounded organelle;mitochondrial inner membrane;mitochondrial membrane;mitochondrial part;mitochondrial ribosome;mitochondrion;non-		Homo sapiens	+
cell part;cytoplasmic part;cytosolic part;cytosolic ribosome;intracellular non-membrane-bounded organelle;intracellular organelle;intracellular organelle part;intracellular part;macromolecular complex;membrane;mitochondrial inner membrane;mitochondrial membrane;mitochondrial part;non-membrane-bounded organelle;organelle;organelle inner membrane;organelle me		Homo sapiens	+
cell part;cytoplasmic part;intracellular membrane-bounded organelle;intracellular organelle;intracellular organelle part;intracellular part;macromolecular complex;membrane;membrane-bounded organelle;mitochondrial inner membrane;mitochondrial membrane;mitochondrial part;mitochondrial small ribosomal subunit;mitochondrion;nuclear part;nucleoplasm;organellar s		Homo sapiens	+
cell part;cytoplasmic part;intracellular organelle part;intracellular part;membrane;membrane-enclosed lumen;mitochondrial intermembrane space;mitochondrial part;organelle envelope lumen;organelle part;plasma membrane	Renin-angiotensin system	Homo sapiens	
cell part;cell projection;centriole;centrosome;cilium;cytoplasmic part;cytoskeletal part;cytosol;intracellular non-membrane-bounded organelle;intracellular organelle;intracellular organelle part;intracellular part;membrane;microtubule organizing center;microtubule organizing center part;non-membrane-bounded organelle;organelle;organelle part;primary cilium		Homo sapiens	+
cell junction;cell part;cytoplasmic membrane-bounded vesicle;cytoplasmic part;cytoplasmic vesicle;intracellular membrane-bounded organelle;intracellular organelle;intracellular part;membrane-bounded organelle;membrane-bounded vesicle;organelle;vesicle		Homo sapiens	+
cell part;chromosomal part;chromosome, centromeric region;condensed chromosome kinetochore;cytoplasmic part;cytosol;intracellular membrane-bounded organelle;intracellular non-membrane-bounded organelle;intracellular organelle;intracellular organelle part;intracellular part;kinetochore;macromolecular complex;membrane;membrane-bounded organelle;Ndc80 c		Homo sapiens	+
cell part;cytoplasmic part;intracellular membrane-bounded organelle;intracellular non-membrane-bounded organelle;intracellular organelle;intracellular organelle part;intracellular part;macromolecular complex;membrane;membrane-bounded organelle;mitochondrial inner membrane;mitochondrial membrane;mitochondrial part;mitochondrial ribosome;mitochondrion;non-		Homo sapiens	+
cell part;cytoplasm;cytoplasmic part;Golgi apparatus;intracellular membrane-bounded organelle;intracellular non-membrane-bounded organelle;intracellular organelle;intracellular organelle part;intracellular part;membrane;me	Ribosome biogenesis in eukaryotes	Homo sapiens	+
cell part;intracellular organelle part;intracellular part;membrane;nuclear membrane;nuclear part;organelle membrane;organelle part		Homo sapiens	+
apoptosome;cell part;cytoplasm;cytoplasmic part;cytoskeleton;cytosolic part;envelope;extracellular membrane-bounded organelle;extracellular organelle;extracellular region;extracellular region part;extracellular vesicular exosome;intracellular membrane-bounded organelle;intracellular non-membrane-bounded organelle;intracellular organelle;intracellular organelle part;ir		Homo sapiens	+
cell part;cytoplasmic membrane-bounded vesicle;cytoplasmic part;cytoplasmic vesicle;cytoplasmic vesicle membrane;cytoplasmic vesicle part;endocytic vesicle;endocytic vesicle membrane;extracellular membrane-bounded organelle;extracellular organelle;extracellular region part;extracellular vesicular exosome;Golgi apparatus;Golgi apparatus part;Golgi cisterna;Gol		Homo sapiens	+
catalytic step 2 spliceosome;cell part;cytoplasm;intracellular organelle part;intracellular part;macromolecular complex;nuclear body;nuclear part;nuclear speck;nucleoplasm part;organelle part;ribonucleoprotein complex;splic	Spliceosome	Homo sapiens	+
cell part;cytoskeletal part;intracellular membrane-bounded organelle;intracellular organelle;intracellular organelle part;intracellular part;macromolecular complex;membrane-bounded organelle;microtubule;nuclear part;nucleop	Wnt signaling pathway	Homo sapiens	+
cell part;cytoplasm;intracellular membrane-bounded organelle;intracellular organelle;intracellular part;membrane-bounded organelle;nucleus;organelle		Homo sapiens	
cell part;cytoplasm;cytoplasmic part;Golgi apparatus;intracellular membrane-bounded organelle;intracellular organelle;intracellular organelle part;intracellular part;membrane;membrane-bounded organelle;nuclear part;nucleoplasm;nucleus;organelle;organelle part;plasma membrane		Homo sapiens	+
cell part;cytoplasm;extracellular membrane-bounded organelle;extracellular organelle;extracellular region part;extracellular vesicular exosome;intracellular part;membrane;membrane-bounded organelle;membrane-bounded vesicle;organelle;plasma membrane;vesicle		Homo sapiens	+
cell part;cytoplasm;cytoplasmic part;cytoskeletal part;extracellular membrane-bounded organelle;extracellular organelle;extracellular region part;extracellular vesicular exosome;intracellular membrane-bounded organelle;intra	Protein processing in endoplasmic reticulum	Homo sapiens	+
cell part;cytoplasmic part;cytosol;intracellular membrane-bounded organelle;intracellular organelle;intracellular part;membrane-bounded organelle;organelle	Drug metabolism - other enzymes;Pyrimidine metabolism	Homo sapiens	
cell part;cytoplasm;intracellular membrane-bounded organelle;intracellular organelle;intracellular organelle part;intracellular part;macromolecular complex;membrane;membrane-bounded organelle;nuclear part;nucleoplasm;nucleus;organelle;organelle part;ribonucleoprotein complex;spliceosomal complex		Homo sapiens	+
cell part;chromatin remodeling complex;cytoplasmic part;Golgi apparatus;Golgi apparatus part;histone methyltransferase complex;intracellular membrane-bounded organelle;intracellular organelle;intracellular organelle part;intracellular part;macromolecular complex;membrane-bounded organelle;methyltransferase complex;nuclear part;nucleoplasm;nucleoplasm part;nu		Homo sapiens	
cell part;intracellular membrane-bounded organelle;intracellular organelle;intracellular organelle part;intracellular part;membrane-bounded organelle;nuclear body;nuclear part;nuclear speck;nucleoplasm part;nucleus;organelle;organelle part		Homo sapiens	+
CCR4-NOT complex;cell part;chromatin;chromosomal part;cytoplasm;cytoplasmic part;cytoskeleton;cytosol;heterochromatin;intracellular membrane-bounded organelle;intracellular non-membrane-bounded organelle;intracellular organelle;intracellular organelle part;intracellular part;macromolecular complex;membrane;membrane-bounded organelle;non-membrane-bou		Homo sapiens	+
cell part;cytoplasm;intracellular membrane-bounded organelle;intracellular organelle;intracellular organelle part;intracellular part;membrane-bounded organelle;nuclear part;nucleoplasm;nucleus;organelle;organelle part	Ubiquitin mediated proteolysis	Homo sapiens	
		Homo sapiens	+
cell part;chloride channel complex;extracellular membrane-bounded organelle;extracellular organelle;extracellular region part;extracellular vesicular exosome;ion channel complex;macromolecular complex;membrane;membrane part;membrane-bounded organelle;membrane-bounded vesicle;organelle;plasma membrane;protein complex;vesicle		Homo sapiens	+
cell part;intracellular non-membrane-bounded organelle;intracellular organelle;intracellular organelle part;intracellular part;macromolecular complex;non-membrane-bounded organelle;nuclear part;nucleolar part;nucleolus;nucleoplasm;organelle;organelle part;PeBoW complex;preribosome;preribosome, large subunit precursor;protein complex;ribonucleoprotein complex		Homo sapiens	
cell part;cytoplasmic membrane-bounded vesicle;cytoplasmic part;cytoplasmic vesicle;Golgi apparatus;integral to membrane;intracellular membrane-bounded organelle;intracellular organelle;intracellular organelle part;intracellular part;intrinsic to membrane;membrane;membrane part;membrane-bounded organelle;membrane-bounded vesicle;nuclear part;nucleoplasm;		Homo sapiens	+
cell part;intracellular membrane-bounded organelle;intracellular non-membrane-bounded organelle;intracellular organelle;intracellular organelle part;intracellular part;membrane-bounded organelle;non-membrane-bounded organelle;nuclear part;nucleolus;nucleus;organelle;organelle part		Homo sapiens	+
cell part;cytoplasm;intracellular non-membrane-bounded organelle;intracellular organelle;intracellular organelle part;intracellular part;membrane;non-membrane-bounded organelle;nuclear part;nucleolus;organelle;organelle part		Homo sapiens	+
cell part;DNA-directed RNA polymerase complex;DNA-directed RNA polymerase I complex;intracellular membrane-bounded organelle;intracellular non-membrane-bounded organelle;intracellular organelle;intracellular organe	Purine metabolism;Pyrimidine metabolism;RNA polymerase	Homo sapiens	+
Cdc73/Paf1 complex;cell part;chromatin;chromosomal part;cytoplasm;cytoplasmic part;intracellular membrane-bounded organelle;intracellular organelle;intracellular organelle part;intracellular part;macromolecular complex;r	RNA degradation	Homo sapiens	
acrosomal vesicle;cell part;cytoplasmic membrane-bounded vesicle;cytoplasmic part;cytoplasmic vesicle;intracellular membrane-bounded organelle;intracellular organelle;intracellular part;macromolecular complex;membrane-bounded organelle;membrane-bounded vesicle;mitochondrion;nucleus;organelle;perinuclear region of cytoplasm;ribonucleoprotein complex;stor		Homo sapiens	+
cell part;cytoplasm;cytoplasmic part;intracellular membrane-bounded organelle;intracellular organelle;intracellular part;membrane-bounded organelle;mitochondrion;nucleus;organelle		Homo sapiens	+
cell part;cytoplasm;cytoplasmic part;cytosol;intracellular membrane-bounded organelle;intracellular organelle;intracellular part;membrane-bounded organelle;nucleus;organelle	Pathways in cancer;Renal cell carcinoma	Homo sapiens	+
cell part;intracellular membrane-bounded organelle;intracellular organelle;intracellular part;membrane-bounded organelle;nucleus;organelle		Homo sapiens	+
extracellular membrane-bounded organelle;extracellular organelle;extracellular region part;extracellular space;extracellular vesicular exosome;membrane-bounded organelle;membrane-bounded vesicle;organelle;vesicle		Homo sapiens	+
cell junction;cell part;clathrin-coated vesicle;coated vesicle;cytoplasmic membrane-bounded vesicle;cytoplasmic part;cytoplasmic vesicle;integral to membrane;integral to mitochondrial membrane;integral to organelle membrane;intracellular membrane-bounded organelle;intracellular organelle;intracellular organelle part;intracellular part;intrinsic to membrane;intrinsic to		Homo sapiens	+
cell part;cytoplasm;extracellular membrane-bounded organelle;extracellular organelle;extracellular region part;extracellular vesicular exosome;intracellular part;membrane-bounded organelle;membrane-bounded vesicle;organelle;vesicle		Homo sapiens	+
cell part;cytoplasmic part;integral to membrane;intracellular membrane-bounded organelle;intracellular organelle;intracellular organelle part;intracellular part;intrinsic to membrane;membrane;membrane part;membrane-bounded organelle;mitochondrial inner membrane;mitochondrial membrane;mitochondrial part;mitochondrion;organelle;organelle inner membrane;organ		Homo sapiens	+
cell part;cytoplasm;cytoplasmic part;cytosol;intracellular part	RNA transport	Homo sapiens	
cell part;cytoplasmic part;intracellular membrane-bounded organelle;intracellular organelle;intracellular part;membrane-bounded organelle;mitochondrion;organelle		Homo sapiens	+
cell part;intracellular membrane-bounded organelle;intracellular non-membrane-bounded organelle;intracellular organelle;intracellular organelle part;intracellular part;membrane;membrane-bounded organelle;non-membrane-	Ribosome biogenesis in eukaryotes	Homo sapiens	
cell part;cell projection;cell projection part;ciliary rootlet;cilium part;cytoplasmic part;cytoskeletal part;cytosol;intracellular organelle part;intracellular part;kinesin complex;kinesin I complex;macromolecular complex;membrane;microtubule;microtubule associated complex;neuron projection;organelle part;protein complex		Homo sapiens	+
cell part;cytoplasm;intracellular part		Homo sapiens	+
aggresome;cell part;inclusion body;intracellular membrane-bounded organelle;intracellular non-membrane-bounded organelle;intracellular organelle;intracellular organelle part;intracellular part;membrane;membrane-bounded	Ribosome biogenesis in eukaryotes;RNA degradation	Homo sapiens	+
cell part;intracellular membrane-bounded organelle;intracellular organelle;intracellular organelle part;intracellular part;macromolecular complex;membrane-bounded organelle;nuclear body;nuclear part;nuclear speck;nucleoplasm part;nucleus;organelle;organelle part;ribonucleoprotein complex		Homo sapiens	+
acrosomal vesicle;cell division site part;cell part;centralspindlin complex;cleavage furrow;cytoplasmic membrane-bounded vesicle;cytoplasmic part;cytoplasmic vesicle;cytoskeletal part;cytosol;extracellular membrane-bounded organelle;extracellular organelle;extracellular region part;extracellular vesicular exosome;extrinsic to internal side of plasma membrane;extrinsic		Homo sapiens	+
cell part;intracellular;intracellular membrane-bounded organelle;intracellular organelle;intracellular part;membrane-bounded organelle;nucleus;organelle	mRNA surveillance pathway	Homo sapiens	+
cell part;cytoplasm;intracellular non-membrane-bounded organelle;intracellular organelle;intracellular organelle part;intracellular part;membrane;non-membrane-bounded organelle;nuclear part;nucleolus;organelle;organelle part		Homo sapiens	
cell part;cytoplasmic part;endoplasmic reticulum part;integral to membrane;integral to plasma membrane;intracellular organelle part;intracellular part;intrinsic to membrane;intrinsic to plasma membrane;macromolecular complex;membrane;membrane part;oligosaccharyltransferase complex;organelle part;plasma membrane;plasma membrane part;protein complex		Homo sapiens	+
cell part;cytoplasmic part;endoplasmic reticulum membrane;endoplasmic reticulum part;endoplasmic reticulum-Golgi intermediate compartment;extracellular membrane-bounded organelle;extracellular organelle;extracellular region part;extracellular vesicular exosome;Golgi apparatus;Golgi apparatus part;Golgi membrane;intracellular membrane-bounded organelle;intra		Homo sapiens	+
cell part;cytoplasmic part;extracellular region part;extracellular space;intracellular membrane-bounded organelle;intracellular non-membrane-bounded organelle;intracellular organelle;intracellular organelle part;intracellular part;macromolecular complex;membrane;membrane-bounded organelle;mitochondrial inner membrane;mitochondrial membrane;mitochondrial part;r		Homo sapiens	
cell part;extracellular membrane-bounded organelle;extracellular organelle;extracellular region part;extracellular vesicular exosome;intracellular membrane-bounded organelle;intracellular organelle;intracellular part;membrane-bounded organelle;membrane-bounded vesicle;nucleus;organelle;vesicle		Homo sapiens	+
cell part;extracellular region part;extracellular space;intracellular membrane-bounded organelle;intracellular organelle;intracellular organelle part;intracellular part;membrane-bounded organelle;nuclear part;nucleoplasm;nucleus;organelle;organelle part		Homo sapiens	+

cell part;cytoplasm;intracellular membrane-bounded organelle;intracellular organelle;intracellular part;membrane-bounded organelle;nucleus;organelle		Homo sapiens	+
cell part;cell surface;cytoplasmic part;endoplasmic reticulum lumen;endoplasmic reticulum part;extracellular matrix;extracellular membrane-bounded organelle;extracellular organelle;extracellular region;extracellular region pa	Basal cell carcinoma;Hedgehog signaling pathway;Melanogenesis;Pathways in cancer;Wnt signaling pathway	Homo sapiens	+
cell part;cytoplasm;cytoplasmic part;cytosol;intracellular membrane-bounded organelle;intracellular organelle;intracellular organelle lumen;intracellular organelle part;intracellular part;membrane-bounded organelle;membrane-enclosed lumen;mitochondrial matrix;mitochondrial part;mitochondrion;nucleus;organelle;organelle lumen;organelle part		Homo sapiens	
Atg12-Atg5-Atg16 complex;autophagic vacuole;axoneme;cell part;cell projection part;cytoplasm;cytoplasmic part;endoplasmic reticulum part;ER-mitochondrion membrane contact site;intracellular membrane-bounded orgar	Regulation of autophagy;RIG-I-like receptor signaling pathway;Shigellosis	Homo sapiens	+
cell part;cytoplasm;intracellular organelle part;intracellular part;nuclear part;nucleoplasm;organelle part		Homo sapiens	+
cell part;cytoplasmic part;early endosome membrane;endoplasmic reticulum;endosomal part;endosome membrane;extracellular membrane-bounded organelle;extracellular organelle;extracellular region part;extracellular vesi	Endocytosis	Homo sapiens	+
cell part;cell projection;integral to membrane;intracellular part;intrinsic to membrane;membrane;membrane part;microvillus;organelle part;plasma membrane;stereocilium		Homo sapiens	+
cell part;cytoplasm;cytoplasmic membrane-bounded vesicle;cytoplasmic part;cytoplasmic vesicle;endosomal part;endosome;endosome membrane;intracellular membrane-bounded organelle;intracellular organelle;intracellular organelle part;intracellular part;late endosome;late endosome membrane;lysosomal membrane;lysosome;lytic vacuole;membrane;membrane-bo		Homo sapiens	
cell part;cytoplasmic membrane-bounded vesicle;cytoplasmic part;cytoplasmic vesicle;endocytic vesicle;endosomal part;endosome;endosome membrane;HOPS complex;intracellular membrane-bounded organelle;intracellular organelle;intracellular organelle part;intracellular part;late endosome;late endosome membrane;lysosomal membrane;lysosome;lytic vacuole;ma		Homo sapiens	
cell part;cell projection;cytoplasm;extracellular membrane-bounded organelle;extracellular organelle;extracellular region part;extracellular vesicular exosome;intracellular membrane-bounded organelle;intracellular organelle;intracellular part;lamellipodium;membrane-bounded organelle;membrane-bounded vesicle;nucleus;organelle;vesicle		Homo sapiens	+
actin cytoskeleton;adherens junction;anchoring junction;cell junction;cell part;cell-substrate adherens junction;cell-substrate junction;cytoplasm;cytoplasmic part;cytoskeletal part;cytoskeleton;focal adhesion;intracellular membrane-bounded organelle;intracellular non-membrane-bounded organelle;intracellular organelle;intracellular organelle part;intracellular part;membr		Homo sapiens	
cell part;cytoplasm;extracellular membrane-bounded organelle;extracellular organelle;extracellular region part;extracellular vesicular exosome;intracellular part;membrane-bounded organelle;membrane-bounded vesicle;orgal	Oocyte meiosis	Homo sapiens	
catalytic step 2 spliceosome;cell part;intracellular organelle part;intracellular part;macromolecular complex;nuclear part;organelle part;ribonucleoprotein complex;spliceosomal complex		Homo sapiens	+
cell part;cytoplasm;intracellular membrane-bounded organelle;intracellular organelle;intracellular part;membrane;membrane-bounded organelle;nucleus;organelle;plasma membrane	MAPK signaling pathway	Homo sapiens	+
cell part;cytoplasm;intracellular part;macromolecular complex;membrane;plasma membrane;protein complex		Homo sapiens	+
cell part;extracellular region part;extracellular space;intracellular membrane-bounded organelle;intracellular organelle;intracellular part;membrane-bounded organelle;nucleus;organelle		Homo sapiens	
cell part;cytoplasmic part;cytosol;intracellular part		Homo sapiens	
cell part;chromosomal part;chromosome, telomeric region;cytoplasm;cytoplasmic part;cytosol;extracellular membrane-bounded organelle;extracellular organelle;extracellular region part;extracellular vesicular exosome;intrac	RNA degradation	Homo sapiens	
cell part;cytoplasmic part;extracellular membrane-bounded organelle;extracellular organelle;extracellular region part;extracellular vesicular exosome;intracellular organelle lumen;intracellular organelle part;intracellular part;me	Oxidative phosphorylation	Homo sapiens	+
cell junction;cell part;cytoplasmic part;intracellular membrane-bounded organelle;intracellular non-membrane-bounded organelle;intracellular organelle;intracellular organelle part;intracellular part;macromolecular complex;membrane;membrane-bounded organelle;mitochondrial inner membrane;mitochondrial membrane;mitochondrial part;mitochondrion;non-membrane-		Homo sapiens	+
anchoring junction;catalytic step 2 spliceosome;cell junction;cell part;cell-cell junction;cytoplasm;cytoskeletal part;desmosome;intermediate filament;intracellular organelle part;intracellular part;macromolecular complex;mem	mRNA surveillance pathway;RNA transport	Homo sapiens	
cell part;integral to membrane;intrinsic to membrane;membrane part		Homo sapiens	
extracellular membrane-bounded organelle;extracellular organelle;extracellular region part;extracellular vesicular exosome;membrane-bounded organelle;membrane-bounded vesicle;organelle;vesicle		Homo sapiens	
cell part;cytoplasmic part;endoplasmic reticulum;endoplasmic reticulum membrane;endoplasmic reticulum part;integral to membrane;intracellular membrane-bounded organelle;intracellular non-membrane-bounded organelle;intracellular organelle;intracellular organelle part;intracellular part;intrinsic to membrane;membrane;membrane part;membrane-bounded organelle		Homo sapiens	+
cell part;cytoplasmic part;Golgi apparatus;Golgi apparatus part;Golgi membrane;integral to membrane;intracellular membrane-bounded organelle;intracellular organelle;intracellular organelle part;intracellular part;intrinsic to membrane;membrane;membrane part;membrane-bounded organelle;mitochondrion;organelle;organelle membrane;organelle part		Homo sapiens	
actin cytoskeleton;cell part;cytoplasm;cytoskeleton;endomembrane system;intracellular non-membrane-bounded organelle;intracellular organelle;intracellular part;membrane;microtubule cytoskeleton;non-membrane-bounded organelle;organelle;plasma membrane		Homo sapiens	
cell part;cytoplasm;cytoplasmic membrane-bounded vesicle;cytoplasmic part;cytoplasmic vesicle;early endosome;endosome;extracellular membrane-bounded organelle;extracellular organelle;extracellular region part;extracellular vesicular exosome;intracellular membrane-bounded organelle;intracellular organelle;intracellular organelle part;intracellular part;membrane-b		Homo sapiens	+
cell part;cytoplasm;cytoplasmic part;Golgi apparatus;intracellular membrane-bounded organelle;intracellular organelle;intracellular organelle part;intracellular part;membrane-bounded organelle;nuclear part;nucleoplasm;organelle;organelle part;perinuclear region of cytoplasm		Homo sapiens	
cell part;chromosomal part;cytoplasm;cytoplasmic part;cytosol;intracellular membrane-bounded organelle;intracellular organelle;intracellular organelle part;intracellular part;macromolecular complex;membrane-bounded organelle;MIS12/MIND type complex;nucleus;organelle;organelle part;protein complex		Homo sapiens	+
cell part;cytoplasm;intracellular part		Homo sapiens	+
cell part;cytoplasm;cytoplasmic part;cytosol;endosomal part;endosome;endosome membrane;ESCRT III complex;extracellular membrane-bounded organelle;extracellular organelle;extracellular region part;extracellular vesicu	Endocytosis	Homo sapiens	+
cell part;cytoplasmic part;endoplasmic reticulum;intracellular membrane-bounded organelle;intracellular organelle;intracellular part;membrane;membrane-bounded organelle;organelle	Other types of O-glycan biosynthesis	Homo sapiens	+
cell part;cytoplasm;intracellular part		Homo sapiens	+
cell part;extracellular membrane-bounded organelle;extracellular organelle;extracellular region;extracellular region part;extracellular space;extracellular vesicular exosome;membrane;membrane-bounded organelle;membrane-bounded vesicle;organelle;plasma membrane;vesicle		Homo sapiens	+
cell part;cytoplasmic part;cytosol;endosome;Golgi apparatus;Golgi apparatus part;Golgi cisterna;Golgi cisterna membrane;Golgi membrane;intracellular membrane-bounded organelle;intracellular organelle;intracellular organelle part;intracellular part;membrane;membrane-bounded organelle;membrane-enclosed lumen;mitochondrial intermembrane space;mitochondrial		Homo sapiens	+
cell part;cytoplasm;cytoskeletal part;extracellular membrane-bounded organelle;extracellular organelle;extracellular region part;extracellular vesicular exosome;intracellular organelle part;intracellular part;macromolecular com	Gap junction;Pathogenic Escherichia coli infection;Phagosome	Homo sapiens	+
cell part;cytoplasmic part;cytoskeleton;cytosol;extrinsic to membrane;intracellular non-membrane-bounded organelle;intracellular organelle;intracellular part;membrane;membrane part;non-membrane-bounded organelle;orga	Tight junction	Homo sapiens	
cell part;cytoplasmic part;extracellular membrane-bounded organelle;extracellular organelle;extracellular region part;extracellular vesicular exosome;Golgi apparatus part;Golgi membrane;intracellular organelle part;intracellular part;membrane;membrane-bounded organelle;membrane-bounded vesicle;organelle;organelle membrane;organelle part;vesicle		Homo sapiens	+
cell part;cytoplasm;histone methyltransferase complex;intracellular membrane-bounded organelle;intracellular non-membrane-bounded organelle;intracellular organelle;intracellular organelle part;intracellular part;macromolecular complex;membrane-bounded organelle;methyltransferase complex;MLL1 complex;non-membrane-bounded organelle;nuclear part;nucleolus;		Homo sapiens	+
cell part;cytoplasmic part;cytosol;endoplasmic reticulum part;intracellular organelle part;intracellular part;membrane;nuclear membrane;nuclear part;organelle membrane;organelle part;perinuclear endoplasmic reticulum;plasma membrane		Homo sapiens	+
cell part;cytoplasmic part;early endosome membrane;endosomal part;endosome membrane;extracellular membrane-bounded organelle;extracellular organelle;extracellular region part;extracellular vesicular exosome;intracell	Endocytosis	Homo sapiens	+
cell part;cytoplasm;cytoplasmic part;endoplasmic reticulum membrane;endoplasmic reticulum part;integral to membrane;intracellular membrane-bounded organelle;intracellular organelle;intracellular organelle part;intracellula	N-Glycan biosynthesis;Various types of N-glycan biosynthesis	Homo sapiens	+
cell part;extracellular region;integral to membrane;intrinsic to membrane;membrane;membrane part;plasma membrane		Homo sapiens	+
cell part;intracellular membrane-bounded organelle;intracellular non-membrane-bounded organelle;intracellular organelle;intracellular organelle part;intracellular part;membrane-bounded organelle;non-membrane-bounded organelle;nuclear part;nucleolus;nucleoplasm;nucleus;organelle;organelle part		Homo sapiens	+
cell part;chromosome;condensed chromosome;condensed nuclear chromosome;CURI complex;cytoplasmic part;intracellular membrane-bounded organelle;intracellular non-membrane-bounded organelle;intracellular organe	Ribosome biogenesis in eukaryotes	Homo sapiens	+
cell part;cytoplasmic part;endoplasmic reticulum;intracellular membrane-bounded organelle;intracellular organelle;intracellular part;membrane-bounded organelle;nucleus;organelle		Homo sapiens	
		Homo sapiens	
cell part;cytoplasm;intracellular membrane-bounded organelle;intracellular organelle;intracellular organelle part;intracellular part;membrane-bounded organelle;nuclear part;nucleoplasm;nucleus;organelle;organelle part		Homo sapiens	+
cell part;cytoplasmic part;cytosol;intracellular part		Homo sapiens	+
cell part;cytoplasm;intracellular part		Homo sapiens	
cell part;intracellular membrane-bounded organelle;intracellular non-membrane-bounded organelle;intracellular organelle;intracellular organelle part;intracellular part;membrane-bounded organelle;non-membrane-bounded organelle;nuclear part;nucleolus;nucleus;organelle;organelle part		Homo sapiens	+
cell part;cytoplasm;intracellular organelle part;intracellular part;nuclear part;nucleoplasm;organelle part		Homo sapiens	+
cell part;cytoplasm;intracellular organelle part;intracellular part;nuclear part;nucleoplasm;organelle part		Homo sapiens	+
		Homo sapiens	+
cell part;intracellular membrane-bounded organelle;intracellular organelle;intracellular part;membrane-bounded organelle;nucleus;organelle		Homo sapiens	+
cell part;cytoplasmic part;cytosol;Golgi apparatus part;Golgi membrane;integral to membrane;intracellular membrane-bounded organelle;intracellular organelle;intracellular organelle part;intracellular part;intrinsic to membran	Arachidonic acid metabolism	Homo sapiens	+
Cajal body;cell body;cell part;cytoplasmic part;cytosol;intracellular membrane-bounded organelle;intracellular non-membrane-bounded organelle;intracellular organelle;intracellular organelle part;intracellular part;membrane-b	RNA transport	Homo sapiens	+
cell part;cytoplasm;intracellular organelle part;intracellular part;nuclear part;nucleoplasm;organelle part	Ubiquitin mediated proteolysis	Homo sapiens	+
cell part;cell projection;cytoplasmic part;dendrite;intracellular membrane-bounded organelle;intracellular organelle;intracellular part;membrane-bounded organelle;mitochondrion;neuron projection;nucleus;organelle	Geraniol degradation;Naphthalene degradation	Homo sapiens	+
cell part;cytoplasmic part;cytosol;extracellular membrane-bounded organelle;extracellular organelle;extracellular region part;extracellular vesicular exosome;intracellular part;membrane-bounded organelle;membrane-bounde	Cell cycle - yeast	Homo sapiens	+
		Homo sapiens	+
cell division site part;cell junction;cell part;cell-cell junction;centralspindlin complex;cleavage furrow;cytoplasm;cytoplasmic part;cytoskeletal part;cytosol;intracellular membrane-bounded organelle;intracellular non-membrane-bounded organelle;intracellular organelle;intracellular organelle part;intracellular part;macromolecular complex;membrane;membrane-bounded or		Homo sapiens	+
cell part;membrane		Homo sapiens	+
cell part;cytoplasmic part;Golgi apparatus;Golgi apparatus part;Golgi membrane;intracellular membrane-bounded organelle;intracellular organelle;intracellular organelle part;intracellular part;membrane;membrane-bounded organelle;organelle;organelle membrane;organelle part		Homo sapiens	+
cell part;cytoplasm;intracellular membrane-bounded organelle;intracellular organelle;intracellular part;membrane;membrane-bounded organelle;nucleus;organelle;plasma membrane		Homo sapiens	+
cell part;cytoplasmic part;integral to membrane;intracellular membrane-bounded organelle;intracellular organelle;intracellular organelle part;intracellular part;intrinsic to membrane;membrane;membrane part;membrane-bounded organelle;mitochondrial inner membrane;mitochondrial membrane;mitochondrial part;mitochondrion;nuclear part;nucleoplasm;organelle;organe		Homo sapiens	+
cell part;membrane		Homo sapiens	+
cell part;cytoplasmic part;integral to membrane;intracellular membrane-bounded organelle;intracellular organelle;intracellular organelle part;intracellular part;intrinsic to membrane;membrane;membrane part;membrane-bounded organelle;mitochondrial inner membrane;mitochondrial membrane;mitochondrial part;mitochondrion;organelle;organelle inner membrane;organ		Homo sapiens	+
cell part;cytoplasmic part;Golgi apparatus part;Golgi membrane;Golgi transport complex;intracellular organelle part;intracellular part;macromolecular complex;membrane;organelle membrane;organelle part;protein complex		Homo sapiens	+
cell part;cytoplasm;intracellular organelle part;intracellular part;macromolecular complex;nuclear part;nucleoplasm;organelle part;protein complex;signalosome		Homo sapiens	+
cell part;cytoplasm;Elongator holoenzyme complex;histone acetyltransferase complex;intracellular non-membrane-bounded organelle;intracellular organelle;intracellular organelle part;intracellular part;macromolecular complex;non-membrane-bounded organelle;nuclear part;nucleolus;nucleoplasm part;organelle;organelle part;protein complex;transcription elongation fac		Homo sapiens	+
		Homo sapiens	+
cell part;cytoplasmic part;cytosol;extracellular membrane-bounded organelle;extracellular organelle;extracellular region part;extracellular vesicular exosome;intracellular part;membrane-bounded organelle;membrane-bounde	Pantothenate and CoA biosynthesis	Homo sapiens	
cell part;cytoplasmic part;extracellular membrane-bounded organelle;extracellular organelle;extracellular region part;extracellular vesicular exosome;intracellular organelle part;intracellular part;lysosomal membrane;membran	Chemokine signaling pathway	Homo sapiens	+
cell part;cytoplasm;cytoplasmic part;cytosol;intracellular membrane-bounded organelle;intracellular organelle;intracellular organelle part;intracellular part;macromolecular complex;membrane-bounded organelle;nuclear part;r	RNA transport	Homo sapiens	+
cell part;cytoplasmic part;intracellular membrane-bounded organelle;intracellular organelle;intracellular organelle lumen;intracellular organelle part;intracellular part;membrane-bounded organelle;membrane-enclosed lumen;mitochondrial matrix;mitochondrial part;mitochondrion;organelle;organelle lumen;organelle part		Homo sapiens	+
cell part;cytoplasmic part;extracellular membrane-bounded organelle;extracellular organelle;extracellular region part;extracellular vesicular exosome;intracellular membrane-bounded organelle;intracellular organelle;intracellular organelle part;intracellular part;membrane-bounded organelle;membrane-bounded vesicle;mitochondrion;nuclear part;nucleoplasm;nucleus;orga		Homo sapiens	+
basement membrane;cell part;extracellular matrix part;extracellular region part;membrane;plasma membrane	Axon guidance	Homo sapiens	+
beta-catenin destruction complex;cell body;cell part;cell projection;cytoplasm;cytoplasmic part;extracellular membrane-bounded organelle;extracellular organelle;extracellular region part;extracellular vesicular exosome;intrac	Wnt signaling pathway	Homo sapiens	+
cell part;cytoplasm;cytoplasmic part;EGO complex;intracellular membrane-bounded organelle;intracellular organelle;intracellular organelle part;intracellular part;lysosome;lytic vacuole;macromolecular complex;membrane part;membrane-bounded organelle;nuclear part;nucleoplasm;nucleus;organelle;organelle part;protein complex;vacuolar part;vacuole		Homo sapiens	
cell part;cytoplasmic part;endoplasmic reticulum;endoplasmic reticulum membrane;endoplasmic reticulum part;intracellular membrane-bounded organelle;intracellular organelle;intracellular organelle part;intracellular part;lysosomal membrane;membrane;membrane part;membrane-bounded organelle;mitochondrion;nucleus;organelle;organelle membrane;organelle part;v		Homo sapiens	+
cell part;cytoplasmic part;early endosome membrane;endosomal part;endosome membrane;Golgi apparatus;Golgi apparatus part;Golgi membrane;integral to membrane;intracellular membrane-bounded organelle;intracellular organelle;intracellular organelle part;intracellular part;intrinsic to membrane;late endosome membrane;lysosomal membrane;membrane;membrar		Homo sapiens	+
cell part;cytoplasm;cytoskeletal part;intracellular non-membrane-bounded organelle;intracellular organelle;intracellular organelle part;intracellular part;macromolecular complex;membrane;microtubule;mitotic spindle;non-membrane-bounded organelle;organelle;organelle part;protein complex;spindle		Homo sapiens	+
cell part;cytoplasmic part;extracellular membrane-bounded organelle;extracellular organelle;extracellular region part;extracellular vesicular exosome;intracellular membrane-bounded organelle;intracellular organelle;intracellular part;membrane-bounded organelle;membrane-bounded vesicle;mitochondrion;organelle;vesicle		Homo sapiens	+
cell part;chromatin;chromosomal part;heterochromatin;intracellular membrane-bounded organelle;intracellular organelle;intracellular organelle part;intracellular part;macromolecular complex;membrane-bounded organelle;nuclear chromatin;nuclear chromosome part;nuclear part;nucleoplasm;nucleus;organelle;organelle part;PcG protein complex;PRC1 complex;protein c		Homo sapiens	+
cell part;cytoplasmic part;cytosol;intracellular membrane-bounded organelle;intracellular organelle;intracellular organelle lumen;intracellular organelle part;intracellular part;membrane-bounded organelle;membrane-enclosed l	Valine, leucine and isoleucine degradation	Homo sapiens	+
actin cytoskeleton;cell part;cytoskeleton;extracellular region part;extracellular space;intracellular membrane-bounded organelle;intracellular non-membrane-bounded organelle;intracellular organelle;intracellular organelle part;intracellular part;membrane-bounded organelle;non-membrane-bounded organelle;nuclear part;nucleoplasm;nucleus;organelle;organelle part		Homo sapiens	+
cell part;cytoplasmic mRNA processing body;cytoplasmic part;cytosol;extracellular region part;extracellular space;intracellular non-membrane-bounded organelle;intracellular organelle;intracellular part;macromolecular complex;non-membrane-bounded organelle;organelle;ribonucleoprotein complex;RNA granule		Homo sapiens	+
catalytic step 2 spliceosome;cell part;cytoplasm;cytoplasmic part;Golgi apparatus;intracellular membrane-bounded organelle;intracellular organelle;intracellular organelle part;intracellular part;macromolecular complex;membrane-bounded organelle;nuclear body;nuclear part;nuclear speck;nucleoplasm;nucleoplasm part;organelle;organelle part;ribonucleoprotein comple		Homo sapiens	+
cell part;histone methyltransferase complex;intracellular membrane-bounded organelle;intracellular organelle;intracellular organelle part;intracellular part;macromolecular complex;membrane-bounded organelle;methyltransfei	Wnt signaling pathway	Homo sapiens	+
cell part;cytoplasm;intracellular membrane-bounded organelle;intracellular organelle;intracellular part;membrane-bounded organelle;nucleus;organelle		Homo sapiens	
cell part;cytoplasmic part;endoplasmic reticulum lumen;endoplasmic reticulum part;intracellular organelle lumen;intracellular organelle part;intracellular part;membrane;membrane-enclosed lumen;organelle lumen;organelle part		Homo sapiens	+
catalytic step 2 spliceosome;cell part;intracellular membrane-bounded organelle;intracellular organelle;intracellular organelle part;intracellular part;macromolecular complex;membrane;membrane-bounded organelle;nuclear p	Spliceosome	Homo sapiens	+
cell part;cytoplasm;extracellular matrix;extracellular membrane-bounded organelle;extracellular organelle;extracellular region part;extracellular vesicular exosome;integral to membrane;intracellular part;intrinsic to membrane;membrane part;membrane-bounded organelle;membrane-bounded vesicle;organelle;proteinaceous extracellular matrix;vesicle		Homo sapiens	+
cell part;cytoplasmic part;endoplasmic reticulum membrane;endoplasmic reticulum part;Golgi apparatus part;Golgi membrane;integral to membrane;intracellular membrane-bounded organelle;intracellular organelle;intracellul	Protein processing in endoplasmic reticulum	Homo sapiens	
cell part;cytoplasm;cytoskeleton;extracellular region part;intercellular bridge;intracellular membrane-bounded organelle;intracellular non-membrane-bounded organelle;intracellular organelle;intracellular organelle part;intracellular part;macromolecular complex;membrane;membrane-bounded organelle;microtubule cytoskeleton;non-membrane-bounded organelle;nuclear		Homo sapiens	+
cell part;cytoplasmic part;endoplasmic reticulum membrane;endoplasmic reticulum part;integral to membrane;intracellular organelle part;intracellular part;intrinsic to membrane;membrane;membrane part;organelle membrane;organelle part		Homo sapiens	+
cell junction;cell part;cell projection;clathrin-coated vesicle;coated vesicle;cytoplasm;cytoplasmic membrane-bounded vesicle;cytoplasmic part;cytoplasmic vesicle;cytoplasmic vesicle membrane;cytoplasmic vesicle part;cytoskeletal part;dendrite;Golgi apparatus;Golgi apparatus part;Golgi membrane;Golgi-associated vesicle;Golgi-associated vesicle membrane;intrace		Homo sapiens	+
cell part;cytoplasmic part;intracellular membrane-bounded organelle;intracellular organelle;intracellular organelle part;intracellular part;large ribosomal subunit;macromolecular complex;membrane;membrane-bounded organelle;mitochondrial inner membrane;mitochondrial large ribosomal subunit;mitochondrial membrane;mitochondrial part;mitochondrion;organellar larg		Homo sapiens	
cell part;chromosome;condensed chromosome;condensed nuclear chromosome;cytoplasmic part;cytoskeletal part;early endosome;endomembrane system;endosomal part;endosome;ESCRT III complex;extracellular membrane-bounded organelle;extracellular organelle;extracellular region part;extracellular vesicular exosome;intracellular membrane-bounded organelle;i		Homo sapiens	+
cell part;integral to membrane;intrinsic to membrane;membrane part		Homo sapiens	+
cell part;cell surface;extracellular membrane-bounded organelle;extracellular organelle;extracellular region part;extracellular vesicular exosome;integral to membrane;intrinsic to membrane;membrane;membrane part;membrane-bounded organelle;membrane-bounded vesicle;organelle;vesicle		Homo sapiens	+
cell part;cytoplasm;intracellular part;membrane;plasma membrane		Homo sapiens	+
cell part;cytoplasm;cytoplasmic part;cytosol;Golgi apparatus;Golgi apparatus part;Golgi membrane;intracellular membrane-bounded organelle;intracellular organelle;intracellular organelle part;intracellular part;membrane;mer	Endocytosis	Homo sapiens	+
cell part;cytoplasmic part;endoplasmic reticulum part;endoplasmic reticulum tubular network;extracellular membrane-bounded organelle;extracellular organelle;extracellular region part;extracellular vesicular exosome;Golgi apparatus;intracellular;intracellular membrane-bounded organelle;intracellular organelle;intracellular organelle part;intracellular part;membrane;memb		Homo sapiens	
cell part;cytoplasm;intracellular membrane-bounded organelle;intracellular organelle;intracellular part;membrane-bounded organelle;nucleus;organelle		Homo sapiens	+
cell part;cytoplasmic part;cytosol;endosomal part;endosome membrane;extracellular membrane-bounded organelle;extracellular organelle;extracellular region part;extracellular vesicular exosome;intracellular organelle part;in	Endocytosis	Homo sapiens	+
cell part;cytoplasmic part;intracellular membrane-bounded organelle;intracellular organelle;intracellular organelle lumen;intracellular organelle part;intracellular part;membrane-bounded organelle;membrane-enclosed lumen;m	Aminoacyl-tRNA biosynthesis	Homo sapiens	+
cell part;cytoplasmic part;intracellular non-membrane-bounded organelle;intracellular organelle;intracellular organelle part;intracellular part;macromolecular complex;membrane;mitochondrial inner membrane;mitochondrial membrane;mitochondrial part;non-membrane-bounded organelle;organelle;organelle inner membrane;organelle membrane;organelle part;ribonucleo		Homo sapiens	+
cell part;cell projection;centrosome;cilium;cytoplasm;cytoplasmic dynein complex;cytoplasmic part;cytoskeletal part;dynein complex;intracellular non-membrane-bounded organelle;intracellular organelle;intracellular organelle part;intracellular part;macromolecular complex;membrane;microtubule;microtubule associated complex;microtubule organizing center;non-memb		Homo sapiens	+
cell part;ER membrane protein complex;integral to membrane;intrinsic to membrane;macromolecular complex;membrane part;protein complex		Homo sapiens	+
cell part;chromatin;chromosomal part;cytoplasm;cytoplasmic exosome (RNase complex);cytoplasmic part;cytosol;exosome (RNase complex);intracellular membrane-bounded organelle;intracellular non-membrane-bounded	RNA degradation	Homo sapiens	+
box H/ACA RNP complex;Cajal body;cell part;intracellular non-membrane-bounded organelle;intracellular organelle;intracellular organelle part;intracellular part;macromolecular complex;non-membrane-bounded organelle;nu	Ribosome biogenesis in eukaryotes	Homo sapiens	+
cell part;cytoplasm;EKC/KEOPS complex;intracellular membrane-bounded organelle;intracellular organelle;intracellular part;macromolecular complex;membrane-bounded organelle;nucleus;organelle;protein complex		Homo sapiens	
cell part;cytoplasmic part;cytoskeletal part;cytosol;extracellular membrane-bounded organelle;extracellular organelle;extracellular region part;extracellular vesicular exosome;intracellular membrane-bounded organelle;intracellular non-membrane-bounded organelle;intracellular organelle;intracellular organelle part;intracellular part;membrane-bounded organelle;membran		Homo sapiens	
cell part;cytoplasm;intracellular part;membrane;plasma membrane		Homo sapiens	
cell part;intracellular organelle part;intracellular part;macromolecular complex;nuclear part;organelle part;ribonucleoprotein complex;small nuclear ribonucleoprotein complex;U1 snRNP		Homo sapiens	+
BBSome;cell part;cytoplasmic part;cytosol;intracellular part;macromolecular complex;protein complex		Homo sapiens	+
cell part;intracellular membrane-bounded organelle;intracellular non-membrane-bounded organelle;intracellular organelle;intracellular organelle part;intracellular part;membrane-bounded organelle;non-membrane-bounded organelle;nuclear part;nucleolus;nucleus;organelle;organelle part		Homo sapiens	+
cell part;cytoplasmic part;cytosol;intracellular;intracellular part	Fructose and mannose metabolism	Homo sapiens	
cell body;cell part;cell projection;cytoplasmic part;endoplasmic reticulum;endoplasmic reticulum part;envelope;extracellular membrane-bounded organelle;extracellular organelle;extracellular region part;extracellular vesicular exosome;integral to endoplasmic reticulum membrane;integral to membrane;integral to organelle membrane;intracellular;intracellular membrane-b		Homo sapiens	+
cell part;DNA-directed RNA polymerase II, holoenzyme;intracellular membrane-bounded organelle;intracellular organelle;intracellular organelle part;intracellular part;macromolecular complex;membrane-bounded organelle;nuclear part;nucleoplasm part;nucleus;organelle;organelle part;protein complex		Homo sapiens	
cell part;cytoplasmic part;extracellular membrane-bounded organelle;extracellular organelle;extracellular region part;extracellular vesicular exosome;intracellular membrane-bounded organelle;intracellular organelle;intracellular part;membrane-bounded organelle;membrane-bounded vesicle;mitochondrion;organelle;vesicle		Homo sapiens	+
cell part;centrosome;cytoplasm;cytoplasmic part;cytoskeletal part;extracellular membrane-bounded organelle;extracellular organelle;extracellular region part;extracellular vesicular exosome;intracellular membrane-bounded c	Alanine, aspartate and glutamate metabolism	Homo sapiens	+
adherens junction;anchoring junction;apical junction complex;cell junction;cell part;cell-cell adherens junction;cell-cell junction;integral to membrane;intrinsic to membrane;membrane;membrane part;plasma membrane;posts	Adherens junction;Cell adhesion molecules (CAMs)	Homo sapiens	+
cell part;chromatin;chromosomal part;cytoplasm;cytoplasmic exosome (RNase complex);cytoplasmic part;cytosol;exosome (RNase complex);intracellular membrane-bounded organelle;intracellular non-membrane-bounded	RNA degradation	Homo sapiens	
axon;cell part;cell projection;cytoplasm;cytoskeletal part;intracellular organelle part;intracellular part;kinesin complex;macromolecular complex;microtubule;microtubule associated complex;neuron projection;organelle part;protein complex		Homo sapiens	+
actin cytoskeleton;actomyosin contractile ring;cell cortex part;cell division site part;cell part;contractile ring;cytoplasmic part;cytoskeletal part;cytoskeleton;intracellular membrane-bounded organelle;intracellular non-membrane-bounded organelle;intracellular organelle;intracellular organelle part;intracellular part;membrane-bounded organelle;non-membrane-bounded or		Homo sapiens	
cell part;cytoplasm;cytoplasmic part;cytosol;extracellular membrane-bounded organelle;extracellular organelle;extracellular region part;extracellular vesicular exosome;intracellular part;membrane-bounded organelle;membrane-bounded vesicle;organelle;vesicle		Homo sapiens	

actin filament;cell part;cytoplasm;cytoskeletal part;intracellular organelle part;intracellular part;macromolecular complex;organelle part;protein complex		Homo sapiens	+
cell part;cytoplasmic part;cytoskeletal part;endosome;Golgi apparatus part;intracellular membrane-bounded organelle;intracellular organelle;intracellular organelle part;intracellular part;membrane;membrane-bounded organe	Ubiquitin mediated proteolysis	Homo sapiens	+
actin cytoskeleton;actin filament bundle;actomyosin;adherens junction;anchoring junction;cell junction;cell part;cell projection;cell-substrate adherens junction;cell-substrate junction;cytoplasm;cytoskeletal part;cytoskeleton;focal adhesion;intracellular membrane-bounded organelle;intracellular non-membrane-bounded organelle;intracellular organelle;intracellular organe		Homo sapiens	+
cell part;cytoplasm;cytoplasmic part;cytosol;intracellular membrane-bounded organelle;intracellular organelle;intracellular organelle part;intracellular part;membrane-bounded organelle;nuclear part;nucleoplasm;organelle;orga	Carbon fixation pathways in prokaryotes;Glycolysis / Gluconeogenesis;Methane metabolism;Propanoate metabolism;Pyruvate metabolism	Homo sapiens	+
CD40 receptor complex;cell part;cytoplasmic part;cytosol;internal side of plasma membrane;intracellular membrane-bounded organelle;intracellular organelle;intracellular organelle part;intracellular part;macromolecular complex;membrane part;membrane-bounded organelle;membrane-enclosed lumen;mitochondrial intermembrane space;mitochondrial part;mitochondri		Homo sapiens	+
cell part;cytoplasm;intracellular membrane-bounded organelle;intracellular non-membrane-bounded organelle;intracellular organelle;intracellular organelle part;intracellular part;membrane;membrane-bounded organelle;non-membrane-bounded organelle;nuclear part;nucleolus;nucleoplasm;nucleus;organelle;organelle part		Homo sapiens	+
cell part;cytoplasmic part;endoplasmic reticulum;extracellular membrane-bounded organelle;extracellular organelle;extracellular region part;extracellular vesicular exosome;Golgi apparatus;intracellular membrane-bounded o	Protein processing in endoplasmic reticulum	Homo sapiens	+
cell part;cytoplasm;cytoplasmic part;cytosol;extracellular membrane-bounded organelle;extracellular organelle;extracellular region part;extracellular vesicular exosome;intracellular part;membrane-bounded organelle;membra	Amino sugar and nucleotide sugar metabolism	Homo sapiens	+
cell part;cytoplasm;intracellular membrane-bounded organelle;intracellular organelle;intracellular organelle part;intracellular part;membrane-bounded organelle;nuclear part;nucleoplasm;nucleus;organelle;organelle part	Endocytosis	Homo sapiens	
cell part;cytoplasm;cytoplasmic part;cytosol;eukaryotic translation initiation factor 2B complex;intracellular part;macromolecular complex;protein complex	RNA transport	Homo sapiens	+
cell part;centrosome;cytoplasm;cytoplasmic part;cytoskeletal part;intracellular membrane-bounded organelle;intracellular non-membrane-bounded organelle;intracellular organelle;intracellular organelle part;intracellular part;macromolecular complex;membrane-bounded organelle;microtubule organizing center;non-membrane-bounded organelle;nuclear part;nucleoplasn		Homo sapiens	+
cell part;cytoplasmic part;cytosol;intracellular membrane-bounded organelle;intracellular organelle;intracellular part;membrane-bounded organelle;mitochondrion;organelle	Pyrimidine metabolism	Homo sapiens	+
Ada2/Gcn5/Ada3 transcription activator complex;cell part;chromosomal part;DNA polymerase complex;epsilon DNA polymerase complex;histone acetyltransferase complex;intracellular membrane-bounded organelle;intrace	Base excision repair;DNA replication;Nucleotide excision repair;Purine metabolism;Pyrimidine metabolism	Homo sapiens	+
cell part;chromatin accessibility complex;chromatin remodeling complex;chromosomal part;DNA polymerase complex;epsilon DNA polymerase complex;intracellular organelle part;intracellular part;macromolecular complex;nuclear chromosome part;nuclear part;organelle part;protein complex		Homo sapiens	+
cell part;centrosome;cytoplasm;cytoplasmic part;cytoskeletal part;envelope;intracellular membrane-bounded organelle;intracellular non-membrane-bounded organelle;intracellular organelle;intracellular organelle part;intracell	RNA transport	Homo sapiens	+
ACF complex;cell part;chromatin accessibility complex;chromatin assembly complex;chromatin remodeling complex;chromosome;intracellular non-membrane-bounded organelle;intracellular organelle;intracellular organelle part;intracellular part;macromolecular complex;non-membrane-bounded organelle;nuclear chromosome;nuclear part;organelle;organelle part;protei		Homo sapiens	+
cell part;cell projection;cell projection part;cytoplasm;dendritic spine;intracellular part;macromolecular complex;membrane;neuron projection;neuron spine;protein complex		Homo sapiens	
extracellular region;organelle;vesicle		Homo sapiens	+
cell part;cytoplasm;cytoplasmic part;cytosol;endoplasmic reticulum membrane;endoplasmic reticulum part;intracellular membrane-bounded organelle;intracellular organelle;intracellular organelle part;intracellular part;membra	Protein processing in endoplasmic reticulum	Homo sapiens	+
cell part;cytoplasmic part;cytosol;extracellular membrane-bounded organelle;extracellular organelle;extracellular region;extracellular region part;extracellular vesicular exosome;intracellular membrane-bounded organelle;intracellular organelle;intracellular part;membrane-bounded organelle;membrane-bounded vesicle;mitochondrion;organelle;vesicle		Homo sapiens	+
cell part;cytoplasm;intracellular membrane-bounded organelle;intracellular organelle;intracellular part;membrane-bounded organelle;nucleus;organelle	Atrazine degradation	Homo sapiens	+
cell part;intracellular membrane-bounded organelle;intracellular non-membrane-bounded organelle;intracellular organelle;intracellular organelle part;intracellular part;membrane-bounded organelle;non-membrane-bounded organelle;nuclear part;nucleolus;nucleus;organelle;organelle part		Homo sapiens	+
cell part;cytoplasmic part;intracellular organelle part;intracellular part;large ribosomal subunit;macromolecular complex;membrane;mitochondrial inner membrane;mitochondrial large ribosomal subunit;mitochondrial membrane;mitochondrial part;organellar large ribosomal subunit;organelle inner membrane;organelle membrane;organelle part;ribonucleoprotein complex		Homo sapiens	
cell part;cytoplasmic part;cytosol;extracellular membrane-bounded organelle;extracellular organelle;extracellular region part;extracellular vesicular exosome;intracellular part;membrane-bounded organelle;membrane-bounde	Fructose and mannose metabolism	Homo sapiens	+
cell part;cytoplasmic part;endoplasmic reticulum membrane;endoplasmic reticulum part;extracellular membrane-bounded organelle;extracellular organelle;extracellular region part;extracellular vesicular exosome;integral to membrane;intracellular organelle part;intracellular part;intrinsic to membrane;membrane;membrane part;membrane-bounded organelle;membrane-b		Homo sapiens	
actin cytoskeleton;cell part;cytoplasmic part;cytoskeleton;cytosol;intracellular membrane-bounded organelle;intracellular non-membrane-bounded organelle;intracellular organelle;intracellular part;membrane-bounded organe	Focal adhesion;Leukocyte transendothelial migration;Regulation of actin cytoskeleton	Homo sapiens	+
		Homo sapiens	+
cell part;cytoplasmic part;integral to membrane;intracellular organelle part;intracellular part;intrinsic to membrane;membrane;membrane part;mitochondrial membrane;mitochondrial part;organelle membrane;organelle part;plasma membrane		Homo sapiens	+
cell part;centromeric heterochromatin;chromatin;chromosomal part;chromosome, centromeric region;heterochromatin;intracellular membrane-bounded organelle;intracellular organelle;intracellular organelle part;intracellular part;membrane-bounded organelle;nucleus;organelle;organelle part		Homo sapiens	+
cell part;centrosome;cytoplasmic part;cytoskeletal part;cytosol;intracellular non-membrane-bounded organelle;intracellular organelle;intracellular organelle part;intracellular part;kinesin complex;macromolecular complex;membrane;microtubule;microtubule associated complex;microtubule organizing center;non-membrane-bounded organelle;organelle;organelle part;plus		Homo sapiens	+
basal part of cell;cell junction;cell part;cytoplasm;cytoplasmic part;cytosol;intracellular part;membrane;plasma membrane;postsynaptic membrane;synapse part;synaptic membrane		Homo sapiens	+
cell part;cytoplasm;cytoplasmic part;cytosol;intracellular part;macromolecular complex;membrane;phenylalanine-tRNA ligase complex;protein complex	Aminoacyl-tRNA biosynthesis	Homo sapiens	+
cell part;cytoplasm;cytoplasmic part;cytosol;intracellular membrane-bounded organelle;intracellular organelle;intracellular organelle lumen;intracellular organelle part;intracellular part;membrane-bounded organelle;membrane	Aminoacyl-tRNA biosynthesis;Valine, leucine and isoleucine biosynthesis	Homo sapiens	+
cell part;cytoplasmic part;cytosol;intracellular membrane-bounded organelle;intracellular organelle;intracellular part;membrane-bounded organelle;nucleus;organelle	Regulation of actin cytoskeleton	Homo sapiens	+
cell part;cytoplasmic part;cytoplasmic ubiquitin ligase complex;cytosol;intracellular part;macromolecular complex;protein complex;ubiquitin ligase complex	Regulation of autophagy	Homo sapiens	
cell part;chromatin;chromosomal part;chromosome;chromosome, centromeric region;cytoplasmic part;cytosol;intracellular membrane-bounded organelle;intracellular non-membrane-bounded organelle;intracellular organelle;intracellular organelle part;intracellular part;membrane-bounded organelle;non-membrane-bounded organelle;nuclear part;nucleoplasm;nucleus;or		Homo sapiens	+
cell part;chromosomal part;condensin complex;cytoplasm;cytoplasmic part;cytosol;intracellular membrane-bounded organelle;intracellular organelle;intracellular organelle part;intracellular part;macromolecular complex;mem	Cell cycle - yeast	Homo sapiens	+
alpha-amino-3-hydroxy-5-methyl-4-isoxazolepropionic acid selective glutamate receptor complex;cell part;cytoplasmic part;endoplasmic reticulum membrane;endoplasmic reticulum part;Golgi apparatus;Golgi apparatus part;Golgi membrane;integral to endoplasmic reticulum membrane;integral to membrane;integral to organelle membrane;intracellular membrane-boun		Homo sapiens	+
cell part;centrosome;cytoplasm;cytoplasmic part;cytoskeletal part;extracellular membrane-bounded organelle;extracellular organelle;extracellular region part;extracellular vesicular exosome;intracellular non-membrane-bounded organelle;intracellular organelle;intracellular organelle part;intracellular part;membrane;membrane-bounded organelle;membrane-bounded vesi		Homo sapiens	+
cell part;cytoplasmic part;cytosol;extracellular membrane-bounded organelle;extracellular organelle;extracellular region part;extracellular vesicular exosome;intracellular part;membrane-bounded organelle;membrane-bounded vesicle;organelle;vesicle		Homo sapiens	+
cell part;intracellular organelle part;intracellular part;nuclear part;nucleoplasm;organelle part		Homo sapiens	
		Homo sapiens	+
cell part;cytoplasmic part;intracellular membrane-bounded organelle;intracellular organelle;intracellular organelle part;intracellular part;membrane;membrane-bounded organelle;microbody;microbody membrane;microbody p	Peroxisome	Homo sapiens	+
cell part;cytoplasmic part;cytosol;intracellular membrane-bounded organelle;intracellular organelle;intracellular part;membrane-bounded organelle;mitochondrion;organelle		Homo sapiens	
basolateral plasma membrane;cell junction;cell part;cell projection;cell-cell junction;cytoplasm;cytoskeletal part;extracellular membrane-bounded organelle;extracellular organelle;extracellular region part;extracellular vesicular exosome;intracellular organelle part;intracellular part;macromolecular complex;membrane;membrane part;membrane-bounded organelle;membra		Homo sapiens	+
cell part;cytoplasm;intracellular membrane-bounded organelle;intracellular non-membrane-bounded organelle;intracellular organelle;intracellular organelle part;intracellular part;macromolecular complex;membrane-bounded organelle;non-membrane-bounded organelle;nuclear part;nucleolus;nucleus;organelle;organelle part;protein complex		Homo sapiens	+
cell part;membrane		Homo sapiens	+
cell part;cell projection;cilium;extracellular membrane-bounded organelle;extracellular organelle;extracellular region part;extracellular vesicular exosome;membrane-bounded organelle;membrane-bounded vesicle;organelle;vesicle		Homo sapiens	
		Homo sapiens	+
apical plasma membrane;cell part;cytoplasmic part;cytoplasmic vesicle membrane;cytoplasmic vesicle part;endoplasmic reticulum;Golgi apparatus;integral to membrane;intracellular membrane-bounded organelle;intracellular organelle;intracellular organelle part;intracellular part;intrinsic to membrane;membrane;membrane part;membrane-bounded organelle;organelle;o		Homo sapiens	
actin filament bundle;actomyosin;axon;cell junction;cell part;cell projection;cell projection part;cytoplasm;cytoskeletal part;dendritic spine;extracellular membrane-bounded organelle;extracellular organelle;extracellular region part;extracellular vesicular exosome;intracellular organelle part;intracellular part;membrane-bounded organelle;membrane-bounded vesicle;neuron		Homo sapiens	
actin cytoskeleton;adherens junction;anchoring junction;cell junction;cell part;cell-substrate adherens junction;cell-substrate junction;contractile fiber part;cytoplasm;cytoplasmic part;cytoskeleton;cytosol;focal adhesion;intra	Focal adhesion	Homo sapiens	
		Homo sapiens	
cell part;cytoplasm;intracellular membrane-bounded organelle;intracellular organelle;intracellular organelle part;intracellular part;membrane;membrane-bounded organelle;nuclear part;nucleoplasm;nucleus;organelle;organelle part;plasma membrane		Homo sapiens	+
cell part;cytoplasmic part;integral to membrane;intracellular membrane-bounded organelle;intracellular non-membrane-bounded organelle;intracellular organelle;intracellular organelle part;intracellular part;intrinsic to membrane;membrane;membrane part;membrane-bounded organelle;mitochondrial inner membrane;mitochondrial membrane;mitochondrial nucleoid;mitoc		Homo sapiens	+
cell part;cytoplasm;cytoplasmic part;cytoskeletal part;endosomal part;endosome membrane;extracellular membrane-bounded organelle;extracellular organelle;extracellular region part;extracellular vesicular exosome;intracellular organelle part;intracellular part;late endosome membrane;lysosomal membrane;membrane;membrane-bounded organelle;membrane-bounded		Homo sapiens	+
cell part;integral to membrane;intrinsic to membrane;membrane part		Homo sapiens	+
cell part;cytoplasmic part;cytosol;intracellular membrane-bounded organelle;intracellular non-membrane-bounded organelle;intracellular organelle;intracellular organelle part;intracellular part;membrane;membrane-bounded organelle;non-membrane-bounded organelle;nuclear part;nucleolus;nucleoplasm;nucleus;organelle;organelle part;plasma membrane		Homo sapiens	+
cell part;cytoplasm;intracellular non-membrane-bounded organelle;intracellular organelle;intracellular organelle part;intracellular part;membrane;non-membrane-bounded organelle;nuclear part;nucleolus;organelle;organelle part		Homo sapiens	+
cell part;cytoplasmic part;intracellular membrane-bounded organelle;intracellular organelle;intracellular part;membrane-bounded organelle;mitochondrion;organelle		Homo sapiens	+
cell part;clathrin coat;clathrin vesicle coat;coated pit;cytoplasmic part;cytoplasmic vesicle part;intracellular;intracellular organelle part;intracellular part;macromolecular complex;membrane;membrane coat;membrane part;organelle part;plasma membrane;protein complex;vesicle coat		Homo sapiens	+
cell part;intracellular non-membrane-bounded organelle;intracellular organelle;intracellular organelle part;intracellular part;macromolecular complex;non-membrane-bounded organelle;nuclear part;nucleolus;organelle;organell	Ribosome biogenesis in eukaryotes	Homo sapiens	+
catalytic step 2 spliceosome;cell part;cytoplasm;intracellular membrane-bounded organelle;intracellular organelle;intracellular organelle part;intracellular part;macromolecular complex;membrane-bounded organelle;nuclear p	Spliceosome	Homo sapiens	
		Homo sapiens	
cell part;cytoplasmic part;intracellular membrane-bounded organelle;intracellular organelle;intracellular organelle part;intracellular part;membrane-bounded organelle;mitochondrion;nuclear part;nucleoplasm;organelle;organelle part		Homo sapiens	
cell part;intracellular organelle part;intracellular part;nuclear part;nucleoplasm;organelle part		Homo sapiens	
cell part;intracellular part;macromolecular complex;pre-snoRNP complex;ribonucleoprotein complex		Homo sapiens	+
cell part;intracellular non-membrane-bounded organelle;intracellular organelle;intracellular organelle part;intracellular part;membrane;non-membrane-bounded organelle;nuclear part;nucleolus;nucleoplasm;organelle;organelle part;plasma membrane		Homo sapiens	+
extracellular membrane-bounded organelle;extracellular organelle;extracellular region part;extracellular vesicular exosome;membrane-bounded organelle;membrane-bounded vesicle;organelle;vesicle		Homo sapiens	+
BRCA1-A complex;BRISC complex;cell part;cytoplasm;intracellular membrane-bounded organelle;intracellular organelle;intracellular organelle part;intracellular part;macromolecular complex;membrane-bounded organelle;nuclear part;nucleoplasm;nucleus;organelle;organelle part;protein complex		Homo sapiens	+
cell part;cytoplasm;intracellular membrane-bounded organelle;intracellular organelle;intracellular part;membrane-bounded organelle;nucleus;organelle		Homo sapiens	+
Cajal body;cell part;cytoplasm;intracellular non-membrane-bounded organelle;intracellular organelle;intracellular organelle part;intracellular part;macromolecular complex;non-membrane-bounded organelle;nuclear body;nucl	Ribosome biogenesis in eukaryotes	Homo sapiens	
cell part;cytoplasmic part;endosome;intracellular membrane-bounded organelle;intracellular organelle;intracellular part;membrane;membrane-bounded organelle;mitochondrion;organelle		Homo sapiens	+
cell part;cytoplasmic part;intracellular organelle lumen;intracellular organelle part;intracellular part;membrane-enclosed lumen;mitochondrial matrix;mitochondrial part;nuclear part;nucleoplasm;organelle lumen;organelle part		Homo sapiens	
cell part;cytoplasm;intracellular membrane-bounded organelle;intracellular organelle;intracellular part;membrane-bounded organelle;nucleus;organelle		Homo sapiens	
cell part;intracellular membrane-bounded organelle;intracellular non-membrane-bounded organelle;intracellular organelle;intracellular organelle part;intracellular part;membrane-bounded organelle;non-membrane-bounded organelle;nuclear part;nucleolus;nucleus;organelle;organelle part		Homo sapiens	
cell part;cytoplasmic part;Golgi apparatus;integral to membrane;intracellular membrane-bounded organelle;intracellular organelle;intracellular part;intrinsic to membrane;membrane;membrane part;membrane-bounded organelle;organelle		Homo sapiens	+
cell part;cytoplasm;cytoplasmic part;extracellular membrane-bounded organelle;extracellular organelle;extracellular region part;extracellular vesicular exosome;intracellular membrane-bounded organelle;intracellular organelle;intracellular organelle part;intracellular part;membrane;membrane-bounded organelle;membrane-bounded vesicle;mitochondrial inner membrane;		Homo sapiens	+
cell part;intracellular organelle part;intracellular part;macromolecular complex;mediator complex;nuclear part;nucleoplasm;nucleoplasm part;organelle part;protein complex		Homo sapiens	
cell part;cytoplasm;cytoplasmic part;endoplasmic reticulum;intracellular membrane-bounded organelle;intracellular organelle;intracellular part;membrane-bounded organelle;mitochondrion;organelle		Homo sapiens	+
cell part;cytoplasmic part;endoplasmic reticulum;endoplasmic reticulum membrane;endoplasmic reticulum part;Golgi apparatus;Golgi apparatus part;Golgi membrane;integral to membrane;intracellular membrane-bounded o	Sphingolipid metabolism	Homo sapiens	+
		Homo sapiens	+
cell part;integral to membrane;intrinsic to membrane;membrane part		Homo sapiens	+
cell part;cytoplasmic part;intracellular membrane-bounded organelle;intracellular organelle;intracellular part;membrane-bounded organelle;mitochondrion;nucleus;organelle		Homo sapiens	+
cell part;cytoplasm;granular component;intracellular membrane-bounded organelle;intracellular non-membrane-bounded organelle;intracellular organelle;intracellular organelle part;intracellular part;membrane-bounded organelle;non-membrane-bounded organelle;nuclear part;nucleolar part;nucleolus;nucleoplasm;nucleus;organelle;organelle part		Homo sapiens	+
cell part;integral to membrane;intrinsic to membrane;membrane;membrane part	Protein processing in endoplasmic reticulum	Homo sapiens	+
box H/ACA RNP complex;box H/ACA snoRNP complex;Cajal body;cell part;intracellular membrane-bounded organelle;intracellular non-membrane-bounded organelle;intracellular organelle;intracellular organelle part;intracell	Ribosome biogenesis in eukaryotes	Homo sapiens	+
cell part;cytoplasm;extracellular membrane-bounded organelle;extracellular organelle;extracellular region part;extracellular vesicular exosome;intracellular organelle part;intracellular part;membrane;membrane-bounded organelle;membrane-bounded vesicle;nuclear part;nucleoplasm;organelle;organelle part;plasma membrane;vesicle		Homo sapiens	+
cell part;chromosomal part;chromosome;chromosome, telomeric region;cytoplasm;envelope;intracellular membrane-bounded organelle;intracellular non-membrane-bounded organelle;intracellular organelle;intracellular organelle part;intracellular part;macromolecular complex;membrane-bounded organelle;non-membrane-bounded organelle;nuclear chromosome;nuclea		Homo sapiens	
cell part;cell projection;cytoplasm;cytoplasmic part;cytoskeleton;cytosol;filopodium;intracellular non-membrane-bounded organelle;intracellular organelle;intracellular part;lamellipodium;macromolecular complex;non-membra	Regulation of actin cytoskeleton	Homo sapiens	+
cell part;cytoplasm;intracellular membrane-bounded organelle;intracellular non-membrane-bounded organelle;intracellular organelle;intracellular organelle part;intracellular part;membrane-bounded organelle;non-membrane-bounded organelle;nuclear part;nucleolus;nucleoplasm;nucleus;organelle;organelle part		Homo sapiens	+
cell part;intracellular non-membrane-bounded organelle;intracellular organelle;intracellular organelle part;intracellular part;macromolecular complex;non-membrane-bounded organelle;nuclear part;nucleolar part;nucleolus;org	Ribosome biogenesis in eukaryotes	Homo sapiens	+
cell part;cytoplasm;intracellular membrane-bounded organelle;intracellular organelle;intracellular part;membrane-bounded organelle;nucleus;organelle	MAPK signaling pathway;Tight junction	Homo sapiens	
cell part;cytoplasmic part;endoplasmic reticulum membrane;endoplasmic reticulum part;integral to membrane;intracellular organelle part;intracellular part;intrinsic to membrane;membrane;membrane part;organelle membrane;organelle part		Homo sapiens	
cell part;contractile fiber part;cytoplasmic part;cytoskeletal part;intracellular organelle part;intracellular part;organelle part;striated muscle thin filament		Homo sapiens	+
cell part;cytoplasmic part;endoplasmic reticulum;endoplasmic reticulum lumen;endoplasmic reticulum part;endoplasmic reticulum-Golgi intermediate compartment;extracellular membrane-bounded organelle;extracellular org	Protein processing in endoplasmic reticulum	Homo sapiens	+
cell part;cytoplasmic part;endoplasmic reticulum;endoplasmic reticulum membrane;endoplasmic reticulum part;integral to endoplasmic reticulum membrane;integral to membrane;integral to organelle membrane;intracellular	Biosynthesis of unsaturated fatty acids	Homo sapiens	+
cell part;cytoplasmic part;cytosol;endoplasmic reticulum;endoplasmic reticulum lumen;endoplasmic reticulum membrane;endoplasmic reticulum part;extracellular membrane-bounded organelle;extracellular organelle;extracellular region;extracellular region part;extracellular space;extracellular vesicular exosome;integral to membrane;intracellular membrane-bounded org		Homo sapiens	+
cell part;cytoplasmic part;extracellular membrane-bounded organelle;extracellular organelle;extracellular region part;extracellular vesicular exosome;integral to membrane;intracellular membrane-bounded organelle;intracellular organelle;intracellular organelle part;intracellular part;intrinsic to membrane;membrane;membrane part;membrane-bounded organelle;membrane		Homo sapiens	+
cell part;cytoplasm;intracellular part;membrane;plasma membrane		Homo sapiens	+
cell part;cytoplasm;intracellular membrane-bounded organelle;intracellular organelle;intracellular organelle part;intracellular part;membrane-bounded organelle;nuclear part;nucleoplasm;nucleus;organelle;organelle part		Homo sapiens	+
axon;cell part;cell projection;cell projection part;CRD-mediated mRNA stability complex;cytoplasm;cytoplasmic part;cytosol;dendritic spine;filopodium;growth cone;intracellular membrane-bounded organelle;intracellular non-membrane-bounded organelle;intracellular organelle;intracellular part;lamellipodium;macromolecular complex;membrane-bounded organelle;neuro		Homo sapiens	
cell part;cytoplasmic part;integral to membrane;intracellular;intracellular membrane-bounded organelle;intracellular organelle;intracellular organelle part;intracellular part;intrinsic to membrane;membrane;membrane part;membrane-bounded organelle;mitochondrial inner membrane;mitochondrial membrane;mitochondrial part;mitochondrion;organelle;organelle inner mem		Homo sapiens	+
	Protein processing in endoplasmic reticulum	Homo sapiens	
cell part;cytoplasmic part;cytosol;extracellular membrane-bounded organelle;extracellular organelle;extracellular region part;extracellular vesicular exosome;intracellular;intracellular membrane-bounded organelle;intracellular	Cysteine and methionine metabolism	Homo sapiens	
caveola;cell part;cytoplasmic part;cytoplasmic vesicle;cytoplasmic vesicle membrane;cytoplasmic vesicle part;envelope;extracellular membrane-bounded organelle;extracellular organelle;extracellular region part;extracellular vesicular exosome;integral to membrane;intracellular membrane-bounded organelle;intracellular organelle;intracellular organelle part;intracellular p		Homo sapiens	+
adherens junction;anchoring junction;cell junction;cell part;cell-substrate adherens junction;cell-substrate junction;cytoplasm;cytoplasmic membrane-bounded vesicle;cytoplasmic part;cytoplasmic vesicle;endocytic vesicle;e	Endocytosis	Homo sapiens	+
caveola;cell part;cytoplasmic part;endosomal part;endosome membrane;extracellular membrane-bounded organelle;extracellular organelle;extracellular region part;extracellular vesicular exosome;intracellular membrane-bou	Endocytosis	Homo sapiens	+
cell part;integral to membrane;integral to plasma membrane;intrinsic to membrane;intrinsic to plasma membrane;membrane;membrane part;plasma membrane;plasma membrane part	Olfactory transduction	Homo sapiens	+
cell part;cytoplasmic part;cytoskeletal part;cytosol;intermediate filament;intracellular membrane-bounded organelle;intracellular organelle;intracellular organelle part;intracellular part;macromolecular complex;membrane-bounded organelle;nucleus;organelle;organelle part;protein complex		Homo sapiens	+
cell part;cell projection part;contractile fiber part;cytoplasmic part;cytoskeletal part;growth cone;intracellular organelle part;intracellular part;organelle part;site of polarized growth;striated muscle thin filament		Homo sapiens	
cell part;cytoplasm;intracellular membrane-bounded organelle;intracellular organelle;intracellular part;membrane;membrane-bounded organelle;nucleus;organelle		Homo sapiens	
cell part;cytoplasm;extracellular region part;extracellular space;intracellular membrane-bounded organelle;intracellular organelle;intracellular part;membrane-bounded organelle;nucleus;organelle		Homo sapiens	+
cell part;extracellular membrane-bounded organelle;extracellular organelle;extracellular region part;extracellular vesicular exosome;integral to membrane;intrinsic to membrane;membrane;membrane part;membrane-bounded organelle;membrane-bounded vesicle;organelle;plasma membrane;vesicle		Homo sapiens	+
cell part;cytoplasmic part;cytosol;endosomal part;endosome membrane;extracellular membrane-bounded organelle;extracellular organelle;extracellular region part;extracellular vesicular exosome;intracellular membrane-bour	Endocytosis	Homo sapiens	+
cell part;cytoplasmic membrane-bounded vesicle;cytoplasmic part;cytoplasmic vesicle;intracellular membrane-bounded organelle;intracellular organelle;intracellular part;membrane-bounded organelle;membrane-bounded vesicle;nucleus;organelle;vesicle		Homo sapiens	+
catalytic step 2 spliceosome;cell part;intracellular membrane-bounded organelle;intracellular organelle;intracellular organelle part;intracellular part;macromolecular complex;membrane-bounded organelle;nuclear part;nucleop	Spliceosome	Homo sapiens	+
cell part;cytoplasmic part;intracellular membrane-bounded organelle;intracellular organelle;intracellular organelle part;intracellular part;large ribosomal subunit;macromolecular complex;membrane;membrane-bounded organelle;mitochondrial inner membrane;mitochondrial large ribosomal subunit;mitochondrial membrane;mitochondrial part;mitochondrion;organellar larg		Homo sapiens	
cell part;cytoplasm;intracellular membrane-bounded organelle;intracellular non-membrane-bounded organelle;intracellular organelle;intracellular organelle part;intracellular part;membrane-bounded organelle;non-membrane-bounded organelle;nuclear part;nucleolus;nucleus;organelle;organelle part		Homo sapiens	+
adherens junction;anchoring junction;cell junction;cell part;cell-substrate adherens junction;cell-substrate junction;cytoplasmic part;endoplasmic reticulum;endoplasmic reticulum membrane;endoplasmic reticulum part;focal adhesion;integral to membrane;intracellular membrane-bounded organelle;intracellular organelle;intracellular organelle part;intracellular part;intrinsi		Homo sapiens	
cell part;cytoplasm;cytoplasmic part;extracellular membrane-bounded organelle;extracellular organelle;extracellular region part;extracellular vesicular exosome;integral to membrane;intracellular membrane-bounded organelle	Alzheimer's disease;Huntington's disease;Oxidative phosphorylation;Parkinson's disease	Homo sapiens	
		Homo sapiens	+
actin cytoskeleton;cell cortex;cell part;cytoplasm;cytoplasmic part;cytoskeleton;intracellular membrane-bounded organelle;intracellular non-membrane-bounded organelle;intracellular organelle;intracellular organelle part;intracellular part;membrane-bounded organelle;mitochondrion;non-membrane-bounded organelle;nuclear part;nucleoplasm;nucleus;organelle;organell		Homo sapiens	
cell part;cytoplasmic part;cytoskeleton;endoplasmic reticulum;endoplasmic reticulum membrane;endoplasmic reticulum part;Golgi apparatus part;Golgi membrane;integral to membrane;intracellular membrane-bounded orga	Tight junction	Homo sapiens	+
		Homo sapiens	+
cell part;clathrin-coated vesicle;coated pit;coated vesicle;cytoplasm;cytoplasmic membrane-bounded vesicle;cytoplasmic part;cytoplasmic vesicle;extracellular membrane-bounded organelle;extracellular organelle;extracellular region part;extracellular vesicular exosome;intracellular membrane-bounded organelle;intracellular organelle;intracellular part;membrane part;m		Homo sapiens	+
cell part;cytoplasm;cytoskeleton;intracellular non-membrane-bounded organelle;intracellular organelle;intracellular part;non-membrane-bounded organelle;organelle		Homo sapiens	+
cell part;cytoplasm;intracellular part		Homo sapiens	+
cell part;chromosomal part;chromosome, centromeric core region;cytoplasmic part;cytoskeletal part;cytosol;intracellular non-membrane-bounded organelle;intracellular organelle;intracellular organelle part;intracellular part;macromolecular complex;microtubule;midbody;non-membrane-bounded organelle;nuclear part;nucleolus;organelle;organelle part;protein complex		Homo sapiens	

cell part;cytoplasm;extracellular membrane-bounded organelle;extracellular organelle;extracellular region part;extracellular vesicular exosome;intracellular membrane-bounded organelle;intracellular organelle;intracellular part;membrane;membrane-bounded organelle;membrane-bounded vesicle;nucleus;organelle;vesicle		Homo sapiens	+
cell part;centrosome;cytoplasm;cytoplasmic part;cytoskeletal part;cytoskeleton;intracellular non-membrane-bounded organelle;intracellular organelle;intracellular organelle part;intracellular part;microtubule cytoskeleton;microtubule organizing center;non-membrane-bounded organelle;nuclear part;nucleolus;nucleoplasm;organelle;organelle part		Homo sapiens	
cell part;cyclin-dependent protein kinase holoenzyme complex;intracellular membrane-bounded organelle;intracellular organelle;intracellular organelle part;intracellular part;macromolecular complex;membrane-bounded organelle;nuclear cyclin-dependent protein kinase holoenzyme complex;nuclear part;nucleus;organelle;organelle part;protein complex		Homo sapiens	+
cell part;cell surface;cytoplasmic part;endoplasmic reticulum membrane;endoplasmic reticulum part;Golgi apparatus;integral to membrane;intracellular membrane-bounded organelle;intracellular organelle;intracellular organelle part;intracellular part;intrinsic to membrane;membrane;membrane part;membrane-bounded organelle;organelle;organelle membrane;organelle p		Homo sapiens	+
actin cytoskeleton;cell part;cytoplasm;cytoskeleton;intracellular non-membrane-bounded organelle;intracellular organelle;intracellular part;non-membrane-bounded organelle;organelle		Homo sapiens	+
cell part;extracellular membrane-bounded organelle;extracellular organelle;extracellular region part;extracellular vesicular exosome;membrane;membrane-bounded organelle;membrane-bounded vesicle;organelle;vesicle		Homo sapiens	+
cell part;cytoplasmic part;endoplasmic reticulum;endoplasmic reticulum part;integral to endoplasmic reticulum membrane;integral to membrane;integral to organelle membrane;intracellular membrane-bounded organelle;intra	Protein processing in endoplasmic reticulum	Homo sapiens	+
cell part;intracellular organelle part;intracellular part;macromolecular complex;membrane;mRNA cleavage and polyadenylation specificity factor complex;mRNA cleavage factor complex;nuclear part;nucleoplasm;nucleoplasn	mRNA surveillance pathway	Homo sapiens	+
cell part;cytoplasm;cytoplasmic part;cytosol;intracellular membrane-bounded organelle;intracellular organelle;intracellular part;membrane-bounded organelle;nucleus;organelle	Aminoacyl-tRNA biosynthesis;Valine, leucine and isoleucine biosynthesis	Homo sapiens	+
cell part;cytoplasm;intracellular membrane-bounded organelle;intracellular organelle;intracellular organelle part;intracellular part;membrane-bounded organelle;nuclear part;nucleoplasm;nucleus;organelle;organelle part		Homo sapiens	
cell part;cytoplasm;intracellular organelle part;intracellular part;nuclear body;nuclear part;nuclear speck;nucleoplasm;nucleoplasm part;organelle part		Homo sapiens	+
cell part;cytoplasmic part;early endosome;endosomal part;endosome;endosome membrane;extracellular membrane-bounded organelle;extracellular organelle;extracellular region part;extracellular vesicular exosome;intracellular membrane-bounded organelle;intracellular organelle;intracellular organelle part;intracellular part;lysosomal membrane;macropinosome;membra		Homo sapiens	
cell part;cytoplasmic part;extracellular membrane-bounded organelle;extracellular organelle;extracellular region part;extracellular vesicular exosome;intracellular membrane-bounded organelle;intracellular organelle;intracellula	Citrate cycle (TCA cycle);Propanoate metabolism	Homo sapiens	+
cell part;cytoplasmic part;cytosol;intracellular part	Purine metabolism	Homo sapiens	
cell part;cytoplasmic part;endoplasmic reticulum;endoplasmic reticulum membrane;endoplasmic reticulum part;Golgi apparatus part;Golgi membrane;integral to membrane;intracellular membrane-bounded organelle;intracell	Phagosome;SNARE interactions in vesicular transport	Homo sapiens	+
cell body;cell part;cell projection;cytoplasm;cytoplasmic part;cytosol;dendrite;extracellular region part;extracellular space;intracellular part;membrane;neuron projection;neuronal cell body;perinuclear region of cytoplasm;plasma membrane		Homo sapiens	+
cell body;cell part;cell projection;cytoplasmic part;cytosol;dendrite;intracellular part;membrane;neuron projection;neuronal cell body		Homo sapiens	+
cell part;clathrin coat;clathrin coat of coated pit;cytoplasmic part;intracellular membrane-bounded organelle;intracellular organelle;intracellular part;macromolecular complex;membrane;membrane coat;membrane part;membrane-bounded organelle;nucleus;organelle;plasma membrane;plasma membrane part;protein complex		Homo sapiens	+
cell part;cytoplasmic part;cytosol;intracellular membrane-bounded organelle;intracellular organelle;intracellular organelle part;intracellular part;macromolecular complex;membrane-bounded organelle;nuclear part;nucleoplasn	Ubiquitin mediated proteolysis	Homo sapiens	+
cell part;COPI vesicle coat;cytoplasmic part;cytoplasmic vesicle part;cytosol;Golgi apparatus part;intracellular organelle part;intracellular part;macromolecular complex;membrane coat;membrane part;organelle part;protein complex;vesicle coat		Homo sapiens	
cell part;Cul2-RING ubiquitin ligase complex;Cul3-RING ubiquitin ligase complex;CUL4 RING ubiquitin ligase complex;Cul5-RING ubiquitin ligase complex;Cul7-RING ubiquitin ligase complex;cullin-RING ubiquitin ligase com	Ubiquitin mediated proteolysis	Homo sapiens	+
adherens junction;anchoring junction;cell junction;cell part;cell-substrate adherens junction;cell-substrate junction;focal adhesion;integral to membrane;intrinsic to membrane;membrane;membrane part	ko05152;Phagosome	Homo sapiens	+
actin filament;cell part;cytoskeletal part;extracellular membrane-bounded organelle;extracellular organelle;extracellular region part;extracellular vesicular exosome;extrinsic to internal side of plasma membrane;extrinsic to me	Chemokine signaling pathway;MAPK signaling pathway;Regulation of actin cytoskeleton	Homo sapiens	+
cell part;cytoplasmic part;endoplasmic reticulum;endoplasmic reticulum membrane;endoplasmic reticulum part;integral to membrane;intracellular membrane-bounded organelle;intracellular organelle;intracellular organelle pa	Steroid biosynthesis	Homo sapiens	+
aggresome;axon;caveola;cell leading edge;cell part;cell projection;chromatin remodeling complex;cytoplasm;cytoplasmic microtubule;cytoplasmic part;cytoskeletal part;cytosol;dendrite;histone deacetylase complex;inclusion body;intracellular membrane-bounded organelle;intracellular organelle;intracellular organelle part;intracellular part;macromolecular complex;meml		Homo sapiens	+
cell part;cytoplasm;cytoplasmic part;cytosol;early endosome;endosomal part;endosome;endosome membrane;extracellular membrane-bounded organelle;extracellular organelle;extracellular region part;extracellular vesicular exosome;intracellular membrane-bounded organelle;intracellular organelle;intracellular organelle part;intracellular part;late endosome;macromolec		Homo sapiens	
cell part;cytoplasm;cytoplasmic part;cytosol;eukaryotic 43S preinitiation complex;eukaryotic 48S preinitiation complex;eukaryotic translation initiation factor 3 complex;extracellular membrane-bounded organelle;extracellular organelle;extracellular region part;extracellular vesicular exosome;intracellular membrane-bounded organelle;intracellular organelle;intracellular pa		Homo sapiens	+
cell part;cytoplasm;cytoplasmic part;cytosol;extracellular membrane-bounded organelle;extracellular organelle;extracellular region part;extracellular vesicular exosome;intracellular organelle lumen;intracellular organelle part;ii	Glyoxylate and dicarboxylate metabolism;Pyruvate metabolism	Homo sapiens	+
cell part;cytoplasmic part;endoplasmic reticulum;extracellular membrane-bounded organelle;extracellular organelle;extracellular region part;extracellular space;extracellular vesicular exosome;intracellular membrane-boundec	Lysosome	Homo sapiens	+
cell part;cytoplasmic part;endoplasmic reticulum;endoplasmic reticulum lumen;endoplasmic reticulum part;intracellular membrane-bounded organelle;intracellular organelle;intracellular organelle lumen;intracellular organelle p	Protein processing in endoplasmic reticulum	Homo sapiens	+
cell part;cytoplasmic part;cytosol;intracellular membrane-bounded organelle;intracellular organelle;intracellular organelle part;intracellular part;macromolecular complex;membrane-bounded organelle;nuclear part;nucleoplasn	Ubiquitin mediated proteolysis	Homo sapiens	+
cell part;chromatin remodeling complex;chromosomal part;H4/H2A histone acetyltransferase complex;histone acetyltransferase complex;histone deacetylase complex;intracellular organelle part;intracellular part;macromolecular complex;NuA4 histone acetyltransferase complex;nuclear chromosome part;nuclear part;nucleoplasm;nucleoplasm part;organelle part;protein		Homo sapiens	+
cell part;cytoplasmic part;cytosol;inclusion body;intracellular organelle part;intracellular part;macromolecular complex;membrane part;nuclear body;nuclear inclusion body;nuclear part;nuclear pore;nuclear speck;nucleoplasn	mRNA surveillance pathway;Ribosome biogenesis in eukaryotes;RNA transport	Homo sapiens	
cell part;cytoplasmic part;cytoplasmic ubiquitin ligase complex;endoplasmic reticulum membrane;endoplasmic reticulum part;ER ubiquitin ligase complex;Hrd1p ubiquitin ligase complex;integral to membrane;intracellular org	Protein processing in endoplasmic reticulum	Homo sapiens	+
cell part;cytoplasm;extracellular membrane-bounded organelle;extracellular organelle;extracellular region part;extracellular vesicular exosome;intracellular part;membrane;membrane-bounded organelle;membrane-bounded vesicle;organelle;vesicle		Homo sapiens	
cell part;cytoplasmic part;integral to membrane;intracellular membrane-bounded organelle;intracellular organelle;intracellular organelle part;intracellular part;intrinsic to membrane;membrane;membrane part;membrane-bounc	Proximal tubule bicarbonate reclamation	Homo sapiens	+
cell part;cytoplasmic part;extracellular membrane-bounded organelle;extracellular organelle;extracellular region part;extracellular vesicular exosome;intracellular organelle part;intracellular part;macromolecular complex;memb	Alzheimer's disease;Cardiac muscle contraction;Huntington's disease;Oxidative phosphorylation;Parkinson's disease	Homo sapiens	+
adherens junction;anchoring junction;cell junction;cell part;cell-cell junction;cytoplasm;cytoplasmic part;cytosol;intracellular organelle part;intracellular part;membrane;nuclear part;nucleoplasm;occluding junction;organelle pa	Tight junction;Vibrio cholerae infection	Homo sapiens	
cell cortex;cell junction;cell part;cell-cell junction;chromosome;condensed chromosome;condensed nuclear chromosome;cytoplasm;cytoplasmic part;cytoskeleton;cytosol;intracellular non-membrane-bounded organelle;intracellular organelle;intracellular organelle part;intracellular part;membrane;non-membrane-bounded organelle;nuclear chromosome;nuclear part;nucl		Homo sapiens	+
ATP-binding cassette (ABC) transporter complex;cell part;cytoplasmic part;envelope;intracellular organelle part;intracellular part;macromolecular complex;membrane;membrane part;mitochondrial envelope;mitochondrial part;organelle envelope;organelle part;protein complex		Homo sapiens	+
adherens junction;anchoring junction;cell junction;cell part;cell-substrate adherens junction;cell-substrate junction;cytoplasm;focal adhesion;intracellular membrane-bounded organelle;intracellular organelle;intracellular part;macromolecular complex;membrane;membrane-bounded organelle;nucleus;organelle;plasma membrane;protein complex		Homo sapiens	
adherens junction;anchoring junction;cell junction;cell part;cell-substrate adherens junction;cell-substrate junction;cytoplasm;cytoplasmic mRNA processing body;cytoplasmic part;focal adhesion;intracellular membrane-bounded organelle;intracellular non-membrane-bounded organelle;intracellular organelle;intracellular part;macromolecular complex;membrane-bounde		Homo sapiens	+
cell part;cytoplasmic part;cytosol;endoplasmic reticulum;endoplasmic reticulum membrane;endoplasmic reticulum part;integral to membrane;intracellular membrane-bounded organelle;intracellular organelle;intracellular orga	Protein export;Protein processing in endoplasmic reticulum	Homo sapiens	
cell part;intracellular membrane-bounded organelle;intracellular organelle;intracellular part;membrane-bounded organelle;nucleus;organelle		Homo sapiens	+
cell part;cytoplasm;extracellular membrane-bounded organelle;extracellular organelle;extracellular region part;extracellular vesicular exosome;intracellular part;membrane-bounded organelle;membrane-bounded vesicle;organelle;vesicle		Homo sapiens	+
cell part;cytoplasmic part;integral to membrane;integral to mitochondrial membrane;integral to mitochondrial outer membrane;integral to organelle membrane;intracellular organelle part;intracellular part;intrinsic to membrane;intrinsic to mitochondrial outer membrane;intrinsic to organelle membrane;membrane part;mitochondrial membrane part;mitochondrial part;organe		Homo sapiens	
cell part;cell projection;cytoplasm;intracellular membrane-bounded organelle;intracellular organelle;intracellular part;lamellipodium;membrane;membrane-bounded organelle;nucleus;organelle;plasma membrane		Homo sapiens	
cell part;chromosomal part;chromosome;chromosome, telomeric region;condensed chromosome;condensed nuclear chromosome;cytoplasmic part;endosomal part;endosome membrane;envelope;integral to membrane;integral to nuclear inner membrane;integral to organelle membrane;intracellular non-membrane-bounded organelle;intracellular organelle;intracellular o		Homo sapiens	+
actin cytoskeleton;actin filament bundle;actomyosin;adherens junction;anchoring junction;cell division site part;cell junction;cell part;cell-substrate adherens junction;cell-substrate junction;cleavage furrow;cytoplasm;cytoskeletal part;cytoskeleton;focal adhesion;intracellular non-membrane-bounded organelle;intracellular organelle;intracellular organelle part;intracellular p		Homo sapiens	+
adherens junction;anchoring junction;cell junction;cell part;cell-substrate adherens junction;cell-substrate junction;cytoplasm;cytoplasmic part;cytosol;endoplasmic reticulum;focal adhesion;intracellular membrane-bounded c	Protein export	Homo sapiens	+
		Homo sapiens	+
cell part;cytoplasm;cytoplasmic part;cytosol;endosomal part;endosome membrane;intracellular organelle part;intracellular part;membrane;organelle membrane;organelle part	Cytosolic DNA-sensing pathway;Hepatitis C;Measles;RIG-I-like receptor signaling pathway;Toll-like receptor signaling pathway	Homo sapiens	
actin cytoskeleton;actin filament bundle;actomyosin;cell part;cytoplasm;cytoplasmic part;cytoskeletal part;cytoskeleton;intracellular non-membrane-bounded organelle;intracellular organelle;intracellular organelle part;intracellular part;macromolecular complex;microtubule;non-membrane-bounded organelle;organelle;organelle part;perinuclear region of cytoplasm;protein		Homo sapiens	
cell part;cytoplasm;intracellular membrane-bounded organelle;intracellular organelle;intracellular part;membrane;membrane-bounded organelle;nucleus;organelle;plasma membrane	Protein processing in endoplasmic reticulum	Homo sapiens	+
cell part;cytoplasmic part;extracellular membrane-bounded organelle;extracellular organelle;extracellular region part;extracellular vesicular exosome;intracellular membrane-bounded organelle;intracellular organelle;intracellular organelle part;intracellular part;lysosome;lytic vacuole;macromolecular complex;membrane;membrane-bounded organelle;membrane-bounded v		Homo sapiens	+
basement membrane;cell part;cytoplasm;cytoplasmic part;cytoplasmic vesicle;extracellular matrix part;extracellular region part;intracellular organelle;intracellular part;organelle;vesicle		Homo sapiens	+
cell part;cytoplasmic membrane-bounded vesicle;cytoplasmic part;cytoplasmic vesicle;cytosol;extracellular membrane-bounded organelle;extracellular organelle;extracellular region part;extracellular vesicular exosome;Golgi apparatus;intracellular membrane-bounded organelle;intracellular organelle;intracellular part;lysosome;lytic vacuole;membrane-bounded organelle		Homo sapiens	+
cell part;cytoplasmic part;endoplasmic reticulum membrane;endoplasmic reticulum part;integral to membrane;intracellular organelle part;intracellular part;intrinsic to membrane;membrane;membrane part;organelle membrane;organelle part		Homo sapiens	
cell part;cytoplasmic part;extracellular membrane-bounded organelle;extracellular organelle;extracellular region part;extracellular vesicular exosome;integral to membrane;intracellular membrane-bounded organelle;intracellul	Amino sugar and nucleotide sugar metabolism	Homo sapiens	
cell part;cytoplasm;cytoplasmic part;cytosolic part;extracellular membrane-bounded organelle;extracellular organelle;extracellular region part;extracellular vesicular exosome;intracellular membrane-bounded organelle;intracellular non-membrane-bounded organelle;intracellular organelle;intracellular organelle part;intracellular part;macromolecular complex;membrane-bo		Homo sapiens	+
cell part;cytoplasmic part;Golgi apparatus;intracellular membrane-bounded organelle;intracellular organelle;intracellular organelle part;intracellular part;macromolecular complex;membrane-bounded organelle;mitochondrion;r	Spliceosome	Homo sapiens	+
cell cortex;cell part;cell projection;cytoplasmic part;endomembrane system;intracellular organelle part;intracellular part;lamellipodium;membrane;nuclear part;nucleoplasm;organelle part;perinuclear region of cytoplasm		Homo sapiens	
cell part;cytoplasmic part;cytosol;extracellular membrane-bounded organelle;extracellular organelle;extracellular region part;extracellular vesicular exosome;intracellular membrane-bounded organelle;intracellular organelle;int	Cysteine and methionine metabolism	Homo sapiens	
cell part;cytoplasm;cytoplasmic part;intracellular membrane-bounded organelle;intracellular organelle;intracellular organelle part;intracellular part;macromolecular complex;membrane;membrane part;membrane-bounded orga	Alzheimer's disease;Huntington's disease;Oxidative phosphorylation;Parkinson's disease	Homo sapiens	+
cell part;cytoplasm;cytoplasmic part;cytosol;eukaryotic translation initiation factor 2B complex;intracellular part;macromolecular complex;protein complex	RNA transport	Homo sapiens	
cell part;cytoplasmic part;cytosol;extracellular membrane-bounded organelle;extracellular organelle;extracellular region part;extracellular vesicular exosome;intracellular organelle part;intracellular part;lysosomal membrane;m	Epithelial cell signaling in Helicobacter pylori infection;ko05152;Lysosome;Oxidative phosphorylation;Phagosome;Rheumatoid arthritis;Vibrio chole	Homo sapiens	
cell junction;cell part;clathrin-coated vesicle;coated vesicle;cytoplasmic membrane-bounded vesicle;cytoplasmic part;cytoplasmic vesicle;Golgi apparatus;integral to membrane;intracellular membrane-bounded organelle;intracellular organelle;intracellular part;intrinsic to membrane;membrane;membrane part;membrane-bounded organelle;membrane-bounded vesicle;or		Homo sapiens	+
cell part;cytoplasm;intracellular membrane-bounded organelle;intracellular organelle;intracellular part;membrane-bounded organelle;nucleus;organelle		Homo sapiens	+
extracellular membrane-bounded organelle;extracellular organelle;extracellular region part;extracellular vesicular exosome;macromolecular complex;membrane-bounded organelle;membrane-bounded vesicle;organelle;protein complex;vesicle		Homo sapiens	
cell part;cytoplasm;intracellular membrane-bounded organelle;intracellular organelle;intracellular organelle part;intracellular part;macromolecular complex;membrane part;membrane-bounded organelle;nuclear part;nuclear pore;nucleus;organelle;organelle part;pore complex;protein complex		Homo sapiens	
cell part;cytoplasmic part;cytoplasmic vesicle part;GARP complex;Golgi apparatus;Golgi apparatus part;integral to membrane;intracellular membrane-bounded organelle;intracellular organelle;intracellular organelle part;intracellular part;intrinsic to membrane;macromolecular complex;membrane part;membrane-bounded organelle;organelle;organelle part;protein complex		Homo sapiens	+
cell part;centromeric heterochromatin;chromatin;chromosomal part;chromosome;condensed chromosome;heterochromatin;intracellular membrane-bounded organelle;intracellular non-membrane-bounded organelle;intracellular organelle;intracellular organelle part;intracellular part;membrane-bounded organelle;non-membrane-bounded organelle;nucleus;organelle;orgar		Homo sapiens	+
cell part;cell surface;cytoplasmic part;intracellular membrane-bounded organelle;intracellular organelle;intracellular part;membrane-bounded organelle;mitochondrion;organelle		Homo sapiens	
cell part;cytoplasmic part;intracellular membrane-bounded organelle;intracellular organelle;intracellular organelle lumen;intracellular organelle part;intracellular part;membrane-bounded organelle;membrane-enclosed lumen;m	Pyrimidine metabolism	Homo sapiens	
cell part;cytoplasmic part;early endosome;endosome;intracellular membrane-bounded organelle;intracellular organelle;intracellular part;membrane-bounded organelle;organelle;recycling endosome;vesicle		Homo sapiens	+
actin cytoskeleton;cell part;cytoplasm;cytoplasmic part;cytoskeleton;extracellular membrane-bounded organelle;extracellular organelle;extracellular region part;extracellular vesicular exosome;intracellular membrane-bounded organelle;intracellular non-membrane-bounded organelle;intracellular organelle;intracellular organelle part;intracellular part;membrane;membrane-		Homo sapiens	+
extracellular membrane-bounded organelle;extracellular organelle;extracellular region part;extracellular vesicular exosome;membrane-bounded organelle;membrane-bounded vesicle;organelle;vesicle	Amino sugar and nucleotide sugar metabolism	Homo sapiens	+
cell part;cytoplasmic part;intracellular membrane-bounded organelle;intracellular organelle;intracellular organelle lumen;intracellular organelle part;intracellular part;membrane-bounded organelle;membrane-enclosed lumen;m	Peroxisome	Homo sapiens	+
cell part;cytoplasmic part;intracellular membrane-bounded organelle;intracellular organelle;intracellular part;macromolecular complex;membrane-bounded organelle;methyltransferase complex;nucleus;organelle;protein complex;tRNA (m1A) methyltransferase complex;tRNA methyltransferase complex		Homo sapiens	+
cell part;cytoplasmic part;integral to membrane;integral to plasma membrane;intracellular membrane-bounded organelle;intracellular organelle;intracellular organelle part;intracellular part;intrinsic to membrane;intrinsic to plasma membrane;membrane;membrane part;membrane-bounded organelle;mitochondrial inner membrane;mitochondrial membrane;mitochondrial pa		Homo sapiens	+
cell cortex;cell junction;cell part;cell projection;clathrin coated vesicle membrane;coated vesicle membrane;cytoplasm;cytoplasmic part;cytoplasmic vesicle membrane;cytoplasmic vesicle part;cytoskeletal part;cytosol;dendrite;extracellular membrane-bounded organelle;extracellular organelle;extracellular region part;extracellular vesicular exosome;Golgi apparatus part;		Homo sapiens	
cell part;centrosome;cytoplasm;cytoplasmic dynein complex;cytoplasmic part;cytoskeletal part;cytosol;dynactin complex;dynein complex;intracellular membrane-bounded organelle;intracellular non-membrane-bounded orga	Huntington's disease;Vasopressin-regulated water reabsorption	Homo sapiens	
anaphase-promoting complex;cell part;cullin-RING ubiquitin ligase complex;cytoplasmic part;cytosol;intracellular;intracellular organelle part;intracellular part;macromolecular complex;nuclear part;nuclear ubiquitin ligase com	Cell cycle;Cell cycle - yeast;Meiosis - yeast;Oocyte meiosis;Progesterone-mediated oocyte maturation;Ubiquitin mediated proteolysis	Homo sapiens	+
anaphase-promoting complex;cell part;cullin-RING ubiquitin ligase complex;cytoplasmic part;cytosol;intracellular membrane-bounded organelle;intracellular organelle;intracellular organelle part;intracellular part;macromolecu	Cell cycle;Cell cycle - yeast;Meiosis - yeast;Oocyte meiosis;Progesterone-mediated oocyte maturation;Ubiquitin mediated proteolysis	Homo sapiens	
cell part;cytoplasmic part;cytoskeleton;extrinsic to membrane;extrinsic to plasma membrane;intracellular non-membrane-bounded organelle;intracellular organelle;intracellular organelle part;intracellular part;membrane;membrane part;membrane raft;membrane-enclosed lumen;mitochondrial inner membrane;mitochondrial intermembrane space;mitochondrial membrane		Homo sapiens	
cell part;cytoplasm;cytoplasmic part;cytosol;early endosome;endosomal part;endosome;endosome membrane;ESCRT I complex;extracellular membrane-bounded organelle;extracellular organelle;extracellular region part;ext	Endocytosis	Homo sapiens	+
cell part;cytoplasmic part;cytosol;intracellular organelle part;intracellular part;macromolecular complex;nuclear part;organelle part;ribonucleoprotein complex;small nucleolar ribonucleoprotein complex;spliceosomal complex	RNA degradation;Spliceosome	Homo sapiens	+
cell part;intracellular membrane-bounded organelle;intracellular organelle;intracellular part;membrane-bounded organelle;nucleus;organelle		Homo sapiens	+
cell part;intracellular membrane-bounded organelle;intracellular organelle;intracellular part;membrane-bounded organelle;nucleus;organelle		Homo sapiens	+
cell part;cytoplasm;intracellular membrane-bounded organelle;intracellular non-membrane-bounded organelle;intracellular organelle;intracellular organelle part;intracellular part;membrane;membrane-bounded organelle;non-membrane-bounded organelle;nuclear membrane;nuclear part;nucleolus;nucleoplasm;nucleus;organelle;organelle membrane;organelle part		Homo sapiens	+
cell part;intracellular organelle part;intracellular part;macromolecular complex;mRNA cleavage and polyadenylation specificity factor complex;mRNA cleavage factor complex;nuclear part;nucleoplasm;nucleoplasm part;orgar	mRNA surveillance pathway	Homo sapiens	
cell part;cytoplasm;cytoplasmic part;cytosol;early endosome membrane;endosomal part;endosome membrane;extracellular membrane-bounded organelle;extracellular organelle;extracellular region part;extracellular vesicula	Colorectal cancer;Pathways in cancer	Homo sapiens	+
cell part;cytoplasm;cytoskeletal part;extracellular membrane-bounded organelle;extracellular organelle;extracellular region part;extracellular vesicular exosome;intracellular membrane-bounded organelle;intracellular organelle	Base excision repair	Homo sapiens	
cell part;cytoplasmic part;cytosol;extracellular membrane-bounded organelle;extracellular organelle;extracellular region part;extracellular vesicular exosome;intracellular;intracellular membrane-bounded organelle;intracellular	Purine metabolism	Homo sapiens	+
catalytic step 2 spliceosome;cell part;intracellular membrane-bounded organelle;intracellular organelle;intracellular organelle part;intracellular part;macromolecular complex;membrane-bounded organelle;nuclear part;nucleus;organelle;organelle part;ribonucleoprotein complex;spliceosomal complex		Homo sapiens	+
cell part;intracellular organelle part;intracellular part;macromolecular complex;nuclear part;nucleoplasm;nucleoplasm part;organelle part;protein complex;transcription factor complex;transcription factor TFIIIC complex		Homo sapiens	+
cell part;cytoplasm;cytoplasmic part;endosome;extracellular membrane-bounded organelle;extracellular organelle;extracellular region part;extracellular vesicular exosome;intracellular membrane-bounded organelle;intracellular organelle;intracellular organelle part;intracellular part;membrane;membrane-bounded organelle;membrane-bounded vesicle;nuclear part;nucleop		Homo sapiens	+
cell part;cytoplasm;cytoplasmic part;cytosol;intracellular membrane-bounded organelle;intracellular non-membrane-bounded organelle;intracellular organelle;intracellular organelle part;intracellular part;membrane;membrane-	mRNA surveillance pathway;RNA transport;Spliceosome	Homo sapiens	+
cell part;cytoplasm;intracellular organelle part;intracellular part;macromolecular complex;membrane;membrane part;nuclear membrane;nuclear part;nuclear pore;nucleoplasm;organelle membrane;organelle part;pore complex	RNA transport	Homo sapiens	
cell part;cytoplasm;intracellular non-membrane-bounded organelle;intracellular organelle;intracellular organelle part;intracellular part;membrane;non-membrane-bounded organelle;nuclear part;nucleolus;organelle;organelle part;plasma membrane		Homo sapiens	+
adherens junction;anchoring junction;cell division site part;cell junction;cell part;cell-substrate adherens junction;cell-substrate junction;cleavage furrow;cytoplasmic part;cytoplasmic vesicle membrane;cytoplasmic vesicle part;endoplasmic reticulum membrane;endoplasmic reticulum part;endosome;extracellular membrane-bounded organelle;extracellular organelle;extra		Homo sapiens	+
actin cytoskeleton;cell part;cell projection;cytoplasmic membrane-bounded vesicle;cytoplasmic part;cytoplasmic vesicle;cytoplasmic vesicle membrane;cytoplasmic vesicle part;cytoskeleton;early endosome;endocytic vesicl	Endocytosis	Homo sapiens	+
cell part;cytoplasmic part;cytosol;extracellular membrane-bounded organelle;extracellular organelle;extracellular region part;extracellular vesicular exosome;intracellular organelle part;intracellular part;macromolecular comple	Antigen processing and presentation;Proteasome	Homo sapiens	+
blood microparticle;cell part;cytoplasm;extracellular region part;intracellular membrane-bounded organelle;intracellular organelle;intracellular organelle part;intracellular part;membrane-bounded organelle;nuclear part;nucleoplasm;nucleus;organelle;organelle part		Homo sapiens	+
autophagic vacuole;cell part;cytoplasm;cytoplasmic membrane-bounded vesicle;cytoplasmic part;cytoplasmic vesicle;cytoplasmic vesicle membrane;cytoplasmic vesicle part;endocytic vesicle;endocytic vesicle membrane;e	Hedgehog signaling pathway	Homo sapiens	+
cell part;cytoplasm;intracellular part;membrane;plasma membrane		Homo sapiens	
cell part;cytoplasmic part;cytosol;intracellular part;membrane	Endocytosis;Fc gamma R-mediated phagocytosis	Homo sapiens	+
cell junction;cell part;cell-cell junction;external side of plasma membrane;extracellular region;integral to membrane;intrinsic to membrane;membrane part;plasma membrane part		Homo sapiens	+
catalytic step 2 spliceosome;cell part;intracellular organelle part;intracellular part;macromolecular complex;nuclear part;organelle part;ribonucleoprotein complex;spliceosomal complex	Spliceosome	Homo sapiens	+
cell part;cytoplasm;intracellular membrane-bounded organelle;intracellular organelle;intracellular part;membrane-bounded organelle;nucleus;organelle		Homo sapiens	+
actin cytoskeleton;adherens junction;anchoring junction;cell junction;cell part;cell projection;cell-substrate adherens junction;cell-substrate junction;cytoplasm;cytoskeleton;flotillin complex;focal adhesion;intracellular non-membrane-bounded organelle;intracellular organelle;intracellular part;lamellipodium;lateral plasma membrane;macromolecular complex;membrane pa		Homo sapiens	+
axon hillock;axon part;cell part;cell projection part;cytoplasm;cytoskeletal part;cytoskeleton;intracellular membrane-bounded organelle;intracellular non-membrane-bounded organelle;intracellular organelle;intracellular organelle part;intracellular part;macromolecular complex;membrane-bounded organelle;microtubule;microtubule cytoskeleton;non-membrane-bounded c		Homo sapiens	+
cell part;intracellular membrane-bounded organelle;intracellular organelle;intracellular part;membrane-bounded organelle;nucleus;organelle	Ribosome biogenesis in eukaryotes	Homo sapiens	
cell part;cytoplasm;intracellular membrane-bounded organelle;intracellular organelle;intracellular organelle part;intracellular part;membrane-bounded organelle;nuclear matrix;nuclear part;nucleoplasm;nucleus;organelle;organelle part		Homo sapiens	+
cell part;intracellular organelle part;intracellular part;nuclear part;nucleoplasm;organelle part		Homo sapiens	
cell part;cytoplasmic part;endoplasmic reticulum;endoplasmic reticulum membrane;endoplasmic reticulum part;Golgi apparatus part;Golgi membrane;integral to membrane;intracellular membrane-bounded organelle;intracellular organelle;intracellular organelle part;intracellular part;intrinsic to membrane;membrane;membrane part;membrane-bounded organelle;organelle		Homo sapiens	+
apical part of cell;axon;cell body;cell cortex;cell part;cell projection;cell projection membrane;cell projection part;clathrin coated vesicle membrane;coated pit;coated vesicle membrane;cytoplasm;cytoplasmic membrane-bounded vesicle;cytoplasmic part;cytoplasmic vesicle;cytoplasmic vesicle membrane;cytoplasmic vesicle part;cytoskeletal part;cytosol;DNA-directed I		Homo sapiens	
cell part;cytoplasm;envelope;extracellular membrane-bounded organelle;extracellular organelle;extracellular region part;extracellular vesicular exosome;intracellular non-membrane-bounded organelle;intracellular organelle;intracellular organelle part;intracellular part;macromolecular complex;membrane;membrane part;membrane-bounded organelle;membrane-bounded		Homo sapiens	+
catalytic step 1 spliceosome;catalytic step 2 spliceosome;cell part;chromosomal part;cytoplasm;cytoplasmic part;cytoskeletal part;intracellular membrane-bounded organelle;intracellular non-membrane-bounded organelle;ir	Spliceosome;Ubiquitin mediated proteolysis	Homo sapiens	+
cell part;cytoplasmic part;early endosome;endosome;extracellular membrane-bounded organelle;extracellular organelle;extracellular region part;extracellular vesicular exosome;extrinsic to membrane;intracellular membrane-bounded organelle;intracellular organelle;intracellular part;membrane part;membrane-bounded organelle;membrane-bounded vesicle;organelle;ves		Homo sapiens	
cell part;cytoplasm;intracellular part		Homo sapiens	+
cell part;cytoplasm;cytoplasmic part;cytoskeleton;cytosol;endoplasmic reticulum;extracellular membrane-bounded organelle;extracellular organelle;extracellular region part;extracellular vesicular exosome;inclusion body;inter	Protein processing in endoplasmic reticulum;Ubiquitin mediated proteolysis	Homo sapiens	+
adherens junction;anchoring junction;caveola;cell junction;cell part;cell projection membrane;cell projection part;cell-cell junction;cell-substrate adherens junction;cell-substrate junction;cytoplasm;cytoplasmic part;cytoplasmic vesicle membrane;cytoplasmic vesicle part;cytoskeleton;cytosol;early endosome;endosomal part;endosome;endosome membrane;extracellular		Homo sapiens	+
cell part;membrane		Homo sapiens	
cell part;cytoplasm;cytoplasmic membrane-bounded vesicle;cytoplasmic part;cytoplasmic vesicle;early endosome membrane;endosomal part;endosome membrane;extrinsic to membrane;intracellular;intracellular membrane-bounded organelle;intracellular organelle;intracellular organelle part;intracellular part;macromolecular complex;membrane;membrane part;membra		Homo sapiens	+
cell part;cytoplasmic part;endoplasmic reticulum part;integral to endoplasmic reticulum membrane;integral to membrane;integral to organelle membrane;intracellular organelle part;intracellular part;intrinsic to endoplasmic ret	Protein processing in endoplasmic reticulum	Homo sapiens	+
cell part;cytoplasmic part;cytosol;extracellular membrane-bounded organelle;extracellular organelle;extracellular region part;extracellular vesicular exosome;intracellular membrane-bounded organelle;intracellular organelle;int	Proteasome	Homo sapiens	+
CD95 death-inducing signaling complex;Cdc48p-Npl4p-Ufd1p AAA ATPase complex;cell part;cytoplasmic part;cytosol;death-inducing signaling complex;endoplasmic reticulum part;envelope;intracellular membrane-bounded organelle;intracellular organelle;intracellular organelle part;intracellular part;macromolecular complex;membrane part;membrane-bounded organe		Homo sapiens	+
adherens junction;anchoring junction;cell junction;cell part;cell surface;cell-substrate adherens junction;cell-substrate junction;centrosome;cytoplasmic part;cytoskeletal part;extracellular membrane-bounded organelle;extracellular organelle;extracellular region;extracellular region part;extracellular vesicular exosome;focal adhesion;integral to membrane;integral to plasma		Homo sapiens	+

cell part;cytoplasm;intracellular membrane-bounded organelle;intracellular non-membrane-bounded organelle;intracellular organelle;intracellular organelle part;intracellular part;membrane-bounded organelle;non-membrane-bounded organelle;nuclear part;nucleolus;nucleus;organelle;organelle part		Homo sapiens	+
cell part;cytoplasm;intracellular membrane-bounded organelle;intracellular organelle;intracellular organelle part;intracellular part;macromolecular complex;membrane-bounded organelle;nuclear part;nucleoplasm;nucleus;organelle;organelle part;protein complex;signalosome		Homo sapiens	+
cell part;intracellular membrane-bounded organelle;intracellular non-membrane-bounded organelle;intracellular organelle;intracellular organelle part;intracellular part;macromolecular complex;membrane;membrane-bounded	Ribosome biogenesis in eukaryotes	Homo sapiens	
cell part;chromosome;cytoplasm;cytoplasmic part;cytoskeleton;cytosol;Golgi apparatus part;Golgi stack;intermediate filament cytoskeleton;intracellular non-membrane-bounded organelle;intracellular organelle;intracellular o	Protein processing in endoplasmic reticulum	Homo sapiens	+
		Homo sapiens	+
cell part;cytoplasmic part;integral to membrane;integral to plasma membrane;intracellular organelle part;intracellular part;intrinsic to membrane;intrinsic to plasma membrane;lysosomal membrane;membrane;membrane part;organelle membrane;organelle part;plasma membrane;plasma membrane part;vacuolar membrane;vacuolar part		Homo sapiens	
cell part;coated pit;cytoplasmic part;endosomal part;endosome lumen;Golgi apparatus part;Golgi membrane;intracellular organelle lumen;intracellular organelle part;intracellular part;macromolecular complex;membrane;mem	Lysosome	Homo sapiens	+
actin cytoskeleton;cell part;cell projection membrane;cell projection part;cytoplasm;cytoplasmic part;cytoskeletal part;cytoskeleton;Golgi apparatus;intracellular membrane-bounded organelle;intracellular non-membrane-bounded organelle;intracellular organelle;intracellular organelle part;intracellular part;leading edge membrane;macromolecular complex;membrane;mer		Homo sapiens	+
acrosomal vesicle;cell part;cell projection;centriolar satellite;centriole;centrosome;cytoplasmic membrane-bounded vesicle;cytoplasmic part;cytoplasmic vesicle;cytoskeletal part;cytoskeleton;cytosol;extracellular region part;intercellular bridge;intracellular membrane-bounded organelle;intracellular non-membrane-bounded organelle;intracellular organelle;intracellular orç		Homo sapiens	+
cell part;cytoplasm;intracellular part		Homo sapiens	
cell part;intracellular membrane-bounded organelle;intracellular organelle;intracellular organelle part;intracellular part;membrane-bounded organelle;nuclear part;nucleoplasm;nucleus;organelle;organelle part		Homo sapiens	+
cell part;cytoplasmic mRNA processing body;cytoplasmic part;cytosol;intracellular non-membrane-bounded organelle;intracellular organelle;intracellular part;macromolecular complex;non-membrane-bounded organelle;organelle;ribonucleoprotein complex;RNA granule		Homo sapiens	
cell cortex part;cell part;cell projection part;centriolar satellite;cytoplasmic part;cytoskeletal part;cytosol;exocyst;growth cone membrane;intracellular organelle part;intracellular part;macromolecular complex;membrane;memb	Insulin signaling pathway	Homo sapiens	+
		Homo sapiens	+
cell part;intracellular membrane-bounded organelle;intracellular organelle;intracellular part;membrane-bounded organelle;nucleus;organelle		Homo sapiens	+
cell part;cell surface;cytoplasmic part;cytoskeleton;endoplasmic reticulum;integral to membrane;integral to plasma membrane;intracellular membrane-bounded organelle;intracellular non-membrane-bounded organelle;intracellular organelle;intracellular part;intrinsic to membrane;intrinsic to plasma membrane;membrane;membrane part;membrane-bounded organelle;noi		Homo sapiens	+
Cajal body;catalytic step 2 spliceosome;cell part;intracellular organelle part;intracellular part;macromolecular complex;nuclear body;nuclear part;nuclear speck;nucleoplasm;nucleoplasm part;organelle part;ribonucleoprotein complex;spliceosomal complex		Homo sapiens	+
cell part;cytoplasm;extracellular membrane-bounded organelle;extracellular organelle;extracellular region part;extracellular vesicular exosome;intracellular membrane-bounded organelle;intracellular non-membrane-bounded organelle;intracellular organelle;intracellular organelle part;intracellular part;macromolecular complex;membrane;membrane-bounded organelle;mer		Homo sapiens	+
basement membrane;cell part;chromatin;chromosomal part;chromosome;chromosome, centromeric region;cohesin complex;cohesin core heterodimer;cytoplasm;cytoplasmic part;cytoskeletal part;cytosol;extracellular matri›	Cell cycle;Cell cycle - yeast;Meiosis - yeast;Oocyte meiosis	Homo sapiens	
cell part;cytoplasm;cytoplasmic part;cytosol;endosomal part;endosome membrane;ESCRT III complex;extracellular membrane-bounded organelle;extracellular organelle;extracellular region part;extracellular vesicular exosom	Endocytosis	Homo sapiens	+
cell part;cytoplasm;cytoplasmic part;cytosol;envelope;intracellular organelle part;intracellular part;nuclear envelope;nuclear part;organelle envelope;organelle part	Fructose and mannose metabolism	Homo sapiens	+
cell part;cytoplasm;cytoplasmic part;cytosol;intracellular part	Amino sugar and nucleotide sugar metabolism	Homo sapiens	
cell part;cytoplasm;cytoplasmic part;cytoskeletal part;cytosol;intracellular membrane-bounded organelle;intracellular organelle;intracellular organelle part;intracellular part;macromolecular complex;membrane-bounded organelle;microtubule organizing center;nuclear part;nucleoplasm;nucleus;organelle;organelle part;perinuclear region of cytoplasm;protein complex;tRNA		Homo sapiens	+
cell part;chromatin;chromatin remodeling complex;chromosomal part;cytoplasm;DNA helicase complex;euchromatin;extracellular membrane-bounded organelle;extracellular organelle;extracellular region part;extracellular vesicular exosome;H4/H2A histone acetyltransferase complex;histone acetyltransferase complex;histone methyltransferase complex;Ino80 complex;i		Homo sapiens	
cell part;cytoplasm;cytoplasmic part;cytoskeletal part;extracellular membrane-bounded organelle;extracellular organelle;extracellular region part;extracellular vesicular exosome;intracellular membrane-bounded organelle;intracellular non-membrane-bounded organelle;intracellular organelle;intracellular organelle lumen;intracellular organelle part;intracellular part;macromo		Homo sapiens	+
cell part;cytoplasmic part;eukaryotic 43S preinitiation complex;eukaryotic 48S preinitiation complex;eukaryotic translation initiation factor 3 complex;fibrillar center;intracellular organelle part;intracellular part;macromolecular complex;membrane;nuclear part;nucleolar part;nucleoplasm;organelle part;protein complex;ribonucleoprotein complex;translation preinitiation com		Homo sapiens	+
extracellular membrane-bounded organelle;extracellular organelle;extracellular region part;extracellular vesicular exosome;membrane-bounded organelle;membrane-bounded vesicle;organelle;vesicle	Protein processing in endoplasmic reticulum	Homo sapiens	+
cell part;chromatin remodeling complex;chromosomal part;cytoplasm;cytoplasmic part;cytoskeletal part;DNA helicase complex;extracellular membrane-bounded organelle;extracellular organelle;extracellular region part;extra	Wnt signaling pathway	Homo sapiens	+
cell part;cytoplasm;cytoplasmic part;cytoskeletal part;cytosol;intracellular organelle part;intracellular part;macromolecular complex;microtubule;nuclear part;nucleoplasm;organelle part;protein complex		Homo sapiens	+
cell part;cytoplasmic part;extracellular membrane-bounded organelle;extracellular organelle;extracellular region part;extracellular vesicular exosome;intracellular membrane-bounded organelle;intracellular organelle;intracellula	Calcium signaling pathway;Huntington's disease;Parkinson's disease	Homo sapiens	+
actin cytoskeleton;cell part;contractile fiber part;cytoplasmic part;cytoskeleton;extracellular membrane-bounded organelle;extracellular organelle;extracellular region part;extracellular space;extracellular vesicular exosome;I b	Axon guidance;Fc gamma R-mediated phagocytosis;Regulation of actin cytoskeleton	Homo sapiens	+
cell part;cytoplasmic part;endoplasmic reticulum membrane;endoplasmic reticulum part;endoplasmic reticulum-Golgi intermediate compartment membrane;Golgi apparatus;integral to membrane;intracellular membrane-bounded organelle;intracellular organelle;intracellular organelle part;intracellular part;intrinsic to membrane;membrane;membrane part;membrane-boun		Homo sapiens	
cell part;cytoplasm;cytoplasmic part;cytosol;intracellular part;macromolecular complex;membrane;phenylalanine-tRNA ligase complex;protein complex	Aminoacyl-tRNA biosynthesis	Homo sapiens	
cell part;cytoplasm;intracellular part;macromolecular complex;membrane;polysome;ribonucleoprotein complex		Homo sapiens	+
adherens junction;anchoring junction;cell junction;cell part;cell projection membrane;cell projection part;cell-substrate adherens junction;cell-substrate junction;cytoplasmic part;cytosol;extracellular membrane-bounded orga	Regulation of actin cytoskeleton	Homo sapiens	+
cell part;cytoplasmic part;endoplasmic reticulum;integral to membrane;integral to plasma membrane;intracellular membrane-bounded organelle;intracellular organelle;intracellular part;intrinsic to membrane;intrinsic to plasma membrane;membrane part;membrane-bounded organelle;organelle;plasma membrane part		Homo sapiens	+
		Homo sapiens	+
apical plasma membrane;cell junction;cell part;cell-cell junction;cytoplasmic part;cytoplasmic vesicle;cytosol;endosome;intracellular membrane-bounded organelle;intracellular organelle;intracellular part;membrane part;membrane-bounded organelle;occluding junction;organelle;plasma membrane part;recycling endosome;tight junction;vesicle		Homo sapiens	+
cell part;cytoplasmic exosome (RNase complex);cytoplasmic part;cytosol;exosome (RNase complex);intracellular organelle part;intracellular part;macromolecular complex;membrane;nuclear exosome (RNase complex);nucle	RNA degradation	Homo sapiens	+
cell part;cytoplasmic part;extracellular membrane-bounded organelle;extracellular organelle;extracellular region part;extracellular vesicular exosome;intracellular;intracellular membrane-bounded organelle;intracellular organel	Drug metabolism - cytochrome P450;Glutathione metabolism;Metabolism of xenobiotics by cytochrome P450;Peroxisome	Homo sapiens	+
cell part;cytoplasmic part;intracellular membrane-bounded organelle;intracellular organelle;intracellular organelle part;intracellular part;macromolecular complex;membrane;membrane-bounded organelle;mitochondrial inner membrane;mitochondrial membrane;mitochondrial part;mitochondrial small ribosomal subunit;mitochondrion;organellar small ribosomal subunit;org		Homo sapiens	
cell part;cytoplasmic part;integral to membrane;integral to mitochondrial inner membrane;integral to mitochondrial membrane;integral to organelle membrane;intracellular membrane-bounded organelle;intracellular organelle;intracellular organelle part;intracellular part;intrinsic to membrane;intrinsic to mitochondrial inner membrane;intrinsic to organelle membrane;membr		Homo sapiens	+
cell part;cytoplasm;intracellular membrane-bounded organelle;intracellular non-membrane-bounded organelle;intracellular organelle;intracellular organelle part;intracellular part;membrane;membrane-bounded organelle;non-membrane-bounded organelle;nuclear part;nucleolus;nucleus;organelle;organelle part		Homo sapiens	
		Homo sapiens	+
cell part;cytoplasmic part;intracellular membrane-bounded organelle;intracellular non-membrane-bounded organelle;intracellular organelle;intracellular organelle part;intracellular part;membrane-bounded organelle;mitochondrial nucleoid;mitochondrial part;mitochondrion;non-membrane-bounded organelle;nucleoid;nucleus;organelle;organelle part		Homo sapiens	+
AP-type membrane coat adaptor complex;cell part;clathrin adaptor complex;cytoplasmic part;cytoplasmic vesicle membrane;cytoplasmic vesicle part;Golgi apparatus;intracellular membrane-bounded organelle;intracellular c	Lysosome	Homo sapiens	+
cell part;integral to membrane;integral to nuclear inner membrane;integral to organelle membrane;intracellular organelle part;intracellular part;intrinsic to membrane;intrinsic to nuclear inner membrane;intrinsic to organelle membrane;membrane;membrane part;nuclear inner membrane;nuclear membrane;nuclear membrane part;nuclear part;organelle inner membrane;org		Homo sapiens	+
cell part;cytoplasmic mRNA processing body;cytoplasmic part;cytosol;extracellular membrane-bounded organelle;extracellular organelle;extracellular region part;extracellular vesicular exosome;intracellular non-membrane-bounded organelle;intracellular organelle;intracellular part;macromolecular complex;membrane-bounded organelle;membrane-bounded vesicle;non-		Homo sapiens	+
cell part;extracellular membrane-bounded organelle;extracellular organelle;extracellular region part;extracellular vesicular exosome;intracellular membrane-bounded organelle;intracellular organelle;intracellular organelle part;intracellular part;macromolecular complex;mediator complex;membrane-bounded organelle;membrane-bounded vesicle;nuclear part;nucleoplasm;r		Homo sapiens	+
cell part;cytoplasm;intracellular membrane-bounded organelle;intracellular organelle;intracellular organelle part;intracellular part;membrane-bounded organelle;nuclear body;nuclear part;nuclear speck;nucleoplasm;nucleoplas	Spliceosome	Homo sapiens	+
box C/D snoRNP complex;Cajal body;cell part;cytoplasm;intracellular membrane-bounded organelle;intracellular non-membrane-bounded organelle;intracellular organelle;intracellular organelle part;intracellular part;macromo	Ribosome biogenesis in eukaryotes	Homo sapiens	+
cell part;chromosomal part;cytoplasm;cytoplasmic part;cytosol;intracellular membrane-bounded organelle;intracellular non-membrane-bounded organelle;intracellular organelle;intracellular organelle part;intracellular part;kine	NOD-like receptor signaling pathway;Plant-pathogen interaction	Homo sapiens	+
cell part;cytoplasmic part;intracellular membrane-bounded organelle;intracellular organelle;intracellular part;membrane-bounded organelle;mitochondrion;organelle		Homo sapiens	+
cell part;cytoplasmic part;intracellular membrane-bounded organelle;intracellular organelle;intracellular part;membrane-bounded organelle;mitochondrion;organelle		Homo sapiens	
cell part;cytoplasm;cytoplasmic part;cytosol;Golgi apparatus part;Golgi membrane;intracellular membrane-bounded organelle;intracellular organelle;intracellular organelle part;intracellular part;membrane;membrane-bounded organelle;nuclear part;nucleoplasm;nucleus;organelle;organelle membrane;organelle part		Homo sapiens	+
cell part;integral to membrane;intrinsic to membrane;membrane part		Homo sapiens	+
catalytic step 2 spliceosome;cell part;cytoplasmic part;cytosol;intracellular organelle part;intracellular part;macromolecular complex;nuclear part;nucleoplasm;organelle part;ribonucleoprotein complex;spliceosomal complex	RNA degradation;Spliceosome	Homo sapiens	+
autophagic vacuole membrane;cell part;cytoplasm;cytoplasmic part;extracellular membrane-bounded organelle;extracellular organelle;extracellular region part;extracellular vesicular exosome;Golgi apparatus part;Golgi mem	Endocytosis	Homo sapiens	+
cell part;cytoplasmic part;cytosol;extracellular membrane-bounded organelle;extracellular organelle;extracellular region part;extracellular vesicular exosome;intracellular part;membrane-bounded organelle;membrane-bounde	mTOR signaling pathway	Homo sapiens	
cell part;intracellular organelle part;intracellular part;macromolecular complex;nuclear body;nuclear part;nuclear speck;nucleoplasm part;organelle part;ribonucleoprotein complex;small nuclear ribonucleoprotein complex;U1 snRNP		Homo sapiens	+
cell part;cytoplasmic part;intracellular membrane-bounded organelle;intracellular organelle;intracellular organelle part;intracellular part;macromolecular complex;membrane-bounded organelle;mitochondrial part;mitochondrial small ribosomal subunit;mitochondrion;organellar small ribosomal subunit;organelle;organelle part;ribonucleoprotein complex;small ribosomal sub		Homo sapiens	+
cell part;cytoplasm;cytoskeletal part;intracellular membrane-bounded organelle;intracellular non-membrane-bounded organelle;intracellular organelle;intracellular organelle part;intracellular part;membrane-bounded organelle	Ribosome biogenesis in eukaryotes	Homo sapiens	+
cell part;cytoplasm;cytoplasmic part;cytosol;exosome (RNase complex);intracellular membrane-bounded organelle;intracellular non-membrane-bounded organelle;intracellular organelle;intracellular organelle part;intracellular	RNA degradation	Homo sapiens	
cell part;COPI vesicle coat;COPII vesicle coat;cytoplasmic part;cytoplasmic vesicle part;endoplasmic reticulum;endoplasmic reticulum membrane;endoplasmic reticulum part;endoplasmic reticulum-Golgi intermediate compartment;endoplasmic reticulum-Golgi intermediate compartment membrane;extracellular membrane-bounded organelle;extracellular organelle;extra		Homo sapiens	+
cell part;intracellular organelle part;intracellular part;macromolecular complex;nuclear part;nucleoplasm;organelle part;ribonucleoprotein complex;spliceosomal complex;U12-type spliceosomal complex	Spliceosome	Homo sapiens	+
cell part;cytoplasmic part;intracellular non-membrane-bounded organelle;intracellular organelle;intracellular organelle part;intracellular part;macromolecular complex;membrane;mitochondrial inner membrane;mitochondrial membrane;mitochondrial part;mitochondrial ribosome;non-membrane-bounded organelle;organellar ribosome;organelle;organelle inner membrane;o		Homo sapiens	
adherens junction;anchoring junction;cell junction;cell part;cell-substrate adherens junction;cell-substrate junction;cytoplasmic part;focal adhesion;intracellular membrane-bounded organelle;intracellular non-membrane-boun	Ribosome biogenesis in eukaryotes	Homo sapiens	+
cell part;cytoplasmic part;intracellular membrane-bounded organelle;intracellular non-membrane-bounded organelle;intracellular organelle;intracellular organelle part;intracellular part;membrane-bounded organelle;mitochondrion;non-membrane-bounded organelle;nuclear part;nucleolus;nucleus;organelle;organelle part		Homo sapiens	
cell part;intracellular membrane-bounded organelle;intracellular non-membrane-bounded organelle;intracellular organelle;intracellular organelle part;intracellular part;membrane-bounded organelle;non-membrane-bounded organelle;nuclear part;nucleolus;nucleus;organelle;organelle part		Homo sapiens	+
catalytic step 2 spliceosome;cell part;extracellular membrane-bounded organelle;extracellular organelle;extracellular region part;extracellular vesicular exosome;intracellular organelle part;intracellular part;macromolecular cor	Spliceosome	Homo sapiens	+
extracellular membrane-bounded organelle;extracellular organelle;extracellular region part;extracellular vesicular exosome;membrane-bounded organelle;membrane-bounded vesicle;organelle;vesicle		Homo sapiens	
Cajal body;cell part;intracellular organelle part;intracellular part;membrane;nuclear body;nuclear part;nucleoplasm;nucleoplasm part;organelle part		Homo sapiens	+
cell part;cytoplasmic part;cytoskeleton;intermediate filament cytoskeleton;intracellular membrane-bounded organelle;intracellular non-membrane-bounded organelle;intracellular organelle;intracellular organelle part;intracellular part;macromolecular complex;membrane;membrane-bounded organelle;mitochondrial inner membrane;mitochondrial membrane;mitochondrial		Homo sapiens	+
cell part;cytoplasmic part;cytosol;intracellular membrane-bounded organelle;intracellular organelle;intracellular part;membrane;membrane-bounded organelle;mitochondrion;organelle		Homo sapiens	
cell part;cytoplasmic membrane-bounded vesicle;cytoplasmic part;cytoplasmic vesicle;cytosol;endosomal part;endosome membrane;ESCRT III complex;extracellular membrane-bounded organelle;extracellular organelle;ext	Endocytosis	Homo sapiens	
cell part;cytoplasm;cytoplasmic part;cytosol;intracellular membrane-bounded organelle;intracellular organelle;intracellular organelle part;intracellular part;macromolecular complex;membrane;membrane-bounded organelle;nu	RNA transport	Homo sapiens	+
cell part;cytoplasm;cytoplasmic part;endoplasmic reticulum membrane;endoplasmic reticulum part;envelope;intracellular membrane-bounded organelle;intracellular organelle;intracellular organelle part;intracellular part;macromolecular complex;membrane;membrane part;membrane-bounded organelle;nuclear envelope;nuclear part;nucleus;organelle;organelle envelope;(		Homo sapiens	
cell part;centrosome;cytoplasmic part;cytoskeletal part;cytosol;intracellular non-membrane-bounded organelle;intracellular organelle;intracellular organelle part;intracellular part;macromolecular complex;microtubule associated complex;microtubule organizing center;non-membrane-bounded organelle;organelle;organelle part;protein complex		Homo sapiens	+
cell part;intracellular membrane-bounded organelle;intracellular organelle;intracellular part;membrane-bounded organelle;nucleus;organelle		Homo sapiens	+
cell part;intracellular membrane-bounded organelle;intracellular non-membrane-bounded organelle;intracellular organelle;intracellular organelle part;intracellular part;membrane-bounded organelle;non-membrane-bounded organelle;nuclear part;nucleolus;nucleoplasm;nucleus;organelle;organelle part		Homo sapiens	+
cell part;cytoplasm;cytoplasmic part;cytosol;cytosolic large ribosomal subunit;intracellular non-membrane-bounded organelle;intracellular organelle;intracellular organelle part;intracellular part;large ribosomal subunit;macrom	Ribosome	Homo sapiens	+
cell part;intracellular membrane-bounded organelle;intracellular non-membrane-bounded organelle;intracellular organelle;intracellular organelle part;intracellular part;macromolecular complex;membrane-bounded organelle;non-membrane-bounded organelle;nuclear body;nuclear part;nuclear speck;nucleolus;nucleoplasm;nucleoplasm part;organelle;organelle part;protein		Homo sapiens	+
cell part;intracellular;intracellular membrane-bounded organelle;intracellular organelle;intracellular organelle part;intracellular part;membrane;membrane-bounded organelle;nuclear part;nucleoplasm;nucleus;organelle;organelle part;plasma membrane		Homo sapiens	+
cell part;extracellular membrane-bounded organelle;extracellular organelle;extracellular region part;extracellular vesicular exosome;membrane;membrane-bounded organelle;membrane-bounded vesicle;organelle;vesicle	mRNA surveillance pathway	Homo sapiens	
adherens junction;anchoring junction;cell junction;cell part;cell projection;cell projection membrane;cell projection part;cell surface;cell-cell junction;cell-substrate adherens junction;cell-substrate junction;cytoplasm;cytoplasr	Focal adhesion	Homo sapiens	
cell part;chromatin remodeling complex;chromosomal part;cytoplasmic part;Golgi apparatus;H4/H2A histone acetyltransferase complex;histone acetyltransferase complex;intracellular membrane-bounded organelle;intracellular organelle;intracellular organelle part;intracellular part;macromolecular complex;membrane-bounded organelle;NuA4 histone acetyltransferase c		Homo sapiens	+
cell part;cytoplasm;intracellular membrane-bounded organelle;intracellular organelle;intracellular part;membrane-bounded organelle;nucleus;organelle		Homo sapiens	
cell junction;cell part;cell projection;cell projection part;cytoplasm;cytoplasmic part;cytoskeleton;cytosol;dendrite;dendritic spine;extrinsic to internal side of plasma membrane;extrinsic to membrane;extrinsic to plasma membrane;filopodium;intracellular non-membrane-bounded organelle;intracellular organelle;intracellular part;membrane part;neuron projection;neuron s		Homo sapiens	+
actin cytoskeleton;adherens junction;anchoring junction;cell junction;cell part;cell projection;cell-cell adherens junction;cell-cell junction;cell-substrate adherens junction;cell-substrate junction;cytoplasm;cytoskeleton;fascia a	Focal adhesion	Homo sapiens	+
cell part;cytoplasmic part;cytosol;intracellular part;membrane;plasma membrane	Adipocytokine signaling pathway;Aldosterone-regulated sodium reabsorption;Insulin signaling pathway;Neurotrophin signaling pathway;Type II dia	Homo sapiens	+
actomyosin;cell body;cell part;cell projection;cell projection part;cilium;cytoplasm;cytoplasmic membrane-bounded vesicle;cytoplasmic part;cytoplasmic vesicle;cytoskeletal part;cytosol;extracellular membrane-bounded organelle;extracellular organelle;extracellular region part;extracellular vesicular exosome;filopodium tip;Golgi apparatus;growth cone;insulin-responsive		Homo sapiens	+
basement membrane;cell part;chromosome;extracellular matrix part;extracellular region part;extracellular space;intracellular membrane-bounded organelle;intracellular non-membrane-bounded organelle;intracellular organelle;intracellular organelle part;intracellular part;membrane;membrane-bounded organelle;non-membrane-bounded organelle;nuclear part;nucleoplasi		Homo sapiens	+
cell part;cytoplasm;intracellular part;membrane;plasma membrane		Homo sapiens	+
adherens junction;anchoring junction;cell junction;cell part;cell-substrate adherens junction;cell-substrate junction;cytoplasmic membrane-bounded vesicle lumen;cytoplasmic part;cytoplasmic vesicle part;endocytic vesicle l	Protein processing in endoplasmic reticulum	Homo sapiens	+
cell part;cytoplasmic part;endoplasmic reticulum;endoplasmic reticulum part;integral to endoplasmic reticulum membrane;integral to membrane;integral to organelle membrane;intracellular membrane-bounded organelle;intracellular organelle;intracellular organelle part;intracellular part;intrinsic to endoplasmic reticulum membrane;intrinsic to membrane;intrinsic to organe		Homo sapiens	
cell part;chromosomal part;chromosome, telomeric region;cytoplasm;intracellular;intracellular membrane-bounded organelle;intracellular organelle;intracellular organelle part;intracellular part;macromolecular complex;membrane;membrane-bounded organelle;nucleus;organelle;organelle part;protein complex;TORC1 complex;TORC2 complex		Homo sapiens	+
cell part;cytoplasm;cytoplasmic part;cytoskeletal part;histone methyltransferase complex;intracellular non-membrane-bounded organelle;intracellular organelle;intracellular organelle part;intracellular part;macromolecular complex;membrane;methyltransferase complex;microtubule organizing center;MLL1 complex;non-membrane-bounded organelle;nuclear part;nucleolu		Homo sapiens	+
cell part;cytoplasmic part;integral to membrane;intracellular membrane-bounded organelle;intracellular organelle;intracellular organelle part;intracellular part;intrinsic to membrane;membrane;membrane part;membrane-bounded organelle;mitochondrial inner membrane;mitochondrial membrane;mitochondrial part;mitochondrion;organelle;organelle inner membrane;organ		Homo sapiens	+
cell part;cytoplasmic part;cytosol;intracellular organelle part;intracellular part;macromolecular complex;nuclear part;organelle part;ribonucleoprotein complex;small nuclear ribonucleoprotein complex;spliceosomal complex;U	RNA degradation;Spliceosome	Homo sapiens	
cell part;cytoplasm;intracellular;intracellular membrane-bounded organelle;intracellular organelle;intracellular part;membrane-bounded organelle;nucleus;organelle		Homo sapiens	+
cell part;cytoplasmic part;extracellular membrane-bounded organelle;extracellular organelle;extracellular region part;extracellular vesicular exosome;integral to membrane;intracellular membrane-bounded organelle;intracellular organelle;intracellular organelle part;intracellular part;intrinsic to membrane;macromolecular complex;membrane;membrane part;membrane-bou		Homo sapiens	
cell part;membrane		Homo sapiens	+
		Homo sapiens	
cell part;intracellular membrane-bounded organelle;intracellular organelle;intracellular organelle part;intracellular part;membrane-bounded organelle;nuclear part;nucleoplasm;nucleus;organelle;organelle part		Homo sapiens	+
cell part;cytoplasmic mRNA processing body;cytoplasmic part;intracellular non-membrane-bounded organelle;intracellular organelle;intracellular part;macromolecular complex;non-membrane-bounded organelle;organelle;ribonucleoprotein complex;RNA granule		Homo sapiens	+
cell part;chromosome;intracellular membrane-bounded organelle;intracellular non-membrane-bounded organelle;intracellular organelle;intracellular organelle part;intracellular part;membrane-bounded organelle;non-membrane-bounded organelle;nuclear part;nucleoplasm;nucleus;organelle;organelle part		Homo sapiens	+
cell part;cytoplasmic part;extracellular region part;extracellular space;intracellular organelle part;intracellular part;macromolecular complex;membrane;mitochondrial inner membrane;mitochondrial intermembrane space protein transporter complex;mitochondrial membrane;mitochondrial part;organelle inner membrane;organelle membrane;organelle part;protein complex		Homo sapiens	
cell part;chromatin remodeling complex;chromosomal part;cytoplasmic part;cytosol;DNA helicase complex;Ino80 complex;intracellular membrane-bounded organelle;intracellular organelle;intracellular organelle part;intracellular part;macromolecular complex;membrane-bounded organelle;nuclear chromosome part;nuclear part;nucleus;organelle;organelle part;proteasor		Homo sapiens	+
actin cytoskeleton;cell junction;cell part;cell projection;cell-cell junction;cytoplasm;cytoskeletal part;cytoskeleton;extracellular membrane-bounded organelle;extracellular organelle;extracellular region part;extracellular vesicul	Bacterial invasion of epithelial cells	Homo sapiens	
cell part;cytoplasmic part;cytosol;extracellular membrane-bounded organelle;extracellular organelle;extracellular region part;extracellular vesicular exosome;intracellular organelle part;intracellular part;lysosomal membrane;m	Collecting duct acid secretion;Epithelial cell signaling in Helicobacter pylori infection;Oxidative phosphorylation;Phagosome;Rheumatoid arthritis;\	Homo sapiens	
cell part;cytoplasm;intracellular membrane-bounded organelle;intracellular organelle;intracellular part;membrane-bounded organelle;nucleus;organelle		Homo sapiens	
cell part;cytoplasmic part;intracellular membrane-bounded organelle;intracellular non-membrane-bounded organelle;intracellular organelle;intracellular organelle part;intracellular part;macromolecular complex;membrane;membrane-bounded organelle;mitochondrial inner membrane;mitochondrial intermembrane space protein transporter complex;mitochondrial membrar		Homo sapiens	+
cell part;cytoplasm;cytoplasmic microtubule;cytoplasmic part;cytoskeletal part;endoplasmic reticulum membrane;endoplasmic reticulum part;integral to membrane;intracellular organelle part;intracellular part;intrinsic to mem	Protein export	Homo sapiens	+
cell part;cytoplasm;cytoplasmic part;extracellular membrane-bounded organelle;extracellular organelle;extracellular region part;extracellular vesicular exosome;intracellular membrane-bounded organelle;intracellular organelle	Amino sugar and nucleotide sugar metabolism;Fructose and mannose metabolism	Homo sapiens	
actomyosin;cell junction;cell leading edge;cell part;cell-cell junction;cytoplasm;cytoskeletal part;cytoskeleton;extracellular membrane-bounded organelle;extracellular organelle;extracellular region part;extracellular vesicular exosome;intracellular non-membrane-bounded organelle;intracellular organelle;intracellular organelle part;intracellular part;membrane;membrane-bo		Homo sapiens	+
catalytic step 2 spliceosome;cell part;cytoplasm;cytoplasmic part;cytosol;exon-exon junction complex;intracellular membrane-bounded organelle;intracellular organelle;intracellular organelle part;intracellular part;macromolec	mRNA surveillance pathway;RNA transport;Spliceosome	Homo sapiens	+
cell part;chromatin;chromosomal part;cytoplasm;intracellular membrane-bounded organelle;intracellular organelle;intracellular organelle part;intracellular part;macromolecular complex;membrane;membrane-bounded organel	Neurotrophin signaling pathway	Homo sapiens	+
cell part;cell projection;clathrin-coated vesicle;coated vesicle;cytoplasm;cytoplasmic membrane-bounded vesicle;cytoplasmic part;cytoplasmic vesicle;cytoplasmic vesicle membrane;cytoplasmic vesicle part;endosome;extracellular membrane-bounded organelle;extracellular organelle;extracellular region part;extracellular vesicular exosome;extrinsic to internal side of p		Homo sapiens	+
cell part;cytoplasmic part;early endosome membrane;endosomal part;endosome membrane;extrinsic to membrane;intracellular membrane-bounded organelle;intracellular organelle;intracellular organelle part;intracellular part;membrane;membrane part;membrane-bounded organelle;organelle;organelle membrane;organelle part		Homo sapiens	+
cell part;cell projection;cytoplasmic part;cytoplasmic vesicle membrane;cytoplasmic vesicle part;early endosome membrane;endosomal part;endosome membrane;extrinsic to endosome membrane;extrinsic to internal side of plasma membrane;extrinsic to membrane;extrinsic to organelle membrane;extrinsic to plasma membrane;intracellular organelle part;intracellular		Homo sapiens	
cell part;centrosome;cytoplasm;cytoplasmic part;cytoskeletal part;intracellular membrane-bounded organelle;intracellular non-membrane-bounded organelle;intracellular organelle;intracellular organelle part;intracellular part;membrane-bounded organelle;microtubule organizing center;non-membrane-bounded organelle;nucleus;organelle;organelle part;spindle pole centr		Homo sapiens	
cell part;cytoplasmic part;intracellular membrane-bounded organelle;intracellular organelle;intracellular organelle part;intracellular part;membrane-bounded organelle;nuclear part;nucleoplasm;nucleus;organelle;organelle part;perinuclear region of cytoplasm		Homo sapiens	+
cell part;chromatin;chromosomal part;cytoplasmic part;intracellular membrane-bounded organelle;intracellular non-membrane-bounded organelle;intracellular organelle;intracellular organelle part;intracellular part;macromolecular complex;membrane-bounded organelle;mitochondrion;non-membrane-bounded organelle;nuclear chromatin;nuclear chromosome part;nuclea		Homo sapiens	+
		Homo sapiens	+
cell part;cytoplasm;cytoplasmic part;cytosol;extracellular membrane-bounded organelle;extracellular organelle;extracellular region part;extracellular vesicular exosome;intracellular part;membrane-bounded organelle;membra	Glycine, serine and threonine metabolism;Methane metabolism;Vitamin B6 metabolism	Homo sapiens	
cell part;intracellular membrane-bounded organelle;intracellular organelle;intracellular organelle part;intracellular part;macromolecular complex;membrane;membrane-bounded organelle;nuclear body;nuclear matrix;nuclear pa	Notch signaling pathway	Homo sapiens	+
cell part;cell surface;integral to membrane;intrinsic to membrane;membrane;membrane part;presynaptic membrane;synapse part;synaptic membrane		Homo sapiens	+
cell part;cytoskeletal part;intracellular membrane-bounded organelle;intracellular non-membrane-bounded organelle;intracellular organelle;intracellular organelle part;intracellular part;membrane-bounded organelle;non-membrane-bounded organelle;nuclear part;nucleolus;nucleus;organelle;organelle part;spindle		Homo sapiens	
cell part;cytoplasmic part;endoplasmic reticulum membrane;endoplasmic reticulum part;integral to membrane;intracellular organelle part;intracellular part;intrinsic to membrane;membrane;membrane part;organelle membrane	N-Glycan biosynthesis	Homo sapiens	+

cell junction;cell part;cytoplasmic part;intracellular membrane-bounded organelle;intracellular organelle;intracellular organelle part;intracellular part;macromolecular complex;membrane;membrane-bounded organelle;mitochondrial inner membrane;mitochondrial membrane;mitochondrial part;mitochondrial small ribosomal subunit;mitochondrion;nuclear part;nucleoplasm		Homo sapiens	
cell part;COPI vesicle coat;cytoplasmic part;cytoplasmic vesicle part;cytosol;Golgi apparatus part;Golgi membrane;intracellular organelle part;intracellular part;macromolecular complex;membrane;membrane coat;membrane part;organelle membrane;organelle part;protein complex;vesicle coat		Homo sapiens	+
cell part;cytoplasmic part;endoplasmic reticulum membrane;endoplasmic reticulum part;extracellular membrane-bounded organelle;extracellular organelle;extracellular region part;extracellular vesicular exosome;integral to membrane;intracellular organelle part;intracellular part;intrinsic to membrane;membrane;membrane part;membrane-bounded organelle;membrane-b		Homo sapiens	
cell part;cytoplasmic part;endoplasmic reticulum lumen;endoplasmic reticulum membrane;endoplasmic reticulum part;intracellular organelle lumen;intracellular organelle part;intracellular part;membrane;membrane part;membrane-enclosed lumen;organelle lumen;organelle membrane;organelle part		Homo sapiens	+
actin cytoskeleton;apical part of cell;cell junction;cell part;cell projection;cell surface;cell-cell junction;centrosome;chloride channel complex;cytoplasm;cytoplasmic part;cytoplasmic vesicle membrane;cytoplasmic vesicle part;cytoskeletal part;cytoskeleton;cytosol;extracellular membrane-bounded organelle;extracellular organelle;extracellular region part;extracellular ves		Homo sapiens	+
cell part;cytoplasm;cytoplasmic part;cytosol;intracellular membrane-bounded organelle;intracellular non-membrane-bounded organelle;intracellular organelle;intracellular organelle lumen;intracellular organelle part;intracellula	Sulfur relay system;Thiamine metabolism	Homo sapiens	
cell part;centrosome;cytoplasm;cytoplasmic part;cytoskeletal part;intracellular non-membrane-bounded organelle;intracellular organelle;intracellular organelle part;intracellular part;microtubule organizing center;non-membrar	RNA transport	Homo sapiens	+
cell part;cytoplasmic part;endoplasmic reticulum membrane;endoplasmic reticulum part;integral to endoplasmic reticulum membrane;integral to membrane;integral to organelle membrane;intracellular organelle part;intracellu	Protein export	Homo sapiens	
collagen;extracellular matrix;extracellular matrix part;extracellular membrane-bounded organelle;extracellular organelle;extracellular region;extracellular region part;extracellular vesicular exosome;membrane-bounded organelle;membrane-bounded vesicle;organelle;proteinaceous extracellular matrix;vesicle		Homo sapiens	+
cell part;cytoplasmic part;extracellular membrane-bounded organelle;extracellular organelle;extracellular region part;extracellular vesicular exosome;integral to membrane;intracellular membrane-bounded organelle;intracellular organelle;intracellular organelle part;intracellular part;intrinsic to membrane;membrane;membrane part;membrane-bounded organelle;membrane		Homo sapiens	+
cell part;cytoplasm;cytoplasmic part;cytosol;Golgi apparatus;Golgi apparatus part;Golgi membrane;intracellular membrane-bounded organelle;intracellular non-membrane-bounded organelle;intracellular organelle;intracellular organelle part;intracellular part;macromolecular complex;membrane;membrane-bounded organelle;non-membrane-bounded organelle;nuclear ma		Homo sapiens	+
actin cytoskeleton;cell part;centrosome;chromosomal part;condensed chromosome kinetochore;cytoplasm;cytoplasmic part;cytoskeletal part;cytoskeleton;cytosol;intracellular membrane-bounded organelle;intracellular non-	Cell cycle;Progesterone-mediated oocyte maturation	Homo sapiens	+
cell part;cytoplasm;cytoplasmic part;cytosol;extracellular membrane-bounded organelle;extracellular organelle;extracellular region part;extracellular vesicular exosome;intracellular membrane-bounded organelle;intracellular non-membrane-bounded organelle;intracellular organelle;intracellular organelle part;intracellular part;membrane;membrane-bounded organelle;mem		Homo sapiens	+
cell part;membrane		Homo sapiens	+
cell part;centrosome;chromosomal part;condensed chromosome kinetochore;cytoplasm;cytoplasmic dynein complex;cytoplasmic part;cytoskeletal part;dynein complex;intracellular non-membrane-bounded organelle;intrace	Phagosome;Vasopressin-regulated water reabsorption	Homo sapiens	
cell part;cytoplasmic part;intracellular membrane-bounded organelle;intracellular organelle;intracellular part;membrane-bounded organelle;mitochondrion;nucleus;organelle		Homo sapiens	
cell part;coated pit;cytoplasm;intracellular membrane-bounded organelle;intracellular organelle;intracellular part;membrane;membrane part;membrane-bounded organelle;nucleus;organelle;plasma membrane	Endocytosis	Homo sapiens	+
extracellular membrane-bounded organelle;extracellular organelle;extracellular region part;extracellular vesicular exosome;membrane-bounded organelle;membrane-bounded vesicle;organelle;vesicle		Homo sapiens	
cell part;cytoplasm;cytoplasmic part;cytoskeletal part;cytosol;cytosolic part;IkappaB kinase complex;intracellular;intracellular membrane-bounded organelle;intracellular non-membrane-bounded organelle;intracellular organe	Acute myeloid leukemia;Adipocytokine signaling pathway;Apoptosis;B cell receptor signaling pathway;Chagas disease (American trypanosomiasis	Homo sapiens	
cell part;cytoplasm;cytoplasmic part;cytoskeleton;cytosol;intracellular membrane-bounded organelle;intracellular non-membrane-bounded organelle;intracellular organelle;intracellular part;membrane-bounded organelle;non-membrane-bounded organelle;nucleus;organelle		Homo sapiens	+
cell part;cytoplasmic part;extracellular membrane-bounded organelle;extracellular organelle;extracellular region part;extracellular vesicular exosome;intracellular membrane-bounded organelle;intracellular organelle;intracellula	Alzheimer's disease;Huntington's disease;Oxidative phosphorylation;Parkinson's disease	Homo sapiens	
cell part;cytoplasmic part;intracellular organelle part;intracellular part;membrane;mitochondrial inner membrane;mitochondrial membrane;mitochondrial part;organelle inner membrane;organelle membrane;organelle part		Homo sapiens	+
actin cytoskeleton;cell junction;cell part;cell projection;cell-cell junction;cytoplasmic part;cytoskeleton;cytosol;early endosome;endosome;extracellular membrane-bounded organelle;extracellular organelle;extracellular region	Adherens junction;Bacterial invasion of epithelial cells;Fc gamma R-mediated phagocytosis;Regulation of actin cytoskeleton;Shigellosis	Homo sapiens	
cell part;cytoplasm;cytoplasmic part;cytosol;intracellular membrane-bounded organelle;intracellular organelle;intracellular organelle part;intracellular part;membrane-bounded organelle;nuclear part;nucleoplasm;nucleus;organelle;organelle part		Homo sapiens	+
actin cytoskeleton;cell part;cytoplasm;cytoskeleton;intracellular non-membrane-bounded organelle;intracellular organelle;intracellular organelle part;intracellular part;macromolecular complex;non-membrane-bounded organelle;nuclear part;nucleoplasm;nucleoplasm part;organelle;organelle part;protein complex;ribonucleoprotein complex;spliceosomal complex;transcrip		Homo sapiens	+
cell part;cis-Golgi network;coated vesicle membrane;cytoplasm;cytoplasmic part;cytoplasmic vesicle membrane;cytoplasmic vesicle part;endoplasmic reticulum exit site;endoplasmic reticulum part;endoplasmic reticulum-Golgi intermediate compartment;ER to Golgi transport vesicle membrane;Golgi apparatus part;intracellular membrane-bounded organelle;intracellula		Homo sapiens	+

N: Protein Probability	N: Combined Total Peptides	N: Combined Spectral Count	N: Combined Unique Spectral Count	N: Combined Total Spectral Count	N: Top Peptide Probability	N: Protein Length	N: -Log ANOVA p value	N: ANOVA q-value	T: Protein	T: Gene	T: Protein ID
1	5	55	0	55	0.999	181	2.1747	0.00060749	sp|A0A024RBG1|NUD4B_HUMAN	NUDT4B	A0A024RBG1
1	16	371	0	371	0.999	268	1.69807	0.00221899	sp|A0A0B4J2D5|GAL3B_HUMAN	GATD3B	A0A0B4J2D5
1	29	791	791	791	0.999	1052	0.51272	0.113914	sp|A0AVT1|UBA6_HUMAN	UBA6	A0AVT1
1	29	400	400	400	0.999	921	1.45102	0.00451354	sp|A0FGR8|ESYT2_HUMAN	ESYT2	A0FGR8
1	18	307	307	307	0.999	632	0.0292754	0.776388	sp|A1L0T0|HACL2_HUMAN	ILVBL	A1L0T0
1	14	139	125	139	0.999	911	0.608678	0.0811718	sp|A1X283|SPD2B_HUMAN	SH3PXD2B	A1X283
1	26	439	439	439	0.999	2376	1.18276	0.0111982	sp|A5YKK6|CNOT1_HUMAN	CNOT1	A5YKK6
1	4	45	0	45	0.999	125	0.858584	0.0348637	sp|A6NCE7|MP3B2_HUMAN	MAP1LC3B2	A6NCE7
1	11	292	292	292	0.999	321	1.43757	0.00473343	sp|A6NDG6|PGP_HUMAN	PGP	A6NDG6
1	23	145	145	145	0.999	2005	3.70947	0	sp|A6NHR9|SMHD1_HUMAN	SMCHD1	A6NHR9
1	5	120	4	120	0.999	1229	0.0261721	0.792146	sp|A8CG34|P121C_HUMAN	POM121C	A8CG34
1	5	104	0	104	0.999	76	2.31271	0.000453669	sp|A8MWD9|RUXGL_HUMAN	SNRPGP15	A8MWD9
1	11	480	0	480	0.999	2078	3.30098	3.76028E-05	sp|E9PAV3|NACAM_HUMAN	NACA	E9PAV3
1	5	107	107	107	0.999	123	1.37355	0.00585328	sp|E9PRG8|CK098_HUMAN	C11orf98	E9PRG8
1	5	73	73	73	0.999	103	2.26981	0.000481508	sp|L0R6Q1|S35U4_HUMAN	SLC35A4	L0R6Q1
1	6	138	138	138	0.999	360	1.25103	0.00893999	sp|O00115|DNS2A_HUMAN	DNASE2	O00115
1	25	448	448	448	0.999	658	1.31831	0.00711698	sp|O00116|ADAS_HUMAN	AGPS	O00116
1	27	363	363	983	0.999	427	3.51424	2.01258E-05	sp|O00148|DX39A_HUMAN	DDX39A	O00148
1	25	748	748	748	0.999	329	1.53046	0.00351942	sp|O00151|PDLI1_HUMAN	PDLIM1	O00151
1	23	456	368	456	0.999	380	0.374784	0.183214	sp|O00154|BACH_HUMAN	ACOT7	O00154
1	68	1212	1180	1242	0.999	1063	8.09157	0	sp|O00159|MYO1C_HUMAN	MYO1C	O00159
1	19	514	514	514	0.999	330	0.834749	0.0376946	sp|O00170|AIP_HUMAN	AIP	O00170
1	12	138	138	138	0.999	669	0.743747	0.0516086	sp|O00178|GTPB1_HUMAN	GTPBP1	O00178
1	14	263	263	263	0.999	592	8.94779	0	sp|O00186|STXB3_HUMAN	STXBP3	O00186
1	11	328	328	328	0.999	183	5.25832	0	sp|O00193|SMAP_HUMAN	SMAP	O00193
1	23	266	215	266	0.999	1094	2.11508	0.000705567	sp|O00203|AP3B1_HUMAN	AP3B1	O00203
1	10	136	136	136	0.999	210	0.704657	0.0587472	sp|O00217|NDUS8_HUMAN	NDUFS8	O00217
1	31	1082	1082	1082	0.999	422	0.537347	0.104079	sp|O00231|PSD11_HUMAN	PSMD11	O00231
1	28	839	839	839	0.999	456	1.67094	0.00238809	sp|O00232|PSD12_HUMAN	PSMD12	O00232
1	10	333	333	333	0.999	223	1.91229	0.00118879	sp|O00233|PSMD9_HUMAN	PSMD9	O00233
1	8	314	314	314	0.999	68	0.690172	0.0617832	sp|O00244|ATOX1_HUMAN	ATOX1	O00244
1	14	322	322	322	0.999	195	3.05027	6.69456E-05	sp|O00264|PGRC1_HUMAN	PGRMC1	O00264
1	25	346	346	346	0.999	1087	1.8785	0.00131976	sp|O00267|SPT5H_HUMAN	SUPT5H	O00267
1	17	260	260	260	0.999	331	0.418815	0.157255	sp|O00273|DFFA_HUMAN	DFFA	O00273
1	14	56	56	56	0.999	1037	0.106113	0.524465	sp|O00291|HIP1_HUMAN	HIP1	O00291
1	26	1057	1057	1057	0.999	241	2.73417	0.000170909	sp|O00299|CLIC1_HUMAN	CLIC1	O00299
1	13	519	519	519	0.999	357	1.25368	0.00887339	sp|O00303|EIF3F_HUMAN	EIF3F	O00303
1	13	241	241	241	0.999	501	2.66308	0.000192476	sp|O00330|ODPX_HUMAN	PDHX	O00330
1	44	1371	1317	1371	0.999	1097	3.1768	5.30973E-05	sp|O00410|IPO5_HUMAN	IPO5	O00410
1	9	230	230	230	0.999	153	0.954658	0.0249672	sp|O00422|SAP18_HUMAN	SAP18	O00422
1	22	606	457	606	0.999	579	1.52377	0.0035684	sp|O00425|IF2B3_HUMAN	IGF2BP3	O00425
1	42	794	794	794	0.999	736	0.597404	0.084479	sp|O00429|DNM1L_HUMAN	DNM1L	O00429
1	7	64	64	64	0.999	366	0.750094	0.0504228	sp|O00442|RTCA_HUMAN	RTCA	O00442
1	8	119	119	119	0.999	1686	2.57115	0.000266223	sp|O00443|P3C2A_HUMAN	PIK3C2A	O00443
1	13	210	210	210	0.999	696	0.853337	0.0354951	sp|O00461|GOLI4_HUMAN	GOLIM4	O00461
1	33	347	347	347	0.999	2068	5.39571	0	sp|O00468|AGRIN_HUMAN	AGRN	O00468
1	43	942	942	942	0.999	737	1.2784	0.00818791	sp|O00469|PLOD2_HUMAN	PLOD2	O00469
1	13	130	130	130	0.999	708	1.38477	0.00563619	sp|O00471|EXOC5_HUMAN	EXOC5	O00471
1	8	259	259	259	0.999	81	2.69498	0.000184725	sp|O00483|NDUA4_HUMAN	NDUFA4	O00483
1	13	284	284	284	0.999	310	2.22554	0.000518047	sp|O00487|PSDE_HUMAN	PSMD14	O00487
1	16	314	263	314	0.999	593	0.290598	0.248633	sp|O00499|BIN1_HUMAN	BIN1	O00499
1	11	277	162	277	0.999	521	1.66621	0.00241144	sp|O00505|IMA4_HUMAN	KPNA3	O00505
1	24	530	530	530	0.999	588	1.59913	0.00294444	sp|O00541|PESC_HUMAN	PES 1.00	O00541
1	23	324	324	324	0.999	298	7.32405	0	sp|O00560|SDCB1_HUMAN	SDCBP	O00560
1	7	99	99	99	0.999	681	1.92917	0.00116113	sp|O00566|MPP10_HUMAN	MPHOSPH10	O00566
1	24	475	475	475	0.999	594	0.901503	0.0300024	sp|O00567|NOP56_HUMAN	NOP56	O00567
1	55	1709	743	1768	0.999	662	2.64435	0.000203976	sp|O00571|DDX3X_HUMAN	DDX3X	O00571
1	8	104	104	104	0.999	558	3.77927	0	sp|O00592|PODXL_HUMAN	PODXL	O00592
1	28	143	143	143	0.999	381	6.82103	0	sp|O00622|CCN1_HUMAN	CCN1	O00622
1	13	99	99	214	0.999	521	0.350796	0.200096	sp|O00629|IMA3_HUMAN	KPNA4	O00629
1	9	230	230	230	0.999	305	0.649866	0.0707052	sp|O00743|PPP6_HUMAN	PPP6C	O00743
1	10	133	133	133	0.999	1011	1.21259	0.0101627	sp|O00754|MA2B1_HUMAN	MAN2B1	O00754
1	6	69	69	69	0.999	179	3.4655	1.98511E-05	sp|O00762|UBE2C_HUMAN	UBE2C	O00762
1	14	341	341	341	0.999	312	0.0872864	0.57056	sp|O00764|PDXK_HUMAN	PDXK	O00764
1	11	51	51	51	0.999	2285	1.91133	0.00119221	sp|O14497|ARI1A_HUMAN	ARID1A	O14497
1	3	59	59	59	0.999	444	1.41188	0.00516199	sp|O14524|NEMP1_HUMAN	NEMP1	O14524
1	4	11	11	11	0.999	160	2.66678	0.00018964	sp|O14558|HSPB6_HUMAN	HSPB6	O14558
1	17	53	53	53	0.999	2027	0.679896	0.0638184	sp|O14578|CTRO_HUMAN	CIT	O14578
1	13	530	530	530	0.999	308	4.84202	0	sp|O14579|COPE_HUMAN	COPE	O14579
1	20	251	251	251	0.999	1153	2.47608	0.000303757	sp|O14617|AP3D1_HUMAN	AP3D1	O14617
1	8	291	291	291	0.999	274	1.69154	0.00225042	sp|O14618|CCS_HUMAN	CCS	O14618
1	6	31	31	31	0.999	336	4.04515	0	sp|O14657|TOR1B_HUMAN	TOR1B	O14657
1	15	74	74	74	0.999	748	6.3715	0	sp|O14672|ADA10_HUMAN	ADAM10	O14672
1	8	259	259	259	0.999	152	2.44344	0.000332549	sp|O14684|PTGES_HUMAN	PTGES	O14684
1	3	39	39	39	0.999	213	0.873732	0.0330796	sp|O14735|CDIPT_HUMAN	CDIPT	O14735
1	23	648	648	648	0.999	125	2.76306	0.000144177	sp|O14737|PDCD5_HUMAN	PDCD5	O14737
1	21	580	580	580	0.999	637	0.546496	0.100892	sp|O14744|ANM5_HUMAN	PRMT5	O14744
1	11	257	257	257	0.999	358	3.94139	0	sp|O14745|NHRF1_HUMAN	NHERF1	O14745
1	11	34	33	34	0.999	1132	5.52226	0	sp|O14746|TERT_HUMAN	TERT	O14746
1	8	80	80	80	0.999	440	1.36314	0.00608077	sp|O14763|TR10B_HUMAN	TNFRSF10B	O14763
1	3	13	1	13	0.999	477	1.7238	0.0020462	sp|O14770|MEIS2_HUMAN	MEIS2	O14770
1	5	115	115	115	0.999	563	1.3573	0.0061785	sp|O14773|TPP1_HUMAN	TPP1	O14773
1	29	565	557	565	0.999	1098	3.85728	0	sp|O14776|TCRG1_HUMAN	TCERG1	O14776
1	7	47	47	47	0.999	642	2.06554	0.000802104	sp|O14777|NDC80_HUMAN	NDC80	O14777
1	15	73	73	73	0.999	923	2.91416	0.000125245	sp|O14786|NRP1_HUMAN	NRP1	O14786
0.9995	3	7	7	7	0.9956	238	3.16924	5.28053E-05	sp|O14817|TSN4_HUMAN	TSPAN4	O14817
1	26	786	517	786	0.999	248	4.05735	0	sp|O14818|PSA7_HUMAN	PSMA7	O14818
1	12	331	331	331	0.999	347	2.16078	0.000623198	sp|O14828|SCAM3_HUMAN	SCAMP3	O14828
1	11	53	51	53	0.999	490	3.99793	0	sp|O14879|IFIT3_HUMAN	IFIT3	O14879
1	5	300	300	300	0.999	152	0.37673	0.181909	sp|O14880|MGST3_HUMAN	MGST3	O14880
1	6	124	124	124	0.999	333	1.76279	0.00180564	sp|O14908|GIPC1_HUMAN	GIPC1	O14908
1	16	224	224	224	0.999	419	2.10317	0.000731154	sp|O14929|HAT1_HUMAN	HAT1	O14929
1	3	75	75	75	0.999	153	2.07317	0.000785761	sp|O14933|UB2L6_HUMAN	UBE2L6	O14933
1	12	172	172	172	0.999	926	3.36575	2.39808E-05	sp|O14936|CSKP_HUMAN	CASK	O14936
1	11	358	358	358	0.999	82	5.55351	0	sp|O14949|QCR8_HUMAN	UQCRQ	O14949
1	13	500	0	500	0.999	172	2.50078	0.000291262	sp|O14950|ML12B_HUMAN	MYL12B	O14950
1	17	376	376	376	0.999	777	6.01582	0	sp|O14964|HGS_HUMAN	HGS	O14964
1	8	31	31	31	0.999	403	0.546648	0.100868	sp|O14965|AURKA_HUMAN	AURKA	O14965
1	7	74	74	102	0.999	610	2.11486	0.000705094	sp|O14967|CLGN_HUMAN	CLGN	O14967
1	28	455	454	455	0.999	1030	2.93081	0.000125984	sp|O14974|MYPT1_HUMAN	PPP1R12A	O14974
1	16	95	95	95	0.999	1311	1.6062	0.00291358	sp|O14976|GAK_HUMAN	GAK	O14976
1	23	770	770	1032	0.999	420	3.22871	0.000054732	sp|O14979|HNRDL_HUMAN	HNRNPDL	O14979
1	36	916	916	916	0.999	1071	1.26762	0.00846026	sp|O14980|XPO1_HUMAN	XPO1	O14980
1	5	95	95	95	0.999	1369	0.908636	0.0292575	sp|O15013|ARHGA_HUMAN	ARHGEF10	O15013
1	39	567	567	567	0.999	2357	0.713565	0.0571979	sp|O15027|SC16A_HUMAN	SEC16A	O15027
1	28	302	301	302	0.999	1838	8.16186	0	sp|O15031|PLXB2_HUMAN	PLXNB2	O15031
1	18	434	434	434	0.999	1029	3.76532	0	sp|O15042|SR140_HUMAN	U2SURP	O15042
1	42	1200	1200	1200	0.999	1338	1.88367	0.0012973	sp|O15067|PUR4_HUMAN	PFAS	O15067
0.9999	2	104	104	104	0.999	133	0.291119	0.248192	sp|O15116|LSM1_HUMAN	LSM 1.00	O15116
1	15	169	169	169	0.999	1278	0.617601	0.0788988	sp|O15118|NPC1_HUMAN	NPC1	O15118
1	4	44	44	44	0.999	323	0.715272	0.0568792	sp|O15121|DEGS1_HUMAN	DEGS1	O15121
1	9	90	90	93	0.999	338	6.79845	0	sp|O15126|SCAM1_HUMAN	SCAMP1	O15126
1	6	126	126	126	0.999	329	1.46571	0.00427218	sp|O15127|SCAM2_HUMAN	SCAMP2	O15127
1	28	1224	1219	1224	0.999	372	0.611146	0.0804246	sp|O15143|ARC1B_HUMAN	ARPC1B	O15143
1	25	940	940	940	0.999	300	1.44249	0.00465	sp|O15144|ARPC2_HUMAN	ARPC2	O15144
1	14	487	487	487	0.999	178	3.18637	5.34521E-05	sp|O15145|ARPC3_HUMAN	ARPC3	O15145
1	5	17	17	17	0.9987	318	8.60125	0	sp|O15162|PLS1_HUMAN	PLSCR1	O15162
1	9	211	211	211	0.999	223	2.36869	0.000408781	sp|O15173|PGRC2_HUMAN	PGRMC2	O15173
1	13	416	416	416	0.999	129	2.5704	0.000269103	sp|O15212|PFD6_HUMAN	PFDN6	O15212
1	37	422	422	422	0.999	689	1.83954	0.00147406	sp|O15231|ZN185_HUMAN	ZNF185	O15231
1	4	125	125	125	0.999	138	2.36476	0.000417234	sp|O15235|RT12_HUMAN	MRPS12	O15235
1	6	223	223	223	0.999	196	3.86811	0	sp|O15258|RER1_HUMAN	RER1	O15258
1	13	281	281	281	0.999	473	0.391217	0.173129	sp|O15269|SPTC1_HUMAN	SPTLC1	O15269
1	12	126	80	126	0.999	562	1.46354	0.00430398	sp|O15270|SPTC2_HUMAN	SPTLC2	O15270
1	10	119	119	119	0.999	1046	0.933597	0.026856	sp|O15294|OGT1_HUMAN	OGT	O15294
1	28	647	647	652	0.999	200	3.39276	2.41838E-05	sp|O15347|HMGB3_HUMAN	HMGB3	O15347

1	22	710	710	710	0.999	546	2.3391	0.00043811	sp|O15355|PPM1G_HUMAN	PPM1G	O15355
1	26	274	243	274	0.999	1258	1.19084	0.010917	sp|O15357|SHIP2_HUMAN	INPPL1	O15357
1	23	962	962	962	0.999	548	0.355358	0.196972	sp|O15371|EIF3D_HUMAN	EIF3D	O15371
1	18	404	404	404	0.999	352	1.91396	0.0011878	sp|O15372|EIF3H_HUMAN	EIF3H	O15372
1	10	205	205	205	0.999	392	1.80927	0.00159157	sp|O15382|BCAT2_HUMAN	BCAT2	O15382
1	3	11	11	11	0.999	142	1.12338	0.0138049	sp|O15392|BIRC5_HUMAN	BIRC5	O15392
1	7	141	141	141	0.999	261	0.799617	0.042488	sp|O15400|STX7_HUMAN	STX7	O15400
1	13	572	572	572	0.999	465	6.6921	0	sp|O15427|MOT4_HUMAN	SLC16A3	O15427
1	5	94	94	94	0.999	510	3.17612	5.29801E-05	sp|O15446|RPA34_HUMAN	POLR1G	O15446
1	33	545	545	545	0.999	535	4.4001	0	sp|O15460|P4HA2_HUMAN	P4HA2	O15460
1	8	41	41	41	0.999	640	6.20887	0	sp|O15484|CAN5_HUMAN	CAPN5	O15484
1	11	129	129	129	0.999	198	1.39632	0.00541475	sp|O15498|YKT6_HUMAN	YKT6	O15498
1	15	384	376	384	0.999	151	2.38731	0.000386503	sp|O15511|ARPC5_HUMAN	ARPC5	O15511
1	4	22	22	22	0.999	142	0.742799	0.0517802	sp|O15514|RPB4_HUMAN	POLR2D	O15514
1	5	152	152	152	0.999	448	2.63137	0.000209262	sp|O15533|TPSN_HUMAN	TAPBP	O15533
1	3	47	47	47	0.999	343	1.23454	0.00944712	sp|O15541|R113A_HUMAN	RNF113A	O15541
1	48	1436	1382	1436	0.999	795	1.49753	0.00386932	sp|O43143|DHX15_HUMAN	DHX15	O43143
1	13	110	110	110	0.999	476	1.69982	0.00221253	sp|O43148|MCES_HUMAN	RNMT	O43148
1	6	49	49	49	0.999	2961	2.97281	9.68718E-05	sp|O43149|ZZEF1_HUMAN	ZZEF1	O43149
1	6	154	154	154	0.999	150	3.02545	7.02479E-05	sp|O43169|CYB5B_HUMAN	CYB5B	O43169
1	10	298	298	298	0.999	522	0.0687303	0.624131	sp|O43172|PRP4_HUMAN	PRPF4	O43172
1	40	1487	1487	1487	0.999	533	3.88866	0	sp|O43175|SERA_HUMAN	PHGDH	O43175
1	22	324	323	324	0.999	492	0.104424	0.528211	sp|O43237|DC1L2_HUMAN	DYNC1LI2	O43237
1	36	1063	1063	1063	0.999	534	0.0611105	0.648559	sp|O43242|PSMD3_HUMAN	PSMD3	O43242
1	16	356	331	356	0.999	624	1.0229	0.0197108	sp|O43252|PAPS1_HUMAN	PAPSS1	O43252
1	12	139	139	139	0.999	779	2.73946	0.00016773	sp|O43264|ZW10_HUMAN	ZW10	O43264
1	21	451	451	451	0.999	800	1.7845	0.00169374	sp|O43290|SNUT1_HUMAN	SART1	O43290
1	8	68	68	68	0.999	454	0.520828	0.11066	sp|O43293|DAPK3_HUMAN	DAPK3	O43293
1	5	29	29	29	0.9989	461	0.880603	0.0323337	sp|O43294|TGFI1_HUMAN	TGFB1I1	O43294
1	7	65	65	65	0.999	606	1.82997	0.00150118	sp|O43318|M3K7_HUMAN	MAP3K7	O43318
1	9	354	354	354	0.999	174	2.12833	0.000679703	sp|O43324|MCA3_HUMAN	EEF1E1	O43324
1	3	33	30	33	0.999	540	3.08973	0.000059448	sp|O43353|RIPK2_HUMAN	RIPK2	O43353
1	40	756	756	1579	0.999	633	4.5382	0	sp|O43390|HNRPR_HUMAN	HNRNPR	O43390
1	22	352	352	352	0.999	683	0.437611	0.146969	sp|O43395|PRPF3_HUMAN	PRPF3	O43395
1	13	444	444	444	0.999	289	2.40995	0.00035969	sp|O43396|TXNL1_HUMAN	TXNL1	O43396
1	17	618	618	618	0.999	206	2.57582	0.000253545	sp|O43399|TPD54_HUMAN	TPD52L2	O43399
1	7	142	142	142	0.999	210	0.273896	0.264953	sp|O43402|EMC8_HUMAN	EMC8	O43402
1	6	36	36	36	0.999	364	3.09707	5.53191E-05	sp|O43427|FIBP_HUMAN	FIBP	O43427
1	27	164	147	621	0.999	1585	0.451968	0.140247	sp|O43432|IF4G3_HUMAN	EIF4G3	O43432
1	10	128	128	128	0.999	177	0.390681	0.173422	sp|O43447|PPIH_HUMAN	PPIH	O43447
1	11	298	156	298	0.999	359	3.96894	0	sp|O43488|ARK72_HUMAN	AKR7A2	O43488
1	32	333	269	333	0.999	1005	8.3148	0	sp|O43491|E41L2_HUMAN	EPB41L2	O43491
1	4	23	23	23	0.999	437	0.86074	0.0345821	sp|O43493|TGON2_HUMAN	TGOLN2	O43493
1	12	187	187	187	0.999	198	1.90516	0.00120727	sp|O43583|DENR_HUMAN	DENR	O43583
1	19	272	272	272	0.999	962	1.40504	0.00525625	sp|O43592|XPOT_HUMAN	XPOT	O43592
1	9	301	301	301	0.999	174	1.55997	0.00327827	sp|O43598|DNPH1_HUMAN	DNPH1	O43598
1	18	325	325	325	0.999	452	0.205698	0.344652	sp|O43615|TIM44_HUMAN	TIMM44	O43615
1	6	191	191	191	0.999	180	1.30912	0.00737189	sp|O43617|TPPC3_HUMAN	TRAPPC3	O43617
1	14	206	202	206	0.999	222	3.21355	5.39933E-05	sp|O43633|CHM2A_HUMAN	CHMP2A	O43633
1	3	77	77	77	0.999	245	3.10707	5.13919E-05	sp|O43657|TSN6_HUMAN	TSPAN6	O43657
1	4	106	106	106	0.999	514	2.744	0.000164534	sp|O43660|PLRG1_HUMAN	PLRG1	O43660
1	10	35	35	35	0.999	620	0.97544	0.0232321	sp|O43663|PRC1_HUMAN	PRC1	O43663
1	8	76	76	76	0.999	181	0.78777	0.044246	sp|O43665|RGS10_HUMAN	RGS10	O43665
1	8	199	199	199	0.999	478	0.593957	0.0853982	sp|O43670|ZN207_HUMAN	ZNF207	O43670
1	6	169	169	169	0.999	189	0.0384158	0.734551	sp|O43674|NDUB5_HUMAN	NDUFB5	O43674
1	5	98	98	98	0.999	99	2.33975	0.000432432	sp|O43678|NDUA2_HUMAN	NDUFA2	O43678
1	12	397	397	397	0.999	348	1.37017	0.00590467	sp|O43681|GET3_HUMAN	GET3	O43681
1	17	662	662	662	0.999	328	1.33719	0.006647	sp|O43684|BUB3_HUMAN	BUB3	O43684
1	100	3750	2337	3750	0.999	911	6.34235	0	sp|O43707|ACTN4_HUMAN	ACTN4	O43707
1	12	208	208	208	0.999	755	0.491342	0.12241	sp|O43719|HTSF1_HUMAN	HTATSF1	O43719
1	20	369	369	369	0.999	822	1.00965	0.0205791	sp|O43747|AP1G1_HUMAN	AP1G1	O43747
1	8	184	184	184	0.999	313	1.35916	0.00615858	sp|O43765|SGTA_HUMAN	SGTA	O43765
1	7	187	95	187	0.999	121	3.08493	0.000059322	sp|O43768|ENSA_HUMAN	ENSA	O43768
1	29	955	955	955	0.999	548	1.41079	0.00518504	sp|O43776|SYNC_HUMAN	NARS1	O43776
1	65	1345	1285	1375	0.999	1136	8.22177	0	sp|O43795|MYO1B_HUMAN	MYO1B	O43795
1	8	342	342	342	0.999	227	3.02409	7.01754E-05	sp|O43809|CPSF5_HUMAN	NUDT21	O43809
1	15	334	334	334	0.999	399	0.266597	0.272373	sp|O43813|LANC1_HUMAN	LANCL1	O43813
1	16	208	208	208	0.999	475	0.264724	0.274242	sp|O43818|U3IP2_HUMAN	RRP9	O43818
1	11	272	272	272	0.999	692	1.58963	0.00302076	sp|O43823|AKAP8_HUMAN	AKAP8	O43823
1	18	351	351	351	0.999	385	1.2089	0.0102916	sp|O43837|IDH3B_HUMAN	IDH3B	O43837
1	20	274	274	274	0.999	1151	0.843067	0.0366541	sp|O43847|NRDC_HUMAN	NRDC	O43847
1	23	648	648	648	0.999	315	6.77898	0	sp|O43852|CALU_HUMAN	CALU	O43852
1	16	57	57	57	0.999	480	2.38151	0.000397858	sp|O43854|EDIL3_HUMAN	EDIL3	O43854
1	22	281	121	350	0.999	530	2.14798	0.000648501	sp|O43865|SAHH2_HUMAN	AHCYL1	O43865
1	7	148	148	148	0.999	106	1.07096	0.0166025	sp|O43920|NDUS5_HUMAN	NDUFS5	O43920
1	12	108	73	108	0.999	631	1.84646	0.00145024	sp|O60216|RAD21_HUMAN	RAD21	O60216
1	6	222	222	222	0.999	97	0.896666	0.0305183	sp|O60220|TIM8A_HUMAN	TIMM8A	O60220
1	27	343	343	397	0.999	1041	0.0684179	0.624965	sp|O60231|DHX16_HUMAN	DHX16	O60231
1	4	91	91	91	0.999	199	2.57938	0.000247906	sp|O60232|ZNRD2_HUMAN	ZNRD2	O60232
1	17	381	277	381	0.999	369	0.824259	0.0391572	sp|O60256|KPRB_HUMAN	PRPSAP2	O60256
1	46	1047	763	1047	0.999	1052	0.712379	0.0573768	sp|O60264|SMCA5_HUMAN	SMARCA5	O60264
1	17	220	189	220	0.999	1321	1.04647	0.0180932	sp|O60271|JIP4_HUMAN	SPAG9	O60271
1	15	59	59	60	0.999	1485	0.706816	0.0583149	sp|O60306|AQR_HUMAN	AQR	O60306
1	35	661	661	661	0.999	960	0.499319	0.119224	sp|O60313|OPA1_HUMAN	OPA1	O60313
1	12	70	43	70	0.999	1816	2.3865	0.000386207	sp|O60333|KIF1B_HUMAN	KIF1B	O60333
1	8	131	131	131	0.999	852	0.848727	0.035959	sp|O60341|KDM1A_HUMAN	KDM1A	O60341
1	12	175	175	175	0.999	496	1.35595	0.00620307	sp|O60443|GSDME_HUMAN	GSDME	O60443
1	33	590	590	680	0.999	711	1.18718	0.0110401	sp|O60488|ACSL4_HUMAN	ACSL4	O60488
1	13	266	264	266	0.999	162	0.715291	0.0568946	sp|O60493|SNX3_HUMAN	SNX3	O60493
1	18	210	210	210	0.999	916	0.195258	0.358761	sp|O60502|OGA_HUMAN	OGA	O60502
1	43	1922	1099	1922	0.999	623	5.16918	0	sp|O60506|HNRPQ_HUMAN	SYNCRIP	O60506
1	8	68	68	68	0.999	579	1.72929	0.0020226	sp|O60508|PRP17_HUMAN	CDC40	O60508
1	7	52	52	106	0.999	1105	2.27707	0.000477091	sp|O60518|RNBP6_HUMAN	RANBP6	O60518
1	16	255	255	255	0.999	372	1.423	0.00499257	sp|O60547|GMDS_HUMAN	GMDS	O60547
1	12	125	90	125	0.999	184	9.15346	0	sp|O60565|GREM1_HUMAN	GREM1	O60565
1	31	602	602	602	0.999	738	2.32152	0.000452381	sp|O60568|PLOD3_HUMAN	PLOD3	O60568
1	4	111	111	111	0.999	245	0.960054	0.0245493	sp|O60573|IF4E2_HUMAN	EIF4E2	O60573
1	56	1030	1015	1030	0.999	1272	0.182679	0.377672	sp|O60610|DIAP1_HUMAN	DIAPH1	O60610
1	34	1224	1224	1224	0.999	434	5.18167	0	sp|O60664|PLIN3_HUMAN	PLIN3	O60664
1	13	137	75	195	0.999	536	5.89584	0	sp|O60684|IMA7_HUMAN	KPNA6	O60684
1	36	767	767	767	0.999	494	0.196498	0.357045	sp|O60701|UGDH_HUMAN	UGDH	O60701
1	5	55	55	55	0.999	386	2.57796	0.000254181	sp|O60711|LPXN_HUMAN	LPXN	O60711
1	52	1045	1040	1045	0.999	968	5.58015	0	sp|O60716|CTND1_HUMAN	CTNND1	O60716
1	18	331	323	331	0.999	519	0.157732	0.418097	sp|O60749|SNX2_HUMAN	SNX2	O60749
1	7	184	184	184	0.999	260	0.895942	0.0305928	sp|O60762|DPM1_HUMAN	DPM1	O60762
1	34	792	792	792	0.999	962	1.00697	0.0207896	sp|O60763|USO1_HUMAN	USO1	O60763
1	10	101	101	101	0.999	492	0.55617	0.0974205	sp|O60784|TOM1_HUMAN	TOM1	O60784
1	7	126	126	126	0.999	627	1.84825	0.00144635	sp|O60826|CCD22_HUMAN	CCDC22	O60826
1	6	166	166	166	0.999	265	2.88308	0.000127907	sp|O60828|PQBP1_HUMAN	PQBP1	O60828
1	5	85	85	85	0.999	178	1.18273	0.011195	sp|O60831|PRAF2_HUMAN	PRAF2	O60831
1	19	374	374	374	0.999	514	0.250111	0.290076	sp|O60832|DKC1_HUMAN	DKC1	O60832
1	33	739	739	739	0.999	1220	1.75065	0.00189003	sp|O60841|IF2P_HUMAN	EIF5B	O60841
1	16	374	374	374	0.999	148	6.20044	0	sp|O60869|EDF1_HUMAN	EDF1	O60869
1	8	106	106	106	0.999	1101	1.37672	0.00578637	sp|O60879|DIAP2_HUMAN	DIAPH2	O60879
1	16	353	353	353	0.999	412	0.618432	0.0787216	sp|O60884|DNJA2_HUMAN	DNAJA2	O60884
1	13	201	116	201	0.999	1362	0.957182	0.0247517	sp|O60885|BRD4_HUMAN	BRD4	O60885
1	13	421	421	421	0.999	122	4.73448	0	sp|O60925|PFD1_HUMAN	PFDN1	O60925
1	9	86	86	86	0.999	754	2.2318	0.000518519	sp|O60934|NBN_HUMAN	NBN	O60934
1	9	121	121	121	0.999	752	3.00211	8.16327E-05	sp|O75027|ABCB7_HUMAN	ABCB7	O75027
1	16	114	38	114	0.999	1071	0.851705	0.0356776	sp|O75044|SRGP2_HUMAN	SRGAP2	O75044
1	60	2457	2457	2457	0.999	606	1.94006	0.00112415	sp|O75083|WDR1_HUMAN	WDR1	O75083
1	35	319	257	319	0.999	1388	0.153367	0.425358	sp|O75116|ROCK2_HUMAN	ROCK2	O75116
1	15	92	92	123	0.999	1294	2.13703	0.000660799	sp|O75122|CLAP2_HUMAN	CLASP2	O75122
1	26	580	551	580	0.999	537	6.24858	0	sp|O75131|CPNE3_HUMAN	CPNE3	O75131
1	12	88	88	88	0.999	1068	2.03017	0.000877986	sp|O75146|HIP1R_HUMAN	HIP1R	O75146
1	18	283	283	283	0.999	1001	0.207884	0.341793	sp|O75150|BRE1B_HUMAN	RNF40	O75150
1	11	131	25	131	0.999	810	0.105012	0.527093	sp|O75152|ZC11A_HUMAN	ZC3H11A	O75152
1	40	696	658	696	0.999	1309	0.506356	0.116333	sp|O75153|CLU_HUMAN	CLUH	O75153
1	20	204	204	204	0.999	2243	1.17779	0.0113893	sp|O75165|DJC13_HUMAN	DNAJC13	O75165

1	10	124	124	124	0.999	753	0.0758616	0.60267	sp|O75175|CNOT3_HUMAN	CNOT3	O75175
1	11	119	105	119	0.999	2603	0.753921	0.0497614	sp|O75179|ANR17_HUMAN	ANKRD17	O75179
1	13	295	295	295	0.999	188	3.01758	7.42268E-05	sp|O75223|GGCT_HUMAN	GGCT	O75223
1	15	456	456	456	0.999	463	1.70165	0.00219709	sp|O75306|NDUS2_HUMAN	NDUFS2	O75306
1	16	147	147	147	0.999	459	0.946222	0.0257327	sp|O75312|ZPR1_HUMAN	ZPR1	O75312
1	9	222	222	283	0.999	286	0.370422	0.186118	sp|O75323|NIPS2_HUMAN	NIPSNAP2	O75323
1	4	18	18	18	0.999	724	1.01815	0.0200101	sp|O75330|HMMR_HUMAN	HMMR	O75330
1	10	287	287	287	0.999	191	1.53537	0.00347998	sp|O75340|PDCD6_HUMAN	PDCD6	O75340
1	20	870	870	870	0.999	108	4.7948	0	sp|O75347|TBCA_HUMAN	TBCA	O75347
1	11	296	249	296	0.999	118	1.40548	0.00525159	sp|O75348|VATG1_HUMAN	ATP6V1G1	O75348
1	18	332	275	332	0.999	444	0.643555	0.0722829	sp|O75351|VPS4B_HUMAN	VPS4B	O75351
1	20	435	386	435	0.999	369	0.964147	0.0241911	sp|O75367|H2AY_HUMAN	MACROH2A1	O75367
1	171	4011	3967	4490	0.999	2602	3.03941	6.65973E-05	sp|O75369|FLNB_HUMAN	FLNB	O75369
1	4	82	82	82	0.999	377	1.0606	0.0172379	sp|O75381|PEX14_HUMAN	PEX14	O75381
1	20	529	529	529	0.999	466	7.05235	0	sp|O75390|CISY_HUMAN	CS	O75390
1	16	647	647	647	0.999	215	5.92885	0	sp|O75396|SC22B_HUMAN	SEC22B	O75396
1	21	438	420	438	0.999	957	0.389504	0.174097	sp|O75400|PR40A_HUMAN	PRPF40A	O75400
1	10	102	102	103	0.999	805	1.94259	0.00111455	sp|O75410|TACC1_HUMAN	TACC1	O75410
1	10	159	159	159	0.999	263	0.38579	0.176484	sp|O75431|MTX2_HUMAN	MTX2	O75431
1	15	338	337	338	0.999	327	1.72327	0.0020518	sp|O75436|VP26A_HUMAN	VPS26A	O75436
1	18	398	398	490	0.999	489	0.397945	0.169148	sp|O75439|MPPB_HUMAN	PMPCB	O75439
1	24	394	394	411	0.999	530	6.21947	0	sp|O75475|PSIP1_HUMAN	PSIP1	O75475
1	14	405	142	405	0.999	348	4.07544	0	sp|O75477|ERLN1_HUMAN	ERLIN1	O75477
1	11	213	213	213	0.999	264	0.210821	0.337947	sp|O75489|NDUS3_HUMAN	NDUFS3	O75489
1	12	268	242	268	0.999	262	1.2058	0.0103799	sp|O75494|SRS10_HUMAN	SRSF10	O75494
1	4	26	26	26	0.999	207	3.34886	2.38379E-05	sp|O75508|CLD11_HUMAN	CLDN11	O75508
1	12	331	331	331	0.999	394	1.34841	0.00637398	sp|O75521|ECI2_HUMAN	ECI2	O75521
1	12	410	410	410	0.999	89	1.49006	0.00396134	sp|O75531|BAF_HUMAN	BANF1	O75531
1	70	1780	1780	1780	0.999	1304	1.78438	0.00169507	sp|O75533|SF3B1_HUMAN	SF3B1	O75533
1	40	839	839	839	0.999	798	5.91958	0	sp|O75534|CSDE1_HUMAN	CSDE1	O75534
1	7	168	168	168	0.999	313	1.37947	0.0057345	sp|O75569|PRKRA_HUMAN	PRKRA	O75569
1	7	243	243	243	0.999	230	0.283351	0.255392	sp|O75608|LYPA1_HUMAN	LYPLA1	O75608
1	71	1366	1356	1366	0.999	2136	1.59645	0.00295893	sp|O75643|U520_HUMAN	SNRNP200	O75643
1	13	184	184	184	0.999	272	0.279848	0.258631	sp|O75663|TIPRL_HUMAN	TIPRL	O75663
1	11	119	119	119	0.999	361	1.41982	0.00503411	sp|O75683|SURF6_HUMAN	SURF6	O75683
1	7	68	68	127	0.999	479	3.11161	5.15021E-05	sp|O75688|PPM1B_HUMAN	PPM1B	O75688
1	26	149	149	149	0.999	2785	1.0562	0.0174643	sp|O75691|UTP20_HUMAN	UTP20	O75691
1	34	568	568	568	0.999	1391	2.0846	0.000771372	sp|O75694|NU155_HUMAN	NUP155	O75694
1	13	110	110	110	0.999	350	4.70534	0	sp|O75695|XRP2_HUMAN	RP2	O75695
1	20	193	193	193	0.999	1129	0.707216	0.0582569	sp|O75717|WDHD1_HUMAN	WDHD1	O75717
1	8	151	150	151	0.999	401	0.201134	0.350741	sp|O75718|CRTAP_HUMAN	CRTAP	O75718
1	3	55	55	55	0.999	350	1.65252	0.00252712	sp|O75787|RENR_HUMAN	ATP6AP2	O75787
1	6	50	50	50	0.999	336	1.56172	0.0032687	sp|O75794|CD123_HUMAN	CDC123	O75794
1	6	88	88	88	0.999	140	1.62166	0.00278919	sp|O75817|POP7_HUMAN	POP7	O75817
1	18	622	622	622	0.999	320	2.99632	8.12183E-05	sp|O75821|EIF3G_HUMAN	EIF3G	O75821
1	9	209	209	209	0.999	258	1.6326	0.00270511	sp|O75822|EIF3J_HUMAN	EIF3J	O75822
1	11	97	97	365	0.999	277	0.551125	0.0992325	sp|O75828|CBR3_HUMAN	CBR3	O75828
1	7	220	220	220	0.999	226	0.551111	0.0992003	sp|O75832|PSD10_HUMAN	PSMD10	O75832
1	11	353	353	353	0.999	475	2.72388	0.000172973	sp|O75844|FACE1_HUMAN	ZMPSTE24	O75844
1	32	931	818	931	0.999	414	1.57961	0.0031248	sp|O75874|IDHC_HUMAN	IDH1	O75874
1	4	167	167	167	0.999	301	3.17335	5.29217E-05	sp|O75880|SCO1_HUMAN	SCO1	O75880
1	4	93	93	93	0.999	186	0.0614702	0.647503	sp|O75884|RBBP9_HUMAN	RBBP9	O75884
1	10	205	205	205	0.999	188	2.26381	0.000483008	sp|O75915|PRAF3_HUMAN	ARL6IP5	O75915
1	12	196	196	196	0.999	225	5.16147	0	sp|O75934|SPF27_HUMAN	BCAS2	O75934
1	11	145	145	145	0.999	186	2.83286	0.000132827	sp|O75935|DCTN3_HUMAN	DCTN3	O75935
1	19	386	386	386	0.999	253	0.503183	0.117685	sp|O75937|DNJC8_HUMAN	DNAJC8	O75937
1	5	129	129	129	0.999	238	2.24485	0.000501433	sp|O75940|SPF30_HUMAN	SMNDC1	O75940
1	14	513	513	513	0.999	161	4.34016	0	sp|O75947|ATP5H_HUMAN	ATP5PD	O75947
0.9997	2	3	3	3	0.9985	239	2.86732	0.000126679	sp|O75954|TSN9_HUMAN	TSPAN9	O75954
1	21	209	209	209	0.999	427	8.31827	0	sp|O75955|FLOT1_HUMAN	FLOT1	O75955
1	17	88	80	88	0.999	3097	0.938176	0.0264662	sp|O75962|TRIO_HUMAN	TRIO	O75962
1	6	153	145	153	0.999	103	1.49093	0.00395115	sp|O75964|ATP5L_HUMAN	ATP5MG	O75964
1	29	188	188	188	0.999	1380	1.82933	0.001505	sp|O75976|CBPD_HUMAN	CPD	O75976
1	11	251	251	251	0.999	335	2.8191	0.000131827	sp|O76003|GLRX3_HUMAN	GLRX3	O76003
1	35	838	838	838	0.999	490	1.04014	0.018452	sp|O76021|RL1D1_HUMAN	RSL1D1	O76021
0.9998	3	8	8	8	0.999	302	4.17265	0	sp|O76061|STC2_HUMAN	STC2	O76061
1	8	142	142	142	0.999	339	0.43386	0.148948	sp|O76071|CIAO1_HUMAN	CIAO1	O76071
1	25	300	300	300	0.999	671	2.28506	0.000467153	sp|O76094|SRP72_HUMAN	SRP72	O76094
1	10	102	102	127	0.999	285	3.6136	0	sp|O94760|DDAH1_HUMAN	DDAH1	O94760
1	4	61	61	61	0.999	535	2.32817	0.000447761	sp|O94763|RMP_HUMAN	URI1	O94763
1	35	861	661	861	0.999	668	1.8362	0.00148174	sp|O94776|MTA2_HUMAN	MTA2	O94776
1	26	331	331	385	0.999	968	2.1025	0.000733333	sp|O94804|STK10_HUMAN	STK10	O94804
1	10	102	102	199	0.999	682	5.44684	0	sp|O94808|GFPT2_HUMAN	GFPT2	O94808
1	12	32	28	32	0.999	1529	6.70825	0	sp|O94813|SLIT2_HUMAN	SLIT2	O94813
1	10	131	131	131	0.999	1766	1.38505	0.00563504	sp|O94822|LTN1_HUMAN	LTN1	O94822
1	26	556	556	556	0.999	608	3.66582	0	sp|O94826|TOM70_HUMAN	TOMM70	O94826
1	22	152	150	182	0.999	1006	4.69225	0	sp|O94832|MYO1D_HUMAN	MYO1D	O94832
1	16	163	163	177	0.999	1032	1.63707	0.00268412	sp|O94855|SC24D_HUMAN	SEC24D	O94855
1	19	299	299	299	0.999	794	2.65927	0.000195633	sp|O94874|UFL1_HUMAN	UFL1	O94874
1	4	8	8	8	0.9987	1247	0.902927	0.0298711	sp|O94885|SASH1_HUMAN	SASH1	O94885
1	5	124	124	124	0.999	489	0.118918	0.494909	sp|O94888|UBXN7_HUMAN	UBXN7	O94888
1	7	53	53	53	0.999	275	0.61428	0.0797231	sp|O94903|PLPHP_HUMAN	PLPBP	O94903
1	15	129	129	392	0.999	339	2.87596	0.00012729	sp|O94905|ERLN2_HUMAN	ERLIN2	O94905
1	45	678	678	678	0.999	941	0.553818	0.098269	sp|O94906|PRP6_HUMAN	PRPF6	O94906
1	8	39	32	39	0.999	266	8.29994	0	sp|O94907|DKK1_HUMAN	DKK 1.00	O94907
1	13	73	73	73	0.999	3013	1.53116	0.00351063	sp|O94915|FRYL_HUMAN	FRYL	O94915
1	21	356	356	356	0.999	669	3.01372	7.40741E-05	sp|O94925|GLSK_HUMAN	GLS	O94925
1	30	265	265	642	0.999	939	2.02258	0.000893179	sp|O94973|AP2A2_HUMAN	AP2A2	O94973
1	43	1069	1031	1069	0.999	1220	2.71642	0.000175627	sp|O94979|SC31A_HUMAN	SEC31A	O94979
1	11	175	173	175	0.999	359	0.272237	0.266598	sp|O94992|HEXI1_HUMAN	HEXIM1	O94992
1	5	19	19	19	0.999	398	2.37754	0.000408537	sp|O95067|CCNB2_HUMAN	CCNB2	O95067
1	23	220	220	220	0.999	2799	2.10873	0.000713904	sp|O95071|UBR5_HUMAN	UBR5	O95071
1	5	13	13	13	0.9975	383	5.80161	0	sp|O95084|PRS23_HUMAN	PRSS23	O95084
1	9	98	83	98	0.999	1147	0.617436	0.0789094	sp|O95104|SCAF4_HUMAN	SCAF4	O95104
1	5	94	94	94	0.999	128	1.90305	0.00121792	sp|O95139|NDUB6_HUMAN	NDUFB6	O95139
1	10	84	84	84	0.999	757	1.91055	0.00119077	sp|O95140|MFN2_HUMAN	MFN2	O95140
1	6	47	47	47	0.999	360	2.43727	0.000334375	sp|O95149|SPN1_HUMAN	SNUPN	O95149
1	10	149	149	149	0.999	1302	1.74047	0.00195105	sp|O95155|UBE4B_HUMAN	UBE4B	O95155
1	7	196	196	196	0.999	310	1.14259	0.0129159	sp|O95159|ZFPL1_HUMAN	ZFPL1	O95159
1	20	316	316	316	0.999	1332	0.377983	0.181225	sp|O95163|ELP1_HUMAN	ELP1	O95163
1	7	182	182	182	0.999	129	2.69155	0.00019115	sp|O95168|NDUB4_HUMAN	NDUFB4	O95168
1	6	202	202	202	0.999	186	2.45288	0.000321513	sp|O95169|NDUB8_HUMAN	NDUFB8	O95169
1	9	186	143	186	0.999	1032	2.2227	0.000522968	sp|O95197|RTN3_HUMAN	RTN3	O95197
1	22	456	456	456	0.999	739	1.57905	0.00312951	sp|O95202|LETM1_HUMAN	LETM1	O95202
1	13	288	288	288	0.999	330	2.88178	0.000127783	sp|O95218|ZRAB2_HUMAN	ZRANB2	O95218
1	3	99	99	99	0.999	450	2.17195	0.000611342	sp|O95219|SNX4_HUMAN	SNX4	O95219
1	14	170	170	172	0.999	432	0.194674	0.359452	sp|O95232|LC7L3_HUMAN	LUC7L3	O95232
1	7	16	16	16	0.999	890	3.85764	0	sp|O95235|KI20A_HUMAN	KIF20A	O95235
1	15	108	48	108	0.999	1232	1.10014	0.0149797	sp|O95239|KIF4A_HUMAN	KIF4A	O95239
1	5	62	62	62	0.999	250	1.23534	0.00941508	sp|O95249|GOSR1_HUMAN	GOSR1	O95249
1	16	398	398	399	0.999	243	1.68997	0.00226474	sp|O95292|VAPB_HUMAN	VAPB	O95292
1	5	78	78	78	0.999	269	7.76994	0	sp|O95297|MPZL1_HUMAN	MPZL1	O95297
1	16	287	287	287	0.999	355	0.57419	0.0914637	sp|O95299|NDUAA_HUMAN	NDUFA10	O95299
1	13	214	164	216	0.999	570	2.20394	0.000560617	sp|O95302|FKBP9_HUMAN	FKBP9	O95302
1	11	343	343	343	0.999	258	4.64569	0	sp|O95336|6PGL_HUMAN	PGLS	O95336
1	26	285	285	310	0.999	614	2.37202	0.000410023	sp|O95340|PAPS2_HUMAN	PAPSS2	O95340
1	46	711	711	711	0.999	1197	1.93628	0.0011344	sp|O95347|SMC2_HUMAN	SMC2	O95347
1	10	96	96	96	0.999	703	0.149172	0.433123	sp|O95352|ATG7_HUMAN	ATG7	O95352
1	13	193	137	193	0.999	564	1.51887	0.00361895	sp|O95361|TRI16_HUMAN	TRIM16	O95361
1	8	202	202	202	0.999	231	4.82429	0	sp|O95372|LYPA2_HUMAN	LYPLA2	O95372
1	38	754	754	754	0.999	1038	0.287104	0.251895	sp|O95373|IPO7_HUMAN	IPO7	O95373
1	7	103	103	103	0.999	493	1.05788	0.0173742	sp|O95376|ARI2_HUMAN	ARIH2	O95376
1	18	447	447	447	0.999	542	0.711648	0.057479	sp|O95394|AGM1_HUMAN	PGM3	O95394
1	5	91	91	91	0.999	341	1.60618	0.00291202	sp|O95400|CD2B2_HUMAN	CD2BP2	O95400
1	18	546	546	546	0.999	338	2.41502	0.000351477	sp|O95433|AHSA1_HUMAN	AHSA1	O95433
1	7	99	99	99	0.999	288	0.817782	0.0400529	sp|O95456|PSMG1_HUMAN	PSMG1	O95456
1	12	217	217	217	0.999	568	0.731083	0.053822	sp|O95470|SGPL1_HUMAN	SGPL1	O95470
1	6	62	62	62	0.999	260	2.01111	0.000904792	sp|O95478|NSA2_HUMAN	NSA2	O95478
1	19	60	60	60	0.999	791	1.86626	0.00136918	sp|O95479|G6PE_HUMAN	H6PD	O95479

1	13	186	147	186	0.999	1093	0.570722	0.0925724	sp|O95486|SC24A_HUMAN	SEC24A	O95486
1	11	65	65	104	0.999	1268	1.5149	0.00365914	sp|O95487|SC24B_HUMAN	SEC24B	O95487
1	4	20	20	20	0.999	513	4.75508	0	sp|O95497|VNN1_HUMAN	VNN 1.00	O95497
1	11	481	481	481	0.999	254	1.16065	0.0121057	sp|O95571|ETHE1_HUMAN	ETHE1	O95571
1	34	779	689	779	0.999	720	0.594067	0.0853973	sp|O95573|ACSL3_HUMAN	ACSL3	O95573
1	6	117	117	117	0.999	424	0.480082	0.127353	sp|O95630|STABP_HUMAN	STAMBP	O95630
1	10	165	165	165	0.999	621	1.50062	0.00382199	sp|O95671|ASML_HUMAN	ASMTL	O95671
1	10	46	10	173	0.999	219	1.56079	0.00327789	sp|O95716|RAB3D_HUMAN	RAB3D	O95716
1	13	245	245	245	0.999	258	2.13846	0.000661247	sp|O95721|SNP29_HUMAN	SNAP29	O95721
1	10	182	161	182	0.999	527	0.250065	0.290041	sp|O95747|OXSR1_HUMAN	OXSR1	O95747
1	48	890	890	1107	0.999	839	0.82714	0.0387806	sp|O95757|HS74L_HUMAN	HSPA4L	O95757
1	14	145	145	386	0.999	552	0.794263	0.0432345	sp|O95758|PTBP3_HUMAN	PTBP3	O95758
1	4	163	163	163	0.999	96	2.21486	0.000543662	sp|O95777|LSM8_HUMAN	LSM 8.00	O95777
1	39	814	437	814	0.999	977	4.24704	0	sp|O95782|AP2A1_HUMAN	AP2A1	O95782
1	18	270	241	270	0.999	577	1.25193	0.00891842	sp|O95793|STAU1_HUMAN	STAU1	O95793
1	6	109	109	109	0.999	387	1.44231	0.00465167	sp|O95801|TTC4_HUMAN	TTC4	O95801
1	18	211	211	211	0.999	425	2.49338	0.000296774	sp|O95810|CAVN2_HUMAN	CAVIN2	O95810
1	19	355	355	355	0.999	211	0.337219	0.20968	sp|O95816|BAG2_HUMAN	BAG2	O95816
1	19	227	227	227	0.999	575	2.08256	0.00077351	sp|O95817|BAG3_HUMAN	BAG3	O95817
1	32	417	346	417	0.999	1239	4.01112	0	sp|O95819|M4K4_HUMAN	MAP4K4	O95819
1	5	132	132	132	0.999	349	0.614863	0.0796319	sp|O95825|QORL1_HUMAN	CRYZL1	O95825
1	33	787	787	787	0.999	613	0.80643	0.0415273	sp|O95831|AIFM1_HUMAN	AIFM1	O95831
1	12	258	258	258	0.999	649	2.62739	0.000218991	sp|O95834|EMAL2_HUMAN	EML2	O95834
1	23	724	724	724	0.999	308	0.912202	0.0288921	sp|O95861|BPNT1_HUMAN	BPNT1	O95861
1	14	147	122	147	0.999	285	5.68605	0	sp|O95865|DDAH2_HUMAN	DDAH2	O95865
1	7	348	348	348	0.999	172	6.45196	0	sp|O95881|TXD12_HUMAN	TXNDC12	O95881
1	7	10	10	10	0.9975	443	4.56747	0	sp|O95967|FBLN4_HUMAN	EFEMP2	O95967
1	6	88	85	88	0.999	291	1.65536	0.00250877	sp|O95983|MBD3_HUMAN	MBD3	O95983
1	5	41	41	54	0.999	172	1.98981	0.000973451	sp|O95989|NUDT3_HUMAN	NUDT3	O95989
1	8	55	55	55	0.999	233	2.54116	0.000278917	sp|O95999|BCL10_HUMAN	BCL10	O95999
1	9	236	236	236	0.999	172	1.71865	0.00208099	sp|O96000|NDUBA_HUMAN	NDUFB10	O96000
1	9	159	159	159	0.999	669	2.30829	0.000455621	sp|O96005|CLPT1_HUMAN	CLPTM1	O96005
1	10	219	219	219	0.999	361	2.30815	0.000455285	sp|O96008|TOM40_HUMAN	TOMM40	O96008
1	17	238	235	238	0.999	591	1.00476	0.0209273	sp|O96013|PAK4_HUMAN	PAK4	O96013
1	11	193	138	193	0.999	429	3.56145	5.16796E-06	sp|O96019|ACL6A_HUMAN	ACTL6A	O96019
1	6	165	165	165	0.999	134	0.705402	0.058608	sp|P00167|CYB5_HUMAN	CYB5A	P00167
1	55	3524	3017	3524	0.999	332	4.69012	0	sp|P00338|LDHA_HUMAN	LDHA	P00338
1	48	1671	277	1671	0.999	558	5.35347	0	sp|P00367|DHE3_HUMAN	GLUD1	P00367
1	6	127	69	127	0.999	187	0.133768	0.463116	sp|P00374|DYR_HUMAN	DHFR	P00374
1	15	507	507	507	0.999	301	2.032	0.000871364	sp|P00387|NB5R3_HUMAN	CYB5R3	P00387
1	16	356	356	356	0.999	522	2.40099	0.000367568	sp|P00390|GSHR_HUMAN	GSR	P00390
1	9	319	319	319	0.999	227	5.19203	0	sp|P00403|COX2_HUMAN	MT-CO2	P00403
1	16	1067	1067	1067	0.999	154	1.69273	0.00224722	sp|P00441|SODC_HUMAN	SOD1	P00441
1	5	15	13	15	0.9947	732	2.00554	0.000915816	sp|P00488|F13A_HUMAN	F13A1	P00488
1	18	672	672	672	0.999	289	2.59503	0.000243449	sp|P00491|PNPH_HUMAN	PNP	P00491
1	12	560	499	560	0.999	218	3.56365	5.18135E-06	sp|P00492|HPRT_HUMAN	HPRT1	P00492
1	28	584	584	584	0.999	430	1.8806	0.00130935	sp|P00505|AATM_HUMAN	GOT2	P00505
1	32	734	734	734	0.999	1210	8.35015	0	sp|P00533|EGFR_HUMAN	EGFR	P00533
1	69	4297	3892	4297	0.999	417	1.22614	0.00970602	sp|P00558|PGK1_HUMAN	PGK 1.00	P00558
1	27	714	714	714	0.999	194	1.34771	0.00638241	sp|P00568|KAD1_HUMAN	AK1	P00568
1	11	65	65	65	0.999	622	5.04655	0	sp|P00734|THRB_HUMAN	F2	P00734
1	7	14	13	14	0.999	705	5.63274	0	sp|P00736|C1R_HUMAN	C1R	P00736
1	5	26	26	26	0.9987	488	6.7957	0	sp|P00742|FA10_HUMAN	F10	P00742
1	7	53	52	53	0.999	810	5.95136	0	sp|P00747|PLMN_HUMAN	PLG	P00747
1	18	98	98	98	0.999	431	10.7732	0	sp|P00749|UROK_HUMAN	PLAU	P00749
1	5	66	66	66	0.999	562	2.01702	0.000906872	sp|P00750|TPA_HUMAN	PLAT	P00750
1	9	153	153	153	0.999	363	1.18774	0.0110334	sp|P00813|ADA_HUMAN	ADA	P00813
0.9999	2	54	54	54	0.999	226	0.458946	0.137103	sp|P00846|ATP6_HUMAN	MT-ATP6	P00846
1	55	1451	1414	1451	0.999	412	1.06266	0.0171114	sp|P00966|ASSY_HUMAN	ASS1	P00966
1	11	73	73	73	0.999	464	6.13419	0	sp|P01008|ANT3_HUMAN	SERPINC1	P01008
1	21	510	368	510	0.999	1474	4.30884	0	sp|P01023|A2MG_HUMAN	A2M	P01023
1	26	278	278	278	0.999	1663	1.67384	0.00236775	sp|P01024|CO3_HUMAN	C3	P01024
1	8	41	41	41	0.9989	1676	4.88204	0	sp|P01031|CO5_HUMAN	C5	P01031
1	3	44	44	44	0.999	207	1.91072	0.00119149	sp|P01033|TIMP1_HUMAN	TIMP1	P01033
1	12	50	14	160	0.999	189	4.59737	0	sp|P01111|RASN_HUMAN	NRAS	P01111
1	11	275	108	275	0.999	189	10.7293	0	sp|P01116|RASK_HUMAN	KRAS	P01116
1	17	169	160	169	0.999	860	2.98525	8.50202E-05	sp|P01130|LDLR_HUMAN	LDLR	P01130
1	12	436	272	436	0.999	362	7.19609	0	sp|P01889|HLAB_HUMAN	HLA-B	P01889
0.9949	8	168	2	298	0.999	362	1.88906	0.00127721	sp|P01893|HLAH_HUMAN	HLA-H	P01893
1	2	27	0	27	0.9973	147	4.58267	0	sp|P02042|HBD_HUMAN	HBD	P02042
1	43	280	277	280	0.999	1464	2.68407	0.000190813	sp|P02452|CO1A1_HUMAN	COL1A1	P02452
1	8	36	36	36	0.999	1466	0.00888493	0.89932	sp|P02461|CO3A1_HUMAN	COL3A1	P02461
1	46	616	287	1328	0.999	472	1.89494	0.00125211	sp|P02533|K1C14_HUMAN	KRT14	P02533
1	53	808	67	1050	0.999	564	5.02297	0	sp|P02538|K2C6A_HUMAN	KRT6A	P02538
1	106	4424	4371	4424	0.999	664	4.85794	0	sp|P02545|LMNA_HUMAN	LMNA	P02545
1	10	57	57	57	0.999	317	4.73656	0	sp|P02649|APOE_HUMAN	APOE	P02649
1	7	29	29	29	0.9989	491	4.00156	0	sp|P02675|FIBB_HUMAN	FGB	P02675
1	3	18	18	18	0.999	559	5.58151	0	sp|P02748|CO9_HUMAN	C9	P02748
1	5	71	71	71	0.999	345	2.78277	0.000141791	sp|P02749|APOH_HUMAN	APOH	P02749
1	95	687	687	687	0.999	2477	4.53888	0	sp|P02751|FINC_HUMAN	FN1	P02751
1	6	81	81	81	0.999	201	6.08919	0	sp|P02753|RET4_HUMAN	RBP4	P02753
1	4	338	338	338	0.999	367	7.29001	0	sp|P02765|FETUA_HUMAN	AHSG	P02765
1	10	109	109	109	0.999	609	3.85637	0	sp|P02771|FETA_HUMAN	AFP	P02771
1	13	251	251	251	0.999	474	6.53672	0	sp|P02774|VTDB_HUMAN	GC	P02774
1	48	1264	1181	1264	0.999	760	2.09913	0.00073551	sp|P02786|TFR1_HUMAN	TFRC	P02786
1	4	143	143	143	0.999	698	5.03443	0	sp|P02787|TRFE_HUMAN	TF	P02787
1	9	211	211	211	0.999	710	5.02466	0	sp|P02788|TRFL_HUMAN	LTF	P02788
1	14	1019	579	1019	0.999	61	2.38741	0.0003868	sp|P02795|MT2_HUMAN	MT2A	P02795
0.9999	4	10	10	10	0.999	625	3.7778	0	sp|P03951|FA11_HUMAN	F11	P03951
1	20	63	63	63	0.999	469	2.95635	0.00010757	sp|P03956|MMP1_HUMAN	MMP1	P03956
1	8	102	102	102	0.999	478	2.99746	8.13835E-05	sp|P04004|VTNC_HUMAN	VTN	P04004
1	31	710	710	710	0.999	527	0.684894	0.0627569	sp|P04040|CATA_HUMAN	CAT	P04040
1	7	82	68	82	0.999	648	0.331822	0.213705	sp|P04049|RAF1_HUMAN	RAF1	P04049
1	7	59	59	59	0.999	536	4.10087	0	sp|P04062|GBA1_HUMAN	GBA1	P04062
1	8	76	76	76	0.999	466	4.63726	0	sp|P04066|FUCO_HUMAN	FUCA1	P04066
1	56	3663	479	3663	0.999	364	3.62244	0	sp|P04075|ALDOA_HUMAN	ALDOA	P04075
1	6	172	172	172	0.999	98	1.00205	0.021172	sp|P04080|CYTB_HUMAN	CSTB	P04080
1	60	2701	2680	2701	0.999	346	5.10591	0	sp|P04083|ANXA1_HUMAN	ANXA1	P04083
1	8	17	17	21	0.999	4563	0.166548	0.403309	sp|P04114|APOB_HUMAN	APOB	P04114
1	10	206	206	206	0.999	222	0.641343	0.0728469	sp|P04179|SODM_HUMAN	SOD2	P04179
1	27	688	688	688	0.999	439	4.04063	0	sp|P04181|OAT_HUMAN	OAT	P04181
1	7	142	142	142	0.999	234	4.90816	0	sp|P04183|KITH_HUMAN	TK1	P04183
1	4	16	16	16	0.999	161	5.43665	0	sp|P04216|THY1_HUMAN	THY1	P04216
0.9948	3	47	47	47	0.9957	495	3.45757	1.97287E-05	sp|P04217|A1BG_HUMAN	A1BG	P04217
1	51	1118	1033	1206	0.999	644	3.62252	0	sp|P04264|K2C1_HUMAN	KRT1	P04264
1	60	7618	7289	7618	0.999	335	0.122731	0.486462	sp|P04406|G3P_HUMAN	GAPDH	P04406
1	9	96	96	96	0.999	464	1.17693	0.0114146	sp|P04424|ARLY_HUMAN	ASL	P04424
1	22	366	289	654	0.999	365	8.4679	0	sp|P04439|HLAA_HUMAN	HLA-A	P04439
1	20	614	582	614	0.999	268	1.04077	0.018426	sp|P04632|CPNS1_HUMAN	CAPNS1	P04632
1	19	108	108	108	0.999	393	3.6978	0	sp|P04637|P53_HUMAN	TP53	P04637
1	11	254	81	516	0.999	61	1.11377	0.0142651	sp|P04732|MT1E_HUMAN	MT1E	P04732
1	20	1178	1178	1178	0.999	205	1.96253	0.00106367	sp|P04792|HSPB1_HUMAN	HSPB1	P04792
1	7	102	102	102	0.999	313	2.13246	0.00067027	sp|P04818|TYSY_HUMAN	TYMS	P04818
1	54	2010	2010	2010	0.999	607	5.8366	0	sp|P04843|RPN1_HUMAN	RPN1	P04843
1	18	631	631	631	0.999	631	9.75855	0	sp|P04844|RPN2_HUMAN	RPN2	P04844
1	29	936	533	936	0.999	355	9.19323	0	sp|P04899|GNAI2_HUMAN	GNAI2	P04899
1	15	330	0	877	0.999	130	3.9426	0	sp|P04908|H2A1B_HUMAN	H2AC4	P04908
1	65	2279	1542	2279	0.999	1023	7.3805	0	sp|P05023|AT1A1_HUMAN	ATP1A1	P05023
1	19	247	247	247	0.999	303	3.22453	5.44218E-05	sp|P05026|AT1B1_HUMAN	ATP1B1	P05026
1	10	51	51	68	0.999	770	2.15611	0.000629706	sp|P05067|A4_HUMAN	APP	P05067
1	16	84	76	145	0.999	517	0.794164	0.0432379	sp|P05091|ALDH2_HUMAN	ALDH2	P05091
1	21	104	104	104	0.999	788	7.00349	0	sp|P05106|ITB3_HUMAN	ITGB3	P05106
1	3	72	72	72	0.999	100	0.580023	0.0896445	sp|P05114|HMGN1_HUMAN	HMGN1	P05114
1	17	108	108	108	0.999	415	3.14319	5.21173E-05	sp|P05120|PAI2_HUMAN	SERPINB2	P05120
1	18	117	117	117	0.999	402	1.21555	0.0100811	sp|P05121|PAI1_HUMAN	SERPINE1	P05121
1	43	2018	525	2018	0.999	298	4.09346	0	sp|P05141|ADT2_HUMAN	SLC25A5	P05141
1	5	180	180	180	0.999	165	5.25786	0	sp|P05161|ISG15_HUMAN	ISG15	P05161
1	32	1046	1046	1046	0.999	315	1.46952	0.00424053	sp|P05198|IF2A_HUMAN	EIF2S1	P05198
1	9	604	568	604	0.999	90	0.933383	0.0268564	sp|P05204|HMGN2_HUMAN	HMGN2	P05204

1	21	296	296	296	0.999	532	9.88687	0	sp|P05362|ICAM1_HUMAN	ICAM1	P05362
1	11	635	635	635	0.999	115	1.09198	0.0153517	sp|P05387|RLA2_HUMAN	RPLP2	P05387
1	23	782	345	782	0.999	317	3.42528	1.96319E-05	sp|P05388|RLA0_HUMAN	RPLP0	P05388
1	4	29	29	29	0.999	202	3.42687	0.000019656	sp|P05452|TETN_HUMAN	CLEC3B	P05452
1	36	1251	1251	1251	0.999	408	1.70836	0.00213677	sp|P05455|LA_HUMAN	SSB	P05455
0.9991	2	16	16	16	0.9989	415	1.77558	0.00174335	sp|P05543|THBG_HUMAN	SERPINA7	P05543
1	5	54	54	54	0.999	499	4.67978	0	sp|P05546|HEP2_HUMAN	SERPIND1	P05546
1	43	2016	1950	2016	0.999	798	8.48116	0	sp|P05556|ITB1_HUMAN	ITGB1	P05556
1	44	1221	1184	1288	0.999	430	1.2894	0.00786934	sp|P05783|K1C18_HUMAN	KRT18	P05783
1	63	1553	1267	1698	0.999	483	1.74884	0.00191195	sp|P05787|K2C8_HUMAN	KRT8	P05787
1	11	28	28	28	0.999	1499	2.49612	0.000297014	sp|P05997|CO5A2_HUMAN	COL5A2	P05997
1	9	206	206	206	0.999	367	0.0995426	0.539838	sp|P06132|DCUP_HUMAN	UROD	P06132
1	6	51	51	51	0.999	429	4.00457	0	sp|P06280|AGAL_HUMAN	GLA	P06280
1	45	643	643	643	0.999	782	2.25478	0.000486331	sp|P06396|GELS_HUMAN	GSN	P06396
1	7	427	427	427	0.999	111	6.23297	0	sp|P06454|PTMA_HUMAN	PTMA	P06454
1	22	629	491	629	0.999	297	3.12953	5.19481E-05	sp|P06493|CDK1_HUMAN	CDK1	P06493
1	41	2467	2467	2467	0.999	529	4.13477	0	sp|P06576|ATPB_HUMAN	ATP5F1B	P06576
1	15	717	717	717	0.999	90	5.45747	0	sp|P06703|S10A6_HUMAN	S100A6	P06703
1	13	416	411	416	0.999	217	0.893545	0.0308608	sp|P06730|IF4E_HUMAN	EIF4E	P06730
1	79	6854	6470	6854	0.999	434	0.941437	0.0261689	sp|P06733|ENOA_HUMAN	ENO1	P06733
1	50	996	996	1261	0.999	847	0.379509	0.180317	sp|P06737|PYGL_HUMAN	PYGL	P06737
1	71	2970	2911	2970	0.999	558	6.11468	0	sp|P06744|G6PI_HUMAN	GPI	P06744
1	40	2810	2755	2810	0.999	294	8.72428	0	sp|P06748|NPM_HUMAN	NPM1	P06748
1	34	623	553	2008	0.999	285	4.92696	0	sp|P06753|TPM3_HUMAN	TPM3	P06753
1	35	598	598	598	0.999	1048	10.7263	0	sp|P06756|ITAV_HUMAN	ITGAV	P06756
1	13	114	114	154	0.999	529	0.833384	0.0378724	sp|P06865|HEXA_HUMAN	HEXA	P06865
1	14	97	97	97	0.999	398	3.79313	0	sp|P07093|GDN_HUMAN	SERPINE2	P07093
1	23	736	736	736	0.999	455	3.30611	3.77358E-05	sp|P07099|HYEP_HUMAN	EPHX1	P07099
1	31	1552	1552	1851	0.999	334	4.81344	0	sp|P07195|LDHB_HUMAN	LDHB	P07195
1	9	296	296	296	0.999	203	0.180839	0.380545	sp|P07203|GPX1_HUMAN	GPX1	P07203
1	70	3344	3344	3344	0.999	508	10.8676	0	sp|P07237|PDIA1_HUMAN	P4HB	P07237
1	12	407	407	407	0.999	194	3.1641	5.26894E-05	sp|P07305|H10_HUMAN	H1-0	P07305
1	24	558	558	558	0.999	412	5.99279	0	sp|P07339|CATD_HUMAN	CTSD	P07339
1	75	4421	1025	4421	0.999	339	7.77148	0	sp|P07355|ANXA2_HUMAN	ANXA2	P07355
1	40	814	814	815	0.999	714	1.08292	0.0158659	sp|P07384|CAN1_HUMAN	CAPN1	P07384
1	51	6821	1074	6821	0.999	444	3.81357	0	sp|P07437|TBB5_HUMAN	TUBB	P07437
1	20	648	648	648	0.999	524	5.67604	0	sp|P07602|SAP_HUMAN	PSAP	P07602
1	24	489	449	489	0.999	556	4.33468	0	sp|P07686|HEXB_HUMAN	HEXB	P07686
1	6	46	8	46	0.999	333	1.00008	0.0212955	sp|P07711|CATL1_HUMAN	CTSL	P07711
1	24	2748	2748	2748	0.999	140	1.6149	0.00283927	sp|P07737|PROF1_HUMAN	PFN1	P07737
1	11	246	246	246	0.999	180	0.694663	0.0607905	sp|P07741|APT_HUMAN	APRT	P07741
1	117	4147	4147	4147	0.999	1512	2.03043	0.000878553	sp|P07814|SYEP_HUMAN	EPRS1	P07814
1	23	760	760	760	0.999	339	5.60007	0	sp|P07858|CATB_HUMAN	CTSB	P07858
1	98	3979	3477	9117	0.999	732	4.70803	0	sp|P07900|HS90A_HUMAN	HSP90AA1	P07900
1	41	1650	1008	1650	0.999	306	3.22724	5.45455E-05	sp|P07910|HNRPC_HUMAN	HNRNPC	P07910
1	12	520	405	520	0.999	91	2.13237	0.000669818	sp|P07919|QCR6_HUMAN	UQCRH	P07919
1	49	467	467	467	0.999	1786	1.60462	0.00291065	sp|P07942|LAMB1_HUMAN	LAMB1	P07942
1	10	164	102	302	0.999	543	5.4986	0	sp|P07947|YES_HUMAN	YES1	P07947
1	45	426	263	1960	0.999	284	6.0528	0	sp|P07951|TPM2_HUMAN	TPM2	P07951
1	34	1100	1100	1100	0.999	510	0.454192	0.139253	sp|P07954|FUMH_HUMAN	FH	P07954
1	53	811	721	811	0.999	1170	5.91807	0	sp|P07996|TSP1_HUMAN	THBS1	P07996
1	24	118	118	118	0.999	1366	2.95341	0.000111332	sp|P08123|CO1A2_HUMAN	COL1A2	P08123
1	81	1328	1328	1328	0.999	673	7.85624	0	sp|P08133|ANXA6_HUMAN	ANXA6	P08133
1	16	292	217	676	0.999	193	2.11684	0.000701613	sp|P08134|RHOC_HUMAN	RHOC	P08134
1	18	290	290	290	0.999	381	6.88744	0	sp|P08174|DAF_HUMAN	CD55	P08174
1	31	1451	1451	1451	0.999	630	8.86494	0	sp|P08195|4F2_HUMAN	SLC3A2	P08195
1	30	607	607	753	0.999	780	0.338997	0.208407	sp|P08237|PFKAM_HUMAN	PFKM	P08237
1	109	9991	2932	10048	0.999	724	6.31469	0	sp|P08238|HS90B_HUMAN	HSP90AB1	P08238
1	23	322	298	322	0.999	638	5.63866	0	sp|P08240|SRPRA_HUMAN	SRPRA	P08240
1	19	292	292	292	0.999	561	1.21938	0.00995131	sp|P08243|ASNS_HUMAN	ASNS	P08243
1	6	9	9	9	0.9989	660	4.39849	0	sp|P08253|MMP2_HUMAN	MMP2	P08253
1	7	103	103	103	0.999	361	0.207673	0.341972	sp|P08397|HEM3_HUMAN	HMBS	P08397
1	21	76	76	76	0.999	750	7.41669	0	sp|P08473|NEP_HUMAN	MME	P08473
1	30	791	637	791	0.999	390	3.82973	0	sp|P08559|ODPA_HUMAN	PDHA1	P08559
1	14	419	419	419	0.999	325	5.29897	0	sp|P08574|CY1_HUMAN	CYC1	P08574
1	11	244	244	303	0.999	225	2.96899	9.64824E-05	sp|P08579|RU2B_HUMAN	SNRPB2	P08579
1	34	1050	1050	1050	0.999	437	0.849544	0.0358613	sp|P08621|RU17_HUMAN	SNRNP70	P08621
1	24	567	567	567	0.999	1049	8.16901	0	sp|P08648|ITA5_HUMAN	ITGA5	P08648
1	99	4493	3941	4493	0.999	466	9.00699	0	sp|P08670|VIME_HUMAN	VIM	P08670
0.9999	2	22	22	22	0.999	491	6.03754	0	sp|P08697|A2AP_HUMAN	SERPINF2	P08697
1	23	852	852	852	0.999	135	2.19874	0.000561453	sp|P08708|RS17_HUMAN	RPS17	P08708
1	37	184	134	503	0.999	400	0.225174	0.319923	sp|P08727|K1C19_HUMAN	KRT19	P08727
1	53	1309	1195	1632	0.999	469	2.4781	0.0003008	sp|P08729|K2C7_HUMAN	KRT7	P08729
1	25	205	205	562	0.999	354	9.60393	0	sp|P08754|GNAI3_HUMAN	GNAI3	P08754
1	54	1659	1659	1659	0.999	320	4.20754	0	sp|P08758|ANXA5_HUMAN	ANXA5	P08758
1	31	59	59	788	0.999	473	4.41179	0	sp|P08779|K1C16_HUMAN	KRT16	P08779
1	23	1317	333	1317	0.999	295	4.32404	0	sp|P08865|RSSA_HUMAN	RPSA	P08865
1	6	107	107	107	0.999	238	3.9901	0	sp|P08962|CD63_HUMAN	CD63	P08962
1	10	252	252	252	0.999	348	0.0264333	0.79096	sp|P09001|RM03_HUMAN	MRPL3	P09001
1	12	358	299	358	0.999	282	0.66801	0.0663048	sp|P09012|SNRPA_HUMAN	SNRPA	P09012
1	5	49	49	49	0.999	288	2.06068	0.000811024	sp|P09038|FGF2_HUMAN	FGF2	P09038
1	30	349	330	597	0.999	434	0.485647	0.124729	sp|P09104|ENOG_HUMAN	ENO2	P09104
1	8	178	178	178	0.999	424	0.455827	0.138624	sp|P09110|THIK_HUMAN	ACAA1	P09110
1	8	112	112	112	0.999	144	0.917725	0.0283398	sp|P09132|SRP19_HUMAN	SRP19	P09132
1	16	1157	1157	1157	0.999	210	3.18467	5.33927E-05	sp|P09211|GSTP1_HUMAN	GSTP1	P09211
1	7	256	256	256	0.999	159	0.681357	0.0635153	sp|P09234|RU1C_HUMAN	SNRPC	P09234
1	19	1481	1481	1481	0.999	135	4.15888	0	sp|P09382|LEG1_HUMAN	LGALS1	P09382
1	6	208	208	208	0.999	244	0.158922	0.415986	sp|P09417|DHPR_HUMAN	QDPR	P09417
1	37	2103	855	2103	0.999	215	6.62525	0	sp|P09429|HMGB1_HUMAN	HMGB1	P09429
1	7	42	42	42	0.999	303	0.193708	0.360831	sp|P09486|SPRC_HUMAN	SPARC	P09486
1	41	383	383	1679	0.999	284	2.18581	0.000586518	sp|P09493|TPM1_HUMAN	TPM1	P09493
1	16	635	570	635	0.999	248	1.62628	0.00275339	sp|P09496|CLCA_HUMAN	CLTA	P09496
1	21	474	474	474	0.999	229	4.3195	0	sp|P09497|CLCB_HUMAN	CLTB	P09497
1	34	738	738	738	0.999	319	9.76803	0	sp|P09525|ANXA4_HUMAN	ANXA4	P09525
1	29	610	610	610	0.999	421	8.88082	0	sp|P09543|CN37_HUMAN	CNP	P09543
1	19	54	50	113	0.999	1106	8.47953	0	sp|P09619|PGFRB_HUMAN	PDGFRB	P09619
1	32	1070	1070	1070	0.999	509	3.37894	2.40964E-05	sp|P09622|DLDH_HUMAN	DLD	P09622
1	47	2253	467	2757	0.999	372	5.92967	0	sp|P09651|ROA1_HUMAN	HNRNPA1	P09651
1	17	301	301	301	0.999	255	1.82205	0.00152962	sp|P09661|RU2A_HUMAN	SNRPA1	P09661
1	8	325	325	325	0.999	75	6.4956	0	sp|P09669|COX6C_HUMAN	COX6C	P09669
1	86	3037	3037	3037	0.999	1014	2.42437	0.000339564	sp|P09874|PARP1_HUMAN	PARP1	P09874
1	15	157	157	157	0.999	1462	3.01284	7.39979E-05	sp|P09884|DPOLA_HUMAN	POLA1	P09884
1	5	32	32	34	0.999	472	2.19533	0.000566248	sp|P09913|IFIT2_HUMAN	IFIT2	P09913
1	8	86	86	86	0.999	478	3.59942	0	sp|P09914|IFIT1_HUMAN	IFIT1	P09914
1	18	140	140	140	0.999	223	5.95814	0	sp|P09936|UCHL1_HUMAN	UCHL1	P09936
1	36	1334	1334	1334	0.999	611	1.65814	0.0024904	sp|P09960|LKHA4_HUMAN	LTA4H	P09960
1	37	493	485	1132	0.999	364	4.18557	0	sp|P09972|ALDOC_HUMAN	ALDOC	P09972
1	15	96	0	96	0.999	1744	5.93852	0	sp|P0C0L4|CO4A_HUMAN	C4A	P0C0L4
1	10	196	0	435	0.999	128	0.284611	0.254334	sp|P0C0S5|H2AZ_HUMAN	H2AZ1	P0C0S5
1	15	1050	0	1050	0.999	130	2.8864	0.000128031	sp|P0C0S8|H2A1_HUMAN	H2AC11	P0C0S8
1	66	2671	0	3190	0.999	641	1.62215	0.00278571	sp|P0DMV8|HS71A_HUMAN	HSPA1A	P0DMV8
1	11	367	0	367	0.999	149	4.67657	0	sp|P0DP23|CALM1_HUMAN	CALM1	P0DP23
1	7	34	0	34	0.999	511	4.71578	0	sp|P0DTE7|AMY1B_HUMAN	AMY1B	P0DTE7
1	19	342	342	342	0.999	538	0.0834417	0.580613	sp|P10155|RO60_HUMAN	RO60	P10155
1	16	202	202	202	0.999	952	0.144077	0.442977	sp|P10253|LYAG_HUMAN	GAA	P10253
1	14	146	146	164	0.999	218	10.0981	0	sp|P10301|RRAS_HUMAN	RRAS	P10301
1	14	104	104	383	0.999	366	4.6614	0	sp|P10321|HLAC_HUMAN	HLA-C	P10321
1	32	743	81	1683	0.999	219	7.978	0	sp|P10412|H14_HUMAN	H1-4	P10412
1	16	341	341	341	0.999	647	2.89534	0.000128155	sp|P10515|ODP2_HUMAN	DLAT	P10515
1	13	651	651	651	0.999	105	4.55059	0	sp|P10599|THIO_HUMAN	TXN	P10599
1	7	300	300	300	0.999	129	4.27873	0	sp|P10606|COX5B_HUMAN	COX5B	P10606
1	5	87	87	87	0.999	480	2.01292	0.000904548	sp|P10619|PPGB_HUMAN	CTSA	P10619
1	10	242	242	242	0.999	155	3.52089	2.01765E-05	sp|P10620|MGST1_HUMAN	MGST1	P10620
1	7	35	35	35	0.999	843	5.02854	0	sp|P10643|CO7_HUMAN	C7	P10643
1	17	299	229	299	0.999	381	0.738426	0.0525085	sp|P10644|KAP0_HUMAN	PRKAR1A	P10644
1	7	41	41	41	0.999	304	4.47057	0	sp|P10646|TFPI1_HUMAN	TFPI	P10646
1	23	627	627	627	0.999	282	3.39276	2.42131E-05	sp|P10768|ESTD_HUMAN	ESD	P10768
1	80	4025	4025	4025	0.999	573	11.7403	0	sp|P10809|CH60_HUMAN	HSPD1	P10809
1	5	14	14	14	0.9989	449	3.20314	5.36913E-05	sp|P10909|CLUS_HUMAN	CLU	P10909

1	77	3373	3312	3538	0.999	654	12.654	0	sp|P11021|BIP_HUMAN	HSPA5	P11021
1	44	693	693	693	0.999	1609	1.6659	0.00241231	sp|P11047|LAMC1_HUMAN	LAMC1	P11047
1	7	98	98	98	0.999	423	2.20627	0.000558596	sp|P11117|PPAL_HUMAN	ACP2	P11117
1	75	4114	2837	4114	0.999	646	1.37606	0.0058029	sp|P11142|HSP7C_HUMAN	HSPA8	P11142
1	11	386	386	386	0.999	492	9.48446	0	sp|P11166|GTR1_HUMAN	SLC2A1	P11166
1	7	100	17	100	0.999	496	4.65069	0	sp|P11169|GTR3_HUMAN	SLC2A3	P11169
1	21	479	479	479	0.999	480	4.56689	0	sp|P11172|UMPS_HUMAN	UMPS	P11172
1	17	765	765	765	0.999	359	8.4639	0	sp|P11177|ODPB_HUMAN	PDHB	P11177
1	77	1653	1272	1653	0.999	843	1.08626	0.015649	sp|P11216|PYGB_HUMAN	PYGB	P11216
1	11	452	291	452	0.999	206	9.04745	0	sp|P11233|RALA_HUMAN	RALA	P11233
1	10	84	84	245	0.999	206	5.22825	0	sp|P11234|RALB_HUMAN	RALB	P11234
1	11	365	365	365	0.999	417	4.50614	0	sp|P11279|LAMP1_HUMAN	LAMP1	P11279
1	23	557	557	557	0.999	421	0.41914	0.157131	sp|P11310|ACADM_HUMAN	ACADM	P11310
1	49	1209	1051	1209	0.999	765	1.74413	0.00193497	sp|P11387|TOP1_HUMAN	TOP 1.00	P11387
1	36	426	307	426	0.999	1531	0.904118	0.0297412	sp|P11388|TOP2A_HUMAN	TOP2A	P11388
1	49	1413	1413	1413	0.999	515	7.02245	0	sp|P11413|G6PD_HUMAN	G6PD	P11413
1	4	73	73	73	0.999	157	1.11256	0.0143222	sp|P11441|UBL4A_HUMAN	UBL4A	P11441
1	49	800	800	800	0.999	1178	0.988235	0.0222167	sp|P11498|PYC_HUMAN	PC	P11498
1	65	2687	2589	2687	0.999	935	0.594282	0.085362	sp|P11586|C1TC_HUMAN	MTHFD1	P11586
1	74	1058	1058	1058	0.999	2491	0.0100094	0.890701	sp|P11717|MPRI_HUMAN	IGF2R	P11717
1	24	704	704	704	0.999	374	0.540671	0.102946	sp|P11766|ADHX_HUMAN	ADH5	P11766
1	10	158	158	296	0.999	303	2.45274	0.00032126	sp|P11802|CDK4_HUMAN	CDK4	P11802
1	24	204	166	490	0.999	318	0.0400855	0.727536	sp|P11908|PRPS2_HUMAN	PRPS2	P11908
1	47	1629	327	1629	0.999	636	0.48726	0.124007	sp|P11940|PABP1_HUMAN	PABPC1	P11940
1	19	969	969	969	0.999	261	5.41531	0	sp|P12004|PCNA_HUMAN	PCNA	P12004
1	41	1118	871	1118	0.999	509	1.38922	0.00555317	sp|P12081|HARS1_HUMAN	HARS1	P12081
1	7	8	8	24	0.999	1806	1.43107	0.00486282	sp|P12107|COBA1_HUMAN	COL11A1	P12107
1	36	256	256	256	0.999	1028	7.47746	0	sp|P12109|CO6A1_HUMAN	COL6A1	P12109
1	40	227	227	227	0.999	1019	6.93472	0	sp|P12110|CO6A2_HUMAN	COL6A2	P12110
1	136	896	896	896	0.999	3177	6.82683	0	sp|P12111|CO6A3_HUMAN	COL6A3	P12111
1	44	515	377	2006	0.999	298	5.42038	0	sp|P12236|ADT3_HUMAN	SLC25A6	P12236
1	15	41	41	41	0.9987	2224	5.77505	0	sp|P12259|FA5_HUMAN	F5	P12259
1	40	1289	1169	1289	0.999	514	4.61941	0	sp|P12268|IMDH2_HUMAN	IMPDH2	P12268
1	133	2847	2806	2847	0.999	2363	4.39941	0	sp|P12270|TPR_HUMAN	TPR	P12270
1	28	582	565	582	0.999	381	0.191998	0.363461	sp|P12277|KCRB_HUMAN	CKB	P12277
1	48	972	972	972	0.999	323	2.75749	0.000147194	sp|P12429|ANXA3_HUMAN	ANXA3	P12429
1	14	223	223	223	0.999	445	0.711096	0.0575497	sp|P12694|ODBA_HUMAN	BCKDHA	P12694
1	79	1573	1570	2907	0.999	892	7.2582	0	sp|P12814|ACTN1_HUMAN	ACTN1	P12814
1	10	158	158	158	0.999	493	4.09654	0	sp|P12955|PEPD_HUMAN	PEPD	P12955
1	51	2006	2006	2006	0.999	609	2.51742	0.00028385	sp|P12956|XRCC6_HUMAN	XRCC6	P12956
1	51	1795	1795	1795	0.999	732	6.6751	0	sp|P13010|XRCC5_HUMAN	XRCC5	P13010
1	15	595	595	595	0.999	169	5.51602	0	sp|P13073|COX41_HUMAN	COX4I1	P13073
0.9999	2	21	14	21	0.999	125	4.32383	0	sp|P13164|IFM1_HUMAN	IFITM1	P13164
1	7	262	262	262	0.999	410	1.2995	0.00757885	sp|P13473|LAMP2_HUMAN	LAMP2	P13473
1	35	1127	1127	1127	0.999	461	5.58785	0	sp|P13489|RINI_HUMAN	RNH1	P13489
1	10	19	11	19	0.999	986	5.26028	0	sp|P13497|BMP1_HUMAN	BMP1	P13497
0.9998	3	9	9	9	0.999	858	4.23352	0	sp|P13591|NCAM1_HUMAN	NCAM1	P13591
1	16	53	53	53	0.999	1032	8.36568	0	sp|P13612|ITA4_HUMAN	ITGA4	P13612
1	89	5887	5625	5887	0.999	858	10.7417	0	sp|P13639|EF2_HUMAN	EEF2	P13639
1	37	641	526	958	0.999	584	2.02312	0.000888603	sp|P13645|K1C10_HUMAN	KRT10	P13645
1	45	594	506	929	0.999	590	3.31831	2.83353E-05	sp|P13647|K2C5_HUMAN	KRT5	P13647
1	60	2131	2131	2131	0.999	645	5.76558	0	sp|P13667|PDIA4_HUMAN	PDIA4	P13667
1	31	521	521	521	0.999	534	2.00336	0.000924841	sp|P13674|P4HA1_HUMAN	P4HA1	P13674
1	17	1085	716	1085	0.999	172	8.34629	0	sp|P13693|TCTP_HUMAN	TPT1	P13693
1	5	47	47	47	0.999	295	1.74245	0.00194188	sp|P13726|TF_HUMAN	F3	P13726
1	21	222	222	285	0.999	627	3.75751	0	sp|P13796|PLSL_HUMAN	LCP1	P13796
1	39	1126	1007	1126	0.999	630	4.87343	0	sp|P13797|PLST_HUMAN	PLS3	P13797
1	23	560	560	560	0.999	732	1.71032	0.00212121	sp|P13798|ACPH_HUMAN	APEH	P13798
1	22	535	535	535	0.999	333	3.66791	0	sp|P13804|ETFA_HUMAN	ETFA	P13804
1	11	154	153	154	0.999	737	0.608236	0.0812893	sp|P13807|GYS1_HUMAN	GYS1	P13807
1	21	676	658	676	0.999	404	2.39699	0.000376543	sp|P13861|KAP2_HUMAN	PRKAR2A	P13861
1	9	216	216	216	0.999	249	1.74533	0.00193033	sp|P13984|T2FB_HUMAN	GTF2F2	P13984
1	7	469	469	469	0.999	128	6.86879	0	sp|P13987|CD59_HUMAN	CD59	P13987
1	9	244	244	244	0.999	350	1.74907	0.00191185	sp|P13995|MTDC_HUMAN	MTHFD2	P13995
1	9	366	366	366	0.999	115	0.488657	0.123478	sp|P14174|MIF_HUMAN	MIF	P14174
1	4	156	156	156	0.999	185	3.52984	2.02532E-05	sp|P14209|CD99_HUMAN	CD99	P14209
1	28	1052	1052	1052	0.999	528	7.98089	0	sp|P14314|GLU2B_HUMAN	PRKCSH	P14314
1	20	711	711	711	0.999	419	2.09659	0.000736702	sp|P14324|FPPS_HUMAN	FDPS	P14324
1	18	88	88	88	0.999	1247	5.51225	0	sp|P14543|NID1_HUMAN	NID1	P14543
1	14	416	355	477	0.999	325	2.87758	0.000127413	sp|P14550|AK1A1_HUMAN	AKR1A1	P14550
1	95	6447	6231	6447	0.999	531	0.177612	0.385611	sp|P14618|KPYM_HUMAN	PKM	P14618
1	86	4247	3596	4500	0.999	803	13.1511	0	sp|P14625|ENPL_HUMAN	HSP90B1	P14625
1	9	118	118	118	0.999	433	1.40512	0.00525686	sp|P14635|CCNB1_HUMAN	CCNB1	P14635
1	12	319	17	319	0.999	240	0.654639	0.0695046	sp|P14678|RSMB_HUMAN	SNRPB	P14678
1	27	375	375	375	0.999	1019	0.072278	0.613163	sp|P14735|IDE_HUMAN	IDE	P14735
1	9	229	229	229	0.999	86	1.78166	0.00171164	sp|P14854|CX6B1_HUMAN	COX6B1	P14854
1	41	1447	1351	1447	0.999	589	3.03647	7.06861E-05	sp|P14866|HNRPL_HUMAN	HNRNPL	P14866
1	44	1354	1354	1354	0.999	501	0.730961	0.0538213	sp|P14868|SYDC_HUMAN	DARS1	P14868
1	31	802	712	802	0.999	745	5.38346	0	sp|P14923|PLAK_HUMAN	JUP	P14923
1	11	253	253	253	0.999	111	0.20256	0.348732	sp|P14927|QCR7_HUMAN	UQCRB	P14927
1	23	262	262	262	0.999	373	1.49813	0.00386517	sp|P15104|GLNA_HUMAN	GLUL	P15104
1	26	1122	1002	1122	0.999	316	4.70661	0	sp|P15121|ALDR_HUMAN	AKR1B1	P15121
1	49	1194	1194	1194	0.999	967	9.36442	0	sp|P15144|AMPN_HUMAN	ANPEP	P15144
1	8	99	99	99	0.999	417	6.41044	0	sp|P15151|PVR_HUMAN	PVR	P15151
1	29	874	330	874	0.999	499	0.190578	0.365614	sp|P15170|ERF3A_HUMAN	GSPT1	P15170
1	5	91	62	91	0.999	398	0.350636	0.200152	sp|P15291|B4GT1_HUMAN	B4GALT1	P15291
1	70	2317	2086	3169	0.999	586	4.27403	0	sp|P15311|EZRI_HUMAN	EZR	P15311
1	12	211	211	211	0.999	230	2.86049	0.000130144	sp|P15374|UCHL3_HUMAN	UCHL3	P15374
1	3	61	61	61	0.999	326	1.5402	0.00344138	sp|P15408|FOSL2_HUMAN	FOSL2	P15408
1	18	208	208	895	0.999	152	0.400642	0.167497	sp|P15531|NDKA_HUMAN	NME1	P15531
1	20	901	901	901	0.999	274	7.61764	0	sp|P15559|NQO1_HUMAN	NQO1	P15559
1	9	204	204	204	0.999	552	2.23237	0.000516037	sp|P15586|GNS_HUMAN	GNS	P15586
1	30	1110	1110	1110	0.999	293	1.07522	0.0163358	sp|P15880|RS2_HUMAN	RPS2	P15880
1	14	261	261	261	0.999	270	2.07692	0.000782034	sp|P15927|RFA2_HUMAN	RPA2	P15927
1	6	187	187	187	0.999	63	0.85073	0.0357789	sp|P15954|COX7C_HUMAN	COX7C	P15954
1	5	24	24	24	0.999	220	2.43868	0.000335423	sp|P16035|TIMP2_HUMAN	TIMP2	P16035
1	18	713	713	713	0.999	742	8.72872	0	sp|P16070|CD44_HUMAN	CD44	P16070
1	17	50	49	493	0.999	143	2.25979	0.000484848	sp|P16104|H2AX_HUMAN	H2AX	P16104
1	26	1020	752	1020	0.999	277	0.456653	0.138251	sp|P16152|CBR1_HUMAN	CBR1	P16152
1	10	95	55	95	0.999	377	1.13943	0.0130594	sp|P16333|NCK1_HUMAN	NCK1	P16333
1	27	1236	716	1236	0.999	226	3.91507	0	sp|P16401|H15_HUMAN	H1-5	P16401
1	33	106	106	1611	0.999	213	0.889839	0.0312821	sp|P16403|H12_HUMAN	H1-2	P16403
1	26	340	340	340	0.999	677	1.6072	0.00291241	sp|P16435|NCPR_HUMAN	POR	P16435
1	55	1852	1517	1852	0.999	1042	4.66439	0	sp|P16615|AT2A2_HUMAN	ATP2A2	P16615
1	12	235	235	235	0.999	419	0.811117	0.0409116	sp|P16930|FAAA_HUMAN	FAH	P16930
1	36	1746	1268	1746	0.999	149	4.46455	0	sp|P16949|STMN1_HUMAN	STMN1	P16949
1	15	130	130	568	0.999	372	0.407389	0.163878	sp|P16989|YBOX3_HUMAN	YBX3	P16989
1	13	307	307	307	0.999	107	0.96556	0.0240627	sp|P17096|HMGA1_HUMAN	HMGA1	P17096
1	27	595	595	595	0.999	413	2.94762	0.000114965	sp|P17174|AATC_HUMAN	GOT1	P17174
1	20	149	120	149	0.999	672	3.46851	1.98758E-05	sp|P17252|KPCA_HUMAN	PRKCA	P17252
1	8	164	120	164	0.999	347	1.18719	0.011045	sp|P17275|JUNB_HUMAN	JUNB	P17275
1	41	809	809	809	0.999	1181	7.5823	0	sp|P17301|ITA2_HUMAN	ITGA2	P17301
1	4	16	16	16	0.999	382	2.57505	0.000253333	sp|P17302|CXA1_HUMAN	GJA1	P17302
1	19	182	182	182	0.999	764	2.72455	0.000173285	sp|P17480|UBF1_HUMAN	UBTF	P17480
1	4	67	67	67	0.999	137	0.180032	0.381771	sp|P17568|NDUB7_HUMAN	NDUFB7	P17568
1	14	250	76	250	0.999	351	0.555186	0.0977119	sp|P17612|KAPCA_HUMAN	PRKACA	P17612
1	43	1441	1440	1441	0.999	700	0.610655	0.0805568	sp|P17655|CAN2_HUMAN	CAPN2	P17655
1	32	673	670	673	0.999	591	3.32637	2.36967E-05	sp|P17812|PYRG1_HUMAN	CTPS1	P17812
1	7	102	102	102	0.999	658	7.63587	0	sp|P17813|EGLN_HUMAN	ENG	P17813
1	64	1211	1211	1922	0.999	614	1.01029	0.0205358	sp|P17844|DDX5_HUMAN	DDX5	P17844
1	29	556	551	771	0.999	780	0.722759	0.0554251	sp|P17858|PFKAL_HUMAN	PFKL	P17858
1	8	182	182	182	0.999	193	0.28048	0.258046	sp|P17900|SAP3_HUMAN	GM2A	P17900
1	14	458	458	458	0.999	250	0.878175	0.032566	sp|P17931|LEG3_HUMAN	LGALS3	P17931
1	36	777	777	777	0.999	439	2.43695	0.000334114	sp|P17980|PRS6A_HUMAN	PSMC3	P17980
1	50	2273	2273	2273	0.999	556	3.28242	4.65658E-05	sp|P17987|TCPA_HUMAN	TCP1	P17987
1	20	500	500	500	0.999	435	0.795903	0.0430605	sp|P18031|PTN1_HUMAN	PTPN1	P18031
1	24	845	845	845	0.999	110	1.49172	0.00393483	sp|P18077|RL35A_HUMAN	RPL35A	P18077
1	23	180	180	180	0.999	799	1.38681	0.00561287	sp|P18084|ITB5_HUMAN	ITGB5	P18084
1	10	137	137	322	0.999	180	3.50202	2.00753E-05	sp|P18085|ARF4_HUMAN	ARF4	P18085

1	48	1822	1822	1822	0.999	248	2.76222	0.000144044	sp|P18124|RL7_HUMAN	RPL7	P18124
1	82	2699	2699	2699	0.999	1134	6.24059	0	sp|P18206|VINC_HUMAN	VCL	P18206
1	20	368	368	368	0.999	2426	3.63944	0	sp|P18583|SON_HUMAN	SON	P18583
1	7	200	200	200	0.999	380	0.73002	0.053997	sp|P18615|NELFE_HUMAN	NELFE	P18615
1	33	1379	1330	1379	0.999	184	5.10751	0	sp|P18621|RL17_HUMAN	RPL17	P18621
1	38	2306	916	2306	0.999	254	2.60445	0.000231096	sp|P18669|PGAM1_HUMAN	PGAM1	P18669
1	14	399	399	399	0.999	421	6.92063	0	sp|P18754|RCC1_HUMAN	RCC1	P18754
0.9999	5	111	23	111	0.999	271	0.305324	0.235733	sp|P18846|ATF1_HUMAN	ATF1	P18846
1	8	59	59	59	0.999	919	0.477594	0.128415	sp|P18858|DNLI1_HUMAN	LIG1	P18858
1	7	167	167	167	0.999	108	3.08469	5.92593E-05	sp|P18859|ATP5J_HUMAN	ATP5PF	P18859
1	10	140	140	140	0.999	633	4.30785	0	sp|P18887|XRCC1_HUMAN	XRCC1	P18887
1	5	34	34	34	0.999	906	1.76751	0.00177983	sp|P19022|CADH2_HUMAN	CDH2	P19022
1	9	191	191	191	0.999	1290	2.32156	0.000452718	sp|P19174|PLCG1_HUMAN	PLCG1	P19174
1	75	2793	2793	2793	0.999	710	3.41772	1.95599E-05	sp|P19338|NUCL_HUMAN	NCL	P19338
1	59	1487	1261	1487	0.999	917	1.90975	0.0011949	sp|P19367|HXK1_HUMAN	HK1	P19367
1	4	76	76	76	0.999	275	3.25855	4.61361E-05	sp|P19387|RPB3_HUMAN	POLR2C	P19387
1	6	99	99	99	0.999	210	0.481076	0.126881	sp|P19388|RPAB1_HUMAN	POLR2E	P19388
1	6	90	90	90	0.999	782	1.56627	0.00324011	sp|P19447|ERCC3_HUMAN	ERCC3	P19447
1	7	132	132	132	0.999	551	0.224851	0.320298	sp|P19525|E2AK2_HUMAN	EIF2AK2	P19525
1	16	588	588	588	0.999	302	1.42928	0.0048869	sp|P19623|SPEE_HUMAN	SRM	P19623
1	13	371	371	392	0.999	350	5.2881	0	sp|P19784|CSK22_HUMAN	CSNK2A2	P19784
1	16	245	245	245	0.999	946	7.47177	0	sp|P19823|ITIH2_HUMAN	ITIH2	P19823
1	10	62	62	64	0.999	911	5.06887	0	sp|P19827|ITIH1_HUMAN	ITIH1	P19827
1	15	168	168	168	0.999	968	2.65913	0.000195462	sp|P19838|NFKB1_HUMAN	NFKB1	P19838
1	10	22	22	22	0.999	344	6.91056	0	sp|P19883|FST_HUMAN	FST	P19883
1	37	319	288	570	0.999	1220	4.76988	0	sp|P20020|AT2B1_HUMAN	ATP2B1	P20020
1	23	591	591	591	0.999	333	2.65464	0.000201915	sp|P20042|IF2B_HUMAN	EIF2S2	P20042
1	28	786	785	786	0.999	488	3.78121	0	sp|P20073|ANXA7_HUMAN	ANXA7	P20073
1	10	405	405	405	0.999	206	3.53831	2.03822E-05	sp|P20290|BTF3_HUMAN	BTF3	P20290
1	12	17	17	125	0.999	219	1.80749	0.00159439	sp|P20337|RAB3B_HUMAN	RAB3B	P20337
1	8	134	134	355	0.999	215	1.56317	0.0032641	sp|P20339|RAB5A_HUMAN	RAB5A	P20339
1	12	122	10	227	0.999	208	6.45135	0	sp|P20340|RAB6A_HUMAN	RAB6A	P20340
1	16	28	23	77	0.999	662	4.31536	0	sp|P20591|MX1_HUMAN	MX1	P20591
1	15	554	554	554	0.999	241	1.61401	0.00284852	sp|P20618|PSB1_HUMAN	PSMB1	P20618
1	14	310	310	310	0.999	277	2.16701	0.00061708	sp|P20645|MPRD_HUMAN	M6PR	P20645
1	9	388	388	388	0.999	150	7.67063	0	sp|P20674|COX5A_HUMAN	COX5A	P20674
1	72	2493	2041	2546	0.999	586	5.11963	0	sp|P20700|LMNB1_HUMAN	LMNB1	P20700
1	9	28	28	170	0.999	1482	2.10335	0.000731642	sp|P20742|PZP_HUMAN	PZP	P20742
1	4	14	14	14	0.9961	298	2.6656	0.000192813	sp|P20774|MIME_HUMAN	OGN	P20774
1	27	812	812	812	0.999	708	2.529	0.000281506	sp|P20810|ICAL_HUMAN	CAST	P20810
1	14	60	60	180	0.999	514	1.32002	0.0070576	sp|P20839|IMDH1_HUMAN	IMPDH1	P20839
1	20	186	171	187	0.999	1838	0.560826	0.095915	sp|P20908|CO5A1_HUMAN	COL5A1	P20908
1	14	114	114	114	0.999	1047	1.62665	0.00275054	sp|P20936|RASA1_HUMAN	RASA1	P20936
1	8	476	476	476	0.999	102	2.8665	0.000126558	sp|P20962|PTMS_HUMAN	PTMS	P20962
1	8	122	0	122	0.999	795	0.348964	0.201378	sp|P21127|CD11B_HUMAN	CDK11B	P21127
1	23	394	337	394	0.999	225	2.05811	0.000819372	sp|P21266|GSTM3_HUMAN	GSTM3	P21266
1	14	351	146	351	0.999	511	4.47175	0	sp|P21281|VATB2_HUMAN	ATP6V1B2	P21281
1	17	311	311	311	0.999	382	0.00820397	0.90472	sp|P21283|VATC1_HUMAN	ATP6V1C1	P21283
1	25	809	807	809	0.999	193	2.54485	0.000276316	sp|P21291|CSRP1_HUMAN	CSRP1	P21291
1	156	5366	4781	5366	0.999	2647	1.24677	0.00906472	sp|P21333|FLNA_HUMAN	FLNA	P21333
1	40	895	888	895	0.999	889	1.47033	0.00422851	sp|P21399|ACOHC_HUMAN	ACO1	P21399
1	32	389	389	389	0.999	574	9.85978	0	sp|P21589|5NTD_HUMAN	NT5E	P21589
1	34	1747	1630	1747	0.999	283	5.31067	0	sp|P21796|VDAC1_HUMAN	VDAC1	P21796
1	21	523	519	523	0.999	280	4.0475	0	sp|P21912|SDHB_HUMAN	SDHB	P21912
1	7	112	112	112	0.999	228	3.61401	0	sp|P21926|CD9_HUMAN	CD9	P21926
1	10	301	301	301	0.999	271	5.26619	0	sp|P21964|COMT_HUMAN	COMT	P21964
1	58	827	809	827	0.999	687	5.37395	0	sp|P21980|TGM2_HUMAN	TGM2	P21980
1	9	165	165	165	0.999	750	1.93883	0.00112593	sp|P22033|MUTA_HUMAN	MMUT	P22033
1	22	417	417	417	0.999	807	0.015981	0.849256	sp|P22059|OSBP1_HUMAN	OSBP	P22059
1	14	386	386	386	0.999	227	3.84694	0	sp|P22061|PIMT_HUMAN	PCMT1	P22061
1	17	672	631	672	0.999	321	0.3182	0.224782	sp|P22087|FBRL_HUMAN	FBL	P22087
1	23	41	24	1326	0.999	263	3.40235	1.94411E-05	sp|P22090|RS4Y1_HUMAN	RPS4Y1	P22090
1	46	1480	1480	1480	0.999	1010	3.00286	0.000081716	sp|P22102|PUR2_HUMAN	GART	P22102
1	40	1310	1310	1310	0.999	425	1.85583	0.00140476	sp|P22234|PUR6_HUMAN	PAICS	P22234
1	11	249	249	249	0.999	547	4.1899	0	sp|P22307|SCP2_HUMAN	SCP2	P22307
1	62	2982	2982	2982	0.999	1058	0.988211	0.0222075	sp|P22314|UBA1_HUMAN	UBA1	P22314
1	18	1086	303	1086	0.999	152	5.18406	0	sp|P22392|NDKB_HUMAN	NME2	P22392
1	33	133	133	133	0.999	925	0.85547	0.0352415	sp|P22413|ENPP1_HUMAN	ENPP1	P22413
1	14	207	207	207	0.999	491	1.13742	0.0131476	sp|P22570|ADRO_HUMAN	FDXR	P22570
1	51	3740	3236	3740	0.999	353	8.31888	0	sp|P22626|ROA2_HUMAN	HNRNPA2B1	P22626
1	10	56	54	56	0.999	906	1.70846	0.00213797	sp|P22681|CBL_HUMAN	CBL	P22681
1	24	900	900	900	0.999	453	2.68216	0.000190476	sp|P22695|QCR2_HUMAN	UQCRC2	P22695
1	9	180	180	180	0.999	423	1.09924	0.0150164	sp|P22830|HEMH_HUMAN	FECH	P22830
1	9	94	94	94	0.999	703	2.82427	0.000132075	sp|P23142|FBLN1_HUMAN	FBLN1	P23142
1	30	639	581	639	0.999	301	0.153769	0.424841	sp|P23193|TCEA1_HUMAN	TCEA1	P23193
1	7	51	51	51	0.999	599	0.682818	0.0631589	sp|P23219|PGH1_HUMAN	PTGS1	P23219
1	37	756	756	756	0.999	1130	10.3058	0	sp|P23229|ITA6_HUMAN	ITGA6	P23229
1	73	2878	2734	2878	0.999	707	2.37778	0.000408848	sp|P23246|SFPQ_HUMAN	SFPQ	P23246
1	12	306	105	306	0.999	451	2.23839	0.000511794	sp|P23258|TBG1_HUMAN	TUBG1	P23258
1	28	1412	1412	1412	0.999	216	10.2241	0	sp|P23284|PPIB_HUMAN	PPIB	P23284
1	14	306	275	306	0.999	584	0.318169	0.224736	sp|P23368|MAOM_HUMAN	ME2	P23368
1	31	924	924	924	0.999	471	2.58486	0.000248739	sp|P23381|SYWC_HUMAN	WARS1	P23381
1	34	2070	2070	2070	0.999	243	1.43012	0.00487543	sp|P23396|RS3_HUMAN	RPS3	P23396
1	5	23	23	23	0.999	1154	1.41687	0.00508049	sp|P23458|JAK1_HUMAN	JAK1	P23458
1	12	25	25	425	0.999	879	3.55476	1.03226E-05	sp|P23497|SP100_HUMAN	SP100	P23497
1	34	1977	1908	1977	0.999	432	1.25768	0.00872727	sp|P23526|SAHH_HUMAN	AHCY	P23526
1	27	1935	1208	1935	0.999	166	4.09401	0	sp|P23528|COF1_HUMAN	CFL1	P23528
1	52	1248	1248	1248	0.999	611	3.92433	0	sp|P23588|IF4B_HUMAN	EIF4B	P23588
1	31	588	331	588	0.999	1241	7.12339	0	sp|P23634|AT2B4_HUMAN	ATP2B4	P23634
1	19	267	267	267	0.999	735	4.13704	0	sp|P23743|DGKA_HUMAN	DGKA	P23743
1	18	296	296	296	0.999	658	1.8414	0.00147107	sp|P23786|CPT2_HUMAN	CPT2	P23786
1	18	339	339	339	0.999	212	0.566117	0.0941313	sp|P23919|KTHY_HUMAN	DTYMK	P23919
1	35	912	912	912	0.999	792	2.40592	0.000365325	sp|P23921|RIR1_HUMAN	RRM1	P23921
1	16	655	655	732	0.999	225	3.73902	0	sp|P24534|EF1B_HUMAN	EEF1B2	P24534
1	20	536	536	536	0.999	256	3.90528	0	sp|P24539|AT5F1_HUMAN	ATP5PB	P24539
1	10	18	18	18	0.999	272	6.6482	0	sp|P24593|IBP5_HUMAN	IGFBP5	P24593
1	8	208	208	208	0.999	158	0.474264	0.129961	sp|P24666|PPAC_HUMAN	ACP1	P24666
1	31	771	771	771	0.999	427	2.76646	0.000144578	sp|P24752|THIL_HUMAN	ACAT1	P24752
1	70	312	312	312	0.999	2201	5.01149	0	sp|P24821|TENA_HUMAN	TNC	P24821
1	10	66	66	369	0.999	172	2.29712	0.000457814	sp|P24844|MYL9_HUMAN	MYL9	P24844
1	29	144	144	144	0.999	1970	0.217117	0.32986	sp|P24928|RPB1_HUMAN	POLR2A	P24928
1	11	164	163	302	0.999	298	0.610528	0.080571	sp|P24941|CDK2_HUMAN	CDK2	P24941
1	48	1089	1089	1089	0.999	808	2.07092	0.000792627	sp|P25205|MCM3_HUMAN	MCM3	P25205
1	15	415	415	415	0.999	297	0.807407	0.0413607	sp|P25325|THTM_HUMAN	MPST	P25325
1	19	960	960	960	0.999	132	6.38925	0	sp|P25398|RS12_HUMAN	RPS12	P25398
1	7	86	86	97	0.999	801	1.37728	0.00578229	sp|P25440|BRD2_HUMAN	BRD2	P25440
1	13	347	138	347	0.999	414	1.01537	0.0201845	sp|P25490|TYY1_HUMAN	YY1	P25490
1	13	332	332	332	0.999	340	0.823374	0.0392574	sp|P25685|DNJB1_HUMAN	DNAJB1	P25685
1	66	2478	2478	2478	0.999	553	0.0358411	0.745542	sp|P25705|ATPA_HUMAN	ATP5F1A	P25705
1	3	15	15	15	0.999	331	1.22064	0.00990433	sp|P25774|CATS_HUMAN	CTSS	P25774
1	33	1453	1453	1453	0.999	263	0.348424	0.201678	sp|P25786|PSA1_HUMAN	PSMA1	P25786
1	12	415	415	415	0.999	234	0.614466	0.0796928	sp|P25787|PSA2_HUMAN	PSMA2	P25787
1	23	1040	1040	1040	0.999	255	1.84129	0.00147021	sp|P25788|PSA3_HUMAN	PSMA3	P25788
1	20	687	687	687	0.999	261	0.943324	0.0259739	sp|P25789|PSA4_HUMAN	PSMA4	P25789
1	25	715	715	715	0.999	1051	11.0131	0	sp|P26006|ITA3_HUMAN	ITGA3	P26006
1	16	63	63	63	0.999	381	6.86401	0	sp|P26022|PTX3_HUMAN	PTX3	P26022
1	88	4464	3386	4464	0.999	577	3.68887	0	sp|P26038|MOES_HUMAN	MSN	P26038
1	17	542	542	542	0.999	483	0.901309	0.0300056	sp|P26196|DDX6_HUMAN	DDX6	P26196
1	51	735	735	735	0.999	1616	2.6353	0.00020654	sp|P26358|DNMT1_HUMAN	DNMT1	P26358
1	16	516	516	516	0.999	475	3.25034	5.51091E-05	sp|P26368|U2AF2_HUMAN	U2AF2	P26368
1	37	1518	1518	1518	0.999	211	3.90296	0	sp|P26373|RL13_HUMAN	RPL13	P26373
1	11	200	200	200	0.999	426	0.429295	0.151438	sp|P26440|IVD_HUMAN	IVD	P26440
1	13	500	500	500	0.999	101	1.58328	0.0030941	sp|P26447|S10A4_HUMAN	S100A4	P26447
1	30	970	970	1307	0.999	209	3.76306	0	sp|P26583|HMGB2_HUMAN	HMGB2	P26583
1	35	1287	1046	1287	0.999	557	7.00361	0	sp|P26599|PTBP1_HUMAN	PTBP1	P26599
1	62	1874	1649	1874	0.999	723	0.685937	0.0625911	sp|P26639|SYTC_HUMAN	TARS1	P26639
1	69	1998	1998	1998	0.999	1264	0.403434	0.166036	sp|P26640|SYVC_HUMAN	VARS1	P26640
1	40	2080	2080	2080	0.999	437	3.00501	7.77892E-05	sp|P26641|EF1G_HUMAN	EEF1G	P26641
1	9	272	272	272	0.999	142	1.72413	0.00204059	sp|P26885|FKBP2_HUMAN	FKBP2	P26885

1	18	570	521	570	0.999	288	11.0768	0	sp|P27105|STOM_HUMAN	STOM	P27105
1	36	1059	1059	1575	0.999	245	2.49704	0.000297254	sp|P27348|1433T_HUMAN	YWHAQ	P27348
1	18	105	89	261	0.999	379	0.256523	0.283047	sp|P27361|MK03_HUMAN	MAPK3	P27361
1	13	20	20	20	0.9989	766	4.48939	0	sp|P27487|DPP4_HUMAN	DPP4	P27487
1	34	1639	1034	1639	0.999	214	5.90557	0	sp|P27635|RL10_HUMAN	RPL10	P27635
1	23	425	425	425	0.999	616	2.11655	0.000701142	sp|P27694|RFA1_HUMAN	RPA1	P27694
1	24	780	780	780	0.999	318	6.51179	0	sp|P27695|APEX1_HUMAN	APEX1	P27695
1	6	33	33	33	0.999	267	3.49072	0.000020025	sp|P27701|CD82_HUMAN	CD82	P27701
1	7	83	83	83	0.999	260	2.77881	0.000141395	sp|P27707|DCK_HUMAN	DCK	P27707
1	69	1510	1510	1611	0.999	2225	0.655423	0.0693133	sp|P27708|PYR1_HUMAN	CAD	P27708
1	33	1994	1994	1994	0.999	417	9.95224	0	sp|P27797|CALR_HUMAN	CALR	P27797
1	63	1676	1675	1676	0.999	1152	4.12623	0	sp|P27816|MAP4_HUMAN	MAP4	P27816
1	38	1519	1491	1519	0.999	592	8.08686	0	sp|P27824|CALX_HUMAN	CANX	P27824
1	5	125	125	125	0.999	276	0.169722	0.398242	sp|P28062|PSB8_HUMAN	PSMB8	P28062
1	7	67	67	67	0.999	219	0.240116	0.301354	sp|P28065|PSB9_HUMAN	PSMB9	P28065
1	14	512	512	512	0.999	241	0.203498	0.347465	sp|P28066|PSA5_HUMAN	PSMA5	P28066
1	8	254	254	254	0.999	264	1.55738	0.00331056	sp|P28070|PSB4_HUMAN	PSMB4	P28070
1	8	392	392	392	0.999	239	2.63166	0.000209442	sp|P28072|PSB6_HUMAN	PSMB6	P28072
1	10	434	434	434	0.999	263	0.809773	0.0410674	sp|P28074|PSB5_HUMAN	PSMB5	P28074
1	15	370	370	370	0.999	659	0.261385	0.277543	sp|P28288|ABCD3_HUMAN	ABCD3	P28288
1	7	47	47	47	0.999	417	0.539807	0.103286	sp|P28300|LYOX_HUMAN	LOX	P28300
1	34	591	591	591	0.999	727	1.53913	0.00344196	sp|P28331|NDUS1_HUMAN	NDUFS1	P28331
1	29	369	369	422	0.999	1107	0.886189	0.0317038	sp|P28340|DPOD1_HUMAN	POLD1	P28340
1	27	57	57	341	0.999	1054	4.88419	0	sp|P28370|SMCA1_HUMAN	SMARCA1	P28370
1	24	627	471	627	0.999	360	1.20906	0.0102944	sp|P28482|MK01_HUMAN	MAPK1	P28482
1	9	55	5	55	0.999	533	2.89678	0.00012828	sp|P28702|RXRB_HUMAN	RXRB	P28702
1	17	314	314	314	0.999	593	1.06525	0.0169265	sp|P28799|GRN_HUMAN	GRN	P28799
1	15	117	117	117	0.999	519	4.00962	0	sp|P28838|AMPL_HUMAN	LAP3	P28838
1	30	467	467	467	0.999	1249	2.33062	0.000442451	sp|P29144|TPP2_HUMAN	TPP2	P29144
1	15	317	317	317	0.999	277	0.720125	0.0559143	sp|P29218|IMPA1_HUMAN	IMPA1	P29218
1	4	5	5	5	0.9983	349	4.83577	0	sp|P29279|CCN2_HUMAN	CCN2	P29279
1	34	239	184	239	0.999	976	11.3922	0	sp|P29317|EPHA2_HUMAN	EPHA2	P29317
1	11	51	50	106	0.999	1055	3.29964	3.75147E-05	sp|P29323|EPHB2_HUMAN	EPHB2	P29323
1	8	103	103	103	0.999	298	0.937342	0.0265358	sp|P29372|3MG_HUMAN	MPG	P29372
1	11	135	98	135	0.999	138	0.959436	0.0245836	sp|P29373|RABP2_HUMAN	CRABP2	P29373
1	67	2857	2795	2857	0.999	623	5.15983	0	sp|P29401|TKT_HUMAN	TKT	P29401
1	15	153	125	153	0.999	404	1.1284	0.013599	sp|P29466|CASP1_HUMAN	CASP1	P29466
1	29	521	521	521	0.999	882	5.55136	0	sp|P29590|PML_HUMAN	PML	P29590
1	31	1399	1322	1399	0.999	281	7.09301	0	sp|P29692|EF1D_HUMAN	EEF1D	P29692
1	10	293	292	293	0.999	332	7.48859	0	sp|P29966|MARCS_HUMAN	MARCKS	P29966
1	10	66	55	151	0.999	359	8.95851	0	sp|P29992|GNA11_HUMAN	GNA11	P29992
1	15	635	635	635	0.999	261	5.15829	0	sp|P30040|ERP29_HUMAN	ERP29	P30040
1	33	1432	1432	1432	0.999	224	5.12267	0	sp|P30041|PRDX6_HUMAN	PRDX6	P30041
1	12	310	310	310	0.999	206	6.03122	0	sp|P30043|BLVRB_HUMAN	BLVRB	P30043
1	11	443	443	443	0.999	214	1.27889	0.00816566	sp|P30044|PRDX5_HUMAN	PRDX5	P30044
1	11	519	10	519	0.999	118	3.60039	0	sp|P30046|DOPD_HUMAN	DDT	P30046
1	18	777	777	777	0.999	256	6.20401	0	sp|P30048|PRDX3_HUMAN	PRDX3	P30048
1	5	156	156	156	0.999	168	1.65229	0.00252655	sp|P30049|ATPD_HUMAN	ATP5F1D	P30049
1	18	820	820	820	0.999	165	3.42772	1.96802E-05	sp|P30050|RL12_HUMAN	RPL12	P30050
1	17	427	427	427	0.999	290	4.84682	0	sp|P30084|ECHM_HUMAN	ECHS1	P30084
1	19	759	759	759	0.999	196	2.57107	0.000266002	sp|P30085|KCY_HUMAN	CMPK1	P30085
1	16	778	778	778	0.999	187	0.556004	0.0974539	sp|P30086|PEBP1_HUMAN	PEBP1	P30086
1	67	2947	2947	2947	0.999	505	11.2586	0	sp|P30101|PDIA3_HUMAN	PDIA3	P30101
1	33	1112	955	1112	0.999	589	3.16968	5.28634E-05	sp|P30153|2AAA_HUMAN	PPP2R1A	P30153
1	16	146	146	303	0.999	601	1.30387	0.00745966	sp|P30154|2AAB_HUMAN	PPP2R1B	P30154
1	15	427	409	456	0.999	207	5.9525	0	sp|P30405|PPIF_HUMAN	PPIF	P30405
1	6	109	109	109	0.999	1462	1.66961	0.0023978	sp|P30414|NKTR_HUMAN	NKTR	P30414
1	14	420	382	420	0.999	496	2.16245	0.000624055	sp|P30419|NMT1_HUMAN	NMT1	P30419
1	12	256	256	256	0.999	316	0.461478	0.135912	sp|P30519|HMOX2_HUMAN	HMOX2	P30519
1	22	502	463	502	0.999	456	0.928559	0.0273225	sp|P30520|PURA2_HUMAN	ADSS2	P30520
1	7	75	75	75	0.999	894	3.91043	0	sp|P30530|UFO_HUMAN	AXL	P30530
1	18	395	395	395	0.999	357	1.92709	0.00115899	sp|P30533|AMRP_HUMAN	LRPAP1	P30533
1	4	132	132	132	0.9989	169	0.601738	0.0831572	sp|P30536|TSPO_HUMAN	TSPO	P30536
1	24	603	603	603	0.999	484	0.0927193	0.55678	sp|P30566|PUR8_HUMAN	ADSL	P30566
1	25	228	210	228	0.999	1438	0.557071	0.0971043	sp|P30622|CLIP1_HUMAN	CLIP1	P30622
1	12	538	538	538	0.999	198	4.4117	0	sp|P30626|SORCN_HUMAN	SRI	P30626
1	26	741	704	741	0.999	379	2.3742	0.000407605	sp|P30740|ILEU_HUMAN	SERPINB1	P30740
1	7	170	170	170	0.999	629	5.71919	0	sp|P30825|CTR1_HUMAN	SLC7A1	P30825
1	24	452	349	452	0.999	517	0.428448	0.151888	sp|P30837|AL1B1_HUMAN	ALDH1B1	P30837
1	37	460	460	460	0.999	1174	0.443156	0.144267	sp|P30876|RPB2_HUMAN	POLR2B	P30876
1	39	1372	1372	1372	0.999	664	0.721207	0.0557413	sp|P31040|SDHA_HUMAN	SDHA	P31040
1	11	53	53	143	0.999	461	2.67715	0.000190308	sp|P31146|COR1A_HUMAN	CORO1A	P31146
1	27	294	294	716	0.999	447	0.961624	0.0244163	sp|P31150|GDIA_HUMAN	GDI1	P31150
1	29	806	582	806	0.999	395	1.42864	0.0049063	sp|P31153|METK2_HUMAN	MAT2A	P31153
1	138	2718	2617	2718	0.999	1500	0.345076	0.203895	sp|P31327|CPSM_HUMAN	CPS1	P31327
1	16	201	188	201	0.999	389	2.09648	0.000736213	sp|P31350|RIR2_HUMAN	RRM2	P31350
1	21	938	938	938	0.999	397	0.804315	0.0417735	sp|P31689|DNJA1_HUMAN	DNAJA1	P31689
1	13	175	88	175	0.999	480	0.949974	0.0253977	sp|P31749|AKT1_HUMAN	AKT1	P31749
1	9	38	38	125	0.999	481	1.0533	0.0176577	sp|P31751|AKT2_HUMAN	AKT2	P31751
1	28	1285	1193	1285	0.999	480	9.49862	0	sp|P31930|QCR1_HUMAN	UQCRC1	P31930
1	8	121	121	121	0.999	336	0.328245	0.216483	sp|P31937|3HIDH_HUMAN	HIBADH	P31937
1	35	1192	1192	1192	0.999	592	3.97167	0	sp|P31939|PUR9_HUMAN	ATIC	P31939
1	20	568	568	568	0.999	346	6.97738	0	sp|P31942|HNRH3_HUMAN	HNRNPH3	P31942
1	40	1403	763	1403	0.999	449	3.87448	0	sp|P31943|HNRH1_HUMAN	HNRNPH1	P31943
1	37	985	893	1714	0.999	246	0.99729	0.021511	sp|P31946|1433B_HUMAN	YWHAB	P31946
1	77	2668	2668	2668	0.999	543	6.69217	0	sp|P31948|STIP1_HUMAN	STIP1	P31948
1	16	762	762	762	0.999	105	4.15573	0	sp|P31949|S10AB_HUMAN	S100A11	P31949
1	15	100	100	100	0.999	1257	1.30489	0.00744815	sp|P32004|L1CAM_HUMAN	L1CAM	P32004
1	23	994	994	1090	0.999	198	1.64014	0.00264481	sp|P32119|PRDX2_HUMAN	PRDX2	P32119
1	10	224	224	224	0.999	178	3.63764	0	sp|P32321|DCTD_HUMAN	DCTD	P32321
1	14	228	218	228	0.999	319	2.03	0.000877419	sp|P32322|P5CR1_HUMAN	PYCR1	P32322
1	17	243	156	243	0.999	592	1.54266	0.00343245	sp|P32455|GBP1_HUMAN	GBP1	P32455
1	23	1002	1002	1002	0.999	192	1.22233	0.00983476	sp|P32969|RL9_HUMAN	RPL9	P32969
1	28	512	475	512	0.999	698	0.0950563	0.55088	sp|P33121|ACSL1_HUMAN	ACSL1	P33121
1	65	1456	1217	1456	0.999	963	0.619105	0.0785591	sp|P33176|KINH_HUMAN	KIF5B	P33176
1	14	208	158	208	0.999	577	3.40857	1.94647E-05	sp|P33240|CSTF2_HUMAN	CSTF2	P33240
1	13	452	452	452	0.999	252	3.41278	1.95122E-05	sp|P33316|DUT_HUMAN	DUT	P33316
1	14	202	150	202	0.999	1531	6.77908	0	sp|P33527|MRP1_HUMAN	ABCC1	P33527
0.9999	2	13	13	13	0.999	79	1.41943	0.0050415	sp|P33552|CKS2_HUMAN	CKS2	P33552
1	25	611	0	1339	0.999	126	1.53093	0.00351295	sp|P33778|H2B1B_HUMAN	H2BC3	P33778
1	7	35	35	35	0.999	857	4.07656	0	sp|P33981|TTK_HUMAN	TTK	P33981
1	59	1335	1280	1335	0.999	863	5.37213	0	sp|P33991|MCM4_HUMAN	MCM4	P33991
1	54	1344	1344	1344	0.999	734	4.58111	0	sp|P33992|MCM5_HUMAN	MCM5	P33992
1	58	1129	1129	1129	0.999	719	0.958847	0.0246376	sp|P33993|MCM7_HUMAN	MCM7	P33993
1	10	182	182	183	0.999	483	1.40071	0.00535291	sp|P34896|GLYC_HUMAN	SHMT1	P34896
1	39	1625	1624	1625	0.999	504	5.22608	0	sp|P34897|GLYM_HUMAN	SHMT2	P34897
1	88	2777	2637	2777	0.999	840	7.30265	0	sp|P34932|HSP74_HUMAN	HSPA4	P34932
1	9	112	112	112	0.999	423	0.489485	0.123172	sp|P34949|MPI_HUMAN	MPI	P34949
1	9	80	80	80	0.999	558	0.849901	0.0358312	sp|P35052|GPC1_HUMAN	GPC1	P35052
1	7	349	349	349	0.999	140	2.59231	0.000242833	sp|P35080|PROF2_HUMAN	PFN2	P35080
1	45	1238	940	1238	0.999	906	5.32812	0	sp|P35221|CTNA1_HUMAN	CTNNA1	P35221
1	30	581	581	671	0.999	781	4.66701	0	sp|P35222|CTNB1_HUMAN	CTNNB1	P35222
1	26	957	957	957	0.999	272	5.4899	0	sp|P35232|PHB1_HUMAN	PHB1	P35232
1	23	695	645	695	0.999	376	2.72584	0.000173599	sp|P35237|SPB6_HUMAN	SERPINB6	P35237
1	56	684	683	1928	0.999	583	1.27605	0.00824108	sp|P35241|RADI_HUMAN	RDX	P35241
1	6	124	124	124	0.999	121	3.56462	5.18807E-06	sp|P35244|RFA3_HUMAN	RPA3	P35244
1	16	337	337	337	0.999	363	0.324451	0.219641	sp|P35249|RFC4_HUMAN	RFC4	P35249
1	12	148	148	148	0.999	354	1.03784	0.0186154	sp|P35250|RFC2_HUMAN	RFC2	P35250
1	16	123	123	123	0.999	1148	2.59156	0.000242628	sp|P35251|RFC1_HUMAN	RFC1	P35251
1	13	370	243	370	0.999	128	2.26424	0.000483357	sp|P35268|RL22_HUMAN	RPL22	P35268
1	15	280	280	280	0.999	517	2.01227	0.00090653	sp|P35269|T2FA_HUMAN	GTF2F1	P35269
1	10	58	58	58	0.999	604	2.15672	0.000630137	sp|P35354|PGH2_HUMAN	PTGS2	P35354
1	24	43	43	133	0.999	1172	7.97951	0	sp|P35442|TSP2_HUMAN	THBS2	P35442
1	8	46	31	46	0.999	961	5.29345	0	sp|P35443|TSP4_HUMAN	THBS4	P35443
1	17	220	220	220	0.999	551	1.30547	0.0074385	sp|P35520|CBS_HUMAN	CBS	P35520
1	38	590	590	646	0.999	623	5.20456	0	sp|P35527|K1C9_HUMAN	KRT9	P35527
1	45	193	188	193	0.999	2871	5.72272	0	sp|P35555|FBN1_HUMAN	FBN1	P35555
1	29	119	119	124	0.999	2912	2.71726	0.000175785	sp|P35556|FBN2_HUMAN	FBN2	P35556
1	23	257	257	257	0.999	1532	1.32481	0.00692955	sp|P35573|GDE_HUMAN	AGL	P35573

1	281	12000	9852	12000	0.999	1960	4.21495	0	sp|P35579|MYH9_HUMAN	MYH9	P35579
1	129	1734	1652	3323	0.999	1976	2.1451	0.000653061	sp|P35580|MYH10_HUMAN	MYH10	P35580
1	56	1195	1195	1195	0.999	906	5.17074	0	sp|P35606|COPB2_HUMAN	COPB2	P35606
1	11	170	170	170	0.999	550	1.95331	0.00108571	sp|P35610|SOAT1_HUMAN	SOAT1	P35610
1	10	98	97	98	0.999	737	0.713623	0.0572058	sp|P35611|ADDA_HUMAN	ADD1	P35611
1	15	637	637	637	0.999	385	7.38146	0	sp|P35613|BASI_HUMAN	BSG	P35613
1	10	20	20	20	0.999	211	6.72864	0	sp|P35625|TIMP3_HUMAN	TIMP3	P35625
1	21	747	677	747	0.999	526	0.955858	0.0248741	sp|P35637|FUS_HUMAN	FUS	P35637
1	23	329	329	329	0.999	2090	2.11616	0.000700671	sp|P35658|NU214_HUMAN	NUP214	P35658
1	26	758	758	758	0.999	375	2.69411	0.000187943	sp|P35659|DEK_HUMAN	DEK	P35659
1	9	125	125	125	0.999	106	3.09909	5.53781E-05	sp|P35754|GLRX1_HUMAN	GLRX	P35754
1	9	145	86	145	0.999	382	0.881052	0.0323068	sp|P35813|PPM1A_HUMAN	PPM1A	P35813
1	41	460	444	705	0.999	639	3.98526	0	sp|P35908|K22E_HUMAN	KRT2	P35908
1	7	173	121	173	0.999	325	0.272889	0.266048	sp|P35914|HMGCL_HUMAN	HMGCL	P35914
1	33	824	824	933	0.999	433	2.60481	0.000231489	sp|P35998|PRS7_HUMAN	PSMC2	P35998
1	6	134	134	134	0.999	182	0.784112	0.0447528	sp|P36405|ARL3_HUMAN	ARL 3.00	P36405
1	27	387	387	639	0.999	400	1.41667	0.00507799	sp|P36507|MP2K2_HUMAN	MAP2K2	P36507
1	25	557	557	557	0.999	298	4.88862	0	sp|P36542|ATPG_HUMAN	ATP5F1C	P36542
1	10	178	114	178	0.999	226	0.116868	0.499509	sp|P36543|VATE1_HUMAN	ATP6V1E1	P36543
1	18	274	274	274	0.999	454	0.295819	0.244103	sp|P36551|HEM6_HUMAN	CPOX	P36551
1	66	2785	2785	2785	0.999	427	3.2276	5.46697E-05	sp|P36578|RL4_HUMAN	RPL4	P36578
1	46	1032	1032	1032	0.999	959	8.6019	0	sp|P36776|LONM_HUMAN	LONP1	P36776
1	40	939	939	939	0.999	562	0.176262	0.387678	sp|P36871|PGM1_HUMAN	PGM1	P36871
1	15	73	73	665	0.999	323	0.737611	0.0526475	sp|P36873|PP1G_HUMAN	PPP1CC	P36873
1	17	406	406	406	0.999	607	1.09886	0.015019	sp|P36915|GNL1_HUMAN	GNL1	P36915
1	6	42	42	42	0.999	418	5.05116	0	sp|P36955|PEDF_HUMAN	SERPINF1	P36955
1	19	627	627	627	0.999	453	2.57787	0.000253968	sp|P36957|ODO2_HUMAN	DLST	P36957
1	6	117	117	117	0.999	197	0.343299	0.205191	sp|P36969|GPX4_HUMAN	GPX4	P36969
1	19	782	782	782	0.999	136	2.07667	0.000781518	sp|P37108|SRP14_HUMAN	SRP14	P37108
1	6	124	124	124	0.999	522	0.380625	0.179738	sp|P37198|NUP62_HUMAN	NUP62	P37198
1	11	117	22	117	0.999	193	8.10033	0	sp|P37235|HPCL1_HUMAN	HPCAL1	P37235
1	20	254	254	254	0.999	417	0.215889	0.331329	sp|P37268|FDFT_HUMAN	FDFT1	P37268
1	25	1243	1167	1243	0.999	199	2.31389	0.000454006	sp|P37802|TAGL2_HUMAN	TAGLN2	P37802
1	31	1586	1586	1586	0.999	337	8.12592	0	sp|P37837|TALDO_HUMAN	TALDO1	P37837
1	23	754	754	754	0.999	255	1.76329	0.00180437	sp|P38117|ETFB_HUMAN	ETFB	P38117
1	60	2210	718	2210	0.999	391	4.10225	0	sp|P38159|RBMX_HUMAN	RBMX	P38159
1	25	757	757	757	0.999	617	3.16723	5.27473E-05	sp|P38606|VATA_HUMAN	ATP6V1A	P38606
1	72	2520	2520	2576	0.999	679	3.84381	0	sp|P38646|GRP75_HUMAN	HSPA9	P38646
1	31	859	859	1220	0.999	411	2.5067	0.000285946	sp|P38919|IF4A3_HUMAN	EIF4A3	P38919
1	36	1689	1689	1689	0.999	145	3.36399	2.39234E-05	sp|P39019|RS19_HUMAN	RPS19	P39019
1	78	3229	2902	3229	0.999	403	3.21961	5.42373E-05	sp|P39023|RL3_HUMAN	RPL3	P39023
1	4	14	14	14	0.9987	1754	6.26126	0	sp|P39060|COIA1_HUMAN	COL18A1	P39060
1	18	584	584	584	0.999	456	5.23148	0	sp|P39656|OST48_HUMAN	DDOST	P39656
1	21	819	740	819	0.999	249	3.82891	0	sp|P39687|AN32A_HUMAN	ANP32A	P39687
1	36	907	907	907	0.999	380	1.45375	0.00446386	sp|P39748|FEN1_HUMAN	FEN1	P39748
1	20	641	641	641	0.999	348	2.01103	0.000904215	sp|P40121|CAPG_HUMAN	CAPG	P40121
1	9	94	94	154	0.999	477	1.55198	0.00336302	sp|P40123|CAP2_HUMAN	CAP2	P40123
1	26	689	626	689	0.999	546	1.99846	0.000944762	sp|P40222|TXLNA_HUMAN	TXLNA	P40222
1	41	1985	1595	1985	0.999	531	8.07203	0	sp|P40227|TCPZ_HUMAN	CCT6A	P40227
1	23	723	723	723	0.999	264	3.89528	0	sp|P40261|NNMT_HUMAN	NNMT	P40261
1	7	48	48	48	0.999	273	1.92512	0.00116248	sp|P40306|PSB10_HUMAN	PSMB10	P40306
1	33	1135	1000	1135	0.999	203	1.38074	0.00570708	sp|P40429|RL13A_HUMAN	RPL13A	P40429
1	5	88	88	88	0.999	181	0.353944	0.197909	sp|P40616|ARL1_HUMAN	ARL 1.00	P40616
1	25	653	653	653	0.999	770	0.975016	0.0232655	sp|P40763|STAT3_HUMAN	STAT3	P40763
1	28	1114	1114	1114	0.999	334	2.51235	0.000283388	sp|P40925|MDHC_HUMAN	MDH1	P40925
1	38	1703	1703	1703	0.999	338	8.38301	0	sp|P40926|MDHM_HUMAN	MDH2	P40926
1	11	248	248	248	0.999	340	0.794771	0.0431834	sp|P40937|RFC5_HUMAN	RFC5	P40937
1	16	194	194	194	0.999	356	0.308273	0.233155	sp|P40938|RFC3_HUMAN	RFC3	P40938
1	57	1251	1251	1251	0.999	763	4.36669	0	sp|P40939|ECHA_HUMAN	HADHA	P40939
1	39	1717	320	1717	0.999	472	1.07754	0.0162162	sp|P41091|IF2G_HUMAN	EIF2S3	P41091
1	8	75	75	75	0.999	584	3.35833	2.38949E-05	sp|P41214|EIF2D_HUMAN	EIF2D	P41214
1	14	94	49	94	0.999	380	6.72089	0	sp|P41221|WNT5A_HUMAN	WNT5A	P41221
1	13	203	203	203	0.999	144	0.51709	0.112141	sp|P41223|BUD31_HUMAN	BUD31	P41223
1	14	536	372	536	0.999	235	0.733363	0.0534128	sp|P41227|NAA10_HUMAN	NAA10	P41227
1	4	115	80	115	0.999	205	2.1721	0.000611765	sp|P41236|IPP2_HUMAN	PPP1R2	P41236
1	11	125	113	125	0.999	450	1.53159	0.00350804	sp|P41240|CSK_HUMAN	CSK	P41240
1	49	1709	1709	1709	0.999	739	3.12034	5.17799E-05	sp|P41250|GARS_HUMAN	GARS1	P41250
1	69	1635	1635	1635	0.999	1262	2.25061	0.000487805	sp|P41252|SYIC_HUMAN	IARS1	P41252
1	4	209	35	209	0.999	113	2.66577	0.000192982	sp|P41567|EIF1_HUMAN	EIF1	P41567
1	8	115	81	115	0.999	596	1.76182	0.00181964	sp|P41743|KPCI_HUMAN	PRKCI	P41743
1	9	221	221	221	0.999	302	0.196677	0.356854	sp|P42126|ECI1_HUMAN	ECI1	P42126
1	34	425	425	942	0.999	694	3.4089	1.94884E-05	sp|P42166|LAP2A_HUMAN	TMPO	P42166
1	30	1122	605	1122	0.999	454	4.45241	0	sp|P42167|LAP2B_HUMAN	TMPO	P42167
1	29	485	485	485	0.999	750	3.91792	0	sp|P42224|STAT1_HUMAN	STAT1	P42224
1	12	119	119	119	0.999	847	1.81314	0.00157562	sp|P42226|STAT6_HUMAN	STAT6	P42226
1	24	444	444	444	0.999	1042	1.85902	0.00139928	sp|P42285|MTREX_HUMAN	MTREX	P42285
1	23	522	116	522	0.999	323	0.74046	0.0521833	sp|P42330|AK1C3_HUMAN	AKR1C3	P42330
1	39	471	471	471	0.999	2549	2.91249	0.000125	sp|P42345|MTOR_HUMAN	MTOR	P42345
1	7	140	113	140	0.999	277	1.69721	0.00222669	sp|P42574|CASP3_HUMAN	CASP3	P42574
1	10	303	121	303	0.999	84	3.25365	5.05747E-05	sp|P42677|RS27_HUMAN	RPS27	P42677
1	78	1897	1871	1897	0.999	1394	3.36518	2.39521E-05	sp|P42704|LPPRC_HUMAN	LRPPRC	P42704
1	25	689	689	689	0.999	397	0.674483	0.0649713	sp|P42765|THIM_HUMAN	ACAA2	P42765
1	13	520	520	520	0.999	123	2.0394	0.000855476	sp|P42766|RL35_HUMAN	RPL35	P42766
1	12	142	93	142	0.999	156	2.80472	0.000135211	sp|P42771|CDN2A_HUMAN	CDKN2A	P42771
1	6	154	154	154	0.999	496	0.449629	0.141276	sp|P42785|PCP_HUMAN	PRCP	P42785
1	10	95	95	95	0.999	3142	1.37391	0.00584838	sp|P42858|HD_HUMAN	HTT	P42858
1	14	100	100	100	0.999	770	3.84666	0	sp|P42892|ECE1_HUMAN	ECE1	P42892
1	22	462	462	599	0.999	410	0.268073	0.270908	sp|P43034|LIS1_HUMAN	PAFAH1B1	P43034
1	13	63	63	63	0.999	646	8.84894	0	sp|P43121|MUC18_HUMAN	MCAM	P43121
1	50	1476	1476	1476	0.999	847	5.37794	0	sp|P43243|MATR3_HUMAN	MATR3	P43243
1	30	438	438	438	0.999	934	1.60718	0.00291085	sp|P43246|MSH2_HUMAN	MSH2	P43246
1	35	782	782	782	0.999	727	3.20494	5.38117E-05	sp|P43304|GPDM_HUMAN	GPD2	P43304
0.9999	2	84	84	84	0.999	286	3.4466	1.97044E-05	sp|P43307|SSRA_HUMAN	SSR1	P43307
1	16	1085	1085	1085	0.999	201	6.24312	0	sp|P43487|RANG_HUMAN	RANBP1	P43487
1	51	1200	1200	1200	0.999	491	2.43847	0.000335161	sp|P43490|NAMPT_HUMAN	NAMPT	P43490
1	4	42	42	42	0.998	599	6.19325	0	sp|P43652|AFAM_HUMAN	AFM	P43652
1	21	1004	895	1004	0.999	418	4.00528	0	sp|P43686|PRS6B_HUMAN	PSMC4	P43686
1	14	300	300	300	0.999	325	2.13864	0.000661695	sp|P43897|EFTS_HUMAN	TSFM	P43897
1	6	117	117	117	0.999	212	3.25658	5.06912E-05	sp|P45877|PPIC_HUMAN	PPIC	P45877
1	19	1021	1021	1021	0.999	294	5.79317	0	sp|P45880|VDAC2_HUMAN	VDAC2	P45880
1	15	386	386	482	0.999	191	3.71385	0	sp|P45973|CBX5_HUMAN	CBX5	P45973
1	38	786	786	786	0.999	858	2.5071	0.000286179	sp|P45974|UBP5_HUMAN	USP5	P45974
1	6	57	40	108	0.999	424	0.000938491	0.983752	sp|P45984|MK09_HUMAN	MAPK9	P45984
1	76	852	852	852	0.999	3256	2.4352	0.000336711	sp|P46013|KI67_HUMAN	MKI67	P46013
0.9979	4	31	5	31	0.999	1223	0.337268	0.209712	sp|P46020|KPB1_HUMAN	PHKA1	P46020
1	25	696	696	696	0.999	587	0.287019	0.251913	sp|P46060|RAGP1_HUMAN	RANGAP1	P46060
1	32	881	831	881	0.999	649	2.78087	0.000141659	sp|P46063|RECQ1_HUMAN	RECQL	P46063
1	27	690	690	690	0.999	812	0.591084	0.0863119	sp|P46087|NOP2_HUMAN	NOP2	P46087
1	11	172	172	172	0.999	304	0.552973	0.0985632	sp|P46108|CRK_HUMAN	CRK	P46108
1	16	261	261	261	0.999	1132	2.00457	0.000920331	sp|P46379|BAG6_HUMAN	BAG6	P46379
1	29	491	491	491	0.999	744	0.197092	0.356421	sp|P46459|NSF_HUMAN	NSF	P46459
1	10	146	80	146	0.999	347	0.220515	0.325577	sp|P46734|MP2K3_HUMAN	MAP2K3	P46734
1	6	90	90	90	0.999	316	1.04092	0.018422	sp|P46736|BRCC3_HUMAN	BRCC3	P46736
1	15	556	556	556	0.999	148	2.20031	0.000562238	sp|P46776|RL27A_HUMAN	RPL27A	P46776
1	48	2210	2210	2210	0.999	297	2.03824	0.000862694	sp|P46777|RL5_HUMAN	RPL5	P46777
1	29	1388	1388	1388	0.999	160	4.26216	0	sp|P46778|RL21_HUMAN	RPL21	P46778
1	27	1462	1462	1462	0.999	137	3.98712	0	sp|P46779|RL28_HUMAN	RPL28	P46779
1	41	1726	1726	1726	0.999	194	1.85295	0.00142212	sp|P46781|RS9_HUMAN	RPS9	P46781
1	32	1141	1141	1141	0.999	204	2.379	0.000406728	sp|P46782|RS5_HUMAN	RPS5	P46782
1	22	1248	1036	1249	0.999	165	4.20836	0	sp|P46783|RS10_HUMAN	RPS10	P46783
1	42	361	346	361	0.999	2468	3.71118	0	sp|P46821|MAP1B_HUMAN	MAP1B	P46821
1	14	464	426	464	0.999	289	0.957828	0.0247113	sp|P46926|GNPI1_HUMAN	GNPDA1	P46926
1	14	319	319	319	0.999	504	1.87564	0.00133094	sp|P46937|YAP1_HUMAN	YAP1	P46937
1	44	305	272	305	0.999	3433	2.58194	0.000248322	sp|P46939|UTRN_HUMAN	UTRN	P46939
1	99	2883	2697	2883	0.999	1657	4.42121	0	sp|P46940|IQGA1_HUMAN	IQGAP1	P46940
1	5	48	48	48	0.999	350	0.441298	0.145121	sp|P46976|GLYG_HUMAN	GYG1	P46976
1	20	478	478	478	0.999	705	2.1519	0.00063388	sp|P46977|STT3A_HUMAN	STT3A	P46977
1	3	52	52	52	0.999	123	1.24334	0.00916029	sp|P47224|MSS4_HUMAN	RABIF	P47224
1	17	379	379	656	0.999	286	1.97853	0.00100566	sp|P47755|CAZA2_HUMAN	CAPZA2	P47755

1	29	975	975	975	0.999	272	4.07085	0	sp|P47756|CAPZB_HUMAN	CAPZB	P47756
1	15	796	311	796	0.999	144	1.6611	0.00247033	sp|P47813|IF1AX_HUMAN	EIF1AX	P47813
1	42	1189	1189	1189	0.999	775	1.98741	0.000978562	sp|P47897|SYQ_HUMAN	QARS1	P47897
1	18	596	596	596	0.999	159	1.41999	0.00503117	sp|P47914|RL29_HUMAN	RPL29	P47914
1	13	341	122	341	0.999	274	3.39421	2.42424E-05	sp|P47985|UCRI_HUMAN	UQCRFS1	P47985
1	14	492	492	492	0.999	213	3.72187	0	sp|P48047|ATPO_HUMAN	ATP5PO	P48047
1	26	316	192	316	0.999	325	4.90281	0	sp|P48059|LIMS1_HUMAN	LIMS1	P48059
1	4	15	15	15	0.9983	93	4.22234	0	sp|P48061|SDF1_HUMAN	CXCL12	P48061
1	32	454	454	454	0.999	710	0.4698	0.132038	sp|P48147|PPCE_HUMAN	PREP	P48147
1	13	185	185	185	0.999	572	1.56708	0.00323548	sp|P48163|MAOX_HUMAN	ME1	P48163
1	9	45	45	45	0.999	235	6.02889	0	sp|P48307|TFPI2_HUMAN	TFPI2	P48307
1	31	837	837	874	0.999	511	4.44968	0	sp|P48444|COPD_HUMAN	ARCN1	P48444
1	20	356	356	356	0.999	732	0.557602	0.0969661	sp|P48449|LSS_HUMAN	LSS	P48449
1	6	230	230	230	0.999	274	0.454543	0.139129	sp|P48507|GSH0_HUMAN	GCLM	P48507
1	13	304	268	304	0.999	253	3.22161	5.43601E-05	sp|P48509|CD151_HUMAN	CD151	P48509
1	10	304	304	304	0.999	350	2.43737	0.000334636	sp|P48556|PSMD8_HUMAN	PSMD8	P48556
1	22	324	324	380	0.999	2157	3.12251	5.18919E-05	sp|P48634|PRC2A_HUMAN	PRRC2A	P48634
1	13	126	126	126	0.999	474	1.39422	0.00546094	sp|P48637|GSHB_HUMAN	GSS	P48637
1	56	1843	1843	1956	0.999	541	4.03598	0	sp|P48643|TCPE_HUMAN	CCT5	P48643
1	35	161	161	161	0.999	1621	2.11704	0.000702557	sp|P48681|NEST_HUMAN	NES	P48681
1	5	21	21	21	0.999	471	0.0965977	0.546986	sp|P48723|HSP13_HUMAN	HSPA13	P48723
1	14	252	178	252	0.999	337	1.50099	0.0038219	sp|P48729|KC1A_HUMAN	CSNK1A1	P48729
1	19	371	371	484	0.999	452	4.77365	0	sp|P48735|IDHP_HUMAN	IDH2	P48735
1	20	495	446	495	0.999	271	4.872	0	sp|P48739|PIPNB_HUMAN	PITPNB	P48739
1	14	114	99	114	0.999	835	4.18864	0	sp|P48960|AGRE5_HUMAN	ADGRE5	P48960
1	10	70	70	70	0.999	469	1.17412	0.0115239	sp|P49005|DPOD2_HUMAN	POLD2	P49005
1	9	171	171	172	0.999	195	3.29176	4.21546E-05	sp|P49006|MRP_HUMAN	MARCKSL1	P49006
1	25	764	764	764	0.999	591	0.333702	0.212418	sp|P49023|PAXI_HUMAN	PXN	P49023
1	9	79	53	79	0.999	400	0.612301	0.0801454	sp|P49137|MAPK2_HUMAN	MAPKAPK2	P49137
1	26	808	808	808	0.999	494	0.712352	0.0573583	sp|P49189|AL9A1_HUMAN	ALDH9A1	P49189
1	23	750	750	750	0.999	117	4.25931	0	sp|P49207|RL34_HUMAN	RPL34	P49207
1	22	817	817	817	0.999	510	7.75811	0	sp|P49257|LMAN1_HUMAN	LMAN1	P49257
1	32	1036	1036	1036	0.999	788	4.26534	0	sp|P49321|NASP_HUMAN	NASP	P49321
1	178	5910	5857	5910	0.999	2511	1.48578	0.00402438	sp|P49327|FAS_HUMAN	FASN	P49327
1	5	106	106	106	0.999	369	1.04327	0.0182802	sp|P49366|DHYS_HUMAN	DHPS	P49366
1	49	2126	2116	2126	0.999	545	1.04682	0.0180772	sp|P49368|TCPG_HUMAN	CCT3	P49368
1	13	207	207	207	0.999	292	1.27047	0.00839778	sp|P49406|RM19_HUMAN	MRPL19	P49406
1	35	1401	1401	1401	0.999	455	4.90221	0	sp|P49411|EFTU_HUMAN	TUFM	P49411
1	28	836	836	836	0.999	539	1.9357	0.00113617	sp|P49419|AL7A1_HUMAN	ALDH7A1	P49419
1	9	34	34	34	0.999	3114	2.25764	0.000486681	sp|P49454|CENPF_HUMAN	CENPF	P49454
1	14	423	423	423	0.999	86	2.36715	0.000414523	sp|P49458|SRP09_HUMAN	SRP9	P49458
1	11	210	209	210	0.999	367	5.75009	0	sp|P49585|PCY1A_HUMAN	PCYT1A	P49585
1	52	1625	1625	1625	0.999	968	2.58783	0.000248949	sp|P49588|SYAC_HUMAN	AARS1	P49588
1	31	704	704	704	0.999	748	2.89978	0.00012853	sp|P49589|SYCC_HUMAN	CARS1	P49589
1	36	691	691	691	0.999	514	0.474033	0.129987	sp|P49591|SYSC_HUMAN	SARS1	P49591
1	5	111	111	111	0.999	454	0.447654	0.14216	sp|P49593|PPM1F_HUMAN	PPM1F	P49593
1	7	104	104	104	0.999	420	1.27615	0.00824304	sp|P49642|PRI1_HUMAN	PRIM1	P49642
1	9	73	73	73	0.999	509	2.68301	0.000190644	sp|P49643|PRI2_HUMAN	PRIM2	P49643
1	7	66	65	66	0.999	377	0.799881	0.0424643	sp|P49662|CASP4_HUMAN	CASP4	P49662
1	10	187	123	187	0.999	727	0.758032	0.0491013	sp|P49711|CTCF_HUMAN	CTCF	P49711
1	8	382	382	382	0.999	205	0.684734	0.0627431	sp|P49720|PSB3_HUMAN	PSMB3	P49720
1	16	576	576	576	0.999	201	5.9043	0	sp|P49721|PSB2_HUMAN	PSMB2	P49721
1	51	1297	1297	1297	0.999	904	2.31735	0.000454343	sp|P49736|MCM2_HUMAN	MCM2	P49736
1	7	24	24	39	0.999	757	3.03497	7.06127E-05	sp|P49747|COMP_HUMAN	COMP	P49747
1	24	687	687	687	0.999	655	5.53122	0	sp|P49748|ACADV_HUMAN	ACADVL	P49748
1	21	206	206	206	0.999	2146	0.114936	0.503635	sp|P49750|YLPM1_HUMAN	YLPM1	P49750
1	12	200	5	200	0.999	483	4.2374	0	sp|P49753|ACOT2_HUMAN	ACOT2	P49753
1	17	564	564	564	0.999	219	7.41603	0	sp|P49755|TMEDA_HUMAN	TMED10	P49755
1	30	644	644	644	0.999	843	2.634	0.000209802	sp|P49756|RBM25_HUMAN	RBM25	P49756
1	10	104	72	104	0.999	651	1.84467	0.0014524	sp|P49757|NUMB_HUMAN	NUMB	P49757
1	15	189	189	189	0.999	351	1.16862	0.0117442	sp|P49770|EI2BB_HUMAN	EIF2B2	P49770
1	17	1116	1116	1116	0.999	126	0.431872	0.149992	sp|P49773|HINT1_HUMAN	HINT1	P49773
1	32	462	462	462	0.999	1475	0.364778	0.190018	sp|P49790|NU153_HUMAN	NUP153	P49790
1	98	1673	1144	1673	0.999	3224	0.700874	0.0595771	sp|P49792|RBP2_HUMAN	RANBP2	P49792
1	19	333	333	333	0.999	464	0.954379	0.0249865	sp|P49821|NDUV1_HUMAN	NDUFV1	P49821
1	8	89	89	270	0.999	483	0.661861	0.0677756	sp|P49840|GSK3A_HUMAN	GSK3A	P49840
1	12	317	136	317	0.999	420	0.0449037	0.707951	sp|P49841|GSK3B_HUMAN	GSK3B	P49841
1	15	244	244	244	0.999	561	1.44416	0.00462494	sp|P49902|5NTC_HUMAN	NT5C2	P49902
1	9	256	256	256	0.999	392	0.761779	0.0485023	sp|P49903|SPS1_HUMAN	SEPHS1	P49903
1	36	1023	1023	1023	0.999	693	2.84616	0.00013346	sp|P49915|GUAA_HUMAN	GMPS	P49915
1	16	362	362	362	0.999	708	1.2162	0.010064	sp|P49959|MRE11_HUMAN	MRE11	P49959
1	22	603	603	603	0.999	366	4.96133	0	sp|P50213|IDH3A_HUMAN	IDH3A	P50213
1	6	149	149	149	0.999	77	3.01204	7.38462E-05	sp|P50238|CRIP1_HUMAN	CRIP1	P50238
1	19	100	99	100	0.999	582	7.31916	0	sp|P50281|MMP14_HUMAN	MMP14	P50281
1	7	79	79	79	0.999	477	1.00471	0.0209269	sp|P50336|PPOX_HUMAN	PPOX	P50336
1	53	1736	1314	1736	0.999	445	4.68624	0	sp|P50395|GDIB_HUMAN	GDI2	P50395
1	10	311	311	311	0.999	254	5.74267	0	sp|P50402|EMD_HUMAN	EMD	P50402
1	20	470	470	470	0.999	773	4.10189	0	sp|P50416|CPT1A_HUMAN	CPT1A	P50416
1	15	174	146	261	0.999	374	1.26644	0.00847993	sp|P50452|SPB8_HUMAN	SERPINB8	P50452
1	20	69	69	134	0.999	376	1.4675	0.00426236	sp|P50453|SPB9_HUMAN	SERPINB9	P50453
1	36	1305	1305	1305	0.999	418	3.52358	2.02276E-05	sp|P50454|SERPH_HUMAN	SERPINH1	P50454
1	15	178	178	178	0.999	330	1.95167	0.00108933	sp|P50479|PDLI4_HUMAN	PDLIM4	P50479
1	28	699	116	699	0.999	369	0.7671	0.0476087	sp|P50502|F10A1_HUMAN	ST13	P50502
1	29	727	727	727	0.999	380	2.21813	0.000530699	sp|P50552|VASP_HUMAN	VASP	P50552
1	32	738	372	738	0.999	870	1.32014	0.00705716	sp|P50570|DYN2_HUMAN	DNM2	P50570
1	20	570	570	570	0.999	478	2.59718	0.000244068	sp|P50579|MAP2_HUMAN	METAP2	P50579
1	5	121	121	121	0.999	147	1.37336	0.00585432	sp|P50583|AP4A_HUMAN	NUDT2	P50583
1	6	66	53	66	0.999	346	2.20915	0.000556962	sp|P50613|CDK7_HUMAN	CDK7	P50613
1	14	91	91	91	0.999	2209	4.21487	0	sp|P50748|KNTC1_HUMAN	KNTC1	P50748
1	31	436	436	436	0.999	2863	5.84762	0	sp|P50851|LRBA_HUMAN	LRBA	P50851
1	14	151	151	151	0.999	628	0.316265	0.226191	sp|P50895|BCAM_HUMAN	BCAM	P50895
1	6	239	239	239	0.999	306	3.29017	4.21053E-05	sp|P50897|PPT1_HUMAN	PPT1	P50897
1	32	1177	1177	1177	0.999	215	3.06891	5.87618E-05	sp|P50914|RL14_HUMAN	RPL14	P50914
1	61	2787	2787	2787	0.999	548	6.42164	0	sp|P50990|TCPQ_HUMAN	CCT8	P50990
1	51	2234	2121	2234	0.999	539	5.6588	0	sp|P50991|TCPD_HUMAN	CCT4	P50991
1	30	600	575	601	0.999	505	9.81206	0	sp|P50995|ANX11_HUMAN	ANXA11	P50995
1	8	101	66	101	0.999	745	0.45005	0.141103	sp|P51003|PAPOA_HUMAN	PAPOLA	P51003
1	25	587	401	587	0.999	621	6.23357	0	sp|P51114|FXR1_HUMAN	FXR1	P51114
1	13	244	244	430	0.999	673	2.05002	0.000836482	sp|P51116|FXR2_HUMAN	FXR2	P51116
1	12	522	301	522	0.999	216	2.72607	0.000173756	sp|P51148|RAB5C_HUMAN	RAB5C	P51148
1	18	627	627	627	0.999	207	5.36835	0	sp|P51149|RAB7A_HUMAN	RAB7A	P51149
1	3	60	32	60	0.999	201	1.65233	0.00252574	sp|P51151|RAB9A_HUMAN	RAB9A	P51151
1	11	295	295	494	0.999	203	1.10906	0.0145054	sp|P51153|RAB13_HUMAN	RAB13	P51153
1	7	118	80	118	0.999	221	3.46237	1.98265E-05	sp|P51159|RB27A_HUMAN	RAB27A	P51159
1	6	109	109	109	0.999	102	1.94245	0.00111386	sp|P51397|DAP1_HUMAN	DAP	P51397
0.9998	2	56	56	56	0.999	185	0.407112	0.163989	sp|P51452|DUS3_HUMAN	DUSP3	P51452
1	29	302	209	302	0.999	1647	0.659585	0.06828	sp|P51532|SMCA4_HUMAN	SMARCA4	P51532
1	6	144	144	144	0.999	393	0.166232	0.403641	sp|P51553|IDH3G_HUMAN	IDH3G	P51553
1	20	230	230	230	0.999	392	4.46239	0	sp|P51570|GALK1_HUMAN	GALK1	P51570
1	10	347	347	347	0.999	173	4.11527	0	sp|P51571|SSRD_HUMAN	SSR4	P51571
1	23	646	646	646	0.999	246	0.512439	0.114013	sp|P51572|BAP31_HUMAN	BCAP31	P51572
1	7	120	120	120	0.999	245	5.34492	0	sp|P51580|TPMT_HUMAN	TPMT	P51580
1	28	413	411	413	0.999	2035	1.86267	0.00138507	sp|P51610|HCFC1_HUMAN	HCFC1	P51610
1	18	320	291	320	0.999	485	4.17479	0	sp|P51648|AL3A2_HUMAN	ALDH3A2	P51648
1	33	682	682	682	0.999	736	2.25324	0.000488506	sp|P51659|DHB4_HUMAN	HSD17B4	P51659
1	13	471	471	471	0.999	324	0.365268	0.189685	sp|P51665|PSMD7_HUMAN	PSMD7	P51665
1	24	257	256	362	0.999	740	1.23247	0.00952813	sp|P51812|KS6A3_HUMAN	RPS6KA3	P51812
1	28	883	865	883	0.999	240	5.68016	0	sp|P51858|HDGF_HUMAN	HDGF	P51858
1	7	54	54	54	0.9989	338	7.34975	0	sp|P51884|LUM_HUMAN	LUM	P51884
1	7	110	110	110	0.999	323	1.76615	0.00179263	sp|P51946|CCNH_HUMAN	CCNH	P51946
1	5	90	90	90	0.999	309	1.32886	0.00685687	sp|P51948|MAT1_HUMAN	MNAT1	P51948
1	10	197	197	197	0.999	172	0.861565	0.0345238	sp|P51970|NDUA8_HUMAN	NDUFA8	P51970
1	36	1815	1815	2068	0.999	378	3.90328	0	sp|P51991|ROA3_HUMAN	HNRNPA3	P51991
1	32	1254	1254	1254	0.999	483	1.29695	0.00765388	sp|P52209|6PGD_HUMAN	PGD	P52209
1	95	4335	4242	4335	0.999	730	6.58297	0	sp|P52272|HNRPM_HUMAN	HNRNPM	P52272
1	17	350	350	350	0.999	529	2.38987	0.000380952	sp|P52292|IMA1_HUMAN	KPNA2	P52292
1	13	313	212	313	0.999	538	4.76959	0	sp|P52294|IMA5_HUMAN	KPNA1	P52294
1	9	178	136	178	0.999	156	0.56899	0.0931558	sp|P52298|NCBP2_HUMAN	NCBP2	P52298
1	12	256	200	256	0.999	607	0.512219	0.114079	sp|P52306|GDS1_HUMAN	RAP1GDS1	P52306

1	19	1228	1228	1228	0.999	204	4.08092	0	sp|P52565|GDIR1_HUMAN	ARHGDIA	P52565
1	18	268	268	268	0.999	201	0.108951	0.517673	sp|P52566|GDIR2_HUMAN	ARHGDIB	P52566
1	8	126	126	126	0.999	562	0.921028	0.02806	sp|P52594|AGFG1_HUMAN	AGFG1	P52594
1	25	717	717	850	0.999	415	5.83081	0	sp|P52597|HNRPF_HUMAN	HNRNPF	P52597
1	7	14	13	14	0.999	851	1.26719	0.00846353	sp|P52630|STAT2_HUMAN	STAT2	P52630
1	32	444	441	444	0.999	1360	0.453662	0.139439	sp|P52701|MSH6_HUMAN	MSH6	P52701
1	18	195	195	195	0.999	1056	2.46725	0.000317712	sp|P52732|KIF11_HUMAN	KIF11	P52732
1	17	221	221	221	0.999	878	0.511762	0.114226	sp|P52735|VAV2_HUMAN	VAV2	P52735
1	17	779	779	779	0.999	366	2.1136	0.000703679	sp|P52788|SPSY_HUMAN	SMS	P52788
1	35	408	408	570	0.999	917	0.758956	0.048947	sp|P52789|HXK2_HUMAN	HK2	P52789
1	12	303	303	303	0.999	198	2.32315	0.000453393	sp|P52815|RM12_HUMAN	MRPL12	P52815
1	5	36	36	36	0.999	247	5.03518	0	sp|P52823|STC1_HUMAN	STC1	P52823
1	31	740	740	740	0.999	689	0.255556	0.283982	sp|P52888|THOP1_HUMAN	THOP1	P52888
1	25	994	717	994	0.999	286	2.75692	0.000150597	sp|P52907|CAZA1_HUMAN	CAPZA1	P52907
1	4	55	55	55	0.999	109	1.931	0.00114954	sp|P52926|HMGA2_HUMAN	HMGA2	P52926
1	12	207	207	207	0.999	208	2.99902	8.14664E-05	sp|P52943|CRIP2_HUMAN	CRIP2	P52943
1	25	368	368	368	0.999	1817	0.402272	0.166619	sp|P52948|NUP98_HUMAN	NUP98	P52948
1	22	427	427	427	0.999	296	1.35175	0.00630622	sp|P53004|BIEA_HUMAN	BLVRA	P53004
1	18	427	427	427	0.999	311	1.75315	0.00186819	sp|P53007|TXTP_HUMAN	SLC25A1	P53007
1	16	332	332	332	0.999	499	1.16952	0.0117052	sp|P53041|PPP5_HUMAN	PPP5C	P53041
1	5	18	18	18	0.999	603	2.04311	0.000855101	sp|P53350|PLK1_HUMAN	PLK1	P53350
1	7	124	124	124	0.999	341	0.859365	0.0347697	sp|P53365|ARFP2_HUMAN	ARFIP2	P53365
1	9	74	74	74	0.999	373	2.01192	0.00090595	sp|P53367|ARFP1_HUMAN	ARFIP1	P53367
1	9	335	335	335	0.999	320	0.706948	0.0583038	sp|P53384|NUBP1_HUMAN	NUBP1	P53384
1	64	2742	2742	2742	0.999	1101	0.352249	0.199082	sp|P53396|ACLY_HUMAN	ACLY	P53396
1	18	496	496	496	0.999	386	7.09763	0	sp|P53582|MAP11_HUMAN	METAP1	P53582
1	14	317	317	317	0.999	346	2.29514	0.000457143	sp|P53597|SUCA_HUMAN	SUCLG1	P53597
1	4	103	103	103	0.999	331	0.437865	0.146844	sp|P53611|PGTB2_HUMAN	RABGGTB	P53611
1	33	881	881	881	0.999	953	3.59423	0	sp|P53618|COPB_HUMAN	COPB1	P53618
1	82	2381	2381	2381	0.999	1224	2.61143	0.000232082	sp|P53621|COPA_HUMAN	COPA	P53621
1	13	431	431	431	0.999	463	1.51512	0.00365896	sp|P53634|CATC_HUMAN	CTSC	P53634
1	4	101	101	101	0.999	142	5.42675	0	sp|P53680|AP2S1_HUMAN	AP2S1	P53680
1	10	257	257	257	0.999	268	2.01127	0.000905371	sp|P53701|CCHL_HUMAN	HCCS	P53701
1	4	71	71	71	0.999	180	3.84198	0	sp|P53801|PTTG_HUMAN	PTTG1IP	P53801
1	15	93	93	93	0.999	917	1.91882	0.00117359	sp|P53814|SMTN_HUMAN	SMTN	P53814
1	8	280	280	280	0.999	500	1.78431	0.00169409	sp|P53985|MOT1_HUMAN	SLC16A1	P53985
1	7	105	105	105	0.999	364	5.47904	0	sp|P53990|IST1_HUMAN	IST1	P53990
1	22	410	396	410	0.999	1094	1.61248	0.00286268	sp|P53992|SC24C_HUMAN	SEC24C	P53992
1	17	512	512	512	0.999	127	3.62134	0	sp|P53999|TCP4_HUMAN	SUB1	P53999
1	3	55	55	55	0.999	237	3.00364	8.17996E-05	sp|P54105|ICLN_HUMAN	CLNS1A	P54105
1	40	1298	1298	1298	0.999	660	0.437018	0.147194	sp|P54136|SYRC_HUMAN	RARS1	P54136
1	6	14	14	14	0.999	1103	6.03939	0	sp|P54289|CA2D1_HUMAN	CACNA2D1	P54289
1	54	1564	1564	1564	0.999	528	1.87897	0.00131576	sp|P54577|SYYC_HUMAN	YARS1	P54577
1	25	617	617	617	0.999	494	2.35267	0.000431047	sp|P54578|UBP14_HUMAN	USP14	P54578
1	43	83	78	1235	0.999	639	2.94926	0.000115079	sp|P54652|HSP72_HUMAN	HSPA2	P54652
1	14	239	239	239	0.999	279	6.89092	0	sp|P54709|AT1B3_HUMAN	ATP1B3	P54709
1	11	249	196	249	0.999	409	4.12033	0	sp|P54727|RD23B_HUMAN	RAD23B	P54727
1	24	714	714	714	0.999	239	6.47404	0	sp|P54819|KAD2_HUMAN	AK2	P54819
1	36	1380	1380	1380	0.999	795	1.91373	0.00118708	sp|P54886|P5CS_HUMAN	ALDH18A1	P54886
1	12	249	226	249	0.999	295	2.46709	0.00031746	sp|P54920|SNAA_HUMAN	NAPA	P54920
1	21	242	242	242	0.999	431	2.16739	0.000617505	sp|P55010|IF5_HUMAN	EIF5	P55010
1	14	368	349	368	0.999	377	3.64122	0	sp|P55036|PSMD4_HUMAN	PSMD4	P55036
1	12	166	166	232	0.999	364	0.405479	0.164884	sp|P55039|DRG2_HUMAN	DRG2	P55039
0.9969	2	10	10	10	0.9989	493	3.33371	2.37812E-05	sp|P55058|PLTP_HUMAN	PLTP	P55058
1	48	1421	1421	1421	0.999	971	6.60798	0	sp|P55060|XPO2_HUMAN	CSE1L	P55060
0.997	2	24	24	24	0.999	237	4.41818	0	sp|P55061|BI1_HUMAN	TMBIM6	P55061
1	74	2850	2850	2850	0.999	806	5.11454	0	sp|P55072|TERA_HUMAN	VCP	P55072
1	11	170	170	170	0.999	439	0.156883	0.419579	sp|P55081|MFAP1_HUMAN	MFAP1	P55081
1	37	985	985	985	0.999	474	3.3033	3.76914E-05	sp|P55084|ECHB_HUMAN	HADHB	P55084
1	21	435	435	435	0.999	182	4.75063	0	sp|P55145|MANF_HUMAN	MANF	P55145
1	27	174	174	174	0.999	1824	0.0668822	0.629684	sp|P55196|AFAD_HUMAN	AFDN	P55196
1	18	753	581	753	0.999	391	2.6259	0.000218803	sp|P55209|NP1L1_HUMAN	NAP1L1	P55209
1	8	61	61	61	0.999	303	1.19268	0.0108541	sp|P55210|CASP7_HUMAN	CASP7	P55210
1	15	398	398	398	0.999	362	0.441749	0.144899	sp|P55263|ADK_HUMAN	ADK	P55263
1	48	1205	1205	1205	0.999	1226	1.46456	0.00427996	sp|P55265|DSRAD_HUMAN	ADAR	P55265
1	15	87	87	87	0.999	1798	4.94107	0	sp|P55268|LAMB2_HUMAN	LAMB2	P55268
1	7	34	34	34	0.999	796	0.0244954	0.800631	sp|P55287|CAD11_HUMAN	CDH11	P55287
1	5	60	60	60	0.999	713	7.49253	0	sp|P55290|CAD13_HUMAN	CDH13	P55290
1	14	459	459	459	0.999	322	1.8532	0.00142296	sp|P55735|SEC13_HUMAN	SEC13	P55735
1	9	163	163	163	0.999	128	0.465935	0.133915	sp|P55769|NH2L1_HUMAN	SNU13	P55769
1	52	1654	1014	1654	0.999	919	1.73317	0.00201134	sp|P55786|PSA_HUMAN	NPEPPS	P55786
1	32	325	325	947	0.999	449	3.27104	4.64037E-05	sp|P55795|HNRH2_HUMAN	HNRNPH2	P55795
1	27	466	425	466	0.999	520	1.15171	0.0125248	sp|P55809|SCOT1_HUMAN	OXCT1	P55809
1	46	1241	1241	1241	0.999	814	2.85586	0.000129895	sp|P55884|EIF3B_HUMAN	EIF3B	P55884
1	7	159	159	159	0.999	195	2.63798	0.000203273	sp|P55957|BID_HUMAN	BID	P55957
1	3	97	97	97	0.999	94	5.29598	0	sp|P56134|ATPK_HUMAN	ATP5MF	P56134
1	12	228	228	228	0.999	461	0.05041	0.686661	sp|P56182|RRP1_HUMAN	RRP1	P56182
1	45	1466	1466	1466	0.999	900	2.28374	0.00046973	sp|P56192|SYMC_HUMAN	MARS1	P56192
1	27	292	292	292	0.999	1179	8.38806	0	sp|P56199|ITA1_HUMAN	ITGA1	P56199
1	5	88	88	180	0.999	112	0.385852	0.176498	sp|P56211|ARP19_HUMAN	ARPP19	P56211
1	6	195	31	195	0.999	51	2.64671	0.000204506	sp|P56381|ATP5E_HUMAN	ATP5F1E	P56381
1	7	173	173	173	0.999	69	4.19208	0	sp|P56385|ATP5I_HUMAN	ATP5ME	P56385
1	7	295	295	295	0.999	245	5.28928	0	sp|P56537|IF6_HUMAN	EIF6	P56537
1	17	389	174	389	0.999	445	3.47741	0.000019975	sp|P56545|CTBP2_HUMAN	CTBP2	P56545
1	4	63	63	63	0.999	128	2.89754	0.000128405	sp|P56556|NDUA6_HUMAN	NDUFA6	P56556
1	5	85	66	85	0.999	870	2.82622	0.000132325	sp|P56945|BCAR1_HUMAN	BCAR1	P56945
1	25	1324	2	1324	0.999	126	2.95167	0.000111221	sp|P57053|H2BFS_HUMAN	H2BC12L	P57053
1	7	185	185	185	0.999	247	1.59061	0.00301384	sp|P57088|TMM33_HUMAN	TMEM33	P57088
1	5	163	163	163	0.999	145	3.07475	5.89474E-05	sp|P57105|SYJ2B_HUMAN	SYNJ2BP	P57105
1	6	89	17	89	0.999	1058	3.23163	5.47945E-05	sp|P57678|GEMI4_HUMAN	GEMIN4	P57678
1	10	114	114	114	0.999	925	1.95386	0.0010839	sp|P57737|CORO7_HUMAN	CORO7	P57737
1	20	533	533	533	0.999	925	2.56903	0.00026888	sp|P57740|NU107_HUMAN	NUP107	P57740
1	11	226	226	226	0.999	596	0.312296	0.22949	sp|P57772|SELB_HUMAN	EEFSEC	P57772
1	6	234	234	234	0.999	118	0.908069	0.0293134	sp|P58546|MTPN_HUMAN	MTPN	P58546
1	19	283	283	283	0.999	168	1.47837	0.00410137	sp|P59998|ARPC4_HUMAN	ARPC4	P59998
1	6	76	76	76	0.999	236	7.68842	0	sp|P60033|CD81_HUMAN	CD81	P60033
1	33	1812	1812	1812	0.999	249	2.48411	0.000298077	sp|P60174|TPIS_HUMAN	TPI1	P60174
1	26	687	687	687	0.999	445	1.17498	0.0115007	sp|P60228|EIF3E_HUMAN	EIF3E	P60228
1	7	267	267	267	0.999	96	5.53841	0	sp|P60468|SC61B_HUMAN	SEC61B	P60468
1	12	150	150	211	0.999	307	4.12402	0	sp|P60510|PP4C_HUMAN	PPP4C	P60510
1	4	123	123	123	0.999	79	0.252299	0.287677	sp|P60602|ROMO1_HUMAN	ROMO1	P60602
1	4	79	79	79	0.999	165	2.41626	0.000351751	sp|P60604|UB2G2_HUMAN	UBE2G2	P60604
1	18	1193	927	1193	0.999	151	4.12819	0	sp|P60660|MYL6_HUMAN	MYL6	P60660
1	57	8145	24	8145	0.999	375	2.50245	0.000291971	sp|P60709|ACTB_HUMAN	ACTB	P60709
1	48	2018	914	2018	0.999	406	6.08978	0	sp|P60842|IF4A1_HUMAN	EIF4A1	P60842
1	22	921	921	921	0.999	119	2.96496	9.60961E-05	sp|P60866|RS20_HUMAN	RPS20	P60866
1	25	519	163	519	0.999	318	0.654291	0.0695801	sp|P60891|PRPS1_HUMAN	PRPS1	P60891
1	28	1050	1050	1050	0.999	246	2.69633	0.000184889	sp|P60900|PSA6_HUMAN	PSMA6	P60900
1	13	634	634	634	0.999	97	6.99429	0	sp|P60903|S10AA_HUMAN	S100A10	P60903
1	15	665	529	665	0.999	191	6.85083	0	sp|P60953|CDC42_HUMAN	CDC42	P60953
1	24	795	795	1200	0.999	165	3.08535	5.93849E-05	sp|P60981|DEST_HUMAN	DSTN	P60981
1	11	281	208	281	0.999	142	2.66209	0.000195804	sp|P60983|GMFB_HUMAN	GMFB	P60983
1	19	417	262	791	0.999	207	8.00883	0	sp|P61006|RAB8A_HUMAN	RAB8A	P61006
1	9	219	219	219	0.999	180	1.72792	0.00202483	sp|P61009|SPCS3_HUMAN	SPCS3	P61009
1	19	466	466	466	0.999	504	3.78133	0	sp|P61011|SRP54_HUMAN	SRP54	P61011
1	13	463	216	463	0.999	212	0.26372	0.275101	sp|P61019|RAB2A_HUMAN	RAB2A	P61019
1	7	107	107	300	0.999	215	0.78481	0.0446396	sp|P61020|RAB5B_HUMAN	RAB5B	P61020
1	4	103	103	103	0.999	79	1.52798	0.00353647	sp|P61024|CKS1_HUMAN	CKS1B	P61024
1	19	596	427	722	0.999	200	7.02148	0	sp|P61026|RAB10_HUMAN	RAB10	P61026
1	5	379	40	379	0.999	147	1.68055	0.00234017	sp|P61077|UB2D3_HUMAN	UBE2D3	P61077
1	12	256	256	256	0.999	183	3.78502	0	sp|P61081|UBC12_HUMAN	UBE2M	P61081
1	9	259	259	259	0.999	200	0.541906	0.102543	sp|P61086|UBE2K_HUMAN	UBE2K	P61086
1	10	556	293	556	0.999	152	4.10711	0	sp|P61088|UBE2N_HUMAN	UBE2N	P61088
1	19	468	468	635	0.999	215	2.50129	0.000291498	sp|P61106|RAB14_HUMAN	RAB14	P61106
1	36	1387	1225	1387	0.999	418	5.09004	0	sp|P61158|ARP3_HUMAN	ACTR3	P61158
1	33	1007	1007	1007	0.999	394	1.80843	0.00159298	sp|P61160|ARP2_HUMAN	ACTR2	P61160
1	17	627	333	627	0.999	376	0.382784	0.178436	sp|P61163|ACTZ_HUMAN	ACTR1A	P61163
1	2	76	76	76	0.999	79	2.82291	0.000131951	sp|P61165|TM258_HUMAN	TMEM258	P61165
1	16	316	316	316	0.999	443	1.28308	0.00806151	sp|P61201|CSN2_HUMAN	COPS2	P61201

1	9	470	0	470	0.999	181	0.506983	0.116113	sp|P61204|ARF3_HUMAN	ARF3	P61204
1	34	855	855	855	0.999	599	2.62935	0.000215753	sp|P61221|ABCE1_HUMAN	ABCE1	P61221
1	13	91	0	372	0.999	184	9.0278	0	sp|P61224|RAP1B_HUMAN	RAP1B	P61224
1	62	2997	2997	2997	0.999	264	3.85837	0	sp|P61247|RS3A_HUMAN	RPS3A	P61247
1	31	1503	451	1503	0.999	145	1.37828	0.00575496	sp|P61254|RL26_HUMAN	RPL26	P61254
1	17	545	545	545	0.999	254	4.73429	0	sp|P61289|PSME3_HUMAN	PSME3	P61289
1	31	1303	1303	1303	0.999	204	4.27172	0	sp|P61313|RL15_HUMAN	RPL15	P61313
1	13	392	0	392	0.999	146	4.86325	0	sp|P61326|MGN_HUMAN	MAGOH	P61326
1	24	995	995	995	0.999	136	3.65761	0	sp|P61353|RL27_HUMAN	RPL27	P61353
1	5	59	57	59	0.999	351	1.56952	0.00321353	sp|P61421|VA0D1_HUMAN	ATP6V0D1	P61421
1	14	532	414	532	0.999	92	4.16843	0	sp|P61513|RL37A_HUMAN	RPL37A	P61513
1	17	697	313	772	0.999	193	4.04834	0	sp|P61586|RHOA_HUMAN	RHOA	P61586
1	4	12	12	12	0.999	244	3.72248	0	sp|P61587|RND3_HUMAN	RND3	P61587
1	28	1402	1402	1402	0.999	102	7.67066	0	sp|P61604|CH10_HUMAN	HSPE1	P61604
1	13	493	252	493	0.999	476	5.97384	0	sp|P61619|S61A1_HUMAN	SEC61A1	P61619
1	18	337	337	337	0.999	197	2.67514	0.000190141	sp|P61758|PFD3_HUMAN	VBP1	P61758
1	19	166	166	168	0.999	594	0.141986	0.446704	sp|P61764|STXB1_HUMAN	STXBP1	P61764
1	8	379	379	379	0.999	119	4.37162	0	sp|P61769|B2MG_HUMAN	B2M	P61769
1	3	56	56	56	0.999	113	4.24512	0	sp|P61803|DAD1_HUMAN	DAD1	P61803
1	12	127	127	127	0.999	151	0.369881	0.186436	sp|P61916|NPC2_HUMAN	NPC2	P61916
1	6	154	154	154	0.999	177	2.47081	0.000308917	sp|P61923|COPZ1_HUMAN	COPZ1	P61923
1	17	329	329	329	0.999	97	1.34452	0.00645081	sp|P61927|RL37_HUMAN	RPL37	P61927
1	9	284	195	284	0.999	95	2.72555	0.000173442	sp|P61956|SUMO2_HUMAN	SUMO2	P61956
1	5	98	98	98	0.999	342	0.993654	0.0217663	sp|P61962|DCAF7_HUMAN	DCAF7	P61962
1	5	58	58	58	0.999	334	0.455876	0.138644	sp|P61964|WDR5_HUMAN	WDR5	P61964
1	6	242	242	242	0.999	127	0.224004	0.32123	sp|P61970|NTF2_HUMAN	NUTF2	P61970
1	41	2255	2255	2255	0.999	463	5.5601	0	sp|P61978|HNRPK_HUMAN	HNRNPK	P61978
1	41	1530	1210	1929	0.999	247	0.322509	0.221173	sp|P61981|1433G_HUMAN	YWHAG	P61981
1	14	174	156	174	0.999	204	7.43312	0	sp|P62070|RRAS2_HUMAN	RRAS2	P62070
1	5	90	90	90	0.999	90	1.0007	0.0212544	sp|P62072|TIM10_HUMAN	TIMM10	P62072
0.9998	2	5	5	5	0.999	268	3.74865	0	sp|P62079|TSN5_HUMAN	TSPAN5	P62079
1	34	1243	1243	1243	0.999	194	1.81302	0.00157469	sp|P62081|RS7_HUMAN	RPS7	P62081
1	17	818	226	818	0.999	330	4.10453	0	sp|P62136|PP1A_HUMAN	PPP1CA	P62136
1	17	174	174	627	0.999	327	2.91397	0.000125122	sp|P62140|PP1B_HUMAN	PPP1CB	P62140
1	29	850	850	959	0.999	440	0.558644	0.0966115	sp|P62191|PRS4_HUMAN	PSMC1	P62191
1	32	878	878	987	0.999	406	1.33002	0.00682732	sp|P62195|PRS8_HUMAN	PSMC5	P62195
1	27	1526	1526	1526	0.999	208	3.68962	0	sp|P62241|RS8_HUMAN	RPS8	P62241
1	17	978	978	978	0.999	130	3.3539	2.38663E-05	sp|P62244|RS15A_HUMAN	RPS15A	P62244
1	28	1483	1483	1483	0.999	146	1.43207	0.00484776	sp|P62249|RS16_HUMAN	RPS16	P62249
1	35	1477	1477	1625	0.999	255	0.901826	0.0299911	sp|P62258|1433E_HUMAN	YWHAE	P62258
1	14	634	634	634	0.999	151	7.64057	0	sp|P62263|RS14_HUMAN	RPS14	P62263
1	16	668	668	668	0.999	143	1.46714	0.00426425	sp|P62266|RS23_HUMAN	RPS23	P62266
1	32	1388	1388	1388	0.999	152	1.32985	0.00683167	sp|P62269|RS18_HUMAN	RPS18	P62269
1	14	338	338	338	0.999	56	2.46161	0.00031962	sp|P62273|RS29_HUMAN	RPS29	P62273
1	26	1098	1098	1098	0.999	151	2.24194	0.0005068	sp|P62277|RS13_HUMAN	RPS13	P62277
1	33	1614	1614	1614	0.999	158	1.96274	0.00106433	sp|P62280|RS11_HUMAN	RPS11	P62280
1	4	132	132	132	0.999	92	1.91416	0.00118853	sp|P62304|RUXE_HUMAN	SNRPE	P62304
1	5	72	72	72	0.999	86	5.39006	0	sp|P62306|RUXF_HUMAN	SNRPF	P62306
1	6	150	150	150	0.999	102	0.997866	0.021475	sp|P62310|LSM3_HUMAN	LSM 3.00	P62310
1	6	153	153	153	0.999	80	1.77666	0.00173973	sp|P62312|LSM6_HUMAN	LSM 6.00	P62312
1	5	149	149	149	0.999	119	0.403912	0.165805	sp|P62314|SMD1_HUMAN	SNRPD1	P62314
1	15	431	431	431	0.999	118	0.380428	0.17983	sp|P62316|SMD2_HUMAN	SNRPD2	P62316
1	13	629	629	629	0.999	126	0.829898	0.0383469	sp|P62318|SMD3_HUMAN	SNRPD3	P62318
1	9	496	496	510	0.999	44	0.644152	0.0721634	sp|P62328|TYB4_HUMAN	TMSB4X	P62328
1	10	190	190	190	0.999	175	1.11255	0.0143177	sp|P62330|ARF6_HUMAN	ARF6	P62330
1	31	1003	1003	1003	0.999	389	0.135317	0.460015	sp|P62333|PRS10_HUMAN	PSMC6	P62333
1	42	1988	1988	1988	0.999	266	2.35298	0.000431698	sp|P62424|RL7A_HUMAN	RPL7A	P62424
0.9992	21	84	4	814	0.999	216	5.12722	0	sp|P62491|RB11A_HUMAN	RAB11A	P62491
1	17	380	380	380	0.999	437	1.51088	0.00370462	sp|P62495|ERF1_HUMAN	ETF1	P62495
1	19	805	805	805	0.999	177	1.27728	0.0082342	sp|P62633|CNBP_HUMAN	CNBP	P62633
1	39	2288	702	2288	0.999	263	2.20289	0.000562631	sp|P62701|RS4X_HUMAN	RPS4X	P62701
1	14	896	896	896	0.999	156	2.08678	0.000761273	sp|P62750|RL23A_HUMAN	RPL23A	P62750
1	33	1449	1449	1449	0.999	249	4.90084	0	sp|P62753|RS6_HUMAN	RPS6	P62753
1	22	1020	1019	1020	0.999	103	2.83686	0.000132953	sp|P62805|H4_HUMAN	H4C1	P62805
1	25	365	365	995	0.999	205	4.08379	0	sp|P62820|RAB1A_HUMAN	RAB1A	P62820
1	29	1606	1606	1606	0.999	216	0.0564711	0.664549	sp|P62826|RAN_HUMAN	RAN	P62826
1	19	785	785	785	0.999	140	4.64718	0	sp|P62829|RL23_HUMAN	RPL23	P62829
1	11	348	67	348	0.999	184	8.11977	0	sp|P62834|RAP1A_HUMAN	RAP1A	P62834
1	8	231	231	231	0.999	145	4.62136	0	sp|P62841|RS15_HUMAN	RPS15	P62841
1	19	552	552	552	0.999	133	3.12128	5.18359E-05	sp|P62847|RS24_HUMAN	RPS24	P62847
1	23	819	819	819	0.999	125	3.53147	2.03046E-05	sp|P62851|RS25_HUMAN	RPS25	P62851
1	7	126	49	126	0.999	115	0.513833	0.113451	sp|P62854|RS26_HUMAN	RPS26	P62854
1	17	764	764	764	0.999	69	2.71494	0.00017547	sp|P62857|RS28_HUMAN	RPS28	P62857
1	5	217	217	217	0.999	133	7.4355	0	sp|P62861|RS30_HUMAN	FAU	P62861
1	26	722	438	722	0.999	340	9.05732	0	sp|P62873|GBB1_HUMAN	GNB1	P62873
1	5	132	132	132	0.999	108	0.265923	0.272996	sp|P62877|RBX1_HUMAN	RBX1	P62877
1	25	408	297	692	0.999	340	8.76933	0	sp|P62879|GBB2_HUMAN	GNB2	P62879
1	19	1087	1087	1087	0.999	115	2.09796	0.000737192	sp|P62888|RL30_HUMAN	RPL30	P62888
1	4	79	1	79	0.999	51	5.15046	0	sp|P62891|RL39_HUMAN	RPL39	P62891
1	24	744	744	744	0.999	125	2.64655	0.000204329	sp|P62899|RL31_HUMAN	RPL31	P62899
1	33	1438	1438	1438	0.999	217	5.52103	0	sp|P62906|RL10A_HUMAN	RPL10A	P62906
1	24	1045	1045	1045	0.999	135	1.37288	0.00586217	sp|P62910|RL32_HUMAN	RPL32	P62910
1	29	1364	1364	1364	0.999	178	2.90717	0.000124878	sp|P62913|RL11_HUMAN	RPL11	P62913
1	40	1769	1769	1769	0.999	257	3.88185	0	sp|P62917|RL8_HUMAN	RPL8	P62917
1	29	3233	2945	3233	0.999	165	0.626226	0.0766674	sp|P62937|PPIA_HUMAN	PPIA	P62937
1	12	280	214	280	0.999	108	1.46488	0.00428212	sp|P62942|FKB1A_HUMAN	FKBP1A	P62942
1	21	733	395	733	0.999	156	5.36366	0	sp|P62979|RS27A_HUMAN	RPS27A	P62979
1	14	281	281	619	0.999	128	1.54949	0.00338462	sp|P62987|RL40_HUMAN	UBA52	P62987
1	12	221	221	221	0.999	217	2.71398	0.000175313	sp|P62993|GRB2_HUMAN	GRB2	P62993
1	24	751	671	751	0.999	288	1.03213	0.0190021	sp|P62995|TRA2B_HUMAN	TRA2B	P62995
1	13	539	156	675	0.999	192	8.24132	0	sp|P63000|RAC1_HUMAN	RAC1	P63000
1	33	1014	517	1014	0.999	937	3.14767	5.25164E-05	sp|P63010|AP2B1_HUMAN	AP2B1	P63010
1	23	415	11	440	0.999	394	7.0641	0	sp|P63092|GNAS2_HUMAN	GNAS	P63092
1	23	81	81	484	0.999	354	1.9861	0.00098175	sp|P63096|GNAI1_HUMAN	GNAI1	P63096
1	39	2575	2003	2575	0.999	245	0.734321	0.0532447	sp|P63104|1433Z_HUMAN	YWHAZ	P63104
1	19	438	186	438	0.999	447	1.96351	0.00106633	sp|P63151|2ABA_HUMAN	PPP2R2A	P63151
1	8	238	122	238	0.999	101	4.9753	0	sp|P63165|SUMO1_HUMAN	SUMO1	P63165
1	9	360	127	360	0.999	89	4.21053	0	sp|P63167|DYL1_HUMAN	DYNLL1	P63167
1	6	407	407	407	0.999	70	3.90648	0	sp|P63173|RL38_HUMAN	RPL38	P63173
1	11	243	243	243	0.999	163	2.14593	0.000650783	sp|P63208|SKP1_HUMAN	SKP1	P63208
1	4	143	104	143	0.999	68	8.78708	0	sp|P63218|GBG5_HUMAN	GNG5	P63218
1	17	864	864	864	0.999	83	5.66245	0	sp|P63220|RS21_HUMAN	RPS21	P63220
1	20	1605	75	1605	0.999	154	7.07231	0	sp|P63241|IF5A1_HUMAN	EIF5A	P63241
1	28	1205	1205	1205	0.999	317	2.25477	0.000485981	sp|P63244|RACK1_HUMAN	RACK1	P63244
1	5	120	120	120	0.999	117	0.00653229	0.918668	sp|P63272|SPT4H_HUMAN	SUPT4H1	P63272
1	10	644	630	644	0.999	44	1.43935	0.00470025	sp|P63313|TYB10_HUMAN	TMSB10	P63313
1	28	988	147	988	0.999	309	0.574928	0.0912515	sp|P67775|PP2AA_HUMAN	PPP2CA	P67775
1	20	1118	680	1118	0.999	324	0.248136	0.292198	sp|P67809|YBOX1_HUMAN	YBX1	P67809
1	13	382	193	382	0.999	179	2.53448	0.000278689	sp|P67812|SC11A_HUMAN	SEC11A	P67812
1	6	165	165	165	0.999	215	0.853037	0.0355065	sp|P67870|CSK2B_HUMAN	CSNK2B	P67870
1	48	2865	1067	2865	0.999	248	5.99876	0	sp|P67936|TPM4_HUMAN	TPM4	P67936
1	41	1001	0	5874	0.999	377	3.37151	2.40385E-05	sp|P68032|ACTC_HUMAN	ACTC1	P68032
1	12	164	18	164	0.999	154	2.13352	0.000671177	sp|P68036|UB2L3_HUMAN	UBE2L3	P68036
1	63	6701	75	6701	0.999	462	4.0096	0	sp|P68104|EF1A1_HUMAN	EEF1A1	P68104
1	50	1964	481	4198	0.999	448	1.41077	0.00518249	sp|P68366|TBA4A_HUMAN	TUBA4A	P68366
1	50	1046	188	6641	0.999	445	4.50018	0	sp|P68371|TBB4B_HUMAN	TUBB4B	P68371
1	19	651	113	651	0.999	391	1.63022	0.00272313	sp|P68400|CSK21_HUMAN	CSNK2A1	P68400
1	11	274	274	274	0.999	229	3.14655	5.24017E-05	sp|P68402|PA1B2_HUMAN	PAFAH1B2	P68402
1	25	583	0	583	0.999	1222	2.70668	0.000178412	sp|P69849|NOMO3_HUMAN	NOMO3	P69849
1	8	355	288	355	0.999	142	4.11667	0	sp|P69905|HBA_HUMAN	HBA2	P69905
1	7	112	81	112	0.999	339	0.879537	0.0324148	sp|P78318|IGBP1_HUMAN	IGBP1	P78318
1	14	239	239	239	0.999	225	1.43876	0.00471386	sp|P78330|SERB_HUMAN	PSPH	P78330
1	5	37	37	47	0.999	1123	6.1138	0	sp|P78332|RBM6_HUMAN	RBM6	P78332
1	39	898	898	898	0.999	907	2.82609	0.0001322	sp|P78344|IF4G2_HUMAN	EIF4G2	P78344
1	10	144	144	144	0.999	268	0.264393	0.274461	sp|P78346|RPP30_HUMAN	RPP30	P78346
1	45	910	909	910	0.999	998	0.831675	0.0381001	sp|P78347|GTF2I_HUMAN	GTF2I	P78347
1	6	35	35	35	0.999	1384	0.496983	0.120151	sp|P78357|CNTP1_HUMAN	CNTNAP1	P78357
1	55	2660	2660	2660	0.999	535	2.46332	0.000319873	sp|P78371|TCPB_HUMAN	CCT2	P78371
1	13	250	250	250	0.999	368	2.29011	0.000468521	sp|P78406|RAE1L_HUMAN	RAE1	P78406

1	22	758	758	758	0.999	241	4.76769	0	sp|P78417|GSTO1_HUMAN	GSTO1	P78417
1	274	7829	7829	7829	0.999	4128	5.13553	0	sp|P78527|PRKDC_HUMAN	PRKDC	P78527
1	12	19	19	19	0.999	464	5.61714	0	sp|P78539|SRPX_HUMAN	SRPX	P78539
1	14	69	69	84	0.999	2803	2.90083	0.000128655	sp|P78559|MAP1A_HUMAN	MAP1A	P78559
1	12	239	239	679	0.999	61	0.345521	0.203587	sp|P80297|MT1X_HUMAN	MT1X	P80297
1	15	244	244	264	0.999	420	1.96059	0.00106351	sp|P80303|NUCB2_HUMAN	NUCB2	P80303
1	21	729	729	729	0.999	227	8.93501	0	sp|P80723|BASP1_HUMAN	BASP1	P80723
1	5	63	63	63	0.999	110	4.56605	0	sp|P81605|DCD_HUMAN	DCD	P81605
1	5	18	18	18	0.999	1093	1.30378	0.00745992	sp|P82094|TMF1_HUMAN	TMF1	P82094
1	14	142	142	142	0.999	360	1.51865	0.00362326	sp|P82650|RT22_HUMAN	MRPS22	P82650
1	6	113	113	113	0.999	173	1.12022	0.0139739	sp|P82663|RT25_HUMAN	MRPS25	P82663
1	14	262	262	262	0.999	430	0.518791	0.111468	sp|P82675|RT05_HUMAN	MRPS5	P82675
1	4	125	125	125	0.999	194	3.59626	0	sp|P82912|RT11_HUMAN	MRPS11	P82912
1	14	458	458	458	0.999	218	0.719819	0.0559609	sp|P82930|RT34_HUMAN	MRPS34	P82930
1	15	317	317	317	0.999	396	0.993734	0.0217687	sp|P82933|RT09_HUMAN	MRPS9	P82933
1	20	612	612	612	0.999	210	2.65239	0.000201739	sp|P82979|SARNP_HUMAN	SARNP	P82979
1	6	84	84	84	0.999	547	1.69443	0.0022408	sp|P83111|LACTB_HUMAN	LACTB	P83111
1	32	1095	1095	1095	0.999	157	4.56823	0	sp|P83731|RL24_HUMAN	RPL24	P83731
1	22	900	160	900	0.999	106	4.06849	0	sp|P83881|RL36A_HUMAN	RPL36A	P83881
1	7	252	252	360	0.999	185	3.23954	5.49828E-05	sp|P83916|CBX1_HUMAN	CBX1	P83916
1	8	92	92	277	0.999	180	1.5183	0.00362757	sp|P84085|ARF5_HUMAN	ARF5	P84085
1	10	503	503	503	0.999	104	5.85646	0	sp|P84090|ERH_HUMAN	ERH	P84090
1	9	161	161	297	0.999	191	6.04552	0	sp|P84095|RHOG_HUMAN	RHOG	P84095
1	35	1238	1238	1238	0.999	196	2.16249	0.000624484	sp|P84098|RL19_HUMAN	RPL19	P84098
1	9	90	90	90	0.999	59	0.415169	0.159288	sp|P84101|SERF2_HUMAN	SERF2	P84101
1	15	802	802	802	0.999	164	1.26192	0.00860405	sp|P84103|SRSF3_HUMAN	SRSF3	P84103
1	9	304	256	304	0.999	733	0.455693	0.138641	sp|P85037|FOXK1_HUMAN	FOXK1	P85037
1	13	59	59	59	0.999	770	1.93801	0.00112523	sp|P98082|DAB2_HUMAN	DAB2	P98082
1	68	414	414	414	0.999	4391	6.73416	0	sp|P98160|PGBM_HUMAN	HSPG2	P98160
1	23	289	248	289	0.999	930	3.26984	4.63499E-05	sp|P98175|RBM10_HUMAN	RBM10	P98175
1	12	551	551	551	0.999	157	3.55045	2.05656E-05	sp|P98179|RBM3_HUMAN	RBM3	P98179
1	18	482	482	482	0.999	246	1.77977	0.00171991	sp|Q00059|TFAM_HUMAN	TFAM	Q00059
1	24	1201	1201	1201	0.999	362	6.76146	0	sp|Q00325|S25A3_HUMAN	SLC25A3	Q00325
1	85	1314	1314	1314	0.999	1268	5.77434	0	sp|Q00341|VIGLN_HUMAN	HDLBP	Q00341
1	12	238	238	246	0.999	322	2.15066	0.000638908	sp|Q00577|PURA_HUMAN	PURA	Q00577
1	6	124	124	124	0.999	391	1.67568	0.00236464	sp|Q00587|BORG5_HUMAN	CDC42EP1	Q00587
1	135	4895	3655	4895	0.999	1675	1.00576	0.0208692	sp|Q00610|CLH1_HUMAN	CLTC	Q00610
1	22	243	243	243	0.999	900	0.475926	0.129111	sp|Q00653|NFKB2_HUMAN	NFKB2	Q00653
1	19	630	630	630	0.999	224	2.59102	0.000242424	sp|Q00688|FKBP3_HUMAN	FKBP3	Q00688
1	12	347	347	347	0.999	189	1.91508	0.00118754	sp|Q00765|REEP5_HUMAN	REEP5	Q00765
1	22	681	681	681	0.999	357	0.322753	0.221025	sp|Q00796|DHSO_HUMAN	SORD	Q00796
1	72	4195	4195	4195	0.999	825	6.00921	0	sp|Q00839|HNRPU_HUMAN	HNRNPU	Q00839
1	11	476	448	476	0.999	240	1.06213	0.0171399	sp|Q01081|U2AF1_HUMAN	U2AF1	Q01081
1	103	2059	1766	2059	0.999	2364	2.07773	0.000779907	sp|Q01082|SPTB2_HUMAN	SPTBN1	Q01082
1	7	189	59	189	0.999	375	2.74179	0.000164234	sp|Q01085|TIAR_HUMAN	TIAL1	Q01085
1	25	1127	542	1127	0.999	290	1.17758	0.0113895	sp|Q01105|SET_HUMAN	SET	Q01105
1	15	741	594	741	0.999	221	0.369525	0.186642	sp|Q01130|SRSF2_HUMAN	SRSF2	Q01130
1	18	153	152	153	0.999	879	0.215997	0.331264	sp|Q01433|AMPD2_HUMAN	AMPD2	Q01433
1	23	1098	930	1098	0.999	135	0.82171	0.0394842	sp|Q01469|FABP5_HUMAN	FABP5	Q01469
1	47	1595	1535	1595	0.999	475	4.25744	0	sp|Q01518|CAP1_HUMAN	CAP1	Q01518
1	14	139	136	139	0.999	520	1.17505	0.011504	sp|Q01581|HMCS1_HUMAN	HMGCS1	Q01581
1	7	399	301	399	0.999	507	9.36087	0	sp|Q01650|LAT1_HUMAN	SLC7A5	Q01650
1	23	433	433	433	0.999	885	0.680104	0.0637799	sp|Q01780|EXOSX_HUMAN	EXOSC10	Q01780
1	7	58	57	58	0.999	1114	1.92756	0.00115971	sp|Q01804|OTUD4_HUMAN	OTUD4	Q01804
1	51	2042	1813	2042	0.999	784	1.56907	0.00321395	sp|Q01813|PFKAP_HUMAN	PFKP	Q01813
1	9	224	224	224	0.999	656	0.0193307	0.828885	sp|Q01844|EWS_HUMAN	EWSR1	Q01844
1	23	408	408	408	0.999	1234	0.292285	0.247207	sp|Q01970|PLCB3_HUMAN	PLCB3	Q01970
1	24	420	402	420	0.999	201	1.60613	0.0029126	sp|Q01995|TAGL_HUMAN	TAGLN	Q01995
1	6	58	58	58	0.999	395	2.15258	0.000632011	sp|Q02127|PYRD_HUMAN	DHODH	Q02127
1	37	884	732	884	0.999	1023	5.00679	0	sp|Q02218|ODO1_HUMAN	OGDH	Q02218
0.9999	5	43	43	43	0.9953	2701	0.46309	0.135266	sp|Q02224|CENPE_HUMAN	CENPE	Q02224
1	15	108	108	108	0.999	960	1.52122	0.00359381	sp|Q02241|KIF23_HUMAN	KIF23	Q02241
1	4	59	59	59	0.999	535	1.6243	0.00277356	sp|Q02252|MMSA_HUMAN	ALDH6A1	Q02252
1	12	66	65	66	0.999	2944	3.04965	6.68058E-05	sp|Q02388|CO7A1_HUMAN	COL7A1	Q02388
1	5	117	57	117	0.999	781	2.65214	0.000201389	sp|Q02447|SP3_HUMAN	SP3	Q02447
1	23	662	242	1177	0.999	215	9.35776	0	sp|Q02539|H11_HUMAN	H1-1	Q02539
1	34	1370	1370	1370	0.999	176	3.93031	0	sp|Q02543|RL18A_HUMAN	RPL18A	Q02543
1	23	776	524	776	0.999	393	0.88372	0.0319728	sp|Q02750|MP2K1_HUMAN	MAP2K1	Q02750
1	38	1595	1595	1595	0.999	459	7.31323	0	sp|Q02790|FKBP4_HUMAN	FKBP4	Q02790
1	31	789	761	789	0.999	727	0.719038	0.0561006	sp|Q02809|PLOD1_HUMAN	PLOD1	Q02809
1	26	440	420	440	0.999	461	1.69289	0.00224401	sp|Q02818|NUCB1_HUMAN	NUCB1	Q02818
1	38	2121	2121	2121	0.999	288	2.7278	0.000174071	sp|Q02878|RL6_HUMAN	RPL6	Q02878
1	51	666	666	666	0.999	1782	0.861192	0.0345355	sp|Q02952|AKA12_HUMAN	AKAP12	Q02952
1	20	398	398	398	0.999	314	2.57763	0.000253756	sp|Q02978|M2OM_HUMAN	SLC25A11	Q02978
1	39	256	218	256	0.999	7570	1.52315	0.00357688	sp|Q03001|DYST_HUMAN	DST	Q03001
1	12	359	332	359	0.999	178	1.55552	0.00332004	sp|Q03135|CAV1_HUMAN	CAV1	Q03135
1	19	382	382	382	0.999	408	0.0859343	0.573818	sp|Q03154|ACY1_HUMAN	ACY1	Q03154
1	55	936	936	1441	0.999	620	3.28705	4.66744E-05	sp|Q03252|LMNB2_HUMAN	LMNB2	Q03252
1	12	213	213	213	0.999	335	7.30618	0	sp|Q03405|UPAR_HUMAN	PLAUR	Q03405
1	6	69	69	69	0.999	748	0.34563	0.203637	sp|Q03518|TAP1_HUMAN	TAP1	Q03518
1	8	114	114	114	0.999	686	1.27598	0.00823726	sp|Q03519|TAP2_HUMAN	TAP2	Q03519
1	18	250	250	250	0.999	1054	3.65201	0	sp|Q03701|CEBPZ_HUMAN	CEBPZ	Q03701
1	11	280	280	280	0.999	551	1.68964	0.00226348	sp|Q04206|TF65_HUMAN	RELA	Q04206
1	15	399	399	399	0.999	297	2.26188	0.000482659	sp|Q04323|UBXN1_HUMAN	UBXN1	Q04323
1	38	1046	1046	1046	0.999	702	1.56117	0.00326909	sp|Q04446|GLGB_HUMAN	GBE1	Q04446
1	74	2303	1846	2303	0.999	1599	3.20087	5.35714E-05	sp|Q04637|IF4G1_HUMAN	EIF4G1	Q04637
1	54	1871	1079	1938	0.999	432	3.76189	0	sp|Q04695|K1C17_HUMAN	KRT17	Q04695
1	17	136	131	136	0.999	2471	6.33839	0	sp|Q04721|NOTC2_HUMAN	NOTCH2	Q04721
1	7	76	24	76	0.999	772	2.13022	0.000674764	sp|Q04726|TLE3_HUMAN	TLE3	Q04726
1	3	11	11	11	0.999	655	2.21493	0.000544045	sp|Q04756|HGFA_HUMAN	HGFAC	Q04756
1	15	644	644	644	0.999	184	1.44863	0.0045491	sp|Q04760|LGUL_HUMAN	GLO1	Q04760
1	11	223	223	223	0.999	148	3.32639	2.37248E-05	sp|Q04837|SSBP_HUMAN	SSBP1	Q04837
1	33	958	958	1486	0.999	246	3.60131	0	sp|Q04917|1433F_HUMAN	YWHAH	Q04917
1	16	208	208	208	0.999	431	0.290206	0.248968	sp|Q05048|CSTF1_HUMAN	CSTF1	Q05048
1	5	48	48	48	0.999	875	0.704462	0.0587671	sp|Q05086|UBE3A_HUMAN	UBE3A	Q05086
1	25	199	181	524	0.999	864	0.934139	0.0268164	sp|Q05193|DYN1_HUMAN	DNM1	Q05193
1	19	168	168	168	0.999	780	0.660888	0.0679853	sp|Q05209|PTN12_HUMAN	PTPN12	Q05209
1	25	276	257	276	0.999	1052	2.64971	0.000201214	sp|Q05397|FAK1_HUMAN	PTK2	Q05397
1	9	298	298	298	0.999	484	0.815841	0.0403252	sp|Q05519|SRS11_HUMAN	SRSF11	Q05519
1	9	54	54	59	0.999	676	2.17229	0.000609418	sp|Q05655|KPCD_HUMAN	PRKCD	Q05655
1	47	1232	1232	1232	0.999	793	4.15857	0	sp|Q05682|CALD1_HUMAN	CALD1	Q05682
1	6	97	97	97	0.999	587	3.37589	2.40674E-05	sp|Q05932|FOLC_HUMAN	FPGS	Q05932
1	14	153	153	153	0.999	890	2.96587	9.61924E-05	sp|Q06033|ITIH3_HUMAN	ITIH3	Q06033
1	23	545	545	545	0.999	593	0.153441	0.425324	sp|Q06124|PTN11_HUMAN	PTPN11	Q06124
1	17	271	271	271	0.999	517	0.918402	0.028285	sp|Q06203|PUR1_HUMAN	PPAT	Q06203
1	32	765	668	765	0.999	699	1.55728	0.00330882	sp|Q06210|GFPT1_HUMAN	GFPT1	Q06210
1	9	95	95	95	0.999	439	0.120274	0.491999	sp|Q06265|EXOS9_HUMAN	EXOSC9	Q06265
1	17	819	819	819	0.999	249	2.78292	0.000141923	sp|Q06323|PSME1_HUMAN	PSME1	Q06323
1	10	101	84	101	0.999	763	0.921697	0.027987	sp|Q06481|APLP2_HUMAN	APLP2	Q06481
1	17	402	402	458	0.999	632	0.387183	0.17563	sp|Q06787|FMR1_HUMAN	FMR1	Q06787
1	4	17	17	17	0.999	376	3.93142	0	sp|Q06828|FMOD_HUMAN	FMOD	Q06828
1	33	2394	1984	2394	0.999	199	6.2236	0	sp|Q06830|PRDX1_HUMAN	PRDX1	Q06830
1	22	794	794	794	0.999	188	1.27721	0.00823409	sp|Q07020|RL18_HUMAN	RPL18	Q07020
1	65	2249	2212	2249	0.999	602	7.99525	0	sp|Q07065|CKAP4_HUMAN	CKAP4	Q07065
1	36	453	453	503	0.999	1748	1.62254	0.00278722	sp|Q07157|ZO1_HUMAN	TJP1	Q07157
1	21	659	462	659	0.999	443	2.20014	0.000561845	sp|Q07666|KHDR1_HUMAN	KHDRBS1	Q07666
1	29	604	315	604	0.999	573	0.377747	0.18129	sp|Q07866|KLC1_HUMAN	KLC1	Q07866
1	80	820	816	820	0.999	4544	4.0702	0	sp|Q07954|LRP1_HUMAN	LRP1	Q07954
1	33	962	942	962	0.999	248	2.24782	0.000496057	sp|Q07955|SRSF1_HUMAN	SRSF1	Q07955
1	21	461	461	461	0.999	439	2.47305	0.000306464	sp|Q07960|RHG01_HUMAN	ARHGAP1	Q07960
1	17	278	278	437	0.999	494	1.46811	0.0042524	sp|Q08170|SRSF4_HUMAN	SRSF4	Q08170
1	19	299	125	299	0.999	521	0.348731	0.201489	sp|Q08209|PP2BA_HUMAN	PPP3CA	Q08209
1	77	2853	2853	2853	0.999	1270	2.22295	0.000523338	sp|Q08211|DHX9_HUMAN	DHX9	Q08211
1	7	121	121	121	0.999	329	0.72303	0.0553948	sp|Q08257|QOR_HUMAN	CRYZ	Q08257
1	15	150	150	150	0.999	1498	2.01411	0.000905128	sp|Q08378|GOGA3_HUMAN	GOLGA3	Q08378
1	27	262	262	262	0.999	1002	1.96175	0.00106168	sp|Q08379|GOGA2_HUMAN	GOLGA2	Q08379
1	20	321	321	321	0.999	585	7.83274	0	sp|Q08380|LG3BP_HUMAN	LGALS3BP	Q08380
1	25	205	205	205	0.999	387	10.9462	0	sp|Q08431|MFGM_HUMAN	MFGE8	Q08431
1	3	99	99	99	0.999	323	0.752914	0.0499294	sp|Q08722|CD47_HUMAN	CD47	Q08722

1	16	290	290	319	0.999	370	0.739698	0.0522846	sp|Q08752|PPID_HUMAN	PPID	Q08752
1	35	946	946	946	0.999	709	3.54676	2.05392E-05	sp|Q08945|SSRP1_HUMAN	SSRP1	Q08945
1	14	168	168	168	0.999	891	1.39692	0.00540762	sp|Q08AF3|SLFN5_HUMAN	SLFN5	Q08AF3
1	7	159	159	159	0.999	782	1.74298	0.00193843	sp|Q08AM6|VAC14_HUMAN	VAC14	Q08AM6
1	48	1157	1157	1157	0.999	767	0.685382	0.0626617	sp|Q08J23|NSUN2_HUMAN	NSUN2	Q08J23
1	27	455	455	998	0.999	425	5.01205	0	sp|Q09028|RBBP4_HUMAN	RBBP4	Q09028
1	28	625	625	625	0.999	790	0.911713	0.0289257	sp|Q09161|NCBP1_HUMAN	NCBP1	Q09161
1	324	7199	7188	7199	0.999	5890	5.66214	0	sp|Q09666|AHNK_HUMAN	AHNAK	Q09666
1	12	325	325	325	0.999	509	3.74163	0	sp|Q0VDF9|HSP7E_HUMAN	HSPA14	Q0VDF9
1	6	9	9	9	0.9987	675	0.535663	0.104665	sp|Q0ZGT2|NEXN_HUMAN	NEXN	Q0ZGT2
1	29	740	740	740	0.999	571	0.29747	0.2427	sp|Q10471|GALT2_HUMAN	GALNT2	Q10471
1	33	379	379	876	0.999	949	0.918247	0.028293	sp|Q10567|AP1B1_HUMAN	AP1B1	Q10567
1	17	185	185	185	0.999	1443	1.50231	0.00380738	sp|Q10570|CPSF1_HUMAN	CPSF1	Q10570
1	4	10	10	10	0.9977	318	6.11623	0	sp|Q10588|BST1_HUMAN	BST1	Q10588
1	5	54	54	54	0.999	180	2.26706	0.000480811	sp|Q10589|BST2_HUMAN	BST2	Q10589
1	20	686	686	686	0.999	525	2.35173	0.000430723	sp|Q10713|MPPA_HUMAN	PMPCA	Q10713
1	13	454	454	454	0.999	414	3.07232	5.88235E-05	sp|Q12765|SCRN1_HUMAN	SCRN1	Q12765
1	17	214	214	214	0.999	1159	0.0583694	0.657933	sp|Q12768|WASC5_HUMAN	WASHC5	Q12768
1	21	112	112	112	0.999	1436	1.2589	0.00869701	sp|Q12769|NU160_HUMAN	NUP160	Q12769
1	18	264	264	264	0.999	808	3.05764	6.28272E-05	sp|Q12788|TBL3_HUMAN	TBL3	Q12788
1	18	601	489	601	0.999	350	1.18641	0.0110694	sp|Q12792|TWF1_HUMAN	TWF1	Q12792
1	38	870	870	870	0.999	758	2.39475	0.000379045	sp|Q12797|ASPH_HUMAN	ASPH	Q12797
1	17	144	144	144	0.999	2813	0.425923	0.153168	sp|Q12802|AKP13_HUMAN	AKAP13	Q12802
1	5	14	14	14	0.999	493	3.2187	0.000054115	sp|Q12805|FBLN3_HUMAN	EFEMP1	Q12805
1	3	28	28	28	0.999	499	0.0428199	0.715827	sp|Q12834|CDC20_HUMAN	CDC20	Q12834
1	14	45	45	45	0.999	308	0.664441	0.0671533	sp|Q12841|FSTL1_HUMAN	FSTL1	Q12841
1	6	88	88	88	0.999	297	0.317629	0.225052	sp|Q12846|STX4_HUMAN	STX4	Q12846
1	11	157	157	157	0.999	480	1.30774	0.00740414	sp|Q12849|GRSF1_HUMAN	GRSF1	Q12849
1	4	16	16	16	0.999	1018	4.28353	0	sp|Q12860|CNTN1_HUMAN	CNTN1	Q12860
1	20	33	32	341	0.999	2000	3.48608	0.00002	sp|Q12873|CHD3_HUMAN	CHD3	Q12873
1	27	743	743	743	0.999	501	3.8032	0	sp|Q12874|SF3A3_HUMAN	SF3A3	Q12874
1	17	110	110	110	0.999	1025	3.14548	5.22876E-05	sp|Q12882|DPYD_HUMAN	DPYD	Q12882
1	29	94	94	94	0.999	760	3.13393	5.20043E-05	sp|Q12884|SEPR_HUMAN	FAP	Q12884
1	17	197	197	197	0.999	1972	0.219429	0.326864	sp|Q12888|TP53B_HUMAN	TP53BP1	Q12888
1	18	587	587	587	0.999	312	4.65604	0	sp|Q12904|AIMP1_HUMAN	AIMP1	Q12904
1	17	704	704	704	0.999	390	2.49155	0.000296296	sp|Q12905|ILF2_HUMAN	ILF2	Q12905
1	43	1705	1580	1705	0.999	894	6.71051	0	sp|Q12906|ILF3_HUMAN	ILF3	Q12906
1	20	548	548	548	0.999	356	4.27265	0	sp|Q12907|LMAN2_HUMAN	LMAN2	Q12907
1	6	12	12	12	0.999	822	1.14818	0.0126754	sp|Q12929|EPS8_HUMAN	EPS8	Q12929
1	42	892	892	953	0.999	704	2.32142	0.000452045	sp|Q12931|TRAP1_HUMAN	TRAP1	Q12931
1	9	73	73	73	0.999	501	0.332998	0.212843	sp|Q12933|TRAF2_HUMAN	TRAF2	Q12933
1	35	487	448	487	0.999	1108	1.99317	0.000970849	sp|Q12965|MYO1E_HUMAN	MYO1E	Q12965
1	8	98	98	98	0.999	351	0.382664	0.178457	sp|Q12972|PP1R8_HUMAN	PPP1R8	Q12972
1	5	85	15	85	0.999	167	2.28576	0.000467495	sp|Q12974|TP4A2_HUMAN	PTP4A2	Q12974
1	12	57	40	57	0.999	859	1.85157	0.00143078	sp|Q12979|ABR_HUMAN	ABR	Q12979
1	13	224	224	224	0.999	717	0.0895387	0.564642	sp|Q12996|CSTF3_HUMAN	CSTF3	Q12996
1	25	714	714	714	0.999	328	0.448125	0.141946	sp|Q13011|ECH1_HUMAN	ECH1	Q13011
1	8	80	70	80	0.999	487	0.735707	0.0530344	sp|Q13043|STK4_HUMAN	STK4	Q13043
1	32	496	496	496	0.999	1269	0.185826	0.37274	sp|Q13045|FLII_HUMAN	FLII	Q13045
1	13	299	299	299	0.999	564	0.758218	0.049091	sp|Q13057|COASY_HUMAN	COASY	Q13057
1	47	746	676	746	0.999	2346	2.16679	0.000616655	sp|Q13085|ACACA_HUMAN	ACACA	Q13085
1	23	513	513	513	0.999	491	0.756059	0.0494053	sp|Q13098|CSN1_HUMAN	GPS1	Q13098
1	22	302	266	302	0.999	557	4.37863	0	sp|Q13123|RED_HUMAN	IK	Q13123
1	17	268	268	268	0.999	283	4.38131	0	sp|Q13126|MTAP_HUMAN	MTAP	Q13126
1	19	186	179	186	0.999	559	0.889146	0.0313608	sp|Q13131|AAPK1_HUMAN	PRKAA1	Q13131
1	23	156	151	156	0.999	1202	0.841678	0.0368189	sp|Q13136|LIPA1_HUMAN	PPFIA1	Q13136
1	18	514	514	514	0.999	414	2.01708	0.000907455	sp|Q13148|TADBP_HUMAN	TARDBP	Q13148
1	25	597	597	764	0.999	305	3.36721	2.40096E-05	sp|Q13151|ROA0_HUMAN	HNRNPA0	Q13151
1	13	495	495	495	0.999	320	1.22726	0.00966697	sp|Q13155|AIMP2_HUMAN	AIMP2	Q13155
1	16	537	537	851	0.999	271	5.10425	0	sp|Q13162|PRDX4_HUMAN	PRDX4	Q13162
1	25	690	422	690	0.999	524	1.52717	0.00353695	sp|Q13177|PAK2_HUMAN	PAK2	Q13177
1	15	759	555	759	0.999	183	3.6466	0	sp|Q13185|CBX3_HUMAN	CBX3	Q13185
1	7	56	56	56	0.999	355	0.00752755	0.909953	sp|Q13190|STX5_HUMAN	STX5	Q13190
1	34	995	995	995	0.999	908	0.617238	0.0789357	sp|Q13200|PSMD2_HUMAN	PSMD2	Q13200
1	5	13	13	13	0.9981	749	3.7201	0	sp|Q13214|SEM3B_HUMAN	SEMA3B	Q13214
1	22	302	302	302	0.999	504	5.95228	0	sp|Q13217|DNJC3_HUMAN	DNAJC3	Q13217
1	11	118	118	118	0.999	472	2.78962	0.000142322	sp|Q13228|SBP1_HUMAN	SELENBP1	Q13228
1	18	344	344	364	0.999	221	3.41866	1.95838E-05	sp|Q13242|SRSF9_HUMAN	SRSF9	Q13242
1	13	169	169	234	0.999	272	3.20301	5.36313E-05	sp|Q13243|SRSF5_HUMAN	SRSF5	Q13243
1	20	709	550	709	0.999	344	2.1483	0.000646217	sp|Q13247|SRSF6_HUMAN	SRSF6	Q13247
1	8	120	120	120	0.999	205	1.57305	0.00319237	sp|Q13257|MD2L1_HUMAN	MAD2L1	Q13257
1	56	2086	2086	2086	0.999	835	3.5387	2.04082E-05	sp|Q13263|TIF1B_HUMAN	TRIM28	Q13263
1	28	1401	1262	1401	0.999	466	7.49944	0	sp|Q13283|G3BP1_HUMAN	G3BP1	Q13283
1	23	171	171	171	0.999	1070	6.02192	0	sp|Q13308|PTK7_HUMAN	PTK7	Q13308
1	29	431	431	1001	0.999	644	1.71218	0.0021191	sp|Q13310|PABP4_HUMAN	PABPC4	Q13310
1	6	96	96	96	0.999	482	1.47072	0.00422054	sp|Q13325|IFIT5_HUMAN	IFIT5	Q13325
1	11	103	86	246	0.999	715	2.22983	0.000519886	sp|Q13330|MTA1_HUMAN	MTA1	Q13330
1	25	873	873	873	0.999	325	8.77431	0	sp|Q13347|EIF3I_HUMAN	EIF3I	Q13347
1	13	146	146	361	0.999	440	0.614688	0.079658	sp|Q13363|CTBP1_HUMAN	CTBP1	Q13363
0.9999	2	43	43	43	0.999	301	2.78843	0.000142189	sp|Q13371|PHLP_HUMAN	PDCL	Q13371
1	11	117	117	319	0.999	147	0.59693	0.0845878	sp|Q13404|UB2V1_HUMAN	UBE2V1	Q13404
1	21	520	508	520	0.999	638	3.82041	0	sp|Q13409|DC1I2_HUMAN	DYNC1I2	Q13409
1	33	621	621	621	0.999	452	3.42072	1.96078E-05	sp|Q13418|ILK_HUMAN	ILK	Q13418
1	34	535	535	535	0.999	1086	1.44115	0.004669	sp|Q13423|NNTM_HUMAN	NNT	Q13423
1	12	68	49	68	0.999	540	4.389	0	sp|Q13425|SNTB2_HUMAN	SNTB2	Q13425
1	16	266	266	266	0.999	754	1.99007	0.000974067	sp|Q13427|PPIG_HUMAN	PPIG	Q13427
1	60	1518	1518	1518	0.999	1488	1.32682	0.00689825	sp|Q13428|TCOF_HUMAN	TCOF1	Q13428
1	39	1416	1416	1416	0.999	895	2.30267	0.000459499	sp|Q13435|SF3B2_HUMAN	SF3B2	Q13435
1	5	79	79	79	0.999	667	0.817701	0.0400514	sp|Q13438|OS9_HUMAN	OS9	Q13438
1	20	83	83	83	0.999	2230	0.303181	0.237649	sp|Q13439|GOGA4_HUMAN	GOLGA4	Q13439
1	18	581	581	581	0.999	181	3.9508	0	sp|Q13442|HAP28_HUMAN	PDAP1	Q13442
1	13	206	206	206	0.999	819	7.8561	0	sp|Q13443|ADAM9_HUMAN	ADAM9	Q13443
1	48	1100	1100	1100	0.999	457	1.73868	0.00196248	sp|Q13451|FKBP5_HUMAN	FKBP5	Q13451
1	17	118	116	118	0.999	2157	1.33479	0.00669834	sp|Q13459|MYO9B_HUMAN	MYO9B	Q13459
1	28	108	108	170	0.999	1354	2.30183	0.000459161	sp|Q13464|ROCK1_HUMAN	ROCK1	Q13464
1	15	309	254	309	0.999	652	1.22474	0.00973882	sp|Q13492|PICAL_HUMAN	PICALM	Q13492
1	13	465	465	465	0.999	440	7.75411	0	sp|Q13501|SQSTM_HUMAN	SQSTM1	Q13501
1	8	141	141	141	0.999	466	1.15224	0.0125112	sp|Q13505|MTX1_HUMAN	MTX1	Q13505
1	40	544	544	4186	0.999	450	2.71379	0.000175156	sp|Q13509|TBB3_HUMAN	TUBB3	Q13509
1	19	353	353	353	0.999	1007	0.606595	0.0817701	sp|Q13523|PRP4B_HUMAN	PRPF4B	Q13523
1	11	519	453	519	0.999	163	1.39696	0.00540831	sp|Q13526|PIN1_HUMAN	PIN1	Q13526
1	5	114	114	114	0.999	118	1.38378	0.00565437	sp|Q13541|4EBP1_HUMAN	EIF4EBP1	Q13541
1	13	123	123	123	0.999	671	1.80239	0.00161538	sp|Q13546|RIPK1_HUMAN	RIPK1	Q13546
1	18	472	294	472	0.999	482	3.27272	4.64576E-05	sp|Q13547|HDAC1_HUMAN	HDAC1	Q13547
1	14	133	106	133	0.999	499	1.07548	0.016329	sp|Q13557|KCC2D_HUMAN	CAMK2D	Q13557
1	26	639	639	639	0.999	401	2.25802	0.000487032	sp|Q13561|DCTN2_HUMAN	DCTN2	Q13561
1	14	247	247	247	0.999	534	1.17578	0.0114683	sp|Q13564|ULA1_HUMAN	NAE1	Q13564
1	19	366	366	366	0.999	536	2.07107	0.000793149	sp|Q13573|SNW1_HUMAN	SNW1	Q13573
1	17	173	173	253	0.999	282	0.159188	0.415645	sp|Q13595|TRA2A_HUMAN	TRA2A	Q13595
1	15	254	254	262	0.999	522	0.408645	0.163241	sp|Q13596|SNX1_HUMAN	SNX1	Q13596
1	9	106	106	106	0.999	381	0.68938	0.0619318	sp|Q13601|KRR1_HUMAN	KRR1	Q13601
1	10	226	226	226	0.999	501	0.0595766	0.653691	sp|Q13610|PWP1_HUMAN	PWP1	Q13610
1	19	392	392	392	0.999	776	0.625153	0.0769587	sp|Q13616|CUL1_HUMAN	CUL1	Q13616
1	22	268	268	268	0.999	745	1.30108	0.00754848	sp|Q13617|CUL2_HUMAN	CUL2	Q13617
1	20	383	353	383	0.999	768	2.92029	0.000125613	sp|Q13618|CUL3_HUMAN	CUL3	Q13618
1	30	188	188	364	0.999	759	0.296068	0.243944	sp|Q13619|CUL4A_HUMAN	CUL4A	Q13619
1	39	654	478	654	0.999	913	2.45183	0.000320755	sp|Q13620|CUL4B_HUMAN	CUL4B	Q13620
1	16	362	362	362	0.999	321	1.31676	0.00716305	sp|Q13630|FCL_HUMAN	GFUS	Q13630
1	8	80	77	80	0.999	225	0.853255	0.0354921	sp|Q13637|RAB32_HUMAN	RAB32	Q13637
1	7	50	50	50	0.999	420	6.04327	0	sp|Q13641|TPBG_HUMAN	TPBG	Q13641
1	17	239	239	239	0.999	323	1.51613	0.00364627	sp|Q13642|FHL1_HUMAN	FHL1	Q13642
1	9	207	207	207	0.999	280	0.108178	0.519431	sp|Q13643|FHL3_HUMAN	FHL3	Q13643
1	7	83	83	83	0.999	434	1.74746	0.00191543	sp|Q13685|AAMP_HUMAN	AAMP	Q13685
1	20	387	387	387	0.999	837	0.968938	0.0237688	sp|Q13724|MOGS_HUMAN	MOGS	Q13724
1	15	287	287	287	0.999	583	7.87106	0	sp|Q13740|CD166_HUMAN	ALCAM	Q13740
1	79	1239	1239	1239	0.999	2472	3.4018	1.94175E-05	sp|Q13813|SPTN1_HUMAN	SPTAN1	Q13813
1	16	168	168	168	0.999	731	2.40619	0.000365608	sp|Q13823|NOG2_HUMAN	GNL2	Q13823
1	34	1202	582	1202	0.999	428	6.34313	0	sp|Q13838|DX39B_HUMAN	DDX39B	Q13838
1	9	122	122	122	0.999	455	2.72652	0.000173913	sp|Q13867|BLMH_HUMAN	BLMH	Q13867

1	11	134	134	134	0.999	293	0.0230518	0.808001	sp|Q13868|EXOS2_HUMAN	EXOSC2	Q13868
1	40	241	59	5182	0.999	445	4.10809	0	sp|Q13885|TBB2A_HUMAN	TUBB2A	Q13885
1	13	195	195	195	0.999	437	0.245083	0.295838	sp|Q13895|BYST_HUMAN	BYSL	Q13895
1	10	169	130	169	0.999	227	0.353807	0.19795	sp|Q13907|IDI1_HUMAN	IDI1	Q13907
1	7	108	108	108	0.999	182	1.10555	0.014684	sp|Q13951|PEBB_HUMAN	CBFB	Q13951
1	58	1069	1069	1069	0.999	2032	1.97783	0.00100754	sp|Q14008|CKAP5_HUMAN	CKAP5	Q14008
1	17	522	522	522	0.999	172	1.7133	0.0021113	sp|Q14011|CIRBP_HUMAN	CIRBP	Q14011
1	18	736	736	736	0.999	142	1.91134	0.00119294	sp|Q14019|COTL1_HUMAN	COTL1	Q14019
1	2	88	88	88	0.999	63	0.101943	0.533954	sp|Q14061|COX17_HUMAN	COX17	Q14061
1	34	1384	1122	1384	0.999	355	3.82371	0	sp|Q14103|HNRPD_HUMAN	HNRNPD	Q14103
1	14	272	272	272	0.999	478	2.04163	0.000853991	sp|Q14108|SCRB2_HUMAN	SCARB2	Q14108
1	15	35	34	35	0.999	1375	4.43419	0	sp|Q14112|NID2_HUMAN	NID2	Q14112
1	17	296	296	296	0.999	746	0.28145	0.257177	sp|Q14137|BOP1_HUMAN	BOP 1.00	Q14137
1	5	67	67	67	0.999	1066	0.647171	0.0713474	sp|Q14139|UBE4A_HUMAN	UBE4A	Q14139
1	5	120	120	120	0.999	624	4.23613	0	sp|Q14145|KEAP1_HUMAN	KEAP1	Q14145
1	33	209	209	459	0.999	953	1.26036	0.00864795	sp|Q14151|SAFB2_HUMAN	SAFB2	Q14151
1	109	4022	4019	4022	0.999	1382	2.72366	0.000172817	sp|Q14152|EIF3A_HUMAN	EIF3A	Q14152
1	9	143	143	143	0.999	803	0.938567	0.0264329	sp|Q14155|ARHG7_HUMAN	ARHGEF7	Q14155
1	30	973	973	973	0.999	1087	2.15674	0.000630569	sp|Q14157|UBP2L_HUMAN	UBAP2L	Q14157
1	33	560	560	560	0.999	1630	1.98228	0.00100126	sp|Q14160|SCRIB_HUMAN	SCRIB	Q14160
1	9	119	63	119	0.999	759	3.00008	8.15494E-05	sp|Q14161|GIT2_HUMAN	GIT2	Q14161
1	12	176	176	176	0.999	292	0.441815	0.144918	sp|Q14165|MLEC_HUMAN	MLEC	Q14165
1	31	651	651	651	0.999	644	1.00587	0.0208679	sp|Q14166|TTL12_HUMAN	TTLL12	Q14166
1	30	657	657	657	0.999	279	0.454639	0.139136	sp|Q14192|FHL2_HUMAN	FHL2	Q14192
1	19	221	203	428	0.999	570	1.22526	0.00971971	sp|Q14195|DPYL3_HUMAN	DPYSL3	Q14195
1	7	109	109	109	0.999	206	3.32344	2.36407E-05	sp|Q14197|ICT1_HUMAN	MRPL58	Q14197
1	41	656	656	656	0.999	1278	1.52746	0.00353878	sp|Q14203|DCTN1_HUMAN	DCTN1	Q14203
1	267	7478	7478	7478	0.999	4646	0.0984287	0.542421	sp|Q14204|DYHC1_HUMAN	DYNC1H1	Q14204
1	6	140	140	140	0.999	305	0.588472	0.0871677	sp|Q14232|EI2BA_HUMAN	EIF2B1	Q14232
1	28	101	101	1205	0.999	407	1.19945	0.0106285	sp|Q14240|IF4A2_HUMAN	EIF4A2	Q14240
1	14	100	100	100	0.999	798	2.20311	0.000563025	sp|Q14241|ELOA1_HUMAN	ELOA	Q14241
1	57	1806	1806	1806	0.999	550	5.86632	0	sp|Q14247|SRC8_HUMAN	CTTN	Q14247
1	13	159	159	159	0.999	428	4.44811	0	sp|Q14254|FLOT2_HUMAN	FLOT2	Q14254
1	7	177	177	177	0.999	317	2.33576	0.000437453	sp|Q14257|RCN2_HUMAN	RCN2	Q14257
1	30	907	907	907	0.999	630	5.25054	0	sp|Q14258|TRI25_HUMAN	TRIM25	Q14258
1	65	476	476	1006	0.999	2725	5.61849	0	sp|Q14315|FLNC_HUMAN	FLNC	Q14315
1	8	148	148	148	0.999	412	1.17639	0.0114468	sp|Q14318|FKBP8_HUMAN	FKBP8	Q14318
1	22	354	306	354	0.999	339	4.79505	0	sp|Q14320|FA50A_HUMAN	FAM50A	Q14320
1	5	37	37	37	0.999	258	0.952145	0.0251843	sp|Q14331|FRG1_HUMAN	FRG1	Q14331
0.9999	6	38	4	38	0.9989	565	8.44328	0	sp|Q14332|FZD2_HUMAN	FZD2	Q14332
1	10	153	68	170	0.999	377	8.6608	0	sp|Q14344|GNA13_HUMAN	GNA13	Q14344
1	3	67	67	67	0.999	236	0.537661	0.104018	sp|Q14353|GAMT_HUMAN	GAMT	Q14353
1	13	267	267	267	0.999	348	1.45049	0.0045193	sp|Q14376|GALE_HUMAN	GALE	Q14376
1	27	948	948	948	0.999	709	0.782578	0.0450084	sp|Q14444|CAPR1_HUMAN	CAPRIN1	Q14444
1	16	315	256	315	0.999	530	1.84578	0.00145175	sp|Q14498|RBM39_HUMAN	RBM39	Q14498
1	4	14	14	14	0.999	560	4.60054	0	sp|Q14520|HABP2_HUMAN	HABP2	Q14520
1	11	192	192	192	0.999	1009	1.08628	0.0156523	sp|Q14527|HLTF_HUMAN	HLTF	Q14527
1	11	226	226	226	0.999	519	2.60875	0.000231884	sp|Q14554|PDIA5_HUMAN	PDIA5	Q14554
1	10	215	215	319	0.999	356	0.227988	0.316671	sp|Q14558|KPRA_HUMAN	PRPSAP1	Q14558
1	53	1274	1274	1274	0.999	821	5.9067	0	sp|Q14566|MCM6_HUMAN	MCM6	Q14566
1	59	858	798	858	0.999	2671	0.259775	0.279306	sp|Q14573|ITPR3_HUMAN	ITPR3	Q14573
1	5	39	39	39	0.999	930	5.74006	0	sp|Q14624|ITIH4_HUMAN	ITIH4	Q14624
1	4	101	101	101	0.999	427	1.15826	0.0122111	sp|Q14653|IRF3_HUMAN	IRF3	Q14653
1	20	210	210	210	0.999	1992	1.2577	0.00873128	sp|Q14669|TRIPC_HUMAN	TRIP12	Q14669
1	13	330	266	330	0.999	1186	0.932762	0.0269106	sp|Q14671|PUM1_HUMAN	PUM1	Q14671
1	15	168	168	168	0.999	2089	0.944695	0.0258578	sp|Q14676|MDC1_HUMAN	MDC 1.00	Q14676
1	17	459	446	459	0.999	625	0.442514	0.144585	sp|Q14677|EPN4_HUMAN	CLINT1	Q14677
1	60	942	923	942	0.999	1233	0.334998	0.211475	sp|Q14683|SMC1A_HUMAN	SMC1A	Q14683
1	19	274	274	274	0.999	758	0.734769	0.0531711	sp|Q14684|RRP1B_HUMAN	RRP1B	Q14684
1	6	42	38	42	0.999	1571	1.56954	0.00321523	sp|Q14689|DIP2A_HUMAN	DIP2A	Q14689
1	15	239	239	239	0.999	1282	1.69467	0.00224205	sp|Q14692|BMS1_HUMAN	BMS1	Q14692
1	15	192	192	192	0.999	798	1.59806	0.00295355	sp|Q14694|UBP10_HUMAN	USP10	Q14694
1	7	318	318	318	0.999	234	4.69263	0	sp|Q14696|MESD_HUMAN	MESD	Q14696
1	66	2591	2591	2591	0.999	944	9.11373	0	sp|Q14697|GANAB_HUMAN	GANAB	Q14697
1	12	129	129	129	0.999	578	9.06191	0	sp|Q14699|RFTN1_HUMAN	RFTN1	Q14699
1	11	111	101	151	0.999	602	0.668851	0.0661293	sp|Q14738|2A5D_HUMAN	PPP2R5D	Q14738
1	18	543	543	543	0.999	615	5.37632	0	sp|Q14739|LBR_HUMAN	LBR	Q14739
1	9	102	102	102	0.999	738	2.997	8.13008E-05	sp|Q14746|COG2_HUMAN	COG2	Q14746
1	52	1272	1272	1272	0.999	893	3.81325	0	sp|Q14764|MVP_HUMAN	MVP	Q14764
1	11	28	26	28	0.999	1721	4.97861	0	sp|Q14766|LTBP1_HUMAN	LTBP1	Q14766
1	35	254	254	254	0.999	3259	0.78579	0.0445236	sp|Q14789|GOGB1_HUMAN	GOLGB1	Q14789
1	8	110	110	110	0.999	479	2.49996	0.00029426	sp|Q14790|CASP8_HUMAN	CASP8	Q14790
1	52	1146	798	1146	0.999	1912	4.00354	0	sp|Q14839|CHD4_HUMAN	CHD4	Q14839
1	33	1246	1246	1246	0.999	261	5.63514	0	sp|Q14847|LASP1_HUMAN	LASP1	Q14847
1	15	383	383	383	0.999	329	2.33621	0.000437781	sp|Q14914|PTGR1_HUMAN	PTGR1	Q14914
1	16	305	305	305	0.999	1978	4.31379	0	sp|Q14966|ZN638_HUMAN	ZNF638	Q14966
1	45	1751	1751	1751	0.999	876	6.18353	0	sp|Q14974|IMB1_HUMAN	KPNB1	Q14974
1	33	614	614	614	0.999	699	0.901339	0.0300129	sp|Q14978|NOLC1_HUMAN	NOLC1	Q14978
1	132	2602	2600	2602	0.999	2115	3.92706	0	sp|Q14980|NUMA1_HUMAN	NUMA1	Q14980
1	9	119	119	119	0.999	1843	0.443653	0.144125	sp|Q14997|PSME4_HUMAN	PSME4	Q14997
1	27	524	524	524	0.999	1478	0.720279	0.0559068	sp|Q14C86|GAPD1_HUMAN	GAPVD1	Q14C86
1	17	228	228	228	0.999	972	1.23643	0.00938608	sp|Q14CX7|NAA25_HUMAN	NAA25	Q14CX7
1	23	457	419	457	0.999	741	2.46047	0.000319368	sp|Q15003|CND2_HUMAN	NCAPH	Q15003
1	2	20	20	20	0.999	111	3.47625	1.99501E-05	sp|Q15004|PAF15_HUMAN	PCLAF	Q15004
1	17	403	403	403	0.999	226	4.94737	0	sp|Q15005|SPCS2_HUMAN	SPCS2	Q15005
1	9	167	167	167	0.999	297	4.95944	0	sp|Q15006|EMC2_HUMAN	EMC2	Q15006
1	6	167	167	167	0.999	396	0.275856	0.262816	sp|Q15007|FL2D_HUMAN	WTAP	Q15007
1	24	503	503	503	0.999	389	0.256261	0.283212	sp|Q15008|PSMD6_HUMAN	PSMD6	Q15008
1	5	81	81	81	0.999	288	3.5691	5.19481E-06	sp|Q15014|MO4L2_HUMAN	MORF4L2	Q15014
1	8	67	67	67	0.999	415	1.5364	0.00346306	sp|Q15018|ABRX2_HUMAN	ABRAXAS2	Q15018
1	24	790	790	790	0.999	361	1.41005	0.00519174	sp|Q15019|SEPT2_HUMAN	SEPTIN2	Q15019
1	32	688	688	688	0.999	963	0.711372	0.0575189	sp|Q15020|SART3_HUMAN	SART3	Q15020
1	26	388	387	388	0.999	1401	0.697699	0.0602158	sp|Q15021|CND1_HUMAN	NCAPD2	Q15021
1	10	152	152	152	0.999	291	1.17075	0.0116659	sp|Q15024|EXOS7_HUMAN	EXOSC7	Q15024
0.9999	1	113	113	113	0.9989	740	3.08254	5.91966E-05	sp|Q15027|ACAP1_HUMAN	ACAP1	Q15027
1	43	1083	1083	1345	0.999	972	3.02984	7.04663E-05	sp|Q15029|U5S1_HUMAN	EFTUD2	Q15029
1	8	197	197	197	0.999	203	3.97949	0	sp|Q15041|AR6P1_HUMAN	ARL6IP1	Q15041
1	24	416	416	416	0.999	981	1.28157	0.00809502	sp|Q15042|RB3GP_HUMAN	RAB3GAP1	Q15042
1	3	22	22	22	0.999	492	1.7748	0.00174596	sp|Q15043|S39AE_HUMAN	SLC39A14	Q15043
1	32	1099	1099	1099	0.999	597	3.22698	5.44835E-05	sp|Q15046|SYK_HUMAN	KARS1	Q15046
1	19	316	316	316	0.999	365	0.341228	0.206617	sp|Q15050|RRS1_HUMAN	RRS1	Q15050
1	19	933	933	933	0.999	248	3.54397	2.05128E-05	sp|Q15056|IF4H_HUMAN	EIF4H	Q15056
1	17	70	70	70	0.999	836	4.08757	0	sp|Q15063|POSTN_HUMAN	POSTN	Q15063
1	14	113	113	113	0.999	660	0.445776	0.143047	sp|Q15067|ACOX1_HUMAN	ACOX1	Q15067
1	46	570	570	570	0.999	1411	0.202406	0.348856	sp|Q15075|EEA1_HUMAN	EEA1	Q15075
1	32	1410	1410	1410	0.999	440	5.49677	0	sp|Q15084|PDIA6_HUMAN	PDIA6	Q15084
1	15	751	751	751	0.999	231	2.22625	0.000518781	sp|Q15102|PA1B3_HUMAN	PAFAH1B3	Q15102
1	8	64	64	64	0.999	449	3.74375	0	sp|Q15113|PCOC1_HUMAN	PCOLCE	Q15113
1	10	194	194	194	0.999	130	4.00879	0	sp|Q15121|PEA15_HUMAN	PEA15	Q15121
1	3	189	189	189	0.999	230	3.14327	5.21739E-05	sp|Q15125|EBP_HUMAN	EBP	Q15125
1	14	339	339	339	0.999	192	0.895879	0.0305901	sp|Q15126|PMVK_HUMAN	PMVK	Q15126
1	512	10698	10363	10698	0.999	4684	3.30027	3.75587E-05	sp|Q15149|PLEC_HUMAN	PLEC	Q15149
1	18	186	186	209	0.999	2024	3.0286	7.03934E-05	sp|Q15154|PCM1_HUMAN	PCM1	Q15154
1	25	1191	1126	1191	0.999	289	3.64396	0	sp|Q15181|IPYR_HUMAN	PPA1	Q15181
1	17	799	799	799	0.999	160	4.54422	0	sp|Q15185|TEBP_HUMAN	PTGES3	Q15185
1	66	2139	2136	2283	0.999	471	4.65928	0	sp|Q15233|NONO_HUMAN	NONO	Q15233
1	10	205	205	205	0.999	358	1.73137	0.00201357	sp|Q15257|PTPA_HUMAN	PTPA	Q15257
1	10	112	111	112	0.999	1439	1.60727	0.00291398	sp|Q15262|PTPRK_HUMAN	PTPRK	Q15262
1	11	141	141	141	0.999	919	2.77065	0.000144712	sp|Q15269|PWP2_HUMAN	PWP2	Q15269
1	5	68	68	68	0.999	862	0.977155	0.0230986	sp|Q15276|RABE1_HUMAN	RABEP1	Q15276
1	16	256	214	459	0.999	201	8.0698	0	sp|Q15286|RAB35_HUMAN	RAB35	Q15286
1	7	238	238	238	0.999	305	4.73798	0	sp|Q15287|RNPS1_HUMAN	RNPS1	Q15287
1	6	100	100	100	0.999	538	0.774282	0.0463896	sp|Q15291|RBBP5_HUMAN	RBBP5	Q15291
1	15	483	483	483	0.999	331	5.80484	0	sp|Q15293|RCN1_HUMAN	RCN1	Q15293
1	6	102	102	102	0.999	655	2.73571	0.000171065	sp|Q15311|RBP1_HUMAN	RALBP1	Q15311
1	11	194	194	194	0.999	1064	3.5798	0	sp|Q15334|L2GL1_HUMAN	LLGL1	Q15334
1	7	197	197	197	0.999	201	5.32627	0	sp|Q15363|TMED2_HUMAN	TMED2	Q15363
1	24	1027	655	1027	0.999	356	8.70337	0	sp|Q15365|PCBP1_HUMAN	PCBP1	Q15365
1	22	530	512	902	0.999	365	6.11811	0	sp|Q15366|PCBP2_HUMAN	PCBP2	Q15366

1	10	299	299	299	0.999	112	5.8768	0	sp|Q15369|ELOC_HUMAN	ELOC	Q15369
1	10	361	361	361	0.999	118	2.97791	9.29293E-05	sp|Q15370|ELOB_HUMAN	ELOB	Q15370
1	8	180	180	180	0.999	184	0.468269	0.132764	sp|Q15382|RHEB_HUMAN	RHEB	Q15382
1	13	379	379	379	0.999	516	3.0059	7.78689E-05	sp|Q15392|DHC24_HUMAN	DHCR24	Q15392
1	47	1279	1279	1279	0.999	1217	2.45152	0.000320503	sp|Q15393|SF3B3_HUMAN	SF3B3	Q15393
1	12	121	121	121	0.999	648	4.3441	0	sp|Q15397|PUM3_HUMAN	PUM3	Q15397
1	11	98	98	98	0.999	846	0.292947	0.246708	sp|Q15398|DLGP5_HUMAN	DLGAP5	Q15398
1	11	262	262	262	0.999	277	0.333039	0.212885	sp|Q15404|RSU1_HUMAN	RSU1	Q15404
1	17	187	187	223	0.999	329	7.14859	0	sp|Q15417|CNN3_HUMAN	CNN3	Q15417
1	25	431	286	431	0.999	735	0.104439	0.528346	sp|Q15418|KS6A1_HUMAN	RPS6KA1	Q15418
1	46	827	577	827	0.999	915	2.4362	0.000336973	sp|Q15424|SAFB1_HUMAN	SAFB	Q15424
1	13	494	494	494	0.999	464	3.67374	0	sp|Q15428|SF3A2_HUMAN	SF3A2	Q15428
1	6	60	4	60	0.999	407	0.701173	0.0595337	sp|Q15434|RBMS2_HUMAN	RBMS2	Q15434
1	10	126	126	126	0.999	360	0.635668	0.0742108	sp|Q15435|PP1R7_HUMAN	PPP1R7	Q15435
1	33	665	650	665	0.999	765	3.6772	0	sp|Q15436|SC23A_HUMAN	SEC23A	Q15436
1	21	312	312	327	0.999	767	0.44355	0.144128	sp|Q15437|SC23B_HUMAN	SEC23B	Q15437
1	34	780	779	780	0.999	793	2.73886	0.00017122	sp|Q15459|SF3A1_HUMAN	SF3A1	Q15459
1	5	76	76	76	0.999	200	2.00785	0.000911295	sp|Q15528|MED22_HUMAN	MED22	Q15528
1	12	81	80	81	0.999	327	2.56078	0.00026799	sp|Q15555|MARE2_HUMAN	MAPRE2	Q15555
1	43	482	482	482	0.999	683	7.07687	0	sp|Q15582|BGH3_HUMAN	TGFBI	Q15582
1	12	358	358	358	0.999	228	0.943379	0.0259747	sp|Q15631|TSN_HUMAN	TSN	Q15631
1	17	557	557	557	0.999	639	5.20759	0	sp|Q15637|SF01_HUMAN	SF1	Q15637
1	17	350	350	350	0.999	601	1.72918	0.00202146	sp|Q15642|CIP4_HUMAN	TRIP10	Q15642
1	17	89	89	89	0.999	1979	0.220272	0.325812	sp|Q15643|TRIPB_HUMAN	TRIP11	Q15643
1	12	199	199	199	0.999	432	1.48703	0.00400813	sp|Q15645|PCH2_HUMAN	TRIP13	Q15645
1	6	28	28	28	0.999	514	1.27131	0.00837205	sp|Q15646|OASL_HUMAN	OASL	Q15646
1	17	534	534	534	0.999	476	1.90709	0.00120631	sp|Q15654|TRIP6_HUMAN	TRIP6	Q15654
1	7	84	84	84	0.999	1187	3.24577	5.50459E-05	sp|Q15678|PTN14_HUMAN	PTPN14	Q15678
1	20	891	891	891	0.999	268	2.82749	0.000132576	sp|Q15691|MARE1_HUMAN	MAPRE1	Q15691
1	24	671	588	671	0.999	326	4.42017	0	sp|Q15717|ELAV1_HUMAN	ELAVL1	Q15717
1	20	278	278	278	0.999	373	0.342986	0.205402	sp|Q15738|NSDHL_HUMAN	NSDHL	Q15738
1	30	111	111	162	0.999	1914	1.09922	0.0150099	sp|Q15746|MYLK_HUMAN	MYLK	Q15746
1	10	414	414	414	0.999	541	8.19215	0	sp|Q15758|AAAT_HUMAN	SLC1A5	Q15758
1	15	307	307	307	0.999	309	0.87028	0.0334493	sp|Q15785|TOM34_HUMAN	TOMM34	Q15785
1	6	35	19	35	0.999	467	1.62012	0.00279417	sp|Q15796|SMAD2_HUMAN	SMAD2	Q15796
1	18	303	251	303	0.999	527	0.395801	0.170564	sp|Q15813|TBCE_HUMAN	TBCE	Q15813
1	9	95	95	97	0.999	346	0.671393	0.0656548	sp|Q15814|TBCC_HUMAN	TBCC	Q15814
1	13	341	139	341	0.999	145	3.04307	6.66667E-05	sp|Q15819|UB2V2_HUMAN	UBE2V2	Q15819
1	21	256	254	256	0.999	593	0.679033	0.0639631	sp|Q15833|STXB2_HUMAN	STXBP2	Q15833
1	8	252	99	252	0.999	100	0.848858	0.0359559	sp|Q15836|VAMP3_HUMAN	VAMP3	Q15836
1	7	308	308	308	0.999	81	2.56616	0.000268434	sp|Q15843|NEDD8_HUMAN	NEDD8	Q15843
1	22	739	9	819	0.999	218	6.98115	0	sp|Q15907|RB11B_HUMAN	RAB11B	Q15907
1	3	44	44	44	0.999	746	2.97824	8.89788E-05	sp|Q15910|EZH2_HUMAN	EZH2	Q15910
1	25	774	774	774	0.999	572	6.24403	0	sp|Q15942|ZYX_HUMAN	ZYX	Q15942
1	36	1217	1217	1217	0.999	437	1.54881	0.00338703	sp|Q16181|SEPT7_HUMAN	SEPTIN7	Q16181
1	9	159	159	159	0.999	407	1.73999	0.00194994	sp|Q16186|ADRM1_HUMAN	ADRM1	Q16186
1	16	440	440	440	0.999	474	0.698712	0.059997	sp|Q16204|CCDC6_HUMAN	CCDC6	Q16204
1	28	683	636	683	0.999	522	2.21741	0.000533145	sp|Q16222|UAP1_HUMAN	UAP1	Q16222
1	11	156	149	156	0.999	282	1.55229	0.00335639	sp|Q16270|IBP7_HUMAN	IGFBP7	Q16270
1	23	76	76	76	0.999	1823	4.27226	0	sp|Q16363|LAMA4_HUMAN	LAMA4	Q16363
1	10	12	12	12	0.999	746	3.1073	5.14469E-05	sp|Q16394|EXT1_HUMAN	EXT1	Q16394
1	20	416	416	416	0.999	504	1.43422	0.0048004	sp|Q16401|PSMD5_HUMAN	PSMD5	Q16401
1	16	86	86	90	0.999	942	3.10009	5.54963E-05	sp|Q16512|PKN1_HUMAN	PKN1	Q16512
1	13	227	223	227	0.999	984	0.495511	0.120662	sp|Q16513|PKN2_HUMAN	PKN2	Q16513
1	13	329	329	331	0.999	193	0.810248	0.0410074	sp|Q16527|CSRP2_HUMAN	CSRP2	Q16527
1	51	1495	1495	1495	0.999	1140	5.55531	0	sp|Q16531|DDB1_HUMAN	DDB1	Q16531
1	12	255	204	255	0.999	360	0.415786	0.158951	sp|Q16539|MK14_HUMAN	MAPK14	Q16539
1	31	864	864	864	0.999	378	2.967	9.63855E-05	sp|Q16543|CDC37_HUMAN	CDC37	Q16543
1	33	1200	937	1200	0.999	572	1.96739	0.00104446	sp|Q16555|DPYL2_HUMAN	DPYSL2	Q16555
1	32	1219	676	1219	0.999	425	4.96399	0	sp|Q16576|RBBP7_HUMAN	RBBP7	Q16576
1	5	23	23	23	0.999	540	6.51546	0	sp|Q16610|ECM1_HUMAN	ECM1	Q16610
1	17	529	529	529	0.999	238	1.26364	0.00856985	sp|Q16629|SRSF7_HUMAN	SRSF7	Q16629
1	12	295	295	295	0.999	551	0.759592	0.0488589	sp|Q16630|CPSF6_HUMAN	CPSF6	Q16630
1	23	394	394	394	0.999	649	2.26984	0.000481858	sp|Q16643|DREB_HUMAN	DBN1	Q16643
1	54	2371	2371	2371	0.999	493	4.90642	0	sp|Q16658|FSCN1_HUMAN	FSCN1	Q16658
1	38	748	693	748	0.999	785	2.56009	0.000267769	sp|Q16666|IF16_HUMAN	IFI16	Q16666
1	21	544	544	544	0.999	335	3.23796	5.49199E-05	sp|Q16698|DECR_HUMAN	DECR1	Q16698
1	9	114	114	114	0.999	1144	0.519964	0.110978	sp|Q16706|MA2A1_HUMAN	MAN2A1	Q16706
1	7	109	109	109	0.999	116	0.341912	0.206197	sp|Q16718|NDUA5_HUMAN	NDUFA5	Q16718
1	9	216	213	216	0.999	297	1.03352	0.0189169	sp|Q16762|THTR_HUMAN	TST	Q16762
1	4	7	7	7	0.999	222	1.89738	0.00123355	sp|Q16763|UBE2S_HUMAN	UBE2S	Q16763
1	7	211	211	211	0.999	197	1.10515	0.0146915	sp|Q16774|KGUA_HUMAN	GUK1	Q16774
1	9	181	181	181	0.999	308	1.05742	0.0173917	sp|Q16775|GLO2_HUMAN	HAGH	Q16775
1	11	194	194	194	0.999	377	1.80682	0.00159579	sp|Q16795|NDUA9_HUMAN	NDUFA9	Q16795
1	27	384	384	384	0.999	640	5.42339	0	sp|Q16822|PCKGM_HUMAN	PCK2	Q16822
1	9	158	158	158	0.999	310	3.21461	5.40541E-05	sp|Q16831|UPP1_HUMAN	UPP1	Q16831
1	25	683	683	683	0.999	314	2.65564	0.000198606	sp|Q16836|HCDH_HUMAN	HADH	Q16836
1	11	151	151	151	0.999	509	1.03763	0.0186312	sp|Q16850|CP51A_HUMAN	CYP51A1	Q16850
1	34	788	788	788	0.999	508	1.63404	0.00269352	sp|Q16851|UGPA_HUMAN	UGP2	Q16851
1	34	793	782	793	0.999	649	6.4281	0	sp|Q16881|TRXR1_HUMAN	TXNRD1	Q16881
1	63	1477	1477	1477	0.999	758	3.70301	0	sp|Q16891|MIC60_HUMAN	IMMT	Q16891
1	44	1372	1372	1372	0.999	747	4.813	0	sp|Q1KMD3|HNRL2_HUMAN	HNRNPUL2	Q1KMD3
1	21	358	358	358	0.999	1249	0.17529	0.389142	sp|Q27J81|INF2_HUMAN	INF2	Q27J81
1	35	644	621	644	0.999	1337	0.0336799	0.755449	sp|Q29RF7|PDS5A_HUMAN	PDS5A	Q29RF7
1	4	122	117	122	0.999	961	1.63513	0.00269281	sp|Q2M2I8|AAK1_HUMAN	AAK1	Q2M2I8
1	11	174	174	174	0.999	1173	0.563479	0.0949614	sp|Q2M389|WASC4_HUMAN	WASHC4	Q2M389
1	8	63	63	63	0.999	1250	2.23833	0.000511429	sp|Q2NKX8|ERC6L_HUMAN	ERCC6L	Q2NKX8
1	16	253	253	253	0.999	804	1.13217	0.01342	sp|Q2NL82|TSR1_HUMAN	TSR1	Q2NL82
1	7	97	97	97	0.999	776	0.0558897	0.666416	sp|Q2TAL8|QRIC1_HUMAN	QRICH1	Q2TAL8
1	15	276	276	276	0.999	513	1.74894	0.00191076	sp|Q2TAY7|SMU1_HUMAN	SMU1	Q2TAY7
1	4	35	35	35	0.999	1938	2.16923	0.000616448	sp|Q2TAZ0|ATG2A_HUMAN	ATG2A	Q2TAZ0
0.9999	2	42	42	42	0.999	348	3.11557	5.16129E-05	sp|Q30201|HFE_HUMAN	HFE	Q30201
1	25	393	393	393	0.999	808	3.25395	5.06329E-05	sp|Q32MZ4|LRRF1_HUMAN	LRRFIP1	Q32MZ4
1	17	264	264	264	0.999	736	4.52845	0	sp|Q32P28|P3H1_HUMAN	P3H1	Q32P28
1	24	189	189	192	0.999	841	0.32947	0.215508	sp|Q3KQU3|MA7D1_HUMAN	MAP7D1	Q3KQU3
1	10	70	70	117	0.999	507	2.20649	0.000558989	sp|Q3KQV9|UAP1L_HUMAN	UAP1L1	Q3KQV9
1	17	309	309	309	0.999	575	1.84917	0.00144484	sp|Q3LXA3|TKFC_HUMAN	TKFC	Q3LXA3
1	6	17	17	17	0.999	195	2.25141	0.000488155	sp|Q3MHD2|LSM12_HUMAN	LSM 12.00	Q3MHD2
1	28	339	272	339	0.999	923	1.1873	0.0110481	sp|Q3SY69|AL1L2_HUMAN	ALDH1L2	Q3SY69
1	8	16	11	16	0.999	1871	2.48251	0.000297838	sp|Q3V6T2|GRDN_HUMAN	CCDC88A	Q3V6T2
1	17	366	366	366	0.999	353	0.0381255	0.735623	sp|Q3ZCQ8|TIM50_HUMAN	TIMM50	Q3ZCQ8
1	8	93	93	93	0.999	553	2.3846	0.000392037	sp|Q49A26|GLYR1_HUMAN	GLYR1	Q49A26
1	3	33	33	33	0.999	808	1.63553	0.00269428	sp|Q4KMP7|TB10B_HUMAN	TBC1D10B	Q4KMP7
1	10	127	127	127	0.999	910	7.81691	0	sp|Q4KMQ2|ANO6_HUMAN	ANO6	Q4KMQ2
1	6	27	27	27	0.999	1135	0.119862	0.492859	sp|Q4L180|FIL1L_HUMAN	FILIP1L	Q4L180
1	16	167	167	167	0.999	841	3.3882	2.41255E-05	sp|Q4V328|GRAP1_HUMAN	GRIPAP1	Q4V328
1	5	17	17	17	0.999	283	2.85191	0.000129771	sp|Q4V9L6|TM119_HUMAN	TMEM119	Q4V9L6
1	5	296	296	296	0.999	144	0.752833	0.0499271	sp|Q4VC31|MIX23_HUMAN	MIX23	Q4VC31
1	13	295	210	295	0.999	433	2.35283	0.000431373	sp|Q52LJ0|FA98B_HUMAN	FAM98B	Q52LJ0
1	3	29	29	29	0.999	1261	0.426585	0.152876	sp|Q52LW3|RHG29_HUMAN	ARHGAP29	Q52LW3
1	13	273	273	273	0.999	469	1.75961	0.00182778	sp|Q53EL6|PDCD4_HUMAN	PDCD4	Q53EL6
1	14	175	175	175	0.999	1204	0.235313	0.307299	sp|Q53EP0|FND3B_HUMAN	FNDC3B	Q53EP0
1	8	17	17	17	0.999	464	3.15871	5.25739E-05	sp|Q53EZ4|CEP55_HUMAN	CEP55	Q53EZ4
1	8	30	30	30	0.999	620	1.5737	0.00318982	sp|Q53F19|NCBP3_HUMAN	NCBP3	Q53F19
1	6	205	205	205	0.999	312	0.121231	0.48987	sp|Q53GQ0|DHB12_HUMAN	HSD17B12	Q53GQ0
1	15	256	256	256	0.999	565	0.37215	0.184887	sp|Q53GS9|SNUT2_HUMAN	USP39	Q53GS9
1	9	100	100	100	0.999	288	0.0658629	0.63289	sp|Q53H82|LACB2_HUMAN	LACTB2	Q53H82
1	11	124	124	124	0.999	274	1.046	0.0181076	sp|Q53H96|P5CR3_HUMAN	PYCR3	Q53H96
0.9997	2	8	8	8	0.999	286	7.38892	0	sp|Q53TN4|CYBR1_HUMAN	CYBRD1	Q53TN4
1	8	103	103	103	0.999	631	0.965661	0.0240644	sp|Q567U6|CCD93_HUMAN	CCDC93	Q567U6
1	7	83	83	500	0.999	418	4.15311	0	sp|Q58FG1|HS904_HUMAN	HSP90AA4P	Q58FG1
1	2	17	17	17	0.999	314	2.83174	0.000132701	sp|Q5BJH7|YIF1B_HUMAN	YIF1B	Q5BJH7
1	22	332	332	332	0.999	582	1.5662	0.0032384	sp|Q5BKZ1|ZN326_HUMAN	ZNF326	Q5BKZ1
1	6	49	49	49	0.999	860	1.69876	0.00221353	sp|Q5C9Z4|NOM1_HUMAN	NOM1	Q5C9Z4
1	19	215	215	215	0.999	1057	6.0128	0	sp|Q5GLZ8|HERC4_HUMAN	HERC4	Q5GLZ8
1	4	69	0	69	0.999	206	0.735633	0.0530295	sp|Q5H9L2|TCAL5_HUMAN	TCEAL5	Q5H9L2
1	9	190	189	190	0.999	873	0.977994	0.0230419	sp|Q5H9R7|PP6R3_HUMAN	PPP6R3	Q5H9R7
1	5	134	134	134	0.999	236	0.58498	0.0881613	sp|Q5HYI8|RABL3_HUMAN	RABL3	Q5HYI8
1	11	90	90	90	0.999	523	2.16784	0.000618785	sp|Q5JPH6|SYEM_HUMAN	EARS2	Q5JPH6

1	21	172	172	172	0.999	1907	0.969034	0.023772	sp|Q5JRA6|TGO1_HUMAN	MIA3	Q5JRA6
1	38	512	512	512	0.999	1037	1.99513	0.000959391	sp|Q5JRX3|PREP_HUMAN	PITRM1	Q5JRX3
1	12	69	69	131	0.999	2229	4.55966	0	sp|Q5JSZ5|PRC2B_HUMAN	PRRC2B	Q5JSZ5
1	36	527	526	527	0.999	1297	0.695074	0.060748	sp|Q5JTH9|RRP12_HUMAN	RRP12	Q5JTH9
1	20	567	567	567	0.999	583	2.6949	0.000184561	sp|Q5JTV8|TOIP1_HUMAN	TOR1AIP1	Q5JTV8
1	15	268	268	268	0.999	985	0.63663	0.074008	sp|Q5JTZ9|SYAM_HUMAN	AARS2	Q5JTZ9
1	7	90	90	90	0.999	399	1.89012	0.00127798	sp|Q5JVF3|PCID2_HUMAN	PCID2	Q5JVF3
1	4	13	13	13	0.999	742	2.04621	0.000845254	sp|Q5KU26|COL12_HUMAN	COLEC12	Q5KU26
1	13	119	119	121	0.999	1068	3.55054	0.000020592	sp|Q5M775|CYTSB_HUMAN	SPECC1	Q5M775
0.9999	2	70	70	70	0.999	344	1.87179	0.00134689	sp|Q5MNZ6|WIPI3_HUMAN	WDR45B	Q5MNZ6
1	9	211	211	211	0.999	272	3.18132	5.32151E-05	sp|Q5RKV6|EXOS6_HUMAN	EXOSC6	Q5RKV6
1	12	76	76	76	0.999	1749	0.957614	0.0247159	sp|Q5SRE5|NU188_HUMAN	NUP188	Q5SRE5
1	35	763	763	763	0.999	553	2.70139	0.000181656	sp|Q5SSJ5|HP1B3_HUMAN	HP1BP3	Q5SSJ5
1	35	517	446	517	0.999	1584	0.949086	0.0254838	sp|Q5SW79|CE170_HUMAN	CEP170	Q5SW79
1	5	110	110	110	0.999	702	1.94726	0.00109789	sp|Q5SY16|NOL9_HUMAN	NOL9	Q5SY16
1	5	70	70	70	0.999	605	1.38531	0.00563194	sp|Q5T0N5|FBP1L_HUMAN	FNBP1L	Q5T0N5
1	16	204	204	204	0.999	1219	1.76278	0.0018046	sp|Q5T1M5|FKB15_HUMAN	FKBP15	Q5T1M5
1	11	69	69	71	0.999	1668	0.174143	0.391001	sp|Q5T200|ZC3HD_HUMAN	ZC3H13	Q5T200
1	64	971	971	971	0.999	5183	0.0400836	0.727326	sp|Q5T4S7|UBR4_HUMAN	UBR4	Q5T4S7
1	15	97	97	97	0.999	1943	1.88296	0.00129892	sp|Q5T5P2|SKT_HUMAN	KIAA1217	Q5T5P2
1	5	111	111	111	0.999	1119	0.282823	0.255791	sp|Q5T6F2|UBAP2_HUMAN	UBAP2	Q5T6F2
1	9	116	116	116	0.999	341	0.373566	0.183971	sp|Q5T6V5|QNG1_HUMAN	QNG1	Q5T6V5
1	5	7	7	7	0.9988	579	4.06445	0	sp|Q5T749|KPRP_HUMAN	KPRP	Q5T749
1	17	158	158	158	0.999	1007	1.06213	0.0171326	sp|Q5T8P6|RBM26_HUMAN	RBM26	Q5T8P6
1	36	110	75	991	0.999	648	1.1113	0.0143809	sp|Q5T9A4|ATD3B_HUMAN	ATAD3B	Q5T9A4
1	3	47	47	47	0.999	541	1.33811	0.00663619	sp|Q5T9L3|WLS_HUMAN	WLS	Q5T9L3
1	4	84	84	84	0.999	328	1.14011	0.0130446	sp|Q5TC12|ATPF1_HUMAN	ATPAF1	Q5TC12
1	12	468	15	468	0.999	136	1.68733	0.00229079	sp|Q5TEC6|H37_HUMAN	H3-7	Q5TEC6
1	9	136	136	136	0.999	455	1.80655	0.00159486	sp|Q5TFE4|NT5D1_HUMAN	NT5DC1	Q5TFE4
1	12	69	69	69	0.999	2472	0.74336	0.051671	sp|Q5UIP0|RIF1_HUMAN	RIF1	Q5UIP0
1	5	29	11	29	0.999	837	1.96199	0.00106234	sp|Q5VSL9|STRP1_HUMAN	STRIP1	Q5VSL9
1	7	62	61	62	0.999	975	0.964767	0.0241417	sp|Q5VTR2|BRE1A_HUMAN	RNF20	Q5VTR2
1	10	176	176	176	0.999	411	0.875684	0.0328685	sp|Q5VW32|BROX_HUMAN	BROX	Q5VW32
1	6	62	62	62	0.999	1801	5.54751	0	sp|Q5VW36|FOCAD_HUMAN	FOCAD	Q5VW36
1	41	838	838	838	0.999	1845	0.416869	0.15837	sp|Q5VYK3|ECM29_HUMAN	ECPAS	Q5VYK3
1	3	98	98	98	0.999	725	2.06047	0.000809961	sp|Q5VZE5|NAA35_HUMAN	NAA35	Q5VZE5
1	7	51	43	51	0.999	765	2.92868	0.00012586	sp|Q5W0V3|FHI2A_HUMAN	FHIP2A	Q5W0V3
1	3	59	59	59	0.999	1314	1.34348	0.00648282	sp|Q5XPI4|RN123_HUMAN	RNF123	Q5XPI4
1	3	39	39	39	0.999	534	5.77034	0	sp|Q5ZPR3|CD276_HUMAN	CD276	Q5ZPR3
1	32	137	137	137	0.999	5207	2.3945	0.000378753	sp|Q63HN8|RN213_HUMAN	RNF213	Q63HN8
0.9985	3	25	25	25	0.999	1409	1.80368	0.00161027	sp|Q63HR2|TENS2_HUMAN	TNS2	Q63HR2
1	14	116	116	116	0.999	851	0.809298	0.0411165	sp|Q63ZY3|KANK2_HUMAN	KANK2	Q63ZY3
1	16	216	48	216	0.999	1341	0.724705	0.0550873	sp|Q641Q2|WAC2A_HUMAN	WASHC2A	Q641Q2
1	12	118	118	118	0.999	838	2.16191	0.000623626	sp|Q658Y4|F91A1_HUMAN	FAM91A1	Q658Y4
1	8	132	89	132	0.999	1250	1.60478	0.00291221	sp|Q66K14|TBC9B_HUMAN	TBC1D9B	Q66K14
1	8	101	101	101	0.999	1059	2.09869	0.000737683	sp|Q66K74|MAP1S_HUMAN	MAP1S	Q66K74
1	9	172	172	172	0.999	237	0.9305	0.0271532	sp|Q66PJ3|AR6P4_HUMAN	ARL6IP4	Q66PJ3
1	10	135	135	135	0.999	756	2.00053	0.000935197	sp|Q68CQ4|UTP25_HUMAN	UTP25	Q68CQ4
1	11	107	107	107	0.999	1445	2.36203	0.000416918	sp|Q68CZ2|TENS3_HUMAN	TNS3	Q68CZ2
1	12	170	170	170	0.999	1043	0.408235	0.163456	sp|Q68E01|INT3_HUMAN	INTS3	Q68E01
1	9	84	60	84	0.999	881	0.439685	0.145917	sp|Q68EM7|RHG17_HUMAN	ARHGAP17	Q68EM7
1	7	93	93	93	0.999	538	0.345603	0.203587	sp|Q69YN2|C19L1_HUMAN	CWF19L1	Q69YN2
1	6	18	18	18	0.9989	1812	0.784813	0.0446567	sp|Q69YN4|VIR_HUMAN	VIRMA	Q69YN4
1	5	18	18	18	0.999	276	2.02806	0.000876289	sp|Q6BCY4|NB5R2_HUMAN	CYB5R2	Q6BCY4
1	5	116	116	116	0.999	382	0.871318	0.0333307	sp|Q6DD87|ZN787_HUMAN	ZNF787	Q6DD87
1	21	609	608	609	0.999	541	0.0989243	0.541287	sp|Q6DD88|ATLA3_HUMAN	ATL3	Q6DD88
1	5	62	62	62	0.999	255	1.35622	0.00620134	sp|Q6DKI1|RL7L_HUMAN	RPL7L1	Q6DKI1
1	5	70	70	70	0.999	435	0.22299	0.322293	sp|Q6DKJ4|NXN_HUMAN	NXN	Q6DKJ4
1	6	53	53	53	0.999	673	2.65227	0.000201564	sp|Q6EMK4|VASN_HUMAN	VASN	Q6EMK4
1	13	307	307	307	0.999	312	0.501702	0.11826	sp|Q6FI81|CPIN1_HUMAN	CIAPIN1	Q6FI81
1	4	33	33	33	0.999	208	3.39248	2.41546E-05	sp|Q6FIF0|ZFAN6_HUMAN	ZFAND6	Q6FIF0
1	3	35	35	35	0.999	204	2.34759	0.000430075	sp|Q6I9Y2|THOC7_HUMAN	THOC7	Q6I9Y2
1	12	146	146	146	0.999	826	0.0913812	0.560252	sp|Q6IA86|ELP2_HUMAN	ELP2	Q6IA86
1	7	84	84	84	0.999	325	3.07344	5.88854E-05	sp|Q6IAN0|DRS7B_HUMAN	DHRS7B	Q6IAN0
1	19	392	392	504	0.999	349	2.70195	0.000181818	sp|Q6IBS0|TWF2_HUMAN	TWF2	Q6IBS0
1	15	204	204	204	0.999	609	0.0960039	0.548393	sp|Q6L8Q7|PDE12_HUMAN	PDE12	Q6L8Q7
1	24	519	519	519	0.999	477	1.7962	0.00164377	sp|Q6NUK1|SCMC1_HUMAN	SLC25A24	Q6NUK1
1	6	9	9	10	0.999	255	4.01609	0	sp|Q6NUK4|REEP3_HUMAN	REEP3	Q6NUK4
1	15	259	259	259	0.999	610	2.51372	0.000283619	sp|Q6NUM9|RETST_HUMAN	RETSAT	Q6NUM9
1	8	127	127	127	0.999	689	0.0193532	0.828995	sp|Q6NUQ4|TM214_HUMAN	TMEM214	Q6NUQ4
1	11	335	335	335	0.999	386	0.184956	0.374028	sp|Q6NVY1|HIBCH_HUMAN	HIBCH	Q6NVY1
1	8	127	127	127	0.999	501	1.27419	0.00828717	sp|Q6NXE6|ARMC6_HUMAN	ARMC6	Q6NXE6
1	21	266	266	266	0.999	613	0.295737	0.244095	sp|Q6NYC8|PPR18_HUMAN	PPP1R18	Q6NYC8
1	34	636	636	636	0.999	390	5.97931	0	sp|Q6NZI2|CAVN1_HUMAN	CAVIN1	Q6NZI2
1	9	160	160	160	0.999	707	0.507446	0.115919	sp|Q6NZY4|ZCHC8_HUMAN	ZCCHC8	Q6NZY4
1	15	190	190	190	0.999	531	1.24193	0.0092071	sp|Q6P1J9|CDC73_HUMAN	CDC73	Q6P1J9
1	15	137	137	137	0.999	951	2.10431	0.000732131	sp|Q6P1N0|C2D1A_HUMAN	CC2D1A	Q6P1N0
1	22	442	442	442	0.999	1401	1.6814	0.00233925	sp|Q6P2E9|EDC4_HUMAN	EDC4	Q6P2E9
1	84	2108	2108	2108	0.999	2335	2.69706	0.000185053	sp|Q6P2Q9|PRP8_HUMAN	PRPF8	Q6P2Q9
1	10	102	102	102	0.999	843	0.918843	0.0282658	sp|Q6P3X3|TTC27_HUMAN	TTC27	Q6P3X3
1	9	75	75	202	0.999	122	0.517908	0.111845	sp|Q6P5R6|RL22L_HUMAN	RPL22L1	Q6P5R6
1	7	111	111	111	0.999	394	1.36719	0.00599519	sp|Q6P6C2|ALKB5_HUMAN	ALKBH5	Q6P6C2
1	17	274	267	274	0.999	788	2.52485	0.000284314	sp|Q6P996|PDXD1_HUMAN	PDXDC1	Q6P996
1	10	117	117	117	0.999	456	3.11941	5.17241E-05	sp|Q6P9B6|MEAK7_HUMAN	MEAK7	Q6P9B6
1	14	188	188	188	0.999	1173	0.571214	0.0924719	sp|Q6PD62|CTR9_HUMAN	CTR9	Q6PD62
1	7	80	6	80	0.999	1433	1.14781	0.0126927	sp|Q6PFW1|VIP1_HUMAN	PPIP5K1	Q6PFW1
1	13	110	110	110	0.999	1564	1.84543	0.00145325	sp|Q6PGP7|SKI3_HUMAN	SKIC3	Q6PGP7
1	22	523	523	523	0.999	645	3.82501	0	sp|Q6PI48|SYDM_HUMAN	DARS2	Q6PI48
1	9	198	198	198	0.999	408	0.462536	0.135401	sp|Q6PIU2|NCEH1_HUMAN	NCEH1	Q6PIU2
1	21	251	251	251	0.999	736	0.0441652	0.710465	sp|Q6PJT7|ZC3HE_HUMAN	ZC3H14	Q6PJT7
1	7	54	54	54	0.999	289	0.926484	0.0275016	sp|Q6PK04|CC137_HUMAN	CCDC137	Q6PK04
1	38	527	527	527	0.999	1096	1.08744	0.0155816	sp|Q6PKG0|LARP1_HUMAN	LARP1	Q6PKG0
1	18	140	140	140	0.999	1708	1.59663	0.00296051	sp|Q6R327|RICTR_HUMAN	RICTOR	Q6R327
1	24	408	408	506	0.999	978	0.0570649	0.662573	sp|Q6UB35|C1TM_HUMAN	MTHFD1L	Q6UB35
1	10	219	219	219	0.999	594	0.0238988	0.8036	sp|Q6UN15|FIP1_HUMAN	FIP1L1	Q6UN15
1	59	563	563	563	0.999	2322	1.83336	0.001495	sp|Q6UVK1|CSPG4_HUMAN	CSPG4	Q6UVK1
1	5	14	14	14	0.999	613	4.79343	0	sp|Q6UVY6|MOXD1_HUMAN	MOXD1	Q6UVY6
1	4	93	93	93	0.999	189	0.224848	0.320198	sp|Q6UW68|TM205_HUMAN	TMEM205	Q6UW68
1	4	46	46	46	0.999	472	2.58136	0.000248114	sp|Q6UX04|CWC27_HUMAN	CWC27	Q6UX04
1	6	77	77	77	0.999	353	1.57252	0.00319111	sp|Q6UXH1|CREL2_HUMAN	CRELD2	Q6UXH1
1	3	3	3	3	0.999	406	1.45541	0.00443015	sp|Q6UXH8|CCBE1_HUMAN	CCBE1	Q6UXH8
1	10	164	164	164	0.999	313	1.19349	0.0108316	sp|Q6UXN9|WDR82_HUMAN	WDR82	Q6UXN9
1	6	123	123	123	0.999	268	2.78365	0.000142056	sp|Q6UXV4|MIC27_HUMAN	APOOL	Q6UXV4
1	5	5	5	5	0.999	1074	4.45985	0	sp|Q6UY14|ATL4_HUMAN	ADAMTSL4	Q6UY14
1	24	223	223	223	0.999	1025	0.000791079	0.985757	sp|Q6WCQ1|MPRIP_HUMAN	MPRIP	Q6WCQ1
1	11	404	404	404	0.999	538	0.317663	0.225091	sp|Q6XQN6|PNCB_HUMAN	NAPRT	Q6XQN6
1	14	72	72	72	0.999	1577	0.495985	0.120551	sp|Q6XZF7|DNMBP_HUMAN	DNMBP	Q6XZF7
1	21	233	233	238	0.999	1299	0.488971	0.123387	sp|Q6Y7W6|GGYF2_HUMAN	GIGYF2	Q6Y7W6
1	18	212	212	212	0.999	1445	4.07537	0	sp|Q6YHK3|CD109_HUMAN	CD109	Q6YHK3
0.926	1	77	77	77	0.998	380	3.19085	5.35117E-05	sp|Q6YI46|TMM64_HUMAN	TMEM64	Q6YI46
1	14	257	257	257	0.999	418	1.21551	0.0100783	sp|Q6YN16|HSDL2_HUMAN	HSDL2	Q6YN16
1	6	117	87	117	0.999	454	1.35905	0.00615562	sp|Q6YP21|KAT3_HUMAN	KYAT3	Q6YP21
1	10	85	85	85	0.999	911	2.46606	0.000316957	sp|Q6ZNB6|NFXL1_HUMAN	NFXL1	Q6ZNB6
1	8	73	73	73	0.999	698	2.05571	0.000821452	sp|Q6ZRP7|QSOX2_HUMAN	QSOX2	Q6ZRP7
1	5	11	11	11	0.999	1223	1.74429	0.00193607	sp|Q6ZS17|RIPR1_HUMAN	RIPOR1	Q6ZS17
1	3	54	54	54	0.999	355	0.268032	0.270866	sp|Q6ZSR9|YJ005_HUMAN		Q6ZSR9
1	7	81	81	87	0.999	915	1.3331	0.00674703	sp|Q6ZXV5|TMTC3_HUMAN	TMTC3	Q6ZXV5
1	13	89	89	89	0.999	1250	3.53991	2.04604E-05	sp|Q70E73|RAPH1_HUMAN	RAPH1	Q70E73
1	16	224	224	224	0.999	350	2.29732	0.00045815	sp|Q70UQ0|IKIP_HUMAN	IKBIP	Q70UQ0
1	20	281	9	734	0.999	136	4.93573	0	sp|Q71DI3|H32_HUMAN	H3C15	Q71DI3
1	9	30	30	46	0.999	724	1.98801	0.000979798	sp|Q71RC2|LARP4_HUMAN	LARP4	Q71RC2
0.9998	2	9	9	9	0.9987	1427	2.72429	0.000173129	sp|Q76LX8|ATS13_HUMAN	ADAMTS13	Q76LX8
1	5	23	18	23	0.999	527	2.37832	0.000406107	sp|Q7KYR7|BT2A1_HUMAN	BTN2A1	Q7KYR7
1	18	269	269	269	0.999	1726	0.416164	0.158778	sp|Q7KZ85|SPT6H_HUMAN	SUPT6H	Q7KZ85
1	79	3313	3313	3313	0.999	910	5.46174	0	sp|Q7KZF4|SND1_HUMAN	SND1	Q7KZF4
1	11	160	94	160	0.999	788	0.669258	0.066036	sp|Q7KZI7|MARK2_HUMAN	MARK2	Q7KZI7
1	45	1021	1021	1021	0.999	1031	1.32406	0.0069414	sp|Q7L014|DDX46_HUMAN	DDX46	Q7L014
1	15	316	316	316	0.999	403	1.30136	0.00754077	sp|Q7L0Y3|TM10C_HUMAN	TRMT10C	Q7L0Y3
1	27	884	827	884	0.999	419	1.82513	0.00152437	sp|Q7L1Q6|5MP2_HUMAN	BZW1	Q7L1Q6

1	32	413	413	413	0.999	1194	1.3676	0.00598845	sp|Q7L2E3|DHX30_HUMAN	DHX30	Q7L2E3
1	15	377	377	377	0.999	374	1.35607	0.0062022	sp|Q7L2H7|EIF3M_HUMAN	EIF3M	Q7L2H7
1	11	273	273	273	0.999	689	1.96347	0.00106567	sp|Q7L2J0|MEPCE_HUMAN	MEPCE	Q7L2J0
1	4	48	1	48	0.999	313	1.11839	0.0140635	sp|Q7L523|RRAGA_HUMAN	RRAGA	Q7L523
1	43	868	423	868	0.999	1253	8.275	0	sp|Q7L576|CYFP1_HUMAN	CYFIP1	Q7L576
1	10	239	239	239	0.999	327	1.2011	0.01057	sp|Q7L5N1|CSN6_HUMAN	COPS6	Q7L5N1
1	6	115	115	115	0.999	544	1.47438	0.00416405	sp|Q7L5N7|PCAT2_HUMAN	LPCAT2	Q7L5N7
1	4	21	21	21	0.999	396	3.2835	0.00004662	sp|Q7L5Y9|MAEA_HUMAN	MAEA	Q7L5Y9
1	17	199	197	199	0.999	1761	0.0111871	0.881907	sp|Q7LBC6|KDM3B_HUMAN	KDM3B	Q7LBC6
1	15	326	326	326	0.999	199	5.62003	0	sp|Q7LBR1|CHM1B_HUMAN	CHMP1B	Q7LBR1
1	12	194	194	194	0.999	110	1.53186	0.00350571	sp|Q7RTV0|PHF5A_HUMAN	PHF5A	Q7RTV0
1	38	595	595	595	0.999	902	0.712392	0.0573968	sp|Q7Z2W4|ZCCHV_HUMAN	ZC3HAV1	Q7Z2W4
1	18	299	299	299	0.999	1120	2.14911	0.000643929	sp|Q7Z2Z2|EFL1_HUMAN	EFL1	Q7Z2Z2
1	12	104	104	104	0.999	507	0.466647	0.13359	sp|Q7Z3B4|NUP54_HUMAN	NUP54	Q7Z3B4
1	11	101	101	101	0.999	1410	3.20877	5.39326E-05	sp|Q7Z3K3|POGZ_HUMAN	POGZ	Q7Z3K3
1	6	75	75	75	0.999	1717	0.890457	0.0312289	sp|Q7Z3U7|MON2_HUMAN	MON2	Q7Z3U7
1	8	42	29	166	0.999	459	3.34152	2.38095E-05	sp|Q7Z3Y8|K1C27_HUMAN	KRT27	Q7Z3Y8
1	15	142	142	142	0.999	695	0.850635	0.0357711	sp|Q7Z417|NUFP2_HUMAN	NUFIP2	Q7Z417
1	4	65	65	65	0.999	152	3.09072	5.95112E-05	sp|Q7Z422|SZRD1_HUMAN	SZRD1	Q7Z422
1	3	47	47	47	0.999	540	1.89475	0.00125136	sp|Q7Z434|MAVS_HUMAN	MAVS	Q7Z434
1	20	265	234	265	0.999	1538	1.19582	0.0107489	sp|Q7Z460|CLAP1_HUMAN	CLASP1	Q7Z460
1	6	56	56	56	0.999	507	1.90103	0.00122538	sp|Q7Z4H8|PLGT3_HUMAN	POGLUT3	Q7Z4H8
1	12	10	10	134	0.999	341	2.87232	0.000126923	sp|Q7Z4I7|LIMS2_HUMAN	LIMS2	Q7Z4I7
1	6	84	84	84	0.999	680	1.92519	0.00116319	sp|Q7Z4Q2|HEAT3_HUMAN	HEATR3	Q7Z4Q2
0.9999	2	30	30	30	0.999	191	0.74241	0.0518394	sp|Q7Z4R8|CF120_HUMAN	C6orf120	Q7Z4R8
1	19	279	279	296	0.999	671	2.06657	0.000797368	sp|Q7Z4V5|HDGR2_HUMAN	HDGFL2	Q7Z4V5
1	9	67	67	67	0.999	244	3.33209	0.000023753	sp|Q7Z4W1|DCXR_HUMAN	DCXR	Q7Z4W1
1	4	19	19	19	0.999	137	5.00296	0	sp|Q7Z5G4|GOGA7_HUMAN	GOLGA7	Q7Z5G4
0.9865	2	111	111	111	0.9957	357	4.76771	0	sp|Q7Z5H4|VN1R5_HUMAN	VN1R5	Q7Z5H4
1	8	80	80	80	0.999	226	2.46766	0.000317965	sp|Q7Z6K5|ARPIN_HUMAN	ARPIN	Q7Z6K5
1	115	1726	1726	1726	0.999	4374	0.300361	0.240149	sp|Q7Z6Z7|HUWE1_HUMAN	HUWE1	Q7Z6Z7
1	10	93	12	93	0.999	585	0.269523	0.269434	sp|Q7Z739|YTHD3_HUMAN	YTHDF3	Q7Z739
1	12	208	208	265	0.999	227	3.14615	5.23446E-05	sp|Q7Z7H5|TMED4_HUMAN	TMED4	Q7Z7H5
1	4	48	48	48	0.999	106	1.6055	0.00291104	sp|Q7Z7K0|COXM1_HUMAN	CMC1	Q7Z7K0
1	5	8	7	8	0.9976	901	0.287827	0.251244	sp|Q7Z7L1|SLN11_HUMAN	SLFN11	Q7Z7L1
1	10	37	37	37	0.999	1253	1.84401	0.0014554	sp|Q86SQ0|PHLB2_HUMAN	PHLDB2	Q86SQ0
1	4	125	125	125	0.999	157	1.55171	0.00336335	sp|Q86SX6|GLRX5_HUMAN	GLRX5	Q86SX6
1	4	70	69	70	0.999	277	1.37105	0.00589403	sp|Q86T03|PP4P1_HUMAN	PIP4P1	Q86T03
1	8	98	98	98	0.999	770	4.38536	0	sp|Q86TB9|PATL1_HUMAN	PATL1	Q86TB9
1	19	175	175	175	0.999	863	0.195012	0.35904	sp|Q86TI2|DPP9_HUMAN	DPP9	Q86TI2
1	7	334	245	334	0.999	306	2.30606	0.000454613	sp|Q86U42|PABP2_HUMAN	PABPN1	Q86U42
1	19	204	204	204	0.999	1689	1.29075	0.00783754	sp|Q86U86|PB1_HUMAN	PBRM1	Q86U86
1	22	754	754	754	0.999	582	4.21588	0	sp|Q86UE4|LYRIC_HUMAN	MTDH	Q86UE4
1	86	1806	1806	1806	0.999	1357	1.99401	0.000968928	sp|Q86UP2|KTN1_HUMAN	KTN1	Q86UP2
1	8	63	63	63	0.999	1035	2.52729	0.000281276	sp|Q86UV5|UBP48_HUMAN	USP48	Q86UV5
1	7	18	18	18	0.9989	667	3.47098	1.99253E-05	sp|Q86UX7|URP2_HUMAN	FERMT3	Q86UX7
1	8	156	156	156	0.999	672	0.685542	0.0626627	sp|Q86V21|AACS_HUMAN	AACS	Q86V21
1	25	236	236	236	0.999	1076	1.03349	0.0189106	sp|Q86V48|LUZP1_HUMAN	LUZP1	Q86V48
1	20	627	627	627	0.999	257	4.71135	0	sp|Q86V81|THOC4_HUMAN	ALYREF	Q86V81
1	12	22	22	106	0.999	1631	4.24448	0	sp|Q86VI3|IQGA3_HUMAN	IQGAP3	Q86VI3
1	15	199	199	199	0.999	953	1.45542	0.00443238	sp|Q86VM9|ZCH18_HUMAN	ZC3H18	Q86VM9
1	53	1458	1283	1458	0.999	1230	2.56506	0.000268212	sp|Q86VP6|CAND1_HUMAN	CAND1	Q86VP6
1	7	59	59	59	0.999	718	0.658888	0.0684469	sp|Q86VS8|HOOK3_HUMAN	HOOK3	Q86VS8
1	7	21	20	21	0.999	417	0.618389	0.0787004	sp|Q86VY4|TSYL5_HUMAN	TSPYL5	Q86VY4
1	17	160	158	160	0.999	1011	0.0848829	0.576491	sp|Q86W92|LIPB1_HUMAN	PPFIBP1	Q86W92
1	3	77	77	77	0.999	139	0.828577	0.0385584	sp|Q86WQ0|NR2CA_HUMAN	NR2C2AP	Q86WQ0
1	5	30	30	30	0.999	208	0.734038	0.0532699	sp|Q86WR0|CCD25_HUMAN	CCDC25	Q86WR0
1	5	50	50	50	0.999	379	1.21989	0.00993415	sp|Q86WV6|STING_HUMAN	STING1	Q86WV6
1	15	362	362	362	0.999	608	1.0642	0.0169889	sp|Q86X55|CARM1_HUMAN	CARM1	Q86X55
1	11	186	186	186	0.999	327	0.114344	0.504894	sp|Q86X76|NIT1_HUMAN	NIT1	Q86X76
1	6	42	42	42	0.999	1143	0.815495	0.0403217	sp|Q86XI2|CNDG2_HUMAN	NCAPG2	Q86XI2
1	16	110	110	110	0.999	938	0.525899	0.108674	sp|Q86XL3|ANKL2_HUMAN	ANKLE2	Q86XL3
1	23	500	500	500	0.999	938	0.585094	0.0881599	sp|Q86XP3|DDX42_HUMAN	DDX42	Q86XP3
1	10	116	116	116	0.999	545	2.34065	0.000432757	sp|Q86XZ4|SPAS2_HUMAN	SPATS2	Q86XZ4
1	11	175	175	175	0.999	855	2.08221	0.000775645	sp|Q86Y56|DAAF5_HUMAN	DNAAF5	Q86Y56
1	8	120	120	120	0.999	276	0.449203	0.141437	sp|Q86Y82|STX12_HUMAN	STX12	Q86Y82
1	4	55	51	55	0.999	675	1.17156	0.0116393	sp|Q86YR5|GPSM1_HUMAN	GPSM1	Q86YR5
1	9	62	62	187	0.999	212	2.04794	0.000838542	sp|Q86YS6|RAB43_HUMAN	RAB43	Q86YS6
1	12	96	96	96	0.999	2850	2.85651	0.000130019	sp|Q86YZ3|HORN_HUMAN	HRNR	Q86YZ3
1	9	49	49	50	0.999	584	0.637626	0.0738856	sp|Q8IU81|I2BP1_HUMAN	IRF2BP1	Q8IU81
1	24	295	286	295	0.999	1116	1.38417	0.00564546	sp|Q8IUD2|RB6I2_HUMAN	ERC1	Q8IUD2
1	3	82	82	82	0.999	188	2.93351	0.000126108	sp|Q8IUR0|TPPC5_HUMAN	TRAPPC5	Q8IUR0
1	9	79	79	79	0.999	490	2.56005	0.000267547	sp|Q8IV08|PLD3_HUMAN	PLD3	Q8IV08
1	6	102	102	102	0.999	349	2.06403	0.000806303	sp|Q8IV48|ERI1_HUMAN	ERI1	Q8IV48
1	74	528	528	531	0.999	5795	1.75534	0.00185657	sp|Q8IVF2|AHNK2_HUMAN	AHNAK2	Q8IVF2
1	8	18	18	18	0.999	736	1.31459	0.00722222	sp|Q8IVL6|P3H3_HUMAN	P3H3	Q8IVL6
1	12	135	135	135	0.999	306	0.356237	0.196339	sp|Q8IVM0|CCD50_HUMAN	CCDC50	Q8IVM0
1	10	55	55	55	0.999	706	7.96873	0	sp|Q8IWA5|CTL2_HUMAN	SLC44A2	Q8IWA5
1	4	47	47	47	0.999	547	1.95502	0.00108276	sp|Q8IWB1|IPRI_HUMAN	ITPRIP	Q8IWB1
1	9	55	55	58	0.999	876	0.813193	0.0406959	sp|Q8IWC1|MA7D3_HUMAN	MAP7D3	Q8IWC1
1	20	191	191	191	0.999	563	4.00447	0	sp|Q8IWE2|NXP20_HUMAN	FAM114A1	Q8IWE2
1	5	88	88	88	0.999	971	0.861717	0.0345232	sp|Q8IWR0|Z3H7A_HUMAN	ZC3H7A	Q8IWR0
1	19	375	375	375	0.999	365	0.67038	0.0658631	sp|Q8IWS0|PHF6_HUMAN	PHF6	Q8IWS0
1	4	45	45	45	0.999	846	2.93828	0.00012253	sp|Q8IWW6|RHG12_HUMAN	ARHGAP12	Q8IWW6
1	11	147	147	147	0.999	916	2.93466	0.000122288	sp|Q8IWX8|CHERP_HUMAN	CHERP	Q8IWX8
1	12	57	57	71	0.999	2542	1.24924	0.00898765	sp|Q8IWZ3|ANKH1_HUMAN	ANKHD1	Q8IWZ3
1	3	10	10	10	0.999	645	2.37112	0.000409712	sp|Q8IWZ8|SUGP1_HUMAN	SUGP1	Q8IWZ8
1	5	38	38	38	0.999	1082	2.37581	0.000407915	sp|Q8IX01|SUGP2_HUMAN	SUGP2	Q8IX01
1	25	585	582	585	0.999	1150	0.4522	0.140166	sp|Q8IX12|CCAR1_HUMAN	CCAR1	Q8IX12
1	12	255	255	255	0.999	793	1.07424	0.0163904	sp|Q8IXB1|DJC10_HUMAN	DNAJC10	Q8IXB1
1	6	112	112	112	0.999	590	3.08214	5.91341E-05	sp|Q8IXH7|NELFD_HUMAN	NELFCD	Q8IXH7
1	12	220	199	220	0.999	618	0.851305	0.0357251	sp|Q8IXI1|MIRO2_HUMAN	RHOT2	Q8IXI1
0.9999	2	65	65	65	0.999	194	2.23587	0.000511064	sp|Q8IXQ3|CI040_HUMAN	C9orf40	Q8IXQ3
1	6	109	109	109	0.999	1001	0.836205	0.0375094	sp|Q8IXT5|RB12B_HUMAN	RBM12B	Q8IXT5
1	14	95	95	95	0.999	1375	2.15245	0.000634313	sp|Q8IY17|PLPL6_HUMAN	PNPLA6	Q8IY17
1	6	122	122	122	0.999	606	1.4126	0.00515468	sp|Q8IY67|RAVR1_HUMAN	RAVER1	Q8IY67
1	14	213	213	213	0.999	847	0.812762	0.0407345	sp|Q8IY81|SPB1_HUMAN	FTSJ3	Q8IY81
1	15	238	223	238	0.999	904	0.155324	0.422169	sp|Q8IYB3|SRRM1_HUMAN	SRRM1	Q8IYB3
1	11	138	138	138	0.999	725	0.136277	0.458213	sp|Q8IYI6|EXOC8_HUMAN	EXOC8	Q8IYI6
1	5	43	43	43	0.999	634	2.98911	8.10537E-05	sp|Q8IYS2|K2013_HUMAN	KIAA2013	Q8IYS2
1	4	70	70	70	0.999	434	1.46885	0.00424444	sp|Q8IYU8|MICU2_HUMAN	MICU2	Q8IYU8
1	3	11	11	11	0.999	590	3.18302	5.33333E-05	sp|Q8IZ07|AN13A_HUMAN	ANKRD13A	Q8IZ07
1	6	85	85	85	0.999	545	2.03663	0.000867314	sp|Q8IZ73|RUSD2_HUMAN	RPUSD2	Q8IZ73
1	19	209	209	209	0.999	802	4.13427	0	sp|Q8IZ83|A16A1_HUMAN	ALDH16A1	Q8IZ83
1	12	131	131	131	0.999	1130	0.774354	0.0463997	sp|Q8IZL8|PELP1_HUMAN	PELP1	Q8IZL8
1	15	275	215	275	0.999	508	1.26731	0.00846374	sp|Q8IZP0|ABI1_HUMAN	ABI1	Q8IZP0
1	10	90	90	90	0.999	666	0.251196	0.288852	sp|Q8N0X7|SPART_HUMAN	SPART	Q8N0X7
1	6	38	38	38	0.999	1335	0.656108	0.0691571	sp|Q8N122|RPTOR_HUMAN	RPTOR	Q8N122
1	30	727	727	727	0.999	923	2.08158	0.000775132	sp|Q8N163|CCAR2_HUMAN	CCAR2	Q8N163
1	4	97	97	97	0.999	169	2.04737	0.000840599	sp|Q8N183|NDUF2_HUMAN	NDUFAF2	Q8N183
1	37	823	823	823	0.999	819	3.18199	5.32741E-05	sp|Q8N1F7|NUP93_HUMAN	NUP93	Q8N1F7
1	11	128	128	128	0.999	835	1.27388	0.0082963	sp|Q8N1G2|CMTR1_HUMAN	CMTR1	Q8N1G2
1	31	702	702	702	0.999	583	0.227723	0.316785	sp|Q8N1G4|LRC47_HUMAN	LRRC47	Q8N1G4
1	3	31	31	31	0.999	530	1.40028	0.00536008	sp|Q8N2G8|GHDC_HUMAN	GHDC	Q8N2G8
1	8	73	73	73	0.999	398	0.612835	0.0800216	sp|Q8N2K0|ABD12_HUMAN	ABHD12	Q8N2K0
0.9999	3	6	6	6	0.999	1624	1.38304	0.00566133	sp|Q8N2S1|LTBP4_HUMAN	LTBP4	Q8N2S1
1	8	99	99	99	0.999	663	0.70193	0.0593741	sp|Q8N392|RHG18_HUMAN	ARHGAP18	Q8N392
1	29	403	403	403	0.999	2202	2.59873	0.000241087	sp|Q8N3C0|ASCC3_HUMAN	ASCC3	Q8N3C0
1	12	110	109	110	0.999	1523	2.27072	0.000479303	sp|Q8N3D4|EH1L1_HUMAN	EHBP1L1	Q8N3D4
1	12	149	149	149	0.999	863	1.44291	0.00464865	sp|Q8N3F8|MILK1_HUMAN	MICALL1	Q8N3F8
1	21	270	268	270	0.999	1231	2.14342	0.000657162	sp|Q8N3U4|STAG2_HUMAN	STAG2	Q8N3U4
1	7	54	54	54	0.999	314	4.99231	0	sp|Q8N474|SFRP1_HUMAN	SFRP1	Q8N474
1	20	107	107	177	0.999	1332	2.06051	0.000810492	sp|Q8N4C8|MINK1_HUMAN	MINK1	Q8N4C8
1	4	126	126	126	0.999	131	3.53513	2.03304E-05	sp|Q8N4V1|EMC5_HUMAN	MMGT1	Q8N4V1
1	8	109	109	109	0.999	542	1.68335	0.00232723	sp|Q8N543|OGFD1_HUMAN	OGFOD1	Q8N543
1	6	27	27	27	0.999	730	0.426799	0.152811	sp|Q8N556|AFAP1_HUMAN	AFAP1	Q8N556
0.9886	2	4	4	4	0.9946	433	3.73874	0	sp|Q8N5I2|ARRD1_HUMAN	ARRDC1	Q8N5I2
1	8	138	138	138	0.999	135	0.214452	0.333129	sp|Q8N5K1|CISD2_HUMAN	CISD2	Q8N5K1
1	5	97	97	97	0.999	171	0.912726	0.0288553	sp|Q8N5M4|TTC9C_HUMAN	TTC9C	Q8N5M4

1	17	393	393	393	0.999	471	2.76433	0.000144311	sp|Q8N684|CPSF7_HUMAN	CPSF7	Q8N684
1	9	13	13	13	0.9981	1762	2.93688	0.000122409	sp|Q8N6G6|ATL1_HUMAN	ADAMTSL1	Q8N6G6
1	15	227	227	227	0.999	521	0.112165	0.509943	sp|Q8N6H7|ARFG2_HUMAN	ARFGAP2	Q8N6H7
1	7	138	138	138	0.999	293	1.95029	0.00109113	sp|Q8N6M0|OTU6B_HUMAN	OTUD6B	Q8N6M0
1	9	102	102	102	0.999	699	1.96917	0.00104326	sp|Q8N6R0|EFNMT_HUMAN	METTL13	Q8N6R0
1	16	246	246	246	0.999	406	0.458477	0.137321	sp|Q8N6T3|ARFG1_HUMAN	ARFGAP1	Q8N6T3
1	18	266	266	266	0.999	993	1.14752	0.0126924	sp|Q8N766|EMC1_HUMAN	EMC1	Q8N766
1	10	101	101	101	0.999	531	3.86639	0	sp|Q8N7H5|PAF1_HUMAN	PAF1	Q8N7H5
1	22	604	596	604	0.999	591	2.64407	0.000203624	sp|Q8N8S7|ENAH_HUMAN	ENAH	Q8N8S7
1	7	46	46	46	0.999	893	1.99306	0.000970234	sp|Q8NB90|AFG2A_HUMAN	AFG2A	Q8NB90
1	9	110	110	110	0.999	726	1.07933	0.0161013	sp|Q8NBF2|NHLC2_HUMAN	NHLRC2	Q8NBF2
1	8	141	141	141	0.999	491	7.13981	0	sp|Q8NBI5|S43A3_HUMAN	SLC43A3	Q8NBI5
1	10	63	63	63	0.999	401	2.55109	0.000276543	sp|Q8NBJ4|GOLM1_HUMAN	GOLM1	Q8NBJ4
1	35	789	759	789	0.999	622	4.92949	0	sp|Q8NBJ5|GT251_HUMAN	COLGALT1	Q8NBJ5
1	8	112	112	112	0.999	301	0.89731	0.0304357	sp|Q8NBJ7|SUMF2_HUMAN	SUMF2	Q8NBJ7
1	5	82	82	82	0.999	392	2.50132	0.000291734	sp|Q8NBL1|PGLT1_HUMAN	POGLUT1	Q8NBL1
1	3	76	76	76	0.999	344	0.837845	0.0373469	sp|Q8NBM4|UBAC2_HUMAN	UBAC2	Q8NBM4
1	9	23	23	23	0.999	300	5.38617	0	sp|Q8NBQ5|DHB11_HUMAN	HSD17B11	Q8NBQ5
1	28	959	959	959	0.999	432	5.36767	0	sp|Q8NBS9|TXND5_HUMAN	TXNDC5	Q8NBS9
1	5	56	56	56	0.999	197	1.992	0.00096962	sp|Q8NBT2|SPC24_HUMAN	SPC24	Q8NBT2
1	9	192	192	192	0.999	361	0.901717	0.0299887	sp|Q8NBU5|ATAD1_HUMAN	ATAD1	Q8NBU5
1	5	92	92	92	0.999	429	0.462751	0.135322	sp|Q8NBX0|SCPDL_HUMAN	SCCPDH	Q8NBX0
1	30	1724	1724	1724	0.999	408	3.71214	0	sp|Q8NC51|SERB1_HUMAN	SERBP1	Q8NC51
1	16	209	209	209	0.999	503	0.0796473	0.591526	sp|Q8NC56|LEMD2_HUMAN	LEMD2	Q8NC56
1	12	74	74	159	0.999	518	0.163394	0.408409	sp|Q8NCA5|FA98A_HUMAN	FAM98A	Q8NCA5
0.9999	4	23	23	23	0.9967	376	5.14549	0	sp|Q8NCH0|CHSTE_HUMAN	CHST14	Q8NCH0
1	13	156	156	156	0.999	879	1.35867	0.00615651	sp|Q8NCN5|PDPR_HUMAN	PDPR	Q8NCN5
1	13	436	436	436	0.999	288	2.80178	0.000135084	sp|Q8NCW5|NNRE_HUMAN	NAXE	Q8NCW5
1	34	680	674	680	0.999	845	1.13858	0.0130922	sp|Q8NE71|ABCF1_HUMAN	ABCF1	Q8NE71
1	8	141	141	141	0.999	351	0.568376	0.0933187	sp|Q8NE86|MCU_HUMAN	MCU	Q8NE86
1	5	28	28	28	0.999	672	2.08959	0.000753816	sp|Q8NEM2|SHCBP_HUMAN	SHCBP1	Q8NEM2
1	3	105	105	105	0.999	376	1.59617	0.00295791	sp|Q8NEW0|ZNT7_HUMAN	SLC30A7	Q8NEW0
1	7	86	86	86	0.999	458	1.85962	0.00139773	sp|Q8NEY8|PPHLN_HUMAN	PPHLN1	Q8NEY8
1	3	53	53	53	0.999	397	1.22803	0.00965686	sp|Q8NEZ2|VP37A_HUMAN	VPS37A	Q8NEZ2
1	11	171	171	171	0.999	534	0.648638	0.0709905	sp|Q8NF37|PCAT1_HUMAN	LPCAT1	Q8NF37
1	10	85	35	85	0.999	3051	0.227874	0.316698	sp|Q8NFC6|BD1L1_HUMAN	BOD1L1	Q8NFC6
1	5	67	67	67	0.999	380	1.43162	0.00484933	sp|Q8NFH3|NUP43_HUMAN	NUP43	Q8NFH3
1	9	121	121	121	0.999	326	1.02597	0.0194163	sp|Q8NFH4|NUP37_HUMAN	NUP37	Q8NFH4
1	7	143	143	143	0.999	326	1.76921	0.00177163	sp|Q8NFH5|NUP35_HUMAN	NUP35	Q8NFH5
1	4	17	17	17	0.9989	357	6.04232	0	sp|Q8NFJ5|RAI3_HUMAN	GPRC5A	Q8NFJ5
1	12	294	294	294	0.999	470	1.50003	0.00382618	sp|Q8NFQ8|TOIP2_HUMAN	TOR1AIP2	Q8NFQ8
1	8	172	172	172	0.999	315	0.0485331	0.694029	sp|Q8NFV4|ABHDB_HUMAN	ABHD11	Q8NFV4
1	10	112	112	112	0.999	434	0.579915	0.089661	sp|Q8NFW8|NEUA_HUMAN	CMAS	Q8NFW8
1	3	9	9	9	0.999	210	2.71879	0.000175943	sp|Q8NFX7|STXB6_HUMAN	STXBP6	Q8NFX7
1	15	190	190	190	0.999	589	0.462824	0.135328	sp|Q8NHP8|PLBL2_HUMAN	PLBD2	Q8NHP8
1	4	37	37	37	0.999	600	1.52469	0.00356818	sp|Q8NHQ9|DDX55_HUMAN	DDX55	Q8NHQ9
1	6	58	58	58	0.999	660	0.588334	0.0871737	sp|Q8NHV4|NEDD1_HUMAN	NEDD1	Q8NHV4
1	13	212	212	212	0.999	1593	1.49208	0.00393476	sp|Q8NI27|THOC2_HUMAN	THOC2	Q8NI27
1	15	194	194	194	0.999	951	5.86312	0	sp|Q8NI36|WDR36_HUMAN	WDR36	Q8NI36
0.9999	2	12	12	12	0.999	63	3.22055	5.42986E-05	sp|Q8TAD7|OCC1_HUMAN	OCC1	Q8TAD7
1	19	208	208	371	0.999	1214	1.64497	0.00260033	sp|Q8TAQ2|SMRC2_HUMAN	SMARCC2	Q8TAQ2
1	23	466	466	466	0.999	608	0.0364175	0.742998	sp|Q8TAT6|NPL4_HUMAN	NPLOC4	Q8TAT6
1	4	62	62	62	0.999	745	0.483552	0.125757	sp|Q8TB52|FBX30_HUMAN	FBXO30	Q8TB52
1	4	56	56	56	0.999	432	0.219146	0.32716	sp|Q8TB61|S35B2_HUMAN	SLC35B2	Q8TB61
1	10	35	35	35	0.999	731	0.822522	0.0393747	sp|Q8TBA6|GOGA5_HUMAN	GOLGA5	Q8TBA6
1	14	257	257	257	0.999	463	0.789706	0.0439231	sp|Q8TBC4|UBA3_HUMAN	UBA3	Q8TBC4
1	8	105	105	105	0.999	691	0.836492	0.037498	sp|Q8TC07|TBC15_HUMAN	TBC1D15	Q8TC07
1	13	173	148	173	0.999	318	0.798164	0.0427217	sp|Q8TC12|RDH11_HUMAN	RDH11	Q8TC12
1	15	128	128	128	0.999	905	0.377368	0.181515	sp|Q8TCG1|CIP2A_HUMAN	CIP2A	Q8TCG1
1	20	378	378	378	0.999	826	1.23565	0.00941027	sp|Q8TCJ2|STT3B_HUMAN	STT3B	Q8TCJ2
1	24	313	313	313	0.999	783	0.678506	0.0640516	sp|Q8TCS8|PNPT1_HUMAN	PNPT1	Q8TCS8
1	7	131	131	131	0.999	979	0.139042	0.452559	sp|Q8TD19|NEK9_HUMAN	NEK9	Q8TD19
1	7	97	97	97	0.999	740	1.38445	0.00563848	sp|Q8TDB6|DTX3L_HUMAN	DTX3L	Q8TDB6
1	13	181	181	181	0.999	881	0.834931	0.0376843	sp|Q8TDD1|DDX54_HUMAN	DDX54	Q8TDD1
1	19	468	468	468	0.999	353	3.2275	5.46075E-05	sp|Q8TDN6|BRX1_HUMAN	BRIX1	Q8TDN6
1	8	102	90	102	0.999	302	1.01745	0.0200436	sp|Q8TDX7|NEK7_HUMAN	NEK7	Q8TDX7
1	8	35	35	35	0.999	1067	0.471501	0.1312	sp|Q8TDZ2|MICA1_HUMAN	MICAL1	Q8TDZ2
1	7	46	46	46	0.999	209	2.8369	0.00013308	sp|Q8TED1|GPX8_HUMAN	GPX8	Q8TED1
1	28	416	414	416	0.999	1508	0.871626	0.0333026	sp|Q8TEQ6|GEMI5_HUMAN	GEMIN5	Q8TEQ6
1	20	185	185	185	0.999	1081	1.09683	0.0151251	sp|Q8TEX9|IPO4_HUMAN	IPO4	Q8TEX9
1	14	197	197	197	0.999	950	0.0867782	0.571775	sp|Q8TF05|PP4R1_HUMAN	PPP4R1	Q8TF05
1	4	96	96	96	0.999	366	0.00779176	0.907862	sp|Q8WTS6|SETD7_HUMAN	SETD7	Q8WTS6
1	16	124	124	124	0.999	800	2.14354	0.000657609	sp|Q8WTT2|NOC3L_HUMAN	NOC3L	Q8WTT2
1	5	71	71	71	0.999	552	5.82064	0	sp|Q8WTV0|SCRB1_HUMAN	SCARB1	Q8WTV0
1	14	322	322	322	0.999	426	3.21961	5.41761E-05	sp|Q8WU90|ZC3HF_HUMAN	ZC3H15	Q8WU90
1	6	14	14	14	0.999	492	1.53334	0.0035013	sp|Q8WUA2|PPIL4_HUMAN	PPIL4	Q8WUA2
1	17	301	301	301	0.999	828	1.76437	0.00180081	sp|Q8WUF5|IASPP_HUMAN	PPP1R13L	Q8WUF5
1	9	66	62	66	0.999	952	0.368418	0.187431	sp|Q8WUI4|HDAC7_HUMAN	HDAC7	Q8WUI4
1	12	25	25	25	0.9989	1361	8.88427	0	sp|Q8WUJ3|CEMIP_HUMAN	CEMIP	Q8WUJ3
1	24	264	264	264	0.999	1156	0.88314	0.0320512	sp|Q8WUM0|NU133_HUMAN	NUP133	Q8WUM0
1	55	1513	1513	1513	0.999	868	6.21148	0	sp|Q8WUM4|PDC6I_HUMAN	PDCD6IP	Q8WUM4
1	18	248	248	248	0.999	373	1.40691	0.00524104	sp|Q8WUP2|FBLI1_HUMAN	FBLIM1	Q8WUP2
1	6	74	74	74	0.999	75	1.82537	0.00152291	sp|Q8WUW1|BRK1_HUMAN	BRK1	Q8WUW1
1	10	198	198	198	0.999	208	0.128343	0.474045	sp|Q8WUY1|THEM6_HUMAN	THEM6	Q8WUY1
1	7	40	39	40	0.999	401	1.63066	0.00272243	sp|Q8WV24|PHLA1_HUMAN	PHLDA1	Q8WV24
0.9999	2	8	8	8	0.999	249	4.46388	0	sp|Q8WV92|MITD1_HUMAN	MITD1	Q8WV92
1	14	158	157	158	0.999	666	0.403248	0.166105	sp|Q8WVC0|LEO1_HUMAN	LEO1	Q8WVC0
1	8	98	98	98	0.999	157	0.636074	0.074104	sp|Q8WVJ2|NUDC2_HUMAN	NUDCD2	Q8WVJ2
1	15	213	213	213	0.999	642	2.59296	0.000243038	sp|Q8WVM8|SCFD1_HUMAN	SCFD1	Q8WVM8
1	7	78	78	78	0.999	318	2.73242	0.000170599	sp|Q8WVY7|UBCP1_HUMAN	UBLCP1	Q8WVY7
1	20	767	767	767	0.999	178	4.83376	0	sp|Q8WW12|PCNP_HUMAN	PCNP	Q8WW12
1	38	325	325	325	0.999	1683	1.89146	0.00127634	sp|Q8WWI1|LMO7_HUMAN	LMO7	Q8WWI1
1	7	45	45	45	0.999	657	7.28715	0	sp|Q8WWI5|CTL1_HUMAN	SLC44A1	Q8WWI5
1	6	48	48	48	0.999	683	1.21092	0.0102272	sp|Q8WWK9|CKAP2_HUMAN	CKAP2	Q8WWK9
1	33	777	739	777	0.999	1075	2.16753	0.000617931	sp|Q8WWM7|ATX2L_HUMAN	ATXN2L	Q8WWM7
1	16	284	284	284	0.999	499	0.362228	0.191864	sp|Q8WWY3|PRP31_HUMAN	PRPF31	Q8WWY3
1	15	253	253	253	0.999	580	1.63571	0.00269575	sp|Q8WX92|NELFB_HUMAN	NELFB	Q8WX92
1	37	474	447	474	0.999	1383	1.37711	0.0057795	sp|Q8WX93|PALLD_HUMAN	PALLD	Q8WX93
1	3	108	108	108	0.999	905	2.49281	0.000296535	sp|Q8WXE9|STON2_HUMAN	STON2	Q8WXE9
1	31	401	401	420	0.999	523	1.68841	0.00228	sp|Q8WXF1|PSPC1_HUMAN	PSPC1	Q8WXF1
1	78	576	525	576	0.999	6885	0.0481774	0.695242	sp|Q8WXH0|SYNE2_HUMAN	SYNE2	Q8WXH0
1	17	158	158	158	0.999	593	1.20481	0.0104075	sp|Q8WXI9|P66B_HUMAN	GATAD2B	Q8WXI9
1	13	248	248	248	0.999	260	0.696889	0.0603778	sp|Q8WXX5|DNJC9_HUMAN	DNAJC9	Q8WXX5
1	15	394	394	394	0.999	563	0.101733	0.534326	sp|Q8WYA6|CTBL1_HUMAN	CTNNBL1	Q8WYA6
1	13	147	147	147	0.999	2266	2.1879	0.000587335	sp|Q8WYP5|ELYS_HUMAN	AHCTF1	Q8WYP5
1	9	128	128	128	0.999	623	3.56176	5.17464E-06	sp|Q8WZA9|IRGQ_HUMAN	IRGQ	Q8WZA9
1	41	1109	1109	1109	0.999	740	1.34073	0.00656435	sp|Q92499|DDX1_HUMAN	DDX1	Q92499
1	3	71	71	71	0.999	469	0.143894	0.443172	sp|Q92504|S39A7_HUMAN	SLC39A7	Q92504
1	10	177	177	177	0.999	227	2.37749	0.000408225	sp|Q92520|FAM3C_HUMAN	FAM3C	Q92520
1	20	357	357	357	0.999	213	1.51465	0.00366342	sp|Q92522|H1X_HUMAN	H1-10	Q92522
1	8	118	118	118	0.999	271	2.95634	0.000107463	sp|Q92530|PSMF1_HUMAN	PSMF1	Q92530
1	21	176	176	176	0.999	1859	0.330653	0.214585	sp|Q92538|GBF1_HUMAN	GBF1	Q92538
1	9	108	108	108	0.999	710	0.843167	0.0366512	sp|Q92541|RTF1_HUMAN	RTF1	Q92541
1	6	94	94	94	0.999	709	1.67966	0.00234109	sp|Q92542|NICA_HUMAN	NCSTN	Q92542
1	8	219	219	219	0.999	642	0.463099	0.13531	sp|Q92544|TM9S4_HUMAN	TM9SF4	Q92544
1	17	390	390	390	0.999	414	0.564084	0.0947668	sp|Q92552|RT27_HUMAN	MRPS27	Q92552
1	10	117	117	117	0.999	508	1.4028	0.00529285	sp|Q92575|UBXN4_HUMAN	UBXN4	Q92575
1	18	167	167	167	0.999	2039	1.98766	0.00097918	sp|Q92576|PHF3_HUMAN	PHF3	Q92576
1	25	1262	1262	1262	0.999	394	2.29603	0.000457478	sp|Q92597|NDRG1_HUMAN	NDRG1	Q92597
1	57	1380	1245	1520	0.999	858	6.63961	0	sp|Q92598|HS105_HUMAN	HSPH1	Q92598
1	18	212	212	366	0.999	483	2.82633	0.00013245	sp|Q92599|SEPT8_HUMAN	SEPTIN8	Q92599
1	5	123	123	123	0.999	299	1.2139	0.0101305	sp|Q92600|CNOT9_HUMAN	CNOT9	Q92600
1	8	99	99	99	0.999	795	1.61597	0.00283432	sp|Q92609|TBCD5_HUMAN	TBC1D5	Q92609
1	28	266	266	266	0.999	2054	0.493638	0.12144	sp|Q92614|MY18A_HUMAN	MYO18A	Q92614
1	11	83	67	83	0.999	738	1.77221	0.00176572	sp|Q92615|LAR4B_HUMAN	LARP4B	Q92615
1	113	2427	2427	2427	0.999	2671	1.38585	0.00562494	sp|Q92616|GCN1_HUMAN	GCN1	Q92616
1	14	157	157	157	0.999	1227	3.8626	0	sp|Q92620|PRP16_HUMAN	DHX38	Q92620
1	34	445	445	445	0.999	2012	1.7858	0.00168641	sp|Q92621|NU205_HUMAN	NUP205	Q92621
1	79	416	416	416	0.999	1479	9.62995	0	sp|Q92626|PXDN_HUMAN	PXDN	Q92626

0.9963	2	3	3	3	0.9947	364	2.68452	0.000190981	sp|Q92633|LPAR1_HUMAN	LPAR1	Q92633
1	18	415	415	442	0.999	251	7.6772	0	sp|Q92688|AN32B_HUMAN	ANP32B	Q92688
1	6	96	96	96	0.999	538	6.74918	0	sp|Q92692|NECT2_HUMAN	NECTIN2	Q92692
1	11	244	244	244	0.999	400	1.2917	0.00782617	sp|Q92734|TFG_HUMAN	TFG	Q92734
1	7	18	18	18	0.9989	480	3.20449	5.37514E-05	sp|Q92743|HTRA1_HUMAN	HTRA1	Q92743
1	23	578	578	583	0.999	370	0.269162	0.269789	sp|Q92747|ARC1A_HUMAN	ARPC1A	Q92747
1	17	260	260	438	0.999	488	1.45876	0.0043662	sp|Q92769|HDAC2_HUMAN	HDAC2	Q92769
1	6	93	86	93	0.999	391	0.964844	0.0241434	sp|Q92785|REQU_HUMAN	DPF2	Q92785
1	7	15	12	15	0.999	2442	2.05388	0.000825065	sp|Q92793|CBP_HUMAN	CREBBP	Q92793
1	10	78	78	78	0.999	1274	0.793656	0.0432982	sp|Q92797|SYMPK_HUMAN	SYMPK	Q92797
1	19	558	558	628	0.999	592	1.66644	0.00241277	sp|Q92804|RBP56_HUMAN	TAF15	Q92804
1	6	9	7	9	0.9975	552	2.91899	0.00012549	sp|Q92819|HYAS2_HUMAN	HAS2	Q92819
1	4	171	171	171	0.999	318	2.14461	0.000652617	sp|Q92820|GGH_HUMAN	GGH	Q92820
1	66	2238	1527	2238	0.999	729	1.33613	0.00666857	sp|Q92841|DDX17_HUMAN	DDX17	Q92841
1	52	873	873	873	0.999	1312	0.508665	0.115443	sp|Q92878|RAD50_HUMAN	RAD50	Q92878
1	17	515	406	515	0.999	486	2.26728	0.000481159	sp|Q92879|CELF1_HUMAN	CELF1	Q92879
1	8	125	125	125	0.999	214	1.33412	0.00671795	sp|Q92882|OSTF1_HUMAN	OSTF1	Q92882
1	29	560	560	560	0.999	912	2.23003	0.000520256	sp|Q92888|ARHG1_HUMAN	ARHGEF1	Q92888
1	7	158	158	158	0.999	307	0.235367	0.30731	sp|Q92890|UFD1_HUMAN	UFD1	Q92890
1	51	944	944	944	0.999	1179	0.0377099	0.737386	sp|Q92896|GSLG1_HUMAN	GLG1	Q92896
1	38	700	631	700	0.999	1129	0.558688	0.0966329	sp|Q92900|RENT1_HUMAN	UPF1	Q92900
1	9	240	240	240	0.999	334	0.773934	0.0464153	sp|Q92905|CSN5_HUMAN	COPS5	Q92905
1	19	204	204	204	0.999	476	1.06018	0.017251	sp|Q92917|GPKOW_HUMAN	GPKOW	Q92917
1	28	484	321	484	0.999	1105	1.2506	0.00895238	sp|Q92922|SMRC1_HUMAN	SMARCC1	Q92922
1	12	190	167	190	0.999	531	0.129618	0.471422	sp|Q92925|SMRD2_HUMAN	SMARCD2	Q92925
1	16	68	68	554	0.999	207	4.74033	0	sp|Q92930|RAB8B_HUMAN	RAB8B	Q92930
1	54	1709	1496	1709	0.999	711	4.7677	0	sp|Q92945|FUBP2_HUMAN	KHSRP	Q92945
1	22	736	465	736	0.999	898	2.1678	0.000618357	sp|Q92973|TNPO1_HUMAN	TNPO1	Q92973
1	36	517	517	517	0.999	986	0.815831	0.0403095	sp|Q92974|ARHG2_HUMAN	ARHGEF2	Q92974
1	3	40	40	40	0.999	594	2.09493	0.000743692	sp|Q92990|GLMN_HUMAN	GLMN	Q92990
1	42	662	447	662	0.999	2554	0.062646	0.643536	sp|Q93008|USP9X_HUMAN	USP9X	Q93008
1	27	561	561	561	0.999	1102	0.213094	0.334896	sp|Q93009|UBP7_HUMAN	USP7	Q93009
1	10	134	134	134	0.999	780	1.04873	0.0179567	sp|Q93034|CUL5_HUMAN	CUL5	Q93034
1	10	83	83	83	0.999	837	0.785995	0.0445131	sp|Q93050|VPP1_HUMAN	ATP6V0A1	Q93050
1	21	377	377	377	0.999	612	0.266172	0.272774	sp|Q93052|LPP_HUMAN	LPP	Q93052
1	3	4	2	4	0.999	196	3.93806	0	sp|Q93062|RBPMS_HUMAN	RBPMS	Q93062
1	13	183	183	183	0.999	411	1.07931	0.0160944	sp|Q969G3|SMCE1_HUMAN	SMARCE1	Q969G3
1	17	203	203	203	0.999	261	1.67313	0.00236514	sp|Q969G5|CAVN3_HUMAN	CAVIN3	Q969G5
1	9	384	384	384	0.999	173	5.84629	0	sp|Q969H8|MYDGF_HUMAN	MYDGF	Q969H8
1	12	91	91	91	0.999	578	0.585865	0.0879643	sp|Q969N2|PIGT_HUMAN	PIGT	Q969N2
1	17	101	101	101	0.999	613	7.27394	0	sp|Q969P0|IGSF8_HUMAN	IGSF8	Q969P0
1	21	126	126	866	0.999	106	0.578923	0.0899494	sp|Q969Q0|RL36L_HUMAN	RPL36AL	Q969Q0
1	10	163	163	163	0.999	477	1.2893	0.00786754	sp|Q969S3|ZN622_HUMAN	ZNF622	Q969S3
1	7	77	77	77	0.999	779	0.811972	0.0408223	sp|Q969S9|RRF2M_HUMAN	GFM2	Q969S9
1	7	149	149	149	0.999	264	2.45449	0.000321767	sp|Q969U7|PSMG2_HUMAN	PSMG2	Q969U7
1	15	447	447	447	0.999	563	3.62924	0	sp|Q969V3|NCLN_HUMAN	NCLN	Q969V3
1	12	309	309	309	0.999	290	0.650729	0.0704912	sp|Q969X5|ERGI1_HUMAN	ERGIC1	Q969X5
1	16	233	233	233	0.999	631	0.182036	0.378655	sp|Q969Z0|FAKD4_HUMAN	TBRG4	Q969Z0
1	9	339	339	339	0.999	154	3.30276	3.76471E-05	sp|Q96A26|F162A_HUMAN	FAM162A	Q96A26
1	22	535	535	535	0.999	483	2.54352	0.000279376	sp|Q96A33|CCD47_HUMAN	CCDC47	Q96A33
1	12	209	209	209	0.999	352	6.277	0	sp|Q96A49|SYAP1_HUMAN	SYAP1	Q96A49
1	17	168	168	168	0.999	974	2.50862	0.000283157	sp|Q96A65|EXOC4_HUMAN	EXOC4	Q96A65
1	4	114	114	114	0.999	205	2.1169	0.000702085	sp|Q96AB3|ISOC2_HUMAN	ISOC2	Q96AB3
1	27	365	359	365	0.999	680	3.72187	0	sp|Q96AC1|FERM2_HUMAN	FERMT2	Q96AC1
1	45	1177	1034	1390	0.999	644	5.5697	0	sp|Q96AE4|FUBP1_HUMAN	FUBP1	Q96AE4
1	29	875	875	875	0.999	307	5.92181	0	sp|Q96AG4|LRC59_HUMAN	LRRC59	Q96AG4
1	8	247	245	247	0.999	228	0.204552	0.346209	sp|Q96AT9|RPE_HUMAN	RPE	Q96AT9
1	8	112	112	112	0.999	596	0.510205	0.114831	sp|Q96AX1|VP33A_HUMAN	VPS33A	Q96AX1
1	18	317	312	317	0.999	582	6.05727	0	sp|Q96AY3|FKB10_HUMAN	FKBP10	Q96AY3
1	5	82	82	82	0.999	276	0.334614	0.211726	sp|Q96B26|EXOS8_HUMAN	EXOSC8	Q96B26
0.9999	2	73	73	73	0.999	74	0.557869	0.0968758	sp|Q96B49|TOM6_HUMAN	TOMM6	Q96B49
1	21	271	271	271	0.999	665	1.1026	0.0148512	sp|Q96B97|SH3K1_HUMAN	SH3KBP1	Q96B97
0.9999	2	23	23	23	0.999	459	1.40893	0.00520491	sp|Q96BH1|RNF25_HUMAN	RNF25	Q96BH1
1	6	69	69	69	0.999	306	1.33546	0.00668251	sp|Q96BJ3|AIDA_HUMAN	AIDA	Q96BJ3
1	10	100	100	100	0.999	352	1.51542	0.00365673	sp|Q96BN8|OTUL_HUMAN	OTULIN	Q96BN8
1	9	186	186	186	0.999	231	0.156498	0.420163	sp|Q96BR5|COA7_HUMAN	COA7	Q96BR5
0.9999	3	8	8	8	0.999	2186	1.01489	0.0201977	sp|Q96BY6|DOC10_HUMAN	DOCK10	Q96BY6
1	9	273	273	273	0.999	138	0.849903	0.0358454	sp|Q96C01|F136A_HUMAN	FAM136A	Q96C01
1	19	488	457	488	0.999	240	0.599448	0.0838837	sp|Q96C19|EFHD2_HUMAN	EFHD2	Q96C19
1	5	64	64	64	0.999	342	0.852746	0.0355399	sp|Q96C23|GALM_HUMAN	GALM	Q96C23
1	15	228	228	238	0.999	320	4.81352	0	sp|Q96C36|P5CR2_HUMAN	PYCR2	Q96C36
1	5	184	184	184	0.999	337	0.78568	0.0445234	sp|Q96C86|DCPS_HUMAN	DCPS	Q96C86
1	7	217	172	217	0.999	147	7.4039	0	sp|Q96C90|PP14B_HUMAN	PPP1R14B	Q96C90
1	9	109	109	109	0.999	615	0.511616	0.114251	sp|Q96CM8|ACSF2_HUMAN	ACSF2	Q96CM8
1	10	175	175	175	0.999	140	0.0849478	0.576476	sp|Q96CP2|FWCH2_HUMAN	FLYWCH2	Q96CP2
1	16	389	389	389	0.999	445	2.41042	0.000356866	sp|Q96CS3|FAF2_HUMAN	FAF2	Q96CS3
1	17	381	381	381	0.999	223	0.53743	0.104074	sp|Q96CT7|CC124_HUMAN	CCDC124	Q96CT7
1	15	139	139	139	0.999	577	1.8251	0.00152347	sp|Q96CV9|OPTN_HUMAN	OPTN	Q96CV9
1	28	741	741	741	0.999	435	4.87284	0	sp|Q96CW1|AP2M1_HUMAN	AP2M1	Q96CW1
1	13	246	246	246	0.999	907	0.103507	0.530377	sp|Q96CW5|GCP3_HUMAN	TUBGCP3	Q96CW5
1	15	89	89	89	0.999	328	4.05534	0	sp|Q96D15|RCN3_HUMAN	RCN3	Q96D15
1	8	178	178	178	0.999	503	2.18134	0.000597222	sp|Q96D46|NMD3_HUMAN	NMD3	Q96D46
1	10	129	95	129	0.999	314	1.2506	0.00894828	sp|Q96DB5|RMD1_HUMAN	RMDN1	Q96DB5
1	14	299	299	299	0.999	245	3.9946	0	sp|Q96DG6|CMBL_HUMAN	CMBL	Q96DG6
1	12	224	224	224	0.999	357	2.71953	0.000176101	sp|Q96DI7|SNR40_HUMAN	SNRNP40	Q96DI7
1	15	198	198	198	0.999	380	1.25295	0.0088895	sp|Q96DV4|RM38_HUMAN	MRPL38	Q96DV4
1	12	194	194	194	0.999	262	1.20597	0.0103828	sp|Q96E11|RRFM_HUMAN	MRRF	Q96E11
1	5	6	6	6	0.999	605	2.26147	0.00048231	sp|Q96EA4|SPDLY_HUMAN	SPDL1	Q96EA4
1	5	73	73	73	0.999	424	2.72358	0.000172662	sp|Q96EB1|ELP4_HUMAN	ELP4	Q96EB1
1	6	33	33	33	0.999	747	3.52287	0.000020202	sp|Q96EB6|SIR1_HUMAN	SIRT1	Q96EB6
1	15	231	231	231	0.999	360	1.8825	0.00130054	sp|Q96EE3|SEH1_HUMAN	SEH1L	Q96EE3
1	9	98	98	98	0.999	621	2.84351	0.000133206	sp|Q96EK5|KBP_HUMAN	KIFBP	Q96EK5
1	5	116	116	116	0.999	167	0.34794	0.201986	sp|Q96EL2|RT24_HUMAN	MRPS24	Q96EL2
1	5	116	116	116	0.999	112	0.392867	0.172256	sp|Q96EL3|RM53_HUMAN	MRPL53	Q96EL3
1	10	259	259	259	0.999	407	2.47238	0.000305976	sp|Q96EP5|DAZP1_HUMAN	DAZAP1	Q96EP5
1	6	82	82	82	0.999	411	1.73304	0.0020102	sp|Q96ER9|MITOK_HUMAN	CCDC51	Q96ER9
1	6	51	51	51	0.999	480	0.249303	0.290858	sp|Q96EY1|DNJA3_HUMAN	DNAJA3	Q96EY1
1	12	178	178	178	0.999	689	0.366163	0.189048	sp|Q96EY7|PTCD3_HUMAN	PTCD3	Q96EY7
1	9	69	69	69	0.999	508	2.97112	9.67742E-05	sp|Q96F86|EDC3_HUMAN	EDC3	Q96F86
1	14	516	516	516	0.999	103	1.8105	0.00158782	sp|Q96FQ6|S10AG_HUMAN	S100A16	Q96FQ6
1	7	82	82	82	0.999	657	0.29014	0.248948	sp|Q96FV9|THOC1_HUMAN	THOC1	Q96FV9
1	14	508	508	508	0.999	271	0.729286	0.0541379	sp|Q96FW1|OTUB1_HUMAN	OTUB1	Q96FW1
1	4	91	91	91	0.999	289	0.242174	0.299197	sp|Q96FX7|TRM61_HUMAN	TRMT61A	Q96FX7
1	29	628	627	628	0.999	612	1.79372	0.00165349	sp|Q96G03|PGM2_HUMAN	PGM2	Q96G03
1	8	256	256	256	0.999	380	0.961634	0.0244263	sp|Q96G23|CERS2_HUMAN	CERS2	Q96G23
1	16	146	146	146	0.999	650	3.46187	0.000019802	sp|Q96G46|DUS3L_HUMAN	DUS3L	Q96G46
1	4	49	49	49	0.999	475	2.41412	0.000351204	sp|Q96GA3|LTV1_HUMAN	LTV1	Q96GA3
1	9	81	81	81	0.999	329	1.60226	0.00293048	sp|Q96GA7|SDSL_HUMAN	SDSL	Q96GA7
1	3	66	66	66	0.999	212	2.8142	0.000135466	sp|Q96GC5|RM48_HUMAN	MRPL48	Q96GC5
1	3	176	176	176	0.999	406	1.06469	0.0169586	sp|Q96GC9|VMP1_HUMAN	VMP1	Q96GC9
1	5	35	35	35	0.999	344	0.873858	0.03308	sp|Q96GD4|AURKB_HUMAN	AURKB	Q96GD4
1	7	167	167	167	0.999	510	1.97661	0.00100627	sp|Q96GM8|TOE1_HUMAN	TOE1	Q96GM8
1	16	217	217	217	0.999	796	0.546459	0.100868	sp|Q96GQ7|DDX27_HUMAN	DDX27	Q96GQ7
1	7	100	100	100	0.999	300	2.32269	0.000453055	sp|Q96H79|ZCCHL_HUMAN	ZC3HAV1L	Q96H79
1	5	50	50	50	0.999	1378	2.94677	0.000118812	sp|Q96HA7|TONSL_HUMAN	TONSL	Q96HA7
1	31	491	491	491	0.999	596	6.29496	0	sp|Q96HC4|PDLI5_HUMAN	PDLIM5	Q96HC4
1	29	667	594	667	0.999	468	2.25862	0.000487383	sp|Q96HE7|ERO1A_HUMAN	ERO1A	Q96HE7
1	15	492	435	492	0.999	289	2.05498	0.000823529	sp|Q96HS1|PGAM5_HUMAN	PGAM5	Q96HS1
1	7	91	91	92	0.999	314	0.6362	0.0740989	sp|Q96HY6|DDRGK_HUMAN	DDRGK1	Q96HY6
1	41	958	958	1239	0.999	572	3.76781	0	sp|Q96I24|FUBP3_HUMAN	FUBP3	Q96I24
1	12	160	160	160	0.999	401	1.4832	0.00405477	sp|Q96I25|SPF45_HUMAN	RBM17	Q96I25
1	23	450	450	450	0.999	432	0.836979	0.0374374	sp|Q96I99|SUCB2_HUMAN	SUCLG2	Q96I99
1	11	131	131	131	0.999	420	0.102765	0.531973	sp|Q96IJ6|GMPPA_HUMAN	GMPPA	Q96IJ6
1	7	264	264	264	0.999	210	0.500851	0.118613	sp|Q96IU4|ABHEB_HUMAN	ABHD14B	Q96IU4
1	8	132	132	132	0.999	340	0.450465	0.14095	sp|Q96IZ0|PAWR_HUMAN	PAWR	Q96IZ0
1	3	10	10	10	0.999	757	1.1626	0.0120141	sp|Q96J84|KIRR1_HUMAN	KIRREL1	Q96J84
1	11	156	156	156	0.999	506	1.98654	0.000979849	sp|Q96JB5|CK5P3_HUMAN	CDK5RAP3	Q96JB5
1	12	50	50	50	0.999	1222	2.0142	0.000905709	sp|Q96JH7|VCIP1_HUMAN	VCPIP1	Q96JH7
1	8	44	28	44	0.999	720	3.71037	0	sp|Q96JJ3|ELMO2_HUMAN	ELMO2	Q96JJ3

1	11	245	245	245	0.999	454	0.0526644	0.678332	sp|Q96JJ7|TMX3_HUMAN	TMX3	Q96JJ7
1	9	78	78	78	0.999	812	1.95442	0.00108458	sp|Q96JM3|CHAP1_HUMAN	CHAMP1	Q96JM3
1	9	203	203	203	0.999	570	0.211086	0.337695	sp|Q96JP5|ZFP91_HUMAN	ZFP91	Q96JP5
1	4	46	46	46	0.999	352	1.24789	0.00903514	sp|Q96JY6|PDLI2_HUMAN	PDLIM2	Q96JY6
1	6	206	206	206	0.999	158	2.75707	0.000150735	sp|Q96K17|BT3L4_HUMAN	BTF3L4	Q96K17
1	5	64	64	101	0.999	471	0.994611	0.0217079	sp|Q96K21|ANCHR_HUMAN	ZFYVE19	Q96K21
1	12	159	159	159	0.999	1375	1.04398	0.0182506	sp|Q96K76|UBP47_HUMAN	USP47	Q96K76
1	12	178	178	178	0.999	322	4.24172	0	sp|Q96KB5|TOPK_HUMAN	PBK	Q96KB5
1	18	267	267	267	0.999	808	0.948387	0.025529	sp|Q96KG9|SCYL1_HUMAN	SCYL1	Q96KG9
1	6	82	82	82	0.999	924	1.16759	0.0117795	sp|Q96KP1|EXOC2_HUMAN	EXOC2	Q96KP1
1	20	516	516	516	0.999	475	2.94637	0.000118694	sp|Q96KP4|CNDP2_HUMAN	CNDP2	Q96KP4
1	8	84	84	84	0.999	1210	1.27673	0.00823955	sp|Q96KQ7|EHMT2_HUMAN	EHMT2	Q96KQ7
1	22	291	291	306	0.999	1074	0.64845	0.0710303	sp|Q96KR1|ZFR_HUMAN	ZFR	Q96KR1
1	8	235	235	235	0.999	541	2.55701	0.000270627	sp|Q96L92|SNX27_HUMAN	SNX27	Q96L92
1	5	37	37	37	0.999	313	1.53126	0.00351245	sp|Q96LJ7|DHRS1_HUMAN	DHRS1	Q96LJ7
1	8	260	260	260	0.999	445	2.56799	0.000268657	sp|Q96M27|PRRC1_HUMAN	PRRC1	Q96M27
1	16	240	240	240	0.999	727	0.664903	0.0670473	sp|Q96MU7|YTDC1_HUMAN	YTHDC1	Q96MU7
1	5	177	177	177	0.999	224	0.129658	0.471471	sp|Q96MW1|CCD43_HUMAN	CCDC43	Q96MW1
1	8	163	163	163	0.999	472	4.59201	0	sp|Q96N66|MBOA7_HUMAN	MBOAT7	Q96N66
1	25	209	176	209	0.999	2140	0.223371	0.321863	sp|Q96N67|DOCK7_HUMAN	DOCK7	Q96N67
1	6	123	123	123	0.999	199	0.15984	0.41455	sp|Q96NC0|ZMAT2_HUMAN	ZMAT2	Q96NC0
0.9941	3	32	32	34	0.9948	1537	0.241399	0.299939	sp|Q96NW7|LRRC7_HUMAN	LRRC7	Q96NW7
1	8	55	36	73	0.999	429	2.00326	0.000926798	sp|Q96P11|NSUN5_HUMAN	NSUN5	Q96P11
1	11	115	115	115	0.999	1450	1.05761	0.017394	sp|Q96P48|ARAP1_HUMAN	ARAP1	Q96P48
1	25	599	599	599	0.999	1041	3.02664	7.03206E-05	sp|Q96P70|IPO9_HUMAN	IPO9	Q96P70
1	27	661	661	661	0.999	669	2.5968	0.000243861	sp|Q96PK6|RBM14_HUMAN	RBM14	Q96PK6
1	8	165	165	165	0.999	341	3.57345	5.20156E-06	sp|Q96PU8|QKI_HUMAN	QKI	Q96PU8
1	16	164	164	164	0.999	661	3.01706	7.41504E-05	sp|Q96PZ0|PUS7_HUMAN	PUS7	Q96PZ0
1	10	74	58	74	0.999	3661	0.802149	0.0421051	sp|Q96Q15|SMG1_HUMAN	SMG1	Q96Q15
1	4	50	50	50	0.999	506	3.14705	0.000052459	sp|Q96QD8|S38A2_HUMAN	SLC38A2	Q96QD8
1	36	1255	1255	1255	0.999	796	0.604604	0.0823411	sp|Q96QK1|VPS35_HUMAN	VPS35	Q96QK1
1	13	250	224	250	0.999	312	0.189687	0.366878	sp|Q96QR8|PURB_HUMAN	PURB	Q96QR8
1	8	57	57	57	0.999	1125	1.09225	0.015348	sp|Q96QU8|XPO6_HUMAN	XPO6	Q96QU8
1	7	29	29	29	0.999	1193	3.14383	5.22307E-05	sp|Q96R06|SPAG5_HUMAN	SPAG5	Q96R06
1	5	8	8	8	0.999	628	1.43104	0.00486041	sp|Q96RF0|SNX18_HUMAN	SNX18	Q96RF0
1	2	70	70	70	0.999	788	1.01833	0.0200101	sp|Q96RN5|MED15_HUMAN	MED15	Q96RN5
1	19	391	391	391	0.999	751	0.839166	0.0371799	sp|Q96RP9|EFGM_HUMAN	GFM1	Q96RP9
1	17	318	318	318	0.999	583	0.495654	0.120636	sp|Q96RS6|NUDC1_HUMAN	NUDCD1	Q96RS6
1	14	133	131	133	0.999	1412	4.00255	0	sp|Q96RT1|ERBIN_HUMAN	ERBIN	Q96RT1
1	5	55	55	55	0.997	5635	6.37863	0	sp|Q96RW7|HMCN1_HUMAN	HMCN1	Q96RW7
1	3	25	25	25	0.999	204	0.571179	0.0924476	sp|Q96S19|MTL26_HUMAN	METTL26	Q96S19
1	5	41	41	41	0.999	729	0.278808	0.259672	sp|Q96S59|RANB9_HUMAN	RANBP9	Q96S59
1	11	117	117	117	0.999	817	1.39409	0.00546218	sp|Q96SB3|NEB2_HUMAN	PPP1R9B	Q96SB3
1	17	262	200	262	0.999	655	0.580519	0.0895068	sp|Q96SB4|SRPK1_HUMAN	SRPK1	Q96SB4
1	3	52	52	52	0.999	561	1.2448	0.00912255	sp|Q96SK2|TM209_HUMAN	TMEM209	Q96SK2
1	10	152	152	152	0.999	819	1.02757	0.0192814	sp|Q96ST2|IWS1_HUMAN	IWS1	Q96ST2
1	18	121	121	121	0.999	1273	0.636892	0.0739811	sp|Q96ST3|SIN3A_HUMAN	SIN3A	Q96ST3
1	19	296	245	296	0.999	977	0.215347	0.331982	sp|Q96T37|RBM15_HUMAN	RBM15	Q96T37
1	22	205	203	205	0.999	708	0.315678	0.22662	sp|Q96T51|RUFY1_HUMAN	RUFY1	Q96T51
1	8	102	102	102	0.999	1030	3.20783	5.38721E-05	sp|Q96T76|MMS19_HUMAN	MMS19	Q96T76
1	28	472	418	472	0.999	793	2.37004	0.000409091	sp|Q96T88|UHRF1_HUMAN	UHRF1	Q96T88
1	46	1406	1406	1406	0.999	746	3.70494	0	sp|Q96TA1|NIBA2_HUMAN	NIBAN2	Q96TA1
0.9999	3	43	41	43	0.999	682	1.25625	0.00876732	sp|Q99081|HTF4_HUMAN	TCF12	Q99081
1	5	215	215	215	0.999	103	1.45486	0.004444	sp|Q99417|MYCBP_HUMAN	MYCBP	Q99417
1	17	503	503	503	0.999	244	2.87115	0.000126801	sp|Q99426|TBCB_HUMAN	TBCB	Q99426
1	9	260	260	260	0.999	277	2.4866	0.000298555	sp|Q99436|PSB7_HUMAN	PSMB7	Q99436
1	24	729	693	729	0.999	309	2.63318	0.000209622	sp|Q99439|CNN2_HUMAN	CNN2	Q99439
1	6	173	173	173	0.999	399	1.11826	0.0140661	sp|Q99442|SEC62_HUMAN	SEC62	Q99442
1	9	81	81	81	0.999	389	1.15742	0.0122567	sp|Q99447|PCY2_HUMAN	PCYT2	Q99447
1	35	551	551	551	0.999	802	1.06897	0.0167117	sp|Q99459|CDC5L_HUMAN	CDC5L	Q99459
1	30	771	771	771	0.999	953	0.709218	0.0578744	sp|Q99460|PSMD1_HUMAN	PSMD1	Q99460
1	8	57	57	57	0.999	211	1.78715	0.00167809	sp|Q99470|SDF2_HUMAN	SDF2	Q99470
1	9	338	338	338	0.999	154	2.6928	0.00019132	sp|Q99471|PFD5_HUMAN	PFDN5	Q99471
1	6	200	42	200	0.999	336	0.867818	0.0337522	sp|Q99496|RING2_HUMAN	RNF2	Q99496
1	25	1567	1567	1567	0.999	189	6.7657	0	sp|Q99497|PARK7_HUMAN	PARK7	Q99497
1	17	499	499	499	0.999	393	2.73363	0.000170754	sp|Q99536|VAT1_HUMAN	VAT1	Q99536
1	4	92	92	92	0.999	433	2.76088	0.000143911	sp|Q99538|LGMN_HUMAN	LGMN	Q99538
1	6	68	68	68	0.999	437	5.94661	0	sp|Q99541|PLIN2_HUMAN	PLIN2	Q99541
1	11	211	211	211	0.999	621	2.6047	0.000231293	sp|Q99543|DNJC2_HUMAN	DNAJC2	Q99543
1	5	52	52	52	0.999	160	3.82343	0	sp|Q99547|MPH6_HUMAN	MPHOSPH6	Q99547
1	18	216	216	216	0.999	741	1.73344	0.00200908	sp|Q99567|NUP88_HUMAN	NUP88	Q99567
1	26	433	433	433	0.999	1024	1.9523	0.00108752	sp|Q99575|POP1_HUMAN	POP1	Q99575
1	10	257	257	257	0.999	98	4.19052	0	sp|Q99584|S10AD_HUMAN	S100A13	Q99584
1	10	198	198	198	0.999	1463	1.24894	0.00899452	sp|Q99590|SCAFB_HUMAN	SCAF11	Q99590
0.9999	6	50	3	50	0.999	435	2.13962	0.000662144	sp|Q99594|TEAD3_HUMAN	TEAD3	Q99594
1	12	286	286	286	0.999	290	1.01087	0.0204975	sp|Q99598|TSNAX_HUMAN	TSNAX	Q99598
1	55	1805	152	1805	0.999	913	0.927536	0.0274251	sp|Q99613|EIF3C_HUMAN	EIF3C	Q99613
1	11	266	266	266	0.999	292	0.0629332	0.64273	sp|Q99614|TTC1_HUMAN	TTC1	Q99614
1	26	424	424	424	0.999	494	2.57479	0.000253122	sp|Q99615|DNJC7_HUMAN	DNAJC7	Q99615
1	39	1438	1438	1438	0.999	299	3.64602	0	sp|Q99623|PHB2_HUMAN	PHB2	Q99623
1	3	123	123	123	0.999	209	0.63695	0.0739949	sp|Q99627|CSN8_HUMAN	COPS8	Q99627
1	4	11	11	11	0.999	342	0.991706	0.0219194	sp|Q99633|PRP18_HUMAN	PRPF18	Q99633
1	13	167	95	167	0.999	725	2.62007	0.000225448	sp|Q99661|KIF2C_HUMAN	KIF2C	Q99661
1	4	42	42	42	0.999	303	1.65833	0.00248738	sp|Q99685|MGLL_HUMAN	MGLL	Q99685
1	16	203	203	241	0.999	1313	1.24032	0.00925205	sp|Q99700|ATX2_HUMAN	ATXN2	Q99700
1	16	132	132	132	0.999	1265	2.96118	9.58084E-05	sp|Q99707|METH_HUMAN	MTR	Q99707
1	11	396	396	396	0.999	261	3.79142	0	sp|Q99714|HCD2_HUMAN	HSD17B10	Q99714
1	54	178	176	178	0.999	3063	4.12858	0	sp|Q99715|COCA1_HUMAN	COL12A1	Q99715
1	24	875	875	995	0.999	332	3.05869	6.28931E-05	sp|Q99729|ROAA_HUMAN	HNRNPAB	Q99729
0.9977	2	38	38	38	0.9987	161	7.16425	0	sp|Q99732|LITAF_HUMAN	LITAF	Q99732
1	22	485	485	657	0.999	375	5.49627	0	sp|Q99733|NP1L4_HUMAN	NAP1L4	Q99733
1	11	82	82	82	0.999	312	2.24175	0.000506438	sp|Q99747|SNAG_HUMAN	NAPG	Q99747
1	37	737	737	737	0.999	780	5.65188	0	sp|Q99798|ACON_HUMAN	ACO2	Q99798
1	11	218	218	218	0.999	663	0.445945	0.142997	sp|Q99805|TM9S2_HUMAN	TM9SF2	Q99805
1	5	96	96	96	0.999	456	5.74037	0	sp|Q99808|S29A1_HUMAN	SLC29A1	Q99808
0.9966	2	10	10	10	0.999	253	3.32489	2.36686E-05	sp|Q99811|PRRX2_HUMAN	PRRX2	Q99811
1	6	49	49	49	0.999	390	2.67215	0.000189807	sp|Q99816|TS101_HUMAN	TSG101	Q99816
1	17	506	506	506	0.999	537	6.03463	0	sp|Q99829|CPNE1_HUMAN	CPNE1	Q99829
1	53	1718	1718	1718	0.999	543	5.18672	0	sp|Q99832|TCPH_HUMAN	CCT7	Q99832
1	16	313	313	313	0.999	306	1.38591	0.00562768	sp|Q99848|EBP2_HUMAN	EBNA1BP2	Q99848
1	18	438	423	438	0.999	371	3.04845	6.67362E-05	sp|Q99873|ANM1_HUMAN	PRMT1	Q99873
1	6	84	84	84	0.999	283	0.413097	0.160554	sp|Q99943|PLCA_HUMAN	AGPAT1	Q99943
1	18	396	332	396	0.999	368	0.291179	0.248212	sp|Q99961|SH3G1_HUMAN	SH3GL1	Q99961
1	25	49	49	49	0.999	751	6.38461	0	sp|Q99985|SEM3C_HUMAN	SEMA3C	Q99985
1	9	180	180	180	0.999	396	1.31533	0.00719548	sp|Q99986|VRK1_HUMAN	VRK1	Q99986
1	7	37	37	37	0.999	308	5.38651	0	sp|Q99988|GDF15_HUMAN	GDF15	Q99988
1	14	412	351	412	0.999	284	1.54601	0.00341035	sp|Q9BPW8|NIPS1_HUMAN	NIPSNAP1	Q9BPW8
1	14	202	202	202	0.999	1015	2.28334	0.000469388	sp|Q9BPX3|CND3_HUMAN	NCAPG	Q9BPX3
1	9	114	114	114	0.999	476	1.03044	0.0191192	sp|Q9BPX6|MICU1_HUMAN	MICU1	Q9BPX6
1	18	146	146	210	0.999	737	0.595605	0.0850018	sp|Q9BQ39|DDX50_HUMAN	DDX50	Q9BQ39
1	16	297	297	297	0.999	826	1.12819	0.0136	sp|Q9BQ52|RNZ2_HUMAN	ELAC2	Q9BQ52
1	8	197	197	197	0.999	176	4.56814	0	sp|Q9BQ61|TRIR_HUMAN	TRIR	Q9BQ61
1	7	286	286	286	0.999	446	1.0313	0.0190598	sp|Q9BQ67|GRWD1_HUMAN	GRWD1	Q9BQ67
1	7	135	135	135	0.999	325	0.978454	0.023005	sp|Q9BQ69|MACD1_HUMAN	MACROD1	Q9BQ69
1	4	89	89	89	0.999	676	1.28505	0.00800186	sp|Q9BQ70|TCF25_HUMAN	TCF25	Q9BQ70
1	6	68	68	68	0.999	279	1.30696	0.00742132	sp|Q9BQ75|CMS1_HUMAN	CMSS1	Q9BQ75
1	7	215	215	215	0.999	342	0.0977122	0.544208	sp|Q9BQA1|MEP50_HUMAN	WDR77	Q9BQA1
1	52	4064	308	4064	0.999	449	0.62036	0.0782607	sp|Q9BQE3|TBA1C_HUMAN	TUBA1C	Q9BQE3
1	10	173	173	173	0.999	337	0.478009	0.1282	sp|Q9BQE5|APOL2_HUMAN	APOL2	Q9BQE5
1	56	1319	1319	1319	0.999	1328	3.08169	5.90717E-05	sp|Q9BQG0|MBB1A_HUMAN	MYBBP1A	Q9BQG0
1	11	73	73	73	0.999	1478	0.679404	0.0639085	sp|Q9BQS8|FYCO1_HUMAN	FYCO1	Q9BQS8
1	25	620	529	626	0.999	489	0.844584	0.0364862	sp|Q9BR76|COR1B_HUMAN	CORO1B	Q9BR76
1	13	299	299	299	0.999	123	4.55766	0	sp|Q9BRA2|TXD17_HUMAN	TXNDC17	Q9BRA2
1	4	73	73	73	0.999	176	1.18671	0.011053	sp|Q9BRG1|VPS25_HUMAN	VPS25	Q9BRG1
1	9	140	140	140	0.999	306	0.239688	0.30183	sp|Q9BRJ2|RM45_HUMAN	MRPL45	Q9BRJ2
1	17	219	219	219	0.999	362	0.383638	0.177933	sp|Q9BRK5|CAB45_HUMAN	SDF4	Q9BRK5
1	4	44	44	44	0.999	392	0.916985	0.0284286	sp|Q9BRP4|PAAF1_HUMAN	PAAF1	Q9BRP4
1	15	421	421	421	0.999	204	2.30274	0.000459838	sp|Q9BRP8|PYM1_HUMAN	PYM1	Q9BRP8
1	4	46	46	46	0.999	373	0.478891	0.127898	sp|Q9BRQ8|FSP1_HUMAN	AIFM2	Q9BRQ8

1	10	220	220	220	0.999	497	1.53961	0.00343958	sp|Q9BRR6|ADPGK_HUMAN	ADPGK	Q9BRR6
1	5	117	117	117	0.999	126	0.89178	0.0310553	sp|Q9BRT2|UQCC2_HUMAN	UQCC2	Q9BRT2
1	12	262	262	262	0.999	385	2.80511	0.000135338	sp|Q9BRX2|PELO_HUMAN	PELO	Q9BRX2
1	4	50	50	50	0.999	216	1.8036	0.00160933	sp|Q9BRX5|PSF3_HUMAN	GINS3	Q9BRX5
1	11	58	55	58	0.999	755	2.1334	0.000670723	sp|Q9BRZ2|TRI56_HUMAN	TRIM56	Q9BRZ2
1	21	708	571	708	0.999	406	1.31715	0.00715512	sp|Q9BS26|ERP44_HUMAN	ERP44	Q9BS26
1	7	142	142	142	0.999	190	0.885625	0.0317487	sp|Q9BSD7|NTPCR_HUMAN	NTPCR	Q9BSD7
1	11	161	161	161	0.999	297	0.695027	0.0607402	sp|Q9BSH4|TACO1_HUMAN	TACO1	Q9BSH4
1	8	131	131	131	0.999	902	1.31999	0.00705805	sp|Q9BSJ2|GCP2_HUMAN	TUBGCP2	Q9BSJ2
1	36	976	976	976	0.999	1104	3.92551	0	sp|Q9BSJ8|ESYT1_HUMAN	ESYT1	Q9BSJ8
1	6	113	113	113	0.999	278	0.976059	0.0231967	sp|Q9BT09|CNPY3_HUMAN	CNPY3	Q9BT09
1	3	70	70	70	0.999	464	1.5347	0.00348857	sp|Q9BT22|ALG1_HUMAN	ALG1	Q9BT22
1	5	111	111	111	0.999	410	0.763565	0.0482138	sp|Q9BT25|HAUS8_HUMAN	HAUS8	Q9BT25
1	18	361	361	361	0.999	406	0.264687	0.274195	sp|Q9BT78|CSN4_HUMAN	COPS4	Q9BT78
1	29	296	296	296	0.999	2240	3.10022	5.55556E-05	sp|Q9BTC0|DIDO1_HUMAN	DIDO1	Q9BTC0
1	11	90	90	200	0.999	594	1.85052	0.00143383	sp|Q9BTC8|MTA3_HUMAN	MTA3	Q9BTC8
1	15	305	305	305	0.999	642	0.764219	0.0480978	sp|Q9BTE3|MCMBP_HUMAN	MCMBP	Q9BTE3
1	10	274	274	274	0.999	268	1.52287	0.00357916	sp|Q9BTT0|AN32E_HUMAN	ANP32E	Q9BTT0
1	8	126	126	126	0.999	479	1.47109	0.0042166	sp|Q9BTU6|P4K2A_HUMAN	PI4K2A	Q9BTU6
1	15	282	282	282	0.999	400	3.63468	0	sp|Q9BTV4|TMM43_HUMAN	TMEM43	Q9BTV4
1	3	33	33	33	0.999	496	2.2818	0.000474873	sp|Q9BTV5|FSD1_HUMAN	FSD1	Q9BTV5
1	14	284	284	284	0.999	1192	2.05334	0.000827136	sp|Q9BTW9|TBCD_HUMAN	TBCD	Q9BTW9
1	10	75	75	75	0.999	390	0.643599	0.0722991	sp|Q9BTY7|HGH1_HUMAN	HGH1	Q9BTY7
1	4	54	52	54	0.999	278	0.716658	0.0566021	sp|Q9BTZ2|DHRS4_HUMAN	DHRS4	Q9BTZ2
1	7	210	210	210	0.999	707	2.64434	0.0002038	sp|Q9BU23|LMF2_HUMAN	LMF2	Q9BU23
1	6	97	97	97	0.999	263	0.613737	0.0798227	sp|Q9BU76|MMTA2_HUMAN	MMTAG2	Q9BU76
1	35	1201	876	2571	0.999	446	6.17614	0	sp|Q9BUF5|TBB6_HUMAN	TUBB6	Q9BUF5
1	34	713	713	713	0.999	856	0.847032	0.0361543	sp|Q9BUJ2|HNRL1_HUMAN	HNRNPUL1	Q9BUJ2
1	11	244	244	244	0.999	212	0.831997	0.0380649	sp|Q9BUL8|PDC10_HUMAN	PDCD10	Q9BUL8
1	8	118	118	118	0.999	242	2.17437	0.000607069	sp|Q9BUP3|HTAI2_HUMAN	HTATIP2	Q9BUP3
1	40	788	788	788	0.999	820	1.2488	0.00899406	sp|Q9BUQ8|DDX23_HUMAN	DDX23	Q9BUQ8
1	5	111	111	111	0.999	198	6.3958	0	sp|Q9BUR5|MIC26_HUMAN	APOO	Q9BUR5
1	6	32	32	32	0.999	245	0.39874	0.168698	sp|Q9BUT1|DHRS6_HUMAN	BDH2	Q9BUT1
1	12	384	384	384	0.999	369	1.32577	0.00691958	sp|Q9BV20|MTNA_HUMAN	MRI1	Q9BV20
1	9	214	214	214	0.999	432	0.500527	0.118705	sp|Q9BV38|WDR18_HUMAN	WDR18	Q9BV38
1	12	97	97	97	0.999	507	2.96591	9.62889E-05	sp|Q9BV44|THUM3_HUMAN	THUMPD3	Q9BV44
1	8	66	66	66	0.999	179	2.05494	0.000825604	sp|Q9BV57|MTND_HUMAN	ADI1	Q9BV57
1	4	109	109	109	0.999	311	2.44566	0.00033281	sp|Q9BV68|RN126_HUMAN	RNF126	Q9BV68
1	8	47	47	47	0.999	373	1.45169	0.00449975	sp|Q9BV79|MECR_HUMAN	MECR	Q9BV79
1	6	211	211	211	0.999	223	1.07597	0.016307	sp|Q9BV86|NTM1A_HUMAN	NTMT1	Q9BV86
1	5	179	179	179	0.999	243	3.88766	0	sp|Q9BVC6|TM109_HUMAN	TMEM109	Q9BVC6
1	11	134	134	134	0.999	233	0.284394	0.254469	sp|Q9BVG4|PBDC1_HUMAN	PBDC1	Q9BVG4
1	6	171	171	171	0.999	516	2.95856	0.000103689	sp|Q9BVI4|NOC4L_HUMAN	NOC4L	Q9BVI4
1	13	499	442	499	0.999	235	4.90726	0	sp|Q9BVK6|TMED9_HUMAN	TMED9	Q9BVK6
1	16	363	363	363	0.999	549	0.29123	0.248232	sp|Q9BVP2|GNL3_HUMAN	GNL3	Q9BVP2
1	4	31	31	31	0.999	552	0.236491	0.305865	sp|Q9BVS4|RIOK2_HUMAN	RIOK2	Q9BVS4
1	17	227	227	227	0.999	656	0.0930296	0.556105	sp|Q9BW27|NUP85_HUMAN	NUP85	Q9BW27
1	4	31	31	31	0.999	186	2.48525	0.000298316	sp|Q9BW83|IFT27_HUMAN	IFT27	Q9BW83
1	18	194	194	240	0.999	718	2.47589	0.000303514	sp|Q9BW92|SYTM_HUMAN	TARS2	Q9BW92
1	22	422	422	422	0.999	397	1.4256	0.00495738	sp|Q9BWD1|THIC_HUMAN	ACAT2	Q9BWD1
1	19	321	131	321	0.999	364	2.59379	0.000243243	sp|Q9BWF3|RBM4_HUMAN	RBM4	Q9BWF3
1	5	155	155	155	0.999	86	1.70657	0.00215126	sp|Q9BWJ5|SF3B5_HUMAN	SF3B5	Q9BWJ5
1	13	172	172	250	0.999	321	0.350625	0.200095	sp|Q9BWM7|SFXN3_HUMAN	SFXN3	Q9BWM7
1	4	63	63	63	0.999	393	1.93407	0.00114039	sp|Q9BWS9|CHID1_HUMAN	CHID1	Q9BWS9
1	9	97	97	97	0.999	796	1.21106	0.0102228	sp|Q9BWU0|NADAP_HUMAN	SLC4A1AP	Q9BWU0
1	4	18	18	18	0.999	385	0.686175	0.0625669	sp|Q9BX40|LS14B_HUMAN	LSM14B	Q9BX40
1	7	78	78	78	0.999	163	1.54484	0.0034232	sp|Q9BX68|HINT2_HUMAN	HINT2	Q9BX68
1	8	175	116	175	0.999	764	2.35128	0.000430399	sp|Q9BXB5|OSB10_HUMAN	OSBPL10	Q9BXB5
1	10	108	108	108	0.999	653	1.43017	0.00487587	sp|Q9BXF6|RFIP5_HUMAN	RAB11FIP5	Q9BXF6
1	5	13	13	13	0.999	246	2.0643	0.000806833	sp|Q9BXJ4|C1QT3_HUMAN	C1QTNF3	Q9BXJ4
1	40	1029	911	1029	0.999	866	0.142111	0.446562	sp|Q9BXJ9|NAA15_HUMAN	NAA15	Q9BXJ9
1	41	1230	1230	1230	0.999	876	1.34099	0.00655222	sp|Q9BXP5|SRRT_HUMAN	SRRT	Q9BXP5
1	10	95	95	95	0.999	403	0.256383	0.28313	sp|Q9BXR0|TGT_HUMAN	QTRT1	Q9BXR0
1	15	567	464	567	0.999	423	0.678328	0.0640914	sp|Q9BXS5|AP1M1_HUMAN	AP1M1	Q9BXS5
1	9	64	64	64	0.999	441	1.54149	0.00343691	sp|Q9BXS6|NUSAP_HUMAN	NUSAP1	Q9BXS6
1	13	267	267	267	0.999	423	0.0469435	0.699799	sp|Q9BXW7|HDHD5_HUMAN	HDHD5	Q9BXW7
1	9	119	119	119	0.999	300	3.11883	5.16685E-05	sp|Q9BXY0|MAK16_HUMAN	MAK16	Q9BXY0
1	7	108	108	108	0.999	194	0.178416	0.38437	sp|Q9BY32|ITPA_HUMAN	ITPA	Q9BY32
1	11	115	115	115	0.999	306	0.320162	0.223093	sp|Q9BY42|RTF2_HUMAN	RTF2	Q9BY42
1	25	629	629	629	0.999	585	1.29568	0.00768957	sp|Q9BY44|EIF2A_HUMAN	EIF2A	Q9BY44
1	11	161	161	161	0.999	421	0.918427	0.0282949	sp|Q9BY77|PDIP3_HUMAN	POLDIP3	Q9BY77
1	14	60	60	60	0.999	575	1.15389	0.01242	sp|Q9BYC5|FUT8_HUMAN	FUT8	Q9BYC5
1	3	118	118	118	0.999	149	1.77547	0.00174235	sp|Q9BYC9|RM20_HUMAN	MRPL20	Q9BYC9
1	7	189	189	189	0.999	178	0.873597	0.0330792	sp|Q9BYD1|RM13_HUMAN	MRPL13	Q9BYD1
1	8	141	141	141	0.999	267	2.48714	0.000298795	sp|Q9BYD2|RM09_HUMAN	MRPL9	Q9BYD2
1	6	91	91	91	0.999	311	1.15858	0.0122024	sp|Q9BYD3|RM04_HUMAN	MRPL4	Q9BYD3
1	14	321	321	321	0.999	205	1.2151	0.0100936	sp|Q9BYN8|RT26_HUMAN	MRPS26	Q9BYN8
1	19	160	160	160	0.999	704	0.242143	0.299135	sp|Q9BYT8|NEUL_HUMAN	NLN	Q9BYT8
1	3	39	39	39	0.999	373	1.14194	0.012954	sp|Q9BYV8|CEP41_HUMAN	CEP41	Q9BYV8
1	12	162	162	162	0.999	928	1.79616	0.00164282	sp|Q9BYX2|TBD2A_HUMAN	TBC1D2	Q9BYX2
1	8	22	22	22	0.999	464	3.70534	0	sp|Q9BZD4|NUF2_HUMAN	NUF2	Q9BZD4
1	12	186	186	186	0.999	423	1.34425	0.0064611	sp|Q9BZE1|RM37_HUMAN	MRPL37	Q9BZE1
1	21	458	458	458	0.999	634	1.69638	0.00222768	sp|Q9BZE4|GTPB4_HUMAN	GTPBP4	Q9BZE4
1	11	160	160	160	0.999	889	1.92007	0.00117575	sp|Q9BZF1|OSBL8_HUMAN	OSBPL8	Q9BZF1
1	22	73	73	73	0.999	1416	1.97725	0.00100691	sp|Q9BZF9|UACA_HUMAN	UACA	Q9BZF9
1	6	117	117	117	0.999	259	2.47266	0.00030622	sp|Q9BZG1|RAB34_HUMAN	RAB34	Q9BZG1
1	7	24	24	24	0.999	848	2.39231	0.000378171	sp|Q9BZJ0|CRNL1_HUMAN	CRNKL1	Q9BZJ0
1	6	60	47	60	0.999	514	1.05466	0.0175531	sp|Q9BZK7|TBL1R_HUMAN	TBL1XR1	Q9BZK7
1	6	87	87	87	0.999	73	0.315502	0.226717	sp|Q9BZL1|UBL5_HUMAN	UBL5	Q9BZL1
1	6	71	35	71	0.999	878	1.19323	0.0108393	sp|Q9BZL6|KPCD2_HUMAN	PRKD2	Q9BZL6
1	21	231	231	231	0.999	928	8.85142	0	sp|Q9BZQ8|NIBA1_HUMAN	NIBAN1	Q9BZQ8
1	6	74	74	74	0.999	441	3.51817	2.01511E-05	sp|Q9BZV1|UBXN6_HUMAN	UBXN6	Q9BZV1
1	7	119	118	119	0.999	261	0.209109	0.340125	sp|Q9BZX2|UCK2_HUMAN	UCK2	Q9BZX2
1	20	420	420	420	0.999	524	4.13867	0	sp|Q9BZZ5|API5_HUMAN	API5	Q9BZZ5
1	7	170	170	170	0.999	99	0.173574	0.391854	sp|Q9C005|DPY30_HUMAN	DPY30	Q9C005
1	9	85	85	85	0.999	505	0.994764	0.0217086	sp|Q9C0B1|FTO_HUMAN	FTO	Q9C0B1
1	50	894	894	894	0.999	1729	1.73603	0.00197955	sp|Q9C0C2|TB182_HUMAN	TNKS1BP1	Q9C0C2
1	17	191	191	191	0.999	1292	0.231695	0.311784	sp|Q9C0C9|UBE2O_HUMAN	UBE2O	Q9C0C9
1	3	56	56	56	0.999	744	2.32993	0.000442121	sp|Q9C0D3|ZY11B_HUMAN	ZYG11B	Q9C0D3
1	3	29	29	29	0.999	523	5.65363	0	sp|Q9C0H2|TTYH3_HUMAN	TTYH3	Q9C0H2
1	14	211	211	211	0.999	423	0.0861142	0.573501	sp|Q9GZL7|WDR12_HUMAN	WDR12	Q9GZL7
1	5	42	42	42	0.999	350	1.67354	0.00236645	sp|Q9GZM5|YIPF3_HUMAN	YIPF3	Q9GZM5
1	11	110	110	110	0.999	422	1.35215	0.00629775	sp|Q9GZR2|REXO4_HUMAN	REXO4	Q9GZR2
1	15	93	93	93	0.999	859	1.23166	0.00954921	sp|Q9GZR7|DDX24_HUMAN	DDX24	Q9GZR7
1	12	152	152	152	0.999	419	1.44263	0.00465033	sp|Q9GZS1|RPA49_HUMAN	POLR1E	Q9GZS1
1	8	161	161	161	0.999	305	0.044456	0.709492	sp|Q9GZS3|SKI8_HUMAN	SKIC8	Q9GZS3
1	8	244	244	244	0.999	109	1.11109	0.014399	sp|Q9GZT3|SLIRP_HUMAN	SLIRP	Q9GZT3
1	3	145	91	145	0.999	377	1.20329	0.0104816	sp|Q9GZT8|NIF3L_HUMAN	NIF3L1	Q9GZT8
1	10	108	108	108	0.999	426	0.89508	0.0306822	sp|Q9GZT9|EGLN1_HUMAN	EGLN1	Q9GZT9
1	9	168	168	168	0.999	254	0.872855	0.0331599	sp|Q9GZU8|PIP30_HUMAN	PSME3IP1	Q9GZU8
0.9999	2	10	10	10	0.9988	223	0.790449	0.0438123	sp|Q9GZX9|TWSG1_HUMAN	TWSG1	Q9GZX9
1	5	122	122	122	0.999	342	0.767478	0.0475657	sp|Q9GZY8|MFF_HUMAN	MFF	Q9GZY8
1	8	218	218	218	0.999	169	3.94128	0	sp|Q9GZZ1|NAA50_HUMAN	NAA50	Q9GZZ1
1	6	67	67	67	0.999	195	1.71194	0.00211791	sp|Q9H061|T126A_HUMAN	TMEM126A	Q9H061
1	9	188	188	188	0.999	479	0.545349	0.101274	sp|Q9H074|PAIP1_HUMAN	PAIP1	Q9H074
1	17	314	314	314	0.999	707	3.26427	4.62428E-05	sp|Q9H078|CLPB_HUMAN	CLPB	Q9H078
1	32	480	480	480	0.999	1025	0.510092	0.114837	sp|Q9H0A0|NAT10_HUMAN	NAT10	Q9H0A0
1	22	152	152	383	0.999	622	1.61351	0.0028556	sp|Q9H0B6|KLC2_HUMAN	KLC2	Q9H0B6
1	17	174	174	174	0.999	392	0.805417	0.0416473	sp|Q9H0C8|ILKAP_HUMAN	ILKAP	Q9H0C8
1	29	658	658	658	0.999	950	1.61705	0.00282289	sp|Q9H0D6|XRN2_HUMAN	XRN2	Q9H0D6
1	4	49	49	49	0.999	558	2.74241	0.000164384	sp|Q9H0G5|NSRP1_HUMAN	NSRP1	Q9H0G5
1	12	67	67	67	0.999	632	2.1598	0.000625514	sp|Q9H0H5|RGAP1_HUMAN	RACGAP1	Q9H0H5
1	14	139	139	189	0.999	616	1.63115	0.00271522	sp|Q9H0L4|CSTFT_HUMAN	CSTF2T	Q9H0L4
1	8	165	165	165	0.999	455	0.318092	0.224749	sp|Q9H0S4|DDX47_HUMAN	DDX47	Q9H0S4
1	7	210	210	210	0.999	335	1.72545	0.00203723	sp|Q9H0U3|MAGT1_HUMAN	MAGT1	Q9H0U3
1	26	1054	230	1054	0.999	201	2.87344	0.000127168	sp|Q9H0U4|RAB1B_HUMAN	RAB1B	Q9H0U4
1	5	114	114	114	0.999	180	0.351725	0.199436	sp|Q9H0U6|RM18_HUMAN	MRPL18	Q9H0U6
1	8	150	150	150	0.999	315	2.0426	0.000854545	sp|Q9H0W9|CK054_HUMAN	C11orf54	Q9H0W9
1	10	47	47	49	0.999	796	1.63922	0.00265429	sp|Q9H1B7|I2BPL_HUMAN	IRF2BPL	Q9H1B7

1	16	616	616	616	0.999	243	3.73001	0	sp|Q9H1E3|NUCKS_HUMAN	NUCKS1	Q9H1E3
1	11	9	9	54	0.9989	359	3.95116	0	sp|Q9H1J7|WNT5B_HUMAN	WNT5B	Q9H1J7
1	7	121	121	121	0.999	167	0.379457	0.180282	sp|Q9H1K1|ISCU_HUMAN	ISCU	Q9H1K1
1	4	127	127	127	0.999	275	5.8541	0	sp|Q9H1Y0|ATG5_HUMAN	ATG5	Q9H1Y0
1	5	27	27	27	0.999	485	3.59889	0	sp|Q9H1Z4|WDR13_HUMAN	WDR13	Q9H1Z4
1	22	356	285	413	0.999	541	7.38173	0	sp|Q9H223|EHD4_HUMAN	EHD4	Q9H223
0.9929	2	32	32	32	0.9966	3354	0.956991	0.0247629	sp|Q9H251|CAD23_HUMAN	CDH23	Q9H251
1	7	141	141	141	0.999	617	0.633385	0.0748013	sp|Q9H267|VP33B_HUMAN	VPS33B	Q9H267
1	7	65	65	65	0.999	941	0.2039	0.346989	sp|Q9H270|VPS11_HUMAN	VPS11	Q9H270
1	11	453	453	453	0.999	93	3.26244	4.61894E-05	sp|Q9H299|SH3L3_HUMAN	SH3BGRL3	Q9H299
1	18	149	149	149	0.999	2365	0.564404	0.0946813	sp|Q9H2D6|TARA_HUMAN	TRIOBP	Q9H2D6
1	44	655	599	655	0.999	1235	0.198192	0.354851	sp|Q9H2G2|SLK_HUMAN	SLK	Q9H2G2
1	7	77	77	77	0.999	161	3.53806	2.03562E-05	sp|Q9H2H8|PPIL3_HUMAN	PPIL3	Q9H2H8
1	5	55	55	55	0.999	898	2.59757	0.000244275	sp|Q9H2K8|TAOK3_HUMAN	TAOK3	Q9H2K8
1	24	551	546	551	0.999	1393	1.15206	0.012518	sp|Q9H2M9|RBGPR_HUMAN	RAB3GAP2	Q9H2M9
1	13	218	218	218	0.999	1102	0.474157	0.129963	sp|Q9H2P0|ADNP_HUMAN	ADNP	Q9H2P0
0.9998	2	69	69	69	0.999	285	0.508654	0.115408	sp|Q9H2P9|DPH5_HUMAN	DPH5	Q9H2P9
1	19	256	228	256	0.999	1008	0.629497	0.0758026	sp|Q9H2U1|DHX36_HUMAN	DHX36	Q9H2U1
1	21	354	354	419	0.999	334	0.796622	0.0429753	sp|Q9H2U2|IPYR2_HUMAN	PPA2	Q9H2U2
1	10	301	301	301	0.999	279	1.24965	0.00898262	sp|Q9H2W6|RM46_HUMAN	MRPL46	Q9H2W6
1	30	619	619	619	0.999	717	0.63038	0.0755717	sp|Q9H307|PININ_HUMAN	PNN	Q9H307
1	3	86	86	86	0.999	879	0.406771	0.164149	sp|Q9H330|TM245_HUMAN	TMEM245	Q9H330
1	10	507	507	507	0.999	86	0.522737	0.109957	sp|Q9H3K6|BOLA2_HUMAN	BOLA2	Q9H3K6
1	6	118	118	118	0.999	280	1.67728	0.00235398	sp|Q9H3N1|TMX1_HUMAN	TMX1	Q9H3N1
1	10	203	203	206	0.999	528	0.435322	0.148147	sp|Q9H3P7|GCP60_HUMAN	ACBD3	Q9H3P7
1	10	163	163	163	0.999	356	0.305808	0.235363	sp|Q9H3Q1|BORG4_HUMAN	CDC42EP4	Q9H3Q1
1	18	145	145	145	0.999	1636	2.76613	0.000144444	sp|Q9H3S7|PTN23_HUMAN	PTPN23	Q9H3S7
1	34	572	572	572	0.999	944	0.0474669	0.697839	sp|Q9H3U1|UN45A_HUMAN	UNC45A	Q9H3U1
1	5	41	41	41	0.999	356	2.88021	0.000127536	sp|Q9H410|DSN1_HUMAN	DSN1	Q9H410
1	5	20	20	20	0.999	327	1.13301	0.0133892	sp|Q9H425|CA198_HUMAN	C1orf198	Q9H425
1	14	512	458	512	0.999	224	3.69759	0	sp|Q9H444|CHM4B_HUMAN	CHMP4B	Q9H444
1	14	240	240	240	0.999	388	1.83846	0.00147319	sp|Q9H488|OFUT1_HUMAN	POFUT1	Q9H488
1	14	159	136	159	0.999	2382	2.22695	0.000519517	sp|Q9H4A3|WNK1_HUMAN	WNK1	Q9H4A3
1	38	979	979	979	0.999	650	2.3043	0.000457227	sp|Q9H4A4|AMPB_HUMAN	RNPEP	Q9H4A4
1	12	204	204	204	0.999	298	1.91922	0.00117431	sp|Q9H4A6|GOLP3_HUMAN	GOLPH3	Q9H4A6
1	15	58	58	821	0.999	451	4.73757	0	sp|Q9H4B7|TBB1_HUMAN	TUBB1	Q9H4B7
1	16	123	122	142	0.999	881	0.687189	0.0623365	sp|Q9H4G0|E41L1_HUMAN	EPB41L1	Q9H4G0
0.9999	2	13	13	13	0.999	154	4.05024	0	sp|Q9H4G4|GAPR1_HUMAN	GLIPR2	Q9H4G4
1	7	69	69	69	0.999	574	3.46009	1.97775E-05	sp|Q9H4L4|SENP3_HUMAN	SENP3	Q9H4L4
1	10	128	101	128	0.999	887	2.1141	0.00070415	sp|Q9H4L5|OSBL3_HUMAN	OSBPL3	Q9H4L5
1	37	736	522	736	0.999	534	5.36998	0	sp|Q9H4M9|EHD1_HUMAN	EHD1	Q9H4M9
1	6	47	47	47	0.999	416	2.87287	0.000127045	sp|Q9H553|ALG2_HUMAN	ALG2	Q9H553
1	19	213	213	213	0.999	836	5.36146	0	sp|Q9H5V8|CDCP1_HUMAN	CDCP1	Q9H5V8
1	9	93	93	93	0.999	360	2.50583	0.000285714	sp|Q9H6F5|CCD86_HUMAN	CCDC86	Q9H6F5
1	12	183	183	183	0.999	1146	1.17017	0.0116821	sp|Q9H6R4|NOL6_HUMAN	NOL6	Q9H6R4
1	12	157	157	157	0.999	1430	0.223464	0.321828	sp|Q9H6S0|YTDC2_HUMAN	YTHDC2	Q9H6S0
1	9	88	88	88	0.999	665	0.631165	0.0753827	sp|Q9H6T3|RPAP3_HUMAN	RPAP3	Q9H6T3
1	7	47	47	47	0.999	567	3.1035	5.13369E-05	sp|Q9H6Z4|RANB3_HUMAN	RANBP3	Q9H6Z4
1	8	193	193	193	0.999	170	1.27643	0.0082413	sp|Q9H773|DCTP1_HUMAN	DCTPP1	Q9H773
1	11	136	119	136	0.999	454	0.487831	0.123795	sp|Q9H788|SH24A_HUMAN	SH2D4A	Q9H788
1	21	281	281	281	0.999	306	1.312	0.00728504	sp|Q9H7B2|RPF2_HUMAN	RPF2	Q9H7B2
1	3	62	62	62	0.999	428	0.82044	0.0396464	sp|Q9H7B4|SMYD3_HUMAN	SMYD3	Q9H7B4
1	13	177	177	177	0.999	661	0.907917	0.0293258	sp|Q9H7D7|WDR26_HUMAN	WDR26	Q9H7D7
1	9	143	143	143	0.999	229	1.73323	0.00201021	sp|Q9H7E9|CH033_HUMAN	C8orf33	Q9H7E9
1	6	27	17	27	0.999	1312	1.28086	0.00810987	sp|Q9H7N4|SFR19_HUMAN	SCAF1	Q9H7N4
1	17	336	336	336	0.999	377	0.830617	0.0382587	sp|Q9H7Z7|PGES2_HUMAN	PTGES2	Q9H7Z7
1	8	104	104	104	0.999	394	2.06018	0.00081205	sp|Q9H814|PHAX_HUMAN	PHAX	Q9H814
1	16	222	222	222	0.999	354	1.25907	0.00869365	sp|Q9H832|UBE2Z_HUMAN	UBE2Z	Q9H832
1	18	432	432	432	0.999	621	1.90126	0.00122612	sp|Q9H845|ACAD9_HUMAN	ACAD9	Q9H845
0.9999	6	188	49	188	0.999	216	0.812631	0.0407482	sp|Q9H8S9|MOB1A_HUMAN	MOB1A	Q9H8S9
0.9998	3	18	18	18	0.999	227	2.65766	0.000195292	sp|Q9H8U3|ZFAN3_HUMAN	ZFAND3	Q9H8U3
1	3	35	35	35	0.999	914	3.7587	0	sp|Q9H8V3|ECT2_HUMAN	ECT2	Q9H8V3
1	8	59	59	59	0.999	726	1.88708	0.00129087	sp|Q9H8Y5|ANKZ1_HUMAN	ANKZF1	Q9H8Y5
1	8	210	210	210	0.999	452	3.11237	5.15575E-05	sp|Q9H8Y8|GORS2_HUMAN	GORASP2	Q9H8Y8
1	19	580	580	580	0.999	190	2.43839	0.000334898	sp|Q9H910|JUPI2_HUMAN	JPT2	Q9H910
1	10	179	106	179	0.999	323	1.04749	0.0180475	sp|Q9H936|GHC1_HUMAN	SLC25A22	Q9H936
1	9	49	49	49	0.999	602	1.98888	0.00097789	sp|Q9H9A6|LRC40_HUMAN	LRRC40	Q9H9A6
1	22	731	653	731	0.999	322	4.55833	0	sp|Q9H9B4|SFXN1_HUMAN	SFXN1	Q9H9B4
1	7	60	60	60	0.999	785	1.63116	0.00271669	sp|Q9H9E3|COG4_HUMAN	COG4	Q9H9E3
1	5	83	83	83	0.999	264	2.67331	0.000189974	sp|Q9H9Q2|CSN7B_HUMAN	COPS7B	Q9H9Q2
1	9	103	103	103	0.999	547	1.54325	0.00343215	sp|Q9H9T3|ELP3_HUMAN	ELP3	Q9H9T3
1	13	166	166	166	0.999	309	2.11947	0.000694949	sp|Q9HA64|KT3K_HUMAN	FN3KRP	Q9HA64
1	10	135	135	135	0.999	311	0.0673269	0.628443	sp|Q9HAB8|PPCS_HUMAN	PPCS	Q9HAB8
1	20	130	130	448	0.999	340	4.47056	0	sp|Q9HAV0|GBB4_HUMAN	GNB4	Q9HAV0
1	24	340	340	340	0.999	1204	1.66761	0.00241189	sp|Q9HAV4|XPO5_HUMAN	XPO5	Q9HAV4
1	16	479	479	479	0.999	217	2.29735	0.000458486	sp|Q9HAV7|GRPE1_HUMAN	GRPEL1	Q9HAV7
1	11	641	641	641	0.999	376	1.38457	0.00563928	sp|Q9HB07|MYG1_HUMAN	MYG1	Q9HB07
1	3	15	15	15	0.999	628	2.63063	0.000209083	sp|Q9HB63|NET4_HUMAN	NTN4	Q9HB63
1	24	921	921	921	0.999	228	2.91494	0.000125367	sp|Q9HB71|CYBP_HUMAN	CACYBP	Q9HB71
1	4	43	10	43	0.999	399	0.372659	0.184586	sp|Q9HB90|RRAGC_HUMAN	RRAGC	Q9HB90
1	5	70	70	70	0.999	336	1.37767	0.0057696	sp|Q9HBH5|RDH14_HUMAN	RDH14	Q9HBH5
1	5	69	69	69	0.999	324	0.832755	0.0379609	sp|Q9HC07|TM165_HUMAN	TMEM165	Q9HC07
1	19	162	162	162	0.999	981	1.03862	0.0185693	sp|Q9HC35|EMAL4_HUMAN	EML4	Q9HC35
1	11	283	283	283	0.999	313	3.27689	4.65116E-05	sp|Q9HC38|GLOD4_HUMAN	GLOD4	Q9HC38
1	5	77	77	77	0.999	389	1.91986	0.00117503	sp|Q9HC52|CBX8_HUMAN	CBX8	Q9HC52
1	13	155	155	155	0.999	563	2.4912	0.000296058	sp|Q9HCC0|MCCB_HUMAN	MCCC2	Q9HCC0
1	10	163	163	163	0.999	579	2.96442	0.000096	sp|Q9HCD5|NCOA5_HUMAN	NCOA5	Q9HCD5
1	22	288	288	288	0.999	1003	1.61188	0.00286975	sp|Q9HCE1|MOV10_HUMAN	MOV10	Q9HCE1
1	8	98	98	98	0.999	908	1.01522	0.0201878	sp|Q9HCG8|CWC22_HUMAN	CWC22	Q9HCG8
1	8	97	89	97	0.999	2581	3.28893	4.20561E-05	sp|Q9HCK8|CHD8_HUMAN	CHD8	Q9HCK8
1	3	75	75	75	0.999	374	0.395559	0.170652	sp|Q9HCN4|GPN1_HUMAN	GPN1	Q9HCN4
1	7	233	233	233	0.999	221	2.54162	0.000279146	sp|Q9HCN8|SDF2L_HUMAN	SDF2L1	Q9HCN8
1	10	147	147	147	0.999	855	3.6314	0	sp|Q9HCS7|SYF1_HUMAN	XAB2	Q9HCS7
1	6	12	12	12	0.999	757	3.7014	0	sp|Q9HCU0|CD248_HUMAN	CD248	Q9HCU0
1	4	76	76	76	0.999	417	0.354016	0.197916	sp|Q9HCU5|PREB_HUMAN	PREB	Q9HCU5
1	10	95	95	95	0.999	224	1.08753	0.0155814	sp|Q9HD15|SRA1_HUMAN	SRA1	Q9HD15
1	14	98	98	98	0.999	1204	2.55451	0.000273702	sp|Q9HD20|AT131_HUMAN	ATP13A1	Q9HD20
1	12	113	113	113	0.999	462	0.97605	0.0231871	sp|Q9HD26|GOPC_HUMAN	GOPC	Q9HD26
1	8	178	178	178	0.999	250	0.583796	0.0884665	sp|Q9HD33|RM47_HUMAN	MRPL47	Q9HD33
1	15	293	293	293	0.999	196	4.78817	0	sp|Q9HD42|CHM1A_HUMAN	CHMP1A	Q9HD42
1	7	326	326	326	0.999	589	1.9213	0.00117402	sp|Q9HD45|TM9S3_HUMAN	TM9SF3	Q9HD45
1	19	572	572	572	0.999	416	0.937458	0.0265369	sp|Q9HDC9|APMAP_HUMAN	APMAP	Q9HDC9
1	5	76	76	76	0.999	1121	1.85082	0.0014323	sp|Q9NNW5|WDR6_HUMAN	WDR6	Q9NNW5
1	13	246	246	246	0.999	516	1.73007	0.00202148	sp|Q9NP61|ARFG3_HUMAN	ARFGAP3	Q9NP61
1	9	87	87	87	0.999	206	0.246351	0.29433	sp|Q9NP72|RAB18_HUMAN	RAB18	Q9NP72
1	4	60	60	60	0.999	194	2.17689	0.000605135	sp|Q9NP77|SSU72_HUMAN	SSU72	Q9NP77
1	11	268	268	268	0.999	307	0.85591	0.0352016	sp|Q9NP79|VTA1_HUMAN	VTA1	Q9NP79
1	13	180	180	180	0.999	518	1.25848	0.0087114	sp|Q9NP81|SYSM_HUMAN	SARS2	Q9NP81
1	7	91	91	96	0.999	439	1.05111	0.0178031	sp|Q9NP92|RT30_HUMAN	MRPS30	Q9NP92
1	6	224	97	224	0.999	96	2.99172	8.11359E-05	sp|Q9NP97|DLRB1_HUMAN	DYNLRB1	Q9NP97
1	6	162	162	162	0.999	242	2.37839	0.000406417	sp|Q9NPA0|EMC7_HUMAN	EMC7	Q9NPA0
1	6	107	107	107	0.999	245	0.774724	0.0463439	sp|Q9NPD3|EXOS4_HUMAN	EXOSC4	Q9NPD3
1	3	100	100	100	0.999	64	2.07395	0.00078628	sp|Q9NPE3|NOP10_HUMAN	NOP10	Q9NPE3
1	11	82	82	82	0.999	335	0.743821	0.0516115	sp|Q9NPF4|OSGEP_HUMAN	OSGEP	Q9NPF4
0.9999	2	103	103	103	0.999	140	0.401446	0.167021	sp|Q9NPJ3|ACO13_HUMAN	ACOT13	Q9NPJ3
1	10	107	106	107	0.999	531	0.599003	0.08401	sp|Q9NPQ8|RIC8A_HUMAN	RIC8A	Q9NPQ8
1	13	100	100	419	0.999	371	1.89369	0.00125783	sp|Q9NQ29|LUC7L_HUMAN	LUC7L	Q9NQ29
1	8	139	139	139	0.999	299	2.37083	0.000409401	sp|Q9NQ48|LZTL1_HUMAN	LZTFL1	Q9NQ48
1	11	205	205	205	0.999	473	0.794808	0.0431908	sp|Q9NQ55|SSF1_HUMAN	PPAN	Q9NQ55
1	9	231	231	231	0.999	270	0.22743	0.317062	sp|Q9NQ88|TIGAR_HUMAN	TIGAR	Q9NQ88
1	12	472	472	472	0.999	1192	2.12036	0.000695418	sp|Q9NQC3|RTN4_HUMAN	RTN4	Q9NQC3
1	13	193	166	193	0.999	326	0.492079	0.122124	sp|Q9NQG5|RPR1B_HUMAN	RPRD1B	Q9NQG5
1	5	52	52	56	0.999	507	3.08114	5.90095E-05	sp|Q9NQH7|XPP3_HUMAN	XPNPEP3	Q9NQH7
1	17	282	282	282	0.999	276	2.74593	0.000161172	sp|Q9NQR4|NIT2_HUMAN	NIT2	Q9NQR4
0.9996	2	9	9	9	0.999	549	1.27003	0.00840684	sp|Q9NQS3|NECT3_HUMAN	NECTIN3	Q9NQS3
1	6	100	100	100	0.999	235	0.149706	0.43221	sp|Q9NQT4|EXOS5_HUMAN	EXOSC5	Q9NQT4
1	12	122	111	122	0.999	1826	1.68819	0.00228096	sp|Q9NQT8|KI13B_HUMAN	KIF13B	Q9NQT8
1	38	374	374	374	0.999	1124	0.437397	0.147031	sp|Q9NQW6|ANLN_HUMAN	ANLN	Q9NQW6
1	25	686	634	686	0.999	623	0.426164	0.15307	sp|Q9NQW7|XPP1_HUMAN	XPNPEP1	Q9NQW7

1	3	24	24	24	0.999	253	4.24089	0	sp|Q9NQY0|BIN3_HUMAN	BIN3	Q9NQY0
1	16	95	91	95	0.999	4857	1.60086	0.0029396	sp|Q9NR09|BIRC6_HUMAN	BIRC6	Q9NR09
1	27	787	787	787	0.999	457	2.30022	0.000458824	sp|Q9NR12|PDLI7_HUMAN	PDLIM7	Q9NR12
1	17	178	178	178	0.999	701	1.55313	0.00334557	sp|Q9NR19|ACSA_HUMAN	ACSS2	Q9NR19
1	6	78	78	78	0.999	239	0.923761	0.0277744	sp|Q9NR28|DBLOH_HUMAN	DIABLO	Q9NR28
1	54	1284	1220	1284	0.999	783	5.28319	0	sp|Q9NR30|DDX21_HUMAN	DDX21	Q9NR30
1	9	203	135	203	0.999	198	2.39665	0.000376253	sp|Q9NR31|SAR1A_HUMAN	SAR1A	Q9NR31
1	22	733	733	733	0.999	359	1.07556	0.0163343	sp|Q9NR45|SIAS_HUMAN	NANS	Q9NR45
1	16	157	157	158	0.999	395	0.331045	0.2143	sp|Q9NR46|SHLB2_HUMAN	SH3GLB2	Q9NR46
1	12	258	258	258	0.999	452	1.48611	0.00402236	sp|Q9NR50|EI2BG_HUMAN	EIF2B3	Q9NR50
1	13	328	143	328	0.999	388	2.59637	0.000243655	sp|Q9NR56|MBNL1_HUMAN	MBNL1	Q9NR56
1	6	45	45	48	0.999	586	0.836481	0.0374833	sp|Q9NRF8|PYRG2_HUMAN	CTPS2	Q9NRF8
1	3	60	60	60	0.999	147	1.59619	0.00295949	sp|Q9NRF9|DPOE3_HUMAN	POLE3	Q9NRF9
1	3	77	77	77	0.999	131	3.47054	1.99005E-05	sp|Q9NRG0|CHRC1_HUMAN	CHRAC1	Q9NRG0
1	6	96	96	96	0.999	546	1.90358	0.00121381	sp|Q9NRG9|AAAS_HUMAN	AAAS	Q9NRG9
1	11	108	108	108	0.999	1556	1.43574	0.00477291	sp|Q9NRL2|BAZ1A_HUMAN	BAZ1A	Q9NRL2
0.9978	4	65	44	65	0.999	753	0.321272	0.222242	sp|Q9NRL3|STRN4_HUMAN	STRN4	Q9NRL3
1	12	43	43	43	0.9989	406	6.32026	0	sp|Q9NRN5|OLFL3_HUMAN	OLFML3	Q9NRN5
1	8	84	46	143	0.999	601	1.58268	0.00310733	sp|Q9NRR5|UBQL4_HUMAN	UBQLN4	Q9NRR5
1	8	119	119	119	0.999	189	3.17754	5.31561E-05	sp|Q9NRV9|HEBP1_HUMAN	HEBP1	Q9NRV9
1	4	108	74	108	0.999	190	2.32054	0.000451709	sp|Q9NRW3|ABC3C_HUMAN	APOBEC3C	Q9NRW3
1	9	120	114	120	0.999	252	1.49942	0.00383853	sp|Q9NRX1|PNO1_HUMAN	PNO1	Q9NRX1
1	10	150	150	150	0.999	175	0.653364	0.0697989	sp|Q9NRX2|RM17_HUMAN	MRPL17	Q9NRX2
1	11	450	450	450	0.999	125	2.21256	0.000551724	sp|Q9NRX4|PHP14_HUMAN	PHPT1	Q9NRX4
1	2	65	65	65	0.999	453	0.691382	0.0615284	sp|Q9NRX5|SERC1_HUMAN	SERINC1	Q9NRX5
1	8	22	22	22	0.999	1499	3.26751	4.62963E-05	sp|Q9NRY4|RHG35_HUMAN	ARHGAP35	Q9NRY4
1	7	37	37	37	0.999	505	1.48334	0.00405479	sp|Q9NRY5|F1142_HUMAN	FAM114A2	Q9NRY5
1	5	143	143	143	0.999	295	2.31188	0.000453333	sp|Q9NRY6|PLS3_HUMAN	PLSCR3	Q9NRY6
1	7	37	37	37	0.999	838	0.988367	0.0222159	sp|Q9NRZ9|HELLS_HUMAN	HELLS	Q9NRZ9
1	11	32	32	32	0.999	1388	1.74085	0.00194989	sp|Q9NS87|KIF15_HUMAN	KIF15	Q9NS87
1	12	60	57	60	0.999	361	1.90301	0.00121718	sp|Q9NSC5|HOME3_HUMAN	HOMER3	Q9NSC5
1	27	755	755	755	0.999	589	1.86809	0.00136282	sp|Q9NSD9|SYFB_HUMAN	FARSB	Q9NSD9
1	54	1106	1106	1106	0.999	1012	2.84592	0.000133333	sp|Q9NSE4|SYIM_HUMAN	IARS2	Q9NSE4
1	11	26	26	31	0.999	1193	1.23131	0.00955757	sp|Q9NSV4|DIAP3_HUMAN	DIAPH3	Q9NSV4
1	9	142	142	142	0.999	314	0.464282	0.134736	sp|Q9NT62|ATG3_HUMAN	ATG3	Q9NT62
1	23	365	365	388	0.999	1447	1.94013	0.00112485	sp|Q9NTI5|PDS5B_HUMAN	PDS5B	Q9NTI5
1	53	979	979	979	0.999	1288	3.68669	0	sp|Q9NTJ3|SMC4_HUMAN	SMC4	Q9NTJ3
1	18	318	318	318	0.999	587	0.760599	0.0487035	sp|Q9NTJ5|SAC1_HUMAN	SACM1L	Q9NTJ5
1	22	802	802	802	0.999	396	0.938854	0.0264095	sp|Q9NTK5|OLA1_HUMAN	OLA1	Q9NTK5
1	7	137	137	137	0.999	307	1.0582	0.0173697	sp|Q9NTX5|ECHD1_HUMAN	ECHDC1	Q9NTX5
1	10	218	218	218	0.999	932	0.497402	0.120022	sp|Q9NTZ6|RBM12_HUMAN	RBM12	Q9NTZ6
1	3	41	41	41	0.999	403	2.7915	0.000138707	sp|Q9NUD5|ZCHC3_HUMAN	ZCCHC3	Q9NUD5
1	7	129	129	129	0.999	292	1.62688	0.00274986	sp|Q9NUI1|DECR2_HUMAN	DECR2	Q9NUI1
1	7	232	232	232	0.999	306	0.62214	0.0778029	sp|Q9NUJ1|ABHDA_HUMAN	ABHD10	Q9NUJ1
1	5	41	39	41	0.999	197	1.08958	0.0154709	sp|Q9NUP9|LIN7C_HUMAN	LIN7C	Q9NUP9
1	17	197	197	197	0.999	558	2.23144	0.000518149	sp|Q9NUQ6|SPS2L_HUMAN	SPATS2L	Q9NUQ6
1	10	140	140	140	0.999	709	4.07493	0	sp|Q9NUQ8|ABCF3_HUMAN	ABCF3	Q9NUQ8
1	18	607	541	607	0.999	324	0.0336041	0.755602	sp|Q9NUQ9|CYRIB_HUMAN	CYRIB	Q9NUQ9
1	7	64	64	64	0.999	699	4.00061	0	sp|Q9NUY8|TBC23_HUMAN	TBC1D23	Q9NUY8
1	4	57	12	57	0.999	361	0.402716	0.166388	sp|Q9NV96|CC50A_HUMAN	TMEM30A	Q9NV96
1	25	554	398	554	0.999	429	0.283279	0.255384	sp|Q9NVA2|SEP11_HUMAN	SEPTIN11	Q9NVA2
1	13	291	211	291	0.999	372	0.166384	0.403495	sp|Q9NVD7|PARVA_HUMAN	PARVA	Q9NVD7
1	9	124	124	124	0.999	400	0.619794	0.0783916	sp|Q9NVG8|TBC13_HUMAN	TBC1D13	Q9NVG8
1	11	101	101	101	0.999	1328	3.16232	5.26316E-05	sp|Q9NVI1|FANCI_HUMAN	FANCI	Q9NVI1
1	44	1208	292	1208	0.999	634	3.92275	0	sp|Q9NVI7|ATD3A_HUMAN	ATAD3A	Q9NVI7
1	8	129	71	129	0.999	186	2.7808	0.000141527	sp|Q9NVJ2|ARL8B_HUMAN	ARL8B	Q9NVJ2
1	3	14	11	14	0.999	165	3.41518	0.000019536	sp|Q9NVM1|EVA1B_HUMAN	EVA1B	Q9NVM1
1	4	55	55	55	0.999	692	2.28957	0.000468178	sp|Q9NVM4|ANM7_HUMAN	PRMT7	Q9NVM4
1	26	533	533	533	0.999	670	3.78802	0	sp|Q9NVP1|DDX18_HUMAN	DDX18	Q9NVP1
1	6	109	109	109	0.999	282	1.62167	0.0027907	sp|Q9NVT9|ARMC1_HUMAN	ARMC1	Q9NVT9
1	7	102	102	102	0.999	263	1.18327	0.0111819	sp|Q9NVZ3|NECP2_HUMAN	NECAP2	Q9NVZ3
1	22	225	225	225	0.999	759	3.49876	2.00501E-05	sp|Q9NW13|RBM28_HUMAN	RBM28	Q9NW13
1	11	249	249	249	0.999	420	0.341567	0.206392	sp|Q9NW64|RBM22_HUMAN	RBM22	Q9NW64
1	15	252	252	252	0.999	654	0.637508	0.073882	sp|Q9NW82|WDR70_HUMAN	WDR70	Q9NW82
1	12	166	166	166	0.999	273	0.199987	0.352332	sp|Q9NWB6|ARGL1_HUMAN	ARGLU1	Q9NWB6
1	22	293	293	299	0.999	1034	0.26152	0.277471	sp|Q9NWH9|SLTM_HUMAN	SLTM	Q9NWH9
1	4	73	73	73	0.999	290	2.33351	0.00044012	sp|Q9NWS0|PIHD1_HUMAN	PIH1D1	Q9NWS0
1	10	146	146	146	0.999	392	1.23121	0.00955868	sp|Q9NWT1|PK1IP_HUMAN	PAK1IP1	Q9NWT1
1	9	222	222	222	0.999	160	2.92262	0.000125737	sp|Q9NWV4|CZIB_HUMAN	CZIB	Q9NWV4
1	8	185	185	185	0.999	329	3.92613	0	sp|Q9NWV8|BABA1_HUMAN	BABAM1	Q9NWV8
0.9999	2	61	61	61	0.999	183	1.24677	0.00906886	sp|Q9NX08|COMD8_HUMAN	COMMD8	Q9NX08
1	6	188	188	188	0.999	153	0.586764	0.087707	sp|Q9NX24|NHP2_HUMAN	NHP2	Q9NX24
1	12	282	282	282	0.999	245	5.04073	0	sp|Q9NX40|OCAD1_HUMAN	OCIAD1	Q9NX40
1	6	68	68	68	0.999	363	0.116314	0.500669	sp|Q9NX46|ADPRS_HUMAN	ADPRS	Q9NX46
1	4	233	233	233	0.999	121	0.390605	0.173416	sp|Q9NX55|HYPK_HUMAN	HYPK	Q9NX55
1	30	422	422	422	0.999	379	0.401936	0.166763	sp|Q9NX58|LYAR_HUMAN	LYAR	Q9NX58
1	6	104	104	104	0.999	359	1.44158	0.00466533	sp|Q9NX62|IMPA3_HUMAN	BPNT2	Q9NX62
1	21	575	575	575	0.999	227	6.12131	0	sp|Q9NX63|MIC19_HUMAN	CHCHD3	Q9NX63
1	3	82	82	82	0.999	200	0.0883114	0.567855	sp|Q9NX70|MED29_HUMAN	MED29	Q9NX70
1	6	59	49	59	0.999	493	0.755356	0.0494975	sp|Q9NX74|DUS2L_HUMAN	DUS2	Q9NX74
1	9	135	135	135	0.999	866	3.14177	5.20607E-05	sp|Q9NXE4|NSMA3_HUMAN	SMPD4	Q9NXE4
1	11	183	183	183	0.999	353	1.53008	0.0035176	sp|Q9NXG2|THUM1_HUMAN	THUMPD1	Q9NXG2
1	4	26	26	26	0.999	423	2.70943	0.000175	sp|Q9NXH8|TOR4A_HUMAN	TOR4A	Q9NXH8
1	16	250	250	250	0.999	659	1.32796	0.00687689	sp|Q9NXH9|TRM1_HUMAN	TRMT1	Q9NXH9
1	7	61	61	61	0.999	580	1.12282	0.0138267	sp|Q9NXV6|CARF_HUMAN	CDKN2AIP	Q9NXV6
1	8	51	51	51	0.999	375	1.18142	0.0112345	sp|Q9NXW2|DJB12_HUMAN	DNAJB12	Q9NXW2
1	6	160	160	160	0.999	217	1.31263	0.00727153	sp|Q9NY12|GAR1_HUMAN	GAR1	Q9NY12
1	25	727	727	727	0.999	737	1.16121	0.0120846	sp|Q9NY33|DPP3_HUMAN	DPP3	Q9NY33
1	6	18	18	18	0.999	399	0.281284	0.2573	sp|Q9NYB0|TE2IP_HUMAN	TERF2IP	Q9NYB0
1	9	91	91	151	0.999	513	1.73155	0.00201471	sp|Q9NYB9|ABI2_HUMAN	ABI2	Q9NYB9
1	39	904	904	904	0.999	920	2.64627	0.000204152	sp|Q9NYF8|BCLF1_HUMAN	BCLAF1	Q9NYF8
1	11	102	102	102	0.999	597	3.58955	0	sp|Q9NYH9|UTP6_HUMAN	UTP6	Q9NYH9
1	16	133	133	133	0.999	800	0.40747	0.163871	sp|Q9NYL2|M3K20_HUMAN	MAP3K20	Q9NYL2
1	3	9	9	9	0.999	201	0.571375	0.0924479	sp|Q9NYL4|FKB11_HUMAN	FKBP11	Q9NYL4
1	17	602	558	602	0.999	352	5.74994	0	sp|Q9NYL9|TMOD3_HUMAN	TMOD3	Q9NYL9
1	48	750	727	750	0.999	1555	1.26548	0.00850554	sp|Q9NYU2|UGGG1_HUMAN	UGGT1	Q9NYU2
1	17	532	532	532	0.999	308	2.46677	0.000317209	sp|Q9NZ01|TECR_HUMAN	TECR	Q9NZ01
1	16	131	131	131	0.999	941	4.29964	0	sp|Q9NZ08|ERAP1_HUMAN	ERAP1	Q9NZ08
1	9	125	125	125	0.999	108	2.40256	0.00036476	sp|Q9NZ45|CISD1_HUMAN	CISD1	Q9NZ45
1	27	583	576	583	0.999	1118	1.11362	0.0142676	sp|Q9NZB2|F120A_HUMAN	FAM120A	Q9NZB2
1	8	130	73	140	0.999	540	0.880452	0.0323416	sp|Q9NZI7|UBIP1_HUMAN	UBP1	Q9NZI7
1	17	100	100	249	0.999	577	0.293206	0.246511	sp|Q9NZI8|IF2B1_HUMAN	IGF2BP1	Q9NZI8
1	9	171	171	171	0.999	389	1.30065	0.00754869	sp|Q9NZJ7|MTCH1_HUMAN	MTCH1	Q9NZJ7
1	14	320	320	320	0.999	359	0.732809	0.0534953	sp|Q9NZL4|HPBP1_HUMAN	HSPBP1	Q9NZL4
1	14	380	380	380	0.999	334	0.490872	0.122583	sp|Q9NZL9|MAT2B_HUMAN	MAT2B	Q9NZL9
1	115	2023	1990	2023	0.999	2061	7.58347	0	sp|Q9NZM1|MYOF_HUMAN	MYOF	Q9NZM1
1	20	5	5	293	0.9987	535	4.06209	0	sp|Q9NZN3|EHD3_HUMAN	EHD3	Q9NZN3
1	31	500	480	562	0.999	543	7.71789	0	sp|Q9NZN4|EHD2_HUMAN	EHD2	Q9NZN4
0.9165	1	49	49	49	0.9977	309	1.27799	0.00819898	sp|Q9NZP5|O5AC2_HUMAN	OR5AC2	Q9NZP5
1	10	103	103	103	0.999	722	2.33106	0.000439791	sp|Q9NZQ3|SPN90_HUMAN	NCKIPSD	Q9NZQ3
0.9998	4	82	82	126	0.9971	351	0.380975	0.179619	sp|Q9NZR1|TMOD2_HUMAN	TMOD2	Q9NZR1
1	23	369	369	369	0.999	677	0.209315	0.339945	sp|Q9NZT2|OGFR_HUMAN	OGFR	Q9NZT2
1	10	37	37	37	0.999	365	0.88418	0.0319328	sp|Q9NZU5|LMCD1_HUMAN	LMCD1	Q9NZU5
1	5	18	18	18	0.999	1036	4.22587	0	sp|Q9NZV1|CRIM1_HUMAN	CRIM1	Q9NZV1
1	8	92	92	92	0.999	219	1.09757	0.0150868	sp|Q9NZZ3|CHMP5_HUMAN	CHMP5	Q9NZZ3
1	3	27	27	27	0.999	198	2.22596	0.000518414	sp|Q9P000|COMD9_HUMAN	COMMD9	Q9P000
1	10	92	92	92	0.999	229	2.51764	0.000284082	sp|Q9P013|CWC15_HUMAN	CWC15	Q9P013
1	9	81	81	81	0.999	296	0.405164	0.165035	sp|Q9P015|RM15_HUMAN	MRPL15	Q9P015
1	10	147	147	147	0.999	225	1.54479	0.00342141	sp|Q9P016|THYN1_HUMAN	THYN1	Q9P016
1	10	128	128	128	0.999	362	0.196843	0.356701	sp|Q9P035|HACD3_HUMAN	HACD3	Q9P035
1	8	165	165	165	0.999	144	0.481634	0.126677	sp|Q9P0J0|NDUAD_HUMAN	NDUFA13	Q9P0J0
1	5	111	111	111	0.999	381	1.47538	0.004154	sp|Q9P0J7|KCMF1_HUMAN	KCMF1	Q9P0J7
1	28	272	271	272	0.999	980	0.495876	0.120573	sp|Q9P0K7|RAI14_HUMAN	RAI14	Q9P0K7
1	28	999	998	999	0.999	249	4.9767	0	sp|Q9P0L0|VAPA_HUMAN	VAPA	Q9P0L0
1	2	18	18	18	0.999	153	1.60799	0.00290479	sp|Q9P0P0|RN181_HUMAN	RNF181	Q9P0P0
1	6	17	17	17	0.999	963	1.40848	0.00520825	sp|Q9P0V3|SH3B4_HUMAN	SH3BP4	Q9P0V3
1	14	299	299	376	0.999	454	0.774169	0.0463917	sp|Q9P0V9|SEP10_HUMAN	SEPTIN10	Q9P0V9
0.9992	4	20	20	20	0.999	2028	2.74592	0.000161025	sp|Q9P219|DAPLE_HUMAN	CCDC88C	Q9P219
1	34	891	891	891	0.999	522	0.379708	0.180234	sp|Q9P258|RCC2_HUMAN	RCC2	Q9P258

1	13	133	132	133	0.999	1576	3.45873	1.97531E-05	sp|Q9P265|DIP2B_HUMAN	DIP2B	Q9P265
1	7	32	32	32	0.999	581	0.0898777	0.563896	sp|Q9P270|SLAI2_HUMAN	SLAIN2	Q9P270
1	10	188	188	188	0.999	314	2.00162	0.000933842	sp|Q9P287|BCCIP_HUMAN	BCCIP	Q9P287
1	29	351	351	351	0.999	879	7.23779	0	sp|Q9P2B2|FPRP_HUMAN	PTGFRN	Q9P2B2
1	7	32	32	32	0.999	639	1.37327	0.00585342	sp|Q9P2B4|CT2NL_HUMAN	CTTNBP2NL	Q9P2B4
1	5	26	26	26	0.999	2071	2.18868	0.000582173	sp|Q9P2D3|HTR5B_HUMAN	HEATR5B	Q9P2D3
1	118	2496	2456	2496	0.999	1410	3.04985	6.68757E-05	sp|Q9P2E9|RRBP1_HUMAN	RRBP1	Q9P2E9
1	9	168	168	168	0.999	782	1.49207	0.00393276	sp|Q9P2I0|CPSF2_HUMAN	CPSF2	Q9P2I0
1	66	1496	1496	1496	0.999	1176	1.13421	0.0133287	sp|Q9P2J5|SYLC_HUMAN	LARS1	Q9P2J5
1	6	19	19	112	0.999	600	0.152629	0.426709	sp|Q9P2K5|MYEF2_HUMAN	MYEF2	Q9P2K5
1	12	146	146	146	0.999	1060	1.23567	0.00941455	sp|Q9P2N5|RBM27_HUMAN	RBM27	Q9P2N5
1	12	163	163	163	0.999	1169	0.708939	0.0579126	sp|Q9P2R3|ANFY1_HUMAN	ANKFY1	Q9P2R3
1	24	343	343	343	0.999	463	1.29106	0.00782812	sp|Q9P2R7|SUCB1_HUMAN	SUCLA2	Q9P2R7
1	8	147	99	147	0.999	348	0.292388	0.247192	sp|Q9P2T1|GMPR2_HUMAN	GMPR2	Q9P2T1
1	4	102	102	102	0.999	335	1.94116	0.00111565	sp|Q9P2W9|STX18_HUMAN	STX18	Q9P2W9
1	13	256	256	256	0.999	475	0.795063	0.0431719	sp|Q9UBB4|ATX10_HUMAN	ATXN10	Q9UBB4
1	3	66	66	66	0.999	729	1.86065	0.00139857	sp|Q9UBB6|NCDN_HUMAN	NCDN	Q9UBB6
1	9	143	143	143	0.999	864	0.992805	0.0218321	sp|Q9UBC2|EP15R_HUMAN	EPS15L1	Q9UBC2
1	25	800	800	800	0.999	346	6.2801	0	sp|Q9UBE0|SAE1_HUMAN	SAE1	Q9UBE0
1	20	116	116	169	0.999	871	0.0302766	0.771542	sp|Q9UBF2|COPG2_HUMAN	COPG2	Q9UBF2
1	4	111	111	111	0.999	113	0.951336	0.0252493	sp|Q9UBF6|RBX2_HUMAN	RNF7	Q9UBF6
1	20	116	116	116	0.999	1479	1.01754	0.020052	sp|Q9UBG0|MRC2_HUMAN	MRC2	Q9UBG0
1	9	421	421	421	0.999	72	10.8128	0	sp|Q9UBI6|GBG12_HUMAN	GNG12	Q9UBI6
1	10	198	198	198	0.999	475	0.909279	0.0292111	sp|Q9UBM7|DHCR7_HUMAN	DHCR7	Q9UBM7
1	12	99	99	99	0.999	1215	0.973472	0.0234083	sp|Q9UBN7|HDAC6_HUMAN	HDAC6	Q9UBN7
1	7	250	250	250	0.999	182	0.131107	0.468664	sp|Q9UBQ0|VPS29_HUMAN	VPS29	Q9UBQ0
1	5	158	158	158	0.999	218	1.75376	0.0018578	sp|Q9UBQ5|EIF3K_HUMAN	EIF3K	Q9UBQ5
1	13	436	436	436	0.999	328	1.65882	0.00248874	sp|Q9UBQ7|GRHPR_HUMAN	GRHPR	Q9UBQ7
1	13	415	415	415	0.999	303	0.786814	0.0443949	sp|Q9UBR2|CATZ_HUMAN	CTSZ	Q9UBR2
1	19	537	537	537	0.999	358	7.10304	0	sp|Q9UBS4|DJB11_HUMAN	DNAJB11	Q9UBS4
1	32	779	779	779	0.999	640	1.24848	0.00900822	sp|Q9UBT2|SAE2_HUMAN	UBA2	Q9UBT2
1	8	51	51	51	0.999	362	1.42058	0.00502376	sp|Q9UBU8|MO4L1_HUMAN	MORF4L1	Q9UBU8
1	13	156	156	156	0.999	619	0.171205	0.395736	sp|Q9UBU9|NXF1_HUMAN	NXF1	Q9UBU9
1	8	122	122	122	0.999	794	1.48549	0.00402843	sp|Q9UBV2|SE1L1_HUMAN	SEL1L	Q9UBV2
1	4	76	76	76	0.999	284	0.550373	0.0994656	sp|Q9UBV8|PEF1_HUMAN	PEF1	Q9UBV8
1	10	189	189	189	0.999	287	4.09747	0	sp|Q9UBX3|DIC_HUMAN	SLC25A10	Q9UBX3
1	5	195	195	195	0.999	63	1.06704	0.0168363	sp|Q9UDW1|QCR9_HUMAN	UQCR10	Q9UDW1
1	47	583	533	583	0.999	1190	0.440713	0.145362	sp|Q9UDY2|ZO2_HUMAN	TJP2	Q9UDY2
1	9	56	56	56	0.999	706	1.38068	0.00570431	sp|Q9UEY8|ADDG_HUMAN	ADD3	Q9UEY8
1	26	439	439	439	0.999	623	3.83352	0	sp|Q9UG63|ABCF2_HUMAN	ABCF2	Q9UG63
1	37	1400	1400	1400	0.999	421	0.206564	0.343482	sp|Q9UGI8|TES_HUMAN	TES	Q9UGI8
1	7	34	34	34	0.999	676	1.49211	0.00393677	sp|Q9UGP4|LIMD1_HUMAN	LIMD1	Q9UGP4
1	12	277	277	277	0.999	760	0.674335	0.0649753	sp|Q9UGP8|SEC63_HUMAN	SEC63	Q9UGP8
1	7	28	28	28	0.999	977	1.5878	0.00304255	sp|Q9UGR2|Z3H7B_HUMAN	ZC3H7B	Q9UGR2
1	10	200	200	200	0.999	375	0.757903	0.049107	sp|Q9UGV2|NDRG3_HUMAN	NDRG3	Q9UGV2
1	6	55	55	55	0.999	379	0.0906777	0.561851	sp|Q9UH62|ARMX3_HUMAN	ARMCX3	Q9UH62
1	27	414	412	414	0.999	585	0.564576	0.094644	sp|Q9UH65|SWP70_HUMAN	SWAP70	Q9UH65
1	17	196	193	196	0.999	717	2.4132	0.000350932	sp|Q9UH99|SUN2_HUMAN	SUN2	Q9UH99
1	50	756	715	756	0.999	759	2.57862	0.000247699	sp|Q9UHB6|LIMA1_HUMAN	LIMA1	Q9UHB6
1	30	496	496	496	0.999	627	2.01432	0.00090629	sp|Q9UHB9|SRP68_HUMAN	SRP68	Q9UHB9
1	20	358	358	358	0.999	332	1.34668	0.0064042	sp|Q9UHD1|CHRD1_HUMAN	CHORDC1	Q9UHD1
1	8	127	103	127	0.999	729	0.0524041	0.679091	sp|Q9UHD2|TBK1_HUMAN	TBK1	Q9UHD2
1	58	1878	1878	1878	0.999	586	0.58362	0.0884934	sp|Q9UHD8|SEPT9_HUMAN	SEPTIN9	Q9UHD8
1	6	206	23	206	0.999	624	3.53129	2.02788E-05	sp|Q9UHD9|UBQL2_HUMAN	UBQLN2	Q9UHD9
1	15	183	183	183	0.999	505	0.756636	0.0493364	sp|Q9UHG3|PCYOX_HUMAN	PCYOX1	Q9UHG3
0.9975	2	2	2	2	0.9988	967	5.38181	0	sp|Q9UHI8|ATS1_HUMAN	ADAMTS1	Q9UHI8
1	11	187	187	187	0.999	492	0.815821	0.0402938	sp|Q9UHL4|DPP2_HUMAN	DPP7	Q9UHL4
1	9	86	86	86	0.999	241	0.115418	0.50261	sp|Q9UHQ4|BAP29_HUMAN	BCAP29	Q9UHQ4
1	12	280	280	280	0.999	305	0.168191	0.400653	sp|Q9UHQ9|NB5R1_HUMAN	CYB5R1	Q9UHQ9
1	10	401	401	401	0.999	154	2.77478	0.000141264	sp|Q9UHV9|PFD2_HUMAN	PFDN2	Q9UHV9
1	27	800	800	800	0.999	559	3.17665	5.30387E-05	sp|Q9UHX1|PUF60_HUMAN	PUF60	Q9UHX1
1	6	97	97	97	0.999	535	0.306466	0.234769	sp|Q9UHY1|NRBP_HUMAN	NRBP1	Q9UHY1
1	6	175	175	175	0.999	261	0.0791726	0.592796	sp|Q9UHY7|ENOPH_HUMAN	ENOPH1	Q9UHY7
1	4	76	76	76	0.999	145	3.03462	7.05394E-05	sp|Q9UI09|NDUAC_HUMAN	NDUFA12	Q9UI09
1	14	193	193	193	0.999	523	0.684871	0.0627367	sp|Q9UI10|EI2BD_HUMAN	EIF2B4	Q9UI10
1	10	238	238	238	0.999	483	0.169228	0.398949	sp|Q9UI12|VATH_HUMAN	ATP6V1H	Q9UI12
1	4	56	56	56	0.999	185	2.45707	0.000318863	sp|Q9UI14|PRAF1_HUMAN	RABAC1	Q9UI14
1	6	123	123	123	0.999	975	2.48963	0.000299035	sp|Q9UI26|IPO11_HUMAN	IPO11	Q9UI26
1	6	101	101	101	0.999	125	0.622959	0.0775658	sp|Q9UI30|TR112_HUMAN	TRMT112	Q9UI30
1	22	565	565	565	0.999	1087	0.0726366	0.612225	sp|Q9UIA9|XPO7_HUMAN	XPO7	Q9UIA9
1	5	61	61	61	0.999	782	2.22678	0.000519149	sp|Q9UID3|VPS51_HUMAN	VPS51	Q9UID3
1	35	345	345	345	0.999	1483	1.08444	0.0157575	sp|Q9UIG0|BAZ1B_HUMAN	BAZ1B	Q9UIG0
1	18	447	408	447	0.999	106	0.272799	0.266084	sp|Q9UII2|ATIF1_HUMAN	ATP5IF1	Q9UII2
1	13	238	238	238	0.999	227	0.204495	0.34619	sp|Q9UIJ7|KAD3_HUMAN	AK3	Q9UIJ7
1	7	22	22	22	0.999	491	2.96326	9.59041E-05	sp|Q9UJ41|RABX5_HUMAN	RABGEF1	Q9UJ41
1	5	71	71	71	0.999	235	1.73585	0.00197842	sp|Q9UJ68|MSRA_HUMAN	MSRA	Q9UJ68
1	12	152	152	152	0.999	344	2.02918	0.000876854	sp|Q9UJ70|NAGK_HUMAN	NAGK	Q9UJ70
1	9	101	101	101	0.999	578	2.19559	0.000566643	sp|Q9UJ83|HACL1_HUMAN	HACL1	Q9UJ83
1	7	150	150	150	0.999	497	0.838781	0.037225	sp|Q9UJA5|TRM6_HUMAN	TRMT6	Q9UJA5
1	18	559	501	559	0.999	675	1.33837	0.00663364	sp|Q9UJS0|S2513_HUMAN	SLC25A13	Q9UJS0
1	22	729	729	729	0.999	430	0.621234	0.0780254	sp|Q9UJU6|DBNL_HUMAN	DBNL	Q9UJU6
1	8	173	173	173	0.999	460	0.175978	0.388076	sp|Q9UJW0|DCTN4_HUMAN	DCTN4	Q9UJW0
1	9	112	112	112	0.999	597	1.0432	0.0182758	sp|Q9UJX2|CDC23_HUMAN	CDC23	Q9UJX2
1	11	142	142	142	0.999	565	0.0913293	0.560218	sp|Q9UJX3|APC7_HUMAN	ANAPC7	Q9UJX3
1	14	359	359	359	0.999	356	0.673517	0.0651458	sp|Q9UJZ1|STML2_HUMAN	STOML2	Q9UJZ1
1	7	233	233	233	0.999	221	5.62354	0	sp|Q9UK41|VPS28_HUMAN	VPS28	Q9UK41
1	7	198	189	198	0.999	103	2.77253	0.000141133	sp|Q9UK45|LSM7_HUMAN	LSM 7.00	Q9UK45
1	10	256	256	256	0.999	544	2.75783	0.00014733	sp|Q9UK59|DBR1_HUMAN	DBR1	Q9UK59
1	12	355	355	355	0.999	154	0.756108	0.0494119	sp|Q9UK76|JUPI1_HUMAN	JPT1	Q9UK76
1	16	392	392	392	0.999	239	4.5321	0	sp|Q9UKD2|MRT4_HUMAN	MRTO4	Q9UKD2
1	11	151	151	151	0.999	684	0.379074	0.180526	sp|Q9UKF6|CPSF3_HUMAN	CPSF3	Q9UKF6
1	19	320	319	320	0.999	709	2.30799	0.000454948	sp|Q9UKG1|DP13A_HUMAN	APPL1	Q9UKG1
1	18	171	171	171	0.999	1724	0.18841	0.368711	sp|Q9UKK3|PARP4_HUMAN	PARP4	Q9UKK3
1	8	335	335	335	0.999	219	2.37391	0.000407295	sp|Q9UKK9|NUDT5_HUMAN	NUDT5	Q9UKK9
1	26	655	557	655	0.999	306	6.32941	0	sp|Q9UKM9|RALY_HUMAN	RALY	Q9UKM9
1	18	230	230	230	0.999	822	1.17326	0.0115648	sp|Q9UKN8|TF3C4_HUMAN	GTF3C4	Q9UKN8
1	18	365	365	365	0.999	424	1.21305	0.0101511	sp|Q9UKS6|PACN3_HUMAN	PACSIN3	Q9UKS6
1	51	1038	1038	1038	0.999	1341	4.11848	0	sp|Q9UKV3|ACINU_HUMAN	ACIN1	Q9UKV3
1	14	281	281	281	0.999	468	0.380972	0.179563	sp|Q9UKX7|NUP50_HUMAN	NUP50	Q9UKX7
1	11	317	317	317	0.999	258	3.53881	2.04342E-05	sp|Q9UKY7|CDV3_HUMAN	CDV3	Q9UKY7
1	7	207	207	207	0.999	225	0.847685	0.036074	sp|Q9UL25|RAB21_HUMAN	RAB21	Q9UL25
1	6	76	69	76	0.999	194	1.57579	0.00316393	sp|Q9UL26|RB22A_HUMAN	RAB22A	Q9UL26
1	19	672	672	672	0.999	239	7.67775	0	sp|Q9UL46|PSME2_HUMAN	PSME2	Q9UL46
1	4	60	60	60	0.999	485	3.01249	0.000073922	sp|Q9ULA0|DNPEP_HUMAN	DNPEP	Q9ULA0
1	7	57	57	57	0.999	237	5.04714	0	sp|Q9ULC3|RAB23_HUMAN	RAB23	Q9ULC3
1	12	261	115	261	0.999	181	0.669294	0.0660412	sp|Q9ULC4|MCTS1_HUMAN	MCTS1	Q9ULC4
1	8	44	44	44	0.999	1129	2.58272	0.000248531	sp|Q9ULH1|ASAP1_HUMAN	ASAP1	Q9ULH1
1	5	14	14	14	0.999	1381	6.32558	0	sp|Q9ULI3|HEG1_HUMAN	HEG1	Q9ULI3
1	5	35	35	35	0.999	285	1.89872	0.00123188	sp|Q9ULR0|ISY1_HUMAN	ISY1	Q9ULR0
1	21	117	117	117	0.999	2610	2.45764	0.000319115	sp|Q9ULT8|HECD1_HUMAN	HECTD1	Q9ULT8
1	34	1027	894	1027	0.999	474	1.52032	0.00360021	sp|Q9ULV4|COR1C_HUMAN	CORO1C	Q9ULV4
1	14	56	56	56	0.999	747	0.758711	0.0489726	sp|Q9ULW0|TPX2_HUMAN	TPX2	Q9ULW0
1	13	297	297	297	0.999	412	0.584278	0.0883339	sp|Q9ULX3|NOB1_HUMAN	NOB1	Q9ULX3
1	13	203	203	203	0.999	646	1.98704	0.000977946	sp|Q9ULX6|AKP8L_HUMAN	AKAP8L	Q9ULX6
0.9999	6	75	54	75	0.999	164	0.154296	0.42393	sp|Q9ULX9|MAFF_HUMAN	MAFF	Q9ULX9
1	9	289	289	289	0.999	239	2.64148	0.000203448	sp|Q9UM00|TMCO1_HUMAN	TMCO1	Q9UM00
1	40	268	268	268	0.999	1294	0.619354	0.078508	sp|Q9UM54|MYO6_HUMAN	MYO6	Q9UM54
1	19	399	43	399	0.999	479	1.44045	0.00467864	sp|Q9UMR2|DD19B_HUMAN	DDX19B	Q9UMR2
1	29	957	957	957	0.999	504	3.23482	5.48571E-05	sp|Q9UMS4|PRP19_HUMAN	PRPF19	Q9UMS4
1	13	180	180	182	0.999	162	0.298095	0.242232	sp|Q9UMY4|SNX12_HUMAN	SNX12	Q9UMY4
1	18	292	292	431	0.999	482	3.8631	0	sp|Q9UN86|G3BP2_HUMAN	G3BP2	Q9UN86
1	18	362	362	362	0.999	303	2.00129	0.000933249	sp|Q9UNE7|CHIP_HUMAN	STUB1	Q9UNE7
1	25	500	500	500	0.999	486	2.75925	0.000147465	sp|Q9UNF0|PACN2_HUMAN	PACSIN2	Q9UNF0
1	31	404	369	404	0.999	606	0.0138906	0.862914	sp|Q9UNF1|MAGD2_HUMAN	MAGED2	Q9UNF1
1	25	588	554	588	0.999	406	2.55291	0.000273476	sp|Q9UNH7|SNX6_HUMAN	SNX6	Q9UNH7
1	7	146	146	146	0.999	185	1.41273	0.00515582	sp|Q9UNL2|SSRG_HUMAN	SSR3	Q9UNL2
1	25	828	828	828	0.999	376	1.23115	0.00955435	sp|Q9UNM6|PSD13_HUMAN	PSMD13	Q9UNM6
1	8	90	90	90	0.999	650	2.75005	0.000157654	sp|Q9UNN5|FAF1_HUMAN	FAF1	Q9UNN5
1	6	117	117	117	0.999	238	2.46804	0.000318218	sp|Q9UNN8|EPCR_HUMAN	PROCR	Q9UNN8

1	4	21	21	21	0.999	313	2.20549	0.000558205	sp|Q9UNQ2|DIM1_HUMAN	DIMT1	Q9UNQ2
1	18	325	325	325	0.999	423	3.8002	0	sp|Q9UNS2|CSN3_HUMAN	COPS3	Q9UNS2
1	12	155	155	155	0.999	943	0.689476	0.061934	sp|Q9UNX4|WDR3_HUMAN	WDR3	Q9UNX4
1	22	405	405	405	0.999	370	1.3283	0.00686878	sp|Q9UNZ2|NSF1C_HUMAN	NSFL1C	Q9UNZ2
1	7	120	120	120	0.999	99	2.39415	0.000378462	sp|Q9UNZ5|L10K_HUMAN	C19orf53	Q9UNZ5
1	13	114	93	114	0.999	1085	0.296819	0.243251	sp|Q9UP95|S12A4_HUMAN	SLC12A4	Q9UP95
0.9998	3	27	27	27	0.999	1137	4.3715	0	sp|Q9UPM8|AP4E1_HUMAN	AP4E1	Q9UPM8
1	100	739	392	806	0.999	7388	0.811537	0.040867	sp|Q9UPN3|MACF1_HUMAN	MACF1	Q9UPN3
1	9	23	23	23	0.9989	1083	2.4523	0.000321007	sp|Q9UPN4|CP131_HUMAN	CEP131	Q9UPN4
1	7	170	170	170	0.999	881	0.243886	0.297139	sp|Q9UPN7|PP6R1_HUMAN	PPP6R1	Q9UPN7
1	6	58	55	58	0.999	1127	2.27833	0.000477438	sp|Q9UPN9|TRI33_HUMAN	TRIM33	Q9UPN9
1	7	53	53	53	0.999	1833	0.358339	0.194815	sp|Q9UPQ9|TNR6B_HUMAN	TNRC6B	Q9UPQ9
1	10	73	73	73	0.999	735	1.10615	0.0146609	sp|Q9UPT5|EXOC7_HUMAN	EXOC7	Q9UPT5
1	20	133	133	148	0.999	2620	3.89847	0	sp|Q9UPU5|UBP24_HUMAN	USP24	Q9UPU5
1	3	38	26	38	0.999	622	2.40333	0.000365043	sp|Q9UPW0|FOXJ3_HUMAN	FOXJ3	Q9UPW0
0.9985	2	32	32	32	0.999	501	2.53131	0.000281736	sp|Q9UPY5|XCT_HUMAN	SLC7A11	Q9UPY5
1	66	1887	1887	1887	0.999	2752	4.87215	0	sp|Q9UQ35|SRRM2_HUMAN	SRRM2	Q9UQ35
1	39	1647	1647	1647	0.999	394	2.96974	9.66767E-05	sp|Q9UQ80|PA2G4_HUMAN	PA2G4	Q9UQ80
1	57	833	833	833	0.999	1217	0.025219	0.79694	sp|Q9UQE7|SMC3_HUMAN	SMC3	Q9UQE7
1	16	371	368	371	0.999	213	6.04639	0	sp|Q9UQN3|CHM2B_HUMAN	CHMP2B	Q9UQN3
1	6	24	21	24	0.999	621	1.26197	0.00860801	sp|Q9Y217|MTMR6_HUMAN	MTMR6	Q9Y217
1	12	69	69	69	0.999	722	0.502559	0.117917	sp|Q9Y223|GLCNE_HUMAN	GNE	Q9Y223
1	7	146	146	146	0.999	244	4.3524	0	sp|Q9Y224|RTRAF_HUMAN	RTRAF	Q9Y224
1	44	1050	1050	1050	0.999	463	0.330093	0.215021	sp|Q9Y230|RUVB2_HUMAN	RUVBL2	Q9Y230
1	8	239	239	239	0.999	131	1.55864	0.00329968	sp|Q9Y237|PIN4_HUMAN	PIN4	Q9Y237
1	22	709	709	709	0.999	564	1.13996	0.0130407	sp|Q9Y262|EIF3L_HUMAN	EIF3L	Q9Y262
1	17	270	270	270	0.999	795	1.33737	0.00665017	sp|Q9Y263|PLAP_HUMAN	PLAA	Q9Y263
1	33	1159	1159	1159	0.999	456	1.6781	0.00234864	sp|Q9Y265|RUVB1_HUMAN	RUVBL1	Q9Y265
1	31	819	819	819	0.999	331	5.18912	0	sp|Q9Y266|NUDC_HUMAN	NUDC	Q9Y266
1	20	515	515	632	0.999	283	4.28581	0	sp|Q9Y277|VDAC3_HUMAN	VDAC3	Q9Y277
1	16	197	197	924	0.999	166	1.05517	0.0175351	sp|Q9Y281|COF2_HUMAN	CFL2	Q9Y281
1	6	114	114	114	0.999	383	0.163674	0.408001	sp|Q9Y282|ERGI3_HUMAN	ERGIC3	Q9Y282
1	28	591	591	591	0.999	508	0.687891	0.0622143	sp|Q9Y285|SYFA_HUMAN	FARSA	Q9Y285
1	17	407	341	407	0.999	367	1.0822	0.0159055	sp|Q9Y295|DRG1_HUMAN	DRG1	Q9Y295
1	31	498	497	498	0.999	1128	2.20739	0.000556571	sp|Q9Y2A7|NCKP1_HUMAN	NCKAP1	Q9Y2A7
1	13	392	392	392	0.999	182	5.85028	0	sp|Q9Y2B0|CNPY2_HUMAN	CNPY2	Q9Y2B0
1	19	235	235	235	0.999	859	3.68112	0	sp|Q9Y2D5|AKAP2_HUMAN	AKAP2	Q9Y2D5
1	4	8	8	8	0.999	779	1.10623	0.0146603	sp|Q9Y2J4|AMOL2_HUMAN	AMOTL2	Q9Y2J4
1	22	361	361	361	0.999	958	1.24716	0.00906204	sp|Q9Y2L1|RRP44_HUMAN	DIS3	Q9Y2L1
1	17	482	482	482	0.999	226	2.88045	0.00012766	sp|Q9Y2Q3|GSTK1_HUMAN	GSTK1	Q9Y2Q3
1	10	265	265	265	0.999	187	0.504891	0.116961	sp|Q9Y2Q9|RT28_HUMAN	MRPS28	Q9Y2Q9
1	4	123	123	123	0.999	106	1.96773	0.00104511	sp|Q9Y2R0|COA3_HUMAN	COA3	Q9Y2R0
1	10	89	89	89	0.999	599	0.298786	0.241577	sp|Q9Y2R4|DDX52_HUMAN	DDX52	Q9Y2R4
1	5	130	115	130	0.999	64	1.19979	0.0106136	sp|Q9Y2S6|TMA7_HUMAN	TMA7	Q9Y2S6
1	11	140	140	140	0.999	368	1.7318	0.00201586	sp|Q9Y2S7|PDIP2_HUMAN	POLDIP2	Q9Y2S7
1	9	241	182	241	0.999	418	0.8222	0.0394108	sp|Q9Y2T2|AP3M1_HUMAN	AP3M1	Q9Y2T2
1	10	135	135	135	0.999	911	1.64913	0.00254923	sp|Q9Y2U8|MAN1_HUMAN	LEMD3	Q9Y2U8
1	10	502	502	502	0.999	147	2.11454	0.000704622	sp|Q9Y2V2|CHSP1_HUMAN	CARHSP1	Q9Y2V2
1	39	901	901	901	0.999	955	1.00159	0.0211965	sp|Q9Y2W1|TR150_HUMAN	THRAP3	Q9Y2W1
1	18	219	219	219	0.999	641	4.39349	0	sp|Q9Y2W2|WBP11_HUMAN	WBP11	Q9Y2W2
1	17	411	411	411	0.999	529	1.96237	0.00106301	sp|Q9Y2X3|NOP58_HUMAN	NOP58	Q9Y2X3
1	12	339	339	339	0.999	365	1.67703	0.0023571	sp|Q9Y2Z0|SGT1_HUMAN	SUGT1	Q9Y2Z0
1	4	21	21	21	0.999	717	3.09926	5.54371E-05	sp|Q9Y2Z2|MTO1_HUMAN	MTO1	Q9Y2Z2
1	23	527	527	527	0.999	439	0.443473	0.144137	sp|Q9Y305|ACOT9_HUMAN	ACOT9	Q9Y305
1	9	231	231	231	0.999	301	1.41269	0.00515525	sp|Q9Y314|NOSIP_HUMAN	NOSIP	Q9Y314
1	4	131	131	131	0.999	296	1.40597	0.00524828	sp|Q9Y320|TMX2_HUMAN	TMX2	Q9Y320
1	6	55	55	55	0.999	95	4.04403	0	sp|Q9Y333|LSM2_HUMAN	LSM 2.00	Q9Y333
1	15	200	199	200	0.999	365	2.27423	0.000476744	sp|Q9Y371|SHLB1_HUMAN	SH3GLB1	Q9Y371
1	20	343	300	343	0.999	341	0.391502	0.173026	sp|Q9Y376|CAB39_HUMAN	CAB39	Q9Y376
1	28	1037	718	1037	0.999	392	0.878895	0.0324848	sp|Q9Y383|LC7L2_HUMAN	LUC7L2	Q9Y383
1	11	303	303	303	0.999	296	1.50212	0.00380748	sp|Q9Y399|RT02_HUMAN	MRPS2	Q9Y399
1	20	403	403	403	0.999	250	1.78523	0.00169472	sp|Q9Y3A5|SBDS_HUMAN	SBDS	Q9Y3A5
1	6	73	73	73	0.999	195	0.28362	0.255209	sp|Q9Y3B2|EXOS1_HUMAN	EXOSC1	Q9Y3B2
1	9	155	155	155	0.999	224	1.15956	0.012162	sp|Q9Y3B3|TMED7_HUMAN	TMED7	Q9Y3B3
1	8	216	216	216	0.999	125	0.987164	0.0222961	sp|Q9Y3B4|SF3B6_HUMAN	SF3B6	Q9Y3B4
1	9	163	163	163	0.999	192	0.188601	0.368476	sp|Q9Y3B7|RM11_HUMAN	MRPL11	Q9Y3B7
1	7	141	141	141	0.999	237	1.22448	0.00974345	sp|Q9Y3B8|ORN_HUMAN	REXO2	Q9Y3B8
1	10	261	261	261	0.999	282	0.130259	0.47032	sp|Q9Y3B9|RRP15_HUMAN	RRP15	Q9Y3B9
1	10	177	177	177	0.999	178	2.96925	9.65795E-05	sp|Q9Y3C1|NOP16_HUMAN	NOP16	Q9Y3C1
1	8	319	319	319	0.999	166	1.49417	0.00390816	sp|Q9Y3C6|PPIL1_HUMAN	PPIL1	Q9Y3C6
1	10	260	260	260	0.999	167	0.161787	0.41112	sp|Q9Y3C8|UFC1_HUMAN	UFC1	Q9Y3C8
1	3	60	60	60	0.999	172	1.35651	0.00619472	sp|Q9Y3D8|KAD6_HUMAN	AK6	Q9Y3D8
1	7	64	64	64	0.999	190	1.55558	0.00332178	sp|Q9Y3D9|RT23_HUMAN	MRPS23	Q9Y3D9
1	8	212	212	212	0.999	179	0.711083	0.0575312	sp|Q9Y3E5|PTH2_HUMAN	PTRH2	Q9Y3E5
1	6	54	54	54	0.999	222	0.5954	0.0850358	sp|Q9Y3E7|CHMP3_HUMAN	CHMP3	Q9Y3E7
1	17	709	709	709	0.999	350	7.33676	0	sp|Q9Y3F4|STRAP_HUMAN	STRAP	Q9Y3F4
1	27	678	678	678	0.999	505	0.478329	0.128085	sp|Q9Y3I0|RTCB_HUMAN	RTCB	Q9Y3I0
1	11	119	101	119	0.999	1069	1.17055	0.0116661	sp|Q9Y3P9|RBGP1_HUMAN	RABGAP1	Q9Y3P9
1	3	92	92	92	0.999	395	2.053	0.000829205	sp|Q9Y3Q8|T22D4_HUMAN	TSC22D4	Q9Y3Q8
1	16	97	97	97	0.999	749	2.86216	0.000130268	sp|Q9Y3T9|NOC2L_HUMAN	NOC2L	Q9Y3T9
1	18	849	849	849	0.999	105	4.33575	0	sp|Q9Y3U8|RL36_HUMAN	RPL36	Q9Y3U8
1	11	224	224	224	0.999	248	4.20549	0	sp|Q9Y3Y2|CHTOP_HUMAN	CHTOP	Q9Y3Y2
1	9	98	98	98	0.999	626	1.02831	0.0192407	sp|Q9Y3Z3|SAMH1_HUMAN	SAMHD1	Q9Y3Z3
1	15	308	308	308	0.999	684	0.450817	0.140781	sp|Q9Y450|HBS1L_HUMAN	HBS1L	Q9Y450
1	183	5761	5103	5761	0.999	2541	0.481362	0.126778	sp|Q9Y490|TLN1_HUMAN	TLN1	Q9Y490
1	16	74	74	74	0.999	3859	2.66415	0.000192644	sp|Q9Y4A5|TRRAP_HUMAN	TRRAP	Q9Y4A5
1	8	152	103	152	0.999	981	0.539792	0.103255	sp|Q9Y4E8|UBP15_HUMAN	USP15	Q9Y4E8
1	9	127	127	127	0.999	1045	2.23283	0.000516405	sp|Q9Y4F1|FARP1_HUMAN	FARP1	Q9Y4F1
1	28	16	16	723	0.999	2542	1.90613	0.00120558	sp|Q9Y4G6|TLN2_HUMAN	TLN2	Q9Y4G6
1	11	44	44	44	0.999	1338	2.04061	0.000856031	sp|Q9Y4H2|IRS2_HUMAN	IRS2	Q9Y4H2
1	7	46	46	76	0.999	1855	1.02142	0.0198067	sp|Q9Y4I1|MYO5A_HUMAN	MYO5A	Q9Y4I1
1	21	168	168	168	0.999	774	2.60676	0.000231687	sp|Q9Y4K0|LOXL2_HUMAN	LOXL2	Q9Y4K0
1	8	58	58	58	0.999	846	2.23027	0.000520626	sp|Q9Y4K4|M4K5_HUMAN	MAP4K5	Q9Y4K4
1	47	1110	1098	1110	0.999	999	5.16188	0	sp|Q9Y4L1|HYOU1_HUMAN	HYOU1	Q9Y4L1
1	21	381	381	381	0.999	447	0.406252	0.164425	sp|Q9Y4P3|TBL2_HUMAN	TBL2	Q9Y4P3
1	7	91	91	91	0.999	837	1.41615	0.00508535	sp|Q9Y4R8|TELO2_HUMAN	TELO2	Q9Y4R8
1	11	96	96	96	0.999	734	1.6387	0.00265502	sp|Q9Y4W2|LAS1L_HUMAN	LAS1L	Q9Y4W2
1	28	437	437	437	0.999	797	2.28869	0.000467836	sp|Q9Y4W6|AFG32_HUMAN	AFG3L2	Q9Y4W6
1	8	322	322	322	0.999	139	0.578171	0.0901964	sp|Q9Y4Z0|LSM4_HUMAN	LSM 4.00	Q9Y4Z0
1	13	184	184	184	0.999	228	2.19091	0.000579791	sp|Q9Y508|RN114_HUMAN	RNF114	Q9Y508
1	15	156	156	156	0.999	469	0.478398	0.128094	sp|Q9Y512|SAM50_HUMAN	SAMM50	Q9Y512
1	43	635	579	635	0.999	2896	3.50297	2.01005E-05	sp|Q9Y520|PRC2C_HUMAN	PRRC2C	Q9Y520
1	18	438	438	438	0.999	386	0.613461	0.0798617	sp|Q9Y570|PPME1_HUMAN	PPME1	Q9Y570
1	3	55	55	55	0.999	266	1.91458	0.00118681	sp|Q9Y580|RBM7_HUMAN	RBM7	Q9Y580
1	6	72	72	89	0.999	579	1.14502	0.0128077	sp|Q9Y5A9|YTHD2_HUMAN	YTHDF2	Q9Y5A9
1	54	1411	1411	1411	0.999	1047	3.54102	2.04866E-05	sp|Q9Y5B9|SP16H_HUMAN	SUPT16H	Q9Y5B9
1	5	96	96	96	0.999	83	0.373851	0.183841	sp|Q9Y5J9|TIM8B_HUMAN	TIMM8B	Q9Y5J9
1	14	356	356	356	0.999	329	2.18619	0.000586926	sp|Q9Y5K5|UCHL5_HUMAN	UCHL5	Q9Y5K5
1	17	339	339	339	0.999	639	0.0211482	0.818715	sp|Q9Y5K6|CD2AP_HUMAN	CD2AP	Q9Y5K6
1	9	132	132	132	0.999	247	0.177177	0.38625	sp|Q9Y5K8|VATD_HUMAN	ATP6V1D	Q9Y5K8
1	11	109	109	109	0.999	923	0.347264	0.202421	sp|Q9Y5L0|TNPO3_HUMAN	TNPO3	Q9Y5L0
1	5	185	185	185	0.999	95	0.88072	0.0323339	sp|Q9Y5L4|TIM13_HUMAN	TIMM13	Q9Y5L4
1	12	400	400	400	0.999	271	1.96294	0.001065	sp|Q9Y5M8|SRPRB_HUMAN	SRPRB	Q9Y5M8
1	5	47	47	47	0.999	360	0.125053	0.481309	sp|Q9Y5P6|GMPPB_HUMAN	GMPPB	Q9Y5P6
1	31	194	128	229	0.999	1711	0.962989	0.0243063	sp|Q9Y5S2|MRCKB_HUMAN	CDC42BPB	Q9Y5S2
1	8	275	275	275	0.999	174	3.55417	1.03093E-05	sp|Q9Y5S9|RBM8A_HUMAN	RBM8A	Q9Y5S9
1	4	10	10	10	0.999	778	1.50587	0.00376422	sp|Q9Y5V3|MAGD1_HUMAN	MAGED1	Q9Y5V3
1	12	131	131	131	0.999	595	3.69403	0	sp|Q9Y5X1|SNX9_HUMAN	SNX9	Q9Y5X1
1	5	60	60	60	0.999	465	0.966623	0.0239917	sp|Q9Y5X2|SNX8_HUMAN	SNX8	Q9Y5X2
1	16	361	361	361	0.999	404	0.0742408	0.607418	sp|Q9Y5X3|SNX5_HUMAN	SNX5	Q9Y5X3
1	4	130	130	130	0.999	271	0.476848	0.12872	sp|Q9Y5Y2|NUBP2_HUMAN	NUBP2	Q9Y5Y2
1	3	48	47	48	0.999	127	1.79811	0.00163541	sp|Q9Y605|MOFA1_HUMAN	MRFAP1	Q9Y605
1	16	253	253	253	0.999	427	1.41987	0.00503264	sp|Q9Y606|PUS1_HUMAN	PUS1	Q9Y606
1	10	86	86	86	0.999	721	1.57284	0.0031928	sp|Q9Y608|LRRF2_HUMAN	LRRFIP2	Q9Y608
1	26	859	859	859	0.999	370	0.669379	0.0660478	sp|Q9Y617|SERC_HUMAN	PSAT1	Q9Y617
1	11	78	78	78	0.999	2514	4.17787	0	sp|Q9Y618|NCOR2_HUMAN	NCOR2	Q9Y618
1	8	250	250	250	0.999	398	7.82245	0	sp|Q9Y639|NPTN_HUMAN	NPTN	Q9Y639
1	5	66	66	66	0.999	262	0.254005	0.285704	sp|Q9Y657|SPIN1_HUMAN	SPIN1	Q9Y657
1	9	164	164	164	0.999	324	1.20643	0.0103857	sp|Q9Y673|ALG5_HUMAN	ALG5	Q9Y673

1	7	98	98	98	0.999	258	0.146838	0.437489	sp|Q9Y676|RT18B_HUMAN	MRPS18B	Q9Y676
1	37	929	876	929	0.999	874	3.64498	0	sp|Q9Y678|COPG1_HUMAN	COPG1	Q9Y678
1	6	73	73	73	0.999	410	0.52172	0.110332	sp|Q9Y679|AUP1_HUMAN	AUP1	Q9Y679
1	3	28	28	28	0.999	222	0.974306	0.0233237	sp|Q9Y680|FKBP7_HUMAN	FKBP7	Q9Y680
1	20	790	790	790	0.999	253	2.02167	0.000895177	sp|Q9Y696|CLIC4_HUMAN	CLIC4	Q9Y696
1	11	135	135	135	0.999	457	0.392542	0.17243	sp|Q9Y697|NFS1_HUMAN	NFS1	Q9Y697
1	16	88	88	88	0.999	838	1.572	0.00319365	sp|Q9Y6A5|TACC3_HUMAN	TACC3	Q9Y6A5
0.9999	1	92	92	92	0.999	169	0.481772	0.126639	sp|Q9Y6A9|SPCS1_HUMAN	SPCS1	Q9Y6A9
1	35	143	143	143	0.999	1016	8.08184	0	sp|Q9Y6C2|EMIL1_HUMAN	EMILIN1	Q9Y6C2
1	12	515	515	515	0.999	303	1.92809	0.00116042	sp|Q9Y6C9|MTCH2_HUMAN	MTCH2	Q9Y6C9
1	11	129	59	129	0.999	1849	2.0343	0.00086934	sp|Q9Y6D6|BIG1_HUMAN	ARFGEF1	Q9Y6D6
1	11	40	40	40	0.999	718	1.70151	0.00219586	sp|Q9Y6D9|MD1L1_HUMAN	MAD1L1	Q9Y6D9
1	8	153	98	153	0.999	443	2.75586	0.000154128	sp|Q9Y6E0|STK24_HUMAN	STK24	Q9Y6E0
1	19	320	320	377	0.999	419	1.26346	0.00856959	sp|Q9Y6E2|5MP1_HUMAN	BZW2	Q9Y6E2
1	18	248	248	249	0.999	523	0.102846	0.531919	sp|Q9Y6G9|DC1L1_HUMAN	DYNC1LI1	Q9Y6G9
1	6	158	42	158	0.999	151	0.666661	0.0666432	sp|Q9Y6H1|CHCH2_HUMAN	CHCHD2	Q9Y6H1
1	5	76	65	76	0.999	576	2.31173	0.000455959	sp|Q9Y6I3|EPN1_HUMAN	EPN1	Q9Y6I3
1	4	72	72	72	0.999	313	0.0698925	0.620578	sp|Q9Y6I9|TX264_HUMAN	TEX264	Q9Y6I9
1	9	198	198	198	0.999	419	0.459872	0.136689	sp|Q9Y6K9|NEMO_HUMAN	IKBKG	Q9Y6K9
1	21	330	330	381	0.999	599	2.21922	0.000525424	sp|Q9Y6M1|IF2B2_HUMAN	IGF2BP2	Q9Y6M1
1	10	183	183	183	0.999	179	0.4097	0.162618	sp|Q9Y6M9|NDUB9_HUMAN	NDUFB9	Q9Y6M9
1	34	778	778	778	0.999	450	1.84442	0.0014539	sp|Q9Y6N5|SQOR_HUMAN	SQOR	Q9Y6N5
1	14	178	178	178	0.999	498	0.411891	0.161236	sp|Q9Y6W5|WASF2_HUMAN	WASF2	Q9Y6W5
1	11	160	159	160	0.999	1032	1.85859	0.00139845	sp|Q9Y6X9|MORC2_HUMAN	MORC2	Q9Y6X9
1	13	130	130	130	0.999	642	1.72142	0.00205965	sp|Q9Y6Y0|NS1BP_HUMAN	IVNS1ABP	Q9Y6Y0
1	16	403	403	403	0.999	1000	1.60498	0.00291377	sp|Q9Y6Y8|S23IP_HUMAN	SEC23IP	Q9Y6Y8

T: Description	T: Entry Name	T: Indistinguishable Proteins	T: Gene name
Diphosphoinositol polyphosphate phosphohydrolase NUDT4B	NUD4B_HUMAN	sp|Q9NZJ9|NUDT4_HUMAN	NUDT4
Putative glutamine amidotransferase-like class 1 domain-containing protein 3B, mitochondrial	GAL3B_HUMAN	sp|P0DPI2|GAL3A_HUMAN	
Ubiquitin-like modifier-activating enzyme 6	UBA6_HUMAN		UBA6
Extended synaptotagmin-2	ESYT2_HUMAN		ESYT2
2-hydroxyacyl-CoA lyase 2	HACL2_HUMAN		ILVBL
SH3 and PX domain-containing protein 2B	SPD2B_HUMAN		SH3PXD2B
CCR4-NOT transcription complex subunit 1	CNOT1_HUMAN		CNOT1
Microtubule-associated proteins 1A/1B light chain 3 beta 2	MP3B2_HUMAN	sp|Q9GZQ8|MLP3B_HUMAN	MAP1LC3B2
Glycerol-3-phosphate phosphatase	PGP_HUMAN		PGP
Structural maintenance of chromosomes flexible hinge domain-containing protein 1	SMHD1_HUMAN		SMCHD1
Nuclear envelope pore membrane protein POM 121C	P121C_HUMAN		POM121C
Putative small nuclear ribonucleoprotein G-like protein 15	RUXGL_HUMAN	sp|P62308|RUXG_HUMAN	SNRPGP15
Nascent polypeptide-associated complex subunit alpha, muscle-specific form	NACAM_HUMAN	sp|Q13765|NACA_HUMAN	NACA
Uncharacterized protein C11orf98	CK098_HUMAN		C11orf98
SLC35A4 upstream open reading frame protein	S35U4_HUMAN		SLC35A4
Deoxyribonuclease-2-alpha	DNS2A_HUMAN		DNASE2
Alkyldihydroxyacetonephosphate synthase, peroxisomal	ADAS_HUMAN		AGPS
ATP-dependent RNA helicase DDX39A	DX39A_HUMAN		DDX39A
PDZ and LIM domain protein 1	PDLI1_HUMAN		PDLIM1
Cytosolic acyl coenzyme A thioester hydrolase	BACH_HUMAN		ACOT7
Unconventional myosin-Ic	MYO1C_HUMAN		MYO1C
AH receptor-interacting protein	AIP_HUMAN		AIP
GTP-binding protein 1	GTPB1_HUMAN		GTPBP1
Syntaxin-binding protein 3	STXB3_HUMAN		STXBP3
Small acidic protein	SMAP_HUMAN		SMAP
AP-3 complex subunit beta-1	AP3B1_HUMAN		AP3B1
NADH dehydrogenase [ubiquinone] iron-sulfur protein 8, mitochondrial	NDUS8_HUMAN		NDUFS8
26S proteasome non-ATPase regulatory subunit 11	PSD11_HUMAN		PSMD11
26S proteasome non-ATPase regulatory subunit 12	PSD12_HUMAN		PSMD12
26S proteasome non-ATPase regulatory subunit 9	PSMD9_HUMAN		PSMD9
Copper transport protein ATOX1	ATOX1_HUMAN		ATOX1
Membrane-associated progesterone receptor component 1	PGRC1_HUMAN		PGRMC1
Transcription elongation factor SPT5	SPT5H_HUMAN		SUPT5H
DNA fragmentation factor subunit alpha	DFFA_HUMAN		DFFA
Huntingtin-interacting protein 1	HIP1_HUMAN		HIP1
Chloride intracellular channel protein 1	CLIC1_HUMAN		CLIC1
Eukaryotic translation initiation factor 3 subunit F	EIF3F_HUMAN		EIF3F
Pyruvate dehydrogenase protein X component, mitochondrial	ODPX_HUMAN		PDHX
Importin-5	IPO5_HUMAN		IPO5
Histone deacetylase complex subunit SAP18	SAP18_HUMAN		SAP18
Insulin-like growth factor 2 mRNA-binding protein 3	IF2B3_HUMAN		IGF2BP3
Dynamin-1-like protein	DNM1L_HUMAN		DNM1L
RNA 3'-terminal phosphate cyclase	RTCA_HUMAN		RTCA
Phosphatidylinositol 4-phosphate 3-kinase C2 domain-containing subunit alpha	P3C2A_HUMAN		PIK3C2A
Golgi integral membrane protein 4	GOLI4_HUMAN		GOLIM4
Agrin	AGRIN_HUMAN		AGRN
Procollagen-lysine,2-oxoglutarate 5-dioxygenase 2	PLOD2_HUMAN		PLOD2
Exocyst complex component 5	EXOC5_HUMAN		EXOC5
Cytochrome c oxidase subunit NDUFA4	NDUA4_HUMAN		NDUFA4
26S proteasome non-ATPase regulatory subunit 14	PSDE_HUMAN		PSMD14
Myc box-dependent-interacting protein 1	BIN1_HUMAN		BIN1
Importin subunit alpha-4	IMA4_HUMAN		KPNA3
Pescadillo homolog	PESC_HUMAN		PES 1.00
Syntenin-1	SDCB1_HUMAN		SDCBP
U3 small nucleolar ribonucleoprotein protein MPP10	MPP10_HUMAN		MPHOSPH10
Nucleolar protein 56	NOP56_HUMAN		NOP56
ATP-dependent RNA helicase DDX3X	DDX3X_HUMAN		DDX3X
Podocalyxin	PODXL_HUMAN		PODXL
CCN family member 1	CCN1_HUMAN		CYR61
Importin subunit alpha-3	IMA3_HUMAN		KPNA4
Serine/threonine-protein phosphatase 6 catalytic subunit	PPP6_HUMAN		PPP6C
Lysosomal alpha-mannosidase	MA2B1_HUMAN		MAN2B1
Ubiquitin-conjugating enzyme E2 C	UBE2C_HUMAN		UBE2C
Pyridoxal kinase	PDXK_HUMAN		PDXK
AT-rich interactive domain-containing protein 1A	ARI1A_HUMAN		ARID1A
Nuclear envelope integral membrane protein 1	NEMP1_HUMAN		TMEM194A
Heat shock protein beta-6	HSPB6_HUMAN		HSPB6
Citron Rho-interacting kinase	CTRO_HUMAN		CIT
Coatomer subunit epsilon	COPE_HUMAN		COPE
AP-3 complex subunit delta-1	AP3D1_HUMAN		AP3D1
Copper chaperone for superoxide dismutase	CCS_HUMAN		CCS
Torsin-1B	TOR1B_HUMAN		TOR1B
Disintegrin and metalloproteinase domain-containing protein 10	ADA10_HUMAN		ADAM10
Prostaglandin E synthase	PTGES_HUMAN		PTGES
CDP-diacylglycerol--inositol 3-phosphatidyltransferase	CDIPT_HUMAN		CDIPT
Programmed cell death protein 5	PDCD5_HUMAN		PDCD5
Protein arginine N-methyltransferase 5	ANM5_HUMAN		PRMT5
Na(+)/H(+) exchange regulatory cofactor NHE-RF1	NHRF1_HUMAN		SLC9A3R1
Telomerase reverse transcriptase	TERT_HUMAN		TERT
Tumor necrosis factor receptor superfamily member 10B	TR10B_HUMAN		TNFRSF10B
Homeobox protein Meis2	MEIS2_HUMAN		MEIS2
Tripeptidyl-peptidase 1	TPP1_HUMAN		TPP1
Transcription elongation regulator 1	TCRG1_HUMAN		TCERG1
Kinetochore protein NDC80 homolog	NDC80_HUMAN		NDC80
Neuropilin-1	NRP1_HUMAN		NRP1
Tetraspanin-4	TSN4_HUMAN		TSPAN4
Proteasome subunit alpha type-7	PSA7_HUMAN		PSMA7
Secretory carrier-associated membrane protein 3	SCAM3_HUMAN		SCAMP3
Interferon-induced protein with tetratricopeptide repeats 3	IFIT3_HUMAN		IFIT3
Glutathione S-transferase 3, mitochondrial	MGST3_HUMAN		MGST3
PDZ domain-containing protein GIPC1	GIPC1_HUMAN		GIPC1
Histone acetyltransferase type B catalytic subunit	HAT1_HUMAN		HAT1
Ubiquitin/ISG15-conjugating enzyme E2 L6	UB2L6_HUMAN		UBE2L6
Peripheral plasma membrane protein CASK	CSKP_HUMAN		CASK
Cytochrome b-c1 complex subunit 8	QCR8_HUMAN		UQCRQ
Myosin regulatory light chain 12B	ML12B_HUMAN	sp|P19105|ML12A_HUMAN	MYL12B
Hepatocyte growth factor-regulated tyrosine kinase substrate	HGS_HUMAN		HGS
Aurora kinase A	AURKA_HUMAN		AURKA
Calmegin	CLGN_HUMAN		CLGN
Protein phosphatase 1 regulatory subunit 12A	MYPT1_HUMAN		PPP1R12A
Cyclin-G-associated kinase	GAK_HUMAN		GAK
Heterogeneous nuclear ribonucleoprotein D-like	HNRDL_HUMAN		HNRNPDL
Exportin-1	XPO1_HUMAN		XPO1
Rho guanine nucleotide exchange factor 10	ARHGA_HUMAN		ARHGEF10
Protein transport protein Sec16A	SC16A_HUMAN		SEC16A
Plexin-B2	PLXB2_HUMAN		PLXNB2
U2 snRNP-associated SURP motif-containing protein	SR140_HUMAN		U2SURP
Phosphoribosylformylglycinamidine synthase	PUR4_HUMAN		PFAS
U6 snRNA-associated Sm-like protein LSm1	LSM1_HUMAN		LSM 1.00
NPC intracellular cholesterol transporter 1	NPC1_HUMAN		NPC1
Sphingolipid delta(4)-desaturase DES1	DEGS1_HUMAN		DEGS1
Secretory carrier-associated membrane protein 1	SCAM1_HUMAN		SCAMP1
Secretory carrier-associated membrane protein 2	SCAM2_HUMAN		SCAMP2
Actin-related protein 2/3 complex subunit 1B	ARC1B_HUMAN		ARPC1B
Actin-related protein 2/3 complex subunit 2	ARPC2_HUMAN		ARPC2
Actin-related protein 2/3 complex subunit 3	ARPC3_HUMAN		ARPC3
Phospholipid scramblase 1	PLS1_HUMAN		PLSCR1
Membrane-associated progesterone receptor component 2	PGRC2_HUMAN		PGRMC2
Prefoldin subunit 6	PFD6_HUMAN		PFDN6
Zinc finger protein 185	ZN185_HUMAN		ZNF185
Small ribosomal subunit protein uS12m	RT12_HUMAN		MRPS12
Protein RER1	RER1_HUMAN		RER1
Serine palmitoyltransferase 1	SPTC1_HUMAN		SPTLC1
Serine palmitoyltransferase 2	SPTC2_HUMAN		SPTLC2
UDP-N-acetylglucosamine--peptide N-acetylglucosaminyltransferase 110 kDa subunit	OGT1_HUMAN		OGT
High mobility group protein B3	HMGB3_HUMAN		HMGB3

Protein phosphatase 1G	PPM1G_HUMAN		PPM1G
Phosphatidylinositol 3,4,5-trisphosphate 5-phosphatase 2	SHIP2_HUMAN		INPPL1
Eukaryotic translation initiation factor 3 subunit D	EIF3D_HUMAN		EIF3D
Eukaryotic translation initiation factor 3 subunit H	EIF3H_HUMAN		EIF3H
Branched-chain-amino-acid aminotransferase, mitochondrial	BCAT2_HUMAN		BCAT2
Baculoviral IAP repeat-containing protein 5	BIRC5_HUMAN		BIRC5
Syntaxin-7	STX7_HUMAN		STX7
Monocarboxylate transporter 4	MOT4_HUMAN		SLC16A3
DNA-directed RNA polymerase I subunit RPA34	RPA34_HUMAN		CD3EAP
Prolyl 4-hydroxylase subunit alpha-2	P4HA2_HUMAN		P4HA2
Calpain-5	CAN5_HUMAN		CAPN5
Synaptobrevin homolog YKT6	YKT6_HUMAN		YKT6
Actin-related protein 2/3 complex subunit 5	ARPC5_HUMAN		ARPC5
DNA-directed RNA polymerase II subunit RPB4	RPB4_HUMAN		POLR2D
Tapasin	TPSN_HUMAN		TAPBP
E3 ubiquitin-protein ligase RNF113A	R113A_HUMAN		RNF113A
ATP-dependent RNA helicase DHX15	DHX15_HUMAN		DHX15
mRNA cap guanine-N7 methyltransferase	MCES_HUMAN		RNMT
Zinc finger ZZ-type and EF-hand domain-containing protein 1	ZZEF1_HUMAN		ZZEF1
Cytochrome b5 type B	CYB5B_HUMAN		CYB5B
U4/U6 small nuclear ribonucleoprotein Prp4	PRP4_HUMAN		PRPF4
D-3-phosphoglycerate dehydrogenase	SERA_HUMAN		PHGDH
Cytoplasmic dynein 1 light intermediate chain 2	DC1L2_HUMAN		DYNC1LI2
26S proteasome non-ATPase regulatory subunit 3	PSMD3_HUMAN		PSMD3
Bifunctional 3'-phosphoadenosine 5'-phosphosulfate synthase 1	PAPS1_HUMAN		PAPSS1
Centromere/kinetochore protein zw10 homolog	ZW10_HUMAN		ZW10
U4/U6.U5 tri-snRNP-associated protein 1	SNUT1_HUMAN		SART1
Death-associated protein kinase 3	DAPK3_HUMAN		DAPK3
Transforming growth factor beta-1-induced transcript 1 protein	TGFI1_HUMAN		TGFB1I1
Mitogen-activated protein kinase kinase kinase 7	M3K7_HUMAN		MAP3K7
Eukaryotic translation elongation factor 1 epsilon-1	MCA3_HUMAN		EEF1E1
Receptor-interacting serine/threonine-protein kinase 2	RIPK2_HUMAN		RIPK2
Heterogeneous nuclear ribonucleoprotein R	HNRPR_HUMAN		HNRNPR
U4/U6 small nuclear ribonucleoprotein Prp3	PRPF3_HUMAN		PRPF3
Thioredoxin-like protein 1	TXNL1_HUMAN		TXNL1
Tumor protein D54	TPD54_HUMAN		TPD52L2
ER membrane protein complex subunit 8	EMC8_HUMAN		EMC8
Acidic fibroblast growth factor intracellular-binding protein	FIBP_HUMAN		FIBP
Eukaryotic translation initiation factor 4 gamma 3	IF4G3_HUMAN		EIF4G3
Peptidyl-prolyl cis-trans isomerase H	PPIH_HUMAN		PPIH
Aflatoxin B1 aldehyde reductase member 2	ARK72_HUMAN		AKR7A2
Band 4.1-like protein 2	E41L2_HUMAN		EPB41L2
Trans-Golgi network integral membrane protein 2	TGON2_HUMAN		TGOLN2
Density-regulated protein	DENR_HUMAN		DENR
Exportin-T	XPOT_HUMAN		XPOT
2'-deoxynucleoside 5'-phosphate N-hydrolase 1	DNPH1_HUMAN		DNPH1
Mitochondrial import inner membrane translocase subunit TIM44	TIM44_HUMAN		TIMM44
Trafficking protein particle complex subunit 3	TPPC3_HUMAN		TRAPPC3
Charged multivesicular body protein 2a	CHM2A_HUMAN		CHMP2A
Tetraspanin-6	TSN6_HUMAN		TSPAN6
Pleiotropic regulator 1	PLRG1_HUMAN		PLRG1
Protein regulator of cytokinesis 1	PRC1_HUMAN		PRC1
Regulator of G-protein signaling 10	RGS10_HUMAN		RGS10
BUB3-interacting and GLEBS motif-containing protein ZNF207	ZN207_HUMAN		ZNF207
NADH dehydrogenase [ubiquinone] 1 beta subcomplex subunit 5, mitochondrial	NDUB5_HUMAN		NDUFB5
NADH dehydrogenase [ubiquinone] 1 alpha subcomplex subunit 2	NDUA2_HUMAN		NDUFA2
ATPase GET3	GET3_HUMAN		ASNA1
Mitotic checkpoint protein BUB3	BUB3_HUMAN		BUB3
Alpha-actinin-4	ACTN4_HUMAN		ACTN4
HIV Tat-specific factor 1	HTSF1_HUMAN		HTATSF1
AP-1 complex subunit gamma-1	AP1G1_HUMAN		AP1G1
Small glutamine-rich tetratricopeptide repeat-containing protein alpha	SGTA_HUMAN		SGTA
Alpha-endosulfine	ENSA_HUMAN		ENSA
Asparagine--tRNA ligase, cytoplasmic	SYNC_HUMAN		NARS
Unconventional myosin-Ib	MYO1B_HUMAN		MYO1B
Cleavage and polyadenylation specificity factor subunit 5	CPSF5_HUMAN		NUDT21
Glutathione S-transferase LANCL1	LANC1_HUMAN		LANCL1
U3 small nucleolar RNA-interacting protein 2	U3IP2_HUMAN		RRP9
A-kinase anchor protein 8	AKAP8_HUMAN		AKAP8
Isocitrate dehydrogenase [NAD] subunit beta, mitochondrial	IDH3B_HUMAN		IDH3B
Nardilysin	NRDC_HUMAN		NRD1
Calumenin	CALU_HUMAN		CALU
EGF-like repeat and discoidin I-like domain-containing protein 3	EDIL3_HUMAN		EDIL3
S-adenosylhomocysteine hydrolase-like protein 1	SAHH2_HUMAN		AHCYL1
NADH dehydrogenase [ubiquinone] iron-sulfur protein 5	NDUS5_HUMAN		NDUFS5
Double-strand-break repair protein rad21 homolog	RAD21_HUMAN		RAD21
Mitochondrial import inner membrane translocase subunit Tim8 A	TIM8A_HUMAN		TIMM8A
Pre-mRNA-splicing factor ATP-dependent RNA helicase DHX16	DHX16_HUMAN		DHX16
Protein ZNRD2	ZNRD2_HUMAN		SSSCA1
Phosphoribosyl pyrophosphate synthase-associated protein 2	KPRB_HUMAN		PRPSAP2
SWI/SNF-related matrix-associated actin-dependent regulator of chromatin subfamily A memb	SMCA5_HUMAN		SMARCA5
C-Jun-amino-terminal kinase-interacting protein 4	JIP4_HUMAN		SPAG9
RNA helicase aquarius	AQR_HUMAN		AQR
Dynamin-like 120 kDa protein, mitochondrial	OPA1_HUMAN		OPA1
Kinesin-like protein KIF1B	KIF1B_HUMAN		KIF1B
Lysine-specific histone demethylase 1A	KDM1A_HUMAN		KDM1A
Gasdermin-E	GSDME_HUMAN		DFNA5
Long-chain-fatty-acid--CoA ligase 4	ACSL4_HUMAN		ACSL4
Sorting nexin-3	SNX3_HUMAN		SNX3
Protein O-GlcNAcase	OGA_HUMAN		MGEA5
Heterogeneous nuclear ribonucleoprotein Q	HNRPQ_HUMAN		SYNCRIP
Pre-mRNA-processing factor 17	PRP17_HUMAN		CDC40
Ran-binding protein 6	RNBP6_HUMAN		RANBP6
GDP-mannose 4,6 dehydratase	GMDS_HUMAN		GMDS
Gremlin-1	GREM1_HUMAN		GREM1
Multifunctional procollagen lysine hydroxylase and glycosyltransferase LH3	PLOD3_HUMAN		PLOD3
Eukaryotic translation initiation factor 4E type 2	IF4E2_HUMAN		EIF4E2
Protein diaphanous homolog 1	DIAP1_HUMAN		DIAPH1
Perilipin-3	PLIN3_HUMAN		PLIN3
Importin subunit alpha-7	IMA7_HUMAN		KPNA6
UDP-glucose 6-dehydrogenase	UGDH_HUMAN		UGDH
Leupaxin	LPXN_HUMAN		LPXN
Catenin delta-1	CTND1_HUMAN		CTNND1
Sorting nexin-2	SNX2_HUMAN		SNX2
Dolichol-phosphate mannosyltransferase subunit 1	DPM1_HUMAN		DPM1
General vesicular transport factor p115	USO1_HUMAN		USO1
Target of Myb1 membrane trafficking protein	TOM1_HUMAN		TOM1
Coiled-coil domain-containing protein 22	CCD22_HUMAN		CCDC22
Polyglutamine-binding protein 1	PQBP1_HUMAN		PQBP1
PRA1 family protein 2	PRAF2_HUMAN		PRAF2
H/ACA ribonucleoprotein complex subunit DKC1	DKC1_HUMAN		DKC1
Eukaryotic translation initiation factor 5B	IF2P_HUMAN		EIF5B
Endothelial differentiation-related factor 1	EDF1_HUMAN		EDF1
Protein diaphanous homolog 2	DIAP2_HUMAN		DIAPH2
DnaJ homolog subfamily A member 2	DNJA2_HUMAN		DNAJA2
Bromodomain-containing protein 4	BRD4_HUMAN		BRD4
Prefoldin subunit 1	PFD1_HUMAN		PFDN1
Nibrin	NBN_HUMAN		NBN
Iron-sulfur clusters transporter ABCB7, mitochondrial	ABCB7_HUMAN		ABCB7
SLIT-ROBO Rho GTPase-activating protein 2	SRGP2_HUMAN		SRGAP2
WD repeat-containing protein 1	WDR1_HUMAN		WDR1
Rho-associated protein kinase 2	ROCK2_HUMAN		ROCK2
CLIP-associating protein 2	CLAP2_HUMAN		CLASP2
Copine-3	CPNE3_HUMAN		CPNE3
Huntingtin-interacting protein 1-related protein	HIP1R_HUMAN		HIP1R
E3 ubiquitin-protein ligase BRE1B	BRE1B_HUMAN		RNF40
Zinc finger CCCH domain-containing protein 11A	ZC11A_HUMAN		ZC3H11A
Clustered mitochondria protein homolog	CLU_HUMAN		CLUH
DnaJ homolog subfamily C member 13	DJC13_HUMAN		DNAJC13

CCR4-NOT transcription complex subunit 3	CNOT3_HUMAN		CNOT3
Ankyrin repeat domain-containing protein 17	ANR17_HUMAN		ANKRD17
Gamma-glutamylcyclotransferase	GGCT_HUMAN		GGCT
NADH dehydrogenase [ubiquinone] iron-sulfur protein 2, mitochondrial	NDUS2_HUMAN		NDUFS2
Zinc finger protein ZPR1	ZPR1_HUMAN		ZPR1
Protein NipSnap homolog 2	NIPS2_HUMAN		GBAS
Hyaluronan mediated motility receptor	HMMR_HUMAN		HMMR
Programmed cell death protein 6	PDCD6_HUMAN		PDCD6
Tubulin-specific chaperone A	TBCA_HUMAN		TBCA
V-type proton ATPase subunit G 1	VATG1_HUMAN		ATP6V1G1
Vacuolar protein sorting-associated protein 4B	VPS4B_HUMAN		VPS4B
Core histone macro-H2A.1	H2AY_HUMAN		H2AFY
Filamin-B	FLNB_HUMAN		FLNB
Peroxisomal membrane protein PEX14	PEX14_HUMAN		PEX14
Citrate synthase, mitochondrial	CISY_HUMAN		CS
Vesicle-trafficking protein SEC22b	SC22B_HUMAN		SEC22B
Pre-mRNA-processing factor 40 homolog A	PR40A_HUMAN		PRPF40A
Transforming acidic coiled-coil-containing protein 1	TACC1_HUMAN		TACC1
Metaxin-2	MTX2_HUMAN		MTX2
Vacuolar protein sorting-associated protein 26A	VP26A_HUMAN		VPS26A
Mitochondrial-processing peptidase subunit beta	MPPB_HUMAN		PMPCB
PC4 and SFRS1-interacting protein	PSIP1_HUMAN		PSIP1
Erlin-1	ERLN1_HUMAN		ERLIN1
NADH dehydrogenase [ubiquinone] iron-sulfur protein 3, mitochondrial	NDUS3_HUMAN		NDUFS3
Serine/arginine-rich splicing factor 10	SRS10_HUMAN		SRSF10
Claudin-11	CLD11_HUMAN		CLDN11
Enoyl-CoA delta isomerase 2	ECI2_HUMAN		ECI2
Barrier-to-autointegration factor	BAF_HUMAN		BANF1
Splicing factor 3B subunit 1	SF3B1_HUMAN		SF3B1
Cold shock domain-containing protein E1	CSDE1_HUMAN		CSDE1
Interferon-inducible double-stranded RNA-dependent protein kinase activator A	PRKRA_HUMAN		PRKRA
Acyl-protein thioesterase 1	LYPA1_HUMAN		LYPLA1
U5 small nuclear ribonucleoprotein 200 kDa helicase	U520_HUMAN		SNRNP200
TIP41-like protein	TIPRL_HUMAN		TIPRL
Surfeit locus protein 6	SURF6_HUMAN		SURF6
Protein phosphatase 1B	PPM1B_HUMAN		PPM1B
Small subunit processome component 20 homolog	UTP20_HUMAN		UTP20
Nuclear pore complex protein Nup155	NU155_HUMAN		NUP155
Protein XRP2	XRP2_HUMAN		RP2
WD repeat and HMG-box DNA-binding protein 1	WDHD1_HUMAN		WDHD1
Cartilage-associated protein	CRTAP_HUMAN		CRTAP
Renin receptor	RENR_HUMAN		ATP6AP2
Cell division cycle protein 123 homolog	CD123_HUMAN		CDC123
Ribonuclease P protein subunit p20	POP7_HUMAN		POP7
Eukaryotic translation initiation factor 3 subunit G	EIF3G_HUMAN		EIF3G
Eukaryotic translation initiation factor 3 subunit J	EIF3J_HUMAN		EIF3J
Carbonyl reductase [NADPH] 3	CBR3_HUMAN		CBR3
26S proteasome non-ATPase regulatory subunit 10	PSD10_HUMAN		PSMD10
CAAX prenyl protease 1 homolog	FACE1_HUMAN		ZMPSTE24
Isocitrate dehydrogenase [NADP] cytoplasmic	IDHC_HUMAN		IDH1
Protein SCO1 homolog, mitochondrial	SCO1_HUMAN		SCO1
Serine hydrolase RBBP9	RBBP9_HUMAN		RBBP9
PRA1 family protein 3	PRAF3_HUMAN		ARL6IP5
Pre-mRNA-splicing factor SPF27	SPF27_HUMAN		BCAS2
Dynactin subunit 3	DCTN3_HUMAN		DCTN3
DnaJ homolog subfamily C member 8	DNJC8_HUMAN		DNAJC8
Survival of motor neuron-related-splicing factor 30	SPF30_HUMAN		SMNDC1
ATP synthase subunit d, mitochondrial	ATP5H_HUMAN		ATP5H
Tetraspanin-9	TSN9_HUMAN		TSPAN9
Flotillin-1	FLOT1_HUMAN		FLOT1
Triple functional domain protein	TRIO_HUMAN		TRIO
ATP synthase subunit g, mitochondrial	ATP5L_HUMAN		ATP5L
Carboxypeptidase D	CBPD_HUMAN		CPD
Glutaredoxin-3	GLRX3_HUMAN		GLRX3
Ribosomal L1 domain-containing protein 1	RL1D1_HUMAN		RSL1D1
Stanniocalcin-2	STC2_HUMAN		STC2
Probable cytosolic iron-sulfur protein assembly protein CIAO1	CIAO1_HUMAN		CIAO1
Signal recognition particle subunit SRP72	SRP72_HUMAN		SRP72
N(G),N(G)-dimethylarginine dimethylaminohydrolase 1	DDAH1_HUMAN		DDAH1
Unconventional prefoldin RPB5 interactor 1	RMP_HUMAN		URI1
Metastasis-associated protein MTA2	MTA2_HUMAN		MTA2
Serine/threonine-protein kinase 10	STK10_HUMAN		STK10
Glutamine--fructose-6-phosphate aminotransferase [isomerizing] 2	GFPT2_HUMAN		GFPT2
Slit homolog 2 protein	SLIT2_HUMAN		SLIT2
E3 ubiquitin-protein ligase listerin	LTN1_HUMAN		LTN1
Mitochondrial import receptor subunit TOM70	TOM70_HUMAN		TOMM70A
Unconventional myosin-Id	MYO1D_HUMAN		MYO1D
Protein transport protein Sec24D	SC24D_HUMAN		SEC24D
E3 UFM1-protein ligase 1	UFL1_HUMAN		UFL1
SAM and SH3 domain-containing protein 1	SASH1_HUMAN		SASH1
UBX domain-containing protein 7	UBXN7_HUMAN		UBXN7
Pyridoxal phosphate homeostasis protein	PLPHP_HUMAN		PROSC
Erlin-2	ERLN2_HUMAN		ERLIN2
Pre-mRNA-processing factor 6	PRP6_HUMAN		PRPF6
Dickkopf-related protein 1	DKK1_HUMAN		DKK 1.00
Protein furry homolog-like	FRYL_HUMAN		FRYL
Glutaminase kidney isoform, mitochondrial	GLSK_HUMAN		GLS
AP-2 complex subunit alpha-2	AP2A2_HUMAN		AP2A2
Protein transport protein Sec31A	SC31A_HUMAN		SEC31A
Protein HEXIM1	HEXI1_HUMAN		HEXIM1
G2/mitotic-specific cyclin-B2	CCNB2_HUMAN		CCNB2
E3 ubiquitin-protein ligase UBR5	UBR5_HUMAN		UBR5
Serine protease 23	PRS23_HUMAN		PRSS23
SR-related and CTD-associated factor 4	SCAF4_HUMAN		SCAF4
NADH dehydrogenase [ubiquinone] 1 beta subcomplex subunit 6	NDUB6_HUMAN		NDUFB6
Mitofusin-2	MFN2_HUMAN		MFN2
Snurportin-1	SPN1_HUMAN		SNUPN
Ubiquitin conjugation factor E4 B	UBE4B_HUMAN		UBE4B
Zinc finger protein-like 1	ZFPL1_HUMAN		ZFPL1
Elongator complex protein 1	ELP1_HUMAN		IKBKAP
NADH dehydrogenase [ubiquinone] 1 beta subcomplex subunit 4	NDUB4_HUMAN		NDUFB4
NADH dehydrogenase [ubiquinone] 1 beta subcomplex subunit 8, mitochondrial	NDUB8_HUMAN		NDUFB8
Reticulon-3	RTN3_HUMAN		RTN3
Mitochondrial proton/calcium exchanger protein	LETM1_HUMAN		LETM1
Zinc finger Ran-binding domain-containing protein 2	ZRAB2_HUMAN		ZRANB2
Sorting nexin-4	SNX4_HUMAN		SNX4
Luc7-like protein 3	LC7L3_HUMAN		LUC7L3
Kinesin-like protein KIF20A	KI20A_HUMAN		KIF20A
Chromosome-associated kinesin KIF4A	KIF4A_HUMAN		KIF4A
Golgi SNAP receptor complex member 1	GOSR1_HUMAN		GOSR1
Vesicle-associated membrane protein-associated protein B/C	VAPB_HUMAN		VAPB
Myelin protein zero-like protein 1	MPZL1_HUMAN		MPZL1
NADH dehydrogenase [ubiquinone] 1 alpha subcomplex subunit 10, mitochondrial	NDUAA_HUMAN		NDUFA10
Peptidyl-prolyl cis-trans isomerase FKBP9	FKBP9_HUMAN		FKBP9
6-phosphogluconolactonase	6PGL_HUMAN		PGLS
Bifunctional 3'-phosphoadenosine 5'-phosphosulfate synthase 2	PAPS2_HUMAN		PAPSS2
Structural maintenance of chromosomes protein 2	SMC2_HUMAN		SMC2
Ubiquitin-like modifier-activating enzyme ATG7	ATG7_HUMAN		ATG7
Tripartite motif-containing protein 16	TRI16_HUMAN		TRIM16
Acyl-protein thioesterase 2	LYPA2_HUMAN		LYPLA2
Importin-7	IPO7_HUMAN		IPO7
E3 ubiquitin-protein ligase ARIH2	ARI2_HUMAN		ARIH2
Phosphoacetylglucosamine mutase	AGM1_HUMAN		PGM3
CD2 antigen cytoplasmic tail-binding protein 2	CD2B2_HUMAN		CD2BP2
Activator of 90 kDa heat shock protein ATPase homolog 1	AHSA1_HUMAN		AHSA1
Proteasome assembly chaperone 1	PSMG1_HUMAN		PSMG1
Sphingosine-1-phosphate lyase 1	SGPL1_HUMAN		SGPL1
Ribosome biogenesis protein NSA2 homolog	NSA2_HUMAN		NSA2
GDH/6PGL endoplasmic bifunctional protein	G6PE_HUMAN		H6PD

Protein transport protein Sec24A	SC24A_HUMAN		SEC24A
Protein transport protein Sec24B	SC24B_HUMAN		SEC24B
Pantetheinase	VNN1_HUMAN		VNN 1.00
Persulfide dioxygenase ETHE1, mitochondrial	ETHE1_HUMAN		ETHE1
Fatty acid CoA ligase Acsl3	ACSL3_HUMAN		ACSL3
STAM-binding protein	STABP_HUMAN		STAMBP
Probable bifunctional dTTP/UTP pyrophosphatase/methyltransferase protein	ASML_HUMAN		ASMTL
Ras-related protein Rab-3D	RAB3D_HUMAN		RAB3D
Synaptosomal-associated protein 29	SNP29_HUMAN		SNAP29
Serine/threonine-protein kinase OSR1	OXSR1_HUMAN		OXSR1
Heat shock 70 kDa protein 4L	HS74L_HUMAN		HSPA4L
Polypyrimidine tract-binding protein 3	PTBP3_HUMAN		PTBP3
U6 snRNA-associated Sm-like protein LSm8	LSM8_HUMAN		LSM 8.00
AP-2 complex subunit alpha-1	AP2A1_HUMAN		AP2A1
Double-stranded RNA-binding protein Staufen homolog 1	STAU1_HUMAN		STAU1
Tetratricopeptide repeat protein 4	TTC4_HUMAN		TTC4
Caveolae-associated protein 2	CAVN2_HUMAN		SDPR
BAG family molecular chaperone regulator 2	BAG2_HUMAN		BAG2
BAG family molecular chaperone regulator 3	BAG3_HUMAN		BAG3
Mitogen-activated protein kinase kinase kinase kinase 4	M4K4_HUMAN		MAP4K4
Quinone oxidoreductase-like protein 1	QORL1_HUMAN		CRYZL1
Apoptosis-inducing factor 1, mitochondrial	AIFM1_HUMAN		AIFM1
Echinoderm microtubule-associated protein-like 2	EMAL2_HUMAN		EML2
3'(2'),5'-bisphosphate nucleotidase 1	BPNT1_HUMAN		BPNT1
N(G),N(G)-dimethylarginine dimethylaminohydrolase 2	DDAH2_HUMAN		DDAH2
Thioredoxin domain-containing protein 12	TXD12_HUMAN		TXNDC12
EGF-containing fibulin-like extracellular matrix protein 2	FBLN4_HUMAN		EFEMP2
Methyl-CpG-binding domain protein 3	MBD3_HUMAN		MBD3
Diphosphoinositol polyphosphate phosphohydrolase 1	NUDT3_HUMAN		NUDT3
B-cell lymphoma/leukemia 10	BCL10_HUMAN		BCL10
NADH dehydrogenase [ubiquinone] 1 beta subcomplex subunit 10	NDUBA_HUMAN		NDUFB10
Putative lipid scramblase CLPTM1	CLPT1_HUMAN		CLPTM1
Mitochondrial import receptor subunit TOM40 homolog	TOM40_HUMAN		TOMM40
Serine/threonine-protein kinase PAK 4	PAK4_HUMAN		PAK4
Actin-like protein 6A	ACL6A_HUMAN		ACTL6A
Cytochrome b5	CYB5_HUMAN		CYB5A
L-lactate dehydrogenase A chain	LDHA_HUMAN		LDHA
Glutamate dehydrogenase 1, mitochondrial	DHE3_HUMAN		GLUD1
Dihydrofolate reductase	DYR_HUMAN		DHFR
NADH-cytochrome b5 reductase 3	NB5R3_HUMAN		CYB5R3
Glutathione reductase, mitochondrial	GSHR_HUMAN		GSR
Cytochrome c oxidase subunit 2	COX2_HUMAN		MT-CO2
Superoxide dismutase [Cu-Zn]	SODC_HUMAN		SOD1
Coagulation factor XIII A chain	F13A_HUMAN		F13A1
Purine nucleoside phosphorylase	PNPH_HUMAN		PNP
Hypoxanthine-guanine phosphoribosyltransferase	HPRT_HUMAN		HPRT1
Aspartate aminotransferase, mitochondrial	AATM_HUMAN		GOT2
Epidermal growth factor receptor	EGFR_HUMAN		EGFR
Phosphoglycerate kinase 1	PGK1_HUMAN		PGK 1.00
Adenylate kinase isoenzyme 1	KAD1_HUMAN		AK1
Prothrombin	THRB_HUMAN		F2
Complement C1r subcomponent	C1R_HUMAN		C1R
Coagulation factor X	FA10_HUMAN		F10
Plasminogen	PLMN_HUMAN		PLG
Urokinase-type plasminogen activator	UROK_HUMAN		PLAU
Tissue-type plasminogen activator	TPA_HUMAN		PLAT
Adenosine deaminase	ADA_HUMAN		ADA
ATP synthase subunit a	ATP6_HUMAN		MT-ATP6
Argininosuccinate synthase	ASSY_HUMAN		ASS1
Antithrombin-III	ANT3_HUMAN		SERPINC1
Alpha-2-macroglobulin	A2MG_HUMAN		A2M
Complement C3	CO3_HUMAN		C3
Complement C5	CO5_HUMAN		C5
Metalloproteinase inhibitor 1	TIMP1_HUMAN		TIMP1
GTPase NRas	RASN_HUMAN		NRAS
GTPase KRas	RASK_HUMAN		KRAS
Low-density lipoprotein receptor	LDLR_HUMAN		LDLR
HLA class I histocompatibility antigen, B alpha chain	HLAB_HUMAN		HLA-B
Putative HLA class I histocompatibility antigen, alpha chain H	HLAH_HUMAN		HLA-H
Hemoglobin subunit delta	HBD_HUMAN	sp|P68871|HBB_HUMAN	HBD
Collagen alpha-1(I) chain	CO1A1_HUMAN		COL1A1
Collagen alpha-1(III) chain	CO3A1_HUMAN		COL3A1
Keratin, type I cytoskeletal 14	K1C14_HUMAN		KRT14
Keratin, type II cytoskeletal 6A	K2C6A_HUMAN		KRT6A
Prelamin-A/C	LMNA_HUMAN		LMNA
Apolipoprotein E	APOE_HUMAN		APOE
Fibrinogen beta chain	FIBB_HUMAN		FGB
Complement component C9	CO9_HUMAN		C9
Beta-2-glycoprotein 1	APOH_HUMAN		APOH
Fibronectin	FINC_HUMAN		FN1
Retinol-binding protein 4	RET4_HUMAN		RBP4
Alpha-2-HS-glycoprotein	FETUA_HUMAN		AHSG
Alpha-fetoprotein	FETA_HUMAN		AFP
Vitamin D-binding protein	VTDB_HUMAN		GC
Transferrin receptor protein 1	TFR1_HUMAN		TFRC
Serotransferrin	TRFE_HUMAN		TF
Lactotransferrin	TRFL_HUMAN		LTF
Metallothionein-2	MT2_HUMAN		MT2A
Coagulation factor XI	FA11_HUMAN		F11
Interstitial collagenase	MMP1_HUMAN		MMP1
Vitronectin	VTNC_HUMAN		VTN
Catalase	CATA_HUMAN		CAT
RAF proto-oncogene serine/threonine-protein kinase	RAF1_HUMAN		RAF1
Lysosomal acid glucosylceramidase	GBA1_HUMAN		GBA
Tissue alpha-L-fucosidase	FUCO_HUMAN		FUCA1
Fructose-bisphosphate aldolase A	ALDOA_HUMAN		ALDOA
Cystatin-B	CYTB_HUMAN		CSTB
Annexin A1	ANXA1_HUMAN		ANXA1
Apolipoprotein B-100	APOB_HUMAN		APOB
Superoxide dismutase [Mn], mitochondrial	SODM_HUMAN		SOD2
Ornithine aminotransferase, mitochondrial	OAT_HUMAN		OAT
Thymidine kinase, cytosolic	KITH_HUMAN		TK1
Thy-1 membrane glycoprotein	THY1_HUMAN		THY1
Alpha-1B-glycoprotein	A1BG_HUMAN		A1BG
Keratin, type II cytoskeletal 1	K2C1_HUMAN		KRT1
Glyceraldehyde-3-phosphate dehydrogenase	G3P_HUMAN		GAPDH
Argininosuccinate lyase	ARLY_HUMAN		ASL
HLA class I histocompatibility antigen, A alpha chain	HLAA_HUMAN		HLA-A
Calpain small subunit 1	CPNS1_HUMAN		CAPNS1
Cellular tumor antigen p53	P53_HUMAN		TP53
Metallothionein-1E	MT1E_HUMAN		MT1E
Heat shock protein beta-1	HSPB1_HUMAN		HSPB1
Thymidylate synthase	TYSY_HUMAN		TYMS
Dolichyl-diphosphooligosaccharide--protein glycosyltransferase subunit 1	RPN1_HUMAN		RPN1
Dolichyl-diphosphooligosaccharide--protein glycosyltransferase subunit 2	RPN2_HUMAN		RPN2
Guanine nucleotide-binding protein G(i) subunit alpha-2	GNAI2_HUMAN		GNAI2
Histone H2A type 1-B/E	H2A1B_HUMAN	sp|Q7L7L0|H2A3_HUMAN	HIST1H2AB
Sodium/potassium-transporting ATPase subunit alpha-1	AT1A1_HUMAN		ATP1A1
Sodium/potassium-transporting ATPase subunit beta-1	AT1B1_HUMAN		ATP1B1
Amyloid-beta precursor protein	A4_HUMAN		APP
Aldehyde dehydrogenase, mitochondrial	ALDH2_HUMAN		ALDH2
Integrin beta-3	ITB3_HUMAN		ITGB3
Non-histone chromosomal protein HMG-14	HMGN1_HUMAN		HMGN1
Plasminogen activator inhibitor 2	PAI2_HUMAN		SERPINB2
Plasminogen activator inhibitor 1	PAI1_HUMAN		SERPINE1
ADP/ATP translocase 2	ADT2_HUMAN		SLC25A5
Ubiquitin-like protein ISG15	ISG15_HUMAN		ISG15
Eukaryotic translation initiation factor 2 subunit 1	IF2A_HUMAN		EIF2S1
Non-histone chromosomal protein HMG-17	HMGN2_HUMAN		HMGN2

<table>
<tr><td>Intercellular adhesion molecule 1</td><td>ICAM1_HUMAN</td><td></td><td>ICAM1</td></tr>
<tr><td>Large ribosomal subunit protein P2</td><td>RLA2_HUMAN</td><td></td><td>RPLP2</td></tr>
<tr><td>Large ribosomal subunit protein uL10</td><td>RLA0_HUMAN</td><td></td><td>RPLP0</td></tr>
<tr><td>Tetranectin</td><td>TETN_HUMAN</td><td></td><td>CLEC3B</td></tr>
<tr><td>Lupus La protein</td><td>LA_HUMAN</td><td></td><td>SSB</td></tr>
<tr><td>Thyroxine-binding globulin</td><td>THBG_HUMAN</td><td></td><td>SERPINA7</td></tr>
<tr><td>Heparin cofactor 2</td><td>HEP2_HUMAN</td><td></td><td>SERPIND1</td></tr>
<tr><td>Integrin beta-1</td><td>ITB1_HUMAN</td><td></td><td>ITGB1</td></tr>
<tr><td>Keratin, type I cytoskeletal 18</td><td>K1C18_HUMAN</td><td></td><td>KRT18</td></tr>
<tr><td>Keratin, type II cytoskeletal 8</td><td>K2C8_HUMAN</td><td></td><td>KRT8</td></tr>
<tr><td>Collagen alpha-2(V) chain</td><td>CO5A2_HUMAN</td><td></td><td>COL5A2</td></tr>
<tr><td>Uroporphyrinogen decarboxylase</td><td>DCUP_HUMAN</td><td></td><td>UROD</td></tr>
<tr><td>Alpha-galactosidase A</td><td>AGAL_HUMAN</td><td></td><td>GLA</td></tr>
<tr><td>Gelsolin</td><td>GELS_HUMAN</td><td></td><td>GSN</td></tr>
<tr><td>Prothymosin alpha</td><td>PTMA_HUMAN</td><td></td><td>PTMA</td></tr>
<tr><td>Cyclin-dependent kinase 1</td><td>CDK1_HUMAN</td><td></td><td>CDK1</td></tr>
<tr><td>ATP synthase subunit beta, mitochondrial</td><td>ATPB_HUMAN</td><td></td><td>ATP5B</td></tr>
<tr><td>Protein S100-A6</td><td>S10A6_HUMAN</td><td></td><td>S100A6</td></tr>
<tr><td>Eukaryotic translation initiation factor 4E</td><td>IF4E_HUMAN</td><td></td><td>EIF4E</td></tr>
<tr><td>Alpha-enolase</td><td>ENOA_HUMAN</td><td></td><td>ENO1</td></tr>
<tr><td>Glycogen phosphorylase, liver form</td><td>PYGL_HUMAN</td><td></td><td>PYGL</td></tr>
<tr><td>Glucose-6-phosphate isomerase</td><td>G6PI_HUMAN</td><td></td><td>GPI</td></tr>
<tr><td>Nucleophosmin</td><td>NPM_HUMAN</td><td></td><td>NPM1</td></tr>
<tr><td>Tropomyosin alpha-3 chain</td><td>TPM3_HUMAN</td><td></td><td>TPM3</td></tr>
<tr><td>Integrin alpha-V</td><td>ITAV_HUMAN</td><td></td><td>ITGAV</td></tr>
<tr><td>Beta-hexosaminidase subunit alpha</td><td>HEXA_HUMAN</td><td></td><td>HEXA</td></tr>
<tr><td>Glia-derived nexin</td><td>GDN_HUMAN</td><td></td><td>SERPINE2</td></tr>
<tr><td>Epoxide hydrolase 1</td><td>HYEP_HUMAN</td><td></td><td>EPHX1</td></tr>
<tr><td>L-lactate dehydrogenase B chain</td><td>LDHB_HUMAN</td><td></td><td>LDHB</td></tr>
<tr><td>Glutathione peroxidase 1</td><td>GPX1_HUMAN</td><td></td><td>GPX1</td></tr>
<tr><td>Protein disulfide-isomerase</td><td>PDIA1_HUMAN</td><td></td><td>P4HB</td></tr>
<tr><td>Histone H1.0</td><td>H10_HUMAN</td><td></td><td>H1F0</td></tr>
<tr><td>Cathepsin D</td><td>CATD_HUMAN</td><td></td><td>CTSD</td></tr>
<tr><td>Annexin A2</td><td>ANXA2_HUMAN</td><td></td><td>ANXA2</td></tr>
<tr><td>Calpain-1 catalytic subunit</td><td>CAN1_HUMAN</td><td></td><td>CAPN1</td></tr>
<tr><td>Tubulin beta chain</td><td>TBB5_HUMAN</td><td></td><td>TUBB</td></tr>
<tr><td>Prosaposin</td><td>SAP_HUMAN</td><td></td><td>PSAP</td></tr>
<tr><td>Beta-hexosaminidase subunit beta</td><td>HEXB_HUMAN</td><td></td><td>HEXB</td></tr>
<tr><td>Procathepsin L</td><td>CATL1_HUMAN</td><td></td><td>CTSL</td></tr>
<tr><td>Profilin-1</td><td>PROF1_HUMAN</td><td></td><td>PFN1</td></tr>
<tr><td>Adenine phosphoribosyltransferase</td><td>APT_HUMAN</td><td></td><td>APRT</td></tr>
<tr><td>Bifunctional glutamate/proline--tRNA ligase</td><td>SYEP_HUMAN</td><td></td><td>EPRS</td></tr>
<tr><td>Cathepsin B</td><td>CATB_HUMAN</td><td></td><td>CTSB</td></tr>
<tr><td>Heat shock protein HSP 90-alpha</td><td>HS90A_HUMAN</td><td></td><td>HSP90AA1</td></tr>
<tr><td>Heterogeneous nuclear ribonucleoproteins C1/C2</td><td>HNRPC_HUMAN</td><td></td><td>HNRNPC</td></tr>
<tr><td>Cytochrome b-c1 complex subunit 6, mitochondrial</td><td>QCR6_HUMAN</td><td></td><td>UQCRH</td></tr>
<tr><td>Laminin subunit beta-1</td><td>LAMB1_HUMAN</td><td></td><td>LAMB1</td></tr>
<tr><td>Tyrosine-protein kinase Yes</td><td>YES_HUMAN</td><td></td><td>YES1</td></tr>
<tr><td>Tropomyosin beta chain</td><td>TPM2_HUMAN</td><td></td><td>TPM2</td></tr>
<tr><td>Fumarate hydratase, mitochondrial</td><td>FUMH_HUMAN</td><td></td><td>FH</td></tr>
<tr><td>Thrombospondin-1</td><td>TSP1_HUMAN</td><td></td><td>THBS1</td></tr>
<tr><td>Collagen alpha-2(I) chain</td><td>CO1A2_HUMAN</td><td></td><td>COL1A2</td></tr>
<tr><td>Annexin A6</td><td>ANXA6_HUMAN</td><td></td><td>ANXA6</td></tr>
<tr><td>Rho-related GTP-binding protein RhoC</td><td>RHOC_HUMAN</td><td></td><td>RHOC</td></tr>
<tr><td>Complement decay-accelerating factor</td><td>DAF_HUMAN</td><td></td><td>CD55</td></tr>
<tr><td>4F2 cell-surface antigen heavy chain</td><td>4F2_HUMAN</td><td></td><td>SLC3A2</td></tr>
<tr><td>ATP-dependent 6-phosphofructokinase, muscle type</td><td>PFKAM_HUMAN</td><td></td><td>PFKM</td></tr>
<tr><td>Heat shock protein HSP 90-beta</td><td>HS90B_HUMAN</td><td></td><td>HSP90AB1</td></tr>
<tr><td>Signal recognition particle receptor subunit alpha</td><td>SRPRA_HUMAN</td><td></td><td>SRPR</td></tr>
<tr><td>Asparagine synthetase [glutamine-hydrolyzing]</td><td>ASNS_HUMAN</td><td></td><td>ASNS</td></tr>
<tr><td>72 kDa type IV collagenase</td><td>MMP2_HUMAN</td><td></td><td>MMP2</td></tr>
<tr><td>Porphobilinogen deaminase</td><td>HEM3_HUMAN</td><td></td><td>HMBS</td></tr>
<tr><td>Neprilysin</td><td>NEP_HUMAN</td><td></td><td>MME</td></tr>
<tr><td>Pyruvate dehydrogenase E1 component subunit alpha, somatic form, mitochondrial</td><td>ODPA_HUMAN</td><td></td><td>PDHA1</td></tr>
<tr><td>Cytochrome c1, heme protein, mitochondrial</td><td>CY1_HUMAN</td><td></td><td>CYC1</td></tr>
<tr><td>U2 small nuclear ribonucleoprotein B''</td><td>RU2B_HUMAN</td><td></td><td>SNRPB2</td></tr>
<tr><td>U1 small nuclear ribonucleoprotein 70 kDa</td><td>RU17_HUMAN</td><td></td><td>SNRNP70</td></tr>
<tr><td>Integrin alpha-5</td><td>ITA5_HUMAN</td><td></td><td>ITGA5</td></tr>
<tr><td>Vimentin</td><td>VIME_HUMAN</td><td></td><td>VIM</td></tr>
<tr><td>Alpha-2-antiplasmin</td><td>A2AP_HUMAN</td><td></td><td>SERPINF2</td></tr>
<tr><td>Small ribosomal subunit protein eS17</td><td>RS17_HUMAN</td><td></td><td>RPS17</td></tr>
<tr><td>Keratin, type I cytoskeletal 19</td><td>K1C19_HUMAN</td><td></td><td>KRT19</td></tr>
<tr><td>Keratin, type II cytoskeletal 7</td><td>K2C7_HUMAN</td><td></td><td>KRT7</td></tr>
<tr><td>Guanine nucleotide-binding protein G(i) subunit alpha-3</td><td>GNAI3_HUMAN</td><td></td><td>GNAI3</td></tr>
<tr><td>Annexin A5</td><td>ANXA5_HUMAN</td><td></td><td>ANXA5</td></tr>
<tr><td>Keratin, type I cytoskeletal 16</td><td>K1C16_HUMAN</td><td></td><td>KRT16</td></tr>
<tr><td>Small ribosomal subunit protein uS2</td><td>RSSA_HUMAN</td><td></td><td>RPSA</td></tr>
<tr><td>CD63 antigen</td><td>CD63_HUMAN</td><td></td><td>CD63</td></tr>
<tr><td>Large ribosomal subunit protein uL3m</td><td>RM03_HUMAN</td><td></td><td>MRPL3</td></tr>
<tr><td>U1 small nuclear ribonucleoprotein A</td><td>SNRPA_HUMAN</td><td></td><td>SNRPA</td></tr>
<tr><td>Fibroblast growth factor 2</td><td>FGF2_HUMAN</td><td></td><td>FGF2</td></tr>
<tr><td>Gamma-enolase</td><td>ENOG_HUMAN</td><td></td><td>ENO2</td></tr>
<tr><td>3-ketoacyl-CoA thiolase, peroxisomal</td><td>THIK_HUMAN</td><td></td><td>ACAA1</td></tr>
<tr><td>Signal recognition particle 19 kDa protein</td><td>SRP19_HUMAN</td><td></td><td>SRP19</td></tr>
<tr><td>Glutathione S-transferase P</td><td>GSTP1_HUMAN</td><td></td><td>GSTP1</td></tr>
<tr><td>U1 small nuclear ribonucleoprotein C</td><td>RU1C_HUMAN</td><td></td><td>SNRPC</td></tr>
<tr><td>Galectin-1</td><td>LEG1_HUMAN</td><td></td><td>LGALS1</td></tr>
<tr><td>Dihydropteridine reductase</td><td>DHPR_HUMAN</td><td></td><td>QDPR</td></tr>
<tr><td>High mobility group protein B1</td><td>HMGB1_HUMAN</td><td></td><td>HMGB1</td></tr>
<tr><td>SPARC</td><td>SPRC_HUMAN</td><td></td><td>SPARC</td></tr>
<tr><td>Tropomyosin alpha-1 chain</td><td>TPM1_HUMAN</td><td></td><td>TPM1</td></tr>
<tr><td>Clathrin light chain A</td><td>CLCA_HUMAN</td><td></td><td>CLTA</td></tr>
<tr><td>Clathrin light chain B</td><td>CLCB_HUMAN</td><td></td><td>CLTB</td></tr>
<tr><td>Annexin A4</td><td>ANXA4_HUMAN</td><td></td><td>ANXA4</td></tr>
<tr><td>2',3'-cyclic-nucleotide 3'-phosphodiesterase</td><td>CN37_HUMAN</td><td></td><td>CNP</td></tr>
<tr><td>Platelet-derived growth factor receptor beta</td><td>PGFRB_HUMAN</td><td></td><td>PDGFRB</td></tr>
<tr><td>Dihydrolipoyl dehydrogenase, mitochondrial</td><td>DLDH_HUMAN</td><td></td><td>DLD</td></tr>
<tr><td>Heterogeneous nuclear ribonucleoprotein A1</td><td>ROA1_HUMAN</td><td></td><td>HNRNPA1</td></tr>
<tr><td>U2 small nuclear ribonucleoprotein A'</td><td>RU2A_HUMAN</td><td></td><td>SNRPA1</td></tr>
<tr><td>Cytochrome c oxidase subunit 6C</td><td>COX6C_HUMAN</td><td></td><td>COX6C</td></tr>
<tr><td>Poly [ADP-ribose] polymerase 1</td><td>PARP1_HUMAN</td><td></td><td>PARP1</td></tr>
<tr><td>DNA polymerase alpha catalytic subunit</td><td>DPOLA_HUMAN</td><td></td><td>POLA1</td></tr>
<tr><td>Interferon-induced protein with tetratricopeptide repeats 2</td><td>IFIT2_HUMAN</td><td></td><td>IFIT2</td></tr>
<tr><td>Interferon-induced protein with tetratricopeptide repeats 1</td><td>IFIT1_HUMAN</td><td></td><td>IFIT1</td></tr>
<tr><td>Ubiquitin carboxyl-terminal hydrolase isozyme L1</td><td>UCHL1_HUMAN</td><td></td><td>UCHL1</td></tr>
<tr><td>Leukotriene A-4 hydrolase</td><td>LKHA4_HUMAN</td><td></td><td>LTA4H</td></tr>
<tr><td>Fructose-bisphosphate aldolase C</td><td>ALDOC_HUMAN</td><td></td><td>ALDOC</td></tr>
<tr><td>Complement C4-A</td><td>CO4A_HUMAN</td><td>sp|P0C0L5|CO4B_HUMAN</td><td>C4A</td></tr>
<tr><td>Histone H2A.Z</td><td>H2AZ_HUMAN</td><td>sp|Q71UI9|H2AV_HUMAN</td><td>H2AFZ</td></tr>
<tr><td>Histone H2A type 1</td><td>H2A1_HUMAN</td><td>sp|Q6FI13|H2A2A_HUMAN, sp|</td><td>HIST1H2AG</td></tr>
<tr><td>Heat shock 70 kDa protein 1A</td><td>HS71A_HUMAN</td><td>sp|P0DMV9|HS71B_HUMAN</td><td>HSPA1A</td></tr>
<tr><td>Calmodulin-1</td><td>CALM1_HUMAN</td><td>sp|P0DP24|CALM2_HUMAN, sp|P0DP25|CALM</td><td></td></tr>
<tr><td>Alpha-amylase 1B</td><td>AMY1B_HUMAN</td><td>sp|P0DTE8|AMY1C_HUMAN, sp|P0DUB6|AMY1</td><td></td></tr>
<tr><td>RNA-binding protein RO60</td><td>RO60_HUMAN</td><td></td><td>TROVE2</td></tr>
<tr><td>Lysosomal alpha-glucosidase</td><td>LYAG_HUMAN</td><td></td><td>GAA</td></tr>
<tr><td>Ras-related protein R-Ras</td><td>RRAS_HUMAN</td><td></td><td>RRAS</td></tr>
<tr><td>HLA class I histocompatibility antigen, C alpha chain</td><td>HLAC_HUMAN</td><td></td><td>HLA-C</td></tr>
<tr><td>Histone H1.4</td><td>H14_HUMAN</td><td></td><td>HIST1H1E</td></tr>
<tr><td>Dihydrolipoyllysine-residue acetyltransferase component of pyruvate dehydrogenase complex,</td><td>ODP2_HUMAN</td><td></td><td>DLAT</td></tr>
<tr><td>Thioredoxin</td><td>THIO_HUMAN</td><td></td><td>TXN</td></tr>
<tr><td>Cytochrome c oxidase subunit 5B, mitochondrial</td><td>COX5B_HUMAN</td><td></td><td>COX5B</td></tr>
<tr><td>Lysosomal protective protein</td><td>PPGB_HUMAN</td><td></td><td>CTSA</td></tr>
<tr><td>Microsomal glutathione S-transferase 1</td><td>MGST1_HUMAN</td><td></td><td>MGST1</td></tr>
<tr><td>Complement component C7</td><td>CO7_HUMAN</td><td></td><td>C7</td></tr>
<tr><td>cAMP-dependent protein kinase type I-alpha regulatory subunit</td><td>KAP0_HUMAN</td><td></td><td>PRKAR1A</td></tr>
<tr><td>Tissue factor pathway inhibitor</td><td>TFPI1_HUMAN</td><td></td><td>TFPI</td></tr>
<tr><td>S-formylglutathione hydrolase</td><td>ESTD_HUMAN</td><td></td><td>ESD</td></tr>
<tr><td>60 kDa heat shock protein, mitochondrial</td><td>CH60_HUMAN</td><td></td><td>HSPD1</td></tr>
<tr><td>Clusterin</td><td>CLUS_HUMAN</td><td></td><td>CLU</td></tr>
</table>

Endoplasmic reticulum chaperone BiP	BIP_HUMAN		HSPA5
Laminin subunit gamma-1	LAMC1_HUMAN		LAMC1
Lysosomal acid phosphatase	PPAL_HUMAN		ACP2
Heat shock cognate 71 kDa protein	HSP7C_HUMAN		HSPA8
Solute carrier family 2, facilitated glucose transporter member 1	GTR1_HUMAN		SLC2A1
Solute carrier family 2, facilitated glucose transporter member 3	GTR3_HUMAN		SLC2A3
Uridine 5'-monophosphate synthase	UMPS_HUMAN		UMPS
Pyruvate dehydrogenase E1 component subunit beta, mitochondrial	ODPB_HUMAN		PDHB
Glycogen phosphorylase, brain form	PYGB_HUMAN		PYGB
Ras-related protein Ral-A	RALA_HUMAN		RALA
Ras-related protein Ral-B	RALB_HUMAN		RALB
Lysosome-associated membrane glycoprotein 1	LAMP1_HUMAN		LAMP1
Medium-chain specific acyl-CoA dehydrogenase, mitochondrial	ACADM_HUMAN		ACADM
DNA topoisomerase 1	TOP1_HUMAN		TOP 1.00
DNA topoisomerase 2-alpha	TOP2A_HUMAN		TOP2A
Glucose-6-phosphate 1-dehydrogenase	G6PD_HUMAN		G6PD
Ubiquitin-like protein 4A	UBL4A_HUMAN		UBL4A
Pyruvate carboxylase, mitochondrial	PYC_HUMAN		PC
C-1-tetrahydrofolate synthase, cytoplasmic	C1TC_HUMAN		MTHFD1
Cation-independent mannose-6-phosphate receptor	MPRI_HUMAN		IGF2R
Alcohol dehydrogenase class-3	ADHX_HUMAN		ADH5
Cyclin-dependent kinase 4	CDK4_HUMAN		CDK4
Ribose-phosphate pyrophosphokinase 2	PRPS2_HUMAN		PRPS2
Polyadenylate-binding protein 1	PABP1_HUMAN		PABPC1
Proliferating cell nuclear antigen	PCNA_HUMAN		PCNA
Histidine--tRNA ligase, cytoplasmic	HARS1_HUMAN		HARS
Collagen alpha-1(XI) chain	COBA1_HUMAN		COL11A1
Collagen alpha-1(VI) chain	CO6A1_HUMAN		COL6A1
Collagen alpha-2(VI) chain	CO6A2_HUMAN		COL6A2
Collagen alpha-3(VI) chain	CO6A3_HUMAN		COL6A3
ADP/ATP translocase 3	ADT3_HUMAN		SLC25A6
Coagulation factor V	FA5_HUMAN		F5
Inosine-5'-monophosphate dehydrogenase 2	IMDH2_HUMAN		IMPDH2
Nucleoprotein TPR	TPR_HUMAN		TPR
Creatine kinase B-type	KCRB_HUMAN		CKB
Annexin A3	ANXA3_HUMAN		ANXA3
2-oxoisovalerate dehydrogenase subunit alpha, mitochondrial	ODBA_HUMAN		BCKDHA
Alpha-actinin-1	ACTN1_HUMAN		ACTN1
Xaa-Pro dipeptidase	PEPD_HUMAN		PEPD
X-ray repair cross-complementing protein 6	XRCC6_HUMAN		XRCC6
X-ray repair cross-complementing protein 5	XRCC5_HUMAN		XRCC5
Cytochrome c oxidase subunit 4 isoform 1, mitochondrial	COX41_HUMAN		COX4I1
Interferon-induced transmembrane protein 1	IFM1_HUMAN		IFITM1
Lysosome-associated membrane glycoprotein 2	LAMP2_HUMAN		LAMP2
Ribonuclease inhibitor	RINI_HUMAN		RNH1
Bone morphogenetic protein 1	BMP1_HUMAN		BMP1
Neural cell adhesion molecule 1	NCAM1_HUMAN		NCAM1
Integrin alpha-4	ITA4_HUMAN		ITGA4
Elongation factor 2	EF2_HUMAN		EEF2
Keratin, type I cytoskeletal 10	K1C10_HUMAN		KRT10
Keratin, type II cytoskeletal 5	K2C5_HUMAN		KRT5
Protein disulfide-isomerase A4	PDIA4_HUMAN		PDIA4
Prolyl 4-hydroxylase subunit alpha-1	P4HA1_HUMAN		P4HA1
Translationally-controlled tumor protein	TCTP_HUMAN		TPT1
Tissue factor	TF_HUMAN		F3
Plastin-2	PLSL_HUMAN		LCP1
Plastin-3	PLST_HUMAN		PLS3
Acylamino-acid-releasing enzyme	ACPH_HUMAN		APEH
Electron transfer flavoprotein subunit alpha, mitochondrial	ETFA_HUMAN		ETFA
Glycogen [starch] synthase, muscle	GYS1_HUMAN		GYS1
cAMP-dependent protein kinase type II-alpha regulatory subunit	KAP2_HUMAN		PRKAR2A
General transcription factor IIF subunit 2	T2FB_HUMAN		GTF2F2
CD59 glycoprotein	CD59_HUMAN		CD59
Bifunctional methylenetetrahydrofolate dehydrogenase/cyclohydrolase, mitochondrial	MTDC_HUMAN		MTHFD2
Macrophage migration inhibitory factor	MIF_HUMAN		MIF
CD99 antigen	CD99_HUMAN		CD99
Glucosidase 2 subunit beta	GLU2B_HUMAN		PRKCSH
Farnesyl pyrophosphate synthase	FPPS_HUMAN		FDPS
Nidogen-1	NID1_HUMAN		NID1
Aldo-keto reductase family 1 member A1	AK1A1_HUMAN		AKR1A1
Pyruvate kinase PKM	KPYM_HUMAN		PKM
Endoplasmin	ENPL_HUMAN		HSP90B1
G2/mitotic-specific cyclin-B1	CCNB1_HUMAN		CCNB1
Small nuclear ribonucleoprotein-associated proteins B and B'	RSMB_HUMAN		SNRPB
Insulin-degrading enzyme	IDE_HUMAN		IDE
Cytochrome c oxidase subunit 6B1	CX6B1_HUMAN		COX6B1
Heterogeneous nuclear ribonucleoprotein L	HNRPL_HUMAN		HNRNPL
Aspartate--tRNA ligase, cytoplasmic	SYDC_HUMAN		DARS
Junction plakoglobin	PLAK_HUMAN		JUP
Cytochrome b-c1 complex subunit 7	QCR7_HUMAN		UQCRB
Glutamine synthetase	GLNA_HUMAN		GLUL
Aldo-keto reductase family 1 member B1	ALDR_HUMAN		AKR1B1
Aminopeptidase N	AMPN_HUMAN		ANPEP
Poliovirus receptor	PVR_HUMAN		PVR
Eukaryotic peptide chain release factor GTP-binding subunit ERF3A	ERF3A_HUMAN		GSPT1
Beta-1,4-galactosyltransferase 1	B4GT1_HUMAN		B4GALT1
Ezrin	EZRI_HUMAN		EZR
Ubiquitin carboxyl-terminal hydrolase isozyme L3	UCHL3_HUMAN		UCHL3
Fos-related antigen 2	FOSL2_HUMAN		FOSL2
Nucleoside diphosphate kinase A	NDKA_HUMAN		NME1
NAD(P)H dehydrogenase [quinone] 1	NQO1_HUMAN		NQO1
N-acetylglucosamine-6-sulfatase	GNS_HUMAN		GNS
Small ribosomal subunit protein uS5	RS2_HUMAN		RPS2
Replication protein A 32 kDa subunit	RFA2_HUMAN		RPA2
Cytochrome c oxidase subunit 7C, mitochondrial	COX7C_HUMAN		COX7C
Metalloproteinase inhibitor 2	TIMP2_HUMAN		TIMP2
CD44 antigen	CD44_HUMAN		CD44
Histone H2AX	H2AX_HUMAN		H2AFX
Carbonyl reductase [NADPH] 1	CBR1_HUMAN		CBR1
Cytoplasmic protein NCK1	NCK1_HUMAN		NCK1
Histone H1.5	H15_HUMAN		HIST1H1B
Histone H1.2	H12_HUMAN		HIST1H1C
NADPH--cytochrome P450 reductase	NCPR_HUMAN		POR
Sarcoplasmic/endoplasmic reticulum calcium ATPase 2	AT2A2_HUMAN		ATP2A2
Fumarylacetoacetase	FAAA_HUMAN		FAH
Stathmin	STMN1_HUMAN		STMN1
Y-box-binding protein 3	YBOX3_HUMAN		YBX3
High mobility group protein HMG-I/HMG-Y	HMGA1_HUMAN		HMGA1
Aspartate aminotransferase, cytoplasmic	AATC_HUMAN		GOT1
Protein kinase C alpha type	KPCA_HUMAN		PRKCA
Transcription factor JunB	JUNB_HUMAN		JUNB
Integrin alpha-2	ITA2_HUMAN		ITGA2
Gap junction alpha-1 protein	CXA1_HUMAN		GJA1
Nucleolar transcription factor 1	UBF1_HUMAN		UBTF
NADH dehydrogenase [ubiquinone] 1 beta subcomplex subunit 7	NDUB7_HUMAN		NDUFB7
cAMP-dependent protein kinase catalytic subunit alpha	KAPCA_HUMAN		PRKACA
Calpain-2 catalytic subunit	CAN2_HUMAN		CAPN2
CTP synthase 1	PYRG1_HUMAN		CTPS1
Endoglin	EGLN_HUMAN		ENG
Probable ATP-dependent RNA helicase DDX5	DDX5_HUMAN		DDX5
ATP-dependent 6-phosphofructokinase, liver type	PFKAL_HUMAN		PFKL
Ganglioside GM2 activator	SAP3_HUMAN		GM2A
Galectin-3	LEG3_HUMAN		LGALS3
26S proteasome regulatory subunit 6A	PRS6A_HUMAN		PSMC3
T-complex protein 1 subunit alpha	TCPA_HUMAN		TCP1
Tyrosine-protein phosphatase non-receptor type 1	PTN1_HUMAN		PTPN1
Large ribosomal subunit protein eL33	RL35A_HUMAN		RPL35A
Integrin beta-5	ITB5_HUMAN		ITGB5
ADP-ribosylation factor 4	ARF4_HUMAN		ARF4

Large ribosomal subunit protein uL30	RL7_HUMAN		RPL7
Vinculin	VINC_HUMAN		VCL
Protein SON	SON_HUMAN		SON
Negative elongation factor E	NELFE_HUMAN		NELFE
Large ribosomal subunit protein uL22	RL17_HUMAN		RPL17
Phosphoglycerate mutase 1	PGAM1_HUMAN		PGAM1
Regulator of chromosome condensation	RCC1_HUMAN		RCC1
Cyclic AMP-dependent transcription factor ATF-1	ATF1_HUMAN		ATF1
DNA ligase 1	DNLI1_HUMAN		LIG1
ATP synthase-coupling factor 6, mitochondrial	ATP5J_HUMAN		ATP5J
DNA repair protein XRCC1	XRCC1_HUMAN		XRCC1
Cadherin-2	CADH2_HUMAN		CDH2
1-phosphatidylinositol 4,5-bisphosphate phosphodiesterase gamma-1	PLCG1_HUMAN		PLCG1
Nucleolin	NUCL_HUMAN		NCL
Hexokinase-1	HXK1_HUMAN		HK1
DNA-directed RNA polymerase II subunit RPB3	RPB3_HUMAN		POLR2C
DNA-directed RNA polymerases I, II, and III subunit RPABC1	RPAB1_HUMAN		POLR2E
General transcription and DNA repair factor IIH helicase subunit XPB	ERCC3_HUMAN		ERCC3
Interferon-induced, double-stranded RNA-activated protein kinase	E2AK2_HUMAN		EIF2AK2
Spermidine synthase	SPEE_HUMAN		SRM
Casein kinase II subunit alpha'	CSK22_HUMAN		CSNK2A2
Inter-alpha-trypsin inhibitor heavy chain H2	ITIH2_HUMAN		ITIH2
Inter-alpha-trypsin inhibitor heavy chain H1	ITIH1_HUMAN		ITIH1
Nuclear factor NF-kappa-B p105 subunit	NFKB1_HUMAN		NFKB1
Follistatin	FST_HUMAN		FST
Plasma membrane calcium-transporting ATPase 1	AT2B1_HUMAN		ATP2B1
Eukaryotic translation initiation factor 2 subunit 2	IF2B_HUMAN		EIF2S2
Annexin A7	ANXA7_HUMAN		ANXA7
Transcription factor BTF3	BTF3_HUMAN		BTF3
Ras-related protein Rab-3B	RAB3B_HUMAN		RAB3B
Ras-related protein Rab-5A	RAB5A_HUMAN		RAB5A
Ras-related protein Rab-6A	RAB6A_HUMAN		RAB6A
Interferon-induced GTP-binding protein Mx1	MX1_HUMAN		MX1
Proteasome subunit beta type-1	PSB1_HUMAN		PSMB1
Cation-dependent mannose-6-phosphate receptor	MPRD_HUMAN		M6PR
Cytochrome c oxidase subunit 5A, mitochondrial	COX5A_HUMAN		COX5A
Lamin-B1	LMNB1_HUMAN		LMNB1
Pregnancy zone protein	PZP_HUMAN		PZP
Mimecan	MIME_HUMAN		OGN
Calpastatin	ICAL_HUMAN		CAST
Inosine-5'-monophosphate dehydrogenase 1	IMDH1_HUMAN		IMPDH1
Collagen alpha-1(V) chain	CO5A1_HUMAN		COL5A1
Ras GTPase-activating protein 1	RASA1_HUMAN		RASA1
Parathymosin	PTMS_HUMAN		PTMS
Cyclin-dependent kinase 11B	CD11B_HUMAN	sp|Q9UQ88|CD11A_HUMAN	CDK11B
Glutathione S-transferase Mu 3	GSTM3_HUMAN		GSTM3
V-type proton ATPase subunit B, brain isoform	VATB2_HUMAN		ATP6V1B2
V-type proton ATPase subunit C 1	VATC1_HUMAN		ATP6V1C1
Cysteine and glycine-rich protein 1	CSRP1_HUMAN		CSRP1
Filamin-A	FLNA_HUMAN		FLNA
Cytoplasmic aconitate hydratase	ACOHC_HUMAN		ACO1
5'-nucleotidase	5NTD_HUMAN		NT5E
Voltage-dependent anion-selective channel protein 1	VDAC1_HUMAN		VDAC1
Succinate dehydrogenase [ubiquinone] iron-sulfur subunit, mitochondrial	SDHB_HUMAN		SDHB
CD9 antigen	CD9_HUMAN		CD9
Catechol O-methyltransferase	COMT_HUMAN		COMT
Protein-glutamine gamma-glutamyltransferase 2	TGM2_HUMAN		TGM2
Methylmalonyl-CoA mutase, mitochondrial	MUTA_HUMAN		MUT
Oxysterol-binding protein 1	OSBP1_HUMAN		OSBP
Protein-L-isoaspartate(D-aspartate) O-methyltransferase	PIMT_HUMAN		PCMT1
rRNA 2'-O-methyltransferase fibrillarin	FBRL_HUMAN		FBL
Small ribosomal subunit protein eS4, Y isoform 1	RS4Y1_HUMAN		RPS4Y1
Trifunctional purine biosynthetic protein adenosine-3	PUR2_HUMAN		GART
Bifunctional phosphoribosylaminoimidazole carboxylase/phosphoribosylaminoimidazole succir	PUR6_HUMAN		PAICS
Sterol carrier protein 2	SCP2_HUMAN		SCP2
Ubiquitin-like modifier-activating enzyme 1	UBA1_HUMAN		UBA1
Nucleoside diphosphate kinase B	NDKB_HUMAN		NME2
Ectonucleotide pyrophosphatase/phosphodiesterase family member 1	ENPP1_HUMAN		ENPP1
NADPH:adrenodoxin oxidoreductase, mitochondrial	ADRO_HUMAN		FDXR
Heterogeneous nuclear ribonucleoproteins A2/B1	ROA2_HUMAN		HNRNPA2B1
E3 ubiquitin-protein ligase CBL	CBL_HUMAN		CBL
Cytochrome b-c1 complex subunit 2, mitochondrial	QCR2_HUMAN		UQCRC2
Ferrochelatase, mitochondrial	HEMH_HUMAN		FECH
Fibulin-1	FBLN1_HUMAN		FBLN1
Transcription elongation factor A protein 1	TCEA1_HUMAN		TCEA1
Prostaglandin G/H synthase 1	PGH1_HUMAN		PTGS1
Integrin alpha-6	ITA6_HUMAN		ITGA6
Splicing factor, proline- and glutamine-rich	SFPQ_HUMAN		SFPQ
Tubulin gamma-1 chain	TBG1_HUMAN		TUBG1
Peptidyl-prolyl cis-trans isomerase B	PPIB_HUMAN		PPIB
NAD-dependent malic enzyme, mitochondrial	MAOM_HUMAN		ME2
Tryptophan--tRNA ligase, cytoplasmic	SYWC_HUMAN		WARS
Small ribosomal subunit protein uS3	RS3_HUMAN		RPS3
Tyrosine-protein kinase JAK1	JAK1_HUMAN		JAK1
Nuclear autoantigen Sp-100	SP100_HUMAN		SP100
Adenosylhomocysteinase	SAHH_HUMAN		AHCY
Cofilin-1	COF1_HUMAN		CFL1
Eukaryotic translation initiation factor 4B	IF4B_HUMAN		EIF4B
Plasma membrane calcium-transporting ATPase 4	AT2B4_HUMAN		ATP2B4
Diacylglycerol kinase alpha	DGKA_HUMAN		DGKA
Carnitine O-palmitoyltransferase 2, mitochondrial	CPT2_HUMAN		CPT2
Thymidylate kinase	KTHY_HUMAN		DTYMK
Ribonucleoside-diphosphate reductase large subunit	RIR1_HUMAN		RRM1
Elongation factor 1-beta	EF1B_HUMAN		EEF1B2
ATP synthase F(0) complex subunit B1, mitochondrial	AT5F1_HUMAN		ATP5F1
Insulin-like growth factor-binding protein 5	IBP5_HUMAN		IGFBP5
Low molecular weight phosphotyrosine protein phosphatase	PPAC_HUMAN		ACP1
Acetyl-CoA acetyltransferase, mitochondrial	THIL_HUMAN		ACAT1
Tenascin	TENA_HUMAN		TNC
Myosin regulatory light polypeptide 9	MYL9_HUMAN		MYL9
DNA-directed RNA polymerase II subunit RPB1	RPB1_HUMAN		POLR2A
Cyclin-dependent kinase 2	CDK2_HUMAN		CDK2
DNA replication licensing factor MCM3	MCM3_HUMAN		MCM3
3-mercaptopyruvate sulfurtransferase	THTM_HUMAN		MPST
Small ribosomal subunit protein eS12	RS12_HUMAN		RPS12
Bromodomain-containing protein 2	BRD2_HUMAN		BRD2
Transcriptional repressor protein YY1	TYY1_HUMAN		YY1
DnaJ homolog subfamily B member 1	DNJB1_HUMAN		DNAJB1
ATP synthase subunit alpha, mitochondrial	ATPA_HUMAN		ATP5A1
Cathepsin S	CATS_HUMAN		CTSS
Proteasome subunit alpha type-1	PSA1_HUMAN		PSMA1
Proteasome subunit alpha type-2	PSA2_HUMAN		PSMA2
Proteasome subunit alpha type-3	PSA3_HUMAN		PSMA3
Proteasome subunit alpha type-4	PSA4_HUMAN		PSMA4
Integrin alpha-3	ITA3_HUMAN		ITGA3
Pentraxin-related protein PTX3	PTX3_HUMAN		PTX3
Moesin	MOES_HUMAN		MSN
Probable ATP-dependent RNA helicase DDX6	DDX6_HUMAN		DDX6
DNA (cytosine-5)-methyltransferase 1	DNMT1_HUMAN		DNMT1
Splicing factor U2AF 65 kDa subunit	U2AF2_HUMAN		U2AF2
Large ribosomal subunit protein eL13	RL13_HUMAN		RPL13
Isovaleryl-CoA dehydrogenase, mitochondrial	IVD_HUMAN		IVD
Protein S100-A4	S10A4_HUMAN		S100A4
High mobility group protein B2	HMGB2_HUMAN		HMGB2
Polypyrimidine tract-binding protein 1	PTBP1_HUMAN		PTBP1
Threonine--tRNA ligase 1, cytoplasmic	SYTC_HUMAN		TARS
Valine--tRNA ligase	SYVC_HUMAN		VARS
Elongation factor 1-gamma	EF1G_HUMAN		EEF1G
Peptidyl-prolyl cis-trans isomerase FKBP2	FKBP2_HUMAN		FKBP2

Stomatin	STOM_HUMAN		STOM
14-3-3 protein theta	1433T_HUMAN		YWHAQ
Mitogen-activated protein kinase 3	MK03_HUMAN		MAPK3
Dipeptidyl peptidase 4	DPP4_HUMAN		DPP4
Large ribosomal subunit protein uL16	RL10_HUMAN		RPL10
Replication protein A 70 kDa DNA-binding subunit	RFA1_HUMAN		RPA1
DNA-(apurinic or apyrimidinic site) endonuclease	APEX1_HUMAN		APEX1
CD82 antigen	CD82_HUMAN		CD82
Deoxycytidine kinase	DCK_HUMAN		DCK
CAD protein	PYR1_HUMAN		CAD
Calreticulin	CALR_HUMAN		CALR
Microtubule-associated protein 4	MAP4_HUMAN		MAP4
Calnexin	CALX_HUMAN		CANX
Proteasome subunit beta type-8	PSB8_HUMAN		PSMB8
Proteasome subunit beta type-9	PSB9_HUMAN		PSMB9
Proteasome subunit alpha type-5	PSA5_HUMAN		PSMA5
Proteasome subunit beta type-4	PSB4_HUMAN		PSMB4
Proteasome subunit beta type-6	PSB6_HUMAN		PSMB6
Proteasome subunit beta type-5	PSB5_HUMAN		PSMB5
ATP-binding cassette sub-family D member 3	ABCD3_HUMAN		ABCD3
Protein-lysine 6-oxidase	LYOX_HUMAN		LOX
NADH-ubiquinone oxidoreductase 75 kDa subunit, mitochondrial	NDUS1_HUMAN		NDUFS1
DNA polymerase delta catalytic subunit	DPOD1_HUMAN		POLD1
Probable global transcription activator SNF2L1	SMCA1_HUMAN		SMARCA1
Mitogen-activated protein kinase 1	MK01_HUMAN		MAPK1
Retinoic acid receptor RXR-beta	RXRB_HUMAN		RXRB
Progranulin	GRN_HUMAN		GRN
Cytosol aminopeptidase	AMPL_HUMAN		LAP3
Tripeptidyl-peptidase 2	TPP2_HUMAN		TPP2
Inositol monophosphatase 1	IMPA1_HUMAN		IMPA1
CCN family member 2	CCN2_HUMAN		CTGF
Ephrin type-A receptor 2	EPHA2_HUMAN		EPHA2
Ephrin type-B receptor 2	EPHB2_HUMAN		EPHB2
DNA-3-methyladenine glycosylase	3MG_HUMAN		MPG
Cellular retinoic acid-binding protein 2	RABP2_HUMAN		CRABP2
Transketolase	TKT_HUMAN		TKT
Caspase-1	CASP1_HUMAN		CASP1
Protein PML	PML_HUMAN		PML
Elongation factor 1-delta	EF1D_HUMAN		EEF1D
Myristoylated alanine-rich C-kinase substrate	MARCS_HUMAN		MARCKS
Guanine nucleotide-binding protein subunit alpha-11	GNA11_HUMAN		GNA11
Endoplasmic reticulum resident protein 29	ERP29_HUMAN		ERP29
Peroxiredoxin-6	PRDX6_HUMAN		PRDX6
Flavin reductase (NADPH)	BLVRB_HUMAN		BLVRB
Peroxiredoxin-5, mitochondrial	PRDX5_HUMAN		PRDX5
D-dopachrome decarboxylase	DOPD_HUMAN		DDT
Thioredoxin-dependent peroxide reductase, mitochondrial	PRDX3_HUMAN		PRDX3
ATP synthase subunit delta, mitochondrial	ATPD_HUMAN		ATP5D
Large ribosomal subunit protein uL11	RL12_HUMAN		RPL12
Enoyl-CoA hydratase, mitochondrial	ECHM_HUMAN		ECHS1
UMP-CMP kinase	KCY_HUMAN		CMPK1
Phosphatidylethanolamine-binding protein 1	PEBP1_HUMAN		PEBP1
Protein disulfide-isomerase A3	PDIA3_HUMAN		PDIA3
Serine/threonine-protein phosphatase 2A 65 kDa regulatory subunit A alpha isoform	2AAA_HUMAN		PPP2R1A
Serine/threonine-protein phosphatase 2A 65 kDa regulatory subunit A beta isoform	2AAB_HUMAN		PPP2R1B
Peptidyl-prolyl cis-trans isomerase F, mitochondrial	PPIF_HUMAN		PPIF
NK-tumor recognition protein	NKTR_HUMAN		NKTR
Glycylpeptide N-tetradecanoyltransferase 1	NMT1_HUMAN		NMT1
Heme oxygenase 2	HMOX2_HUMAN		HMOX2
Adenylosuccinate synthetase isozyme 2	PURA2_HUMAN		ADSS
Tyrosine-protein kinase receptor UFO	UFO_HUMAN		AXL
Alpha-2-macroglobulin receptor-associated protein	AMRP_HUMAN		LRPAP1
Translocator protein	TSPO_HUMAN		TSPO
Adenylosuccinate lyase	PUR8_HUMAN		ADSL
CAP-Gly domain-containing linker protein 1	CLIP1_HUMAN		CLIP1
Sorcin	SORCN_HUMAN		SRI
Leukocyte elastase inhibitor	ILEU_HUMAN		SERPINB1
High affinity cationic amino acid transporter 1	CTR1_HUMAN		SLC7A1
Aldehyde dehydrogenase X, mitochondrial	AL1B1_HUMAN		ALDH1B1
DNA-directed RNA polymerase II subunit RPB2	RPB2_HUMAN		POLR2B
Succinate dehydrogenase [ubiquinone] flavoprotein subunit, mitochondrial	SDHA_HUMAN		SDHA
Coronin-1A	COR1A_HUMAN		CORO1A
Rab GDP dissociation inhibitor alpha	GDIA_HUMAN		GDI1
S-adenosylmethionine synthase isoform type-2	METK2_HUMAN		MAT2A
Carbamoyl-phosphate synthase [ammonia], mitochondrial	CPSM_HUMAN		CPS1
Ribonucleoside-diphosphate reductase subunit M2	RIR2_HUMAN		RRM2
DnaJ homolog subfamily A member 1	DNJA1_HUMAN		DNAJA1
RAC-alpha serine/threonine-protein kinase	AKT1_HUMAN		AKT1
RAC-beta serine/threonine-protein kinase	AKT2_HUMAN		AKT2
Cytochrome b-c1 complex subunit 1, mitochondrial	QCR1_HUMAN		UQCRC1
3-hydroxyisobutyrate dehydrogenase, mitochondrial	3HIDH_HUMAN		HIBADH
Bifunctional purine biosynthesis protein ATIC	PUR9_HUMAN		ATIC
Heterogeneous nuclear ribonucleoprotein H3	HNRH3_HUMAN		HNRNPH3
Heterogeneous nuclear ribonucleoprotein H	HNRH1_HUMAN		HNRNPH1
14-3-3 protein beta/alpha	1433B_HUMAN		YWHAB
Stress-induced-phosphoprotein 1	STIP1_HUMAN		STIP1
Protein S100-A11	S10AB_HUMAN		S100A11
Neural cell adhesion molecule L1	L1CAM_HUMAN		L1CAM
Peroxiredoxin-2	PRDX2_HUMAN		PRDX2
Deoxycytidylate deaminase	DCTD_HUMAN		DCTD
Pyrroline-5-carboxylate reductase 1, mitochondrial	P5CR1_HUMAN		PYCR1
Guanylate-binding protein 1	GBP1_HUMAN		GBP1
Large ribosomal subunit protein uL6	RL9_HUMAN		RPL9
Long-chain-fatty-acid--CoA ligase 1	ACSL1_HUMAN		ACSL1
Kinesin-1 heavy chain	KINH_HUMAN		KIF5B
Cleavage stimulation factor subunit 2	CSTF2_HUMAN		CSTF2
Deoxyuridine 5'-triphosphate nucleotidohydrolase, mitochondrial	DUT_HUMAN		DUT
Multidrug resistance-associated protein 1	MRP1_HUMAN		ABCC1
Cyclin-dependent kinases regulatory subunit 2	CKS2_HUMAN		CKS2
Histone H2B type 1-B	H2B1B_HUMAN		HIST1H2BB
Dual specificity protein kinase TTK	TTK_HUMAN		TTK
DNA replication licensing factor MCM4	MCM4_HUMAN		MCM4
DNA replication licensing factor MCM5	MCM5_HUMAN		MCM5
DNA replication licensing factor MCM7	MCM7_HUMAN		MCM7
Serine hydroxymethyltransferase, cytosolic	GLYC_HUMAN		SHMT1
Serine hydroxymethyltransferase, mitochondrial	GLYM_HUMAN		SHMT2
Heat shock 70 kDa protein 4	HSP74_HUMAN		HSPA4
Mannose-6-phosphate isomerase	MPI_HUMAN		MPI
Glypican-1	GPC1_HUMAN		GPC1
Profilin-2	PROF2_HUMAN		PFN2
Catenin alpha-1	CTNA1_HUMAN		CTNNA1
Catenin beta-1	CTNB1_HUMAN		CTNNB1
Prohibitin 1	PHB1_HUMAN		PHB
Serpin B6	SPB6_HUMAN		SERPINB6
Radixin	RADI_HUMAN		RDX
Replication protein A 14 kDa subunit	RFA3_HUMAN		RPA3
Replication factor C subunit 4	RFC4_HUMAN		RFC4
Replication factor C subunit 2	RFC2_HUMAN		RFC2
Replication factor C subunit 1	RFC1_HUMAN		RFC1
Large ribosomal subunit protein eL22	RL22_HUMAN		RPL22
General transcription factor IIF subunit 1	T2FA_HUMAN		GTF2F1
Prostaglandin G/H synthase 2	PGH2_HUMAN		PTGS2
Thrombospondin-2	TSP2_HUMAN		THBS2
Thrombospondin-4	TSP4_HUMAN		THBS4
Cystathionine beta-synthase	CBS_HUMAN		CBS
Keratin, type I cytoskeletal 9	K1C9_HUMAN		KRT9
Fibrillin-1	FBN1_HUMAN		FBN1
Fibrillin-2	FBN2_HUMAN		FBN2
Glycogen debranching enzyme	GDE_HUMAN		AGL

Myosin-9	MYH9_HUMAN		MYH9
Myosin-10	MYH10_HUMAN		MYH10
Coatomer subunit beta'	COPB2_HUMAN		COPB2
Sterol O-acyltransferase 1	SOAT1_HUMAN		SOAT1
Alpha-adducin	ADDA_HUMAN		ADD1
Basigin	BASI_HUMAN		BSG
Metalloproteinase inhibitor 3	TIMP3_HUMAN		TIMP3
RNA-binding protein FUS	FUS_HUMAN		FUS
Nuclear pore complex protein Nup214	NU214_HUMAN		NUP214
Protein DEK	DEK_HUMAN		DEK
Glutaredoxin-1	GLRX1_HUMAN		GLRX
Protein phosphatase 1A	PPM1A_HUMAN		PPM1A
Keratin, type II cytoskeletal 2 epidermal	K22E_HUMAN		KRT2
Hydroxymethylglutaryl-CoA lyase, mitochondrial	HMGCL_HUMAN		HMGCL
26S proteasome regulatory subunit 7	PRS7_HUMAN		PSMC2
ADP-ribosylation factor-like protein 3	ARL3_HUMAN		ARL 3.00
Dual specificity mitogen-activated protein kinase kinase 2	MP2K2_HUMAN		MAP2K2
ATP synthase subunit gamma, mitochondrial	ATPG_HUMAN		ATP5C1
V-type proton ATPase subunit E 1	VATE1_HUMAN		ATP6V1E1
Oxygen-dependent coproporphyrinogen-III oxidase, mitochondrial	HEM6_HUMAN		CPOX
Large ribosomal subunit protein uL4	RL4_HUMAN		RPL4
Lon protease homolog, mitochondrial	LONM_HUMAN		LONP1
Phosphoglucomutase-1	PGM1_HUMAN		PGM1
Serine/threonine-protein phosphatase PP1-gamma catalytic subunit	PP1G_HUMAN		PPP1CC
Guanine nucleotide-binding protein-like 1	GNL1_HUMAN		GNL1
Pigment epithelium-derived factor	PEDF_HUMAN		SERPINF1
Dihydrolipoyllysine-residue succinyltransferase component of 2-oxoglutarate dehydrogenase c	ODO2_HUMAN		DLST
Phospholipid hydroperoxide glutathione peroxidase	GPX4_HUMAN		GPX4
Signal recognition particle 14 kDa protein	SRP14_HUMAN		SRP14
Nuclear pore glycoprotein p62	NUP62_HUMAN		NUP62
Hippocalcin-like protein 1	HPCL1_HUMAN		HPCAL1
Squalene synthase	FDFT_HUMAN		FDFT1
Transgelin-2	TAGL2_HUMAN		TAGLN2
Transaldolase	TALDO_HUMAN		TALDO1
Electron transfer flavoprotein subunit beta	ETFB_HUMAN		ETFB
RNA-binding motif protein, X chromosome	RBMX_HUMAN		RBMX
V-type proton ATPase catalytic subunit A	VATA_HUMAN		ATP6V1A
Stress-70 protein, mitochondrial	GRP75_HUMAN		HSPA9
Eukaryotic initiation factor 4A-III	IF4A3_HUMAN		EIF4A3
Small ribosomal subunit protein eS19	RS19_HUMAN		RPS19
Large ribosomal subunit protein uL3	RL3_HUMAN		RPL3
Collagen alpha-1(XVIII) chain	COIA1_HUMAN		COL18A1
Dolichyl-diphosphooligosaccharide--protein glycosyltransferase 48 kDa subunit	OST48_HUMAN		DDOST
Acidic leucine-rich nuclear phosphoprotein 32 family member A	AN32A_HUMAN		ANP32A
Flap endonuclease 1	FEN1_HUMAN		FEN1
Macrophage-capping protein	CAPG_HUMAN		CAPG
Adenylyl cyclase-associated protein 2	CAP2_HUMAN		CAP2
Alpha-taxilin	TXLNA_HUMAN		TXLNA
T-complex protein 1 subunit zeta	TCPZ_HUMAN		CCT6A
Nicotinamide N-methyltransferase	NNMT_HUMAN		NNMT
Proteasome subunit beta type-10	PSB10_HUMAN		PSMB10
Large ribosomal subunit protein uL13	RL13A_HUMAN		RPL13A
ADP-ribosylation factor-like protein 1	ARL1_HUMAN		ARL 1.00
Signal transducer and activator of transcription 3	STAT3_HUMAN		STAT3
Malate dehydrogenase, cytoplasmic	MDHC_HUMAN		MDH1
Malate dehydrogenase, mitochondrial	MDHM_HUMAN		MDH2
Replication factor C subunit 5	RFC5_HUMAN		RFC5
Replication factor C subunit 3	RFC3_HUMAN		RFC3
Trifunctional enzyme subunit alpha, mitochondrial	ECHA_HUMAN		HADHA
Eukaryotic translation initiation factor 2 subunit 3	IF2G_HUMAN		EIF2S3
Eukaryotic translation initiation factor 2D	EIF2D_HUMAN		EIF2D
Protein Wnt-5a	WNT5A_HUMAN		WNT5A
Protein BUD31 homolog	BUD31_HUMAN		BUD31
N-alpha-acetyltransferase 10	NAA10_HUMAN		NAA10
Protein phosphatase inhibitor 2	IPP2_HUMAN		PPP1R2
Tyrosine-protein kinase CSK	CSK_HUMAN		CSK
Glycine--tRNA ligase	GARS_HUMAN		GARS
Isoleucine--tRNA ligase, cytoplasmic	SYIC_HUMAN		IARS
Eukaryotic translation initiation factor 1	EIF1_HUMAN		EIF1
Protein kinase C iota type	KPCI_HUMAN		PRKCI
Enoyl-CoA delta isomerase 1, mitochondrial	ECI1_HUMAN		ECI1
Lamina-associated polypeptide 2, isoform alpha	LAP2A_HUMAN		TMPO
Lamina-associated polypeptide 2, isoforms beta/gamma	LAP2B_HUMAN		TMPO
Signal transducer and activator of transcription 1-alpha/beta	STAT1_HUMAN		STAT1
Signal transducer and activator of transcription 6	STAT6_HUMAN		STAT6
Exosome RNA helicase MTR4	MTREX_HUMAN		SKIV2L2
Aldo-keto reductase family 1 member C3	AK1C3_HUMAN		AKR1C3
Serine/threonine-protein kinase mTOR	MTOR_HUMAN		MTOR
Caspase-3	CASP3_HUMAN		CASP3
Small ribosomal subunit protein eS27	RS27_HUMAN		RPS27
Leucine-rich PPR motif-containing protein, mitochondrial	LPPRC_HUMAN		LRPPRC
3-ketoacyl-CoA thiolase, mitochondrial	THIM_HUMAN		ACAA2
Large ribosomal subunit protein uL29	RL35_HUMAN		RPL35
Cyclin-dependent kinase inhibitor 2A	CDN2A_HUMAN		CDKN2A
Lysosomal Pro-X carboxypeptidase	PCP_HUMAN		PRCP
Huntingtin	HD_HUMAN		HTT
Endothelin-converting enzyme 1	ECE1_HUMAN		ECE1
Platelet-activating factor acetylhydrolase IB subunit beta	LIS1_HUMAN		PAFAH1B1
Cell surface glycoprotein MUC18	MUC18_HUMAN		MCAM
Matrin-3	MATR3_HUMAN		MATR3
DNA mismatch repair protein Msh2	MSH2_HUMAN		MSH2
Glycerol-3-phosphate dehydrogenase, mitochondrial	GPDM_HUMAN		GPD2
Translocon-associated protein subunit alpha	SSRA_HUMAN		SSR1
Ran-specific GTPase-activating protein	RANG_HUMAN		RANBP1
Nicotinamide phosphoribosyltransferase	NAMPT_HUMAN		NAMPT
Afamin	AFAM_HUMAN		AFM
26S proteasome regulatory subunit 6B	PRS6B_HUMAN		PSMC4
Elongation factor Ts, mitochondrial	EFTS_HUMAN		TSFM
Peptidyl-prolyl cis-trans isomerase C	PPIC_HUMAN		PPIC
Voltage-dependent anion-selective channel protein 2	VDAC2_HUMAN		VDAC2
Chromobox protein homolog 5	CBX5_HUMAN		CBX5
Ubiquitin carboxyl-terminal hydrolase 5	UBP5_HUMAN		USP5
Mitogen-activated protein kinase 9	MK09_HUMAN		MAPK9
Proliferation marker protein Ki-67	KI67_HUMAN		MKI67
Phosphorylase b kinase regulatory subunit alpha, skeletal muscle isoform	KPB1_HUMAN		PHKA1
Ran GTPase-activating protein 1	RAGP1_HUMAN		RANGAP1
ATP-dependent DNA helicase Q1	RECQ1_HUMAN		RECQL
Probable 28S rRNA (cytosine(4447)-C(5))-methyltransferase	NOP2_HUMAN		NOP2
Adapter molecule crk	CRK_HUMAN		CRK
Large proline-rich protein BAG6	BAG6_HUMAN		BAG6
Vesicle-fusing ATPase	NSF_HUMAN		NSF
Dual specificity mitogen-activated protein kinase kinase 3	MP2K3_HUMAN		MAP2K3
Lys-63-specific deubiquitinase BRCC36	BRCC3_HUMAN		BRCC3
Large ribosomal subunit protein uL15	RL27A_HUMAN		RPL27A
Large ribosomal subunit protein uL18	RL5_HUMAN		RPL5
Large ribosomal subunit protein eL21	RL21_HUMAN		RPL21
Large ribosomal subunit protein eL28	RL28_HUMAN		RPL28
Small ribosomal subunit protein uS4	RS9_HUMAN		RPS9
Small ribosomal subunit protein uS7	RS5_HUMAN		RPS5
Small ribosomal subunit protein eS10	RS10_HUMAN		RPS10
Microtubule-associated protein 1B	MAP1B_HUMAN		MAP1B
Glucosamine-6-phosphate isomerase 1	GNPI1_HUMAN		GNPDA1
Transcriptional coactivator YAP1	YAP1_HUMAN		YAP1
Utrophin	UTRN_HUMAN		UTRN
Ras GTPase-activating-like protein IQGAP1	IQGA1_HUMAN		IQGAP1
Glycogenin-1	GLYG_HUMAN		GYG1
Dolichyl-diphosphooligosaccharide--protein glycosyltransferase subunit STT3A	STT3A_HUMAN		STT3A
Guanine nucleotide exchange factor MSS4	MSS4_HUMAN		RABIF
F-actin-capping protein subunit alpha-2	CAZA2_HUMAN		CAPZA2

F-actin-capping protein subunit beta	CAPZB_HUMAN		CAPZB
Eukaryotic translation initiation factor 1A, X-chromosomal	IF1AX_HUMAN		EIF1AX
Glutamine--tRNA ligase	SYQ_HUMAN		QARS
Large ribosomal subunit protein eL29	RL29_HUMAN		RPL29
Cytochrome b-c1 complex subunit Rieske, mitochondrial	UCRI_HUMAN		UQCRFS1
ATP synthase subunit O, mitochondrial	ATPO_HUMAN		ATP5O
LIM and senescent cell antigen-like-containing domain protein 1	LIMS1_HUMAN		LIMS1
Stromal cell-derived factor 1	SDF1_HUMAN		CXCL12
Prolyl endopeptidase	PPCE_HUMAN		PREP
NADP-dependent malic enzyme	MAOX_HUMAN		ME1
Tissue factor pathway inhibitor 2	TFPI2_HUMAN		TFPI2
Coatomer subunit delta	COPD_HUMAN		ARCN1
Lanosterol synthase	LSS_HUMAN		LSS
Glutamate--cysteine ligase regulatory subunit	GSH0_HUMAN		GCLM
CD151 antigen	CD151_HUMAN		CD151
26S proteasome non-ATPase regulatory subunit 8	PSMD8_HUMAN		PSMD8
Protein PRRC2A	PRC2A_HUMAN		PRRC2A
Glutathione synthetase	GSHB_HUMAN		GSS
T-complex protein 1 subunit epsilon	TCPE_HUMAN		CCT5
Nestin	NEST_HUMAN		NES
Heat shock 70 kDa protein 13	HSP13_HUMAN		HSPA13
Casein kinase I isoform alpha	KC1A_HUMAN		CSNK1A1
Isocitrate dehydrogenase [NADP], mitochondrial	IDHP_HUMAN		IDH2
Phosphatidylinositol transfer protein beta isoform	PIPNB_HUMAN		PITPNB
Adhesion G protein-coupled receptor E5	AGRE5_HUMAN		CD97
DNA polymerase delta subunit 2	DPOD2_HUMAN		POLD2
MARCKS-related protein	MRP_HUMAN		MARCKSL1
Paxillin	PAXI_HUMAN		PXN
MAP kinase-activated protein kinase 2	MAPK2_HUMAN		MAPKAPK2
4-trimethylaminobutyraldehyde dehydrogenase	AL9A1_HUMAN		ALDH9A1
Large ribosomal subunit protein eL34	RL34_HUMAN		RPL34
Protein ERGIC-53	LMAN1_HUMAN		LMAN1
Nuclear autoantigenic sperm protein	NASP_HUMAN		NASP
Fatty acid synthase	FAS_HUMAN		FASN
Deoxyhypusine synthase	DHYS_HUMAN		DHPS
T-complex protein 1 subunit gamma	TCPG_HUMAN		CCT3
Large ribosomal subunit protein bL19m	RM19_HUMAN		MRPL19
Elongation factor Tu, mitochondrial	EFTU_HUMAN		TUFM
Alpha-aminoadipic semialdehyde dehydrogenase	AL7A1_HUMAN		ALDH7A1
Centromere protein F	CENPF_HUMAN		CENPF
Signal recognition particle 9 kDa protein	SRP09_HUMAN		SRP9
Choline-phosphate cytidylyltransferase A	PCY1A_HUMAN		PCYT1A
Alanine--tRNA ligase, cytoplasmic	SYAC_HUMAN		AARS
Cysteine--tRNA ligase, cytoplasmic	SYCC_HUMAN		CARS
Serine--tRNA ligase, cytoplasmic	SYSC_HUMAN		SARS
Protein phosphatase 1F	PPM1F_HUMAN		PPM1F
DNA primase small subunit	PRI1_HUMAN		PRIM1
DNA primase large subunit	PRI2_HUMAN		PRIM2
Caspase-4	CASP4_HUMAN		CASP4
Transcriptional repressor CTCF	CTCF_HUMAN		CTCF
Proteasome subunit beta type-3	PSB3_HUMAN		PSMB3
Proteasome subunit beta type-2	PSB2_HUMAN		PSMB2
DNA replication licensing factor MCM2	MCM2_HUMAN		MCM2
Cartilage oligomeric matrix protein	COMP_HUMAN		COMP
Very long-chain specific acyl-CoA dehydrogenase, mitochondrial	ACADV_HUMAN		ACADVL
YLP motif-containing protein 1	YLPM1_HUMAN		YLPM1
Acyl-coenzyme A thioesterase 2, mitochondrial	ACOT2_HUMAN		ACOT2
Transmembrane emp24 domain-containing protein 10	TMEDA_HUMAN		TMED10
RNA-binding protein 25	RBM25_HUMAN		RBM25
Protein numb homolog	NUMB_HUMAN		NUMB
Translation initiation factor eIF-2B subunit beta	EI2BB_HUMAN		EIF2B2
Adenosine 5'-monophosphoramidase HINT1	HINT1_HUMAN		HINT1
Nuclear pore complex protein Nup153	NU153_HUMAN		NUP153
E3 SUMO-protein ligase RanBP2	RBP2_HUMAN		RANBP2
NADH dehydrogenase [ubiquinone] flavoprotein 1, mitochondrial	NDUV1_HUMAN		NDUFV1
Glycogen synthase kinase-3 alpha	GSK3A_HUMAN		GSK3A
Glycogen synthase kinase-3 beta	GSK3B_HUMAN		GSK3B
Cytosolic purine 5'-nucleotidase	5NTC_HUMAN		NT5C2
Selenide, water dikinase 1	SPS1_HUMAN		SEPHS1
GMP synthase [glutamine-hydrolyzing]	GUAA_HUMAN		GMPS
Double-strand break repair protein MRE11	MRE11_HUMAN		MRE11A
Isocitrate dehydrogenase [NAD] subunit alpha, mitochondrial	IDH3A_HUMAN		IDH3A
Cysteine-rich protein 1	CRIP1_HUMAN		CRIP1
Matrix metalloproteinase-14	MMP14_HUMAN		MMP14
Protoporphyrinogen oxidase	PPOX_HUMAN		PPOX
Rab GDP dissociation inhibitor beta	GDIB_HUMAN		GDI2
Emerin	EMD_HUMAN		EMD
Carnitine O-palmitoyltransferase 1, liver isoform	CPT1A_HUMAN		CPT1A
Serpin B8	SPB8_HUMAN		SERPINB8
Serpin B9	SPB9_HUMAN		SERPINB9
Serpin H1	SERPH_HUMAN		SERPINH1
PDZ and LIM domain protein 4	PDLI4_HUMAN		PDLIM4
Hsc70-interacting protein	F10A1_HUMAN		ST13
Vasodilator-stimulated phosphoprotein	VASP_HUMAN		VASP
Dynamin-2	DYN2_HUMAN		DNM2
Methionine aminopeptidase 2	MAP2_HUMAN		METAP2
Bis(5'-nucleosyl)-tetraphosphatase [asymmetrical]	AP4A_HUMAN		NUDT2
Cyclin-dependent kinase 7	CDK7_HUMAN		CDK7
Kinetochore-associated protein 1	KNTC1_HUMAN		KNTC1
Lipopolysaccharide-responsive and beige-like anchor protein	LRBA_HUMAN		LRBA
Basal cell adhesion molecule	BCAM_HUMAN		BCAM
Palmitoyl-protein thioesterase 1	PPT1_HUMAN		PPT1
Large ribosomal subunit protein eL14	RL14_HUMAN		RPL14
T-complex protein 1 subunit theta	TCPQ_HUMAN		CCT8
T-complex protein 1 subunit delta	TCPD_HUMAN		CCT4
Annexin A11	ANX11_HUMAN		ANXA11
Poly(A) polymerase alpha	PAPOA_HUMAN		PAPOLA
RNA-binding protein FXR1	FXR1_HUMAN		FXR1
RNA-binding protein FXR2	FXR2_HUMAN		FXR2
Ras-related protein Rab-5C	RAB5C_HUMAN		RAB5C
Ras-related protein Rab-7a	RAB7A_HUMAN		RAB7A
Ras-related protein Rab-9A	RAB9A_HUMAN		RAB9A
Ras-related protein Rab-13	RAB13_HUMAN		RAB13
Ras-related protein Rab-27A	RB27A_HUMAN		RAB27A
Death-associated protein 1	DAP1_HUMAN		DAP
Dual specificity protein phosphatase 3	DUS3_HUMAN		DUSP3
Transcription activator BRG1	SMCA4_HUMAN		SMARCA4
Isocitrate dehydrogenase [NAD] subunit gamma, mitochondrial	IDH3G_HUMAN		IDH3G
Galactokinase	GALK1_HUMAN		GALK1
Translocon-associated protein subunit delta	SSRD_HUMAN		SSR4
B-cell receptor-associated protein 31	BAP31_HUMAN		BCAP31
Thiopurine S-methyltransferase	TPMT_HUMAN		TPMT
Host cell factor 1	HCFC1_HUMAN		HCFC1
Aldehyde dehydrogenase family 3 member A2	AL3A2_HUMAN		ALDH3A2
Peroxisomal multifunctional enzyme type 2	DHB4_HUMAN		HSD17B4
26S proteasome non-ATPase regulatory subunit 7	PSMD7_HUMAN		PSMD7
Ribosomal protein S6 kinase alpha-3	KS6A3_HUMAN		RPS6KA3
Hepatoma-derived growth factor	HDGF_HUMAN		HDGF
Lumican	LUM_HUMAN		LUM
Cyclin-H	CCNH_HUMAN		CCNH
CDK-activating kinase assembly factor MAT1	MAT1_HUMAN		MNAT1
NADH dehydrogenase [ubiquinone] 1 alpha subcomplex subunit 8	NDUA8_HUMAN		NDUFA8
Heterogeneous nuclear ribonucleoprotein A3	ROA3_HUMAN		HNRNPA3
6-phosphogluconate dehydrogenase, decarboxylating	6PGD_HUMAN		PGD
Heterogeneous nuclear ribonucleoprotein M	HNRPM_HUMAN		HNRNPM
Importin subunit alpha-1	IMA1_HUMAN		KPNA2
Importin subunit alpha-5	IMA5_HUMAN		KPNA1
Nuclear cap-binding protein subunit 2	NCBP2_HUMAN		NCBP2
Rap1 GTPase-GDP dissociation stimulator 1	GDS1_HUMAN		RAP1GDS1

Rho GDP-dissociation inhibitor 1	GDIR1_HUMAN		ARHGDIA
Rho GDP-dissociation inhibitor 2	GDIR2_HUMAN		ARHGDIB
Arf-GAP domain and FG repeat-containing protein 1	AGFG1_HUMAN		AGFG1
Heterogeneous nuclear ribonucleoprotein F	HNRPF_HUMAN		HNRNPF
Signal transducer and activator of transcription 2	STAT2_HUMAN		STAT2
DNA mismatch repair protein Msh6	MSH6_HUMAN		MSH6
Kinesin-like protein KIF11	KIF11_HUMAN		KIF11
Guanine nucleotide exchange factor VAV2	VAV2_HUMAN		VAV2
Spermine synthase	SPSY_HUMAN		SMS
Hexokinase-2	HXK2_HUMAN		HK2
Large ribosomal subunit protein bL12m	RM12_HUMAN		MRPL12
Stanniocalcin-1	STC1_HUMAN		STC1
Thimet oligopeptidase	THOP1_HUMAN		THOP1
F-actin-capping protein subunit alpha-1	CAZA1_HUMAN		CAPZA1
High mobility group protein HMGI-C	HMGA2_HUMAN		HMGA2
Cysteine-rich protein 2	CRIP2_HUMAN		CRIP2
Nuclear pore complex protein Nup98-Nup96	NUP98_HUMAN		NUP98
Biliverdin reductase A	BIEA_HUMAN		BLVRA
Tricarboxylate transport protein, mitochondrial	TXTP_HUMAN		SLC25A1
Serine/threonine-protein phosphatase 5	PPP5_HUMAN		PPP5C
Serine/threonine-protein kinase PLK1	PLK1_HUMAN		PLK1
Arfaptin-2	ARFP2_HUMAN		ARFIP2
Arfaptin-1	ARFP1_HUMAN		ARFIP1
Cytosolic Fe-S cluster assembly factor NUBP1	NUBP1_HUMAN		NUBP1
ATP-citrate synthase	ACLY_HUMAN		ACLY
Methionine aminopeptidase 1	MAP11_HUMAN		METAP1
Succinate--CoA ligase [ADP/GDP-forming] subunit alpha, mitochondrial	SUCA_HUMAN		SUCLG1
Geranylgeranyl transferase type-2 subunit beta	PGTB2_HUMAN		RABGGTB
Coatomer subunit beta	COPB_HUMAN		COPB1
Coatomer subunit alpha	COPA_HUMAN		COPA
Dipeptidyl peptidase 1	CATC_HUMAN		CTSC
AP-2 complex subunit sigma	AP2S1_HUMAN		AP2S1
Holocytochrome c-type synthase	CCHL_HUMAN		HCCS
Pituitary tumor-transforming gene 1 protein-interacting protein	PTTG_HUMAN		PTTG1IP
Smoothelin	SMTN_HUMAN		SMTN
Monocarboxylate transporter 1	MOT1_HUMAN		SLC16A1
IST1 homolog	IST1_HUMAN		IST1
Protein transport protein Sec24C	SC24C_HUMAN		SEC24C
Activated RNA polymerase II transcriptional coactivator p15	TCP4_HUMAN		SUB1
Methylosome subunit pICln	ICLN_HUMAN		CLNS1A
Arginine--tRNA ligase, cytoplasmic	SYRC_HUMAN		RARS
Voltage-dependent calcium channel subunit alpha-2/delta-1	CA2D1_HUMAN		CACNA2D1
Tyrosine--tRNA ligase, cytoplasmic	SYYC_HUMAN		YARS
Ubiquitin carboxyl-terminal hydrolase 14	UBP14_HUMAN		USP14
Heat shock-related 70 kDa protein 2	HSP72_HUMAN		HSPA2
Sodium/potassium-transporting ATPase subunit beta-3	AT1B3_HUMAN		ATP1B3
UV excision repair protein RAD23 homolog B	RD23B_HUMAN		RAD23B
Adenylate kinase 2, mitochondrial	KAD2_HUMAN		AK2
Delta-1-pyrroline-5-carboxylate synthase	P5CS_HUMAN		ALDH18A1
Alpha-soluble NSF attachment protein	SNAA_HUMAN		NAPA
Eukaryotic translation initiation factor 5	IF5_HUMAN		EIF5
26S proteasome non-ATPase regulatory subunit 4	PSMD4_HUMAN		PSMD4
Developmentally-regulated GTP-binding protein 2	DRG2_HUMAN		DRG2
Phospholipid transfer protein	PLTP_HUMAN		PLTP
Exportin-2	XPO2_HUMAN		CSE1L
Bax inhibitor 1	BI1_HUMAN		TMBIM6
Transitional endoplasmic reticulum ATPase	TERA_HUMAN		VCP
Microfibrillar-associated protein 1	MFAP1_HUMAN		MFAP1
Trifunctional enzyme subunit beta, mitochondrial	ECHB_HUMAN		HADHB
Mesencephalic astrocyte-derived neurotrophic factor	MANF_HUMAN		MANF
Afadin	AFAD_HUMAN		MLLT4
Nucleosome assembly protein 1-like 1	NP1L1_HUMAN		NAP1L1
Caspase-7	CASP7_HUMAN		CASP7
Adenosine kinase	ADK_HUMAN		ADK
Double-stranded RNA-specific adenosine deaminase	DSRAD_HUMAN		ADAR
Laminin subunit beta-2	LAMB2_HUMAN		LAMB2
Cadherin-11	CAD11_HUMAN		CDH11
Cadherin-13	CAD13_HUMAN		CDH13
Protein SEC13 homolog	SEC13_HUMAN		SEC13
NHP2-like protein 1	NH2L1_HUMAN		NHP2L1
Puromycin-sensitive aminopeptidase	PSA_HUMAN		NPEPPS
Heterogeneous nuclear ribonucleoprotein H2	HNRH2_HUMAN		HNRNPH2
Succinyl-CoA:3-ketoacid coenzyme A transferase 1, mitochondrial	SCOT1_HUMAN		OXCT1
Eukaryotic translation initiation factor 3 subunit B	EIF3B_HUMAN		EIF3B
BH3-interacting domain death agonist	BID_HUMAN		BID
ATP synthase subunit f, mitochondrial	ATPK_HUMAN		ATP5J2
Ribosomal RNA processing protein 1 homolog A	RRP1_HUMAN		RRP1
Methionine--tRNA ligase, cytoplasmic	SYMC_HUMAN		MARS
Integrin alpha-1	ITA1_HUMAN		ITGA1
cAMP-regulated phosphoprotein 19	ARP19_HUMAN		ARPP19
ATP synthase subunit epsilon, mitochondrial	ATP5E_HUMAN		ATP5E
ATP synthase subunit e, mitochondrial	ATP5I_HUMAN		ATP5I
Eukaryotic translation initiation factor 6	IF6_HUMAN		EIF6
C-terminal-binding protein 2	CTBP2_HUMAN		CTBP2
NADH dehydrogenase [ubiquinone] 1 alpha subcomplex subunit 6	NDUA6_HUMAN		NDUFA6
Breast cancer anti-estrogen resistance protein 1	BCAR1_HUMAN		BCAR1
Histone H2B type F-S	H2BFS_HUMAN		H2BFS
Transmembrane protein 33	TMM33_HUMAN		TMEM33
Synaptojanin-2-binding protein	SYJ2B_HUMAN		SYNJ2BP
Gem-associated protein 4	GEMI4_HUMAN		GEMIN4
Coronin-7	CORO7_HUMAN		CORO7
Nuclear pore complex protein Nup107	NU107_HUMAN		NUP107
Selenocysteine-specific elongation factor	SELB_HUMAN		EEFSEC
Myotrophin	MTPN_HUMAN		MTPN
Actin-related protein 2/3 complex subunit 4	ARPC4_HUMAN		ARPC4
CD81 antigen	CD81_HUMAN		CD81
Triosephosphate isomerase	TPIS_HUMAN		TPI1
Eukaryotic translation initiation factor 3 subunit E	EIF3E_HUMAN		EIF3E
Protein transport protein Sec61 subunit beta	SC61B_HUMAN		SEC61B
Serine/threonine-protein phosphatase 4 catalytic subunit	PP4C_HUMAN		PPP4C
Reactive oxygen species modulator 1	ROMO1_HUMAN		ROMO1
Ubiquitin-conjugating enzyme E2 G2	UB2G2_HUMAN		UBE2G2
Myosin light polypeptide 6	MYL6_HUMAN		MYL6
Actin, cytoplasmic 1	ACTB_HUMAN		ACTB
Eukaryotic initiation factor 4A-I	IF4A1_HUMAN		EIF4A1
Small ribosomal subunit protein uS10	RS20_HUMAN		RPS20
Ribose-phosphate pyrophosphokinase 1	PRPS1_HUMAN		PRPS1
Proteasome subunit alpha type-6	PSA6_HUMAN		PSMA6
Protein S100-A10	S10AA_HUMAN		S100A10
Cell division control protein 42 homolog	CDC42_HUMAN		CDC42
Destrin	DEST_HUMAN		DSTN
Glia maturation factor beta	GMFB_HUMAN		GMFB
Ras-related protein Rab-8A	RAB8A_HUMAN		RAB8A
Signal peptidase complex subunit 3	SPCS3_HUMAN		SPCS3
Signal recognition particle subunit SRP54	SRP54_HUMAN		SRP54
Ras-related protein Rab-2A	RAB2A_HUMAN		RAB2A
Ras-related protein Rab-5B	RAB5B_HUMAN		RAB5B
Cyclin-dependent kinases regulatory subunit 1	CKS1_HUMAN		CKS1B
Ras-related protein Rab-10	RAB10_HUMAN		RAB10
Ubiquitin-conjugating enzyme E2 D3	UB2D3_HUMAN		UBE2D3
NEDD8-conjugating enzyme Ubc12	UBC12_HUMAN		UBE2M
Ubiquitin-conjugating enzyme E2 K	UBE2K_HUMAN		UBE2K
Ubiquitin-conjugating enzyme E2 N	UBE2N_HUMAN		UBE2N
Ras-related protein Rab-14	RAB14_HUMAN		RAB14
Actin-related protein 3	ARP3_HUMAN		ACTR3
Actin-related protein 2	ARP2_HUMAN		ACTR2
Alpha-centractin	ACTZ_HUMAN		ACTR1A
Transmembrane protein 258	TM258_HUMAN		TMEM258
COP9 signalosome complex subunit 2	CSN2_HUMAN		COPS2

<table>
<tr><td>ADP-ribosylation factor 3</td><td>ARF3_HUMAN</td><td>sp|P84077|ARF1_HUMAN</td><td>ARF3</td></tr>
<tr><td>ATP-binding cassette sub-family E member 1</td><td>ABCE1_HUMAN</td><td></td><td>ABCE1</td></tr>
<tr><td>Ras-related protein Rap-1b</td><td>RAP1B_HUMAN</td><td></td><td>RAP1B</td></tr>
<tr><td>Small ribosomal subunit protein eS1</td><td>RS3A_HUMAN</td><td></td><td>RPS3A</td></tr>
<tr><td>Large ribosomal subunit protein uL24</td><td>RL26_HUMAN</td><td></td><td>RPL26</td></tr>
<tr><td>Proteasome activator complex subunit 3</td><td>PSME3_HUMAN</td><td></td><td>PSME3</td></tr>
<tr><td>Large ribosomal subunit protein eL15</td><td>RL15_HUMAN</td><td></td><td>RPL15</td></tr>
<tr><td>Protein mago nashi homolog</td><td>MGN_HUMAN</td><td>sp|Q96A72|MGN2_HUMAN</td><td>MAGOH</td></tr>
<tr><td>Large ribosomal subunit protein eL27</td><td>RL27_HUMAN</td><td></td><td>RPL27</td></tr>
<tr><td>V-type proton ATPase subunit d 1</td><td>VA0D1_HUMAN</td><td></td><td>ATP6V0D1</td></tr>
<tr><td>Large ribosomal subunit protein eL43</td><td>RL37A_HUMAN</td><td></td><td>RPL37A</td></tr>
<tr><td>Transforming protein RhoA</td><td>RHOA_HUMAN</td><td></td><td>RHOA</td></tr>
<tr><td>Rho-related GTP-binding protein RhoE</td><td>RND3_HUMAN</td><td></td><td>RND3</td></tr>
<tr><td>10 kDa heat shock protein, mitochondrial</td><td>CH10_HUMAN</td><td></td><td>HSPE1</td></tr>
<tr><td>Protein transport protein Sec61 subunit alpha isoform 1</td><td>S61A1_HUMAN</td><td></td><td>SEC61A1</td></tr>
<tr><td>Prefoldin subunit 3</td><td>PFD3_HUMAN</td><td></td><td>VBP1</td></tr>
<tr><td>Syntaxin-binding protein 1</td><td>STXB1_HUMAN</td><td></td><td>STXBP1</td></tr>
<tr><td>Beta-2-microglobulin</td><td>B2MG_HUMAN</td><td></td><td>B2M</td></tr>
<tr><td>Dolichyl-diphosphooligosaccharide--protein glycosyltransferase subunit DAD1</td><td>DAD1_HUMAN</td><td></td><td>DAD1</td></tr>
<tr><td>NPC intracellular cholesterol transporter 2</td><td>NPC2_HUMAN</td><td></td><td>NPC2</td></tr>
<tr><td>Coatomer subunit zeta-1</td><td>COPZ1_HUMAN</td><td></td><td>COPZ1</td></tr>
<tr><td>Large ribosomal subunit protein eL37</td><td>RL37_HUMAN</td><td></td><td>RPL37</td></tr>
<tr><td>Small ubiquitin-related modifier 2</td><td>SUMO2_HUMAN</td><td></td><td>SUMO2</td></tr>
<tr><td>DDB1- and CUL4-associated factor 7</td><td>DCAF7_HUMAN</td><td></td><td>DCAF7</td></tr>
<tr><td>WD repeat-containing protein 5</td><td>WDR5_HUMAN</td><td></td><td>WDR5</td></tr>
<tr><td>Nuclear transport factor 2</td><td>NTF2_HUMAN</td><td></td><td>NUTF2</td></tr>
<tr><td>Heterogeneous nuclear ribonucleoprotein K</td><td>HNRPK_HUMAN</td><td></td><td>HNRNPK</td></tr>
<tr><td>14-3-3 protein gamma</td><td>1433G_HUMAN</td><td></td><td>YWHAG</td></tr>
<tr><td>Ras-related protein R-Ras2</td><td>RRAS2_HUMAN</td><td></td><td>RRAS2</td></tr>
<tr><td>Mitochondrial import inner membrane translocase subunit Tim10</td><td>TIM10_HUMAN</td><td></td><td>TIMM10</td></tr>
<tr><td>Tetraspanin-5</td><td>TSN5_HUMAN</td><td></td><td>TSPAN5</td></tr>
<tr><td>Small ribosomal subunit protein eS7</td><td>RS7_HUMAN</td><td></td><td>RPS7</td></tr>
<tr><td>Serine/threonine-protein phosphatase PP1-alpha catalytic subunit</td><td>PP1A_HUMAN</td><td></td><td>PPP1CA</td></tr>
<tr><td>Serine/threonine-protein phosphatase PP1-beta catalytic subunit</td><td>PP1B_HUMAN</td><td></td><td>PPP1CB</td></tr>
<tr><td>26S proteasome regulatory subunit 4</td><td>PRS4_HUMAN</td><td></td><td>PSMC1</td></tr>
<tr><td>26S proteasome regulatory subunit 8</td><td>PRS8_HUMAN</td><td></td><td>PSMC5</td></tr>
<tr><td>Small ribosomal subunit protein eS8</td><td>RS8_HUMAN</td><td></td><td>RPS8</td></tr>
<tr><td>Small ribosomal subunit protein uS8</td><td>RS15A_HUMAN</td><td></td><td>RPS15A</td></tr>
<tr><td>Small ribosomal subunit protein uS9</td><td>RS16_HUMAN</td><td></td><td>RPS16</td></tr>
<tr><td>14-3-3 protein epsilon</td><td>1433E_HUMAN</td><td></td><td>YWHAE</td></tr>
<tr><td>Small ribosomal subunit protein uS11</td><td>RS14_HUMAN</td><td></td><td>RPS14</td></tr>
<tr><td>Small ribosomal subunit protein uS12</td><td>RS23_HUMAN</td><td></td><td>RPS23</td></tr>
<tr><td>Small ribosomal subunit protein uS13</td><td>RS18_HUMAN</td><td></td><td>RPS18</td></tr>
<tr><td>Small ribosomal subunit protein uS14</td><td>RS29_HUMAN</td><td></td><td>RPS29</td></tr>
<tr><td>Small ribosomal subunit protein uS15</td><td>RS13_HUMAN</td><td></td><td>RPS13</td></tr>
<tr><td>Small ribosomal subunit protein uS17</td><td>RS11_HUMAN</td><td></td><td>RPS11</td></tr>
<tr><td>Small nuclear ribonucleoprotein E</td><td>RUXE_HUMAN</td><td></td><td>SNRPE</td></tr>
<tr><td>Small nuclear ribonucleoprotein F</td><td>RUXF_HUMAN</td><td></td><td>SNRPF</td></tr>
<tr><td>U6 snRNA-associated Sm-like protein LSm3</td><td>LSM3_HUMAN</td><td></td><td>LSM 3.00</td></tr>
<tr><td>U6 snRNA-associated Sm-like protein LSm6</td><td>LSM6_HUMAN</td><td></td><td>LSM 6.00</td></tr>
<tr><td>Small nuclear ribonucleoprotein Sm D1</td><td>SMD1_HUMAN</td><td></td><td>SNRPD1</td></tr>
<tr><td>Small nuclear ribonucleoprotein Sm D2</td><td>SMD2_HUMAN</td><td></td><td>SNRPD2</td></tr>
<tr><td>Small nuclear ribonucleoprotein Sm D3</td><td>SMD3_HUMAN</td><td></td><td>SNRPD3</td></tr>
<tr><td>Thymosin beta-4</td><td>TYB4_HUMAN</td><td></td><td>TMSB4X</td></tr>
<tr><td>ADP-ribosylation factor 6</td><td>ARF6_HUMAN</td><td></td><td>ARF6</td></tr>
<tr><td>26S proteasome regulatory subunit 10B</td><td>PRS10_HUMAN</td><td></td><td>PSMC6</td></tr>
<tr><td>Large ribosomal subunit protein eL8</td><td>RL7A_HUMAN</td><td></td><td>RPL7A</td></tr>
<tr><td>Ras-related protein Rab-11A</td><td>RB11A_HUMAN</td><td></td><td>RAB11A</td></tr>
<tr><td>Eukaryotic peptide chain release factor subunit 1</td><td>ERF1_HUMAN</td><td></td><td>ETF1</td></tr>
<tr><td>CCHC-type zinc finger nucleic acid binding protein</td><td>CNBP_HUMAN</td><td></td><td>CNBP</td></tr>
<tr><td>Small ribosomal subunit protein eS4, X isoform</td><td>RS4X_HUMAN</td><td></td><td>RPS4X</td></tr>
<tr><td>Large ribosomal subunit protein uL23</td><td>RL23A_HUMAN</td><td></td><td>RPL23A</td></tr>
<tr><td>Small ribosomal subunit protein eS6</td><td>RS6_HUMAN</td><td></td><td>RPS6</td></tr>
<tr><td>Histone H4</td><td>H4_HUMAN</td><td></td><td>HIST1H4A</td></tr>
<tr><td>Ras-related protein Rab-1A</td><td>RAB1A_HUMAN</td><td></td><td>RAB1A</td></tr>
<tr><td>GTP-binding nuclear protein Ran</td><td>RAN_HUMAN</td><td></td><td>RAN</td></tr>
<tr><td>Large ribosomal subunit protein uL14</td><td>RL23_HUMAN</td><td></td><td>RPL23</td></tr>
<tr><td>Ras-related protein Rap-1A</td><td>RAP1A_HUMAN</td><td></td><td>RAP1A</td></tr>
<tr><td>Small ribosomal subunit protein uS19</td><td>RS15_HUMAN</td><td></td><td>RPS15</td></tr>
<tr><td>Small ribosomal subunit protein eS24</td><td>RS24_HUMAN</td><td></td><td>RPS24</td></tr>
<tr><td>Small ribosomal subunit protein eS25</td><td>RS25_HUMAN</td><td></td><td>RPS25</td></tr>
<tr><td>Small ribosomal subunit protein eS26</td><td>RS26_HUMAN</td><td></td><td>RPS26</td></tr>
<tr><td>Small ribosomal subunit protein eS28</td><td>RS28_HUMAN</td><td></td><td>RPS28</td></tr>
<tr><td>Ubiquitin-like FUBI-ribosomal protein eS30 fusion protein</td><td>RS30_HUMAN</td><td></td><td>FAU</td></tr>
<tr><td>Guanine nucleotide-binding protein G(I)/G(S)/G(T) subunit beta-1</td><td>GBB1_HUMAN</td><td></td><td>GNB1</td></tr>
<tr><td>E3 ubiquitin-protein ligase RBX1</td><td>RBX1_HUMAN</td><td></td><td>RBX1</td></tr>
<tr><td>Guanine nucleotide-binding protein G(I)/G(S)/G(T) subunit beta-2</td><td>GBB2_HUMAN</td><td></td><td>GNB2</td></tr>
<tr><td>Large ribosomal subunit protein eL30</td><td>RL30_HUMAN</td><td></td><td>RPL30</td></tr>
<tr><td>Large ribosomal subunit protein eL39</td><td>RL39_HUMAN</td><td></td><td>RPL39</td></tr>
<tr><td>Large ribosomal subunit protein eL31</td><td>RL31_HUMAN</td><td></td><td>RPL31</td></tr>
<tr><td>Large ribosomal subunit protein uL1</td><td>RL10A_HUMAN</td><td></td><td>RPL10A</td></tr>
<tr><td>Large ribosomal subunit protein eL32</td><td>RL32_HUMAN</td><td></td><td>RPL32</td></tr>
<tr><td>Large ribosomal subunit protein uL5</td><td>RL11_HUMAN</td><td></td><td>RPL11</td></tr>
<tr><td>Large ribosomal subunit protein uL2</td><td>RL8_HUMAN</td><td></td><td>RPL8</td></tr>
<tr><td>Peptidyl-prolyl cis-trans isomerase A</td><td>PPIA_HUMAN</td><td></td><td>PPIA</td></tr>
<tr><td>Peptidyl-prolyl cis-trans isomerase FKBP1A</td><td>FKB1A_HUMAN</td><td></td><td>FKBP1A</td></tr>
<tr><td>Ubiquitin-40S ribosomal protein S27a</td><td>RS27A_HUMAN</td><td></td><td>RPS27A</td></tr>
<tr><td>Ubiquitin-ribosomal protein eL40 fusion protein</td><td>RL40_HUMAN</td><td></td><td>UBA52</td></tr>
<tr><td>Growth factor receptor-bound protein 2</td><td>GRB2_HUMAN</td><td></td><td>GRB2</td></tr>
<tr><td>Transformer-2 protein homolog beta</td><td>TRA2B_HUMAN</td><td></td><td>TRA2B</td></tr>
<tr><td>Ras-related C3 botulinum toxin substrate 1</td><td>RAC1_HUMAN</td><td></td><td>RAC1</td></tr>
<tr><td>AP-2 complex subunit beta</td><td>AP2B1_HUMAN</td><td></td><td>AP2B1</td></tr>
<tr><td>Guanine nucleotide-binding protein G(s) subunit alpha isoforms short</td><td>GNAS2_HUMAN</td><td></td><td>GNAS</td></tr>
<tr><td>Guanine nucleotide-binding protein G(i) subunit alpha-1</td><td>GNAI1_HUMAN</td><td></td><td>GNAI1</td></tr>
<tr><td>14-3-3 protein zeta/delta</td><td>1433Z_HUMAN</td><td></td><td>YWHAZ</td></tr>
<tr><td>Serine/threonine-protein phosphatase 2A 55 kDa regulatory subunit B alpha isoform</td><td>2ABA_HUMAN</td><td></td><td>PPP2R2A</td></tr>
<tr><td>Small ubiquitin-related modifier 1</td><td>SUMO1_HUMAN</td><td></td><td>SUMO1</td></tr>
<tr><td>Dynein light chain 1, cytoplasmic</td><td>DYL1_HUMAN</td><td></td><td>DYNLL1</td></tr>
<tr><td>Large ribosomal subunit protein eL38</td><td>RL38_HUMAN</td><td></td><td>RPL38</td></tr>
<tr><td>S-phase kinase-associated protein 1</td><td>SKP1_HUMAN</td><td></td><td>SKP1</td></tr>
<tr><td>Guanine nucleotide-binding protein G(I)/G(S)/G(O) subunit gamma-5</td><td>GBG5_HUMAN</td><td></td><td>GNG5</td></tr>
<tr><td>Small ribosomal subunit protein eS21</td><td>RS21_HUMAN</td><td></td><td>RPS21</td></tr>
<tr><td>Eukaryotic translation initiation factor 5A-1</td><td>IF5A1_HUMAN</td><td></td><td>EIF5A</td></tr>
<tr><td>Small ribosomal subunit protein RACK1</td><td>RACK1_HUMAN</td><td></td><td>GNB2L1</td></tr>
<tr><td>Transcription elongation factor SPT4</td><td>SPT4H_HUMAN</td><td></td><td>SUPT4H1</td></tr>
<tr><td>Thymosin beta-10</td><td>TYB10_HUMAN</td><td></td><td>TMSB10</td></tr>
<tr><td>Serine/threonine-protein phosphatase 2A catalytic subunit alpha isoform</td><td>PP2AA_HUMAN</td><td></td><td>PPP2CA</td></tr>
<tr><td>Y-box-binding protein 1</td><td>YBOX1_HUMAN</td><td></td><td>YBX1</td></tr>
<tr><td>Signal peptidase complex catalytic subunit SEC11A</td><td>SC11A_HUMAN</td><td></td><td>SEC11A</td></tr>
<tr><td>Casein kinase II subunit beta</td><td>CSK2B_HUMAN</td><td></td><td>CSNK2B</td></tr>
<tr><td>Tropomyosin alpha-4 chain</td><td>TPM4_HUMAN</td><td></td><td>TPM4</td></tr>
<tr><td>Actin, alpha cardiac muscle 1</td><td>ACTC_HUMAN</td><td></td><td>ACTC1</td></tr>
<tr><td>Ubiquitin-conjugating enzyme E2 L3</td><td>UB2L3_HUMAN</td><td></td><td>UBE2L3</td></tr>
<tr><td>Elongation factor 1-alpha 1</td><td>EF1A1_HUMAN</td><td></td><td>EEF1A1</td></tr>
<tr><td>Tubulin alpha-4A chain</td><td>TBA4A_HUMAN</td><td></td><td>TUBA4A</td></tr>
<tr><td>Tubulin beta-4B chain</td><td>TBB4B_HUMAN</td><td></td><td>TUBB4B</td></tr>
<tr><td>Casein kinase II subunit alpha</td><td>CSK21_HUMAN</td><td></td><td>CSNK2A1</td></tr>
<tr><td>Platelet-activating factor acetylhydrolase IB subunit alpha2</td><td>PA1B2_HUMAN</td><td></td><td>PAFAH1B2</td></tr>
<tr><td>BOS complex subunit NOMO3</td><td>NOMO3_HUMAN</td><td>sp|Q5JPE7|NOMO2_HUMAN</td><td>NOMO3</td></tr>
<tr><td>Hemoglobin subunit alpha</td><td>HBA_HUMAN</td><td></td><td>HBA1</td></tr>
<tr><td>Immunoglobulin-binding protein 1</td><td>IGBP1_HUMAN</td><td></td><td>IGBP1</td></tr>
<tr><td>Phosphoserine phosphatase</td><td>SERB_HUMAN</td><td></td><td>PSPH</td></tr>
<tr><td>RNA-binding protein 6</td><td>RBM6_HUMAN</td><td></td><td>RBM6</td></tr>
<tr><td>Eukaryotic translation initiation factor 4 gamma 2</td><td>IF4G2_HUMAN</td><td></td><td>EIF4G2</td></tr>
<tr><td>Ribonuclease P protein subunit p30</td><td>RPP30_HUMAN</td><td></td><td>RPP30</td></tr>
<tr><td>General transcription factor II-I</td><td>GTF2I_HUMAN</td><td></td><td>GTF2I</td></tr>
<tr><td>Contactin-associated protein 1</td><td>CNTP1_HUMAN</td><td></td><td>CNTNAP1</td></tr>
<tr><td>T-complex protein 1 subunit beta</td><td>TCPB_HUMAN</td><td></td><td>CCT2</td></tr>
<tr><td>mRNA export factor RAE1</td><td>RAE1L_HUMAN</td><td></td><td>RAE1</td></tr>
</table>

Glutathione S-transferase omega-1	GSTO1_HUMAN		GSTO1
DNA-dependent protein kinase catalytic subunit	PRKDC_HUMAN		PRKDC
Sushi repeat-containing protein SRPX	SRPX_HUMAN		SRPX
Microtubule-associated protein 1A	MAP1A_HUMAN		MAP1A
Metallothionein-1X	MT1X_HUMAN		MT1X
Nucleobindin-2	NUCB2_HUMAN		NUCB2
Brain acid soluble protein 1	BASP1_HUMAN		BASP1
Dermcidin	DCD_HUMAN		DCD
TATA element modulatory factor	TMF1_HUMAN		TMF1
Small ribosomal subunit protein mS22	RT22_HUMAN		MRPS22
Small ribosomal subunit protein mS25	RT25_HUMAN		MRPS25
Small ribosomal subunit protein uS5m	RT05_HUMAN		MRPS5
Small ribosomal subunit protein uS11m	RT11_HUMAN		MRPS11
Small ribosomal subunit protein mS34	RT34_HUMAN		MRPS34
Small ribosomal subunit protein uS9m	RT09_HUMAN		MRPS9
SAP domain-containing ribonucleoprotein	SARNP_HUMAN		SARNP
Serine beta-lactamase-like protein LACTB, mitochondrial	LACTB_HUMAN		LACTB
Large ribosomal subunit protein eL24	RL24_HUMAN		RPL24
Large ribosomal subunit protein eL42	RL36A_HUMAN		RPL36A
Chromobox protein homolog 1	CBX1_HUMAN		CBX1
ADP-ribosylation factor 5	ARF5_HUMAN		ARF5
Enhancer of rudimentary homolog	ERH_HUMAN		ERH
Rho-related GTP-binding protein RhoG	RHOG_HUMAN		RHOG
Large ribosomal subunit protein eL19	RL19_HUMAN		RPL19
Small EDRK-rich factor 2	SERF2_HUMAN		SERF2
Serine/arginine-rich splicing factor 3	SRSF3_HUMAN		SRSF3
Forkhead box protein K1	FOXK1_HUMAN		FOXK1
Disabled homolog 2	DAB2_HUMAN		DAB2
Basement membrane-specific heparan sulfate proteoglycan core protein	PGBM_HUMAN		HSPG2
RNA-binding protein 10	RBM10_HUMAN		RBM10
RNA-binding protein 3	RBM3_HUMAN		RBM3
Transcription factor A, mitochondrial	TFAM_HUMAN		TFAM
Solute carrier family 25 member 3	S25A3_HUMAN		SLC25A3
Vigilin	VIGLN_HUMAN		HDLBP
Transcriptional activator protein Pur-alpha	PURA_HUMAN		PURA
Cdc42 effector protein 1	BORG5_HUMAN		CDC42EP1
Clathrin heavy chain 1	CLH1_HUMAN		CLTC
Nuclear factor NF-kappa-B p100 subunit	NFKB2_HUMAN		NFKB2
Peptidyl-prolyl cis-trans isomerase FKBP3	FKBP3_HUMAN		FKBP3
Receptor expression-enhancing protein 5	REEP5_HUMAN		REEP5
Sorbitol dehydrogenase	DHSO_HUMAN		SORD
Heterogeneous nuclear ribonucleoprotein U	HNRPU_HUMAN		HNRNPU
Splicing factor U2AF 35 kDa subunit	U2AF1_HUMAN		U2AF1
Spectrin beta chain, non-erythrocytic 1	SPTB2_HUMAN		SPTBN1
Nucleolysin TIAR	TIAR_HUMAN		TIAL1
Protein SET	SET_HUMAN		SET
Serine/arginine-rich splicing factor 2	SRSF2_HUMAN		SRSF2
AMP deaminase 2	AMPD2_HUMAN		AMPD2
Fatty acid-binding protein 5	FABP5_HUMAN		FABP5
Adenylyl cyclase-associated protein 1	CAP1_HUMAN		CAP1
Hydroxymethylglutaryl-CoA synthase, cytoplasmic	HMCS1_HUMAN		HMGCS1
Large neutral amino acids transporter small subunit 1	LAT1_HUMAN		SLC7A5
Exosome component 10	EXOSX_HUMAN		EXOSC10
OTU domain-containing protein 4	OTUD4_HUMAN		OTUD4
ATP-dependent 6-phosphofructokinase, platelet type	PFKAP_HUMAN		PFKP
RNA-binding protein EWS	EWS_HUMAN		EWSR1
1-phosphatidylinositol 4,5-bisphosphate phosphodiesterase beta-3	PLCB3_HUMAN		PLCB3
Transgelin	TAGL_HUMAN		TAGLN
Dihydroorotate dehydrogenase (quinone), mitochondrial	PYRD_HUMAN		DHODH
2-oxoglutarate dehydrogenase complex component E1	ODO1_HUMAN		OGDH
Centromere-associated protein E	CENPE_HUMAN		CENPE
Kinesin-like protein KIF23	KIF23_HUMAN		KIF23
Methylmalonate-semialdehyde dehydrogenase [acylating], mitochondrial	MMSA_HUMAN		ALDH6A1
Collagen alpha-1(VII) chain	CO7A1_HUMAN		COL7A1
Transcription factor Sp3	SP3_HUMAN		SP3
Histone H1.1	H11_HUMAN		HIST1H1A
Large ribosomal subunit protein eL20	RL18A_HUMAN		RPL18A
Dual specificity mitogen-activated protein kinase kinase 1	MP2K1_HUMAN		MAP2K1
Peptidyl-prolyl cis-trans isomerase FKBP4	FKBP4_HUMAN		FKBP4
Procollagen-lysine,2-oxoglutarate 5-dioxygenase 1	PLOD1_HUMAN		PLOD1
Nucleobindin-1	NUCB1_HUMAN		NUCB1
Large ribosomal subunit protein eL6	RL6_HUMAN		RPL6
A-kinase anchor protein 12	AKA12_HUMAN		AKAP12
Mitochondrial 2-oxoglutarate/malate carrier protein	M2OM_HUMAN		SLC25A11
Dystonin	DYST_HUMAN		DST
Caveolin-1	CAV1_HUMAN		CAV1
Aminoacylase-1	ACY1_HUMAN		ACY1
Lamin-B2	LMNB2_HUMAN		LMNB2
Urokinase plasminogen activator surface receptor	UPAR_HUMAN		PLAUR
Antigen peptide transporter 1	TAP1_HUMAN		TAP1
Antigen peptide transporter 2	TAP2_HUMAN		TAP2
CCAAT/enhancer-binding protein zeta	CEBPZ_HUMAN		CEBPZ
Transcription factor p65	TF65_HUMAN		RELA
UBX domain-containing protein 1	UBXN1_HUMAN		UBXN1
1,4-alpha-glucan-branching enzyme	GLGB_HUMAN		GBE1
Eukaryotic translation initiation factor 4 gamma 1	IF4G1_HUMAN		EIF4G1
Keratin, type I cytoskeletal 17	K1C17_HUMAN		KRT17
Neurogenic locus notch homolog protein 2	NOTC2_HUMAN		NOTCH2
Transducin-like enhancer protein 3	TLE3_HUMAN		TLE3
Hepatocyte growth factor activator	HGFA_HUMAN		HGFAC
Lactoylglutathione lyase	LGUL_HUMAN		GLO1
Single-stranded DNA-binding protein, mitochondrial	SSBP_HUMAN		SSBP1
14-3-3 protein eta	1433F_HUMAN		YWHAH
Cleavage stimulation factor subunit 1	CSTF1_HUMAN		CSTF1
Ubiquitin-protein ligase E3A	UBE3A_HUMAN		UBE3A
Dynamin-1	DYN1_HUMAN		DNM1
Tyrosine-protein phosphatase non-receptor type 12	PTN12_HUMAN		PTPN12
Focal adhesion kinase 1	FAK1_HUMAN		PTK2
Serine/arginine-rich splicing factor 11	SRS11_HUMAN		SRSF11
Protein kinase C delta type	KPCD_HUMAN		PRKCD
Caldesmon	CALD1_HUMAN		CALD1
Folylpolyglutamate synthase, mitochondrial	FOLC_HUMAN		FPGS
Inter-alpha-trypsin inhibitor heavy chain H3	ITIH3_HUMAN		ITIH3
Tyrosine-protein phosphatase non-receptor type 11	PTN11_HUMAN		PTPN11
Amidophosphoribosyltransferase	PUR1_HUMAN		PPAT
Glutamine--fructose-6-phosphate aminotransferase [isomerizing] 1	GFPT1_HUMAN		GFPT1
Exosome complex component RRP45	EXOS9_HUMAN		EXOSC9
Proteasome activator complex subunit 1	PSME1_HUMAN		PSME1
Amyloid beta precursor like protein 2	APLP2_HUMAN		APLP2
Fragile X messenger ribonucleoprotein 1	FMR1_HUMAN		FMR1
Fibromodulin	FMOD_HUMAN		FMOD
Peroxiredoxin-1	PRDX1_HUMAN		PRDX1
Large ribosomal subunit protein eL18	RL18_HUMAN		RPL18
Cytoskeleton-associated protein 4	CKAP4_HUMAN		CKAP4
Tight junction protein ZO-1	ZO1_HUMAN		TJP1
KH domain-containing, RNA-binding, signal transduction-associated protein 1	KHDR1_HUMAN		KHDRBS1
Kinesin light chain 1	KLC1_HUMAN		KLC1
Prolow-density lipoprotein receptor-related protein 1	LRP1_HUMAN		LRP1
Serine/arginine-rich splicing factor 1	SRSF1_HUMAN		SRSF1
Rho GTPase-activating protein 1	RHG01_HUMAN		ARHGAP1
Serine/arginine-rich splicing factor 4	SRSF4_HUMAN		SRSF4
Protein phosphatase 3 catalytic subunit alpha	PP2BA_HUMAN		PPP3CA
ATP-dependent RNA helicase A	DHX9_HUMAN		DHX9
Quinone oxidoreductase	QOR_HUMAN		CRYZ
Golgin subfamily A member 3	GOGA3_HUMAN		GOLGA3
Golgin subfamily A member 2	GOGA2_HUMAN		GOLGA2
Galectin-3-binding protein	LG3BP_HUMAN		LGALS3BP
Lactadherin	MFGM_HUMAN		MFGE8
Leukocyte surface antigen CD47	CD47_HUMAN		CD47

Peptidyl-prolyl cis-trans isomerase D	PPID_HUMAN		PPID
FACT complex subunit SSRP1	SSRP1_HUMAN		SSRP1
Schlafen family member 5	SLFN5_HUMAN		SLFN5
Protein VAC14 homolog	VAC14_HUMAN		VAC14
RNA cytosine C(5)-methyltransferase NSUN2	NSUN2_HUMAN		NSUN2
Histone-binding protein RBBP4	RBBP4_HUMAN		RBBP4
Nuclear cap-binding protein subunit 1	NCBP1_HUMAN		NCBP1
Neuroblast differentiation-associated protein AHNAK	AHNK_HUMAN		AHNAK
Heat shock 70 kDa protein 14	HSP7E_HUMAN		HSPA14
Nexilin	NEXN_HUMAN		NEXN
Polypeptide N-acetylgalactosaminyltransferase 2	GALT2_HUMAN		GALNT2
AP-1 complex subunit beta-1	AP1B1_HUMAN		AP1B1
Cleavage and polyadenylation specificity factor subunit 1	CPSF1_HUMAN		CPSF1
ADP-ribosyl cyclase/cyclic ADP-ribose hydrolase 2	BST1_HUMAN		BST1
Bone marrow stromal antigen 2	BST2_HUMAN		BST2
Mitochondrial-processing peptidase subunit alpha	MPPA_HUMAN		PMPCA
Secernin-1	SCRN1_HUMAN		SCRN1
WASH complex subunit 5	WASC5_HUMAN		KIAA0196
Nuclear pore complex protein Nup160	NU160_HUMAN		NUP160
Transducin beta-like protein 3	TBL3_HUMAN		TBL3
Twinfilin-1	TWF1_HUMAN		TWF1
Aspartyl/asparaginyl beta-hydroxylase	ASPH_HUMAN		ASPH
A-kinase anchor protein 13	AKP13_HUMAN		AKAP13
EGF-containing fibulin-like extracellular matrix protein 1	FBLN3_HUMAN		EFEMP1
Cell division cycle protein 20 homolog	CDC20_HUMAN		CDC20
Follistatin-related protein 1	FSTL1_HUMAN		FSTL1
Syntaxin-4	STX4_HUMAN		STX4
G-rich sequence factor 1	GRSF1_HUMAN		GRSF1
Contactin-1	CNTN1_HUMAN		CNTN1
Chromodomain-helicase-DNA-binding protein 3	CHD3_HUMAN		CHD3
Splicing factor 3A subunit 3	SF3A3_HUMAN		SF3A3
Dihydropyrimidine dehydrogenase [NADP(+)]	DPYD_HUMAN		DPYD
Prolyl endopeptidase FAP	SEPR_HUMAN		FAP
TP53-binding protein 1	TP53B_HUMAN		TP53BP1
Aminoacyl tRNA synthase complex-interacting multifunctional protein 1	AIMP1_HUMAN		AIMP1
Interleukin enhancer-binding factor 2	ILF2_HUMAN		ILF2
Interleukin enhancer-binding factor 3	ILF3_HUMAN		ILF3
Vesicular integral-membrane protein VIP36	LMAN2_HUMAN		LMAN2
Epidermal growth factor receptor kinase substrate 8	EPS8_HUMAN		EPS8
Heat shock protein 75 kDa, mitochondrial	TRAP1_HUMAN		TRAP1
TNF receptor-associated factor 2	TRAF2_HUMAN		TRAF2
Unconventional myosin-Ie	MYO1E_HUMAN		MYO1E
Nuclear inhibitor of protein phosphatase 1	PP1R8_HUMAN		PPP1R8
Protein tyrosine phosphatase type IVA 2	TP4A2_HUMAN		PTP4A2
Active breakpoint cluster region-related protein	ABR_HUMAN		ABR
Cleavage stimulation factor subunit 3	CSTF3_HUMAN		CSTF3
Delta(3,5)-Delta(2,4)-dienoyl-CoA isomerase, mitochondrial	ECH1_HUMAN		ECH1
Serine/threonine-protein kinase 4	STK4_HUMAN		STK4
Protein flightless-1 homolog	FLII_HUMAN		FLII
Bifunctional coenzyme A synthase	COASY_HUMAN		COASY
Acetyl-CoA carboxylase 1	ACACA_HUMAN		ACACA
COP9 signalosome complex subunit 1	CSN1_HUMAN		GPS1
Protein Red	RED_HUMAN		IK
S-methyl-5'-thioadenosine phosphorylase	MTAP_HUMAN		MTAP
5'-AMP-activated protein kinase catalytic subunit alpha-1	AAPK1_HUMAN		PRKAA1
Liprin-alpha-1	LIPA1_HUMAN		PPFIA1
TAR DNA-binding protein 43	TADBP_HUMAN		TARDBP
Heterogeneous nuclear ribonucleoprotein A0	ROA0_HUMAN		HNRNPA0
Aminoacyl tRNA synthase complex-interacting multifunctional protein 2	AIMP2_HUMAN		AIMP2
Peroxiredoxin-4	PRDX4_HUMAN		PRDX4
Serine/threonine-protein kinase PAK 2	PAK2_HUMAN		PAK2
Chromobox protein homolog 3	CBX3_HUMAN		CBX3
Syntaxin-5	STX5_HUMAN		STX5
26S proteasome non-ATPase regulatory subunit 2	PSMD2_HUMAN		PSMD2
Semaphorin-3B	SEM3B_HUMAN		SEMA3B
DnaJ homolog subfamily C member 3	DNJC3_HUMAN		DNAJC3
Methanethiol oxidase	SBP1_HUMAN		SELENBP1
Serine/arginine-rich splicing factor 9	SRSF9_HUMAN		SRSF9
Serine/arginine-rich splicing factor 5	SRSF5_HUMAN		SRSF5
Serine/arginine-rich splicing factor 6	SRSF6_HUMAN		SRSF6
Mitotic spindle assembly checkpoint protein MAD2A	MD2L1_HUMAN		MAD2L1
Transcription intermediary factor 1-beta	TIF1B_HUMAN		TRIM28
Ras GTPase-activating protein-binding protein 1	G3BP1_HUMAN		G3BP1
Inactive tyrosine-protein kinase 7	PTK7_HUMAN		PTK7
Polyadenylate-binding protein 4	PABP4_HUMAN		PABPC4
Interferon-induced protein with tetratricopeptide repeats 5	IFIT5_HUMAN		IFIT5
Metastasis-associated protein MTA1	MTA1_HUMAN		MTA1
Eukaryotic translation initiation factor 3 subunit I	EIF3I_HUMAN		EIF3I
C-terminal-binding protein 1	CTBP1_HUMAN		CTBP1
Phosducin-like protein	PHLP_HUMAN		PDCL
Ubiquitin-conjugating enzyme E2 variant 1	UB2V1_HUMAN		UBE2V1
Cytoplasmic dynein 1 intermediate chain 2	DC1I2_HUMAN		DYNC1I2
Integrin-linked protein kinase	ILK_HUMAN		ILK
NAD(P) transhydrogenase, mitochondrial	NNTM_HUMAN		NNT
Beta-2-syntrophin	SNTB2_HUMAN		SNTB2
Peptidyl-prolyl cis-trans isomerase G	PPIG_HUMAN		PPIG
Treacle protein	TCOF_HUMAN		TCOF1
Splicing factor 3B subunit 2	SF3B2_HUMAN		SF3B2
Protein OS-9	OS9_HUMAN		OS9
Golgin subfamily A member 4	GOGA4_HUMAN		GOLGA4
28 kDa heat- and acid-stable phosphoprotein	HAP28_HUMAN		PDAP1
Disintegrin and metalloproteinase domain-containing protein 9	ADAM9_HUMAN		ADAM9
Peptidyl-prolyl cis-trans isomerase FKBP5	FKBP5_HUMAN		FKBP5
Unconventional myosin-IXb	MYO9B_HUMAN		MYO9B
Rho-associated protein kinase 1	ROCK1_HUMAN		ROCK1
Phosphatidylinositol-binding clathrin assembly protein	PICAL_HUMAN		PICALM
Sequestosome-1	SQSTM_HUMAN		SQSTM1
Metaxin-1	MTX1_HUMAN		MTX1
Tubulin beta-3 chain	TBB3_HUMAN		TUBB3
Serine/threonine-protein kinase PRP4 homolog	PRP4B_HUMAN		PRPF4B
Peptidyl-prolyl cis-trans isomerase NIMA-interacting 1	PIN1_HUMAN		PIN1
Eukaryotic translation initiation factor 4E-binding protein 1	4EBP1_HUMAN		EIF4EBP1
Receptor-interacting serine/threonine-protein kinase 1	RIPK1_HUMAN		RIPK1
Histone deacetylase 1	HDAC1_HUMAN		HDAC1
Calcium/calmodulin-dependent protein kinase type II subunit delta	KCC2D_HUMAN		CAMK2D
Dynactin subunit 2	DCTN2_HUMAN		DCTN2
NEDD8-activating enzyme E1 regulatory subunit	ULA1_HUMAN		NAE1
SNW domain-containing protein 1	SNW1_HUMAN		SNW1
Transformer-2 protein homolog alpha	TRA2A_HUMAN		TRA2A
Sorting nexin-1	SNX1_HUMAN		SNX1
KRR1 small subunit processome component homolog	KRR1_HUMAN		KRR1
Periodic tryptophan protein 1 homolog	PWP1_HUMAN		PWP1
Cullin-1	CUL1_HUMAN		CUL1
Cullin-2	CUL2_HUMAN		CUL2
Cullin-3	CUL3_HUMAN		CUL3
Cullin-4A	CUL4A_HUMAN		CUL4A
Cullin-4B	CUL4B_HUMAN		CUL4B
GDP-L-fucose synthase	FCL_HUMAN		TSTA3
Ras-related protein Rab-32	RAB32_HUMAN		RAB32
Trophoblast glycoprotein	TPBG_HUMAN		TPBG
Four and a half LIM domains protein 1	FHL1_HUMAN		FHL1
Four and a half LIM domains protein 3	FHL3_HUMAN		FHL3
Angio-associated migratory cell protein	AAMP_HUMAN		AAMP
Mannosyl-oligosaccharide glucosidase	MOGS_HUMAN		MOGS
CD166 antigen	CD166_HUMAN		ALCAM
Spectrin alpha chain, non-erythrocytic 1	SPTN1_HUMAN		SPTAN1
Nucleolar GTP-binding protein 2	NOG2_HUMAN		GNL2
Spliceosome RNA helicase DDX39B	DX39B_HUMAN		DDX39B
Bleomycin hydrolase	BLMH_HUMAN		BLMH

Exosome complex component RRP4	EXOS2_HUMAN		EXOSC2
Tubulin beta-2A chain	TBB2A_HUMAN		TUBB2A
Bystin	BYST_HUMAN		BYSL
Isopentenyl-diphosphate Delta-isomerase 1	IDI1_HUMAN		IDI1
Core-binding factor subunit beta	PEBB_HUMAN		CBFB
Cytoskeleton-associated protein 5	CKAP5_HUMAN		CKAP5
Cold-inducible RNA-binding protein	CIRBP_HUMAN		CIRBP
Coactosin-like protein	COTL1_HUMAN		COTL1
Cytochrome c oxidase copper chaperone	COX17_HUMAN		COX17
Heterogeneous nuclear ribonucleoprotein D0	HNRPD_HUMAN		HNRNPD
Lysosome membrane protein 2	SCRB2_HUMAN		SCARB2
Nidogen-2	NID2_HUMAN		NID2
Ribosome biogenesis protein BOP1	BOP1_HUMAN		BOP 1.00
Ubiquitin conjugation factor E4 A	UBE4A_HUMAN		UBE4A
Kelch-like ECH-associated protein 1	KEAP1_HUMAN		KEAP1
Scaffold attachment factor B2	SAFB2_HUMAN		SAFB2
Eukaryotic translation initiation factor 3 subunit A	EIF3A_HUMAN		EIF3A
Rho guanine nucleotide exchange factor 7	ARHG7_HUMAN		ARHGEF7
Ubiquitin-associated protein 2-like	UBP2L_HUMAN		UBAP2L
Protein scribble homolog	SCRIB_HUMAN		SCRIB
ARF GTPase-activating protein GIT2	GIT2_HUMAN		GIT2
Malectin	MLEC_HUMAN		MLEC
Tubulin--tyrosine ligase-like protein 12	TTL12_HUMAN		TTLL12
Four and a half LIM domains protein 2	FHL2_HUMAN		FHL2
Dihydropyrimidinase-related protein 3	DPYL3_HUMAN		DPYSL3
Large ribosomal subunit protein mL62	ICT1_HUMAN		ICT1
Dynactin subunit 1	DCTN1_HUMAN		DCTN1
Cytoplasmic dynein 1 heavy chain 1	DYHC1_HUMAN		DYNC1H1
Translation initiation factor eIF-2B subunit alpha	EI2BA_HUMAN		EIF2B1
Eukaryotic initiation factor 4A-II	IF4A2_HUMAN		EIF4A2
Elongin-A	ELOA1_HUMAN		TCEB3
Src substrate cortactin	SRC8_HUMAN		CTTN
Flotillin-2	FLOT2_HUMAN		FLOT2
Reticulocalbin-2	RCN2_HUMAN		RCN2
E3 ubiquitin/ISG15 ligase TRIM25	TRI25_HUMAN		TRIM25
Filamin-C	FLNC_HUMAN		FLNC
Peptidyl-prolyl cis-trans isomerase FKBP8	FKBP8_HUMAN		FKBP8
Protein FAM50A	FA50A_HUMAN		FAM50A
Protein FRG1	FRG1_HUMAN		FRG1
Frizzled-2	FZD2_HUMAN		FZD2
Guanine nucleotide-binding protein subunit alpha-13	GNA13_HUMAN		GNA13
Guanidinoacetate N-methyltransferase	GAMT_HUMAN		GAMT
UDP-glucose 4-epimerase	GALE_HUMAN		GALE
Caprin-1	CAPR1_HUMAN		CAPRIN1
RNA-binding protein 39	RBM39_HUMAN		RBM39
Hyaluronan-binding protein 2	HABP2_HUMAN		HABP2
Helicase-like transcription factor	HLTF_HUMAN		HLTF
Protein disulfide-isomerase A5	PDIA5_HUMAN		PDIA5
Phosphoribosyl pyrophosphate synthase-associated protein 1	KPRA_HUMAN		PRPSAP1
DNA replication licensing factor MCM6	MCM6_HUMAN		MCM6
Inositol 1,4,5-trisphosphate receptor type 3	ITPR3_HUMAN		ITPR3
Inter-alpha-trypsin inhibitor heavy chain H4	ITIH4_HUMAN		ITIH4
Interferon regulatory factor 3	IRF3_HUMAN		IRF3
E3 ubiquitin-protein ligase TRIP12	TRIPC_HUMAN		TRIP12
Pumilio homolog 1	PUM1_HUMAN		PUM1
Mediator of DNA damage checkpoint protein 1	MDC1_HUMAN		MDC 1.00
Clathrin interactor 1	EPN4_HUMAN		CLINT1
Structural maintenance of chromosomes protein 1A	SMC1A_HUMAN		SMC1A
Ribosomal RNA processing protein 1 homolog B	RRP1B_HUMAN		RRP1B
Disco-interacting protein 2 homolog A	DIP2A_HUMAN		DIP2A
Ribosome biogenesis protein BMS1 homolog	BMS1_HUMAN		BMS1
Ubiquitin carboxyl-terminal hydrolase 10	UBP10_HUMAN		USP10
LRP chaperone MESD	MESD_HUMAN		MESDC2
Neutral alpha-glucosidase AB	GANAB_HUMAN		GANAB
Raftlin	RFTN1_HUMAN		RFTN1
Serine/threonine-protein phosphatase 2A 56 kDa regulatory subunit delta isoform	2A5D_HUMAN		PPP2R5D
Delta(14)-sterol reductase LBR	LBR_HUMAN		LBR
Conserved oligomeric Golgi complex subunit 2	COG2_HUMAN		COG2
Major vault protein	MVP_HUMAN		MVP
Latent-transforming growth factor beta-binding protein 1	LTBP1_HUMAN		LTBP1
Golgin subfamily B member 1	GOGB1_HUMAN		GOLGB1
Caspase-8	CASP8_HUMAN		CASP8
Chromodomain-helicase-DNA-binding protein 4	CHD4_HUMAN		CHD4
LIM and SH3 domain protein 1	LASP1_HUMAN		LASP1
Prostaglandin reductase 1	PTGR1_HUMAN		PTGR1
Zinc finger protein 638	ZN638_HUMAN		ZNF638
Importin subunit beta-1	IMB1_HUMAN		KPNB1
Nucleolar and coiled-body phosphoprotein 1	NOLC1_HUMAN		NOLC1
Nuclear mitotic apparatus protein 1	NUMA1_HUMAN		NUMA1
Proteasome activator complex subunit 4	PSME4_HUMAN		PSME4
GTPase-activating protein and VPS9 domain-containing protein 1	GAPD1_HUMAN		GAPVD1
N-alpha-acetyltransferase 25, NatB auxiliary subunit	NAA25_HUMAN		NAA25
Condensin complex subunit 2	CND2_HUMAN		NCAPH
PCNA-associated factor	PAF15_HUMAN		KIAA0101
Signal peptidase complex subunit 2	SPCS2_HUMAN		SPCS2
ER membrane protein complex subunit 2	EMC2_HUMAN		EMC2
Pre-mRNA-splicing regulator WTAP	FL2D_HUMAN		WTAP
26S proteasome non-ATPase regulatory subunit 6	PSMD6_HUMAN		PSMD6
Mortality factor 4-like protein 2	MO4L2_HUMAN		MORF4L2
BRISC complex subunit Abraxas 2	ABRX2_HUMAN		FAM175B
Septin-2	SEPT2_HUMAN		SEPT2
Squamous cell carcinoma antigen recognized by T-cells 3	SART3_HUMAN		SART3
Condensin complex subunit 1	CND1_HUMAN		NCAPD2
Exosome complex component RRP42	EXOS7_HUMAN		EXOSC7
Arf-GAP with coiled-coil, ANK repeat and PH domain-containing protein 1	ACAP1_HUMAN		ACAP1
116 kDa U5 small nuclear ribonucleoprotein component	U5S1_HUMAN		EFTUD2
ADP-ribosylation factor-like protein 6-interacting protein 1	AR6P1_HUMAN		ARL6IP1
Rab3 GTPase-activating protein catalytic subunit	RB3GP_HUMAN		RAB3GAP1
Metal cation symporter ZIP14	S39AE_HUMAN		SLC39A14
Lysine--tRNA ligase	SYK_HUMAN		KARS
Ribosome biogenesis regulatory protein homolog	RRS1_HUMAN		RRS1
Eukaryotic translation initiation factor 4H	IF4H_HUMAN		EIF4H
Periostin	POSTN_HUMAN		POSTN
Peroxisomal acyl-coenzyme A oxidase 1	ACOX1_HUMAN		ACOX1
Early endosome antigen 1	EEA1_HUMAN		EEA1
Protein disulfide-isomerase A6	PDIA6_HUMAN		PDIA6
Platelet-activating factor acetylhydrolase IB subunit alpha1	PA1B3_HUMAN		PAFAH1B3
Procollagen C-endopeptidase enhancer 1	PCOC1_HUMAN		PCOLCE
Astrocytic phosphoprotein PEA-15	PEA15_HUMAN		PEA15
3-beta-hydroxysteroid-Delta(8),Delta(7)-isomerase	EBP_HUMAN		EBP
Phosphomevalonate kinase	PMVK_HUMAN		PMVK
Plectin	PLEC_HUMAN		PLEC
Pericentriolar material 1 protein	PCM1_HUMAN		PCM1
Inorganic pyrophosphatase	IPYR_HUMAN		PPA1
Prostaglandin E synthase 3	TEBP_HUMAN		PTGES3
Non-POU domain-containing octamer-binding protein	NONO_HUMAN		NONO
Serine/threonine-protein phosphatase 2A activator	PTPA_HUMAN		PPP2R4
Receptor-type tyrosine-protein phosphatase kappa	PTPRK_HUMAN		PTPRK
Periodic tryptophan protein 2 homolog	PWP2_HUMAN		PWP2
Rab GTPase-binding effector protein 1	RABE1_HUMAN		RABEP1
Ras-related protein Rab-35	RAB35_HUMAN		RAB35
RNA-binding protein with serine-rich domain 1	RNPS1_HUMAN		RNPS1
Retinoblastoma-binding protein 5	RBBP5_HUMAN		RBBP5
Reticulocalbin-1	RCN1_HUMAN		RCN1
RalA-binding protein 1	RBP1_HUMAN		RALBP1
Lethal(2) giant larvae protein homolog 1	L2GL1_HUMAN		LLGL1
Transmembrane emp24 domain-containing protein 2	TMED2_HUMAN		TMED2
Poly(rC)-binding protein 1	PCBP1_HUMAN		PCBP1
Poly(rC)-binding protein 2	PCBP2_HUMAN		PCBP2

Elongin-C	ELOC_HUMAN		TCEB1
Elongin-B	ELOB_HUMAN		TCEB2
GTP-binding protein Rheb	RHEB_HUMAN		RHEB
Delta(24)-sterol reductase	DHC24_HUMAN		DHCR24
Splicing factor 3B subunit 3	SF3B3_HUMAN		SF3B3
Pumilio homolog 3	PUM3_HUMAN		KIAA0020
Disks large-associated protein 5	DLGP5_HUMAN		DLGAP5
Ras suppressor protein 1	RSU1_HUMAN		RSU1
Calponin-3	CNN3_HUMAN		CNN3
Ribosomal protein S6 kinase alpha-1	KS6A1_HUMAN		RPS6KA1
Scaffold attachment factor B1	SAFB1_HUMAN		SAFB
Splicing factor 3A subunit 2	SF3A2_HUMAN		SF3A2
RNA-binding motif, single-stranded-interacting protein 2	RBMS2_HUMAN		RBMS2
Protein phosphatase 1 regulatory subunit 7	PP1R7_HUMAN		PPP1R7
Protein transport protein Sec23A	SC23A_HUMAN		SEC23A
Protein transport protein Sec23B	SC23B_HUMAN		SEC23B
Splicing factor 3A subunit 1	SF3A1_HUMAN		SF3A1
Mediator of RNA polymerase II transcription subunit 22	MED22_HUMAN		MED22
Microtubule-associated protein RP/EB family member 2	MARE2_HUMAN		MAPRE2
Transforming growth factor-beta-induced protein ig-h3	BGH3_HUMAN		TGFBI
Translin	TSN_HUMAN		TSN
Splicing factor 1	SF01_HUMAN		SF1
Cdc42-interacting protein 4	CIP4_HUMAN		TRIP10
Thyroid receptor-interacting protein 11	TRIPB_HUMAN		TRIP11
Pachytene checkpoint protein 2 homolog	PCH2_HUMAN		TRIP13
2'-5'-oligoadenylate synthase-like protein	OASL_HUMAN		OASL
Thyroid receptor-interacting protein 6	TRIP6_HUMAN		TRIP6
Tyrosine-protein phosphatase non-receptor type 14	PTN14_HUMAN		PTPN14
Microtubule-associated protein RP/EB family member 1	MARE1_HUMAN		MAPRE1
ELAV-like protein 1	ELAV1_HUMAN		ELAVL1
Sterol-4-alpha-carboxylate 3-dehydrogenase, decarboxylating	NSDHL_HUMAN		NSDHL
Myosin light chain kinase, smooth muscle	MYLK_HUMAN		MYLK
Neutral amino acid transporter B(0)	AAAT_HUMAN		SLC1A5
Mitochondrial import receptor subunit TOM34	TOM34_HUMAN		TOMM34
Mothers against decapentaplegic homolog 2	SMAD2_HUMAN		SMAD2
Tubulin-specific chaperone E	TBCE_HUMAN		TBCE
Tubulin-specific chaperone C	TBCC_HUMAN		TBCC
Ubiquitin-conjugating enzyme E2 variant 2	UB2V2_HUMAN		UBE2V2
Syntaxin-binding protein 2	STXB2_HUMAN		STXBP2
Vesicle-associated membrane protein 3	VAMP3_HUMAN		VAMP3
NEDD8	NEDD8_HUMAN		NEDD8
Ras-related protein Rab-11B	RB11B_HUMAN		RAB11B
Histone-lysine N-methyltransferase EZH2	EZH2_HUMAN		EZH2
Zyxin	ZYX_HUMAN		ZYX
Septin-7	SEPT7_HUMAN		SEPT7
Proteasomal ubiquitin receptor ADRM1	ADRM1_HUMAN		ADRM1
Coiled-coil domain-containing protein 6	CCDC6_HUMAN		CCDC6
UDP-N-acetylhexosamine pyrophosphorylase	UAP1_HUMAN		UAP1
Insulin-like growth factor-binding protein 7	IBP7_HUMAN		IGFBP7
Laminin subunit alpha-4	LAMA4_HUMAN		LAMA4
Exostosin-1	EXT1_HUMAN		EXT1
26S proteasome non-ATPase regulatory subunit 5	PSMD5_HUMAN		PSMD5
Serine/threonine-protein kinase N1	PKN1_HUMAN		PKN1
Serine/threonine-protein kinase N2	PKN2_HUMAN		PKN2
Cysteine and glycine-rich protein 2	CSRP2_HUMAN		CSRP2
DNA damage-binding protein 1	DDB1_HUMAN		DDB1
Mitogen-activated protein kinase 14	MK14_HUMAN		MAPK14
Hsp90 co-chaperone Cdc37	CDC37_HUMAN		CDC37
Dihydropyrimidinase-related protein 2	DPYL2_HUMAN		DPYSL2
Histone-binding protein RBBP7	RBBP7_HUMAN		RBBP7
Extracellular matrix protein 1	ECM1_HUMAN		ECM1
Serine/arginine-rich splicing factor 7	SRSF7_HUMAN		SRSF7
Cleavage and polyadenylation specificity factor subunit 6	CPSF6_HUMAN		CPSF6
Drebrin	DREB_HUMAN		DBN1
Fascin	FSCN1_HUMAN		FSCN1
Gamma-interferon-inducible protein 16	IF16_HUMAN		IFI16
2,4-dienoyl-CoA reductase [(3E)-enoyl-CoA-producing], mitochondrial	DECR_HUMAN		DECR1
Alpha-mannosidase 2	MA2A1_HUMAN		MAN2A1
NADH dehydrogenase [ubiquinone] 1 alpha subcomplex subunit 5	NDUA5_HUMAN		NDUFA5
Thiosulfate sulfurtransferase	THTR_HUMAN		TST
Ubiquitin-conjugating enzyme E2 S	UBE2S_HUMAN		UBE2S
Guanylate kinase	KGUA_HUMAN		GUK1
Hydroxyacylglutathione hydrolase, mitochondrial	GLO2_HUMAN		HAGH
NADH dehydrogenase [ubiquinone] 1 alpha subcomplex subunit 9, mitochondrial	NDUA9_HUMAN		NDUFA9
Phosphoenolpyruvate carboxykinase [GTP], mitochondrial	PCKGM_HUMAN		PCK2
Uridine phosphorylase 1	UPP1_HUMAN		UPP1
Hydroxyacyl-coenzyme A dehydrogenase, mitochondrial	HCDH_HUMAN		HADH
Lanosterol 14-alpha demethylase	CP51A_HUMAN		CYP51A1
UTP--glucose-1-phosphate uridylyltransferase	UGPA_HUMAN		UGP2
Thioredoxin reductase 1, cytoplasmic	TRXR1_HUMAN		TXNRD1
MICOS complex subunit MIC60	MIC60_HUMAN		IMMT
Heterogeneous nuclear ribonucleoprotein U-like protein 2	HNRL2_HUMAN		HNRNPUL2
Inverted formin-2	INF2_HUMAN		INF2
Sister chromatid cohesion protein PDS5 homolog A	PDS5A_HUMAN		PDS5A
AP2-associated protein kinase 1	AAK1_HUMAN		AAK1
WASH complex subunit 4	WASC4_HUMAN		KIAA1033
DNA excision repair protein ERCC-6-like	ERC6L_HUMAN		ERCC6L
Pre-rRNA-processing protein TSR1 homolog	TSR1_HUMAN		TSR1
Transcriptional regulator QRICH1	QRIC1_HUMAN		QRICH1
WD40 repeat-containing protein SMU1	SMU1_HUMAN		SMU1
Autophagy-related protein 2 homolog A	ATG2A_HUMAN		ATG2A
Hereditary hemochromatosis protein	HFE_HUMAN		HFE
Leucine-rich repeat flightless-interacting protein 1	LRRF1_HUMAN		LRRFIP1
Prolyl 3-hydroxylase 1	P3H1_HUMAN		LEPRE1
MAP7 domain-containing protein 1	MA7D1_HUMAN		MAP7D1
UDP-N-acetylhexosamine pyrophosphorylase-like protein 1	UAP1L_HUMAN		UAP1L1
Triokinase/FMN cyclase	TKFC_HUMAN		DAK
Protein LSM12	LSM12_HUMAN		LSM 12.00
Mitochondrial 10-formyltetrahydrofolate dehydrogenase	AL1L2_HUMAN		ALDH1L2
Girdin	GRDN_HUMAN		CCDC88A
Mitochondrial import inner membrane translocase subunit TIM50	TIM50_HUMAN		TIMM50
Cytokine-like nuclear factor N-PAC	GLYR1_HUMAN		GLYR1
TBC1 domain family member 10B	TB10B_HUMAN		TBC1D10B
Anoctamin-6	ANO6_HUMAN		ANO6
Filamin A-interacting protein 1-like	FIL1L_HUMAN		FILIP1L
GRIP1-associated protein 1	GRAP1_HUMAN		GRIPAP1
Transmembrane protein 119	TM119_HUMAN		TMEM119
Protein MIX23	MIX23_HUMAN		CCDC58
Protein FAM98B	FA98B_HUMAN		FAM98B
Rho GTPase-activating protein 29	RHG29_HUMAN		ARHGAP29
Programmed cell death protein 4	PDCD4_HUMAN		PDCD4
Fibronectin type III domain-containing protein 3B	FND3B_HUMAN		FNDC3B
Centrosomal protein of 55 kDa	CEP55_HUMAN		CEP55
Nuclear cap-binding protein subunit 3	NCBP3_HUMAN		C17orf85
Very-long-chain 3-oxoacyl-CoA reductase	DHB12_HUMAN		HSD17B12
U4/U6.U5 tri-snRNP-associated protein 2	SNUT2_HUMAN		USP39
Endoribonuclease LACTB2	LACB2_HUMAN		LACTB2
Pyrroline-5-carboxylate reductase 3	P5CR3_HUMAN		PYCRL
Plasma membrane ascorbate-dependent reductase CYBRD1	CYBR1_HUMAN		CYBRD1
Coiled-coil domain-containing protein 93	CCD93_HUMAN		CCDC93
Putative heat shock protein HSP 90-alpha A4	HS904_HUMAN		HSP90AA4P
Protein YIF1B	YIF1B_HUMAN		YIF1B
DBIRD complex subunit ZNF326	ZN326_HUMAN		ZNF326
Nucleolar MIF4G domain-containing protein 1	NOM1_HUMAN		NOM1
Probable E3 ubiquitin-protein ligase HERC4	HERC4_HUMAN		HERC4
Transcription elongation factor A protein-like 5	TCAL5_HUMAN	sp|Q969E4|TCAL3_HUMAN	TCEAL5
Serine/threonine-protein phosphatase 6 regulatory subunit 3	PP6R3_HUMAN		PPP6R3
Rab-like protein 3	RABL3_HUMAN		RABL3
Probable glutamate--tRNA ligase, mitochondrial	SYEM_HUMAN		EARS2

Transport and Golgi organization protein 1 homolog	TGO1_HUMAN		MIA3
Presequence protease, mitochondrial	PREP_HUMAN		PITRM1
Protein PRRC2B	PRC2B_HUMAN		PRRC2B
RRP12-like protein	RRP12_HUMAN		RRP12
Torsin-1A-interacting protein 1	TOIP1_HUMAN		TOR1AIP1
Alanine--tRNA ligase, mitochondrial	SYAM_HUMAN		AARS2
PCI domain-containing protein 2	PCID2_HUMAN		PCID2
Collectin-12	COL12_HUMAN		COLEC12
Cytospin-B	CYTSB_HUMAN		SPECC1
WD repeat domain phosphoinositide-interacting protein 3	WIPI3_HUMAN		WDR45B
Exosome complex component MTR3	EXOS6_HUMAN		EXOSC6
Nucleoporin NUP188	NU188_HUMAN		NUP188
Heterochromatin protein 1-binding protein 3	HP1B3_HUMAN		HP1BP3
Centrosomal protein of 170 kDa	CE170_HUMAN		CEP170
Polynucleotide 5'-hydroxyl-kinase NOL9	NOL9_HUMAN		NOL9
Formin-binding protein 1-like	FBP1L_HUMAN		FNBP1L
FK506-binding protein 15	FKB15_HUMAN		FKBP15
Zinc finger CCCH domain-containing protein 13	ZC3HD_HUMAN		ZC3H13
E3 ubiquitin-protein ligase UBR4	UBR4_HUMAN		UBR4
Sickle tail protein homolog	SKT_HUMAN		KIAA1217
Ubiquitin-associated protein 2	UBAP2_HUMAN		UBAP2
Queuosine 5'-phosphate N-glycosylase/hydrolase	QNG1_HUMAN		C9orf64
Keratinocyte proline-rich protein	KPRP_HUMAN		KPRP
RNA-binding protein 26	RBM26_HUMAN		RBM26
ATPase family AAA domain-containing protein 3B	ATD3B_HUMAN		ATAD3B
Protein wntless homolog	WLS_HUMAN		WLS
ATP synthase mitochondrial F1 complex assembly factor 1	ATPF1_HUMAN		ATPAF1
Histone H3-7	H37_HUMAN		HIST2H3PS2
5'-nucleotidase domain-containing protein 1	NT5D1_HUMAN		NT5DC1
Telomere-associated protein RIF1	RIF1_HUMAN		RIF1
Striatin-interacting protein 1	STRP1_HUMAN		STRIP1
E3 ubiquitin-protein ligase BRE1A	BRE1A_HUMAN		RNF20
BRO1 domain-containing protein BROX	BROX_HUMAN		BROX
Focadhesin	FOCAD_HUMAN		FOCAD
Proteasome adapter and scaffold protein ECM29	ECM29_HUMAN		ECM29
N-alpha-acetyltransferase 35, NatC auxiliary subunit	NAA35_HUMAN		NAA35
FHF complex subunit HOOK interacting protein 2A	FHI2A_HUMAN		FAM160B1
E3 ubiquitin-protein ligase RNF123	RN123_HUMAN		RNF123
CD276 antigen	CD276_HUMAN		CD276
E3 ubiquitin-protein ligase RNF213	RN213_HUMAN		RNF213
Tensin-2	TENS2_HUMAN		TNS2
KN motif and ankyrin repeat domain-containing protein 2	KANK2_HUMAN		KANK2
WASH complex subunit 2A	WAC2A_HUMAN		FAM21A
Protein FAM91A1	F91A1_HUMAN		FAM91A1
TBC1 domain family member 9B	TBC9B_HUMAN		TBC1D9B
Microtubule-associated protein 1S	MAP1S_HUMAN		MAP1S
ADP-ribosylation factor-like protein 6-interacting protein 4	AR6P4_HUMAN		ARL6IP4
U3 small nucleolar RNA-associated protein 25 homolog	UTP25_HUMAN		DIEXF
Tensin-3	TENS3_HUMAN		TNS3
Integrator complex subunit 3	INT3_HUMAN		INTS3
Rho GTPase-activating protein 17	RHG17_HUMAN		ARHGAP17
CWF19-like protein 1	C19L1_HUMAN		CWF19L1
Protein virilizer homolog	VIR_HUMAN		KIAA1429
NADH-cytochrome b5 reductase 2	NB5R2_HUMAN		CYB5R2
Zinc finger protein 787	ZN787_HUMAN		ZNF787
Atlastin-3	ATLA3_HUMAN		ATL3
Ribosomal protein uL30-like	RL7L_HUMAN		RPL7L1
Nucleoredoxin	NXN_HUMAN		NXN
Vasorin	VASN_HUMAN		VASN
Anamorsin	CPIN1_HUMAN		CIAPIN1
AN1-type zinc finger protein 6	ZFAN6_HUMAN		ZFAND6
THO complex subunit 7 homolog	THOC7_HUMAN		THOC7
Elongator complex protein 2	ELP2_HUMAN		ELP2
Dehydrogenase/reductase SDR family member 7B	DRS7B_HUMAN		DHRS7B
Twinfilin-2	TWF2_HUMAN		TWF2
2',5'-phosphodiesterase 12	PDE12_HUMAN		PDE12
Mitochondrial adenyl nucleotide antiporter SLC25A24	SCMC1_HUMAN		SLC25A24
Receptor expression-enhancing protein 3	REEP3_HUMAN		REEP3
All-trans-retinol 13,14-reductase	RETST_HUMAN		RETSAT
Transmembrane protein 214	TM214_HUMAN		TMEM214
3-hydroxyisobutyryl-CoA hydrolase, mitochondrial	HIBCH_HUMAN		HIBCH
Armadillo repeat-containing protein 6	ARMC6_HUMAN		ARMC6
Phostensin	PPR18_HUMAN		PPP1R18
Caveolae-associated protein 1	CAVN1_HUMAN		PTRF
Zinc finger CCHC domain-containing protein 8	ZCHC8_HUMAN		ZCCHC8
Parafibromin	CDC73_HUMAN		CDC73
Coiled-coil and C2 domain-containing protein 1A	C2D1A_HUMAN		CC2D1A
Enhancer of mRNA-decapping protein 4	EDC4_HUMAN		EDC4
Pre-mRNA-processing-splicing factor 8	PRP8_HUMAN		PRPF8
Tetratricopeptide repeat protein 27	TTC27_HUMAN		TTC27
Ribosomal protein eL22-like	RL22L_HUMAN		RPL22L1
RNA demethylase ALKBH5	ALKB5_HUMAN		ALKBH5
Pyridoxal-dependent decarboxylase domain-containing protein 1	PDXD1_HUMAN		PDXDC1
MTOR-associated protein MEAK7	MEAK7_HUMAN		TLDC1
RNA polymerase-associated protein CTR9 homolog	CTR9_HUMAN		CTR9
Inositol hexakisphosphate and diphosphoinositol-pentakisphosphate kinase 1	VIP1_HUMAN		PPIP5K1
Superkiller complex protein 3	SKI3_HUMAN		TTC37
Aspartate--tRNA ligase, mitochondrial	SYDM_HUMAN		DARS2
Neutral cholesterol ester hydrolase 1	NCEH1_HUMAN		NCEH1
Zinc finger CCCH domain-containing protein 14	ZC3HE_HUMAN		ZC3H14
Coiled-coil domain-containing protein 137	CC137_HUMAN		CCDC137
La-related protein 1	LARP1_HUMAN		LARP1
Rapamycin-insensitive companion of mTOR	RICTR_HUMAN		RICTOR
Monofunctional C1-tetrahydrofolate synthase, mitochondrial	C1TM_HUMAN		MTHFD1L
Pre-mRNA 3'-end-processing factor FIP1	FIP1_HUMAN		FIP1L1
Chondroitin sulfate proteoglycan 4	CSPG4_HUMAN		CSPG4
DBH-like monooxygenase protein 1	MOXD1_HUMAN		MOXD1
Transmembrane protein 205	TM205_HUMAN		TMEM205
Spliceosome-associated protein CWC27 homolog	CWC27_HUMAN		CWC27
Protein disulfide isomerase CRELD2	CREL2_HUMAN		CRELD2
Collagen and calcium-binding EGF domain-containing protein 1	CCBE1_HUMAN		CCBE1
WD repeat-containing protein 82	WDR82_HUMAN		WDR82
MICOS complex subunit MIC27	MIC27_HUMAN		APOOL
ADAMTS-like protein 4	ATL4_HUMAN		ADAMTSL4
Myosin phosphatase Rho-interacting protein	MPRIP_HUMAN		MPRIP
Nicotinate phosphoribosyltransferase	PNCB_HUMAN		NAPRT
Dynamin-binding protein	DNMBP_HUMAN		DNMBP
GRB10-interacting GYF protein 2	GGYF2_HUMAN		GIGYF2
CD109 antigen	CD109_HUMAN		CD109
Transmembrane protein 64	TMM64_HUMAN		TMEM64
Hydroxysteroid dehydrogenase-like protein 2	HSDL2_HUMAN		HSDL2
Kynurenine--oxoglutarate transaminase 3	KAT3_HUMAN		CCBL2
NF-X1-type zinc finger protein NFXL1	NFXL1_HUMAN		NFXL1
Sulfhydryl oxidase 2	QSOX2_HUMAN		QSOX2
Rho family-interacting cell polarization regulator 1	RIPR1_HUMAN		FAM65A
Uncharacterized protein FLJ45252	YJ005_HUMAN		
Protein O-mannosyl-transferase TMTC3	TMTC3_HUMAN		TMTC3
Ras-associated and pleckstrin homology domains-containing protein 1	RAPH1_HUMAN		RAPH1
Inhibitor of nuclear factor kappa-B kinase-interacting protein	IKIP_HUMAN		IKBIP
Histone H3.2	H32_HUMAN		HIST2H3A
La-related protein 4	LARP4_HUMAN		LARP4
A disintegrin and metalloproteinase with thrombospondin motifs 13	ATS13_HUMAN		ADAMTS13
Butyrophilin subfamily 2 member A1	BT2A1_HUMAN		BTN2A1
Transcription elongation factor SPT6	SPT6H_HUMAN		SUPT6H
Staphylococcal nuclease domain-containing protein 1	SND1_HUMAN		SND1
Serine/threonine-protein kinase MARK2	MARK2_HUMAN		MARK2
Probable ATP-dependent RNA helicase DDX46	DDX46_HUMAN		DDX46
tRNA methyltransferase 10 homolog C	TM10C_HUMAN		TRMT10C
eIF5-mimic protein 2	5MP2_HUMAN		BZW1

ATP-dependent RNA helicase DHX30	DHX30_HUMAN		DHX30
Eukaryotic translation initiation factor 3 subunit M	EIF3M_HUMAN		EIF3M
7SK snRNA methylphosphate capping enzyme	MEPCE_HUMAN		MEPCE
Ras-related GTP-binding protein A	RRAGA_HUMAN		RRAGA
Cytoplasmic FMR1-interacting protein 1	CYFP1_HUMAN		CYFIP1
COP9 signalosome complex subunit 6	CSN6_HUMAN		COPS6
Lysophosphatidylcholine acyltransferase 2	PCAT2_HUMAN		LPCAT2
E3 ubiquitin-protein transferase MAEA	MAEA_HUMAN		MAEA
Lysine-specific demethylase 3B	KDM3B_HUMAN		KDM3B
Charged multivesicular body protein 1b	CHM1B_HUMAN		CHMP1B
PHD finger-like domain-containing protein 5A	PHF5A_HUMAN		PHF5A
Zinc finger CCCH-type antiviral protein 1	ZCCHV_HUMAN		ZC3HAV1
Elongation factor-like GTPase 1	EFL1_HUMAN		EFTUD1
Nucleoporin p54	NUP54_HUMAN		NUP54
Pogo transposable element with ZNF domain	POGZ_HUMAN		POGZ
Protein MON2 homolog	MON2_HUMAN		MON2
Keratin, type I cytoskeletal 27	K1C27_HUMAN		KRT27
FMR1-interacting protein NUFIP2	NUFP2_HUMAN		NUFIP2
SUZ domain-containing protein 1	SZRD1_HUMAN		SZRD1
Mitochondrial antiviral-signaling protein	MAVS_HUMAN		MAVS
CLIP-associating protein 1	CLAP1_HUMAN		CLASP1
Protein O-glucosyltransferase 3	PLGT3_HUMAN		KDELC2
LIM and senescent cell antigen-like-containing domain protein 2	LIMS2_HUMAN		LIMS2
HEAT repeat-containing protein 3	HEAT3_HUMAN		HEATR3
UPF0669 protein C6orf120	CF120_HUMAN		C6orf120
Hepatoma-derived growth factor-related protein 2	HDGR2_HUMAN		HDGFRP2
L-xylulose reductase	DCXR_HUMAN		DCXR
Golgin subfamily A member 7	GOGA7_HUMAN		GOLGA7
Vomeronasal type-1 receptor 5	VN1R5_HUMAN		VN1R5
Arpin	ARPIN_HUMAN		ARPIN
E3 ubiquitin-protein ligase HUWE1	HUWE1_HUMAN		HUWE1
YTH domain-containing family protein 3	YTHD3_HUMAN		YTHDF3
Transmembrane emp24 domain-containing protein 4	TMED4_HUMAN		TMED4
COX assembly mitochondrial protein homolog	COXM1_HUMAN		CMC1
Schlafen family member 11	SLN11_HUMAN		SLFN11
Pleckstrin homology-like domain family B member 2	PHLB2_HUMAN		PHLDB2
Glutaredoxin-related protein 5, mitochondrial	GLRX5_HUMAN		GLRX5
Type 1 phosphatidylinositol 4,5-bisphosphate 4-phosphatase	PP4P1_HUMAN		TMEM55B
Protein PAT1 homolog 1	PATL1_HUMAN		PATL1
Dipeptidyl peptidase 9	DPP9_HUMAN		DPP9
Polyadenylate-binding protein 2	PABP2_HUMAN		PABPN1
Protein polybromo-1	PB1_HUMAN		PBRM1
Protein LYRIC	LYRIC_HUMAN		MTDH
Kinectin	KTN1_HUMAN		KTN1
Ubiquitin carboxyl-terminal hydrolase 48	UBP48_HUMAN		USP48
Fermitin family homolog 3	URP2_HUMAN		FERMT3
Acetoacetyl-CoA synthetase	AACS_HUMAN		AACS
Leucine zipper protein 1	LUZP1_HUMAN		LUZP1
THO complex subunit 4	THOC4_HUMAN		ALYREF
Ras GTPase-activating-like protein IQGAP3	IQGA3_HUMAN		IQGAP3
Zinc finger CCCH domain-containing protein 18	ZCH18_HUMAN		ZC3H18
Cullin-associated NEDD8-dissociated protein 1	CAND1_HUMAN		CAND1
Protein Hook homolog 3	HOOK3_HUMAN		HOOK3
Testis-specific Y-encoded-like protein 5	TSYL5_HUMAN		TSPYL5
Liprin-beta-1	LIPB1_HUMAN		PPFIBP1
Nuclear receptor 2C2-associated protein	NR2CA_HUMAN		NR2C2AP
Coiled-coil domain-containing protein 25	CCD25_HUMAN		CCDC25
Stimulator of interferon genes protein	STING_HUMAN		TMEM173
Histone-arginine methyltransferase CARM1	CARM1_HUMAN		CARM1
Deaminated glutathione amidase	NIT1_HUMAN		NIT1
Condensin-2 complex subunit G2	CNDG2_HUMAN		NCAPG2
Ankyrin repeat and LEM domain-containing protein 2	ANKL2_HUMAN		ANKLE2
ATP-dependent RNA helicase DDX42	DDX42_HUMAN		DDX42
Spermatogenesis-associated serine-rich protein 2	SPAS2_HUMAN		SPATS2
Dynein axonemal assembly factor 5	DAAF5_HUMAN		DNAAF5
Syntaxin-12	STX12_HUMAN		STX12
G-protein-signaling modulator 1	GPSM1_HUMAN		GPSM1
Ras-related protein Rab-43	RAB43_HUMAN		RAB43
Hornerin	HORN_HUMAN		HRNR
Interferon regulatory factor 2-binding protein 1	I2BP1_HUMAN		IRF2BP1
ELKS/Rab6-interacting/CAST family member 1	RB6I2_HUMAN		ERC1
Trafficking protein particle complex subunit 5	TPPC5_HUMAN		TRAPPC5
5'-3' exonuclease PLD3	PLD3_HUMAN		PLD3
3'-5' exoribonuclease 1	ERI1_HUMAN		ERI1
Protein AHNAK2	AHNK2_HUMAN		AHNAK2
Prolyl 3-hydroxylase 3	P3H3_HUMAN		LEPREL2
Coiled-coil domain-containing protein 50	CCD50_HUMAN		CCDC50
Choline transporter-like protein 2	CTL2_HUMAN		SLC44A2
Inositol 1,4,5-trisphosphate receptor-interacting protein	IPRI_HUMAN		ITPRIP
MAP7 domain-containing protein 3	MA7D3_HUMAN		MAP7D3
Protein NOXP20	NXP20_HUMAN		FAM114A1
Zinc finger CCCH domain-containing protein 7A	Z3H7A_HUMAN		ZC3H7A
PHD finger protein 6	PHF6_HUMAN		PHF6
Rho GTPase-activating protein 12	RHG12_HUMAN		ARHGAP12
Calcium homeostasis endoplasmic reticulum protein	CHERP_HUMAN		CHERP
Ankyrin repeat and KH domain-containing protein 1	ANKH1_HUMAN		ANKHD1
SURP and G-patch domain-containing protein 1	SUGP1_HUMAN		SUGP1
SURP and G-patch domain-containing protein 2	SUGP2_HUMAN		SUGP2
Cell division cycle and apoptosis regulator protein 1	CCAR1_HUMAN		CCAR1
DnaJ homolog subfamily C member 10	DJC10_HUMAN		DNAJC10
Negative elongation factor C/D	NELFD_HUMAN		NELFCD
Mitochondrial Rho GTPase 2	MIRO2_HUMAN		RHOT2
Uncharacterized protein C9orf40	CI040_HUMAN		C9orf40
RNA-binding protein 12B	RB12B_HUMAN		RBM12B
Patatin-like phospholipase domain-containing protein 6	PLPL6_HUMAN		PNPLA6
Ribonucleoprotein PTB-binding 1	RAVR1_HUMAN		RAVER1
pre-rRNA 2'-O-ribose RNA methyltransferase FTSJ3	SPB1_HUMAN		FTSJ3
Serine/arginine repetitive matrix protein 1	SRRM1_HUMAN		SRRM1
Exocyst complex component 8	EXOC8_HUMAN		EXOC8
Uncharacterized protein KIAA2013	K2013_HUMAN		KIAA2013
Calcium uptake protein 2, mitochondrial	MICU2_HUMAN		MICU2
Ankyrin repeat domain-containing protein 13A	AN13A_HUMAN		ANKRD13A
Pseudouridylate synthase RPUSD2	RUSD2_HUMAN		RPUSD2
Aldehyde dehydrogenase family 16 member A1	A16A1_HUMAN		ALDH16A1
Proline-, glutamic acid- and leucine-rich protein 1	PELP1_HUMAN		PELP1
Abl interactor 1	ABI1_HUMAN		ABI1
Spartin	SPART_HUMAN		SPG20
Regulatory-associated protein of mTOR	RPTOR_HUMAN		RPTOR
Cell cycle and apoptosis regulator protein 2	CCAR2_HUMAN		CCAR2
NADH dehydrogenase [ubiquinone] 1 alpha subcomplex assembly factor 2	NDUF2_HUMAN		NDUFAF2
Nuclear pore complex protein Nup93	NUP93_HUMAN		NUP93
Cap-specific mRNA (nucleoside-2'-O-)-methyltransferase 1	CMTR1_HUMAN		CMTR1
Leucine-rich repeat-containing protein 47	LRC47_HUMAN		LRRC47
GH3 domain-containing protein	GHDC_HUMAN		GHDC
Lysophosphatidylserine lipase ABHD12	ABD12_HUMAN		ABHD12
Latent-transforming growth factor beta-binding protein 4	LTBP4_HUMAN		LTBP4
Rho GTPase-activating protein 18	RHG18_HUMAN		ARHGAP18
Activating signal cointegrator 1 complex subunit 3	ASCC3_HUMAN		ASCC3
EH domain-binding protein 1-like protein 1	EH1L1_HUMAN		EHBP1L1
MICAL-like protein 1	MILK1_HUMAN		MICALL1
Cohesin subunit SA-2	STAG2_HUMAN		STAG2
Secreted frizzled-related protein 1	SFRP1_HUMAN		SFRP1
Misshapen-like kinase 1	MINK1_HUMAN		MINK1
ER membrane protein complex subunit 5	EMC5_HUMAN		MMGT1
Prolyl 3-hydroxylase OGFOD1	OGFD1_HUMAN		OGFOD1
Actin filament-associated protein 1	AFAP1_HUMAN		AFAP1
Arrestin domain-containing protein 1	ARRD1_HUMAN		ARRDC1
CDGSH iron-sulfur domain-containing protein 2	CISD2_HUMAN		CISD2
Tetratricopeptide repeat protein 9C	TTC9C_HUMAN		TTC9C

Cleavage and polyadenylation specificity factor subunit 7	CPSF7_HUMAN		CPSF7
ADAMTS-like protein 1	ATL1_HUMAN		ADAMTSL1
ADP-ribosylation factor GTPase-activating protein 2	ARFG2_HUMAN		ARFGAP2
Deubiquitinase OTUD6B	OTU6B_HUMAN		OTUD6B
eEF1A lysine and N-terminal methyltransferase	EFNMT_HUMAN		METTL13
ADP-ribosylation factor GTPase-activating protein 1	ARFG1_HUMAN		ARFGAP1
ER membrane protein complex subunit 1	EMC1_HUMAN		EMC1
RNA polymerase II-associated factor 1 homolog	PAF1_HUMAN		PAF1
Protein enabled homolog	ENAH_HUMAN		ENAH
ATPase family gene 2 protein homolog A	AFG2A_HUMAN		SPATA5
NHL repeat-containing protein 2	NHLC2_HUMAN		NHLRC2
Equilibrative nucleobase transporter 1	S43A3_HUMAN		SLC43A3
Golgi membrane protein 1	GOLM1_HUMAN		GOLM1
Procollagen galactosyltransferase 1	GT251_HUMAN		COLGALT1
Inactive C-alpha-formylglycine-generating enzyme 2	SUMF2_HUMAN		SUMF2
Protein O-glucosyltransferase 1	PGLT1_HUMAN		POGLUT1
Ubiquitin-associated domain-containing protein 2	UBAC2_HUMAN		UBAC2
Estradiol 17-beta-dehydrogenase 11	DHB11_HUMAN		HSD17B11
Thioredoxin domain-containing protein 5	TXND5_HUMAN		TXNDC5
Kinetochore protein Spc24	SPC24_HUMAN		SPC24
Outer mitochondrial transmembrane helix translocase	ATAD1_HUMAN		ATAD1
Saccharopine dehydrogenase-like oxidoreductase	SCPDL_HUMAN		SCCPDH
SERPINE1 mRNA-binding protein 1	SERB1_HUMAN		SERBP1
LEM domain-containing protein 2	LEMD2_HUMAN		LEMD2
Protein FAM98A	FA98A_HUMAN		FAM98A
Carbohydrate sulfotransferase 14	CHSTE_HUMAN		CHST14
Pyruvate dehydrogenase phosphatase regulatory subunit, mitochondrial	PDPR_HUMAN		PDPR
NAD(P)H-hydrate epimerase	NNRE_HUMAN		APOA1BP
ATP-binding cassette sub-family F member 1	ABCF1_HUMAN		ABCF1
Calcium uniporter protein, mitochondrial	MCU_HUMAN		MCU
SHC SH2 domain-binding protein 1	SHCBP_HUMAN		SHCBP1
Zinc transporter 7	ZNT7_HUMAN		SLC30A7
Periphilin-1	PPHLN_HUMAN		PPHLN1
Vacuolar protein sorting-associated protein 37A	VP37A_HUMAN		VPS37A
Lysophosphatidylcholine acyltransferase 1	PCAT1_HUMAN		LPCAT1
Biorientation of chromosomes in cell division protein 1-like 1	BD1L1_HUMAN		BOD1L1
Nucleoporin Nup43	NUP43_HUMAN		NUP43
Nucleoporin Nup37	NUP37_HUMAN		NUP37
Nucleoporin NUP35	NUP35_HUMAN		NUP35
Retinoic acid-induced protein 3	RAI3_HUMAN		GPRC5A
Torsin-1A-interacting protein 2	TOIP2_HUMAN		TOR1AIP2
Protein ABHD11	ABHDB_HUMAN		ABHD11
N-acylneuraminate cytidylyltransferase	NEUA_HUMAN		CMAS
Syntaxin-binding protein 6	STXB6_HUMAN		STXBP6
Putative phospholipase B-like 2	PLBL2_HUMAN		PLBD2
ATP-dependent RNA helicase DDX55	DDX55_HUMAN		DDX55
Protein NEDD1	NEDD1_HUMAN		NEDD1
THO complex subunit 2	THOC2_HUMAN		THOC2
WD repeat-containing protein 36	WDR36_HUMAN		WDR36
Overexpressed in colon carcinoma 1 protein	OCC1_HUMAN		OCC1
SWI/SNF complex subunit SMARCC2	SMRC2_HUMAN		SMARCC2
Nuclear protein localization protein 4 homolog	NPL4_HUMAN		NPLOC4
F-box only protein 30	FBX30_HUMAN		FBXO30
Adenosine 3'-phospho 5'-phosphosulfate transporter 1	S35B2_HUMAN		SLC35B2
Golgin subfamily A member 5	GOGA5_HUMAN		GOLGA5
NEDD8-activating enzyme E1 catalytic subunit	UBA3_HUMAN		UBA3
TBC1 domain family member 15	TBC15_HUMAN		TBC1D15
Retinol dehydrogenase 11	RDH11_HUMAN		RDH11
Protein CIP2A	CIP2A_HUMAN		KIAA1524
Dolichyl-diphosphooligosaccharide--protein glycosyltransferase subunit STT3B	STT3B_HUMAN		STT3B
Polyribonucleotide nucleotidyltransferase 1, mitochondrial	PNPT1_HUMAN		PNPT1
Serine/threonine-protein kinase Nek9	NEK9_HUMAN		NEK9
E3 ubiquitin-protein ligase DTX3L	DTX3L_HUMAN		DTX3L
ATP-dependent RNA helicase DDX54	DDX54_HUMAN		DDX54
Ribosome biogenesis protein BRX1 homolog	BRX1_HUMAN		BRIX1
Serine/threonine-protein kinase Nek7	NEK7_HUMAN		NEK7
[F-actin]-monooxygenase MICAL1	MICA1_HUMAN		MICAL1
Probable glutathione peroxidase 8	GPX8_HUMAN		GPX8
Gem-associated protein 5	GEMI5_HUMAN		GEMIN5
Importin-4	IPO4_HUMAN		IPO4
Serine/threonine-protein phosphatase 4 regulatory subunit 1	PP4R1_HUMAN		PPP4R1
Histone-lysine N-methyltransferase SETD7	SETD7_HUMAN		SETD7
Nucleolar complex protein 3 homolog	NOC3L_HUMAN		NOC3L
Scavenger receptor class B member 1	SCRB1_HUMAN		SCARB1
Zinc finger CCCH domain-containing protein 15	ZC3HF_HUMAN		ZC3H15
Peptidyl-prolyl cis-trans isomerase-like 4	PPIL4_HUMAN		PPIL4
RelA-associated inhibitor	IASPP_HUMAN		PPP1R13L
Histone deacetylase 7	HDAC7_HUMAN		HDAC7
Cell migration-inducing and hyaluronan-binding protein	CEMIP_HUMAN		CEMIP
Nuclear pore complex protein Nup133	NU133_HUMAN		NUP133
Programmed cell death 6-interacting protein	PDC6I_HUMAN		PDCD6IP
Filamin-binding LIM protein 1	FBLI1_HUMAN		FBLIM1
Protein BRICK1	BRK1_HUMAN		BRK1
Protein THEM6	THEM6_HUMAN		THEM6
Pleckstrin homology-like domain family A member 1	PHLA1_HUMAN		PHLDA1
MIT domain-containing protein 1	MITD1_HUMAN		MITD1
RNA polymerase-associated protein LEO1	LEO1_HUMAN		LEO1
NudC domain-containing protein 2	NUDC2_HUMAN		NUDCD2
Sec1 family domain-containing protein 1	SCFD1_HUMAN		SCFD1
Ubiquitin-like domain-containing CTD phosphatase 1	UBCP1_HUMAN		UBLCP1
PEST proteolytic signal-containing nuclear protein	PCNP_HUMAN		PCNP
LIM domain only protein 7	LMO7_HUMAN		LMO7
Choline transporter-like protein 1	CTL1_HUMAN		SLC44A1
Cytoskeleton-associated protein 2	CKAP2_HUMAN		CKAP2
Ataxin-2-like protein	ATX2L_HUMAN		ATXN2L
U4/U6 small nuclear ribonucleoprotein Prp31	PRP31_HUMAN		PRPF31
Negative elongation factor B	NELFB_HUMAN		NELFB
Palladin	PALLD_HUMAN		PALLD
Stonin-2	STON2_HUMAN		STON2
Paraspeckle component 1	PSPC1_HUMAN		PSPC1
Nesprin-2	SYNE2_HUMAN		SYNE2
Transcriptional repressor p66-beta	P66B_HUMAN		GATAD2B
DnaJ homolog subfamily C member 9	DNJC9_HUMAN		DNAJC9
Beta-catenin-like protein 1	CTBL1_HUMAN		CTNNBL1
Protein ELYS	ELYS_HUMAN		AHCTF1
Immunity-related GTPase family Q protein	IRGQ_HUMAN		IRGQ
ATP-dependent RNA helicase DDX1	DDX1_HUMAN		DDX1
Zinc transporter SLC39A7	S39A7_HUMAN		SLC39A7
Protein FAM3C	FAM3C_HUMAN		FAM3C
Histone H1.10	H1X_HUMAN		H1FX
Proteasome inhibitor PI31 subunit	PSMF1_HUMAN		PSMF1
Golgi-specific brefeldin A-resistance guanine nucleotide exchange factor 1	GBF1_HUMAN		GBF1
RNA polymerase-associated protein RTF1 homolog	RTF1_HUMAN		RTF1
Nicastrin	NICA_HUMAN		NCSTN
Transmembrane 9 superfamily member 4	TM9S4_HUMAN		TM9SF4
Small ribosomal subunit protein mS27	RT27_HUMAN		MRPS27
UBX domain-containing protein 4	UBXN4_HUMAN		UBXN4
PHD finger protein 3	PHF3_HUMAN		PHF3
Protein NDRG1	NDRG1_HUMAN		NDRG1
Heat shock protein 105 kDa	HS105_HUMAN		HSPH1
Septin-8	SEPT8_HUMAN		SEPT8
CCR4-NOT transcription complex subunit 9	CNOT9_HUMAN		RQCD1
TBC1 domain family member 5	TBCD5_HUMAN		TBC1D5
Unconventional myosin-XVIIIa	MY18A_HUMAN		MYO18A
La-related protein 4B	LAR4B_HUMAN		LARP4B
Stalled ribosome sensor GCN1	GCN1_HUMAN		GCN1L1
Pre-mRNA-splicing factor ATP-dependent RNA helicase PRP16	PRP16_HUMAN		DHX38
Nuclear pore complex protein Nup205	NU205_HUMAN		NUP205
Peroxidasin homolog	PXDN_HUMAN		PXDN

Lysophosphatidic acid receptor 1	LPAR1_HUMAN		LPAR1
Acidic leucine-rich nuclear phosphoprotein 32 family member B	AN32B_HUMAN		ANP32B
Nectin-2	NECT2_HUMAN		PVRL2
Protein TFG	TFG_HUMAN		TFG
Serine protease HTRA1	HTRA1_HUMAN		HTRA1
Actin-related protein 2/3 complex subunit 1A	ARC1A_HUMAN		ARPC1A
Histone deacetylase 2	HDAC2_HUMAN		HDAC2
Zinc finger protein ubi-d4	REQU_HUMAN		DPF2
CREB-binding protein	CBP_HUMAN		CREBBP
Symplekin	SYMPK_HUMAN		SYMPK
TATA-binding protein-associated factor 2N	RBP56_HUMAN		TAF15
Hyaluronan synthase 2	HYAS2_HUMAN		HAS2
Gamma-glutamyl hydrolase	GGH_HUMAN		GGH
Probable ATP-dependent RNA helicase DDX17	DDX17_HUMAN		DDX17
DNA repair protein RAD50	RAD50_HUMAN		RAD50
CUGBP Elav-like family member 1	CELF1_HUMAN		CELF1
Osteoclast-stimulating factor 1	OSTF1_HUMAN		OSTF1
Rho guanine nucleotide exchange factor 1	ARHG1_HUMAN		ARHGEF1
Ubiquitin recognition factor in ER-associated degradation protein 1	UFD1_HUMAN		UFD1L
Golgi apparatus protein 1	GSLG1_HUMAN		GLG1
Regulator of nonsense transcripts 1	RENT1_HUMAN		UPF1
COP9 signalosome complex subunit 5	CSN5_HUMAN		COPS5
G-patch domain and KOW motifs-containing protein	GPKOW_HUMAN		GPKOW
SWI/SNF complex subunit SMARCC1	SMRC1_HUMAN		SMARCC1
SWI/SNF-related matrix-associated actin-dependent regulator of chromatin subfamily D memb	SMRD2_HUMAN		SMARCD2
Ras-related protein Rab-8B	RAB8B_HUMAN		RAB8B
Far upstream element-binding protein 2	FUBP2_HUMAN		KHSRP
Transportin-1	TNPO1_HUMAN		TNPO1
Rho guanine nucleotide exchange factor 2	ARHG2_HUMAN		ARHGEF2
Glomulin	GLMN_HUMAN		GLMN
Probable ubiquitin carboxyl-terminal hydrolase FAF-X	USP9X_HUMAN		USP9X
Ubiquitin carboxyl-terminal hydrolase 7	UBP7_HUMAN		USP7
Cullin-5	CUL5_HUMAN		CUL5
V-type proton ATPase 116 kDa subunit a 1	VPP1_HUMAN		ATP6V0A1
Lipoma-preferred partner	LPP_HUMAN		LPP
RNA-binding protein with multiple splicing	RBPMS_HUMAN		RBPMS
SWI/SNF-related matrix-associated actin-dependent regulator of chromatin subfamily E memb	SMCE1_HUMAN		SMARCE1
Caveolae-associated protein 3	CAVN3_HUMAN		PRKCDBP
Myeloid-derived growth factor	MYDGF_HUMAN		MYDGF
GPI transamidase component PIG-T	PIGT_HUMAN		PIGT
Immunoglobulin superfamily member 8	IGSF8_HUMAN		IGSF8
Ribosomal protein eL42-like	RL36L_HUMAN		RPL36AL
Cytoplasmic 60S subunit biogenesis factor ZNF622	ZN622_HUMAN		ZNF622
Ribosome-releasing factor 2, mitochondrial	RRF2M_HUMAN		GFM2
Proteasome assembly chaperone 2	PSMG2_HUMAN		PSMG2
BOS complex subunit NCLN	NCLN_HUMAN		NCLN
Endoplasmic reticulum-Golgi intermediate compartment protein 1	ERGI1_HUMAN		ERGIC1
FAST kinase domain-containing protein 4	FAKD4_HUMAN		TBRG4
Protein FAM162A	F162A_HUMAN		FAM162A
PAT complex subunit CCDC47	CCD47_HUMAN		CCDC47
Synapse-associated protein 1	SYAP1_HUMAN		SYAP1
Exocyst complex component 4	EXOC4_HUMAN		EXOC4
Isochorismatase domain-containing protein 2	ISOC2_HUMAN		ISOC2
Fermitin family homolog 2	FERM2_HUMAN		FERMT2
Far upstream element-binding protein 1	FUBP1_HUMAN		FUBP1
Leucine-rich repeat-containing protein 59	LRC59_HUMAN		LRRC59
Ribulose-phosphate 3-epimerase	RPE_HUMAN		RPE
Vacuolar protein sorting-associated protein 33A	VP33A_HUMAN		VPS33A
Peptidyl-prolyl cis-trans isomerase FKBP10	FKB10_HUMAN		FKBP10
Exosome complex component RRP43	EXOS8_HUMAN		EXOSC8
Mitochondrial import receptor subunit TOM6 homolog	TOM6_HUMAN		TOMM6
SH3 domain-containing kinase-binding protein 1	SH3K1_HUMAN		SH3KBP1
E3 ubiquitin-protein ligase RNF25	RNF25_HUMAN		RNF25
Axin interactor, dorsalization-associated protein	AIDA_HUMAN		AIDA
Ubiquitin thioesterase otulin	OTUL_HUMAN		OTULIN
Cytochrome c oxidase assembly factor 7	COA7_HUMAN		COA7
Dedicator of cytokinesis protein 10	DOC10_HUMAN		DOCK10
Protein FAM136A	F136A_HUMAN		FAM136A
EF-hand domain-containing protein D2	EFHD2_HUMAN		EFHD2
Galactose mutarotase	GALM_HUMAN		GALM
Pyrroline-5-carboxylate reductase 2	P5CR2_HUMAN		PYCR2
m7GpppX diphosphatase	DCPS_HUMAN		DCPS
Protein phosphatase 1 regulatory subunit 14B	PP14B_HUMAN		PPP1R14B
Medium-chain acyl-CoA ligase ACSF2, mitochondrial	ACSF2_HUMAN		ACSF2
FLYWCH family member 2	FWCH2_HUMAN		FLYWCH2
FAS-associated factor 2	FAF2_HUMAN		FAF2
Coiled-coil domain-containing protein 124	CC124_HUMAN		CCDC124
Optineurin	OPTN_HUMAN		OPTN
AP-2 complex subunit mu	AP2M1_HUMAN		AP2M1
Gamma-tubulin complex component 3	GCP3_HUMAN		TUBGCP3
Reticulocalbin-3	RCN3_HUMAN		RCN3
60S ribosomal export protein NMD3	NMD3_HUMAN		NMD3
Regulator of microtubule dynamics protein 1	RMD1_HUMAN		RMDN1
Carboxymethylenebutenolidase homolog	CMBL_HUMAN		CMBL
U5 small nuclear ribonucleoprotein 40 kDa protein	SNR40_HUMAN		SNRNP40
Large ribosomal subunit protein mL38	RM38_HUMAN		MRPL38
Ribosome-recycling factor, mitochondrial	RRFM_HUMAN		MRRF
Protein Spindly	SPDLY_HUMAN		SPDL1
Elongator complex protein 4	ELP4_HUMAN		ELP4
NAD-dependent protein deacetylase sirtuin-1	SIR1_HUMAN		SIRT1
Nucleoporin SEH1	SEH1_HUMAN		SEH1L
KIF-binding protein	KBP_HUMAN		KIAA1279
Small ribosomal subunit protein uS3m	RT24_HUMAN		MRPS24
Large ribosomal subunit protein mL53	RM53_HUMAN		MRPL53
DAZ-associated protein 1	DAZP1_HUMAN		DAZAP1
Mitochondrial potassium channel	MITOK_HUMAN		CCDC51
DnaJ homolog subfamily A member 3, mitochondrial	DNJA3_HUMAN		DNAJA3
Small ribosomal subunit protein mS39	PTCD3_HUMAN		PTCD3
Enhancer of mRNA-decapping protein 3	EDC3_HUMAN		EDC3
Protein S100-A16	S10AG_HUMAN		S100A16
THO complex subunit 1	THOC1_HUMAN		THOC1
Ubiquitin thioesterase OTUB1	OTUB1_HUMAN		OTUB1
tRNA (adenine(58)-N(1))-methyltransferase catalytic subunit TRMT61A	TRM61_HUMAN		TRMT61A
Phosphopentomutase	PGM2_HUMAN		PGM2
Ceramide synthase 2	CERS2_HUMAN		CERS2
tRNA-dihydrouridine(47) synthase [NAD(P)(+)]-like	DUS3L_HUMAN		DUS3L
Protein LTV1 homolog	LTV1_HUMAN		LTV1
Serine dehydratase-like	SDSL_HUMAN		SDSL
Large ribosomal subunit protein mL48	RM48_HUMAN		MRPL48
Vacuole membrane protein 1	VMP1_HUMAN		VMP1
Aurora kinase B	AURKB_HUMAN		AURKB
Target of EGR1 protein 1	TOE1_HUMAN		TOE1
Probable ATP-dependent RNA helicase DDX27	DDX27_HUMAN		DDX27
Zinc finger CCCH-type antiviral protein 1-like	ZCCHL_HUMAN		ZC3HAV1L
Tonsoku-like protein	TONSL_HUMAN		TONSL
PDZ and LIM domain protein 5	PDLI5_HUMAN		PDLIM5
ERO1-like protein alpha	ERO1A_HUMAN		ERO1L
Serine/threonine-protein phosphatase PGAM5, mitochondrial	PGAM5_HUMAN		PGAM5
DDRGK domain-containing protein 1	DDRGK_HUMAN		DDRGK1
Far upstream element-binding protein 3	FUBP3_HUMAN		FUBP3
Splicing factor 45	SPF45_HUMAN		RBM17
Succinate--CoA ligase [GDP-forming] subunit beta, mitochondrial	SUCB2_HUMAN		SUCLG2
Mannose-1-phosphate guanyltransferase alpha	GMPPA_HUMAN		GMPPA
Putative protein-lysine deacylase ABHD14B	ABHEB_HUMAN		ABHD14B
PRKC apoptosis WT1 regulator protein	PAWR_HUMAN		PAWR
Kin of IRRE-like protein 1	KIRR1_HUMAN		KIRREL
CDK5 regulatory subunit-associated protein 3	CK5P3_HUMAN		CDK5RAP3
Deubiquitinating protein VCPIP1	VCIP1_HUMAN		VCPIP1
Engulfment and cell motility protein 2	ELMO2_HUMAN		ELMO2

Protein disulfide-isomerase TMX3	TMX3_HUMAN		TMX3
Chromosome alignment-maintaining phosphoprotein 1	CHAP1_HUMAN		CHAMP1
E3 ubiquitin-protein ligase ZFP91	ZFP91_HUMAN		ZFP91
PDZ and LIM domain protein 2	PDLI2_HUMAN		PDLIM2
Transcription factor BTF3 homolog 4	BT3L4_HUMAN		BTF3L4
Abscission/NoCut checkpoint regulator	ANCHR_HUMAN		ZFYVE19
Ubiquitin carboxyl-terminal hydrolase 47	UBP47_HUMAN		USP47
Lymphokine-activated killer T-cell-originated protein kinase	TOPK_HUMAN		PBK
N-terminal kinase-like protein	SCYL1_HUMAN		SCYL1
Exocyst complex component 2	EXOC2_HUMAN		EXOC2
Cytosolic non-specific dipeptidase	CNDP2_HUMAN		CNDP2
Histone-lysine N-methyltransferase EHMT2	EHMT2_HUMAN		EHMT2
Zinc finger RNA-binding protein	ZFR_HUMAN		ZFR
Sorting nexin-27	SNX27_HUMAN		SNX27
Dehydrogenase/reductase SDR family member 1	DHRS1_HUMAN		DHRS1
Protein PRRC1	PRRC1_HUMAN		PRRC1
YTH domain-containing protein 1	YTDC1_HUMAN		YTHDC1
Coiled-coil domain-containing protein 43	CCD43_HUMAN		CCDC43
Lysophospholipid acyltransferase 7	MBOA7_HUMAN		MBOAT7
Dedicator of cytokinesis protein 7	DOCK7_HUMAN		DOCK7
Zinc finger matrin-type protein 2	ZMAT2_HUMAN		ZMAT2
Leucine-rich repeat-containing protein 7	LRRC7_HUMAN		LRRC7
28S rRNA (cytosine-C(5))-methyltransferase	NSUN5_HUMAN		NSUN5
Arf-GAP with Rho-GAP domain, ANK repeat and PH domain-containing protein 1	ARAP1_HUMAN		ARAP1
Importin-9	IPO9_HUMAN		IPO9
RNA-binding protein 14	RBM14_HUMAN		RBM14
KH domain-containing RNA-binding protein QKI	QKI_HUMAN		QKI
Pseudouridylate synthase 7 homolog	PUS7_HUMAN		PUS7
Serine/threonine-protein kinase SMG1	SMG1_HUMAN		SMG1
Sodium-coupled neutral amino acid symporter 2	S38A2_HUMAN		SLC38A2
Vacuolar protein sorting-associated protein 35	VPS35_HUMAN		VPS35
Transcriptional activator protein Pur-beta	PURB_HUMAN		PURB
Exportin-6	XPO6_HUMAN		XPO6
Sperm-associated antigen 5	SPAG5_HUMAN		SPAG5
Sorting nexin-18	SNX18_HUMAN		SNX18
Mediator of RNA polymerase II transcription subunit 15	MED15_HUMAN		MED15
Elongation factor G, mitochondrial	EFGM_HUMAN		GFM1
NudC domain-containing protein 1	NUDC1_HUMAN		NUDCD1
Erbin	ERBIN_HUMAN		ERBB2IP
Hemicentin-1	HMCN1_HUMAN		HMCN1
Methyltransferase-like 26	MTL26_HUMAN		C16orf13
Ran-binding protein 9	RANB9_HUMAN		RANBP9
Neurabin-2	NEB2_HUMAN		PPP1R9B
SRSF protein kinase 1	SRPK1_HUMAN		SRPK1
Transmembrane protein 209	TM209_HUMAN		TMEM209
Protein IWS1 homolog	IWS1_HUMAN		IWS1
Paired amphipathic helix protein Sin3a	SIN3A_HUMAN		SIN3A
RNA-binding protein 15	RBM15_HUMAN		RBM15
RUN and FYVE domain-containing protein 1	RUFY1_HUMAN		RUFY1
MMS19 nucleotide excision repair protein homolog	MMS19_HUMAN		MMS19
E3 ubiquitin-protein ligase UHRF1	UHRF1_HUMAN		UHRF1
Protein Niban 2	NIBA2_HUMAN		FAM129B
Transcription factor 12	HTF4_HUMAN		TCF12
c-Myc-binding protein	MYCBP_HUMAN		MYCBP
Tubulin-folding cofactor B	TBCB_HUMAN		TBCB
Proteasome subunit beta type-7	PSB7_HUMAN		PSMB7
Calponin-2	CNN2_HUMAN		CNN2
Translocation protein SEC62	SEC62_HUMAN		SEC62
Ethanolamine-phosphate cytidylyltransferase	PCY2_HUMAN		PCYT2
Cell division cycle 5-like protein	CDC5L_HUMAN		CDC5L
26S proteasome non-ATPase regulatory subunit 1	PSMD1_HUMAN		PSMD1
Stromal cell-derived factor 2	SDF2_HUMAN		SDF2
Prefoldin subunit 5	PFD5_HUMAN		PFDN5
E3 ubiquitin-protein ligase RING2	RING2_HUMAN		RNF2
Parkinson disease protein 7	PARK7_HUMAN		PARK7
Synaptic vesicle membrane protein VAT-1 homolog	VAT1_HUMAN		VAT1
Legumain	LGMN_HUMAN		LGMN
Perilipin-2	PLIN2_HUMAN		PLIN2
DnaJ homolog subfamily C member 2	DNJC2_HUMAN		DNAJC2
M-phase phosphoprotein 6	MPH6_HUMAN		MPHOSPH6
Nuclear pore complex protein Nup88	NUP88_HUMAN		NUP88
Ribonucleases P/MRP protein subunit POP1	POP1_HUMAN		POP1
Protein S100-A13	S10AD_HUMAN		S100A13
Protein SCAF11	SCAFB_HUMAN		SCAF11
Transcriptional enhancer factor TEF-5	TEAD3_HUMAN		TEAD3
Translin-associated protein X	TSNAX_HUMAN		TSNAX
Eukaryotic translation initiation factor 3 subunit C	EIF3C_HUMAN		EIF3C
Tetratricopeptide repeat protein 1	TTC1_HUMAN		TTC1
DnaJ homolog subfamily C member 7	DNJC7_HUMAN		DNAJC7
Prohibitin-2	PHB2_HUMAN		PHB2
COP9 signalosome complex subunit 8	CSN8_HUMAN		COPS8
Pre-mRNA-splicing factor 18	PRP18_HUMAN		PRPF18
Kinesin-like protein KIF2C	KIF2C_HUMAN		KIF2C
Monoglyceride lipase	MGLL_HUMAN		MGLL
Ataxin-2	ATX2_HUMAN		ATXN2
Methionine synthase	METH_HUMAN		MTR
3-hydroxyacyl-CoA dehydrogenase type-2	HCD2_HUMAN		HSD17B10
Collagen alpha-1(XII) chain	COCA1_HUMAN		COL12A1
Heterogeneous nuclear ribonucleoprotein A/B	ROAA_HUMAN		HNRNPAB
Lipopolysaccharide-induced tumor necrosis factor-alpha factor	LITAF_HUMAN		LITAF
Nucleosome assembly protein 1-like 4	NP1L4_HUMAN		NAP1L4
Gamma-soluble NSF attachment protein	SNAG_HUMAN		NAPG
Aconitate hydratase, mitochondrial	ACON_HUMAN		ACO2
Transmembrane 9 superfamily member 2	TM9S2_HUMAN		TM9SF2
Equilibrative nucleoside transporter 1	S29A1_HUMAN		SLC29A1
Paired mesoderm homeobox protein 2	PRRX2_HUMAN		PRRX2
Tumor susceptibility gene 101 protein	TS101_HUMAN		TSG101
Copine-1	CPNE1_HUMAN		CPNE1
T-complex protein 1 subunit eta	TCPH_HUMAN		CCT7
Probable rRNA-processing protein EBP2	EBP2_HUMAN		EBNA1BP2
Protein arginine N-methyltransferase 1	ANM1_HUMAN		PRMT1
1-acyl-sn-glycerol-3-phosphate acyltransferase alpha	PLCA_HUMAN		AGPAT1
Endophilin-A2	SH3G1_HUMAN		SH3GL1
Semaphorin-3C	SEM3C_HUMAN		SEMA3C
Serine/threonine-protein kinase VRK1	VRK1_HUMAN		VRK1
Growth/differentiation factor 15	GDF15_HUMAN		GDF15
Protein NipSnap homolog 1	NIPS1_HUMAN		NIPSNAP1
Condensin complex subunit 3	CND3_HUMAN		NCAPG
Calcium uptake protein 1, mitochondrial	MICU1_HUMAN		MICU1
ATP-dependent RNA helicase DDX50	DDX50_HUMAN		DDX50
Zinc phosphodiesterase ELAC protein 2	RNZ2_HUMAN		ELAC2
Telomerase RNA component interacting RNase	TRIR_HUMAN		C19orf43
Glutamate-rich WD repeat-containing protein 1	GRWD1_HUMAN		GRWD1
ADP-ribose glycohydrolase MACROD1	MACD1_HUMAN		MACROD1
Ribosome quality control complex subunit TCF25	TCF25_HUMAN		TCF25
Protein CMSS1	CMS1_HUMAN		CMSS1
Methylosome protein WDR77	MEP50_HUMAN		WDR77
Tubulin alpha-1C chain	TBA1C_HUMAN		TUBA1C
Apolipoprotein L2	APOL2_HUMAN		APOL2
Myb-binding protein 1A	MBB1A_HUMAN		MYBBP1A
FYVE and coiled-coil domain-containing protein 1	FYCO1_HUMAN		FYCO1
Coronin-1B	COR1B_HUMAN		CORO1B
Thioredoxin domain-containing protein 17	TXD17_HUMAN		TXNDC17
Vacuolar protein-sorting-associated protein 25	VPS25_HUMAN		VPS25
Large ribosomal subunit protein mL45	RM45_HUMAN		MRPL45
45 kDa calcium-binding protein	CAB45_HUMAN		SDF4
Proteasomal ATPase-associated factor 1	PAAF1_HUMAN		PAAF1
Partner of Y14 and mago	PYM1_HUMAN		WIBG
Ferroptosis suppressor protein 1	FSP1_HUMAN		AIFM2

ADP-dependent glucokinase	ADPGK_HUMAN		ADPGK
Ubiquinol-cytochrome-c reductase complex assembly factor 2	UQCC2_HUMAN		UQCC2
Protein pelota homolog	PELO_HUMAN		PELO
DNA replication complex GINS protein PSF3	PSF3_HUMAN		GINS3
E3 ubiquitin-protein ligase TRIM56	TRI56_HUMAN		TRIM56
Endoplasmic reticulum resident protein 44	ERP44_HUMAN		ERP44
Cancer-related nucleoside-triphosphatase	NTPCR_HUMAN		NTPCR
Translational activator of cytochrome c oxidase 1	TACO1_HUMAN		TACO1
Gamma-tubulin complex component 2	GCP2_HUMAN		TUBGCP2
Extended synaptotagmin-1	ESYT1_HUMAN		ESYT1
Protein canopy homolog 3	CNPY3_HUMAN		CNPY3
Chitobiosyldiphosphodolichol beta-mannosyltransferase	ALG1_HUMAN		ALG1
HAUS augmin-like complex subunit 8	HAUS8_HUMAN		HAUS8
COP9 signalosome complex subunit 4	CSN4_HUMAN		COPS4
Death-inducer obliterator 1	DIDO1_HUMAN		DIDO1
Metastasis-associated protein MTA3	MTA3_HUMAN		MTA3
Mini-chromosome maintenance complex-binding protein	MCMBP_HUMAN		MCMBP
Acidic leucine-rich nuclear phosphoprotein 32 family member E	AN32E_HUMAN		ANP32E
Phosphatidylinositol 4-kinase type 2-alpha	P4K2A_HUMAN		PI4K2A
Transmembrane protein 43	TMM43_HUMAN		TMEM43
Fibronectin type III and SPRY domain-containing protein 1	FSD1_HUMAN		FSD1
Tubulin-specific chaperone D	TBCD_HUMAN		TBCD
Protein HGH1 homolog	HGH1_HUMAN		HGH1
Dehydrogenase/reductase SDR family member 4	DHRS4_HUMAN		DHRS4
Lipase maturation factor 2	LMF2_HUMAN		LMF2
Multiple myeloma tumor-associated protein 2	MMTA2_HUMAN		MMTAG2
Tubulin beta-6 chain	TBB6_HUMAN		TUBB6
Heterogeneous nuclear ribonucleoprotein U-like protein 1	HNRL1_HUMAN		HNRNPUL1
Programmed cell death protein 10	PDC10_HUMAN		PDCD10
Oxidoreductase HTATIP2	HTAI2_HUMAN		HTATIP2
Probable ATP-dependent RNA helicase DDX23	DDX23_HUMAN		DDX23
MICOS complex subunit MIC26	MIC26_HUMAN		APOO
Dehydrogenase/reductase SDR family member 6	DHRS6_HUMAN		BDH2
Methylthioribose-1-phosphate isomerase	MTNA_HUMAN		MRI1
WD repeat-containing protein 18	WDR18_HUMAN		WDR18
tRNA (guanine(6)-N2)-methyltransferase THUMP3	THUM3_HUMAN		THUMPD3
Acireductone dioxygenase	MTND_HUMAN		ADI1
E3 ubiquitin-protein ligase RNF126	RN126_HUMAN		RNF126
Enoyl-[acyl-carrier-protein] reductase, mitochondrial	MECR_HUMAN		MECR
N-terminal Xaa-Pro-Lys N-methyltransferase 1	NTM1A_HUMAN		NTMT1
Transmembrane protein 109	TM109_HUMAN		TMEM109
Protein PBDC1	PBDC1_HUMAN		PBDC1
Nucleolar complex protein 4 homolog	NOC4L_HUMAN		NOC4L
Transmembrane emp24 domain-containing protein 9	TMED9_HUMAN		TMED9
Guanine nucleotide-binding protein-like 3	GNL3_HUMAN		GNL3
Serine/threonine-protein kinase RIO2	RIOK2_HUMAN		RIOK2
Nuclear pore complex protein Nup85	NUP85_HUMAN		NUP85
Intraflagellar transport protein 27 homolog	IFT27_HUMAN		IFT27
Threonine--tRNA ligase, mitochondrial	SYTM_HUMAN		TARS2
Acetyl-CoA acetyltransferase, cytosolic	THIC_HUMAN		ACAT2
RNA-binding protein 4	RBM4_HUMAN		RBM4
Splicing factor 3B subunit 5	SF3B5_HUMAN		SF3B5
Sideroflexin-3	SFXN3_HUMAN		SFXN3
Chitinase domain-containing protein 1	CHID1_HUMAN		CHID1
Kanadaptin	NADAP_HUMAN		SLC4A1AP
Protein LSM14 homolog B	LS14B_HUMAN		LSM14B
Adenosine 5'-monophosphoramidase HINT2	HINT2_HUMAN		HINT2
Oxysterol-binding protein-related protein 10	OSB10_HUMAN		OSBPL10
Rab11 family-interacting protein 5	RFIP5_HUMAN		RAB11FIP5
Complement C1q tumor necrosis factor-related protein 3	C1QT3_HUMAN		C1QTNF3
N-alpha-acetyltransferase 15, NatA auxiliary subunit	NAA15_HUMAN		NAA15
Serrate RNA effector molecule homolog	SRRT_HUMAN		SRRT
Queuine tRNA-ribosyltransferase catalytic subunit 1	TGT_HUMAN		QTRT1
AP-1 complex subunit mu-1	AP1M1_HUMAN		AP1M1
Nucleolar and spindle-associated protein 1	NUSAP_HUMAN		NUSAP1
Haloacid dehalogenase-like hydrolase domain-containing 5	HDHD5_HUMAN		CECR5
Protein MAK16 homolog	MAK16_HUMAN		MAK16
Inosine triphosphate pyrophosphatase	ITPA_HUMAN		ITPA
Replication termination factor 2	RTF2_HUMAN		RTFDC1
Eukaryotic translation initiation factor 2A	EIF2A_HUMAN		EIF2A
Polymerase delta-interacting protein 3	PDIP3_HUMAN		POLDIP3
Alpha-(1,6)-fucosyltransferase	FUT8_HUMAN		FUT8
Large ribosomal subunit protein bL20m	RM20_HUMAN		MRPL20
Large ribosomal subunit protein uL13m	RM13_HUMAN		MRPL13
Large ribosomal subunit protein bL9m	RM09_HUMAN		MRPL9
Large ribosomal subunit protein uL4m	RM04_HUMAN		MRPL4
Small ribosomal subunit protein mS26	RT26_HUMAN		MRPS26
Neurolysin, mitochondrial	NEUL_HUMAN		NLN
Centrosomal protein of 41 kDa	CEP41_HUMAN		CEP41
TBC1 domain family member 2A	TBD2A_HUMAN		TBC1D2
Kinetochore protein Nuf2	NUF2_HUMAN		NUF2
Large ribosomal subunit protein mL37	RM37_HUMAN		MRPL37
GTP-binding protein 4	GTPB4_HUMAN		GTPBP4
Oxysterol-binding protein-related protein 8	OSBL8_HUMAN		OSBPL8
Uveal autoantigen with coiled-coil domains and ankyrin repeats	UACA_HUMAN		UACA
Ras-related protein Rab-34	RAB34_HUMAN		RAB34
Crooked neck-like protein 1	CRNL1_HUMAN		CRNKL1
F-box-like/WD repeat-containing protein TBL1XR1	TBL1R_HUMAN		TBL1XR1
Ubiquitin-like protein 5	UBL5_HUMAN		UBL5
Serine/threonine-protein kinase D2	KPCD2_HUMAN		PRKD2
Protein Niban 1	NIBA1_HUMAN		FAM129A
UBX domain-containing protein 6	UBXN6_HUMAN		UBXN6
Uridine-cytidine kinase 2	UCK2_HUMAN		UCK2
Apoptosis inhibitor 5	API5_HUMAN		API5
Protein dpy-30 homolog	DPY30_HUMAN		DPY30
Alpha-ketoglutarate-dependent dioxygenase FTO	FTO_HUMAN		FTO
182 kDa tankyrase-1-binding protein	TB182_HUMAN		TNKS1BP1
(E3-independent) E2 ubiquitin-conjugating enzyme	UBE2O_HUMAN		UBE2O
Protein zyg-11 homolog B	ZY11B_HUMAN		ZYG11B
Protein tweety homolog 3	TTYH3_HUMAN		TTYH3
Ribosome biogenesis protein WDR12	WDR12_HUMAN		WDR12
Protein YIPF3	YIPF3_HUMAN		YIPF3
RNA exonuclease 4	REXO4_HUMAN		REXO4
ATP-dependent RNA helicase DDX24	DDX24_HUMAN		DDX24
DNA-directed RNA polymerase I subunit RPA49	RPA49_HUMAN		POLR1E
Superkiller complex protein 8	SKI8_HUMAN		WDR61
SRA stem-loop-interacting RNA-binding protein, mitochondrial	SLIRP_HUMAN		SLIRP
NIF3-like protein 1	NIF3L_HUMAN		NIF3L1
Egl nine homolog 1	EGLN1_HUMAN		EGLN1
PSME3-interacting protein	PIP30_HUMAN		FAM192A
Twisted gastrulation protein homolog 1	TWSG1_HUMAN		TWSG1
Mitochondrial fission factor	MFF_HUMAN		MFF
N-alpha-acetyltransferase 50	NAA50_HUMAN		NAA50
Transmembrane protein 126A	T126A_HUMAN		TMEM126A
Polyadenylate-binding protein-interacting protein 1	PAIP1_HUMAN		PAIP1
Mitochondrial disaggregase	CLPB_HUMAN		CLPB
RNA cytidine acetyltransferase	NAT10_HUMAN		NAT10
Kinesin light chain 2	KLC2_HUMAN		KLC2
Integrin-linked kinase-associated serine/threonine phosphatase 2C	ILKAP_HUMAN		ILKAP
5'-3' exoribonuclease 2	XRN2_HUMAN		XRN2
Nuclear speckle splicing regulatory protein 1	NSRP1_HUMAN		NSRP1
Rac GTPase-activating protein 1	RGAP1_HUMAN		RACGAP1
Cleavage stimulation factor subunit 2 tau variant	CSTFT_HUMAN		CSTF2T
Probable ATP-dependent RNA helicase DDX47	DDX47_HUMAN		DDX47
Magnesium transporter protein 1	MAGT1_HUMAN		MAGT1
Ras-related protein Rab-1B	RAB1B_HUMAN		RAB1B
Large ribosomal subunit protein uL18m	RM18_HUMAN		MRPL18
Ester hydrolase C11orf54	CK054_HUMAN		C11orf54
Probable E3 ubiquitin-protein ligase IRF2BPL	I2BPL_HUMAN		IRF2BPL

Nuclear ubiquitous casein and cyclin-dependent kinase substrate 1	NUCKS_HUMAN		NUCKS1
Protein Wnt-5b	WNT5B_HUMAN		WNT5B
Iron-sulfur cluster assembly enzyme ISCU	ISCU_HUMAN		ISCU
Autophagy protein 5	ATG5_HUMAN		ATG5
WD repeat-containing protein 13	WDR13_HUMAN		WDR13
EH domain-containing protein 4	EHD4_HUMAN		EHD4
Cadherin-23	CAD23_HUMAN		CDH23
Vacuolar protein sorting-associated protein 33B	VP33B_HUMAN		VPS33B
Vacuolar protein sorting-associated protein 11 homolog	VPS11_HUMAN		VPS11
SH3 domain-binding glutamic acid-rich-like protein 3	SH3L3_HUMAN		SH3BGRL3
TRIO and F-actin-binding protein	TARA_HUMAN		TRIOBP
STE20-like serine/threonine-protein kinase	SLK_HUMAN		SLK
Peptidyl-prolyl cis-trans isomerase-like 3	PPIL3_HUMAN		PPIL3
Serine/threonine-protein kinase TAO3	TAOK3_HUMAN		TAOK3
Rab3 GTPase-activating protein non-catalytic subunit	RBGPR_HUMAN		RAB3GAP2
Activity-dependent neuroprotector homeobox protein	ADNP_HUMAN		ADNP
Diphthine methyl ester synthase	DPH5_HUMAN		DPH5
ATP-dependent DNA/RNA helicase DHX36	DHX36_HUMAN		DHX36
Inorganic pyrophosphatase 2, mitochondrial	IPYR2_HUMAN		PPA2
Large ribosomal subunit protein mL46	RM46_HUMAN		MRPL46
Pinin	PININ_HUMAN		PNN
Transmembrane protein 245	TM245_HUMAN		TMEM245
BolA-like protein 2	BOLA2_HUMAN		BOLA2
Thioredoxin-related transmembrane protein 1	TMX1_HUMAN		TMX1
Golgi resident protein GCP60	GCP60_HUMAN		ACBD3
Cdc42 effector protein 4	BORG4_HUMAN		CDC42EP4
Tyrosine-protein phosphatase non-receptor type 23	PTN23_HUMAN		PTPN23
Protein unc-45 homolog A	UN45A_HUMAN		UNC45A
Kinetochore-associated protein DSN1 homolog	DSN1_HUMAN		DSN1
Uncharacterized protein C1orf198	CA198_HUMAN		C1orf198
Charged multivesicular body protein 4b	CHM4B_HUMAN		CHMP4B
GDP-fucose protein O-fucosyltransferase 1	OFUT1_HUMAN		POFUT1
Serine/threonine-protein kinase WNK1	WNK1_HUMAN		WNK1
Aminopeptidase B	AMPB_HUMAN		RNPEP
Golgi phosphoprotein 3	GOLP3_HUMAN		GOLPH3
Tubulin beta-1 chain	TBB1_HUMAN		TUBB1
Band 4.1-like protein 1	E41L1_HUMAN		EPB41L1
Golgi-associated plant pathogenesis-related protein 1	GAPR1_HUMAN		GLIPR2
Sentrin-specific protease 3	SENP3_HUMAN		SENP3
Oxysterol-binding protein-related protein 3	OSBL3_HUMAN		OSBPL3
EH domain-containing protein 1	EHD1_HUMAN		EHD1
Alpha-1,3/1,6-mannosyltransferase ALG2	ALG2_HUMAN		ALG2
CUB domain-containing protein 1	CDCP1_HUMAN		CDCP1
Coiled-coil domain-containing protein 86	CCD86_HUMAN		CCDC86
Nucleolar protein 6	NOL6_HUMAN		NOL6
3'-5' RNA helicase YTHDC2	YTDC2_HUMAN		YTHDC2
RNA polymerase II-associated protein 3	RPAP3_HUMAN		RPAP3
Ran-binding protein 3	RANB3_HUMAN		RANBP3
dCTP pyrophosphatase 1	DCTP1_HUMAN		DCTPP1
SH2 domain-containing protein 4A	SH24A_HUMAN		SH2D4A
Ribosome production factor 2 homolog	RPF2_HUMAN		RPF2
Histone-lysine N-methyltransferase SMYD3	SMYD3_HUMAN		SMYD3
WD repeat-containing protein 26	WDR26_HUMAN		WDR26
UPF0488 protein C8orf33	CH033_HUMAN		C8orf33
Splicing factor, arginine/serine-rich 19	SFR19_HUMAN		SCAF1
Prostaglandin E synthase 2	PGES2_HUMAN		PTGES2
Phosphorylated adapter RNA export protein	PHAX_HUMAN		PHAX
Ubiquitin-conjugating enzyme E2 Z	UBE2Z_HUMAN		UBE2Z
Complex I assembly factor ACAD9, mitochondrial	ACAD9_HUMAN		ACAD9
MOB kinase activator 1A	MOB1A_HUMAN		MOB1A
AN1-type zinc finger protein 3	ZFAN3_HUMAN		ZFAND3
Protein ECT2	ECT2_HUMAN		ECT2
tRNA endonuclease ANKZF1	ANKZ1_HUMAN		ANKZF1
Golgi reassembly-stacking protein 2	GORS2_HUMAN		GORASP2
Jupiter microtubule associated homolog 2	JUPI2_HUMAN		HN1L
Mitochondrial glutamate carrier 1	GHC1_HUMAN		SLC25A22
Leucine-rich repeat-containing protein 40	LRC40_HUMAN		LRRC40
Sideroflexin-1	SFXN1_HUMAN		SFXN1
Conserved oligomeric Golgi complex subunit 4	COG4_HUMAN		COG4
COP9 signalosome complex subunit 7b	CSN7B_HUMAN		COPS7B
Elongator complex protein 3	ELP3_HUMAN		ELP3
Ketosamine-3-kinase	KT3K_HUMAN		FN3KRP
Phosphopantothenate--cysteine ligase	PPCS_HUMAN		PPCS
Guanine nucleotide-binding protein subunit beta-4	GBB4_HUMAN		GNB4
Exportin-5	XPO5_HUMAN		XPO5
GrpE protein homolog 1, mitochondrial	GRPE1_HUMAN		GRPEL1
MYG1 exonuclease	MYG1_HUMAN		C12orf10
Netrin-4	NET4_HUMAN		NTN4
Calcyclin-binding protein	CYBP_HUMAN		CACYBP
Ras-related GTP-binding protein C	RRAGC_HUMAN		RRAGC
Retinol dehydrogenase 14	RDH14_HUMAN		RDH14
Transmembrane protein 165	TM165_HUMAN		TMEM165
Echinoderm microtubule-associated protein-like 4	EMAL4_HUMAN		EML4
Glyoxalase domain-containing protein 4	GLOD4_HUMAN		GLOD4
Chromobox protein homolog 8	CBX8_HUMAN		CBX8
Methylcrotonoyl-CoA carboxylase beta chain, mitochondrial	MCCB_HUMAN		MCCC2
Nuclear receptor coactivator 5	NCOA5_HUMAN		NCOA5
Helicase MOV-10	MOV10_HUMAN		MOV10
Pre-mRNA-splicing factor CWC22 homolog	CWC22_HUMAN		CWC22
Chromodomain-helicase-DNA-binding protein 8	CHD8_HUMAN		CHD8
GPN-loop GTPase 1	GPN1_HUMAN		GPN1
Stromal cell-derived factor 2-like protein 1	SDF2L_HUMAN		SDF2L1
Pre-mRNA-splicing factor SYF1	SYF1_HUMAN		XAB2
Endosialin	CD248_HUMAN		CD248
Prolactin regulatory element-binding protein	PREB_HUMAN		PREB
Steroid receptor RNA activator 1	SRA1_HUMAN		SRA1
Endoplasmic reticulum transmembrane helix translocase	AT131_HUMAN		ATP13A1
Golgi-associated PDZ and coiled-coil motif-containing protein	GOPC_HUMAN		GOPC
Large ribosomal subunit protein uL29m	RM47_HUMAN		MRPL47
Charged multivesicular body protein 1a	CHM1A_HUMAN		CHMP1A
Transmembrane 9 superfamily member 3	TM9S3_HUMAN		TM9SF3
Adipocyte plasma membrane-associated protein	APMAP_HUMAN		APMAP
WD repeat-containing protein 6	WDR6_HUMAN		WDR6
ADP-ribosylation factor GTPase-activating protein 3	ARFG3_HUMAN		ARFGAP3
Ras-related protein Rab-18	RAB18_HUMAN		RAB18
RNA polymerase II subunit A C-terminal domain phosphatase SSU72	SSU72_HUMAN		SSU72
Vacuolar protein sorting-associated protein VTA1 homolog	VTA1_HUMAN		VTA1
Serine--tRNA ligase, mitochondrial	SYSM_HUMAN		SARS2
Large ribosomal subunit protein mL65	RT30_HUMAN		MRPS30
Dynein light chain roadblock-type 1	DLRB1_HUMAN		DYNLRB1
ER membrane protein complex subunit 7	EMC7_HUMAN		EMC7
Exosome complex component RRP41	EXOS4_HUMAN		EXOSC4
H/ACA ribonucleoprotein complex subunit 3	NOP10_HUMAN		NOP10
tRNA N6-adenosine threonylcarbamoyltransferase	OSGEP_HUMAN		OSGEP
Acyl-coenzyme A thioesterase 13	ACO13_HUMAN		ACOT13
Synembryn-A	RIC8A_HUMAN		RIC8A
Putative RNA-binding protein Luc7-like 1	LUC7L_HUMAN		LUC7L
Leucine zipper transcription factor-like protein 1	LZTL1_HUMAN		LZTFL1
Suppressor of SWI4 1 homolog	SSF1_HUMAN		PPAN
Fructose-2,6-bisphosphatase TIGAR	TIGAR_HUMAN		TIGAR
Reticulon-4	RTN4_HUMAN		RTN4
Regulation of nuclear pre-mRNA domain-containing protein 1B	RPR1B_HUMAN		RPRD1B
Xaa-Pro aminopeptidase 3	XPP3_HUMAN		XPNPEP3
Omega-amidase NIT2	NIT2_HUMAN		NIT2
Nectin-3	NECT3_HUMAN		PVRL3
Exosome complex component RRP46	EXOS5_HUMAN		EXOSC5
Kinesin-like protein KIF13B	KI13B_HUMAN		KIF13B
Anillin	ANLN_HUMAN		ANLN
Xaa-Pro aminopeptidase 1	XPP1_HUMAN		XPNPEP1

Bridging integrator 3	BIN3_HUMAN		BIN3
Baculoviral IAP repeat-containing protein 6	BIRC6_HUMAN		BIRC6
PDZ and LIM domain protein 7	PDLI7_HUMAN		PDLIM7
Acetyl-coenzyme A synthetase, cytoplasmic	ACSA_HUMAN		ACSS2
Diablo IAP-binding mitochondrial protein	DBLOH_HUMAN		DIABLO
Nucleolar RNA helicase 2	DDX21_HUMAN		DDX21
GTP-binding protein SAR1a	SAR1A_HUMAN		SAR1A
Sialic acid synthase	SIAS_HUMAN		NANS
Endophilin-B2	SHLB2_HUMAN		SH3GLB2
Translation initiation factor eIF-2B subunit gamma	EI2BG_HUMAN		EIF2B3
Muscleblind-like protein 1	MBNL1_HUMAN		MBNL1
CTP synthase 2	PYRG2_HUMAN		CTPS2
DNA polymerase epsilon subunit 3	DPOE3_HUMAN		POLE3
Chromatin accessibility complex protein 1	CHRC1_HUMAN		CHRAC1
Aladin	AAAS_HUMAN		AAAS
Bromodomain adjacent to zinc finger domain protein 1A	BAZ1A_HUMAN		BAZ1A
Striatin-4	STRN4_HUMAN		STRN4
Olfactomedin-like protein 3	OLFL3_HUMAN		OLFML3
Ubiquilin-4	UBQL4_HUMAN		UBQLN4
Heme-binding protein 1	HEBP1_HUMAN		HEBP1
DNA dC->dU-editing enzyme APOBEC-3C	ABC3C_HUMAN		APOBEC3C
RNA-binding protein PNO1	PNO1_HUMAN		PNO1
Large ribosomal subunit protein bL17m	RM17_HUMAN		MRPL17
14 kDa phosphohistidine phosphatase	PHP14_HUMAN		PHPT1
Serine incorporator 1	SERC1_HUMAN		SERINC1
Rho GTPase-activating protein 35	RHG35_HUMAN		ARHGAP35
Protein FAM114A2	F1142_HUMAN		FAM114A2
Phospholipid scramblase 3	PLS3_HUMAN		PLSCR3
Lymphoid-specific helicase	HELLS_HUMAN		HELLS
Kinesin-like protein KIF15	KIF15_HUMAN		KIF15
Homer protein homolog 3	HOME3_HUMAN		HOMER3
Phenylalanine--tRNA ligase beta subunit	SYFB_HUMAN		FARSB
Isoleucine--tRNA ligase, mitochondrial	SYIM_HUMAN		IARS2
Protein diaphanous homolog 3	DIAP3_HUMAN		DIAPH3
Ubiquitin-like-conjugating enzyme ATG3	ATG3_HUMAN		ATG3
Sister chromatid cohesion protein PDS5 homolog B	PDS5B_HUMAN		PDS5B
Structural maintenance of chromosomes protein 4	SMC4_HUMAN		SMC4
Phosphatidylinositol-3-phosphatase SAC1	SAC1_HUMAN		SACM1L
Obg-like ATPase 1	OLA1_HUMAN		OLA1
Ethylmalonyl-CoA decarboxylase	ECHD1_HUMAN		ECHDC1
RNA-binding protein 12	RBM12_HUMAN		RBM12
Zinc finger CCHC domain-containing protein 3	ZCHC3_HUMAN		ZCCHC3
Peroxisomal 2,4-dienoyl-CoA reductase [(3E)-enoyl-CoA-producing]	DECR2_HUMAN		DECR2
Palmitoyl-protein thioesterase ABHD10, mitochondrial	ABHDA_HUMAN		ABHD10
Protein lin-7 homolog C	LIN7C_HUMAN		LIN7C
SPATS2-like protein	SPS2L_HUMAN		SPATS2L
ATP-binding cassette sub-family F member 3	ABCF3_HUMAN		ABCF3
CYFIP-related Rac1 interactor B	CYRIB_HUMAN		FAM49B
TBC1 domain family member 23	TBC23_HUMAN		TBC1D23
Cell cycle control protein 50A	CC50A_HUMAN		TMEM30A
Septin-11	SEP11_HUMAN		SEPT11
Alpha-parvin	PARVA_HUMAN		PARVA
TBC1 domain family member 13	TBC13_HUMAN		TBC1D13
Fanconi anemia group I protein	FANCI_HUMAN		FANCI
ATPase family AAA domain-containing protein 3A	ATD3A_HUMAN		ATAD3A
ADP-ribosylation factor-like protein 8B	ARL8B_HUMAN		ARL8B
Protein eva-1 homolog B	EVA1B_HUMAN		EVA1B
Protein arginine N-methyltransferase 7	ANM7_HUMAN		PRMT7
ATP-dependent RNA helicase DDX18	DDX18_HUMAN		DDX18
Armadillo repeat-containing protein 1	ARMC1_HUMAN		ARMC1
Adaptin ear-binding coat-associated protein 2	NECP2_HUMAN		NECAP2
RNA-binding protein 28	RBM28_HUMAN		RBM28
Pre-mRNA-splicing factor RBM22	RBM22_HUMAN		RBM22
WD repeat-containing protein 70	WDR70_HUMAN		WDR70
Arginine and glutamate-rich protein 1	ARGL1_HUMAN		ARGLU1
SAFB-like transcription modulator	SLTM_HUMAN		SLTM
PIH1 domain-containing protein 1	PIHD1_HUMAN		PIH1D1
p21-activated protein kinase-interacting protein 1	PK1IP_HUMAN		PAK1IP1
CXXC motif containing zinc binding protein	CZIB_HUMAN		C1orf123
BRISC and BRCA1-A complex member 1	BABA1_HUMAN		BABAM1
COMM domain-containing protein 8	COMD8_HUMAN		COMMD8
H/ACA ribonucleoprotein complex subunit 2	NHP2_HUMAN		NHP2
OCIA domain-containing protein 1	OCAD1_HUMAN		OCIAD1
ADP-ribosylhydrolase ARH3	ADPRS_HUMAN		ADPRHL2
Huntingtin-interacting protein K	HYPK_HUMAN		HYPK
Cell growth-regulating nucleolar protein	LYAR_HUMAN		LYAR
Golgi-resident adenosine 3',5'-bisphosphate 3'-phosphatase	IMPA3_HUMAN		IMPAD1
MICOS complex subunit MIC19	MIC19_HUMAN		CHCHD3
Mediator of RNA polymerase II transcription subunit 29	MED29_HUMAN		MED29
tRNA-dihydrouridine(20) synthase [NAD(P)+]-like	DUS2L_HUMAN		DUS2
Sphingomyelin phosphodiesterase 4	NSMA3_HUMAN		SMPD4
THUMP domain-containing protein 1	THUM1_HUMAN		THUMPD1
Torsin-4A	TOR4A_HUMAN		TOR4A
tRNA (guanine(26)-N(2))-dimethyltransferase	TRM1_HUMAN		TRMT1
CDKN2A-interacting protein	CARF_HUMAN		CDKN2AIP
DnaJ homolog subfamily B member 12	DJB12_HUMAN		DNAJB12
H/ACA ribonucleoprotein complex subunit 1	GAR1_HUMAN		GAR1
Dipeptidyl peptidase 3	DPP3_HUMAN		DPP3
Telomeric repeat-binding factor 2-interacting protein 1	TE2IP_HUMAN		TERF2IP
Abl interactor 2	ABI2_HUMAN		ABI2
Bcl-2-associated transcription factor 1	BCLF1_HUMAN		BCLAF1
U3 small nucleolar RNA-associated protein 6 homolog	UTP6_HUMAN		UTP6
Mitogen-activated protein kinase kinase kinase 20	M3K20_HUMAN		ZAK
Peptidyl-prolyl cis-trans isomerase FKBP11	FKB11_HUMAN		FKBP11
Tropomodulin-3	TMOD3_HUMAN		TMOD3
UDP-glucose:glycoprotein glucosyltransferase 1	UGGG1_HUMAN		UGGT1
Very-long-chain enoyl-CoA reductase	TECR_HUMAN		TECR
Endoplasmic reticulum aminopeptidase 1	ERAP1_HUMAN		ERAP1
CDGSH iron-sulfur domain-containing protein 1	CISD1_HUMAN		CISD1
Constitutive coactivator of PPAR-gamma-like protein 1	F120A_HUMAN		FAM120A
Upstream-binding protein 1	UBIP1_HUMAN		UBP1
Insulin-like growth factor 2 mRNA-binding protein 1	IF2B1_HUMAN		IGF2BP1
Mitochondrial carrier homolog 1	MTCH1_HUMAN		MTCH1
Hsp70-binding protein 1	HPBP1_HUMAN		HSPBP1
Methionine adenosyltransferase 2 subunit beta	MAT2B_HUMAN		MAT2B
Myoferlin	MYOF_HUMAN		MYOF
EH domain-containing protein 3	EHD3_HUMAN		EHD3
EH domain-containing protein 2	EHD2_HUMAN		EHD2
Olfactory receptor 5AC2	O5AC2_HUMAN		OR5AC2
NCK-interacting protein with SH3 domain	SPN90_HUMAN		NCKIPSD
Tropomodulin-2	TMOD2_HUMAN		TMOD2
Opioid growth factor receptor	OGFR_HUMAN		OGFR
LIM and cysteine-rich domains protein 1	LMCD1_HUMAN		LMCD1
Cysteine-rich motor neuron 1 protein	CRIM1_HUMAN		CRIM1
Charged multivesicular body protein 5	CHMP5_HUMAN		CHMP5
COMM domain-containing protein 9	COMD9_HUMAN		COMMD9
Spliceosome-associated protein CWC15 homolog	CWC15_HUMAN		CWC15
Large ribosomal subunit protein uL15m	RM15_HUMAN		MRPL15
Thymocyte nuclear protein 1	THYN1_HUMAN		THYN1
Very-long-chain (3R)-3-hydroxyacyl-CoA dehydratase 3	HACD3_HUMAN		HACD3
NADH dehydrogenase [ubiquinone] 1 alpha subcomplex subunit 13	NDUAD_HUMAN		NDUFA13
E3 ubiquitin-protein ligase KCMF1	KCMF1_HUMAN		KCMF1
Ankycorbin	RAI14_HUMAN		RAI14
Vesicle-associated membrane protein-associated protein A	VAPA_HUMAN		VAPA
E3 ubiquitin-protein ligase RNF181	RN181_HUMAN		RNF181
SH3 domain-binding protein 4	SH3B4_HUMAN		SH3BP4
Septin-10	SEP10_HUMAN		SEPT10
Protein Daple	DAPLE_HUMAN		CCDC88C
Protein RCC2	RCC2_HUMAN		RCC2

Disco-interacting protein 2 homolog B	DIP2B_HUMAN		DIP2B
SLAIN motif-containing protein 2	SLAI2_HUMAN		SLAIN2
BRCA2 and CDKN1A-interacting protein	BCCIP_HUMAN		BCCIP
Prostaglandin F2 receptor negative regulator	FPRP_HUMAN		PTGFRN
CTTNBP2 N-terminal-like protein	CT2NL_HUMAN		CTTNBP2NL
HEAT repeat-containing protein 5B	HTR5B_HUMAN		HEATR5B
Ribosome-binding protein 1	RRBP1_HUMAN		RRBP1
Cleavage and polyadenylation specificity factor subunit 2	CPSF2_HUMAN		CPSF2
Leucine--tRNA ligase, cytoplasmic	SYLC_HUMAN		LARS
Myelin expression factor 2	MYEF2_HUMAN		MYEF2
RNA-binding protein 27	RBM27_HUMAN		RBM27
Rabankyrin-5	ANFY1_HUMAN		ANKFY1
Succinate--CoA ligase [ADP-forming] subunit beta, mitochondrial	SUCB1_HUMAN		SUCLA2
GMP reductase 2	GMPR2_HUMAN		GMPR2
Syntaxin-18	STX18_HUMAN		STX18
Ataxin-10	ATX10_HUMAN		ATXN10
Neurochondrin	NCDN_HUMAN		NCDN
Epidermal growth factor receptor substrate 15-like 1	EP15R_HUMAN		EPS15L1
SUMO-activating enzyme subunit 1	SAE1_HUMAN		SAE1
Coatomer subunit gamma-2	COPG2_HUMAN		COPG2
RING-box protein 2	RBX2_HUMAN		RNF7
C-type mannose receptor 2	MRC2_HUMAN		MRC2
Guanine nucleotide-binding protein G(I)/G(S)/G(O) subunit gamma-12	GBG12_HUMAN		GNG12
7-dehydrocholesterol reductase	DHCR7_HUMAN		DHCR7
Histone deacetylase 6	HDAC6_HUMAN		HDAC6
Vacuolar protein sorting-associated protein 29	VPS29_HUMAN		VPS29
Eukaryotic translation initiation factor 3 subunit K	EIF3K_HUMAN		EIF3K
Glyoxylate reductase/hydroxypyruvate reductase	GRHPR_HUMAN		GRHPR
Cathepsin Z	CATZ_HUMAN		CTSZ
DnaJ homolog subfamily B member 11	DJB11_HUMAN		DNAJB11
SUMO-activating enzyme subunit 2	SAE2_HUMAN		UBA2
Mortality factor 4-like protein 1	MO4L1_HUMAN		MORF4L1
Nuclear RNA export factor 1	NXF1_HUMAN		NXF1
Protein sel-1 homolog 1	SE1L1_HUMAN		SEL1L
Peflin	PEF1_HUMAN		PEF1
Mitochondrial dicarboxylate carrier	DIC_HUMAN		SLC25A10
Cytochrome b-c1 complex subunit 9	QCR9_HUMAN		UQCR10
Tight junction protein ZO-2	ZO2_HUMAN		TJP2
Gamma-adducin	ADDG_HUMAN		ADD3
ATP-binding cassette sub-family F member 2	ABCF2_HUMAN		ABCF2
Testin	TES_HUMAN		TES
LIM domain-containing protein 1	LIMD1_HUMAN		LIMD1
Translocation protein SEC63 homolog	SEC63_HUMAN		SEC63
Zinc finger CCCH domain-containing protein 7B	Z3H7B_HUMAN		ZC3H7B
Protein NDRG3	NDRG3_HUMAN		NDRG3
Armadillo repeat-containing X-linked protein 3	ARMX3_HUMAN		ARMCX3
Switch-associated protein 70	SWP70_HUMAN		SWAP70
SUN domain-containing protein 2	SUN2_HUMAN		SUN2
LIM domain and actin-binding protein 1	LIMA1_HUMAN		LIMA1
Signal recognition particle subunit SRP68	SRP68_HUMAN		SRP68
Cysteine and histidine-rich domain-containing protein 1	CHRD1_HUMAN		CHORDC1
Serine/threonine-protein kinase TBK1	TBK1_HUMAN		TBK1
Septin-9	SEPT9_HUMAN		SEPT9
Ubiquilin-2	UBQL2_HUMAN		UBQLN2
Prenylcysteine oxidase 1	PCYOX_HUMAN		PCYOX1
A disintegrin and metalloproteinase with thrombospondin motifs 1	ATS1_HUMAN		ADAMTS1
Dipeptidyl peptidase 2	DPP2_HUMAN		DPP7
B-cell receptor-associated protein 29	BAP29_HUMAN		BCAP29
NADH-cytochrome b5 reductase 1	NB5R1_HUMAN		CYB5R1
Prefoldin subunit 2	PFD2_HUMAN		PFDN2
Poly(U)-binding-splicing factor PUF60	PUF60_HUMAN		PUF60
Nuclear receptor-binding protein	NRBP_HUMAN		NRBP1
Enolase-phosphatase E1	ENOPH_HUMAN		ENOPH1
NADH dehydrogenase [ubiquinone] 1 alpha subcomplex subunit 12	NDUAC_HUMAN		NDUFA12
Translation initiation factor eIF-2B subunit delta	EI2BD_HUMAN		EIF2B4
V-type proton ATPase subunit H	VATH_HUMAN		ATP6V1H
Prenylated Rab acceptor protein 1	PRAF1_HUMAN		RABAC1
Importin-11	IPO11_HUMAN		IPO11
Multifunctional methyltransferase subunit TRM112-like protein	TR112_HUMAN		TRMT112
Exportin-7	XPO7_HUMAN		XPO7
Vacuolar protein sorting-associated protein 51 homolog	VPS51_HUMAN		VPS51
Tyrosine-protein kinase BAZ1B	BAZ1B_HUMAN		BAZ1B
ATPase inhibitor, mitochondrial	ATIF1_HUMAN		ATPIF1
GTP:AMP phosphotransferase AK3, mitochondrial	KAD3_HUMAN		AK3
Rab5 GDP/GTP exchange factor	RABX5_HUMAN		RABGEF1
Mitochondrial peptide methionine sulfoxide reductase	MSRA_HUMAN		MSRA
N-acetyl-D-glucosamine kinase	NAGK_HUMAN		NAGK
2-hydroxyacyl-CoA lyase 1	HACL1_HUMAN		HACL1
tRNA (adenine(58)-N(1))-methyltransferase non-catalytic subunit TRM6	TRM6_HUMAN		TRMT6
Electrogenic aspartate/glutamate antiporter SLC25A13, mitochondrial	S2513_HUMAN		SLC25A13
Drebrin-like protein	DBNL_HUMAN		DBNL
Dynactin subunit 4	DCTN4_HUMAN		DCTN4
Cell division cycle protein 23 homolog	CDC23_HUMAN		CDC23
Anaphase-promoting complex subunit 7	APC7_HUMAN		ANAPC7
Stomatin-like protein 2, mitochondrial	STML2_HUMAN		STOML2
Vacuolar protein sorting-associated protein 28 homolog	VPS28_HUMAN		VPS28
U6 snRNA-associated Sm-like protein LSm7	LSM7_HUMAN		LSM 7.00
Lariat debranching enzyme	DBR1_HUMAN		DBR1
Jupiter microtubule associated homolog 1	JUPI1_HUMAN		HN1
mRNA turnover protein 4 homolog	MRT4_HUMAN		MRTO4
Cleavage and polyadenylation specificity factor subunit 3	CPSF3_HUMAN		CPSF3
DCC-interacting protein 13-alpha	DP13A_HUMAN		APPL1
Protein mono-ADP-ribosyltransferase PARP4	PARP4_HUMAN		PARP4
ADP-sugar pyrophosphatase	NUDT5_HUMAN		NUDT5
RNA-binding protein Raly	RALY_HUMAN		RALY
General transcription factor 3C polypeptide 4	TF3C4_HUMAN		GTF3C4
Protein kinase C and casein kinase substrate in neurons protein 3	PACN3_HUMAN		PACSIN3
Apoptotic chromatin condensation inducer in the nucleus	ACINU_HUMAN		ACIN1
Nuclear pore complex protein Nup50	NUP50_HUMAN		NUP50
Protein CDV3 homolog	CDV3_HUMAN		CDV3
Ras-related protein Rab-21	RAB21_HUMAN		RAB21
Ras-related protein Rab-22A	RB22A_HUMAN		RAB22A
Proteasome activator complex subunit 2	PSME2_HUMAN		PSME2
Aspartyl aminopeptidase	DNPEP_HUMAN		DNPEP
Ras-related protein Rab-23	RAB23_HUMAN		RAB23
Malignant T-cell-amplified sequence 1	MCTS1_HUMAN		MCTS1
Arf-GAP with SH3 domain, ANK repeat and PH domain-containing protein 1	ASAP1_HUMAN		ASAP1
Protein HEG homolog 1	HEG1_HUMAN		HEG1
Pre-mRNA-splicing factor ISY1 homolog	ISY1_HUMAN		ISY1
E3 ubiquitin-protein ligase HECTD1	HECD1_HUMAN		HECTD1
Coronin-1C	COR1C_HUMAN		CORO1C
Targeting protein for Xklp2	TPX2_HUMAN		TPX2
RNA-binding protein NOB1	NOB1_HUMAN		NOB1
A-kinase anchor protein 8-like	AKP8L_HUMAN		AKAP8L
Transcription factor MafF	MAFF_HUMAN		MAFF
Calcium load-activated calcium channel	TMCO1_HUMAN		TMCO1
Unconventional myosin-VI	MYO6_HUMAN		MYO6
ATP-dependent RNA helicase DDX19B	DD19B_HUMAN		DDX19B
Pre-mRNA-processing factor 19	PRP19_HUMAN		PRPF19
Sorting nexin-12	SNX12_HUMAN		SNX12
Ras GTPase-activating protein-binding protein 2	G3BP2_HUMAN		G3BP2
E3 ubiquitin-protein ligase CHIP	CHIP_HUMAN		STUB1
Protein kinase C and casein kinase substrate in neurons protein 2	PACN2_HUMAN		PACSIN2
Melanoma-associated antigen D2	MAGD2_HUMAN		MAGED2
Sorting nexin-6	SNX6_HUMAN		SNX6
Translocon-associated protein subunit gamma	SSRG_HUMAN		SSR3
26S proteasome non-ATPase regulatory subunit 13	PSD13_HUMAN		PSMD13
FAS-associated factor 1	FAF1_HUMAN		FAF1
Endothelial protein C receptor	EPCR_HUMAN		PROCR

Probable dimethyladenosine transferase	DIM1_HUMAN		DIMT1
COP9 signalosome complex subunit 3	CSN3_HUMAN		COPS3
WD repeat-containing protein 3	WDR3_HUMAN		WDR3
NSFL1 cofactor p47	NSF1C_HUMAN		NSFL1C
Leydig cell tumor 10 kDa protein homolog	L10K_HUMAN		C19orf53
Solute carrier family 12 member 4	S12A4_HUMAN		SLC12A4
AP-4 complex subunit epsilon-1	AP4E1_HUMAN		AP4E1
Microtubule-actin cross-linking factor 1, isoforms 1/2/3/4/5	MACF1_HUMAN		MACF1
Centrosomal protein of 131 kDa	CP131_HUMAN		CEP131
Serine/threonine-protein phosphatase 6 regulatory subunit 1	PP6R1_HUMAN		PPP6R1
E3 ubiquitin-protein ligase TRIM33	TRI33_HUMAN		TRIM33
Trinucleotide repeat-containing gene 6B protein	TNR6B_HUMAN		TNRC6B
Exocyst complex component 7	EXOC7_HUMAN		EXOC7
Ubiquitin carboxyl-terminal hydrolase 24	UBP24_HUMAN		USP24
Forkhead box protein J3	FOXJ3_HUMAN		FOXJ3
Cystine/glutamate transporter	XCT_HUMAN		SLC7A11
Serine/arginine repetitive matrix protein 2	SRRM2_HUMAN		SRRM2
Proliferation-associated protein 2G4	PA2G4_HUMAN		PA2G4
Structural maintenance of chromosomes protein 3	SMC3_HUMAN		SMC3
Charged multivesicular body protein 2b	CHM2B_HUMAN		CHMP2B
Myotubularin-related protein 6	MTMR6_HUMAN		MTMR6
Bifunctional UDP-N-acetylglucosamine 2-epimerase/N-acetylmannosamine kinase	GLCNE_HUMAN		GNE
RNA transcription, translation and transport factor protein	RTRAF_HUMAN		C14orf166
RuvB-like 2	RUVB2_HUMAN		RUVBL2
Peptidyl-prolyl cis-trans isomerase NIMA-interacting 4	PIN4_HUMAN		PIN4
Eukaryotic translation initiation factor 3 subunit L	EIF3L_HUMAN		EIF3L
Phospholipase A-2-activating protein	PLAP_HUMAN		PLAA
RuvB-like 1	RUVB1_HUMAN		RUVBL1
Nuclear migration protein nudC	NUDC_HUMAN		NUDC
Voltage-dependent anion-selective channel protein 3	VDAC3_HUMAN		VDAC3
Cofilin-2	COF2_HUMAN		CFL2
Endoplasmic reticulum-Golgi intermediate compartment protein 3	ERGI3_HUMAN		ERGIC3
Phenylalanine--tRNA ligase alpha subunit	SYFA_HUMAN		FARSA
Developmentally-regulated GTP-binding protein 1	DRG1_HUMAN		DRG1
Nck-associated protein 1	NCKP1_HUMAN		NCKAP1
Protein canopy homolog 2	CNPY2_HUMAN		CNPY2
A-kinase anchor protein 2	AKAP2_HUMAN		AKAP2
Angiomotin-like protein 2	AMOL2_HUMAN		AMOTL2
Exosome complex exonuclease RRP44	RRP44_HUMAN		DIS3
Glutathione S-transferase kappa 1	GSTK1_HUMAN		GSTK1
Small ribosomal subunit protein bS1m	RT28_HUMAN		MRPS28
Cytochrome c oxidase assembly factor 3 homolog, mitochondrial	COA3_HUMAN		COA3
Probable ATP-dependent RNA helicase DDX52	DDX52_HUMAN		DDX52
Translation machinery-associated protein 7	TMA7_HUMAN		TMA7
Polymerase delta-interacting protein 2	PDIP2_HUMAN		POLDIP2
AP-3 complex subunit mu-1	AP3M1_HUMAN		AP3M1
Inner nuclear membrane protein Man1	MAN1_HUMAN		LEMD3
Calcium-regulated heat-stable protein 1	CHSP1_HUMAN		CARHSP1
Thyroid hormone receptor-associated protein 3	TR150_HUMAN		THRAP3
WW domain-binding protein 11	WBP11_HUMAN		WBP11
Nucleolar protein 58	NOP58_HUMAN		NOP58
Protein SGT1 homolog	SGT1_HUMAN		SUGT1
Protein MTO1 homolog, mitochondrial	MTO1_HUMAN		MTO1
Acyl-coenzyme A thioesterase 9, mitochondrial	ACOT9_HUMAN		ACOT9
Nitric oxide synthase-interacting protein	NOSIP_HUMAN		NOSIP
Thioredoxin-related transmembrane protein 2	TMX2_HUMAN		TMX2
U6 snRNA-associated Sm-like protein LSm2	LSM2_HUMAN		LSM 2.00
Endophilin-B1	SHLB1_HUMAN		SH3GLB1
Calcium-binding protein 39	CAB39_HUMAN		CAB39
Putative RNA-binding protein Luc7-like 2	LC7L2_HUMAN		LUC7L2
Small ribosomal subunit protein uS2m	RT02_HUMAN		MRPS2
Ribosome maturation protein SBDS	SBDS_HUMAN		SBDS
Exosome complex component CSL4	EXOS1_HUMAN		EXOSC1
Transmembrane emp24 domain-containing protein 7	TMED7_HUMAN		TMED7
Splicing factor 3B subunit 6	SF3B6_HUMAN		SF3B6
Large ribosomal subunit protein uL11m	RM11_HUMAN		MRPL11
Oligoribonuclease, mitochondrial	ORN_HUMAN		REXO2
RRP15-like protein	RRP15_HUMAN		RRP15
Nucleolar protein 16	NOP16_HUMAN		NOP16
Peptidyl-prolyl cis-trans isomerase-like 1	PPIL1_HUMAN		PPIL1
Ubiquitin-fold modifier-conjugating enzyme 1	UFC1_HUMAN		UFC1
Adenylate kinase isoenzyme 6	KAD6_HUMAN		AK6
Small ribosomal subunit protein mS23	RT23_HUMAN		MRPS23
Peptidyl-tRNA hydrolase 2, mitochondrial	PTH2_HUMAN		PTRH2
Charged multivesicular body protein 3	CHMP3_HUMAN		CHMP3
Serine-threonine kinase receptor-associated protein	STRAP_HUMAN		STRAP
RNA-splicing ligase RtcB homolog	RTCB_HUMAN		RTCB
Rab GTPase-activating protein 1	RBGP1_HUMAN		RABGAP1
TSC22 domain family protein 4	T22D4_HUMAN		TSC22D4
Nucleolar complex protein 2 homolog	NOC2L_HUMAN		NOC2L
Large ribosomal subunit protein eL36	RL36_HUMAN		RPL36
Chromatin target of PRMT1 protein	CHTOP_HUMAN		CHTOP
Deoxynucleoside triphosphate triphosphohydrolase SAMHD1	SAMH1_HUMAN		SAMHD1
HBS1-like protein	HBS1L_HUMAN		HBS1L
Talin-1	TLN1_HUMAN		TLN1
Transformation/transcription domain-associated protein	TRRAP_HUMAN		TRRAP
Ubiquitin carboxyl-terminal hydrolase 15	UBP15_HUMAN		USP15
FERM, ARHGEF and pleckstrin domain-containing protein 1	FARP1_HUMAN		FARP1
Talin-2	TLN2_HUMAN		TLN2
Insulin receptor substrate 2	IRS2_HUMAN		IRS2
Unconventional myosin-Va	MYO5A_HUMAN		MYO5A
Lysyl oxidase homolog 2	LOXL2_HUMAN		LOXL2
Mitogen-activated protein kinase kinase kinase kinase 5	M4K5_HUMAN		MAP4K5
Hypoxia up-regulated protein 1	HYOU1_HUMAN		HYOU1
Transducin beta-like protein 2	TBL2_HUMAN		TBL2
Telomere length regulation protein TEL2 homolog	TELO2_HUMAN		TELO2
Ribosomal biogenesis protein LAS1L	LAS1L_HUMAN		LAS1L
AFG3-like protein 2	AFG32_HUMAN		AFG3L2
U6 snRNA-associated Sm-like protein LSm4	LSM4_HUMAN		LSM 4.00
E3 ubiquitin-protein ligase RNF114	RN114_HUMAN		RNF114
Sorting and assembly machinery component 50 homolog	SAM50_HUMAN		SAMM50
Protein PRRC2C	PRC2C_HUMAN		PRRC2C
Protein phosphatase methylesterase 1	PPME1_HUMAN		PPME1
RNA-binding protein 7	RBM7_HUMAN		RBM7
YTH domain-containing family protein 2	YTHD2_HUMAN		YTHDF2
FACT complex subunit SPT16	SP16H_HUMAN		SUPT16H
Mitochondrial import inner membrane translocase subunit Tim8 B	TIM8B_HUMAN		TIMM8B
Ubiquitin carboxyl-terminal hydrolase isozyme L5	UCHL5_HUMAN		UCHL5
CD2-associated protein	CD2AP_HUMAN		CD2AP
V-type proton ATPase subunit D	VATD_HUMAN		ATP6V1D
Transportin-3	TNPO3_HUMAN		TNPO3
Mitochondrial import inner membrane translocase subunit Tim13	TIM13_HUMAN		TIMM13
Signal recognition particle receptor subunit beta	SRPRB_HUMAN		SRPRB
Mannose-1-phosphate guanyltransferase beta	GMPPB_HUMAN		GMPPB
Serine/threonine-protein kinase MRCK beta	MRCKB_HUMAN		CDC42BPB
RNA-binding protein 8A	RBM8A_HUMAN		RBM8A
Melanoma-associated antigen D1	MAGD1_HUMAN		MAGED1
Sorting nexin-9	SNX9_HUMAN		SNX9
Sorting nexin-8	SNX8_HUMAN		SNX8
Sorting nexin-5	SNX5_HUMAN		SNX5
Cytosolic Fe-S cluster assembly factor NUBP2	NUBP2_HUMAN		NUBP2
MORF4 family-associated protein 1	MOFA1_HUMAN		MRFAP1
Pseudouridylate synthase 1 homolog	PUS1_HUMAN		PUS1
Leucine-rich repeat flightless-interacting protein 2	LRRF2_HUMAN		LRRFIP2
Phosphoserine aminotransferase	SERC_HUMAN		PSAT1
Nuclear receptor corepressor 2	NCOR2_HUMAN		NCOR2
Neuroplastin	NPTN_HUMAN		NPTN
Spindlin-1	SPIN1_HUMAN		SPIN1
Dolichyl-phosphate beta-glucosyltransferase	ALG5_HUMAN		ALG5

Small ribosomal subunit protein mS40	RT18B_HUMAN		MRPS18B
Coatomer subunit gamma-1	COPG1_HUMAN		COPG1
Lipid droplet-regulating VLDL assembly factor AUP1	AUP1_HUMAN		AUP1
Peptidyl-prolyl cis-trans isomerase FKBP7	FKBP7_HUMAN		FKBP7
Chloride intracellular channel protein 4	CLIC4_HUMAN		CLIC4
Cysteine desulfurase	NFS1_HUMAN		NFS1
Transforming acidic coiled-coil-containing protein 3	TACC3_HUMAN		TACC3
Signal peptidase complex subunit 1	SPCS1_HUMAN		SPCS1
EMILIN-1	EMIL1_HUMAN		EMILIN1
Mitochondrial carrier homolog 2	MTCH2_HUMAN		MTCH2
Brefeldin A-inhibited guanine nucleotide-exchange protein 1	BIG1_HUMAN		ARFGEF1
Mitotic spindle assembly checkpoint protein MAD1	MD1L1_HUMAN		MAD1L1
Serine/threonine-protein kinase 24	STK24_HUMAN		STK24
eIF5-mimic protein 1	5MP1_HUMAN		BZW2
Cytoplasmic dynein 1 light intermediate chain 1	DC1L1_HUMAN		DYNC1LI1
Coiled-coil-helix-coiled-coil-helix domain-containing protein 2	CHCH2_HUMAN		CHCHD2
Epsin-1	EPN1_HUMAN		EPN1
Testis-expressed protein 264	TX264_HUMAN		TEX264
NF-kappa-B essential modulator	NEMO_HUMAN		IKBKG
Insulin-like growth factor 2 mRNA-binding protein 2	IF2B2_HUMAN		IGF2BP2
NADH dehydrogenase [ubiquinone] 1 beta subcomplex subunit 9	NDUB9_HUMAN		NDUFB9
Sulfide:quinone oxidoreductase, mitochondrial	SQOR_HUMAN		SQRDL
Actin-binding protein WASF2	WASF2_HUMAN		WASF2
ATPase MORC2	MORC2_HUMAN		MORC2
Influenza virus NS1A-binding protein	NS1BP_HUMAN		IVNS1ABP
SEC23-interacting protein	S23IP_HUMAN		SEC23IP
